# Integrative Transcriptomic and Network Analysis of Shared Osteo-Immune Regulatory Programs in Postmenopausal Osteoporosis and Osteosarcoma Within Central Mexican Cohorts

**DOI:** 10.3390/cimb48070747

**Published:** 2026-07-22

**Authors:** Rogelio Frank Jiménez-Ortega, Aldo Hugo de la Cruz-Montoya, Nelly Patiño, Rafael Velázquez-Cruz, Alberto Hidalgo-Bravo

**Affiliations:** 1 Servicio de Medicina Genómica, Instituto Nacional de Rehabilitación Luis Guillermo Ibarra Ibarra (INRLGII), Mexico City 14389, Mexico; rfrankjo@gmail.com (R.F.J.-O.); audelacm@gmail.com (A.H.d.l.C.-M.); 2 Unidad de Citometría de Flujo (UCiF), Instituto Nacional de Medicina Genómica (INMEGEN), Mexico City 14610, Mexico; lnpatino@inmegen.gob.mx; 3 Laboratorio de Genómica del Metabolismo Óseo, Instituto Nacional de Medicina Genómica (INMEGEN), Mexico City 14610, Mexico; rvelazquez@inmegen.gob.mx

**Keywords:** osteoporosis, osteosarcoma, osteoimmunology, transcriptomics, pathway integration

## Abstract

Osteoporosis (OP) and osteosarcoma (OS) are biologically distinct skeletal disorders that share dysregulated bone remodeling, inflammatory signaling, and microenvironmental interactions. This study performed an integrative transcriptomic analysis to identify shared osteoimmune regulatory programs in circulating monocytes from postmenopausal women with OP and OS tumors from Central Mexican cohorts. Two independent RNA-seq cohorts were analyzed separately and then integrated: circulating monocytes from postmenopausal women with OP and controls (7 OP and 7 controls), and donor-matched OS tissues (7 tumors and 7 healthy bone samples). Differential expression, module-based filtering, pathway enrichment, cross-cohort functional integration, directional concordance, and targeted interaction network analyses were performed. The OP cohort showed 169 differentially expressed genes, whereas the OS cohort showed 2135 genes. Module-based filtering retained 82 genes in OP and 278 in OS, with only six genes directly shared. However, pathway-level integration identified convergent signals involving PI3K-Akt, HIF-1 signaling, lipid and atherosclerosis, phagosome, endoplasmic reticulum protein processing, focal adhesion, proteoglycans in cancer, and cancer-related pathways. Directional analysis of 27 shared pathway-associated genes revealed predominantly discordant regulation, with CTNNB1 emerging as a central network node. These findings suggest that OP and OS converge through specific osteoimmune pathways rather than through a uniform shared gene program.

## 1. Introduction

Osteoporosis (OP) and osteosarcoma (OS) are clinically distinct skeletal disorders that both involve significant alterations in bone remodeling, inflammatory signaling, and tissue homeostasis. OP is a systemic skeletal disease defined by reduced bone strength and increased fracture risk, representing a major health burden in aging populations, including Mexico. In contrast, OS is the most common primary malignant bone tumor, characterized by aggressive local growth, early metastatic potential, and poor outcomes in advanced stages. Despite differences in age distribution, clinical behavior, and biological context, both disorders are associated with dysregulated interactions among bone cells, immune pathways, and extracellular matrix remodeling [1,2,3,4].

Postmenopausal OP is increasingly recognized as an osteo-immune disorder rather than solely an endocrine condition. Estrogen deficiency promotes a pro-resorptive inflammatory environment, favoring osteoclast formation and bone loss. Circulating monocytes are particularly relevant in this context, as they originate from the monocyte/macrophage lineage that produces osteoclast precursors and reflect systemic signals linked to pathological bone resorption. Recent studies indicate that monocyte composition and monocyte-derived transcriptomic signatures are associated with OP and bone mineral density (BMD) variation, supporting their value as a biologically informative compartment for molecular analyses [5,6,7,8].

OS is no longer regarded only as a tumor of malignant osteoblastic cells. Current evidence demonstrates that its progression is strongly influenced by the tumor immune microenvironment, including immune infiltration patterns, stromal interactions, and extracellular matrix remodeling. These components shape tumor growth, metastatic behavior, and treatment response, indicating that complex osteo-immune regulatory processes also govern OS [4,9,10].

Consequently, despite the involvement of different biological compartments, a comparative analysis of circulating monocytes from postmenopausal OP and OS tumors is biologically justified to identify convergent regulatory programs related to inflammation, bone remodeling, and immune–stromal crosstalk [4,9,10]. Methodologically, integrative transcriptomic analysis combined with pathway- and interaction-based network approaches provides an appropriate framework for this objective. Differential expression analysis identifies genes and pathways associated with each condition, while cross-cohort functional integration defines shared biological programs beyond direct gene overlap. This systems-level strategy is particularly useful in complex diseases, where common mechanisms may be distributed across pathways rather than confined to individual genes.

Accordingly, this study conducted an integrative transcriptomic analysis to identify shared osteo-immune regulatory programs in circulating monocytes derived from postmenopausal women with OP and OS tumors in Central Mexican cohorts. By comparing these two biologically distinct yet bone-related contexts, the analysis aimed to define convergent molecular pathways and candidate shared regulators that may link bone fragility, osteoclast-related immune activity, and bone malignancy [4,9,10,11].

## 2. Materials and Methods

### 2.1. Study Design and Analytical Overview

An integrative comparative transcriptomic analysis was performed using two independent RNA-seq cohorts from Central Mexico. The analytical framework combined differential expression profiling, cross-cohort integration, functional enrichment, and network-based interaction analyses to identify shared osteo-immune regulatory programs between postmenopausal OP and OS.

### 2.2. OP and OS Cohorts and Transcriptomic Datasets

The OP RNA-seq cohort comprised 14 unrelated postmenopausal women from the Mexican Health Worker Cohort Study (MHWCS) [12], including 7 non-osteoporotic controls and 7 women with OP. Participant recruitment, eligibility criteria, BMD assessment, informed consent procedures, monocyte isolation, and the experimental workflow used to generate the circulating monocyte transcriptomic profiles analyzed here were based on previously published methodology [13]. In the present study, these profiles were used to represent the systemic osteo-immune compartment associated with postmenopausal OP, thereby defining this cohort as the OP component within the integrative comparative framework. This cohort study was approved by the ethics committees of the Mexican Institute of Social Security (IMSS; approval No. 12CEI 09 006 14) and the National Institute of Genomic Medicine (INMEGEN; approval No. 266-17/2016/I).

The OS cohort comprised patients treated at the Instituto Nacional de Rehabilitación “Luis Guillermo Ibarra Ibarra” (INRLGII), Mexico City, Mexico. Tumor tissue and tumor-free healthy bone tissue were obtained from seven patients diagnosed with osteosarcoma who underwent surgical resection at INRLGII. From each patient, tumor tissue and tumor-free healthy bone tissue were collected, resulting in 14 RNA-seq tissue samples. The healthy bone samples were obtained from the resection margins, and the absence of tumor activity was confirmed by histopathological examination. All clinicopathologic variables corresponded to donor-level characteristics, and the paired design was retained in the downstream differential expression analysis. This cohort was approved by the Ethics Committee of the Instituto Nacional de Rehabilitación “Luis Guillermo Ibarra Ibarra” (approval No. 29/15).

Both cohorts were derived from institutions located in Central Mexico. The OP cohort was obtained from the Mexican Health Worker Cohort Study in Cuernavaca, Morelos, and has been previously described as comprising Mexican-Mestizo postmenopausal women. The OS cohort was obtained from patients treated at the Instituto Nacional de Rehabilitación “Luis Guillermo Ibarra Ibarra” in Mexico City. Individual-level self-reported ethnicity and genetic ancestry estimates were not available for all participants. Therefore, because of the small sample size and the lack of ancestry-informative data, all analyses were performed without stratification or adjustment for ethnic background. Accordingly, the findings should be interpreted as being derived from Central Mexican cohorts and not as representative of all ethnic or genetic backgrounds in Mexico.

RNA-seq library preparation and sequencing were performed using the same workflow for both cohorts. Total RNA was obtained from CD14+ circulating monocytes in the OP cohort and from tumor tissue and matched healthy bone tissue in the OS cohort. Total RNA was purified using the miRNeasy Mini Kit (QIAGEN GmbH, Hilden, Germany), and RNA quantity and integrity were evaluated using a Qubit 3.0 Fluorometer (Invitrogen, Thermo Fisher Scientific Inc., Waltham, MA, USA) and a 2100 Bioanalyzer (Agilent Technologies, Inc., Santa Clara, CA, USA), respectively. cDNA libraries were constructed from 30 ng of RNA using the SMARTer small RNA-Seq Kit for Illumina (Clontech Laboratories, Inc., Palo Alto, CA, USA), according to the manufacturer’s protocol. Briefly, input total RNA was polyadenylated, cDNA synthesis was performed, adapter sequences were incorporated, Illumina adapters were added during PCR amplification, and cDNA was size-selected using Agencourt AMPure XP beads (Beckman Coulter, Inc., Brea, CA, USA). Purified cDNA libraries were sequenced for 50 cycles on an Illumina NextSeq 500 platform (Illumina, Inc., San Diego, CA, USA). The corresponding raw RNA-seq datasets were deposited in the NCBI Gene Expression Omnibus (GEO) under accession numbers GSE337508 for the OP cohort and GSE337651 for the OS cohort.

### 2.3. Differential Expression Analysis

After retrieval of the raw RNA-seq count matrices for both cohorts (Supplementary Tables S1 and S2), all downstream transcriptomic analyses were performed independently, as the OP and OS datasets represented distinct biological compartments and study designs. Differential expression analysis was performed in R v4.6.1 (R Foundation for Statistical Computing, Vienna, Austria) using the edgeR and limma packages. Genes with very low expression were filtered before normalization, retaining genes with at least 1 count per million in at least three libraries. Read count normalization was performed using the trimmed mean of M-values (TMM) method implemented in edgeR, followed by voomWithQualityWeights transformation in limma to model the mean–variance relationship of RNA-seq count data while accounting for sample-level quality weights. In the OP cohort, differential expression analysis compared circulating monocyte profiles between non-osteoporotic and osteoporotic postmenopausal women using a two-group design matrix. In the OS cohort, differential expression analysis compared OS tumor tissues with matched healthy bone tissues from the same individuals using a paired design that included donor identity as a blocking factor. Gene-wise linear models were fitted with limma, and empirical Bayes moderation was applied to obtain moderated statistics. Genes were considered differentially expressed if they met a nominal *p*-value < 0.05 and an absolute log fold-change (logFC) > 0.5. *p*-values were also adjusted for multiple testing using the Benjamini–Hochberg false discovery rate procedure and are reported in the supplementary differential expression tables. Full differential expression outputs for the OP and OS cohorts are provided in Supplementary Tables S3 and S4, respectively.

### 2.4. Exploratory Transcriptomic Analysis

**Figure 1:**
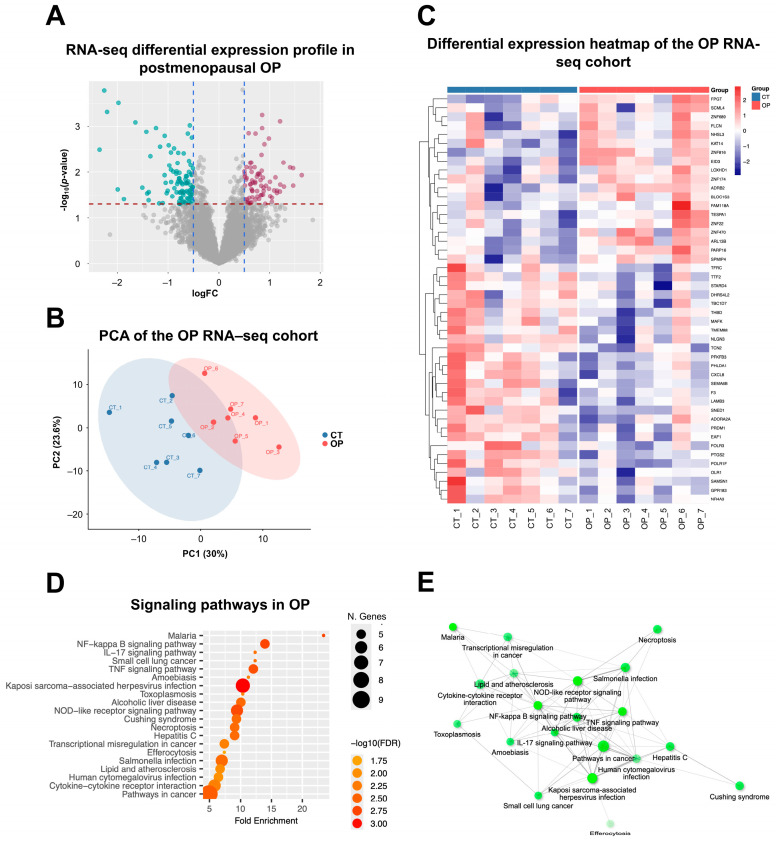
Differential expression profile, exploratory transcriptomic structure, and pathway-level functional organization of the postmenopausal OP RNA-seq cohort. (**A**) Volcano plot showing differentially expressed genes identified between circulating monocytes from non-osteoporotic controls (CT) and women with postmenopausal osteoporosis (OP), using nominal *p* < 0.05 and |logFC| > 0.5 as significance thresholds. (**B**) Principal component analysis (PCA) based on the OP differentially expressed gene matrix, showing partial separation between CT and OP samples along PC1 (30.0%) and PC2 (23.6%); the shaded region indicates the CT group. (**C**) Heatmap showing 46 OP differentially expressed genes selected using a stricter threshold, nominal *p* < 0.01 and |logFC| > 0.5, after row-wise scaling and unsupervised hierarchical clustering, showing group-associated expression patterns between CT and OP samples. The processed OP expression matrix and differential expression output are provided in Supplementary Tables S1 and S3, respectively. (**D**) Dot plot of enriched signaling pathways identified from the module-filtered OP gene set, summarizing fold enrichment, number of genes per term, and significance level. (**E**) Pathway-overlap network derived from the same module-filtered gene set, showing the interconnected functional structure of enriched terms, with inflammatory and immune-related pathways forming the main network core. CT, non-osteoporotic controls; OP, postmenopausal osteoporosis.

**Figure 2:**
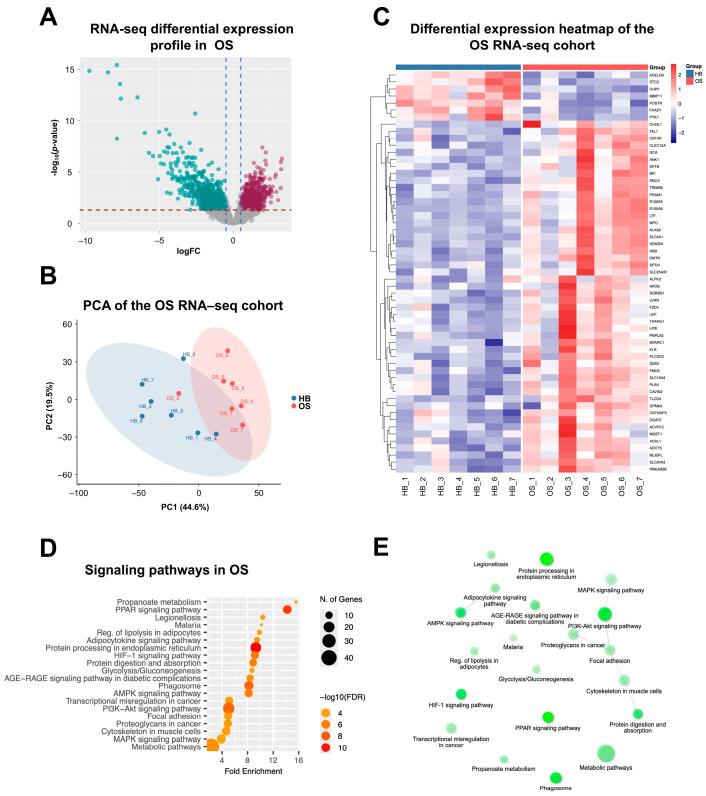
Differential expression profile, exploratory transcriptomic structure, and pathway-level functional organization of the OS RNA-seq cohort. (**A**) Volcano plot showing the differentially expressed genes identified between OS tumor tissue and matched healthy bone tissue (HB), using nominal *p* < 0.05 and |logFC| > 0.5 as significance thresholds. (**B**) PCA based on the OS differentially expressed gene matrix, showing clear separation between HB and OS samples along PC1 (44.6%) and PC2 (19.5%). (**C**) Heatmap of 57 OS differentially expressed genes selected using a stricter threshold, nominal p < 1 × 10−5 and |logFC| > 2.0, after row-wise scaling and unsupervised hierarchical clustering, showing distinct expression patterns between OS tumor tissue and matched healthy bone samples. The processed OS expression matrix and differential expression output are provided in Supplementary Tables S2 and S4, respectively. (**D**) Dot plot of enriched signaling pathways identified from the module-filtered OS gene set, summarizing fold enrichment, number of genes per term, and significance level. (**E**) Pathway-overlap network derived from the same module-filtered gene set, showing the interconnected functional structure of the enriched terms, with signaling, metabolic, adhesion, and stress-response pathways forming the main network core. HB: matched healthy bone; OS, osteosarcoma.

Principal component analysis (PCA) was performed separately for the OP and OS cohorts using expression matrices restricted to differentially expressed genes (DEGs) to determine whether the DEGs captured cohort-specific sample structure. For visualization purposes, the heatmaps shown in Figure 1C and Figure 2C were regenerated using cohort-specific, stricter display thresholds to improve gene-label readability. Figure 1C was restricted to 46 OP differentially expressed genes selected using nominal *p* < 0.01 and |logFC| > 0.5, whereas Figure 2C was restricted to 57 OS differentially expressed genes selected using nominal *p* < 1 × 10^−5^ and |logFC| > 2.0. The full DEG sets were maintained for differential expression summaries and downstream analyses. Heatmaps were generated after row-wise scaling, and samples were organized by unsupervised hierarchical clustering to visualize expression patterns, transcriptomic separation, and internal consistency within each cohort. To further refine the DEG lists to a more interpretable biological core, a module-based filtering step was applied independently to each cohort. In this context, a module was operationally defined as a functionally coherent subset of DEGs that appeared recurrently across overlapping enriched signaling terms rather than as a genome-wide co-expression module. Genes were retained when they consistently mapped to connected pathway clusters representing shared biological processes within each dataset. The resulting module-filtered gene sets were subsequently analyzed in ShinyGO v0.85 (South Dakota State University, Brookings, SD, USA) to identify enriched signaling pathways and visualize pathway-overlap networks. Enrichment was summarized through dot plots and term-overlap maps to highlight biologically connected signaling modules in each cohort.

### 2.5. Shared Pathway-Associated Functional Network Analysis

To identify molecular features common to both skeletal disorders, module-filtered gene sets from the OP and OS cohorts were compared at the gene-symbol level using overlap analysis. The intersecting and cohort-specific gene sets were visualized using a Venn diagram. To determine whether the shared component reflected biologically coherent convergence rather than a simple numerical overlap, the combined gene sets were re-evaluated through pathway enrichment analysis in ShinyGO v0.85. This integrative step assessed whether the pathways represented by the shared and cohort-associated genes remained consistent with those identified in the individual cohort analyses. Enrichment results were summarized as a dot plot of shared signaling pathways. A gene–pathway association network was constructed to visualize the functional relationships between enriched terms and their contributing genes across both conditions. As this network was derived from pathway membership and functional overlap, it was interpreted as an integrative functional interaction map rather than a formal gene co-expression network.

### 2.6. Directional Concordance Analysis of Shared Pathway Genes

An extended directional concordance analysis was performed to evaluate cross-cohort transcriptional consistency. Shared pathway-associated genes identified through cross-cohort functional integration in ShinyGO were compared using logFC values from the independent OP and OS differential expression analyses. Contrast orientation was harmonized so that positive values indicated higher expression in disease relative to control in both cohorts. Genes were classified into concordant or discordant directional categories, and their distribution was summarized using bar and scatter plots.

### 2.7. Targeted Gene Interaction Network Analysis

To further examine the organizational structure of the shared gene space, a targeted gene interaction network was constructed in Cytoscape using the shared pathway-associated genes identified after cross-cohort integration and extended concordance analysis. This analysis aimed to determine whether the shared genes formed a non-random connected structure and to identify the principal connected components within the network. Network topology was summarized by reporting the number of nodes, the number of edges, the average node degree, average local clustering coefficient, expected number of edges, and the corresponding enrichment *p*-value. Previously prioritized genes were highlighted to facilitate their visualization within the final network.

### 2.8. Statistical Analysis and Software

All statistical analyses and visualizations were performed in R. Differential expression analysis was conducted using edgeR v3.18.1 (Walter and Eliza Hall Institute of Medical Research, Parkville, VIC, Australia) for low-expression filtering and TMM normalization, and limma v3.32.7 (Walter and Eliza Hall Institute of Medical Research, Parkville, VIC, Australia) for voomWithQualityWeights transformation, linear modeling, and empirical Bayes moderation. Data import and handling were performed using readxl v1.5.0 (Posit Software, PBC, Boston, MA, USA) and tidyverse v2.0.0 (Posit Software, PBC, Boston, MA, USA). PCA plots were generated using the prcomp function in base R and visualized with ggplot2 v4.0.3 ggplot2 version 4.0.3 (Posit Software, PBC, Boston, MA, USA). Heatmaps were generated using pheatmap v1.0.13 (Raivo Kolde, University of Tartu, Tartu, Estonia) after log2(x + 1) transformation and row-wise scaling. Gene overlap analyses were performed using VennDiagram v1.8.2 (Hanbo Chen and Paul C. Boutros, Ontario Institute for Cancer Research, Toronto, ON, Canada), and directional concordance plots were generated using ggplot2. Pathway enrichment analyses and term-overlap networks were generated in ShinyGO v0.85 (South Dakota State University, Brookings, SD, USA). The targeted gene interaction network was constructed and visualized in Cytoscape v3.10.4 (Cytoscape Consortium, San Diego, CA, USA). Differential expression analyses were conducted independently for each cohort according to its study design, with significance thresholds applied as defined at each analytical step to support biological interpretation and manuscript-ready visualization.

## 3. Results

In the OP RNA-seq cohort, both study groups included seven postmenopausal women. No statistically significant differences were observed between non-osteoporotic and osteoporotic participants regarding age, height, weight, body mass index, or blood glucose levels (*p* > 0.05). As expected, the osteoporotic group showed markedly lower hip BMD than the non-osteoporotic group (0.67 ± 0.01 vs. 0.98 ± 0.06 g/cm^2^, *p* < 0.001), together with a substantially lower hip T-score (−2.61 ± 0.11 vs. −0.21 ± 0.50, *p* < 0.001), confirming the densitometric distinction between the groups (Table 1).

The OS RNA-seq cohort comprised seven patients. As stated in the Methods section, both tumor tissue and healthy tissue were derived from the same individual. Therefore, a total of 14 tissue samples—seven OS tumor samples and seven healthy bone tissues—were analyzed. Patients had a mean age of 21.57 ± 7.00 years, and 42.9% were female. The femur was the predominant primary tumor site (85.7%), whereas one case arose in the tibia (14.3%). During follow-up, metastatic disease occurred in 5 of 7 patients (71.4%) and relapse occurred in 4 of 7 patients (57.1%) (Table 2).

The OP cohort showed a discrete yet organized differential expression pattern between non-osteoporotic controls and women with OP (Figure 1A). A total of 93 downregulated and 76 upregulated genes were identified, indicating bidirectional transcriptional changes in circulating monocytes. PCA based on the OP differentially expressed gene (DEG) matrix demonstrated partial separation between the groups, with PC1 and PC2 explaining 30.0% and 23.6% of the variance, respectively (Figure 1B). Although some overlap remained, control and OP samples occupied distinct regions in the PCA space. This trend was consistent with the revised OP heatmap, which was restricted to 46 differentially expressed genes selected using a stricter visualization threshold and revealed group-associated expression patterns between both groups (Figure 1C). After module-based filtering, 82 genes were retained in the postmenopausal OP cohort. Functional enrichment analysis indicated that these genes were primarily associated with inflammatory and immune-related pathways, including NF-κB, TNF, IL-17, NOD-like receptor signaling, cytokine–cytokine receptor interaction, and lipid and atherosclerosis pathways (Figure 1D). Additional enriched terms, such as efferocytosis, necroptosis, and transcriptional misregulation in cancer, suggested broader inflammatory and stress-response processes. The pathway-overlap network demonstrated that these pathways formed an interconnected structure, with cytokine signaling and innate immune activation at the center of the network (Figure 1E).

The OS cohort demonstrated a broader and more pronounced differential expression profile than the OP cohort, with more extreme logFC values and a greater number of significant DEGs, including 1087 upregulated and 1048 downregulated genes (Figure 2A).

This pattern aligns with the marked transcriptional divergence between OS tissue and healthy bone. PCA showed clearer separation in OS than in OP, with PC1 and PC2 explaining 44.6% and 19.5% of the variance, respectively (Figure 2B). Most healthy bone and OS samples were distributed in distinct regions of the PCA space.

The revised OS heatmap, restricted to 57 differentially expressed genes selected using a stricter visualization threshold, supported this result by showing large-scale expression differences and consistent clustering of OS tumor samples and matched healthy bone samples (Figure 2C). Module-based filtering retained 278 genes in the OS cohort.

These genes were enriched in pathways related to tumor signaling and metabolic reprogramming, including PI3K-Akt, MAPK, PPAR, AMPK, and HIF-1 signaling pathways, as well as AGE-RAGE signaling, focal adhesion, proteoglycans in cancer, phagosome, glycolysis/gluconeogenesis, and metabolic pathways (Figure 2D). The pathway network showed a connected structure in which signaling, metabolic, adhesion, and stress-response pathways converged (Figure 2E).

**Figure 3:**
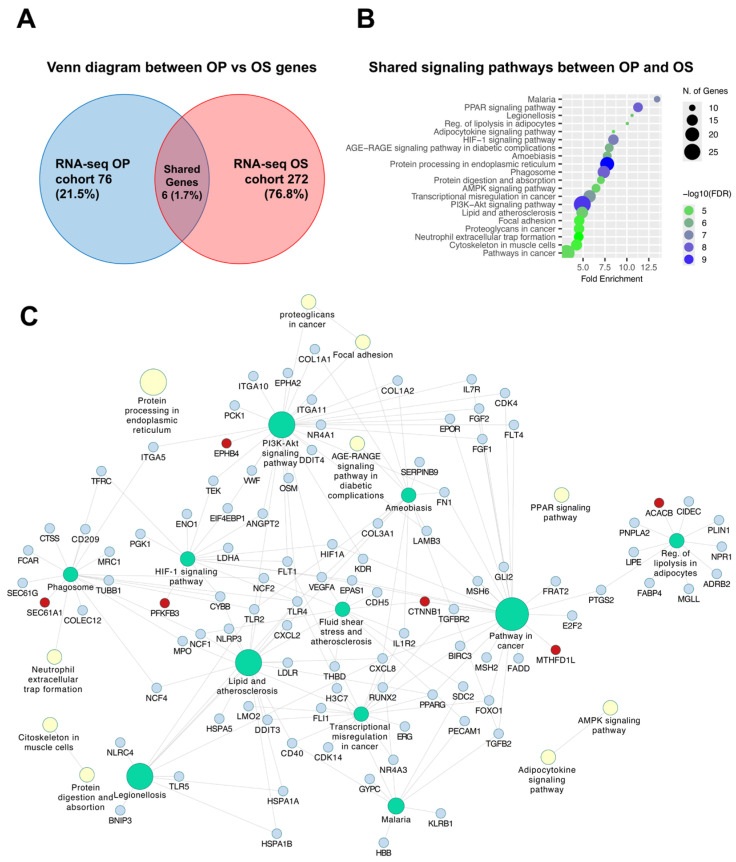
Cross-cohort integration and shared gene–pathway functional network between postmenopausal OP and OS. (**A**) Venn diagram showing the overlap between the module-filtered gene sets derived independently from the OP and OS RNA-seq cohorts, identifying six genes shared directly, alongside 76 genes unique to OP and 272 genes unique to OS. (**B**) Dot plot summarizing the signaling pathways shared between both cohorts after cross-cohort functional integration, showing fold enrichment, the number of genes contributing to each term, and the significance level. (**C**) Integrated gene–pathway interaction map of the broader shared functional space between OP and OS, showing the relationships between enriched shared signaling pathways and their associated genes. Overall, the figure shows that although direct gene-level overlap was limited, both cohorts converged on a broader shared functional space linked to inflammatory, metabolic, stress-response, and microenvironment-related processes. The shared pathway-associated gene lists and thematic gene sets used for the cross-cohort integration are provided in Supplementary Table S5.

After module-based filtering, cross-cohort comparison showed limited gene-level overlap between OP and OS. Most genes were cohort-specific, with 76 unique to OP and 272 unique to OS, whereas only six genes were shared (Figure 3A), indicating largely distinct transcriptomic profiles. Despite this limited overlap, pathway-level integration revealed convergent biological signals across both conditions. Shared enrichment terms included PI3K-Akt and HIF-1 signaling, lipid and atherosclerosis, phagosome, protein processing in the endoplasmic reticulum, focal adhesion, proteoglycans in cancer, and pathways in cancer (Figure 3B), suggesting convergence of inflammatory, metabolic, stress-response, and microenvironment-related processes. The integrated gene–pathway network showed that these shared signals formed a connected functional structure rather than isolated enrichment events. Highly connected hubs included pathways in cancer, PI3K-Akt signaling, HIF-1 signaling, lipid and atherosclerosis, phagosome, and transcriptional misregulation in cancer (Figure 3C).

To extend the comparison beyond the six directly shared genes, a broader set of 27 shared pathway-associated genes derived from cross-cohort functional integration was analyzed. Discordant regulation predominated between OP and OS (Supplementary Figure S1): 18 of 27 genes (66.7%) showed opposite expression directions, whereas 9 out of 27 (33.3%) showed concordant expression. The most common pattern was downregulation in OP and upregulation in OS (11 genes), followed by upregulation in OP and downregulation in OS (7 genes). These findings suggest that the shared pathway-level signal across both conditions was mainly driven by opposing gene-level expression patterns.

**Figure 4:**
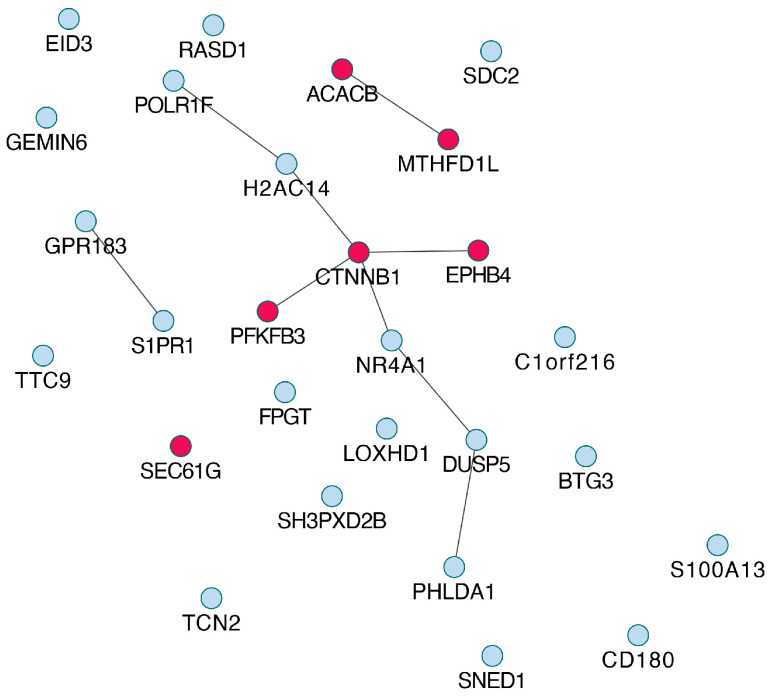
Targeted gene interaction network of the shared pathway-associated genes. Targeted interaction analysis of the shared pathway-associated genes identified a limited, fragmented network with 27 nodes and 9 edges, an average node degree of 0.667, and an average local clustering coefficient of 0.296. The network retained more interactions than expected by chance (expected number of edges = 4; enrichment *p* = 0.02), supporting a non-random organization of the shared gene space. The largest connected component was centered on *CTNNB1*, which was linked to *PFKFB3*, *EPHB4*, *NR4A1*, and *H2AC14* and extended through secondary connections involving *H2AC14*–*POLR1F* and *NR4A1*–*DUSP5*–*PHLDA1*. Smaller connected components included the *ACACB*–*MTHFD1L* and *GPR183*–*S1PR1* pairs, whereas several genes remained isolated. Previously prioritized shared genes are highlighted in red. The 27 shared pathway-associated genes used for the targeted interaction network are provided in Supplementary Table S5, and the corresponding R source code is provided in Supplementary Materials Codes.

The targeted network analysis of the shared pathway-associated genes showed a limited and fragmented interaction structure (27 nodes, 9 edges; average node degree = 0.667; average local clustering coefficient = 0.296), although the network retained more interactions than expected by chance (expected edges = 4; enrichment *p* = 0.02) (Figure 4). The largest connected component was organized around *CTNNB1*, which was linked to *PFKFB3*, *EPHB4*, *NR4A1*, and *H2AC14*, with secondary connections extending through *H2AC14*–*POLR1F* and *NR4A1*–*DUSP5*–*PHLDA1*. Smaller components included the *ACACB*–*MTHFD1L* and *GPR183*–*S1PR1* pairs. In contrast, several genes, including *SEC61G*, *GEMIN6*, *SDC2*, *RASD1*, *CD180*, *S100A13*, *EID3*, *TCN2*, *SNED1*, *BTG3*, *C1orf216*, *FPGT*, *LOXHD1*, and *SH3PXD2B*, remained isolated. Among the prioritized genes, *CTNNB1*, *ACACB*, *MTHFD1L*, *EPHB4*, and *PFKFB3* were located within connected components, whereas *SEC61G* remained disconnected.

## 4. Discussion

The integrative analysis showed that direct gene-level overlap between postmenopausal osteoporosis and osteosarcoma is limited, while convergence becomes more apparent at the pathway level. This finding indicates that these disorders do not share a broad transcriptomic identity but instead overlap within a restricted set of osteoimmune programs involving inflammatory signaling, metabolic adaptation, hypoxia-related responses, and extracellular interactions. This interpretation aligns with recent transcriptomic studies conducted on postmenopausal osteoporosis, which highlight immune-cell disequilibrium, particularly among monocyte populations and the broader bone immune environment. Similarly, contemporary osteosarcoma literature increasingly supports the view that tumor behavior is strongly influenced by immune, stromal, and matrix-dependent microenvironmental cues [8,14,15,16].

The derivation of the osteoporosis signal from circulating monocytes is particularly significant. Monocytes serve not only as osteoclast precursors but also as active regulators of inflammatory tone and bone resorption. Recent single-cell studies suggest that postmenopausal osteoporosis is more closely associated with shifts in monocyte subset composition and immune-cell ecosystem structure than with uniform bulk transcriptional changes.

Accordingly, the identification of shared pathways, such as the phagosome, PI3K-Akt, HIF-1 signaling, and protein processing in the endoplasmic reticulum, likely reflects conserved stress-adaptive and immune-regulatory mechanisms rather than tissue-specific marker genes. On the other hand, in osteosarcoma, analogous processes occur within a specialized bone tumor microenvironment, where hypoxia, immune evasion, macrophage polarization, angiogenesis, and extracellular matrix remodeling contribute to invasion, metastatic behavior, and treatment resistance [15,16,17,18].

The observed network structure further supports this interpretation. The organization of the largest connected component around CTNNB1 is biologically significant, as β-catenin plays a central role at the intersection of bone homeostasis, osteogenic differentiation, cell adhesion, and tumor-associated signaling. Recent studies emphasize that Wnt/β-catenin signaling is a key regulatory axis for bone formation and skeletal maintenance, and in osteosarcoma, it is increasingly associated with aggressive phenotypes and interactions with the extracellular matrix. Additionally, the enrichment of focal adhesion and proteoglycan-related processes is consistent with evidence that osteosarcoma aggressiveness is strongly influenced by matrix organization, adhesion signaling, and mechanotransduction. In contrast, in bone biology, these systems affect lineage commitment, osteoblast–osteoclast coupling, and tissue remodeling [17,19,20,21,22,23].

However, the limited direct overlap of six genes and the fragmented topology of the targeted interaction network bring awareness for interpreting the shared signature as a single, robust pan-skeletal program. A more measured interpretation is that osteoporosis and osteosarcoma converge within selected regulatory circuits while maintaining strong compartment-specific transcriptional architecture, as expected given the comparison of peripheral immune cells with tumor tissue. This also clarifies why the extended directional concordance analysis across pathway-associated genes was informative: it captured biological alignment that a strict intersection would underestimate. These findings are methodologically consistent with emerging multi-omics and single-cell studies, which demonstrate that both postmenopausal bone loss and osteosarcoma are shaped by heterogeneous cell states and context-dependent signaling rather than by a uniform molecular core [24,25,26,27,28].

This study presents both strengths and limitations. A primary strength is its integrative analytical design, which enabled the identification of shared osteoimmune regulatory themes between osteoporosis and osteosarcoma despite their biological and tissue-specific differences. By combining differential expression analysis, pathway-level integration, extended directional concordance, and targeted interaction network analysis, the study identified biologically meaningful convergence beyond the limited direct gene-level overlap. This framework enhanced the interpretability of cross-cohort comparisons and highlighted prioritized pathways related to bone remodeling, inflammatory signaling, hypoxia, and focal adhesion, providing a relevant basis for future translational and mechanistic studies. Nonetheless, several limitations should be acknowledged, including the small cohort sizes, biological independence, derivation from different tissue compartments, and the absence of functional validation of the shared signature in external datasets or experimental models. These limitations, however, also justify the integrative strategy employed, as it emphasizes reproducible biological themes over potentially unstable gene-level overlap. Future research should validate the prioritized genes and pathways in independent cohorts, incorporate single-cell or spatial transcriptomic approaches to clarify cell-type contributions, and assess whether CTNNB1-centered, hypoxia-related, or focal adhesion-associated programs can distinguish clinically relevant phenotypes in bone loss and bone malignancy.

## 5. Conclusions

Postmenopausal OP and OS appear to converge not through a uniform shared gene program but through a restricted set of osteoimmune pathways related to inflammation, hypoxia, metabolic adaptation, and extracellular interactions. Despite the biological differences between circulating monocytes and the heterogeneous tumor tissue, this integrative approach identified a coherent functional space in which CTNNB1-centered connectivity may serve as a key organizing feature. These findings support pathway-level integration as an effective strategy for uncovering biologically meaningful links across distinct skeletal disorders and provide a foundation for future studies to validate shared regulators with potential mechanistic and translational relevance.

## Figures and Tables

**Table 1:** Clinical and densitometric characteristics.

Variable	Non-Osteoporotic *n* = 7	Osteoporotic *n* = 7	*p*-Value
Age (years)	68.86 ± 0.89	70.43 ± 3.20	0.269
Height (cm)	154.70 ± 2.02	150.60 ± 1.80	0.168
Weight (kg)	56.53 ± 2.05	50.96 ± 3.31	0.197
BMI (kg/m^2^)	23.77 ± 1.19	23.47 ± 3.38	0.871
BGL (mg/dL)	123.3 ± 34.96	100.90 ± 13.85	0.220
Hip BMD (g/cm^2^)	0.98 ± 0.06	0.67 ± 0.01	<0.001
Hip t-score (SD)	−0.21 ± 0.50	−2.61 ± 0.11	<0.001

BMI: Body Mass Index; BGL: Blood glucose level; BMD: Bone Mineral Density; SD: Standard deviation. Note. Data are presented as mean ± SD.

**Table 2:** Donor-level clinicopathologic characteristics of the paired osteosarcoma RNA-seq cohort.

Variable	Paired Osteosarcoma Cohort (*n* = 7 Patients; 14 Tissues)
RNA-seq tissue design, *n* (%)	7 matched pairs (100.0)
Matched healthy bone tissue (HB), *n* (%)	7 (100.0)
Osteosarcoma tumor tissue (OS), *n* (%)	7 (100.0)
Age (years)	21.57 ± 7.00
Female sex, *n* (%)	3 (42.9)
Primary site in femur, *n* (%)	6 (85.7)
Primary site in tibia, *n* (%)	1 (14.3)
Other primary sites, *n* (%)	0 (0.0)
Metastatic disease at any time, *n*/*N* (%)	5/7 (71.4)
Relapse at any time, *n*/*N* (%)	4/7 (57.1)

Data are presented as mean ± SD or *n* (%), as appropriate. RNA-seq was performed on paired osteosarcoma tumor tissue and matched healthy bone tissue obtained from the same donors. All clinicopathologic variables correspond to donor-level characteristics; therefore, between-group statistical comparisons are not applicable for this paired design. HB, healthy bone; OS, osteosarcoma.

## Data Availability

Raw RNA-seq data have been deposited in the NCBI Gene Expression Omnibus (GEO) under accession numbers GSE337508 for the OP cohort and GSE337651 for the OS cohort. The GEO records are currently approved and held as private during peer review and will be released upon publication or no later than 3 July 2028. Individual-level clinical data are not publicly available due to ethical and privacy restrictions involving human participants and archived tissue specimens. Processed data supporting the analyses, including expression matrices, differential expression outputs, module-filtered gene sets, shared pathway-associated gene lists, and the R source code used for preprocessing, differential expression analysis, visualization, pathway enrichment summaries, directional concordance analysis, and network-related analyses, have been deposited in Zenodo and are publicly available at https://doi.org/10.5281/zenodo.21076230. Additional information may be made available by the corresponding author upon reasonable request and subject to institutional and ethical approvals.

## Supplementary Materials

The following supporting information can be downloaded at: https://www.mdpi.com/article/10.3390/cimb48070747/s1.

## Abbreviations

The following abbreviations are used in this manuscript:

AGE-RAGE	Advanced glycation end products–receptor for advanced glycation end products
AMPK	AMP-activated protein kinase
BMD	Bone mineral density
BMI	Body mass index
CT	Non-osteoporotic controls
DEGs	Differentially expressed genes
HB	Matched healthy bone
HIF-1	Hypoxia-inducible factor 1
IL-17	Interleukin 17
IMSS	Mexican Institute of Social Security
INMEGEN	National Institute of Genomic Medicine
INRLGII	Instituto Nacional de Rehabilitación “Luis Guillermo Ibarra Ibarra”
logFC	Log fold-change
MAPK	Mitogen-activated protein kinase
MHWCS	Mexican Health Worker Cohort Study
NF-κB	Nuclear factor kappa B
NOD	Nucleotide-binding oligomerization domain
OP	Osteoporosis
OS	Osteosarcoma
PC1	Principal component 1
PC2	Principal component 2
PCA	Principal component analysis
PI3K-Akt	Phosphoinositide 3-kinase–protein kinase B
PPAR	Peroxisome proliferator-activated receptor
RNA-seq	RNA sequencing
TNF	Tumor necrosis factor
