# Supplementary material for: Integrative Transcriptomic and Network Analysis of Shared Osteo-Immune Regulatory Programs in Postmenopausal Osteoporosis and Osteosarcoma Within Central Mexican Cohorts
Source: Curr Issues Mol Biol. 2026 Jul 22;48(7):747. doi: 10.3390/cimb48070747 (PMC13409484; doi:10.3390/cimb48070747)
Supplement: Supplementary file 1 [file cimb-48-00747-s001.zip › cimb-4375466 Figure S1.pdf]

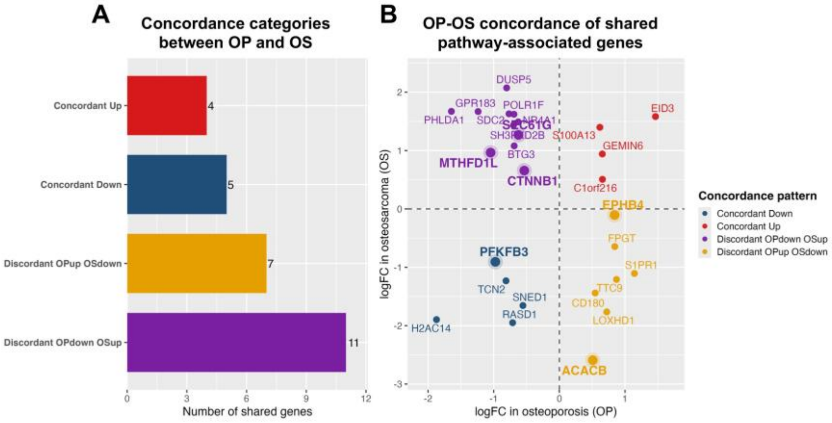

Figure S1. Directional concordance analysis of shared pathway-associated genes and R scripts used for preprocessing, differential expression analysis, visualization, pathway enrichment summaries, directional concordance analysis, and network-related analyses.
