# Supplementary material for: Integrative Transcriptomic and Network Analysis of Shared Osteo-Immune Regulatory Programs in Postmenopausal Osteoporosis and Osteosarcoma Within Central Mexican Cohorts
Source: Curr Issues Mol Biol. 2026 Jul 22;48(7):747. doi: 10.3390/cimb48070747 (PMC13409484; doi:10.3390/cimb48070747)
Supplement: Supplementary file 1 [file cimb-48-00747-s001.zip › Table S3 OP differential expression output.pdf]

Gene_symbol	ID_CT_1	ID_CT_2	ID_CT_3	ID_CT_4	ID_CT_5	ID_CT_6
OLR1	149.245707	8.75882426	237.015236	205.815289	77.6992671	21.935338
SEMA6B	46.8912543	17.6194479	6.19569569	28.480666	23.2482035	4.85120112
F3	162.029394	24.3445716	31.1720023	34.7997828	9.48877127	27.8771255
PHLDA1	346.919117	63.5610732	100.836566	184.706286	113.579562	43.2547271
TTF2	160.618285	123.272609	101.36641	74.0868465	124.09655	84.6434779
GPR183	777.858253	155.853086	237.739399	419.882541	321.499947	183.765761
LAMB3	101.364925	35.9225049	46.8802034	25.5367579	21.911325	43.3402257
DHRS4L2	79.725	102.765441	22.7269858	46.2949199	63.74333	52.2978002
CXCL8	9125.39188	3116.32403	5804.55879	6121.80054	5210.6235	3388.30655
TBC1D7	62.7688157	72.4832362	23.7703553	46.3870647	51.0422775	44.11638
THBD	157.389254	96.2164479	29.1529576	58.4235169	92.9136585	41.0000074
PTGS2	2359.65107	814.486755	2078.91623	2038.54292	1790.81381	1432.44962
STARD4	128.963287	48.8245342	47.4110355	42.3866216	68.0175929	58.2507895
FOLR3	26.9781101	27.6190704	226.212954	212.293881	12.7309213	36.2338125
NR4A3	588.9288	152.242073	333.74931	317.681421	297.150216	238.593835
PRDM1	1157.93624	593.93685	699.369391	568.279321	551.916688	738.553053
POLR1F	215.916713	116.767474	174.966719	125.242876	118.342485	124.65802
ADORA2A	160.07519	72.6418042	68.1884282	98.059037	120.17896	51.0828653
TFRC	918.568853	430.094629	279.484427	359.243845	554.686338	384.617838
TCN2	120.642734	106.903131	66.901619	49.7822433	43.3542317	64.4554069
SNED1	28.5755597	40.9614664	15.8674562	25.0963477	26.2865773	21.6715205
PFKFB3	5224.16197	3059.85434	1476.54577	2836.4683	1917.09793	1474.84436
MAFK	152.952918	130.274936	74.3897291	98.7507846	149.606128	72.2935066
TMEM88	48.774056	19.7519432	12.7164957	15.7611385	16.7158912	11.8508255
NLGN3	25.4423045	23.1373074	7.29008657	20.3446353	16.7423926	19.3155803
SAMSN1	1373.95835	321.899277	398.617714	521.642207	396.432392	345.786265
EAF1	598.742787	521.092886	366.687372	347.05035	520.914835	438.767202
ADRB2	45.7638658	56.3054709	14.2589512	29.5260915	25.0394282	48.356362
ZNF816	6.41492185	9.15907014	11.9376509	9.37101661	11.6959141	10.271978
PARP16	18.76495	22.5206132	8.67379039	10.5799461	20.9034824	10.5963105
ZNF689	41.3714101	81.1091709	18.2981813	33.2671888	42.4648711	53.6100516
LOXHD1	83.6802263	151.806256	58.5649232	33.8952585	103.020832	126.995257
TESPA1	42.4827806	75.4887824	43.2277169	64.4627425	57.9665222	41.5881844
BLOC1S3	21.6423475	40.7284985	5.65764947	14.1412279	20.2790787	21.9557585
ZNF174	23.1454932	18.9560136	6.53362699	5.33770948	19.2324713	11.5225919
ZNF470	7.5245768	9.52455706	7.55527791	3.39130314	10.5904819	5.51999481
FLCN	94.8505113	176.272361	41.0135557	43.4533219	114.298721	95.8359825
NHSL3	25.9716525	46.3317272	19.1606995	23.4196222	22.548951	20.7337726
ZNF22	31.1601149	51.2281903	30.989889	19.4193463	30.3255979	27.6581666
FAM118A	76.3396122	153.451924	66.1872733	55.7892948	88.0154139	98.3537571
SCML4	10.4686488	6.97393282	1.17598731	1.95103541	9.9060467	3.5147592
SPMIP4	48.9151622	33.9357845	12.4366043	29.2261541	49.8575889	23.047089
EID3	2.03685794	6.16723872	1.89708244	0	2.13552595	9.16850577

FPGT	21.0572705	16.6153542	17.4623482	18.5259964	29.9549979	34.907135
KAT14	42.7020253	85.8683103	54.5728683	38.7575615	29.4397669	45.5503173
ARL13B	42.3114647	40.3517597	24.665985	27.1417765	36.8096541	33.7138367

ID_CT_7	ID_OP_1	ID_OP_2	ID_OP_3	ID_OP_4	ID_OP_5	ID_OP_6
55.1392627	6.9191888	27.7271685	0	33.0126478	28.9592645	24.0279594
1.12216376	7.66107697	4.6241772	2.35797344	3.95061893	7.10292368	6.03219323
0	3.50300045	13.5708561	2.4701746	9.77952628	8.56464118	18.7053088
33.40096	12.8666644	37.7828999	15.3227738	36.6122957	50.1934508	104.931125
136.441243	77.8623213	82.5324989	55.4806087	84.240782	49.3617523	123.822005
151.017223	67.074701	188.270831	81.6390541	207.401512	74.3897578	183.731037
3.03294469	9.89742316	15.9355104	2.59586099	14.7114004	18.8609196	26.6917087
86.8852616	51.6105853	40.6844512	23.133746	54.0493069	33.2366647	68.8272957
2184.0851	956.595915	2912.2619	2072.82515	2125.68257	2865.26044	3666.22125
44.7178717	36.0607347	40.2478644	23.4246766	35.3346776	16.3362457	60.6523725
77.733973	38.0816782	37.9748222	11.391664	25.7285778	40.9194509	65.9301082
520.915062	310.103175	1116.09793	505.349012	603.583046	828.324128	709.924979
75.4131166	46.7066941	57.6674127	16.7415153	46.7979606	7.27199577	50.750343
10.1040074	32.2510871	17.9542688	5.33321534	2.95680635	16.3229053	41.2461755
143.690329	126.025293	197.8468	87.1988157	178.830153	128.08122	209.371037
313.778761	206.866353	535.134357	169.828693	447.238064	234.845735	558.644488
98.2574144	88.0592988	115.743305	69.3339463	62.8965468	67.2288348	91.7106742
10.3152483	2.84071525	9.6832935	5.73185322	62.2076785	33.3665022	189.115745
286.505682	357.441839	357.851397	249.131666	266.139913	169.535043	417.652907
58.1214393	43.4616929	21.0299418	17.1484441	64.3630014	47.5363231	53.3338331
18.5611695	10.828313	15.7946412	14.0439673	14.0252102	13.7572775	24.2368194
534.30313	448.569353	986.892225	853.609459	893.482277	1528.82511	1770.79872
95.2352405	87.1689873	61.6060975	42.0957292	81.0814726	67.3893873	124.058861
32.9443017	6.75296226	9.18561497	2.90395304	19.0985469	9.78621491	23.7769653
19.2593308	5.02812085	11.8228942	1.96580248	14.576081	5.98972946	21.9519827
220.194508	262.211217	353.071137	130.574906	407.822098	285.097939	252.046222
189.880065	204.453505	335.194774	150.93724	377.316602	160.221659	456.440435
36.3939376	33.4125417	55.5307923	72.2254213	61.9382908	63.7281225	67.0784008
5.73778413	27.4582536	27.1413738	24.9693843	14.435584	10.1838424	23.50971
9.302753	18.6667833	15.5849615	20.3836231	25.8644855	25.9878926	47.8607276
38.8149617	79.9081849	61.2572627	40.1331941	55.0825715	45.2927901	104.624432
47.9862096	74.8452887	142.414609	116.544774	118.342535	106.746632	206.91902
16.5365316	49.2539347	58.2895196	67.0038891	68.7441249	48.0892143	213.82683
19.4574277	32.9055658	29.332688	67.4075242	19.6238863	21.7779817	61.0698138
5.77981588	16.7510503	27.1428894	20.0789393	14.1283063	18.3767088	27.4107671
3.86364204	15.1386115	6.55791196	28.1957567	16.9251762	4.39462782	21.6505566
69.0525118	201.929651	123.81343	73.6927729	95.3063821	90.5574926	202.525763
9.60382248	45.2448124	38.3785109	22.9072009	29.9087411	22.1093553	57.9624436
15.525238	35.306724	32.7240688	33.8117153	43.9889017	34.7476301	73.9099818
65.3202676	95.8271286	80.7919863	100.123972	79.2646248	96.6041354	399.730883
5.93632802	23.7688962	9.66850066	0	9.86991555	4.02880013	27.3380603
22.1219972	46.5532639	35.4518124	55.706819	41.9352936	32.5898783	62.8722229
0	19.1633274	16.9859349	6.26318476	9.19551109	2.04693562	8.00371623

28.1255112	35.6361941	33.6058399	32.1373289	28.9654045	21.9795714	54.003218
28.7003774	76.1683428	60.4306066	56.8507664	48.7475735	49.0285946	75.2320443
26.419774	41.7723596	48.7560205	58.376791	67.2771605	32.8941885	57.233079

ID_OP_7
27.2807411
3.72978276
6.35176489
26.1652632
71.5363345
109.023413
13.9211412
36.098526
1842.17874
36.5264065
55.932011
967.989245
36.4215469
8.4289708
204.878803
414.729555
91.1927155
52.2595577
339.285224
20.3467074
20.5021793
1040.78107
78.141096
14.9512784
10.4330115
220.681713
320.639552
52.8188246
20.1326079
24.7784592
52.723768
118.807976
140.388828
30.4738379
20.5119793
14.8168952
118.902251
43.2841374
61.0652288
133.173814
22.6343476
50.8594807
10.8205718

47.3960057
61.4007425
53.3259648
