# Supplementary material for: Integrative Transcriptomic and Network Analysis of Shared Osteo-Immune Regulatory Programs in Postmenopausal Osteoporosis and Osteosarcoma Within Central Mexican Cohorts
Source: Curr Issues Mol Biol. 2026 Jul 22;48(7):747. doi: 10.3390/cimb48070747 (PMC13409484; doi:10.3390/cimb48070747)
Supplement: Supplementary file 1 [file cimb-48-00747-s001.zip › Table S4 OS differential expression output .pdf]

Gene_symbol	ID_HB_1	ID_HB_2	ID_HB_3	ID_HB_4	ID_HB_5	ID_HB_6
S100A9	0.35020258	2.20871824	0.96565412	2.05400109	0.3433502	1.35193518
S100A8	0	0	0	0.40753607	0	0.44692057
SLC4A1	0.29914967	3.23315093	0.50081409	2.45059054	0.45876636	0.39966834
LTF	0.50749761	5.37657025	0	1.59205986	0	0.36106792
HBB	21.8259833	30.0122317	1.44490815	12.3124187	16.4612039	1.84240802
MPO	0.23020275	2.36128705	0.61608141	1.67034599	0.39507432	0
ACSL1	60.114714	186.672033	64.2978287	38.9338127	59.5627551	72.4556132
TRIM58	0.95020211	9.1324627	3.10084221	5.29095893	1.8500867	3.83625203
SLC19A3	8.10603907	6.61002972	0	4.26826915	0.99269577	0.43549288
ALAS2	0.39193295	0.97441682	0.87046913	2.19743762	1.5190425	0
DMTN	3.11392248	4.6402655	0.80401364	2.06570733	2.51247364	1.20925009
HEMGN	0	2.64616667	0	3.55400744	1.79866447	0
ADCY5	3.64175775	7.94692622	9.98398624	0.85251632	0.39133964	3.56464965
PRAM1	0.50313526	3.66120887	2.53480838	1.88319833	0.98222819	1.02552817
ANK1	11.8834946	15.7972525	11.3016759	2.6564452	3.89608179	9.68830852
TLCD4	4.53446658	2.14474436	3.81620772	3.31971978	1.07420115	6.45117219
SPTB	19.944309	10.0237787	3.50411332	11.4488269	2.61759321	8.66727289
CNTNAP3	5.52294929	32.6885686	22.8140011	8.16489966	4.3313221	5.11937968
LEP	5.85565685	9.90361187	0.4813045	4.20760206	0.73440635	1.16813586
DGAT2	4.12131255	26.5923301	15.0809897	8.35398996	3.40023129	3.94711612
KDELR3	15.661426	21.6086122	32.1584247	22.9800514	21.1103506	75.9964132
G0S2	57.3908736	108.883626	3.83833785	32.6357434	29.1067449	5.26481023
SORBS1	69.1276297	87.3096356	42.7939548	33.6353091	6.88785413	13.0405627
SPTA1	1.73984189	4.28507786	1.39776565	8.48120533	28.8301332	0.16780924
LVRN	10.2940389	8.47510737	5.47376086	1.8443142	1.2768999	0.42293081
PLIN1	54.6387558	107.449719	1.22092151	15.0814158	1.57624334	1.05875784
BPI	0.39161043	0	2.16593016	2.12670244	1.80920247	0
MLXIPL	1.61689084	2.52206833	10.1356181	2.7751411	1.71445648	0.24894882
SLC24A3	2.71810247	2.37143868	10.3975937	1.27052676	0.37849009	0.62425214
ACVR1C	4.39079409	10.2350445	9.73160638	6.70497319	8.81093335	2.26096468
PRKAR2B	27.4969889	36.5915852	74.4318926	20.4731662	7.85030657	8.7453147
GPM6A	2.09943573	3.19621095	5.48546935	2.35412608	1.82924759	5.91336147
STC2	9.16863865	13.2322288	5.71955176	12.2324358	27.6493808	118.692038
SNCA	7.93781722	10.7914564	16.9431718	13.2719426	5.46225504	2.41426286
FHAD1	46.657126	114.894712	143.047788	30.6321338	156.236363	255.624996
LIPE	10.0856485	13.5525457	2.89571752	5.32514411	4.03175772	2.51406995
POSTN	3292.90818	1343.0425	759.527954	594.072631	812.396277	2122.94813
SLC25A37	13.9120685	51.9679635	38.5829429	15.4234525	41.4002207	15.8337339
CHI3L1	3.5203552	0.94163842	0	1.63669039	1.15949036	0.4170925
GCA	11.6695337	33.3074317	26.6386969	12.7610363	16.1807877	10.3500594
CSF3R	7.54296965	53.9095098	3.3664616	8.42060108	14.2190625	10.2337638
APOB	0.44922681	4.53708958	0.42027218	8.05190017	1.39204108	0.6231168
KLB	2.37210441	2.39924679	0.47177167	5.39573801	2.19547082	0

CAVIN2	35.9810504	55.0094966	5.11204823	20.2571394	9.55230584	3.8286445
TRARG1	8.42398233	5.32182015	0	2.94404358	0.76373719	0.66470345
CHPF	52.4143854	26.7181039	38.6659381	11.3166586	27.1042545	81.4513567
TAL1	2.32769726	14.9506776	1.77522409	4.5513761	2.63594557	2.83902081
FZD4	48.3857797	153.382034	14.3294829	36.5217997	28.9005905	19.3401739
PNPLA2	16.8412754	51.8130781	21.3090726	15.4011504	12.6216698	12.4994767
MTARC1	10.0729883	11.580557	10.7001122	10.4246577	9.95422245	0.38866312
FMO2	4.3901082	40.9149553	1.28020977	25.2588574	4.21789844	1.09253535
PTK7	112.083849	185.814098	254.003887	27.8850883	634.204123	2530.73604
MGST1	7.83160409	8.33327881	21.3846459	4.00859186	8.73632322	6.08261424
MMP11	23.2677357	14.6657572	18.781844	5.31535681	40.979349	19.2498441
PLCXD3	2.90252183	0	0	1.90719629	1.16875675	0
CLEC12A	6.99515844	9.77208328	0.56624112	7.88200176	1.23833589	1.66995115
ALPK3	32.8370141	83.7688737	18.3658701	21.2462877	45.1446919	22.8492557

ID_HB_7	ID_OS_1	ID_OS_2	ID_OS_3	ID_OS_4	ID_OS_5	ID_OS_6
7.7600574	62.3617383	6.93412581	12.126088	988.163887	41.5485312	336.225588
1.6082829	18.9656032	1.14118623	4.63876	522.468667	21.6638994	180.438966
0	30.0073078	3.11304871	24.4801199	1522.31398	42.9691054	186.509802
0.98603037	51.9425952	2.72673622	11.1643468	796.995831	45.5771617	393.4624
47.7654091	231.116354	18.3292637	354.354452	4933.18364	314.993697	1195.04664
3.55238636	47.7335678	0.32047835	5.55165418	608.600186	37.7167065	226.027591
59.0734245	290.633656	178.964807	1044.19975	251.812908	370.178643	304.267206
6.26783783	16.7854012	14.5399803	13.4856132	198.237438	13.2473149	70.7080498
4.31291132	24.0711369	2.95474719	350.182927	49.5199489	169.209297	86.2029778
1.11822495	10.4475811	1.14208471	23.324654	338.427491	13.36376	55.6585761
4.14820288	13.0696816	4.38043214	39.0522075	161.484354	18.6924977	15.431351
1.74408553	22.0474272	1.83832686	13.5740428	865.679723	30.7239856	137.648355
0.9082491	16.6248063	6.86531834	98.057066	16.3549624	26.0402949	24.046188
3.10689138	17.584994	2.13916574	3.61161737	44.1281227	4.11751645	33.0370389
5.9033875	16.7565553	18.5402022	9.40125612	625.134707	25.6348688	47.8733002
4.36811651	3.39048966	18.094011	61.677917	87.9058121	22.2256498	16.1656315
7.1946715	18.5799255	85.8017715	11.0164656	690.980672	28.2999779	76.2007257
2.09106386	26.9814087	23.8055187	52.162292	15.9459378	46.9501564	24.3446247
3.95486467	21.6938054	4.48981321	469.598506	18.3116514	65.4695359	18.4318354
1.76438081	59.9121062	12.0584555	314.374147	18.6134921	46.7211283	30.9915934
107.928869	3.022618	10.2464325	6.19512092	2.06185842	8.60092892	13.3324387
17.1514793	1667.31279	63.8995715	1433.88176	74.1382133	336.072652	199.137321
22.3818413	332.185954	46.8280807	2448.26333	169.963161	617.153573	195.744125
4.91821574	31.7923885	2.17882765	10.7129532	1657.26725	64.6485499	140.811357
5.25872459	22.4693308	5.14132449	386.933138	39.806203	234.566018	47.9997043
5.25560492	482.77147	27.4493033	3490.4307	205.659427	714.894965	320.269039
1.01130605	8.22594263	2.79480864	1.71229582	125.032671	4.0140549	90.4644928
3.98439383	15.8906139	5.0573712	76.3870201	7.10844807	39.8705363	29.5442264
0.92527099	15.441641	12.9037569	47.2185511	14.6750947	12.7906937	18.6057663
3.8419796	27.5614933	11.3062	229.13088	26.4345612	60.9078978	8.73908792
21.225026	83.0852553	56.634156	603.97436	155.92944	110.787342	80.1526666
0.92391782	0	5.11296868	67.2358375	9.95631867	33.6945284	22.9740012
65.5816422	0.88484277	6.54675511	0.54004568	1.937281	2.15005817	1.83446695
3.68308042	32.4860345	14.1599656	30.9110135	293.809273	17.8641261	92.4866912
45.0338834	8.78652469	42.9899034	8.50212149	7.68299528	10.8044722	22.1590696
6.16862162	10.1409332	4.62992744	554.165994	26.4109539	68.7920758	14.7403708
762.106565	346.232947	403.977161	50.5361769	42.4750519	44.2511058	244.799926
49.2337479	76.520603	35.5195036	47.6994684	522.151337	74.287311	85.072176
0.90432695	810.734906	3.65048482	6.78538612	57.0476689	2.27251322	12.236837
16.1031951	14.1962227	36.1144681	40.874107	238.383401	31.0813666	90.6617686
10.3176101	24.3094463	18.0193283	30.6178959	255.768366	43.7114737	100.595098
8.21614584	4.5253659	2.38395667	858.501215	20.2348959	66.6070944	50.3129828
0.88059101	29.4949374	1.51855051	225.652343	11.294815	58.7275326	11.1834312

12.7828602	97.0366809	41.3600375	283.058506	120.492564	193.801061	67.7654998
1.91881117	22.0412674	1.87795939	539.522052	16.2105435	55.7847723	18.0203892
153.276797	9.94957936	13.52161	13.6039755	4.86871109	6.81409302	4.69059598
0	7.43776966	2.26040806	34.5597947	122.675634	46.2728444	24.6037779
39.4379463	157.179212	59.7141576	1024.67716	90.5326181	316.616884	145.493364
31.9397309	53.2744922	15.6427537	463.317328	52.3740866	80.6345601	58.9182257
17.4617301	34.7286153	7.02720138	222.747471	59.0887121	135.55942	35.3830292
0	71.1496703	17.2211731	734.220252	74.073431	264.154268	175.884106
188.540094	34.8022243	165.914061	19.6324304	12.1275817	31.9841535	28.8585342
22.8995033	41.1374419	26.8523743	277.19386	17.7035839	52.0955549	20.6685971
55.7833301	2.59823734	11.7516368	3.57256425	1.72569328	2.60787556	5.83681712
1.87092726	27.5331279	0.40024986	39.7556041	10.9884149	55.9858557	8.22177044
2.95249392	5.07356693	6.82625754	23.3737022	121.454626	24.5343633	102.821235
143.377256	73.8667174	107.776347	456.395972	44.0244015	198.721971	139.292351

ID_OS_7
799.483161
349.33879
361.772445
485.406553
1628.88159
229.81692
121.166775
66.8887026
24.807896
83.3973321
41.3080144
270.467894
8.78154323
26.6996999
204.105686
25.4558797
215.583897
23.5390113
6.06302751
22.8515571
5.00930504
122.810424
72.0480846
495.421932
12.3909997
120.929108
58.3082772
5.44505209
4.23603481
7.71700391
54.5811144
13.3304836
3.00740196
93.7608144
9.58098237
23.1902817
26.5595659
170.240949
25.8328123
93.8957192
127.741754
16.0092484
7.01810019

68.0414196
8.4983974
2.82414998
36.821167
63.0275161
34.3900981
33.2166067
75.98337
18.2413483
8.52009355
3.43804406
8.34275778
37.933532
47.3466311
