# Supplementary material for: Integrative Transcriptomic and Network Analysis of Shared Osteo-Immune Regulatory Programs in Postmenopausal Osteoporosis and Osteosarcoma Within Central Mexican Cohorts
Source: Curr Issues Mol Biol. 2026 Jul 22;48(7):747. doi: 10.3390/cimb48070747 (PMC13409484; doi:10.3390/cimb48070747)
Supplement: Supplementary file 1 [file cimb-48-00747-s001.zip › Table S5 shared pathway-associated gene lists.pdf]

LOXHD1
EID3
FPGT
ACACB
CD180
GEMIN6
S100A13
C1orf216
S1PR1
TTC9
PHLDA1
GPR183
POLR1F
TCN2
SNED1
PFKFB3
DUSP5
MTHFD1L
NR4A1
BTG3
SDC2
CTNNB1
SH3PXD2B
SEC61G
H2AC14
RASD1
