# Supplementary material for: Integrative Transcriptomic and Network Analysis of Shared Osteo-Immune Regulatory Programs in Postmenopausal Osteoporosis and Osteosarcoma Within Central Mexican Cohorts
Source: Curr Issues Mol Biol. 2026 Jul 22;48(7):747. doi: 10.3390/cimb48070747 (PMC13409484; doi:10.3390/cimb48070747)
Supplement: Supplementary file 1 [file Table S1 processed OP RNA-seq expression matrix.pdf › Table S1 processed OP RNA-seq expression matrix.pdf]

Transcript	Gene_ID	Gene_name	ID_1	ID_2	ID_3	ID_4
ENSG000000	ARF5	protein_codin	145.870367	165.687874	101.656443	76.9682396
ENSG000000	M6PR	protein_codin	429.644332	702.189575	222.710338	319.371027
ENSG000001	ESRRA	protein_codin	0	88.9074087	41.6428873	47.1799642
ENSG000000	FKBP4	protein_codin	102.011642	158.029943	67.5305244	66.767162
ENSG000000	CYP26B1	protein_codin	0	0	0.79983745	0
ENSG000000	NDUFAF7	protein_codin	14.1704071	34.9413364	16.5863086	9.96927087
ENSG000000	FUCA2	protein_codin	119.919026	162.840646	61.4027749	80.3888018
ENSG000000	DBNDD1	protein_codin	0	0	0	0
ENSG000000	HS3ST1	protein_codin	1.02539398	5.10157593	0	0
ENSG000000	SEMA3F	protein_codin	0	0	0	0
ENSG000000	CFTR	protein_codin	0	0	0	0
ENSG000000	CYP51A1	protein_codin	64.9935828	69.4588013	0	25.7102734
ENSG000000	USP28	protein_codin	0	0	0	38.9540859
ENSG000000	STPG1	protein_codin	1.90698808	3.69718912	0	0.95431799
ENSG000000	NIPAL3	protein_codin	15.2871057	17.6104855	6.25214218	2.83204823
ENSG000000	TMEM176A	protein_codin	21.2444298	24.2011139	89.3556407	103.29689
ENSG000000	SLC7A2	protein_codin	0	0	0	0
ENSG000000	HSPB6	protein_codin	0	0	0	0
ENSG000000	ZNF195	protein_codin	0	0	0	0
ENSG000000	PDK4	protein_codin	271.211651	348.703647	253.290834	304.758997
ENSG000000	CCL26	protein_codin	0	0	0	0
ENSG000000	USH1C	protein_codin	0	0	0	0
ENSG000000	RALA	protein_codin	119.551897	86.2028615	47.3398622	98.2040092
ENSG000000	BCAP29	protein_codin	60.7869283	58.1608095	48.1235796	46.9440966
ENSG000000	BAIAP2L1	protein_codin	0.94246725	1.53014938	0	0
ENSG000000	CACNG3	protein_codin	0	0	0	0
ENSG000000	TMEM132A	protein_codin	0	0	0	0
ENSG000000	DVL2	protein_codin	23.0410725	39.0511073	10.9570109	0
ENSG000000	GGCT	protein_codin	4.58225007	3.17048098	0	9.74552966
ENSG000000	RPAP3	protein_codin	104.734437	43.3443264	49.6117787	29.6686193
ENSG000000	IFRD1	protein_codin	0	57.8317448	9.97425358	6.02354662
ENSG000000	UPP2	protein_codin	0	0	0	0
ENSG000000	PRSS21	protein_codin	0	8.77576816	0	0
ENSG000000	HOXA11	protein_codin	0	0	0	0
ENSG000000	CX3CL1	protein_codin	0	0	0	0
ENSG000001	PRR5	protein_codin	0	0	0	0
ENSG000000	TRAPPC6A	protein_codin	0	6.53365257	2.8142798	2.02881256
ENSG000000	SPATA20	protein_codin	33.8841113	0	25.5583962	35.3569378
ENSG000000	CEACAM7	protein_codin	0	0	0	0
ENSG000000	CD79B	protein_codin	12.0327616	0	4.58736793	0
ENSG000000	RHBDD2	protein_codin	22.9326671	22.6673917	5.89697627	38.0312785
ENSG000000	RPUSD1	protein_codin	17.9638399	24.7954803	5.57459166	34.5604837
ENSG000000	TSR3	protein_codin	116.707645	144.109917	72.1206992	55.4056972

ENSG000000	OSBPL7	protein_codin	13.660522	36.610962	23.477315	22.9743519
ENSG000000	ARHGAP33	protein_codin	0	0	0	0
ENSG000000	NDUFAB1	protein_codin	63.0710095	97.9051736	38.2223584	51.9860696
ENSG000000	YBX2	protein_codin	0	0	0	0
ENSG000000	PDK2	protein_codin	0	20.984643	11.4507774	0
ENSG000000	ITGA3	protein_codin	0.89978468	0	0	0
ENSG000000	KRT33A	protein_codin	0	0	0	0
ENSG000000	LRRC23	protein_codin	0	0	7.44296306	2.91714639
ENSG000000	IL32	protein_codin	2.7046247	0	0	0
ENSG000000	TFAP2D	protein_codin	0.89395685	0	0	0
ENSG000000	SPRTN	protein_codin	16.1567137	0	0.73483747	13.7173969
ENSG000000	CRY1	protein_codin	23.6698131	41.3468229	9.65193436	21.2845969
ENSG000000	MAPK8IP2	protein_codin	0	0	0	0
ENSG000000	PGLYRP1	protein_codin	0	0	0	0
ENSG000000	STARD3NL	protein_codin	117.485612	122.415919	77.6317964	67.202361
ENSG000000	CAMK1G	protein_codin	0	0	0	0
ENSG000000	CD9	protein_codin	80.4263412	270.527751	93.4866816	111.463179
ENSG000000	CD74	protein_codin	815.471587	1079.97094	478.086494	639.795208
ENSG000000	RPS20	protein_codin	1258.55054	1713.37949	631.959083	846.136553
ENSG000000	DYRK4	protein_codin	3.63089733	0	1.43846273	2.07071174
ENSG000000	FAM76A	protein_codin	0	2.76283298	0	0
ENSG000000	MGST1	protein_codin	0	0	0	0
ENSG000000	CPA1	protein_codin	0	0	0	0
ENSG000000	SYPL1	protein_codin	0	0	0	0
ENSG000000	RANBP9	protein_codin	311.114407	405.559911	207.195847	252.472744
ENSG000000	CD4	protein_codin	1367.02165	2299.69698	745.578534	1113.31066
ENSG000000	PLEKHG6	protein_codin	0	0	0	0
ENSG000000	SS18L2	protein_codin	0	4.75701289	0	5.88634786
ENSG000000	VPS13D	protein_codin	0	0	0	0
ENSG000000	TSPAN9	protein_codin	17.470565	170.695485	114.641677	45.9790316
ENSG000001	CYP4F2	protein_codin	0	0	0	0
ENSG000000	QPCTL	protein_codin	0	0	14.850587	0
ENSG000000	HIVEP2	protein_codin	0	2.75120022	7.85224056	0
ENSG000000	PPP5C	protein_codin	88.3591517	91.1183832	57.3702559	52.9335502
ENSG000002	NME1	protein_codin	0	0	0	0
ENSG000000	UBR7	protein_codin	86.7345594	114.476589	48.8138855	58.5355134
ENSG000000	MAP4K5	protein_codin	0	0	11.2740469	0
ENSG000002	INMT	protein_codin	2.02495347	0	0	0
ENSG000000	ERCC1	protein_codin	8.38274395	23.9933283	0	0
ENSG000000	ABHD5	protein_codin	0	0	8.01196229	0
ENSG000000	GPRC5A	protein_codin	2.29654613	0	0	0
ENSG000000	HEBP1	protein_codin	121.497263	150.70512	42.5240452	71.5944468
ENSG000000	DNASE1L1	protein_codin	0	3.80395275	20.3974623	4.52217304
ENSG000000	COX15	protein_codin	56.1871921	79.7096623	25.128379	44.3479955

ENSG0000007	MS4A12	protein_codin	0	0	0	0
ENSG000000	RGPD5	protein_codin	0	0	57.1482962	38.2690735
ENSG000000	XYLT2	protein_codin	79.3779282	90.9111403	75.3876337	33.9746336
ENSG000000	FTSJ1	protein_codin	0	7.14296049	2.65846251	0
ENSG000000	SCTR	protein_codin	0	0	0	0
ENSG000000	RALBP1	protein_codin	0	117.821335	193.007086	0
ENSG000000	PSD	protein_codin	0	0	0	0
ENSG000000	SYT13	protein_codin	0	0	0	0
ENSG000000	SNAI2	protein_codin	0	0	0	0
ENSG0000007	VDAC3	protein_codin	213.449593	381.121418	162.113624	152.823189
ENSG000000	SLC7A9	protein_codin	0	0	0	0
ENSG000000	GABRA1	protein_codin	0	0	0	0
ENSG000000	RTF2	protein_codin	55.2893497	98.9342484	42.7686082	0
ENSG000000	SLC45A4	protein_codin	0	32.0101699	8.72112717	7.28631572
ENSG000000	RB1CC1	protein_codin	495.686525	262.46998	0	0
ENSG000000	AKAP11	protein_codin	334.62369	189.55619	262.58874	217.566284
ENSG000000	STRAP	protein_codin	0	3.76033623	0	9.21142291
ENSG000000	PIGQ	protein_codin	0	41.5848787	0	0
ENSG0000007	CDH17	protein_codin	2.12839105	0	0	0
ENSG000000	RNASET2	protein_codin	162.706157	102.605531	44.847846	88.9341799
ENSG000000	B4GALT7	protein_codin	17.3379926	26.8988991	7.41522515	7.83086936
ENSG000000	SLC39A9	protein_codin	0	9.16036609	28.5820499	22.5938453
ENSG000000	FAM13B	protein_codin	222.383438	186.934825	134.294072	167.320423
ENSG000000	RANBP3	protein_codin	0	0	0	15.2122213
ENSG000000	CHPF2	protein_codin	121.238385	184.63874	58.2554622	123.932886
ENSG000000	GABARAPL2	protein_codin	270.84933	497.054618	69.3768945	243.96206
ENSG000000	MYOC	protein_codin	0	0	0	0
ENSG000000	FAM136A	protein_codin	39.893416	70.999639	56.6210525	52.9692967
ENSG000000	NSMAF	protein_codin	121.01638	179.616355	86.0917466	83.6466297
ENSG000000	OTC	protein_codin	0	0	0	0
ENSG000000	TTC17	protein_codin	130.796846	104.800477	113.351727	64.3950946
ENSG000000	HOXC8	protein_codin	0	0	0	0
ENSG000000	MRI1	protein_codin	43.5967925	59.7229399	18.737228	0
ENSG000000	BOD1L1	protein_codin	838.973188	486.895916	413.281078	900.418401
ENSG000000	TARBP1	protein_codin	62.9373016	71.7209623	34.9976681	33.4309196
ENSG000000	RIPOR1	protein_codin	0	262.026074	56.4225854	110.185482
ENSG000000	BEST2	protein_codin	0.96382783	0.96778412	0	0
ENSG000000	RTN4R	protein_codin	0	0	0	0
ENSG000000	PSMA4	protein_codin	25.5148072	12.3122929	8.25128131	9.79670555
ENSG000000	RIPOR3	protein_codin	0	0	0	0
ENSG000000	SPATA7	protein_codin	0	0	0	0
ENSG000000	ZPBP	protein_codin	0	0	0	0
ENSG000000	CTNS	protein_codin	33.2818894	32.150556	17.5562537	3.71553673
ENSG000000	LCP2	protein_codin	612.893778	471.049018	279.011609	326.351948

ENSG000000	GOPC	protein_codin	93.9804323	53.5744992	69.6770205	63.1496347
ENSG000000	DCN	protein_codin	0	0	0	0
ENSG000000	TNFRSF17	protein_codin	8.37714097	16.1539033	50.8949567	3.39622353
ENSG000000	MRPS10	protein_codin	42.4202982	80.7996971	19.7600462	53.9753033
ENSG000000	GRN	protein_codin	2925.90214	2695.27472	718.812114	1782.28325
ENSG000000	THAP3	protein_codin	17.3156358	17.3731337	0	20.7215132
ENSG000000	VAMP3	protein_codin	354.111868	436.346734	133.84832	196.016468
ENSG000000	UTS2	protein_codin	0	0	0	1.26556985
ENSG000000	RCN1	protein_codin	77.348255	110.570005	45.4934493	50.6956407
ENSG000000	RFC2	protein_codin	26.8828993	38.5980014	24.4224167	19.8962926
ENSG000000	PPP1R3F	protein_codin	5.4886561	13.1656083	0	4.55771914
ENSG000000	NEXMIF	protein_codin	0	0	0	0
ENSG000000	ARHGEF5	protein_codin	0	16.4918728	7.74929993	0
ENSG000000	NFE2L3	protein_codin	18.0526458	143.391211	43.5474751	80.9669836
ENSG000000	TNIP3	protein_codin	0	1.05083946	0	0
ENSG000000	SIKE1	protein_codin	91.5851619	31.9653365	47.767316	54.5708607
ENSG000000	TLL1	protein_codin	3.70072832	0	0	0
ENSG000000	CBLN4	protein_codin	0	0	0	0
ENSG000000	CLDN11	protein_codin	0	0	0	0
ENSG000000	FAM168A	protein_codin	53.1204211	0	0	32.3819298
ENSG000000	RELT	protein_codin	326.870628	488.579885	183.945043	219.290934
ENSG000000	CDC27	protein_codin	94.0185328	53.4254068	46.6408929	87.2229278
ENSG000000	PKP2	protein_codin	0	0	0	0
ENSG000001	IL1RAP	protein_codin	33.4606568	0	18.5487828	44.6584265
ENSG000000	YIPF1	protein_codin	47.2312053	77.4471076	19.8254118	40.1306876
ENSG000001	ADCK2	protein_codin	25.6074322	44.4054339	6.71937963	20.3028727
ENSG000000	SLC2A3	protein_codin	0	0	0	0
ENSG000001	ENPP2	protein_codin	10.7015549	0	0	0
ENSG000000	CTDP1	protein_codin	92.0729676	98.2090852	0	17.9623234
ENSG000000	STYK1	protein_codin	1.09037725	0	0.84077298	0
ENSG000000	GNA11	protein_codin	14.8928044	32.0879296	12.5438508	18.6300176
ENSG000000	CREB3L3	protein_codin	0	0	0	0
ENSG000000	PIGV	protein_codin	0	0	0	0
ENSG000000	HDAC7	protein_codin	39.0365175	0	0	0
ENSG000000	MRPS35	protein_codin	107.342892	157.112543	110.097019	99.5487276
ENSG000000	BTN3A1	protein_codin	0	0	0	0
ENSG000000	APPBP2	protein_codin	48.2015168	46.3558816	25.8177224	42.8382647
ENSG000000	RPL18	protein_codin	407.027991	243.211388	116.648668	141.549067
ENSG000000	CA11	protein_codin	2.94467123	0.99366963	1.01765184	1.85733548
ENSG000000	ISOC2	protein_codin	0	3.04214726	0	8.92691485
ENSG000000	EPN1	protein_codin	127.700042	209.034117	40.6632768	81.2225243
ENSG000000	CD22	protein_codin	0	0	0	0
ENSG000000	GSC2	protein_codin	0	0	0	0
ENSG000000	ZFY	protein_codin	0	0	0	0

ENSG000000	HDHD5	protein_codin	0	0	0	0
ENSG000000	KCNQ1	protein_codin	82.1313601	190.350186	0	58.9287628
ENSG000000	TRIB2	protein_codin	11.0939514	31.0446948	19.9114437	11.3387855
ENSG000000	OTUD5	protein_codin	9.27771595	50.1902136	0	0
ENSG000000	GPKOW	protein_codin	62.8526477	102.056149	53.0627272	57.4592028
ENSG000000	AQR	protein_codin	125.430443	55.5439319	70.2405121	77.9749874
ENSG000001	KLK13	protein_codin	0	0	0	0
ENSG000001	KLK14	protein_codin	0	0	0	0
ENSG000000	ST6GALNAC1	protein_codin	0	0	0	0
ENSG000000	MBD3	protein_codin	9.35427145	26.9414366	13.1179762	22.9634283
ENSG000000	LMCD1	protein_codin	0	0	0	1.11823968
ENSG000000	PSMC4	protein_codin	141.398302	139.583736	86.6234469	67.1288349
ENSG000000	FNDC8	protein_codin	0	0	0	0
ENSG000000	CAMKK1	protein_codin	3.76233993	1.44846788	0	0
ENSG000000	FAM50A	protein_codin	12.4662186	0	4.07874288	2.29991003
ENSG000000	ACAP1	protein_codin	53.9934998	90.5990388	35.3868673	54.8486841
ENSG000000	DERL2	protein_codin	55.7018954	39.8647509	18.1112725	45.4544589
ENSG000000	NOX3	protein_codin	0	0	0	0
ENSG000000	ANO8	protein_codin	71.9736882	89.6496944	33.2584863	35.2814943
ENSG000001	KDM4B	protein_codin	74.0650652	113.462255	25.1489116	47.0036822
ENSG000000	PANX2	protein_codin	9.68198892	17.7427748	0	6.39381436
ENSG000000	ICAM3	protein_codin	300.790432	425.300343	243.240625	248.620362
ENSG000000	CAMSAP3	protein_codin	0	0	0.81301192	0
ENSG000000	CTTNBP2	protein_codin	0	0	0	0
ENSG000000	ACTL6B	protein_codin	0	0	0	0
ENSG000001	FAM135B	protein_codin	0	0	0	0
ENSG000000	CIC	protein_codin	6.24481525	82.5547965	4.81744807	3.65970166
ENSG000000	KIF22	protein_codin	0	0	0	4.04029912
ENSG000000	PRSS22	protein_codin	0	0	0	0
ENSG000000	CEACAM1	protein_codin	0	0	0	0
ENSG000000	YTHDC2	protein_codin	202.648441	122.348497	95.557817	81.883463
ENSG000000	TMEM161A	protein_codin	0	3.69025483	0	0
ENSG000000	BCAR1	protein_codin	0	0	0	0
ENSG000000	FOXJ2	protein_codin	278.51674	351.028522	182.466299	152.520508
ENSG000000	TNFRSF1A	protein_codin	302.914596	315.990073	57.50322	213.321192
ENSG000000	GOLGA5	protein_codin	163.366695	153.011239	118.556441	153.356452
ENSG000000	METTL22	protein_codin	38.1200554	47.2224727	28.1760942	30.7591611
ENSG000000	CELSR3	protein_codin	14.8705367	14.9121393	4.48411812	6.86649887
ENSG000000	PPP2R5B	protein_codin	54.3688015	52.480796	22.3474796	32.4556254
ENSG000000	PYGM	protein_codin	0	5.93795167	0	0
ENSG000000	BCL3	protein_codin	669.678986	461.360628	129.794244	265.702038
ENSG000000	KCNAB2	protein_codin	0	0	0	0
ENSG000000	PIGB	protein_codin	7.46949421	19.9171968	11.2355659	32.1355388
ENSG000000	PDZD4	protein_codin	0	0	2.24102315	0

ENSG000001	NPIPB4	protein_codin	0	10.0990943	7.26005723	0
ENSG000000	PRLH	protein_codin	0	0	0	0
ENSG000000	REEP1	protein_codin	0	2.96934507	0	0
ENSG000000	FSTL3	protein_codin	0	0	0	7.80692823
ENSG000000	EPHA8	protein_codin	0	0	0	0
ENSG000000	TRIP13	protein_codin	0	5.2388914	23.5049127	0
ENSG000000	P4HA2	protein_codin	0	0	0	0
ENSG000000	VASH1	protein_codin	171.84652	307.496988	59.9304246	207.495488
ENSG000000	PDCD2	protein_codin	0	0	0	0
ENSG000000	LLGL2	protein_codin	0	0	0	0
ENSG000001	KRT14	protein_codin	0	0	0	0
ENSG000001	KRT20	protein_codin	0	0	0	0
ENSG000000	ARHGEF10L	protein_codin	0	0	38.8520677	0
ENSG000000	SPP2	protein_codin	0	0	0	0
ENSG000000	HSD17B10	protein_codin	47.6191029	70.576159	38.2802478	24.2831208
ENSG000000	FGF4	protein_codin	0	0	0	0
ENSG000000	RGS11	protein_codin	0	0	0	0
ENSG000000	NMRK2	protein_codin	0	0	0	0
ENSG000001	MASP1	protein_codin	0	0	1.27187461	0
ENSG000000	ST6GAL1	protein_codin	0	90.1855736	7.92498346	71.1502068
ENSG000000	TIMM21	protein_codin	3.25262137	9.58451585	0	1.7088012
ENSG000000	BPIFB2	protein_codin	0	0	0	0
ENSG000000	REXO1	protein_codin	324.128905	298.621325	136.526758	152.31473
ENSG000000	MKRN2	protein_codin	56.7443174	71.2689306	39.4479885	47.9058538
ENSG000000	GPATCH1	protein_codin	71.6121147	75.8905919	38.9923533	38.5514113
ENSG000000	IL4R	protein_codin	223.986253	0	0	81.4208879
ENSG000000	KEAP1	protein_codin	54.0701279	45.0337617	0	29.8535438
ENSG000000	P2RY10	protein_codin	19.2484748	13.4119282	20.5462171	1.68416298
ENSG000000	TNS1	protein_codin	148.058798	0	0	0
ENSG000000	NGFR	protein_codin	0	1.68221468	2.13576259	0
ENSG000000	NTN1	protein_codin	0	0	0	0.84670982
ENSG000000	ISOC1	protein_codin	13.6275771	45.7485724	18.1432482	11.9461
ENSG000000	KLF6	protein_codin	0	0	0	0
ENSG000000	TRO	protein_codin	0	0	0	0
ENSG000000	MNT	protein_codin	317.618901	372.809464	108.013742	223.653722
ENSG000000	LAPTM4A	protein_codin	236.170914	283.654636	67.7560653	198.163092
ENSG000000	ADAM7	protein_codin	0	0	0	0
ENSG000000	ASNS	protein_codin	0	0	0	0
ENSG000000	PTPN18	protein_codin	660.455018	973.752279	287.157697	445.150944
ENSG000000	DRD4	protein_codin	6.56551337	0	1.80950286	0
ENSG000000	SCT	protein_codin	0	0	0	0
ENSG000000	ALDH3A2	protein_codin	0	0	0	40.5894444
ENSG000000	STK10	protein_codin	2508.41051	3250.06688	963.63022	1514.47884
ENSG000000	ALPK1	protein_codin	0	0	0	0

ENSG000000	TBX21	protein_codin	0.97213519	4.69008669	1.92881103	0
ENSG000000	MRPS34	protein_codin	31.9855751	59.8182347	26.333538	42.156367
ENSG000000	CA12	protein_codin	0	0	0	0
ENSG000001	MAP2K5	protein_codin	65.7683124	71.6454331	28.7676037	57.0790114
ENSG000000	TIGAR	protein_codin	65.052591	38.121989	29.074649	32.967771
ENSG000000	FGF20	protein_codin	0	0	0	0
ENSG000000	MTMR7	protein_codin	0	0	2.4169013	0
ENSG000000	CPB2	protein_codin	0	0	0	0
ENSG000000	FAM107B	protein_codin	6.41493435	2.77607854	8.24981384	3.05929485
ENSG000000	CDK13	protein_codin	25.5579943	46.034455	32.6274362	58.7142946
ENSG000000	TSPAN32	protein_codin	0	22.9450765	0	16.2176624
ENSG000000	FAR2	protein_codin	0	3.874721	0	8.43585166
ENSG000000	TRAM2	protein_codin	110.987873	122.79604	146.304369	50.5508304
ENSG000000	CLDN18	protein_codin	0	1.11448347	0	0
ENSG000000	ROPN1	protein_codin	0	0	0	0
ENSG000000	NDUFB4	protein_codin	35.7262418	78.3631255	29.3120657	30.9056595
ENSG000000	HEATR6	protein_codin	87.7015197	78.5878591	32.2751396	39.3493897
ENSG000000	ERLEC1	protein_codin	32.930949	0	80.4371942	50.8097651
ENSG000001	CLIC5	protein_codin	0	0	0	0
ENSG000000	TMEM131	protein_codin	555.759798	510.305179	268.178933	473.671106
ENSG000001	ARPP21	protein_codin	0	0	0	0
ENSG000000	CEACAM21	protein_codin	0	0	0	0
ENSG000000	TMEM38A	protein_codin	8.91800103	21.3768472	5.71871332	5.02859047
ENSG000000	PVR	protein_codin	0	0	36.7379871	0
ENSG000001	ZNF222	protein_codin	6.95479228	5.80530804	1.12949185	0
ENSG000001	PSG6	protein_codin	0	0	0	0
ENSG000000	ACTR6	protein_codin	20.8808479	23.7853555	0	0
ENSG000000	SLC25A3	protein_codin	61.325251	53.5360698	0	24.6818679
ENSG000000	NR1H4	protein_codin	0	0	0	0
ENSG000000	FAP	protein_codin	0	0	0	0
ENSG000000	NFKB2	protein_codin	133.272592	75.7525216	11.8249254	77.6190912
ENSG000000	MUSK	protein_codin	0	0	0.89785731	0.94948465
ENSG000000	DMRT3	protein_codin	0	0	0	0
ENSG000000	OSBPL6	protein_codin	0	0	0	0
ENSG000000	CCN5	protein_codin	0	0	0	0
ENSG000000	CTSA	protein_codin	209.638578	342.630679	108.186107	158.63533
ENSG000001	ANKRD16	protein_codin	4.49749069	0	5.53802107	0
ENSG000001	GAL3ST2	protein_codin	0	0.81495554	0	0
ENSG000000	BLTP3A	protein_codin	191.111661	225.870105	181.858148	203.607039
ENSG000000	OSTM1	protein_codin	0	76.6696618	88.5978318	30.2833434
ENSG000000	IMPG2	protein_codin	3.03738394	4.06044246	0	2.06424837
ENSG000000	ACTN1	protein_codin	1131.11805	2609.70119	1183.37478	952.471653
ENSG000002	NAIP	protein_codin	562.633312	395.365509	225.042408	615.255662
ENSG000000	DELE1	protein_codin	41.5641594	122.840574	74.1949152	27.0729569

ENSG000000	SLC13A1	protein_codin	0	0	0	0
ENSG000000	PCDHB4	protein_codin	0	0	0	0
ENSG000001	PCDHB2	protein_codin	0	0	0	0
ENSG000000	IFT25	protein_codin	24.6733349	11.9900717	19.2374676	9.93739113
ENSG000000	STRADB	protein_codin	34.3417834	43.1355763	10.1336867	24.6391152
ENSG000000	FAM135A	protein_codin	76.4902592	52.755508	0	15.1953581
ENSG000000	DLG3	protein_codin	0	0	0	0
ENSG000000	SEMA5B	protein_codin	0	0	0	0
ENSG000000	SNAP91	protein_codin	0	0	0	0
ENSG000000	PLOD1	protein_codin	215.613582	331.46137	95.4543182	151.561625
ENSG000000	TDRD3	protein_codin	3.1185513	9.67800755	0	10.0828016
ENSG000000	OXCT1	protein_codin	22.2630904	12.2788282	24.848613	7.67784353
ENSG000000	ZNF324	protein_codin	62.7423734	65.9769538	30.5355491	25.5751203
ENSG000000	ZNF416	protein_codin	3.96660317	16.7687336	1.98692032	2.99059776
ENSG000000	RPS5	protein_codin	318.643265	567.385921	214.724297	207.174107
ENSG000000	FAM234B	protein_codin	19.0257633	21.4693562	10.8520235	15.4004879
ENSG000000	PILRA	protein_codin	390.089707	593.592289	197.309498	314.7555
ENSG000000	RRN3	protein_codin	170.236271	120.497662	77.2079052	94.5249598
ENSG000001	MOGAT2	protein_codin	0	0	0	0
ENSG000000	CHERP	protein_codin	6.87998768	101.705426	11.4306804	0
ENSG000001	AQP2	protein_codin	0	0	0	0
ENSG000000	DIMT1	protein_codin	0	0	5.72064793	0
ENSG000000	EIF2AK1	protein_codin	840.358662	1779.58338	548.231288	756.048776
ENSG000000	NME8	protein_codin	33.294492	0	8.45618276	3.85960631
ENSG000000	EPDR1	protein_codin	4.97711422	16.4825121	5.00060947	4.87974901
ENSG000000	MRPL28	protein_codin	54.1022593	52.5554862	14.0911056	23.3177887
ENSG000000	HBQ1	protein_codin	3.53623666	4.39811506	0	2.41696828
ENSG000000	CEACAM6	protein_codin	0	0	0	0
ENSG000000	RBM22	protein_codin	209.591821	235.419805	105.617599	140.185674
ENSG000000	HSD17B2	protein_codin	0	0	0	0
ENSG000000	MAP2	protein_codin	0	0	0	0
ENSG000000	ZW10	protein_codin	38.6404642	60.1770855	4.87258642	33.8943779
ENSG000001	ITGB4	protein_codin	0	1.53284052	0	0
ENSG000000	PPP1R15A	protein_codin	8013.71314	5904.29527	4430.83229	2843.51609
ENSG000000	TRIP6	protein_codin	16.0523096	13.2276704	0	15.2003737
ENSG000000	ADAM11	protein_codin	0	0	0	0
ENSG000000	LAMP2	protein_codin	76.0145215	55.844671	28.5164827	55.8996749
ENSG000001	SLC22A4	protein_codin	44.3569456	51.9172948	11.4781016	34.6234491
ENSG000000	CETP	protein_codin	9.28248865	12.6456498	9.72978205	0
ENSG000000	MT3	protein_codin	0	0	0	0
ENSG000000	PTHLH	protein_codin	0	0	0	0
ENSG000000	TFAP2C	protein_codin	0	0	0	0
ENSG000000	SULT2B1	protein_codin	0	0	0	0
ENSG000001	EPS8L1	protein_codin	0	0	0	0

ENSG000001	CACNB4	protein_codin	0	0	0	0
ENSG000001	NOL4L	protein_codin	0	1.08384958	0	0
ENSG000000	DNMT3B	protein_codin	0	0	0	0
ENSG000000	REM1	protein_codin	0	0	0	0
ENSG000000	PDRG1	protein_codin	10.0752636	19.8532493	13.537562	4.01796903
ENSG000000	EPB41L1	protein_codin	0	0	0	0
ENSG000000	PPP1R13B	protein_codin	8.27475226	9.04625385	0	0
ENSG000001	TGM6	protein_codin	0	0	0	0
ENSG000001	RALGAPA2	protein_codin	692.076598	377.071915	283.208573	259.106687
ENSG000000	RPL6	protein_codin	2287.41267	3126.36935	1816.19652	1745.87814
ENSG000000	ESF1	protein_codin	0	0	0	0
ENSG000000	SLC8B1	protein_codin	0	2.43768318	0	0
ENSG000000	TMEM230	protein_codin	128.030915	181.557127	69.9633491	104.223807
ENSG000000	OAS1	protein_codin	205.1008	61.8119292	0	0
ENSG000000	SIRT4	protein_codin	0	0	0	0
ENSG000000	TRMT6	protein_codin	111.890981	86.7024556	18.7126246	68.148859
ENSG000002	HAUS5	protein_codin	0	9.28991182	29.4081762	1.84148851
ENSG000000	UQCRC1	protein_codin	245.253387	298.642409	70.5840079	160.070457
ENSG000000	GMIP	protein_codin	469.56727	630.734102	285.745248	380.340311
ENSG000000	LAG3	protein_codin	0.86714878	0.82477788	0	0
ENSG000000	MLF2	protein_codin	136.434354	232.496111	29.3203944	112.24775
ENSG000000	OTUB2	protein_codin	2.98628618	6.7252309	0	1.9875633
ENSG000000	LTBP4	protein_codin	0	0	0	0
ENSG000000	TFAP4	protein_codin	14.6923602	31.0120304	12.1105671	16.4811006
ENSG000000	PDCD7	protein_codin	92.9725417	110.832913	33.2555629	59.0543783
ENSG000000	SPG21	protein_codin	209.212277	275.710296	37.5206202	142.000905
ENSG000000	CHRD	protein_codin	0	0	0	0
ENSG000000	FLT3LG	protein_codin	0	0	0	6.17668883
ENSG000000	GNPTG	protein_codin	45.6652802	65.0104647	25.3157385	7.90653223
ENSG000000	GOLGA3	protein_codin	0	0	0	0
ENSG000000	CD209	protein_codin	0	0	0	0
ENSG000000	EFNB1	protein_codin	76.1053207	132.791386	47.0375203	44.0381201
ENSG000000	GLG1	protein_codin	0	9.49774285	6.20740033	0
ENSG000000	PLEKHG2	protein_codin	311.920156	80.7462898	209.027417	98.3108905
ENSG000000	DLL3	protein_codin	0	0	0	0
ENSG000000	NAT14	protein_codin	0	2.24273978	0	0
ENSG000001	AASDH	protein_codin	0	32.9003352	16.8346887	0
ENSG000000	LAMB4	protein_codin	0	0	0	0
ENSG000000	DLD	protein_codin	82.4841437	48.3906262	39.9778786	45.9269682
ENSG000000	ABCC6	protein_codin	15.8627495	33.7930419	14.9157992	9.62392063
ENSG000000	CMTM6	protein_codin	807.051482	796.659152	415.728388	654.466287
ENSG000000	APOH	protein_codin	0	0	0	0
ENSG000000	SPAG7	protein_codin	165.999228	282.195732	148.541317	166.886215
ENSG000000	ESR1	protein_codin	6.42553456	4.28871359	0	0

ENSG000000!	RGS17	protein_codin	1.80441561	0	0	0
ENSG000000!	TMEM101	protein_codin	28.9621137	73.8644721	21.2528292	21.023315
ENSG000000!	CCDC80	protein_codin	0	1.30760803	6.50217711	0
ENSG000000!	CMA1	protein_codin	0	0	0	0
ENSG000000!	PSME1	protein_codin	1044.91898	1456.81253	458.827478	786.959523
ENSG000000!	HAUS4	protein_codin	0	0	0	26.5059368
ENSG000000!	CEBPE	protein_codin	1.95595111	0.94902513	2.53907151	0.91169186
ENSG000000!	OSGEP	protein_codin	31.9327318	38.0442198	0	8.0217374
ENSG000000!	SLC22A17	protein_codin	0	0	0	0
ENSG000000!	G2E3	protein_codin	34.6305167	17.3285894	12.0919404	11.1015269
ENSG000000!	TGM1	protein_codin	0	4.47366454	1.83186851	0
ENSG000000!	TBX15	protein_codin	0	0	0	0
ENSG000000!	TEKT2	protein_codin	0.91046831	2.72738361	1.38797531	1.83112044
ENSG000000!	UNC13D	protein_codin	466.400663	756.770303	321.881834	302.128403
ENSG000000!	COMT	protein_codin	0	0	0	0
ENSG000000!	XYLB	protein_codin	0	13.4305848	8.38586789	0
ENSG000000!	OR1I1	protein_codin	0	0	0	0
ENSG000001!	ADH7	protein_codin	0	0	0	0
ENSG000001	ADH1A	protein_codin	0	0	0	0
ENSG000001	KRT23	protein_codin	0	1.68627178	0	0
ENSG000000!	CDC6	protein_codin	0	0	9.74014789	0
ENSG000000!	AAAS	protein_codin	46.6292374	47.8377582	23.6479937	0
ENSG000000!	CBX5	protein_codin	127.464674	113.596929	137.449054	61.4440611
ENSG000000	KLHL20	protein_codin	45.2341376	30.5848073	18.422963	38.9567328
ENSG000000!	FMO2	protein_codin	0	0	0	0
ENSG000000!	DHPS	protein_codin	52.859453	82.9704249	19.5894696	58.5465845
ENSG000001	RAB26	protein_codin	0	0	0	0
ENSG000001	PTBP3	protein_codin	0	9.95316006	0	0
ENSG000000!	PSMD5	protein_codin	73.0469317	63.8949384	25.27557	22.9519264
ENSG000000!	NANS	protein_codin	185.574691	292.827772	107.048385	145.017629
ENSG000000!	SEMA4G	protein_codin	0	0	0	0
ENSG000000!	TPSD1	protein_codin	0	0	0	0
ENSG000001	GSTA3	protein_codin	0	0	0	0
ENSG000001	MAPK13	protein_codin	30.6975127	9.22146225	13.7544222	10.3162094
ENSG000000!	TMEM14A	protein_codin	0	2.66827967	1.14637959	1.31678436
ENSG000002	BAG6	protein_codin	0	0	0	0
ENSG000002	PRRT1	protein_codin	0	0	0	0
ENSG000002	LST1	protein_codin	1.766657	4.01656697	0	3.8502766
ENSG000000!	ZNF184	protein_codin	0	17.5540664	16.3508412	0
ENSG000000	VCL	protein_codin	2229.22662	6003.06483	2570.84952	2157.85248
ENSG000000!	SIRT1	protein_codin	99.4042177	104.336428	68.9891763	63.2264136
ENSG000000	TGFBR3	protein_codin	8.59257584	0	4.1331044	0
ENSG000000!	TMED1	protein_codin	21.1278367	22.7909254	11.3413801	8.80155268
ENSG000000!	ERMP1	protein_codin	0	0	13.7146934	14.7330192

ENSG000000[illegible]	MZF1	protein_codin	0	10.0860761	0	0
ENSG000000[illegible]	OCEL1	protein_codin	5.51858404	30.8617049	0	8.08372939
ENSG000000[illegible]	PSMD8	protein_codin	0	0	0	0
ENSG000000[illegible]	STX1B	protein_codin	4.79158183	3.85566975	2.39534614	0
ENSG000000[illegible]	BCL7C	protein_codin	34.6324135	40.7274507	25.4912636	31.5638551
ENSG000000[illegible]	EFNA2	protein_codin	0	0	0	0
ENSG000000[illegible]	ATP5F1D	protein_codin	59.657442	52.7757278	0	10.0872674
ENSG000000[illegible]	CBARP	protein_codin	0	0	0	0
ENSG000000[illegible]	PCDH11Y	protein_codin	0	0	0	0
ENSG000000[illegible]	FGF22	protein_codin	0	0	0	0
ENSG000000[illegible]	SMIM24	protein_codin	0	0	0	0
ENSG000000[illegible]	IGFALS	protein_codin	0	0.88113757	0	0
ENSG000000[illegible]	MARCHF2	protein_codin	175.786048	276.804092	155.998198	124.600347
ENSG000000[illegible]	NDUFB7	protein_codin	92.5855736	112.720299	69.841726	58.8526999
ENSG000000[illegible]	TECR	protein_codin	0	62.331793	38.7969946	22.6184862
ENSG000000[illegible]	TIMM13	protein_codin	29.6598442	67.3138482	14.7079955	27.2037022
ENSG000000[illegible]	CDC34	protein_codin	83.3351452	148.027739	26.8289546	68.550995
ENSG000000[illegible]	MISP	protein_codin	0	0.67550241	0	0
ENSG000000[illegible]	POLR2E	protein_codin	115.312952	146.994949	41.7142431	64.640369
ENSG000000[illegible]	GADD45B	protein_codin	811.821972	951.853255	524.855883	420.097641
ENSG000000[illegible]	MADCAM1	protein_codin	0	0	0	0
ENSG000001[illegible]	MAPK12	protein_codin	0	1.34694427	0	0
ENSG000000[illegible]	SERPIND1	protein_codin	0	0	0	0
ENSG000000[illegible]	SNAP29	protein_codin	281.331261	382.854131	245.985722	306.997562
ENSG000001[illegible]	THAP7	protein_codin	0	10.3372886	0	11.1387226
ENSG000000[illegible]	MMP11	protein_codin	0	0	0	0
ENSG000002[illegible]	MIF	protein_codin	105.068062	165.24408	66.3309522	63.4914062
ENSG000000[illegible]	DDTL	protein_codin	4.44698649	3.510739	2.29675599	1.08917961
ENSG000000[illegible]	OSM	protein_codin	140.745854	110.163171	115.118662	166.02515
ENSG000000[illegible]	TBC1D10A	protein_codin	48.3033617	49.0741116	22.7544049	8.31273513
ENSG000000[illegible]	SF3A1	protein_codin	744.524782	1093.52078	534.533659	549.089007
ENSG000001[illegible]	USP18	protein_codin	7.38613958	9.48705317	3.72529607	0
ENSG000000[illegible]	RNF215	protein_codin	0	0	4.48574125	0
ENSG000001[illegible]	SEC14L3	protein_codin	0	0	0	0
ENSG000001[illegible]	SNRPD3	protein_codin	175.348915	222.732423	93.3702582	104.954827
ENSG000001[illegible]	MAPK1	protein_codin	603.582448	739.976556	250.550433	426.607968
ENSG000001[illegible]	TCN2	protein_codin	0	73.315667	58.2274919	45.5179974
ENSG000001[illegible]	CRYBB3	protein_codin	0	0	0	0
ENSG000001[illegible]	MORC2	protein_codin	25.2313074	40.5668796	25.717148	25.2612427
ENSG000001[illegible]	SLC25A1	protein_codin	63.8210935	74.1875109	38.3620077	37.0472745
ENSG000001[illegible]	PLA2G3	protein_codin	0	0.89638078	0	0
ENSG000001[illegible]	LGALS2	protein_codin	160.7242	112.637393	103.000103	292.288794
ENSG000002[illegible]	PDXP	protein_codin	9.90096168	13.4010214	2.06433676	8.73897809
ENSG000001[illegible]	ASPHD2	protein_codin	31.2641535	57.8870995	19.6423524	37.7783428

ENSG000001	LGALS1	protein_codin	406.044723	389.27996	106.232887	236.463306
ENSG000001	PIK3IP1	protein_codin	27.1969871	41.4179318	24.2196935	21.4772522
ENSG000001	SRRD	protein_codin	45.6689364	67.2194773	24.9941594	20.2571765
ENSG000001	PATZ1	protein_codin	9.73623625	19.7903145	1.71630364	0
ENSG000001	ANKRD54	protein_codin	20.8492862	17.9922128	5.16779295	11.513456
ENSG000001	SNU13	protein_codin	27.4987309	21.8196025	0	7.82851981
ENSG000001	MICALL1	protein_codin	37.6439482	70.3140199	5.71324524	9.4263927
ENSG000001	CENPM	protein_codin	2.04259137	0	0	0
ENSG000001	KDELR3	protein_codin	2.28055337	0	0	0
ENSG000001	DDX17	protein_codin	947.834415	912.933102	494.368166	590.513108
ENSG000001	DMC1	protein_codin	0	0	1.56261289	0
ENSG000001	HSCB	protein_codin	16.4719323	0	0	11.3422929
ENSG000001	CBY1	protein_codin	0	0	0	0
ENSG000001	TOMM22	protein_codin	162.58366	240.143514	95.2625664	127.997141
ENSG000001	RSPH14	protein_codin	0	0	0.84910361	0
ENSG000001	XBP1	protein_codin	254.548382	419.800348	301.157377	230.70695
ENSG000001	RTCB	protein_codin	110.412051	163.793053	65.5267254	82.3256944
ENSG000001	JOSD1	protein_codin	242.521106	121.776472	139.740627	107.466919
ENSG000001	GTPBP1	protein_codin	283.370162	281.200049	176.208298	141.096002
ENSG000001	PPP6R2	protein_codin	14.6910916	63.0607943	0	0
ENSG000001	DNAL4	protein_codin	24.7327031	22.6438778	9.28401669	8.31479288
ENSG000001	C22orf31	protein_codin	0	0	0	0
ENSG000001	MIOX	protein_codin	0	0	0	0
ENSG000001	LMF2	protein_codin	0	0	0	14.7046582
ENSG000001	CBX6	protein_codin	26.8546394	16.5345133	3.11798049	11.604511
ENSG000001	RHBDD3	protein_codin	0	7.54910784	0	4.29054483
ENSG000002	APOBEC3D	protein_codin	16.5869544	12.4457862	15.267161	13.8225252
ENSG000001	RASL10A	protein_codin	3.96286421	0	0.78001838	1.98166435
ENSG000001	HMGXB4	protein_codin	86.5297804	154.332939	62.2624592	76.7180808
ENSG000001	BIK	protein_codin	3.01828461	2.84080314	3.75091521	0
ENSG000001	HMOX1	protein_codin	336.648622	324.551455	129.065504	221.513999
ENSG000001	NIPSNAP1	protein_codin	28.9647134	58.0543917	19.4883181	28.9119552
ENSG000001	MCM5	protein_codin	95.1206362	53.7644656	0	12.650334
ENSG000001	ARSA	protein_codin	61.2539501	69.8157569	20.9078249	17.4175061
ENSG000001	RASD2	protein_codin	0.92722578	1.84479158	0.88519337	1.81637145
ENSG000001	TTLL12	protein_codin	126.350502	164.956396	50.266774	78.9771861
ENSG000001	CBX7	protein_codin	50.5979956	65.7334148	28.8096783	36.3709252
ENSG000001	ACR	protein_codin	0	0	0	0.90158707
ENSG000001	CABP7	protein_codin	4.61991441	10.2190891	1.1251108	0
ENSG000001	RPL3	protein_codin	1944.80977	3388.83431	1699.64204	1540.3191
ENSG000001	SYNGR1	protein_codin	0	0	0	0
ENSG000001	TAB1	protein_codin	114.23469	127.790959	43.886934	53.2919276
ENSG000001	PNPLA5	protein_codin	0	0	0	0
ENSG000001	PNPLA3	protein_codin	0	0	0	0

ENSG000001	MYH9	protein_codin	15374.5998	27618.6217	18347.3273	12958.0813
ENSG000001	TXN2	protein_codin	103.776226	158.80665	42.6461177	65.0314671
ENSG000001	FOXRED2	protein_codin	0	0	0	0
ENSG000001	EIF3D	protein_codin	552.582711	821.470253	232.213406	355.715978
ENSG000002	ADSL	protein_codin	36.7588252	72.4144464	71.0281596	40.0487301
ENSG000001	PVALB	protein_codin	52.7563204	0	0	0
ENSG000001	UPK3A	protein_codin	2.87933674	10.5717671	0	0
ENSG000001	FAM118A	protein_codin	0	0	0	0
ENSG000001	ST13	protein_codin	482.782038	648.84644	234.163256	413.679611
ENSG000001	IL2RB	protein_codin	0	0	0	0
ENSG000001	RBX1	protein_codin	46.6065874	78.7754431	35.7473953	24.7599132
ENSG000001	L3MBTL2	protein_codin	77.5146842	111.891121	47.3264859	31.4393504
ENSG000001	CHADL	protein_codin	3.78041485	7.52427983	1.85854367	0.87530441
ENSG000001	PHF5A	protein_codin	73.3436709	104.609879	33.0226169	52.0276549
ENSG000001	ACO2	protein_codin	166.794777	259.260229	107.862296	108.019408
ENSG000001	PMM1	protein_codin	38.2085607	37.9576721	12.2032905	25.0871598
ENSG000001	CERK	protein_codin	173.241992	296.001514	90.5485958	124.964781
ENSG000001	BRD1	protein_codin	0	73.3322992	0	37.9012378
ENSG000001	ZBED4	protein_codin	171.0596	258.121776	78.7776502	136.002915
ENSG000001	HDAC10	protein_codin	91.1708642	82.6302313	30.0111063	31.8881105
ENSG000001	RIPK3	protein_codin	42.6866996	33.6045189	11.2461782	31.361484
ENSG000000	PAPOLA	protein_codin	580.698159	422.036224	382.442875	487.825852
ENSG000000	HSP90AA1	protein_codin	2405.45494	2097.31708	2315.68657	1952.26318
ENSG000000	NID2	protein_codin	0	14.5638738	7.8368854	0
ENSG000000	MARK3	protein_codin	82.7446155	144.453089	66.1327	127.682375
ENSG000000	SNAPC1	protein_codin	77.0369675	48.3493068	52.8609096	49.3092033
ENSG000000	SUPT16H	protein_codin	530.971913	536.624167	437.943175	444.723858
ENSG000001	ABHD4	protein_codin	2.53272341	1.55143141	0	0
ENSG000001	FKBP3	protein_codin	0	2.19045988	1.99473747	0
ENSG000001	CTSG	protein_codin	2.69147536	2.59991424	2.75252987	0
ENSG000001	GZMH	protein_codin	9.55785082	4.58981856	0.89341962	0
ENSG000001	GZMB	protein_codin	6.49611671	41.4792309	10.6756317	0
ENSG000001	PRMT5	protein_codin	0	0	0	0
ENSG000001	COCH	protein_codin	0	0	0	0
ENSG000001	AP4S1	protein_codin	10.0239674	0	0	5.44504919
ENSG000001	POLE2	protein_codin	0	17.3591244	0	0
ENSG000001	SOS2	protein_codin	456.237909	262.067956	177.084398	319.982906
ENSG000001	CDKL1	protein_codin	0	0	0	1.97992645
ENSG000001	PYGL	protein_codin	550.252116	1071.80764	327.282664	452.525181
ENSG000002	ENSG000002	processed_ps	0	1.09793661	0	0
ENSG000001	GNPNAT1	protein_codin	10.3500123	16.7421067	7.77044426	0
ENSG000001	CDKN3	protein_codin	0	0	0	0
ENSG000001	CNIH1	protein_codin	54.0303926	40.6689448	28.4543531	32.8791396
ENSG000001	CGRRF1	protein_codin	48.9087975	21.8556628	19.7832862	36.4740631

ENSG000001	ATP6V1D	protein_codin	65.1468702	73.8816135	41.1259342	79.2716829
ENSG000001	CCDC198	protein_codin	0	0	0	0
ENSG000001	PLEK2	protein_codin	0	0	0	0
ENSG000001	LRRC74A	protein_codin	0	0	0	0
ENSG000001	PIGH	protein_codin	18.420583	29.0647762	12.9025413	13.0503337
ENSG000001	PSMA3	protein_codin	59.1158497	70.1364112	74.5982628	55.097067
ENSG000001	VTI1B	protein_codin	62.7705679	123.644068	40.4546173	63.0131375
ENSG000001	TIMM9	protein_codin	0	0	10.6508404	0
ENSG000001	GSTZ1	protein_codin	12.4267271	19.8219739	0	0
ENSG000001	TMED8	protein_codin	227.771721	251.823377	183.862987	143.353028
ENSG000001	SAMD15	protein_codin	8.07053975	11.0354706	8.45509859	5.11690032
ENSG000001	AHSA1	protein_codin	72.65211	185.488855	90.5877896	104.242654
ENSG000001	ISM2	protein_codin	0	0	0	0
ENSG000001	SPTLC2	protein_codin	994.491497	753.273624	383.472985	511.525905
ENSG000001	RIN3	protein_codin	1467.60068	1790.38472	506.290076	626.286268
ENSG000001	ALKBH1	protein_codin	16.8348237	26.6949535	11.9829724	9.83777628
ENSG000001	CHGA	protein_codin	0	0	0	0
ENSG000001	DHRS7	protein_codin	2.80597404	0	2.1776564	2.82402793
ENSG000001	SIX4	protein_codin	0	1.26061696	0	0
ENSG000001	CEP128	protein_codin	11.5088669	6.46036063	0	21.4390629
ENSG000001	ERH	protein_codin	0	0	0	0
ENSG000001	SLC10A1	protein_codin	1.08110274	1.05748223	0	0
ENSG000001	EIF5	protein_codin	561.430023	321.021131	251.5676	229.042261
ENSG000001	SLC8A3	protein_codin	0	0	0	0
ENSG000001	ZFYVE21	protein_codin	45.5304789	25.3680448	12.858933	22.2530641
ENSG000001	BDKRB1	protein_codin	0	0	0	0
ENSG000001	VRK1	protein_codin	0	0	0	0
ENSG000001	PAPLN	protein_codin	4.50474723	0	0	0
ENSG000001	APEX1	protein_codin	105.312738	190.960236	53.4694394	60.5987932
ENSG000001	PABPN1	protein_codin	580.060314	938.676956	241.095964	352.780249
ENSG000001	EFS	protein_codin	0	0	1.0292527	0
ENSG000001	ARHGAP5	protein_codin	0	0	2.78361377	0
ENSG000001	CINP	protein_codin	10.4265023	22.418625	0	12.5855167
ENSG000001	SRP54	protein_codin	66.8461468	0	14.1427433	0
ENSG000001	PCK2	protein_codin	82.1526141	86.8628881	48.6166197	48.6533004
ENSG000001	NFKBIA	protein_codin	17778.4348	15151.6214	5458.54481	9489.50412
ENSG000001	EMC9	protein_codin	0	1.61275028	2.04684136	0
ENSG000001	PSME2	protein_codin	164.908056	321.09574	168.009062	102.693031
ENSG000001	BRMS1L	protein_codin	16.6485286	4.29599304	10.7325004	6.77746045
ENSG000001	PNN	protein_codin	1091.17496	890.929755	948.488079	769.028219
ENSG000001	RABGGTA	protein_codin	45.0007487	77.3551846	37.0533543	37.7065195
ENSG000000	CYP24A1	protein_codin	0	0	0	0
ENSG000001	PTPRA	protein_codin	0	0	9.23680233	0
ENSG000000	NSFL1C	protein_codin	145.152271	115.665037	30.8013252	82.7590754

ENSG000000:	ZFP64	protein_codin	44.9844249	0	0	0
ENSG000000(	SIRPG	protein_codin	2.73969882	0	0.80947114	0
ENSG000001(	PYGB	protein_codin	257.781658	438.084528	206.300249	167.915179
ENSG000001(	PROCR	protein_codin	1.92796032	3.75225777	0.90670329	0.94774482
ENSG000001(	MYBL2	protein_codin	22.9256155	29.5894915	163.661334	8.86865719
ENSG000001(	R3HDML	protein_codin	0	0	0	0
ENSG000001(	PABPC1L	protein_codin	0	0	0	0
ENSG000001(	PABPC1L	protein_codin	0	0	0	0
ENSG000001(	PABPC1L	protein_codin	0	0	0	0
ENSG000001(	SALL4	protein_codin	0	0	0.9678512	0
ENSG000001(	CSTF1	protein_codin	5.18775893	9.40950769	8.42713834	18.1505588
ENSG000001(	TPD52L2	protein_codin	0	0	0	0
ENSG000001(	CTSZ	protein_codin	732.566122	1648.31143	542.661496	719.768062
ENSG000001(	TUBB1	protein_codin	7547.51306	18122.8584	7072.18636	6723.27234
ENSG000001(	SLCO4A1	protein_codin	0	0	3.84174998	0
ENSG000001(	TCFL5	protein_codin	2.48209929	0	5.90539066	0
ENSG000001(	BIRC7	protein_codin	0	0	0	0
ENSG000001(	UBOX5	protein_codin	44.2666791	60.2508869	25.7130611	27.9779579
ENSG000001(	EEF1A2	protein_codin	0	0	0	0
ENSG000001(	PTK6	protein_codin	0	0	0	0
ENSG000001:	SRMS	protein_codin	0.85982648	1.69991785	0.82117614	0
ENSG000001(	ADISSP	protein_codin	0	6.83059756	0	2.37584006
ENSG000001(	TRIB3	protein_codin	14.5824622	10.1809117	0	7.10343727
ENSG000001(	CSNK2A1	protein_codin	25.9099267	28.5856495	16.2874107	0
ENSG000001	MACROD2	protein_codin	0	0	0	0
ENSG000001(	SLC52A3	protein_codin	0	0	0	0
ENSG000001(	RSPO4	protein_codin	0	0	0	0
ENSG000001(	PROKR2	protein_codin	3.17358482	0	0	0
ENSG000001(	FERMT1	protein_codin	0	0	0	0
ENSG000001(	PDYN	protein_codin	0	0	0	0
ENSG000001(	TM9SF4	protein_codin	70.2017437	0	0	79.1121273
ENSG000001(	TLDC2	protein_codin	0	0	0	0
ENSG000001(	SNTA1	protein_codin	1.86903936	0.92391338	1.86902069	0
ENSG000001(	OXT	protein_codin	0	0	0	0
ENSG000001(	PXMP4	protein_codin	0	7.38840932	0	0
ENSG000001(	CHMP4B	protein_codin	857.194463	1183.71671	627.70186	597.80853
ENSG000001:	LBP	protein_codin	0	0	0	0.92931224
ENSG000001(	SLC32A1	protein_codin	1.70861204	0	0	0
ENSG000001(	CST4	protein_codin	0	0	0.90780923	0
ENSG000001(	WFDC2	protein_codin	0	0	0	0
ENSG000001(	AHCY	protein_codin	87.413413	118.489401	66.2122272	57.9822797
ENSG000001(	SPINT3	protein_codin	0	0	0	0
ENSG000001(	PIGU	protein_codin	6.07388772	9.21580933	0	8.22002033
ENSG000001(	ACOT8	protein_codin	20.2130606	50.2044939	0	9.67998183

ENSG000001	APMAP	protein_codin	194.323529	269.561005	84.7856606	141.70638
ENSG000000	TXNL1	protein_codin	17.3098456	15.2150742	15.8863413	15.5664561
ENSG000001	MYL12A	protein_codin	2265.47992	4135.67981	1587.33112	1586.20553
ENSG000001	RNF125	protein_codin	178.201116	154.702865	51.2671111	105.968986
ENSG000001	POLI	protein_codin	0	13.1956767	0	42.4447515
ENSG000000	NOX1	protein_codin	0	0	0	0
ENSG000000	ARSD	protein_codin	13.3902105	25.6289273	11.8154124	16.2267943
ENSG000002	TAF9	protein_codin	53.6095222	38.221702	7.01913555	51.8845
ENSG000000	IDH3G	protein_codin	143.119244	217.217068	33.8649501	129.260263
ENSG000000	SLC25A43	protein_codin	28.3193815	41.3595202	33.1575452	19.209784
ENSG000001	H2BW1	protein_codin	0	0	0	0
ENSG000001	MXRA5	protein_codin	0	0.91057604	0.9724149	0.93002411
ENSG000001	VSIG1	protein_codin	1.04557606	3.19164949	10.4313569	0
ENSG000001	PSMD10	protein_codin	38.1084582	45.6748303	7.9878903	27.5648869
ENSG000001	STS	protein_codin	0	0	0	0
ENSG000001	PGRMC1	protein_codin	692.91562	1003.89517	664.486633	451.740779
ENSG000001	RHOXF1	protein_codin	0	0	0	0
ENSG000001	NXT2	protein_codin	0	0	0	0
ENSG000001	GUCY2F	protein_codin	0	0	0	0
ENSG000001	ATP1B4	protein_codin	0	0	0	0
ENSG000001	TLR8	protein_codin	331.990046	456.806098	93.0588919	348.275012
ENSG000001	WDR13	protein_codin	58.1325012	51.8868814	24.4287567	26.7445135
ENSG000001	PAGE4	protein_codin	0	0	0	0
ENSG000001	GLRA2	protein_codin	0	0	0	0
ENSG000001	STAG2	protein_codin	53.5397073	66.2182794	63.5512577	43.5435736
ENSG000001	F9	protein_codin	0	0	0	0
ENSG000001	ABCD1	protein_codin	220.593742	269.319537	73.1438478	137.158171
ENSG000000	BCORL1	protein_codin	5.8629795	0	0	0
ENSG000001	KCND1	protein_codin	6.64117684	15.5407383	11.2667881	6.87581884
ENSG000001	SLC25A14	protein_codin	0	0	0	0
ENSG000001	FMR1	protein_codin	0	0	0	0
ENSG000001	PQBP1	protein_codin	32.1040116	37.9343542	0	21.1406913
ENSG000001	PCSK1N	protein_codin	0	0	0	0
ENSG000001	RAB40AL	protein_codin	0.91324325	0	0.88560157	0
ENSG000001	GPR50	protein_codin	0	0	0	0
ENSG000000	HUWE1	protein_codin	138.891414	95.6566269	117.24758	51.2804465
ENSG000001	RP2	protein_codin	480.3943	399.24327	126.122644	342.000162
ENSG000001	USP11	protein_codin	0	0	0	0
ENSG000001	HTATSF1	protein_codin	150.237505	202.39524	212.525843	158.72485
ENSG000001	TIMP1	protein_codin	375.649183	567.936694	110.739297	307.714752
ENSG000001	PIN4	protein_codin	0	2.85607859	0	0
ENSG000001	ITIH6	protein_codin	0	0	0	0
ENSG000001	MAGED2	protein_codin	7.65540838	11.1319038	14.3010093	22.5950697
ENSG000001	GLA	protein_codin	55.6200253	96.1322478	41.2291285	52.5100604

ENSG0000007	ATP12A	protein_codin	0	0	0	0
ENSG0000001	NDFIP2	protein_codin	0	0	0	0
ENSG0000001	MLNR	protein_codin	0	0	0	0
ENSG0000001	ACP5	protein_codin	7.01327435	7.23497245	0	0
ENSG0000001	ARHGEF7	protein_codin	0	4.37491844	0	0
ENSG0000001	SGCG	protein_codin	0	0	0	0
ENSG0000001	DHRS12	protein_codin	7.06718295	21.6793978	3.02115189	0
ENSG0000001	OLFM4	protein_codin	0	0	0	0
ENSG0000001	ACSM2A	protein_codin	0	0	0	0
ENSG0000000	NTHL1	protein_codin	11.0875713	17.5822584	0	0
ENSG0000000	ZNF263	protein_codin	0	59.3779654	39.667914	29.0476074
ENSG0000000	MMP2	protein_codin	0	0	0	0
ENSG0000001	CHD9	protein_codin	0	0	0	0
ENSG0000001	ZNF205	protein_codin	22.7176352	0	2.2600188	6.08282886
ENSG0000000	ORC6	protein_codin	0	6.73543332	0	0
ENSG0000001	PHAF1	protein_codin	99.5607929	118.602454	41.2160517	57.8177823
ENSG0000001	CORO1A	protein_codin	1560.85339	2249.03316	358.960588	1042.63383
ENSG0000001	MT4	protein_codin	0	0	0	0
ENSG0000001	LYRM1	protein_codin	3.70424126	15.416354	5.96631958	0
ENSG0000001	NUTF2	protein_codin	0	4.45021982	8.25096724	16.6170672
ENSG0000001	CBLN1	protein_codin	0	0	0	0
ENSG0000001	ARL2BP	protein_codin	93.1729493	131.939233	54.5059295	64.327167
ENSG0000001	PLLP	protein_codin	0	0	0	0
ENSG0000001	CCL22	protein_codin	3.16453686	1.04325646	1.78058152	0
ENSG0000001	DHODH	protein_codin	12.2358825	11.6904997	3.79847111	0
ENSG0000001	CCL17	protein_codin	0	0	0	0
ENSG0000001	ACD	protein_codin	0	0	0	2.09537262
ENSG0000001	POLR2C	protein_codin	118.271908	147.334544	45.9076266	72.0632463
ENSG0000001	PARD6A	protein_codin	0	0	0	0
ENSG0000001	MMP15	protein_codin	0	1.82178135	4.14504067	0
ENSG0000001	USB1	protein_codin	62.1524167	75.1206462	0	44.4109549
ENSG0000001	CFAP263	protein_codin	0	0	0	0
ENSG0000001	PRSS54	protein_codin	0	0	0	0
ENSG0000001	NME3	protein_codin	20.2921576	21.8263998	3.75805179	11.3177589
ENSG0000001	PSMD7	protein_codin	333.372471	544.246311	230.226549	314.378019
ENSG0000001	SETD6	protein_codin	20.6530322	22.7802102	0	8.09828494
ENSG0000001	SLC38A7	protein_codin	51.5676037	95.5241628	19.9707469	30.9360823
ENSG0000001	HAS3	protein_codin	1.72308577	0	0.83544422	0
ENSG0000001	SMPD3	protein_codin	7.89116633	27.083092	23.7024867	0
ENSG0000001	SLC7A6	protein_codin	0	89.8610753	0	0
ENSG0000001	PLA2G15	protein_codin	40.2788697	57.7091156	19.2235145	27.8853832
ENSG0000001	FA2H	protein_codin	0	0	0	0
ENSG0000001	CMC2	protein_codin	0	7.02195242	4.46223509	0
ENSG0000001	PDIA2	protein_codin	0	0	0	0

ENSG000002	ARHGDIG	protein_codin	0	0	0	0
ENSG000001	MPG	protein_codin	1.52993829	4.04878224	0	0
ENSG000001	HSDL1	protein_codin	58.5545845	88.170574	30.3109613	54.1626888
ENSG000001	WFDC1	protein_codin	0.86423787	3.30772818	0	0
ENSG000001	USP10	protein_codin	264.736438	298.50552	116.33687	150.35096
ENSG000001	TSC2	protein_codin	0	0	0	0
ENSG000001	ZNF500	protein_codin	9.65262474	31.9554791	10.2915048	13.1529682
ENSG000001	NME4	protein_codin	28.1106837	52.0944001	38.2248999	20.3460777
ENSG000002	DECR2	protein_codin	7.5557334	0	0	3.74889
ENSG000001	ANTKMT	protein_codin	0	3.51060198	2.45235679	10.6423281
ENSG000001	METRN	protein_codin	0	0	0	0
ENSG000001	STUB1	protein_codin	114.411416	145.15687	46.9392198	61.115113
ENSG000001	RHBDL1	protein_codin	0	0	0	0
ENSG000001	MEFV	protein_codin	739.551967	934.264999	381.899058	521.761021
ENSG000001	CRYM	protein_codin	0	0	0	0
ENSG000001	CAPN15	protein_codin	141.386128	213.946555	86.6734011	83.9518475
ENSG000001	AQP8	protein_codin	0	0	0	0
ENSG000001	USP31	protein_codin	100.410782	129.542205	86.6000646	51.1959908
ENSG000001	HMOX2	protein_codin	0	0	0	0
ENSG000001	TOX3	protein_codin	0	0	0	0
ENSG000001	QPRT	protein_codin	0	1.43564829	0	0
ENSG000001	MAZ	protein_codin	130.410633	281.901618	119.139414	92.3605017
ENSG000001	CDIPT	protein_codin	58.5423821	121.77832	34.3109096	33.7023857
ENSG000001	BCKDK	protein_codin	182.219003	261.358131	54.8551097	108.14125
ENSG000001	KAT8	protein_codin	85.6029859	196.678443	51.1881158	75.4573111
ENSG000001	TMC5	protein_codin	0	0	0	0
ENSG000001	SLC6A2	protein_codin	0	0	0	0
ENSG000001	KNOP1	protein_codin	15.311184	32.6163279	24.5621655	22.6658002
ENSG000001	MGA	protein_codin	213.055827	89.4113898	190.481127	155.649887
ENSG000001	AQP9	protein_codin	938.76702	556.903787	284.441494	719.315269
ENSG000001	CSK	protein_codin	328.028708	576.867045	50.4837026	162.435672
ENSG000001	MTFMT	protein_codin	10.8749603	7.35114606	11.7656584	9.40069084
ENSG000001	RASL12	protein_codin	0	0	0	0
ENSG000001	CTSH	protein_codin	0	0	0	0
ENSG000001	CEMIP	protein_codin	0	13.624445	7.20204478	2.96231647
ENSG000001	EHD4	protein_codin	305.511785	304.008343	118.268466	238.974528
ENSG000001	TGM5	protein_codin	0	0	0	0
ENSG000001	GABPB1	protein_codin	50.4540537	50.6322279	36.6486268	32.0203444
ENSG000001	SCG3	protein_codin	0	0	0	0
ENSG000001	DNAJC17	protein_codin	17.7395317	31.5143863	4.42250363	9.53527787
ENSG000001	RHOV	protein_codin	0	0	0	0
ENSG000001	VPS18	protein_codin	134.563533	185.800502	50.567749	80.4459032
ENSG000001	OIP5	protein_codin	0	0	2.0051567	1.29329901
ENSG000001	BLOC1S6	protein_codin	206.4785	111.366914	128.37537	174.127309

ENSG000000	EXTL3	protein_codin	0	0	0	0
ENSG000000	FDFT1	protein_codin	199.09164	232.392504	56.2129567	86.2824181
ENSG000001	AGO2	protein_codin	285.608583	313.76393	184.043866	130.853547
ENSG000000	PAG1	protein_codin	344.615467	175.764995	137.916809	187.095817
ENSG000000	TG	protein_codin	6.70887898	6.70062077	0	1.8963703
ENSG000001	BRF2	protein_codin	7.78928888	20.0383134	4.45884217	13.4937082
ENSG000001	ZFAND1	protein_codin	90.5754086	58.5359588	0	0
ENSG000001	RP1	protein_codin	0	0	0	0
ENSG000001	RIPK2	protein_codin	481.53309	163.046688	296.837629	289.81284
ENSG000001	CPQ	protein_codin	255.184113	250.229832	89.4471249	160.702541
ENSG000001	DECR1	protein_codin	10.1388524	2.41924175	5.35997053	11.7473035
ENSG000001	SFRP1	protein_codin	0	0	0	0
ENSG000001	PLAT	protein_codin	0	0	0	0
ENSG000001	DKK4	protein_codin	0	0	0	0
ENSG000001	GDAP1	protein_codin	0	0	0	0
ENSG000001	PHF20L1	protein_codin	244.988614	162.267032	224.354918	189.332322
ENSG000001	EIF3E	protein_codin	0	0	0	0
ENSG000001	EMC2	protein_codin	10.903469	19.3425456	11.2408908	31.6616241
ENSG000001	CCN4	protein_codin	0	0	0	0
ENSG000001	STMN2	protein_codin	0	0	1.43753456	0
ENSG000001	TRPS1	protein_codin	130.510208	101.495336	124.289083	56.7750344
ENSG000001	CHRAC1	protein_codin	159.896712	158.410526	41.3631227	96.7859489
ENSG000001	NCALD	protein_codin	0	2.08642656	0	0
ENSG000001	GML	protein_codin	0	0	0	0
ENSG000001	UBR5	protein_codin	0	92.0096297	165.481528	0
ENSG000001	PYCR3	protein_codin	4.57512837	4.56495158	0	2.65122958
ENSG000001	MTMR9	protein_codin	98.3171542	34.5116113	41.2187817	46.4767098
ENSG000001	DCTN6	protein_codin	54.5389429	32.0438975	22.3325712	15.0520229
ENSG000001	GSR	protein_codin	295.658242	485.279116	167.79337	277.142956
ENSG000001	TNFRSF10A	protein_codin	51.4426289	83.0643731	24.7199917	20.1126818
ENSG000001	PPP2CB	protein_codin	51.9646411	102.170339	29.0468075	50.6992724
ENSG000001	NEFM	protein_codin	0	0	0	0
ENSG000001	KCTD9	protein_codin	80.5847444	54.495927	51.6292523	44.4425727
ENSG000000	HOMER3	protein_codin	0	0	0	0
ENSG000000	CNOT3	protein_codin	103.460502	149.370187	80.2627402	116.877273
ENSG000000	EXOSC5	protein_codin	28.8378221	25.1807969	15.2856949	12.5054558
ENSG000000	PNPLA6	protein_codin	0	25.3197753	0	0
ENSG000000	PLAUR	protein_codin	718.934186	521.052899	397.547953	444.086344
ENSG000000	PAF1	protein_codin	194.171917	291.718544	91.7035031	151.408859
ENSG000000	STXBP2	protein_codin	539.873606	688.099378	235.854777	428.393184
ENSG000001	CYP4F3	protein_codin	0	0	3.68861086	8.55410531
ENSG000002	ZNF432	protein_codin	35.9876839	0	26.9427503	15.5670198
ENSG000001	ZNF180	protein_codin	0	2.71919507	0	0
ENSG000001	ZNF780B	protein_codin	5.40806505	9.78218489	8.72274744	8.58975261

ENSG000001	MAN2B1	protein_codin	43.187128	55.3536013	10.7506598	9.38997935
ENSG000001	TULP2	protein_codin	0	0	0	0
ENSG000001	DHDH	protein_codin	0	0	0	0
ENSG000001	RUVBL2	protein_codin	0	0	0	0
ENSG000001	ECH1	protein_codin	202.554845	315.111483	114.113314	185.152091
ENSG000001	HNRNPL	protein_codin	152.718095	231.731209	37.7020566	125.347032
ENSG000001	SARS2	protein_codin	0	30.6454477	12.8042024	0
ENSG000001	KCNA7	protein_codin	0	0.93290567	0	0
ENSG000001	SNRNP70	protein_codin	91.1788817	190.023863	58.982663	0
ENSG000001	RELB	protein_codin	253.560518	234.217439	47.1180494	143.720667
ENSG000001	CLASRP	protein_codin	88.9739046	155.290299	17.6595898	84.1456669
ENSG000001	LIN7B	protein_codin	0	8.11709848	2.99420956	3.64794384
ENSG000001	PPP1R37	protein_codin	7.90963485	33.9034925	16.6205461	11.7885156
ENSG000001	FCGRT	protein_codin	625.480591	755.684979	85.8276041	376.316591
ENSG000001	CKM	protein_codin	0.88521611	0.90360658	0	2.72548526
ENSG000001	PEX11G	protein_codin	0.88866003	0	1.5773857	0
ENSG000001	SLC17A7	protein_codin	0	0	0	0
ENSG000001	RNASEH2A	protein_codin	19.8939977	34.1194671	13.771891	7.70891122
ENSG000001	SF3A2	protein_codin	148.387913	214.938109	97.7603186	91.8438225
ENSG000001	AMH	protein_codin	1.77847247	0	0.83404095	0
ENSG000001	DKKL1	protein_codin	0	0	0	0
ENSG000001	TRMT1	protein_codin	54.3007714	11.6959982	23.9367513	0
ENSG000001	RETN	protein_codin	26.754386	44.0458273	0	28.9987374
ENSG000001	RSPH6A	protein_codin	0	1.33269323	0	0
ENSG000001	TBC1D17	protein_codin	0	19.9737639	14.8123556	20.0090759
ENSG000001	YJU2B	protein_codin	24.7340927	0	11.6742475	23.9710692
ENSG000001	TLE5	protein_codin	0	49.0912032	0	15.0409919
ENSG000001	SGTA	protein_codin	33.4124095	93.6996058	48.540561	43.959114
ENSG000002	ENSG000002	lncRNA	0	0	0	0
ENSG000001	SNAPC2	protein_codin	17.138962	27.0086591	0	9.91861285
ENSG000001	C19orf53	protein_codin	0	0	0	0
ENSG000001	FIZ1	protein_codin	10.1368514	10.8921156	2.58684348	8.45328868
ENSG000001	C19orf44	protein_codin	0	6.12191499	0	0
ENSG000001	CYP4F2	protein_codin	0	0	0	0
ENSG000001	EPHX3	protein_codin	0	0	0	0
ENSG000001	ZNF419	protein_codin	0	0	0	0
ENSG000001	SLC1A6	protein_codin	0	0	0	0
ENSG000001	POP4	protein_codin	0	0	0	0
ENSG000001	PDCD5	protein_codin	1.0403718	0	0	0
ENSG000001	LGALS13	protein_codin	0	0	0	0
ENSG000001	FBL	protein_codin	0	67.7962061	35.2899039	40.7521199
ENSG000001	CLC	protein_codin	6.65914431	11.5398897	1.52901955	0
ENSG000001	CCNP	protein_codin	0	0	0	0
ENSG000001	EBI3	protein_codin	0.88245822	0.88052999	0	0

ENSG000001	TBCB	protein_codin	109.546109	95.2865401	51.8150707	45.0800551
ENSG000001	FSD1	protein_codin	0	0	0	0
ENSG000001	POLR2I	protein_codin	3.66129158	5.06920408	0	0
ENSG000001	APLP1	protein_codin	0	0	0	0
ENSG000001	CACTIN	protein_codin	0	14.8131044	0	0
ENSG000000	ARHGAP33	protein_codin	0	0	0	0
ENSG000001	CCDC9	protein_codin	162.753133	156.764374	89.5828907	94.5332833
ENSG000001	TGFB1	protein_codin	3100.90399	4656.11204	1593.46366	1760.49312
ENSG000001	DMAC2	protein_codin	32.5449872	45.5957864	20.1990259	13.132955
ENSG000001	CEACAM4	protein_codin	39.2348146	39.6257756	18.5603968	10.9169404
ENSG000001	PLIN3	protein_codin	261.429414	395.967873	124.904793	226.820078
ENSG000001	CD79A	protein_codin	27.5512283	17.4293872	35.7214965	6.54120124
ENSG000001	LIM2	protein_codin	0	0	0	0
ENSG000001	RPS19	protein_codin	959.423053	1292.75776	502.399033	575.490403
ENSG000001	NKG7	protein_codin	9.39373577	18.8176148	4.07145929	5.73763747
ENSG000001	ICAM5	protein_codin	5.69128987	0	0	0
ENSG000001	CEACAM5	protein_codin	0.9781615	0	0	0
ENSG000001	CRX	protein_codin	0	0	0	0
ENSG000001	SULT2A1	protein_codin	0	0.96915708	0	0
ENSG000001	CDC37	protein_codin	348.246079	544.59767	169.050023	288.16468
ENSG000001	RABAC1	protein_codin	77.9111543	63.6108632	16.6949714	43.1013157
ENSG000001	CNFN	protein_codin	2.90538662	0	0	0
ENSG000001	ZNRF4	protein_codin	0	0	0	0
ENSG000001	HAS1	protein_codin	0	0	0	0
ENSG000001	RAB3D	protein_codin	858.803039	976.594615	227.218279	551.001856
ENSG000001	DBP	protein_codin	8.15300914	65.6012727	13.803805	5.93326088
ENSG000001	EPOR	protein_codin	30.7471599	57.690402	27.5069602	13.6852607
ENSG000001	RASIP1	protein_codin	0	0	0	0
ENSG000001	FGF21	protein_codin	0	0	0	0
ENSG000001	WDR83OS	protein_codin	0	0	0	0
ENSG000001	CACNG7	protein_codin	0	0	0	0
ENSG000001	GCDH	protein_codin	0	25.6879253	0	0
ENSG000001	DNASE2	protein_codin	58.351784	109.887441	44.5498581	43.4439095
ENSG000001	LENG1	protein_codin	42.263144	89.8648312	32.1792833	29.4578674
ENSG000001	RPL18A	protein_codin	1298.82859	2484.49052	411.490819	1049.74183
ENSG000001	SLC5A5	protein_codin	0.96046108	0	0	0
ENSG000001	KCNN1	protein_codin	2.64674098	0	0	0
ENSG000001	ARRDC2	protein_codin	22.2090555	105.800907	14.5777339	72.9931489
ENSG000001	PIK3R2	protein_codin	121.895862	0	0	0
ENSG000001	RAB3A	protein_codin	10.8604298	16.4969135	8.97746425	7.77714258
ENSG000002	PSENEN	protein_codin	26.5470666	28.4878815	0	0
ENSG000001	COMP	protein_codin	0	0	0	0
ENSG000001	UPK1A	protein_codin	0	0	0	1.06223978
ENSG000001	TMEM147	protein_codin	43.4698603	57.4024571	10.6634808	27.4810381

ENSG000001	GAPDHS	protein_codin	0	0	0	0
ENSG000001	HAMP	protein_codin	0	0	0	0
ENSG000001	USF2	protein_codin	236.136892	351.501635	0	190.098528
ENSG000001	KXD1	protein_codin	147.964096	242.859347	42.1747815	71.644586
ENSG000001	FKBP8	protein_codin	146.295029	269.371282	68.7672966	108.043871
ENSG000001	ERF	protein_codin	81.9314357	58.2952002	0	43.7196806
ENSG000001	GSK3A	protein_codin	87.0230385	136.956192	0	42.3914711
ENSG000001	ZNF574	protein_codin	0	0	0	0
ENSG000001	SIPA1L3	protein_codin	228.028326	346.870989	194.932255	178.03111
ENSG000001	CADM4	protein_codin	5.89534645	9.71327718	5.49503027	2.83993881
ENSG000000	PON1	protein_codin	0	0	0	0
ENSG000002	CYP3A43	protein_codin	0	0	0	0
ENSG000002	ABCF2-H2BK1	protein_codin	59.8152536	182.078261	100.924103	67.1179515
ENSG000000	HGF	protein_codin	33.8247546	30.7509259	26.6390633	19.4558576
ENSG000002	TSPAN11-AS1	lncRNA	0	0	0	0
ENSG000000	LAMB1	protein_codin	0	0	0	0
ENSG000000	UBE2D4	protein_codin	39.7869743	39.2243664	26.825225	26.9566455
ENSG000000	WNT16	protein_codin	0	0	0	0
ENSG000001	CPA2	protein_codin	0	0	0	0
ENSG000001	CPA4	protein_codin	0	0	0	0
ENSG000001	GTPBP10	protein_codin	0	15.3737232	14.967003	6.58561609
ENSG000001	TFPI2	protein_codin	2.56409368	7.66835297	2.49962555	2.63561852
ENSG000001	BET1	protein_codin	0	16.4097084	22.9232713	3.49108098
ENSG000001	NAMPT	protein_codin	0	0	0	0
ENSG000001	POLR1F	protein_codin	215.916713	108.307748	174.966719	125.242876
ENSG000001	PON2	protein_codin	0	14.7360809	0	13.9821897
ENSG000001	ITGB8	protein_codin	2.15125374	2.08924336	3.16048382	0
ENSG000001	HBP1	protein_codin	85.877782	87.9538537	10.7161227	131.073223
ENSG000001	SP4	protein_codin	11.4206358	0	0	25.6762508
ENSG000001	CBLL1	protein_codin	28.4998608	65.8189193	30.1077442	21.4695386
ENSG000001	PALS2	protein_codin	8.16014409	0	7.59057473	0
ENSG000001	OGDH	protein_codin	409.603625	344.156024	217.7513	191.384292
ENSG000001	NPVF	protein_codin	0	0	0	0
ENSG000001	H2AZ2	protein_codin	195.637725	207.696435	155.889194	154.253175
ENSG000001	CAV2	protein_codin	15.3040664	21.1944223	7.86784086	14.3334804
ENSG000001	HOXA2	protein_codin	0	0.90805068	0	0
ENSG000001	LFNG	protein_codin	348.764694	304.28855	96.2079372	141.674554
ENSG000001	HOXA5	protein_codin	14.2512155	5.12450674	9.26438434	7.67769628
ENSG000001	HOXA6	protein_codin	0	0	0	0
ENSG000001	TSPAN12	protein_codin	0	0	0	0
ENSG000001	EVX1	protein_codin	0	0	0	0
ENSG000001	CHN2	protein_codin	0	61.8076096	31.2990406	73.8365282
ENSG000001	ABHD11	protein_codin	0	16.7860413	4.75251478	0
ENSG000001	FKBP14	protein_codin	14.5948886	8.55077079	13.2647748	13.0182721

ENSG000001	STX1A	protein_codin	2.29791793	0	0	0
ENSG000001	NOD1	protein_codin	56.569478	88.8385044	14.2012991	38.4113561
ENSG000001	CCL24	protein_codin	0	0	0	2.73868714
ENSG000001	BUD31	protein_codin	13.1153266	52.315416	13.1043885	9.37242223
ENSG000001	CYP3A5	protein_codin	0	0	0	0
ENSG000001	SNX8	protein_codin	34.006867	30.5985574	18.9575504	7.95849814
ENSG000001	WASL	protein_codin	109.873522	105.384619	44.8086689	107.090528
ENSG000001	HYAL4	protein_codin	0	0	0	0
ENSG000001	SPAM1	protein_codin	0	0	0	0
ENSG000001	AIMP2	protein_codin	49.0332653	60.2877989	13.0870073	8.6916966
ENSG000001	TFR2	protein_codin	0	0	0	0
ENSG000001	MOSPD3	protein_codin	0	0	0	0
ENSG000001	PCOLCE	protein_codin	0	0	0	0
ENSG000001	RBM28	protein_codin	111.669273	134.958593	56.9007549	61.7571155
ENSG000001	IRS3P	transcribed_p	0.89052141	0	0	0
ENSG000001	LSM5	protein_codin	1.98697629	10.8471569	0.78016743	0
ENSG000001	SERPINE1	protein_codin	67.7412817	86.6692784	45.7991319	33.1743012
ENSG000001	MOGAT3	protein_codin	0	0	0	0
ENSG000001	C1GALT1	protein_codin	21.9998845	43.9980444	34.5752292	29.1531267
ENSG000001	PLOD3	protein_codin	91.8391977	123.146922	37.9093839	32.9985305
ENSG000001	RPA3	protein_codin	7.10587004	3.54752451	0	2.29032249
ENSG000002	FIS1	protein_codin	74.9613457	119.974218	44.9438818	66.6344606
ENSG000001	GLCCI1	protein_codin	52.4233676	36.8105186	46.6865541	20.1008095
ENSG000001	MYL10	protein_codin	0	0	0	0
ENSG000001	NRF1	protein_codin	52.4847475	31.838637	16.1535043	13.1202493
ENSG000001	CEP41	protein_codin	7.11220952	4.17627256	0	6.92506395
ENSG000001	ZNF862	protein_codin	46.9517658	62.6214925	21.7155433	25.9977925
ENSG000001	MEST	protein_codin	3.57765838	0	0	0
ENSG000001	RARRES2	protein_codin	0	0	0	0
ENSG000002	YAE1	protein_codin	0	1.54222664	0	0
ENSG000001	GIMAP2	protein_codin	192.130471	217.819851	138.512024	154.800607
ENSG000001	PSMA2	protein_codin	84.2205039	92.4459993	26.3855993	70.837674
ENSG000001	MRPL32	protein_codin	32.9020005	85.9387923	67.3193864	50.2817909
ENSG000001	COA1	protein_codin	7.05525765	17.4396872	0	0
ENSG000001	AEBP1	protein_codin	0	12.239356	13.7206718	0
ENSG000001	POLD2	protein_codin	0	0	0	0
ENSG000001	MYL7	protein_codin	0	0	0	0
ENSG000001	BCL7B	protein_codin	265.285575	271.88954	83.336293	101.237038
ENSG000001	YKT6	protein_codin	69.3955261	96.3144933	76.1897866	48.5959
ENSG000001	CLIP2	protein_codin	56.0211978	175.485132	70.8948545	43.6352085
ENSG000000	PTGS1	protein_codin	0	0	0	0
ENSG000000	ZNF510	protein_codin	53.0766945	30.4938594	33.0158551	0
ENSG000002	ZNF688	protein_codin	27.2816571	25.5615793	12.484896	14.5326523
ENSG000000	CHMP5	protein_codin	104.218299	181.168973	103.071499	65.5954415

ENSG000001	SPATA6L	protein_codin	0	0	0	0
ENSG000001	FKTN	protein_codin	0	4.62047091	0	0
ENSG000001	SEC61B	protein_codin	58.7855027	98.8678688	44.1103624	61.0822923
ENSG000001	C5	protein_codin	65.6494678	10.4186353	0	19.2978732
ENSG000001	AKNA	protein_codin	17.8395144	0	0	13.3080832
ENSG000001	TNFSF8	protein_codin	197.663025	220.877346	158.017324	155.938251
ENSG000001	AK1	protein_codin	0	0	0	0
ENSG000001	RLN1	protein_codin	0	0	0	0
ENSG000001	PLGRKT	protein_codin	5.03218286	26.7625357	10.1245762	11.8713744
ENSG000001	TBC1D13	protein_codin	0	0	0	0
ENSG000001	ELAVL2	protein_codin	0	0	0	0
ENSG000001	EDF1	protein_codin	484.438117	782.789127	289.027627	351.067531
ENSG000001	SETX	protein_codin	1792.93132	1463.05886	1172.93787	1501.99168
ENSG000002	VIM	protein_codin	12282.2793	10272.1884	2773.89921	4837.79238
ENSG000000	BLNK	protein_codin	0	7.50081009	29.3480498	1.50270946
ENSG000000	CYP26A1	protein_codin	0	0	0	0
ENSG000001	ATE1	protein_codin	0	0	0	0
ENSG000001	CDH23	protein_codin	79.6075386	66.0560413	44.5811046	23.0807418
ENSG000001	CCSER2	protein_codin	21.8518305	0	0	0
ENSG000001	ACTA2	protein_codin	0	0	0	0
ENSG000001	PITRM1	protein_codin	0	0	31.9148928	0
ENSG000001	STN1	protein_codin	19.791272	0	11.5661231	0
ENSG000001	DNAJC12	protein_codin	0	0	0	0
ENSG000001	PPIF	protein_codin	372.813032	403.654171	189.414008	217.58896
ENSG000001	TBC1D12	protein_codin	123.958312	96.1657673	45.0695617	81.2022073
ENSG000000	MPO	protein_codin	35.0732534	25.2135401	23.7572036	21.4886437
ENSG000000	ST6GALNAC2	protein_codin	6.60522314	28.1068878	11.5425946	3.7755766
ENSG000000	DHX33	protein_codin	57.2890109	106.992686	28.6811233	25.1572088
ENSG000000	UTP18	protein_codin	127.388246	162.72495	73.8469596	82.1484047
ENSG000000	SLC25A39	protein_codin	0	18.6473461	19.7915681	10.8694366
ENSG000000	P2RX5	protein_codin	14.5497992	18.7784394	31.547982	0
ENSG000001	EPX	protein_codin	0.91709305	0	0	2.74035003
ENSG000001	CRYBA1	protein_codin	0	0	0	0
ENSG000001	NUFIP2	protein_codin	731.901262	499.009803	416.485006	531.606487
ENSG000001	GIT1	protein_codin	176.629675	210.371994	55.3895531	76.8670296
ENSG000001	RPL19	protein_codin	4693.18129	7345.15484	2847.63647	4042.91328
ENSG000001	RUNDC3A	protein_codin	0.82435011	0	0.91934721	0
ENSG000001	CSF3	protein_codin	0	0	0	0
ENSG000002	SUPT4H1	protein_codin	132.557453	177.956119	60.6339666	80.1615815
ENSG000001	WNT3	protein_codin	0	0	0	0
ENSG000001	SHPK	protein_codin	16.4806842	14.6239337	24.7033738	23.8607432
ENSG000002	TAX1BP3	protein_codin	106.459063	184.784134	69.9010233	77.7757088
ENSG000001	P2RX1	protein_codin	107.919459	243.101913	35.5845725	65.1622192
ENSG000001	KRT37	protein_codin	0	0	0	0

ENSG000001	GOSR2	protein_codin	0	0	0	0
ENSG000001	PNPO	protein_codin	0	0	0	0
ENSG000001	TVP23C	protein_codin	3.80560858	11.6230535	2.92747105	3.31851267
ENSG000001	RPS6KB1	protein_codin	0	59.9614127	0	69.5536612
ENSG000001	ZNF29P	unprocessed_	0	0	0	0
ENSG000001	CBX1	protein_codin	149.831242	204.649796	90.2412406	95.4448273
ENSG000001	PIGL	protein_codin	16.7515671	0	14.5411774	0
ENSG000001	GALK1	protein_codin	56.2171314	82.5879976	29.9509674	0
ENSG000001	HOXB6	protein_codin	0	0	0.45610935	0
ENSG000001	PFN1	protein_codin	1883.14818	2258.66181	571.657104	1211.74654
ENSG000001	SLC25A11	protein_codin	89.5129655	116.472613	28.3082903	49.3866483
ENSG000001	RASD1	protein_codin	8.75235943	16.9424253	13.5050561	0
ENSG000001	NUP88	protein_codin	0	0	0	0
ENSG000001	C1QBP	protein_codin	117.358178	197.551639	36.3485478	41.2451655
ENSG000001	CPD	protein_codin	739.436659	339.62941	258.135737	555.299006
ENSG000001	GOSR1	protein_codin	65.1784883	80.1596331	44.228966	41.7632397
ENSG000001	CCDC47	protein_codin	164.396722	232.316586	112.679891	213.789721
ENSG000001	MED31	protein_codin	14.33525	1.38891342	6.47594886	3.41502118
ENSG000001	DRG2	protein_codin	58.9503667	74.1416403	17.044794	50.1498618
ENSG000001	AKAP10	protein_codin	213.408515	213.974919	100.625765	157.664176
ENSG000001	ALDH3A1	protein_codin	0	0	0	0
ENSG000001	SMARCD2	protein_codin	122.288506	139.018491	0	54.2961364
ENSG000002	PRR29	protein_codin	0.80426603	0	0	0
ENSG000001	SYNGR2	protein_codin	342.407331	373.723292	111.781072	198.553021
ENSG000001	DDX5	protein_codin	0	0	0	0
ENSG000001	ASIC2	protein_codin	0	0	0	0
ENSG000001	CCL2	protein_codin	2.70765066	0	0	0
ENSG000001	CCL1	protein_codin	0	0	1.1420855	0
ENSG000001	CCL13	protein_codin	0	0	0	0
ENSG000001	PEX12	protein_codin	1.19479175	0	0	0
ENSG000001	KRT32	protein_codin	0	0	0	0
ENSG000001	KAT2A	protein_codin	21.2319625	32.7046124	0	9.91249792
ENSG000001	NAGLU	protein_codin	46.9063455	58.8937935	16.7994979	38.0546147
ENSG000001	HSD17B1	protein_codin	3.24111481	0.91506434	0.82834573	1.7902809
ENSG000001	ABI3	protein_codin	84.583383	181.587233	57.0318865	72.0756625
ENSG000001	COL1A1	protein_codin	0	0.78354123	0	0
ENSG000001	MRPL27	protein_codin	22.7351419	21.488225	17.8015429	0
ENSG000001	LRRC59	protein_codin	356.354302	447.688292	218.403371	253.159414
ENSG000001	HDAC5	protein_codin	91.6471851	21.562166	12.9380949	49.886487
ENSG000001	PPY	protein_codin	0	0	0	0
ENSG000001	DUSP3	protein_codin	286.068209	432.222261	208.849725	255.025898
ENSG000001	CACNG1	protein_codin	0	0	0	0
ENSG000001	HLF	protein_codin	0	2.29710023	2.00482723	0
ENSG000001	EFNB3	protein_codin	0	0	0	0

ENSG000001	FAM20A	protein_codin	0	0	0	0
ENSG000001	RANGRF	protein_codin	2.77373781	0	9.86591015	8.18501762
ENSG000001	RCVRN	protein_codin	7.50988967	2.32688964	1.16601433	3.87808627
ENSG000001	MYH1	protein_codin	0	3.09537677	0	0
ENSG000001	VTN	protein_codin	0	0	0	0
ENSG000001	TNFAIP1	protein_codin	45.2995279	37.7689038	16.3209615	34.2453908
ENSG000001	TMEM97	protein_codin	0	0	2.9610642	0
ENSG000001	FOXN1	protein_codin	0	0.79344085	0	1.55151962
ENSG000001	ALDOC	protein_codin	9.87328009	17.4133055	5.92285941	0
ENSG000000	CD38	protein_codin	59.4335758	48.1055497	175.974339	58.842026
ENSG000000	IBSP	protein_codin	0	0	0	0
ENSG000000	LAP3	protein_codin	0	0	0	0
ENSG000001	CXCL6	protein_codin	0	0	0	0
ENSG000000	JADE1	protein_codin	105.493165	66.9942131	58.6917862	62.8548686
ENSG000000	RUFY3	protein_codin	93.6361388	36.401127	37.7905872	44.7077211
ENSG000000	AFM	protein_codin	0	0	0	0
ENSG000000	AFP	protein_codin	0	0	0	0
ENSG000001	PHOX2B	protein_codin	0	0	0	0
ENSG000001	GNRHR	protein_codin	0	0	0	0
ENSG000001	CWH43	protein_codin	0	0	0	0
ENSG000001	SULT1E1	protein_codin	0	0	0	0
ENSG000001	SMR3A	protein_codin	0	0	0	0
ENSG000001	LAMTOR3	protein_codin	9.13823693	26.9125974	0	29.6296448
ENSG000001	PF4V1	protein_codin	60.0271902	261.863078	111.391777	45.7240699
ENSG000001	NFKB1	protein_codin	195.43413	216.904152	47.7552695	117.353855
ENSG000001	KLHL2	protein_codin	101.03815	37.5792134	41.5446413	48.5463084
ENSG000001	IL2	protein_codin	0	0	0	0
ENSG000001	WFS1	protein_codin	5.46464125	0	6.26164262	0
ENSG000001	GAR1	protein_codin	0	14.9102987	0	0
ENSG000001	FRG1	protein_codin	165.549718	159.528974	99.0402284	109.841756
ENSG000001	CLNK	protein_codin	3.29562895	0	0	1.12008636
ENSG000001	FAM149A	protein_codin	0	0	0	0
ENSG000000	SPA17	protein_codin	2.57799069	15.4216649	1.92680735	0
ENSG000000	CD82	protein_codin	4.97453193	0	0	46.8832322
ENSG000001	LDHA	protein_codin	45.7156768	0	0	0
ENSG000000	SPI1	protein_codin	399.66784	518.847144	220.706266	322.768258
ENSG000000	PLEKHB1	protein_codin	0	0	0	0
ENSG000001	CRYAB	protein_codin	0	0	0	0
ENSG000001	CTSC	protein_codin	167.422169	247.042754	87.5286788	102.110973
ENSG000001	ZPR1	protein_codin	89.6813324	55.0269378	48.2179219	35.6552921
ENSG000001	CRTAM	protein_codin	0	0	0	0
ENSG000001	JHY	protein_codin	24.1518552	11.7406173	9.45919215	7.51754342
ENSG000001	HSPA8	protein_codin	0	0	972.073983	0
ENSG000001	DTX4	protein_codin	27.0705412	38.8745636	14.0037899	8.19879848

ENSG000001	UNC93B1	protein_codin	607.49838	679.178012	202.813802	347.059522
ENSG000001	PUS3	protein_codin	0	0	7.0611312	20.0438495
ENSG000001	ST3GAL4	protein_codin	0	0	0	0
ENSG000001	SF1	protein_codin	0	0	0	0
ENSG000001	CCND1	protein_codin	1.00833506	13.7856007	5.9299767	1.95744634
ENSG000001	CCDC86	protein_codin	33.1740932	80.6732533	27.1206046	18.5340842
ENSG000001	PRPF19	protein_codin	81.2303588	148.328847	42.4113937	67.4099583
ENSG000001	TMEM109	protein_codin	72.9945174	144.585715	34.2259609	54.3312725
ENSG000001	ANAPC15	protein_codin	73.944123	91.5852046	33.6009498	82.6548724
ENSG000001	PANX1	protein_codin	178.386048	217.631552	118.572143	114.352004
ENSG000001	APOA5	protein_codin	0	0	0	0
ENSG000001	APOC3	protein_codin	0	0	0	0
ENSG000001	IL10RA	protein_codin	155.842904	286.246624	170.535412	0
ENSG000001	GALNT18	protein_codin	0	0	0	0
ENSG000001	BIRC2	protein_codin	291.907161	144.432001	193.370089	215.432971
ENSG000001	PDHX	protein_codin	64.0414773	58.5173213	54.452697	61.0890354
ENSG000001	SLC15A3	protein_codin	0	0	0	0
ENSG000001	SCGB2A2	protein_codin	0	0	0	0
ENSG000000	DGAT2	protein_codin	21.8461792	20.9011624	12.04911	5.58396573
ENSG000001	C11orf58	protein_codin	277.937131	225.890089	165.636748	248.593413
ENSG000001	RPS13	protein_codin	0	4.94392793	17.2883994	0
ENSG000000	YBX3	protein_codin	467.170519	413.378814	161.526187	225.455434
ENSG000000	DDX11	protein_codin	0	0	0	0
ENSG000000	SART3	protein_codin	21.4304688	0	0	0
ENSG000000	ZNF268	protein_codin	26.9875605	0	0	19.8238461
ENSG000000	RPLP0	protein_codin	66.2968195	92.5871658	43.0142873	41.0992906
ENSG000000	PXN	protein_codin	0	0	0	0
ENSG000000	SLC25A3	protein_codin	0	0	0	0
ENSG000000	RAD52	protein_codin	0	18.561878	0	5.97982995
ENSG000000	POLR3B	protein_codin	35.6388342	37.7231727	21.5704208	0
ENSG000001	PPFIBP1	protein_codin	11.0837478	0	0	6.71893926
ENSG000001	CD69	protein_codin	32.4667558	8.26210066	36.6457323	6.79438183
ENSG000001	PRDM4	protein_codin	122.00935	134.923876	90.4004515	58.3103323
ENSG000001	CLEC2B	protein_codin	162.570519	146.42881	136.457503	108.894829
ENSG000001	SELPLG	protein_codin	52.7051873	76.1171532	20.9391382	10.3790343
ENSG000001	ASIC1	protein_codin	0	0	0	0
ENSG000001	DAO	protein_codin	0	0	0	0
ENSG000001	KCTD10	protein_codin	80.9482901	199.360984	32.6178839	79.8900216
ENSG000001	MLEC	protein_codin	174.583088	365.223419	224.308947	147.728133
ENSG000001	MVK	protein_codin	0	5.33941297	0	0
ENSG000001	CSRNP2	protein_codin	39.9482863	49.1447309	23.2535281	27.9503129
ENSG000001	IL23A	protein_codin	11.8523831	23.1623966	17.1928946	5.61693427
ENSG000001	SYT10	protein_codin	0	0	1.46457811	0
ENSG000001	CYP27B1	protein_codin	0	0	0.90561721	0

ENSG000001	MYF6	protein_codin	0	0	0	0
ENSG000001	MYF5	protein_codin	0	0	0	0
ENSG000001	GLI1	protein_codin	0	0	0	0
ENSG000001	PPM1H	protein_codin	40.9482097	60.4699191	14.750926	30.7284406
ENSG000001	LTA4H	protein_codin	904.348705	1875.40661	372.146171	752.920573
ENSG000001	ELK3	protein_codin	324.369076	403.999697	191.2285	263.87047
ENSG000001	ITFG2	protein_codin	48.6323504	95.6321507	26.9905817	31.9164367
ENSG000001	PRR4	protein_codin	0	0	0	0
ENSG000001	PARP11	protein_codin	0	0	0	0
ENSG000001	ARPC3	protein_codin	2062.23081	3694.4462	1209.07033	2148.57709
ENSG000001	GPN3	protein_codin	9.87210578	8.53475112	10.3663554	13.352183
ENSG000001	FGF6	protein_codin	0	0	0	0.8087457
ENSG000001	MYL2	protein_codin	0	0	0	0
ENSG000001	RAD51AP1	protein_codin	0	0	0	0
ENSG000001	AKAP3	protein_codin	0	0	0	0
ENSG000001	DUSP16	protein_codin	0	0	3.66293452	0
ENSG000001	CREBL2	protein_codin	282.402903	268.71979	125.565327	168.235747
ENSG000001	CDKN1B	protein_codin	349.403382	282.497805	138.82326	258.072621
ENSG000001	GPRC5D	protein_codin	1.96406661	3.02094185	19.3174578	2.05309344
ENSG000001	SCNN1A	protein_codin	0	0	0	0
ENSG000001	LTBR	protein_codin	319.648804	548.085608	154.029123	315.604822
ENSG000001	OGFOD2	protein_codin	0	0	0	5.88689498
ENSG000001	OAS3	protein_codin	127.345312	440.51085	0	0
ENSG000001	ART4	protein_codin	0	0	2.52751657	0
ENSG000001	MGP	protein_codin	0	0	0	0
ENSG000001	ARHGDIB	protein_codin	2281.94834	3529.75387	1214.55149	1799.43808
ENSG000001	GTF2H3	protein_codin	0	0	0	0
ENSG000001	RERGL	protein_codin	0	0	0	0
ENSG000001	ENDOU	protein_codin	2.71552001	0	0	0
ENSG000001	VDR	protein_codin	0	0	0	0
ENSG000001	FZD10	protein_codin	0	0.91324762	0	0
ENSG000001	IL26	protein_codin	0	0	0	0
ENSG000001	IFNG	protein_codin	0	0	0	0
ENSG000001	NUP107	protein_codin	8.69258035	5.00393002	4.2946987	0
ENSG000001	CNOT2	protein_codin	132.712493	153.550295	88.649453	85.8705454
ENSG000001	TIMELESS	protein_codin	29.0127572	37.8515521	50.3165532	25.186291
ENSG000001	KRR1	protein_codin	67.0424063	32.7826397	50.7205058	66.3261528
ENSG000001	MRPL51	protein_codin	54.1081683	63.7573683	32.6322741	22.8127552
ENSG000001	GAPDH	protein_codin	4602.73199	7426.91822	1606.32574	3851.99508
ENSG000001	ACRBP	protein_codin	61.7666418	64.4584827	31.4155154	44.7001047
ENSG000001	COPS7A	protein_codin	0	0	0	0
ENSG000001	GNB3	protein_codin	0	0	0	0
ENSG000001	CDCA3	protein_codin	0	0	0	0
ENSG000001	CHPT1	protein_codin	64.7925128	105.685981	3.35098293	49.471012

ENSG000001	USP5	protein_codin	0	50.6009088	7.41058815	0
ENSG000001	TPI1	protein_codin	0	0	0	0
ENSG000001	ENO2	protein_codin	51.6223853	29.2107936	18.3502721	25.6887248
ENSG000001	C12orf57	protein_codin	18.0285793	17.4476953	0	0
ENSG000001	APOBEC1	protein_codin	0	0	0	0
ENSG000001	NANOG	protein_codin	1.1580809	0	0	0
ENSG000001	GOLT1B	protein_codin	28.9129532	12.7704163	14.8271113	19.305574
ENSG000001	PRKAB1	protein_codin	38.9821853	94.1804629	11.4701743	22.0332406
ENSG000001	CMAS	protein_codin	0	43.2188211	26.7279907	26.2114116
ENSG000001	HCFC2	protein_codin	47.9462315	44.79469	14.1826697	29.397177
ENSG000001	CLEC4A	protein_codin	125.643341	173.692031	66.7749528	122.091816
ENSG000001	AICDA	protein_codin	0	0	0	0
ENSG000001	RAB35	protein_codin	142.958145	204.072078	85.0688474	101.1744
ENSG000001	COX6A1	protein_codin	137.357088	165.930788	53.3697764	78.476174
ENSG000002	GATC	protein_codin	0	0	0	0
ENSG000001	RFX4	protein_codin	0	0	0	0
ENSG000001	SRSF9	protein_codin	163.820407	274.078663	69.556885	145.796206
ENSG000001	FGFR1OP2	protein_codin	36.1179883	18.8515318	36.2782041	28.0283233
ENSG000001	KLRB1	protein_codin	5.14181475	9.23025805	2.9791539	0
ENSG000000	ZNF76	protein_codin	9.23368859	7.4430753	0	6.9203331
ENSG000000	IYD	protein_codin	0	0	0	0
ENSG000002	LINC03033	lncRNA	6.28570625	0	0	2.13727955
ENSG000000	FYN	protein_codin	93.7271131	44.6491322	0	28.0265985
ENSG000001	RSPH4A	protein_codin	0	0	0	0
ENSG000001	TMEM14C	protein_codin	75.8578801	98.7853138	32.9588048	46.8233615
ENSG000001	SMIM8	protein_codin	0	9.13251917	6.42892853	11.8475921
ENSG000001	ADTRP	protein_codin	0	0	0	0
ENSG000001	ASF1A	protein_codin	21.1597914	30.8406313	14.6252099	15.672388
ENSG000001	HINT3	protein_codin	112.740998	76.8273689	26.9690189	80.5712447
ENSG000001	NCOA7	protein_codin	0	50.3847835	79.5874225	28.4559357
ENSG000001	ULBP1	protein_codin	0	2.95167179	0	1.02649814
ENSG000002	SLC44A4	protein_codin	0	0	0	0
ENSG000001	FBXO5	protein_codin	14.2091172	2.85652506	24.0407263	0
ENSG000001	OPRM1	protein_codin	0	0	0	0
ENSG000001	FANCE	protein_codin	0	0	0	0
ENSG000001	TULP1	protein_codin	0	0	0	0
ENSG000001	MAPK14	protein_codin	297.32582	421.117518	178.17542	250.439252
ENSG000001	MAPK14	protein_codin	34.6227309	21.8925514	55.5696448	12.2092391
ENSG000001	STK38	protein_codin	666.8184	1009.05973	414.265597	535.235485
ENSG000002	HLA-DOA	protein_codin	48.9901258	69.1141877	41.6103054	25.0528674
ENSG000001	MCM3	protein_codin	0	0	0	0
ENSG000001	RNF8	protein_codin	63.2114834	73.2544205	41.9674186	34.4005784
ENSG000001	SAYSD1	protein_codin	19.6060418	26.963417	3.69066566	0
ENSG000001	KIF6	protein_codin	0	0	0	0

ENSG000001	CAP2	protein_codin	0	0	0	0
ENSG000001	GPR63	protein_codin	1.5305316	1.5138435	0	0
ENSG000001	FBXL4	protein_codin	0	30.4349467	10.5652738	16.4993991
ENSG000001	GPLD1	protein_codin	2.11103974	1.06677245	5.86116552	0
ENSG000001	ACOT13	protein_codin	27.796511	34.3060205	24.0497085	21.3751979
ENSG000001	RPS12	protein_codin	1015.98887	1708.60724	623.698444	867.933369
ENSG000001	B3GAT2	protein_codin	0	0	0	0
ENSG000001	GMNN	protein_codin	5.95685135	2.07009363	1.69236378	0
ENSG000001	SNX3	protein_codin	585.685807	1055.35332	370.584101	479.328666
ENSG000002:	LINC01702	lncRNA	0	0	0	0
ENSG000001	ZBTB24	protein_codin	0	0	41.294255	0
ENSG000001	FIG4	protein_codin	0	76.9821091	0	36.1545429
ENSG000001	ADGRG6	protein_codin	0	0	0	0
ENSG000001	UNC93A	protein_codin	0	0	0	0
ENSG000001	C6orf118	protein_codin	0	0	0	0
ENSG000001	MDFI	protein_codin	0	0	0	0
ENSG000001	TFEB	protein_codin	0	0	0	0
ENSG000001	BYSL	protein_codin	10.5999849	13.900605	10.1094693	5.61749884
ENSG000001	TBP	protein_codin	18.3570441	27.0813643	0	0
ENSG000001	GUCA1B	protein_codin	1.99228341	1.97006397	2.16792586	0
ENSG000001	PRPH2	protein_codin	0.93418364	0.94051467	0	0
ENSG000001	PPP2R5D	protein_codin	0	0	0	0
ENSG000001	MRPL2	protein_codin	0	6.97418632	8.77584311	0
ENSG000001	PTK7	protein_codin	0	0	0	0
ENSG000001	PTK7	protein_codin	0	0	0	11.9547782
ENSG000001	DNPH1	protein_codin	26.9374523	25.8782177	6.18778481	8.26881043
ENSG000001	EXOC2	protein_codin	133.99284	153.176484	62.1981136	119.422983
ENSG000001	TMEM30A	protein_codin	506.924685	379.991241	225.900965	480.663662
ENSG000001	VEGFA	protein_codin	0	0	17.2179784	3.01837172
ENSG000001'	H3P16	processed_ps	8.70658959	17.3897858	5.71982632	0
ENSG000001	TTK	protein_codin	0	0	0	0
ENSG000001	CCN6	protein_codin	0	0	0	0
ENSG000001	LAMA4	protein_codin	0	0	0	0
ENSG000001	ENPP5	protein_codin	0	0	0	0
ENSG000001	LY86	protein_codin	183.904093	279.763694	86.4703217	124.448912
ENSG000001	PRSS16	protein_codin	0	0	0	0
ENSG000001	MEP1A	protein_codin	0	0	0	0
ENSG000000:	MTREX	protein_codin	205.08574	167.578287	129.441188	137.160817
ENSG000000	ISL1	protein_codin	0	0	0	0
ENSG000000	SLC6A7	protein_codin	13.6934709	14.6111707	4.86357731	5.74319648
ENSG000001	HARS2	protein_codin	50.0839649	55.8099673	0	0
ENSG000001	NUDT12	protein_codin	0	5.94505575	1.27786378	3.49275325
ENSG000001	TENT4A	protein_codin	188.061256	247.570017	57.935196	124.901057
ENSG000001	GHR	protein_codin	0	0	0	0

ENSG000001	DAP	protein_codin	275.821408	545.786231	213.332357	238.69693
ENSG000001	BRD8	protein_codin	15.9765496	0	0	20.8505722
ENSG000001	MRPS30	protein_codin	23.6133494	15.4573334	12.3504879	11.6800395
ENSG000001	HBEGF	protein_codin	374.778128	138.423572	340.672028	208.068854
ENSG000001	LOX	protein_codin	6.37749081	7.62238265	25.1738064	0
ENSG000001	GZMK	protein_codin	2.43685166	5.01644642	0	0
ENSG000001	CDH9	protein_codin	0	0	0	0
ENSG000001	SPARC	protein_codin	816.891259	1124.41638	355.819053	538.301524
ENSG000001	HAND1	protein_codin	0	0	0	0
ENSG000001	PCDHB3	protein_codin	0	0	0.72976891	0
ENSG000001	PCDHB5	protein_codin	0	0	0	0
ENSG000001	PCDHB6	protein_codin	0	0	0	0
ENSG000001	PCDHB7	protein_codin	0	0	0	0
ENSG000001	PCDHB15	protein_codin	0	0	0	0
ENSG000001	GRM6	protein_codin	0	0	0	0
ENSG000001	THG1L	protein_codin	35.9226807	71.7834953	22.0055915	28.9058448
ENSG000001	IL12B	protein_codin	0	0.9699153	0	0
ENSG000001	BTNL8	protein_codin	0	0	0	0
ENSG000001	TTC1	protein_codin	65.026869	0	0	0
ENSG000000	C1QTNF3	protein_codin	3.7675028	0	0	0
ENSG000001	IRX4	protein_codin	0	0	0	0
ENSG000001	LNPEP	protein_codin	630.052471	275.076095	287.88989	382.573185
ENSG000002	RAD1P1	processed_ps	1.01958494	0	0	0
ENSG000001	AGXT2	protein_codin	0	0	0	0
ENSG000001	PRLR	protein_codin	0	0	0	0
ENSG000001	IL4	protein_codin	0	0	0	0
ENSG000001	IL5	protein_codin	0	0	0	0
ENSG000001	ST8SIA4	protein_codin	256.182745	144.478552	119.943337	219.569498
ENSG000001	PCDH12	protein_codin	9.04364796	11.7576766	7.24577858	4.95787296
ENSG000001	NUP155	protein_codin	76.2892737	90.6715328	45.7861524	41.7422218
ENSG000001	PPP2CA	protein_codin	54.8536995	33.0469558	58.0765004	32.6765191
ENSG000001	NR3C1	protein_codin	0	0	7.05226612	0
ENSG000001	C5orf15	protein_codin	126.95852	143.241191	75.8503221	81.954729
ENSG000001	TRIM23	protein_codin	42.2595355	15.4494004	7.56221682	17.2805512
ENSG000001	TRAPPC13	protein_codin	40.2179957	0	18.5227969	0
ENSG000001	RARS1	protein_codin	91.824135	121.810601	57.0077499	61.4470338
ENSG000001	CDX1	protein_codin	0	0	0	0
ENSG000001	BNIP1	protein_codin	3.78661945	1.91768377	1.61371293	4.12676668
ENSG000000	SLC7A14	protein_codin	0	0	0	0
ENSG000000	SEMA3G	protein_codin	2.85420715	2.82170822	2.28325561	2.7728149
ENSG000000	ZMYND10	protein_codin	0	0	0	0
ENSG000000	LTF	protein_codin	0	0	0	0
ENSG000000	MLH1	protein_codin	0	0	0	0
ENSG000001	EHHADH	protein_codin	8.2716453	0	0	7.25098855

ENSG000001	CRBN	protein_codin	87.4370735	89.4226429	0	0
ENSG000001	HRG	protein_codin	0	0	0	0
ENSG000001	BCL6	protein_codin	71.8493215	0	0	0
ENSG000001	FAM162A	protein_codin	3.69594356	18.3259264	18.165845	4.47172905
ENSG000001	RBP2	protein_codin	0	0	0	0
ENSG000001	PFKFB4	protein_codin	117.055299	140.718916	57.7229546	81.8984313
ENSG000001	HES1	protein_codin	18.5218415	0	6.53010659	0
ENSG000001	ECT2	protein_codin	31.8065277	0	0	24.6327436
ENSG000001	GNAT1	protein_codin	0	0	0	0
ENSG000001	TUSC2	protein_codin	40.0860224	57.4012659	18.1351855	18.4006997
ENSG000001	NPRL2	protein_codin	0	0	0	0
ENSG000001	CYB561D2	protein_codin	8.45011666	0	7.78074275	25.833681
ENSG000001	CEP15	protein_codin	11.1949857	5.31702283	6.42239002	3.42792606
ENSG000001	GNB4	protein_codin	366.972928	240.163966	146.343807	256.047384
ENSG000001	MORC1	protein_codin	18.9333385	33.9466872	11.8348369	15.2270825
ENSG000001	UMPS	protein_codin	11.7707109	9.72176181	8.34212151	7.24225714
ENSG000001	ABTB1	protein_codin	131.992784	170.655355	77.045168	122.641601
ENSG000001	KLHL18	protein_codin	155.068031	239.148999	78.1524388	82.609921
ENSG000001	HEMK1	protein_codin	26.4768438	28.727747	21.6398382	0
ENSG000001	RRP9	protein_codin	10.9277434	21.4985004	12.4029072	4.85579251
ENSG000001	AADAC	protein_codin	0	0	0	0
ENSG000001	EIF1B	protein_codin	145.913884	155.452504	33.3365159	73.2582775
ENSG000001	ZBTB47	protein_codin	0	0	0	0
ENSG000001	TNNC1	protein_codin	0	0	0	0
ENSG000001	NKTR	protein_codin	450.016981	0	0	641.480131
ENSG000001	SPCS1	protein_codin	96.2684376	164.573095	32.8060529	82.8505473
ENSG000001	NEK4	protein_codin	104.389381	139.932952	55.2741416	107.909058
ENSG000000	LDAF1	protein_codin	0	13.6547106	0	0
ENSG000000	WDFY1	protein_codin	323.946593	355.506734	171.390106	316.252597
ENSG000000	EIF2AK2	protein_codin	0	17.5968645	0	0
ENSG000000	CPS1	protein_codin	0	0	0	0
ENSG000000	DAZAP1	protein_codin	104.166163	153.62492	57.4887965	108.957183
ENSG000000	DDX1	protein_codin	0	138.12074	107.156877	120.207172
ENSG000000	FKBP7	protein_codin	3.32900614	0	0	0
ENSG000000	HEATR5B	protein_codin	261.390778	193.690731	118.552626	124.707747
ENSG000000	MDH1	protein_codin	87.3085623	105.24447	60.8549166	51.9624252
ENSG000000	MAPRE3	protein_codin	9.9592862	16.3191191	12.179613	4.65115269
ENSG000000	TMSB10	protein_codin	4682.45405	5905.30067	2313.33958	3101.46746
ENSG000000	MSH2	protein_codin	0	0	20.0002958	0
ENSG000000	NCK2	protein_codin	217.263703	686.586902	218.722943	264.014309
ENSG000000	TFPI	protein_codin	95.0393283	65.9650832	47.8597015	29.3130164
ENSG000000	NDUFS1	protein_codin	141.339243	124.672371	87.8409223	96.655322
ENSG000000	SLC11A1	protein_codin	538.23663	487.930264	153.767066	174.056732
ENSG000000	APOB	protein_codin	0	1.0257716	0	0

ENSG000001	RTKN	protein_codin	0	0.90934794	1.10924023	0
ENSG000001	INO80B	protein_codin	53.3356725	80.4482841	23.2895128	24.3447033
ENSG000001	TTL	protein_codin	125.929484	103.718786	56.0250194	60.0573738
ENSG000001	FAHD2A	protein_codin	15.6860654	29.4640879	14.9030905	25.0148795
ENSG000001	SF3B6	protein_codin	61.3709056	121.39896	64.414668	62.7200157
ENSG000001	CENPA	protein_codin	0	0	5.64437242	0
ENSG000001	SLC30A3	protein_codin	0	0	0	0
ENSG000001	MPV17	protein_codin	0	0	0	19.054359
ENSG000001	NRBP1	protein_codin	41.0688819	97.8711073	20.6984486	83.3598838
ENSG000001	ITGA4	protein_codin	0	0	0	0
ENSG000001	SNX17	protein_codin	189.939033	331.356954	76.6809859	84.959497
ENSG000001	REEP6	protein_codin	1.90097198	7.7185294	0.75089954	2.81314623
ENSG000001	APC2	protein_codin	2.51877542	0	1.52272044	0
ENSG000001	RPS15	protein_codin	42.3256216	63.9934348	5.46644341	0
ENSG000001	GCA	protein_codin	180.378601	178.140583	78.7746478	154.133179
ENSG000002	WBP1	protein_codin	0	21.7007068	11.7865075	0
ENSG000001	TTC31	protein_codin	0	0	32.3198497	40.008657
ENSG000001	NDUFS7	protein_codin	62.685811	110.794912	26.7258195	53.5240489
ENSG000001	PCGF1	protein_codin	14.024797	24.1736428	4.37396314	0
ENSG000001	TLX2	protein_codin	0	0	0	0
ENSG000001	DOK1	protein_codin	65.0934382	58.614875	0	0
ENSG000001	POLE4	protein_codin	4.68513989	0	0	0
ENSG000001	ACADL	protein_codin	0	0	0	0
ENSG000001	EVA1A	protein_codin	0	0	0	0
ENSG000001	LANCL1	protein_codin	2.60787793	0	0	0
ENSG000001	REG1A	protein_codin	0	0	0	0
ENSG000001	FANCL	protein_codin	0	0	0	0
ENSG000001	IGFBP2	protein_codin	26.8410387	33.9413786	22.7369755	7.29338194
ENSG000001	IGFBP5	protein_codin	0	0	0	0
ENSG000001	KCNJ13	protein_codin	0	0	0	0
ENSG000002	CCT4P2	processed_ps	0	0	0	0
ENSG000001	GGCX	protein_codin	26.265508	29.5218598	15.3915274	14.1830808
ENSG000001	NEU2	protein_codin	0	0	0	0
ENSG000001	MOB4	protein_codin	0	0	0	0
ENSG000001	HSPE1	protein_codin	35.5121213	49.5027912	36.2590348	27.2370126
ENSG000001	WNT6	protein_codin	0	4.81994735	1.40664889	0
ENSG000001	IL1RL1	protein_codin	0	0	0	1.16240237
ENSG000001	IL18R1	protein_codin	6.40919261	0	2.60866348	0
ENSG000001	SLC9A2	protein_codin	0	0	0	0
ENSG000001	AZU1	protein_codin	6.10515564	10.7983009	0	0.88188049
ENSG000001	PPP1R7	protein_codin	0	106.536808	27.1694467	0
ENSG000001	PASK	protein_codin	0	0	10.0945323	0
ENSG000001	PROC	protein_codin	0	0	0	0
ENSG000001	ID2	protein_codin	70.4525014	39.1065887	12.3751676	12.9644184

ENSG000001	ODC1	protein_codin	223.787263	668.50429	293.513335	259.494529
ENSG000001	PLEKHB2	protein_codin	0	28.7730057	0	0
ENSG000001	GREB1	protein_codin	1.1349848	0	0	0
ENSG000001	GORASP2	protein_codin	91.3969438	132.493459	69.6920433	47.6609689
ENSG000001	CEBPZ	protein_codin	171.535664	124.499229	141.682749	119.825922
ENSG000001	PRKD3	protein_codin	234.373762	195.315749	86.8426302	246.30444
ENSG000001	RMDN2	protein_codin	0	4.35968986	0	1.48103373
ENSG000001	DLX2	protein_codin	0	0	0	0
ENSG000001	SLC1A4	protein_codin	26.5281686	46.1862911	64.5866355	10.8723494
ENSG000001	ORC2	protein_codin	53.0770797	57.4074645	18.6010291	49.0691747
ENSG000001	COX7A2L	protein_codin	114.867425	146.265524	28.7192899	58.5704365
ENSG000002	PPP3R1	protein_codin	745.730153	1099.03539	410.633408	567.801314
ENSG000001	PLEK	protein_codin	4402.95854	4528.83992	2255.09157	2671.83063
ENSG000001	PRTN3	protein_codin	0.93397171	0.97718529	2.13478982	0.94343267
ENSG000001	KISS1R	protein_codin	0	0	0	0
ENSG000001	GRIN3B	protein_codin	0.90550956	0.92026302	0.73745473	0
ENSG000001	VAX2	protein_codin	0	0	0	0
ENSG000001	ATP6V1B1	protein_codin	0	0	0	0
ENSG000001	MSH6	protein_codin	0	0	0	0
ENSG000001	PLEKHA3	protein_codin	89.5940293	65.836933	44.0903563	53.9808104
ENSG000001	SPR	protein_codin	0	11.1526984	0	2.73132733
ENSG000000	FAM76A	protein_codin	6.70032219	0	11.618254	0
ENSG000000	ENO1	protein_codin	1275.68277	2037.01243	550.078356	1173.7413
ENSG000002	MMP23A	transcribed_u	0	0	0	0
ENSG000000	CDC7	protein_codin	7.42100585	9.01074392	5.09859362	9.23276894
ENSG000000	SYDE2	protein_codin	0	0	0	0
ENSG000000	SARS1	protein_codin	243.345651	417.037751	204.20652	197.593632
ENSG000000	CLCA1	protein_codin	0	0	0	0
ENSG000001	BCL9	protein_codin	26.1280815	49.8224712	40.5401845	0
ENSG000001	TPSG1	protein_codin	0	0	0	0
ENSG000001	ANGPTL1	protein_codin	0	0	0	0
ENSG000001	TCEANC2	protein_codin	68.8143239	117.922252	66.7889274	54.0065122
ENSG000001	TMEM59	protein_codin	112.603207	71.7233154	34.2035807	63.8468181
ENSG000001	RPL22	protein_codin	333.146458	494.038635	159.362777	304.101198
ENSG000001	OPRD1	protein_codin	0	0	0	0
ENSG000001	WDR77	protein_codin	34.4595366	36.7536512	23.2832286	17.1005436
ENSG000001	RNF19B	protein_codin	211.802496	294.504273	36.5707044	128.277313
ENSG000001	DLGAP3	protein_codin	6.05673779	9.92048084	3.62168386	1.91774294
ENSG000001	C1orf21	protein_codin	8.97017439	20.019562	14.3286957	4.01316248
ENSG000001	MAD2L2	protein_codin	0	4.92668481	0	3.5705043
ENSG000001	MFN2	protein_codin	341.602628	619.533371	373.104411	132.622453
ENSG000001	MIIP	protein_codin	73.4445878	107.12389	48.8059851	41.2330477
ENSG000001	SLC35D1	protein_codin	50.5181777	32.9410443	37.0833613	24.2587996
ENSG000001	PRAMEF10	protein_codin	0	0	0	0

ENSG000002	PRAMEF4	protein_codin	0	0	0	0
ENSG000001	PRDM2	protein_codin	63.9897012	0	19.2685134	0
ENSG000001	RGS2	protein_codin	1547.95005	1026.03532	1383.28183	1631.07826
ENSG000002	DENND1B	protein_codin	0	0	0	0
ENSG000001	WARS2	protein_codin	12.9546147	29.6302708	0	11.0661575
ENSG000001	OSCP1	protein_codin	0	0	1.09491989	0
ENSG000001	NT5C1A	protein_codin	2.01167803	0.99380377	1.13129082	0
ENSG000001	KDM5B	protein_codin	0	0	0	0
ENSG000000	AKR7A2	protein_codin	51.7570686	83.3665789	16.4064962	40.5017672
ENSG000001	GBP3	protein_codin	33.1194718	18.9869529	24.5950471	16.7507152
ENSG000001	RAB29	protein_codin	3.23586542	0	0	0
ENSG000001	CD160	protein_codin	0.94433043	0	1.23586073	0
ENSG000001	HMGCL	protein_codin	0	0	0	0
ENSG000001	APH1A	protein_codin	2.54299603	1.5939263	0.54460017	5.2301584
ENSG000001	P3H1	protein_codin	0	15.2517752	17.755929	14.8896207
ENSG000001	EBNA1BP2	protein_codin	70.3181675	85.2896645	53.5154846	39.9730226
ENSG000001	ATP6V0B	protein_codin	0	0	1.72249586	0
ENSG000001	SLC19A2	protein_codin	0	0	0	0
ENSG000001	SELL	protein_codin	1131.86075	1860.40886	393.693054	710.666095
ENSG000001	FMO6P	transcribed_u	0	0	0	0
ENSG000001	VAMP4	protein_codin	66.4926198	37.1825091	22.963892	54.6606797
ENSG000001	GRHL3	protein_codin	0	0	0	0
ENSG000001	SYF2	protein_codin	532.161267	523.756673	344.167104	436.994947
ENSG000001	DHDDS	protein_codin	31.0285159	61.224349	0	27.490463
ENSG000001	PPP1R8	protein_codin	4.78669711	15.0499039	0	1.5331305
ENSG000001	SLC5A9	protein_codin	0	0	0	0
ENSG000001	CTSD	protein_codin	1173.61506	1598.96857	270.454775	943.495483
ENSG000001	COLEC11	protein_codin	0	0	0	0
ENSG000001	STAG1	protein_codin	0	0	0	13.49313
ENSG000001	A4GNT	protein_codin	0	0	0	0
ENSG000001	MMP8	protein_codin	1.13635645	0	0	0
ENSG000001	APOA1	protein_codin	0	0	1.10752227	0
ENSG000001	SLC8A2	protein_codin	0	0	0	0
ENSG000001	NR5A2	protein_codin	0	0	2.31070611	2.28179909
ENSG000001	CAMSAP2	protein_codin	0	0	20.4817649	0
ENSG000000	FCGR2B	protein_codin	61.9207162	24.6579276	48.9961907	0
ENSG000001	FCRLA	protein_codin	0	0	0	0
ENSG000001	EEF1B2	protein_codin	28.79265	18.1265685	17.5451343	4.44558083
ENSG000001	ATIC	protein_codin	112.14449	71.1169656	27.3778289	25.3346118
ENSG000001	TNP1	protein_codin	0	0	0	0
ENSG000001	FASTKD2	protein_codin	30.8192433	0	4.95620804	11.6626379
ENSG000001	TTR	protein_codin	0	0	0	0
ENSG000001	B4GALT6	protein_codin	0	0	0	0
ENSG000000	DOP1A	protein_codin	38.1896262	0	7.56097384	12.2949704

ENSG000001	FILIP1	protein_codin	0	0	0	0
ENSG000001	CASP8AP2	protein_codin	0	0	17.6748002	98.0059069
ENSG000001	UBE3D	protein_codin	0	2.02851496	0	0
ENSG000001	SPACA1	protein_codin	0	0	0	0
ENSG000001	SGIP1	protein_codin	0	0	0	0
ENSG000001	TBPL1	protein_codin	15.3265355	20.2712112	6.01988822	6.27456532
ENSG000001	ZC2HC1B	protein_codin	0	0	0	0
ENSG000001	FBXO30	protein_codin	62.5505731	49.6672451	34.2845775	41.5461976
ENSG000001	ADAT2	protein_codin	0	14.8667354	15.3306147	3.37452953
ENSG000001	TNFAIP3	protein_codin	0	0	0	0
ENSG000001	SGK1	protein_codin	502.020561	226.423291	171.308194	295.395369
ENSG000001	TCF21	protein_codin	0.95707378	0	0	0
ENSG000001	PMFBP1	protein_codin	10.4540969	0	0	5.42962683
ENSG000001	MED28	protein_codin	196.983018	276.58272	126.214399	111.275045
ENSG000002	RNF103	protein_codin	59.2043429	47.1888601	10.5427055	45.2037768
ENSG000001	MYL12B	protein_codin	867.442789	1120.93553	436.04753	694.887664
ENSG000001	ARMC2	protein_codin	8.47584195	12.3068117	6.30429304	0
ENSG000001	GHRH	protein_codin	0	0	0	0
ENSG000001	RPN2	protein_codin	302.887494	672.66782	403.743974	269.793028
ENSG000001	MTCL2	protein_codin	324.242952	419.836091	177.888255	166.155496
ENSG000001	PKD2	protein_codin	39.7272196	55.6110794	19.9729836	37.8342084
ENSG000001	ABCG2	protein_codin	0	0	0	0
ENSG000001	SPP1	protein_codin	0	0	0	0
ENSG000001	STBD1	protein_codin	6.97586024	0	4.57734671	5.01856441
ENSG000001	ADH6	protein_codin	0	0	0	0
ENSG000001	CCNI	protein_codin	1183.17732	1608.606	585.593503	716.038355
ENSG000001	RARRES1	protein_codin	0	0	0	0
ENSG000001	RAB3GAP2	protein_codin	0	0	0	0
ENSG000001	LDAH	protein_codin	0	0	0	0
ENSG000001	FGF23	protein_codin	0	1.01039502	0	4.13476646
ENSG000001	OTUD4P1	processed_ps	2.04134489	2.06718338	0	0
ENSG000001	ELL2	protein_codin	246.58541	92.2072265	297.845263	86.0209307
ENSG000001	GLRX	protein_codin	287.966445	459.739873	182.211254	240.753145
ENSG000001	NDUFB3	protein_codin	46.7809722	67.216509	30.1417475	47.5370634
ENSG000001	ZFAND5	protein_codin	0	0	0	0
ENSG000001	GDA	protein_codin	0	0	0	0
ENSG000001	ECRG4	protein_codin	0	0	0	0
ENSG000001	YWHAQ	protein_codin	336.923326	476.190594	188.795872	232.269253
ENSG000001	ITGB1BP1	protein_codin	0	28.689081	0	0
ENSG000001	CPSF3	protein_codin	156.303451	112.885474	88.203094	117.338921
ENSG000001	PIGZ	protein_codin	1.01833433	3.09331804	0	0
ENSG000001	DDX55	protein_codin	45.1436059	80.4788387	37.1333116	48.6807178
ENSG000001	CCDC92	protein_codin	93.6777796	120.744555	87.193871	60.2899031
ENSG000001	LGALS8	protein_codin	10.6324773	9.66207982	0	9.15112489

ENSG000001	FKBP15	protein_codin	0	0	94.7967342	30.5868681
ENSG000001	HDHD3	protein_codin	0	0	4.53179817	0
ENSG000001	ALG2	protein_codin	33.070365	0	7.60481743	7.43439549
ENSG000001	APC2	protein_codin	0	0	0	0
ENSG000001	VPS4B	protein_codin	165.672064	134.565114	81.1644288	124.667664
ENSG000002	SERPINB10	protein_codin	3.03502837	10.2861235	11.3788287	16.8044893
ENSG000001	GSC	protein_codin	0	0	0	0.71884894
ENSG000000	ADCK1	protein_codin	3.62297377	0	0	0
ENSG000001	PGF	protein_codin	0	0	0	0
ENSG000001	IFI27L2	protein_codin	26.1948353	36.8530163	17.9908566	22.8583328
ENSG000001	NEK9	protein_codin	18.6083569	15.6063244	14.7408203	11.8115878
ENSG000001	ACYP1	protein_codin	0	0	2.44507886	0
ENSG000001	IFT43	protein_codin	0	0	0	0
ENSG000001	NPC2	protein_codin	0	3.59916059	0	5.4298689
ENSG000001	IRF2BPL	protein_codin	150.019081	180.520479	44.2331071	140.226137
ENSG000001	ACOT2	protein_codin	0	0	0	0
ENSG000001	FLVCR2	protein_codin	181.301594	138.366391	41.3541578	95.3810281
ENSG000001	DLST	protein_codin	15.089915	3.68806147	0	0
ENSG000001	TGFB3	protein_codin	0	3.66568353	2.24541242	3.51792821
ENSG000001	ZC2HC1C	protein_codin	0	0	0	0
ENSG000001	SLIRP	protein_codin	39.1365924	0	23.3802049	0
ENSG000001	PAPOLG	protein_codin	25.4938535	39.4606163	19.631638	33.9707734
ENSG000001	TP53I3	protein_codin	6.58105555	31.3768578	0	13.3021267
ENSG000001	RHOQ	protein_codin	401.25616	269.884571	128.387521	222.167779
ENSG000001	TMEM214	protein_codin	110.42138	140.817584	63.4174649	86.3061858
ENSG000001	ATAD2B	protein_codin	502.635781	457.474253	256.059578	380.601909
ENSG000001	FAM98A	protein_codin	0	0	18.7318341	0
ENSG000001	YIPF4	protein_codin	178.003627	212.867504	88.1297721	102.259758
ENSG000001	AFTPH	protein_codin	170.122034	222.202882	159.9335	165.88636
ENSG000001	LGALSL	protein_codin	102.386562	291.502289	71.0037236	131.660805
ENSG000001	CRIPT	protein_codin	68.9297703	33.381537	32.2633056	42.7280219
ENSG000001	ADGRB3	protein_codin	0	0	0	0
ENSG000001	MFSD13A	protein_codin	39.9571681	47.0764243	10.2435146	16.3534965
ENSG000001	SLF2	protein_codin	132.508199	102.968085	94.2813399	91.441999
ENSG000001	LIPF	protein_codin	0	0	0	0
ENSG000001	PPP1R3C	protein_codin	0	0	0	0
ENSG000001	MXI1	protein_codin	20.415639	61.1791906	0	0
ENSG000001	PRLHR	protein_codin	0	0	0	0
ENSG000001	KCNIP2	protein_codin	0	0	0	1.51481242
ENSG000001	HOXB8	protein_codin	0	0	0	0
ENSG000001	HOXB5	protein_codin	0	0	0	0.90271664
ENSG000002	HOXB7	protein_codin	0	0.79958885	0	0
ENSG000001	HOXB1	protein_codin	0	0	0	0
ENSG000001	DUSP1	protein_codin	13435.7412	13968.8233	4020.1578	9688.28128

ENSG000001:	PANK3	protein_codin	469.026687	204.668485	266.829017	267.911713
ENSG000001:	MSX2	protein_codin	0	0	0	1.08889818
ENSG000001:	INSL4	protein_codin	0	0	0	0
ENSG000002	IFNA7	protein_codin	0	1.66369258	0	0
ENSG000001:	LRP11	protein_codin	4.23976531	5.39864216	0	1.99638567
ENSG000001:	CCDC170	protein_codin	46.0157196	20.7196784	15.1925206	33.1803956
ENSG000001:	ARAP3	protein_codin	102.747967	48.2088247	27.6889444	78.4480321
ENSG000001:	PCDHB8	protein_codin	0	0	0	0
ENSG000001:	PCDHB10	protein_codin	0	0	0	0
ENSG000001:	PCDHB14	protein_codin	0	0	0	0
ENSG000001:	PCDHB12	protein_codin	0	0	0	0
ENSG000001:	SLC25A2	protein_codin	0	0	0	1.09824327
ENSG000001	PRRX1	protein_codin	1.24154187	0	1.1947658	1.27033728
ENSG000001:	TNN	protein_codin	0	0	0	0
ENSG000001:	TTLL2	protein_codin	0	0	0	0
ENSG000001:	MSANTD2	protein_codin	7.63187424	0	0	2.72322839
ENSG000001:	PDZD11	protein_codin	6.45191032	16.9957533	4.76397158	6.61695152
ENSG000001:	NUDCD1	protein_codin	18.887395	13.6043643	13.3375626	0
ENSG000001:	CCDC77	protein_codin	0	0	0	0
ENSG000001:	MTRF1	protein_codin	0	0	4.97393808	0
ENSG000002	CCDC169	protein_codin	0	0	0	0
ENSG000002	CCDC169	protein_codin	0	0	0	0
ENSG000001:	UFM1	protein_codin	266.11755	162.040853	190.1333	165.105471
ENSG000001:	ELF1	protein_codin	563.615546	559.23461	367.701612	380.161167
ENSG000001:	ALG5	protein_codin	17.856862	15.0818036	0	0
ENSG000001:	EXOSC8	protein_codin	0	0	0	0
ENSG000001:	FAM53C	protein_codin	167.478302	212.669975	100.099057	104.942469
ENSG000001:	MYOT	protein_codin	0	0	0	0
ENSG000001:	EGR1	protein_codin	1942.65294	1797.82275	979.985742	959.437278
ENSG000000	PFN2	protein_codin	0	0	0	0
ENSG000001:	SERP1	protein_codin	615.861687	596.758151	271.730493	509.229908
ENSG000001:	MTERF2	protein_codin	0	0	4.06314829	0
ENSG000001:	NFYB	protein_codin	24.4914729	0	20.1791167	21.688098
ENSG000001:	WASHC3	protein_codin	11.9841312	49.5666117	52.7579188	10.2157504
ENSG000001	FZD3	protein_codin	0	0	9.71340968	0
ENSG000001	SLC39A14	protein_codin	0	0	0	0
ENSG000001:	DUSP4	protein_codin	0	2.88299479	3.45345171	5.83365659
ENSG000001:	DUSP4	protein_codin	13.7487648	0	0	0
ENSG000001:	SORBS3	protein_codin	43.3161036	47.1695162	0	24.5951277
ENSG000001:	CHRNA2	protein_codin	0	2.81062704	0	1.66293931
ENSG000001:	PPP3CC	protein_codin	20.1696894	40.2010431	14.2897577	32.0622788
ENSG000001:	RNF170	protein_codin	0	0	0	0
ENSG000001:	TARDBP	protein_codin	112.646597	99.7533436	52.2701253	82.5765389
ENSG000001:	PRAMEF2	protein_codin	0	0	0	0

ENSG000001	RDH10	protein_codin	22.7521557	15.0623787	12.5104123	26.0344114
ENSG000001	LUC7L3	protein_codin	244.796307	323.024056	233.931667	231.885863
ENSG000001	DLX4	protein_codin	0	0.92574434	0	0
ENSG000001	COIL	protein_codin	66.536933	114.904113	51.1322565	48.6165624
ENSG000001	TBX2	protein_codin	0	0	0	0
ENSG000001	SLC35B1	protein_codin	52.2001936	0	0	0
ENSG000001	TBX4	protein_codin	0	0	0	0
ENSG000001	TEX14	protein_codin	6.61561655	0	0	0
ENSG000001	FAM117A	protein_codin	110.322335	209.43309	95.3040408	74.957703
ENSG000001	NCAPH	protein_codin	1.43654712	1.43855977	1.73507697	0
ENSG000001	TMEM131L	protein_codin	252.559167	212.110925	148.868866	154.655877
ENSG000001	MND1	protein_codin	0	0	6.24008576	0
ENSG000001	ZNF141	protein_codin	25.5826786	29.7147591	25.8888777	21.5294174
ENSG000001	TSHZ3	protein_codin	133.559172	225.592104	114.719855	141.548904
ENSG000001	TAS2R8	protein_codin	0	0	1.24447131	0
ENSG000001	PLBD1	protein_codin	1188.92742	2179.66694	485.092755	1233.39228
ENSG000002	KLRK1	protein_codin	19.4327548	5.07014097	0	2.07973692
ENSG000001	TAS2R10	protein_codin	0	2.79816396	1.1499218	0
ENSG000002	PRB1	protein_codin	0	0	0	0
ENSG000001	PYROXD1	protein_codin	65.4730676	38.2255369	30.9163037	25.4764457
ENSG000001	IAPP	protein_codin	0	0	0	0
ENSG000001	KCNJ8	protein_codin	0	0	0	0
ENSG000001	TAS2R7	protein_codin	0	0	0	0
ENSG000001	TAS2R9	protein_codin	0	0	0	0
ENSG000001	ZNF549	protein_codin	17.0974656	0	0	16.3057478
ENSG000001	ZSCAN18	protein_codin	0	0	0	0
ENSG000001	ZNF211	protein_codin	0	12.2102446	1.79104613	10.1315945
ENSG000001	TFG	protein_codin	0	64.37196	0	0
ENSG000001	KALRN	protein_codin	0	0	0	0
ENSG000001	NAA50	protein_codin	182.222339	109.49848	82.6596967	116.644413
ENSG000000	PAX6	protein_codin	0	0	0	0
ENSG000001	MAPK8IP1	protein_codin	4.59526894	0	1.73030024	0.89098296
ENSG000001	PEX16	protein_codin	0	0	0	0
ENSG000001	DEPDC7	protein_codin	0	6.34030867	0	2.15253958
ENSG000001	CAT	protein_codin	468.166616	1002.91131	366.863802	563.85413
ENSG000000	ACHE	protein_codin	0	0	0	0
ENSG000001	FBXO24	protein_codin	0	0	0	0
ENSG000001	GJB6	protein_codin	0	0	0	0
ENSG000001	GJA3	protein_codin	1.14326712	0	0	0
ENSG000001	GHSR	protein_codin	0	0	0	0
ENSG000001	TNFSF10	protein_codin	450.409268	352.904988	357.402306	453.622744
ENSG000001	SLITRK3	protein_codin	0	0	0	0
ENSG000001	CPXM2	protein_codin	2.61741843	0.88537494	0	0
ENSG000002	GSTM2	protein_codin	0	0	17.5842211	0

ENSG000002	ADORA3	protein_codin	3.14781973	7.33416581	0	1.03567211
ENSG000001	GTDC1	protein_codin	0	0	0	0
ENSG000001	CXCR4	protein_codin	1217.90878	480.152517	245.717608	201.66756
ENSG000001	ACVR2A	protein_codin	41.1250539	0	17.5916683	20.1508621
ENSG000001	POLK	protein_codin	59.9414852	62.1201471	77.9093236	37.8441656
ENSG000001	FLT3	protein_codin	81.0375453	71.7445419	18.3167185	26.3389067
ENSG000001	RASL11A	protein_codin	27.1643896	12.9927619	3.51440163	10.2071471
ENSG000001	FYTTD1	protein_codin	157.191712	145.981105	96.6141939	156.590344
ENSG000001	MTERF4	protein_codin	43.1063705	0	0	31.2655979
ENSG000001	MRPS2	protein_codin	40.2561259	62.6494483	10.455312	0
ENSG000001	MYOG	protein_codin	0	0	0	0
ENSG000001	COPA	protein_codin	116.840427	370.691292	0	99.484142
ENSG000001	PRM2	protein_codin	0	0	0	0
ENSG000001	OPN4	protein_codin	0	0	0	0
ENSG000001	AHR	protein_codin	922.700165	395.170096	467.241828	560.752023
ENSG000001	SCRN1	protein_codin	167.427357	166.335827	94.7297855	135.335714
ENSG000000	ETV1	protein_codin	0	0	0	0
ENSG000001	BBS9	protein_codin	37.3741963	0	0	0
ENSG000001	OCM	protein_codin	0	0	0	0
ENSG000001	EEPD1	protein_codin	99.1458858	79.9133079	34.5140324	74.6032444
ENSG000001	KIAA0087	lncRNA	2.29265197	0	0	0
ENSG000001	WIPF3	protein_codin	0	0	0	0
ENSG000001	NPY	protein_codin	0	0	0	0
ENSG000001	HOXA7	protein_codin	0	1.13148475	0.85044429	0
ENSG000001	INHBA	protein_codin	11.7464939	1.26780341	15.0398224	17.9555204
ENSG000001	FKBP9	protein_codin	41.8112113	33.9970872	6.75564927	0
ENSG000001	POLM	protein_codin	44.7208743	54.8008665	23.5438576	0
ENSG000001	RAMP3	protein_codin	3.68012287	6.53368748	0	0.89900535
ENSG000001	MRM2	protein_codin	0	69.9724336	41.0641538	29.1444239
ENSG000001	TWIST1	protein_codin	0	0.94033241	0	0
ENSG000001	SLC25A51	protein_codin	26.8051502	20.0907748	10.5056988	20.5809939
ENSG000001	CLTA	protein_codin	0	0	0	0
ENSG000001	TAF1L	protein_codin	0.9668674	0.96159332	0.95345849	2.97567489
ENSG000001	PHF24	protein_codin	0	0.80447487	0	0
ENSG000001	DNAI1	protein_codin	0	0	0	0
ENSG000001	DCAF10	protein_codin	0	0	32.9219372	0
ENSG000001	ZC3HAV1	protein_codin	447.246527	405.357127	254.290502	385.641958
ENSG000001	AKR1D1	protein_codin	0	0	0	0
ENSG000001	NEUROG3	protein_codin	0	0	0	0
ENSG000001	SRGN	protein_codin	3588.45398	3466.93365	1346.4785	3137.32057
ENSG000001	EGR2	protein_codin	87.1754159	61.9867214	351.915837	59.0939424
ENSG000001	FAM149B1	protein_codin	43.6256784	43.0449859	17.6570289	21.651424
ENSG000000	UNG	protein_codin	4.51943557	7.86976901	0	0
ENSG000000	DYNLL1	protein_codin	203.883587	387.019117	162.030962	202.782893

ENSG000001:	IFT81	protein_codin	0	0	0	3.24486018
ENSG000001:	ACADS	protein_codin	23.8423041	30.6976936	0	19.6189793
ENSG000001:	HVCN1	protein_codin	231.128781	349.580399	86.5084268	201.381321
ENSG000001:	RNF11	protein_codin	645.220805	1362.5304	641.732648	505.421273
ENSG000001:	BHLHE41	protein_codin	3.47263178	4.1014967	26.3884531	2.27276069
ENSG000001:	SSPN	protein_codin	0	0	0	0
ENSG000001:	ITPR2	protein_codin	16.8782459	11.2153729	15.2977609	11.5281666
ENSG000001	STX10	protein_codin	27.7395145	81.1407843	47.92114	0
ENSG000001:	DDX39A	protein_codin	68.663171	109.641641	52.7742604	76.5041409
ENSG000001:	PKN1	protein_codin	294.831866	237.605327	0	123.912528
ENSG000001:	TRIR	protein_codin	324.662094	451.285447	155.994243	234.569147
ENSG000001:	ADGRE5	protein_codin	78.0465323	112.434879	63.6253912	75.0632528
ENSG000001!	ZNF442	protein_codin	1.83580091	2.786528	0	0
ENSG000001	KLHL24	protein_codin	220.259208	127.362653	79.1200096	85.9842167
ENSG000001:	CCDC70	protein_codin	0.64662995	0	0	0
ENSG000001:	EBPL	protein_codin	13.2300283	24.5934513	1.9991636	0
ENSG000001:	ATP7B	protein_codin	0	0	0	0
ENSG000001:	ZC3H13	protein_codin	154.418499	51.5741303	36.3660739	145.344647
ENSG000001:	CENPK	protein_codin	16.4745782	0	11.6884621	7.9237716
ENSG000001:	NEUROD4	protein_codin	0	0	1.10599219	0
ENSG000001:	PFDN5	protein_codin	18.0607249	27.9450736	16.3494254	0
ENSG000001:	ORMDL2	protein_codin	16.6886003	15.9793048	8.84846605	4.50219504
ENSG000001:	NR4A1	protein_codin	373.062515	214.278866	161.937768	188.420678
ENSG000001:	PDE1B	protein_codin	52.7431462	15.5015757	28.6450708	38.7537047
ENSG000001:	HOXC13	protein_codin	0	0	0	0
ENSG000001:	LRP1	protein_codin	8776.17268	11086.5545	3933.00051	5033.34449
ENSG000001:	HOXC11	protein_codin	0	0	0	0
ENSG000001:	HOXC12	protein_codin	0	0	0	0
ENSG000001!	HOXC6	protein_codin	0	0	0	0
ENSG000001:	SMUG1	protein_codin	11.0336927	3.4718046	0	0
ENSG000001:	TYRL	transcribed_u	0	0	0	0
ENSG000001	FAAH	protein_codin	34.1228602	50.2139547	20.3634326	22.9779307
ENSG000001	RSRP1	protein_codin	0	0	0	20.812546
ENSG000001:	IL13RA2	protein_codin	0	0	0	0
ENSG000001	LAMA4	protein_codin	0	0	0	0
ENSG000001:	COL10A1	protein_codin	6.9909883	0	13.2549094	11.8284255
ENSG000000:	SEC61A1	protein_codin	147.444757	393.162359	172.504605	54.2767862
ENSG000001!	TCEAL3	protein_codin	10.5958415	43.9026368	11.2298933	8.37254327
ENSG000001:	RAB9B	protein_codin	0	4.11442797	1.35109622	0
ENSG000001:	NRK	protein_codin	0	0	0	0
ENSG000001:	MAGEA9	protein_codin	0	0	0	0
ENSG000001:	RAB9A	protein_codin	3.55297422	0	0	1.0651215
ENSG000000	RIF1	protein_codin	254.026626	123.939639	0	0
ENSG000001:	TTC21B	protein_codin	63.8289587	0	32.7107243	0

ENSG000001:	NMI	protein_codin	187.229342	227.78072	90.5024833	175.909784
ENSG000001:	TNFAIP6	protein_codin	92.3964067	116.898261	60.2743699	45.2818269
ENSG000001:	ACVR1C	protein_codin	0	0	2.82470057	0
ENSG000001:	SLC36A1	protein_codin	135.516646	164.1212	81.7728603	67.6820113
ENSG000001:	BATF3	protein_codin	40.4293154	47.1930788	13.7390116	12.4698547
ENSG000001:	KCNJ2	protein_codin	125.100499	80.1549825	15.2222499	67.7485899
ENSG000001:	EXOSC9	protein_codin	71.0859614	65.881699	32.8834064	41.9248196
ENSG000001:	PLA2G12A	protein_codin	194.433281	220.834196	250.753483	146.68539
ENSG000000!	LTBP4	protein_codin	17.9236715	0	0	0
ENSG000000	SNRPA	protein_codin	144.609684	159.514424	49.5808584	49.5468926
ENSG000001:	B9D2	protein_codin	14.1012063	13.5923616	2.93062787	10.5750951
ENSG000001:	COQ8B	protein_codin	0	0	0	0
ENSG000001:	C4BPB	protein_codin	0	0	0	0
ENSG000002	ZNF468	protein_codin	44.0210805	5.3077865	26.1973322	20.6530445
ENSG000001	ZNF415	protein_codin	0	0	0	0
ENSG000002	ZNF350	protein_codin	35.6828681	43.2082177	23.5432859	28.553438
ENSG000001:	RAB38	protein_codin	4.17132104	4.36005534	6.52490687	4.39114572
ENSG000001:	GPR83	protein_codin	0	1.038118	0	1.0504037
ENSG000002	HAUS3	protein_codin	17.6729917	0	0	24.748334
ENSG000001:	CHPF	protein_codin	1.68919063	8.7699997	0	0
ENSG000001:	INHA	protein_codin	0	0	0	0
ENSG000001:	FAM124B	protein_codin	0	0	1.24774341	1.94677405
ENSG000001	SP140L	protein_codin	0	0	0	0
ENSG000001:	ENKD1	protein_codin	9.21528812	1.74433474	7.81370464	8.45416704
ENSG000001	UBE2C	protein_codin	0	0	0	0
ENSG000000	SLC35C2	protein_codin	29.5144278	0	0	0
ENSG000001	ACTR5	protein_codin	35.4284114	40.537143	19.0114348	20.5416055
ENSG000001:	MC3R	protein_codin	0	0	0	0
ENSG000001:	GCNT7	protein_codin	0	0	0	0
ENSG000001:	CTCFL	protein_codin	0	0	0	0
ENSG000002	SYS1	protein_codin	96.2975414	122.001834	37.270353	57.4262213
ENSG000001:	PI3	protein_codin	0	0	0.60690885	0
ENSG000001:	WFDC3	protein_codin	0	0	0	0
ENSG000001:	SLC12A5	protein_codin	0	0	0	0
ENSG000001:	ARHGAP40	protein_codin	0	0	0	0
ENSG000001:	ATP5F1E	protein_codin	913.721253	1230.7879	465.236809	688.405082
ENSG000001:	PLCG1	protein_codin	17.8998107	20.8564916	0	10.8656805
ENSG000001:	SRSF6	protein_codin	765.851153	810.438455	331.626776	469.649019
ENSG000001:	RAB22A	protein_codin	133.858693	145.207133	48.3016826	88.4324385
ENSG000001:	PTGIS	protein_codin	0	0	0	0
ENSG000001:	CDH26	protein_codin	0	0	0	0
ENSG000001:	SNAI1	protein_codin	35.4636317	32.8848	18.8184689	18.8990549
ENSG000001:	MOCS3	protein_codin	17.9499274	35.1724425	9.35788554	10.175111
ENSG000001:	RNF114	protein_codin	126.325075	108.092861	68.1050982	99.3064325

ENSG000002	ENSG000002	lncRNA	0	0	0	0
ENSG000001	MAGEA10	protein_codin	0	0	0	0
ENSG000001	PEPD	protein_codin	94.3312428	146.918029	0	0
ENSG000001	IL9R	protein_codin	0	0	2.46589912	0
ENSG000001	NAGK	protein_codin	82.4326171	90.7215631	33.5378903	75.0115477
ENSG000001	MCEE	protein_codin	8.88424406	12.1075503	13.9514711	3.67212374
ENSG000001	PAIP2B	protein_codin	3.98678639	9.87264227	7.30960331	6.04083149
ENSG000001	SNRNP27	protein_codin	70.7218077	100.869028	48.0899947	64.5182049
ENSG000001	MPHOSPH10	protein_codin	105.961204	135.496738	121.285716	90.8061227
ENSG000001	IL17C	protein_codin	0	0	0	0
ENSG000000	LIPE	protein_codin	0	0	0	0
ENSG000001	PSG9	protein_codin	0	0	0	0
ENSG000002	PSG4	protein_codin	0	0	0	0
ENSG000002	PSG1	protein_codin	0	0	0	0
ENSG000001	HIF3A	protein_codin	0	0	0	0
ENSG000001	HIF3A	protein_codin	0	0	0	0
ENSG000001	IRGC	protein_codin	0	0.79141908	0	0
ENSG000002	PINLYP	protein_codin	0	0	0	0
ENSG000001	LYPD3	protein_codin	11.9550447	8.32766975	5.2057136	0
ENSG000001	CEACAM8	protein_codin	0	0	0	0
ENSG000002	RNF39	protein_codin	0	0	0	0
ENSG000001	DST	protein_codin	0	65.8279436	0	38.0029648
ENSG000001	FBXO9	protein_codin	0	0	0	0
ENSG000001	PACSIN1	protein_codin	0	0	0	0
ENSG000001	RRP36	protein_codin	104.865493	171.708351	48.8095988	82.3415269
ENSG000001	BTN1A1	protein_codin	0	0	0.996108	0
ENSG000001	BTN3A3	protein_codin	98.1500772	212.419277	90.0509829	89.7553929
ENSG000001	SNRPC	protein_codin	141.643341	263.008004	76.4430778	106.671387
ENSG000001	SLC17A1	protein_codin	0	0	0	0
ENSG000001	ABCC10	protein_codin	0	0	0	0
ENSG000001	H1-3	protein_codin	215.404768	266.129017	360.503015	167.485961
ENSG000002	H4C6	protein_codin	34.0266849	52.2731529	35.6564725	47.9471466
ENSG000001	PEX6	protein_codin	0	0	0	0
ENSG000001	UNC5CL	protein_codin	0	0	0	0
ENSG000001	AARS2	protein_codin	47.6577607	77.2814154	45.7024534	29.4185928
ENSG000001	H1-1	protein_codin	0	0	0.7171613	1.63164215
ENSG000001	ZNF391	protein_codin	1.57619218	0	0.99537653	0
ENSG000001	OR2B6	protein_codin	0	2.3372226	0.99420772	2.3699852
ENSG000001	TCP11	protein_codin	0	0	0	0
ENSG000001	APOBEC2	protein_codin	0	0	0	0
ENSG000001	KLHDC3	protein_codin	19.8961218	17.5108569	1.58937721	8.82332322
ENSG000001	TREM1	protein_codin	1304.61037	645.544556	391.305264	765.5884
ENSG000001	MEA1	protein_codin	0	0	0	1.31464184
ENSG000001	COL21A1	protein_codin	0	0	0	0

ENSG000001	CDKN1A	protein_codin	675.896639	521.246428	137.3203	300.359362
ENSG000001	SOX4	protein_codin	151.6617	347.590971	58.0565052	185.166441
ENSG000001	CPNE5	protein_codin	0	0	0	0
ENSG000001	SSR1	protein_codin	103.996362	109.249532	112.871539	99.5604076
ENSG000001	NRN1	protein_codin	0	0	0	0
ENSG000001	ATXN1	protein_codin	0	388.249496	206.486747	51.3774006
ENSG000001	DEK	protein_codin	0	0	5.0045308	61.5311942
ENSG000001	BLOC1S5	protein_codin	4.43677045	20.3838086	25.1351269	0
ENSG000001	OPN5	protein_codin	0	0	0	0
ENSG000001	LRRFIP1	protein_codin	1913.21944	618.053843	928.10682	821.310844
ENSG000001	ENSG000001	lncRNA	0.8147673	0	0	0.79961773
ENSG000001	EREG	protein_codin	1593.75656	264.041656	561.265236	1017.88671
ENSG000001	TRIM51	protein_codin	0	0	0	0
ENSG000001	MYRF-AS1	lncRNA	0	0	0	0
ENSG000001	SCGB1D2	protein_codin	0	0	0	0
ENSG000001	SCGB2A1	protein_codin	0	0	0	0
ENSG000001	EMC3	protein_codin	110.5065	183.866725	78.0923066	63.1288669
ENSG000001	SH3TC1	protein_codin	81.2776623	100.586655	0	0
ENSG000001	KATNAL2	protein_codin	0	3.24872288	0	0
ENSG000001	BBS2	protein_codin	0	0	0	0
ENSG000001	MT2A	protein_codin	198.650462	50.8600941	26.7175125	35.6115203
ENSG000001	GOT2	protein_codin	73.6703382	118.342569	50.780455	49.8821914
ENSG000000	SPHK2	protein_codin	0	14.3369433	3.4764289	0
ENSG000001	PIWIL1	protein_codin	0	0	0	0
ENSG000001	RAP2A	protein_codin	134.027274	85.8279566	93.6298423	88.6721009
ENSG000001	SLC10A2	protein_codin	1.1580755	0	0	0
ENSG000001	HROB	protein_codin	0	0	0	0
ENSG000001	SLC22A5	protein_codin	9.13447896	0	0	0
ENSG000001	IRF1	protein_codin	1357.22355	1270.60194	615.714695	539.190399
ENSG000001	DMAC2L	protein_codin	0	0	0	0
ENSG000001	BMP4	protein_codin	0	0	0	0
ENSG000001	PTGER2	protein_codin	401.208563	173.21464	84.5737158	159.485878
ENSG000002	RPS29	protein_codin	851.09967	1280.30027	169.723621	755.369556
ENSG000001	SOX9	protein_codin	0	0	0	0
ENSG000001	MYH2	protein_codin	0	0	0	0
ENSG000001	MRPS7	protein_codin	41.0983067	111.224707	30.2511167	39.6644287
ENSG000001	ARMC7	protein_codin	32.605074	56.2884909	13.4650244	13.5077549
ENSG000001	NUP85	protein_codin	17.2085086	0	0	0
ENSG000001	MIF4GD	protein_codin	4.24891164	5.39061731	0	0
ENSG000001	NT5C	protein_codin	0	37.4282956	0	2.88668339
ENSG000001	MSTO1	protein_codin	14.5456006	0	13.3066403	8.06043566
ENSG000001	MBOAT7	protein_codin	137.907372	225.326111	50.559281	124.366078
ENSG000001	EPS8L1	protein_codin	0	2.24303038	0	0
ENSG000002	LILRB3	protein_codin	172.862572	190.702203	69.4043502	71.1442611

ENSG000002	LILRA6	protein_codin	20.5183077	127.326416	44.7742774	145.683329
ENSG000001	ZBTB46	protein_codin	14.1504725	28.4151097	0	4.47842603
ENSG000001	SLC35F5	protein_codin	162.723423	95.8363272	113.878174	146.807855
ENSG000001	INSIG2	protein_codin	34.6760433	17.348435	0	18.8403775
ENSG000001	PSD4	protein_codin	98.5433126	131.484424	53.7924438	35.6964823
ENSG000001	PSPN	protein_codin	0	0	0	0
ENSG000002	ENSG000002	processed_ps	1.02014299	1.03547984	0	0
ENSG000001	ALKBH7	protein_codin	41.978663	75.0154628	22.062222	37.2918098
ENSG000001	CLPP	protein_codin	33.1694522	21.2004185	0	2.8024619
ENSG000001	TNFSF9	protein_codin	19.4891894	12.9377541	7.63802147	11.930392
ENSG000001	THOC2	protein_codin	366.263125	53.1295173	23.1831936	189.584765
ENSG000001	RPL23	protein_codin	0	0	0	0
ENSG000002	STRADA	protein_codin	0	0	0	0
ENSG000001	CD70	protein_codin	0	0	0	0
ENSG000001	C3	protein_codin	34.581229	33.4218085	1.46842988	21.3604442
ENSG000001	SH2D3A	protein_codin	1.95459047	2.55575619	0	0
ENSG000001	TNFSF14	protein_codin	47.1661617	0	6.32233298	25.6917289
ENSG000001	RTN2	protein_codin	0	0	0	0
ENSG000001	EML2	protein_codin	24.4113944	39.2534931	13.3436242	24.0364992
ENSG000001	VASP	protein_codin	1176.61201	1519.04519	213.106414	705.290061
ENSG000001	SYMPK	protein_codin	0	93.7202284	52.6649247	0
ENSG000000	CFAP61	protein_codin	0	0	0	0
ENSG000001	CDC25B	protein_codin	101.895867	48.9994412	77.3471823	29.1184977
ENSG000001	GNRH2	protein_codin	0	0	0	0
ENSG000001	CD93	protein_codin	2540.72696	2456.15199	1019.83504	1966.64828
ENSG000001	CST8	protein_codin	0	0	0	0
ENSG000001	PSMF1	protein_codin	73.9597301	131.763665	40.3160616	69.0106536
ENSG000001	CSTL1	protein_codin	0	0	0	0
ENSG000001	TMX4	protein_codin	725.276891	544.764016	282.682597	435.254062
ENSG000001	RRBP1	protein_codin	0	0	722.069206	220.055331
ENSG000001	DSTN	protein_codin	25.9028099	26.2775187	24.9831674	39.2301543
ENSG000001	LAMP5	protein_codin	3.96493311	14.3846575	17.9501004	9.18909655
ENSG000001	SNRPB2	protein_codin	9.89355586	10.6453173	0	4.81299451
ENSG000001	TCF15	protein_codin	0	0	0	0
ENSG000001	OTOR	protein_codin	0	0	1.19391401	0
ENSG000001	BANF2	protein_codin	0	0	0	0
ENSG000001	FAM110A	protein_codin	0	0	0	0
ENSG000002	SCRT2	protein_codin	0	0	0	0
ENSG000001	DEFB129	protein_codin	0	0	0	0
ENSG000002	RAD21L1	protein_codin	0	0	0	0
ENSG000001	TLE6	protein_codin	0	0	0	0
ENSG000001	S1PR4	protein_codin	61.4773244	71.40105	12.538117	55.3464291
ENSG000001	NCLN	protein_codin	171.515295	165.653795	59.5413607	79.5586288
ENSG000001	CITED1	protein_codin	0	0	0	0

ENSG000001:	ANKHD1	protein_codin	2.58381875	2.18917014	11.9228621	0
ENSG000000:	PITHD1	protein_codin	54.7279057	100.299657	46.707332	50.8956967
ENSG000001:	MAX	protein_codin	19.3779347	36.6901958	16.3277062	17.9873718
ENSG000002:	FNTB	protein_codin	74.3150074	87.115676	14.1669611	23.5986159
ENSG000001:	ARMCX5	protein_codin	4.28210425	0	0	0
ENSG000001:	MMP24	protein_codin	1.90929774	0.93668379	1.07266318	0
ENSG000001:	NECAB3	protein_codin	0	0	0	0
ENSG000001:	RALY	protein_codin	503.683662	672.649458	289.517587	332.582925
ENSG000001:	C20orf173	protein_codin	0	0	0	0
ENSG000001:	BPIFB9P	transcribed_u	0	0	0	0
ENSG000001:	PLAGL2	protein_codin	536.996175	542.670027	194.950683	297.776428
ENSG000001:	AGO3	protein_codin	28.7112594	43.4443396	33.0081849	30.6929293
ENSG000001:	UROD	protein_codin	58.0835793	76.4745727	14.8883024	34.6061388
ENSG000002!	ENSG000002!	lncRNA	0	15.0020181	3.59606818	0
ENSG000001:	KLC1	protein_codin	0	0	26.8021703	0
ENSG000001:	PCID2	protein_codin	0	0	0	4.66373323
ENSG000001:	SLURP1	protein_codin	0	0	0	0.79611635
ENSG000001:	LRFN3	protein_codin	0	0	0	0
ENSG000001:	IGFLR1	protein_codin	36.381035	53.9442141	19.660659	35.8328869
ENSG000001:	CAPNS1	protein_codin	170.456355	224.690893	42.5185527	57.9030096
ENSG000001:	PDCD2L	protein_codin	0	0	5.22102617	5.42005101
ENSG000001:	UBA2	protein_codin	22.4125382	50.72326	14.7357101	28.651196
ENSG000001:	FFAR2	protein_codin	0	0	0	0
ENSG000001:	HCST	protein_codin	82.4460574	126.304618	38.662421	67.2205016
ENSG000001:	FFAR1	protein_codin	61.1762958	21.5347356	17.0337244	12.85113
ENSG000001'	KRT13	protein_codin	0	0	0	0
ENSG000001!	KRT35	protein_codin	0	0	0	0
ENSG000001'	KRT38	protein_codin	0	0	0	0
ENSG000001:	CCR7	protein_codin	2.06341454	0.98385455	0.83674802	0
ENSG000001'	KRT9	protein_codin	0	0	0	0
ENSG000001:	NR1D1	protein_codin	39.5547882	49.8719201	3.84334449	11.0531405
ENSG000002	ARL2	protein_codin	9.48208473	44.0623848	17.7750379	19.510624
ENSG000001:	BCL2L12	protein_codin	2.72242599	0	4.90353877	0
ENSG000001:	BCL2L12	protein_codin	0	0	0	3.34426077
ENSG000001:	RRAS	protein_codin	143.745944	146.863471	21.4915074	70.0487912
ENSG000001:	PRRG2	protein_codin	0	0	0.88938081	0.80185294
ENSG000001:	TSKS	protein_codin	5.29420813	0	0	6.7186196
ENSG000001(	NOP53	protein_codin	681.363213	869.491321	434.362339	463.618243
ENSG000001:	FLRT1	protein_codin	0	0	0	0
ENSG000001:	SBDS	protein_codin	109.896998	223.98343	0	66.4210326
ENSG000001:	CSN1S1	protein_codin	0	0	0	0
ENSG000001:	STATH	protein_codin	0	0	0	0
ENSG000001:	HTN1	protein_codin	0	0	0	0
ENSG000000(	IFI35	protein_codin	0	0	12.3530784	11.4080943

ENSG000001	MLX	protein_codin	4.00873557	8.25709107	0	0
ENSG000001	WNK4	protein_codin	0	0	0	0
ENSG000002	DNASE1	protein_codin	0	0	0	0
ENSG000001	TRAP1	protein_codin	77.6543425	121.283749	62.0739131	57.7918713
ENSG000001	SUGP1	protein_codin	0	80.8424931	56.2288392	0
ENSG000001	DDX49	protein_codin	89.9250664	97.9896165	41.0281917	40.3327738
ENSG000001	GDF1	protein_codin	0	0	0	0
ENSG000001	SDF2	protein_codin	10.2232772	10.2835328	13.2525901	16.6709378
ENSG000001	NSRP1	protein_codin	242.130796	147.183436	135.919094	82.4540668
ENSG000001	AHDC1	protein_codin	43.61242	48.3730771	14.8146667	21.6511247
ENSG000001	SLC35A2	protein_codin	0	0	0	9.99415993
ENSG000001	PQBP1	protein_codin	0	0	0	0
ENSG000001	CFP	protein_codin	490.996863	658.420915	439.894691	361.18691
ENSG000001	ELK1	protein_codin	69.5560225	64.3185899	45.2346566	48.9712916
ENSG000001	ATG14	protein_codin	151.233887	175.292079	84.4675554	89.2521181
ENSG000001	DLGAP5	protein_codin	1.36098466	2.9219465	26.5138828	1.58888884
ENSG000001	L3HYPDH	protein_codin	8.96618971	16.059745	8.55242904	8.75273439
ENSG000001	HSPA2	protein_codin	14.3935177	9.4308237	4.98460005	8.73224171
ENSG000001	TBPL2	protein_codin	0	0	0	0
ENSG000001	SGPP1	protein_codin	37.8033796	36.9220379	20.4095413	9.96008038
ENSG000001	PLEKHG3	protein_codin	0	74.5151784	0	0
ENSG000001	EVI2A	protein_codin	39.3639561	75.7955378	85.9321852	81.7330761
ENSG000001	OMG	protein_codin	0	0	0	0
ENSG000001	AIF1L	protein_codin	0	0	0	0
ENSG000001	FAM78A	protein_codin	58.2054533	57.0134349	8.51727213	5.48156255
ENSG000001	CTAG2	protein_codin	0	0	0	0
ENSG000000	MAGEC2	protein_codin	0	0	0	0
ENSG000001	CANX	protein_codin	411.735287	197.197461	84.3343719	370.735542
ENSG000001	PYCARD	protein_codin	367.250277	504.726842	118.949312	266.174634
ENSG000001	COX7C	protein_codin	448.542069	688.629678	199.229624	316.128885
ENSG000000	PHPT1	protein_codin	46.0790278	57.8630252	30.5160835	27.025507
ENSG000001	TRAF2	protein_codin	20.1374479	21.8630424	17.0815422	4.3541744
ENSG000001	ABHD8	protein_codin	16.0190755	39.10796	7.94798259	27.5424809
ENSG000001	HELB	protein_codin	32.916298	41.9002786	28.532036	31.4223736
ENSG000001	TSPAN8	protein_codin	0	0	0	0
ENSG000001	RAB3IP	protein_codin	21.0348658	0	0	1.48450962
ENSG000001	YEATS4	protein_codin	2.86096464	23.2346223	15.0926015	0
ENSG000000	NDUFB2	protein_codin	40.6775227	48.2895713	23.2382788	21.6984643
ENSG000001	TAS2R3	protein_codin	0	0	0	0
ENSG000001	TAS2R4	protein_codin	4.42952284	0	3.28174839	0
ENSG000001	TAS2R5	protein_codin	1.03640769	0	0.75928824	1.03744184
ENSG000001	ZNF777	protein_codin	63.1242392	122.929859	38.005437	49.6770276
ENSG000001	IDUA	protein_codin	0	16.3424981	20.0619944	31.6266952
ENSG000001	ZNF317	protein_codin	0	173.512299	41.2908443	117.890943

ENSG000001:	PIN1	protein_codin	102.497523	120.278326	44.6881461	56.775212
ENSG000001:	FBXL12	protein_codin	0	28.8482731	0	0
ENSG000001	KIF17	protein_codin	0	0	0	0
ENSG000001	PLA2G2C	protein_codin	0	0	0	0
ENSG000001	CYP4F11	protein_codin	0	0	0	0
ENSG000001:	SIN3B	protein_codin	190.971255	441.742766	151.60297	196.374044
ENSG000001:	EPS15L1	protein_codin	0	0	0	37.1651442
ENSG000001:	KLF2	protein_codin	936.576029	854.39513	246.594485	450.624065
ENSG000001:	OR7C2	protein_codin	0	0	0	0
ENSG000001:	OR7C1	protein_codin	0	0	0	0
ENSG000001:	F2RL3	protein_codin	47.4380545	103.55647	45.1265112	29.9083332
ENSG000001	HCFC1R1	protein_codin	32.3176235	45.2394055	16.5987565	8.36458156
ENSG000002	JPT2	protein_codin	52.8816413	97.7996951	52.6726577	48.7192515
ENSG000000:	UNKL	protein_codin	0	27.6818173	0	0
ENSG000001:	GFER	protein_codin	18.2897558	31.3623305	18.6117246	11.1322937
ENSG000001:	SYNGR3	protein_codin	0	0	0	0
ENSG000001	RAB40C	protein_codin	25.8135251	72.9979532	0	47.4453424
ENSG000001:	WDR24	protein_codin	0	0	0	0
ENSG000001:	GNG13	protein_codin	0	0	0	0
ENSG000001:	TUBBP1	transcribed_p	0	0	1.93606163	0
ENSG000002:	ZNF10	protein_codin	1.61187128	0	4.86906656	2.28739539
ENSG000001:	TICAM1	protein_codin	229.045328	231.754852	94.1567985	116.085446
ENSG000001	MON1B	protein_codin	246.361749	353.909614	107.30873	164.942489
ENSG000001:	METTL25	protein_codin	0	17.6137039	4.78302276	0
ENSG000001	EIF3K	protein_codin	411.484183	629.810706	189.440116	290.904331
ENSG000001:	EMC6	protein_codin	42.6745082	77.8231395	9.17609948	46.4918181
ENSG000001:	OR1E2	protein_codin	0	0	0	0
ENSG000001	CACTIN	protein_codin	0	58.8577244	29.4368497	0
ENSG000001:	TUBA4A	protein_codin	310.186623	722.668851	315.301943	227.317444
ENSG000001:	VIL1	protein_codin	40.1379245	143.503323	26.3581262	30.8401736
ENSG000001:	AAMP	protein_codin	56.4581318	0	0	0
ENSG000001:	PNKD	protein_codin	20.2191092	28.4387235	10.4358717	12.384656
ENSG000001:	TNFRSF19	protein_codin	0	0	0	0
ENSG000000	PHTF2	protein_codin	0	0	0	0
ENSG000001	HSPB1	protein_codin	35.2023253	66.0092131	44.0509753	45.5846374
ENSG000001:	GNG11	protein_codin	400.43548	1505.41164	485.489802	485.326399
ENSG000001:	SEM1	protein_codin	89.9473607	93.4066258	81.4469065	65.1225066
ENSG000001:	GNGT1	protein_codin	0	0	0	0
ENSG000001:	PTPN12	protein_codin	974.454803	1156.58613	1025.54143	802.595827
ENSG000001:	FGL2	protein_codin	4104.28892	2004.38545	2355.74983	3121.12871
ENSG000001:	STYXL1	protein_codin	0	0	0	15.7939719
ENSG000001:	PEX1	protein_codin	14.4489182	42.3694837	0	0
ENSG000001:	LRFN1	protein_codin	21.9877375	22.3993985	7.20214756	10.7812812
ENSG000001:	SPINK2	protein_codin	0	2.54530971	5.08303915	0

ENSG000001:	RASL11B	protein_codin	0	0	0	0
ENSG000001:	TUBGCP6	protein_codin	144.624229	158.444196	59.6805775	57.6370286
ENSG000001:	DGCR6L	protein_codin	45.0668493	103.52732	58.7933071	37.0542215
ENSG000001(	NCF4	protein_codin	248.608608	324.764548	98.5926638	165.867175
ENSG000001(	CYTH4	protein_codin	469.549731	534.314627	148.57607	262.302959
ENSG000001(	GCAT	protein_codin	1.78632567	1.07076884	0	1.77212632
ENSG000001(	SGSM3	protein_codin	92.6976541	147.195347	52.761488	34.7933905
ENSG000001(	SEZ6L	protein_codin	0	0	10.3973968	0
ENSG000001:	VPREB3	protein_codin	0	0	0	0
ENSG000001:	SDF2L1	protein_codin	22.8542423	45.5302237	33.6069902	19.8425033
ENSG000001:	YWHAH	protein_codin	777.045393	1511.84859	675.762662	608.149561
ENSG000002:	RFPL1S	lncRNA	0	0	0	0
ENSG000001:	RFPL2	protein_codin	0	0	0	0
ENSG000001:	A4GALT	protein_codin	0	0	0	0
ENSG000001:	RFPL3	protein_codin	0	0	0	0
ENSG000001:	CDC42EP1	protein_codin	34.9544097	29.4621021	20.434399	48.9104937
ENSG000001:	MCHR1	protein_codin	0	0	0	0
ENSG000001:	GALR3	protein_codin	0	0	0	0
ENSG000001:	TST	protein_codin	0	57.5239066	13.4502107	29.8556012
ENSG000001:	APOL5	protein_codin	0	0	0	0
ENSG000001:	IGLL1	protein_codin	0	0.82681731	2.88745476	0
ENSG000001:	CCDC117	protein_codin	87.9822496	137.862343	62.0779282	62.214541
ENSG000001:	APOL2	protein_codin	6.79213373	81.0575592	0	0
ENSG000001:	RAC2	protein_codin	744.58736	1091.83219	387.433235	550.511442
ENSG000001:	LIF	protein_codin	21.5016425	0	4.6380651	12.0381096
ENSG000001:	C22orf23	protein_codin	0	0	0	0
ENSG000001:	APOBEC3A	protein_codin	52.0525261	511.945215	111.615898	238.898186
ENSG000001:	EMC4	protein_codin	6.05465922	4.9191735	11.4699776	2.48298686
ENSG000001(	PMPCB	protein_codin	0	25.0543206	0	28.9383836
ENSG000001(	DNAJC2	protein_codin	0	0	106.63274	0
ENSG000001:	TAS2R16	protein_codin	0	0	0	0
ENSG000001:	ATP6V1F	protein_codin	398.154306	735.842061	142.400489	296.054042
ENSG000001:	LSM8	protein_codin	38.3713845	28.3701468	13.990577	18.6008293
ENSG000001:	VGF	protein_codin	0	0	0	0
ENSG000001:	STRIP2	protein_codin	8.50824982	7.77238834	13.0672685	0
ENSG000001:	DNAJB9	protein_codin	74.7369106	45.1763596	60.8880305	82.0146201
ENSG000001:	LRRC4	protein_codin	17.8038675	28.8763207	8.76959707	10.8251594
ENSG000001:	CALU	protein_codin	0	0	0	0
ENSG000001:	SMO	protein_codin	2.27950063	1.82646012	0	1.8035132
ENSG000001:	LRRC17	protein_codin	0	0	0	0
ENSG000001:	OPN1SW	protein_codin	0	0	0	0
ENSG000000(	SIRT2	protein_codin	116.855546	138.018571	69.308333	62.4523987
ENSG000001:	HOXD3	protein_codin	0	0	0	0
ENSG000001:	MTX2	protein_codin	14.9291153	24.774249	12.5103468	0

ENSG000001	HOXD9	protein_codin	0	0	0	0
ENSG000001	HOXD10	protein_codin	0.90182847	1.83512436	0	0
ENSG000001	HOXD11	protein_codin	0	0	0	0
ENSG000001	ARHGAP22	protein_codin	0	0	0	4.3843776
ENSG000000	PIAS1	protein_codin	294.019396	281.007634	188.048512	273.530836
ENSG000000	SNAP23	protein_codin	721.807929	936.235361	340.935217	504.215023
ENSG000001	TMOD2	protein_codin	283.163682	122.709174	127.199813	160.585438
ENSG000001	SLTM	protein_codin	35.757203	22.6695651	200.405738	32.2974546
ENSG000001	DLL4	protein_codin	0	0	0	0
ENSG000001	ALDH1A2	protein_codin	0	0	0	0
ENSG000001	KNSTRN	protein_codin	0	0	0	0
ENSG000001	SERF2	protein_codin	528.9971	873.781387	443.834685	472.432368
ENSG000001	CLN6	protein_codin	39.1920647	61.3776943	25.9105697	20.6267973
ENSG000001	ARPP19	protein_codin	164.961412	109.544651	108.17516	109.934561
ENSG000001	VPS13C	protein_codin	792.356985	414.763715	322.526251	503.568858
ENSG000001	ISLR	protein_codin	0	0	0	0
ENSG000001	THAP10	protein_codin	0	1.32565128	0	0
ENSG000001	AMOTL2	protein_codin	0	0	0	0
ENSG000001	ACKR4	protein_codin	0	0	0	0
ENSG000001	MBD4	protein_codin	0	0	0	0
ENSG000001	COPB1	protein_codin	102.667398	131.591134	40.6113344	87.3839378
ENSG000001	MYOD1	protein_codin	0	0	0	0
ENSG000001	TPH1	protein_codin	0	0	3.90437263	0
ENSG000001	E2F8	protein_codin	0	0	0	0
ENSG000001	SOX15	protein_codin	0	0	0	0
ENSG000001	PIMREG	protein_codin	0	0	0	0
ENSG000001	USP6	protein_codin	0	0	0	0
ENSG000001	ZNF232	protein_codin	0	0	0	0
ENSG000001	AIPL1	protein_codin	0	0	0	0
ENSG000001	CD68	protein_codin	2597.0083	4353.62211	1100.20231	2112.12773
ENSG000001	TXNDC17	protein_codin	3.51924034	3.99911835	1.83392564	10.2614634
ENSG000001	ATP1B2	protein_codin	0	6.10239333	0.71745713	0
ENSG000001	FXR2	protein_codin	54.3103363	75.7249534	27.1559779	45.5114712
ENSG000001	MPDU1	protein_codin	28.9275561	53.2174765	13.3001666	8.75814719
ENSG000001	CCN4	protein_codin	0	0	0	0
ENSG000001	DNAAF11	protein_codin	7.94612381	7.158994	2.65825001	14.8797694
ENSG000002	QTRT1	protein_codin	5.85059296	0	0	0
ENSG000001	ILF3	protein_codin	0	27.7301992	0	0
ENSG000001	AP1M2	protein_codin	0	19.7985593	0	2.94769404
ENSG000001	ERI1	protein_codin	29.0146482	42.1052288	22.9157855	25.3964146
ENSG000001	CLEC11A	protein_codin	22.9701168	23.3050225	13.861319	12.6502535
ENSG000001	KLK12	protein_codin	0	0	0	0
ENSG000001	SIGLEC9	protein_codin	166.599514	271.660492	58.3019632	128.397055
ENSG000002	ENSG000002	protein_codin	0	0	0	0

ENSG000001	NFATC4	protein_codin	0	0	0	0
ENSG000001	PRORP	protein_codin	0	0	0	0
ENSG000000	CMA1	protein_codin	0	0.78700209	0	0
ENSG000000	GEMIN2	protein_codin	10.0164595	0	0	0
ENSG000001	DHRS2	protein_codin	0.82844499	0	0	0
ENSG000001	BCL2L2	protein_codin	110.516731	136.551242	0	63.3287092
ENSG000001	PARP2	protein_codin	0	0	5.16492264	8.24892231
ENSG000001	FOXA1	protein_codin	0	0	0	0
ENSG000001	EAPP	protein_codin	132.298178	131.481797	83.5206298	85.8463536
ENSG000001	EGLN3	protein_codin	40.6406726	69.0012594	12.7058004	17.3380829
ENSG000001	PIP4P1	protein_codin	12.7442991	45.5414302	0	10.0335231
ENSG000001	NEDD8	protein_codin	178.509175	249.558519	142.938698	123.065031
ENSG000001	DAD1	protein_codin	181.358513	193.389701	83.6010443	96.3701137
ENSG000001	CDO1	protein_codin	0	0	0	0
ENSG000001	RAP1B	protein_codin	270.165178	69.4014777	51.38463	74.3935281
ENSG000001	ADGRE1	protein_codin	111.402316	119.785901	43.7180668	95.847975
ENSG000001	AANAT	protein_codin	0.83901112	0	0	0
ENSG000001	ARHGEF6	protein_codin	583.359412	311.9785	390.837333	551.73443
ENSG000001	ART1	protein_codin	0	0	0	0
ENSG000001	CHRNA10	protein_codin	0	1.89754802	0	1.10563899
ENSG000001	TTTY1B	lncRNA	0	0	0	0
ENSG000001	RPS4Y1	protein_codin	0	0	0	0
ENSG000001	VCY1B	protein_codin	0	0	0	0
ENSG000001	VCY	protein_codin	0	0	0	0
ENSG000002	RBMY1J	protein_codin	0	0	0	0
ENSG000001	CDY2A	protein_codin	0	0	0	0
ENSG000000	DAZL	protein_codin	0	0	0	0
ENSG000001	MAPK8IP3	protein_codin	357.461292	326.204428	192.177895	268.561517
ENSG000000	MKNK2	protein_codin	455.229755	500.687664	99.6747764	256.525675
ENSG000001	KLF16	protein_codin	50.7234902	65.1458571	33.7959138	29.9246769
ENSG000001	PGAP6	protein_codin	61.7695568	139.599715	0	51.7352282
ENSG000002	INS	protein_codin	0	0	0	0
ENSG000001	ABHD17A	protein_codin	0	0	43.4117391	0
ENSG000001	SALL1	protein_codin	0	0	0	0
ENSG000001	ZC3H14	protein_codin	32.4726821	6.45569967	4.21572599	11.6219389
ENSG000000	MYO16	protein_codin	0	0	0	0
ENSG000000	LMAN1	protein_codin	194.757607	118.711496	231.69534	123.343133
ENSG000000	NUP37	protein_codin	0	13.4207916	0	0
ENSG000001	DMXL2	protein_codin	1151.19479	744.13458	324.395493	433.589702
ENSG000001	DSC2	protein_codin	7.45533427	9.08129601	1.59107495	1.70401869
ENSG000000	ANGEL1	protein_codin	17.0909795	0	0	0
ENSG000000	SAMD4A	protein_codin	36.7467062	40.9898832	25.2431288	6.61198601
ENSG000000	CNGB1	protein_codin	0	0	0	0
ENSG000001	CDADC1	protein_codin	26.6070493	29.6152564	11.609862	13.3408716

ENSG000001	NALCN	protein_codin	0	0	0	0
ENSG000001	VPS35L	protein_codin	0	0	0	0
ENSG000001	DOCK7	protein_codin	0	0	0	0
ENSG000002	CORO7	protein_codin	255.78532	338.721003	79.8580528	115.931434
ENSG000001	SEC14L5	protein_codin	105.073616	318.624709	146.559788	93.3855096
ENSG000000	CEP170B	protein_codin	0	0	0	0
ENSG000000	CLSPN	protein_codin	0	0	0	0
ENSG000001	PBX4	protein_codin	5.40392236	13.9190452	6.16633331	0
ENSG000001	DHX40	protein_codin	136.611122	76.617391	52.6781562	110.665612
ENSG000001	DTWD1	protein_codin	0	0	0	0
ENSG000001	MEGF8	protein_codin	58.9661319	55.1030794	16.3961134	0
ENSG000001	ZNF221	protein_codin	0	0	0	0
ENSG000000	HCN2	protein_codin	5.90973791	3.0784773	1.33846247	0.99363256
ENSG000000	WDR18	protein_codin	0	0	0	0
ENSG000001	IGSF21	protein_codin	0	0	0	0
ENSG000001	INTS2	protein_codin	0	27.9028123	17.0747415	0
ENSG000001	KHNYN	protein_codin	277.818267	89.4997239	134.292188	0
ENSG000000	CERS4	protein_codin	10.7003128	31.6796272	0	0
ENSG000001	ESRP2	protein_codin	0	0	0	0
ENSG000001	LILRA1	protein_codin	409.569267	454.561898	152.647846	345.028484
ENSG000002	LILRA2	protein_codin	70.1201675	114.641667	35.7281934	59.5178201
ENSG000002	LILRA2	protein_codin	113.365709	153.729593	34.6481164	71.376986
ENSG000000	TUBG2	protein_codin	3.88454123	22.1136097	6.29807797	5.2932301
ENSG000001	TUBG1	protein_codin	15.7646216	21.1430564	36.1130555	0
ENSG000001	CFHR4	protein_codin	0	0	0	0
ENSG000001	RPS16	protein_codin	906.922021	1406.75145	308.074474	648.814777
ENSG000001	MAST1	protein_codin	1.73675926	0	1.47617312	0
ENSG000001	PLPPR2	protein_codin	135.838548	138.106927	25.5344905	95.4028984
ENSG000001	SULT1C2	protein_codin	0	0	0	0
ENSG000001	NCAPG	protein_codin	4.26242713	6.62604149	39.4790378	5.61720761
ENSG000001	RABGAP1L	protein_codin	212.3457	175.023535	196.765291	150.948276
ENSG000001	ESYT2	protein_codin	751.596203	0	0	0
ENSG000001	ALOX12	protein_codin	208.108161	394.932151	240.53474	194.829265
ENSG000001	FBXO44	protein_codin	3.86177244	0	0	0
ENSG000001	FBXO44	protein_codin	5.81083653	0	0	0
ENSG000001	UGT2A3	protein_codin	0	0	0	0
ENSG000000	ADAMTS2	protein_codin	9.10817089	0	0	0
ENSG000001	CIAO3	protein_codin	8.98715109	20.7972308	7.54351545	0
ENSG000001	HBA2	protein_codin	0	0	0	0
ENSG000000	TRNT1	protein_codin	0	0	32.2749386	18.7202976
ENSG000001	PDGFRL	protein_codin	0	0	0	0
ENSG000000	DHX29	protein_codin	238.928914	0	0	0
ENSG000001	DHX58	protein_codin	56.018639	76.7846379	37.9322323	29.7394653
ENSG000001	KRT12	protein_codin	0	0	0	0

ENSG00000(	KRT31	protein_codin	0	0	0	0
ENSG00000(	KRT33B	protein_codin	0	0	0	0
ENSG000001	PCCB	protein_codin	35.452308	66.9626729	49.7320639	33.298986
ENSG000001	ARFGEF3	protein_codin	3.11425037	1.02964155	0	0
ENSG00000(	USP40	protein_codin	0	0	0	0
ENSG00000(	VPS50	protein_codin	23.5449312	0	11.2049918	6.52454377
ENSG000001	SNX4	protein_codin	47.0733129	92.8037441	26.5299183	33.9838559
ENSG000001	ROPN1B	protein_codin	0	0	0	0
ENSG00000(	SPAG1	protein_codin	0	0	21.6263415	0
ENSG00000(	RRM2B	protein_codin	140.551137	108.526945	84.3751596	86.2766589
ENSG00000(	RHOBTB2	protein_codin	38.2767906	35.9063768	0	17.4589645
ENSG00000(	RAF1	protein_codin	0	0	0	22.3980597
ENSG00000(	TDRD1	protein_codin	0	0	0	0
ENSG00000(	MED17	protein_codin	6.47539476	7.65689939	1.75716348	15.6823257
ENSG00000(	RIC1	protein_codin	27.060255	13.024278	15.1456795	7.9826221
ENSG00000(	SCML2	protein_codin	4.39592779	0	0	0
ENSG00000(	NXPE1	protein_codin	0	0	1.15369438	0
ENSG00000(	TSG101	protein_codin	122.829917	143.597699	41.7514669	59.9195338
ENSG00000(	CARD10	protein_codin	0.76424887	0	0	0
ENSG00000(	KIAA0930	protein_codin	0	0	0	46.5537383
ENSG00000(	DHX35	protein_codin	0	74.0795331	16.3378234	26.9604547
ENSG00000(	TRPC4AP	protein_codin	335.242973	368.457228	131.26931	176.025293
ENSG00000(	TYMP	protein_codin	707.973461	1033.8804	96.5002123	604.233157
ENSG00000(	DNAAF9	protein_codin	200.956048	165.875612	71.4518216	78.6931091
ENSG00000(	ELN	protein_codin	0	0	0	0
ENSG00000(	FKBP6	protein_codin	0	0	0	0
ENSG000001	CUL9	protein_codin	115.201497	228.504986	75.1139184	84.5618961
ENSG00000(	ACTR3C	protein_codin	0	0	0	0
ENSG00000(	PCDHGA12	protein_codin	0	0	0.89975925	0
ENSG00000(	PCDHGC5	protein_codin	0	0	0	0
ENSG000001	TMCO6	protein_codin	0	0	3.76920549	0
ENSG00000(	NDUFA2	protein_codin	64.4945039	58.3595465	38.2096354	33.0458293
ENSG000001	UBE4A	protein_codin	477.000693	367.322953	266.382263	407.706767
ENSG00000(	POLDIP3	protein_codin	0	175.0304	22.0422965	61.1281724
ENSG00000(	MICAL3	protein_codin	0	0	0	1.96168097
ENSG00000(	TRMT2A	protein_codin	44.3961381	0	0	0
ENSG00000(	ESS2	protein_codin	3.12888955	18.9872478	5.08199958	5.58127652
ENSG00000(	MYH13	protein_codin	0	0	0	0
ENSG00000(	ZSCAN9	protein_codin	0	16.236313	0	14.2347378
ENSG00000(	ZKSCAN3	protein_codin	2.82588702	19.180658	2.88244482	2.88326861
ENSG00000(	MICB	protein_codin	0	0	0	0
ENSG00000(	KRT5	protein_codin	0.91664257	2.45340057	20.9891636	0
ENSG00000(	KRT1	protein_codin	0.95701187	0	0	0
ENSG00000(	KRT75	protein_codin	0	0	0	0

ENSG000001	KRT6C	protein_codin	0	0	0	0
ENSG000001	KRT6B	protein_codin	0	0	0	0
ENSG000001	DPF2	protein_codin	0	32.9458274	0	0
ENSG000001	GAMT	protein_codin	1.46696048	2.03769639	1.39305682	0
ENSG000001	GALNT8	protein_codin	0	1.08218202	1.05472557	1.01738226
ENSG000001	KCNA5	protein_codin	0	0.9582343	3.46451177	0
ENSG000001	CRACR2A	protein_codin	27.6146303	28.3558974	10.2685482	19.7344435
ENSG000001	PSMG3	protein_codin	0	0	0	0
ENSG000001	STARD8	protein_codin	0	0	0	0
ENSG000001	NALF2	protein_codin	0	0	0.93513351	0
ENSG000001	ECSIT	protein_codin	0	0	0	0
ENSG000001	LDLR	protein_codin	282.909645	268.909735	37.5941741	79.6391881
ENSG000001	ELOF1	protein_codin	41.9771618	61.1740394	26.6901847	24.6692604
ENSG000001	ANGPTL8	protein_codin	0	0	0	0
ENSG000001	CNN1	protein_codin	0	24.6560415	20.9562687	0
ENSG000001	CDC16	protein_codin	0	0	0	20.4599209
ENSG000001	CDC16	protein_codin	0	55.2154508	28.3218997	20.6014616
ENSG000001	ZSCAN10	protein_codin	0	0	0	0
ENSG000001	NECTIN2	protein_codin	63.4667512	70.7880394	16.9023064	27.3008109
ENSG000001	NECTIN2	protein_codin	47.5281103	84.000994	25.3924392	13.2453785
ENSG000001	APOE	protein_codin	2.74800214	2.70986254	0	0.84734505
ENSG000001	TOMM40	protein_codin	0	12.9321893	21.2075489	6.94906828
ENSG000002	APOC2	protein_codin	0	0	0	0
ENSG000001	ALLC	protein_codin	0	0.83105563	0	0
ENSG000001	GADD45G	protein_codin	3.77374209	3.78165538	3.1654822	0
ENSG000001	XPO7	protein_codin	371.067899	562.910746	276.841658	272.581919
ENSG000001	ACE2	protein_codin	0	0	0	0
ENSG000002	INF2	protein_codin	0	89.6353737	31.1237865	25.9085216
ENSG000001	FAM98C	protein_codin	11.9204152	18.4149108	11.4300107	10.5115675
ENSG000001	SAFB2	protein_codin	434.863021	758.25607	438.235331	342.341589
ENSG000001	NCAN	protein_codin	0	0	0	0
ENSG000001	PLVAP	protein_codin	1.95735652	2.50952769	10.2471034	0.97531075
ENSG000001	BST2	protein_codin	289.524833	509.446338	142.412092	200.486882
ENSG000001	NSUN5	protein_codin	3.07709201	4.5213204	6.05018115	1.58140985
ENSG000001	SLC27A1	protein_codin	112.602622	168.540513	32.6470855	62.3307543
ENSG000001	USHBP1	protein_codin	0	0	0	0
ENSG000001	COLGALT1	protein_codin	746.587195	859.573826	315.596967	439.7395
ENSG000001	MRPL34	protein_codin	15.2218352	54.8378072	12.6410626	14.1748286
ENSG000001	PGLS	protein_codin	158.691786	223.184796	32.7100879	116.425763
ENSG000001	LSM7	protein_codin	22.8599276	32.6209351	21.4172047	24.7558069
ENSG000001	MAS1	protein_codin	0	2.44809962	0.92222474	2.12766049
ENSG000001	MLLT1	protein_codin	312.665047	369.573202	132.635956	169.199601
ENSG000001	FUT5	protein_codin	0	0	0	0
ENSG000001	BMP15	protein_codin	0	0	0	0

ENSG000001:	ACTN4	protein_codin	544.488019	724.094238	290.355487	420.45374
ENSG000001:	NDUFA10	protein_codin	0	0	0	0
ENSG000001!	ZNF655	protein_codin	27.3622026	37.6886174	0	39.8576577
ENSG000001:	EPO	protein_codin	0	0	0	0
ENSG000001:	CACNG6	protein_codin	0	0	0	0
ENSG000001:	ZSWIM6	protein_codin	549.265371	606.311515	239.015656	341.792775
ENSG000002:	SCO2	protein_codin	32.3879946	5.79975829	0	4.43845919
ENSG000001(	ZNF764	protein_codin	6.20301282	17.5897422	6.67993586	0
ENSG000001(	ZNF747	protein_codin	17.351368	26.3737881	8.06778865	1.6637942
ENSG000001:	PXDN	protein_codin	0	1.88758175	0.86897374	0
ENSG000001:	GDF15	protein_codin	2.40772587	0	0	0
ENSG000001:	PGPEP1	protein_codin	0	11.840763	0	4.3490224
ENSG000001:	LSM4	protein_codin	58.5555674	92.4323972	47.0552394	47.8831904
ENSG000001:	JUND	protein_codin	1547.48366	1473.77997	418.151563	836.599202
ENSG000001:	HRC	protein_codin	0	0	0	0
ENSG000001:	TRPM4	protein_codin	0	5.47575241	0	2.5247838
ENSG000001:	ZNF557	protein_codin	29.0326139	50.7332467	15.0772834	36.8078912
ENSG000001:	OLFM1	protein_codin	5.22065049	6.97665081	0	0
ENSG000001(	NUMBL	protein_codin	11.8903475	13.9359557	0	6.53849515
ENSG000001:	TNNI2	protein_codin	0	39.059944	0	23.5890962
ENSG000002!	CYP2G1P	lncRNA	0.93669206	0	0	0
ENSG000001:	ATXN10	protein_codin	124.19687	125.947146	26.8318445	102.109736
ENSG000001:	TUBGCP2	protein_codin	3.63603638	0	0	0
ENSG000001:	CALY	protein_codin	0	0	0	0
ENSG000001:	CYP2E1	protein_codin	0	0	0	0
ENSG000001:	HBZ	protein_codin	0	0	0	0
ENSG000001:	MNX1	protein_codin	0	0	0	0
ENSG000001:	ZNF337	protein_codin	6.72061346	19.6231477	0	0
ENSG000002:	ALKBH6	protein_codin	0	0	0	0
ENSG000001:	CEP85	protein_codin	0	0	0	0
ENSG000001:	TAF4	protein_codin	122.441198	127.529398	37.1878393	61.3120828
ENSG000001:	GATA5	protein_codin	0	0	0	0
ENSG000001:	RBBP8NL	protein_codin	0	0	0	0
ENSG000001:	LAMA5	protein_codin	2.04404376	9.06678013	24.1567635	0
ENSG000001:	ADRM1	protein_codin	76.5000668	133.721481	81.5360589	98.9050581
ENSG000001:	PRDM12	protein_codin	0	0	0	0
ENSG000001:	UBE2M	protein_codin	71.8391417	126.227137	50.3651298	65.9487005
ENSG000001:	TRIM28	protein_codin	119.737612	118.104215	23.1510979	30.0333656
ENSG000001:	YIPF2	protein_codin	31.0805923	46.8260432	0	25.0239352
ENSG000001:	EIF2S3	protein_codin	1154.46847	1293.15562	594.025464	818.494122
ENSG000001:	TMEM160	protein_codin	40.3233266	42.3437349	8.10440743	20.603103
ENSG000001:	ZC3H4	protein_codin	228.987405	413.301618	150.735689	176.294831
ENSG000001:	GMFG	protein_codin	0	15.8028805	0	7.44802586
ENSG000001:	MAP3K10	protein_codin	46.7218012	51.9183875	14.6928128	19.1814822

ENSG000001	SESN2	protein_codin	39.7522474	41.6768314	20.7564704	54.0899408
ENSG000001	CCDC62	protein_codin	0	0	0	1.56273557
ENSG000001	HIP1R	protein_codin	4.73778946	3.1646887	13.6481241	0
ENSG000001	MRPL4	protein_codin	69.5000122	72.9499048	24.8262032	12.1019681
ENSG000001	PPAN	protein_codin	12.4659711	26.6422514	22.3886843	14.5664412
ENSG000001	EIF3G	protein_codin	373.249241	653.025991	144.861552	300.984801
ENSG000001	ANGPTL6	protein_codin	3.28927985	0	0	0
ENSG000001	SHFL	protein_codin	35.79825	74.6919964	0	25.9607445
ENSG000001	ZNF426	protein_codin	56.0796426	36.3601489	19.0488117	23.9453566
ENSG000001	SLC6A8	protein_codin	7.9155797	4.07423802	0.86474496	8.68683352
ENSG000001	ZNF331	protein_codin	2.68776305	4.81085029	0	0
ENSG000001	ZNF236	protein_codin	144.748064	141.861275	73.9026519	125.385392
ENSG000001	SLC7A10	protein_codin	2.79303137	0	0	0
ENSG000001	LRP3	protein_codin	59.781965	89.3416391	24.0897728	33.4854455
ENSG000001	MTRFR	protein_codin	6.75221977	14.1801323	0	6.0520705
ENSG000001	GRWD1	protein_codin	30.7943362	76.4516246	28.5488385	16.0928514
ENSG000001	NOL11	protein_codin	72.7795084	70.8594284	0	41.6086508
ENSG000001	UBE4B	protein_codin	83.3236803	160.772845	117.407163	96.8921481
ENSG000001	PKDREJ	protein_codin	0	2.13172152	0	0
ENSG000001	NUTM2F	protein_codin	0	0	0	0
ENSG000001	SLC35D2	protein_codin	69.087819	64.559985	28.0970475	40.2037531
ENSG000002	TXLNGY	lncRNA	0	0	0	0
ENSG000001	PPIL4	protein_codin	229.61458	170.700739	147.534214	149.338969
ENSG000001	AKAP12	protein_codin	0	17.8320202	0	0
ENSG000001	LATS1	protein_codin	51.615627	83.6744583	55.6216708	53.3950196
ENSG000001	BPIFB1	protein_codin	0	0	0	0
ENSG000001	BPIFA2	protein_codin	0	0	0	0
ENSG000001	RBM39	protein_codin	0	0	0	0
ENSG000001	DEFB118	protein_codin	0	0	0	0
ENSG000001	ACSS2	protein_codin	0	0	0	0
ENSG000001	EDA2R	protein_codin	0	0	0	0
ENSG000001	ARHGEF9	protein_codin	0	0	0	0
ENSG000001	GFAP	protein_codin	0	6.0102539	0	0
ENSG000001	HIGD1B	protein_codin	0	0	0	0
ENSG000001	ATP6V1E1	protein_codin	194.533509	286.808452	84.705818	143.059581
ENSG000001	CCL25	protein_codin	0	0	0	0
ENSG000001	COX4I1	protein_codin	915.981345	1266.91667	450.588568	556.086252
ENSG000001	EMC8	protein_codin	14.8989527	52.7699977	6.99054442	20.4688032
ENSG000001	GSE1	protein_codin	685.89952	839.47081	407.2408	340.694005
ENSG000002	C16orf95	protein_codin	4.75924908	0	0	0
ENSG000001	GINS2	protein_codin	1.03852385	3.14787427	6.79640049	0
ENSG000002	TTTY11	lncRNA	0	0	0	0
ENSG000001	F12	protein_codin	0	0	0	0
ENSG000001	NFATC1	protein_codin	67.7040323	0	0	16.9286479

ENSG000001	IDO1	protein_codin	0	0	0	0
ENSG000001	ABCB7	protein_codin	69.6608056	0	10.0332821	26.5377737
ENSG000001	HAUS8	protein_codin	10.7949899	17.5323763	4.53200522	5.37070279
ENSG000001	ADGRE3	protein_codin	25.1083807	89.2292948	24.9048425	73.3679729
ENSG000001	MRPS25	protein_codin	0	0	0	0
ENSG000001	TBC1D5	protein_codin	69.6898881	50.437605	4.13043066	18.786648
ENSG000001	CAPN7	protein_codin	148.184844	110.262881	72.1738613	94.7229222
ENSG000001	C3orf20	protein_codin	0	0	0	0.84035327
ENSG000001	RBSN	protein_codin	0	0	50.8693939	0
ENSG000001	NAPSA	protein_codin	0	0	0	0
ENSG000001	NR1H2	protein_codin	300.366715	460.745761	168.35681	261.557899
ENSG000001	PDLIM4	protein_codin	0	0	0	0
ENSG000001	GFPT2	protein_codin	0	0	0	0
ENSG000001	RPL27	protein_codin	1713.1718	2698.69736	618.410624	1339.87062
ENSG000001	PSMC3IP	protein_codin	0	0	0	3.7291425
ENSG000001	VPS25	protein_codin	63.4896081	67.7828612	31.5249078	38.9582718
ENSG000001	RAMP2	protein_codin	0	0	0	0
ENSG000001	AOC2	protein_codin	0	3.06773277	0	0
ENSG000001	G6PC1	protein_codin	0	0	0	0
ENSG000002	PCDHAC1	protein_codin	0	0	0	0
ENSG000002	PCDHGA3	protein_codin	0	0	0	0
ENSG000001	NDFIP1	protein_codin	368.653403	448.382047	145.350572	271.651898
ENSG000001	TTTY6B	lncRNA	0	0	0	0
ENSG000001	EXOC4	protein_codin	157.638567	180.379948	82.4972129	127.826152
ENSG000001	PPFIA1	protein_codin	105.487023	334.446704	141.642316	161.654902
ENSG000000	FLYWCH1	protein_codin	21.2219522	79.9768651	7.49033919	23.7204511
ENSG000001	TMEM204	protein_codin	0	10.9749994	6.33688902	3.59551965
ENSG000001	THOC6	protein_codin	0	4.75635652	0	15.5561215
ENSG000001	BARX1	protein_codin	0	0	0	0
ENSG000001	WDR44	protein_codin	200.495102	239.352159	122.140038	130.93095
ENSG000001	CKMT2	protein_codin	0	0	0	0
ENSG000001	ZCCHC9	protein_codin	13.3348611	12.6032921	10.1296357	11.7803174
ENSG000001	KRT15	protein_codin	0	0	0	0
ENSG000001	TNS4	protein_codin	0.86289863	0.85194816	0	0
ENSG000001	RARA	protein_codin	297.984585	358.895877	178.025587	118.133415
ENSG000001	KRTAP9-8	protein_codin	0	0	0	0
ENSG000001	PPP1R1B	protein_codin	0	0	0	0
ENSG000001	FMO5	protein_codin	35.5340577	45.1332076	18.570821	33.8696606
ENSG000001	PRKAB2	protein_codin	79.6480821	149.954862	70.2179205	57.2209773
ENSG000000	FUS	protein_codin	0	0	0	0
ENSG000002	ENSG000002	lncRNA	0	0	0	0
ENSG000001	FSHB	protein_codin	0	0	0	0
ENSG000001	ZNF132	protein_codin	0	7.58544045	0	5.79424285
ENSG000001	USP29	protein_codin	0	0	0	0

ENSG000001	ZNF211	protein_codin	8.59405404	0	0	0
ENSG000001	CHSY1	protein_codin	231.35277	265.656157	95.0950659	176.726227
ENSG000001	SNRPA1	protein_codin	34.5782131	21.1076676	40.481729	24.4431544
ENSG000001	NR0B2	protein_codin	0	0	0	0
ENSG000001	LIN28A	protein_codin	0	0	0	0
ENSG000001	ADCY7	protein_codin	569.433389	994.371641	260.825181	509.394641
ENSG000001	THAP1	protein_codin	32.6795904	54.5577835	23.4341888	36.8930457
ENSG000001	RHPN2	protein_codin	0	1.02094771	0	0
ENSG000001	FAAP24	protein_codin	0	0	0	0
ENSG000001	LRRC9	protein_codin	0	0	0	0
ENSG000001	ACTR10	protein_codin	25.2796662	40.8983834	7.27768993	19.5780542
ENSG000001	GCH1	protein_codin	0	153.857279	0	0
ENSG000001	LGALS3	protein_codin	557.538802	629.809469	150.550328	271.32984
ENSG000001	UBE2L2	processed_ps	0	0	0	0
ENSG000001	PODNL1	protein_codin	0	0.90565714	0	0
ENSG000001	ZNF700	protein_codin	0	7.70567491	5.15309454	47.9968388
ENSG000001	DNAJB1	protein_codin	130.479914	217.659271	82.6719618	94.8123946
ENSG000001	ZSWIM4	protein_codin	0	0	0	0
ENSG000001	RFX1	protein_codin	123.542105	158.11835	36.7397731	67.0869243
ENSG000001	DCAF15	protein_codin	96.4130419	133.979986	36.7013364	52.6404032
ENSG000001	SDC1	protein_codin	2.81734826	2.82099707	0	0
ENSG000001	TRIM21	protein_codin	99.8246296	238.004205	75.2756933	95.5309495
ENSG000000	WDR7	protein_codin	15.463473	48.3677454	0	177.929384
ENSG000001	SMARCC1	protein_codin	255.410841	241.204789	159.450781	174.192272
ENSG000001	SLC6A11	protein_codin	0	3.14705281	0	0
ENSG000001	NUP210	protein_codin	349.759625	635.487621	301.672025	277.142326
ENSG000001	HSD17B7	protein_codin	0	13.3088918	11.1565955	10.8743228
ENSG000001	EMILIN2	protein_codin	2616.31154	3130.73927	1180.8862	1750.52559
ENSG000001	DNHD1	protein_codin	89.2440132	90.6085781	49.8960101	39.9464675
ENSG000001	ARFIP2	protein_codin	0	0	0	0
ENSG000001	RRP8	protein_codin	59.2356131	94.797949	47.5407517	54.9681896
ENSG000001	TIMM10B	protein_codin	158.207522	201.972453	80.2592735	92.6343916
ENSG000001	EFR3A	protein_codin	241.755282	178.807238	99.2451238	254.251312
ENSG000002	OC90	protein_codin	0	0	0	0
ENSG000001	PTCD3	protein_codin	136.14733	109.471093	89.0949127	85.9873163
ENSG000001	IMMT	protein_codin	34.2009292	0	0	0
ENSG000001	MRPL35	protein_codin	9.04285055	0	0	0
ENSG000001	IQCA1	protein_codin	0	0	0	0
ENSG000001	ILKAP	protein_codin	0	26.0111891	16.1518122	35.7377028
ENSG000001	PER2	protein_codin	0	0	46.5611926	0
ENSG000001	RAMP1	protein_codin	4.60052409	6.37309499	3.01115522	0
ENSG000001	SCLY	protein_codin	11.0673509	17.2776344	0	0
ENSG000001	PTPRE	protein_codin	86.8683788	56.2829956	9.40975155	12.4291975
ENSG000001	CARD6	protein_codin	99.3130009	179.427674	52.65232	90.7600793

ENSG000001:	RAP1GAP2	protein_codin	145.101723	33.3401622	0	0
ENSG000001:	MYBBP1A	protein_codin	0	0	10.2026348	0
ENSG000001:	RPA1	protein_codin	145.580609	154.204883	84.2357677	112.897943
ENSG000001:	SERPINF1	protein_codin	7.26041817	4.03052163	17.509217	0
ENSG000002:	ENSG000002:	processed_ps	0	0	0	0
ENSG000001:	EEFSEC	protein_codin	56.9094055	62.9889731	15.6505306	27.3650366
ENSG000001:	TMEM128	protein_codin	4.42573077	6.01941742	4.24880607	6.00230388
ENSG000001:	COQ3	protein_codin	0	9.47704172	3.65189899	0
ENSG000001:	POPDC3	protein_codin	0	0	0	0
ENSG000001:	LANCL2	protein_codin	36.3526099	52.6802842	13.2284507	19.7824471
ENSG000001:	GRSF1	protein_codin	35.3545194	54.7915011	20.4318596	46.5088508
ENSG000001:	JCHAIN	protein_codin	766.286435	516.618254	2941.25866	226.513358
ENSG000001:	UTP3	protein_codin	154.84188	203.796998	123.894644	114.808713
ENSG000001:	WBP2	protein_codin	464.317186	672.768988	160.460487	294.591717
ENSG000001:	H3-3B	protein_codin	1581.6638	1489.52697	639.953796	923.450552
ENSG000001:	TRIM47	protein_codin	2.38845658	0	0	0
ENSG000001:	ZRANB2	protein_codin	42.5211169	32.4010436	29.6679165	57.6851595
ENSG000001:	KDM6B	protein_codin	1188.45437	475.358706	70.5930193	562.620462
ENSG000001	ASGR2	protein_codin	279.48392	335.507966	145.078538	157.706241
ENSG000001:	SLC52A1	protein_codin	0	0	0	0
ENSG000001:	GUCY2D	protein_codin	0	5.63709669	1.61439866	0.91181649
ENSG000001:	CLEC10A	protein_codin	6.90432954	60.3272635	14.5934883	23.6770123
ENSG000001:	RIDA	protein_codin	14.4716444	6.13955773	8.86996492	7.12040068
ENSG000001:	MATN2	protein_codin	0	0	0	0
ENSG000001	BRD8	protein_codin	0	0	0	1.85435525
ENSG000001:	REEP2	protein_codin	0	0	0	0
ENSG000001:	PCBD2	protein_codin	33.9672239	23.8569063	17.3750473	20.1597735
ENSG000001:	ERAL1	protein_codin	41.9017303	29.1809266	9.34030882	32.4499047
ENSG000001:	NIP7	protein_codin	18.5654469	30.4785114	0	2.47971872
ENSG000001:	NIP7	protein_codin	0	12.0236043	5.5427055	7.33793681
ENSG000001:	TERF2	protein_codin	113.153049	137.104794	40.7052425	32.5298372
ENSG000001:	VPS4A	protein_codin	0	0	0	0
ENSG000001	JAG1	protein_codin	45.9875725	49.9864947	20.2234173	32.5621737
ENSG000001:	HSPA12B	protein_codin	0.78864844	0	0	0
ENSG000001:	SNAP25	protein_codin	0	0	0	0
ENSG000001:	BTBD3	protein_codin	42.548468	41.4772603	0	10.3658653
ENSG000001:	NXT1	protein_codin	52.118573	36.937661	14.68573	17.8066223
ENSG000001:	RIN2	protein_codin	305.461522	200.897364	92.7926303	129.415264
ENSG000001:	SSTR4	protein_codin	0	0	0	0
ENSG000001:	CRP	protein_codin	0	0	0.9914385	0
ENSG000001:	HAPLN2	protein_codin	0	0	0	0
ENSG000001:	APCS	protein_codin	0	0	1.37775278	0
ENSG000001:	IGHMBP2	protein_codin	35.2393393	40.3945054	9.51402876	33.8840344
ENSG000001:	ACY3	protein_codin	0	0.82139666	0	0

ENSG000001	TESMIN	protein_codin	0	0	3.72848152	0
ENSG000001	DPH2	protein_codin	14.600421	29.8131987	0	0
ENSG000001	PABPC1L	protein_codin	0	1.82660119	2.18461173	5.27699343
ENSG000001	ZSWIM3	protein_codin	3.95418418	8.70944724	3.88571474	1.99549686
ENSG000001	OSER1	protein_codin	152.188131	235.082252	106.233375	156.818981
ENSG000001	SERINC3	protein_codin	10.3711712	15.15077	5.87477988	0
ENSG000002	KCNK15-AS1	lncRNA	1.59720581	0	0	0.97046235
ENSG000001	DMGDH	protein_codin	0	0	0	0
ENSG000001	BHMT2	protein_codin	0	0	0	0
ENSG000001	AP3B1	protein_codin	138.482435	0	0	221.150224
ENSG000001	ZBED3	protein_codin	16.8222735	56.740085	13.0395508	29.8281157
ENSG000001	SYT4	protein_codin	0	0	0	0
ENSG000001	SLC14A2	protein_codin	0	0	2.97357141	0
ENSG000001	NMUR2	protein_codin	0	0	0	0
ENSG000001	PDE6A	protein_codin	0	0	0	0
ENSG000001	MTUS2	protein_codin	0	0	0	0
ENSG000001	USPL1	protein_codin	81.9530408	30.9604792	63.2456547	46.4048609
ENSG000001	XPO4	protein_codin	138.468578	90.5678039	94.0070334	91.1952213
ENSG000001	HMGB1P5	transcribed_p	0	0	0	0
ENSG000001	RNF17	protein_codin	0	0	0	0
ENSG000001	RNF17	protein_codin	0	0	0	0
ENSG000001	RNF17	protein_codin	0	0	0	0
ENSG000001	CHRM3	protein_codin	2.21203838	2.23267124	0	2.29596292
ENSG000002	MYH4	protein_codin	1.59559087	0	0	0
ENSG000001	PEMT	protein_codin	22.0399388	30.6826699	5.73713395	10.2912904
ENSG000001	SCO1	protein_codin	59.8511114	152.839982	47.123841	48.3045656
ENSG000001	CHI3L1	protein_codin	0	0.90663947	1.00741435	0
ENSG000001	MYBPH	protein_codin	0	0	0	0
ENSG000001	CHIT1	protein_codin	0	0	0	0
ENSG000001	LGR6	protein_codin	0	0	0	0
ENSG000001	DCLK1	protein_codin	0	0	0	0
ENSG000001	CCNA1	protein_codin	0	0	0	0
ENSG000001	RFXAP	protein_codin	4.75581568	13.2940276	7.34856471	8.00945649
ENSG000001	STARD13	protein_codin	0	0	0	0
ENSG000001	MORC4	protein_codin	7.92511911	0	0	0
ENSG000001	RNF128	protein_codin	0	0	0	0
ENSG000002	GNG5P1	processed_ps	0	0	0	0
ENSG000001	PCDH19	protein_codin	0	0	0	0
ENSG000001	MTNAP1	protein_codin	8.86576401	0	5.38462186	5.96055444
ENSG000001	SLC39A11	protein_codin	26.3137011	0	0	0
ENSG000001	BTBD2	protein_codin	113.395156	218.392923	89.7893033	105.660728
ENSG000001	KMT5C	protein_codin	14.4230977	16.4825708	0	7.40334599
ENSG000001	ZNF414	protein_codin	36.2028789	14.1771189	0	6.51980342
ENSG000001	PDE6B	protein_codin	6.37731742	0	0	3.03596544

ENSG000001:	HSPBP1	protein_codin	0	0	0	0
ENSG000001:	CSNK1G2	protein_codin	372.083777	382.159686	87.7870614	169.62505
ENSG000001	RTTN	protein_codin	0	0	15.740362	0
ENSG000001:	MACROD1	protein_codin	0	3.17016354	0	0
ENSG000001:	LGALS12	protein_codin	0	0	0	0
ENSG000001:	PLAAT4	protein_codin	67.3951557	72.4554964	58.740759	44.9060641
ENSG000001:	PLAAT2	protein_codin	0.96745396	2.84963562	22.7398627	1.9094155
ENSG000001!	ZNF679	protein_codin	0	0	0	0
ENSG000001:	CEP20	protein_codin	77.8578692	69.5663324	0	49.4325063
ENSG000001:	MED10	protein_codin	36.8602102	54.8513063	33.6870125	27.253231
ENSG000001(	CCDC134	protein_codin	171.864974	134.139123	60.577855	75.150879
ENSG000001:	SLC2A11	protein_codin	0	0	0	0
ENSG000001:	SEC14L4	protein_codin	0	0	0	0
ENSG000002	PI4KA	protein_codin	593.003388	764.551432	300.660837	358.487223
ENSG000002!	ENSG000002!	lncRNA	0	0	0	0
ENSG000001:	GIMAP4	protein_codin	722.809691	1155.91247	285.244907	737.306035
ENSG000001:	MKRN1	protein_codin	561.260545	947.435403	286.517662	512.149554
ENSG000001:	ACTR3B	protein_codin	13.2368221	39.7945767	21.1007141	17.2357545
ENSG000001:	NTS	protein_codin	0	0	0	0
ENSG000001:	BTG1	protein_codin	1941.55044	1243.7571	664.789736	1294.89785
ENSG000001:	DYDC2	protein_codin	0	0	0	0
ENSG000001:	TMTC1	protein_codin	0	0	0	0
ENSG000001:	KRAS	protein_codin	41.0279003	0	0	23.0099575
ENSG000001:	IPO8	protein_codin	192.435166	148.59827	86.6376999	160.992308
ENSG000001:	SPINK5	protein_codin	0	0	0	0
ENSG000001	PMP2	protein_codin	0	0	0	0
ENSG000001	FABP4	protein_codin	0	0	0	0
ENSG000001:	IMPA1	protein_codin	24.2081087	9.70847733	6.29246239	18.143243
ENSG000001:	E2F5	protein_codin	1.50514515	0	1.34968859	0
ENSG000001:	CCDC59	protein_codin	66.1809691	22.9692282	0	34.3638567
ENSG000001:	LYVE1	protein_codin	0	0	0	0
ENSG000001:	SBF2	protein_codin	0	44.5340589	0	0
ENSG000001:	MICAL2	protein_codin	0	48.1394759	6.99020325	0
ENSG000001:	RRAS2	protein_codin	0	0	0	0
ENSG000001:	TEX15	protein_codin	1.21077653	0	4.92124587	2.49706275
ENSG000001:	SARAF	protein_codin	565.957113	685.878206	295.989805	389.177081
ENSG000001:	RNF122	protein_codin	21.8330571	24.4439183	14.1460099	8.92007325
ENSG000001:	DUSP26	protein_codin	0	0	0	0
ENSG000001:	ERG28	protein_codin	5.04287775	17.9193829	6.51305377	8.19782985
ENSG000001:	DGLUCY	protein_codin	153.697828	236.917989	11.3346817	75.1410227
ENSG000001:	UNC79	protein_codin	0.97409077	0.97578426	0	0
ENSG000001:	CATSPERB	protein_codin	0	1.93369773	4.24741303	5.2272319
ENSG000001:	VRTN	protein_codin	0	0.82965029	0	0
ENSG000002	SYNJ2BP	protein_codin	114.70082	90.5659252	49.986127	111.983535

ENSG000001	TTC9	protein_codin	59.8226109	17.0232791	6.7515181	15.9816026
ENSG000001	MED6	protein_codin	47.6446318	35.5437034	16.791458	30.8201902
ENSG000001	EIF2S1	protein_codin	89.9431797	109.120166	57.6250698	45.3765213
ENSG000001	ADAM20	protein_codin	0	0	0	2.41904641
ENSG000001	ELP3	protein_codin	46.5942437	63.5368633	30.353298	17.1278871
ENSG000001	PEBP4	protein_codin	0	0	0	0
ENSG000001	ADAMDEC1	protein_codin	7.51635104	7.47790339	0	10.9862062
ENSG000001	CTIF	protein_codin	134.806323	157.483226	64.3890543	71.7506679
ENSG000001	MRO	protein_codin	0	0	3.19242553	0
ENSG000001	MBD2	protein_codin	727.777803	850.147555	320.779534	492.170396
ENSG000001	IER3IP1	protein_codin	58.6442865	40.6194736	49.0766228	31.4224683
ENSG000001	KGD4	protein_codin	31.3032409	42.7802667	15.8128388	9.31115683
ENSG000001	CCNB1	protein_codin	6.15121763	0	0	0
ENSG000001	CDK7	protein_codin	0	0	17.2338211	0
ENSG000001	CD180	protein_codin	69.2761137	103.836498	49.5567998	67.8032829
ENSG000000	IL5RA	protein_codin	0	0	3.33995885	0
ENSG000001	IRAK2	protein_codin	155.824529	80.195486	57.5931371	42.7911436
ENSG000001	CAMK1	protein_codin	159.295099	247.661567	66.7821608	59.1602164
ENSG000001	VHL	protein_codin	223.738654	353.995887	109.05396	171.888866
ENSG000001	BHLHE40	protein_codin	950.155683	735.925566	193.794667	514.253396
ENSG000001	ARL8B	protein_codin	491.112631	461.821706	149.511156	352.507738
ENSG000001	EDEM1	protein_codin	434.904248	409.914957	304.25373	261.64542
ENSG000001	CHL1	protein_codin	0	0	0	0
ENSG000001	DPH6	protein_codin	12.8121893	8.07970902	8.84396145	0
ENSG000001	KATNBL1	protein_codin	34.8225604	30.2282189	24.1107569	11.9779734
ENSG000001	EMC7	protein_codin	82.3111355	103.480222	34.2649042	61.6853447
ENSG000001	TRPM1	protein_codin	0	0	0	0
ENSG000001	AMPD2	protein_codin	93.0636029	97.8334887	44.6707039	11.2838366
ENSG000001	REG4	protein_codin	0	0	0	0
ENSG000001	TSHB	protein_codin	0	0	0	0
ENSG000001	GSTM5	protein_codin	0	3.6629162	1.70191468	0
ENSG000001	GSTM3	protein_codin	9.10181682	8.18743955	5.87671231	0
ENSG000001	SORT1	protein_codin	495.728378	812.157422	109.528286	419.633669
ENSG000001	WNT2B	protein_codin	19.549525	6.73578912	2.54234955	3.02007151
ENSG000001	LAMTOR5	protein_codin	5.1358956	9.89585272	1.11128204	7.45772479
ENSG000001	NOTCH2	protein_codin	3285.74883	3598.957	1311.98946	2050.64402
ENSG000001	TRIM45	protein_codin	0	2.7339365	0	0
ENSG000001	CD101	protein_codin	28.9466084	197.511848	34.7917511	31.0570413
ENSG000002	VTCN1P1	processed_ps	0	0	0	0
ENSG000001	AP4B1	protein_codin	0	0	0	0
ENSG000001	PPHLN1	protein_codin	0	3.9126008	5.31380416	0
ENSG000001	FKBP11	protein_codin	0	12.5818852	0	1.17711452
ENSG000001	ARF3	protein_codin	435.499985	653.360718	195.701139	296.740376
ENSG000001	TMEM106C	protein_codin	10.8418249	0	16.3106895	0

ENSG000001:	SLC38A2	protein_codin	382.007901	190.841497	181.231255	221.783723
ENSG000002:	PLEKHA8P1	lncRNA	0	35.1625485	0	0
ENSG000001:	KIDINS220	protein_codin	0	0	0	0
ENSG000001:	LPIN1	protein_codin	30.9024022	0	59.0938647	7.53798028
ENSG000001:	CMPK2	protein_codin	60.2257423	92.0065148	0	49.7899178
ENSG000001:	SAA2	protein_codin	0	0	0	0
ENSG000001:	ANO3	protein_codin	0	0	1.2650671	0
ENSG000001:	FST	protein_codin	0	0	0	0
ENSG000001:	CFHR5	protein_codin	0	0	0	0
ENSG000001:	ERN2	protein_codin	0	0	0	0
ENSG000000:	NEDD4L	protein_codin	0	0	0	0
ENSG000001:	RAX	protein_codin	0	0.81161099	0	0
ENSG000001:	NARS1	protein_codin	365.836272	381.407912	178.069642	308.248182
ENSG000001:	GRP	protein_codin	0	0	0	0
ENSG000001:	RELCH	protein_codin	0	0	80.353769	23.7443098
ENSG000001:	IL2RA	protein_codin	0	0	0	0
ENSG000001:	CCNH	protein_codin	27.0134396	0	26.2989022	32.7025235
ENSG000001:	HRH4	protein_codin	0	1.62586815	0.69224104	1.22549538
ENSG000001:	CABLES1	protein_codin	0	5.31377523	0	0
ENSG000001:	EMP1	protein_codin	86.6578806	7.82682298	4.76748278	11.3311213
ENSG000001:	RERG	protein_codin	0	0	0	0
ENSG000001:	SLCO1B1	protein_codin	0	0	0	0
ENSG000001:	SPX	protein_codin	2.81305127	0	1.95339899	2.37185524
ENSG000001:	DDB2	protein_codin	24.2243812	40.5316691	34.0137191	21.7435518
ENSG000001:	ACP2	protein_codin	0	0	0	0
ENSG000000:	FOLH1	protein_codin	0	0	0	0
ENSG000001:	RTL8C	protein_codin	90.2101793	117.145155	30.812633	43.6556144
ENSG000001:	RAB33A	protein_codin	2.93412763	4.1662585	1.71730336	1.0525081
ENSG000002	RBMX2P2	processed_ps	0	0	0	0
ENSG000001:	MTNR1B	protein_codin	0	0	0	0
ENSG000001:	PUM1	protein_codin	138.714286	76.9130478	126.913708	0
ENSG000001:	SPOCD1	protein_codin	5.45083399	92.107391	37.5280516	24.8203835
ENSG000001:	PHC2	protein_codin	0	0	0	0
ENSG000001:	TUT4	protein_codin	33.6346018	20.673174	20.7676241	14.9440311
ENSG000001:	PRPF38A	protein_codin	238.638044	198.311761	182.57798	177.314002
ENSG000001:	DSG3	protein_codin	3.40868892	1.14122965	0	1.17710842
ENSG000001:	RNF138	protein_codin	29.5967261	29.4545115	26.8548977	21.9535845
ENSG000001:	DSG1	protein_codin	0	0	0	0
ENSG000001:	DSC1	protein_codin	0	0	0	0
ENSG000001:	DSC1	protein_codin	0	0	0	0
ENSG000001:	FHOD3	protein_codin	0	0	0	0
ENSG000001:	DAGLA	protein_codin	63.6164564	37.7246153	0	0
ENSG000001:	TIMM10	protein_codin	21.28537	37.0223814	13.3107848	18.1130613
ENSG000001:	AHNAK	protein_codin	75.9227629	45.867464	18.0295924	21.5108771

ENSG000001	CBLIF	protein_codin	0	0	0	0
ENSG000001	APLNR	protein_codin	0	0	0	0
ENSG000001	FADS2	protein_codin	0	9.01999182	22.2718933	0
ENSG000001	TMEM258	protein_codin	0	4.70828553	0	2.91642622
ENSG000001	TCN1	protein_codin	1.47872044	3.28561851	3.31611893	1.42740286
ENSG000001	CEP135	protein_codin	69.017331	60.5262888	103.709413	53.5124639
ENSG000001	PDGFRA	protein_codin	13.9132018	15.8868995	9.68846627	2.66512067
ENSG000001	BIVM	protein_codin	0	9.96768291	10.5696444	17.4272385
ENSG000001	CARS2	protein_codin	137.716415	187.66753	78.0282311	106.997206
ENSG000001	ADAMTS8	protein_codin	0	0.84204087	0	0
ENSG000001	KLB	protein_codin	0	0	0	2.42986646
ENSG000000	SEMA6A	protein_codin	0	0	0	0
ENSG000001	APC	protein_codin	0	0	0	0
ENSG000001	NREP	protein_codin	15.5331769	62.0508034	14.7842665	0
ENSG000001	UBQLN1	protein_codin	80.9719037	181.838899	46.9592246	102.842336
ENSG000001	ANXA1	protein_codin	2518.6989	1378.63065	1212.2403	1315.74913
ENSG000001	GOLM1	protein_codin	0	4.60043967	27.2076203	0
ENSG000001	ENTREP1	protein_codin	0	0	0	0
ENSG000001	ADAM19	protein_codin	276.514341	265.468867	263.265153	197.939626
ENSG000001	CCNJL	protein_codin	0	0	2.79853802	0
ENSG000001	USP30	protein_codin	28.357368	47.0164445	26.251684	12.4968262
ENSG000001	SDS	protein_codin	0.93778605	0	1.76592199	0
ENSG000001	MSI1	protein_codin	0.97575274	0.97708038	0	0.95275643
ENSG000001	HNF1A	protein_codin	0	0	0	0
ENSG000001	TBX3	protein_codin	0	0	0	0
ENSG000001	OASL	protein_codin	0	14.5050764	7.62421129	7.68460993
ENSG000001	HRK	protein_codin	3.14716907	1.04333447	0	0
ENSG000001	RNFT2	protein_codin	0	3.94901956	5.0975208	0
ENSG000001	DTX1	protein_codin	0.78499963	0	0	0
ENSG000001	TRAFD1	protein_codin	2.89374787	25.8392826	6.01837918	7.16443334
ENSG000000	ASB4	protein_codin	0	0	0	0
ENSG000001	GSAP	protein_codin	160.232225	176.583618	89.8219938	114.228903
ENSG000001	OCM2	protein_codin	0	0	0	0
ENSG000002	UPK3B	protein_codin	0	0	0.89245519	0
ENSG000001	TMEM243	protein_codin	21.8368672	33.5180268	14.4337535	26.2667382
ENSG000001	GTPBP10	protein_codin	8.04268162	0	0	13.9359878
ENSG000001	TMEM60	protein_codin	7.94531408	24.9800698	3.40960181	9.12455923
ENSG000001	PNPLA8	protein_codin	69.9591124	23.3686024	41.6477203	78.9179432
ENSG000001	HILPDA	protein_codin	14.5124337	0	3.70938883	0
ENSG000001	RINT1	protein_codin	26.9253433	24.2508801	23.6089395	0
ENSG000000	KMT2E	protein_codin	359.286342	242.48977	223.876573	354.88814
ENSG000001	BACH2	protein_codin	7.04933208	7.08157008	5.13603084	0
ENSG000001	KHDC1	protein_codin	0	0	0	0
ENSG000001	CEP162	protein_codin	0	0	0	0

ENSG000001	NT5E	protein_codin	3.37197126	1.36651834	5.49231342	2.3504462
ENSG000001	MRAP2	protein_codin	1.0482253	1.08695497	1.20001632	2.18709888
ENSG000001	AKIRIN2	protein_codin	613.935754	715.318423	226.339413	360.72135
ENSG000001	ORC3	protein_codin	29.30532	0	8.10329249	0
ENSG000001	LMO2	protein_codin	21.6890261	34.2856439	12.9500346	46.897412
ENSG000001	NAT10	protein_codin	99.2144867	208.492815	72.5393243	79.7560623
ENSG000001	EHF	protein_codin	0	1.0910303	0	1.38155785
ENSG000001	ELF5	protein_codin	0	0	0	0
ENSG000001	PRRG4	protein_codin	130.826756	144.264118	74.3129312	148.489426
ENSG000000	METTL1	protein_codin	0	0	1.96612426	0
ENSG000001	CD63	protein_codin	364.092383	380.569615	37.464632	164.220302
ENSG000001	PRPH	protein_codin	0	0	0	0
ENSG000001	AVIL	protein_codin	11.1692581	5.84827661	2.73334092	0
ENSG000001	AMHR2	protein_codin	0	0	0	0
ENSG000001	LACRT	protein_codin	0	0	0	0
ENSG000001	GDF11	protein_codin	7.03736343	16.9353973	1.40538387	2.00167082
ENSG000001	ITGA7	protein_codin	0	0	0	0
ENSG000001	FAM186B	protein_codin	0	0	0	0
ENSG000001	RDH5	protein_codin	0	0	0	0
ENSG000001	AGAP2	protein_codin	35.1856216	58.1639619	30.0708256	38.2910455
ENSG000002	ENSG000002	protein_codin	0	0	0	0
ENSG000001	KRT85	protein_codin	0	0	0	0
ENSG000001	CDK4	protein_codin	20.0564144	38.7024911	15.7388763	12.4681743
ENSG000001	PPP1R1A	protein_codin	0.73507161	0	0	0
ENSG000001	TROAP	protein_codin	0	0	0	0
ENSG000001	TSPAN31	protein_codin	21.9493201	27.7942	14.9361226	12.6321741
ENSG000001	TFCP2	protein_codin	71.1436452	168.04548	85.6725136	66.738011
ENSG000001	PAN2	protein_codin	0	0	45.0072485	10.7241558
ENSG000001	ESPL1	protein_codin	14.1968761	0	14.6013412	3.3324545
ENSG000001	ZC3H10	protein_codin	57.772369	67.5119221	35.7670866	43.3033387
ENSG000001	KRT84	protein_codin	0	0	0	0
ENSG000001	ACVR1B	protein_codin	95.7819536	139.934385	37.6547204	78.2121262
ENSG000001	OS9	protein_codin	0	0	0	0
ENSG000001	KRT82	protein_codin	0	0	0	0
ENSG000001	KCNH3	protein_codin	160.163045	63.3016096	9.04117953	0
ENSG000001	PKIB	protein_codin	0	0	0	0
ENSG000001	TAAR5	protein_codin	0	0	0	0
ENSG000001	NMBR	protein_codin	0	1.32593499	0	0
ENSG000001	SMPD2	protein_codin	28.2990261	26.528853	9.77062041	17.2001962
ENSG000001	REPS1	protein_codin	38.4423483	32.608645	22.2796247	6.84491914
ENSG000002	ENSG000002	unprocessed_	0	0	0	0
ENSG000001	HTRA2	protein_codin	0	2.79630352	0	0
ENSG000001	PRADC1	protein_codin	10.8366969	13.6635626	7.57271037	2.92649437
ENSG000001	CCT7	protein_codin	173.77811	253.355546	120.587469	100.955736

ENSG000001	RAB11FIP5	protein_codin	7.18116712	3.86402279	0	0
ENSG000001	SMYD5	protein_codin	0	0	0	0
ENSG000001	DYSF	protein_codin	160.290041	89.0628944	83.199564	67.3752299
ENSG000002	MRPL53	protein_codin	77.3002351	77.1503664	29.5748537	42.9167115
ENSG000001	EMX1	protein_codin	0.86150959	0	0	0
ENSG000001	KCNMB4	protein_codin	10.5517395	13.1679271	3.51870859	8.04886769
ENSG000001	GNS	protein_codin	1274.16037	1168.54533	168.20385	845.336207
ENSG000001	MDM2	protein_codin	0	0	4.46052194	0
ENSG000001	MDM2	protein_codin	663.848721	314.35034	283.11574	228.664605
ENSG000001	KLHL36	protein_codin	29.161638	21.7845333	0	22.3563389
ENSG000001	BCO1	protein_codin	0	0	0	0
ENSG000001	MPHOSPH6	protein_codin	48.6826474	64.4254264	74.6341387	46.6215742
ENSG000002	TMEM231	protein_codin	0	6.16125022	0	1.05143473
ENSG000001	NDRG4	protein_codin	0	0	0	0
ENSG000001	DYNC1LI2	protein_codin	269.869036	224.420527	143.238317	149.017305
ENSG000001	FBXL8	protein_codin	0	0	2.86946532	4.28207128
ENSG000001	FHOD1	protein_codin	156.169083	219.985254	106.308865	100.410657
ENSG000001	CCDC102A	protein_codin	3.86477501	3.91708268	3.49361715	0.94705427
ENSG000001	PCNX2	protein_codin	53.988176	47.2071242	30.3738626	33.3194493
ENSG000001	URB2	protein_codin	17.7423097	42.967394	12.8077549	12.0088964
ENSG000001	TAF5L	protein_codin	42.8622318	116.084881	20.7495926	47.4078201
ENSG000001	STX6	protein_codin	177.748624	288.648166	103.622753	127.103508
ENSG000001	RGS8	protein_codin	0	0	0	1.15026328
ENSG000001	NPL	protein_codin	0	27.0860805	0	61.8017792
ENSG000001	LAMC1	protein_codin	45.7531467	80.2565575	89.3919719	32.9350374
ENSG000001	PNKD	protein_codin	155.185059	304.258449	79.3393115	96.9554188
ENSG000001	SP110	protein_codin	215.899787	116.694759	141.494901	179.922421
ENSG000001	SP110	protein_codin	139.644538	299.145379	163.98008	275.889031
ENSG000001	MRPL44	protein_codin	41.775935	97.7289122	39.2031153	60.7803318
ENSG000001	CHRND	protein_codin	0	0	0	0
ENSG000001	PAX3	protein_codin	0	0	0	0
ENSG000001	DOCK10	protein_codin	162.257079	0	0	155.716985
ENSG000001	TTLL4	protein_codin	0	120.107763	0	66.9309239
ENSG000001	USP37	protein_codin	42.8407681	28.543843	35.5288509	10.3824184
ENSG000001	HTR2B	protein_codin	1.33052045	0	0	0
ENSG000001	SLC19A3	protein_codin	0	0	0	0
ENSG000001	SERPINE2	protein_codin	0	13.0363885	0	8.7078083
ENSG000001	WNT10A	protein_codin	0	0.87152201	0	0
ENSG000001	TMBIM1	protein_codin	321.996391	465.312718	138.540391	126.874662
ENSG000001	CYP27A1	protein_codin	251.863919	283.77873	103.064461	274.887015
ENSG000001	EIF4E2	protein_codin	257.243875	327.384658	102.320617	143.592828
ENSG000001	CAB39	protein_codin	502.514311	575.964768	307.342407	372.227602
ENSG000001	COX5B	protein_codin	325.898782	470.601261	149.716264	242.171837
ENSG000001	REV1	protein_codin	99.392559	104.011343	53.1806411	88.1734836

ENSG000001:	MFSD9	protein_codin	16.1343897	20.5607565	9.70454395	4.30584424
ENSG000001:	TMEM127	protein_codin	382.034651	0	0	0
ENSG000001:	EDAR	protein_codin	0	0	0	0
ENSG000001:	MRPS9	protein_codin	61.2066448	92.9255177	70.0963597	37.2088887
ENSG000001:	GPR45	protein_codin	0	0	0	0
ENSG000001:	C2orf49	protein_codin	90.5858016	33.1156412	13.554696	38.8420546
ENSG000001!	ANKRD36B	protein_codin	0	0	0	15.7202092
ENSG000001:	EPC2	protein_codin	89.0306579	129.701718	54.1646987	74.2047501
ENSG000001:	ALDH1L2	protein_codin	0	0	4.7913499	0
ENSG000001:	USP44	protein_codin	0	0	0	0
ENSG000001:	PLXNC1	protein_codin	1270.16683	1069.49693	352.900642	1108.43375
ENSG000001:	APPL2	protein_codin	0	60.6027809	17.567797	61.6111588
ENSG000001:	DRAM1	protein_codin	106.108542	173.321759	65.0477988	113.99932
ENSG000001:	SLC41A2	protein_codin	10.6896697	1.5985681	7.40019356	0
ENSG000002	TPTE2P4	transcribed_u	0	1.42761981	0	0
ENSG000001:	CKAP2	protein_codin	0	0	72.4103671	0
ENSG000001:	THSD1	protein_codin	0	0	0	0
ENSG000001:	RCBTB1	protein_codin	0	0	0	0
ENSG000001:	MED4	protein_codin	110.259333	115.554094	54.5869146	63.3452171
ENSG000002	RPL13AP9	processed_ps	0	0	1.17245287	0
ENSG000001:	NUDT15	protein_codin	24.5303936	26.1218328	7.43550703	19.6674936
ENSG000001(	PLEKHA8	protein_codin	1.73523613	0	1.3193363	0
ENSG000000:	CAMK2B	protein_codin	0	0	0	0
ENSG000001:	SPDYE1	protein_codin	0	0	0	0
ENSG000001:	CHST12	protein_codin	0	0	71.0494284	0
ENSG000001:	IGF2BP3	protein_codin	42.5723613	55.7355576	32.9023237	15.680847
ENSG000001:	GPNMB	protein_codin	0	9.0987026	0	6.97916675
ENSG000000(	ABCB5	protein_codin	0	0	0	0
ENSG000001:	KDELR2	protein_codin	133.059478	168.049773	71.1753429	104.532688
ENSG000001:	NUP42	protein_codin	20.375614	0	0	21.4049772
ENSG000001:	IL6	protein_codin	77.5311845	7.42837931	22.2277111	32.511849
ENSG000001:	BZW2	protein_codin	44.9646404	30.8820892	37.7436664	18.813127
ENSG000001:	TBRG4	protein_codin	15.0016472	34.9262445	0	0
ENSG000001:	DDX56	protein_codin	50.6567179	81.4973233	18.6187886	0
ENSG000001:	HUS1	protein_codin	24.2052467	4.65761587	18.2993136	29.0848557
ENSG000001:	CCM2	protein_codin	37.1872418	76.2347493	35.8076223	48.1834059
ENSG000001:	MYO1G	protein_codin	878.228306	1116.36961	275.694091	306.548753
ENSG000001:	TTYH3	protein_codin	864.683494	859.283391	168.714342	330.303369
ENSG000001:	CIDEB	protein_codin	164.469474	235.869257	55.0356348	117.221489
ENSG000001:	TTC5	protein_codin	32.6240822	54.2999677	18.2444871	13.9648983
ENSG000001:	NKX2-8	protein_codin	0	0	0	0
ENSG000001(	ACSBG1	protein_codin	8.72658101	7.10414981	14.307634	9.24228243
ENSG000001:	MTHFS	protein_codin	27.7180278	44.7908099	6.14574574	22.4819708
ENSG000001:	ABHD17C	protein_codin	0	0	0	0

ENSG000001:	IREB2	protein_codin	273.360898	112.497081	117.198723	213.239301
ENSG000001:	ALPK3	protein_codin	11.113777	6.95272251	8.78855141	8.87136638
ENSG000001:	TM6SF1	protein_codin	0	0	0	0
ENSG000001:	CIB2	protein_codin	1.77285243	3.63221213	0	0
ENSG000001:	CALCOCO2	protein_codin	312.735712	235.823166	90.7705614	167.043768
ENSG000001:	RSAD1	protein_codin	75.6368945	114.267192	47.5717146	40.5411307
ENSG000001:	NMT1	protein_codin	0	0	0	0
ENSG000001:	SRSF1	protein_codin	206.405697	87.6940385	118.884333	159.648003
ENSG000001:	VEZF1	protein_codin	55.3928164	18.8054102	56.9523221	116.969007
ENSG000001:	CHAD	protein_codin	0	0	0	0
ENSG000001:	TACO1	protein_codin	19.5268402	31.8736163	3.53885552	14.4848262
ENSG000001:	TEX2	protein_codin	0	0	0	0
ENSG000002(	CSHL1	protein_codin	0	0	0	0
ENSG000001:	LIMD2	protein_codin	167.500924	268.396569	105.821672	120.207098
ENSG000001:	BRIP1	protein_codin	0	0	0	0
ENSG000001:	KAT7	protein_codin	131.076399	152.865284	33.0648582	59.0523984
ENSG000001:	RTP4	protein_codin	9.86567135	7.88499285	4.0982481	4.96996589
ENSG000001:	NDUFB5	protein_codin	33.2827987	17.7004665	20.8670191	0
ENSG000001:	MRPL47	protein_codin	0	3.89919489	0	0
ENSG000001:	TRA2B	protein_codin	12.6291512	40.5266734	0	4.6813204
ENSG000001:	MARCHF7	protein_codin	164.032358	94.9740872	99.6481497	97.7592049
ENSG000002·	CD302	protein_codin	691.253852	493.182017	363.247499	597.984201
ENSG000001:	GALNT5	protein_codin	3.67848509	0	0	0
ENSG000001:	TANK	protein_codin	98.820823	156.751065	121.10574	136.648577
ENSG000001:	BLK	protein_codin	0	12.691918	15.2568165	0
ENSG000001:	SKIL	protein_codin	129.459064	0	38.5654676	68.1466679
ENSG000001:	KCTD3	protein_codin	57.9011454	21.8516357	43.2730326	29.7805671
ENSG000001:	ZNG1B	protein_codin	9.97931714	12.7830572	0	0
ENSG000001:	IL36G	protein_codin	0	0	0	0
ENSG000001:	IL1RN	protein_codin	11.1452318	0	0	0
ENSG000001:	IL36A	protein_codin	0	0	0	0
ENSG000001:	IL36B	protein_codin	0	0	0	0
ENSG000001:	CFC1	protein_codin	0	0	0	0
ENSG000001:	CCDC115	protein_codin	67.375361	83.1044806	30.8847295	48.652081
ENSG000001:	SAP130	protein_codin	23.0375354	47.1479713	45.9229814	48.950222
ENSG000001:	SAP130	protein_codin	35.5725023	42.1326424	52.6123461	46.8189298
ENSG000001:	BIN1	protein_codin	0	0	0	0
ENSG000001:	IMP4	protein_codin	0	19.5562943	19.4706664	10.2859524
ENSG000001:	HS6ST1	protein_codin	121.675194	134.574099	60.4145945	90.5662512
ENSG000001:	UGGT1	protein_codin	342.995381	401.565871	212.576475	215.524395
ENSG000001:	GYPC	protein_codin	47.9601609	77.9905603	33.7922857	44.7075888
ENSG000001:	GAD2	protein_codin	0	0	0	0
ENSG000001:	PALM2AKAP2	protein_codin	0	0	0	0
ENSG000001:	LRRC8A	protein_codin	0	19.5674846	0	0

ENSG000001	ECPAS	protein_codin	39.3767842	35.5517971	0	0
ENSG000001	TOR1B	protein_codin	64.2997884	114.721208	40.9102136	56.581559
ENSG000001	RALGPS1	protein_codin	0	20.16059	6.3519623	0
ENSG000001	OR1J1	protein_codin	0	0	0	0
ENSG000001	OR13C9	protein_codin	0	0	0	0
ENSG000001	TMOD1	protein_codin	10.0042704	5.6729163	0	0
ENSG000001	DAB2IP	protein_codin	0.83125633	0	1.83732015	0
ENSG000001	SLC31A2	protein_codin	324.665487	289.722391	122.996402	133.233468
ENSG000001	ZNF189	protein_codin	35.6744054	0	0	0
ENSG000002	ORM1	protein_codin	0.99543558	2.78828683	0	3.04463768
ENSG000001	STX17	protein_codin	117.417021	72.5506322	51.5958727	57.7566787
ENSG000001	BAAT	protein_codin	0	0	0	0
ENSG000001	GABBR2	protein_codin	0.93459062	0	0	0
ENSG000001	HEMGN	protein_codin	248.507783	162.369229	139.948521	8.92431294
ENSG000001	PSMB7	protein_codin	130.934258	216.047744	99.6304539	103.745347
ENSG000001	RABEPK	protein_codin	3.21109217	0	0	10.7584647
ENSG000001	OR1L4	protein_codin	0	0	0	0
ENSG000001	PDCL	protein_codin	57.3738076	59.7027175	23.1523824	49.9381393
ENSG000001	CTSV	protein_codin	0	2.65118389	0	0
ENSG000001	ARPC5L	protein_codin	26.4421751	42.0144574	23.1997443	17.9863044
ENSG000001	ENPP2	protein_codin	0	0	0	0
ENSG000001	DERL1	protein_codin	100.712619	103.345078	23.449521	80.1704589
ENSG000001	MYC	protein_codin	0	2.1452571	0	0
ENSG000001	CCN3	protein_codin	1.10169024	1.09461008	0	1.0836794
ENSG000001	IFNA6	protein_codin	0	0	0	0
ENSG000001	RANBP6	protein_codin	98.8364615	79.2452736	58.8785091	70.1115755
ENSG000001	RNF38	protein_codin	0	0	0	0
ENSG000001	CCL21	protein_codin	0	0	0	0
ENSG000001	SIT1	protein_codin	0	2.51961568	2.15468692	0
ENSG000001	DMRT2	protein_codin	0	0	0	0
ENSG000002	CCL27	protein_codin	0	0	0	0
ENSG000001	DCTN3	protein_codin	46.8773064	77.5864895	27.5162715	37.1011506
ENSG000001	CD72	protein_codin	0	4.10964722	3.12090414	1.43848944
ENSG000001	HINT2	protein_codin	14.0006756	18.3067318	10.3745826	8.23302443
ENSG000001	RIPOR2	protein_codin	0	0	0	0
ENSG000001	KLC4	protein_codin	17.4536904	8.48168107	0	3.87422219
ENSG000001	KIF13A	protein_codin	477.193182	416.508819	182.399688	320.115605
ENSG000001	GMPR	protein_codin	107.272537	243.598242	122.286041	77.1828799
ENSG000001	TMEM63B	protein_codin	78.5462329	83.9315753	28.166952	52.3905594
ENSG000001	FRS3	protein_codin	13.6955189	0	5.36921809	7.37221674
ENSG000001	TTBK1	protein_codin	0	0.76856486	0	0.74642294
ENSG000001	TJAP1	protein_codin	0	0	0	0
ENSG000001	TINAG	protein_codin	0	0	0	0
ENSG000001	GCM1	protein_codin	0	1.0362942	0	1.05140242

ENSG000001	RIPK1	protein_codin	14.178105	22.6445348	8.65968168	7.01629489
ENSG000001	TUBB2B	protein_codin	0	0	0	0
ENSG000002	PSORS1C2	protein_codin	0	0	0	0.73975997
ENSG000002	C6orf15	protein_codin	0	0	0	0
ENSG000002	MRPS18B	protein_codin	48.0437667	109.316488	40.249806	44.4475158
ENSG000001	IER3	protein_codin	1169.95675	976.939158	297.914837	610.553184
ENSG000002	PSORS1C1	protein_codin	0	0	0	0
ENSG000001	PGBD1	protein_codin	0	0	0	0
ENSG000002	GTF2H4	protein_codin	5.2990471	13.0409032	6.10033238	5.27533514
ENSG000002	POU5F1	protein_codin	0	0	0	0
ENSG000001	CLPS	protein_codin	0	0	0	0
ENSG000001	RNF144B	protein_codin	1040.51977	820.136071	294.256818	491.530581
ENSG000002	HLA-F	protein_codin	376.125868	375.255309	85.582703	128.605384
ENSG000001	NRM	protein_codin	0	0	0	0
ENSG000001	FAM8A1	protein_codin	110.305732	43.5040762	62.0856164	58.0497644
ENSG000001	C6orf52	protein_codin	0	0	0	0
ENSG000001	FGFBP2	protein_codin	0.93059764	4.62950277	0	0
ENSG000001	TLR2	protein_codin	667.868682	235.196067	198.539666	281.446068
ENSG000001	THAP12	protein_codin	261.382632	205.0122	64.4300143	253.924489
ENSG000001	ANKRD42	protein_codin	0	0	0	16.1942026
ENSG000001	CCDC90B	protein_codin	0	0	3.08902887	3.8378506
ENSG000001	RAB30	protein_codin	0	0	0	0
ENSG000001	CREBZF	protein_codin	0	0	0	0
ENSG000001	LRRC32	protein_codin	3.52662154	17.4713975	9.97778505	15.1362074
ENSG000001	MRPL15	protein_codin	39.0207566	43.5494638	12.0153557	15.2702572
ENSG000001	PI15	protein_codin	0	0	0	0
ENSG000001	TTPA	protein_codin	0	0	0	0
ENSG000001	GGH	protein_codin	0	0	35.4326056	5.69072346
ENSG000001	SLCO5A1	protein_codin	0	0	1.69046221	0
ENSG000001	SULF1	protein_codin	0	0	0	0
ENSG000001	TGS1	protein_codin	60.3014949	118.479236	53.1344056	89.1970196
ENSG000001	SDCBP	protein_codin	298.851288	173.672927	0	200.539845
ENSG000000	USP2	protein_codin	2.58812372	0	0	0
ENSG000001	HTR3B	protein_codin	1.07551797	1.06708528	0	0
ENSG000001	SORL1	protein_codin	2010.39976	2092.49438	964.59989	1521.10467
ENSG000001	BUD13	protein_codin	54.8702784	116.23297	37.2032543	45.1649273
ENSG000001	MMP7	protein_codin	0	0	0	0
ENSG000001	MMP20	protein_codin	0	0	0	0
ENSG000001	MMP27	protein_codin	0	0	0	0
ENSG000001	DCUN1D5	protein_codin	33.308037	15.2085136	23.6332152	14.4114423
ENSG000002	FDXACB1	protein_codin	5.28113088	6.72446816	4.68935435	2.14641887
ENSG000001	POU2F3	protein_codin	0	0	0	0
ENSG000001	FDX1	protein_codin	57.6339555	64.6744245	38.8288283	55.8243747
ENSG000001	CFAP68	protein_codin	3.17431284	8.38118197	3.08781116	2.31578616

ENSG000001	FXYD6	protein_codin	0	0	0	0
ENSG000001	ARHGAP20	protein_codin	0	0	0	0
ENSG000001	FXYD2	protein_codin	0	0	0	0
ENSG000001	MMP13	protein_codin	0	0	0	0
ENSG000001	CASP5	protein_codin	0	0	0	0
ENSG000001	ALKBH8	protein_codin	0	0	0	3.02869662
ENSG000001	UNC13C	protein_codin	0	1.11756608	2.2460198	0
ENSG000001	SQOR	protein_codin	355.613016	435.088363	170.597825	277.792294
ENSG000001	CTDSPL2	protein_codin	154.081219	110.576557	74.5564894	135.219436
ENSG000001	THBS1	protein_codin	3663.84761	10260.046	5809.54619	3803.71933
ENSG000001	NUSAP1	protein_codin	0	26.1449915	14.4569926	3.63119472
ENSG000001	NDUFAF1	protein_codin	10.9337244	0	0	11.1257957
ENSG000001	KIF23	protein_codin	0	0	0	0
ENSG000002	SPESP1-NOX	protein_codin	0	0	0	0
ENSG000001	HAUS2	protein_codin	33.92223	39.6408938	13.8033332	23.0085562
ENSG000001	PARP6	protein_codin	0	0	0	0
ENSG000001	RPLP1	protein_codin	2313.45797	3722.04289	1328.64259	1778.00345
ENSG000001	LRRC49	protein_codin	0	0	2.0106391	0
ENSG000001	TUBGCP4	protein_codin	6.7631236	0	0	0
ENSG000001	RMDN3	protein_codin	0	9.95856959	0	0
ENSG000001	ITPKA	protein_codin	0	0	0	0
ENSG000001	PLCB2	protein_codin	0	374.164256	231.013726	260.311907
ENSG000001	TMEM62	protein_codin	24.2339698	26.5449083	9.57300232	18.6628377
ENSG000001	PAK6	protein_codin	0	0	0	0
ENSG000001	ADAM10	protein_codin	519.673929	260.541268	252.173045	393.016109
ENSG000001	BCL2L10	protein_codin	0	0	0	0
ENSG000001	RSL24D1	protein_codin	179.457649	188.810671	96.5376764	164.550749
ENSG000001	GCHFR	protein_codin	5.82219711	9.34810725	9.39928438	10.7534971
ENSG000001	MNS1	protein_codin	0	4.24677168	2.76304533	1.09144863
ENSG000001	BCAR3	protein_codin	1.17883629	2.7602314	0	0
ENSG000001	TTLL7	protein_codin	0	0	7.1184395	0
ENSG000001	FNBP1L	protein_codin	92.5155639	92.9930167	75.1635225	54.7986681
ENSG000001	KYAT3	protein_codin	69.0228329	59.6262137	0	0
ENSG000001	ARHGAP29	protein_codin	22.0655216	4.41317819	3.79108067	6.3641646
ENSG000001	RTCA	protein_codin	86.3148322	86.7867815	55.9085527	36.7111001
ENSG000000	CEP68	protein_codin	0	0	0	0
ENSG000001	IFT172	protein_codin	0	0	0	0
ENSG000001	SELENOI	protein_codin	39.0801684	24.683918	33.9928182	21.7415338
ENSG000001	KHK	protein_codin	0	0	0	0
ENSG000001	KHK	protein_codin	0	7.77603182	4.54968519	0
ENSG000001	ADCY3	protein_codin	0	5.77117872	0	0
ENSG000001	PNPT1	protein_codin	0	20.1517863	9.1213151	25.8205335
ENSG000001	DYNC2LI1	protein_codin	6.50574401	5.89045832	4.14799101	0
ENSG000001	ACTR2	protein_codin	1644.57884	1201.15032	880.397934	1376.29062

ENSG000001:	PREB	protein_codin	72.7248245	118.660658	63.0899673	81.9064898
ENSG000001:	PREPL	protein_codin	0	0	13.1246755	23.8075319
ENSG000001:	SLC3A1	protein_codin	0	0	0	1.11728609
ENSG000001:	SIX3	protein_codin	0	0	0	0
ENSG000001:	CENPO	protein_codin	0	0	0	0
ENSG000001:	LRPPRC	protein_codin	58.6255831	0	15.0865326	7.66293651
ENSG000001:	CYP2C9	protein_codin	0	0	0	0
ENSG000001:	LOXL4	protein_codin	0	0	0	0
ENSG000001:	BTBD16	protein_codin	0	0	0	0
ENSG000001:	KIF11	protein_codin	0	0	46.6370097	0
ENSG000001:	TACC2	protein_codin	0	0	0	0
ENSG000001:	CALHM2	protein_codin	32.1514911	41.7034789	9.35456671	0
ENSG000001:	ARL3	protein_codin	69.0432383	60.6914396	22.7262779	25.306162
ENSG000001:	KIF20B	protein_codin	48.9542535	4.28950917	95.8134424	33.5463322
ENSG000001:	EXOC6	protein_codin	61.6572982	36.6932762	50.0651141	32.123935
ENSG000001:	SHTN1	protein_codin	0	0	0	0
ENSG000000	FGFR3	protein_codin	0	0	0	0
ENSG000001:	DBR1	protein_codin	0	24.4824035	23.3750314	0
ENSG000001	TOPBP1	protein_codin	245.036888	176.31948	152.148346	178.224187
ENSG000001:	DNAJC13	protein_codin	627.455009	350.717194	399.228356	621.417831
ENSG000001:	GPR87	protein_codin	0	0	0	0
ENSG000002	SLFN12L	protein_codin	0	17.5085837	0	3.26721065
ENSG000001	SATB2	protein_codin	0	0	0	0
ENSG000001:	AOX1	protein_codin	0	0	0	0
ENSG000001	ERBB4	protein_codin	0	0	0	0
ENSG000001:	BARD1	protein_codin	20.10219	3.02644031	7.66811847	13.1673167
ENSG000001:	MSTN	protein_codin	0	0	0	0
ENSG000001:	ASNSD1	protein_codin	47.4756338	40.4749581	19.2453303	25.4817552
ENSG000001:	METTL5	protein_codin	0	1.68615212	0	0
ENSG000001:	SSB	protein_codin	288.801054	275.216541	276.598069	258.726244
ENSG000001:	CDK15	protein_codin	0	0	0	0
ENSG000001:	PPIG	protein_codin	0	0	0	0
ENSG000001:	HECW2	protein_codin	0	0	0	0
ENSG000001	CRYGB	protein_codin	0	0	0	0
ENSG000001:	CHRNA1	protein_codin	0	0	0	0
ENSG000001:	WDR12	protein_codin	0	50.9961277	0	0
ENSG000001:	ABI2	protein_codin	0	0	0	0
ENSG000001:	ABI2	protein_codin	39.5358316	47.4725597	53.434418	27.2700089
ENSG000001:	ABI2	protein_codin	5.29535984	0	0	0
ENSG000001:	ITGAV	protein_codin	0	0	0	0
ENSG000001:	SLC40A1	protein_codin	725.427439	1230.47828	284.773871	504.307665
ENSG000001:	SLC35A5	protein_codin	34.185634	29.5264475	18.9032496	18.3437439
ENSG000001	COL8A1	protein_codin	0	0	0	0
ENSG000001:	SLC49A4	protein_codin	114.393262	119.756088	45.7643619	76.8583381

ENSG000001	GUCA1C	protein_codin	0	0	0	0
ENSG000001	CCDC54	protein_codin	0	0	0	0
ENSG000001	COX17	protein_codin	94.0916611	153.266536	82.8103453	59.6491976
ENSG000000	WBP11	protein_codin	327.902984	335.432502	149.859607	241.764507
ENSG000001	ATF7IP	protein_codin	349.735227	225.476256	167.489516	241.60059
ENSG000000	LMO3	protein_codin	0	0	0	0
ENSG000000	GUCY2C	protein_codin	2.02518454	1.03072672	0	0
ENSG000000	EPYC	protein_codin	0	0	0	0
ENSG000001	TSPAN11	protein_codin	0	0	0	0
ENSG000000	BMAL2	protein_codin	0	7.34251823	0	0
ENSG000000	TRHDE	protein_codin	0	9.05835428	5.20167722	9.3602053
ENSG000000	OSBPL8	protein_codin	0	40.8197056	25.4000177	0
ENSG000001	SLC16A7	protein_codin	0	0	55.9274106	86.7719143
ENSG000000	INTS13	protein_codin	44.9514424	55.7336002	42.7540429	48.4233631
ENSG000000	BCAT1	protein_codin	56.154277	17.6471569	26.4498905	81.567405
ENSG000001	GYS2	protein_codin	0	2.21212592	0	0
ENSG000001	SLCO1B3	protein_codin	0	0	0	0
ENSG000001	ST8SIA1	protein_codin	0	0	0	0
ENSG000000	ABCC9	protein_codin	7.83536929	1.08864031	5.23928378	2.61500405
ENSG000000	ABCC9	protein_codin	0	0	0	0
ENSG000001	LIN7A	protein_codin	7.89830437	19.7249027	0	0
ENSG000000	SYT1	protein_codin	0	0	0	0
ENSG000001	ACSS3	protein_codin	0	0	0	0
ENSG000000	PPP1R12A	protein_codin	0	0	0	0
ENSG000000	HAL	protein_codin	0	0	0	0
ENSG000001	TMPO	protein_codin	119.104306	109.499636	39.7477955	88.8628313
ENSG000000	CDK17	protein_codin	87.4694391	102.364036	72.1668588	55.9094915
ENSG000001	METAP2	protein_codin	0	0	0	0
ENSG000000	TMCC3	protein_codin	66.1181219	114.377498	29.9035844	28.3486011
ENSG000000	IRAK3	protein_codin	1953.21305	1048.30757	576.547462	965.00499
ENSG000001	RXYLT1	protein_codin	18.2937069	0	7.06836316	0
ENSG000001	KIAA0586	protein_codin	0	0	0	0
ENSG000000	MNAT1	protein_codin	0	0	0	2.90244494
ENSG000001	TRMT5	protein_codin	13.1570628	13.383283	7.23232139	12.7971019
ENSG000000	FERRY3	protein_codin	44.6120583	30.384141	26.2601239	34.4745806
ENSG000001	PRMT8	protein_codin	0	0	0	0
ENSG000001	CCND2	protein_codin	0	74.1581456	157.6677	0
ENSG000000	RAB21	protein_codin	326.48528	208.996672	146.910919	249.099085
ENSG000001	PTPRB	protein_codin	0	0	0	0
ENSG000000	LYZ	protein_codin	24554.0462	32463.8636	16129.2234	20723.1375
ENSG000001	ENTREP2	protein_codin	0	0	0	0
ENSG000001	LIPG	protein_codin	1.17316787	0	0	1.19087362
ENSG000000	TGDS	protein_codin	4.22291263	9.71924103	11.658974	6.33134186
ENSG000000	FOXN3	protein_codin	89.6908524	193.960481	100.567703	64.2476773

ENSG000001(	PSMC1	protein_codin	14.5223912	20.6482085	7.24224733	12.8463685
ENSG00000(	GALC	protein_codin	144.47635	105.850987	51.9502778	93.3200175
ENSG000001	PPWD1	protein_codin	87.1618729	0	51.4890308	0
ENSG00000(	PEBP1	protein_codin	76.9998681	113.879901	109.691696	63.914223
ENSG000001	SPRING1	protein_codin	77.5528343	91.4459776	51.4339893	61.1452239
ENSG00000(	MOCOS	protein_codin	2.16545312	1.05902629	0	1.08451174
ENSG00000(	ZNF24	protein_codin	13.3597054	48.199075	0	31.2094372
ENSG00000(	ZNF397	protein_codin	0	0	0	0
ENSG00000(	PZP	protein_codin	0	0	0	0
ENSG00000(	CLEC2D	protein_codin	0	0	0	0
ENSG00000(	CLEC2D	protein_codin	0	0	20.1318256	0
ENSG00000(	LRP6	protein_codin	0	0	0	0
ENSG000001	YPEL5	protein_codin	631.366713	764.615619	507.314531	524.840998
ENSG000001	LMNB1	protein_codin	212.294163	283.681216	95.5929937	306.462487
ENSG00000(	SNCAIP	protein_codin	0	0	0	0
ENSG00000(	SNCAIP	protein_codin	0	0	0	0
ENSG00000(	SNX24	protein_codin	0	11.5706462	0	5.36077168
ENSG00000(	HS3ST2	protein_codin	0	0	0	0
ENSG00000(	REXO5	protein_codin	0	0	0	0
ENSG00000(	XYLT1	protein_codin	359.949924	283.024461	105.157399	123.916496
ENSG00000(	DNAH3	protein_codin	0	0.91536076	0	0
ENSG000000	LDAF1	protein_codin	26.6701334	46.5509802	14.6981717	19.1617245
ENSG00000(	NUP133	protein_codin	107.759518	131.976315	79.0064482	94.1322791
ENSG000001	CORO1C	protein_codin	888.901027	1266.75732	431.24354	687.114029
ENSG00000(	NUAK1	protein_codin	0	0	0	0
ENSG000001	VWF	protein_codin	153.719849	153.24356	130.622937	80.5186229
ENSG00000(	ENSG00000(	protein_codin	8.30805606	2.54342685	0	0
ENSG000001	LPCAT3	protein_codin	66.7846966	77.1798322	4.28725544	48.940954
ENSG000001	MRPS27	protein_codin	44.3530568	0	28.7115065	21.9219098
ENSG000001	CERT1	protein_codin	0	0	0	0
ENSG00000(	HEXB	protein_codin	267.518828	317.04228	134.041157	153.904891
ENSG00000(	KLHL5	protein_codin	0	22.2987359	0	0
ENSG00000(	KLHL5	protein_codin	0	7.97807043	0	0
ENSG00000(	UBE2K	protein_codin	152.298678	102.130496	77.048064	106.02063
ENSG00000(	LIAS	protein_codin	0	0	0	0
ENSG00000(	N4BP2	protein_codin	164.486344	59.2588707	115.677753	75.1198485
ENSG00000(	KLF3	protein_codin	583.520553	562.867311	284.788423	429.966365
ENSG00000(	TBC1D1	protein_codin	66.1007019	116.741962	13.2322515	64.9804242
ENSG00000(	RSBN1	protein_codin	0	0	0	0
ENSG00000(	CSDE1	protein_codin	176.669378	599.123879	214.565792	79.8595403
ENSG000001	CASQ2	protein_codin	0	0	0	0
ENSG00000(	PACC1	protein_codin	35.0756441	59.1193508	24.4559352	26.9778661
ENSG00000(	HHAT	protein_codin	0	0	12.0338005	4.68781227
ENSG00000(	PPP2R5A	protein_codin	177.026004	229.187149	59.112794	142.90214

ENSG000000	TRAF5	protein_codin	0	15.039087	0	0
ENSG000001	HSD11B1	protein_codin	0.81797583	0	0	0
ENSG000000	PPP2R3C	protein_codin	103.116712	89.786291	62.119486	75.971143
ENSG000001	PSMA6	protein_codin	322.639079	376.021671	197.197926	122.543312
ENSG000001	REEP5	protein_codin	0	0	0	1.13616889
ENSG000001	MAN2A1	protein_codin	397.409243	274.257922	287.785973	286.623836
ENSG000001	EPB41L4A	protein_codin	4.10629477	3.16324103	0	1.19624779
ENSG000001	ENOX1	protein_codin	0	2.84143749	1.20745853	0
ENSG000001	TSC22D1	protein_codin	591.935584	1671.34246	520.955547	707.992398
ENSG000001	DGKH	protein_codin	58.672904	29.6641734	66.7497021	43.1756731
ENSG000001	USP22	protein_codin	521.829105	733.093632	173.058945	360.074722
ENSG000001	B9D1	protein_codin	0	0	0	0
ENSG000001	SPECC1	protein_codin	0	0	0	0
ENSG000000	MSMO1	protein_codin	6.94220842	4.61114523	5.99190534	4.73954346
ENSG000001	PALLD	protein_codin	5.81631777	1.48073977	0	0
ENSG000001	ICE2	protein_codin	75.5270694	0	48.2341361	0
ENSG000000	RORA	protein_codin	0	0	0	0
ENSG000000	GPATCH2L	protein_codin	119.451828	212.814982	102.016269	65.6179121
ENSG000001	SNW1	protein_codin	358.608269	434.1721	204.311879	217.020098
ENSG000000	POMT2	protein_codin	0	0	0	0
ENSG000001	ROCK2	protein_codin	34.3388878	40.2773426	22.1302177	25.1737583
ENSG000001	MIB1	protein_codin	201.302434	128.136403	103.941913	154.656677
ENSG000000	TMEM260	protein_codin	30.6965227	4.83579279	2.99217941	0
ENSG000000	AP5M1	protein_codin	49.2868591	44.1898049	25.5982135	25.8672417
ENSG000001	ZNF430	protein_codin	42.3947077	29.9212372	31.6851067	34.0806184
ENSG000000	MAST4	protein_codin	33.9092369	62.097538	87.4164055	35.2309512
ENSG000000	IPO5	protein_codin	0	0	27.0205761	12.9432231
ENSG000000	PALB2	protein_codin	0	0	0	0
ENSG000000	KATNIP	protein_codin	196.542714	199.914182	87.8178026	74.8773932
ENSG000000	DSG2	protein_codin	0	0	0	0
ENSG000001	NOL4	protein_codin	0	0	0	0
ENSG000001	RNF138	protein_codin	158.034283	78.4615265	59.8499321	125.091832
ENSG000001	LPIN2	protein_codin	83.311093	96.199966	38.2302585	0
ENSG000000	NDC80	protein_codin	0.99643612	3.087838	24.5712907	1.05671527
ENSG000000	THOC1	protein_codin	0	0	19.8265287	0
ENSG000001	USP14	protein_codin	161.744594	65.3350709	67.7506052	50.4687223
ENSG000001	MYOM1	protein_codin	35.2593818	85.7371086	75.3066727	58.195412
ENSG000001	HERC2	protein_codin	275.205194	432.198843	299.327013	144.975312
ENSG000000	DPEP1	protein_codin	0	0	0	0
ENSG000001	SLC7A5	protein_codin	361.332412	157.751942	129.007003	141.167969
ENSG000000	CYBA	protein_codin	792.500774	1150.37277	298.912832	648.172412
ENSG000001	ARL1	protein_codin	16.4196457	56.6151485	16.2514268	24.9392552
ENSG000001	UTP20	protein_codin	86.2151115	74.9405605	67.4490342	40.3039119
ENSG000000	COX10	protein_codin	38.0407352	51.7207813	21.0595434	21.0998777

ENSG000002	FBXW10B	protein_codin	0	0	0	0
ENSG000000	SREBF1	protein_codin	72.0652777	53.9104432	15.887617	8.30795253
ENSG000000	TTC19	protein_codin	43.2868125	39.3055366	8.31710251	31.4795959
ENSG000002	ENSG000002	lncRNA	3.85606055	5.00824216	13.2445349	0
ENSG000002	TNFRSF13B	protein_codin	0	6.46980513	44.5891102	0
ENSG000001	STX2	protein_codin	0	44.8261799	0	0
ENSG000001	ADGRD1	protein_codin	26.5016789	55.672043	21.1995723	31.8415763
ENSG000000	RIMBP2	protein_codin	0	0	0	0
ENSG000000	CLEC16A	protein_codin	3.20004233	22.9622285	0	6.88332191
ENSG000001	BFAR	protein_codin	44.6165676	77.6895483	31.0492817	44.7606217
ENSG000001	CPPED1	protein_codin	0	0	0	0
ENSG000001	KPNA3	protein_codin	250.19132	175.928902	146.616276	164.817838
ENSG000000	SFSWAP	protein_codin	293.715969	419.25287	178.121614	241.129893
ENSG000000	PALS1	protein_codin	25.9143097	0	0	40.7846539
ENSG000001	CDK2AP1	protein_codin	47.5077112	134.176731	35.9583426	70.0432769
ENSG000000	SCARB1	protein_codin	0	30.4222241	0	0
ENSG000000	L2HGDH	protein_codin	0	0	0	0
ENSG000000	RTRAF	protein_codin	98.0167145	68.6872678	47.9856903	73.8444294
ENSG000001	SLC6A4	protein_codin	0	6.61781242	0	0
ENSG000001	UTP6	protein_codin	60.8014307	6.72480257	27.2037759	73.0681477
ENSG000001	PSMD11	protein_codin	104.859566	127.753031	29.2210618	53.4408987
ENSG000000	TMEM98	protein_codin	0	0	0	0
ENSG000001	BLMH	protein_codin	42.8195903	66.0431077	27.3013906	42.5599942
ENSG000001	TAOK1	protein_codin	1292.33256	670.763335	634.794687	761.747952
ENSG000000	BTBD1	protein_codin	221.773983	205.599866	112.256688	142.80426
ENSG000001	AP3B2	protein_codin	0	0	0	0
ENSG000001	CUX2	protein_codin	2.00627489	14.9266682	19.735593	1.95640693
ENSG000001	RASAL1	protein_codin	0	0	0	0
ENSG000000	LHX5	protein_codin	1.6102477	0	0	0
ENSG000001	ALDH2	protein_codin	152.976625	424.923358	157.918673	157.74316
ENSG000000	ERP29	protein_codin	424.866742	632.26647	280.21419	364.945238
ENSG000000	ANKRD13A	protein_codin	435.113842	519.92761	207.956994	250.378972
ENSG000001	TRPV4	protein_codin	0	0	6.88549758	6.32753598
ENSG000001	RBM19	protein_codin	118.558399	239.993906	139.655878	84.7675978
ENSG000001	NAA25	protein_codin	23.8822747	4.20217639	5.03520328	14.2331217
ENSG000000	ZFAND6	protein_codin	64.9443549	92.2628311	38.0180488	34.615021
ENSG000001	CHRNB4	protein_codin	0	0	0	0
ENSG000001	FAH	protein_codin	0	0	19.6625384	0
ENSG000001	MESD	protein_codin	43.4776873	80.3463252	78.7105131	49.3097069
ENSG000000	CDH1	protein_codin	0	0	0	0
ENSG000000	AARS1	protein_codin	0	0	102.415246	0
ENSG000001	VAC14	protein_codin	207.314166	204.716709	66.8547946	98.766207
ENSG000001	TANGO6	protein_codin	145.763788	211.853604	83.7230752	104.09362
ENSG000000	ARG2	protein_codin	27.9136668	46.4227409	16.4178214	27.5895757

ENSG000001	TM9SF1	protein_codin	0	0	19.6752832	0
ENSG000001	IL17B	protein_codin	0	0	0	0
ENSG000000	NDST1	protein_codin	300.274492	507.020587	198.939061	219.938143
ENSG000001	CSNK1A1	protein_codin	0	0	0	0
ENSG000001	PDGFRB	protein_codin	0	1.98525859	0	2.83536012
ENSG000000	FAT2	protein_codin	0.92178269	0	0	0.92673364
ENSG000001	CYSTM1	protein_codin	23.0074483	16.7788157	10.3964328	16.6781341
ENSG000001	PFDN1	protein_codin	86.7807311	122.332482	64.2551497	100.290633
ENSG000001	PSMD9	protein_codin	0	4.53216657	0	7.61248238
ENSG000000	ANAPC5	protein_codin	169.042384	311.03276	189.23749	139.759515
ENSG000001	BCL7A	protein_codin	6.74370206	11.9072177	9.48122497	1.98912025
ENSG000000	P2RX7	protein_codin	3.63642215	0	7.47719889	0
ENSG000001	CIT	protein_codin	0	0	0	5.35854183
ENSG000000	ATG2B	protein_codin	0	0	12.823275	0
ENSG000000	CYP46A1	protein_codin	0	1.66351005	0	0
ENSG000000	GNB5	protein_codin	20.7561346	18.2698197	8.50555442	0
ENSG000001	MYO5C	protein_codin	0	0	4.81717286	0
ENSG000000	AP4E1	protein_codin	99.2831714	46.266136	47.9106912	45.8253472
ENSG000000	ATOSA	protein_codin	0	42.9873138	63.370225	0
ENSG000000	MAPK6	protein_codin	416.92367	80.1817195	206.505241	205.136264
ENSG000001	SECISBP2L	protein_codin	41.4875311	40.2968632	0	85.9325006
ENSG000001	SPPL2A	protein_codin	107.129891	89.8842985	28.4993104	54.1599287
ENSG000001	GLCE	protein_codin	54.4437267	93.8679254	36.8676415	56.258172
ENSG000001	CORO2B	protein_codin	0	0	0	0
ENSG000001	THSD4	protein_codin	0	0	0	0
ENSG000001	SPG11	protein_codin	0	0	163.442607	178.38213
ENSG000001	SLC30A4	protein_codin	5.2072571	4.23831657	5.09390225	1.35971552
ENSG000001	EIF3J	protein_codin	119.888729	82.9704552	73.5657171	75.8801832
ENSG000000	HACD3	protein_codin	20.5677133	32.4291935	47.6974565	21.6236164
ENSG000001	APH1B	protein_codin	57.8231027	47.0439949	12.8936019	12.5047133
ENSG000001	AAGAB	protein_codin	87.234575	92.6578263	30.301047	51.3050512
ENSG000000	TIPIN	protein_codin	0	0	11.3967617	0
ENSG000001	CILP	protein_codin	0.97373996	0	2.07256802	0.98304793
ENSG000001	TRIP4	protein_codin	127.25748	135.311743	45.1122685	102.446537
ENSG000001	PARP16	protein_codin	17.7698416	0	0	0
ENSG000000	SNX1	protein_codin	87.4803417	137.285202	48.1048184	138.405619
ENSG000001	RAB11A	protein_codin	260.073917	322.244083	153.632734	187.981835
ENSG000000	DAPK2	protein_codin	6.72029745	0	0	0
ENSG000000	SLC24A1	protein_codin	0	0	7.71512225	29.0371635
ENSG000001	LACTB	protein_codin	182.249453	225.775494	88.9920133	157.118754
ENSG000001	PRPF40B	protein_codin	0	0	0	0
ENSG000001	CCNT1	protein_codin	34.1031705	68.9244403	61.7498151	0
ENSG000000	NEO1	protein_codin	0	0	5.24233044	0
ENSG000001	HCN4	protein_codin	0	0	0	0

ENSG000001:	SEMA7A	protein_codin	8.23020132	0	6.40309036	0
ENSG000001:	LOXL1	protein_codin	0	0.83308815	0	0
ENSG000000:	FLT4	protein_codin	0	0	0	0
ENSG000001	FAF2	protein_codin	149.877152	201.045487	104.830639	116.134348
ENSG000000'	CDHR2	protein_codin	0	0	0	0
ENSG000001	RNF130	protein_codin	14.2409102	0	0	6.99864316
ENSG000001	CNOT6	protein_codin	198.796206	143.7703	99.7932078	122.435139
ENSG000001:	MCF2L	protein_codin	0	4.98701314	2.97561009	0
ENSG000001:	TUBGCP3	protein_codin	142.677078	205.966214	42.5398202	118.805463
ENSG000001	RBM25	protein_codin	307.526881	87.5945758	116.733221	171.422265
ENSG000001	LTBP2	protein_codin	0	6.22925948	2.99997835	3.66441749
ENSG000001	VSX2	protein_codin	0	0	0	0
ENSG000001·	SERPINA10	protein_codin	0	0	0	0
ENSG000000	SPAG9	protein_codin	191.063274	128.496616	49.6746998	70.6577694
ENSG000001	SGCA	protein_codin	0	0	0	0
ENSG000001	MARS1	protein_codin	171.701445	162.764876	94.0740914	50.1749854
ENSG000001	ATP5F1B	protein_codin	470.117011	756.648325	369.4261	381.481254
ENSG000000'	RBMS2	protein_codin	145.002067	112.338851	56.4060255	89.808959
ENSG000001:	IKZF4	protein_codin	0	16.413227	18.892897	0
ENSG000001	PTGES3	protein_codin	348.356919	554.412674	210.217023	442.975419
ENSG000000'	PIK3C3	protein_codin	170.399993	156.303232	138.692398	159.018015
ENSG000001	MEOX2	protein_codin	0	0	0	0
ENSG000001	PHF3	protein_codin	189.480183	107.893283	78.3818609	126.571805
ENSG000001	SLC11A2	protein_codin	0	0	0	0
ENSG000001:	ATF1	protein_codin	41.0593626	64.3882597	40.4735914	61.8783756
ENSG000000:	LETMD1	protein_codin	0	51.570717	11.2657714	27.8426935
ENSG000000	EIF4B	protein_codin	1616.88896	2129.0981	707.538123	1281.14357
ENSG000000	CALCOCO1	protein_codin	21.1877434	0	4.48923563	0
ENSG000001	COPZ1	protein_codin	91.2159698	121.924954	43.4218111	81.8198845
ENSG000001	MMD	protein_codin	528.050695	782.085039	396.398412	409.919578
ENSG000001	TSPAN13	protein_codin	3.942161	15.3073993	20.7915744	5.08064288
ENSG000001	TTC23	protein_codin	6.37994175	0	0	0
ENSG000001:	NUP153	protein_codin	415.833508	356.871225	189.698257	244.126798
ENSG000000	FECH	protein_codin	0	24.7665471	4.86837527	7.17368367
ENSG000000·	RAB27B	protein_codin	981.589359	999.572159	759.562933	577.314091
ENSG000001	ZDHHC2	protein_codin	205.754698	128.99948	83.7962684	157.390308
ENSG000000:	MSR1	protein_codin	0	0	0	0
ENSG000001:	MTUS1	protein_codin	0	0	0	0
ENSG000000:	CLXN	protein_codin	0	1.2750862	0	0
ENSG000001	MCM4	protein_codin	0	0	0	0
ENSG000001	ERICH1	protein_codin	343.473614	469.50036	253.28508	256.694749
ENSG000000:	MYOM2	protein_codin	0	0	0.59964752	0
ENSG000001:	TWSG1	protein_codin	66.6824815	57.361968	38.7777698	46.5314482
ENSG000000:	SEH1L	protein_codin	38.3252306	65.0305544	20.92212	27.3901386

ENSG000001	ANKRD12	protein_codin	221.446114	72.2914444	94.5069713	88.0674212
ENSG000001	CEP76	protein_codin	0	15.0533056	14.6367783	15.5012448
ENSG000001	RBL2	protein_codin	502.217573	552.416601	258.622822	380.394391
ENSG000000	LPCAT2	protein_codin	171.732369	134.693937	42.4252169	183.806876
ENSG000001	RPGRIP1L	protein_codin	0	0	0	0
ENSG000000	CACNG4	protein_codin	0	0	0	0
ENSG000000	WIPI1	protein_codin	66.8120144	122.766997	46.8479908	51.7268754
ENSG000001	WDR59	protein_codin	32.6405179	55.3818904	27.8971909	21.6523988
ENSG000000	MTFR1	protein_codin	24.107721	14.0210227	10.0586146	20.1227577
ENSG000000	CDH19	protein_codin	0	0	0	0
ENSG000001	SMAD7	protein_codin	33.5858163	44.6268369	1.28261464	15.4992677
ENSG000001	SMAD2	protein_codin	105.144328	63.769325	53.5079558	39.5342343
ENSG000001	RNMT	protein_codin	0	0	0	0
ENSG000001	DNAJB6	protein_codin	171.891107	349.056885	58.463309	213.502693
ENSG000001	VIPR2	protein_codin	0.93584174	0	0.86279613	1.02411458
ENSG000000	KCNH2	protein_codin	0	0	0	0
ENSG000001	RHEB	protein_codin	71.6963769	77.6976927	27.3669937	44.8472666
ENSG000000	SMARCD3	protein_codin	51.0005616	0	75.1725655	15.1136219
ENSG000000	KMT2C	protein_codin	80.547624	0	0	0
ENSG000000	PSMB1	protein_codin	206.143763	273.589368	96.2317997	161.031698
ENSG000001	RBFA	protein_codin	13.4081531	14.8491903	17.7636405	0
ENSG000001	ADNP2	protein_codin	143.407321	123.505074	64.9304465	107.358527
ENSG000001	CRISPLD1	protein_codin	0	3.4925853	0	1.20618303
ENSG000001	TRPA1	protein_codin	0	0	0	0
ENSG000001	CSPP1	protein_codin	0	0	0	0
ENSG000000	TRAM1	protein_codin	343.852538	337.709301	272.349768	355.054392
ENSG000000	ARFGEF1	protein_codin	628.302521	456.996579	350.372341	486.347877
ENSG000001	ANXA13	protein_codin	0	0	0	0
ENSG000000	TMED2	protein_codin	323.775243	600.3275	276.408958	409.871728
ENSG000000	ARHGAP28	protein_codin	0	0	0	0
ENSG000001	RIMS2	protein_codin	0	0	0	0
ENSG000000	EML1	protein_codin	0	0	0	0
ENSG000001	YY1	protein_codin	498.849541	737.771298	225.763637	367.785602
ENSG000000	RCOR1	protein_codin	453.151968	623.936067	221.454991	389.300818
ENSG000001	MOB3B	protein_codin	32.321457	30.9211526	15.6421924	16.3794868
ENSG000001	ZNF175	protein_codin	28.4823118	21.3465977	22.3235741	8.08945892
ENSG000001	CD33	protein_codin	138.989827	350.239227	107.806825	209.226667
ENSG000001	PIH1D1	protein_codin	72.6074255	78.2601418	38.205063	64.9523251
ENSG000002	ENSG000002	protein_codin	0	0	0	0
ENSG000001	SCPEP1	protein_codin	426.601462	673.756831	245.630939	301.34807
ENSG000001	LPO	protein_codin	0	0	0	0
ENSG000000	VMP1	protein_codin	0	0	0	0
ENSG000000	PRR11	protein_codin	48.0777504	70.6519555	82.922796	20.6512063
ENSG000001	TRIM37	protein_codin	23.6427832	0	61.3406623	60.4257643

ENSG000001:	PKMYT1	protein_codin	0	0	0	0
ENSG000001(	LMF1	protein_codin	29.8890406	1.82192949	0	11.9140939
ENSG000000!	NUBP2	protein_codin	38.6097765	32.1200502	15.9154235	36.4751569
ENSG000000(	PKD1	protein_codin	182.808342	230.225718	0	68.5651336
ENSG000000!	RAB11FIP3	protein_codin	16.8740681	57.5839692	0	7.45874068
ENSG000001(	ELOB	protein_codin	61.3232816	90.3133957	21.7174666	51.2747781
ENSG000001:	CHTF18	protein_codin	6.09104503	11.4140518	10.1109538	0
ENSG000000(	RHBDF1	protein_codin	0	0	0	0
ENSG000001(	CLCN7	protein_codin	0	44.9570629	50.8845139	12.9042604
ENSG000001(	TELO2	protein_codin	60.0306383	95.7749276	25.8264513	35.9392992
ENSG000001(	AXIN1	protein_codin	200.826873	196.137405	127.061441	50.7542108
ENSG000000(	LIG3	protein_codin	0	14.9584863	0	0
ENSG000001	RPE65	protein_codin	0	0	0	0
ENSG000000:	IL12RB2	protein_codin	0	0	0	0
ENSG000001	ANKRD13C	protein_codin	46.1031078	22.5126659	15.0318307	30.7527793
ENSG000001	WLS	protein_codin	40.8449486	85.0377018	10.9067227	16.6684168
ENSG000001(	SLC1A1	protein_codin	0	0	0	1.2361238
ENSG000002:	ENSG000002:	lncRNA	0	0	0	0
ENSG000001·	FAM86B2	protein_codin	0	0	0	0
ENSG000001:	GLIS2	protein_codin	0	0	0	0
ENSG000000(	CREBBP	protein_codin	739.368147	943.550767	312.360184	410.481921
ENSG000001(	MGRN1	protein_codin	408.499329	493.849088	154.986144	204.229081
ENSG000000:	ALG1	protein_codin	9.68478025	6.62055832	2.11280943	5.29579002
ENSG000001(	DNAJA3	protein_codin	14.4488256	3.7430461	0	7.22453984
ENSG000001	UBN1	protein_codin	226.218975	330.416258	67.8166106	167.004225
ENSG000001(	N4BP1	protein_codin	117.122541	138.536987	33.3532078	104.387049
ENSG000001(	WSB1	protein_codin	145.761481	161.41044	72.4288947	104.002199
ENSG0000007	TRAF4	protein_codin	7.25057871	10.2229465	7.22805174	0
ENSG0000007	TRAF4	protein_codin	0	0	0	0
ENSG000001(	RGS9	protein_codin	11.1484931	12.4446611	0	10.1513176
ENSG000000(	ITGA2B	protein_codin	758.670374	2227.84413	1101.19664	735.373461
ENSG000001:	MAPT	protein_codin	0	0	0	0
ENSG000000(	DHX8	protein_codin	297.369438	276.794354	105.847465	112.592318
ENSG000000(	SLC4A1	protein_codin	0	0	0	0
ENSG000001:	KANSL1	protein_codin	27.9822911	5.19040274	3.24950499	25.5904185
ENSG000001(	CRISPLD2	protein_codin	492.329631	645.039431	82.2521075	528.963159
ENSG000001(	FOXF1	protein_codin	0	0	0	0
ENSG000001(	COTL1	protein_codin	5713.40199	8423.01073	2422.48682	4427.90938
ENSG000000(	ATP2C2	protein_codin	0	0	0	0
ENSG000001(	MLYCD	protein_codin	132.888531	190.35211	86.865061	70.250946
ENSG000001(	METTL2B	protein_codin	0	51.1909619	0	25.2945136
ENSG000001(	SMURF2	protein_codin	43.645124	56.7156833	23.3808359	14.3584938
ENSG000000(	GLP2R	protein_codin	0	0	0	0
ENSG000000(	DNAH9	protein_codin	0	0	0	0

ENSG000000	ARHGAP44	protein_codin	0	0	0	0
ENSG000001	CHD5	protein_codin	0	0	1.17336143	0
ENSG000002	ERP44	protein_codin	0	0	113.424386	0
ENSG000001	INVS	protein_codin	19.3491757	0	24.4802124	0
ENSG000001	INVS	protein_codin	71.4827641	72.7617761	53.9976196	50.9956274
ENSG000001	GINS1	protein_codin	0	0	0	0
ENSG000000	SLC12A2	protein_codin	12.79671	26.229103	40.4084502	16.1542394
ENSG000001	SLC27A6	protein_codin	0	0	0	0
ENSG000001	FBN2	protein_codin	197.566293	205.980532	76.3964083	142.329629
ENSG000000	NLRP1	protein_codin	0	82.2289952	0	128.382093
ENSG000001	RNF167	protein_codin	56.5284151	119.590403	16.6221289	76.0274902
ENSG000000	PITPNM3	protein_codin	0	0	0	0
ENSG000001	ISM1	protein_codin	0.95876591	0	0	0
ENSG000000	GNAO1	protein_codin	0	1.8080831	1.39440181	0
ENSG000000	GNAO1	protein_codin	10.8227532	17.4190723	16.9762145	0
ENSG000000	CFAP20	protein_codin	37.705238	44.595987	13.0971964	6.27832186
ENSG000001	MT2P1	processed_ps	0	0	0	0
ENSG000000	SLC12A3	protein_codin	0	0	0	0
ENSG000000	CSNK2A2	protein_codin	64.7048067	103.669357	42.7132448	43.9109461
ENSG000000	COQ9	protein_codin	42.2962952	35.9156313	12.9478734	29.8203697
ENSG000001	NLRC5	protein_codin	0	274.562476	226.33824	73.1246967
ENSG000000	SRCAP	protein_codin	0	0	0	0
ENSG000000	SETD1A	protein_codin	0	424.187719	0	0
ENSG000000	HSD3B7	protein_codin	0	5.41383495	0	1.28565786
ENSG000001	ZNF629	protein_codin	69.1437935	134.577634	28.2206282	51.9085551
ENSG000000	PTPN3	protein_codin	0	0	0	0
ENSG000001	SEC23B	protein_codin	5.54643929	20.6911369	0	5.75782109
ENSG000001	PCSK2	protein_codin	0	0	0	0
ENSG000000	DZANK1	protein_codin	0	0	0	0
ENSG000001	OGN	protein_codin	0	0	0	0
ENSG000000	SPTLC1	protein_codin	203.218578	183.783206	89.4481034	155.110194
ENSG000001	CHCHD3	protein_codin	135.694339	201.51778	114.916689	83.5989521
ENSG000000	ZC3H3	protein_codin	184.701171	206.348619	56.3886082	112.663384
ENSG000001	GSDMD	protein_codin	131.754399	244.40313	81.0883832	104.731108
ENSG000001	RPL8	protein_codin	476.745735	576.377443	142.81424	324.512987
ENSG000001	DENND3	protein_codin	0	0	0	0
ENSG000001	DOK5	protein_codin	0	0	0	0
ENSG000001	TTPAL	protein_codin	35.8357865	55.8052233	0	17.1268116
ENSG000000	ADA2	protein_codin	155.942791	127.826772	0	0
ENSG000000	CECR2	protein_codin	0	0	0	0
ENSG000001	NHERF1	protein_codin	176.56571	320.918792	119.400959	161.56047
ENSG000001	CHST8	protein_codin	0	0	0	0
ENSG000001	ATP4A	protein_codin	0	0	0	0
ENSG000001	KIRREL2	protein_codin	0	0	0	0

ENSG000001	HPN	protein_codin	0	0	0	0
ENSG000000	TYROBP	protein_codin	650.26401	730.291885	129.238329	472.703137
ENSG000000	ZBTB32	protein_codin	0	0	15.4956254	0
ENSG000001	SCN1B	protein_codin	0	0	0	0
ENSG000001	RBM42	protein_codin	185.993846	176.378216	66.8873023	98.5625148
ENSG000001	VAMP7	protein_codin	34.8387314	0	0	6.21036299
ENSG000001	CCNE1	protein_codin	5.03890069	0	15.9489161	0
ENSG000001	BPNT2	protein_codin	201.208846	219.793716	134.677426	185.037225
ENSG000001	RAB2A	protein_codin	103.769912	120.121818	47.3767476	97.0554012
ENSG000000	ANOS1	protein_codin	4.42518876	1.1150736	1.10675106	3.76232243
ENSG000000	ITCH	protein_codin	0	0	0	0
ENSG000001	HCK	protein_codin	21.5623776	49.9850205	4.55883366	5.6739068
ENSG000001	CCM2L	protein_codin	0	0	0	0
ENSG000001	CDKN2C	protein_codin	5.66364178	7.30107284	0	0
ENSG000000	TTC39A	protein_codin	0	0	0	0
ENSG000000	TTC39A	protein_codin	0.94131444	2.77320575	0	0
ENSG000001	ACIN1	protein_codin	0	0	0	0
ENSG000001	AJUBA	protein_codin	0	1.02040358	0	0
ENSG000001	TEP1	protein_codin	528.607553	639.165667	290.973455	271.615843
ENSG000002	ENSG000002	processed_ps	0	0	0	0
ENSG000001	CDH20	protein_codin	0	0	0	0
ENSG000000	PHLPP1	protein_codin	44.3187466	62.765959	16.8916655	49.1830444
ENSG000000	FBLN1	protein_codin	0	0	0	0
ENSG000001	EFCAB6	protein_codin	0	0	0	0
ENSG000000	CELSR1	protein_codin	0	0	0	0
ENSG000001	PIK3R3	protein_codin	7.69305992	0	6.81141013	0
ENSG000001	PRDX1	protein_codin	1.7867062	4.34901689	7.9374223	8.01197333
ENSG000000	RPS6KA6	protein_codin	1.28579282	26.6034512	2.34624059	0
ENSG000001	POF1B	protein_codin	0	0	0	0
ENSG000000	PGS1	protein_codin	109.565441	62.7686186	40.8347783	55.3115061
ENSG000001	QRICH2	protein_codin	0	0	0	0
ENSG000000	TIMP2	protein_codin	755.248401	1206.1962	262.886515	522.373552
ENSG000001	LGALS3BP	protein_codin	73.7488454	30.534809	29.1472219	0
ENSG000000	MOV10L1	protein_codin	0	0	0	0
ENSG000002	SHANK3	protein_codin	6.13283932	8.11075516	8.83376419	0
ENSG000000	UPF1	protein_codin	0	388.483668	163.67081	171.634009
ENSG000001	PDE4C	protein_codin	0	0	0	1.77265339
ENSG000001	ELL	protein_codin	209.612671	123.271297	65.9951515	67.1491988
ENSG000000	MAST3	protein_codin	0	109.09804	129.778128	83.528231
ENSG000001	COPE	protein_codin	386.998307	518.631809	257.969813	281.667646
ENSG000001	MAU2	protein_codin	190.269549	201.203473	91.2120655	117.280181
ENSG000001	MAU2	protein_codin	21.860979	23.7655869	15.284294	22.5674712
ENSG000001	TMEM59L	protein_codin	0	0	0	0.82004702
ENSG000000	KLHL13	protein_codin	0	0	0	0

ENSG000001	RBFOX2	protein_codin	0	0	0	0
ENSG000000	PAK3	protein_codin	0	0	0	0
ENSG000000	TRPC5	protein_codin	0	0	0	0
ENSG000000	MID2	protein_codin	1.27579209	0	0	0
ENSG000001	AMMECR1	protein_codin	0	27.0009298	17.893277	25.9654063
ENSG000001	PRKX	protein_codin	358.855428	440.885509	177.240495	204.808902
ENSG000000	RRAGB	protein_codin	0	12.1699368	0	0
ENSG000000	HUWE1	protein_codin	0	750.705893	694.575482	436.645805
ENSG000000	MAMLD1	protein_codin	0	0	0	0
ENSG000001	BPI	protein_codin	0	0	0	0
ENSG000001	SLA2	protein_codin	149.798464	389.686924	182.91866	99.334088
ENSG000000	MYH7B	protein_codin	0	2.66925511	1.22193678	1.75567627
ENSG000001	SAMHD1	protein_codin	0	0	0	0
ENSG000001	RALGAPB	protein_codin	81.3097984	0	0	11.7160129
ENSG000000	XRCC1	protein_codin	120.852387	175.727928	81.9254675	60.7597313
ENSG000000	PAFAH1B3	protein_codin	0	2.09848321	2.52934545	4.0281922
ENSG000000	MARK4	protein_codin	107.425919	144.005095	30.6556173	43.4128521
ENSG000002	ZNF225	protein_codin	14.8524465	5.35666873	0	3.35462877
ENSG000001	GRIK5	protein_codin	0	0	0	0
ENSG000001	SIM1	protein_codin	0	0	1.16131337	0
ENSG000000	HACE1	protein_codin	32.9245186	0	25.3627817	26.7263741
ENSG000001	ST3GAL3	protein_codin	0	0	0	0
ENSG000000	ATRN	protein_codin	161.584924	147.138609	67.3048423	73.5829358
ENSG000001	SIRPB1	protein_codin	81.362789	0	0	51.365118
ENSG000001	CNPY4	protein_codin	15.2965257	28.5697224	10.9480666	13.404215
ENSG000001	RASA4	protein_codin	241.483088	376.608713	89.4751744	70.1559056
ENSG000001	ZSCAN25	protein_codin	13.5568654	0	8.10053045	0
ENSG000002	ARPC1A	protein_codin	165.386851	194.981632	42.0703657	90.6755683
ENSG000000	MYDGF	protein_codin	51.0249045	96.2133984	60.7879926	48.4812155
ENSG000001	MAP2K2	protein_codin	376.484922	562.192067	118.080433	229.949026
ENSG000000	HMG20B	protein_codin	0	0	0	4.22490804
ENSG000000	TLE2	protein_codin	0	0	0	0
ENSG000000	GNA15	protein_codin	391.273671	417.888489	158.422998	215.434695
ENSG000001	DPP9	protein_codin	142.223258	160.307174	85.4192836	103.633698
ENSG000001	ZFR2	protein_codin	0	0	0	0
ENSG000001	YJU2	protein_codin	109.770357	134.538618	49.9251668	56.2931166
ENSG000001	PTPRS	protein_codin	47.3028789	136.858435	93.6792386	29.2320442
ENSG000000	TCF3	protein_codin	23.0911214	53.0143902	0	0
ENSG000000	MPND	protein_codin	0	0	0	0
ENSG000000	ANKRD24	protein_codin	2.35682755	0	0	0
ENSG000001	PIAS4	protein_codin	103.266108	134.070103	45.6219588	86.6293213
ENSG000001	ZMYND8	protein_codin	0	0	0	0
ENSG000001	CSE1L	protein_codin	97.3605169	47.6763997	61.4242755	49.4449519
ENSG000001	ZNF330	protein_codin	72.4980174	114.295802	55.4165351	76.0813894

ENSG000001	INPP4B	protein_codin	1.90203267	0	5.69642347	5.93665561
ENSG000001	GAB1	protein_codin	46.1312929	65.6772461	47.0630789	24.1169107
ENSG000001	GAB1	protein_codin	13.8736605	0	17.3052401	14.2960488
ENSG000001	UCP1	protein_codin	0	0	0	0
ENSG000001	NOMO3	protein_codin	0	0	0	0
ENSG000001	MAPK3	protein_codin	105.650903	157.203724	45.6531981	46.3115937
ENSG000001	EEF2K	protein_codin	71.6498993	90.8173037	55.6278784	41.5527304
ENSG000002	NXF5	lncRNA	0	0	0	0
ENSG000001	SYTL4	protein_codin	35.7391474	122.909728	80.4156922	50.8326326
ENSG000001	DHTKD1	protein_codin	106.682656	99.377411	93.1039558	71.7602388
ENSG000001	OPTN	protein_codin	0	0	11.4987343	0
ENSG000001	PHYH	protein_codin	16.8713844	30.3605459	0	18.4087809
ENSG000000	CRISP3	protein_codin	0	0	0	0
ENSG000001	AKAP7	protein_codin	0	0	0	0
ENSG000001	SORCS1	protein_codin	0	0	0	0
ENSG000001	MAP3K8	protein_codin	28.4418359	10.4082435	5.23733238	0
ENSG000001	EPC1	protein_codin	37.0952299	103.925477	32.4267956	0
ENSG000001	MTPAP	protein_codin	77.9765809	97.4953286	68.0361502	70.1292863
ENSG000001	LDB3	protein_codin	0	0	0	0
ENSG000000	SCARF1	protein_codin	65.2128703	97.4820532	27.9967363	0
ENSG000000	SMG6	protein_codin	241.772606	263.604392	133.358843	138.322841
ENSG000001	ASPA	protein_codin	0	1.2131677	0	1.2412893
ENSG000001	DPH1	protein_codin	0	22.6721004	0	0
ENSG000001	DPH1	protein_codin	0	0	0	0
ENSG000000	ITGAE	protein_codin	1.03307216	1.09233275	0.99718535	3.30344791
ENSG000001	PLD2	protein_codin	0	145.587482	14.5315995	59.7741901
ENSG000001	METTL16	protein_codin	40.339476	98.053998	40.097159	45.4909308
ENSG000000	SLC27A5	protein_codin	0	0	0	0
ENSG000000	ABCA7	protein_codin	225.475197	245.77434	39.4472767	100.034747
ENSG000000	ZNF264	protein_codin	97.2941885	157.25103	54.1601277	79.4176779
ENSG000000	CNN2	protein_codin	657.353957	994.340487	291.958531	298.658704
ENSG000001	A1BG	protein_codin	0	0	0	0
ENSG000001	CCDC6	protein_codin	418.048526	294.642736	164.201124	313.266819
ENSG000001	BICC1	protein_codin	0	0	0	1.18063663
ENSG000000	GGT5	protein_codin	2.5855828	0.85780187	0	2.48096736
ENSG000001	RAB36	protein_codin	3.96084691	1.9478743	14.1346161	0
ENSG000000	CABIN1	protein_codin	0	321.478271	0	151.727229
ENSG000000	SMARCB1	protein_codin	24.9561552	97.625657	87.2413607	43.2761095
ENSG000000	PRKCQ	protein_codin	25.8468038	34.7980629	16.9444707	25.7572379
ENSG000001	AKR1C4	protein_codin	0	0	1.33431184	0
ENSG000000	WDR37	protein_codin	33.0100448	0	0	12.0512646
ENSG000000	SLC17A6	protein_codin	0	0	0	0
ENSG000001	CAPZA1	protein_codin	779.500746	722.288247	406.646017	649.233448
ENSG000000	PALMD	protein_codin	0	0	0	0.81428205

ENSG000001	PLPPR5	protein_codin	0	0	0	0
ENSG000001	KIF18A	protein_codin	2.13175258	7.19466065	18.0393221	2.07774622
ENSG000001	BBOX1	protein_codin	0	0	0	0
ENSG000000	MSH4	protein_codin	0	0	1.87280356	2.34248284
ENSG000000	DGCR2	protein_codin	86.5357924	252.452313	0	123.210906
ENSG000000	CDC45	protein_codin	0	0	0	0
ENSG000000	UFD1	protein_codin	75.0798557	77.2280382	27.9921242	26.058418
ENSG000000	MED15	protein_codin	154.966626	189.153366	23.5975255	82.0241774
ENSG000000	ARVCF	protein_codin	0	0	3.24231688	0
ENSG000001	HIRA	protein_codin	89.4310785	184.539036	91.5249198	87.5908707
ENSG000001	PPM1F	protein_codin	662.17685	674.766102	299.988632	351.729643
ENSG000001	UBE2R2	protein_codin	644.72182	811.779027	254.683377	432.996855
ENSG000001	SYP	protein_codin	0	0	0	0
ENSG000001	ACTR3	protein_codin	757.346277	647.963492	383.894354	569.117991
ENSG000000	DDX18	protein_codin	199.116804	159.308515	120.891696	136.828232
ENSG000002	ARFGAP3	protein_codin	186.818729	218.540056	95.6665892	104.644302
ENSG000001	PACSIN2	protein_codin	365.852535	605.870182	148.796506	298.016024
ENSG000001	EP300	protein_codin	737.626022	679.976139	168.386781	183.787575
ENSG000001	SLC25A17	protein_codin	0	0	1.45306027	0
ENSG000001	DESI1	protein_codin	141.061168	184.685979	79.9892071	80.865775
ENSG000001	NOVA2	protein_codin	0	0	0	0
ENSG000001	PLEKHA4	protein_codin	0	2.69334264	0	0
ENSG000001	FAM83E	protein_codin	0	0	0.91328981	0
ENSG000001	MREG	protein_codin	2.08047284	5.35861658	3.94330315	0
ENSG000001	GRIN2D	protein_codin	5.79670876	7.10235396	2.72601649	0
ENSG000000	AP2S1	protein_codin	107.682089	174.348366	36.1661616	56.6349117
ENSG000001	LIG1	protein_codin	0	0	0	0
ENSG000001	OPA3	protein_codin	54.7691936	82.0555362	40.3295862	51.7422287
ENSG000001	GYS1	protein_codin	0	0	0	0
ENSG000000	EHD2	protein_codin	8.7285376	11.8049432	4.99032917	0
ENSG000000	HSD17B14	protein_codin	0	0	0	0
ENSG000000	STRN4	protein_codin	297.877195	558.484594	205.870548	184.452333
ENSG000000	GIPR	protein_codin	0	0	0	0
ENSG000001	LY9	protein_codin	9.58769425	0	0	3.58976197
ENSG000000	CLNS1A	protein_codin	10.0330662	16.3959273	0	0
ENSG000001	P2RX3	protein_codin	0	0	0	0
ENSG000000	NOX4	protein_codin	0	0	0	0
ENSG000000	TYR	protein_codin	0	0	0	0
ENSG000001	IL37	protein_codin	0	0	0	0
ENSG000001	POLR1B	protein_codin	0	25.3874559	31.6491382	0
ENSG000001	PAX8	protein_codin	0	0	0	0
ENSG000001	PAX8	protein_codin	0	0	0	0
ENSG000001	IL1A	protein_codin	89.7640276	33.0684261	21.8851471	50.8879311
ENSG000001	IL1B	protein_codin	8988.75394	2286.95262	2166.50505	2370.44615

ENSG000001	TCF25	protein_codin	100.443441	82.2588174	0	78.0878724
ENSG000001	TCF25	protein_codin	32.9323685	115.912259	20.3531145	27.3557644
ENSG000001	ZNF541	protein_codin	0	0	0	0
ENSG000001	NAPA	protein_codin	286.70513	365.115045	98.101744	174.261657
ENSG000000	EED	protein_codin	0	0	0	0
ENSG000001	HNRNPUL1	protein_codin	2.88262564	3.07078014	0	14.9856744
ENSG000000	BLVRB	protein_codin	192.882623	279.026355	66.4485169	137.514955
ENSG000002	MIA	protein_codin	0	0	0	0
ENSG000000	ITPKC	protein_codin	60.212188	91.355485	17.748217	0
ENSG000000	KCNK6	protein_codin	374.674969	625.052667	226.384389	239.64811
ENSG000001	BRD4	protein_codin	691.688576	255.073481	407.208557	366.123804
ENSG000001	IL27RA	protein_codin	338.004686	456.213878	77.6404549	222.244478
ENSG000000	WIZ	protein_codin	4.78430743	17.2027382	0	0
ENSG000001	ASF1B	protein_codin	1.43100371	16.1200408	12.3569744	3.34811128
ENSG000001	ILVBL	protein_codin	0	26.9749116	0	14.3363416
ENSG000001	FAM32A	protein_codin	318.055946	661.801747	182.953691	277.543454
ENSG000000	NOTCH3	protein_codin	1.99133547	5.83035811	2.55702528	1.89900185
ENSG000001	MED26	protein_codin	67.0957209	89.4981935	35.7704058	51.3230819
ENSG000000	CD44	protein_codin	6010.99831	6455.50331	1775.65833	3329.00571
ENSG000001	COMMD9	protein_codin	84.5215223	76.6039867	21.7259555	49.6635715
ENSG000001	C9	protein_codin	0	0	0	0
ENSG000001	LIFR	protein_codin	0	0	1.23422775	0
ENSG000000	C6	protein_codin	0	0.99283881	0	0
ENSG000001	PRKCG	protein_codin	0	0	0	0
ENSG000001	PPP1R12C	protein_codin	298.021279	406.664942	139.477024	172.670371
ENSG000000	PTPRH	protein_codin	0	0	0	0
ENSG000000	NLRP2	protein_codin	0	0	11.4386947	0
ENSG000000	ALG6	protein_codin	3.64878495	10.8820722	1.71707514	1.96017814
ENSG000001	DNAJC6	protein_codin	0	0	0	7.29804643
ENSG000001	WDR11	protein_codin	167.780867	242.667562	96.7503528	167.503611
ENSG000000	PGR	protein_codin	0	0	0	0
ENSG000000	BIRC3	protein_codin	274.766668	89.307526	39.479164	91.8101625
ENSG000001	CEP126	protein_codin	4.36424182	0	7.36691047	2.28444177
ENSG000002	CROCCP3	lncRNA	22.7467037	23.436058	1.63396749	3.35433735
ENSG000001	SLC10A3	protein_codin	73.2348487	77.422617	37.8363521	0
ENSG000000	ATP2B3	protein_codin	0	0	0	0
ENSG000001	LUC7L2	protein_codin	89.0695465	0	127.144404	0
ENSG000000	PARP12	protein_codin	166.876035	220.956177	39.1772668	67.9445294
ENSG000001	P4HA1	protein_codin	0	11.3465189	11.3018865	0
ENSG000001	VPS26A	protein_codin	24.5616173	2.30948091	10.6664338	6.84091767
ENSG000001	PALD1	protein_codin	0	0	0	0
ENSG000000	APLP2	protein_codin	0	1508.92699	496.061243	1073.22706
ENSG000001	DDX25	protein_codin	0	0	0	0
ENSG000000	CDON	protein_codin	1.93370266	0	0	0

ENSG000001	FOXRED1	protein_codin	0	0	0	0
ENSG000001	DCPS	protein_codin	45.8774846	80.7311852	71.8112494	31.0726923
ENSG000001	SIAE	protein_codin	0	2.53330368	0	0
ENSG000001	LCN1	protein_codin	0	0	0	0
ENSG000001	KCNT1	protein_codin	0	0	0	0
ENSG000001	BARHL1	protein_codin	0	0	0	0
ENSG000001	DBH	protein_codin	0	0	0	0
ENSG000001	ARID3A	protein_codin	329.955231	407.478131	179.290115	188.591518
ENSG000001	ELANE	protein_codin	5.75299111	5.93898539	3.160019	0.9863569
ENSG000000	MTIF2	protein_codin	86.7621456	72.7384119	15.0314634	44.7801823
ENSG000001	CCDC88A	protein_codin	47.5833729	0	0	0
ENSG000001	ASB3	protein_codin	31.4139381	48.1434582	43.6401504	33.9695245
ENSG000001	TANC1	protein_codin	0	4.76790475	7.67393218	1.95981331
ENSG000000	LY75	protein_codin	160.130153	68.2535091	104.379269	128.571579
ENSG000001	ACVR1	protein_codin	5.48713569	17.4732549	30.7530916	0
ENSG000001	CD81	protein_codin	82.1500538	93.1333082	15.26735	31.7373905
ENSG000000	TOLLIP	protein_codin	0	0	0	25.146347
ENSG000000	OSBPL5	protein_codin	0	0	23.329525	40.8521464
ENSG000001	CNRIP1	protein_codin	0	2.6505192	0	0
ENSG000001	PNO1	protein_codin	33.391341	61.7782221	25.7234222	21.401285
ENSG000001	TAF1B	protein_codin	42.8734131	45.0693945	36.5541725	23.2244584
ENSG000001	CNTN3	protein_codin	13.521158	26.9811707	7.23635368	13.0651148
ENSG000001	PDZRN3	protein_codin	0	0	0	0
ENSG000000	CHRDL2	protein_codin	0	0	0	0
ENSG000001	SPCS2	protein_codin	61.7733273	59.7746144	63.6263458	39.9007673
ENSG000001	ARHGEF17	protein_codin	8.87075075	11.8281595	3.66554433	4.83349392
ENSG000000	POLD3	protein_codin	134.014233	151.993408	83.2287958	99.8079914
ENSG000001	SELP	protein_codin	208.859722	292.85673	193.547516	117.768913
ENSG000000	SUCO	protein_codin	137.23003	63.4447601	25.260407	97.6609413
ENSG000000	SNRNP40	protein_codin	84.5809193	127.574514	48.4317385	55.8137805
ENSG000001	DNAJC8	protein_codin	311.909002	551.624776	225.554115	312.1409
ENSG000001	MECR	protein_codin	0	0	0	0
ENSG000001	TFCP2L1	protein_codin	8.02409041	4.40946	4.86140551	0
ENSG000000	PTPN4	protein_codin	41.1968805	31.3768041	31.6540346	33.5586416
ENSG000000	CLASP1	protein_codin	12.7468414	0	8.59519183	6.32404701
ENSG000001	EPB41L5	protein_codin	0	2.29512398	2.05290464	0
ENSG000001	LHX4	protein_codin	0	1.03936251	0	0
ENSG000001	FAM20B	protein_codin	209.326265	243.08606	136.430521	111.907112
ENSG000001	EPAS1	protein_codin	10.8557997	9.15469514	7.59196514	11.9287061
ENSG000001	EPCAM	protein_codin	0	1.29385874	0	0
ENSG000000	SRBD1	protein_codin	95.4588877	108.649726	96.9043836	71.0509136
ENSG000000	SDF4	protein_codin	28.5434014	38.4962483	0	4.8815842
ENSG000001	SGO1	protein_codin	0	0	10.292014	0
ENSG000001	KAT2B	protein_codin	236.401462	192.444728	159.86008	157.647408

ENSG000001	FNBP4	protein_codin	463.218833	355.77296	182.412047	202.428501
ENSG000002	NDUFS3	protein_codin	41.4217262	46.0016463	23.7609708	19.6617947
ENSG000001	ACCS	protein_codin	20.3783591	65.2285552	2.8445214	4.88143937
ENSG000000	CHMP2B	protein_codin	96.2046359	111.565996	76.3150886	123.884495
ENSG000001	EIF2AK4	protein_codin	359.680517	403.006334	300.399177	207.519147
ENSG000000	WDR76	protein_codin	4.56739989	7.61976169	8.62526683	9.76000678
ENSG000000	TYRO3	protein_codin	2.22149375	0	0	0
ENSG000000	LTK	protein_codin	0	0	0	0
ENSG000000	TP53BP1	protein_codin	0	0	0	0
ENSG000001	ZNF106	protein_codin	49.7568069	54.5929527	0	45.5402746
ENSG000001	SLC25A12	protein_codin	0	12.635444	31.2124959	0
ENSG000001	AKT3	protein_codin	0	0	0	0
ENSG000001	DESI2	protein_codin	6.1705468	0	0	1.8354946
ENSG000001	GREB1	protein_codin	0	0	0	0
ENSG000000	SYT7	protein_codin	0	0	0	0
ENSG000001	OSBP	protein_codin	372.722108	370.612452	225.752434	239.323578
ENSG000001	ZC2HC1A	protein_codin	0	0	0	0
ENSG000001	IL7	protein_codin	0	0	0	0
ENSG000001	CHMP3	protein_codin	235.365394	362.674584	81.1774151	141.799827
ENSG000000	POLR1A	protein_codin	113.181367	147.755116	78.9564401	56.0790299
ENSG000001	GNLY	protein_codin	13.2092215	23.0265574	0	0
ENSG000001	VAMP8	protein_codin	199.782243	290.364316	102.927213	135.943984
ENSG000000	CAPG	protein_codin	357.668078	530.197269	43.4029504	227.50212
ENSG000000	MAP4K3	protein_codin	62.907032	68.0649836	28.4927608	49.9040711
ENSG000001	RWDD3	protein_codin	0	0	0	0
ENSG000001	RND3	protein_codin	0	0	0	0
ENSG000000	USE1	protein_codin	26.7542282	52.8438048	19.9007656	26.3371533
ENSG000001	STAM2	protein_codin	119.315968	88.5251555	62.9007073	75.4572711
ENSG000001	GRB14	protein_codin	0	0	0	0
ENSG000001	STRN	protein_codin	415.334452	211.909803	209.711215	270.070994
ENSG000001	CHIC2	protein_codin	144.35182	137.134965	53.0480464	93.1177124
ENSG000001	KDR	protein_codin	0	0	0	0
ENSG000000	LNX1	protein_codin	0	0	0	0
ENSG000001	TNFRSF8	protein_codin	0	0	0	0
ENSG000000	KIF1B	protein_codin	99.6595925	185.954179	0	94.2777658
ENSG000000	PKP1	protein_codin	0	0	0	0
ENSG000000	STK17B	protein_codin	423.566765	232.328281	270.565822	0
ENSG000001	GTF3C3	protein_codin	5.528548	46.5831432	17.3853746	44.2672033
ENSG000001	COQ10B	protein_codin	134.340629	110.988327	50.8669708	81.0670938
ENSG000001	PEX5L	protein_codin	0	0	0	0
ENSG000000	USP13	protein_codin	0	0	0	0
ENSG000001	PIK3CA	protein_codin	100.506734	50.9454841	28.8899349	40.4942845
ENSG000001	MFN1	protein_codin	66.1940873	0	0	20.1062265
ENSG000001	TAF12	protein_codin	12.5846434	13.5407331	9.33156395	0

ENSG000001	MAN1C1	protein_codin	0	33.6003327	15.1232503	0
ENSG000000	SLC9A1	protein_codin	145.510387	297.48639	86.5034488	125.036502
ENSG000000	GATB	protein_codin	11.3700553	58.8124672	0	0
ENSG000001	EHBP1	protein_codin	19.2440193	0	47.9198787	16.1173035
ENSG000001	SLC7A6OS	protein_codin	59.0962212	135.29294	49.624767	36.9534374
ENSG000002	CKLF	protein_codin	27.7531149	110.329641	19.6217726	48.901799
ENSG000002	LCAT	protein_codin	5.29611783	10.0083409	6.39137579	7.54624452
ENSG000001	CTCF	protein_codin	139.955637	318.206654	115.921268	77.2922564
ENSG000000	CDH3	protein_codin	0	0	0	0
ENSG000001	IFT46	protein_codin	5.43789126	0	3.09406487	3.82137735
ENSG000001	IFT46	protein_codin	21.0119059	20.9034309	0	0
ENSG000001	NECTIN1	protein_codin	40.9688232	85.4237665	15.324708	22.1116711
ENSG000001	SC5D	protein_codin	14.5434151	4.67035031	9.56997791	5.70831571
ENSG000000	ARCN1	protein_codin	289.929021	446.973307	146.628208	306.488983
ENSG000001	TREH	protein_codin	0	0	0	0
ENSG000001	UPK2	protein_codin	0	0	0	0
ENSG000001	CBL	protein_codin	417.214251	567.563514	231.421983	329.736474
ENSG000000	MCAM	protein_codin	0	0	0	0
ENSG000001	TECTA	protein_codin	0	0	0	0
ENSG000000	GPC1	protein_codin	0	0	0	0.78957059
ENSG000000	FARP2	protein_codin	15.5040993	7.43458244	13.8382279	7.29914489
ENSG000001	SLC5A7	protein_codin	0	0	0	0
ENSG000000	NGEF	protein_codin	0	0	0	0
ENSG000000	SP100	protein_codin	50.359085	75.4211953	82.6981033	57.5500086
ENSG000000	DGKD	protein_codin	541.593897	586.63266	233.426845	189.99698
ENSG000001	EFHD1	protein_codin	0	0	0	0
ENSG000000	DNAJC10	protein_codin	0	0	168.859427	0
ENSG000001	TUBB4A	protein_codin	0	0	0	0
ENSG000000	MCOLN1	protein_codin	47.2540673	59.4001509	17.5427249	49.8434245
ENSG000001	GPR108	protein_codin	106.619411	219.201174	48.8070922	93.7053454
ENSG000001	SLC25A23	protein_codin	0	0	0	0
ENSG000001	DGUOK	protein_codin	0	0	5.96015994	8.67226795
ENSG000001	LOXL3	protein_codin	0	15.2528878	26.1593294	31.7592957
ENSG000000	ITGA6	protein_codin	0	0	0	0
ENSG000001	HAT1	protein_codin	84.722336	56.7497043	39.4963209	43.1364938
ENSG000001	ATF2	protein_codin	104.740734	64.7619978	0	61.312924
ENSG000001	GPSM2	protein_codin	0	0	0	5.5409312
ENSG000000	SLC25A24	protein_codin	170.986364	116.393702	75.1322588	115.611714
ENSG000000	LAMC2	protein_codin	0	0.92854711	0	0
ENSG000001	OSGEPL1	protein_codin	4.6527887	3.30478662	5.36314286	0
ENSG000000	MCM6	protein_codin	59.3286484	156.620662	67.8916411	47.994841
ENSG000000	CCNT2	protein_codin	64.3113485	37.5625785	0	25.1848097
ENSG000001	RAB3GAP1	protein_codin	0	0	0	15.7369695
ENSG000001	ZRANB3	protein_codin	12.1603511	0	7.01857329	5.66890975

ENSG000000	R3HDM1	protein_codin	0	0	0	17.7758767
ENSG000001	DARS1	protein_codin	174.912205	195.398423	123.960934	189.978021
ENSG000001	LCT	protein_codin	0	0	0	0.97550211
ENSG000000	AGPS	protein_codin	95.1174886	0	10.1664202	86.4224981
ENSG000001	ORC4	protein_codin	17.814187	0	41.3252596	0
ENSG000001	KYNU	protein_codin	131.003225	66.3584293	41.7250257	46.3612737
ENSG000000	ERO1B	protein_codin	6.10408516	1.3837632	8.26215645	5.55597502
ENSG000000	ARID4B	protein_codin	382.928399	84.0602671	0	0
ENSG000001	NID1	protein_codin	201.965431	559.278405	170.489907	121.35328
ENSG000001	CYTIP	protein_codin	1425.45589	515.076051	412.279336	556.243166
ENSG000000	MUL1	protein_codin	51.4872798	75.6370579	26.1098466	44.1961625
ENSG000000	CAPZB	protein_codin	919.310563	1453.97227	197.984223	757.564445
ENSG000000	CAPZB	protein_codin	37.419215	17.8680557	11.4438194	8.59708201
ENSG000001	ECE1	protein_codin	140.27428	201.262525	0	51.6231589
ENSG000000	EIF4G3	protein_codin	93.5600033	295.237086	601.912906	308.467209
ENSG000001	NMU	protein_codin	0	0	0	0
ENSG000001	PPAT	protein_codin	5.67509458	6.14669642	18.1174112	6.22040797
ENSG000001	PAICS	protein_codin	0	13.3216765	0	5.91149291
ENSG000001	SRD5A3	protein_codin	0	18.2911822	0	0
ENSG000001	CRACD	protein_codin	0	0	0	0
ENSG000000	NOA1	protein_codin	95.6897796	155.582902	55.1363231	66.99743
ENSG000001	POPDC2	protein_codin	0	0	0	0
ENSG000000	POLQ	protein_codin	19.172164	18.3666135	35.6792883	10.4778326
ENSG000001	UPK1B	protein_codin	0	0	0	0
ENSG000000	GSK3B	protein_codin	318.00179	214.876858	77.4046651	205.892303
ENSG000001	TIMMDC1	protein_codin	0	31.8859571	0	0
ENSG000000	ARHGAP31	protein_codin	623.745405	647.8469	219.317775	287.39723
ENSG000001	CD80	protein_codin	4.66691157	2.11327714	3.83979186	0
ENSG000001	CHST10	protein_codin	5.81591618	7.68292005	0.90561914	0.97458806
ENSG000001	PDCL3	protein_codin	36.4912867	53.2898899	51.6193291	48.2696873
ENSG000001	TXNDC9	protein_codin	18.4143864	29.3267935	27.686431	32.2630065
ENSG000001	IL1RL2	protein_codin	3.99060066	0	0.91666451	0
ENSG000000	RPL31	protein_codin	445.740757	613.506616	246.721257	335.425809
ENSG000001	IL18RAP	protein_codin	0	0	0	0
ENSG000001	GFM1	protein_codin	24.2960351	50.3008168	32.4470172	66.4420236
ENSG000000	LXN	protein_codin	17.7949968	27.0296546	12.0197662	10.1561897
ENSG000001	MFSD1	protein_codin	0	0	0	0
ENSG000000	CASP8	protein_codin	0	0	0	0
ENSG000000	ALS2	protein_codin	0	0	0	0
ENSG000000	NOP58	protein_codin	201.511984	153.094905	173.799177	122.67403
ENSG000001	OCIAD1	protein_codin	28.839234	37.7440994	17.1899427	27.6946082
ENSG000001	SLAIN2	protein_codin	145.422019	119.637529	67.3773934	94.2379378
ENSG000000	TXK	protein_codin	0	2.82215102	0	0
ENSG000001	GABRA4	protein_codin	0	0	0	1.68385789

ENSG0000007	TOP2B	protein_codin	95.7534245	59.1342094	34.786128	75.6316913
ENSG0000010	YWHAE	protein_codin	621.703138	675.821424	171.721031	451.674688
ENSG0000013	ARHGAP24	protein_codin	19.5141499	7.62959604	2.39762976	0
ENSG0000013	FAM13A	protein_codin	2.85378613	7.95062053	0	2.79413226
ENSG0000013	HERC6	protein_codin	21.9146251	21.230198	0	26.354546
ENSG0000013	HERC5	protein_codin	96.5903006	64.5815641	56.699532	56.9888821
ENSG0000013	PCDH10	protein_codin	0	0	0	0
ENSG0000013	NDST4	protein_codin	0	0	0	0
ENSG0000014	ANK2	protein_codin	0	0	0	0
ENSG0000013	ZGRF1	protein_codin	0	0	0	0
ENSG000001	CRYGD	protein_codin	0	0	0	0
ENSG000001	ADAM23	protein_codin	4.83584556	2.46256225	0	0
ENSG000001	PIKFYVE	protein_codin	447.444428	239.681376	256.655348	304.292813
ENSG000001	BCHE	protein_codin	0	0	0	0
ENSG0000009	SI	protein_codin	0	0	0	0
ENSG0000023	SLC19A4P	lncRNA	0	0	0	0
ENSG0000013	COPS4	protein_codin	105.598386	66.6435285	38.52401	71.5013385
ENSG0000013	PRKG2	protein_codin	4.32158916	0	6.24860112	0
ENSG0000013	RASGEF1B	protein_codin	0	39.7492103	0	56.2356903
ENSG0000013	SEC31A	protein_codin	0	0	0	0
ENSG0000013	GPAT3	protein_codin	9.05074642	25.0250403	9.54941096	52.7003739
ENSG0000003	CUL3	protein_codin	102.834681	26.6900801	30.0638764	57.9033169
ENSG0000006	GUCY1B1	protein_codin	252.633297	305.121116	159.649864	120.273382
ENSG0000012	GRIA2	protein_codin	0	0	0	0
ENSG0000010	GLRB	protein_codin	0	0	0	0
ENSG0000010	RAPGEF2	protein_codin	58.7863676	17.6093326	29.6655286	33.2385622
ENSG0000005	FNIP2	protein_codin	181.497644	109.648227	39.5240793	98.6267316
ENSG000001	C2orf42	protein_codin	0	20.0693348	0	0
ENSG0000007	ADD2	protein_codin	1.63050985	6.76653639	2.33553078	0
ENSG000001	PCYOX1	protein_codin	0	0	20.9653917	0
ENSG0000005	MXD1	protein_codin	1270.62699	808.442167	604.688302	698.189561
ENSG0000007	ZNF638	protein_codin	383.16113	459.665039	236.143328	448.112216
ENSG0000012	ATP8A1	protein_codin	144.883336	158.296072	135.169674	112.651044
ENSG000000	SLC30A9	protein_codin	89.412915	55.1584244	34.9317135	45.0971008
ENSG0000010	TMEM33	protein_codin	27.5656813	38.6606759	8.33855259	18.7936459
ENSG0000009	SEC22C	protein_codin	74.7140324	63.9528932	40.0080703	30.425382
ENSG0000004	CDH10	protein_codin	0	0	0	0
ENSG000001	CD86	protein_codin	8.73617453	10.3117445	20.3466819	0
ENSG0000012	CSTA	protein_codin	290.169055	411.235852	121.090766	222.092142
ENSG0000008	SLC4A4	protein_codin	0	0	0	0
ENSG0000013	FGF2	protein_codin	0	0	0	2.8746363
ENSG0000013	BBS7	protein_codin	11.023918	16.1179615	6.09050773	23.4755401
ENSG0000013	BLTP1	protein_codin	214.100092	83.5190234	83.9668178	35.5315777
ENSG000001	RBBP5	protein_codin	37.5616544	36.2139769	59.5712639	48.2809617

ENSG000001	IFT57	protein_codin	58.2397267	32.6237737	50.0427744	39.4365331
ENSG000001	FRMD4A	protein_codin	0	0	0	0
ENSG000001	UBE2S	protein_codin	31.2197959	47.3157231	20.6604809	28.9244683
ENSG000001	GZMM	protein_codin	0	0.85166207	0	0
ENSG000001	SHC2	protein_codin	0	0	0	0
ENSG000000	PHRF1	protein_codin	438.67449	616.697518	246.145089	272.432219
ENSG000000	PALM	protein_codin	0	1.35071806	0	0
ENSG000000	IL11	protein_codin	0	0	0	0
ENSG000001	BMPR1B	protein_codin	0	0	0	0
ENSG000001	RAP1GDS1	protein_codin	25.6341863	0	41.366733	0
ENSG000000	AGA	protein_codin	19.3186016	52.0524444	26.8309075	31.8580461
ENSG000001	NEIL3	protein_codin	20.9788553	62.5190247	21.4978297	28.5076171
ENSG000001	RAB17	protein_codin	0	0	0	0
ENSG000001	MLPH	protein_codin	0	0	0	0
ENSG000000	ASB1	protein_codin	28.6806489	48.7132371	11.5103516	20.4231234
ENSG000000	CP	protein_codin	0	0	0	0
ENSG000001	WNT5A	protein_codin	0	1.28522451	0	0
ENSG000001	THRA	protein_codin	21.501909	23.4495578	11.848536	0
ENSG000001	CNTNAP1	protein_codin	0	0	0	0
ENSG000001	PSMD3	protein_codin	165.915183	270.400367	120.027763	136.427887
ENSG000000	SMARCE1	protein_codin	26.3759921	30.4433789	0	0
ENSG000001	RAPGEFL1	protein_codin	0	6.56143184	1.33712677	0
ENSG000001	CASC3	protein_codin	295.935188	381.904602	132.220209	163.128567
ENSG000000	ATP6V0A1	protein_codin	39.1844755	82.1926153	50.3392656	0
ENSG000001	KRT24	protein_codin	0	0	0	0
ENSG000001	STAT3	protein_codin	34.6928063	163.313407	236.246719	129.142447
ENSG000001	FBXL20	protein_codin	287.425551	329.294951	178.114776	136.715171
ENSG000000	KCNH4	protein_codin	0	0	0	0
ENSG000001	NNT	protein_codin	0	0	0	12.6730565
ENSG000000	FGF10	protein_codin	0	1.17287177	0	1.17779347
ENSG000001	MTRR	protein_codin	7.31114492	0	0	0
ENSG000001	FASTKD3	protein_codin	1.91833973	13.1740981	5.05126773	4.47272586
ENSG000000	NSUN2	protein_codin	122.981172	163.997106	140.491573	76.735324
ENSG000000	MECOM	protein_codin	0	0	0	0
ENSG000001	LRRC31	protein_codin	0	0	0	0
ENSG000001	SERPINI2	protein_codin	0	0	0	0
ENSG000001	UFSP2	protein_codin	11.5016247	22.992336	3.8556718	7.49588072
ENSG000001	KLKB1	protein_codin	0	0	0	0
ENSG000000	F11	protein_codin	0	0	0	0
ENSG000001	SNX25	protein_codin	0	0	0	0
ENSG000001	FNDC4	protein_codin	0	0	0	0
ENSG000000	CAD	protein_codin	35.7594122	51.2103674	45.64506	14.9819518
ENSG000001	POMC	protein_codin	0	0	0	0
ENSG000001	DNMT3A	protein_codin	17.6429658	0	0	0

ENSG000000	RAB10	protein_codin	510.082992	701.290548	165.838113	460.788848
ENSG000001	DNAJC27	protein_codin	13.2620642	0	8.25806316	11.9916067
ENSG000000	KIF3C	protein_codin	59.3850307	123.713979	47.0833315	0
ENSG000000	FOSL2	protein_codin	1812.14038	1680.07636	771.865019	1149.15601
ENSG000000	GCKR	protein_codin	0	0	0	0
ENSG000001	GPN1	protein_codin	18.7073865	26.1405485	7.975732	6.9827931
ENSG000000	EFR3B	protein_codin	2.39054864	0	0	0
ENSG000001	GTF3C2	protein_codin	98.6496268	182.459203	83.701903	86.4784675
ENSG000001	CSPG5	protein_codin	0	0	0	0
ENSG000000	TMEM40	protein_codin	1.45283079	6.94035839	0	0
ENSG000000	TP63	protein_codin	0	8.85632449	19.9092181	0
ENSG000001	CLDN16	protein_codin	1.32127086	2.60229882	3.1904332	8.10054475
ENSG000001	PLAAT1	protein_codin	0	23.336085	0	0.94696298
ENSG000001	ITGA9	protein_codin	2.89053114	9.87042966	11.4041369	3.2234608
ENSG000001	FGFRL1	protein_codin	53.4556216	0	24.6208807	12.6863645
ENSG000000	MAEA	protein_codin	20.7090384	17.78827	0	0
ENSG000000	ADD1	protein_codin	28.9385935	0	0	0
ENSG000001	TMEM175	protein_codin	0	17.3846705	24.7489705	0
ENSG000000	PLPP1	protein_codin	0	0	0	0
ENSG000000	GPBP1	protein_codin	0	0	0	0
ENSG000001	SLC2A9	protein_codin	48.0109162	62.0532692	34.3709301	39.1473081
ENSG000000	WDR1	protein_codin	18.108296	11.4640233	121.599408	0
ENSG000001	MMRN1	protein_codin	119.883535	294.477022	148.279661	73.7870958
ENSG000001	PDE5A	protein_codin	81.5989841	215.050239	132.206924	90.843375
ENSG000001	PRDM5	protein_codin	0	0	2.27325561	7.3688943
ENSG000001	TRPC3	protein_codin	0	0	0	1.18217649
ENSG000001	MIER2	protein_codin	125.952321	108.119435	29.4219452	57.4056703
ENSG000001	LYL1	protein_codin	218.072278	378.239787	129.184424	163.281257
ENSG000000	HOOK2	protein_codin	25.6793191	26.6283209	12.3158959	10.0706197
ENSG000000	COL5A3	protein_codin	0	0	0	0
ENSG000000	ICAM1	protein_codin	1632.49149	979.529347	603.571094	555.615681
ENSG000001	OLFM2	protein_codin	0	0	0	0
ENSG000001	KLF1	protein_codin	0	4.66305834	0.7755236	0
ENSG000000	RIMS1	protein_codin	0	0	0	0
ENSG000001	C3orf52	protein_codin	0	12.0227275	0	0
ENSG000000	SIDT1	protein_codin	0	0	26.8500536	0
ENSG000000	PI4K2B	protein_codin	75.282371	63.7050188	24.2701792	53.086702
ENSG000001	TBC1D19	protein_codin	12.6719228	4.20139907	4.29627475	0
ENSG000001	PPARGC1A	protein_codin	0	1.54682208	1.80722162	7.80205911
ENSG000000	SEL1L3	protein_codin	0	0	108.710796	0
ENSG000001	F13A1	protein_codin	3667.18322	7945.68219	4532.90554	3526.64231
ENSG000001	RIOK1	protein_codin	0	57.6431116	33.260273	18.3512414
ENSG000001	NUP54	protein_codin	48.2288018	0	35.1257989	41.1965362
ENSG000001	CXCL9	protein_codin	8.16230653	17.2229416	3.63729927	10.4374554

ENSG000001	SEPTIN11	protein_codin	198.959988	58.3301827	128.133943	99.0558932
ENSG000001	SCARB2	protein_codin	380.35804	302.106561	0	324.200502
ENSG000001	USO1	protein_codin	35.0324098	0	46.2464161	7.67284203
ENSG000001	ANXA3	protein_codin	0	2.28085745	0	0
ENSG000001	ARSB	protein_codin	240.434978	370.649629	76.5096437	144.994453
ENSG000001	PDE8B	protein_codin	2.18437274	0	0	0
ENSG000000	RAD18	protein_codin	33.6625835	14.3978093	11.673	11.8572895
ENSG000001	SLC12A7	protein_codin	609.777939	664.047714	109.002104	310.452951
ENSG000000	SDHA	protein_codin	240.577068	226.642551	133.778644	123.650634
ENSG000002	PDCD6	protein_codin	82.1746905	173.742302	16.7485986	44.8982564
ENSG000001	MTMR12	protein_codin	125.075847	118.492874	63.1726754	36.2758461
ENSG000001	CEP72	protein_codin	2.95234965	16.6083846	8.44474133	3.91649533
ENSG000000	SLC9A3	protein_codin	2.053757	0	0	0
ENSG000001	XRN1	protein_codin	197.611638	125.88518	160.509947	46.6028446
ENSG000001	GRK7	protein_codin	0	0	0	0
ENSG000001	GRPEL1	protein_codin	64.5895249	93.1145533	31.741403	69.8514035
ENSG000000	EVC	protein_codin	7.9844711	33.5701331	2.8295479	17.8435013
ENSG000001	LMAN2L	protein_codin	15.9399654	32.2772279	12.7229661	13.0720906
ENSG000000	MGAT4A	protein_codin	0	0	0	0
ENSG000001	ZAP70	protein_codin	0	0	0	0
ENSG000000	PPP2R3A	protein_codin	1.4546999	6.49179788	2.01927868	2.94872603
ENSG000001	CEP70	protein_codin	6.67016899	0	0	4.00575162
ENSG000002	ACAD11	protein_codin	20.1067271	0	30.4579646	26.2862805
ENSG000000	UBA5	protein_codin	0	0	0	0
ENSG000000	ASTE1	protein_codin	0	15.5758088	10.6082795	5.98922747
ENSG000000	CDV3	protein_codin	627.472299	700.942037	286.117093	542.813129
ENSG000001	MRPL3	protein_codin	71.0227316	115.972641	45.5847764	52.0070219
ENSG000001	GALNT7	protein_codin	98.0162708	64.6555568	46.7853944	75.7862296
ENSG000000	SOX30	protein_codin	0	0	0	0
ENSG000001	GCNT2	protein_codin	16.9931815	26.7931809	2.59101453	12.7806591
ENSG000001	BST1	protein_codin	179.376317	379.99755	74.4927432	145.37842
ENSG000000	FAM184B	protein_codin	0	0	0	0
ENSG000000	DGKG	protein_codin	130.038819	159.108497	82.8148284	98.815741
ENSG000000	MAP3K13	protein_codin	1.13505184	3.32566803	10.7163525	0
ENSG000000	DNAJB11	protein_codin	102.895154	197.118432	220.360831	130.30052
ENSG000000	FETUB	protein_codin	0	0	0	0
ENSG000000	DEPDC1B	protein_codin	5.65555512	0	13.8496756	0
ENSG000000	ERCC8	protein_codin	0	0	0	0
ENSG000001	SSR3	protein_codin	265.5232	300.499085	261.705611	200.129455
ENSG000000	MGLL	protein_codin	103.037575	110.971624	155.419993	57.6997898
ENSG000000	MCM2	protein_codin	7.40020707	27.8407291	46.0572176	4.81887838
ENSG000000	RAB7A	protein_codin	517.34991	738.768013	142.036335	278.144842
ENSG000001	CFAP92	protein_codin	1.36514858	2.52432664	3.04527908	0
ENSG000000	ZFR	protein_codin	793.280466	744.862272	450.367547	566.988943

ENSG000001	GOLPH3	protein_codin	238.045937	346.569783	125.518224	216.17408
ENSG000001	CDH6	protein_codin	0	1.60442316	1.30150736	0
ENSG000001	SUB1	protein_codin	257.948368	166.298546	267.431593	287.865125
ENSG000001	NPR3	protein_codin	0	18.5563489	32.7566411	0
ENSG000000	VCAN	protein_codin	9012.6326	6762.19144	9154.97861	9114.14053
ENSG000001	RASGRF2	protein_codin	0	6.46911162	1.66127131	4.25233238
ENSG000001	MSH3	protein_codin	169.428805	56.693246	43.0994916	73.486595
ENSG000001	CPEB4	protein_codin	373.290388	389.190564	253.953302	333.284042
ENSG000001	STC2	protein_codin	0	0	0	0
ENSG000001	ATP6V0E1	protein_codin	139.225509	298.955928	91.9402236	209.646072
ENSG000000	FBXW11	protein_codin	0	0	0	0
ENSG000000	THOC3	protein_codin	33.8920183	46.543564	0	42.0153329
ENSG000000	RPL26L1	protein_codin	34.5204367	105.594995	21.4939002	39.6470155
ENSG000000	DNAH5	protein_codin	0	0	0	0
ENSG000000	WDR70	protein_codin	45.7930865	63.6767864	6.89257885	30.2833372
ENSG000000	RAI14	protein_codin	0	0	0	0
ENSG000001	TARS1	protein_codin	130.218908	182.978139	104.822701	124.880506
ENSG000000	SLC1A3	protein_codin	0	0	0	0
ENSG000001	AMBP	protein_codin	0	0	0	0
ENSG000000	WHRN	protein_codin	0	0	0	0
ENSG000001	COBL	protein_codin	0	0	0	0
ENSG000001	ARRDC3	protein_codin	281.029012	295.126155	226.128341	212.876644
ENSG000001	SLF1	protein_codin	39.1370626	31.275553	12.6380219	15.4250105
ENSG000001	CENPE	protein_codin	0	24.6844003	126.687468	0
ENSG000001	TET2	protein_codin	606.612723	499.175014	678.246889	597.504439
ENSG000002	ARHGEF38	protein_codin	0	0	0	0
ENSG000001	ENPEP	protein_codin	0	2.47599271	1.14606307	0
ENSG000001	CASP6	protein_codin	33.2617168	36.4640555	18.0513942	4.90105965
ENSG000001	LEF1	protein_codin	3.38617394	0	0	0
ENSG000001	EGF	protein_codin	118.96165	62.2577935	41.646618	20.8724165
ENSG000001	PAPSS1	protein_codin	233.315501	210.771814	116.504479	129.852747
ENSG000001	SEC24B	protein_codin	144.182628	79.4758493	65.6534403	134.167592
ENSG000001	MCM8	protein_codin	7.95480276	0	0	0
ENSG000001	NME5	protein_codin	0	0	0	0
ENSG000001	PAIP2	protein_codin	775.490475	841.189343	459.465252	597.126563
ENSG000001	SIL1	protein_codin	0	1.80668984	0	0
ENSG000000	IPCEF1	protein_codin	0	0	0	0
ENSG000001	ANKRD7	protein_codin	0	0	0	0
ENSG000000	MRPL22	protein_codin	5.04135076	0	0	1.58730715
ENSG000001	IQCG	protein_codin	44.3035784	32.2096285	28.6303145	15.6639753
ENSG000000	LSG1	protein_codin	111.928173	144.640457	68.6018639	94.2415005
ENSG000002	ENSG000002	processed_ps	0	0	0	0
ENSG000000	PCNP	protein_codin	157.027647	112.690638	40.737621	83.5250226
ENSG000000	ST3GAL6	protein_codin	0	0	0	0

ENSG00000(	RBM27	protein_codin	322.786681	251.204471	162.681557	213.639002
ENSG000001	JAKMIP2	protein_codin	0	0	0	0
ENSG000001	SHOC2	protein_codin	0	0	0	8.3270889
ENSG000001	TLE4	protein_codin	38.0595968	28.3441613	0	27.578538
ENSG000001	WWC1	protein_codin	4.22344281	0	9.27796139	0
ENSG00000(	GABRP	protein_codin	0	0	0	0
ENSG00000(	SPDL1	protein_codin	13.442544	13.8287326	44.8389654	4.56678861
ENSG000001	MINDY4	protein_codin	1.89356341	3.7935675	1.08892593	2.89858075
ENSG000001	SSBP1	protein_codin	101.646159	157.676878	90.7863313	96.8762339
ENSG000001	TRPV5	protein_codin	0	0	0	0
ENSG000001	ABCB6	protein_codin	0	0	0	5.82383104
ENSG000001	PECR	protein_codin	7.90137901	13.8402611	10.5609465	14.5038719
ENSG000002	VDAC1	protein_codin	44.3026508	26.1942994	45.5346867	0
ENSG00000(	CDKL3	protein_codin	0	0	0	0
ENSG000001	UBE2B	protein_codin	105.734363	118.426673	39.7611026	77.7941855
ENSG00000(	PITX1	protein_codin	0	0	1.32942047	0
ENSG00000(	FSTL4	protein_codin	7.94175876	11.1586742	3.79710552	0
ENSG00000(	AFF4	protein_codin	578.766864	248.26069	269.940666	310.967709
ENSG000001	KCTD20	protein_codin	0	0	0	0
ENSG000001	ZMIZ2	protein_codin	38.8164485	99.0038578	38.4437284	14.6633702
ENSG00000(	CUL7	protein_codin	57.3380191	0	0	23.5259963
ENSG000001	MED20	protein_codin	43.6638392	86.3986283	15.0700842	23.7536627
ENSG000001	XPO5	protein_codin	90.3856268	121.581553	64.8366427	57.6608984
ENSG000001	SRF	protein_codin	203.846818	294.882732	98.5226773	154.036777
ENSG00000(	SEMA3C	protein_codin	80.868912	20.5213971	37.5679838	118.727479
ENSG00000(	SEMA3A	protein_codin	2.55028298	1.18130004	0	0
ENSG00000(	NRP1	protein_codin	0	0	8.93406068	0
ENSG000001	CCNY	protein_codin	0	0	0	0
ENSG000001	APBA1	protein_codin	14.0276476	19.0611971	12.7799543	10.1647887
ENSG000001	PIP5K1B	protein_codin	10.3541422	20.531931	0	3.28760962
ENSG00000(	TNPO3	protein_codin	127.829766	268.397014	128.205614	0
ENSG000001	TAX1BP1	protein_codin	520.837577	457.673535	266.552569	426.730151
ENSG000001	CPVL	protein_codin	1645.6826	1853.74354	609.357504	1367.73257
ENSG000001	HIBADH	protein_codin	40.4091039	49.7319371	31.9283025	26.7118828
ENSG000001	UGT2B10	protein_codin	0	0	0	0
ENSG00000(	STAP1	protein_codin	0	2.04546455	13.3449606	0
ENSG00000(	ADGRF5	protein_codin	0	0	0	0
ENSG00000(	POLB	protein_codin	14.8088739	0	0	0
ENSG000001	SLC17A2	protein_codin	0	0	0	0
ENSG000001	WWP1	protein_codin	59.7168428	33.1516345	123.191003	91.9878327
ENSG000001	CALB1	protein_codin	0	0	3.2779977	0
ENSG000001	NBN	protein_codin	0	0	0	0
ENSG00000(	ST7	protein_codin	11.6507455	26.5217503	2.03011031	0
ENSG000001	TFEC	protein_codin	287.650189	201.51096	157.845643	282.436452

ENSG000001(	WNT2	protein_codin	0	0	0	0
ENSG000001:	ANXA11	protein_codin	456.249616	718.80729	258.483778	289.287384
ENSG000001(	TSPAN14	protein_codin	25.5544604	36.0769408	22.0187342	27.2367148
ENSG000002:	ENSG000002:	processed_ps	0	0	0	0
ENSG000001	NRXN2	protein_codin	0	0	0	0
ENSG000001:	MYRF	protein_codin	0	0	0	0
ENSG000001:	PRDX5	protein_codin	152.107824	281.913478	128.726192	121.650633
ENSG000000	POLA2	protein_codin	0	3.05612477	18.7708726	0
ENSG000001·	B3GAT3	protein_codin	33.0453721	42.9107308	14.0522634	26.8384821
ENSG000000	MGST2	protein_codin	28.8868116	40.3318545	0	32.6236512
ENSG000001!	ADH4	protein_codin	0	0	0	0
ENSG000001:	MTTP	protein_codin	0	0	0	0
ENSG000001(	BLVRA	protein_codin	244.545403	312.063712	124.590847	153.730344
ENSG000000	KIF9	protein_codin	0	0	4.00518149	0
ENSG000000	LARS2	protein_codin	0	0	0	6.17178053
ENSG000000(	RHOA	protein_codin	0	0	0	0
ENSG000001	USP4	protein_codin	203.85875	194.985356	65.41962	96.0384133
ENSG000000	PTPN23	protein_codin	0	150.531769	14.2338115	119.405585
ENSG000001	PRKAR2A	protein_codin	188.506535	197.746999	93.7226722	129.016642
ENSG000000	EXOSC7	protein_codin	0	15.307393	10.2410074	6.64186661
ENSG000001	SCAP	protein_codin	100.721995	138.921011	55.5392673	70.2574687
ENSG000000	OPRK1	protein_codin	0	0	0	0
ENSG000000(	ICA1	protein_codin	0	19.3795425	0	0
ENSG000001	ABCC5	protein_codin	79.3447646	110.419227	0	0
ENSG000001	CLCN2	protein_codin	0	0	0	0
ENSG000000	MCCC1	protein_codin	45.2313678	85.5193541	56.0210229	35.7540817
ENSG000000	LAMP3	protein_codin	2.22067351	0	0	0
ENSG000001	WASF1	protein_codin	0	0	0	0
ENSG000001:	AHI1	protein_codin	0	0	0	0
ENSG000001	ALDH8A1	protein_codin	0	0	0	0
ENSG000001(	UBXN8	protein_codin	6.53781336	19.4767319	10.5106735	0
ENSG000001:	VAPB	protein_codin	14.6424902	12.8353012	7.36144545	10.4453385
ENSG000000	GNAS	protein_codin	369.484234	724.712645	259.744326	258.471574
ENSG000001:	PMEPA1	protein_codin	0	0	0	0
ENSG000001(	PON3	protein_codin	0	0	0	0
ENSG000000(	SLC25A13	protein_codin	53.1533081	49.2771625	22.0733202	25.8460244
ENSG000001(	NPTX2	protein_codin	0	0	0	0
ENSG000001	PPP6R3	protein_codin	0	0	0	0
ENSG000001	PPP6R3	protein_codin	19.4304543	18.6546695	0	39.5378059
ENSG000001	CPT1A	protein_codin	397.609991	600.401956	228.75615	362.13425
ENSG000000(	GAL	protein_codin	0	0	0	0
ENSG000001	FBXO3	protein_codin	0	0	0	0
ENSG000001	KIAA1549L	protein_codin	0	0	0	0
ENSG000001(	NSMF	protein_codin	85.7201862	67.9348422	29.4620889	34.7235945

ENSG0000007	RPS6KA2	protein_codin	30.4316539	17.4263436	0	10.7424455
ENSG000001	TCIRG1	protein_codin	452.230658	648.598826	208.660133	353.434672
ENSG000001	CHKA	protein_codin	34.4137957	43.5912851	7.2270007	24.8462494
ENSG000001	ADAM18	protein_codin	0	0	0	0
ENSG000001	ADAM2	protein_codin	0	0	0	0
ENSG000000	ANK1	protein_codin	3.9621315	3.90025095	0	4.00947754
ENSG000000	KAT6A	protein_codin	400.074722	188.201868	103.732052	305.252449
ENSG000000	PRKAR2B	protein_codin	432.762947	845.678701	824.994238	348.028416
ENSG000001	DUS4L	protein_codin	8.30996141	0	0	0
ENSG000000	ABCB4	protein_codin	0	0	0	0
ENSG000000	ABCB1	protein_codin	0	0	0	0
ENSG000000	ADAM22	protein_codin	0	0	0	0
ENSG000000	DBF4	protein_codin	6.35089262	12.5863574	0	0
ENSG0000007	SRI	protein_codin	69.6356979	52.0947371	10.4370017	42.089578
ENSG000001	RBM48	protein_codin	11.3235166	29.0267613	15.0501198	5.02866883
ENSG000001	CDK6	protein_codin	87.4045786	0	86.4160635	95.9805649
ENSG000000	CDK14	protein_codin	4.17739217	7.96812467	0	0
ENSG000000	ANKIB1	protein_codin	167.562625	125.015199	107.904433	105.5678
ENSG000000	VPS41	protein_codin	40.2007064	30.9713908	62.3396412	0
ENSG000000	ANLN	protein_codin	12.0827423	8.98802828	7.29247974	16.0277553
ENSG000001	EIF4H	protein_codin	0	0	0	0
ENSG000000	GTF2IRD1	protein_codin	0	0	0	0
ENSG0000007	BUD23	protein_codin	62.7996606	70.9854027	58.5521792	50.2311138
ENSG000000	ADAM28	protein_codin	16.2987337	19.593606	30.2663505	25.633158
ENSG000000	STMN4	protein_codin	0	0	0	0
ENSG000001	DMTN	protein_codin	0	0	0	0
ENSG000000	LZTS1	protein_codin	0	0	0	0
ENSG000001	R3HCC1	protein_codin	38.491725	73.5235671	34.0326544	37.334928
ENSG000001	SH2D4A	protein_codin	0.88919389	1.79118288	0	0
ENSG000000	SLC18A1	protein_codin	0	0	0	0
ENSG000001	PDLIM2	protein_codin	17.3591226	22.9009138	8.04523696	15.8517235
ENSG000001	FSCN3	protein_codin	0	0.97160529	0	0
ENSG000000	ZNF800	protein_codin	135.834316	123.235419	39.7307128	98.2465874
ENSG0000007	ACAT1	protein_codin	53.0245637	0	36.4689595	18.0809667
ENSG000001	ELMOD1	protein_codin	0	0	0	0
ENSG000001	EXPH5	protein_codin	0	4.22774707	0	7.53413509
ENSG000001	ADAP1	protein_codin	152.856025	86.4905134	56.0080545	62.7503371
ENSG000001	PMS2	protein_codin	0	0	0	0
ENSG000000	MAD1L1	protein_codin	167.386074	168.186698	102.01674	53.3510587
ENSG000002	GET4	protein_codin	67.1043717	55.0920871	26.3138162	51.1651541
ENSG000002	RUFY2	protein_codin	11.194989	11.2701014	9.24838056	13.5338173
ENSG000000	HNRNPH3	protein_codin	266.793073	228.613352	106.302534	215.631361
ENSG000001	SLC25A16	protein_codin	17.0840843	9.27914329	5.24108257	2.38608326
ENSG000000	CCAR1	protein_codin	247.337579	0	134.119301	0

ENSG0000007	REXO2	protein_codin	29.441705	22.3877059	35.7298818	12.4429221
ENSG000002	RBM7P1	processed_ps	0	0	0	0
ENSG000001	SQLE	protein_codin	66.0095526	43.1973676	13.0255997	44.4608847
ENSG000001	SNX19	protein_codin	393.564564	486.358709	182.251686	235.378146
ENSG0000007	BRINP1	protein_codin	0	0.8925804	0	0
ENSG000001	MAPKAP1	protein_codin	0	0	0	0
ENSG000001	GTF2H1	protein_codin	64.5607077	67.5872858	34.4989228	78.0382552
ENSG000001	SERGEF	protein_codin	30.7401321	27.3973522	10.4088669	15.8724205
ENSG000001	CSRP3	protein_codin	0	0	0	0
ENSG000001	KCNC1	protein_codin	0	0	0	0
ENSG000000	PIK3C2A	protein_codin	6.71634947	0	0	31.280062
ENSG000000	FHIP1B	protein_codin	58.2986672	0	32.7464145	28.7159499
ENSG000001	RNF141	protein_codin	191.252269	65.0419386	46.0909915	80.1338217
ENSG000001	HPX	protein_codin	0.87959661	0	0.77032177	0
ENSG000001	IDE	protein_codin	6.92894126	15.1130791	6.08995611	0
ENSG000000	BTAF1	protein_codin	625.26541	378.752891	437.827773	422.867129
ENSG000001	CCNJ	protein_codin	0	0	0	0
ENSG000001	TCTN3	protein_codin	0	0	0	0
ENSG000001	CPEB3	protein_codin	31.4110255	34.5732225	7.19003111	25.6413936
ENSG000002	DAXX	protein_codin	17.7848239	40.7794038	0	42.9240581
ENSG000000	MLN	protein_codin	0	0	0	0
ENSG000000	GLT8D1	protein_codin	8.13657146	26.4274946	11.6282043	5.24652896
ENSG000000	RBM6	protein_codin	453.600943	407.039837	375.689154	210.50315
ENSG000001	TMEM115	protein_codin	67.9108355	78.5368908	26.8676945	45.9248275
ENSG000001	GNAI2	protein_codin	30.4235953	98.3273866	37.5551444	44.8415188
ENSG000001	HYAL1	protein_codin	0	0	0	0
ENSG000000	DOCK3	protein_codin	1.96093546	3.90168007	0	0
ENSG000000	CACNA2D2	protein_codin	0	0	0	0
ENSG000000	ITIH4	protein_codin	14.709313	30.9621863	26.831036	23.6377173
ENSG000001	SLIT1	protein_codin	4.81867926	4.74381263	0	1.88061789
ENSG000001	SFRP5	protein_codin	0	0	0	0
ENSG000001	GMEB2	protein_codin	64.2381776	75.0218729	10.2512938	7.28712901
ENSG000001	GID8	protein_codin	205.425718	261.060224	121.809545	119.706865
ENSG000001	DIDO1	protein_codin	0	0	0	0
ENSG000001	SLC2A4RG	protein_codin	59.8436535	33.250396	30.051714	22.0737708
ENSG000002	PCMTD2	protein_codin	3.35236565	3.122008	1.87327773	6.09985469
ENSG000001	PRPF6	protein_codin	459.68766	752.033977	234.641342	361.414924
ENSG000001	TIMP3	protein_codin	0	2.01555295	10.3463692	3.03926969
ENSG000001	SLC5A4	protein_codin	0	0	0	0
ENSG000001	FBXO7	protein_codin	80.8302082	177.146621	11.1197246	61.371042
ENSG000001	SLC5A1	protein_codin	0	0	0	0
ENSG000002	PISD	protein_codin	0	0	0	0
ENSG000001	EIF2B2	protein_codin	30.0154366	22.6376764	13.1316939	5.36882906
ENSG000001	RNF185	protein_codin	0	0	0	0

ENSG000001	TTLL1	protein_codin	11.5807949	24.0090553	3.41366541	16.2784487
ENSG000002	MTFP1	protein_codin	7.97656333	0	1.64729423	0
ENSG000001	PATZ1	protein_codin	0	0	0	33.3777363
ENSG000001	TEF	protein_codin	11.5755227	26.7696689	10.0499098	2.18184851
ENSG000001	B4GALNT3	protein_codin	0	3.82223727	0	0
ENSG000001	PDE6H	protein_codin	4.01443991	5.87699308	0.97172612	2.9845389
ENSG000001	ERP27	protein_codin	33.4567637	18.9126553	11.8163268	13.4570697
ENSG000001	ETV6	protein_codin	0	0	0	0
ENSG000001	BCL2L14	protein_codin	0	0	0	0
ENSG000001	GABARAPL1	protein_codin	102.380768	4.98880255	100.791137	0
ENSG000000	DNM1L	protein_codin	0	0	0	0
ENSG000001	ALG10	protein_codin	7.54019038	5.18858138	12.5816897	2.01950192
ENSG000000	BMAL2	protein_codin	0	5.90001187	0	0
ENSG000001	PLCZ1	protein_codin	0	0	0	0
ENSG000001	AEBP2	protein_codin	62.4722836	46.2496191	38.1951797	5.12258474
ENSG000001	SLCO1C1	protein_codin	0	0	0	0
ENSG000001	ETNK1	protein_codin	165.408819	116.984178	68.0433454	148.548554
ENSG000001	ZCRB1	protein_codin	0	0	0	0
ENSG000001	TMEM117	protein_codin	0	0	0	0
ENSG000001	C1RL	protein_codin	58.8797635	111.130376	42.2684675	62.3402644
ENSG000001	NDUFA9	protein_codin	39.5660124	27.7778908	20.6674359	27.0868447
ENSG000001	CLSTN3	protein_codin	30.7426818	0	17.1898021	28.934445
ENSG000001	KLRG1	protein_codin	0	2.91344707	0	0
ENSG000001	TAPBPL	protein_codin	69.0560816	81.0165869	70.6520807	56.7619643
ENSG000001	CD27	protein_codin	7.39253745	16.1711632	49.2963529	0
ENSG000001	RBP5	protein_codin	0	0	0	0
ENSG000001	PEX5	protein_codin	36.9695064	51.6148182	13.3279585	24.8045762
ENSG000001	PEX5	protein_codin	0	0	0	0
ENSG000001	SLC38A4	protein_codin	0	0	0	0
ENSG000001	AMIGO2	protein_codin	2.68882167	6.50584478	5.75602375	0
ENSG000001	SCAF11	protein_codin	35.9657759	24.8734904	19.0785455	17.9334901
ENSG000001	ANP32D	protein_codin	0	0	1.00526759	0
ENSG000001	LLPH	protein_codin	59.0957866	49.9750403	33.4863953	33.1254148
ENSG000001	MARCHF9	protein_codin	10.183427	13.264463	1.82721893	4.71093434
ENSG000001	INHBE	protein_codin	0	0.90442301	0	0
ENSG000001	GLIPR1	protein_codin	1136.08417	631.451504	513.745382	776.099097
ENSG000001	BEST3	protein_codin	0	0	0	0
ENSG000001	PHLDA1	protein_codin	10.4608381	0	0	0
ENSG000001	TMEM19	protein_codin	38.2879888	35.3594701	35.0081451	41.894598
ENSG000001	LGR5	protein_codin	0	0	0	0
ENSG000001	CPSF6	protein_codin	0	17.3754668	0	0
ENSG000000	SLC6A15	protein_codin	0	0	0	0
ENSG000001	TMTC3	protein_codin	32.016269	19.2789969	20.8749783	15.5838987
ENSG000001	LUM	protein_codin	0	0	0	0

ENSG000001	KERA	protein_codin	0	0	0	0
ENSG000001	TMPO	protein_codin	214.271746	179.624335	67.1738978	123.256627
ENSG000001	SNRPF	protein_codin	70.6397663	147.646445	48.312813	68.7428191
ENSG000001	AMDHD1	protein_codin	0	0	4.15406295	4.64218353
ENSG000001	NEDD1	protein_codin	19.8996768	0	0	22.1678186
ENSG000001	SYCP3	protein_codin	0	0	0	0
ENSG000001	ASCL1	protein_codin	0	0	0.69825784	0
ENSG000002	GOLGA2P5	transcribed_u	0	0	0	0.75614906
ENSG000001	GAS2L3	protein_codin	0	6.33234852	0	2.92231512
ENSG000001	SLC15A4	protein_codin	70.4880789	183.620933	35.8066259	81.7389654
ENSG000001	TDG	protein_codin	0	0	0	9.75806915
ENSG000000	CHFR	protein_codin	0	0	0	0
ENSG000001	SLC46A3	protein_codin	19.3111723	29.5643823	16.7926999	26.6409899
ENSG000001	CDK2	protein_codin	0	42.6606688	6.62604128	19.4402316
ENSG000001	SUOX	protein_codin	16.3217377	54.486179	0	12.8653971
ENSG000001	SLC39A5	protein_codin	0	0	0	0
ENSG000001	CCDC65	protein_codin	0	0	0	0
ENSG000001	TARBP2	protein_codin	8.32655896	26.2603416	10.7360069	0
ENSG000001	GPR84	protein_codin	34.6541763	19.6408507	13.1441827	0
ENSG000001	NPFF	protein_codin	0	0	0	0
ENSG000001	NABP2	protein_codin	21.4319533	24.4960273	14.1987707	9.75769382
ENSG000001	SMARCC2	protein_codin	401.155021	587.928533	100.449323	226.424753
ENSG000002	N4BP2L2	protein_codin	105.005842	53.9198707	37.0316014	51.9504326
ENSG000001	MAP3K12	protein_codin	20.0226869	0	0	0
ENSG000001	ITGB7	protein_codin	46.6305753	95.5260813	116.797479	0
ENSG000001	CSAD	protein_codin	0	55.0023544	0	0
ENSG000000	ERBB3	protein_codin	0	0	0	0
ENSG000001	LMBR1L	protein_codin	1.40330193	0	3.07070519	0
ENSG000001	MYG1	protein_codin	0	0	0	0
ENSG000001	ESYT1	protein_codin	5.47683526	55.9757503	15.4923592	0
ENSG000001	TMBIM6	protein_codin	1343.41182	1943.74495	629.053035	992.683405
ENSG000001	ANKRD52	protein_codin	109.706784	117.148842	84.8536314	43.1562
ENSG000001	KRT71	protein_codin	0	0	0	0
ENSG000001	RB1	protein_codin	180.409203	183.716627	0	116.043171
ENSG000001	DIABLO	protein_codin	18.985749	33.2749498	22.6504392	0
ENSG000001	SBNO1	protein_codin	416.160524	189.559904	157.28405	211.123443
ENSG000001	VPS33A	protein_codin	40.0474264	94.9544806	0	36.8328787
ENSG000001	VPS37B	protein_codin	190.79956	267.282593	99.9955148	132.166224
ENSG000001	RHOF	protein_codin	109.023846	159.414196	74.3072563	51.5018213
ENSG000001	DIAPH3	protein_codin	0	0	0	0
ENSG000001	SLAIN1	protein_codin	0	0	0	0
ENSG000001	RBM26	protein_codin	316.583836	265.04457	281.008268	211.137051
ENSG000000	PXN	protein_codin	105.582226	73.1328442	27.7025514	141.977269
ENSG000001	SRRM4	protein_codin	0	0	0	0

ENSG000001:	METTL21C	protein_codin	0	0	0	0
ENSG000001:	RNF113B	protein_codin	0	0	0	0
ENSG000001:	ZIC5	protein_codin	0	1.22729655	0	0
ENSG000001:	RAB20	protein_codin	182.597749	168.981061	98.065061	97.3745131
ENSG000000:	ANKRD10	protein_codin	58.062205	158.281532	46.180213	47.1599758
ENSG000001:	TTC6	protein_codin	0	0	0	0
ENSG000001:	SSTR1	protein_codin	0	0	0	0
ENSG000001:	CDH24	protein_codin	0	0	0	0
ENSG000001:	REM2	protein_codin	1.98460362	4.92206632	1.43767939	2.99276473
ENSG000001:	CBLN3	protein_codin	6.86332964	5.93474294	1.01769483	0.94640488
ENSG000000!	TINF2	protein_codin	43.0672881	41.561462	34.7540596	0
ENSG000001!	NOP9	protein_codin	100.406755	212.358585	69.5546669	73.4547498
ENSG000001:	FITM1	protein_codin	1.99996791	0	1.9324721	0
ENSG000001:	FANCM	protein_codin	0	0	0	0
ENSG000000:	L2HGDH	protein_codin	11.1611568	4.01432304	3.63517511	3.0603426
ENSG000001:	PELI2	protein_codin	505.950265	435.75585	245.0065	345.820954
ENSG000001:	RTN1	protein_codin	319.656179	418.788302	174.111655	251.759334
ENSG000001:	ARMH4	protein_codin	0	3.95686811	2.35678325	0
ENSG000001:	SLC38A6	protein_codin	0	0	0	0
ENSG000001:	RDH12	protein_codin	0	0	0	0
ENSG000001:	RAB15	protein_codin	0	0	0	0
ENSG000001·	WDR89	protein_codin	0	0	0	0
ENSG000001·	ESR2	protein_codin	0	0	0	0
ENSG000001·	GPR65	protein_codin	121.589708	125.324098	78.8795423	140.688406
ENSG000001·	PTGR2	protein_codin	1.69664505	8.09570639	0	1.37522603
ENSG000001·	JDP2	protein_codin	308.895923	232.721773	81.5475017	111.061654
ENSG000001·	AK7	protein_codin	0	0.96531704	0	0
ENSG000001·	FAM181A	protein_codin	0	0	0	0
ENSG000001	ITPK1	protein_codin	78.7129388	20.1346493	0	34.0049435
ENSG000001·	FBLN5	protein_codin	0	0	0	0
ENSG000001	TRIP11	protein_codin	115.511773	219.667918	98.0465508	68.6986461
ENSG000001:	EMC4	protein_codin	74.9881267	105.907858	71.3284007	48.6165802
ENSG000001·	DUOXA1	protein_codin	0	0	0	0
ENSG000001:	ZNF280D	protein_codin	95.4607745	66.3821589	46.9277199	86.8243191
ENSG000001·	TCF12	protein_codin	194.235961	114.582131	114.316088	190.787677
ENSG000001·	MFAP1	protein_codin	78.5139069	157.133187	65.6498282	67.4889753
ENSG000001·	SORD	protein_codin	13.6425243	32.8407171	0	9.43097716
ENSG000001	MYEF2	protein_codin	2.97052947	5.10915767	0	0
ENSG000001·	LYSMD2	protein_codin	113.262622	143.005397	49.5294988	73.5683849
ENSG000001·	SLC27A2	protein_codin	0	5.36895715	0	0
ENSG000001·	FGF7	protein_codin	1.33508592	1.35222864	1.56200794	1.37967365
ENSG000001·	HDC	protein_codin	0	5.65841079	0	1.02894723
ENSG000002	MYZAP	protein_codin	98.7511507	42.8823207	31.6411765	52.231339
ENSG000000:	RAD51	protein_codin	0	0	0	0

ENSG000001	GTF2A2	protein_codin	0	21.0844912	10.7613317	0
ENSG000001	SRP14	protein_codin	481.477736	773.868187	236.701198	381.723442
ENSG000001	DISP2	protein_codin	3.01477334	0	4.01848307	0.9953382
ENSG000001	TTBK2	protein_codin	304.050994	164.093303	129.030653	168.935975
ENSG000001	ANP32A	protein_codin	65.7764948	133.332799	233.430304	0
ENSG000001	COMMD4	protein_codin	13.0717202	23.9881694	0	8.15895796
ENSG000001	UBE2Q2	protein_codin	134.047808	86.8793391	34.042188	63.3395111
ENSG000001	ETFA	protein_codin	76.0993311	182.825708	124.199721	31.6518346
ENSG000001	BCL2A1	protein_codin	1150.3354	476.14824	667.182662	613.963376
ENSG000001	TSPAN3	protein_codin	18.1011795	8.01941118	3.03821934	12.1341098
ENSG000001	SKIC8	protein_codin	56.5708711	83.9984766	41.8726997	43.0657499
ENSG000001	MAN2C1	protein_codin	94.7991792	132.666662	0	89.0935394
ENSG000001	TLNRD1	protein_codin	167.99386	223.012473	70.2540806	95.7393548
ENSG000001	TPM1	protein_codin	0	0	0	0
ENSG000001	ARRDC4	protein_codin	226.61476	208.597656	121.662302	157.607509
ENSG000001	PIF1	protein_codin	0	0	0	0
ENSG000001	USP3	protein_codin	0	0	0	0
ENSG000001	CYP11A1	protein_codin	0	0	0	0
ENSG000001	BBS4	protein_codin	0	0	6.17434163	18.6674837
ENSG000001	PML	protein_codin	0	93.3710155	0	0
ENSG000001	PML	protein_codin	0	58.0170036	0	0
ENSG000001	ADAMTS17	protein_codin	1.81989852	0	0	0
ENSG000001	CCDC33	protein_codin	0	0	0	0
ENSG000002	HEXA	protein_codin	33.4528141	38.7166422	12.7933689	5.92447758
ENSG000001	SCAMP2	protein_codin	202.962401	307.716245	82.3765272	179.095512
ENSG000001	RHCG	protein_codin	0	0	0	0
ENSG000001	POLG	protein_codin	0	46.4508683	0	0
ENSG000001	RLBP1	protein_codin	0	0	0	0
ENSG000001	WDR93	protein_codin	0	0	0	0
ENSG000001	TICRR	protein_codin	0	0	0	0
ENSG000001	DET1	protein_codin	0	0	6.40589744	0
ENSG000001	MFGE8	protein_codin	0	0	0	0
ENSG000001	MFGE8	protein_codin	0	0	0	0
ENSG000001	ZNF710	protein_codin	191.140613	328.489664	83.4248494	108.741353
ENSG000001	ST8SIA2	protein_codin	1.90229704	0	0	0
ENSG000001	FURIN	protein_codin	0	0	48.7353137	0
ENSG000001	IQGAP1	protein_codin	5024.13701	2462.81614	2281.90431	3687.94674
ENSG000001	CRTC3	protein_codin	127.073583	0	0	0
ENSG000001	EFL1	protein_codin	72.4696578	112.266568	98.5268255	36.4362648
ENSG000001	SEC11A	protein_codin	308.381788	522.349621	111.931103	308.243959
ENSG000001	SEPTIN12	protein_codin	0	0	0.95594792	0
ENSG000001	ABAT	protein_codin	82.1787481	158.992226	104.711394	70.0298659
ENSG000001	PMM2	protein_codin	63.6333878	47.3512855	27.9258049	38.8311257
ENSG000001	PRSS36	protein_codin	0	0	0	0

ENSG000001	ITGAX	protein_codin	572.027351	1007.78219	247.286665	458.361628
ENSG000001	ARMC5	protein_codin	92.260216	40.7113694	0	0
ENSG000001	FTO	protein_codin	0	67.6112019	23.3843236	26.0533856
ENSG000001	UQCRC2	protein_codin	211.654981	284.663263	113.671229	140.840843
ENSG000001	CDR2	protein_codin	44.9614026	37.9570947	29.0160185	22.0327393
ENSG000001	IGSF6	protein_codin	768.397109	1342.81759	440.616973	783.105814
ENSG000001	NKD1	protein_codin	4.93780042	3.91209287	0	0
ENSG000001	DHX38	protein_codin	321.694209	487.796266	249.868681	288.578937
ENSG000001	TXNL4B	protein_codin	33.1234589	64.5949725	27.4184677	28.2983486
ENSG000001	MARVELD3	protein_codin	0	0	1.09197899	0
ENSG000001	ZFHX3	protein_codin	1063.53767	936.977783	445.083607	646.833927
ENSG000001	NUDT7	protein_codin	0	0	0	0
ENSG000001	CMTM2	protein_codin	2.17817772	10.7443519	0	0
ENSG000001	CDH11	protein_codin	0	0	0	0
ENSG000001	MAP1LC3B	protein_codin	422.308929	567.703563	287.771782	292.58893
ENSG000001	CDH13	protein_codin	0	0	0	0
ENSG000001	ZCCHC14	protein_codin	42.0680703	36.0628605	0	0
ENSG000001	ADAD2	protein_codin	0	0	0	0
ENSG000001	IRF8	protein_codin	0	133.212853	0	107.443316
ENSG000001	ZNF174	protein_codin	19.4407151	0	6.53362699	0
ENSG000001	RPL3L	protein_codin	0	0.69617675	0	0
ENSG000001	NDUFB10	protein_codin	201.800424	342.24103	197.58858	139.628354
ENSG000001	PDPK1	protein_codin	0	48.0301213	6.67610763	0
ENSG000001	DEF8	protein_codin	0	11.2161791	0	0
ENSG000001	CBFA2T3	protein_codin	109.459558	164.521201	53.7425523	75.6925966
ENSG000001	GALNS	protein_codin	77.9035713	130.348536	44.951962	58.0543372
ENSG000001	GAS8	protein_codin	0	0	0	0
ENSG000001	SPG7	protein_codin	0	0	0	0
ENSG000001	MED9	protein_codin	26.4265922	41.2264369	21.55358	15.6682825
ENSG000001	NCOR1	protein_codin	139.981727	36.2565828	34.3460808	38.9629545
ENSG000001	COPS3	protein_codin	113.577606	143.43775	44.8399066	0
ENSG000001	GID4	protein_codin	37.7232952	52.8755582	13.7841936	17.7213634
ENSG000001	CPNE7	protein_codin	0	0	0	0
ENSG000001	PHF12	protein_codin	16.9396679	63.7166463	4.50930202	10.0214875
ENSG000000	PIGS	protein_codin	16.5035336	0	0	37.6274837
ENSG000001	NEK8	protein_codin	21.2587946	7.07483728	5.13204897	8.20655818
ENSG000001	DPEP3	protein_codin	9.46477239	0	0	0
ENSG000001	DPEP2	protein_codin	4.3592841	3.4684339	3.19177068	3.93741036
ENSG000001	GFOD2	protein_codin	3.98792756	3.01786026	2.65348841	11.3436974
ENSG000001	NOB1	protein_codin	94.3259563	163.607539	58.4590849	75.3144413
ENSG000001	PRPSAP2	protein_codin	0	54.0866557	13.6772374	0
ENSG000001	B9D1	protein_codin	0	0	0	0
ENSG000001	UNC45B	protein_codin	0	0	0	0
ENSG000000	TSPOAP1	protein_codin	32.7992046	0	0	0

ENSG000001	PCTP	protein_codin	97.322858	197.411742	41.7149742	104.807016
ENSG000001	OR4D1	protein_codin	3.1346296	0	0	0
ENSG000001	KIF2B	protein_codin	0.89461311	0	0	0
ENSG000000	EPN3	protein_codin	0	0	0	0
ENSG000001	MTNAP1	protein_codin	0	0	0	14.443128
ENSG000001	TOB1	protein_codin	0	0	0	0
ENSG000001	SPATA22	protein_codin	0	0	0	0
ENSG000001	SGSM2	protein_codin	129.603729	255.716544	89.3371874	91.1519196
ENSG000001	LRRC46	protein_codin	1.65657069	0	0.82127759	0
ENSG000001	SSH2	protein_codin	1060.25178	739.926506	301.684945	511.249579
ENSG000001	RHBDL3	protein_codin	0	0	0	0
ENSG000001	SPACA3	protein_codin	0	0	1.1239301	0
ENSG000001	ABCA8	protein_codin	0	0	0	0
ENSG000001	ABCA10	protein_codin	0	0	0	0
ENSG000001	MPP2	protein_codin	0.90655298	0	0	0
ENSG000001	G6PC3	protein_codin	33.2892326	0	0	26.5907595
ENSG000001	CLTC	protein_codin	246.539417	36.0199314	62.5885751	121.811322
ENSG000001	CHCT1	protein_codin	0	0	0	0
ENSG000001	SS18	protein_codin	28.3641658	8.57930421	11.2515126	8.85716099
ENSG000001	SS18	protein_codin	0	0	0	0
ENSG000001	CDH2	protein_codin	0	0	0	0
ENSG000001	TAF4B	protein_codin	48.3798258	36.424822	4.09134859	27.9151067
ENSG000001	AFG3L2	protein_codin	73.7623982	218.023354	26.1818234	150.15694
ENSG000001	IMPA2	protein_codin	120.019243	211.496854	56.1374101	112.076139
ENSG000001	GNAL	protein_codin	0	0	0	0
ENSG000001	SLC39A6	protein_codin	90.1691094	114.723232	49.0265905	81.3591308
ENSG000001	DTNA	protein_codin	0	0	0	0
ENSG000001	C18orf21	protein_codin	6.39459947	5.71545895	0	0
ENSG000001	GALNT1	protein_codin	242.469842	95.6334428	105.03406	162.495268
ENSG000001	ASXL3	protein_codin	0	0	0	0
ENSG000001	ADCYAP1	protein_codin	0	0	0	0
ENSG000001	MEP1B	protein_codin	0	0	0	0
ENSG000001	SLC25A52	protein_codin	0	0	0	0
ENSG000001	GAREM1	protein_codin	0	0	0	0
ENSG000001	ESCO1	protein_codin	149.48624	125.096581	129.194351	110.455412
ENSG000001	GATA6	protein_codin	0	0	0	0
ENSG000000	LAMA3	protein_codin	0	0	0	0
ENSG000001	GREB1L	protein_codin	0	0	0	0
ENSG000001	RMC1	protein_codin	82.2811465	150.460983	75.7822025	80.5527375
ENSG000001	NPC1	protein_codin	76.2666511	106.98592	42.186485	40.4060328
ENSG000001	MYH10	protein_codin	0	0	0	0
ENSG000001	ARRB2	protein_codin	999.511851	1208.1668	371.193992	531.743527
ENSG000000	NLRP1	protein_codin	0	50.6562533	0	0
ENSG000001	ZMYND15	protein_codin	0	0	0	0

ENSG000001	SAT2	protein_codin	54.788386	73.3526279	19.808483	39.5374255
ENSG000001	ASGR1	protein_codin	59.0756797	60.1611583	15.0156127	42.70818
ENSG000001	PIK3R5	protein_codin	0	0	0	0
ENSG000001	TP53	protein_codin	0	161.071382	0	93.6577084
ENSG000001	CCDC40	protein_codin	0	0	0	0
ENSG000001	ARHGDIA	protein_codin	855.457007	977.767804	300.147212	459.035636
ENSG000001	TTYH2	protein_codin	113.246163	208.138382	38.6082141	81.2001366
ENSG000001	RAB40B	protein_codin	0	3.35962123	1.89603729	0
ENSG000001	CSNK1D	protein_codin	2.87553025	6.57897074	0	0
ENSG000001	FN3KRP	protein_codin	27.5453731	51.2176692	2.35919799	26.3475818
ENSG000001	TRIM65	protein_codin	30.7838218	49.0631767	13.1108793	16.3978921
ENSG000001	CBX8	protein_codin	3.08195876	12.730756	7.50179145	4.02958765
ENSG000001	SECTM1	protein_codin	594.133041	539.465877	127.857907	281.772281
ENSG000001	RNF157	protein_codin	0	0	0	0
ENSG000001	CEP131	protein_codin	13.517368	30.2149794	39.5450356	0
ENSG000001	ZNF750	protein_codin	0	0	0	0
ENSG000002	ENSG000002	processed_ps	0	0	0	0
ENSG000001	CBX4	protein_codin	275.633952	385.617251	206.574447	243.931492
ENSG000001	CBX2	protein_codin	0	0	0	0
ENSG000001	ARK2C	protein_codin	0	0	0	0
ENSG000001	DYM	protein_codin	67.5121891	66.4104193	21.0622734	60.1735832
ENSG000001	ELAC1	protein_codin	5.03917362	14.9499435	6.59931915	0
ENSG000001	MBD1	protein_codin	0	0	0	0
ENSG000001	MBD1	protein_codin	16.0335879	0	0	0
ENSG000001	TNFRSF11A	protein_codin	2.48818354	3.54400733	3.00431068	1.02263572
ENSG000001	SERPINB13	protein_codin	0	0	0	0
ENSG000001	SERPINB12	protein_codin	0	0	0	0
ENSG000001	ZCCHC2	protein_codin	38.7172424	50.9888535	15.4690198	49.7799366
ENSG000001	CBLN2	protein_codin	0	0	0	0
ENSG000001	PMAIP1	protein_codin	0	17.217528	0	0
ENSG000001	ERBB2	protein_codin	0	0	0	0
ENSG000001	KRT10	protein_codin	57.5322692	56.3744198	13.1424275	42.9281748
ENSG000001	PNMT	protein_codin	0	1.86869159	0	0
ENSG000001	ARL5C	protein_codin	0	0	0	0
ENSG000001	IGFBP4	protein_codin	0.92976681	1.8530207	0	0
ENSG000001	TXNL4A	protein_codin	40.6552722	80.9817891	22.3569983	39.1310981
ENSG000001	AKAP8	protein_codin	32.4903432	0	0	0
ENSG000001	CYP4F22	protein_codin	0	1.73522145	0	0
ENSG000001	MISP3	protein_codin	10.2841246	15.0618356	9.64511792	7.74720934
ENSG000001	SAMD1	protein_codin	0	2.76835431	1.16726857	0
ENSG000001	SLC39A3	protein_codin	48.1477057	60.3381382	18.7810747	19.3963086
ENSG000001	PLPP2	protein_codin	0	0	0	0
ENSG000001	ZNF544	protein_codin	0	0	9.34295869	8.74417153
ENSG000001	ZIM3	protein_codin	0	0	0	0

ENSG000001	PRDM15	protein_codin	0	0	0	0
ENSG000001	FEM1A	protein_codin	132.534362	230.186551	78.5828557	105.248624
ENSG000001	CIB3	protein_codin	0	0	0	0
ENSG000002	CALR3	protein_codin	0	0	0	0
ENSG000001	SH3GL1	protein_codin	184.614357	229.358992	93.2634577	131.119565
ENSG000001	PGPEP1	protein_codin	112.358014	136.66085	59.741226	59.6118032
ENSG000001	ARMC6	protein_codin	0	2.88967615	0	0
ENSG000001	DMRTC2	protein_codin	0	0	0	0
ENSG000001	CCDC97	protein_codin	131.526926	164.563923	75.4622505	92.6459146
ENSG000001	ZNF45	protein_codin	26.4441777	15.7075048	12.9115115	19.4233225
ENSG000002	BCKDHA	protein_codin	142.015891	220.277328	72.8823663	104.826436
ENSG000001	ZFP14	protein_codin	26.2601216	16.7105114	15.4015011	3.10763343
ENSG000002	ZNF155	protein_codin	13.8493271	2.84574535	3.30740022	12.7178478
ENSG000001	SSBP4	protein_codin	87.7508383	83.3009734	48.3389953	35.6436021
ENSG000001	SMG9	protein_codin	68.8550442	55.5643705	18.4284995	17.968972
ENSG000001	PSG9	protein_codin	0	0	0	0
ENSG000001	HUNK	protein_codin	0	0	0	0
ENSG000001	LRRC56	protein_codin	4.72628133	1.87347725	4.27320594	0.9002253
ENSG000001	IFNAR1	protein_codin	188.158025	82.9956041	49.5381469	188.969278
ENSG000001	SOD1	protein_codin	174.012491	238.727218	91.473381	117.283583
ENSG000001	SIK1	protein_codin	4.10390121	4.86032183	0	14.1343539
ENSG000001	DNMT3L	protein_codin	0	0	0	0
ENSG000001	SCYL1	protein_codin	0	0	0	0
ENSG000001	IL19	protein_codin	0	1.91767564	0	0.95922174
ENSG000001	EMP3	protein_codin	275.183681	291.879431	62.9835798	119.043557
ENSG000001	DMWD	protein_codin	26.0522657	5.70519141	4.66074007	13.8975626
ENSG000001	SAE1	protein_codin	64.535199	62.3520299	28.9152728	49.8057298
ENSG000001	BCAM	protein_codin	0	0	0	0
ENSG000001	NTN5	protein_codin	0	0	0	0
ENSG000001	GEMIN7	protein_codin	10.1077335	18.2302617	4.46198677	5.75814527
ENSG000000	WDR62	protein_codin	0	0	0	0
ENSG000002	FXYD7	protein_codin	0	0	0.73023068	0
ENSG000001	ADAMTS10	protein_codin	0	0	0	0
ENSG000001	SLC6A3	protein_codin	0	0	0	0
ENSG000001	RNPEPL1	protein_codin	207.77425	216.596832	65.5243728	137.375454
ENSG000001	CAPN10	protein_codin	4.58603494	0	0	0
ENSG000001	CAPN10	protein_codin	0	0	0	0
ENSG000002	KIR3DL2	protein_codin	0	0	0.83691182	0.95186592
ENSG000002	ZNF702P	lncRNA	0	0	0	0
ENSG000001	ZNF581	protein_codin	29.7246313	21.9004377	2.46591292	8.99882667
ENSG000001	LILRB4	protein_codin	0	0	0	0
ENSG000001	ZNF816	protein_codin	0	0	0	0
ENSG000001	CACNG8	protein_codin	5.01923528	3.98425455	9.2272422	0.98542809
ENSG000000	EPN1	protein_codin	129.916825	118.413356	66.1052428	68.3087662

ENSG000002	LILRA6	protein_codin	0	0	4.50479917	0
ENSG000001	TIMM29	protein_codin	21.6913108	18.9548519	10.5626314	6.94710206
ENSG000001	FBN3	protein_codin	0	0	0	0
ENSG000001	ECSIT	protein_codin	0	49.9506384	9.83440082	0
ENSG000001	EVI5L	protein_codin	46.1057929	97.5354544	38.8786791	55.061996
ENSG000001	TIMM44	protein_codin	19.8284257	27.1099726	39.1084505	20.7825493
ENSG000001	TM4SF5	protein_codin	0	0	0	0
ENSG000001	SLC47A1	protein_codin	6.45703802	7.42390817	0.95676913	3.25364534
ENSG000001	KLHDC4	protein_codin	41.2289079	42.4761116	0	33.9857154
ENSG000001	PSMB6	protein_codin	109.043563	134.104888	58.1113869	59.5781206
ENSG000001	GPR32	protein_codin	0	0	0	0
ENSG000001	ACP4	protein_codin	0	0	0.86530526	0
ENSG000001	ZNF473	protein_codin	23.2105023	0	0	0
ENSG000001	RPS11	protein_codin	1916.48989	3430.41923	1144.79405	1651.20143
ENSG000001	PTH2	protein_codin	0	0	0	0
ENSG000002	SPIB	protein_codin	0	0	0	0
ENSG000001	IGLON5	protein_codin	0.92606011	0.93302539	0.99344913	0
ENSG000001	RCN3	protein_codin	102.982621	81.7885496	56.6122858	35.2491382
ENSG000001	ZNF614	protein_codin	18.1784455	24.9783756	15.8053684	9.49058719
ENSG000002	ESPNP	transcribed_u	0	0	0	0
ENSG000001	WRAP73	protein_codin	53.2765141	43.4592424	14.2984093	10.1273892
ENSG000001	CFAP74	protein_codin	0	0	0	0
ENSG000001	PRDM16	protein_codin	0.8526503	0	0	0
ENSG000001	ARHGEF19	protein_codin	0	7.73907139	0	1.38050677
ENSG000001	PGD	protein_codin	1987.92365	3693.97803	831.70358	2143.91682
ENSG000001	SH3BGRL3	protein_codin	1804.22677	2679.78776	869.991242	1331.79283
ENSG000001	IL22RA1	protein_codin	0	0	0	0
ENSG000001	ZNF593	protein_codin	0	0	4.3475648	4.20113831
ENSG000001	C1orf216	protein_codin	19.1708537	27.4362486	22.1689587	6.83173709
ENSG000001	EVA1B	protein_codin	0	0	0	0
ENSG000001	PLK4	protein_codin	9.94317384	0	0	0
ENSG000001	FCN3	protein_codin	0	0	0	0
ENSG000001	ITGB3BP	protein_codin	40.6899946	43.5592204	0	23.4162359
ENSG000001	TINAGL1	protein_codin	0	0	0	0
ENSG000001	MOB3C	protein_codin	0	66.6776768	0	81.2874555
ENSG000001	CYP4B1	protein_codin	0	0	0	0
ENSG000001	SLC44A3	protein_codin	0	0	0	0
ENSG000001	FNBP1L	protein_codin	0	16.071429	0	0
ENSG000001	CTTNBP2NL	protein_codin	53.1795318	35.2747351	24.0991667	44.8622951
ENSG000001	PSMA5	protein_codin	34.9517873	59.2119429	9.68306325	42.6214781
ENSG000001	CD53	protein_codin	571.478268	1077.57125	270.821743	418.033065
ENSG000001	PROK1	protein_codin	0	0	0	0
ENSG000001	CELSR2	protein_codin	29.1433067	37.3721208	9.932683	4.8971229
ENSG000001	GPR161	protein_codin	0	0	0	0

ENSG000001	MPC2	protein_codin	0	0	0	0
ENSG000002	SFT2D2	protein_codin	469.029872	617.118123	300.219594	331.360936
ENSG000001	STYXL2	protein_codin	0.70523731	0	0	0
ENSG000001	POU2F1	protein_codin	0	0	0	0
ENSG000001	ILDR2	protein_codin	0	6.09286698	2.93166455	7.53357518
ENSG000001	FCGR2A	protein_codin	746.430433	607.52515	440.062705	464.941676
ENSG000001	NUF2	protein_codin	0	0	0	0
ENSG000001	PRCC	protein_codin	140.036196	250.516532	81.0202884	115.198383
ENSG000001	FCRL4	protein_codin	0	0	0	0
ENSG000001	TOR1AIP1	protein_codin	9.37511302	8.20865654	7.23423737	59.2131004
ENSG000001	HMCN1	protein_codin	1.02488318	2.0503422	4.19718207	0
ENSG000001	PRUNE1	protein_codin	67.0509387	200.266062	35.4194289	56.543715
ENSG000001	SF3B4	protein_codin	155.506493	190.948857	83.2153227	118.263209
ENSG000001	CGN	protein_codin	0.87256951	0	0	0
ENSG000001	S100A11	protein_codin	2830.9597	3003.19379	807.041009	1896.07906
ENSG000001	SETDB1	protein_codin	0	186.86381	0	0
ENSG000001	ADAMTSL4	protein_codin	55.952298	77.8689849	0	0
ENSG000001	CTSK	protein_codin	0	0	0	0
ENSG000001	CERS2	protein_codin	7.32147681	4.5545965	0	5.27846739
ENSG000001	ENSA	protein_codin	148.039632	122.835096	120.241147	95.501906
ENSG000001	POGZ	protein_codin	0	47.5075914	13.8854406	52.6092511
ENSG000001	GOLPH3L	protein_codin	31.8166678	11.790135	26.9572264	15.3986866
ENSG000001	KCNH1	protein_codin	0	0	0	0
ENSG000001	EIF2D	protein_codin	67.8022081	122.525098	48.6670576	34.8565183
ENSG000001	VASH2	protein_codin	0	3.59448277	0	0
ENSG000001	CRNN	protein_codin	0.62051543	0	0	0
ENSG000001	ADAM15	protein_codin	124.688233	0	95.0447158	68.3978927
ENSG000001	JTB	protein_codin	151.945075	301.043889	91.791356	132.982398
ENSG000001	TPM3	protein_codin	0	0	0	0
ENSG000001	NUP210L	protein_codin	4.01864571	6.16183921	1.08286757	2.08555326
ENSG000001	SLC27A3	protein_codin	14.2328076	41.2709038	5.88366007	22.2337663
ENSG000001	UBAP2L	protein_codin	44.4278609	110.937142	2.83056253	34.7361788
ENSG000001	GON4L	protein_codin	73.6734974	133.941158	45.699166	43.8880327
ENSG000001	CREB3L4	protein_codin	0	0	0	0
ENSG000001	KCNN3	protein_codin	0	2.06887576	3.51752629	2.38908323
ENSG000001	CAPN9	protein_codin	0	0	0	0
ENSG000001	ACP1	protein_codin	74.3764803	167.15263	72.5577364	96.9281115
ENSG000001	ACP1	protein_codin	53.652568	102.913259	33.1035653	20.5473565
ENSG000001	SDE2	protein_codin	286.546064	227.493109	135.732304	165.677716
ENSG000001	ARF1	protein_codin	1401.35059	2275.07779	813.978102	1001.34862
ENSG000001	ITPKB	protein_codin	0	0	0	0
ENSG000001	CNIH3	protein_codin	0	0	0	0
ENSG000002	LEFTY1	protein_codin	6.70361334	13.4847197	0	2.78776429
ENSG000001	C1orf35	protein_codin	34.3789866	80.2260864	36.2259188	32.5958532

ENSG000001	LBR	protein_codin	258.193052	415.182399	169.92742	342.20361
ENSG000001	WNT9A	protein_codin	0	3.06653417	0	0
ENSG000001	EPHX1	protein_codin	22.8051744	57.5207617	20.4942641	18.2024643
ENSG000001	REN	protein_codin	0	1.83399893	0	0
ENSG000001	SOX13	protein_codin	0	0	0	0.70985964
ENSG000001	PPFIA4	protein_codin	0	1.5712923	0	0
ENSG000001	PLEKHA6	protein_codin	0	0	0	0
ENSG000001	ARL8A	protein_codin	177.240343	197.463028	71.1887617	103.722482
ENSG000001	OSR1	protein_codin	0	0	0	0
ENSG000001	GDF7	protein_codin	2.00777484	1.98931323	0	0
ENSG000001	PDIA6	protein_codin	375.490609	622.263303	552.65613	328.123332
ENSG000001	RHOB	protein_codin	520.819259	719.225275	358.823809	444.893462
ENSG000001	ATP6V1C2	protein_codin	0	0	0	0
ENSG000001	HNRNPLL	protein_codin	22.6012488	30.1924722	11.1875203	28.1196268
ENSG000001	GALM	protein_codin	15.3604174	25.0181138	16.1174778	18.3595115
ENSG000001	RPL21	protein_codin	24.8683862	8.94887552	6.63577453	4.12578498
ENSG000001	ABCG8	protein_codin	0	0	0	0
ENSG000001	CALM2	protein_codin	1017.40965	775.249487	403.153085	514.472902
ENSG000001	RPS27A	protein_codin	2285.06352	3758.5881	716.188322	2044.33873
ENSG000001	WDPCP	protein_codin	6.37995539	5.16747159	0	5.3713052
ENSG000001	VPS54	protein_codin	131.008474	49.6061917	34.9117462	79.4544162
ENSG000001	REG3G	protein_codin	0	0	0	0
ENSG000001	ETAA1	protein_codin	38.1872813	15.4774989	25.0594522	24.4802288
ENSG000001	SNRPG	protein_codin	69.275204	85.0898443	37.838079	55.561286
ENSG000001	CLEC4F	protein_codin	0	0	2.73069668	0
ENSG000001	MEIS1	protein_codin	9.89275508	43.5555402	0	24.2486835
ENSG000001	OTOF	protein_codin	0	0	0	0
ENSG000001	TRIM43	protein_codin	0	0	0	0
ENSG000001	CIAO1	protein_codin	99.2211533	99.2431288	59.7141343	43.3908162
ENSG000001	MRPS5	protein_codin	61.1356531	79.7511592	26.1372754	42.7694239
ENSG000001	ANKRD53	protein_codin	0	1.62789408	0	0
ENSG000001	TPRKB	protein_codin	38.953322	34.158486	25.719203	26.8398012
ENSG000001	NAT8	protein_codin	0	0	0	0
ENSG000001	EXOC6B	protein_codin	42.5289733	43.5343176	26.0184109	34.71812
ENSG000001	RTKN	protein_codin	0	0	0	0
ENSG000001	SFXN5	protein_codin	80.7712435	78.1140317	39.6951895	68.1634697
ENSG000001	TEX261	protein_codin	80.4670951	159.641311	42.3735838	70.6014067
ENSG000001	DUSP11	protein_codin	35.2577389	0	0	0
ENSG000001	SULT1C4	protein_codin	3.31370704	8.60200095	4.70913099	0
ENSG000000	RGPD5	protein_codin	0	0	0	0
ENSG000001	MALL	protein_codin	0	0	5.43268636	0
ENSG000001	RALB	protein_codin	404.32675	683.831925	202.642607	322.696739
ENSG000001	C1QL2	protein_codin	0	0	0	0
ENSG000001	TMEM177	protein_codin	0	2.89924634	0	0

ENSG000001	SLC20A1	protein_codin	327.730889	354.303463	67.8223423	174.799445
ENSG000001	FBLN7	protein_codin	0	0	0	0
ENSG000001	SNRPA1P1	processed_ps	0	2.42436012	0	0
ENSG000001	CNGA3	protein_codin	0	0	0	0
ENSG000001	FAHD2B	protein_codin	0	0.93586154	3.7927708	1.01248424
ENSG000001	UBXN4	protein_codin	765.317811	555.035466	432.225715	573.912248
ENSG000001	NXPH2	protein_codin	0	0	0	0
ENSG000001	GPR17	protein_codin	0	0	0	0
ENSG000001	POLR2D	protein_codin	41.351453	75.2937701	42.1850768	13.6465424
ENSG000001	AMMECR1L	protein_codin	33.6516781	53.4232181	0	20.1005672
ENSG000001	SLC4A10	protein_codin	0	0	0	0
ENSG000001	SCRN3	protein_codin	21.3221819	9.04866791	8.40933058	33.9977573
ENSG000001	WIPF1	protein_codin	38.7974169	35.5949939	80.0198646	0
ENSG000001	LNPK	protein_codin	100.75528	48.8916549	55.3408234	72.6054969
ENSG000001	TMEFF2	protein_codin	0	0	0	0
ENSG000001	UBR3	protein_codin	331.131225	214.211134	172.718821	228.46328
ENSG000002	KLHL23	protein_codin	0	0	0	0
ENSG000001	METTL21A	protein_codin	0	23.7598328	0	0
ENSG000001	UNC80	protein_codin	0	0	0	0
ENSG000001	PTH2R	protein_codin	0	0	0	0
ENSG000001	NRP2	protein_codin	5.28182999	12.5057561	1.72630917	0
ENSG000001	CPO	protein_codin	0	0	0	0
ENSG000000	CASP10	protein_codin	3.04767438	3.56108775	0	2.85113504
ENSG000001	ABCA12	protein_codin	0	0	0	0
ENSG000001	SUMF1	protein_codin	81.4899048	103.184467	33.4578319	61.7876571
ENSG000001	NYAP2	protein_codin	0	1.2140356	0	0
ENSG000001	ACKR3	protein_codin	63.9543774	9.73349468	16.8917913	8.94302843
ENSG000001	UBE2F	protein_codin	61.2076226	107.007555	41.01845	62.5013304
ENSG000001	HES6	protein_codin	5.61715221	1.30142301	0	1.71476065
ENSG000001	ANKMY1	protein_codin	0	0	0	0
ENSG000001	PPP1R7	protein_codin	5.32414638	7.74990626	5.06256137	7.88532851
ENSG000001	DIS3L2	protein_codin	15.4327134	18.1990438	19.3351623	27.5946949
ENSG000001	CPNE9	protein_codin	0	0	1.00356155	0
ENSG000001	TAMM41	protein_codin	0	0	0	14.9939494
ENSG000001	VGLL4	protein_codin	2.95663956	0	0	0
ENSG000001	RAB5A	protein_codin	151.32179	145.117318	77.4788277	104.920845
ENSG000001	RETREG2	protein_codin	28.7957117	69.2459896	62.9283441	28.7347732
ENSG000001	CTDSP1	protein_codin	69.7025742	219.860264	52.9330624	84.785548
ENSG000001	SLC4A3	protein_codin	0	0	0	0
ENSG000001	CNOT9	protein_codin	54.2834213	104.373545	52.402246	48.5371734
ENSG000001	MARCHF4	protein_codin	0	0.8303316	0.84512356	0
ENSG000001	DNPEP	protein_codin	0	73.2835446	20.7485189	21.6088879
ENSG000001	PNKD	protein_codin	15.1438896	0	0	6.65716421
ENSG000001	DYNC1LI1	protein_codin	176.078378	149.528081	120.058561	115.98409

ENSG000001	RBMS3	protein_codin	0	0	0	0
ENSG000001	GASK1A	protein_codin	0	0	0	0
ENSG000001	CSRNP1	protein_codin	991.861467	637.417885	442.955805	579.248521
ENSG000000	SEC22C	protein_codin	9.59877378	0	0	0
ENSG000001	SLC22A14	protein_codin	0	0	0	0
ENSG000001	CTDSPL	protein_codin	81.8418203	223.289556	92.4535063	94.843736
ENSG000001	STAC	protein_codin	2.76045202	0	0	0
ENSG000001	IQSEC1	protein_codin	152.640623	69.8904233	28.4605358	49.5093731
ENSG000001	RPL32	protein_codin	932.328491	1773.22207	477.757155	977.894688
ENSG000001	ARL6IP5	protein_codin	861.188773	1045.96482	313.438285	521.556664
ENSG000001	LRIG1	protein_codin	22.1226372	17.4817393	7.67218723	0
ENSG000000	ITIH1	protein_codin	0	0	0	0
ENSG000001	LRTM1	protein_codin	0	0	0	0
ENSG000002	CNPY2	protein_codin	18.9848273	64.4448173	18.9264188	0
ENSG000001	LIMD1	protein_codin	144.022043	222.053491	128.986076	104.044165
ENSG000001	ZKSCAN7	protein_codin	4.21091647	0	0	0
ENSG000001	COL8A1	protein_codin	0	0.81726904	1.0259286	0
ENSG000001	NXPE3	protein_codin	139.022031	208.450893	55.3151647	109.833266
ENSG000001	ADGRG7	protein_codin	0	0	0	0
ENSG000001	MYH15	protein_codin	0	0	0	0
ENSG000001	ABHD10	protein_codin	34.1426956	75.8082346	23.8651796	39.7535576
ENSG000001	TAGLN3	protein_codin	0	0	0	0
ENSG000001	PLA1A	protein_codin	0	0	0	0
ENSG000001	RABL3	protein_codin	10.3683332	12.055078	11.5545921	2.99504309
ENSG000001	CFAP91	protein_codin	0	0	0	0
ENSG000001	BOC	protein_codin	0	0.81512324	0	0
ENSG000001	ATP6V1A	protein_codin	478.771837	455.238474	255.165816	525.216696
ENSG000002	ENSG000002	lncRNA	0	0	0	0
ENSG000001	MED12L	protein_codin	17.1195691	0	43.3452382	0
ENSG000001	EIF2A	protein_codin	42.1819337	158.807308	86.0333132	181.570469
ENSG000001	ALDH1L1	protein_codin	0	0	0	0
ENSG000001	RNF7	protein_codin	118.088407	99.1569896	57.2513811	62.2982358
ENSG000001	TRPC1	protein_codin	0	0	0	0
ENSG000002	ISY1	protein_codin	83.8257076	110.119314	0	65.4903878
ENSG000001	FTH1	protein_codin	11818.0334	10836.5964	1168.55378	7039.17742
ENSG000001	RUBCN	protein_codin	120.131637	126.180902	18.0687143	45.7655604
ENSG000001	AMT	protein_codin	0	0	0	0
ENSG000001	TCTA	protein_codin	55.8869716	88.3504739	35.9488035	34.1229188
ENSG000002	ABHD14A	protein_codin	0	6.54508618	2.72642451	1.83310184
ENSG000001	NICN1	protein_codin	12.7870693	0	10.1982442	23.3301378
ENSG000001	UCN2	protein_codin	0	0	0	0
ENSG000001	STXBP5L	protein_codin	0	0	0	0
ENSG000001	EAF2	protein_codin	41.661918	40.9277032	49.341013	32.3447128
ENSG000001	ILDR1	protein_codin	0	0	0	0

ENSG000001	TM4SF19	protein_codin	0	0	0	0
ENSG000001	SLIT2	protein_codin	0	0	0	0
ENSG000001	AHSG	protein_codin	0	0	0	0
ENSG000001	VWA5B2	protein_codin	0	0	0	0
ENSG000001	DGKQ	protein_codin	17.2090083	95.8321214	0	46.9187094
ENSG000001	CENPC	protein_codin	64.2795658	23.7844324	23.173251	42.4257481
ENSG000001	EPHA5	protein_codin	0	0	0	0
ENSG000001	CORIN	protein_codin	0	0	0	0
ENSG000001	ATP10D	protein_codin	124.434931	152.072016	110.653061	146.037135
ENSG000001	OCIAD2	protein_codin	0	6.76738327	9.38995608	3.81585248
ENSG000001	SLC10A4	protein_codin	0	0	0	0
ENSG000001	SLC10A6	protein_codin	0	0	0.90415758	0
ENSG000001	SCD5	protein_codin	0	1.418311	0	0
ENSG000001	ENOPH1	protein_codin	57.8448611	87.2177637	13.9190418	39.7095398
ENSG000001	CABS1	protein_codin	0	0	0	0
ENSG000001	GC	protein_codin	0	0	0	0
ENSG000001	TRMT10A	protein_codin	0	5.89318205	4.40144568	3.20637482
ENSG000001	KLHL8	protein_codin	98.594659	90.5525965	80.1401173	70.6719803
ENSG000001	PYURF	protein_codin	156.600122	285.000286	80.8226867	131.124222
ENSG000001	TBCK	protein_codin	0	0	0	7.34246749
ENSG000001	CISD2	protein_codin	3.22491741	7.30824704	11.9671026	15.0856678
ENSG000001	DDIT4L	protein_codin	0	0	0	0
ENSG000001	AP1AR	protein_codin	5.65798879	12.9998324	5.1553005	23.6323587
ENSG000001	AFG2A	protein_codin	20.4556223	19.2727313	14.0483257	11.4129671
ENSG000001	FABP2	protein_codin	0	0	0	0
ENSG000001	CCNA2	protein_codin	1.05052643	0	20.6580977	1.0517101
ENSG000001	USP53	protein_codin	0	0	12.4714351	0
ENSG000001	SETD7	protein_codin	378.118011	341.187693	131.443169	199.614255
ENSG000001	NAF1	protein_codin	25.9978766	25.3619636	0	15.437842
ENSG000001	MARCHF1	protein_codin	457.334612	300.66284	199.271221	341.820545
ENSG000001	SFRP2	protein_codin	0	0	0	0
ENSG000001	RPS3A	protein_codin	2815.04221	3849.31686	1674.77528	2337.12114
ENSG000001	PDGFC	protein_codin	43.8829751	141.232235	39.055408	63.0371102
ENSG000001	GLRA3	protein_codin	0	0	0	1.66802468
ENSG000001	ROPN1L	protein_codin	0	2.73706585	0	0
ENSG000001	NDUFS6	protein_codin	59.6275687	95.1799375	44.2341353	43.535449
ENSG000001	MARCHF6	protein_codin	167.338983	130.129024	91.2585177	90.9062129
ENSG000001	NKD2	protein_codin	0	0	0	0
ENSG000001	CDH18	protein_codin	0	0	0	0
ENSG000001	ADAMTS16	protein_codin	0	0	0	0
ENSG000001	SRD5A1	protein_codin	38.5526847	79.8066315	17.4122543	18.7085491
ENSG000001	MYO10	protein_codin	0	0	0	0
ENSG000001	OTULINL	protein_codin	511.772225	612.86049	266.82505	434.864642
ENSG000001	RPL37	protein_codin	138.988487	169.070176	118.013082	151.320825

ENSG000001	SKP2	protein_codin	5.37526843	14.5752271	0	10.446958
ENSG000001	SKP2	protein_codin	0	0	0	0
ENSG000001	OSMR	protein_codin	0	0	0	0
ENSG000001	UGT3A1	protein_codin	0	0	1.13957436	0
ENSG000001	PLK2	protein_codin	69.1164284	32.1346418	60.8714291	18.5283377
ENSG000001	GZMA	protein_codin	1.9687044	9.98039712	2.65460217	1.03599106
ENSG000001	PELO	protein_codin	21.9431921	20.7167846	6.01008319	11.9177851
ENSG000001	TRIM23	protein_codin	0	8.93432388	9.04270038	0
ENSG000001	HAPLN1	protein_codin	0	0	0	0
ENSG000001	BHMT	protein_codin	0	0	0	0
ENSG000001	ANKRD31	protein_codin	0	3.28133778	1.73248385	0
ENSG000001	IQGAP2	protein_codin	2067.96899	1111.32815	1119.78627	1252.48496
ENSG000001	CRHBP	protein_codin	3.21675988	10.9576537	3.12732729	2.1320824
ENSG000001	RASA1	protein_codin	86.7043601	53.1752733	0	59.4135157
ENSG000001	LIX1	protein_codin	0	0	0	0
ENSG000001	GTF2H2	protein_codin	0	0	15.5743593	5.86318703
ENSG000001	SPATA9	protein_codin	0	0	0	0
ENSG000001	TNFAIP8	protein_codin	48.3553238	84.4705833	22.5301675	57.1616267
ENSG000001	FEM1C	protein_codin	205.558922	98.7552403	93.0128024	134.901271
ENSG000001	COMMD10	protein_codin	46.3121659	34.1670394	12.636135	0
ENSG000001	MEGF10	protein_codin	0	0	0	0
ENSG000001	ADAMTS19	protein_codin	0	0	0	0
ENSG000001	YIPF5	protein_codin	65.1328499	67.4911406	38.1841277	49.3900039
ENSG000001	ARHGAP26	protein_codin	230.974994	165.555281	0	0
ENSG000001	LECT2	protein_codin	0	0	0	0
ENSG000001	SLC25A48	protein_codin	0	0	0	0
ENSG000001	CDKN2AIPNLI	processed_ps	0	0	0	0
ENSG000001	IL9	protein_codin	0	0	0	0
ENSG000001	TIMD4	protein_codin	0	0	0	0
ENSG000001	RNF145	protein_codin	0	0	0	0
ENSG000001	GABRA6	protein_codin	0	0	0	0
ENSG000001	GABRB2	protein_codin	0	0	0	0
ENSG000001	SPINK7	protein_codin	0	0	0	0
ENSG000001	PCYOX1L	protein_codin	80.0897685	120.278451	47.1493308	48.8734621
ENSG000001	GLRA1	protein_codin	0	0	0	0
ENSG000001	ZNF300	protein_codin	0	0	4.74442014	0
ENSG000001	N4BP3	protein_codin	2.90553157	0.94329046	0	0.9207867
ENSG000001	NHP2	protein_codin	29.9715548	82.0366493	11.2003835	16.7501154
ENSG000000	ADAMTS2	protein_codin	10.2608236	0	0	0.92351181
ENSG000001	FGF18	protein_codin	0	0	0	0
ENSG000001	KCNMB1	protein_codin	26.5157503	47.6351245	15.6078873	16.7989713
ENSG000001	MYLK4	protein_codin	0	0	0	6.68695107
ENSG000001	FAM217A	protein_codin	0	0	0	0
ENSG000001	FARS2	protein_codin	52.6189224	71.2655989	20.6181158	28.6791789

ENSG000001	CDKAL1	protein_codin	100.523204	73.1181155	49.6458117	77.0097908
ENSG000001	PSD2	protein_codin	1.8899558	0	0.96187056	0
ENSG000001	LRRTM2	protein_codin	5.74527941	2.32915545	0	4.74343909
ENSG000001	ZMAT2	protein_codin	444.343105	714.834602	202.674755	412.723346
ENSG000001	GFRA3	protein_codin	0	0	0	0
ENSG000001	MRS2	protein_codin	0	0	0	0
ENSG000001	H2BC1	protein_codin	1.73268586	0	0	0
ENSG000001	KAAG1	lncRNA	0.92089724	0	0	1.85025307
ENSG000001	TRIM7	protein_codin	0	30.432495	7.72130681	15.0826866
ENSG000001	HIGD2A	protein_codin	119.084156	203.820009	30.2843558	89.792328
ENSG000001	PLA2G7	protein_codin	82.2920644	94.8753272	8.0287631	207.331394
ENSG000001	RNF44	protein_codin	354.703562	438.267789	141.892232	245.696994
ENSG000001	MMUT	protein_codin	55.4555747	32.1622894	27.9925475	49.9922977
ENSG000002	ENSG000002	lncRNA	0	0	0	0
ENSG000001	ABT1	protein_codin	65.3643906	103.291672	30.6841136	33.4504976
ENSG000001	PPP1R18	protein_codin	584.553631	818.901922	98.8413715	498.567303
ENSG000001	DAAM2	protein_codin	0	0	0	0
ENSG000001	PRIM2	protein_codin	7.13036821	0	0	0
ENSG000001	MLIP	protein_codin	0	0	0	0
ENSG000001	HMGCLL1	protein_codin	0	0	0	0
ENSG000001	SCUBE3	protein_codin	0.97987171	1.93458615	0.98445625	1.93788708
ENSG000001	FGD2	protein_codin	228.785156	173.493481	0	152.795533
ENSG000001	ANO7	protein_codin	0	0	0	0
ENSG000001	CRIP3	protein_codin	0	0	0	0
ENSG000001	NFKBIE	protein_codin	0	0	10.373203	0
ENSG000001	CYP39A1	protein_codin	0	0	0	0
ENSG000001	PHIP	protein_codin	167.779719	49.0030272	35.0108286	87.844862
ENSG000001	HMGN3	protein_codin	57.5499386	80.9654794	87.9999797	34.8532834
ENSG000001	MMS22L	protein_codin	0	0	0	0
ENSG000001	PM20D2	protein_codin	11.0941284	19.1947147	29.8200127	19.0815588
ENSG000001	CD164	protein_codin	0	0	0	0
ENSG000001	TBC1D32	protein_codin	0	0	0	0
ENSG000001	CLVS2	protein_codin	0	0	0	0
ENSG000001	GPR6	protein_codin	0	0	0	0
ENSG000001	TAAR2	protein_codin	0	0	0	0
ENSG000001	ARG1	protein_codin	0	0	0	0
ENSG000001	TAAR6	protein_codin	0	0	0	0
ENSG000001	TAAR8	protein_codin	0	0	0	0
ENSG000001	TAAR1	protein_codin	0	0	0	0
ENSG000002	VNN3P	lncRNA	6.09060385	0	0	0
ENSG000001	SLC18B1	protein_codin	19.2440103	44.4653001	13.4538209	19.3901171
ENSG000001	SLC2A12	protein_codin	1.00210831	2.04382776	0.90749566	1.04171945
ENSG000001	SHPRH	protein_codin	23.8610965	4.84151625	29.2315392	13.657449
ENSG000001	AIG1	protein_codin	1.26694495	0	0	0

ENSG000001	TIAM2	protein_codin	4.06542516	1.11619499	0	0
ENSG000001	QKI	protein_codin	0	131.031848	0	26.3974484
ENSG000001	PNLDC1	protein_codin	0	0	0	0
ENSG000001	SLC22A3	protein_codin	0	1.15391483	0	0
ENSG000001	VWDE	protein_codin	2.49052824	0	0	0
ENSG000001	GNA12	protein_codin	201.092723	253.211811	121.840006	152.51334
ENSG000001	ESYT2	protein_codin	5.98233715	39.4486132	29.5621504	11.1806409
ENSG000000	GGCT	protein_codin	16.7141341	50.683958	0	0
ENSG000001	FERD3L	protein_codin	0	0	0	0
ENSG000001	EGFR	protein_codin	0	0	0	0
ENSG000001	CDCA5	protein_codin	0	1.3056943	6.56036767	0
ENSG000001	IGFBP1	protein_codin	0	0	0	0
ENSG000002	KCTD7	protein_codin	0	0	0	0
ENSG000002	PMS2P14	transcribed_u	0	0	0	0
ENSG000001	SSC4D	protein_codin	2.53455819	0	0	0
ENSG000001	POMZP3	protein_codin	0	0	2.78641708	0
ENSG000000	PHTF2	protein_codin	0	5.72076287	0	0
ENSG000002	FKBP6P2	lncRNA	0	0	0	0
ENSG000001	CCT6A	protein_codin	340.644385	393.527468	306.381194	235.62621
ENSG000001	PSPH	protein_codin	0	0	0	0
ENSG000001	SUMF2	protein_codin	0	0	0	0
ENSG000000	LAT2	protein_codin	144.520676	63.4938514	113.153941	190.363213
ENSG000001	ASB15	protein_codin	0	0	0	0
ENSG000001	GIGYF1	protein_codin	87.9127772	85.2301135	24.9336274	42.2114537
ENSG000001	AGBL3	protein_codin	0	2.25882293	0	0
ENSG000001	STRA8	protein_codin	0	0	0	0
ENSG000001	ZC3HAV1L	protein_codin	16.4857203	16.2925466	11.8822151	15.3349431
ENSG000001	TMEM140	protein_codin	222.114112	380.333177	105.195297	200.389632
ENSG000001	EPHA1	protein_codin	0	0	0	0
ENSG000001	NOM1	protein_codin	114.834277	147.683949	62.4028096	58.4091749
ENSG000001	ASB10	protein_codin	0	0	0	0
ENSG000001	NLGN4X	protein_codin	0	0	0	0
ENSG000001	DENND2A	protein_codin	0	0	0	0
ENSG000001	TMEM47	protein_codin	0	0	0	0
ENSG000001	SPIN2B	protein_codin	0	12.352076	4.88262104	2.01399857
ENSG000001	CCNB3	protein_codin	0	0	2.11179206	2.75273468
ENSG000001	CDK16	protein_codin	0	0	0	0
ENSG000002	ZNF630	protein_codin	0	0	0	0
ENSG000001	CHST7	protein_codin	65.6503369	49.3121271	9.01907192	36.452641
ENSG000001	NDUFB11	protein_codin	19.8849445	0	0	0
ENSG000001	RAB41	protein_codin	0	0	0	0
ENSG000001	TAF1	protein_codin	0	0	0	0
ENSG000001	NONO	protein_codin	193.344564	178.688688	174.969166	0
ENSG000001	EBP	protein_codin	0	27.2650288	8.14360803	0

ENSG000001	AWAT2	protein_codin	0	0	0	0
ENSG000001	SNX12	protein_codin	0	0	0	0
ENSG000001	IL2RG	protein_codin	29.5723244	20.490117	22.5697418	6.56408852
ENSG000001	ZNF711	protein_codin	3.61057948	6.19700944	5.16557199	0
ENSG000001	CPXCR1	protein_codin	0	0	0	0
ENSG000001	SYTL4	protein_codin	0	9.83204256	0	0
ENSG000001	RIPPLY1	protein_codin	0	0	0	0
ENSG000001	TBC1D8B	protein_codin	3.7119667	0	0	0
ENSG000001	FRMPD3	protein_codin	0	0	0	0
ENSG000001	HTR2C	protein_codin	0	0	0	0
ENSG000001	UPF3B	protein_codin	0	0	96.406816	0
ENSG000001	DOCK11	protein_codin	930.961076	780.223683	530.098491	554.665914
ENSG000001	DOCK11	protein_codin	259.277393	96.557888	0	163.824509
ENSG000001	ARHGAP36	protein_codin	0	0	0	0
ENSG000001	GPR119	protein_codin	0	0	0	0
ENSG000001	CT55	protein_codin	0	0	0	0
ENSG000001	MFHAS1	protein_codin	86.381061	88.6537021	28.81334	47.9216808
ENSG000001	DLC1	protein_codin	7.3787578	4.37516501	19.3153456	3.7858752
ENSG000001	FBXO25	protein_codin	0	0	0	0
ENSG000001	MAGEA4	protein_codin	0	0	0	0
ENSG000000	SLC18A1	protein_codin	0	0	0	0
ENSG000001	ATP6V1B2	protein_codin	284.781224	612.011654	183.365324	235.453832
ENSG000001	ADRA1A	protein_codin	0	0	0	0
ENSG000001	CHRNA6	protein_codin	0	0	0	0
ENSG000001	GNRH1	protein_codin	0	0	1.11001019	1.05654005
ENSG000001	BIN3	protein_codin	42.2219166	61.9318213	23.8780542	35.0438378
ENSG000001	DOK2	protein_codin	443.985487	636.238235	141.96116	284.375737
ENSG000001	TNFRSF10B	protein_codin	316.514497	401.423873	112.823886	204.460263
ENSG000001	DOCK5	protein_codin	1580.07837	1155.163	632.383598	921.266691
ENSG000001	STAR	protein_codin	0	0	0	0.76132181
ENSG000001	ST18	protein_codin	3.12820518	1.03364699	2.46623711	0
ENSG000001	RGS20	protein_codin	0	0	0	0
ENSG000001	TACC1	protein_codin	121.844252	14.258588	62.603842	12.4005255
ENSG000001	GINS4	protein_codin	4.98935775	3.31866617	8.78321867	2.09210025
ENSG000001	ARMC1	protein_codin	27.7950069	55.5616388	28.1751394	36.9703202
ENSG000001	DNAJC5B	protein_codin	0	3.35909547	0	0
ENSG000001	CRH	protein_codin	0	0	0	0
ENSG000001	TRIM55	protein_codin	0	0.81540841	2.39776452	0
ENSG000001	ADHFE1	protein_codin	0	0	0	0
ENSG000001	MRPS28	protein_codin	0	16.5613783	0	7.41954233
ENSG000001	LACTB2	protein_codin	28.7705334	43.8739563	7.09676429	23.5577447
ENSG000001	PRDM14	protein_codin	0	0	0	0
ENSG000001	TERF1	protein_codin	0	0	0	24.0466943
ENSG000001	TERF1	protein_codin	4.92696111	0	0	0

ENSG000001	SLC26A7	protein_codin	0	0	0	0
ENSG000001	PSKH2	protein_codin	0	0	0	0
ENSG000001	SYBU	protein_codin	0	0	0	0
ENSG000001	LRP12	protein_codin	5.46021322	0	3.49440223	0
ENSG000001	RSPO2	protein_codin	0	0	0	0
ENSG000001	EIF3H	protein_codin	518.918399	770.140758	156.712021	377.478567
ENSG000001	NDUFB9	protein_codin	165.944376	279.701725	97.2016828	126.406342
ENSG000001	TATDN1	protein_codin	0	12.7177403	0	11.5600456
ENSG000001	FAM83A	protein_codin	0	0	0	0
ENSG000001	C8orf76	protein_codin	37.1748254	53.4126213	22.7656735	20.1808667
ENSG000001	GSDMC	protein_codin	0	0	0	0
ENSG000001	FAM135B	protein_codin	0	0.90515295	1.00646519	0
ENSG000001	TTTY7	lncRNA	0	0	0	0
ENSG000001	TTTY7B	lncRNA	0	0	0	0
ENSG000001	ZNF16	protein_codin	0	0	0	0
ENSG000001	ARHGAP39	protein_codin	0	1.49957557	0	0
ENSG000001	SLC39A4	protein_codin	0	0	0	0
ENSG000001	UHRF2	protein_codin	58.1503153	68.1912536	44.1946192	0
ENSG000001	RIC1	protein_codin	100.999595	76.7405439	54.0919287	71.3167189
ENSG000001	PLIN2	protein_codin	191.994149	252.008443	84.6943431	167.164068
ENSG000001	CDKN2B	protein_codin	22.4411268	37.0803683	26.6628695	5.18808278
ENSG000001	IFNA1	protein_codin	0	0	0	0
ENSG000001	ADAMTSL1	protein_codin	0	0	0	0
ENSG000001	IFNK	protein_codin	0	0	0	0
ENSG000001	FBXO10	protein_codin	5.14255085	4.93634082	0	0
ENSG000001	CNTNAP3B	protein_codin	0	0	0	0
ENSG000001	SIGMAR1	protein_codin	42.8932991	113.678325	15.4230015	0
ENSG000001	CEP78	protein_codin	0	0	12.5293519	0
ENSG000001	NTRK2	protein_codin	0	0	0	0
ENSG000000	TUT7	protein_codin	34.8575739	109.070372	123.264615	189.941811
ENSG000000	FAM120A	protein_codin	552.098416	808.154496	349.906552	410.14805
ENSG000001	AOPEP	protein_codin	25.8956067	5.07821041	9.57998243	5.29936324
ENSG000001	OR13C4	protein_codin	0	0	0	0
ENSG000001	ZNF462	protein_codin	0	5.86115282	7.291367	1.01003776
ENSG000001	OR1K1	protein_codin	0	0	0	0
ENSG000001	OLFM1	protein_codin	0	0	0	1.23679694
ENSG000001	ASB6	protein_codin	50.5824997	130.078097	39.6670666	35.251351
ENSG000001	ASB6	protein_codin	0	0	0	0
ENSG000001	PTGES2	protein_codin	0	0	0	0
ENSG000001	CIZ1	protein_codin	18.3743785	0	0	0
ENSG000001	LCN2	protein_codin	15.9078301	98.9770269	32.1581662	27.7147251
ENSG000001	PAEP	protein_codin	0	0	0	0
ENSG000001	IDI2	protein_codin	0	0	0	0.9272049
ENSG000001	LCN9	protein_codin	0	0	0	0

ENSG000001	PNPLA7	protein_codin	6.53067046	2.5956964	0.95178528	10.9090138
ENSG000001	SEC16A	protein_codin	0	0	0	0
ENSG000001	DPH7	protein_codin	25.9589643	23.0500359	11.8764462	16.8758516
ENSG000001	CACNA1B	protein_codin	0	0.84396811	0	0
ENSG000001	NACC2	protein_codin	412.105828	214.853629	160.093695	393.568042
ENSG000001	PROSER2	protein_codin	31.3122071	89.6639773	46.8498054	43.7932475
ENSG000001	USP6NL	protein_codin	0	0	6.69488915	0
ENSG000001	MINDY3	protein_codin	34.9515516	30.0952664	9.42979451	12.0072482
ENSG000002	CCDC7	protein_codin	0	0	0	0
ENSG000001	NRBF2	protein_codin	48.1430331	193.147836	53.4418419	61.8549131
ENSG000001	TMEM26	protein_codin	0	0	0	0
ENSG000001	PBLD	protein_codin	6.80210551	3.53421789	0	1.06876122
ENSG000001	HERC4	protein_codin	18.4858071	12.1663014	17.8516254	0
ENSG000001	GLUD1	protein_codin	368.816226	440.961297	66.7144578	293.166277
ENSG000001	HTR7	protein_codin	3.07761986	5.22794037	0	0
ENSG000001	RPP30	protein_codin	0	0	0	0
ENSG000001	ADD3	protein_codin	424.5205	350.885825	230.892572	305.872963
ENSG000001	VAX1	protein_codin	0	0	0	1.00917396
ENSG000001	NPFFR1	protein_codin	0	3.08743793	0.98374188	2.06823342
ENSG000001	TCF7L2	protein_codin	0	37.2166522	0	86.5937219
ENSG000000	SORBS1	protein_codin	0	0	0	0
ENSG000001	FUOM	protein_codin	12.5861826	16.6949411	0	12.7905781
ENSG000001	PAOX	protein_codin	0	16.7571525	0	6.41541517
ENSG000001	CFAP43	protein_codin	0	2.0111932	0	0
ENSG000001	PPRC1	protein_codin	48.0618055	117.576233	65.7260089	41.9333302
ENSG000001	ITPRIP	protein_codin	0	0	188.937463	0
ENSG000001	BCCIP	protein_codin	84.6103354	66.3868687	57.4385022	88.1082569
ENSG000001	BTBD10	protein_codin	19.5674137	0	0	18.5052292
ENSG000001	ADM	protein_codin	88.3796697	60.7839679	12.8900242	29.9110967
ENSG000001	GAS2	protein_codin	0	0	0	0
ENSG000001	LIN7C	protein_codin	124.369814	78.9008309	91.0226823	89.242296
ENSG000001	LRRC4C	protein_codin	0	0	7.63185997	8.38301337
ENSG000001	IMMP1L	protein_codin	0	0	0	0
ENSG000001	SAA4	protein_codin	0	0	0	0
ENSG000001	CARS1	protein_codin	130.970113	149.690745	45.4341448	106.214815
ENSG000001	PGAP2	protein_codin	0	0	0	0
ENSG000001	TUT1	protein_codin	5.17693914	0	3.9995575	0
ENSG000001	SCGB1A1	protein_codin	0	0	0	0
ENSG000001	TRIM6	protein_codin	0	0	2.55121097	0
ENSG000001	ZNF214	protein_codin	0	0	0	0
ENSG000001	TNNT3	protein_codin	0	0	0	0
ENSG000001	ZNF215	protein_codin	0	12.2507961	0	0
ENSG000001	HSD17B12	protein_codin	58.8015316	38.8759538	47.3073318	60.7349783
ENSG000001	SLC1A2	protein_codin	0	0	0	0

ENSG000000:	CD44	protein_codin	65.487765	58.6714038	48.7205777	24.133405
ENSG000000:	CD44	protein_codin	0	2.48451307	0	0
ENSG000001	GLYAT	protein_codin	0	0	0	0
ENSG000001	SERPING1	protein_codin	44.3211231	13.0653974	45.289517	41.2081104
ENSG000001	OR5F1	protein_codin	0	0	0	0
ENSG000001	SSRP1	protein_codin	282.238774	405.043391	197.20189	208.349647
ENSG000002	TMX2	protein_codin	30.8163009	23.9200922	13.9711099	13.0273502
ENSG000001	SLC43A1	protein_codin	0	0	0	0
ENSG000001	CSTPP1	protein_codin	3.64522477	14.6057029	0	7.75752599
ENSG000001	HIKESHI	protein_codin	29.3123869	25.7360564	5.53322546	16.8315623
ENSG000001	SESN3	protein_codin	0	0	0	0
ENSG000001	ENDOD1	protein_codin	396.019483	863.16993	611.435712	381.422148
ENSG000001	CCDC82	protein_codin	0	0	0	0
ENSG000000	ACER3	protein_codin	13.2997142	0	7.43366408	3.2795345
ENSG000001	TENM4	protein_codin	18.5525648	55.0897201	2.96206691	0
ENSG000001	PAK1	protein_codin	0	0	0	0
ENSG000001	RPS3	protein_codin	0	0	0	19.3927753
ENSG000001	ZC3H12C	protein_codin	0	8.2627447	32.9082705	11.0591805
ENSG000001	C11orf52	protein_codin	0	0	0	0
ENSG000001	NPAT	protein_codin	81.9794641	110.782436	51.2218807	32.9979213
ENSG000001	ATM	protein_codin	0	0	0	0
ENSG000001	AASDHPPT	protein_codin	73.601159	55.7654971	29.9656756	48.3161359
ENSG000001	PLCB4	protein_codin	0	0	0	0
ENSG000001	LAMTOR1	protein_codin	453.04048	691.460287	294.192304	337.242956
ENSG000001	ST14	protein_codin	147.417964	262.16778	57.2510603	143.521834
ENSG000000	APLP2	protein_codin	48.9886806	68.5721917	46.0873481	0
ENSG000001	GGTLC1	protein_codin	0	0	0	0.92893836
ENSG000000	ZNF343	protein_codin	40.7280989	20.6107134	23.9178813	0
ENSG000001	OVOL2	protein_codin	0	0	0	0
ENSG000000	SMOX	protein_codin	0	0	14.355705	0
ENSG000001	MTA2	protein_codin	222.474691	333.273351	42.4375534	73.1881584
ENSG000001	TMEM138	protein_codin	5.61367344	6.09731132	0	0
ENSG000002	FADS3	protein_codin	11.3967719	20.2156096	0	6.909986
ENSG000001	ROM1	protein_codin	0	0	0.97123832	0
ENSG000001	MYRF	protein_codin	0	0	0	0
ENSG000001	FADS2	protein_codin	27.1188201	33.0971313	0	8.97188418
ENSG000001	EML3	protein_codin	77.6891595	73.5687209	0	34.5274623
ENSG000001	INCENP	protein_codin	95.8334486	56.5241836	114.912195	38.7976168
ENSG000001	ZP1	protein_codin	0	0	0	0.91861645
ENSG000001	OOSP2	protein_codin	0	0	0	0
ENSG000001	WDR74	protein_codin	0	0	4.1758702	13.7623417
ENSG000001	MS4A3	protein_codin	0	0	0	0
ENSG000001	PLCH2	protein_codin	0	0	0	0
ENSG000001	FRG1BP	transcribed_u	0	0	0	0

ENSG000001	NINL	protein_codin	0	0	0	0
ENSG000001	MS4A2	protein_codin	11.8120196	2.72942223	0	1.59861288
ENSG000001	BSCL2	protein_codin	0	0	0	0
ENSG000001	EI24	protein_codin	32.3846133	40.5680244	13.0241926	15.4913309
ENSG000001	CHEK1	protein_codin	3.25605984	0	0	0
ENSG000001	FEZ1	protein_codin	0	0	0	0
ENSG000001	ESAM	protein_codin	139.400518	185.455562	57.1421901	68.2058609
ENSG000001	CEP164	protein_codin	159.115417	159.151491	50.1893989	121.434827
ENSG000001	MPZL2	protein_codin	23.3629691	27.3768311	4.35411489	16.1911363
ENSG000001	SCN2B	protein_codin	0	0	0	0
ENSG000001	MPZL3	protein_codin	42.4672829	56.8387778	29.0027056	17.7805127
ENSG000001	SIDT2	protein_codin	0	0	3.73427397	0
ENSG000001	TAGLN	protein_codin	8.46986016	32.1755528	26.5863719	0
ENSG000001	DUSP15	protein_codin	0	0	0	0
ENSG000001	COMMD7	protein_codin	145.340059	170.290641	58.8656685	93.6610487
ENSG000001	MYL9	protein_codin	102.998446	485.511721	746.484604	125.346604
ENSG000001	KIAA1755	protein_codin	0	0	0	0
ENSG000001	SLC13A3	protein_codin	0	0	0	0
ENSG000001	OCSTAMP	protein_codin	0	0	0	0
ENSG000001	MTCL2	protein_codin	20.0923655	16.539469	25.7177611	20.3837934
ENSG000001	PIGT	protein_codin	0	0	0	0
ENSG000001	PIGT	protein_codin	62.559668	151.116421	0	83.5599348
ENSG000001	ERGIC3	protein_codin	8.83756579	9.76124599	6.76509602	1.07843537
ENSG000001	SPINT4	protein_codin	0	0	0	0
ENSG000002	ENSG000002	lncRNA	0	0	0	0
ENSG000001	LSM14B	protein_codin	136.506334	217.240505	74.6620736	110.228349
ENSG000001	LSM14B	protein_codin	0	0	0	0
ENSG000001	CABLES2	protein_codin	40.1342315	55.8575484	13.935315	15.7159467
ENSG000001	AIP	protein_codin	102.067541	211.148871	96.2280836	62.5756793
ENSG000001	LTO1	protein_codin	0	0	5.65267491	0
ENSG000001	GPHA2	protein_codin	0	0	0	0
ENSG000001	SLC22A9	protein_codin	0	0	0	0
ENSG000001	NUDT22	protein_codin	25.3585989	35.8520648	7.27017981	18.0981913
ENSG000001	FERMT3	protein_codin	67.7880007	276.298977	84.2425827	83.1893604
ENSG000001	PLCB3	protein_codin	173.232036	298.785328	111.909344	116.348007
ENSG000001	MRPL49	protein_codin	7.06564024	27.9286031	36.9609922	0
ENSG000000	CAPN1	protein_codin	722.372936	1147.27709	194.117941	541.06941
ENSG000001	CDC42EP2	protein_codin	74.0763652	62.0137579	8.72066223	23.9450484
ENSG000001	FAU	protein_codin	26.7116701	21.2125725	8.5716889	18.5658175
ENSG000001	TM7SF2	protein_codin	0	0	0	0
ENSG000001	VPS51	protein_codin	78.6907767	154.762835	0	16.4133927
ENSG000001	TBX6	protein_codin	0	0	0	2.51284793
ENSG000001	PPP4C	protein_codin	201.505507	301.525528	51.8170304	159.076569
ENSG000001	TLCD3B	protein_codin	0	0	0	0

ENSG000001	HIRIP3	protein_codin	18.4683411	34.1444969	13.1830637	21.9223328
ENSG000001	TAOK2	protein_codin	62.0751427	121.697617	39.207906	52.3336804
ENSG000001	TMEM219	protein_codin	87.4255268	116.649682	42.5565075	39.9301214
ENSG000001	MMP10	protein_codin	0	1.13446515	0	0
ENSG000001	CNKSR2	protein_codin	0	0	0	0
ENSG000000	CD44	protein_codin	17.4147739	12.1807658	17.7302741	2.11316266
ENSG000001	CNTN5	protein_codin	0	0	0	0
ENSG000001	SIRPB1	protein_codin	237.601068	0	0	0
ENSG000001	DUSP6	protein_codin	692.840155	567.786447	148.002399	295.73285
ENSG000001	KLRF1	protein_codin	0	0	0	2.32645616
ENSG000000	YBX3	protein_codin	250.825087	311.743748	152.617781	158.728313
ENSG000002	PRB4	protein_codin	0	0	0	0
ENSG000001	OR8K1	protein_codin	0	0	0	0
ENSG000001	OR5M9	protein_codin	0	0	0	0
ENSG000001	CTF1	protein_codin	0	0	0	0
ENSG000001	CWC15	protein_codin	77.1016301	100.994983	60.7502611	80.9746732
ENSG000001	ARID5B	protein_codin	0	73.9969002	0	114.951828
ENSG000001	BLTP3B	protein_codin	407.812037	269.940301	218.716729	289.863994
ENSG000001	TMEM218	protein_codin	0	0	0	0
ENSG000001	KIAA1328	protein_codin	15.6941027	0	0	4.15676468
ENSG000001	DHRS12	protein_codin	0	1.13823418	0	1.22295431
ENSG000001	FAM124A	protein_codin	0	0	0	0
ENSG000001	MIA2	protein_codin	0	0	0	0
ENSG000001	MIA2	protein_codin	0	31.259548	0	60.0999592
ENSG000001	HNMT	protein_codin	34.2512756	50.968742	35.3146574	37.2728337
ENSG000001	HNMT	protein_codin	51.1575675	43.9968176	13.5884087	50.6492482
ENSG000001	SPOPL	protein_codin	325.125303	116.554325	159.137295	193.888253
ENSG000001	LYPD6B	protein_codin	0	0	0	0
ENSG000001	PDCD4	protein_codin	264.403106	117.462576	246.716158	173.35658
ENSG000001	ADRA2A	protein_codin	2.01452768	41.5567061	23.7765048	10.4056491
ENSG000001	GPM6A	protein_codin	0	0	0	0
ENSG000001	WDR17	protein_codin	23.3855234	0	7.99991514	0
ENSG000001	SPATA4	protein_codin	0	0	0	0
ENSG000001	CD226	protein_codin	341.683138	398.444353	280.874114	185.886308
ENSG000001	DLG2	protein_codin	0	0	0	0
ENSG000001	CCDC83	protein_codin	0	0	0	0
ENSG000001	PRSS23	protein_codin	1.39551636	2.31189647	0	0
ENSG000001	MTMR12	protein_codin	4.45442249	0	0	0
ENSG000001	PPP1R1C	protein_codin	0	0	0	0
ENSG000001	POU2AF2	protein_codin	0	0	0	0
ENSG000001	CCT5	protein_codin	19.1738374	19.5293434	4.35097443	18.1955124
ENSG000001	ATPSCKMT	protein_codin	0	0	6.23724919	0
ENSG000001	DOCK1	protein_codin	26.6353265	46.4539339	16.4896328	24.8501564
ENSG000001	DLAT	protein_codin	26.7524159	0	0	52.692181

ENSG000001	PIH1D2	protein_codin	0	0	0	0
ENSG000001	NKAPD1	protein_codin	0	0	0	0
ENSG000001	IL18	protein_codin	30.7095381	56.5223567	0	0
ENSG000001	TEX12	protein_codin	0	3.24628346	0	0
ENSG000001	PTS	protein_codin	0	17.6900748	6.01954849	2.18995202
ENSG000001	USP15	protein_codin	609.987155	278.06382	309.276224	351.51957
ENSG000001	SLC5A12	protein_codin	0	0	0	0
ENSG000001	FREM2	protein_codin	0	0	1.08962283	0
ENSG000001	CRIM1	protein_codin	13.9222376	12.7645919	14.1837519	19.3423294
ENSG000001	SEC24D	protein_codin	157.507348	172.883191	94.0561648	120.8292
ENSG000001	DENR	protein_codin	163.263941	89.6248586	104.289084	103.507796
ENSG000001	ABCB9	protein_codin	0	0	0	0
ENSG000000	PITPNM2	protein_codin	68.8654617	99.5047583	49.8267387	49.2418182
ENSG000001	RILPL2	protein_codin	398.107153	511.314289	168.975697	293.053642
ENSG000000	TRNT1	protein_codin	0	0	0	0
ENSG000001	TKTL2	protein_codin	4.26304688	2.16998313	2.19566352	2.20414224
ENSG000001	PRSS53	protein_codin	0	0	0	0
ENSG000001	SLC7A11	protein_codin	6.1488416	5.11182493	20.9260524	4.18578553
ENSG000001	NOCT	protein_codin	177.911923	50.2915317	108.383893	88.0331037
ENSG000001	CACNA2D4	protein_codin	6.28054871	11.5079296	0	0
ENSG000001	DCP1B	protein_codin	25.3135033	20.2714315	18.9697554	10.7607666
ENSG000001	KCNA6	protein_codin	0	0	0	0
ENSG000001	THRB	protein_codin	0	0	0	0
ENSG000001	NGLY1	protein_codin	0	13.1801343	0	0
ENSG000001	NGLY1	protein_codin	0	0	0	0
ENSG000001	OXSM	protein_codin	1.30120745	5.38083023	0	2.65756335
ENSG000001	LDHC	protein_codin	0	0	0	0
ENSG000001	LDHAL6A	protein_codin	0	0	0	0
ENSG000001	TMEM86A	protein_codin	15.9990377	12.1701209	14.4617344	10.9394094
ENSG000001	NOPCHAP1	protein_codin	10.5790826	16.1228257	13.24765	8.55304026
ENSG000001	TMEM263	protein_codin	11.4966747	0	27.961868	0
ENSG000001	ABTB3	protein_codin	83.0122589	221.276629	98.3793919	6.69192166
ENSG000001	ANK3	protein_codin	0	0	2.00166784	0
ENSG000001	FAM53B	protein_codin	37.3088109	56.8065904	14.4954881	14.5253381
ENSG000001	PLBD2	protein_codin	266.341897	300.018781	66.3945011	163.218553
ENSG000001	SLC2A13	protein_codin	33.875116	14.1373968	18.428919	13.7389419
ENSG000001	GXYLT1	protein_codin	12.8418214	13.7468829	1.49585885	8.94567954
ENSG000001	DIP2C	protein_codin	0	3.89424741	0	0
ENSG000001	EIF4E	protein_codin	0	0	4.43534738	0
ENSG000001	DSC2	protein_codin	0	0	0	0
ENSG000000	MAT2B	protein_codin	0	6.52944473	0	0
ENSG000001	AKAP6	protein_codin	4.4371979	2.44703854	1.53508886	0
ENSG000001	FAM177A1	protein_codin	55.6289614	38.8189243	29.7269489	32.5808793
ENSG000001	TMEM18	protein_codin	22.4134772	29.0916912	7.32488612	17.1241831

ENSG000001	THRSP	protein_codin	0	0	0	0
ENSG000001	NDUFC2	protein_codin	5.35443704	14.0615025	9.87066149	12.3318421
ENSG000001	NARS2	protein_codin	0	0	0	0
ENSG000001	MYCN	protein_codin	0	11.5000133	4.87425337	0
ENSG000001	MSGN1	protein_codin	0	0	0	0.86670087
ENSG000001	NUBPL	protein_codin	9.07464803	4.3705801	5.25404479	0
ENSG000001	ATP6V1G3	protein_codin	0	0	0	0
ENSG000001	FER	protein_codin	108.208885	44.3193668	78.4155765	79.3149222
ENSG000000	NRXN3	protein_codin	0	1.24629856	0.99632817	0
ENSG000001	CEP128	protein_codin	40.7820202	66.7087888	229.930311	20.2519825
ENSG000001	CDC123	protein_codin	113.492429	138.16118	61.3308382	60.8661782
ENSG000001	SCLT1	protein_codin	301.409834	467.741988	211.585397	242.937686
ENSG000001	C4orf33	protein_codin	34.8159908	11.2983297	22.735271	6.53477136
ENSG000001	SLC25A31	protein_codin	0	0	0	0
ENSG000001	KHDRBS2	protein_codin	0	0	0	0
ENSG000001	PTPRO	protein_codin	0	0	0	0
ENSG000001	EPS8	protein_codin	0	0	0	0
ENSG000001	ACAD8	protein_codin	24.2948221	13.637708	10.474632	18.4457487
ENSG000001	VPS26B	protein_codin	188.597359	263.233223	66.8782314	84.2283918
ENSG000001	QDPR	protein_codin	11.0102665	42.3006528	0	30.4815191
ENSG000001	MUC15	protein_codin	0	0	0	0
ENSG000001	QTRT2	protein_codin	85.9635438	0	17.0052895	62.3147691
ENSG000001	CGNL1	protein_codin	0	0.82252581	0	0
ENSG000001	POU4F2	protein_codin	0	0	0	0
ENSG000001	PIGF	protein_codin	13.0916826	5.19489396	0	9.78719749
ENSG000001	PPP1R21	protein_codin	0	0	0	0
ENSG000001	WDR35	protein_codin	4.33821809	9.46537689	11.4934286	14.0307294
ENSG000001	MFSD6	protein_codin	0	95.9255959	0	146.025433
ENSG000001	ASAP2	protein_codin	119.855043	194.866037	94.4238576	112.222755
ENSG000001	FLI1	protein_codin	4.25205438	51.5716458	23.3958061	7.56543755
ENSG000000	BARX2	protein_codin	0	0	0	0
ENSG000001	TMEM45B	protein_codin	1.05703799	0	0	0
ENSG000001	CENPU	protein_codin	3.63047822	7.26943785	5.47435677	10.4639723
ENSG000001	ACSL1	protein_codin	118.414252	0	0	0
ENSG000001	SLC25A4	protein_codin	0	2.14816172	2.33156702	0
ENSG000001	AMN1	protein_codin	2.32918492	13.6278916	0	3.56220688
ENSG000001	VWA8	protein_codin	21.1699241	7.58876267	0	0
ENSG000001	NBAS	protein_codin	240.264607	246.205056	169.944909	158.414968
ENSG000001	ZNF385D	protein_codin	6.33530892	7.36366408	10.506692	16.5566742
ENSG000001	GUF1	protein_codin	54.4397845	43.6139004	42.2324526	45.1373961
ENSG000001	CCDC175	protein_codin	57.1524739	52.2917552	0	0
ENSG000001	PABPC3	protein_codin	7.66998902	0	0	0
ENSG000001	ATP8A2	protein_codin	0	0	0	0
ENSG000001	FBXO4	protein_codin	0	0	7.90493414	9.10004437

ENSG000001	PARP8	protein_codin	202.406835	91.1367805	67.8336191	95.1374718
ENSG000001	NVL	protein_codin	0	29.833155	0	0
ENSG000001	GLT1D1	protein_codin	9.45495461	17.9005994	10.5415728	11.4703184
ENSG000001	FBXW7	protein_codin	150.892596	21.1545473	44.3435252	39.4178908
ENSG000001	RBM46	protein_codin	0	0	0	0
ENSG000000	TXNDC16	protein_codin	81.1156263	54.374992	0	0
ENSG000001	KANSL1L	protein_codin	23.0166966	14.9404622	6.31061911	8.78192395
ENSG000001	MCHR2	protein_codin	0	0	0	0
ENSG000001	EPHA4	protein_codin	0	0	3.55398404	0
ENSG000001	FARSB	protein_codin	48.5206529	49.8172285	37.3107436	25.0725779
ENSG000001	KCNE4	protein_codin	0	0	0	0
ENSG000001	TNFSF4	protein_codin	0	3.11052956	3.09912489	3.43236865
ENSG000001	MZT2B	protein_codin	37.9638914	49.2770894	26.3064175	26.7640337
ENSG000001	ASTN1	protein_codin	0	1.74184185	0	0
ENSG000001	CFC1B	protein_codin	0	0	0	0
ENSG000001	MGAT5	protein_codin	0	0	0	12.316335
ENSG000001	TMEM163	protein_codin	3.1307809	2.42005641	1.70133134	2.15322866
ENSG000001	MED13L	protein_codin	768.676662	737.460607	548.068253	513.229468
ENSG000001	GPATCH11	protein_codin	19.1939051	0	20.0808903	0
ENSG000001	HSPB8	protein_codin	0	0	0	0
ENSG000001	GEMIN6	protein_codin	6.65135987	15.8424405	5.4946323	5.95316366
ENSG000001	TMEM178A	protein_codin	0	0	0	0
ENSG000001	OBI1	protein_codin	35.3589857	41.2358478	21.9073063	34.6886941
ENSG000001	ZC3H13	protein_codin	152.808868	301.691809	455.768241	326.637355
ENSG000001	CYSLTR2	protein_codin	0	37.8278671	12.1755103	0
ENSG000001	GRID2	protein_codin	0	0	0	0
ENSG000001	ARL11	protein_codin	103.54204	206.296526	70.7041297	84.1617377
ENSG000001	ARL14EP	protein_codin	30.9472745	36.9381948	20.3090548	19.522545
ENSG000001	EPG5	protein_codin	0	0	0	0
ENSG000001	ATP5F1A	protein_codin	0	397.733167	0	220.571811
ENSG000001	HAUS1	protein_codin	50.7030676	24.9882178	22.8183315	23.8799721
ENSG000001	SPC25	protein_codin	1.22010105	3.53784924	1.66936573	0
ENSG000001	G6PC2	protein_codin	0	0	0	0
ENSG000001	PDK1	protein_codin	131.786465	112.572474	78.34707	80.9756065
ENSG000001	PTH	protein_codin	0	0	0	0
ENSG000001	PDE3B	protein_codin	79.9320955	91.2731197	91.6153808	46.7141698
ENSG000001	FASTKD3	protein_codin	3.36915674	0	0	7.69284693
ENSG000001	TCF7L1	protein_codin	0	0.92757396	0.69265767	0
ENSG000001	CRYGC	protein_codin	0	0	0	0
ENSG000001	KCNK13	protein_codin	1.98281076	8.062598	1.53763871	5.93202565
ENSG000001	UHMK1	protein_codin	7.85289404	34.7065579	9.31881428	5.17402271
ENSG000001	ATG10	protein_codin	0	14.0540576	6.4515051	14.5898442
ENSG000001	FAM151B	protein_codin	19.0977276	8.40702867	5.81169246	1.46716242
ENSG000001	GUCY1A2	protein_codin	0	0	0	0

ENSG000001	CWF19L2	protein_codin	177.774377	153.035793	159.976196	153.308473
ENSG000001	HOMER1	protein_codin	0	0	0	0
ENSG000001	XRCC4	protein_codin	14.9992316	0	0	0
ENSG000000	ANKRD44	protein_codin	285.195686	316.25911	159.269324	324.373885
ENSG000002	MARS2	protein_codin	5.86538916	10.6795004	8.64553904	5.00879094
ENSG000001	BOLL	protein_codin	0	0	0	0
ENSG000001	ZNF547	protein_codin	2.25220914	0	2.30832981	0
ENSG000001	ZNF304	protein_codin	0	0	0	16.4743708
ENSG000001	ZNF773	protein_codin	0	0	0	0
ENSG000002	ZNF551	protein_codin	38.0598334	32.5951448	15.7393866	16.6980913
ENSG000001	ZNF256	protein_codin	0	0	0	5.87517632
ENSG000001	ZSCAN1	protein_codin	0	0	0	0
ENSG000001	CACNB2	protein_codin	0	0	0	0
ENSG000001	USP12	protein_codin	404.080546	350.210275	243.256356	295.72947
ENSG000001	CAMK4	protein_codin	3.97953603	3.29100058	3.90125486	2.07407813
ENSG000001	TRIM36	protein_codin	6.51965128	0	5.04030877	0
ENSG000002	TMED7-TICAM	protein_codin	12.9511507	13.1198772	4.35277395	3.81700364
ENSG000001	ZFP36L2	protein_codin	2400.35904	2224.04756	494.705362	1075.95309
ENSG000001	FLT1	protein_codin	4.98348623	5.17314879	8.13020547	3.84825278
ENSG000001	PLEKHH2	protein_codin	0	0	0	0
ENSG000001	PPM1B	protein_codin	87.8182429	88.7130763	32.0642327	85.5634945
ENSG000001	YAP1	protein_codin	0	0	0	0
ENSG000001	IGSF10	protein_codin	2.96035341	5.96188021	5.71742458	3.03598562
ENSG000001	SPEF2	protein_codin	9.50492069	0	0	0
ENSG000001	SPARCL1	protein_codin	0	0	0	0
ENSG000001	DMP1	protein_codin	0	0	0	0
ENSG000001	MBNL1	protein_codin	0	48.3828072	0	0
ENSG000001	MBNL1	protein_codin	69.5237832	0	14.4876391	97.0238955
ENSG000001	GRIA4	protein_codin	0	0	0	0
ENSG000001	UGT3A2	protein_codin	0	0	0	0
ENSG000001	NADK2	protein_codin	0	11.1751845	0	15.602043
ENSG000001	NIPBL	protein_codin	256.767836	158.455237	0	147.446016
ENSG000001	GADL1	protein_codin	0	0	0	0
ENSG000001	GPD1L	protein_codin	55.9780198	72.1021195	42.1209245	0
ENSG000001	OTX1	protein_codin	0	0	0.62930756	0
ENSG000001	GJA1	protein_codin	0	0	0	0
ENSG000000	GMCL1	protein_codin	48.8880656	39.7374891	28.2490214	52.1564194
ENSG000001	CCNO	protein_codin	0	0	0	0
ENSG000001	TIA1	protein_codin	0	50.4476109	40.0818377	48.4138037
ENSG000001	SLC30A6	protein_codin	54.2982959	0	4.10756132	34.7640935
ENSG000002	ITGA1	protein_codin	0	0	0	3.98183965
ENSG000000	JADE2	protein_codin	6.7199017	0	33.1393655	0
ENSG000001	CATSPER3	protein_codin	0	1.02529365	0	0
ENSG000000	WASHC2A	protein_codin	452.139726	431.921979	217.027234	290.830248

ENSG000001	DYNLT5	protein_codin	6.36035885	0	11.3659256	4.0625763
ENSG000001	LIPA	protein_codin	0	0	0	0
ENSG000001	BMP3	protein_codin	0	0	0	1.34721428
ENSG000001	HHEX	protein_codin	116.398872	270.091618	59.330644	178.370779
ENSG000001	GRM1	protein_codin	0	0	0	0
ENSG000001	GGPS1	protein_codin	12.1545573	4.53948527	0	10.0702193
ENSG000001	ADAMTS18	protein_codin	0	0	0	0
ENSG000001	ZNF117	protein_codin	31.3952345	0	0	0
ENSG000001	RAB3C	protein_codin	4.44815068	4.40095553	1.96547545	2.52292896
ENSG000001	LMNTD1	protein_codin	0	1.03891806	0	0
ENSG000001	RASSF8	protein_codin	0	0	5.11553192	0
ENSG000001	RAD17	protein_codin	0	0	15.5158827	0
ENSG000001	MED21	protein_codin	101.915211	61.7190197	55.8742074	59.1264064
ENSG000001	PLOD2	protein_codin	0	0	0	0
ENSG000001	STK32B	protein_codin	3.91593483	3.94492394	2.91801506	10.8913812
ENSG000001	JAKMIP1	protein_codin	0	0	0	0
ENSG000001	ZIC1	protein_codin	0	0	0	0
ENSG000001	ADGRA3	protein_codin	1.06623261	6.10027498	3.07485634	0
ENSG000001	CPB1	protein_codin	0	0	0	0
ENSG000002	SREK1IP1P1	processed_ps	3.00195457	0.94452235	0	0
ENSG000001	MR1	protein_codin	12.0787507	28.3079121	16.7639854	10.2090924
ENSG000001	SRP19	protein_codin	20.3377818	51.0591169	21.5076393	24.2318218
ENSG000001	CENPH	protein_codin	5.47589189	11.3678432	8.8479785	0
ENSG000001	TEKT5	protein_codin	0	0	0	0
ENSG000001	NUBP1	protein_codin	65.8201669	141.811497	36.1722232	60.1016848
ENSG000001	TXNDC11	protein_codin	271.079449	309.163515	143.430661	162.261427
ENSG000001	NPIPA6	protein_codin	0	77.5506513	56.9221165	0
ENSG000000	RIOK2	protein_codin	13.8535064	27.177886	6.63182477	23.5952294
ENSG000002	ENSG000002	processed_ps	0	0	0	0
ENSG000001	CETN3	protein_codin	0	2.324771	0	3.08393705
ENSG000001	SMARCA5	protein_codin	678.811516	558.790618	502.713588	521.550411
ENSG000001	SYCP2L	protein_codin	3.19997876	0	13.6576223	0
ENSG000001	BMP6	protein_codin	269.690274	563.612141	115.865876	165.99613
ENSG000001	UXS1	protein_codin	153.262794	354.284282	113.288563	166.569933
ENSG000001	RASSF3	protein_codin	58.240758	106.928342	109.56417	33.9144962
ENSG000001	HNRNPU	protein_codin	563.584955	165.026621	228.37246	180.920144
ENSG000001	RANBP2	protein_codin	1166.05539	615.355351	821.5365	822.014075
ENSG000001	TMEM87B	protein_codin	172.629056	185.222708	95.7065662	136.961523
ENSG000001	OR14K1	protein_codin	0	0	0	0
ENSG000001	PTPRR	protein_codin	0	0	0	0
ENSG000001	CCDC148	protein_codin	0	0	0	0
ENSG000001	PLA2R1	protein_codin	0	0	0	0
ENSG000001	ITGB6	protein_codin	0	0	0	0
ENSG000001	SCN3A	protein_codin	0	0	0	0

ENSG000001	SCN2A	protein_codin	0	0	0	0
ENSG000001	FEZF2	protein_codin	0	0	0	0
ENSG000001	CADPS	protein_codin	0	0	0	0
ENSG000001	CD96	protein_codin	5.85210757	8.18910174	0	1.0335537
ENSG000001	ATG3	protein_codin	466.494224	703.12376	94.3011819	488.243387
ENSG000000	ADGRF5	protein_codin	0	0	0	0
ENSG000001	ADGRF1	protein_codin	0	0	0	0
ENSG000001	ADGRF4	protein_codin	0	0	0	0
ENSG000001	FRMD1	protein_codin	0	0	0	0
ENSG000001	TRAPPC8	protein_codin	15.5974192	12.7521715	8.81622633	13.1770046
ENSG000001	FAM81B	protein_codin	3.35696619	50.6647817	0	6.52582201
ENSG000001	DTNA	protein_codin	0	5.53524776	0	0
ENSG000001	INO80C	protein_codin	2.28232585	4.17066025	0	0
ENSG000001	LPCAT1	protein_codin	436.62061	314.078773	127.88366	180.780549
ENSG000001	PLEKHG4B	protein_codin	0	1.71860739	0	0
ENSG000001	NMRAL1	protein_codin	81.9825013	55.6115642	23.9291239	56.2337377
ENSG000001	ZDHHC11	protein_codin	1.31194785	0	0.97909303	0
ENSG000001	UBALD1	protein_codin	22.7339951	60.6621156	4.08061088	16.2168178
ENSG000001	LYSET	protein_codin	15.5093686	13.5716676	4.48353933	9.37364832
ENSG000001	TEX29	protein_codin	5.53606404	1.81016653	0	0
ENSG000001	SPACA7	protein_codin	0	0	0	0
ENSG000001	FBXL2	protein_codin	0	0	0	0
ENSG000001	UBP1	protein_codin	32.1953195	0	0	0
ENSG000001	UBP1	protein_codin	67.1125109	77.7537112	28.2310422	100.294928
ENSG000001	RMND5A	protein_codin	379.575533	526.655066	264.664458	400.298706
ENSG000001	CD8A	protein_codin	7.79490051	2.78075057	0	0
ENSG000001	RPIA	protein_codin	58.2036741	66.9092702	39.8075457	35.1360095
ENSG000001	VILL	protein_codin	0	0	0	0
ENSG000000	SERPINB3	protein_codin	0	0	0	0
ENSG000001	HGD	protein_codin	17.9542434	18.5139866	7.66569445	5.8651011
ENSG000001	GTF2E1	protein_codin	23.0029581	51.4744332	22.2052711	26.2125235
ENSG000001	CFDP1	protein_codin	154.823115	293.9595	99.4759325	170.829824
ENSG000001	TGIF2LX	protein_codin	0.6918907	0	0	0
ENSG000001	SPMIP4	protein_codin	28.7663357	30.137651	12.4366043	7.14129658
ENSG000001	TMPRSS11D	protein_codin	0	0	0	0
ENSG000002	HOXA10	protein_codin	0	4.59681061	0	0
ENSG000001	JAZF1	protein_codin	154.285078	162.974151	16.4285379	69.5223092
ENSG000001	KCNJ16	protein_codin	0	0	0	0
ENSG000001	TRIP12	protein_codin	0	0	0	0
ENSG000001	FBXO36	protein_codin	2.15621356	1.11232294	0	0
ENSG000000	PGM3	protein_codin	0	0	0	0
ENSG000001	CEBPG	protein_codin	100.924029	98.4660763	44.9066829	137.420275
ENSG000001	KCTD15	protein_codin	6.99762322	11.1384038	0	0
ENSG000001	MCOLN2	protein_codin	0	0	0	0

ENSG000001	DDAH1	protein_codin	6.91215475	0	8.40486464	0
ENSG000001	ERBIN	protein_codin	100.657463	69.5286784	42.2565288	104.204611
ENSG000001	SREK1	protein_codin	0	0	0	0
ENSG000002	CLCA3P	lncRNA	0	0	0	0
ENSG000001	DGKE	protein_codin	41.5030893	4.00475906	17.6200744	22.0637475
ENSG000001	MSI2	protein_codin	88.8819663	41.1453861	12.4631131	47.2088215
ENSG000001	HS3ST3A1	protein_codin	1.96507036	0	0	0
ENSG000001	GDPD1	protein_codin	5.45090592	0	2.09456077	8.21991837
ENSG000001	SEMA3D	protein_codin	2.71096795	0	0	0
ENSG000001	ASB17	protein_codin	0	0	0	0
ENSG000001	GRAP	protein_codin	8.91278773	20.5835151	2.22864028	11.0341338
ENSG000001	MAX	protein_codin	0	0	0	0
ENSG000001	IMPACT	protein_codin	65.4100751	51.8499815	46.7271141	56.124377
ENSG000001	THY1	protein_codin	0	0	0	0
ENSG000001	C16orf74	protein_codin	0	0	0	1.62476197
ENSG000001	TBCEL	protein_codin	0	13.8181119	0	0
ENSG000001	JPH3	protein_codin	0	0	0	0
ENSG000001	ANKH	protein_codin	38.7686621	56.9289225	20.5991383	9.83679834
ENSG000001	UBASH3B	protein_codin	146.384856	176.071246	111.33646	130.251053
ENSG000001	OTULIN	protein_codin	241.566329	231.297754	149.852034	140.949107
ENSG000001	OR8A1	protein_codin	0	0	0	0
ENSG000001	PANX3	protein_codin	0	0	0	0
ENSG000001	TBRG1	protein_codin	97.5291983	0	59.8662667	0
ENSG000001	NRGN	protein_codin	2281.02881	5505.07694	1548.80226	1849.26291
ENSG000001	GPR15	protein_codin	17.26266	5.2650576	82.814983	0
ENSG000001	TOMM70	protein_codin	87.1001054	82.9782818	60.6743339	76.8553533
ENSG000001	ABI3BP	protein_codin	0	0	0	0
ENSG000001	CC2D1B	protein_codin	71.1518279	82.8834038	22.0531709	35.186659
ENSG000001	CERS3	protein_codin	0	0	0	0
ENSG000001	PRKCA	protein_codin	10.3271746	0	0	0
ENSG000002	ENSG000002	lncRNA	0	0	1.05729554	0
ENSG000001	ABCA6	protein_codin	0	4.42786686	3.98675799	10.726835
ENSG000001	C4orf19	protein_codin	0	0	0	0
ENSG000001	UCHL1	protein_codin	0	0	0	0
ENSG000001	DISP1	protein_codin	0	0	0	0
ENSG000001	TNIK	protein_codin	0	0	0	0
ENSG000001	FAM167A	protein_codin	1.96192225	1.93192653	2.10364629	0.96482393
ENSG000001	NEIL2	protein_codin	2.06000706	3.59906734	0	1.80127562
ENSG000001	ATP8B4	protein_codin	0	58.2705864	42.7974045	64.2889391
ENSG000001	WNT3A	protein_codin	0	0	0	0
ENSG000001	OBSCN	protein_codin	7.00059168	16.1303943	0	0
ENSG000001	TRIM11	protein_codin	33.7478929	55.208023	0	0
ENSG000001	ENAH	protein_codin	0	0	0	0
ENSG000001	PPP1R3A	protein_codin	0	0	0	0

ENSG000001	PPP1R3A	protein_codin	0	0	0	0
ENSG000001	CCSAP	protein_codin	0	0	0	35.7333225
ENSG000001	ASZ1	protein_codin	0	0	0	0
ENSG000001	SH3RF1	protein_codin	32.3961982	49.7668501	42.1368773	33.3221345
ENSG000001	OSCAR	protein_codin	47.6774024	174.377584	10.5209446	56.8416589
ENSG000002	KLHL41	protein_codin	0	1.31872235	0	0
ENSG000001	GPR26	protein_codin	0	0	0	1.01996253
ENSG000000	DHX32	protein_codin	41.0715408	90.0255309	13.3066592	25.4725869
ENSG000001	C10orf90	protein_codin	0	0	0	0
ENSG000001	OLA1	protein_codin	12.9125838	23.3619112	18.0685987	13.585594
ENSG000001	ATP5MC3	protein_codin	34.0231627	30.0846845	36.561259	31.4807535
ENSG000001	PDLIM3	protein_codin	0	0	0	0
ENSG000001	PDLIM3	protein_codin	0	0	0	0
ENSG000001	PDLIM3	protein_codin	0	0	0	0
ENSG000001	SORBS2	protein_codin	0	0	0	0
ENSG000001	ELOC	protein_codin	0	24.7384266	5.05321642	0
ENSG000001	LY96	protein_codin	43.9525592	51.1088724	28.0835898	44.6135422
ENSG000001	TMSB4Y	protein_codin	37.8190789	63.1872107	31.76296	42.2165605
ENSG000001	CXADR	protein_codin	0	0	0	0
ENSG000001	C21orf91	protein_codin	64.3068988	31.867574	48.8567794	57.1623375
ENSG000001	TMPRSS15	protein_codin	0	0	0	0
ENSG000001	NCAM2	protein_codin	0	0	0	0
ENSG000001	SEL1L2	protein_codin	0	0	0	0
ENSG000001	RABGEF1	protein_codin	104.404237	76.516543	54.9596437	38.8376474
ENSG000001	ATP5PF	protein_codin	102.284126	201.432452	96.8889046	102.575315
ENSG000001	ADAMTS1	protein_codin	0	0	0	0
ENSG000001	ADAMTS5	protein_codin	6.92189197	32.9850956	2.5533091	17.9028778
ENSG000001	TSEN2	protein_codin	0	0	0	0
ENSG000001	SLFN13	protein_codin	16.2670615	0	2.54043293	0
ENSG000001	WNT7A	protein_codin	0	0	0.95088477	0
ENSG000001	XPC	protein_codin	218.073227	276.695221	58.9403816	165.672643
ENSG000001	MYO5B	protein_codin	0	0	0	0
ENSG000001	FGD5	protein_codin	0	0	0	0
ENSG000001	FLCN	protein_codin	73.6447728	137.6564	27.8049807	36.5226153
ENSG000001	DPH3	protein_codin	0	0	0	15.2863415
ENSG000001	OXNAD1	protein_codin	16.6461242	0	0	10.2183928
ENSG000001	ACAA2	protein_codin	9.98729226	17.2936372	1.52740049	38.8069402
ENSG000001	CXXC1	protein_codin	0	69.5436601	44.5140233	62.5850431
ENSG000001	SKA1	protein_codin	0	0	12.9150852	0
ENSG000001	PPP4R1	protein_codin	78.162747	111.116831	39.4117771	36.7815694
ENSG000001	CCDC144BP	transcribed_u	0	71.7581225	72.7201216	0
ENSG000001	USP43	protein_codin	0	0	0	0
ENSG000001	SAMD14	protein_codin	0	0	0	0
ENSG000001	RAB6B	protein_codin	30.0536439	76.2332656	102.933829	23.1738594

ENSG000001	ABCC3	protein_codin	61.0959157	83.0437192	108.574387	8.2404275
ENSG000001	ANKRD40	protein_codin	195.687063	248.620788	147.511265	132.617647
ENSG000001	CA10	protein_codin	0	0	0	0
ENSG000002	ZNF286B	lncRNA	0	4.37634904	0	0
ENSG000001	VOPP1	protein_codin	162.447596	218.250432	190.8548	55.3369922
ENSG000002	MRPS17	protein_codin	30.6884967	21.3553407	16.4062426	18.5616157
ENSG000001	DKK2	protein_codin	1.15512286	0	0.92024271	0
ENSG000001	CA2	protein_codin	178.029024	207.009303	140.687773	107.982665
ENSG000001	CA3	protein_codin	0	0	1.17069774	0
ENSG000001	UNC93B2	transcribed_p	0.85832914	0	0	0.81067998
ENSG000001	ATP6V0D2	protein_codin	0	0	0	0
ENSG000001	AK9	protein_codin	0	3.94810924	14.1595436	0
ENSG000001	ERCC3	protein_codin	66.7487834	70.2834932	59.1655537	68.6079274
ENSG000001	ODF1	protein_codin	0	0	0	0
ENSG000001	KLF10	protein_codin	2528.06099	3515.07639	1142.46584	1708.75853
ENSG000001	PIP4P2	protein_codin	105.558201	90.340894	41.5076158	55.9006357
ENSG000001	OTUD6B	protein_codin	59.4628078	0	25.008895	0
ENSG000001	AGPAT5	protein_codin	27.9442028	7.15978829	9.71833116	19.9069874
ENSG000000	MAN2B2	protein_codin	209.710364	450.412933	119.388823	124.913986
ENSG000001	OR4K1	protein_codin	0	1.29323225	0	0
ENSG000001	MARVELD1	protein_codin	129.640958	241.164384	54.9206189	79.7556768
ENSG000001	HSPA13	protein_codin	78.8973524	55.9314246	72.1655158	47.5570041
ENSG000001	SAMSN1	protein_codin	0	0	0	0
ENSG000001	USP25	protein_codin	689.977437	455.007711	290.368055	412.414046
ENSG000001	USP25	protein_codin	130.685886	59.0746892	114.768388	112.541785
ENSG000001	GRAMD2B	protein_codin	0	0	0	0
ENSG000001	C16orf87	protein_codin	43.4874457	47.113414	21.3119506	53.9879601
ENSG000001	RHOC	protein_codin	1.46029182	0	1.6087214	0
ENSG000001	LONP2	protein_codin	242.859319	278.720259	163.141306	145.468431
ENSG000001	TRIM74	protein_codin	0	0	0	0
ENSG000001	NIFK	protein_codin	37.9820786	51.2271857	23.9409358	8.90173172
ENSG000001	OXA1L	protein_codin	138.882225	274.62814	0	64.3793845
ENSG000001	SLC7A7	protein_codin	85.7765817	37.3563181	39.5071487	55.6844285
ENSG000001	CCDC146	protein_codin	26.6255298	24.1269282	32.8615039	17.8005994
ENSG000000	GEMIN5	protein_codin	40.7770193	80.5887697	32.2004168	35.3581287
ENSG000001	MAGEC1	protein_codin	0	0	0.775731	0.96876094
ENSG000001	CNOT8	protein_codin	316.587038	358.340964	110.489129	199.47677
ENSG000001	GRIA1	protein_codin	0	0	0	0
ENSG000001	BOD1	protein_codin	21.3539173	30.4405055	5.35264408	10.5869645
ENSG000001	LRGUK	protein_codin	4.79227032	0	0.91027558	1.09129637
ENSG000000	AKR1B1	protein_codin	39.7752308	93.4902586	43.780712	35.5802673
ENSG000001	SETD9	protein_codin	10.4462122	5.93187137	0	0
ENSG000001	NUP205	protein_codin	145.013304	131.140772	90.8405827	116.38644
ENSG000001	CYP2C18	protein_codin	0	0	0	0

ENSG000000	FIRRM	protein_codin	0	0	0	0
ENSG000001	XAGE2	protein_codin	0	0	0	0
ENSG000001	PDE11A	protein_codin	0	2.3085658	0	1.18994296
ENSG000001	RBM45	protein_codin	23.2102869	19.5478266	10.3119094	10.7157536
ENSG000001	PDIA4	protein_codin	106.004392	152.927715	93.0399712	87.97325
ENSG000001	KDM8	protein_codin	10.6093833	6.05182976	2.79380427	8.54662493
ENSG000001	BANP	protein_codin	0	48.6241158	0	0
ENSG000001	OTOA	protein_codin	0	0	0	0
ENSG000002	PPIL3	protein_codin	0	0	0	0
ENSG000001	HYCC2	protein_codin	0	8.89503189	2.47753642	0
ENSG000000	CASP10	protein_codin	166.260495	191.236205	120.463834	87.6436162
ENSG000001	FLACC1	protein_codin	0	0	0	0
ENSG000001	HSD3BP1	transcribed_u	0	0	0	0
ENSG000001	C2CD6	protein_codin	0	1.19096585	0	0
ENSG000001	TMEM237	protein_codin	0	0	0	0
ENSG000001	FZD7	protein_codin	1.99907278	6.40178322	2.2977993	1.01358573
ENSG000001	DEPTOR	protein_codin	1.99779156	8.74093374	4.23330781	1.99435861
ENSG000001	SLC26A2	protein_codin	58.6455348	48.3407011	46.5398904	32.6845462
ENSG000001	CSF1R	protein_codin	0	0	0	0
ENSG000001	LSM11	protein_codin	9.9765312	13.9850317	7.10505807	6.00287467
ENSG000001	MED7	protein_codin	5.17801029	29.2353754	20.9296863	5.76194715
ENSG000001	SLC24A2	protein_codin	0	1.16110972	0	1.19307048
ENSG000001	TRIM42	protein_codin	0	0	0	0
ENSG000001	PXYLP1	protein_codin	14.3081759	33.4657898	0	13.2028318
ENSG000001	ADCY8	protein_codin	0	0	0	0
ENSG000001	RASA2	protein_codin	160.417297	108.089846	92.8783472	126.593528
ENSG000000	ATP1B3	protein_codin	172.034548	132.732525	69.1980751	62.9784414
ENSG000001	RAET1L	protein_codin	0	0	0	0
ENSG000001	SMC2	protein_codin	23.3457586	0	0	0
ENSG000001	TMBIM4	protein_codin	17.0033962	0	19.8055429	9.25204722
ENSG000001	VBP1	protein_codin	72.0002747	47.9143734	44.3757543	45.9324783
ENSG000001	AFF2	protein_codin	0	0	0	0
ENSG000001	VAMP7	protein_codin	260.576794	226.554496	143.133666	184.333023
ENSG000001	KIF5A	protein_codin	0	0	0.93378756	0
ENSG000001	NAT2	protein_codin	0	0	0	0
ENSG000001	MAGEA8	protein_codin	0	0	0	0
ENSG000001	PSD3	protein_codin	0	0	0	0
ENSG000002	ARHGEF25	protein_codin	0	0	0	0
ENSG000001	MIDEAS	protein_codin	0	0	0	0
ENSG000001	FAM161B	protein_codin	0	16.6447739	0	10.0304659
ENSG000001	GNAQ	protein_codin	1173.24562	989.58207	696.375172	788.985787
ENSG000001	WIF1	protein_codin	0	0	0	0
ENSG000001	UGT2A1	protein_codin	0	0	0	0
ENSG000001	MMP16	protein_codin	0	0	0	0

ENSG000001	ADK	protein_codin	0	0	0	0
ENSG000001	KCNMA1	protein_codin	0	0	0	0
ENSG000001	KCNMA1	protein_codin	7.55152518	0	0	0
ENSG000001	BATF	protein_codin	34.709159	47.9170603	27.0747118	29.8112258
ENSG000001	DCK	protein_codin	166.613153	227.469288	107.846843	161.634987
ENSG000001	TTLL5	protein_codin	0	0	0	7.81223596
ENSG000001	ADAMTS3	protein_codin	0	0	0	1.19521156
ENSG000001	CFAP418	protein_codin	3.15415448	7.31041773	5.20751569	3.22109554
ENSG000001	DRAM2	protein_codin	44.1194224	30.8133934	1.49951982	16.0086333
ENSG000001	STOM	protein_codin	383.382822	661.217887	323.180344	334.613722
ENSG000001	PPEF2	protein_codin	3.14744421	1.94457995	0	0
ENSG000001	CFAP161	protein_codin	9.90144545	17.0027756	16.3840975	8.12549221
ENSG000001	NAAA	protein_codin	97.7013603	232.286196	28.8741519	81.6842957
ENSG000001	ADAMTSL3	protein_codin	3.00870343	0	2.25023151	0
ENSG000001	SLC28A1	protein_codin	0	0	0	0
ENSG000001	CXCL13	protein_codin	0	0	0	0
ENSG000001	WHAMM	protein_codin	167.022723	200.551789	70.3419459	116.042085
ENSG000001	RWDD2B	protein_codin	0	0	8.47437448	10.283445
ENSG000001	CCT8	protein_codin	384.694209	419.310403	248.114528	274.488415
ENSG000001	NAA11	protein_codin	0	0	0	0
ENSG000001	BACH1	protein_codin	1004.83299	540.382896	356.042017	636.064399
ENSG000001	CLDN17	protein_codin	0	0	0	0
ENSG000001	TSPAN7	protein_codin	0	0	0	0
ENSG000001	TIAM1	protein_codin	117.418092	0	0	0
ENSG000001	SCAF4	protein_codin	232.134451	227.235007	134.510677	179.193275
ENSG000001	GGTLC1	protein_codin	0	0	0	0
ENSG000001	ANKRD9	protein_codin	15.6847	42.6970147	18.0308813	25.5872988
ENSG000001	ATP5MJ	protein_codin	124.4159	246.411314	66.9308453	85.2384859
ENSG000001	FUT6	protein_codin	0	0	0	0
ENSG000001	PCDH1	protein_codin	1.73917376	0	0	0
ENSG000001	GDF6	protein_codin	0	0	0	0
ENSG000001	UQCRB	protein_codin	30.2066784	16.9705555	14.5594598	27.2903225
ENSG000001	MTERF3	protein_codin	7.43203239	7.40829098	13.0237349	6.04596495
ENSG000001	RPL30	protein_codin	863.188739	1529.59311	232.158725	747.496296
ENSG000001	KCNS2	protein_codin	1.15413569	0	0	0
ENSG000001	TYSND1	protein_codin	19.9128498	27.9945379	3.60477408	12.2672132
ENSG000001	CD109	protein_codin	0	10.8679586	0	0
ENSG000001	NODAL	protein_codin	0	0	0	0
ENSG000001	PRG3	protein_codin	0	0	0	0
ENSG000001	KIF6	protein_codin	0	0	0	0
ENSG000001	UBE2L6	protein_codin	238.939888	409.273057	128.241498	184.898321
ENSG000001	ZDHHC5	protein_codin	260.206159	357.266662	149.284655	178.854416
ENSG000001	PARP6	protein_codin	0	0	0	59.6432186
ENSG000001	CELF6	protein_codin	0	0	0	0

ENSG000001	ZFAND3	protein_codin	409.952886	550.394209	317.355713	309.920752
ENSG000001	KAT6B	protein_codin	216.92639	153.735116	27.4555377	0
ENSG000001	RAB11FIP1	protein_codin	956.777405	661.273272	252.071009	415.185104
ENSG000001	UNC5D	protein_codin	0	0	0	0
ENSG000001	GLYATL2	protein_codin	0	0	0	0
ENSG000001	AIFM1	protein_codin	0	85.0619189	0	25.4550946
ENSG000001	BAG4	protein_codin	178.484714	218.502188	87.2420313	135.292778
ENSG000001	TBC1D31	protein_codin	12.6019409	0	0	0
ENSG000001	NTAQ1	protein_codin	4.13656476	0	0	0
ENSG000001	ATAD2	protein_codin	71.6781424	78.5676944	77.253614	61.4592934
ENSG000001	FBXO32	protein_codin	0	4.06423511	0	0
ENSG000001	NSMCE2	protein_codin	45.6828298	0	0	16.9295415
ENSG000001	ZNF689	protein_codin	37.4879284	79.2436483	16.4494797	29.0296507
ENSG000001	PRR14	protein_codin	26.6840826	0	0	0
ENSG000001	FBRS	protein_codin	207.351838	203.418792	144.296267	89.8301871
ENSG000001	FRRS1	protein_codin	0	10.1339294	0	0
ENSG000001	SASS6	protein_codin	19.1156448	9.11525175	22.4819141	11.1568463
ENSG000001	COX6A2	protein_codin	0	0	0	0
ENSG000001	BCKDK	protein_codin	0	0	0	0
ENSG000001	ZIC3	protein_codin	0	0	0	0
ENSG000001	MALSU1	protein_codin	0	2.0960316	3.72826435	2.41468259
ENSG000001	VPS8	protein_codin	217.450504	230.182736	181.344243	157.995884
ENSG000001	LHFPL4	protein_codin	0	0	0	0
ENSG000001	B3GNT7	protein_codin	1.88849585	0.93313417	0	1.80928104
ENSG000001	MPV17L	protein_codin	9.25841967	2.32060099	1.25359186	0
ENSG000001	BUB1B	protein_codin	0	0	40.337709	0
ENSG000001	PDE6D	protein_codin	23.5034335	41.5933089	20.5268718	13.6805724
ENSG000001	KNG1	protein_codin	0	0	0	0
ENSG000001	SST	protein_codin	0	0	0	0
ENSG000001	FANCD2	protein_codin	3.09209667	4.80072759	0	13.8672607
ENSG000001	TATDN2	protein_codin	5.42925768	46.6137085	0	17.0960261
ENSG000001	GHRL	protein_codin	3.2668837	1.68630419	0	0
ENSG000001	NOMO1	protein_codin	173.869332	243.247598	139.527182	96.8859771
ENSG000001	EXOG	protein_codin	26.3539	36.9050489	20.2570404	3.66041896
ENSG000001	HMBOX1	protein_codin	82.7257418	120.868936	52.5184928	118.504828
ENSG000001	NTAN1	protein_codin	54.4168187	59.6528384	75.0412566	32.344337
ENSG000001	NMNAT2	protein_codin	0.99696556	0.99193367	1.9592061	1.02031031
ENSG000002	TSPY8	protein_codin	0	0	0	0
ENSG000001	ZFYVE9	protein_codin	0	0	0	0
ENSG000001	LYZL4	protein_codin	0	0	0	0
ENSG000001	FCHO2	protein_codin	0	0	0	10.3514181
ENSG000001	SLC6A1	protein_codin	0	0	0	0
ENSG000001	RBPMS	protein_codin	0	0	0	0
ENSG000001	TMEM171	protein_codin	0	0	0	0

ENSG000001	KLHL40	protein_codin	0	1.79303452	0	0.9134629
ENSG000001	TIMP4	protein_codin	0	0	0	0
ENSG000001	PPARG	protein_codin	0	0	0	0
ENSG000001	NRG1	protein_codin	0	0	0	0
ENSG000000	ABCF2	protein_codin	67.196535	93.1057719	37.5426338	43.4658825
ENSG000001	ODR4	protein_codin	146.0141	66.1961336	133.735271	97.1637352
ENSG000001	PRKAG2	protein_codin	0	0	0	0
ENSG000002	CYB5RL	protein_codin	0	0	0	0
ENSG000001	HTR5A	protein_codin	0	0	1.05686457	0
ENSG000001	STEAP2	protein_codin	0	0	0	0
ENSG000001	RBM33	protein_codin	263.945073	431.976674	49.1175698	171.918432
ENSG000001	TSSK4	protein_codin	0	0	0	0
ENSG000001	CLDN12	protein_codin	0	0	0	0
ENSG000001	FZD1	protein_codin	86.0632979	153.016772	34.1932814	95.8689928
ENSG000001	HMGCR	protein_codin	0	0	31.0932721	76.5363439
ENSG000001	GATAD1	protein_codin	139.020456	133.529467	48.6805739	89.0579453
ENSG000001	IPPK	protein_codin	23.7173707	15.3145821	20.9791575	10.4205663
ENSG000002	THTPA	protein_codin	51.9044632	60.8321463	18.0573203	27.9561805
ENSG000002	PDF	protein_codin	9.03733893	12.7055054	6.38774116	4.2013031
ENSG000001	TMED6	protein_codin	0	0	0	0
ENSG000001	CLEC18A	protein_codin	0	0	0	3.16398076
ENSG000000	PDPR	protein_codin	151.22945	324.214838	88.1223059	145.057654
ENSG000001	ARMC12	protein_codin	0	0	0	0
ENSG000001	DDX19B	protein_codin	119.577791	87.3975292	59.3619849	39.5560679
ENSG000001	FCSK	protein_codin	16.7188423	31.0820558	13.5266927	21.7717863
ENSG000002	MDP1	protein_codin	10.1471536	16.0945395	0	0
ENSG000001	IL34	protein_codin	0	0	0	0
ENSG000001	DHRS1	protein_codin	20.5422267	46.8335654	3.80848698	13.0394144
ENSG000001	KIT	protein_codin	0	0	0	0
ENSG000001	CACNA1D	protein_codin	0	0	0	0
ENSG000000	IL17RB	protein_codin	0	0	0	0
ENSG000001	HYDIN	protein_codin	0	0	0	0
ENSG000001	ZNF19	protein_codin	3.55087542	6.08784155	0	3.10214872
ENSG000001	CCNB2	protein_codin	2.01197483	0	9.2279441	1.84428978
ENSG000001	ERC2	protein_codin	0	0	0	0
ENSG000001	FAM81A	protein_codin	1.06196098	5.37419901	7.21099138	0
ENSG000001	MYO1E	protein_codin	144.406298	161.917773	69.5997002	86.0849415
ENSG000001	APPL1	protein_codin	366.558987	136.250687	146.008131	180.11739
ENSG000001	ERG	protein_codin	0	11.8985467	0	0
ENSG000002	HMGN1	protein_codin	0	0	0	0
ENSG000001	LCA5L	protein_codin	0	0	0	0
ENSG000000	PREX2	protein_codin	1.87652846	0	3.50520872	0
ENSG000001	TMEM164	protein_codin	0	0	35.0247242	0
ENSG000001	MX1	protein_codin	38.9569506	23.583813	0	0

ENSG000002	SPATS1	protein_codin	0	0	0	0
ENSG000001	TPM1	protein_codin	0	0	0	0
ENSG000001	TAB3	protein_codin	186.259578	0	0	93.1566101
ENSG000001	SLC38A10	protein_codin	62.1404496	47.2058253	0	47.1707595
ENSG000001	DMD	protein_codin	0	0	0	0
ENSG000001	C9orf43	protein_codin	0	2.86599326	0	0
ENSG000001	ZNF618	protein_codin	30.952442	54.7807045	36.0713579	31.3830379
ENSG000001	DGKI	protein_codin	0	0	0	0
ENSG000001	TMEM268	protein_codin	125.104247	227.02072	88.3542826	78.598132
ENSG000001	SVOPL	protein_codin	0	0	0	0
ENSG000001	ASTN2	protein_codin	0	0	0	0
ENSG000001	COQ5	protein_codin	17.6526965	55.5454124	18.5171319	29.9204855
ENSG000001	KLHL29	protein_codin	0	0	0	0
ENSG000000	NCOA1	protein_codin	324.133459	224.981109	0	110.793652
ENSG000001	BRAF	protein_codin	0	0	0	0
ENSG000001	PSMG3	protein_codin	11.000184	19.3169536	9.84921569	16.2043421
ENSG000001	CABP1	protein_codin	0	0	0	0
ENSG000001	DTNB	protein_codin	0	0	0	0
ENSG000001	FMNL2	protein_codin	30.7407171	23.8963789	19.7296729	5.09583869
ENSG000001	DPYSL5	protein_codin	0	0	0	0
ENSG000001	DRC1	protein_codin	0	0	0	0
ENSG000001	RAB28	protein_codin	0	2.26248746	0	0
ENSG000001	MEGF11	protein_codin	0	0	0	0
ENSG000001	C12orf43	protein_codin	3.52802818	20.3750415	2.85772692	1.8470485
ENSG000001	PEX10	protein_codin	0	45.8597313	15.4892741	0
ENSG000001	OLFML2A	protein_codin	0	0	0	0.97787511
ENSG000001	WIPI2	protein_codin	63.7382762	140.702836	19.8404709	61.7268231
ENSG000002	SSX2B	protein_codin	0	0	0	0
ENSG000001	SMAD6	protein_codin	2.34930849	4.56800718	0	1.92356753
ENSG000001	CIB4	protein_codin	0	0	0	0
ENSG000001	KRTCAP3	protein_codin	0	0	0	0
ENSG000001	CFAP251	protein_codin	0	0	4.72478742	0
ENSG000001	MRPL17	protein_codin	17.0857475	40.5201868	4.11064524	22.9807873
ENSG000001	DUSP2	protein_codin	0	0	0	0
ENSG000001	PTPDC1	protein_codin	0	0	0	0
ENSG000001	ERCC6L2	protein_codin	22.9638811	10.2925637	2.73227433	15.546462
ENSG000001	NCK1	protein_codin	89.0292553	34.5745869	0	46.9531283
ENSG000001	HPD	protein_codin	0	1.51506963	0	0.78173419
ENSG000001	RHPN1	protein_codin	0	0	0	0
ENSG000001	ZNF782	protein_codin	0	10.0861057	18.4207358	0
ENSG000001	FANCC	protein_codin	7.16258933	0	0	0
ENSG000001	MRAS	protein_codin	22.1503421	49.0975155	18.756048	24.0985719
ENSG000001	ABHD3	protein_codin	0	21.3441502	25.0074615	34.4615963
ENSG000001	ESYT3	protein_codin	0	0	0	0

ENSG000000	PIK3CB	protein_codin	134.574304	0	0	0
ENSG000001	TENT5B	protein_codin	0	0	0.64357069	0
ENSG000001	LRRFIP1	protein_codin	3732.81486	1534.52877	1198.88056	1847.15496
ENSG000001	ACTR1B	protein_codin	82.5433201	116.68217	34.263381	40.236214
ENSG000001	RHBDL2	protein_codin	0	0	0	0
ENSG000002	PCDHAC2	protein_codin	0	0	0	0
ENSG000002	PCDHA13	protein_codin	0	0	0	0
ENSG000001	PLS3	protein_codin	0	0	0	0
ENSG000001	SHROOM4	protein_codin	32.9290764	36.02961	34.2989435	11.6351365
ENSG000001	H2BC5	protein_codin	72.6825963	190.014081	53.7539516	32.7786714
ENSG000001	MITD1	protein_codin	95.0988511	157.360397	90.5876165	85.7808312
ENSG000000	BTN3A1	protein_codin	247.310689	381.327548	111.334972	164.016546
ENSG000001	EIF5B	protein_codin	545.263266	424.808894	656.393591	477.218642
ENSG000001	TMSB15A	protein_codin	0	9.76827205	0.99495199	0
ENSG000001	CNOT11	protein_codin	82.8299737	128.310031	41.9210811	78.6054101
ENSG000001	CATIP	protein_codin	1.48637348	0	0	0
ENSG000001	NRG2	protein_codin	0	0	0	0
ENSG000000	ACSM3	protein_codin	0	0	6.62237161	0
ENSG000001	NRG2	protein_codin	0	1.59371793	0	0
ENSG000001	CD1A	protein_codin	0	6.20105101	1.557607	5.27106355
ENSG000001	SPATA2	protein_codin	19.1930385	24.2477425	23.0411322	36.3920095
ENSG000001	HMHB1	protein_codin	0	0	0	0
ENSG000001	NCF1	protein_codin	944.969569	1153.35365	95.4987969	421.821077
ENSG000001	LRRC51	protein_codin	0	6.47908194	4.05552797	0
ENSG000001	PPP1R9A	protein_codin	4.46792577	4.51486854	0	0
ENSG000001	ZFAND2B	protein_codin	0	7.00540352	0	0
ENSG000000	NPC1L1	protein_codin	0	0	0	0
ENSG000001	SLCO2B1	protein_codin	0	0	0	0
ENSG000001	TMED4	protein_codin	16.2043692	22.3996012	11.7734391	7.01779239
ENSG000001	PAGE5	protein_codin	0	0	0	0
ENSG000001	OBSL1	protein_codin	2.69374911	0	4.15220691	0
ENSG000001	PKD1L1	protein_codin	0	0	0	0
ENSG000001	ELK4	protein_codin	28.8869469	53.0039791	37.0613261	19.1428761
ENSG000001	SLAMF8	protein_codin	18.2380271	27.5577347	3.23485392	6.93724913
ENSG000001	OR10J6P	unprocessed_	0	0	0	0
ENSG000000	ANK1	protein_codin	27.0224804	0	0	0
ENSG000001	CDH15	protein_codin	0	0	0	0
ENSG000001	NBL1	protein_codin	0	0	0	0
ENSG000001	HTR6	protein_codin	0.99200924	0	0	0.94479027
ENSG000002	ENSG000002	protein_codin	0	0	0	0
ENSG000001	ZSCAN23	protein_codin	0	0	0	0
ENSG000001	SPATA2L	protein_codin	28.8228852	20.8650542	22.2532716	23.007131
ENSG000001	VWA5B1	protein_codin	0	0	0	0
ENSG000001	ZNF276	protein_codin	105.704296	0	73.7252503	60.3976161

ENSG000001	NPM2	protein_codin	0	0	0	0
ENSG000001	USP21	protein_codin	0	0	0	20.234515
ENSG000001	SLC45A1	protein_codin	0	0	0	1.59994988
ENSG000002	ENSG000002	lncRNA	0	0	0	0
ENSG000001	MACF1	protein_codin	1109.09398	587.721363	602.77492	678.36793
ENSG000001	FCER1G	protein_codin	959.226743	966.503111	329.303669	536.757837
ENSG000001	FHIP2B	protein_codin	64.1278958	44.9474531	42.7885125	21.9532779
ENSG000001	SLC39A14	protein_codin	0	0	5.41007589	0
ENSG000001	WFDC8	protein_codin	0	0	0	0
ENSG000001	CHRNB3	protein_codin	0	0	0	0
ENSG000001	PPP3CC	protein_codin	54.6397788	28.5155492	6.94081726	7.83113915
ENSG000001	ARHGAP17	protein_codin	99.9371324	116.232501	57.3299978	66.4631913
ENSG000002	C8orf58	protein_codin	15.0619049	21.6559793	0	3.24655848
ENSG000001	WNT9B	protein_codin	0	1.89317097	0	0
ENSG000001	SEC16A	protein_codin	20.4911179	101.513185	47.3273313	23.4332363
ENSG000001	CACHD1	protein_codin	0	0	0	0
ENSG000001	SLC25A37	protein_codin	51.069379	28.779333	20.8966498	46.6330664
ENSG000002	TMEM141	protein_codin	16.6598211	28.4811417	6.98020404	16.6789272
ENSG000001	CELA3A	protein_codin	0	0	0	0
ENSG000001	MIS18A	protein_codin	10.1818872	6.36834483	6.10657725	8.1439714
ENSG000001	CFAP298	protein_codin	6.48046481	1.80663016	2.60572113	3.02568986
ENSG000001	KPNB1	protein_codin	148.088776	303.495588	90.7730074	141.993084
ENSG000001	WNT4	protein_codin	0	0	0	0
ENSG000001	PAXBP1	protein_codin	36.6293677	14.3964596	8.11627036	24.5834269
ENSG000002	IL10RB	protein_codin	124.759687	244.122565	31.416929	100.956323
ENSG000001	MRPL10	protein_codin	0	3.80102088	2.11757728	4.12074614
ENSG000001	SLC12A6	protein_codin	273.715545	288.443127	160.975846	189.668666
ENSG000001	SCRN2	protein_codin	18.2563681	22.9050856	0	0
ENSG000001	IFNGR2	protein_codin	931.191856	968.514252	162.775658	764.343735
ENSG000001	NCOA5	protein_codin	72.6233502	117.58209	48.8044683	61.416215
ENSG000002	MIR6501	miRNA	0	0	0	0
ENSG000002	CRYZL1	protein_codin	3.38849381	25.8979779	21.9549416	8.73578566
ENSG000000	ELMO2	protein_codin	0	0	51.8874225	0
ENSG000001	STC1	protein_codin	0	0	0	0
ENSG000000	OGFR	protein_codin	537.784447	759.593549	255.008547	399.840942
ENSG000001	PRAC1	protein_codin	0	0	0	0
ENSG000001	HOXB13	protein_codin	0	0	0	0
ENSG000002	ATP5PO	protein_codin	131.184483	216.283328	77.1650386	108.528396
ENSG000001	KCNE2	protein_codin	0	0	0	0
ENSG000001	SLC13A3	protein_codin	0	0	0	0
ENSG000001	SNF8	protein_codin	60.5959766	67.6963561	0	26.73978
ENSG000001	IGF2BP1	protein_codin	0	0	0	0
ENSG000001	CBR1	protein_codin	75.8082682	133.256507	43.4397136	60.2757964
ENSG000001	CBR3	protein_codin	0.95588634	0.9789109	0	3.91149854

ENSG000001	CIART	protein_codin	0	0	0	0
ENSG000001	GJD2	protein_codin	0	0	0	0
ENSG000002	ENSG000002	lncRNA	0	0	0	0
ENSG000001	ACTC1	protein_codin	0	0	0	0
ENSG000002	C2orf81	protein_codin	0	0	0	1.10410292
ENSG000001	SIM2	protein_codin	0	0	0	0
ENSG000001	GFRA4	protein_codin	0	0	0	0
ENSG000001	CCDC142	protein_codin	4.81122286	0	0	0
ENSG000000	PPP1R12B	protein_codin	0	0	0	0
ENSG000001	MCAT	protein_codin	7.66595907	12.3139901	0	1.16225509
ENSG000000	PKD2L2	protein_codin	1.35825446	0	0	0
ENSG000001	GOLGA6A	protein_codin	3.62879005	0.89240632	1.33007889	0
ENSG000001	SCUBE1	protein_codin	0	0	0	0
ENSG000001	ARHGAP27	protein_codin	8.21416833	0	0	0
ENSG000001	PLA2G4D	protein_codin	0	0	0	0
ENSG000001	PLA2G4F	protein_codin	0	0	0	0
ENSG000001	P3H3	protein_codin	0	0	0	3.24548968
ENSG000001	RFX5	protein_codin	32.7159422	62.1790484	0	70.9366415
ENSG000001	M1AP	protein_codin	0	0	2.29711744	2.67230331
ENSG000001	PSMB4	protein_codin	127.823584	212.026579	17.7768883	70.2904904
ENSG000001	BTG2	protein_codin	1393.44852	1888.05763	1056.47586	849.179527
ENSG000001	IRX6	protein_codin	0	0	0	0
ENSG000001	CES5A	protein_codin	0	0	0	0
ENSG000001	HK2	protein_codin	594.769632	683.578232	154.574222	337.534783
ENSG000001	CELF3	protein_codin	0	2.01215516	0	0
ENSG000001	CELF3	protein_codin	0	0	0	0
ENSG000001	ALDH4A1	protein_codin	0	0	0	0
ENSG000001	STARD9	protein_codin	19.4364543	20.0417862	27.2197936	5.82319208
ENSG000001	AMFR	protein_codin	299.059222	469.939506	132.223885	209.878678
ENSG000001	UBR1	protein_codin	95.5198124	82.9487421	76.9435962	62.7688436
ENSG000001	MED8	protein_codin	9.00568861	0	16.0400876	6.88668637
ENSG000001	RGL4	protein_codin	0	0	0	0
ENSG000002	SPRR2C	transcribed_u	0	0	0	0
ENSG000002	MT1A	protein_codin	0	0	0	0
ENSG000001	PGLYRP3	protein_codin	0	0	0	0
ENSG000000	DERL3	protein_codin	3.30941919	14.3046151	29.7204504	8.1517382
ENSG000001	ISL2	protein_codin	0	1.80636207	5.34918953	3.59097221
ENSG000001	GSTT2B	protein_codin	0	0	0	0.94080134
ENSG000001	CPNE2	protein_codin	132.979235	148.619936	18.0595215	74.9252524
ENSG000001	GPBP1L1	protein_codin	149.36556	92.3049185	174.300068	94.442992
ENSG000001	NAE1	protein_codin	34.084482	33.3204388	16.8468546	42.8087557
ENSG000000	CLEC2D	protein_codin	85.4784643	43.2861897	48.0808018	37.8193637
ENSG000000	CBFB	protein_codin	0	0	0	29.788651
ENSG000001	ACE	protein_codin	0	0	0	0

ENSG000001	ACE	protein_codin	0	0	0	0
ENSG000001	UROC1	protein_codin	0	0	0	0
ENSG000001	SPMIP8	protein_codin	0	0	0	0
ENSG000001	MATCAP1	protein_codin	0	7.64742269	16.7937692	16.4523113
ENSG000001	SHF	protein_codin	2.36512655	3.12796522	0	0
ENSG000001	SPON2	protein_codin	0	4.63862323	0.73076711	0
ENSG000001	CHCHD6	protein_codin	3.9012928	0	2.67795946	0
ENSG000001	CTBP1	protein_codin	120.580179	62.8278124	23.6213487	79.4023182
ENSG000001	TPPP3	protein_codin	11.4661734	0	0	0
ENSG000002	ANKRD18B	protein_codin	0	0	0	0
ENSG000001	ATP6V0D1	protein_codin	282.149317	430.668004	87.6031617	192.566771
ENSG000001	AGRP	protein_codin	0	0	0	0
ENSG000001	ZFYVE28	protein_codin	30.0366371	28.342209	9.18618041	2.52175261
ENSG000001	PIP	protein_codin	0	0	0	0
ENSG000001	PSKH1	protein_codin	46.5548347	66.3529731	24.6930972	31.7488466
ENSG000001	VPS53	protein_codin	0	0	0	0
ENSG000001	ABR	protein_codin	233.674565	248.954469	70.4728075	128.404228
ENSG000001	TCAF2P1	transcribed_u	0	0	0	0
ENSG000001	ZNF235	protein_codin	17.855877	0	12.4141765	15.5888365
ENSG000001	TNFRSF13C	protein_codin	7.00596512	5.94264148	1.86537786	0
ENSG000001	DMPK	protein_codin	0	0	0	0
ENSG000001	PRKD2	protein_codin	63.9079306	102.999842	13.6874501	67.2921034
ENSG000001	PTGIR	protein_codin	111.929567	154.64332	34.2326088	47.9334896
ENSG000001	CALM3	protein_codin	453.343682	797.076457	356.2345	396.491875
ENSG000001	HIF3A	protein_codin	0	0	0	0
ENSG000001	IQCC	protein_codin	0	0	10.1307967	0
ENSG000002	LINC02418	lncRNA	0	0	0	0
ENSG000001	SSU72	protein_codin	316.046647	397.957694	81.0364694	220.114134
ENSG000001	TRIM62	protein_codin	60.3829211	85.6099448	34.8230768	60.9787837
ENSG000000	AP1M1	protein_codin	53.0967453	107.220391	18.0778672	16.888708
ENSG000001	CPAMD8	protein_codin	0	3.8850837	4.44092791	0
ENSG000001	NR2F6	protein_codin	17.6430999	18.9964266	6.97200461	4.63524289
ENSG000001	MIX23	protein_codin	0	10.7198294	0	5.22145616
ENSG000001	KALRN	protein_codin	6.12577793	0	4.19255253	0
ENSG000001	HAPLN4	protein_codin	0	0	0	0
ENSG000001	CILP2	protein_codin	0	1.61675064	0	1.54767441
ENSG000001	ATP13A1	protein_codin	0	0	0	0
ENSG000001	TFF2	protein_codin	0	0	0	0
ENSG000001	TFF1	protein_codin	0	0	0	0
ENSG000001	UBASH3A	protein_codin	0	0	0	0
ENSG000001	RSPH1	protein_codin	0	0	0	0
ENSG000001	PDE9A	protein_codin	0	0	0	0
ENSG000001	PKNOX1	protein_codin	25.8908152	32.5707024	0	18.1239778
ENSG000001	U2AF1	protein_codin	0	92.8182634	0	70.1948331

ENSG000001(	CRYAA	protein_codin	0	0	0	0
ENSG000001(	HSF2BP	protein_codin	0	0	0	0.98323623
ENSG000001(	PDXK	protein_codin	221.478526	143.749265	83.5717382	130.985501
ENSG000001(	CSTB	protein_codin	152.329012	239.060893	44.4694423	124.812311
ENSG000001(	AGPAT3	protein_codin	166.146021	250.059223	41.9011387	110.247226
ENSG000001(	TRAPPC10	protein_codin	209.382288	267.336016	96.0841765	125.069286
ENSG000002	PWP2	protein_codin	0	2.91021374	0	16.053653
ENSG000001(	GATD3	protein_codin	7.80725518	0	3.22262795	0
ENSG000001(	AIRE	protein_codin	0.78072568	0	0	0
ENSG000001(	LRRC3	protein_codin	6.98039294	6.82762653	4.20515033	6.63897499
ENSG000001!	ZNF583	protein_codin	10.2790915	6.85703644	0	8.06134957
ENSG000001:	KIR2DL1	protein_codin	0	0	0	0
ENSG000001(	SLX9	protein_codin	7.40406794	22.0820032	13.6686424	8.48742084
ENSG000001(	FTCD	protein_codin	0	0	0	0
ENSG000001(	SPATC1L	protein_codin	0	0	0	0
ENSG000001(	MCM3AP	protein_codin	572.131656	602.647196	359.446868	336.713968
ENSG000001(	C21orf58	protein_codin	2.72910462	21.8383945	5.62093146	0
ENSG000001(	S100B	protein_codin	0	5.1192049	0	2.05461108
ENSG000001(	PRMT2	protein_codin	14.3045424	59.5002321	41.8596801	24.1113293
ENSG000002:	SIGLEC12	protein_codin	0	0	0	5.13265604
ENSG000001(	CLDND2	protein_codin	0	0	0	0
ENSG000001(	CACFD1	protein_codin	4.45969196	18.3290259	1.09616791	5.51928526
ENSG000001:	KLK8	protein_codin	0	0	0	0
ENSG000001(	FCN2	protein_codin	0	0	0	0
ENSG000001	ZNF626	protein_codin	0	6.5506492	0	0
ENSG000001	PSG9	protein_codin	0	0	0	0
ENSG000002:	LILRA4	protein_codin	0	25.4354066	27.0725682	1.90962843
ENSG000001!	CYP2A7	protein_codin	0	0	0	0
ENSG000001(	GPSM1	protein_codin	0	0	0	0
ENSG000001(	HIPK4	protein_codin	0.8517297	0	0	0
ENSG000001(	PRX	protein_codin	0	0	0	0
ENSG000001(	ST6GALNAC6	protein_codin	0	7.9456928	5.76243594	0
ENSG000001(	SHKBP1	protein_codin	387.254583	529.337372	97.9273206	243.11648
ENSG000002	KIR3DL3	protein_codin	0	0	0	0
ENSG000001	NCR1	protein_codin	0	0	3.97296785	0
ENSG000001(	RDH13	protein_codin	3.35885388	2.19258405	0	0
ENSG000001(	ZER1	protein_codin	137.393478	209.689807	64.720184	96.5094368
ENSG000001(	TNNT1	protein_codin	10.9703853	0	0	0
ENSG000001(	PKN3	protein_codin	1.1185415	7.61135352	4.22340559	3.69681152
ENSG000001(	TMEM190	protein_codin	0	0	0	0
ENSG000001	NLRP8	protein_codin	0	0	0	0
ENSG000001(	MED27	protein_codin	22.4362887	31.645583	14.6501313	18.6946954
ENSG000001(	JAML	protein_codin	0	0	4.22599781	0
ENSG000001!	ZNF667	protein_codin	0	3.22117393	0	0

ENSG000001:	FXYD2	protein_codin	0	0	0	0
ENSG000001	TLCD1	protein_codin	0	0	0	0
ENSG000002	TMEM199	protein_codin	54.70249	85.4330046	24.8544294	44.5837374
ENSG000001	SAFB	protein_codin	83.9980029	94.103385	36.6687641	21.9006631
ENSG000001	PSG6	protein_codin	0	0	0	0
ENSG000001	PHLDB3	protein_codin	0	8.48134544	0	0
ENSG000001	CD3G	protein_codin	0	0	0	0
ENSG000001	ETHE1	protein_codin	54.1098454	57.8900255	11.1443454	38.8186759
ENSG000001	S100A1	protein_codin	0	0	0	0
ENSG000001	CXCR5	protein_codin	1.91556622	2.82909351	1.93169261	0
ENSG000001	ZBTB7B	protein_codin	309.465508	457.92119	243.609121	233.130008
ENSG000001	FLAD1	protein_codin	23.4686389	59.16082	17.4442607	25.0139667
ENSG000001	NLRX1	protein_codin	0	8.53457194	22.8141374	0
ENSG000001	UBE2Q1	protein_codin	0	45.5072924	0	36.9361812
ENSG000001	ANO10	protein_codin	20.2180735	0	0	0
ENSG000001	RUSC1	protein_codin	14.2364655	0	4.74282506	0
ENSG000001	PAQR6	protein_codin	0	0	3.44270195	0
ENSG000001	CCR5	protein_codin	21.0714898	45.688779	7.87457762	12.5382331
ENSG000001	CCDC12	protein_codin	0	0	0	0
ENSG000001	MYL3	protein_codin	0	0	0	0
ENSG000001	PPP1R35	protein_codin	14.15453	25.7323177	3.99760998	0
ENSG000001	PEAR1	protein_codin	0	124.080032	0	0
ENSG000000	RNF126	protein_codin	93.6786475	115.05822	21.1268709	56.9597072
ENSG000002	GPC2	protein_codin	0	0	0.8571943	0
ENSG000001	DBN1	protein_codin	0	0	0	0
ENSG000001	ZNF3	protein_codin	0	0	0	0
ENSG000001	AZGP1	protein_codin	0	0	0	0
ENSG000001	FGFR4	protein_codin	0.82823069	0	0	0
ENSG000001	CYP11B1	protein_codin	0	0	0	0
ENSG000001	LY6K	protein_codin	0	2.04805822	1.95408369	0
ENSG000001	NACC1	protein_codin	125.531983	199.595586	22.1557844	130.744605
ENSG000001	HK3	protein_codin	937.309816	1177.57263	292.732822	605.015747
ENSG000001	IER2	protein_codin	1940.17491	4284.86586	596.131594	1571.85148
ENSG000001	ZSCAN21	protein_codin	19.085605	31.4497354	12.7135945	13.1169264
ENSG000002	ATP5MF	protein_codin	81.1972873	105.293186	56.1651988	70.8518409
ENSG000001	CPSF4	protein_codin	39.197278	19.2296176	0	0
ENSG000001	PTCD1	protein_codin	35.8491725	61.464668	19.3661975	22.425006
ENSG000001	LY6E	protein_codin	73.8559351	104.927945	88.93232	94.400694
ENSG000001	VPS28	protein_codin	45.3123918	122.407255	0	83.8749173
ENSG000001	PTGER1	protein_codin	0	0	0.52504624	0
ENSG000001	LRRC14	protein_codin	40.8928039	57.4426723	17.6466697	22.8629986
ENSG000001	ZNF333	protein_codin	52.2796731	94.7534396	4.81817874	39.9996332
ENSG000002	CUX1	protein_codin	391.376931	0	0	0
ENSG000002	CUX1	protein_codin	185.253183	0	113.412581	133.69039

ENSG0000010	PPP1R16A	protein_codin	0	0	0	0
ENSG0000015	ZNF251	protein_codin	21.8407742	17.77098	6.92729113	5.672562
ENSG0000010	ALKBH4	protein_codin	12.8209192	25.1407391	5.27085702	2.88339089
ENSG0000010	TEKTL1	protein_codin	0	0	0	0
ENSG0000011	ABHD17A	protein_codin	179.007966	254.924639	26.1447399	110.30407
ENSG0000015	ZNF250	protein_codin	21.0570832	0	0	4.04289672
ENSG0000010	MRNIP	protein_codin	0	0	0	0
ENSG0000010	MGAT4B	protein_codin	246.423146	504.651436	128.849415	214.131949
ENSG000002	LTC4S	protein_codin	2.52769285	1.61200487	0	0
ENSG0000010	MAML1	protein_codin	432.822363	503.438459	170.560966	252.299827
ENSG0000010	PGLYRP2	protein_codin	0	0	0	0
ENSG0000000	POLR2J	protein_codin	221.521254	364.97339	122.907354	162.914077
ENSG0000010	LRWD1	protein_codin	10.8004276	18.1004954	11.7345136	6.43831568
ENSG0000010	SCGB3A1	protein_codin	0	0	0	0.69510207
ENSG0000010	PSMC2	protein_codin	93.5693342	116.442611	77.295441	127.064407
ENSG0000010	CELF5	protein_codin	0	0	0	0
ENSG0000010	USP41P	transcribed_u	0	0	0	0
ENSG0000009	MED15	protein_codin	123.167733	332.612726	46.2712506	119.012168
ENSG0000002	TUBA3FP	protein_codin	0	0	0	0
ENSG0000010	YDJC	protein_codin	0	13.4278055	0	12.055089
ENSG0000010	CCDC116	protein_codin	0	0.76612614	0	0
ENSG0000004	DCUN1D1	protein_codin	133.598259	94.2466033	59.9159483	101.781818
ENSG0000010	AP2M1	protein_codin	660.123102	761.168037	240.870491	430.807295
ENSG0000010	ABCF3	protein_codin	0	0	0	0
ENSG0000010	PCYT1A	protein_codin	71.4496759	49.1675791	6.62753129	34.2609654
ENSG0000015	ZNF585A	protein_codin	0	0	0	0
ENSG0000020	FBXO17	protein_codin	0	0	0	0
ENSG0000010	FBXO27	protein_codin	0	0	0	0
ENSG0000010	U2AF1L4	protein_codin	3.56759748	0	0	0
ENSG0000010	THAP8	protein_codin	6.80492935	19.1133299	8.24397877	0
ENSG000002	HBE1	protein_codin	0	0	0	0
ENSG0000002	HBD	protein_codin	0	0	0	0
ENSG0000010	COX7A1	protein_codin	0	0	0	0
ENSG0000010	ZNF382	protein_codin	0	2.15919525	1.46868281	0
ENSG0000010	IKZF3	protein_codin	0	0	0	0
ENSG0000010	GRIN2C	protein_codin	0	0	0	0
ENSG0000010	FDXR	protein_codin	0	0	0	0
ENSG0000010	SAP30BP	protein_codin	11.3522316	6.19302932	9.51187544	3.26827541
ENSG0000010	ACOX1	protein_codin	253.313464	293.284961	95.8941606	159.482933
ENSG0000010	CYGB	protein_codin	0	0	0	0
ENSG0000010	CABP5	protein_codin	59.7158466	163.808847	47.6749713	17.0409115
ENSG0000010	TMEM143	protein_codin	9.59590987	9.14350408	5.52188138	0
ENSG0000008	BAX	protein_codin	3.56789045	15.3681345	2.89427148	10.7746245
ENSG0000010	KLHL10	protein_codin	0	0	0	0

ENSG000001	KRT8	protein_codin	0	0	0	0
ENSG000001	STAT5B	protein_codin	269.429453	530.819082	211.012826	293.778539
ENSG000001	HCRT	protein_codin	0.73331843	0.74780716	0	0
ENSG000000	PLEKHH3	protein_codin	0	0	0	0
ENSG000001	ALDH16A1	protein_codin	59.7012069	103.217206	37.5196568	60.2811021
ENSG000001	PSME3	protein_codin	0	0	0	0
ENSG000001	DCD	protein_codin	0	0	0	0
ENSG000001	NCKAP1L	protein_codin	934.419352	978.205736	451.069017	508.036166
ENSG000001	ITGA5	protein_codin	770.766913	1005.41366	287.429358	424.292495
ENSG000001	CD300LG	protein_codin	0	0	0	0
ENSG000001	NAGS	protein_codin	0	0	0	0
ENSG000001	IZUMO2	protein_codin	0	0	0	0
ENSG000001	LSM12	protein_codin	140.436731	246.081425	60.841271	91.2645776
ENSG000001	ASB16	protein_codin	0	0	0	0
ENSG000000	MYL6	protein_codin	0	36.6938694	35.4613063	0
ENSG000001	SHANK1	protein_codin	0	1.15488609	0	0
ENSG000001	FAM171A2	protein_codin	0	0	0	0
ENSG000001	ZNF613	protein_codin	7.57549842	2.69551152	8.57930043	0
ENSG000001	SDR9C7	protein_codin	0	0	0	0
ENSG000001	KRT86	protein_codin	0	0	0	0
ENSG000001	WNT1	protein_codin	0.94667245	0	0.70459039	0
ENSG000001	AQP5	protein_codin	0	0	0	0
ENSG000002	C6orf136	protein_codin	0	9.17963644	0	0
ENSG000001	LARP4	protein_codin	0	41.2373122	12.2211271	15.9433556
ENSG000001	CELA1	protein_codin	0.97387227	1.85596195	1.84217102	0
ENSG000001	ZNF562	protein_codin	0	0	0	0
ENSG000001	TAMALIN	protein_codin	398.21179	118.222553	82.6306193	102.879535
ENSG000001	KRT83	protein_codin	0	0	0	0
ENSG000000	PDE4A	protein_codin	50.60858	64.0222225	18.1922497	22.7156548
ENSG000001	SYCE2	protein_codin	1.64378409	0.8282861	0	0
ENSG000001	ZNF563	protein_codin	1.02098322	3.10125618	1.20070749	1.02176208
ENSG000001	KRT72	protein_codin	0	0	0	0
ENSG000001	SYNGAP1	protein_codin	0	0	0	3.32810699
ENSG000001	IP6K3	protein_codin	0	0	0	0
ENSG000001	LEMD2	protein_codin	84.3135828	90.5560126	17.5455976	32.4998036
ENSG000001	ALOX15	protein_codin	0	0.95726223	0	0
ENSG000001	ZNF653	protein_codin	0	19.1743637	0	6.82239186
ENSG000001	MED11	protein_codin	0	18.5053017	0	10.0252787
ENSG000001	CXCL16	protein_codin	136.127298	211.972364	0	68.9581644
ENSG000001	SLC13A5	protein_codin	0	0	0	0
ENSG000001	BCL6B	protein_codin	0	0	0	0
ENSG000002	TNFSF12	protein_codin	28.5036475	0	15.3280292	53.7932767
ENSG000002	TNFSF12-TNF	protein_codin	72.1579906	113.568517	64.5736259	0
ENSG000001	FGF11	protein_codin	0	0	0	0

ENSG000001	EIF4A1	protein_codin	779.799427	1081.16839	398.989497	498.992931
ENSG000001	CCDC42	protein_codin	0	0	0	0
ENSG000001	PRSS33	protein_codin	0	0	0	0
ENSG000001	POLR3K	protein_codin	13.9753194	43.0859123	11.6623818	11.1125663
ENSG000001	SNRNP25	protein_codin	13.7558615	31.7604538	29.4459347	16.4912078
ENSG000000	LUC7L	protein_codin	0	0	8.54375514	0
ENSG000000	PIGQ	protein_codin	0	0	0	0
ENSG000001	WDR90	protein_codin	6.00709167	0	0	0
ENSG000001	SSRP1	protein_codin	4.32384609	18.7471121	3.81904339	0
ENSG000001	WDR24	protein_codin	32.7132822	59.4711756	15.2145738	25.6648575
ENSG000001	CCDC78	protein_codin	0	0	0	0
ENSG000000	SOX8	protein_codin	0	0	0	0
ENSG000001	SSTR5	protein_codin	0	0	0	0
ENSG000002	PTX4	protein_codin	0	0	0	0
ENSG000000	CRAMP1	protein_codin	0	0	0	0
ENSG000001	CCNF	protein_codin	2.96373628	9.78323687	9.17746881	6.78843799
ENSG000001	AMDHD2	protein_codin	0	28.2784017	0	0
ENSG000001	NTN3	protein_codin	0	0	0	0
ENSG000001	PAQR4	protein_codin	11.5635963	35.3714615	0	0
ENSG000001	FLYWCH2	protein_codin	0	0	0	0
ENSG000001	SLX4	protein_codin	95.6834821	196.371269	62.4109384	87.1680644
ENSG000001	ADCY9	protein_codin	216.674092	367.790263	86.6555121	126.935657
ENSG000001	CLPB	protein_codin	0	0	0	119.625316
ENSG000001	NEU3	protein_codin	112.081806	133.588668	63.2221224	39.6825143
ENSG000001	MAP4K2	protein_codin	36.2277427	25.923507	28.8247474	14.4149782
ENSG000001	CYB561A3	protein_codin	50.6956156	86.2112804	0	0
ENSG000001	GNG3	protein_codin	0	0.86132673	0	0
ENSG000001	UBXN1	protein_codin	0	0	14.0841774	28.4197141
ENSG000002	NCKIPSD	protein_codin	0	0	0	0
ENSG000001	TTC9C	protein_codin	1.63000479	0	0	0
ENSG000001	TAF6L	protein_codin	17.1695862	28.9953778	6.3285508	15.5043204
ENSG000001	NXF1	protein_codin	221.598524	240.079841	55.1673732	155.397915
ENSG000001	STX5	protein_codin	63.7906857	101.439181	56.5096405	0
ENSG000001	SLC25A45	protein_codin	0	0	0	0
ENSG000001	RPL29	protein_codin	1198.12635	2129.62288	864.98414	979.2751
ENSG000002	DCP1A	protein_codin	26.7058065	50.199718	21.5939867	18.6216078
ENSG000001	SPINDOC	protein_codin	9.18847651	21.3706897	6.87430698	3.96789219
ENSG000001	SYVN1	protein_codin	75.4722426	73.4785618	46.3741813	71.1683902
ENSG000001	ZFPL1	protein_codin	31.8842735	40.0905872	14.3113237	15.4403587
ENSG000001	CABP2	protein_codin	0	0	0	0
ENSG000001	LRP5	protein_codin	0	15.1422666	0	0
ENSG000001	TPCN2	protein_codin	134.452184	131.651569	32.7725803	64.6429896
ENSG000001	FGF19	protein_codin	0	0	0	0
ENSG000001	CYP4A22	protein_codin	0	0	0	0

ENSG000001	PDZK1IP1	protein_codin	19.2910095	137.389313	28.7636983	92.2246577
ENSG000001	TAL1	protein_codin	18.107787	0	0	0
ENSG000001	ZYG11B	protein_codin	233.884596	143.749127	107.216157	124.014645
ENSG000001	CZIB	protein_codin	31.1545189	59.8293797	12.1282842	33.8406268
ENSG000001	USP24	protein_codin	613.324648	225.389742	323.240049	343.594395
ENSG000000	DNAJC11	protein_codin	29.1095012	61.0016287	29.808584	0
ENSG000001	GMEB1	protein_codin	48.9693816	72.5410757	0	29.4811458
ENSG000001	RHCE	protein_codin	1.13668442	0	5.57582847	0
ENSG000001	RAVER2	protein_codin	0	0	0	0
ENSG000001	RBP7	protein_codin	97.1326075	253.104306	72.9528057	94.6161982
ENSG000001	KNCN	protein_codin	0.80178137	0	0	0
ENSG000001	SLC25A34	protein_codin	10.6448194	9.84406505	2.97699471	1.90651305
ENSG000002	DISP3	protein_codin	0	0	0.74044468	0
ENSG000001	DRAXIN	protein_codin	4.96921944	1.98137148	1.07818922	0
ENSG000001	PDPN	protein_codin	0	0	0	0
ENSG000001	LAPTM5	protein_codin	3419.66201	4525.83616	1453.22025	2606.64162
ENSG000001	AZIN2	protein_codin	0	2.24674434	0	0
ENSG000001	NHSL3	protein_codin	0	0	0	0
ENSG000001	TMCO4	protein_codin	38.3564241	27.9777863	26.4778931	10.7094612
ENSG000001	MEGF6	protein_codin	0	0	0	0
ENSG000001	CCDC27	protein_codin	0	0	0	0
ENSG000001	DOCK6	protein_codin	8.0183838	0	0	0
ENSG000001	FUBP1	protein_codin	98.5893909	263.533751	179.514153	131.433439
ENSG000001	LRRC53	protein_codin	0	1.16413961	0	0
ENSG000001	LHX8	protein_codin	0	0	0	0
ENSG000001	NTNG1	protein_codin	0	1.23188637	0	0
ENSG000001	C1orf52	protein_codin	82.6362598	60.8595337	4.01873028	4.40258901
ENSG000001	DNAI3	protein_codin	0.96721999	0	0	0
ENSG000002	GBP7	protein_codin	0	1.92456264	0	0
ENSG000001	ODF2L	protein_codin	17.7512412	0	0	0
ENSG000001	GFI1	protein_codin	6.80416574	0	0	2.89230709
ENSG000002	LINC02802	lncRNA	0	0	0	0
ENSG000001	AGL	protein_codin	0	0	0	0
ENSG000001	KCNT2	protein_codin	0	0	0	0
ENSG000001	VCAM1	protein_codin	0	0	0	0
ENSG000000	ASPM	protein_codin	0	0	0	7.43494572
ENSG000002	DENND1B	protein_codin	29.9248221	10.1875456	41.7566854	24.1155316
ENSG000002	DENND1B	protein_codin	0	0	0	0
ENSG000001	ZNF281	protein_codin	297.805892	138.037901	107.659548	38.5083188
ENSG000001	ARPC5	protein_codin	239.525493	488.87813	219.873199	208.580314
ENSG000001	ZNF496	protein_codin	0	18.3324813	45.0550132	35.7610132
ENSG000001	NCSTN	protein_codin	156.467917	326.501274	64.3386568	171.914715
ENSG000001	OLFML2B	protein_codin	24.0485471	5.34488429	0	0
ENSG000001	FCRLA	protein_codin	0	0	0	0

ENSG000001	C1orf74	protein_codin	3.13344798	4.22759381	0	1.06016745
ENSG000001	LMX1A	protein_codin	0	0	0	0
ENSG000001	LRRC52	protein_codin	0	0	0	0
ENSG000001	GARIN4	protein_codin	7.96373246	1.00708297	1.40794982	3.01977633
ENSG000001	TDRD5	protein_codin	0	0	0	0
ENSG000001	RGSL1	protein_codin	0	0	0	0
ENSG000001	NMNAT2	protein_codin	0	0	0	0
ENSG000001	C1orf115	protein_codin	32.4778862	40.5027261	11.748601	10.938938
ENSG000001	BAZ2B	protein_codin	102.393068	64.1788318	83.7969123	61.6625248
ENSG000002	STPG4	protein_codin	3.62842653	0	5.33046185	0
ENSG000001	PPP1R21	protein_codin	106.038731	139.144913	111.599972	57.2476975
ENSG000001	LHCGR	protein_codin	0	0	0	0
ENSG000001	PKDCC	protein_codin	0	0	0	0
ENSG000001	HAAO	protein_codin	30.8764099	51.5875049	14.396492	28.5401248
ENSG000001	MAPKAPK2	protein_codin	86.0666606	148.493886	55.4412013	56.1131818
ENSG000001	IL24	protein_codin	0	0	0	0
ENSG000001	CR1L	protein_codin	0	4.20969615	2.42514235	1.07037412
ENSG000001	CAPN2	protein_codin	1610.90303	1780.918	399.79683	679.712584
ENSG000001	MRPL55	protein_codin	0	5.97514814	16.9329926	0
ENSG000001	OBSCN-AS1	lncRNA	0	0	0	0
ENSG000001	REL	protein_codin	0	0	36.5793085	0
ENSG000001	PEX13	protein_codin	36.9161367	49.9901245	17.0563246	34.3831796
ENSG000001	SANBR	protein_codin	12.308851	3.5408184	0	0
ENSG000001	TRIM17	protein_codin	0	0	0	0
ENSG000001	RFTN2	protein_codin	0	0	0	0
ENSG000000	SPRTN	protein_codin	19.8457558	15.5864764	10.149617	23.3635036
ENSG000001	DISC1	protein_codin	0	0	0	0
ENSG000001	LINC01931	lncRNA	0	0	0	0
ENSG000001	CAPN13	protein_codin	0	0	0	0
ENSG000001	LRRTM1	protein_codin	0	0	0	0
ENSG000001	MEMO1	protein_codin	64.0061558	27.409025	11.3555632	25.8782293
ENSG000001	DPY30	protein_codin	1.4248262	1.46331068	0	0
ENSG000001	MAIP1	protein_codin	0	0	0	0
ENSG000001	KCNF1	protein_codin	0	0	0	0
ENSG000001	SLC66A3	protein_codin	67.502863	61.967717	23.0257017	51.2847724
ENSG000001	ARL5A	protein_codin	251.80426	88.2543641	124.189445	168.571867
ENSG000001	LRATD1	protein_codin	0	0	0	0
ENSG000000	ARHGAP15	protein_codin	125.643632	84.2077012	100.986119	124.379468
ENSG000001	KCNJ3	protein_codin	0	0	0	0
ENSG000001	NEUROD1	protein_codin	0	0	0	0
ENSG000001	PRORSD1P	transcribed_u	0	0	0	0
ENSG000001	FRZB	protein_codin	0	0	0	0
ENSG000001	NUP35	protein_codin	0	7.11047022	0	0
ENSG000002	DNAAF10	protein_codin	9.60186314	8.28796122	3.59780968	4.81742406

ENSG000001	CCDC138	protein_codin	0	0	0	0
ENSG000001	ZSWIM2	protein_codin	0	0	1.46264443	0
ENSG000001	FBXO41	protein_codin	0	76.0804791	50.6473726	0
ENSG000001	WDCP	protein_codin	0	9.568239	0	0
ENSG000001	FAM228A	protein_codin	0	0	0	0.87974667
ENSG000001	VSNL1	protein_codin	0	0	0	0
ENSG000001	CCDC74A	protein_codin	0	0	0	0
ENSG000001	ANKRD30BL	protein_codin	0	0	0	0
ENSG000001	SLC16A14	protein_codin	0	4.97767287	1.99361631	0
ENSG000001	TEKT4	protein_codin	0	0	0	0
ENSG000001	EN1	protein_codin	0	0	0	0
ENSG000001	ZNF514	protein_codin	3.13728066	5.30220544	6.05881779	0
ENSG000001	SERPINB8	protein_codin	0	20.2822093	0	0
ENSG000001	SPATA18	protein_codin	0	0	0	0
ENSG000001	CFAP221	protein_codin	0	0	0	0
ENSG000001	KCNIP3	protein_codin	0	0	0	0
ENSG000001	INHBB	protein_codin	0	1.89939721	0	0.94546825
ENSG000000	CCNT2	protein_codin	0	0	32.0791955	0
ENSG000001	BBS5	protein_codin	0	0	0	0
ENSG000001	ANKRD36C	protein_codin	172.583075	93.4588014	194.313042	100.958926
ENSG000001	HPGDS	protein_codin	2.08726463	10.529273	2.91589186	4.17886797
ENSG000001	PDHA2	protein_codin	0	0	0	0
ENSG000001	STPG2	protein_codin	0	2.08973383	0.74791089	0
ENSG000001	SLC9A4	protein_codin	0	0	1.01256704	0
ENSG000001	PACRGL	protein_codin	0	0	0	0
ENSG000001	BNIPL	protein_codin	0	0	0	0
ENSG000001	C1QTNF7	protein_codin	0	0	0	0
ENSG000001	CHAC2	protein_codin	1.10199147	3.28656376	7.97755562	1.10715583
ENSG000001	TMOD4	protein_codin	2.65767117	0	0	0
ENSG000001	RNF149	protein_codin	657.122096	660.723482	197.71768	301.128149
ENSG000001	IWS1	protein_codin	189.029957	318.749358	202.325882	154.804711
ENSG000001	CDC42EP3	protein_codin	1077.29742	910.080263	310.438996	791.086319
ENSG000001	BOLA3	protein_codin	0	0	0	0
ENSG000001	SPRR3	protein_codin	0	0	0	0
ENSG000001	BMP10	protein_codin	2.2277131	1.13796751	0	4.56814766
ENSG000001	TGFA	protein_codin	1.97154826	22.7175461	13.3348727	7.95752956
ENSG000001	CPT1C	protein_codin	0	0	0	0
ENSG000001	MERTK	protein_codin	109.132811	22.0312782	19.1079489	32.9112
ENSG000001	CCNYL1	protein_codin	163.13747	113.296265	75.8649685	111.518691
ENSG000001	FZD5	protein_codin	18.8298142	17.0250466	11.5256554	17.6693807
ENSG000001	NPPC	protein_codin	0	0	0	0
ENSG000001	GNPDA2	protein_codin	0	11.4550995	4.17314311	13.177104
ENSG000001	GABRG1	protein_codin	0	0	0	0
ENSG000001	ALPG	protein_codin	0	0	0	0

ENSG000001	GABRB1	protein_codin	0	0	0	0
ENSG000001	NIPAL1	protein_codin	0	0	0	0
ENSG000001	ALPI	protein_codin	0	0	0	0
ENSG000001	HNRNPDL	protein_codin	28.2925907	36.6747613	0	26.224341
ENSG000001	HELQ	protein_codin	34.281579	0	24.3232561	22.6951667
ENSG000001	MRPS18C	protein_codin	40.1857118	77.6079292	12.8544013	16.9922955
ENSG000002	ENSG000002	processed_ps	0	0	0	0
ENSG000001	CHN1	protein_codin	0	0	0	0
ENSG000001	GPR155	protein_codin	5.46943651	0	2.5830132	0
ENSG000001	CLDN1	protein_codin	0	0	0	0
ENSG000001	FLAD1	protein_codin	0	0	0	0
ENSG000001	DCST1	protein_codin	0	0	0	0
ENSG000001	ATP13A4	protein_codin	1.72859008	0	0	0
ENSG000001	LINC01116	lncRNA	0	0	0	0
ENSG000001	COL6A3	protein_codin	0	0	2.86035233	0
ENSG000001	YY1AP1	protein_codin	0	0	0	0
ENSG000001	TAFA4	protein_codin	0	0	0	0
ENSG000001	EOGT	protein_codin	0	0	18.0831659	19.2335976
ENSG000001	POGLUT1	protein_codin	47.2354624	56.3779321	15.0343537	63.5242351
ENSG000001	CCKAR	protein_codin	0	0	0	0
ENSG000001	IGFN1	protein_codin	0	0	0	0
ENSG000001	ATP1A1	protein_codin	483.806291	946.892094	169.949734	381.435861
ENSG000001	SLC15A2	protein_codin	0	6.78377588	0	0
ENSG000001	EIF4E3	protein_codin	193.608186	327.317531	86.7990603	200.065133
ENSG000001	PROK2	protein_codin	55.9982146	48.3797997	6.48564669	35.0438025
ENSG000001	TEX55	protein_codin	0	0	0	0
ENSG000001	LRRC58	protein_codin	196.829696	101.981914	109.165074	122.526915
ENSG000001	FSTL1	protein_codin	92.2163485	105.461041	0	37.0594811
ENSG000001	RNPEP	protein_codin	466.614527	478.610845	109.785025	266.522872
ENSG000001	PDCL2	protein_codin	0	0	0	0
ENSG000001	TMEM169	protein_codin	0	0	0	3.37764316
ENSG000001	AMPD3	protein_codin	0	0	0	0
ENSG000001	IGFBP7	protein_codin	104.968888	116.640246	23.256266	84.2958303
ENSG000001	KRTCAP2	protein_codin	51.671549	54.8392857	28.8575413	30.796034
ENSG000001	CXCR1	protein_codin	1.02331932	5.12217192	1.65821269	3.16355389
ENSG000001	ARPC2	protein_codin	44.6247197	80.6407807	9.26188743	36.8053646
ENSG000001	CCT3	protein_codin	195.898389	333.387255	149.982608	156.565129
ENSG000001	TMEM79	protein_codin	0	0	2.60317644	0
ENSG000001	CNOT9	protein_codin	26.0690006	74.5305038	22.9588027	24.1743918
ENSG000001	SSR2	protein_codin	214.531752	372.458823	129.900496	127.919789
ENSG000001	RNF25	protein_codin	20.982494	32.3777751	14.3542481	18.7074923
ENSG000001	PPFIA4	protein_codin	0	0	0	0
ENSG000001	STK36	protein_codin	0	0	0	0
ENSG000000	PTPRN	protein_codin	0	0	0	0

ENSG000001	NEK10	protein_codin	0	0	0	0
ENSG000001	FEV	protein_codin	0	0	0	0
ENSG000001	CRYBA2	protein_codin	0	0	0	0
ENSG000001	CFAP65	protein_codin	0	0	0	0
ENSG000001	IHH	protein_codin	1.89308413	0.968445	0.87777657	0
ENSG00000	SLC4A7	protein_codin	0	0	0	0
ENSG000002	SLC23A3	protein_codin	0	0	0	0
ENSG000001	EOMES	protein_codin	0	2.97142655	0	0
ENSG000001	CIP2A	protein_codin	0	0	0	0
ENSG000001	AZI2	protein_codin	44.7005928	30.2199261	14.2982817	52.6958264
ENSG000001	ABCB6	protein_codin	0	16.5332908	0	0
ENSG000001	TGFBR2	protein_codin	106.545233	360.112834	69.5278517	74.0764992
ENSG000001	RETNLB	protein_codin	0	0	0	0
ENSG000001	TRAT1	protein_codin	0	0	0	0
ENSG000001	HDAC11	protein_codin	3.57587485	0	0	0
ENSG000001	GLB1L	protein_codin	4.75241866	23.8217074	0	0
ENSG000001	FBLN2	protein_codin	26.7499034	0	0	0
ENSG000001	FBLN2	protein_codin	0	0	1.03016187	0
ENSG000001	TUBA1A	protein_codin	2.36436329	15.7342994	0	11.2781409
ENSG000001	CHCHD4	protein_codin	0	2.46328094	2.22730372	0
ENSG000001	STT3B	protein_codin	326.531477	266.852414	124.853898	176.196858
ENSG000001	SERPINI1	protein_codin	2.38210777	4.77228233	0	0
ENSG000001	PRKCI	protein_codin	83.8887615	33.9409771	44.7659588	52.1495674
ENSG00000	RETSAT	protein_codin	40.6951688	54.1220804	28.9582458	20.0800238
ENSG000001	IFI16	protein_codin	0	157.064804	0	152.199419
ENSG000001	EIF5A2	protein_codin	5.91050043	4.73440733	2.00744131	1.2088767
ENSG000001	EFHB	protein_codin	0	0	0	0
ENSG000001	RPL22L1	protein_codin	3.42568634	3.26363016	3.85904412	1.62564386
ENSG000001	FABP1	protein_codin	0	0	0	0
ENSG000001	PPM1L	protein_codin	0	0	0	0
ENSG000001	ABI2	protein_codin	7.67606864	4.29569587	1.36703687	0
ENSG000001	CTLA4	protein_codin	0	0	0	0
ENSG000001	CD200R1	protein_codin	0	3.00583768	7.484222	0
ENSG000001	GTPBP8	protein_codin	0	0	0	0
ENSG000002	CFAP44	protein_codin	0	24.1784784	17.3821047	0
ENSG000001	SPICE1	protein_codin	23.5611925	31.9418338	12.7290477	32.5646962
ENSG000001	PTPRG	protein_codin	4.5794659	3.21145074	16.7334999	0
ENSG000001	CCDC191	protein_codin	20.5970416	16.6356822	26.5286081	23.8799276
ENSG000001	DRD3	protein_codin	0	0	0	0
ENSG000001	NKX6-1	protein_codin	0	0	0	1.54998879
ENSG000001	CDS1	protein_codin	1.1557249	2.34687362	0	0
ENSG000001	WDFY3	protein_codin	789.973212	556.181068	335.150142	584.080542
ENSG000001	COX18	protein_codin	21.3002574	15.0526093	12.2799217	0
ENSG000001	SYNPR	protein_codin	0	0	0	0

ENSG000001	C3orf49	protein_codin	0	1.0164278	0	0
ENSG000001	ALB	protein_codin	0	0	0	0
ENSG000001	C4orf36	protein_codin	0	0	0	0
ENSG000001	THOC7	protein_codin	113.182456	101.660952	94.0475711	79.064269
ENSG000001	ATXN7	protein_codin	0	0	0	0
ENSG000001	PSMD6	protein_codin	161.566512	188.406354	107.592718	154.048855
ENSG000001	PRICKLE2	protein_codin	0	0	0	0
ENSG000001	ADAMTS9	protein_codin	0	0	0	0
ENSG000001	PPM1K	protein_codin	0	0	12.7462256	10.1633607
ENSG000001	ERICH6	protein_codin	0	0	0	0
ENSG000001	CLRN1	protein_codin	0	0	0	0
ENSG000001	GMPS	protein_codin	95.8255048	122.657478	54.0533999	63.0804269
ENSG000001	TIPARP	protein_codin	91.0761168	49.748562	25.941504	28.2653176
ENSG000001	CCNL1	protein_codin	0	0	0	0
ENSG000001	CCNL1	protein_codin	189.394427	38.0853841	54.6488014	73.3390103
ENSG000001	PTX3	protein_codin	128.878274	125.326677	73.7928065	65.6144652
ENSG000001	RSRC1	protein_codin	60.7936965	68.1106801	0	90.5493885
ENSG000001	HESX1	protein_codin	5.52322868	3.37361604	0	0
ENSG000001	SLMAP	protein_codin	0	0	0	0
ENSG000001	SLMAP	protein_codin	78.9490169	20.996093	63.5002733	0
ENSG000001	RPL9	protein_codin	0	1116.25272	0	0
ENSG000001	FLNB	protein_codin	0	0	0	0
ENSG000001	SMIM14	protein_codin	119.766936	136.595414	78.0155206	114.721786
ENSG000001	RPP14	protein_codin	0	18.8691581	0	18.2754079
ENSG000001	ABHD6	protein_codin	3.0231452	13.1431493	2.02465058	7.67382785
ENSG000001	CFAP20DC	protein_codin	0	0	0	0
ENSG000001	RBM47	protein_codin	447.905409	442.437152	160.350079	301.609148
ENSG000001	APBB2	protein_codin	0	0	0	0
ENSG000001	IL17RC	protein_codin	0	0	0	0
ENSG000001	PRRT3	protein_codin	6.70377006	0	0	1.98335687
ENSG000000	SYN1	protein_codin	2.68686156	0	0	0
ENSG000001	CAND2	protein_codin	0	0	0.84256624	0
ENSG000001	PCOLCE2	protein_codin	0	0	0	0
ENSG000001	MTMR14	protein_codin	100.904413	167.578644	136.86203	89.6024698
ENSG000001	TTC14	protein_codin	234.1491	178.575697	98.3388906	98.8137018
ENSG000001	CXCL3	protein_codin	251.804809	26.899712	82.5832981	27.7619915
ENSG000001	CXCL5	protein_codin	189.69042	411.422588	196.222945	157.896799
ENSG000001	PPBP	protein_codin	1761.56342	3824.09631	1714.46431	1353.73374
ENSG000001	PF4	protein_codin	1211.61041	3137.62229	1433.27225	690.73694
ENSG000000	CXCL2	protein_codin	286.159603	127.391312	92.4074792	114.161591
ENSG000001	SHROOM3	protein_codin	0	0	0	0
ENSG000001	CPA3	protein_codin	6.02731464	1.50027986	7.18198908	2.11904745
ENSG000001	GYG1	protein_codin	146.587967	193.870563	64.0341931	108.403704
ENSG000001	HPS3	protein_codin	128.880324	86.1799077	28.0761813	122.951067

ENSG000001	TM4SF18	protein_codin	1.51683439	0	0	0
ENSG000001	SNRK	protein_codin	398.170321	350.544097	172.535539	273.43111
ENSG000001	ZNF502	protein_codin	7.35524367	0	0	0
ENSG000001	ZNF35	protein_codin	6.09137401	23.7390499	0	9.71996776
ENSG000001	TCF23	protein_codin	0	0.97219579	0	0
ENSG000001	DNAJC5G	protein_codin	0	0	0	0
ENSG000001	TRIM54	protein_codin	0	0	0	0
ENSG000001	UCN	protein_codin	1.90840398	1.87945826	0.9853372	0.87858094
ENSG000002	MRPL33	protein_codin	54.7051242	49.6168048	29.6427204	15.5593572
ENSG000001	KIAA1143	protein_codin	106.659237	109.209758	72.7199782	86.1747438
ENSG000002	PPP1CB	protein_codin	439.862037	345.76376	159.3009	239.116903
ENSG000001	TGM4	protein_codin	0	0	0	0
ENSG000001	WDR43	protein_codin	0	0	0	0
ENSG000001	ZDHHC3	protein_codin	91.5191922	141.677146	38.0714565	81.8326699
ENSG000001	CDCP1	protein_codin	6.93778928	16.4900341	6.10632514	2.14081553
ENSG000001	CLEC3B	protein_codin	0	0	0	0
ENSG000001	LZTFL1	protein_codin	0	0	29.1874301	0
ENSG000001	FYCO1	protein_codin	377.537894	511.396254	267.62618	249.478431
ENSG000001	CCR1	protein_codin	255.124597	474.790157	166.594391	213.269437
ENSG000001	RTP3	protein_codin	0	0	0	0
ENSG000001	LRRC2	protein_codin	0	0	0	0
ENSG000002	CRIPTO	protein_codin	0	0	0	0
ENSG000001	ELP6	protein_codin	8.70766716	30.6219181	20.8455754	14.6156798
ENSG000001	DTX3L	protein_codin	589.608871	703.200043	365.935679	490.61771
ENSG000000	ITGB5	protein_codin	122.258699	337.835563	166.466867	202.33075
ENSG000001	NMNAT3	protein_codin	0	7.13181961	0	0
ENSG000001	TPRA1	protein_codin	0	19.6322348	9.26658257	0
ENSG000001	MEAF6	protein_codin	75.5690403	94.1058393	22.2352344	27.6596567
ENSG000001	SNIP1	protein_codin	30.3354685	58.3227496	19.8120318	19.397358
ENSG000001	DNALI1	protein_codin	0	0	0	0
ENSG000001	OSBPL11	protein_codin	386.961331	358.173516	184.085091	274.713193
ENSG000001	KLF15	protein_codin	0	0	0	0
ENSG000001	CAMK2N2	protein_codin	0	0	0	0
ENSG000001	LIPH	protein_codin	22.865476	51.4280021	21.8844191	3.95564029
ENSG000001	TMEM41A	protein_codin	4.55954816	0	0	0
ENSG000001	RPN1	protein_codin	547.369527	934.492632	595.491764	489.757243
ENSG000001	SENP2	protein_codin	63.4122668	75.5504114	50.1226761	21.1857313
ENSG000001	IFT122	protein_codin	0	0	0	0
ENSG000001	IGF2BP2-AS1	lncRNA	0	1.826374	0	0
ENSG000001	RHO	protein_codin	0	0	0	0
ENSG000001	RFC4	protein_codin	0	5.7573921	2.735755	0
ENSG000001	RPL39L	protein_codin	2.96235117	0	4.73666117	0
ENSG000001	MASP1	protein_codin	0	0	0	0
ENSG000001	BAP1	protein_codin	48.3624406	81.7275281	0	27.0364035

ENSG000001	RFT1	protein_codin	38.7274323	45.9666242	27.2127876	17.6860704
ENSG000001	PBRM1	protein_codin	0	0	0	0
ENSG000001	ARHGEF3	protein_codin	222.121873	429.266462	165.391366	101.322203
ENSG000001	IL17RD	protein_codin	0	0	0.95552967	0
ENSG000001	LRPAP1	protein_codin	412.940234	642.41965	257.584777	283.381988
ENSG000001	ZDHHC19	protein_codin	0	0	0	0
ENSG000001	SLC51A	protein_codin	0	0	0	0
ENSG000001	UBXN7	protein_codin	484.316995	231.268854	191.444328	270.71036
ENSG000001	PIGX	protein_codin	0	0	0	0
ENSG000001	RUBCN	protein_codin	102.517401	208.708765	170.267694	108.055504
ENSG000001	MELTF	protein_codin	2.84328754	0.94744024	0.85937191	1.79905698
ENSG000001	MELTF	protein_codin	13.1488871	0	2.60047509	1.91464245
ENSG000001	OTOP1	protein_codin	0.93351691	0	0	0
ENSG000001	S100P	protein_codin	0	0	0	8.59030474
ENSG000001	EXO5	protein_codin	0	0	0	0
ENSG000001	CLDN19	protein_codin	0	0	0	0
ENSG000001	P3H1	protein_codin	78.9043319	72.8712377	0	0
ENSG000001	CAMK2D	protein_codin	60.2368936	76.4290032	31.2607416	42.9667915
ENSG000001	METAP1	protein_codin	93.6785653	103.19518	58.8170019	64.4993851
ENSG000001	ADH5	protein_codin	48.2881262	87.8034607	5.27879632	81.6288768
ENSG000000	DAPP1	protein_codin	28.2306665	58.4676582	14.1965625	17.0078449
ENSG000001	H2AZ1	protein_codin	113.70408	150.351507	80.704586	93.3234289
ENSG000001	EMCN	protein_codin	0	0	0	0
ENSG000001	SLC9B1	protein_codin	0	0	0	0
ENSG000001	BDH2	protein_codin	5.41920342	4.60507839	3.376267	17.5490262
ENSG000001	PGRMC2	protein_codin	50.8301886	53.5408888	43.7374495	27.6284347
ENSG000001	CAMP	protein_codin	0	0	0	0
ENSG000001	FBXW12	protein_codin	0	0	0	0
ENSG000001	PLXNB1	protein_codin	11.246643	16.3995924	9.16446602	6.41026958
ENSG000001	SHISA5	protein_codin	100.233529	124.080951	22.5266896	54.9561104
ENSG000001	PRKAR2A	protein_codin	0	0	0	0
ENSG000001	IHO1	protein_codin	0.85384821	1.66540984	0	0
ENSG000001	BSN	protein_codin	0	3.47426223	2.14989465	0
ENSG000001	APEH	protein_codin	20.8395123	147.253917	0	59.8597049
ENSG000001	HSPA4L	protein_codin	0	0	1.38454677	3.97490977
ENSG000001	MFSD8	protein_codin	0	0	0	0
ENSG000001	CAMKV	protein_codin	0	0	0	0
ENSG000001	MON1A	protein_codin	2.86498993	11.4778061	5.35795434	9.8383656
ENSG000001	MST1R	protein_codin	0	0	0	0
ENSG000001	GRM2	protein_codin	0	0	0	0
ENSG000001	POC1A	protein_codin	0	0	0	0
ENSG000001	ETNPPL	protein_codin	0	0	0	0
ENSG000001	PPM1M	protein_codin	40.235902	75.5219788	29.5354804	14.3973979
ENSG000001	WDR82	protein_codin	329.421039	508.112057	182.984612	191.234493

ENSG000001(	PRSS12	protein_codin	0	0	0	0
ENSG000001(	NDST3	protein_codin	2.4256041	8.65541704	6.33929709	0
ENSG000001(	HMGB2	protein_codin	301.930967	532.473256	151.956262	226.818487
ENSG000001(	SAP30	protein_codin	211.549937	209.219112	106.479876	79.1408651
ENSG000001(	SCRG1	protein_codin	0	0	0	0
ENSG000001(	MAD2L1	protein_codin	4.77518254	3.89062355	5.64478963	0
ENSG000001(	ANXA5	protein_codin	1312.36519	1705.64441	483.063283	597.59439
ENSG000001(	ADAD1	protein_codin	0	0	0	0
ENSG000001(	GUCY1A1	protein_codin	0	0	0	0
ENSG000001(	CEP44	protein_codin	18.4422359	25.5869434	0	30.5813505
ENSG000001(	HPGD	protein_codin	3.72727837	13.6479886	10.0036268	0
ENSG000001(	HPGD	protein_codin	27.2706954	10.0442144	15.9058886	14.2285959
ENSG000001(	ASB5	protein_codin	0	0	0	0
ENSG000001:	GRIA2	protein_codin	0	0	0	0
ENSG000001(	TMEM144	protein_codin	34.7736363	30.3028401	0	37.9614519
ENSG000001(	GASK1B	protein_codin	85.4703574	171.364607	103.717664	313.673054
ENSG000001(	NPY1R	protein_codin	0	0	0	0
ENSG000001(	NAA15	protein_codin	113.876557	50.6806725	44.6626316	66.8165883
ENSG000001(	IL15	protein_codin	69.5281263	29.7912368	14.5688983	16.7906885
ENSG000001'	DCLK2	protein_codin	0	1.50549937	0	0
ENSG000001(	FBXW7	protein_codin	29.7361124	4.19554048	47.1282562	13.458663
ENSG000001(	ICE1	protein_codin	349.879766	220.98411	249.411724	261.496935
ENSG000001(	HHIP	protein_codin	0	0	0	0
ENSG000001(	ABCE1	protein_codin	122.641261	108.254711	93.5743812	67.3682906
ENSG000001(	LSM6	protein_codin	18.4731922	12.7094486	4.54937367	7.94070312
ENSG000001(	TMEM184C	protein_codin	99.6769507	80.6518975	44.6779579	81.1705806
ENSG000001(	ITGA2	protein_codin	18.4313678	48.4489766	39.8303728	12.5355532
ENSG000001(	SLC45A2	protein_codin	0	0	0	0
ENSG000001(	EDIL3	protein_codin	0	0	0	0
ENSG000001(	TMEM161B	protein_codin	0	0	0	0
ENSG000001(	NDUFAF2	protein_codin	31.6194025	61.7373051	28.3386874	32.604144
ENSG000001(	ZNF474	protein_codin	0	0	0	0
ENSG000001(	LMBRD2	protein_codin	63.5290922	51.3506475	25.5883576	52.6571392
ENSG000001(	RANBP3L	protein_codin	0	0	0	0
ENSG000001(	RNF180	protein_codin	0	0	1.12673884	0
ENSG000001(	STARD4	protein_codin	72.0713514	33.1362765	32.1602797	21.6960346
ENSG000001(	F2RL2	protein_codin	0	0	0	0
ENSG000001(	PGGT1B	protein_codin	5.21761518	0	0	2.20244663
ENSG000001(	ANKRD33B	protein_codin	162.672534	300.378229	188.995607	112.758034
ENSG000001(	CMBL	protein_codin	0	6.98331636	6.77798289	1.29202346
ENSG000001(	C5orf63	protein_codin	2.27134154	0	0	0
ENSG000001(	PRRC1	protein_codin	72.8206781	104.955147	44.9532195	71.9271761
ENSG000001(	RPS23	protein_codin	75.787374	74.4542134	54.3537345	58.9510146
ENSG000001(	F2RL1	protein_codin	26.5363851	37.9215085	15.7515112	25.4054867

ENSG000001	WDR41	protein_codin	80.070805	39.1192726	37.4286972	62.8282364
ENSG000001	PRDM9	protein_codin	0	0	0	0.72284847
ENSG000001	NDUFS4	protein_codin	122.691607	162.618809	85.193619	102.053052
ENSG000001	SCGB3A2	protein_codin	0	0	0	0
ENSG000001	SPINK1	protein_codin	0	0	0	0
ENSG000001	FBXO38	protein_codin	87.38809	58.9853344	13.346804	15.9668751
ENSG000001	TCERG1	protein_codin	14.6523358	0	0	75.3234994
ENSG000001	AFAP1L1	protein_codin	0	0	0	0
ENSG000001	CDC20B	protein_codin	0	0	0	0
ENSG000001	GPX8	protein_codin	0	0	0	0
ENSG000001	TIGD6	protein_codin	0	8.48166947	0	1.62630813
ENSG000001	SPZ1	protein_codin	0	0	0	0
ENSG000001	ENPP6	protein_codin	0	1.1006135	0	0
ENSG000001	CAGE1	protein_codin	2.9468982	0	1.03401497	2.00787687
ENSG000001	ERAP1	protein_codin	15.4196803	17.6543257	13.2719007	9.71734976
ENSG000001	MAP1B	protein_codin	52.9163351	237.796862	102.96599	17.6059647
ENSG000001	TMEM174	protein_codin	0	0	0	0
ENSG000001	CARTPT	protein_codin	0	0	0	0
ENSG000001	RICTOR	protein_codin	397.332026	0	79.6340669	0
ENSG000001	TENT2	protein_codin	0	0	0	8.74754391
ENSG000001	ANKRA2	protein_codin	40.4312605	50.7135454	23.3477362	34.8805803
ENSG000001	UBLCP1	protein_codin	106.949146	70.2921673	72.1269342	51.9608778
ENSG000001	UTP15	protein_codin	0	11.9361825	6.04639196	0
ENSG000002	ARHGEF28	protein_codin	0	0	0	0
ENSG000001	TLR3	protein_codin	0	0	0	0
ENSG000002	ARHGEF28	protein_codin	0.95422776	0	0	0
ENSG000001	PRKAA1	protein_codin	1.9125456	0	0	0
ENSG000001	NSA2	protein_codin	44.6274604	29.1109632	0	51.7530452
ENSG000001	GFM2	protein_codin	30.4654837	25.2124964	8.97774395	52.8284204
ENSG000001	FBXO4	protein_codin	10.3947211	0	0	0
ENSG000001	CCDC127	protein_codin	24.1566372	41.4370881	11.6693656	13.801116
ENSG000001	FOXQ1	protein_codin	0	1.00638332	0.70341027	1.91983619
ENSG000001	NKD2	protein_codin	0	0	0	0
ENSG000001	RAPGEF6	protein_codin	0	0	0	0
ENSG000001	TNFRSF21	protein_codin	63.333634	39.1821553	27.9902875	20.6825446
ENSG000002	ENSG000002	lncRNA	0	0	0	0
ENSG000001	ACSL6	protein_codin	0	0	0	0
ENSG000001	IL3	protein_codin	0	0	0	0
ENSG000001	CSF2	protein_codin	0	0	0	0
ENSG000001	SEPTIN8	protein_codin	0	0	0	0
ENSG000001	GDF9	protein_codin	0	0	0	0
ENSG000001	LEAP2	protein_codin	0	0	0	0
ENSG000002	CFAP206	protein_codin	0	0	0	0
ENSG000001	TLX3	protein_codin	0	0	0	0

ENSG000001	NPM1	protein_codin	503.325993	652.622011	0	202.291656
ENSG000001	ADGRG6	protein_codin	0	0	0	3.54616234
ENSG000000	FBXW11	protein_codin	54.5976169	113.651513	37.9077067	48.8522872
ENSG000001	TBXT	protein_codin	0	0	0	0
ENSG000001	CREBRF	protein_codin	261.174604	164.299524	137.206971	202.321359
ENSG000001	DCBLD1	protein_codin	7.18388963	20.0271336	8.62733138	8.07724926
ENSG000001	TMEM200A	protein_codin	0	0	0	0
ENSG000001	IL22RA2	protein_codin	0	0	0	0
ENSG000001	SPMIP7	protein_codin	0	0	0	0
ENSG000001	H2AC1	protein_codin	0	0	0	0
ENSG000001	IL31RA	protein_codin	0	12.1330888	0	0
ENSG000000	SCIN	protein_codin	0	5.98407913	2.65401702	0
ENSG000001	DAGLB	protein_codin	54.0892737	158.53877	12.4280202	74.0286688
ENSG000001	MATCAP2	protein_codin	0	2.76712941	0	0
ENSG000001	TRA2A	protein_codin	67.9799688	50.9418244	49.9639915	88.1940281
ENSG000001	GALNT10	protein_codin	166.772996	251.688477	135.825308	96.0981165
ENSG000001	SAP30L	protein_codin	45.2845291	99.9677974	21.2388573	57.7670442
ENSG000001	MYOZ3	protein_codin	0	0	0	0
ENSG000001	COG5	protein_codin	143.745911	124.026776	0	107.153232
ENSG000001	NEUROD6	protein_codin	0	0	0	0
ENSG000001	BMT2	protein_codin	67.2250207	56.3156716	34.0308993	64.2509385
ENSG000001	GPR85	protein_codin	0	0	1.39926513	0
ENSG000001	SLU7	protein_codin	426.929962	274.488398	275.95853	291.82571
ENSG000001	CAMLG	protein_codin	14.687008	10.4954524	16.0961606	43.3198146
ENSG000001	RP9	protein_codin	48.4176622	109.769038	36.2247907	45.3573963
ENSG000001	FBXL21P	transcribed_u	0	0	0	0
ENSG000001	BMPER	protein_codin	0	0	0	0
ENSG000001	SMAD5-AS1	lncRNA	0	0	0	0
ENSG000001	RELL2	protein_codin	2.58136588	0	0	0
ENSG000001	ANKHD1	protein_codin	111.928504	33.2589827	67.2493278	43.0949512
ENSG000001	HSPA9	protein_codin	0	0	0	0
ENSG000001	RSPH10B2	protein_codin	0	0	0	0
ENSG000001	SLC29A4	protein_codin	0	0	0	0
ENSG000001	TEX47	protein_codin	0	0	0	0
ENSG000001	STEAP1	protein_codin	0	0	0	0
ENSG000001	FABP5	protein_codin	29.6755326	34.4849565	71.3272856	9.99621834
ENSG000001	SHH	protein_codin	0	0	0	0
ENSG000001	CHMP4C	protein_codin	0.9990451	8.09002795	1.09233623	3.03167785
ENSG000001	FNDC1	protein_codin	0	0	0	0
ENSG000001	COL1A2	protein_codin	0	0	0	0
ENSG000001	CASD1	protein_codin	31.7922321	14.6974236	17.9028474	6.48241563
ENSG000001	PGAM2	protein_codin	1.67298682	0	0	0
ENSG000001	PLG	protein_codin	0	0	0	0
ENSG000001	BRI3	protein_codin	149.905173	261.636569	20.6065694	105.04403

ENSG000001	LMTK2	protein_codin	320.827111	328.483192	203.224866	234.604689
ENSG000001	XKR6	protein_codin	3.01539338	2.57097394	1.73047558	3.05000343
ENSG000001	SLC35G3	protein_codin	0	0	0	0
ENSG000001	RGS20	protein_codin	0	0	0	0
ENSG000001	SOX17	protein_codin	0	0.92304185	0	0
ENSG000001	ADCY1	protein_codin	0	0	0	0
ENSG000001	C8orf48	protein_codin	1.01244322	0	0	0
ENSG000001	SUN3	protein_codin	0	0	0	0
ENSG000001	RAD21	protein_codin	473.026348	402.136016	291.53506	391.290205
ENSG000001	MED30	protein_codin	14.1590286	42.202891	8.79960964	20.5540474
ENSG000001	TNFRSF11B	protein_codin	0	0	0	0
ENSG000001	SBSPON	protein_codin	0	0	0	0
ENSG000002	ENSG000002	transcribed_u	0	0	0	0
ENSG000001	PHKG1	protein_codin	3.39739046	8.96390844	2.29420811	0
ENSG000001	EN2	protein_codin	0	0	0	0
ENSG000001	KCNV1	protein_codin	0	0	0	0
ENSG000001	CSMD3	protein_codin	0	0	0	0
ENSG000001	SPIDR	protein_codin	151.206329	102.664452	71.2043794	54.4875982
ENSG000001	ORC5	protein_codin	20.3027764	36.6266338	9.48811127	0
ENSG000001	DEFA4	protein_codin	0	0	0	0
ENSG000001	DEFA6	protein_codin	0	0	0	0
ENSG000001	OSGIN2	protein_codin	0	0	0	0
ENSG000001	DEFB1	protein_codin	0	0	0	0
ENSG000001	DNAAF5	protein_codin	23.9565078	59.3880608	22.7506759	26.3825504
ENSG000001	OXR1	protein_codin	0	0	0	0
ENSG000001	ANGPT1	protein_codin	0	47.3062805	0	0
ENSG000001	TMEM74	protein_codin	1.33071945	0	0	0
ENSG000001	GPER1	protein_codin	0	0	1.13222004	0
ENSG000001	TMEM184A	protein_codin	0	0	0	0
ENSG000001	MTUS1	protein_codin	0	0	0	0
ENSG000001	NOS3	protein_codin	0	0	0	0
ENSG000001	SPAG11B	protein_codin	0	0	0	0
ENSG000001	ABCB8	protein_codin	0	0	0	0
ENSG000001	MICALL2	protein_codin	8.23409205	13.7186011	3.04321625	0
ENSG000002	ASIC3	protein_codin	0	0	0	0
ENSG000001	CDK5	protein_codin	0	0	0	0
ENSG000001	SLC7A13	protein_codin	0	0	0	0
ENSG000001	FASTK	protein_codin	21.8754085	0	0	10.3101154
ENSG000001	TMUB1	protein_codin	24.7737836	18.8230425	6.64577835	16.0639726
ENSG000001	FMC1	protein_codin	1.62188286	7.41101963	0	0
ENSG000001	GBX1	protein_codin	0	0	0	0
ENSG000001	PHAX	protein_codin	11.5685616	16.3085894	14.2301889	13.3234387
ENSG000001	COX6C	protein_codin	0	134.776864	0	0
ENSG000001	OSR2	protein_codin	0	0	0	0

ENSG000001(	BAALC	protein_codin	0.92403756	0	0.99848981	0
ENSG000001(	DCAF13	protein_codin	0	0	0	25.6140073
ENSG000001(	DCSTAMP	protein_codin	6.39637451	6.42378541	2.78031973	0
ENSG000001(	VIRMA	protein_codin	182.090123	192.198989	100.725814	123.74602
ENSG000001!	RAD54B	protein_codin	0	0	0	2.427963
ENSG000001(	GEM	protein_codin	0	2.05229597	4.24284644	0
ENSG000001(	PDP1	protein_codin	345.251754	312.606951	169.681513	215.247688
ENSG000001(	RPP25L	protein_codin	0	1.68117009	3.60423265	0
ENSG000001:	TTC39B	protein_codin	0	0	21.9428724	0
ENSG000001(	FAM219A	protein_codin	0	0	0	0
ENSG000001(	SPMIP6	protein_codin	0	0	0	0
ENSG000001(	MYORG	protein_codin	4.98846255	1.01609281	0	0
ENSG000001(	TMEM65	protein_codin	175.687637	103.805883	69.45315	115.3103
ENSG000001(	UBAP1	protein_codin	225.844018	199.074437	169.567746	153.115656
ENSG000001(	CNTNAP3	protein_codin	1.25050464	0	3.24699745	0
ENSG000000!	HSD3B7	protein_codin	13.2752735	5.80304319	0	0
ENSG000001(	NFIL3	protein_codin	826.536201	442.816158	507.934071	548.674915
ENSG000001(	LETM2	protein_codin	0	0	0	0
ENSG000001(	ZMAT4	protein_codin	0	0	0	0
ENSG000001(	CPA6	protein_codin	0	0	0	0
ENSG000001(	TMC1	protein_codin	0	0	0	0
ENSG000001(	ALDH1A1	protein_codin	326.536516	681.34039	287.43375	229.56094
ENSG000001:	CCDC136	protein_codin	1.67627735	0	0	4.86637323
ENSG000001(	KDM1B	protein_codin	0	23.7734877	34.3542482	7.02949046
ENSG000001(	KIF27	protein_codin	16.971589	2.86294269	13.7316633	15.3336412
ENSG000001(	SSMEM1	protein_codin	0	0	0	0
ENSG000001(	COL14A1	protein_codin	0	0	0	0
ENSG000001(	ZHX1	protein_codin	22.8058527	0	0	32.5426428
ENSG000001(	CFAP47	protein_codin	0	0	0	0
ENSG000001(	METTL27	protein_codin	0	0	0	0
ENSG000001.	SYTL5	protein_codin	0	0	0	0
ENSG000001(	RNF183	protein_codin	0	0	0	0
ENSG000001(	VEGFD	protein_codin	0	0	0	0
ENSG000001.	MRRF	protein_codin	0	9.9637934	0	0
ENSG000001(	OR1Q1	protein_codin	0	0	0	0
ENSG000001(	GAPVD1	protein_codin	167.76561	56.2185523	60.9497092	61.6197608
ENSG000001(	WNK2	protein_codin	2.99594836	0	0	0
ENSG000001(	HDX	protein_codin	0	7.50869747	7.5754388	0
ENSG000001.	AOPEP	protein_codin	0	0	2.56546036	5.94676746
ENSG000001(	AQP7	protein_codin	0	0	0	0
ENSG000001(	NOL6	protein_codin	54.663268	58.6530551	16.8784125	24.8934053
ENSG000001(	AQP3	protein_codin	3.78056983	5.21449217	73.1725851	5.71088349
ENSG000001(	TRMT10B	protein_codin	13.6860546	14.2787841	19.5456563	0
ENSG000001(	PIGO	protein_codin	0	0	0	0

ENSG000001	ARMC3	protein_codin	0	2.32010141	0	0
ENSG000001	MELK	protein_codin	0	0	0	0
ENSG000001	EEF1A2	protein_codin	0	0	0	0
ENSG000001	DEUP1	protein_codin	0	0	0	0
ENSG000001	HECTD2	protein_codin	0	0	0	0
ENSG000001	SLC7A3	protein_codin	0	0	0	0
ENSG000001	PABIR2	protein_codin	46.8258631	62.7867818	82.7885806	42.3720201
ENSG000001	FBXO33	protein_codin	192.786213	176.283993	92.9716892	105.397291
ENSG000002	METTL21EP	protein_codin	0	0	0	0
ENSG000001	LRFN5	protein_codin	0	0	0	0
ENSG000001	WDFY2	protein_codin	901.002911	460.254741	226.302971	343.902558
ENSG000001	SPTSSA	protein_codin	89.8637126	138.072679	61.1821997	73.8251837
ENSG000001	WRN	protein_codin	11.2816566	12.6457455	9.85153452	10.6203339
ENSG000001	CFL2	protein_codin	25.0808177	8.81068963	0	0
ENSG000001	TSHR	protein_codin	0	0	2.27996157	0
ENSG000001	GTF2A1	protein_codin	5.2919626	0	5.60673215	4.58432139
ENSG000001	SSX7	protein_codin	0	0.86324318	0	0
ENSG000001	KRBOX4	protein_codin	12.371802	23.6735153	0	7.19334293
ENSG000001	PGM2L1	protein_codin	29.5146078	12.3614153	27.8894787	12.5039656
ENSG000001	FOLR2	protein_codin	20.0486786	6.91799966	14.380205	9.76321695
ENSG000001	INPPL1	protein_codin	631.40906	844.757886	370.183736	378.388473
ENSG000001	PHOX2A	protein_codin	0	0	0	0
ENSG000001	CRYL1	protein_codin	25.1991181	101.958495	43.4632955	49.1634405
ENSG000001	HEPACAM	protein_codin	0	0	0	0
ENSG000001	SKA3	protein_codin	0	0	0	0
ENSG000001	PCDH11X	protein_codin	0	1.05862837	0	0
ENSG000001	PCF11	protein_codin	9.10461142	36.7440968	7.88620057	3.26011452
ENSG000001	PKNOX2	protein_codin	0	0	0	0.82199175
ENSG000001	RPL10L	protein_codin	0	0	0	0
ENSG000001	LRR1	protein_codin	10.5994992	8.43039995	6.22323174	9.80559048
ENSG000001	RPL36AL	protein_codin	647.61507	564.522122	511.92292	567.917375
ENSG000001	DNAAF2	protein_codin	15.5311816	0	5.3967498	2.67688407
ENSG000001	DEPP1	protein_codin	2.95734904	6.71457795	15.6329672	1.87130085
ENSG000001	MAGEC3	protein_codin	0	0	0	0
ENSG000001	ZNF22	protein_codin	31.1601149	51.2281903	30.989889	19.4193463
ENSG000001	KLHDC2	protein_codin	10.4572756	19.0161229	6.19179283	15.9764753
ENSG000001	NEMF	protein_codin	29.6763529	26.5864394	37.9320132	52.7311274
ENSG000001	ARF6	protein_codin	448.890325	722.778319	220.162496	471.089701
ENSG000001	RPUSD4	protein_codin	37.6365893	48.9484646	24.3290288	29.0732996
ENSG000001	TMEM63C	protein_codin	11.9652152	14.693469	5.14423615	3.92603606
ENSG000001	NGB	protein_codin	0	0	0	0
ENSG000001	TRIM9	protein_codin	0	0	0	0
ENSG000001	AKR1E2	protein_codin	0	0	0	2.53727784
ENSG000001	RXFP2	protein_codin	0	0	0	0

ENSG000001(	SSX3	protein_codin	0	0	0	0
ENSG000001:	NAA30	protein_codin	0	0	0	0
ENSG000000(	SEC61A2	protein_codin	0	0	1.47470207	0
ENSG000001(	OXGR1	protein_codin	1.22358706	1.25795397	2.21355858	1.29064416
ENSG000001(	VSTM4	protein_codin	0	0	0	0
ENSG000001(	SOHLH1	protein_codin	0	0	0	0
ENSG000001(	VDAC2	protein_codin	0	0	0	0
ENSG000001(	ABRAXAS2	protein_codin	53.2719513	61.2001189	31.6038884	44.113204
ENSG000001(	PRDX3	protein_codin	186.742433	189.056733	84.3598607	148.042861
ENSG000001'	CLEC7A	protein_codin	159.474935	157.572856	118.728341	101.336646
ENSG000001(	CLEC1B	protein_codin	96.9341737	125.196198	65.0750922	52.5583077
ENSG000001(	TMEM52B	protein_codin	0	0	2.78949624	3.79762071
ENSG000001(	SNAPC4	protein_codin	34.9968393	0	26.8692948	0
ENSG000001(	ENTR1	protein_codin	9.65887723	5.35451067	4.27017082	7.04430278
ENSG000001(	FRMD7	protein_codin	0	0	0	0
ENSG000001(	AK8	protein_codin	0	0	0.97412015	0
ENSG000001(	TSC1	protein_codin	0	0	0	0
ENSG000001(	HPRT1	protein_codin	56.180956	69.2657292	32.6789693	45.500445
ENSG000000	TSPAN17	protein_codin	0	8.5141308	6.21576644	1.67043424
ENSG000001:	BCL2L14	protein_codin	0	0	3.88045502	0
ENSG000001:	KIAA1191	protein_codin	103.817094	166.497525	59.5537546	87.7214609
ENSG000001(	BORCS5	protein_codin	0	1.72945529	0	0
ENSG000001	DUSP16	protein_codin	14.5291082	15.1493959	9.38687809	12.3060254
ENSG000001(	ZMYND19	protein_codin	25.9188738	26.9376867	9.77475143	7.40838578
ENSG000001(	STOX1	protein_codin	0	0	0	0
ENSG000001:	JAKMIP3	protein_codin	0	0	0	0
ENSG000001:	SARDH	protein_codin	0	0	0	0
ENSG000001(	STK32C	protein_codin	46.6986447	50.8127747	15.4053129	25.9677175
ENSG000001(	OR4K2	protein_codin	0	0	0	0
ENSG000001:	HK1	protein_codin	8.0885645	42.2023982	81.2929128	31.6169658
ENSG000001(	SLC39A2	protein_codin	0	0	0	0
ENSG000001(	NDRG2	protein_codin	0	0	0	0
ENSG000001(	NDRG2	protein_codin	0	0	0	0
ENSG000001(	RNASE7	protein_codin	0	0	0	0
ENSG000001(	ARHGEF40	protein_codin	446.524423	318.608159	134.422122	158.487279
ENSG000001(	C12orf50	protein_codin	0	0	0	0
ENSG000001(	PPP1R36	protein_codin	0	1.04461264	0	0
ENSG000001(	VWA2	protein_codin	0	0	0	0
ENSG000001(	METTL3	protein_codin	0	85.569293	25.0240936	0
ENSG000001(	ERICH6B	protein_codin	0	0	0	0
ENSG000001:	GAS1	protein_codin	4.00322076	5.0886949	0.78631303	0
ENSG000001(	TRUB1	protein_codin	12.1475127	26.6609186	18.6659623	11.9628271
ENSG000000(	WAPL	protein_codin	738.348708	591.845199	485.063895	518.872615
ENSG000001:	SHLD2	protein_codin	169.785697	23.2760312	82.042717	84.4841989

ENSG000001	SHLD2	protein_codin	32.6076055	83.0073964	7.44951186	0
ENSG000001	ANKRD2	protein_codin	0	0	0	0
ENSG000001	ARHGAP42	protein_codin	0	0	35.0266074	3.60987461
ENSG000001	ISCA2	protein_codin	6.10399716	11.9752808	2.05555178	9.74632372
ENSG000001	OTOGL	protein_codin	0	0	2.65154311	0
ENSG000001	TTLL5	protein_codin	57.5043757	90.9226544	45.3765545	56.9163683
ENSG000001	PACSIN3	protein_codin	0	0	0	0
ENSG000001	SERPINA4	protein_codin	0	0	0	0
ENSG000001	SERPINA9	protein_codin	0	0	0	0
ENSG000001	PSMC3	protein_codin	0	0	0	46.649888
ENSG000001	RAPSN	protein_codin	0	0	0	0
ENSG000001	DGLUCY	protein_codin	0	0	0	0
ENSG000001	CPSF2	protein_codin	241.079696	267.167489	143.820097	190.584184
ENSG000001	SMCO2	protein_codin	0	0	0	1.06920756
ENSG000001	CAPRIN2	protein_codin	15.5522836	0	0	5.67161644
ENSG000001	MOAP1	protein_codin	90.348244	80.8524101	49.8692462	40.4213269
ENSG000000	BTBD7	protein_codin	7.02111678	13.9196254	7.57939844	20.856619
ENSG000001	LRRK2	protein_codin	587.182088	420.862309	211.538199	398.400918
ENSG000001	CLMN	protein_codin	486.794787	536.505974	196.013577	281.441499
ENSG000001	PDZRN4	protein_codin	0	0	0	0
ENSG000001	SLC6A5	protein_codin	0	0	0	0
ENSG000001	NELL1	protein_codin	0	0	0	0
ENSG000001	PTER	protein_codin	7.09084848	0	0	0
ENSG000001	SMCO4	protein_codin	69.3213502	117.513638	33.4007965	78.6474902
ENSG000001	KCNC2	protein_codin	0	0	0	0
ENSG000001	R3HCC1L	protein_codin	74.9726371	31.8021114	42.0192191	51.2881755
ENSG000001	PIWIL4	protein_codin	13.4846108	30.9994861	21.9547873	23.378749
ENSG000001	AMOTL1	protein_codin	0	0.99649949	0	0
ENSG000001	LIPC	protein_codin	0	16.3926117	0	0
ENSG000001	TCP11L2	protein_codin	78.0302113	93.2024833	71.9003485	0
ENSG000001	SPRED1	protein_codin	16.4632216	15.563972	9.02381309	11.1133403
ENSG000001	C14orf93	protein_codin	6.34363014	12.6738866	0	0
ENSG000001	GPR176	protein_codin	0	0	0	1.82483208
ENSG000001	JAM3	protein_codin	124.156381	278.24493	303.149457	221.495193
ENSG000001	BCCIP	protein_codin	14.5056973	16.7514222	15.344113	14.7223046
ENSG000001	WDR20	protein_codin	0	0	0	0
ENSG000000	VPS35	protein_codin	139.087679	107.650151	112.389727	94.3621834
ENSG000001	SPATA19	protein_codin	0	0	0	0
ENSG000001	AMN	protein_codin	0	1.66735883	0	0
ENSG000001	IKBIP	protein_codin	52.5129445	46.3792113	16.1683741	32.6437946
ENSG000001	FOXN4	protein_codin	0	0	0	0
ENSG000001	HIF1AN	protein_codin	160.954772	171.982825	93.6455856	77.585782
ENSG000001	ADAMTS15	protein_codin	0	0	0	0
ENSG000001	NDUFB8	protein_codin	0	0	0	0

ENSG000001(	PHKB	protein_codin	218.880765	128.770328	198.989669	153.362011
ENSG000001(	ZFYVE19	protein_codin	0	0	0	0
ENSG000001(	PPP1R14D	protein_codin	0	0	0	0
ENSG000001(	AVPR1A	protein_codin	74.3154075	49.7812051	53.4498456	29.6826185
ENSG000000:	MRPL43	protein_codin	11.519851	27.6615005	0	11.0367755
ENSG000001(	DEPDC4	protein_codin	0	0	0	0
ENSG000001(	C16orf78	protein_codin	0.81798398	0.81897298	1.12435606	0.82481879
ENSG000001:	HEATR3	protein_codin	76.227927	65.7964657	50.6368593	40.0340276
ENSG000001(	LRTM2	protein_codin	0	0	0	0
ENSG000002:	BRD7P5	processed_ps	0	0.83605379	0	0
ENSG000001(	TRMT61A	protein_codin	0	0	0	0
ENSG000001(	BAG5	protein_codin	166.34545	165.980793	80.7511453	121.830974
ENSG000001(	POLL	protein_codin	15.4577094	43.5663104	0	0
ENSG000001(	LARP6	protein_codin	0	0	0	0
ENSG000001(	ZNF319	protein_codin	84.2081475	135.216419	23.7014484	62.9917771
ENSG000001(	HPS6	protein_codin	47.7055303	106.248715	22.9910657	48.7195787
ENSG000000:	TTC17	protein_codin	301.231548	324.303585	152.39776	271.286684
ENSG000001:	MDM2	protein_codin	0	0	0	0
ENSG000001(	COPS2	protein_codin	138.559606	87.4135392	109.601279	90.3889309
ENSG000001(	GABRB3	protein_codin	0	0	0	0
ENSG000000:	PLEKHA5	protein_codin	0	0	0	29.6086339
ENSG000001(	TBATA	protein_codin	0	0	0	0
ENSG000001:	CHODL	protein_codin	0	0	0	0
ENSG000001(	SGPL1	protein_codin	0	0	0	0
ENSG000001(	PCBD1	protein_codin	8.77679491	13.9984775	5.00442251	9.50550378
ENSG000001(	CCT2	protein_codin	92.6977134	197.058584	145.592462	112.729087
ENSG000001:	TMEM132B	protein_codin	0	0	0	0
ENSG000001	GNPTAB	protein_codin	275.045141	160.195111	155.362486	288.341161
ENSG000001(	DNAAF8	protein_codin	0	0	0	0
ENSG000001(	SCN3B	protein_codin	0	0	0	0
ENSG000001(	COX11	protein_codin	0	9.91665176	0	10.5310822
ENSG000001(	FAM227B	protein_codin	0	0	0	0
ENSG000001:	CLDN10	protein_codin	1.34190429	0	0	0
ENSG000001(	CYYR1	protein_codin	0	2.33669039	3.29264067	0
ENSG000001:	CDH8	protein_codin	0	0	0	0
ENSG000001(	MYRFL	protein_codin	0	0	0	0
ENSG000002'	BORCS7-ASM	protein_codin	5.37701191	4.65353903	4.00100712	1.0806843
ENSG000000'	ACAT1	protein_codin	1.80263352	5.58876924	0	4.55332065
ENSG000001(	CDH23	protein_codin	12.1446439	5.46615655	5.28337832	5.33614418
ENSG000001(	C2	protein_codin	52.0134305	36.7130583	11.2878723	17.9891199
ENSG000001(	ANAPC16	protein_codin	9.51413077	78.2221721	5.27389317	15.5314839
ENSG000001(	APBB1	protein_codin	0	0	0	0
ENSG000001(	TRIM44	protein_codin	772.040695	832.075874	544.185264	555.827331
ENSG000001(	CCDC182	protein_codin	0	0	0	0

ENSG000001(	ILK	protein_codin	208.200854	391.008356	54.5154825	131.162328
ENSG000001(	TAF10	protein_codin	50.9332535	68.0866437	30.6437904	14.2412752
ENSG000001(	TPP1	protein_codin	937.437481	1186.84769	437.194411	737.181228
ENSG000001(	MSS51	protein_codin	16.0854214	18.6779271	0	7.10524449
ENSG000001(	CYB5A	protein_codin	0	5.94222795	6.14021474	0
ENSG000001(	RAG1	protein_codin	0	0	0	0
ENSG000001(	DCHS1	protein_codin	38.0978355	33.3898969	12.0711487	6.74005635
ENSG000001(	POTED	protein_codin	0	0	0	0.98279788
ENSG000001(	OR10A5	protein_codin	0	0	0	0
ENSG000001(	OR2D2	protein_codin	0	0	0	0
ENSG000002(	PCMTD2	protein_codin	7.26475061	21.5920271	4.25372802	8.67455072
ENSG000001:	NLRP14	protein_codin	0	0	3.97829149	0
ENSG000001(	PPFIBP2	protein_codin	59.6337569	75.4215531	53.9056086	0
ENSG000001(	CYB5R2	protein_codin	0	2.86320778	0	0
ENSG000001!	SERPINB2	protein_codin	10.1971163	22.5068732	22.1699739	43.3161974
ENSG000001(	GARRE1	protein_codin	64.9874467	85.1808212	34.1350329	45.4069377
ENSG000001(	TUB	protein_codin	0	0	0	0
ENSG000001(	OR5P1P	unprocessed_	0	0	0	0
ENSG000001(	IDH3A	protein_codin	6.9900459	0	2.78247426	0
ENSG000001(	CRABP1	protein_codin	0	0	0	0
ENSG000002	AMD1P4	transcribed_p	0	0	0	0
ENSG000000(	CTDP1	protein_codin	1.99417643	0	3.60098326	1.99774317
ENSG000001(	RNF169	protein_codin	568.271802	611.030433	315.991564	392.236389
ENSG000001(	CHRNA5	protein_codin	0	0	0	0
ENSG000001(	CENPN	protein_codin	5.75799535	8.10806735	1.01776793	1.39053216
ENSG000001(	ATMIN	protein_codin	173.507047	139.303314	62.5523177	114.100035
ENSG000001(	AKIP1	protein_codin	0	14.3991148	0	10.0237206
ENSG000001(	C16orf46	protein_codin	2.57473864	0	2.20733464	0
ENSG000001(	TMEM41B	protein_codin	0	0	0	0
ENSG000001(	PKD1L2	protein_codin	0	0	0	0
ENSG000001(	LEO1	protein_codin	67.4411739	107.816018	97.3117912	65.9651703
ENSG000001(	ZNF143	protein_codin	0	0	14.6812421	0
ENSG000001(	TMX3	protein_codin	160.519033	69.5986814	61.8248181	115.17794
ENSG000001(	MFAP4	protein_codin	0	0	0	1.06443288
ENSG000001(	MAPK7	protein_codin	0	0	0	9.82457906
ENSG000001(	WEE1	protein_codin	2.72805098	0	0	0
ENSG000001:	ALG8	protein_codin	0	0	0	0
ENSG000001(	HDGFL3	protein_codin	6.15359229	4.50527029	4.73580551	6.74933356
ENSG000002:	POLR2M	protein_codin	102.749124	91.0915809	54.1436576	99.4581694
ENSG000001(	NDST2	protein_codin	95.1610761	127.104572	0	65.1027371
ENSG000001(	CLEC3A	protein_codin	0	0	0	0
ENSG000001(	CLEC4E	protein_codin	177.387125	184.319856	62.480587	127.034707
ENSG000001(	CLEC4D	protein_codin	201.356322	155.358994	70.9303644	140.447575
ENSG000001(	ZNF3	protein_codin	0	32.3300566	41.4150649	0

ENSG0000010	HSBP1P2	processed_ps	0	0	0	0
ENSG0000010	RIMKLB	protein_codin	0	0	0	0
ENSG000002	ZNF407	protein_codin	42.979148	74.4030611	18.8364608	21.7134764
ENSG0000010	BEAN1	protein_codin	0	0	0	0.81120692
ENSG0000010	TK2	protein_codin	0	0	30.319466	0
ENSG0000010	A2ML1	protein_codin	0	0	0	0
ENSG0000010	TMED3	protein_codin	40.7101337	78.4740487	45.3181267	42.3796926
ENSG0000010	SLC38A8	protein_codin	0.93678533	0	0	0
ENSG0000010	SEC11C	protein_codin	3.4069004	13.4569532	7.90864391	0
ENSG0000010	CPLX4	protein_codin	0	0	0	0
ENSG0000010	GALR1	protein_codin	1.19531134	1.18685457	0	0
ENSG0000010	IQCD	protein_codin	6.40876367	8.28308896	0	4.36689714
ENSG0000010	CENPV	protein_codin	0	0	0	0
ENSG0000010	CDH16	protein_codin	0	0	0	0
ENSG0000010	RRAD	protein_codin	7.86322054	5.80518625	4.99067457	5.66627846
ENSG0000010	MC4R	protein_codin	0	0	1.18807039	0
ENSG0000010	HSP90B1	protein_codin	701.903992	615.879688	2321.92774	827.620692
ENSG000002	ENSG000002	processed_ps	0	0	1.24385018	0
ENSG000001	SLC9A5	protein_codin	0	0	0	0
ENSG000000	NCAPH2	protein_codin	10.5157643	9.15761129	5.25208895	0
ENSG0000010	PPP1R16B	protein_codin	13.0037484	46.1661212	69.3103296	37.6385156
ENSG000001	VWA3A	protein_codin	1.63015722	0	1.01213601	0
ENSG0000010	CHRFAM7A	protein_codin	10.1761077	12.1823523	0	0
ENSG000000	POLR3E	protein_codin	62.0898238	98.5731611	19.8142049	50.9308841
ENSG000001	MMP3	protein_codin	0	0	0	0
ENSG0000010	TVP23A	protein_codin	0	0	0	0
ENSG000001	ZNF211	protein_codin	0	0	0	0
ENSG0000010	TMPRSS5	protein_codin	0	0	0	0
ENSG0000010	COG1	protein_codin	54.7826395	78.4133198	47.0792641	48.1370987
ENSG000001	FAM199X	protein_codin	0	0	6.64511709	0
ENSG0000010	ZNF592	protein_codin	0	0	0	0
ENSG000001	MARVELD3	protein_codin	0	0	0	0.8274377
ENSG0000010	GOLM2	protein_codin	308.642533	237.692121	95.0742236	179.05912
ENSG0000010	HTR3A	protein_codin	0	1.88994217	0	0
ENSG0000010	NNMT	protein_codin	0	0	0	0
ENSG000001	SERINC4	protein_codin	0.92759787	0	0	0
ENSG0000010	SLFN5	protein_codin	103.989428	74.4007652	73.9558225	93.0691844
ENSG0000010	AP1G1	protein_codin	241.781692	277.56383	195.860603	149.927137
ENSG000001	ERI2	protein_codin	0	1.90794412	0	0
ENSG0000010	BMERB1	protein_codin	0	0	0	0
ENSG0000010	SAAL1	protein_codin	0	6.88169407	0	0
ENSG0000010	YPEL4	protein_codin	0	0	0	0
ENSG0000010	PPIB	protein_codin	391.346314	817.578202	435.308676	289.615576
ENSG000001	FANCI	protein_codin	0	0	0	0

ENSG000001	CIAO2A	protein_codin	170.490681	240.669787	121.321581	88.1047316
ENSG000001	PCLAF	protein_codin	1.09288477	1.26857133	3.31830345	1.12329461
ENSG000001	MYH11	protein_codin	0	0	0	0
ENSG000001	UEVLD	protein_codin	0	4.56518786	0	0
ENSG000001	LDHD	protein_codin	0	19.9725464	0	5.45278985
ENSG000001	PLIN1	protein_codin	0	0	0	0
ENSG000001	PEX11A	protein_codin	2.08219128	3.16233757	3.3902949	5.32978451
ENSG000001	MESP1	protein_codin	0	0.84489918	0	0
ENSG000001	ANPEP	protein_codin	1482.63673	1924.6905	354.825207	735.390295
ENSG000001	SCNN1G	protein_codin	0	0	0	0
ENSG000001	RBPMS2	protein_codin	21.5186146	13.1218159	10.3089561	0
ENSG000001	GLYATL1	protein_codin	0	0	0	0
ENSG000001	TERF2IP	protein_codin	328.075778	448.452471	203.397139	222.101155
ENSG000001	DCTN5	protein_codin	46.6900935	74.8554416	38.2075652	19.9509091
ENSG000001	C18orf54	protein_codin	0	0	0	0
ENSG000001	PLK1	protein_codin	0	3.63271451	25.4330116	2.64814178
ENSG000001	GPR182	protein_codin	0	0	0	0
ENSG000001	ZBTB39	protein_codin	47.9509964	70.0330528	28.876826	55.671234
ENSG000001	CACNG2	protein_codin	0	0	0	0
ENSG000001	CLPX	protein_codin	82.7623169	78.776196	35.5658209	61.0414764
ENSG000001	TAC3	protein_codin	0	0	0	0
ENSG000001	CHP2	protein_codin	0	0	0	0
ENSG000001	MYO1A	protein_codin	7.34631308	0	2.83756663	5.60910684
ENSG000001	OR4D6	protein_codin	0	0	0	0
ENSG000001	NEMP1	protein_codin	24.9247835	42.4402145	23.4039021	18.1941614
ENSG000001	NAB2	protein_codin	0	32.6988604	10.9324061	0
ENSG000001	STAT6	protein_codin	853.512164	1423.93336	535.021796	706.888416
ENSG000000	DPP8	protein_codin	172.258665	75.5969926	45.9685351	121.559214
ENSG000001	ATP23	protein_codin	6.05105892	0	8.33870558	4.96910989
ENSG000001	PATL1	protein_codin	330.938966	416.624274	163.152819	231.548881
ENSG000001	MRPL16	protein_codin	88.6890765	120.584302	96.5792173	55.357019
ENSG000001	MIR202HG	lncRNA	0	0	0	0
ENSG000001	SCG5	protein_codin	0	0	0	0
ENSG000001	AGFG2	protein_codin	0	2.78229677	0	0
ENSG000001	NYAP1	protein_codin	0	0	0	0
ENSG000001	TSC22D4	protein_codin	276.338241	322.683947	127.134089	175.377009
ENSG000001	MS4A6E	protein_codin	0	0	0	0
ENSG000001	MS4A7	protein_codin	110.49554	38.5591718	32.0657788	0
ENSG000001	MS4A14	protein_codin	0	6.63352154	17.6431247	0
ENSG000001	MS4A5	protein_codin	0	0	0	0
ENSG000001	EEF1AKMT3	protein_codin	3.393084	10.9915475	3.48305609	13.7364504
ENSG000001	CCNDBP1	protein_codin	24.470954	40.1998848	11.9376808	4.10747977
ENSG000001	MS4A8	protein_codin	0	0	0	0
ENSG000001	CCDC178	protein_codin	0	0	0	0

ENSG000001(	MAP1A	protein_codin	307.529744	906.507092	914.563879	328.448676
ENSG000001(	AKTIP	protein_codin	6.97947619	4.40484203	0	6.837772
ENSG000001(	MAPRE2	protein_codin	358.355444	674.699121	273.102253	332.756772
ENSG000001(	EVA1C	protein_codin	14.9638021	17.4703438	0	0
ENSG000002	TCP10L	protein_codin	0	0	0	0
ENSG000001	CFAP298	protein_codin	45.9588444	56.2996456	6.50039362	26.3349243
ENSG000001	SON	protein_codin	596.105528	515.15158	214.621461	408.4903
ENSG000002	CKMT1B	protein_codin	0	0	0	0
ENSG000001(	PDIA3	protein_codin	57.4557392	118.689516	29.0413979	33.9594376
ENSG000001(	NUDT21	protein_codin	168.125051	180.835832	117.98441	132.532764
ENSG000000	HERPUD1	protein_codin	45.6600979	107.848749	0	17.8801093
ENSG000001(	NAT16	protein_codin	0	0	0	0
ENSG000001	RUNX1	protein_codin	47.5454567	45.3600519	45.9990031	30.0956154
ENSG000001	BPIFC	protein_codin	0	0	0	0
ENSG000000	TPX2	protein_codin	5.88796301	13.7542695	31.5600859	4.99584291
ENSG000001(	B4GALNT2	protein_codin	0	0	0	0
ENSG000001(	GNGT2	protein_codin	0	0	3.84934417	0
ENSG000001(	PHB1	protein_codin	46.6993747	47.4803549	0	34.5187579
ENSG000001(	SNRPD1	protein_codin	25.0655949	60.3944408	26.6182934	25.2675277
ENSG000001	LRSAM1	protein_codin	0	0	0	0
ENSG000001(	PIP5KL1	protein_codin	0	0	0	0
ENSG000001(	TMEM92	protein_codin	0	0	0	0
ENSG000001(	EEIG1	protein_codin	0	0	0	0
ENSG000001(	ACSF2	protein_codin	30.7454395	62.2388625	17.9764685	7.3816989
ENSG000001(	COQ4	protein_codin	17.5469504	24.512983	11.5974933	9.35792252
ENSG000001(	SLC27A4	protein_codin	38.6843666	57.5296079	29.6539482	23.2560575
ENSG000001(	ANKRD40CL	protein_codin	0	0	0	0
ENSG000001	DYNLRB1	protein_codin	2.12927946	2.26199308	1.2068364	1.06796851
ENSG000001	TRPM2	protein_codin	0	33.6129354	0	11.6145617
ENSG000001	TRPM2	protein_codin	0	0	19.9838063	10.0109172
ENSG000001(	TBC1D21	protein_codin	0	0	0	0
ENSG000001	COL6A2	protein_codin	5.11901686	0	0	0
ENSG000001(	ZNF85	protein_codin	0	0	0	0
ENSG000001(	PRR15L	protein_codin	0	0	0	0
ENSG000001(	GPRC5B	protein_codin	0	0	0.84984278	0
ENSG000001(	CRK	protein_codin	42.8953076	66.477341	0	47.5081057
ENSG000001(	C16orf92	protein_codin	0	0	0	0
ENSG000001(	GOLGA6C	protein_codin	0	0	0	0
ENSG000001(	TBC1D2B	protein_codin	177.383223	255.469353	142.146686	159.572135
ENSG000001(	NOD2	protein_codin	68.9271878	0	0	92.0949761
ENSG000001(	SNX20	protein_codin	28.1080651	50.4361727	0	21.5556633
ENSG000001(	LOXHD1	protein_codin	0	0	0	0
ENSG000001(	HDHD2	protein_codin	53.0721896	87.9719392	45.0045556	34.5612803
ENSG000001(	ZNF91	protein_codin	52.522275	48.7232027	105.078254	54.8342621

ENSG000000[illegible]	GCN1	protein_codin	250.123472	330.592196	214.831328	111.425214
ENSG000001[illegible]	RNF214	protein_codin	22.1600636	0	3.09240682	3.60329563
ENSG000001[illegible]	MED1	protein_codin	316.591615	291.59915	212.855037	206.216638
ENSG000001[illegible]	PGAP3	protein_codin	0	19.4733985	0	7.98507836
ENSG000001[illegible]	ENGASE	protein_codin	15.0108134	42.9370514	19.534067	10.7590748
ENSG000001[illegible]	ATP5MG	protein_codin	276.039127	409.652071	168.236354	170.464937
ENSG000001[illegible]	CD3D	protein_codin	0	1.14492802	0	0
ENSG000001[illegible]	TEPSIN	protein_codin	0	13.2438096	8.71489182	0
ENSG000001[illegible]	PGAP2	protein_codin	0	19.4313431	0	0
ENSG000001[illegible]	STIM1	protein_codin	208.065744	346.91681	0	106.841219
ENSG000001[illegible]	RRM1	protein_codin	47.6416755	54.9183494	102.06164	25.6996769
ENSG000001[illegible]	TRIM68	protein_codin	10.8828612	22.342466	0	0
ENSG000001[illegible]	MMP26	protein_codin	0	0	0	0
ENSG000001[illegible]	OR51B5	protein_codin	0	0	0	0
ENSG000001[illegible]	OR51Q1	protein_codin	0	0	0	0
ENSG000001[illegible]	FN3K	protein_codin	3.48991462	12.4464113	4.50437053	2.97481903
ENSG000001[illegible]	PRRT2	protein_codin	0	4.58281547	0	0
ENSG000001[illegible]	ZNF428	protein_codin	9.49687401	24.5349861	12.0596383	7.32402881
ENSG000001[illegible]	ZNF226	protein_codin	8.93486344	5.31971405	0	0
ENSG000001[illegible]	PRR14	protein_codin	63.6920058	120.713608	56.6823879	48.1079952
ENSG000000[illegible]	MARK4	protein_codin	0	0	0	0
ENSG000001[illegible]	ZNF668	protein_codin	38.2822326	52.8652819	15.9360503	20.2214394
ENSG000001[illegible]	ZNF646	protein_codin	73.7964952	216.01082	47.8159831	90.6169382
ENSG000001[illegible]	VKORC1	protein_codin	0	0	0	0
ENSG000000	ERCC1	protein_codin	120.660964	147.992704	55.2735868	42.2026588
ENSG000001[illegible]	HIF3A	protein_codin	0	0	0	0
ENSG000001[illegible]	ZNF267	protein_codin	409.153507	246.320263	174.024327	273.296761
ENSG000001[illegible]	GNG8	protein_codin	0	4.49114462	2.73328438	0
ENSG000001[illegible]	DACT3	protein_codin	0	0	0	0
ENSG000001[illegible]	BBC3	protein_codin	0	0	0	0
ENSG000001[illegible]	USP32	protein_codin	603.345178	217.675233	211.29303	284.096308
ENSG000001[illegible]	CA4	protein_codin	0	0	0	0
ENSG000001[illegible]	SMG8	protein_codin	8.8368312	42.2913788	12.8237838	15.8860538
ENSG000001[illegible]	TPM4	protein_codin	0	0	0	0
ENSG000001[illegible]	RAB8A	protein_codin	902.173582	1789.12388	447.077694	803.964599
ENSG000001[illegible]	MIDN	protein_codin	704.48756	779.016704	179.991707	505.006524
ENSG000001	PCSK4	protein_codin	0	0	0	0
ENSG000001[illegible]	JSRP1	protein_codin	0	2.68886374	9.58656554	0.84184953
ENSG000001[illegible]	KLHL26	protein_codin	23.5482237	31.8055516	5.83452094	25.7958138
ENSG000001[illegible]	KRT17P2	transcribed_u	0	0	0	0
ENSG000001[illegible]	JPH3	protein_codin	0	0	0	0
ENSG000001[illegible]	ZC3H18	protein_codin	233.951725	359.297178	145.32886	150.732673
ENSG000001[illegible]	MVD	protein_codin	19.1354801	13.5462134	10.690521	8.6111512
ENSG000001[illegible]	PIEZO1	protein_codin	784.630254	1014.42864	369.353978	341.513658

ENSG000001	CDT1	protein_codin	2.86721296	4.77701141	7.62924373	0.91707042
ENSG000001	TRAPPC2L	protein_codin	5.49796927	8.48646802	7.9426939	0
ENSG000001	ANKRD11	protein_codin	564.156816	932.252221	228.627013	665.190584
ENSG000001	SPATA33	protein_codin	0	6.48910848	1.892265	1.83438663
ENSG000001	UNC119	protein_codin	51.9561442	42.7239756	0	10.6447276
ENSG000001	RSKR	protein_codin	8.13714803	6.154355	0	0
ENSG000001	PROCA1	protein_codin	0	0	19.4726769	6.50371213
ENSG000001	ZNF641	protein_codin	33.9870468	135.008495	43.3423272	33.9891769
ENSG000001	RAB34	protein_codin	0	0	0	18.4611243
ENSG000001	LALBA	protein_codin	0	0	0	0
ENSG000001	CACNB3	protein_codin	0	0	0	0
ENSG000001	TP53I13	protein_codin	23.4394479	24.0481751	13.2652255	29.2943113
ENSG000001	WNT10B	protein_codin	0	0	0	0
ENSG000001	KMT2D	protein_codin	499.387889	0	0	187.623922
ENSG000001	RHEBL1	protein_codin	0	0	0	0
ENSG000001	TUBA1A	protein_codin	367.498674	311.142569	194.887976	221.043551
ENSG000001	TUBA1C	protein_codin	23.571918	47.3056471	22.7784881	28.2625965
ENSG000001	ZNF524	protein_codin	38.1492245	56.2558999	11.2820768	35.3100657
ENSG000001	ZNF534	protein_codin	0	0	0	0
ENSG000001	ZNF701	protein_codin	11.0012408	30.1091143	7.31027084	0
ENSG000001	ZNF83	protein_codin	0	0	0	0
ENSG000002	CYP2A6	protein_codin	0	0	0	0
ENSG000001	CYP2A7	protein_codin	0	0	0	0
ENSG000001	GPD1	protein_codin	0	0	0	0
ENSG000002	LIN37	protein_codin	6.66289871	28.3273365	0	6.5828529
ENSG000001	PROSER3	protein_codin	0	9.96935893	4.91901161	0
ENSG000002	PRODH2	protein_codin	0	0	0	0
ENSG000001	AXL	protein_codin	0	0	0	0
ENSG000000	DIP2B	protein_codin	365.364672	318.040758	199.023591	295.843419
ENSG000001	DMAC2	protein_codin	0	0	0	1.91064211
ENSG000001	ANKRD33	protein_codin	0	0	0	0
ENSG000001	TTYH1	protein_codin	0	0	0	0
ENSG000001	CDC42EP5	protein_codin	0	0	0	0
ENSG000001	LAIR2	protein_codin	0	0.979055	0	0
ENSG000001	TMEM145	protein_codin	0	1.76517868	0	0
ENSG000001	ZNF526	protein_codin	33.8157256	41.9924228	14.173381	23.4092635
ENSG000001	GRIK5	protein_codin	2.41196097	7.32714723	2.60146603	0
ENSG000001	PPP1R14A	protein_codin	17.2892683	28.2854868	8.42760591	11.8802686
ENSG000001	SPINT2	protein_codin	143.533193	224.848402	74.4877026	79.157883
ENSG000001	C19orf33	protein_codin	15.0189887	69.399658	1.47810871	5.19963423
ENSG000001	PSCA	protein_codin	0	0	0	0
ENSG000001	LY6D	protein_codin	0	0	0	0
ENSG000001	DAPK3	protein_codin	50.8767511	68.6385405	22.2285868	30.6458292
ENSG000001	TMIGD2	protein_codin	0	0	0	0

ENSG000001	CHAF1A	protein_codin	118.440775	129.562433	58.653854	82.2805183
ENSG000001	UBXN6	protein_codin	169.675399	330.996295	68.5399321	178.210842
ENSG000001	PLIN4	protein_codin	0	0	0	0
ENSG000001	NLRP4	protein_codin	0	0	0	0
ENSG000001	SLC39A4	protein_codin	0	0	0	0
ENSG000000	ZNF582	protein_codin	8.32132785	0	2.97753296	1.04025577
ENSG000001	ZFP28	protein_codin	6.03529575	6.01435795	4.46944893	3.03110617
ENSG000001	RECQL4	protein_codin	1.42226291	0	0	0
ENSG000001	TLCD3A	protein_codin	7.41217315	3.1497616	0	0
ENSG000001	MFSD3	protein_codin	3.05849388	10.4115495	0.89077018	1.89583023
ENSG000001	GLOD4	protein_codin	0	0	0	0
ENSG000001	GLOD4	protein_codin	50.2309897	114.924108	38.6914383	34.6373278
ENSG000001	KIFC2	protein_codin	0	60.0328499	0	0
ENSG000001	SLC43A2	protein_codin	788.046872	606.101583	253.628548	333.332257
ENSG000001	RILP	protein_codin	4.4081807	36.1506138	5.07152743	18.6353443
ENSG000001	TSR1	protein_codin	95.1113532	138.779349	80.6177281	58.1546341
ENSG000001	TRPV3	protein_codin	0	0	0	0
ENSG000001	HSD11B1L	protein_codin	0	0	0	0
ENSG000001	CYB5D2	protein_codin	24.6957106	46.6115223	0	32.5209577
ENSG000001	GGT6	protein_codin	0	0	0	0
ENSG000001	PELP1	protein_codin	13.7081002	0	0	51.3612141
ENSG000001	ZNF577	protein_codin	0	0	0	0
ENSG000002	CGB1	protein_codin	0	0	0	0
ENSG000001	CGB5	protein_codin	0	0	0	0
ENSG000001	KLK1	protein_codin	1.8495781	0	0	0
ENSG000001	ACER1	protein_codin	0	0	0	0
ENSG000001	OTUB1	protein_codin	44.6447964	34.641718	16.8445955	12.9532526
ENSG000001	SLC25A23	protein_codin	0	5.59421722	19.7656288	2.84126731
ENSG000001	ANGPTL4	protein_codin	0	0	0	0
ENSG000002	NDUFA7	protein_codin	39.249379	41.7568339	7.87393584	25.8626182
ENSG000001	CD320	protein_codin	0	0	13.3792438	0
ENSG000001	RCOR2	protein_codin	0	0	0	0
ENSG000001	SPRYD3	protein_codin	82.2048552	102.390203	49.7784027	48.4552647
ENSG000001	IGFBP6	protein_codin	0	0	0	0
ENSG000001	SOAT2	protein_codin	0	0	0	0
ENSG000001	ZNF558	protein_codin	37.9073912	21.8645606	8.90791803	24.4670892
ENSG000001	ZNF560	protein_codin	0	0	0	0
ENSG000001	CDK2AP2	protein_codin	58.1383717	83.7820156	50.8643865	3.99111773
ENSG000001	NUDT8	protein_codin	0	0	0	0
ENSG000001	PRDX2	protein_codin	47.2390113	44.4143321	21.8272418	24.4200891
ENSG000001	OR5I1	protein_codin	0	0	0	0
ENSG000001	ZNF443	protein_codin	4.08842586	11.6099404	0	8.64494903
ENSG000001	CD300LF	protein_codin	8.16392839	114.597536	36.6026859	54.4792257
ENSG000001	MRPL58	protein_codin	28.1879262	52.1722588	23.9705283	27.8149355

ENSG000001	ATP5PD	protein_codin	178.644166	247.951822	125.109696	106.100215
ENSG000001	TMEM88	protein_codin	48.774056	19.7519432	12.7164957	15.7611385
ENSG000001	EVPL	protein_codin	0	1.4344019	0	0
ENSG000001	ACOX1	protein_codin	0	0	21.1991233	0
ENSG000001	MGAT5B	protein_codin	0	0	0	0
ENSG000000	BIRC5	protein_codin	0	0	12.1905445	0
ENSG000001	TK1	protein_codin	0.94510304	1.74182172	14.3640531	2.74638709
ENSG000001	CYP7A1	protein_codin	1.22160295	0	1.14919162	0
ENSG000001	KRT16	protein_codin	0	0	0	0
ENSG000001	KRT27	protein_codin	0	0	0	0
ENSG000001	GSDMA	protein_codin	0.90542085	0	0	0
ENSG000001	KRT10-AS1	lncRNA	0	0	0	0
ENSG000001	GHDC	protein_codin	4.72093732	46.8155951	0	0
ENSG000001	FAM234A	protein_codin	0	7.17956469	3.35221886	0
ENSG000001	FAM234A	protein_codin	0	0	0	0
ENSG000002	LINC00671	lncRNA	0	0	0	0
ENSG000001	METTL26	protein_codin	0	32.4795575	9.81506633	9.75687255
ENSG000001	SOST	protein_codin	0	0	0	0
ENSG000000	UNKL	protein_codin	28.4298568	21.3448934	10.8108495	13.5477507
ENSG000001	SPSB3	protein_codin	0	0	0	0
ENSG000001	MLST8	protein_codin	0	3.03427728	0	0
ENSG000001	E4F1	protein_codin	45.229647	80.3288381	47.17231	31.5381822
ENSG000001	ECI1	protein_codin	57.4295201	68.7370218	15.7880752	21.7075024
ENSG000002	RNPS1	protein_codin	0	0	0	0
ENSG000001	ABCA3	protein_codin	0	33.5906944	14.1673033	0
ENSG000001	KCTD5	protein_codin	58.4189623	96.600511	30.9882774	54.4085325
ENSG000001	SRRM2	protein_codin	5146.96267	4973.44507	1330.45842	2770.51806
ENSG000001	ZNF597	protein_codin	13.1580802	24.338718	7.07435314	14.4968155
ENSG000001	SDHAF2	protein_codin	0	27.1274758	26.2228865	0
ENSG000001	DDB1	protein_codin	219.310589	188.46872	187.074693	213.86313
ENSG000001	VPS37C	protein_codin	58.6718363	89.2616687	20.1961096	20.0232856
ENSG000001	VWCE	protein_codin	0	0	0	0.82184495
ENSG000001	RAB3IL1	protein_codin	0	0	0	0
ENSG000001	ASRGL1	protein_codin	25.0354421	53.4468801	13.8954666	16.1755926
ENSG000001	BSCL2	protein_codin	0	0	1.00983575	3.2547062
ENSG000002	HNRNPUL2	protein_codin	366.755006	496.282748	221.07728	185.6615
ENSG000001	POLR2G	protein_codin	45.7783047	79.1323748	37.7807805	36.1428902
ENSG000001	PLAAT5	protein_codin	0	0	0	0
ENSG000000	ACAA1	protein_codin	19.3225132	0	0	9.00677395
ENSG000001	RPSA	protein_codin	321.438108	148.689709	116.307437	62.9034386
ENSG000001	ENTPD3	protein_codin	0	1.95263347	1.09726662	0
ENSG000001	ULK4	protein_codin	39.6139454	32.35368	21.7739211	21.439079
ENSG000001	FADD	protein_codin	46.0288073	115.511339	23.3694698	41.3480664
ENSG000000	CTTN	protein_codin	34.8066984	59.3251422	93.6433732	29.1750782

ENSG000001	LTBP3	protein_codin	0	0	0	0
ENSG000002	ARL2-SNX15	protein_codin	0	0	0	0
ENSG000001	BATF2	protein_codin	0	17.3510622	5.64390327	0
ENSG000001	SLC22A11	protein_codin	0	0	0	0
ENSG000001	NRXN2	protein_codin	0	0	0	0
ENSG000001	MAJIN	protein_codin	0	0	0	0
ENSG000001	CCDC88B	protein_codin	19.6628607	0	0	0
ENSG000001	SCARA3	protein_codin	0	0	0	0
ENSG000001	PBK	protein_codin	0	0	4.66985779	0
ENSG000001	PNOC	protein_codin	0	0	0	0
ENSG000001	MSANTD4	protein_codin	35.6356699	17.6715084	14.5457334	11.2205858
ENSG000001	TNRC6B	protein_codin	323.478604	266.28572	113.553002	253.914523
ENSG000001	UBXN1	protein_codin	218.059722	331.690065	35.2652609	100.081987
ENSG000001	FBXW10	protein_codin	0	0	0	0
ENSG000001	NXNL1	protein_codin	0	0	0	0
ENSG000001	STEAP4	protein_codin	140.203597	128.830125	64.9043356	179.454909
ENSG000001	TADA3	protein_codin	236.885175	407.665055	187.705537	154.921407
ENSG000001	LRRC28	protein_codin	19.0883529	31.1356947	18.84078	22.2508207
ENSG000001	B3GNT2	protein_codin	103.708036	89.8720502	46.5099984	89.1975928
ENSG000001	PYGO1	protein_codin	0	0	0	1.84372604
ENSG000001	OGG1	protein_codin	0	0	0	0
ENSG000001	HSPB3	protein_codin	0.94042596	4.79337227	3.04851717	1.96502525
ENSG000001	IRX1	protein_codin	0	0	0	0
ENSG000001	OGG1	protein_codin	24.4903734	0	0	0
ENSG000001	CD14	protein_codin	968.950111	783.339666	183.175607	730.715415
ENSG000001	ZNF467	protein_codin	552.335228	596.572678	155.096182	270.066987
ENSG000001	SLAMF1	protein_codin	8.00497969	5.30322517	10.8535071	3.07005405
ENSG000001	OGG1	protein_codin	0	20.5720695	0	0
ENSG000001	OR5A2	protein_codin	0	0	0	0
ENSG000001	IRX2	protein_codin	0	0	0	0
ENSG000001	DNAJC18	protein_codin	9.48491852	7.10240403	5.50585445	1.13632162
ENSG000001	FGB	protein_codin	1.14121915	0	0	0
ENSG000000	SESN1	protein_codin	4.32181523	11.31695	2.23033662	8.31479225
ENSG000001	PLRG1	protein_codin	18.0822396	0	0	19.1926865
ENSG000001	PIEZO2	protein_codin	0	0	0	0
ENSG000001	SMAD1	protein_codin	44.8967806	12.5535181	4.00022989	27.0456549
ENSG000001	SPATA24	protein_codin	0	0	0	0
ENSG000001	LKAAEAR1	protein_codin	0	0	0	0
ENSG000001	ZNF280A	protein_codin	0	0.98910758	0	0
ENSG000001	NHLH1	protein_codin	4.94642855	3.8575904	1.80324516	0
ENSG000001	FUT9	protein_codin	6.4609531	0	0	0
ENSG000001	PCSK9	protein_codin	0	0	0	0
ENSG000001	OR8I2	protein_codin	0	0	0	0
ENSG000001	MZB1	protein_codin	35.2920396	33.4463683	174.510583	0

ENSG000001	KCND3	protein_codin	92.6432073	126.478803	60.1492231	0
ENSG000001	IRF2BP1	protein_codin	32.3285752	53.1853773	17.5539854	23.4202951
ENSG000001	NUDT9	protein_codin	31.4285441	37.1726887	11.5202163	4.97537852
ENSG000001	DCLK2	protein_codin	0	0	1.67759478	0
ENSG000001	FOXA3	protein_codin	0	0	0	0
ENSG000001	UBB	protein_codin	1045.91797	1587.13311	622.980742	728.682434
ENSG000001	RBKS	protein_codin	0	3.16010843	1.03972218	3.99351938
ENSG000001	SDC2	protein_codin	6.71746379	7.59253082	1.35952669	1.92261393
ENSG000001	TPM3	protein_codin	46.2275408	73.9198138	49.4445532	0
ENSG000001	ZC3H14	protein_codin	41.7729355	0	0	0
ENSG000001	HSD17B13	protein_codin	3.29059321	0	2.99329801	7.93518369
ENSG000001	LGALS9	protein_codin	19.1343898	1.99423941	4.72560469	11.0193811
ENSG000001	THAP9	protein_codin	14.801274	10.8395442	10.2004542	0
ENSG000001	DDX19A	protein_codin	130.064302	149.330332	70.9491988	52.5766577
ENSG000001	DEFB4A	protein_codin	0	0	0	0
ENSG000001	FAM151A	protein_codin	0	0	0	0
ENSG000001	PDGFD	protein_codin	0	0	0	0
ENSG000001	DDI1	protein_codin	0	0	0.87918191	0
ENSG000001	GAA	protein_codin	0	0	0	0
ENSG000001	OR8U1	protein_codin	0	0	0	0
ENSG000001	HNRNPR	protein_codin	0	0	0	0
ENSG000001	VPREB1	protein_codin	0	0	0	0
ENSG000001	ELOVL6	protein_codin	0	0	0	0
ENSG000001	ZNF804A	protein_codin	16.2315072	16.3153688	9.78416376	24.019973
ENSG000001	ITGB1	protein_codin	450.648599	1285.9133	265.257997	832.903506
ENSG000001	FNTA	protein_codin	114.134905	114.972951	52.1049472	74.4448145
ENSG000001	VSTM2A	protein_codin	0	0	0	0
ENSG000001	LUZP1	protein_codin	103.91672	127.785784	49.4306306	74.736109
ENSG000000	RFX3	protein_codin	3.741767	0	7.78455737	0
ENSG000001	AHSP	protein_codin	0	0	0	0
ENSG000001	WDR48	protein_codin	145.999166	130.825189	66.378084	86.6369014
ENSG000002	CCDC7	protein_codin	1.16905247	0	0	0
ENSG000001	MN1	protein_codin	64.3293363	46.0597812	10.1914357	16.9983012
ENSG000001	ING2	protein_codin	67.7369361	72.6271714	36.3054907	46.4335857
ENSG000001	SCN11A	protein_codin	3.20582131	0	0	0
ENSG000001	BFSP2	protein_codin	0	0	0	0
ENSG000001	ZNF217	protein_codin	0	121.588958	166.650353	233.573057
ENSG000001	CANT1	protein_codin	133.192296	254.099573	81.2739321	138.224609
ENSG000001	ITGB2	protein_codin	62.4010711	23.6982111	0	17.860371
ENSG000001	CDKN2AIP	protein_codin	7.47054625	40.9111734	21.9818599	10.5335374
ENSG000001	ENC1	protein_codin	36.1575878	73.9949137	10.3803641	41.2352966
ENSG000001	COPRS	protein_codin	25.329265	33.6032828	0	10.659517
ENSG000000	MPHOSPH9	protein_codin	20.5081648	64.1418775	46.4067054	29.2387265
ENSG000001	NME6	protein_codin	0	1.11094761	0	0

ENSG000001	GABARAP	protein_codin	373.501223	473.858799	20.0933452	202.427974
ENSG000001	TMEM42	protein_codin	11.0639311	10.244175	0	5.68860794
ENSG000000	SMARCA2	protein_codin	0	0	0	0
ENSG000001	KIF5B	protein_codin	1507.46426	1045.70688	972.058311	1114.26783
ENSG000002	TMEM256	protein_codin	37.422857	44.4029704	18.2142531	19.0453517
ENSG000001	TRIM8	protein_codin	131.61786	134.286566	54.4829405	35.5801402
ENSG000001	TRIAP1	protein_codin	0	1.88907621	2.5679775	0
ENSG000001	DMRT2	protein_codin	0	0	0	0
ENSG000000	KARS1	protein_codin	317.281905	455.964041	205.220433	189.739975
ENSG000001	CKAP2L	protein_codin	12.2718804	6.38761834	31.4112948	3.16553362
ENSG000001	PDILT	protein_codin	0	0	0	0
ENSG000001	PTGER4	protein_codin	453.572893	388.654916	97.8772983	218.968841
ENSG000001	MCC	protein_codin	0	3.10877901	16.2685341	2.11895364
ENSG000001	KCNAB1	protein_codin	1.17860591	0	0	0
ENSG000001	HTRA4	protein_codin	0	1.09576135	0	0
ENSG000001	CLCC1	protein_codin	0	0	0	0
ENSG000001	MTCH2	protein_codin	58.2361011	122.090649	46.3213175	53.3123252
ENSG000001	CDC25A	protein_codin	0	0	0	0
ENSG000001	C1QTNF4	protein_codin	3.7908816	0	0.63000288	0
ENSG000001	SF3B3	protein_codin	175.654585	225.190193	117.060968	116.486184
ENSG000001	CXXC5	protein_codin	74.5558804	153.794367	54.0198093	103.494616
ENSG000001	CLIP1	protein_codin	0	0	0	0
ENSG000001	PYM1	protein_codin	0	12.0353296	22.2717164	0
ENSG000001	VAT1L	protein_codin	0	0	0	0
ENSG000001	ABR	protein_codin	92.8889244	98.7138462	41.1426647	0
ENSG000000	ABCC8	protein_codin	0	0	0	0
ENSG000001	MYRIP	protein_codin	0	0	0	0
ENSG000001	GPR82	protein_codin	1.31364391	0	0	0
ENSG000001	DESI2	protein_codin	74.8272825	84.4448776	44.162349	54.1527192
ENSG000001	GP2	protein_codin	0	0	0	0
ENSG000001	RGPD8	protein_codin	123.300388	0	0	67.0040045
ENSG000001	OR5B12	protein_codin	0	0	0	0
ENSG000001	OR5B2	protein_codin	0	0	0	0
ENSG000001	NEUROD2	protein_codin	2.7572489	0	0	2.70264806
ENSG000001	KYAT1	protein_codin	0	0	0	0
ENSG000001	ZNF25	protein_codin	121.045679	57.1448688	45.0359605	61.9362446
ENSG000002	OR6Q1	protein_codin	0	0	0	0
ENSG000001	CDK5R2	protein_codin	0	0	0	0
ENSG000001	CALB2	protein_codin	0	0	0	0
ENSG000001	SCAMP3	protein_codin	8.03016687	64.4892155	6.61596563	43.8182042
ENSG000001	P2RY12	protein_codin	132.71704	147.30615	60.4578369	91.698605
ENSG000001	ITPR1	protein_codin	0	0	0	0
ENSG000001	PRSS27	protein_codin	1.82717774	0.91307247	2.12304622	0
ENSG000001	TRH	protein_codin	0	2.36810506	0	0

ENSG000001	OR2K2	protein_codin	0	0	0	0
ENSG000001	SLC25A33	protein_codin	29.7352124	49.9802784	14.729271	16.3125985
ENSG000001	ALKBH3	protein_codin	23.8043715	23.1155279	6.47726882	6.94143017
ENSG000001	CLP1	protein_codin	0	36.067504	0	17.1030462
ENSG000001	PDHB	protein_codin	123.463048	144.445339	38.3864944	86.2166151
ENSG000001	JUNB	protein_codin	7053.72503	8528.72386	2964.57786	5794.9006
ENSG000001	ANKRD49	protein_codin	0	0	0	0
ENSG000001	BUB1	protein_codin	0	0	65.9245942	0
ENSG000000	CTNNA1	protein_codin	728.409997	1046.09475	399.553036	529.886021
ENSG000001	NUDCD2	protein_codin	8.86104965	0	1.06677971	8.11825342
ENSG000001	PXK	protein_codin	0	0	8.11966658	4.04237162
ENSG000001	TTC36	protein_codin	0.83492983	1.66236444	0.85763281	0
ENSG000001	LETM1	protein_codin	181.421829	261.696751	98.4779913	124.57592
ENSG000001	MRGPRX1	protein_codin	0	0	0	0
ENSG000001	PALM2AKAP2	protein_codin	0	0	0	0
ENSG000001	AURKC	protein_codin	0	0	0	0
ENSG000001	SPRY3	protein_codin	31.289288	0	0	0
ENSG000000	FRYL	protein_codin	33.8724872	5.20279873	13.3448737	7.82962234
ENSG000001	ACOX2	protein_codin	0	0	0	0
ENSG000001	STARD5	protein_codin	18.8528064	2.78852682	9.32273033	1.19487205
ENSG000001	INSR	protein_codin	121.457554	139.9344	35.0149208	67.3865595
ENSG000001	ZNF561	protein_codin	0	37.7833303	0	12.5073728
ENSG000001	ZCCHC4	protein_codin	21.6026203	6.26465013	8.31464688	9.9981493
ENSG000001	UBTF	protein_codin	19.2559318	0	0	0
ENSG000001	RPS9	protein_codin	630.780091	412.922451	132.586162	335.014688
ENSG000001	KCNK3	protein_codin	0	0	0	0
ENSG000000	ACHE	protein_codin	0	0	0	0
ENSG000001	TEX13B	protein_codin	0	0.76549107	0	0
ENSG000001	NLGN2	protein_codin	3.05387997	1.69090344	0	0
ENSG000001	TSEN34	protein_codin	16.3377636	24.302736	12.4135722	38.7370382
ENSG000001	GARIN3	protein_codin	0	0	0	0
ENSG000001	GSX1	protein_codin	0	0	0	0
ENSG000001	SLFNL1	protein_codin	0	0	0	0
ENSG000001	DLG4	protein_codin	162.141981	200.083823	84.1742267	110.600847
ENSG000001	AMDHD2	protein_codin	0	20.8973895	33.2084728	27.2838983
ENSG000001	PYDC1	protein_codin	0	0	0	0
ENSG000001	POLR1D	protein_codin	73.3447175	114.867711	52.516079	40.5511275
ENSG000001	IL16	protein_codin	115.814416	241.536706	70.6498752	83.686907
ENSG000001	ZBTB46	protein_codin	11.4973795	0	8.04164624	4.00249366
ENSG000001	CEBPB	protein_codin	1398.46664	1640.00748	334.977141	841.676119
ENSG000001	KCNK9	protein_codin	0.91867954	0	0.5878621	0
ENSG000000	ARHGAP6	protein_codin	0	0	0	0
ENSG000001	CTRB2	protein_codin	0	0	0	0
ENSG000001	OR8J1	protein_codin	0	0	0	0

ENSG000001	COL22A1	protein_codin	0	0	0	0
ENSG000001	CSNK1G1	protein_codin	168.764086	172.864516	74.3641963	93.687844
ENSG000001	ENTREP1	protein_codin	0	0	0	0
ENSG000001	DONSON	protein_codin	15.7909773	18.1507188	0	0
ENSG000001	SIX2	protein_codin	0	0	0.72738589	0
ENSG000000	RFXANK	protein_codin	0	0	0	15.2040936
ENSG000001	ARL13B	protein_codin	0	0	0	0
ENSG000001	DONSON	protein_codin	3.95217828	0	0	0
ENSG000001	IL7R	protein_codin	37.7165184	56.5229358	19.5620364	7.35587104
ENSG000001	LMAN2	protein_codin	260.048218	378.12148	238.122564	204.039112
ENSG000001	ARHGAP42	protein_codin	0	0	0	0
ENSG000001	SHLD1	protein_codin	6.70540782	3.1947934	0	0
ENSG000001	COL8A2	protein_codin	0	40.0417006	0	0
ENSG000001	MDM1	protein_codin	23.9261703	0	0	0
ENSG000001	POP7	protein_codin	23.520914	35.1187021	14.2588588	25.1443427
ENSG000001	NETO2	protein_codin	36.4316812	23.986699	7.65417576	20.4347274
ENSG000001	NSG2	protein_codin	0	0	0	0
ENSG000001	PPDPFL	protein_codin	0	0	0	0
ENSG000001	PRELID1	protein_codin	660.59533	937.330578	420.471957	408.137926
ENSG000001	GNB2	protein_codin	298.702567	356.22115	91.0525461	167.95799
ENSG000001	NRTN	protein_codin	0	0	0	0
ENSG000001	EMB	protein_codin	388.965877	239.057676	160.475413	297.50674
ENSG000001	FUT3	protein_codin	0	0	0	0
ENSG000001	GLOD5	protein_codin	0	0	0	0
ENSG000001	HCN1	protein_codin	0	0	0	0
ENSG000001	EIF2AK3	protein_codin	183.082802	65.5644982	99.9672201	34.3028415
ENSG000001	RAB24	protein_codin	89.7139044	0	59.4739583	75.7569267
ENSG000001	SPMIP9	protein_codin	0	0.88686599	0	0
ENSG000001	RAB24	protein_codin	0	0	0	0
ENSG000001	TMEM129	protein_codin	0	0	11.5612169	3.91526662
ENSG000001	HIPK3	protein_codin	1143.02136	674.231649	402.122633	718.220017
ENSG000001	PA2G4	protein_codin	186.272306	247.641785	96.2809605	173.484823
ENSG000001	ZSCAN2	protein_codin	0	0	0.91336415	0
ENSG000001	MMADHC	protein_codin	64.2549929	95.7612753	47.875204	47.5167475
ENSG000001	OR2B2	protein_codin	1.20512978	0	0	0
ENSG000001	ARNT2	protein_codin	0	0	0	0
ENSG000001	CES3	protein_codin	0	0	0	0
ENSG000001	SLC50A1	protein_codin	0	0	0	0
ENSG000001	SCN9A	protein_codin	0	0	0	0
ENSG000001	TCTN2	protein_codin	0	0	0	0
ENSG000000	MRC2	protein_codin	0	1.18346026	0	0
ENSG000001	SHCBP1	protein_codin	11.4892579	1.68253544	33.2691482	8.54502593
ENSG000001	MECP2	protein_codin	0	0	0	0
ENSG000001	SCN1A	protein_codin	0	0	0	0

ENSG00000009	MAEA	protein_codin	0	171.896941	10.1131763	70.4759263
ENSG0000019	HOXC4	protein_codin	2.36923159	0	0	0
ENSG0000010	BRD3	protein_codin	517.667954	862.040867	233.338739	410.695943
ENSG0000000	SIRPG	protein_codin	0	0	0	0
ENSG0000017	TRABD	protein_codin	28.1973105	51.3563011	68.1271974	10.1152117
ENSG0000010	ARF4	protein_codin	231.355788	290.850956	101.982095	188.283537
ENSG0000018	HOXC9	protein_codin	0	0	0	0
ENSG0000010	METTL15	protein_codin	29.9958046	0	13.8045216	0
ENSG0000018	HOXC10	protein_codin	0	0	0	0
ENSG0000017	METTL18	protein_codin	2.45159591	2.24670631	6.59544476	0
ENSG0000010	BTD	protein_codin	2.83647478	0	2.0597004	1.68717325
ENSG0000014	SCAPER	protein_codin	51.4416527	41.4429323	25.8747468	13.1745167
ENSG0000017	ACYP2	protein_codin	0	0	1.09502116	6.19487946
ENSG0000012	PDS5A	protein_codin	761.665634	455.333385	407.203223	509.536646
ENSG0000017	RNF139	protein_codin	159.099895	0	0	0
ENSG0000017	NDUFA3	protein_codin	0	0	0	0
ENSG0000017	FOS	protein_codin	15066.0741	11955.251	5244.66653	12651.0772
ENSG0000010	CRTC2	protein_codin	26.856678	12.0948559	0	27.8413581
ENSG0000017	TMED10	protein_codin	218.985311	331.121329	161.538231	255.722934
ENSG0000010	PCBP1	protein_codin	1410.21447	1931.34325	712.723216	994.388663
ENSG0000010	ZNF30	protein_codin	1.0691005	3.19990078	0	0
ENSG0000010	KCNJ4	protein_codin	0.83411671	0.84092538	0	0
ENSG0000010	THAP11	protein_codin	105.537009	124.636402	25.5391645	75.1113964
ENSG0000014	AUH	protein_codin	33.3944384	16.1252522	0	0
ENSG0000007	MARK3	protein_codin	0	0	0	6.26484037
ENSG0000017	CAMTA1	protein_codin	5.10069075	5.45979686	2.87497253	4.12345168
ENSG0000017	MRAP	protein_codin	0	0	0	0
ENSG0000008	RFX2	protein_codin	188.28042	41.3341748	23.8155376	0
ENSG0000013	DTNB	protein_codin	0	0	0	0
ENSG0000010	ZEB2	protein_codin	70.0990449	106.353367	0	0
ENSG0000014	ARHGAP17	protein_codin	178.298555	218.007737	98.3005815	128.976159
ENSG0000013	CCDC174	protein_codin	0	30.5448189	6.64780849	2.51326037
ENSG0000017	CHST11	protein_codin	176.042513	393.596855	106.07191	61.1204388
ENSG0000017	DCDC1	protein_codin	0	0	0	0
ENSG0000010	NFU1	protein_codin	12.3260642	8.55360405	0	0
ENSG0000010	ANTXR1	protein_codin	0	2.05224908	0	1.04226088
ENSG0000017	FGGY	protein_codin	0	0	8.97444048	0
ENSG0000010	PRY	protein_codin	0	0	0	0
ENSG0000017	TRAPPC1	protein_codin	74.8041608	89.0971306	71.1641332	59.3505222
ENSG0000010	SLC38A11	protein_codin	0	0	0	0
ENSG0000013	KREMEN2	protein_codin	0	0	0	0
ENSG0000017	SDR16C5	protein_codin	0	0	0	0
ENSG0000020	LST1	protein_codin	43.6948542	28.9043707	11.6645832	0
ENSG0000017	HMGN1P35	processed_ps	0	0	0	0

ENSG000001	CHCHD7	protein_codin	36.348563	45.4816528	44.6722184	0
ENSG000001	RBMY1F	protein_codin	0	0	0	0
ENSG000001	N6AMT1	protein_codin	0	0	0	3.51746144
ENSG000001	PROKR1	protein_codin	1.04203502	0	0	0
ENSG000001	KCNAB3	protein_codin	12.9690475	4.95731632	1.81410437	6.94210506
ENSG000001	APLF	protein_codin	84.967024	168.273858	48.9340641	50.2356799
ENSG000001	PRY2	protein_codin	0	0	0	0
ENSG000001	ZNF296	protein_codin	21.7045269	49.1669232	9.0253282	19.4970381
ENSG000001	EYA1	protein_codin	0	0	0	0
ENSG000001	REPS2	protein_codin	0	0	49.2286445	55.7373192
ENSG000001	SP7	protein_codin	0	0	0	0
ENSG000001	WDR87	protein_codin	0.81120645	0	0	0
ENSG000001	MCM7	protein_codin	83.6972137	106.201334	129.285244	22.741559
ENSG000002	ATP6V1G2	protein_codin	0	0	0	0
ENSG000001	SYCE1	protein_codin	0	0	0	0
ENSG000001	COPS6	protein_codin	79.1202949	157.977931	41.5415445	75.0677197
ENSG000002	H2AC8	protein_codin	154.909723	387.457132	231.14043	148.664989
ENSG000001	GPR37	protein_codin	0	0	0	0
ENSG000002	RBMY2FP	lncRNA	0	0	0	0
ENSG000001	HAS2	protein_codin	0	0	0	0
ENSG000001	CAVIN3	protein_codin	5.13070807	10.0623178	0	3.51639875
ENSG000001	ANKK1	protein_codin	0	0	0	0
ENSG000002	EGLN2	protein_codin	157.636457	228.155552	90.8092287	70.1882585
ENSG000000	ARAP2	protein_codin	237.435299	157.281085	148.942919	214.425465
ENSG000001	TSPY14P	unprocessed_	0	0	0	0
ENSG000001	GPRIN1	protein_codin	3.81144724	10.5162303	3.07743831	2.79878771
ENSG000000	JMJD6	protein_codin	0	0	0	0
ENSG000001	POLR1C	protein_codin	0	0	3.07159674	0
ENSG000001	ZNF439	protein_codin	7.01359641	21.7230699	0	0
ENSG000001	HS1BP3	protein_codin	100.025837	109.789606	34.9678923	66.3394449
ENSG000001	AGAP1	protein_codin	2.87661178	1.12631226	0	0
ENSG000001	HINT1	protein_codin	99.759268	120.403331	42.226957	61.4069999
ENSG000001	KLK7	protein_codin	0	0	0	0
ENSG000001	ORMDL3	protein_codin	74.2451438	103.747023	131.136925	49.7807495
ENSG000001	NTRK2	protein_codin	0	0	0	0
ENSG000001	KBTBD2	protein_codin	252.855606	259.683126	143.444892	132.439146
ENSG000001	DTWD2	protein_codin	11.9601523	14.0911179	8.65282785	0
ENSG000001	ZNF440	protein_codin	40.7666024	21.894792	23.1682985	23.4746797
ENSG000001	RIOX1	protein_codin	19.5025154	55.2629895	14.243032	22.8561108
ENSG000001	CPLX1	protein_codin	0	0	0	0
ENSG000001	VAV1	protein_codin	0	0	50.6018666	0
ENSG000001	NT5C1B	protein_codin	0	0	0	0
ENSG000001	CLEC7A	protein_codin	0	0	0	0
ENSG000001	KCNS3	protein_codin	0	0	0	1.06434487

ENSG000001	OR7A2P	transcribed_u	0	0	0	0
ENSG000001	ZNF565	protein_codin	6.86711806	5.45688173	2.05919706	0
ENSG000001	AFAP1L2	protein_codin	13.0665671	29.4000986	24.2276738	18.6449074
ENSG000001	TTBK1	protein_codin	0	0	0	0
ENSG000001	CAVIN2	protein_codin	4395.08716	6393.69393	5161.98281	3117.01653
ENSG000001	MYO1B	protein_codin	0	0	0	0
ENSG000000	ADCYAP1R1	protein_codin	0	0	0	0
ENSG000001	C15orf40	protein_codin	21.7758303	29.5227022	13.1765463	7.56003313
ENSG000001	FAM110A	protein_codin	9.75268044	0	0	0
ENSG000001	RAMAC	protein_codin	38.1823877	39.1046401	15.6706911	18.8914023
ENSG000001	FRMD3	protein_codin	116.772697	211.430477	9.90048711	28.3852868
ENSG000000	GIPR	protein_codin	0	0	0.65294284	0
ENSG000001	H1-4	protein_codin	965.946886	1146.95733	985.507125	735.009722
ENSG000001	ADORA2B	protein_codin	11.3082989	10.5395791	4.35645908	7.93101634
ENSG000001	HOMER2	protein_codin	35.9833783	23.2541383	7.81443329	0
ENSG000001	LINC00208	lncRNA	0	0	0	0
ENSG000001	RAB40A	protein_codin	0	0	0	0
ENSG000001	ZNF420	protein_codin	0	0	7.79351612	0
ENSG000001	GPR25	protein_codin	1.04210301	0	2.89114354	0.97922144
ENSG000000	SEC61A2	protein_codin	3.11312847	3.29649976	5.26401698	1.37958697
ENSG000001	SPACA5B	protein_codin	2.23212175	0	0	0
ENSG000001	C11orf24	protein_codin	60.7150596	81.8887188	38.455142	34.9985487
ENSG000001	ANKS3	protein_codin	0	0	0	0
ENSG000001	HSPB2	protein_codin	0	0	0	0
ENSG000001	NUDT16L1	protein_codin	0	42.0316712	11.7683108	9.77791105
ENSG000001	ATP5ME	protein_codin	128.71996	191.35164	106.749215	83.2018203
ENSG000001	RPAP1	protein_codin	31.7781836	29.7835911	23.441145	25.9146895
ENSG000001	MACROH2A1	protein_codin	96.7469117	147.684021	13.5234601	5.8807663
ENSG000001	TIGD4	protein_codin	0	3.53974155	1.27887462	1.20630418
ENSG000001	PPP4R4	protein_codin	4.31040898	0	0	0
ENSG000001	SPACA5	protein_codin	0	0	0	0
ENSG000001	CLEC12A	protein_codin	277.918288	304.759341	47.2169805	64.573182
ENSG000001	KMT5B	protein_codin	27.3067995	35.3911236	2.16318858	13.0048649
ENSG000001	KCTD19	protein_codin	0	1.66733191	1.05424855	0
ENSG000001	DRD5	protein_codin	0	0	0	0
ENSG000001	TMC7	protein_codin	0	0	0	0
ENSG000001	TMEM154	protein_codin	271.398054	241.248574	128.318665	207.478397
ENSG000002	DISP3	protein_codin	0	0	0	0
ENSG000001	PAM	protein_codin	0	0	0	0
ENSG000001	GPR22	protein_codin	1.52106136	0	0	0
ENSG000001	GPR27	protein_codin	25.6715932	36.8473538	3.16967047	20.3841581
ENSG000001	ARL6IP1	protein_codin	171.953434	239.667369	113.888057	180.689006
ENSG000001	OR5AU1	protein_codin	0	0	0	0
ENSG000001	FSHR	protein_codin	0	0	0	0

ENSG000001	MIR31HG	lncRNA	0	0	0	0
ENSG000001	NUDT6	protein_codin	4.33552842	4.57604376	0	0
ENSG000000	ELOVL5	protein_codin	324.826684	429.419746	170.688705	296.365719
ENSG000001	NKRF	protein_codin	24.1821915	43.1569187	11.5358876	0
ENSG000001	EXOSC10	protein_codin	0	0	20.0601311	0
ENSG000001	SLC6A19	protein_codin	0	0	0	0
ENSG000001	SRGAP2C	protein_codin	0	0	0	0
ENSG000001	UTF1	protein_codin	5.37820014	3.02694167	0	1.7523257
ENSG000001	MRM3	protein_codin	37.4098652	56.2617865	12.4873086	23.5690887
ENSG000001	CDKN2A	protein_codin	0	0	0	0
ENSG000001	SOX7	protein_codin	0	0	0	0.9310734
ENSG000001	CRYGA	protein_codin	0	0	0	0
ENSG000001	IL13	protein_codin	1.96782374	0	0	0
ENSG000001	TMEM126A	protein_codin	16.6192238	32.5331295	0	7.44878286
ENSG000001	RGPD3	protein_codin	7.02812545	7.47024387	1.8595286	3.28980938
ENSG000001	INO80E	protein_codin	0	7.14354558	1.76007016	1.68892787
ENSG000001	C8orf74	protein_codin	0	0	0	0
ENSG000001	LDB2	protein_codin	0	0	0	0
ENSG000001	INSL5	protein_codin	0	0	0	0
ENSG000001	PRDM10	protein_codin	0	0	0	52.9239532
ENSG000000	ZFX	protein_codin	0	0	0	0
ENSG000001	ECEL1	protein_codin	0	0	0	0
ENSG000001	CXCR6	protein_codin	0	0	0	0
ENSG000001	RRM2	protein_codin	0	10.0333825	0	0
ENSG000001	TM4SF20	protein_codin	1.43579329	0	1.29895365	0
ENSG000000	PLEKHG6	protein_codin	0	0	0	0
ENSG000001	MFF	protein_codin	0	0	4.0252185	12.1488952
ENSG000001	PEX6	protein_codin	29.8855274	65.6220849	34.6808732	18.5223748
ENSG000001	KNDC1	protein_codin	3.12237409	3.75465106	0	0
ENSG000001	KRT78	protein_codin	0	0	0	0
ENSG000001	TTC39C	protein_codin	21.723686	0	8.26154677	20.4960956
ENSG000001	CTNND2	protein_codin	0	0	0	0
ENSG000001	RNASE2	protein_codin	215.892093	162.704469	40.9144818	206.432329
ENSG000001	COL3A1	protein_codin	0	0	0	0
ENSG000001	RNASE3	protein_codin	10.3766415	6.0378483	0	3.08143307
ENSG000001	OR1F1	protein_codin	0	0	0	0
ENSG000001	XPO6	protein_codin	176.338419	348.088114	47.3988991	75.2020302
ENSG000001	C1GALT1C1	protein_codin	26.9293167	48.519269	34.0202278	33.7016855
ENSG000001	PTCRA	protein_codin	8.88498595	11.4304862	9.51864855	6.94868057
ENSG000001	RNASE6	protein_codin	192.611505	242.560548	85.824922	164.984066
ENSG000001	RGL1	protein_codin	2.59887023	0	4.72064428	0
ENSG000001	FAM171B	protein_codin	3.21756469	0	2.15751426	2.23703609
ENSG000001	CST5	protein_codin	0	0	0	0
ENSG000000	AAMDC	protein_codin	0	0	0	1.68732438

ENSG000001	OR1L6	protein_codin	0	0	0	0
ENSG000001	CST2	protein_codin	0	0	0	0.76305201
ENSG000000	DBNDD1	protein_codin	0	0	0	0
ENSG000001	RPL7L1	protein_codin	0	14.9058057	0	0
ENSG000001	VASN	protein_codin	0	0.90262867	1.07216561	0
ENSG000001	PCNX1	protein_codin	243.603967	195.174032	106.312403	171.718101
ENSG000001	FPR1	protein_codin	854.144213	1070.39655	193.596933	708.540713
ENSG000001	CST1	protein_codin	0	0	0	0
ENSG000002	LINC02449	lncRNA	0	0	0	0
ENSG000001	AMOT	protein_codin	0	7.5247287	0	0
ENSG000001	SHE	protein_codin	8.07407824	14.1210985	1.83785098	0.99242255
ENSG000001	MAP6	protein_codin	0	0	0	0
ENSG000000	GLP2R	protein_codin	0	0	0	0
ENSG000001	DCC	protein_codin	0.9583385	0	0	0
ENSG000001	LONRF3	protein_codin	21.03873	0	0	0
ENSG000001	SRP9	protein_codin	223.144909	197.60222	137.736657	158.367944
ENSG000001	NANP	protein_codin	13.1893213	12.1399482	9.65223224	9.28542095
ENSG000001	HSFY2	protein_codin	0	0	0	0
ENSG000001	TMEM208	protein_codin	31.0191258	38.2112151	19.7980958	28.2909568
ENSG000001	PGK2	protein_codin	0	0	0	0
ENSG000001	PAFAH1B2	protein_codin	11.4901221	0	19.6963955	0
ENSG000001	OR1L3	protein_codin	0	0	0	0
ENSG000001	JPT1	protein_codin	0	6.06561949	1.69762589	6.57988165
ENSG000001	TPST1	protein_codin	0	13.0525284	0	0
ENSG000001	HSPA4	protein_codin	348.227452	356.281139	292.360826	322.512711
ENSG000001	UQCRFS1	protein_codin	171.464663	262.581185	91.2865709	138.287434
ENSG000001	ASL	protein_codin	15.795292	0	24.0433205	19.656598
ENSG000001	TPD52L1	protein_codin	0	0	0	0
ENSG000001	OR1N1	protein_codin	0	0	0	0
ENSG000001	TACR3	protein_codin	0	1.38403532	0	1.41004589
ENSG000001	SNAP25	protein_codin	0	0	1.25500034	0
ENSG000001	MUC7	protein_codin	0	0	1.20848081	0
ENSG000001	GUSB	protein_codin	185.85905	247.693011	78.5186619	137.786909
ENSG000001	SLFN12	protein_codin	0	0	0	0
ENSG000001	SMR3B	protein_codin	0	0	0	0
ENSG000001	LRATD2	protein_codin	4.46262797	4.53457765	0	0
ENSG000001	IL17D	protein_codin	0	0	0.58424247	0
ENSG000001	ZSCAN32	protein_codin	9.65340584	0	0	0
ENSG000002	SUGT1P3	lncRNA	0	0	0	0
ENSG000001	OR2C1	protein_codin	0	0	0	0
ENSG000001	HES4	protein_codin	0	0.86733626	0	0
ENSG000001	CSN3	protein_codin	0	0	0	0
ENSG000001	PPIH	protein_codin	14.9205912	16.8529734	6.0667224	5.16975714
ENSG000001	SIK2	protein_codin	180.477743	237.204859	82.7861112	82.9833987

ENSG000001	COL4A3	protein_codin	0	0	0	0
ENSG000001	PRPF8	protein_codin	1249.00922	1686.88588	773.031005	975.192978
ENSG000001	CHSY3	protein_codin	0	0	0	0.95161184
ENSG000001	ADH1B	protein_codin	0	0	0	0
ENSG000001	OR4K15	protein_codin	0	0	0	0
ENSG000001	REG1B	protein_codin	0	0	0	0
ENSG000001	P2RY1	protein_codin	100.942851	111.81484	86.1585618	61.0510246
ENSG000001	COMMD5	protein_codin	0	15.3610221	19.3188116	0
ENSG000001	ZNHIT1	protein_codin	90.2583671	154.379337	37.3873644	84.2900038
ENSG000001	UGT2B4	protein_codin	0	0	0	0
ENSG000001	NINJ2	protein_codin	6.10446167	11.3261383	1.92061314	4.82546161
ENSG000002	MUC12	protein_codin	0	0	0	0
ENSG000001	IRS1	protein_codin	108.442533	132.772786	116.15375	40.8677736
ENSG000001	GAP43	protein_codin	0	0	0	0
ENSG000001	YEATS2	protein_codin	196.460014	292.742217	173.097444	128.388251
ENSG000001	RAD17	protein_codin	0	0	0	0
ENSG000001	UGT1A6	protein_codin	0	0	0	0
ENSG000001	NMUR1	protein_codin	0.97288345	3.93163981	0	0
ENSG000001	OR2AT4	protein_codin	0	0	0	0
ENSG000001	REG3A	protein_codin	0	0	0	0
ENSG000001	ESCO2	protein_codin	0	7.07909346	7.88073568	0
ENSG000001	C22orf15	protein_codin	0	0	0	0
ENSG000002	UGT1A1	protein_codin	0	0	0	0
ENSG000001	STIP1	protein_codin	155.5844	336.739715	148.586163	134.338
ENSG000001	UGT2B7	protein_codin	0	0	0	0
ENSG000000	SIRT6	protein_codin	0	0	0	0
ENSG000001	PWWP2B	protein_codin	159.235045	140.874552	14.3782427	46.7211672
ENSG000001	ODAD2	protein_codin	0	0	0	0
ENSG000002	FAM138C	lncRNA	0	0	0	0
ENSG000001	TACR1	protein_codin	0	0	0	0
ENSG000001	TUB	protein_codin	0	0	0	0
ENSG000001	HDAC3	protein_codin	114.723756	167.809026	62.9909121	68.1711057
ENSG000001	EFCAB3	protein_codin	0	0	0	0
ENSG000001	BOLA2B	protein_codin	1.21171711	2.53352569	0	4.02003906
ENSG000001	HTR1E	protein_codin	0	1.02299803	1.13980085	0
ENSG000001	S1PR1	protein_codin	2.7454704	8.58699779	8.8326189	0
ENSG000001	TM4SF4	protein_codin	0	0	0	0
ENSG000000	TMPRSS11E	protein_codin	0	0	0	0
ENSG000001	TRIM35	protein_codin	47.9143625	104.535885	30.1828302	28.9755493
ENSG000001	TM4SF1	protein_codin	1.22857451	18.8736726	6.29250984	0
ENSG000001	EPB41L4A	protein_codin	0	0	2.93690256	0
ENSG000001	MGAT2	protein_codin	73.225239	142.161431	50.4739022	64.0354823
ENSG000001	PTAFR	protein_codin	0	0	0	0
ENSG000001	SCG2	protein_codin	0	0	0	0

ENSG000001	FGF13	protein_codin	0	0	1.11427905	1.44201073
ENSG000001	MINAR1	protein_codin	0	0	0	0
ENSG000001	GRM5	protein_codin	0	0.91897447	0	0
ENSG000001	OR7G3	protein_codin	0	0	0	0
ENSG000001	GRM5	protein_codin	0	0	0	0
ENSG000001	VXN	protein_codin	0	0	0	0
ENSG000001	SEMA4C	protein_codin	0	0	3.59268998	2.79535676
ENSG000001	WFDC13	protein_codin	0	0	0	0
ENSG000001	USP47	protein_codin	2.94481168	0	0	0
ENSG000002	ANKRD39P1	processed_ps	0	0	0	0
ENSG000001	TMEM135	protein_codin	27.6049876	21.5829801	26.0052255	29.4673479
ENSG000001	CNNM3	protein_codin	61.7422037	86.1621363	23.2022908	41.1571188
ENSG000001	SMYD4	protein_codin	30.7615757	42.1021282	25.1117283	16.4167565
ENSG000001	FRMPD2	protein_codin	0	0	0	0
ENSG000002	TWF2	protein_codin	316.764058	460.455688	129.053519	274.238051
ENSG000001	RBMX2	protein_codin	115.025914	154.437394	66.6049164	64.1586642
ENSG000001	LAMB2	protein_codin	37.5640744	59.172455	23.5801261	21.3254027
ENSG000001	RTKN	protein_codin	0	0	0	0
ENSG000001	AFG2B	protein_codin	8.55129672	25.0542269	0	0
ENSG000001	ZNF100	protein_codin	21.0690012	27.8773828	19.9000565	15.3868648
ENSG000001	NPNT	protein_codin	0	0	0	0
ENSG000001	IL12A	protein_codin	3.04318471	0	0	0
ENSG000001	FXR1	protein_codin	15.8796831	51.7319083	24.9794073	42.2966735
ENSG000001	CD2BP2	protein_codin	44.6889378	91.6160115	37.2817477	42.7105932
ENSG000001	KRT74	protein_codin	0	0	0	0
ENSG000002	AKR1C7P	transcribed_u	0	0	0	0
ENSG000001	MBD3L1	protein_codin	0	0	0	0
ENSG000001	RAB33B	protein_codin	48.2100354	56.008419	0	31.1488103
ENSG000001	SIGLEC7	protein_codin	30.6493835	0	0	0
ENSG000001	DEGS2	protein_codin	0	0	0	1.70589449
ENSG000001	TBL2	protein_codin	27.6498066	36.4464759	7.32504944	1.70708394
ENSG000001	LCMT2	protein_codin	4.7736658	16.6462948	0	4.33265778
ENSG000001	GLYCTK	protein_codin	0	0	0	0
ENSG000001	FANCA	protein_codin	15.7238273	19.3380927	9.82489851	16.3353066
ENSG000001	FAM88B	lncRNA	0	0	0	0
ENSG000001	TET2	protein_codin	438.161491	171.409614	163.850733	295.664089
ENSG000001	PATE1	protein_codin	0	0	0	0
ENSG000001	CERS6	protein_codin	236.412706	159.892612	166.675621	130.48982
ENSG000001	KRT73	protein_codin	0	0.91673128	0	0
ENSG000001	FAM98B	protein_codin	0	0	0	0
ENSG000001	OR5E1P	transcribed_u	0	0	0	0
ENSG000001	CAMK2G	protein_codin	0	0	0	0
ENSG000001	PHACTR2	protein_codin	0	0	0	0
ENSG000001	CEP89	protein_codin	8.72146326	9.54917307	16.2585856	4.10843985

ENSG000001[illegible]	RNFT1	protein_codin	0	0	26.5890604	0
ENSG000001[illegible]	DTYMK	protein_codin	15.5473807	20.5417638	14.6451963	7.66914955
ENSG000001[illegible]	CYCS	protein_codin	309.915476	301.196183	148.569976	191.76898
ENSG000001[illegible]	TSPAN5	protein_codin	0	27.1267747	10.6027708	0
ENSG000001[illegible]	TET3	protein_codin	0	0	61.4968008	20.1111884
ENSG000001[illegible]	SLC9C1	protein_codin	0	0	0	0
ENSG000001[illegible]	PRND	protein_codin	0	1.27114751	0	0
ENSG000001[illegible]	CENPN	protein_codin	2.61595758	10.8386016	10.2120966	6.8815853
ENSG000001[illegible]	FEZ2	protein_codin	0	0	0	12.1323166
ENSG000001[illegible]	EMCN	protein_codin	4.99186256	0	0	0
ENSG000000[illegible]	VPS50	protein_codin	12.785679	20.3627876	0	15.2369757
ENSG000001[illegible]	CCL11	protein_codin	0	0	0	0
ENSG000001[illegible]	BCR	protein_codin	154.788767	388.84496	138.596863	177.487989
ENSG000001[illegible]	GTSF1	protein_codin	6.02388237	0	4.2893311	0
ENSG000001[illegible]	KLF11	protein_codin	291.83578	433.390214	168.728499	268.700619
ENSG000001[illegible]	FEN1	protein_codin	16.3076562	35.566304	48.2941062	12.2112429
ENSG000001[illegible]	OR10H2	protein_codin	0	0	0	0
ENSG000001[illegible]	ONECUT1	protein_codin	0	1.05784177	0	0
ENSG000001[illegible]	DYNLRB2	protein_codin	0	0	0	0
ENSG000001[illegible]	PPM1D	protein_codin	0	0	0	21.4730299
ENSG000001[illegible]	RNF187	protein_codin	214.922315	340.14309	71.2659459	149.24758
ENSG000001[illegible]	MTBP	protein_codin	3.5028642	0	8.71360217	8.91193018
ENSG000000[illegible]	SMOX	protein_codin	82.5712719	235.608586	100.916082	28.9976444
ENSG000001[illegible]	MMGT1	protein_codin	84.11795	30.7520261	45.6602711	84.3688783
ENSG000001[illegible]	SCAND1	protein_codin	95.1419751	159.371576	25.0499322	68.13828
ENSG000001[illegible]	ADRB2	protein_codin	45.7638658	56.3054709	14.2589512	29.5260915
ENSG000001[illegible]	SLN	protein_codin	0	0	0	0
ENSG000001[illegible]	MBOAT2	protein_codin	29.2497996	16.4266167	34.2765633	30.0716406
ENSG000001[illegible]	ZNF239	protein_codin	0	0	0	0
ENSG000001[illegible]	MGMT	protein_codin	20.1823051	47.824467	13.7190535	9.06908703
ENSG000001[illegible]	ATOH1	protein_codin	0	0	0	0
ENSG000001[illegible]	DDX54	protein_codin	166.647033	135.847045	177.480269	94.5953733
ENSG000001[illegible]	LSM3	protein_codin	87.1464868	140.780747	75.4387408	63.2471727
ENSG000001[illegible]	USP19	protein_codin	0	0	0	0
ENSG000001[illegible]	DPYD	protein_codin	93.1376238	47.4184661	31.7937571	46.1212218
ENSG000001[illegible]	PTPRE	protein_codin	1929.50144	1899.25884	1013.1577	1364.13205
ENSG000001[illegible]	TEFM	protein_codin	3.66501583	0	0	0
ENSG000001[illegible]	PTGDR	protein_codin	0	9.68687742	6.25480623	3.28391927
ENSG000001[illegible]	LRP8	protein_codin	55.9047665	32.4771867	37.1685015	25.4751837
ENSG000001[illegible]	ASXL1	protein_codin	0	0	91.2252937	0
ENSG000001[illegible]	MT1E	protein_codin	9.0735796	1.26459997	0	0.98988671
ENSG000001[illegible]	ANKRD27	protein_codin	132.952475	171.610091	93.2042199	67.9743653
ENSG000001[illegible]	CHRNB1	protein_codin	11.1793204	17.115649	4.11539872	2.04954039
ENSG000001[illegible]	ISG20	protein_codin	15.331315	0	7.53713321	0

ENSG000001	TMEM43	protein_codin	226.642368	323.867353	102.799068	137.563293
ENSG000001	KCNG3	protein_codin	0	0	0	0
ENSG000001	TXNDC2	protein_codin	0	0	0	0
ENSG000001	TRIM56	protein_codin	278.519424	357.947928	164.452833	161.52613
ENSG000001	SOX14	protein_codin	0	0	0	0
ENSG000000	GNAS	protein_codin	0	0	0	0
ENSG000001	FSTL5	protein_codin	0	0	0	0
ENSG000001	HSPBAP1	protein_codin	30.5750494	51.8272449	22.1618601	38.5784814
ENSG000001	ALCAM	protein_codin	83.9725083	37.7491712	0	82.3743879
ENSG000001	TRMT61B	protein_codin	17.9118214	20.1701412	7.63134414	15.5168843
ENSG000001	KCNS1	protein_codin	0	0	0	0
ENSG000000	GNAS	protein_codin	0	0	0	0
ENSG000001	SNX7	protein_codin	0	0	0	0
ENSG000001	QARS1	protein_codin	434.142917	543.9118	195.161935	244.935502
ENSG000002	TMEM276	protein_codin	0	8.30961232	0	0
ENSG000001	CSDC2	protein_codin	0	0.78521867	0	0
ENSG000001	MUC17	protein_codin	0	0	0	0
ENSG000001	PRKCE	protein_codin	106.242668	103.051541	29.6768135	67.24664
ENSG000001	MTM1	protein_codin	0	0	0	0
ENSG000001	ZPLD1	protein_codin	0	1.32625528	0	0
ENSG000001	USP42	protein_codin	56.8666582	121.676592	65.8859783	102.420814
ENSG000001	MRPL13	protein_codin	20.8551675	36.0992396	23.4006182	21.8256707
ENSG000001	CBR4	protein_codin	32.2358633	39.5025309	26.1427356	40.9281104
ENSG000001	STX18	protein_codin	80.6577122	59.4976982	0	58.1096242
ENSG000001	SCGB1D1	protein_codin	0	0	0	0
ENSG000001	CHST14	protein_codin	4.3422584	7.45956891	10.3998658	9.23303483
ENSG000001	MLKL	protein_codin	34.0585969	37.5080096	33.9187956	30.2776632
ENSG000001	RER1	protein_codin	0	3.21327868	0	0
ENSG000001	ST3GAL5	protein_codin	0	0	0	0
ENSG000001	FOXI1	protein_codin	0	0	0	0.84115825
ENSG000000	IBTK	protein_codin	288.04853	99.453667	213.090952	179.649919
ENSG000001	KRTAP1-1	protein_codin	0	0	0	0
ENSG000001	ATOH8	protein_codin	24.1652758	10.411929	7.98384327	0
ENSG000001	STK32A	protein_codin	0	0	0	0
ENSG000001	REEP4	protein_codin	85.718527	129.634705	37.1923414	56.5014391
ENSG000001	SLC26A5	protein_codin	0	0	0	0
ENSG000001	ZNF608	protein_codin	0	2.86390878	13.7525955	2.24885751
ENSG000001	LGI3	protein_codin	0	0	0	0
ENSG000001	GBX2	protein_codin	0	0	0	0
ENSG000001	RETREG1	protein_codin	0	0	0	0
ENSG000002	PSG11	protein_codin	0	0	0	0
ENSG000001	HOXD4	protein_codin	0	0	0	0
ENSG000001	MTCL1	protein_codin	0	0	0	0
ENSG000001	C2orf68	protein_codin	189.509549	252.48897	80.6195335	106.583931

ENSG000001	ZNF396	protein_codin	0	0	0	0
ENSG000001	BMP1	protein_codin	0	0	0	0
ENSG000001	TMEM150A	protein_codin	0	0	0	0
ENSG000001	STX8	protein_codin	84.8897762	124.948195	59.0821971	73.7379783
ENSG000001	RNF181	protein_codin	107.056192	89.5228253	22.0359025	65.2520307
ENSG000002	ETV5	protein_codin	0	0	0	0
ENSG000001	BPTF	protein_codin	266.364441	280.433032	51.9790645	211.978124
ENSG000001	VAMP5	protein_codin	71.4031789	73.2591284	34.628423	49.3907119
ENSG000001	BMP1	protein_codin	0	0	0	0
ENSG000001	TBCA	protein_codin	1.90774289	0	3.53052518	4.13469213
ENSG000001	DPY19L2	protein_codin	0	0	0	3.34625387
ENSG000001	LRG1	protein_codin	94.5004823	40.9327627	11.2834437	76.5242197
ENSG000001	BAALC	protein_codin	0	0	0	0
ENSG000001	NMRAL2P	transcribed_u	0	0	0	0
ENSG000000	SORBS1	protein_codin	0	0	11.1185962	0
ENSG000001	TMEM37	protein_codin	0	0	0	0
ENSG000001	OTP	protein_codin	0	0	0	0
ENSG000001	POLR3D	protein_codin	49.6349438	51.0535134	10.7172618	43.1292263
ENSG000001	MAT2A	protein_codin	226.715404	414.504398	161.454274	158.866514
ENSG000001	PPIC	protein_codin	0	5.66026215	0	0
ENSG000002	ATP6V1E2	protein_codin	7.03614049	1.92626658	0	0
ENSG000001	ARMC10	protein_codin	0	2.12306595	3.14490718	1.87182387
ENSG000001	PIGF	protein_codin	0	2.75067907	14.3414966	7.01203735
ENSG000001	CEP120	protein_codin	66.787225	0	0	14.3211726
ENSG000001	TMEM192	protein_codin	52.9304891	54.5211031	29.3459928	36.0094963
ENSG000001	CEP120	protein_codin	0	53.6847308	83.9735001	75.0584422
ENSG000001	PRL	protein_codin	0.86819793	0	0	0
ENSG000001	SLC22A25	protein_codin	0	0	0	0
ENSG000001	ZNF778	protein_codin	4.12254691	35.1661967	0	22.7825559
ENSG000001	SOCS5	protein_codin	46.9008438	0	4.45361182	0
ENSG000001	FOXD4L1	protein_codin	0	0	0	1.00901587
ENSG000001	PSG8	protein_codin	0	0	0	0
ENSG000002	ENSG000002	lncRNA	0	0	0	0
ENSG000001	ROBO4	protein_codin	0	5.2508039	0	0
ENSG000001	PCDHB1	protein_codin	0	0	0	1.28522228
ENSG000001	HAS3	protein_codin	0	0	0	5.06305464
ENSG000001	ZNF672	protein_codin	82.3940439	186.34043	60.0503881	61.9460472
ENSG000002	DERPC	protein_codin	0	0	6.26021421	0
ENSG000001	TMC6	protein_codin	0	0	0	0
ENSG000002	PCDHGC4	protein_codin	0	0	0	0
ENSG000001	ZNF692	protein_codin	10.8615844	29.0256834	0	8.25889299
ENSG000001	CXCL10	protein_codin	9.02330952	28.1207792	14.7041821	10.2651332
ENSG000001	CDY1	protein_codin	0	0	0	0
ENSG000001	CXCL11	protein_codin	3.13490388	0	0	4.92085654

ENSG000001	UBE2E1	protein_codin	112.482022	168.100855	59.8596869	124.282081
ENSG000001	RPL36P14	processed_ps	0	0.75746492	0	0
ENSG000001	CYBC1	protein_codin	97.8580315	176.864782	0	85.8629764
ENSG000001	KRT28	protein_codin	0	0	0	0
ENSG000000	PTGER3	protein_codin	0	5.98311372	0.98933265	0
ENSG000001	ADRA1B	protein_codin	0	0	0	0
ENSG000001	RASA4B	protein_codin	16.1591928	135.174575	19.6324291	0
ENSG000001	LRRC45	protein_codin	53.341365	53.7144345	32.526944	24.1321603
ENSG000001	SGO1	protein_codin	0	0	0	0
ENSG000001	CENPX	protein_codin	2.42522789	13.2769868	0	0
ENSG000001	LRRC8E	protein_codin	0	0	0	0
ENSG000001	CDCA7	protein_codin	0	0	0	8.16491367
ENSG000001	ASPSCR1	protein_codin	3.46524206	3.87205725	0	0
ENSG000001	AVEN	protein_codin	14.7675643	20.203368	27.7049097	6.82115399
ENSG000001	LINC01620	lncRNA	0	0	0	0
ENSG000001	RBFA	protein_codin	5.09148654	9.58466785	10.95403	4.97662839
ENSG000001	ASPSCR1	protein_codin	44.5597445	67.282694	34.0888683	20.5642247
ENSG000001	FASN	protein_codin	108.919848	139.683443	0	41.6781335
ENSG000002	NFS1	protein_codin	3.15456707	0	0	0
ENSG000001	RCAN2	protein_codin	0	0	0	0
ENSG000001	NPTX1	protein_codin	0	0	0	0
ENSG000001	GFRA2	protein_codin	0	0	0	1.17697985
ENSG000001	DUS1L	protein_codin	42.5483651	116.847929	27.8692639	74.3133232
ENSG000001	RNF150	protein_codin	0	0	0	0
ENSG000001	RPTOR	protein_codin	142.580709	229.721359	57.5557528	98.8477827
ENSG000001	BLTP1	protein_codin	0	0	0	0
ENSG000001	NEGR1	protein_codin	5.62234022	0	8.99632439	11.1783943
ENSG000001	GPS1	protein_codin	0	0	65.4196056	0
ENSG000001	OR8B12	protein_codin	0	0	0	0
ENSG000001	PAIP1	protein_codin	26.4033802	33.3771576	0	21.6456136
ENSG000001	B4GALT6	protein_codin	29.1077539	33.5765059	14.010607	20.5596046
ENSG000002	CSPG4P2Y	lncRNA	0	0	0	0
ENSG000001	FAM83B	protein_codin	1.27541651	1.28739075	0	0
ENSG000001	C5orf34	protein_codin	0	0	1.09117368	0
ENSG000001	DCXR	protein_codin	11.1231653	41.4291423	7.47272419	21.0655341
ENSG000002	COG8	protein_codin	45.9019261	56.7001267	34.8927398	35.1987576
ENSG000001	CDY1B	protein_codin	0	0	0	0
ENSG000000	LNX1	protein_codin	0	0	0	0
ENSG000001	RAC3	protein_codin	0	1.79993604	0	0
ENSG000001	CHRNA7	protein_codin	0	0	0	12.937688
ENSG000001	RBMXL2	protein_codin	0	0	0	0
ENSG000001	ZNF57	protein_codin	0	17.6888233	0	8.01980067
ENSG000001	ZNF584	protein_codin	0	0	0	0
ENSG000001	FEM1B	protein_codin	256.328017	178.490731	87.4979563	185.992793

ENSG000001(	NTSR2	protein_codin	0	0	0	0
ENSG000001	FIP1L1	protein_codin	80.9593964	91.4498214	0	41.2838245
ENSG000001	ZNF131	protein_codin	0	0	0	0
ENSG000001	GON7	protein_codin	16.9159895	33.6908755	15.4160337	15.1828825
ENSG000001(	CHRM1	protein_codin	0	0	0	0
ENSG000001(	MPLKIP	protein_codin	54.101361	85.1242881	23.3691376	51.2672816
ENSG000001(	ANKMY2	protein_codin	30.3952591	42.7508054	18.8524173	26.6425492
ENSG000001	PAH	protein_codin	0	0	0	0
ENSG000001!	OR10G6	protein_codin	0	0	0	0
ENSG000001	C7orf33	protein_codin	0	0	0	0
ENSG000001	USP38	protein_codin	0	51.253465	0	0
ENSG000001	OR4D5	protein_codin	0	0	0	0
ENSG000000	IGF1	protein_codin	0.83876197	2.4424335	6.8891436	1.18354303
ENSG000001(	OTUD7A	protein_codin	0.98684595	1.94697315	0	0
ENSG000001	PWWP2A	protein_codin	70.6703837	108.461045	49.3490246	56.1541354
ENSG000001	SOSTDC1	protein_codin	0	1.37443129	0	0
ENSG000001(	NT5DC2	protein_codin	0	0	0	0
ENSG000001(	AXIN2	protein_codin	0	0	0	0
ENSG000001	NSUN4	protein_codin	61.1898176	61.1957439	26.9290627	31.504475
ENSG000001(	SPRR1B	protein_codin	0	0	0	0
ENSG000001(	MAP2K1	protein_codin	0	202.349188	0	48.5243539
ENSG000001(	UQCC5	protein_codin	4.90290203	3.76015025	0	2.5539292
ENSG000001	GTPBP2	protein_codin	0	26.978379	32.3060995	0
ENSG000001:	P4HA1	protein_codin	0	0	0	0
ENSG000001	GTPBP2	protein_codin	285.190936	426.608149	156.914496	189.316513
ENSG000001	MYOZ2	protein_codin	0	0	0	0
ENSG000001	RALGAPA1	protein_codin	0	33.370284	9.81295727	92.1539044
ENSG000000	CACNG5	protein_codin	0	0	0	0
ENSG000001	SYNPO2	protein_codin	0	0	0	0
ENSG000001	LDHAL6B	protein_codin	0	0	0	0
ENSG000001(	KLF13	protein_codin	1222.09052	1611.61265	466.035402	665.398388
ENSG000001(	COG7	protein_codin	30.9985162	84.5811168	21.5764831	29.6001006
ENSG000002(	ENSG000002(	processed_ps	0	0	0	0
ENSG000001(	MTNR1A	protein_codin	0	0	0	0
ENSG000002:	OR51A10P	unitary_pseuc	0	0	0	0
ENSG000001(	INSM2	protein_codin	0	0	0.54903088	0
ENSG000001:	USP8	protein_codin	0	0	0	75.0949119
ENSG000001	TSNARE1	protein_codin	0	0	0	0
ENSG000002	GIMAP1	protein_codin	197.751977	317.878416	76.084948	159.859974
ENSG000002:	LRTOMT	protein_codin	0	0	0	0
ENSG000001(	ABHD15	protein_codin	30.3276118	56.5857713	35.2136968	31.5538763
ENSG000001(	TMEM87A	protein_codin	9.79211619	5.79228011	10.3860068	0
ENSG000001	DNAJB7	protein_codin	0	0	0	0
ENSG000001	SUCLG2	protein_codin	124.670357	165.255305	63.7086886	71.0253658

ENSG000001	E2F6	protein_codin	0	0	0	0
ENSG000002	SUCLG2P2	processed_ps	0	0	0	0
ENSG000001	JHY	protein_codin	0	3.58092746	0	0
ENSG000000	PLPP1	protein_codin	4.28334609	0	2.20842565	4.23734645
ENSG000001	COPS9	protein_codin	0	0	0	0
ENSG000001	GIMAP8	protein_codin	248.172342	524.231807	133.124687	164.836211
ENSG000001	HEXIM2	protein_codin	0	4.84077754	4.14492028	0
ENSG000001	SYVN1	protein_codin	0	0	0	0
ENSG000001	PDCD6IP	protein_codin	372.612841	176.171069	66.3633115	269.731923
ENSG000001	PATJ	protein_codin	0	0	0	0
ENSG000001	NDUFA10	protein_codin	0	8.8329326	0	0
ENSG000001	MRPL39	protein_codin	7.37690667	0	6.14657782	3.87614093
ENSG000000	PHTF2	protein_codin	57.9673626	52.0328326	44.7925636	37.735693
ENSG000001	SCNN1B	protein_codin	0	0	0	0
ENSG000001	ANTXR2	protein_codin	0	5.61960548	0	0
ENSG000000	USH2A	protein_codin	0	0	1.09558016	0
ENSG000001	ZNF138	protein_codin	0	0	0	0
ENSG000001	HTRA3	protein_codin	0	2.52884782	0	0
ENSG000002	PCDHA10	protein_codin	0	0	0	0
ENSG000001	GLB1	protein_codin	265.165563	295.985301	99.849555	118.128189
ENSG000002	SLC26A6	protein_codin	0	0	0	0
ENSG000001	DDIT4	protein_codin	680.454007	366.373725	73.1121069	213.971137
ENSG000001	TMEM223	protein_codin	13.9218341	30.0850235	7.43271757	3.9606265
ENSG000001	CLCN5	protein_codin	0	0	3.10204265	0
ENSG000001	GLB1	protein_codin	0	0	0	0
ENSG000000	SLCO1A2	protein_codin	0	0	0	0
ENSG000001	OR52A5	protein_codin	0	0	0	0
ENSG000001	HSFY1	protein_codin	0	0	0	0
ENSG000001	GP9	protein_codin	189.335449	310.386394	176.815158	113.893333
ENSG000001	RBM33	protein_codin	179.795144	220.04745	176.650127	135.582515
ENSG000001	CXCL8	protein_codin	5999.36133	909.711101	3235.29387	3756.52343
ENSG000001	MRPL4	protein_codin	0	0	0	15.7436149
ENSG000001	PARM1	protein_codin	13.0562372	12.1620439	65.709008	5.12445911
ENSG000001	CNTNAP4	protein_codin	0	0	0	0
ENSG000001	FAM90A1	protein_codin	0	0	0	0.8973107
ENSG000001	RASSF6	protein_codin	0	0	12.1381526	0
ENSG000001	ZNF33A	protein_codin	11.9759305	39.5242994	0	29.2362344
ENSG000001	MORN4	protein_codin	0	0	0	0
ENSG000001	CDKL2	protein_codin	0	0	0	0
ENSG000001	ZIK1	protein_codin	3.98772384	0	0	0
ENSG000001	CCDC126	protein_codin	25.9259069	22.2913273	4.43831113	0
ENSG000001	MAB21L4	protein_codin	0	0	0	0
ENSG000001	ACSM1	protein_codin	0	0	0	1.78997685
ENSG000001	AGXT	protein_codin	0	0	0	0

ENSG000001	FAM161A	protein_codin	4.03251865	0	0	0
ENSG000001	ANKRD2	protein_codin	0	0	0	0
ENSG000001	CMTM8	protein_codin	0	0	0	0
ENSG000001	ZBBX	protein_codin	0	0	0	0
ENSG000001	MRFAP1L2	protein_codin	0	0	0	1.66524151
ENSG000001	PANK1	protein_codin	3.94506844	6.3553627	4.02563393	3.16296682
ENSG000001	KIAA1217	protein_codin	0	0	0	0
ENSG000000	MAGI3	protein_codin	0	10.5878629	3.4234043	0
ENSG000001	SHANK2-AS3	lncRNA	0	0	0	0
ENSG000001	OR10V1	protein_codin	0	0	0	0
ENSG000001	AKNA	protein_codin	767.315721	807.704959	267.891708	430.994418
ENSG000001	CAVIN4	protein_codin	6.09768948	0	4.46335493	2.03536787
ENSG000001	CCDC110	protein_codin	0	0	0	0
ENSG000001	GOT1L1	protein_codin	0	0	0	0
ENSG000001	HOOK3	protein_codin	609.165289	331.149789	338.292192	489.778232
ENSG000001	ACOT12	protein_codin	0	0	0	0
ENSG000001	YWHAG	protein_codin	624.317467	1007.33596	361.389692	552.700652
ENSG000001	HARS1	protein_codin	0	11.3884898	0	0
ENSG000001	ZNF556	protein_codin	0	1.14893465	0	0
ENSG000001	C3AR1	protein_codin	253.766631	105.541043	39.2957267	83.601254
ENSG000001	NKIRAS2	protein_codin	0	8.75315196	5.54960121	1.82376081
ENSG000001	MCRIP2	protein_codin	10.0412821	13.8244855	1.52592635	6.57455266
ENSG000001	ZNF17	protein_codin	0	0	0	9.46134832
ENSG000001	KIAA0232	protein_codin	23.7161031	0	0	0
ENSG000001	SYNPO	protein_codin	0	0.81021808	0	0
ENSG000001	ATP9B	protein_codin	0	121.500494	0	44.0942643
ENSG000001	BCL2L1	protein_codin	178.701358	155.108497	0	0
ENSG000001	SEC23A	protein_codin	119.98641	127.170718	73.692436	118.164064
ENSG000001	KHDRBS1	protein_codin	0	5.45853399	0	0
ENSG000001	NAT1	protein_codin	12.0488134	0	2.39115002	6.46833773
ENSG000001	PPID	protein_codin	84.3265834	133.020223	49.9013269	67.4110456
ENSG000000	MLLT10	protein_codin	0	28.2173184	0	0
ENSG000001	CDC40	protein_codin	304.446367	162.454079	133.430922	192.601057
ENSG000001	ETFDH	protein_codin	24.1992114	39.5753424	0	0
ENSG000001	THOP1	protein_codin	6.35092714	16.4830511	0	1.99143936
ENSG000001	CYTL1	protein_codin	0	2.99981502	6.00400057	0
ENSG000001	ZBTB5	protein_codin	78.4229681	59.9919447	34.1235009	35.1649859
ENSG000001	LGALS4	protein_codin	1.678472	0	0	0
ENSG000001	RXFP1	protein_codin	0	0	0	0
ENSG000001	TVP23B	protein_codin	38.3292217	48.5133622	17.1831521	42.328115
ENSG000001	ZRSR2	protein_codin	302.159184	378.406516	197.887754	183.683188
ENSG000001	CALML6	protein_codin	0	1.3255092	0	0
ENSG000001	ASCC2	protein_codin	72.4559432	166.254391	20.3118075	65.5085785
ENSG000001	AFF1	protein_codin	179.869032	187.675771	213.644016	261.642002

ENSG000001	RBM23	protein_codin	0	0	0	10.9623504
ENSG000001	CARNS1	protein_codin	4.68248664	0	0	0
ENSG000001	MGAT1	protein_codin	304.201627	439.270194	119.580025	144.768205
ENSG000001	OR2Y1	protein_codin	0	0	0	0
ENSG000001	NABP1	protein_codin	0	0	0	0
ENSG000001	RPL15	protein_codin	1174.42676	1450.49216	482.278158	941.785379
ENSG000001	STARD6	protein_codin	0	0	0	0
ENSG000001	HPCAL1	protein_codin	101.459448	210.606089	15.5711097	0
ENSG000001	NABP1	protein_codin	125.591369	113.253336	81.5911715	113.247961
ENSG000001	HAVCR2	protein_codin	64.6102596	0	0	0
ENSG000002	TRIM40	protein_codin	0	0	0	0
ENSG000000	AIFM2	protein_codin	5.98512202	8.53646719	12.7296216	1.00465592
ENSG000001	SRP68	protein_codin	164.771948	227.491477	146.655522	112.707238
ENSG000001	ADCY6	protein_codin	16.0795217	0	0	0
ENSG000001	CTSW	protein_codin	30.0505384	29.4764295	0	38.5421473
ENSG000001	CYB5B	protein_codin	3.2397555	5.30239042	0	0
ENSG000000	THAP3	protein_codin	0	0	3.12230915	0
ENSG000001	ZWILCH	protein_codin	0	0	19.3289454	9.1998213
ENSG000000	ADAT1	protein_codin	20.1649517	0	19.7598122	0
ENSG000001	MCL1	protein_codin	332.35245	113.816575	338.572377	119.704797
ENSG000002	CRYGS	protein_codin	5.08834991	0	0.96999815	0
ENSG000002	FNIP1	protein_codin	5.88310573	0	0	0
ENSG000001	ARR3	protein_codin	0	0	0	0
ENSG000001	RPL4	protein_codin	279.579412	401.503023	148.570999	277.320561
ENSG000002	FNIP1	protein_codin	247.017535	181.842781	116.597319	129.45213
ENSG000001	WFDC5	protein_codin	0	0	0	0
ENSG000001	FOXP4	protein_codin	0	57.2322617	0	28.939211
ENSG000001	SNAPC5	protein_codin	8.12634688	19.8653512	11.1177694	4.94588057
ENSG000001	RAD9A	protein_codin	0	12.2558861	9.30905131	0
ENSG000001	EFEMP2	protein_codin	0	8.58895834	0	3.14891742
ENSG000001	RAPGEF6	protein_codin	77.0112202	41.9045509	45.0473999	30.9374479
ENSG000001	MRPS12	protein_codin	27.6528978	33.1710765	10.6677845	11.9401125
ENSG000001	PTDSS2	protein_codin	9.71888089	33.4479997	7.2329878	5.18611998
ENSG000001	TMEM134	protein_codin	0	5.98638124	0	0
ENSG000001	DDX23	protein_codin	302.193232	480.142103	217.727855	246.31764
ENSG000001	SLC16A13	protein_codin	3.02911168	7.42691122	1.51718554	4.89792068
ENSG000001	ZNF471	protein_codin	5.45607317	0	0	2.17937302
ENSG000000	DLEC1	protein_codin	0	0	0	0
ENSG000001	FRG2C	protein_codin	0	0	1.16448292	0
ENSG000001	CHST1	protein_codin	0	0	0.70179364	0
ENSG000001	SLC37A2	protein_codin	0	0	0	0
ENSG000001	UNC93B3	unprocessed_	0	0	0	0
ENSG000000	PTCD2	protein_codin	0	0	4.57004424	3.99296711
ENSG000001	EMILIN2	protein_codin	5.57132951	5.77389395	2.13070058	10.1551526

ENSG000001	THAP2	protein_codin	21.2666707	21.3644953	12.1130703	17.3281941
ENSG000001	RAPH1	protein_codin	2.11254279	0	0	0
ENSG000001	ZNF80	protein_codin	0	0	0	1.02741634
ENSG000001	METTL5	protein_codin	0	0	0	0
ENSG000001	SPMIP3	protein_codin	0	0	0	0
ENSG000001	CCNE2	protein_codin	2.67198461	0	1.98291438	0
ENSG000001	MUS81	protein_codin	65.5676177	91.0296434	28.3200029	31.7600042
ENSG000001	SSH3	protein_codin	9.19242669	43.9014466	6.77493677	13.1635971
ENSG000001	DSG4	protein_codin	0	0	0	0
ENSG000001	FAM53A	protein_codin	0	0	0	0
ENSG000001	H2AZ2	protein_codin	71.737711	108.038656	61.7873148	68.682511
ENSG000001	PHYKPL	protein_codin	35.0549866	80.2521387	14.629765	20.9596513
ENSG000001	NUP93	protein_codin	0	0	0	3.39999679
ENSG000001	HMGCLL1	protein_codin	0	0	0	0
ENSG000001	CFL1	protein_codin	1473.58848	1780.63451	400.422527	936.248247
ENSG000001	ELF4	protein_codin	274.818921	415.123214	55.8556942	138.193336
ENSG000002	PCDHGC3	protein_codin	0	2.87368101	0	0
ENSG000001	CRYBG2	protein_codin	0.76029958	0	0.9312531	0
ENSG000001	PDE7B	protein_codin	24.6174262	8.29629379	5.67373551	6.39479448
ENSG000001	PLG	protein_codin	0	0	0	0
ENSG000001	COQ10A	protein_codin	0	0	0	3.09645623
ENSG000001	CCDC63	protein_codin	0	0	0	0
ENSG000001	TP53I11	protein_codin	5.22530803	0	0	0
ENSG000001	IQCK	protein_codin	0	0	0	0
ENSG000001	BRSK2	protein_codin	0	0	0	0
ENSG000001	RNASE8	protein_codin	0	0	0	0
ENSG000001	RGMB	protein_codin	0	0	0	0
ENSG000001	CRB3	protein_codin	0	0	0	0
ENSG000001	PPM1E	protein_codin	1.15042493	1.14410343	0	0
ENSG000001	KCNG4	protein_codin	0	0	0.88941503	0
ENSG000001	MSRB3	protein_codin	61.2856226	0	26.5234213	0
ENSG000001	PSMA8	protein_codin	0	0	0	0
ENSG000000	USP48	protein_codin	78.9154959	57.0350459	46.8586399	25.5851817
ENSG000001	FBXO22	protein_codin	47.5377407	45.7591	29.2761838	27.5122195
ENSG000001	TLCD3A	protein_codin	0	0	0	0
ENSG000001	SEC16B	protein_codin	6.04865064	0	9.81662125	0
ENSG000001	MS4A10	protein_codin	2.18771653	2.18583182	0	0
ENSG000001	TENT5D	protein_codin	0	0	0	0
ENSG000001	SSH3	protein_codin	0	0	2.3011358	0
ENSG000001	GOLT1A	protein_codin	0	0	0	0
ENSG000001	PROP1	protein_codin	0	0	0	0
ENSG000000	GEMIN2	protein_codin	0	0	8.67012652	0
ENSG000001	LEMD3	protein_codin	135.775848	165.189071	69.6217121	98.6466061
ENSG000001	SNX32	protein_codin	0	0	0	0

ENSG000001	CLDN15	protein_codin	0	0	0	0
ENSG000002	ENSG000002	lncRNA	0	0	0	0
ENSG000001	PDLIM2	protein_codin	0	3.77351284	4.5379819	3.18292314
ENSG000000	PIGS	protein_codin	67.112376	142.557835	30.8869588	31.7030388
ENSG000001	DHX36	protein_codin	52.8641621	61.6003554	12.5190602	73.2950616
ENSG000001	PLA2G1B	protein_codin	0	1.09625532	0	0
ENSG000000	LTBP4	protein_codin	0	0	0	0
ENSG000001	GMPPB	protein_codin	0	0	21.7350758	0
ENSG000001	SLFN11	protein_codin	112.756719	198.669546	64.4209031	0
ENSG000001	WDR49	protein_codin	0	6.312698	0	0
ENSG000001	DUSP6	protein_codin	27.4302822	135.522617	46.4250968	0
ENSG000001	GMPPB	protein_codin	20.8547034	42.0498763	0	18.7426588
ENSG000001	CASP3	protein_codin	13.1016367	61.6652282	24.430361	17.8255699
ENSG000001	MAPK7	protein_codin	42.9171351	29.2247936	16.3970211	0
ENSG000001	RNASEH2C	protein_codin	0	36.7747566	15.6596914	6.71064442
ENSG000001	AOC3	protein_codin	0	0	0	0
ENSG000001	TUT1	protein_codin	10.7331216	4.3742862	12.1708003	0
ENSG000001	ANKRD13D	protein_codin	75.7679321	67.4259532	0	42.7181293
ENSG000001	ATAD1	protein_codin	5.60307518	9.38519496	2.10527772	1.98566902
ENSG000001	SLC17A3	protein_codin	0	0	0	0
ENSG000001	MUC4	protein_codin	0	0	0	0
ENSG000002	DUSP13A	protein_codin	0	0	0	0
ENSG000001	LRRN3	protein_codin	0	0	0	0
ENSG000001	LRRFIP1	protein_codin	222.92768	74.1953894	41.3948427	80.9889616
ENSG000000	RSF1	protein_codin	130.154902	69.5841211	62.6068761	96.4810693
ENSG000001	STOX2	protein_codin	4.08959691	0	0.91132747	1.09362154
ENSG000001	CSPG4	protein_codin	0.86016491	0	0.91702131	0
ENSG000001	CCAR2	protein_codin	148.210576	192.859748	112.484737	86.5233885
ENSG000001	APOBEC3F	protein_codin	53.477583	42.8265906	33.0910139	30.4047121
ENSG000001	SNX33	protein_codin	73.6454374	108.1102	35.7341554	31.4105551
ENSG000001	LYG1	protein_codin	0	4.0361262	2.45572514	1.02713652
ENSG000001	PDZRN3	protein_codin	0	0	0	0
ENSG000001	LGR6	protein_codin	0	0	0	0
ENSG000001	TMOD3	protein_codin	605.431311	480.134885	412.42214	418.267955
ENSG000001	SNUPN	protein_codin	41.663704	69.2642324	39.2201572	33.0947981
ENSG000001	GRK2	protein_codin	1528.9273	1966.41755	737.498493	830.087175
ENSG000001	MIR4435-2HG	lncRNA	0	0	0	0
ENSG000001	CD200R1	protein_codin	0	0	0	13.3520283
ENSG000001	EVX2	protein_codin	0	0	0	1.99984874
ENSG000001	SNTG2	protein_codin	0	0	0	0
ENSG000001	RELA	protein_codin	36.6000727	15.4396171	3.15649313	15.8538639
ENSG000001	APOBEC4	protein_codin	0	0	0	0
ENSG000001	ATAD3B	protein_codin	0	0	0	3.02061145
ENSG000001	ZNF699	protein_codin	29.1353688	20.9032831	7.78579605	18.197479

ENSG000001	BCL2L11	protein_codin	0	21.8364362	0	0
ENSG000001	MARCHF3	protein_codin	0	3.93173997	0.79636939	4.0674614
ENSG000001	ATP2A2	protein_codin	117.711925	52.7359299	0	65.6880242
ENSG000001	ABCD2	protein_codin	6.37013501	0	5.89463264	0
ENSG000000	PRKACA	protein_codin	858.674598	953.164005	273.91382	507.880661
ENSG000001	OR7D4	protein_codin	0	0	0	0
ENSG000001	ZNF622	protein_codin	116.538339	236.360227	94.091494	122.201112
ENSG000001	PSMD1	protein_codin	177.861911	120.491073	103.651478	0
ENSG000001	ISX	protein_codin	0	0	0	0
ENSG000001	NGLY1	protein_codin	7.00073028	0	0	0
ENSG000001	SEZ6L2	protein_codin	0	0	0	0
ENSG000001	BCL2L14	protein_codin	0	0	0	0
ENSG000002	AP1G2	protein_codin	9.46064258	9.30913982	6.95799107	2.40274344
ENSG000000	BTK	protein_codin	266.631034	412.222355	0	0
ENSG000000	NFYC	protein_codin	30.6004365	50.5367267	0	0
ENSG000001	DHX37	protein_codin	82.8400869	97.7138478	44.0356533	57.6524735
ENSG000001	ALG10B	protein_codin	23.32937	8.43797252	14.6139299	5.96872395
ENSG000000	NPFFR2	protein_codin	0	0	0	0
ENSG000001	ASPHD1	protein_codin	0	0	0	0
ENSG000001	KCTD13	protein_codin	0	0	0	0
ENSG000001	COP1	protein_codin	110.637185	50.7671837	9.42144334	34.6704151
ENSG000001	TRMT112	protein_codin	0	0	0	0
ENSG000001	DAG1	protein_codin	82.7972706	115.440845	18.692074	32.4820144
ENSG000001	KDM2A	protein_codin	16.0770432	14.4841551	0	48.9828182
ENSG000001	MUCL1	protein_codin	0	0	0	0
ENSG000001	FBXW10	protein_codin	0	0	0	0
ENSG000001	MLKL	protein_codin	328.135663	442.673443	176.274862	242.730946
ENSG000001	ZNF596	protein_codin	0	0	0	0
ENSG000002	PCMTD2	protein_codin	159.745117	100.724577	69.1788611	66.5260208
ENSG000001	RHOD	protein_codin	0	0	0	0
ENSG000001	ADCK5	protein_codin	15.7849426	0	6.26056601	18.8911879
ENSG000001	PIKFYVE	protein_codin	9.4863756	0	0	9.4677022
ENSG000001	LEP	protein_codin	0	0	0	0.9991985
ENSG000000	RUNX3	protein_codin	274.478047	310.179758	67.8126024	109.088949
ENSG000001	EGFL7	protein_codin	5.28494808	10.254124	2.49383065	0
ENSG000001	TAOK2	protein_codin	65.8407743	109.774281	35.33483	45.9528241
ENSG000001	LKAAEAR1	protein_codin	0	0	0	0
ENSG000001	GCSAM	protein_codin	1.27450533	6.58050588	4.24951959	0
ENSG000001	TUBB8B	protein_codin	0	3.27592048	0	0
ENSG000000	CYP2W1	protein_codin	0	0	0	0
ENSG000000	U2AF2	protein_codin	156.835103	253.201501	125.677105	106.185857
ENSG000001	OR10H5	protein_codin	0	0	0	0
ENSG000001	PHYHD1	protein_codin	0	0	0	0
ENSG000001	SIPA1L2	protein_codin	2.25957316	0	2.15541972	0

ENSG000001	MYEOV	protein_codin	0	0	7.35337113	0
ENSG000001	NDUFA11	protein_codin	73.989931	98.5138257	24.7891702	63.577055
ENSG000001	C11orf86	protein_codin	0	0	0	0
ENSG000001	CCDC106	protein_codin	1.39060629	0	3.02118329	1.94937686
ENSG000001	TLR10	protein_codin	0	0	0	0
ENSG000001	TLR1	protein_codin	58.4370429	95.3926972	18.5836428	43.9816206
ENSG000001	ACAD9	protein_codin	47.1026243	62.957064	0	49.8248701
ENSG000001	CKS1B	protein_codin	5.47650092	5.43810677	0	6.56990057
ENSG000001	DBN1	protein_codin	72.6087019	77.2880239	7.10999999	0
ENSG000001	PTPN7	protein_codin	2.13997878	4.80009614	7.6550515	0
ENSG000001	GOLIM4	protein_codin	276.734496	240.646115	394.683387	258.299142
ENSG000000	BAD	protein_codin	34.0440738	54.9220377	13.6983578	36.5480096
ENSG000001	UBXN2A	protein_codin	52.7183073	74.3460543	40.2782091	34.6604013
ENSG000001	CTBP2	protein_codin	43.9615363	25.4908041	9.58165555	8.39817237
ENSG000001	CEP290	protein_codin	0	0	32.6147162	0
ENSG000000	REST	protein_codin	410.949399	271.583732	327.598731	116.680083
ENSG000001	CENPS	protein_codin	4.30147131	10.1509613	0	1.08832734
ENSG000001	PBLD	protein_codin	0	0	12.7684611	0
ENSG000001	C12orf76	protein_codin	0	0	0	0
ENSG000001	LCLAT1	protein_codin	0	0	0	0
ENSG000001	ZHX3	protein_codin	0	0	0	0
ENSG000001	DUS3L	protein_codin	25.7604669	35.74653	7.34640264	13.5954035
ENSG000001	SLC2A9	protein_codin	0	0	0	0
ENSG000001	ZGRF1	protein_codin	0	0	23.3146599	0
ENSG000001	PTMS	protein_codin	29.631524	59.3699246	20.7518076	21.2768841
ENSG000000	GPBP1	protein_codin	39.6943692	16.316518	15.2395725	6.14436441
ENSG000001	MRGPRF	protein_codin	0	0.80450702	0	0
ENSG000001	MAP3K11	protein_codin	468.208759	618.837882	194.370504	329.283801
ENSG000001	MRGPRD	protein_codin	0	0	0	0
ENSG000001	VPS26C	protein_codin	101.323601	169.243791	74.2760932	82.2192693
ENSG000001	TMEM9B	protein_codin	30.4306425	0	0	0
ENSG000001	PSME3IP1	protein_codin	163.473174	167.119408	30.6164446	138.153234
ENSG000001	OR6M1	protein_codin	0	0	0	0
ENSG000000	CRTAC1	protein_codin	0	0	0	0
ENSG000001	GRB7	protein_codin	0	0	0	0
ENSG000000	RORA	protein_codin	0	0	0	0
ENSG000001	CATSPERD	protein_codin	0	0	0	0
ENSG000001	NRIP3	protein_codin	0	0	4.36702583	0
ENSG000001	P2RY14	protein_codin	9.18078224	3.42856462	12.6756506	3.2326929
ENSG000001	GPR171	protein_codin	0	1.53153468	0	0
ENSG000001	WDR81	protein_codin	0	0	0	0
ENSG000003	CAST	protein_codin	0	0	0	0
ENSG000001	PTGR1	protein_codin	0	2.89771181	0	3.36660055
ENSG000001	MXRA8	protein_codin	0	0	0	0

ENSG000002	ENSG000002	unprocessed_	0	0	0	0
ENSG000000	HFE	protein_codin	0	0	0	0
ENSG000001	ZNF483	protein_codin	0	1.34781134	0	2.76352572
ENSG000001	MED12L	protein_codin	0	0	0	0
ENSG000001	PPARGC1B	protein_codin	155.589438	232.014379	47.8450191	87.9066521
ENSG000001	ETFB	protein_codin	73.1143867	150.165245	85.9446676	76.3269499
ENSG000001	TPT1	protein_codin	1441.20573	2643.49952	2870.29296	1254.26287
ENSG000001	SCUBE2	protein_codin	0	0	2.04307766	0
ENSG000001	EEF1A1	protein_codin	304.670627	0	0	0
ENSG000001	PPM1J	protein_codin	7.5522414	2.72113676	3.84037703	0
ENSG000000	SLC6A15	protein_codin	0	0	0	0
ENSG000001	XCR1	protein_codin	4.88154765	21.6802911	4.59580181	4.9890811
ENSG000001	EHBP1L1	protein_codin	1668.44298	1908.23732	660.991335	902.449371
ENSG000001	SNURFL	processed_ps	0	0	0	0
ENSG000001	ATP6V1G3	protein_codin	0	0	0	0
ENSG000001	EEF2	protein_codin	7999.7647	13610.5139	4241.25169	6316.44267
ENSG000001	ZMIZ2	protein_codin	114.843339	98.7100267	43.6260982	38.8898662
ENSG000001	PPP1R14B	protein_codin	64.8504967	83.9046724	73.9103084	27.0582596
ENSG000001	MICOS13	protein_codin	25.0175524	31.2073495	12.8569552	14.6144279
ENSG000001	ZNRD2	protein_codin	36.6993415	44.8716805	32.3299656	30.0237894
ENSG000001	GRIK1-AS1	lncRNA	0	0	0	0
ENSG000001	ARID5B	protein_codin	42.7374605	0	69.3635145	26.4026651
ENSG000000	MKNK2	protein_codin	126.358786	155.365605	97.9035854	107.436843
ENSG000002	MRPL38	protein_codin	43.3946721	79.6231169	41.4020239	26.5012969
ENSG000001	AKIP1	protein_codin	0	0	1.71359674	0
ENSG000001	FKBP2	protein_codin	54.1379825	77.1196063	34.3380148	33.7310419
ENSG000001	AKIP1	protein_codin	0	21.7629883	0	6.65903692
ENSG000001	BRSK1	protein_codin	0	0	0	0
ENSG000001	KLRC4	protein_codin	0	0	0	0
ENSG000001	MOB1B	protein_codin	465.837449	329.369878	339.92048	373.206714
ENSG000001	SH3RF3	protein_codin	7.00183446	17.0134927	4.2131976	12.7547652
ENSG000001	VEGFB	protein_codin	9.60237456	10.0952823	11.8878722	0
ENSG000001	POLR1G	protein_codin	12.2366001	33.3992056	0	0
ENSG000001	RETREG3	protein_codin	130.969554	232.367352	72.9298489	74.3025521
ENSG000001	ANAPC10	protein_codin	14.2248719	38.4918754	11.9557974	23.791612
ENSG000001	KLF7	protein_codin	493.532244	239.286626	142.084951	237.283204
ENSG000001	NWD2	protein_codin	0	0	1.09253016	0
ENSG000001	MZT2A	protein_codin	10.4129836	10.2061733	13.5837567	2.55344143
ENSG000001	ATG4D	protein_codin	10.9416065	9.14761385	10.5475483	0
ENSG000000	GSDMB	protein_codin	0	0	0	0
ENSG000001	ADORA1	protein_codin	0	0	0	0
ENSG000001	IQCA1	protein_codin	0	0	0	0
ENSG000001	B4GALT2	protein_codin	0	0	0	0
ENSG000001	HADH	protein_codin	22.378169	48.1717402	14.5441899	21.6986126

ENSG000001	CGGBP1	protein_codin	171.248531	179.350988	59.9208109	180.271567
ENSG000001	SLC26A7	protein_codin	0	0	0	0
ENSG000001	OLR1	protein_codin	88.8137442	5.92180859	113.952522	95.8674587
ENSG000002	LIME1	protein_codin	0	0	34.2794957	0
ENSG000001	SUSD5	protein_codin	0	0	0	0
ENSG000001	OR4B1	protein_codin	0	0	0	0
ENSG000001	CHST2	protein_codin	160.640412	166.759912	69.3351801	93.2862625
ENSG000001	PPARG	protein_codin	0	0	0	0
ENSG000001	ATP5MK	protein_codin	78.6226573	114.491224	51.0093649	51.2166414
ENSG000000	DNASE1L1	protein_codin	41.692596	0	0	14.5828327
ENSG000001	MRPL57	protein_codin	59.5023797	66.9255357	29.2093487	40.3215599
ENSG000001	LRFN4	protein_codin	18.1347433	13.6723203	3.20083477	8.53725471
ENSG000001	FAM86B2	protein_codin	0	0	0	0
ENSG000001	APTX	protein_codin	0	0	0	0
ENSG000001	OR1L1	protein_codin	0	0	0	0
ENSG000001	PDE4D	protein_codin	0	0	0	0
ENSG000001	RCE1	protein_codin	0	0	0	0
ENSG000001	PTPN2	protein_codin	2.78293555	0	0	0
ENSG000001	LMAN1L	protein_codin	0	0	0	0
ENSG000001	INHBC	protein_codin	1.02130874	0	0	0
ENSG000001	OR10P1	protein_codin	2.09079947	0	0	0
ENSG000001	KRT2	protein_codin	0	0	0	0
ENSG000001	ZNF680	protein_codin	21.491896	26.149298	12.9445744	14.3658601
ENSG000001	FCRLA	protein_codin	0	0	0	0
ENSG000001	AP1AR	protein_codin	41.2338061	34.3551289	14.6293476	38.5221835
ENSG000001	TMEM255A	protein_codin	0	0	0	0
ENSG000001	GRAMD2A	protein_codin	0	0	0	0
ENSG000001	FAM241A	protein_codin	58.1725005	44.2477758	17.9357687	41.8410322
ENSG000001	RIC3	protein_codin	0	0	0	0
ENSG000001	RND1	protein_codin	6.09190979	0	0.91278837	0
ENSG000001	KLHL3	protein_codin	0	2.87242502	5.84472946	1.41936761
ENSG000001	HSPA6	protein_codin	28.6219155	130.97927	35.8514898	47.2184058
ENSG000001	TOPAZ1	protein_codin	0	0	0	0
ENSG000002	FAUP4	lncRNA	0	0	0	0
ENSG000001	TMEM234	protein_codin	12.2858338	0	0	21.5383464
ENSG000002	TRIM59	protein_codin	20.2925415	9.22812193	5.58693383	10.000716
ENSG000001	COL14A1	protein_codin	0	0	0	1.1183557
ENSG000001	NARF	protein_codin	11.614031	1.47905295	0	1.5239276
ENSG000001	UTP23	protein_codin	47.3894849	26.9670341	13.338184	51.1776778
ENSG000001	HSFY1	protein_codin	0	0	0	0
ENSG000001	SPIRE1	protein_codin	0	0	0	0
ENSG000001	GSTM1	protein_codin	41.2130331	0	0	0
ENSG000001	GGA2	protein_codin	208.351145	279.932276	120.600722	136.219949
ENSG000001	PGAP3	protein_codin	0	0	0	0

ENSG000001	GCC2	protein_codin	0	88.1631205	77.0911434	0
ENSG000002	SMG1P1	transcribed_u	0	12.1443285	0	0
ENSG000001	ABLIM3	protein_codin	0	0	0	0
ENSG000001	ENSG000001	lncRNA	0	0	0	0
ENSG000001	IRF3	protein_codin	0	0	0	0
ENSG000001	ADCY5	protein_codin	0	0	0.76449117	0
ENSG000001	TIGD3	protein_codin	8.78487698	8.81627811	2.87156174	2.87867616
ENSG000001	CD36	protein_codin	0	0	0	0
ENSG000001	TCAP	protein_codin	0	0.87245756	0	0
ENSG000000	ATP2A3	protein_codin	18.403752	152.292193	57.7224622	30.4679571
ENSG000002	CSHL1	protein_codin	0	0	0	0
ENSG000001	HYDIN	protein_codin	0	1.54261196	0	0.78590072
ENSG000002	ZNF407	protein_codin	0	0	0	0
ENSG000001	FBXW8	protein_codin	12.384717	31.0653445	13.3346257	5.38923176
ENSG000001	TRMT10C	protein_codin	20.9930335	25.0178189	10.4953137	32.6582249
ENSG000001	GPR148	protein_codin	0	0	0	0
ENSG000001	DAW1	protein_codin	0	0	0	0
ENSG000001	SEC22A	protein_codin	0	3.83214042	0	6.72892951
ENSG000001	DAPL1	protein_codin	0	0	0	0
ENSG000001	ACO1	protein_codin	26.5123815	20.3918956	13.0361795	23.9666933
ENSG000000	CFLAR	protein_codin	1508.84805	951.391458	858.642633	902.120296
ENSG000002	NAXD	protein_codin	0	0	10.401828	0
ENSG000001	KLK10	protein_codin	0	0	0	0
ENSG000001	CLOCK	protein_codin	0	0	56.5304901	0
ENSG000001	GLE1	protein_codin	128.633336	135.162139	0	19.2398927
ENSG000001	MPPE1	protein_codin	14.2266304	18.1659209	4.54120924	26.5992391
ENSG000001	NDST2	protein_codin	0	0	47.3792937	0
ENSG000001	BAALC	protein_codin	0	6.46950381	0	2.31805557
ENSG000001	TPMT	protein_codin	79.8833582	98.5734127	43.597638	76.8686184
ENSG000001	MAL	protein_codin	0	0	0	0
ENSG000001	DAB2IP	protein_codin	0	0	0	0
ENSG000001	MSL2	protein_codin	154.410666	215.317458	41.6127265	122.116725
ENSG000001	SPTBN2	protein_codin	0	0	0	0
ENSG000001	CLEC7A	protein_codin	23.3430559	49.9497512	38.3889006	23.4774114
ENSG000001	SP3	protein_codin	267.055591	147.4483	172.916015	195.418927
ENSG000001	ATP6V0A4	protein_codin	0	0	0	0
ENSG000001	MIR9-1HG	lncRNA	0	0	0	0
ENSG000000	TSPAN17	protein_codin	43.9410098	0	15.5408323	21.2182333
ENSG000001	DSEL	protein_codin	8.20415688	9.20714464	22.1917961	2.09923627
ENSG000001	RBM4B	protein_codin	16.4959859	24.6255087	6.21384437	0
ENSG000000	HLTF	protein_codin	27.201089	36.2234892	0	17.9464354
ENSG000001	CYP2S1	protein_codin	117.72419	89.0678803	22.951531	71.7254948
ENSG000001	RPL12P13	unprocessed_	1.01864282	2.02025016	3.08933684	2.97213274
ENSG000001	TADA2B	protein_codin	93.7076092	139.539389	54.6363802	44.090714

ENSG000001	AGFG1	protein_codin	708.853727	171.066487	205.312678	358.800105
ENSG000001	CFAP184	protein_codin	4.85782969	10.8211643	5.52409058	8.60481968
ENSG000001	RBM4	protein_codin	0	0	0	30.7257374
ENSG000001	PRLR	protein_codin	0	0	0	0
ENSG000001	LIPT2	protein_codin	1.02270399	10.4629695	1.12292042	2.08508717
ENSG000000	SNCB	protein_codin	0	0	0	0
ENSG000001	PSMD2	protein_codin	318.519775	398.915243	187.701592	231.766781
ENSG000001	LGI4	protein_codin	2.51578657	0	4.64237449	0
ENSG000001	TFDP3	protein_codin	0	0	0	0
ENSG000001	ATP8B3	protein_codin	0	2.35294733	0	0
ENSG000001	KCNE3	protein_codin	335.32266	626.644579	130.00701	311.241807
ENSG000000	MATK	protein_codin	0	0	0	0
ENSG000002	RBM14	protein_codin	176.069679	163.192002	65.5585915	97.9317511
ENSG000000	PSMC5	protein_codin	154.873747	250.450069	95.7849248	103.175937
ENSG000001	KLK6	protein_codin	0	0	0	0
ENSG000001	ZCCHC12	protein_codin	0	0	0	0
ENSG000001	CHRNA9	protein_codin	0	0	0	0
ENSG000001	CTDSPL	protein_codin	0	1.28974898	0	0
ENSG000001	CCS	protein_codin	0	0	0	0
ENSG000001	CYP7B1	protein_codin	0	2.74663419	1.10950917	0
ENSG000001	INSM1	protein_codin	1.01463115	0	0	0
ENSG000002	CCDC13	protein_codin	0	0	0	0
ENSG000001	OR10AD1	protein_codin	0	0	0	0
ENSG000002	TTLL3	protein_codin	0	0	0	0
ENSG000001	ARV1	protein_codin	23.555429	0	4.41632706	18.4710422
ENSG000001	VANGL1	protein_codin	7.76214531	11.0739494	8.94865083	0
ENSG000000	ALAS1	protein_codin	50.4974076	115.142838	4.50939928	52.3036232
ENSG000000	PDE8A	protein_codin	0	0	0	0
ENSG000000	LRCH4	protein_codin	195.927602	155.57654	72.5830471	107.898808
ENSG000000	VPS41	protein_codin	330.861781	239.525345	121.262968	267.608683
ENSG000001	DPP10	protein_codin	0	0	0	0
ENSG000001	CTSF	protein_codin	0	0	0	0
ENSG000001	NSUN5	protein_codin	0	0	4.23588704	1.49500825
ENSG000002	EIF4EBP3	protein_codin	16.0519716	35.8125997	1.85545335	9.29666056
ENSG000001	PIGG	protein_codin	0	34.3922279	0	24.9175382
ENSG000001	ARHGAP32	protein_codin	0	68.5951854	82.222226	75.9866921
ENSG000000	TBX5	protein_codin	0	0	0	0
ENSG000001	CCDC14	protein_codin	0	0	0	0
ENSG000001	GPRC6A	protein_codin	0	0	0	0
ENSG000001	PARP15	protein_codin	0	0	0	0
ENSG000000	GP6	protein_codin	0	62.1503013	0	0
ENSG000001	XXYLT1	protein_codin	83.4533536	102.065251	13.3881405	55.7751001
ENSG000001	ARL10	protein_codin	7.87562646	6.04499923	4.73244101	1.01203239
ENSG000001	METTL18	protein_codin	0	0	0	2.35673131

ENSG000001	CPED1	protein_codin	88.3858845	23.1273141	32.6562695	24.3607126
ENSG000001	SNED1	protein_codin	19.2804445	30.2296295	10.7996711	16.9271831
ENSG000001	AGR3	protein_codin	0	0	0	0
ENSG000000	HHATL	protein_codin	0	0	0	0
ENSG000001	CLTB	protein_codin	0	8.90461094	0	0
ENSG000001	VCPIP1	protein_codin	326.104705	318.442817	167.003265	260.870998
ENSG000001	TNKS	protein_codin	113.945656	78.5834425	44.1084653	38.6744294
ENSG000001	HCFC1	protein_codin	745.422901	1065.45775	346.039864	527.038389
ENSG000001	ZDHHC24	protein_codin	25.5586071	36.6210522	14.0243918	19.6763202
ENSG000001	CASP2	protein_codin	125.484263	143.754109	44.8944197	95.4763431
ENSG000001	NAA20	protein_codin	5.52708829	9.83689182	0	6.86231618
ENSG000001	TBC1D8B	protein_codin	0	0	0	1.67558769
ENSG000001	GPD2	protein_codin	4.81590112	0	0	0
ENSG000001	PPP1R3B	protein_codin	60.1786603	56.1278727	20.8389314	47.071016
ENSG000001	CCDC74B	protein_codin	0	0	0	0
ENSG000001	UCP2	protein_codin	121.084687	92.8347158	25.9896921	41.3075524
ENSG000001	SLC39A1	protein_codin	0	0	0	0
ENSG000001	RFNG	protein_codin	21.0566594	56.8369231	15.9001409	23.1675517
ENSG000001	MEPCE	protein_codin	0	0	0	0
ENSG000001	CELF1	protein_codin	79.4101659	57.2585112	37.4489476	55.5124743
ENSG000001	CBY2	protein_codin	0	0	0	0
ENSG000001	TRPV1	protein_codin	0	0	0	1.5342409
ENSG000001	SUGT1	protein_codin	160.740941	47.3982058	47.4031275	71.1163796
ENSG000001	DDX31	protein_codin	0	0	0	0
ENSG000002	FAM86B3P	lncRNA	1.87996691	0	0	0
ENSG000001	PHOSPHO1	protein_codin	0	0	0	0
ENSG000001	SPSB4	protein_codin	0	0	0	0
ENSG000001	COA1	protein_codin	0	0	1.87990897	2.28549898
ENSG000001	PAAF1	protein_codin	11.4561159	10.3464925	3.49194009	0
ENSG000001	SLC5A6	protein_codin	0	19.2146777	10.9937248	19.8824591
ENSG000001	TERT	protein_codin	0	0	0	0
ENSG000001	FAM131A	protein_codin	26.6719519	29.1959985	0	0
ENSG000001	WNT5B	protein_codin	9.86293597	21.2118003	4.234395	4.88134901
ENSG000001	ZNHIT2	protein_codin	1.34509176	5.66000612	0	0
ENSG000001	RPL10P2	processed_ps	0	0	0	0
ENSG000001	DCAKD	protein_codin	0	0	0	0
ENSG000001	CYB561D1	protein_codin	0	0	0	0
ENSG000001	SULT1B1	protein_codin	106.173617	56.1708402	44.0996127	73.2212565
ENSG000001	MRPL48	protein_codin	8.18080168	39.1351857	13.1412722	0
ENSG000001	NEFH	protein_codin	24.7356578	30.6839864	11.0969543	4.96031558
ENSG000001	CYP4A11	protein_codin	0	0	0	0
ENSG000001	RAB6A	protein_codin	186.473833	120.171012	59.8733747	56.8243133
ENSG000002	SPESP1	protein_codin	0	1.13175548	1.11514303	1.97980725
ENSG000001	ADCY4	protein_codin	0	0	3.43599341	0

ENSG000001	SETD6	protein_codin	0	0	8.09887086	0
ENSG000001	JUP	protein_codin	52.1717431	156.661109	81.8768996	34.6854276
ENSG000001	CFAP70	protein_codin	0	4.37939961	4.57044217	0
ENSG000001	TRAM1L1	protein_codin	0	0	0	0
ENSG000001	ELMO1	protein_codin	172.673398	125.418485	69.9704912	103.595065
ENSG000002	STAG3L5P-PV	lncRNA	22.2519257	46.5662989	14.3319706	0
ENSG000001	FANCI	protein_codin	0	0	36.6662801	0
ENSG000001	MRPS22	protein_codin	14.1446337	36.5106839	3.99579749	13.9741839
ENSG000001	HAP1	protein_codin	0	0	0	0
ENSG000001	CD164	protein_codin	536.415974	402.553411	188.58321	377.742849
ENSG000001	REP15	protein_codin	0	1.0185442	0	0
ENSG000001	MAPK14	protein_codin	0	0	0	0
ENSG000001	KCNJ5-AS1	lncRNA	0	0	0	0
ENSG000001	RASGRP1	protein_codin	0	0	0	0
ENSG000001	MIS18BP1	protein_codin	660.065405	641.015844	476.191679	485.509938
ENSG000001	MAPK10	protein_codin	0	0	0	0
ENSG000001	ADAM17	protein_codin	244.083131	276.070686	136.715384	166.756128
ENSG000001	ZBTB21	protein_codin	83.3936492	49.5300021	46.7201136	42.4642309
ENSG000001	CD300A	protein_codin	0	0	0	0
ENSG000001	CD34	protein_codin	1.44664574	4.89866009	9.73189865	3.05836766
ENSG000001	UGT8	protein_codin	0	0	0	0
ENSG000001	EIF1	protein_codin	77.2464397	66.0497508	17.5141918	0
ENSG000001	POMZP3	protein_codin	7.61379918	36.3912832	0	0
ENSG000000	ANKRD10	protein_codin	294.581277	207.181625	159.58993	196.320784
ENSG000001	DTD2	protein_codin	17.7229965	27.9701141	12.3420417	16.531679
ENSG000000	TNS1	protein_codin	0	0	0	0
ENSG000001	SLC25A30	protein_codin	0	4.46227879	1.90622829	0
ENSG000001	IQCB1	protein_codin	65.7784187	61.8162406	71.0081112	54.6808308
ENSG000001	NEURL3	protein_codin	0	0	0	0
ENSG000001	ZNF621	protein_codin	0	0	2.62136442	0
ENSG000001	MYO1H	protein_codin	0	0	0.79718479	0
ENSG000001	IQCF1	protein_codin	0	0	0	0
ENSG000001	TMEM51-AS1	lncRNA	0	0	0	0
ENSG000001	EVC2	protein_codin	0	0	0	0
ENSG000001	TBC1D16	protein_codin	19.5750268	22.097364	3.73977426	10.7321065
ENSG000001	SLCO2A1	protein_codin	4.86968933	5.68515406	0.93178398	0
ENSG000001	HDLBP	protein_codin	189.629756	249.815292	46.8390582	68.8906879
ENSG000001	CBX2	protein_codin	0.95662985	2.91497597	5.2164124	0.90930933
ENSG000001	SLCO4C1	protein_codin	27.7872544	26.9493535	20.1585184	37.0095186
ENSG000001	CDC20	protein_codin	0	0	16.8351293	0
ENSG000002	CCPG1	protein_codin	146.786238	86.1674264	141.016528	116.236401
ENSG000000	LRRC7	protein_codin	0	0	0	0
ENSG000001	DPY19L1	protein_codin	0	0	0	0
ENSG000001	SFT2D3	protein_codin	40.3944067	54.1140663	12.6806079	16.9519965

ENSG000001	TMEM52	protein_codin	1.69192925	1.67067329	0	0.77041404
ENSG000001	TTLL9	protein_codin	0	0	0.78717361	0
ENSG000001	MRPL11	protein_codin	14.8819122	11.1539763	20.6000069	30.6153449
ENSG000000	BMAL2	protein_codin	0	8.86286148	0	0
ENSG000001	TERF1P1	processed_ps	0	0	0	0
ENSG000001	SPNS1	protein_codin	23.3801203	0	16.9162493	29.8068583
ENSG000001	DNAI2	protein_codin	0	1.50878101	0	0
ENSG000001	ZNF738	protein_codin	1.4135	7.38583131	7.37496909	10.3639058
ENSG000001	MADD	protein_codin	0	0	0	0
ENSG000001	NCALD	protein_codin	0	0	3.65181461	0
ENSG000001	NPAS4	protein_codin	0	0	0	0
ENSG000001	MOBP	protein_codin	0	0	0	0
ENSG000001	ZNF497	protein_codin	3.39691834	0	0	0
ENSG000001	ZNF431	protein_codin	24.2910028	38.1035078	65.7558976	45.2584337
ENSG000001	APBB1	protein_codin	0	9.70090987	0	0
ENSG000001	CARHSP1	protein_codin	24.4670461	16.6020849	6.02122115	12.5174612
ENSG000001	PRDM2	protein_codin	81.1724438	152.57432	43.3569399	68.3039048
ENSG000001	ENSG000001	lncRNA	0	0	0	0
ENSG000001	PCP2	protein_codin	1.56555425	12.6182326	2.61197797	6.66204768
ENSG000001	CSRP2	protein_codin	1.01472537	0	0	0
ENSG000001	DMXL1	protein_codin	161.253251	86.5355432	65.7091195	82.5193457
ENSG000001	BOD1	protein_codin	6.71069713	17.1894388	2.01036504	0
ENSG000001	DNAL1	protein_codin	0	2.03594267	5.17527633	0
ENSG000001	PARL	protein_codin	86.0278821	100.524353	0	30.8053522
ENSG000001	GPR151	protein_codin	0	0	0	0
ENSG000001	PCSK1	protein_codin	0	0	0	0
ENSG000001	RPL38	protein_codin	276.718575	492.762402	94.9451024	293.026623
ENSG000000	KMT2E	protein_codin	255.489552	78.5927255	13.6556632	0
ENSG000001	ARMH3	protein_codin	10.6206432	12.6218803	10.2077382	12.9040035
ENSG000001	SLC19A1	protein_codin	73.9675328	97.5942084	40.8928634	69.1516124
ENSG000001	HEG1	protein_codin	29.2565537	55.621768	8.94636943	30.0805772
ENSG000001	DENND6A	protein_codin	220.392045	201.300438	87.4067912	93.4570516
ENSG000001	PPP2R1B	protein_codin	0	0	1.86184821	0
ENSG000001	P2RY2	protein_codin	0	0	0	9.67280787
ENSG000001	ZFYVE21	protein_codin	27.2975791	58.9222067	14.6854729	17.1570572
ENSG000001	ACOT1	protein_codin	1.10447035	0	0	0
ENSG000000	DPYSL2	protein_codin	1769.53832	1835.09471	436.579038	664.513004
ENSG000001	TNS3	protein_codin	496.192514	684.754872	222.283191	364.050424
ENSG000001	SLC29A2	protein_codin	0	7.02515655	0	0
ENSG000002	VN1R4	protein_codin	0	0	0	0
ENSG000001	FCHSD2	protein_codin	0	0	0	0
ENSG000001	HOXB9	protein_codin	0	0	0	0
ENSG000001	PDE12	protein_codin	116.373041	78.6953914	51.8390743	51.2422157
ENSG000001	B4GAT1	protein_codin	5.90797071	5.96980953	3.93110514	1.95309659

ENSG000000	CNGB1	protein_codin	0	0	0	0
ENSG000001	HRAS	protein_codin	22.3336408	24.3401039	0	11.9458211
ENSG000001	LRCH1	protein_codin	125.250759	105.029673	45.4605251	70.6290932
ENSG000001	DNAH12	protein_codin	1.07237029	0	0	0
ENSG000001	KRT17	protein_codin	0	0	0	0
ENSG000001	ZNF706	protein_codin	74.8587901	61.7281815	19.6562515	40.4465022
ENSG000001	GLUL	protein_codin	100.646678	186.012602	63.2932179	91.6027858
ENSG000000	CD84	protein_codin	0	0	0	0
ENSG000000	TEAD2	protein_codin	0	0	0	0
ENSG000001	INTS6	protein_codin	47.76846	45.5463981	41.4572905	38.876623
ENSG000002	GPR162	protein_codin	29.0851789	33.419484	21.120806	16.3079302
ENSG000001	MARCHF10	protein_codin	0	0	0	0
ENSG000001	ZMYND8	protein_codin	0	0	20.1455809	0
ENSG000001	MAP9	protein_codin	0	0	0	0
ENSG000001	AKT2	protein_codin	0	0	0	0
ENSG000001	ABCC12	protein_codin	0	0	0	0
ENSG000001	TTC9B	protein_codin	0	0	0	0
ENSG000001	WASHC4	protein_codin	20.2739004	0	1.82129998	5.21972728
ENSG000001	RIN1	protein_codin	29.9341579	28.2852414	24.0455501	11.0485944
ENSG000001	IL37	protein_codin	0	0	0	0
ENSG000001	CD248	protein_codin	0	0	0	0
ENSG000001	ZNF624	protein_codin	24.826546	18.4045827	18.7910742	23.7392269
ENSG000001	GNPDA1	protein_codin	33.368734	53.9023129	0	14.4231218
ENSG000001	HERPUD2	protein_codin	179.296196	194.523615	59.4588742	87.5939622
ENSG000001	LSM1	protein_codin	74.8154725	116.125711	71.2731362	50.504032
ENSG000001	OR52W1	protein_codin	0	0	0	0
ENSG000001	RFESD	protein_codin	6.38988321	5.57147467	0	0
ENSG000000	SOX30	protein_codin	0	0	0	0
ENSG000001	WFIKKN2	protein_codin	0	0	0	0
ENSG000001	ARHGAP12	protein_codin	0	0	0	0
ENSG000001	MTRES1	protein_codin	7.96714619	21.0088955	0	7.17460429
ENSG000001	DEPDC7	protein_codin	0	0	0	0
ENSG000001	ZBTB4	protein_codin	41.7878719	125.230784	11.4064722	56.7049992
ENSG000001	HPSE	protein_codin	194.348797	281.851284	99.2968942	141.464512
ENSG000001	RNF26	protein_codin	43.0497179	98.0187721	25.1336193	39.3880702
ENSG000001	ZMAT3	protein_codin	148.099501	104.970156	110.415595	118.070471
ENSG000001	MDM2	protein_codin	17.3636667	13.8359315	0	0
ENSG000001	ZNF490	protein_codin	36.994342	71.2900029	29.6049914	36.4857352
ENSG000001	SLC22A8	protein_codin	0	0	0	0
ENSG000001	CNIH2	protein_codin	0	1.98647933	0	0.93510531
ENSG000001	PLAC8L1	protein_codin	0	0	1.0405939	2.17666523
ENSG000001	DMAC2L	protein_codin	0	0	0	0
ENSG000001	COQ2	protein_codin	3.74382101	1.41273254	0	0
ENSG000001	COQ2	protein_codin	16.2480196	0	0	0

ENSG000001	RAB1B	protein_codin	396.341967	674.371302	243.285399	266.46244
ENSG000001	RAG2	protein_codin	0	0	0	0
ENSG000001	HMGA1	protein_codin	143.709132	213.992983	132.485177	117.703417
ENSG000001	IMPA1	protein_codin	0	8.59865981	0	6.60679768
ENSG000001	RABL6	protein_codin	282.977904	532.986934	217.697912	238.404258
ENSG000000	METTL2A	protein_codin	7.54063137	14.8177354	8.21295085	8.02866153
ENSG000001	PLAC8	protein_codin	64.401355	29.7681886	13.7234746	89.1900897
ENSG000001	ADGRF3	protein_codin	0	0	0.97300467	0.88510424
ENSG000001	DBI	protein_codin	0	5.27035078	0	0
ENSG000001	RPL13	protein_codin	13.1171284	26.4151336	7.57342826	23.2651727
ENSG000001	ISCA1	protein_codin	21.7516418	55.6319734	23.6513284	22.2493136
ENSG000001	RPL21	protein_codin	890.428068	1384.94612	338.299838	731.035287
ENSG000001	GABRB3	protein_codin	0	0	0	0
ENSG000000	CYLD	protein_codin	65.479261	0	0	0
ENSG000001	PPARD	protein_codin	0	0	0	0
ENSG000001	YY1AP1	protein_codin	33.5435519	0	0	4.41831544
ENSG000001	FGFBP3	protein_codin	7.88575963	7.97635958	0.87865275	0
ENSG000001	EDN3	protein_codin	0	0	0	0
ENSG000001	ENGASE	protein_codin	0	63.3108878	14.8113848	37.8561716
ENSG000001	MLC1	protein_codin	34.4285403	31.6106539	13.5336675	17.3644526
ENSG000001	SH3PXD2B	protein_codin	129.363978	53.9511592	25.5772658	46.4844926
ENSG000001	LSP1	protein_codin	2209.33805	2045.38447	827.763746	2113.04433
ENSG000001	ANKS4B	protein_codin	0	0	0	0
ENSG000001	ODAPH	protein_codin	0	0	0	0
ENSG000001	HOXB3	protein_codin	0	0	0	0
ENSG000001	FBXO45	protein_codin	16.2598818	18.4515862	4.73230348	1.81817738
ENSG000001	FBXO31	protein_codin	3.16817335	6.88795031	10.0401731	12.8248139
ENSG000000	ATP9A	protein_codin	52.9122736	165.595442	88.7946128	48.9478859
ENSG000001	THAP6	protein_codin	0	5.08021661	0	0
ENSG000002	AGAP6	protein_codin	3.79739493	0	0	0
ENSG000001	UBQLN3	protein_codin	1.9546128	0	0	0
ENSG000001	C1QTNF1	protein_codin	0.85281227	1.67657912	0	0
ENSG000001	ADPGK	protein_codin	46.8998523	41.0992142	0	0
ENSG000001	UQCRH	protein_codin	202.555721	391.801727	258.9594	207.091752
ENSG000001	HECTD4	protein_codin	71.9040786	27.4533387	36.9482034	26.4463119
ENSG000001	WDR74	protein_codin	18.0645387	14.8619912	3.74407434	0
ENSG000001	IL1RL1	protein_codin	0	0	0	0
ENSG000002	PRSS1	protein_codin	0	0	0	0
ENSG000000	RBFOX1	protein_codin	0	0	0	0
ENSG000001	HIGD2B	protein_codin	0	0	0	0
ENSG000001	TRHR	protein_codin	0	0	0	0
ENSG000001	ZNF408	protein_codin	11.4635514	30.3225476	3.66812721	10.4599131
ENSG000001	PDP2	protein_codin	10.4832518	7.57174485	6.40232549	2.05562624
ENSG000001	PPP1R8	protein_codin	23.8112604	53.2201736	27.1598254	28.1467354

ENSG000001:	SEC31A	protein_codin	0	0	0	49.3291007
ENSG000001	FAM170B	protein_codin	0	0	0	0
ENSG000001	SSX5	protein_codin	0	0	0	0
ENSG000001	OXSR1	protein_codin	307.572983	282.759984	166.067659	188.38424
ENSG000001	SNX31	protein_codin	0	0	0	0
ENSG000002	AQP1	protein_codin	0	0	0	0
ENSG000001	RNF32	protein_codin	0	0	0	0
ENSG000002	ENSG000002	lncRNA	0	0	0	0
ENSG000001	COMMD1	protein_codin	48.9182196	68.4839039	33.342191	47.7967267
ENSG000002	ENSG000002	protein_codin	0	0	1.43147223	0
ENSG000001	MMP14	protein_codin	32.6809948	65.5836382	9.62729689	21.9970947
ENSG000001	SLC22A13	protein_codin	0	0	0	0
ENSG000001	PRG2	protein_codin	1.01602008	7.13210125	0.95175082	0
ENSG000001	TCP11	protein_codin	0	0	0	0
ENSG000001	HSPB7	protein_codin	0	0	0	0
ENSG000001	MRPL52	protein_codin	0	0	1.76843696	0
ENSG000001	ISCU	protein_codin	189.464862	298.023332	134.090779	108.411866
ENSG000001	ERCC4	protein_codin	30.7491526	21.6317238	15.7996461	12.1395806
ENSG000001	F2	protein_codin	0	0	0	0
ENSG000002	ATXN7L3P1	processed_ps	0	0	0	0
ENSG000001	TLR8	protein_codin	168.652361	280.716622	111.0623	256.188988
ENSG000001	KICS2	protein_codin	0	6.25808582	7.980975	1.76385525
ENSG000001	TWNK	protein_codin	6.83666564	0	7.10802592	2.99400319
ENSG000001	ZNF507	protein_codin	0	0	0	0
ENSG000001	TRIB1	protein_codin	459.699035	226.698391	336.581433	278.746544
ENSG000001	MOS	protein_codin	0	0	0	0
ENSG000001	CCL19	protein_codin	0	0	0	0
ENSG000001	KRAS	protein_codin	93.6334145	93.6895448	93.505174	93.3916026
ENSG000000	SCFD1	protein_codin	4.30971868	0	0	0
ENSG000001	NIPAL4	protein_codin	6.81568528	4.81295928	4.04056325	5.82242238
ENSG000001	ABRA	protein_codin	0	0	0	0
ENSG000001	ARHGAP1	protein_codin	321.556759	418.223428	136.961825	168.316345
ENSG000001	GLS2	protein_codin	0	0	0	0
ENSG000001	NRF1	protein_codin	0	0	0	0
ENSG000000	IL5RA	protein_codin	0	0	0	0
ENSG000002	GPR199P	lncRNA	0	0	0	3.34291169
ENSG000001	GAL3ST3	protein_codin	0	0	0.90758897	0
ENSG000000	USP36	protein_codin	0	42.2412507	76.6256629	34.6355421
ENSG000000	CYP3A43	protein_codin	0	0	0	0
ENSG000001	ENSG000001	lncRNA	0.97575965	0	0	0
ENSG000001	ZNF417	protein_codin	65.7950223	40.7206097	27.2084298	31.8703562
ENSG000001	AKNA	protein_codin	469.90637	672.351891	441.144203	317.346121
ENSG000001	RPS14	protein_codin	0	2.50917532	0	3.44197701
ENSG000001	OXR1	protein_codin	258.562085	188.603929	167.249686	183.050945

ENSG000001:	MEN1	protein_codin	0	0	0	0
ENSG000001'	ADGRE1	protein_codin	133.937396	81.7884221	0	0
ENSG000001'	CKAP5	protein_codin	231.499158	220.776021	275.121636	220.122833
ENSG000001'	CTU2	protein_codin	0	4.95962422	2.55960306	0
ENSG000001	NSD2	protein_codin	0	0	0	0
ENSG000001'	MED16	protein_codin	0	0	0	0
ENSG000001'	CATSPER1	protein_codin	12.4331849	13.0015092	2.05467255	13.2407317
ENSG000002	CPT1B	protein_codin	0	24.5788096	0	0
ENSG000001	GAPVD1	protein_codin	92.7908656	150.550595	46.8694741	75.6345784
ENSG000002!	DPY19L2P2	lncRNA	0	0	0	0
ENSG000001'	CST6	protein_codin	0	0	0	0
ENSG000001'	CMKLR1	protein_codin	14.4899212	105.324055	20.822454	21.3760019
ENSG000002	KRT25	protein_codin	0	0	0	0
ENSG000001:	NFE2	protein_codin	116.016172	268.382227	140.254891	78.3738544
ENSG000001'	NLRP6	protein_codin	0	1.7969493	0	0
ENSG000002:	FBLIM1P2	processed_ps	0	0	0	0
ENSG000001'	BANF1	protein_codin	29.4151937	51.2501102	17.2259188	9.11012698
ENSG000001'	RSRC1	protein_codin	0	0	0	0
ENSG000001'	TMEM70	protein_codin	49.3039296	74.9003831	20.6332633	19.0404039
ENSG000001	PHF19	protein_codin	17.5789118	24.4290078	11.2855233	8.65597288
ENSG000000	MSL3	protein_codin	58.2954659	77.5680078	62.930005	66.9230627
ENSG000001	MINDY1	protein_codin	50.7446869	33.2759013	2.57243987	15.9140509
ENSG000001'	FAM3C2P	transcribed_p	9.40097839	12.620947	30.4626151	13.856718
ENSG000001'	MCTP1	protein_codin	177.26874	207.843237	112.486037	82.1079108
ENSG000001'	GLIS1	protein_codin	0	0	0	0
ENSG000001'	EIF1AD	protein_codin	17.2652877	28.1734217	7.49099876	0
ENSG000001:	HP1BP3	protein_codin	333.620174	387.615348	122.151232	182.404583
ENSG000001	NAGPA	protein_codin	80.9024626	98.9812485	0	53.9959407
ENSG000001	DCAF6	protein_codin	87.2005936	164.823923	100.184118	78.4437783
ENSG000000!	CATSPERG	protein_codin	0	0	0	0
ENSG000001	PMP22	protein_codin	0	0	0	0
ENSG000001:	STX16	protein_codin	0	0	0	0
ENSG000001'	SPATA31G1	protein_codin	0	0	0	0
ENSG000001	FOLR1	protein_codin	0	0	0	0
ENSG000001'	RTP1	protein_codin	0	0	0	0
ENSG000001'	OR5J2	protein_codin	0	0	0	0
ENSG000001'	COX6B1P3	processed_ps	0	0	0	0
ENSG000001:	DNAJB5	protein_codin	28.4470482	17.2228867	2.98256042	0
ENSG000001:	ELF5	protein_codin	0	0	0	0
ENSG000001'	OR10AG1	protein_codin	0	0	0	0
ENSG000001'	FNDC9	protein_codin	0	0	0	0
ENSG000001	KCNK10	protein_codin	0	2.09775448	0	0
ENSG000001	PFKM	protein_codin	0	0	0	0
ENSG000000'	SPEG	protein_codin	0	0	0	0

ENSG000001	AMZ1	protein_codin	0	0	0	0
ENSG000001	RUNDC3B	protein_codin	3.06257126	0	0	0
ENSG000001	RACGAP1	protein_codin	0	0	0	0
ENSG000001	TBC1D10C	protein_codin	0	0	0	0
ENSG000001	SART1	protein_codin	360.445253	594.521964	205.997513	278.725716
ENSG000002	PGA5	protein_codin	2.03400852	0	0	0
ENSG000001	CAMSAP1	protein_codin	0	0	0	0
ENSG000000	CLCN6	protein_codin	0	0	23.2014828	0
ENSG000001	POLD4	protein_codin	46.6944262	18.0183121	28.7332838	29.6383003
ENSG000001	SWSAP1	protein_codin	11.1350748	16.8804773	10.351538	0
ENSG000001	DNAH7	protein_codin	4.42617105	0	0	0
ENSG000001	DST	protein_codin	0	0	261.384317	145.87953
ENSG000001	CLCF1	protein_codin	32.801098	32.1807052	17.8019521	20.1754297
ENSG000002	PSG1	protein_codin	0	0	0	0
ENSG000001	GLIPR1L1	protein_codin	0	0	0	0
ENSG000001	GPR152	protein_codin	0	0	0.62997046	0
ENSG000001	FGF5	protein_codin	0	0	0	0
ENSG000001	MACROH2A1	protein_codin	176.092172	391.368605	109.421854	115.749011
ENSG000002	MRPL46	protein_codin	5.36173839	33.9665807	7.8331107	6.89610706
ENSG000001	COL6A5	protein_codin	0	0	0	0
ENSG000001	HOXC5	protein_codin	0	0	0	0
ENSG000001	PEAK1	protein_codin	0	117.150629	0	0
ENSG000001	RMI2	protein_codin	0	3.4572966	0	2.32064164
ENSG000001	VWC2L	protein_codin	0	0	0	0
ENSG000001	PRM1	protein_codin	0	0	0	0
ENSG000001	DRAP1	protein_codin	115.708398	223.75042	119.04993	89.5172373
ENSG000001	NR1D2	protein_codin	202.638224	103.444669	72.7045652	100.884199
ENSG000001	B3GAT1	protein_codin	0	0	0	0
ENSG000001	BTN2A1	protein_codin	0	0	0	0
ENSG000001	CHMP2A	protein_codin	408.113255	643.787699	286.188707	346.211051
ENSG000001	PODN	protein_codin	0	0	0	0
ENSG000001	RESF1	protein_codin	972.407213	733.679902	699.039662	697.20923
ENSG000001	FOSL1	protein_codin	64.3728593	6.970608	20.5533968	31.9488989
ENSG000001	CCDC85B	protein_codin	42.6674506	91.8488903	29.0727971	27.6368736
ENSG000001	TNFRSF10D	protein_codin	51.3514067	64.5539895	16.0412019	33.3780627
ENSG000001	PABPC5	protein_codin	2.94075585	0	0	0
ENSG000001	RPS6KB2	protein_codin	77.7672443	101.430085	36.1742303	55.0829468
ENSG000001	USP15	protein_codin	249.366494	117.192739	99.9364195	114.298693
ENSG000001	FAM174A	protein_codin	0	0	6.27917855	0
ENSG000001	CD7	protein_codin	0	2.4123652	0	0
ENSG000001	YPEL2	protein_codin	455.525427	505.06373	212.146788	264.969754
ENSG000000	ENTPD2	protein_codin	0	0	0	0
ENSG000001	TNP2	protein_codin	0	0	0	0
ENSG000001	ENSAP3	processed_ps	0	0	0	0

ENSG000002	HS3ST5	protein_codin	0	3.41363742	0	0
ENSG000001	GUK1	protein_codin	24.3648442	73.4636531	39.6493671	25.7912051
ENSG000001	CGREF1	protein_codin	0	0	0	0
ENSG000002	GNRHR2	lncRNA	0	0	0	0
ENSG000001	GFM1	protein_codin	0	0	3.34916272	1.42701725
ENSG000001	TCHP	protein_codin	0	19.1623514	77.9081719	0
ENSG000000	AURKA	protein_codin	0	0	0	0
ENSG000002	C1orf167	protein_codin	0	0	1.18518768	0
ENSG000001	TMEM168	protein_codin	32.2192327	26.7671395	37.5258397	15.8405872
ENSG000001	RNF152	protein_codin	3.29059274	0	3.24371371	3.33141444
ENSG000001	RNF166	protein_codin	349.704397	419.678411	122.443142	235.474094
ENSG000001	HR	protein_codin	0	0.7491413	0	0
ENSG000001	LSMEM1	protein_codin	0	2.59152892	21.1297243	0
ENSG000000	GPATCH2L	protein_codin	70.0196677	76.3576316	37.4843577	39.9241245
ENSG000001	MED25	protein_codin	130.087018	130.21748	50.5195861	66.2460923
ENSG000002	KRT18P60	processed_ps	0	0	0	0
ENSG000001	GJC3	protein_codin	0	0	0	0
ENSG000001	KDM3A	protein_codin	0	0	0	0
ENSG000001	AGGF1	protein_codin	133.960613	166.255505	56.9338055	140.715696
ENSG000001	PNPLA1	protein_codin	0	0	0	0
ENSG000001	XIAPP3	processed_ps	6.12273577	0	1.13524731	0
ENSG000000	ZBTB11	protein_codin	0	23.7536966	21.8015697	22.2042896
ENSG000001	KCNRG	protein_codin	0	4.12247231	2.64301003	0
ENSG000001	DOK3	protein_codin	53.8182219	6.68640022	0	0
ENSG000001	JAM2	protein_codin	0	1.05213665	1.93296404	0
ENSG000001	SIAH2	protein_codin	288.671856	436.274776	125.396238	197.906979
ENSG000001	KRI1	protein_codin	0	0	0	39.9516786
ENSG000001	TPT1P10	processed_ps	6.8586109	5.04652358	6.3470014	1.03108878
ENSG000001	TPM3	protein_codin	54.1303319	35.5385742	10.0151154	30.8366893
ENSG000001	TUBA3E	protein_codin	0	0	0	0
ENSG000001	PPP1CA	protein_codin	0	0	0	0
ENSG000001	CDK4	protein_codin	0	0	0	0
ENSG000000	EXD2	protein_codin	0	0	0	0
ENSG000001	BACE1	protein_codin	30.3269683	103.350441	14.3804097	16.3490195
ENSG000001	PRCP	protein_codin	33.3632583	20.4563795	3.74845173	0
ENSG000001	OR8H1	protein_codin	0	0	0	0
ENSG000001	PARP10	protein_codin	127.247105	129.037542	69.9091919	103.992064
ENSG000002	ARNILA	lncRNA	0.99121411	1.97794931	1.18387541	0.9739747
ENSG000001	OR5T3	protein_codin	0	0	0	0
ENSG000001	ZNF227	protein_codin	29.2303305	22.2893493	16.187173	0
ENSG000001	PTH1R	protein_codin	0	0	0	0
ENSG000001	SEC16A	protein_codin	180.336941	560.084121	0	302.444835
ENSG000001	OGFOD3	protein_codin	0	13.2954562	1.03622912	18.559104
ENSG000001	PARP10	protein_codin	0	3.47137463	0	0

ENSG000001	LINC00471	lncRNA	0	0	0	0
ENSG000000	CADPS2	protein_codin	0	0	0	0
ENSG000001	FOXG1	protein_codin	0	0	0	0
ENSG000001	GPR137	protein_codin	0	0	2.84226408	4.72262537
ENSG000001	RHBDF2	protein_codin	6.29585423	0	0	7.6281071
ENSG000001	SEC16A	protein_codin	0	0	0	0
ENSG000001	ARHGAP45	protein_codin	1559.56628	2280.27412	637.02933	1013.99777
ENSG000000	RPLP0	protein_codin	178.444899	273.31337	52.0761824	111.163419
ENSG000001	ATOX1	protein_codin	10.7846044	8.34173938	16.1948146	19.4334482
ENSG000001	ZNF41	protein_codin	0	28.2928944	23.9925029	19.8355333
ENSG000001	SRSF7	protein_codin	42.9963573	58.0906855	82.1313131	0
ENSG000001	VDAC2	protein_codin	0	20.4725349	0	3.17724992
ENSG000001	UTS2R	protein_codin	2.00307661	4.15032522	5.44161823	3.96927879
ENSG000001	DVL3	protein_codin	263.13533	309.875635	111.609912	170.010501
ENSG000001	ISG20L2	protein_codin	96.9205124	110.251227	59.5969038	40.4486484
ENSG000001	CHST12	protein_codin	0	0	0	0
ENSG000001	C7	protein_codin	0	0	0	0
ENSG000001	HOXD8	protein_codin	0	0	0	0
ENSG000001	INTS14	protein_codin	32.0960419	23.6602177	0	0
ENSG000001	PFN4	protein_codin	0	0	3.01663554	0
ENSG000001	CHMP7	protein_codin	69.4847408	0	5.56456769	0
ENSG000001	FBXL13	protein_codin	0	0	0	0
ENSG000001	KRT80	protein_codin	0	1.95827456	0	0
ENSG000001	TMEM25	protein_codin	0	0	0	0
ENSG000000	PHF21B	protein_codin	0	0	0	0
ENSG000001	MAK	protein_codin	0	0	0	0
ENSG000001	TRIP10	protein_codin	0	4.37306221	0	0
ENSG000001	DHRS4	protein_codin	64.6938202	70.4212718	32.7451465	45.3472198
ENSG000001	OR4D11	protein_codin	0	0	0	0
ENSG000001	GRINA	protein_codin	151.493901	153.042282	35.6186755	95.4933131
ENSG000001	TRIP10	protein_codin	41.2898085	36.1200456	15.636846	15.9690891
ENSG000000	TACC3	protein_codin	244.004489	89.6991583	0	0
ENSG000002	TERB1	protein_codin	0	0	0	0
ENSG000000	SPAG6	protein_codin	0	0	0	0
ENSG000001	BTF3L4	protein_codin	52.1375163	49.6520783	14.4660687	31.7176241
ENSG000001	NEUROG2	protein_codin	0	0	0	0
ENSG000002	ENSG000002	transcribed_u	0	0	0	0
ENSG000001	SERTAD2	protein_codin	506.600062	566.79418	263.666087	467.107198
ENSG000001	CLASP2	protein_codin	23.6339316	0	0	0
ENSG000001	PUF60	protein_codin	0	0	0	0
ENSG000000	OSBPL3	protein_codin	85.6756345	60.5904298	35.6682053	15.7279936
ENSG000001	TAF7	protein_codin	161.738947	396.044606	85.7430108	137.986382
ENSG000000	MLXIPL	protein_codin	0	0	0	0
ENSG000001	RMND5B	protein_codin	43.0375295	56.4740094	28.2761937	0

ENSG000001	ASTN2	protein_codin	0	0	0	0.93581176
ENSG000001	CDK5R1	protein_codin	20.3787589	36.38598	5.715609	4.82328907
ENSG000001	NDUFS7	protein_codin	0	0	0	0
ENSG000000	CD6	protein_codin	2.05028077	7.10718505	0	0
ENSG000001	PDE4DIP	protein_codin	26.703598	74.5238339	30.6534747	22.3111408
ENSG000001	ATP2A2	protein_codin	116.814342	65.2595896	53.3863959	56.8393986
ENSG000001	RPA2	protein_codin	0	6.41820113	9.35833841	2.10060776
ENSG000001	ZNF135	protein_codin	0	0	0	0
ENSG000001	OR8K5	protein_codin	0	0	0	0
ENSG000001	SH2D4B	protein_codin	0	0	0	0
ENSG000002	C8orf82	protein_codin	7.95394439	3.38684146	0	4.44216871
ENSG000001	NDUFS8	protein_codin	43.8659901	85.9845748	22.2956681	26.6173959
ENSG000001	OR8H3	protein_codin	0	0	0	0
ENSG000001	MPRIP	protein_codin	0	57.7009152	110.776258	79.2531125
ENSG000001	PITPNA	protein_codin	244.895914	261.027739	84.8012039	122.420168
ENSG000001	LINC00670	lncRNA	0	0	0	0
ENSG000001	OR8H2	protein_codin	0	0	0	0
ENSG000001	TUFM	protein_codin	369.899975	562.781734	233.71076	281.376685
ENSG000001	THAP5	protein_codin	9.91214722	15.011616	0	4.60402153
ENSG000001	SUV39H2	protein_codin	0	0	0	0
ENSG000000	DNAJC25	protein_codin	5.20532513	7.10255181	11.566967	0
ENSG000001	GIMAP7	protein_codin	136.665714	448.575453	63.7566331	115.6764
ENSG000001	OR5J1P	unprocessed_	0	0	0	0
ENSG000001	POC1B	protein_codin	40.4459239	65.878489	51.9790799	20.0985506
ENSG000001	CFAP47	protein_codin	0	0	0	0
ENSG000001	ING5	protein_codin	28.1079624	20.4421058	6.31913844	9.90456678
ENSG000002	ENSG000002	lncRNA	0	0	0	0
ENSG000001	ZNF821	protein_codin	10.1624524	3.7055161	1.98634952	0
ENSG000001	AP4S1	protein_codin	3.54713556	1.17998898	0.96109873	1.14569375
ENSG000001	AQP11	protein_codin	0	0	0	0
ENSG000001	ANKAR	protein_codin	0	0	0	0
ENSG000001	NFKBIB	protein_codin	31.7479374	29.5668068	0	21.8637714
ENSG000001	GMPPA	protein_codin	0	0	0	0
ENSG000001	CD163L1	protein_codin	14.7359605	0	6.28759292	0
ENSG000001	GNAI2	protein_codin	3532.10865	5767.68951	1879.23624	2696.90775
ENSG000001	MRPS23	protein_codin	45.1972965	47.6319991	31.8406545	18.3258107
ENSG000001	MCMDC2	protein_codin	0	0	0	0
ENSG000001	EPSTI1	protein_codin	267.026195	232.325903	108.2708	125.661064
ENSG000001	FZR1	protein_codin	0	0	0	0
ENSG000001	EPSTI1	protein_codin	0	2.92450156	0	3.76297695
ENSG000001	LGALS9	protein_codin	55.7380102	267.876464	53.2374716	102.593096
ENSG000000	LAMA3	protein_codin	0	0	0	0
ENSG000001	DSCC1	protein_codin	0	0	4.10345216	0
ENSG000001	C2CD3	protein_codin	5.8338708	0	0	0

ENSG000001	ARHGEF2	protein_codin	164.396064	373.695513	186.887333	169.675629
ENSG000001	COL26A1	protein_codin	0	0	0	0
ENSG000001	PNMA8A	protein_codin	0	0	0	0
ENSG000001	ARHGEF2	protein_codin	0	0	13.0042498	0
ENSG000001	ACAD10	protein_codin	52.1265939	0	17.9512997	13.7845044
ENSG000001	EBF1	protein_codin	6.88089514	0	0	1.51026207
ENSG000001	DENND2B	protein_codin	0	3.18446164	0	0
ENSG000000	CASP10	protein_codin	0	0	3.00885249	0
ENSG000001	ZDHHC7	protein_codin	607.387956	998.651541	212.093157	411.875716
ENSG000001	OSBPL2	protein_codin	142.725505	226.25025	68.3969147	65.1191941
ENSG000000	NOS2	protein_codin	0	0	0	0
ENSG000001	FLJ13224	lncRNA	3.8989054	1.9901969	1.57208491	0.95983952
ENSG000001	ARMC10P1	processed_ps	0	0	0	0
ENSG000001	PRODH	protein_codin	0	0	0	0.86404703
ENSG000001	RPA3P1	processed_ps	0	0	0	0
ENSG000001	FAM20C	protein_codin	129.613201	154.950143	22.1185808	91.6849422
ENSG000000	FAS	protein_codin	67.8219962	38.2332755	15.1342473	23.3142597
ENSG000000	FUZ	protein_codin	0	8.25110646	0	0
ENSG000001	DALRD3	protein_codin	5.68185211	19.7847874	7.90613893	1.67025322
ENSG000001	RCAN1	protein_codin	51.3299301	46.2120951	0	41.2705127
ENSG000001	EIF3J-DT	lncRNA	0	0	3.32554448	4.09849633
ENSG000001	FBXO34	protein_codin	76.6693756	162.082223	47.7606026	44.8470075
ENSG000001	GPR89A	protein_codin	14.3341542	0	3.10853864	11.2170203
ENSG000001	STX4	protein_codin	61.0400881	116.702153	24.481038	46.774628
ENSG000001	FAM216B	protein_codin	0	0	0	0
ENSG000000	LIMCH1	protein_codin	0	0	0	0
ENSG000000	MKS1	protein_codin	0	0	0	0
ENSG000001	ENSG000001	lncRNA	0	0	0	0
ENSG000001	AKAP17A	protein_codin	469.033651	564.237466	276.014368	310.646353
ENSG000001	HNRNPD	protein_codin	102.400177	55.2962865	32.1303424	49.3372032
ENSG000001	TRDMT1	protein_codin	10.5159167	0	0	0
ENSG000001	OR5AN1	protein_codin	0	0	0	0
ENSG000002	FAM86EP	lncRNA	0	0	0	0
ENSG000000	GNAS	protein_codin	0	0	0	0
ENSG000002	ZNF321P	lncRNA	6.05606124	13.6390521	5.55190759	0
ENSG000001	ZNF606-AS1	lncRNA	0	0	0	0
ENSG000001	RGS5	protein_codin	0	0	0	0
ENSG000001	TEX44	protein_codin	0.76776047	0.76333188	0	0
ENSG000001	B3GALNT2	protein_codin	2.1485461	5.58359752	0	5.58818319
ENSG000001	ZNF436	protein_codin	10.3758565	0	0	10.5504095
ENSG000001	BHLHA15	protein_codin	0	2.33551112	11.7800123	0
ENSG000001	CSNK1D	protein_codin	79.4198869	192.779206	53.7311651	0
ENSG000001	UCP3	protein_codin	7.41947171	3.90120011	6.87199387	3.89922432
ENSG000001	ENSG000001	lncRNA	0	0	0	0

ENSG000001:	DDX54	protein_codin	115.650395	299.575219	0	101.601989
ENSG000001	IFT43	protein_codin	2.76730454	0	0	0.89159508
ENSG000001:	SLAIN1	protein_codin	0	0	0	0
ENSG000001	BICRAL	protein_codin	324.991968	379.141473	155.734094	174.212228
ENSG000001	H2AC6	protein_codin	1096.77457	2288.16606	635.202451	1073.89434
ENSG000002	CTAGE9	protein_codin	0	0	0	1.00411428
ENSG000001	BTBD9	protein_codin	0	0	0	0
ENSG000001	CHAF1B	protein_codin	0	12.1328011	6.60516074	0
ENSG000001	CYP11B1	protein_codin	0	0	0	0
ENSG000001	RELL1	protein_codin	74.2226891	54.7839502	31.4824453	22.8770415
ENSG000000	TMEM40	protein_codin	103.332502	228.918819	50.2688864	92.0080811
ENSG000001	AKAP1	protein_codin	0	0	19.394404	0
ENSG000001	STAT2	protein_codin	336.279866	672.765399	113.764573	155.573315
ENSG000001	COX8A	protein_codin	109.028176	141.778465	14.6460497	74.2320939
ENSG000001	SLC35C1	protein_codin	58.2665762	71.5775458	20.0758286	17.528297
ENSG000001	RPL27A	protein_codin	61.8134908	122.493849	35.9920998	39.8356176
ENSG000001	GPHB5	protein_codin	0	0	0	0
ENSG000001	CCT6B	protein_codin	5.31175981	4.35508837	0	0
ENSG000001	ASAH1	protein_codin	0	0	0	0
ENSG000001	CLEC18C	protein_codin	0	0	0	0
ENSG000001	GPR89B	protein_codin	0	0	7.61647194	0
ENSG000001	GAK	protein_codin	454.744996	639.58488	262.79299	265.251784
ENSG000001	LUZP1	protein_codin	55.2942355	73.1343676	74.0213976	61.0643622
ENSG000001	MEIS2	protein_codin	0	0	0	0
ENSG000002	PRKDC	protein_codin	706.069169	391.737268	337.662073	230.742905
ENSG000000	ANK1	protein_codin	0	0	0	0
ENSG000001	BBS12	protein_codin	8.18492615	7.21421069	5.5641431	3.14661448
ENSG000001	PHLDA2	protein_codin	23.3456157	19.6425309	6.03245494	3.9666563
ENSG000001	ZNF575	protein_codin	0	2.55112133	0	0
ENSG000001	ZBTB12BP	processed_ps	0	0	0	0
ENSG000001	KCNMB3	protein_codin	0	2.29928051	9.71540579	0
ENSG000002	ENSG000002	lncRNA	0	0	0	0
ENSG000001	TNS2	protein_codin	0	0	0	0
ENSG000001	SLC2A2	protein_codin	0	0	0	0
ENSG000001	MRGPRX4	protein_codin	0	0	0	0
ENSG000001	RPS2P28	processed_ps	0.93864282	0	0	0
ENSG000001	TMEM94	protein_codin	0	0	65.7497805	0
ENSG000001	OR4C6	protein_codin	0	0	0	0
ENSG000001	SENP7	protein_codin	0	0	0	0
ENSG000000	NOP14	protein_codin	179.296974	296.281523	185.654594	172.660729
ENSG000001	DEFB104A	protein_codin	0	0	0	0
ENSG000001	TNS2	protein_codin	0	0	0	0
ENSG000001	MRPL48	protein_codin	3.07196053	0	1.19930905	0
ENSG000000	RNF4	protein_codin	90.4731872	132.864162	24.3393595	77.0160423

ENSG000000	ARHGAP28	protein_codin	0	0	0	0
ENSG000001	SPECC1L	protein_codin	0	0	36.6917318	57.6767729
ENSG000001	H2BC4	protein_codin	22.0862093	45.1645591	16.6404673	48.9033433
ENSG000001	AGTRAP	protein_codin	142.174143	167.461921	0	124.311355
ENSG000000	PPP2R2C	protein_codin	0	0	0	0
ENSG000000	ADAMTS6	protein_codin	0	0	0	0
ENSG000001	CKS2	protein_codin	6.05252139	23.1433441	11.4575271	10.6568084
ENSG000001	DEFB103A	protein_codin	0	0	0	0
ENSG000001	KDM3B	protein_codin	653.084578	1050.70503	520.05049	546.966582
ENSG000001	ZNG1A	protein_codin	0	57.3763645	0	0
ENSG000001	C19orf18	protein_codin	0	0.96630276	1.03252179	0.9960846
ENSG000001	DPM2	protein_codin	8.50352404	2.54089844	4.30892778	8.20200965
ENSG000001	ZHX2	protein_codin	65.6371576	67.2208762	23.0107554	43.2366227
ENSG000001	NHP2	protein_codin	13.9090288	0	0	0
ENSG000001	DNAJC28	protein_codin	0	0	0	0
ENSG000001	NEPRO	protein_codin	33.5612406	0	12.9363018	20.5182471
ENSG000001	CD300LB	protein_codin	52.7886784	103.786831	70.5348427	46.2900245
ENSG000001	FUT7	protein_codin	18.7420831	21.5294279	3.29994777	14.0159949
ENSG000001	UTP4	protein_codin	51.0292597	66.6581679	16.30704	32.5098719
ENSG000001	ZMYM3	protein_codin	94.2539167	149.320167	67.6027204	60.3855848
ENSG000001	P2RX4	protein_codin	305.993451	189.50731	125.475407	102.443623
ENSG000001	LILRB2	protein_codin	1766.52465	2079.10161	528.547778	1165.2057
ENSG000001	SAMD4B	protein_codin	174.449556	93.7779432	65.7840447	83.6532635
ENSG000001	TLCD5	protein_codin	0	0	0	0
ENSG000001	LINC02880	lncRNA	0	0	0	0
ENSG000001	IGSF8	protein_codin	19.7268107	36.4459465	0	0
ENSG000001	CSRNP3	protein_codin	0	0	0	0
ENSG000001	MKRN3	protein_codin	0	0.97406993	0.98378124	0
ENSG000001	SUMO2	protein_codin	119.697462	209.089459	72.0177583	108.203762
ENSG000001	PALM2AKAP2	protein_codin	0	3.75194153	0	0
ENSG000001	ZNF620	protein_codin	0	8.14904973	0	0
ENSG000001	ZNF77	protein_codin	5.95461863	8.02204205	13.2288198	8.95400067
ENSG000001	IQCF1	protein_codin	0	0	0	0
ENSG000001	CRHR1	protein_codin	0	0	0	0
ENSG000001	TPD52L3	protein_codin	0	0	0	0
ENSG000001	MITF	protein_codin	0	0	26.6427686	0
ENSG000001	BORCS5	protein_codin	46.7187004	38.4612822	28.9915469	27.2001804
ENSG000002	CDPF1	protein_codin	5.97219672	12.0769477	0	0
ENSG000001	YES1	protein_codin	0	0	21.3236755	0
ENSG000001	HCLS1	protein_codin	1431.9542	2194.19189	373.82455	1061.27592
ENSG000001	EXOC3L1	protein_codin	0	0	0	0
ENSG000001	MITF	protein_codin	0	0	0	0
ENSG000001	R3HCC1L	protein_codin	0	44.0431588	58.1987482	0
ENSG000000	POLR2B	protein_codin	232.795834	179.420364	114.099255	155.720915

ENSG000001	FARSA	protein_codin	74.2916067	70.2552851	0	55.294678
ENSG000001	ZMYM4	protein_codin	335.306389	321.611677	212.986415	265.372758
ENSG000001	SUPT6H	protein_codin	530.339591	524.816011	218.220842	302.080822
ENSG000001	NSUN3	protein_codin	37.8651616	35.7943993	37.2093209	34.864935
ENSG000001	DHFR2	protein_codin	0	21.2757873	0	0
ENSG000001	BVES	protein_codin	0	0	1.25359613	0
ENSG000001	OR4C15	protein_codin	0	0	0	0
ENSG000000	WASHC2A	protein_codin	0	0	118.600176	77.4568099
ENSG000001	PFAS	protein_codin	31.3577166	18.7900647	17.5720623	18.5156656
ENSG000001	FHAD1	protein_codin	0	0	0	0
ENSG000000	SLC13A2	protein_codin	0	0	0	0
ENSG000001	KCNH6	protein_codin	0	0	0	0
ENSG000001	SNX14	protein_codin	0	0	0	0
ENSG000001	HBS1L	protein_codin	0	0	3.23629643	0
ENSG000001	UBXN11	protein_codin	56.5013128	71.7782895	36.9185405	32.9068791
ENSG000001	ZNF619	protein_codin	0	0	0	0
ENSG000001	QNG1	protein_codin	0	0	0	0
ENSG000001	OR52B3P	unitary_pseuc	0	0	0	0
ENSG000001	OR4A16	protein_codin	0	0	0	0
ENSG000001	SAMD12	protein_codin	0	1.11662768	6.60569444	0
ENSG000000	EPN2	protein_codin	0	0	0	0
ENSG000000	ARHGAP33	protein_codin	7.21913732	0	2.61534494	0
ENSG000001	LINS1	protein_codin	9.44686641	21.3862566	0	4.88292138
ENSG000001	NEUROG1	protein_codin	0	0	0	0
ENSG000000	RECQL	protein_codin	0	0	1.48434797	0
ENSG000001	SULT1A1	protein_codin	296.839343	357.413284	109.177877	166.3343
ENSG000001	CD55	protein_codin	0	14.5763999	0	0
ENSG000001	TTC12	protein_codin	0	0	0	0
ENSG000001	SKA3	protein_codin	0	0	10.3522289	0
ENSG000001	WDR75	protein_codin	29.1029752	29.0560513	22.2007598	18.041171
ENSG000002	PINX1	protein_codin	0	21.2853523	0	7.02265238
ENSG000001	COPG1	protein_codin	266.450476	421.418678	233.397084	213.385802
ENSG000000	SH2D3C	protein_codin	232.091555	353.507405	133.676838	179.661595
ENSG000001	PLD5	protein_codin	0	0	0	0
ENSG000002	MSTO2P	transcribed_u	0	0	0	0
ENSG000001	AMBRA1	protein_codin	50.3775856	0	0	0
ENSG000001	IMP3	protein_codin	8.74649053	5.56653453	0	1.24727312
ENSG000001	SLIT1	protein_codin	0	0	0	0
ENSG000001	TLN1	protein_codin	11770.4246	22039.9566	13713.4925	7864.05403
ENSG000001	ANXA2R	protein_codin	19.1963834	19.3368922	3.99951232	5.56071408
ENSG000001	LPCAT4	protein_codin	13.6656999	8.66090722	2.93233141	3.92424408
ENSG000001	BDNF	protein_codin	0	0	0	0
ENSG000001	PENK	protein_codin	0	0	0	0
ENSG000001	HNRNPA0	protein_codin	219.678664	278.366866	107.67981	137.275675

ENSG000001	ANXA2R-OT1	lncRNA	0	0	0	0
ENSG000001	PRIMPOL	protein_codin	40.2829924	0	0	5.89189368
ENSG000001	LGALS7B	protein_codin	0	0	0	0
ENSG000001	EXD1	protein_codin	0	0	0	0
ENSG000001	DBF4B	protein_codin	4.97640826	3.41249943	6.01587221	1.22064367
ENSG000001	EXOC3	protein_codin	0	300.307624	0	0
ENSG000000	OSBPL6	protein_codin	0	0	0	0
ENSG000001	GPR88	protein_codin	0	1.10154116	2.1914907	2.15640663
ENSG000001	TNIP1	protein_codin	107.014717	0	0	0
ENSG000001	CNTD1	protein_codin	0	0	1.15442232	0
ENSG000001	TRIM41	protein_codin	51.3886657	66.740377	34.2063137	37.2714087
ENSG000001	RHOT2	protein_codin	152.243069	114.226205	56.5921943	25.0080067
ENSG000001	ST8SIA5	protein_codin	0	0	0	0
ENSG000001	TARDBP	protein_codin	0	56.4002345	34.659582	21.5914052
ENSG000001	STX19	protein_codin	0	0	0	0
ENSG000001	EDC3	protein_codin	33.0341677	44.5095612	18.0201148	18.982673
ENSG000002	SRSF8CP	transcribed_p	0	0	0	0
ENSG000001	LEO1	protein_codin	0	0	0	0
ENSG000001	FLAD1	protein_codin	20.0200376	0	0	0
ENSG000002	NAIP	protein_codin	160.751722	100.795311	61.0519579	65.0391914
ENSG000001	RAB43	protein_codin	0	0	0	0
ENSG000002	PRSS50	protein_codin	0	0.9147955	1.8524907	1.78478008
ENSG000000	TNRC6A	protein_codin	344.861741	246.510145	97.2374408	114.531915
ENSG000001	GARIN1B	protein_codin	0	0	0	0
ENSG000001	SERTM1	protein_codin	0	0	0	0
ENSG000001	STK33	protein_codin	0	0	0	0
ENSG000001	RNASEH1	protein_codin	102.638358	177.668085	57.2506699	54.9917563
ENSG000000	ADGRA2	protein_codin	29.2411362	69.3530961	19.0116066	0
ENSG000001	CALML3	protein_codin	0	0	0.6332161	0
ENSG000000	RFFL	protein_codin	0	0	0	0
ENSG000000	CHDH	protein_codin	0	1.9944608	0	0
ENSG000000	KIF26A	protein_codin	0	0	0	0
ENSG000001	GPHN	protein_codin	0	6.23997736	0	0
ENSG000001	ASAP2	protein_codin	155.022876	287.375759	255.710387	70.7293713
ENSG000001	MMP1	protein_codin	5.01971224	12.1614383	8.12737355	1.03442215
ENSG000000	SPAST	protein_codin	0	0	3.45134124	15.9095961
ENSG000001	CCDC43	protein_codin	83.2504194	107.25407	58.9973036	32.3151511
ENSG000001	RTKN2	protein_codin	0	1.27334096	0	0
ENSG000002	ENSG000002	lncRNA	0	0	0	0
ENSG000001	TENM3-AS1	lncRNA	0	0	0	0
ENSG000001	IFT22	protein_codin	0	4.1978624	0	0
ENSG000001	FZD2	protein_codin	21.0121101	58.8818804	8.50132916	26.9105517
ENSG000001	CLEC1A	protein_codin	6.71816954	0	0	0
ENSG000001	DAZ3	protein_codin	0	0	0	0

ENSG000001	ARSJ	protein_codin	0	1.10202568	0	0
ENSG000001	PLEKHF2	protein_codin	198.414342	157.0601	56.3686186	90.162543
ENSG000000	ANAPC4	protein_codin	64.2419264	65.3688891	38.8731484	47.4924634
ENSG000001	RPS6	protein_codin	1007.50095	0	861.935027	786.596756
ENSG000001	PLXDC1	protein_codin	0	2.32653168	0	0
ENSG000001	ODAD1	protein_codin	0	0	0	0
ENSG000001	MEN1	protein_codin	19.3275976	0	0	0
ENSG000001	TNIP2	protein_codin	213.846554	344.443091	140.925861	152.711658
ENSG000002	ZNF487	protein_codin	0	24.5402253	0	10.3430746
ENSG000001	WSB2	protein_codin	50.8576459	229.403709	40.9794714	63.3017942
ENSG000001	OR2A4	protein_codin	0	0	0	1.13943514
ENSG000001	DTNA	protein_codin	0	0	0	0
ENSG000001	GCSH	protein_codin	9.11507313	0	3.30693144	0
ENSG000001	ZNF354C	protein_codin	15.3449837	6.19195194	14.2282128	8.29937857
ENSG000001	USP20	protein_codin	7.95062893	34.4766902	28.9908937	34.9704395
ENSG000001	CIMAP1D	protein_codin	0	0	0	0
ENSG000002	TUBB3	protein_codin	0	0	0	0.86939962
ENSG000000	MPP4	protein_codin	0	0	0	0
ENSG000001	OR11H6	protein_codin	0	0	0	0
ENSG000000	AQP6	protein_codin	0	0	0	0
ENSG000000	CNOT4	protein_codin	49.7549774	43.0102993	27.9714553	0
ENSG000000	CDC42	protein_codin	79.613501	66.7505518	24.1955811	11.1461306
ENSG000000	RRP12	protein_codin	0	0	0	0
ENSG000001	MRPL1	protein_codin	10.7380732	15.9947642	2.27265185	15.0691254
ENSG000001	LDLRAD3	protein_codin	77.879926	81.5243879	36.2687237	46.9668978
ENSG000001	ADGRE2	protein_codin	505.287164	733.408311	163.925135	326.003124
ENSG000000	NCAPD2	protein_codin	193.478501	162.358344	116.858536	59.0939062
ENSG000001	ARL6IP4	protein_codin	292.240118	452.980318	83.7377512	212.389587
ENSG000000	CHFR	protein_codin	39.7630008	23.5425387	0	0
ENSG000000	MED29	protein_codin	147.300618	114.139016	0	108.127097
ENSG000000	CD209	protein_codin	0	0	0	0
ENSG000000	PDS5B	protein_codin	355.915921	166.878929	206.536289	226.396201
ENSG000000	CD209	protein_codin	17.0316885	17.0575104	0.83984321	7.8652846
ENSG000002	ENSG000002	protein_codin	0	0	0	0
ENSG000001	ACRV1	protein_codin	0	0	0	0
ENSG000002	NEURL4	protein_codin	0	53.1056194	12.5266094	27.7752435
ENSG000001	RPUSD2	protein_codin	3.90439605	8.50678323	7.19735587	0
ENSG000001	CCL25	protein_codin	0	0	0	0
ENSG000001	ELMOD3	protein_codin	0	0	0	0
ENSG000001	TSC22D3	protein_codin	0	21.7952147	0	9.78179786
ENSG000001	DLGAP1	protein_codin	0	0	0	0
ENSG000001	ZNF843	protein_codin	0	0	0	0
ENSG000001	OR4L1	protein_codin	0	0	1.5444963	0
ENSG000001	DIPK2A	protein_codin	46.8591231	60.9086241	16.6389192	65.0957264

ENSG000001	LINC00324	lncRNA	795.513583	842.475914	257.188319	974.92983
ENSG000000	CD200	protein_codin	0	0	0	0
ENSG000001	ABCD3	protein_codin	1.7976172	2.87404305	0	3.36140169
ENSG000001	ARPC2	protein_codin	1139.17449	1504.23913	371.343062	715.209625
ENSG000001	RPL7A	protein_codin	23.4050633	24.9561253	3.02157465	0
ENSG000001	LSM10	protein_codin	50.5661287	79.4389148	23.560086	72.9740645
ENSG000001	RPL5	protein_codin	1.85172642	3.40917859	1.85362378	5.37318609
ENSG000001	ITGA11	protein_codin	0	0	0	0
ENSG000001	MDH2	protein_codin	187.26772	242.50748	86.1514129	116.522642
ENSG000001	WDR90	protein_codin	0	0	0	0
ENSG000001	HEPHL1	protein_codin	0	0	0	0
ENSG000001	ZMAT4	protein_codin	0	0	0	0
ENSG000002	PYY2	transcribed_u	0	0.85527332	0	0
ENSG000001	SNAP47	protein_codin	0	0	0	0
ENSG000001	CPZ	protein_codin	0	0	0	0
ENSG000001	TCIM	protein_codin	0	0	0	0
ENSG000001	METAP1D	protein_codin	1.98607049	5.0185874	3.22141624	4.0840412
ENSG000001	PHF20L1	protein_codin	30.9952656	0	0	0
ENSG000001	ZNF114	protein_codin	0	0	0	0
ENSG000000	TFE3	protein_codin	243.912976	437.712	181.425508	202.851876
ENSG000000	ING3	protein_codin	2.23713094	9.01395591	5.60419138	2.31855725
ENSG000001	ROCK2	protein_codin	322.047778	125.75066	208.687519	121.485246
ENSG000001	ABHD14B	protein_codin	0	0	0	0
ENSG000001	SLC41A3	protein_codin	0	0	0	0
ENSG000001	RBP7	protein_codin	13.5650332	13.9671204	0	6.21799019
ENSG000001	ADAD2	protein_codin	1.43192234	0	0	0
ENSG000001	DMAP1	protein_codin	33.9573162	61.7678731	0	34.6944231
ENSG000001	OR4K5	protein_codin	0	0	0	0
ENSG000001	YOD1	protein_codin	94.6858802	43.4300491	0	70.8680378
ENSG000001	FGF13	protein_codin	0	0	0	0
ENSG000001	GDPD4	protein_codin	0	0	0	0
ENSG000000	WNK1	protein_codin	197.225079	143.591877	0	0
ENSG000001	OR4N2	protein_codin	0	0	0	0
ENSG000001	LYSMD3	protein_codin	82.5224674	37.5170984	36.784401	37.2791814
ENSG000001	OR4M1	protein_codin	0	0	0	1.32684546
ENSG000001	TRIM55	protein_codin	0	0	0	0
ENSG000001	OS9	protein_codin	703.473447	1153.04638	360.517575	435.770015
ENSG000002	PPP2R2A	protein_codin	0	0	0	0
ENSG000001	KCND3	protein_codin	0	0	0	0
ENSG000001	ASB2	protein_codin	0	0	0	2.64529524
ENSG000001	SH3PXD2A	protein_codin	0	0	0	0
ENSG000001	C16orf89	protein_codin	0	0	0	0
ENSG000000	PKN2	protein_codin	145.611483	169.020288	65.0962368	67.5243351
ENSG000001	WRAP53	protein_codin	29.4845714	19.3031042	3.26008172	14.7345999

ENSG000001	TUBA8	protein_codin	92.8062337	253.285607	147.080607	47.4809697
ENSG000001	ZNF518A	protein_codin	97.3731391	65.3769586	16.2135482	57.0343003
ENSG000001	SMCO3	protein_codin	0	0	1.42260149	0
ENSG000001	EXOSC4	protein_codin	19.1140216	25.4743659	14.4523955	7.5999566
ENSG000001	ZFP91	protein_codin	687.980565	772.941873	456.710704	533.557931
ENSG000001	MCM9	protein_codin	15.2836903	8.70633666	3.57638103	2.49858152
ENSG000002	RPTN	protein_codin	0	0	0	0
ENSG000001	MAP7D3	protein_codin	29.1063946	13.3600465	28.5192847	36.0756312
ENSG000001	TDRD6	protein_codin	0	0	0	0
ENSG000001	NKIRAS2	protein_codin	0	1.77871571	0	0
ENSG000001	RPS4X	protein_codin	2349.72693	3882.34581	1666.11192	1978.32478
ENSG000001	SPATA9	protein_codin	0	0	0	0
ENSG000002	SCAMP4	protein_codin	107.827777	116.361755	25.1362944	0
ENSG000001	HNF4A	protein_codin	0	0	0	0
ENSG000001	PCDHB9	protein_codin	1.12389563	0	0	0
ENSG000001	LINC02724	lncRNA	0	14.6385363	3.54521968	0
ENSG000001	LCN15	protein_codin	0	0	0	0
ENSG000001	ENSG000001	lncRNA	0	0	0	0
ENSG000001	NDUFAF4	protein_codin	1.82235084	4.70443418	3.14246855	1.05171747
ENSG000001	MAP7D1	protein_codin	565.292098	381.182287	140.234707	158.451047
ENSG000001	LARP4B	protein_codin	410.838985	516.863887	187.789158	294.315347
ENSG000001	CYP8B1	protein_codin	0	0	0	0
ENSG000000	RGS11	protein_codin	0	0	0	0
ENSG000001	DEFB104B	protein_codin	0	0	0	1.56762904
ENSG000001	GCNT2	protein_codin	17.10129	19.1769694	3.0945459	8.60423816
ENSG000001	SLC2A11	protein_codin	6.32392355	8.03737164	5.87783024	0
ENSG000001	CSH1	protein_codin	0	0	0	0
ENSG000001	AURKB	protein_codin	0	0	0	0
ENSG000000	PDIA5	protein_codin	27.9970803	93.7788735	53.2588183	15.9696571
ENSG000001	LILRB5	protein_codin	0	0	0	0
ENSG000001	PRIMA1	protein_codin	0	0	0	0
ENSG000001	KCNG2	protein_codin	2.80744298	0	0	0
ENSG000001	BCAT2	protein_codin	5.06298326	14.634022	0	5.80560195
ENSG000001	ZNF786	protein_codin	8.87291493	37.5858321	8.03325826	9.41420585
ENSG000001	EEF1A1	protein_codin	0	0	0	0
ENSG000001	ANO1	protein_codin	0	0	0	0
ENSG000001	PRKAG1	protein_codin	0	0	0	0
ENSG000001	LPA	protein_codin	0	0.89959558	0	0
ENSG000001	CLK4	protein_codin	0	30.9852289	51.3115155	0
ENSG000001	MCM9	protein_codin	97.2235441	77.1950802	54.502954	99.2843667
ENSG000001	UNCX	protein_codin	0	0	0	0
ENSG000001	LNX2	protein_codin	18.8999698	0	25.745674	0
ENSG000001	SLC12A4	protein_codin	65.9881453	72.8974791	23.9847819	38.8380976
ENSG000001	CCNG2	protein_codin	42.4836103	60.2926608	14.3441834	48.9426293

ENSG000001	SYTL2	protein_codin	0	0	8.39158469	0
ENSG000001	ZNF133	protein_codin	0	0	0	0
ENSG000001	KCNA2	protein_codin	0	0	10.9788275	0
ENSG000001	SEMA6D	protein_codin	0	0	0	0
ENSG000002	OR2AE1	protein_codin	1.12959635	0	0	0
ENSG000001	C11orf42	protein_codin	0	0	0	0
ENSG000001	ICOS	protein_codin	0	0	0	0
ENSG000001	CCDC89	protein_codin	0	2.16052814	0	1.0808022
ENSG000001	SOX6	protein_codin	0	3.58947982	0	0
ENSG000001	SIGLECL1	protein_codin	0	0	0	0
ENSG000001	CLU	protein_codin	294.889863	589.452212	237.581621	236.125885
ENSG000001	USF3	protein_codin	326.037645	152.79556	209.025734	321.43389
ENSG000001	FAM89B	protein_codin	11.2202209	20.1240703	0	6.5309772
ENSG000000	AHRR	protein_codin	0	0	0	0
ENSG000001	UGDH	protein_codin	0	45.3826541	30.1505865	34.7666417
ENSG000001	TMEM107	protein_codin	0	0	0	0
ENSG000001	LRRC31	protein_codin	0	0	0	0
ENSG000001	ZNF540	protein_codin	0	0	0	0
ENSG000001	LSMEM2	protein_codin	0	0.86221521	0	0
ENSG000001	CALR	protein_codin	1240.44677	2112.63166	0	1115.14828
ENSG000001	ZBTB8A	protein_codin	2.61693101	0	0.88390934	0
ENSG000001	TTC9C	protein_codin	18.8628731	28.0046857	7.36493277	8.39684181
ENSG000001	ABHD1	protein_codin	0	0	0	0
ENSG000001	KRBOX5	protein_codin	0	0	0	3.08404749
ENSG000001	ZFAND2A	protein_codin	18.0742083	78.3238824	17.5235465	0
ENSG000001	OR52B1P	unprocessed_	0	0	0	0
ENSG000002	VAMP2	protein_codin	126.844301	141.516572	49.2873253	50.2869445
ENSG000002	VN1R76P	unprocessed_	0	0	0	0
ENSG000001	OR56B3P	unprocessed_	0	0	0	0
ENSG000000	AACS	protein_codin	14.3991748	40.440364	16.6689022	14.3447763
ENSG000001	OR56B4	protein_codin	0	0	0	0
ENSG000001	NPHP1	protein_codin	0	0	0	1.11333365
ENSG000000	AACS	protein_codin	2.56522885	2.75064269	0	2.5277601
ENSG000001	SLC9A9	protein_codin	148.419512	130.058141	98.0306422	92.7231468
ENSG000001	CCDC184	protein_codin	0	0	0	0
ENSG000001	PANK2	protein_codin	63.6451028	36.7946518	9.8086507	35.8087628
ENSG000001	TESPA1	protein_codin	0	18.9747045	0	0
ENSG000001	STK25	protein_codin	19.3364347	8.5271913	8.80749926	0
ENSG000001	HNRNPH2	protein_codin	558.689072	416.161528	237.81562	416.395482
ENSG000001	DNAJC7	protein_codin	0	6.40017038	0	0
ENSG000001	ZSCAN26	protein_codin	15.3839727	0	3.79834506	5.138521
ENSG000001	NSUN7	protein_codin	3.28179907	1.68330694	1.6517821	0
ENSG000001	DLC1	protein_codin	0	0	0	0
ENSG000001	MBLAC2	protein_codin	2.99859515	18.1096667	11.812449	2.58984566

ENSG000001	GRB2	protein_codin	0	44.934619	0	0
ENSG000001	FBN1	protein_codin	0	0	0	0
ENSG000000	GSK3B	protein_codin	0	40.1944029	60.7894509	10.9454926
ENSG000001	CDK1	protein_codin	0	3.41668549	9.55298147	3.48987736
ENSG000001	SNAPC5	protein_codin	7.15228765	8.29991613	0	0
ENSG000000	DEF6	protein_codin	308.621669	373.726331	163.077295	138.709882
ENSG000000	IL20RA	protein_codin	0	0	0	0
ENSG000001	PMAIP1	protein_codin	122.329537	65.9414073	72.0171583	82.7170875
ENSG000001	HNF4A	protein_codin	0	0	0	0
ENSG000001	SHMT1	protein_codin	51.955663	31.9739048	19.9831465	0
ENSG000001	PTPN13	protein_codin	0	6.1443684	0	2.10151565
ENSG000000	GPM6B	protein_codin	0	0	0	0
ENSG000001	MTX1	protein_codin	17.8201964	13.8147104	0	8.09819983
ENSG000001	BIN1	protein_codin	0	0	0	0
ENSG000001	CCZ1B	protein_codin	345.691524	351.699122	254.436235	130.192481
ENSG000001	TSPAN16	protein_codin	0	0	0	0
ENSG000001	EFCAB11	protein_codin	1.76629515	2.82643865	0	0
ENSG000001	PUS10	protein_codin	8.6787733	0	6.8574652	0
ENSG000001	RHOJ	protein_codin	0	0	0	0
ENSG000000	APBA3	protein_codin	29.3408381	32.0128804	11.8330629	12.2135246
ENSG000001	VRK3	protein_codin	25.6970547	40.3846486	0	22.3706582
ENSG000001	OR10Q1	protein_codin	0	0	0	0
ENSG000002	LINC01503	lncRNA	14.8011748	16.1205056	3.98072077	4.8538174
ENSG000001	AP3S1	protein_codin	128.521597	142.413067	71.7591879	101.169202
ENSG000001	OXTR	protein_codin	17.0122474	35.9702027	19.328953	10.1477723
ENSG000001	CABP1	protein_codin	0	0	0	0
ENSG000001	GRB2	protein_codin	1138.23821	1402.3733	474.248383	810.432256
ENSG000001	ENSG000001	lncRNA	2.9672311	0	0	0
ENSG000001	ALX1	protein_codin	0	0	0	0
ENSG000001	PNMA1	protein_codin	158.529857	381.124196	109.230775	188.568739
ENSG000001	LLGL1	protein_codin	44.0181513	50.4272357	22.1676065	25.0061858
ENSG000001	MINDY2	protein_codin	24.8694676	0	0	0
ENSG000001	NCAM1	protein_codin	8.75362287	2.39414292	1.03634375	3.09540611
ENSG000001	KLHDC4	protein_codin	2.28842704	0	0	0
ENSG000001	RAD23A	protein_codin	49.3841323	60.2668847	14.8736012	25.9500053
ENSG000001	TRIM25	protein_codin	337.756817	480.850403	181.673566	209.928845
ENSG000000	TRIT1	protein_codin	0	0	0	0
ENSG000000	STOML1	protein_codin	0	0	0	0
ENSG000000	SLC7A8	protein_codin	0	0	0	0
ENSG000000	STOML1	protein_codin	0	6.33449255	0.94062516	0
ENSG000000	MAP2K3	protein_codin	4.69118896	0	7.81624077	0
ENSG000001	KLC2	protein_codin	10.5263057	0	0	0
ENSG000001	TRAPPC14	protein_codin	41.6962179	68.416076	4.60640426	37.4999062
ENSG000001	GADD45GIP1	protein_codin	82.4873396	130.119528	54.2035195	66.9465983

ENSG0000010	CACFD1	protein_codin	0	0	0	0
ENSG0000015	ZNF569	protein_codin	0	12.8099144	0	0
ENSG0000013	LYPLA1	protein_codin	244.141343	118.399387	0	85.8377719
ENSG0000029	ENSG0000029	lncRNA	0	0	0	0
ENSG0000018	PLAG1	protein_codin	6.28142088	5.23863913	20.7483212	7.22664685
ENSG0000014	NSD3	protein_codin	218.312096	270.739934	89.4022777	217.61439
ENSG0000017	RBM44	protein_codin	0	0	0	0
ENSG000001	SMAP1	protein_codin	131.486419	166.169446	79.7808338	142.203728
ENSG0000017	CTDP1-DT	lncRNA	0.67038238	0.66954436	0	0
ENSG0000017	FOXR1	protein_codin	0	0	0	0
ENSG0000017	ZBED2	protein_codin	0	1.00942382	0	0
ENSG0000017	LINC02873	lncRNA	0	0	0	0
ENSG0000010	MATCAP2	protein_codin	0	0	0	0
ENSG0000014	NSD3	protein_codin	193.717566	76.6216911	99.8735015	99.5944687
ENSG0000018	OR52N2	protein_codin	0	0	1.18239962	0
ENSG0000014	MVB12A	protein_codin	23.1474312	44.7707284	2.38456507	17.1202417
ENSG0000017	BAK1P1	processed_ps	0	0.98717071	2.09273743	1.01663372
ENSG0000017	SGF29	protein_codin	36.516422	83.502886	35.440346	34.8784658
ENSG0000017	DAND5	protein_codin	0	0	0	0
ENSG0000013	CHTF18	protein_codin	0	0	0	0
ENSG0000000	DNAJA2	protein_codin	329.860278	558.499573	184.399352	333.332576
ENSG0000017	CES2	protein_codin	132.002378	107.54699	38.2025143	74.1628165
ENSG0000018	OR52N5	protein_codin	0	0	0	0
ENSG0000017	PARL	protein_codin	19.3076526	0	43.2816642	33.6261386
ENSG0000017	VCX2	protein_codin	0	0	0	0
ENSG0000017	DLGAP1-AS1	lncRNA	3.43392888	0	0	0
ENSG000001	PDE4D	protein_codin	34.6531391	38.1751062	31.8976184	35.3020286
ENSG0000018	OR56B1	protein_codin	2.6407387	1.34205898	0	0
ENSG0000017	SPEN-AS1	lncRNA	8.29033895	23.9877979	2.62190456	6.10952219
ENSG0000013	ASCC1	protein_codin	0	25.2740505	24.2653559	0
ENSG0000010	ARHGEF7	protein_codin	0	0	0	0
ENSG0000013	LRCH2	protein_codin	0	0	0	0
ENSG0000012	CNOT1	protein_codin	446.724859	472.383991	349.387277	312.769822
ENSG000001	SLC4A3	protein_codin	0	0	0	0
ENSG0000013	ASCC1	protein_codin	25.0387072	25.7336235	0	0
ENSG0000017	MSRA	protein_codin	25.5629242	80.9715562	17.9143719	42.06672
ENSG0000012	ZNF776	protein_codin	48.3230391	39.0041594	58.1716009	19.522819
ENSG0000010	EEF1D	protein_codin	123.723528	129.850721	31.9269244	72.8602454
ENSG0000010	HOXA3	protein_codin	0	2.91454933	0	0.93896196
ENSG0000007	TOLLIP	protein_codin	153.486137	275.016198	84.4348119	101.540967
ENSG0000017	EGR3	protein_codin	285.722987	170.55591	293.204138	158.431594
ENSG0000018	ZNF559	protein_codin	12.3641261	0	9.96748941	14.0946727
ENSG0000014	AFF3	protein_codin	0	31.9502999	27.6615568	14.1674957
ENSG0000013	MPPE1	protein_codin	14.2906915	0	0	0

ENSG000001	ZNF554	protein_codin	13.2567898	11.9284882	0	0
ENSG000001	MPPE1	protein_codin	4.82132221	4.22499428	0	3.39869774
ENSG000001	SEPTIN4	protein_codin	0	0	9.63627534	0
ENSG000001	SETDB2	protein_codin	0	0	0	0
ENSG000001	SEPTIN4	protein_codin	0	0	0	0
ENSG000002	PVRIG	protein_codin	0	0	0	0
ENSG000001	PER1	protein_codin	1418.9586	698.830081	404.69678	272.700257
ENSG000001	DNAJC14	protein_codin	0	0	0	0
ENSG000001	MIR7-3HG	lncRNA	0	0	0	0
ENSG000000	STAG3	protein_codin	0	0	0	0
ENSG000000	EYA2	protein_codin	0	0	0	0
ENSG000002	INSL3	protein_codin	3.85005324	0	0	0
ENSG000001	CD300LG	protein_codin	0	0	0	0
ENSG000001	ODF2L	protein_codin	0	6.8166215	13.4988983	11.1422906
ENSG000000	SEZ6	protein_codin	0	0	0	0
ENSG000001	PFKFB3	protein_codin	0	0	0	0
ENSG000001	SOBP	protein_codin	0	0	0	0
ENSG000000	BID	protein_codin	166.955524	158.650339	75.5991082	64.7401505
ENSG000000	ICA1	protein_codin	0	17.043915	7.97680708	0
ENSG000001	AP1B1	protein_codin	102.390743	145.755768	0	0
ENSG000001	SLC2A4	protein_codin	0	0	0	0
ENSG000001	SSUH2	protein_codin	0	0	0	0
ENSG000001	SPOCK2	protein_codin	0	0	0	0
ENSG000001	TRAPPC5	protein_codin	6.15252823	0	0	14.6603207
ENSG000001	GLYATL1	protein_codin	0	0	0	0
ENSG000001	HRH3	protein_codin	0	0	0	0
ENSG000000	ZNF671	protein_codin	14.8583742	24.3637191	0	8.81539384
ENSG000001	GEN1	protein_codin	0	0	34.4503295	22.9644994
ENSG000000	RABGAP1	protein_codin	5.16070508	10.8845334	1.48620166	2.79963768
ENSG000001	CEP112	protein_codin	0	0	0	0
ENSG000001	MAMDC4	protein_codin	0	0	0	4.31468815
ENSG000001	ACSF3	protein_codin	19.6584403	0	0	0
ENSG000001	LRRN4CL	protein_codin	0	0	0.93664441	0
ENSG000001	TRPT1	protein_codin	0	0	0	0
ENSG000001	KANK4	protein_codin	0	0	0	0
ENSG000001	PXMP2	protein_codin	2.9456906	3.00054187	0	4.9973908
ENSG000001	RAB23	protein_codin	0	9.64928419	0	0
ENSG000001	NTRK3	protein_codin	0	0	0	0
ENSG000001	MTG1	protein_codin	10.4920711	15.7233856	11.480917	9.84880217
ENSG000001	RANBP10	protein_codin	100.277814	150.623434	52.5116943	71.5624657
ENSG000000	PRSS8	protein_codin	0.90187409	0	0	0
ENSG000001	TLE3	protein_codin	28.5720735	0	9.00895178	46.5494049
ENSG000001	TPM1	protein_codin	0	8.22968581	0	0
ENSG000000	MRPS24	protein_codin	34.907224	45.6025723	27.6931754	26.0606397

ENSG000001	ZMYM6	protein_codin	0	0	0	0
ENSG000002	SLURP2	protein_codin	0	0	0	0
ENSG000001	CERS5	protein_codin	25.9662132	24.9990802	0	0
ENSG000001	MID1	protein_codin	0	0	0	0
ENSG000002	PGAM5	protein_codin	5.09209261	2.51629105	3.41748475	12.642848
ENSG000001	PPHLN1	protein_codin	0	0	0	0
ENSG000001	FRMD8	protein_codin	106.884363	189.239137	68.9547231	96.517313
ENSG000001	TTC39C	protein_codin	41.9005339	136.297681	32.8890432	140.446891
ENSG000001	SIX5	protein_codin	0	0	0	0
ENSG000000	BCL2L13	protein_codin	60.4299398	101.894679	24.5266392	49.6668095
ENSG000001	DISC1	protein_codin	4.37517301	1.06185491	0	2.1730919
ENSG000001	S100Z	protein_codin	5.53034964	11.285703	7.16292774	22.8660735
ENSG000001	FXYD2	protein_codin	0.85690013	0	1.02801097	0
ENSG000001	LINC02363	lncRNA	1.05027693	1.97115857	3.80657355	1.02373748
ENSG000001	MAB21L2	protein_codin	0	0	0	0
ENSG000001	RTN4	protein_codin	488.732207	605.968194	112.710497	302.3815
ENSG000001	RGS3	protein_codin	0	0	0	0
ENSG000001	PSMG2	protein_codin	51.4857175	63.2298496	0	24.0278198
ENSG000001	PROM2	protein_codin	0	0	0	0
ENSG000001	PCNX4	protein_codin	0	0	36.5250776	15.1976567
ENSG000001	C2orf73	protein_codin	0	0	0	0
ENSG000001	TMEM11	protein_codin	24.0196942	44.6478294	14.2432986	24.6855055
ENSG000001	SIGLEC7	protein_codin	54.4079066	114.967904	25.1431795	51.7694507
ENSG000001	NOSTRIN	protein_codin	0	0	1.04338488	0
ENSG000001	ENSG000001	lncRNA	0	0	0	0
ENSG000001	CAPZA3	protein_codin	0	0	0	0
ENSG000001	PROM2	protein_codin	0	0	0	0
ENSG000001	DEFB124	protein_codin	0	0	0	0
ENSG000002	CPNE1	protein_codin	0	0	38.6699305	27.0652097
ENSG000001	FBXO46	protein_codin	54.5925245	58.9305274	20.6038653	33.8686773
ENSG000001	ASB8	protein_codin	82.1880579	159.380153	64.1714829	83.0432808
ENSG000001	TUBB6	protein_codin	95.247504	81.2173659	72.1805527	14.1322341
ENSG000001	AK5	protein_codin	0	0	0	0
ENSG000001	CDHR3	protein_codin	0	0	0	0
ENSG000000	SLC35C2	protein_codin	0	0	0	15.0936467
ENSG000001	RRH	protein_codin	0	0	0	0
ENSG000001	WSCD1	protein_codin	0	0	0	0
ENSG000001	HOPX	protein_codin	0	9.51053178	0	0
ENSG000001	UGT2B17	protein_codin	0	0	7.29348351	0
ENSG000001	DRICH1	protein_codin	1.88236626	6.51073836	1.33208885	1.9180217
ENSG000001	IL1RAP	protein_codin	0	0	0	0
ENSG000001	SOHLH2	protein_codin	0	0	1.35314979	0
ENSG000000	NOS1	protein_codin	0.92391225	0	0	0
ENSG000001	HADHB	protein_codin	196.915687	206.742949	39.2370376	150.099756

ENSG000001	TSPYL6	protein_codin	0	0	0	0
ENSG000001	FJX1	protein_codin	0	0	0	0
ENSG000001	HES7	protein_codin	0	0	0	0
ENSG000001	TACC1	protein_codin	438.693802	354.490085	146.379001	312.781707
ENSG000001	AMOTL1	protein_codin	57.5204369	0	0	35.5659132
ENSG000001	OR2D3	protein_codin	0	0	0	0
ENSG000001	OR14C36	protein_codin	0	0	0	0
ENSG000001	ATG4C	protein_codin	13.3630095	15.9474234	4.69343628	30.0432387
ENSG000001	HNRNPCL1	protein_codin	0	0.92476546	0.91018912	0
ENSG000001	NME9	protein_codin	0.99976138	0	0	0
ENSG000000	SLC25A5	protein_codin	411.70381	551.397343	133.135558	236.235072
ENSG000000	HFE	protein_codin	0	0	0	0
ENSG000001	MAGEF1	protein_codin	27.175821	70.4938042	20.2554514	5.85481547
ENSG000001	SPAG11B	protein_codin	0	0	0	0
ENSG000002	BAG1P1	processed_ps	0	0	0	0
ENSG000001	RECQL5	protein_codin	26.9119482	46.3447497	0	0
ENSG000000	TTC27	protein_codin	28.7666569	51.0487406	40.6364458	17.5605721
ENSG000001	L3MBTL4	protein_codin	3.2138205	7.7084196	17.910668	16.6810323
ENSG000001	KCTD1	protein_codin	0	0	0	0
ENSG000000	MYO3B	protein_codin	0	0	0	0
ENSG000001	COX14	protein_codin	0	1.73212119	0	4.7295878
ENSG000001	CCDC34	protein_codin	0	0	1.95095945	0
ENSG000001	NKPD1	protein_codin	0.93465153	0	0	0
ENSG000001	RNF32	protein_codin	1.08145145	0	0	0
ENSG000000	KDM5D	protein_codin	0	0	0	0
ENSG000001	OR2M7	protein_codin	0	0	0	0
ENSG000000	RAP1GAP	protein_codin	0	0	0.51964914	0
ENSG000001	PDLIM5	protein_codin	239.937843	210.945635	120.189373	157.149865
ENSG000001	SLC5A10	protein_codin	0	0	0	0
ENSG000001	FDCSP	protein_codin	0	0	0	0
ENSG000000	GRAMD1A	protein_codin	0	0	0	0
ENSG000001	CA8	protein_codin	0	0	0	0
ENSG000001	CC2D1A	protein_codin	152.537623	143.305322	52.4022924	63.9365191
ENSG000001	VPS39	protein_codin	211.497863	254.370068	125.597814	157.883812
ENSG000001	PDLIM5	protein_codin	22.7970536	0	0	0
ENSG000000	LASP1	protein_codin	773.205778	1087.80713	441.386019	659.302452
ENSG000002	GANC	protein_codin	130.478966	136.097662	44.7559115	74.2796026
ENSG000001	OR2T33	protein_codin	0	0	0	0
ENSG000000	CAPN3	protein_codin	0	0	0	0
ENSG000000	USH1C	protein_codin	0	0	0	0
ENSG000001	RNF168	protein_codin	237.207616	329.890847	121.473678	141.137183
ENSG000001	IL11RA	protein_codin	0	0	0	0
ENSG000001	OR4F17	protein_codin	0	0	0	0
ENSG000002	PAM16	protein_codin	0	4.94345329	0	0

ENSG000001	MICU3	protein_codin	0	3.15991661	1.05246431	0
ENSG000001	NFATC1	protein_codin	0	54.7794521	32.766723	0
ENSG000001	UBE2N	protein_codin	76.3987658	39.8123086	12.1035261	42.6940077
ENSG000001	SYTL1	protein_codin	0	75.4087487	26.8884643	29.9567472
ENSG000000	CRAT	protein_codin	50.8153331	0	0	0
ENSG000001	MAMSTR	protein_codin	0	0	0	0
ENSG000001	TOM1L2	protein_codin	0	0	0	0
ENSG000002	RTEL1	protein_codin	0	6.47438762	0	12.4839625
ENSG000001	OR2M1P	unprocessed_	0	0	0	0
ENSG000000	DERL3	protein_codin	4.04279246	6.41405837	5.40852746	0
ENSG000001	ZNF101	protein_codin	0	0	0	2.11711915
ENSG000000	CLSPN	protein_codin	0	1.7730399	11.9782984	4.42475826
ENSG000001	DEFB103B	protein_codin	0	0	0	0
ENSG000001	GINS3	protein_codin	0	0	0	3.78754652
ENSG000001	EDEM3	protein_codin	212.574648	117.027882	0	171.872788
ENSG000001	DEFB4B	protein_codin	0	0	0	0
ENSG000001	GRHPR	protein_codin	75.8419067	145.892261	36.4032871	75.7871952
ENSG000001	GREM2	protein_codin	0	0	0	0
ENSG000001	OVCH1	protein_codin	0	0	0	0
ENSG000001	WDFY3-AS2	lncRNA	0	0	0	0
ENSG000001	TPRKB	protein_codin	0	0	0	1.83597574
ENSG000001	PLCZ1	protein_codin	0	0	0	0
ENSG000001	DGKZ	protein_codin	0	0	0	0
ENSG000001	ZSCAN4	protein_codin	0	0	0	0
ENSG000001	RBM24	protein_codin	0	0	0	0
ENSG000001	MYO1D	protein_codin	11.8584549	13.7605267	51.0547408	3.82560776
ENSG000001	NBPF3	protein_codin	14.8010531	30.0953525	12.9395749	7.28659867
ENSG000001	PKD2L1	protein_codin	0	0.8473894	0	0
ENSG000001	C3orf22	protein_codin	0	0	0	0
ENSG000001	ALOXE3	protein_codin	0	0	0	0
ENSG000001	SAMD9L	protein_codin	410.372503	136.948408	204.907769	183.594869
ENSG000001	C18orf32	protein_codin	17.9788155	19.8994373	8.18277877	7.58599187
ENSG000001	OR2AJ1	protein_codin	0	0	0	0
ENSG000001	LMO7DN	lncRNA	0	0	0	0
ENSG000001	RORC	protein_codin	0	0	0	0
ENSG000001	NBPF3	protein_codin	0	0	0	17.6426538
ENSG000001	PTPRA	protein_codin	0	64.7555993	0	0
ENSG000001	MRTFB	protein_codin	0	0	0	17.3065242
ENSG000002	TSTD1	protein_codin	0	1.70269219	0	0
ENSG000001	NINJ2-AS1	lncRNA	31.6656755	0	21.0099242	20.0190743
ENSG000001	PPFIBP1	protein_codin	0	7.6824924	5.25119718	0
ENSG000001	ZC3H14	protein_codin	2.74534732	0	0	0
ENSG000001	BBS1	protein_codin	12.7518532	32.3185795	13.1518072	0
ENSG000001	C5orf46	protein_codin	0	0	0	0

ENSG000001	LRR1	protein_codin	0	1.81064588	0	0
ENSG000000	MRPL43	protein_codin	4.58301423	2.43006137	1.94215916	1.24553786
ENSG000001	FUT6	protein_codin	0	0	0	0
ENSG000001	GLTP	protein_codin	261.29315	325.864577	101.153915	193.429091
ENSG000001	HTR3C	protein_codin	0	0	0	0
ENSG000001	CTAGE10P	processed_ps	0	0.88201033	0	0
ENSG000001	ANKRD23	protein_codin	0	0	0	0
ENSG000000	MRPL43	protein_codin	46.1115112	44.8361251	0	36.212469
ENSG000001	SLBP	protein_codin	0	9.18424428	54.1491419	0
ENSG000001	ZWINT	protein_codin	0	0	0	0
ENSG000001	NDUFV2	protein_codin	120.190656	210.127224	104.001736	86.6998126
ENSG000001	BOK	protein_codin	0.80743531	0	0	0
ENSG000001	WASHC5	protein_codin	253.57864	109.318902	94.4867225	172.919653
ENSG000001	CD276	protein_codin	0	0	0	0
ENSG000001	LINC01559	lncRNA	0	1.02232957	0	0
ENSG000001	TMC8	protein_codin	175.311947	289.97977	103.3078	128.857807
ENSG000001	SYCN	protein_codin	0	0	0	0
ENSG000001	ZNF366	protein_codin	14.805361	48.7468795	9.05014554	18.9132202
ENSG000001	CD276	protein_codin	0	0	0	0
ENSG000001	SLCO3A1	protein_codin	580.968695	399.960853	124.29243	318.456032
ENSG000001	GAPT	protein_codin	159.123814	111.340299	0	97.9296181
ENSG000001	PEX7	protein_codin	4.16913867	0	0	4.66616933
ENSG000001	MBIP	protein_codin	26.9481546	0	24.1045392	0
ENSG000001	MET	protein_codin	0	0	0	0
ENSG000001	ZNF185	protein_codin	0	0	0	0
ENSG000001	CXCR2	protein_codin	17.1178721	33.0525339	14.1422581	21.5756895
ENSG000001	EML4	protein_codin	275.501459	172.603527	100.711991	91.2003623
ENSG000000	NFX1	protein_codin	75.4572128	64.1555001	47.6772438	43.8861263
ENSG000001	ERICH5	protein_codin	0	0	0	0
ENSG000001	ZNF185	protein_codin	25.6695373	162.150139	172.248988	94.7684111
ENSG000001	NACAP2	processed_ps	0.92092499	0	0	0
ENSG000000	ABL1	protein_codin	0	15.1976608	12.8109464	0
ENSG000001	SFTPC	protein_codin	0	0	0.76720617	0
ENSG000001	EPS8L2	protein_codin	0	0	0	0
ENSG000001	HID1	protein_codin	0	0	0.64421724	0
ENSG000000	MEOX1	protein_codin	0	0	0	0
ENSG000001	FKRP	protein_codin	28.0105374	44.7644165	10.7496285	10.6637929
ENSG000001	SLC25A42	protein_codin	6.95540933	17.545406	9.63841087	3.40428523
ENSG000001	A2M	protein_codin	0.93700089	0	1.41095397	0
ENSG000000	PABPC1	protein_codin	2501.23467	2379.60595	198.838546	1509.43507
ENSG000000	RHBDD2	protein_codin	39.2725334	33.1926421	0	10.9880984
ENSG000001	FIBIN	protein_codin	0	0	0	0
ENSG000001	MAP6D1	protein_codin	5.77752552	5.8902451	3.17006019	0
ENSG000001	CA5B	protein_codin	114.011351	92.8998143	33.0524012	45.1708991

ENSG000001	ORAI3	protein_codin	62.1805562	117.84873	31.1223209	48.1778998
ENSG000001	OR10D3	protein_codin	0	0	0	0
ENSG000001	SLC38A9	protein_codin	0	0	0	0
ENSG000002	ENSG000002	protein_codin	0	0	0	0
ENSG000001	EIF4E1B	protein_codin	0	0	0	0
ENSG000001	B3GNT3	protein_codin	0	0	0	0
ENSG000001	ANKFN1	protein_codin	0	0	0	0
ENSG000001	AVL9	protein_codin	164.035552	121.427035	45.1576265	104.970907
ENSG000001	ATXN7L1	protein_codin	0	8.37609202	0	0
ENSG000001	SHOC1	protein_codin	0	0	0	0
ENSG000001	LRRC75B	protein_codin	0	0	0	0.82807711
ENSG000001	FOXP1	protein_codin	0	0	0	0
ENSG000001	PAQR4	protein_codin	41.0376569	37.5070828	21.5955405	16.0777762
ENSG000001	HDDC2	protein_codin	0	20.8064224	0	0
ENSG000001	FOXP1	protein_codin	0	0	0	0
ENSG000001	SYNGR1	protein_codin	0	19.4792666	0	0
ENSG000001	ST6GALNAC5	protein_codin	1.01594958	0	0	0
ENSG000000	LTBP4	protein_codin	0	0	0	0
ENSG000001	ZFP3	protein_codin	27.1920035	22.7953729	7.96976982	12.0643782
ENSG000001	IGSF3	protein_codin	0	0	0	3.1928319
ENSG000001	ZFYVE1	protein_codin	50.5876916	0	0	0
ENSG000001	SYT9	protein_codin	0	0	0	0
ENSG000001	C3orf38	protein_codin	66.0990673	80.6321351	36.2278495	52.3161199
ENSG000001	C1orf131	protein_codin	11.3012739	2.03534874	0	4.5643069
ENSG000001	CYC1	protein_codin	113.127199	139.777174	47.7024928	73.6053379
ENSG000000	ANKRD24	protein_codin	0	0	10.3665076	0
ENSG000001	NRIP1	protein_codin	735.206645	422.595126	427.454753	571.745147
ENSG000001	SWAP70	protein_codin	195.054967	80.0041528	84.1968052	187.14636
ENSG000001	ALS2CL	protein_codin	7.38042035	8.9508627	6.96781387	6.76411186
ENSG000001	INTS3	protein_codin	110.840264	143.990187	55.6836367	113.815722
ENSG000001	PTPN6	protein_codin	1255.03941	1464.88431	425.666367	727.748846
ENSG000001	CTXN1	protein_codin	0	0.79457563	0	0
ENSG000001	CTDNEP1	protein_codin	11.0406418	11.1706981	3.44716651	7.50636987
ENSG000001	ATAT1	protein_codin	0	7.19752566	0	4.01328086
ENSG000001	GEMIN4	protein_codin	29.6062726	73.1113644	34.5085027	24.5280141
ENSG000001	TALDO1	protein_codin	672.432342	1489.7887	480.257539	658.095663
ENSG000001	TMEM184A	protein_codin	0	0	0	0
ENSG000001	SLC25A20	protein_codin	41.9561212	91.1738694	29.6543551	59.9204099
ENSG000001	TMEM184A	protein_codin	1.51354741	0	0	0
ENSG000001	NADSYN1	protein_codin	158.445851	211.267685	100.992003	127.677977
ENSG000001	ATAT1	protein_codin	0	3.79137094	0	0
ENSG000001	ALG2	protein_codin	0	25.2888883	18.4092281	0
ENSG000001	SH3BGRL3	protein_codin	21.9094087	26.7331229	1.50236043	17.3261291
ENSG000001	WFIKKN1	protein_codin	2.63600881	0	0.88662969	0

ENSG000001:	RNF170	protein_codin	0	0	0	0
ENSG000001	C14orf119	protein_codin	18.4058587	41.5283398	24.0820031	25.8346183
ENSG000001	MLXIP	protein_codin	752.580254	967.216097	444.398919	316.217375
ENSG000002	ZBTB48	protein_codin	4.96546565	0	0	0
ENSG000001	NAP1L4P1	processed_ps	87.7461646	36.3732637	63.9460614	46.8579952
ENSG000001	PSAPL1	protein_codin	0	0	0	0
ENSG000001:	RNF170	protein_codin	2.17515915	1.00903883	0	1.0986995
ENSG000001:	TBC1D15	protein_codin	0	0	0	0
ENSG000001:	RALGPS1	protein_codin	0	0	0	0
ENSG000001	ZBTB34	protein_codin	227.869471	231.325125	134.648574	110.276229
ENSG000001	KLHL11	protein_codin	52.0469148	38.5545111	31.2301515	29.9494632
ENSG000001	FBF1	protein_codin	10.4335909	0	10.9858745	3.59204233
ENSG000001	APOL1	protein_codin	10.7774417	0	10.0767951	0
ENSG000001	DNAJB8	protein_codin	0	0	0	0
ENSG000001	CES5A	protein_codin	0	0	0	0
ENSG000000	TRO	protein_codin	0	0	0	0
ENSG000001	RAPH1	protein_codin	77.750965	43.7838509	16.7855579	24.0088769
ENSG000001	TMEM39A	protein_codin	24.4985184	19.4365598	21.0398042	10.6720129
ENSG000000	TTC7A	protein_codin	582.811757	551.855527	134.489288	198.378987
ENSG000001	DIS3L	protein_codin	0	0	0	0
ENSG000001	MPDZ	protein_codin	0	0	0	0
ENSG000001	F2R	protein_codin	308.778162	289.141109	199.049225	121.467209
ENSG000001	DIS3L	protein_codin	33.1127354	115.274644	38.1168639	0
ENSG000001	MPDZ	protein_codin	0	0	0	0
ENSG000001	LRRC42	protein_codin	0	5.60217722	0	0
ENSG000001	KCNK10	protein_codin	0	0	8.41446632	0
ENSG000001	SHISA3	protein_codin	0	0	0.9224164	0
ENSG000001:	GFRA4	protein_codin	0	0	0	0
ENSG000001	PRDX1	protein_codin	124.714816	215.960705	71.0681362	102.583995
ENSG000001	LURAP1L	protein_codin	3.13330776	10.7117111	0	1.05691601
ENSG000000	TAC1	protein_codin	0	0	0	0
ENSG000001	GORASP1	protein_codin	0	0	0	0
ENSG000001	DCTPP1	protein_codin	7.69965768	0	3.25066039	0
ENSG000001	ZNF572	protein_codin	2.07335139	1.03463746	0	2.05606545
ENSG000001	UBASH3A	protein_codin	0	0	0	0
ENSG000001	ZNF771	protein_codin	0	0	1.71808539	0
ENSG000001	SERINC4	protein_codin	0	0	0	0
ENSG000001	LRRN1	protein_codin	1.0970579	0	1.8107527	0
ENSG000000	P3H2	protein_codin	0	0	0	0
ENSG000001	IGSF22	protein_codin	0	0	0	0
ENSG000001	CHST13	protein_codin	13.4801422	20.7476745	5.0015844	1.85513049
ENSG000001	ETV4	protein_codin	0	0	0	0
ENSG000001	STK17A	protein_codin	33.9820953	28.9203016	38.7126916	12.0003393
ENSG000001:	ELL3	protein_codin	0	2.56986559	0	0

ENSG000001	IL17RA	protein_codin	2284.38547	3851.33763	941.454841	1805.631
ENSG000001	ZDHHC22	protein_codin	0	0	0	0
ENSG000001	UBE2O	protein_codin	212.469576	425.875401	215.710601	146.833059
ENSG000001	ITPRID1	protein_codin	0	0	0	0.79946734
ENSG000001	WDTC1	protein_codin	98.6208779	85.3964036	0	0
ENSG000001	ETS1	protein_codin	86.2523941	77.2698509	86.0952582	11.3042036
ENSG000001	LCLAT1	protein_codin	2.58790127	2.44187027	1.88486677	0
ENSG000000	KARS1	protein_codin	0	44.4462087	0	0
ENSG000002	TEX35	protein_codin	0	0	0	0
ENSG000001	SHISA2	protein_codin	0	0	0	0
ENSG000001	OR6B2	protein_codin	0	0	0	0
ENSG000001	SLC35E3	protein_codin	69.7592377	38.3458658	31.4264343	31.5663676
ENSG000001	CCDC93	protein_codin	163.073218	98.0745944	55.6654811	13.8973728
ENSG000001	PCK1	protein_codin	0.98464743	0	0	0
ENSG000001	RUFY1	protein_codin	127.723516	148.991348	184.440788	134.615325
ENSG000001	SORBS2	protein_codin	0	0	0	0
ENSG000001	OTOS	protein_codin	0	0	0.66227716	0
ENSG000001	MCFD2	protein_codin	149.622168	178.383194	86.0799444	103.013862
ENSG000001	SORBS2	protein_codin	0	0	0	0
ENSG000001	OSBPL1A	protein_codin	69.5181086	80.1701794	35.0437365	0
ENSG000001	KREMEN2	protein_codin	0	0	0	0
ENSG000001	TMUB2	protein_codin	62.4190131	78.6780798	18.5360401	0
ENSG000001	TFAP2A	protein_codin	0	0	0	0
ENSG000001	ADGRL2	protein_codin	0	0	0	0
ENSG000001	CPNE7	protein_codin	0	0	0	0
ENSG000001	RPP40	protein_codin	0	2.09404175	0	0
ENSG000001	SCD5	protein_codin	3.04803618	10.7489999	3.20846082	4.97349829
ENSG000000	MRI1	protein_codin	6.25769443	32.3689356	6.82549629	24.7275199
ENSG000001	NT5DC1	protein_codin	40.0119771	35.9353218	14.514096	42.8733731
ENSG000001	KRBA1	protein_codin	0	0	19.6639853	0
ENSG000001	ZFPM1	protein_codin	48.0308837	63.269792	26.7380752	25.158075
ENSG000001	TMEM31	protein_codin	0	0	0	0
ENSG000001	FARP1	protein_codin	0	3.9226741	2.74119828	1.02845595
ENSG000001	ANKDD1A	protein_codin	12.1829531	0	0	10.393709
ENSG000000	ARID1B	protein_codin	0	4.48281696	0	0
ENSG000001	KLK12	protein_codin	0	0	0	0
ENSG000001	HTR1F	protein_codin	7.49097681	1.0716819	8.38854525	2.17751099
ENSG000000	PKM	protein_codin	52.6643651	71.7018387	19.9658591	23.1082348
ENSG000001	ZNF479	protein_codin	0	0	0	0
ENSG000001	EPHA10	protein_codin	0	0	0	0
ENSG000001	LRRC37A3	protein_codin	0	0	29.796381	9.59136115
ENSG000001	FMN2	protein_codin	0	0	0	0
ENSG000001	TMCO5A	protein_codin	0	0	0	0
ENSG000001	ARHGEF18	protein_codin	46.1615375	0	0	14.7464237

ENSG000001	DCAF4L2	protein_codin	0	0	0	0
ENSG000001	SLC24A3-AS1	lncRNA	0	0	0	0
ENSG000001	TMTC1	protein_codin	10.4472873	0	2.1110827	0
ENSG000001	KIDINS220	protein_codin	3.27458025	11.3388472	19.7188366	11.2689955
ENSG000001	PRR15	protein_codin	0	0.81268677	0	0
ENSG000001	LINC01699	lncRNA	0	0	0	0
ENSG000001	RBBP6	protein_codin	437.013637	395.688453	167.805241	414.771837
ENSG000001	KLK11	protein_codin	0	0	0	0
ENSG000001	FUBP3	protein_codin	98.5982814	143.927954	50.2014453	73.9536997
ENSG000001	KLK11	protein_codin	0	0	0	0
ENSG000002	OR4A5	protein_codin	0	0	0	0
ENSG000001	ENSG000001	lncRNA	0	0	0	0
ENSG000001	B4GALT3	protein_codin	23.6256994	44.9858669	30.8082042	26.0753459
ENSG000001	ZNF384	protein_codin	0	97.2042362	0	22.6457052
ENSG000001	EPC1	protein_codin	0	0	0	0
ENSG000002	ZNF611	protein_codin	0	0	0	0
ENSG000001	VKORC1	protein_codin	0	0	0	14.8344518
ENSG000001	NECTIN3	protein_codin	0	0	0	0
ENSG000001	OR4C5	protein_codin	0	0	0	0
ENSG000001	ENSG000001	lncRNA	0	0	0	0
ENSG000001	RPL21	protein_codin	18.2225722	33.5525708	12.0846814	5.27820244
ENSG000001	DYRK2	protein_codin	2.09311275	0	0	0
ENSG000001	MARCHF8	protein_codin	165.352264	249.513013	101.112911	96.1369184
ENSG000001	GPR35	protein_codin	7.48524381	0	0	0
ENSG000001	ZNF140	protein_codin	0	0	0	1.22006693
ENSG000001	SHROOM1	protein_codin	0	0	0	0
ENSG000001	OR4C3	protein_codin	0	0	0	0
ENSG000001	GATD1	protein_codin	37.9737185	99.6618318	20.7935153	35.94213
ENSG000001	METTL4	protein_codin	0	5.74024495	13.6268899	16.7287259
ENSG000001	AIFM1	protein_codin	0	0	0	0
ENSG000001	RNF213	protein_codin	395.390686	526.642566	212.721793	220.272697
ENSG000001	MOB3C	protein_codin	128.474174	253.467031	141.627123	30.6818406
ENSG000001	MACIR	protein_codin	11.6636574	25.8443447	5.95315132	5.91737167
ENSG000001	RABGGTB	protein_codin	10.146019	0	1.83018027	6.04337997
ENSG000001	RNF157	protein_codin	0	0	1.63031872	0
ENSG000000	SLC4A8	protein_codin	0	5.60145755	0	0
ENSG000001	C2orf69	protein_codin	77.5267412	40.3723243	32.3522577	57.3459942
ENSG000001	HROB	protein_codin	0	0	0	0
ENSG000000	IFT88	protein_codin	18.3327152	7.82192047	0	0
ENSG000001	OR4S1	protein_codin	0	0	0	0
ENSG000001	OR2G3	protein_codin	0	0	0	0
ENSG000001	CNGB3	protein_codin	0	0	0	0
ENSG000001	SCAP	protein_codin	0	0	0	0
ENSG000001	HSPH1	protein_codin	0	0	0	0

ENSG000000	ITGA3	protein_codin	0	0	3.3133428	0
ENSG000001	ARL4D	protein_codin	1.91977744	1.8790119	0	0
ENSG000001	OR4X1	protein_codin	0	0	0	0
ENSG000001	PRPF18	protein_codin	14.8764291	32.9597262	46.366281	30.4826207
ENSG000001	IL17RD	protein_codin	0	0	0	0
ENSG000001	GIT2	protein_codin	111.930161	240.610343	78.999192	125.39497
ENSG000001	OR2G2	protein_codin	0	0	0	0
ENSG000002	PSG11	protein_codin	0	0	0	0
ENSG000001	CLIP4	protein_codin	6.51750475	0	0	12.3230412
ENSG000001	METRNL	protein_codin	318.146862	222.466538	53.0864541	105.421719
ENSG000000	LMO3	protein_codin	0	0	0	0
ENSG000001	ZNF454	protein_codin	0	0	0	0
ENSG000002	PMF1-BGLAP	protein_codin	0	0	1.20015487	0
ENSG000000	PRTFDC1	protein_codin	11.2128161	22.8500578	10.0717623	6.37197619
ENSG000001	DDI2	protein_codin	0	3.16028335	11.8860685	7.39258321
ENSG000001	ZNF48	protein_codin	0	0	0	0
ENSG000001	SGIP1	protein_codin	0	0	0	0
ENSG000001	TENT5A	protein_codin	226.948141	200.738887	126.898222	141.081831
ENSG000001	FGF8	protein_codin	0	0	0	0
ENSG000000	PITPNM2	protein_codin	111.956145	266.264739	141.72009	39.7049164
ENSG000002	LINC00301	lncRNA	0	0	0	0
ENSG000001	RBPMS	protein_codin	0	0	0	0
ENSG000001	ATRIP	protein_codin	14.6580171	31.1164947	22.6286424	6.50854256
ENSG000001	ITGB2	protein_codin	1186.43687	1216.57207	164.359736	650.164038
ENSG000001	ZDHHC20	protein_codin	59.9426682	62.6895442	27.1767466	101.673326
ENSG000002	RNPS1	protein_codin	163.154048	298.310845	177.996022	146.112585
ENSG000001	SLC25A22	protein_codin	24.8355421	7.34874207	8.9972157	13.2562488
ENSG000001	NHLH2	protein_codin	0	0	0	0
ENSG000001	TFEC	protein_codin	0	7.77399979	18.4519093	8.20085269
ENSG000001	FOXL1	protein_codin	0	0	0	0
ENSG000001	NEK6	protein_codin	233.813535	245.471003	116.500639	187.397709
ENSG000001	KBTBD11	protein_codin	91.2926717	151.277968	47.4847254	91.7485268
ENSG000001	LRRC37A	protein_codin	0	5.68464374	0	0
ENSG000002	ASPRV1	protein_codin	0	0	2.02684223	0.95096295
ENSG000001	DNAI7	protein_codin	0	0	0	0
ENSG000001	RRS1	protein_codin	41.1707705	67.2573787	35.0669286	44.6732605
ENSG000000	SCAMP1	protein_codin	53.3946574	35.5303441	32.6085748	58.0207278
ENSG000000	AGPAT4	protein_codin	264.800789	117.449312	79.5307791	63.2761184
ENSG000001	HYAL1	protein_codin	0	0	0	1.75853014
ENSG000001	PCDH15	protein_codin	0	0	0	0
ENSG000001	TAGLN2	protein_codin	329.171714	182.906076	822.6456	113.627541
ENSG000000	PHF23	protein_codin	0	117.843544	0	34.0023193
ENSG000001	ENSG000001	lncRNA	0	0	0	0
ENSG000002	PAGR1	protein_codin	106.144446	151.941145	42.1919823	39.1597604

ENSG000001	OLFML3	protein_codin	0	0	0	0.86861705
ENSG000000	STEEP1	protein_codin	4.24585502	14.9581875	0	0
ENSG000001	INPP5K	protein_codin	0	11.1955548	6.63801815	2.61083802
ENSG000001	EZH2	protein_codin	5.54751361	0	5.01602082	0
ENSG000001	SLC25A19	protein_codin	0	0	4.14063185	0
ENSG000001	ITPRID2	protein_codin	156.193351	198.856317	87.4944802	102.271576
ENSG000001	RPL23P2	transcribed_p	0	0	0.82761192	0
ENSG000001	FAM230I	lncRNA	0	0	0	0
ENSG000001	KIF1A	protein_codin	0	0	0	0
ENSG000000	BCKDHB	protein_codin	9.36432713	0	6.46493172	4.43671906
ENSG000001	IQCK	protein_codin	0	0	0	0
ENSG000000	ELN	protein_codin	0	0	0	0
ENSG000002	MTHFD2P5	processed_ps	0	0	0	0
ENSG000001	AMIGO3	protein_codin	0	0	6.63703261	5.80475058
ENSG000001	CARF	protein_codin	0	0	0	0
ENSG000001	ZNF121	protein_codin	138.691032	100.763034	90.6948911	85.4364117
ENSG000001	GLIPR1L2	protein_codin	0	0	0	0
ENSG000000	TP63	protein_codin	0	0	0	0
ENSG000001	B3GALNT1	protein_codin	0	0	0	0
ENSG000001	SCRIB	protein_codin	81.6239481	168.874424	77.2551665	108.001807
ENSG000001	CIDEA	protein_codin	0	0	0	0
ENSG000000	ELN	protein_codin	0	0	0	0
ENSG000002	CFHR1	protein_codin	0	0	0	0
ENSG000001	CCDC65	protein_codin	0	0	0	0
ENSG000001	SLC16A8	protein_codin	0	0	0	0
ENSG000001	TMEM270	protein_codin	0	0	0	0
ENSG000001	MBOAT4	protein_codin	0	0	0	0
ENSG000001	GPS1	protein_codin	4.74309763	27.6199179	5.28380303	40.8939131
ENSG000001	ZNF596	protein_codin	0	3.42662815	2.45967219	0
ENSG000001	ANO6	protein_codin	999.815486	1206.03824	745.228887	886.937919
ENSG000001	KDF1	protein_codin	0	0	0	0
ENSG000001	POLE	protein_codin	0	154.488739	0	89.491808
ENSG000001	RAB39A	protein_codin	25.2766052	16.9611201	11.598371	8.17350721
ENSG000001	PACS1	protein_codin	444.290179	701.293371	238.357562	276.162349
ENSG000001	ZNF655	protein_codin	16.0062345	36.4526592	0	27.3142056
ENSG000001	ANKRD30B	protein_codin	0	0	0	0
ENSG000001	CHORDC1	protein_codin	55.8644701	44.0132418	5.20114528	19.2762301
ENSG000001	PIANP	protein_codin	0	0	0	0
ENSG000000	ZDHHC8	protein_codin	78.8261926	70.580222	26.7630756	41.9252264
ENSG000001	ZNF236	protein_codin	109.290563	63.456267	47.934996	37.1138719
ENSG000001	ZNRF1	protein_codin	23.1354153	11.5072629	2.45001348	0
ENSG000001	NQO1	protein_codin	3.27002704	0	0	0
ENSG000001	EHD1	protein_codin	458.973107	276.372841	193.235843	252.03262
ENSG000001	FAIM2	protein_codin	0.88858119	0	0	0

ENSG000001	AKAP5	protein_codin	0	0	0.88095766	0
ENSG000001	ITFG1	protein_codin	163.874213	171.347797	95.1165304	128.369727
ENSG000001	IL15	protein_codin	23.4269178	10.7539923	18.337344	19.6690836
ENSG000001	CHRM2	protein_codin	0	0	0	0
ENSG000001	KYAT1	protein_codin	0	0	0	0
ENSG000001	SSBP2	protein_codin	55.2110665	33.4202981	28.0656521	27.0775359
ENSG000001	RBMX	protein_codin	282.593122	326.786767	152.738706	267.498437
ENSG000001	INPPL1	protein_codin	7.32887416	1.48353371	0	2.32747635
ENSG000001	PIK3R1	protein_codin	40.4481397	3.17798083	0	8.6620229
ENSG000001	DUS3L	protein_codin	0	0	0	0
ENSG000001	DNASE1L2	protein_codin	0	0	0	0
ENSG000001	TSPY2	protein_codin	0	0	0	0
ENSG000001	OR6S1	protein_codin	0	0	0	0
ENSG000001	GLS	protein_codin	0	0	0	0
ENSG000001	PELI3	protein_codin	51.4566921	67.8600571	17.0965946	21.1115166
ENSG000001	ADIPOQ	protein_codin	0	0	0	0
ENSG000001	LRIG3	protein_codin	0	0	0	0
ENSG000002	ADAM20P1	transcribed_u	1.06207682	1.06980243	4.03944588	0
ENSG000001	UEVLD	protein_codin	0	0	0	0
ENSG000001	COL9A1	protein_codin	0	0	0	0
ENSG000001	MAGOHB	protein_codin	9.84118749	8.89481301	2.52071636	4.119052
ENSG000001	ARL14	protein_codin	0	0	0	0
ENSG000001	BLOC1S4	protein_codin	7.66326061	22.7234151	2.19213159	10.0564577
ENSG000001	KIF1C	protein_codin	276.133692	465.270897	165.95403	178.426998
ENSG000001	NECAP1P1	processed_ps	0	0	0	0
ENSG000001	NFATC2IP	protein_codin	190.318901	251.034483	106.96674	113.27917
ENSG000001	DAB2	protein_codin	485.486235	725.828547	255.309478	346.399862
ENSG000001	KLK8	protein_codin	0	0	0	0
ENSG000001	MRFAP1L1	protein_codin	52.6174111	119.409771	56.7122174	41.378973
ENSG000001	AAR2	protein_codin	40.2678898	60.2252282	0	20.2802971
ENSG000001	SSR4	protein_codin	4.4571109	7.48413509	0	0
ENSG000001	B3GNTL1	protein_codin	29.1398765	18.6120805	15.9083439	10.1112434
ENSG000002	HBA1	protein_codin	8.28799244	51.4284938	1.39094784	5.4004892
ENSG000001	SMCHD1	protein_codin	707.680342	186.872861	361.816248	383.062268
ENSG000001	RNF144A	protein_codin	10.3393076	17.2980851	4.98300944	0
ENSG000000	CLPTM1L	protein_codin	108.558973	171.740696	20.9300312	60.068911
ENSG000001	OR4F21	protein_codin	0	0	0	0
ENSG000001	MRFAP1	protein_codin	2.67999044	8.04424498	4.57678424	2.22457991
ENSG000001	MRGPRF	protein_codin	0	0	0	0
ENSG000002	FMN1	protein_codin	102.234688	32.5720509	10.1207777	24.3807552
ENSG000001	PCSK7	protein_codin	56.7274746	44.6055999	4.85887099	72.2829063
ENSG000000	CIRBP	protein_codin	47.915589	107.367241	14.1562185	43.8757491
ENSG000001	CRTAP	protein_codin	511.106243	667.07443	203.794061	432.250231
ENSG000001	SPTBN5	protein_codin	50.3001226	43.2841781	9.94655879	6.76024099

ENSG000001	RCN2	protein_codin	20.8534268	21.7050016	22.1345062	19.2289362
ENSG000001	SEC14L4	protein_codin	0	0	0	0
ENSG000001	ZEB1	protein_codin	0	0	28.2804357	0
ENSG000001	IRX5	protein_codin	0	0	0	0
ENSG000001	PITPNB	protein_codin	39.9652073	121.011406	0	0
ENSG000000	IGSF9B	protein_codin	0	1.32319341	0	0
ENSG000001	VWF	protein_codin	0	0	0	0
ENSG000000	GCFC2	protein_codin	0	9.58008464	0	12.3012714
ENSG000001	PRPF31	protein_codin	195.458035	296.3226	134.540145	177.049011
ENSG000000	ENPP4	protein_codin	29.9455432	4.11417126	23.8056804	11.5483119
ENSG000001	VN1R1	protein_codin	0	2.44269756	1.1014452	0
ENSG000000	NR1H4	protein_codin	0	0	0	0
ENSG000002	PLXNA4	protein_codin	7.83445232	37.6513611	0	0
ENSG000001	TOP3A	protein_codin	89.0108353	232.75889	79.9228754	88.9932255
ENSG000001	RTL3	protein_codin	0	0	0	0
ENSG000001	DNMT3A	protein_codin	17.3880499	38.7510234	33.0478407	26.6183261
ENSG000001	PRORP	protein_codin	0	5.33926855	0	1.90488125
ENSG000000	HCCS	protein_codin	0	0	5.56406893	0
ENSG000000	DPP8	protein_codin	0	16.8572325	12.6955263	29.7209052
ENSG000002	DNAAF4	protein_codin	0	0	0	0
ENSG000001	RPLP2	protein_codin	3044.37101	4652.09927	1156.64351	2064.811
ENSG000001	HACL1	protein_codin	41.3012471	39.9379109	0	15.485324
ENSG000001	TMTC2	protein_codin	0	0	0	0
ENSG000001	SEC14L4	protein_codin	0	0	0	0
ENSG000001	LINC00304	lncRNA	0	0	0	1.68728073
ENSG000001	TGIF2LY	protein_codin	0	0	0	0
ENSG000000	ZNF207	protein_codin	0	0	0	16.2685265
ENSG000002	MTRF1LP1	processed_ps	0	0	0	0
ENSG000001	LEPROTL1	protein_codin	90.7301576	150.388946	41.2048683	69.6463337
ENSG000001	OR6T1	protein_codin	0	0	0	0
ENSG000000	TUBA3D	protein_codin	0	0	0	0
ENSG000001	NCBP2	protein_codin	34.6797309	26.4816828	0	53.6793103
ENSG000001	D2HGDH	protein_codin	0	0	0	5.44952211
ENSG000000	NUDC	protein_codin	202.006144	324.53384	150.517725	157.754267
ENSG000001	SGPP2	protein_codin	3.22739113	1.0866739	0.93677815	0
ENSG000001	BAIAP2	protein_codin	24.0065923	80.9691795	0	15.7180148
ENSG000002	H3P14	transcribed_p	4.44729352	18.7305777	5.78357776	10.6763861
ENSG000001	ZNF610	protein_codin	1.00825046	1.99480037	0	0
ENSG000001	ATG16L2	protein_codin	414.290579	537.041217	177.864972	245.154072
ENSG000001	BAIAP2	protein_codin	0	3.49171081	0	0
ENSG000001	TOMM5	protein_codin	65.0662745	121.526511	42.3542491	43.4459131
ENSG000001	DSCAML1	protein_codin	0	0	0	0
ENSG000001	FOLR2	protein_codin	1.99964381	2.18601211	0	0
ENSG000001	TMEM214	protein_codin	0	0	42.238609	0

ENSG000001	HIGD1A	protein_codin	43.6486832	26.9403074	4.94008408	39.8774283
ENSG000001	PRDM16-DT	lncRNA	0	0	0	0
ENSG000001	SENP3	protein_codin	80.9900305	96.3795039	40.9534806	46.2546704
ENSG000001	FTMT	protein_codin	1.63664975	0	0	0
ENSG000000	ME2	protein_codin	81.1497546	40.9391862	38.9046011	31.9494985
ENSG000000	CYBRD1	protein_codin	113.171836	155.037715	91.7635349	185.68056
ENSG000001	OR8D4	protein_codin	0	0	0	0
ENSG000000	CLK1	protein_codin	0	0	17.1523568	0
ENSG000000	YBX1	protein_codin	2041.83535	3011.54292	1551.76938	1644.8207
ENSG000001	SEPTIN1	protein_codin	21.5237044	34.8223489	64.5743407	13.7179579
ENSG000001	CCDC33	protein_codin	0	0	0	0
ENSG000001	RNF151	protein_codin	0	0	0	0
ENSG000001	TCP1	protein_codin	125.160022	115.420999	72.0926846	71.4762033
ENSG000001	PRDM16-DT	lncRNA	0	0	0	0
ENSG000001	GCC1	protein_codin	112.112919	178.792506	88.929395	83.2581027
ENSG000001	ENSG000001	processed_ps	0.94168567	3.69932651	0	0
ENSG000001	AMER3	protein_codin	0	0.84889539	0	0
ENSG000001	SIGLEC8	protein_codin	0	0	0	0
ENSG000001	DUOX1	protein_codin	0	0	0	0
ENSG000001	RAB8B	protein_codin	404.82937	304.309362	175.230419	390.20975
ENSG000001	SFXN1	protein_codin	31.1073462	17.8702221	17.0484709	10.3989896
ENSG000001	XRRA1	protein_codin	0	0	26.1306764	0
ENSG000001	PDE1C	protein_codin	0	0	0	0
ENSG000001	DNAJC4	protein_codin	41.2031359	48.0499842	23.2468542	26.272995
ENSG000001	ZBTB38	protein_codin	0	0	47.8687422	0
ENSG000001	HYDIN	protein_codin	0	0	0	0
ENSG000001	ENSG000001	transcribed_p	0.80159908	0	0	0
ENSG000001	KIAA1549L	protein_codin	0	0	0	0
ENSG000001	SLC25A41	protein_codin	0	0	0	0
ENSG000001	ERCC6L2-AS1	lncRNA	0	5.31485517	0	0
ENSG000001	PPIP5K2	protein_codin	68.7031786	48.8141113	16.7310625	25.5014258
ENSG000001	OR52E2	protein_codin	0	0	0	0
ENSG000001	OAZ3	protein_codin	2.59593166	0	0	0
ENSG000001	FBXO39	protein_codin	3.85219812	0	0	0.99361159
ENSG000001	ZNF543	protein_codin	8.11714248	32.2973944	13.2865936	14.3643168
ENSG000001	PINK1	protein_codin	115.735002	180.995276	54.2144831	109.074793
ENSG000001	PLD6	protein_codin	3.00797174	8.12545223	7.47221887	6.95220764
ENSG000001	FKBP10	protein_codin	0	0	0	0
ENSG000001	TTLL11	protein_codin	4.79162588	25.8998873	6.13963106	9.5161485
ENSG000001	CATSPER2	protein_codin	0	0	0	0
ENSG000001	IP6K1	protein_codin	64.6614767	41.1628954	0	0
ENSG000001	DYNAP	protein_codin	0	0	0	0
ENSG000001	GLDC	protein_codin	12.7175139	10.7091183	52.8524694	0
ENSG000001	PHYHIP	protein_codin	0	0	0	0

ENSG000001	CASKIN2	protein_codin	0	0	0	0
ENSG000001	TMEM132E	protein_codin	0	1.67861749	0	0
ENSG000001	GJD4	protein_codin	0	0	0	0
ENSG000001	GPR137C	protein_codin	7.60911181	0	0	0
ENSG000001	THAP4	protein_codin	0	0	0	0
ENSG000001	STXBP5	protein_codin	91.5029039	32.9133874	40.2096321	20.0653322
ENSG000001	DNAJC4	protein_codin	17.0907776	0	15.0706233	0
ENSG000001	SEPTIN4	protein_codin	0	0	0	0.83595209
ENSG000001	VSIG10	protein_codin	0	0	0	0.94459879
ENSG000001	B3GNT8	protein_codin	0	13.8145988	0	2.56426399
ENSG000000	STAB1	protein_codin	1012.02129	1248.83233	342.987359	659.831137
ENSG000001	PRKCB	protein_codin	483.986018	757.658189	545.089624	414.487729
ENSG000001	C14orf39	protein_codin	0	0	0	0
ENSG000001	CNPY1	protein_codin	0	0	0	0
ENSG000001	AURKAIP1	protein_codin	0	14.5413766	0	25.9761474
ENSG000001	PHETA2	protein_codin	6.03789981	17.4626133	6.39305251	3.78817284
ENSG000000	MAT2B	protein_codin	128.884001	101.268902	34.9125059	73.126583
ENSG000001	TPPP2	protein_codin	0	0	0	0
ENSG000001	SPACA4	protein_codin	0	0	0	0
ENSG000001	CA13	protein_codin	21.8340378	19.1643835	15.620041	10.1976003
ENSG000000	SPAG5	protein_codin	28.3921303	26.8060559	65.6608524	22.0499158
ENSG000001	RBIS	protein_codin	0	0	0	0
ENSG000002	RBMXL1	protein_codin	0	0	0	0
ENSG000001	LMO7	protein_codin	0	0	19.5451749	0
ENSG000001	LINC00469	lncRNA	0	0.85156273	0	0
ENSG000001	BOLL	protein_codin	0	0	0	0
ENSG000001	UBE2D3	protein_codin	0	31.0455125	38.2432193	0
ENSG000002	P2RY11	protein_codin	10.4297551	14.7443754	19.6374912	7.70643122
ENSG000001	GVQW3	protein_codin	1.2947569	2.49544001	0	0
ENSG000001	ACBD4	protein_codin	8.10284762	0	13.0787204	0
ENSG000001	ULK1	protein_codin	406.489454	392.397071	107.000906	212.776832
ENSG000001	BHLHE22	protein_codin	1.00945366	0	0	0
ENSG000001	TMEM108	protein_codin	0	0	0	0
ENSG000000	PIGQ	protein_codin	28.7456106	0	4.69914743	36.4506664
ENSG000001	BPTF	protein_codin	0	0	0	0
ENSG000001	CCDC71	protein_codin	34.7425295	56.6428351	15.1713784	24.9489949
ENSG000001	VARS2	protein_codin	22.7583112	0	0	0
ENSG000001	SPATS2	protein_codin	17.8586338	0	42.8378104	16.2516671
ENSG000001	GLYR1	protein_codin	177.380067	373.103215	125.069646	189.900974
ENSG000001	SLC14A1	protein_codin	0	0	0	0
ENSG000001	CLIC2	protein_codin	2.56669424	2.70175362	0	6.20011514
ENSG000001	FCRL6	protein_codin	0	0	0	0
ENSG000001	PAK4	protein_codin	7.91998449	16.6523115	6.03016877	0
ENSG000001	ABRAXAS1	protein_codin	25.1796708	18.5872914	17.3254042	10.1465269

ENSG000001	CRTC1	protein_codin	42.9278685	36.7905246	40.8486341	51.6427017
ENSG000000	NAALAD2	protein_codin	0	0	0	0
ENSG000001	ATAD5	protein_codin	13.47753	31.5579447	33.3897693	11.7898851
ENSG000001	COQ7	protein_codin	1.52803101	25.4138727	6.10561126	1.79822048
ENSG000001	SOX11	protein_codin	0	0	0	0
ENSG000001	CD151	protein_codin	0	23.8491203	6.39505529	13.8121264
ENSG000001	TM6SF1	protein_codin	54.9734961	65.0771726	26.1087689	36.4987819
ENSG000001	POLR2L	protein_codin	113.331551	78.3232247	45.7311484	47.7980641
ENSG000001	SET	protein_codin	416.019531	540.471398	191.206315	269.379049
ENSG000001	TSHZ1	protein_codin	42.2054324	60.1852866	10.391139	28.9232283
ENSG000000	ROGDI	protein_codin	187.760281	250.395461	73.1407019	64.7281428
ENSG000001	OR51T1	protein_codin	0	0	0	0
ENSG000000	EHD3	protein_codin	270.675957	556.79489	357.141999	252.626526
ENSG000002	OR51H1	lncRNA	0	0	0	0
ENSG000001	PUS1	protein_codin	17.589355	0	5.97916931	7.82666335
ENSG000001	DEFB114	protein_codin	0	0	0	0
ENSG000001	BPNT1	protein_codin	0	0	0	0
ENSG000001	PPP2R1A	protein_codin	88.493065	73.5497527	42.160562	71.8360864
ENSG000001	NIPSNAP2	protein_codin	178.349201	86.792952	78.6709686	124.523752
ENSG000000	PCBP4	protein_codin	0	0	0	0
ENSG000001	OR51S1	protein_codin	0	0	0	0
ENSG000001	OR10H4	protein_codin	0	0	0	0
ENSG000001	OR51F2	protein_codin	0	0	0.99109634	0
ENSG000001	TRIM72	protein_codin	4.0137428	3.00756866	1.98004638	0
ENSG000001	TSPYL5	protein_codin	10.9552225	8.10834169	2.71061686	5.97305502
ENSG000001	FAM133A	protein_codin	0	0	0	0
ENSG000001	FHL2	protein_codin	0	0	0	0
ENSG000001	LTBP3	protein_codin	0	45.4998576	14.8882286	0
ENSG000000	IL12RB1	protein_codin	20.3156235	16.2313689	3.09857082	10.8688977
ENSG000001	NPEPPS	protein_codin	151.56809	259.787459	0	0
ENSG000000	HSD17B6	protein_codin	0	0	0	0
ENSG000001	NOP2	protein_codin	0	0	0	0
ENSG000001	TPRX1	protein_codin	0	0	0	0
ENSG000001	DNPEP	protein_codin	0	5.25045057	0	0
ENSG000001	RPP25	protein_codin	7.85305323	20.6271422	7.0558838	11.4174966
ENSG000001	PANK1	protein_codin	0	0	0	0
ENSG000001	RPL10A	protein_codin	678.968858	1006.25765	478.303604	543.598386
ENSG000001	NUDT4B	protein_codin	103.135841	105.529782	69.3456604	82.0803266
ENSG000000	SMC1A	protein_codin	767.715328	847.122687	520.776651	468.203173
ENSG000001	MYPOP	protein_codin	52.8466738	57.1623609	13.2037733	25.9457249
ENSG000001	HECW1-IT1	lncRNA	0	0	0	0
ENSG000001	SLC49A3	protein_codin	14.819748	18.2127428	0	11.316608
ENSG000001	FLJ40194	lncRNA	3.07531576	0	0	0
ENSG000001	XPNPEP1	protein_codin	12.2167894	30.2324527	3.90870144	4.3283098

ENSG000001	GPR62	protein_codin	0	0	0	0
ENSG000000	UBA6	protein_codin	181.30782	104.598459	103.274081	124.972811
ENSG000001	FAM210A	protein_codin	0	0	0	0
ENSG000001	TULP1	protein_codin	0	0	0	0
ENSG000001	MAPK3	protein_codin	0	0	0	0
ENSG000000	FAM3A	protein_codin	8.30346588	0	0	0
ENSG000002	TNFSF12	protein_codin	0	0	21.194068	0
ENSG000001	KLHL7	protein_codin	7.8782707	6.89457984	0	4.33645484
ENSG000001	SV2C	protein_codin	0	0	0	0
ENSG000001	OR7A5	protein_codin	0	0	0	0
ENSG000001	CD244	protein_codin	23.1616446	38.1416621	11.5822636	2.59823353
ENSG000001	SSNA1	protein_codin	53.2915911	79.4957512	35.1228549	43.9907008
ENSG000001	WDR33	protein_codin	260.119742	328.172482	178.837807	139.628664
ENSG000001	IFT20	protein_codin	0	0	0	0
ENSG000001	RPRML	protein_codin	0	0	0	0
ENSG000001	WDR20	protein_codin	14.0681972	13.5996307	5.28982825	7.21805617
ENSG000001	PTGR3	protein_codin	101.49619	55.3019465	21.3005111	58.551236
ENSG000001	HORMAD1	protein_codin	0	3.87346006	0	0
ENSG000000	PNKP	protein_codin	33.8580688	85.173694	0	29.7119556
ENSG000001	COX5A	protein_codin	63.6958978	101.43593	38.3995364	46.0193144
ENSG000001	GCNT4	protein_codin	0	0	0	0
ENSG000001	EEA1	protein_codin	548.1985	380.570626	356.350066	439.232131
ENSG000001	EGFLAM	protein_codin	0	0	0	0
ENSG000002	GCC2-AS1	lncRNA	0	0	0	0
ENSG000001	SERTAD3	protein_codin	60.4937984	88.634119	22.1105833	26.9444989
ENSG000001	ZBTB7A	protein_codin	766.160538	1067.09295	262.4197	532.309618
ENSG000001	CDC20B	protein_codin	0	0.8599429	0.99191532	0
ENSG000001	PRMT9	protein_codin	31.4377116	16.3199619	25.0134842	27.1699566
ENSG000001	MAF1	protein_codin	262.488282	427.93669	144.849639	179.295056
ENSG000001	ZNF354B	protein_codin	30.670076	34.1665842	18.4241203	14.1095643
ENSG000001	KCTD2	protein_codin	0	105.179015	55.9411876	66.7620082
ENSG000001	EIF2S3B	protein_codin	0	4.30747984	0	5.66925968
ENSG000001	FAM124A	protein_codin	0	0.95744045	0	0.95733227
ENSG000002	ENSG000002	lncRNA	0	0	0	0
ENSG000001	COL12A1	protein_codin	0	0	0	0
ENSG000001	INPP1	protein_codin	6.5455882	36.8850788	5.83014226	17.3082046
ENSG000001	CCDC168	protein_codin	5.95451872	2.98344415	8.94839831	6.18655245
ENSG000000	SF3B2	protein_codin	831.427408	1180.95051	627.196525	571.72848
ENSG000001	DDX10	protein_codin	74.1018495	119.220203	114.900119	112.931743
ENSG000000	WNT11	protein_codin	9.26621229	9.01113886	5.77085275	6.10601951
ENSG000001	PLEKHD1	protein_codin	0.97375953	10.3564358	3.18711124	0.96155101
ENSG000001	MMP19	protein_codin	3.25612155	0	0	0
ENSG000001	PRR18	protein_codin	0	0	0	0
ENSG000000	MFAP3	protein_codin	0	0	0	8.17975208

ENSG000001	OR1E1	protein_codin	0	0	0	0
ENSG000001	SH2B1	protein_codin	0	7.15585937	0	4.17454587
ENSG000001	BASP1	protein_codin	255.788637	353.841908	290.775097	317.571264
ENSG000001	RUVBL1	protein_codin	38.0413404	90.2692866	50.4668825	21.1125913
ENSG000001	CEP295NL	protein_codin	124.291726	123.006155	46.6737925	58.2691367
ENSG000001	CAMK2G	protein_codin	0	0	4.21999986	0
ENSG000001	OR52D1	protein_codin	0	0	0	0
ENSG000001	SUZ12	protein_codin	217.77261	167.824524	108.906186	157.798682
ENSG000001	OR52H1	protein_codin	0	0	0	0
ENSG000001	PHF8	protein_codin	0	0	0	28.1815472
ENSG000002	DIO1	protein_codin	0	0	0	0
ENSG000001	CAMK2G	protein_codin	113.060105	169.223197	36.6878061	107.127535
ENSG000001	MSI2	protein_codin	0	0	0	9.45474209
ENSG000001	NHERF4	protein_codin	0	0	0	0
ENSG000001	EPM2AIP1	protein_codin	201.958569	142.810096	77.2158247	106.010373
ENSG000001	NCL	protein_codin	145.789	85.461669	72.6109192	61.1785774
ENSG000001	ZNF660	protein_codin	9.4716014	0	4.16387282	1.12799054
ENSG000001	ZNF18	protein_codin	20.6695275	0	3.90691122	14.7835763
ENSG000001	MICOS10	protein_codin	30.9538251	15.1333185	5.62125368	15.499919
ENSG000001	ZYX	protein_codin	2153.69887	3105.10571	1134.52741	1378.93783
ENSG000001	NDUFV1	protein_codin	74.6311724	159.73618	81.7955202	66.4860197
ENSG000002	SELENOWP1	processed_ps	0	0	0	0
ENSG000001	PLEC	protein_codin	1280.65789	1114.10253	285.160565	472.95677
ENSG000000	ATOSB	protein_codin	157.51206	185.151151	67.1239843	100.284377
ENSG000001	ZCCHC7	protein_codin	95.1048424	122.109773	38.0602629	74.1182823
ENSG000001	ZNF584	protein_codin	0	0	0	0
ENSG000001	B4GALNT3	protein_codin	0	0	0	0
ENSG000001	MYL11	protein_codin	0	0	0	0
ENSG000001	CD46	protein_codin	59.0192552	0	5.8848153	34.0191566
ENSG000001	E2F7	protein_codin	0	0	0	0
ENSG000001	SEC24A	protein_codin	24.9940393	21.1021538	13.9848265	21.3257624
ENSG000001	KCNH5	protein_codin	0	0	0	0
ENSG000001	NAPG	protein_codin	31.3446593	81.3775849	0	19.3602159
ENSG000000	ACADVL	protein_codin	18.2894345	0	6.08438698	15.8082586
ENSG000001	RTBDN	protein_codin	0	0	0	0
ENSG000001	TMC6	protein_codin	0	0	0	0
ENSG000001	CD46	protein_codin	0	0	0	0
ENSG000001	ZNF335	protein_codin	186.085075	251.736245	141.212396	145.396286
ENSG000001	AMBN	protein_codin	0	0	0	0
ENSG000001	ABITRAM	protein_codin	35.9139864	32.2158031	16.9503119	28.4928971
ENSG000001	HIC1	protein_codin	13.6529697	2.97570489	0	0
ENSG000001	MAZ	protein_codin	155.477935	112.588679	0	0
ENSG000001	BANK1	protein_codin	65.7211813	14.2389624	48.516294	0
ENSG000001	UACA	protein_codin	59.9698482	7.71289671	12.258661	9.47402685

ENSG000001	FOXJ1	protein_codin	0	3.37196156	0	0.81090285
ENSG000001	ANKRD29	protein_codin	0	0	0	0
ENSG000001	PODXL	protein_codin	0	0	0	1.76474242
ENSG000001	RPS15A	protein_codin	25.1638107	50.2866345	21.2466955	20.2888185
ENSG000001	WDR45	protein_codin	0	0	0	0
ENSG000000	CDH7	protein_codin	0	0	0	0
ENSG000001	RRP7A	protein_codin	89.2126757	152.459261	39.752109	28.994484
ENSG000001	MIEF2	protein_codin	6.79520383	13.3745154	0	0
ENSG000001	CACNA1F	protein_codin	0	0	0	0
ENSG000001	DUOXA2	protein_codin	0	0	0	0
ENSG000001	ZNRF2	protein_codin	158.47311	165.052899	66.8568232	107.938792
ENSG000001	DYRK1B	protein_codin	27.0666556	27.6404346	0	0
ENSG000001	GPR4	protein_codin	0	0	0	0
ENSG000001	PPP3CA	protein_codin	164.798031	0	33.5047123	50.7517095
ENSG000001	OPA3	protein_codin	3.74585527	1.90125753	0	0.93523061
ENSG000001	NAP1L5	protein_codin	5.22157179	3.06893892	1.2659593	3.128548
ENSG000000	AGBL5	protein_codin	27.2574727	64.8310043	28.2308015	17.793894
ENSG000001	LCP1	protein_codin	7209.2881	8455.31614	3202.03993	5244.59272
ENSG000001	LRRC61	protein_codin	0	0	0	0
ENSG000001	SPNS1	protein_codin	0	0	0	31.199183
ENSG000001	TSPEAR	protein_codin	0	0	0	0
ENSG000000	SULT2B1	protein_codin	0	0	0	0
ENSG000002	SPATS1	protein_codin	0	0	0	0
ENSG000001	CYP11B2	protein_codin	0	0	0	0
ENSG000001	NTRK2	protein_codin	0	0	0	0
ENSG000000	ATP11B	protein_codin	346.733266	172.03194	186.169177	185.053623
ENSG000001	TMEM67	protein_codin	0	0	0	0
ENSG000001	TPM3	protein_codin	0	0	0	0
ENSG000001	OR3A4P	transcribed_u	0	0	0	0
ENSG000001	ZNF491	protein_codin	1.0074026	5.10108502	0	6.18408143
ENSG000001	PHF21A	protein_codin	0	149.105751	0	0
ENSG000001	GCAT	protein_codin	0	0	0	0
ENSG000001	TMEM102	protein_codin	0	4.41278001	0	6.97872346
ENSG000001	EIF3M	protein_codin	13.5481386	21.0204517	2.32651388	0
ENSG000001	TMEM78	lncRNA	0	0	0	2.54475604
ENSG000001	TYMS	protein_codin	0	0	8.78185502	0
ENSG000001	PRR7	protein_codin	11.6215385	0	0	8.54128828
ENSG000001	TYMS	protein_codin	0	3.116123	13.5017194	1.94205439
ENSG000001	TMEM63B	protein_codin	0	0	0	0
ENSG000001	TYMS	protein_codin	5.11414194	11.4185158	24.3207198	0
ENSG000001	INTS11	protein_codin	0	0	0	0
ENSG000001	LRSAM1	protein_codin	0	0	0	0
ENSG000001	MOB4	protein_codin	52.7569525	61.3166771	41.0540833	26.6206626
ENSG000001	CLDN22	protein_codin	0	0	0	0

ENSG000002	GH1	protein_codin	0	0	0	0
ENSG000001	C10orf67	protein_codin	0	0	0	0
ENSG000001	SLC14A2	protein_codin	0	0	0	0
ENSG000001	RPL7A	protein_codin	1861.73927	2843.53819	1058.72891	1171.38183
ENSG000001	SLC17A8	protein_codin	0	0	1.38493848	0
ENSG000001	SMARCD2	protein_codin	0	9.54757573	99.3233616	0
ENSG000001	ANKZF1	protein_codin	44.2374751	0	54.3949695	0
ENSG000001	LINC00305	lncRNA	0	0	0	0
ENSG000001	PIPOX	protein_codin	4.40551508	0	0	0.88785931
ENSG000001	NR2C2	protein_codin	0	14.1550525	9.98996365	0
ENSG000001	SPHK1	protein_codin	2.55817533	22.3278047	4.66204787	16.2581313
ENSG000001	MTUS2-AS1	lncRNA	0	0	0	0
ENSG000001	SHLD2P3	transcribed_u	2.05319164	0	0	0
ENSG000001	OR3A1	protein_codin	0	0	0	0
ENSG000001	ME3	protein_codin	0	0	0	0
ENSG000001	RPH3AL	protein_codin	0	0	0	0
ENSG000001	HIF1A	protein_codin	10.7802425	0	5.80097927	0
ENSG000001	LRRC57	protein_codin	0	0	3.33397734	9.89661887
ENSG000001	CPT1C	protein_codin	0	0	0	0
ENSG000001	MTMR4	protein_codin	106.703155	179.062271	77.5939983	85.9678686
ENSG000001	SENP5	protein_codin	169.728681	141.079089	83.6421405	105.120673
ENSG000001	POGLUT3	protein_codin	0	0	0	0
ENSG000001	DIRAS1	protein_codin	0.93198947	8.39665347	2.86373287	0
ENSG000001	ACSS1	protein_codin	135.669145	166.862568	85.2577906	69.7626842
ENSG000000	CASP8	protein_codin	0	49.1235125	0	11.6960985
ENSG000002	TMSB4XP4	processed_ps	19.7181308	37.760856	9.65606034	13.836708
ENSG000001	TMEM45A	protein_codin	1.67839695	0	0	3.38601232
ENSG000001	RAP2B	protein_codin	903.322868	1400.97038	435.745686	755.987128
ENSG000002	PLEKHO2	protein_codin	235.079839	445.111373	169.900286	140.212033
ENSG000001	FBXO9	protein_codin	129.310972	166.658957	46.7783965	80.7554326
ENSG000001	MRPS31	protein_codin	29.4468082	56.9101495	41.1825739	33.9400476
ENSG000001	ELMOD2	protein_codin	34.1938384	17.0507276	26.1686059	26.8885482
ENSG000001	WIPF2	protein_codin	272.084452	341.764556	144.858319	196.997444
ENSG000001	CHID1	protein_codin	7.47533331	0	10.4022633	1.39334431
ENSG000001	PHKB	protein_codin	0	0	32.0875053	0
ENSG000001	PPM1M	protein_codin	145.940637	188.078872	49.1763798	112.054683
ENSG000001	PHYKPL	protein_codin	0	0	0	0
ENSG000001	KMT5B	protein_codin	0	0	2.6201292	0
ENSG000001	MTMR3	protein_codin	185.219599	101.170658	116.863388	178.402741
ENSG000001	PLAAT3	protein_codin	0	2.84524694	4.05369394	1.54616434
ENSG000001	GRIA2	protein_codin	0	0	0	0
ENSG000001	WDR97	protein_codin	2.76145237	0	3.80107652	2.43093697
ENSG000001	METAP2	protein_codin	53.556499	33.3607038	28.0699211	48.4366298
ENSG000000	TCOF1	protein_codin	0	0	0	0

ENSG000001	DDHD1	protein_codin	0	4.56597888	0	7.70130795
ENSG000001	C19orf12	protein_codin	10.990581	20.2185528	0	0
ENSG000001	SPEM1	protein_codin	0	0	0	0
ENSG000001	CBLL2	protein_codin	0	0	0	0
ENSG000000	NUCB2	protein_codin	62.7120463	104.653627	195.033748	103.200697
ENSG000002	PFN2-AS1	lncRNA	0	0	0	0
ENSG000001	DEGS1	protein_codin	94.4314734	178.071245	67.5212744	84.7618897
ENSG000001	USP39	protein_codin	32.5055435	149.64221	59.2963046	29.3907792
ENSG000000	LRRC23	protein_codin	0	0	0	0
ENSG000001	ZNF513	protein_codin	44.8808661	60.9018621	29.6747733	36.3771449
ENSG000001	ARMC10	protein_codin	25.2293178	42.9217643	11.3636364	0
ENSG000001	ARMC10	protein_codin	2.93948994	4.85031248	3.28449197	12.5464384
ENSG000001	MPI	protein_codin	0	11.4937191	7.73788938	0
ENSG000001	CDC25C	protein_codin	1.65754767	0	0	0
ENSG000001	HNRNPCL4	protein_codin	0	0	0	0
ENSG000001	MYCBPAP	protein_codin	0	0	0	0
ENSG000001	HSF5	protein_codin	1.04706944	5.29484364	0.98531087	2.09706194
ENSG000001	COG4	protein_codin	0	0	0	0
ENSG000001	GYS1	protein_codin	101.310926	220.341488	57.0776815	79.1068545
ENSG000001	TYMSOS	lncRNA	0	0	0	0
ENSG000000	GAS7	protein_codin	338.693562	35.6110429	0	0
ENSG000001	CDK19	protein_codin	70.2587957	0	0	10.320876
ENSG000001	TRIM73	protein_codin	0	0	0	0
ENSG000001	SH3TC2	protein_codin	49.1960915	156.682057	20.2900503	46.4401767
ENSG000001	CPN2	protein_codin	0	0	0	0
ENSG000001	TSFM	protein_codin	11.8415144	0	11.9295357	12.5118074
ENSG000001	SRRM1	protein_codin	0	0	0	0
ENSG000001	NDRG1	protein_codin	116.536948	222.875357	17.9736195	71.0514597
ENSG000001	SNRNP200	protein_codin	865.133155	973.560337	655.885394	636.405585
ENSG000001	ENDOV	protein_codin	0	5.90240792	4.70826242	0
ENSG000001	HTR1A	protein_codin	0	0	0	0
ENSG000000	PXN	protein_codin	0	0	28.5739056	0
ENSG000001	PIP4K2A	protein_codin	2.25841887	0	0	0
ENSG000001	CD37	protein_codin	1022.85423	1516.58096	189.301104	678.92217
ENSG000001	FN1	protein_codin	0	0	0	0
ENSG000001	ANAPC2	protein_codin	49.6737569	73.4786722	28.0418678	28.2521758
ENSG000000	MRE11	protein_codin	124.315051	82.5809056	61.6356811	61.4287573
ENSG000001	LGALS8	protein_codin	0	0	0	0
ENSG000001	STRA6	protein_codin	0	0	0	0
ENSG000001	STXBP6	protein_codin	0	0	0	0
ENSG000001	CSTF3	protein_codin	42.4554099	48.7989846	39.5021826	27.0332365
ENSG000001	EIF4A2	protein_codin	0	0	0	0
ENSG000000	MRE11	protein_codin	7.85260955	38.2828089	0	40.084023
ENSG000001	TAF1D	protein_codin	238.634817	306.676552	228.996187	278.50953

ENSG00000(	ST7	protein_codin	0	0	0	0
ENSG000001(	ENO3	protein_codin	0	4.25490362	0	0
ENSG000001'	ST8SIA3	protein_codin	3.31021905	1.11445722	1.08128414	0
ENSG000001(	PRX	protein_codin	7.95704791	0	0	6.05405019
ENSG000001(	SERPINF2	protein_codin	0	0	0	0
ENSG000001'	GYPA	protein_codin	0	0	0	0
ENSG000001:	KCNJ1	protein_codin	0	0	0	0
ENSG000001'	RFLNA	protein_codin	0	0	0	0
ENSG000001(	KLK4	protein_codin	0	0	0	0
ENSG000001.	ZNF687	protein_codin	31.007696	41.5527728	3.69324697	3.81811785
ENSG00000'	CAPN6	protein_codin	0	0.97721996	0	1.00886262
ENSG000001!	CYP2B6	protein_codin	0	0	0	0
ENSG000002	IRF9	protein_codin	0	132.135044	67.6261841	60.6523115
ENSG000001:	TDRP	protein_codin	43.5947429	72.7934306	39.560618	21.0707664
ENSG00000(	PLXND1	protein_codin	1246.55439	1318.94351	245.481609	585.844446
ENSG000001:	MAP1S	protein_codin	135.324019	229.271795	68.6209047	96.0048471
ENSG00000!	RNF31	protein_codin	0	0	0	0
ENSG000001'	CD28	protein_codin	0	22.3053126	0	0
ENSG000001(	DNMBP	protein_codin	256.973923	194.511552	82.2967673	128.359642
ENSG000001.	NLRP12	protein_codin	149.260515	0	83.5434852	97.1397384
ENSG000001:	FANCB	protein_codin	29.70507	21.5215797	0	0
ENSG000001:	PCNX2	protein_codin	0	0	0	5.19933369
ENSG000001:	TH	protein_codin	0	0	0	0
ENSG000001.	THNSL2	protein_codin	0	0	0	0
ENSG000001:	ZCCHC10	protein_codin	0	24.4502068	4.38372353	18.9845611
ENSG000001(	SAMD3	protein_codin	0	0	0	0
ENSG000001'	B3GNT4	protein_codin	0	0	0	0
ENSG000001	SLC25A36	protein_codin	8.59787144	0	6.00154845	0
ENSG000001:	MBNL1	protein_codin	55.4548895	21.5681553	18.7301204	31.9391907
ENSG000001(	GRK3	protein_codin	813.1686	816.64022	398.174214	505.081462
ENSG000001:	MBNL1	protein_codin	2262.27225	1288.078	663.77006	1213.65078
ENSG00000'	MAP4K4	protein_codin	224.373512	205.084904	43.8448742	107.785326
ENSG000001.	SIDT2	protein_codin	215.916252	313.032432	8.39975064	85.4073949
ENSG000001'	CALCB	protein_codin	0	0	0	0
ENSG000001'	CRLF3	protein_codin	229.386615	322.438207	166.811384	173.826829
ENSG000001:	CNDP2	protein_codin	19.6441588	5.04585414	0	6.79016237
ENSG000001'	TRAPPC12	protein_codin	152.771781	243.778196	59.8495631	77.0723723
ENSG000001'	CIITA	protein_codin	989.049462	1341.73202	591.863204	384.374684
ENSG000001'	LGALS9B	protein_codin	0	0	0	0
ENSG000001:	EDNRA	protein_codin	0	0	0	0
ENSG000001:	CNDP2	protein_codin	40.8519086	12.5078925	26.0298909	0
ENSG000001(	ZKSCAN1	protein_codin	304.792185	315.901917	242.901708	253.873971
ENSG000001(	CCDC149	protein_codin	54.1706499	29.5774653	12.6375832	30.1514637
ENSG000001(	MYEF2	protein_codin	4.96791744	0	2.12298015	0

ENSG000001	NIN	protein_codin	0	0	0	32.8690789
ENSG000001	FARS2	protein_codin	0	0	0	0
ENSG000001	GLIS3	protein_codin	0	0	0	0
ENSG000001	DDX39A	protein_codin	0	0	0	0
ENSG000001	GARIN6	protein_codin	0	0	0	0
ENSG000001	RNF128	protein_codin	0	0	0	0
ENSG000001	DCUN1D3	protein_codin	62.9752306	34.6910484	15.5872737	31.756949
ENSG000001	RNF227	protein_codin	0	4.27363996	0	6.25711184
ENSG000001	AGO3	protein_codin	30.9133664	11.6639688	10.1330874	31.2529061
ENSG000001	CCDC121	protein_codin	0	0	2.50798797	4.20290627
ENSG000001	PRMT5	protein_codin	26.3951436	91.6326507	30.5727074	26.7479827
ENSG000000	PPIE	protein_codin	10.2739231	32.7921001	6.58437828	7.29272045
ENSG000001	H1-8	protein_codin	0	0	0	0
ENSG000001	WFDC11	protein_codin	0	0	0	0
ENSG000000	BAIAP3	protein_codin	9.52769955	0	0	0
ENSG000001	ZNF875	protein_codin	0	0	7.3737867	0
ENSG000001	SLC34A1	protein_codin	0	0	0	0
ENSG000000	DTX2	protein_codin	35.3411572	0	6.64164675	0
ENSG000001	ACTL9	protein_codin	0	0	0	0
ENSG000001	RCHY1	protein_codin	30.1723283	18.7090638	22.7641398	18.3579113
ENSG000001	SYNE4	protein_codin	0	0	0	0
ENSG000001	TTTY14	lncRNA	0	0	0	0
ENSG000001	SAGE1	protein_codin	0	0	0	0
ENSG000001	VEGFA	protein_codin	0	0	0	0
ENSG000001	MTURN	protein_codin	651.900876	1376.16352	871.592301	521.63504
ENSG000000	HSPA5	protein_codin	0	0	0	0
ENSG000001	ZNF283	protein_codin	0	0	0	0
ENSG000001	COQ8B	protein_codin	0	0	0	0
ENSG000001	DPY19L2	protein_codin	0	6.27425777	5.61859507	0
ENSG000001	MTURN	protein_codin	192.234697	145.145263	199.376705	147.506445
ENSG000000	PSEN1	protein_codin	3.29382628	0	0	0
ENSG000001	SH3GL3	protein_codin	0	0	2.70486329	0
ENSG000001	CIZ1	protein_codin	0	0	0	0
ENSG000001	USP9X	protein_codin	0	0	0	0
ENSG000001	USHBP1	protein_codin	0	0	0	0
ENSG000002	EEF1AKMT4	protein_codin	6.06650659	6.58731998	2.40331974	0.96731723
ENSG000001	ANO5	protein_codin	0	0	0	0
ENSG000001	ATF7IP2	protein_codin	0	0	8.09253462	0
ENSG000001	MYO5B	protein_codin	0	0	0	0
ENSG000001	GALNT14	protein_codin	0	0	0	0
ENSG000001	ZNF410	protein_codin	78.608869	136.671479	35.8714458	86.1549957
ENSG000001	LILRB1	protein_codin	876.347708	771.560002	264.975933	436.672894
ENSG000001	MBOAT1	protein_codin	220.30391	259.927276	86.6915108	88.1634129
ENSG000001	REDIC1	protein_codin	0	0	0	0

ENSG000002	ENSG000002	lncRNA	0	0	0	0
ENSG000001	CACNB2	protein_codin	0	0	0	0
ENSG000001	CYP4F12	protein_codin	0	0	0.8267775	0
ENSG000001	SLC6A18	protein_codin	0	0	0	0
ENSG000001	NLRC3	protein_codin	0	0	0	0
ENSG000001	CD19	protein_codin	0	0	0	0
ENSG000001	FAM193A	protein_codin	0	0	0	0
ENSG000001	FAM187B	protein_codin	2.62665672	0	0	0.85453434
ENSG000001	SSH2	protein_codin	202.262526	224.206863	35.6594209	66.3033647
ENSG000001	SAV1	protein_codin	116.944601	175.037131	75.2410766	85.7681326
ENSG000001	PPP1R42	protein_codin	0	0	0	0
ENSG000001	PRPSAP1	protein_codin	29.3941851	53.4101964	26.5011299	15.8573334
ENSG000001	RNF40	protein_codin	62.3642331	0	0	50.950293
ENSG000001	RNF135	protein_codin	52.862509	55.3837277	14.9220339	16.7438994
ENSG000001	TRPM8	protein_codin	0	0	0	0
ENSG000001	ASCC3	protein_codin	17.4943853	15.5924659	0	0
ENSG000001	IQUB	protein_codin	3.35583465	5.29829816	3.74219823	0
ENSG000001	ASCC3	protein_codin	0	4.97661616	3.53613007	0
ENSG000001	SCN4B	protein_codin	0	0	0	0
ENSG000001	GLULP4	processed_ps	0	1.02941504	0	0
ENSG000001	MC5R	protein_codin	0	0	0	0
ENSG000001	TAF7L	protein_codin	0	0	0	0
ENSG000001	DIAPH2	protein_codin	167.867981	81.5859735	58.5038734	122.283307
ENSG000001	SCAPER	protein_codin	64.5709412	0	75.0846117	43.9077887
ENSG000001	CREG2	protein_codin	0	0	0	0
ENSG000002	DDR1	protein_codin	0	0	0	0
ENSG000001	CAMKK2	protein_codin	0	0	0	0
ENSG000001	RALGPS2	protein_codin	0	0	0	0
ENSG000000	MRPS33	protein_codin	4.33429449	8.02523858	3.40264294	5.5689796
ENSG000000	PIAS2	protein_codin	36.7957391	22.7266824	14.4404253	17.0782071
ENSG000001	IDSP1	unprocessed_	1.24488309	0	0	0
ENSG000002	PPT2	protein_codin	0	18.2027962	0	0
ENSG000001	MED14	protein_codin	183.984253	144.493117	91.5132934	122.973726
ENSG000001	PLSCR3	protein_codin	23.6438165	4.85320557	3.9900637	0
ENSG000001	VPS37A	protein_codin	65.0256382	53.3570328	12.6010847	40.802582
ENSG000001	FCAMR	protein_codin	0	0	0	0
ENSG000001	ARID1A	protein_codin	615.496665	669.527154	146.036551	268.681467
ENSG000001	PRPF3	protein_codin	199.946116	278.201888	170.838047	146.890546
ENSG000001	FCAMR	protein_codin	0	0	0	0
ENSG000001	LRRC51	protein_codin	0	1.77863214	0	0
ENSG000001	YARS2	protein_codin	7.93677985	19.7627217	11.5053309	0
ENSG000000	METTL1	protein_codin	0	5.93717343	0	0
ENSG000001	NUPR1	protein_codin	0.96130858	0	1.33109311	0
ENSG000001	GTPBP3	protein_codin	13.3856768	0	8.74056463	14.5909143

ENSG000001:	GRHL1	protein_codin	41.564043	65.7408662	31.9293184	26.2617716
ENSG000001:	CHCHD5	protein_codin	10.0106106	12.4424087	0	9.59976663
ENSG000001	SPATA31C2	protein_codin	0	0	0	0
ENSG000001	NUP98	protein_codin	15.3099533	0	0	0
ENSG000000	LIMS2	protein_codin	0	0	0	0
ENSG000001	VPS37D	protein_codin	0	0	0	0
ENSG000001:	CD164	protein_codin	10.1682618	0	15.0810846	27.1955146
ENSG000000:	SMARCA2	protein_codin	2.43109819	0	0	3.01308054
ENSG000001	SLC22A1	protein_codin	0	0	0	0
ENSG000001:	DYNC1I1	protein_codin	0	0	0	0
ENSG000001	AQP10	protein_codin	17.9903379	42.7889785	29.0200311	9.75540678
ENSG000001:	PSMG4	protein_codin	0	0	0	0
ENSG000001	LINC00314	lncRNA	0	0	0	0
ENSG000000	CRMP1	protein_codin	0	0	0	0
ENSG000001	RPL10P16	transcribed_p	8.82391597	18.3589256	5.12711204	15.599496
ENSG000001	AHCYL2	protein_codin	31.0391002	60.6262416	0	0
ENSG000001:	NKX2-6	protein_codin	0	0	0	0
ENSG000001	IL6-AS1	lncRNA	1.11050165	0	0	1.18957074
ENSG000001	MTSS1	protein_codin	151.903641	27.8883018	0	0
ENSG000002	ENSG000002	lncRNA	0	0	0	0
ENSG000000	PCM1	protein_codin	60.0949802	25.260967	16.9015704	5.23159056
ENSG000001	SLITRK5	protein_codin	1.48549275	2.97139995	0	1.59875789
ENSG000001:	MIF4GD	protein_codin	58.1248495	119.165055	66.3184504	33.4662286
ENSG000001	HPS1	protein_codin	0	0	0	0
ENSG000001:	TTC29	protein_codin	0	0	0	0
ENSG000001	HMGCS1	protein_codin	14.9173257	0	0	3.08063941
ENSG000001	CIMAP1A	protein_codin	0	0	0	0
ENSG000001	FOXN3P1	processed_ps	0.82834848	0	0	0.81267899
ENSG000001	VIPR1	protein_codin	97.3950572	142.210613	49.3476435	33.698497
ENSG000001	ENSG000001	transcribed_p	0	1.6543556	0	0
ENSG000001	CYTH2	protein_codin	0	0	0	0
ENSG000001:	ZBTB2	protein_codin	105.913982	109.685855	48.3246735	76.9291233
ENSG000001	BET1L	protein_codin	24.089078	19.2238409	0	3.01391074
ENSG000001	ENTHD1	protein_codin	0	1.0673285	1.01251707	0
ENSG000001	CHMP6	protein_codin	74.6838465	133.912688	38.8522787	51.3975264
ENSG000001	GGA1	protein_codin	97.7355191	0	0	0
ENSG000001	C14orf28	protein_codin	0	0	0	6.09679813
ENSG000000	ANGPT2	protein_codin	0	0	0	0
ENSG000001	RIC8A	protein_codin	0	0	0	0
ENSG000001	CEP295	protein_codin	179.14018	153.060991	191.099418	134.06097
ENSG000001	PCTP	protein_codin	9.33121574	43.7197675	0	0
ENSG000001	ATXN2L	protein_codin	26.2500939	12.8863582	0	131.165782
ENSG000001	ZNF7	protein_codin	0	0	0	0
ENSG000000	CUL1	protein_codin	119.961067	360.9564	152.365865	156.841987

ENSG0000010	CFAP410	protein_codin	0	13.4636278	18.918664	11.863774
ENSG0000010	C8orf34	protein_codin	0	0	0	0
ENSG0000014	PLCB3	protein_codin	0	0	0	0
ENSG0000013	WDFY4	protein_codin	993.383945	1298.67106	435.076166	522.667433
ENSG0000017	CBX3P1	processed_ps	0	0	0	0
ENSG0000017	MAGEB18	protein_codin	0	0.91526501	0	0
ENSG0000010	MTHFD2L	protein_codin	0	0	0	0
ENSG0000013	KLHL36	protein_codin	17.1991127	15.5269926	0	0
ENSG0000014	FBXW5	protein_codin	230.733413	434.948774	44.7340932	158.282027
ENSG0000010	MIEF1	protein_codin	80.9458296	139.081664	85.2341519	37.1840038
ENSG0000015	CDK20	protein_codin	0	0	0	0
ENSG0000002	HMGB3	protein_codin	27.5214663	14.20626	23.0980579	11.0101449
ENSG000002	DYNLT2B	protein_codin	0	0	0	0
ENSG0000010	NELL1	protein_codin	0	1.08551581	0	0
ENSG0000010	KLK2	protein_codin	0	0	0	0
ENSG0000008	IPO11	protein_codin	0	0	30.6833891	0
ENSG0000017	LMNB2	protein_codin	130.271128	121.544595	66.8991808	57.2448782
ENSG000002	ZNF580	protein_codin	10.2764757	29.5715908	7.73453766	0
ENSG0000017	STARD5	protein_codin	0	0	0	0
ENSG000002	EEF1A1P16	processed_ps	15.0603832	11.1970869	7.2614029	11.0278971
ENSG0000017	ZNF784	protein_codin	14.1397594	17.9342752	9.07250194	12.3733628
ENSG0000008	C5orf22	protein_codin	52.545269	41.3202983	0	35.072442
ENSG0000014	DIS3L2	protein_codin	0	57.7046454	31.7491013	20.6769519
ENSG0000028	ENSG0000028	lncRNA	0	0	0	0
ENSG0000018	SOX2	protein_codin	0	0	0.81438025	0
ENSG0000029	ENSG0000029	lncRNA	1.20257838	0	0	0
ENSG0000018	SLC47A2	protein_codin	0	0	0	0
ENSG0000017	HASPIN	protein_codin	4.85976744	7.69348153	8.51423794	1.93257635
ENSG000002	ZNF579	protein_codin	8.84700641	14.1374358	6.23205893	12.1630747
ENSG0000010	SCNN1D	protein_codin	0	1.46557171	0	0
ENSG0000010	UBE2I	protein_codin	0	0	0	0
ENSG0000017	PACS2	protein_codin	30.7747717	94.7154059	15.2926999	0
ENSG0000008	PGR	protein_codin	0	1.18543433	0	0
ENSG0000017	MED16	protein_codin	61.4362479	0	25.3387532	0
ENSG0000010	LARGE2	protein_codin	0	0	0	0
ENSG0000010	CEP57	protein_codin	46.0457506	13.6197185	12.4098041	44.4579671
ENSG0000009	HNRNPM	protein_codin	616.657125	794.867401	333.411898	451.25789
ENSG0000017	MSC	protein_codin	3.95154856	1.19197702	8.15447837	1.99271887
ENSG0000010	CEP57	protein_codin	0	30.2585638	0	0
ENSG000001	CTNNAL1	protein_codin	8.93077105	23.0166044	50.9440879	24.0179168
ENSG0000002	PGA3	protein_codin	0	1.87684429	0	0
ENSG0000000	IL32	protein_codin	0	0	0	0
ENSG000001	RAD1	protein_codin	20.5631459	0	3.13181098	8.48528812
ENSG0000018	RABGAP1L	protein_codin	9.0611034	0	0	0

ENSG0000007	AMPH	protein_codin	6.57852177	1.82017834	0	0
ENSG000001	SHQ1	protein_codin	27.0613222	32.3902684	0	25.141686
ENSG000001	P2RY13	protein_codin	197.144112	261.02558	89.3176037	158.32062
ENSG000001	RPL23P6	processed_ps	7.81596302	5.63771955	0	5.96492883
ENSG000001	CLGN	protein_codin	11.535927	1.0648458	1.1726957	3.20151023
ENSG000001	SPINK6	protein_codin	0	0	0	0
ENSG000001	MARVELD2	protein_codin	0	0	0	0
ENSG000001	C11orf71	protein_codin	0	0	0	0
ENSG000001	PPM1A	protein_codin	0	0	239.014473	0
ENSG000001	SPATA31E1	protein_codin	0	0.72643414	0	0
ENSG000001	CABP4	protein_codin	3.97582642	2.99651564	1.37641535	1.97670606
ENSG000001	PPM1A	protein_codin	114.184746	90.7992906	55.4224214	82.548024
ENSG000001	ZNRF3-AS1	lncRNA	0	0	0	0
ENSG000003	CAST	protein_codin	0	0	73.874221	0
ENSG000001	YLPM1	protein_codin	504.304502	594.303822	144.662161	358.421466
ENSG000001	LACC1	protein_codin	104.702892	61.1285704	44.0013423	53.133044
ENSG000001	OR52K2	protein_codin	2.05437331	3.09011272	2.93627458	5.86933516
ENSG000001	KIAA0319L	protein_codin	0	0	0	0
ENSG000001	CYP4F8	protein_codin	0	0	0	0
ENSG000001	CEP152	protein_codin	17.2792114	5.07455439	82.4580818	0
ENSG000001	PRKRA	protein_codin	0	54.7555766	0	0
ENSG000001	TUBD1	protein_codin	0	0	0	0
ENSG000001	ORMDL1	protein_codin	34.7232404	36.7789091	28.348765	0
ENSG000001	BBX	protein_codin	211.437424	155.81509	160.125048	168.444646
ENSG000001	ENSG000001	lncRNA	0	0	0	0
ENSG000001	MRPS11	protein_codin	28.4463705	31.2446382	15.9888076	11.8930517
ENSG000001	DMRTA1	protein_codin	0	0	0	0
ENSG000001	RMI1	protein_codin	0	24.0915966	0	0
ENSG000001	SNX22	protein_codin	0	0	0	0
ENSG000000	ASB4	protein_codin	0	0	0	0
ENSG000001	FLNC	protein_codin	0	0	0	0
ENSG000001	CLVS1	protein_codin	0	0	0	0
ENSG000001	PRDM7	protein_codin	0.86357028	0	0	0
ENSG000001	RPRM	protein_codin	0.8183105	0	0	0
ENSG000002	NEU4	protein_codin	0	0	0	0
ENSG000001	FRAS1	protein_codin	0	0	0	0
ENSG000001	HAO2	protein_codin	0	0	0	0
ENSG000000	RNF10	protein_codin	413.470033	427.031737	81.6603536	203.254748
ENSG000000	CLEC2D	protein_codin	7.96010923	0	0	0
ENSG000001	MEIOB	protein_codin	0	0	0	0
ENSG000001	CEP192	protein_codin	77.3039925	51.8454551	16.5455435	19.4867387
ENSG000001	CCZ1	protein_codin	126.212513	185.974324	40.0792877	167.304319
ENSG000001	IQCE	protein_codin	0	30.7864886	0	28.0601812
ENSG000001	VENTX	protein_codin	125.981838	89.4370661	38.0426445	23.9957985

ENSG000001	KLHL38	protein_codin	0	0	0	0
ENSG000001	IQCE	protein_codin	0	0	0	11.8479432
ENSG000001	WFDC9	protein_codin	0	0	0	0
ENSG000001	KLK3	protein_codin	0	0	0	0
ENSG000001	HMGB1	protein_codin	573.944884	701.071753	284.462751	417.447918
ENSG000001	OAZ2	protein_codin	135.697998	311.975686	85.9236976	135.388491
ENSG000001	DCAF11	protein_codin	0	0	0	0
ENSG000001	UPB1	protein_codin	27.4329285	14.2953645	9.57551544	14.1595481
ENSG000001	ARHGEF4	protein_codin	0	0	0	0
ENSG000001	SLC4A1AP	protein_codin	146.588523	226.446923	93.0878509	0
ENSG000001	NIM1K	protein_codin	0	0	0	0
ENSG000001	GRTP1	protein_codin	0	0	1.31984966	0
ENSG000001	MAF	protein_codin	11.2376343	13.0067216	0	0
ENSG000001	ZNF561	protein_codin	9.08308253	25.4117759	6.52421508	10.4902976
ENSG000001	KIF15	protein_codin	7.91806645	0	29.3356557	15.3171392
ENSG000001	C11orf16	protein_codin	0	0	0	0
ENSG000001	NOL4L	protein_codin	9.96699382	6.70407521	0	4.09344249
ENSG000001	FAXDC2	protein_codin	203.071926	452.661084	285.112777	169.444333
ENSG000001	RPL21	protein_codin	0	0	0	0
ENSG000001	ISCA1	protein_codin	3.89097902	1.56213069	0	1.43121492
ENSG000001	ENTR1P2	processed_ps	0	0	0	0.80430051
ENSG000001	RNF185	protein_codin	0	78.5866905	29.3768368	28.1430721
ENSG000001	MROH1	protein_codin	83.4796203	109.462859	29.2488536	0
ENSG000001	GHRHR	protein_codin	0	0	0	0
ENSG000001	NFKBIZ	protein_codin	0	4.14874679	0	0
ENSG000001	HSD11B2	protein_codin	0	0	0	0
ENSG000001	GTPBP6	protein_codin	110.248213	137.253438	35.4925128	86.2784175
ENSG000001	CD300LF	protein_codin	159.159588	148.3901	90.0586546	92.3474873
ENSG000001	NFKBIZ	protein_codin	7454.13089	3951.61162	979.740017	3545.48118
ENSG000001	TRAF7	protein_codin	211.047828	230.123525	69.1992621	94.0492434
ENSG000001	SLC22A24	protein_codin	0	0	0	0
ENSG000001	PORCN	protein_codin	0	0	0	0
ENSG000002	ABCF1	protein_codin	0	0	0	0
ENSG000000	SCMH1	protein_codin	0	0	0	0
ENSG000001	TAC4	protein_codin	0	0	0	0
ENSG000001	AHCTF1	protein_codin	56.780916	43.4060696	65.1616057	139.559596
ENSG000001	SLC37A3	protein_codin	0	22.9274532	14.2838098	13.4021947
ENSG000001	LCE1D	protein_codin	0	0	0	0
ENSG000002	ENSG000002	lncRNA	12.0243852	4.08944986	9.00510622	5.01015216
ENSG000001	ITLN1	protein_codin	0	0	0	0
ENSG000001	OR10A7	protein_codin	0	0	0	0
ENSG000001	THOC6	protein_codin	41.9481677	43.6924854	3.55232852	5.70434363
ENSG000001	TLK2	protein_codin	39.4464336	50.1625005	32.5919279	56.0033185
ENSG000001	VGLL2	protein_codin	0	0	0	0

ENSG000001	SNX18	protein_codin	3.1206854	0	0	8.8493153
ENSG000001	LIN28A	protein_codin	1.11093539	0	0	0
ENSG000001	EID2B	protein_codin	6.0966552	8.85577941	3.50962956	2.00290284
ENSG000000	PIBF1	protein_codin	68.300077	0	46.3870714	11.8415536
ENSG000002	PTPRCAP	protein_codin	30.7122769	25.8170444	67.5324686	5.12565108
ENSG000001	CCNC	protein_codin	0	0	8.87051555	3.08809541
ENSG000001	ACOT4	protein_codin	0	0	0	0
ENSG000001	CCR8	protein_codin	0	0	0	0
ENSG000001	SGSH	protein_codin	0	109.437272	21.6510299	66.1530659
ENSG000002	KIR3DL2	protein_codin	0	0	0	0
ENSG000000	FGFR1	protein_codin	0	23.204306	0	0
ENSG000002	ENSG000002	lncRNA	0	0	0	0
ENSG000001	CUL4A	protein_codin	0	43.2080655	70.0644461	0
ENSG000001	ADORA2A-AS	lncRNA	34.5994801	11.8844001	19.8625943	13.5405953
ENSG000001	WASHC3	protein_codin	1.95340127	0	0	8.19285138
ENSG000001	ZNF639	protein_codin	0	0	0	0
ENSG000002	POLR2J4	lncRNA	29.3912818	34.2961507	20.3844114	18.1411193
ENSG000001	RWDD4	protein_codin	37.2492161	13.7433438	16.640363	16.6961599
ENSG000001	SLC36A4	protein_codin	66.0299128	52.0974139	42.5059855	100.722581
ENSG000001	ITM2C	protein_codin	0	0	0	0
ENSG000001	RIIAD1	protein_codin	0	0	0	0
ENSG000001	ITM2C	protein_codin	49.2171382	71.8478803	170.404345	32.6660243
ENSG000001	CRELD1	protein_codin	0	0	0	0
ENSG000001	PEG3	protein_codin	0	0	0	0
ENSG000001	TMEM176B	protein_codin	142.34484	81.4166601	197.348804	354.255944
ENSG000001	ARL6IP6	protein_codin	0	16.6608304	5.47161955	11.2753369
ENSG000000	IFT80	protein_codin	37.1716207	18.0573356	19.9781476	8.48542597
ENSG000000	SSH1	protein_codin	3.95010129	4.07040059	1.81367073	0
ENSG000001	C3orf80	protein_codin	0	4.47676165	0	1.10230023
ENSG000000	SSH1	protein_codin	239.173839	255.952828	130.807931	67.9606734
ENSG000001	VNN2	protein_codin	212.972465	225.070751	146.694863	361.151515
ENSG000001	B3GNT5	protein_codin	148.560423	20.0156902	63.6589431	43.1331131
ENSG000001	OR9K1P	transcribed_u	0	0	0	0
ENSG000001	GDNF	protein_codin	0	0	0	0
ENSG000001	COX6B2	protein_codin	0	0	0	0
ENSG000001	PRNT	lncRNA	0	0	0	0
ENSG000001	KIR3DL1	protein_codin	0	0	0	0
ENSG000001	SPAG11A	protein_codin	0	0	0	0
ENSG000001	GPR139	protein_codin	0	0	0	0
ENSG000001	KDSR	protein_codin	0	0	0	0
ENSG000000	TNFRSF12A	protein_codin	0	0	0	0
ENSG000001	C4orf17	protein_codin	0	0	0	0
ENSG000001	RPL24P4	processed_ps	0.90504099	2.68394659	0	1.82455568
ENSG000001	MAGED1	protein_codin	0	49.8884449	0	0

ENSG000001	CCDC66	protein_codin	27.9462742	0	0	0
ENSG000001	GGA1	protein_codin	24.8997559	13.2814643	6.73913754	7.15799567
ENSG000000	VSIG2	protein_codin	0	0	0.9317103	0
ENSG000001	ZBTB33	protein_codin	132.783955	80.9310151	33.8663818	69.3630633
ENSG000001	SPAG11A	protein_codin	0	0	0	0
ENSG000001	PLEKHJ1	protein_codin	87.8631944	114.534449	47.1742691	42.8251689
ENSG000001	TNNI3K	protein_codin	0	0	0	0
ENSG000001	LARP1B	protein_codin	4.31475936	0	0	0
ENSG000001	ZNF609	protein_codin	621.545934	812.861018	371.377822	412.869459
ENSG000001	TMEM150B	protein_codin	29.0427891	60.7899152	6.02784983	21.832786
ENSG000001	ERGIC1	protein_codin	0	0	0	5.80309601
ENSG000001	PTPMT1	protein_codin	23.2518128	45.1102415	22.5604955	33.7099935
ENSG000001	ERICH3	protein_codin	0	0	0	0
ENSG000001	SUMO4	protein_codin	18.0153294	1.92149966	7.8790228	7.61235993
ENSG000001	PTPMT1	protein_codin	36.4490641	54.1400663	28.4997256	12.4940681
ENSG000001	ZFAS1	lncRNA	0	3.3227951	0	0
ENSG000000	ABCC9	protein_codin	0	0	0	0
ENSG000001	ABLIM3	protein_codin	120.698591	370.542943	338.986867	134.686854
ENSG000001	RIT2	protein_codin	0	0	0	0
ENSG000001	VWDE	protein_codin	0	0	0	0
ENSG000001	AATK	protein_codin	0	25.9419992	0	47.5321835
ENSG000001	GSTM4	protein_codin	0	8.98529549	6.57937928	0
ENSG000001	FAM87B	lncRNA	0	0.95724418	0	0.98676255
ENSG000001	ATP13A2	protein_codin	62.2332872	103.995595	0	12.8405464
ENSG000001	HARBI1	protein_codin	0	13.6481522	3.02056075	0.99493081
ENSG000001	IMPDH2	protein_codin	38.8992427	144.271629	75.7677727	0
ENSG000001	CYP4F11	protein_codin	0	0	0	0
ENSG000001	TRIML2	protein_codin	0	0	0	0
ENSG000001	ZNF518B	protein_codin	73.9527015	87.3925123	69.5623865	65.2146191
ENSG000001	LENG8	protein_codin	259.549923	361.402114	139.324642	143.221235
ENSG000001	APOLD1	protein_codin	0	0	0	0
ENSG000001	FHL5	protein_codin	0	0	0	0
ENSG000001	ZKSCAN5	protein_codin	0	0	0	0
ENSG000001	EDDM3B	protein_codin	0	0	0	0
ENSG000001	ETV3	protein_codin	107.545418	43.4417932	35.9330637	33.3497405
ENSG000001	ACAP2	protein_codin	837.475471	462.07514	344.391706	661.038935
ENSG000001	YME1L1	protein_codin	55.3309464	0	64.777921	84.0464669
ENSG000001	ZNF274	protein_codin	0	0	0	16.2645115
ENSG000001	DNAI3	protein_codin	0	0	0	0
ENSG000000	CHRNA3	protein_codin	0	0	0	0
ENSG000001	ATP10B	protein_codin	0	0	0	0
ENSG000001	DCAF8	protein_codin	16.3012611	61.5621897	0	54.6964728
ENSG000000	DCBLD2	protein_codin	7.23424717	7.05177739	0	0
ENSG000001	EDDM3A	protein_codin	0	0	0	0

ENSG000001(	BRSK1	protein_codin	0.68926129	0	0	0
ENSG000001!	SULT1C2	protein_codin	0	0	0	0
ENSG000001	OR5AK2	protein_codin	0	0	0	0
ENSG000001	KLK15	protein_codin	0	0	0	0
ENSG000000	DCBLD2	protein_codin	0	0	3.28875322	0
ENSG000001	ZFP42	protein_codin	0	0	0	0
ENSG000001	STK11	protein_codin	0	134.767776	0	52.5784842
ENSG000001	LCORL	protein_codin	38.2071024	0	0	12.8378443
ENSG000001	GSX2	protein_codin	0	0	0	0
ENSG000001	NDUFAF3	protein_codin	34.7657121	38.164758	24.4431003	13.1779167
ENSG000001	ADGB	protein_codin	0	0	0	0
ENSG000001	NDUFAF3	protein_codin	26.3906469	57.2287765	15.7080497	27.2769879
ENSG000001	ADGB	protein_codin	0	0	1.1462295	0
ENSG000000	DKK3	protein_codin	0	0	0	0
ENSG000001	SSBP3	protein_codin	0	2.97036929	0	3.60961705
ENSG000001	NPR3	protein_codin	2.82660798	0	0	7.23557286
ENSG000001(	HACD1	protein_codin	0	1.15978249	0	1.10533209
ENSG000001(	TAGAP	protein_codin	23.9957164	83.3028564	0	0
ENSG000001	ENSG000001	processed_ps	0	0	0	0
ENSG000002!	TPRXL	lncRNA	0	0	0	0
ENSG000001	KLHDC3	protein_codin	82.8062698	142.340406	47.3910776	64.787078
ENSG000002!	TMEM277P	lncRNA	0	0	0	0
ENSG000001	CCDC57	protein_codin	69.1567236	87.1381377	27.8381337	41.3589728
ENSG000001	VIPAS39	protein_codin	0	4.97462826	0	0
ENSG000001	FLII	protein_codin	1059.35021	1617.51458	615.329508	718.242196
ENSG000001	AHI1	protein_codin	0	0	0	0
ENSG000001	SEC16B	protein_codin	0	0	0	0
ENSG000001	PSD3	protein_codin	84.5557947	58.1390219	96.5478706	35.6581176
ENSG000001	TMEM86B	protein_codin	4.90955793	5.10044334	0.82992252	5.57738067
ENSG000001	NOC2L	protein_codin	133.873133	179.179349	44.3094609	97.6750379
ENSG000001(	CLRN1	protein_codin	0	0	0	1.51969533
ENSG000001	CARM1	protein_codin	150.083342	168.230221	67.8869117	76.4105058
ENSG000001	SEPTIN7P13	transcribed_u	0	0	0	0
ENSG000000	MARCO	protein_codin	37.2767883	191.568519	45.4766595	153.27137
ENSG000001	TBC1D31	protein_codin	0	46.203404	11.3469669	0
ENSG000001	NR2F1	protein_codin	0	0	0	0
ENSG000001	PAK2	protein_codin	1242.31163	1297.34272	530.265057	850.695235
ENSG000001(	TLE5	protein_codin	422.152561	676.928159	247.628918	255.379301
ENSG000001	RPAIN	protein_codin	0	0	0	0
ENSG000001(	RBBP8	protein_codin	218.26295	103.687571	110.356896	121.474746
ENSG000001	PRM3	protein_codin	0	0	0	0
ENSG000001	TIMM22	protein_codin	15.8226047	43.7110849	10.3907452	32.518137
ENSG000001	GALK2	protein_codin	0	20.886146	0	0
ENSG000001	ZSCAN2	protein_codin	0	12.9042006	4.28833328	0

ENSG000001	CABYR	protein_codin	0	0	0	0
ENSG000001	FGFR1OP2	protein_codin	210.001224	184.475958	108.363813	144.505663
ENSG000002	RPL21P70	processed_ps	0	0	0	0
ENSG000001	OR5M8	protein_codin	0	0	0	0
ENSG000001	FAM9B	protein_codin	0	0	0	0
ENSG000001	CETN1	protein_codin	0	0	0	0
ENSG000001	RUSF1	protein_codin	30.5482145	75.2602566	36.964096	41.8908573
ENSG000001	MMS19	protein_codin	2.90969905	0	0	0
ENSG000001	ATP10B	protein_codin	0	0	0	0
ENSG000001	GBA1	protein_codin	0	0	0	0
ENSG000001	TMEM151A	protein_codin	0.82581673	0	0	0
ENSG000001	SLC16A6	protein_codin	73.4979459	0	73.540442	0
ENSG000001	GOLGA8R	protein_codin	39.6316353	38.75	20.73984	41.2425043
ENSG000001	PTPN2	protein_codin	220.885482	311.824868	60.0396518	264.213163
ENSG000001	SENP6	protein_codin	0	0	0	0
ENSG000001	AK4	protein_codin	0	11.046085	0	7.94083529
ENSG000001	KHDRBS1	protein_codin	643.102817	885.277958	217.46044	479.62911
ENSG000001	EXOSC3	protein_codin	15.3361391	22.637764	1.36574725	0
ENSG000001	NETO1	protein_codin	0	0	0	0
ENSG000000	RRN3	protein_codin	0	0	0	0
ENSG000000	EED	protein_codin	0	20.1536613	17.1310041	0
ENSG000001	CLEC4M	protein_codin	0	0	0	0
ENSG000001	CDCA8	protein_codin	3.93127343	1.97954774	19.6316627	1.99842451
ENSG000001	OR6C70	protein_codin	0	0	0	0
ENSG000001	FAM186A	protein_codin	0.96964787	0	0	0
ENSG000000	SEPHS1	protein_codin	59.9886728	35.5565771	12.1711486	0
ENSG000001	CCDC107	protein_codin	0	0	0	0
ENSG000001	FAM174B	protein_codin	0	0	0	0
ENSG000001	BRF1	protein_codin	0	0	0	0
ENSG000000	GGT5	protein_codin	0	0	0	0
ENSG000001	SMAD3	protein_codin	519.277314	396.070968	124.281897	220.148509
ENSG000001	TANGO2	protein_codin	29.8529368	47.7524582	13.3375157	19.0380023
ENSG000002	XKR4	protein_codin	1.06423644	2.11810132	0.89361512	1.08483886
ENSG000001	PIEZO1	protein_codin	0	0	0	0
ENSG000001	IL36B	protein_codin	0	0	0	0
ENSG000001	C11orf87	protein_codin	0	0	2.52098601	0
ENSG000001	PRR14L	protein_codin	558.633103	448.845294	372.905648	395.099403
ENSG000001	BOLA3	protein_codin	5.96263314	8.28934956	7.62875397	3.06828729
ENSG000001	ADI1	protein_codin	208.678283	390.806907	155.242478	258.934097
ENSG000001	MIPOL1	protein_codin	0	5.49831086	0	1.61554129
ENSG000001	KPNA7	protein_codin	0	0	0	0
ENSG000001	SP1	protein_codin	485.395276	263.102848	213.03908	190.643978
ENSG000001	SLC25A18	protein_codin	0	0	0	0
ENSG000001	FANCF	protein_codin	20.0694083	29.1645062	4.52037015	9.0619031

ENSG000001	TNFAIP8L1	protein_codin	107.962783	126.436879	31.8338041	45.2268162
ENSG000001	DNAH8	protein_codin	0	0	0	0
ENSG000001	CBFA2T3	protein_codin	98.211636	120.276944	56.9893106	33.6290621
ENSG000001	UBALD2	protein_codin	397.049048	460.165968	210.326882	293.051347
ENSG000001	TOB2	protein_codin	156.97796	262.618496	96.8097568	120.34795
ENSG000002	PSG3	protein_codin	0	0	0	0
ENSG000001	AGPAT3	protein_codin	0	0	0	0
ENSG000001	JMJD1C	protein_codin	73.3260621	23.6515865	70.1544853	49.5471592
ENSG000001	DZIP1L	protein_codin	5.22725218	9.40095898	3.89802342	0
ENSG000001	IFNLR1	protein_codin	3.85920108	2.8646879	16.0586477	0
ENSG000001	TNFAIP8L3	protein_codin	0	0	0	0
ENSG000000	BRAP	protein_codin	37.3912491	52.6427566	41.6463332	21.0038926
ENSG000001	CCDC60	protein_codin	0	0	0	0
ENSG000001	MCAT	protein_codin	0	8.18806025	5.89026229	1.61968038
ENSG000001	ATP11C	protein_codin	0	0	0	29.2606009
ENSG000001	RWDD4	protein_codin	0	0	0	0
ENSG000001	PDXK	protein_codin	11.3097721	17.0784753	10.2112849	2.92714493
ENSG000001	IFNLR1	protein_codin	0	0	0	0
ENSG000001	NT5DC4	protein_codin	0	0	0	0
ENSG000001	OXCT2	protein_codin	11.3600838	10.6130192	8.51485494	0
ENSG000001	FOXP1	protein_codin	52.349149	29.2069107	8.01637858	19.7423526
ENSG000001	MC2R	protein_codin	0	2.01274592	0	2.04964057
ENSG000000	EYA2	protein_codin	0	0	0	0
ENSG000001	TRAK1	protein_codin	61.347797	6.76941002	0	0
ENSG000001	ZNF84	protein_codin	0	0	0	0
ENSG000001	FAM110C	protein_codin	0	0	0	0
ENSG000001	FUT10	protein_codin	21.3651906	7.26888162	5.43644828	9.25254449
ENSG000001	COL10A1	protein_codin	0	0	0	0
ENSG000001	SERPINA7	protein_codin	0	0	0	0
ENSG000001	SERHL2	protein_codin	0	0	0	0
ENSG000001	PARPBP	protein_codin	0	0	0	0
ENSG000001	USP45	protein_codin	0	0	0	0
ENSG000001	RNF123	protein_codin	77.2820137	95.5810082	26.1658362	35.8816704
ENSG000001	CACNA1C	protein_codin	0	0	0	0
ENSG000001	BTNL9	protein_codin	0	0	0	0
ENSG000001	SIX6	protein_codin	0	1.01106251	0	0
ENSG000001	CFD	protein_codin	218.038477	512.43867	117.452005	153.175958
ENSG000001	ACSM6	protein_codin	0	0	0	0
ENSG000002	KRT81	protein_codin	0	0	0.92303783	0
ENSG000001	ADGRF4	protein_codin	0	0	0	0
ENSG000001	PLB1	protein_codin	60.0129774	42.8971174	30.3648525	32.9270123
ENSG000000	RASSF1	protein_codin	21.3978676	18.5173707	21.3112529	24.3543986
ENSG000001	NDUFA12	protein_codin	50.1052907	43.5889712	15.3838825	34.4497442
ENSG000001	GAS6	protein_codin	27.0583985	31.3398688	23.5888138	13.5933409

ENSG000001	GRIK1	protein_codin	0	0	0	0
ENSG000001	FAAP100	protein_codin	57.9267268	26.7318277	24.0853921	32.2096844
ENSG000001	PLPP2	protein_codin	0	0	0	0
ENSG000000	YAF2	protein_codin	2.43312078	7.9678164	5.89410495	0
ENSG000001	DNAH14	protein_codin	0	0	0	0
ENSG000001	FFAR3	protein_codin	18.3682514	0	0.93422315	1.78874759
ENSG000001	DOLPP1	protein_codin	0	0	0	0
ENSG000001	KREMEN1	protein_codin	0	2.52900307	1.5591731	0
ENSG000001	HAPSTR1	protein_codin	163.980544	220.805913	76.8830635	138.403556
ENSG000001	ZNF704	protein_codin	0	0	0	1.13556814
ENSG000002	ENSG000002	lncRNA	4.44369451	0	9.32885645	0
ENSG000002	DEFA3	protein_codin	0	0	0	0
ENSG000000	FBLN1	protein_codin	0	0	0	0
ENSG000001	KCNK12	protein_codin	0.97966636	0	0	1.95471322
ENSG000001	CXorf38	protein_codin	245.82861	290.771493	103.878771	208.238257
ENSG000001	ADAMTSL1	protein_codin	0	0	0	0
ENSG000002	FUNDC2P1	processed_ps	0	0.9117212	0	0.95209376
ENSG000001	TUBB	protein_codin	599.752784	1035.91094	404.800628	467.134021
ENSG000001	AFMID	protein_codin	0	0	0	0
ENSG000000	PHF7	protein_codin	13.6275364	13.1051915	0	14.4856951
ENSG000001	LRIT3	protein_codin	0	0	0	0
ENSG000001	ZNF610	protein_codin	0	0	0	0
ENSG000000	EML1	protein_codin	0	0	0	0
ENSG000002	CCDC144NL	lncRNA	0	0	0	0
ENSG000001	OTOL1	protein_codin	0	0.78454444	0	0
ENSG000002	OR6X1	protein_codin	0	0	0	0
ENSG000001	ZAR1	protein_codin	0	0	0	0
ENSG000001	GRID1	protein_codin	0	0	0	1.88216628
ENSG000001	HEXD	protein_codin	61.1829821	52.2678743	0	13.6453674
ENSG000001	SCN5A	protein_codin	0	0	0	0
ENSG000001	DDX53	protein_codin	0	0	1.28585392	0
ENSG000000	RIPOR3	protein_codin	14.9694402	27.0099546	48.7688139	7.77915517
ENSG000001	IGDCC3	protein_codin	0	0	0	0
ENSG000000	CMTM1	protein_codin	0	0	0	0
ENSG000001	CD300LD-AS	lncRNA	3.3344904	0	0	0
ENSG000001	RAB11B	protein_codin	282.942173	439.203171	139.510488	214.243966
ENSG000001	KCNH7	protein_codin	0	0	0	0
ENSG000001	SLC24A3	protein_codin	92.8403314	151.206874	81.7370529	68.950607
ENSG000001	KLHL15	protein_codin	0	0	0	0
ENSG000001	SPRR4	protein_codin	0	0.80070425	0	0
ENSG000001	OR8B8	protein_codin	0	0	0	0
ENSG000001	ZNF71	protein_codin	18.5787779	3.84481949	9.24317837	6.7863565
ENSG000001	GLUD2	protein_codin	3.52201619	1.1385123	1.11668081	1.14150952
ENSG000001	NPIPA1	protein_codin	71.5571375	70.155125	36.2498339	0

ENSG000001	ZKSCAN2	protein_codin	18.7119293	29.0904108	12.820567	27.0542769
ENSG000001	IL22	protein_codin	0	0	0	0
ENSG000002	ZNF807P	unprocessed_	0	0	0	0
ENSG000001	SPSB1	protein_codin	16.9544971	20.8352012	15.0110891	4.34549021
ENSG000001	TASOR2	protein_codin	0	0	0	0
ENSG000001	NLRP7	protein_codin	0	0	0	0
ENSG000000	DNMT3B	protein_codin	0	0	0	0
ENSG000001	OR4G2P	transcribed_u	0	0	0	0
ENSG000001	LGALS9C	protein_codin	14.9248329	3.05387665	3.23245227	2.51466217
ENSG000001	KRT36	protein_codin	1.67066515	0.81691526	0	0
ENSG000001	ATAD1	protein_codin	59.4109777	20.1302109	30.3871683	46.2282521
ENSG000001	TPM3	protein_codin	847.847923	927.468295	252.004467	462.229538
ENSG000001	ZNF85	protein_codin	11.990308	6.40865444	7.50190667	5.2326007
ENSG000001	OR4C46	protein_codin	0	0	0	0
ENSG000001	VTCN1	protein_codin	0	0	0	0
ENSG000001	DENND5A	protein_codin	325.68804	553.625391	118.543176	273.519157
ENSG000001	PLPBP	protein_codin	135.703038	215.884538	81.984416	95.4470386
ENSG000001	ZNF662	protein_codin	0	0	0	0
ENSG000001	LIN9	protein_codin	0	0	0	0
ENSG000001	OR5BS1P	protein_codin	0	0	0	0
ENSG000001	IFITM1	protein_codin	0	0	0	0
ENSG000001	KCNA4	protein_codin	0	0	0	0
ENSG000001	FAM120C	protein_codin	0	15.9454451	12.2381897	7.42154392
ENSG000001	CEP120	protein_codin	0	0	0	0
ENSG000001	ENSG000001	transcribed_u	1.05738839	1.02312942	0	0
ENSG000001	ODF4	protein_codin	0.89143285	0	0	0
ENSG000001	ERMAP	protein_codin	19.3586451	33.3846882	24.9153124	3.06004183
ENSG000001	PAPPA	protein_codin	1.03888554	2.05273962	3.0821989	3.14287424
ENSG000002	PPME1	protein_codin	29.0035317	42.7211864	0	29.5711931
ENSG000001	DSCR4	lncRNA	0	0	0	0
ENSG000001	CRELD2	protein_codin	40.4333416	77.7653452	87.5496688	24.1774703
ENSG000001	TNRC18	protein_codin	0	0	61.7819202	0
ENSG000001	PHKG2	protein_codin	15.8896806	18.0795496	14.8371324	11.8072876
ENSG000001	LRRC14B	protein_codin	0	0	0	0
ENSG000001	ST6GALNAC3	protein_codin	18.3280871	4.88029408	9.56498444	12.9474751
ENSG000001	COL4A5	protein_codin	0	0	0	0
ENSG000000	SLC9A7	protein_codin	8.96268272	22.2273756	22.3415013	0
ENSG000001	ENPP7	protein_codin	0.78316772	0	0	0
ENSG000001	OR6C76	protein_codin	0	0	0	0
ENSG000001	COL7A1	protein_codin	0	0	0	0
ENSG000001	LINC00334	lncRNA	0	0	0	0
ENSG000001	POU3F2	protein_codin	0	0	1.05920162	0
ENSG000001	APRG1	lncRNA	0	0	0	0
ENSG000001	PTRHD1	protein_codin	48.8565573	70.4997572	17.4091526	34.8227666

ENSG000001	RNF135	protein_codin	105.91412	124.198532	14.86495	61.1030644
ENSG000001	SKP1	protein_codin	0	32.2062262	0	16.847442
ENSG000001	BTBD9	protein_codin	18.0590575	19.2066171	0	0
ENSG000002	CATSPERZ	protein_codin	0	0	0	0
ENSG000001	KCNH8	protein_codin	0	3.13250797	0.98205157	1.06971921
ENSG000001	PTGER4P2	transcribed_p	0	0	0	0
ENSG000001	COA3	protein_codin	16.0255334	22.2647852	9.66369641	6.73004881
ENSG000001	CTAG1B	protein_codin	0	0	0	0
ENSG000001	MYT1	protein_codin	0	0	0	0
ENSG000001	RNASE10	protein_codin	0	1.02079454	0	0
ENSG000001	C1QTNF8	protein_codin	0	0	0	0
ENSG000002	CASTOR3P	lncRNA	27.7302146	25.4665398	10.6188543	7.77144227
ENSG000001	IZUMO1R	protein_codin	0	0	0	0
ENSG000001	TTC7B	protein_codin	49.6183294	85.6387135	50.1452343	19.6158615
ENSG000001	GK4P	processed_ps	0	1.79248229	3.51954132	3.75375466
ENSG000001	CCDC144A	protein_codin	0	12.3464177	0	0
ENSG000001	GINS3	protein_codin	0	3.72080199	6.9415153	0
ENSG000001	BRICD5	protein_codin	0	2.82686266	0	2.71268258
ENSG000001	HSD17B13	protein_codin	0	3.69743896	0	0
ENSG000002	RTL10	protein_codin	0	0	0	0
ENSG000001	DNAH14	protein_codin	0	0	0	0
ENSG000001	NRROS	protein_codin	277.915718	396.674505	89.9646498	167.649316
ENSG000001	PPIAP24	processed_ps	0	0	0	0
ENSG000001	LRP2BP	protein_codin	0	0	0	0
ENSG000000	ATP2C1	protein_codin	0	11.1551244	0	0
ENSG000001	AGAP2	protein_codin	21.8080976	28.5444488	0	0
ENSG000001	OR2T29	protein_codin	0	0	0	0
ENSG000001	TNFRSF18	protein_codin	0	0	0	0
ENSG000001	TRMT12	protein_codin	28.8928926	42.8395849	14.6138152	29.8872128
ENSG000001	NLRP10	protein_codin	0	0	0	0
ENSG000001	OR51M1	protein_codin	0	0	0	0
ENSG000001	PCP4	protein_codin	0	0	0	0
ENSG000000	IP6K2	protein_codin	99.1748976	203.450236	73.832636	79.4206968
ENSG000001	SYNM	protein_codin	0	0	0	0
ENSG000001	CIB1	protein_codin	88.8645635	108.498675	0	46.0816419
ENSG000001	ZNF26	protein_codin	29.6215962	22.6225837	21.8766748	20.1115932
ENSG000001	PRSS55	protein_codin	0	0	0	0
ENSG000001	KCNJ15	protein_codin	16.1897061	9.84330589	1.4935864	6.79845018
ENSG000002	TAF9	protein_codin	0	0	0	0
ENSG000001	RHD	protein_codin	0	0	0	0
ENSG000001	SIRT7	protein_codin	55.2496208	101.495573	41.2425289	45.4231279
ENSG000001	ARSI	protein_codin	1.75523201	0	0	0
ENSG000001	CCDC34	protein_codin	3.97207422	6.02769702	9.55277843	0
ENSG000001	DEFB108B	protein_codin	0	0	0	0

ENSG000001	HAX1	protein_codin	61.1234509	109.708328	17.7876378	57.3917546
ENSG000001	CAPS2	protein_codin	0	0	0	0
ENSG000001	COA5	protein_codin	27.6259218	40.6523974	17.3752344	29.3329203
ENSG000001	GAPDHP24	processed_ps	1.06183089	0	0	0
ENSG000000	PPP2R5C	protein_codin	63.2965148	215.619657	40.8949275	104.6781
ENSG000001	BACE2	protein_codin	0	0	0	0
ENSG000000	PTPN21	protein_codin	0	0	0	0
ENSG000000	ANKRD44	protein_codin	35.507664	14.6264754	28.098864	25.3741246
ENSG000001	VMO1	protein_codin	0	0	0	0
ENSG000001	NXPH3	protein_codin	0	0	0	0
ENSG000001	ZNF92	protein_codin	67.1642437	51.4785647	52.0974632	45.0304983
ENSG000001	ZSWIM9	protein_codin	0	0	0	0
ENSG000001	ARMCX2	protein_codin	0	0	0	0
ENSG000001	TMTC4	protein_codin	0	0	0	0
ENSG000001	DHX34	protein_codin	20.3607537	97.6514193	21.1056172	0
ENSG000001	OR2T34	protein_codin	0	0	0	0
ENSG000001	FRMD3	protein_codin	26.2344461	84.997832	17.4236474	34.7896165
ENSG000001	SPDYE4	protein_codin	0	0	0	0
ENSG000001	OR4N4	protein_codin	0	0	0	0
ENSG000001	CLDN6	protein_codin	0	0	0	0
ENSG000001	OTOP3	protein_codin	0	0	0	0
ENSG000001	TTTY8B	lncRNA	0	0	0	0
ENSG000001	WBP2NL	protein_codin	2.29265683	2.59924635	2.58215714	1.16396402
ENSG000001	PAWR	protein_codin	13.0366741	16.4855596	23.0447146	3.55682137
ENSG000001	SH2D7	protein_codin	0	0	0	0
ENSG000001	CNOT10	protein_codin	45.5056657	118.931783	47.3204776	54.2512525
ENSG000001	PPP4R4	protein_codin	2.11064545	0	0	0
ENSG000001	NOP10	protein_codin	53.3975128	71.042997	33.4570859	34.3884706
ENSG000001	FES	protein_codin	429.04235	536.171315	173.074628	297.420526
ENSG000001	CLEC4G	protein_codin	0	0	0	0
ENSG000001	PDE9A	protein_codin	0	0	0	0
ENSG000001	CLRN1	protein_codin	0	0	0	0
ENSG000001	CAPN12	protein_codin	0	0	0	0
ENSG000000	KLHL22	protein_codin	39.738428	80.834295	15.9251656	18.3065795
ENSG000001	HCAR2	protein_codin	186.679618	78.4725073	48.2485141	97.6322678
ENSG000001	OR4F6	protein_codin	0	0	0	0
ENSG000001	TMIGD1	protein_codin	0	0	0	0
ENSG000001	OR1G1	protein_codin	0	0	0	0
ENSG000001	GKN2	protein_codin	0	0	0	0
ENSG000001	GIMAP6	protein_codin	177.115949	319.825784	63.7134465	99.0491707
ENSG000001	CDYL	protein_codin	0	0	0	0
ENSG000000	MCF2L2	protein_codin	0	0	0	0
ENSG000001	FOXK1	protein_codin	274.88591	387.437752	118.58993	141.62193
ENSG000001	C1S	protein_codin	0	0.8889973	0	0

ENSG000001[illegible]	SHMT2	protein_codin	62.4908678	131.821039	70.8358781	81.6601265
ENSG000001[illegible]	THEMIS2	protein_codin	103.984905	71.098969	17.4431876	79.9888018
ENSG000001[illegible]	SYNGR1	protein_codin	10.5066756	10.5521377	9.50605281	3.32558764
ENSG000001[illegible]	KCNIP1	protein_codin	0	0	0	0
ENSG000001[illegible]	SDR42E1	protein_codin	3.33399633	1.24891399	4.45148845	0
ENSG000001[illegible]	GALNT9	protein_codin	0	0	0	0
ENSG000000[illegible]	P2RX7	protein_codin	323.037029	384.737092	107.249819	181.885282
ENSG000001[illegible]	OAF	protein_codin	198.524879	254.275681	69.2926656	111.102055
ENSG000001[illegible]	ZGPAT	protein_codin	0	0	0	0
ENSG000001[illegible]	GALR2	protein_codin	0.96692775	1.02963938	0	0
ENSG000001[illegible]	MANEAL	protein_codin	2.01468711	0	0	0
ENSG000001[illegible]	CALN1	protein_codin	0	0	0	0
ENSG000001[illegible]	UMODL1-AS1	lncRNA	0	0	0	0
ENSG000002[illegible]	NFAM1	protein_codin	872.183507	1314.44096	450.222174	582.132526
ENSG000001[illegible]	TCEAL2	protein_codin	0	0	0	0
ENSG000001[illegible]	NFXL1	protein_codin	0	32.4799846	0	0
ENSG000001[illegible]	SEPTIN9	protein_codin	150.876341	125.043631	56.0619424	43.2707147
ENSG000001	TPO	protein_codin	0	0	0	0
ENSG000001[illegible]	DDX51	protein_codin	2.95063063	3.36272979	1.26050505	1.92034882
ENSG000001[illegible]	SPNS2	protein_codin	0	0	0	2.68132625
ENSG000001[illegible]	RGMA	protein_codin	2.98077139	0	0	1.9827378
ENSG000001[illegible]	RPL35A	protein_codin	4.67239961	0	0	0
ENSG000001[illegible]	RFLNB	protein_codin	351.798822	398.392802	150.438374	341.778953
ENSG000001[illegible]	NFATC1	protein_codin	0	0	0	0
ENSG000001[illegible]	SULT1C3	protein_codin	0	0	0	0
ENSG000001[illegible]	BCAN	protein_codin	0	0	0	0
ENSG000001[illegible]	KRTAP6-1	protein_codin	0	0	0	0
ENSG000001[illegible]	GP1BA	protein_codin	291.757689	440.006097	284.758599	121.21989
ENSG000001[illegible]	UTY	protein_codin	0	0	0	0
ENSG000001[illegible]	HGS	protein_codin	0	0	0	0
ENSG000001[illegible]	DDIAS	protein_codin	0	0	11.5352752	5.00209437
ENSG000001[illegible]	CRIP2	protein_codin	10.9454325	1.441792	0	0
ENSG000001[illegible]	TMEM54	protein_codin	0	0	0	0
ENSG000000[illegible]	LZTS3	protein_codin	0	10.9916568	0	0
ENSG000000[illegible]	PLEKHH1	protein_codin	1.89271755	0	0	0
ENSG000001[illegible]	SPPL2C	protein_codin	0	0	0	0
ENSG000001[illegible]	OGFOD3	protein_codin	10.1176391	18.178144	2.10175058	0
ENSG000001[illegible]	NKX2-5	protein_codin	0	0	0	0
ENSG000001[illegible]	FAM181B	protein_codin	0	0	0	0
ENSG000001[illegible]	RIBC1	protein_codin	0	0.62709656	0	0
ENSG000001[illegible]	CCDC137	protein_codin	50.9908853	87.100035	33.3008	24.2006275
ENSG000001[illegible]	ATPAF1	protein_codin	8.2227976	20.7882562	9.70373908	0
ENSG000001[illegible]	AP1S2	protein_codin	412.716824	395.282583	47.6289332	220.944551
ENSG000001[illegible]	RBM10	protein_codin	25.4266672	108.216934	12.7079524	0

ENSG000001	ARL17A	protein_codin	0	0	0	0
ENSG000001	TOP1MT	protein_codin	71.7732799	97.8093246	50.6249492	49.6720405
ENSG000002	EEF1G	protein_codin	2311.74401	3648.15044	1620.90558	1850.62127
ENSG000001	S100A7A	protein_codin	0	0	0	0
ENSG000001	PRSS57	protein_codin	7.99972316	3.97843491	2.88164412	5.60093291
ENSG000001	GRPEL2	protein_codin	14.0478789	15.1366258	16.933845	14.2496195
ENSG000001	NOP56	protein_codin	69.0740902	73.4806156	0	0
ENSG000001	BLZF1	protein_codin	13.089447	4.18870446	0	5.26950921
ENSG000001	ZNF599	protein_codin	4.21609896	0	0	2.14530112
ENSG000001	PGBD2	protein_codin	22.1030107	29.9010553	18.8431501	10.2965847
ENSG000001	OLFML1	protein_codin	0	0	0	0
ENSG000001	TPM2	protein_codin	0	13.2793106	51.7888882	3.59882255
ENSG000001	NUPR2	protein_codin	0	0	0	0
ENSG000001	CYLC1	protein_codin	0.90745958	0	0	0
ENSG000001	TRIM61	protein_codin	0	0	0	0
ENSG000001	YBEY	protein_codin	3.29094234	0	0	0
ENSG000001	GPR39	protein_codin	0	0	0	0
ENSG000002	OR52E6	protein_codin	0	0	0	0
ENSG000001	SETD3	protein_codin	16.4982744	31.5454139	0	0
ENSG000001	RP1L1	protein_codin	0	0	0	0
ENSG000001	MAGEA6	protein_codin	0	0	0	0
ENSG000001	FAM47B	protein_codin	0	0	0	0
ENSG000001	CIMAP1B	protein_codin	39.1399594	19.2699293	0	14.0997969
ENSG000001	HNMT	protein_codin	29.274834	25.7175647	7.30443356	34.679299
ENSG000001	KIAA0825	protein_codin	0	0	0	0
ENSG000002	ATP8B5P	lncRNA	0	0	0	0
ENSG000001	PTP4A3	protein_codin	0	0	0	0
ENSG000001	PDLIM1	protein_codin	426.288	513.855346	283.318684	152.871011
ENSG000001	GAST	protein_codin	0	0	0	0
ENSG000001	PMCH	protein_codin	0	0	0	0
ENSG000001	C16orf54	protein_codin	314.926522	337.766589	170.364367	126.160329
ENSG000001	CRISP1	protein_codin	0	0	0	0
ENSG000001	YIF1B	protein_codin	5.28973678	22.1716627	16.6142289	6.57296323
ENSG000001	MARCKSL1	protein_codin	128.339883	106.356138	43.2001708	51.7983302
ENSG000001	ASAH2	protein_codin	0	0	0	0
ENSG000001	HNRNPH1	protein_codin	0	0	0	0
ENSG000001	OR5P2	protein_codin	0	0	0	0
ENSG000001	C10orf120	protein_codin	0	0	0	0
ENSG000001	PRR23B	protein_codin	0	0	0	0
ENSG000001	LMNTD2	protein_codin	5.89218673	8.26084034	0.97260969	1.25686618
ENSG000001	SRARP	protein_codin	0.96283615	0	0	1.91280387
ENSG000001	DHX36	protein_codin	0	0	64.592477	0
ENSG000001	TRIM69	protein_codin	0	0	0	0
ENSG000001	NPAP1	protein_codin	0	0	0	0

ENSG0000007	CARMIL1	protein_codin	49.8146283	50.3029051	45.2506657	28.2309766
ENSG000001	NPY2R	protein_codin	0	0	0	0
ENSG000002	ADAT3	protein_codin	5.81344244	8.08999714	2.64213502	8.95386308
ENSG000000	MAPK8IP2	protein_codin	0.9276616	3.70022821	0	0
ENSG000001	TEKTIP1	protein_codin	0	0	0.92023653	0
ENSG000000	DZANK1	protein_codin	0	0	0	0
ENSG000002	PSG2	protein_codin	0	0	0	0
ENSG000001	RGPD6	protein_codin	4.82694207	0	0	0
ENSG000001	GNB1L	protein_codin	0	3.88122954	1.45732001	2.83074807
ENSG0000007	NFATC3	protein_codin	0	140.297087	0	70.4006987
ENSG000001	UNC5A	protein_codin	0	0	0	0
ENSG000001	MFSD5	protein_codin	40.6400042	66.8677001	8.176121	24.1572002
ENSG000001	RIPPLY3	protein_codin	0	0	0	0
ENSG000001	NDUFB1	protein_codin	2.22937507	0	1.98507684	0
ENSG000001	TSPO	protein_codin	0	0	0	0
ENSG000001	SNN	protein_codin	816.124907	1896.8641	656.258237	714.865921
ENSG000001	IL20RB	protein_codin	7.86240948	0	0	0
ENSG000001	SERPINA5	protein_codin	0	0	0	0
ENSG000001	GDPGP1	protein_codin	10.116913	13.1485136	0	5.05877504
ENSG000001	CSF1	protein_codin	11.4276156	16.4820992	8.07022543	11.3701999
ENSG000001	CIMIP2C	protein_codin	0	0	0	0
ENSG000001	B3GALT5-AS1	lncRNA	0	0	1.0063295	0
ENSG000001	WBP2NL	protein_codin	0	0	0	0
ENSG000001	KRTAP8-1	protein_codin	0	0	0	0
ENSG000001	C2CD2	protein_codin	0	0	0	0
ENSG000001	DAOA	protein_codin	0	0	0	0
ENSG000002	PEX26	protein_codin	0	0	0	63.7608857
ENSG000001	FNDC1	protein_codin	0	0	0	0
ENSG000001	ZNF775	protein_codin	8.70624399	15.7091996	4.69837166	1.83054754
ENSG000001	PDE4B	protein_codin	0	0	14.3865529	25.1352412
ENSG000001	ZSCAN22	protein_codin	14.8301938	0	0	0
ENSG000001	PIGP	protein_codin	7.58244295	0	0	0
ENSG000001	FBXL7	protein_codin	0.96535312	0.97019651	0	0
ENSG000001	TTTY8	lncRNA	0	0	0	0
ENSG000001	MFSD10	protein_codin	5.16924689	21.3775142	8.25989952	2.69106667
ENSG000000	ACSM2B	protein_codin	0	0	0	0
ENSG000001	TBX1	protein_codin	0	0	0	0
ENSG000001	MEX3B	protein_codin	16.6106731	17.5552436	6.67700745	9.93608827
ENSG000001	IL25	protein_codin	0	0	0	0
ENSG000001	C6orf58	protein_codin	0	0	0	0
ENSG000001	IRX3	protein_codin	0	2.9947341	0.65770391	0
ENSG000001	HDHD5-AS1	lncRNA	0	0	0	0
ENSG000002	MBD3L5	protein_codin	0	0	0	0
ENSG000001	TRIM49	protein_codin	0	0	0	0

ENSG000001!	FAM43A	protein_codin	32.6252183	88.9194132	15.9007953	20.2553968
ENSG000001!	MRGPRX2	protein_codin	0	0	0	0
ENSG000001!	SLC16A5	protein_codin	3.39948994	27.3469072	9.0418419	10.8891255
ENSG000001!	GPR132	protein_codin	152.60233	139.156913	42.9353705	55.4776457
ENSG000001!	FREM3	protein_codin	0.94042357	0.95313446	0	0
ENSG000001!	TMCC2	protein_codin	0	0	0	0
ENSG000001!	MFSD6L	protein_codin	0.97305592	3.91374511	3.1851397	0.9535386
ENSG000001!	RPS27AP20	processed_ps	0.80325025	0.85265772	0.83902493	0.82010631
ENSG000001!	MRPL11	protein_codin	17.5293115	12.4026717	0	0
ENSG000001!	ALDH1A3	protein_codin	0	0	0	0
ENSG000001!	LPAR5	protein_codin	27.3680966	90.003823	40.7333526	28.9545212
ENSG000001!	TRIM64B	protein_codin	2.02053245	0	0	0
ENSG000001!	PYCR1	protein_codin	0	0	0	0
ENSG000001!	CSH1	protein_codin	0	0	0	0
ENSG000001!	TEDC1	protein_codin	7.88234119	2.93865383	0	0
ENSG000001!	CNGA2	protein_codin	0	0	0	0
ENSG000001!	CALHM1	protein_codin	0.9204291	1.8469907	0	0
ENSG000001!	IST1	protein_codin	0	0	0	0
ENSG000001!	GDF3	protein_codin	0	0	0.70318506	0
ENSG000002!	POM121L7P	processed_ps	0	0	0	0
ENSG000001!	LRRC36	protein_codin	0	0	0	0
ENSG000001!	MOB2	protein_codin	50.4954745	76.2940032	8.93666613	26.3782676
ENSG000001!	B4GALNT4	protein_codin	1.56537759	0	0	0
ENSG000001!	USP45	protein_codin	2.31016191	0	9.80649448	0
ENSG000001!	SIVA1	protein_codin	10.4000215	15.2763194	3.04816857	7.8685074
ENSG000001!	GTF3C6	protein_codin	120.060454	144.317713	138.447756	82.2579961
ENSG000001!	ATAT1	protein_codin	0	0	0	0
ENSG000001!	SLC52A2	protein_codin	25.4613997	12.2312271	0	3.98046413
ENSG000001!	NEXN	protein_codin	689.632857	471.003359	236.712122	494.776019
ENSG000001!	DLG2	protein_codin	0	0	0	0
ENSG000002!	ZNF722	protein_codin	0	0	0	0
ENSG000001!	SETD2	protein_codin	33.1568508	161.719465	17.1419768	11.4971089
ENSG000001!	UQCR10	protein_codin	113.160239	136.274917	51.1043893	77.9134919
ENSG000001!	BRCC3	protein_codin	100.020315	57.390447	0	41.4033612
ENSG000002!	SLC6A10P	lncRNA	0	0	1.91811908	0
ENSG000001!	ADRA2C	protein_codin	0	0	0	0
ENSG000001!	IDH2	protein_codin	63.6495292	121.308331	88.832493	77.479185
ENSG000001!	HOXB2	protein_codin	7.02820685	0	1.87386908	6.84222901
ENSG000000!	AKR7A2	protein_codin	8.38602972	14.600686	10.0837621	12.6905305
ENSG000001!	ATAT1	protein_codin	0	0	0	0
ENSG000001!	CEND1	protein_codin	0	0	1.85992419	0
ENSG000001!	TMEM44	protein_codin	0	0	0	0
ENSG000001!	RXFP3	protein_codin	0	0	0	0
ENSG000002!	ZNF616	protein_codin	0	4.36060243	0	0

ENSG000001	EIF4ENIF1	protein_codin	134.423049	100.026475	78.9592339	65.8533738
ENSG000001	MRPL54	protein_codin	54.8395225	61.0051101	20.0352152	31.5036103
ENSG000001	SKA2	protein_codin	43.6120237	65.2773969	31.255392	26.8891466
ENSG000001	ZNF696	protein_codin	9.1206955	8.86305526	5.93112323	2.92631096
ENSG000001	FAM87A	lncRNA	0	0	0	0
ENSG000001	TRAPPC6B	protein_codin	19.8240865	0	0	21.0512588
ENSG000001	ARMCX2	protein_codin	2.91012038	3.84241589	0	0
ENSG000002	OR4C50P	unprocessed_	0	0	0	0
ENSG000001	DMBT1	protein_codin	0	0	0	0
ENSG000002	ENSG000002	protein_codin	0	0	2.72743547	0
ENSG000001	ZNF570	protein_codin	19.5774128	4.86458757	7.22646675	18.6175492
ENSG000001	SAMD14	protein_codin	29.4233869	80.253213	78.9288697	36.1716628
ENSG000001	SLX1B	protein_codin	279.109104	308.367211	50.5337133	90.6747841
ENSG000001	TPM3	protein_codin	394.451153	760.609429	374.31329	288.950142
ENSG000001	CABCOCO1	protein_codin	0	0	0	0
ENSG000001	LMLN	protein_codin	16.6130406	4.76786082	15.2708224	12.5701691
ENSG000001	SPATC1L	protein_codin	6.74715132	13.5550802	1.76970876	3.72070584
ENSG000001	ST3GAL3	protein_codin	0	0	0	5.19356714
ENSG000001	ZDHHC23	protein_codin	0	0	0	0
ENSG000000	ADA2	protein_codin	0	11.1170448	0	0
ENSG000002	CRIP1	protein_codin	154.694273	51.6505483	26.185461	19.6963237
ENSG000001	ZKSCAN8	protein_codin	111.953927	71.4779209	94.1268874	52.5937031
ENSG000001	IRF7	protein_codin	0	0	0	48.5391882
ENSG000001	DHRS7C	protein_codin	0	0	0	0
ENSG000001	PPP1R27	protein_codin	0	0	0	0
ENSG000000	MRTO4	protein_codin	30.6777627	51.8802199	29.1897692	10.9135734
ENSG000001	SV2B	protein_codin	3.39350319	1.13986781	0	0
ENSG000001	GTF2H2	protein_codin	20.5742892	0	0	0
ENSG000001	F8	protein_codin	0	4.75559846	0	7.72950218
ENSG000001	SMIM10	protein_codin	8.1864633	2.0782995	4.13281447	3.14843697
ENSG000000	SLC12A1	protein_codin	0	0	0	0
ENSG000001	CTHRC1	protein_codin	0	0	0	0
ENSG000001	CTNNA3	protein_codin	0	0	0	0
ENSG000001	WDR4	protein_codin	1.83909898	14.9563182	0	0
ENSG000001	RGPD6	protein_codin	0	41.2748353	0	19.3329742
ENSG000001	BACE2	protein_codin	3.1313597	1.36283943	2.87533919	0
ENSG000001	HDDC3	protein_codin	0	0	0	0
ENSG000001	TTTY13	lncRNA	0	0	0	0
ENSG000001	ATP6V0A2	protein_codin	78.088283	90.2646423	38.2120849	59.9991089
ENSG000001	ACTRT3	protein_codin	0	0	2.36992217	0
ENSG000001	VMA21	protein_codin	119.460547	117.891891	50.5420152	83.1333853
ENSG000001	IGLL1	protein_codin	0	0	0	0
ENSG000001	ENSG000001	lncRNA	0	0	0	1.05415139
ENSG000001	EP400	protein_codin	2.04821997	6.51592254	2.51053975	0

ENSG000001	CREB3L2	protein_codin	260.585594	340.571651	205.322351	216.248515
ENSG000001	C1QTNF12	protein_codin	0.80582251	1.58595046	0	0
ENSG000001	EPHB3	protein_codin	8.74265961	19.2630271	0	6.64690439
ENSG000001	ATP6V0C	protein_codin	669.168832	942.312869	126.960866	506.972941
ENSG000001	TUBA8	protein_codin	0	0	2.994534	15.2759885
ENSG000001	RCAN2	protein_codin	0	0	0	0
ENSG000001	OPRM1	protein_codin	6.40453281	1.27773133	4.86584694	10.1796345
ENSG000001	CYP2A13	protein_codin	0	0	0	0
ENSG000001	MT1E	protein_codin	0	0	0	0
ENSG000001	PRKCD	protein_codin	419.66807	522.332755	178.952485	139.865697
ENSG000001	KPNA2	protein_codin	29.7078027	23.0761301	82.4827838	53.7334277
ENSG000001	TBX18	protein_codin	0	0	0	0
ENSG000001	ADAMTSL5	protein_codin	0	0	0.90318219	0
ENSG000002	TSPEAR-AS1	lncRNA	0	0	0	0
ENSG000002	CNN2P12	processed_ps	0	0	0	0.79250473
ENSG000002	PCAT6	lncRNA	0	0	0	0
ENSG000001	CHD3	protein_codin	0	0	148.601035	0
ENSG000001	PRR14L	protein_codin	2.54977245	0	0	0
ENSG000001	SLC5A2	protein_codin	0	0	0	0
ENSG000001	OR2T10	protein_codin	0	0	0	0
ENSG000001	ZNF397	protein_codin	42.3861217	41.4986414	38.9983706	29.1014135
ENSG000001	DDX41	protein_codin	178.110719	273.00234	77.0567472	140.101385
ENSG000001	TGIF1	protein_codin	20.0564339	12.1737206	8.03164228	10.6206533
ENSG000001	GJC1	protein_codin	0	0	0	0
ENSG000001	WFDC10B	protein_codin	0	0	0	0
ENSG000001	NOMO2	protein_codin	0	0	0	0
ENSG000001	ENSG000001	lncRNA	10.3603887	4.0535899	0	0
ENSG000001	CD86	protein_codin	327.46345	482.016628	372.344378	386.36615
ENSG000000	SLC22A16	protein_codin	0	5.59103039	0	0
ENSG000001	LINC01547	lncRNA	0	0	0	0
ENSG000001	KRT79	protein_codin	0	0	0	0
ENSG000001	TPM3P8	processed_ps	3.00343704	2.10288404	8.23794948	1.0379853
ENSG000001	CDCA2	protein_codin	0	0	14.3368693	0
ENSG000001	INTS5	protein_codin	19.3441257	49.0783403	16.3151258	17.9392474
ENSG000001	RGPD8	protein_codin	37.337166	0	26.5670418	0
ENSG000001	NOC4L	protein_codin	35.5998208	40.3779331	16.225232	19.3423342
ENSG000001	DEFA5	protein_codin	0	0	0	0
ENSG000002	HBG1	protein_codin	0	0	0	0
ENSG000001	ZNF470	protein_codin	6.31744934	9.52455706	7.55527791	0
ENSG000001	FBXO21	protein_codin	35.1073641	24.4935655	0	0
ENSG000002	INF2	protein_codin	18.4211102	66.855969	0	0
ENSG000002	LYRM4	protein_codin	21.0731327	28.2703124	15.2338997	18.110877
ENSG000001	MAPK11	protein_codin	0	0	0	0
ENSG000001	TMCC2	protein_codin	51.9818612	0	0	0

ENSG000001	TLCD2	protein_codin	16.3314176	17.4481125	3.55032463	2.07252607
ENSG000001	NR2C1	protein_codin	0	0	0	7.10785904
ENSG000001	GRIN2A	protein_codin	0	0	0	0
ENSG000001	CMTM4	protein_codin	0	0	7.57032286	6.54877256
ENSG000000	RARB	protein_codin	0	0	0	0
ENSG000001	WDR5B	protein_codin	15.0528343	12.0739275	6.94354749	7.16736103
ENSG000000	ZNF195	protein_codin	0	0	0	0
ENSG000001	ZFP41	protein_codin	7.3405334	26.38448	16.6230394	16.8292584
ENSG000001	HHIPL1	protein_codin	0	0	0	0
ENSG000001	MX2	protein_codin	329.63305	400.539954	90.1722062	217.483901
ENSG000001	KDELR1	protein_codin	125.098166	251.528338	91.4937052	119.829181
ENSG000002	KRT6A	protein_codin	0	0.92550324	0	0.93250274
ENSG000001	OR56A4	protein_codin	0	0	0	0
ENSG000001	RBBP7	protein_codin	6.27888136	10.3541821	15.323263	7.90008092
ENSG000001	ANKRD11	protein_codin	0	0	296.018601	341.084447
ENSG000001	HNRNPA1	protein_codin	808.013945	1263.93536	878.623332	640.138332
ENSG000001	FLRT2	protein_codin	0	4.39404741	0	3.02620975
ENSG000001	TMEM95	protein_codin	0	0	0	0
ENSG000001	SLC37A4	protein_codin	0	11.7377963	6.64476947	0
ENSG000001	CD300C	protein_codin	92.6178043	112.770589	22.419141	15.6956603
ENSG000001	STING1	protein_codin	104.062145	184.613714	53.4186684	81.398946
ENSG000001	PRMT3	protein_codin	3.10749148	0	7.59553677	3.83506924
ENSG000001	KRTAP20-2	protein_codin	0	0	0	0
ENSG000001	EYS	protein_codin	0	0	0	0
ENSG000001	ALG12	protein_codin	48.3939857	80.9332104	33.3427344	46.4483317
ENSG000001	TPT1-AS1	lncRNA	5.34745691	5.6804521	1.55730349	3.46103953
ENSG000001	C12orf60	protein_codin	0	0	0	0
ENSG000001	ANKRD17	protein_codin	204.553539	177.354408	150.81118	144.288391
ENSG000001	RAB11FIP1	protein_codin	380.929061	620.265647	177.380783	350.83406
ENSG000001	NR4A3	protein_codin	0	0	0	0
ENSG000001	RAB28	protein_codin	12.6400716	34.0466786	11.5908681	26.1850906
ENSG000000	SUGP2	protein_codin	0	9.95608974	3.65191788	0
ENSG000001	TMEM89	protein_codin	0.94680632	0	0	0
ENSG000001	SOCS3	protein_codin	2368.92489	1706.4923	569.249024	1734.50577
ENSG000001	ADSS1	protein_codin	6.86571281	2.89614621	3.40651689	0
ENSG000002	PRAMEF7	protein_codin	0	0	0	0
ENSG000000	KCNH2	protein_codin	0	1.32571471	0	0
ENSG000001	SULT4A1	protein_codin	0	0	0	0
ENSG000001	ADAP2	protein_codin	202.010077	201.951264	61.339365	111.01985
ENSG000001	BEST3	protein_codin	0	0	0	0
ENSG000000	DNAJA1	protein_codin	821.436482	854.153905	549.355703	568.09763
ENSG000001	MAGI1	protein_codin	0	0.91671743	0	0
ENSG000001	TREX2	protein_codin	0	0	0	0
ENSG000001	TUBB	protein_codin	0	0	0	0

ENSG000001	KANK3	protein_codin	0.79171115	0	0	0
ENSG000001	EVI2B	protein_codin	2387.41068	2258.79844	1435.85505	2127.63642
ENSG000001	PTTG1IP	protein_codin	550.007363	801.061295	224.281161	423.908932
ENSG000001	UBE2G2	protein_codin	0	13.2694358	0	0
ENSG000001	SNX20	protein_codin	169.876642	235.938682	71.3028297	119.017745
ENSG000001	LRRC8B	protein_codin	0	0	0	0
ENSG000001	SOX1	protein_codin	0	0	0	0
ENSG000001	CCR4	protein_codin	0	42.8591006	54.1670978	25.2884309
ENSG000001	BAALC	protein_codin	0	0	0	0
ENSG000001	RPS27L	protein_codin	8.90292238	38.0383507	9.26138993	0
ENSG000002	RPL9P7	processed_ps	3.96587221	1.75808232	1.5002989	0
ENSG000001	BOLA2	protein_codin	35.8533801	22.9753538	0	24.4710299
ENSG000001	COPG2	protein_codin	2.35375814	0	0	0
ENSG000001	ANKRD23	protein_codin	0	0	0	0
ENSG000001	CDK10	protein_codin	0	1.69876271	0	0
ENSG000001	NTF3	protein_codin	0	0	0	0
ENSG000001	TTLL1	protein_codin	0	0	0	0
ENSG000001	EWSR1	protein_codin	59.2237267	35.582626	0	0
ENSG000001	IL3RA	protein_codin	9.86499503	20.3572765	9.36392675	3.79115014
ENSG000001	NRXN1	protein_codin	0	0	0	0
ENSG000001	PDE6G	protein_codin	3.43038616	5.02349135	0	0
ENSG000001	MOSMO	protein_codin	0	0	0	0
ENSG000001	BPY2	protein_codin	0	0	0	0
ENSG000001	INPP5J	protein_codin	0	0	0	0
ENSG000001	PRMT3	protein_codin	59.8063514	33.139201	32.695151	20.0219575
ENSG000001	REC114	protein_codin	0	0	0	0
ENSG000001	LINC01405	lncRNA	0	0	0	0
ENSG000001	TRIOBP	protein_codin	0	2.00814279	0	0
ENSG000001	CYP2F1	protein_codin	0	0	0	0
ENSG000001	KCND2	protein_codin	0	0	0	0
ENSG000001	H2AC21	protein_codin	84.2130498	116.011347	41.1322725	44.0911306
ENSG000001	ID2	protein_codin	5.53679212	3.06264401	0	12.4115422
ENSG000001	NPLOC4	protein_codin	254.101283	428.048931	126.64095	181.068608
ENSG000001	CLSTN3	protein_codin	0	0	0	0
ENSG000001	ENKUR	protein_codin	89.4843576	91.9373775	33.4502264	50.9589447
ENSG000001	ZNF479	protein_codin	0	0.91692781	0	0
ENSG000001	PDGFB	protein_codin	40.677957	40.7520097	14.8742594	4.3181402
ENSG000001	SLC9B1P1	unprocessed_	0	0	0	0
ENSG000001	CSTF2T	protein_codin	110.087243	170.647226	59.4244358	113.232403
ENSG000001	DUT	protein_codin	16.8822781	46.5064851	1.57365482	16.4041436
ENSG000001	FBLN7	protein_codin	0	0	0	0
ENSG000001	MAGEA2B	protein_codin	0	0	0	0
ENSG000001	CLN8	protein_codin	41.6791042	73.5998169	29.3745034	18.1931665
ENSG000001	DLK1	protein_codin	0	0	0	0

ENSG000001	RTN4RL1	protein_codin	3.73360423	1.87447861	0	1.82688442
ENSG000001	C11orf54	protein_codin	26.5192223	0	0	0
ENSG000001	DMTF1	protein_codin	0	0	0	0
ENSG000001	LRATD1	protein_codin	0	0	0	0
ENSG000001	GLRX3	protein_codin	47.2785862	59.752918	36.1387726	29.1613701
ENSG000001	ACP7	protein_codin	0	0	0	0
ENSG000001	GLTPD2	protein_codin	1.76407472	0.84505514	0	0
ENSG000001	RPL21P132	processed_ps	0	0	0	0
ENSG000001	MORF4L1	protein_codin	47.7508385	4.47350554	24.5181516	31.5197087
ENSG000001	FBXW4	protein_codin	7.01844978	17.4435572	15.9609809	9.86716281
ENSG000001	PCYT2	protein_codin	0	0	0	0
ENSG000001	ASCL2	protein_codin	28.9466086	46.026679	7.24812467	16.8125672
ENSG000001	SLC25A21	protein_codin	0	0	0	0
ENSG000002	ACTE1P	lncRNA	0	0	0	0
ENSG000001	RPH3AL	protein_codin	0	0	0	0
ENSG000001	DDX59	protein_codin	119.322377	111.026607	97.7293166	90.261952
ENSG000001	MTA1	protein_codin	211.927556	95.7650029	0	124.673508
ENSG000001	PURA	protein_codin	94.1348319	65.6586424	43.3114634	52.9981773
ENSG000001	GLRX5	protein_codin	32.1498867	65.3147318	15.2576576	27.1994769
ENSG000001	FILIP1L	protein_codin	0	0	0	0
ENSG000001	IKZF1	protein_codin	316.354318	621.368128	55.5659205	203.523269
ENSG000001	SNORC	protein_codin	0	0	0	0
ENSG000001	POFUT2	protein_codin	23.8063181	5.02777373	10.0549808	2.02971195
ENSG000001	HS3ST4	protein_codin	0	0	0	0
ENSG000001	CPNE8	protein_codin	245.102565	120.110343	66.8714144	151.702525
ENSG000002	ENSG000002	lncRNA	0	5.14035701	0	0
ENSG000001	POMK	protein_codin	14.6080246	0	8.38576814	15.3453512
ENSG000001	H2AC20	protein_codin	618.502066	1175.85614	431.292725	453.346777
ENSG000001	EPB41L5	protein_codin	0	0	0	2.77367736
ENSG000001	UTY	protein_codin	0	0	0	0
ENSG000001	ZNF789	protein_codin	4.91647584	3.72774017	6.55285686	4.87731475
ENSG000002	ENSG000002	lncRNA	0	0	0	0
ENSG000001	RBM43	protein_codin	16.0488838	39.2218221	11.8680185	15.6142605
ENSG000001	OTOP2	protein_codin	0	2.36140126	0	0
ENSG000001	XKR3	protein_codin	0	0	0	0
ENSG000001	CLCNKA	protein_codin	0	0	0	0
ENSG000001	C8orf33	protein_codin	35.5787698	80.4737669	32.0452372	27.0043124
ENSG000001	NPIPA6	protein_codin	23.1304526	30.0724564	32.3021519	17.3172557
ENSG000001	TMEM121B	protein_codin	105.881018	97.7052364	7.27192484	32.2509055
ENSG000001	H1-5	protein_codin	38.1377641	59.4725046	361.399729	38.5980468
ENSG000001	DGCR6	protein_codin	10.6596986	3.14240493	0	0
ENSG000001	LRRC28	protein_codin	0	0	0	0
ENSG000001	TAB1	protein_codin	0	0	0	0
ENSG000001	TRAIP	protein_codin	0	4.05656696	0	0

ENSG000001	DRG1	protein_codin	33.6208399	78.3514419	32.9643657	33.629744
ENSG000001	HOXD1	protein_codin	0	0	0	0
ENSG000001	CD8B	protein_codin	0	0	0	0
ENSG000001	BEST3	protein_codin	0	0	0	0
ENSG000001	SMDT1	protein_codin	38.2111897	44.1728671	20.0486979	25.8538063
ENSG000001	P4HB	protein_codin	328.974097	458.655204	168.265401	401.669395
ENSG000001	H3C13	protein_codin	3.56928223	2.08863644	8.66377628	0.89772817
ENSG000001	EFCAB13	protein_codin	67.2204768	108.463711	18.0295936	58.8916195
ENSG000001	FMNL1	protein_codin	159.661796	328.728427	27.6043579	94.5342747
ENSG000001	NGRN	protein_codin	56.5278005	77.6088377	16.1157125	15.3352763
ENSG000001	RAI2	protein_codin	0	0	0	0
ENSG000001	EEF1A1	protein_codin	58.0673476	66.7016809	15.5549266	45.1614571
ENSG000001	SLC25A10	protein_codin	0	0	0	0
ENSG000000	CROT	protein_codin	5.03905286	6.67928888	10.1979888	0
ENSG000001	NR2C2AP	protein_codin	2.04494134	8.72116368	4.50037127	1.53681224
ENSG000002	RHEX	protein_codin	0	0	0	0
ENSG000001	MEIS3	protein_codin	0	0	0	0
ENSG000001	PKP3	protein_codin	0	0	0	0
ENSG000001	SLC6A17	protein_codin	0	0	0	0
ENSG000001	ZNF703	protein_codin	135.986376	203.901998	25.6131166	102.247531
ENSG000001	PSMG1	protein_codin	11.8249839	11.2430099	10.3996432	12.3558612
ENSG000001	CADM1	protein_codin	0	0	0	0
ENSG000002	AHCYP5	processed_ps	0	0	0	0
ENSG000001	CALCA	protein_codin	0	0	0	0
ENSG000001	DUSP8	protein_codin	0	7.63304763	0	0
ENSG000001	BMP8A	protein_codin	1.04382307	0	0.96022737	0
ENSG000001	BGN	protein_codin	0	0	0	0
ENSG000001	P4HA3	protein_codin	0	0	0	0
ENSG000001	SYNDIG1L	protein_codin	0	0	0	0
ENSG000001	PCARE	protein_codin	0	0.78168857	0	0
ENSG000001	EIF3C	protein_codin	1629.88813	2756.48147	1211.25764	1436.24148
ENSG000001	DSE	protein_codin	177.026245	114.847685	36.5334944	126.224157
ENSG000001	SPAG16	protein_codin	4.55189332	0	0	0
ENSG000001	TBK1	protein_codin	0	0	0	0
ENSG000001	ZNF605	protein_codin	0	4.43902614	5.39554088	0
ENSG000001	OLFML2A	protein_codin	0	0	1.65596424	0
ENSG000001	KCNJ12	protein_codin	0	0	0	0
ENSG000001	LIMK2	protein_codin	70.7272562	103.222446	40.3409096	57.116063
ENSG000002	FAM187A	protein_codin	0	0	0	0
ENSG000001	ZNF438	protein_codin	54.2673561	0	0	6.51270385
ENSG000001	RSRC2	protein_codin	57.990176	49.1218224	18.8750513	29.8775798
ENSG000001	SLC2A3P2	processed_ps	1.07959898	1.05586084	0	4.4032866
ENSG000001	SS18L1	protein_codin	75.2450612	47.721576	20.6597076	21.6767849
ENSG000001	IMMP2L	protein_codin	8.84440646	0	7.00692428	0

ENSG000001	SETD3	protein_codin	311.866428	439.986213	137.185764	251.302485
ENSG000001	CSF3	protein_codin	0	0	0	0
ENSG000001	RNLS	protein_codin	4.02589197	8.64363871	4.43799076	2.4714342
ENSG000001	SPATA32	protein_codin	0	0	1.00812603	0
ENSG000001	JAG2	protein_codin	8.32276674	0	0	0
ENSG000001	TTTY14	lncRNA	0	0	0	0
ENSG000002	ZNF345	protein_codin	0	0	6.41212831	11.8983808
ENSG000001	UPP1	protein_codin	17.3691406	15.0428607	1.76393925	0
ENSG000001	DEXI	protein_codin	3.5678386	0	0.85595547	5.55597057
ENSG000001	KRT7	protein_codin	0	0	0	0
ENSG000000	RANBP1	protein_codin	107.534798	211.510792	82.162256	58.7432873
ENSG000000	FTL	protein_codin	5013.89065	7442.27576	1806.18023	4384.61464
ENSG000001	PCSK6	protein_codin	9.60138106	0	0	0
ENSG000001	TRBV21OR9-2	TR_V_pseudo	0	0	0	0
ENSG000001	CNTN2	protein_codin	0	0	0	0
ENSG000001	SELENOF	protein_codin	223.503998	232.811944	109.71731	125.333348
ENSG000001	ACSM5	protein_codin	0	0	0	0
ENSG000002	PPIEL	lncRNA	0	1.64441271	0	0
ENSG000001	SLC35D3	protein_codin	10.1014555	23.0795764	11.4752215	8.32179872
ENSG000001	SPRR3	protein_codin	0	0	0	0
ENSG000001	ENSG000001	processed_ps	0	0	0	0
ENSG000001	GLUL	protein_codin	16.1149244	13.4207954	4.40123836	11.1253498
ENSG000000	DGKA	protein_codin	0	20.4819773	0	0
ENSG000001	CNOT10	protein_codin	28.8621595	0	0	0
ENSG000001	FBXL6	protein_codin	27.3304632	23.916524	0	0
ENSG000001	OPCML	protein_codin	0	0	0	0
ENSG000001	PTCH1	protein_codin	0	4.11060067	0	0
ENSG000001	PAXBP1	protein_codin	0	21.0044874	0	0
ENSG000002	CYTOR	lncRNA	38.1741319	73.8409375	30.0065482	25.2632862
ENSG000001	C14orf180	protein_codin	0	0	0	0
ENSG000001	PRKD1	protein_codin	0	0	0	0
ENSG000001	RPL29P16	processed_ps	0	0	0.93952991	1.56780786
ENSG000001	IFNL2	protein_codin	0	0	0	0
ENSG000001	DPP6	protein_codin	0	0	0	0
ENSG000001	LINC00482	lncRNA	11.707707	2.5668124	0	7.64061561
ENSG000002	C1QTNF9	protein_codin	0	0	0	0
ENSG000001	SOCS1	protein_codin	3.2473867	5.73263413	0	0.92986913
ENSG000001	EWSR1	protein_codin	3.98386416	4.91851307	26.1027658	25.1828639
ENSG000001	OR51J1	unprocessed_	1.25961333	0	0	2.6044361
ENSG000001	CEP63	protein_codin	0	0	0	0
ENSG000001	EWSR1	protein_codin	0	34.6500978	0	0
ENSG000001	EXOC7	protein_codin	220.005415	269.741751	153.194807	85.9912143
ENSG000001	PHF6	protein_codin	58.9969059	0	0	49.5090147
ENSG000000	WSCD2	protein_codin	0	0	0	0

ENSG000002	KRT17P5	transcribed_u	0	0	0	0
ENSG000001	DAB1	protein_codin	0	0	0	0
ENSG000001	EPGN	protein_codin	0	0	0	0
ENSG000001	SRPRA	protein_codin	339.393155	557.156697	266.396277	259.910117
ENSG000000	ZC3H11A	protein_codin	26.6564661	0	12.9891756	22.0526131
ENSG000001	KIF21B	protein_codin	0	0	0	0
ENSG000001	SETD4	protein_codin	19.3614232	5.26866159	0	0
ENSG000001	VCF2	protein_codin	0	0	0	0
ENSG000001	KCNH7	protein_codin	0	0	0	0
ENSG000001	CIMAP1C	protein_codin	0.91620564	0	0.99902135	0.89893395
ENSG000001	TMPRSS2	protein_codin	0	0	0	0
ENSG000001	TMT1A	protein_codin	206.976815	404.094049	156.033805	200.758543
ENSG000001	PTPRM	protein_codin	0	0	0	0
ENSG000001	WASHC4	protein_codin	726.652113	282.507207	213.055743	254.196043
ENSG000001	ROBO2	protein_codin	0	0	0	0
ENSG000001	VDAC2	protein_codin	251.196985	367.307604	155.057627	173.177976
ENSG000000	YBX1	protein_codin	67.9414061	87.7261876	84.0194209	0
ENSG000001	AP2A2	protein_codin	45.2725382	0	17.8649535	0
ENSG000001	C2CD4C	protein_codin	0	0	0	0
ENSG000001	OR4F15	protein_codin	0	0	0	0
ENSG000001	CSGALNACT1	protein_codin	38.2779753	12.7897078	0	0
ENSG000001	OR52L1	protein_codin	0	0	0	0
ENSG000001	GPRASP2	protein_codin	0.98468674	3.76854883	4.22403764	1.95680051
ENSG000001	RPL11P4	processed_ps	0	0	0	0
ENSG000001	CSGALNACT2	processed_ps	0	1.12860477	0	0
ENSG000001	PIWIL3	protein_codin	0	0	0	0
ENSG000001	CHST6	protein_codin	0	2.08428604	0	0
ENSG000001	SNAI3	protein_codin	19.25229	33.2355405	13.643031	10.4796061
ENSG000001	C6orf120	protein_codin	95.1943958	91.1888759	50.9784588	73.0041031
ENSG000001	PRAMEF1	protein_codin	0	0	0	0
ENSG000001	RGS19	protein_codin	139.089067	210.267321	50.6569097	44.6565202
ENSG000001	ZNF816	protein_codin	0	0	0	0
ENSG000001	NSMCE3	protein_codin	74.7911357	167.335026	51.1991277	60.4998237
ENSG000001	FBLIM1	protein_codin	0	0	0	0
ENSG000001	ZFP1	protein_codin	17.3544124	0	0	9.47378714
ENSG000001	EMILIN3	protein_codin	0.83402146	0	0	0
ENSG000001	MRGPRG	protein_codin	0	0	0	0
ENSG000001	ZNF534	protein_codin	0.9569981	0.94571894	0	0
ENSG000001	DGAT1	protein_codin	0	9.07852031	0	9.71263439
ENSG000001	LIMS1	protein_codin	261.324048	295.523363	136.284549	125.272171
ENSG000001	JRKL	protein_codin	31.5606953	32.8277592	22.4676666	39.79143
ENSG000001	WT1	protein_codin	0	0	0	0
ENSG000001	ENSG000001	processed_ps	0	0	0	1.199787
ENSG000002	RPL23AP57	processed_ps	0	5.96210802	0	1.64486669

ENSG000001	FIGLA	protein_codin	0	0	0	0
ENSG000002	MT1H	protein_codin	0	0	0	0
ENSG000001	SPACDR	protein_codin	1.72417773	0	0	0.98216892
ENSG000001	KRTAP11-1	protein_codin	0	0	0	0
ENSG000001	NIBAN3	protein_codin	0	0	2.4055569	0
ENSG000001	DDX28	protein_codin	55.6901701	61.6138164	12.2819314	29.9134712
ENSG000002	SPDYE3	protein_codin	0	0	0	0
ENSG000001	SHC4	protein_codin	0	0	0	0
ENSG000001	KRT76	protein_codin	0	0	0	0
ENSG000001	GPR19	protein_codin	0	0	0.78949866	0
ENSG000001	TCEAL7	protein_codin	0	0	0	0
ENSG000001	MARCHF11	protein_codin	0	0	0	0
ENSG000000	POLR1H	protein_codin	6.72863796	0	0	0
ENSG000001	CCR10	protein_codin	2.9216362	0	1.45530375	0
ENSG000001	CYB5D1	protein_codin	45.6572772	25.217443	29.8348879	6.56208429
ENSG000001	LINC00313	lncRNA	0	0	0	0
ENSG000002	ENSG000002	lncRNA	22.5537977	8.00499964	12.9635225	0
ENSG000000	RNF215	protein_codin	6.93901633	7.77443909	5.99302665	1.01536181
ENSG000001	FGF7P2	transcribed_u	0	0	0	0
ENSG000001	EP400	protein_codin	65.5989691	24.7195084	58.5458189	11.8261926
ENSG000001	SEMA4B	protein_codin	124.505973	221.944138	40.8728226	57.1875578
ENSG000001	HEXIM1	protein_codin	90.4562088	220.823765	92.3154295	78.1238715
ENSG000001	HOXB4	protein_codin	2.03234451	1.0079947	4.59564161	2.98437142
ENSG000001	APRG1	lncRNA	3.52520187	0	0	2.55719695
ENSG000001	PLA2G6	protein_codin	0	0	0	0
ENSG000001	RIPK4	protein_codin	0.90862523	0	0	0
ENSG000001	TRIML1	protein_codin	0	0	0	0
ENSG000001	BAIAP2L2	protein_codin	0	0.7807891	0	0
ENSG000001	PTGDR2	protein_codin	3.94138913	8.96213592	1.40789109	1.98380363
ENSG000001	IL1R2	protein_codin	16.6685293	0	0	0
ENSG000001	LAMP1	protein_codin	625.813441	1117.99351	304.717266	472.515974
ENSG000001	FLJ40288	lncRNA	0	0	0	0
ENSG000002	ELOA2	protein_codin	0	0	0	0
ENSG000001	GPR173	protein_codin	1.9475398	1.91952273	0	0
ENSG000001	OSBP2	protein_codin	7.27870034	0	0	0
ENSG000001	LINC00158	lncRNA	0	0	0	0
ENSG000001	ZNF484	protein_codin	0	12.0868333	0	20.4063299
ENSG000001	DCUN1D2	protein_codin	17.0520502	0	0	0
ENSG000001	TMED9	protein_codin	198.653907	305.256165	132.958582	144.096853
ENSG000002	NXF5	lncRNA	0	0	0	0
ENSG000001	TRAK2	protein_codin	271.33876	261.183717	161.303174	173.620095
ENSG000001	ST3GAL3	protein_codin	0	0	0	0
ENSG000001	WDR53	protein_codin	6.09389593	8.37412426	7.05454163	4.95138144
ENSG000001	LMLN	protein_codin	0	0	0	0

ENSG000001	TMPRSS11B	protein_codin	0	0	0	0
ENSG000001	FAM133A	protein_codin	0	0	0	0
ENSG000002	OR5K1	protein_codin	0	0	0	0
ENSG000001	OR4M2B	protein_codin	0	0	0	0
ENSG000001	MXI1	protein_codin	92.4774146	86.9604204	35.5392432	47.5440565
ENSG000001	ANXA2	protein_codin	6.69170873	0	0	7.35417801
ENSG000001	TRIM49B	protein_codin	0	0	0	0
ENSG000001	RLIM	protein_codin	455.526882	274.12021	295.789727	267.648922
ENSG000001	TAS2R60	protein_codin	0	0	0	2.55072985
ENSG000000	CMTM1	protein_codin	0	0	0	0
ENSG000001	DBX2	protein_codin	1.02984759	0	0	0
ENSG000001	TBL3	protein_codin	0	0	0	4.41453977
ENSG000001	XPOT	protein_codin	175.457035	197.605838	123.680652	159.515325
ENSG000001	TBX1	protein_codin	0	0	0	0
ENSG000001	URAD	protein_codin	0	0	0	0
ENSG000001	SIGIRR	protein_codin	0	0	0	0
ENSG000001	LYSMD4	protein_codin	0	2.94203698	0	0
ENSG000001	SIMC1	protein_codin	1.31717945	1.3665542	0	0
ENSG000001	KLHDC8B	protein_codin	10.0645397	25.5511581	19.2065101	12.5738642
ENSG000001	STAC3	protein_codin	0	0	0	49.5645838
ENSG000001	ASB7	protein_codin	66.4689186	76.4817214	52.6432152	45.0734382
ENSG000001	SLC22A10	protein_codin	0	0	0	0
ENSG000001	GH2	protein_codin	0	0	0	0
ENSG000001	AEN	protein_codin	17.9288797	24.8591114	24.8755754	14.8042502
ENSG000001	HOATZ	protein_codin	0	0	0	0
ENSG000000	SERPINB3	protein_codin	0	0	0	0
ENSG000001	NOG	protein_codin	0.98864588	1.96331876	0.87599897	0.93810095
ENSG000001	ANO9	protein_codin	0	0	0	0
ENSG000001	PHF12	protein_codin	199.633027	245.226434	142.162722	150.618011
ENSG000001	NLRP9	protein_codin	0	0	0	0
ENSG000001	SLC8A1	protein_codin	296.329935	87.8576653	113.912268	161.891407
ENSG000001	COLEC10	protein_codin	1.24364467	0	0	0
ENSG000001	VSTM4	protein_codin	1.0298359	0	0	1.0347704
ENSG000001	ZNF530	protein_codin	0	0	0	0
ENSG000001	SPATA31A6	protein_codin	0.86661363	0	0	0
ENSG000001	TUBA1B	protein_codin	3.42581072	49.3172695	8.13017074	5.08557381
ENSG000001	SUMO3	protein_codin	267.699587	442.321065	162.16091	232.395119
ENSG000001	BET1L	protein_codin	0	0	0	0
ENSG000001	FAM133CP	processed_ps	0	3.56548846	4.73365441	1.08434034
ENSG000001	MACC1	protein_codin	2.55821357	0	0	0
ENSG000001	ENSG000001	lncRNA	0	0	0	0
ENSG000001	CYP2U1	protein_codin	10.0848199	14.5046772	19.0340983	5.10898957
ENSG000000	GEMIN8	protein_codin	4.97702348	0	6.49132188	0
ENSG000001	CRADD	protein_codin	5.74908608	14.4782657	15.1739949	6.0037331

ENSG000001	CDHR1	protein_codin	0.90251185	0	0	0
ENSG000001	SPATA45	protein_codin	0	0	0	0
ENSG000000	LMO3	protein_codin	0	0	0	0
ENSG000001	ENSG000001	lncRNA	1.00720728	0	0	0
ENSG000001	FAM43B	protein_codin	0	0	0	0
ENSG000001	PLEKHA7	protein_codin	0	0	0	2.67170168
ENSG000001	IZUMO1	protein_codin	0	0.91809756	0	0
ENSG000001	RFX6	protein_codin	0	1.02989581	0	0
ENSG000001	OGFRP1	lncRNA	0	14.9895228	0	0
ENSG000001	SLIT3	protein_codin	0	0	0	0
ENSG000002	RPL17-C18or	protein_codin	0	0	0	0
ENSG000001	ADSS1	protein_codin	0	0	0	0
ENSG000000	PRKCH	protein_codin	40.5382013	38.7376491	14.9294204	24.2466175
ENSG000001	APOL4	protein_codin	5.83131786	24.1860557	0	0
ENSG000001	YIPF7	protein_codin	0	0	0	0
ENSG000001	PHACTR1	protein_codin	0	0	0	0
ENSG000000	CMTM1	protein_codin	0	0	0	0
ENSG000001	NR2C1	protein_codin	11.4932397	27.9234334	4.13524043	4.77741428
ENSG000001	TNFAIP2	protein_codin	1526.59135	3888.56689	713.35733	2595.91656
ENSG000001	JAKMIP2	protein_codin	2.59499798	0	0	0
ENSG000001	EEF1AKMT3	protein_codin	0	0	0	0
ENSG000001	LYG2	protein_codin	0	0	0	0
ENSG000001	DGAT2L6	protein_codin	0	0.93839744	0	0
ENSG000001	MTMR3	protein_codin	263.53609	94.8912702	0	0
ENSG000001	TPRN	protein_codin	0	0	0	0
ENSG000001	MAD2L1	protein_codin	0	0	17.4128006	0
ENSG000001	TMEM186	protein_codin	7.90305332	8.82945524	1.21083592	3.00370305
ENSG000001	MGAT1	protein_codin	0	0	0	0
ENSG000001	LCK	protein_codin	0	0	0	0
ENSG000001	PSMF1	protein_codin	158.948943	295.456618	116.359952	126.224446
ENSG000001	PP2D1	protein_codin	0	0	0	0
ENSG000001	TSKU	protein_codin	0	0	0	0
ENSG000001	MAGEA11	protein_codin	1.8035954	0	0	0
ENSG000001	UXT	protein_codin	81.9923682	134.799634	46.6967048	65.812732
ENSG000001	IQCF2	protein_codin	0	0	0	0
ENSG000001	FIGN	protein_codin	2.39035818	1.1962739	0	1.35844693
ENSG000001	MRPL40	protein_codin	54.1957571	87.9641914	49.3933475	51.743241
ENSG000001	SMTN	protein_codin	33.9404139	33.5023898	62.0340348	0
ENSG000001	NDUFV1-DT	lncRNA	1.69933746	0	0	1.25667609
ENSG000001	DCAF4L1	protein_codin	4.5837608	0	4.16172552	0
ENSG000001	PAX8-AS1	lncRNA	0	0	0	0
ENSG000001	TRIM50	protein_codin	0	0	0	0
ENSG000002	H2AC14	protein_codin	29.656769	39.2168713	338.146106	11.6986407
ENSG000000	ACAA1	protein_codin	118.494793	203.606253	34.5196697	72.0317938

ENSG000001	TAS1R1	protein_codin	0	0	0	0
ENSG000001	TMEM259	protein_codin	0	78.9684893	0	14.963939
ENSG000001	COPB2	protein_codin	319.649253	481.206987	245.198347	251.511155
ENSG000001	ZNF804B	protein_codin	0	0	0	0
ENSG000001	PDE8B	protein_codin	0	3.87547523	0	1.97947591
ENSG000001	ZNF583	protein_codin	2.19145331	0	4.97196275	5.95877159
ENSG000001	TM2D3	protein_codin	0	0	0	0
ENSG000001	ZSCAN30	protein_codin	14.7519623	0	0	2.89881909
ENSG000001	GPRIN3	protein_codin	34.8303967	19.6055423	17.7915732	35.2715048
ENSG000001	TSEN54	protein_codin	0	3.75854505	0	0
ENSG000001	ING1	protein_codin	94.830834	151.454931	36.9493608	64.1820133
ENSG000001	BRWD1	protein_codin	49.8172989	44.5851739	0	64.943389
ENSG000001	C18orf21	protein_codin	9.27493698	0	0	6.48688033
ENSG000001	CSPG4P4Y	transcribed_u	0	0	0	0
ENSG000001	AHNAK2	protein_codin	0	0	0	0
ENSG000001	CCDC172	protein_codin	0	0	0	0
ENSG000001	TSSC4	protein_codin	10.8823412	34.8706876	0	0
ENSG000001	EFNA5	protein_codin	0	1.169728	0	4.692232
ENSG000001	ANKRD45	protein_codin	1.18654064	0	0	0
ENSG000001	IGIP	protein_codin	1.00009579	2.02449657	3.74988818	1.00746107
ENSG000002	TMED7-TICAM	protein_codin	0	0	0	0
ENSG000001	RNF32-DT	lncRNA	0	3.51866139	1.71516415	0
ENSG000001	INKA1	protein_codin	20.4424807	21.4314744	12.3587107	9.84661504
ENSG000002	OLIG2	protein_codin	0	0	0.55632227	0
ENSG000000	SELE	protein_codin	0	0	0	0
ENSG000001	VPS33B	protein_codin	19.377875	64.9637357	24.6514187	0
ENSG000001	NPB	protein_codin	0	0	0	0
ENSG000002	SPDYE8	protein_codin	0	0	0	0
ENSG000001	EWSR1	protein_codin	0	0	0	0
ENSG000001	RIOX2	protein_codin	0	4.21014455	0	0
ENSG000001	FAM83F	protein_codin	3.00322224	5.92292514	3.0242869	2.98932079
ENSG000001	POLR2F	protein_codin	0	0	0	0
ENSG000001	ABHD17B	protein_codin	43.6100055	46.0803491	15.1700314	20.3274714
ENSG000001	PIF1	protein_codin	0	0	3.11959415	0
ENSG000001	S1PR5	protein_codin	0	0	0	0
ENSG000002	POLR2J2	protein_codin	15.2962365	43.4297467	6.85614583	2.72394652
ENSG000001	TEX19	protein_codin	0	0	0	0
ENSG000001	PPFIA2	protein_codin	0	0	0	0
ENSG000001	TMEM179B	protein_codin	53.5317164	99.0988813	21.9238043	36.7195421
ENSG000001	STAC2	protein_codin	0	0	0	0
ENSG000001	APOBEC3B	protein_codin	0	0	0	0
ENSG000001	ADGRF3	protein_codin	0	0	0	0
ENSG000001	KNTC1	protein_codin	43.7878376	50.7510967	58.0548465	16.6522455
ENSG000001	MAFA	protein_codin	0.91492979	0.93588743	0	0

ENSG000002	ENSG000002	lncRNA	0	0	1.60427324	0
ENSG000000	METTL2A	protein_codin	1.99260704	8.50407946	5.711628	7.21270287
ENSG000001	UBA7	protein_codin	350.841784	453.399163	205.765848	215.379573
ENSG000001	SREBF2-AS1	lncRNA	13.730547	28.5593849	4.99273837	5.95590169
ENSG000001	ADGRG3	protein_codin	0	0	0	0
ENSG000001	CLN3	protein_codin	0	0	0	0
ENSG000001	PGP	protein_codin	37.492564	69.6706117	18.9452922	41.1784297
ENSG000001	BSG	protein_codin	8.86055375	9.4536061	1.92577643	2.38523082
ENSG000001	RESP18	protein_codin	0	0	0	0
ENSG000001	SCN5A	protein_codin	0	0	0	0
ENSG000001	GALNT17	protein_codin	0	0	0	0
ENSG000001	RALY	protein_codin	90.5830596	95.9211697	42.3193479	83.4759802
ENSG000001	EP400	protein_codin	0	0	0	0
ENSG000001	PIGA	protein_codin	0	40.9439002	28.5256105	0
ENSG000001	MCEMP1	protein_codin	159.359036	180.076064	45.459911	94.6464316
ENSG000000	TNK2	protein_codin	8.32668133	18.2999013	16.2782622	33.5244319
ENSG000001	TTC32	protein_codin	10.1566633	18.5227306	5.92795789	10.5990767
ENSG000001	LIMK2	protein_codin	0	0	0	0
ENSG000001	LSAMP	protein_codin	0	0	0	0
ENSG000001	IFNL1	protein_codin	0	0	0	0
ENSG000001	TUBB2A	protein_codin	0	21.9179291	29.4391962	31.8198687
ENSG000001	SH3BGR	protein_codin	0	0	0	0
ENSG000001	POU6F1	protein_codin	14.2552672	18.2922204	0	0
ENSG000001	BEX5	protein_codin	1.08814836	2.178253	2.96744638	0
ENSG000001	MED12	protein_codin	0	0	0	0
ENSG000001	RPL14P6	processed_ps	0.8211164	0	0	0
ENSG000000	HMG20B	protein_codin	182.873888	459.821172	205.227128	133.047405
ENSG000001	ENSG000001	lncRNA	0	0	0	0
ENSG000001	RNF212	protein_codin	0	1.3991774	0	0
ENSG000001	PMS2P6	transcribed_u	0	0	0	0
ENSG000002	MRPL12	protein_codin	47.3865473	55.7660481	23.3864958	18.8571183
ENSG000001	ARHGEF37	protein_codin	0	0	0	0
ENSG000001	BCL2	protein_codin	2.03077229	2.65822479	0	0
ENSG000001	TH	protein_codin	0	0	0	0
ENSG000000	PCDH11Y	protein_codin	0	2.1576425	0	0
ENSG000001	C12orf56	protein_codin	0	0	0	0
ENSG000001	TCF12	protein_codin	0	29.0785436	0	0
ENSG000001	ANKS1B	protein_codin	0	0	0	0
ENSG000001	GABRG3	protein_codin	0	0	0	0
ENSG000001	CRACR2A	protein_codin	30.1581141	44.2942192	20.7381101	18.5928537
ENSG000002	KCTD11	protein_codin	19.2534682	25.189746	5.07763554	0
ENSG000001	NUTM1	protein_codin	0	0	0	0
ENSG000001	H1-10	protein_codin	1440.34577	1354.88184	324.846378	560.890055
ENSG000001	NAA38	protein_codin	30.4863704	31.3412469	10.4763933	15.0805839

ENSG000001	CLASP2	protein_codin	11.5300257	0	3.94035501	0
ENSG000002	USP17L2	protein_codin	0	0	0	0
ENSG000001	UGT3A1	protein_codin	0	1.14991058	0	0
ENSG000001	TRARG1	protein_codin	0	0	0	0
ENSG000001	RPL7P26	processed_ps	3.05718407	1.01963796	0	7.23038543
ENSG000002	KRTAP4-8	protein_codin	0	0	0	0
ENSG000001	NELL2	protein_codin	0	0	0	0
ENSG000001	MBD3L3	protein_codin	0	0	0	0
ENSG000002	EGFL8	protein_codin	0	4.84940455	2.65625381	0
ENSG000001	TPH2	protein_codin	0	0	0	0
ENSG000001	PRKCZ-AS1	lncRNA	0.9178157	2.70442986	0	0
ENSG000001	CCDC87	protein_codin	0	0	0	0.86745505
ENSG000001	CASP9	protein_codin	0	24.6785915	11.9336001	18.9240154
ENSG000001	SPEM2	protein_codin	0	0	0	0
ENSG000001	NELFA	protein_codin	18.5973536	0	0	0
ENSG000002	LCE3C	protein_codin	0	0	0	0
ENSG000000	GP6	protein_codin	0	0	0	0
ENSG000001	PCLO	protein_codin	1.97250183	0	0	0
ENSG000001	KRTAP21-2	protein_codin	0	0	0	0
ENSG000001	SPTBN1	protein_codin	277.066237	423.287089	480.529286	66.6691143
ENSG000001	UNC80	protein_codin	0	0	0	0
ENSG000001	NME9	protein_codin	0	0	0	0
ENSG000001	CAAP1	protein_codin	50.6040257	31.1454807	32.7719622	11.574995
ENSG000001	BCDIN3D	protein_codin	2.03042234	7.99974789	2.6072751	4.1440761
ENSG000001	CISD1	protein_codin	13.9862655	12.0328823	2.68558723	10.2017646
ENSG000001	SPIN2B	protein_codin	8.81952423	4.17412895	0	13.5916045
ENSG000001	RHOT1	protein_codin	125.644441	149.885561	123.270247	62.6288238
ENSG000001	ZNF677	protein_codin	3.90767311	0	0	0
ENSG000001	C2CD5	protein_codin	101.249255	104.915198	82.6069144	96.9462256
ENSG000002	ARHGEF25	protein_codin	0	0	0	0
ENSG000001	OR5D18	protein_codin	0	0	0	0
ENSG000001	ZNF568	protein_codin	0	3.94521525	0	0
ENSG000001	POLR1B	protein_codin	0	0	0	0
ENSG000001	APAF1	protein_codin	0	0	0	0
ENSG000001	FAM9C	protein_codin	0	1.58211528	0	0
ENSG000001	ACTL7A	protein_codin	0	0	0	0
ENSG000001	GSAP	protein_codin	88.0327014	113.121432	79.6983103	84.3256687
ENSG000001	OIT3	protein_codin	0	0	0	0
ENSG000001	EMID1	protein_codin	0	0	0	0
ENSG000001	AKR1E2	protein_codin	0	0	0	0
ENSG000001	RGS7BP	protein_codin	0	0	0	0
ENSG000001	PRODH	protein_codin	0	0	0	0
ENSG000001	CPEB4	protein_codin	204.602947	0	152.9864	59.4177086
ENSG000001	DMRTC1B	protein_codin	0	0	0	0

ENSG000001	KRTAP19-6	protein_codin	0	0	0	0
ENSG000001	F3	protein_codin	120.03061	24.3445716	19.0294535	26.9241287
ENSG000001	GNAL	protein_codin	0	0	0	0
ENSG000001	KRTAP19-2	protein_codin	0	0	0	0
ENSG000001	SPDYA	protein_codin	0	0	0	0
ENSG000001	KRTAP19-4	protein_codin	0	0	0	0
ENSG000001	SHOX	protein_codin	0	0	0	0
ENSG000001	RASA3	protein_codin	723.333798	1048.08924	477.83984	414.119556
ENSG000002	KRTAP19-3	protein_codin	0	0	0	0
ENSG000001	KRTAP15-1	protein_codin	0	0	0	0
ENSG000001	KRTAP13-4	protein_codin	0	0	0	0
ENSG000001	ZNF197	protein_codin	0	0	0	0
ENSG000001	TAL2	protein_codin	0	0	0	0
ENSG000001	HOMER1	protein_codin	6.13678573	5.08825008	6.49735163	4.12170245
ENSG000001	ZNF761	protein_codin	0	0	19.2640914	17.312397
ENSG000001	AZI2	protein_codin	59.5773871	53.6490913	0	40.7606488
ENSG000002	KRTAP9-4	protein_codin	0	0	0	0
ENSG000001	ZNF529	protein_codin	15.3087483	12.0175868	6.91302328	0
ENSG000001	ENSG000001	unprocessed_	0	0	0	0
ENSG000001	SREK1	protein_codin	101.546766	51.7837586	40.4447456	94.9619895
ENSG000001	TEX38	protein_codin	0	0	0	0
ENSG000001	C2CD3	protein_codin	0	0	0	0
ENSG000001	HTR3D	protein_codin	0	0	0	0
ENSG000001	RFTN1	protein_codin	173.823601	257.838972	134.713456	128.828005
ENSG000001	FGF3	protein_codin	0	0	0	0
ENSG000000	HDAC6	protein_codin	0	0	0	0
ENSG000001	ZSCAN2	protein_codin	0	1.42216421	3.44636175	0
ENSG000001	MIR22HG	lncRNA	20.9985821	0	10.1993981	0
ENSG000001	TMEM52B	protein_codin	1.70247381	1.94505129	1.44695289	2.87302389
ENSG000001	KRTAP19-5	protein_codin	1.18308223	0	0	0
ENSG000001	KRTAP23-1	protein_codin	0	0	0	0
ENSG000001	POLR3C	protein_codin	49.589004	63.2182293	59.9111977	38.9969094
ENSG000002	TCP10L	protein_codin	0	0	0	0
ENSG000001	LYPD6	protein_codin	0	0	0	1.22728244
ENSG000001	RGS9BP	protein_codin	0	2.76968441	0.7826313	0
ENSG000001	UBL4B	protein_codin	0	0	0	0
ENSG000001	ZNF749	protein_codin	8.05014321	3.47140328	2.18496869	1.02606537
ENSG000001	PPFIA3	protein_codin	0	0	0	0
ENSG000001	PPP4R1L	transcribed_u	0	0	0	0
ENSG000000	EML1	protein_codin	0	0	0	0
ENSG000001	CYP4Z1	protein_codin	0	0	0	0
ENSG000001	ZNF347	protein_codin	13.0228325	1.857121	11.7767713	6.35689843
ENSG000001	KRTAP17-1	protein_codin	0	0	0	0
ENSG000001	KIF27	protein_codin	41.5360703	9.40863579	0	7.84991926

ENSG000001	RPS6KA4	protein_codin	186.56343	192.402959	63.7054527	158.851994
ENSG000001	MPPED1	protein_codin	0	0	0	0
ENSG000001	ARAP1	protein_codin	927.715886	1082.83362	370.15363	448.621658
ENSG000001	ZNF20	protein_codin	7.23242962	0	3.10677428	0
ENSG000001	CDC42BPA	protein_codin	0	0	0	0
ENSG000001	DLST	protein_codin	239.943771	280.488169	115.794002	158.092273
ENSG000001	C1QL4	protein_codin	0	0	0	0
ENSG000001	DNAJB14	protein_codin	0	26.3973314	0	33.2541688
ENSG000001	EDARADD	protein_codin	0	0	0	0
ENSG000001	MAPT	protein_codin	0	0	0	0
ENSG000001	ZNF555	protein_codin	8.45389777	15.9716611	10.7483922	8.12148811
ENSG000001	KPNA4	protein_codin	95.3669543	0	0	0
ENSG000001	SYNE3	protein_codin	0	0	0	0
ENSG000001	TAS2R42	protein_codin	0	0	0	0
ENSG000001	DYNC2H1	protein_codin	0	0	0	0
ENSG000001	TRDN	protein_codin	0	0	0	0
ENSG000001	LCE5A	protein_codin	0	0	0	0
ENSG000001	TTF1	protein_codin	77.357489	58.980341	61.2774625	45.2979186
ENSG000001	AP1S3	protein_codin	0	0	0	0
ENSG000001	TCP11L1	protein_codin	65.2003252	101.79538	31.0958361	35.7139502
ENSG000001	SLC51B	protein_codin	0	0	0	0
ENSG000001	ANGPTL5	protein_codin	0	0	0	0
ENSG000001	CELF5	protein_codin	0	0	0.6186688	0
ENSG000001	TPGS2	protein_codin	34.6882925	86.5862985	30.5260771	15.1735092
ENSG000001	ADGRA3	protein_codin	0	0	0	4.59051405
ENSG000001	SOWAHB	protein_codin	0	0.87172126	0	0
ENSG000001	IFTAP	protein_codin	15.6629576	0	0	15.9535486
ENSG000001	TEAD1	protein_codin	0	0	0	0
ENSG000001	PTBP3	protein_codin	10.2355374	30.2705751	0	12.4390133
ENSG000001	SPATA12	protein_codin	0	0	0	0
ENSG000001	ARMH4	protein_codin	0	2.27826223	0	0
ENSG000001	ARID2	protein_codin	173.689934	56.1852798	66.6973074	90.6211163
ENSG000001	MT1B	protein_codin	0	0	0	0
ENSG000002	UPK3B	protein_codin	0	0.88998479	0	0
ENSG000001	MT1F	protein_codin	5.383139	0	0	0
ENSG000001	PNRC2	protein_codin	386.433963	419.169718	218.612733	291.794323
ENSG000001	USP16	protein_codin	0	0	37.4635082	0
ENSG000001	AKAP14	protein_codin	0	0	0	0
ENSG000001	TXNRD2	protein_codin	41.1404288	39.3362298	0	30.4446107
ENSG000001	MEGF8	protein_codin	0	0	0	0
ENSG000002	LCE1F	protein_codin	0	0	0	0
ENSG000000	TRMT11	protein_codin	19.03096	0	0	11.5021315
ENSG000001	QRFPR	protein_codin	0	0	0	0
ENSG000001	ARGFX	protein_codin	0	0	0	0

ENSG000002	ENSG000002	lncRNA	0	0	0	0
ENSG000001	DEFB128	protein_codin	0	0	0	0
ENSG000001	TMLHE	protein_codin	69.0627004	24.320472	29.2330273	45.2049918
ENSG000001	PDCD1	protein_codin	0	0	0	0
ENSG000001	PTPRB	protein_codin	0	0	0	0
ENSG000001	CENATAC	protein_codin	27.5131677	18.0266856	9.28568323	14.9064733
ENSG000001	TRIM7	protein_codin	31.8018396	12.4986607	0	0
ENSG000001	GPR141	protein_codin	62.2727217	60.3889103	33.5382925	43.7753693
ENSG000001	TACC2	protein_codin	0	0	0	0
ENSG000001	OR10T2	protein_codin	0	0	0	0
ENSG000001	ATP6V1FP1	processed_ps	0	0	0	0
ENSG000001	ABCC5	protein_codin	35.3456735	19.8248471	47.333456	71.9057423
ENSG000000	CCDC88C	protein_codin	4.28096443	5.29382109	4.79863588	4.91975341
ENSG000001	CEP41	protein_codin	0	0	0	0
ENSG000001	PSMB5	protein_codin	0	0	0	0
ENSG000001	PDE2A	protein_codin	8.14889952	35.9104808	15.5618039	0
ENSG000001	C17orf58	protein_codin	0	0	0	0
ENSG000001	TMEM150A	protein_codin	20.157885	39.5917612	8.63336478	0
ENSG000001	ERCC6L	protein_codin	0	2.01907226	0	4.19802442
ENSG000001	PDZD8	protein_codin	395.941459	243.976267	230.646797	288.973893
ENSG000001	TREM1	protein_codin	42.1916004	31.6032426	19.2642322	23.6050857
ENSG000001	PFDN5	protein_codin	618.60521	989.70717	331.688301	479.126834
ENSG000001	PRDX2	protein_codin	0	0	0	0
ENSG000002	S1PR3	protein_codin	27.164792	0	44.3424938	56.1088957
ENSG000001	WDR86	protein_codin	0	0	0	0
ENSG000001	PRMT2	protein_codin	8.09607956	5.07881002	4.89199408	0
ENSG000001	TREX2	protein_codin	0	0	2.44179893	0
ENSG000001	COL4A6	protein_codin	0	0	0	0
ENSG000001	SLC25A23	protein_codin	1.69392334	0	0	0
ENSG000001	ZMIZ1	protein_codin	1430.54021	1268.42824	461.467729	665.403921
ENSG000001	NUDT17	protein_codin	1.97703146	5.73109107	4.76518942	1.93734433
ENSG000001	ZNF410	protein_codin	0	0	0	0
ENSG000001	NDEL1	protein_codin	82.7843444	60.0210669	19.5511214	60.3514989
ENSG000002	FMN1	protein_codin	0	0	0	0
ENSG000001	BTLA	protein_codin	4.34437312	5.24076895	31.8750045	1.08175339
ENSG000001	REEP4	protein_codin	0	0	0	0
ENSG000001	SLX4IP	protein_codin	65.7093415	66.390121	38.7279633	33.781882
ENSG000001	SPNS1	protein_codin	12.222229	50.0045169	0	6.5844217
ENSG000001	POFUT2	protein_codin	0	0	0	0
ENSG000000	PPP2R3A	protein_codin	0	1.70387328	0	0
ENSG000001	KCNK18	protein_codin	0	0	0	0
ENSG000001	KLC1	protein_codin	0	0	0	0
ENSG000000	ZDHHC8	protein_codin	29.0968978	27.85578	0	0
ENSG000001	FMR1	protein_codin	5.12964727	6.22135724	7.76354411	0

ENSG000001	ZNF480	protein_codin	0	0	0	0
ENSG000001	LINC00207	lncRNA	0	0	0	0
ENSG000001	TMEM212	protein_codin	0	0	0	0
ENSG000001	TAC4	protein_codin	0	0	0	0
ENSG000002	ENSG000002	processed_ps	0	0	0	0
ENSG000001	COQ6	protein_codin	0	12.0979451	0	5.66606574
ENSG000001	FSD2	protein_codin	1.08628657	0	0	0
ENSG000002	GSTA1	protein_codin	0	2.06612523	0	0
ENSG000001	CARMIL2	protein_codin	0	0	0	0
ENSG000001	ZACN	protein_codin	0	0	0	0
ENSG000002	ZNF726	protein_codin	0	0	1.17631884	0
ENSG000001	INO80C	protein_codin	14.6750121	9.12153888	8.36139123	1.80783149
ENSG000002	PRAMEF14	protein_codin	0	0	0	0
ENSG000001	TERT	protein_codin	0	0	0	0
ENSG000001	CCKBR	protein_codin	0	0	0	0
ENSG000001	TRIM46	protein_codin	0	0	0	0
ENSG000001	DCUN1D4	protein_codin	36.3568178	0	0	19.4783137
ENSG000001	CYP2R1	protein_codin	5.53674231	13.8450699	0	1.11966971
ENSG000001	DHRSX	protein_codin	52.3952565	88.5793087	39.9426161	44.8771683
ENSG000001	CHGA	protein_codin	0	0	0	0
ENSG000001	CHP1	protein_codin	473.840723	600.953469	179.93016	351.019499
ENSG000001	PLCD1	protein_codin	0	0	0	0
ENSG000001	KRTAP10-8	protein_codin	0	0	0	0
ENSG000002	KRTAP20-1	protein_codin	0	0	0	0
ENSG000001	TMEM68	protein_codin	0	0	0	0
ENSG000002	HLA-F	protein_codin	132.938785	235.309324	0	0
ENSG000002	KRTAP10-11	protein_codin	0	0	0	0
ENSG000001	RPS19BP1	protein_codin	102.730295	182.767678	77.7433645	56.8993312
ENSG000001	DUSP18	protein_codin	18.7960673	89.7186682	0	0
ENSG000001	KRTAP22-1	protein_codin	0	0	0	0
ENSG000001	CCK	protein_codin	0	0	0	0
ENSG000001	PLCH1	protein_codin	73.5874258	30.5711385	56.7984541	0
ENSG000001	TRAPPC11	protein_codin	244.17117	161.515415	132.258413	201.204335
ENSG000001	ENTPD5	protein_codin	21.1418085	30.0547718	27.5451379	16.4412814
ENSG000000	HSP90AA1	protein_codin	0	0	3.0503856	0
ENSG000001	FAM91A1	protein_codin	335.422386	161.096807	153.794504	213.36694
ENSG000001	SERPINA11	protein_codin	0	0	0	0
ENSG000001	ZSCAN25	protein_codin	19.4577869	0	0	0
ENSG000001	FUNDC2P3	processed_ps	0	0	0	0
ENSG000001	AP4S1	protein_codin	0	0	0	0
ENSG000001	OR13F1	protein_codin	0	0	0	0
ENSG000001	C9orf85	protein_codin	23.628392	15.7783936	6.60867776	8.36860028
ENSG000001	EMSY	protein_codin	44.3202929	31.8347128	23.6707751	0
ENSG000000	PPP2R5C	protein_codin	0	0	0	0

ENSG000001	PRELID2	protein_codin	0	0	0	0
ENSG000000	BTBD7	protein_codin	64.351776	85.4547427	41.2533785	69.4749939
ENSG000001	PLK5	protein_codin	0	0	0	0
ENSG000001	DEFB105A	protein_codin	0	0	1.21535377	0
ENSG000001	NEXN	protein_codin	0	0	0	0
ENSG000001	FOXD2	protein_codin	0.90792751	1.82868811	0	0
ENSG000001	BCL9L	protein_codin	2.10791349	12.6026618	0	8.048049
ENSG000002	STARD10	protein_codin	23.5956534	24.395067	0	11.1944914
ENSG000001	CTBP2	protein_codin	24.550596	5.73534241	7.04621855	0
ENSG000001	HPDL	protein_codin	0.89464014	0	0	0
ENSG000001	C2orf76	protein_codin	6.9385263	15.7253928	1.17956374	4.38554462
ENSG000001	TMEM262	protein_codin	0	0	0	0
ENSG000001	PGAM1	protein_codin	684.420822	914.736736	355.457096	480.170933
ENSG000001	TMPRSS11A	protein_codin	0	0	0	0
ENSG000001	CMC1	protein_codin	1.10703657	0	0	0
ENSG000002	KRTAP19-7	protein_codin	0	0	0	0
ENSG000001	FSCN2	protein_codin	0.82614432	1.66725423	1.38567378	0
ENSG000001	OR10J5	protein_codin	0	0	0	0
ENSG000001	LRCH3	protein_codin	73.5685846	137.081676	31.6544495	118.246043
ENSG000001	LGMN	protein_codin	5.01822402	12.9490362	0	0
ENSG000001	DEFB131A	protein_codin	0	0	0	0
ENSG000000	KMT2E	protein_codin	0	0	0	100.440012
ENSG000001	TMEM216	protein_codin	12.3007482	17.3871718	0	3.73662355
ENSG000001	RAX	protein_codin	0	0	0	0
ENSG000001	TPM1	protein_codin	20.1356814	17.4786524	0	0
ENSG000001	KRTAP6-2	protein_codin	0	0	0	0
ENSG000001	CELF4	protein_codin	0	0	0	0
ENSG000001	OR10H1	protein_codin	0	0	0	0
ENSG000001	GGN	protein_codin	0	2.46967977	0	0
ENSG000001	PPIP5K1	protein_codin	0	0	43.5849206	0
ENSG000001	ZNF81	protein_codin	0	0	0	0
ENSG000001	SF1	protein_codin	32.0396048	47.9770208	0	13.950075
ENSG000001	RSBN1L	protein_codin	166.509257	174.969105	60.2101818	111.075841
ENSG000001	PARVA	protein_codin	0	0	0	0
ENSG000001	NF2	protein_codin	0	0	0	0
ENSG000001	LRRC37A3	protein_codin	0	0	1.20167839	1.12523175
ENSG000001	KTN1	protein_codin	12.5794795	6.68108537	4.18279542	9.94605163
ENSG000001	EMC10	protein_codin	63.870194	53.4022803	25.8639501	16.758147
ENSG000001	NAA20	protein_codin	52.0757827	57.9557902	17.1556701	36.9401595
ENSG000001	CMTM7	protein_codin	83.7883985	146.132581	24.8264763	84.3843156
ENSG000001	RREB1	protein_codin	0	0	0	0
ENSG000001	HEATR4	protein_codin	0	1.6612669	0	0
ENSG000001	LRRC70	protein_codin	11.9236585	0	0	5.0523213
ENSG000001	ITM2C	protein_codin	0	21.8386423	102.258199	0

ENSG000001	PPP1CC	protein_codin	236.030437	217.046708	116.113747	166.751034
ENSG000001	C3orf70	protein_codin	0	0	1.01888027	0
ENSG000001	H1-7	protein_codin	0.87978104	0	0	0.84403292
ENSG000001	TPI1P3	transcribed_p	0	0	0	0
ENSG000001	DEFB107A	protein_codin	0	0	0	0
ENSG000001	PPIL2	protein_codin	19.0599424	5.20283623	7.72222831	6.79828194
ENSG000001	OR13C8	protein_codin	0	0	0	0
ENSG000001	UBE2C	protein_codin	0	0	0	0
ENSG000001	RASSF6	protein_codin	0	0	0	0
ENSG000001	FOXE3	protein_codin	0.96153382	0	0	0
ENSG000001	KDM4D	protein_codin	0	0	0.97522816	0
ENSG000001	ZNF480	protein_codin	4.86418434	5.4900584	0	2.54743441
ENSG000001	KRTAP21-1	protein_codin	0	0	0	0
ENSG000001	RTN4RL2	protein_codin	1.87130551	0.94238354	0	0
ENSG000000	MARK3	protein_codin	0	0	0	0
ENSG000001	FAT4	protein_codin	0	6.14268294	0	4.21158938
ENSG000002	ZNF486	protein_codin	5.08951768	12.7838361	3.09390264	6.07187206
ENSG000001	CCIN	protein_codin	21.6187371	19.2287176	3.87491848	5.07345156
ENSG000001	LCE1A	protein_codin	0	0	0	0
ENSG000001	DHRS4L2	protein_codin	40.0344009	45.9988379	0	12.2473231
ENSG000001	GATA4	protein_codin	0	0	0	0
ENSG000001	KTN1-AS1	lncRNA	0	0	0	0
ENSG000001	EXOC7	protein_codin	72.4831292	88.3563875	54.3543626	0
ENSG000001	ETNK1	protein_codin	0	0	7.99089521	0
ENSG000001	FMNL3	protein_codin	66.0598677	137.525937	74.4773115	25.0207759
ENSG000001	ERLIN2	protein_codin	0	16.3078271	0	0
ENSG000001	ANKRD37	protein_codin	5.06305896	6.70231092	0	3.65177245
ENSG000001	TMSB4XP2	unprocessed_	42.9636516	118.008762	30.2405543	53.9169249
ENSG000000	PKM	protein_codin	1833.95923	2975.32192	1111.60642	1513.6061
ENSG000001	CDKN3	protein_codin	0	0	9.88866548	0
ENSG000001	CCDC73	protein_codin	0	4.50418153	0	0
ENSG000001	DEFB106A	protein_codin	0	0	0	0
ENSG000001	POLM	protein_codin	0	0	0	5.8670777
ENSG000000	TESC	protein_codin	48.1378272	66.2407208	11.7586223	28.0354127
ENSG000001	IGFN1	protein_codin	0	0	0	0
ENSG000001	PES1	protein_codin	0	18.8870909	0	0
ENSG000001	PTRH1	protein_codin	0	0.96648355	0	0
ENSG000001	SLC36A3	protein_codin	0	0	0	0
ENSG000002	OR4C12	protein_codin	0	0	0	0
ENSG000001	SLC36A2	protein_codin	0	0	0	0
ENSG000001	CYP27C1	protein_codin	0	0	0	0
ENSG000001	INTU	protein_codin	7.49374759	0	1.30565511	4.7562976
ENSG000001	FOXK2	protein_codin	97.2746208	177.20924	47.1336635	46.6925281
ENSG000001	ZFAND4	protein_codin	15.5767029	9.48956039	7.00533828	2.53612266

ENSG000001	WDR20	protein_codin	0	2.58948377	0	0
ENSG000000	MAP4	protein_codin	0	0	0	0
ENSG000001	PITPNB	protein_codin	85.4519014	0	108.405374	68.6736711
ENSG000001	NHP2P1	processed_ps	0	0	0	0
ENSG000001	GNG2	protein_codin	12.3893796	2.55419962	2.62116166	16.4636645
ENSG000001	CFAP144	protein_codin	0	0	0	0
ENSG000001	FAM170A	protein_codin	0	0	0	0
ENSG000001	ATP4B	protein_codin	0	0	0	0
ENSG000001	SESTD1	protein_codin	11.0161518	37.4807288	49.7147684	0
ENSG000001	WDR25	protein_codin	0	0	0	16.9492464
ENSG000002	HBB	protein_codin	3.19108061	26.5357699	0	0
ENSG000001	OR1J2	protein_codin	0	0	0	0
ENSG000001	HTR3E	protein_codin	0	0	0	0
ENSG000001	PIP5K1C	protein_codin	180.4816	201.441385	64.3668772	144.423678
ENSG000001	ZNRF1	protein_codin	9.18079808	22.5144718	0	7.22131232
ENSG000001	WASF3	protein_codin	7.54010929	70.2946207	0	3.22148896
ENSG000001	UNC5B	protein_codin	0	0	0	3.53768895
ENSG000001	TCFL5	protein_codin	35.5676217	33.4699345	11.6380987	24.0419429
ENSG000001	GIT1	protein_codin	6.32687025	0	0	0
ENSG000001	SPATA2L	protein_codin	16.0033596	9.85732792	0	0
ENSG000001	AGAP3	protein_codin	6.87795736	4.54713887	0	1.75888946
ENSG000001	TBX10	protein_codin	0	0	0	0
ENSG000000	RC3H2	protein_codin	8.25907413	25.8006265	3.87343412	1.80618963
ENSG000002	LPAL2	lncRNA	0	0	0	0
ENSG000001	TMEM17	protein_codin	7.85445988	0	1.11723171	0
ENSG000001	NIBAN3	protein_codin	0	0	0	0
ENSG000001	OR9Q1	protein_codin	0	0	0	0
ENSG000001	KLHDC10	protein_codin	56.3597313	72.8413884	33.9732589	44.5657195
ENSG000001	KLK13	protein_codin	0	0	0	0
ENSG000001	RIC3	protein_codin	0	0	0	0
ENSG000001	SELENOV	protein_codin	0	0	0	0
ENSG000001	ST3GAL3	protein_codin	0	0	0	0
ENSG000001	ARL13B	protein_codin	8.41648007	0	0	0
ENSG000001	PDE9A	protein_codin	0	0	0	0
ENSG000001	GLDN	protein_codin	0	0	0	0
ENSG000001	ACVR1C	protein_codin	0	0	0	0
ENSG000001	TRABD2A	protein_codin	0	0	0	0
ENSG000001	TMEM104	protein_codin	137.945451	225.949651	85.6158473	107.057297
ENSG000001	SLC10A7	protein_codin	0	21.7804468	0	0
ENSG000001	MYO18B	protein_codin	0	0	0	0
ENSG000000	KCNQ1	protein_codin	82.4467084	180.759694	90.6174944	69.5068394
ENSG000002	LMLN-AS1	lncRNA	3.03237152	4.36170834	0	0
ENSG000001	DEFB106B	protein_codin	0	0	0	0
ENSG000001	ADGRD1	protein_codin	0	0	0	0

ENSG000001	TYSND1	protein_codin	0	0	0	6.33753028
ENSG000001	SPATA21	protein_codin	0	0	0	0
ENSG000001	RNF220	protein_codin	17.1087635	20.6771358	8.00402904	17.4387005
ENSG000001	GPATCH8	protein_codin	125.538541	108.179037	95.3221206	6.75196604
ENSG000001	CCT6A	protein_codin	12.6972893	17.5837961	0	0
ENSG000001	MAGED4B	protein_codin	0	0	0	0
ENSG000002	ENSG000002	lncRNA	0	0	0	1.64226413
ENSG000001	SF3B1	protein_codin	1998.28576	1228.60393	1061.75332	1339.85697
ENSG000001	TPCN1	protein_codin	156.889199	437.657021	205.646823	201.63434
ENSG000001	DEFB105B	protein_codin	0	0	0	0
ENSG000001	PDE9A	protein_codin	0	0	0	0
ENSG000001	ATP5IF1	protein_codin	227.676759	265.321091	149.260196	146.969663
ENSG000001	ENSG000001	protein_codin	5.35526117	11.2770204	0	5.46288957
ENSG000001	VSTM2B	protein_codin	0	0	0	0
ENSG000001	PRR30	protein_codin	0	0	0.76379792	0
ENSG000001	PLA2G6	protein_codin	0	0	0	0
ENSG000001	GHRL	protein_codin	0	7.404568	0	6.14829347
ENSG000001	CSMD1	protein_codin	0	17.3678962	0	15.9421733
ENSG000001	KRT26	protein_codin	0	0.93859225	0	0
ENSG000001	SAMD7	protein_codin	0	0	0	0
ENSG000001	SLITRK2	protein_codin	0	0	0	0
ENSG000001	UGT2B28	protein_codin	0	0	0	0
ENSG000000	PPP2R2C	protein_codin	0	0	0	0
ENSG000001	OR5D14	protein_codin	0	0	0	0
ENSG000002	AMACR	protein_codin	0	4.79044879	0	10.597951
ENSG000001	VWCE	protein_codin	0	0	0	0
ENSG000001	ENSG000001	lncRNA	0.85205015	0	1.04698152	0
ENSG000001	CFAP73	protein_codin	0	0	0	0
ENSG000001	VSIG10L	protein_codin	0	0	0	0
ENSG000001	GABRA5	protein_codin	0	0	0	0
ENSG000001	TCF20	protein_codin	123.898238	157.811144	121.616296	145.620669
ENSG000001	LCE3B	protein_codin	0	0	0	0
ENSG000001	KATNA1	protein_codin	0	0	0	0
ENSG000001	KATNA1	protein_codin	28.0819697	0	4.97899066	16.0078658
ENSG000002	ZNF436-AS1	lncRNA	0.97808953	0	0.94264639	0.98057038
ENSG000000	ANK1	protein_codin	0	0	0	0
ENSG000001	DPPA4	protein_codin	1.0466933	11.2664658	4.64728987	0
ENSG000001	ANKRD46	protein_codin	0	0	5.67475866	9.61644163
ENSG000001	BCL2A1	protein_codin	36.1416418	21.1598936	33.4824327	36.9276311
ENSG000002	GPR142	protein_codin	0	0.82624526	0	0
ENSG000000	RORA	protein_codin	12.8061391	3.68398952	4.73277031	1.11233797
ENSG000001	LCE3A	protein_codin	0	0	0	0
ENSG000001	GRK1	protein_codin	0	0	0	0
ENSG000001	NPAS2	protein_codin	0	0	0	0

ENSG000001	SUGCT	protein_codin	0	0	0	0
ENSG000001	GGTLC1	protein_codin	0	0	0	0
ENSG000001	ATP5F1C	protein_codin	46.0417559	83.6879373	0	0
ENSG000001	BCL11A	protein_codin	0	36.8788991	0	8.8845207
ENSG000001	SULT1A2	protein_codin	11.0905422	9.3146663	0	8.20088029
ENSG000000	TDP1	protein_codin	23.5218286	27.3498822	29.8603492	19.3481278
ENSG000001	TNRC6B	protein_codin	417.393122	265.833161	203.591131	195.481994
ENSG000001	LOXHD1	protein_codin	60.3185947	114.669846	48.4016552	17.0227104
ENSG000000	NRXN3	protein_codin	0	0	0	3.6545289
ENSG000000	SLK	protein_codin	957.048546	595.447606	446.183747	690.901113
ENSG000001	ACTR8	protein_codin	47.5058446	72.1990874	37.6914771	37.5973194
ENSG000001	CENPA	protein_codin	0	0	0	0
ENSG000001	SLC44A2	protein_codin	0	0	7.54751185	0
ENSG000001	APOBEC3B	protein_codin	11.8372395	5.34556553	41.5783458	19.480013
ENSG000001	CACNA1C	protein_codin	0	0	0	0
ENSG000001	UNC119	protein_codin	111.537454	146.129439	51.8419741	78.6216932
ENSG000001	CDKN2D	protein_codin	5.42840762	0	63.7974188	0
ENSG000001	WFDC10B	protein_codin	0	0	0	0
ENSG000001	F11R	protein_codin	0	3.83074012	0	0
ENSG000000	CENPQ	protein_codin	17.5113225	15.7940588	13.8961526	12.7892541
ENSG000001	LMO1	protein_codin	0	0	0	0
ENSG000001	ST6GALNAC4	protein_codin	11.7844441	26.3613466	21.4479714	9.09338739
ENSG000001	CDC42EP4	protein_codin	81.3278647	63.6091552	14.0006516	50.9267091
ENSG000001	UBE2E2	protein_codin	0	20.2002198	0	0
ENSG000001	ZNF354A	protein_codin	56.3209654	37.4175501	37.5347969	36.5373851
ENSG000000	ZNF446	protein_codin	8.15947426	0	0	0
ENSG000001	CRISP1	protein_codin	0	0	0	0
ENSG000001	PAQR6	protein_codin	0	10.8060004	0	0
ENSG000001	ALAS2	protein_codin	0	0	0	0
ENSG000001	RAD51D	protein_codin	0	0	0	0
ENSG000001	GRB10	protein_codin	28.2069785	8.59342212	0	14.7643928
ENSG000001	DACT1	protein_codin	0	4.08878203	0	0
ENSG000001	ZNF212	protein_codin	41.1455835	29.0489215	12.9544694	9.74635073
ENSG000000	SLC6A16	protein_codin	0	0	0.63979321	0
ENSG000001	PSMF1	protein_codin	50.0217125	58.2895257	27.2937462	23.5860832
ENSG000001	SERHL2	protein_codin	0	0	0	0
ENSG000001	UXT	protein_codin	3.99413363	5.22271221	3.37878078	1.73142531
ENSG000001	IQCH	protein_codin	0	0	0	0
ENSG000001	BTF3	protein_codin	606.288317	724.123259	332.998529	410.429518
ENSG000000	FGFR1	protein_codin	0	0	0	0
ENSG000001	RASGEF1B	protein_codin	114.850112	0	28.5019478	4.20363076
ENSG000001	TP53I3	protein_codin	53.5200443	15.2340741	5.63302897	4.56002873
ENSG000002	ENSG00000	protein_codin	0	0	0	0
ENSG000001	ZBTB16	protein_codin	61.8604184	0	37.7454301	0

ENSG000001	ZDHHC4	protein_codin	12.9950206	26.3913907	2.22073521	0
ENSG000001	TARS3	protein_codin	3.96981328	50.5454188	30.5145373	23.4342734
ENSG000001	UBA1	protein_codin	323.717996	205.465985	324.041597	194.344897
ENSG000001	ARL6	protein_codin	0	0	0	0
ENSG000001	OVOL1	protein_codin	0	0	0	0
ENSG000001	ELF4	protein_codin	380.89459	397.894392	191.698008	200.541617
ENSG000001	NCKAP5L	protein_codin	215.852366	298.389832	112.976735	116.296553
ENSG000001	TUBA1B	protein_codin	524.260286	919.46438	640.960057	500.71621
ENSG000001	PNRC1	protein_codin	3952.70034	4247.49642	1446.74472	2538.82443
ENSG000001	DEDD2	protein_codin	0	26.3408461	0	0
ENSG000000	HNRNPC	protein_codin	208.373003	135.666744	104.373352	81.8684476
ENSG000001	HDAC5	protein_codin	0	6.95040987	0	0
ENSG000001	PANK2	protein_codin	190.733784	331.460387	94.3749892	181.660389
ENSG000001	TOR2A	protein_codin	0	19.0827083	0	0
ENSG000001	FRMD1	protein_codin	0	0	0	0
ENSG000001	KIR2DL1	protein_codin	1.01197612	0	0	0
ENSG000000	ZNF532	protein_codin	0	0	0	0
ENSG000000	DDX3Y	protein_codin	1.68596707	0	0	0
ENSG000001	RAB6A	protein_codin	331.975485	488.042253	153.81614	283.200602
ENSG000001	URGCP	protein_codin	18.1540102	20.3581035	10.6735887	6.91763473
ENSG000001	TNNI1	protein_codin	0	0	0	0
ENSG000001	RNF24	protein_codin	19.62571	45.4983972	0	48.3108581
ENSG000002	NME1	protein_codin	0	0	0	0
ENSG000001	FGG	protein_codin	0	0	0	0
ENSG000001	NINL	protein_codin	0	0	0	0
ENSG000000	OGFOD1	protein_codin	27.0122932	19.2905037	7.66872635	33.8368468
ENSG000001	ASXL2	protein_codin	408.87459	352.134271	265.794265	262.012192
ENSG000001	NLRP3	protein_codin	2057.81818	697.915666	467.546962	713.517409
ENSG000001	IL17F	protein_codin	0	0.97538684	0	0
ENSG000001	ARL17A	protein_codin	53.9856692	101.182037	64.469081	14.2357628
ENSG000001	PCGF5	protein_codin	107.2825	51.7618225	8.41433162	39.0384359
ENSG000001	ZNF548	protein_codin	29.5006365	40.6116299	0	16.7509884
ENSG000002	SARNP	protein_codin	214.208656	215.331226	138.916714	137.725304
ENSG000001	THEM6	protein_codin	5.07163228	17.0711279	6.10531054	4.72506845
ENSG000001	ZNF202	protein_codin	25.6280136	0	0	0
ENSG000001	RAD54B	protein_codin	0	0	0	0
ENSG000001	HTR7	protein_codin	0	0	2.47978142	10.5245844
ENSG000001	KIAA0930	protein_codin	273.771687	484.95098	159.454383	228.461757
ENSG000001	KLRD1	protein_codin	0	0	0	0
ENSG000001	RGN	protein_codin	0	0	0	0
ENSG000002	STRADA	protein_codin	0	0	0	0
ENSG000001	LIMK1	protein_codin	135.393778	165.460806	69.1591161	72.3792193
ENSG000000	TRAF5	protein_codin	0	0	0	0
ENSG000001	AKT3	protein_codin	0	0	0	0

ENSG000001	HMG20A	protein_codin	43.8567093	50.1782424	37.7441316	29.4495413
ENSG000001	CDCA4	protein_codin	42.5053277	39.1736151	25.5721856	33.5903941
ENSG000001	MRPL37	protein_codin	8.55290423	0	0	0
ENSG000001	SLC22A8	protein_codin	0	0	0	0
ENSG000001	LIPA	protein_codin	253.310955	596.038529	169.866632	315.991854
ENSG000000	NDUFAF7	protein_codin	0	0	10.6665816	3.13534317
ENSG000001	CHST5	protein_codin	0	0	0	0
ENSG000001	MTDH	protein_codin	448.811619	273.965478	351.680126	330.512664
ENSG000001	SNTB2	protein_codin	143.643136	97.48226	36.4265874	51.6192259
ENSG000000	HEPH	protein_codin	0	0	0	0
ENSG000002	SRA1	protein_codin	144.776958	118.727781	66.5948341	45.1125059
ENSG000001	SYNM	protein_codin	80.94661	170.24341	206.959559	28.4624731
ENSG000001	TMEM39B	protein_codin	0	0	0	0
ENSG000001	MRPL55	protein_codin	0	0	0	0
ENSG000001	HYAL3	protein_codin	0	13.7283438	0	3.81318252
ENSG000001	STARD3	protein_codin	44.4228328	75.1771045	41.3840957	53.2863955
ENSG000001	LARP1	protein_codin	38.3006402	0	0	20.8323351
ENSG000000	CMTM1	protein_codin	0	0	0	0
ENSG000000	ZXDC	protein_codin	99.8685101	103.231667	49.8905167	63.664191
ENSG000001	KLK5	protein_codin	0	0	0	0
ENSG000001	SPAG17	protein_codin	2.8476714	0	1.23091435	0.98826891
ENSG000001	SPTY2D1	protein_codin	203.540887	148.147811	146.731989	189.277508
ENSG000001	LRAT	protein_codin	1.34234233	0	0	0
ENSG000001	CYP3A7	protein_codin	0	0	0	0
ENSG000000	ACP3	protein_codin	111.220945	125.787967	72.4173959	101.976306
ENSG000000	DNAAF6	protein_codin	0	0	0	0
ENSG000001	TESK1	protein_codin	47.4515254	53.6200214	41.9041492	48.4552852
ENSG000001	PLEKHA8P1	transcribed_p	0	11.1081241	12.8217958	0
ENSG000001	CYP3A4	protein_codin	0	0	0	0
ENSG000001	CEP170	protein_codin	296.764585	167.370652	190.196584	231.778748
ENSG000001	AP3S2	protein_codin	63.2355714	139.292707	30.7212808	52.1502096
ENSG000000	TBXAS1	protein_codin	0	0	0	0
ENSG000001	SERPINB7	protein_codin	0	0	0	0
ENSG000001	ACOT9	protein_codin	17.3340317	31.3785255	34.4657123	34.8670009
ENSG000001	GGT7	protein_codin	9.59868378	17.4216868	0	7.56210196
ENSG000001	ZFP36L1	protein_codin	132.190205	158.415901	34.9242807	61.8310865
ENSG000001	EPPIN	protein_codin	0	0	0	0
ENSG000001	RMND1	protein_codin	0	0	0	11.817024
ENSG000001	EIF5A	protein_codin	24.4397501	38.003738	0	11.0582817
ENSG000000	CDC14A	protein_codin	132.324539	87.8618317	25.886515	58.7678142
ENSG000001	ZFYVE19	protein_codin	17.4682002	0	3.78431359	0
ENSG000001	EIF5A	protein_codin	496.609557	581.917632	215.382585	265.157709
ENSG000001	SLC22A12	protein_codin	0	0	0	0
ENSG000001	GNL3L	protein_codin	0	0	0	27.9029456

ENSG000001	PIK3R1	protein_codin	18.6921008	19.0222236	22.4932027	4.21443575
ENSG000000	NFKB2	protein_codin	0	0	0	0
ENSG000000	ARHGAP10	protein_codin	137.833957	265.295391	112.859635	100.65516
ENSG000001	SCAI	protein_codin	25.5349163	9.65448356	16.0738018	10.4507511
ENSG000000	MFSD11	protein_codin	0	3.26258413	3.24033142	0
ENSG000001	ZP3	protein_codin	17.2231421	0	7.64923119	5.27962531
ENSG000001	MRPL55	protein_codin	9.35084933	3.40059633	2.03298499	3.28818264
ENSG000000	FGFR2	protein_codin	0	0	0	0
ENSG000001	CIDEB	protein_codin	26.748287	5.11689448	21.401951	33.8748343
ENSG000001	ZNF576	protein_codin	16.9075587	10.9820101	6.89296576	4.14443522
ENSG000001	DNAJB2	protein_codin	0	12.1234489	0	0
ENSG000000	MMP25	protein_codin	0	102.153716	0	45.4775101
ENSG000001	PBLD	protein_codin	21.4147856	0	0	0
ENSG000001	CUL4B	protein_codin	67.8731282	107.778277	68.1884671	0
ENSG000000	EPHA3	protein_codin	0	1.20558829	0	0
ENSG000001	LINC03040	protein_codin	2.59661283	0	4.95041793	0
ENSG000000	IFFO1	protein_codin	0	0	0	0
ENSG000002	ZNF324B	protein_codin	9.70444507	0	8.0742441	9.24467896
ENSG000001	PNPLA2	protein_codin	328.663458	397.511594	93.9546882	210.748229
ENSG000001	RNASEH2B	protein_codin	0	0	0	0
ENSG000000	HFE	protein_codin	0	0	0	0
ENSG000001	PPP2R2B	protein_codin	0	0	0	0
ENSG000001	GCK	protein_codin	0	0	0	0
ENSG000000	SLC39A9	protein_codin	147.701987	193.91753	62.2197899	131.525177
ENSG000001	HLCS	protein_codin	0	0	0	0
ENSG000001	CDK20	protein_codin	0	0	0	0
ENSG000001	TMEM217	protein_codin	0	0	0	0
ENSG000001	AGAP1	protein_codin	0	0	0	0
ENSG000001	PLSCR2	protein_codin	0	0	0	0
ENSG000000	LRRFIP2	protein_codin	18.1772256	2.4961906	4.58673932	7.24218818
ENSG000001	TMEM97	protein_codin	5.6193181	2.13413241	0	0
ENSG000000	ASAP3	protein_codin	0	0	0	0
ENSG000001	ZC3H14	protein_codin	0	0	0	0
ENSG000001	ROMO1	protein_codin	4.10835306	1.15203664	0	0
ENSG000001	C2CD2L	protein_codin	0	16.8468281	0	0
ENSG000002	TRIM16	protein_codin	16.7201914	16.6558955	0	0
ENSG000001	SEC23B	protein_codin	19.4902406	64.4160017	16.2746484	44.2455266
ENSG000001	ZNF687	protein_codin	172.962321	125.435284	80.1435902	96.7050224
ENSG000001	HORMAD2	protein_codin	0	0	0	0
ENSG000001	RGS12	protein_codin	0	0	0	1.88880701
ENSG000001	SLC25A26	protein_codin	0	0	0	0
ENSG000000	SEL1L	protein_codin	480.244539	325.312612	341.731727	351.612653
ENSG000000	HDHD5	protein_codin	59.9196865	82.7436178	20.6425369	50.0873131
ENSG000001	EGFLAM	protein_codin	0	0	0	0

ENSG000000	MOXD1	protein_codin	3.56209796	0	0	0
ENSG000001	HJV	protein_codin	0	0	0	0
ENSG000001	ZCCHC7	protein_codin	86.9524715	89.4615222	75.3897014	81.2170336
ENSG000000	DAZAP1	protein_codin	5.37024035	0	0	6.48983991
ENSG000001	BRIX1	protein_codin	20.3375854	34.8365844	40.0928117	26.9859383
ENSG000002	TAFA5	protein_codin	0	0	0	0
ENSG000001	BVES	protein_codin	0	0	0	0
ENSG000002	SSX2	protein_codin	0	0	0	0
ENSG000001	ATXN2L	protein_codin	20.0775645	173.924383	0	0
ENSG000000	RAB27A	protein_codin	143.915255	70.9902047	87.6436371	141.209156
ENSG000001	SDC3	protein_codin	0	2.4115824	0	0
ENSG000001	CLDN2	protein_codin	0	0	0	0
ENSG000002	ANG	protein_codin	6.95528278	0	6.94570287	4.43098198
ENSG000001	DHX15	protein_codin	349.364995	255.08311	187.697044	215.328619
ENSG000001	CAPS2	protein_codin	0	0	0	0
ENSG000000	FNDC3B	protein_codin	21.3630195	0	15.1300549	20.7128893
ENSG000001	DRC7	protein_codin	7.75805483	6.86980465	5.3659297	5.37618084
ENSG000001	FCRLB	protein_codin	0	0	0	0
ENSG000001	CIDEC	protein_codin	0	0	0	0
ENSG000001	CCDC88A	protein_codin	0	0	0	0
ENSG000001	PAX3	protein_codin	0	0	0	0
ENSG000002	CSH2	protein_codin	0	0	0	0
ENSG000001	ATG101	protein_codin	52.5640436	42.6091646	20.1274183	40.4373204
ENSG000001	KRT13	protein_codin	0	0	0	0
ENSG000000	UNG	protein_codin	0	0	0	0
ENSG000001	OPRL1	protein_codin	34.9995683	42.6996291	7.17649269	13.5225157
ENSG000001	NXN	protein_codin	0	0	2.66024205	0
ENSG000001	HPS4	protein_codin	0	0	0	0
ENSG000001	LCK	protein_codin	0	0	0	0
ENSG000001	GDPD5	protein_codin	34.047308	60.1201689	4.63384048	20.7696808
ENSG000001	SNCA	protein_codin	0	0	0	0
ENSG000002	LINC01123	lncRNA	0	0	0	0
ENSG000001	HBG2	protein_codin	0	0.86579592	55.124097	12.0914186
ENSG000001	SKAP1	protein_codin	6.21364959	0	0	0
ENSG000001	FN1	protein_codin	0	0	0	0
ENSG000001	ZNF385B	protein_codin	0	0	0	0
ENSG000001	HIP1	protein_codin	274.182075	161.725545	73.2469189	167.537545
ENSG000001	LUZP2	protein_codin	0	0	0	0
ENSG000001	STARD13	protein_codin	4.40503915	0	0	0
ENSG000001	ATF3	protein_codin	3.43751428	0	23.5821381	3.46262136
ENSG000001	MIER3	protein_codin	0	0	0	0
ENSG000001	MID1IP1	protein_codin	81.1717687	117.003714	21.0594624	10.360987
ENSG000002	ARHGAP8	protein_codin	0	0	0	0
ENSG000001	MYADM	protein_codin	0	0	1.19311573	0

ENSG000002	ZNF224	protein_codin	99.3085664	57.9243904	58.6847958	40.3793539
ENSG000002	AGPAT1	protein_codin	121.721472	0	0	0
ENSG000001	PRR5	protein_codin	2.26230559	0	0	0
ENSG000001	PRELID3A	protein_codin	0	0	0	0
ENSG000002	SLC26A6	protein_codin	75.3883794	65.6343284	12.1223836	46.9846052
ENSG000000	SEC62	protein_codin	142.228262	113.123171	78.5560828	112.528883
ENSG000001	ATP5MK	protein_codin	0	0	0	0
ENSG000001	PTDSS1	protein_codin	217.406617	418.485924	121.667929	257.107785
ENSG000001	AGTPBP1	protein_codin	0	0	0	0
ENSG000001	HEXD	protein_codin	9.54385344	8.56852057	5.48547137	0
ENSG000000	SUGP2	protein_codin	31.9698284	0	0	16.9661153
ENSG000001	PACRG	protein_codin	0	0	0	0
ENSG000001	OPRM1	protein_codin	0	0	0	0
ENSG000000	EPB41L2	protein_codin	0	0	0	0
ENSG000001	ZNF444	protein_codin	12.7748525	0	0	11.3273511
ENSG000001	C8orf34	protein_codin	0	0	0	0
ENSG000002	CELA3B	protein_codin	0	0	0	0
ENSG000001	MRPL35	protein_codin	13.6758928	16.2339988	5.10470427	8.23825969
ENSG000001	MFF	protein_codin	4.52077345	0	5.83529117	0
ENSG000001	SH2B1	protein_codin	23.0960618	39.4679122	0	10.2455748
ENSG000001	UGP2	protein_codin	24.0111432	40.0054202	5.67999761	29.7328992
ENSG000001	IL18BP	protein_codin	0	0	0	0
ENSG000001	NECAP2	protein_codin	321.915277	537.125239	72.9527092	171.45509
ENSG000000	TBC1D22A	protein_codin	80.0997034	61.3082822	23.6666364	25.9855817
ENSG000001	HIF1A	protein_codin	932.927974	552.804531	398.250144	777.780949
ENSG000000	EZR	protein_codin	98.4780741	17.2148201	21.7394285	85.8204435
ENSG000001	CAMKK2	protein_codin	10.8761777	0	0	0
ENSG000001	NUDT4	protein_codin	0	27.8532133	12.8997811	46.3284247
ENSG000001	MXD4	protein_codin	148.777774	268.560366	77.2899375	115.859349
ENSG000001	CTBP2	protein_codin	81.1086199	80.3528655	30.8349811	57.6417719
ENSG000001	AZIN1	protein_codin	258.108284	85.1589288	142.581986	166.053018
ENSG000001	WFDC3	protein_codin	0	0	0	0
ENSG000001	PCGF6	protein_codin	0	0	0	0
ENSG000001	SCARA3	protein_codin	0	0	0	0
ENSG000001	CXCL14	protein_codin	0	0	0	0
ENSG000000	RBBP9	protein_codin	24.0491613	41.7722812	15.1405661	20.3816519
ENSG000001	CDR2L	protein_codin	6.72864108	31.6660225	7.148712	4.57285921
ENSG000001	P2RX4	protein_codin	0	0	0	0
ENSG000000	STPG1	protein_codin	0	0	0	0
ENSG000000	TNPO1	protein_codin	0	0	39.8578347	56.6169711
ENSG000000	STARD7	protein_codin	473.03886	806.353236	256.960844	370.962212
ENSG000001	COL2A1	protein_codin	0	0	0	0
ENSG000001	PBRM1	protein_codin	0	0	0	0
ENSG000001	ATF4	protein_codin	201.337963	232.235654	58.7795377	90.6981787

ENSG000001	FAM53B	protein_codin	440.674677	442.862327	143.169056	171.866057
ENSG000001	BAG5	protein_codin	35.6603744	31.0795492	32.6178396	36.6985022
ENSG000001	CRYGN	protein_codin	0	0	0	0
ENSG000001	NPHP3	protein_codin	50.1250174	18.4550416	23.283517	0
ENSG000001	ABHD12B	protein_codin	0	0	0	0
ENSG000001	OBSCN-AS1	lncRNA	0	0	0	0
ENSG000001	LRRC8D	protein_codin	237.882977	346.240219	40.3789037	126.768805
ENSG000000	MSL3	protein_codin	71.0104624	54.8313378	33.7361103	41.8259264
ENSG000001	DGKH	protein_codin	14.2064828	0	0	0
ENSG000001	PCID2	protein_codin	23.2971456	12.2715328	0	10.7850107
ENSG000000	LUC7L	protein_codin	7.69180296	18.5513239	5.8283766	9.07255041
ENSG000001	FGD3	protein_codin	0	0	0	0
ENSG000001	SEC13	protein_codin	0	0	0	14.2506213
ENSG000001	CCDC68	protein_codin	0	0	1.89579855	0
ENSG000001	KCNE1	protein_codin	0	9.26699919	4.71683622	0
ENSG000001	NAB1	protein_codin	39.3378052	31.2079793	32.5379971	40.3882901
ENSG000001	WTAP	protein_codin	153.910023	53.8971594	76.16016	109.512571
ENSG000001	CLPTM1	protein_codin	214.976693	351.672067	69.3089038	169.785778
ENSG000001	ZNF644	protein_codin	247.536942	236.939291	272.324664	187.312531
ENSG000001	PPARD	protein_codin	5.70745926	6.30486178	4.39064445	0
ENSG000000	ZNF112	protein_codin	0	1.81710742	0	0
ENSG000002	SOD2	protein_codin	625.298559	650.517384	427.954539	434.521188
ENSG000000	ARHGAP6	protein_codin	1.6313485	0	0	0
ENSG000001	SERPINA9	protein_codin	0	0	0	0
ENSG000001	LRRC71	protein_codin	0	0	0	0
ENSG000001	RAD51C	protein_codin	0	0	0	0
ENSG000001	ZNF226	protein_codin	11.1809331	0	0	9.96355118
ENSG000001	NIPA1	protein_codin	14.7515998	38.3318739	51.3686638	11.6003944
ENSG000001	NIPA2	protein_codin	26.6023666	4.03982844	0	5.78809666
ENSG000002	PRAMENP	lncRNA	0	0	1.06435729	0
ENSG000001	AVPR2	protein_codin	0	0	0	0
ENSG000001	ITPRIP	protein_codin	816.960786	797.807471	325.651202	424.34139
ENSG000001	FIP1L1	protein_codin	0	0	0	52.7831326
ENSG000000	SIRT6	protein_codin	13.7077401	48.6132905	6.17396464	2.58051915
ENSG000000	SCMH1	protein_codin	0	0	4.57907221	3.95361233
ENSG000002	SSX2	protein_codin	0	0	0	0
ENSG000000	NRIP2	protein_codin	0	0.91165397	0	0
ENSG000000	IGF1	protein_codin	6.54821841	2.52222638	41.2998471	6.27519407
ENSG000002	DXO	protein_codin	8.32219387	0	7.58312537	0
ENSG000001	RTN4	protein_codin	0	0	0	21.6362183
ENSG000001	KIAA1958	protein_codin	31.0209686	12.0949432	16.5698635	31.2917564
ENSG000001	MPP7	protein_codin	0	0	0	0
ENSG000001	PPP2R5E	protein_codin	157.866726	78.4543351	31.2587157	111.029679
ENSG000001	ADORA2A	protein_codin	0	0	0	46.771354

ENSG000001	ZFYVE27	protein_codin	9.20560497	0	21.4551545	19.3280092
ENSG000001	TSPO	protein_codin	200.797861	291.856971	70.0547553	189.877254
ENSG000001	POLR3H	protein_codin	0	3.03007616	7.8890933	0
ENSG000000	CAPN3	protein_codin	0	1.77714729	2.2999706	0
ENSG000001	EBAG9	protein_codin	0	0	0	0
ENSG000000	LZTS3	protein_codin	0	0	0.96753903	3.28099239
ENSG000001	RSPH10B	protein_codin	0	0	0	0
ENSG000001	SMUG1	protein_codin	0	0	0	0
ENSG000001	MUC1	protein_codin	0	0	0	0
ENSG000000	BCL2L13	protein_codin	0	10.6401419	2.01841276	0
ENSG000001	AP1S1	protein_codin	23.2091075	33.5584975	14.5836124	9.17414089
ENSG000001	CBX3	protein_codin	5.96580175	3.81651924	3.30152996	1.72745092
ENSG000001	EDRF1	protein_codin	0	77.2642371	0	0
ENSG000001	GSG1	protein_codin	0	1.97469926	1.89840897	0
ENSG000000	CHAT	protein_codin	0	0	0	0
ENSG000000	CREM	protein_codin	0	0	0	0
ENSG000001	PRP4K	protein_codin	189.98238	76.9869464	181.843178	60.3755304
ENSG000001	AADAT	protein_codin	0	0	1.15584144	0
ENSG000000	ARHGEF1	protein_codin	0	0	0	0
ENSG000001	MED19	protein_codin	0	2.99086186	0	1.89135737
ENSG000001	ZNF394	protein_codin	192.198866	124.355235	58.787734	61.9302679
ENSG000001	YIF1B	protein_codin	0	38.4646073	31.288968	22.0263629
ENSG000001	SINHCAF	protein_codin	49.4799853	0	28.0319625	49.6987571
ENSG000001	PWWP3B	protein_codin	0	0	0	0
ENSG000001	TTC33	protein_codin	58.3099005	92.0170899	56.410813	61.4927676
ENSG000001	FGF1	protein_codin	0	0	0	0
ENSG000001	AKAP1	protein_codin	39.6936777	81.1874157	21.3128212	33.2103986
ENSG000001	DTX3	protein_codin	0	0	0	0
ENSG000001	PTPA	protein_codin	0	73.2145185	0	17.2811729
ENSG000000	TASP1	protein_codin	25.3602706	14.968335	0	0
ENSG000001	GHRHR	protein_codin	0	0	0	0
ENSG000001	CARINH	lncRNA	73.2618186	103.724872	46.7493555	25.3300693
ENSG000001	MGAT4B	protein_codin	37.6616025	67.6396047	56.6779412	47.7992173
ENSG000001	SUPT7L	protein_codin	50.0003397	36.1049989	14.4710039	13.5813171
ENSG000001	MASP1	protein_codin	0	0	0	0
ENSG000001	PLS1	protein_codin	0	0	0	0
ENSG000002	ENSG000002	lncRNA	76.9411651	50.7934397	15.4768619	14.570252
ENSG000001	AQP12A	protein_codin	0	0	0	0
ENSG000001	ZNF664	protein_codin	38.0717648	199.696925	121.776529	185.493881
ENSG000001	STIL	protein_codin	0	0	0	0
ENSG000001	GALNT16	protein_codin	0	0	0	0
ENSG000000	C6	protein_codin	0	0	0	0
ENSG000000	SPTLC1	protein_codin	0	2.75709105	0	0
ENSG000001	C1QTNF6	protein_codin	1.41356084	0	0	0

ENSG000001	SGCD	protein_codin	0	0	0	0
ENSG000001	SUV39H1	protein_codin	0	0	17.5069417	0
ENSG000000	ZC3H15	protein_codin	441.794409	383.637282	275.191735	346.430197
ENSG000001	ZBTB49	protein_codin	26.6853399	15.1287207	0	14.4773709
ENSG000001	HOPX	protein_codin	1.14899516	0	0	0
ENSG000001	ADORA1	protein_codin	0	0	0	0
ENSG000001	PPHLN1	protein_codin	25.9004853	23.0836706	19.383137	21.066661
ENSG000001	RERE	protein_codin	86.7342495	83.0484281	126.457258	23.0140876
ENSG000001	MS4A4A	protein_codin	0	0	0	33.1226905
ENSG000001	AIRE	protein_codin	0	0	0	0
ENSG000000	RHOBTB1	protein_codin	167.194063	604.574359	218.096115	38.1693716
ENSG000001	MS4A15	protein_codin	0	0	0	0
ENSG000002	AIF1	protein_codin	348.611844	371.788742	43.670745	182.846981
ENSG000001	HEY1	protein_codin	0	0	0	0
ENSG000001	PHF20L1	protein_codin	0	0	0	0
ENSG000001	IL21R	protein_codin	4.86607484	6.63286196	2.73952641	0.94982406
ENSG000001	EDN3	protein_codin	0	0	0	0
ENSG000001	NR1I2	protein_codin	0	0	0	0
ENSG000001	PYCR1	protein_codin	0	0	0	0
ENSG000001	PACSIN2	protein_codin	36.9414814	72.6070089	0	0
ENSG000001	ZMYM5	protein_codin	30.2168103	16.4635552	18.5698074	18.0468243
ENSG000001	HNRNPF	protein_codin	237.855405	709.709033	413.670986	379.570567
ENSG000001	KLHL25	protein_codin	3.41925601	6.38760954	1.49522283	3.53381743
ENSG000001	STX3	protein_codin	119.548467	118.594163	68.8303473	74.6833189
ENSG000000	CABIN1	protein_codin	141.681284	86.3328895	54.2646773	22.3957052
ENSG000001	ZC4H2	protein_codin	0	0	3.14042567	0
ENSG000001	TSPAN16	protein_codin	0	0	0	0
ENSG000001	ZNF420	protein_codin	3.5241618	11.523468	0	1.38424812
ENSG000002	CCDC183	protein_codin	4.32038013	3.90741881	6.16267652	0
ENSG000000	ZFYVE16	protein_codin	135.150182	74.9509226	137.79811	77.231793
ENSG000001	ZNF385A	protein_codin	0	39.1723954	6.41874667	5.99988378
ENSG000001	SLITRK4	protein_codin	13.2842578	14.0869433	17.4000814	31.6961023
ENSG000001	MAPK15	protein_codin	0	0	0	0
ENSG000000	ELAC2	protein_codin	49.6350159	55.988335	54.3232494	71.6284983
ENSG000001	PLCB1	protein_codin	98.2614864	39.0961279	0	48.0012949
ENSG000001	FBXO40	protein_codin	9.93855191	9.93302987	6.63123074	4.01689121
ENSG000000	MEF2A	protein_codin	0	0	0	0
ENSG000001	LIMS1	protein_codin	1.69287066	0	0	1.83553731
ENSG000001	C5orf24	protein_codin	0	0	0	0
ENSG000001	RUNDC3B	protein_codin	0	0	0	0
ENSG000000	EPB41L1	protein_codin	0	0	0	0
ENSG000000	PUM2	protein_codin	115.553546	0	0	0
ENSG000001	SLA	protein_codin	459.786245	512.573017	161.279918	214.599611
ENSG000001	CMTR2	protein_codin	0	0	0	0

ENSG000000	NOS1	protein_codin	0	0	0	0
ENSG000001	TTC8	protein_codin	0	0	0	0
ENSG000002	ENSG000002	protein_codin	5.61561719	19.4215251	18.1100385	0
ENSG000001	GZF1	protein_codin	187.577782	159.468034	113.4731	133.384658
ENSG000001	ISYNA1	protein_codin	0	0	0	0
ENSG000001	NQO2	protein_codin	16.7840417	18.3968538	11.4307954	0
ENSG000001	KPTN	protein_codin	5.08543777	0	0.99764394	0
ENSG000001	ENOX2	protein_codin	0	0	0	4.51240963
ENSG000001	UBE2D3	protein_codin	0	0	0	0
ENSG000001	SPRYD4	protein_codin	43.0043078	41.9058634	24.6330906	19.0602456
ENSG000001	MRPL30	protein_codin	37.6420764	59.9160886	20.7178213	30.4316107
ENSG000001	CAGE1	protein_codin	0	0	0	0
ENSG000001	PHF8	protein_codin	5.98382602	39.5783524	0	33.1839064
ENSG000001	RBM39	protein_codin	0	0	0	0
ENSG000001	EME1	protein_codin	0	0	0	0
ENSG000000	APLP2	protein_codin	1285.27413	1691.81024	0	677.801593
ENSG000001	RAB28	protein_codin	10.742983	11.064947	0	6.08764003
ENSG000001	LBR	protein_codin	0	0	0	0
ENSG000001	CDIN1	protein_codin	0	0	0	0
ENSG000001	ANKRD19P	transcribed_u	0	0	0	0
ENSG000001	PRIM1	protein_codin	0	14.8485342	0	0
ENSG000001	ECPAS	protein_codin	329.034969	400.565225	328.485349	413.458021
ENSG000001	UGT2B15	protein_codin	0	0	0	0
ENSG000001	UBQLN2	protein_codin	187.235779	235.780534	99.613608	142.022673
ENSG000001	ZNF600	protein_codin	0	0	0	0
ENSG000002	ENSG000002	lncRNA	0	0	0	0
ENSG000000	SLC23A2	protein_codin	182.087007	234.41316	100.895568	100.689027
ENSG000003	CAST	protein_codin	0	0	0	0
ENSG000001	TRIM69	protein_codin	0	0	0	0
ENSG000001	ERAS	protein_codin	0	0	0	0
ENSG000002	TMEM167B	protein_codin	176.769761	173.858196	72.3971893	159.790778
ENSG000001	TPM3P7	processed_ps	12.6752904	7.91666166	1.57877163	4.92755442
ENSG000001	PEAR1	protein_codin	103.483108	110.211953	76.498761	67.8717002
ENSG000001	LRFN2	protein_codin	0	0	0	0
ENSG000001	CCDC157	protein_codin	0	0	0	0
ENSG000000	ANGPT2	protein_codin	0.94236738	0	0	0
ENSG000001	TAGAP	protein_codin	190.623683	239.831577	106.575416	132.923485
ENSG000002	MFSD2B	protein_codin	0	33.5580968	28.9170939	6.78605315
ENSG000000	ADCY2	protein_codin	1.03012828	0	0	0
ENSG000001	CSMD2	protein_codin	0	0	0	0
ENSG000001	CHODL	protein_codin	0	0	0	0
ENSG000002	SNURF	protein_codin	0	0	0	0
ENSG000001	FBLL1	protein_codin	0	0	0	0
ENSG000001	AURKAIP1	protein_codin	88.6796824	135.157583	52.8350416	63.8537574

ENSG000000	FBXL19	protein_codin	44.2812556	74.7673947	0	12.7162762
ENSG000001	KCNJ5	protein_codin	0	0	0	0
ENSG000001	OTUD6A	protein_codin	0	0	0	0
ENSG000001	FAM78B	protein_codin	0	0	0	0
ENSG000001	DMBT1	protein_codin	0	0	0	0
ENSG000001	CPM	protein_codin	0	0	0	0
ENSG000001	RFLNA	protein_codin	0	0	0	0
ENSG000001	TAF13	protein_codin	27.5390227	43.1205209	30.8752079	19.18063
ENSG000002	PRKDC	protein_codin	226.443708	373.940422	162.56766	397.131034
ENSG000001	FIBP	protein_codin	25.0776205	25.1886803	0	18.4033872
ENSG000001	AURKAIP1	protein_codin	0	1.58266889	0	0
ENSG000001	VSTM1	protein_codin	27.7183094	0	14.202736	43.4573991
ENSG000001	RMDN3	protein_codin	31.1868379	29.0847824	8.96870937	0
ENSG000001	H1-6	protein_codin	2.57438188	3.44326597	2.03809992	0.87064265
ENSG000001	SLPI	protein_codin	0	0	0	0
ENSG000001	FOCAD	protein_codin	35.4346939	50.3120999	45.9292685	35.5367031
ENSG000000	CLUL1	protein_codin	0	0	0	0
ENSG000001	CDK5RAP3	protein_codin	114.266748	178.060848	150.134098	68.0773947
ENSG000001	METTL26	protein_codin	7.37565803	3.00954638	0.90740423	0
ENSG000002	SNRPEP3	processed_ps	0	1.04055059	1.09542439	1.12226735
ENSG000001	QPCT	protein_codin	126.024024	176.207788	48.0619271	140.663937
ENSG000000	NUDCD3	protein_codin	1.60294509	1.76195205	0	1.54700905
ENSG000000	ACACB	protein_codin	0	0	0	0
ENSG000001	GLS	protein_codin	128.809285	125.264416	96.7968794	105.298648
ENSG000001	CA7	protein_codin	0	0	0	0
ENSG000001	FAIM	protein_codin	0	0	0	2.23217155
ENSG000001	NAA40	protein_codin	36.4506688	13.0361501	21.5664592	17.0670855
ENSG000000	PALM	protein_codin	0	0	0	0
ENSG000001	ING1	protein_codin	31.3031279	0	75.4579598	32.7137525
ENSG000001	ARHGEF3	protein_codin	0	0	0	0
ENSG000001	USP37	protein_codin	10.6782642	6.34130824	0	0
ENSG000001	PRKN	protein_codin	0	0	0	0
ENSG000000	TREM2	protein_codin	0	0	0	0
ENSG000001	CHST4	protein_codin	0	0	0	0
ENSG000001	MAFF	protein_codin	205.059387	32.9451908	30.5719312	15.7146852
ENSG000001	DUSP29	protein_codin	0	0	0	0
ENSG000001	NR4A3	protein_codin	293.788299	117.29927	132.635342	191.343469
ENSG000001	DPYSL4	protein_codin	0	1.92418508	0	0
ENSG000001	ZFTA	protein_codin	0	0	0	0
ENSG000001	SPRED3	protein_codin	0.91857439	0	0	0
ENSG000001	SHANK2	protein_codin	0.91525682	0	0	0
ENSG000001	GJB5	protein_codin	0	0	0	0
ENSG000001	SCOC	protein_codin	0	0	0	20.5761961
ENSG000000	SNX10	protein_codin	413.274112	238.014479	211.560427	257.626179

ENSG000001	TREX2	protein_codin	0	0	0	0
ENSG000001	MLPH	protein_codin	0	0	0	0
ENSG000001	MATCAP2	protein_codin	0	0	0	0
ENSG000001	DYNLRB2	protein_codin	0	0	0	0
ENSG000001	HPS1	protein_codin	0	0	0	0
ENSG000001	PSMD10	protein_codin	4.87679209	4.53707767	2.83470734	1.33286919
ENSG000001	SCNN1D	protein_codin	1.09436011	0	0	0
ENSG000001	TRPV2	protein_codin	121.681729	137.05687	52.5642179	41.9627805
ENSG000001	RARG	protein_codin	0	25.4272913	0	0
ENSG000001	MEIS2	protein_codin	0	0	0	0
ENSG000000	ZFX	protein_codin	60.3807543	53.2918197	112.17602	94.449846
ENSG000001	NPY5R	protein_codin	0	0	0	0
ENSG000002	ENSG000002	lncRNA	0	0	1.23076422	0
ENSG000001	OTOF	protein_codin	0	0	0	0
ENSG000001	MCF2	protein_codin	0	0	0	0
ENSG000001	FAM180A	protein_codin	0	0	0	0
ENSG000002	SPDYE19P	unprocessed_	0	0	0	0
ENSG000001	KLHL17	protein_codin	16.4076354	12.8594322	3.48456675	9.36268282
ENSG000002	CRCP	protein_codin	0	51.7094365	33.4006667	0
ENSG000000	GSTO2	protein_codin	0	0	0	0
ENSG000001	SRSF10	protein_codin	0	30.2225352	20.2642713	0
ENSG000001	SLC28A1	protein_codin	0	0	0	0
ENSG000001	SAXO4	protein_codin	0	0	0	8.9308137
ENSG000001	OR13H1	protein_codin	0	0	0	0
ENSG000001	MBOAT7	protein_codin	0	0	0	6.29929313
ENSG000001	SLC25A15	protein_codin	15.5127798	0	0	0
ENSG000001	ACIN1	protein_codin	0	0	2.53142082	0
ENSG000001	XRCC4	protein_codin	14.2726032	35.7078969	34.7278097	4.74133831
ENSG000001	ZNF81	protein_codin	96.8261742	46.4954374	49.4270297	51.4929801
ENSG000001	PARK7	protein_codin	106.383775	167.908359	34.5434283	94.4075268
ENSG000001	NF2	protein_codin	0	113.376967	52.9812804	0
ENSG000001	ZFP92	protein_codin	17.8439721	23.8199161	4.67162022	8.89952954
ENSG000001	VWA1	protein_codin	0.80457081	0.84685653	0	0
ENSG000001	ATP5MC2	protein_codin	0	0	2.83834879	0
ENSG000001	SLC3A2	protein_codin	56.4996762	109.343571	38.9021156	157.98618
ENSG000001	ANKRD34B	protein_codin	18.2799279	0	1.85097806	1.04864893
ENSG000001	MUC1	protein_codin	0	0	0	0
ENSG000001	TEKT1	protein_codin	0	0	0	0
ENSG000001	TEKT3	protein_codin	0	0	0	0
ENSG000001	TRIM2	protein_codin	0	0	0	0
ENSG000001	MAOA	protein_codin	0	0	0	0
ENSG000001	FAM86FP	transcribed_u	0	0	0	0
ENSG000001	DCBLD1	protein_codin	8.17717071	22.1651123	0	6.02210921
ENSG000001	LFNG	protein_codin	0	0	0	0

ENSG000001	TBCCD1	protein_codin	27.422407	37.44945	15.7348392	0
ENSG000001	SATB1	protein_codin	174.193673	121.901978	45.0633984	107.424319
ENSG000001	SCNN1A	protein_codin	0	0	2.40262447	0
ENSG000001	TPST2	protein_codin	98.6653345	165.576608	45.8512572	80.1650529
ENSG000001	PARVB	protein_codin	84.3598346	103.204781	37.5752758	36.9300209
ENSG000001	MTSS2	protein_codin	35.7939115	62.5342944	27.6785806	33.1343035
ENSG000000	GNAS	protein_codin	41.0918868	57.2511716	24.4389304	21.2138507
ENSG000001	TNFSF13	protein_codin	436.955685	593.78899	28.242971	388.05856
ENSG000001	DYRK1A	protein_codin	83.7061342	148.377766	97.043117	27.0741207
ENSG000002	TBC1D3P4	unprocessed_	0	0	0	0
ENSG000001	IMPDH1	protein_codin	0	0	0	0
ENSG000001	CRTC1	protein_codin	49.4079611	97.6416329	0	14.8911926
ENSG000000	ESR1	protein_codin	0	0	0	0
ENSG000001	RGS12	protein_codin	0	0	0	0
ENSG000001	DNAJB12	protein_codin	161.732155	110.273935	25.0041545	88.6210112
ENSG000000	ATP9A	protein_codin	0	0	0	7.31594273
ENSG000001	EIF4EBP1	protein_codin	159.674905	243.888988	61.1890502	101.779709
ENSG000001	PLET1	protein_codin	0	0.90169825	0	0
ENSG000001	NCR1	protein_codin	1.4566545	0	0	3.41258586
ENSG000001	TPSAB1	protein_codin	0	0	0	0
ENSG000001	RTN3	protein_codin	0	0	0	0
ENSG000001	CATSPER4	protein_codin	0	0	0	0
ENSG000001	OLFM3	protein_codin	0	0	3.90435974	2.68040346
ENSG000001	SMTNL2	protein_codin	0	0	0	0
ENSG000001	PCDH8	protein_codin	0	0	0	0
ENSG000000	TEAD3	protein_codin	0	0.80073022	0	0
ENSG000001	HELT	protein_codin	0	0	0	0
ENSG000001	HSFY1	protein_codin	0	0	0	0
ENSG000000	METTL24	protein_codin	2.35145749	3.55259936	0.94789748	0
ENSG000001	MAP3K15	protein_codin	0	0	0	0
ENSG000000	RUNX3	protein_codin	0	0	0	0
ENSG000001	FAM184A	protein_codin	0	0	0	0
ENSG000001	DFFB	protein_codin	3.48255114	20.6710914	3.64968049	0
ENSG000002	CLEC12B	protein_codin	45.5561353	16.181117	8.20347545	15.3837341
ENSG000001	KRTDAP	protein_codin	0	0	0	0
ENSG000001	EDA	protein_codin	0	0	0	0
ENSG000001	RNASE9	protein_codin	0	0	0	0
ENSG000001	MYOM3	protein_codin	0	0	0	0
ENSG000001	PSPC1	protein_codin	173.404984	92.3910637	52.824579	95.9860723
ENSG000001	GAL3ST1	protein_codin	0	0	0	0
ENSG000001	IGHV1OR15-9	IG_V_gene	2.13924422	0	0	0
ENSG000002	SYT3	protein_codin	0	0	0	0
ENSG000001	PRRC2C	protein_codin	404.487953	618.076944	65.2462065	0
ENSG000001	PHF8	protein_codin	52.5032529	48.5959727	21.1930743	13.965014

ENSG000001	TRPC4	protein_codin	0	0	0	0
ENSG000001	CUZD1	protein_codin	1.16684184	0	0	0
ENSG000000	DTNBP1	protein_codin	17.7947488	7.92086806	10.2360383	12.0026728
ENSG000001	ZC3H12B	protein_codin	8.0395797	2.56995996	6.11761562	6.18153347
ENSG000001	PTGES2	protein_codin	0	71.5085327	30.9229586	0
ENSG000001	LRP1	protein_codin	6.92519475	15.9056198	0	0
ENSG000000	NEDD4	protein_codin	0	7.60528309	2.84797976	0
ENSG000001	TSPY13P	transcribed_u	0	0	0	0
ENSG000001	NCR3LG1	protein_codin	96.410005	63.0239576	21.8219227	37.4303141
ENSG000001	TRIM9	protein_codin	0.86280086	0.84104879	0	0
ENSG000001	RPL14	protein_codin	94.0733019	170.478821	48.8965001	90.6559783
ENSG000002	SULT1A3	protein_codin	0	214.1139	0	0
ENSG000001	PAIP1	protein_codin	5.31709805	0	0	0
ENSG000001	ZNF721	protein_codin	60.3123999	40.0693128	50.3351923	28.4213876
ENSG000001	USP9Y	protein_codin	0	0	0	0
ENSG000000	MAP3K20	protein_codin	188.360046	69.6420158	79.5785684	146.497365
ENSG000001	COMMD4	protein_codin	0	0	0	4.94926143
ENSG000001	LRRC25	protein_codin	226.861993	417.876108	94.6439345	191.460534
ENSG000001	CFAP36	protein_codin	5.58887503	3.14629838	0	0
ENSG000001	SMIM15	protein_codin	46.3617067	27.0089533	10.8116577	29.0239066
ENSG000002	ENSG000002	lncRNA	0.97508707	1.83005088	0	0
ENSG000001	CYP2C18	protein_codin	0	0	0	0
ENSG000001	MAP3K7CL	protein_codin	0	0	0	0
ENSG000001	LINC01116	lncRNA	0	0	0	3.30771757
ENSG000001	DUSP21	protein_codin	0	0	0	0
ENSG000000	TSPAN32	protein_codin	0	3.42414816	0	0
ENSG000001	CRHR1	protein_codin	0	0	0	0
ENSG000001	KLHL7	protein_codin	2.05714446	0	2.2814138	0
ENSG000000	PLAUR	protein_codin	91.2026394	90.0505851	54.3097421	64.5368696
ENSG000001	RHOC	protein_codin	67.4213142	126.435265	38.8404149	44.5610051
ENSG000000	PHF20	protein_codin	0	0	2.30310873	4.70949031
ENSG000000	TFPI	protein_codin	19.9300282	59.7097099	47.8375241	21.7445747
ENSG000001	NOSIP	protein_codin	60.1308822	94.1259261	34.2811313	40.5874954
ENSG000002	UBE2Q2P1	lncRNA	7.33822051	1.14154404	0	0
ENSG000001	CERKL	protein_codin	0	0	0	0
ENSG000001	CLYBL	protein_codin	2.77954235	0	0	0
ENSG000001	ATAD3A	protein_codin	0	0	45.6597706	0
ENSG000002	TBC1D3P2	lncRNA	0	0	0	0
ENSG000000	ING3	protein_codin	38.7792515	45.1058982	44.6628945	49.1502433
ENSG000000	SMOX	protein_codin	79.0566388	157.56137	23.8718735	34.1720731
ENSG000001	NANOS3	protein_codin	0	0	0	0
ENSG000001	C9orf153	protein_codin	0	0	0	0
ENSG000001	CRISP2	protein_codin	1.27862107	0	0	0
ENSG000001	FOXR2	protein_codin	0	0	0	0

ENSG000001	C1QTNF1	protein_codin	0	0	0	0
ENSG000001	DEFB119	protein_codin	0	0	0	0
ENSG000001	IFI6	protein_codin	0	3.32792317	3.04224912	0
ENSG000001	NUDT6	protein_codin	0	0	0	3.97010809
ENSG000001	ABHD12	protein_codin	30.0544369	43.8956908	15.7543371	28.6788083
ENSG000001	PDLIM2	protein_codin	0	0	0	0
ENSG000001	NLGN4Y	protein_codin	0	0	0	0
ENSG000001	NRN1L	protein_codin	0	1.23414674	0	0
ENSG000001	CMTM5	protein_codin	0	0	0	0
ENSG000001	YBEY	protein_codin	0	0	0	0
ENSG000001	PHF10	protein_codin	0	18.0429381	0	23.8875264
ENSG000001	TCEA2	protein_codin	0	0	0	0
ENSG000001	FPR3	protein_codin	26.2686268	43.5437778	11.4334387	8.6704098
ENSG000001	SLC25A14	protein_codin	0	0	0	0
ENSG000001	FBXL14	protein_codin	52.8201682	71.9724007	22.8495887	38.4817362
ENSG000001	SPRY1	protein_codin	22.7685812	9.83955916	0	0
ENSG000001	ZNF449	protein_codin	36.1892292	8.99817749	12.4913836	10.1590252
ENSG000001	MUC4	protein_codin	0	0	0	0
ENSG000001	HAVCR1	protein_codin	3.68250364	4.26751842	0	1.38728588
ENSG000000	NIPAL3	protein_codin	6.03991477	3.64318318	6.41574904	3.1756987
ENSG000000	DLGAP4	protein_codin	0	0	1.58461133	0
ENSG000001	CDK5RAP1	protein_codin	0	0	0	0
ENSG000001	OASL	protein_codin	0	0	8.49944611	0
ENSG000001	SFN	protein_codin	2.73002775	1.80686433	0	0.86799945
ENSG000002	ZNF564	protein_codin	68.3027786	107.364327	34.0307497	62.0536697
ENSG000001	SH2D4B	protein_codin	1.96764486	0.96766109	0	0
ENSG000001	MYB	protein_codin	0	0	0	0
ENSG000001	ZNF621	protein_codin	122.847657	87.9796267	65.1612851	56.5885014
ENSG000001	SOCS4	protein_codin	0	0	0	0
ENSG000001	ITGA4	protein_codin	8.97640904	13.3463751	7.55329055	5.00113479
ENSG000001	POLL	protein_codin	4.84254837	12.8003307	7.51835722	3.9921681
ENSG000001	SIGLEC10	protein_codin	61.0742938	63.3096099	23.6399692	51.5226273
ENSG000001	AMTN	protein_codin	0	0	0	0
ENSG000001	FCRL6	protein_codin	0	0.80666429	0	1.61882628
ENSG000001	DFFB	protein_codin	0	0	0	0
ENSG000001	DYNLT4	protein_codin	0	0	0	0
ENSG000001	RAP1GDS1	protein_codin	0	0	0	0
ENSG000000	NEO1	protein_codin	0	0	0	0
ENSG000001	RPGR	protein_codin	139.43873	39.0506317	71.1222017	35.8909118
ENSG000001	PIK3AP1	protein_codin	1186.0619	1068.87946	376.253962	844.50983
ENSG000001	SEC24C	protein_codin	35.5855302	101.529376	120.69794	0
ENSG000001	ENSG000001	unprocessed_	0	0	0	0
ENSG000001	METTL17	protein_codin	0	9.39582002	11.2445289	0
ENSG000001	TMEM130	protein_codin	0	0	0	0

ENSG000001(	TAS1R3	protein_codin	4.54412964	0.90443421	1.8338327	0
ENSG000001'	PURG	protein_codin	0	0	0	0
ENSG000001(	SDC3	protein_codin	19.2641897	44.0745402	25.1594994	24.7970679
ENSG000001:	SRFBP1	protein_codin	49.1813456	41.4997038	35.0268289	45.0905124
ENSG000001:	ANP32B	protein_codin	968.581058	1649.29018	1165.11739	815.896898
ENSG000000(	CASP8	protein_codin	182.63788	217.660349	179.453149	103.982973
ENSG000000(	ZNF76	protein_codin	63.7124253	21.4162294	7.16611755	30.8675237
ENSG000001(	YIF1B	protein_codin	6.91953953	5.36289258	0	0
ENSG000000:	DOCK9	protein_codin	0	0	0	0
ENSG000001:	NUDT14	protein_codin	2.74029925	0	0	2.0802435
ENSG000001(	ZDHHC3	protein_codin	64.8755797	81.2307686	21.6983327	22.7557397
ENSG000001(	HSD11B1L	protein_codin	0	0	0	0
ENSG000001:	LITAF	protein_codin	92.5163859	40.6986413	21.5680579	33.3979963
ENSG000001:	LRRC17	protein_codin	0	0	0	0
ENSG000001	SRSF3	protein_codin	0	0	0	0
ENSG000000'	TRNT1	protein_codin	0	9.588721	0	0
ENSG000000(	CSDE1	protein_codin	0	0	264.635166	351.117457
ENSG000001'	SLC26A5	protein_codin	0	0	0	0
ENSG000001:	ABCB7	protein_codin	0	0	31.5300149	21.4011167
ENSG000000:	ERMP1	protein_codin	0	0	0	0
ENSG000001:	DCHS2	protein_codin	0	0	0	0
ENSG000001(	GFI1B	protein_codin	109.25911	354.533177	207.856378	84.1046737
ENSG000001:	WNT7B	protein_codin	0	0	0	0
ENSG000001:	SAXO2	protein_codin	2.66022185	0	0	0
ENSG000001(	YPEL1	protein_codin	5.95339111	9.1268371	5.8054107	1.36213363
ENSG000001(	RPS16	protein_codin	21.4626928	14.0580197	2.4130711	12.5316281
ENSG000001'	LRRC37A3	protein_codin	1.91621484	1.84207756	1.31664106	0
ENSG000001(	OTX2	protein_codin	0	0	0	0
ENSG000001:	GJB4	protein_codin	0	0	0	0
ENSG000001:	VMAC	protein_codin	33.6021863	47.1741305	30.8752962	13.1997095
ENSG000001(	RIOK3	protein_codin	571.879423	692.655968	341.356793	486.596382
ENSG000002	PTCHD4	protein_codin	1.25101034	0	2.41616036	0
ENSG000000(	PHKA1	protein_codin	0	0	0	0
ENSG000001(	CCNY	protein_codin	16.3423723	59.4136324	0	0
ENSG000001:	ZNF573	protein_codin	3.51483728	0	0	0
ENSG000001	DCAF17	protein_codin	0	0	0	0
ENSG000001:	PRMT7	protein_codin	0	5.99737636	6.81896164	0
ENSG000001	OGG1	protein_codin	2.84415976	0	0	2.54075818
ENSG000001:	MYO1B	protein_codin	0	0	0	0
ENSG000001:	RNF17	protein_codin	0	0	0	0
ENSG000001:	GLUL	protein_codin	374.952891	339.012795	299.751783	174.676585
ENSG000002(	LST1	protein_codin	21.0489865	9.31195313	4.86761514	6.05874047
ENSG000001:	ZCCHC13	protein_codin	0	0	0	0
ENSG000002:	ENSG000002:	processed_ps	0	0	0	0

ENSG000001	MROH7	protein_codin	0	0	0	0
ENSG000001	S100A13	protein_codin	0	0	0	0
ENSG000001	PODNL1	protein_codin	0	0	0	0
ENSG000001	CLIC5	protein_codin	1.02844388	0	0.96400153	0
ENSG000001	NR4A2	protein_codin	1009.39662	224.636824	543.326334	420.789217
ENSG000001	STAMBP	protein_codin	0	0	0	0
ENSG000001	MSL1	protein_codin	88.572888	140.662674	72.7567008	0
ENSG000000	SCARB1	protein_codin	30.8241991	0	0	0
ENSG000001	HSPE1P11	processed_ps	8.74611005	3.02274866	2.81353176	3.93732308
ENSG000001	GRM8	protein_codin	0	0	0	0
ENSG000000	BAZ1B	protein_codin	309.293586	238.220492	233.337018	147.368079
ENSG000001	OTOF	protein_codin	0	0.89438646	0	0
ENSG000001	NDUFA4	protein_codin	108.945513	135.573848	33.1978766	93.9909852
ENSG000001	IL22RA2	protein_codin	0	0	0	0
ENSG000002	TTLL13	protein_codin	0	0	0	0
ENSG000000	ALDH3A2	protein_codin	0	0	0	0
ENSG000001	SERF2	protein_codin	94.3251932	129.347598	24.077329	52.8550933
ENSG000002	RLIMP1	processed_ps	0.92650618	0.94464475	0	0
ENSG000002	ZNF254	protein_codin	1.96902056	0	0	0
ENSG000001	ENSA	protein_codin	0	0	0	0
ENSG000001	UBC	protein_codin	8664.8761	11418.1218	3885.52359	5475.29642
ENSG000001	ZNF587	protein_codin	67.5545281	144.681206	64.5373869	60.7862235
ENSG000001	ZNF189	protein_codin	0	16.5522881	0	0
ENSG000001	DMP1	protein_codin	0	0	0	0
ENSG000001	POLDIP3	protein_codin	0	0	0	0
ENSG000001	DMKN	protein_codin	0	0	0	0
ENSG000001	SERINC1	protein_codin	596.162033	308.457904	264.297408	415.753063
ENSG000001	CSMD3	protein_codin	0	1.22185941	0	0
ENSG000000	PDE8A	protein_codin	0	0	0	0
ENSG000001	PRDM8	protein_codin	6.95607742	8.05975922	0	0
ENSG000000	BRPF3	protein_codin	0	0	0	0
ENSG000001	OLFM1	protein_codin	0	0	0	0
ENSG000001	ARL4C	protein_codin	83.5370681	26.1475107	14.1584962	58.7101797
ENSG000001	GALNT15	protein_codin	0	0	0	0
ENSG000000	SLC7A8	protein_codin	0	0	0	0
ENSG000001	NUDT1	protein_codin	1.30042131	0	0	6.84709215
ENSG000001	DUSP15	protein_codin	0	0	0	0
ENSG000001	ANKRD6	protein_codin	0	0	0	8.95923499
ENSG000000	NECAP1	protein_codin	0	45.1681345	0	10.5771562
ENSG000000	NLRP2	protein_codin	0	0	0	0
ENSG000001	DNAJC12	protein_codin	0	0	0	0
ENSG000001	SLC35F4	protein_codin	0	0	0	0
ENSG000001	DNAJB13	protein_codin	0	0	0	0
ENSG000001	CLEC2A	protein_codin	0	0	0	0

ENSG000001[illegible]	SEMA4F	protein_codin	0	0	0	0
ENSG000001[illegible]	BTG3	protein_codin	0	0	0	0
ENSG000001[illegible]	LRRC43	protein_codin	0	0.77228845	0	0
ENSG000001[illegible]	DAB2	protein_codin	0	0	0	0
ENSG000000	ETV7	protein_codin	0	0	0	3.69070873
ENSG000000[illegible]	CHAT	protein_codin	0	0	0	0
ENSG000000[illegible]	ICA1	protein_codin	0	0	0	0
ENSG000001[illegible]	H2BC11	protein_codin	22.0977087	77.2554569	57.823359	36.4969847
ENSG000001[illegible]	CFAP410	protein_codin	29.343483	30.8052172	0	0
ENSG000001[illegible]	KSR2	protein_codin	0	0	0	0
ENSG000001[illegible]	BORCS7	protein_codin	86.0450666	69.4576094	39.5204868	55.0944469
ENSG000001[illegible]	NMNAT3	protein_codin	0	0	0	0
ENSG000001[illegible]	CEP83	protein_codin	19.3625317	19.9729704	0	0
ENSG000001[illegible]	ELSPBP1	protein_codin	0	0	0	0
ENSG000001[illegible]	ZNF28	protein_codin	0	0	0	0
ENSG000001[illegible]	CLEC17A	protein_codin	0.86659456	0	0	0
ENSG000001[illegible]	NCCRP1	protein_codin	1.00944158	0	0	1.00024639
ENSG000001[illegible]	MEFV	protein_codin	171.372133	451.706943	0	124.018235
ENSG000001[illegible]	USP54	protein_codin	0	0	0	0
ENSG000001[illegible]	SEMA4D	protein_codin	0	0	0	0
ENSG000001[illegible]	USP47	protein_codin	5.70519734	5.44276391	10.5846994	0
ENSG000001[illegible]	IFITM3P3	processed_ps	1.65531459	0	0	0
ENSG000000[illegible]	SLC24A1	protein_codin	20.6037163	6.03262152	0	0
ENSG000001[illegible]	HMGB1	protein_codin	571.177072	702.070873	511.092447	507.275645
ENSG000001[illegible]	MARCHF1	protein_codin	75.7307214	97.5142614	60.8485392	49.4173597
ENSG000001[illegible]	NFASC	protein_codin	0	0	0	0
ENSG000001[illegible]	RBPMS	protein_codin	0	0	0.33936312	0
ENSG000001[illegible]	CCNYL1	protein_codin	0	13.6343297	0	6.21674718
ENSG000001[illegible]	SECISBP2	protein_codin	156.487538	78.8381739	63.5836313	8.4274705
ENSG000001[illegible]	ZNF292	protein_codin	202.148508	143.069957	135.343685	110.986805
ENSG000001[illegible]	ZNF493	protein_codin	0	0	0	0
ENSG000001[illegible]	TSSK4	protein_codin	0	0	0	0
ENSG000001[illegible]	VHLL	protein_codin	0	0	0	0
ENSG000002[illegible]	KIR2DS4	protein_codin	1.8012609	0	0	0
ENSG000001[illegible]	ENY2	protein_codin	4.80421891	9.21631832	0	9.03704521
ENSG000001[illegible]	MRAP	protein_codin	0	0	0	0
ENSG000001[illegible]	WFDC2	protein_codin	0	0	0	0
ENSG000001[illegible]	ZNF648	protein_codin	0	0	0.91446917	0
ENSG000001[illegible]	USP1	protein_codin	38.543744	31.7910325	0	6.40568143
ENSG000001[illegible]	CLEC18B	protein_codin	0	0	0	0
ENSG000001[illegible]	OLR1	protein_codin	0	0	2.55618412	2.42377141
ENSG000001[illegible]	ZNF695	protein_codin	0	0	0	1.22543022
ENSG000001[illegible]	SDCBP2	protein_codin	0	0	2.80738788	6.26793833
ENSG000001[illegible]	HMX2	protein_codin	0	0	0	0

ENSG000001	KCNJ11	protein_codin	0	0	0	0
ENSG000001	EIF4G2	protein_codin	1134.27552	796.089311	304.948483	607.117568
ENSG000001	MYMK	protein_codin	0	0	0	0
ENSG000001	RTN3	protein_codin	34.4074495	133.347251	42.4227288	56.5460099
ENSG000001	MBD1	protein_codin	32.2237564	22.5333384	21.9396525	14.1261743
ENSG000001	ABCA9	protein_codin	0	0	0	0
ENSG000001	DOK1	protein_codin	0	42.9725868	37.2084865	43.1304531
ENSG000001	CSRP1	protein_codin	168.356503	198.728246	91.4100816	101.750196
ENSG000000	SLC26A3	protein_codin	0	0	0	0
ENSG000001	SPAM1	protein_codin	0	0	0	0
ENSG000001	TMC1	protein_codin	0	0	0	0
ENSG000001	AIDA	protein_codin	123.574983	94.7476797	68.4471324	76.8062362
ENSG000000	KRIT1	protein_codin	0	0	0	0
ENSG000001	FPR2	protein_codin	9.82125376	0	0	9.58901752
ENSG000000	TRAF3IP2	protein_codin	0	15.004677	0	0
ENSG000002	NCR3	protein_codin	0	0	0	0
ENSG000001	FASLG	protein_codin	0	0	0	0
ENSG000002	GLI4	protein_codin	19.3512448	0	0	0
ENSG000002	ENSG000002	misc_RNA	0	0	0	0
ENSG000001	RET	protein_codin	0	9.41553164	0	0
ENSG000001	PLCH1	protein_codin	0	0	0	18.4568343
ENSG000001	STYXL1	protein_codin	0	0	0	0
ENSG000001	KCTD21	protein_codin	22.5425066	47.0096508	12.3485265	11.3744524
ENSG000001	UBE3B	protein_codin	0	0	0	0
ENSG000001	PARD3	protein_codin	0	97.0890627	100.659912	53.4885293
ENSG000001	MIOS	protein_codin	15.7628822	24.697171	0	18.3030259
ENSG000001	DOK7	protein_codin	0	0	0	0
ENSG000001	NANOS1	protein_codin	0	0	0	0
ENSG000000	PLAUR	protein_codin	300.037733	217.438008	304.350675	78.1057116
ENSG000000	OFD1	protein_codin	44.4635782	51.157243	0	0
ENSG000001	DOK4	protein_codin	2.70962721	0	0	0
ENSG000000	FGFR3	protein_codin	0	0	0	0
ENSG000001	RNF133	protein_codin	0	0	0	0
ENSG000001	ENOSF1	protein_codin	0	0	6.53062189	4.99445735
ENSG000001	GPT2	protein_codin	7.02716027	9.93103895	2.94532039	2.92953943
ENSG000000	SP100	protein_codin	893.977067	419.383047	517.636087	618.268387
ENSG000001	WASH6P	protein_codin	0	0	0	0
ENSG000002	IFITM10	protein_codin	9.62637051	0	6.35604047	1.13524942
ENSG000002	SLC25A51P4	lncRNA	0	0	0	0
ENSG000001	INTS12	protein_codin	32.3484196	39.6851971	0	20.8328866
ENSG000001	C5orf47	protein_codin	0	0	1.33464403	0
ENSG000000	GAB2	protein_codin	448.287669	197.569536	335.432341	340.947437
ENSG000000	CYP2W1	protein_codin	0	0	0	0
ENSG000000	VRK2	protein_codin	62.7051	24.5961825	0	72.912979

ENSG000001	TSPAN18	protein_codin	118.189442	346.856887	159.856147	108.964096
ENSG000001	LIAS	protein_codin	0	20.2735887	3.72040659	0
ENSG000001	HIRA	protein_codin	41.9353867	29.4379656	0	0
ENSG000001	C3orf33	protein_codin	0	0	0	0
ENSG000001	HRH3	protein_codin	0	0	0	0
ENSG000001	ZNF69	protein_codin	0	2.75563208	1.95848521	3.14696735
ENSG000000	ETV7	protein_codin	0	0	0	0
ENSG000001	PAQR6	protein_codin	10.2683544	0	1.18261657	0
ENSG000001	BTNL8	protein_codin	0	0	0	0
ENSG000000	ERCC1	protein_codin	18.4219846	22.7584452	7.94891392	13.4209473
ENSG000002	ENSG000002	lncRNA	7.70936801	4.5320265	0	1.76181074
ENSG000001	PSMD10	protein_codin	0	0	19.8503996	0
ENSG000000	MEF2C	protein_codin	0	0	8.38629577	0
ENSG000001	GLRA3	protein_codin	0	0	1.18215079	0
ENSG000001	SERHL2	protein_codin	0	0	0	0
ENSG000001	LGALS12	protein_codin	0	0	0	26.6120177
ENSG000002	GOLGA8IP	lncRNA	0	0	0	0
ENSG000001	NAALADL1	protein_codin	0	0	0	0
ENSG000001	FBXO38	protein_codin	68.7874673	64.4433713	26.7870087	64.0181425
ENSG000001	RASSF4	protein_codin	278.319264	381.591865	184.417843	137.084859
ENSG000001	MMP13	protein_codin	0	0	0	0
ENSG000001	ZNF326	protein_codin	133.042767	47.9517285	54.5956952	70.8254456
ENSG000001	SPAG8	protein_codin	0	1.70585571	0	0
ENSG000000	DYNC1I2	protein_codin	134.448403	95.0823825	0	0
ENSG000002	PKD1P6-NPIP	lncRNA	0	1.23806318	0	0
ENSG000001	TMEM201	protein_codin	7.85815686	8.67479938	6.62793873	3.65188019
ENSG000001	SLC37A3	protein_codin	0	0	0	0
ENSG000002	UBE2V1	protein_codin	0	0	0	0
ENSG000001	KCNK7	protein_codin	0	0	0	0
ENSG000001	SH2D6	protein_codin	0	0	0	0
ENSG000001	CFAP70	protein_codin	0	0	0	0
ENSG000002	ENSG000002	lncRNA	0	0	0	0
ENSG000000	FOLH1	protein_codin	0	0	0	0
ENSG000001	ADGRG5	protein_codin	0	0	0	0
ENSG000001	CTSL	protein_codin	0	0	0	0
ENSG000001	NDUFV3	protein_codin	81.6055581	94.3358518	16.1545628	44.8622269
ENSG000001	KRTAP24-1	protein_codin	0	0	0	0
ENSG000001	ZDHHC14	protein_codin	0	0	0	0
ENSG000000	CFAP61	protein_codin	0	0	0	0
ENSG000001	TMEM135	protein_codin	0	0	20.0837796	0
ENSG000002	SOX18	protein_codin	0.92414935	0	0	0
ENSG000001	XRRA1	protein_codin	0	35.2176851	39.9490126	27.1662652
ENSG000001	C17orf99	protein_codin	0	0	0	0
ENSG000001	INSIG1	protein_codin	185.385502	387.610557	84.509513	154.081802

ENSG000001	XIRP1	protein_codin	0	0	0	0
ENSG000001	DOCK7	protein_codin	0	0	33.8875501	0
ENSG000001	TMEM201	protein_codin	18.6831623	24.3721367	6.08509826	13.6892584
ENSG000001	TUBB4B	protein_codin	328.553991	436.46506	219.877671	214.590253
ENSG000001	PRDX6	protein_codin	500.373119	1105.27678	398.414875	323.357823
ENSG000001	NRG2	protein_codin	0	0	0	0
ENSG000001	ATXN2L	protein_codin	319.428028	462.763049	53.145015	73.7136727
ENSG000001	H3-5	protein_codin	4.97978055	3.31067537	0	0
ENSG000001	NUP43	protein_codin	38.891629	95.8457718	29.1401345	24.2174699
ENSG000001	RAB37	protein_codin	0	0	0	0
ENSG000001	LIN54	protein_codin	26.1347017	39.5246958	14.8160032	0
ENSG000002	SIRPB3P	transcribed_u	0	0	0.8235154	0
ENSG000001	RTL4	protein_codin	0	0	1.07501874	0
ENSG000001	PTAR1	protein_codin	194.857252	30.9690666	122.731194	114.2419
ENSG000001	CPSF7	protein_codin	0	8.66666877	0	0
ENSG000001	GSPT2	protein_codin	5.03552359	10.1553492	9.28298314	5.24165948
ENSG000002	ENSG000002	lncRNA	0	0	0	0
ENSG000001	FSCB	protein_codin	0	0	0	0
ENSG000000	DOCK9	protein_codin	1.78468323	0	1.73516374	0
ENSG000001	CAP1	protein_codin	0	104.360672	0	0
ENSG000002	UPK3BL1	protein_codin	148.073092	115.318679	23.9496389	45.2393856
ENSG000001	GFRAL	protein_codin	0	0	0	0
ENSG000001	GTF2F2	protein_codin	93.0110637	67.3015906	34.7959617	74.9688688
ENSG000001	SYNE4	protein_codin	0	0.79021775	0	0
ENSG000001	HIPK1	protein_codin	13.3919515	0	31.5735572	0
ENSG000001	ZSCAN16	protein_codin	23.8112783	25.5639589	16.0082827	10.8322691
ENSG000001	NAPRT	protein_codin	0	49.2671265	4.76205453	0
ENSG000000	DLGAP4	protein_codin	41.3386234	63.6868349	19.8189844	49.3895643
ENSG000001	LINC00265	lncRNA	0	0	0	0
ENSG000000	TPX2	protein_codin	0	0	13.0837914	0
ENSG000001	UTS2B	protein_codin	0	0	0	0
ENSG000001	CYB5A	protein_codin	2.81428707	1.56245617	0	0
ENSG000001	MIA3	protein_codin	204.32262	236.173865	144.060489	167.472806
ENSG000001	FAM86B1	protein_codin	0	0	0	0
ENSG000001	VAPA	protein_codin	87.5998931	92.4344117	52.1242217	50.5264404
ENSG000001	MEIS2	protein_codin	0	0	0	0
ENSG000001	SIGLEC8	protein_codin	0	0	0	0
ENSG000001	LIMK2	protein_codin	48.9594087	0	0	0
ENSG000001	FOXN2	protein_codin	302.433813	225.397414	147.970975	213.049426
ENSG000001	ACOXL	protein_codin	0	0	1.11102482	0
ENSG000001	UBE2L6	protein_codin	5.17650884	17.2233208	5.19652057	12.9299794
ENSG000000	RANBP3	protein_codin	99.014444	104.894409	48.9304784	60.4860102
ENSG000001	ADGRG2	protein_codin	0	0	0	0
ENSG000002	ENSG000002	lncRNA	0	0	0	0

ENSG000001	LHX6	protein_codin	0	0	0	0
ENSG000002	NCOA4P2	processed_ps	0	0	0	0
ENSG000001	GRIA1	protein_codin	0	0	0	0
ENSG000000	SLC4A4	protein_codin	0	0	2.30646571	0
ENSG000001	SOCS2	protein_codin	0	0	0	0
ENSG000001	PTGES	protein_codin	15.0097553	5.90370772	5.5164322	0.96907229
ENSG000001	BRAT1	protein_codin	96.3035095	109.17401	30.1989084	40.7680351
ENSG000001	CCDC30	protein_codin	0	6.37051127	0	5.33190264
ENSG000001	CCDC144A	protein_codin	0	0	1.08962931	0
ENSG000001	ZNF746	protein_codin	0	179.583135	41.5925793	36.0608412
ENSG000001	C2orf88	protein_codin	870.203163	1118.03013	769.064195	679.701774
ENSG000001	SHBG	protein_codin	0	0	0	0
ENSG000001	BCLAF3	protein_codin	4.41081885	6.2272973	4.58221515	4.51296994
ENSG000001	IFT27	protein_codin	0	0	0	0
ENSG000001	PAQR9	protein_codin	2.57111237	0	1.10484406	1.32001276
ENSG000001	PDE4D	protein_codin	128.316197	87.6139131	0	0
ENSG000001	GOLGB1	protein_codin	0	0	0	0
ENSG000001	CPED1	protein_codin	12.5131773	14.0802344	6.65712399	8.71880552
ENSG000001	BRCC3	protein_codin	0	0	9.14570778	10.6107387
ENSG000001	RRP1B	protein_codin	142.232852	198.177031	116.746775	94.876334
ENSG000001	NHLRC1	protein_codin	0	2.98146141	0	0
ENSG000001	VWC2	protein_codin	1.42516439	2.83837109	0	0
ENSG000000	ATXN3	protein_codin	35.1840561	18.1075748	42.0822459	0
ENSG000000	SYN1	protein_codin	2.87452172	4.90731141	3.18875167	6.61648206
ENSG000002	CDK11B	protein_codin	0	90.4566607	0	0
ENSG000002	ENSG000002	processed_ps	0	0	0	0
ENSG000001	ZNF124	protein_codin	78.3630403	63.4022214	39.5176244	62.359849
ENSG000001	SERPING1	protein_codin	0	0	0	0
ENSG000001	MOCS1	protein_codin	0	0.86110442	0	0
ENSG000001	PPP1R9A	protein_codin	0	0	0	3.44970304
ENSG000001	ENSG000001	transcribed_p	0	0	0	0
ENSG000001	PBX1	protein_codin	0	3.47505065	4.18879061	2.15839211
ENSG000001	KCNK10	protein_codin	1.10486858	1.72418563	0	0
ENSG000001	FAM86MP	transcribed_u	0	0	0	0
ENSG000001	RASEF	protein_codin	6.47109128	2.35565991	0.73840813	2.04648088
ENSG000002	TCL1B	protein_codin	0	0	0	0
ENSG000001	RILPL1	protein_codin	0	0	0	0
ENSG000001	EYA1	protein_codin	0	0	0	0
ENSG000001	CLPB	protein_codin	0	0	0	12.0317003
ENSG000000	ADGRL1	protein_codin	0	0	0	0
ENSG000001	PAPLN	protein_codin	0	1.93579489	0	0
ENSG000001	DNMT1	protein_codin	163.500977	0	18.1708679	189.737865
ENSG000001	SLC2A14	protein_codin	0	0	0	0
ENSG000002	OR1J4	protein_codin	0	0	0	0

ENSG000001	IL19	protein_codin	0	0	0	0
ENSG000001	PPP1CC	protein_codin	0	41.0073717	28.7029952	0
ENSG000001	ENSG000001	lncRNA	0	0	0	0
ENSG000001	OBP2A	protein_codin	0	0	0	0
ENSG000001	SLC26A9	protein_codin	0	0	0	0
ENSG000001	SPATA31F3	protein_codin	0	0	0	0
ENSG000001	PALM3	protein_codin	0	0.6669057	0	0
ENSG000000	ACSL4	protein_codin	0	0	0	0
ENSG000001	PFKM	protein_codin	0	0	0	0
ENSG000001	LRRIQ4	protein_codin	0	0	0	0
ENSG000001	GPR18	protein_codin	4.95721318	5.17816414	2.35669429	0
ENSG000000	PKP2	protein_codin	40.7522118	24.2888883	4.66691849	10.9564111
ENSG000000	ARHGAP44	protein_codin	0	0	0	0
ENSG000001	TERB2	protein_codin	0	0	0	0
ENSG000001	CCNG1	protein_codin	186.797378	169.081834	161.261403	137.385146
ENSG000000	CDK13	protein_codin	141.107971	190.183951	49.8008954	147.62254
ENSG000001	RECQL5	protein_codin	0	0	0	0
ENSG000001	CDC25B	protein_codin	0	0	0	10.8755889
ENSG000001	DST	protein_codin	41.0034881	26.2348492	78.6442861	36.3316669
ENSG000001	TRERF1	protein_codin	0	0	0	0
ENSG000001	NLRP7	protein_codin	0	0	0	0
ENSG000001	TBC1D16	protein_codin	0	0	0	0
ENSG000001	RHCE	protein_codin	0	0	0	0
ENSG000001	PLEKHG5	protein_codin	0	0	0	0
ENSG000001	HM13	protein_codin	303.958717	445.747456	158.280344	232.058956
ENSG000000	IDS	protein_codin	478.384016	404.717289	243.472875	216.816386
ENSG000001	H1-0	protein_codin	595.432955	297.897307	276.227603	247.109417
ENSG000001	ALOX15P2	unprocessed_	0	0.94934388	0	0
ENSG000001	CRYZ	protein_codin	8.39361309	0	1.92729882	0
ENSG000001	NVL	protein_codin	0	0	0	0
ENSG000000	IP6K2	protein_codin	0	4.29813191	0	0
ENSG000001	PGLYRP2	protein_codin	0	0	0	0.85164832
ENSG000001	PPIL4	protein_codin	32.7833217	24.8273333	9.88382718	24.0495352
ENSG000001	NECTIN1	protein_codin	0	5.37861719	3.4443453	0
ENSG000001	PNCK	protein_codin	0	0	0	0
ENSG000001	TC2N	protein_codin	83.002271	106.737623	102.157258	73.8898115
ENSG000001	RNFT1P3	unprocessed_	0	0	0	0
ENSG000001	RNASE1	protein_codin	5.7327376	0	0	0
ENSG000000	WNK1	protein_codin	157.609496	0	41.1074672	192.94122
ENSG000001	ZNF169	protein_codin	15.0508766	0	11.9048765	0
ENSG000001	SENP8	protein_codin	0	3.55713592	0	0
ENSG000001	HNRNPA1	protein_codin	64.2399578	55.884005	67.2947508	55.7004927
ENSG000000	EXOC5	protein_codin	24.4328364	21.1326402	0	12.8127053
ENSG000000	FBLN1	protein_codin	0	0	0	0

ENSG000001(	SUN1	protein_codin	43.756834	44.9551728	14.6768439	13.4239343
ENSG000002	GOLGA2P6	unprocessed_	0	0	0	0
ENSG000001(	PTK2	protein_codin	0	0	0	0
ENSG000000(	SPHK2	protein_codin	9.32309498	17.5170571	0	6.34169962
ENSG000001(	BROX	protein_codin	0	0	11.7530035	0
ENSG000001	KLRG2	protein_codin	0	0	0	0
ENSG000001	MCCC2	protein_codin	0	0	0	0
ENSG000001	EXD3	protein_codin	0	7.63927402	4.25454087	0
ENSG000001	STAU1	protein_codin	40.124571	63.1063978	0	0
ENSG000001	FAM131A	protein_codin	7.98041382	22.9756481	0	12.59927
ENSG000001	CLDN4	protein_codin	0	0	0	0
ENSG000001	ZNF780A	protein_codin	0	0	0	0
ENSG000001	PAQR5	protein_codin	0	0	0	0
ENSG000001	ACER2	protein_codin	27.2902629	90.6580979	19.2467321	39.4586812
ENSG000001(	ANKRD33	protein_codin	0	0	0	0
ENSG000001	MAP2K5	protein_codin	1.27823111	4.23427154	4.16065423	3.77208729
ENSG000001	PDE8B	protein_codin	0	0	0	0
ENSG000001	ADIPOR1	protein_codin	639.87988	1150.17417	317.787535	545.318344
ENSG000001(	ICAM4	protein_codin	0	0	0	0
ENSG000001(	TUBD1	protein_codin	0	0	0	0
ENSG000001	PKHD1	protein_codin	0	0	0	0
ENSG000001	IL1F10	protein_codin	0	0	0	0
ENSG000001	LINC00910	lncRNA	2.88053254	0	1.24365614	0
ENSG000001	POMT1	protein_codin	0	34.1888903	0	0
ENSG000001	H2AP	protein_codin	0	0	0	0
ENSG000001	H2AC7	protein_codin	36.6769794	52.9812878	94.1768276	53.5194058
ENSG000002	CDK11B	protein_codin	98.7661312	129.710571	95.2928225	85.3828866
ENSG000001	ART3	protein_codin	0	0	0	0
ENSG000000	TNC	protein_codin	0	0	0	0
ENSG000001(	POP5	protein_codin	2.75894993	11.0684269	0	1.78091978
ENSG000001	LCN10	protein_codin	0	0	0	0
ENSG000001	SYCP2L	protein_codin	0	2.57594228	0	0
ENSG000001(	ANKRD55	protein_codin	0	0	0	5.7663335
ENSG000001(	CAV1	protein_codin	0	0	0	3.74610003
ENSG000001	TDP2	protein_codin	38.1823428	66.399658	0	14.2294715
ENSG000001	TRIM60	protein_codin	0	0	0	0
ENSG000001	SAMD11	protein_codin	0	0	0	0
ENSG000001	ANAPC1	protein_codin	67.4128446	108.009344	57.4676804	45.5373828
ENSG000001(	BSDC1	protein_codin	41.9048822	43.3669527	21.0839607	24.6383401
ENSG000002(	SERPINB4	protein_codin	0	0	0	0
ENSG000001(	BEND7	protein_codin	0	0	0	0
ENSG000000	RNF19A	protein_codin	147.995727	91.0983129	114.306946	103.136301
ENSG000001	ESR2	protein_codin	0	0	1.90468649	0
ENSG000001	GATA2	protein_codin	91.605072	103.093692	42.4589656	41.7827127

ENSG000001	IL4I1	protein_codin	0	0	0	0
ENSG000000	MCOLN3	protein_codin	0	0	0	0
ENSG000001	MPST	protein_codin	0	8.65734814	0	0
ENSG000000	SLC25A40	protein_codin	85.9205329	88.5368072	35.41148	77.7397618
ENSG000001	POPDC2	protein_codin	5.75948366	0	4.29104987	0
ENSG000001	TMEM221	protein_codin	0	0	0	0
ENSG000001	GMPPA	protein_codin	0	0	0	0
ENSG000001	RBM33	protein_codin	0	0	0	0
ENSG000001	SRSF10	protein_codin	8.95136201	6.83999501	0	6.52850678
ENSG000001	B4GALNT1	protein_codin	0	0	0	0
ENSG000001	FCF1	protein_codin	41.0846526	52.2478909	24.8754397	36.5638673
ENSG000001	ZNF606	protein_codin	0	0	0	0
ENSG000001	NBR1	protein_codin	8.51804739	0	0	14.2392503
ENSG000001	NIPAL2	protein_codin	5.37868835	4.29626292	5.82711276	7.79443468
ENSG000001	TSTD2	protein_codin	51.2079048	38.4094434	13.822039	20.4670862
ENSG000001	DNM1	protein_codin	0	0	0	0
ENSG000000	CLDND1	protein_codin	20.7948331	20.3236145	0	0
ENSG000000	MKNK1	protein_codin	19.282764	0	15.138812	0
ENSG000001	MGAT3	protein_codin	8.01180568	5.93477572	11.2985652	4.81429167
ENSG000001	ARMCX3	protein_codin	10.327835	3.43787346	5.81984884	2.84646093
ENSG000001	ZNF823	protein_codin	4.00074941	11.4612996	0	8.36699421
ENSG000001	HAP1	protein_codin	0	0.79555322	0	0
ENSG000001	TOX2	protein_codin	0	4.32747227	0	1.67682023
ENSG000001	SIMC1	protein_codin	0	0	0	0
ENSG000002	LCN6	protein_codin	0	0	0	0
ENSG000001	FAM72A	protein_codin	4.3173633	5.64015563	0	0
ENSG000000	ADCK1	protein_codin	0	18.2749776	3.54897943	3.78750975
ENSG000001	FAM222B	protein_codin	0	0	0	0
ENSG000000	CFLAR	protein_codin	0	0	0	0
ENSG000001	CFL2	protein_codin	0	0	6.6876282	5.05650876
ENSG000001	SERPINA12	protein_codin	0	0	0	0
ENSG000000	LIMA1	protein_codin	0	0	0	0
ENSG000001	METTL23	protein_codin	15.283477	0	6.13187049	0
ENSG000001	RTL6	protein_codin	64.0739616	82.2592217	21.9900154	39.3357325
ENSG000001	SH2B3	protein_codin	1449.5724	1623.34512	582.313356	758.126371
ENSG000001	DLK1	protein_codin	0	0	3.19469324	0
ENSG000001	ENO4	protein_codin	0	0	0	0
ENSG000001	FBXL5	protein_codin	442.829526	321.882934	140.79058	454.16192
ENSG000001	C2orf80	protein_codin	0	0	0	0
ENSG000001	PERM1	protein_codin	0	0	0	0
ENSG000001	NANOS2	protein_codin	0	0	0	0
ENSG000001	DPM3	protein_codin	0	4.84493396	0	0
ENSG000002	TP53TG3B	protein_codin	0	0	0	0
ENSG000001	RTN3	protein_codin	44.3915467	100.257658	8.02548769	133.809169

ENSG0000007	GLI2	protein_codin	0	0	0	0
ENSG0000019	SPG7	protein_codin	26.6432598	0	9.82159139	2.83293367
ENSG0000017	KAT5	protein_codin	51.8038103	105.984543	0	0
ENSG0000017	KLHL6	protein_codin	239.186357	203.449293	40.4388052	124.110091
ENSG0000018	BRWD1	protein_codin	56.4795781	66.7656851	17.3701389	34.8114315
ENSG0000014	RHBDD1	protein_codin	176.776178	156.228578	76.7507094	83.8523179
ENSG0000016	TEX264	protein_codin	0	77.5909297	23.2451073	28.9078101
ENSG0000018	NOXA1	protein_codin	6.99252626	3.38945308	6.48673778	0
ENSG0000012	RAP1B	protein_codin	4.55388822	0	16.3610764	31.4826259
ENSG0000017	CORO1B	protein_codin	9.61170825	30.5458128	9.76896389	22.104203
ENSG0000005	ATP2B4	protein_codin	0	0	0	0
ENSG0000018	PTMA	protein_codin	940.602831	1394.71349	892.262806	767.35963
ENSG0000014	TPM3	protein_codin	0	0	0	0
ENSG0000017	ZDHHC14	protein_codin	0	0	3.17640057	0
ENSG0000000	NFYA	protein_codin	163.796465	173.271048	100.259191	116.409992
ENSG0000016	GOLGA8EP	transcribed_u	0	0	0	0
ENSG0000013	CAPRIN1	protein_codin	172.257764	254.147266	211.051105	160.421398
ENSG0000017	LBX2	protein_codin	0	0	0	0
ENSG000001	NECTIN1	protein_codin	0	0	0	0
ENSG0000018	NPIPB4	protein_codin	55.2404531	30.9453494	17.792056	68.0322134
ENSG0000016	UBXN8	protein_codin	0	0	0	0
ENSG0000018	VCX	protein_codin	0	0.55386176	0	0
ENSG0000013	SLCO2B1	protein_codin	0	0	0	0
ENSG0000016	HAGHL	protein_codin	0	0	0	0
ENSG0000018	IGFL3	protein_codin	0	0	0	0
ENSG0000013	SKOR1	protein_codin	1.46626143	2.92221665	3.02460426	5.10516139
ENSG0000012	FLRT3	protein_codin	0	0	0	0
ENSG0000018	TRAK1	protein_codin	27.0325773	0	85.9756388	0
ENSG0000018	HMGB1	protein_codin	170.049621	60.3725044	136.907878	108.565159
ENSG0000000	NADK	protein_codin	0	138.354207	19.6774684	0
ENSG0000016	NEK10	protein_codin	0	3.03140805	0	0
ENSG0000010	MEST	protein_codin	14.7203526	65.3761576	27.3835446	0
ENSG0000018	BRI3BP	protein_codin	70.4322758	136.558709	37.8340349	70.5118368
ENSG0000018	CCDC66	protein_codin	37.8574646	37.147135	35.1397883	39.8404852
ENSG0000010	SGK2	protein_codin	0	0	0	0
ENSG0000018	SLC24A5	protein_codin	0	0	0	0
ENSG0000009	SYDE2	protein_codin	0	0	1.2246235	0
ENSG0000007	FGFR1	protein_codin	0	0	0	0
ENSG0000013	NABP2	protein_codin	1.69959464	0	0	0
ENSG0000018	ZKSCAN3	protein_codin	3.23153125	0	0	4.08676844
ENSG0000010	TRIM14	protein_codin	182.901271	238.875962	64.2116403	165.522745
ENSG0000010	C14orf93	protein_codin	0	0	0	1.99371302
ENSG0000005	SYNE2	protein_codin	0	0	0	0
ENSG0000014	PARN	protein_codin	0	31.7770464	46.1667915	38.9035961

ENSG000001	TPM3	protein_codin	0	0	0	0
ENSG000001	ATF3	protein_codin	193.127653	159.941599	137.868598	93.1009103
ENSG000001	TREML4	protein_codin	0	0	0	0
ENSG000001	INSR	protein_codin	217.728097	335.918413	101.324511	166.031416
ENSG000001	MIA2	protein_codin	0	7.7725765	12.8739435	3.10320857
ENSG000001	CBLC	protein_codin	0	0	0	0
ENSG000001	LCTL	protein_codin	0	0	0	0
ENSG000001	ABCA2	protein_codin	0	78.6823093	34.5880076	41.0330544
ENSG000001	ATP7A	protein_codin	26.7993609	0	0	0
ENSG000001	DCAF5	protein_codin	225.408753	389.699754	89.4277306	39.9230157
ENSG000001	PDE4B	protein_codin	2.85060544	6.78099106	0	0
ENSG000000	KCNAB2	protein_codin	0	0	96.9183274	0
ENSG000001	CDH5	protein_codin	0	1.79472139	0	5.42427262
ENSG000001	NAPEPLD	protein_codin	12.0044013	5.3669009	0	0
ENSG000001	THYN1	protein_codin	0	0	11.8300164	0
ENSG000001	LMO7	protein_codin	0	13.2463497	0	0
ENSG000001	ING4	protein_codin	0	0	3.91191104	13.3195024
ENSG000001	CFAP65	protein_codin	0	0	0	0
ENSG000001	FOXO4	protein_codin	0	29.799511	0	0
ENSG000001	TMEM248	protein_codin	328.51015	390.923478	149.220559	219.421889
ENSG000000	CFLAR	protein_codin	0	2.29230364	0	0
ENSG000001	SERPINA6	protein_codin	0	0	0	0
ENSG000000	FERMT2	protein_codin	0	0	0	0
ENSG000002	TMEM179	protein_codin	0	0	0	0
ENSG000001	GRM8	protein_codin	0	0	0	0
ENSG000001	MAP3K7CL	protein_codin	2.77380784	15.6973315	0	13.8523001
ENSG000000	TNFRSF12A	protein_codin	0	0	0	2.34390055
ENSG000001	CLUAP1	protein_codin	0	0	0	0
ENSG000001	PAX4	protein_codin	0	0	0	0
ENSG000001	GP2	protein_codin	0	0	0	0
ENSG000001	MAX	protein_codin	0	0	0	2.98608118
ENSG000002	SPDYE2	protein_codin	0	0	0	0
ENSG000001	ANKFY1	protein_codin	289.388345	486.247534	150.24713	220.487253
ENSG000001	LRRC37B	protein_codin	6.26594414	0	0	12.8911259
ENSG000001	ATP13A2	protein_codin	6.42125287	0	0	0
ENSG000000	MAP4K3	protein_codin	0	0	0	0
ENSG000001	MYL1	protein_codin	0	0	0	0
ENSG000001	TMEM202	protein_codin	0	0	0	0
ENSG000001	TAF1C	protein_codin	0	0	0	0
ENSG000001	DCTN3	protein_codin	0	3.37608332	0	0
ENSG000001	PPA2	protein_codin	3.0636986	6.70891523	0	14.863953
ENSG000001	SEMA6C	protein_codin	4.39300567	0	0	5.08467664
ENSG000001	PEDS1-UBE2\	protein_codin	0	35.1518752	0	0
ENSG000001	GNA14	protein_codin	0.98849629	1.01266456	1.55942993	0

ENSG000001!	VN1R2	protein_codin	0	0	0	0
ENSG000001:	MPRIP	protein_codin	116.364158	264.905883	150.32442	122.309069
ENSG000001:	HEPACAM2	protein_codin	0	0	0	0
ENSG000001·	ASTN2	protein_codin	0	0	0.8972776	0
ENSG000001:	MESP2	protein_codin	0	0	0	0
ENSG000001:	PMEPA1	protein_codin	0	10.3608048	0	0
ENSG000002!	ENSG000002!	lncRNA	0	0	0	0
ENSG000001:	PRR19	protein_codin	0	0	0	0
ENSG000001:	MIA2	protein_codin	0	27.675802	0	17.0431605
ENSG000002:	RFPL4AL1	protein_codin	0	0	1.17158926	0.94542173
ENSG000001:	ANKS1B	protein_codin	0	0	0	0
ENSG000001:	TRIM28	protein_codin	0	129.27384	20.6064509	16.6513295
ENSG000001	RAD1	protein_codin	0	12.7804539	6.40569705	7.60691172
ENSG000000	SCIN	protein_codin	0	0	0	0
ENSG000001·	WDR31	protein_codin	1.64943443	6.0279704	0	0
ENSG000001:	DNER	protein_codin	2.15380919	1.09644975	0.98432316	0
ENSG000000	CD22	protein_codin	0	0	0	0
ENSG000000	JARID2	protein_codin	1362.70243	1315.81056	721.010662	814.755106
ENSG000001·	FAM163A	protein_codin	0	0	0	0
ENSG000001:	SSUH2	protein_codin	0	0	0	0
ENSG000001	COL22A1	protein_codin	0	0	0	0
ENSG000001:	KRT77	protein_codin	0	0	0	0
ENSG000002·	CDK11B	protein_codin	0	45.4291454	13.0538963	14.2362383
ENSG000001!	ZKSCAN7	protein_codin	0	0	0	0
ENSG000001	CRHR2	protein_codin	0	0	0	0
ENSG000001:	TRIM29	protein_codin	0	0	0	0
ENSG000001:	TNIK	protein_codin	0	0	0	0
ENSG000002!	ENSG000002!	lncRNA	0	0	0	0
ENSG000002	ENSG000002	processed_ps	0	0.87024767	0	0
ENSG000000	DPP8	protein_codin	50.1198833	33.3031197	30.5334466	22.1024362
ENSG000001	TSPAN14	protein_codin	0	0	0	0
ENSG000001:	TAF9B	protein_codin	11.0907598	7.24439347	13.2636493	9.27232268
ENSG000001	C6orf132	protein_codin	0	0	0	0
ENSG000001:	LINC01164	lncRNA	0	0	0	0
ENSG000001:	TMEM220	protein_codin	4.56801739	1.37979279	3.33079383	0
ENSG000001	LGALS8	protein_codin	33.3875852	164.076357	95.4081599	58.5468143
ENSG000001	ZBTB8OS	protein_codin	0	0	0	0
ENSG000001	CEP97	protein_codin	0	0	0	0
ENSG000001·	DLX1	protein_codin	0	0	0	0
ENSG000001:	SBK1	protein_codin	0.96051127	2.87603923	0	0.9184664
ENSG000001	MYB	protein_codin	0	0	0	0
ENSG000001	CXCL17	protein_codin	0	0	0	0
ENSG000001·	NFIC	protein_codin	25.4151661	0	0	0
ENSG000000:	RABEP1	protein_codin	120.178209	183.965031	123.200555	90.4364727

ENSG000003	CAST	protein_codin	22.3469066	26.8487547	0	0
ENSG000000	EPB41L3	protein_codin	35.505071	0	2.92557067	11.1153702
ENSG000001	CCR6	protein_codin	0	0	0	0
ENSG000001	ABLIM2	protein_codin	0	0	0	0
ENSG000001	LAMTOR4	protein_codin	119.408879	174.282719	79.3880294	82.4560016
ENSG000002	COL11A2	protein_codin	0	3.04756669	0	0
ENSG000001	PCDHB13	protein_codin	0	0	0	0
ENSG000001	DALRD3	protein_codin	24.4612683	0	0	3.67682654
ENSG000001	CCDC62	protein_codin	1.94844109	2.9885163	2.22273313	1.63786646
ENSG000001	SYT8	protein_codin	0	0	0	0
ENSG000002	OR2S2	protein_codin	0	0	0	0
ENSG000001	ZNF70	protein_codin	27.8980605	37.3901389	33.1254088	11.7876118
ENSG000001	PPP2R1B	protein_codin	14.8311288	20.670536	0	0
ENSG000001	BBC3	protein_codin	0	0	0	0
ENSG000001	RAB36	protein_codin	0	0	0	0
ENSG000001	CNTNAP3B	protein_codin	0	0	0	0
ENSG000000	NADK	protein_codin	528.340965	810.772608	178.975976	509.751218
ENSG000001	SLC24A2	protein_codin	0	0	0	0
ENSG000000	IKZF2	protein_codin	7.03676233	0	0	0
ENSG000001	POM121L15P	transcribed_p	0	0	0	0
ENSG000001	BMX	protein_codin	0	0	0	0
ENSG000001	CIR1	protein_codin	493.317019	332.110397	268.344395	274.09776
ENSG000001	GTF3C5	protein_codin	0	0	0	0
ENSG000001	CTSL	protein_codin	7.13545374	10.1558569	0	11.5601939
ENSG000001	CCDC30	protein_codin	0	0	0	0
ENSG000001	PPP1R17	protein_codin	0	2.09497785	0	2.61411569
ENSG000001	TRIM14	protein_codin	0	0	0	14.5349315
ENSG000001	BABAM2	protein_codin	0	12.0296972	0	4.72968385
ENSG000001	RXFP1	protein_codin	0	0	0	0
ENSG000001	RHD	protein_codin	0	0	0	0
ENSG000001	KCNQ5	protein_codin	0	0	0	0
ENSG000001	FBLN5	protein_codin	0	0	0	0
ENSG000001	PEAK3	protein_codin	23.3347369	39.3195702	7.22140321	24.3522988
ENSG000001	SAMD11	protein_codin	0	0	0	0
ENSG000000	MRPL43	protein_codin	0	1.75651681	0	2.25337439
ENSG000001	HNRNPA1P61	processed_ps	0	0	0	0
ENSG000000	PMS1	protein_codin	0	0	0	0
ENSG000001	RASSF6	protein_codin	0	0	0	0
ENSG000001	PTPRR	protein_codin	0	0	0	0
ENSG000001	PDPK1	protein_codin	373.388158	439.217144	154.347528	181.689513
ENSG000001	IFNAR2	protein_codin	0	16.4958784	11.2070826	0
ENSG000001	NBPF3	protein_codin	7.84122894	0	24.9531951	0
ENSG000000	CREM	protein_codin	0	0	0	0
ENSG000001	CLDN14	protein_codin	0	0	0	0

ENSG000000	BID	protein_codin	1.85680462	1.79041562	0	0
ENSG000000	TBC1D2	protein_codin	101.582687	71.0438734	0	0
ENSG000001	OBP2A	protein_codin	0	0	0	0
ENSG000001	AMPD2	protein_codin	0	0	0	0
ENSG000001	IFNAR2	protein_codin	0	0	0	0
ENSG000001	COX8C	protein_codin	0	1.42705257	0	0
ENSG000002	EMP2	protein_codin	1.06098814	0	0	1.08900892
ENSG000001	JMJD7-PLA2G	protein_codin	14.9791859	0	0	0
ENSG000000	HUWE1	protein_codin	1134.54265	871.835888	0	504.758302
ENSG000001	DPY30	protein_codin	37.0688546	63.6579853	29.511884	31.2126911
ENSG000001	DPH5	protein_codin	0	0	0	13.7245172
ENSG000001	TTN	protein_codin	0	0	0	0
ENSG000001	DPY19L3	protein_codin	48.4662954	23.1544359	31.0886944	24.1528998
ENSG000001	NRXN1	protein_codin	0	0	0	0
ENSG000001	TMEM91	protein_codin	0	0	0	0
ENSG000001	SUMF2	protein_codin	0	0	37.758063	0
ENSG000001	UBE2L3	protein_codin	106.95429	117.306246	14.0625221	81.312592
ENSG000000	COBLL1	protein_codin	0	0	6.53686264	0
ENSG000001	KCNK7	protein_codin	0	0	0	0
ENSG000001	SSX2IP	protein_codin	119.463314	202.13928	139.267664	92.487984
ENSG000000	IGBP1	protein_codin	17.3172402	46.5670321	0	0
ENSG000001	CLEC14A	protein_codin	0	0	0	0
ENSG000001	PKN1	protein_codin	332.636931	606.624108	244.491747	269.904938
ENSG000001	ISM2	protein_codin	0	0	0	0
ENSG000001	COL18A1	protein_codin	0	0	12.45763	7.2088307
ENSG000001	IKBKB	protein_codin	0	0	6.70124462	0
ENSG000001	SLC20A2	protein_codin	0	35.9728779	0	0
ENSG000001	JPH1	protein_codin	0	0	0	0
ENSG000001	SP6	protein_codin	0	0	0	0
ENSG000001	SMPD1	protein_codin	0	84.9636276	20.8552118	46.7447661
ENSG000000	CECR2	protein_codin	0	0	0	0
ENSG000001	AFF2	protein_codin	8.2058193	23.2897305	13.1122345	13.5012267
ENSG000001	NHSL1	protein_codin	2.53789576	0	1.97881499	0
ENSG000001	AKAP7	protein_codin	0	1.55213264	1.95927714	6.06650981
ENSG000001	JPH2	protein_codin	0	0	0	0
ENSG000001	BCOR	protein_codin	0	0	0	0
ENSG000001	GJA4	protein_codin	0	0	0	0
ENSG000001	PBX3	protein_codin	62.3244881	0	46.5982135	0
ENSG000001	KCNJ14	protein_codin	0	0	0	0.9405165
ENSG000001	RBPJ	protein_codin	14.2962778	0	13.9796164	0
ENSG000000	POU2F2	protein_codin	178.559623	198.205877	0	68.6277897
ENSG000000	TMEM230	protein_codin	0	0	7.77639149	0
ENSG000001	LMX1A	protein_codin	0	0	0	0
ENSG000002	ADSL	protein_codin	46.8131216	38.8520438	22.0580905	24.9783855

ENSG000002	GOLGA8B	protein_codin	0	0	0	0
ENSG000001	OAS2	protein_codin	14.9978411	11.1115285	6.82447274	0
ENSG000001	CSRNP3	protein_codin	0	0	0	0
ENSG000001	RBPJ	protein_codin	108.544802	24.6831708	0	0
ENSG000001	ASCL4	protein_codin	0	0	0	0
ENSG000001	PDE8B	protein_codin	0	0	0	0
ENSG000000	NADK	protein_codin	5.09700398	11.5227214	0	0
ENSG000001	DCAKD	protein_codin	0	0	0	0
ENSG000001	DCDC1	protein_codin	0	0	0	0
ENSG000001	ATP13A5	protein_codin	0	0	0	0
ENSG000002	GH1	protein_codin	0	0	0	0
ENSG000001	SERINC3	protein_codin	609.502845	875.279787	335.755029	557.800447
ENSG000002	KIR2DL3	protein_codin	0	0	0	0
ENSG000001	ASTL	protein_codin	110.203455	28.1331363	43.9866029	31.5592545
ENSG000001	MASTL	protein_codin	49.4766232	52.7089003	39.737839	18.9925175
ENSG000001	DIABLO	protein_codin	0	0	0	0
ENSG000001	CCDC83	protein_codin	1.07480759	0	0	0
ENSG000001	INSIG1	protein_codin	0	0	0	3.37122808
ENSG000001	FRMD4A	protein_codin	0	0	0	0
ENSG000001	BIRC7	protein_codin	0	0	0	0
ENSG000001	SNRNP48	protein_codin	59.1193448	49.6108863	33.8916831	48.6587711
ENSG000001	EYS	protein_codin	0	0	0	0
ENSG000001	RAB5IF	protein_codin	2.35791833	0	0	2.25138844
ENSG000001	ZNF341	protein_codin	27.3138313	0	0	3.78329622
ENSG000001	PLSCR1	protein_codin	111.92583	90.0899228	50.823795	149.143625
ENSG000001	NR3C2	protein_codin	0	0	0	0
ENSG000001	TSHR	protein_codin	0	0	0	0
ENSG000001	ASCC1	protein_codin	0	0	0	0
ENSG000001	BRWD1	protein_codin	314.342912	169.470853	198.731402	211.81964
ENSG000000	HAUS4	protein_codin	28.0997568	37.7636099	0	0
ENSG000000	ALDH3B1	protein_codin	342.977665	410.867297	81.6940048	187.312985
ENSG000001	TMPPE	protein_codin	31.8612338	35.7037439	7.28872266	22.6731564
ENSG000001	CMTM5	protein_codin	12.2890538	39.2754343	12.7057982	3.36477962
ENSG000001	DUSP18	protein_codin	0	0	0	1.70984654
ENSG000001	PODXL2	protein_codin	0	1.78568685	2.19648578	0.87725359
ENSG000001	MUC1	protein_codin	0	0	0	0
ENSG000001	ZNF79	protein_codin	13.053405	15.901845	21.0391299	17.9265444
ENSG000001	UBE3B	protein_codin	152.095802	256.371305	81.3492301	86.9494543
ENSG000001	IKBIP	protein_codin	2.66851823	16.5540012	2.77536909	20.1094422
ENSG000001	RTN1	protein_codin	0	7.00929688	4.10353786	0
ENSG000001	JAK1	protein_codin	639.033653	1244.60739	592.120615	520.827087
ENSG000001	C2orf66	protein_codin	0	0	12.8419256	1.63384112
ENSG000001	PANK1	protein_codin	1.45474658	0	0	0
ENSG000001	AGMO	protein_codin	0	0	0	0

ENSG000001	OTOG	protein_codin	0	0	1.02161769	0
ENSG000001	ZBTB42	protein_codin	10.6859721	9.81743205	3.68766948	4.76960646
ENSG000001	IL1RAP	protein_codin	0	0	0	0
ENSG000000	ZNF207	protein_codin	16.8588677	33.8484518	17.734208	13.2052576
ENSG000001	NAB2	protein_codin	12.0750888	0	0	9.60831928
ENSG000001	PPP3CB	protein_codin	26.1678754	26.0550769	17.6951553	11.1731946
ENSG000001	GDAP1L1	protein_codin	0	0	0	0
ENSG000000	MKNK1	protein_codin	0	0	0	0
ENSG000001	CYBC1	protein_codin	0	0	0	0
ENSG000001	BPTF	protein_codin	0	0	0	0
ENSG000001	SLC9A3-OT1	lncRNA	6.51077958	9.94583071	5.17232566	0
ENSG000001	NYX	protein_codin	0	0	0	0
ENSG000001	TMEM120B	protein_codin	0	0	0	0
ENSG000002	RAB1AP1	processed_ps	19.6592545	15.9577021	9.92995052	17.809182
ENSG000002	RUFY2	protein_codin	16.0352598	19.6371154	12.6607076	14.8113593
ENSG000001	DUSP19	protein_codin	0	6.95330688	0	0
ENSG000001	RGS3	protein_codin	0	0	0	0
ENSG000001	OR14I1	protein_codin	0	0	0	0
ENSG000001	TMTC4	protein_codin	0	0	16.0957362	0
ENSG000001	FOXM1	protein_codin	0	0	0	0
ENSG000001	SPMAP2	protein_codin	0	0	0	0
ENSG000001	SNX21	protein_codin	0	0	0	0
ENSG000000	HIPK2	protein_codin	83.8690711	326.345587	198.536088	171.206454
ENSG000001	GPRC5C	protein_codin	1.63409334	0	0	0
ENSG000001	TCAIM	protein_codin	0	0	17.0515102	10.8601687
ENSG000001	ZNF12	protein_codin	0	45.7702548	51.9626015	24.3329868
ENSG000000	INPP5A	protein_codin	0	21.2624272	0	0
ENSG000002	ENSG000002	lncRNA	0	0	0	0
ENSG000001	SOX12	protein_codin	23.7139853	58.2732111	18.6256619	13.6654167
ENSG000001	CAMK2D	protein_codin	0	0	0	0
ENSG000001	SNRPD2	protein_codin	83.3738171	129.387087	71.2918639	79.2997423
ENSG000000	FUT8	protein_codin	61.6164851	94.9677036	109.769599	51.0518245
ENSG000000	MAP2K3	protein_codin	558.634236	616.719232	90.3166988	324.28222
ENSG000001	C19orf12	protein_codin	0	0	0	0
ENSG000001	C15orf61	protein_codin	9.59044795	11.7370979	2.76788396	6.26282515
ENSG000001	ZBTB8OSP1	processed_ps	0	0	0	0
ENSG000001	RBM38	protein_codin	0	16.7779754	16.0047596	0
ENSG000001	RPL32P36	processed_ps	0	0	0	0
ENSG000001	NPR2	protein_codin	0	0	0	0
ENSG000001	ATP13A4	protein_codin	18.801508	6.78008116	17.3685005	0
ENSG000001	TP53INP1	protein_codin	81.751774	73.9673478	105.816463	72.1859243
ENSG000001	WDR20	protein_codin	56.25242	64.741897	0	0
ENSG000000	CBFA2T2	protein_codin	0	53.4863923	0	15.8235223
ENSG000001	CDC42BPG	protein_codin	7.88777415	3.85273146	2.48297028	3.78137779

ENSG000001(	CARMIL3	protein_codin	0.89294562	4.41723288	3.97982157	0
ENSG000001	PKLR	protein_codin	0	0	0	0
ENSG000001	TMEM215	protein_codin	0	0	0	0
ENSG000001	SUSD6	protein_codin	867.343956	997.711028	346.634077	437.481773
ENSG000001	SDHC	protein_codin	0	0	0	0
ENSG000001	LY6H	protein_codin	0	0	0	0
ENSG000001	MXRA8	protein_codin	0	0	0	0
ENSG000001	NEXN	protein_codin	0	0	334.12021	0
ENSG000001	LINC00319	lncRNA	0	0	0	0
ENSG000001	AUTS2	protein_codin	0	0	17.8156183	0
ENSG000001	DUSP9	protein_codin	0	0	0	0
ENSG000001	PROZ	protein_codin	0	0	0	0
ENSG000001	SYDE1	protein_codin	0	0	0	0
ENSG000000	VCAN	protein_codin	0	0	0	0
ENSG000001	ERBB4	protein_codin	0	0	0	0
ENSG000001	ZDHHC3	protein_codin	14.6709725	10.7932401	6.9321694	7.17109438
ENSG000000	CFLAR	protein_codin	51.0161371	95.9796923	19.0161398	7.37881783
ENSG000001	BTBD8	protein_codin	0	6.21843314	0	0
ENSG000001	PAQR3	protein_codin	0	0	0	0
ENSG000002	TBC1D3P3	unprocessed_	0	0	0	0
ENSG000001	FOXD4L3	protein_codin	0	0	0	0
ENSG000000	SPTB	protein_codin	0	0	0	0
ENSG000000	PRSS3	protein_codin	0	0	0	0
ENSG000000	TCF7	protein_codin	2.86517365	0	0	0
ENSG000001	BTNL3	protein_codin	0.9669113	0	0	0
ENSG000001	WFDC2	protein_codin	0	0	0	0
ENSG000001	SCGB1C1	protein_codin	4.42105298	6.29521029	2.16921666	3.67454127
ENSG000000	FXYD5	protein_codin	43.7314141	48.7240436	18.4659494	44.2067652
ENSG000001	CFAP54	protein_codin	0	0	0	0
ENSG000001	FAM230E	lncRNA	0	0	0	0
ENSG000002	PKP4-AS1	lncRNA	0	0	0	0
ENSG000001	LRRC39	protein_codin	0	3.93768471	0	0
ENSG000000	NLRC4	protein_codin	15.457028	12.8472616	0	0
ENSG000001	ST3GAL2	protein_codin	140.050166	231.174904	81.5918699	153.701431
ENSG000001	EGFR	protein_codin	0	0	0	0
ENSG000001	MADD	protein_codin	172.528773	155.414402	9.9070752	61.8576079
ENSG000001	NLRP13	protein_codin	0	0	0	0
ENSG000001	PPCDC	protein_codin	30.8023996	35.3263662	27.8567002	14.7567871
ENSG000000	EPB41L3	protein_codin	0	0	0	0
ENSG000000	CD82	protein_codin	17.9988035	37.550325	17.3693631	11.3426216
ENSG000001	SUSD4	protein_codin	0	0	0	0
ENSG000000	BCAT1	protein_codin	0	11.8342911	17.1001052	0
ENSG000001	GSDME	protein_codin	0	0	0	0
ENSG000002	PSG5	protein_codin	0	0	0	0

ENSG000002	ENSG000002	lncRNA	0	0	0	0
ENSG000001	HSD11B1L	protein_codin	0	0	0	0
ENSG000001	MAGEH1	protein_codin	10.9545283	19.042781	12.3805789	7.21406295
ENSG000001	EIF4G1	protein_codin	0	87.08191	0	7.79872362
ENSG000001	RAP2C	protein_codin	259.30781	185.066791	104.835546	136.204869
ENSG000001	SMAD4	protein_codin	0	0	0	0
ENSG000001	ENSG000001	lncRNA	0	0	0	0
ENSG000002	ENSG000002	lncRNA	0	0	0	0.82207654
ENSG000001	TTN	protein_codin	28.5633732	0	59.4652045	11.2267205
ENSG000001	EZHIP	protein_codin	0	0	0	0
ENSG000000	HEPH	protein_codin	0	0	0	0
ENSG000001	SPATA13	protein_codin	476.365677	336.534714	211.362425	312.936091
ENSG000001	PABIR2	protein_codin	56.6218367	116.557809	20.2287999	30.8266213
ENSG000001	C3orf62	protein_codin	132.944644	68.22108	43.5235773	44.7742801
ENSG000001	SNX18	protein_codin	183.375235	185.599349	65.0289975	162.453305
ENSG000001	MCM7	protein_codin	0	0	0	0
ENSG000000	TM7SF3	protein_codin	100.683101	136.760328	46.5959917	53.8429854
ENSG000001	BSX	protein_codin	0	0	0	0
ENSG000001	CFAP77	protein_codin	0	0	0	0
ENSG000001	DAP3	protein_codin	65.3807368	0	65.2583302	40.7279073
ENSG000001	NELFB	protein_codin	125.62485	146.203137	65.7535326	74.1588466
ENSG000002	LYPD4	protein_codin	0	0	0	0
ENSG000001	ESPNL	protein_codin	0	0	0	0
ENSG000001	EPB41	protein_codin	0	0	0	0
ENSG000001	SCNN1B	protein_codin	0	0	0	0.83264788
ENSG000001	ALKBH2	protein_codin	0	0	0	0
ENSG000001	QRFP	protein_codin	0	0	0	0
ENSG000002	ENSG000002	lncRNA	0	0	0	0
ENSG000001	UBE4B	protein_codin	208.196243	231.389221	54.7477213	117.307165
ENSG000001	NEMP2	protein_codin	0	0	0	1.1827502
ENSG000001	UBE2D3	protein_codin	169.597884	93.4346891	42.5473892	50.9323964
ENSG000001	PRELP	protein_codin	0	0	0	0
ENSG000000	MAPK9	protein_codin	8.23436511	5.60165287	0	0
ENSG000000	CTNNA2	protein_codin	0	0	0	0
ENSG000001	B3GALT5	protein_codin	0	0	0	0
ENSG000001	BACH2	protein_codin	0	0	0	0
ENSG000002	SDHAP3	lncRNA	0	0	0	0
ENSG000001	CD300LF	protein_codin	0	43.1550658	32.8685418	0
ENSG000001	SYCE1	protein_codin	0	0	0	0
ENSG000001	PRDM2	protein_codin	0	49.1607968	67.5319498	23.1261314
ENSG000001	HLA-DQA1	protein_codin	24.3057387	94.4033563	0	57.6738523
ENSG000001	TBC1D7	protein_codin	0	0	0	0
ENSG000001	CTSL	protein_codin	24.1097697	0	0	0
ENSG000001	INTS8	protein_codin	20.9896264	0	26.2012886	0

ENSG000000	SLC6A13	protein_codin	0	0	0	0
ENSG000001	IFT56	protein_codin	0	0	0	0
ENSG000001	KCNIP2	protein_codin	0	0	0	0
ENSG000000	VCAN	protein_codin	13472.4167	5053.5841	3208.15781	10261.7128
ENSG000001	RIC3	protein_codin	0	0	0	0
ENSG000000	ST7L	protein_codin	0	14.6075573	0	0
ENSG000001	CAV2	protein_codin	0	0	16.400413	0
ENSG000001	RTP5	protein_codin	0	0	0	0
ENSG000001	DUSP28	protein_codin	0.96064409	3.86049003	0	0
ENSG000001	DPYSL3	protein_codin	0.96328377	0	0	0
ENSG000000	SLC12A2	protein_codin	0	0	0	0
ENSG000001	LYPLA1	protein_codin	0	31.278529	14.1158776	0
ENSG000001	MAFK	protein_codin	120.153896	102.953568	51.1803775	70.5328708
ENSG000000	ATG5	protein_codin	0	0	0	0
ENSG000001	KRTAP4-5	protein_codin	0	0	0	0
ENSG000001	CCDC18	protein_codin	47.5707509	23.0494348	19.982992	55.7345435
ENSG000001	SRSF10	protein_codin	69.0928253	39.2409087	33.4988527	34.3365966
ENSG000001	MUC1	protein_codin	0	0	0	0
ENSG000001	CLCN1	protein_codin	0	0	0	0
ENSG000001	VAV3	protein_codin	47.4647341	49.6643712	66.8821072	36.2534341
ENSG000001	RPS2	protein_codin	1402.48358	2547.99444	294.215057	1035.70208
ENSG000001	APOD	protein_codin	0	0	0	0
ENSG000001	ASB7	protein_codin	11.4878473	32.2270127	0	23.2134238
ENSG000000	FERMT2	protein_codin	0	0	0	0
ENSG000001	ZNF772	protein_codin	0	0	0	0
ENSG000001	N4BP2L1	protein_codin	26.3172491	25.868814	2.18685819	2.31334453
ENSG000000	NT5C2	protein_codin	41.3448118	57.3759278	15.1793135	0
ENSG000001	TRIP12	protein_codin	0	0	0	1.31413651
ENSG000001	IDO2	protein_codin	0	0	0	0
ENSG000001	SIGLEC6	protein_codin	0	0	0	0
ENSG000001	LRRC41	protein_codin	0	14.5330032	0	0
ENSG000001	B3GLCT	protein_codin	16.1638665	17.3923101	7.65221615	13.5460781
ENSG000001	TMPO	protein_codin	29.4007077	38.8321206	17.2679651	23.2462537
ENSG000001	TFEB	protein_codin	2.58078627	8.15560637	4.35287757	0
ENSG000002	LRRD1	protein_codin	0	0	0	0
ENSG000001	CHIA	protein_codin	0	0	0	0
ENSG000001	ZNF791	protein_codin	98.0186656	99.3555832	81.325798	58.7921335
ENSG000001	PTBP3	protein_codin	63.1644523	59.063551	17.2362074	32.3916167
ENSG000000	ZNF195	protein_codin	0	0	0	4.42009253
ENSG000001	MYOCD	protein_codin	0	0	0	0
ENSG000000	SEMA6A	protein_codin	0	0	3.46795831	1.02782157
ENSG000001	FAM98C	protein_codin	0	0	0	0
ENSG000001	CDHR4	protein_codin	0	2.04396604	0	0
ENSG000001	DMPK	protein_codin	0	0	0	0

ENSG000002	FDX2	protein_codin	0	0	0	0
ENSG000001	E2F1	protein_codin	7.01280722	44.4669177	26.5336824	4.92527428
ENSG000001	ZNF669	protein_codin	0	0	0	0
ENSG000001	TLK2	protein_codin	46.448824	45.3791721	15.804494	0
ENSG000001	OR6C74	protein_codin	0	4.21826683	0	0
ENSG000001	HHIPL2	protein_codin	0	0	1.88962677	0
ENSG000001	MBTPS1	protein_codin	284.044277	459.8196	137.517894	217.213648
ENSG000001	CD55	protein_codin	26.4910133	14.293058	13.7726753	6.22336032
ENSG000001	ZFAND5	protein_codin	17.8044591	13.528584	0	0
ENSG000001	FYB2	protein_codin	0	0	0	0
ENSG000001	TADA3	protein_codin	7.64616565	0	8.44906187	0
ENSG000001	DICER1	protein_codin	118.231616	254.674496	80.1128575	316.247327
ENSG000001	LRRC66	protein_codin	0	0	0.95078512	0
ENSG000001	ZNF34	protein_codin	16.518454	0	0	13.7636867
ENSG000001	LYAR	protein_codin	159.797013	0	21.8884999	0
ENSG000001	SHISA6	protein_codin	0	0	0	0
ENSG000000	HOXA9	protein_codin	0	3.27391001	0	3.31783965
ENSG000001	TCEA2	protein_codin	9.89140815	14.2260907	0	0
ENSG000001	ZNF177	protein_codin	1.04624934	0	3.69183444	3.08301132
ENSG000001	NHSL1	protein_codin	42.3973933	41.1574992	12.545466	19.5843282
ENSG000001	CSMD3	protein_codin	0	0	0	0
ENSG000001	CASKIN1	protein_codin	0.77909629	0.7682792	0	0
ENSG000001	POTEH	protein_codin	0	0	0	0
ENSG000001	DMD	protein_codin	0	0	0	0
ENSG000001	ATG4A	protein_codin	0	0	0	0
ENSG000001	TRIM24	protein_codin	57.0426082	55.4854929	33.7327928	52.608167
ENSG000001	PDXK	protein_codin	37.9947711	47.1167448	0	13.062389
ENSG000001	RELN	protein_codin	0	0	0	0
ENSG000001	ATP7A	protein_codin	0	0	0	0
ENSG000001	C1orf162	protein_codin	188.814926	326.123788	112.246254	195.863291
ENSG000001	TP53BP2	protein_codin	394.561773	327.39516	74.3110612	281.642243
ENSG000001	RXFP1	protein_codin	0	0	1.11790364	0
ENSG000001	THNSL2	protein_codin	0	0	0	0
ENSG000001	P4HTM	protein_codin	0	0	9.54964054	0
ENSG000001	USF2	protein_codin	223.395197	434.635923	240.829039	122.321196
ENSG000001	EFHC2	protein_codin	0	0	0	0
ENSG000002	DOC2B	protein_codin	0.69112083	0	0	0
ENSG000001	CXCL12	protein_codin	0	0	0	0
ENSG000001	PRYP3	unprocessed_	0	0	0	0
ENSG000001	STX10	protein_codin	0	0	0	0
ENSG000001	FAM111B	protein_codin	0	0	0	0
ENSG000001	ZNF540	protein_codin	0	0	1.3186574	0
ENSG000001	MBNL2	protein_codin	104.845957	130.225031	121.710872	87.9555382
ENSG000001	SLC35E4	protein_codin	15.4578577	11.5977797	1.93843406	9.91231531

ENSG000002	ATP6V0A1	protein_codin	0	0	0	0
ENSG000001	RASAL3	protein_codin	160.857532	264.51993	70.4406283	117.568224
ENSG000001	MEAK7	protein_codin	16.7504889	19.6453364	8.87474002	8.00639436
ENSG000001	EXT2	protein_codin	0	0	0	0
ENSG000001	GGA1	protein_codin	0	110.641989	41.3882141	56.6378925
ENSG000001	UBTF	protein_codin	41.5610622	0	0	29.737634
ENSG000001	TMEM203	protein_codin	40.2398205	59.039997	13.9887331	28.2445543
ENSG000001	DGKZ	protein_codin	0	0	0	0
ENSG000001	H1-2	protein_codin	475.931213	579.317114	692.076195	358.930644
ENSG000001	NKAPL	protein_codin	5.02712927	5.18709832	8.9655968	4.18264242
ENSG000001	ABCG1	protein_codin	0	0	0	0
ENSG000001	RBPJL	protein_codin	0	0	0	0
ENSG000000	NTN4	protein_codin	0	0	0	0
ENSG000001	PDCD1	protein_codin	0	0.77880929	0	0
ENSG000001	SEZ6L	protein_codin	8.08879215	0	0	0
ENSG000002	LRRC69	protein_codin	0	0	0	0
ENSG000000	NOX4	protein_codin	0	0	0	0
ENSG000001	MED22	protein_codin	19.8717552	27.9762583	0	0
ENSG000000	CLDN18	protein_codin	0	0	0	0
ENSG000000	TSPOAP1	protein_codin	66.7282772	134.49932	190.685643	105.185943
ENSG000000	WNT8B	protein_codin	0.92858166	0	0	0
ENSG000002	PKD1P6	transcribed_u	14.7021309	12.2561375	2.28171474	7.39360512
ENSG000001	LRRK2	protein_codin	230.286086	87.3745	64.3021009	187.312094
ENSG000001	NPM1P14	processed_ps	0	1.15462259	1.50982888	0
ENSG000001	ACOT11	protein_codin	4.62546394	3.04126879	0	4.21384944
ENSG000001	DAPL1	protein_codin	0	0	0	0
ENSG000001	CDYL	protein_codin	34.9302743	0	0	0
ENSG000001	VIPAS39	protein_codin	0	0	0	0
ENSG000001	ZNF93	protein_codin	9.32013896	11.0959513	7.30713436	9.09938947
ENSG000001	ZNF678	protein_codin	21.5865791	20.4262087	19.9892136	26.4091426
ENSG000001	SUGT1	protein_codin	73.0959782	96.5661708	64.8565085	66.8032328
ENSG000001	DNAJA4	protein_codin	0	15.9880237	6.71778753	0
ENSG000001	NR3C1	protein_codin	17.2703653	40.3033126	28.8993235	23.3916019
ENSG000000	SP140	protein_codin	8.63244726	3.08139504	14.1917143	0
ENSG000001	MCRS1	protein_codin	37.251693	92.9981026	19.9325472	30.3309496
ENSG000001	MROH8	protein_codin	0	0	0	0
ENSG000001	ICMT	protein_codin	45.8656609	77.5581582	16.8348475	33.9538025
ENSG000001	UBAP2L	protein_codin	0	295.709135	0	34.8669816
ENSG000001	RGS3	protein_codin	0	0	0	0
ENSG000001	PYCR2	protein_codin	148.074478	243.98477	145.061052	154.881765
ENSG000001	TGIF1	protein_codin	12.4212728	0	2.58939346	23.5998069
ENSG000001	ASH2L	protein_codin	63.4568337	107.192282	33.7023508	71.6791595
ENSG000001	TCF12	protein_codin	0	0	0	0
ENSG000001	SNTN	protein_codin	0	0	0	0

ENSG000001	SUSD4	protein_codin	0	0	0	0
ENSG000001	PSMA8	protein_codin	0	0	0	0
ENSG000001	CAV3	protein_codin	0	0	0	0
ENSG000001	RGS6	protein_codin	0	17.2838791	0	9.08025583
ENSG000002	ENSG000002	lncRNA	10.8369894	38.2933314	5.63667697	18.3176249
ENSG000001	CCDC141	protein_codin	0	3.19239593	0	0
ENSG000001	FOXO3	protein_codin	369.263186	291.314518	0	239.184866
ENSG000001	PLCH2	protein_codin	0	0	0	0
ENSG000001	ELK1	protein_codin	0	0	0	0
ENSG000001	IL18BP	protein_codin	0	0	0	0
ENSG000001	SOWAHD	protein_codin	11.5604938	21.8816107	4.74522648	9.45514639
ENSG000001	CYP1A2	protein_codin	0	0	0	0
ENSG000001	CORO2A	protein_codin	0	0	17.3406174	0
ENSG000002	CPTP	protein_codin	34.7445758	37.7360796	11.9168453	19.4178833
ENSG000001	OSGIN1	protein_codin	0	0	0	0
ENSG000001	KBTBD12	protein_codin	0	0	0	0
ENSG000001	C19orf67	protein_codin	0	0	0	0
ENSG000001	P2RX2	protein_codin	0	0	0	0
ENSG000001	ZNF763	protein_codin	0	5.19289375	0	0
ENSG000001	FGD6	protein_codin	105.791629	55.014688	37.1753748	89.0552491
ENSG000001	FAM25A	protein_codin	0	0	0	0
ENSG000001	MS4A4A	protein_codin	0	16.6944142	6.3282231	0
ENSG000001	CEP41	protein_codin	0	0	0	4.25052312
ENSG000001	HMGCR	protein_codin	82.4302187	80.4789485	29.6095286	0
ENSG000001	ZNF136	protein_codin	31.9099099	0	36.6193213	16.758386
ENSG000001	SEPTIN6	protein_codin	0	0	0	0
ENSG000001	NUDT1	protein_codin	1.19596218	0	0	0
ENSG000001	RPS21	protein_codin	550.565367	931.38156	286.376326	435.218165
ENSG000001	PLEKHM3	protein_codin	0	0	0	0
ENSG000001	DIPK1C	protein_codin	0	0	0	0
ENSG000001	MIR3667HG	lncRNA	15.4568485	4.32720871	6.32887573	2.01280214
ENSG000001	RNF222	protein_codin	0	0	0	0
ENSG000001	CASZ1	protein_codin	0	0	0	2.96227737
ENSG000000	FXYD3	protein_codin	0	0	0	0
ENSG000001	PXMP4	protein_codin	0	0	0	0
ENSG000001	SUSD4	protein_codin	2.54382549	0	1.78455428	0
ENSG000002	FAM32BP	processed_ps	0	0	0	0
ENSG000001	PPM1G	protein_codin	344.741192	577.186728	250.306902	261.467088
ENSG000001	VDAC2	protein_codin	0	0	0	0
ENSG000001	RAPGEF5	protein_codin	0	0	0	0
ENSG000001	HM13	protein_codin	24.7439773	27.0864175	0	28.6901884
ENSG000001	SPINT1	protein_codin	11.1992168	19.2697064	0	0
ENSG000001	RPL28	protein_codin	49.7805839	90.0394655	38.1548213	21.1286207
ENSG000001	LRRC27	protein_codin	0	8.77198614	0	0

ENSG000002	ZNF880	protein_codin	0	0	0	0
ENSG000001	IL6R	protein_codin	25.3193199	5.40839054	21.9091081	0
ENSG000002	NPIPA7	protein_codin	0	7.70522458	0	0
ENSG000001	TAF6	protein_codin	0	0	0	0
ENSG000001	DYRK2	protein_codin	75.4491889	67.3694563	49.0906618	62.6907596
ENSG000001	ZNF14	protein_codin	23.5769986	12.6927947	8.52666292	8.70638553
ENSG000001	CACNA1C	protein_codin	0	0	0	0
ENSG000001	WARS1	protein_codin	165.954095	505.928203	127.813697	295.582961
ENSG000000	SIRPG	protein_codin	0	0	0	0
ENSG000002	COX19	protein_codin	131.293463	184.275359	48.5002134	59.5571823
ENSG000000	SYNE2	protein_codin	102.209114	44.387265	165.673249	15.3848622
ENSG000000	HERPUD1	protein_codin	9.35513326	3.04482983	7.09064273	0
ENSG000001	ENTPD8	protein_codin	0	0	0	0
ENSG000001	SPRY4	protein_codin	0	0	0	0
ENSG000001	CT55	protein_codin	0	0	0	0
ENSG000000	TRIO	protein_codin	0	4.25661776	0	0
ENSG000001	GRAP2	protein_codin	478.862472	1123.50945	780.221889	403.294278
ENSG000000	CACNB1	protein_codin	0	0	0	0
ENSG000001	ZCCHC17	protein_codin	0	66.8763372	0	0
ENSG000001	CARM1	protein_codin	0	8.25512586	0	6.02507362
ENSG000000	YTHDC1	protein_codin	81.909749	20.3620712	36.9415867	54.727874
ENSG000001	SBK2	protein_codin	0	0	0	0
ENSG000002	AKT1S1	protein_codin	17.6095237	12.2330404	0	9.19206067
ENSG000001	AFDN	protein_codin	0	0	0	0
ENSG000000	CBFA2T2	protein_codin	5.99256154	2.23652421	1.87319898	0
ENSG000000	TRIO	protein_codin	102.06754	372.184615	84.1253929	216.519069
ENSG000001	MST1R	protein_codin	0	0	0	0
ENSG000001	ILDR1	protein_codin	0	0	0	0
ENSG000001	FHL2	protein_codin	0	0	0	2.34907138
ENSG000002	ZNF550	protein_codin	6.32422887	10.449074	9.04209519	0
ENSG000001	CARD14	protein_codin	0	0	0	0
ENSG000000	RNF13	protein_codin	42.5709407	28.1098218	4.97472781	13.0336442
ENSG000001	ERFE	protein_codin	0	0	0	0
ENSG000001	OR7D2	protein_codin	0	0	0	0
ENSG000001	FGF8	protein_codin	0	0	0	0
ENSG000001	CDC25B	protein_codin	0	0	62.505408	0
ENSG000001	GAD1	protein_codin	0	0	0	0
ENSG000000	POU1F1	protein_codin	0	0	0	0
ENSG000001	STX2	protein_codin	0	4.532787	0	0
ENSG000001	ABCB9	protein_codin	0	0	0	0
ENSG000001	RGS20	protein_codin	0	0	0	0
ENSG000002	ZSCAN31	protein_codin	0	0	0	0
ENSG000001	CCDC38	protein_codin	0	0	0	0
ENSG000001	ESR2	protein_codin	0	0	0	0

ENSG000001	MAPT	protein_codin	0	0	0	0
ENSG000001	TAF3	protein_codin	197.64979	237.360471	124.918326	116.991011
ENSG000001	C15orf48	protein_codin	13.835693	8.98201475	8.12414855	21.1211999
ENSG000001	KRBOX4	protein_codin	6.89384412	6.63064116	5.4670397	6.22053227
ENSG000001	TEX11	protein_codin	0	0	0	0
ENSG000001	STARD13	protein_codin	0	0	0	0
ENSG000001	ZMAT5	protein_codin	39.2753638	76.747968	17.9990713	34.6927918
ENSG000001	C15orf62	protein_codin	0.97052179	0	2.23864659	1.86848325
ENSG000001	RUFY4	protein_codin	0	0	0	0
ENSG000001	TRPC6	protein_codin	11.3096983	16.6857477	19.4435392	10.8722724
ENSG000001	ICOSLG	protein_codin	5.43219186	24.2557963	5.84957395	10.720222
ENSG000001	KPNA1	protein_codin	81.6800094	80.1072479	72.8958151	81.2927647
ENSG000001	DMBT1	protein_codin	0	0	0	0
ENSG000001	XBP1	protein_codin	143.287501	159.212883	96.2971928	52.8336749
ENSG000000	CREM	protein_codin	0	0	0	0
ENSG000001	C1orf52	protein_codin	16.6049512	19.0880084	0	14.8493679
ENSG000001	OLA1	protein_codin	49.5491053	34.0324312	0	67.5646265
ENSG000000	RBL1	protein_codin	42.3611835	46.8021241	36.0341394	35.3232741
ENSG000002	KRTAP1-3	protein_codin	0	0	0	0
ENSG000000	CA12	protein_codin	0	0	0	0
ENSG000001	ADGRE3	protein_codin	3.4764655	4.04339451	0	2.94529788
ENSG000001	ATG4B	protein_codin	21.185957	15.7045449	0	0
ENSG000001	ASB11	protein_codin	0	0	0	0
ENSG000001	CC2D2B	protein_codin	0	0	0	0
ENSG000001	ZNF197	protein_codin	41.7392273	0	25.4763213	6.25066744
ENSG000000	CEACAM1	protein_codin	0	0	0	0
ENSG000001	TRIOBP	protein_codin	0	0	0	0
ENSG000001	EVC2	protein_codin	0	0	0	0
ENSG000000	NPFFR2	protein_codin	0	0	0	0
ENSG000001	ALKAL2	protein_codin	0	2.18855027	0	0
ENSG000001	GRAPL	protein_codin	0	0	0	0
ENSG000001	INTS15	protein_codin	69.9567248	123.266372	33.5690127	34.8102882
ENSG000000	IYD	protein_codin	0	0	0	0
ENSG000002	SLC35F6	protein_codin	205.790127	233.264603	92.0315148	89.4390383
ENSG000001	ZNF395	protein_codin	143.396228	156.302489	45.288994	56.4972479
ENSG000001	ANKRD36P1	transcribed_u	0	0	0	0
ENSG000000	KCNQ2	protein_codin	0	0	0	0
ENSG000001	NOVA1	protein_codin	0	0	0	0
ENSG000001	LARP7	protein_codin	99.8501334	84.2878195	95.6308262	141.262077
ENSG000001	DUSP22	protein_codin	65.6749453	77.9480766	18.9270321	44.4821844
ENSG000001	TMEM198	protein_codin	1.79743759	0	0	0
ENSG000001	TMEM234	protein_codin	0	9.20766159	0	0
ENSG000000	KCNQ2	protein_codin	0	0	0	0
ENSG000000	DGKG	protein_codin	14.4561933	65.2149051	0	0

ENSG000001	PAX3	protein_codin	0	0	0	0
ENSG000001	OR5W2	protein_codin	0	0	0	0
ENSG000001	ABCB10	protein_codin	86.0434646	59.8970217	38.6814205	28.8229021
ENSG000001	ZNF66	protein_codin	22.3606942	12.1300295	31.0323828	19.1970478
ENSG000001	NR6A1	protein_codin	15.7445019	0	5.78787737	0
ENSG000001	PRR27	protein_codin	0	0	0	0
ENSG000001	SH3BP4	protein_codin	0	0	0	4.98397944
ENSG000001	PIGC	protein_codin	24.4236242	29.9215201	21.7748392	19.1032148
ENSG000001	RCBTB2	protein_codin	98.0015148	88.881506	83.5331171	101.241625
ENSG000000	DTNBP1	protein_codin	113.369686	159.08888	88.2540693	0
ENSG000001	TPD52L3	protein_codin	0	0	0	0
ENSG000001	ATP5PD	protein_codin	1.50145965	11.7112567	0	0
ENSG000000	CDC42	protein_codin	172.12546	134.957498	157.784891	72.7229524
ENSG000001	CEACAM3	protein_codin	0	0	0	0
ENSG000001	FZD9	protein_codin	0	0.87204695	0	0
ENSG000001	EGFR	protein_codin	0	0	0	0
ENSG000001	LIPI	protein_codin	0	0	0	0
ENSG000001	TNNT3	protein_codin	0	0	0	0
ENSG000001	TPRG1L	protein_codin	24.074152	0	0	0
ENSG000001	KLHL33	protein_codin	0	0	0	0
ENSG000001	NKX2-3	protein_codin	1.64838144	1.66773025	0.68710024	0
ENSG000001	RSRC2	protein_codin	0	0	0	0
ENSG000000	FLYWCH1	protein_codin	0	0	0	0
ENSG000001	SRR	protein_codin	5.05312481	7.68032544	4.82773438	2.09054147
ENSG000001	ECM2	protein_codin	0	0	0	0
ENSG000001	TMEM156	protein_codin	0	1.17625522	0	0
ENSG000001	LEPR	protein_codin	0	0	0	0
ENSG000001	S100A14	protein_codin	0	0	0	0
ENSG000002	SLX1B-SULT1	lncRNA	75.9157841	55.5427684	7.28689776	17.6361574
ENSG000001	GDNF	protein_codin	0	0	0	0
ENSG000001	DROSHA	protein_codin	0	0	50.9697474	5.76646295
ENSG000001	SMARCA4	protein_codin	0	0	0	0
ENSG000001	SGCA	protein_codin	0	0	0	0
ENSG000001	OGG1	protein_codin	10.0496681	34.1166455	5.64507881	17.3487158
ENSG000001	PLEKHG7	protein_codin	0	0	3.34608819	0
ENSG000001	DEFB108F	unprocessed_	0	0	0	0
ENSG000002	LINC02929	lncRNA	0	0	0	0
ENSG000001	MRRF	protein_codin	40.6809694	25.6129838	27.8160679	21.56088
ENSG000001	LDLRAD2	protein_codin	0.95240957	5.63877821	3.48587032	1.83346511
ENSG000002	UGT1A10	protein_codin	0	0	0	0
ENSG000001	ZFAND4	protein_codin	11.0506737	0	7.46956549	11.7248177
ENSG000001	UNC119B	protein_codin	24.3906869	41.3503061	13.9054501	12.6559253
ENSG000001	SPHKAP	protein_codin	0	0.95082319	0	0
ENSG000001	RTN2	protein_codin	24.4486363	20.1617241	0	0

ENSG000001	MCPH1	protein_codin	30.2541831	15.3644608	35.2564917	21.6467496
ENSG000000	MAP3K14	protein_codin	158.768769	221.085592	78.6379195	89.6866033
ENSG000001	RUNX1	protein_codin	282.004041	382.266512	76.3729396	180.92244
ENSG000002	GLI4	protein_codin	0	0	0	0
ENSG000002	ENSG000002	lncRNA	0	0	0	0
ENSG000001	POMGNT2	protein_codin	0	0	0	0
ENSG000001	PCNX2	protein_codin	3.56775032	6.85760814	8.62963105	0
ENSG000001	RPS10	protein_codin	0	22.6478092	0	2.98996518
ENSG000001	EIF4ENIF1	protein_codin	0	0	0	0
ENSG000001	AK5	protein_codin	0	0	0	0
ENSG000001	NR3C2	protein_codin	0	0	1.81074246	0
ENSG000001	SMC4	protein_codin	0	38.3500815	0	34.1731898
ENSG000001	HMGN3	protein_codin	40.4087894	75.6703889	54.9839588	48.4668589
ENSG000001	ABL2	protein_codin	0	0	0	0
ENSG000001	SERPINB13	protein_codin	0	0	0	0
ENSG000001	RGS12	protein_codin	0	0	0	0
ENSG000002	KRT18P24	processed_ps	1.48758001	0	0	0
ENSG000001	OR7E12P	transcribed_u	0	0	0	0
ENSG000001	GLYAT	protein_codin	0	0	0	0
ENSG000001	RPL10	protein_codin	26.9932163	45.2090957	16.4331091	23.1198528
ENSG000000	SIGLEC1	protein_codin	144.307573	129.697519	61.8054647	52.4658879
ENSG000001	INSIG1	protein_codin	0	54.7272862	19.8864535	25.6445128
ENSG000001	CCDC15	protein_codin	0	23.3091125	24.576636	19.1937288
ENSG000001	FRMD6	protein_codin	4.65819819	0	0	0
ENSG000001	PDZK1	protein_codin	0	0	0	0
ENSG000001	BABAM2	protein_codin	3.30315184	0	0	0
ENSG000001	CIMIP2A	protein_codin	0	0	0	0
ENSG000001	DHRS2	protein_codin	0	0	0	0
ENSG000001	SNX21	protein_codin	6.43405443	0	16.9252361	0
ENSG000001	RBM38	protein_codin	0	0	0	0
ENSG000000	TFAP2B	protein_codin	0	0	0	0
ENSG000001	LYSMD4	protein_codin	0	0	0	0
ENSG000001	RAB5IF	protein_codin	145.45595	249.134024	60.7116577	122.991726
ENSG000002	SPATA31D1	protein_codin	0	0	0	0
ENSG000001	ZNF174	protein_codin	3.70477814	10.6909066	0	0
ENSG000001	TPM4	protein_codin	20.7999836	42.2683	21.7940132	11.0596043
ENSG000001	KLRD1	protein_codin	0	0	0	0
ENSG000001	USP7	protein_codin	284.166568	280.030956	46.3365835	133.977713
ENSG000001	EFHB	protein_codin	0	0	0	0
ENSG000001	BSPH1	protein_codin	0	0	0	0
ENSG000002	MRPL20	protein_codin	85.9567598	97.2901671	51.522094	65.9616214
ENSG000001	OR7E25P	transcribed_u	0	0	0	0
ENSG000001	SYNGR4	protein_codin	0	0	0	0
ENSG000001	ENG	protein_codin	63.0745981	66.7717211	0	0

ENSG000001:	GTDC1	protein_codin	0	0	0	0
ENSG000001	SRP72P2	processed_ps	0	0	0	0
ENSG000001:	ZDHHC7	protein_codin	101.623481	202.563088	50.3127821	161.201099
ENSG000001:	PUS7L	protein_codin	101.212946	52.5900287	55.4923713	62.8856437
ENSG000001	ASPSCR1	protein_codin	0	15.9366441	0	0
ENSG000001	LARP6	protein_codin	0	0	0	0
ENSG000001	PCDH18	protein_codin	0	0	0	0
ENSG000001	ANAPC11	protein_codin	17.0603213	18.7706729	0	8.57804892
ENSG000001	TNNI3	protein_codin	0	0	0	0
ENSG000001	ZNF833P	lncRNA	0	0	0	0
ENSG000001	TSLP	protein_codin	0	0	0	0
ENSG000001	MAP3K2	protein_codin	95.2670962	82.2388822	55.8607328	49.7315889
ENSG000000	NTN4	protein_codin	0	0	0	1.19001659
ENSG000001	NNT	protein_codin	40.2046702	29.5416548	54.3610596	34.8543325
ENSG000000	SMARCB1	protein_codin	8.99640306	0	0	0
ENSG000001	MIA3	protein_codin	153.392078	127.447893	165.929526	201.029026
ENSG000001	BPGM	protein_codin	21.9777183	22.7763602	7.74593054	16.2678178
ENSG000001	ZNF107	protein_codin	0	0	0	0
ENSG000000	CD6	protein_codin	0	0	3.56870958	0
ENSG000001	CPSF4L	protein_codin	0	0.71562978	0	0
ENSG000001	ARHGAP12	protein_codin	72.6211762	63.9025415	48.0695872	119.741318
ENSG000001	CLCN2	protein_codin	0	0	0	0
ENSG000001	SLC15A5	protein_codin	1.73852542	0	0	0
ENSG000000	TBC1D23	protein_codin	54.537216	0	17.7455142	16.9266307
ENSG000000	CUL3	protein_codin	44.7814271	37.7280579	10.6288915	40.7097823
ENSG000001	FLI1	protein_codin	0	0	0	0
ENSG000000	PVR	protein_codin	16.0142605	62.1871657	0	0
ENSG000001	FAM221A	protein_codin	0	0	0	0
ENSG000000	PDE4A	protein_codin	0	0	0	0
ENSG000001	ZNF433	protein_codin	0	0	0	0
ENSG000001	MPPE1	protein_codin	20.2980603	58.7641531	21.8307656	0
ENSG000001	SRSF10	protein_codin	0	0	24.309892	25.1480594
ENSG000000	LAT2	protein_codin	89.9146552	40.1553038	0	8.57953254
ENSG000001	GYG1	protein_codin	11.481266	3.33699964	5.28864438	0
ENSG000001	CLK3	protein_codin	0	11.4872775	6.28077656	4.45858604
ENSG000001	LYPD1	protein_codin	0	0	0	0
ENSG000001	SNCA	protein_codin	0	0	0	0
ENSG000000	KCNQ1	protein_codin	0	0	0	0
ENSG000000	AP3D1	protein_codin	0	0	0	0
ENSG000001	ZNF85	protein_codin	0	0	0	0
ENSG000002	RSC1A1	protein_codin	31.025925	14.2967783	21.3801841	18.7236853
ENSG000001	HSPD1	protein_codin	0	0	0	0
ENSG000001	OR52B6	protein_codin	0	0	0	0
ENSG000001	SLC2A11	protein_codin	0	0	0	0

ENSG000001	BCAP31	protein_codin	281.615169	505.439085	148.436996	181.380413
ENSG000001	TRADD	protein_codin	29.35736	60.3082047	11.0095443	29.3719828
ENSG000001	ADRB3	protein_codin	0	0	0	0
ENSG000001	TPRG1	protein_codin	0	0	0	0
ENSG000001	CORO6	protein_codin	0	0	0	0
ENSG000001	LIN28B	protein_codin	0	1.4590943	0	0
ENSG000001	MRPS5	protein_codin	42.818137	23.6288428	0	4.93793076
ENSG000001	DPPA3	protein_codin	0	0	0	0
ENSG000001	RNF41	protein_codin	76.7009722	58.9259886	0	0
ENSG000001	THUMPD3	protein_codin	10.0502129	23.4585284	0	9.97902868
ENSG000001	TBC1D28	protein_codin	0	0	0	0
ENSG000000	FOXN3	protein_codin	303.622442	160.794513	163.981383	213.894031
ENSG000001	ZAR1L	protein_codin	0	0	0	0
ENSG000001	LINC00943	lncRNA	0	0	0	0
ENSG000000	MLXIPL	protein_codin	0	0	0	0
ENSG000001	THAP1	protein_codin	12.3673879	4.59007949	8.44679338	0
ENSG000001	ARHGAP5	protein_codin	43.7578051	43.4161608	19.7437654	23.7114658
ENSG000001	CTSB	protein_codin	0	67.8807742	8.53961107	14.8755385
ENSG000001	PRICKLE1	protein_codin	18.1410646	2.50341823	15.9750692	14.3711839
ENSG000001	TGIF1	protein_codin	0	0	0	0
ENSG000001	PLEC	protein_codin	3843.23262	2918.16795	1263.79188	1040.12345
ENSG000001	IDH1	protein_codin	132.60649	231.367164	81.5765043	96.0546199
ENSG000001	CCDC78	protein_codin	0	0.95587811	0	0
ENSG000001	GRK4	protein_codin	0	0	0	0
ENSG000001	PTK7	protein_codin	0	0	0	0
ENSG000001	GFM2	protein_codin	0	0	44.6302611	0
ENSG000001	PPM1B	protein_codin	2.09445982	5.02650807	2.72888758	0
ENSG000001	AKR1E2	protein_codin	0	0	0	0
ENSG000001	SEC24C	protein_codin	116.176437	143.677329	25.6504936	104.289395
ENSG000001	RSU1	protein_codin	362.772296	1155.17878	273.70126	485.952958
ENSG000001	RAD17	protein_codin	0	0	0	0
ENSG000000	SKAP2	protein_codin	709.236974	685.024157	588.526058	593.112728
ENSG000001	NPTN	protein_codin	135.031495	198.994784	33.2909506	111.090148
ENSG000001	TM7SF2	protein_codin	0	0	0	0
ENSG000001	COL12A1	protein_codin	0	0	0	0
ENSG000000	BAX	protein_codin	10.9583346	59.711738	12.971585	26.5946629
ENSG000002	LTB4R	protein_codin	0	36.075802	2.98202357	17.9139081
ENSG000001	RAD51D	protein_codin	24.2409113	0	10.0755419	10.853293
ENSG000002	CSH2	protein_codin	0	0	0	0
ENSG000001	GCK	protein_codin	0	0	0	0
ENSG000001	BNC1	protein_codin	0	0	0	0
ENSG000001	VHL	protein_codin	62.3273543	46.0207927	13.487571	35.6188934
ENSG000001	NARF	protein_codin	31.3018005	75.3221728	25.9589579	53.2063544
ENSG000001	GIPC1	protein_codin	0	9.4146137	10.1326872	0

ENSG000001:	MBNL2	protein_codin	76.633733	0	0	54.2653166
ENSG000002	CKLF	protein_codin	61.5229572	0	0	0
ENSG000001:	CPEB2	protein_codin	0	0	0	0
ENSG000000!	CREM	protein_codin	11.7742456	8.77431679	0	5.18357363
ENSG000001	UBE2G2	protein_codin	314.426245	287.444371	103.238412	91.268831
ENSG000001:	STAT5A	protein_codin	0	0	0	0
ENSG000000	PTPRU	protein_codin	0	0	0	0
ENSG000001:	BCL11B	protein_codin	9.29609005	0	0	0
ENSG000001	ACTG2	protein_codin	0	0	0	0
ENSG000001:	CLTA	protein_codin	420.768998	643.599421	76.687826	262.222934
ENSG000001	WDR35	protein_codin	0	0	0	0
ENSG000001:	SLX1A	protein_codin	0	0	0	0
ENSG000001	KIR2DL4	protein_codin	0	0	0	0
ENSG000000!	WAC	protein_codin	151.066789	39.696979	29.0100097	48.0428892
ENSG000001	TMEM130	protein_codin	0	0	0	0
ENSG000000	APLP2	protein_codin	6.10340177	6.87623625	0	12.7092112
ENSG000000	HDAC4	protein_codin	26.4692725	110.838427	53.5926612	0
ENSG000001	CASP7	protein_codin	9.71140914	0	0	0
ENSG000001:	SDSL	protein_codin	0	0	0	0
ENSG000001:	CENPL	protein_codin	0	0	0	0
ENSG000001	SGK3	protein_codin	0	108.585684	99.6192429	94.3241108
ENSG000000	NISCH	protein_codin	0	0	104.724561	155.192424
ENSG000001	PAM	protein_codin	4.67244409	0	6.16217266	0
ENSG000001	MALT1	protein_codin	91.8302956	27.0109393	61.7784437	12.7590134
ENSG000001	FERMT3	protein_codin	1093.30542	2219.00733	838.98627	905.878263
ENSG000001	MS4A1	protein_codin	0	0	0	0
ENSG000001	ATG4A	protein_codin	0	0	0	0
ENSG000001	ATF2	protein_codin	7.8630186	0	59.0158763	0
ENSG000001	ZDHHC16	protein_codin	32.4690179	0	0	0
ENSG000001	NLRP12	protein_codin	3.41914895	0	0	0
ENSG000001	RBM10	protein_codin	0	0	0	80.7306485
ENSG000001	GOLM2	protein_codin	156.458805	73.8002775	0	125.090537
ENSG000001	CLTB	protein_codin	230.860915	293.392971	102.1104	153.097085
ENSG000001	ZNF274	protein_codin	30.239085	0	5.23674119	0
ENSG000001	MAGEA8	protein_codin	0.7421527	0	0	0
ENSG000001	RBPJ	protein_codin	0	12.1538073	0	2.00888782
ENSG000001!	KRTAP4-6	protein_codin	0	0	0	0
ENSG000001	TRIM3	protein_codin	25.2439494	23.0261317	3.59247284	13.1703651
ENSG000001:	UPF3B	protein_codin	140.511532	138.332873	60.2658325	136.063392
ENSG000001	LILRB5	protein_codin	0	0	0	0
ENSG000000:	SPO11	protein_codin	0	0	0	0
ENSG000001:	YAP1	protein_codin	0	0	0	0
ENSG000001	CERS2	protein_codin	18.4205696	0	0	10.5706365
ENSG000000	P2RX5	protein_codin	0	0	20.2295798	0

ENSG000001	TPO	protein_codin	0	0	0	0
ENSG000001	FCAR	protein_codin	0	0	0	0
ENSG000001	LPAR6	protein_codin	2.0679141	0	0	0
ENSG000001	SNX16	protein_codin	0	16.8134914	0	0
ENSG000001	NOL10	protein_codin	0	0	0	0
ENSG000001	PPL	protein_codin	0	0	0	0
ENSG000001	CSNK1G3	protein_codin	38.3192156	0	0	46.9185926
ENSG000001	CLEC4A	protein_codin	2.8201061	32.4415223	1.69780933	0
ENSG000001	TLK2	protein_codin	50.9399253	43.4537126	14.6687172	31.9247061
ENSG000001	PDE8B	protein_codin	0	0	1.832607	0
ENSG000001	PTK2B	protein_codin	531.97217	528.971095	312.786009	325.970112
ENSG000000	ARID1B	protein_codin	75.8529084	90.7473507	11.3102509	8.79432248
ENSG000001	TJP1	protein_codin	0	0	0	0
ENSG000000	EXOC1	protein_codin	43.6991224	7.50341073	0	0
ENSG000001	TUBD1	protein_codin	5.56913693	9.72476633	1.12490515	0
ENSG000000	MADCAM1	protein_codin	0	0	0	0
ENSG000001	MUC4	protein_codin	0	0	0	0
ENSG000000	SRPK1	protein_codin	9.59609344	11.1578373	12.55243	3.92560714
ENSG000001	RNF6	protein_codin	113.841009	120.807335	107.580201	53.2588374
ENSG000001	EIF4G1	protein_codin	144.831279	133.071396	19.1912825	135.830171
ENSG000001	FLNC	protein_codin	0	0	0	0
ENSG000000	GANAB	protein_codin	39.1205687	44.7194931	0	19.7336521
ENSG000000	NFATC3	protein_codin	198.44499	59.1004246	0	0
ENSG000000	IGF2BP2	protein_codin	92.488415	220.17347	99.7008237	112.319679
ENSG000001	FAM47A	protein_codin	0	0	0	0
ENSG000001	TCF7L2	protein_codin	14.4515659	0	0	4.4023187
ENSG000000	NNAT	protein_codin	0	0	0	0
ENSG000001	GATA3	protein_codin	0	12.0668241	3.69768693	0
ENSG000001	RAB5C	protein_codin	469.22434	578.029367	198.061245	287.659823
ENSG000000	DLEC1	protein_codin	2.34608396	9.86533273	2.42152821	0
ENSG000001	BIN1	protein_codin	0	0	0	4.98860611
ENSG000001	OSTF1	protein_codin	348.033969	347.194383	164.002465	261.120501
ENSG000001	IKZF3	protein_codin	0	0	0	0
ENSG000001	ARID3B	protein_codin	191.247727	187.892169	123.684005	128.554937
ENSG000001	CHST15	protein_codin	347.405081	359.209766	134.027376	216.185141
ENSG000001	TPD52L2	protein_codin	346.426032	460.842437	147.201581	249.422735
ENSG000001	XAGE3	protein_codin	0	0	0	0
ENSG000000	MTMR2	protein_codin	6.14875554	0	0	0
ENSG000001	TTC8	protein_codin	13.7105721	11.0495763	0	0
ENSG000000	MYLK	protein_codin	0	0	0	0
ENSG000000	CTTN	protein_codin	341.863893	912.233992	381.327539	373.02649
ENSG000000	UBE2A	protein_codin	0	15.1882729	0	0
ENSG000002	ENSG000002	lncRNA	0	0	0	0
ENSG000001	ARRB2	protein_codin	129.32223	102.049343	45.2054057	37.1344916

ENSG000000	F7	protein_codin	0	0	0	0
ENSG000001	SNX14	protein_codin	0	0	0	0
ENSG000001	NUDT2	protein_codin	21.8218171	49.9453475	0	0
ENSG000002	LILRB3	protein_codin	5.77921637	26.3640762	14.8539551	0
ENSG000001	SNRPN	protein_codin	51.826705	138.775413	72.9900589	0
ENSG000001	CDK5RAP1	protein_codin	52.7894613	0	16.2496671	37.7914084
ENSG000001	AIFM1	protein_codin	0	13.7638483	0	24.6705974
ENSG000000	TBL1Y	protein_codin	0	0	0	0
ENSG000000	CLCN6	protein_codin	128.185207	138.466116	32.4134676	85.9992884
ENSG000000	FXYD3	protein_codin	0	0.96604388	0	0
ENSG000001	RHCE	protein_codin	0	0	0	0
ENSG000001	DRD2	protein_codin	0	0	0	0
ENSG000001	DDIT3	protein_codin	0	0	9.43721787	0
ENSG000001	SIGLEC6	protein_codin	0	0	0	0
ENSG000001	TSPAN3	protein_codin	13.8399976	0	0	0
ENSG000002	FBXO16	protein_codin	0	0	0	0
ENSG000002	TSPAN4	protein_codin	0	0	0	0
ENSG000001	RBM23	protein_codin	6.40383381	0	7.34714535	0
ENSG000001	ABCB9	protein_codin	0	0	0	0
ENSG000000	CBFA2T2	protein_codin	19.3634087	80.3713523	0	0
ENSG000001	NPAS3	protein_codin	0	0	0	0
ENSG000001	ECM1	protein_codin	0	0	0	0
ENSG000001	KIR2DL4	protein_codin	0	0	0	0
ENSG000000	SMOX	protein_codin	0	37.3863032	6.52377618	20.4560099
ENSG000001	DHRS7B	protein_codin	2.50726699	11.8529325	6.8925255	2.15230232
ENSG000002	CSHL1	protein_codin	0	0	0	0
ENSG000002	ZFP64	protein_codin	0	49.0795061	0	25.3996914
ENSG000001	E2F3	protein_codin	67.3358879	127.693688	48.2069226	69.5613536
ENSG000001	ALDH1A3	protein_codin	0	0	0	0
ENSG000001	ANTXR2	protein_codin	36.5887017	0	8.03841053	6.59166918
ENSG000001	FCER2	protein_codin	0	0	0	0
ENSG000001	IKZF1	protein_codin	0	0	0	0
ENSG000001	ATRIP	protein_codin	1.251543	4.89975305	2.63162919	3.75383544
ENSG000001	CD99L2	protein_codin	20.7519466	10.5018972	9.81923784	8.28507486
ENSG000001	FGF8	protein_codin	0	0	0	0
ENSG000001	BRME1	protein_codin	0	1.33911697	0	0
ENSG000001	XAF1	protein_codin	0	0	0	0
ENSG000001	TMPRSS6	protein_codin	0	0	0	0
ENSG000001	SLC41A3	protein_codin	1.78944403	0	0	3.27117425
ENSG000001	MYL9	protein_codin	255.855034	1753.34808	1938.44011	332.422523
ENSG000001	APP	protein_codin	0	120.038074	0	0
ENSG000001	CTH	protein_codin	0	2.89719217	0	1.94222532
ENSG000001	IL36RN	protein_codin	0	0	0	0
ENSG000000	CASP10	protein_codin	0	58.8463075	25.8319275	0

ENSG000001:	ABI1	protein_codin	15.7518638	3.73094779	13.1671159	3.83064603
ENSG000001(	MLX	protein_codin	56.4831131	31.7304724	0	20.6518856
ENSG000001:	SLC4A5	protein_codin	0	0	0	0
ENSG000001'	UBE2E1	protein_codin	0	34.1854217	0	0
ENSG000000(	TAC1	protein_codin	0	0	0	0
ENSG000001(	IKZF3	protein_codin	48.1632383	40.5882651	160.513274	25.6084279
ENSG000001	PARD3	protein_codin	10.0442082	29.0613307	7.32334996	0
ENSG000001(	SPMAP2	protein_codin	0	0	0	0
ENSG000002:	IL6STP1	transcribed_p	1.3369525	0	0	0
ENSG000001'	BSG	protein_codin	3.19550328	0	0	0
ENSG000001	PAM	protein_codin	0	3.37561268	0	4.53734876
ENSG000001'	SEZ6L2	protein_codin	0	0	0	0
ENSG000001	TPO	protein_codin	0	0	0	0
ENSG000000'	DLG1	protein_codin	0	0	0	0
ENSG000001:	CACNG6	protein_codin	0	0	0	0
ENSG000000:	CAMK2B	protein_codin	0	0	0	0
ENSG000001:	KNL1	protein_codin	0	3.81412942	0	0
ENSG000000(	FGFR2	protein_codin	0	0	0	0
ENSG000001:	JAG2	protein_codin	0	0	0	2.70870859
ENSG000000	PHF7	protein_codin	0	1.22763472	3.93886045	0
ENSG000001:	ITGA7	protein_codin	0	0	0	0
ENSG000001	CAMKK2	protein_codin	0	0	0	0
ENSG000001:	SYNE1	protein_codin	0	0	0	0
ENSG000001	PTPA	protein_codin	0	0	0	0
ENSG000001(	COG5	protein_codin	0	0	75.615795	0
ENSG000001'	KRCC1	protein_codin	132.134371	153.555889	83.2473777	112.989156
ENSG000001	RNF175	protein_codin	0	1.0721501	0	0
ENSG000001:	SIVA1	protein_codin	5.14411311	12.7201982	4.68532173	18.2586455
ENSG000001:	ZNF546	protein_codin	5.46997035	2.265794	3.69143202	0
ENSG000001(	YY1AP1	protein_codin	0	0	8.61639383	0
ENSG000001:	DZIP1	protein_codin	0	0	0	0
ENSG000001	KCNQ4	protein_codin	0	0	0	0
ENSG000001	GPR107	protein_codin	232.956856	214.619845	127.084177	148.558191
ENSG000001'	R3HDM2	protein_codin	0	0	0	0
ENSG000001	TMEM44	protein_codin	0	0	0	0
ENSG000001:	PSAT1	protein_codin	0	0	0	1.06242397
ENSG000001:	KLC4	protein_codin	18.1841541	14.7841684	23.4017527	15.3851418
ENSG000000:	CAMK2B	protein_codin	0	0	0	0
ENSG000001:	CRHR1	protein_codin	0	0	0	0
ENSG000001(	IFTAP	protein_codin	4.16757099	5.56225661	0	3.65000263
ENSG000001:	PMEPA1	protein_codin	0	0	0	0
ENSG000000'	ZFYVE26	protein_codin	0	0	0	0
ENSG000001	RABGAP1L	protein_codin	0	116.527151	196.701957	0
ENSG000001(	PPP1R14A	protein_codin	0	0.92543495	0	0

ENSG000001!	RNU1-75P	snRNA	0	0	0	0
ENSG000001:	ZNF644	protein_codin	0	0	0	0
ENSG000001	IFT122	protein_codin	0	0	0	0
ENSG000001:	ZNF211	protein_codin	11.991558	19.501821	0	0
ENSG000001	IL23R	protein_codin	0	0	0	0
ENSG000001	LARP4	protein_codin	41.2653705	0	32.0666307	0
ENSG000000	AURKA	protein_codin	0	0	0	0
ENSG000001	STOM	protein_codin	76.2003147	151.194657	109.363114	111.081367
ENSG000001:	MKKS	protein_codin	0	0	0	0
ENSG000001	UBE2J2	protein_codin	0	0	0	0
ENSG000001:	CSTL1	protein_codin	0	0	0	0
ENSG000001	COL6A3	protein_codin	0	0	0	0
ENSG000000	KITLG	protein_codin	0	0	0	0
ENSG000001	EIF2B4	protein_codin	60.2553835	110.229219	48.0709881	31.7799632
ENSG000001:	STAU1	protein_codin	63.5140878	0	121.788085	0
ENSG000000	ATG16L1	protein_codin	11.7448565	0	0	0
ENSG000001	ZNF419	protein_codin	0	0	0	0
ENSG000000	MAPK9	protein_codin	23.9871102	28.5980407	16.2701159	4.6756979
ENSG000001:	SMARCC2	protein_codin	0	0	0	33.0019747
ENSG000001:	NOVA1	protein_codin	0	0	0	0
ENSG000001	SUPT6H	protein_codin	456.305237	503.828012	491.928422	321.721463
ENSG000001:	RPL36	protein_codin	560.951671	736.506967	215.229741	235.126749
ENSG000002	CHMP4A	protein_codin	125.367213	194.082479	83.6722767	95.826186
ENSG000000	ANK1	protein_codin	84.8038805	303.091807	166.739197	138.804125
ENSG000001	EPB41	protein_codin	0	177.960607	120.80679	0
ENSG000002	MIR133A2	miRNA	0	0	0	0
ENSG000000	ARHGEF1	protein_codin	0	0	0	0
ENSG000001	MAGED2	protein_codin	50.760364	80.7569622	29.8720849	16.8706166
ENSG000001	LRP8	protein_codin	0	0	0	0
ENSG000001	SMTN	protein_codin	0	0	0	0
ENSG000001	ADAM2	protein_codin	0	0	0	0
ENSG000001:	ALDH1A2	protein_codin	0	0	0	0
ENSG000001	GUCY1A2	protein_codin	0	0	0	0
ENSG000001	CACNA1C	protein_codin	0	0	0	0
ENSG000001!	ZNF334	protein_codin	0	0	0	0
ENSG000001	LSR	protein_codin	0	0	0	0
ENSG000001	GRIN2C	protein_codin	0	0	0	0
ENSG000001	CLCN3	protein_codin	52.7803211	39.6671692	61.9363944	9.87806388
ENSG000001	SMG7	protein_codin	275.752622	227.039823	156.52416	159.597972
ENSG000000	CNTN1	protein_codin	0	0	0	0
ENSG000001:	HMGA1	protein_codin	0	9.17093554	0	0
ENSG000001:	KLK8	protein_codin	0	0	0	0
ENSG000001	LRRC15	protein_codin	0	0	0	0
ENSG000001:	SPOP	protein_codin	58.1723783	7.11266293	5.88486123	7.79455397

ENSG000001	ST3GAL3	protein_codin	0	27.3978629	0	0
ENSG000000	NUP50	protein_codin	250.591677	388.81521	238.7927	223.696275
ENSG000000	RNF14	protein_codin	0	0	11.472176	15.8291033
ENSG000001	SLC28A2	protein_codin	0	1.17476455	0	0
ENSG000001	VCAM1	protein_codin	0	0	0	0
ENSG000001	BACE2	protein_codin	0	0	0	0
ENSG000001	TRAPPC6B	protein_codin	22.8081838	16.7909618	27.2948101	0
ENSG000000	EPN2	protein_codin	0	0	0	0
ENSG000000	MAP4K4	protein_codin	185.549864	520.987222	517.850621	367.983449
ENSG000001	CDCA7	protein_codin	2.37632808	0	0	0
ENSG000002	HGH1	protein_codin	20.9483697	41.4179393	15.846622	0
ENSG000000	ERC1	protein_codin	0	0	0	0
ENSG000001	TNFRSF10B	protein_codin	0	41.0391061	25.1755609	18.7366974
ENSG000001	PIDD1	protein_codin	13.3891725	0	10.21681	0
ENSG000001	SSX5	protein_codin	0	0	0	0
ENSG000000	HAUS4	protein_codin	0	0	0	0
ENSG000001	AZIN1	protein_codin	364.913725	461.819953	103.053116	237.547187
ENSG000001	CAB39L	protein_codin	0	0	0	0
ENSG000001	CD5	protein_codin	15.6906314	3.88089797	0.99152981	0.96259482
ENSG000001	ABCG1	protein_codin	0	0	0	0
ENSG000001	KLRC1	protein_codin	0	0	0	0
ENSG000001	KLC1	protein_codin	0	14.0554327	0	0
ENSG000000	ASIC4	protein_codin	0	0	0	0
ENSG000000	PTPN18	protein_codin	31.9032479	0	0	7.13815447
ENSG000000	RBM5	protein_codin	410.421875	613.353084	190.44014	204.865092
ENSG000001	SLC5A11	protein_codin	0	0	0	0
ENSG000001	KIRREL2	protein_codin	0	0	0	0
ENSG000001	HAP1	protein_codin	0	0	0	0
ENSG000001	ENSG000001	transcribed_u	0	0	0	0
ENSG000001	KLHDC4	protein_codin	9.6682331	15.3566637	13.4609715	11.0359213
ENSG000000	WAC	protein_codin	223.066353	296.219647	127.586505	253.795226
ENSG000001	SLC22A18	protein_codin	0	3.95757596	0	0
ENSG000001	PRDX5	protein_codin	0	0	1.38318004	8.01207476
ENSG000001	SLC49A3	protein_codin	4.56546149	6.71322295	0	0
ENSG000001	MBD1	protein_codin	0	0	0	0
ENSG000001	TM2D3	protein_codin	0	18.0380942	0	14.9570954
ENSG000001	FGF8	protein_codin	0	0	0	0
ENSG000001	NSD1	protein_codin	297.987727	210.342733	313.958931	211.39633
ENSG000001	MINK1	protein_codin	0	0	0	0
ENSG000001	MIA2	protein_codin	224.567885	160.14973	123.968761	61.7943975
ENSG000001	RBBP6	protein_codin	0	0	121.369468	0
ENSG000001	UBOX5	protein_codin	8.25769939	23.9145027	2.98751796	0
ENSG000001	RAC1	protein_codin	485.800908	644.40557	171.555335	328.266932
ENSG000000	ANK1	protein_codin	0	0.71201066	0	1.38155494

ENSG000000:	OSBPL5	protein_codin	98.886993	91.9746371	49.0891621	35.4074452
ENSG000001	CAMTA2	protein_codin	176.269881	211.371152	93.2392713	166.909015
ENSG000001	NLRP3	protein_codin	135.107356	103.534248	39.6938775	93.0829322
ENSG000000	MYL6	protein_codin	0	16.7282493	2.26651024	3.67259232
ENSG000001	RGS7	protein_codin	0	0	0	0
ENSG000001	TRAF6	protein_codin	0	27.5615504	23.6634562	20.5279782
ENSG000001	PAM	protein_codin	53.5248667	73.7885879	19.0925831	12.3599456
ENSG000001	IMPDH1	protein_codin	394.19253	623.382207	115.795319	162.357897
ENSG000001	PTPA	protein_codin	0	37.431894	9.0159474	17.4155148
ENSG000001	MEF2D	protein_codin	208.328183	233.589703	38.3271001	127.995266
ENSG000001	RBPJ	protein_codin	0	0	0	12.4468392
ENSG000001	TOM1L1	protein_codin	0	2.09639638	0	0
ENSG000000	UBE3C	protein_codin	262.256505	337.548721	148.26138	199.227169
ENSG000001	TJP2	protein_codin	26.5296959	19.5126691	39.44526	7.18162173
ENSG000001	DGUOK	protein_codin	22.5025851	13.1340486	0	0
ENSG000001	PTN	protein_codin	0	0	0	0
ENSG000000	WDR54	protein_codin	5.55098185	5.55644868	3.38347589	0
ENSG000001	LAIR1	protein_codin	0	0	9.82972439	8.99947263
ENSG000001	TPD52L2	protein_codin	0	0	12.6931508	0
ENSG000001	MRPL55	protein_codin	0	0	2.62729042	0
ENSG000001	CACNA1H	protein_codin	0	0	0	0
ENSG000001	PRLR	protein_codin	0	4.02291204	0	0
ENSG000001	CLCC1	protein_codin	0	0	0	0
ENSG000000	DNMT3B	protein_codin	5.71871226	0	8.06524766	0
ENSG000001	CHEK2	protein_codin	0	28.7936117	0	0
ENSG000001	ACVR1C	protein_codin	0	0	0	0
ENSG000000	NCOA1	protein_codin	774.252071	400.741097	155.759068	172.306297
ENSG000002	TNFRSF25	protein_codin	0	0	0	0
ENSG000000	CAMKK1	protein_codin	60.898927	0	11.1639194	15.6652144
ENSG000001	C8orf34	protein_codin	0	0	0	0
ENSG000001	BMX	protein_codin	0	0	0	0
ENSG000001	BTG3	protein_codin	22.4144677	15.7010079	8.96257401	21.6395621
ENSG000001	GPAM	protein_codin	10.7637387	11.2739561	12.3709567	7.23039429
ENSG000001	RNF111	protein_codin	80.9742786	0	0	0
ENSG000003	CAST	protein_codin	0	16.4406576	0	0
ENSG000001	STRBP	protein_codin	15.3595024	16.8130793	12.2833008	8.44361231
ENSG000001	SEC31A	protein_codin	0	126.083004	0	202.719804
ENSG000000	FTSJ1	protein_codin	63.700536	84.7545187	59.4483075	41.9957282
ENSG000001	IFT122	protein_codin	0	0	0	0
ENSG000000	CNN2	protein_codin	18.3885062	0	15.5888149	60.0330833
ENSG000001	TRPC6	protein_codin	0	13.2950381	0	0
ENSG000001	IKZF3	protein_codin	0	0	0	0
ENSG000000	REXO5	protein_codin	0	0	0	0
ENSG000001	CRHR2	protein_codin	0	0	0	0

ENSG000001	HELLS	protein_codin	0	0	0	0
ENSG000001	RPL35	protein_codin	954.404175	1539.38794	491.963019	684.936796
ENSG000000	ARID4A	protein_codin	15.2897602	20.2886038	0	0
ENSG000001	SSBP4	protein_codin	69.6958044	64.6906783	0	27.4835978
ENSG000001	GATD3	protein_codin	0	0	0	0
ENSG000000	ACSL4	protein_codin	110.186055	27.5962962	62.5755697	90.7818055
ENSG000000	SMARCE1	protein_codin	18.1803816	0	5.16483531	23.0297296
ENSG000002	DNAAF4	protein_codin	0	0	0	0
ENSG000001	KLC1	protein_codin	67.8811738	59.9990546	21.3649048	35.0411432
ENSG000001	VPS39	protein_codin	73.6765922	114.883072	81.4628207	55.2573686
ENSG000001	ERGIC3	protein_codin	244.472672	314.87449	114.282398	155.767365
ENSG000001	CASP9	protein_codin	25.9728907	43.2064293	15.9923505	16.9089962
ENSG000000	PTPRC	protein_codin	370.927362	214.439312	75.1486042	200.563952
ENSG000001	ZDHHC1	protein_codin	22.3075221	15.9476274	3.94055984	3.45132977
ENSG000001	EXO1	protein_codin	0	0	12.7821296	0
ENSG000001	CCNB3	protein_codin	0	0	0	0
ENSG000001	SENP7	protein_codin	26.3275658	82.7690181	55.2460492	20.995592
ENSG000001	CDH26	protein_codin	10.7945986	4.70330124	0	4.88534597
ENSG000000	CAMK2A	protein_codin	0	0	0	0
ENSG000000	CHRNA3	protein_codin	0	0	0	0
ENSG000001	IRF7	protein_codin	0	4.60954428	28.737272	0
ENSG000001	POLDIP3	protein_codin	134.565094	61.0600927	83.8121444	72.2778084
ENSG000001	CLEC1B	protein_codin	34.5526598	66.8179713	89.3781279	16.6751618
ENSG000001	PPA2	protein_codin	0	0	1.37455583	0
ENSG000001	PAX8	protein_codin	0	6.86728644	1.12594931	0
ENSG000001	CISH	protein_codin	16.2695235	43.8956126	0	15.2426662
ENSG000001	SLC23A1	protein_codin	1.02999705	0.99650646	0	0
ENSG000001	CHRNA1	protein_codin	0	0	0	0
ENSG000001	BIN1	protein_codin	39.9272287	62.2115674	0	14.5394032
ENSG000000	CNTN1	protein_codin	0	2.9503323	0	0
ENSG000000	CREM	protein_codin	0	0	5.46974029	0
ENSG000001	SNCG	protein_codin	0	0	0	0
ENSG000001	P2RX2	protein_codin	0	0	0	0
ENSG000001	MDM2	protein_codin	0	0	0	0
ENSG000001	WSB1	protein_codin	0	0	0	0
ENSG000001	DYRK1B	protein_codin	0	11.485942	21.968814	2.58110661
ENSG000000	MAP3K4	protein_codin	0	26.5628508	0	0
ENSG000001	ADARB1	protein_codin	16.5596953	8.0046689	0	0
ENSG000001	RBMS1	protein_codin	90.9877154	42.8596832	45.2823869	39.9071753
ENSG000001	KCNIP2	protein_codin	0	0	0	2.6704119
ENSG000002	ENSG000002	lncRNA	0	0	0	0.72795337
ENSG000001	C7orf57	protein_codin	0	0	0	0
ENSG000001	SBF1	protein_codin	0	83.7646765	0	0
ENSG000001	AMELX	protein_codin	0	0	0	0

ENSG000001	ALDH5A1	protein_codin	0	1.97276001	0	0
ENSG000001	ZPBP2	protein_codin	0	0	0	0
ENSG000000	HNRNPM	protein_codin	3.53450575	0	0	0
ENSG000001	APOBEC3H	protein_codin	0	0	0	0
ENSG000001	CKB	protein_codin	6.578144	0	0	4.5077082
ENSG000001	PTPRO	protein_codin	0	0	0	0
ENSG000001	DHX30	protein_codin	0	0	0	0
ENSG000001	PROS1	protein_codin	0	0	0	7.04074287
ENSG000000	CHRD	protein_codin	0	0	0	0
ENSG000001	APP	protein_codin	0	44.3353411	0	44.8795679
ENSG000002	ENSG000002	lncRNA	35.3034367	50.6925487	28.2487053	19.6579163
ENSG000001	DTNA	protein_codin	0	0	0	0
ENSG000001	NDRG3	protein_codin	93.1099928	144.726933	47.0555146	57.9761875
ENSG000001	ADNP	protein_codin	82.4588151	121.853154	119.235287	60.1020067
ENSG000001	ALDH3B2	protein_codin	0	0	0	0
ENSG000001	TEX14	protein_codin	0	12.7238103	12.0600212	15.8228688
ENSG000000	GNAS	protein_codin	0.86884581	0	0	0
ENSG000000	PTBP1	protein_codin	195.099374	345.52562	76.4097796	128.740921
ENSG000001	PFKL	protein_codin	360.864085	645.069005	142.955575	229.855232
ENSG000001	NUDT13	protein_codin	10.3303634	0	1.15765972	0
ENSG000001	TRIM4	protein_codin	10.9432085	128.713724	20.0059602	41.3242858
ENSG000002	ASIC3	protein_codin	0	0	0	0
ENSG000001	COLEC11	protein_codin	0	0	0	0
ENSG000001	PDE9A	protein_codin	0	0	0	0
ENSG000001	TGM5	protein_codin	0	0	0	0
ENSG000001	PTP4A3	protein_codin	0	0	0	0
ENSG000000	DOP1A	protein_codin	145.161873	111.365426	99.6055904	75.4301004
ENSG000000	WDR3	protein_codin	77.0141106	108.780452	51.249106	57.4830494
ENSG000001	TBX3	protein_codin	0	0	2.60860242	5.62769623
ENSG000001	PUF60	protein_codin	167.469285	229.132502	86.5962356	135.068522
ENSG000001	IL22RA2	protein_codin	0	0	0	0
ENSG000000	ARID4B	protein_codin	0	0	0	0
ENSG000001	HPS5	protein_codin	72.1454777	50.5350618	40.9464506	33.7702099
ENSG000000	NFATC3	protein_codin	0	93.8961833	124.677293	92.4474513
ENSG000001	RLIM	protein_codin	224.559818	110.782103	115.844617	136.090663
ENSG000001	TNFSF13	protein_codin	24.4077665	26.7377803	0	8.91414574
ENSG000001	MADD	protein_codin	0	0	0	0
ENSG000001	PTK7	protein_codin	0	0	0	0
ENSG000001	AGTR1	protein_codin	0	0	0	0
ENSG000001	THSD1	protein_codin	0	0	0	0
ENSG000001	B2M	protein_codin	131.103423	366.888993	134.874725	150.916033
ENSG000001	H2AZ2	protein_codin	22.244532	40.355183	29.9044297	0
ENSG000001	ALDH8A1	protein_codin	0	0	0	0
ENSG000001	AKT1	protein_codin	0	0	0	0

ENSG000001	UBE2D3	protein_codin	0	0	0	0
ENSG000001	APOL3	protein_codin	30.9488065	13.5684442	2.63338089	23.4483515
ENSG000001	RHCE	protein_codin	0	0	0	0
ENSG000001	ART3	protein_codin	0	0	0	0
ENSG000001	GSTM1	protein_codin	0	0	0	0
ENSG000001	CNBD2	protein_codin	0	0	0	0
ENSG000001	SNX3	protein_codin	40.8403017	89.1505303	23.4105468	43.6044331
ENSG000001	RREB1	protein_codin	139.085491	0	0	165.091715
ENSG000001	NXPH4	protein_codin	0	0.82472869	0	0
ENSG000001	UBE2J2	protein_codin	27.2948527	0	0	11.4545395
ENSG000001	RHCE	protein_codin	0	0	0	0
ENSG000001	IFT122	protein_codin	0	0	0	0
ENSG000001	OPRL1	protein_codin	0	0	0	0
ENSG000001	CD8B	protein_codin	0	0	0	0
ENSG000001	CFAP36	protein_codin	27.6517429	37.9435705	24.9786682	25.8758575
ENSG000001	ADGRG5	protein_codin	5.92204402	8.1817955	13.5481049	1.94191532
ENSG000001	PELI3	protein_codin	3.07375773	0	2.82820484	0
ENSG000001	EPB41	protein_codin	0	46.2847465	77.9121693	0
ENSG000000	ATP2B3	protein_codin	0	0	0	0
ENSG000001	POFUT2	protein_codin	0	41.1730948	6.76000847	20.4902088
ENSG000001	C8orf34	protein_codin	0	0	0	0
ENSG000001	CTNNB1	protein_codin	0	175.291808	140.312667	0
ENSG000001	CLIC6	protein_codin	0	0	0	0
ENSG000001	HDAC10	protein_codin	0	0	0	0
ENSG000001	UBA3	protein_codin	26.6930466	0	0	0
ENSG000001	FCRLA	protein_codin	0	0	2.06984953	0
ENSG000001	MIR494	miRNA	0	0	0	0
ENSG000001	LEPR	protein_codin	13.1321224	0	4.45721509	8.79138495
ENSG000001	BPIFB6	protein_codin	0	0	0	0
ENSG000001	CALB2	protein_codin	0	0	0	0
ENSG000001	GSTZ1	protein_codin	1.9555949	2.82044677	0	0
ENSG000002	CEP43	protein_codin	0	0	0	0
ENSG000000	CDHR5	protein_codin	0	0	0	0
ENSG000000	EXOC1	protein_codin	66.2110319	101.673252	75.7835234	76.1094855
ENSG000000	MYLIP	protein_codin	16.0450224	17.4484202	18.3348317	15.667442
ENSG000001	MUC4	protein_codin	0	0	0	0
ENSG000001	ZNF683	protein_codin	0	0	0	0
ENSG000001	HNRNPDL	protein_codin	19.7225684	60.907213	88.1628648	37.8313024
ENSG000001	KDM5C	protein_codin	0	1.60582095	0.94351143	0
ENSG000001	POP1	protein_codin	10.7571411	18.291019	7.42658985	12.8029317
ENSG000001	KCNMB3	protein_codin	0	0	0	0
ENSG000000	RFC1	protein_codin	86.319197	21.6457894	146.57685	117.993812
ENSG000001	UQCC1	protein_codin	16.2375666	0	0	0
ENSG000000	TBX5	protein_codin	0	0	0	0.94836696

ENSG000001	CMTM7	protein_codin	30.8616268	0	0	8.3676865
ENSG000000	SMARCA2	protein_codin	390.613688	544.607986	0	164.955719
ENSG000001	CSNK2A1	protein_codin	53.0420004	83.8313824	0	100.601201
ENSG000000	CAPN3	protein_codin	12.5163028	0	0	0
ENSG000001	GALNT14	protein_codin	0	0	3.52179252	0
ENSG000001	P2RY6	protein_codin	3.01842582	0	0	0
ENSG000000	SNAP23	protein_codin	10.2561949	16.339243	11.9905914	5.98743178
ENSG000001	CDK5RAP2	protein_codin	0	0	0	0
ENSG000001	PIP5K1A	protein_codin	63.6385905	133.195728	0	55.8395088
ENSG000001	IL37	protein_codin	0	0	0	0
ENSG000001	MAL	protein_codin	0	0	0	0
ENSG000000	CD79B	protein_codin	3.92998574	0	0	1.19621936
ENSG000001	IQCB1	protein_codin	0	0	0	2.15067307
ENSG000001	ARHGEF10	protein_codin	0	0	6.99922302	0
ENSG000000	MYNN	protein_codin	32.6138407	42.3608405	23.1923703	31.5218112
ENSG000001	SCEL	protein_codin	0	0	0	0
ENSG000000	PPEF1	protein_codin	0	0	0	0
ENSG000001	NAV2	protein_codin	0	4.55286591	0	0
ENSG000001	COPE	protein_codin	13.3633572	0	0	0
ENSG000001	MFF	protein_codin	0	0	0	21.9993539
ENSG000001	CLEC7A	protein_codin	2.81112772	0	1.44555245	0
ENSG000001	TCF7L2	protein_codin	10.2311906	6.75511994	3.62921904	12.9594236
ENSG000001	ARMC9	protein_codin	0	0	0	0
ENSG000001	NFAT5	protein_codin	0	0	0	34.1241356
ENSG000001	PARD3B	protein_codin	0	2.4577515	0	0
ENSG000001	PKIG	protein_codin	0	0	0	0
ENSG000001	CCR6	protein_codin	0	0	0	0
ENSG000001	COG3	protein_codin	101.193776	112.418905	60.6599059	79.7658508
ENSG000001	DTNB	protein_codin	0	0	0	0
ENSG000000	HFE	protein_codin	0	0	7.9562905	0
ENSG000001	ADAM33	protein_codin	0	0	0	0
ENSG000001	NDUFB6	protein_codin	0	14.3173914	4.32663029	0
ENSG000001	IL4	protein_codin	0	0	0	0
ENSG000000	ARID1B	protein_codin	201.014791	124.512088	0	0
ENSG000001	TAF1A	protein_codin	0	8.79681953	0	0
ENSG000001	SAMM50	protein_codin	84.5316909	95.4324685	43.9538012	52.9197993
ENSG000001	NASP	protein_codin	15.4498421	0	0	0
ENSG000001	COPS7B	protein_codin	0	0	0	0
ENSG000001	TRIM55	protein_codin	0	0	0	0
ENSG000001	P2RX2	protein_codin	0	0	0	0
ENSG000000	BIRC5	protein_codin	0	0	18.2067413	0
ENSG000001	MDM2	protein_codin	0	0	0	56.0223471
ENSG000000	ARHGAP4	protein_codin	677.177001	918.870214	304.532436	383.239255
ENSG000001	CACNA1D	protein_codin	0	0	0	0

ENSG000001	CILK1	protein_codin	0	0	0	0
ENSG000000	PTBP1	protein_codin	145.510404	0	107.821027	0
ENSG000001	CNTN6	protein_codin	0	0	0	0
ENSG000001	DOC2A	protein_codin	0	2.84301469	0	0
ENSG000001	SMAD9	protein_codin	0	0	0	0
ENSG000001	RNF38	protein_codin	10.5188426	0	28.5998956	27.6606501
ENSG000001	ENTREP3	protein_codin	38.0462525	34.4738061	9.05224534	2.58482619
ENSG000001	FSIP1	protein_codin	8.36009611	2.12065186	8.71106945	1.31859808
ENSG000001	KCNC2	protein_codin	0	0	0	0
ENSG000001	DUOXA2	protein_codin	0	0	0	0
ENSG000000	PPP2R5C	protein_codin	63.0963871	0	17.7166481	0
ENSG000001	ALDH6A1	protein_codin	0	0	0	0
ENSG000001	KLRD1	protein_codin	0	0	0	1.49064733
ENSG000001	FBXO25	protein_codin	11.4702234	13.1012572	8.65436778	18.2041487
ENSG000000	ACADVL	protein_codin	0	10.8071543	0	0
ENSG000001	SEPTIN7	protein_codin	66.518247	13.7541028	75.5670797	0
ENSG000001	FCN2	protein_codin	0	0	0	0
ENSG000000	PRKACA	protein_codin	0	0	0	0
ENSG000000	POU1F1	protein_codin	0	0	0	0
ENSG000001	C7orf25	protein_codin	0	22.1313355	0	5.27066887
ENSG000001	UBE2D3	protein_codin	19.460725	7.70564406	5.53827961	9.40216703
ENSG000000	DDX11	protein_codin	0	0	0	0
ENSG000001	ANO10	protein_codin	25.7037606	11.2277775	0	0
ENSG000001	EIF4G1	protein_codin	0	299.390573	340.158247	0
ENSG000000	TAC1	protein_codin	0	0	0	0
ENSG000000	MARK2	protein_codin	24.0266816	41.7273113	33.9778888	6.4384365
ENSG000001	ELP2	protein_codin	0	0	23.2605264	0
ENSG000001	PDAP1	protein_codin	158.77642	313.599193	140.859359	175.880792
ENSG000001	SPACA9	protein_codin	0	4.94422591	0	0
ENSG000001	GRHL3	protein_codin	0	0	0	0
ENSG000001	OR9A2	protein_codin	0	0	0	0
ENSG000001	PAX3	protein_codin	0	0	0	0
ENSG000001	SEZ6L2	protein_codin	0	0	0	0
ENSG000001	IKZF3	protein_codin	0	0	0	0
ENSG000001	PARD3	protein_codin	0	0	0	0
ENSG000001	PRSS37	protein_codin	0	0	0	0
ENSG000000	PILRA	protein_codin	165.593689	181.577131	67.2913625	119.969582
ENSG000001	C1orf43	protein_codin	148.059261	198.341784	0	104.839023
ENSG000001	PYCARD	protein_codin	70.1652982	69.4669248	30.3609856	27.1224599
ENSG000000	DDT	protein_codin	0	0	0	0
ENSG000001	SUPT20H	protein_codin	0	17.6276682	0	16.8089033
ENSG000001	CASP2	protein_codin	0	0	19.1170441	0
ENSG000001	ELP4	protein_codin	0	11.5802602	0	0
ENSG000001	SLC47A2	protein_codin	0	0	0	0

ENSG000001	CLEC12A	protein_codin	100.664104	62.4277109	83.3171472	94.3393369
ENSG000001	LDHB	protein_codin	110.168312	175.644863	129.284228	117.486541
ENSG000001	SLC25A10	protein_codin	0	3.89906799	0	1.46097994
ENSG000001	RGS3	protein_codin	0	0	0	1.60508634
ENSG000001	SEC13	protein_codin	168.952802	287.493844	184.611204	133.961577
ENSG000001	FCRLA	protein_codin	0	0	0	0
ENSG000001	ATR	protein_codin	0	33.3368392	0	0
ENSG000001	INPP5K	protein_codin	22.0365219	47.414348	22.5694411	28.6559291
ENSG000000	TNC	protein_codin	0	0	0	0
ENSG000001	MAPKAP1	protein_codin	0	0	0	0
ENSG000001	H2AZ2	protein_codin	10.4731696	11.7013856	6.61902476	27.6041327
ENSG000001	TSC2	protein_codin	0	0	0	0
ENSG000001	HINFP	protein_codin	25.2975142	41.2299097	12.7897197	0
ENSG000001	NCR1	protein_codin	3.99980078	0	0	0
ENSG000001	NDRG2	protein_codin	0	0	0	0
ENSG000000	CYTH3	protein_codin	36.0176021	59.6405848	29.7389999	32.0576884
ENSG000000	CAMK2B	protein_codin	0	0	0	0
ENSG000001	CDH26	protein_codin	0	0	4.77946369	0
ENSG000000	MAP4K4	protein_codin	328.211756	435.401825	269.644666	425.205075
ENSG000001	THBS4	protein_codin	0	0	0	0
ENSG000001	SNRNP35	protein_codin	0	0	0	0
ENSG000000	STMN4	protein_codin	0	0	0	0
ENSG000000	MSR1	protein_codin	18.4392522	1.50668473	2.70875491	12.4522362
ENSG000001	GRIN1	protein_codin	0	0	0	0
ENSG000001	FOXP2	protein_codin	0	0	0	0
ENSG000001	BBS9	protein_codin	0	0	0	0
ENSG000001	EZH2	protein_codin	0	11.3450161	0	0
ENSG000001	FADS1	protein_codin	127.404887	246.158795	60.465396	82.710232
ENSG000001	GNAI1	protein_codin	8.54478969	0	0	0
ENSG000001	SPATA16	protein_codin	0	0	0	0
ENSG000000	CAMK2A	protein_codin	0	0	0	0
ENSG000001	AFDN	protein_codin	0	0	0	0
ENSG000001	RHOG	protein_codin	484.687267	630.952337	205.241057	353.56494
ENSG000001	ODF2	protein_codin	0	0	0	54.3837629
ENSG000001	ST3GAL3	protein_codin	4.95976686	31.2503539	0	0
ENSG000001	GNAT2	protein_codin	0.92738496	0	0	0
ENSG000000	WASHC2A	protein_codin	519.801236	239.206656	133.951021	90.7564938
ENSG000001	COPE	protein_codin	0	0	0	0
ENSG000001	USP25	protein_codin	0	0	0	0
ENSG000001	MRPL10	protein_codin	63.8308981	90.6273158	35.8497484	42.3983812
ENSG000001	TAS1R1	protein_codin	0	0	0	0
ENSG000002	CKLF	protein_codin	23.1541936	17.2608703	12.6388498	16.7201005
ENSG000001	PARD3B	protein_codin	0	0	0	0
ENSG000001	NMD3	protein_codin	118.610721	50.0384802	88.2666586	87.0638372

ENSG000001	CABP1	protein_codin	0	0	0	0
ENSG000001	SMN1	protein_codin	0	0	0	0
ENSG000001	NPTN	protein_codin	83.5093717	105.22708	53.3619511	24.4966291
ENSG000001	ZFP30	protein_codin	0	0	0	0
ENSG000001	P2RX2	protein_codin	0	0	0	0
ENSG000001	NASP	protein_codin	157.420843	166.285634	185.661331	128.406948
ENSG000001	CDC25A	protein_codin	1.96195796	0	9.65045192	0
ENSG000001	MTMR14	protein_codin	163.237651	166.78811	0	95.0224
ENSG000001	CNTFR	protein_codin	0	0	0	0
ENSG000001	HABP2	protein_codin	0	0	0	0
ENSG000000	ACP3	protein_codin	94.3085614	169.763141	53.9786035	103.522461
ENSG000001	SMPD4	protein_codin	72.4129241	0	34.8128847	0
ENSG000001	CAMK2G	protein_codin	100.645524	118.551771	67.9169035	63.5335376
ENSG000000	PLD1	protein_codin	76.822748	73.458991	40.0151507	61.8445916
ENSG000002	FXYD1	protein_codin	0	0	0	0
ENSG000000	CS	protein_codin	238.442114	302.596591	130.318931	193.526736
ENSG000001	EFS	protein_codin	0	0	0	0
ENSG000001	SLC66A2	protein_codin	13.7789015	2.7679847	1.71298241	3.64061636
ENSG000002	GH1	protein_codin	0	0	0	0
ENSG000001	ELP2	protein_codin	0	16.9118331	0	0
ENSG000001	TPD52L2	protein_codin	0	0	0	0
ENSG000001	N6AMT1	protein_codin	3.38212003	2.24681163	0	0
ENSG000001	SPAG11A	protein_codin	0	0	0	0
ENSG000001	PDE1A	protein_codin	0	0	0	0
ENSG000001	PPA2	protein_codin	75.9173521	99.2919436	44.5177552	28.959354
ENSG000001	BNIP1	protein_codin	18.4507857	30.3761777	10.9483463	12.6121186
ENSG000001	UPF3A	protein_codin	0	73.3469124	0	0
ENSG000001	MTMR3	protein_codin	204.337157	165.157191	71.8010813	126.287932
ENSG000001	PFDN5	protein_codin	100.439135	137.909691	38.6929204	90.2339052
ENSG000001	DPYS	protein_codin	0	0	1.14245292	0
ENSG000001	SLAIN1	protein_codin	0	0	0	0
ENSG000000	CD59	protein_codin	0	10.8970419	0	0
ENSG000000	CHAT	protein_codin	0	0	0	0
ENSG000001	MAPT	protein_codin	0	0	0	0
ENSG000000	FYB1	protein_codin	0	0	0	0
ENSG000001	SLX1B	protein_codin	14.7737039	45.6472597	11.0321386	14.500156
ENSG000001	GSG1	protein_codin	0	0	0	0
ENSG000000	EED	protein_codin	0	8.75163685	0	6.27893061
ENSG000001	BIN1	protein_codin	0	0	0	0
ENSG000000	DHPS	protein_codin	0	0	2.39082993	0
ENSG000001	PTPN11	protein_codin	53.0023776	175.533298	155.844212	63.7933929
ENSG000001	IKZF3	protein_codin	0	0	0	0
ENSG000001	TRAF3	protein_codin	0	0	0	0
ENSG000001	TOR1A	protein_codin	150.874441	255.681058	64.7801212	126.453582

ENSG00000(	NRCAM	protein_codin	0	0	0	0
ENSG000001	DNAH12	protein_codin	0	0	0	1.29889331
ENSG000002	TNFRSF25	protein_codin	0	0	0	0
ENSG000001	IAH1	protein_codin	0	8.46384619	0	0
ENSG000000	ZNF37A	protein_codin	51.0653703	8.28148734	17.154521	0
ENSG000000	FAM114A2	protein_codin	14.3412671	4.0636809	7.10008287	16.674305
ENSG000001	OSCAR	protein_codin	6.89523718	22.7276409	0	0
ENSG000000	IFT88	protein_codin	37.9123778	18.2877439	20.8476257	17.5399685
ENSG000001	NKX2-4	protein_codin	0	0	0	0
ENSG000001	AKR1A1	protein_codin	37.7487595	0	22.5514227	0
ENSG000001	HNRNPK	protein_codin	0	0	0	0
ENSG000001	LAIR2	protein_codin	1.00033843	0	0.97770722	0
ENSG000001	USP4	protein_codin	325.98122	397.821454	225.010991	227.14251
ENSG000001	MTERF1	protein_codin	13.2170031	13.7431175	6.15061315	0
ENSG000000	SLC4A4	protein_codin	0	0	0	0
ENSG000001	PATZ1	protein_codin	41.8639978	32.7838834	28.8088505	0
ENSG000000	FGFR2	protein_codin	0	0	0	0
ENSG000001	TRIM41	protein_codin	0	0	0	0
ENSG000001	SMC6	protein_codin	100.440186	40.6162376	56.3153388	86.2183951
ENSG000002	TNFRSF25	protein_codin	2.59547995	0	0	0
ENSG000001	NUMA1	protein_codin	0	5.89241753	0	0
ENSG000001	NPM1	protein_codin	0	0	0	0
ENSG000001	DGCR8	protein_codin	51.1850796	107.109168	40.953162	0
ENSG000000	CD6	protein_codin	0	0	0	0
ENSG000000	ATP2A3	protein_codin	0	0	20.5273534	0
ENSG000001	ACLY	protein_codin	95.9861439	227.043329	82.3925704	139.029413
ENSG000001	ADAMTS12	protein_codin	0	0	0	0
ENSG000001	PLAT	protein_codin	0	0	0.91755135	0
ENSG000001	TCF7L2	protein_codin	62.7132904	0	0	0
ENSG000002	NUP62	protein_codin	243.139523	306.735443	120.366161	184.366167
ENSG000001	SNX15	protein_codin	1.31831814	1.25646978	0	3.45145021
ENSG000000	ELMO2	protein_codin	0	0	0	0
ENSG000001	RGN	protein_codin	0	0	0	0
ENSG000002	MIR6073	miRNA	0	0	0	0
ENSG000001	STAG3L4	transcribed_u	0	0	0	0
ENSG000001	ACAN	protein_codin	0	1.70387636	0	0
ENSG000001	CACNB2	protein_codin	0	0	0	0
ENSG000001	XRCC3	protein_codin	0	0	6.96926425	0
ENSG000000	RFC2	protein_codin	11.612381	5.68139807	0	0
ENSG000001	SLC37A1	protein_codin	82.651786	54.5274586	44.4882142	28.7066697
ENSG000001	FMNL3	protein_codin	27.693999	86.2692057	24.8834257	36.98452
ENSG000000	NRDC	protein_codin	659.091422	549.801169	212.414678	349.882657
ENSG000001	CBS	protein_codin	0	0	0	0
ENSG000001	IL37	protein_codin	0	0	0	0

ENSG000001:	SLC43A3	protein_codin	0	0	0	0
ENSG000000	TRPC7	protein_codin	0	0	0	0
ENSG000000	AP2S1	protein_codin	2.0512784	0	0	0
ENSG000001	PPOX	protein_codin	15.072349	0	0	0
ENSG000001	HTRA2	protein_codin	0	0	0	0
ENSG000001	LGALS8	protein_codin	0	0	0	0
ENSG000001	MITF	protein_codin	78.1537356	67.0978529	48.9872823	84.5035275
ENSG000001	PROSER1	protein_codin	0	143.394432	0	0
ENSG000001	SPNS1	protein_codin	0	0	0	0
ENSG000001	ZNF385A	protein_codin	0	0	0	0
ENSG000002	DTNB-AS1	lncRNA	0	0.87533144	0	0
ENSG000000	MTMR2	protein_codin	0	23.1041235	0	0
ENSG000001	HNRNPD	protein_codin	0	46.2903897	0	0
ENSG000001	PSMD13	protein_codin	8.23309296	0	0	0
ENSG000001	CFAP100	protein_codin	0	0	0	0
ENSG000001	UTP4	protein_codin	0	0	0	0
ENSG000001	THYN1	protein_codin	2.88062291	13.09889	13.6607897	14.9412991
ENSG000001	KIF23	protein_codin	0	11.6209986	21.0037288	0
ENSG000001	TCL6	lncRNA	0	0	0	0
ENSG000001	APOL4	protein_codin	0	0	0	0
ENSG000001	IGDCC4	protein_codin	0	0	0	0
ENSG000000	HFE	protein_codin	0	0	0	0
ENSG000002	CPNE1	protein_codin	0	7.57565881	0	0
ENSG000001	CYB5R3	protein_codin	32.8643783	0	46.0516747	52.5166242
ENSG000001	MPI	protein_codin	0	6.86289784	1.83858463	2.56112926
ENSG000001	P2RX2	protein_codin	0	0	0	0
ENSG000001	ZMYND8	protein_codin	0	128.277645	77.9114485	60.0352283
ENSG000001	ATP2B2	protein_codin	0	0	0	0
ENSG000001	PTTG1	protein_codin	1.72099964	0	27.3160205	0
ENSG000001	PRDX5	protein_codin	2.06586236	3.37440307	0	0
ENSG000001	TOR3A	protein_codin	0	0	0	0
ENSG000001	MYL1	protein_codin	0	0	0	0
ENSG000001	HNRNPUL1	protein_codin	51.0568032	119.19145	66.7018081	26.6933835
ENSG000001	HPS5	protein_codin	0	6.89677521	0	10.9695302
ENSG000001	ASS1	protein_codin	0	0	0	0
ENSG000001	TPD52L2	protein_codin	0	6.06213816	0	42.3680815
ENSG000001	RIPK4	protein_codin	0	0	0	0
ENSG000001	RAPSN	protein_codin	4.57984411	0	0	0.97148211
ENSG000001	ACVR2B	protein_codin	14.7804573	5.03190868	13.9061155	5.04229721
ENSG000001	BNIP1	protein_codin	0	0	0	0
ENSG000000	KCNAB2	protein_codin	0	0	0	0
ENSG000001	CACNG6	protein_codin	0.89686458	0.86133764	0	0
ENSG000001	VGLL2	protein_codin	0	0	0	0
ENSG000001	UBE2C	protein_codin	6.86971887	0	0	0

ENSG000001	CD8A	protein_codin	0	0	0	0
ENSG000000	CEACAM1	protein_codin	0	0	0	0
ENSG000001	CHIA	protein_codin	0	0	0	0
ENSG000001	RAD51AP1	protein_codin	0	0	0	0
ENSG000001	CLEC4A	protein_codin	0	3.50760391	3.30707051	0
ENSG000001	SPTBN4	protein_codin	0	0	0	0
ENSG000001	ZDHHC16	protein_codin	0	21.6827739	14.8470705	0
ENSG000001	ZC3H7B	protein_codin	218.276513	363.615279	143.119401	141.407858
ENSG000001	CFAP52	protein_codin	0	0	0	0
ENSG000001	RHBDL1	protein_codin	0.96268622	0.97422517	1.91540292	0
ENSG000001	FBXO25	protein_codin	34.5831184	50.413048	18.3167027	18.6323188
ENSG000001	MKLN1	protein_codin	271.895531	92.7022895	103.96189	133.652589
ENSG000001	CD96	protein_codin	0	0	7.79613013	0
ENSG000001	ABHD2	protein_codin	934.026848	989.289628	309.081484	786.80645
ENSG000002	PRR5-ARHGA	protein_codin	0	0	0.88390611	0
ENSG000001	EIF4G1	protein_codin	0	18.9140293	0	0
ENSG000001	TAC4	protein_codin	0	0	0	0
ENSG000001	TBL1XR1	protein_codin	99.4701135	27.826527	0	60.2537546
ENSG000000	CD44	protein_codin	11.6256071	36.3886954	0	0
ENSG000000	CACNA1G	protein_codin	0	0	0	0
ENSG000000	PKP1	protein_codin	0	0	0	0
ENSG000001	BIN1	protein_codin	0	0	0	0
ENSG000001	CRHR1	protein_codin	0	0	0	0
ENSG000001	SEC61G	protein_codin	76.254519	69.7088677	39.486056	48.1682916
ENSG000001	BTN2A2	protein_codin	0	0	0	0
ENSG000001	CAPN10	protein_codin	0	0	0	5.08152248
ENSG000001	FAM184A	protein_codin	0	0	0	0
ENSG000001	TEX9	protein_codin	0	0	9.84002558	5.48898323
ENSG000000	FGFR3	protein_codin	0	0	0.71708168	0
ENSG000001	TH	protein_codin	0	0	0	0
ENSG000001	Y_RNA	misc_RNA	0	0	0	0
ENSG000001	PTK7	protein_codin	14.0500946	0	0	0
ENSG000001	OGG1	protein_codin	0	0	0	3.21521061
ENSG000002	DDX47	protein_codin	26.7109901	0	0	0
ENSG000001	MRPL39	protein_codin	14.7767613	25.8912428	15.792682	0
ENSG000001	PKIA	protein_codin	11.1178962	11.1855078	18.4549512	3.46444766
ENSG000001	TAF1A	protein_codin	5.35716732	0	10.0621983	0
ENSG000001	KAT5	protein_codin	40.9277354	87.1901572	0	0
ENSG000001	CASP6	protein_codin	0	0	0	0
ENSG000001	RPL7	protein_codin	1303.34172	1323.93363	609.912109	1047.60413
ENSG000000	BRCA1	protein_codin	0	0	0	0
ENSG000001	ZNF383	protein_codin	0	7.84112838	0	0
ENSG000001	MAL	protein_codin	1.0606808	0	0	0
ENSG000001	CTSB	protein_codin	0	226.324861	75.2584991	0

ENSG000001[illegible]	PROK2	protein_codin	10.5877187	2.27875975	2.93380472	2.82486105
ENSG000001[illegible]	KCNIP2	protein_codin	0	0	0	0
ENSG000000[illegible]	NSFL1C	protein_codin	0	0	0	0
ENSG000001[illegible]	POLR2K	protein_codin	35.3937254	37.4721796	18.1023816	30.9513612
ENSG000001[illegible]	ST3GAL3	protein_codin	0	0	0	0
ENSG000001[illegible]	ABHD12B	protein_codin	0	0	0	0
ENSG000000	HFE	protein_codin	0	0	0	0
ENSG000001[illegible]	CSN2	protein_codin	0	0	0	0
ENSG000001[illegible]	NOL6	protein_codin	19.2697192	25.7408853	19.8944813	13.1676354
ENSG000001[illegible]	KLHDC4	protein_codin	0	0	0	0
ENSG000001[illegible]	KCTD14	protein_codin	0	2.12996649	1.02265408	0
ENSG000001[illegible]	PHYHD1	protein_codin	0	0	2.73125604	0
ENSG000000[illegible]	CAMK2B	protein_codin	0	0	0	0
ENSG000001[illegible]	AADAT	protein_codin	0	0	0	1.18049592
ENSG000001[illegible]	ACLY	protein_codin	87.2859906	139.450977	58.2568195	51.1748789
ENSG000000[illegible]	NFYA	protein_codin	95.0437476	130.86366	71.1579786	57.086313
ENSG000001[illegible]	ARPC5L	protein_codin	10.0010653	8.19181828	4.24304601	1.41462479
ENSG000001[illegible]	PAK5	protein_codin	0	0	0	0
ENSG000001[illegible]	IL37	protein_codin	0	0	0	0
ENSG000001[illegible]	CLEC7A	protein_codin	226.909354	298.945887	0	117.021801
ENSG000001[illegible]	ANKS6	protein_codin	5.29234473	6.49224464	2.89400604	0
ENSG000001[illegible]	YWHAZ	protein_codin	941.513934	732.995835	294.608271	508.804558
ENSG000000[illegible]	GDE1	protein_codin	202.737079	278.41198	94.7419578	135.04002
ENSG000001[illegible]	PARD6G	protein_codin	1.95783614	9.8438338	11.1859237	8.88784896
ENSG000001	CREB1	protein_codin	12.7388616	6.71846796	2.00050443	10.5131885
ENSG000001[illegible]	NSD2	protein_codin	0	0	0	0
ENSG000001[illegible]	SLC6A20	protein_codin	0	0	0.94386441	1.11983071
ENSG000001[illegible]	TRIM55	protein_codin	0	0	0	0
ENSG000001[illegible]	PTPN2	protein_codin	0	0	0	5.19321881
ENSG000000[illegible]	BCLAF1	protein_codin	0	0	0	21.1397737
ENSG000001[illegible]	MTMR14	protein_codin	186.594272	243.891376	34.0745126	154.905755
ENSG000000	CD74	protein_codin	10037.9233	18399.3679	8012.64316	8839.83894
ENSG000001[illegible]	MFF	protein_codin	0	0	0	0
ENSG000001[illegible]	HNRNPD	protein_codin	171.062147	424.916094	118.228169	249.216017
ENSG000001[illegible]	USP15	protein_codin	38.665823	67.2657141	21.9528059	60.7426972
ENSG000001[illegible]	CDK10	protein_codin	23.8980063	38.892754	0	20.8119084
ENSG000001[illegible]	RAI1	protein_codin	137.044742	204.891995	67.3576182	89.5079766
ENSG000001	SKP1	protein_codin	210.011505	195.86012	88.3831264	106.019883
ENSG000000[illegible]	COL11A1	protein_codin	0	0	0	0
ENSG000001[illegible]	GABRB2	protein_codin	0	0	0	0
ENSG000001[illegible]	LMBR1	protein_codin	50.6522519	21.8568804	20.9163002	16.9262276
ENSG000001[illegible]	SIGMAR1	protein_codin	0	0	0	36.5950615
ENSG000001[illegible]	SPPL3	protein_codin	107.444653	148.491365	42.7250787	77.7282645
ENSG000001[illegible]	ATP6V0A4	protein_codin	0	0	0	0

ENSG000001	DDX4	protein_codin	0	0	0	0
ENSG000000	MAP2K4	protein_codin	43.2079531	71.5117339	42.634699	36.9649593
ENSG000001	ARFGAP1	protein_codin	0	0	0	0
ENSG000001	DIABLO	protein_codin	3.20222529	3.99008621	0	0
ENSG000001	BSG	protein_codin	403.265821	721.685746	170.148077	335.61973
ENSG000001	COL6A3	protein_codin	0	9.91891397	0	0
ENSG000001	MRPS11	protein_codin	0	0	0	0
ENSG000001	FOSB	protein_codin	1932.86227	180.720091	285.314327	814.302201
ENSG000001	ARFIP1	protein_codin	43.3102503	55.1616598	0	0
ENSG000000	CAMK2B	protein_codin	0	0	0	0
ENSG000001	NPHS1	protein_codin	0	0	0	0
ENSG000000	GYG2	protein_codin	0	0	0	0
ENSG000001	RBCK1	protein_codin	0	0	0	11.339007
ENSG000001	ACAP3	protein_codin	0	0	0	0
ENSG000001	CHIA	protein_codin	0	0	0	0
ENSG000001	RAB34	protein_codin	0	0	0	0
ENSG000001	CCNB1IP1	protein_codin	0	12.1270545	0	7.23746989
ENSG000001	YWHAB	protein_codin	1007.91967	928.871613	421.662581	730.647001
ENSG000001	CREB3	protein_codin	23.9079231	48.1244145	26.5714191	16.3487978
ENSG000001	SERPINB8	protein_codin	154.464616	72.6107001	71.800465	90.8005967
ENSG000001	RNF38	protein_codin	187.099019	50.2868048	17.1222653	45.7999656
ENSG000001	FCAR	protein_codin	0	0	0	0
ENSG000001	ESR2	protein_codin	0	1.90629485	0	0.98580642
ENSG000001	DCAF4	protein_codin	11.1261077	0	0	0
ENSG000001	ABCC11	protein_codin	0	0	0	0
ENSG000001	SNX16	protein_codin	20.6263696	0	0	18.030827
ENSG000001	RBAK	protein_codin	10.7691688	21.2756753	0	0
ENSG000000	HSP90AB1	protein_codin	94.599822	493.683087	270.454574	99.5074047
ENSG000001	OCA2	protein_codin	0	0	0	0
ENSG000001	SIGLEC10	protein_codin	66.5059355	45.1557267	35.8760197	0
ENSG000001	FASTK	protein_codin	0	0	0	0
ENSG000000	DNMT3B	protein_codin	0	0	0	0
ENSG000000	HMMR	protein_codin	0	0	0	0
ENSG000001	NRF1	protein_codin	0	0	0	0
ENSG000001	NF2	protein_codin	0	0	0	0
ENSG000001	CABP2	protein_codin	0	0	0	0
ENSG000001	MBD1	protein_codin	0	0	0	0
ENSG000001	MATN4	protein_codin	0	0	0	0
ENSG000001	SLC23A1	protein_codin	0	0	0	0
ENSG000001	RNU1-15P	snRNA	0	0	0	0
ENSG000001	ZDHHC16	protein_codin	0	0	0	0
ENSG000001	EIF4H	protein_codin	592.62496	931.101907	244.14096	457.210701
ENSG000000	RASGRP2	protein_codin	0	0	0	0
ENSG000001	PML	protein_codin	45.9830093	29.8748739	0	0

ENSG000001	SLC13A4	protein_codin	0	0	0	0
ENSG000001	PLTP	protein_codin	0	0	0	2.5240278
ENSG000001	CDK2	protein_codin	13.9145764	0	15.6267985	0
ENSG000001	TMEM25	protein_codin	0	0	0	0
ENSG000001	PID1	protein_codin	13.7220252	21.9896401	2.74785154	8.79332274
ENSG000000	BRCA1	protein_codin	0	0	0	0
ENSG000001	MAL	protein_codin	0	0	0	0
ENSG000001	CAPN10	protein_codin	0	0	10.9066961	0
ENSG000001	SHMT1	protein_codin	0	0	0	8.06226203
ENSG000000	CWF19L1	protein_codin	35.7589246	54.654819	23.9830788	28.9973686
ENSG000000	SORBS1	protein_codin	0	0	0	0
ENSG000001	IL1RN	protein_codin	10.9348094	12.1951698	10.8846976	0
ENSG000001	C1QTNF1	protein_codin	0	0	0	0
ENSG000001	PPA2	protein_codin	0	0	0	0
ENSG000001	SLC12A9	protein_codin	48.7362932	0	0	0
ENSG000001	GPX4	protein_codin	0	69.6728289	0	30.8013874
ENSG000001	SELENON	protein_codin	0	0	0	0
ENSG000001	NSD1	protein_codin	38.4309385	101.212378	0	54.1668693
ENSG000001	SLC12A6	protein_codin	23.2889032	58.0321536	0	0
ENSG000001	DDX21	protein_codin	494.463717	213.555824	283.307014	323.172615
ENSG000001	KALRN	protein_codin	0	3.24419428	0	1.0462979
ENSG000001	DNAI7	protein_codin	0	3.09786493	0	2.12053309
ENSG000000	PLEKHB1	protein_codin	0	0	0	0
ENSG000000	BORCS8-MEF	protein_codin	32.3307528	22.3712036	0	14.6432353
ENSG000001	APP	protein_codin	0	0	0	0
ENSG000001	SVIP	protein_codin	100.915021	159.053595	87.1422896	122.878458
ENSG000001	VMO1	protein_codin	0	1.81976493	0	0
ENSG000001	TBC1D20	protein_codin	0	0	0	0
ENSG000001	DPAGT1	protein_codin	0	0	0	0
ENSG000001	KANSL3	protein_codin	178.450413	0	56.0826786	115.097699
ENSG000001	PRKAA1	protein_codin	63.9712727	115.824688	92.5234446	103.350409
ENSG000001	MAGI2	protein_codin	0	0	7.37825412	0
ENSG000001	UCKL1	protein_codin	62.970598	90.510379	29.2767017	19.0576597
ENSG000001	REG4	protein_codin	0	0	0	0
ENSG000001	DUSP19	protein_codin	6.04897381	11.4592015	13.6566379	5.15104736
ENSG000000	HGF	protein_codin	0	0	0	0
ENSG000001	DMPK	protein_codin	0	0	0	0
ENSG000001	SEPTIN6	protein_codin	249.549744	499.416209	415.234444	245.47877
ENSG000001	ETFB	protein_codin	9.60542902	7.06033532	3.83135652	5.38740098
ENSG000001	SETDB2	protein_codin	0	0	0	6.63486772
ENSG000001	TRIM4	protein_codin	79.5390424	68.7078391	31.3735367	42.0010044
ENSG000001	NOXO1	protein_codin	0	0	0	0
ENSG000001	NDUFV3	protein_codin	87.3162627	235.495005	85.6254463	99.1506187
ENSG000001	TAP1	protein_codin	157.884507	341.14222	0	0

ENSG000001	RHOT1	protein_codin	23.5418627	6.6763335	27.5442348	0
ENSG000001	OSCP1	protein_codin	0.9061925	0	0	0
ENSG000001	SMARCAD1	protein_codin	28.7276152	0	8.20282737	12.8470025
ENSG000001	IMPDH1	protein_codin	0	12.6327603	0	16.2922703
ENSG000001	ACSL5	protein_codin	94.7217796	0	0	0
ENSG000001	PKIB	protein_codin	0	0	0	0
ENSG000001	PLA2G4C	protein_codin	0	0	0	0
ENSG000001	RPL32P21	processed_ps	0	0	0	0
ENSG000001	EPPIN	protein_codin	0	0	0	0
ENSG000001	DDX46	protein_codin	345.892869	254.744621	286.089944	268.2841
ENSG000001	TMEM25	protein_codin	0	0	0	0
ENSG000001	DENND5B	protein_codin	0	0	0	0
ENSG000001	GATD1	protein_codin	0	0	0	0
ENSG000001	FBXO2	protein_codin	0	0	0.74515472	0
ENSG000001	NAMPT	protein_codin	5.46996993	2.15149426	1.89501108	5.47757951
ENSG000001	DDX55	protein_codin	1.91873579	1.72103831	5.54400715	0
ENSG000001	AP2A1	protein_codin	581.532524	591.699675	141.286192	269.016823
ENSG000002	ENSG000002	lncRNA	0	0	0	0
ENSG000001	BPIFA1	protein_codin	0	0	0	0
ENSG000001	PPTC7	protein_codin	361.393713	239.981987	133.466341	165.237187
ENSG000001	SYT5	protein_codin	0	0	0	0
ENSG000002	ZNF805	protein_codin	42.6715638	39.4162814	58.5004264	27.1117152
ENSG000001	KRT72	protein_codin	0	0	0	0
ENSG000001	RAD17	protein_codin	0	0	39.268902	0
ENSG000001	ITPK1	protein_codin	0	34.9365829	16.0711717	12.3864802
ENSG000001	DUS1L	protein_codin	0	0	0	0
ENSG000001	HRCT1	protein_codin	1.77232469	0	0	0
ENSG000001	TRERF1	protein_codin	37.366253	135.608287	72.1725502	92.093923
ENSG000001	S100A4	protein_codin	17.9292259	16.7802706	0	0
ENSG000002	TSPEAR-AS1	lncRNA	0	0	0	0
ENSG000000	HDAC7	protein_codin	191.17122	293.857038	89.0377588	105.676697
ENSG000000	CRKL	protein_codin	476.339806	757.997482	267.21325	295.796017
ENSG000000	ZNF112	protein_codin	0	0	0	0
ENSG000000	SLC11A1	protein_codin	122.322242	286.97472	0	212.675204
ENSG000001	KCNMA1	protein_codin	0	0	0	0
ENSG000000	GNAS	protein_codin	60.5117691	187.551444	105.638265	69.4769936
ENSG000001	SYF2	protein_codin	0	3.96705909	3.57667159	7.01684217
ENSG000001	PRPF40A	protein_codin	31.3377413	5.8616532	19.4343007	5.76232418
ENSG000001	NRDE2	protein_codin	140.857888	182.773773	53.3531674	98.3134279
ENSG000001	HNF4G	protein_codin	0	0	0	1.18884437
ENSG000001	COPS8	protein_codin	54.5225371	14.8149344	34.3715994	41.6181898
ENSG000001	RFPL1	protein_codin	0	0	0	0
ENSG000001	C9orf163	lncRNA	0	1.73153436	0	0
ENSG000001	ZNF774	protein_codin	0	0	1.12138018	0.92978272

ENSG000000!	LRRFIP2	protein_codin	22.1329741	56.7684322	18.516211	18.8218966
ENSG000001:	KANK4	protein_codin	0	0	0	0
ENSG000001:	MUTYH	protein_codin	0	0	0	0
ENSG000002:	STMND1	protein_codin	0	0	0	0
ENSG000001(	KIAA0586	protein_codin	0	24.5428678	0	0
ENSG000001:	MYPN	protein_codin	0	0	0	0
ENSG000001·	ARNT	protein_codin	67.8845254	0	20.312663	68.4614504
ENSG000000!	CD209	protein_codin	0	0	0	0
ENSG000001	APBB3	protein_codin	0	0	6.79915738	21.2147389
ENSG000000(	MEF2A	protein_codin	117.599571	31.7906956	0	30.3736015
ENSG000000!	NLRP1	protein_codin	0	0	0	0
ENSG000001:	LRP8	protein_codin	0	9.07472886	0	0
ENSG000001:	SEPTIN6	protein_codin	0	0	0	0
ENSG000000:	RNH1	protein_codin	51.7231334	0	0	32.7064
ENSG000001(	C11orf54	protein_codin	26.8753295	22.6387085	6.77422713	4.46971246
ENSG000001:	FAM78B	protein_codin	0	0	0	0
ENSG000002(	ENSG000002(	lncRNA	0	0	0	0
ENSG000001'	ELP5	protein_codin	0	0	0	3.94547295
ENSG000001(	LRRIQ3	protein_codin	0	0	0	0
ENSG000001!	ENSG000001!	lncRNA	0	0	0	1.83467767
ENSG000002·	FBXW10B	protein_codin	1.006292	0	0.94528869	0
ENSG000001:	ZYX	protein_codin	13.272987	48.9782396	0	0
ENSG000001(	NFAT5	protein_codin	72.3764418	0	0	214.431576
ENSG000000'	TP73	protein_codin	0	0	0	0
ENSG000001(	TTC8	protein_codin	0	0	0	0
ENSG000001:	CEP63	protein_codin	0	0	0	0
ENSG000001!	ATG7	protein_codin	0	118.019962	55.235249	0
ENSG000002!	CRYBB2P1	lncRNA	4.78722601	5.57223883	3.70336611	2.17276662
ENSG000001!	TCF4	protein_codin	8.69489746	0	0	0
ENSG000001	IRAG2	protein_codin	71.8232148	0	0	0
ENSG000001!	IPO4	protein_codin	0	33.533654	27.1068742	0
ENSG000000:	BAX	protein_codin	2.57251586	0	0	0
ENSG000001(	VPS72	protein_codin	0	0	0	0
ENSG000001(	WDR91	protein_codin	3.37557549	0	0	0
ENSG000001'	SPATA31G1	protein_codin	0	0	0.99006514	0
ENSG000001(	RBM3	protein_codin	208.67041	331.989556	161.785474	178.041789
ENSG000001!	ADAMTSL2	protein_codin	0	0	0	0
ENSG000001(	KANK1	protein_codin	0	0	0	0
ENSG000001!	DDRGK1	protein_codin	117.650204	135.987123	59.8890532	58.638268
ENSG000001	MAK	protein_codin	0	0	0	16.9038815
ENSG000001:	RTN3	protein_codin	0	0	0	4.92119931
ENSG000001:	MAP2K5	protein_codin	0	2.10124319	0	0
ENSG000000:	TRPM3	protein_codin	0	0	0	0
ENSG000001(	MFF	protein_codin	58.3068471	82.6210286	54.4463133	24.8356285

ENSG000001	VPS54	protein_codin	3.1484805	8.8042151	8.47290693	0
ENSG000001	RPE	protein_codin	38.9096216	0	15.8326909	0
ENSG000001	PDGFA	protein_codin	77.336596	138.240099	29.8407322	0
ENSG000002	H2AB2	protein_codin	3.36188484	0	0	0.79554497
ENSG000001	ENSG000001	lncRNA	0	0	0	0
ENSG000002	CTSL3P	lncRNA	0	0	0	0
ENSG000000	ARHGEF1	protein_codin	459.607598	700.342	420.209353	232.798184
ENSG000001	SCN8A	protein_codin	0	0	0	0
ENSG000001	SMOC2	protein_codin	0	0	0	0
ENSG000001	CAPN9	protein_codin	0	0	0	0
ENSG000002	ENSG000002	lncRNA	0	0	0	0
ENSG000001	MBP	protein_codin	0	0	0	0
ENSG000001	RMDN2	protein_codin	33.4374836	42.8610731	22.4327726	0
ENSG000001	ANXA6	protein_codin	331.981961	528.27944	0	267.89811
ENSG000001	COL13A1	protein_codin	0	0	0	0
ENSG000001	ADRA1A	protein_codin	0	0	0	0
ENSG000001	FILIP1L	protein_codin	0	0	0	0
ENSG000001	TP53I11	protein_codin	0.87372651	0	0.77130817	0
ENSG000001	CKAP5	protein_codin	0	0	0	0
ENSG000002	SDHAP1	lncRNA	0	0	0	0
ENSG000001	TEDC1	protein_codin	0	0	0	0
ENSG000001	AK5	protein_codin	0	0	0	0
ENSG000001	MAP7	protein_codin	0	0	0	2.37909968
ENSG000001	TWIST1	protein_codin	0	0	0	0
ENSG000001	MED23	protein_codin	31.7100008	19.5197299	6.26174323	31.1701766
ENSG000001	ITPR1	protein_codin	0	0	0	55.1349728
ENSG000001	STYX	protein_codin	69.7855577	73.9830338	28.7068725	104.088185
ENSG000001	LBHD1	protein_codin	9.61791858	11.2227378	1.74531156	9.55328003
ENSG000001	PLEC	protein_codin	636.799527	863.467005	0	317.969394
ENSG000001	ZBTB45	protein_codin	0	0	0	0
ENSG000001	CYP3A4	protein_codin	0	0	0	0
ENSG000000	ZNF195	protein_codin	25.1085569	21.0797602	0	14.7887267
ENSG000000	TP63	protein_codin	0	0	0	0
ENSG000001	TYW5	protein_codin	29.0883765	13.8958392	10.9720147	6.41418734
ENSG000000	MLXIPL	protein_codin	0	0	0	0
ENSG000001	SLC37A2	protein_codin	0	0	20.7621159	0
ENSG000000	THRAP3	protein_codin	221.629958	266.588836	83.918322	213.380907
ENSG000000	ERO1B	protein_codin	0	0	7.8496663	19.6161257
ENSG000000	CRISP3	protein_codin	0	0	0	0
ENSG000001	HKDC1	protein_codin	0	0	0.91151783	0
ENSG000001	RPS6KL1	protein_codin	0	0	1.10122611	0
ENSG000001	CLEC4C	protein_codin	0	0	0	0
ENSG000001	TRDMT1	protein_codin	45.0118802	10.6994495	11.1401886	20.2321815
ENSG000001	HMGA2	protein_codin	0	0	0	0

ENSG000001	OCA2	protein_codin	0	0	0	0
ENSG000001	STK31	protein_codin	0	0	0	0
ENSG000001	HRG-AS1	lncRNA	0	1.01585665	0	0
ENSG000001	WNK3	protein_codin	0	0	0	0
ENSG000001	NUTM2G	protein_codin	0	1.56840863	0	0
ENSG000000	FYN	protein_codin	0	0	0	0
ENSG000001	ACSL5	protein_codin	0	0	0	0
ENSG000001	ZNF44	protein_codin	0	0	0	0
ENSG000000	LMO3	protein_codin	0	0	0	0
ENSG000001	DIDO1	protein_codin	161.740815	164.44246	103.316976	0
ENSG000001	ELOVL2	protein_codin	0	0	0	0
ENSG000001	HNRNPA2B1	protein_codin	0	0	0	0
ENSG000001	BMF	protein_codin	73.6100955	139.282034	7.43568839	180.859369
ENSG000001	IGSF11	protein_codin	0	0	0	0
ENSG000001	SYNE1	protein_codin	8.47894782	0	15.5638601	0
ENSG000001	AKAP12	protein_codin	0	0	0	0
ENSG000001	CALML4	protein_codin	2.77126965	0	1.31009299	1.03215347
ENSG000001	HCFC1R1	protein_codin	3.03259869	7.32327888	1.77563619	0
ENSG000001	DNHD1	protein_codin	28.6877354	24.7253576	7.57395681	17.6703662
ENSG000002	IGHV3OR16-9	IG_V_gene	3.09166636	0.93493361	0	0
ENSG000001	YY1AP1	protein_codin	0	8.13178268	0	0
ENSG000001	PES1	protein_codin	138.328589	191.15571	97.0890337	59.4750089
ENSG000000	HNRNPH3	protein_codin	42.1342763	55.4900564	77.0125487	63.067701
ENSG000001	ZNF589	protein_codin	88.6324865	92.0980442	69.5556786	0
ENSG000001	ACAP3	protein_codin	144.059086	164.44779	69.3388688	91.0400485
ENSG000001	ENSA	protein_codin	4.19671367	13.0684468	7.44358703	0
ENSG000001	STK26	protein_codin	0	0	0	0
ENSG000001	HEY1	protein_codin	0	0	0	0
ENSG000001	SND1	protein_codin	564.509018	911.330112	454.580032	514.058806
ENSG000002	UGT1A9	protein_codin	0	0	0	0
ENSG000001	SEMA6D	protein_codin	0	0	0	0
ENSG000001	ENSG000001	lncRNA	1.86770317	3.2665148	3.99647547	2.24356856
ENSG000001	TTC3	protein_codin	36.0815122	19.5413093	0	33.9637118
ENSG000001	ANKRD18CP	transcribed_u	0	0	0	0
ENSG000001	GPSM1	protein_codin	0	13.3080613	6.84496173	8.93132959
ENSG000001	CCDC81	protein_codin	0	0	3.08158135	0
ENSG000001	PCDHB11	protein_codin	0	0	0	0
ENSG000000	CREM	protein_codin	0	0	0	0
ENSG000001	CRYBA4	protein_codin	0	0	0	0
ENSG000001	PGAP1	protein_codin	26.916046	0	6.68683283	11.978496
ENSG000001	PLAGL1	protein_codin	0	0	0	0
ENSG000001	GPT	protein_codin	0	0	0	0
ENSG000001	UBE2Q2P1	transcribed_u	0	0	0	0
ENSG000000	SLC22A17	protein_codin	0	0	0	0

ENSG000001	ALDH9A1	protein_codin	88.7217069	165.075406	42.7426914	101.704715
ENSG000001	WLS	protein_codin	10.8390112	51.7645759	0	29.9880841
ENSG000001	FN1	protein_codin	0	0	0	0
ENSG000001	ADGRG2	protein_codin	0	0	0	0
ENSG000001	BPNT1	protein_codin	0	0	7.70990342	5.31319256
ENSG000001	RPL15	protein_codin	17.6316216	63.3712728	53.6863462	4.63289091
ENSG000001	FAR1	protein_codin	62.8403839	81.2794571	34.8408134	143.282709
ENSG000001	NKX2-1	protein_codin	0	0	0	0
ENSG000001	GABPA	protein_codin	47.736356	24.292569	85.7395225	62.7717887
ENSG000002	CYP3A43	protein_codin	0	0	0	0
ENSG000000	NRDC	protein_codin	0	0	0	84.40594
ENSG000001	SERF1A	protein_codin	18.9658058	5.35728839	0	7.44987154
ENSG000001	EPHA5	protein_codin	0	0	0	0
ENSG000000	FMO1	protein_codin	0	0	0	0
ENSG000001	ARMCX4	protein_codin	0	0	0	0
ENSG000001	CD46	protein_codin	299.157734	163.40976	0	0
ENSG000002	TRIM6-TRIM3	protein_codin	0	0	0	0
ENSG000000	MOV10L1	protein_codin	0	0	0	0
ENSG000001	KLRF1	protein_codin	0	0	0	0
ENSG000000	AK2	protein_codin	37.522814	0	0	0
ENSG000002	NAA80	protein_codin	0	0	0	0
ENSG000001	AXIN1	protein_codin	0	110.400155	0	83.2718107
ENSG000001	RAD17	protein_codin	0	11.4155129	0	47.6862945
ENSG000000	RABL2B	protein_codin	0	20.245422	0	0
ENSG000001	BMP1	protein_codin	0	0	0	0
ENSG000000	CTSA	protein_codin	38.7496511	63.8342639	41.3472396	15.9632831
ENSG000001	TMEM205	protein_codin	24.2065936	23.1804131	17.6970612	7.71905863
ENSG000001	SLC25A26	protein_codin	0	0	0	0
ENSG000001	SLC39A13	protein_codin	12.1448253	25.2233534	0	0
ENSG000001	SLC38A6	protein_codin	5.47559354	13.8963271	0	0
ENSG000001	EGFLAM	protein_codin	0	0	0	0
ENSG000001	VKORC1	protein_codin	13.3544625	5.38631011	2.24219118	0
ENSG000000	CELF2	protein_codin	55.784098	144.305413	191.748479	83.0244185
ENSG000001	LSR	protein_codin	4.71392848	0	8.66168238	0
ENSG000000	SMG6	protein_codin	0	0	0	0.9922223
ENSG000001	PER1	protein_codin	0	0	4.32178223	0
ENSG000001	TMEM207	protein_codin	0	0	0	0
ENSG000001	MIA3	protein_codin	0	0	0	0
ENSG000001	ANAPC13	protein_codin	43.4382791	39.9915727	24.7632925	42.0749923
ENSG000000	WAC	protein_codin	160.926819	108.769284	44.9923504	103.235871
ENSG000001	SCARA5	protein_codin	0	0	0	0
ENSG000002	HBZP1	unprocessed_	0	0	0	0
ENSG000001	SUV39H2	protein_codin	0	0	0	0
ENSG000002	CRYZL2P-SEC	lncRNA	2.12587685	0	0	0

ENSG000001	PNRC1	protein_codin	177.195317	306.32385	103.129331	97.9182574
ENSG000001	OR5K4	protein_codin	0	0	0	0
ENSG000001	PITX2	protein_codin	0	0	0	0
ENSG000001	LUC7L2	protein_codin	40.7675418	136.474143	19.4247955	128.149009
ENSG000002	CYP21A1P	transcribed_u	0	0	0	0
ENSG000001	BPIFA2	protein_codin	0	0	0	0
ENSG000001	PIP4K2C	protein_codin	46.4395324	0	11.9992374	26.8804369
ENSG000001	PLSCR4	protein_codin	0	12.9742116	0	0
ENSG000001	FMOD	protein_codin	0	1.92552522	0	1.9764251
ENSG000001	ATG7	protein_codin	138.023066	189.804482	39.9998649	100.631756
ENSG000001	ZNF649	protein_codin	10.0552362	16.9709226	0	6.04417861
ENSG000001	PLEC	protein_codin	1259.25434	909.939397	568.678538	392.516175
ENSG000001	ZNF43	protein_codin	28.5058885	0	0	0
ENSG000001	PDE5A	protein_codin	238.697171	454.096304	275.683521	314.926971
ENSG000001	IL31RA	protein_codin	0	0	0	0
ENSG000001	MYL4	protein_codin	0	0	0	0
ENSG000001	ACAP3	protein_codin	0	0	0	0
ENSG000000	KIF16B	protein_codin	89.4133386	87.1711948	91.840879	69.5725227
ENSG000001	FCN3	protein_codin	0	0	0	0
ENSG000000	CACNA1G	protein_codin	0	0	0	0
ENSG000001	ENTPD6	protein_codin	14.4098107	34.014372	9.61905755	0
ENSG000001	DDX4	protein_codin	0	0	0	0
ENSG000001	LARGE1	protein_codin	0	0	2.33284206	0
ENSG000001	PTPA	protein_codin	43.6464711	0	0	0
ENSG000001	BRDT	protein_codin	0	0	0	0
ENSG000001	SEMA4A	protein_codin	329.180087	313.781028	165.439093	0
ENSG000001	H2BW2	protein_codin	0	0	0	0
ENSG000001	GLRA2	protein_codin	0	0	0	0
ENSG000001	BANP	protein_codin	0	0	0	0
ENSG000001	INCA1	protein_codin	0	0	0	0
ENSG000000	BCL2L13	protein_codin	0	0	0	0
ENSG000001	NET1	protein_codin	10.9905217	6.68807237	5.39555868	6.21056642
ENSG000001	ASGR2	protein_codin	108.076406	13.1817102	0	39.2507299
ENSG000001	DNAJC4	protein_codin	2.6733801	0	1.08799948	0
ENSG000001	ATRNL1	protein_codin	0	0	0	0
ENSG000001	TSGA10	protein_codin	0	0	0	0
ENSG000001	H4C11	protein_codin	65.537537	114.777743	33.8036997	56.7971823
ENSG000001	NUMB	protein_codin	0	0	0	0
ENSG000001	NEIL1	protein_codin	0	0	0	0
ENSG000001	EIF5A	protein_codin	9.56112604	17.4177807	4.3480391	8.9157757
ENSG000001	BBS9	protein_codin	0	0	0	0
ENSG000001	HTT	protein_codin	250.238478	348.796406	118.660875	219.78246
ENSG000001	KCTD6	protein_codin	6.07835272	2.38966894	8.8324766	0
ENSG000001	PITX2	protein_codin	0	0	0	0

ENSG000001	KLHL28	protein_codin	15.1009275	9.13439041	4.83904105	0
ENSG000000	EIF3I	protein_codin	0	0	0	0
ENSG000002	STIMATE	protein_codin	233.507194	194.036704	35.8385201	101.020932
ENSG000001	C5AR1	protein_codin	2099.77566	998.531252	347.976814	705.09413
ENSG000001	SRGAP1	protein_codin	21.6882142	7.24727659	2.45039747	13.4590711
ENSG000001	GPAA1	protein_codin	0	56.3486936	0	0
ENSG000002	ZNF736	protein_codin	13.5440273	31.5414778	8.95797141	12.40642
ENSG000000	ENTPD2	protein_codin	0	0	0	0
ENSG000001	GPBP1L1	protein_codin	400.354126	559.953493	105.001691	206.203547
ENSG000001	BLM	protein_codin	17.1921206	0	0	0
ENSG000001	ZNF565	protein_codin	0	0	0	0
ENSG000000	LIMS2	protein_codin	0.87094259	0	0	0
ENSG000001	ITSN2	protein_codin	91.1840776	4.91912324	16.3898718	38.945058
ENSG000000	BTBD7	protein_codin	19.0460688	34.3739505	0	0
ENSG000001	SCN8A	protein_codin	0	0	0	0
ENSG000000	GPM6B	protein_codin	0	4.09700937	0	0
ENSG000002	MTFP1	protein_codin	0	12.079091	0	0
ENSG000001	ZNF160	protein_codin	1.33335048	2.59673556	0	2.22355174
ENSG000001	CD99L2	protein_codin	16.8789238	0	10.8007328	0
ENSG000001	MRPL52	protein_codin	18.8786469	11.5747822	4.15203859	3.10931714
ENSG000001	ITGB2	protein_codin	0	0	0	0
ENSG000001	VPS13B	protein_codin	0	0	0	0
ENSG000000	TBL1Y	protein_codin	0	0	0	0
ENSG000000	BCAT1	protein_codin	3.77278642	0	0	0
ENSG000000	LTK	protein_codin	3.76339682	4.22446526	6.95673431	0
ENSG000001	EYA4	protein_codin	2.56392687	0	0	0
ENSG000001	BEGAIN	protein_codin	0	0	0	0
ENSG000001	ATG10	protein_codin	2.47860737	0	0	0
ENSG000001	SPART	protein_codin	0	0	0	0
ENSG000002	MIR548XHG	lncRNA	0	0	0	0
ENSG000001	C6orf89	protein_codin	36.136744	29.9233098	29.1109712	13.8329569
ENSG000001	MSRB3	protein_codin	0	100.269723	21.6270167	47.9687911
ENSG000001	KCNQ5	protein_codin	0	0	14.9828942	0
ENSG000001	SEC31A	protein_codin	0	0	0	0
ENSG000001	AQP10	protein_codin	0	0	0	0
ENSG000001	ZNF607	protein_codin	2.20695372	0	1.26373566	0
ENSG000001	EOLA2	protein_codin	0	0	7.29684202	16.6453154
ENSG000001	POLR3H	protein_codin	55.3562801	0	0	28.8311959
ENSG000001	FOXN4	protein_codin	0	0	0	0
ENSG000000	CECR2	protein_codin	0	0	0	0
ENSG000001	MAGEA11	protein_codin	0	0	0	0
ENSG000000	ATP6V1H	protein_codin	3.49699978	9.91940797	0	3.2541331
ENSG000001	FAM72B	protein_codin	0	7.04354701	2.06041734	0
ENSG000001	HMBOX1	protein_codin	0	0	0	0

ENSG000001	PTPRD	protein_codin	0	0	0	0
ENSG000001	TBC1D9B	protein_codin	608.368147	776.64694	256.858225	275.121901
ENSG000001	OCLN	protein_codin	0	0	0	0
ENSG000001	SYNCRIP	protein_codin	276.309669	79.7332903	55.0839969	182.024686
ENSG000000	PAX2	protein_codin	0	0	0	0
ENSG000001	NUP98	protein_codin	0	0	0	0
ENSG000001	AP3M1	protein_codin	57.2841999	53.3575551	27.7579339	29.230441
ENSG000001	KEL	protein_codin	4.38498489	0	3.79711483	1.72235912
ENSG000001	OR5H2	protein_codin	0	0	0	0
ENSG000002	FTLP6	processed_ps	0	0	0	0
ENSG000000	FAS	protein_codin	0	0	0	0
ENSG000001	MINK1	protein_codin	410.509479	828.922864	339.889008	371.883827
ENSG000001	OR2L5	protein_codin	0	0	0	0
ENSG000000	MSR1	protein_codin	0	0	0	0
ENSG000001	AK9	protein_codin	1.61157518	0	3.74893528	0
ENSG000001	APCDD1	protein_codin	13.9393758	26.7608824	11.4509484	16.7433941
ENSG000001	EYA4	protein_codin	0	0	0	0
ENSG000001	ANK3	protein_codin	0	0	0	0
ENSG000001	GRM1	protein_codin	0	0	0	0
ENSG000001	TDRD7	protein_codin	137.725102	106.43158	60.6854007	76.9958699
ENSG000001	DNAJA3	protein_codin	24.4459406	50.4262643	37.4556884	18.2499935
ENSG000001	GMPR2	protein_codin	0	46.4329535	0	4.46008973
ENSG000001	LINGO1	protein_codin	0	0	0	0
ENSG000001	NPHP1	protein_codin	0.86622803	0	0	0
ENSG000001	ANO1	protein_codin	0	0	0	0
ENSG000001	DNM3	protein_codin	0	42.2569722	24.7690116	0
ENSG000001	EBF3	protein_codin	0	0	0	0
ENSG000001	GIT2	protein_codin	93.4256719	185.032636	0	227.521959
ENSG000001	CHCHD7	protein_codin	20.7630888	0	0	22.3718365
ENSG000001	THSD4	protein_codin	0	0.96018216	0	0
ENSG000001	MORN3	protein_codin	0	0.99761851	1.27980477	0.9934847
ENSG000001	WARS1	protein_codin	0	14.6219842	2.21827591	0
ENSG000001	ZFYVE19	protein_codin	0	9.35617277	0	1.81695748
ENSG000001	OR10D1P	unprocessed_	0	0	0	0
ENSG000001	ITGB1BP1	protein_codin	12.7253893	5.48693682	10.8743143	3.96296206
ENSG000000	MYH7	protein_codin	0	0	0	0
ENSG000001	CD47	protein_codin	235.43698	181.365145	89.7876248	98.2981877
ENSG000001	MIER1	protein_codin	248.89936	127.631978	179.492512	179.527909
ENSG000001	GATA3-AS1	lncRNA	0	0	0	0
ENSG000001	PGBD2	protein_codin	8.27685815	2.41008188	0	0
ENSG000001	SHTN1	protein_codin	0	63.0297977	61.0482709	40.0629204
ENSG000001	ZCCHC10	protein_codin	4.51885474	0	18.3336443	0
ENSG000001	SYT17	protein_codin	0	1.56717613	0	0
ENSG000001	SCAMP3	protein_codin	0	0	4.82637193	0

ENSG000001	SPATA22	protein_codin	0	0	0	0
ENSG000001	BOC	protein_codin	0	0	0	0
ENSG000001	RNF220	protein_codin	0	0	0	0
ENSG000001	GABRA5	protein_codin	0	0	0	0
ENSG000001	SUSD1	protein_codin	0	0	0	0
ENSG000001	MFSD12	protein_codin	107.619139	121.335312	35.7515313	66.2354182
ENSG000001	CCDC69	protein_codin	1070.27257	1309.94844	546.888294	712.129161
ENSG000001	GFRA1	protein_codin	0	0	0	0
ENSG000001	SAP30BP	protein_codin	0	0	0	0
ENSG000001	EFEMP1	protein_codin	0	0	0	0
ENSG000001	COLCA1	lncRNA	0	0	0	0
ENSG000000	ARID4A	protein_codin	384.146436	218.689013	200.217382	198.983611
ENSG000001	CSF2RA	protein_codin	0	0	0	0
ENSG000001	MFSD10	protein_codin	33.1097205	33.8872611	0	0
ENSG000001	TMEM116	protein_codin	0	0	0	0
ENSG000000	ERC1	protein_codin	0	0	0	0
ENSG000000	NUDCD3	protein_codin	94.2540943	222.29804	76.4816815	83.3521963
ENSG000000	LAMC3	protein_codin	0	0	0	0.85475304
ENSG000001	KRTAP13-1	protein_codin	0	0	0	0
ENSG000001	MBNL1	protein_codin	0	0	0	0
ENSG000002	ZNF512	protein_codin	0	0	0	0
ENSG000001	P3H4	protein_codin	0	0	0	0
ENSG000002	NFAM1	protein_codin	124.885169	149.764394	55.8300849	43.1054034
ENSG000001	GRK6	protein_codin	444.521366	331.214094	115.898342	209.320935
ENSG000001	RBPJ	protein_codin	210.102012	124.151979	110.790665	187.020586
ENSG000001	CLN3	protein_codin	0	0	0	0
ENSG000001	RIC8B	protein_codin	0	9.45295066	0	0
ENSG000001	COL18A1	protein_codin	0	0	0	0
ENSG000001	RYR1	protein_codin	0	0	0	0
ENSG000001	FADS2	protein_codin	0	0	0	0
ENSG000001	VANGL1	protein_codin	18.0740901	47.6204955	16.8444299	0
ENSG000001	LTO1	protein_codin	0	0	0	0
ENSG000001	TXNL4A	protein_codin	3.45774861	0	0	0
ENSG000001	PLEKHA4	protein_codin	0	0	0	0
ENSG000001	LMX1B	protein_codin	0	0	0	0
ENSG000001	MUTYH	protein_codin	0	0	0	0
ENSG000001	YY1AP1	protein_codin	0	0	0	0
ENSG000001	COMMD9	protein_codin	17.4629677	23.3227115	14.8555523	17.725206
ENSG000002	PDE4C	protein_codin	0	0	0	0
ENSG000001	ZNF493	protein_codin	6.23962034	14.8164427	0	8.15614301
ENSG000001	CARD11	protein_codin	0	0	0	0
ENSG000002	ENSG000002	processed_ps	0	0	0	0
ENSG000001	RGS6	protein_codin	0	0	11.5868356	0
ENSG000000	OPHN1	protein_codin	58.4359389	57.1497512	58.5138139	28.6489172

ENSG000001[illegible]	C2CD4A	protein_codin	0	0	0	0
ENSG000001[illegible]	FCAR	protein_codin	661.857518	448.773082	219.964894	275.653252
ENSG000000	DPF1	protein_codin	0	0	0	0
ENSG000001[illegible]	DHCR7	protein_codin	0	0	0	0
ENSG000001[illegible]	TBCD	protein_codin	0	0	3.4235418	0
ENSG000001[illegible]	SPNS3	protein_codin	0	0	0	0
ENSG000002[illegible]	AFG3L1P	lncRNA	0	0	0	0
ENSG000001[illegible]	ZNF536	protein_codin	0	0	0	0
ENSG000001[illegible]	TRRAP	protein_codin	270.466733	364.739937	85.3125165	228.62825
ENSG000001[illegible]	NHERF4	protein_codin	0	0	0	0
ENSG000001[illegible]	TPRA1	protein_codin	112.733359	69.7190275	20.17971	37.9601084
ENSG000001[illegible]	HTR3A	protein_codin	0	0	0	0
ENSG000001[illegible]	ZNF140	protein_codin	0	0	0	0
ENSG000001[illegible]	CLK2	protein_codin	0	0	11.8517872	14.5477168
ENSG000001[illegible]	NEK5	protein_codin	12.0414043	4.14497444	1.27359769	1.11916322
ENSG000001[illegible]	PDLIM7	protein_codin	16.531075	14.0984186	5.11328436	8.94485065
ENSG000001	SLC26A8	protein_codin	0	0	0	0
ENSG000001[illegible]	CFAP70	protein_codin	0	0	0	0
ENSG000000[illegible]	SYNJ2	protein_codin	38.0981357	45.9410304	12.9312052	13.8806769
ENSG000001[illegible]	TRIM17	protein_codin	0	0	0	0
ENSG000001[illegible]	COX7B2	protein_codin	0	0	0	0
ENSG000001[illegible]	ANKRD35	protein_codin	0	0	0	0
ENSG000001[illegible]	DEFB107B	protein_codin	0	0	0	0
ENSG000001[illegible]	STIP1	protein_codin	0	0	0	0
ENSG000001[illegible]	IFFO2	protein_codin	0	1.54070428	0	0
ENSG000001[illegible]	MORC4	protein_codin	0	5.69710341	9.52817207	3.8170829
ENSG000001[illegible]	KRT39	protein_codin	0	0	0	0
ENSG000000[illegible]	FBXL3	protein_codin	205.081479	157.389931	119.973049	196.691497
ENSG000001[illegible]	UBE2H	protein_codin	247.157671	433.231476	209.673891	182.56142
ENSG000001[illegible]	TLR4	protein_codin	1093.45418	730.262122	391.281329	789.241879
ENSG000001[illegible]	RAB11FIP2	protein_codin	48.0410267	6.22935875	30.6493835	5.25970374
ENSG000001[illegible]	TASOR	protein_codin	6.64002073	0	0	0
ENSG000001[illegible]	MRTFA	protein_codin	176.537934	175.47818	108.455373	99.0780725
ENSG000001[illegible]	OPRL1	protein_codin	0	24.5559653	12.7011518	0
ENSG000001[illegible]	ZNF397	protein_codin	1.60259533	2.40422752	9.44361863	1.20983477
ENSG000001[illegible]	HOXA1	protein_codin	0	3.02806791	1.55286941	1.03644289
ENSG000001[illegible]	SORBS2	protein_codin	0	0	0	0
ENSG000001	PMFBP1	protein_codin	0	0	2.87953135	0
ENSG000000[illegible]	RBFOX1	protein_codin	0	0	0	0
ENSG000000[illegible]	ADAM11	protein_codin	0.90471848	1.82698176	0	0
ENSG000001[illegible]	XIAP	protein_codin	0	0	0	0
ENSG000000[illegible]	PHACTR3	protein_codin	0	18.5889035	0	0
ENSG000002[illegible]	ZNF253	protein_codin	0	0	0	4.50173402
ENSG000001[illegible]	VAT1	protein_codin	61.3648209	97.6502151	31.0035413	63.6484379

ENSG000001	TMEM18	protein_codin	0	0	0	0
ENSG000001	KCNIP2	protein_codin	0	0	0	0
ENSG000001	PLEKHA7	protein_codin	0	0	0	0
ENSG000000	YTHDC1	protein_codin	617.119409	380.989582	559.776385	482.078866
ENSG000000	DNM2	protein_codin	0	0	0	0
ENSG000001	MBD6	protein_codin	370.868428	334.44251	132.95569	161.289956
ENSG000001	RSL1D1	protein_codin	135.191214	179.088898	89.4822006	153.501367
ENSG000001	PRMT2	protein_codin	146.171284	276.035046	139.145555	101.570386
ENSG000001	FNBP1	protein_codin	67.6574642	0	108.061718	0
ENSG000001	STRN3	protein_codin	52.8800576	44.5064461	21.1250415	72.1837248
ENSG000001	ZNF44	protein_codin	0	0	10.9992325	0
ENSG000001	IFT70A	protein_codin	18.406854	21.1203006	4.61775814	6.53004808
ENSG000001	CLEC12A	protein_codin	201.395022	0	206.560445	371.315937
ENSG000001	COA4	protein_codin	35.0135272	50.0171467	22.3665388	19.053809
ENSG000001	SFXN4	protein_codin	0	22.9237373	11.4726619	0
ENSG000001	ADAMTS13	protein_codin	0	0	0	0
ENSG000001	UBAC2	protein_codin	0	10.8174314	10.1431769	25.9405396
ENSG000001	PCNX3	protein_codin	195.057293	281.274581	88.5930231	144.055621
ENSG000000	TBC1D22A	protein_codin	0	0	0	0
ENSG000001	PLGLB1	protein_codin	0	0	0	4.11692198
ENSG000001	RET	protein_codin	0	2.55625373	5.03308056	3.7262021
ENSG000001	TNFRSF14	protein_codin	189.855945	252.86351	97.6086885	97.5355313
ENSG000001	TCF7L2	protein_codin	8.46409201	9.6256958	1.99569648	0
ENSG000002	ENSG000002	processed_ps	0	0	0	0
ENSG000001	NAALADL1	protein_codin	0	0	0	0
ENSG000001	TRPM8	protein_codin	0	0	0	0
ENSG000001	THOC2	protein_codin	258.863285	266.81594	388.634748	297.646268
ENSG000001	AIDA	protein_codin	0	0	0	0
ENSG000001	RPL23A	protein_codin	0	74.4020147	42.8341456	0
ENSG000000	ADD2	protein_codin	0	0	0	0
ENSG000000	FAS	protein_codin	0	0	0	0
ENSG000002	OR4F14P	unprocessed_	0	0	0	0
ENSG000001	NDUFA5	protein_codin	0	0	8.36151754	4.08257023
ENSG000001	GBP4	protein_codin	365.911361	400.921935	296.811426	278.595104
ENSG000001	ZC3H7A	protein_codin	279.500321	293.268314	82.7428992	207.559251
ENSG000001	PRPF39	protein_codin	6.26249479	0	4.96535205	0
ENSG000001	VPS13A	protein_codin	0	0	0	0
ENSG000001	PER2	protein_codin	0	0	0	0
ENSG000001	ARHGEF4	protein_codin	0	0	0	0
ENSG000001	ZNF384	protein_codin	104.303632	53.9542685	74.016301	46.2063369
ENSG000001	SLC17A5	protein_codin	33.6978048	51.5479168	13.4890478	29.5186724
ENSG000001	MLH3	protein_codin	0	67.2709583	0	19.6916773
ENSG000001	TRPC4	protein_codin	0	0	0	0
ENSG000000	STAU2	protein_codin	7.91102114	18.3021021	0	0

ENSG000001!	OR11L1	protein_codin	0	0	0	0
ENSG0000017	LRRC20	protein_codin	0	0	0	0
ENSG000001	MXRA7	protein_codin	4.58219242	1.17256611	4.46738001	3.29152027
ENSG000001	COMMD6	protein_codin	38.6425685	74.6893588	14.7654645	25.8553289
ENSG000001	UBE2I	protein_codin	0	0	0	0
ENSG000001!	CSF2RA	protein_codin	3.0319783	0	0	2.65940161
ENSG000001	PDHA1	protein_codin	0	13.7633436	3.94189548	0
ENSG000001	TUT4	protein_codin	4.49657545	0	0	2.08910991
ENSG000001	ART3	protein_codin	0	0	0	0
ENSG000001!	SERPINA1	protein_codin	0	0	0	0
ENSG0000007	SREBF1	protein_codin	8.99156995	0	0	0
ENSG000001	CLEC9A	protein_codin	0	2.98292699	3.56058276	1.95621465
ENSG0000017	ZNF483	protein_codin	0.91166729	0	0	0
ENSG000001!	MIB2	protein_codin	0	0	0	17.5830971
ENSG000001	FAM227A	protein_codin	0	0	0	0
ENSG000002	ERCC6	protein_codin	43.388198	0	18.3921674	16.2565139
ENSG000001!	HNRNPAB	protein_codin	0	0	0	0
ENSG000001!	GARS1-DT	lncRNA	122.8604	110.684791	65.0802237	56.7960257
ENSG000001	MMS19	protein_codin	17.1295887	24.5058696	0	0
ENSG000001!	PDLIM7	protein_codin	179.971149	191.843516	95.0258823	72.9434678
ENSG000000	ADD1	protein_codin	66.3266976	6.8018257	20.1633352	0
ENSG000001	LIPF	protein_codin	0	0	0	0
ENSG000001!	C1D	protein_codin	0	0	7.88942878	0
ENSG000000!	PCBP4	protein_codin	0	0	0	0
ENSG000001	CAB39L	protein_codin	0	0	0	0
ENSG000001	DBI	protein_codin	230.709478	344.966632	111.813683	187.947427
ENSG000001	WWOX	protein_codin	0	0	6.94576025	0
ENSG000001!	SVIL	protein_codin	0	0	0	0
ENSG000001	WDR88	protein_codin	0	0	0	2.01902281
ENSG000002	MSRB3-AS1	lncRNA	0	0	0	0
ENSG000001!	STK31	protein_codin	0	0	0	0
ENSG0000017	MLF1	protein_codin	0	0	0	0
ENSG0000017	GPR160	protein_codin	32.7915639	38.0593222	16.4872883	47.8616514
ENSG000001	ZNF507	protein_codin	0	0	2.88285909	0
ENSG000001	PLS3	protein_codin	0	0	0	0
ENSG000001!	GTF2E2	protein_codin	54.5990619	83.6813849	39.6890895	23.3543833
ENSG000001	NLGN4Y	protein_codin	0	0	0	0
ENSG000002	HP	protein_codin	7.61102392	0	8.09907948	6.31611445
ENSG000001!	LINC02291	lncRNA	0	0	0	0
ENSG000000	DTNBP1	protein_codin	0	0	0	69.7506218
ENSG000002!	ENSG000002!	lncRNA	0	0	0	0
ENSG000001	LCK	protein_codin	0	0	0	0
ENSG000001	PRELID3B	protein_codin	40.1504052	43.0842064	45.3260524	58.7373782
ENSG000001	ATP5MC1	protein_codin	31.5329159	38.9679401	12.647072	18.4694541

ENSG000001	SLC25A46	protein_codin	38.0101442	45.9056442	35.3468383	31.9429066
ENSG000001	SH3PXD2A	protein_codin	0	0	0	0
ENSG000001	TRIM4	protein_codin	16.0256123	0	17.2715738	0
ENSG000001	TCAF1	protein_codin	0	0	0	0
ENSG000000	MFSD11	protein_codin	0	0	0	3.75973243
ENSG000001	ADAM15	protein_codin	95.8311829	141.261531	30.4405718	26.2726614
ENSG000001	STX16	protein_codin	167.668694	238.967207	97.6450669	91.5703813
ENSG000001	GRK6	protein_codin	10.7197492	4.74014794	0	0
ENSG000001	PORCN	protein_codin	0	0	0	0
ENSG000001	TAT	protein_codin	0	0	0	0
ENSG000001	PPIA	protein_codin	564.157962	923.868021	499.906464	464.601041
ENSG000001	ZGPAT	protein_codin	48.6966338	0	34.9204123	85.4143551
ENSG000001	ZMYND8	protein_codin	0	0	0	0
ENSG000002	GABBR1	protein_codin	26.3293553	3.20551386	3.40713109	2.57797712
ENSG000001	MIER1	protein_codin	19.1900744	4.49756297	3.54086045	1.91843741
ENSG000001	FRMD8	protein_codin	0	0	0	0
ENSG000001	DDX19B	protein_codin	34.8306397	144.167426	69.2108238	56.382463
ENSG000001	MBP	protein_codin	0	3.15959318	0	0
ENSG000001	TCF7L2	protein_codin	0	0	0	0
ENSG000001	SEMA6D	protein_codin	0	0	0	0
ENSG000001	STK39	protein_codin	62.2499617	26.7528023	21.0108436	27.9346805
ENSG000001	RTN3	protein_codin	0	0	4.98578391	12.8874081
ENSG000001	WWP2	protein_codin	0	0	36.6251737	0
ENSG000001	FN1	protein_codin	0	0	0	0
ENSG000001	ENSG000001	lncRNA	0	0	0.91323062	0
ENSG000001	MAPK8IP3	protein_codin	71.7122569	84.7243465	0	0
ENSG000001	LCOR	protein_codin	0	0	0	0
ENSG000002	TRIM13	protein_codin	0	0	0	0
ENSG000001	BTG4	protein_codin	0	0	0	0
ENSG000001	LINC01565	lncRNA	0	4.82870217	1.89132547	0.96406889
ENSG000002	NPIPB2	protein_codin	10.5911812	2.71096811	6.86804926	4.40168233
ENSG000001	SIRPA	protein_codin	1033.54486	1343.32861	429.587176	462.722058
ENSG000001	MMP23B	protein_codin	0	2.64265839	0	0
ENSG000001	SPEF2	protein_codin	0	0	0	0
ENSG000001	JPT1	protein_codin	16.8203033	9.73631963	0	6.10672464
ENSG000001	GRHL3	protein_codin	0	0	0	0
ENSG000002	LINC01967	lncRNA	1.99286762	0	0	1.95963967
ENSG000001	HNRNPF	protein_codin	26.4232806	0	60.0953781	0
ENSG000000	MYO9A	protein_codin	140.668527	43.074284	94.6039062	66.3493355
ENSG000000	JKAMP	protein_codin	0	17.5414754	0	0
ENSG000000	PHLDB1	protein_codin	0	0	0	0
ENSG000001	ARFIP1	protein_codin	0	7.6444821	9.72040064	17.02863
ENSG000001	TCF4	protein_codin	0	0	0	0
ENSG000001	CYP20A1	protein_codin	104.624099	138.992726	83.7355189	69.5082178

ENSG000001	ADD3	protein_codin	273.625653	330.032758	346.712518	305.134181
ENSG000001	LAMB3	protein_codin	0	2.61177711	2.04616833	0
ENSG000001	COL27A1	protein_codin	0	0	0	0
ENSG000001	EFCAB6	protein_codin	0	0	0	0
ENSG000000	NCDN	protein_codin	0	24.6573467	12.9992315	0
ENSG000002	NPEPL1	protein_codin	153.120324	176.452531	78.9775871	102.45541
ENSG000001	ARSA	protein_codin	0	13.8057575	0	21.0594388
ENSG000002	ARHGAP8	protein_codin	0	0	0	0
ENSG000001	PTPRT	protein_codin	0	0	1.02228188	0
ENSG000001	TJP1	protein_codin	0	0	0	0
ENSG000001	ZNF44	protein_codin	58.0321953	46.2922621	0	21.9715643
ENSG000001	LIPC	protein_codin	5.76032979	17.9851059	4.03751233	7.1265427
ENSG000001	ACSL5	protein_codin	58.1062856	167.759022	128.88443	73.7597663
ENSG000001	NOXO1	protein_codin	0	0	0	0
ENSG000001	SUOX	protein_codin	16.7630526	0	0	4.68206058
ENSG000001	PSMD12	protein_codin	32.0589853	27.3240719	7.27524449	19.4433618
ENSG000001	TRAPPC3L	protein_codin	56.4610627	32.7186556	33.1440674	25.4392674
ENSG000002	OR8B4	protein_codin	0	0	0	0
ENSG000001	ST3GAL4	protein_codin	3.17804136	3.17959195	0	0
ENSG000001	ENSG000001	lncRNA	0	0.82082528	0	0.8308015
ENSG000000	ANO2	protein_codin	3.9100586	0	0	0
ENSG000001	CHKA	protein_codin	19.0965865	22.2411345	5.82533716	1.9928119
ENSG000001	UVRAG	protein_codin	206.0799	112.74716	52.1358451	92.3647669
ENSG000001	ACMSD	protein_codin	0	0	0	0
ENSG000001	NPAS3	protein_codin	0	0	0	0
ENSG000001	RAC1	protein_codin	182.238139	229.656485	61.9811702	119.971207
ENSG000000	RNF14	protein_codin	0	0	0	0
ENSG000001	CDKL1	protein_codin	0	0	0	0
ENSG000001	PTPRA	protein_codin	78.8317523	188.727299	78.1359286	105.802129
ENSG000000	SH3YL1	protein_codin	0	0	0	0
ENSG000001	PXK	protein_codin	145.495921	199.722352	75.2532493	155.226296
ENSG000002	NPIPB5	protein_codin	3.37523257	4.56960917	1.1750087	2.43299759
ENSG000001	KATNAL2	protein_codin	0	0	0	0
ENSG000001	FBRS	protein_codin	388.792073	534.045615	136.00777	312.702956
ENSG000001	SUN5	protein_codin	0	0	0	0
ENSG000001	NF1	protein_codin	0	0	0	0
ENSG000001	ZNF527	protein_codin	0	0	0	0
ENSG000000	GTF3C1	protein_codin	0	187.521938	255.845289	232.395323
ENSG000001	SUPT20H	protein_codin	0	0	0	0
ENSG000000	RNH1	protein_codin	0	0	0	0
ENSG000001	ACBD7	protein_codin	0	0	0	0
ENSG000001	CDCA7L	protein_codin	0	0	0	0
ENSG000001	CENPL	protein_codin	0	0	0	0
ENSG000000	CDK11A	protein_codin	0	33.6954948	0	18.5405848

ENSG000001	SLC39A1	protein_codin	76.5386348	96.8700098	49.3516678	55.0457253
ENSG000001	ENTPD4	protein_codin	14.1169996	64.9209459	18.966113	0
ENSG000000	FGFR1	protein_codin	0	0	4.44635844	0
ENSG000001	RBM38	protein_codin	207.116912	384.221434	154.712251	163.282306
ENSG000001	HMGA2-AS1	lncRNA	5.09099162	2.18008243	8.02116901	2.08391675
ENSG000001	FRMD6	protein_codin	0	0	0	0
ENSG000000	DCX	protein_codin	0	0	0	0
ENSG000001	CDC16	protein_codin	109.369756	64.4799232	45.2536562	0
ENSG000000	FGFR2	protein_codin	0	0	0	0
ENSG000001	CDAN1	protein_codin	14.7829893	40.7577045	0	9.07142286
ENSG000000	UBA5	protein_codin	2.05659962	10.3888924	9.43240472	2.75892598
ENSG000001	AKAP9	protein_codin	0	0	0	0
ENSG000001	RANGAP1	protein_codin	69.3405299	152.193748	81.1549978	47.5894811
ENSG000001	G3BP1	protein_codin	0	0	0	0
ENSG000002	ENSG000002	lncRNA	0	0.94460542	3.80207025	0
ENSG000001	CEMIP	protein_codin	0	0	0	0
ENSG000001	SDAD1	protein_codin	120.074636	103.772473	14.41444	40.7101188
ENSG000001	LHX8	protein_codin	0	0	0	0
ENSG000000	AMPH	protein_codin	0	0	0	0
ENSG000000	MED24	protein_codin	0	0	0	0
ENSG000001	CXADR	protein_codin	0	0	0	0
ENSG000001	SMOC2	protein_codin	0	0	0	0
ENSG000001	RBCK1	protein_codin	20.8371525	13.9272185	5.95476478	10.5210676
ENSG000001	JAML	protein_codin	528.115128	1027.34026	220.460545	495.78538
ENSG000001	TMPRSS11F	protein_codin	0	0	0	0
ENSG000001	OR2B4P	transcribed_u	0	0	0	0
ENSG000001	NUMB	protein_codin	0	0	0	0
ENSG000001	PXDNL	protein_codin	0	1.87660456	0	0
ENSG000000	JPH4	protein_codin	13.0605566	9.72480465	2.61455166	2.92718617
ENSG000001	EEF1A1	protein_codin	11.7271409	46.8634871	0	0
ENSG000001	PAOX	protein_codin	0	0	0	0
ENSG000001	NCAPG2	protein_codin	0	3.7258753	19.6668959	0
ENSG000001	SPACA9	protein_codin	0	0	1.81825555	2.95753965
ENSG000001	GALNT6	protein_codin	0	33.5330087	0	0
ENSG000001	ZNF770	protein_codin	89.3426302	115.891772	74.8565514	74.3088808
ENSG000001	ZNF614	protein_codin	0	0	0	0
ENSG000000	PLD1	protein_codin	16.2170233	0	0	16.2269722
ENSG000000	SH3BP2	protein_codin	0	0	0	0
ENSG000001	MYO5A	protein_codin	0	0	0	0
ENSG000001	PAK1	protein_codin	18.4678591	48.8000389	0	25.6804031
ENSG000001	PLEC	protein_codin	0	234.343017	111.768968	56.3219343
ENSG000002	ZNF876P	lncRNA	2.4032725	0	0	0
ENSG000001	KPNA5	protein_codin	21.1368974	0	33.678484	0
ENSG000001	UPF2	protein_codin	0	6.98090156	0	0

ENSG000001	AREL1	protein_codin	175.139681	206.91551	21.1212194	138.229677
ENSG000000	PICALM	protein_codin	0	52.0237022	13.7421995	35.8357868
ENSG000000	PUS7	protein_codin	0	49.6086637	34.0453793	0
ENSG000001	RXRA	protein_codin	6.1362192	17.4746849	21.0666866	21.3539189
ENSG000001	TMEM91	protein_codin	0	0	0	0
ENSG000001	BTN3A2	protein_codin	22.5877895	112.165441	0	19.6662636
ENSG000001	ORAI2	protein_codin	70.5756957	232.779635	102.049142	37.7477477
ENSG000001	SPRED2	protein_codin	22.7272691	22.5581534	3.97659201	12.0999243
ENSG000001	ODAD3	protein_codin	0	0	0	0
ENSG000001	LSS	protein_codin	0	0	0	6.20884355
ENSG000001	ARIH2	protein_codin	113.542909	126.037298	47.3642084	48.4872384
ENSG000001	PITPNM1	protein_codin	211.585991	190.686773	118.015782	0
ENSG000000	ACOX3	protein_codin	28.0988505	41.0857164	0	5.51074859
ENSG000001	RAB19	protein_codin	0	0	0	0
ENSG000001	CARD11	protein_codin	0	0	6.49689272	0
ENSG000000	IGBP1	protein_codin	372.41767	471.964126	212.469638	251.374314
ENSG000001	RAP1A	protein_codin	463.362932	453.761198	300.024537	337.69415
ENSG000001	ATF7IP2	protein_codin	11.1437209	0	0	0
ENSG000001	MPG	protein_codin	73.7167684	92.0205954	34.169363	51.35256
ENSG000000	DLL3	protein_codin	0	0.93912968	0	0
ENSG000001	PTPRD	protein_codin	0	0	0	0
ENSG000001	TBC1D7	protein_codin	0	0	0	0
ENSG000001	HYLS1	protein_codin	0	0	0	0
ENSG000001	NRBP1	protein_codin	1.41231185	0	0	0
ENSG000001	MYOM1	protein_codin	11.6722428	16.1255526	3.66328273	0
ENSG000001	SEMA4D	protein_codin	0	0	0	0
ENSG000001	THRB	protein_codin	0	32.7162188	0	9.80991081
ENSG000001	ARHGEF26	protein_codin	0	0	0	0
ENSG000001	NUDT10	protein_codin	3.17599517	3.1416651	3.12078111	0
ENSG000001	SOWAHC	protein_codin	26.3942288	30.8343688	16.2664748	27.9155432
ENSG000001	UBE2C	protein_codin	0	4.94537189	0	3.12092619
ENSG000001	ZNF140	protein_codin	6.44395987	0	0.4526684	0
ENSG000001	ASPH	protein_codin	23.6678303	0	0	0
ENSG000002	EML6	protein_codin	0	13.6954223	27.2416954	1.48260592
ENSG000000	NEDD4L	protein_codin	0	0	0	10.4464704
ENSG000001	WDR45	protein_codin	0	0	0	0
ENSG000001	RPS26	protein_codin	7.30821953	0	0	0
ENSG000000	FAM168A	protein_codin	198.181142	318.15063	161.813166	146.580511
ENSG000001	H3C4	protein_codin	9.96018645	39.0372899	69.9341515	31.5571072
ENSG000000	BAX	protein_codin	0	0	0	3.21119857
ENSG000001	GPI	protein_codin	167.650317	131.463056	40.811527	103.530893
ENSG000000	SPATA20	protein_codin	40.3780327	48.5629572	6.77832334	0
ENSG000000	BCKDHB	protein_codin	7.83165114	15.8213605	0	7.50192162
ENSG000001	STOML2	protein_codin	48.2020477	61.419821	33.154342	6.32374673

ENSG000001(	PIGR	protein_codin	0.97689926	0.87017858	0	0
ENSG000002	ENSG000002	lncRNA	0	0	0	1.59661431
ENSG000001	ADPRHL1	protein_codin	0	1.18720449	0	1.78350229
ENSG000001	GABRA2	protein_codin	0	0	0	0
ENSG000001(	PLD3	protein_codin	7.26898045	7.16923575	0	0
ENSG000001	GMNN	protein_codin	0	0	0	18.4421495
ENSG000000	PPIE	protein_codin	18.9126072	52.4393786	0	0
ENSG000001	AADACL2	protein_codin	0	0	0	0
ENSG000001	ADAM33	protein_codin	1.70544648	0	0	0
ENSG000001	ZNG1A	protein_codin	0	0	0	0
ENSG000001	CD34	protein_codin	6.55375186	15.8361879	8.27382175	0
ENSG000001	SAA1	protein_codin	0	0	0	0
ENSG000001	ENSA	protein_codin	0	0	0	0
ENSG000002	H2BC7	protein_codin	36.6087578	63.5721814	77.4813858	28.7991776
ENSG000001	OSCAR	protein_codin	0	0	0	0
ENSG000000	CHRD	protein_codin	0	0	0	0
ENSG000001(	FABP7	protein_codin	0	0	0	0
ENSG000001	CCDC25	protein_codin	0	0	0	0
ENSG000001	LEKR1	protein_codin	0	0.99851992	0	0
ENSG000001	C6orf132	protein_codin	0	0	0	0
ENSG000001(	RSPO1	protein_codin	0	0	0	0
ENSG000001	MAN1A2	protein_codin	844.507802	1196.6871	626.548779	640.690513
ENSG000001	ZNF300P1	transcribed_u	0	0	0	0
ENSG000002	Metazoa_SRP	misc_RNA	0	0	0	0
ENSG000001	WFDC11	protein_codin	0	0	0	0
ENSG000001(	MEGF6	protein_codin	0	0	0	0
ENSG000001	COG6	protein_codin	0	0	0	0
ENSG000001	SON	protein_codin	1798.02344	3103.66022	1511.389	1645.05638
ENSG000001	INS-IGF2	protein_codin	0	0	0	0
ENSG000000	SPATA7	protein_codin	0	3.03181116	10.096184	0
ENSG000001	ZNF772	protein_codin	5.92008112	12.886541	0	11.9375321
ENSG000002	TSGA13	protein_codin	0	0.81568708	0	0
ENSG000001(	ADAMTS13	protein_codin	0	0	0	0
ENSG000001	APOLD1	protein_codin	0	0	2.67548295	0
ENSG000001	GABRG2	protein_codin	0	0	0	0
ENSG000000	PTGER3	protein_codin	0	0	0	0
ENSG000001	DTNB	protein_codin	0	6.84150324	10.7652705	7.04964175
ENSG000001	ADGRG2	protein_codin	0	0	0	0
ENSG000001	PEX14	protein_codin	53.5478457	56.3787354	16.2598844	48.0985918
ENSG000001	ABCC11	protein_codin	0	0	0	0
ENSG000001	SLC30A10	protein_codin	0	0	0	0
ENSG000001	ARC	protein_codin	0	1.65810283	0.94812951	0.7916483
ENSG000001	NRARP	protein_codin	9.21130662	5.30901706	1.24111892	7.21171181
ENSG000001(	NAALADL1	protein_codin	0	0	0	0

ENSG000000	KDM1A	protein_codin	35.0946756	0	0	0
ENSG000001	OSCP1	protein_codin	0	0	0	0
ENSG000000	GANAB	protein_codin	443.299049	502.031667	262.537614	287.308366
ENSG000001	KCNIP2	protein_codin	0	0	1.71684579	0
ENSG000001	ARHGEF12	protein_codin	330.923755	568.282925	583.911085	200.886111
ENSG000000	SESN1	protein_codin	31.6994743	20.0616333	7.54564249	17.7409149
ENSG000001	JTB	protein_codin	18.4933271	0	5.27903661	2.41705543
ENSG000001	ATN1	protein_codin	100.794213	136.680419	37.1135905	55.7108408
ENSG000001	FDPS	protein_codin	0	0	0	0
ENSG000001	BDNF	protein_codin	0	0	4.06544277	0
ENSG000001	MAGEA1	protein_codin	0	0	0	0
ENSG000001	TMEM259	protein_codin	228.323164	265.550352	45.6062297	156.581691
ENSG000000	PGC	protein_codin	0	0	0	0
ENSG000002	ENSG000002	lncRNA	0	0	0	0
ENSG000001	HNRNPA2B1	protein_codin	0	0	0	0
ENSG000001	ELP5	protein_codin	0	0	0	0
ENSG000001	FLVCR1-DT	lncRNA	0	0	0	0
ENSG000001	SEM1	protein_codin	0	0	0	0
ENSG000001	SEPTIN10	protein_codin	0	0	0	0
ENSG000001	S100PBP	protein_codin	15.8638221	4.29894304	4.68089723	6.90668344
ENSG000001	PPP4R2	protein_codin	213.369471	126.109342	123.18993	148.10118
ENSG000000	FOLH1	protein_codin	0	0	0	0
ENSG000001	RSPO3	protein_codin	0	3.56190694	0	3.56299298
ENSG000001	ATP5F1C	protein_codin	131.804982	171.74435	121.108213	121.985303
ENSG000001	BTN2A2	protein_codin	35.636487	49.4461329	25.5264074	16.9565569
ENSG000000	MYO16	protein_codin	0	0	0	0
ENSG000001	CNNM1	protein_codin	0	0.89218778	0	0
ENSG000001	NUDT1	protein_codin	18.4531467	28.8343547	20.6649067	3.49003637
ENSG000000	MYNN	protein_codin	0	0	11.998073	0
ENSG000001	SANBR	protein_codin	0	13.2563622	0	7.84994793
ENSG000001	SIAH1	protein_codin	55.6690191	42.8494966	16.201612	21.3674576
ENSG000001	ZNF790	protein_codin	3.16760586	5.37315952	3.70350955	0
ENSG000001	RORC	protein_codin	0	0	0	0
ENSG000000	ATP2B4	protein_codin	0	1.11596254	0	0
ENSG000001	HNRNPH1	protein_codin	27.1597122	45.8910576	34.0333482	29.2210709
ENSG000001	SLC39A8	protein_codin	0	0	0	0
ENSG000001	APBB3	protein_codin	0	0	0	0
ENSG000001	XXYLT1	protein_codin	1.81005002	2.73855362	0	0
ENSG000001	HVCN1	protein_codin	0	6.87098692	0	4.06886583
ENSG000001	MAMSTR	protein_codin	0	0	0	0
ENSG000001	NDRG4	protein_codin	0	0	0	0
ENSG000001	TMEM217	protein_codin	0	0	0	0
ENSG000001	CRB3	protein_codin	0	0	0	0
ENSG000001	PIK3R4	protein_codin	74.9393799	109.589215	54.2588986	87.0246958

ENSG0000007	PPP1R12B	protein_codin	0	0	10.8879659	29.4425908
ENSG000001	PIWIL2	protein_codin	0	0	0	0
ENSG000001	SLC26A5	protein_codin	0	0	0	0
ENSG000001	PCYT1B	protein_codin	12.1397836	50.6936189	26.2958301	35.2676591
ENSG000001	NACA	protein_codin	1.36064259	1.39106665	0	1.40483712
ENSG000001	PBRM1	protein_codin	138.436284	93.9245299	25.1401111	174.692409
ENSG000001	TNNT1	protein_codin	0	0	0	0
ENSG000001	TAF7L	protein_codin	3.05366912	0	0	1.06496661
ENSG0000007	APBB1IP	protein_codin	59.446445	55.7846559	24.772534	36.0907206
ENSG000001	CCDC88B	protein_codin	440.092813	648.768502	359.422388	251.317199
ENSG000001	MAGEB10	protein_codin	0	0	0	0
ENSG000001	EDRF1	protein_codin	0	0	58.5819807	0
ENSG000001	ARL4A	protein_codin	22.8678619	15.0541767	11.5554486	15.5900089
ENSG000000	ITGAL	protein_codin	1014.66082	2045.8143	476.71463	566.033521
ENSG000001	RNF146	protein_codin	0	0	12.7105981	45.4107526
ENSG000000	SMARCD3	protein_codin	88.3539855	197.76737	0	94.8501234
ENSG000001	SPTBN1	protein_codin	321.276574	625.869397	465.019	127.262608
ENSG000002	RTEL1	protein_codin	0	9.50524549	0	0
ENSG000002	HBM	protein_codin	0	0	0	0
ENSG000001	PPP1R26	protein_codin	10.8948061	32.3433102	18.9884882	0
ENSG000001	NRG1	protein_codin	0	0	0	7.26436341
ENSG000001	ARMCX2	protein_codin	0	0	0	0
ENSG000001	SMAD2	protein_codin	0	107.239654	0	91.3614652
ENSG000001	BLTP3B	protein_codin	59.8911847	38.5058992	35.968478	19.4936249
ENSG000001	TBC1D9B	protein_codin	7.7681945	23.5868923	0	18.1584409
ENSG000001	B4GALT2	protein_codin	0	0	3.74459332	0
ENSG000001	TMEM71	protein_codin	0	20.2152546	0	29.6684125
ENSG0000007	ACADVL	protein_codin	283.707277	574.841525	190.819347	187.611785
ENSG000000	MYLIP	protein_codin	126.309331	130.685621	69.3713846	90.7609145
ENSG000001	BCL11A	protein_codin	0	0	0	4.45789165
ENSG000001	GOLGA4	protein_codin	0	0	0	0
ENSG000001	NHEJ1	protein_codin	40.6929239	52.3841326	33.832664	17.0516752
ENSG000001	C10orf90	protein_codin	0	0	0	0
ENSG000000	VEZT	protein_codin	2.82134919	6.52088928	9.37981716	3.0604238
ENSG000001	CACNA2D1	protein_codin	0	0	0	0
ENSG000001	TNPO2	protein_codin	59.2543325	57.6031036	27.327063	42.4347366
ENSG000001	TNFRSF10C	protein_codin	23.9393795	59.419398	6.95563754	18.3497976
ENSG000002	ATP10A	protein_codin	2.22412433	8.95248704	0	0
ENSG000001	PCED1A	protein_codin	0	0	0	6.41814804
ENSG000002	TNFRSF25	protein_codin	0	3.95617745	0	0
ENSG000002	PCDHA7	protein_codin	0	0	0	0
ENSG000001	BICD2	protein_codin	345.672983	387.845203	161.061014	166.986421
ENSG000001	GYPC	protein_codin	52.5103781	38.1666914	21.6019122	19.4915497
ENSG000001	FIGNL1	protein_codin	0	16.7571907	0	7.89058026

ENSG000001	RLIG1	protein_codin	0	15.2755121	3.49152654	0
ENSG000001	SASH3	protein_codin	820.403264	1165.84979	468.785653	548.669985
ENSG000000	IFFO1	protein_codin	87.9432947	160.668779	26.7304503	60.3961716
ENSG000001	IL27	protein_codin	0	1.80907504	1.04331725	0.85005873
ENSG000001	NBR2	lncRNA	0	19.1351543	0	0
ENSG000001	PARVG	protein_codin	388.329407	577.168398	202.540517	234.200342
ENSG000000	EIF4G3	protein_codin	0	0	0	49.7202839
ENSG000000	CREM	protein_codin	0	0	10.7609685	0
ENSG000001	NEK11	protein_codin	0	0	0	0
ENSG000001	POTEE	protein_codin	8.43670821	0	0	10.6777493
ENSG000001	SLC8A3	protein_codin	17.0133168	110.572176	32.8111038	32.2440457
ENSG000001	ABCD4	protein_codin	24.5953705	39.8408508	6.00193614	10.5822235
ENSG000001	SLITRK4	protein_codin	0	0	0	0
ENSG000001	ZNF708	protein_codin	17.7817544	16.4111897	33.6523531	23.3274155
ENSG000001	NCL	protein_codin	29.1486916	0	640.497495	0
ENSG000000	CDK11A	protein_codin	2.09393439	0	0	0
ENSG000000	RAB18	protein_codin	0	0	0	0
ENSG000000	GPM6B	protein_codin	0	0	0	0
ENSG000000	PTBP1	protein_codin	0	300.089238	0	108.057906
ENSG000001	H2BC12	protein_codin	107.32208	275.651473	155.045401	179.250185
ENSG000001	ADAM15	protein_codin	0	0	0	0
ENSG000001	IGF2R	protein_codin	1418.31825	1377.26391	487.074485	725.896531
ENSG000001	TXNDC11	protein_codin	0	0	88.4897921	0
ENSG000001	ZNF585A	protein_codin	0	8.79213263	2.36466977	2.15051993
ENSG000001	ARG1	protein_codin	0	0	0	0
ENSG000001	CLCC1	protein_codin	0	0	41.7383448	0
ENSG000001	CILK1	protein_codin	5.36079779	0	0	0
ENSG000001	SPINK14	protein_codin	0	0	0	0
ENSG000001	PICK1	protein_codin	27.275667	36.993865	0	16.9549603
ENSG000001	CALM1	protein_codin	399.660867	521.213735	229.189435	283.336285
ENSG000001	PAQR6	protein_codin	0	6.68814344	0	1.4125172
ENSG000001	KLRG1	protein_codin	0	0	3.22002043	4.35072758
ENSG000001	RNF19B	protein_codin	245.084386	257.103569	101.503297	138.933765
ENSG000001	SCRIB	protein_codin	50.828186	22.5957482	17.1970884	0
ENSG000001	MFNG	protein_codin	105.680988	200.910028	79.3635909	90.2051475
ENSG000001	GUCA2A	protein_codin	0	0	0	0
ENSG000002	ZNF254	protein_codin	0	14.1659332	0	0
ENSG000001	ADPRH	protein_codin	7.94390449	14.4989473	15.6016363	0
ENSG000001	ZBTB14	protein_codin	0	0	0	0
ENSG000001	CHD4	protein_codin	43.5941975	204.630119	0	0
ENSG000002	AHSA2P	lncRNA	5.57680937	12.8563969	3.23060032	0
ENSG000001	MED27	protein_codin	0	0	0	0
ENSG000001	DMD	protein_codin	0	0	0	0
ENSG000000	ELN	protein_codin	0	0	0	0

ENSG000001	CAVIN1	protein_codin	2.93483169	11.7470744	6.9420334	4.8369945
ENSG000001	PEX2	protein_codin	14.0098728	25.1374131	13.6959903	17.6204419
ENSG000001	OSBPL1A	protein_codin	0	0	0	18.0493028
ENSG000000	RASSF1	protein_codin	10.1280764	0	0	0
ENSG000002	ENSG000002	lncRNA	0	0	0	0
ENSG000002	ENSG000002	lncRNA	0	0	0	0
ENSG000001	CAPN10	protein_codin	0	0	0	0
ENSG000001	RAB4B	protein_codin	72.6014817	102.845201	26.8953805	37.3347769
ENSG000001	SLC30A6	protein_codin	0	0	0	0
ENSG000001	RNF38	protein_codin	16.9653944	5.35212496	0	0
ENSG000001	CFAP43	protein_codin	0	0	0	0
ENSG000002	NUP210P1	lncRNA	0	0	0	0
ENSG000001	LMO7	protein_codin	14.067524	0	0	0
ENSG000001	HNRNPF	protein_codin	137.325869	210.717475	58.4450838	68.1615696
ENSG000001	MOB3A	protein_codin	1229.14773	1407.95414	373.152407	755.22564
ENSG000001	DCTD	protein_codin	40.5032322	68.6906526	30.2090459	43.6321833
ENSG000001	PRODH	protein_codin	0	0	2.1175485	0
ENSG000001	ECE1	protein_codin	85.3004245	42.3770345	49.177994	65.8751768
ENSG000001	ANK2	protein_codin	0	0	0	0
ENSG000000	OSBPL6	protein_codin	0	1.34507966	0	0
ENSG000001	AGTPBP1	protein_codin	774.14173	470.459494	427.237988	643.695153
ENSG000002	ZHX1-C8orf7	protein_codin	6.78695427	2.33110665	6.33855263	0
ENSG000001	ELAVL4	protein_codin	0	0	0	0
ENSG000001	ATP2A1	protein_codin	0	0	0	0
ENSG000001	TRIM38	protein_codin	753.853493	764.109133	468.450068	575.962901
ENSG000001	ITPR1	protein_codin	298.030735	354.878161	288.027713	426.948698
ENSG000001	UBXN11	protein_codin	0	215.737263	0	0
ENSG000001	ACSL6	protein_codin	0	0	1.34572726	0
ENSG000000	ADIPOR2	protein_codin	165.526322	271.650208	157.36398	141.124522
ENSG000001	LRBA	protein_codin	278.745806	138.427593	115.775658	154.747839
ENSG000000	KITLG	protein_codin	0	0	0	0
ENSG000001	NRP2	protein_codin	0	0	0	1.92327186
ENSG000001	ZGPAT	protein_codin	66.611911	51.0321899	62.7724065	6.77239898
ENSG000001	OCRL	protein_codin	63.2606087	63.1143795	24.0263816	48.6805925
ENSG000000	SPAG9	protein_codin	374.613598	284.889961	234.633999	36.3260076
ENSG000001	MCRS1	protein_codin	0	0	0	0
ENSG000002	ENSG000002	processed_ps	0	0	0	0
ENSG000001	NELL1	protein_codin	0	0	0	0
ENSG000001	XPNPEP3	protein_codin	41.8376454	37.1739113	28.8552809	38.9268931
ENSG000001	PSMD12	protein_codin	36.1631589	21.7887247	9.00421618	24.46522
ENSG000001	SPMIP10	protein_codin	0	0	0	0
ENSG000001	SPOCK3	protein_codin	0	0	0	10.0990862
ENSG000002	MYBPHL	protein_codin	0	0	0	0
ENSG000001	DYNLRB1	protein_codin	106.828136	197.467687	42.0864609	84.5677502

ENSG000001	MCU	protein_codin	0	0	0	0
ENSG000001	VPS13B	protein_codin	328.862366	216.524991	132.833199	183.488355
ENSG000001	GM2A	protein_codin	324.67471	499.497142	151.441923	507.761894
ENSG000001	ZDHHC9	protein_codin	6.30705901	5.96970385	1.46420689	6.61687953
ENSG000001	ABTB3	protein_codin	2.36483757	0	0	0
ENSG000001	TCL6	lncRNA	0	0.84991826	0	0
ENSG000001	CEPT1	protein_codin	20.1795855	56.5950809	0	0
ENSG000001	PWWP3B	protein_codin	0	0	0	0
ENSG000001	TOP3B	protein_codin	29.4534681	49.7366424	19.7489447	16.2938972
ENSG000001	ZMYM6	protein_codin	145.500096	55.0883185	74.2389405	61.2152409
ENSG000001	RAET1E	protein_codin	0	1.01653664	0	0
ENSG000001	MPV17	protein_codin	4.0142863	5.14954807	1.09842207	1.21457606
ENSG000001	UBE2D3	protein_codin	0	0	0	0
ENSG000001	BCL11B	protein_codin	9.03277161	21.4548221	2.30478357	0
ENSG000001	PTPA	protein_codin	33.881399	0	0	0
ENSG000001	DOK3	protein_codin	7.84167907	2.9825402	0	2.32340088
ENSG000001	WFDC8	protein_codin	0	0	0	0
ENSG000000	C3orf18	protein_codin	0	0	0	0
ENSG000001	ZFYVE9	protein_codin	0	0	3.1357401	0
ENSG000001	TRAPPC11	protein_codin	0	98.6550253	0	0
ENSG000001	SFPQ	protein_codin	1502.22445	1572.04786	714.931551	1033.37383
ENSG000001	TMEM217	protein_codin	0	0	0	0
ENSG000001	HHIPL1	protein_codin	0	0	0	0
ENSG000001	CDK16	protein_codin	75.0153417	80.7736185	34.0600098	18.0547193
ENSG000001	EXOC7	protein_codin	0	0	0	0
ENSG000001	DCHS2	protein_codin	0	0	0	0
ENSG000001	IRF5	protein_codin	225.254435	338.225852	122.289505	163.754032
ENSG000001	CDC42SE1	protein_codin	620.851042	626.737601	359.55916	267.426584
ENSG000000	DZANK1	protein_codin	0	0	0	0
ENSG000001	TBC1D8B	protein_codin	0	0	9.25486049	3.11041504
ENSG000000	RC3H2	protein_codin	280.169904	0	0	208.721242
ENSG000001	MAP1LC3C	protein_codin	0	0	0	0
ENSG000000	SMARCA2	protein_codin	0	0	0	0
ENSG000000	KCNQ2	protein_codin	0	0	0	0
ENSG000001	COL9A1	protein_codin	0	0	0	0
ENSG000001	ZNF23	protein_codin	0	0	0	0
ENSG000001	ZBTB20	protein_codin	0	0	241.007098	0
ENSG000001	INKA2	protein_codin	163.568214	191.744524	78.7394847	54.648863
ENSG000000	FKBP5	protein_codin	1285.6954	1229.64911	257.178806	475.590287
ENSG000001	GRB10	protein_codin	0	0	0	0
ENSG000002	DBNDD2	protein_codin	0	0	0	0
ENSG000001	SMARCAL1	protein_codin	0	74.5275393	0	0
ENSG000001	REPS2	protein_codin	60.6958254	97.6270349	18.4627011	0
ENSG000001	PAOX	protein_codin	0	0	0	0

ENSG000001	CSF1	protein_codin	0	0	0	0
ENSG000001	ERFE	protein_codin	0	0	0	0
ENSG000001	GFPT1	protein_codin	0	76.2511011	9.73502628	10.6730263
ENSG000001	APAF1	protein_codin	0	0	0	0
ENSG000001	SRPK2	protein_codin	5.48814218	14.3485705	7.48073712	28.8304714
ENSG000001	NUDT13	protein_codin	0	3.34270208	0	5.91532328
ENSG000001	ATP13A1	protein_codin	166.569672	225.875856	108.977324	110.43665
ENSG000001	HDGF	protein_codin	436.878504	980.319992	276.734514	423.442668
ENSG000001	TNIK	protein_codin	0	0	0	0
ENSG000001	ZNF248	protein_codin	16.0209044	0	0	0
ENSG000001	GALP	protein_codin	0	0	0	0
ENSG000001	GET3	protein_codin	112.865352	166.292034	75.5567111	89.5872331
ENSG000002	ENSG000002	lncRNA	5.89291815	0	8.88326951	0
ENSG000000	MYCBP2	protein_codin	0	0	0	0
ENSG000001	DLK2	protein_codin	0	0	1.02862939	0
ENSG000000	FAS	protein_codin	22.9642082	10.0125787	0	7.29073662
ENSG000001	TCAF2	protein_codin	0	0	0	0
ENSG000001	SYNJ1	protein_codin	0	0	0	0
ENSG000000	RTF2	protein_codin	45.651795	25.6703678	43.6605287	31.0915777
ENSG000001	ADGRE5	protein_codin	1456.86655	1276.33993	356.012878	746.047431
ENSG000001	LAGE3	protein_codin	5.83367969	13.548723	1.53055776	6.56207068
ENSG000001	MDGA2	protein_codin	0	0	0	0
ENSG000001	ENTR1	protein_codin	9.77245014	9.19208837	0	0
ENSG000001	PRAMEF8	protein_codin	0	0	0	0
ENSG000001	IFNA10	protein_codin	0	0	0	0
ENSG000001	SIGLEC10	protein_codin	37.2040949	0	0	0
ENSG000001	RTN4	protein_codin	244.484501	0	0	176.417491
ENSG000001	OR5B17	protein_codin	0	0	0	0
ENSG000001	CGB3	protein_codin	0	0	0	0
ENSG000001	RPRD1A	protein_codin	0	0	0	0
ENSG000001	ANAPC11	protein_codin	0	0	0	0
ENSG000001	RICTOR	protein_codin	0	180.867846	134.546624	184.311376
ENSG000001	SMC4	protein_codin	0	0	0	0
ENSG000001	MFN1	protein_codin	0	31.5752779	12.2823122	0
ENSG000001	CCRL2	protein_codin	0	0	0	0
ENSG000001	AKNAD1	protein_codin	0	0	0	0
ENSG000001	ERGIC3	protein_codin	5.05412501	42.1133329	0	18.2064349
ENSG000001	CEACAM3	protein_codin	3.04121481	0	2.96134956	8.5951565
ENSG000001	NCR1	protein_codin	0	3.53199427	0	0
ENSG000001	CYYR1-AS1	lncRNA	0	0	0	0
ENSG000000	MVP	protein_codin	620.624829	593.409111	317.637958	235.385762
ENSG000000	EYA2	protein_codin	0	0	0	0
ENSG000001	LINC01560	lncRNA	6.26228	10.2396797	1.43991502	2.06980082
ENSG000001	ABHD11	protein_codin	0	0	0	0

ENSG000001	CCR3	protein_codin	0	0	0	0
ENSG000001	GIP	protein_codin	0	0	0	0
ENSG000000	USP33	protein_codin	0	146.605825	0	0
ENSG000001	C7orf50	protein_codin	17.4235412	0	14.3597059	0
ENSG000001	ACSL3	protein_codin	0	0	0	0
ENSG000001	CEP350	protein_codin	19.6549042	2.35478274	4.14674152	5.26916336
ENSG000001	SLC29A2	protein_codin	0	0	2.05344248	0
ENSG000001	MOV10	protein_codin	0	13.4250803	8.2421432	0
ENSG000001	RABGAP1L	protein_codin	17.174932	4.75574662	16.2056931	7.01831213
ENSG000001	FRMD4A	protein_codin	0	0	0	0
ENSG000000	SMC1B	protein_codin	0	0	0	0
ENSG000001	IGKV7-3	IG_V_pseudog	0	0	0	0
ENSG000001	CERT1	protein_codin	0	0	0	0
ENSG000002	HOMEZ	protein_codin	36.4330988	79.8639829	14.1696065	35.1709929
ENSG000001	TMEM229B	protein_codin	0	0	23.0268906	0
ENSG000001	TENT4B	protein_codin	8.45739728	0	3.48972273	0
ENSG000001	RABL6	protein_codin	0	0	0	0
ENSG000001	MBNL1	protein_codin	20.0362183	0	24.930297	42.1589239
ENSG000001	ECE2	protein_codin	0	0	0	0
ENSG000001	SSBP3	protein_codin	0	15.9826574	16.5003068	19.1668457
ENSG000001	STRN3	protein_codin	72.4720331	42.628631	54.0030174	5.81437887
ENSG000000	NFE2L1	protein_codin	0	0	0	0
ENSG000001	ACIN1	protein_codin	0	1.68261	5.18352332	1.74776762
ENSG000001	USP6	protein_codin	0	0	0	0
ENSG000002	ARPIN	protein_codin	121.628089	136.875737	30.2963485	62.8439005
ENSG000001	ARID5A	protein_codin	0	0	0	0
ENSG000001	ZNF451	protein_codin	0	0	0	0
ENSG000001	COPS7B	protein_codin	0	0	0	4.07533216
ENSG000001	ZNF43	protein_codin	0	16.2566941	18.7506995	0
ENSG000001	KIR2DL4	protein_codin	0	0	0	0
ENSG000001	HNRNPA1L2	protein_codin	26.9380138	20.6427093	26.6483478	19.6040662
ENSG000001	QRICH1	protein_codin	83.1341993	17.83763	38.2596647	50.3348338
ENSG000001	POP5	protein_codin	14.1992798	25.8304056	14.2868786	20.6410753
ENSG000001	TMEM79	protein_codin	2.98036136	0	0	0
ENSG000001	ZNF74	protein_codin	0	0	4.50027772	5.41369768
ENSG000001	TOR4A	protein_codin	163.989151	215.953316	57.0060894	110.819004
ENSG000001	DTHD1	protein_codin	0	0	0	0
ENSG000002	N4BP2L2	protein_codin	0	0	0	0
ENSG000001	ARMH1	protein_codin	14.2126847	15.2586397	3.61639388	11.8175841
ENSG000001	ZNF92	protein_codin	0	5.23883205	2.53860147	12.1894597
ENSG000001	PCDH1	protein_codin	0	0	0	2.04160198
ENSG000001	FIBP	protein_codin	30.7997551	70.765885	18.4234249	17.8792372
ENSG000001	BET1	protein_codin	7.15207148	6.8009698	2.03566117	7.38854015
ENSG000001	PLN	protein_codin	0	0	0	0

ENSG0000010	RIMKLB	protein_codin	0	2.15092074	0	0
ENSG0000008	TRPM3	protein_codin	0	0	0	0
ENSG0000013	GPALPP1	protein_codin	11.0880262	13.7747429	5.38673387	10.0235706
ENSG0000014	ZFYVE27	protein_codin	0	0	0	0
ENSG0000018	RHD	protein_codin	0	0	0	0
ENSG0000017	ADGRG2	protein_codin	0	0	0	0
ENSG0000019	SPOCK3	protein_codin	0	0	0	0
ENSG0000007	DHRS9	protein_codin	10.2227317	2.90037998	0	0
ENSG0000015	SYCP2	protein_codin	0	0	0	0
ENSG0000006	FGFR2	protein_codin	0	0	0	0
ENSG0000014	CIZ1	protein_codin	0	0	0	0
ENSG000001	FXR1	protein_codin	0	4.60079626	4.51762358	6.38826803
ENSG000001	APBB3	protein_codin	0	0	0	0
ENSG0000018	SETD3	protein_codin	0	0	0	0
ENSG0000014	POC5	protein_codin	0	0	0	0
ENSG0000009	CAPN3	protein_codin	0	21.1660986	7.56914489	10.9569501
ENSG0000017	RABEP2	protein_codin	0	0	3.63348109	0
ENSG0000009	WDR7	protein_codin	274.914535	253.319033	159.970716	107.428914
ENSG0000013	SLC66A2	protein_codin	57.3438579	103.620249	33.0919137	54.721904
ENSG000001	ALDH5A1	protein_codin	0	0	3.42940312	0
ENSG0000014	MAP3K6	protein_codin	44.213445	56.168262	10.2403973	34.6870844
ENSG0000018	SSTR2	protein_codin	1.05962664	1.05570616	0	0
ENSG0000010	AP1B1	protein_codin	313.641762	212.363726	166.785122	110.529827
ENSG0000013	SLC37A4	protein_codin	0	2.35760437	2.5181202	0
ENSG0000019	CTBP1-DT	lncRNA	0	7.82639939	0	0
ENSG0000013	PHF11	protein_codin	0	24.0108759	0	0
ENSG0000008	SEMA5B	protein_codin	0	0	0	0
ENSG0000006	IPO5	protein_codin	71.5162119	16.6043565	0	0
ENSG0000015	UPF2	protein_codin	475.568939	346.609813	395.55881	284.091655
ENSG0000019	OR51D1	protein_codin	0	0	0	0
ENSG0000013	DNAJC14	protein_codin	0	0	0	0
ENSG0000010	BMX	protein_codin	0	0	0	0
ENSG0000010	TMEM170A	protein_codin	0	0	0	30.1254775
ENSG0000010	TAC3	protein_codin	0	0	0	0
ENSG000000	HFE	protein_codin	0	30.4292094	7.28502125	0
ENSG0000013	POT1	protein_codin	0	11.3363126	3.36561351	0
ENSG0000017	LRRC20	protein_codin	0	2.69408401	3.68107775	0
ENSG0000017	CCR9	protein_codin	0	0	0	0
ENSG0000017	FAM219B	protein_codin	28.099362	33.2755166	8.91151283	0
ENSG0000019	PIGN	protein_codin	0	0	0	0
ENSG0000015	ENPP3	protein_codin	0	0	0	0
ENSG0000010	DENND2D	protein_codin	19.7864078	11.20253	15.1987867	4.39277423
ENSG0000009	BRPF3	protein_codin	42.1305389	83.2585445	87.4135499	11.0797145
ENSG0000017	PLEC	protein_codin	371.358607	222.34187	0	156.765477

ENSG000001(	SLC30A7	protein_codin	177.499924	101.453818	101.504527	116.443575
ENSG000000	BRCA1	protein_codin	0	0	0	0
ENSG000001!	SZT2	protein_codin	0	0	0	0
ENSG000001.	ZNF816	protein_codin	0	0	0	0
ENSG000001.	ASAP1	protein_codin	0	0	0	0
ENSG0000001	DLG1	protein_codin	47.2434541	0	0	0
ENSG000000.	ATP2B4	protein_codin	756.875275	611.349161	274.176107	430.14265
ENSG000001!	BCO2	protein_codin	0	0	0	0
ENSG000001!	MIER1	protein_codin	19.0080712	22.5597845	14.19073	9.5999555
ENSG000001!	ASB13	protein_codin	49.1630332	59.1753542	17.5984578	40.0782328
ENSG000000.	MYBPC2	protein_codin	0	0	0	0
ENSG000001.	SAP130	protein_codin	0	0	0	0
ENSG000001	GHR	protein_codin	0	0	0	0
ENSG000001!	DSCR8	lncRNA	0	0	0	0
ENSG000000.	PSEN1	protein_codin	0	394.941472	104.731512	167.830862
ENSG000001	CD46	protein_codin	0	127.546077	0	211.675828
ENSG000001!	GRM7	protein_codin	0	0	0	0
ENSG000001.	DCLRE1C	protein_codin	0	0	0	0
ENSG000001(	TRMT1	protein_codin	34.2060871	72.4609629	0	42.1943413
ENSG000001.	ETFBKMT	protein_codin	2.78419531	5.21071857	2.53599881	2.18350754
ENSG000001.	NTRK3	protein_codin	0	0	0	0
ENSG000001	PRAMEF12	protein_codin	0	0	0	1.03600618
ENSG000001.	CREB5	protein_codin	697.785456	254.611445	227.726206	329.959317
ENSG000001!	SLC6A9	protein_codin	0	0	0	0
ENSG0000011	NEGR1	protein_codin	1.68681931	7.26702743	1.61537718	0
ENSG000001	RTN4	protein_codin	0	0	9.66776654	10.0766307
ENSG000001!	MAFG	protein_codin	191.544964	240.308346	82.5198576	101.430778
ENSG000001.	MCTP2	protein_codin	45.5412345	47.0840095	40.1522076	76.225674
ENSG000001.	GOLGA7	protein_codin	2.90959127	8.03269426	2.43647223	0
ENSG000001.	CHM	protein_codin	74.5895247	39.029531	35.2133833	41.8357915
ENSG000000(	HYAL2	protein_codin	18.9728773	33.9904463	12.2176445	10.3749157
ENSG000001(	DDHD1	protein_codin	15.3978409	27.1434878	0	0
ENSG000001	HHLA2	protein_codin	0	0	0	0
ENSG000000(	CDK11A	protein_codin	0	41.4565445	0	12.3103559
ENSG000002.	HP	protein_codin	0	0	0	0
ENSG000001	UNC50	protein_codin	16.2340411	40.4474552	11.1924526	13.4063348
ENSG000001.	THSD4	protein_codin	0	0	0.9111046	0
ENSG000001.	GJA9	protein_codin	0	0	0	0
ENSG000001!	ZNF98	protein_codin	0	2.36755667	3.64320132	0
ENSG000001(	TXNDC2	protein_codin	5.36568453	3.57911417	2.2771507	4.37940335
ENSG000001(	CCDC103	protein_codin	0	0	0	0
ENSG000001	APOA4	protein_codin	0	0	0	0
ENSG000001	PHTF1	protein_codin	32.2790452	25.0245325	0	14.8666852
ENSG000001	NRP2	protein_codin	0	11.6166681	7.78725767	4.01486728

ENSG000001	RPLP1	protein_codin	807.532046	1857.33453	339.281314	911.115027
ENSG000001	NPIPB9	protein_codin	8.90387763	3.48021743	0	0
ENSG000001	OGA	protein_codin	3.21236352	0	0	0
ENSG000001	NPAS3	protein_codin	0	0	0	0
ENSG000001	SGO2	protein_codin	20.4414469	19.2595505	29.0774159	17.4776315
ENSG000002	RRP7BP	lncRNA	0	8.77500182	0	2.27412291
ENSG000001	CLN3	protein_codin	0	10.6461948	0	0
ENSG000001	COL13A1	protein_codin	0	0	0	0
ENSG000001	NAT9	protein_codin	37.0733611	43.9143987	0	17.1603264
ENSG000001	AMER2	protein_codin	0	0	0	0
ENSG000001	OR8D1	protein_codin	0	0	0	0
ENSG000001	HJV	protein_codin	0	0	0	0
ENSG000001	AIG1	protein_codin	4.12684405	11.3865408	12.3331944	6.28640495
ENSG000001	COPS5	protein_codin	72.0740355	105.806135	45.9208275	64.77295
ENSG000001	SFI1	protein_codin	0	0	0	0
ENSG000001	G3BP2	protein_codin	374.333422	390.683577	247.652508	273.851277
ENSG000001	CLN3	protein_codin	0	0	0	0
ENSG000001	ZNF655	protein_codin	0	0	0	0
ENSG000001	STMN1	protein_codin	3.02473139	0	0	0
ENSG000001	ARL6IP4	protein_codin	3.26521063	0	0	0
ENSG000001	FN1	protein_codin	0	0	0	0
ENSG000001	ADCY6	protein_codin	0	0	0	0
ENSG000002	ENSG000002	lncRNA	0	0	0	0
ENSG000001	LVRN	protein_codin	0	0	2.58947646	0
ENSG000001	MDM1	protein_codin	0	0	0	0
ENSG000002	ENSG000002	lncRNA	0	1.05588	0	0
ENSG000001	SEMA4F	protein_codin	0	0	0	0
ENSG000001	HMX3	protein_codin	0	0	0	0
ENSG000002	LINC01554	lncRNA	0	0	0	0
ENSG000001	RFX4	protein_codin	0	0	0	0
ENSG000001	C19orf47	protein_codin	0	0	0	0
ENSG000001	CDK5RAP1	protein_codin	5.99922747	21.0439858	0	0
ENSG000001	RNF40	protein_codin	102.456823	0	0	86.6809306
ENSG000000	NEDD4L	protein_codin	0	0	0	0
ENSG000001	IFT20	protein_codin	0	0	0	0
ENSG000001	SPSB1	protein_codin	0	0	0	0
ENSG000001	ZBTB44	protein_codin	0	0	0	19.6614079
ENSG000001	ZNF441	protein_codin	8.1383789	10.5281276	13.5350837	11.5952699
ENSG000001	APP	protein_codin	732.76121	1498.1161	518.485014	655.906582
ENSG000002	MAGEA12	protein_codin	0	0	0	0
ENSG000000	RHOBTB1	protein_codin	0	0	0	140.144621
ENSG000002	ASIC3	protein_codin	0	11.6939281	0	0
ENSG000002	ANKRD18B	protein_codin	0	0	0	0
ENSG000001	DDC	protein_codin	0	0	0	0

ENSG000001(	CCNE1	protein_codin	0	4.2433211	0	0
ENSG00000(	TLL2	protein_codin	0	0	0	0
ENSG000001(	CADPS	protein_codin	0	0	0	0
ENSG000001!	SERTAD1	protein_codin	30.8949284	22.8195747	11.3687458	10.9441658
ENSG000001	MAPKAPK3	protein_codin	316.561108	535.003882	207.590442	269.183927
ENSG000001!	ERI2	protein_codin	7.17847378	7.42614993	10.1366546	3.91561541
ENSG000001:	BIN1	protein_codin	0	0	0	0
ENSG000001	ACYP1	protein_codin	0	7.8106168	0	0
ENSG000001:	MIR9-1HG	lncRNA	0	0	0	0
ENSG000001(	NFIA	protein_codin	8.26689853	0	0	0
ENSG000001.	TPM1	protein_codin	0	0	9.54365248	0
ENSG000001(	TMUB2	protein_codin	0	0	2.50109401	0
ENSG000001:	FAM3B	protein_codin	0	0	0	0
ENSG000001	PHF8	protein_codin	4.17916978	0	0	0
ENSG000001	ADGRG2	protein_codin	0	0	0	0
ENSG000001.	ELK4	protein_codin	301.041591	209.9508	146.790391	233.926292
ENSG000001	FEZ2	protein_codin	0	62.5849469	25.2434256	85.7961272
ENSG000001	ANKLE2	protein_codin	159.61171	213.607521	121.083233	127.257722
ENSG000001(	FKTN	protein_codin	22.1219388	0	0	2.80839842
ENSG000001!	BLOC1S6P1	processed_ps	0	0	0	0
ENSG000001:	CCDC32	protein_codin	0	0	0	0
ENSG000002	DDX47	protein_codin	32.4739269	36.8314616	27.4991341	26.901374
ENSG000001(	AIMP1	protein_codin	0	8.62032044	0	0
ENSG000001:	TACC2	protein_codin	0	0	0	0
ENSG000001	AIRIM	protein_codin	8.01666205	0	0	0
ENSG000001	RAD23B	protein_codin	700.765205	959.956871	391.437612	582.748545
ENSG000001!	TUSC1	protein_codin	0.97501955	6.9508833	1.60470541	0.91382481
ENSG000001:	TMCC2	protein_codin	0	0	0	0
ENSG00000:	SYNE2	protein_codin	0	0	0	0
ENSG00000(	NIPAL3	protein_codin	0	0	0	0
ENSG000001:	STX16	protein_codin	73.9144617	53.5810292	0	21.9404514
ENSG000001:	RAD17	protein_codin	0	0	0	0
ENSG000001:	KCNIP2	protein_codin	5.38157438	4.3228134	0	0
ENSG00000(	ST7L	protein_codin	0	0	0	0
ENSG000001:	OXA1L	protein_codin	0	4.8229331	4.4866675	0
ENSG000001!	FAM47C	protein_codin	0	2.26136129	0	0
ENSG000001(	KLK2	protein_codin	0	0	0	0
ENSG000001:	OSBPL2	protein_codin	0	11.2735265	0	0
ENSG000001	SLC4A3	protein_codin	0	0	0.97958602	0
ENSG000001!	GMFB	protein_codin	63.1793405	43.3003813	26.4087681	35.5062428
ENSG000001!	DMD	protein_codin	1.0773237	0	0	0
ENSG000001!	H4C16	protein_codin	8.31512064	13.2764866	13.8714315	4.21905357
ENSG000001	SETMAR	protein_codin	0	11.6156131	0.9015256	6.51391115
ENSG000001:	SEMA6D	protein_codin	0	0	0	0

ENSG000001	ZNF329	protein_codin	0	0	10.3315536	4.05712858
ENSG000000	DCX	protein_codin	0	0	0	0
ENSG000001	RAD9B	protein_codin	0	0	0	0
ENSG000002	ENSG000002	lncRNA	1.03163462	6.08301013	2.32859951	2.93355439
ENSG000001	MAGT1	protein_codin	0	0	0	0
ENSG000001	DAPK1	protein_codin	0	0	0	0
ENSG000000	ASIC4	protein_codin	0	0	0	0
ENSG000001	THOC5	protein_codin	0	26.1450073	0	0
ENSG000000	TRPM3	protein_codin	0	0	0	0
ENSG000001	POTEE	protein_codin	0	0	0	0
ENSG000001	KLF8	protein_codin	0	0	1.37031311	0
ENSG000001	KLHL14	protein_codin	0	0	0	0
ENSG000002	CYP2D7	protein_codin	0	0	0	1.59279124
ENSG000001	NR3C2	protein_codin	4.21857419	0	0	0
ENSG000001	CFAP299	protein_codin	0	0	0	0
ENSG000001	RGR	protein_codin	0	0	0	0
ENSG000000	MPPED2	protein_codin	1.61767806	7.3682297	3.23604793	2.21054033
ENSG000001	OR2L13	protein_codin	0	0	0	0
ENSG000001	PAX5	protein_codin	0	0	0	0
ENSG000001	FHL2	protein_codin	0	0	0	0
ENSG000001	HMGN5	protein_codin	22.1445079	20.8220093	17.2062256	12.4244931
ENSG000001	TOX2	protein_codin	0	0	0	0
ENSG000000	PLOD1	protein_codin	0	0	0	0
ENSG000001	PDE1A	protein_codin	0	0	0	0
ENSG000001	LRRC20	protein_codin	0	0	0	6.2475511
ENSG000001	CNTNAP3	protein_codin	0	0	0	0
ENSG000001	DMRT2	protein_codin	0	0	0	0
ENSG000001	WDSUB1	protein_codin	0	0	0	0
ENSG000001	ZNF346	protein_codin	21.1484663	71.9288643	27.5028912	26.7562925
ENSG000001	ZNF483	protein_codin	1.13909553	0	0	0
ENSG000001	MS4A3	protein_codin	0	0	0	2.39947697
ENSG000001	METTL9	protein_codin	261.262186	306.114535	120.789804	277.483419
ENSG000001	SRSF2	protein_codin	4.4439895	10.1929347	0	0
ENSG000002	S1PR3	protein_codin	60.2698174	132.301081	34.3007439	110.332824
ENSG000001	GRAMD1C	protein_codin	0	0	0	0
ENSG000001	SPTAN1	protein_codin	0	0	0	0
ENSG000000	ITGAL	protein_codin	154.251757	525.526912	138.36704	160.847566
ENSG000000	RNASET2	protein_codin	23.7516749	5.97538867	8.63649677	1.48196816
ENSG000001	CD46	protein_codin	0	0	0	0
ENSG000001	SERPINH1	protein_codin	10.576089	5.73942462	7.512144	2.41528509
ENSG000001	EPHB4	protein_codin	0	0	0	0
ENSG000001	KIR3DL1	protein_codin	0	0.90680954	0	0.93362362
ENSG000001	CHD3	protein_codin	338.069541	380.836855	0	137.75365
ENSG000001	CTSE	protein_codin	0	0	0	0

ENSG0000010	BDH1	protein_codin	0	0	3.82788874	0
ENSG000001	ITPRIP	protein_codin	0	0	0	19.986234
ENSG0000010	CIMAP2	protein_codin	0	0	0	0
ENSG000001	GAD1	protein_codin	0	0	0	0
ENSG000001	RABEP2	protein_codin	39.3416403	68.3842453	46.2509731	30.8048427
ENSG000001	TES	protein_codin	265.256468	307.025695	153.293183	172.575293
ENSG000001	ABHD17AP1	unprocessed_	0	0.95711703	0.70391463	0
ENSG000001	SMARCAL1	protein_codin	72.1588456	0	23.6614122	0
ENSG000001	SRC	protein_codin	0	0	0	0
ENSG000000	DNM1L	protein_codin	0	0	0	0
ENSG000001	GMPPA	protein_codin	13.0710103	14.5675607	0	3.22607997
ENSG000000	WDR37	protein_codin	0	24.7618242	0	8.29539325
ENSG000001	DMAC1	protein_codin	18.1029332	25.0329631	10.7485256	11.2719991
ENSG000001	ENPP3	protein_codin	0	0	4.66013301	0
ENSG000001	TMBIM4	protein_codin	61.4910831	40.8181111	0	0
ENSG000001	ELP2	protein_codin	41.2471434	13.2088249	20.8122454	19.0830964
ENSG000001	ENSG000001	lncRNA	0	4.04181429	1.91643212	0
ENSG000001	PPP1CA	protein_codin	13.0620395	13.9789848	10.9826149	0
ENSG000001	RTP2	protein_codin	0	0	0	0
ENSG000001	DMTN	protein_codin	180.364839	307.740234	113.455342	147.979197
ENSG000000	CACNA1G	protein_codin	0	0	0	0
ENSG0000010	MS4A7	protein_codin	577.867349	252.197033	118.529522	243.360922
ENSG000001	TNKS1BP1	protein_codin	28.9483089	0	0	25.6289629
ENSG000001	LCA5L	protein_codin	0	0	0	0
ENSG000001	NF1	protein_codin	0	76.97499	85.1739945	94.2333598
ENSG000001	TPM1	protein_codin	0	0	106.390862	2.39510321
ENSG000001	HSD17B11	protein_codin	327.99147	358.658582	186.310066	299.146685
ENSG0000010	FCRL1	protein_codin	0	0	0	0
ENSG000001	FAM217B	protein_codin	0	0	0	0
ENSG000002	TAFA5	protein_codin	0	0	0	0
ENSG000001	ZNF100	protein_codin	24.3695819	21.6061333	28.1535259	12.0142318
ENSG000001	SUV39H2	protein_codin	0	0	3.69670876	0
ENSG0000010	ICA1L	protein_codin	0	5.54441982	0	0
ENSG000001	PAK4	protein_codin	0	4.46645079	4.14550919	0
ENSG000000	ZC3HC1	protein_codin	0	0	0	0
ENSG000000	FUT8	protein_codin	6.21896989	39.9387628	30.7759852	0
ENSG000002	ARHGAP19	protein_codin	15.1030105	0	0	30.2152895
ENSG0000010	CX3CR1	protein_codin	177.510755	92.3790167	25.5650625	18.9929911
ENSG000001	PPHLN1	protein_codin	0	66.3189523	0	0
ENSG000001	KCNMB2	protein_codin	0	0	0	0
ENSG000000	SUSD2	protein_codin	6.16578752	4.39052636	3.61304189	1.72420423
ENSG000001	MYOF	protein_codin	60.9938232	0	0	0
ENSG0000010	AKT2	protein_codin	10.7576913	17.018386	0	0
ENSG000001	WDR55	protein_codin	92.6961177	156.639494	42.7454859	43.9098792

ENSG00000019	HNRNPAB	protein_codin	205.645568	383.407042	181.24889	110.902094
ENSG0000007	FRYL	protein_codin	0	172.354843	189.731928	64.9785172
ENSG0000009	CDHR5	protein_codin	0	1.7400169	0	1.67238119
ENSG0000001	USP20	protein_codin	54.9774341	0	0	0
ENSG0000001	RUNX1	protein_codin	160.803659	84.1972043	81.4906196	90.6384239
ENSG0000001	ECHDC2	protein_codin	0	3.69548422	0	0
ENSG0000001	PPIP5K2	protein_codin	0	0	0	0
ENSG0000001	GRIK2	protein_codin	0	0	0	0
ENSG0000001	RHOT1	protein_codin	25.4968471	0	0	0
ENSG0000001	HNRNPLL	protein_codin	0	0	0	0
ENSG0000007	XAB2	protein_codin	156.821658	231.256812	81.4074232	132.676522
ENSG0000001	MIGA2	protein_codin	0	0	0	0
ENSG0000001	MAP3K19	protein_codin	0	0	0	0
ENSG0000001	WTAP	protein_codin	1.99819496	7.95370143	1.58641063	0
ENSG0000001	GRM8	protein_codin	0	0	0	0
ENSG0000002	KNOP1P5	processed_ps	0	0	0	0
ENSG0000001	OSCAR	protein_codin	67.4542956	17.8242146	30.2163723	34.6502585
ENSG0000001	DCAF4	protein_codin	10.0412185	9.59190117	7.29714596	0
ENSG0000001	KIF13A	protein_codin	39.2716543	6.43417737	15.3914044	28.2975985
ENSG0000001	ATL1	protein_codin	0	0	0	0
ENSG0000001	TXNDC15	protein_codin	47.6583414	63.5699251	18.1135116	38.5734347
ENSG0000000	CNOT3	protein_codin	18.6238558	7.05415054	8.77393435	2.80815078
ENSG0000000	COL11A1	protein_codin	0	0	0	0
ENSG0000001	C20orf204	protein_codin	0	0	0	0
ENSG0000007	CEACAM1	protein_codin	0	0	0	0
ENSG0000001	RNF24	protein_codin	308.089581	413.132325	205.108082	237.803596
ENSG0000001	ESRP1	protein_codin	0	0	0	0
ENSG0000001	GDA	protein_codin	0	0	0	0
ENSG0000001	MAX	protein_codin	508.360584	665.747689	20.0720804	377.525979
ENSG0000001	GSTK1	protein_codin	404.540727	632.073461	232.30147	229.348169
ENSG0000001	TEAD4	protein_codin	0	0	0	0
ENSG0000001	DNA2	protein_codin	0	9.16993689	12.7930453	0
ENSG0000000	ZNF343	protein_codin	0	0	0	0
ENSG0000001	ITIH2	protein_codin	0	0	0	0
ENSG0000001	TXLNB	protein_codin	10.9715011	5.96959094	3.06226411	1.37586899
ENSG0000001	KIAA1671	protein_codin	0	0	0	0
ENSG0000001	EPHA2	protein_codin	1.90230231	5.74974995	11.0909307	0
ENSG0000001	OR6C6	protein_codin	0	0	0	0
ENSG0000001	TOMM7	protein_codin	178.061611	240.985838	62.2600299	160.151919
ENSG0000002	POLR2J2	protein_codin	58.7097738	35.4592458	24.1675319	13.4161329
ENSG0000001	ARMC8	protein_codin	0	12.1614052	4.48978707	27.2982675
ENSG0000002	ENSG0000002	lncRNA	0	0	0	0
ENSG0000001	PDE11A	protein_codin	0	0	1.20323182	0
ENSG0000001	PPIAP22	processed_ps	0	18.8309883	2.27679883	5.62034536

ENSG000001	VCF2	protein_codin	2.73607676	0	1.41165446	4.11268285
ENSG000001	AFAP1	protein_codin	0	0	0	0
ENSG000001	ZFHX3	protein_codin	0	0	0	0
ENSG000002	FRG1CP	transcribed_u	0	0	0	0
ENSG000001	TRIM33	protein_codin	286.260024	146.185186	106.529291	189.325322
ENSG000001	STAT4	protein_codin	0	0	0	0
ENSG000001	ZSCAN2	protein_codin	0	0	0	0
ENSG000001	DMAC2L	protein_codin	0	0	2.25149557	0
ENSG000001	POGZ	protein_codin	61.3215206	3.52792875	0	28.8325826
ENSG000001	TRPC4	protein_codin	0	0	0	0
ENSG000002	ENSG000002	lncRNA	0	0	0	0
ENSG000000	CASP8	protein_codin	0	16.3025243	0	18.7673291
ENSG000000	FGFR2	protein_codin	0	0	0	0
ENSG000001	ZNF429	protein_codin	2.03982925	7.65419052	7.80142726	2.13560968
ENSG000000	RAD52	protein_codin	8.88192042	12.6366634	0	3.77183695
ENSG000001	APOL2	protein_codin	72.988805	112.794887	105.614205	73.6226692
ENSG000001	KCNN3	protein_codin	0	0	5.20536343	1.63150173
ENSG000001	BCL11A	protein_codin	0	0	0	3.39054125
ENSG000002	COL6A6	protein_codin	0	0.96005963	0	0
ENSG000002	PSMB10	protein_codin	217.331081	278.942789	92.4866802	196.284502
ENSG000001	PLEKHG1	protein_codin	0	0	0	0
ENSG000001	NRG2	protein_codin	0	0	0	0
ENSG000001	ZNF682	protein_codin	0	0	0	0
ENSG000001	PATE2	protein_codin	0	0	0	0
ENSG000001	SLC6A20	protein_codin	0	0	0	0
ENSG000001	AKAP4	protein_codin	0	0	0	0
ENSG000001	EXO5	protein_codin	0	7.54062418	0	0
ENSG000000	CSDE1	protein_codin	0	0	0	0
ENSG000002	ARHGAP19	protein_codin	100.683389	91.5300823	55.1226401	65.2598347
ENSG000001	PLXNB1	protein_codin	0	0	0	0
ENSG000001	FLNB	protein_codin	140.563207	81.2867529	79.6540311	84.9537998
ENSG000001	PTPN5	protein_codin	0	0	0	0
ENSG000001	ALKAL1	protein_codin	0	0	0	0
ENSG000001	VPS13B	protein_codin	0	0	0	0
ENSG000001	TNFSF11	protein_codin	0	0	0	0
ENSG000000	RAB21	protein_codin	141.23287	133.679665	125.124712	106.802211
ENSG000001	TPD52L2	protein_codin	0	0	0	0
ENSG000001	SIPA1L1	protein_codin	356.008662	319.131831	229.417007	253.339551
ENSG000000	NFIX	protein_codin	0	0	0	0
ENSG000001	EDNRA	protein_codin	0	0	0	0
ENSG000001	PFN3	protein_codin	0	0	0	0
ENSG000001	TMA16	protein_codin	21.7454132	14.7402866	22.8690581	19.5354243
ENSG000001	FIP1L1	protein_codin	142.447602	175.089338	0	38.7888231
ENSG000002	ANKRD36BP1	lncRNA	0	0	0	0

ENSG000001:	MICAL1	protein_codin	49.596749	0	0	0
ENSG000001	APBB3	protein_codin	0	0	0	0
ENSG000002	ZNF781	lncRNA	0	0	0	0
ENSG000001	SCGB1D4	protein_codin	0	0	0	0
ENSG000001	FRMD1	protein_codin	0	0	0	0
ENSG000001	ARNT	protein_codin	304.415433	450.197654	96.0123178	199.541248
ENSG000001	CELF1	protein_codin	103.97729	142.020894	0	69.2482371
ENSG000001	PRKAR1A	protein_codin	283.478102	282.40179	443.300581	0
ENSG000001	ESR2	protein_codin	0	0	0	0
ENSG000001	ADGRE5	protein_codin	177.278148	125.335435	26.3393	47.0165504
ENSG000001	ANKRD17	protein_codin	150.590901	70.1907816	40.1470622	57.4054943
ENSG000001	ZNG1B	protein_codin	0	0	9.5316811	0
ENSG000000	REX1BD	protein_codin	50.0852574	36.2702378	26.0441234	8.76084935
ENSG000002	RPL27AP	processed_ps	0.97373665	0	1.41889201	0.9368453
ENSG000001	GYPE	protein_codin	0	0	0	0
ENSG000001	WDR5	protein_codin	67.0997261	91.7022569	37.9469021	40.2092697
ENSG000001	CYP11A1	protein_codin	0	0	0	0
ENSG000001	ELF2	protein_codin	15.4171512	4.1465657	2.83163685	22.7745092
ENSG000001	ASTN2	protein_codin	1.59301582	1.5406585	0	0
ENSG000001	OR5AC2	protein_codin	0	0	0	0
ENSG000001	GIMAP5	protein_codin	66.5199188	189.370616	30.3280102	45.7479589
ENSG000001	PASK	protein_codin	0	0	0	0
ENSG000002	SPECC1L-AD(	protein_codin	0	40.0546198	0	0
ENSG000001	WARS1	protein_codin	360.313335	463.045646	298.46947	52.6845398
ENSG000001	ZNF707	protein_codin	0	0	6.92912545	0
ENSG000000	SLC4A8	protein_codin	45.3741077	28.5733902	25.9935937	21.9754761
ENSG000001	NAALADL1	protein_codin	0	0	1.40860259	0
ENSG000001	NTRK1	protein_codin	0	0	0	0
ENSG000001	SP110	protein_codin	0	144.009512	0	0
ENSG000001	MAX	protein_codin	372.600087	987.300284	529.526826	248.49957
ENSG000001	UBL5	protein_codin	5.76018556	9.85566329	4.46152456	14.5640807
ENSG000000	FCGR2B	protein_codin	22.9087959	0	0	0
ENSG000001	ENAH	protein_codin	0	0	0	0
ENSG000001	SPATS2L	protein_codin	0	0	0	0
ENSG000001	MRPL42	protein_codin	0	18.0048181	0	0
ENSG000001	SLAIN1	protein_codin	0	0	0	0
ENSG000001	EXT2	protein_codin	0	0	0	0
ENSG000001	SLC4A5	protein_codin	0	0	0	0
ENSG000001	ENTPD4	protein_codin	319.287064	269.3977	160.887349	197.726858
ENSG000001	HELZ	protein_codin	665.600856	386.883769	287.770921	464.744745
ENSG000001	CTNND1	protein_codin	0	0	0	0
ENSG000001	ZXDA	protein_codin	35.3637221	21.1766384	22.7384535	13.666036
ENSG000001	ZNF23	protein_codin	0	0	0	0
ENSG000001	QSOX2	protein_codin	30.0513305	50.680982	10.5587682	19.864665

ENSG000001	ZBTB18	protein_codin	0	0	12.3641193	0
ENSG000000	CAMK2B	protein_codin	0	0	0	0
ENSG000001	UCKL1	protein_codin	7.75234731	3.43341401	5.1644654	2.26213706
ENSG000001	GATAD2A	protein_codin	3.40113859	0	0	8.10710689
ENSG000000	HMMR	protein_codin	0	0	22.2889298	0
ENSG000001	BAZ1A	protein_codin	0	56.4829951	5.02445654	83.6036892
ENSG000001	IPO8	protein_codin	0	0	0	0
ENSG000002	SMIM7	protein_codin	10.3170743	28.7168202	0	6.22769496
ENSG000001	AMPD2	protein_codin	6.38966646	19.1060184	11.6470604	0
ENSG000000	RFX3	protein_codin	14.8893036	14.9436925	11.8517407	3.97814091
ENSG000001	BDP1	protein_codin	338.798351	194.22436	229.329939	202.332538
ENSG000001	ZBTB1	protein_codin	57.2031006	39.2021698	37.0548862	9.13999053
ENSG000001	H2AC13	protein_codin	15.5050976	36.0453498	103.307234	29.2527762
ENSG000001	NLGN3	protein_codin	0	0	0	0
ENSG000001	SMTN	protein_codin	0	0	0	0
ENSG000001	ZNF571	protein_codin	3.17039466	4.92729475	4.28421963	1.05540668
ENSG000001	SKIC3	protein_codin	164.626311	112.603091	0	165.803576
ENSG000002	SLC26A6	protein_codin	0	0	0	0
ENSG000001	GPRACR	lncRNA	0	0.91515402	1.00294345	0
ENSG000000	NPFFR2	protein_codin	0	0	0	0
ENSG000001	FUT4	protein_codin	290.803294	264.771353	104.583663	150.245388
ENSG000001	FZD6	protein_codin	0	0	0	0
ENSG000002	ENSG000002	protein_codin	0	12.8363833	6.9253949	5.15177086
ENSG000001	PRRT2	protein_codin	0	0	0	0
ENSG000001	SYN3	protein_codin	3.92967726	1.05969441	0	0
ENSG000001	IQCH	protein_codin	0	1.23109349	0	0
ENSG000001	PARD3B	protein_codin	0	0	0	0
ENSG000001	PBLD	protein_codin	0	24.0121005	0	14.855591
ENSG000001	SIRPA	protein_codin	47.8455786	211.005752	0	195.468011
ENSG000001	NPSR1-AS1	lncRNA	0	0	0	0
ENSG000001	LINC02991	lncRNA	0	0	0	0
ENSG000001	ACADSB	protein_codin	34.9283615	14.3486042	22.9685832	29.8146485
ENSG000001	CEACAM19	protein_codin	6.50983174	6.9949343	7.56943719	0
ENSG000002	ENSG000002	processed_ps	0	0	0.87989885	0
ENSG000000	CDK11A	protein_codin	0	0	0	0
ENSG000000	FAM76B	protein_codin	12.879785	28.5794556	5.83212158	71.1523992
ENSG000001	FAM3D	protein_codin	1.8011191	0.86987867	0	0
ENSG000000	GNB5	protein_codin	35.6732474	64.6875725	63.7707816	31.7243971
ENSG000001	MRPL19	protein_codin	0	0	0	0
ENSG000001	KLK10	protein_codin	0	0	0	0
ENSG000001	BCAS4	protein_codin	0	3.59686485	0	0
ENSG000001	GTPBP3	protein_codin	0	0	0	0
ENSG000001	STIP1	protein_codin	0	0	0	0
ENSG000001	MICAL1	protein_codin	428.332602	545.132842	157.666276	197.512857

ENSG000001	CLIP1	protein_codin	0	0	0	0
ENSG000001	MANEA	protein_codin	0	17.4077114	47.7433917	0
ENSG000001	CCL20	protein_codin	65.7162015	19.4693728	12.3967498	0
ENSG000001	CNDP1	protein_codin	0	0	0	0
ENSG000001	CAMSAP2	protein_codin	14.1679377	6.6754117	0	18.8423684
ENSG000001	PRDM10	protein_codin	0	0	0	0
ENSG000001	TSKS	protein_codin	0	3.52934269	2.81149225	0
ENSG000001	PNLIPRP1	protein_codin	0	0	0	0
ENSG000000	REV3L	protein_codin	0	0	0	0
ENSG000001	GK2	protein_codin	0	0	0	0
ENSG000001	ABCB8	protein_codin	21.7346193	0	18.3803602	12.9410794
ENSG000001	NOL8	protein_codin	0	0	0	8.13300994
ENSG000001	CCER1	protein_codin	0	0.89326112	0	0
ENSG000001	TMC2	protein_codin	0	0	0	0
ENSG000000	DZANK1	protein_codin	0	0	0	0
ENSG000001	TMEM126B	protein_codin	0	0	4.55460912	2.32933415
ENSG000001	FAM114A1	protein_codin	26.322344	21.6856165	6.95107552	12.867018
ENSG000001	TFEB	protein_codin	2.63088793	0	0	0
ENSG000001	STK33	protein_codin	0	0	0	0
ENSG000001	LPAR1	protein_codin	0	0	0	11.3307536
ENSG000002	RPL41	protein_codin	55.1809789	58.8611226	30.3392774	34.5163066
ENSG000000	MAMLD1	protein_codin	0	0	0	0
ENSG000001	COL20A1	protein_codin	4.35347081	0.86280644	0	0
ENSG000001	DUS2	protein_codin	50.2698954	35.4270824	0	55.9640313
ENSG000001	FHAD1	protein_codin	1.66169151	0	0	0
ENSG000001	VCP	protein_codin	177.764988	209.556777	0	111.96671
ENSG000001	FAM222A	protein_codin	1.78919594	1.77083871	0	0
ENSG000001	R3HDM2	protein_codin	0	0	0	0
ENSG000001	PELI1	protein_codin	2089.38504	709.265472	490.610545	581.340333
ENSG000001	MYPN	protein_codin	0	0	0	0
ENSG000001	APP	protein_codin	346.721786	726.694417	420.001917	309.021975
ENSG000001	DLC1	protein_codin	0	0	0	0
ENSG000001	PDE4D	protein_codin	57.610822	48.2749127	42.2233352	0
ENSG000000	SIRT2	protein_codin	0	0	0	0
ENSG000001	CCNB1IP1	protein_codin	22.9549663	14.9496338	16.4926027	0
ENSG000000	EDC4	protein_codin	128.461614	178.592626	81.5017969	126.750696
ENSG000001	MARCHF5	protein_codin	81.3232551	79.7797039	36.2670485	64.953002
ENSG000000	DPP8	protein_codin	61.8592118	102.495631	22.7947169	83.8157332
ENSG000002	RBMY1E	protein_codin	0	0	0	0
ENSG000001	SETMAR	protein_codin	0	0	0	0
ENSG000001	RAB3GAP2	protein_codin	86.109033	48.4220083	85.052721	61.7645098
ENSG000000	AASS	protein_codin	0	0	0	0
ENSG000001	NUMA1	protein_codin	0	0	0	0
ENSG000001	GGPS1	protein_codin	0	0	0	0

ENSG000001	POTEC	protein_codin	0	0	0	0
ENSG000001	SEPSECS	protein_codin	0	0	0	0
ENSG000001	ANKRD30B	protein_codin	0	0	0	0
ENSG000001	ZNF763	protein_codin	3.73933386	0	6.11486607	9.30589606
ENSG000001	ANKRD46	protein_codin	0	0	0	0
ENSG000001	NCKAP5	protein_codin	0	0	0	0
ENSG000001	SPANXC	protein_codin	0	0	0	0
ENSG000001	PTPA	protein_codin	67.0237254	0	60.7663165	0
ENSG000001	NCOA6	protein_codin	297.606975	411.077675	185.581054	178.920107
ENSG000001	TRAPPC4	protein_codin	0	8.41706872	0	15.3250056
ENSG000001	VTCN1	protein_codin	0	0	0	0
ENSG000001	TGFBR2	protein_codin	127.867403	41.665599	8.49274303	31.3708862
ENSG000001	MAP3K5	protein_codin	333.825297	405.810919	276.90861	376.035711
ENSG000002	RPL32P3	transcribed_u	0	0	0	0
ENSG000001	AKAP9	protein_codin	0	0	241.321187	176.991714
ENSG000001	GABPB1	protein_codin	12.3450686	0	4.5090476	6.65863007
ENSG000001	AP2A1	protein_codin	0	0	0	0
ENSG000001	CYP2D6	protein_codin	0	0	0	0.82108886
ENSG000001	ZNF511	protein_codin	30.129523	31.1763906	10.6032133	4.95598719
ENSG000001	KLHL9	protein_codin	30.3100731	35.9289299	23.5202631	27.0346938
ENSG000001	IL31RA	protein_codin	0	0	0	0
ENSG000001	MTNAP1	protein_codin	0	0	0	0
ENSG000001	ZNF574	protein_codin	38.2945697	55.5232024	26.1727654	21.4667584
ENSG000001	TRPM6	protein_codin	0	0	2.35247572	0
ENSG000001	HYAL3	protein_codin	11.2197118	0	5.40184333	0
ENSG000001	SMARCAD1	protein_codin	40.0449263	89.9224546	61.5904552	59.9316716
ENSG000001	CLEC4M	protein_codin	0	0	0	0
ENSG000001	SSU72	protein_codin	133.387853	155.382303	63.7861489	86.2584025
ENSG000001	PDE3A	protein_codin	91.8869443	46.3187754	73.7005287	15.8890323
ENSG000001	INTS15	protein_codin	2.39689721	0	0	0
ENSG000001	SNHG11	lncRNA	0	0	0	0
ENSG000001	SGMS2	protein_codin	32.5934077	11.6390933	6.86014935	3.7662985
ENSG000001	SHANK1	protein_codin	0	0	1.76886202	3.05090856
ENSG000001	KIR2DL4	protein_codin	0	0	0	0
ENSG000001	SFTA2	protein_codin	0	0	0	0
ENSG000001	NAE1	protein_codin	0	0	0	0
ENSG000000	ST6GALNAC1	protein_codin	0	0	0	0
ENSG000001	AXL	protein_codin	0	0	0	0
ENSG000001	MAP2K6	protein_codin	0	18.9687205	15.3343876	22.4847543
ENSG000001	GNRH2	protein_codin	0	0	0	0
ENSG000000	CACNA1G	protein_codin	0	0	1.42398822	0
ENSG000001	MTHFD2L	protein_codin	0	0	9.04683616	16.6684091
ENSG000002	NOL12	protein_codin	10.4664703	15.9891473	18.1774501	8.87578086
ENSG000001	MLF1	protein_codin	0	0	0	0

ENSG000001	SH3RF2	protein_codin	0	0	0	0
ENSG000000	KCNQ2	protein_codin	0	0	0	0
ENSG000001	NLRC3	protein_codin	0	14.9245932	14.4262224	2.99018392
ENSG000001	PIGK	protein_codin	0	0	2.72559591	0
ENSG000001	GULP1	protein_codin	0	0	0	0
ENSG000001	PPP6R2	protein_codin	17.51002	0	0	16.3146496
ENSG000001	ECE2	protein_codin	0	0	0	0
ENSG000000	ATP2B1	protein_codin	0	0	0	0
ENSG000000	ATP2B3	protein_codin	0	0	0	0
ENSG000001	KLRC1	protein_codin	0	0	0	0
ENSG000001	SYTL2	protein_codin	0	0	0	8.31409085
ENSG000001	WWP2	protein_codin	236.412569	275.726528	88.436294	133.670796
ENSG000001	CD163	protein_codin	296.794956	327.570168	138.35824	166.34503
ENSG000001	ANKRD12	protein_codin	314.671254	237.323899	267.385814	262.616283
ENSG000000	RFX2	protein_codin	0	29.0010769	0	32.3175321
ENSG000001	ESRRG	protein_codin	0	0	0	0
ENSG000002	EEF1A1P4	processed_ps	13.4581024	26.6246293	14.8562575	8.46313049
ENSG000001	NUP98	protein_codin	0	26.6369942	0	0
ENSG000001	AHCYL1	protein_codin	18.8596877	0	0	0
ENSG000001	MAP3K15	protein_codin	0	0	0	0
ENSG000001	SPTSSB	protein_codin	0	0	0	0
ENSG000001	GOLGA8A	protein_codin	14.9221444	4.83057794	80.340793	3.97888502
ENSG000001	ABI1	protein_codin	0	0	0	0
ENSG000001	SUPT5H	protein_codin	21.3759568	0	37.5705309	0
ENSG000001	H2AC11	protein_codin	15.5517338	21.761626	90.6811871	16.2674467
ENSG000001	PIK3CG	protein_codin	52.3063502	12.5033862	46.8951459	7.28632501
ENSG000001	IKZF1	protein_codin	0	0	0	0
ENSG000002	PRICKLE4	protein_codin	0	0	0	0
ENSG000001	FTHL17	protein_codin	0	0	0	0
ENSG000000	RRAGD	protein_codin	0	0	0	0
ENSG000001	FRA10AC1	protein_codin	50.5356956	47.3871455	26.8122286	51.678033
ENSG000001	YY1AP1	protein_codin	0	0	0	0
ENSG000000	ABCB4	protein_codin	0	0	0	0
ENSG000000	DNTTIP2	protein_codin	349.642538	231.915282	415.94639	243.132168
ENSG000001	KIRREL1	protein_codin	0	0	0	0
ENSG000000	POLR3E	protein_codin	0	0	0	6.94020021
ENSG000001	B4GALT4	protein_codin	17.7915664	10.5743182	9.46731296	8.46791962
ENSG000001	TTN	protein_codin	0	0	0	0
ENSG000001	MAPK10	protein_codin	0	0	0	0
ENSG000001	UQCC1	protein_codin	0	0	0	8.27119041
ENSG000001	ELAVL3	protein_codin	0	0	0.89369212	0
ENSG000001	LYPD2	protein_codin	0	0	0	0
ENSG000001	SLC25A29	protein_codin	31.1432414	65.5215624	12.6256029	17.601132
ENSG000001	VSIG10	protein_codin	12.0632954	10.0001347	4.44358662	9.21903695

ENSG000001	PLAC1	protein_codin	0	0	0	0
ENSG000000	SNX11	protein_codin	56.2403626	44.429888	12.1050113	18.9165499
ENSG000002	INGX	transcribed_p	0	0	0	0
ENSG000001	ADAM29	protein_codin	0	0	0	0
ENSG000001	ODF2L	protein_codin	17.0972336	0	13.5446535	0
ENSG000002	ENSG000002	lncRNA	0	0	0	0
ENSG000001	PHF2	protein_codin	293.155607	393.933588	159.112968	176.388021
ENSG000001	FHOD3	protein_codin	0	0	0	0
ENSG000001	CCDC197	protein_codin	0	0	0	0
ENSG000001	MYOF	protein_codin	513.52387	788.420746	197.356979	370.37091
ENSG000001	PDLIM5	protein_codin	32.7197707	21.0484887	14.1218739	11.5044196
ENSG000001	SLC2A10	protein_codin	1.06019433	1.04696038	0	0
ENSG000001	FCAR	protein_codin	0	0	0	0
ENSG000000	BCS1L	protein_codin	0	15.617646	0	0
ENSG000001	PLD3	protein_codin	0	0	0	0
ENSG000000	CTPS2	protein_codin	12.2944037	3.8861866	13.3560506	0
ENSG000001	ADAM15	protein_codin	0	0	6.38512508	0
ENSG000001	PCBP2	protein_codin	157.718544	123.766256	0	0
ENSG000001	SH2B1	protein_codin	0	0	0	0
ENSG000001	ZNF705A	protein_codin	0	0	0	0
ENSG000001	EOLA1	protein_codin	2.60479209	0	11.9304393	4.50438234
ENSG000001	STK40	protein_codin	24.4147977	22.957522	5.23374474	13.3498732
ENSG000001	ANXA10	protein_codin	0	0	0	0
ENSG000001	H3C12	protein_codin	2.07103654	1.12624278	21.1879287	3.31472592
ENSG000001	XRCC6	protein_codin	109.48737	104.063761	43.795693	46.3635585
ENSG000001	CD2AP	protein_codin	131.442506	91.0147747	114.327645	84.0794362
ENSG000001	TPGS1	protein_codin	18.4296275	22.0252841	7.46910747	21.8745643
ENSG000001	MTARC2	protein_codin	0	0	0	0
ENSG000001	AADACL3	protein_codin	0	0	0	0
ENSG000001	CMTM5	protein_codin	0	36.228653	7.4920756	23.6237703
ENSG000001	XRCC2	protein_codin	0	0	0	0
ENSG000001	MYOZ1	protein_codin	0	0	0	0
ENSG000000	FIRRM	protein_codin	31.9551964	12.2898707	0	0
ENSG000000	SLAMF7	protein_codin	0	5.48882679	22.3080774	0
ENSG000001	KLHDC1	protein_codin	24.9360857	17.0964734	0	22.4757074
ENSG000001	TMEM139	protein_codin	0	0	0	0
ENSG000001	PLXNB2	protein_codin	1065.7162	2499.06785	419.837352	966.67299
ENSG000002	ANKRD20A18	transcribed_u	0	0	0	0
ENSG000001	CGB2	protein_codin	0	0	0	0
ENSG000001	SLFNL1	protein_codin	0.89324526	0	1.13577369	0
ENSG000001	OR51A7	protein_codin	0	0	0	0
ENSG000001	DDX42	protein_codin	8.66266028	17.2553189	0	0
ENSG000000	FGFR2	protein_codin	0	0	0	0
ENSG000001	DNAH8	protein_codin	0	2.18304951	0	0

ENSG000001	KLHL14	protein_codin	8.12104414	3.07746576	8.78568619	0
ENSG000001	C6orf89	protein_codin	4.87324999	0	0	0
ENSG000000	ARSF	protein_codin	0	0	0	0
ENSG000001	EDARADD	protein_codin	0	0	0	0
ENSG000000	RASSF1	protein_codin	41.8695297	107.150253	9.72118206	0
ENSG000000	ATXN3	protein_codin	0	0	0	0
ENSG000001	RBFOX2	protein_codin	0	0	0	0
ENSG000001	FGF1	protein_codin	1.11852218	0	0	0
ENSG000001	ARAP1	protein_codin	0	0	0	0
ENSG000000	POLR1H	protein_codin	8.01649282	22.5791672	10.429687	0
ENSG000001	Metazoa_SRP	misc_RNA	0	0	0	0
ENSG000001	DYNC1I1	protein_codin	0	17.5031126	0	0
ENSG000001	EHD1	protein_codin	23.5274352	0	0	0
ENSG000001	TRPV6	protein_codin	1.4255584	0	0	0
ENSG000000	ARCN1	protein_codin	0	9.3241248	0	10.8996772
ENSG000001	CIBAR1	protein_codin	0	0	0	0
ENSG000001	HK1	protein_codin	598.88376	861.373055	100.276919	531.011083
ENSG000001	NUP214	protein_codin	0	0	0	0
ENSG000001	RPE	protein_codin	0	48.3882191	0	40.5263922
ENSG000001	BABAM1	protein_codin	17.649308	48.3992128	20.7973866	9.05938216
ENSG000001	TMC3	protein_codin	0	0	0	0
ENSG000001	FGF17	protein_codin	0	0	0	0
ENSG000001	EFL1	protein_codin	0	0	0	0
ENSG000001	LDLRAD4	protein_codin	0	0	57.1407922	38.7599703
ENSG000001	MYO18A	protein_codin	39.3628197	39.5307183	9.47463933	21.9368027
ENSG000001	WASF1	protein_codin	0	0	0	0
ENSG000001	RGR	protein_codin	0	0	0	0
ENSG000001	CCDC196	protein_codin	0	0	0	0
ENSG000001	YIF1A	protein_codin	4.18086119	0	0	0
ENSG000001	PCBP2	protein_codin	297.531553	642.082584	102.547879	255.069205
ENSG000001	IFT172	protein_codin	0	7.09081317	4.31961704	0
ENSG000001	ZNF33B	protein_codin	71.5925503	51.5562438	56.505066	36.1183937
ENSG000001	C1orf116	protein_codin	14.0205329	53.6822186	18.6723888	8.58708884
ENSG000001	MFAP5	protein_codin	0	0	0	0
ENSG000001	SLC33A1	protein_codin	3.31686702	0	12.2786664	19.62967
ENSG000001	PLGLB2	protein_codin	13.2953451	1.13739093	5.18838282	0
ENSG000001	TCF20	protein_codin	0	0	0	0
ENSG000002	ENSG000002	lncRNA	0	0	1.23802996	0
ENSG000001	APOA1	protein_codin	0	0	0	0
ENSG000001	KRT78	protein_codin	0	0	0	0
ENSG000001	TBX1	protein_codin	0	0	0	0
ENSG000001	SLC25A44	protein_codin	54.0639513	143.097717	45.492838	48.5852402
ENSG000001	WASH6P	protein_codin	41.6467014	66.082852	0	18.4705215
ENSG000001	ATG13	protein_codin	9.2218547	11.9318518	8.65743446	18.7361542

ENSG000001	TRIM3	protein_codin	0	0	0	0
ENSG000001	TECPR2	protein_codin	171.753605	323.532196	134.325058	167.686385
ENSG000001	MPZL1	protein_codin	0	0	0	0
ENSG000001	RUNX2	protein_codin	58.6070205	61.9534753	18.1653024	10.6172979
ENSG000001	LIN9	protein_codin	0.92869793	2.78872451	0	1.79369277
ENSG000001	DNMT1	protein_codin	123.460415	524.021051	141.571233	98.5447768
ENSG000001	MBIP	protein_codin	0	18.5652841	0	0
ENSG000000	ATP6V1H	protein_codin	0	31.4651498	24.0029044	37.1483158
ENSG000001	KCNK5	protein_codin	7.86375634	8.70877898	1.93502848	2.8660156
ENSG000001	BCR	protein_codin	159.915707	154.122066	17.790084	37.0456462
ENSG000001	GTF3C2	protein_codin	5.38763065	0	0	0
ENSG000002	EMP2	protein_codin	2.15935953	2.15841032	0	2.2094982
ENSG000001	UBAP1	protein_codin	0	0	0	0
ENSG000001	CPLX2	protein_codin	0	0	0	0
ENSG000001	GPAT2	protein_codin	0	0	0	0
ENSG000001	SHARPIN	protein_codin	0	21.1615338	4.1337845	21.9251068
ENSG000001	NUMB	protein_codin	0	0	0	0
ENSG000001	HAND2	protein_codin	0	0	0	0
ENSG000001	DSE	protein_codin	288.429182	374.861927	193.798216	205.493937
ENSG000001	PCNT	protein_codin	446.452122	550.812191	417.33279	347.773312
ENSG000001	INPP5D	protein_codin	331.039619	240.796691	131.787091	187.885696
ENSG000001	LFNG	protein_codin	121.909316	51.1841559	4.71490632	42.750601
ENSG000001	AKR1B10	protein_codin	0	0	0	0
ENSG000001	LRP10	protein_codin	338.961692	468.673096	256.830894	237.23924
ENSG000002	AP4M1	protein_codin	0	0	0	0
ENSG000001	OR2T3	protein_codin	0	0	0	0
ENSG000001	HAPLN3	protein_codin	0	0	0	0
ENSG000001	RYR1	protein_codin	0	0	0	0
ENSG000001	TP53	protein_codin	0	0	0	0
ENSG000001	ZSWIM5	protein_codin	0	3.16475746	0.98677492	0
ENSG000000	CBFA2T2	protein_codin	0	0	36.0062115	0
ENSG000001	OR6K2	protein_codin	0	0	0	0
ENSG000002	H2AC17	protein_codin	48.8371611	109.494665	115.320324	51.9794505
ENSG000001	SDHAF2	protein_codin	0	0	0	0
ENSG000001	STX16	protein_codin	0	0	0	0
ENSG000001	CELA2A	protein_codin	0	0	0	0
ENSG000001	LRRC61	protein_codin	5.70986258	4.01584632	0	0
ENSG000001	CBS	protein_codin	0	0	0	0
ENSG000001	TYW1	protein_codin	91.192975	141.053473	75.8615241	0
ENSG000001	BCL11A	protein_codin	0	0	0	0
ENSG000001	SLC39A10	protein_codin	54.1182226	37.9178238	47.2802082	26.0800216
ENSG000000	CFH	protein_codin	0	0	0	0
ENSG000000	ATP2C1	protein_codin	0	0	0	42.7332496
ENSG000001	MBP	protein_codin	0	0	0	0

ENSG000002	RBM12	protein_codin	0	232.808326	79.8183152	0
ENSG000001	OSCAR	protein_codin	0	17.0646399	0	0
ENSG000001	PGLYRP4	protein_codin	0	0	0	0
ENSG000001	ELF3	protein_codin	22.1444508	25.8858201	10.54917	5.94948084
ENSG000000	ZRANB1	protein_codin	212.115873	157.401607	99.7183455	148.59764
ENSG000001	SUPV3L1	protein_codin	22.8656252	28.693097	23.7070646	17.4417954
ENSG000001	FN1	protein_codin	0	0	0	0
ENSG000001	SLC6A12	protein_codin	26.327583	0	0	0
ENSG000001	NDRG3	protein_codin	0	5.78617886	0	0
ENSG000001	NOL4L	protein_codin	108.886003	109.62849	46.3134499	45.3985389
ENSG000001	HIBCH	protein_codin	18.4230616	2.72559069	13.0966667	0
ENSG000001	TRAPPC2	protein_codin	5.76094745	11.4664311	18.001829	0
ENSG000001	RPL37A	protein_codin	82.5264012	95.3284072	10.8026718	58.8265288
ENSG000001	NIF3L1	protein_codin	34.7564403	0	0	21.7793265
ENSG000000	OSBPL6	protein_codin	0	1.88382331	0	0
ENSG000001	NACA3P	processed_ps	32.3077297	15.9023252	8.43651944	10.9106644
ENSG000000	DNM2	protein_codin	0	0	0	0
ENSG000001	OR11H13P	unprocessed_	0	0	0	0
ENSG000001	STYXL1	protein_codin	10.9259518	11.6604566	13.1245047	0
ENSG000001	RNF217	protein_codin	1.96914519	2.55550351	3.93069942	0
ENSG000001	G3BP2	protein_codin	50.760941	122.606551	43.6066737	84.2162063
ENSG000001	IFI16	protein_codin	115.837276	0	51.7187686	0
ENSG000001	ITGB1BP1	protein_codin	14.2945877	19.4251528	6.62822111	19.6220647
ENSG000000	MGST1	protein_codin	2.43289547	0	0	0
ENSG000001	APP	protein_codin	0	0	0	0
ENSG000001	NIPA2	protein_codin	0	0	1.92243106	0
ENSG000001	ZNF138	protein_codin	0	0	0	0
ENSG000000	RGS11	protein_codin	0	0	0	0
ENSG000001	SLC39A14	protein_codin	0	0	10.635299	0
ENSG000001	GRIP1	protein_codin	0	0	0	0
ENSG000001	PHOSPHO2	protein_codin	0	0	0	0
ENSG000001	DSG4	protein_codin	0	0	0	0
ENSG000000	STOML1	protein_codin	3.24190708	0	0	2.43081079
ENSG000002	EFNA4	protein_codin	0	0	0	0
ENSG000001	AKAP12	protein_codin	9.8392841	0	14.6930783	6.14244551
ENSG000001	SPAG11B	protein_codin	0	0	0	0
ENSG000001	COL18A1	protein_codin	0	0	0	0
ENSG000001	WIPF1	protein_codin	0	0	0	0
ENSG000001	TLK1	protein_codin	252.445603	339.520435	457.085862	175.485132
ENSG000001	WDSUB1	protein_codin	20.4565119	45.9258106	16.037107	18.2553234
ENSG000001	ZDHHC14	protein_codin	10.3680512	18.5027946	7.19053188	9.34621513
ENSG000001	SIRT5	protein_codin	0	20.5843469	0	0
ENSG000001	AMZ2	protein_codin	0	0	0	0
ENSG000001	PTPN22	protein_codin	67.8886038	0	129.966829	14.0154626

ENSG000001	MB	protein_codin	0	0	0	0
ENSG000001	ZNF675	protein_codin	14.8226285	17.1686261	16.2444919	20.1890695
ENSG000002	ENSG000002	lncRNA	0	0	0	0
ENSG000001	NPSR1	protein_codin	0	0	0	0
ENSG000001	CEP57L1	protein_codin	0	0	0	0
ENSG000001	PFKM	protein_codin	0	0	0	0
ENSG000001	ZNF808	protein_codin	31.8525377	63.1274259	25.2918707	51.5309898
ENSG000001	SIRPB2	protein_codin	189.327776	355.288242	96.0489184	196.025393
ENSG000001	MDK	protein_codin	0	0	0	0
ENSG000001	MPDU1	protein_codin	0	0	0	0
ENSG000001	ENSG000001	lncRNA	0	0	0	0
ENSG000002	PLXNA4	protein_codin	0	0	94.2192439	3.99815882
ENSG000000	TRAF3IP2	protein_codin	0	0	0	0
ENSG000002	ATP5MF	protein_codin	0	0	0	0
ENSG000001	DMD	protein_codin	0	0	0	0
ENSG000001	LUADT1	lncRNA	0	0	0	0
ENSG000001	RXRG	protein_codin	0	0	0	0
ENSG000001	FOXM1	protein_codin	0	0	0	0
ENSG000001	NT5C1B	protein_codin	0	0	0	0
ENSG000001	NTRK2	protein_codin	0	0	0	0
ENSG000001	ARPC5	protein_codin	214.363318	216.788841	60.2113999	136.648162
ENSG000001	ZMYM1	protein_codin	0	0	0	0
ENSG000002	ENSG000002	processed_ps	1.33943091	0	0	0
ENSG000001	WASHC2C	protein_codin	4.08852159	4.57437538	23.9063417	25.5961557
ENSG000001	TMEM25	protein_codin	0	0	0	0
ENSG000001	TRRAP	protein_codin	149.915853	30.9940128	0	0
ENSG000001	TEAD4	protein_codin	0	0	0	0
ENSG000001	MTCL1	protein_codin	0	0	0	0
ENSG000001	DDA1	protein_codin	31.5611184	44.950924	17.452295	21.8910081
ENSG000002	CSNK1E	protein_codin	0	0	0	22.725857
ENSG000001	ASIC2	protein_codin	0	0	0	0
ENSG000001	SPINK5	protein_codin	0	0	0	0
ENSG000001	ANKRD13C-D	lncRNA	0	0	1.74655629	0
ENSG000001	KCTD18	protein_codin	36.2472581	25.4308923	2.17384467	6.85574397
ENSG000001	PORCN	protein_codin	0	0	0	0
ENSG000001	AK3	protein_codin	10.2816965	24.703989	19.1148679	19.5440338
ENSG000001	CTAG1B	protein_codin	0	0	0	0
ENSG000000	FAM3A	protein_codin	20.9039794	11.3493875	0	35.4843845
ENSG000001	RBM23	protein_codin	122.789221	128.413539	84.949632	94.5213182
ENSG000000	BZW1	protein_codin	0	0	0	0
ENSG000001	PLPPR3	protein_codin	0	0	0	0
ENSG000001	PDLIM7	protein_codin	32.1971863	56.4403916	19.3028658	61.8974867
ENSG000001	ANKRD36B	protein_codin	108.335718	58.0561624	148.658303	24.7212154
ENSG000001	CCDC88B	protein_codin	16.4807589	89.7777248	0	26.386733

ENSG000001	AMZ2	protein_codin	37.6194287	82.0720366	22.5060545	0
ENSG000002	SERHL	lncRNA	0	0	0	0
ENSG000001	ART5	protein_codin	0	0	0	0
ENSG000001	ARHGEF18	protein_codin	32.8680659	67.1009873	23.7328012	15.2613071
ENSG000000	PHACTR3	protein_codin	0	0	0	0
ENSG000000	FRMPD1	protein_codin	0	0	0	0
ENSG000001	PML	protein_codin	0	15.4091772	0	0
ENSG000001	ADGRL2	protein_codin	0	0	0	0
ENSG000001	SULF2	protein_codin	150.660208	249.717189	84.3813894	177.308812
ENSG000000	ATG2B	protein_codin	267.750805	162.756804	141.994801	151.469301
ENSG000000	MPND	protein_codin	7.83117372	7.46131144	0	10.4221904
ENSG000001	KIF19	protein_codin	0	0	0	0
ENSG000001	TP53TG1	lncRNA	0	0	0	0
ENSG000001	IPP	protein_codin	0	6.65964138	5.77898623	3.08576216
ENSG000001	FAM3C	protein_codin	23.3081407	13.1383344	31.3487125	20.4483972
ENSG000001	NDUFB5	protein_codin	9.11431139	36.6734729	0	8.32617029
ENSG000001	PTPRF	protein_codin	24.8459379	17.2643077	0	0
ENSG000001	P2RX4	protein_codin	0	4.12911933	0	20.3907034
ENSG000001	BRMS1	protein_codin	65.7304747	129.688625	30.11667	39.6278056
ENSG000001	OR2L3	protein_codin	0	0	0	0
ENSG000000	MPP4	protein_codin	0	0	0	0
ENSG000001	CCT8L2	protein_codin	0	0	0	0
ENSG000001	ZNF517	protein_codin	2.72960486	2.10571824	0	5.28394207
ENSG000001	APAF1	protein_codin	273.190592	0	0	155.431463
ENSG000001	ZNF135	protein_codin	0	0	0	0
ENSG000001	CCSER2	protein_codin	65.1209073	30.1360988	19.5556021	44.8310114
ENSG000001	ZFYVE27	protein_codin	0	18.6690558	0	0
ENSG000001	SIGLEC6	protein_codin	0	0	0	0
ENSG000000	ATP2A3	protein_codin	0	0	0	0
ENSG000001	CLN3	protein_codin	0	0	0	0
ENSG000001	NRAP	protein_codin	0	0	0	0
ENSG000001	SRSF2	protein_codin	249.088361	349.370578	63.1220604	197.600096
ENSG000001	CRB2	protein_codin	0	0	0	0.95649608
ENSG000001	TCEAL8	protein_codin	10.5015148	29.3708902	18.9602645	17.089655
ENSG000000	SDF4	protein_codin	275.0665	357.723712	105.293359	203.099201
ENSG000001	MCMBP	protein_codin	41.8064292	51.1708027	90.1968936	47.7848635
ENSG000001	HLA-DRB1	protein_codin	1163.79756	2061.00792	766.179077	664.26757
ENSG000001	DHDDS	protein_codin	23.8370458	0	19.5965601	0
ENSG000001	ZNF133	protein_codin	0	0	0	0
ENSG000001	RAI2	protein_codin	0	0	0	0
ENSG000001	ESRRG	protein_codin	0	0	0	0
ENSG000001	KALRN	protein_codin	0	0	0	0
ENSG000001	BRD4	protein_codin	137.794478	124.728124	44.0011512	113.936731
ENSG000001	CLN3	protein_codin	0	0	0	0

ENSG000001	ARRB1	protein_codin	296.511602	496.95472	141.351075	198.365248
ENSG000001	SH2D1A	protein_codin	0	0	0	0
ENSG000001	DCAF12L2	protein_codin	0	0	0	0
ENSG000001	KCTD8	protein_codin	0	0	0	0
ENSG000001	ENTPD6	protein_codin	65.3974459	11.153024	0	0
ENSG000001	DMBX1	protein_codin	0	0	0	0
ENSG000002	NXF4	lncRNA	0	0	0	0
ENSG000001	GAL3ST4	protein_codin	0	0	0	0
ENSG000001	HOXA4	protein_codin	0	1.96735069	0	3.74865245
ENSG000001	PDE4D	protein_codin	0	0	0	4.23127377
ENSG000001	SEPTIN2	protein_codin	33.1841107	0	0	30.9538651
ENSG000000	CD84	protein_codin	8.72474881	12.9084724	4.22384276	5.36281283
ENSG000001	TPK1	protein_codin	46.3256984	46.0313192	23.0880257	12.4060993
ENSG000001	GTPBP4	protein_codin	11.2308057	22.2924869	6.2576559	4.26345297
ENSG000001	PLOD2	protein_codin	0	24.4423644	31.1502501	0
ENSG000001	CDK18	protein_codin	0	0	0	0
ENSG000001	FCER2	protein_codin	10.3226561	6.80139199	0	0
ENSG000001	XRCC6	protein_codin	615.687938	780.324449	366.13246	431.792904
ENSG000001	SPAG16-DT	lncRNA	0	0	0	0
ENSG000001	PYY	protein_codin	0	0	0	0
ENSG000001	KIRREL1	protein_codin	0	0	0	0
ENSG000001	LCE1B	protein_codin	0	0	0	0
ENSG000001	EWSR1	protein_codin	9.65798092	22.7651239	0	0
ENSG000001	ARL9	protein_codin	0	0	0	0
ENSG000000	KMT2C	protein_codin	0.0024783	128.172432	140.65732	0
ENSG000000	NFIX	protein_codin	29.8722818	70.7527018	15.606583	40.6722471
ENSG000001	CACNA1E	protein_codin	0.82054225	0	0	0
ENSG000000	TFRC	protein_codin	0	0	5.65874564	0
ENSG000001	MACF1	protein_codin	0	0	0	0
ENSG000001	ZNF107	protein_codin	0	0	0	0
ENSG000001	SPN	protein_codin	33.1775142	41.2985367	0	14.540647
ENSG000001	CYP2D6	protein_codin	0	0	0.63427721	0
ENSG000001	LIAT1	protein_codin	8.53117067	0	3.79158326	3.42464417
ENSG000001	MAK16	protein_codin	69.1065095	60.7466751	36.1014759	33.590448
ENSG000000	AGBL5	protein_codin	18.6706971	14.4410439	0	7.80052606
ENSG000001	MAGED4B	protein_codin	0	0	0	0
ENSG000001	PPIP5K1	protein_codin	11.2665788	0	0	34.7866215
ENSG000001	CD300A	protein_codin	173.672484	350.451498	108.347442	147.802468
ENSG000000	FGFR2	protein_codin	0	0	0	0
ENSG000001	MYT1	protein_codin	0	0	0	0
ENSG000000	ERGIC2	protein_codin	77.4090976	64.5093426	64.030217	41.4249647
ENSG000001	NPIPA5	protein_codin	58.284752	36.5244984	3.51109142	0
ENSG000001	SEPTIN6	protein_codin	26.7569367	18.0870822	36.7755546	10.1246357
ENSG000000	DDX3Y	protein_codin	0	0	0	0

ENSG000001	ADD3	protein_codin	0	0	4.34209088	0
ENSG000000	ZMYND10	protein_codin	0	0	0	0
ENSG000002	MFRP	protein_codin	0	0	0	0
ENSG000001	SCNN1A	protein_codin	0	0	0	0
ENSG000001	ZNG1C	protein_codin	0	0	0	0
ENSG000001	DYNC1H1	protein_codin	123.108258	96.7011445	0	66.7042124
ENSG000001	ZNF605	protein_codin	2.38032426	0	0	2.31624812
ENSG000001	CDK5RAP2	protein_codin	0	30.4423654	0	0
ENSG000001	FAM118B	protein_codin	4.58419455	2.34683748	2.36241497	0
ENSG000001	KIRREL2	protein_codin	0	0.72864035	0	0
ENSG000002	RTEL1	protein_codin	44.1066208	44.046719	0	25.5816993
ENSG000001	ZNF66	protein_codin	0	0	0	0
ENSG000001	CD46	protein_codin	0	17.725957	59.1041894	0
ENSG000001	OR52M1	protein_codin	0	1.55293375	0	0
ENSG000001	ETS2	protein_codin	27.3313876	9.65827685	8.05191218	9.11377552
ENSG000001	LHFPL5	protein_codin	0	0	0	0
ENSG000001	CTSE	protein_codin	0	0	0	0
ENSG000001	PTGES3L-AAR	protein_codin	1.86765804	0	0	0
ENSG000001	ADGRE2	protein_codin	150.214776	203.858718	39.2479563	104.775709
ENSG000001	CACNA1A	protein_codin	0	0	0	0
ENSG000001	FOXP2	protein_codin	0	0	0	0
ENSG000001	CUEDC1	protein_codin	0	7.23534776	0	0
ENSG000000	MAP4	protein_codin	41.7768772	107.549366	11.5927531	0
ENSG000001	CCDC102B	protein_codin	0	0	4.20044053	0
ENSG000001	APH1A	protein_codin	92.3360753	138.043158	39.2083772	65.467665
ENSG000001	SUPT20H	protein_codin	40.137185	97.2528001	0	42.8397677
ENSG000001	DISP1	protein_codin	0	0	0	0
ENSG000001	F8	protein_codin	6.42146526	10.7460403	26.3602895	3.9330445
ENSG000001	RIOX2	protein_codin	49.3020976	0	0	0
ENSG000002	CUX1	protein_codin	177.783887	893.483373	427.764901	402.841582
ENSG000001	AFAP1	protein_codin	175.250384	188.297256	71.2411482	69.552763
ENSG000001	MSN	protein_codin	5310.40483	7986.18969	2155.91643	4382.56095
ENSG000001	UAP1L1	protein_codin	0	46.0194952	20.4752098	15.0452502
ENSG000001	ATP2B2	protein_codin	0	0	0	0
ENSG000001	PRKAR1B	protein_codin	0	0	0	0
ENSG000001	ADGRG2	protein_codin	0	0	0	0
ENSG000001	VPS13A	protein_codin	8.72110288	13.6160738	26.4731085	17.1221168
ENSG000001	CACNB4	protein_codin	0	0	0	0
ENSG000001	NR4A1	protein_codin	0	3.79709415	0	0
ENSG000001	SUDS3	protein_codin	1.00472617	2.00100996	0	0
ENSG000001	CEP85L	protein_codin	8.381785	0	10.9243821	7.76328858
ENSG000000	SEZ6	protein_codin	0	0	0	0
ENSG000001	RAB5B	protein_codin	46.3791121	37.7745824	8.23280035	20.4718151
ENSG000001	PPIP5K1	protein_codin	0	0	0	0

ENSG00000(	MYLK	protein_codin	23.480852	51.1304355	41.0284336	0
ENSG000001!	BAZ1A	protein_codin	936.794478	746.78361	618.706912	772.404073
ENSG000001!	ZNF860	protein_codin	0	1.10321885	0	0
ENSG00000(	CASS4	protein_codin	0	0	0	8.49706203
ENSG00000(	GATAD2A	protein_codin	117.879396	85.3655889	29.4908322	64.1716598
ENSG00000(	GSDMB	protein_codin	0	0	0	0
ENSG000001!	FLNA	protein_codin	9654.59182	20057.9156	9939.90086	7719.53061
ENSG000001!	C20orf96	protein_codin	15.786363	24.3450895	4.39089702	3.10618354
ENSG00000(	HLA-G	protein_codin	0	0	0	4.79292152
ENSG00000(	CLDN7	protein_codin	0	0	0	2.0766926
ENSG00000(	MAPK8	protein_codin	0	0	0	29.0344638
ENSG000001!	ZNF460	protein_codin	201.216772	139.698014	92.1862968	112.042486
ENSG00000(	LZTS3	protein_codin	6.21743684	0	0	0
ENSG000001!	CLEC4C	protein_codin	0	0	0	0
ENSG00000(	RUNX1T1	protein_codin	0	0	0	0
ENSG00000(	MAP2	protein_codin	0	0	0	0
ENSG00000(	PARP9	protein_codin	5.58306166	13.1368889	30.8370973	0
ENSG000001!	ZNF461	protein_codin	0	0	0	0
ENSG00000(	OR2W3	protein_codin	10.0623677	21.0746557	2.42479803	3.93785329
ENSG00000(	ANKS1A	protein_codin	146.533966	218.139778	64.9251579	57.7060192
ENSG00000(	TIAM2	protein_codin	0	0	0	0
ENSG000001	SLC41A3	protein_codin	22.3895244	33.4775452	18.1176672	12.58904
ENSG000001!	OR5B21	protein_codin	0	0	0	0
ENSG00000(	LRRCC1	protein_codin	35.6323976	0	34.6538435	32.9281304
ENSG00000(	TEX56P	lncRNA	0	0	0	0
ENSG00000(	SPRR2D	protein_codin	0	0	0	0
ENSG00000(	STIL	protein_codin	0	14.3784183	0	0
ENSG000001!	SDHAF3	protein_codin	0	0	0	0
ENSG00000(	CDC16	protein_codin	0	0	0	0
ENSG00000(	HNRNPK	protein_codin	371.113364	558.499853	184.23392	201.228921
ENSG00000(	ZNF317	protein_codin	127.035675	46.467711	18.0569767	0
ENSG000001!	SLC35F1	protein_codin	0	0	0	0
ENSG00000(	TRIM9	protein_codin	0	0	1.61750061	0
ENSG00000(	EIF2B3	protein_codin	13.578142	11.8005314	8.83114112	7.58692763
ENSG000001	NRP2	protein_codin	0	0	0	0
ENSG000001!	SRGAP3	protein_codin	0	0	0	6.32345965
ENSG00000(	CRCP	protein_codin	29.887031	41.2387723	28.2254957	34.0365267
ENSG00000(	MYH10	protein_codin	0	0	0	0
ENSG00000(	UGT1A1	protein_codin	0	0	0	0
ENSG000001!	SLC22A6	protein_codin	0	0	0	0
ENSG000001	OPRM1	protein_codin	0	0	0	0
ENSG000002	SULT1A4	protein_codin	183.412056	0	47.096956	0
ENSG00000(	STAU1	protein_codin	0	234.029045	55.3582597	119.785825
ENSG00000(	DSC3	protein_codin	0	0	0	0

ENSG000001:	MDM2	protein_codin	0	0	2.17873724	0
ENSG000001(	SIN3A	protein_codin	0	0	0	0
ENSG000001!	Metazoa_SRP	misc_RNA	0	0	1.15344413	0
ENSG000001:	PAK4	protein_codin	11.9042888	0	0	12.7660146
ENSG000001(	SYTL3	protein_codin	10.2226701	0	0	0
ENSG000001:	CPNE8	protein_codin	0	2.90350617	0	0
ENSG000001:	PPARGC1B	protein_codin	17.9385206	0	9.34603433	0
ENSG000001!	CLPSL2	protein_codin	0	0	0	0
ENSG000001:	MORF4L2	protein_codin	32.0090403	70.1679464	64.649268	29.3234626
ENSG000001!	PLEKHG4	protein_codin	0	0	0	0
ENSG000001(	NDRG2	protein_codin	0	0	0	0
ENSG000001!	FABP12	protein_codin	0	0	0	0
ENSG000001(	ZNF528	protein_codin	3.69913236	8.480263	0	2.10063069
ENSG000001(	UBE2J2	protein_codin	0	0	0	0
ENSG000001:	COL4A2	protein_codin	0	0	0	0
ENSG000001!	MAN2A2	protein_codin	0	0	87.784565	0
ENSG000001(	PEA15	protein_codin	477.516289	603.93154	187.658089	329.7707
ENSG000001!	KCNRG	protein_codin	3.83923056	0	0	3.09873029
ENSG000001!	NMB	protein_codin	0	0.99782181	0	0
ENSG000001!	NRAP	protein_codin	0	0	0	0
ENSG000000'	KCNQ2	protein_codin	0	0	0	0
ENSG000001:	SPOCD1	protein_codin	0	0	0	0
ENSG000002:	LINC02384	lncRNA	0	0	0	0
ENSG000001	SLC17A2	protein_codin	0	0	0	0
ENSG000001!	MME	protein_codin	0	0	8.39175432	0
ENSG000001'	CCDC144A	protein_codin	0	0	0	0
ENSG000001(	SNTB2	protein_codin	0	0	0	0
ENSG000001:	TRPC6	protein_codin	0	0	0	0
ENSG000001	CLEC4A	protein_codin	19.2901909	27.3898393	0	9.8971158
ENSG000001:	GSTCD	protein_codin	0	6.07801001	0	6.57134962
ENSG000001'	ANGEL2	protein_codin	68.502224	0	0	5.28654895
ENSG000001	CNPPD1	protein_codin	114.348827	183.038073	56.0984571	85.1590401
ENSG000001(	WDR72	protein_codin	0	0	0	0
ENSG000001:	CDC42SE2	protein_codin	514.805234	678.777074	254.156575	356.11222
ENSG000001'	PFKFB3	protein_codin	1160.74351	769.561837	837.474926	752.969855
ENSG000001'	CCDC144A	protein_codin	0	0	0	0
ENSG000001:	PIGP	protein_codin	0	10.0178249	0	6.83511867
ENSG000001!	CES1	protein_codin	19.706295	58.105763	77.1045338	250.561655
ENSG000001!	ENSG000001!	lncRNA	1.1189778	0	0	0
ENSG000001	KIF21B	protein_codin	33.1190699	38.9880509	55.2873273	15.1861914
ENSG000001!	DPP4	protein_codin	0	0	0	0
ENSG000001(	CLIP3	protein_codin	2.83239826	4.72480871	1.10450759	1.87485883
ENSG000001	PLAGL1	protein_codin	11.83788	24.031228	0	20.5960073
ENSG000001!	TOPORS	protein_codin	121.156925	36.3566702	55.2574441	5.9337689

ENSG0000010	PCMTD1	protein_codin	91.4035231	17.4269037	32.9415147	9.78208671
ENSG000001	ETF1	protein_codin	299.439447	270.021077	267.204776	267.777202
ENSG000001	KRTAP26-1	protein_codin	0	0	0	0
ENSG000001	IL16	protein_codin	0	0	1.72585852	0
ENSG000001	TACC2	protein_codin	0	0	0	0
ENSG000001	MMP17	protein_codin	25.7001403	58.9403057	9.84814115	26.5961775
ENSG000001	SCAF1	protein_codin	304.103068	365.359976	97.646011	181.586981
ENSG000001	RRM2	protein_codin	0	0	0	0
ENSG000000	P4HA2	protein_codin	0	2.43446184	0	0
ENSG000001	FAIM	protein_codin	1.67235536	1.65410369	0	4.20214284
ENSG000001	TPPP	protein_codin	7.74632013	9.73925877	2.98804666	0
ENSG000001	VPS29	protein_codin	70.7736946	94.6049525	63.3285797	65.7633016
ENSG000001	NPSR1	protein_codin	0	0	0	0
ENSG000001	SLC6A9	protein_codin	0	0	0	0
ENSG000001	WDHD1	protein_codin	0	0	8.40861215	8.52788653
ENSG000001	FBXL22	protein_codin	0	0.88024694	1.96143116	0
ENSG000001	ANKRD53	protein_codin	0	0	0	2.54119337
ENSG000001	STYXL1	protein_codin	0	0	0	0
ENSG000001	TC2N	protein_codin	77.6977937	90.5250656	45.4895564	0
ENSG000001	MEF2D	protein_codin	170.992035	213.001286	160.40486	108.966389
ENSG000001	ACSS2	protein_codin	131.123578	206.150961	74.8208572	42.0417987
ENSG000001	MACROH2A1	protein_codin	15.8098817	0	0	22.868251
ENSG000001	URI1	protein_codin	46.5101048	22.2502728	59.2374839	60.9200958
ENSG000001	MATN4	protein_codin	0	0	0	0
ENSG000001	PIM3	protein_codin	888.75132	722.515383	176.220769	458.041911
ENSG000001	LONP1	protein_codin	28.0301282	42.7515193	21.6109583	0
ENSG000001	KLK3	protein_codin	0	0	0	0
ENSG000001	EPHB4	protein_codin	0	0	0	0
ENSG000001	ARGLU1	protein_codin	77.6344858	127.798194	188.939846	58.5260342
ENSG000001	DCLK1	protein_codin	0	0	0	0
ENSG000000	WWTR1	protein_codin	0	0	0	0
ENSG000001	ITGB1BP1	protein_codin	10.0282555	0	0	0
ENSG000001	C15orf39	protein_codin	0	0	0	0
ENSG000001	CLCN3	protein_codin	107.7943	115.533445	25.3569224	116.402646
ENSG000001	ZNF148	protein_codin	168.488994	139.590885	56.0147013	127.742684
ENSG000000	PAK3	protein_codin	0	0	0	0
ENSG000001	PCED1A	protein_codin	57.474329	69.7470805	15.1686428	29.6608148
ENSG000001	NAV2	protein_codin	0	0	12.5688326	0
ENSG000001	ATP6AP1-DT	lncRNA	7.99003307	12.9041027	5.02558979	5.87062921
ENSG000001	SLC17A3	protein_codin	0	0	0	0
ENSG000002	TLR9	protein_codin	4.78775553	0	10.9275437	0
ENSG000001	PPP3CB	protein_codin	55.1103889	110.423059	29.69041	49.1889277
ENSG000000	ATG5	protein_codin	0	0	1.48243017	0
ENSG000001	MAP1LC3A	protein_codin	7.27904033	8.36369833	0	0

ENSG000001	FAM153A	protein_codin	0	0	0	0
ENSG000001	SLA2	protein_codin	0	26.4278498	35.1354273	0
ENSG000001	ADAM15	protein_codin	0	0	0	48.2778173
ENSG000000	LGALS14	protein_codin	0	0	0	0
ENSG000001	CSNK1G3	protein_codin	0	23.9726918	27.1755023	30.0517799
ENSG000001	GDPD3	protein_codin	0	0	7.20295485	0
ENSG000000	FUT8	protein_codin	0	4.29958778	0	0
ENSG000001	HTR4	protein_codin	0	0	0	0
ENSG000001	PPARD	protein_codin	30.7426431	84.5852806	28.0810468	21.9144289
ENSG000001	ADARB1	protein_codin	0	0	12.1801459	0
ENSG000001	ZNF711	protein_codin	0	0	0	0
ENSG000000	ZC3HC1	protein_codin	0	0	0	0
ENSG000001	DRC7	protein_codin	0	0	0	0
ENSG000001	SQSTM1	protein_codin	286.275697	831.91962	261.863276	273.762386
ENSG000001	PAQR8	protein_codin	0	0	19.536791	39.6974291
ENSG000001	ZNF567	protein_codin	22.5728073	0	0	0
ENSG000001	CLIC6	protein_codin	0	0	0	0
ENSG000001	MIRLET7BHG	lncRNA	26.3314357	11.8932366	7.53723023	8.51691629
ENSG000001	TTI2	protein_codin	3.75173991	14.0331674	6.61343048	0
ENSG000000	ST7L	protein_codin	15.7887534	0	6.45942651	11.4989951
ENSG000001	RDH16	protein_codin	0	0	1.08069613	0.98935791
ENSG000001	SPATS2L	protein_codin	20.5583781	22.9669877	0	0
ENSG000000	CACNA1G	protein_codin	0	0	0	0
ENSG000001	VKORC1L1	protein_codin	0	0	0	0
ENSG000002	KRTAP12-2	protein_codin	0	0	0	0
ENSG000001	GYPA	protein_codin	0	0	0	0
ENSG000000	MYLK	protein_codin	0	0	0	16.4150769
ENSG000001	TRPM6	protein_codin	0	0	0	0
ENSG000001	SDCBP2	protein_codin	0	0	0	0
ENSG000001	C20orf203	protein_codin	0	0	0	0
ENSG000001	GJA9	protein_codin	0	0	0	0
ENSG000001	HNRNPA2B1	protein_codin	298.501525	352.724525	383.180531	138.204027
ENSG000001	RBBP8	protein_codin	0	0	0	0
ENSG000000	CYB561	protein_codin	0	0	0	10.2446017
ENSG000001	BSCL2	protein_codin	0	0	0	0
ENSG000001	LSR	protein_codin	0	17.6063537	0	3.07068349
ENSG000001	UBAP2	protein_codin	27.5394468	33.5870007	0	0
ENSG000001	GTPBP4	protein_codin	148.726192	119.45453	108.676596	104.588313
ENSG000001	SPP1	protein_codin	0	0	2.68991725	0
ENSG000001	PI4KAP2	transcribed_u	0	0	0	0
ENSG000001	RRBP1	protein_codin	0	0	0	0
ENSG000001	GLT8D2	protein_codin	0	0	0	0
ENSG000001	FAM217B	protein_codin	64.7113678	57.1525932	52.1215505	44.4344651
ENSG000001	C1S	protein_codin	0	0	0	0

ENSG000001	SCYL2	protein_codin	182.77226	172.256666	113.196597	170.316006
ENSG000001	CDK5RAP2	protein_codin	15.0634573	0	0	0
ENSG000000	TRPM3	protein_codin	0	0	0	0
ENSG000001	FAM177B	protein_codin	0	0	0.98651948	0
ENSG000001	RPS24	protein_codin	102.881155	108.954925	33.3662116	73.9732614
ENSG000000	NLE1	protein_codin	0	0	0	0
ENSG000001	ELMO3	protein_codin	0	0	0	0
ENSG000001	SCUBE1	protein_codin	0	1.80646944	0	2.72682715
ENSG000001	ANKHD1	protein_codin	0	0	0	0
ENSG000001	MRPL37	protein_codin	27.112954	70.5912175	10.5383834	20.6307839
ENSG000001	TLK1	protein_codin	0	0	0	13.4047063
ENSG000002	SIGLEC14	protein_codin	393.781673	892.350671	216.810773	447.414569
ENSG000001	GNL3L	protein_codin	270.543686	461.287036	224.48198	212.759093
ENSG000001	RGL1	protein_codin	35.0310813	29.9199249	6.52699251	21.4607479
ENSG000001	COL9A1	protein_codin	0	0	0	0
ENSG000001	NAA60	protein_codin	0	0	0	0
ENSG000001	RGS22	protein_codin	0	0	0	0
ENSG000001	DNAJC5	protein_codin	280.429072	487.87373	87.7042731	223.303243
ENSG000001	NOL8	protein_codin	44.3523732	24.6824033	41.6350215	0
ENSG000001	TTN	protein_codin	1.05303745	0	0	2.04783109
ENSG000001	PRDM10	protein_codin	58.4633377	30.29983	38.1108175	9.28205059
ENSG000002	LAT	protein_codin	0	12.5951389	0	0
ENSG000000	GLI2	protein_codin	0	0	0	0
ENSG000001	EIF3B	protein_codin	58.0841089	136.913145	24.3482575	12.7240999
ENSG000001	SOX10	protein_codin	0	0	0	0
ENSG000001	WDFY4	protein_codin	4.94312219	0	0	0
ENSG000001	LINC00523	lncRNA	0	0	0	0
ENSG000001	MTHFSD	protein_codin	18.5324804	48.9228227	13.3997557	34.6753657
ENSG000001	TRRAP	protein_codin	37.9155602	38.1942076	20.1396358	20.3430211
ENSG000000	ERC1	protein_codin	158.405567	216.50554	79.6914355	0
ENSG000000	NLRC4	protein_codin	0	66.0659486	29.2459263	30.7388933
ENSG000001	DAAM1	protein_codin	256.126803	207.936703	223.860149	126.84038
ENSG000001	ZMYND8	protein_codin	134.477679	0	0	0
ENSG000001	PACRGL	protein_codin	13.5335078	0	0	0
ENSG000001	SEZ6L	protein_codin	22.2818401	25.0615865	0	13.9977489
ENSG000000	AOC1	protein_codin	0	0	0.87059165	0
ENSG000001	ETS2	protein_codin	586.470001	463.560145	334.049275	388.527579
ENSG000001	UBE2Z	protein_codin	164.514384	211.958411	72.8058824	126.708647
ENSG000001	TOMM20L	protein_codin	0	0	0	0
ENSG000001	ZNF219	protein_codin	16.7410716	0	0	10.070321
ENSG000000	ZCWPW1	protein_codin	0	0	0	0
ENSG000001	HS3ST3B1	protein_codin	5.59259125	15.5844548	1.89239908	0
ENSG000001	PPP1R13L	protein_codin	0	0	0	0
ENSG000000	MPC1	protein_codin	67.7063814	108.221676	52.9335004	34.9892575

ENSG000000	CACNA2D2	protein_codin	0	0	0	0
ENSG000001	FGF1	protein_codin	0	0	0	0
ENSG000002	DBNDD2	protein_codin	24.5100028	38.3152914	9.93834771	14.907607
ENSG000000	NCKAP1	protein_codin	89.4912857	40.0825528	3.7227599	0
ENSG000001	CPZ	protein_codin	0	0	0	0
ENSG000001	KCNIP3	protein_codin	0	0	0	0
ENSG000001	AEBP2	protein_codin	37.7654258	21.2098576	17.1234384	10.2311626
ENSG000001	FAM107A	protein_codin	0	0	0	0
ENSG000001	STRBP	protein_codin	6.59292469	36.068586	54.9534266	16.0986451
ENSG000000	PRSS3	protein_codin	0	0	0	0
ENSG000001	GIT2	protein_codin	323.401054	438.591057	190.290866	103.720492
ENSG000001	MYO1C	protein_codin	0	142.620209	51.6219844	0
ENSG000001	CTRB1	protein_codin	0	0	0	0
ENSG000001	DENND4C	protein_codin	0	0	0	0
ENSG000001	ZSCAN12	protein_codin	0	15.2408408	12.2657385	0
ENSG000001	TRDN	protein_codin	0	0	0	0
ENSG000001	TMEM239	protein_codin	0	0	0	0
ENSG000000	GRAMD4	protein_codin	288.729288	244.195974	46.9394519	0
ENSG000001	HAO2	protein_codin	0	0	0	0
ENSG000001	GPAA1	protein_codin	0	0	0	0
ENSG000001	GON4L	protein_codin	0	0	29.268824	0
ENSG000001	WASF3	protein_codin	60.2497293	0	56.2869039	26.6454707
ENSG000002	RBMY1A1	protein_codin	0	0	0	0
ENSG000001	PLEKHS1	protein_codin	0	0	0	0
ENSG000001	MPEG1	protein_codin	5202.0798	7571.48535	3113.47673	5497.67087
ENSG000001	BCO2	protein_codin	0	0	0	0
ENSG000000	WDR47	protein_codin	0	0	0	0
ENSG000001	UBA3	protein_codin	137.387159	152.17877	118.113035	179.657167
ENSG000001	MEPE	protein_codin	0	0	0	0
ENSG000002	MATR3	protein_codin	71.9730884	199.069654	0	79.022629
ENSG000001	GFPT1	protein_codin	66.307517	0	75.3272774	49.8395724
ENSG000001	GSTM3	protein_codin	0	0	0	0
ENSG000000	LAMC3	protein_codin	0	0	0	0
ENSG000001	RFWD3	protein_codin	44.6872827	110.134912	19.3116554	46.9182441
ENSG000000	PUM2	protein_codin	277.371398	276.180921	151.162057	307.551684
ENSG000000	LAT2	protein_codin	0	2.81664701	0	0
ENSG000001	RAB25	protein_codin	0	0	0	0
ENSG000000	ZNF37A	protein_codin	66.971412	26.3627618	31.4334697	61.2071233
ENSG000001	NMRK1	protein_codin	14.2051124	19.8657503	0	15.3362622
ENSG000001	GART	protein_codin	32.9204821	7.87700843	13.3409265	18.5855713
ENSG000001	STAT1	protein_codin	0	0	0	0
ENSG000001	PIK3CD	protein_codin	47.7699817	37.8407159	0	25.0683295
ENSG000001	SPAG11B	protein_codin	0	0	0	0
ENSG000001	ZNF627	protein_codin	15.5759521	26.5648039	8.54150256	0

ENSG000001(	MICU1	protein_codin	209.249831	401.466398	103.293802	152.176326
ENSG000001	CCL28	protein_codin	3.99368489	2.65898446	2.41345708	0
ENSG000001	GPALPP1	protein_codin	0	0	0	19.1895375
ENSG000001	ITPRIPL1	protein_codin	3.15714361	2.06597239	5.00081287	1.05592396
ENSG000001	SMURF1	protein_codin	0	0	0	0
ENSG000001	LRIG2	protein_codin	70.5056545	52.6681102	18.5426594	29.7637823
ENSG000000	MARK2	protein_codin	0	0	0	0
ENSG000001	PPP1R14C	protein_codin	3.08832256	4.14096479	1.09324232	1.0253959
ENSG000001	RASGEF1C	protein_codin	0.82432178	0.84618347	0	0
ENSG000001	SLC34A3	protein_codin	0	0	0	0
ENSG000001	SMC5	protein_codin	468.080901	277.928768	257.145642	291.840034
ENSG000001	YY1AP1	protein_codin	0	6.48407229	5.83640433	0
ENSG000001	ABHD14B	protein_codin	38.9835327	79.6078214	22.0506007	22.172172
ENSG000001	KAZN	protein_codin	0	0	0	0
ENSG000001	KCNN3	protein_codin	0	0	0	3.69587147
ENSG000001	ZWINT	protein_codin	1.46884123	0	7.82322284	0
ENSG000001	OPA1	protein_codin	59.305111	22.4072322	0	0
ENSG000001	DZIP1	protein_codin	0	0	0	0
ENSG000001	IFI6	protein_codin	79.554896	158.202252	44.9422458	73.8287834
ENSG000001	RBM39	protein_codin	231.322421	0	237.893207	104.047826
ENSG000001	POTEF	protein_codin	1.84303653	2.81886231	0	1.91072689
ENSG000001	NF2	protein_codin	0	0	2.19889108	0
ENSG000001	CLK2	protein_codin	0	144.684491	28.3934193	55.1724632
ENSG000001	IQGAP3	protein_codin	0	0	0	0
ENSG000001	MVB12B	protein_codin	49.427784	133.497542	62.6012919	59.4272827
ENSG000001	PAPSS2	protein_codin	33.5602227	48.3051594	24.6184456	6.84012047
ENSG000001	CAPZA2	protein_codin	418.458051	344.298232	228.032601	228.556696
ENSG000001	PRC1	protein_codin	15.7106091	7.13536753	0	0
ENSG000001	PJA2	protein_codin	899.157653	693.734729	665.03059	678.798229
ENSG000001	SLC26A11	protein_codin	0	0	0	17.7745748
ENSG000001	QKI	protein_codin	460.275574	407.664722	74.2057636	174.07684
ENSG000001	LDB1	protein_codin	14.3209097	15.921356	0	2.42690058
ENSG000001	STYXL2	protein_codin	0	0	0	0
ENSG000002	CSAG4	lncRNA	0	0	0	0
ENSG000001	DST	protein_codin	49.7159925	0	64.4722881	58.2680867
ENSG000001	SREBF2	protein_codin	230.209296	343.484247	73.1973833	128.617627
ENSG000001	ASTN2	protein_codin	0	0	0	0
ENSG000000	PTGER3	protein_codin	0	0	0	0
ENSG000001	CSAG1	protein_codin	0	0	0	0
ENSG000000	ATP1A2	protein_codin	0	0	0	0
ENSG000001	DENND4B	protein_codin	397.854229	379.920484	99.5774344	190.420028
ENSG000001	SERBP1	protein_codin	67.7602576	24.8603091	22.6248645	0
ENSG000000	ARHGEF10L	protein_codin	14.1181746	14.8467341	0	0
ENSG000001	RUSC2	protein_codin	58.0321283	141.567322	68.8995929	115.28245

ENSG00000015	MT-ND3	protein_codin	1510.89541	2025.0994	525.824324	1247.26452
ENSG00000015	RASSF9	protein_codin	0	0	0	1.62824025
ENSG00000015	GPRASP3	protein_codin	0	0	0	0
ENSG000001	BTN3A3	protein_codin	0	45.3505084	0	9.7284364
ENSG00000017	RNF34	protein_codin	43.8395627	56.6644338	44.2868009	0
ENSG00000013	TMEM123	protein_codin	51.5670288	28.2655138	3.39503475	30.3635822
ENSG00000015	ATG9A	protein_codin	81.9580812	79.3479164	47.6162912	44.426275
ENSG00000017	AKAP13	protein_codin	2140.808	1499.27284	1340.38076	1091.89897
ENSG00000015	CDC42BPB	protein_codin	97.8095267	150.483674	64.5857601	80.9326346
ENSG000001	ARHGEF2	protein_codin	188.348707	306.67475	0	136.729253
ENSG000001	MXI1	protein_codin	0	0	0	0
ENSG00000013	C8A	protein_codin	0	0	0	0
ENSG00000016	CD300LF	protein_codin	19.1325058	60.2094128	11.0808978	42.3829491
ENSG000001	TRPM6	protein_codin	0	6.11030574	0	0
ENSG00000015	SPOUT1	protein_codin	25.9656868	73.4922626	24.4522514	24.9349683
ENSG00000015	DCAF12	protein_codin	192.932346	297.468479	73.3699471	128.371194
ENSG00000010	HACD1	protein_codin	0	5.10108046	0	0
ENSG00000015	CNOT7	protein_codin	127.33055	117.630253	71.1623568	109.11317
ENSG00000010	BNIPL	protein_codin	1.92046666	0	5.25457644	0
ENSG00000013	TBRG4	protein_codin	8.87226214	22.7558209	0	8.39779968
ENSG00000015	CHAMP1	protein_codin	0	86.4052073	0	0
ENSG00000000	TAF11	protein_codin	51.1515561	0	0	0
ENSG00000000	CTNNA2	protein_codin	0	0	0	0
ENSG00000013	CHD1L	protein_codin	0	0	0	0
ENSG00000008	MAST2	protein_codin	79.6138954	96.1523164	44.6043286	51.6387282
ENSG00000010	BEX3	protein_codin	121.006009	362.343059	103.919989	162.151935
ENSG00000008	PHACTR3	protein_codin	0	0	10.0247612	0.87517073
ENSG00000000	WNT16	protein_codin	0	0	0	0
ENSG00000010	AGL	protein_codin	0	8.09659523	0	0
ENSG00000010	LDLRAD4	protein_codin	0	0	0	0
ENSG00000015	EGFL6	protein_codin	0	0	0	0
ENSG00000010	LMNA	protein_codin	0	0	0	0
ENSG00000015	CD47	protein_codin	40.87486	49.3717004	38.7622249	51.7271417
ENSG00000014	ZNF438	protein_codin	22.7524157	0	0	0
ENSG00000017	CLSTN1	protein_codin	290.056337	510.848967	163.629408	182.344828
ENSG000001	GPX7	protein_codin	0	3.0602859	2.1855479	1.00586923
ENSG00000017	TCEA2	protein_codin	0	0	0	0
ENSG00000010	ATP1B4	protein_codin	0	0	0.95662732	0
ENSG00000015	LRRTM3	protein_codin	0	0	0	0
ENSG00000012	ZNF644	protein_codin	2.4655785	1.97514371	9.18158207	6.44559244
ENSG00000000	CAMK1G	protein_codin	0.90093282	0	0	0
ENSG00000012	ZSCAN20	protein_codin	0	6.15089763	0	5.13254298
ENSG00000025	NXF5	lncRNA	0	0	0	0
ENSG00000015	CTNND1	protein_codin	0	0	0	0

ENSG000002	MT-ND4L	protein_codin	1138.43483	1404.24165	1012.10369	1047.05494
ENSG000001	TOP1	protein_codin	764.119421	968.706431	865.678163	739.410497
ENSG000001	IFT140	protein_codin	0	0	0	0
ENSG000001	FOXJ3	protein_codin	217.458124	164.486634	0	0
ENSG000001	SSBP3-AS1	lncRNA	1.00283291	2.02862441	0	1.01762341
ENSG000001	UBL7	protein_codin	49.5167842	48.4052696	25.9303669	33.3797989
ENSG000001	VWA5A	protein_codin	8.86322184	0	22.562154	16.8062147
ENSG000000	NCKAP1	protein_codin	3.14692667	0	4.60688712	0
ENSG000002	AMY2B	protein_codin	40.7313135	21.0586612	4.99210203	32.9379446
ENSG000001	MAP3K3	protein_codin	545.24478	740.410135	321.288238	368.642817
ENSG000001	POU3F3	protein_codin	0	0	0	0
ENSG000001	ENTREP3	protein_codin	0	14.249048	0	21.681001
ENSG000001	ZFP2	protein_codin	0	0	0	0
ENSG000001	EIF1AY	protein_codin	0	0	0	0
ENSG000001	CTR9	protein_codin	340.386998	304.090096	284.418664	269.195968
ENSG000001	SMURF1	protein_codin	184.340714	158.227563	77.9059255	93.7713841
ENSG000001	LTN1	protein_codin	162.587267	152.151993	126.300645	133.668647
ENSG000001	LDB3	protein_codin	0	0	0	0
ENSG000001	MOCS2	protein_codin	0	0	0	0
ENSG000001	TNNI1	protein_codin	0	1.88454479	0	0
ENSG000001	MT-ND4	protein_codin	19548.6115	24398.2564	14026.0144	14443.4452
ENSG000001	CTNNBL1	protein_codin	289.170438	359.983423	194.947883	185.591538
ENSG000001	DCLRE1A	protein_codin	21.1672056	7.60078665	23.6608363	7.33986363
ENSG000000	ZFP64	protein_codin	5.60200728	2.749208	6.50222185	7.83563533
ENSG000001	GSTZ1	protein_codin	0	0	0	0
ENSG000001	MT-ND1	protein_codin	21840.4659	28828.6587	11706.2561	13585.5639
ENSG000001	CTNND1	protein_codin	0	0	0	0
ENSG000001	ST3GAL3	protein_codin	0	0	0	0
ENSG000001	ESRRG	protein_codin	0	0	0	0
ENSG000001	DZIP1	protein_codin	0	0	0	0
ENSG000001	ST3GAL3	protein_codin	0	0	0	0
ENSG000001	ARHGEF11	protein_codin	0	69.3464633	14.0459868	69.3517051
ENSG000001	KIAA0753	protein_codin	47.3519449	30.6604849	4.20896509	27.5090968
ENSG000000	PHLDB1	protein_codin	0	0	0	0
ENSG000001	KIF21A	protein_codin	0	0	0	0
ENSG000001	CERS2	protein_codin	0	0	0	0
ENSG000001	IGSF1	protein_codin	0	0	0	0
ENSG000001	TOX	protein_codin	2.00463053	14.7617554	11.944206	6.13158566
ENSG000001	TLR10	protein_codin	0	0	0	1.29106365
ENSG000001	HMGN2	protein_codin	474.288823	660.313563	331.414838	419.217719
ENSG000001	ZNF318	protein_codin	476.264329	480.032961	190.561784	414.897871
ENSG000001	DENND10	protein_codin	169.642102	157.99358	60.0300922	130.796906
ENSG000001	IRAG2	protein_codin	62.7297406	0	114.775569	0
ENSG000000	ADGRL1	protein_codin	10.086002	13.8218846	5.29442886	8.76408848

ENSG000001!	RPL12	protein_codin	1702.06178	2589.34106	787.896254	1383.93263
ENSG000001(	NSMCE1	protein_codin	58.5769781	78.5569368	37.510154	45.5704314
ENSG000002	APOBEC3C	protein_codin	244.108758	310.258898	204.914207	241.791174
ENSG000001(	SP8	protein_codin	0	0	0	0
ENSG000001	ST6GALNAC4	protein_codin	0	7.1031916	0	0
ENSG000001!	MTOR	protein_codin	130.028013	370.056078	0	159.289778
ENSG000002	GAGE12B	protein_codin	0	0	0	0
ENSG000001	NF2	protein_codin	0	0	0	0
ENSG000001!	MT-ND2	protein_codin	18153.9942	24257.75	8976.81344	12970.0118
ENSG000001!	TOGARAM1	protein_codin	36.7395244	28.4169024	30.2211163	34.5039441
ENSG000001!	OGA	protein_codin	574.100255	518.953226	394.439253	200.376046
ENSG000001!	MYBPC1	protein_codin	0	0	0	0
ENSG000001!	MAGEE1	protein_codin	0	0.97746256	0	0
ENSG000001!	DMD	protein_codin	0	0	0	0
ENSG000002	PRR5-ARHGA	protein_codin	0	0	0	0
ENSG000001	NRG2	protein_codin	0	0	0	0
ENSG000001!	TGM2	protein_codin	22.6226064	18.3383081	11.3765589	30.698207
ENSG000001	ASTN2	protein_codin	0	0	0	0
ENSG000001	PJA1	protein_codin	6.71757317	0	3.62546546	10.8529377
ENSG000001!	MPHOSPH8	protein_codin	391.436993	326.059168	236.568419	300.734289
ENSG000001!	PHETA1	protein_codin	36.1546289	0	0	0
ENSG000001!	LRRC10	protein_codin	0	1.13795642	0	0
ENSG000001	PSMD9	protein_codin	7.78866908	4.2682595	1.58040744	1.44787199
ENSG000001!	PAX9	protein_codin	0	0	0	0
ENSG000001(	FAM110B	protein_codin	0	0	0	0
ENSG000001(	HPS1	protein_codin	3.56003034	0	0	0
ENSG000000	GLI2	protein_codin	0	0	0	0
ENSG000001	BCL2L11	protein_codin	0	1.07428133	0	0
ENSG000001!	RCSD1	protein_codin	80.3634804	179.593484	2.6230892	92.823478
ENSG000000	FAM135A	protein_codin	0	29.9733007	25.1118727	29.1232475
ENSG000001!	CES1	protein_codin	29.7055365	0	0	0
ENSG000001!	PNP	protein_codin	180.323207	344.005528	169.704623	132.125752
ENSG000000	GAB2	protein_codin	532.307143	1031.2728	242.279794	337.360387
ENSG000000	IGSF9	protein_codin	0	0	0	0
ENSG000001!	OPA1	protein_codin	0	57.5238981	91.2625769	127.119499
ENSG000000	PPEF1	protein_codin	0	0	0	0
ENSG000001	FCRL2	protein_codin	0	0	1.09413468	0
ENSG000001!	ZNF511	protein_codin	65.5861258	73.9968347	15.4281486	49.0788908
ENSG000000!	ACOT7	protein_codin	3.98649458	6.6948622	3.18693677	0
ENSG000001	BECN1	protein_codin	11.4340041	51.9543386	0	78.0049408
ENSG000001!	ZNF521	protein_codin	0	11.8671796	3.81009137	8.95788242
ENSG000001!	ESRRG	protein_codin	0	0	0	0
ENSG000000	CNOT4	protein_codin	0	0	0	0
ENSG000001	SLC25A47	protein_codin	0	0	0	0

ENSG000001	CNKSR1	protein_codin	0	0	0	1.06892472
ENSG000001	MRPL24	protein_codin	10.4025856	53.0012883	18.3942462	3.43941999
ENSG000001	ENSA	protein_codin	0	1.47532297	2.1041226	0
ENSG000002	CRYZL1	protein_codin	0	9.52064846	5.65778263	0
ENSG000001	ECI2	protein_codin	24.6349533	51.8169641	0	17.407245
ENSG000001	BRINP2	protein_codin	0	0	0	0
ENSG000001	SGMS1	protein_codin	0	0	9.70794489	3.18553518
ENSG000000	CDC14A	protein_codin	0	0	0	0
ENSG000001	CLIP2	protein_codin	188.400442	358.541549	142.657896	104.589581
ENSG000001	UBAP2L	protein_codin	0	139.769073	0	0
ENSG000001	SELENON	protein_codin	91.8621193	121.359811	25.2223389	6.77646716
ENSG000001	GRHL3	protein_codin	0	0	0	0
ENSG000001	FICD	protein_codin	0	0	4.40293855	0
ENSG000001	OSBPL9	protein_codin	4.44859327	0	17.3319546	4.75862196
ENSG000001	PJA2	protein_codin	134.007504	0	0	0
ENSG000001	MUC2	protein_codin	1.73866023	0	2.39646173	0
ENSG000000	MAP2	protein_codin	2.92445018	0	0	0
ENSG000000	WNT8A	protein_codin	0	0	0	0
ENSG000001	OSTC	protein_codin	122.10926	156.302099	88.707028	100.378793
ENSG000001	IPO9	protein_codin	227.126032	225.346615	137.027268	128.482402
ENSG000001	KRT19	protein_codin	0	0	0	0
ENSG000001	MT-ND5	protein_codin	13196.1787	21589.8731	7835.24521	10301.6894
ENSG000002	PPT2	protein_codin	0	0	0	0
ENSG000001	MAPK10	protein_codin	0	0	0	0
ENSG000001	CAMTA2	protein_codin	39.1047771	31.7630351	15.869071	0
ENSG000001	RBPJ	protein_codin	1.84813438	0	0	2.07065619
ENSG000001	SLC9A8	protein_codin	5.30682028	0	0	0
ENSG000001	R3HDM4	protein_codin	336.895689	544.893671	191.917236	229.0922
ENSG000001	RPL39	protein_codin	987.388195	1857.54739	467.508824	870.530696
ENSG000001	TOGARAM1	protein_codin	0	0	0	0
ENSG000000	KIFAP3	protein_codin	52.1152698	71.1632578	71.1888573	22.0416548
ENSG000001	ABLIM2	protein_codin	0	0	0	0
ENSG000001	DZIP3	protein_codin	0	0	29.0319231	7.27461276
ENSG000001	ZFP69B	protein_codin	0	0	0	0
ENSG000001	HIPK1	protein_codin	0	0	0	0
ENSG000001	BCAN	protein_codin	0	0	0	0
ENSG000001	NLGN1	protein_codin	0	0	0	0
ENSG000000	CREM	protein_codin	0	0	0	0
ENSG000001	GPRASP1	protein_codin	0	0	0	0
ENSG000001	COL4A5	protein_codin	0	0	0	0
ENSG000001	C1orf174	protein_codin	57.304788	75.6552358	23.3435595	20.4892751
ENSG000001	DLX1	protein_codin	0	0	0	0
ENSG000001	PSMB5	protein_codin	11.3569025	28.6522405	6.14285293	14.9081989
ENSG000001	MFAP3L	protein_codin	567.412403	696.462272	491.857728	353.456167

ENSG000001	GTPBP3	protein_codin	0	1.85325192	0	0
ENSG000001	MT-CO1	protein_codin	47658.2397	70739.022	24491.717	45572.9116
ENSG000001	ZFYVE9	protein_codin	0	3.91929286	0	0
ENSG000001	ARHGAP11A	protein_codin	9.82617757	4.64413428	24.9729634	4.4152984
ENSG000001	ENSA	protein_codin	0	0	0	0
ENSG000001	AKR7A3	protein_codin	1.90545608	2.00705382	0	1.18219773
ENSG000001	TSEN15	protein_codin	0	18.457127	7.07375185	6.38884875
ENSG000001	MAGEB3	protein_codin	0	0	0	0
ENSG000001	ATP11C	protein_codin	60.9066833	41.466822	36.5064147	0
ENSG000001	CLIP1	protein_codin	322.023792	101.746296	171.405005	252.229065
ENSG000001	PCDH11X	protein_codin	0	0	0	0
ENSG000000	ULK2	protein_codin	98.1510212	81.3843009	52.7116397	68.5153198
ENSG000001	TYW1	protein_codin	0	0	0	71.6791289
ENSG000001	SLC26A1	protein_codin	0	0	0	0
ENSG000001	SAXO5	protein_codin	0	0	0	0
ENSG000000	NFE2L1	protein_codin	23.2929672	30.5569736	35.7497825	0
ENSG000001	GRM3	protein_codin	0	0	1.01355934	1.04299557
ENSG000001	LSM14B	protein_codin	17.6656442	10.3280463	2.1664321	0
ENSG000001	NAA35	protein_codin	96.915167	75.6725408	60.4846173	64.055348
ENSG000000	MSL3	protein_codin	0	4.26559849	0	0
ENSG000001	ALPK2	protein_codin	0	1.22791625	0	0
ENSG000001	CALD1	protein_codin	8.42977656	9.71054612	102.69186	0
ENSG000001	NF2	protein_codin	52.3967502	0	0	0
ENSG000001	RUNDC1	protein_codin	50.3598596	137.900666	44.8472833	62.525739
ENSG000001	ANKMY1	protein_codin	11.0041206	1.47151116	3.618262	2.09263966
ENSG000001	WDR88	protein_codin	0	0	0	0
ENSG000001	MT-ND6	protein_codin	83.9851289	219.130165	111.10651	85.3250808
ENSG000000	COMT	protein_codin	0	37.8280414	0	0
ENSG000001	TMEM184B	protein_codin	0	57.2599567	0	12.8069569
ENSG000001	MYBPC1	protein_codin	0	0	0	0
ENSG000001	ST6GAL2	protein_codin	0	0	0	0
ENSG000001	TBCK	protein_codin	8.80002548	0	0	23.463939
ENSG000001	OR10R3P	unprocessed_	0	0	0	0
ENSG000001	MACF1	protein_codin	0	0	0	0
ENSG000000	SRPK1	protein_codin	0	0	0	0
ENSG000001	TIA1	protein_codin	0	0	0	17.0944307
ENSG000000	UTS2	protein_codin	0	0	7.02968484	16.5184606
ENSG000001	L1CAM	protein_codin	0	0.91332201	0	0.81128646
ENSG000001	SLC17A3	protein_codin	0	0	0	0
ENSG000001	BABAM2	protein_codin	0	4.50557065	28.5351754	0
ENSG000001	APOL3	protein_codin	0	10.3781901	0	5.3559161
ENSG000001	OSGIN1	protein_codin	1.47237244	0	0	2.12631434
ENSG000001	ANKRD30A	protein_codin	0	0	0	1.05958742
ENSG000001	OPA1	protein_codin	0	0	0	0

ENSG000001	VAMP1	protein_codin	14.0173261	21.7080781	13.7476927	4.10184233
ENSG000001	TIFA	protein_codin	16.337465	17.387643	17.9054006	0
ENSG000001	GRM1	protein_codin	0	0	0	0
ENSG000001	GPATCH3	protein_codin	46.0234134	95.2666996	34.1226035	28.9426648
ENSG000001	TBKBP1	protein_codin	0	2.97378973	3.5041004	0
ENSG000001	FAM111A	protein_codin	0	0	0	92.6854443
ENSG000001	PCDH11X	protein_codin	0	0	0	0
ENSG000001	DLX1	protein_codin	0	0	0	0
ENSG000001	GJB1	protein_codin	0	0	0	0
ENSG000001	CNTNAP2	protein_codin	0	0	0	0
ENSG000000	E2F2	protein_codin	25.8876249	41.0987159	37.0492956	15.9630022
ENSG000001	SFT2D1	protein_codin	45.3104344	58.1831414	24.4713117	36.4263763
ENSG000001	RAD17	protein_codin	0	0	0	0
ENSG000001	MAP3K3	protein_codin	384.6776	394.539956	52.4450022	174.770684
ENSG000000	METTL13	protein_codin	45.3531322	57.6627497	0	18.7556584
ENSG000001	ABLIM2	protein_codin	0	0	0	0
ENSG000001	MINDY1	protein_codin	22.3726947	26.84375	7.42963969	0
ENSG000001	MT-CO2	protein_codin	7826.07453	8265.93888	3911.36871	6164.5833
ENSG000001	CYB5R3	protein_codin	0	0	0	0
ENSG000001	ISLR2	protein_codin	0	0	0	0
ENSG000001	DMAP1	protein_codin	0	13.4170857	0	0
ENSG000001	ST3GAL3	protein_codin	0	0	0	0
ENSG000001	QKI	protein_codin	398.111528	204.138648	147.503417	273.389864
ENSG000001	RNF121	protein_codin	0	0	23.7764139	31.8818181
ENSG000000	SBNO2	protein_codin	546.784316	727.444676	207.421744	346.029702
ENSG000001	QKI	protein_codin	288.215341	304.566873	110.404308	192.119505
ENSG000001	PCDH7	protein_codin	0	0	0	0
ENSG000001	CD3E	protein_codin	10.1408582	8.21857161	0	0
ENSG000001	ANKRD13C-D	lncRNA	0	4.50413147	0	0
ENSG000001	WDTC1	protein_codin	71.4359852	105.609198	61.8096416	114.516756
ENSG000001	TGFB1I1	protein_codin	0	0	33.9977051	21.8397167
ENSG000001	FOXJ3	protein_codin	0	0	0	0
ENSG000001	PIGO	protein_codin	0	18.6379444	14.9015664	0
ENSG000001	IL1RN	protein_codin	0	0	0	0
ENSG000001	SGMS1	protein_codin	124.526534	117.607472	64.3703297	100.931025
ENSG000000	RNF13	protein_codin	0	41.1326422	0	0
ENSG000001	CIPC	protein_codin	56.1295332	39.1595931	30.4025695	28.9593859
ENSG000001	MT-CYB	protein_codin	8399.84118	10671.9752	4068.87647	6674.24037
ENSG000001	LSR	protein_codin	0	0	0	0
ENSG000000	PAX2	protein_codin	0	0	0	0
ENSG000001	L3MBTL3	protein_codin	49.2111614	37.6131637	25.486761	18.86473
ENSG000001	CELF4	protein_codin	0	0	0	0
ENSG000001	CTNND1	protein_codin	0	0	0	0
ENSG000001	RNF220	protein_codin	13.909162	21.0071289	7.03721504	3.93275806

ENSG000001	ABCG1	protein_codin	40.0496915	14.5568232	16.5797592	10.4010181
ENSG000001	ST6GAL2	protein_codin	0	0	0	0
ENSG000001	SMC3	protein_codin	271.554356	239.90364	238.766574	299.96552
ENSG000001	ZNF485	protein_codin	1.04402221	3.1570901	1.87575599	3.17019356
ENSG000001	ST3GAL3	protein_codin	0	0	0	0
ENSG000001	SMG5	protein_codin	297.171236	582.425248	209.699884	130.404717
ENSG000001	PSMD10	protein_codin	0	4.42134016	0	0
ENSG000000	MAP2K3	protein_codin	26.1361452	0	0	0
ENSG000001	GRIN3A	protein_codin	1.99122174	2.00664961	2.05557339	2.02540705
ENSG000001	ZNF277	protein_codin	44.756585	16.1221916	32.4373152	16.509769
ENSG000000	TRPM3	protein_codin	0	0	0	0
ENSG000001	HORMAD1	protein_codin	5.66131528	0	4.33227058	10.226287
ENSG000001	OPA1	protein_codin	0	0	0	0
ENSG000001	YY1AP1	protein_codin	0	0	0	8.26570156
ENSG000001	BLOC1S2	protein_codin	0	2.88707416	0	0
ENSG000001	ASTN1	protein_codin	0	0	0	0
ENSG000001	FCRL5	protein_codin	0	0	0	3.05567773
ENSG000001	TEDC2	protein_codin	0	0	0	0
ENSG000001	SPRYD7	protein_codin	3.01751445	9.14231891	8.02852955	10.1155527
ENSG000001	XAF1	protein_codin	418.652506	202.225021	276.961024	256.09948
ENSG000000	ST7L	protein_codin	0	0	6.67909257	0
ENSG000000	MOK	protein_codin	0	0	0	0
ENSG000001	PCDH15	protein_codin	0	0	0	0
ENSG000002	MT-ATP8	protein_codin	2716.24532	3607.49366	2068.42695	1726.59954
ENSG000001	EPS8L3	protein_codin	0	0	0	0
ENSG000001	COL6A1	protein_codin	0	0	0	0
ENSG000001	MSRB1	protein_codin	172.516578	381.982587	42.8327041	177.416085
ENSG000001	GMEB1	protein_codin	36.2295792	33.9034198	41.8764222	16.540319
ENSG000002	DCTN1	protein_codin	0	0	0	0
ENSG000001	TSC22D2	protein_codin	246.630984	273.817081	206.768447	145.318771
ENSG000002	KRTAP1-5	protein_codin	0	0	0	0
ENSG000001	TAFA3	protein_codin	0	0	1.89724826	0
ENSG000001	PNMA5	protein_codin	3.15826685	0	0	0
ENSG000001	ILF2	protein_codin	227.88277	417.491932	219.413314	187.640566
ENSG000000	JADE2	protein_codin	11.2761201	33.8570717	6.06538952	39.5369272
ENSG000001	NOS1AP	protein_codin	0	0	0	3.98080609
ENSG000001	MT-ATP6	protein_codin	8632.42456	11357.3719	5954.85651	7064.45246
ENSG000001	SCAMP5	protein_codin	0	0	21.258961	0
ENSG000001	CALD1	protein_codin	98.4495716	140.544151	77.0000515	92.7521305
ENSG000001	GPX6	protein_codin	0	0	0	0
ENSG000001	CELF1	protein_codin	0	0	0	0
ENSG000001	TMEM184B	protein_codin	79.0901197	44.4130157	41.9482636	27.3378271
ENSG000001	OPA1	protein_codin	163.441066	0	0	0
ENSG000001	CMTM3	protein_codin	0	0	0	0

ENSG000001!	ARMCX6	protein_codin	18.7497586	54.8948374	17.5226921	16.8093627
ENSG000001(	ZNF326	protein_codin	6.06530585	14.3401147	3.98742657	11.4250068
ENSG000001(	AGL	protein_codin	62.6858702	24.6777641	30.2970866	40.9986904
ENSG000002(	COL11A2	protein_codin	0	0	0	0
ENSG000002	DIO1	protein_codin	0	0	0	0
ENSG000001(	MAG	protein_codin	0	0	0	0
ENSG000000	PER3	protein_codin	11.9395832	26.7273376	9.73219685	4.0231801
ENSG000001	GOLGA4	protein_codin	0	0	250.585482	413.365091
ENSG000001	GABRG2	protein_codin	0	0	0	0
ENSG000001!	ARHGEF15	protein_codin	0	0	0	0
ENSG000001!	COLGALT2	protein_codin	0	0	0	0
ENSG000002!	GNRHR2	lncRNA	0	0	0	0
ENSG000001(	CD300A	protein_codin	29.2082292	42.7947204	36.0330825	0
ENSG000001	MINDY1	protein_codin	41.2002021	167.756628	43.575224	50.4855782
ENSG000000!	SORBS1	protein_codin	0	0	0	0
ENSG000001!	ZNF277	protein_codin	140.456785	97.7162665	72.9082551	84.1410813
ENSG000001	IFT81	protein_codin	6.67458599	0	0.92864284	0
ENSG000001!	ZNF830	protein_codin	30.234078	55.9709219	26.4243876	33.7127996
ENSG000001	FOXM1	protein_codin	0	0	5.1982237	0
ENSG000001!	SMOC1	protein_codin	0	0	0	0
ENSG000001	ZNF384	protein_codin	0	114.02347	11.9192861	0
ENSG000001	KIF21A	protein_codin	0	0	0	0
ENSG000001	CDY1	protein_codin	0	0	0	0
ENSG000001!	EPS8L3	protein_codin	0	0	0	0
ENSG000001!	CCDC152	protein_codin	5.97179184	6.15708761	0	2.47150765
ENSG000001!	PLXNB3	protein_codin	19.7196702	46.274419	42.4745646	11.2805066
ENSG000001!	SFMBT2	protein_codin	0	0	0	0
ENSG000001(	SLC25A14	protein_codin	0	0	1.97009459	0
ENSG000001!	L1CAM	protein_codin	0	0	0	0
ENSG000001!	KIFBP	protein_codin	0	0	0	0
ENSG000002	CNTF	protein_codin	19.8161439	2.03758172	7.28195149	7.47777842
ENSG000001(	PORCN	protein_codin	0	0	4.46980378	0
ENSG000001	CSNK1G3	protein_codin	41.4728807	10.9927825	17.0759656	24.7838479
ENSG000001	PHF20L1	protein_codin	50.9842077	33.2378961	21.8352093	11.27397
ENSG000001!	ITSN2	protein_codin	675.691562	391.800731	331.414291	373.980492
ENSG000001	ASL	protein_codin	9.4813162	16.7710837	10.8439722	0
ENSG000001!	ASB12	protein_codin	0	0	0	0
ENSG000001	PCGF3	protein_codin	196.151672	236.235241	54.1487887	104.134651
ENSG000001	BRDT	protein_codin	0	0	0	0
ENSG000002	CCDC7	protein_codin	19.3640396	0	0	6.86051573
ENSG000001!	GLMP	protein_codin	42.1292	66.279423	14.2665227	0
ENSG000001!	VEPH1	protein_codin	42.5819289	28.6963849	0	0
ENSG000001!	SHISA4	protein_codin	0	0	0	0
ENSG000000!	PTGS1	protein_codin	864.42695	1706.19614	714.160063	571.552957

ENSG000002	MTCP1	protein_codin	0	0	0	0
ENSG000000	METTL13	protein_codin	0	0	12.8954244	0
ENSG000001	IFI6	protein_codin	43.4754707	23.2001708	0	3.02266965
ENSG000001	SLC39A13	protein_codin	0	0	0	9.6650973
ENSG000001	RAB3IP	protein_codin	0	10.6031892	0	0
ENSG000001	SLC9B2	protein_codin	0	0	0	0
ENSG000002	UBE2WP1	processed_ps	0	0	0	0
ENSG000001	SNX6	protein_codin	0	0	0	19.9167323
ENSG000001	SUCNR1	protein_codin	2.16976454	30.8911936	19.4273036	14.4953391
ENSG000001	MRPL21	protein_codin	57.8550167	83.7544559	25.5212482	38.0230849
ENSG000000	NFE2L1	protein_codin	87.4662051	151.898806	97.3777574	21.2389753
ENSG000002	BAG6	protein_codin	156.330593	246.96009	110.85186	44.5765947
ENSG000001	ENSA	protein_codin	36.2330104	34.5203414	9.30409174	18.3651497
ENSG000000	WHRN	protein_codin	7.3826789	0	0	1.17488366
ENSG000002	ENSG000002	lncRNA	9.47053767	4.445006	0	7.19296981
ENSG000000	CACNA1S	protein_codin	0	0	0	0
ENSG000001	ZNF652	protein_codin	138.073135	270.328089	152.946325	161.576481
ENSG000001	FAN1	protein_codin	0	9.39476509	0	0
ENSG000001	FAM9B	protein_codin	0	0	0	0
ENSG000002	H2AC10P	transcribed_u	0	0	0	0
ENSG000001	DRD2	protein_codin	0	0	0	0
ENSG000002	NOTCH2NLA	protein_codin	598.735183	252.120461	188.249041	381.543007
ENSG000001	C1orf43	protein_codin	0	54.470545	19.2658631	7.57921015
ENSG000002	MRPS6	protein_codin	0	0	0	0
ENSG000001	MT-CO3	protein_codin	10122.4802	11897.1075	6387.64328	6963.22632
ENSG000001	CD247	protein_codin	0	2.45523889	0	0
ENSG000001	FBH1	protein_codin	149.400122	0	93.3300408	60.5254572
ENSG000002	CKLF	protein_codin	3.94940708	11.4180071	0	0
ENSG000001	CNR1	protein_codin	1.30040744	1.32003553	0	0
ENSG000000	PCDH11Y	protein_codin	0	0	0	0
ENSG000001	UTY	protein_codin	0	0	0	0
ENSG000001	GAGE2A	protein_codin	0	0	0	0
ENSG000001	MIRLET7E	miRNA	0	0	0	0
ENSG000001	MIR375	miRNA	0	0	0	0
ENSG000001	MIR30E	miRNA	0	0	0	0
ENSG000001	MIRLET7A2	miRNA	0	0	0	0
ENSG000001	MIR429	miRNA	0	0	0	0
ENSG000002	MIR29A	miRNA	0	0	0	0
ENSG000001	MIR380	miRNA	0	0	0	0
ENSG000001	MIR449A	miRNA	0	0	0	0
ENSG000001	MIR345	miRNA	0	0	0	0
ENSG000002	MIRLET7A3	miRNA	0	0	0	0
ENSG000001	MIR16-2	miRNA	0	0	0	0
ENSG000001	MIR340	miRNA	0	0	0	0

ENSG000001	MIR107	miRNA	0	0	0	0
ENSG000001	MIR448	miRNA	0	0	0	0
ENSG000002	MIR21	miRNA	0	0	0	0
ENSG000001	MIR370	miRNA	0	0	0	0
ENSG000001	MIR412	miRNA	0	0	0	0
ENSG000001	MIR377	miRNA	0	0	0	0
ENSG000001	MIR1-1	miRNA	0	0	0	0
ENSG000001	MIR381	miRNA	0	0	0	0
ENSG000001	MIR339	miRNA	0	0	0	0
ENSG000001	MIR103A2	miRNA	0.89894728	1.00846867	0	0
ENSG000001	MIR369	miRNA	0	0	0	0
ENSG000002	MIR655	miRNA	0	0	0	0
ENSG000001	MIRLET7C	miRNA	0	0	0	0
ENSG000001	MIR371A	miRNA	0	0	0	0
ENSG000001	MIR425	miRNA	0	0	0	0
ENSG000001	MIR103A1	miRNA	0	0	0	0
ENSG000001	MIR219A1	miRNA	0	0	0	0
ENSG000001	MIR210	miRNA	0	0	0	0
ENSG000001	MIR335	miRNA	0	0	0	0
ENSG000001	MIR378A	miRNA	0	0	0	0
ENSG000001	MIR361	miRNA	0	0	0	0
ENSG000001	MIR324	miRNA	0	0	0	0
ENSG000002	MIR302B	miRNA	0	0	0	0
ENSG000001	MIR135B	miRNA	0	0	0	0
ENSG000002	MIR22	miRNA	0	0	0	0
ENSG000001	MIR101-2	miRNA	0	0	0	0
ENSG000002	MIR330	miRNA	0	0	0	0
ENSG000001	MIR323A	miRNA	0	0	0	0
ENSG000002	MIR423	miRNA	0	0	0	0
ENSG000001	MIRLET7F1	miRNA	0	0	0	1.31358637
ENSG000001	MIR26A1	miRNA	0	0	0	0
ENSG000001	MIR129-2	miRNA	0	0	0	0
ENSG000001	MIR133B	miRNA	0	0	0	0
ENSG000001	MIR342	miRNA	0	0	0	0
ENSG000001	MIR148A	miRNA	0	0	0	0
ENSG000001	MIR379	miRNA	0	0	0	0
ENSG000002	MIR202	miRNA	0	0	0	0
ENSG000001	MIR326	miRNA	0	0.77096592	0	0
ENSG000001	MIR410	miRNA	0	0	0	0
ENSG000001	MIR30C2	miRNA	0	0	0	0
ENSG000001	MIR372	miRNA	0	0	0	0
ENSG000002	MIR424	miRNA	0	0	0	0
ENSG000001	MIR302C	miRNA	0	0	0	0
ENSG000001	MIR346	miRNA	0	0	0	0

ENSG000001!	MIR409	miRNA	0	0	0	0
ENSG000001!	MIR411	miRNA	0	0	0	0
ENSG000001!	MIR26B	miRNA	0	0	0	0
ENSG000001!	MIR148B	miRNA	0	0	0	0
ENSG000001!	MIR383	miRNA	0	0	0	0
ENSG000001!	MIR365A	miRNA	0	0	0	0
ENSG000001!	MIR450A1	miRNA	0	0	0	0
ENSG000001!	MIRLET7D	miRNA	0	0	0	0
ENSG000001!	MIR101-1	miRNA	0.88865221	1.00430383	0	0
ENSG000001!	MIR373	miRNA	0	0	0	0
ENSG000001!	MIR302D	miRNA	0	0	0	0
ENSG000002	MIR20A	miRNA	0	0	0	0
ENSG000001!	MIRLET7G	miRNA	0	0	0	0
ENSG000001!	MIR337	miRNA	0	0	0	0
ENSG000001!	MIR30D	miRNA	0	0	0	0
ENSG000001!	MIR422A	miRNA	0	0	0	0
ENSG000001!	MIR208A	miRNA	0	0	0	0
ENSG000001!	MIR96	miRNA	0	0	0	0
ENSG000001!	MIR126	miRNA	0	0	0	0
ENSG000001!	MIRLET7A1	miRNA	0	0	0	0
ENSG000001!	MIR374A	miRNA	0	0	0	0
ENSG000001!	MIR367	miRNA	0	0	0	0
ENSG000001!	MIR331	miRNA	0	0	0	0
ENSG000001!	MIR31	miRNA	0	0	0	0
ENSG000001!	MIRLET7I	miRNA	0	0	0	0
ENSG000002	MIR18A	miRNA	0	0	0	0
ENSG000002	MIR365B	miRNA	0	0	0	0
ENSG000001!	ENSG000001!	snoRNA	0	0	0	0
ENSG000001!	RN7SKP72	misc_RNA	0	1.14853064	0	1.11145753
ENSG000001!	Y_RNA	misc_RNA	0	0	0	0
ENSG000001!	Y_RNA	misc_RNA	0	0	0	0
ENSG000001!	RNA5SP289	rRNA_pseudo	6.65857407	0.97168276	4.38482049	0.89850973
ENSG000001!	Y_RNA	misc_RNA	0	0	0	0
ENSG000001!	Y_RNA	misc_RNA	0	0	0	0
ENSG000001!	RNU105C	snoRNA	0	0	0	0
ENSG000001!	RNU6-1123P	snRNA	0	0	0	0
ENSG000001!	RN7SKP184	misc_RNA	0	0	0	0
ENSG000001!	RNU6-500P	snRNA	1.80903178	0	0	0
ENSG000001!	Y_RNA	misc_RNA	0.90473307	0	0	0
ENSG000001!	Y_RNA	misc_RNA	1.40082239	2.51868074	5.29177854	1.37779437
ENSG000001!	Y_RNA	misc_RNA	3.5723746	7.7270997	2.64512522	4.6196452
ENSG000001!	Y_RNA	misc_RNA	0	0	1.03720386	0.91265668
ENSG000001!	RNU6-50P	snRNA	0	0	0	0
ENSG000001!	ENSG000001!	snoRNA	0	0	0	0

ENSG000001!	RNU6-834P	snRNA	0	0	0	0
ENSG000001!	RNA5SP46	rRNA_pseudo	0	0	0	0
ENSG000001!	Y_RNA	misc_RNA	0	0	0	0
ENSG000001!	Y_RNA	misc_RNA	0	0	0	0
ENSG000001!	RNU6-896P	snRNA	0	0	0	0
ENSG000001!	RNU6-28P	snRNA	20.9759997	7.54233695	8.53493363	14.0079179
ENSG000001!	RNU6-664P	snRNA	0	0	0	0
ENSG000001!	RNU6-874P	snRNA	0	0	0	0
ENSG000001!	Y_RNA	misc_RNA	0	0	0	0
ENSG000001!	SNORA60	snoRNA	6.34374572	10.8053361	5.38959592	16.3999685
ENSG000001!	RNU6-349P	snRNA	0	0	0	0
ENSG000001!	Y_RNA	misc_RNA	0	0	0	0
ENSG000001!	RNA5SP26	rRNA_pseudo	0	0	0	0
ENSG000001!	RNU6-661P	snRNA	0	0	0	0
ENSG000001!	ENSG000001!	snoRNA	0.80048867	0	0	0
ENSG000001!	RNU1-58P	snRNA	0	0	0	0
ENSG000001!	Y_RNA	misc_RNA	2.25810263	0	1.0362401	0.90928169
ENSG000001!	RNU6-1094P	snRNA	7.3801034	0	0.90036421	2.88700813
ENSG000001!	RNU6-502P	snRNA	0	0	0	0
ENSG000001!	Y_RNA	misc_RNA	0	0	4.25792959	2.33802822
ENSG000001!	Y_RNA	misc_RNA	6.66241942	21.860174	6.578198	5.63928942
ENSG000001!	SNORA21	snoRNA	121.733392	230.172884	53.0458524	178.368693
ENSG000001!	RNU6-1312P	snRNA	1.83289763	0	0	0
ENSG000001!	RNA5SP430	rRNA_pseudo	0	0	0	0
ENSG000001!	RNU6-208P	snRNA	0	0	0	0.45278706
ENSG000001!	Y_RNA	misc_RNA	0	0	0.88845657	0
ENSG000001!	RNU6-858P	snRNA	0	0.97471675	0	0
ENSG000001!	RNU6-222P	snRNA	87.5491943	105.827444	32.1202773	85.4829863
ENSG000001!	SNORD115-3	snoRNA	0	0	0	0
ENSG000001!	RNU4-82P	snRNA	0	2.03079346	0	0
ENSG000001!	RNA5SP347	rRNA_pseudo	0	0	0	0
ENSG000001!	RNA5SP52	rRNA_pseudo	1.88598503	3.95424165	1.77629616	1.77769688
ENSG000001!	RN7SKP25	misc_RNA	1.08909455	0	0.82192991	0
ENSG000001!	ENSG000001!	snoRNA	0	0	0	0
ENSG000001!	RNA5SP183	rRNA_pseudo	0	0.99954417	0.88010734	0
ENSG000001!	RNU4-39P	snRNA	0	0	0	0
ENSG000001!	RNU6-1298P	snRNA	0	0	0	0
ENSG000001!	RNU6-230P	snRNA	0	0	0	0
ENSG000001!	Y_RNA	misc_RNA	0	0	0	0
ENSG000001!	Y_RNA	misc_RNA	0.88568859	0	0	0
ENSG000001!	RNU6-204P	snRNA	0	0	0	0
ENSG000001!	RNU5E-1	snRNA	243.260337	364.884127	251.727807	269.481297
ENSG000001!	RNU6-766P	snRNA	0	0	0	0
ENSG000001!	Y_RNA	misc_RNA	0	0	0	0

ENSG000001!	RNA5SP432	rRNA_pseudo	0	0	0	0
ENSG000001!	RNA5S1	rRNA	72018.1787	131496.953	52834.8742	89689.3722
ENSG000001!	RNA5SP273	rRNA_pseudo	0	0	0	0
ENSG000001!	Y_RNA	misc_RNA	4.46554912	4.02504862	0	0
ENSG000001!	RNU6-1115P	snRNA	0	0	0	0
ENSG000001!	RNU1-150P	snRNA	0	0	0	0
ENSG000001!	Y_RNA	misc_RNA	0	2.07643403	1.76302097	0.95315794
ENSG000001!	SNORA63E	snoRNA	1.5610275	6.93444274	0.57402357	3.098686
ENSG000001!	RNA5SP327	rRNA_pseudo	0	0	0	0
ENSG000001!	Y_RNA	misc_RNA	0	1.02700407	0	0.92341466
ENSG000001!	RNU6-1084P	snRNA	1.80921234	3.00656541	0.89346605	0.90557412
ENSG000001!	U3	snoRNA	0	0	0	0
ENSG000001!	RNA5SP453	rRNA_pseudo	0	0	0	0
ENSG000001!	RNU5F-1	snRNA	72.3882934	81.9954074	37.092193	73.6749504
ENSG000001!	Y_RNA	misc_RNA	0	0	0	0
ENSG000001!	RNU6-79P	snRNA	0	0	0	0
ENSG000002'	RNU12	snRNA	774.237342	1120.76055	417.352966	759.769992
ENSG000001!	RNU6-369P	snRNA	0	0	0	0
ENSG000001!	SNORD114-7	snoRNA	0	0	0	0
ENSG000001!	ENSG000001!	snoRNA	0	0	0	0
ENSG000001!	RNU6-600P	snRNA	0	0	0	0.9162821
ENSG000001!	RNA5SP93	rRNA_pseudo	0	0	0.89368042	0
ENSG000001!	Y_RNA	misc_RNA	0	0	0	0
ENSG000001!	RNY4P19	misc_RNA	0.8784472	3.10585799	0	0
ENSG000001!	RNA5SP308	rRNA_pseudo	0	0	0	0
ENSG000001!	SNORA1B	snoRNA	0	0	0	0
ENSG000001!	RNA5SP301	rRNA_pseudo	0	0	0	0
ENSG000001!	Y_RNA	misc_RNA	0.88218416	2.05263535	2.71119593	0.91265668
ENSG000001!	Y_RNA	misc_RNA	0	1.01667955	0	0.90928169
ENSG000001!	SNORD62	snoRNA	0	0	0.79965026	0
ENSG000001!	RNA5SP370	rRNA_pseudo	53.4159802	74.7184046	26.4077416	70.2986651
ENSG000001!	RN7SKP170	misc_RNA	0	0	0	0
ENSG000001!	VTRNA3-1P	misc_RNA	0	0	0	0
ENSG000001!	Y_RNA	misc_RNA	0	0	0	0
ENSG000001!	RNU1-108P	snRNA	0	0	0	0
ENSG000001!	SNORD9	snoRNA	34.6630988	93.5573455	28.0940666	48.7334117
ENSG000002'	SNORA17B	snoRNA	0	0	0	0
ENSG000001!	Y_RNA	misc_RNA	0	5.61615091	0	0.89556092
ENSG000001!	Y_RNA	misc_RNA	0	0	0	0
ENSG000001!	Y_RNA	misc_RNA	30.3821724	44.9441479	32.8582531	20.7451426
ENSG000001!	RNU6-543P	snRNA	0	0	0	0
ENSG000001!	RNU6-845P	snRNA	0	0	0	0
ENSG000001!	RNA5SP276	rRNA_pseudo	0	0	0	0
ENSG000002'	SNORA71	snoRNA	0	0	0	0

ENSG000001!	RNA5SP388	rRNA_pseudo	9.68001888	14.9644275	4.70582393	14.3861315
ENSG000001!	RNA5SP191	rRNA_pseudo	269.479017	350.278659	172.823195	359.669438
ENSG000001!	RNU4-35P	snRNA	0	0	0	0
ENSG000001!	Y_RNA	misc_RNA	0	0	0	0
ENSG000001!	RNU6-1216P	snRNA	0	0	0	0
ENSG000001!	Y_RNA	misc_RNA	8.32062336	25.0032162	15.5432713	7.61557213
ENSG000001!	Y_RNA	misc_RNA	0	0	0	0
ENSG000001!	RNU6-556P	snRNA	0	0	0	0
ENSG000001!	RNU1-62P	snRNA	0	0	0	1.77534504
ENSG000001!	ENSG000001!	snoRNA	0	0	0	0
ENSG000001!	Y_RNA	misc_RNA	0	0	0	0
ENSG000001!	Y_RNA	misc_RNA	0	0	0	0
ENSG000001!	SNORA63	snoRNA	0	0	0	0
ENSG000001!	ENSG000001!	snoRNA	0	0	0	0
ENSG000001!	RN7SKP104	misc_RNA	0	0	0	0
ENSG000001!	Y_RNA	misc_RNA	0	0	0	0
ENSG000001!	SNORA31	snoRNA	95.952893	304.554802	82.4010627	136.133745
ENSG000001!	RNA5SP389	rRNA_pseudo	68.3505458	145.575955	47.7141548	80.8980096
ENSG000001!	RNU6-633P	snRNA	0	0	0	0
ENSG000001!	RNU4-15P	snRNA	0	0	0	0
ENSG000001!	Y_RNA	misc_RNA	0	0	0	0
ENSG000001!	RNU1-70P	snRNA	0	0	0	0.88767252
ENSG000001!	SNORD115-2!	snoRNA	0	0	0	0
ENSG000001!	Y_RNA	misc_RNA	0	0	0	0
ENSG000001!	RNU6-1313P	snRNA	0	0	0	0
ENSG000001!	RNU1-94P	snRNA	0	0	0	0
ENSG000001!	RNU6-247P	snRNA	0	0	0	0
ENSG000001!	RNA5SP110	rRNA_pseudo	0	0	0	0
ENSG000001!	RNA5SP477	rRNA_pseudo	0	0	0	0
ENSG000001!	RNU6-212P	snRNA	0	0	0	0
ENSG000001!	RNU6-235P	snRNA	9.63063739	2.44044123	0	3.36574363
ENSG000001!	Y_RNA	misc_RNA	0	0	0	0
ENSG000001!	Y_RNA	misc_RNA	4.46206269	15.5732572	3.40911379	4.61826515
ENSG000001!	RNU6-386P	snRNA	0	0	0	0
ENSG000001!	RNA5SP226	rRNA_pseudo	2231.27651	5600.00948	2145.75213	2702.69763
ENSG000001!	RNA5SP295	rRNA_pseudo	11.1080749	4.14473997	4.55873554	12.8962167
ENSG000001!	RNU6-462P	snRNA	0	0	0	0
ENSG000001!	Y_RNA	misc_RNA	0	0	0	0
ENSG000001!	RNA5SP305	rRNA_pseudo	0	0	0	0
ENSG000001!	RNU6-315P	snRNA	0	0	0	0
ENSG000001!	Y_RNA	misc_RNA	0	0	0	0
ENSG000001!	RNA5SP195	rRNA_pseudo	5.52179005	2.01303617	0.88308263	0
ENSG000001!	Y_RNA	misc_RNA	0	0	0	0
ENSG000001!	Y_RNA	misc_RNA	7.4396142	14.0513	2.17699324	4.82475719

ENSG000001!	RNU6-545P	snRNA	0	0	0	0
ENSG000001!	SNORA63	snoRNA	0	0	0	0
ENSG000002	RNU1-5P	snRNA	0	0	0	0
ENSG000001!	RNA5SP361	rRNA_pseudo	0	0	0	0
ENSG000001!	RNU6-37P	snRNA	578.235817	0	135.205197	801.418273
ENSG000001!	RNA5SP502	rRNA_pseudo	160.383183	223.011713	150.124027	198.354634
ENSG000001!	Y_RNA	misc_RNA	22.7139109	51.5739656	22.3883802	25.1866741
ENSG000001!	ENSG000001!	snoRNA	0	0	0	0
ENSG000001!	Y_RNA	misc_RNA	0	0	0	0
ENSG000001!	RNU5A-1	snRNA	3932.28742	9240.82856	2707.2368	6593.24907
ENSG000001!	RNU6-228P	snRNA	0	0	0	0
ENSG000001!	ENSG000001!	snoRNA	0	0	0	0.88884811
ENSG000001!	RNA5SP174	rRNA_pseudo	2.79862248	2.96516564	4.4757499	0.91148095
ENSG000001!	SNORD18C	snoRNA	0	0	0	0
ENSG000001!	SNORD114-1	snoRNA	0	0	0	0
ENSG000001!	RNU6-822P	snRNA	0	0	0	0
ENSG000001!	Y_RNA	misc_RNA	0	1.01667955	0	0
ENSG000001!	Y_RNA	misc_RNA	0	0	0	0
ENSG000001!	RNA5SP119	rRNA_pseudo	11.042872	11.0709896	5.29815631	6.29395194
ENSG000001!	Y_RNA	misc_RNA	0	0	0	0
ENSG000001!	RNA5SP321	rRNA_pseudo	0	0	0	0
ENSG000001!	SNORD114-1	snoRNA	0	0	0	0
ENSG000001!	RNU6-1301P	snRNA	4.14809355	3.76020592	0	4.49062969
ENSG000001!	Y_RNA	misc_RNA	6.509535	8.70914679	0.93198685	4.61683618
ENSG000001!	RNU6-859P	snRNA	0	0	0	0
ENSG000001!	RNU6-562P	snRNA	8.19194302	5.54653265	1.2163352	5.14628733
ENSG000001!	RNU6-951P	snRNA	0	0	0	0
ENSG000001!	RNY4P24	misc_RNA	0.8784472	3.10585799	0.90242428	0.94514214
ENSG000001!	RNA5SP124	rRNA_pseudo	0	0	0	0
ENSG000001!	RNA5SP258	rRNA_pseudo	0	0	0	0
ENSG000001!	RN7SKP20	misc_RNA	0	0	0	0
ENSG000001!	RNU6-989P	snRNA	0	0	0	0
ENSG000001!	RNU6-1010P	snRNA	0	0	0	0
ENSG000001!	RNU1-14P	snRNA	17.3334714	12.1445978	16.484575	14.6426057
ENSG000001!	Y_RNA	misc_RNA	0	0	0	0
ENSG000001!	SNORD33	snoRNA	123.650267	328.512743	109.231163	186.309836
ENSG000001!	SNORA63	snoRNA	0	0	0	0
ENSG000001!	RNU4-79P	snRNA	1.89408113	0	0	0
ENSG000001!	Y_RNA	misc_RNA	1.78482515	2.13053131	1.88078687	0.94145449
ENSG000001!	Y_RNA	misc_RNA	0	0	0	0
ENSG000001!	RNA5SP319	rRNA_pseudo	0	0	0	0
ENSG000001!	RN7SKP294	misc_RNA	0	0	0	0
ENSG000001!	RNU4-90P	snRNA	0	0	0	0
ENSG000001!	RNU6-1330P	snRNA	0	0	0	0

ENSG000001!	RNU6-1272P	snRNA	0	0	0	0
ENSG000001!	RNU1-35P	snRNA	0	0	0	0
ENSG000001!	RNU6-1266P	snRNA	2.25000406	4.88017202	1.89685405	0
ENSG000001!	SNORD3G	snoRNA	0	0.93954145	0	0
ENSG000001!	Y_RNA	misc_RNA	7.819714	3.29503705	4.6983205	3.76353843
ENSG000001!	Y_RNA	misc_RNA	0	0	0	0
ENSG000001!	RNU4-21P	snRNA	0	0	0	0
ENSG000001!	SNORD16	snoRNA	103.871823	290.427914	68.6625832	197.207641
ENSG000001!	RNU6-862P	snRNA	0	0	0	0
ENSG000002'	ENSG000002'	snoRNA	0	0	0	0
ENSG000001!	Y_RNA	misc_RNA	0	0	0	0
ENSG000001!	Y_RNA	misc_RNA	52.6666383	136.772725	62.8710702	46.1809227
ENSG000001!	RN7SKP185	misc_RNA	18.5655346	13.8158368	10.2022268	11.6954404
ENSG000001!	RNU1-38P	snRNA	0	0	0	0
ENSG000001!	RN7SKP173	misc_RNA	5.63524414	5.77197237	3.55195074	1.88846362
ENSG000001!	RNU6-1128P	snRNA	0	0	0	0
ENSG000001!	RNU6-446P	snRNA	0	0	0	0
ENSG000001!	Y_RNA	misc_RNA	0	0	0	0
ENSG000001!	RNU6-223P	snRNA	0	0	0	0
ENSG000001!	Y_RNA	misc_RNA	0	1.01506493	0	0.93158155
ENSG000001!	RNU6-170P	snRNA	0	0	0	0
ENSG000001!	Y_RNA	misc_RNA	0	0	0	0
ENSG000001!	RNU4-23P	snRNA	0	0	0	0
ENSG000001!	Y_RNA	misc_RNA	0	0	0	0
ENSG000001!	Y_RNA	misc_RNA	0	0	0	0
ENSG000001!	SNORD115-2	snoRNA	0	0	0	0
ENSG000001!	U8	snoRNA	0	0	0	0
ENSG000001!	Y_RNA	misc_RNA	0	0	0	0
ENSG000001!	Y_RNA	misc_RNA	0	0	0.88796544	0
ENSG000001!	RN7SKP74	misc_RNA	0	0	0	0
ENSG000001!	RNU6-655P	snRNA	0	0	0	0
ENSG000001!	RN7SKP95	misc_RNA	0.9930577	1.02064046	0	0
ENSG000001!	RNU6-1079P	snRNA	0	0	0	0
ENSG000001!	Y_RNA	misc_RNA	0	2.59554253	0	0
ENSG000001!	RNA5SP307	rRNA_pseudo	0.9159114	0	0	0.91282215
ENSG000001!	RNU6-227P	snRNA	4.55155965	3.05058115	0.89919121	2.72784506
ENSG000001!	RNU6-552P	snRNA	0	0	0	0
ENSG000001!	Y_RNA	misc_RNA	0	0	0	0
ENSG000001!	SNORD36A	snoRNA	56.6467364	140.034674	48.1961786	102.470162
ENSG000001!	Y_RNA	misc_RNA	53.6151236	114.33344	50.7964518	49.7834924
ENSG000001!	SNORD104	snoRNA	55.5572609	77.0321983	39.6067916	77.4473196
ENSG000001!	Y_RNA	misc_RNA	0	0	0	0
ENSG000001!	Y_RNA	misc_RNA	0	0	0	0
ENSG000001!	Y_RNA	misc_RNA	0	0	0	0

ENSG000001!	Y_RNA	misc_RNA	0	0	0	0
ENSG000001!	RNU6-363P	snRNA	0	0	0	0
ENSG000001!	RNU6-78P	snRNA	0	0	0	0
ENSG000002'	ENSG000002'	snoRNA	0	0	0	0
ENSG000001!	RNA5SP426	rRNA_pseudo	0	0	0	0
ENSG000001!	RNU1-76P	snRNA	0	0	0	0
ENSG000001!	Y_RNA	misc_RNA	0	0	0	0
ENSG000001!	Y_RNA	misc_RNA	0	0	0	0
ENSG000001!	SNORD115-9	snoRNA	0	0	0	0
ENSG000001!	ENSG000001!	snoRNA	0	0	0	0
ENSG000001!	RNU6-1287P	snRNA	0	0	0	0
ENSG000001!	SNORA52	snoRNA	20.0688392	42.9276408	16.2094031	29.4547194
ENSG000001!	RNA5SP188	rRNA_pseudo	0	0	0	0
ENSG000001!	SNORA80C	snoRNA	0	0.97455536	0	0
ENSG000001!	RNY3P2	misc_RNA	4.46206269	1.05209697	0.88151049	0
ENSG000001!	RNU6-836P	snRNA	0	0	0	0
ENSG000001!	RNU6-451P	snRNA	0	0	0	0
ENSG000001!	RNU6-1233P	snRNA	0	0	0	0
ENSG000001!	RNU6-924P	snRNA	0	0	0	0
ENSG000001!	Y_RNA	misc_RNA	0	0	0	0
ENSG000001!	SNORD114-5	snoRNA	0	0	0	0
ENSG000001!	Y_RNA	misc_RNA	0	0	0	0
ENSG000001!	RNU6-1159P	snRNA	0	0	0	0
ENSG000001!	RNA5SP383	rRNA_pseudo	0.91834436	1.54567149	0.77693869	0
ENSG000001!	RNU1-134P	snRNA	4.88397736	2.87087178	2.65732672	3.59301711
ENSG000001!	RNA5SP491	rRNA_pseudo	0	0	0	0
ENSG000001!	RNA5SP374	rRNA_pseudo	0	0	0	0
ENSG000001!	RNU6-428P	snRNA	0.92103995	2.00509442	0	0
ENSG000001!	RNU6-1260P	snRNA	0	0	0	0
ENSG000001!	ENSG000001!	snoRNA	0	0	0	0
ENSG000001!	RNU6-199P	snRNA	0	0	0	0
ENSG000001!	RNU6-1290P	snRNA	0	0	0	0
ENSG000001!	RN7SKP291	misc_RNA	3.97569131	0.96383702	1.84592629	2.71518254
ENSG000001!	Y_RNA	misc_RNA	0	2.00437743	0.89346605	0
ENSG000001!	SNORD115-2'	snoRNA	0	0	0	0
ENSG000001!	RNU1-47P	snRNA	0	0	0	0
ENSG000001!	RNA5SP150	rRNA	789.329959	1354.76788	549.504931	792.65655
ENSG000001!	Y_RNA	misc_RNA	0	0	0	0
ENSG000001!	RNA5SP358	rRNA_pseudo	20.9323337	29.8845918	18.2082495	26.9752048
ENSG000001!	RNA5SP375	rRNA_pseudo	0	0	0	0
ENSG000001!	RNU1-72P	snRNA	0	0	0	0
ENSG000001!	RNU5F-6P	snRNA	0	0	0	0
ENSG000001!	U3	snoRNA	0	0	0.89080805	0
ENSG000001!	RNU6-490P	snRNA	0	0	0	0

ENSG000001!	U3	snoRNA	0	0	0	0
ENSG000001!	ENSG000001!	snoRNA	0	0	0	0
ENSG000001!	RNU6-1120P	snRNA	0	0	0	0
ENSG000001!	RNU6-582P	snRNA	0	0	0	0.90557412
ENSG000001!	Y_RNA	misc_RNA	0	0	0	0
ENSG000001!	RNU6-495P	snRNA	0	0	0	0
ENSG000001!	Y_RNA	misc_RNA	16.6308253	27.7714259	13.2688801	27.6838279
ENSG000001!	Y_RNA	misc_RNA	0	0	0	0
ENSG000001!	RNU6-942P	snRNA	0.90451589	2.00437743	0.89346605	0
ENSG000001!	RNA5SP452	rRNA_pseudo	0	1.99908835	15.8419317	1.79371205
ENSG000001!	Y_RNA	misc_RNA	0	0	0	0
ENSG000001!	RN7SKP149	misc_RNA	0	0	0	0
ENSG000001!	RNVU1-22	snRNA	2.94813531	4.7867857	1.76807261	3.57582685
ENSG000001!	RNU6-888P	snRNA	0	2.05263535	0.90404856	0.91265668
ENSG000001!	Y_RNA	misc_RNA	0.88879741	0	0	0
ENSG000001!	RN7SKP90	misc_RNA	7.63457349	10.3954025	10.0986167	7.31255002
ENSG000001!	Y_RNA	misc_RNA	0	0	0	0
ENSG000001!	RNU6-330P	snRNA	0	0	0	0
ENSG000001!	RNU6-249P	snRNA	0	0	0	0
ENSG000001!	Y_RNA	misc_RNA	0	0	0	0
ENSG000001!	RNU1-17P	snRNA	0	0.96311719	0	0
ENSG000002	RNA5SP257	rRNA_pseudo	0	0	0.8948672	0
ENSG000001!	ENSG000001!	snoRNA	0	0	0	0
ENSG000001!	Y_RNA	misc_RNA	0	0	0	0
ENSG000001!	Y_RNA	misc_RNA	0	0	0	0
ENSG000001!	Y_RNA	misc_RNA	0	3.11465171	1.76302097	1.84647684
ENSG000001!	RNU6-1273P	snRNA	0	0	0	0
ENSG000001!	RNU6-492P	snRNA	0	0	0	0
ENSG000001!	RNU5B-2P	snRNA	0	0	0	1.79076826
ENSG000001!	Y_RNA	misc_RNA	0	0	1.09422313	0
ENSG000001!	Y_RNA	misc_RNA	0	1.00218871	0	1.81114824
ENSG000001!	SNORD114-1(	snoRNA	0	0	0	0
ENSG000002	RMRP	ribozyme	0	0	0	0
ENSG000001!	RNU4-44P	snRNA	0	1.93267353	0	0
ENSG000001!	RNU6-161P	snRNA	0	0	0	0
ENSG000001!	RNU6-1252P	snRNA	0	0	0	0
ENSG000001!	ENSG000001!	snoRNA	0	0	0	0
ENSG000001!	RNA5SP405	rRNA_pseudo	0	0	0	0
ENSG000001!	RNU1-18P	snRNA	10.0997743	10.5918877	0.89054226	6.21803914
ENSG000001!	RNY1P16	misc_RNA	0	0	0	0
ENSG000001!	SNORD81	snoRNA	0	0	0	0
ENSG000001!	Y_RNA	misc_RNA	0	0	0	0
ENSG000001!	Y_RNA	misc_RNA	0	0	0.8816789	0
ENSG000001!	RN7SKP75	misc_RNA	0	0	0	0

ENSG000001!	SNORD114-1!	snoRNA	0	0	0	0
ENSG000001!	RNU6-653P	snRNA	0	0	0	0
ENSG000001!	Y_RNA	misc_RNA	0	0	0	0
ENSG000001!	RNA5SP443	rRNA	0	0	0	0
ENSG000001!	ENSG000001!	snoRNA	0	0	0	0
ENSG000001!	SNORD115-1	snoRNA	0	0	0	0
ENSG000001!	SNORD1B	snoRNA	9.72103135	13.2003309	7.10573402	18.7400081
ENSG000001!	Y_RNA	misc_RNA	0	0	0	0
ENSG000001!	RNU6-605P	snRNA	0	0	0	0
ENSG000001!	Y_RNA	misc_RNA	0	0	0	0
ENSG000001!	SNORD115-3	snoRNA	0	0	0	0
ENSG000001!	RNU6-797P	snRNA	0	0	0	0
ENSG000001!	RN7SKP243	misc_RNA	2.80939095	2.42464634	0.88389692	0
ENSG000001!	SNORA73	snoRNA	0	0	0.89032248	0
ENSG000001!	Y_RNA	misc_RNA	0	0	0	0
ENSG000001!	RNA5SP264	rRNA_pseudo	0	0	0	0
ENSG000001!	RNU6-486P	snRNA	0	0	0	0
ENSG000001!	VTRNA1-1	vault_RNA	319.623228	1139.93389	350.081675	599.163414
ENSG000001!	RNA5SP145	rRNA_pseudo	6506.60288	11959.6011	3954.97792	7472.70472
ENSG000002	RNU6-986P	snRNA	0	0	0	0
ENSG000002	Y_RNA	misc_RNA	0	0	0	0
ENSG000002	Y_RNA	misc_RNA	2.01237993	0	0.89251976	1.90544107
ENSG000002	RNU6-623P	snRNA	0	1.21738029	0	0
ENSG000002	RNA5SP448	rRNA_pseudo	0.90078679	0	0.88978263	0.90366269
ENSG000002	Y_RNA	misc_RNA	0	0	0	0
ENSG000002	U8	snoRNA	0	0	0	0
ENSG000002	RNA5SP98	rRNA_pseudo	0	0	0	0
ENSG000002	RNU6-642P	snRNA	0	0	0	0
ENSG000002	RNU6-403P	snRNA	0	0	0	0
ENSG000002	RNU4-73P	snRNA	0	0	0	0
ENSG000002	RNA5SP65	rRNA_pseudo	0	0	0	0
ENSG000002	Y_RNA	misc_RNA	0	0	0	0
ENSG000002	Y_RNA	misc_RNA	0	0	0	0
ENSG000002	U3	snoRNA	0	0	0	0
ENSG000002	RN7SKP115	misc_RNA	0	0	0	0.97034776
ENSG000002	RNU6-812P	snRNA	0	0	0	0
ENSG000002	Y_RNA	misc_RNA	0	0	0	0
ENSG000002	ENSG000002	snoRNA	0	0	0	0
ENSG000002	Y_RNA	misc_RNA	0	0	0	0
ENSG000002	RNA5SP218	rRNA_pseudo	0	0	0	0
ENSG000002	Y_RNA	misc_RNA	0	0	0	0
ENSG000002	Y_RNA	misc_RNA	0	0	0	0
ENSG000002	RNU4-58P	snRNA	16.1366279	14.513702	7.89610594	14.3917217
ENSG000002	ENSG000002	snoRNA	0	1.96821498	0	0

ENSG000002	RNY4P9	misc_RNA	1.78443156	9.05177359	3.66736218	1.84606985
ENSG000002	Y_RNA	misc_RNA	0	0	0	0
ENSG000002	RNU4-80P	snRNA	0.94704056	0.96633733	0	0.89810726
ENSG000002	ENSG000002	snoRNA	0	0	0	0
ENSG000002	ENSG000002	snoRNA	0	0	0	0.89968811
ENSG000002	RNA5SP326	rRNA_pseudo	0	0	0	0
ENSG000002	SNORD68	snoRNA	9.64491672	0	17.721771	0
ENSG000002	Y_RNA	misc_RNA	8.93922929	12.0937119	3.17909555	3.569317
ENSG000002	RNU6-433P	snRNA	0	0	0	0.91949833
ENSG000002	SNORA73B	snoRNA	2581.92893	4143.97569	1706.94081	2461.77092
ENSG000002	SNORD114-3	snoRNA	0	0	0	0
ENSG000002	Y_RNA	misc_RNA	0	2.07407724	0	0.91639566
ENSG000002	RN7SKP163	misc_RNA	7.7684938	1.12999103	2.39116005	4.30203499
ENSG000002	RNU6-181P	snRNA	0	0	0	0
ENSG000002	RNU6-1167P	snRNA	0	0	0	0
ENSG000002	RNU6-508P	snRNA	0	0	0	0
ENSG000002	RNU6-252P	snRNA	0	0	0	0
ENSG000002	RNU6-251P	snRNA	0	0	0	0
ENSG000002	RNU6-984P	snRNA	0	0	0	0
ENSG000002	RNU6-1249P	snRNA	14.0950412	11.3247379	7.23123459	15.0025249
ENSG000002	RNU6-774P	snRNA	0	0	0	0
ENSG000002	ENSG000002	snoRNA	0	0	0	0
ENSG000002	ENSG000002	snoRNA	0	0	0	0
ENSG000002	RNA5SP123	rRNA_pseudo	0	0	0	0
ENSG000002	Y_RNA	misc_RNA	0.8989474	1.00846867	1.77593088	0
ENSG000002	Y_RNA	misc_RNA	0.89241238	4.15308295	3.526436	0.92341045
ENSG000002	Y_RNA	misc_RNA	0.94305916	0	0	0
ENSG000002	RN7SKP77	misc_RNA	0	1.18686894	0	0
ENSG000002	RNU6-1021P	snRNA	0	0	0	0
ENSG000002	Y_RNA	misc_RNA	0	0	0	0
ENSG000002	RNY1P10	misc_RNA	0	0	0	0
ENSG000002	RNU6-778P	snRNA	0	0	0	0
ENSG000002	Y_RNA	misc_RNA	0	0	0	0
ENSG000002	RNU6-544P	snRNA	0	0	0	0
ENSG000002	ENSG000002	snoRNA	0	0	0	0
ENSG000002	RN7SKP231	misc_RNA	0	0	0	0
ENSG000002	RNU6-216P	snRNA	8.60112163	13.6897281	3.48421845	9.41607161
ENSG000002	RNU6-23P	snRNA	3.27429465	2.81875269	0.81678175	3.65177138
ENSG000002	RNU5B-1	snRNA	2850.77528	5120.57303	1633.51293	3948.23428
ENSG000002	RN7SKP145	misc_RNA	0	0	0	0
ENSG000002	Y_RNA	misc_RNA	0	0	0	0
ENSG000002	Y_RNA	misc_RNA	7.44660182	11.2849357	8.66564669	7.33154915
ENSG000002	RNA5SP350	rRNA_pseudo	0	1.02504286	1.77691314	0.90231595
ENSG000002	RNU5D-1	snRNA	51.6696772	57.2066029	25.6572355	24.680746

ENSG000002	Y_RNA	misc_RNA	0	2.56579419	0	0
ENSG000002	Y_RNA	misc_RNA	0	3.10572704	0	0
ENSG000002	Y_RNA	misc_RNA	0	0	0	0
ENSG000002	RNU6-1309P	snRNA	0	0	0	0
ENSG000002	RNU1-19P	snRNA	13.3833886	2.87104634	5.31508281	10.474865
ENSG000002	Y_RNA	misc_RNA	0	0	0	0
ENSG000002	Telomerase-v	misc_RNA	33.6988737	48.4913831	10.3127686	29.2341706
ENSG000002	RNU6-238P	snRNA	0	0	0	0
ENSG000002	RNU1-20P	snRNA	0	0	0.88583469	0
ENSG000002	SNORD1C	snoRNA	31.1124815	58.1372466	32.401068	51.077574
ENSG000002	RNU6-832P	snRNA	1.24114385	0	0	0
ENSG000002	U8	snoRNA	0	0	0	0
ENSG000002	RNU1-21P	snRNA	1.50730723	1.22721473	0	0
ENSG000002	RN7SKP211	misc_RNA	0	0	0	0
ENSG000002	Y_RNA	misc_RNA	2.63534124	1.03524235	0	0.94514214
ENSG000002	RNU1-22P	snRNA	6.38910808	3.89152472	1.71965421	4.63068445
ENSG000002	ENSG000002	snoRNA	0	0	0	0
ENSG000002	Y_RNA	misc_RNA	0	2.07643403	0	1.84647684
ENSG000002	Y_RNA	misc_RNA	0	0	0	0
ENSG000002	RNY4P27	misc_RNA	0	0	0.90258482	0
ENSG000002	RNU6-992P	snRNA	0	0	0	0
ENSG000002	SNORD113-6	snoRNA	0	0	0	0
ENSG000002	RNU6-745P	snRNA	0.89258296	0	0	0
ENSG000002	RNU6-839P	snRNA	0.86530273	1.02077812	0	0
ENSG000002	RNU6-697P	snRNA	0	0	0	0
ENSG000002	RNU6-179P	snRNA	0	0	0	0
ENSG000002	U3	snoRNA	0	0	0	0
ENSG000002	RNU6-626P	snRNA	0	0	0	0
ENSG000002	RNA5SP382	rRNA_pseudo	0.93526083	3.2955268	1.97227634	0.80358011
ENSG000002	RNA5SP197	rRNA_pseudo	0	0	0	0
ENSG000002	SNORD115-1	snoRNA	0	0	0	0
ENSG000002	RNU1-23P	snRNA	0	0	0	0
ENSG000002	ENSG000002	snoRNA	0	0	0	0
ENSG000002	SNORA70	snoRNA	2.17514348	3.2086652	0	3.0308635
ENSG000002	RNA5SP133	rRNA_pseudo	0	0	0	0
ENSG000002	Y_RNA	misc_RNA	0	0	0	0
ENSG000002	RN7SKP79	misc_RNA	5.81484267	2.97258768	3.17062578	5.84797463
ENSG000002	RNA5SP263	rRNA_pseudo	4.76021849	5.31317526	5.0172773	8.97191001
ENSG000002	RNU6-254P	snRNA	0	0	0	0
ENSG000002	RNA5SP214	rRNA_pseudo	0	0	0	0
ENSG000002	RNU6-1147P	snRNA	0	0	0	0.90557412
ENSG000002	Y_RNA	misc_RNA	0	0	0	0
ENSG000002	RNU6-529P	snRNA	0	0	0	0
ENSG000002	RNU6-536P	snRNA	0	0	0	0

ENSG000002	Y_RNA	misc_RNA	0	0	0	0
ENSG000002	RNU6-97P	snRNA	0.88879741	0	0	0.91949833
ENSG000002	SNORD35A	snoRNA	10.3250104	12.9759432	9.02840203	9.48097657
ENSG000002	Y_RNA	misc_RNA	0	0	0	0
ENSG000002	Y_RNA	misc_RNA	0	0	0	0
ENSG000002	RNU6-66P	snRNA	0	0	0	0
ENSG000002	RNU6-838P	snRNA	0	0	0.87605146	0.91752101
ENSG000002	RNU4-32P	snRNA	0	0	0	0
ENSG000002	RNA5SP199	rRNA_pseudo	129.748045	188.536921	99.6342372	135.398892
ENSG000002	RNA5SP352	rRNA_pseudo	12.1757908	31.5007004	15.6778702	22.6381107
ENSG000002	SNORD114-1	snoRNA	0	0	0	0
ENSG000002	RNU6-624P	snRNA	0	0	0	0
ENSG000002	Y_RNA	misc_RNA	0	0	0	0
ENSG000002	Y_RNA	misc_RNA	0	1.03679415	0	0
ENSG000002	ENSG000002	snoRNA	0	0	0	0
ENSG000002	Y_RNA	misc_RNA	0	0.95415735	0	0
ENSG000002	RNA5SP393	rRNA_pseudo	0	0	0	0
ENSG000002	ENSG000002	snoRNA	0	0	0	0
ENSG000002	RNU6-527P	snRNA	0.88879741	1.02810291	0	3.67799334
ENSG000002	RNU1-83P	snRNA	3.88075847	3.43929244	1.28579307	5.77727762
ENSG000002	Y_RNA	misc_RNA	0	0	0	0
ENSG000002	RNA5SP483	rRNA_pseudo	0	0	0	0
ENSG000002	RNU6-940P	snRNA	0	0	0	0
ENSG000002	RNU6-1255P	snRNA	0	0.99954447	0	0
ENSG000002	Y_RNA	misc_RNA	4.35896114	2.25302737	0.93300938	0
ENSG000002	Y_RNA	misc_RNA	0	0	0.91656009	0
ENSG000002	RNA5SP215	rRNA_pseudo	6.48760209	8.80806602	4.45369694	2.72502228
ENSG000002	RN7SKP255	misc_RNA	69.5537926	97.5897768	23.918083	67.9334802
ENSG000002	RNA5-8SP7	rRNA_pseudo	0	0	0	0
ENSG000002	Y_RNA	misc_RNA	75.9173787	208.308355	124.333865	76.635235
ENSG000002	SNORD3P1	snoRNA	0	0	0	0
ENSG000002	SNORA63	snoRNA	72.6694063	184.150656	31.1548903	83.3419643
ENSG000002	Y_RNA	misc_RNA	0	0	0	0
ENSG000002	RNA5SP391	rRNA_pseudo	0	0	0	0
ENSG000002	RNA5SP62	rRNA_pseudo	0	0	0	0.91662469
ENSG000002	RNU6-183P	snRNA	0	0	0	0
ENSG000002	Y_RNA	misc_RNA	58.2299677	78.8789786	28.368682	27.0631328
ENSG000002	Y_RNA	misc_RNA	0	0	0.88151049	0
ENSG000002	RNA5SP333	rRNA_pseudo	0.92738234	1.00278154	0.90818522	0
ENSG000002	RNU1-49P	snRNA	0	0	0	0
ENSG000002	RNU1-105P	snRNA	0	0	0	0
ENSG000002	Y_RNA	misc_RNA	0	0	0	0
ENSG000002	RNU6-485P	snRNA	0	0	0	0
ENSG000002	RNU6-1285P	snRNA	0.89049615	0	0	0

ENSG000002	Y_RNA	misc_RNA	0	0	0	0
ENSG000002	SNORA71D	snoRNA	23.3816513	43.9565806	18.5905829	30.8994085
ENSG000002	SNORA72	snoRNA	0	0	0	0
ENSG000002	RNU6-833P	snRNA	0	0	0	0
ENSG000002	RNU6-792P	snRNA	0	0	0	0
ENSG000002	Y_RNA	misc_RNA	1.80870294	1.05210651	0.76564395	0
ENSG000002	RNU6-511P	snRNA	0	0	0	0
ENSG000002	SNORD113-8	snoRNA	0	0	0	0.96291912
ENSG000002	RNU6-666P	snRNA	0	0	0	0
ENSG000002	RNU5E-8P	snRNA	0	0	0	0.91001161
ENSG000002	RNU5E-10P	snRNA	0	0	0	0
ENSG000002	ENSG000002	snoRNA	0	0	0	0
ENSG000002	RNU5B-4P	snRNA	0	0	0	0
ENSG000002	RN7SKP45	misc_RNA	0	0	0	0
ENSG000002	ENSG000002	snoRNA	0	0	0	0
ENSG000002	RNU6-618P	snRNA	0	0	0	0
ENSG000002	RNU6-509P	snRNA	4.56337989	5.18625326	2.72694493	4.958007
ENSG000002	Y_RNA	misc_RNA	0	1.02700407	0	0
ENSG000002	Y_RNA	misc_RNA	0	0	0	0
ENSG000002	RNU6-698P	snRNA	0	0	0	0
ENSG000002	SNORA38B	snoRNA	45.8707725	75.6628723	38.7494195	76.5770452
ENSG000002	Y_RNA	misc_RNA	0	0	0	0
ENSG000002	SNORD115-2	snoRNA	0	0	0	0
ENSG000002	RNU6-1099P	snRNA	0	0	0	0
ENSG000002	SNORD114-2	snoRNA	0	0	0	0
ENSG000002	RNU6-1169P	snRNA	0	0	0	0
ENSG000002	RNA5SP74	rRNA_pseudo	91.4471269	117.212796	63.227597	88.9733343
ENSG000002	RNA5SP346	rRNA_pseudo	0	0	0	0
ENSG000002	SNORD114-2	snoRNA	0	0	0	0
ENSG000002	SNORA63B	snoRNA	0	0	0	0
ENSG000002	Y_RNA	misc_RNA	18.7206926	35.1452778	5.91303019	21.8864234
ENSG000002	Y_RNA	misc_RNA	0	0	0	0
ENSG000002	ENSG000002	snoRNA	0	0	0	0
ENSG000002	RNU6-1155P	snRNA	0	0	0	0.90557412
ENSG000002	Y_RNA	misc_RNA	0	0	0	0
ENSG000002	Y_RNA	misc_RNA	0	0	0	0
ENSG000002	RNU4-81P	snRNA	0	0	0	0
ENSG000002	Y_RNA	misc_RNA	0.89241238	0	0	0
ENSG000002	RNA5-8SP2	rRNA_pseudo	24.275338	14.1925656	9.7993134	16.9539903
ENSG000002	Y_RNA	misc_RNA	0	0	0	0
ENSG000002	RNU6-799P	snRNA	0	0	0	0
ENSG000002	ENSG000002	snoRNA	0	0	0	0
ENSG000002	RNU6-1066P	snRNA	0	0	0	0
ENSG000002	RNU6-1339P	snRNA	0	0	0	0

ENSG000002	RNU6-1085P	snRNA	0	0	0	0
ENSG000002	Y_RNA	misc_RNA	0	0	0	0
ENSG000002	RNU6-1112P	snRNA	0	0	0	0
ENSG000002	RNU6-1097P	snRNA	0	0	0	0
ENSG000002	RNU6-727P	snRNA	25.6540713	26.5109533	9.75245485	27.9266015
ENSG000002	SNORD118	snoRNA	734.565725	801.654196	511.853336	687.348474
ENSG000002	RNA5SP403	rRNA_pseudo	1.83291426	5.07705026	1.75210292	1.83504202
ENSG000002	RNU6-112P	snRNA	0	0	0	0
ENSG000002	RNU6-1125P	snRNA	0	0	0	0
ENSG000002	RN7SKP44	misc_RNA	0	0	0	0
ENSG000002	RNA5SP507	rRNA_pseudo	0	0	0	0
ENSG000002	RN7SKP162	misc_RNA	0	1.36715227	0	0
ENSG000002	SNORD115-4	snoRNA	0	0	0	0
ENSG000002	SNORD114-2	snoRNA	0	0	0	0
ENSG000002	RNU6-1017P	snRNA	0	0	0	0
ENSG000002	RNU6-1244P	snRNA	0.91644882	0	0	0
ENSG000002	Y_RNA	misc_RNA	15.3381112	27.6414447	23.7772866	18.0817144
ENSG000002	SNORD115-1	snoRNA	0	0	0	0
ENSG000002	RNA5SP322	rRNA_pseudo	0	0	0	0
ENSG000002	RN7SKP203	misc_RNA	28.4777514	38.3634556	9.55355913	16.5526902
ENSG000002	U3	snoRNA	0	0	0	0
ENSG000002	Y_RNA	misc_RNA	122.663829	286.756413	133.356238	80.8608052
ENSG000002	RNU6-1205P	snRNA	0.89365585	0	0	0.92341466
ENSG000002	U8	snoRNA	0	0.98856041	0	0
ENSG000002	Y_RNA	misc_RNA	0	0	0	0
ENSG000002	SNORD115-5	snoRNA	0	0	0	0
ENSG000002	Y_RNA	misc_RNA	2.6856594	13.5311963	6.19897267	3.91031748
ENSG000002	Y_RNA	misc_RNA	0	0	0	0
ENSG000002	RNA5SP378	rRNA_pseudo	1.87802479	0	0	0.91540324
ENSG000002	RNU6-1214P	snRNA	0	0	0	0
ENSG000002	Y_RNA	misc_RNA	7.14644981	20.5878887	14.4734518	8.05896225
ENSG000002	RNU6-957P	snRNA	1.77137681	0	0	0
ENSG000002	RNU6-1209P	snRNA	0	0	0	0
ENSG000002	RNY4P14	misc_RNA	0	0	0	0
ENSG000002	RNA5SP457	rRNA_pseudo	0	0	0	0
ENSG000002	RNU6-1331P	snRNA	0	0	0	0
ENSG000002	SNORD35B	snoRNA	4.05430786	22.0276701	6.29114223	12.6651988
ENSG000002	SNORA33	snoRNA	30.1958158	76.3705866	21.2789711	33.6453233
ENSG000002	ENSG000002	snoRNA	0	0	0	0
ENSG000002	RNY4P6	misc_RNA	4.43089757	15.6338864	8.18988663	8.57661523
ENSG000002	U3	snoRNA	0	0	0	0
ENSG000002	RN7SKP176	misc_RNA	0	0	0	6.84450855
ENSG000002	Y_RNA	misc_RNA	1.77692147	0	0	0
ENSG000002	U3	snoRNA	0	0	0	0

ENSG000002	Y_RNA	misc_RNA	0	0	0	0.91971121
ENSG000002	RNU6-137P	snRNA	0	0	0	0
ENSG000002	Y_RNA	misc_RNA	0	0	0	0
ENSG000002	RNU6-1020P	snRNA	0	0	0	0
ENSG000002	RNU6-525P	snRNA	0	0	0	0
ENSG000002	RNU6-103P	snRNA	0	0	0	0
ENSG000002	RNA5SP429	rRNA_pseudo	11180.6261	16392.0289	7613.70169	11171.6591
ENSG000002	RNU6-288P	snRNA	0	0	0	0
ENSG000002	RNU6-640P	snRNA	6.42132701	7.10633599	0	3.65418093
ENSG000002	SNORD115-3	snoRNA	0	0	0	0
ENSG000002	RNU5F-7P	snRNA	0	0	0	0
ENSG000002	RNU6-829P	snRNA	0	0	0	0
ENSG000002	RNU6-1284P	snRNA	0	2.01693735	0.88796544	0
ENSG000002	Y_RNA	misc_RNA	0	0	0.88082171	0
ENSG000002	RNU6-414P	snRNA	2.99022526	2.10869494	0.91644097	0
ENSG000002	Y_RNA	misc_RNA	0	0	0	0
ENSG000002	RNA5SP514	rRNA_pseudo	4.36245049	16.726451	10.884874	20.0477396
ENSG000002	Y_RNA	misc_RNA	0	0	0	0
ENSG000002	SNORD115-3	snoRNA	0	0	0	0
ENSG000002	RNU6-824P	snRNA	0	0	0	0
ENSG000002	RNVU1-33	snRNA	6.95607375	2.87369043	2.66868451	0.88995345
ENSG000002	Y_RNA	misc_RNA	0	0	0	0
ENSG000002	RNA5SP50	rRNA_pseudo	1.84188811	2.09815793	1.79621073	2.27185731
ENSG000002	Y_RNA	misc_RNA	0	0	0	0
ENSG000002	SNORD114-1	snoRNA	0	0	0	0
ENSG000002	Y_RNA	misc_RNA	0	0	0	0
ENSG000002	SNORD114-2	snoRNA	0	0	0	0
ENSG000002	RNA5SP349	rRNA_pseudo	0	0	0	0
ENSG000002	Y_RNA	misc_RNA	0	0	0	0
ENSG000002	Y_RNA	misc_RNA	0	0	0	0
ENSG000002	RNA5SP447	rRNA_pseudo	0	0	0	0
ENSG000002	ENSG000002	snoRNA	0.94908409	0	0	0
ENSG000002	RNU6-1059P	snRNA	0	0	0	0
ENSG000002	SNORD18A	snoRNA	3.2444051	7.40258704	5.28380445	10.811251
ENSG000002	RNA5SP325	rRNA_pseudo	34.7344356	84.591093	20.5708779	44.6750186
ENSG000002	RNY1P11	misc_RNA	0.91980834	1.01246136	0	0.89573321
ENSG000002	Y_RNA	misc_RNA	0	0	0	0
ENSG000002	SNORD113-7	snoRNA	0	0	0	0
ENSG000002	Y_RNA	misc_RNA	0	0	0	0
ENSG000002	SNORD114-2	snoRNA	0	0	0	0
ENSG000002	RNU5F-3P	snRNA	0	0	0	0
ENSG000002	SNORD115-3	snoRNA	0	0	0	0
ENSG000002	RNU6-1210P	snRNA	0	0	0	0
ENSG000002	Y_RNA	misc_RNA	0	0	0	0

ENSG000002	RNU6-226P	snRNA	0	1.00218871	0	0
ENSG000002	RNA5SP114	rRNA_pseudo	0	1.01185413	0	0
ENSG000002	Y_RNA	misc_RNA	0.90854847	0	0	0
ENSG000002	ENSG000002	snoRNA	0	0	0	0
ENSG000002	RNU4-92P	snRNA	0	0	0.88885841	0
ENSG000002	SNORA5B	snoRNA	0.76385625	0	0	0
ENSG000002	SNORD116-1	snoRNA	0	0	0	0
ENSG000002	Y_RNA	misc_RNA	0	0	0	0
ENSG000002	RNU6-1188P	snRNA	0	0	0	0
ENSG000002	RNU6-912P	snRNA	0	0	0	0
ENSG000002	RN7SKP174	misc_RNA	1.06851379	2.03239071	0	0
ENSG000002	RN7SKP160	misc_RNA	7.37035383	0	0	3.6980213
ENSG000002	SNORD18	snoRNA	0	0	0	0
ENSG000002	SNORD115-4	snoRNA	0	0	0	0
ENSG000002	RNU6-263P	snRNA	6.34154925	4.01469852	5.38242717	9.07552336
ENSG000002	RNU6-379P	snRNA	0	0	0	0
ENSG000002	Y_RNA	misc_RNA	0	0	0	0
ENSG000002	RNY3P3	misc_RNA	0	0	0	0
ENSG000002	RNA5SP335	rRNA_pseudo	174.505131	246.842417	131.930774	236.332331
ENSG000002	Y_RNA	misc_RNA	0	0	0	0
ENSG000002	U3	snoRNA	0	0	0	0
ENSG000002	RNU6-674P	snRNA	0	0	0	0
ENSG000002	Y_RNA	misc_RNA	0	0	0.88151049	0
ENSG000002	RNU6-873P	snRNA	0	0	0	0
ENSG000002	ENSG000002	snoRNA	0	0	0	0
ENSG000002	RN7SKP93	misc_RNA	0	0.94662902	0	0
ENSG000002	RNA5SP27	rRNA_pseudo	0	0	0	0
ENSG000002	RNA5SP28	rRNA_pseudo	0	0	0	0
ENSG000002	RNU6-682P	snRNA	0	0	0	0
ENSG000002	Y_RNA	misc_RNA	0.89341316	0	0	0.92427345
ENSG000002	RN7SKP103	misc_RNA	0	0	0	0
ENSG000002	RNA5SP260	rRNA_pseudo	4.81781943	7.30012671	2.47787156	2.79988485
ENSG000002	RNU6-931P	snRNA	0	0	0	0
ENSG000002	SNORD115-8	snoRNA	0	0	0	0
ENSG000002	RNU6-271P	snRNA	0	0	0	0
ENSG000002	RNU1-124P	snRNA	0	0	0	0
ENSG000002	RNU6-194P	snRNA	0	0	0	0
ENSG000002	ENSG000002	snoRNA	0	0	0	0
ENSG000002	Y_RNA	misc_RNA	0	0	0	0
ENSG000002	RNA5SP472	rRNA_pseudo	0	0	0	0
ENSG000002	RNA5SP161	rRNA_pseudo	2041.28231	3404.43329	1265.35186	2634.44743
ENSG000002	Y_RNA	misc_RNA	0.88218416	1.02631768	0	0.91265668
ENSG000002	RNU1-31P	snRNA	0	0	0	0
ENSG000002	Y_RNA	misc_RNA	0	0	0	0

ENSG000002	Y_RNA	misc_RNA	0	0	0	0
ENSG000002	ENSG000002	snoRNA	0	0	0	0
ENSG000002	Y_RNA	misc_RNA	0	0	0	0
ENSG000002	RNA5SP68	rRNA_pseudo	0	0	0	0
ENSG000002	RNU6-236P	snRNA	0	0	0	0
ENSG000002	SNORD115-1	snoRNA	0	0	0	0
ENSG000002	RNU6-884P	snRNA	0	0	0	0
ENSG000002	RN7SKP113	misc_RNA	0	0	0	0
ENSG000002	RNU6-961P	snRNA	0.92726464	3.35150867	0.93699465	1.75395314
ENSG000002	Y_RNA	misc_RNA	0	0	0	0
ENSG000002	Y_RNA	misc_RNA	0	0	0	0
ENSG000002	RNU6-478P	snRNA	0	0	0	0
ENSG000002	RNU6-105P	snRNA	0.91448312	0	0	0
ENSG000002	RN7SKP180	misc_RNA	3.32726455	7.78636591	4.14793913	4.898142
ENSG000002	SNORD8	snoRNA	163.065023	517.922442	134.073448	283.550754
ENSG000002	RNA5SP234	rRNA_pseudo	0	0	0	0
ENSG000002	Y_RNA	misc_RNA	1.83916277	1.01185413	0	0
ENSG000002	RNU1-65P	snRNA	0	0	0	0
ENSG000002	SNORA80A	snoRNA	3.77080539	3.9481997	4.44429203	4.45229282
ENSG000002	RN7SKP250	misc_RNA	2.19455897	2.22212455	0	1.04706312
ENSG000002	RNU4-1	snRNA	1042.94371	2469.20147	1338.20636	1741.59432
ENSG000002	RNU6-753P	snRNA	0	0	0	0
ENSG000002	RNU6-770P	snRNA	0	0	0	0
ENSG000002	RNU1-130P	snRNA	0	0	0	0
ENSG000002	SNORD115-2	snoRNA	0	0	0	0
ENSG000002	RNA5SP275	rRNA_pseudo	0	0	0	0
ENSG000002	RNU1-32P	snRNA	0	0	0	0
ENSG000002	SNORD115-6	snoRNA	0	0	0	0
ENSG000002	RNU6-595P	snRNA	0	0	0	0
ENSG000002	RNU6-1091P	snRNA	0	0	0	0
ENSG000002	SNORA38	snoRNA	5.64904839	16.8006107	10.6286431	9.74029726
ENSG000002	RNU6-899P	snRNA	0	0	0	0
ENSG000002	RNU6-1204P	snRNA	0	0	0	0
ENSG000002	Y_RNA	misc_RNA	0	0	0	0
ENSG000002	SNORD114-2	snoRNA	0	0	0	0
ENSG000002	RNU6-324P	snRNA	0	0	0	0
ENSG000002	Y_RNA	misc_RNA	0	0	0	0
ENSG000002	RN7SKP134	misc_RNA	0	0	0	0
ENSG000002	SNORD36B	snoRNA	19.7961926	36.3972545	16.4255052	24.1585214
ENSG000002	SNORD114-4	snoRNA	0	0	0	0
ENSG000002	Y_RNA	misc_RNA	0	0	0	0
ENSG000002	RNA5SP48	rRNA_pseudo	4.62557969	5.00792084	0.89038329	1.81843584
ENSG000002	RNU6-82P	snRNA	0.90451589	1.00218871	0	0
ENSG000002	Y_RNA	misc_RNA	0	0	0	0

ENSG000002	Y_RNA	misc_RNA	1.94667041	2.17188802	0	4.99058455
ENSG000002	Y_RNA	misc_RNA	0	0	0	0
ENSG000002	SNORD27	snoRNA	143.881866	309.978422	150.694641	231.529969
ENSG000002	RNA5SP499	rRNA_pseudo	0	0	0	0
ENSG000002	Y_RNA	misc_RNA	0.96263454	4.06660676	5.76949268	11.0437175
ENSG000002	Y_RNA	misc_RNA	0	0	0	0
ENSG000002	Y_RNA	misc_RNA	0	0	0	0
ENSG000002	RN7SKP36	misc_RNA	3.30350062	1.89275132	0.6857081	2.8469992
ENSG000002	RNU6-457P	snRNA	0	0	0	0
ENSG000002	RNU6-810P	snRNA	0.90451589	0	0	0
ENSG000002	RNA5SP456	rRNA_pseudo	0	0	0	0
ENSG000002	RNA5SP399	rRNA_pseudo	0	0	0	0
ENSG000002	Y_RNA	misc_RNA	0	0	0	0
ENSG000002	RNU6-340P	snRNA	0	0	0.44673318	0
ENSG000002	RNU6-560P	snRNA	0.8705659	2.05654942	0.88715039	0
ENSG000002	SNORD14E	snoRNA	5.33913313	11.3876655	3.69173592	10.1265226
ENSG000002	RNU6-681P	snRNA	0	0	0	0
ENSG000002	Y_RNA	misc_RNA	0	0	0.88947672	0
ENSG000002	Y_RNA	misc_RNA	0	0	0	0
ENSG000002	RNU1-146P	snRNA	0	0	0	0
ENSG000002	RNU6-598P	snRNA	0	0	0	0
ENSG000002	Y_RNA	misc_RNA	0	0	0	0
ENSG000002	RNU4-13P	snRNA	48.3917877	31.9369293	14.9143644	37.7629322
ENSG000002	RNA5SP99	rRNA_pseudo	25.4643436	49.3727549	19.9950014	36.2292546
ENSG000002	ENSG000002	snoRNA	0	0	0	0
ENSG000002	RNU6-944P	snRNA	0	0	0	0
ENSG000002	RN7SKP245	misc_RNA	0	0	0	0
ENSG000002	ENSG000002	snoRNA	0	0	0	0
ENSG000002	RNU6-1243P	snRNA	0	0	0	0
ENSG000002	RNU6-627P	snRNA	0	0	0	0
ENSG000002	RNU1-42P	snRNA	8.80380754	3.8293548	2.64875757	14.3021286
ENSG000002	RNU6-854P	snRNA	0	0	0	0
ENSG000002	SNORD46	snoRNA	49.7726548	213.845361	63.2116495	127.810268
ENSG000002	RNA5SP435	rRNA_pseudo	19.42064	52.2573811	15.0284634	30.1833847
ENSG000002	RNU6-553P	snRNA	0	0	0.90242428	0
ENSG000002	Y_RNA	misc_RNA	0	0	0	0
ENSG000002	RNU6-1048P	snRNA	0	0	0	0
ENSG000002	RNU6-960P	snRNA	0	0	0	0
ENSG000002	RNU6-1090P	snRNA	1.76416425	0	0	0.91255103
ENSG000002	RNU6-694P	snRNA	0	0	0	0
ENSG000002	RNU6-501P	snRNA	0	1.00846867	0	0
ENSG000002	SNORD115-3:	snoRNA	0	0	0	0
ENSG000002	Y_RNA	misc_RNA	0	0	0	0
ENSG000002	RNU6-801P	snRNA	0	0	0	0

ENSG000002	SNORA74A	snoRNA	62.9710044	85.2200755	50.9179698	60.5225727
ENSG000002	RNU6-289P	snRNA	0	0	0	0
ENSG000002	RN7SKP87	misc_RNA	0	0	0	0
ENSG000002	ENSG000002	snoRNA	0	0	0	0
ENSG000002	RNU5A-8P	snRNA	9.25155477	15.433687	31.2066822	14.9207349
ENSG000002	RNU4-87P	snRNA	0	0	0.91567563	0
ENSG000002	RNU1-7P	snRNA	0	0	0	0
ENSG000002	Y_RNA	misc_RNA	1.78482515	1.03821701	0.88151049	0
ENSG000002	Y_RNA	misc_RNA	0	0	0	0
ENSG000002	SNORA3A	snoRNA	68.6870173	139.235641	74.5537733	105.125593
ENSG000002	RNA5SP493	rRNA_pseudo	0.86812063	1.03881232	1.789597	0.93715531
ENSG000002	RNU6-819P	snRNA	0	0	0	0
ENSG000002	SNORD115-3	snoRNA	0	0	0	0
ENSG000002	ENSG000002	snoRNA	0	0	0	0
ENSG000002	RNVU1-34	snRNA	0	0	0	0
ENSG000002	Y_RNA	misc_RNA	0	0	0	0
ENSG000002	ENSG000002	snoRNA	0	0	0	0
ENSG000002	RNA5SP516	rRNA_pseudo	0	0	0	0
ENSG000002	RNU6-270P	snRNA	0	0	0	0
ENSG000002	ENSG000002	snoRNA	1.88540269	0	0	0
ENSG000002	Y_RNA	misc_RNA	0	0	0	0
ENSG000002	ENSG000002	snoRNA	0	0	0	0
ENSG000002	RN7SKP224	misc_RNA	0.99442482	0.97685229	0.81799777	0
ENSG000002	Y_RNA	misc_RNA	0	0	0	0
ENSG000002	Y_RNA	misc_RNA	0	0	0	0
ENSG000002	RNA5SP232	rRNA_pseudo	0	0	0	0
ENSG000002	RNU6-280P	snRNA	0	0	0	0
ENSG000002	RNU6-374P	snRNA	0	0	0	0
ENSG000002	RNU6-743P	snRNA	0.92813883	2.13682116	0	2.89498071
ENSG000002	RNY3P11	misc_RNA	2.72940993	8.13515776	3.6083069	4.0677206
ENSG000002	SNORD74B	snoRNA	0	0	0	0
ENSG000002	Y_RNA	misc_RNA	0	0	0	0
ENSG000002	RN7SKP107	misc_RNA	0	0	0	0
ENSG000002	RNU6-151P	snRNA	0	0	0	0
ENSG000002	Y_RNA	misc_RNA	0	1.00974371	0	0.91662469
ENSG000002	RNU6-704P	snRNA	0	0	0	0
ENSG000002	RNU1-96P	snRNA	0	0	0	0
ENSG000002	Y_RNA	misc_RNA	0	0	0	0
ENSG000002	RNA5SP469	rRNA_pseudo	0	0	0.89202115	0
ENSG000002	ENSG000002	snoRNA	0	0	0	0
ENSG000002	RNA5SP242	rRNA_pseudo	240.033064	364.65071	196.078614	327.472573
ENSG000002	ENSG000002	snoRNA	0	0	0	0
ENSG000002	RNU6-268P	snRNA	1.80903178	1.00218871	0	0
ENSG000002	Y_RNA	misc_RNA	0	1.03821701	0	0

ENSG000002	Y_RNA	misc_RNA	0	0	0	0
ENSG000002	RNU6-668P	snRNA	0	0	0	0
ENSG000002	RNA5SP336	rRNA_pseudo	12.0438871	31.3904217	14.1564994	10.1109843
ENSG000002	RNU6-461P	snRNA	0	0	0	0
ENSG000002	RN7SKP280	misc_RNA	0	0	0	0
ENSG000002	RNU4-84P	snRNA	0.94929847	1.45349624	0.43819849	1.3476644
ENSG000002	Y_RNA	misc_RNA	0	0	0	0
ENSG000002	Y_RNA	misc_RNA	0	0	0	0
ENSG000002	Y_RNA	misc_RNA	0	0	0	0
ENSG000002	RNU4-51P	snRNA	0.96240457	0	0.88864856	1.7838694
ENSG000002	RNU6-188P	snRNA	0	0	0	0
ENSG000002	RN7SKP214	misc_RNA	0	0.86538363	1.73491141	4.43262927
ENSG000002	RNU6-173P	snRNA	0	0	0	0
ENSG000002	RNA5SP394	rRNA_pseudo	0	0	0	0
ENSG000002	Y_RNA	misc_RNA	0	0	0	0
ENSG000002	RNU6-266P	snRNA	0	0	0	0
ENSG000002	RNA5SP387	rRNA_pseudo	9.41187305	8.82976228	3.37665105	5.91829813
ENSG000002	RNU6-366P	snRNA	0	0	0	0
ENSG000002	RNY1	misc_RNA	2089.62708	5689.61821	1840.14756	2582.06283
ENSG000002	Y_RNA	misc_RNA	0	0	0	0
ENSG000002	RNA5SP245	rRNA_pseudo	0.91883523	0	0	0
ENSG000002	RNU6-647P	snRNA	0	0	0	0
ENSG000002	Y_RNA	misc_RNA	0	0	0	0
ENSG000002	Y_RNA	misc_RNA	0	0	0	0
ENSG000002	RNU1-33P	snRNA	0	0	0	0
ENSG000002	RNY1P12	misc_RNA	0.90879593	8.17832729	1.79545571	1.81720103
ENSG000002	SNORA58B	snoRNA	10.3166084	10.7463383	15.9994513	21.2673813
ENSG000002	ENSG000002	snoRNA	0	0	0	0
ENSG000002	Y_RNA	misc_RNA	0	0	0	0
ENSG000002	RNU6-777P	snRNA	0	0	0	0
ENSG000002	RNU6-353P	snRNA	0	0	0	0
ENSG000002	RN7SKP128	misc_RNA	0	0	0	0
ENSG000002	RNU1-151P	snRNA	0	0	0	0
ENSG000002	SNORD115-4	snoRNA	0	0	0	0
ENSG000002	RNA5SP175	rRNA_pseudo	0	0	0	0
ENSG000002	RNA5SP42	rRNA_pseudo	0	0	0	0
ENSG000002	ENSG000002	snoRNA	0	0	0	0
ENSG000002	RNU6-371P	snRNA	0	0	0	0
ENSG000002	RNU1-24P	snRNA	0	0	0	0
ENSG000002	SNORA62	snoRNA	0	0	0	0
ENSG000002	Y_RNA	misc_RNA	0	0	0	0
ENSG000002	RN7SKP10	misc_RNA	0	0	0	0
ENSG000002	RNU6-454P	snRNA	0	0	0	0
ENSG000002	RNU4-36P	snRNA	0.96099864	0	0.87176797	0

ENSG000002	RNU6-1229P	snRNA	0	0	0	0
ENSG000002	RNA5SP368	rRNA_pseudo	13.6906131	16.8356426	7.15549445	10.0101262
ENSG000002	RNU1-132P	snRNA	0	0	0	0
ENSG000002	RNU6-853P	snRNA	0	0	0	0
ENSG000002	Y_RNA	misc_RNA	0	0	0	0
ENSG000002	RNU6-1322P	snRNA	0	0	0	0
ENSG000002	RNU6-115P	snRNA	32.9225693	34.1782883	4.50722149	22.9200954
ENSG000002	RNU6-670P	snRNA	0	0	0	0
ENSG000002	RNVU1-3	snRNA	0	0	1.75968181	4.45638438
ENSG000002	RNU4-68P	snRNA	1.89408113	0.96633733	0	0.89810726
ENSG000002	RNA5SP202	rRNA_pseudo	1216.94	2587.16424	1266.81387	1766.85788
ENSG000002	RNU4-33P	snRNA	0	0	0	0
ENSG000002	Y_RNA	misc_RNA	0.89221597	0	0	0.92303493
ENSG000002	RNU6-879P	snRNA	0	0	0	0
ENSG000002	ENSG000002	snoRNA	0	0	0	0
ENSG000002	RN7SKP118	misc_RNA	1.83943616	0.95342079	0	0.92112116
ENSG000002	Y_RNA	misc_RNA	0	0	0	0
ENSG000002	Y_RNA	misc_RNA	0	0	0	0
ENSG000002	SNORD42	snoRNA	0	0	0	0
ENSG000002	RNA5SP139	rRNA_pseudo	0	0	0	0
ENSG000002	Y_RNA	misc_RNA	8.27291842	21.9339453	10.6863202	11.3370941
ENSG000002	Y_RNA	misc_RNA	0	0	0	0
ENSG000002	Y_RNA	misc_RNA	0	0	0	0
ENSG000002	RNU4-40P	snRNA	0	1.93742847	0	0
ENSG000002	RNU6-1305P	snRNA	0	1.02810291	0	0
ENSG000002	Y_RNA	misc_RNA	2.6763899	6.39564324	3.80136297	2.98830835
ENSG000002	SNORA63D	snoRNA	4.70754012	5.92962783	2.65716055	9.74029726
ENSG000002	RNU4-50P	snRNA	0.94704056	0	0	0
ENSG000002	Y_RNA	misc_RNA	0	0	0	0
ENSG000002	SNORD114-9	snoRNA	0	0	0	0
ENSG000002	RNU6-978P	snRNA	0	0	0	0
ENSG000002	RNY1P1	misc_RNA	0	0	0	0
ENSG000002	RNU6-654P	snRNA	0	0	0	0
ENSG000002	ENSG000002	snoRNA	0	0	0	0
ENSG000002	SNORD114-1	snoRNA	0	0	0	0
ENSG000002	RNU4-10P	snRNA	0	0	0	0.8919347
ENSG000002	RNU6-990P	snRNA	0	0	0	0
ENSG000002	RNU6-1075P	snRNA	0	0	0	0
ENSG000002	SNORD114-6	snoRNA	0	0	0	0
ENSG000002	SNORD73B	snoRNA	0	0	0	0
ENSG000002	RNU6-755P	snRNA	0	0	0.89919121	0
ENSG000002	RNU1-112P	snRNA	0	0	0	0
ENSG000002	RNU6-776P	snRNA	0	0	0	0
ENSG000002	RNA5SP192	rRNA_pseudo	5.59688407	4.9328162	3.58329417	5.15001538

ENSG000002	Y_RNA	misc_RNA	0	1.02631768	0	0
ENSG000002	Y_RNA	misc_RNA	0	0	0	0
ENSG000002	Y_RNA	misc_RNA	0	0	0	0
ENSG000002	RNA5SP524	rRNA	0	0	0	0
ENSG000002	RN7SKP171	misc_RNA	0	0	0	0
ENSG000002	RNU6-1047P	snRNA	0	0	0	0
ENSG000002	RN7SKP76	misc_RNA	0	0	0	0
ENSG000002	RNU1-34P	snRNA	0	1.26953659	0	0
ENSG000002	RNU6-153P	snRNA	0	0	0	0
ENSG000002	RNU6-1019P	snRNA	0	0	0	0
ENSG000002	RNU4-41P	snRNA	0	0	0	0
ENSG000002	Y_RNA	misc_RNA	0	0	0	0
ENSG000002	RNU6-1164P	snRNA	0	0	0	0
ENSG000002	SNORD115-2	snoRNA	0	0	0	0
ENSG000002	RNA5SP130	rRNA_pseudo	0	0	0.89368042	0
ENSG000002	SNORA65	snoRNA	50.0118199	94.2234512	33.6917047	70.832259
ENSG000002	RNU6-512P	snRNA	0	0	0	0
ENSG000002	Y_RNA	misc_RNA	0	0	0.88169593	0
ENSG000002	Y_RNA	misc_RNA	0	0	0	0
ENSG000002	RNA5SP72	rRNA_pseudo	0	0	0	0
ENSG000002	Y_RNA	misc_RNA	0	0	0	0
ENSG000002	RN7SKP227	misc_RNA	0.97235435	3.00522297	1.24615776	5.90233651
ENSG000002	ENSG000002	snoRNA	0	0	0	0
ENSG000002	RNU4-59P	snRNA	0	0	0	0
ENSG000002	SNORD114-3	snoRNA	0	0	0	0
ENSG000002	RNA5S9	rRNA	5091.4366	8259.24452	3759.85824	5293.08727
ENSG000002	RNU6-361P	snRNA	0	0	0	0
ENSG000002	RNA5SP142	rRNA_pseudo	0	0	0	0
ENSG000002	SNORD115-2	snoRNA	0	0	0	0
ENSG000002	U3	snoRNA	0	0	0	0
ENSG000002	SNORD32B	snoRNA	0	0	0	0
ENSG000002	SNORD115-2	snoRNA	0	0	0	0
ENSG000002	Y_RNA	misc_RNA	0	0	0	0
ENSG000002	Y_RNA	misc_RNA	1.84047876	0	0	0
ENSG000002	RNU6-421P	snRNA	0	0	0	0
ENSG000002	RNU4-38P	snRNA	0	0	0	2.69828993
ENSG000002	Y_RNA	misc_RNA	0.91460128	0	0	0
ENSG000002	U3	snoRNA	0	0	0	0
ENSG000002	RNA5SP69	rRNA_pseudo	0	0	0	0
ENSG000002	RNU105B	snoRNA	3.53032232	0.84423055	1.19309569	0
ENSG000002	Y_RNA	misc_RNA	0	0	0.89202115	0
ENSG000002	Y_RNA	misc_RNA	0	0	0	0
ENSG000002	RNA5SP500	rRNA_pseudo	0	0	0	0
ENSG000002	RN7SKP193	misc_RNA	4.7678125	3.06966226	2.58850793	5.11918032

ENSG000002	RNA5SP433	rRNA_pseudo	0	0	0	0
ENSG000002	Y_RNA	misc_RNA	0	0	0	0
ENSG000002	RN7SKP37	misc_RNA	0	0	2.84977805	0
ENSG000002	Y_RNA	misc_RNA	0	0	0	0
ENSG000002	RNU6-522P	snRNA	37.9610265	14.7289798	5.52024406	13.4023386
ENSG000002	U3	snoRNA	0	0	0	0
ENSG000002	Y_RNA	misc_RNA	0	0	0	0
ENSG000002	Y_RNA	misc_RNA	0	0	0	0
ENSG000002	RNU6-1251P	snRNA	64.8370589	62.449049	23.9554044	46.7528986
ENSG000002	SNORA70	snoRNA	0	0	0	0
ENSG000002	RNY4P23	misc_RNA	0.89221597	2.01150545	0.97122252	0
ENSG000002	RNU4-76P	snRNA	0.94704056	0.96633733	1.75152075	0.89810726
ENSG000002	RNU6-558P	snRNA	0	0	0	0
ENSG000002	ENSG000002	snoRNA	0	0	0	0
ENSG000002	RNU6-1163P	snRNA	0	0	0	0
ENSG000002	SNORA68B	snoRNA	2.81569759	8.60589601	5.73415076	9.62923425
ENSG000002	RNU6-1141P	snRNA	0	0	0	0
ENSG000002	RNU6-1078P	snRNA	0	0	0	0
ENSG000002	SNORA71	snoRNA	0	0	0	0
ENSG000002	RNA5SP140	rRNA_pseudo	0	0	0	0
ENSG000002	RN7SKP257	misc_RNA	0	0	0	0
ENSG000002	U8	snoRNA	0	0	0	0
ENSG000002	SNORD14B	snoRNA	4.472463	12.1162931	11.0590333	25.3865987
ENSG000002	Y_RNA	misc_RNA	0	0	0	0
ENSG000002	ENSG000002	snoRNA	0	0	0	0
ENSG000002	ENSG000002	snoRNA	0	0	0	0
ENSG000002	Y_RNA	misc_RNA	6.88018514	6.06049428	4.38140567	1.83324986
ENSG000002	RNA5SP141	rRNA_pseudo	107.745909	177.389174	83.9946037	153.486877
ENSG000002	RNA5SP204	rRNA_pseudo	0	0	0	0
ENSG000002	RNA5SP512	rRNA_pseudo	0	0	0	0
ENSG000002	Y_RNA	misc_RNA	0	0	0	0
ENSG000002	RN7SKP246	misc_RNA	0	0	0	0
ENSG000002	Y_RNA	misc_RNA	0	0	0	0
ENSG000002	RN7SKP71	misc_RNA	92.0431742	115.505322	18.3141754	54.4847209
ENSG000002	RNU6-1277P	snRNA	0	0	0	0
ENSG000002	Y_RNA	misc_RNA	0	0.99954447	0.89729694	0
ENSG000002	RNU6-335P	snRNA	0	0	0	0
ENSG000002	RNU4-24P	snRNA	0.94908409	0	0.87634893	0
ENSG000002	RNU6-660P	snRNA	0	0	0	0
ENSG000002	RNU4-67P	snRNA	0	0.96633733	0	0
ENSG000002	RNA5SP515	rRNA_pseudo	0	0	0	0
ENSG000002	RNU6-646P	snRNA	0	0	0	0
ENSG000002	Y_RNA	misc_RNA	1.06335202	2.24688424	0	2.07566041
ENSG000002	RNU6-309P	snRNA	0	0	0	0

ENSG000002	RNU6-1082P	snRNA	0	0	0	0
ENSG000002	RNA5SP509	rRNA_pseudo	0	0	0	0
ENSG000002	SNORA63C	snoRNA	0.93627601	0	0.87972409	0
ENSG000002	Y_RNA	misc_RNA	0	0	0	0
ENSG000002	RNU6-1317P	snRNA	0	0	0	0
ENSG000002	SNORA55	snoRNA	21.9149805	36.2250756	20.3697432	21.0963583
ENSG000002	RNU4-4P	snRNA	0	0	0	0
ENSG000002	Y_RNA	misc_RNA	0	0	0	0
ENSG000002	ENSG000002	snoRNA	0	0	0	0
ENSG000002	Y_RNA	misc_RNA	0	0	0	0
ENSG000002	ENSG000002	snoRNA	0	0	0	0
ENSG000002	RNA5SP379	rRNA_pseudo	0.91958115	0	0	0
ENSG000002	RNY4P7	misc_RNA	0.87872533	1.03536094	0	2.83662331
ENSG000002	RNU6-164P	snRNA	0	3.72925952	0	0
ENSG000002	RNA5SP353	rRNA_pseudo	0	1.99334543	0	0
ENSG000002	SNORD115-1	snoRNA	0.88065004	0	0	0
ENSG000002	Y_RNA	misc_RNA	0	0	0	0
ENSG000002	SNORD45B	snoRNA	16.6202167	42.9587713	4.53068762	27.6319924
ENSG000002	Y_RNA	misc_RNA	0.88218416	5.13158905	1.80774343	0
ENSG000002	RNU4-75P	snRNA	0	0	0	0
ENSG000002	RNA5SP78	rRNA_pseudo	0	0	0	0
ENSG000002	RNU1-141P	snRNA	0	0	0	0
ENSG000002	RN7SKP275	misc_RNA	0	0	0	0
ENSG000002	Y_RNA	misc_RNA	181.917251	296.235968	160.173222	134.87091
ENSG000002	RNU6-312P	snRNA	0	0	0	0
ENSG000002	ENSG000002	snoRNA	0	0	0	0
ENSG000002	Y_RNA	misc_RNA	7.06647389	7.29831597	4.78997837	5.12734726
ENSG000002	ENSG000002	snoRNA	0	0	0	0
ENSG000002	Y_RNA	misc_RNA	0	0	0	0
ENSG000002	RN7SKP217	misc_RNA	0	0	0.91607258	0
ENSG000002	Y_RNA	misc_RNA	0	1.03825148	0	0
ENSG000002	SNORA71C	snoRNA	2.91999188	2.02457663	0	4.33831847
ENSG000002	ENSG000002	snoRNA	1.00024931	1.07330758	4.88996298	0
ENSG000002	RNU6-707P	snRNA	92.5141329	70.130658	61.4919411	101.102799
ENSG000002	RNA5SP513	rRNA_pseudo	1.87430229	1.98583992	0.89925393	0.88446837
ENSG000002	RNU6-645P	snRNA	0	0	0	0
ENSG000002	RNA5SP205	rRNA_pseudo	0	0	0	0
ENSG000002	RNU6-450P	snRNA	0.90451589	0	0	0
ENSG000002	RNA5SP478	rRNA_pseudo	0	0	0	0
ENSG000002	Y_RNA	misc_RNA	0	0	0	0
ENSG000002	RNA5SP194	rRNA	0	0	0	0
ENSG000002	RN7SKP237	misc_RNA	0	0.87521946	1.44999034	0
ENSG000002	Y_RNA	misc_RNA	0	0	0	0
ENSG000002	ENSG000002	snoRNA	0	0	0	0

ENSG000002	SNORA62	snoRNA	0	0	0	0
ENSG000002	SNORA16B	snoRNA	0	0	0	0.8987242
ENSG000002	RNU4-85P	snRNA	0	0	0	0
ENSG000002	Y_RNA	misc_RNA	0	0	0	0
ENSG000002	Y_RNA	misc_RNA	0	0	0	0
ENSG000002	Y_RNA	misc_RNA	0	0	0	0
ENSG000002	RNU6-726P	snRNA	0	0	0	0
ENSG000002	Y_RNA	misc_RNA	0	0	0	0
ENSG000002	Y_RNA	misc_RNA	14.8508517	39.0313118	16.2160304	17.2959696
ENSG000002	SNORD114-1	snoRNA	0	0	0	0
ENSG000002	RNVU1-6	snRNA	4.22229357	15.2595626	4.00420576	23.1106598
ENSG000002	RNU6-973P	snRNA	0	0	0	0
ENSG000002	Y_RNA	misc_RNA	0	0	0	0
ENSG000002	RN7SKP50	misc_RNA	0.94132365	0.95571081	0	0.94840751
ENSG000002	Y_RNA	misc_RNA	0	0	0	0
ENSG000002	Y_RNA	misc_RNA	0	3.11539643	1.775246	0
ENSG000002	RNA5SP510	rRNA_pseudo	0	0	0	0
ENSG000002	SNORD114-1	snoRNA	0	0	0	0
ENSG000002	RNU4-56P	snRNA	0	0	0	0
ENSG000002	Y_RNA	misc_RNA	0	0	0	0
ENSG000002	RNU1-93P	snRNA	0	0	0	0
ENSG000002	RNU6-343P	snRNA	0.88879741	0	0	0
ENSG000002	RN7SKP78	misc_RNA	15.1146476	1.05358426	1.62240078	0
ENSG000002	RN7SKP191	misc_RNA	1.21663128	0	0	0
ENSG000002	Y_RNA	misc_RNA	0	0	0	0
ENSG000002	RNU6-593P	snRNA	0	0	0	0
ENSG000002	RNU6-99P	snRNA	0	1.00218871	0	0
ENSG000002	ENSG000002	snoRNA	0	0	0	0
ENSG000002	RNA5SP517	rRNA_pseudo	0.95042658	0	0	0
ENSG000002	RNA5SP132	rRNA_pseudo	0	0	0	0
ENSG000002	Y_RNA	misc_RNA	0	1.00974371	0	0
ENSG000002	RNU6-219P	snRNA	0	0	0	0
ENSG000002	SNORA71A	snoRNA	8.55926491	7.62394188	11.1897875	11.7362473
ENSG000002	RN7SKP124	misc_RNA	3.22043489	0	0	0
ENSG000002	Y_RNA	misc_RNA	0	0	0	0
ENSG000002	RNU6-740P	snRNA	0	0	0	0
ENSG000002	RNU4-16P	snRNA	0	0	0	0
ENSG000002	RNU4-42P	snRNA	0.9600583	0	0	0.89187143
ENSG000002	RNU4-28P	snRNA	0	0	0	0
ENSG000002	RNA5SP84	rRNA_pseudo	0	0	0	0
ENSG000002	RN7SKP15	misc_RNA	0	0	0	0
ENSG000002	RNU6-200P	snRNA	0	0	0	0
ENSG000002	RNU1-91P	snRNA	0.9737271	0	0	0.89362723
ENSG000002	RNA5SP505	rRNA_pseudo	0	0	0	0

ENSG000002	ENSG000002	snoRNA	0	0	0	0
ENSG000002	RNA5SP51	rRNA_pseudo	0	0	0	0
ENSG000002	RNU6-621P	snRNA	0	0	0	0
ENSG000002	RNU6-923P	snRNA	0	0	0	0
ENSG000002	Y_RNA	misc_RNA	6.24921596	10.5729315	6.170574	3.69447577
ENSG000002	Y_RNA	misc_RNA	0	0	0	0
ENSG000002	RNU4-7P	snRNA	23.75375	32.9728504	9.68952809	33.4115322
ENSG000002	Y_RNA	misc_RNA	0	0	0	0
ENSG000002	SNORD115-4	snoRNA	0	0	0	0
ENSG000002	Y_RNA	misc_RNA	0	0	0	0
ENSG000002	RNY4P16	misc_RNA	1.76416425	0	0	0
ENSG000002	RN7SKP28	misc_RNA	0	0	0	0
ENSG000002	RNU6-1187P	snRNA	0	0	0	0
ENSG000002	RNU6-865P	snRNA	0	0	0	0
ENSG000002	SNORA14A	snoRNA	6.67429022	14.6183356	6.14324924	14.3795919
ENSG000002	Y_RNA	misc_RNA	0	1.04745752	0.90912135	0
ENSG000002	RNU4-91P	snRNA	0	0	0	0.91197992
ENSG000002	RNY4P34	misc_RNA	0	1.00928016	0.91653527	0.92149219
ENSG000002	RNU6-7	snRNA	1036.5243	1308.5519	0	801.961105
ENSG000002	RNU6-283P	snRNA	0	0	0	0
ENSG000002	RNU12-2P	snRNA	2.97422622	0	1.52167934	2.73709625
ENSG000002	ENSG000002	snoRNA	0	0	0	0
ENSG000002	RNU6-60P	snRNA	5.46187182	3.6885485	0.89961439	2.72784506
ENSG000002	Y_RNA	misc_RNA	0	0	0	0
ENSG000002	RN7SKP6	misc_RNA	0	0	0	0
ENSG000002	ENSG000002	snoRNA	0	0	0	0
ENSG000002	Y_RNA	misc_RNA	0	0	0	0
ENSG000002	RNA5SP90	rRNA_pseudo	0	0	0	0
ENSG000002	SNORD113-4	snoRNA	0	0	0	0
ENSG000002	U3	snoRNA	0	0	0	0
ENSG000002	SNORD32A	snoRNA	32.0897554	45.9348323	21.2203166	34.3700951
ENSG000002	Y_RNA	misc_RNA	0	0	0	0
ENSG000002	SNORD115-1	snoRNA	0	0	0	0
ENSG000002	Y_RNA	misc_RNA	1.90403714	1.04716749	0.63705036	0
ENSG000002	Y_RNA	misc_RNA	0	0	0	0
ENSG000002	RN7SKP239	misc_RNA	1.11800728	0	0	1.0950419
ENSG000002	RNU6-1107P	snRNA	0	0	0	0
ENSG000002	SNORD114-2	snoRNA	0	0	0	0
ENSG000002	RNY1P2	misc_RNA	0	0	0	0
ENSG000002	RNVU1-24	snRNA	0	0	0	0
ENSG000002	SNORD113-3	snoRNA	0	0	0	0
ENSG000002	ENSG000002	snoRNA	0	0	0	0
ENSG000002	RNA5SP396	rRNA_pseudo	0	0	0	0
ENSG000002	RNU6-818P	snRNA	0	0	0	0

ENSG000002	RNA5SP359	rRNA_pseudo	0	0	0	0
ENSG000002	RNU6-686P	snRNA	0	0	0	0
ENSG000002	ENSG000002	snoRNA	0	0	0	0
ENSG000002	RNU6-1303P	snRNA	0	0	0	0
ENSG000002	RNA5SP125	rRNA_pseudo	0	0	0	0
ENSG000002	RN7SKP133	misc_RNA	0	1.31230269	0	0
ENSG000002	SNORD87	snoRNA	415.583387	1519.29223	502.973705	620.931667
ENSG000002	Y_RNA	misc_RNA	0	0	0	0
ENSG000002	Y_RNA	misc_RNA	0	0	0	0
ENSG000002	RNU6-304P	snRNA	0	0	0	0
ENSG000002	RNA5SP173	rRNA_pseudo	0	0	0	0
ENSG000002	RNA5SP479	rRNA_pseudo	0	1.0064536	0	0
ENSG000002	ENSG000002	snoRNA	0	0	0	0
ENSG000002	RNA5SP160	rRNA_pseudo	0	0	0	0
ENSG000002	RNU1-133P	snRNA	0	0	0	0
ENSG000002	Y_RNA	misc_RNA	0	1.03524218	0	1.90937799
ENSG000002	RNU6-563P	snRNA	0	0	0	0
ENSG000002	RNU6-34P	snRNA	5.57137785	9.39553965	3.32629459	2.89756701
ENSG000002	RNU6-828P	snRNA	0	0	0	0
ENSG000002	RNU6-534P	snRNA	0	0	0	0
ENSG000002	Y_RNA	misc_RNA	0	0	0	0
ENSG000002	RNU6-119P	snRNA	0	0	0	0
ENSG000002	SNORD52	snoRNA	2.20897214	8.2836433	2.57724032	11.3427677
ENSG000002	Y_RNA	misc_RNA	0	0	0	0
ENSG000002	RN7SKP55	misc_RNA	2.83967243	2.80120535	0	0
ENSG000002	RNU6-336P	snRNA	0	0	0	0
ENSG000002	RNA5SP267	rRNA_pseudo	14.9176859	24.038141	11.4913385	23.716683
ENSG000002	RNA5SP302	rRNA_pseudo	0	2.05353487	0.90773118	0
ENSG000002	RNU6-384P	snRNA	0	0	0	0
ENSG000002	SNORA5C	snoRNA	20.3398773	46.9230765	12.5178946	36.7683505
ENSG000002	Y_RNA	misc_RNA	0.91974183	0	0	0
ENSG000002	RNU6-1325P	snRNA	0	0	0	0
ENSG000002	Y_RNA	misc_RNA	0	0	0	0
ENSG000002	RNU6-275P	snRNA	0	0	0	0
ENSG000002	RN7SKP226	misc_RNA	0	0.86643739	0	1.76746308
ENSG000002	SNORD117	snoRNA	15.5366726	27.8872856	9.93032864	17.964323
ENSG000002	Y_RNA	misc_RNA	1.94333913	0	0	0
ENSG000002	Y_RNA	misc_RNA	0	0	0	0
ENSG000002	RNA5SP189	rRNA_pseudo	0	0	0	0
ENSG000002	SNORA63	snoRNA	0	0	0	0
ENSG000002	RN7SKP9	misc_RNA	12.6038012	14.5776186	4.04163182	16.2019286
ENSG000002	RN7SKP130	misc_RNA	0	0	0	0
ENSG000002	RNU6-392P	snRNA	0	0	0	0
ENSG000002	Y_RNA	misc_RNA	0.91644882	0	0	0

ENSG000002	RNU5E-4P	snRNA	21.7267299	39.0949309	22.1394883	22.1328043
ENSG000002	RNU6-1180P	snRNA	166.91496	185.120998	51.3760602	136.263565
ENSG000002	RNU4-8P	snRNA	0.9721111	1.98394845	0.67701389	1.84376482
ENSG000002	ENSG000002	snoRNA	0	0	0	0
ENSG000002	SNORA73A	snoRNA	667.823146	1176.00491	457.18904	731.083073
ENSG000002	U8	snoRNA	0	0	0	0
ENSG000002	U8	snoRNA	0	0	0	0
ENSG000002	SNORA71	snoRNA	0	0	0	0
ENSG000002	RNA5SP488	rRNA_pseudo	0	0	0	0
ENSG000002	RNU6-915P	snRNA	0	0	0	0
ENSG000002	RNU6-526P	snRNA	0	0	0	0
ENSG000002	SNORA73	snoRNA	0	0	0	0
ENSG000002	RNY4P17	misc_RNA	0	0	0	0
ENSG000002	Y_RNA	misc_RNA	0	0	0	0
ENSG000002	RNU4-9P	snRNA	2.84725227	0.96894635	0.87634893	2.69491274
ENSG000002	RNA5SP149	rRNA_pseudo	3700.93062	7473.69375	4362.1454	4330.83968
ENSG000002	SNORD48	snoRNA	4.9767959	24.6300768	7.80472736	19.1261686
ENSG000002	RNU6-1131P	snRNA	0	0	0	0
ENSG000002	RNU6-867P	snRNA	0	0	0	0
ENSG000002	ENSG000002	snoRNA	0	0	0	0
ENSG000002	Y_RNA	misc_RNA	1.81096694	8.01892169	2.6851922	1.81274206
ENSG000002	SNORD115-1	snoRNA	0	0	0	0
ENSG000002	SNORD1A	snoRNA	8.57708353	24.7543167	11.8826499	16.4018428
ENSG000002	SNORD114-3	snoRNA	0	0	0	0
ENSG000002	Y_RNA	misc_RNA	0	0	0	0
ENSG000002	RNA5SP166	rRNA_pseudo	0	0	0	0
ENSG000002	SNORD31B	snoRNA	0	0	0	0
ENSG000002	Y_RNA	misc_RNA	5.34460695	6.05568094	0	2.76478051
ENSG000002	RNU6-702P	snRNA	7.57703736	3.07522624	4.56895938	8.96902312
ENSG000002	RNA5SP331	rRNA_pseudo	0	0	0	0
ENSG000002	Y_RNA	misc_RNA	11.8708494	11.263264	7.82364009	14.5826796
ENSG000002	RNA5SP298	rRNA_pseudo	167.631589	282.496743	138.606074	247.177823
ENSG000002	Y_RNA	misc_RNA	0	0	0	0
ENSG000002	ENSG000002	snoRNA	0	0	0	0
ENSG000002	SNORD50B	snoRNA	6.2630859	16.2355858	7.85202736	4.31193754
ENSG000002	Y_RNA	misc_RNA	0	0	0	0
ENSG000002	Y_RNA	misc_RNA	11.3004896	12.396021	7.37818105	7.94710394
ENSG000002	RNU6-294P	snRNA	0	0	0	0
ENSG000002	RN7SKP178	misc_RNA	1.03467078	1.09597982	0	0
ENSG000002	RNA5SP97	rRNA_pseudo	0	0	0	0
ENSG000002	Y_RNA	misc_RNA	0	0	0	0
ENSG000002	ENSG000002	snoRNA	0.89258296	8.21603434	3.77862407	2.9681187
ENSG000002	Y_RNA	misc_RNA	0	0	0	0
ENSG000002	Y_RNA	misc_RNA	0	0	0	0

ENSG000002	RNU6-967P	snRNA	0	0	0	0
ENSG000002	RN7SKP153	misc_RNA	0	0	0	0
ENSG000002	SNORA72	snoRNA	0	0	0	0
ENSG000002	SNORD114-2	snoRNA	0	0	0	0
ENSG000002	RNY1P13	misc_RNA	0	4.05005677	2.70806365	3.64431078
ENSG000002	RN7SKP48	misc_RNA	30.8737409	29.7632974	12.2839787	28.2805277
ENSG000002	SNORA71E	snoRNA	0	0	0	2.08107266
ENSG000002	SNORD115-3	snoRNA	0	0	0	0
ENSG000002	RNU6-590P	snRNA	0.90451589	0	0	0.90557412
ENSG000002	RNU1-140P	snRNA	0	0	0	0
ENSG000002	RN7SKP189	misc_RNA	0.85821819	0.83607539	0	0
ENSG000002	Y_RNA	misc_RNA	3.52873741	6.15857945	0	2.73797046
ENSG000002	Y_RNA	misc_RNA	0	0	0	0
ENSG000002	RNU6-1022P	snRNA	0	0	0	0
ENSG000002	RNA5SP442	rRNA_pseudo	10.9246273	8.06944297	6.29477509	10.4820783
ENSG000002	RNU1-43P	snRNA	0	0	0	0.97606975
ENSG000002	RNA5SP485	rRNA_pseudo	0	0	0	0
ENSG000002	RNA5SP172	rRNA	25.8340628	15.4311979	6.72518572	23.5571586
ENSG000002	Y_RNA	misc_RNA	0	1.01185413	1.76164342	0
ENSG000002	SNORD115-2	snoRNA	0	0	0	0
ENSG000002	Y_RNA	misc_RNA	0	0	0	0.94514214
ENSG000002	RNA5SP224	rRNA_pseudo	0	0	0	0
ENSG000002	RNA5SP363	rRNA_pseudo	0	0	0	0
ENSG000002	SNORD115-1	snoRNA	0	0	0	0
ENSG000002	SNORA72	snoRNA	0	0	0.88572033	0
ENSG000002	SNORA70	snoRNA	0	0	0	0
ENSG000002	SNORD113-9	snoRNA	0	0	0	0
ENSG000002	RNU6-673P	snRNA	0	0	0	0
ENSG000002	RNY3P1	misc_RNA	73.4957757	192.906805	77.727181	67.8350683
ENSG000002	ENSG000002	snoRNA	0	0	0	0
ENSG000002	RNA5SP126	rRNA_pseudo	0	0	0	0
ENSG000002	Y_RNA	misc_RNA	0	0	0	0
ENSG000002	RNA5-8SP4	rRNA_pseudo	0	0	0	0
ENSG000002	RN7SKP22	misc_RNA	0	0	0	0
ENSG000002	RNA5SP138	rRNA_pseudo	0	0	0	0
ENSG000002	SNORD115-2	snoRNA	0	0	0	0
ENSG000002	SNORA62	snoRNA	0	0	0	0
ENSG000002	Y_RNA	misc_RNA	0	0	0	0
ENSG000002	Y_RNA	misc_RNA	0	0	0	0
ENSG000002	Y_RNA	misc_RNA	0	0	0	0
ENSG000002	Y_RNA	misc_RNA	0	0	0	0
ENSG000002	SNORD115-3	snoRNA	0	0	0	0
ENSG000002	SNORA23	snoRNA	383.532855	781.527746	303.018203	502.386987
ENSG000002	RNA5SP428	rRNA_pseudo	0	0	0	0

ENSG000002	RNU1-36P	snRNA	0	0	0	0
ENSG000002	Y_RNA	misc_RNA	0.89221597	1.00575241	0.91665411	0
ENSG000002	Y_RNA	misc_RNA	1.77800617	0	0	0.91982052
ENSG000002	Y_RNA	misc_RNA	0	1.03821701	0	0.92323821
ENSG000002	RNU6-619P	snRNA	0	0	0	0
ENSG000002	RNU6-806P	snRNA	0	0	0	0
ENSG000002	Y_RNA	misc_RNA	0	2.07643403	0	0
ENSG000002	SNORD81	snoRNA	0	0	0	0
ENSG000002	RNU6-934P	snRNA	0	0	0	0
ENSG000002	RNU6-1240P	snRNA	0	0	0	0
ENSG000002	RNU6-1134P	snRNA	0	0	0	0
ENSG000002	Y_RNA	misc_RNA	0	0	0	0
ENSG000002	RNU6-580P	snRNA	0	0	0	0
ENSG000002	SNORD38A	snoRNA	3.8684968	11.629621	7.11000022	10.5332771
ENSG000002	RNU6-399P	snRNA	0	0	0	0
ENSG000002	Y_RNA	misc_RNA	0	2.07643403	3.52604224	0
ENSG000002	RNU6-215P	snRNA	0	0	0	0
ENSG000002	Y_RNA	misc_RNA	0	0	0	0
ENSG000002	SNORD49B	snoRNA	0	0	0	0
ENSG000002	RNA5SP223	rRNA_pseudo	0	0	0	0
ENSG000002	Y_RNA	misc_RNA	0	0	0	0
ENSG000002	RNA5SP324	rRNA_pseudo	0	0	0	0
ENSG000002	SNORD114-2	snoRNA	0	0	0	0
ENSG000002	RNU6-391P	snRNA	0	0	0	0
ENSG000002	RNU6-382P	snRNA	0	0	0	0
ENSG000002	RNA5SP152	rRNA_pseudo	1.82920257	1.00974371	0	0
ENSG000002	RNA5SP19	rRNA_pseudo	7.24157232	14.9906684	7.20246719	11.7943642
ENSG000002	RN7SKP80	misc_RNA	66.1278274	129.227136	18.4949616	76.5800365
ENSG000002	ENSG000002	snoRNA	0	0	0	0
ENSG000002	RNA5SP455	rRNA_pseudo	0	0	0	0
ENSG000002	RNA5SP79	rRNA_pseudo	0	0	0	0
ENSG000002	RNU4-66P	snRNA	0	0	0	0
ENSG000002	RNU6-1175P	snRNA	0.90451589	3.00656541	0	1.81114824
ENSG000002	Y_RNA	misc_RNA	0	0	0	0
ENSG000002	RNU6-715P	snRNA	0	0	0	0
ENSG000002	Y_RNA	misc_RNA	0	0	0	1.79076826
ENSG000002	Y_RNA	misc_RNA	0	0	0	0
ENSG000002	RNU6-1280P	snRNA	0	0	0	0
ENSG000002	RNU6-290P	snRNA	0	3.28486563	0	0
ENSG000002	RNU6-1306P	snRNA	0	0	0	0
ENSG000002	RNA5SP190	rRNA_pseudo	0	0	0	0
ENSG000002	SNORD58C	snoRNA	11.9107687	27.3508572	13.8199452	33.1545219
ENSG000002	RNU6-1172P	snRNA	0	0	0	0
ENSG000002	RNU6-234P	snRNA	0	0	0	0

ENSG000002	Y_RNA	misc_RNA	0	0	0	0
ENSG000002	RNU4-12P	snRNA	0.94929847	1.45349624	0.43819849	1.3476644
ENSG000002	VTRNA1-2	vault_RNA	8.94708114	41.0407176	50.7873347	33.8690292
ENSG000002	RNU6-690P	snRNA	0	0	0	0
ENSG000002	RNU6-302P	snRNA	0	0	0	0
ENSG000002	RNY4P3	misc_RNA	0	1.00930906	0.9165358	0.92143951
ENSG000002	RNU1-100P	snRNA	0.97679565	0.95695677	0	0
ENSG000002	Y_RNA	misc_RNA	28.0448796	61.5399601	43.6662126	26.7602065
ENSG000002	Y_RNA	misc_RNA	0	1.01185413	0	0.89538413
ENSG000002	SNORD114-1	snoRNA	0	0	0	0
ENSG000002	Y_RNA	misc_RNA	0	0	0	0.92323821
ENSG000002	Y_RNA	misc_RNA	1.81678853	1.99936095	0.89729694	0.91184883
ENSG000002	RNA5SP329	rRNA_pseudo	0	0	0	0
ENSG000002	RNU6-407P	snRNA	0	0	0	0
ENSG000002	RNY4P28	misc_RNA	0	0	0	0
ENSG000002	RNU4-11P	snRNA	0	0	0	0
ENSG000002	RNU6-742P	snRNA	0	0	0	0
ENSG000002	RNU5E-7P	snRNA	0	0	0	0
ENSG000002	RNA5SP117	rRNA_pseudo	0	0	0	0
ENSG000002	RNU1-114P	snRNA	0	0	0	0
ENSG000002	Y_RNA	misc_RNA	0	0	0	0
ENSG000002	RNU6-1327P	snRNA	0	0	0	0
ENSG000002	RNA5-8SP5	rRNA_pseudo	0	0	0	0
ENSG000002	RNA5SP128	rRNA_pseudo	0	0	0	0
ENSG000002	SNORD115-7	snoRNA	0	0	0	0
ENSG000002	Y_RNA	misc_RNA	0	0	0	0
ENSG000002	Y_RNA	misc_RNA	3.18730264	1.19637171	3.12738739	0
ENSG000002	ENSG000002	snoRNA	0	0	0	0
ENSG000002	RNU6-1283P	snRNA	0	0	0	0
ENSG000002	RNU6-497P	snRNA	0	0	0	0
ENSG000002	RNA5SP355	rRNA_pseudo	157.652285	236.928114	112.274169	239.724606
ENSG000002	SNORD115-3	snoRNA	0	0	0	0
ENSG000002	SNORA30B	snoRNA	0	0	0	0
ENSG000002	Y_RNA	misc_RNA	0	0	0	0
ENSG000002	SNORD113-1	snoRNA	0	0	0	0
ENSG000002	RNA5SP427	rRNA_pseudo	0	0	0	0
ENSG000002	Y_RNA	misc_RNA	0	0	0	0
ENSG000002	7SK	misc_RNA	49900.953	78586.0132	15494.5154	42176.1928
ENSG000002	RNU1-115P	snRNA	0	0	0	0
ENSG000002	Y_RNA	misc_RNA	0	0	0	0
ENSG000002	RNU6-1034P	snRNA	0	0	0	0
ENSG000002	RNU6-169P	snRNA	0	0	1.09795619	0
ENSG000002	Y_RNA	misc_RNA	0	1.03821701	0.88151049	0
ENSG000002	RNU1-51P	snRNA	0	0	0	0

ENSG000002	ENSG000002	snoRNA	0	0	0	0
ENSG000002	RN7SKP47	misc_RNA	0	0	0	0
ENSG000002	Y_RNA	misc_RNA	0	0	0	0
ENSG000002	Y_RNA	misc_RNA	0	0	0	0
ENSG000002	RNA5SP240	rRNA_pseudo	0	0	0	0
ENSG000002	RNU6-282P	snRNA	0	0	0	0
ENSG000002	RNU6-1138P	snRNA	0	0	0	0
ENSG000002	ENSG000002	snoRNA	0	0	0.87827977	0
ENSG000002	U3	snoRNA	0	0	0	0
ENSG000002	RNU6-53P	snRNA	0	0	0	0
ENSG000002	RNU6-396P	snRNA	0	0	0	0
ENSG000002	RNU6-737P	snRNA	30.2024697	63.1273799	133.272718	116.561836
ENSG000002	RN7SKP186	misc_RNA	0	0.98191166	0	0
ENSG000002	RNU6-1276P	snRNA	0	0	0	0
ENSG000002	RNU6-728P	snRNA	0	0	0	0
ENSG000002	RNU5E-5P	snRNA	0	0	0	0
ENSG000002	Y_RNA	misc_RNA	0	0	0	0
ENSG000002	SNORD14C	snoRNA	80.3557559	268.000707	91.2983989	173.5495
ENSG000002	Y_RNA	misc_RNA	0	1.01314333	0	0
ENSG000002	Y_RNA	misc_RNA	0	0	0	0
ENSG000002	RNU6-1318P	snRNA	0	0	0	0
ENSG000002	RN7SKP69	misc_RNA	5.7653537	0	0	0
ENSG000002	SNORD115-4	snoRNA	0	0	0	0
ENSG000002	RNA5SP22	rRNA_pseudo	0	0.99699442	0.89202115	0.90505115
ENSG000002	RNA5SP77	rRNA_pseudo	1.91345111	1.01185258	2.39230917	0.91896178
ENSG000002	RNU6-596P	snRNA	0	0	0	0
ENSG000002	U3	snoRNA	0	0	0	0
ENSG000002	U8	snoRNA	0	0	0	0
ENSG000002	SNORD114-1	snoRNA	0	0	0	0
ENSG000002	Y_RNA	misc_RNA	0	0	0.90368164	0
ENSG000002	Y_RNA	misc_RNA	0	0	0	0
ENSG000002	ENSG000002	snoRNA	0	0	0	0
ENSG000002	Y_RNA	misc_RNA	0	1.03821701	1.76302097	0.92323821
ENSG000002	Y_RNA	misc_RNA	1.89763562	7.84832501	0	1.86316309
ENSG000002	RNA5SP235	rRNA_pseudo	0	0	0	0
ENSG000002	ENSG000002	snoRNA	0	0	0	0
ENSG000002	RNU6-117P	snRNA	0	0	0	0
ENSG000002	RNA5SP37	rRNA_pseudo	0	1.99885391	0	0
ENSG000002	SNORD114-2	snoRNA	0	0	0	0
ENSG000002	RNU6-1335P	snRNA	0	0	0	0
ENSG000002	RNY3P12	misc_RNA	0.89241238	0	0	0.92323821
ENSG000002	RNU6-487P	snRNA	199.884116	173.616153	61.7003166	203.115107
ENSG000002	RNU6-1130P	snRNA	0	0	0	0
ENSG000002	Y_RNA	misc_RNA	12.3127314	19.6869204	8.14695844	12.3034391

ENSG000002	RNU6-996P	snRNA	0	0	0	0
ENSG000002	Y_RNA	misc_RNA	0.89381887	9.91382931	3.53488836	2.82210988
ENSG000002	Y_RNA	misc_RNA	0	0	0	0
ENSG000002	RNU1-64P	snRNA	0	0	0	0
ENSG000002	SNORD6	snoRNA	7.26715319	6.21145375	2.69189081	7.51817596
ENSG000002	RNU1-84P	snRNA	0	0	0	0
ENSG000002	Y_RNA	misc_RNA	0	0	0	0
ENSG000002	RNA5SP131	rRNA_pseudo	0	0	0	0
ENSG000002	RNA5SP366	rRNA_pseudo	24.1531341	27.2711936	14.9422666	33.687629
ENSG000002	RNU6-609P	snRNA	0	0	0	0
ENSG000002	RNA5SP167	rRNA_pseudo	0	0	0	0
ENSG000002	Y_RNA	misc_RNA	0	0	0	0
ENSG000002	RNA5SP238	rRNA_pseudo	0	0	0	0
ENSG000002	ENSG000002	snoRNA	0	0	0	0
ENSG000002	RNU6-359P	snRNA	0	0	0	0
ENSG000002	RNU4-45P	snRNA	0	0	0	0
ENSG000002	RNU6-1051P	snRNA	0	0	0	0
ENSG000002	ENSG000002	snoRNA	0.82388009	0.94914908	0	0
ENSG000002	RN7SKP208	misc_RNA	0	0	0	0
ENSG000002	Y_RNA	misc_RNA	0	1.01185413	0	0
ENSG000002	RNU1-16P	snRNA	0.98283952	2.88771476	2.66979002	0.88767252
ENSG000002	RNU6-326P	snRNA	0	0	0	0
ENSG000002	RN7SKP204	misc_RNA	0	0	0	0
ENSG000002	RNY3	misc_RNA	4957.40969	14244.8299	5506.0304	4543.29841
ENSG000002	RNA5SP277	rRNA_pseudo	0	0	0	0
ENSG000002	Y_RNA	misc_RNA	35.534422	82.3345973	43.3926367	38.8406863
ENSG000002	RNU6-652P	snRNA	0	0	0	0
ENSG000002	RN7SKP249	misc_RNA	1.0774788	3.16108138	2.14232493	6.30499625
ENSG000002	Y_RNA	misc_RNA	0.88568859	0	0	0
ENSG000002	SNORA62	snoRNA	162.744541	279.186619	118.432078	212.454432
ENSG000002	Y_RNA	misc_RNA	0	0	0	0
ENSG000002	SNORA62	snoRNA	0	0	0	0
ENSG000002	SNORA25B	snoRNA	0	0	0	0
ENSG000002	SNORA70	snoRNA	0	0	0	0
ENSG000002	RNU1-55P	snRNA	1.95241086	0	2.44527764	0
ENSG000002	Y_RNA	misc_RNA	0	2.07663676	0.88173723	0.92332454
ENSG000002	RNA5SP279	rRNA_pseudo	0	0	0	0
ENSG000002	Y_RNA	misc_RNA	0	0.98656324	0.89582354	0
ENSG000002	RNA5SP211	rRNA_pseudo	108.350418	112.778979	91.1159256	117.812101
ENSG000002	Y_RNA	misc_RNA	0	0	0	0
ENSG000002	SNORA70	snoRNA	0	0	0	0
ENSG000002	RN7SKP292	misc_RNA	0.94806067	3.85166015	3.96133506	0
ENSG000002	RN7SKP1	misc_RNA	0	0	0.68803347	0
ENSG000002	RNU6-61P	snRNA	0	0	0	0

ENSG000002	Y_RNA	misc_RNA	0	2.02370826	0	0
ENSG000002	SNORD82	snoRNA	16.9590776	27.7291159	11.3358722	20.7768311
ENSG000002	Y_RNA	misc_RNA	0	0	0	0
ENSG000002	RN7SKP187	misc_RNA	0	0	2.64049854	0
ENSG000002	Y_RNA	misc_RNA	0	0	0.88151049	0
ENSG000002	RNVU1-21	snRNA	5.87377811	7.63740977	2.12203849	1.32491796
ENSG000002	Y_RNA	misc_RNA	0	0	0	0
ENSG000002	RNA5SP259	rRNA_pseudo	80.5699428	89.83592	57.2121845	94.9857326
ENSG000002	RNY3P13	misc_RNA	2.32947232	3.76113155	3.8123981	2.77164286
ENSG000002	Y_RNA	misc_RNA	0	0	0.90368164	0
ENSG000002	RN7SKP269	misc_RNA	0.98581645	0.98689143	0	0
ENSG000002	Y_RNA	misc_RNA	0	0	0	0
ENSG000002	RNA5SP30	rRNA_pseudo	0	0	0	0.89943008
ENSG000002	RNU6-827P	snRNA	0	0	0	0.92341466
ENSG000002	RNU6-744P	snRNA	0	0	0	0
ENSG000002	RNU6-108P	snRNA	0	0	0	0
ENSG000002	RNU4-48P	snRNA	0	0	0	0
ENSG000002	RNA5SP88	rRNA_pseudo	0	0	0	0
ENSG000002	RNU6-438P	snRNA	0	0	0	0
ENSG000002	RNU6-54P	snRNA	9.5088395	7.2815689	2.2356965	3.19966921
ENSG000002	ENSG000002	snoRNA	0	0	0	0
ENSG000002	Y_RNA	misc_RNA	0	0	0	0
ENSG000002	SNORD42	snoRNA	0	0	0	0
ENSG000002	RNY4P10	misc_RNA	1.75779315	3.10677836	0.90306642	0.94557479
ENSG000002	RNU5E-6P	snRNA	201.799984	287.57297	265.407516	240.349781
ENSG000002	RNU6-669P	snRNA	0	0	0	0
ENSG000002	SNORA63	snoRNA	0	0	0	0
ENSG000002	Y_RNA	misc_RNA	0	0	0	0
ENSG000002	Y_RNA	misc_RNA	0	0	0	0
ENSG000002	RNU4-17P	snRNA	0	0	0	0
ENSG000002	Y_RNA	misc_RNA	11.7661703	9.26070515	7.86193454	8.60584523
ENSG000002	Y_RNA	misc_RNA	0	0	0	0
ENSG000002	RNU4-20P	snRNA	0	0	0	0
ENSG000002	RNA5SP248	rRNA_pseudo	0	0	0	0
ENSG000002	RN7SKP81	misc_RNA	0	0	0.89894667	0
ENSG000002	RNA5SP283	rRNA_pseudo	0	0	0	0
ENSG000002	Y_RNA	misc_RNA	0	0	0	0
ENSG000002	SNORD25	snoRNA	0	8.3480182	4.58730733	0
ENSG000002	RNU6-81P	snRNA	0	0	0	0
ENSG000002	SNORD14	snoRNA	0	1.02453311	0	0
ENSG000002	U3	snoRNA	0	0	0	0
ENSG000002	RNU6-499P	snRNA	0	0	0	0
ENSG000002	RNU6-597P	snRNA	0	0	0	0
ENSG000002	RNU6-716P	snRNA	0	0	0	0

ENSG000002	Y_RNA	misc_RNA	0	0	0	0
ENSG000002	RNU6-898P	snRNA	0	0	0	0
ENSG000002	SNORD116	snoRNA	0	0	0	0
ENSG000002	RNA5SP151	rRNA_pseudo	0	0	0	0
ENSG000002	SNORD34	snoRNA	145.775924	431.18504	161.04936	247.049742
ENSG000002	Y_RNA	misc_RNA	0	0	0	0
ENSG000002	RN7SKP230	misc_RNA	17.8972435	36.3389845	17.1785302	33.511834
ENSG000002	RNU6-805P	snRNA	0	0	0	0
ENSG000002	Y_RNA	misc_RNA	0	0	0	0
ENSG000002	VTRNA1-3	vault_RNA	0	1.02925239	4.4779029	0.97073691
ENSG000002	ENSG000002	snoRNA	0	0	0	0
ENSG000002	Y_RNA	misc_RNA	0	0	0	0
ENSG000002	Y_RNA	misc_RNA	0	0	0.88151049	0
ENSG000002	RNU6-650P	snRNA	0	0	0	0
ENSG000002	SNORD18B	snoRNA	0	0	0	0
ENSG000002	RNU6-395P	snRNA	0	0	0	0
ENSG000002	Y_RNA	misc_RNA	0	5.12692058	0.88920828	0.90360676
ENSG000002	RNU6-1329P	snRNA	36.9329987	28.3911406	31.2864745	46.0431564
ENSG000002	Y_RNA	misc_RNA	9.63848836	10.4763672	8.14167668	6.27621641
ENSG000002	U8	snoRNA	0	0	0	0
ENSG000002	RNU4-2	snRNA	6308.72259	10614.8126	3421.45391	7487.09472
ENSG000002	Y_RNA	misc_RNA	0	0	0	0
ENSG000002	MIR539	miRNA	0	0	0	0
ENSG000002	MIR421	miRNA	0	0	0	0
ENSG000002	MIR146B	miRNA	0	0	0	0
ENSG000002	MIR582	miRNA	0	0	0	0
ENSG000002	MIR488	miRNA	0	0	0	0
ENSG000000	MBTPS2	protein_codin	11.4354243	6.63956899	0.92998881	3.85197546
ENSG000001	SENP7	protein_codin	0	0	1.20639049	0
ENSG000001	ECT2	protein_codin	0	0	0	0
ENSG000001	GART	protein_codin	0	0	0	0
ENSG000002	ENSG000002	lncRNA	0	0	0	0
ENSG000002	ADGRA1-AS1	lncRNA	0	0	0	0
ENSG000002	LIX1L-AS1	lncRNA	0	0	0	0
ENSG000002	ENSG000002	lncRNA	0	0	0	0
ENSG000002	KIAA1671-AS1	lncRNA	0	0	0	0
ENSG000000	RNF10	protein_codin	8.038272	7.88320112	1.45729237	1.28761996
ENSG000002	Metazoa_SRP	misc_RNA	0	0	0	0
ENSG000001	SPATS2L	protein_codin	0	0	0	0
ENSG000001	SCNN1A	protein_codin	0	0	0	0
ENSG000002	SAT1-DT	lncRNA	0	0	0	0
ENSG000002	FAM78B-AS1	lncRNA	0	0	0	0
ENSG000000	MTIF2	protein_codin	1.12949427	0	0	0
ENSG000001	FEZ1	protein_codin	0.91425531	1.77180878	0	1.81086614

ENSG000002	NRIR	lncRNA	3.41800181	0	0.91404309	0
ENSG000002	ENSG000002	lncRNA	0	0	0	0
ENSG000002	ENSG000002	lncRNA	0	0	1.01585188	1.57064648
ENSG000000	TNFRSF1A	protein_codin	9.58277138	14.9786619	4.50628593	0
ENSG000002	ENSG000002	lncRNA	0	0	0	0
ENSG000001	VSTM1	protein_codin	0	0	0	0
ENSG000002	PSG5	protein_codin	0	0	0	0
ENSG000002	LINC01562	lncRNA	3.50117914	0	0	0
ENSG000002	KANTR	protein_codin	2.3368028	0	0	0
ENSG000002	MAP4K3-DT	lncRNA	0	0	0	2.25721688
ENSG000002	ENSG000002	lncRNA	0	1.08193425	0	1.02584003
ENSG000002	ENSG000002	lncRNA	0	0	0	0
ENSG000001	ZNF548	protein_codin	13.6895439	0	10.1534464	0
ENSG000001	NDRG2	protein_codin	0	0	0	0
ENSG000001	STRN3	protein_codin	5.36494443	1.00109366	1.09268899	1.01915261
ENSG000002	LINC01320	lncRNA	0	0.87559428	0	0
ENSG000001	SEPTIN2	protein_codin	12.7940779	1.29534625	3.23302452	1.24052574
ENSG000001	SAXO4	protein_codin	0	1.1433128	2.35406895	0
ENSG000002	STMND1	protein_codin	0	0	0	0
ENSG000002	CSNK1E	protein_codin	1.01473444	2.21543862	1.19976693	3.02609632
ENSG000002	ENSG000002	lncRNA	0	1.12560468	0.96912004	1.12998436
ENSG000002	SHISA5P2	processed_ps	0	0	0	0
ENSG000002	SSU72-AS1	lncRNA	14.2929758	13.3165706	2.19567988	15.8005581
ENSG000002	ENSG000002	lncRNA	0	0	0	0
ENSG000002	Metazoa_SRP	misc_RNA	1.15608926	0	0	0
ENSG000002	PCBP1-AS1	lncRNA	0	0	0	0
ENSG000002	ENSG000002	lncRNA	0	0	0	0
ENSG000001	ECT2	protein_codin	0	0	0	0
ENSG000002	ENSG000002	processed_ps	2.22559123	0	0	0
ENSG000002	LINC01232	lncRNA	0	0	0	0
ENSG000002	ENSG000002	lncRNA	0	0	0	0
ENSG000001	MLXIP	protein_codin	11.4583668	4.48336735	1.39956307	9.7210106
ENSG000002	CHP1P2	transcribed_p	9.06787614	0	0	2.59674103
ENSG000002	ENSG000002	lncRNA	0	0	0	0
ENSG000002	ENSG000002	unprocessed_	0	0	0	0
ENSG000001	RHNO1	protein_codin	0	1.19898495	0	2.18759387
ENSG000000	YBX3	protein_codin	0	0	0	0
ENSG000002	PRORSD1P	lncRNA	0	0	0	0
ENSG000000	YBX3	protein_codin	0	0	0	0
ENSG000001	SLC15A4	protein_codin	52.9879236	36.2214327	6.33654042	4.601143
ENSG000002	HMGB1P39	processed_ps	0	1.77935379	0	0
ENSG000002	LINC01743	lncRNA	0	0	1.14080179	0
ENSG000002	TFAP2A-AS1	lncRNA	0	0	0	0
ENSG000001	LINC00311	lncRNA	0	0	0	0

ENSG000002	RBMS3-AS2	lncRNA	0	0	0	0
ENSG000001	MTRFR	protein_codin	0	0	12.7115771	0
ENSG000000	ANAPC5	protein_codin	5.33300882	3.61271188	3.55671527	4.79331753
ENSG000001	C2CD3	protein_codin	3.47563551	0	0.95676106	0
ENSG000002	DPF3	protein_codin	0	0	0	0
ENSG000001	AKAP7	protein_codin	0	0	4.89745156	0
ENSG000002	ENSG000002	lncRNA	11.3590122	6.05013607	3.20039011	4.87684125
ENSG000001	FOXM1	protein_codin	0	0	1.33691978	0
ENSG000002	SNHG20	lncRNA	6.3673675	16.4792906	3.91633033	1.99667827
ENSG000002	ENSG000002	lncRNA	0	0	0	0
ENSG000000	QPCTL	protein_codin	0	0	0	1.30775014
ENSG000002	Metazoa_SRP	misc_RNA	0	0	0	0
ENSG000001	SH3BP5	protein_codin	9.14839033	0	0	0
ENSG000001	NUMA1	protein_codin	0	0	0	0
ENSG000002	IGBP1-AS1	lncRNA	0	0	0	0
ENSG000001	RAP1GAP2	protein_codin	211.949833	325.400764	111.716572	150.271666
ENSG000002	Metazoa_SRP	misc_RNA	0	0	0	0
ENSG000001	ZPR1	protein_codin	0	0	0	0
ENSG000002	LINC00970	lncRNA	0	0	0	0
ENSG000002	RHOT1P2	processed_ps	1.67192727	0	0.7940512	0
ENSG000002	ENSG000002	lncRNA	1.77057072	0	0	0
ENSG000002	GP1BB	protein_codin	446.293688	1141.80331	363.033437	395.155462
ENSG000001	IHO1	protein_codin	0	0	0	0
ENSG000002	MIR205HG	lncRNA	0	0	0	0
ENSG000001	ATAD2B	protein_codin	10.3992984	2.4004643	9.89270347	2.43555327
ENSG000002	ITGA9-AS1	lncRNA	3.87590439	7.85452695	0	7.28615715
ENSG000001	SHC1	protein_codin	2.3257977	3.92039261	1.11304954	0
ENSG000002	NUTM2A-AS1	lncRNA	0	0	0	0
ENSG000002	ENSG000002	lncRNA	21.2232762	18.7227432	7.84246571	14.4086985
ENSG000002	ENSG000002	processed_ps	0	0	0	0
ENSG000002	ENSG000002	processed_ps	0	0	0	0
ENSG000002	ENSG000002	lncRNA	0	0	0	0
ENSG000001	FOXRED2	protein_codin	3.65823205	0	0	5.644237
ENSG000001	NDUFB6	protein_codin	13.8931116	11.9616292	2.19448457	0
ENSG000001	ZNF692	protein_codin	0	0	0	0
ENSG000001	SH3BP5L	protein_codin	73.2400034	228.728601	58.2092965	81.2215534
ENSG000001	OR2T4	protein_codin	0	0	0	0
ENSG000001	OR2M5	protein_codin	0	0	0	0
ENSG000002	OR2L2	protein_codin	0	0	0	0
ENSG000001	OR2AK2	protein_codin	0	1.34656096	0	0
ENSG000001	TRIM58	protein_codin	668.888503	1014.55863	590.9029	469.34677
ENSG000002	OR14A2	protein_codin	0	0	0	0
ENSG000001	OR2C3	protein_codin	0	2.293674	0	0
ENSG000001	GCSAML	protein_codin	14.9515802	24.3342309	35.6387058	8.59212291

ENSG000001(	GCSAML	protein_codin	0	0	2.01889135	0
ENSG000001(	GCSAML	protein_codin	0	0	0	0
ENSG000002(	OR2W5P	transcribed_u	0	0	0	0
ENSG000001(	NLRP3	protein_codin	280.739387	175.794429	28.2337366	0
ENSG000001	ZNF669	protein_codin	0	0	0	0
ENSG000001	ZNF669	protein_codin	0	0	0	0
ENSG000002	ZNF670	protein_codin	5.77736995	0	0	2.35499885
ENSG000001	ZNF695	protein_codin	0	0	0	0
ENSG000001	ENSG000001	transcribed_u	0	0	0	0
ENSG000001	ZNF141	protein_codin	2.09756843	5.64417323	3.27499424	0
ENSG000001	AHCTF1	protein_codin	392.569048	172.854202	132.062865	28.1706576
ENSG000001	SCCPDH	protein_codin	21.907873	49.0644322	15.8606038	13.1049042
ENSG000001(	CNST	protein_codin	0	0	4.60755847	0
ENSG000001(	CNST	protein_codin	88.7690084	123.998507	21.7159889	70.064987
ENSG000001(	CNST	protein_codin	528.653126	885.074721	361.933856	366.14012
ENSG000001(	TFB2M	protein_codin	13.1263696	27.5141347	9.77335766	16.7279993
ENSG000001	SMYD3	protein_codin	7.89120749	0	2.28883271	0
ENSG000001	SMYD3	protein_codin	0	0	0	0
ENSG000001(	KIF26B	protein_codin	13.8300269	18.5558276	1.77936357	9.84617226
ENSG000002(	EFCAB2	protein_codin	0	9.4846005	0	0
ENSG000002(	EFCAB2	protein_codin	27.127168	13.5261564	3.79656657	6.86893092
ENSG000002(	EFCAB2	protein_codin	19.1927675	13.649588	0	0
ENSG000001	HNRNPU	protein_codin	248.793297	283.968417	108.859029	267.779671
ENSG000001	HNRNPU	protein_codin	99.8890818	44.6434772	88.0699877	64.9836619
ENSG000002(	COX20	protein_codin	3.34556172	9.53087047	0	5.94650436
ENSG000002	COX20P1	processed_ps	0	0	0	0
ENSG000001	CATSPERE	protein_codin	0	0	0	0
ENSG000001	CATSPERE	protein_codin	0	2.75559504	0	0
ENSG000001	CATSPERE	protein_codin	0	0	0	0
ENSG000000	ADSS2	protein_codin	153.396651	145.429422	83.6095351	88.1031021
ENSG000001	SPMIP3	protein_codin	0	0	0	0
ENSG000001	AKT3	protein_codin	23.8041462	43.5917068	28.5957672	0
ENSG000001	AKT3	protein_codin	0	62.1857593	15.230078	0
ENSG000000	SDCCAG8	protein_codin	215.1137	207.62753	229.857787	175.905862
ENSG000001	CEP170	protein_codin	162.406189	155.819624	165.333459	0
ENSG000001	CEP170	protein_codin	0	0	0	0
ENSG000001	CEP170	protein_codin	65.4562826	13.5705834	32.6548903	73.5557473
ENSG000001	PLD5	protein_codin	0	1.21418663	0	0
ENSG000001	EXO1	protein_codin	0	0	0	0
ENSG000001(	WDR64	protein_codin	0	0	0	0
ENSG000002(	CHML	protein_codin	168.317765	126.514619	138.958063	133.250549
ENSG000000	OPN3	protein_codin	9.71691322	17.6831047	4.8278776	9.62799503
ENSG000001	KMO	protein_codin	0	0	0	0
ENSG000001	KMO	protein_codin	0	10.1390664	9.23865218	0

ENSG000001	KMO	protein_codin	13.9969593	19.3677353	11.870583	13.3979992
ENSG000001	KMO	protein_codin	12.4989586	0	0	16.8510897
ENSG000000	FH	protein_codin	54.6086694	48.7793104	46.3388604	47.6756478
ENSG000001	RGS7	protein_codin	0	0	0	0
ENSG000001	RGS7	protein_codin	0	0	0	0
ENSG000001	RGS7	protein_codin	0	0	0	0
ENSG000001	ZP4	protein_codin	0	0	0	0
ENSG000001	RYR2	protein_codin	0	0	0	0
ENSG000001	MTR	protein_codin	0	0	0	0
ENSG000001	MTR	protein_codin	109.566033	116.596567	51.5892511	89.8061616
ENSG000000	ACTN2	protein_codin	0	0	0	0
ENSG000001	HEATR1	protein_codin	1.65254192	0	0	0
ENSG000001	HEATR1	protein_codin	36.3444652	0	0	0
ENSG000001	HEATR1	protein_codin	135.623267	163.471828	122.846048	92.247377
ENSG000001	LGALS8	protein_codin	31.3680927	50.2635765	20.7114175	23.2578154
ENSG000001	LGALS8	protein_codin	283.51957	306.750378	0	189.216442
ENSG000002	ENO1P1	transcribed_p	0	0.92864287	0.99280574	0
ENSG000000	ERO1B	protein_codin	17.4152855	0	11.7453201	5.0941385
ENSG000000	GPR137B	protein_codin	1.02888203	1.38469664	0	0
ENSG000000	GPR137B	protein_codin	21.6561681	18.3017066	12.3406182	0
ENSG000001	NID1	protein_codin	0	229.735831	0	0
ENSG000001	GNG4	protein_codin	0	0	0	0
ENSG000001	GNG4	protein_codin	0	0	0	0
ENSG000001	B3GALNT2	protein_codin	34.2523062	40.9869872	24.8585419	23.6688361
ENSG000002	TBCE	protein_codin	0	0	0	17.7907113
ENSG000000	ARID4B	protein_codin	0	179.238166	227.388242	270.835571
ENSG000001	TOMM20	protein_codin	193.237961	260.990636	109.982811	140.043641
ENSG000001	IRF2BP2	protein_codin	651.818272	652.783037	131.107252	377.188258
ENSG000001	IRF2BP2	protein_codin	1185.62391	1253.87863	491.817513	861.13173
ENSG000001	COA6	protein_codin	8.7009039	3.78933484	0	4.04398484
ENSG000001	COA6	protein_codin	0	7.64130583	0	22.3699355
ENSG000001	COA6	protein_codin	21.3757866	2.16983284	0	0
ENSG000001	SLC35F3	protein_codin	0	0	1.5782384	0
ENSG000001	SLC35F3	protein_codin	0	0	0	0
ENSG000001	KCNK1	protein_codin	0	0	0	0
ENSG000001	KCNK1	protein_codin	0	1.29946569	0	0
ENSG000001	MAP3K21	protein_codin	0	7.89390358	8.76327535	10.3151465
ENSG000001	MAP3K21	protein_codin	7.96692838	0	6.40313485	10.8949632
ENSG000001	MAP3K21	protein_codin	27.1657585	6.91777275	12.7571891	0
ENSG000001	NTPCR	protein_codin	1.10846558	1.19453142	0	0
ENSG000001	NTPCR	protein_codin	4.01529631	8.63585215	1.19988498	11.9572683
ENSG000001	SIPA1L2	protein_codin	10.1416577	0	0	0
ENSG000001	DISC1	protein_codin	0	0	0	0
ENSG000001	DISC1	protein_codin	26.3692437	15.4820218	0	0

ENSG000001	DISC1	protein_codin	36.0570546	0	42.6956956	21.8225626
ENSG000001	DISC1	protein_codin	69.4659129	0	54.3886451	0
ENSG000001	TSNAX	protein_codin	131.793144	64.3178808	59.0210323	110.606465
ENSG000001	EGLN1	protein_codin	224.602638	174.229072	71.8499961	197.111308
ENSG000000	SPRTN	protein_codin	0	0	0	0
ENSG000001	EXOC8	protein_codin	117.020667	104.341174	70.214	104.052741
ENSG000001	GNPAT	protein_codin	138.347482	150.440072	78.6068836	91.0780089
ENSG000001	C1orf131	protein_codin	26.2504856	79.1054052	27.7774171	19.201611
ENSG000001	C1orf131	protein_codin	8.24281231	0	6.18866183	5.04118966
ENSG000001	TRIM67	protein_codin	0	0	0	0
ENSG000001	FAM89A	protein_codin	4.93723246	13.1164394	7.90768981	15.5456219
ENSG000001	ARV1	protein_codin	0	19.8951486	11.836885	0
ENSG000001	TTC13	protein_codin	143.522264	104.103023	77.6934384	92.2688781
ENSG000001	TTC13	protein_codin	0	41.6408938	0	18.4708438
ENSG000001	C1orf198	protein_codin	185.130069	598.079694	230.257909	177.461518
ENSG000001	CAPN9	protein_codin	0.87521862	0	0.75989563	0
ENSG000001	AGT	protein_codin	0	0	0	0
ENSG000001	COG2	protein_codin	10.8403862	0	0	0
ENSG000001	COG2	protein_codin	153.404792	257.756356	97.8952114	120.274988
ENSG000001	OTX1	protein_codin	0	0	0	0
ENSG000001	GALNT2	protein_codin	406.698118	614.002841	232.272146	258.289346
ENSG000001	TAF5L	protein_codin	2.18748093	0.9853853	0	0.820867
ENSG000001	TAF5L	protein_codin	71.9269273	37.9106868	40.9783473	27.6869865
ENSG000000	NUP133	protein_codin	0	0	0	0
ENSG000001	ACTA1	protein_codin	0	0	0	0
ENSG000001	ACTA1	protein_codin	3.67755697	1.90712851	0	0
ENSG000001	CCSAP	protein_codin	15.8395714	0	0	0
ENSG000001	CCSAP	protein_codin	139.363207	187.783008	137.888287	112.760598
ENSG000001	RAB4A	protein_codin	68.2015568	175.527769	77.4179978	88.9519088
ENSG000001	RHOU	protein_codin	122.505089	169.724233	65.5791694	98.1105426
ENSG000001	H2AC25	protein_codin	10.4947236	5.96812801	2.44767756	0
ENSG000001	H3-4	protein_codin	0	0	0	0
ENSG000001	TRIM17	protein_codin	1.62821865	0	0	0
ENSG000001	TRIM17	protein_codin	0	0	0	0
ENSG000001	TRIM11	protein_codin	47.8279271	78.6811412	30.7974842	44.7502231
ENSG000001	OBSCN	protein_codin	0	0	9.67274189	2.9870671
ENSG000001	OBSCN	protein_codin	3.15703059	5.58380172	0	0
ENSG000001	IBA57	protein_codin	62.4884929	57.8208371	39.1087256	23.0180325
ENSG000002	IBA57-DT	lncRNA	0	0	1.10808877	0
ENSG000001	GJC2	protein_codin	1.87704092	0	1.6216465	0.8959671
ENSG000001	GUK1	protein_codin	26.436037	23.0938723	0	0
ENSG000001	GUK1	protein_codin	15.5169043	19.1674979	10.3763388	11.3956392
ENSG000001	GUK1	protein_codin	0	0	0	0
ENSG000001	GUK1	protein_codin	0	0	0	1.11436478

ENSG000001	GUK1	protein_codin	5.3673617	0	0	0
ENSG000001	GUK1	protein_codin	0	0	0	0
ENSG000001	GUK1	protein_codin	0	65.3868022	0	0
ENSG000001	GUK1	protein_codin	0	0	0	1.40540499
ENSG000001	MRPL55	protein_codin	0	0	0	0
ENSG000001	MRPL55	protein_codin	0	0	0	0
ENSG000001	MRPL55	protein_codin	0	0	0	0
ENSG000001	MRPL55	protein_codin	0	0	0	0
ENSG000001	MRPL55	protein_codin	0	0	2.77487143	0
ENSG000001	MRPL55	protein_codin	0	0	0	0
ENSG000001	MRPL55	protein_codin	0	0	0	0
ENSG000001	MRPL55	protein_codin	0	2.2798017	0	0
ENSG000001	MRPL55	protein_codin	0	0	0	0
ENSG000001	MRPL55	protein_codin	0	0	0	0
ENSG000001	MRPL55	protein_codin	0	2.57870477	0	2.14233628
ENSG000001	MRPL55	protein_codin	0	0	0	0
ENSG000001	MRPL55	protein_codin	8.37161581	17.6980844	0	10.9139685
ENSG000001	MRPL55	protein_codin	3.29432559	3.09496584	0	6.09988112
ENSG000001	DLL1	protein_codin	2.80707565	0	0.7994526	0
ENSG000001	PRSS38	protein_codin	0	0	0.98799689	0
ENSG000001	SNAP47	protein_codin	0	0	0	0
ENSG000001	SNAP47	protein_codin	0	0	0	0
ENSG000001	CDC42BPA	protein_codin	49.4733824	49.2078908	42.0454864	0
ENSG000001	CDC42BPA	protein_codin	10.0783306	0	0	0
ENSG000001	CDC42BPA	protein_codin	11.6021604	3.36887969	13.4276036	5.63681565
ENSG000001	CDC42BPA	protein_codin	21.0931533	36.2876298	15.9968912	33.2815554
ENSG000001	ERMARD	protein_codin	28.6174961	21.6219683	28.5333204	10.5369784
ENSG000001	ERMARD	protein_codin	0	0	0	0
ENSG000001	ERMARD	protein_codin	0	0	0	0
ENSG000001	DYNLT2	protein_codin	0	0	0	0
ENSG000001	COQ8A	protein_codin	142.317204	296.053503	113.284976	126.272661
ENSG000001	COQ8A	protein_codin	0	0	0	0
ENSG000002	ENSG000002	protein_codin	6.5241665	25.5911554	0	31.2572262
ENSG000001	PHF10	protein_codin	87.9358466	88.1196569	103.104583	0
ENSG000001	PSEN2	protein_codin	0	0	0	0
ENSG000001	PSEN2	protein_codin	0	0	0	0
ENSG000001	ITPKB	protein_codin	46.2448275	27.3475144	10.3280311	15.0110743
ENSG000001	THBS2	protein_codin	0	0	0	0
ENSG000002	STUM	protein_codin	0.94710255	5.51794473	0	0
ENSG000002	STUM	protein_codin	0	0	0	0
ENSG000001	PARP1	protein_codin	6.60279385	4.16333968	8.12928674	2.13351805
ENSG000001	PARP1	protein_codin	0	4.21986727	0.92012164	0
ENSG000001	PARP1	protein_codin	91.5090889	157.821647	258.178263	157.899572
ENSG000001	DACT2	protein_codin	0	0	0	0

ENSG000001	DACT2	protein_codin	0	0	0	0
ENSG000001	AFDN	protein_codin	20.7948029	0	0	0
ENSG000001	LIN9	protein_codin	0	0	0	0
ENSG000001	AFDN	protein_codin	0	0	23.107111	0
ENSG000001	MIXL1	protein_codin	2.14748337	1.11983733	0	0
ENSG000001	ACBD3	protein_codin	250.776202	342.952296	311.930659	258.240493
ENSG000001	H3-3A	protein_codin	69.4082395	51.6513855	14.8801104	59.9857622
ENSG000001	H3-3A	protein_codin	305.887213	138.29145	93.3464614	134.489802
ENSG000001	H3-3A	protein_codin	1623.00924	2834.8978	554.877029	1421.17134
ENSG000001	H3-3A	protein_codin	1.78560774	17.715848	2.73203187	11.1542599
ENSG000001	LEFTY2	protein_codin	0	0	0.82195345	1.73003339
ENSG000001	AFDN-DT	lncRNA	0	0	0	0
ENSG000002	TCP10L3	lncRNA	0	0	0	0
ENSG000001	UNC93A	protein_codin	0	0	0	0
ENSG000001	UNC93A	protein_codin	0	0	0	0
ENSG000001	GPR31	protein_codin	0	0	0	0
ENSG000001	TMEM63A	protein_codin	129.831372	218.361468	87.8816633	74.519663
ENSG000001	EPHX1	protein_codin	0	0	0	0
ENSG000001	SRP9	protein_codin	0	0	3.62338147	0
ENSG000001	SRP9	protein_codin	16.1473467	27.8378276	16.0776386	32.2372173
ENSG000001	ENAH	protein_codin	2.48818395	0	3.21776095	0
ENSG000001	ENAH	protein_codin	0	0	0	0
ENSG000002	CEP43	protein_codin	3.70252654	6.31283797	2.6561264	4.234066
ENSG000001	DNAH14	protein_codin	0	0	0	0
ENSG000001	DNAH14	protein_codin	0	6.03390223	0	0
ENSG000001	DNAH14	protein_codin	0	0	0	0
ENSG000000	RNASET2	protein_codin	38.3340699	30.3619163	7.04885131	32.2835756
ENSG000001	CNIH4	protein_codin	9.1701226	7.22673808	6.31417338	0
ENSG000001	CNIH4	protein_codin	0	0	0	0
ENSG000001	CNIH4	protein_codin	0	0	0	6.0500631
ENSG000001	CNIH4	protein_codin	32.3193363	66.6543393	16.8722147	33.4767595
ENSG000001	FBXO28	protein_codin	59.6295087	32.8029354	17.7197598	14.356913
ENSG000000	RPS6KA2	protein_codin	0	4.45094939	0	0
ENSG000001	RPS6KA1	protein_codin	0	2.577606	0	0
ENSG000000	MPC1	protein_codin	0	0	6.64987238	0
ENSG000001	TBXT	protein_codin	0	0	0	0
ENSG000002	CAPN8	protein_codin	0	0	0	0
ENSG000002	CAPN8	protein_codin	0	0	0	0
ENSG000001	CCDC185	protein_codin	0.79392763	0.77947942	0	0.74626001
ENSG000001	TBXT	protein_codin	0	0	0.79633551	0
ENSG000001	SUSD4	protein_codin	0	0	0	0
ENSG000001	PDE10A	protein_codin	0	0	0	0
ENSG000001	PACRG	protein_codin	0	0	0	0
ENSG000001	PACRG	protein_codin	0	0	0	0.85595084

ENSG000001	TAF1A	protein_codin	0	0	0	0
ENSG000001	PRKN	protein_codin	0	0	0	1.05733973
ENSG000001	PRKN	protein_codin	0	0	0	0
ENSG000001	PRKN	protein_codin	0	0	0	0
ENSG000001	PRKN	protein_codin	1.35574827	5.82963681	0	0
ENSG000001	PRKN	protein_codin	0	0	2.9977934	0
ENSG000001	DUSP10	protein_codin	100.848884	123.556765	70.0686473	93.228776
ENSG000001	HLX	protein_codin	153.47728	187.388281	78.7398311	208.857117
ENSG000000	AGPAT4	protein_codin	17.2149002	6.8482942	3.72802461	0
ENSG000001	MTARC1	protein_codin	30.5032103	29.1306518	11.1455986	33.6067053
ENSG000000	AGPAT4	protein_codin	0	0	0	0
ENSG000001	MTARC2	protein_codin	0	3.38406459	0	2.27136249
ENSG000001	MARK1	protein_codin	0	0	0	0
ENSG000001	MARK1	protein_codin	0	0	0	0
ENSG000000	MAP3K4	protein_codin	94.7771636	57.8097396	57.9532778	73.9857593
ENSG000000	MAP3K4	protein_codin	0	0	0	0
ENSG000000	IARS2	protein_codin	228.125347	209.635064	93.2204383	134.526762
ENSG000001	EPRS1	protein_codin	325.515512	305.583334	274.506196	306.002834
ENSG000001	PLG	protein_codin	0	0	0	0
ENSG000001	SLC30A10	protein_codin	0	0	0	0
ENSG000001	LYPLAL1	protein_codin	0	18.379826	0	8.09170316
ENSG000001	LYPLAL1	protein_codin	0	0	0	0
ENSG000000	TGFB2	protein_codin	0	0	0	5.57771357
ENSG000000	TGFB2	protein_codin	10.2874015	50.8377772	14.9829477	0
ENSG000000	RRP15	protein_codin	29.8534887	18.7511251	27.5768746	10.0300398
ENSG000001	SPATA17	protein_codin	6.43299311	1.26865392	0	0
ENSG000000	GPATCH2	protein_codin	48.0807566	51.9339246	32.0664189	46.4642438
ENSG000000	GPATCH2	protein_codin	29.916725	14.0515045	19.9053729	6.76669012
ENSG000001	ESRRG	protein_codin	0	0	0	0
ENSG000001	ESRRG	protein_codin	0	0	0	0
ENSG000001	ESRRG	protein_codin	0	0	0	0
ENSG000000	USH2A	protein_codin	0	1.20828887	0	0
ENSG000001	SLC22A2	protein_codin	0	0	0	0
ENSG000001	SLC22A2	protein_codin	0	0	0	0
ENSG000001	CENPF	protein_codin	0	12.5652318	225.784078	0
ENSG000001	PTPN14	protein_codin	2.11181572	0	1.96254236	0
ENSG000001	SMYD2	protein_codin	67.0016851	63.8807903	42.1735934	36.5893425
ENSG000001	PROX1	protein_codin	0	0	0	0
ENSG000001	RPS6KC1	protein_codin	5.01130491	0	0	0
ENSG000001	RPS6KC1	protein_codin	58.4338318	28.9229045	42.8629756	27.422991
ENSG000001	ANGEL2	protein_codin	0	35.0676338	41.6720246	22.2287362
ENSG000001	SLC22A1	protein_codin	2.16700124	0	0	0
ENSG000001	VASH2	protein_codin	0.89760391	0.92134058	3.48831112	0
ENSG000001	VASH2	protein_codin	0	0	3.37095374	1.13472659

ENSG000001	VASH2	protein_codin	0	0	0	0
ENSG000001	VASH2	protein_codin	0	0	0	0
ENSG000001	VASH2	protein_codin	0	0	0	0
ENSG000001	VASH2	protein_codin	0	0	0	0
ENSG000001	FLVCR1	protein_codin	44.5796782	30.5063688	15.1188361	20.539124
ENSG000002	TATDN3	protein_codin	50.124957	0	14.6970208	25.9177397
ENSG000002	TATDN3	protein_codin	0	42.866493	11.7324404	0
ENSG000001	NSL1	protein_codin	23.8179402	23.0347588	18.8421394	19.9271414
ENSG000001	NSL1	protein_codin	80.923083	108.15369	60.5571585	46.0456928
ENSG000001	NSL1	protein_codin	0	0	0	0
ENSG000001	ATF3	protein_codin	0	0	0	0
ENSG000001	ATF3	protein_codin	38.7135789	18.0298517	20.8563337	35.0177835
ENSG000001	ATF3	protein_codin	0	0	9.57915349	1.38297245
ENSG000001	ATF3	protein_codin	0	0	0	0
ENSG000001	NENF	protein_codin	51.2845982	89.6066889	40.5142281	35.0907141
ENSG000001	DTL	protein_codin	0	4.61182919	14.5901017	0
ENSG000001	INTS7	protein_codin	25.1473214	0	0	0
ENSG000001	INTS7	protein_codin	0	0	12.2815266	0
ENSG000001	INTS7	protein_codin	3.59335884	0	15.8835646	24.6777853
ENSG000001	LPGAT1	protein_codin	66.3425148	86.1114886	30.7795745	86.4359411
ENSG000001	LPGAT1	protein_codin	818.323045	427.331287	327.566994	425.778886
ENSG000001	NEK2	protein_codin	0	0	0	0
ENSG000001	NEK2	protein_codin	0	0	6.78552656	0
ENSG000001	SLC30A1	protein_codin	173.207036	189.65527	72.6582665	147.510988
ENSG000001	RD3	protein_codin	0	0	0	0
ENSG000000	TRAF5	protein_codin	8.57317162	0	5.1433088	0
ENSG000001	RCOR3	protein_codin	56.2320428	46.7777679	67.7778864	74.8007097
ENSG000001	RCOR3	protein_codin	23.0413621	35.5747698	11.3055829	11.3469016
ENSG000001	KCNH1	protein_codin	0	0	0	0
ENSG000000	HHAT	protein_codin	0	0	0	0
ENSG000000	HHAT	protein_codin	0	0	0	0
ENSG000000	SERTAD4	protein_codin	0	0	0	0
ENSG000001	SYT14	protein_codin	0	0	0	0
ENSG000001	SYT14	protein_codin	0	0	0	0
ENSG000001	IRF6	protein_codin	0	0	0	0
ENSG000000	TRAF3IP3	protein_codin	103.500886	193.712338	151.698659	89.0129819
ENSG000000	TRAF3IP3	protein_codin	0	30.2066502	0	0
ENSG000000	TRAF3IP3	protein_codin	172.902759	248.541982	132.131145	121.280092
ENSG000000	TRAF3IP3	protein_codin	0	0	0	0
ENSG000001	HSD11B1	protein_codin	0	0	0	0
ENSG000001	HSD11B1	protein_codin	0	0	0	0
ENSG000001	G0S2	protein_codin	8982.26518	6882.84838	5024.42511	4703.96963
ENSG000001	LAMB3	protein_codin	1.89433	0	0	0
ENSG000000	PLXNA2	protein_codin	64.4075976	68.5483859	26.9949322	47.2884482

ENSG000001	MRPL18	protein_codin	34.9786382	111.867608	25.4886896	52.2732383
ENSG000001	CD34	protein_codin	0	5.7031915	0	0
ENSG000001	CD46	protein_codin	182.954093	126.832844	247.175358	0
ENSG000001	CD46	protein_codin	154.209888	0	54.0659265	195.630538
ENSG000001	CD46	protein_codin	0	0	0	0
ENSG000001	ACAT2	protein_codin	60.7804546	54.0072516	25.7146929	26.7948477
ENSG000002	CR1	protein_codin	0	0	0	0
ENSG000002	CR1	protein_codin	0	0	0	0
ENSG000002	CR1	protein_codin	0	4.51728191	0	0
ENSG000002	CR1	protein_codin	0	0	0	0
ENSG000002	CR1	protein_codin	142.81352	113.095546	132.951927	0
ENSG000002	SOD2	protein_codin	0	0	0	0
ENSG000002	SOD2	protein_codin	850.392571	774.774485	455.273272	372.479502
ENSG000001	CR2	protein_codin	0	0	0	0
ENSG000001	CR2	protein_codin	0	0	0	0
ENSG000001	CR2	protein_codin	0	3.27528972	3.94897964	0
ENSG000001	CD55	protein_codin	0	0	2.22883836	6.92182876
ENSG000001	CD55	protein_codin	110.445546	57.1356238	6.31006582	38.4391545
ENSG000001	TAGAP	protein_codin	394.189649	292.315231	180.739792	245.765263
ENSG000001	RSPH3	protein_codin	110.61452	116.541207	38.3841808	76.9044431
ENSG000001	C4BPA	protein_codin	0	0	0	0
ENSG000001	S100B	protein_codin	1.76220815	0	0	0
ENSG000002	LINC02901	lncRNA	0	0	0	0
ENSG000002	LINC02901	lncRNA	0	0	0	0
ENSG000002	RDXP3	processed_ps	0	1.84718175	1.21469651	0.92611926
ENSG000000	EZR	protein_codin	1646.7614	1440.4077	792.031944	741.318332
ENSG000001	C4BPB	protein_codin	0	0	0	0
ENSG000001	C4BPB	protein_codin	0	0	0	0
ENSG000001	PFKFB2	protein_codin	8.39014516	11.7064885	2.92546218	3.75254731
ENSG000001	PFKFB2	protein_codin	36.5395359	73.1089754	45.2956863	24.5495458
ENSG000001	SYTL3	protein_codin	159.673663	59.3186545	18.8622408	121.253094
ENSG000001	YOD1	protein_codin	0	0	77.2091831	0
ENSG000001	DYNLT1	protein_codin	20.5513416	25.1529802	28.260491	0
ENSG000001	DYNLT1	protein_codin	160.685666	48.3459115	37.8809556	67.4179284
ENSG000001	DYNLT1	protein_codin	104.07282	132.826659	32.3535734	53.9831122
ENSG000001	TMEM181	protein_codin	0	0	0	0
ENSG000001	FCMR	protein_codin	15.9568135	14.1119337	9.77301009	0
ENSG000001	IL24	protein_codin	0	0	0	0
ENSG000001	TULP4	protein_codin	47.0306538	88.333371	0	27.4932527
ENSG000001	IL24	protein_codin	0	0	0	0
ENSG000001	IL20	protein_codin	0	0	0	0
ENSG000001	TULP4	protein_codin	160.235782	164.272381	126.738245	99.2186457
ENSG000001	IL20	protein_codin	0	0	0	0
ENSG000001	IL10	protein_codin	11.9516491	0	6.25415778	3.79720044

ENSG000001	SERAC1	protein_codin	0	0	0	0
ENSG000001	MAPKAPK2	protein_codin	455.875174	561.133075	445.853613	302.056569
ENSG000001	DYRK3	protein_codin	0	0	0	0
ENSG000001	DYRK3	protein_codin	0	0	0	0
ENSG000001	DYRK3	protein_codin	2.08215326	8.43336855	3.95250645	1.07201243
ENSG000000	SYNJ2	protein_codin	0	2.81996349	5.24247071	1.83871927
ENSG000000	SYNJ2	protein_codin	6.3630906	3.63855432	0	0
ENSG000001	EIF2D	protein_codin	0	0	0	23.3195245
ENSG000001	IKBKE-AS1	lncRNA	0.92365876	2.69264811	0	0
ENSG000000	SYNJ2	protein_codin	0	0	0	0
ENSG000001	SRGAP2C	protein_codin	370.287269	206.898623	139.8929	234.049026
ENSG000001	AVPR1B	protein_codin	0	0.99174917	2.01610883	0
ENSG000001	FAM72A	protein_codin	0	0	0	0
ENSG000001	FAM72A	protein_codin	0	0	0	0
ENSG000001	SLC26A9	protein_codin	0	0	0	0
ENSG000001	SLC26A9	protein_codin	0	0	0	0
ENSG000001	PM20D1	protein_codin	0	0	0	0
ENSG000001	SLC41A1	protein_codin	7.94378056	12.8558823	13.3901928	2.04235322
ENSG000001	RAB29	protein_codin	80.3370121	102.489139	35.0989934	50.9522058
ENSG000000	NUCKS1	protein_codin	515.710912	661.166252	449.867269	424.520181
ENSG000002	TMEM242	protein_codin	8.7924742	4.56836467	0	7.4487978
ENSG000001	SLC45A3	protein_codin	6.39410577	29.6813779	14.3385588	8.59114245
ENSG000001	MFSD4A	protein_codin	0	0	0	0
ENSG000001	LEMD1	protein_codin	0	0	0	0
ENSG000001	LEMD1	protein_codin	0	0	0	0
ENSG000001	LEMD1	protein_codin	0	0	0	0
ENSG000001	LEMD1	protein_codin	0	0	0	0
ENSG000001	LEMD1	protein_codin	0	0	0	0
ENSG000001	KLHDC8A	protein_codin	0	0	0	0
ENSG000001	KLHDC8A	protein_codin	0	0	0	0
ENSG000001	NUAK2	protein_codin	174.003002	404.249523	78.4798838	180.602545
ENSG000001	TMCC2	protein_codin	0	6.03409865	0	0
ENSG000001	DSTYK	protein_codin	160.184574	180.131124	77.28977	109.781351
ENSG000001	DSTYK	protein_codin	0	9.00737891	0	7.38966095
ENSG000001	RBBP5	protein_codin	35.7545291	78.2926862	12.0873463	0
ENSG000001	CLDN20	protein_codin	0	0	0	0
ENSG000000	TFB1M	protein_codin	6.8690493	14.4530407	0	3.41474538
ENSG000001	TMEM81	protein_codin	1.01930784	4.08518616	0.98642302	2.06993403
ENSG000001	NFASC	protein_codin	0	0	0	0
ENSG000001	LRRN2	protein_codin	0	0	0	0
ENSG000001	LRRN2	protein_codin	0.85646914	0	0	0
ENSG000001	LRRN2	protein_codin	0	0	0	0.7999009
ENSG000002	SCAF8	protein_codin	316.86466	350.707205	247.838523	139.313485
ENSG000001	MDM4	protein_codin	12.6095733	9.72858868	34.0622144	0

ENSG000001	MDM4	protein_codin	5.26655397	3.34675976	1.93841403	0
ENSG000001	MDM4	protein_codin	824.259249	688.785074	368.212561	249.439815
ENSG000001	MDM4	protein_codin	0	0	0	0
ENSG000001	PIK3C2B	protein_codin	0	0	0	0
ENSG000002	SCAF8	protein_codin	181.480725	132.803515	0	169.91384
ENSG000001	PIK3C2B	protein_codin	0	0	16.2611451	0
ENSG000001	PPP1R15B	protein_codin	1702.25541	1109.97621	595.876733	1056.29729
ENSG000001	KISS1	protein_codin	0	0	0	0
ENSG000001	CTSD	protein_codin	0	0	0	0
ENSG000001	ETNK2	protein_codin	0	0	0	0
ENSG000001	ETNK2	protein_codin	0	0	0	0
ENSG000001	ETNK2	protein_codin	0	0	0	0
ENSG000001	SOX13	protein_codin	0	0	1.06792469	0
ENSG000001	SOX13	protein_codin	0	0	0	0
ENSG000001	SOX5	protein_codin	0	1.57824918	0	0
ENSG000001	LINC00303	lncRNA	0	0	0	0
ENSG000001	SNRPE	protein_codin	31.5051572	35.6942981	16.0180582	16.6155124
ENSG000000	ZC3H11A	protein_codin	276.370457	338.233137	305.98027	266.690153
ENSG000000	ZC3H11A	protein_codin	6.37147007	8.17009842	76.6523282	7.37534835
ENSG000000	ZC3H11A	protein_codin	0	0	0	0
ENSG000001	LAX1	protein_codin	0	0	0	0
ENSG000001	LAX1	protein_codin	24.2310983	3.71592288	57.6619092	1.98725906
ENSG000000	IPCEF1	protein_codin	141.192683	109.88095	63.8381776	96.8315692
ENSG000001	OPTC	protein_codin	0	0	0	0
ENSG000000	RGS17	protein_codin	0	0	1.39692003	4.54498343
ENSG000001	CHIT1	protein_codin	0.90095179	0	0	0
ENSG000001	MTRF1L	protein_codin	0	0	0	0
ENSG000001	MTRF1L	protein_codin	0	0	0	0
ENSG000001	MTRF1L	protein_codin	55.1988319	10.2412366	19.468865	20.9891865
ENSG000001	ADORA1	protein_codin	0	0	0	0
ENSG000001	ADORA1	protein_codin	0	0	0	0
ENSG000001	PPFIA4	protein_codin	1.41147405	0	0	0
ENSG000001	FBXO5	protein_codin	0	12.7425152	2.02349589	10.4807001
ENSG000001	TMEM183A	protein_codin	173.595412	241.899081	87.5207282	116.499742
ENSG000001	VIP	protein_codin	0	0	0	0
ENSG000001	VIP	protein_codin	0	0	3.26056483	0
ENSG000001	MYCT1	protein_codin	65.6111906	64.3838787	50.9004289	13.3843125
ENSG000001	SYNE1	protein_codin	26.8916702	40.7361788	0	26.9583618
ENSG000001	CYB5R1	protein_codin	114.702202	204.636847	70.7418149	86.2447336
ENSG000001	SYNE1	protein_codin	49.0423532	0	0	35.4529327
ENSG000001	SYNE1	protein_codin	0	0	0	0
ENSG000001	ADIPOR1	protein_codin	0	0	0	0
ENSG000001	SYNE1	protein_codin	465.453704	128.78139	244.575694	250.647751
ENSG000001	SYNE1	protein_codin	0	0	0	0

ENSG000001	SYNE1	protein_codin	0	0	0	0
ENSG000001	KLHL12	protein_codin	14.4641335	0	0	6.476251
ENSG000001	KLHL12	protein_codin	5.4256936	0	0	0
ENSG000001	KLHL12	protein_codin	80.0981335	159.698442	65.5039396	49.5246181
ENSG000001	RABIF	protein_codin	31.5290387	71.5333607	22.6193548	26.25476
ENSG000001	KDM5B	protein_codin	0	0	4.0710777	7.6123668
ENSG000001	KDM5B	protein_codin	0	0	0	0
ENSG000001	SYT2	protein_codin	0.93676705	0.92145563	0	0
ENSG000001	SYT2	protein_codin	0	0	0	0
ENSG000000	PPP1R12B	protein_codin	0	0	0	1.51079791
ENSG000002	PPP1R12BP1	transcribed_u	0	0	0	0
ENSG000002	ENSG000002	lncRNA	0	0	0	0
ENSG000001	LGR6	protein_codin	0	0	0	0
ENSG000001	PTPN7	protein_codin	4.67528028	3.67239289	0	0
ENSG000001	GPR37L1	protein_codin	0.95378203	0	0	0
ENSG000001	ELF3	protein_codin	0	0	0	0
ENSG000001	ELF3	protein_codin	0	0	0	0
ENSG000001	RNPEP	protein_codin	0	0	0	0
ENSG000001	TIMM17A	protein_codin	51.1925116	38.7961769	25.9566694	33.0215991
ENSG000001	LMOD1	protein_codin	0	0	0	0
ENSG000001	ARMT1	protein_codin	87.0866962	43.6007219	39.5793471	69.9521092
ENSG000001	NAV1	protein_codin	45.5168096	37.6132613	0	0
ENSG000001	NAV1	protein_codin	0	0	0	0
ENSG000001	NAV1	protein_codin	46.5772806	0	0	21.6514373
ENSG000001	CSRP1	protein_codin	7.14550162	13.5955343	0	0
ENSG000001	MTHFD1L	protein_codin	0	0	1.89052145	0
ENSG000001	MTHFD1L	protein_codin	7.18421915	0	0	0
ENSG000001	PHLDA3	protein_codin	0	0	0	0
ENSG000001	PHLDA3	protein_codin	0.9578108	1.89376607	0	0
ENSG000001	TNNI1	protein_codin	0	0	0	0
ENSG000001	LAD1	protein_codin	0	0.73896915	0	0
ENSG000001	TNNT2	protein_codin	0	0	0	0
ENSG000001	TNNT2	protein_codin	0	0	0	0
ENSG000001	MTHFD1L	protein_codin	0	7.61344534	0	11.0056866
ENSG000001	TNNT2	protein_codin	0	0	0	0
ENSG000000	PKP1	protein_codin	0	0	0	0
ENSG000001	PLEKHG1	protein_codin	0	0	0	0
ENSG000001	TMEM9	protein_codin	0	0	0	0
ENSG000001	TMEM9	protein_codin	0	0	0	0
ENSG000001	TMEM9	protein_codin	0	0	0	0
ENSG000001	TMEM9	protein_codin	37.7812261	40.9601249	17.9974792	33.47378
ENSG000000	IYD	protein_codin	0	0	0	0
ENSG000000	CACNA1S	protein_codin	0	0	0	0
ENSG000001	ULBP3	protein_codin	0	2.03276596	0	0

ENSG000001	RAET1L	protein_codin	0	0	0	0
ENSG000001	INAVA	protein_codin	0	0	0	0
ENSG000001	KIF14	protein_codin	0	0	0	0
ENSG000001	ULBP2	protein_codin	0	0	0	0
ENSG000001	ZNF281	protein_codin	75.564055	156.156135	265.308691	252.782324
ENSG000001	ZNF281	protein_codin	426.267705	260.439347	116.192082	393.78595
ENSG000002	LINC00862	lncRNA	0	0	0	0
ENSG000002	LINC00862	lncRNA	0	0	0	0
ENSG000001	NR5A2	protein_codin	0	0	0	0
ENSG000002	RAET1G	protein_codin	0	0	0	0
ENSG000002	RAET1G	protein_codin	0	0	0	0
ENSG000001	NR5A2	protein_codin	0	0	0	0
ENSG000001	RAET1E	protein_codin	0	0	0	0
ENSG000000	PTPRC	protein_codin	146.73326	50.9074407	38.7573265	85.6229347
ENSG000000	PTPRC	protein_codin	71.5808086	143.709201	125.089675	61.4548565
ENSG000001	LRP11	protein_codin	1.79139911	2.72333964	0	0
ENSG000001	WDR45	protein_codin	2.12996378	0	1.85418771	3.01711902
ENSG000001	PCMT1	protein_codin	34.0134852	51.1075571	12.4481828	36.252989
ENSG000000	PTPRC	protein_codin	0	0	0	0
ENSG000001	PCMT1	protein_codin	0	0	0	0
ENSG000001	ATP6V1G3	protein_codin	0	0	0	0
ENSG000001	NEK7	protein_codin	0	0	0	1.52510172
ENSG000001	PCMT1	protein_codin	97.6253434	167.815355	91.3483031	132.317876
ENSG000001	NEK7	protein_codin	252.791898	231.734333	144.149396	207.994523
ENSG000001	LHX9	protein_codin	0	5.35195936	0	0
ENSG000001	LHX9	protein_codin	0	0	0	0.9977108
ENSG000001	LHX9	protein_codin	0	0	0	0
ENSG000001	LHX9	protein_codin	0	0	0	0
ENSG000002	C1orf53	protein_codin	3.26181995	2.04309568	2.73413672	0
ENSG000002	DENND1B	protein_codin	0	0	6.51862744	0
ENSG000001	CRB1	protein_codin	0	0	0	0
ENSG000001	CRB1	protein_codin	0	0	0	0
ENSG000001	CRB1	protein_codin	0	0	0	0
ENSG000001	NUP43	protein_codin	0	0	0	0
ENSG000001	NUP43	protein_codin	2.5079345	0	0	1.55368861
ENSG000001	ZBTB41	protein_codin	46.2637849	33.1962504	33.244905	13.5792106
ENSG000000	ASPM	protein_codin	5.54088871	0	0	0
ENSG000000	ASPM	protein_codin	0	0	102.475935	0
ENSG000001	KATNA1	protein_codin	17.477071	14.8561154	0	0
ENSG000001	F13B	protein_codin	0	1.2456508	0	0
ENSG000000	CFHR2	protein_codin	0	0	0	0
ENSG000001	CFHR4	protein_codin	0	0	0	0
ENSG000000	GINM1	protein_codin	152.663801	131.787413	81.1900115	115.977672
ENSG000000	CFHR2	protein_codin	0	0	0	1.36010451

ENSG000002	CFHR1	protein_codin	0	0	0	0
ENSG000001	CFHR3	protein_codin	0	0	0	0
ENSG000001	CFHR3	protein_codin	0	0	0	0
ENSG000000	CFH	protein_codin	0	0	0	0
ENSG000001	KCNT2	protein_codin	0	0	0	0
ENSG000001	B3GALT2	protein_codin	0	1.33748991	0	0
ENSG000001	CDC73	protein_codin	129.723825	70.375906	85.927593	78.3605846
ENSG000000	GLRX2	protein_codin	0	7.93256547	4.04143638	0
ENSG000000	GLRX2	protein_codin	0	0	0	0
ENSG000001	RO60	protein_codin	86.130872	87.5191207	88.6378454	88.2390848
ENSG000001	RO60	protein_codin	0	0	0	0
ENSG000001	RO60	protein_codin	0	0	12.1517345	4.91989354
ENSG000001	RO60	protein_codin	0	0	0	0
ENSG000001	RO60	protein_codin	6.24565379	0	26.8572224	0
ENSG000001	UCHL5	protein_codin	0	0	0	0
ENSG000001	UCHL5	protein_codin	0	0	0	11.8841156
ENSG000001	UCHL5	protein_codin	12.1384063	0	13.9921359	13.0791804
ENSG000001	UCHL5	protein_codin	22.3979737	39.6941774	0	0
ENSG000001	UCHL5	protein_codin	73.7739656	0	39.9962139	20.4657405
ENSG000001	UCHL5	protein_codin	0	17.8173372	0	0
ENSG000000	TAB2	protein_codin	478.574487	237.066201	215.812265	500.817814
ENSG000000	RGS1	protein_codin	26.3328203	0	30.9837135	22.2252447
ENSG000001	RGS18	protein_codin	2384.43112	3903.42581	1609.05417	1721.84359
ENSG000001	BRINP3	protein_codin	0	0	0	0
ENSG000001	UST	protein_codin	15.5325981	20.2055209	1.99673847	9.07860415
ENSG000001	PLA2G4A	protein_codin	61.3963441	36.4159148	32.6279267	31.3927519
ENSG000001	SASH1	protein_codin	433.438334	168.674971	45.784351	78.2986666
ENSG000000	PTGS2	protein_codin	1976.54062	558.829446	1745.74132	1593.45673
ENSG000001	SASH1	protein_codin	0	0	0	0
ENSG000001	ODR4	protein_codin	0	0	0	0
ENSG000002	SAMD5	protein_codin	0	0	0	0
ENSG000001	STXBP5	protein_codin	0	0	28.8206605	0
ENSG000002	STXBP5-AS1	lncRNA	0	0	0	0
ENSG000000	TPR	protein_codin	1311.33306	823.197426	1090.02389	896.724058
ENSG000001	STXBP5	protein_codin	0	0	0	42.8081396
ENSG000001	STXBP5	protein_codin	0	0	0	0
ENSG000001	PRG4	protein_codin	1.55623424	0	0	0
ENSG000001	PRG4	protein_codin	0	0	0	0
ENSG000001	PRG4	protein_codin	0	0	0	0
ENSG000001	ADGB	protein_codin	0	0	0	0
ENSG000001	RAB32	protein_codin	291.673122	448.348779	194.89682	192.188668
ENSG000001	IVNS1ABP	protein_codin	17.718037	5.22919464	16.1927339	19.0721495
ENSG000001	IVNS1ABP	protein_codin	206.167221	255.40272	86.9630481	143.300449
ENSG000001	SWT1	protein_codin	24.0692871	4.03343834	4.08429419	4.36468584

ENSG000001	SWT1	protein_codin	33.1845966	52.5781608	47.3909533	29.1541898
ENSG000001	SHPRH	protein_codin	95.9819998	123.311562	51.2409556	79.9658954
ENSG000001	TRMT1L	protein_codin	55.3881585	47.2378244	26.7388927	31.6511139
ENSG000001	RNF2	protein_codin	0	15.9194209	5.89494519	0
ENSG000001	RNF2	protein_codin	56.1769393	0	0	0
ENSG000001	NIBAN1	protein_codin	751.274425	947.598741	268.391049	435.699623
ENSG000001	EDEM3	protein_codin	0	0	0	0
ENSG000001	C1orf21	protein_codin	3.53605617	10.1920211	0	0
ENSG000001	TSEN15	protein_codin	0	0	1.70161466	0
ENSG000001	EPM2A	protein_codin	0	0	0	0
ENSG000001	COLGALT2	protein_codin	0	0	0	0
ENSG000001	COLGALT2	protein_codin	2.38676943	0	0	0
ENSG000001	UTRN	protein_codin	0	0	0	0
ENSG000001	UTRN	protein_codin	82.0762451	68.7459643	36.6247493	52.5071018
ENSG000001	UTRN	protein_codin	18.7667815	27.7915719	3.96528932	22.5902825
ENSG000001	ARPC5	protein_codin	184.028285	296.579757	133.383742	180.249756
ENSG000001	NCF2	protein_codin	1058.44193	1638.74343	1812.51359	1686.83003
ENSG000001	NCF2	protein_codin	1058.22588	2302.45954	0	443.104404
ENSG000001	SMG7	protein_codin	67.2144118	0	14.9141929	0
ENSG000001	SMG7	protein_codin	1.27771867	0	3.28114367	1.80196081
ENSG000001	UTRN	protein_codin	3306.66566	1856.91719	1956.01741	2135.77651
ENSG000001	SHCBP1L	protein_codin	0	0	0	0
ENSG000001	DHX9	protein_codin	611.093235	615.503297	444.224967	451.819095
ENSG000001	NPL	protein_codin	0	0	0	0
ENSG000001	NPL	protein_codin	158.518843	91.3512214	52.510079	0
ENSG000001	NPL	protein_codin	5.51040975	16.5292311	3.7991532	5.72623342
ENSG000001	NPL	protein_codin	45.315373	53.8876219	10.0959341	48.1587202
ENSG000001	RGS8	protein_codin	0	0	0	0
ENSG000001	RGS8	protein_codin	0	0	0	0
ENSG000001	RGS16	protein_codin	3.96817775	0	5.75415309	0.97167866
ENSG000001	RNASEL	protein_codin	60.4535137	112.049375	46.4439521	28.3007154
ENSG000001	RGSL1	protein_codin	0	0	0	0
ENSG000002	LINC00272	lncRNA	0	0	0	0
ENSG000002	TEDDM1	protein_codin	0	0	0	0
ENSG000001	STX11	protein_codin	714.742692	775.105064	256.87922	432.806324
ENSG000001	SF3B5	protein_codin	101.757338	137.70528	40.0048435	85.407042
ENSG000001	CACNA1E	protein_codin	0	0	0	0
ENSG000001	PLAGL1	protein_codin	2.08024721	2.3868694	3.65792973	2.16793408
ENSG000001	PLAGL1	protein_codin	45.543648	0	46.5865827	10.4500923
ENSG000001	CACNA1E	protein_codin	0	0	0	0
ENSG000001	PORCN	protein_codin	0	0	0	0
ENSG000001	LTV1	protein_codin	141.500757	140.621578	185.914264	97.3520522
ENSG000001	IER5	protein_codin	935.973816	1693.51433	280.500537	662.192371
ENSG000001	MR1	protein_codin	5.91698881	3.25161403	0	1.48405569

ENSG000001	MR1	protein_codin	0	0	1.21441191	0
ENSG000001	MR1	protein_codin	173.777902	190.863994	88.3682945	145.529067
ENSG000001	PHACTR2	protein_codin	74.5046005	59.6731394	60.2504666	97.6659443
ENSG000001	PHACTR2	protein_codin	108.811873	52.8864554	35.3180097	41.1446211
ENSG000000	FUCA2	protein_codin	0	0	0	0
ENSG000001	KIAA1614	protein_codin	0	1.00538069	0	0
ENSG000001	KIAA1614	protein_codin	0	5.50354795	0	0
ENSG000001	XPR1	protein_codin	123.542334	91.9098517	24.1353927	64.6108718
ENSG000001	XPR1	protein_codin	127.398095	70.8878032	83.9248696	84.2516559
ENSG000000	PEX3	protein_codin	10.5120591	13.9801354	15.5180802	9.10487592
ENSG000000	PEX3	protein_codin	0	0	0	0
ENSG000001	ADAT2	protein_codin	5.93531385	5.50003921	0	0
ENSG000002	ACBD6	protein_codin	89.1577954	118.534579	57.3351071	49.6900258
ENSG000001	AIG1	protein_codin	0	0	4.01343712	0.99361968
ENSG000001	QSOX1	protein_codin	181.03681	259.945016	52.2074406	155.452838
ENSG000001	AIG1	protein_codin	0	1.31201738	0	3.93095845
ENSG000001	QSOX1	protein_codin	199.27901	192.168	104.992831	118.805808
ENSG000000	HIVEP2	protein_codin	0	0	0	3.63519763
ENSG000000	HIVEP2	protein_codin	279.418936	211.69391	173.302519	187.832975
ENSG000001	METTL3	protein_codin	0	0	0	0
ENSG000001	CEP350	protein_codin	384.939889	240.271244	102.629435	422.189549
ENSG000001	ADGRG6	protein_codin	0	5.9765206	0	0
ENSG000001	ADGRG6	protein_codin	4.39587798	0	5.87144756	0
ENSG000001	TOR1AIP2	protein_codin	0	0	10.1666856	11.618419
ENSG000001	TDRD5	protein_codin	0	0	0	0
ENSG000001	NPHS2	protein_codin	0	0	0	0
ENSG000001	NPHS2	protein_codin	0	0	0	0
ENSG000001	AXDND1	protein_codin	0	3.30980579	0	0
ENSG000000	SOAT1	protein_codin	200.072634	229.963196	130.060444	214.088727
ENSG000000	VTA1	protein_codin	15.3185051	27.6438299	29.3246319	4.85186496
ENSG000001	ABL2	protein_codin	0	0	0	0
ENSG000001	TOR3A	protein_codin	0	7.03539717	0	0
ENSG000001	TOR3A	protein_codin	143.510936	184.411876	79.1723202	81.9675939
ENSG000001	ANGPTL1	protein_codin	0	0	0	0
ENSG000000	VTA1	protein_codin	11.4163835	10.4264204	0	7.28279172
ENSG000001	RALGPS2	protein_codin	5.5258903	2.89599381	0	0
ENSG000001	RALGPS2	protein_codin	73.6248818	38.5515154	60.2710323	45.4980613
ENSG000001	RALGPS2	protein_codin	0	0	13.8684725	0
ENSG000002	C1orf220	lncRNA	0	0	0	0
ENSG000002	TEX35	protein_codin	0	0	0	0
ENSG000002	TEX35	protein_codin	0	0	0	0
ENSG000002	TEX35	protein_codin	0	0	0	0
ENSG000002	TEX35	protein_codin	0	0	0	0
ENSG000000	RASAL2	protein_codin	16.110891	13.5306373	0	18.2838384

ENSG000001	CITED2	protein_codin	351.183881	367.300016	94.7555777	272.668049
ENSG000001	TXLNB	protein_codin	0	0	0	0
ENSG000001	ASTN1	protein_codin	0	0	0	0
ENSG000001	HECA	protein_codin	813.680138	518.735239	337.78727	507.802342
ENSG000001	ABRACL	protein_codin	95.8756313	156.711769	37.2665359	70.8437731
ENSG000001	PAPPA2	protein_codin	0	0	0	0
ENSG000001	PAPPA2	protein_codin	2.8206222	1.85785584	0	0
ENSG000001	REPS1	protein_codin	28.7168692	139.637209	30.9003967	0
ENSG000001	COP1	protein_codin	0	0	32.2440052	0
ENSG000001	COP1	protein_codin	0	36.3310148	75.890705	37.6170884
ENSG000001	COP1	protein_codin	203.669899	102.359383	10.1886542	84.3187128
ENSG000001	TNR	protein_codin	0	0	0	0
ENSG000001	MRPS14	protein_codin	0	0	0	0
ENSG000001	CACYBP	protein_codin	79.1088391	70.0578373	77.3987436	0
ENSG000002	ECT2L	protein_codin	0	0	1.22748972	0
ENSG000002	GPR52	protein_codin	9.51848613	5.94494308	11.1491346	4.01666033
ENSG000001	RABGAP1L	protein_codin	0	0	0	0
ENSG000001	RABGAP1L	protein_codin	182.33341	69.365236	63.1109593	138.008138
ENSG000001	RABGAP1L	protein_codin	0	1.2018847	6.26881887	1.25770893
ENSG000001	RC3H1	protein_codin	179.204437	114.932624	126.325609	62.7984536
ENSG000001	RC3H1	protein_codin	661.219947	293.326481	254.592837	428.967305
ENSG000000	HEBP2	protein_codin	63.1161476	125.370757	48.3435285	69.9931479
ENSG000001	SERPINC1	protein_codin	0	0	0	0
ENSG000001	ZBTB37	protein_codin	149.883207	76.0932914	90.1987232	87.8263255
ENSG000001	ZBTB37	protein_codin	0	0	0	0
ENSG000001	ZBTB37	protein_codin	0	13.5825528	0	0
ENSG000001	CENPL	protein_codin	0	0	6.71284738	0
ENSG000001	ANKRD45	protein_codin	0	0	0	0
ENSG000001	SLC9C2	protein_codin	0	1.60098119	0	0
ENSG000002	PRDX6-AS1	lncRNA	4.95442108	1.03094497	1.74850093	0
ENSG000001	TNFSF4	protein_codin	169.728864	297.298547	75.4697424	119.801715
ENSG000001	FASLG	protein_codin	0	0	1.07731229	1.01577427
ENSG000000	SUCO	protein_codin	5.0348991	6.65864361	3.71112932	0
ENSG000001	C1orf105	protein_codin	0	0	0	0
ENSG000001	C1orf105	protein_codin	0	1.83212228	0	0
ENSG000001	C1orf105	protein_codin	0	0	0	0
ENSG000001	PIGC	protein_codin	10.137178	0	0	6.20176757
ENSG000001	DNM3	protein_codin	324.888445	379.17125	242.948182	225.353133
ENSG000001	DNM3	protein_codin	9.92977095	17.3447743	14.0279945	1.6429853
ENSG000001	OLIG3	protein_codin	0	0	0	0
ENSG000000	METTL13	protein_codin	0	0	6.67207987	0
ENSG000000	IFNGR1	protein_codin	514.35975	412.72921	0	291.363231
ENSG000001	VAMP4	protein_codin	12.9259037	18.0569179	0	7.77921031
ENSG000001	PRRC2C	protein_codin	2749.13025	1723.10032	0	1685.07233

ENSG000000	IL20RA	protein_codin	0	0	0	0
ENSG000000	IL20RA	protein_codin	0	0	0	0
ENSG000000	FMO4	protein_codin	5.81439922	4.28635745	4.25863284	0
ENSG000000	FMO1	protein_codin	0	0	0	0
ENSG000002	FMO6P	lncRNA	0	0	0	0
ENSG000000	FMO3	protein_codin	0	0	0	0
ENSG000001	PEX7	protein_codin	0	0	1.77509057	0
ENSG000001	MROH9	protein_codin	0	0	0	0
ENSG000001	MROH9	protein_codin	0	0	0	0
ENSG000001	PRRX1	protein_codin	0	0	0	0
ENSG000001	GORAB	protein_codin	4.94375924	7.03730085	0	0
ENSG000001	GORAB	protein_codin	0	0	0	0
ENSG000002	NTMT2	protein_codin	0	0	0	0
ENSG000000	KIFAP3	protein_codin	0	2.85244044	12.3164384	2.68421438
ENSG000000	KIFAP3	protein_codin	0	0	0	0
ENSG000000	SCYL3	protein_codin	0	0	0	0
ENSG000000	SCYL3	protein_codin	40.2612246	6.80502345	14.6077238	7.92960209
ENSG000000	SCYL3	protein_codin	12.8741865	40.2997604	19.0560687	11.5847032
ENSG000000	SELE	protein_codin	0	0	0	0
ENSG000000	SELE	protein_codin	0	0	1.14966731	0
ENSG000000	SELE	protein_codin	0	0	0	0
ENSG000000	SELE	protein_codin	0	0	0	0
ENSG000001	MTFR2	protein_codin	0	0	0	0
ENSG000001	SELP	protein_codin	2.62957658	8.17245906	0	7.26943987
ENSG000001	SELP	protein_codin	22.936388	106.237119	39.4212671	13.4523351
ENSG000001	SELP	protein_codin	0	0	0	0
ENSG000001	F5	protein_codin	503.602025	500.701349	235.458793	626.156433
ENSG000001	F5	protein_codin	150.791979	89.373939	47.2014252	137.192931
ENSG000001	AHI1	protein_codin	16.1406947	0	13.0283748	0
ENSG000001	AHI1	protein_codin	0	0	0	0
ENSG000001	SLC19A2	protein_codin	0	0	10.039939	0
ENSG000001	CCDC181	protein_codin	0.82747574	0	1.04499981	0
ENSG000001	CCDC181	protein_codin	0	0	0	0
ENSG000001	BLZF1	protein_codin	0	0	3.10284284	1.65870446
ENSG000001	BLZF1	protein_codin	25.9019404	59.6168586	31.0506669	19.673633
ENSG000001	NME7	protein_codin	14.4137519	0	21.307273	6.53277412
ENSG000001	MYB	protein_codin	0	0	0	0
ENSG000001	MYB	protein_codin	34.7670267	0	7.57985747	0
ENSG000001	ATP1B1	protein_codin	0	29.8656872	0	0
ENSG000001	ATP1B1	protein_codin	0	0	0	0
ENSG000001	DPT	protein_codin	0	0	0	0
ENSG000001	XCL1	protein_codin	0	0	0	0
ENSG000001	XCL2	protein_codin	1.1330752	0	0	1.14000009
ENSG000001	HBS1L	protein_codin	9.55166324	0	0	0

ENSG000001	TBX19	protein_codin	0	4.12261786	4.6246561	4.33991567
ENSG000001	HBS1L	protein_codin	58.4634519	35.2868659	45.4158824	48.7595918
ENSG000001	HBS1L	protein_codin	52.7853635	41.609713	55.8336206	18.4112839
ENSG000002	SFT2D2	protein_codin	5.39142445	0	2.01286469	3.94367772
ENSG000001	TIPRL	protein_codin	24.8640887	29.5234433	24.8532023	9.61482076
ENSG000001	TIPRL	protein_codin	54.313069	91.7964174	41.3209794	72.0734962
ENSG000001	GPR161	protein_codin	0	0	0	0
ENSG000001	GPR161	protein_codin	0	0	0	0.86238594
ENSG000001	HBS1L	protein_codin	35.182924	18.7271529	22.4482686	30.5510413
ENSG000001	GPR161	protein_codin	0	0	0	0
ENSG000001	DCAF6	protein_codin	32.7331014	9.34520053	0	12.7373565
ENSG000001	DCAF6	protein_codin	29.7315923	51.3835483	18.5188982	53.4060841
ENSG000001	ALDH8A1	protein_codin	0	3.01371402	0	0
ENSG000001	MPC2	protein_codin	62.2502939	56.3819957	25.1630069	33.9370042
ENSG000001	ALDH8A1	protein_codin	0.89219319	0	2.70804975	0.89194876
ENSG000001	ADCY10	protein_codin	0	0	0	2.19068949
ENSG000001	ADCY10	protein_codin	0	0	0	0
ENSG000001	MPZL1	protein_codin	18.8395437	25.3311516	12.8881718	5.19191607
ENSG000001	RCSD1	protein_codin	748.61392	1013.79014	768.412441	684.024349
ENSG000001	SGK1	protein_codin	28.0759146	50.5936618	23.0959939	32.7423731
ENSG000001	SGK1	protein_codin	45.1079965	121.22977	0	0
ENSG000001	SGK1	protein_codin	0	0	0	0
ENSG000001	POU2F1	protein_codin	0	0	13.4236052	0
ENSG000001	POU2F1	protein_codin	25.0130833	8.26688526	8.87757138	8.53264081
ENSG000001	POU2F1	protein_codin	198.05168	93.9140341	0	110.001813
ENSG000001	GPA33	protein_codin	3.90373764	6.88734812	1.90390282	2.956084
ENSG000000	TBPL1	protein_codin	0	6.25387375	5.76268929	0
ENSG000001	MAEL	protein_codin	0	0	0	0
ENSG000000	TBPL1	protein_codin	26.2597243	36.0257939	9.59392643	23.086441
ENSG000001	MAEL	protein_codin	0	0	0	0
ENSG000001	TADA1	protein_codin	3.10578996	19.363962	12.700163	10.2329422
ENSG000001	POGK	protein_codin	124.952696	192.488583	71.0345289	116.766164
ENSG000001	POGK	protein_codin	214.538042	200.57559	109.320256	94.3152875
ENSG000001	UCK2	protein_codin	28.1272445	20.6681335	8.76185023	10.4650509
ENSG000001	TMCO1	protein_codin	213.97862	252.323142	78.0349067	178.51347
ENSG000001	TCF21	protein_codin	0	0	0	0
ENSG000001	MGST3	protein_codin	0	0	4.57316962	0
ENSG000001	MGST3	protein_codin	0	0	0	0
ENSG000001	MGST3	protein_codin	40.2185252	47.3133979	0	22.1313054
ENSG000001	MGST3	protein_codin	16.9492403	9.17148278	0	12.7761278
ENSG000001	MGST3	protein_codin	12.8025259	20.7108521	19.2156521	19.737409
ENSG000001	LMX1A	protein_codin	0	0	0	0
ENSG000001	PBX1	protein_codin	7.84532274	38.9391634	36.6659949	24.4131323
ENSG000001	NUF2	protein_codin	5.28180793	0	2.158564	0

ENSG000001	RGS5	protein_codin	0	0	0	0
ENSG000001	RGS4	protein_codin	0	0	0	0
ENSG000001	RGS4	protein_codin	0	0	0	0
ENSG000001	RGS4	protein_codin	0	0	0	0
ENSG000001	CCDC190	protein_codin	0	0	0	0
ENSG000001	CCDC190	protein_codin	0	0	0	0
ENSG000001	CCDC190	protein_codin	0	0	0	0
ENSG000001	HSD17B7	protein_codin	0	0	0	0
ENSG000001	SLC18B1	protein_codin	3.28168344	0	0	0
ENSG000001	DDR2	protein_codin	1.08907542	1.09161975	4.63998974	0
ENSG000001	DDR2	protein_codin	0	0	0	0
ENSG000001	UAP1	protein_codin	0	0	44.3564138	0
ENSG000001	UAP1	protein_codin	32.8119521	24.4824154	0	24.2977038
ENSG000002	VNN3P	lncRNA	0	2.11465464	0	20.8562928
ENSG000001	VNN1	protein_codin	184.527814	231.493391	99.3421186	266.066646
ENSG000001	SH2D1B	protein_codin	0	1.23915275	1.15347423	2.52127817
ENSG000001	TAAR2	protein_codin	0	0	0	1.67112665
ENSG000002	ENSG000002	protein_codin	0	0	0	0
ENSG000001	SPATA46	protein_codin	0	0	0	0
ENSG000000	STX7	protein_codin	136.674063	176.368986	77.719481	119.530298
ENSG000001	OLFML2B	protein_codin	0	0	0	0
ENSG000001	OLFML2B	protein_codin	0	3.9518838	4.51635013	4.71953098
ENSG000000	STX7	protein_codin	418.495657	429.980394	236.540768	315.17163
ENSG000001	ATF6	protein_codin	237.13561	116.928747	109.527838	189.420668
ENSG000000	DUSP12	protein_codin	16.9785792	41.8217428	20.844638	24.4673209
ENSG000001	FCRLB	protein_codin	0	0	0	0
ENSG000001	FCRLB	protein_codin	0.8364098	0	0	0
ENSG000001	FCRLB	protein_codin	0	0	0	0
ENSG000001	FCRLB	protein_codin	0	0	2.3773809	0
ENSG000001	FCRLA	protein_codin	0	0	0	0
ENSG000001	FCRLA	protein_codin	0	2.52735143	0	0
ENSG000001	FCRLA	protein_codin	0	0	0	0
ENSG000001	FCRLA	protein_codin	0	0	0	0
ENSG000001	FCRLA	protein_codin	0	0	0	0
ENSG000000	FCGR2B	protein_codin	0	0	15.7825165	0
ENSG000000	MOXD1	protein_codin	0	0	6.03859274	0
ENSG000001	FCGR3B	protein_codin	1.10160495	0	0	0
ENSG000002	FCGR3A	protein_codin	131.546133	0	0	0
ENSG000001	FCGR2A	protein_codin	593.875189	735.901138	415.076379	444.31701
ENSG000001	CFAP126	protein_codin	0	0	0	0
ENSG000001	SDHC	protein_codin	77.6830293	106.428077	35.1209158	49.5594435
ENSG000001	CCN2	protein_codin	2.76575231	0	0	0
ENSG000001	NR1I3	protein_codin	0	0	0	0
ENSG000001	NR1I3	protein_codin	0	0	0	0

ENSG000001	NR1I3	protein_codin	0	0	0	0
ENSG000001	NR1I3	protein_codin	0	0	0	0
ENSG000001	NR1I3	protein_codin	0	0	0	0
ENSG000001	NR1I3	protein_codin	0	0	0	0
ENSG000001	NR1I3	protein_codin	0	0	0	0
ENSG000001	TOMM40L	protein_codin	20.3782489	0	0	0
ENSG000001	TOMM40L	protein_codin	0	77.8377835	55.7371022	59.2250373
ENSG000001	APOA2	protein_codin	0	0	0	0
ENSG000001	FCER1G	protein_codin	152.25141	117.879403	43.6988286	69.7122126
ENSG000001	NDUFS2	protein_codin	0	0	0	0
ENSG000001	ADAMTS4	protein_codin	0	0	0	0
ENSG000001	ADAMTS4	protein_codin	1.02877745	2.0075342	1.0977914	0
ENSG000001	B4GALT3	protein_codin	0	0	0	0
ENSG000001	PPOX	protein_codin	0	0	0	0
ENSG000001	USP21	protein_codin	0	27.1548839	9.3226743	0
ENSG000001	USP21	protein_codin	16.0449921	0	0	0
ENSG000001	UFC1	protein_codin	92.4916035	142.034845	90.2970607	66.8629276
ENSG000001	DEDD	protein_codin	1.90099016	3.47194027	0	6.19973057
ENSG000001	DEDD	protein_codin	74.2569663	106.878713	49.0354328	61.3440416
ENSG000001	NIT1	protein_codin	0	0	0	0
ENSG000001	NIT1	protein_codin	22.719773	28.3823443	18.7847143	10.3051294
ENSG000001	NIT1	protein_codin	34.7253074	54.9049598	14.1880037	31.2347013
ENSG000001	PFDN2	protein_codin	80.0702944	170.492004	113.746478	80.4324883
ENSG000001	KLHDC9	protein_codin	0	0	0	0
ENSG000001	NECTIN4	protein_codin	0	0.97521189	0	0
ENSG000001	ARHGAP30	protein_codin	2231.13019	3357.89173	1150.69378	1662.80445
ENSG000001	ARHGAP30	protein_codin	0	58.3569388	211.720886	21.7266122
ENSG000001	ARHGAP30	protein_codin	2.29552089	0	0	0
ENSG000001	ARHGAP30	protein_codin	4.96810315	0	1.18595784	0
ENSG000001	USF1	protein_codin	0	2.04644279	0	0
ENSG000001	USF1	protein_codin	0	0	1.82667689	2.52754408
ENSG000001	USF1	protein_codin	77.441925	169.969108	51.3629672	92.6577257
ENSG000002	TSTD1	protein_codin	0	0	0	0
ENSG000002	TSTD1	protein_codin	0	9.57623336	4.3111518	2.92712601
ENSG000001	F11R	protein_codin	296.648822	449.734013	186.548826	164.713774
ENSG000001	ITLN2	protein_codin	0	0	0.88322993	0
ENSG000001	CD244	protein_codin	0	0	45.3287043	0
ENSG000001	CD244	protein_codin	162.11717	328.315551	78.797901	139.389712
ENSG000001	LY9	protein_codin	0	0	0	0
ENSG000001	LY9	protein_codin	0	0	0	0
ENSG000001	LY9	protein_codin	2.09467353	2.300996	2.60458508	0
ENSG000000	SLAMF7	protein_codin	0	0	0	0
ENSG000000	SLAMF7	protein_codin	95.393062	114.764695	200.929174	53.1552358
ENSG000001	CD48	protein_codin	14.6385007	14.5700005	3.75505144	7.80252613

ENSG000001	CD48	protein_codin	952.805639	1581.52611	578.280392	701.113021
ENSG000000	CD84	protein_codin	39.9865073	57.7350079	47.8909125	48.9842802
ENSG000000	CD84	protein_codin	0	11.4607921	0	0
ENSG000000	CD84	protein_codin	0	0	0	0
ENSG000001	MED23	protein_codin	0	0	16.44911	13.3445865
ENSG000000	CD84	protein_codin	319.603131	327.443914	266.851399	287.151603
ENSG000001	SLAMF6	protein_codin	0	0	0	0
ENSG000001	SLAMF6	protein_codin	4.9307615	0	0	2.06699854
ENSG000001	MED23	protein_codin	0	0	0	0
ENSG000001	SLAMF6	protein_codin	0	10.0791924	28.4113322	0
ENSG000001	MED23	protein_codin	0	0	0	0
ENSG000001	VANGL2	protein_codin	2.05710415	3.06138985	0	3.07907273
ENSG000001	NCSTN	protein_codin	15.0017291	35.5561986	16.2427429	24.4222092
ENSG000001	MED23	protein_codin	106.304523	85.2715937	64.3966287	60.5950708
ENSG000001	COPA	protein_codin	144.416783	0	0	0
ENSG000001	PEX19	protein_codin	64.9540288	103.45738	25.2691665	49.923514
ENSG000001	DCAF8	protein_codin	0	0	8.13109857	0
ENSG000001	DCAF8	protein_codin	278.860273	248.355929	136.676813	139.955858
ENSG000001	PEA15	protein_codin	35.2497256	30.7921429	24.0897481	2.41469143
ENSG000001	PEA15	protein_codin	9.09567041	37.1312396	8.43079743	7.12586248
ENSG000001	CASQ1	protein_codin	0	0.92332188	0	0
ENSG000001	ATP1A4	protein_codin	0	0	0	0
ENSG000001	IGSF8	protein_codin	13.000409	0	0	10.5745957
ENSG000001	ARG1	protein_codin	0	0	0	0
ENSG000001	KCNJ9	protein_codin	1.62956364	0	0.81000505	0
ENSG000001	PIGM	protein_codin	20.091701	25.4394056	6.4210366	42.2764852
ENSG000001	SLAMF9	protein_codin	0	0	0	0
ENSG000001	SLAMF9	protein_codin	0	0	0	0
ENSG000000	IGSF9	protein_codin	0	0	0	0
ENSG000001	TAGLN2	protein_codin	146.252336	111.861174	45.8472183	79.900925
ENSG000001	TAGLN2	protein_codin	2836.40455	4484.80275	1578.47438	1718.74733
ENSG000002	CFAP45	protein_codin	48.4488477	4.46589582	0	0
ENSG000002	VSIG8	protein_codin	1.29592051	0	0	0
ENSG000002	ENSG000002	protein_codin	0	0	0	0
ENSG000001	SLAMF8	protein_codin	0	0	0	0
ENSG000001	FCRL6	protein_codin	0	0	0	0
ENSG000001	DUSP23	protein_codin	0	0	0	0
ENSG000001	DUSP23	protein_codin	34.9257425	49.119185	14.4848782	22.3904043
ENSG000001	DUSP23	protein_codin	0	8.95315305	0	0
ENSG000001	CRP	protein_codin	0	0	0	0
ENSG000001	CRP	protein_codin	0	0	0	0
ENSG000001	CRP	protein_codin	0	0	0	0
ENSG000001	FCER1A	protein_codin	0	0	0	0
ENSG000001	FCER1A	protein_codin	0	0	33.6124394	9.64386963

ENSG000002	ACKR1	protein_codin	0	0	0	0
ENSG000002	ACKR1	protein_codin	0	0	0	0
ENSG000001	CADM3	protein_codin	0	0	0	0
ENSG000001	CADM3	protein_codin	0	0	0	0
ENSG000000	EPB41L2	protein_codin	0	0	0	3.20867112
ENSG000000	EPB41L2	protein_codin	0	0	0	0
ENSG000001	AIM2	protein_codin	0	0	0	0
ENSG000001	AIM2	protein_codin	12.6175358	19.7305124	52.9352648	22.3410739
ENSG000001	IFI16	protein_codin	250.315839	180.300917	0	292.806001
ENSG000001	IFI16	protein_codin	465.020668	493.45146	613.355234	331.593673
ENSG000001	SAMD3	protein_codin	0	0	4.85157178	0
ENSG000001	PYHIN1	protein_codin	2.50166643	0	0	0
ENSG000001	L3MBTL3	protein_codin	0	0	0	0
ENSG000001	PYHIN1	protein_codin	0	2.90873758	0	0
ENSG000001	L3MBTL3	protein_codin	0	0	0	0
ENSG000001	PYHIN1	protein_codin	0	0	11.0908228	0
ENSG000001	MNDA	protein_codin	2691.85777	3179.1018	1945.26482	2891.74978
ENSG000002	TMEM244	protein_codin	0	0	0	0
ENSG000002	OR6K3	protein_codin	0	0	0	0
ENSG000002	OR6K3	protein_codin	0	0	0	0
ENSG000001	ARHGAP18	protein_codin	510.393091	844.95085	544.895607	408.953683
ENSG000001	CD1E	protein_codin	0	0	0	0
ENSG000001	CD1E	protein_codin	0	1.28800052	0	0
ENSG000001	CD1E	protein_codin	0	0	0	0
ENSG000001	CD1E	protein_codin	0	0	0	0
ENSG000001	CD1E	protein_codin	0	0	0	0
ENSG000001	CD1E	protein_codin	0	0	0	0
ENSG000001	CD1E	protein_codin	2.76990232	1.93352082	1.15330531	3.68536227
ENSG000001	CD1E	protein_codin	5.90530697	0	0	0
ENSG000001	CD1E	protein_codin	0	0	0	0
ENSG000001	CD1E	protein_codin	0	0	0	0
ENSG000001	CD1E	protein_codin	0	0	0	0
ENSG000001	CD1E	protein_codin	0	0	0	0
ENSG000001	CD1B	protein_codin	2.13172938	1.03893734	0	2.12371783
ENSG000001	CD1C	protein_codin	16.4366565	19.3169599	7.81469585	3.46098837
ENSG000001	CD1D	protein_codin	110.345449	290.026916	19.994898	52.3921671
ENSG000001	KIRREL1	protein_codin	0	0	0	0
ENSG000001	KIRREL1	protein_codin	0	0	0	0
ENSG000000	CD5L	protein_codin	0	0	0	0
ENSG000001	FCRL1	protein_codin	0	2.0390049	0	0
ENSG000001	FCRL1	protein_codin	0	0	7.54153187	0
ENSG000001	FCRL2	protein_codin	0	1.08543582	0	2.17605811
ENSG000001	FCRL2	protein_codin	0	0	0	0
ENSG000001	FCRL3	protein_codin	4.03618883	8.09945636	0	0

ENSG0000010	FCRL3	protein_codin	0	0	0	0
ENSG000001	FCRL5	protein_codin	6.22261178	0	15.1242795	4.98584953
ENSG000001	FCRL5	protein_codin	0	0	5.65916954	0
ENSG000001	ETV3	protein_codin	326.14603	321.168754	123.364242	184.982418
ENSG000001	ARHGEF11	protein_codin	660.77957	710.081908	234.065869	391.222237
ENSG000000	INSRR	protein_codin	0	0	0	0
ENSG000001	NTRK1	protein_codin	0	0	0	0
ENSG000000	SH2D2A	protein_codin	3.53696853	4.27299382	1.7567965	0
ENSG000000	SH2D2A	protein_codin	0	0	0	0
ENSG000001	PTPRK	protein_codin	0	0	0	0
ENSG000001	PTPRK	protein_codin	0	0	0	0
ENSG000001	HDGF	protein_codin	0	3.98851308	0	0
ENSG000001	PTPRK	protein_codin	0	0	0	0
ENSG000001	HDGF	protein_codin	41.2888738	150.649403	237.246397	18.793265
ENSG000001	PTPRK	protein_codin	0	0	0	0
ENSG000001	MRPL24	protein_codin	19.0983325	12.6799085	0	9.96972426
ENSG000001	PTPRK	protein_codin	0	0	0	0
ENSG000001	PTPRK	protein_codin	1.0113326	0	0	0
ENSG000001	METTL25B	protein_codin	2.54982243	36.9196541	8.20830617	0
ENSG000001	METTL25B	protein_codin	31.6239684	17.091401	3.95369888	19.7414575
ENSG000001	ISG20L2	protein_codin	30.7465096	79.3071727	7.61083007	27.474374
ENSG000001	CRABP2	protein_codin	5.51768823	3.04337319	0	0
ENSG000001	CRABP2	protein_codin	0	1.25130209	0	3.33338306
ENSG000001	CRABP2	protein_codin	6.62743727	0	4.27763153	0
ENSG000001	NES	protein_codin	0	1.49128288	0	0
ENSG000001	PTPRK	protein_codin	0	0	0	0
ENSG000001	GPATCH4	protein_codin	15.9653628	25.7567393	21.7668829	0
ENSG000001	NAXE	protein_codin	0	0	0	0
ENSG000001	NAXE	protein_codin	0	0	8.4692201	0
ENSG000001	NAXE	protein_codin	11.7786836	34.764051	0	20.1347584
ENSG000001	TTC24	protein_codin	0.97029301	0	10.0614192	0
ENSG000001	MIR9-1HG	lncRNA	0	0	0	0
ENSG000001	MIR9-1HG	lncRNA	0	0	0	0
ENSG000001	THEMIS	protein_codin	0	0	0	0
ENSG000001	THEMIS	protein_codin	0	0	0	0
ENSG000001	TSACC	protein_codin	0	0	0	0
ENSG000001	TSACC	protein_codin	0	0	0	0
ENSG000001	TSACC	protein_codin	0	0	0	0
ENSG000001	TSACC	protein_codin	0	0	0	0
ENSG000001	TSACC	protein_codin	0	0	0	0
ENSG000001	CCT3	protein_codin	0	5.51099545	1.21590978	1.52990068
ENSG000001	CCT3	protein_codin	0	0	0	0
ENSG000001	CCT3	protein_codin	0	0	0	0
ENSG000001	CCT3	protein_codin	0	0	0	0

ENSG000001(	CCT3	protein_codin	0	0	0	0
ENSG000001!	GLMP	protein_codin	0	5.84565438	0	0
ENSG000001(	PAQR6	protein_codin	0	0	0	0
ENSG000002	BGLAP	protein_codin	0	0	0	0
ENSG000001(	PMF1	protein_codin	0	0	0	0
ENSG000002(	PMF1-BGLAP	protein_codin	0	0	0	0
ENSG000001(	PMF1	protein_codin	63.1862954	73.1543784	31.7517511	55.372707
ENSG000001(	PMF1	protein_codin	0	27.4857019	0	3.69123321
ENSG000001	KIAA0408	protein_codin	0	0	0	0
ENSG000001!	SEMA4A	protein_codin	96.8001874	194.323028	21.4868851	117.156588
ENSG000001!	SEMA4A	protein_codin	0	0	0	0
ENSG000001!	SEMA4A	protein_codin	17.3845656	126.621614	40.7132028	115.97586
ENSG000000!	ECHDC1	protein_codin	0	0	0	0
ENSG000000!	ECHDC1	protein_codin	10.4988969	30.6279203	19.910599	0
ENSG000000!	ECHDC1	protein_codin	0	0	0	0
ENSG000000!	ECHDC1	protein_codin	0	0	0	0
ENSG000000!	ECHDC1	protein_codin	0	0	0	0
ENSG000001(	LMNA	protein_codin	0	4.96476247	0	15.7321797
ENSG000001(	LMNA	protein_codin	55.4267337	0	0	6.56454233
ENSG000001(	LMNA	protein_codin	0	0	0	0
ENSG000001(	LMNA	protein_codin	190.539698	149.922033	111.040976	43.6294785
ENSG000001(	LMNA	protein_codin	0	0	0	0
ENSG000001	LAMTOR2	protein_codin	0	0	0	0
ENSG000001	LAMTOR2	protein_codin	0	0	14.1517451	0
ENSG000001	LAMTOR2	protein_codin	53.3051401	83.9356435	11.9718523	44.8821771
ENSG000001(	UBQLN4	protein_codin	82.4221054	127.936703	47.0334654	44.9376658
ENSG000001(	SSR2	protein_codin	25.70152	10.1595006	11.6477311	13.3138741
ENSG000001	RNF146	protein_codin	0	70.5790622	46.3520312	0
ENSG000001	RSPO3	protein_codin	0	0	0	0
ENSG000001	RXFP4	protein_codin	2.00143762	0	0	0.968966
ENSG000001	KHDC4	protein_codin	4.47763822	0	0	10.4545481
ENSG000001	KHDC4	protein_codin	61.36332	37.1971965	39.0396868	9.24750644
ENSG000001	KHDC4	protein_codin	19.5487161	28.2056721	13.9411272	32.8766176
ENSG000001	RIT1	protein_codin	0	0	0	0
ENSG000001	RIT1	protein_codin	324.586707	267.248035	194.210663	137.230088
ENSG000001	SYT11	protein_codin	103.676653	122.245865	61.649328	83.191752
ENSG000002(	CENPW	protein_codin	0	1.91412181	1.21130913	0
ENSG000002(	CENPW	protein_codin	0	0	0	0
ENSG000002(	CENPW	protein_codin	6.11753583	25.8703175	9.97825603	3.346336
ENSG000001(	YY1AP1	protein_codin	0	0	0	0
ENSG000001	GON4L	protein_codin	59.3124431	131.729786	0	0
ENSG000000(	TRMT11	protein_codin	0	0	0	0
ENSG000001	DAP3	protein_codin	109.760092	50.5648734	34.4837695	0
ENSG000001(	YY1AP1	protein_codin	0	0	0	0

ENSG000001[illegible]	YY1AP1	protein_codin	0	0	0	0
ENSG000001[illegible]	MSTO1	protein_codin	0	0	0	0
ENSG000001	ASH1L	protein_codin	0	0	0	31.8278028
ENSG000001[illegible]	RUSC1	protein_codin	7.32627204	2.17018598	0	0
ENSG000001[illegible]	RUSC1	protein_codin	0	19.5782081	0	0
ENSG000001[illegible]	RUSC1	protein_codin	0	0	0	0
ENSG000001[illegible]	RUSC1	protein_codin	0	0	0	0
ENSG000001[illegible]	FDPS	protein_codin	6.39247953	0	0	0
ENSG000001	NCOA7	protein_codin	129.139568	38.6555971	46.7857634	57.0555213
ENSG000001[illegible]	HCN3	protein_codin	3.04060232	0	0.87142747	7.06088776
ENSG000001[illegible]	HEY2	protein_codin	0	0	0	0
ENSG000001[illegible]	HEY2	protein_codin	0	0	0	0
ENSG000001[illegible]	ENTREP3	protein_codin	0	0	0	0
ENSG000001[illegible]	ENTREP3	protein_codin	0	0	0	0
ENSG000001[illegible]	GBA1	protein_codin	114.331254	134.13002	47.204502	126.198838
ENSG000002[illegible]	GBA1LP	lncRNA	0	0	0	0
ENSG000001[illegible]	MTX1	protein_codin	0	0	8.04369788	0
ENSG000001[illegible]	THBS3	protein_codin	31.772279	71.0095898	23.1756285	15.4575945
ENSG000001[illegible]	TRIM46	protein_codin	0	0	0	0
ENSG000001[illegible]	TRIM46	protein_codin	1.64407099	0	0	0
ENSG000001[illegible]	TRIM46	protein_codin	0	0	0	0
ENSG000001	TPD52L1	protein_codin	0	0	0	0
ENSG000001[illegible]	MUC1	protein_codin	0	0	0	0
ENSG000001[illegible]	MUC1	protein_codin	0	0	0	0
ENSG000001[illegible]	MUC1	protein_codin	0	0	0	0
ENSG000001[illegible]	MUC1	protein_codin	0	0	0	0
ENSG000001[illegible]	MUC1	protein_codin	0	0	0	0
ENSG000001[illegible]	MUC1	protein_codin	0	0	0	0
ENSG000001[illegible]	DPM3	protein_codin	0	0	0	4.32001117
ENSG000001[illegible]	DPM3	protein_codin	18.1084698	38.3206285	14.1738481	15.361962
ENSG000001[illegible]	SLC50A1	protein_codin	0	88.4956923	28.3870816	39.2863169
ENSG000001	TPD52L1	protein_codin	0	0	0	0
ENSG000001[illegible]	SLC50A1	protein_codin	32.3936643	67.5276213	0	0
ENSG000001[illegible]	SLC50A1	protein_codin	0	17.0080887	0	0
ENSG000001[illegible]	EFNA1	protein_codin	0	0	0	0
ENSG000001[illegible]	EFNA1	protein_codin	0	0	0	0
ENSG000001[illegible]	EFNA3	protein_codin	2.7949623	4.6131549	0	1.7449514
ENSG000002[illegible]	EFNA4	protein_codin	3.95529038	5.05296302	3.00782097	5.84138233
ENSG000001[illegible]	ADAM15	protein_codin	0	0	0	9.21433074
ENSG000001[illegible]	ADAM15	protein_codin	5.04068988	0	0	0
ENSG000001[illegible]	RNF217	protein_codin	12.5636119	0	2.44718022	0
ENSG000001[illegible]	RNF217	protein_codin	1.11570984	0	0	0
ENSG000001[illegible]	NKAIN2	protein_codin	0	0	0	0
ENSG000001[illegible]	NKAIN2	protein_codin	0	2.34785005	3.42242493	2.34158353

ENSG000001	DCST1	protein_codin	0	0	0	0
ENSG000001	DCST1	protein_codin	0	0	0.76477794	0
ENSG000001	DCST2	protein_codin	0	0.89838279	0.89630838	0
ENSG000001	DCST2	protein_codin	0	0	0.87427812	2.74785189
ENSG000001	ZBTB7B	protein_codin	0	123.469217	0	154.243852
ENSG000001	LENEP	protein_codin	0	0	0	0
ENSG000001	FLAD1	protein_codin	0	2.92791238	0	0
ENSG000001	FLAD1	protein_codin	7.31260839	8.26958733	0	2.76989254
ENSG000001	FLAD1	protein_codin	0	0	0	2.96826213
ENSG000001	FLAD1	protein_codin	8.14264877	2.57204021	0	0
ENSG000001	CKS1B	protein_codin	0	0	0	0
ENSG000001	CLVS2	protein_codin	0	0	0	0
ENSG000001	CKS1B	protein_codin	0	0	6.73342007	0
ENSG000001	SMPDL3A	protein_codin	62.5910609	50.2692273	22.8731239	38.6351328
ENSG000001	FABP7	protein_codin	0	0	0	0
ENSG000001	SHC1	protein_codin	3.73740523	0	0	23.2244391
ENSG000001	PKIB	protein_codin	0	0	0	0
ENSG000001	PKIB	protein_codin	0	0	1.37755864	0
ENSG000001	SHC1	protein_codin	85.7895181	61.5096786	27.6592464	0
ENSG000001	SHC1	protein_codin	25.8302965	0	0	0
ENSG000001	PKIB	protein_codin	0	0	0	0
ENSG000001	SHC1	protein_codin	0	116.705471	39.4141453	61.649151
ENSG000000	HSF2	protein_codin	39.3895236	30.5604341	0	0
ENSG000001	PYGO2	protein_codin	2.60687468	1.07583281	0	0
ENSG000001	PYGO2	protein_codin	50.9185495	93.3657612	26.1484599	32.1370714
ENSG000001	PBXIP1	protein_codin	0	0	0	0
ENSG000001	PBXIP1	protein_codin	262.231666	277.032586	177.759277	156.090817
ENSG000001	TBC1D32	protein_codin	0	1.10178749	0	0
ENSG000001	PBXIP1	protein_codin	0	81.4029676	31.0607471	0
ENSG000001	PMVK	protein_codin	30.9878776	44.0155015	18.2955216	23.1612091
ENSG000001	MAN1A1	protein_codin	561.533753	383.738735	461.09571	348.225258
ENSG000001	ADAR	protein_codin	0	0	0	0
ENSG000001	FAM184A	protein_codin	0	0	0	0
ENSG000001	ADAR	protein_codin	618.405335	853.825375	0	542.793096
ENSG000001	FAM184A	protein_codin	0	0	3.86775666	0
ENSG000001	CHRNB2	protein_codin	0	0	0	0
ENSG000001	MCM9	protein_codin	1.48989877	3.88572279	0	0
ENSG000001	TDRD10	protein_codin	0	0	0	0
ENSG000001	TDRD10	protein_codin	0	0	0	0
ENSG000001	IL6R	protein_codin	648.60967	1124.95323	394.957718	522.11695
ENSG000001	ATP8B2	protein_codin	0	0	0	0
ENSG000001	CEP85L	protein_codin	0	0	0	17.774299
ENSG000001	ATP8B2	protein_codin	33.5399147	32.5410639	61.2432128	0
ENSG000001	ATP8B2	protein_codin	0	0	0	0

ENSG000001	CEP85L	protein_codin	236.514015	197.05034	185.251328	190.074846
ENSG000001	NUS1	protein_codin	220.944646	179.664407	124.30655	182.440338
ENSG000000	GOPC	protein_codin	4.25423859	0	0	13.6836006
ENSG000001	UBAP2L	protein_codin	0	8.8033902	0	0
ENSG000000	ROS1	protein_codin	0	0	0	0
ENSG000000	ROS1	protein_codin	0	1.09423879	0	1.12056627
ENSG000001	C1orf43	protein_codin	0	0	0	0
ENSG000001	SYCE1	protein_codin	0	0	0	0
ENSG000001	C1orf43	protein_codin	8.99395703	1.2909842	5.28086741	3.09496043
ENSG000001	C1orf43	protein_codin	20.9212349	0	0	0
ENSG000001	CYP2E1	protein_codin	0	0	0	0
ENSG000001	C1orf43	protein_codin	91.8358997	150.544931	65.8360225	38.3667467
ENSG000001	CFAP141	protein_codin	0	1.17809756	0	0
ENSG000001	TPM3	protein_codin	4.91312236	9.24338746	2.31605839	28.4333103
ENSG000002	TPM4P2	transcribed_p	3.25173648	8.81429139	15.1721923	7.83517601
ENSG000001	TPM3	protein_codin	0	0	0	0
ENSG000001	TPM3	protein_codin	1107.5855	2308.66713	1179.51363	1089.08351
ENSG000001	TPM3	protein_codin	370.020583	277.961459	53.996622	315.219197
ENSG000001	PAOX	protein_codin	5.63488603	0	9.39595683	0
ENSG000001	TPM3	protein_codin	167.707837	214.348129	31.6416131	135.780882
ENSG000001	ECHS1	protein_codin	95.4387463	135.689369	47.4083686	61.1626663
ENSG000001	GPRC6A	protein_codin	0	0	0	0
ENSG000001	FUOM	protein_codin	7.80274732	0	0	0
ENSG000001	FUOM	protein_codin	8.17971669	18.668503	7.80221987	3.84495969
ENSG000001	NUP210L	protein_codin	0	0	0	0
ENSG000002	ZNF511-PRAF	protein_codin	0	0	0	0
ENSG000001	CALY	protein_codin	0	0	0	0
ENSG000001	FAM162B	protein_codin	0	0	0	0
ENSG000001	CALY	protein_codin	0	0	0	0
ENSG000001	NUP210L	protein_codin	0	0	0	0
ENSG000001	TUBGCP2	protein_codin	34.0518124	28.5143134	17.320789	11.0260277
ENSG000001	KPNA5	protein_codin	21.6606883	6.09731241	15.7682638	19.8173442
ENSG000001	KNDC1	protein_codin	2.08824065	1.23003739	0	0
ENSG000001	ZUP1	protein_codin	0	0	0	0
ENSG000001	RAB13	protein_codin	70.6661332	94.4789617	63.7830019	41.9545547
ENSG000001	ZUP1	protein_codin	43.0063012	41.1521191	21.5508463	27.7970526
ENSG000001	RSPH4A	protein_codin	0.91984787	0	2.80503091	0
ENSG000001	RSPH4A	protein_codin	0	3.75180341	0	1.8950174
ENSG000001	CFAP46	protein_codin	0	0.79171428	0	0.79497434
ENSG000001	CFAP46	protein_codin	0	0	0	0
ENSG000001	JTB	protein_codin	0	25.0621578	0	0
ENSG000001	RWDD1	protein_codin	17.7349743	15.7542987	4.57043339	10.0101855
ENSG000001	NKX6-2	protein_codin	0	0	0	0
ENSG000000	INPP5A	protein_codin	21.2549053	22.6569437	20.7727942	16.2491707

ENSG000000	INPP5A	protein_codin	48.472799	101.309182	17.9853923	26.9744098
ENSG000001	CALHM4	protein_codin	0	0	0	0
ENSG000001	CALHM4	protein_codin	0	0	0	0
ENSG000001	CALHM5	protein_codin	17.207384	54.3852331	27.0552066	16.3512615
ENSG000001	CREB3L4	protein_codin	0	0	0	6.43980311
ENSG000001	CREB3L4	protein_codin	0	0	2.6353308	0
ENSG000001	TRAPPC3L	protein_codin	0	0	0	0
ENSG000001	CREB3L4	protein_codin	0	0	0	0
ENSG000001	CALHM6	protein_codin	0	121.122148	39.4133188	53.8769348
ENSG000001	CALHM6	protein_codin	78.4743943	95.1675347	14.6213544	52.9786495
ENSG000001	CALHM6	protein_codin	98.056224	10.3252593	0	0
ENSG000001	CREB3L4	protein_codin	11.2111211	11.8303977	3.10918669	0
ENSG000001	TSPYL1	protein_codin	229.414806	343.061077	138.411091	177.303971
ENSG000001	LRRC27	protein_codin	0	16.1820745	0	0
ENSG000001	LRRC27	protein_codin	9.50539219	0	0	0
ENSG000001	LRRC27	protein_codin	0	0	0	0
ENSG000001	STK32C	protein_codin	0	1.14920106	1.09276298	0.88383071
ENSG000001	STK32C	protein_codin	1.01112623	0	0	0
ENSG000001	SLC39A1	protein_codin	0	0	2.32232697	0
ENSG000001	STK32C	protein_codin	0	2.3931491	0	1.97382692
ENSG000001	SLC39A1	protein_codin	5.41414807	37.4892708	0	12.4244097
ENSG000001	DPYSL4	protein_codin	0	0	0	0
ENSG000001	CRTC2	protein_codin	19.6438896	22.1235886	0	0
ENSG000001	HDAC2	protein_codin	235.729878	157.034903	183.192285	134.568183
ENSG000001	CRTC2	protein_codin	126.014255	195.881545	59.4379831	88.3125971
ENSG000001	BNIP3	protein_codin	0	37.3534109	19.9314712	5.26723619
ENSG000001	LAMA4	protein_codin	0	0	0	0
ENSG000001	LAMA4	protein_codin	0	0	0	0
ENSG000001	TCERG1L	protein_codin	0	0	0	0
ENSG000001	GLRX3	protein_codin	12.5837806	11.4053875	4.02938227	4.27282586
ENSG000001	DENND4B	protein_codin	123.575277	192.531834	130.487864	95.6035674
ENSG000001	EBF3	protein_codin	0	0	0	0
ENSG000001	MKI67	protein_codin	47.9536413	42.7770632	707.988159	45.8841544
ENSG000001	MKI67	protein_codin	19.5048571	15.7014252	286.97552	15.9011186
ENSG000001	GATAD2B	protein_codin	314.695707	386.213127	138.121447	144.330136
ENSG000002	FAM229B	protein_codin	0	1.14747599	0	1.18391442
ENSG000000	TUBE1	protein_codin	0	8.17632977	0	0
ENSG000001	SLC27A3	protein_codin	0	0	0	0
ENSG000001	SLC27A3	protein_codin	9.51897846	44.5283057	12.3449204	9.39670023
ENSG000000	TUBE1	protein_codin	7.13121422	8.67791529	8.47409945	0
ENSG000001	CCN6	protein_codin	0	0	0	0
ENSG000001	CCN6	protein_codin	0	0	0	0
ENSG000000	FYN	protein_codin	42.1254974	14.6894415	44.674823	0
ENSG000001	INTS3	protein_codin	0	17.5647967	2.77431645	7.86279597

ENSG000001	INTS3	protein_codin	16.32829	0	0	0
ENSG000001	CLRN3	protein_codin	0	0	0	0
ENSG000001	C10orf90	protein_codin	0	0	0	0
ENSG000001	ADAM12	protein_codin	0	3.25389263	5.9256761	1.92179707
ENSG000001	NPR1	protein_codin	0	0	0	0
ENSG000000	FYN	protein_codin	0	0	0	16.6433789
ENSG000001	ADAM12	protein_codin	5.27757509	0	7.97696835	2.11725946
ENSG000001	NPR1	protein_codin	0	1.75735584	0	0
ENSG000001	ILF2	protein_codin	3.26838903	0	0	0
ENSG000000	FYN	protein_codin	398.193054	763.449038	129.627257	443.601693
ENSG000001	ILF2	protein_codin	10.1685401	0	0	0
ENSG000001	SNAPIN	protein_codin	45.1560668	64.6107612	10.1806653	48.1158728
ENSG000001	CHTOP	protein_codin	31.2236653	45.192765	19.8155986	16.9398953
ENSG000001	CHTOP	protein_codin	0	0	0	0
ENSG000002	FANK1	protein_codin	0	0	0	0
ENSG000001	CHTOP	protein_codin	23.7642646	42.1957842	21.3705963	11.8903739
ENSG000002	FANK1	protein_codin	0	0	0	0
ENSG000002	FANK1	protein_codin	0	0	0	0
ENSG000001	CHTOP	protein_codin	0	0	0	0
ENSG000001	S100A1	protein_codin	0	0	0	0
ENSG000001	S100A1	protein_codin	0	0	0	0
ENSG000001	S100A14	protein_codin	0	0	0	0
ENSG000001	S100A14	protein_codin	0	0	0	0
ENSG000001	S100A14	protein_codin	0	0	0	0
ENSG000001	S100A16	protein_codin	0	0	0	0
ENSG000001	S100A16	protein_codin	0	0	0	0
ENSG000001	S100A16	protein_codin	0	0	0	0
ENSG000001	S100A16	protein_codin	0	0	0	0
ENSG000001	S100A2	protein_codin	0	0	0	0
ENSG000001	S100A2	protein_codin	0	0	0	0
ENSG000001	S100A2	protein_codin	0	0	0	0
ENSG000001	S100A2	protein_codin	0	0	0	0
ENSG000001	S100A3	protein_codin	0	0	0	0
ENSG000001	S100A3	protein_codin	0	0	0	0
ENSG000001	S100A4	protein_codin	0	15.3072859	103.086802	4.08736104
ENSG000001	S100A4	protein_codin	20.0373961	50.5756279	16.9992218	26.8418446
ENSG000001	S100A4	protein_codin	1521.47373	1390.84086	224.899891	765.030559
ENSG000001	S100A5	protein_codin	0	0	0	0
ENSG000001	S100A5	protein_codin	2.60060873	0	0	0
ENSG000001	S100A6	protein_codin	3915.12583	4838.90947	1294.92031	2425.03821
ENSG000001	S100A6	protein_codin	3.86611334	5.30381298	2.79249177	1.68614903
ENSG000000	DHX32	protein_codin	9.32921173	2.74112914	12.6331957	10.4845288
ENSG000001	S100A7	protein_codin	0	0	0	0
ENSG000001	S100A7	protein_codin	0	0	0	0

ENSG000001	S100A7L2	unprocessed_	0	0	0	0
ENSG000001	S100A7A	protein_codin	0	0	0	0
ENSG000001	S100A7A	protein_codin	0	0	0	1.00986296
ENSG000000	TRAF3IP2	protein_codin	0	0	0	0
ENSG000000	TRAF3IP2	protein_codin	1.80616738	0	0	0
ENSG000001	S100A8	protein_codin	9.04041378	16.0042501	0	8.68617254
ENSG000001	S100A8	protein_codin	5227.04785	5788.92558	1154.8956	3914.00141
ENSG000000	TRAF3IP2	protein_codin	0	0	0	0
ENSG000000	TRAF3IP2	protein_codin	2.81064212	0	0	0
ENSG000001	S100A12	protein_codin	1028.72683	1056.42349	177.897532	643.636617
ENSG000001	S100A9	protein_codin	14131.165	15133.3289	4331.84954	9324.08821
ENSG000001	PGLYRP4	protein_codin	0	0	1.01663632	0
ENSG000002	LORICRIN	protein_codin	0	0	0	0
ENSG000002	PRR9	protein_codin	0	0	0	0
ENSG000002	LELP1	protein_codin	0	0	0	0
ENSG000001	SPRR2G	protein_codin	0	0	0	0
ENSG000002	SPRR2E	protein_codin	0	0	0	0
ENSG000002	SPRR2E	protein_codin	0	0	0	0
ENSG000001	SPRR2B	protein_codin	0	0	0	0
ENSG000001	SPRR2D	protein_codin	0	0	0	0
ENSG000001	SPRR2D	protein_codin	0	0	0	0
ENSG000001	SPRR2D	protein_codin	0	0	0	0
ENSG000001	BCCIP	protein_codin	13.3796041	26.8084197	26.0767182	6.03200617
ENSG000000	TRAF3IP2	protein_codin	9.46564909	3.71650699	5.54830051	7.88755299
ENSG000001	SPRR1A	protein_codin	0	0	0	0
ENSG000001	IVL	protein_codin	0.6853096	0	0	0
ENSG000001	SMCP	protein_codin	0	0	0	0
ENSG000001	LCE1E	protein_codin	0	0	0	0
ENSG000001	LCE1E	protein_codin	0	0	0	0
ENSG000001	UROS	protein_codin	0	0	0	2.88058337
ENSG000001	KPLCE	protein_codin	0	0	0	0
ENSG000001	LCE4A	protein_codin	0	0	0	0
ENSG000001	UROS	protein_codin	0	2.75160131	1.79517254	3.77752081
ENSG000001	LCE2A	protein_codin	0	0	0	0
ENSG000001	LCE2B	protein_codin	0	0	0	0
ENSG000001	LCE2C	protein_codin	0	0	0	0
ENSG000001	LCE2D	protein_codin	0	0	0	0
ENSG000001	UROS	protein_codin	0	6.41149785	0	0
ENSG000001	LCE3D	protein_codin	0	0	0	0
ENSG000001	LCE3E	protein_codin	0	0	0	0
ENSG000001	CRCT1	protein_codin	0	0	0	0
ENSG000001	UROS	protein_codin	23.3368474	34.2609848	23.0732933	0
ENSG000001	FLG	protein_codin	1.67196708	1.71805255	1.06177852	0
ENSG000001	HRNR	protein_codin	0	1.04686733	0.66439405	0

ENSG000000	REV3L	protein_codin	215.529401	134.076954	133.54707	186.028579
ENSG000001	TCHHL1	protein_codin	1.65995842	0	1.27785028	0
ENSG000001	MMP21	protein_codin	0.89419407	0.90131224	0	0
ENSG000001	S100A10	protein_codin	906.33722	212.460857	274.756728	98.5438208
ENSG000001	S100A10	protein_codin	2231.52942	1928.05207	492.931433	950.244482
ENSG000001	EDRF1	protein_codin	0	0	0	0
ENSG000001	EDRF1	protein_codin	17.3962088	25.6517476	10.4177509	16.5936468
ENSG000001	THEM4	protein_codin	5.03842943	4.47200477	4.37156863	2.29996659
ENSG000001	EDRF1	protein_codin	42.4196899	0	0	0
ENSG000001	THEM5	protein_codin	3.7960413	9.23774474	7.25591837	1.84168162
ENSG000002	LINGO4	protein_codin	0	0	0	0
ENSG000001	TEX36	protein_codin	0	0	0	0
ENSG000001	TDRKH	protein_codin	0	0	0	0
ENSG000001	TDRKH	protein_codin	0	0	4.27320434	0
ENSG000001	TDRKH	protein_codin	12.0743951	0	0	0
ENSG000001	TDRKH	protein_codin	0	0	0	0
ENSG000001	TDRKH	protein_codin	0	0	0	0.95201536
ENSG000001	MRPL9	protein_codin	0	0	7.64040703	4.16112564
ENSG000001	MRPL9	protein_codin	44.7645378	50.2874889	17.7238212	26.5602543
ENSG000002	EEF1AKMT2	protein_codin	0	25.9158663	9.2962028	6.25983436
ENSG000001	SNX27	protein_codin	0	0	0	0
ENSG000001	LHPP	protein_codin	0	0	0	30.4067812
ENSG000001	SNX27	protein_codin	0	0	0	0
ENSG000001	LHPP	protein_codin	46.5396301	83.3168063	35.027936	7.11913044
ENSG000001	SNX27	protein_codin	140.558693	157.867343	67.5379335	69.5336763
ENSG000000	OAT	protein_codin	135.461843	132.399999	61.3873246	108.654349
ENSG000001	MFSD4B	protein_codin	0	0	0	0
ENSG000001	TUFT1	protein_codin	0	10.6711318	0	5.06414664
ENSG000001	TUFT1	protein_codin	0	0	4.39410345	0
ENSG000001	SLC16A10	protein_codin	0	1.49199957	0	0
ENSG000001	SLC16A10	protein_codin	3.92002327	0	2.39720174	5.38099905
ENSG000001	CPXM2	protein_codin	0	0	0	0
ENSG000001	BUB3	protein_codin	44.2302373	68.1743156	24.1344109	14.9262542
ENSG000001	BUB3	protein_codin	6.75141065	3.80635414	2.8138244	0
ENSG000001	POGZ	protein_codin	162.046567	183.24096	92.7653579	80.4067095
ENSG000001	RPF2	protein_codin	16.1467637	19.5402524	6.45784567	6.61724371
ENSG000001	BUB3	protein_codin	28.099717	43.41928	20.3331067	23.6871848
ENSG000001	SELENBP1	protein_codin	0	0	0	0
ENSG000001	ACADSB	protein_codin	0	6.28613281	0	5.67464471
ENSG000001	RFX5	protein_codin	0	12.285436	16.2713036	11.860199
ENSG000001	PI4KB	protein_codin	111.500011	133.839463	77.3296536	0
ENSG000001	PI4KB	protein_codin	0	0	0	0
ENSG000001	PI4KB	protein_codin	210.157922	270.233057	57.8868367	144.512073
ENSG000001	PI4KB	protein_codin	8.03881027	62.301976	46.306961	72.0275481

ENSG000001:	AMD1	protein_codin	0	12.8056005	0	0
ENSG000001:	AMD1	protein_codin	160.737652	301.146717	96.9146564	174.089036
ENSG000001	PSMD4	protein_codin	42.6784017	55.4937919	34.3250612	18.6459736
ENSG000001	PSMD4	protein_codin	376.275545	703.37214	262.722634	290.554641
ENSG000001:	AMD1	protein_codin	318.832304	455.810651	224.68749	254.88286
ENSG000000	IKZF5	protein_codin	125.845414	76.9526578	65.7794484	78.9316239
ENSG000001	PSTK	protein_codin	0	0	0	0
ENSG000001	PIP5K1A	protein_codin	9.20481053	24.0391193	49.2636283	0
ENSG000001	PIP5K1A	protein_codin	0	0	0	0
ENSG000001	C10orf88	protein_codin	7.10853179	0	0	1.42328136
ENSG000001	VPS72	protein_codin	55.218343	151.592971	33.0156384	55.0361048
ENSG000002	FAM24A	protein_codin	0	0	0	0
ENSG000002	C10orf88B	transcribed_p	0	0	0	0
ENSG000002	FAM24B	protein_codin	1.28765007	0	0	2.48326746
ENSG000002	FAM24B	protein_codin	0	0	0	0
ENSG000001	CUZD1	protein_codin	0	0	0	0
ENSG000001	CUZD1	protein_codin	0	0	1.12535757	0
ENSG000001	CUZD1	protein_codin	0	0	0	0
ENSG000001	SCNM1	protein_codin	25.1344647	25.6408639	0	0
ENSG000002	ENSG000002	protein_codin	0	0	0	0
ENSG000001	SCNM1	protein_codin	0	0	0	0
ENSG000001	LYSMD1	protein_codin	1.85649347	1.80319471	0	0
ENSG000001	DMBT1	protein_codin	0	0	0	0
ENSG000001	TNFAIP8L2	protein_codin	68.025528	164.951523	25.8270993	60.5148751
ENSG000001	CDK19	protein_codin	0	24.3492588	12.605094	0
ENSG000001	SEMA6C	protein_codin	0	0	0	0
ENSG000001	SEMA6C	protein_codin	0	0	0	0
ENSG000001	SEMA6C	protein_codin	0	1.86269499	2.11980005	0
ENSG000001	GABPB2	protein_codin	42.3950374	62.8428747	28.4419302	25.2220962
ENSG000001	GABPB2	protein_codin	96.9952691	90.5978281	58.8639684	39.7314455
ENSG000000	SLC22A16	protein_codin	30.393855	0	8.98951839	15.2371543
ENSG000002	MLLT11	protein_codin	5.10420573	14.1739441	4.32340028	3.08253923
ENSG000002	DDO	protein_codin	5.17876917	9.36068432	3.98771229	7.07630742
ENSG000002	DDO	protein_codin	0	10.4537494	0	0
ENSG000001	C1orf56	protein_codin	3.85614199	1.93007411	5.96573599	0.94753442
ENSG000001	CDC40	protein_codin	33.4362294	41.2142023	60.0082324	0
ENSG000001	BNIPL	protein_codin	0	0.98281663	0	1.00230598
ENSG000001	CDC40	protein_codin	0	0	0	0
ENSG000001	PRUNE1	protein_codin	0	32.9620647	33.453156	0
ENSG000001	PRUNE1	protein_codin	7.55798922	3.91249474	0	8.90582972
ENSG000001	PRUNE1	protein_codin	63.2571284	158.749459	112.341846	87.4818703
ENSG000001	PRUNE1	protein_codin	0	0	0	0
ENSG000001	WASF1	protein_codin	0	0	0	0
ENSG000001	FIG4	protein_codin	26.8163375	0	0	0

ENSG000001	ANXA9	protein_codin	3.86324965	1.91856396	2.38688974	2.88428312
ENSG000001	AK9	protein_codin	13.1165345	28.9887997	14.3285243	8.1243959
ENSG000001	CERS2	protein_codin	14.7127279	0	0	0
ENSG000001	CERS2	protein_codin	361.419308	649.064219	204.43412	245.835523
ENSG000001	NUMA1	protein_codin	1.11643177	0	1.43528191	0
ENSG000001	SETDB1	protein_codin	4.82063682	12.2076041	2.58848503	11.3812787
ENSG000001	SETDB1	protein_codin	0	0	0	6.77691181
ENSG000001	SETDB1	protein_codin	2.39613399	9.37691573	0	0
ENSG000002	ENSG000002	lncRNA	0	0	2.20980327	0
ENSG000002	CCDC162P	transcribed_u	0	0	0	0
ENSG000001	CEP57L1	protein_codin	0	0	0	0
ENSG000001	SETDB1	protein_codin	0	0	0	52.1867919
ENSG000001	CEP57L1	protein_codin	0	0	0	5.23795271
ENSG000000	SESN1	protein_codin	4.12834898	3.79926281	3.88845253	5.88227058
ENSG000001	ARMC2	protein_codin	0	0	0	0
ENSG000002	LINC00222	lncRNA	0	0	0	0
ENSG000001	AFG1L	protein_codin	3.83532879	1.73991645	5.89381344	2.20519321
ENSG000001	SNX3	protein_codin	0	10.8112725	8.69120943	2.45352265
ENSG000001	NR2E1	protein_codin	0	0	0	0
ENSG000001	HTRA1	protein_codin	0	0	0	1.85989891
ENSG000001	CTSS	protein_codin	3204.10221	3484.23317	2114.91255	2077.75743
ENSG000001	NR2E1	protein_codin	0	0	0	0
ENSG000001	HORMAD1	protein_codin	16.1000553	6.16139743	5.80451532	4.71272604
ENSG000001	PLEKHA1	protein_codin	5.33793793	0	12.5310394	0
ENSG000001	PLEKHA1	protein_codin	7.03725796	0	0	0
ENSG000001	PLEKHA1	protein_codin	0	0	0	0
ENSG000001	HORMAD1	protein_codin	0	0	0	0
ENSG000001	TACC2	protein_codin	0	0	0	0
ENSG000001	TACC2	protein_codin	0	0	0	0
ENSG000001	TACC2	protein_codin	0	0	0	0
ENSG000001	TACC2	protein_codin	0	0	0	0
ENSG000000	SEC63	protein_codin	335.433234	256.160495	232.790418	279.704771
ENSG000001	TACC2	protein_codin	0	0	0	0
ENSG000001	TACC2	protein_codin	0	0	0	0
ENSG000001	ENSA	protein_codin	237.410041	374.739373	132.174276	135.64713
ENSG000001	ENSA	protein_codin	0	0	0	1.01841936
ENSG000001	NSMCE4A	protein_codin	0	2.5198249	0	0
ENSG000001	SCML4	protein_codin	6.03709027	4.99833638	0	0
ENSG000001	SCML4	protein_codin	0	0	0	0.94269118
ENSG000001	NSMCE4A	protein_codin	84.5932064	94.7981526	19.7983987	56.1863325
ENSG000001	SCML4	protein_codin	0	0	1.17598731	0
ENSG000001	MCL1	protein_codin	2028.30764	727.49766	308.050654	223.084863
ENSG000002	ENSG000002	lncRNA	0	0	0	0.92446417
ENSG000001	PDSS2	protein_codin	7.23278438	8.04884589	0	1.26406205

ENSG000001(	PDSS2	protein_codin	26.909466	46.4471436	21.0691413	30.2722087
ENSG000001	ADAMTSL4	protein_codin	104.760189	92.196658	0	0
ENSG000001	ADAMTSL4	protein_codin	79.9707993	23.7063503	93.7186335	73.0240102
ENSG000001(	ATE1	protein_codin	0	15.0139804	0	0
ENSG000001	ADAMTSL4	protein_codin	6.75249258	7.35258915	5.62668569	19.7293638
ENSG000001	BEND3	protein_codin	8.65077505	8.20269295	5.36652881	3.48748336
ENSG000001(	ATE1	protein_codin	0	0	0	0
ENSG000001	QRSL1	protein_codin	15.7264712	10.0341018	13.9133771	0
ENSG000001	QRSL1	protein_codin	22.5237682	51.4314418	20.8412261	30.3194366
ENSG000001	ECM1	protein_codin	0	10.9062932	0	3.10079255
ENSG000001	ECM1	protein_codin	4.47161048	0	0.9232452	0
ENSG000001	TARS2	protein_codin	23.2657025	33.333738	0	8.31664226
ENSG000001	TARS2	protein_codin	0	4.92027636	2.58178447	2.79401342
ENSG000001	TARS2	protein_codin	0	0	0	0
ENSG000000(	FGFR2	protein_codin	0	0	0	0
ENSG000000(	FGFR2	protein_codin	0	0	0	0
ENSG000000(	FGFR2	protein_codin	0	0	0	0
ENSG000000(	FGFR2	protein_codin	0	0	0	0
ENSG000000(	FGFR2	protein_codin	0	0	0	0
ENSG000001	RTN4IP1	protein_codin	14.6881773	23.8363557	5.27645821	9.17929437
ENSG000001	TARS2	protein_codin	31.8739801	47.7206479	30.0228915	30.1923844
ENSG000001(	RPRD2	protein_codin	5.31002859	14.9090617	0	0
ENSG000001(	RPRD2	protein_codin	176.849432	118.252754	83.6634163	90.1735939
ENSG000000	ATG5	protein_codin	0	0	0	0
ENSG000001	LINC01561	lncRNA	0	0	0	0
ENSG000002(	PLPP4	protein_codin	0	0	0	0
ENSG000001(	SEC23IP	protein_codin	6.17171358	2.29997587	17.441656	2.43284627
ENSG000000	ATG5	protein_codin	44.1814557	24.3633191	0	38.8941922
ENSG000001	MCMBP	protein_codin	133.922967	220.255843	33.877013	154.061037
ENSG000001	INPP5F	protein_codin	1.61725224	1.72434036	0	0
ENSG000001	BAG3	protein_codin	47.5928217	39.3696272	19.7985569	39.7477467
ENSG000001	TIAL1	protein_codin	22.7268934	5.21835792	0	0
ENSG000001	TIAL1	protein_codin	19.3618653	18.2859348	4.24591946	14.9822849
ENSG000000	PRDM1	protein_codin	0	0	0	0
ENSG000000	PRDM1	protein_codin	66.8450272	21.9988142	42.1663089	0
ENSG000001	TIAL1	protein_codin	40.608891	93.8222608	49.8133281	34.5213385
ENSG000001	CIART	protein_codin	0	0	0	0
ENSG000001	CIART	protein_codin	0	0	0	0
ENSG000000	PRDM1	protein_codin	917.546278	441.545296	602.209647	452.579125
ENSG000001	C1orf54	protein_codin	3.66498258	8.35324364	0	3.09526657
ENSG000001	C1orf54	protein_codin	3.834044	24.4799185	6.92450272	10.2532917
ENSG000001	RGS10	protein_codin	48.1975147	233.124883	354.015135	0
ENSG000001	C1orf54	protein_codin	1.18830794	5.09311833	3.31424481	1.16292437
ENSG000001	RGS10	protein_codin	726.961338	1771.93023	265.169668	651.9062

ENSG000001	GRK5	protein_codin	0	0	2.2967977	0
ENSG000001	APH1A	protein_codin	37.9737631	79.7128193	10.5261567	56.9961637
ENSG000000	PREP	protein_codin	91.6629221	128.54811	66.1874331	64.0609085
ENSG000001	CA14	protein_codin	0	0	0	0
ENSG000001	ANP32E	protein_codin	1.06292378	0	0	0
ENSG000001	ANP32E	protein_codin	0	4.3359047	94.3949353	8.5962039
ENSG000002	BVES-AS1	lncRNA	0	0	0	0
ENSG000002	BVES-AS1	lncRNA	0	0	0	0
ENSG000002	LIN28B-AS1	lncRNA	0	0	0	0
ENSG000000	PLEKHO1	protein_codin	1000.35898	1799.54087	730.112749	779.914398
ENSG000000	HACE1	protein_codin	0	18.0313988	0	12.8739625
ENSG000000	PLEKHO1	protein_codin	14.984299	119.284402	10.9405397	28.4071238
ENSG000000	HACE1	protein_codin	16.1092454	2.21458029	1.43414968	9.78715904
ENSG000001	VPS45	protein_codin	0	0	0	0
ENSG000001	SFXN4	protein_codin	0	0	0	0
ENSG000001	GRIK2	protein_codin	0	0	0	0
ENSG000001	GRIK2	protein_codin	0	0	0	0
ENSG000001	GRIK2	protein_codin	0	0	0	0
ENSG000000	MTMR11	protein_codin	0	0	0	0
ENSG000001	ASCC3	protein_codin	34.1581205	22.0898369	30.817637	21.1917348
ENSG000001	EIF3A	protein_codin	2653.53823	2360.87373	1924.681	2489.59214
ENSG000001	SV2A	protein_codin	0	0	0	0
ENSG000001	SV2A	protein_codin	0	7.91783467	0.84555531	1.99584562
ENSG000001	BOLA1	protein_codin	0	0	0	0
ENSG000001	CACUL1	protein_codin	343.553316	199.224169	169.098398	159.941196
ENSG000001	BOLA1	protein_codin	0	0	5.74088985	5.35887328
ENSG000001	BOLA1	protein_codin	4.65455098	6.7988702	1.85057303	0.93856147
ENSG000001	H2BC21	protein_codin	249.344582	528.913541	176.455894	323.693279
ENSG000002	H3C14	protein_codin	0	0	68.948903	0
ENSG000002	H2AC18	protein_codin	0	0	107.286671	156.006687
ENSG000001	ASCC3	protein_codin	177.031772	138.425369	195.520565	109.381849
ENSG000002	H3C10	protein_codin	130.343419	231.406391	50.5813067	201.996092
ENSG000002	H2BC18	protein_codin	59.0347147	82.2152716	90.8910784	57.1829255
ENSG000001	FCGR1A	protein_codin	185.004837	249.833186	55.1250139	146.92567
ENSG000001	FAM204A	protein_codin	5.32451479	0	0	3.26025094
ENSG000002	FAM204BP	processed_ps	2.06273469	0	9.64269066	1.03678022
ENSG000001	FAM204A	protein_codin	174.161821	134.120733	80.992101	83.432991
ENSG000002	FAM72C	protein_codin	0	5.28819145	0	0
ENSG000001	H2BC2P	transcribed_u	0	0	0	0
ENSG000002	FCGR1BP	lncRNA	0	0	0	0
ENSG000001	FAM204A	protein_codin	103.461694	76.2184201	49.84257	57.3559573
ENSG000001	RAB11FIP2	protein_codin	69.4259052	44.871573	42.1936752	58.448499
ENSG000001	VAX1	protein_codin	0	1.80818976	0.53934393	0
ENSG000001	ENO4	protein_codin	0	0	0	0

ENSG000001	SIM1	protein_codin	0	0	0	0
ENSG000001	HSPA12A	protein_codin	0	0	0	0
ENSG000001	SPMIP5	protein_codin	0	0	0	0
ENSG000001	MCHR2	protein_codin	0	0	0	0
ENSG000001	PRDM13	protein_codin	0	0	0	0
ENSG000001	PRDM13	protein_codin	0	0	0	0
ENSG000001	CCNC	protein_codin	1.15798355	2.55573592	3.24649747	6.17427047
ENSG000001	CCNC	protein_codin	0	0	0	0
ENSG000001	PNLIP	protein_codin	0	0	0	0
ENSG000002	NBPF19	protein_codin	707.180136	278.958833	0	339.342483
ENSG000002	PNLIPRP3	protein_codin	0	0	0	0
ENSG000001	USP45	protein_codin	0	0	0	0
ENSG000001	USP45	protein_codin	7.30409558	10.8965856	0	0
ENSG000001	GFRA1	protein_codin	0	0	0	0
ENSG000001	GJA8	protein_codin	0	0	0	0
ENSG000001	GFRA1	protein_codin	0	0	0	0
ENSG000001	PNISR	protein_codin	0	0	0	198.194468
ENSG000001	COQ3	protein_codin	0	0	0	0
ENSG000001	FBXL4	protein_codin	100.743044	38.9093766	70.1532362	53.4744506
ENSG000001	FHIP2A	protein_codin	0	0	1.39561365	0
ENSG000001	FHIP2A	protein_codin	154.47005	118.663229	74.3668462	117.194593
ENSG000001	FHIP2A	protein_codin	11.2709416	6.16413031	9.63802283	8.26626138
ENSG000001	MMS22L	protein_codin	3.97982354	14.5327736	5.04748217	6.26473189
ENSG000000	ABLIM1	protein_codin	13.8347899	19.756881	0	0
ENSG000001	KLHL32	protein_codin	0	0	0	0
ENSG000000	ABLIM1	protein_codin	1.64637694	36.536488	17.0603735	15.7131778
ENSG000001	CHD1L	protein_codin	0	0	0	0
ENSG000001	CHD1L	protein_codin	79.7239172	0	0	0
ENSG000001	KLHL32	protein_codin	2.53910566	0	0	0
ENSG000001	AFAP1L2	protein_codin	6.45613718	70.5341508	0	0
ENSG000001	FMO5	protein_codin	0	12.8533763	12.109012	0
ENSG000000	UFL1	protein_codin	201.97922	151.485722	173.499918	160.376246
ENSG000000	TDRD1	protein_codin	0	0	0	0
ENSG000000	TDRD1	protein_codin	0	0	0	0
ENSG000001	CCDC186	protein_codin	0	0	0	0
ENSG000001	CCDC186	protein_codin	15.5054301	16.8866868	0	13.5555944
ENSG000001	CCDC186	protein_codin	165.401303	58.8186568	147.728316	100.026986
ENSG000001	CD160	protein_codin	0	0	0	0
ENSG000001	MANEA	protein_codin	0	6.28083646	0	0
ENSG000001	POLR3C	protein_codin	10.1223794	0	0	0
ENSG000000	ADRB1	protein_codin	4.05751896	15.1316209	0.58834561	6.2247589
ENSG000001	EPHA7	protein_codin	0	0	0	0
ENSG000001	PIAS3	protein_codin	0	0	0	0
ENSG000001	PIAS3	protein_codin	0	0	0	0

ENSG000001!	NHLRC2	protein_codin	101.02407	80.4103734	51.1406231	97.3650061
ENSG000001:	EPHA7	protein_codin	0	0	0	0
ENSG000001·	ITGA10	protein_codin	0	0	0	0
ENSG000001!	DCLRE1A	protein_codin	0	24.1196847	0	0
ENSG000001:	PEX11B	protein_codin	12.7963591	32.5856077	11.572497	12.0362036
ENSG000002(	RBM8A	protein_codin	88.2597227	129.463549	35.7593508	102.796326
ENSG000001·	PLEKHS1	protein_codin	0	0	0	0
ENSG000001·	PLEKHS1	protein_codin	1.43494639	0	0	0
ENSG000001·	PLEKHS1	protein_codin	0	0	0	0
ENSG000001:	POLR3GL	protein_codin	3.37974353	23.1854865	31.3709406	8.42443325
ENSG000001:	POLR3GL	protein_codin	135.362185	195.79493	112.550459	95.0991462
ENSG000001(	CASP7	protein_codin	0	0	0	0
ENSG000001(	CASP7	protein_codin	28.1364046	44.3799099	45.2764595	43.3764795
ENSG000001:	MAP3K7	protein_codin	18.3638897	12.6494038	5.2570782	17.3864708
ENSG000001(	CASP7	protein_codin	0	6.95181255	16.5580302	0
ENSG000001:	MAP3K7	protein_codin	0	0	0	0
ENSG000001:	MAP3K7	protein_codin	0	0	0	0
ENSG000001:	MAP3K7	protein_codin	0	0	0	0
ENSG000001(	CASP7	protein_codin	0	0	0	0
ENSG000001:	MAP3K7	protein_codin	43.3433842	33.5568734	26.6348125	48.3266581
ENSG000001	PDE4DIP	protein_codin	0	0	0	0
ENSG000001	PDE4DIP	protein_codin	0	0	0	5.92344246
ENSG000001	PDE4DIP	protein_codin	0	0	0	0
ENSG000001:	GJA10	protein_codin	0	0	0	0
ENSG000001	PDE4DIP	protein_codin	0	0	0	0
ENSG000001	PDE4DIP	protein_codin	0	0	0	0
ENSG000001!	NRAP	protein_codin	0	0	0	0
ENSG000001!	NRAP	protein_codin	0	0	0	0
ENSG000001:	SCAF11	protein_codin	597.567773	291.990465	340.196302	418.951257
ENSG000001(	NBPF20	protein_codin	131.258504	12.7116594	0	0
ENSG000002!	FCGR1BP	lncRNA	0	87.4484055	13.8802638	33.5024904
ENSG000002!	FCGR1BP	lncRNA	84.4981228	0	11.9647449	0
ENSG000002:	H2BC19P	transcribed_u	0	0	0	0
ENSG000001·	TCF7L2	protein_codin	0	0	0	0
ENSG000002:	H3P6	processed_ps	310.856133	462.348073	141.093453	141.836274
ENSG000001·	TCF7L2	protein_codin	0	0	0	0
ENSG000001	FAM72B	protein_codin	0	0	0	5.74787984
ENSG000002:	ENSG000002:	lncRNA	0	0	0	0
ENSG000001	MDN1	protein_codin	245.048883	386.899186	279.259344	160.023225
ENSG000001·	TCF7L2	protein_codin	0	13.2676763	4.89047185	0
ENSG000001·	TCF7L2	protein_codin	0	0	0	0
ENSG000001:	ADAM30	protein_codin	0	0	0	0
ENSG000001:	REG4	protein_codin	0	0	0	0
ENSG000000:	ZDHHC6	protein_codin	0	24.1342612	12.9995847	17.3517144

ENSG000000	ZDHHC6	protein_codin	0	0	0	0
ENSG000001	HMGCS2	protein_codin	0	0	0	0
ENSG000000	PHGDH	protein_codin	0	0	0	0
ENSG000001	ANKRD6	protein_codin	0	0	0	0
ENSG000000	PHGDH	protein_codin	0	0	0	0
ENSG000002	HSD3B1	protein_codin	0	0	0	0
ENSG000000	RRAGD	protein_codin	74.8169137	120.225617	22.6943555	64.6962601
ENSG000002	HSD3B2	protein_codin	0	0	0	0
ENSG000001	TECTB	protein_codin	0	0	0	0
ENSG000001	IL9R	protein_codin	0.98552169	1.94945958	1.74031095	0
ENSG000001	GPAM	protein_codin	0	0	5.9244404	0
ENSG000001	WARS2	protein_codin	0	11.5573161	11.997228	0
ENSG000000	TBX15	protein_codin	0.90820852	0	0	0
ENSG000001	TMLHE	protein_codin	63.7201686	61.5139156	30.2676772	24.490781
ENSG000000	WDR3	protein_codin	0	0	0	0
ENSG000001	GDAP2	protein_codin	93.8936509	33.4778953	47.1202817	41.0749949
ENSG000001	GDAP2	protein_codin	225.504388	97.2071096	103.318653	144.319086
ENSG000001	TENT5C	protein_codin	261.954253	471.170752	455.817071	229.748356
ENSG000001	CLIC2	protein_codin	8.48374367	19.1105831	11.464454	15.4164922
ENSG000001	GABRR1	protein_codin	0	0	0	0
ENSG000001	SHOC2	protein_codin	324.74871	276.196588	162.700273	206.55369
ENSG000001	RAB39B	protein_codin	4.94088305	3.02978386	2.05496489	1.01845023
ENSG000001	VTCN1	protein_codin	0	0	0	0
ENSG000001	BRCC3	protein_codin	6.24940482	10.7204198	0	0
ENSG000001	TRIM45	protein_codin	0	3.42534729	0	0
ENSG000001	BRCC3	protein_codin	0	0	27.1822841	12.8282298
ENSG000001	TRIM45	protein_codin	7.64104309	0	5.18376593	2.02578015
ENSG000001	TTF2	protein_codin	82.8470371	69.4029727	47.7810822	39.5462678
ENSG000001	CD101	protein_codin	0	0	0	0
ENSG000001	PNRC1	protein_codin	2.77528576	32.7957124	6.02198243	35.6288068
ENSG000001	RNGTT	protein_codin	0	0	15.6608884	34.6962532
ENSG000002	MTCP1	protein_codin	0	5.41097075	0	0
ENSG000001	CD2	protein_codin	0	0	0	0
ENSG000001	CD2	protein_codin	10.7555129	9.99181151	0	3.02762231
ENSG000001	CMC4	protein_codin	0	0	1.29508665	0
ENSG000001	IGSF3	protein_codin	0	0	0	0
ENSG000001	CMC4	protein_codin	13.4902056	18.1830221	3.98989804	9.57723517
ENSG000001	RNGTT	protein_codin	103.297267	26.423839	35.5623058	48.0768487
ENSG000001	IGSF3	protein_codin	0	1.87541166	0	0
ENSG000001	CD58	protein_codin	15.2904107	18.1845788	2.43995888	7.76346446
ENSG000001	CD58	protein_codin	130.240372	107.835123	44.6276485	71.6834757
ENSG000002	ATP1A1-AS1	lncRNA	0	0	0	0
ENSG000002	ATP1A1-AS1	lncRNA	0	0	0	0
ENSG000001	ATP1A1	protein_codin	40.1421208	21.9642522	35.2781097	37.0780802

ENSG000001	ATP1A1	protein_codin	205.676795	0	139.122594	50.2609155
ENSG000001	FUNDC2	protein_codin	16.627808	28.8355229	12.3651952	14.3288496
ENSG000001	CNR1	protein_codin	0	0	0	0
ENSG000001	MAB21L3	protein_codin	35.7902714	0	0	0
ENSG000001	CNR1	protein_codin	0	0	0	0
ENSG000001	SLC22A15	protein_codin	17.2240785	11.6681362	4.47200778	2.74608902
ENSG000001	SLC22A15	protein_codin	122.276646	145.83172	73.704716	99.1624459
ENSG000002	F8A2	protein_codin	16.2379151	41.2820039	0	4.05524061
ENSG000001	NHLH2	protein_codin	0	0	0	0
ENSG000001	VANGL1	protein_codin	0	0	0	0
ENSG000001	VANGL1	protein_codin	0	0	0	17.2994156
ENSG000001	NGF	protein_codin	0	0	0	0
ENSG000001	TSPAN2	protein_codin	0	0	0	0
ENSG000001	TSPAN2	protein_codin	35.7312119	40.8436571	23.5236634	23.7673898
ENSG000001	SYCP1	protein_codin	0	0	0	0
ENSG000002	RBM20	protein_codin	1.77886155	0	0.96485215	0
ENSG000001	SYCP1	protein_codin	0	0	0	0
ENSG000000	SIKE1	protein_codin	0	0	0	1.75492504
ENSG000000	SIKE1	protein_codin	0	10.3059303	0	0
ENSG000002	SMIM9	protein_codin	0	0	0	0
ENSG000000	CSDE1	protein_codin	67.1103713	41.368604	0	28.0626657
ENSG000001	MPP1	protein_codin	1.42382218	10.536534	0	0
ENSG000001	MPP1	protein_codin	598.867208	1337.71876	289.803209	555.963955
ENSG000002	NRAS	protein_codin	353.052105	175.476495	201.664066	220.634637
ENSG000001	RARS2	protein_codin	0	0	0	0
ENSG000001	AMPD1	protein_codin	0	0	0	0
ENSG000001	BCAS2	protein_codin	47.2803293	32.9839657	36.7834928	39.3568106
ENSG000001	TRIM33	protein_codin	56.9414031	122.161438	60.5580313	52.4248393
ENSG000001	SYT6	protein_codin	0	0	0	0
ENSG000001	DKC1	protein_codin	0	0	0	0
ENSG000001	OLFML3	protein_codin	0	0	0	0
ENSG000001	SLC35A1	protein_codin	48.6352775	50.0636016	26.818414	44.7880124
ENSG000001	HIPK1	protein_codin	4.52781628	0	0	0
ENSG000001	HIPK1	protein_codin	409.402326	222.273362	192.97352	303.411555
ENSG000001	SLC35A1	protein_codin	0	0	14.5459354	0
ENSG000001	SLC35A1	protein_codin	11.2490787	12.4420863	0	22.9061489
ENSG000001	HIPK1	protein_codin	908.739711	714.997429	322.98327	487.360808
ENSG000001	HIPK1	protein_codin	0	177.546645	0	0
ENSG000001	HIPK1	protein_codin	56.9220629	12.0908112	50.286998	0
ENSG000002	CFAP206	protein_codin	0	0	0	0
ENSG000001	AP4B1	protein_codin	0	20.5940423	0	0
ENSG000001	AP4B1	protein_codin	0	0	0	0
ENSG000001	GAB3	protein_codin	7.11955448	25.673694	0	0
ENSG000001	AP4B1	protein_codin	0	0	0	0

ENSG000001	SMIM8	protein_codin	2.10548776	0	5.67770324	0
ENSG000001	AP4B1	protein_codin	48.2448544	0	0	0
ENSG000001	SMIM8	protein_codin	0	0	0	0
ENSG000002	C6orf163	protein_codin	0	0	0	2.05295302
ENSG000001	GAB3	protein_codin	163.641895	70.8274478	0	13.1230252
ENSG000001	GJB7	protein_codin	0	0	0	0
ENSG000001	ZNF292	protein_codin	204.996203	104.390247	74.2551157	82.7708185
ENSG000001	ZNF292	protein_codin	22.0335549	14.734756	9.78237517	21.8000204
ENSG000001	BCL2L15	protein_codin	1.30144188	4.20962827	1.07663079	0
ENSG000000	RSBN1	protein_codin	0	0	0	0
ENSG000001	CGA	protein_codin	0	0	0	0
ENSG000001	DUSP5	protein_codin	208.889282	149.866085	178.606216	120.417436
ENSG000001	CTAG2	protein_codin	0	0	0	0
ENSG000001	SMIM11P1	processed_ps	0	0	0	0
ENSG000001	SMNDC1	protein_codin	77.2337058	142.188265	90.5183096	40.2540021
ENSG000001	PHTF1	protein_codin	0	13.039411	0	0
ENSG000001	PHTF1	protein_codin	20.6352004	0	6.46141021	0
ENSG000001	SMNDC1	protein_codin	102.213095	60.5366841	35.6854227	37.1949577
ENSG000001	PHTF1	protein_codin	0	0	0	0
ENSG000002	SNHG5	lncRNA	0	0	0	20.9381907
ENSG000000	MAGI3	protein_codin	0	0	0	0
ENSG000001	MXI1	protein_codin	0	0	0	0
ENSG000000	MAGI3	protein_codin	16.1406447	0	14.4137806	4.17693057
ENSG000000	MAGI3	protein_codin	0	0	0	0
ENSG000001	G6PD	protein_codin	84.6620237	47.9367034	0	38.1686185
ENSG000001	SYNCRIP	protein_codin	0	0	0	0
ENSG000001	SLC16A1	protein_codin	0	0	0	0
ENSG000001	SNX14	protein_codin	0	0	0	62.8162046
ENSG000001	TAFA3	protein_codin	0.89731396	0	0	0
ENSG000001	RHOC	protein_codin	0	0	0	0
ENSG000001	RHOC	protein_codin	0	0	0	0
ENSG000001	SNX14	protein_codin	0	8.68260732	0	7.71603192
ENSG000001	RHOC	protein_codin	10.3368491	20.0607137	0	0
ENSG000001	RHOC	protein_codin	0	0	0	0
ENSG000001	RHOC	protein_codin	0	0	0	0
ENSG000000	FAM3A	protein_codin	0	0	0	0
ENSG000001	RHOC	protein_codin	20.5100958	21.846832	12.6546362	0
ENSG000000	FAM3A	protein_codin	0	0	0	0
ENSG000001	MOV10	protein_codin	23.0798136	0	0	0
ENSG000001	MOV10	protein_codin	59.1820669	61.4980011	25.1191958	0
ENSG000001	NT5E	protein_codin	0	0	0	0
ENSG000001	SLC10A3	protein_codin	0	80.6567338	9.32248708	23.5437649
ENSG000001	NT5E	protein_codin	0	0	0	0
ENSG000001	UBL4A	protein_codin	0	0	0	0

ENSG000002	ADD3-AS1	lncRNA	0	0	0	0
ENSG000002	ADD3-AS1	lncRNA	1.12150301	0	0	0
ENSG000001	XPNPEP1	protein_codin	0	0	0	0
ENSG000001	UBL4A	protein_codin	110.121294	254.67972	96.7817369	92.8877041
ENSG000001	TBX18	protein_codin	0	0	0	0
ENSG000000	ST7L	protein_codin	0	4.12140964	0	0
ENSG000000	ST7L	protein_codin	0	0	0	0
ENSG000000	ST7L	protein_codin	2.47504564	0	5.31379979	2.36512732
ENSG000000	CYB5R4	protein_codin	82.0381287	65.0861862	29.4162297	74.7768778
ENSG000000	CYB5R4	protein_codin	239.464466	242.81938	161.87617	185.523922
ENSG000001	PLXNA3	protein_codin	47.7758982	35.5837115	21.4349895	6.14864751
ENSG000001	XPNPEP1	protein_codin	0	0	0	0
ENSG000001	WNT2B	protein_codin	0	0	0	0
ENSG000001	WNT2B	protein_codin	6.27350384	3.20133204	4.84335112	4.05263519
ENSG000002	RIPPLY2	protein_codin	0	0	0	0
ENSG000002	RIPPLY2	protein_codin	0	0	0	0
ENSG000000	SNAP91	protein_codin	0	0	0	0
ENSG000000	SNAP91	protein_codin	0	0	0	0
ENSG000000	SNAP91	protein_codin	0	0	0	0
ENSG000001	KCND3	protein_codin	0	0	0	28.3595285
ENSG000001	SORCS1	protein_codin	0	0	0	0
ENSG000001	PRSS35	protein_codin	0	0	0	0
ENSG000001	SORCS3	protein_codin	0	0	0	0
ENSG000000	DDX20	protein_codin	0	0	0	0
ENSG000001	CFAP58	protein_codin	0	0	0	0
ENSG000001	CFAP58	protein_codin	8.74831853	6.87426339	7.09772346	2.01568171
ENSG000000	ME1	protein_codin	37.2850706	35.4212909	20.4009931	16.6900578
ENSG000000	GSTO2	protein_codin	2.33264131	0	0	0
ENSG000001	RAP1A	protein_codin	131.710885	74.9842331	63.7976915	116.439751
ENSG000001	GSTO1	protein_codin	7.40084851	11.3599899	3.92055685	0
ENSG000001	GSTO1	protein_codin	262.394646	425.980648	143.216082	174.956268
ENSG000001	TMIGD3	protein_codin	0	0	0	2.9012
ENSG000001	TMIGD3	protein_codin	0	5.69278256	0	0
ENSG000001	C1orf162	protein_codin	376.44115	587.81133	173.623213	363.905378
ENSG000001	CFAP43	protein_codin	0	0	0	0
ENSG000001	CFAP43	protein_codin	0	0	0	0
ENSG000001	ATP5PB	protein_codin	219.59782	270.024286	63.7561697	150.374372
ENSG000001	ATP5PB	protein_codin	41.635813	11.5933948	2.94107562	0
ENSG000000	RWDD2A	protein_codin	0.97905195	11.8013895	6.47023667	6.05692377
ENSG000001	SFR1	protein_codin	10.9794274	0	0	0
ENSG000001	SFR1	protein_codin	19.5475102	19.9089887	11.1362985	26.1494171
ENSG000000	OVGP1	protein_codin	5.84767107	6.89109848	2.29456521	3.01305234
ENSG000000	COL17A1	protein_codin	0	0	0	0
ENSG000001	CIMAP3	protein_codin	0	0	0	0

ENSG000001	CIMAP3	protein_codin	1.16231459	0	0	1.1554503
ENSG000000	DOP1A	protein_codin	0	0	0	0
ENSG000001	CHIA	protein_codin	0	0	0	0
ENSG000002	CHIAP2	lncRNA	0	0	0	0
ENSG000000	CHI3L2	protein_codin	0	0	0	0
ENSG000001	UBE3D	protein_codin	0	1.35106802	3.98233441	2.00732275
ENSG000001	UBE3D	protein_codin	0	0	0	0
ENSG000000	CHI3L2	protein_codin	0	0	0	0
ENSG000001	TPBG	protein_codin	0	9.02313507	0	0
ENSG000000	IBTK	protein_codin	0	97.4168209	0	49.9928423
ENSG000001	DENND2D	protein_codin	9.4880417	45.7974872	0	10.5529733
ENSG000001	TENT5A	protein_codin	18.6840903	0	0	0
ENSG000000	SLK	protein_codin	182.589376	27.5129291	52.0559784	65.02773
ENSG000001	TENT5A	protein_codin	0	36.9855112	0	51.32025
ENSG000002	PCMTD2	protein_codin	0	45.6392497	29.5339463	19.0796616
ENSG000000	BCKDHB	protein_codin	0	0	2.05943996	0
ENSG000000	ATP6AP1	protein_codin	139.25693	268.295121	51.2532736	116.24623
ENSG000001	LRIF1	protein_codin	21.6228836	10.5530516	3.6050131	7.22829048
ENSG000001	STN1	protein_codin	0	10.4287163	0	17.1996251
ENSG000001	NPBWR2	protein_codin	0	0	0	0
ENSG000001	KCNA3	protein_codin	0	0	0	0
ENSG000001	KCNA2	protein_codin	0	0	0	0
ENSG000001	KCNA10	protein_codin	0	0	0	0
ENSG000001	SH3PXD2A	protein_codin	42.80285	102.454524	61.9494262	25.4208846
ENSG000001	TAFAZZIN	protein_codin	2.47735809	0	0	0
ENSG000001	SLC16A4	protein_codin	0	0	0	0
ENSG000001	NEURL1	protein_codin	9.47700392	27.9921878	3.22648297	8.21146662
ENSG000001	SLC16A4	protein_codin	0	0	0	0
ENSG000001	CALHM3	protein_codin	0	0	0	0
ENSG000001	RBM15	protein_codin	16.2281527	30.1000184	0	0
ENSG000001	KCNC4	protein_codin	20.7739449	10.338746	8.27459464	14.0045233
ENSG000001	CALHM2	protein_codin	61.962488	136.785739	8.26577572	65.4968085
ENSG000001	STRIP1	protein_codin	125.803617	140.7467	57.0054312	62.9348432
ENSG000001	STRIP1	protein_codin	0	0	0	0
ENSG000001	PDCD11	protein_codin	132.193388	200.942351	127.179803	118.761171
ENSG000001	TTK	protein_codin	5.43293811	0	11.4959493	2.91641971
ENSG000001	AHCYL1	protein_codin	312.474122	519.183986	166.355261	236.808371
ENSG000002	NEURL1B	protein_codin	12.9032933	8.74487625	14.3527327	2.90719292
ENSG000001	CSF1	protein_codin	0	0	0	0
ENSG000001	CSF1	protein_codin	0	0	6.68085247	5.56824447
ENSG000001	EPS8L3	protein_codin	0	0	0	0
ENSG000000	DNASE1L1	protein_codin	48.3330885	87.4145894	0	22.5626048
ENSG000000	DNASE1L1	protein_codin	57.8384995	96.7098553	48.8431269	44.7612167
ENSG000000	DNASE1L1	protein_codin	0	0	0	10.9213327

ENSG000001	ATP5MK	protein_codin	6.15231075	0	0	0	
ENSG000001	GSTM5	protein_codin	0	0	0	0	
ENSG000001	GSTM5	protein_codin	0	0	1.50611662	0	
ENSG000001	ATP5MK	protein_codin	0	9.2018823	3.19030355	3.56165126	
ENSG000001	ELOVL4	protein_codin	0	0	0	0	
ENSG000001	RPL10	protein_codin	0	0	0	0	
ENSG000001	GSTM1	protein_codin	0	0	0	0	
ENSG000001	GSTM1	protein_codin	2.16127855	0	0	0	
ENSG000001	ATP5MK	protein_codin	0	9.27531474	2.48377727	11.0560889	
ENSG000002	GSTM2	protein_codin	9.6878603	7.21930636	0	12.5913923	
ENSG000002	GSTM2	protein_codin	0	0	0	0	
ENSG000002	GSTM2	protein_codin	0	0	0	0	
ENSG000001	GSTM4	protein_codin	0	3.80175364	0	0	
ENSG000001	EMD	protein_codin	11.4028724	6.66254546	1.8372409	0	
ENSG000001	GSTM4	protein_codin	10.185213	16.7417571	0	9.87080386	
ENSG000001	SH3BGRL2	protein_codin	974.079274	1411.2179	1653.36584	692.95074	
ENSG000001	TAF5	protein_codin	0	0	0	0	
ENSG000001	AMPD2	protein_codin	0	15.6829248	0	7.45462353	
ENSG000001	EMD	protein_codin	47.7755351	96.6071219	21.4784294	46.1691298	
ENSG000002	ENSG000002	lncRNA	0	0	0	0	
ENSG000001	LCA5	protein_codin	10.6292168	1.96658446	19.728918	0	
ENSG000001	PCGF6	protein_codin	16.0123596	14.5890469	8.30283874	18.0800895	
ENSG000001	INA	protein_codin	0	2.09041959	0.73613553	1.05672795	
ENSG000001	FLNA	protein_codin	1376.02267	2767.12943	1872.79453	1077.63863	
ENSG000000	GNAI3	protein_codin	614.786907	446.975374	275.292345	421.795553	
ENSG000001	FLNA	protein_codin	36.0996164	409.657714	83.6030026	34.1807027	
ENSG000000	NT5C2	protein_codin	0	0	0	5.86201947	
ENSG000001	AMIGO1	protein_codin	0	0	0	0	
ENSG000001	AMIGO1	protein_codin	0	1.93765743	1.01442763	0.96758907	
ENSG000000	MPHOSPH9	protein_codin	0	0	0	0	
ENSG000001	CYB561D1	protein_codin	0	0	0	0	
ENSG000001	ATXN7L2	protein_codin	4.55112573	0	0	0	
ENSG000001	ATXN7L2	protein_codin	0	12.5443784	0	0	
ENSG000001	SYPL2	protein_codin	0	0	0.81470383	0	
ENSG000001	CNNM2	protein_codin	12.7670522	46.1328295	20.7918847	18.3613292	
ENSG000001	CNNM2	protein_codin	46.6077982	40.066654	26.211137	20.7159314	
ENSG000002	AS3MT	protein_codin	0	0	0	0	
ENSG000001	BORCS7	protein_codin	23.4920479	15.8520756	0	10.7215667	
ENSG000001	WBP1L	protein_codin	0	0.839857	1.75459803	0	
ENSG000001	SAMD10	protein_codin	0	0	2.00342193	1.86850628	
ENSG000001	CYP17A1	protein_codin	0	0	0	0	
ENSG000001	ZNF512B	protein_codin	28.8117749	37.0698113	21.3967239	18.8971407	
ENSG000001	WBP1L	protein_codin	21.0945206	0	0	0	
ENSG000001	SFXN2	protein_codin	6.10573586	10.0806524	8.11607967	0	

ENSG000001	SUFU	protein_codin	2.02870025	10.4135057	0	0
ENSG000001	SUFU	protein_codin	91.1612928	158.508337	40.5415086	68.7318993
ENSG000001	PSRC1	protein_codin	0	0	17.3284953	0
ENSG000001	PSRC1	protein_codin	11.1759212	0	0	0
ENSG000001	ACTR1A	protein_codin	218.529583	384.722423	153.021501	141.308871
ENSG000001	PSRC1	protein_codin	0	10.9144686	0	4.44236043
ENSG000001	UCKL1	protein_codin	8.72983168	0	0	0
ENSG000001	PSRC1	protein_codin	18.0110432	33.245844	0	9.55021914
ENSG000000	TKTL1	protein_codin	0	0	0	0
ENSG000000	TKTL1	protein_codin	1.05007814	0	0	0
ENSG000001	ABHD16B	protein_codin	0.94389723	0	0.75499947	0
ENSG000000	SARS1	protein_codin	0	0	0	0
ENSG000001	TPD52L2	protein_codin	26.248571	22.905819	19.9317275	12.7235209
ENSG000001	OPN1MW2	protein_codin	0	0	0	0
ENSG000001	MFSD13A	protein_codin	0	0	0	0
ENSG000001	ELAPOR1	protein_codin	0	0	4.03008472	6.98007918
ENSG000001	CUEDC2	protein_codin	107.64261	109.137744	74.8619188	68.6173923
ENSG000001	ELAPOR1	protein_codin	0	0	0	0
ENSG000001	ELAPOR1	protein_codin	7.41687796	8.7772951	0	0
ENSG000001	IRAK1BP1	protein_codin	2.30623409	0	1.16779954	1.16941652
ENSG000001	CFAP276	protein_codin	0	0	0	0
ENSG000001	CFAP276	protein_codin	0	0	0	0
ENSG000001	HTR1B	protein_codin	0	0	0	0
ENSG000001	CFAP276	protein_codin	0	0	0	0
ENSG000001	CFAP276	protein_codin	0	0	0	0
ENSG000001	IMPG1	protein_codin	0	0	0	0
ENSG000001	OPN1LW	protein_codin	0	0	0	0
ENSG000001	IMPG1	protein_codin	0	0	0	0
ENSG000001	FBXL15	protein_codin	33.8848344	33.5882085	6.27575948	28.23201
ENSG000001	MECP2	protein_codin	0	1.86071934	2.70681463	0
ENSG000000	WDR47	protein_codin	0	14.3764682	40.0801563	0
ENSG000001	IMPG1	protein_codin	0	0	0	0
ENSG000000	WDR47	protein_codin	68.0429254	57.6120567	0	18.3991595
ENSG000000	NFKB2	protein_codin	453.985397	576.470459	297.131577	263.384866
ENSG000001	ZGPAT	protein_codin	0	145.362605	0	0
ENSG000001	CLCC1	protein_codin	0	0	0	0
ENSG000001	CLCC1	protein_codin	0	0	0	0
ENSG000001	CLCC1	protein_codin	0	0	0	0
ENSG000001	IRAK1	protein_codin	0	0	0	3.39022447
ENSG000001	IRAK1	protein_codin	0	0	0	0
ENSG000001	MYO6	protein_codin	0	0	0	0
ENSG000001	CLCC1	protein_codin	0	9.79062403	0	0
ENSG000001	MYO6	protein_codin	0	0	0	0
ENSG000001	IRAK1	protein_codin	307.651387	533.431245	174.573813	217.756674

ENSG000001	TMEM187	protein_codin	8.98685929	6.88905591	5.02519799	3.97265187
ENSG000001	GBF1	protein_codin	46.0095594	0	0	0
ENSG000001	HCFC1	protein_codin	109.76816	196.455595	152.568235	64.8877533
ENSG000001	MYO6	protein_codin	0	0	7.57752257	0
ENSG000002	SPATA42	lncRNA	0	0	0	0
ENSG000001	AKNAD1	protein_codin	0	0	0	0
ENSG000001	AKNAD1	protein_codin	0	0	0	0
ENSG000002	TNFRSF6B	protein_codin	0	0	0	0
ENSG000001	RENBP	protein_codin	12.0791275	56.0263287	0	21.5028465
ENSG000001	AKNAD1	protein_codin	0	0	0	0
ENSG000001	PITX3	protein_codin	0	0	0	0
ENSG000002	RTEL1	protein_codin	0	0	0	0
ENSG000001	ELOVL3	protein_codin	3.86976471	3.88624752	0	0.97923905
ENSG000001	NOLC1	protein_codin	111.247745	0	99.666237	72.0028375
ENSG000001	STXBP3	protein_codin	190.579803	159.9062	118.12248	155.828117
ENSG000001	NAA10	protein_codin	9.18334249	36.6126997	0	33.1448048
ENSG000001	SENP6	protein_codin	131.113814	73.7664349	0	77.1558084
ENSG000001	NAA10	protein_codin	0	0	0	0
ENSG000001	PPRC1	protein_codin	0	0	0	0
ENSG000001	NAA10	protein_codin	0	3.99940367	0	0
ENSG000000	ARHGAP4	protein_codin	19.5033499	13.1473405	0	0
ENSG000001	FNDC7	protein_codin	0	0	0	0
ENSG000002	RTEL1	protein_codin	0	0	0	0
ENSG000001	FILIP1	protein_codin	0	0	0	0
ENSG000001	PRPF38B	protein_codin	528.980144	495.362995	280.947109	395.250245
ENSG000001	PRPF38B	protein_codin	0	0	0	0
ENSG000001	PRPF38B	protein_codin	193.447521	192.852502	166.425234	171.454443
ENSG000000	ARHGAP4	protein_codin	39.1652901	61.8563061	21.7081327	39.3275139
ENSG000001	HENMT1	protein_codin	1.48012013	0	0	0
ENSG000001	HENMT1	protein_codin	8.55791997	0	0	15.1367124
ENSG000001	ARMH3	protein_codin	162.037277	247.53075	73.1655958	108.841915
ENSG000001	EEIG2	protein_codin	295.180816	118.670025	147.280066	139.556403
ENSG000001	NBPF6	protein_codin	0	0	0	0
ENSG000000	SLC25A24	protein_codin	0	2.95518767	1.86557673	0
ENSG000001	KCNIP2	protein_codin	0	0	0	0
ENSG000001	AVPR2	protein_codin	0	0	0	0
ENSG000001	TMEM30A	protein_codin	8.7465098	35.7733632	16.4155107	4.67503192
ENSG000001	STMN3	protein_codin	17.743001	12.7063732	11.3722791	0
ENSG000001	L1CAM	protein_codin	1.01945639	0	0	0
ENSG000001	VAV3	protein_codin	113.171899	118.007156	95.5176977	167.350322
ENSG000001	L1CAM	protein_codin	0	0	0	0
ENSG000001	L1CAM	protein_codin	0	0	0	0
ENSG000001	NTNG1	protein_codin	0	0	0	0
ENSG000001	NTNG1	protein_codin	0	0	0	0

ENSG000001(	NTNG1	protein_codin	0	0	0	0
ENSG000001(	NTNG1	protein_codin	0	0	0	0
ENSG000001(	GMEB2	protein_codin	35.2390513	0	0	9.51046446
ENSG000001(	NTNG1	protein_codin	0	0	0	0
ENSG000001(	NTNG1	protein_codin	0	0	0	0
ENSG000001(	NTNG1	protein_codin	0	0	0	0
ENSG000001(	GMEB2	protein_codin	44.691377	82.6568073	48.7401764	43.6460446
ENSG000001!	PRMT6	protein_codin	17.9309381	22.3620272	8.14944036	16.7864208
ENSG000001	AMY1C	protein_codin	0	0	0	0
ENSG000001	AMY1B	protein_codin	0	0	0	0
ENSG000001	COX7A2	protein_codin	0	0	0	0
ENSG000001	HELZ2	protein_codin	0	0	0	0
ENSG000002	AMY1A	protein_codin	0	0	0	0
ENSG000001	SSR4	protein_codin	0	16.8246425	4.40394635	0
ENSG000001	SSR4	protein_codin	105.733316	172.650704	52.9440838	102.183236
ENSG000001	SSR4	protein_codin	0	9.12265295	0	0
ENSG000001	COX7A2	protein_codin	8.57852615	11.7685387	2.62915666	3.79855352
ENSG000000(	IDH3G	protein_codin	36.7946932	16.5508791	8.45619879	16.7228407
ENSG000000(	IDH3G	protein_codin	0	15.8690938	0	0
ENSG000001!	OGA	protein_codin	256.546705	223.601257	123.482746	168.786886
ENSG000000(	COL11A1	protein_codin	0	0	0	0
ENSG000001	FNDC11	protein_codin	0	1.58384465	0	0
ENSG000001	FNDC11	protein_codin	0	0	0	0
ENSG000001	SRPK3	protein_codin	0	0	0	0
ENSG000001	SRPK3	protein_codin	0	0	0	1.53031873
ENSG000001	OLFM3	protein_codin	1.47514982	0	0	0
ENSG000001	SRPK3	protein_codin	0	0	0	0
ENSG000001	DPH5	protein_codin	0	0	0	0
ENSG000001	SRPK3	protein_codin	0	0	0	0
ENSG000001	DPH5	protein_codin	8.83824424	0	0	0
ENSG000001(	NPM3	protein_codin	4.9089786	5.85483748	8.3606461	1.84880274
ENSG000001(	SLC30A7	protein_codin	5.14835692	0	0	0
ENSG000001(	SLC30A7	protein_codin	0	0	0	16.2063131
ENSG000001(	EXTL2	protein_codin	0	0	0	0
ENSG000001(	EXTL2	protein_codin	2.39056283	3.56727339	5.7476648	1.65191545
ENSG000001(	VCAM1	protein_codin	0	0	0	0
ENSG000001(	VCAM1	protein_codin	0	0	0	0
ENSG000000	CDC14A	protein_codin	0	10.0648295	2.54672699	7.49300469
ENSG000001	RTCA	protein_codin	0	0.98643955	0	0
ENSG000001	RTCA	protein_codin	49.3859711	78.0547656	41.8709588	55.9091723
ENSG000001(	ABCD1	protein_codin	8.4372077	4.29689409	1.17675049	4.58987171
ENSG000001	DBT	protein_codin	9.25502044	6.78512371	0	7.99416836
ENSG000001	DBT	protein_codin	17.7699188	25.1878226	15.5674726	17.0843008
ENSG000001	LRRC39	protein_codin	0	0	7.03372163	0

ENSG000001	LRRC39	protein_codin	7.04037464	0	0	2.99350148
ENSG000001	TRMT13	protein_codin	0	1.99269682	0	0
ENSG000001	TRMT13	protein_codin	24.9065633	35.452628	28.3719812	23.1498729
ENSG000001	PNCK	protein_codin	0	0	0	0
ENSG000001	TRMT13	protein_codin	3.74627533	0	0	0
ENSG000001	PNCK	protein_codin	0	0	0	0
ENSG000001	DPCD	protein_codin	0	0	0	0
ENSG000001	PNCK	protein_codin	0	0	0	0
ENSG000001	DPCD	protein_codin	4.76260302	13.0775529	5.84871174	4.72530625
ENSG000001	MFSD14A	protein_codin	235.757576	236.405828	96.8358011	221.040756
ENSG000001	SLC35A3	protein_codin	0	18.3912189	0	0
ENSG000001	SLC35A3	protein_codin	0	13.9151685	0	0
ENSG000001	AGL	protein_codin	0	0	0	0
ENSG000001	POLL	protein_codin	13.012134	0	7.72740138	0
ENSG000001	AGL	protein_codin	0	0	0	0
ENSG000001	AGL	protein_codin	0	0	0	0
ENSG000001	DUSP9	protein_codin	0	0	0	0
ENSG000001	POLL	protein_codin	0	19.4074839	0	9.61866453
ENSG000001	POLL	protein_codin	0	0	0	0
ENSG000001	POLL	protein_codin	0	0	0	0
ENSG000001	FRRS1	protein_codin	0	0	0	0
ENSG000001	PPDPF	protein_codin	12.1591258	23.6558929	17.4277449	7.73391142
ENSG000001	PPDPF	protein_codin	205.994239	541.736485	254.536054	273.376407
ENSG000001	BTRC	protein_codin	5.78324055	4.18543454	3.8380185	0
ENSG000001	PLPPR4	protein_codin	0	0	0	0
ENSG000001	PLPPR4	protein_codin	0	0	0	0
ENSG000000	ATP2B3	protein_codin	0	0	0	0
ENSG000001	BTRC	protein_codin	45.7652009	72.8616357	36.3776203	40.4646112
ENSG000001	PLPPR5	protein_codin	0	0	0	0
ENSG000001	DPYD	protein_codin	1717.63852	1071.08443	709.693202	1207.20451
ENSG000001	LBX1	protein_codin	0.71459951	0	0	0
ENSG000001	TLX1	protein_codin	0	2.27704853	0	0
ENSG000001	PTBP2	protein_codin	0	0	16.9727172	0
ENSG000001	PTBP2	protein_codin	0	0	0	0
ENSG000001	KAZALD1	protein_codin	0	0	0	0
ENSG000001	RWDD3	protein_codin	12.2316111	10.2978394	22.833678	12.4486704
ENSG000001	TLCD4	protein_codin	0	24.0116056	1.90992361	1.11160602
ENSG000001	ALG14	protein_codin	3.13977113	4.12728621	4.74887602	8.1957969
ENSG000001	CNN3	protein_codin	0	0	0	0
ENSG000001	F3	protein_codin	19.6088986	0	0	0
ENSG000002	HAUS7	protein_codin	0	5.8890368	0.99947804	0
ENSG000002	HAUS7	protein_codin	18.9762731	36.0662669	19.8123344	19.3131226
ENSG000001	TREX2	protein_codin	0	0	0	0
ENSG000001	ABCD3	protein_codin	85.5417077	0	52.0849897	45.460731

ENSG000001	PDZD7	protein_codin	4.45292163	0	0	0
ENSG000001	ARHGAP29	protein_codin	0	2.35974633	0	5.77774822
ENSG000001	LZTS2	protein_codin	10.2565797	17.1596644	7.41761215	3.90146658
ENSG000000	KCNQ2	protein_codin	0	0	0	0
ENSG000001	LZTS2	protein_codin	18.8365054	0	0	0
ENSG000000	KCNQ2	protein_codin	0	0	0	0
ENSG000001	ABCA4	protein_codin	0	0	0	0
ENSG000001	TWNK	protein_codin	0	13.2777664	0	0
ENSG000001	TREX2	protein_codin	0	0	0	0
ENSG000001	TREX2	protein_codin	1.36159635	0	0	0
ENSG000000	MRPL43	protein_codin	0	0	1.571857	0
ENSG000000	MRPL43	protein_codin	0	0	1.61535065	0
ENSG000000	GCLM	protein_codin	61.1242369	53.2938365	49.0515277	45.4409171
ENSG000000	MRPL43	protein_codin	0	0	0	0
ENSG000000	MRPL43	protein_codin	0	0	0	0
ENSG000001	BCAR3	protein_codin	0	0	0	0
ENSG000001	BCAR3	protein_codin	2.46673739	0	11.8117308	0
ENSG000001	BCAR3	protein_codin	0	0	0	0
ENSG000000	ZNF275	protein_codin	29.0599298	41.0913682	0	0
ENSG000000	SEMA4G	protein_codin	1.87238803	0	0	0
ENSG000000	ZNF275	protein_codin	0	0	34.3502001	23.8555258
ENSG000001	FNBP1L	protein_codin	0	0	34.5361747	0
ENSG000002	ENSG000002	lncRNA	0	0	0	0
ENSG000001	CHRNA4	protein_codin	0	0	0	0
ENSG000001	DR1	protein_codin	84.2671253	137.896635	24.4040197	62.2852505
ENSG000001	ZNF185	protein_codin	0	0	0	23.1146914
ENSG000001	SLF2	protein_codin	198.545974	179.268202	111.607921	126.976118
ENSG000001	SLF2	protein_codin	0	0	0	0
ENSG000001	DR1	protein_codin	282.267131	266.17916	162.722974	229.393653
ENSG000001	NSDHL	protein_codin	2.73680611	32.3734209	2.48024131	5.91099365
ENSG000001	ARFGAP1	protein_codin	0	0	0	0
ENSG000001	CCDC18	protein_codin	0	0	0	0
ENSG000001	CETN2	protein_codin	59.0622425	109.176224	57.6496268	52.5835332
ENSG000002	MAGEA3	protein_codin	0	0	0	0
ENSG000001	TMED5	protein_codin	231.327051	202.208044	243.649367	206.242156
ENSG000001	TMED5	protein_codin	297.688509	148.398846	150.770958	222.490438
ENSG000001	ARFGAP1	protein_codin	87.4993224	128.893524	31.6830601	0
ENSG000001	CSAG1	protein_codin	0	0	0	0
ENSG000001	TMED5	protein_codin	144.019182	157.32573	132.202859	131.961758
ENSG000001	CSAG1	protein_codin	0	0	0	0
ENSG000001	MAGEA2B	protein_codin	0	0	0	0
ENSG000000	PAX2	protein_codin	0	0	0	0
ENSG000001	MTF2	protein_codin	81.5394878	0	0	64.3537249
ENSG000001	MTO1	protein_codin	0	0	0	0

ENSG000001	MTF2	protein_codin	26.3212954	33.2281228	48.9294443	41.0553003
ENSG000001	MTO1	protein_codin	7.46192089	26.8617047	26.3127215	7.80779482
ENSG000001	NKAIN4	protein_codin	0	0	0	0
ENSG000001	MTO1	protein_codin	0	18.9976889	0	30.1990836
ENSG000001	DIPK1A	protein_codin	5.94846653	0	12.2834839	0
ENSG000001	NKAIN4	protein_codin	0	0	0	0
ENSG000000	GABRA3	protein_codin	0	1.99239353	0	0
ENSG000001	CGAS	protein_codin	40.0173894	80.0371898	33.5170384	30.317712
ENSG000001	NKAIN4	protein_codin	0	0	0	0
ENSG000001	NKAIN4	protein_codin	0	0	0	0
ENSG000001	CGAS	protein_codin	0	5.21965392	0	0
ENSG000001	NDUFB8	protein_codin	0	1.77747488	0	0
ENSG000001	RPL5	protein_codin	3205.54124	5483.47818	3207.15501	2922.15829
ENSG000001	NDUFB8	protein_codin	4.14389662	1.30419926	0	0
ENSG000001	MAGEA10	protein_codin	0	0	0	0
ENSG000001	GABRE	protein_codin	0	0	0	0
ENSG000000	EVI5	protein_codin	115.499993	10.7608296	39.6969466	131.092305
ENSG000001	GFI1	protein_codin	0	0	0	0
ENSG000001	YTHDF1	protein_codin	13.4764863	18.4337728	0	5.49736499
ENSG000000	DDX43	protein_codin	0	6.02923512	3.88672162	0
ENSG000001	YTHDF1	protein_codin	143.374209	234.679933	88.5772564	126.394491
ENSG000001	MAGEA4	protein_codin	0	0	0	0
ENSG000001	LINC00029	lncRNA	0	0	0	0
ENSG000000	SEC31B	protein_codin	0	18.7643557	0	17.2589335
ENSG000001	BHLHE23	protein_codin	0	0	0	0
ENSG000001	SLC17A9	protein_codin	6.45557319	0	12.3646794	5.60784102
ENSG000001	FATE1	protein_codin	0	0	0	0
ENSG000001	SLC17A9	protein_codin	11.3680056	0	0	5.67201362
ENSG000001	PRRG3	protein_codin	0	0	0	0
ENSG000001	PRRG3	protein_codin	0	0	0	0
ENSG000000	SCD	protein_codin	39.9917803	25.4133875	7.43064725	7.99655915
ENSG000001	PASD1	protein_codin	0	0	0	0
ENSG000002	OOEP	protein_codin	0	0	0	0
ENSG000001	GLMN	protein_codin	12.8493278	8.22740986	16.8861096	11.0918887
ENSG000001	VMA21	protein_codin	0	0	0	0
ENSG000002	OOEP	protein_codin	0	1.79993222	0	0
ENSG000001	DIDO1	protein_codin	0	0	0	0
ENSG000002	KHDC3L	protein_codin	0	0	0	0
ENSG000001	DIDO1	protein_codin	0	0	0	130.72276
ENSG000002	DPPA5	protein_codin	0	0	0	0
ENSG000001	DIDO1	protein_codin	69.1559221	64.8510182	38.7689919	0
ENSG000001	BLOC1S2	protein_codin	24.2200042	41.9246721	31.8582339	34.7894782
ENSG000002	C1orf146	protein_codin	0	0	0	0
ENSG000002	C1orf146	protein_codin	0	0	0	0

ENSG000001	CD99L2	protein_codin	81.9992118	161.599173	31.2770233	54.784686
ENSG000001	BTBD8	protein_codin	0	0	4.16164376	0
ENSG000002	ENSG000002	lncRNA	0	0	0	0
ENSG000001	BTBD8	protein_codin	0	2.76493509	0	0
ENSG000001	EPHX4	protein_codin	0	0	0	0
ENSG000001	KHDC1	protein_codin	0	0	0	0
ENSG000000	MTMR1	protein_codin	14.9831331	5.96549998	9.5712129	1.18370019
ENSG000002	KHDC1L	protein_codin	0	0	0	0
ENSG000001	BRDT	protein_codin	0	0	0	0
ENSG000000	MTMR1	protein_codin	14.3450527	0	10.1279512	2.74081054
ENSG000001	KCNQ5	protein_codin	1.11459264	0	0	0
ENSG000001	MTM1	protein_codin	0	2.31389028	1.03606869	2.47731052
ENSG000001	MTM1	protein_codin	91.2938008	0	0	43.9561014
ENSG000002	CHUK	protein_codin	114.144674	81.1487765	90.5502648	96.8510184
ENSG000001	KCNQ5	protein_codin	0	0	0	0
ENSG000000	TGFBR3	protein_codin	0	0	0	0
ENSG000000	MAMLD1	protein_codin	0	0	0	0
ENSG000001	EOLA2	protein_codin	8.53973868	9.71339033	0	0
ENSG000001	EOLA2	protein_codin	7.07625545	23.215566	0	0
ENSG000001	ERLIN1	protein_codin	13.1920962	0	0	0
ENSG000001	EOLA2	protein_codin	22.9184939	15.4599084	19.6956637	12.9795038
ENSG000001	HSFX1	protein_codin	1.87600417	0.94645074	1.80569485	0
ENSG000001	CPN1	protein_codin	0	0	0	0
ENSG000000	RIMS1	protein_codin	0	0	0	0
ENSG000000	RIMS1	protein_codin	0	0	0	0
ENSG000001	HFM1	protein_codin	0	3.60419577	0	2.47032971
ENSG000000	ABCC2	protein_codin	0	0	0	0
ENSG000001	OGFRL1	protein_codin	1075.8032	494.163105	526.158626	859.478467
ENSG000002	ENSG000002	lncRNA	1.7920395	3.71506038	1.18124353	0
ENSG000001	ZNF644	protein_codin	170.827076	50.4507415	11.5460496	79.0637963
ENSG000000	IDS	protein_codin	78.0802089	93.8399813	13.8032232	44.4435522
ENSG000001	BARHL2	protein_codin	0	0	0	0
ENSG000001	ZNF326	protein_codin	4.89210212	0	0	0
ENSG000001	SMAP1	protein_codin	0	0	0	0
ENSG000002	ENSG000002	protein_codin	0	0	0	0
ENSG000001	LRRC8C	protein_codin	266.94086	101.259281	86.5774502	167.806567
ENSG000001	SMAP1	protein_codin	0	0	0	0
ENSG000001	GBP6	protein_codin	2.37123349	1.05767564	3.55770538	0
ENSG000001	AFF2	protein_codin	15.5834898	0	16.1170059	0
ENSG000001	AFF2	protein_codin	12.2031357	43.5256808	12.7295499	20.0964972
ENSG000001	GBP5	protein_codin	251.396464	337.002985	362.057119	248.414709
ENSG000001	AFF2	protein_codin	2.33623224	0	1.88052343	0
ENSG000000	OGFR	protein_codin	111.156472	171.500151	72.8815501	51.9860555
ENSG000001	GBP2	protein_codin	1263.18887	1442.71385	1080.12262	1010.98028

ENSG000001	FMR1NB	protein_codin	0	0	0	0
ENSG000001	FMR1	protein_codin	0	4.98092571	0	13.9543183
ENSG000001	FMR1	protein_codin	0	0	0	0
ENSG000001	CUTC	protein_codin	2.34418869	0	0	0
ENSG000001	GBP1	protein_codin	85.8538537	262.564094	270.675905	157.373243
ENSG000001	SDHAF4	protein_codin	5.99515099	18.146605	2.93691893	8.33585119
ENSG000001	FMR1	protein_codin	0	0	19.5040022	0
ENSG000001	CUTC	protein_codin	34.2151287	64.8064483	25.0799728	25.8432849
ENSG000001	FMR1	protein_codin	18.1954965	32.6494843	18.4595391	0
ENSG000000	FAM135A	protein_codin	0	0	0	0
ENSG000001	GBP3	protein_codin	83.8044795	64.3715564	0	0
ENSG000001	GBP3	protein_codin	45.4757047	27.4495119	23.9908387	25.5562503
ENSG000000	COX15	protein_codin	41.3551603	33.9714172	26.2857762	23.1874231
ENSG000001	KYAT3	protein_codin	0	0	0	0
ENSG000001	MRGBP	protein_codin	43.3805153	72.7708457	19.8899956	36.8928737
ENSG000001	ENTPD7	protein_codin	46.797565	49.5150441	41.3876238	48.6121397
ENSG000001	SLITRK2	protein_codin	0	1.00925259	0	0
ENSG000001	KYAT3	protein_codin	18.6328676	50.7181749	32.3350919	87.7625879
ENSG000002	SPANXN1	protein_codin	0	0	0	0
ENSG000001	SLC25A28	protein_codin	0	25.0462968	0	0
ENSG000001	COL9A1	protein_codin	0	0	0	0
ENSG000001	GTF2B	protein_codin	99.2103237	159.398791	30.7242448	61.6054953
ENSG000001	NTSR1	protein_codin	71.9389952	62.0819132	5.15780582	23.5592613
ENSG000001	SPANXN3	protein_codin	0	0	0	0
ENSG000001	SPANXN4	protein_codin	0	0	0	0
ENSG000001	SLCO4A1	protein_codin	4.85149362	0	0	0
ENSG000001	GOT1	protein_codin	6.88621172	19.4188476	12.0503824	5.10959218
ENSG000001	CREG1	protein_codin	542.33147	883.271697	226.633032	669.004524
ENSG000000	PKN2	protein_codin	114.485752	87.1114261	108.351307	94.3702318
ENSG000001	SPANXD	protein_codin	0	0	0	0
ENSG000002	SPANXA2	protein_codin	0	0	0	0
ENSG000001	SPANXA1	protein_codin	0	0	0	0
ENSG000001	ENSG000001	lncRNA	0.86556637	0	0	0
ENSG000000	PKN2	protein_codin	277.405358	78.1130508	87.546321	99.9004667
ENSG000001	MIR1-1HG	lncRNA	0	0	0	0
ENSG000001	LDOC1	protein_codin	0	0	0	0
ENSG000002	LINC00632	lncRNA	0	0	0	0
ENSG000001	SOX3	protein_codin	0.88405324	0	0	0
ENSG000002	CXorf66	protein_codin	0	0	0	0
ENSG000001	LMO4	protein_codin	17.1061242	62.779752	38.9936639	19.7202049
ENSG000001	LMO4	protein_codin	86.2374652	94.1826986	29.4014116	65.4025759
ENSG000001	HPSE2	protein_codin	0	0	0	0
ENSG000002	ENSG000002	protein_codin	1.48266117	0	0	0
ENSG000001	HPSE2	protein_codin	0	0	0	0

ENSG000001	HS2ST1	protein_codin	38.0470571	45.3178421	22.8634384	49.9686037
ENSG000001	HS2ST1	protein_codin	0	6.5854851	10.9283222	11.8710499
ENSG000001	HPSE2	protein_codin	0	0	0	0
ENSG000001	SELENOF	protein_codin	0	15.9670768	43.5411381	12.9340798
ENSG000001	ATP11C	protein_codin	0	0	21.3213934	0
ENSG000000	SH3GLB1	protein_codin	361.440896	163.861806	167.794074	230.39509
ENSG000001	RPS21	protein_codin	1.07218897	2.24019516	0	0
ENSG000000	CLCA4	protein_codin	0	0	0	0
ENSG000001	CLCA2	protein_codin	1.17145456	1.16313413	0	0
ENSG000001	ODF2L	protein_codin	0	8.65626009	0	0
ENSG000001	LMBRD1	protein_codin	0	0	0	0
ENSG000001	COL24A1	protein_codin	17.8603425	35.907516	100.239269	21.4474921
ENSG000001	MCF2	protein_codin	0	0	0	0
ENSG000001	ZNHIT6	protein_codin	49.2626323	46.6520734	49.1425356	29.9854345
ENSG000001	PYROXD2	protein_codin	8.60945335	8.13288214	0	2.75689559
ENSG000001	MCF2	protein_codin	0	0	0	0
ENSG000001	R3HCC1L	protein_codin	78.8346097	0	0	39.1443763
ENSG000001	R3HCC1L	protein_codin	0	0	0	0
ENSG000000	MCOLN3	protein_codin	0	0	0	1.34713605
ENSG000000	MCOLN3	protein_codin	0	0	0	0
ENSG000000	CRTAC1	protein_codin	0	0	0	0
ENSG000001	RPS21	protein_codin	0	1.23287421	0	1.23319638
ENSG000001	DNAI3	protein_codin	0	0	0	0
ENSG000000	CRTAC1	protein_codin	0	0	0	0
ENSG000001	ADGRB3	protein_codin	8.24421447	9.35389337	5.86003145	5.35933151
ENSG000001	GOLGA7B	protein_codin	0	1.00797051	0	0
ENSG000001	ZIC3	protein_codin	0	0	0	0
ENSG000001	MCOLN2	protein_codin	1.6127293	0	5.57725886	0
ENSG000001	ZFYVE27	protein_codin	9.43602343	0	0	0
ENSG000001	LPAR3	protein_codin	0	0	0	0
ENSG000001	ZFYVE27	protein_codin	0	26.5989204	0	0
ENSG000001	EYS	protein_codin	0	0	0	0
ENSG000001	ARHGEF6	protein_codin	277.608225	407.078935	174.030282	185.199504
ENSG000001	EYS	protein_codin	0	0	0	0
ENSG000001	ARHGEF6	protein_codin	0	34.370363	0	0
ENSG000001	LINC01555	lncRNA	0	0	0	0
ENSG000001	CTBS	protein_codin	63.2795213	55.5670967	30.1586109	29.6104994
ENSG000001	AVPI1	protein_codin	14.074511	22.7255535	7.48211809	1.96403915
ENSG000001	CD40LG	protein_codin	11.246388	0	0	0
ENSG000001	CD40LG	protein_codin	0	12.1180075	18.9821727	4.29912335
ENSG000001	CTBS	protein_codin	75.7380548	40.2494634	22.1422999	14.5763728
ENSG000001	PI4K2A	protein_codin	140.977463	173.120566	86.7723155	131.347345
ENSG000001	VGLL1	protein_codin	0	0	0	0
ENSG000001	MORN4	protein_codin	0	0	0	0

ENSG000002	C10orf62	protein_codin	0	0	0	0
ENSG000001	GNG5	protein_codin	44.9833376	12.8934791	14.4531892	52.3025463
ENSG000002	HOGA1	protein_codin	0	0	0	0
ENSG000001	GNG5	protein_codin	93.5665056	164.982289	36.5633756	45.1720095
ENSG000002	HOGA1	protein_codin	0	0	0	0
ENSG000002	HOGA1	protein_codin	0	0	0	0.96879115
ENSG000001	BRS3	protein_codin	0	0	0	0
ENSG000002	ENSG000002	protein_codin	0	0	0	0
ENSG000001	ADGRG4	protein_codin	0	0	0	0
ENSG000001	RPF1	protein_codin	86.5544763	92.2112223	55.0622154	57.9067957
ENSG000001	ANKRD2	protein_codin	0	0	0	0
ENSG000001	RPF1	protein_codin	11.8461205	24.1492863	20.0425117	17.6762267
ENSG000001	LGSN	protein_codin	0	0	0	0
ENSG000001	LGSN	protein_codin	0	0	0	0
ENSG000001	FKBP1C	protein_codin	5.581032	7.04826372	1.95073753	4.63431069
ENSG000001	MAP7D3	protein_codin	77.7985967	60.5776619	65.8889744	55.2811365
ENSG000001	MAP7D3	protein_codin	2.12534493	0	5.97577633	0
ENSG000001	DNASE2B	protein_codin	0	0	0	0
ENSG000001	MAP7D3	protein_codin	0	5.38334679	0	0
ENSG000001	UBTD1	protein_codin	37.7244507	58.2418423	20.2542727	49.6962724
ENSG000001	DNASE2B	protein_codin	0	0	0	0
ENSG000002	SAMD13	protein_codin	0	0	0	0
ENSG000002	SAMD13	protein_codin	0	0	0	0
ENSG000002	SAMD13	protein_codin	0	0	0	0
ENSG000002	SAMD13	protein_codin	0	0	0	0
ENSG000002	SAMD13	protein_codin	0	0	0	0
ENSG000000	FHL1	protein_codin	5.02692931	0	4.01117192	0
ENSG000000	FHL1	protein_codin	0	0	0	0
ENSG000001	LAMA5	protein_codin	0	0	0	1.65471282
ENSG000001	PRKACB	protein_codin	8.56225892	10.8440536	11.3641539	0
ENSG000000	FHL1	protein_codin	0	0	0	0
ENSG000001	PRKACB	protein_codin	0	0	4.46112164	0
ENSG000001	PRKACB	protein_codin	213.983138	224.183095	146.581644	154.018741
ENSG000001	PRIM2	protein_codin	0	0	0	0
ENSG000001	PRKACB	protein_codin	8.49536748	0	9.41897681	0
ENSG000001	PRKACB	protein_codin	0	10.6840865	0	7.55376221
ENSG000001	LAMA5	protein_codin	0	0	0	0
ENSG000001	BAG2	protein_codin	7.05078289	6.16177959	10.2716399	1.02980446
ENSG000001	SLC9A6	protein_codin	0	66.6428076	20.954915	43.9592311
ENSG000001	SLC9A6	protein_codin	37.2841737	8.47552526	0	0
ENSG000001	SLC9A6	protein_codin	0	0	1.32029723	0
ENSG000001	ZNF451	protein_codin	2.48194481	0	0	0
ENSG000001	ZNF451	protein_codin	135.594262	73.9484498	83.8385593	67.6202553
ENSG000001	ZNF451	protein_codin	210.509248	129.044783	101.283133	127.391352

ENSG000001	SAGE1	protein_codin	0	0	0	0
ENSG000001	ZNF451	protein_codin	7.59969758	0	7.09294797	0
ENSG000001	ZNF451	protein_codin	0	0	0	0
ENSG000001	ADGRL2	protein_codin	0	0	0	0
ENSG000001	ADGRL2	protein_codin	0	0	0	0
ENSG000001	ADGRL2	protein_codin	0	0	0	0
ENSG000001	ADGRL2	protein_codin	0	0	0	0
ENSG000001	ADGRL2	protein_codin	0	0	0	0
ENSG000001	ADGRL2	protein_codin	0	0	0	0
ENSG000001	ADGRL2	protein_codin	0	0	0	0
ENSG000001	ADGRL2	protein_codin	0	0	0	0
ENSG000001	ADGRL2	protein_codin	0	0	0	0
ENSG000001	KIAA1586	protein_codin	21.5180564	37.2438978	8.59166408	17.4588667
ENSG000001	ADGRL4	protein_codin	0	0	0	0
ENSG000001	BEND6	protein_codin	0	0	0	0
ENSG000001	BEND6	protein_codin	0	0	1.06838597	0
ENSG000001	IFI44	protein_codin	141.35399	79.2656174	107.117275	77.3930963
ENSG000001	BEND6	protein_codin	0	2.30421124	0	0
ENSG000001	IFI44L	protein_codin	162.848798	61.6951388	132.908808	105.657715
ENSG000001	INTS6L	protein_codin	0	0	0	30.5489732
ENSG000001	PTGFR	protein_codin	0	0	0	0
ENSG000001	PTGFR	protein_codin	0	0	0	4.83718834
ENSG000001	PTGFR	protein_codin	2.80746789	2.80824414	2.38474341	0
ENSG000001	GIPC2	protein_codin	0	0	0	0
ENSG000001	ZNF449	protein_codin	1.35125303	1.13661885	0	1.1539388
ENSG000001	DNAJB4	protein_codin	51.1985619	17.1048583	3.14188059	21.525481
ENSG000001	ZNF75D	protein_codin	0	0	0	0
ENSG000001	DST	protein_codin	50.9052353	59.5363902	102.64559	43.7126392
ENSG000001	ZNF75D	protein_codin	63.3886502	70.1985423	47.7441328	47.5664095
ENSG000001	FUBP1	protein_codin	112.546554	135.674541	71.8434855	94.6915461
ENSG000001	FUBP1	protein_codin	174.783106	102.877119	125.185337	128.612476
ENSG000002	RTL8A	protein_codin	27.086052	45.4323337	4.9484068	8.85526393
ENSG000001	MOSPD1	protein_codin	0	0	0	0
ENSG000001	MOSPD1	protein_codin	0	0	0	0
ENSG000001	MMS19	protein_codin	0	0	0	0
ENSG000001	MOSPD1	protein_codin	14.6004854	10.8114018	0	13.0271116
ENSG000001	PABIR3	protein_codin	0	0	0	0
ENSG000001	PABIR3	protein_codin	0	0	0	0
ENSG000001	DST	protein_codin	0	119.885721	0	0
ENSG000001	PABIR2	protein_codin	25.4445942	0	0	0
ENSG000001	MIGA1	protein_codin	0	14.250911	14.225641	0
ENSG000000	USP33	protein_codin	203.717233	222.824236	153.565278	170.829217
ENSG000000	USP33	protein_codin	0	0	0	0
ENSG000000	USP33	protein_codin	231.368359	19.5701188	96.4878654	191.504767

ENSG000000:	ZZZ3	protein_codin	12.6070751	0	0	17.0514155
ENSG000001:	PHF6	protein_codin	4.5780664	3.46132159	0	3.08446777
ENSG000001:	PHF6	protein_codin	4.65111338	4.02789185	0	4.40828191
ENSG000000:	ZZZ3	protein_codin	149.910731	151.215911	152.284717	115.396924
ENSG000001:	PHF6	protein_codin	0	0	0	0
ENSG000002	CCDC160	protein_codin	0	0	0	0
ENSG000001	PIGK	protein_codin	46.7834524	27.689093	29.6366787	35.6673028
ENSG000001:	COL21A1	protein_codin	0	0	0	0
ENSG000001	GPC3	protein_codin	0	0	0	0
ENSG000001:	COL21A1	protein_codin	0	0	0	0
ENSG000001	MTG2	protein_codin	10.7644383	25.7945803	17.43634	6.88938707
ENSG000001:	RABGGTB	protein_codin	0	0	0	0
ENSG000000	GPC4	protein_codin	27.3908573	6.23916674	2.43097538	3.16161084
ENSG000001	BMP5	protein_codin	0	0	0	0
ENSG000001:	USP26	protein_codin	0	0	0	0
ENSG000001	HS6ST2	protein_codin	0	0	0	0
ENSG000001	ACADM	protein_codin	6.51955878	0	0	0
ENSG000001	HS6ST2	protein_codin	0	0	0	0
ENSG000000	MBNL3	protein_codin	0	0	0	0
ENSG000001	ACADM	protein_codin	56.7948194	0	38.2157463	0
ENSG000001	ZDHHC16	protein_codin	6.65330975	0	0	6.75223509
ENSG000000	MBNL3	protein_codin	0	0	2.42666509	2.40251217
ENSG000001	ZDHHC16	protein_codin	0	2.05842858	5.4584393	0
ENSG000001	SS18L1	protein_codin	12.1992975	0	0	17.1510223
ENSG000000	MBNL3	protein_codin	0	0	0	0
ENSG000001	HMGCLL1	protein_codin	0	0	0	0
ENSG000001	HMGCLL1	protein_codin	0	0	0	0
ENSG000000	MBNL3	protein_codin	95.3270077	17.9496134	25.9262168	60.3915742
ENSG000001	ZDHHC16	protein_codin	0	0	14.6266054	22.422066
ENSG000001:	SLC44A5	protein_codin	0	0	1.47676261	0
ENSG000000	MBNL3	protein_codin	142.811631	134.979574	131.922336	153.83212
ENSG000001	PSMA7	protein_codin	21.1939862	25.0665635	4.47843628	8.6855078
ENSG000001:	SLC44A5	protein_codin	0	0	0	0
ENSG000001	PSMA7	protein_codin	11.503029	22.2652641	11.79304	6.10307407
ENSG000001:	HCRTR2	protein_codin	0	0	0	0
ENSG000001:	TINAG	protein_codin	0	0	0	0
ENSG000001	TYW3	protein_codin	54.3945947	48.2861063	26.0894854	41.8771998
ENSG000001:	TINAG	protein_codin	0	0	0	0
ENSG000001	CRYZ	protein_codin	0	0	0	0
ENSG000001	CRYZ	protein_codin	0	2.33961671	0	0
ENSG000001	CRYZ	protein_codin	0	0	0	0
ENSG000001	PSMA7	protein_codin	286.882318	510.435737	190.922654	235.94409
ENSG000001:	RAP2C	protein_codin	0	0	4.83317876	0
ENSG000001	MLIP	protein_codin	0	0	0	0

ENSG000001	MLIP	protein_codin	0	0	0	0
ENSG000001	FRMD7	protein_codin	0	0	1.15304258	0
ENSG000001	LRRC1	protein_codin	0	0	0	0
ENSG000001	EXOSC1	protein_codin	8.78996901	0	2.46950689	10.409928
ENSG000001	EXOSC1	protein_codin	0	0	0	0
ENSG000001	EXOSC1	protein_codin	8.72108326	0	1.70544652	0
ENSG000001	LRRC1	protein_codin	0	7.55617885	2.07398234	1.059509
ENSG000001	TNNI3K	protein_codin	0	0	0	0
ENSG000002	FPGT-TNNI3K	protein_codin	0	4.27209337	0	0
ENSG000002	FPGT	protein_codin	0	0	0	0
ENSG000002	FPGT-TNNI3K	protein_codin	2.77171266	1.89901417	7.68071248	2.8244318
ENSG000002	FPGT	protein_codin	21.0572705	16.6153542	17.4623482	18.5259964
ENSG000002	FPGT-TNNI3K	protein_codin	0	0	0	0
ENSG000001	IGSF1	protein_codin	0	0	0	0
ENSG000001	IGSF1	protein_codin	0	0	0	0
ENSG000001	EXOSC1	protein_codin	21.5219441	69.7444895	16.5063713	16.4839991
ENSG000001	IGSF1	protein_codin	0	0	0	0
ENSG000001	IGSF1	protein_codin	0	0	0	0
ENSG000001	KLHL31	protein_codin	0	0	0	0
ENSG000001	LRRIQ3	protein_codin	0	0	1.74183039	0
ENSG000001	IGSF1	protein_codin	0	0	0	0
ENSG000001	LRRIQ3	protein_codin	4.53533697	0	0	0
ENSG000000	ELOVL5	protein_codin	25.7120179	20.3451939	9.78187835	13.727998
ENSG000001	LSM14B	protein_codin	4.8022338	5.26608176	5.25519344	1.46329293
ENSG000000	ELOVL5	protein_codin	9.77805171	2.83495288	0	0
ENSG000001	ZRANB2	protein_codin	87.7473751	83.6305732	88.3109149	30.8885737
ENSG000001	ARHGAP36	protein_codin	0	0.93607249	0	0
ENSG000001	ARHGAP36	protein_codin	0	0	0	0
ENSG000000	PTGER3	protein_codin	7.74794468	7.7820466	3.26043586	4.71015211
ENSG000001	ENOX2	protein_codin	0	0	0	0
ENSG000000	PTGER3	protein_codin	1.30914285	0	0.83120938	0
ENSG000001	ENOX2	protein_codin	0	33.1515455	0	63.8419544
ENSG000001	CTH	protein_codin	0	0	0	0
ENSG000001	FBXO9	protein_codin	117.498989	131.426656	85.5084184	37.1564015
ENSG000001	ANKRD13C-D	lncRNA	0	0	0	0
ENSG000001	ANKRD13C	protein_codin	50.2606913	27.8628977	20.8876871	25.7623385
ENSG000001	RBMX2	protein_codin	2.10741332	0	0	1.0515131
ENSG000001	SRSF11	protein_codin	142.191669	45.6530935	0	74.8647932
ENSG000001	SRSF11	protein_codin	0	0	22.3817994	0
ENSG000001	SRSF11	protein_codin	74.102792	177.746682	32.8682281	115.021634
ENSG000000	LRRC40	protein_codin	70.039841	35.4225118	17.2036215	34.4147978
ENSG000000	LRRC7	protein_codin	0	0	0	0
ENSG000001	GSTA4	protein_codin	0	0	0	0
ENSG000001	GSTA4	protein_codin	0	0	0	0

ENSG000001	GSTA4	protein_codin	0	0	0	0
ENSG000000	DEPDC1	protein_codin	0	0	12.182418	0
ENSG000001	GSTA3	protein_codin	0	0	0	0
ENSG000001	WLS	protein_codin	0	2.49411473	7.24217942	0
ENSG000001	WLS	protein_codin	0	0	0	0
ENSG000001	WLS	protein_codin	0	0	12.1427297	25.4754261
ENSG000000	ZNF280C	protein_codin	23.557254	9.39227253	11.2895364	16.5755952
ENSG000001	DIRAS3	protein_codin	0	0	0	0
ENSG000001	GNG12	protein_codin	6.50087692	0	1.1894151	0
ENSG000001	GADD45A	protein_codin	0	0	0	0
ENSG000001	GADD45A	protein_codin	29.6504869	41.6126167	24.0794798	11.2338186
ENSG000001	GSTA5	protein_codin	0	0	0	0
ENSG000001	SERBP1	protein_codin	319.607301	491.094623	399.714785	264.759988
ENSG000001	CDH26	protein_codin	2.49569893	2.43661122	3.68329118	3.58300391
ENSG000000	RRP12	protein_codin	270.98751	385.102309	90.5606482	165.235129
ENSG000001	SERBP1	protein_codin	121.758721	98.9962826	86.6656147	93.973848
ENSG000001	SERBP1	protein_codin	271.548979	249.440396	165.760452	192.945013
ENSG000001	PPP1R3D	protein_codin	44.36756	50.909547	11.8167632	28.2265526
ENSG000000	IL12RB2	protein_codin	0	0	0	0
ENSG000001	SYCP2	protein_codin	29.3022197	17.1353826	14.1816414	20.0352534
ENSG000002	C1orf141	protein_codin	0	0	0	0
ENSG000002	C1orf141	protein_codin	0	0	0	0
ENSG000002	C1orf141	protein_codin	0	0	0	0
ENSG000001	MIER1	protein_codin	73.8132483	32.8318922	23.9490513	28.2203648
ENSG000001	MIER1	protein_codin	79.0865954	61.5786271	39.6401372	96.249054
ENSG000000	PHACTR3	protein_codin	4.41221923	0	0	0
ENSG000001	MIER1	protein_codin	0	0	0	0
ENSG000001	MIER1	protein_codin	0	0	0	0
ENSG000001	FRAT2	protein_codin	104.598032	249.684606	26.8576841	92.0322921
ENSG000001	FRAT1	protein_codin	131.078309	141.301574	33.6378047	90.8770526
ENSG000001	DNAI4	protein_codin	5.55222621	14.2348024	0	10.1505589
ENSG000001	DNAI4	protein_codin	0	0	8.45179118	0
ENSG000001	EDN3	protein_codin	0	0	0	0
ENSG000001	DNAI4	protein_codin	0	0	0	0
ENSG000002	ARHGAP19	protein_codin	0	35.3943219	14.7301499	0
ENSG000001	EDN3	protein_codin	0	0	0	0
ENSG000001	ZNF831	protein_codin	4.84493746	8.70247812	1.02507778	1.89910017
ENSG000001	PRELID3B	protein_codin	21.7637092	14.819765	9.52036731	0
ENSG000001	SGIP1	protein_codin	0	0	0	0
ENSG000001	SGIP1	protein_codin	0	0	0	0
ENSG000001	SLIT1	protein_codin	0	0	0	0
ENSG000001	PDE4B	protein_codin	2978.90593	1762.6034	1542.95424	1708.87717
ENSG000001	PDE4B	protein_codin	0	0	1.44182759	0
ENSG000001	LEPR	protein_codin	0	0	0	0

ENSG000001	LEPR	protein_codin	8.40745248	14.008122	16.8303264	0
ENSG000001	LEPR	protein_codin	83.1421814	53.7909666	29.325071	37.4447153
ENSG000001	ZDHHC9	protein_codin	0	12.2723409	3.24267168	0
ENSG000002	LEPROT	protein_codin	306.239992	419.784731	212.911227	216.198153
ENSG000000	EFHC1	protein_codin	7.79203453	10.4638079	14.4459793	0
ENSG000001	DNAJC6	protein_codin	0	26.9733719	0	0
ENSG000001	SLIT1	protein_codin	0	0	1.86872252	0
ENSG000001	RAVER2	protein_codin	23.269664	7.97061677	9.89571489	7.14805865
ENSG000000	GNAS	protein_codin	0	0	0	0
ENSG000001	UBE2U	protein_codin	0	0	0	0
ENSG000001	UBE2U	protein_codin	0	0	0	0
ENSG000001	ROR1	protein_codin	0	0	0	1.06042235
ENSG000001	ROR1	protein_codin	0	0	0	0
ENSG000000	GNAS	protein_codin	13.1159691	49.9107417	170.826218	0
ENSG000000	PGM1	protein_codin	0	5.68162103	0	5.55126459
ENSG000000	PGM1	protein_codin	85.6333692	126.88186	27.8699316	55.9735716
ENSG000000	GNAS	protein_codin	450.142025	877.911409	402.102643	363.046132
ENSG000001	DLEU2L	lncRNA	4.44934808	4.58232254	1.23379012	2.32096963
ENSG000002	EFCAB7	protein_codin	2.13752604	4.08792143	4.23251308	5.28752795
ENSG000001	ITGB3BP	protein_codin	0	26.3367467	0	0
ENSG000000	GNAS	protein_codin	1439.87582	2564.54738	841.063063	1302.87817
ENSG000001	LCOR	protein_codin	0	0	0	0
ENSG000000	GNAS	protein_codin	0	0	0	0
ENSG000000	GNAS	protein_codin	0	0	0.7204151	1.64084781
ENSG000000	GNAS	protein_codin	0	0	0	0
ENSG000000	GNAS	protein_codin	0	0	0	0
ENSG000001	LCOR	protein_codin	303.350379	114.287636	139.985301	169.21064
ENSG000001	XPNPEP2	protein_codin	0	0	0	0
ENSG000001	XPNPEP2	protein_codin	0.92677128	2.76610542	0	0.9211246
ENSG000001	PIK3AP1	protein_codin	22.5317118	147.574719	161.310047	118.666118
ENSG000001	PIK3AP1	protein_codin	258.606777	465.211021	0	0
ENSG000001	OCRL	protein_codin	0	7.24699473	0	0
ENSG000001	FOXD3	protein_codin	0.87563066	0	0	0
ENSG000001	PKHD1	protein_codin	0	2.10794041	0	0
ENSG000001	ATG4C	protein_codin	0	0	0	0
ENSG000001	ATG4C	protein_codin	41.8108702	48.4090711	35.3341203	8.28480531
ENSG000001	SMARCA1	protein_codin	0	0	0	0
ENSG000001	SMARCA1	protein_codin	0	0	0	0
ENSG000001	SMARCA1	protein_codin	4.04113027	1.02254816	1.05978371	0
ENSG000001	ACTRT1	protein_codin	0	0	0	0
ENSG000001	PRR32	protein_codin	0	0	0	0
ENSG000001	DCAF12L1	protein_codin	0	0	0	0
ENSG000001	ANGPTL3	protein_codin	0	0	0	0
ENSG000000	TENM1	protein_codin	0	3.05341775	0.94904966	0

ENSG000001:	STX16	protein_codin	172.716259	71.7378846	100.342117	82.6075274
ENSG000001:	SH2D1A	protein_codin	0	0	0	0
ENSG000001:	STX16	protein_codin	0	86.2960068	0	0
ENSG000000'	TM9SF3	protein_codin	330.180259	213.884066	208.215422	267.431333
ENSG000001	STAG2	protein_codin	506.742827	193.395146	485.354715	259.162292
ENSG000001	STAG2	protein_codin	67.5985666	79.4879344	0	103.793359
ENSG000001	USP1	protein_codin	134.31422	141.803263	151.623334	116.445686
ENSG000002	DEFB110	protein_codin	0	0	0	0
ENSG000001!	APCDD1L	protein_codin	0	0	0	0
ENSG000001:	KANK4	protein_codin	0	0	0	0
ENSG000001:	KANK4	protein_codin	0	0.85212858	0	0
ENSG000001	STAG2	protein_codin	0	251.276418	85.5346103	166.05107
ENSG000001:	PATJ	protein_codin	0	12.0647261	12.5980562	0
ENSG000000!	CRISP3	protein_codin	0	0	0	0
ENSG000001	STAG2	protein_codin	96.881622	0	0	38.9228467
ENSG000001'	MIR4435-2HG	lncRNA	0	0	0	0
ENSG000001:	CIMIP1	protein_codin	0	0	0	0
ENSG000002	ENSG000002	lncRNA	0	0	0	0
ENSG000001!	OPALIN	protein_codin	0	0	0	0
ENSG000001:	ZBP1	protein_codin	0	0	0	0
ENSG000001	DNTT	protein_codin	0	1.77575217	0	0
ENSG000001	RHAG	protein_codin	0	0	1.82930623	0
ENSG000000!	BLNK	protein_codin	0	22.5132024	0	0
ENSG000001	TM2D1	protein_codin	57.3005796	23.9852997	12.9992898	26.5606515
ENSG000001	TM2D1	protein_codin	0	3.36576625	0	3.74164267
ENSG000001	TM2D1	protein_codin	3.85731081	2.09019148	0	11.6913505
ENSG000001	NFIA	protein_codin	0	0	0	0
ENSG000001	NFIA	protein_codin	0	0	0	0
ENSG000001	NFIA	protein_codin	0	0	0	0
ENSG000001	NFIA	protein_codin	0	0	0	1.55674105
ENSG000001	NFIA	protein_codin	0	0	0	0
ENSG000001!	C6orf141	protein_codin	0	0	0	0
ENSG000001:	CTCFL	protein_codin	0	0	0	0
ENSG000002	GLYATL3	protein_codin	0	0	0	0
ENSG000001	XIAP	protein_codin	90.2511342	78.8417794	126.904548	85.2987358
ENSG000001	C1orf87	protein_codin	0	0	0	0
ENSG000001:	CYP2J2	protein_codin	0	0	0	0
ENSG000001:	ENTPD1	protein_codin	687.93176	295.926389	284.113713	305.5537
ENSG000001:	ENTPD1	protein_codin	40.5461144	36.9611835	15.0128714	28.0221262
ENSG000001:	ENTPD1	protein_codin	47.4696134	133.776529	31.8189633	79.913805
ENSG000001:	HOOK1	protein_codin	9.21245904	0	10.8423888	0
ENSG000001	TCTN3	protein_codin	0	8.54232766	1.55278919	0
ENSG000001'	FGGY	protein_codin	0	0	0	0
ENSG000001:	OPN5	protein_codin	0	0	0	0

ENSG000001	FGGY	protein_codin	0	0	0	0
ENSG000001	TCTN3	protein_codin	13.7257394	23.2946028	2.20411162	7.16139786
ENSG000001	FGGY	protein_codin	0	0	0	0
ENSG000001	RBM38	protein_codin	0	0	0	1.46065522
ENSG000001	ADGRF4	protein_codin	0	0	0	0
ENSG000000	ALDH18A1	protein_codin	0	29.8452704	28.8814527	10.2822121
ENSG000001	JUN	protein_codin	1833.10174	4033.69472	1045.07338	1601.28993
ENSG000000	ALDH18A1	protein_codin	18.8869242	0	0	0
ENSG000001	TACSTD2	protein_codin	0	22.1777954	4.70444858	0
ENSG000001	OMA1	protein_codin	61.4140451	76.3382793	39.332945	53.0405541
ENSG000000	SORBS1	protein_codin	0	0	0	0
ENSG000000	SORBS1	protein_codin	0	0	0	0
ENSG000001	DAB1	protein_codin	0	0	0	0
ENSG000001	DAB1	protein_codin	0	0	0	0
ENSG000001	DAB1	protein_codin	0	0	0	0
ENSG000001	DAB1	protein_codin	0	0	1.01512536	0
ENSG000000	C8B	protein_codin	0	0	0	0
ENSG000000	SORBS1	protein_codin	0	0	0	0
ENSG000001	FYB2	protein_codin	0	0	0	0
ENSG000000	SORBS1	protein_codin	0	0	0	0
ENSG000001	RAE1	protein_codin	0	26.8676618	0	0
ENSG000001	ADGRF1	protein_codin	0	0	0	0
ENSG000001	PRKAA2	protein_codin	0	0	0	0
ENSG000000	SORBS1	protein_codin	2.21232035	0	0	0
ENSG000000	SORBS1	protein_codin	0	0	0	0
ENSG000000	SORBS1	protein_codin	0	0	0	0
ENSG000001	VPS37B	protein_codin	34.1691243	14.5739491	29.2691292	21.5183332
ENSG000000	SORBS1	protein_codin	0	0	0	0
ENSG000001	PLPP3	protein_codin	4.15720636	2.07970405	1.95423121	6.41870814
ENSG000001	ADGRF1	protein_codin	0	0	0	0
ENSG000000	SPO11	protein_codin	0	0	0	0
ENSG000000	SPO11	protein_codin	0	0	0	0
ENSG000001	TMEM61	protein_codin	0	0	0	0
ENSG000001	DHCR24	protein_codin	10.5332797	29.8913601	0	1.6747138
ENSG000001	CYP2C8	protein_codin	0	0	0	0
ENSG000001	CIMAP2	protein_codin	0	0	0	0
ENSG000000	TTC22	protein_codin	0	0	0	0
ENSG000000	TTC22	protein_codin	0.9704058	0	3.04005805	0.94041408
ENSG000001	PARS2	protein_codin	3.89511706	7.5959201	2.99959723	3.78273596
ENSG000002	TTC4	protein_codin	35.8097091	45.108125	10.2856213	16.9011485
ENSG000002	TTC4	protein_codin	0	0	0	0
ENSG000001	MROH7	protein_codin	0	0	0	0
ENSG000001	FAM151A	protein_codin	0	0	0	0
ENSG000002	CT47B1	protein_codin	0	0	0	0

ENSG00000017	C1GALT1C1	protein_codin	0	0	0	0
ENSG00000023	MCTS1	protein_codin	0	12.6132066	0	0
ENSG00000010	ACOT11	protein_codin	20.1241233	18.5262946	11.2984199	1.35328216
ENSG00000023	MCTS1	protein_codin	20.0124271	22.4517879	15.8335977	18.9619991
ENSG00000015	SSBP3	protein_codin	39.6437203	89.9282212	0	32.2594323
ENSG00000015	SSBP3	protein_codin	1.82910151	10.0356328	1.86676082	1.75422537
ENSG00000010	CYP2C19	protein_codin	0	0	0	0
ENSG00000015	CUL4B	protein_codin	33.4731062	0	0	0
ENSG00000015	CUL4B	protein_codin	0	0	0	0
ENSG000002	FAM209B	protein_codin	6.2013349	2.313512	2.65122745	3.94139773
ENSG000001	HELLS	protein_codin	3.18761087	4.50386243	17.5211472	1.57067963
ENSG00000012	FAM209A	protein_codin	8.21027913	3.51174853	2.68245773	0
ENSG00000015	CDCP2	protein_codin	0	0	0	0
ENSG000001	TCEANC2	protein_codin	0	4.36874587	0	1.22497507
ENSG000001	HELLS	protein_codin	0	0	0	0
ENSG00000000	LAMP2	protein_codin	351.000137	335.06217	176.117618	290.693289
ENSG000001	TMEM59	protein_codin	39.2577412	78.0822303	34.2748722	36.0914972
ENSG000001	TMEM59	protein_codin	20.6763545	56.9890437	65.2236926	43.1740415
ENSG000001	TMEM59	protein_codin	0	11.8740088	5.76764973	0
ENSG00000015	SLC25A27	protein_codin	0	0	0	3.54912838
ENSG000001	TMEM59	protein_codin	45.655372	62.2147505	23.1397159	49.6286999
ENSG00000012	TMEM255A	protein_codin	1.14818207	4.65049128	1.06400582	2.3403316
ENSG00000008	AURKA	protein_codin	0	0	0	0
ENSG00000014	CACNA1B	protein_codin	0	0	0	0
ENSG00000020	LDLRAD1	protein_codin	0	0	0	0
ENSG00000017	NOC3L	protein_codin	23.4714524	50.5436001	50.0150177	23.7883794
ENSG00000020	LDLRAD1	protein_codin	0	0	0	0
ENSG00000014	CACNA1B	protein_codin	0	0	0	0
ENSG000001	LRRC42	protein_codin	0	0	0	1.36827592
ENSG00000012	TMEM255A	protein_codin	0	0	0	0
ENSG000001	LRRC42	protein_codin	23.4916493	16.0410554	16.7406238	0
ENSG00000014	CACNA1B	protein_codin	0	0	0	0
ENSG00000017	RCAN2	protein_codin	1.16445487	0	0	0
ENSG00000013	PLCE1	protein_codin	0.99651274	2.26461249	0	2.05383306
ENSG00000008	IFT25	protein_codin	0	8.68999331	1.16825469	1.39846469
ENSG00000008	IFT25	protein_codin	0	23.8745217	0	7.84802756
ENSG00000008	IFT25	protein_codin	1.64745261	8.85092621	6.25427867	13.1761628
ENSG00000013	PLCE1	protein_codin	0	0	2.24119473	0
ENSG000001	ENPP5	protein_codin	0	0	0	0
ENSG00000012	FAM210B	protein_codin	121.730629	153.509526	55.9105839	108.569323
ENSG00000013	PLCE1	protein_codin	0	0	0	0
ENSG00000013	RHOXF2	protein_codin	0	0	0	0
ENSG00000020	ENSG00000020	lncRNA	0	0	0	0
ENSG00000018	EHMT1	protein_codin	68.1708416	42.6837818	16.4593472	20.5103929

ENSG000000	YIPF1	protein_codin	3.50716823	0	0	0
ENSG000002	RHOXF2B	protein_codin	0	0	0	0
ENSG000001	SLC35G1	protein_codin	0	0	0	1.39326205
ENSG000001	NKAP	protein_codin	46.3263039	47.8708061	25.3179649	7.90962023
ENSG000001	LGI1	protein_codin	0	0	0	0
ENSG000002	ARRDC1-AS1	lncRNA	0	0	0	0
ENSG000001	LGI1	protein_codin	0	0	0	0
ENSG000001	PFDN4	protein_codin	56.9066125	63.715659	0	40.3745183
ENSG000001	ARRDC1	protein_codin	110.809828	119.110086	38.0549762	80.0247241
ENSG000001	AKAP14	protein_codin	0	0	0.96314212	0
ENSG000001	AKAP14	protein_codin	0	0	0	0
ENSG000000	NDC1	protein_codin	37.8662781	31.3260707	21.5113453	19.5051996
ENSG000001	AKAP14	protein_codin	0	0	0	0
ENSG000001	RUNX2	protein_codin	0	16.5789912	43.9779192	39.9904259
ENSG000000	BCAS1	protein_codin	0	1.56423312	0	0
ENSG000001	RUNX2	protein_codin	0	0	0	0
ENSG000001	NDUFA1	protein_codin	105.151751	155.389161	54.6793837	77.0534781
ENSG000001	RUNX2	protein_codin	0	0	0	0
ENSG000001	RNF113A	protein_codin	60.6737971	136.031726	33.9842219	51.018947
ENSG000001	MRPL41	protein_codin	37.4021596	58.4865584	19.1826101	37.0302212
ENSG000001	DMRTB1	protein_codin	0	0	0	0
ENSG000000	PDE6C	protein_codin	0	0	0	0
ENSG000001	LRP8	protein_codin	0	0	0	0
ENSG000002	WTAPP1	lncRNA	0	0	0	0
ENSG000001	SUPT3H	protein_codin	0	0	0	0
ENSG000001	SUPT3H	protein_codin	3.38533066	4.39336018	2.32349923	3.90700142
ENSG000001	SUPT3H	protein_codin	0	0	0	0
ENSG000001	RBP4	protein_codin	0	0	0	0
ENSG000001	MAGOH	protein_codin	4.01587034	4.982141	0	1.61405835
ENSG000001	RBP4	protein_codin	0	0	0	0
ENSG000001	NSMF	protein_codin	3.92479193	1.2499946	1.1711392	5.45156914
ENSG000001	RBP4	protein_codin	0	0	0	0
ENSG000001	MAGOH	protein_codin	57.7783175	46.5693844	21.8706641	39.3461413
ENSG000001	ZNF217	protein_codin	325.054328	393.711704	0	31.2206338
ENSG000001	NSMF	protein_codin	0	29.3333885	14.3393877	12.7650465
ENSG000001	NSMF	protein_codin	0	12.3511768	0	0
ENSG000001	NSMF	protein_codin	0	5.3043379	0	0
ENSG000001	NSMF	protein_codin	14.7968203	0	3.91881212	0
ENSG000000	CDC5L	protein_codin	449.507192	350.709397	324.310536	285.300427
ENSG000001	SEPTIN8	protein_codin	0	0	0	0
ENSG000001	FFAR4	protein_codin	6.09536564	5.21672833	0	2.11066074
ENSG000001	NSMF	protein_codin	12.5051836	34.5806103	16.9798686	3.93158864
ENSG000001	FFAR4	protein_codin	0	0	0	0
ENSG000001	CEP55	protein_codin	3.12734156	1.42088955	43.0526087	2.18990193

ENSG000001	CPT2	protein_codin	0	57.5350436	20.2612929	0
ENSG000002	ENSG000002	lncRNA	0	0	0	0
ENSG000001	MYOF	protein_codin	1.1798788	1.04075231	4.32410545	0
ENSG000001	MYOF	protein_codin	0	0	0	0
ENSG000001	SLC1A7	protein_codin	0	0	0	0
ENSG000001	SEPTIN8	protein_codin	0	0	0	0
ENSG000001	SLC1A7	protein_codin	0	0	0	0
ENSG000001	TSHZ2	protein_codin	1.04544189	2.08257074	0	0
ENSG000001	PODN	protein_codin	0	0	0	0
ENSG000001	TCTE1	protein_codin	0	0	0.76574103	0
ENSG000001	TCTE1	protein_codin	2.84953701	0	0	0.94969441
ENSG000001	ENTPD8	protein_codin	0	0	0	0
ENSG000001	SCP2	protein_codin	0	0	14.6450525	28.4887544
ENSG000001	SCP2	protein_codin	0	0	0	0
ENSG000001	SCP2	protein_codin	70.776889	48.2671106	10.1891375	0
ENSG000000	ZFP64	protein_codin	0	0	21.6963523	0
ENSG000000	ZFP64	protein_codin	0	0	0	2.63926576
ENSG000001	ECHDC2	protein_codin	0	0	1.78314762	0
ENSG000001	NDOR1	protein_codin	23.0348475	0	20.3452109	0
ENSG000001	ECHDC2	protein_codin	0	0	0	0
ENSG000000	ZFP64	protein_codin	0	0	0	0
ENSG000002	ZYG11A	protein_codin	0	0	2.6466939	0
ENSG000000	CYP26A1	protein_codin	0	0	0	0
ENSG000002	ZYG11A	protein_codin	3.36458162	0	3.5848994	0
ENSG000001	COA7	protein_codin	33.0344684	35.9229734	19.4717292	17.1519005
ENSG000001	SALL4	protein_codin	0	0	0	0
ENSG000001	TUT4	protein_codin	22.0881226	5.1079885	27.3221403	20.1803723
ENSG000001	LRRC26	protein_codin	0	0	0	0
ENSG000001	EXOC6	protein_codin	7.65681893	7.16603797	5.249348	13.8248136
ENSG000001	TUT4	protein_codin	0	3.46487836	0	13.9945166
ENSG000001	GRIN1	protein_codin	0	0	0	0
ENSG000001	GRIN1	protein_codin	0	0	0	0
ENSG000001	EXOC6	protein_codin	0	0	0	0
ENSG000001	GRIN1	protein_codin	0	0	0	0
ENSG000000	HSP90AB1	protein_codin	17.218606	0	6.45612855	20.9539084
ENSG000001	GRIN1	protein_codin	0	0	0	0
ENSG000000	UBE2A	protein_codin	62.2589997	104.026748	26.5936944	0
ENSG000001	GRIN1	protein_codin	0	0	0	0
ENSG000001	GRIN1	protein_codin	0	0	0	0
ENSG000001	GRIN1	protein_codin	0	0	0	0
ENSG000001	NFATC2	protein_codin	19.8189779	78.5553283	0	51.0961154
ENSG000000	ORC1	protein_codin	2.87780095	4.73561325	0	1.95090621
ENSG000000	ORC1	protein_codin	0	0	17.0076642	0
ENSG000000	UBE2A	protein_codin	70.0761627	24.3665826	17.1176358	30.3414786

ENSG000002	KCNG1	protein_codin	0	0	0	0
ENSG000001	DPP7	protein_codin	110.105775	106.551865	32.6518426	27.6202814
ENSG000001	IDE	protein_codin	0	0	0	0
ENSG000000	DPM1	protein_codin	2.88109	5.68326386	5.85599238	6.14951518
ENSG000000	DPM1	protein_codin	2.90433242	0	0	6.80095685
ENSG000001	CC2D1B	protein_codin	40.766553	34.1426575	30.735664	0
ENSG000001	MAN1B1	protein_codin	0	0	0	0
ENSG000000	DPM1	protein_codin	29.5608635	30.2627881	23.3551168	14.7140056
ENSG000001	MAN1B1	protein_codin	0	59.9518858	37.2602467	0
ENSG000001	ZFYVE9	protein_codin	3.28998059	3.46512112	0	0
ENSG000001	NPDC1	protein_codin	0	0	0	0
ENSG000001	NPDC1	protein_codin	3.74879827	5.43421646	1.39070489	0
ENSG000001	ADNP	protein_codin	102.63896	121.352173	72.5489664	40.9059417
ENSG000001	ABCA2	protein_codin	60.183436	0	0	0
ENSG000001	BCAS4	protein_codin	0	1.18152368	0	0
ENSG000001	PARD6B	protein_codin	5.01834057	3.0537883	2.50370527	3.07171797
ENSG000001	KTI12	protein_codin	26.2239034	31.2846413	8.92239657	7.82694938
ENSG000001	PAXX	protein_codin	21.6151261	24.1885477	18.163604	12.2258782
ENSG000001	PTPN1	protein_codin	343.387367	267.891775	182.134188	177.20431
ENSG000001	PTGDS	protein_codin	0	0	0.94356222	0
ENSG000001	PTGDS	protein_codin	0	16.0895262	9.53498166	0
ENSG000001	TXNDC12	protein_codin	101.428123	219.952408	72.8336163	88.2782702
ENSG000001	TNKS2	protein_codin	507.800326	284.63014	251.63381	368.653384
ENSG000001	LONRF3	protein_codin	0	0	0	29.4638632
ENSG000001	LINC02692	lncRNA	0	0	0	0
ENSG000001	LCN12	protein_codin	0	0	0	0
ENSG000001	LCN12	protein_codin	0	0	0	0
ENSG000001	C8G	protein_codin	0	0	1.58948569	0
ENSG000002	LINC01270	lncRNA	0	0	0	0
ENSG000001	IL13RA1	protein_codin	15.0354778	5.57505944	16.7963407	4.39438907
ENSG000000	HSP90AB1	protein_codin	1349.6077	2303.70086	1403.48288	1087.27937
ENSG000001	EDF1	protein_codin	3.93492339	0	3.01558599	0
ENSG000001	EDF1	protein_codin	10.7803098	37.478411	7.77279881	25.386308
ENSG000002	PEDS1	protein_codin	24.877418	0	0	22.3038307
ENSG000002	PEDS1	protein_codin	43.4267614	65.1674824	28.1639955	22.7113488
ENSG000001	RAB3B	protein_codin	1.01845779	2.06711799	1.03947119	1.05101808
ENSG000002	PEDS1	protein_codin	119.163975	125.609056	55.9955368	0
ENSG000002	UBE2V1	protein_codin	0	0	0	0
ENSG000002	PEDS1	protein_codin	23.7823351	30.6501255	8.47942702	19.0816659
ENSG000000	PHPT1	protein_codin	0	0	0	0
ENSG000001	RABL6	protein_codin	55.4251136	0	0	0
ENSG000001	IL13RA1	protein_codin	558.494126	539.884729	174.71218	272.493115
ENSG000001	HECTD2	protein_codin	0	0	0	0
ENSG000001	RABL6	protein_codin	6.00769859	0	0	6.52962537

ENSG000002	UBE2V1	protein_codin	303.991478	359.51063	124.971503	186.387183
ENSG000002	UBE2V1	protein_codin	10.4956569	16.2654298	6.22915553	30.4432327
ENSG000001	HECTD2	protein_codin	2.11045085	13.7588646	2.01739453	2.09175403
ENSG000002	CCDC183	protein_codin	0	0	0	0
ENSG000002	LCN8	protein_codin	0	0	0	0
ENSG000001	DIPK1B	protein_codin	8.63798081	6.47356471	7.38154863	0
ENSG000001	DIPK1B	protein_codin	11.9063478	44.5710336	8.27191066	16.4703858
ENSG000001	AGPAT2	protein_codin	5.11458852	11.5082051	5.01167317	8.50102271
ENSG000001	AGPAT2	protein_codin	130.993376	151.186499	28.014328	86.1461472
ENSG000001	ANKRD1	protein_codin	0	0	0	0
ENSG000001	EGFL7	protein_codin	0	12.8570394	19.0519989	15.2261732
ENSG000001	EGFL7	protein_codin	0	1.32187503	0	0
ENSG000001	RPP30	protein_codin	15.4237475	6.38410475	4.02592252	10.4083118
ENSG000001	SEC16A	protein_codin	0	0	135.637473	0
ENSG000002	OSBPL9P4	processed_ps	0	0.95500151	0	0
ENSG000001	SLC29A1	protein_codin	0	0	0	0
ENSG000001	B4GALT5	protein_codin	335.134958	248.805469	82.789527	172.909606
ENSG000001	INPP5E	protein_codin	29.0063595	23.4749333	0	0
ENSG000001	SLC29A1	protein_codin	0	0	0	0
ENSG000001	OSBPL9	protein_codin	10.3568693	11.7379385	3.61203573	19.646721
ENSG000001	PMPCA	protein_codin	0	83.8275485	23.9052588	0
ENSG000001	HTR7	protein_codin	0	0	0	0
ENSG000001	PMPCA	protein_codin	0	0	0	0
ENSG000001	ENTR1	protein_codin	6.95898661	23.1062732	5.12726957	6.00610058
ENSG000001	SLC29A1	protein_codin	0	0	0	0
ENSG000001	ENTR1	protein_codin	43.918024	84.3123453	24.9831624	42.8841029
ENSG000000	EPS15	protein_codin	19.2071173	18.9049837	11.0305227	6.72224395
ENSG000001	KIF20B	protein_codin	0	0	0	0
ENSG000000	EPS15	protein_codin	6.41409207	7.72263515	0	0
ENSG000001	SLC29A1	protein_codin	0	0	0	0
ENSG000001	CARD9	protein_codin	195.391526	289.324608	80.0979212	95.9082852
ENSG000000	EPS15	protein_codin	300.448819	175.919056	0	102.140219
ENSG000001	CARD9	protein_codin	0	0	0	0
ENSG000002	DNLZ	protein_codin	16.7676394	8.79274706	10.0016473	9.50304228
ENSG000002	DNLZ	protein_codin	0	0	0	0
ENSG000001	SLC29A1	protein_codin	0	0	0	0
ENSG000001	KCNB1	protein_codin	0	1.95516363	0	0
ENSG000001	ZFAS1	lncRNA	0	4.92283495	0	6.40481541
ENSG000001	ZNFX1	protein_codin	74.6827439	75.921264	22.3438326	67.7585129
ENSG000001	LHX3	protein_codin	0	0	0	0
ENSG000000	TTC39A	protein_codin	0	0	0	0
ENSG000001	LHX3	protein_codin	0	0.7823104	0	0
ENSG000000	TTC39A	protein_codin	0	0	0	0
ENSG000001	ZNFX1	protein_codin	115.469929	111.754983	153.009608	33.9408218

ENSG000001	NACC2	protein_codin	131.368535	377.081845	0	0
ENSG000001	ZNFX1	protein_codin	67.1739522	58.6194385	15.8000306	21.4431656
ENSG000001	SLC29A1	protein_codin	0	0	6.03113033	0
ENSG000001	UBAC1	protein_codin	190.214037	298.480585	92.4802932	115.514207
ENSG000001	KCNT1	protein_codin	0	0	0	0
ENSG000002	C1orf185	protein_codin	0	0	0	0
ENSG000001	CDKN2C	protein_codin	4.02974669	5.74591611	0	3.45945475
ENSG000002	GLT6D1	protein_codin	0	0	0	0
ENSG000001	PAEP	protein_codin	0	0	0	0
ENSG000001	PAEP	protein_codin	0	0	0	0
ENSG000001	OBP2A	protein_codin	0	0	0	0
ENSG000001	LCN1	protein_codin	0	0	0	0
ENSG000001	MRPS2	protein_codin	0	0	8.17557609	31.942316
ENSG000001	PIERCE1	protein_codin	0	1.58037292	0	0
ENSG000001	PIERCE1	protein_codin	0	0.78088609	0	0
ENSG000001	SLC16A12	protein_codin	0	0	1.29632103	0
ENSG000001	PIERCE1	protein_codin	0	0	0	0
ENSG000001	STAU1	protein_codin	6.23177479	0	0	7.78566085
ENSG000001	OLFM1	protein_codin	54.9766031	36.1140656	0	0
ENSG000001	IFIT5	protein_codin	43.6045438	29.6144407	19.6164927	26.1445954
ENSG000001	OLFM1	protein_codin	0	0	2.76549905	3.63247416
ENSG000001	OLFM1	protein_codin	0.99133435	0	0	0
ENSG000001	OLFM1	protein_codin	3.59514576	0.89693166	0	1.72352054
ENSG000001	STAU1	protein_codin	0	0	39.0252795	0
ENSG000001	IFIT1	protein_codin	41.1404525	60.0378654	22.7501102	23.8032423
ENSG000000	FCN1	protein_codin	15291.7834	19815.7482	4243.90392	14426.6098
ENSG000002	IFIT1B	protein_codin	0	1.01753095	0	0
ENSG000001	IFIT3	protein_codin	0	45.8301586	15.6544282	0
ENSG000002	COL5A1-AS1	lncRNA	0	0	0	0
ENSG000001	COL5A1	protein_codin	0	0	0	0
ENSG000001	IFIT3	protein_codin	87.660311	39.8110262	39.3710064	0
ENSG000001	ELAVL4	protein_codin	0	0	0	0
ENSG000001	COL5A1	protein_codin	1.83629653	4.46198602	2.64130612	0.87972234
ENSG000001	ELAVL4	protein_codin	0	0	0	0
ENSG000001	WDR44	protein_codin	0	0	0	0
ENSG000001	ELAVL4	protein_codin	0	0	0	0
ENSG000001	ELAVL4	protein_codin	0	0	0	0
ENSG000001	WDR44	protein_codin	13.2394665	0	0	0
ENSG000001	IFIT2	protein_codin	80.00751	56.8767859	0	0
ENSG000001	ELAVL4	protein_codin	0	0	0	0
ENSG000001	STAU1	protein_codin	242.748825	202.231768	0	59.5593649
ENSG000001	BEND5	protein_codin	0	0	3.48398457	0
ENSG000001	BRD3	protein_codin	63.5612415	117.284671	127.02103	0
ENSG000001	BRD4	protein_codin	111.681231	207.043531	52.6920773	89.1391353

ENSG000001	AGBL4	protein_codin	0	0	0	0
ENSG000001	LIPA	protein_codin	19.1171837	58.5914573	0	21.4929264
ENSG000001	AGBL4	protein_codin	0	0	0	0
ENSG000001	AGBL4	protein_codin	0	0	0	0
ENSG000001	SPATA6	protein_codin	0	0	0	0
ENSG000001	BRD3	protein_codin	1.0780719	0	0.81145107	0
ENSG000001	SPATA6	protein_codin	21.9332365	9.57012677	0	0
ENSG000001	SPATA6	protein_codin	75.4512822	0	18.3860789	34.7335537
ENSG000001	WDR44	protein_codin	65.8029801	158.356132	137.297795	91.9301463
ENSG000001	VAV2	protein_codin	10.8350191	0	0	0
ENSG000001	VAV2	protein_codin	135.86249	113.948524	36.3250466	38.8245203
ENSG000001	CH25H	protein_codin	34.5720791	1.18235655	0.73850404	3.56895941
ENSG000001	STAU1	protein_codin	70.17863	88.9360719	26.4419838	125.528997
ENSG000000	FAS	protein_codin	0	0	8.62018229	0
ENSG000001	SARDH	protein_codin	0	0	0	0.91189156
ENSG000001	SARDH	protein_codin	0	0	0	0
ENSG000001	CMPK1	protein_codin	16.156581	60.0737534	5.39497948	43.9624295
ENSG000001	CMPK1	protein_codin	11.1135579	0	0	5.13895615
ENSG000001	SARDH	protein_codin	0	0	0	0
ENSG000001	CMPK1	protein_codin	259.745284	105.163881	91.7787847	207.75148
ENSG000000	KLHL13	protein_codin	0	0	0	0
ENSG000001	STIL	protein_codin	3.4994091	0	0	0
ENSG000000	KLHL13	protein_codin	0	0	0	0
ENSG000000	KLHL13	protein_codin	0	0	0	0
ENSG000001	TAL1	protein_codin	91.184176	460.899151	20.5824702	100.99198
ENSG000001	PDZK1IP1	protein_codin	0	1.81604346	0	0
ENSG000001	CYP4A22	protein_codin	0	0	0	0
ENSG000001	CYP4A22	protein_codin	0	0	0	0
ENSG000001	TMEM63B	protein_codin	0	0	0	0
ENSG000002	CT83	protein_codin	0	0	0	0
ENSG000001	SLC2A6	protein_codin	0	49.6582832	0	27.0983063
ENSG000001	SLC2A6	protein_codin	198.202835	113.65057	37.4134806	118.996588
ENSG000001	CYP4X1	protein_codin	0	0	0	0
ENSG000001	CYP4A11	protein_codin	0	0	0	0
ENSG000001	AGTR2	protein_codin	0	0	0	0
ENSG000001	ADAMTS13	protein_codin	0	0	0	0
ENSG000001	ADAMTS13	protein_codin	0	0	0	0
ENSG000001	ADAMTS13	protein_codin	0	0	0	0.82672162
ENSG000001	ARFGEF2	protein_codin	291.173304	226.701063	197.711052	171.891528
ENSG000001	CYP4B1	protein_codin	0	0	0	0
ENSG000001	LUZP4	protein_codin	0	0	0	0
ENSG000001	LUZP4	protein_codin	0	0	0	0
ENSG000001	STAMBPL1	protein_codin	4.86285658	0	0	2.20824312
ENSG000001	CYP4B1	protein_codin	0	0	0	0

ENSG000001:	STAMBPL1	protein_codin	0	5.11631485	0	9.30838337
ENSG000001:	STAMBPL1	protein_codin	0	0	5.22613496	0
ENSG000001:	STAMBPL1	protein_codin	1.28709979	1.33238391	0	0
ENSG000001(	ADAMTS13	protein_codin	0	0	1.78259013	0
ENSG000001	ANKRD22	protein_codin	22.5123391	12.5747867	4.73135438	15.8023652
ENSG000001	EFCAB14	protein_codin	0	0	0	85.6391467
ENSG000001	REXO4	protein_codin	0	10.7308282	0	0
ENSG000001:	IL13RA2	protein_codin	0	0	0	0
ENSG000002	LIPJ	protein_codin	0	0	0	0
ENSG000001	MOB3C	protein_codin	96.2572017	80.1209867	70.4986842	30.3927116
ENSG000001:	PREX1	protein_codin	2521.12119	2710.9286	1113.8097	1745.90025
ENSG000001	REXO4	protein_codin	42.6912079	107.323482	38.8062363	41.2724369
ENSG000000	MKNK1	protein_codin	0	0	35.7489368	120.279593
ENSG000000	MKNK1	protein_codin	0	0	0	0
ENSG000001	RNLS	protein_codin	10.256915	0	0	5.36010241
ENSG000001	HTR2C	protein_codin	0	0	0	0
ENSG000001	HTR2C	protein_codin	0	0	0	0
ENSG000001	PTEN	protein_codin	0	0	0	0
ENSG000001!	STKLD1	protein_codin	0	0.84998613	0	0
ENSG000001:	AMOT	protein_codin	0	0	0	0
ENSG000001:	AMOT	protein_codin	1.55259993	0	5.22538724	2.0246885
ENSG000001:	AMOT	protein_codin	2.93921263	2.34219177	0	0
ENSG000001	SURF2	protein_codin	28.203088	48.2868135	15.4453821	26.3040605
ENSG000001	LHFPL1	protein_codin	0	0	0	0
ENSG000002	TRPC5OS	protein_codin	0	0	0	0
ENSG000001	SURF1	protein_codin	35.1434673	36.5526351	6.30489358	36.9529888
ENSG000000	RAD54L	protein_codin	0	0	0	0
ENSG000001(	ALG13	protein_codin	46.1372529	12.0031183	9.46788669	20.5496034
ENSG000001	LURAP1	protein_codin	1.01284782	1.01229943	0	0
ENSG000001	SURF4	protein_codin	6.15366397	0	0	0
ENSG000000	POMGNT1	protein_codin	0	0	0	0
ENSG000001	SURF4	protein_codin	217.745968	384.462881	80.9283048	163.076309
ENSG000001	SURF4	protein_codin	0	0	0.86967249	0
ENSG000000	POMGNT1	protein_codin	0	0	0	0
ENSG000000	DCX	protein_codin	0	0	0	0
ENSG000001(	MINPP1	protein_codin	14.1198334	0	0	37.0083946
ENSG000001(	MINPP1	protein_codin	0	62.1926863	22.2586466	8.50659273
ENSG000001:	NCOA3	protein_codin	209.125916	230.899272	458.704233	131.719229
ENSG000001:	NCOA3	protein_codin	789.342166	820.34189	688.109725	248.511787
ENSG000001	MED22	protein_codin	0	2.10993345	0	0
ENSG000001	TSPAN1	protein_codin	0	0	0	0
ENSG000001:	NCOA3	protein_codin	151.380746	0	29.7266212	329.841503
ENSG000001	PIK3R3	protein_codin	0	0	0	0
ENSG000000	PAK3	protein_codin	0	1.48218801	0	0

ENSG000000[illegible]	MAST2	protein_codin	17.2101364	0	0	0
ENSG000000[illegible]	PAK3	protein_codin	0	0	0	0
ENSG000002[illegible]	BMS1P3	transcribed_u	0	0	0	0
ENSG000001[illegible]	ADIRF	protein_codin	0	0	0	0
ENSG000001[illegible]	MRPL14	protein_codin	16.1326985	44.2661507	10.2427966	13.9635844
ENSG000001[illegible]	SNCG	protein_codin	0	0	0	0
ENSG000001[illegible]	SURF6	protein_codin	118.97278	165.158678	86.5554136	80.1888408
ENSG000001[illegible]	ZMYND8	protein_codin	0	0	0	0
ENSG000001[illegible]	TMEM69	protein_codin	50.4568444	62.445816	27.8461652	35.7419182
ENSG000001[illegible]	MMRN2	protein_codin	1.79812061	1.79833243	0	0
ENSG000002[illegible]	LCN1P2	unprocessed_	0	0	0	0
ENSG000001	LCN1P1	unprocessed_	0	0	0	0
ENSG000001[illegible]	OBP2B	protein_codin	0	0	0	0
ENSG000001[illegible]	GBGT1	protein_codin	0	0	0	0
ENSG000001[illegible]	BMPR1A	protein_codin	0	0	0	0
ENSG000001[illegible]	GBGT1	protein_codin	3.65657422	17.5227654	1.85344708	3.83922371
ENSG000001[illegible]	GBGT1	protein_codin	20.6144504	0	0	17.3386419
ENSG000001[illegible]	CHRDL1	protein_codin	0	0	0	0
ENSG000001[illegible]	GBGT1	protein_codin	0	40.2764644	0	0
ENSG000001[illegible]	CCDC17	protein_codin	0	0	0	0
ENSG000001[illegible]	CHRDL1	protein_codin	0	0	0	0
ENSG000001[illegible]	RALGDS	protein_codin	0	0	0	0
ENSG000001[illegible]	RALGDS	protein_codin	86.5458631	0	4.62621137	30.6794587
ENSG000001[illegible]	NASP	protein_codin	0	0	0	0
ENSG000001[illegible]	GNG5B	protein_codin	0	0	0	0
ENSG000001	VEGFA	protein_codin	0	0	0	0
ENSG000001[illegible]	LDB3	protein_codin	0	0	0	0
ENSG000001[illegible]	AMMECR1	protein_codin	0	0	0	0
ENSG000001[illegible]	AMMECR1	protein_codin	0	0	0	0
ENSG000001[illegible]	RALGDS	protein_codin	13.4341231	0	3.97576851	0
ENSG000001	VEGFA	protein_codin	0	0	0	0
ENSG000001[illegible]	LDB3	protein_codin	0	0	0	0
ENSG000001	VEGFA	protein_codin	421.593553	493.489353	173.377342	335.668443
ENSG000001[illegible]	TMEM164	protein_codin	0	69.9301904	0	62.9653233
ENSG000001	AKR1A1	protein_codin	0	0	0	0
ENSG000001[illegible]	OPN4	protein_codin	0	0	0	0
ENSG000001[illegible]	TMEM164	protein_codin	299.057463	470.775692	156.171092	175.752041
ENSG000001[illegible]	TMEM164	protein_codin	67.4744626	23.104278	0	21.4036876
ENSG000000[illegible]	WAPL	protein_codin	5.43734468	0	0	0
ENSG000001	VEGFA	protein_codin	209.057033	162.124342	54.4828898	66.5535351
ENSG000001	PRDX1	protein_codin	0	0	0	0
ENSG000001[illegible]	CEL	protein_codin	6.56678431	4.75759272	3.82858765	3.61422146
ENSG000000[illegible]	TESK2	protein_codin	0	0	0	0
ENSG000000[illegible]	TESK2	protein_codin	0	0	0	0

ENSG000001	CCSER2	protein_codin	100.907732	56.7916137	55.0128823	34.0877992
ENSG000001	TOE1	protein_codin	15.1084105	37.6644976	13.8881075	0
ENSG000001	RGR	protein_codin	0	0	0	0
ENSG000001	GTF3C5	protein_codin	60.4761795	55.2426047	42.1613387	46.8427783
ENSG000001	MUTYH	protein_codin	0	0	0	0
ENSG000001	GTF3C5	protein_codin	22.0371487	19.6933105	11.2355853	9.83846031
ENSG000001	KCNE5	protein_codin	0	0	1.7746396	0.95310155
ENSG000001	TP53RK	protein_codin	0	0	0	0
ENSG000001	NXT2	protein_codin	4.11928544	4.85100773	1.83160293	4.81646918
ENSG000001	MUTYH	protein_codin	0	0	0	0
ENSG000001	LRIT1	protein_codin	0	0	0	0
ENSG000001	NXT2	protein_codin	28.0642192	22.959386	15.5484308	31.8979736
ENSG000001	NXT2	protein_codin	0	0	0	0
ENSG000001	GTF3C5	protein_codin	0	30.8521683	0	0
ENSG000001	MUTYH	protein_codin	0	0	0	0
ENSG000002	LRIT2	protein_codin	0	0	0	2.93603391
ENSG000001	TP53RK	protein_codin	16.9314857	39.3208603	9.16624661	16.8839476
ENSG000001	MUTYH	protein_codin	0	0	0	0
ENSG000000	MRPS18A	protein_codin	28.8249399	33.6188149	17.4383036	15.6795036
ENSG000001	CDHR1	protein_codin	0	0	0	0
ENSG000001	SLC13A3	protein_codin	0	0	0	0
ENSG000001	GFI1B	protein_codin	91.8859539	100.0968	0	44.5932751
ENSG000001	GFI1B	protein_codin	6.61475812	0	0	0
ENSG000001	GPR15LG	protein_codin	0	0	0	0
ENSG000001	IRS4	protein_codin	0	0.99787998	0	0
ENSG000000	MRPS18A	protein_codin	24.138408	47.750631	4.87724433	11.7022874
ENSG000001	GHITM	protein_codin	302.472259	365.714873	187.355058	212.425311
ENSG000001	SPACA9	protein_codin	3.9032531	5.09918161	0	0
ENSG000001	NRG3	protein_codin	0	0	0	0
ENSG000001	NRG3	protein_codin	0	0	0	0
ENSG000001	GTF3C4	protein_codin	69.6680276	97.1054358	43.4637147	55.9063955
ENSG000001	SH2D4B	protein_codin	0	0	0	0
ENSG000001	DDX31	protein_codin	1.57768925	0	2.23750656	0
ENSG000001	TSPAN14	protein_codin	0	12.3305392	2.60540087	0
ENSG000001	TSPAN14	protein_codin	1.87271458	2.18502472	0	0
ENSG000001	TSPAN14	protein_codin	74.177837	108.67057	35.0417552	37.1082
ENSG000001	DDX31	protein_codin	17.4066341	25.1738401	26.0251381	14.9226055
ENSG000001	RSPH9	protein_codin	5.87854352	5.79326551	5.0218476	1.93658058
ENSG000001	TSPAN14	protein_codin	0	0	0	0
ENSG000001	RSPH9	protein_codin	0	0	0	1.91252886
ENSG000001	HECTD3	protein_codin	0	6.61211366	0	2.72039592
ENSG000001	MAD2L1BP	protein_codin	35.2506032	42.2150157	24.0613586	21.1136242
ENSG000001	HECTD3	protein_codin	123.459968	128.668812	46.4243412	46.5459271
ENSG000002	FAM66C	lncRNA	0	0	0	0

ENSG000000	ELMO2	protein_codin	0	0	0	0
ENSG000001	PRXL2A	protein_codin	0	0	0	0
ENSG000000	EIF2B3	protein_codin	0	0	0	0
ENSG000000	EIF2B3	protein_codin	16.5742972	19.0697857	28.519921	7.71377783
ENSG000001	PRXL2A	protein_codin	0	17.1310422	0	0
ENSG000001	PRXL2A	protein_codin	0	0	14.1021227	0
ENSG000001	PRXL2A	protein_codin	10.2287773	0	0	4.21106942
ENSG000001	RAPGEF1	protein_codin	123.67498	98.7028716	8.41087735	87.8040614
ENSG000001	RAPGEF1	protein_codin	108.169165	140.896631	6.46707332	4.91377344
ENSG000001	PTCH2	protein_codin	0.99680133	1.96025469	2.89775027	0.93538032
ENSG000001	RAPGEF1	protein_codin	745.42677	977.859283	472.28737	434.068724
ENSG000001	DYDC2	protein_codin	0	0	0	0
ENSG000001	DYDC2	protein_codin	0	0	0	0
ENSG000001	DYDC2	protein_codin	0	0	0	0
ENSG000001	PLK3	protein_codin	300.105204	0	0	0
ENSG000001	DYDC1	protein_codin	0	0	0	0
ENSG000001	DYDC1	protein_codin	0	0	0	0
ENSG000001	BEST4	protein_codin	2.70394522	0	0	0
ENSG000001	UCK1	protein_codin	0	0	0	0
ENSG000001	RPS8	protein_codin	117.606139	336.509106	245.330215	245.404012
ENSG000001	UCK1	protein_codin	0	0	0	0
ENSG000001	UCK1	protein_codin	4.28101617	0	0	0
ENSG000001	MAT1A	protein_codin	0	0	0	0
ENSG000002	ENSG000002	processed_ps	2.64329169	0	1.11162296	1.8306565
ENSG000001	UCK1	protein_codin	56.0436192	49.0152299	29.3853625	37.8834645
ENSG000001	COL4A6	protein_codin	0	0	0	0
ENSG000001	KIF2C	protein_codin	0	0	0	0
ENSG000001	POMT1	protein_codin	0	0	0	0
ENSG000001	KIF2C	protein_codin	10.3347898	0.91717261	16.0951968	1.89298396
ENSG000001	POLH	protein_codin	22.4435769	16.3181152	7.84450479	14.4391509
ENSG000000	SLC35C2	protein_codin	36.854628	85.0284024	22.465928	20.1979848
ENSG000001	POMT1	protein_codin	0	0	0	0
ENSG000000	SLC35C2	protein_codin	37.9618131	25.7556211	0	0
ENSG000001	ANXA11	protein_codin	95.1098197	94.524992	39.3725438	91.3701337
ENSG000001	ATG4A	protein_codin	33.3290715	48.4294719	23.6306384	14.4942787
ENSG000001	TMEM53	protein_codin	2.94520885	2.01398607	1.46427667	0
ENSG000001	POLH	protein_codin	9.39097702	12.8301393	13.5038977	9.16546069
ENSG000001	TMEM53	protein_codin	0	0	0	0
ENSG000001	TMEM53	protein_codin	0	0	0	0
ENSG000001	TMEM53	protein_codin	0	0	0	0
ENSG000001	TMEM53	protein_codin	3.65669782	0.8787037	1.98756118	1.0024429
ENSG000001	ATG4A	protein_codin	2.01357726	0	4.05742746	0
ENSG000001	ERI3	protein_codin	27.792316	0	9.83390378	14.5701973
ENSG000001	ERI3	protein_codin	14.4844477	35.1301741	14.1389692	13.5719817

ENSG000001	PLPP7	protein_codin	2.87626427	0	0	0
ENSG000001	PLAC9	protein_codin	0	0	0	0
ENSG000001	PLPP7	protein_codin	4.24956547	2.63793965	0	2.60073404
ENSG000001	PLAC9	protein_codin	0	0	0	0
ENSG000001	FAM78A	protein_codin	20.6394192	43.1356668	14.2023635	4.17134746
ENSG000001	PLAC9	protein_codin	0	0	0	0
ENSG000001	FAM78A	protein_codin	142.854118	295.523677	76.8960826	153.88528
ENSG000001	TMEM254	protein_codin	0	0	0	0
ENSG000001	TMEM254	protein_codin	1.78008118	0	0	0
ENSG000001	TMEM254	protein_codin	0	0	0	0
ENSG000001	CD40	protein_codin	0	0	22.5319532	0
ENSG000001	TMEM254	protein_codin	0	2.59102812	0	0
ENSG000001	TMEM254	protein_codin	0	0	12.2046966	0
ENSG000001	CD40	protein_codin	36.0081888	37.2893937	0	15.6498042
ENSG000001	DMAP1	protein_codin	0	0	0	5.42926798
ENSG000001	DMAP1	protein_codin	0	0	0	0
ENSG000001	NCOA5	protein_codin	0	21.4027961	0	0
ENSG000001	SFTPD	protein_codin	0	0	0	0
ENSG000001	PSMD10	protein_codin	0	0	7.72180451	0
ENSG000001	PSMD10	protein_codin	0	0	0	0
ENSG000001	AIF1L	protein_codin	0	0	0	0
ENSG000001	AIF1L	protein_codin	0	0	0	0
ENSG000001	KLF17	protein_codin	0	0	0	0.94446718
ENSG000001	AIF1L	protein_codin	0	0.87662311	0	0
ENSG000001	AIF1L	protein_codin	0	0	0	0
ENSG000001	AIF1L	protein_codin	0	0	0	0
ENSG000001	SLC6A9	protein_codin	0	0	0	0
ENSG000001	SLC6A9	protein_codin	0	0	0	0
ENSG000001	AIF1L	protein_codin	0	0	0	0
ENSG000001	SLC6A9	protein_codin	0	0	0	0
ENSG000001	AIF1L	protein_codin	0	0	0	0
ENSG000001	SLC12A5	protein_codin	0	0	0	0
ENSG000001	CALCOCO2	protein_codin	0	0	0	0
ENSG000001	CCDC24	protein_codin	0	0	0	0
ENSG000001	NUTM2B	protein_codin	0	0	0	0
ENSG000001	NUTM2G	protein_codin	0	0	0	0
ENSG000001	B4GALT2	protein_codin	0	0	0	0.94504319
ENSG000001	SFTPA2	protein_codin	0	0	0	0
ENSG000001	SFTPA2	protein_codin	0	0	0	0
ENSG000001	MMP9	protein_codin	21.5719062	6.95105521	8.56896565	2.95116589
ENSG000001	ZCCHC24	protein_codin	0	0	7.10048864	0
ENSG000001	ZCCHC24	protein_codin	77.4649955	61.207971	26.5450759	38.0086586
ENSG000001	FIBCD1	protein_codin	0	0	0	0
ENSG000001	FIBCD1	protein_codin	0	0	0	0

ENSG000001	IPO13	protein_codin	0	0	0	0
ENSG000001	IPO13	protein_codin	59.9950908	58.9304466	32.4423829	44.1157811
ENSG000001	POLR1C	protein_codin	6.85188041	0	0	0
ENSG000000	ABL1	protein_codin	188.820329	259.666062	76.8864529	122.902113
ENSG000001	EXOSC2	protein_codin	0	0	0	0
ENSG000001	EXOSC2	protein_codin	0	0	12.8481018	0
ENSG000001	EXOSC2	protein_codin	0	0	0	0
ENSG000001	ARTN	protein_codin	0	0	0	0
ENSG000001	EXOSC2	protein_codin	19.2860899	29.7561871	0	10.7535837
ENSG000001	ARTN	protein_codin	0	0	0	0
ENSG000001	RPS24	protein_codin	1356.62717	2570.14495	885.834477	1233.71174
ENSG000001	ST3GAL3	protein_codin	0	0	0	0
ENSG000001	ST3GAL3	protein_codin	0	0	0	0
ENSG000001	ST3GAL3	protein_codin	0	0	0	0
ENSG000001	ST3GAL3	protein_codin	0	0	0	0
ENSG000001	ST3GAL3	protein_codin	0	0	0	0
ENSG000001	ST3GAL3	protein_codin	0	0	0	0
ENSG000001	POLR3A	protein_codin	0	0	31.5575094	0
ENSG000001	ST3GAL3	protein_codin	0	0	18.0619894	0
ENSG000001	ST3GAL3	protein_codin	0	0	0	0
ENSG000001	TSC22D3	protein_codin	0	0	0	0
ENSG000001	TSC22D3	protein_codin	111.0562	196.689804	80.8483534	91.0986397
ENSG000001	TSC22D3	protein_codin	15.7500264	0	0	6.94817164
ENSG000001	ASS1	protein_codin	0	0	0	0
ENSG000002	ENSG000002	lncRNA	0	0	0	0
ENSG000001	TSC22D3	protein_codin	41.4500349	26.2608296	6.1627823	18.0311506
ENSG000001	DLG5	protein_codin	0	37.336104	3.58002577	5.07838258
ENSG000001	ASS1	protein_codin	0	0	0	0
ENSG000001	ASS1	protein_codin	0	0	0	0
ENSG000000	KDM4A	protein_codin	180.989295	267.431513	147.82273	132.301046
ENSG000001	TSC22D3	protein_codin	2287.6969	1726.65389	561.376391	783.191292
ENSG000001	NCS1	protein_codin	0	0	0	0
ENSG000001	KCNMA1	protein_codin	0	0	0	0
ENSG000001	PTPRF	protein_codin	0	0	0	0
ENSG000001	GPR107	protein_codin	0	0	0	0
ENSG000001	PTPRF	protein_codin	0	0	0	0
ENSG000001	KCNMA1	protein_codin	0	0	0	0
ENSG000001	PCIF1	protein_codin	142.935222	211.403565	84.8072041	115.775916
ENSG000001	GPR107	protein_codin	10.2660722	102.683289	34.9987531	0
ENSG000001	YIPF3	protein_codin	0	0	0	0
ENSG000001	PRPS1	protein_codin	19.5618396	21.0571614	0	2.71999566
ENSG000001	PRPS1	protein_codin	0	0	0	0
ENSG000001	PLTP	protein_codin	0	0	0	0
ENSG000001	KCNMA1	protein_codin	0	0	0	0

ENSG000001	YIPF3	protein_codin	130.804101	176.662358	78.855758	95.9218125
ENSG000001	HYI	protein_codin	6.83101012	5.53876742	3.83940608	0
ENSG000001	PRPS1	protein_codin	0	0	0	0
ENSG000001	USP20	protein_codin	0	73.3861199	0	0
ENSG000001	HYI	protein_codin	7.75464871	10.5560415	0	0
ENSG000001	PLTP	protein_codin	6.10236095	0	0	0
ENSG000001	HYI	protein_codin	0	0	0	0
ENSG000001	HYI	protein_codin	0	17.7161492	7.51519258	4.97333354
ENSG000001	HYI	protein_codin	0	0	0	1.70721071
ENSG000001	PRPS1	protein_codin	8.76766551	28.5806869	32.2215447	18.5252785
ENSG000001	KCNMA1	protein_codin	0	0	0	0
ENSG000001	KCNMA1	protein_codin	0	0	0	0
ENSG000002	LRRC73	protein_codin	0.88634987	0.91846206	0	0
ENSG000001	KCNMA1	protein_codin	0	0	0	0
ENSG000001	TJAP1	protein_codin	46.9981074	0	4.55975146	0
ENSG000001	TJAP1	protein_codin	0	0	0	0
ENSG000001	C9orf78	protein_codin	281.120901	337.847342	135.26677	219.028899
ENSG000001	TJAP1	protein_codin	0	0	0	0
ENSG000001	SZT2	protein_codin	6.43294962	9.78632125	5.09288929	1.76689968
ENSG000002	MYCLP1	processed_ps	0	0	0.85089631	0
ENSG000001	TJAP1	protein_codin	67.566517	89.1332507	13.8922002	52.4100155
ENSG000000	DNAAF6	protein_codin	0	0	0	0
ENSG000001	TJAP1	protein_codin	5.82746947	10.2843869	0	3.30965754
ENSG000001	MED8	protein_codin	8.00229304	0	0	0
ENSG000001	MED8	protein_codin	27.1521623	101.910316	15.5863355	32.3109239
ENSG000000	ELOVL1	protein_codin	81.6597392	87.2211261	13.656956	32.6674556
ENSG000000	CTSA	protein_codin	85.4367354	75.9989237	32.4368969	53.5341846
ENSG000001	CDC20	protein_codin	0	0	0	0
ENSG000001	NUP62CL	protein_codin	0	0	2.69102541	0
ENSG000001	PRRX2	protein_codin	0	0	0	0
ENSG000001	MPL	protein_codin	87.8654903	181.696026	79.8352412	67.2699928
ENSG000000	TIE1	protein_codin	0	0	0	0.89252857
ENSG000001	C9orf50	protein_codin	0	0	0	0.75532909
ENSG000000	RBM41	protein_codin	35.9228051	3.23342064	21.423943	5.29055389
ENSG000001	NTMT1	protein_codin	10.5463636	8.95080974	4.67665958	3.19576456
ENSG000001	NTMT1	protein_codin	0	2.14890129	0	0
ENSG000001	NTMT1	protein_codin	0	0	7.47923876	0
ENSG000000	CTSA	protein_codin	0	0	0	0
ENSG000001	DLK2	protein_codin	0	0	0	0
ENSG000001	NTMT1	protein_codin	0	0	0	5.69472804
ENSG000000	RBM41	protein_codin	0	9.68711299	0	30.3985167
ENSG000001	DLK2	protein_codin	0	0	0	0
ENSG000001	TMEM156	protein_codin	0	0	1.13427542	0
ENSG000002	IER5L-AS1	lncRNA	0	0	0	0

ENSG000001	IER5L	protein_codin	28.5605801	70.5523149	5.38475716	36.2199922
ENSG000002	CFAP57	protein_codin	0	0	0	0
ENSG000001	LRMDA	protein_codin	19.6359785	34.2891722	14.5978033	10.5455473
ENSG000001	SLC2A1	protein_codin	0	0	0	0
ENSG000001	ZNF691	protein_codin	0	0	0	0
ENSG000001	ZNF691	protein_codin	0	0	0	0
ENSG000001	ZNF691	protein_codin	0	0	0	0
ENSG000001	ZNF691	protein_codin	0	0	0	0
ENSG000001	ZNF691	protein_codin	0	0	0	0
ENSG000001	ZNF691	protein_codin	0	0	3.98327728	0
ENSG000001	ABCC10	protein_codin	0	4.34002	0	0
ENSG000001	ERMAP	protein_codin	0	0	0	0
ENSG000001	ABCC10	protein_codin	33.6274694	18.5345467	12.0636118	10.6229833
ENSG000001	ERMAP	protein_codin	81.2707838	62.4426372	0	48.6391454
ENSG000001	NEURL2	protein_codin	2.72058737	0	0	0
ENSG000001	SPATA25	protein_codin	0	0	1.0138138	0
ENSG000001	SVBP	protein_codin	43.8343415	133.434754	46.7378137	61.7329726
ENSG000001	SVBP	protein_codin	4.58699924	0	0	1.72577929
ENSG000001	ZSWIM1	protein_codin	49.1949435	57.4758357	35.1006377	18.6247593
ENSG000001	ZNF503	protein_codin	80.098129	65.3142287	17.6348328	29.5055774
ENSG000001	C1orf50	protein_codin	17.8861359	26.9148914	21.6264092	8.59566024
ENSG000001	P3H1	protein_codin	0	0	0	0
ENSG000001	ABCC10	protein_codin	49.1059315	87.473014	31.1042063	24.7682877
ENSG000001	COMTD1	protein_codin	9.49895955	5.76414337	0	2.76432994
ENSG000001	CLDN19	protein_codin	0	0	0	0
ENSG000001	SNX21	protein_codin	0	0	0	0
ENSG000001	SNX21	protein_codin	0	9.33493898	0	0
ENSG000001	RADX	protein_codin	0	0	2.44575986	0
ENSG000001	DOLPP1	protein_codin	14.8952243	23.0897237	8.08195866	0
ENSG000001	SNX21	protein_codin	2.39948142	22.8821175	0	0
ENSG000001	RADX	protein_codin	0	0	0	0
ENSG000001	PPIH	protein_codin	0	0	0	0
ENSG000001	PPIH	protein_codin	0	0	0	0
ENSG000001	PWWP3B	protein_codin	0	0	0	0
ENSG000001	SH3GLB2	protein_codin	0	0	0	14.7150051
ENSG000001	TNNC2	protein_codin	22.96252	52.5504554	17.4814421	9.36667632
ENSG000001	PPCS	protein_codin	118.292645	178.879997	50.2691172	82.5827084
ENSG000001	TNNC2	protein_codin	0	0	0	0
ENSG000001	SH3GLB2	protein_codin	62.8091114	53.290941	32.4379841	33.799341
ENSG000001	PPCS	protein_codin	8.48886186	27.8653313	0	22.5746824
ENSG000001	PPCS	protein_codin	44.4603171	19.0967046	15.3941503	3.40723437
ENSG000001	PPCS	protein_codin	0	0	0	0
ENSG000001	SERPINA7	protein_codin	0	0	0	0
ENSG000001	SH3GLB2	protein_codin	27.2182669	65.7525387	26.2321788	0

ENSG000000	ZMYND12	protein_codin	0	6.38773215	1.55162612	1.07906767
ENSG000001	UBE2C	protein_codin	0	0	7.15351176	0
ENSG000001	CRIP3	protein_codin	0	5.68504313	0	0
ENSG000001	FOXJ3	protein_codin	5.3199883	11.6271557	15.7716723	10.8830306
ENSG000001	FOXJ3	protein_codin	9.55105529	42.4365947	7.89176619	11.1596325
ENSG000001	FOXJ3	protein_codin	0	0	0	0
ENSG000001	SLC22A7	protein_codin	0	0	0	0
ENSG000000	NUP188	protein_codin	187.31182	207.412586	144.898914	121.802659
ENSG000000	GUCA2B	protein_codin	0	0	0	0
ENSG000001	IL1RAPL2	protein_codin	0	0	0	0
ENSG000001	HIVEP3	protein_codin	0	0	0	0
ENSG000001	HIVEP3	protein_codin	0	0	0	0
ENSG000001	SLC22A7	protein_codin	0	0	0	0
ENSG000001	DOLK	protein_codin	6.84475853	20.5583524	2.47255563	4.98235562
ENSG000001	EDN2	protein_codin	0	0	0	0
ENSG000001	ESX1	protein_codin	0	0	0	0
ENSG000001	SLC22A7	protein_codin	0	0	0	0
ENSG000002	FOXO6	protein_codin	0.81664596	2.49468108	0	0
ENSG000001	PHYHD1	protein_codin	0	0	0	0
ENSG000000	SCMH1	protein_codin	19.529819	3.43698888	2.51595164	0
ENSG000000	SCMH1	protein_codin	0	5.90238821	4.11582163	0
ENSG000000	SCMH1	protein_codin	0	0	0	0
ENSG000001	LRRC8A	protein_codin	0	0	0	0
ENSG000001	LRRC8A	protein_codin	125.688186	175.589298	96.368751	130.637374
ENSG000001	SLFNL1	protein_codin	0	0	0	0
ENSG000001	SLFNL1	protein_codin	0	0	0	0
ENSG000001	CTPS1	protein_codin	9.8327098	0	0	0
ENSG000001	DNTTIP1	protein_codin	160.720674	255.227304	94.7137982	128.44532
ENSG000001	TCEAL1	protein_codin	1.78209435	0	0	7.39563922
ENSG000001	TCEAL1	protein_codin	3.12947079	18.2691179	4.40988643	15.7751254
ENSG000001	TCEAL1	protein_codin	20.5511913	16.1834802	8.5540755	0
ENSG000001	TCEAL3	protein_codin	30.0827821	22.3661792	33.0726673	21.8890237
ENSG000001	TCEAL3	protein_codin	0	0	2.42685468	0
ENSG000001	TCEAL4	protein_codin	0	0	0	0
ENSG000001	WFDC3	protein_codin	0	0	0	0
ENSG000001	WFDC3	protein_codin	0	0	0	0
ENSG000001	RAB40A	protein_codin	0	0	0	0
ENSG000001	BEX3	protein_codin	1.69783013	1.82013581	1.54079564	1.69940969
ENSG000001	BEX3	protein_codin	0	0	0	0
ENSG000001	CITED4	protein_codin	37.8722881	50.7175932	3.83682714	37.8478128
ENSG000001	ENDOG	protein_codin	16.5213136	13.2415929	7.00692332	17.7584037
ENSG000001	WFDC10A	protein_codin	0	0	0	0
ENSG000001	BEX3	protein_codin	70.4567406	262.984846	90.5497896	110.406636
ENSG000001	CUL9	protein_codin	0	0	0	0

ENSG000001	TBC1D13	protein_codin	63.0064002	110.094002	44.520433	51.6375108
ENSG000000	NFYC	protein_codin	0	0	0	0
ENSG000000	NFYC	protein_codin	15.227711	0	4.16206131	20.7432242
ENSG000000	NFYC	protein_codin	0	1.65889741	4.32352206	0
ENSG000000	NFYC	protein_codin	0	0	9.18130992	0
ENSG000001	TCEAL9	protein_codin	3.48244569	14.2733415	18.8317545	10.2605663
ENSG000001	TCEAL9	protein_codin	18.8026527	65.0114941	53.3280941	19.6258953
ENSG000001	ZDHHC12	protein_codin	60.2847544	52.3740971	30.1740554	40.7680863
ENSG000001	TCEAL7	protein_codin	0	0	0	0
ENSG000001	ZDHHC12	protein_codin	0	25.4444751	4.37494379	0
ENSG000000	NFYC	protein_codin	7.39908142	2.94555682	0	14.3339699
ENSG000002	WFDC6	protein_codin	0	0	0	0
ENSG000001	BEX2	protein_codin	1.48607921	0	5.03184995	0.94786036
ENSG000001	WFDC2	protein_codin	0	0	0	0
ENSG000001	BEX2	protein_codin	3.25067972	3.65565757	0	0
ENSG000002	TCEAL5	protein_codin	0	0	0	0
ENSG000001	RIMS3	protein_codin	0	0	0	0
ENSG000001	RIMS3	protein_codin	9.97399054	11.8228754	9.57140475	4.96183976
ENSG000001	TCEAL8	protein_codin	54.041021	54.9398691	22.5714951	41.7654171
ENSG000001	SET	protein_codin	486.779163	608.547069	0	359.462574
ENSG000001	SAMD8	protein_codin	70.2619581	54.4905468	19.0667044	22.0473569
ENSG000001	SET	protein_codin	47.6197163	204.965894	209.641743	139.826719
ENSG000001	PIGT	protein_codin	0	0	0	0
ENSG000001	BEX4	protein_codin	0	7.72483432	6.72214278	0
ENSG000001	SET	protein_codin	0	0	0	0
ENSG000001	BEX4	protein_codin	32.7547585	35.1954565	30.2362076	26.343442
ENSG000001	ZNF684	protein_codin	1.17316639	1.50895571	0	1.26125108
ENSG000001	ZNF684	protein_codin	0	0	0	0
ENSG000001	ZNF684	protein_codin	12.9288939	12.7373567	7.82543058	0
ENSG000002	DUSP13A	protein_codin	0	0	0	0
ENSG000001	EXO5	protein_codin	10.6314573	8.57383594	8.96970965	2.03037736
ENSG000001	ZFP69	protein_codin	20.158834	16.3800228	0	8.72177911
ENSG000001	ZFP69	protein_codin	2.45423079	2.37872417	13.9189786	2.55075661
ENSG000000	SMAP2	protein_codin	33.1277549	22.5431766	14.4613484	0
ENSG000002	DBNDD2	protein_codin	0	0	0	0
ENSG000001	KAT6B	protein_codin	10.4009785	10.1965099	15.1847396	24.9700207
ENSG000002	DBNDD2	protein_codin	1.35499058	0	1.62120577	1.53153491
ENSG000001	KAT6B	protein_codin	0	11.8555867	130.156635	16.41691
ENSG000001	DYNC2I2	protein_codin	7.57557856	6.69489806	15.1921717	1.8557571
ENSG000002	DBNDD2	protein_codin	4.58092944	3.242242	0	4.66147159
ENSG000000	SMAP2	protein_codin	2347.03205	1840.231	505.582399	887.026744
ENSG000002	DBNDD2	protein_codin	0	0	0	0
ENSG000002	DBNDD2	protein_codin	0	0	0	0
ENSG000002	DBNDD2	protein_codin	0	0	0	0

ENSG000001	KAT6B	protein_codin	0	0	0	0
ENSG000001	KAT6B	protein_codin	0	0	0	0
ENSG000001	TP53TG5	protein_codin	0	0	0	0
ENSG000002	SYS1	protein_codin	0	0	0	0
ENSG000001	BEX1	protein_codin	1.95143765	11.082193	6.42524439	2.91806742
ENSG000001	SPTAN1	protein_codin	543.729339	774.529057	374.9243	355.406545
ENSG000001	SDC4	protein_codin	126.68022	119.620486	27.3346617	34.7116625
ENSG000001	ADK	protein_codin	62.0967353	0	0	20.8304932
ENSG000001	GPRASP3	protein_codin	0	0	0	0
ENSG000000	COL9A2	protein_codin	0	0	0	0
ENSG000001	SPTAN1	protein_codin	0	0	0	0
ENSG000001	RBPJL	protein_codin	0	0	0	0
ENSG000001	ARMCX5	protein_codin	0	0	0	0
ENSG000001	RBPJL	protein_codin	0	0	0	0
ENSG000001	AP3M1	protein_codin	55.05486	74.9019627	33.338455	54.803478
ENSG000000	COL9A2	protein_codin	33.1402429	63.3656814	0	29.8516405
ENSG000001	MATN4	protein_codin	0	0	0	0
ENSG000000	VCL	protein_codin	883.889415	1735.5286	860.372852	794.488128
ENSG000001	MATN4	protein_codin	0	0	0	0
ENSG000000	ZMPSTE24	protein_codin	63.2626433	77.855532	42.0965308	71.8036573
ENSG000001	PLAU	protein_codin	18.8108596	0	0	4.00110516
ENSG000001	CAMK2G	protein_codin	0	0	0	0
ENSG000001	TMCO2	protein_codin	0	0	0	0
ENSG000001	SEMG2	protein_codin	0.95250862	1.90396336	0	0
ENSG000001	GLE1	protein_codin	0	0	0	0
ENSG000001	RLF	protein_codin	389.274471	187.904869	224.215974	226.673568
ENSG000002	TCEAL6	protein_codin	0	0.80388683	0	0
ENSG000002	TCEAL6	protein_codin	0	0	0	0
ENSG000001	PPT1	protein_codin	0	1.8458748	0	0
ENSG000001	PPT1	protein_codin	0	0	0	0
ENSG000001	TCEAL2	protein_codin	1.37405127	0	0	0
ENSG000001	SEMG1	protein_codin	0	0	0	0.98964829
ENSG000001	ZMAT1	protein_codin	0	0	0	0
ENSG000001	WFDC12	protein_codin	0	0	0	0
ENSG000001	WFDC5	protein_codin	0	0	0	0
ENSG000001	ODF2	protein_codin	0	0	0	0
ENSG000001	CAP1	protein_codin	429.362225	433.935348	289.524724	215.68065
ENSG000001	CAP1	protein_codin	191.032745	304.990497	64.7949841	175.786636
ENSG000001	CAP1	protein_codin	0	124.164815	134.923461	72.3437715
ENSG000001	STK4	protein_codin	212.52314	254.476616	327.264161	163.93853
ENSG000001	CAP1	protein_codin	0	0	0	0
ENSG000002	NXF5	lncRNA	0	0	0	0
ENSG000001	CAP1	protein_codin	1247.60289	1679.98469	579.375863	852.555977
ENSG000001	STK4	protein_codin	942.560625	867.798529	365.005361	780.011681

ENSG000001:	ODF2	protein_codin	110.897812	151.743119	196.058434	63.0956527
ENSG000001:	GNMT	protein_codin	4.86132836	5.7359309	5.03036446	0
ENSG000001(	MFSD2A	protein_codin	12.7000399	0	0	0
ENSG000001(	MFSD2A	protein_codin	41.9953219	54.6483335	8.8612773	0
ENSG00000:	TOMM34	protein_codin	30.5721758	45.6735871	36.4843252	17.2073955
ENSG000001:	ODF2	protein_codin	0	0	0	0
ENSG000001	MYCL	protein_codin	0	5.61507819	1.96638683	1.14746465
ENSG000001	MYCL	protein_codin	33.6932012	154.199234	11.9444407	60.9067722
ENSG00000(	TRIT1	protein_codin	34.412486	0	0	23.4852604
ENSG000001(	PABPC1L	protein_codin	2.51310791	0	3.80658297	0
ENSG000001(	PABPC1L	protein_codin	0	0	0	0
ENSG000001(	PABPC1L	protein_codin	9.2793267	17.1562449	13.420104	7.3601238
ENSG000001(	PABPC1L	protein_codin	0	1.13352823	0	1.89685745
ENSG000001	BMP8B	protein_codin	27.5423255	44.1626844	36.4833079	12.8962803
ENSG000002(	ARMCX7P	transcribed_u	0	0	0	0
ENSG000001:	ARMCX1	protein_codin	8.05221842	8.87636348	3.16543436	11.1510962
ENSG00000(	PPIE	protein_codin	11.1609212	6.53040691	6.12193652	2.22690503
ENSG000001'	CHCHD1	protein_codin	22.036871	34.0986199	18.4913326	18.0555918
ENSG00000(	PPIE	protein_codin	0	0	0	0
ENSG000001:	CNPY3	protein_codin	1027.44545	1514.19701	511.596006	675.299719
ENSG000001'	CHCHD1	protein_codin	0	3.89515822	0	0
ENSG000001(	CERCAM	protein_codin	4.74542204	2.85954842	1.0380074	0
ENSG000001(	YWHAB	protein_codin	415.846076	613.135063	147.919602	309.115458
ENSG000001!	FUT11	protein_codin	34.2635107	90.5005183	22.2748598	43.435064
ENSG000001(	CERCAM	protein_codin	0	0	0	0.85667002
ENSG000001	HPCAL4	protein_codin	2.08127648	0	0	0
ENSG000001(	URM1	protein_codin	0	0	0	0
ENSG000002(	RPL36A	protein_codin	10.7812066	0	58.5613148	0
ENSG000001(	URM1	protein_codin	18.2862753	37.4729352	12.7678955	9.88589182
ENSG000001(	RIMS4	protein_codin	0	0	0	0
ENSG000001(	HEYL	protein_codin	2.73939523	0.90190686	0	0
ENSG000001(	URM1	protein_codin	31.2887973	69.7669354	14.8275029	40.9020218
ENSG00000!	PABPC4	protein_codin	71.9388101	0	0	19.5934369
ENSG00000!	PABPC4	protein_codin	0	0	0	0
ENSG00000!	PABPC4	protein_codin	226.113648	302.933662	45.5531803	153.332309
ENSG000001:	KCNK15	protein_codin	0	0	0	0
ENSG00000!	PABPC4	protein_codin	16.0832933	30.5809586	11.0214368	17.0788633
ENSG00000(	CCN5	protein_codin	0	0	0	0
ENSG00000(	CCN5	protein_codin	0	0	0	0
ENSG000001(	SLC27A4	protein_codin	0	14.0062076	0	0
ENSG000001(	SYNPO2L	protein_codin	0.92978587	1.8309802	0	0.90368938
ENSG000001!	ADA	protein_codin	0	0	20.2790338	0
ENSG000001(	COQ4	protein_codin	4.9347155	4.38063384	3.2246888	1.16410048
ENSG000001:	TBCC	protein_codin	37.5138058	66.7199533	28.5233157	23.4769773

ENSG000001!	TOMM7	protein_codin	1.31791439	5.09037773	0	0
ENSG000001(	PKIG	protein_codin	0	0	0	0
ENSG000001(	PKIG	protein_codin	0	0	0	0
ENSG000001(	PKIG	protein_codin	10.329373	1.97975076	1.63999002	2.17635804
ENSG000001(	TRUB2	protein_codin	27.4792402	61.9595999	31.5918938	19.9857318
ENSG000001(	PKIG	protein_codin	0	0	1.54413517	1.51208937
ENSG000001(	PKIG	protein_codin	0	0	0	0
ENSG000001(	PKIG	protein_codin	2.69593781	36.0377586	25.0947477	13.6121973
ENSG000001'	SWI5	protein_codin	0	0	0	0
ENSG00000O:	UBR2	protein_codin	242.616492	288.579095	140.390667	67.6721593
ENSG00000O:	UBR2	protein_codin	594.008186	455.012708	303.403017	476.365999
ENSG000001:	TIMM8A	protein_codin	10.7661137	5.81553349	6.14488302	5.35822937
ENSG00000O:	UBR2	protein_codin	2.98472708	0	3.10801259	2.24510039
ENSG000001:	TTPAL	protein_codin	0	0	20.6913995	0
ENSG000001:	TTPAL	protein_codin	0	0	0	0
ENSG000001(	TAF7L	protein_codin	0	0	0	0
ENSG000001(	LINC01620	lncRNA	0	0	0	0
ENSG000001(	MSS51	protein_codin	0	0	0	0
ENSG000001:	MACF1	protein_codin	209.545662	0	65.7558321	289.582496
ENSG000001(	DRP2	protein_codin	0	0	1.18090793	0
ENSG000001:	TRERF1	protein_codin	74.7134332	0	0	0
ENSG000001:	ANXA7	protein_codin	67.4061213	76.4787773	52.2202505	39.4653952
ENSG000001(	HNF4A	protein_codin	0	0	0	0
ENSG000001:	ANXA7	protein_codin	302.228465	448.503852	129.141886	227.60099
ENSG000001:	TRERF1	protein_codin	117.643552	134.006518	0	0
ENSG000001(	DNM1	protein_codin	0	19.4495183	10.4799767	0
ENSG000001:	MACF1	protein_codin	532.678972	197.611435	584.893367	409.609152
ENSG000001(	CENPI	protein_codin	0	0	0	0
ENSG000001(	CENPI	protein_codin	0	0	0	0
ENSG000001:	TMEM35A	protein_codin	0	0	0	0
ENSG000001'	TRMT2B	protein_codin	0	0	2.42065955	0
ENSG000001'	TRMT2B	protein_codin	25.9610671	31.7911458	4.16240571	0
ENSG000001'	CIZ1	protein_codin	337.362809	515.934663	301.077349	177.643073
ENSG000001'	TRMT2B	protein_codin	0	0	8.2723132	0
ENSG000001:	MRPS16	protein_codin	2.97875242	0	4.01456984	0
ENSG000001:	MRPS16	protein_codin	86.4002795	96.8350816	46.4563279	44.4642824
ENSG000001'	CIZ1	protein_codin	31.7525917	0	4.93829413	0
ENSG000002	DNAJC9	protein_codin	77.1093428	120.722026	74.1612321	30.7673918
ENSG000001:	GDAP1L1	protein_codin	0	0	0	0
ENSG000001'	ARL13A	protein_codin	0	0	0	0
ENSG000001'	CIZ1	protein_codin	0	52.1478258	0	28.1659536
ENSG000001:	FAM149B1	protein_codin	15.0303089	13.0495913	11.517964	11.4323171
ENSG000001'	XKRX	protein_codin	0	0	0	0
ENSG00000O'	GUCA1A	protein_codin	0	0	0	0

ENSG000000	NOX1	protein_codin	0	0	0	0
ENSG000002	CIMIP3	protein_codin	0	0	0	0
ENSG000000	NOX1	protein_codin	0	0	0	0
ENSG000000	NOX1	protein_codin	0	0	0	0
ENSG000001	NDUFS5	protein_codin	99.6211473	355.041558	88.1075726	84.8954334
ENSG000001	NDUFS5	protein_codin	91.2702209	83.9088162	57.7769635	56.8947885
ENSG000001	OSER1	protein_codin	0	0	0	0
ENSG000001	CSTF2	protein_codin	26.0357153	49.4974839	36.271529	23.8180018
ENSG000001	TAF8	protein_codin	110.401275	111.53994	0	37.8408963
ENSG000001	TAF8	protein_codin	14.6744005	8.6707191	5.65439318	4.84637237
ENSG000001	ECD	protein_codin	121.327933	80.8360529	84.7724262	63.5201782
ENSG000001	JPH2	protein_codin	0	0	1.93597982	0
ENSG000001	SYTL4	protein_codin	0	0	3.48062875	0
ENSG000001	TAF8	protein_codin	0	11.7615512	0	7.76439375
ENSG000001	AKIRIN1	protein_codin	8.81841511	9.34501568	0	3.62394506
ENSG000001	CCND3	protein_codin	9.63761356	2.22043221	3.80750218	2.09666079
ENSG000001	CCND3	protein_codin	0	0	6.84214616	0
ENSG000001	SYTL4	protein_codin	65.7126573	171.976388	73.3732716	56.4121442
ENSG000001	RHBDL2	protein_codin	0	0	0	0
ENSG000001	CCND3	protein_codin	527.855651	880.058945	382.565176	370.589531
ENSG000002	ENSG000002	processed_ps	0	0	0	0
ENSG000001	BBLN	protein_codin	71.9917426	115.951136	39.3703101	51.9959555
ENSG000001	BYSL	protein_codin	0	0	0	0
ENSG000001	NUDT13	protein_codin	0	0	0	0
ENSG000001	LCN2	protein_codin	2.80939156	0	0	0
ENSG000001	TOX2	protein_codin	0	0	0	0
ENSG000001	RRAGC	protein_codin	135.742564	186.998418	65.6015757	128.741471
ENSG000001	GTSF1L	protein_codin	0	1.43789183	0	0
ENSG000001	SRPX2	protein_codin	0	2.0079283	0	0
ENSG000001	GTSF1L	protein_codin	0	0	0	0
ENSG000001	USP49	protein_codin	6.93978758	3.02662807	0	0
ENSG000001	P4HA1	protein_codin	121.988821	80.5975339	23.6797526	64.792355
ENSG000001	USP49	protein_codin	0	3.35812593	2.39411043	5.08009611
ENSG000001	POU3F1	protein_codin	0	0	0	0
ENSG000001	UTP11	protein_codin	42.8493035	89.9717704	32.9461636	49.5971146
ENSG000001	FHL3	protein_codin	0	89.2521797	25.8417349	54.2442506
ENSG000001	LCN2	protein_codin	0	0	0	0
ENSG000001	FRS3	protein_codin	0	27.1929124	0	0
ENSG000001	SF3A3	protein_codin	250.821075	330.197386	256.54247	144.644372
ENSG000000	TSPAN6	protein_codin	0	0	1.24371454	5.99995335
ENSG000002	INPP5B	protein_codin	8.75032648	18.2648608	8.11533905	4.30860211
ENSG000002	INPP5B	protein_codin	62.4535706	51.5452695	19.016292	0
ENSG000000	PGC	protein_codin	0	0	0	0
ENSG000002	INPP5B	protein_codin	0	25.974837	0	0

ENSG000002	INPP5B	protein_codin	92.1791766	136.460288	78.842127	72.7360311
ENSG000001	IFT52	protein_codin	39.9000394	65.2306635	19.1609221	40.3851955
ENSG000000	TNMD	protein_codin	0.95225874	0.9713921	0	0
ENSG000001	PLA2G12B	protein_codin	0	0	0	0
ENSG000001	TFEB	protein_codin	73.6088692	125.12723	52.9035283	82.3523276
ENSG000001	PCDH19	protein_codin	0	0	0	0
ENSG000001	MTF1	protein_codin	341.222647	354.142358	185.455551	263.124451
ENSG000001	IFT52	protein_codin	0	0	0	4.53730584
ENSG000002	RPA4	protein_codin	11.694125	12.0449056	5.58102233	11.2904001
ENSG000001	C1orf122	protein_codin	0	0	0	5.69436392
ENSG000001	C1orf122	protein_codin	1.45130276	7.86793141	0	0
ENSG000001	YRDC	protein_codin	62.5170923	67.463161	33.5024676	25.3761833
ENSG000001	MANEAL	protein_codin	0	0	0	0
ENSG000001	EPHA10	protein_codin	0	0	0	0
ENSG000001	DIAPH2	protein_codin	505.638281	241.728892	174.706591	218.707186
ENSG000001	MDFI	protein_codin	0	0	0	0
ENSG000001	MDFI	protein_codin	0	0	0	0
ENSG000001	MCU	protein_codin	53.0022685	92.1912097	61.6530762	42.0466916
ENSG000001	CDCA8	protein_codin	0	0	4.83415101	0
ENSG000001	FOXP4	protein_codin	0	11.4605064	0	0
ENSG000001	GNL2	protein_codin	311.787865	263.143165	177.613587	196.143018
ENSG000001	FOXP4	protein_codin	39.4900169	0	27.0107798	0
ENSG000001	SLC25A25	protein_codin	7.66456281	0	0	0
ENSG000001	SLC25A25	protein_codin	0	0	0	0
ENSG000001	SLC25A25	protein_codin	78.7992313	64.3547557	16.2808436	37.0238667
ENSG000001	SLC25A25	protein_codin	0	0	0	0
ENSG000001	MEAF6	protein_codin	70.4164484	79.6099028	59.9684147	26.2949766
ENSG000001	MEAF6	protein_codin	30.3336408	25.1946641	21.8018003	27.192253
ENSG000001	MEAF6	protein_codin	39.4566864	83.9059137	0	63.8620449
ENSG000001	SGK2	protein_codin	0	0	0	0
ENSG000001	NAIF1	protein_codin	19.2843394	33.9447725	8.93950708	13.7032078
ENSG000001	NAP1L3	protein_codin	0	2.01407614	5.36726672	0
ENSG000000	NCR2	protein_codin	0	0	0	0
ENSG000001	EEIG1	protein_codin	0	0	15.5715375	0
ENSG000000	NCR2	protein_codin	0	0	0	0
ENSG000001	ZC3H12A	protein_codin	453.032306	542.160864	220.533123	266.383845
ENSG000001	PCDH11X	protein_codin	0	0	0	0
ENSG000000	NCR2	protein_codin	0	0	0	0
ENSG000001	GRIK3	protein_codin	0	0	0	0
ENSG000001	GRIK3	protein_codin	0	0	0	0
ENSG000001	PCDH11X	protein_codin	0	0	0	0
ENSG000001	EEIG1	protein_codin	16.0861187	39.7770489	8.12229047	12.4478839
ENSG000001	PCDH11X	protein_codin	0	0	0	0
ENSG000001	SGK2	protein_codin	0	0	0	0

ENSG000001	CSF3R	protein_codin	25.4754762	49.1849502	63.0468762	37.4553672
ENSG000001	CSF3R	protein_codin	381.679529	513.202786	334.526045	273.099614
ENSG000001	PABPC5	protein_codin	0	0	0	0
ENSG000001	CSF3R	protein_codin	115.017874	271.518641	132.837539	86.8389002
ENSG000001	SPOCK2	protein_codin	23.6706244	23.971884	13.1288046	6.76526548
ENSG000001	DPM2	protein_codin	1.16461733	0	0	0
ENSG000001	CPXCR1	protein_codin	0	0	0	0
ENSG000000	TREM2	protein_codin	0	0.85514521	0	0.83925764
ENSG000001	KLHL4	protein_codin	0	0	0	1.0272887
ENSG000001	CHST3	protein_codin	0	0	0	0
ENSG000001	MRPS15	protein_codin	79.6001384	144.737818	37.1980294	69.949919
ENSG000001	KLHL4	protein_codin	0	0	0	0
ENSG000000	TREM2	protein_codin	0	0	0	0
ENSG000001	DACH2	protein_codin	0	0	0	0
ENSG000001	TREML1	protein_codin	141.411028	484.627495	205.454289	126.438136
ENSG000001	STK40	protein_codin	50.6644739	105.158366	12.3232567	16.3121025
ENSG000001	STK40	protein_codin	9.09624825	0	0	0
ENSG000001	DACH2	protein_codin	0	0	0	0
ENSG000001	STK40	protein_codin	381.810191	651.684409	274.575968	323.749042
ENSG000001	L3MBTL1	protein_codin	0	0	5.59891931	0
ENSG000001	L3MBTL1	protein_codin	0	1.47865297	0	0
ENSG000001	L3MBTL1	protein_codin	0	0	0	0
ENSG000002	SH3D21	protein_codin	1.99751563	0	0	0
ENSG000001	ST6GALNAC6	protein_codin	0	2.89989399	0	0
ENSG000001	ST6GALNAC6	protein_codin	2.92445623	0	0	0
ENSG000001	ST6GALNAC6	protein_codin	25.516931	43.3163106	3.94142505	0
ENSG000001	POF1B	protein_codin	0	0	0.7698571	0
ENSG000001	ST6GALNAC6	protein_codin	0	0	0	11.4727342
ENSG000001	MAP7D1	protein_codin	100.78127	349.686571	174.523558	113.919562
ENSG000001	MAP7D1	protein_codin	427.482797	432.795766	97.5596746	280.666593
ENSG000001	MAP7D1	protein_codin	34.2345979	96.6248074	68.4335176	42.43297
ENSG000001	OARD1	protein_codin	0	0	0	0
ENSG000001	AK1	protein_codin	0	0	0	0
ENSG000001	COL6A5	protein_codin	0	0	0	0
ENSG000001	TSPO2	protein_codin	0	0	0	0
ENSG000000	TRAPPC3	protein_codin	0	0	0	0
ENSG000001	TSPO2	protein_codin	0	0	0	0
ENSG000000	TRAPPC3	protein_codin	4.296563	4.43035976	1.47991323	1.38878715
ENSG000000	TRAPPC3	protein_codin	0	2.4289044	0	0
ENSG000001	UNC5CL	protein_codin	0	0	0	0
ENSG000001	ZNF711	protein_codin	7.57445989	0	7.48618445	0
ENSG000000	TRAPPC3	protein_codin	33.7651659	86.0179645	21.4325041	43.5330168
ENSG000002	TDRG1	lncRNA	0	0	0	0
ENSG000002	LINC00951	lncRNA	0.98461231	0.96717825	0.99090488	0

ENSG000001	APOOL	protein_codin	51.0300426	42.2544587	43.4317076	26.987684
ENSG000001	HDX	protein_codin	30.2041685	0	0	16.4901305
ENSG000001	ADPRS	protein_codin	42.8370221	72.0353736	19.9702906	26.2410139
ENSG000001	MOCS1	protein_codin	0	0	0	0
ENSG000001	PTPRT	protein_codin	0	0	0	0
ENSG000001	PTPRT	protein_codin	0	0	0	0
ENSG000001	MOCS1	protein_codin	0	0	0	0
ENSG000001	SLC29A3	protein_codin	36.6276169	75.8777892	22.8760568	33.3680922
ENSG000001	PTPRT	protein_codin	0	0	0	0
ENSG000001	AGO3	protein_codin	257.732255	123.265686	148.854885	126.359607
ENSG000001	UNC5B	protein_codin	0.95064722	2.85321018	1.03812585	0
ENSG000001	PTPRT	protein_codin	0	0	0	0
ENSG000001	MOCS1	protein_codin	0	0	0	0
ENSG000001	PTPRT	protein_codin	0	0	0	0
ENSG000001	PTPRT	protein_codin	0	0	0	0
ENSG000001	SGPL1	protein_codin	176.707982	264.225216	74.6207383	120.11343
ENSG000001	ENG	protein_codin	0	0	0	0
ENSG000000	AGO1	protein_codin	480.191337	655.654863	351.862422	404.814631
ENSG000000	AGO1	protein_codin	16.546832	0	24.5600905	0
ENSG000001	ADAMTS14	protein_codin	0	0	0	0
ENSG000001	ADAMTS14	protein_codin	0	1.69417228	0	0
ENSG000001	PRF1	protein_codin	16.050037	33.7395432	7.48217847	3.89244958
ENSG000001	AGO4	protein_codin	962.528587	877.943312	400.798437	598.510503
ENSG000001	SH3BGRL	protein_codin	1163.80477	1596.5442	956.082729	1122.57734
ENSG000001	EIF4EBP2	protein_codin	599.202009	877.97014	267.94804	463.923309
ENSG000000	CLSPN	protein_codin	6.27207696	6.90629924	17.9401573	0
ENSG000001	CHD6	protein_codin	55.0904094	38.0704758	14.1108745	15.8196772
ENSG000001	LRRC20	protein_codin	0	0	0	0
ENSG000001	FPGS	protein_codin	0	0	0	0
ENSG000002	ENSG000002	lncRNA	0	0	0	0
ENSG000000	KCNK16	protein_codin	0	0	0	0
ENSG000001	FPGS	protein_codin	0	0	0	0
ENSG000000	KCNK16	protein_codin	0	0	0	0
ENSG000001	PPA1	protein_codin	0	0	5.90718661	0
ENSG000001	KCNK17	protein_codin	0	0	1.31645031	0
ENSG000001	PPA1	protein_codin	84.7470364	104.387756	69.1460611	69.9467437
ENSG000001	CHD6	protein_codin	344.871078	333.687829	249.498956	273.427741
ENSG000001	TFAP2E	protein_codin	4.65029447	0	0	0
ENSG000000	SAR1A	protein_codin	11.8723362	13.9868491	0	6.9470345
ENSG000001	PSMB2	protein_codin	88.4743876	136.619431	53.1562657	79.041742
ENSG000000	SAR1A	protein_codin	200.671603	178.333608	128.19582	148.424716
ENSG000000	SAR1A	protein_codin	0	3.35917444	0	0
ENSG000000	SAR1A	protein_codin	39.0784795	36.6608348	10.2368812	0
ENSG000000	SAR1A	protein_codin	0	0	3.96993817	29.6316587

ENSG000002	NCDN	protein_codin	36.7703251	35.0670423	4.39705054	21.8533128
ENSG000001	FPGS	protein_codin	0	0	0	0
ENSG000004	AIFM2	protein_codin	0	3.1251448	1.68306986	0
ENSG000001	SAYSD1	protein_codin	9.39747713	0	0	18.1964412
ENSG000001	HMGN5	protein_codin	0	0.81630652	0	0
ENSG000002	NCDN	protein_codin	0	3.54269083	0	0
ENSG000000	MACROH2A2	protein_codin	0	8.80865388	0	1.62383046
ENSG000001	GLP1R	protein_codin	0	1.05191562	0	0
ENSG000001	LPIN3	protein_codin	0	0	0	0
ENSG000001	ZHX3	protein_codin	0	0	6.49114075	0
ENSG000001	CDK9	protein_codin	210.983104	263.239574	129.513797	122.373557
ENSG000001	KIAA0319L	protein_codin	0	0	0	0
ENSG000001	PLCG1	protein_codin	0	0	0	0
ENSG000001	BRWD3	protein_codin	627.41048	327.304404	331.17553	477.369432
ENSG000000	SH2D3C	protein_codin	8.07137446	7.59357802	0	4.95727386
ENSG000000	SH2D3C	protein_codin	0	0	0	0
ENSG000001	DNAH8	protein_codin	0	0	0	0
ENSG000001	FAM241B	protein_codin	0	0	0	0
ENSG000001	TOR2A	protein_codin	0	0	0	0
ENSG000001	TOR2A	protein_codin	87.1569151	112.727859	25.6303129	79.2336782
ENSG000000	FARP2	protein_codin	4.84667097	0	4.4181826	0
ENSG000001	TTC16	protein_codin	0	0.76957637	0	0
ENSG000000	TSPAN15	protein_codin	0	0	0	0
ENSG000001	HDLBP	protein_codin	100.615383	144.197029	141.654785	139.226137
ENSG000001	CFAP157	protein_codin	0	0	0	0
ENSG000001	TBX22	protein_codin	0	0	0	0
ENSG000001	CFAP157	protein_codin	0	0	0	0
ENSG000001	TBX22	protein_codin	0	0	0	0
ENSG000000	ITM2A	protein_codin	3.11094731	21.0125964	8.59126693	0
ENSG000001	STXBP1	protein_codin	0	0	0	0
ENSG000001	STXBP1	protein_codin	0	0	0	0
ENSG000001	CYSLTR1	protein_codin	70.4465166	67.1863591	29.3593095	53.7743593
ENSG000000	TACR2	protein_codin	0	0	0	0
ENSG000000	TACR2	protein_codin	0	0	0	0
ENSG000001	NIBAN2	protein_codin	299.528694	363.904024	115.355026	228.457872
ENSG000002	MAFB	protein_codin	2059.32513	1315.4464	389.618989	713.224794
ENSG000001	NIBAN2	protein_codin	37.7845923	9.22738766	0	13.5008562
ENSG000001	PGK1	protein_codin	874.079157	963.973607	283.61046	494.575743
ENSG000001	ANKMY1	protein_codin	0	0	0	0
ENSG000001	LRSAM1	protein_codin	0	0	0	0
ENSG000001	DHX35	protein_codin	0	0	0	0
ENSG000001	LRSAM1	protein_codin	0	0	20.2702187	0
ENSG000001	DHX35	protein_codin	31.9695608	0	0	0
ENSG000002	TRAF3IP1	protein_codin	0	0	0	0

ENSG000001!	ZMYM1	protein_codin	0	0	0	0
ENSG000001!	ZMYM1	protein_codin	0	0	0	0
ENSG000001(	PPP1R16B	protein_codin	0	0	0	0
ENSG000001(	ZMYM6	protein_codin	3.10936469	4.62644374	1.65625617	0
ENSG000001:	COX7B	protein_codin	6.05075369	11.3661478	0	6.89443549
ENSG000001(	MAGT1	protein_codin	0	0	3.66589472	0
ENSG000002	TMEM35B	protein_codin	18.0947513	19.968885	5.59501422	9.92344296
ENSG00000(	ATRX	protein_codin	144.564073	114.416053	92.6589749	92.253849
ENSG000001	ARHGAP40	protein_codin	0	0.74915835	0	0
ENSG000001	DLGAP3	protein_codin	0	0	0	0
ENSG000001	ADIG	protein_codin	0	0	0	0
ENSG000001:	SLC2A8	protein_codin	0	0	0	0
ENSG000001(	PBDC1	protein_codin	11.6575108	14.5541985	0	10.87114
ENSG000001(	PBDC1	protein_codin	69.0948055	80.0668193	48.3114055	56.472391
ENSG000001	MAGEE2	protein_codin	0	0	0	0
ENSG000001:	SLC2A8	protein_codin	0	0	0	0
ENSG000001	GJB3	protein_codin	0	0	0	0
ENSG000001	GLO1	protein_codin	67.4148244	81.5332465	23.8234579	36.6927315
ENSG000001	GJB3	protein_codin	0	0	0	0
ENSG000001(	ZDHHC15	protein_codin	0	1.30692255	1.1654598	0
ENSG00000	SPP2	protein_codin	0	0	0	0
ENSG000001:	SLC2A8	protein_codin	8.15022908	9.19036288	0	0
ENSG00000!	UPRT	protein_codin	0	0	0	0
ENSG000001	C1orf94	protein_codin	0	0	0	0
ENSG000001	CSMD2	protein_codin	0	0	0	1.87750138
ENSG00000!	UPRT	protein_codin	25.7248017	0	7.35056806	0
ENSG000001	CSMD2	protein_codin	0.9065544	0	0	0
ENSG000001	CSMD2	protein_codin	0	0	0	0
ENSG000001	VPS26A	protein_codin	4.31072472	0	2.03620187	7.88328315
ENSG00000!	UPRT	protein_codin	31.4680587	46.8996876	12.4215275	17.0010274
ENSG000002	ENSG000002!	lncRNA	0	0	0	0
ENSG000001:	GARNL3	protein_codin	0	3.52657114	1.19942597	0
ENSG000001:	GARNL3	protein_codin	0	0	0	0
ENSG000001	CSMD2	protein_codin	0	1.8241406	0	0
ENSG000001	ZFAND3	protein_codin	46.596917	56.2889706	20.5560394	17.6318497
ENSG000001	ZFAND3	protein_codin	9.598735	64.1069184	0	0
ENSG000001	ABCB7	protein_codin	38.2593038	56.6093875	0	0
ENSG000001	HJURP	protein_codin	0	0	0	0
ENSG000001	MDGA1	protein_codin	0	1.82497821	0	0
ENSG000001!	TGM2	protein_codin	0	0	0	0
ENSG000001!	CCDC167	protein_codin	7.64364683	16.8137171	21.4303456	6.3717273
ENSG000002	UGT1A4	protein_codin	0	0	0	0
ENSG000001	ZSCAN20	protein_codin	0.94964707	1.72969137	1.08492973	1.88588213
ENSG000002	UGT1A5	protein_codin	0	0	0	0

ENSG000001	ANGPTL2	protein_codin	0	0	0	0
ENSG000001	PHC2	protein_codin	309.272967	279.408421	75.3806592	204.503873
ENSG000001	PHC2	protein_codin	39.7203811	28.8106485	0	1.93149575
ENSG000001	UGT1A6	protein_codin	0	0	0	0
ENSG000001	ANGPTL2	protein_codin	0	0.97369185	0	0
ENSG000002	UGT1A7	protein_codin	0	0	0	0
ENSG000001	ZNF362	protein_codin	0	0	0	45.7983885
ENSG000001	RPRD1B	protein_codin	62.2418135	84.9140693	47.1845432	53.4236698
ENSG000001	RALGPS1	protein_codin	11.5200344	0	0	2.41666565
ENSG000001	RALGPS1	protein_codin	4.91690684	0	1.57656809	0
ENSG000001	RALGPS1	protein_codin	2.5407558	0	0	0
ENSG000001	AZIN2	protein_codin	0	0	4.64925363	0
ENSG000001	AZIN2	protein_codin	0	0	0	0
ENSG000002	UGT1A10	protein_codin	0	0	0	0
ENSG000001	TTI1	protein_codin	57.6239729	73.8025275	50.6889855	51.9215183
ENSG000001	TTI1	protein_codin	0	21.0062583	0	0
ENSG000000	AK2	protein_codin	72.1050373	124.511572	72.4035391	25.8104147
ENSG000002	UGT1A8	protein_codin	0	0	0	0
ENSG000001	CMTR1	protein_codin	126.654739	153.895944	89.7318924	99.6238341
ENSG000001	RNF19B	protein_codin	346.606396	250.452906	197.919551	119.845106
ENSG000001	ZBTB43	protein_codin	113.03591	47.6718204	73.3451565	104.31219
ENSG000001	VSTM2L	protein_codin	0	0	0	0
ENSG000001	VSTM2L	protein_codin	0	0	1.59310799	0
ENSG000001	TMEM54	protein_codin	0	0	0	0
ENSG000001	ZBTB43	protein_codin	234.639009	106.243016	72.5049164	96.0411857
ENSG000002	CTNNBL1P1	processed_ps	0	0	0	0
ENSG000001	HPCA	protein_codin	0	0	0	0
ENSG000001	CTNNBL1	protein_codin	0	0	3.49507782	2.04406241
ENSG000001	FNDC5	protein_codin	0	0	0	0
ENSG000001	CTNNBL1	protein_codin	2.06234178	0	0	1.83390614
ENSG000001	LMX1B	protein_codin	0	0	0	0
ENSG000001	S100PBP	protein_codin	46.2032044	89.6382845	36.8170743	23.0740126
ENSG000001	S100PBP	protein_codin	0	0	0	0
ENSG000001	YARS1	protein_codin	77.4139549	164.066415	141.844508	71.5659042
ENSG000001	RNF8	protein_codin	0	4.42751716	0	2.02467311
ENSG000001	NHSL3	protein_codin	0	0	0	0
ENSG000001	NHSL3	protein_codin	0	0	0	0
ENSG000001	PBX3	protein_codin	0	14.4023307	0	3.08538148
ENSG000001	SYNC	protein_codin	0	1.61576906	0	0
ENSG000001	RBBP4	protein_codin	0	0	19.5730802	0
ENSG000001	PBX3	protein_codin	0	12.1077165	0	8.30048769
ENSG000001	PBX3	protein_codin	15.1897071	113.569316	0	48.0947
ENSG000000	TBC1D22B	protein_codin	46.8932925	68.4389388	21.7222574	35.0892687
ENSG000001	PBX3	protein_codin	3.92864668	0	11.5480354	0

ENSG000001(	RBBP4	protein_codin	195.558325	180.629864	116.180101	106.254658
ENSG000001	MAPKAP1	protein_codin	0	1.74420889	0	0
ENSG000001	MAPKAP1	protein_codin	96.8309825	135.195054	35.2134383	74.2286286
ENSG000001	ZBTB8OS	protein_codin	6.70052789	0	7.30956419	0
ENSG000002(	CHIC1	protein_codin	0	3.29142309	0	3.36461802
ENSG000001	MAPKAP1	protein_codin	18.5943453	35.1062468	4.96598701	3.99587662
ENSG000002(	CHIC1	protein_codin	5.01823923	0	2.88903495	0
ENSG000001	ZBTB8OS	protein_codin	0	0	0	0
ENSG000002(	ENSG000002(	lncRNA	0	0	0	0
ENSG000001:	PIM1	protein_codin	575.93378	239.926947	127.697523	175.139706
ENSG000001(	ZBTB8A	protein_codin	1.61289466	1.6808399	0	2.35961512
ENSG000001	MAPKAP1	protein_codin	0	3.48409645	4.31972507	0
ENSG000001:	CDX4	protein_codin	0	0	0	0
ENSG000001	NAP1L2	protein_codin	0	0	0	0
ENSG000002!	NAP1L6P	lncRNA	1.933313	0	0	0
ENSG000001	PABPC1L2A	protein_codin	0	0	0	0
ENSG000001(	BSDC1	protein_codin	0	0	0	2.10847638
ENSG000001	PABPC1L2B	protein_codin	0	0	0	0
ENSG000000	ATG16L1	protein_codin	0	0	0	0
ENSG000001	DMRTC1B	protein_codin	0	0	0	0
ENSG000001	DMRTC1B	protein_codin	0	0	0	0
ENSG000001(	TSSK3	protein_codin	0	0	0	0
ENSG000001	FGD2	protein_codin	0	0	0	0
ENSG000001(	BLCAP	protein_codin	112.537839	122.642352	25.6921357	63.247122
ENSG000001:	RABEPK	protein_codin	0	0	0	0
ENSG000000(	PHKA1	protein_codin	1.10700696	2.03493784	4.62117297	0
ENSG000000(	PHKA1	protein_codin	0	0	0	0
ENSG000001:	RABEPK	protein_codin	3.47430353	3.58288974	1.20049506	2.27437538
ENSG000000(	PHKA1	protein_codin	0	0	0	0
ENSG000001	PPP6C	protein_codin	171.418926	170.333768	103.239693	145.096052
ENSG000001	HDAC1	protein_codin	159.786118	244.356399	97.827224	119.190996
ENSG000001	SCAI	protein_codin	5.84484076	18.0906577	0	6.17300166
ENSG000001:	GOLGA1	protein_codin	0	0	0	0
ENSG000000(	NGEF	protein_codin	0.8166414	1.3706807	0	0
ENSG000001	HDAC8	protein_codin	0	6.96437068	0	2.73566851
ENSG000001:	GOLGA1	protein_codin	141.020638	187.213209	108.417577	83.5276807
ENSG000001	HDAC8	protein_codin	3.06513049	0	6.47774196	0
ENSG000001	LCK	protein_codin	1.97905133	0	0	0
ENSG000001!	SRC	protein_codin	4.19278655	0	0	0
ENSG000001	HDAC8	protein_codin	0	0	0	0
ENSG000001	HDAC8	protein_codin	0	0	0	0
ENSG000001	LCK	protein_codin	0	0	0	0
ENSG000002(	GIGYF2	protein_codin	0	17.1869909	61.3401	0
ENSG000001!	SRC	protein_codin	0	14.7671241	0	0

ENSG000001	HDAC8	protein_codin	0	0	0	0
ENSG000001	RPL35	protein_codin	5.91607352	23.0586449	17.5401556	13.3466281
ENSG000001	HDAC8	protein_codin	7.29756732	0	6.8319069	3.69286985
ENSG000001	HDAC8	protein_codin	0	0	0	0
ENSG000001	WDR38	protein_codin	0	0	0	0
ENSG000001	SRC	protein_codin	200.117099	294.477525	116.06357	116.896901
ENSG000001	OLFML2A	protein_codin	20.2187609	19.2499895	2.53359729	0
ENSG000001	FAM167B	protein_codin	0	0	0	0
ENSG000001	HDAC8	protein_codin	0	8.927819	0	8.00144318
ENSG000001	NR6A1	protein_codin	0	0	0	0
ENSG000001	DDX50	protein_codin	78.9971509	94.789003	56.7981992	56.6757423
ENSG000000	EIF3I	protein_codin	0	0	0	5.89379319
ENSG000001	NR5A1	protein_codin	0.84720104	0	0	0
ENSG000001	NR5A1	protein_codin	0	0	0	0
ENSG000001	HDAC8	protein_codin	0	0	0	0
ENSG000002	ECEL1P1	transcribed_u	0	0	0	0
ENSG000001	TMEM234	protein_codin	0	0	7.93029835	0
ENSG000001	NEK6	protein_codin	0	4.11314011	0	0
ENSG000001	NEK6	protein_codin	0	18.3884152	9.99566448	4.23204643
ENSG000001	CCDC28B	protein_codin	0	12.713303	3.37988076	0
ENSG000001	NEK6	protein_codin	0	0	0	0
ENSG000002	ENSG000002	lncRNA	6.95118271	7.23371834	6.24915041	0
ENSG000001	MANBAL	protein_codin	3.14037405	19.8427269	16.8274332	0
ENSG000001	MANBAL	protein_codin	0	14.3129081	0	8.45711322
ENSG000001	COPS7B	protein_codin	0	0	0	0
ENSG000000	TXLNA	protein_codin	129.709325	188.513328	36.6708863	116.810979
ENSG000000	TXLNA	protein_codin	133.031601	273.512304	200.513546	84.5462022
ENSG000001	GHRH	protein_codin	0	0	0	0
ENSG000001	LHX2	protein_codin	0	0	0	0
ENSG000001	MTCH1	protein_codin	21.8600085	57.5688599	0	0
ENSG000001	DENND1A	protein_codin	100.631154	98.0668295	28.0147534	23.7375961
ENSG000001	CITED1	protein_codin	0	0	0	0
ENSG000001	DENND1A	protein_codin	106.226633	131.679027	49.6743345	107.311432
ENSG000001	RPN2	protein_codin	103.225049	141.714996	35.1170162	76.0669066
ENSG000001	DENND1A	protein_codin	396.724276	552.140401	220.836192	254.593899
ENSG000000	KPNA6	protein_codin	318.779448	394.597475	159.550313	235.132613
ENSG000001	RPS4X	protein_codin	0	4.18449695	0	0
ENSG000001	MTCH1	protein_codin	105.213763	149.248089	33.0029725	0
ENSG000001	CRB2	protein_codin	0	0	0	0
ENSG000001	RPN2	protein_codin	17.603354	24.0963298	0	0
ENSG000001	PSMD1	protein_codin	0	0	0	0
ENSG000002	C2orf72	protein_codin	0	0	0	0
ENSG000001	TET1	protein_codin	17.0754709	11.1410066	16.1209748	15.4689788
ENSG000000	SP140	protein_codin	0	8.54646941	2.73645844	4.72985543

ENSG000000	RABGAP1	protein_codin	242.223139	295.228246	152.000111	193.797809
ENSG000001	FBXO36	protein_codin	0	0	0	0
ENSG000001	ZBTB26	protein_codin	9.25697627	0	0	0
ENSG000001	ADGRB2	protein_codin	0	0	0	0
ENSG000001	ZBTB26	protein_codin	31.3109811	35.3314438	16.9934866	21.7627677
ENSG000001	ERCC6L	protein_codin	4.18216998	0	6.54021877	0
ENSG000001	ADGRB2	protein_codin	0	0	0	0
ENSG000001	ZBTB6	protein_codin	6.13119336	15.32348	9.66505982	13.5535662
ENSG000001	PIN4	protein_codin	0	0	0	0
ENSG000000	RBL1	protein_codin	17.6571079	8.16805471	11.6308666	2.17618959
ENSG000001	DAW1	protein_codin	0	0	0	0
ENSG000000	COL16A1	protein_codin	0	0	0	0
ENSG000000	COL16A1	protein_codin	0	0	0	0
ENSG000001	PIN4	protein_codin	16.4942117	0	0	18.0064876
ENSG000000	RC3H2	protein_codin	66.0537169	178.845682	208.140988	34.705876
ENSG000001	AGFG1	protein_codin	21.3934206	0	13.7689902	28.8227001
ENSG000000	COL16A1	protein_codin	0	0	0	0
ENSG000001	ATOH7	protein_codin	0	0	0	0
ENSG000001	PI16	protein_codin	0	0.82602301	0	0
ENSG000001	MYPN	protein_codin	0	0	0	0
ENSG000002	NHSL2	protein_codin	46.572878	39.4913086	13.4593474	3.75799484
ENSG000001	OR5C1	protein_codin	0	0	0	0
ENSG000001	OR1L6	protein_codin	0	0	0	0
ENSG000001	C6orf89	protein_codin	3.09085246	0	0	0
ENSG000001	OR1L1	protein_codin	0	0	0	0
ENSG000001	OR1N2	protein_codin	0	0	0	0
ENSG000001	CXCR3	protein_codin	1.97568174	0	0	0
ENSG000001	CXCR3	protein_codin	0	5.83912823	33.1831374	1.9093457
ENSG000001	GCNA	protein_codin	12.1210465	9.13587562	0	0
ENSG000001	GCNA	protein_codin	0	0	8.78552387	5.12174595
ENSG000000	PTGS1	protein_codin	32.8187821	141.9574	33.9203275	33.8491832
ENSG000001	PPIL1	protein_codin	8.11575488	0	2.6633276	0
ENSG000001	HERC4	protein_codin	94.9469497	76.3663243	64.859739	87.2999909
ENSG000001	OGT	protein_codin	0	0	0	0
ENSG000001	PEF1	protein_codin	82.4649879	108.995216	47.796782	57.8719425
ENSG000001	HCRTR1	protein_codin	0	0	0	0
ENSG000001	HCRTR1	protein_codin	0	0	0	0
ENSG000001	SERINC2	protein_codin	34.4144559	33.468436	4.95959582	20.0141606
ENSG000001	SERINC2	protein_codin	0	0	0	0
ENSG000001	CDKN1A	protein_codin	21.6047662	50.3594418	38.3059838	4.27068334
ENSG000001	FABP3	protein_codin	0	0	0	0
ENSG000001	ZCCHC17	protein_codin	119.616096	122.472038	85.8225684	55.415919
ENSG000001	SRSF3	protein_codin	283.267374	203.812203	67.6571731	47.4306637
ENSG000001	OGT	protein_codin	186.961754	198.138181	204.151957	75.5262628

ENSG000000	SNRNP40	protein_codin	3.05395943	0	2.2913838	1.31466895
ENSG000001	MRRF	protein_codin	0	0	0	0
ENSG000001	MRRF	protein_codin	0	0	0	0
ENSG000001	MRRF	protein_codin	0	0	2.20872625	0
ENSG000001	MRRF	protein_codin	7.11127503	5.80592214	2.70225727	2.96001167
ENSG000001	MRRF	protein_codin	19.3053128	27.1156415	21.0050446	17.4636106
ENSG000001	KCTD20	protein_codin	493.788054	886.99117	563.842429	468.249381
ENSG000001	DSN1	protein_codin	0	0	0	0
ENSG000001	CTNNA3	protein_codin	0	0	2.52354046	0
ENSG000000	NKAIN1	protein_codin	0	0	0	0
ENSG000000	ETV7	protein_codin	2.31032738	0	0	0
ENSG000000	ETV7	protein_codin	0	0	0	0
ENSG000001	DSN1	protein_codin	0	0	0	0
ENSG000001	PUM1	protein_codin	0	0	0	0
ENSG000001	PUM1	protein_codin	0	32.1619202	2.82343277	21.4357287
ENSG000001	PUM1	protein_codin	0	224.372603	0	215.508408
ENSG000001	DSN1	protein_codin	20.3864951	0	31.9727123	0
ENSG000001	LHX6	protein_codin	0	0	0	0
ENSG000001	LHX6	protein_codin	0	0	0	0
ENSG000001	REEP3	protein_codin	148.535906	91.7765634	81.4951155	96.4390692
ENSG000001	MAPK13	protein_codin	0	1.9351063	0	0
ENSG000001	SLC4A3	protein_codin	0	0	0	0
ENSG000001	MORN5	protein_codin	0	0	0	0
ENSG000001	MATN1	protein_codin	0	0	0	0
ENSG000001	MAPK13	protein_codin	4.51478428	14.0036498	0	18.2380404
ENSG000001	NDUFA8	protein_codin	20.4821414	34.6289811	15.4206271	19.5606376
ENSG000001	NDRG3	protein_codin	0	0	10.177459	0
ENSG000001	TAF1	protein_codin	0	0	0	0
ENSG000001	TTLL11	protein_codin	0	0	0	0
ENSG000001	TTLL11	protein_codin	0	5.31985077	0	0
ENSG000000	PTPRU	protein_codin	0	0	0	0
ENSG000001	ADO	protein_codin	65.7620526	83.1844417	58.1769856	51.1729038
ENSG000002	LINC02929	lncRNA	0	0	0	0
ENSG000001	RTKN2	protein_codin	0	0	0	0
ENSG000001	TAF1	protein_codin	80.3627335	130.46738	0	0
ENSG000001	MECR	protein_codin	0	11.3806222	6.90004801	2.77294439
ENSG000002	GGTA1	protein_codin	17.0366511	31.4364123	7.40077211	0
ENSG000001	SRSF4	protein_codin	376.846903	601.961464	211.467344	234.968321
ENSG000001	EPB41	protein_codin	90.7002867	52.5765574	0	77.927622
ENSG000001	EPB41	protein_codin	0	0	0	0
ENSG000001	EPB41	protein_codin	9.49449341	22.8436334	7.34327832	13.3602633
ENSG000001	NDRG3	protein_codin	0	0	0	0
ENSG000001	GSN	protein_codin	25.556756	2.4903226	4.58362319	0
ENSG000001	GSN	protein_codin	2.25892973	5.16765419	0	0

ENSG000001	GSN	protein_codin	37.1593823	137.680984	17.2215473	16.2267595
ENSG000001	CDK1	protein_codin	0	0	0	0
ENSG000001	YTHDF2	protein_codin	224.325155	425.384492	158.32357	239.194806
ENSG000001	ANK3	protein_codin	0	0	0	0
ENSG000001	GMEB1	protein_codin	112.830122	124.3268	50.0252995	41.8118613
ENSG000001	GSN	protein_codin	0	6.85362532	0	0
ENSG000001	ANK3	protein_codin	0	0	0	0
ENSG000000	SRPK1	protein_codin	0	0	0	1.61325643
ENSG000001	GSN	protein_codin	19.6739673	10.0415297	0	6.92972766
ENSG000001	TAF12	protein_codin	27.5866919	17.351317	11.6591214	0
ENSG000000	SRPK1	protein_codin	100.182957	144.535421	95.1106471	0
ENSG000001	RAB42	protein_codin	0	0	0	0.92166412
ENSG000001	ANK3	protein_codin	8.8323965	17.3737409	24.001015	0
ENSG000001	ITGB1BP2	protein_codin	3.50246936	2.57567471	0	0.87433766
ENSG000001	TRNAU1AP	protein_codin	55.4736254	39.4039432	15.8062088	12.9856325
ENSG000001	RCC1	protein_codin	2.82571088	0	0	0
ENSG000001	RCC1	protein_codin	16.2187273	5.4160011	10.4427212	0
ENSG000001	RCC1	protein_codin	0	24.6293524	0	8.68961268
ENSG000002	PHACTR4	protein_codin	157.759951	215.647459	103.328646	56.6676633
ENSG000002	PHACTR4	protein_codin	155.743041	187.984602	73.5229485	66.8040763
ENSG000001	RAB14	protein_codin	63.2510432	65.806741	24.2137696	42.4244045
ENSG000001	NONO	protein_codin	110.721646	204.673174	57.3616037	160.51948
ENSG000001	MED18	protein_codin	4.59211966	17.2521625	9.96715757	4.14663159
ENSG000001	CNTRL	protein_codin	130.182271	118.374308	46.5192367	24.9517746
ENSG000001	CNTRL	protein_codin	189.287542	192.115947	44.5346881	87.1067381
ENSG000001	CNTRL	protein_codin	16.2122184	0	0	0
ENSG000001	CNTRL	protein_codin	81.5142436	0	50.4766249	25.2050302
ENSG000001	RAB5IF	protein_codin	0	0	0	0
ENSG000001	CNTRL	protein_codin	200.195518	0	344.69733	245.60816
ENSG000001	NONO	protein_codin	91.2673962	460.894603	0	275.470866
ENSG000001	PTAFR	protein_codin	760.261735	892.75484	219.926922	362.613559
ENSG000002	CLPSL1	protein_codin	0	0	0	0
ENSG000001	EYA3	protein_codin	26.2190258	42.9113424	31.2667411	0
ENSG000001	EYA3	protein_codin	0	0	0	0
ENSG000001	CNTRL	protein_codin	25.7689228	0	0	0
ENSG000001	ARMC12	protein_codin	0	0	0	1.05496081
ENSG000001	ARMC12	protein_codin	1.99916463	2.99563152	1.13022989	0
ENSG000001	EYA3	protein_codin	0	0	0	0
ENSG000001	TGIF2	protein_codin	71.7246045	115.298164	39.2460439	46.0922853
ENSG000001	OBSL1	protein_codin	0	9.24213142	0	0
ENSG000001	TGIF2	protein_codin	0	0	0	0
ENSG000001	OBSL1	protein_codin	0	0	0	0
ENSG000001	PHYHIPL	protein_codin	0	0	0	0
ENSG000001	PHYHIPL	protein_codin	0	0	0	0

ENSG000001	TMEM198	protein_codin	0	0	0	0
ENSG000001	XKR8	protein_codin	18.3823345	0	0	19.1582657
ENSG000001	BICC1	protein_codin	3.12756822	3.1900078	5.59267538	0
ENSG000000	TRAF1	protein_codin	159.816379	100.668764	74.1876593	0
ENSG000001	SMPDL3B	protein_codin	0	0	0	0
ENSG000001	CHPF	protein_codin	0	0	0	1.3151189
ENSG000001	TULP1	protein_codin	0	0	0	0
ENSG000001	SMPDL3B	protein_codin	0	0	0	0
ENSG000001	TFAM	protein_codin	22.00155	20.6996238	11.5054394	0
ENSG000001	PHF19	protein_codin	100.760047	75.5306327	29.6222962	32.4472966
ENSG000001	TFAM	protein_codin	0	0	2.9947845	8.51352065
ENSG000000	PSMD5	protein_codin	3.51488904	0	0	0
ENSG000000	PSMD5	protein_codin	8.04085379	19.4559792	0	7.95027429
ENSG000000	DLGAP4	protein_codin	0	0	0	0
ENSG000001	GMPPA	protein_codin	38.9172096	45.2576467	30.5955773	20.8824185
ENSG000001	RPA2	protein_codin	0	23.4405783	0	0
ENSG000000	UBE2D1	protein_codin	221.412339	283.480421	142.25073	174.427855
ENSG000001	RPA2	protein_codin	32.9185732	101.574255	51.8677108	59.2124151
ENSG000000	DLGAP4	protein_codin	0	0	0	0
ENSG000001	GMPPA	protein_codin	2.32939557	0	0	0
ENSG000000	PSMD5	protein_codin	2.58808512	2.90412692	0	0
ENSG000001	THEMIS2	protein_codin	1407.00244	1974.18999	584.257437	780.39921
ENSG000001	THEMIS2	protein_codin	93.8099973	33.3744315	16.0111821	22.2439251
ENSG000001	THEMIS2	protein_codin	109.989321	30.4959557	10.5699695	47.4098414
ENSG000001	MEGF9	protein_codin	1644.4923	1762.51734	855.254022	1325.21118
ENSG000001	PPP1R8	protein_codin	0	0	2.38046709	0
ENSG000001	AAR2	protein_codin	0	4.32156281	0	0
ENSG000001	CDCA7L	protein_codin	9.40163433	5.90516762	14.8256977	0
ENSG000001	IPMK	protein_codin	202.415581	126.631334	70.0548527	123.133636
ENSG000000	EPB41L1	protein_codin	0	0	0	0
ENSG000001	STX12	protein_codin	200.551671	256.897051	108.496647	169.328325
ENSG000001	ZWINT	protein_codin	0	0	14.0878148	0
ENSG000000	EPB41L1	protein_codin	0	0	0	0
ENSG000000	EPB41L1	protein_codin	0	0	0	0
ENSG000000	FAM76A	protein_codin	24.2971268	0	0	20.8205476
ENSG000000	EPB41L1	protein_codin	0	0	0	0
ENSG000000	ZNF76	protein_codin	0	27.0246283	25.5297716	0
ENSG000000	FAM76A	protein_codin	58.8625872	67.396518	40.7286914	31.742993
ENSG000001	PCDH15	protein_codin	0	0	0	0
ENSG000001	PCDH15	protein_codin	0	0	0	0
ENSG000001	PCDH15	protein_codin	0	0	0	0
ENSG000001	DES	protein_codin	0	0	0	3.24414003
ENSG000001	DNPEP	protein_codin	0	0	0	0
ENSG000000	BRINP1	protein_codin	0	0	0	0

ENSG000001	PCDH15	protein_codin	0	2.42607713	0	0
ENSG000001	DNPEP	protein_codin	33.6729989	0	10.1222287	0
ENSG000001	MBL2	protein_codin	0	0	0	0
ENSG000001	DKK1	protein_codin	0	0	0	0
ENSG000001	DNPEP	protein_codin	0	38.3687	0	0
ENSG000001	CNBD2	protein_codin	0	0	0	0
ENSG000001	TCP11	protein_codin	0	0	0	0
ENSG000001	PRKG1	protein_codin	0	0	14.5400494	4.53884043
ENSG000001	PRKG1	protein_codin	0	0	0	0
ENSG000001	ZMYM3	protein_codin	0	0	0	0
ENSG000001	TCP11	protein_codin	0	0	0	0
ENSG000001	PRKG1	protein_codin	58.0415149	64.5340528	48.1371953	20.1852725
ENSG000001	ZMYM3	protein_codin	0	0	0	0
ENSG000001	ZMYM3	protein_codin	0	0	0	0
ENSG000001	TRIM32	protein_codin	7.31621358	7.18205156	0	1.05941037
ENSG000001	ZMYM3	protein_codin	0	0	0	0
ENSG000001	ASTN2	protein_codin	0	0	0	0
ENSG000001	ZMYM3	protein_codin	0	0	0	0
ENSG000001	SCAND1	protein_codin	44.1103859	55.6275082	10.9909274	27.7987128
ENSG000001	A1CF	protein_codin	0	0	0	0
ENSG000001	A1CF	protein_codin	0	0	0	0
ENSG000001	A1CF	protein_codin	0	0	0	1.33444692
ENSG000001	ZMYM3	protein_codin	4.88908235	0	4.55330897	0
ENSG000000	PHF20	protein_codin	76.3916963	132.835485	66.7743779	69.7059639
ENSG000001	A1CF	protein_codin	0	0	0	0
ENSG000000	FGR	protein_codin	176.318506	175.594881	136.552524	92.9880618
ENSG000000	FGR	protein_codin	780.698709	876.078571	241.805742	452.825028
ENSG000000	FGR	protein_codin	968.993156	1352.60772	181.568625	679.895887
ENSG000002	ASAH2B	protein_codin	0	0	0	0
ENSG000002	ASAH2B	protein_codin	3.6114047	0	0	0
ENSG000001	AHDC1	protein_codin	0	0	0	0
ENSG000000	PHF20	protein_codin	228.355852	224.198792	115.290239	121.345697
ENSG000002	LINC00474	lncRNA	0	0	0	0
ENSG000001	DELEC1	lncRNA	0	0	0	0
ENSG000001	SNRPC	protein_codin	4.79236403	5.47833261	0	0
ENSG000001	SNRPC	protein_codin	18.0719036	16.8372425	0	0
ENSG000001	ILRUN	protein_codin	29.0015035	44.724981	11.288719	15.5487321
ENSG000001	ILRUN	protein_codin	192.878575	287.869338	181.786669	145.072731
ENSG000001	GPR3	protein_codin	0	0	0	0
ENSG000001	CD164L2	protein_codin	0	0	0	0
ENSG000001	ILRUN	protein_codin	48.1488824	47.2633282	23.3395765	20.1407226
ENSG000001	CD164L2	protein_codin	0	0	0	0
ENSG000001	GJB1	protein_codin	0	0	0	0
ENSG000001	CD164L2	protein_codin	0	0	0	0

ENSG000001	SPDEF	protein_codin	0	0	0	0
ENSG000001	RBM39	protein_codin	708.713663	534.961922	284.835865	495.202032
ENSG000001	MAP3K6	protein_codin	7.43134581	0	0	0
ENSG000001	PACSIN1	protein_codin	0.96871455	7.60871324	20.4567723	1.36175518
ENSG000001	TNFSF15	protein_codin	0	0	0	1.03143257
ENSG000001	TNFSF15	protein_codin	4.07104933	7.01293221	5.0103493	4.07950076
ENSG000001	TMEM268	protein_codin	0	0	0	0
ENSG000001	ATP6V1G1	protein_codin	264.166684	340.41719	150.471805	229.280379
ENSG000001	NLGN3	protein_codin	0	0	0	0
ENSG000002	AGAP6	protein_codin	0	0	0	0
ENSG000000	WHRN	protein_codin	0	5.00700044	0.70824427	7.15611279
ENSG000001	ROMO1	protein_codin	0	4.3926036	0.92875922	0
ENSG000001	AKNA	protein_codin	571.213733	988.65652	448.095078	408.262215
ENSG000001	TMEM222	protein_codin	0	30.2512219	13.6966137	0
ENSG000001	ROMO1	protein_codin	33.1949656	51.8665806	12.0908955	24.3599235
ENSG000001	ROMO1	protein_codin	4.58607532	4.7931247	1.98206694	7.01045142
ENSG000001	AKNA	protein_codin	4.39742513	12.378182	6.4279503	0
ENSG000001	MED12	protein_codin	0	0	0	0
ENSG000000	SLC9A1	protein_codin	2.6292468	19.5535746	0.94091593	0
ENSG000002	NFS1	protein_codin	0	0	0	0
ENSG000000	SLC9A1	protein_codin	0	0	0.86746286	5.78073375
ENSG000001	AKNA	protein_codin	0	0	0	0
ENSG000000	SLC9A1	protein_codin	21.2964517	18.6942221	15.7239492	8.92577626
ENSG000002	NFS1	protein_codin	5.72139174	3.44199372	9.37207183	0
ENSG000001	AGAP5	protein_codin	0	0	0	3.05830872
ENSG000001	MED12	protein_codin	0	113.768862	0	0
ENSG000001	OGDHL	protein_codin	0	0	0	0
ENSG000002	RBM12	protein_codin	136.020878	0	0	133.6736
ENSG000001	C10orf53	protein_codin	0	0	0	0
ENSG000001	C10orf53	protein_codin	0	0	0	0
ENSG000001	C10orf53	protein_codin	1.19029308	0	0	0
ENSG000002	RBM12	protein_codin	65.0644483	20.4124324	16.8747568	22.5489492
ENSG000001	SLC18A3	protein_codin	0	2.06811726	0.61564489	1.00353831
ENSG000001	HMGA1	protein_codin	26.1486257	0	3.1960826	4.23372757
ENSG000001	ZNF618	protein_codin	0	0	0	2.4754316
ENSG000001	ZNF618	protein_codin	21.8718298	0	17.5627973	0
ENSG000001	GPN2	protein_codin	4.37674115	0	0	4.07442126
ENSG000001	RGS3	protein_codin	64.6582037	100.54972	0	42.2120444
ENSG000001	GPN2	protein_codin	35.5114519	73.1234858	38.5759781	23.9235436
ENSG000001	RGS3	protein_codin	0	0	0	0
ENSG000001	DRGX	protein_codin	0	0.86754983	0	0
ENSG000001	RGS3	protein_codin	5.48031053	10.3371968	0	0
ENSG000002	ZDHHC18	protein_codin	16.7019229	0	0	0
ENSG000002	ZDHHC18	protein_codin	62.5625975	115.997268	42.3756825	59.2367136

ENSG000001	C10orf71	protein_codin	0	0	0	0
ENSG000000	PIGV	protein_codin	0	2.47809975	0	0
ENSG000002	TMEM273	protein_codin	1.2206652	5.82293829	0.92085797	4.02567295
ENSG000002	TMEM273	protein_codin	15.0163862	20.1330388	0	8.06407536
ENSG000001	ARID1A	protein_codin	63.3093597	99.9349671	74.5988909	37.0300994
ENSG000002	TMEM273	protein_codin	0	0	0	0
ENSG000001	RUFY4	protein_codin	6.86273242	8.96134508	0	0
ENSG000002	TMEM273	protein_codin	4.02916145	0	0	3.75532033
ENSG000001	LRRC18	protein_codin	0	0	0	0
ENSG000001	WDFY4	protein_codin	1.55591565	2.97876162	1.64789721	0
ENSG000001	RPS6KA1	protein_codin	0	108.327302	0	63.4701327
ENSG000001	C9orf43	protein_codin	1.94764899	0	7.6844979	3.08486108
ENSG000001	RPS6KA1	protein_codin	0	18.8278715	0	20.098383
ENSG000001	RPS6KA1	protein_codin	0	0	15.5737665	0
ENSG000001	POLE3	protein_codin	307.222459	557.640423	240.286975	245.376375
ENSG000001	ARHGAP22	protein_codin	0	0	0	2.2867406
ENSG000001	POLE3	protein_codin	0	0	0	0
ENSG000001	ARHGAP22	protein_codin	10.8869356	0	0	0
ENSG000001	MAPK8	protein_codin	9.87216273	0	7.22068061	0
ENSG000001	MAPK8	protein_codin	0	0	0	0
ENSG000001	GRM4	protein_codin	0	0	0	0
ENSG000001	MAPK8	protein_codin	0	57.8255681	24.5948953	0
ENSG000001	HDHD3	protein_codin	4.87706754	9.98176537	0	4.60484972
ENSG000001	GRM4	protein_codin	0	0	0	0
ENSG000001	MAPK8	protein_codin	0	0	0	0
ENSG000001	BSPRY	protein_codin	0	0	0	0.97473331
ENSG000001	DHDDS	protein_codin	16.4814766	10.8920395	7.49468362	3.3097632
ENSG000001	DHDDS	protein_codin	0	0	0	0
ENSG000001	MAPK8	protein_codin	0	0	0	0
ENSG000001	WDR31	protein_codin	0	0	0	0
ENSG000001	PRPF4	protein_codin	64.0193226	128.527447	44.2153118	53.8981939
ENSG000001	PRPF4	protein_codin	32.4279678	13.7161852	7.10819994	7.82162835
ENSG000001	FRMPD2	protein_codin	0	0	0	0
ENSG000001	IL2RG	protein_codin	24.9083014	35.4098271	20.7626827	0
ENSG000001	CDC26	protein_codin	45.3587873	53.1608734	43.6184179	60.909866
ENSG000001	CRYBG2	protein_codin	0	0	0	0
ENSG000001	CRYBG2	protein_codin	0	0	0	0
ENSG000001	CRYBG2	protein_codin	0	0.87280103	0	0
ENSG000001	SLC31A1	protein_codin	276.202203	205.154706	84.968293	132.123065
ENSG000001	CD52	protein_codin	98.8745418	585.47624	93.6431729	301.926471
ENSG000001	UQCC2	protein_codin	9.09035767	10.7381922	0	4.02510352
ENSG000001	UBXN11	protein_codin	0	0	0	0
ENSG000001	UBXN11	protein_codin	43.8672955	80.8884145	39.8425018	36.0329666
ENSG000002	PTPN20	protein_codin	0	0	0	0

ENSG000001	SLC31A2	protein_codin	0	0	0	0
ENSG000001	UBXN11	protein_codin	44.9427234	27.551576	89.0474197	52.9151173
ENSG000001	UBXN11	protein_codin	0	0	0	0
ENSG000001	UBXN11	protein_codin	169.253373	50.9516997	9.84663284	5.20669018
ENSG000001	ZFP37	protein_codin	1.45209424	1.4818387	0	0
ENSG000001	SLC46A2	protein_codin	137.194793	194.03148	26.1726433	86.9888653
ENSG000001	UQCC2	protein_codin	23.967024	34.6560802	15.5175561	12.0276317
ENSG000001	SNX30	protein_codin	553.670162	629.44658	305.495358	394.341872
ENSG000001	INIP	protein_codin	0	2.23984741	2.68451231	0
ENSG000001	INIP	protein_codin	5.38640907	5.37858507	1.98790292	2.51831381
ENSG000001	INIP	protein_codin	106.068828	145.321073	42.840564	87.8690509
ENSG000001	KIAA1958	protein_codin	30.7562343	15.8392739	32.5377935	30.1070306
ENSG000002	CXorf65	protein_codin	0	0	0	0
ENSG000001	CNKSR1	protein_codin	0	0	0	0
ENSG000001	PTBP3	protein_codin	44.8673445	20.8891362	11.5957902	15.1601848
ENSG000001	PTBP3	protein_codin	739.381093	473.786072	444.191647	671.078382
ENSG000001	FOXO4	protein_codin	132.028373	170.872159	59.0170348	87.4823134
ENSG000001	SUSD1	protein_codin	0	0	231.904937	93.7813408
ENSG000001	SUSD1	protein_codin	47.9603343	7.13284185	0	0
ENSG000001	ZNF593	protein_codin	18.6106789	35.6788423	14.5611019	10.7398811
ENSG000001	FAM110D	protein_codin	0	0	0	0
ENSG000001	PDIK1L	protein_codin	0	26.4283019	14.7970649	28.598494
ENSG000001	SUSD1	protein_codin	663.58615	914.304966	163.192458	374.019213
ENSG000001	PDIK1L	protein_codin	38.8614044	5.10636197	0	0
ENSG000001	TRIM63	protein_codin	0	0	0	0
ENSG000000	SPAG4	protein_codin	0	0	0	0
ENSG000001	SNX12	protein_codin	28.0601528	52.5167479	27.1797571	29.7483465
ENSG000001	SLC30A2	protein_codin	0	0	0	0
ENSG000001	SLC30A2	protein_codin	0	0	0	0.94426336
ENSG000001	UGCG	protein_codin	51.1186036	51.3822765	56.3578043	38.7931286
ENSG000001	EXTL1	protein_codin	0.80039212	1.5640273	0	0
ENSG000000	ADD1	protein_codin	0	3.41705507	0	1.98024575
ENSG000001	PAFAH2	protein_codin	29.2319378	53.5345115	24.602889	23.7022717
ENSG000001	SHOC1	protein_codin	0	0	0	0
ENSG000001	PAFAH2	protein_codin	0	0	0	0
ENSG000001	SHOC1	protein_codin	0	0	0	0
ENSG000001	STMN1	protein_codin	0	43.6231368	79.3532706	17.7590251
ENSG000002	GNG10	protein_codin	110.747534	125.901876	52.199442	87.1443032
ENSG000002	DNAJC25-GN	protein_codin	29.12512	14.164	0	5.0047602
ENSG000001	PAQR7	protein_codin	31.7050146	0	17.3515307	0
ENSG000001	AUNIP	protein_codin	0	8.85887689	0	0
ENSG000001	SLC7A3	protein_codin	0	0	0	0
ENSG000001	MTFR1L	protein_codin	0	11.2834117	11.5376819	0
ENSG000001	MTFR1L	protein_codin	0	0	3.36801958	0

ENSG000001	MTFR1L	protein_codin	46.4894673	102.014373	58.0641775	61.0271439
ENSG000002	LRRC37A5P	lncRNA	0	0	0	0
ENSG000001	MTFR1L	protein_codin	3.5984896	15.0239956	0	0
ENSG000001	PTGR1	protein_codin	1.07747446	1.49642714	0	0
ENSG000002	NPY4R	protein_codin	0	0	0	0
ENSG000001	PTGR1	protein_codin	0	0	0	0
ENSG000002	GPRIN2	protein_codin	0	0	0	0
ENSG000001	SELENON	protein_codin	233.840506	363.683128	141.419664	162.826149
ENSG000000	ITPR3	protein_codin	0	0	0	17.8884828
ENSG000002	GPRIN2	protein_codin	0	0	1.11741562	0.98524693
ENSG000000	IKZF2	protein_codin	0	0	6.66152179	0
ENSG000001	TEX11	protein_codin	0	1.0867456	0	0
ENSG000002	SYT15	protein_codin	0	0	0	0
ENSG000002	SYT15	protein_codin	0	0	0	0
ENSG000001	PTGR1	protein_codin	0	0	2.58654197	0
ENSG000000	IKZF2	protein_codin	0	0	0	0
ENSG000002	SYT15	protein_codin	0	0	0	0
ENSG000001	MAN1C1	protein_codin	0	0	0	0
ENSG000001	MAN1C1	protein_codin	58.9050473	45.5992913	12.0430659	25.5269308
ENSG000001	TEX11	protein_codin	0	0	0	0
ENSG000002	BMS1P1	transcribed_u	0	0	0	0
ENSG000001	LDLRAP1	protein_codin	177.556768	490.224683	104.216799	153.66271
ENSG000002	PTPN20	protein_codin	0	0	0	0
ENSG000002	PTPN20	protein_codin	0	0	0	0
ENSG000002	MACO1	protein_codin	92.6046694	74.7158298	61.5028859	34.1163076
ENSG000001	C20orf173	protein_codin	0	0	0	0
ENSG000002	PTPN20	protein_codin	0	0	0	0
ENSG000000	DLG3	protein_codin	3.71161048	0	5.33573084	0
ENSG000001	TMEM50A	protein_codin	211.798718	336.742083	108.441296	170.423066
ENSG000001	WASHC2C	protein_codin	30.2095661	8.34970226	14.3261976	14.1171197
ENSG000000	DLG3	protein_codin	0	0	0	0
ENSG000001	WASHC2C	protein_codin	51.8468663	314.33301	304.766151	284.063506
ENSG000001	ZFAND4	protein_codin	0	0	0	0
ENSG000001	GDF5	protein_codin	0	0	0	0
ENSG000001	ZFAND4	protein_codin	23.5152579	38.7217123	18.1432049	24.4978455
ENSG000001	ZFAND4	protein_codin	0	0	0	0
ENSG000001	GDF5	protein_codin	0.82425655	0	0	0
ENSG000001	ZNF483	protein_codin	2.82280959	0	0	0
ENSG000002	GDF5-AS1	lncRNA	0	0	0	0
ENSG000001	CLIC4	protein_codin	544.492173	587.743332	439.416845	412.425192
ENSG000001	UQCC1	protein_codin	2.42819362	15.6236244	15.8139822	0
ENSG000001	GDPD2	protein_codin	0	0	0	0
ENSG000001	ECPAS	protein_codin	0	0	12.7265691	0
ENSG000001	UQCC1	protein_codin	0	0	0	0

ENSG000001(	UQCC1	protein_codin	0	26.1449257	0	0
ENSG000001:	SRRM1	protein_codin	414.594012	309.167059	250.996784	316.731726
ENSG000000	ALOX5	protein_codin	1007.11769	982.851713	297.628578	537.442007
ENSG000001(	NCMAP	protein_codin	0	0	0	0
ENSG000001	RCAN3	protein_codin	0	0	5.10847809	0
ENSG000001(	UQCC1	protein_codin	0	0	0	0
ENSG000001	RCAN3	protein_codin	108.523986	90.3044952	54.7792046	53.1711816
ENSG000000(	NIPAL3	protein_codin	50.6360532	66.8157891	52.7881845	33.4312265
ENSG000002:	OR13A1	protein_codin	0	0	0	0
ENSG000000!	KIF4A	protein_codin	4.76939481	6.82290804	25.8973957	1.98585671
ENSG000001:	FAM83C	protein_codin	0	0	0	0
ENSG000000(	STPG1	protein_codin	0	0	0	0
ENSG000001:	MDH1B	protein_codin	0	0	0	0
ENSG000001	ADAM23	protein_codin	0	0	0.94686022	0
ENSG000001(	IFNLR1	protein_codin	0	0	0	0
ENSG000001(	IFNLR1	protein_codin	0	0	0	0
ENSG000001(	IFNLR1	protein_codin	0	0	0	0
ENSG000002(	ZDBF2	protein_codin	0	0	6.69061792	0
ENSG000001(	CXCL12	protein_codin	0	0	0	0
ENSG000001'	OR2K2	protein_codin	0	0	0	0
ENSG000001(	CXCL12	protein_codin	0	0	0	0
ENSG000001!	LPAR1	protein_codin	0	0	0	0
ENSG000001!	LPAR1	protein_codin	31.7980308	79.9442217	14.5096285	39.1090945
ENSG000002:	LINC00840	lncRNA	0	0	0	0
ENSG000001(	ZNF32	protein_codin	18.0930177	39.0349145	12.2779031	15.3871897
ENSG000001.	MYOM3	protein_codin	0	0	0	0
ENSG000001!	ZNF485	protein_codin	0	0	0	0
ENSG000002.	EIF6	protein_codin	6.350745	0	1.80876	5.59837089
ENSG000000:	MUSK	protein_codin	0	0	0	0
ENSG000000:	MUSK	protein_codin	0	0	0	0
ENSG000002.	EIF6	protein_codin	5.69774902	0	0	0
ENSG000001!	ZNF239	protein_codin	0	0	0	0
ENSG000000:	MUSK	protein_codin	1.24134049	0	0	0
ENSG000002.	EIF6	protein_codin	46.8306784	74.107314	19.0379037	38.270824
ENSG000001(	SRSF10	protein_codin	0	0	0	0
ENSG000001(	SRSF10	protein_codin	0	9.789738	16.7772493	0
ENSG000001:	PDZD11	protein_codin	1.47629811	0	0	1.61615247
ENSG000001!	RASGEF1A	protein_codin	0	0	0	0
ENSG000002!	ENSG000002!	lncRNA	0	0	0	0
ENSG000001!	RASGEF1A	protein_codin	0.97509168	0.98368938	2.72313186	2.98780689
ENSG000001(	SVEP1	protein_codin	0	3.84267684	0	0
ENSG000001(	CSGALNACT2	protein_codin	1317.94193	664.520173	443.217638	744.455701
ENSG000000:	BAK1	protein_codin	0	44.0651087	0	34.0076403
ENSG000001(	PNRC2	protein_codin	9.38841897	6.08125893	3.08611412	2.6828269

ENSG000001(	SVEP1	protein_codin	0	0	0	1.09345325
ENSG000001	CNR2	protein_codin	3.99641243	1.98989347	3.03912262	0
ENSG000001	RAB41	protein_codin	0	0	0	0
ENSG000002	FUCA1P1	processed_ps	0	0	0	0
ENSG000001	FUCA1	protein_codin	130.043426	243.665285	26.533227	80.6421411
ENSG000001	CD28	protein_codin	37.6860384	2.48515909	7.65909054	5.55591033
ENSG000001	CUTA	protein_codin	0	0	0	0
ENSG000001	HMGCL	protein_codin	0	0	0	0
ENSG000001	HMGCL	protein_codin	5.58809433	17.4393646	0	14.2956223
ENSG000000	EDEM2	protein_codin	5.51955758	0	0	0
ENSG000000	EDEM2	protein_codin	98.0508347	155.812533	68.3072844	80.3591947
ENSG000001	RAPH1	protein_codin	0	0	0	0
ENSG000001	ARR3	protein_codin	0	0	0	0
ENSG000001	CUTA	protein_codin	0	0	0	0
ENSG000001	GALE	protein_codin	15.9600919	10.0447829	1.18050591	5.23820665
ENSG000001	CUTA	protein_codin	12.9813312	13.1358529	3.46233409	13.8495168
ENSG000000	LYPLA2	protein_codin	0	3.6698573	0	0
ENSG000000	LYPLA2	protein_codin	5.52902914	2.10779946	3.34065642	0
ENSG000000	LYPLA2	protein_codin	1.18350465	0	0	0
ENSG000000	LYPLA2	protein_codin	0	0	0	1.69862094
ENSG000002	TXNDC8	protein_codin	1.35941574	0	0	0
ENSG000002	TXNDC8	protein_codin	0	0	0	0
ENSG000002	TXNDC8	protein_codin	0	0	0	0
ENSG000001	PHF1	protein_codin	5.92424993	0	0	3.80823193
ENSG000000	LYPLA2	protein_codin	84.6413345	106.915713	33.8832705	59.2762823
ENSG000001	TXN	protein_codin	0	0	0	3.3490021
ENSG000001	PHF1	protein_codin	0	23.1764363	34.656224	27.8254404
ENSG000001	TXN	protein_codin	89.4372309	99.3565792	41.6716695	57.5071704
ENSG000001	BMS1	protein_codin	288.645329	262.130466	262.047044	165.738096
ENSG000001	P2RY4	protein_codin	0	1.05534516	0.84874896	1.05486724
ENSG000002	RPL12P1	processed_ps	0	0.8877851	0	0.88459755
ENSG000002	AWAT1	protein_codin	0	0	0	0
ENSG000000	PITHD1	protein_codin	0	0	0	0
ENSG000001	PALM2AKAP2	protein_codin	78.810904	138.930413	331.555582	112.302911
ENSG000001	PALM2AKAP2	protein_codin	0	0	0	0
ENSG000001	PALM2AKAP2	protein_codin	0	0	0	0
ENSG000000	PTPN3	protein_codin	0	1.10474375	0	0
ENSG000002	DAXX	protein_codin	107.490213	152.78795	68.4320399	39.8578892
ENSG000001	EDA	protein_codin	0	0	0	0
ENSG000001	RPL11	protein_codin	545.187555	1197.01534	346.791112	512.737009
ENSG000001	EDA	protein_codin	0	0	0	0
ENSG000001	EDA	protein_codin	0	0	0	0
ENSG000001	MDS2	lncRNA	0	1.98856401	0	0
ENSG000000	EPB41L4B	protein_codin	0	0	0	0

ENSG000001	ID3	protein_codin	0	0	0	2.6979383
ENSG000000	EPB41L4B	protein_codin	0	0	0	0
ENSG000001	PJA1	protein_codin	5.90347688	40.2123433	15.2382994	0
ENSG000002	BMPR2	protein_codin	3.50782785	2.63655805	3.83682052	0
ENSG000002	BMPR2	protein_codin	347.915015	208.923156	148.477703	251.755999
ENSG000001	PJA1	protein_codin	0	0	0	0
ENSG000001	PJA1	protein_codin	0	0	0	0
ENSG000001	TMEM245	protein_codin	89.6935653	124.517153	77.2967601	99.938702
ENSG000001	CTNNAL1	protein_codin	0	0	0	0
ENSG000001	CTNNAL1	protein_codin	0	0	0	0
ENSG000001	CTNNAL1	protein_codin	32.4926622	21.9546724	0	0
ENSG000001	STARD8	protein_codin	121.949953	175.892742	80.0497693	74.2912577
ENSG000001	STARD8	protein_codin	0	0	0	6.10942878
ENSG000002	TCEA3	protein_codin	0	0	0	0
ENSG000002	TCEA3	protein_codin	0	0	0	0
ENSG000002	PFDN6	protein_codin	0	11.513276	0	10.6206615
ENSG000002	PFDN6	protein_codin	19.6860408	23.716778	17.7517435	12.1274378
ENSG000001	ZNF436	protein_codin	0	33.20374	14.4959076	0
ENSG000002	PFDN6	protein_codin	6.45370783	21.108607	9.81537741	0
ENSG000001	NARF	protein_codin	34.3023321	0	4.57546625	6.76365311
ENSG000001	HNRNPR	protein_codin	309.677013	328.619496	183.485835	229.61938
ENSG000001	HNRNPR	protein_codin	250.619333	216.947026	174.721263	129.282093
ENSG000002	WDR46	protein_codin	78.0008917	122.3213	35.4704168	72.7113134
ENSG000001	ZNF33A	protein_codin	137.76713	50.4373079	28.1362817	0
ENSG000001	HTR1D	protein_codin	0	0	0	0
ENSG000001	YIPF6	protein_codin	4.10178392	0	0	4.76925562
ENSG000001	ABITRAM	protein_codin	0	6.65182892	0	3.20278529
ENSG000001	EPHB2	protein_codin	0	0	0	0
ENSG000001	EPHB2	protein_codin	18.9452356	30.1509382	8.42859842	10.1561047
ENSG000001	EPHB2	protein_codin	23.0615921	45.6212842	18.2420705	9.83609564
ENSG000001	ZNF25	protein_codin	0	0	25.972123	0
ENSG000001	C1QC	protein_codin	0	0	0	0
ENSG000001	C1QC	protein_codin	0	0	0	0
ENSG000001	C1QC	protein_codin	0	0	0	0
ENSG000000	ZNF510	protein_codin	0	0	0	0
ENSG000001	C1QA	protein_codin	1.78882726	1.52084131	0	0
ENSG000001	YIPF6	protein_codin	0	0	0	0
ENSG000000	EPHA8	protein_codin	0	0	0	0
ENSG000000	ELP1	protein_codin	0	0	0	0
ENSG000001	ZNF248	protein_codin	0	0	0	0
ENSG000000	CASP10	protein_codin	9.49598377	6.37341992	6.94757167	0
ENSG000001	ZBTB40	protein_codin	0	0	0	73.4856772
ENSG000002	RING1	protein_codin	72.402507	94.3759306	65.3780701	37.5609082
ENSG000001	ANKRD30A	protein_codin	0	0	0	0

ENSG000002	HSD17B8	protein_codin	3.69718393	0	3.07476421	1.78562821
ENSG000001	CELA3A	protein_codin	0	0	0	0
ENSG000002	CELA3B	protein_codin	0	0	0	0
ENSG000001	ACTL7B	protein_codin	0	0	0	0
ENSG000001	KLF4	protein_codin	295.715941	167.229783	36.7095126	244.255813
ENSG000001	HSPG2	protein_codin	0	0	0	0
ENSG000001	SLC39A7	protein_codin	0	84.0711087	39.8923696	74.0115011
ENSG000001	HSPG2	protein_codin	0	0	0	0
ENSG000001	SLC39A7	protein_codin	74.1222389	79.1227664	85.5545651	11.5622882
ENSG000002	RXRB	protein_codin	35.2599716	59.3491747	24.1947301	31.6596109
ENSG000002	RXRB	protein_codin	12.219232	33.8172294	0	18.6682139
ENSG000001	ZNF462	protein_codin	0	0	0	0
ENSG000000	TMEM38B	protein_codin	11.5654866	5.01774273	10.6340456	3.30409666
ENSG000001	AR	protein_codin	13.1556454	31.6732325	29.3178221	9.18139551
ENSG000000	TMEM38B	protein_codin	10.8398063	10.167142	0	16.0259354
ENSG000001	FZD8	protein_codin	3.0324859	0	0.79524121	1.9692076
ENSG000001	HSPG2	protein_codin	16.3665242	36.6539659	25.3529683	5.76290672
ENSG000001	AOX1	protein_codin	0	0.95514372	0.91516009	0
ENSG000001	CCNY	protein_codin	611.556389	1032.67265	243.507967	626.332879
ENSG000001	FKTN	protein_codin	0	1.72332891	0	2.44887605
ENSG000001	CCNY	protein_codin	200.266843	170.676232	166.672802	81.6352891
ENSG000001	FSD1L	protein_codin	0	8.21299007	6.42894763	14.2644148
ENSG000002	COL11A2	protein_codin	2.59779496	0	4.48134629	0
ENSG000000	CREM	protein_codin	0	0	0	0
ENSG000001	EDA2R	protein_codin	0	0	0	0
ENSG000000	SLC44A1	protein_codin	232.955007	107.924859	154.821832	122.569977
ENSG000000	CREM	protein_codin	0	0	0	0
ENSG000000	SLC44A1	protein_codin	0	15.723786	0	12.0312458
ENSG000000	SLC44A1	protein_codin	58.3782473	52.2580233	16.7064225	61.1760525
ENSG000000	CREM	protein_codin	5.43602137	2.00088894	2.0630776	1.88763871
ENSG000000	USP48	protein_codin	47.8956838	14.0014871	52.435225	35.8558456
ENSG000000	USP48	protein_codin	125.239611	110.919308	87.3456334	73.8114124
ENSG000001	ABCA1	protein_codin	42.0740482	34.1753707	30.1925939	42.4747233
ENSG000000	CREM	protein_codin	0	0	0	0
ENSG000001	ABCA1	protein_codin	228.267129	62.7624568	127.064117	200.731739
ENSG000001	VSIG4	protein_codin	25.0240432	0	0	0
ENSG000001	PGAP1	protein_codin	0	0	0	0
ENSG000002	OXLD1	protein_codin	17.0719136	26.360637	0	0
ENSG000001	CUL2	protein_codin	0	60.4105578	0	0
ENSG000001	NPLOC4	protein_codin	50.9317289	54.723516	16.144126	29.8493957
ENSG000001	CUL2	protein_codin	0	0	0	10.0572529
ENSG000001	CUL2	protein_codin	0	0	0	0
ENSG000001	CUL2	protein_codin	86.5379392	0	31.2635093	23.0288976
ENSG000001	ACAD8	protein_codin	3.03341359	0	0	0

ENSG000001(	CUL2	protein_codin	0	0	0	20.6652547
ENSG000000'	RAP1GAP	protein_codin	0	0	0	0
ENSG000001	SLC38A10	protein_codin	461.296868	748.353839	304.811892	313.614681
ENSG000000'	RAP1GAP	protein_codin	0	0	0	0
ENSG000001(	NIPSNAP3B	protein_codin	4.96812304	2.68560578	4.32755818	4.09238873
ENSG000000'	RAP1GAP	protein_codin	0	0	0	0
ENSG000000'	RAP1GAP	protein_codin	0	0	0	0
ENSG000001	NIPSNAP3A	protein_codin	74.2844866	91.0586019	28.7898953	57.7404754
ENSG000001	PARD3	protein_codin	0	9.83845796	7.59886099	0
ENSG000001	PARD3	protein_codin	0	0	0	0
ENSG000001	PARD3	protein_codin	0	0	0	0
ENSG000001	OPCML	protein_codin	0	0	0	0
ENSG000002'	OR13C5	protein_codin	0	0	0	0
ENSG000001	CEP131	protein_codin	0	0	0	0
ENSG000001	NTM	protein_codin	0	0	0	0
ENSG000001	NTM	protein_codin	0	0	0	0
ENSG000001	SMC2	protein_codin	24.7327641	39.4307315	70.5068877	33.221204
ENSG000001	PARD3	protein_codin	0	0	66.0709053	0
ENSG000001	PARD3	protein_codin	134.750458	0	0	32.3839378
ENSG000001	PARD3	protein_codin	0	11.1521415	0	0
ENSG000001	NTM	protein_codin	0	0	0	0
ENSG000001	AATK	protein_codin	0	18.5791786	6.08294263	0
ENSG000001	SMC2	protein_codin	0	9.59732707	24.7797116	0
ENSG000001	PARD3	protein_codin	0	0	0	0
ENSG000001	NCOA6	protein_codin	109.998791	65.1572707	48.0614702	71.8916838
ENSG000001	CYLC2	protein_codin	0	0	0	0
ENSG000002	LINC00587	lncRNA	0	0	0	0
ENSG000000	LAS1L	protein_codin	0	0	20.4965186	55.4513517
ENSG000001	PPP3R2	protein_codin	0	0	0	1.07265141
ENSG000000	LAS1L	protein_codin	88.9674312	0	0	0
ENSG000000'	TP53INP2	protein_codin	81.2553109	100.230131	31.938199	0
ENSG000000'	TP53INP2	protein_codin	108.31645	74.5552756	45.489998	92.9413244
ENSG000000	LAS1L	protein_codin	0	146.86702	0	0
ENSG000002	HLA-DOA	protein_codin	3.40564128	2.58973774	0	0
ENSG000000	NRP1	protein_codin	0	0	0	0
ENSG000001	RNF20	protein_codin	0	0	0	0
ENSG000001(	PIGU	protein_codin	0	0	7.86106179	0
ENSG000000	NRP1	protein_codin	0	0	0	0
ENSG000000	NRP1	protein_codin	0	0	0	0
ENSG000000	NRP1	protein_codin	0	0	4.48704005	0
ENSG000002	BRD2	protein_codin	3.61629835	23.375254	2.55816145	3.51525915
ENSG000000	PMS1	protein_codin	0	0	0	0
ENSG000001(	ALPL	protein_codin	0	0	0	0
ENSG000001(	ALPL	protein_codin	0	0	0	0

ENSG000002	BRD2	protein_codin	0	0	0	0
ENSG000001	ALPL	protein_codin	0	0	0	0
ENSG000001	MAP1LC3A	protein_codin	0	0	0	0
ENSG000001	ZC4H2	protein_codin	0	6.01418123	0	6.77924252
ENSG000001	ALPL	protein_codin	0	0	0	0
ENSG000002	HLA-DMA	protein_codin	206.70225	651.944176	125.521707	225.435296
ENSG000001	DYNLRB1	protein_codin	3.36024607	1.39700173	1.15569191	1.36865471
ENSG000001	PGAP4	protein_codin	2.21346802	0	2.06869045	0
ENSG000001	PGAP4	protein_codin	0	8.98479874	0	0
ENSG000001	PGAP4	protein_codin	0	3.33628502	0	0
ENSG000001	MTMR8	protein_codin	3.07019479	6.21486833	0	1.37818525
ENSG000002	PSMB9	protein_codin	125.287576	292.535442	56.4992915	101.550725
ENSG000001	ZNF189	protein_codin	16.8894913	16.2624279	28.6158205	20.0784288
ENSG000000	ITCH	protein_codin	8.96532545	22.284771	12.8617668	0
ENSG000001	MRPL50	protein_codin	40.7603734	48.672094	21.5863595	26.0794286
ENSG000002	COL5A2	protein_codin	0	0	0	0
ENSG000000	NRP1	protein_codin	0	51.1039493	62.0802552	34.0514065
ENSG000001	AMER1	protein_codin	24.3905496	56.0514161	36.1218092	20.0200356
ENSG000001	ARHGEF9	protein_codin	0	0	0	9.22314721
ENSG000001	ARHGEF9	protein_codin	4.58684714	8.45010812	10.5008931	0
ENSG000001	PLPPR1	protein_codin	0	0	0	0
ENSG000000	NRP1	protein_codin	22.3343023	0	23.2404479	0
ENSG000001	CCDC40	protein_codin	0	0	0	0
ENSG000001	CCDC40	protein_codin	6.28709901	2.18765794	1.23167599	0
ENSG000001	ARHGEF9	protein_codin	3.99538314	0	0	0
ENSG000002	TMEFF1	protein_codin	9.68057612	10.4169386	0	0
ENSG000002	PSMB8	protein_codin	26.184939	34.9749483	17.0886938	12.3411795
ENSG000002	PSMB8	protein_codin	407.979332	472.196653	59.6866414	252.115128
ENSG000001	SPIN4	protein_codin	11.8743596	14.3532982	6.56890588	7.29524118
ENSG000000	MSANTD3	protein_codin	0	0	0	0
ENSG000000	MSANTD3	protein_codin	3.6030893	0	0	0
ENSG000001	ZXDB	protein_codin	53.2730699	53.3337265	26.8525259	34.4295279
ENSG000001	ECE1	protein_codin	0	0	0	0
ENSG000002	TAP2	protein_codin	195.294637	264.78436	143.885952	138.210336
ENSG000001	FAAH2	protein_codin	0	0	0.91239443	0
ENSG000001	TEX10	protein_codin	28.6067158	48.7803949	29.4366574	20.5298878
ENSG000001	SPIN2A	protein_codin	0	0	0	0
ENSG000001	ITGAV	protein_codin	0	0	0	0
ENSG000001	SPIN2A	protein_codin	7.26391834	4.13231586	1.58703734	0
ENSG000001	SPIN2B	protein_codin	0	6.23122539	4.5882618	0
ENSG000002	SPIN3	protein_codin	11.0396059	0	12.0437334	0
ENSG000001	INVS	protein_codin	4.19326384	8.199076	3.39525492	14.4250248
ENSG000002	NBDY	protein_codin	64.0827325	56.6875427	20.6467135	29.1332475
ENSG000001	KLF8	protein_codin	0	3.17046296	0	0

ENSG000001	NUP35	protein_codin	5.12294326	2.23488789	0	0
ENSG000000	EIF4G3	protein_codin	3.34882292	5.17326957	3.02220123	1.13832243
ENSG000000	EIF4G3	protein_codin	81.1444281	38.0717551	0	13.5948698
ENSG000002	HLA-DQA2	protein_codin	159.365535	300.092015	33.7343367	11.5222418
ENSG000000	RRAGB	protein_codin	36.3913189	8.70988371	19.8375207	6.86856751
ENSG000001	HLA-DQB1	protein_codin	41.7416509	11.9357846	127.058741	0
ENSG000002	LINC01993	lncRNA	0	0.84259811	0.99694356	0
ENSG000000	BIRC5	protein_codin	1.03735958	0	0	0
ENSG000001	HLA-DQA1	protein_codin	6.71369476	0	0	0
ENSG000002	PAGE3	protein_codin	0	0	0	0
ENSG000001	PAGE5	protein_codin	0	0	0	0
ENSG000001	ASIP	protein_codin	0	0	1.37708356	0
ENSG000001	PAGE5	protein_codin	0	0	0	0
ENSG000002	PAGE2	protein_codin	0	0	0	0
ENSG000001	CERKL	protein_codin	0	0	0	0
ENSG000002	PAGE2	protein_codin	0	0	0	0
ENSG000001	CERKL	protein_codin	0	0	0	0
ENSG000001	CERKL	protein_codin	0	0	0	0
ENSG000002	PAGE2B	protein_codin	0	0	0	0
ENSG000002	PAGE2B	protein_codin	0	0	0	0
ENSG000001	HLA-DRB5	protein_codin	909.125597	1577.44745	1262.1218	547.949674
ENSG000001	MSANTD2	protein_codin	0	0	0	0
ENSG000001	EIF2S2	protein_codin	398.249932	448.202594	324.363056	365.74366
ENSG000002	HLA-DRA	protein_codin	858.983387	1466.96812	691.154444	565.848016
ENSG000002	LINC01973	lncRNA	2.97477858	5.87045178	2.33732712	1.96038942
ENSG000001	APEX2	protein_codin	37.6133916	86.1640213	19.6049553	30.227586
ENSG000001	TGFBR1	protein_codin	0	0	0	0
ENSG000001	PFKFB1	protein_codin	0	0	0	0
ENSG000002	BTNL2	protein_codin	0	0	0	0
ENSG000001	TGFBR1	protein_codin	0	30.6012738	0	0
ENSG000001	MGAT5B	protein_codin	0	0	0	0
ENSG000001	HP1BP3	protein_codin	15.3090026	11.28961	0	20.6557013
ENSG000002	COL15A1	protein_codin	0.85981816	0	0	0
ENSG000001	HP1BP3	protein_codin	42.5186074	57.4965274	18.7269957	20.4705351
ENSG000001	PFKFB1	protein_codin	0	0	0	0
ENSG000001	GALNT12	protein_codin	3.06959148	0	1.98706312	1.05499469
ENSG000002	TSBP1	protein_codin	0	0	0	0
ENSG000001	ANKS6	protein_codin	0	0	0	0
ENSG000000	TRO	protein_codin	0	0	0	0
ENSG000002	NOTCH4	protein_codin	8.24474182	4.50278398	14.2971074	0
ENSG000002	OR10G9	protein_codin	0	0	0	0
ENSG000002	CCDC7	protein_codin	3.94991865	4.08817893	0	0
ENSG000002	TMEM225	protein_codin	0	0	0	0
ENSG000002	CCDC7	protein_codin	0	0	0	0

ENSG000001	SH2D5	protein_codin	1.69454967	0	0	0
ENSG000001	MXRA7	protein_codin	0	0	3.0524203	0
ENSG000002	GPSM3	protein_codin	556.515765	638.76961	249.400353	331.393042
ENSG000000	TRO	protein_codin	0	0	1.75331764	0
ENSG000002	GPSM3	protein_codin	4.09780243	0	0	0
ENSG000001	KIF17	protein_codin	0	2.20406064	0	0
ENSG000002	PBX2	protein_codin	362.834168	520.840921	197.241571	202.499725
ENSG000001	MAGED2	protein_codin	21.2403074	77.975275	20.2161952	38.1921334
ENSG000002	AGER	protein_codin	0	0	0	0
ENSG000002	AGER	protein_codin	0	0	0	0
ENSG000001	MAGED2	protein_codin	57.7708244	0	33.9092367	0
ENSG000001	MAGED2	protein_codin	0	76.3809943	0	0
ENSG000000	TBC1D2	protein_codin	24.1880861	58.1664427	15.3086015	14.0740962
ENSG000000	TBC1D2	protein_codin	0	138.318459	72.3799728	86.8270729
ENSG000000	TBC1D2	protein_codin	0	11.4361714	42.1898809	0
ENSG000002	AGER	protein_codin	4.69736232	8.24132878	0	0
ENSG000001	MAGED2	protein_codin	19.9652807	85.9832874	99.2548344	26.7715732
ENSG000002	AGER	protein_codin	0	0	0	4.55836179
ENSG000002	AGER	protein_codin	0	0	0	0
ENSG000001	CDA	protein_codin	104.434085	144.234415	50.6193907	70.3010474
ENSG000002	AGER	protein_codin	0	0	0	0
ENSG000001	CORO2A	protein_codin	59.4260707	34.9558047	0	40.459016
ENSG000001	CAMK2N1	protein_codin	2.30685157	0	0	0
ENSG000001	VWA5B1	protein_codin	0	0	0	0
ENSG000001	GRIK4	protein_codin	0	0	0	0
ENSG000002	RNF5	protein_codin	78.0734325	113.44704	28.1947938	31.5187624
ENSG000001	TLCD5	protein_codin	0	0	0	0
ENSG000001	TRIM14	protein_codin	0	0	0	0
ENSG000001	UBXN10	protein_codin	3.00705834	3.00182444	1.00101497	0
ENSG000001	PLA2G2F	protein_codin	0	0	0	0
ENSG000002	AGPAT1	protein_codin	0	164.4071	0	0
ENSG000001	PLA2G2D	protein_codin	0	0	0	0
ENSG000002	AGPAT1	protein_codin	137.209369	341.547304	227.4682	171.057896
ENSG000001	PLA2G5	protein_codin	0	0	0	0
ENSG000001	EPC1	protein_codin	91.3412085	76.4078528	52.6052038	69.6258087
ENSG000001	PLA2G2A	protein_codin	0	0	0	0
ENSG000001	RALY	protein_codin	5.98677181	6.74815551	6.41368884	8.0841627
ENSG000001	PLA2G2E	protein_codin	0	0	0	0
ENSG000001	TRMO	protein_codin	0	0	3.11137682	4.08772772
ENSG000001	TRMO	protein_codin	58.9658249	35.1154366	34.8707918	36.5361071
ENSG000001	TRMO	protein_codin	12.7315566	19.2765113	10.4856444	0
ENSG000001	OTUD3	protein_codin	39.2055325	32.9981603	26.8603916	32.639478
ENSG000001	RNF186	protein_codin	0	0	0	0
ENSG000001	FOXE1	protein_codin	0	0	0.71745015	0

ENSG000001(	TMCO4	protein_codin	11.0379859	49.2931958	6.26348034	0
ENSG000001:	XPA	protein_codin	115.049047	119.257133	83.0801928	91.0570432
ENSG000002(	PJVK	protein_codin	0	0	0	0
ENSG000001:	NCBP1	protein_codin	12.0729271	3.73492342	0	0
ENSG000001(	FGD1	protein_codin	0	0	0	0.76733158
ENSG000001:	NBL1	protein_codin	0	0	0	0
ENSG000002:	PPT2	protein_codin	0	0	0	0
ENSG000002:	DRC12	protein_codin	4.94657352	4.40656369	0	0
ENSG000000'	CAPZB	protein_codin	0	0	0	11.8464304
ENSG000002:	PPT2	protein_codin	5.27310789	0	2.625103	0
ENSG000000'	CAPZB	protein_codin	0	44.3012604	0	101.612029
ENSG000001:	NCBP1	protein_codin	121.679336	126.034274	76.1145455	80.103263
ENSG000002(	PRRT1	protein_codin	0	0	0	0
ENSG000001:	TSR2	protein_codin	61.75622	109.28334	41.3842889	41.1896377
ENSG000000:	SLC66A1	protein_codin	0	2.58463497	1.8764213	0
ENSG000000:	SLC66A1	protein_codin	17.3514013	25.6915623	11.7521986	15.3220036
ENSG000002(	FKBPL	protein_codin	3.8564282	4.68763563	0	1.8496392
ENSG000001:	TSTD2	protein_codin	4.09204884	8.23986139	0	4.52126486
ENSG000001:	TSTD2	protein_codin	0	2.7244796	4.75523191	0
ENSG000001:	H2AX	protein_codin	14.0474189	30.0586039	13.0764517	23.1449848
ENSG000001!	WNK3	protein_codin	0	1.13526063	0	0
ENSG000001:	TSTD2	protein_codin	0	5.17882113	8.22239663	0
ENSG000001:	TSTD2	protein_codin	0	7.7713815	11.1406742	3.07474734
ENSG000001:	TMOD1	protein_codin	0	8.86795787	11.3618696	4.23549262
ENSG000001:	FAM120C	protein_codin	19.2007397	16.9315512	6.19020833	8.6162502
ENSG000000(	TBXA2R	protein_codin	41.4929592	107.934694	16.6594371	43.0787442
ENSG000001:	EMC1	protein_codin	0	0	0	0
ENSG000001:	ZNF341	protein_codin	0	41.8955522	13.1330214	17.2106712
ENSG000002	ATF6B	protein_codin	167.118607	337.382411	94.934029	44.9455297
ENSG000002	ATF6B	protein_codin	139.286536	188.15383	97.7272707	186.088265
ENSG000002:	SUGT1P4-STR	lncRNA	1.24357733	2.74880087	2.21928261	1.69500584
ENSG000002:	SUGT1P4-STR	lncRNA	0	0	0	0
ENSG000001:	EMC1	protein_codin	0	94.7663059	26.3065783	25.2589136
ENSG000000!	MAP3K20	protein_codin	55.4907669	75.697044	19.656645	31.2384098
ENSG000002(	SMIM5	protein_codin	34.2426125	101.84019	27.7044345	46.081909
ENSG000001:	UBR4	protein_codin	24.6646812	19.2967227	15.3078877	20.4594839
ENSG000001:	C20orf144	protein_codin	0	0	0	0
ENSG000001:	UBR4	protein_codin	12.1879694	36.2188876	11.9639879	2.86450042
ENSG000001:	UBR4	protein_codin	41.7189421	30.8557357	14.0631772	16.429
ENSG000000'	LLGL2	protein_codin	3.88593793	2.16122996	5.56853258	0
ENSG000000:	ZNF510	protein_codin	0	0	0	21.4588122
ENSG000001:	PRXL2C	protein_codin	38.4886421	77.1785667	23.264027	45.6706283
ENSG000001:	NECAB3	protein_codin	11.9977904	0	2.10163564	1.73973157
ENSG000000:	CDC14B	protein_codin	4.93595215	42.1407683	20.2965444	27.1481105

ENSG000000	CDC14B	protein_codin	151.035582	289.01994	75.6300513	42.7344059
ENSG000000	CDC14B	protein_codin	0	0	7.6464985	0
ENSG000001	TNXB	protein_codin	1.56624994	0	0	0
ENSG000001	ARHGAP12	protein_codin	48.3261576	27.8080403	40.0845495	25.3287391
ENSG000001	TMEM94	protein_codin	0	0	0	0
ENSG000001	HABP4	protein_codin	26.2444071	56.7781095	58.8923944	3.04196425
ENSG000001	ARHGAP12	protein_codin	0	0	0	0
ENSG000001	HABP4	protein_codin	0	0	0	0
ENSG000000	CYBRD1	protein_codin	52.1937692	0	0	0
ENSG000001	UBR4	protein_codin	1743.0182	2212.2708	1237.98695	1421.75777
ENSG000001	DCAF17	protein_codin	0	30.1914687	0	0
ENSG000001	ZNF367	protein_codin	81.3008205	107.450424	48.0624784	38.7905541
ENSG000001	SLC35D2	protein_codin	1.54746266	9.46371135	1.05504813	7.38526351
ENSG000001	METTL8	protein_codin	0	0	0	9.99419452
ENSG000001	SLC35D2	protein_codin	0	12.3112637	0	0
ENSG000001	SLC25A19	protein_codin	8.92607253	0	2.91319326	0
ENSG000001	HSD17B3	protein_codin	0	3.93108752	0	0
ENSG000001	HSD17B3	protein_codin	0	0	0	0
ENSG000001	PYROXD1	protein_codin	0	0	0	0
ENSG000001	PTCH1	protein_codin	0	0	0	0
ENSG000001	GAD1	protein_codin	0	0	0	0
ENSG000000	CBFA2T2	protein_codin	0	0	23.3456088	12.7354339
ENSG000002	SP5	protein_codin	0	0	0	0
ENSG000001	KCTD2	protein_codin	68.5028342	24.1262577	0	0
ENSG000001	PTCH1	protein_codin	0	0	0	0
ENSG000000	HSD17B10	protein_codin	0	0	0	0
ENSG000001	UPF3A	protein_codin	129.773741	35.1319508	23.1305009	78.3084887
ENSG000001	SIK3	protein_codin	178.585924	0	124.734298	0
ENSG000000	HSD17B10	protein_codin	0	6.55302213	0	0
ENSG000001	FANCC	protein_codin	0	0	7.46041871	0
ENSG000001	CDC16	protein_codin	5.02591397	19.976739	10.7644187	0
ENSG000001	ZNF438	protein_codin	0	0	0	0
ENSG000001	AOPEP	protein_codin	0	14.3194626	0	0
ENSG000001	LYZL2	protein_codin	0	0	0	0
ENSG000001	APOA1	protein_codin	0	0	0	0
ENSG000001	MAP3K8	protein_codin	207.112761	112.285307	126.093077	93.0279765
ENSG000001	MAP3K8	protein_codin	172.676811	110.049983	131.826745	65.019485
ENSG000001	APOA1	protein_codin	0	0	0	0
ENSG000001	FBP1	protein_codin	92.3698255	199.205125	52.4106154	137.806085
ENSG000001	RIBC1	protein_codin	0	0	1.93724538	0
ENSG000001	APOA1	protein_codin	0	0	0	0
ENSG000002	STK19	protein_codin	0	0	0	0
ENSG000002	STK19	protein_codin	0	0	0	0
ENSG000001	FBP2	protein_codin	0.89855272	0	0	0

ENSG0000007	SMC1A	protein_codin	0	0	0	0
ENSG000001	ALDH4A1	protein_codin	5.08355828	11.3147227	5.54263674	1.75327095
ENSG000001	MFSD14B	protein_codin	421.773666	288.676349	118.438852	281.956891
ENSG000001	APOC3	protein_codin	0	0	0	0
ENSG000001	TMEM255B	protein_codin	4.82535768	0	0	0
ENSG000002	DXO	protein_codin	0	0	0	0
ENSG000001	NSD1	protein_codin	0	0.89080299	1.29133754	0
ENSG000002	CD300LD	protein_codin	10.4897787	5.6241795	0	2.85351064
ENSG000001	TMEM255B	protein_codin	12.4086007	11.6957146	1.86789753	5.15871112
ENSG000002	DXO	protein_codin	0	0	0	0
ENSG000001	PTPDC1	protein_codin	19.2086597	0	0	0
ENSG000001	G6PC2	protein_codin	0	0	0	0
ENSG000001	IQSEC2	protein_codin	9.78261782	22.1591595	13.7978359	0
ENSG000002	BTBD17	protein_codin	0	0	0	0
ENSG000001	TFDP1	protein_codin	122.776915	145.025738	94.982747	97.5589135
ENSG000001	TAS1R2	protein_codin	0	0	0	0
ENSG000000	PAX7	protein_codin	0	0	0	0
ENSG000001	JCAD	protein_codin	3.95831748	4.84330793	18.3305097	0
ENSG000001	KDM5C	protein_codin	0	0	0	0
ENSG000001	KDM5C	protein_codin	75.4911791	146.369595	163.369853	70.9305986
ENSG000000	FAM120A	protein_codin	16.4002881	7.10818553	8.38604039	20.25645
ENSG000001	TMCO3	protein_codin	26.1758039	0	0	0
ENSG000002	SKIC2	protein_codin	0	0	31.8775631	45.3861519
ENSG000001	SVIL	protein_codin	126.881901	128.001021	30.837339	60.4628065
ENSG000001	SVIL	protein_codin	491.204505	418.675127	248.272724	182.135435
ENSG000001	KDM5C	protein_codin	177.827865	234.285002	0	117.561226
ENSG000001	DCUN1D2	protein_codin	19.1842958	0	0	17.3337237
ENSG000001	SCN1A	protein_codin	0	0	0	0
ENSG000001	ACTL8	protein_codin	0	0	0	0
ENSG000000	ARHGEF10L	protein_codin	370.859404	403.879682	86.709336	212.322742
ENSG000001	FAM120AOS	protein_codin	75.2817036	119.303447	70.9069371	58.9404202
ENSG000001	BPIFA1	protein_codin	0	0	0	0
ENSG000000	ARHGEF10L	protein_codin	0	25.8966829	9.99328319	0
ENSG000001	ADPRHL1	protein_codin	0	0	0	0
ENSG000001	BPIFA1	protein_codin	0	0	0	0
ENSG000002	NELFE	protein_codin	0	0	0	0
ENSG000002	NELFE	protein_codin	102.572974	169.1096	51.5081543	58.3670667
ENSG000001	GRTP1	protein_codin	0	2.00314125	0	0
ENSG000001	GRTP1	protein_codin	1.8762138	0	2.7005439	0
ENSG000001	RCC2	protein_codin	65.9328458	90.018092	102.084164	30.6701167
ENSG000001	RCC2	protein_codin	451.635807	668.23479	211.88002	344.946289
ENSG000001	SCN2A	protein_codin	0	0	0	0
ENSG000001	CUL4A	protein_codin	22.0261043	16.0902424	8.85567157	12.986751
ENSG000001	CUL4A	protein_codin	25.3999779	4.41067807	0	4.07247545

ENSG000001	TSPYL2	protein_codin	44.7786732	64.3971579	66.3271133	34.9652008
ENSG000001	BUD13	protein_codin	0	0	0	0
ENSG000001	NINJ1	protein_codin	786.12572	783.80702	215.310212	470.306856
ENSG000001	PADI4	protein_codin	273.344685	496.739014	160.186037	267.065628
ENSG000001	BPIFA3	protein_codin	0	0	0	0
ENSG000001	PADI4	protein_codin	2.11623033	16.5311898	1.19971553	1.27209363
ENSG000001	BPIFA3	protein_codin	0	0	0	0
ENSG000001	PCID2	protein_codin	17.914795	5.07534497	2.84839034	9.34160737
ENSG000000	COBLL1	protein_codin	0	0	0	0
ENSG000001	PCID2	protein_codin	0	0	0	0
ENSG000001	PADI3	protein_codin	0	0	2.08100477	0
ENSG000001	CARD19	protein_codin	77.8392841	141.885384	48.6352451	111.472933
ENSG000002	BPIFA4P	lncRNA	0	0	0	0
ENSG000001	GPR173	protein_codin	0	0	0	0
ENSG000001	SUSD3	protein_codin	16.6126662	0	0	0
ENSG000001	PADI1	protein_codin	0	0	0	0
ENSG000001	SUSD3	protein_codin	0	43.7520369	19.1086192	20.8093738
ENSG000001	PCID2	protein_codin	0	0	0	0
ENSG000001	NXPE4	protein_codin	0	0	0	0
ENSG000001	PCID2	protein_codin	0	44.9520001	0	0
ENSG000001	PADI2	protein_codin	0	0	0	0
ENSG000001	FGD3	protein_codin	0	0	0	0
ENSG000001	BPIFB4	protein_codin	0	0	0	0
ENSG000001	PADI2	protein_codin	137.438599	194.44803	176.393327	199.757525
ENSG000000	RBM7	protein_codin	91.7332941	64.3818951	38.8874676	69.6924012
ENSG000001	XAGE3	protein_codin	0	0	0	0
ENSG000001	BPIFB3	protein_codin	0	0	0.91487554	0
ENSG000001	ZNF484	protein_codin	0	0	22.5140293	0
ENSG000001	GCG	protein_codin	0	0	0	0
ENSG000001	HTR3A	protein_codin	0	0	0	0
ENSG000001	SDHB	protein_codin	120.917965	111.495163	42.8009055	90.7931182
ENSG000001	LYZL1	protein_codin	0	0	0	0
ENSG000001	XAGE5	protein_codin	0	0	0	0
ENSG000001	XAGE5	protein_codin	0	0	0	0
ENSG000002	SPANXN5	protein_codin	0	0	0	0
ENSG000001	BICD2	protein_codin	614.380299	1150.65283	531.498806	432.524391
ENSG000001	SLC4A10	protein_codin	0	0	0	0
ENSG000001	SUN5	protein_codin	0	0	0	0
ENSG000002	LINC01517	lncRNA	0	0	0	0
ENSG000001	IPPK	protein_codin	27.6457582	25.0884335	0	13.5038319
ENSG000001	SUN5	protein_codin	0	0	0	0
ENSG000002	ZBTB12	protein_codin	0	0.88248064	0	0
ENSG000002	EHMT2	protein_codin	0	10.1390217	18.8424924	0
ENSG000002	EHMT2	protein_codin	27.2201618	56.2362687	45.0938531	0

ENSG000000	BAMBI	protein_codin	10.0776037	11.1630974	11.6184132	4.13545579
ENSG000001	MFAP2	protein_codin	0	0	0	0
ENSG000001	MFAP2	protein_codin	0	0	0	0
ENSG000002	EHMT2	protein_codin	83.4762406	124.93833	0	41.7228843
ENSG000001	ECM2	protein_codin	0	1.18213872	0	0
ENSG000000	CROCC	protein_codin	145.338197	126.196843	41.4757634	28.7505417
ENSG000001	ASPN	protein_codin	0	0	0	0
ENSG000001	ASPN	protein_codin	0	0	0	0
ENSG000001	PROZ	protein_codin	0.92254045	0	0	0
ENSG000002	SDHD	protein_codin	91.6447893	130.979254	43.9424553	73.5439826
ENSG000001	OMD	protein_codin	0	0	1.29406601	0
ENSG000001	F10	protein_codin	0	0	0	0
ENSG000001	FAM20A	protein_codin	0	0.92733996	0	0
ENSG000001	F10	protein_codin	0	0	0	0
ENSG000001	OGN	protein_codin	0	0	0	0
ENSG000002	SLC44A4	protein_codin	0	0	0	0
ENSG000001	MAPRE1	protein_codin	567.723895	764.31584	361.094502	421.323654
ENSG000001	CENPP	protein_codin	1.778811	4.12892957	0	0
ENSG000001	SPATA21	protein_codin	0	0	0	0
ENSG000001	CENPP	protein_codin	0	0	0	0
ENSG000000	F7	protein_codin	0	0	0	0
ENSG000001	CENPP	protein_codin	22.0658361	21.2608219	14.6175801	5.00812932
ENSG000000	FBXO42	protein_codin	139.384245	180.143288	57.9898746	89.9845458
ENSG000002	XAGE1A	protein_codin	1.33389523	0	0	0
ENSG000001	MCF2L	protein_codin	0	0	4.65402653	0
ENSG000001	CPLANE2	protein_codin	1.98948779	4.84926435	0	1.87832066
ENSG000002	XAGE1A	protein_codin	0	0	0	0
ENSG000002	XAGE1A	protein_codin	0	0	0	0
ENSG000001	MCF2L	protein_codin	0	0	0	0
ENSG000001	MCF2L	protein_codin	1.74560269	6.13357486	9.56596079	1.74111908
ENSG000002	LAYN	protein_codin	0	0	0	0
ENSG000002	LAYN	protein_codin	0	0.92894924	0	0
ENSG000002	XAGE1B	protein_codin	0	0	0	0
ENSG000001	HOATZ	protein_codin	0	0	0	0
ENSG000001	MAGED4	protein_codin	0	0	0	0
ENSG000001	IARS1	protein_codin	0	0	0	0
ENSG000000	ATP11A	protein_codin	503.130731	0	197.820387	191.433927
ENSG000002	NEU1	protein_codin	0	30.1763079	0	0
ENSG000002	SNHG32	lncRNA	0	0	0	0
ENSG000002	SNHG32	lncRNA	0	0	0	0
ENSG000002	SNHG32	lncRNA	0	0	0	2.1733579
ENSG000002	SNHG32	lncRNA	6.84101487	14.7038267	4.41513059	9.43453238
ENSG000002	SNHG32	lncRNA	0	0	0	0
ENSG000002	SNHG32	lncRNA	0	0	0	0

ENSG000001!	IARS1	protein_codin	28.8141294	79.4459409	0	0
ENSG000001	ENSG000001	lncRNA	0	0	0	0
ENSG000000	ATP11A	protein_codin	587.213344	997.842481	240.302966	468.370905
ENSG000000	WAC	protein_codin	13.4945069	38.2553978	3.82748509	0
ENSG000001	ZBTB40	protein_codin	322.84016	289.416846	197.501751	92.2017901
ENSG000002	HSPA1B	protein_codin	29.4590228	35.932913	14.0055243	27.3901858
ENSG000002	HSPA1A	protein_codin	470.876027	1274.50957	251.753606	593.948504
ENSG000002	HSPA1L	protein_codin	14.8790468	18.8365022	16.7076465	8.07669586
ENSG000002	LSM2	protein_codin	18.8825583	42.2103612	3.88573902	10.2320016
ENSG000001	FAM131C	protein_codin	0	0	0	0
ENSG000002	VARS1	protein_codin	152.462262	202.932863	101.583987	76.8825911
ENSG000000	WAC	protein_codin	0	0	0	0
ENSG000001	CLCNKB	protein_codin	0	0	0	0
ENSG000001	TUBGCP3	protein_codin	51.7355972	61.8106502	88.0559184	0
ENSG000002	NOL4L-DT	lncRNA	0	0	0	0
ENSG000001	NOL4L	protein_codin	0	0	1.36901024	0
ENSG000001	NOL4L	protein_codin	1.49318909	0	0	0
ENSG000001	CLCNKB	protein_codin	0	0	0	0
ENSG000001	SLC35F2	protein_codin	0	0	0	0
ENSG000001	ASXL1	protein_codin	193.448102	275.522671	7.67008908	98.5332929
ENSG000002	VWA7	protein_codin	0.8888294	0	0	0
ENSG000001	ASXL1	protein_codin	0	12.8553636	6.44471645	3.13238874
ENSG000001	CLCNKA	protein_codin	0	0	0	0
ENSG000001	MAGED1	protein_codin	0	0	0	0
ENSG000001	SPACA7	protein_codin	0	0	0	0
ENSG000001	AXIN2	protein_codin	0	0	0.86506117	0
ENSG000002	MSH5	protein_codin	0	0	0	0
ENSG000001	ROR2	protein_codin	0.82559514	0	0	0
ENSG000001	LDHA	protein_codin	28.140752	34.1548359	15.3921854	19.0730477
ENSG000001	KIF3B	protein_codin	149.509274	162.434247	75.998253	88.1671298
ENSG000002	LINC02337	lncRNA	0	0	0	0
ENSG000001	ROR2	protein_codin	0	0	0	0
ENSG000001	HSPB7	protein_codin	0	0	0.91508573	0
ENSG000001	MPP7	protein_codin	118.062089	128.024575	105.686345	74.0217384
ENSG000001	MAGED1	protein_codin	0	0	0	0
ENSG000001	ARHGEF7	protein_codin	36.9558619	0	19.9070485	16.4643314
ENSG000002	CASP12	protein_codin	0	0	0	0
ENSG000001	POFUT1	protein_codin	0	3.1378647	0	2.34880479
ENSG000001	AUH	protein_codin	20.1943678	20.6854394	14.23464	29.7904048
ENSG000001	MPP7	protein_codin	0	0	0	0
ENSG000001	ZBTB17	protein_codin	8.27074292	4.43759763	0	10.7935451
ENSG000001	RND3	protein_codin	0	0	0	0
ENSG000001	DYNC2H1	protein_codin	36.5245018	15.3045499	16.2570687	34.6001067
ENSG000001	ARHGEF7	protein_codin	124.379503	189.496862	74.4006801	117.215695

ENSG000001	ARHGEF7	protein_codin	0	0	0	0
ENSG000002	MSH5	protein_codin	0	0	0	0
ENSG000001	ARHGEF7	protein_codin	0	20.6927432	0	0
ENSG000001	ZBTB17	protein_codin	88.7560557	98.1394294	52.9841742	42.8694699
ENSG000001	SYK	protein_codin	0	46.700614	0	0
ENSG000001	SYK	protein_codin	0	0	0	0
ENSG000001	POFUT1	protein_codin	122.958341	182.29881	61.3445874	92.0497606
ENSG000002	MSH5	protein_codin	0	0	0	0
ENSG000001	SYK	protein_codin	1048.23611	1177.88904	547.553477	622.078392
ENSG000001	SYK	protein_codin	930.776509	1546.75965	548.139995	645.026287
ENSG000002	MSH5	protein_codin	2.43380251	0	6.68677972	0
ENSG000000	SPEN	protein_codin	1237.86962	1084.1781	539.429443	612.454599
ENSG000001	DIRAS2	protein_codin	0	0	0	0
ENSG000001	FBLIM1	protein_codin	0	0	0	0
ENSG000001	GADD45G	protein_codin	0	0	0	0
ENSG000001	FBLIM1	protein_codin	0	0	0	0
ENSG000001	MAGED1	protein_codin	23.7202805	0	41.2176488	16.0449131
ENSG000001	KYNU	protein_codin	52.4935083	92.6845945	60.1158356	48.9883503
ENSG000001	ING1	protein_codin	15.2314246	74.5836821	8.16236454	24.6039372
ENSG000001	ING1	protein_codin	42.1853017	99.3637253	0	14.6405299
ENSG000002	CLIC1	protein_codin	168.180075	0	0	0
ENSG000002	CLIC1	protein_codin	0	2.07440977	0	0
ENSG000001	CARS2	protein_codin	0	8.63806261	0	0
ENSG000001	TMEM82	protein_codin	0	0	0	0
ENSG000002	CLIC1	protein_codin	349.82722	557.908901	152.225633	280.715689
ENSG000002	DDAH2	protein_codin	0	0	31.0091205	20.2525097
ENSG000002	DDAH2	protein_codin	0	48.9058782	0	0
ENSG000001	MKX	protein_codin	0	0	0	0
ENSG000002	DDAH2	protein_codin	22.9294543	20.5197789	2.77520343	0
ENSG000001	PLEKHM2	protein_codin	220.104803	226.207158	138.243549	153.72012
ENSG000001	PLEKHM2	protein_codin	254.17435	320.343856	82.0918893	64.3657242
ENSG000000	RAB18	protein_codin	0	18.2818867	0	0
ENSG000002	MPIG6B	protein_codin	0	2.1357835	0	0
ENSG000002	MPIG6B	protein_codin	0	0	0	0
ENSG000001	SECISBP2	protein_codin	88.3534351	100.765777	26.4209444	141.040957
ENSG000002	MPIG6B	protein_codin	386.054202	733.709877	449.222487	217.441391
ENSG000002	MPIG6B	protein_codin	3.18154044	0	0	0
ENSG000000	PSMC5	protein_codin	0	10.1813418	0	0
ENSG000002	MPIG6B	protein_codin	0	0	0	0
ENSG000002	LY6G6C	protein_codin	0	0	1.74384868	0
ENSG000001	COL4A1	protein_codin	0	0	0	0
ENSG000002	LY6G6D	protein_codin	0	0	1.34057225	0
ENSG000002	LY6G6D	protein_codin	0	5.71096424	0	0
ENSG000001	AGMAT	protein_codin	1.06719841	0	0.89169289	0

ENSG000001	SHC3	protein_codin	0	0	0	0
ENSG000002	LY6G6F	protein_codin	30.6841676	64.2804318	41.4610102	23.7473175
ENSG000001	SHC3	protein_codin	0	0	0	0
ENSG000001	DNAJC16	protein_codin	0	11.8811418	17.8929537	3.16131788
ENSG000002	STRADA	protein_codin	0	0	0	0
ENSG000001	MAP3K19	protein_codin	0	0	0	0
ENSG000001	MAP3K19	protein_codin	0	0	0	0
ENSG000002	S1PR3	protein_codin	42.437447	114.046656	77.2268463	73.2300525
ENSG000001	DNAJC16	protein_codin	84.4627453	119.08887	0	69.7865263
ENSG000001	DNAJC16	protein_codin	4.41965342	18.2095571	11.4118722	9.89933769
ENSG000002	S1PR3	protein_codin	0	0.87073077	0	1.65496239
ENSG000001	HCK	protein_codin	1386.52507	2057.42105	515.341748	1069.02936
ENSG000001	NXNL2	protein_codin	0	0	0	0
ENSG000001	NXNL2	protein_codin	0	0	0	0
ENSG000001	IRS2	protein_codin	3248.7638	2379.49819	701.808829	1340.74928
ENSG000000	MYO16	protein_codin	0	0	0.99670115	0
ENSG000001	SPIN1	protein_codin	92.1816468	57.413232	41.3481715	73.6477912
ENSG000001	HCK	protein_codin	112.77241	221.706488	46.8767881	109.524577
ENSG000002	LY6G5C	protein_codin	4.92711661	1.72180142	0	0
ENSG000002	LY6G5B	protein_codin	68.0613644	23.7997902	20.7596106	12.949069
ENSG000002	CSNK2B	protein_codin	0	54.108337	110.618581	0
ENSG000002	CSNK2B	protein_codin	0	0	0	0
ENSG000002	ENSG000002	transcribed_p	0	0	0	0
ENSG000001	CDK20	protein_codin	0	7.35746343	0.62888281	2.84291175
ENSG000002	ENSG000002	protein_codin	0	0	0	0
ENSG000002	CSNK2B	protein_codin	185.28884	278.062121	0	160.908561
ENSG000001	CDK20	protein_codin	0	0	0	0
ENSG000002	CSNK2B	protein_codin	0	0	3.08107286	0
ENSG000001	TNFSF13B	protein_codin	314.173577	217.27174	202.427725	329.657895
ENSG000001	ACBD5	protein_codin	0	8.05593288	27.1339701	8.25499459
ENSG000001	CASP9	protein_codin	0	0	0	0
ENSG000002	GPANK1	protein_codin	32.0585052	37.1292818	19.0609532	0
ENSG000001	CTSL	protein_codin	14.8090887	1.55201508	5.19713934	1.99783279
ENSG000002	GPANK1	protein_codin	5.07329586	0	4.08854821	0
ENSG000002	GPANK1	protein_codin	0	12.2573224	0	2.37635081
ENSG000001	ACBD5	protein_codin	0	13.0384977	4.81408867	0
ENSG000001	ABHD13	protein_codin	198.546186	115.435544	91.0067779	122.919286
ENSG000002	GPANK1	protein_codin	0	12.5490212	0	22.7858544
ENSG000001	ACBD5	protein_codin	0	13.8001078	0	0
ENSG000001	ACBD5	protein_codin	0	0	0	82.5241705
ENSG000002	GPANK1	protein_codin	13.8383734	0	8.22390565	0
ENSG000002	CELA2B	protein_codin	0	0	0	0
ENSG000002	C6orf47	protein_codin	70.6236145	108.245172	33.7393772	37.4491772
ENSG000002	NALF1	protein_codin	0	0	0	0

ENSG000002	APOM	protein_codin	1.84232843	0	0	0
ENSG000002	APOM	protein_codin	0	0	0	4.02536807
ENSG000002	APOM	protein_codin	0	2.81096864	0.90645777	0
ENSG000001	TTLL9	protein_codin	0	0	0	0
ENSG000001	TTLL9	protein_codin	0	0	0	0
ENSG000001	ARGLU1	protein_codin	9.42227095	43.854678	58.6850906	23.1802708
ENSG000001	TTLL9	protein_codin	0	0.76359474	0	0
ENSG000001	DAOA	protein_codin	0	0	0	0
ENSG000001	TTLL9	protein_codin	0	0	0	0
ENSG000001	MASTL	protein_codin	3.06778547	6.25318305	0	4.59880253
ENSG000001	CTRC	protein_codin	1.01602333	0	2.20291043	0.89767243
ENSG000000	NAALAD2	protein_codin	0	0	0	2.36626228
ENSG000001	MASTL	protein_codin	40.1537251	22.5389336	6.38564758	12.1264592
ENSG000000	TUT7	protein_codin	39.1203051	50.7364652	130.974658	39.4240055
ENSG000000	TUT7	protein_codin	17.2727727	7.45695965	15.3733437	4.96228442
ENSG000001	CTRC	protein_codin	0	0.85446192	0	0
ENSG000001	ERCC5	protein_codin	22.2079034	16.499833	21.7109124	16.3790959
ENSG000000	TUT7	protein_codin	0	0	0	0
ENSG000001	ERCC5	protein_codin	0	0	0	0
ENSG000000	TUT7	protein_codin	669.105738	627.582694	664.739258	466.481299
ENSG000000	TUT7	protein_codin	731.906953	269.834777	367.239063	257.293864
ENSG000002	BAG6	protein_codin	0	0	0	0
ENSG000001	DUSP15	protein_codin	0	0	0	0
ENSG000002	BAG6	protein_codin	29.2216115	0	0	0
ENSG000001	FOXS1	protein_codin	0	0	0	0
ENSG000000	NOX4	protein_codin	0	0	0	0
ENSG000001	EFHD2	protein_codin	2390.4658	2803.65125	861.980349	1584.6197
ENSG000001	MYLK2	protein_codin	0	0	0	0.69457518
ENSG000002	LINC01854	lncRNA	0.99762551	0	0	0
ENSG000001	ISCA1	protein_codin	86.1659999	177.190618	74.0042167	74.4892258
ENSG000001	NUDT11	protein_codin	4.97358888	0	0	0
ENSG000001	MYLK2	protein_codin	0	0	0	0
ENSG000001	FHAD1	protein_codin	0	0	0	0
ENSG000001	FHAD1	protein_codin	0	0	0	0
ENSG000001	FHAD1	protein_codin	0	0	0	0
ENSG000001	FHAD1	protein_codin	0	0	2.72136583	0
ENSG000001	C9orf153	protein_codin	0	0	0	0
ENSG000001	POGLUT2	protein_codin	0	0	0	0
ENSG000001	NUDT10	protein_codin	0	0	0	0
ENSG000002	PRRC2A	protein_codin	822.882731	867.356287	368.380629	451.914961
ENSG000001	TMEM51	protein_codin	1.65319794	0	0	0
ENSG000001	TMEM51	protein_codin	0	0	5.54745255	1.91356867
ENSG000001	YME1L1	protein_codin	215.785481	140.15424	91.3332732	125.449113
ENSG000001	TEX30	protein_codin	0	7.3525602	0	0

ENSG000001	SHROOM4	protein_codin	6.46594952	10.3779009	24.0629448	0
ENSG000001	TEX30	protein_codin	0	0	4.18376427	0
ENSG000001	TEX30	protein_codin	7.46913276	0	0	3.04607002
ENSG000001	TEX30	protein_codin	0	0	0	0
ENSG000001	KAZN	protein_codin	0.69165551	0	0	0
ENSG000001	TEX30	protein_codin	0	5.61276886	9.55459129	0
ENSG000001	KAZN	protein_codin	0	1.61678218	3.78483181	0
ENSG000001	TEX30	protein_codin	0	0	0	0
ENSG000002	PRRC2A	protein_codin	157.069848	502.104612	204.67416	223.596723
ENSG000001	CCNB3	protein_codin	0	0	0	0
ENSG000001	NAA35	protein_codin	10.7484919	4.28910333	12.1882428	11.066111
ENSG000001	CCNB3	protein_codin	0	0	0	0
ENSG000001	PRDM2	protein_codin	0	0	0	0
ENSG000002	AIF1	protein_codin	41.9319737	84.8902845	110.398566	26.7912559
ENSG000001	TPP2	protein_codin	125.247831	87.0905768	76.0664336	98.3080912
ENSG000001	BCL2L1	protein_codin	0	117.120225	49.9914393	13.9266792
ENSG000001	PDPN	protein_codin	0	0	0	0
ENSG000002	AIF1	protein_codin	1365.32394	1824.32542	495.612211	749.361925
ENSG000001	PDPN	protein_codin	0	0	0	0
ENSG000001	BCL2L1	protein_codin	4.37447543	34.6374235	10.3260714	26.2069171
ENSG000001	AKAP4	protein_codin	0	0	0	0
ENSG000001	TPP2	protein_codin	0	0	0	0
ENSG000002	NCR3	protein_codin	0	0	0	0
ENSG000002	NCR3	protein_codin	0	0	0	0
ENSG000002	NCR3	protein_codin	0	0	0	0
ENSG000001	COX4I2	protein_codin	0	0	0	0
ENSG000001	AGTPBP1	protein_codin	23.0297725	11.5757334	0	7.16435521
ENSG000001	AGTPBP1	protein_codin	27.9991294	31.036692	40.8095047	45.7257801
ENSG000001	AGTPBP1	protein_codin	0	120.931917	0	0
ENSG000001	LRRC38	protein_codin	0	0	0	0
ENSG000002	LST1	protein_codin	8.52648488	2.36355336	0	10.1262275
ENSG000001	ANKRD26	protein_codin	100.337942	37.4879552	0	14.2627241
ENSG000001	CLCN5	protein_codin	41.4154811	73.4482094	32.8771741	53.2548286
ENSG000002	LST1	protein_codin	0	0	0	16.672596
ENSG000002	LST1	protein_codin	211.192541	390.175402	104.786555	134.979599
ENSG000001	CLCN5	protein_codin	112.876729	0	40.3926907	28.1795154
ENSG000002	LST1	protein_codin	5.71308185	8.13971033	0	1.16308654
ENSG000002	LST1	protein_codin	8.20435487	0	3.20861417	0
ENSG000001	TUBD1	protein_codin	0	0	0	5.3905478
ENSG000002	LST1	protein_codin	158.747799	343.569717	46.4218643	166.64535
ENSG000002	PRAMEF17	protein_codin	0	0	0	0
ENSG000002	LST1	protein_codin	0	143.742922	0	102.4245
ENSG000002	PRAMEF19	protein_codin	0	0	0	0
ENSG000001	DLG2	protein_codin	0	0	0	0

ENSG000001	ID1	protein_codin	6.20039444	0	0.68853244	3.67321527
ENSG000001	CLCN5	protein_codin	0	0	2.04833808	0
ENSG000002	LST1	protein_codin	5.0527979	18.553215	2.32316386	5.36938399
ENSG000001	ID1	protein_codin	10.5278199	8.5191985	0	0
ENSG000001	BIN1	protein_codin	0	0	0	0
ENSG000001	FGF14	protein_codin	0	0	0	0
ENSG000001	ABI2	protein_codin	5.75953403	7.74949303	4.46374964	0
ENSG000001	ABI1	protein_codin	85.3492503	0	140.80231	80.3844407
ENSG000001	ABI1	protein_codin	96.8107315	95.0913153	0	95.1096551
ENSG000001	ABI1	protein_codin	0	90.519187	57.9753233	0
ENSG000001	ABI1	protein_codin	0	0	0	0
ENSG000001	PAGE4	protein_codin	0	0	0	0
ENSG000001	ABI1	protein_codin	0	0	0	0
ENSG000001	FGF14	protein_codin	0	1.45304431	0	0
ENSG000002	NFKBIL1	protein_codin	15.8985472	9.65683918	0	0
ENSG000002	NFKBIL1	protein_codin	18.5456746	0	0	7.89141736
ENSG000002	NFKBIL1	protein_codin	0	40.5336716	22.0552151	12.8818633
ENSG000000	PAGE1	protein_codin	0	0	0	0
ENSG000002	ATP6V1G2	protein_codin	0	1.19023368	0	0
ENSG000002	PRAMEF15	protein_codin	0	0	0	0
ENSG000001	ALG8	protein_codin	0	0	0	0
ENSG000001	ABI1	protein_codin	0	0	0	0
ENSG000001	ITGBL1	protein_codin	0	0	0	0
ENSG000001	ABI1	protein_codin	185.375806	101.367594	58.8893006	99.6805762
ENSG000001	ABI1	protein_codin	0	0	0	0
ENSG000001	DDX39B	protein_codin	32.71401	138.314841	28.6672998	45.7221001
ENSG000001	ITGBL1	protein_codin	0	0	0	0
ENSG000002	ATP6V1G2-DI	protein_codin	0	7.52579573	0	0
ENSG000000	PPP1R3F	protein_codin	0	0	0	0
ENSG000002	PRAMEF6	protein_codin	0	0	0	0
ENSG000002	MCCD1	protein_codin	0.72101666	0	0	0
ENSG000000	FOXP3	protein_codin	0	0	0	0
ENSG000000	FOXP3	protein_codin	0	0	0	0
ENSG000001	NALCN	protein_codin	0	0	0	0
ENSG000000	FOXP3	protein_codin	1.65066923	0	0	0
ENSG000001	NTRK2	protein_codin	0	0	0	0
ENSG000001	CFAP107	protein_codin	0	0	0	0
ENSG000001	NTRK2	protein_codin	0	0	0	0
ENSG000001	PDSS1	protein_codin	20.3681932	36.2345586	18.8642038	29.9395393
ENSG000001	GDPD4	protein_codin	0	0	0	0
ENSG000002	AADACL4	protein_codin	0	0	0	0
ENSG000001	CCDC22	protein_codin	88.6224143	154.894267	62.2178409	91.5918164
ENSG000002	HLA-C	protein_codin	5540.22956	9070.60624	4305.7829	5998.16019
ENSG000002	ZNF550	protein_codin	0	0	0	10.5893257

ENSG000001:	ZNF549	protein_codin	0	21.9262066	13.3744897	0
ENSG000001:	TMTC4	protein_codin	19.1669474	21.6677394	0	13.1655244
ENSG000000	APBB1IP	protein_codin	427.880313	854.73934	329.244847	168.793354
ENSG000002	HLA-C	protein_codin	243.534927	261.996219	225.893387	292.692887
ENSG000001	SLC28A3	protein_codin	2.09697386	3.33025526	1.65682596	2.32227411
ENSG000001	GAD2	protein_codin	0	0	0	0
ENSG000001	GGACT	protein_codin	0	0	0	0
ENSG000001	CACNA1F	protein_codin	1.91072292	4.73348557	2.29408396	0.97807158
ENSG000001	TCF19	protein_codin	0	0	0	8.29958386
ENSG000001	HNRNPK	protein_codin	33.2959961	38.2557866	23.637052	9.72167839
ENSG000001	TCF19	protein_codin	0	23.2852037	0	0
ENSG000000	TNFRSF1B	protein_codin	2155.95539	2989.88344	650.361074	1466.67022
ENSG000001	GAD2	protein_codin	0	0	1.13106719	0
ENSG000000	DGAT2	protein_codin	7.65008564	55.139933	0	46.3944141
ENSG000001	HNRNPK	protein_codin	523.758555	313.771898	324.475245	438.777448
ENSG000001	CACNA1F	protein_codin	0	0	0	0
ENSG000002	CCHCR1	protein_codin	32.2766734	25.8945773	0	6.57242961
ENSG000001	PCCA	protein_codin	28.4329189	0	0	23.9188337
ENSG000001	HNRNPK	protein_codin	0	0	0	0
ENSG000001	PCCA	protein_codin	0	31.2800279	0	0
ENSG000001	PCCA	protein_codin	0	0	27.5423181	0
ENSG000002	CDSN	protein_codin	0	0	0	0
ENSG000001	KLHL35	protein_codin	0.94945762	0.95197353	3.23871236	0
ENSG000002	MUC21	protein_codin	0	0	0	0
ENSG000001	CCDC93	protein_codin	178.530047	233.764622	108.277023	175.24229
ENSG000000	MYO3A	protein_codin	0	0	0	0
ENSG000000	MYO3A	protein_codin	0	0	0	0
ENSG000001	SYP	protein_codin	0	0	0	0
ENSG000001	DEFB123	protein_codin	0	0	0	0
ENSG000000	PRICKLE3	protein_codin	8.08512634	7.92656917	0	3.42952132
ENSG000002	DEFB121	protein_codin	0	0	0	0
ENSG000002	DEFB121	protein_codin	0	0	0	0
ENSG000001	DEFB119	protein_codin	0	0	0	0
ENSG000002	GTF2H4	protein_codin	0	0	0	0
ENSG000000	PRICKLE3	protein_codin	0	0	0	0
ENSG000001	DEFB119	protein_codin	0	0	0	0
ENSG000001	PLP2	protein_codin	9.31578397	3.27703392	1.27086971	1.43610178
ENSG000000	CHRDL2	protein_codin	0	0	0	0
ENSG000001	SHISA7	protein_codin	0	0	0	0
ENSG000001	PLP2	protein_codin	438.856335	545.112254	113.75048	281.035207
ENSG000000	CHRDL2	protein_codin	0	0	0	0
ENSG000002	FRG1JP	transcribed_u	0	0	0	0
ENSG000000	ZIC2	protein_codin	0	0	0	0
ENSG000001	QNG1	protein_codin	0	0	0	0

ENSG000001(	QNG1	protein_codin	25.5116399	48.3768927	14.8847799	26.3290595
ENSG000001	GID4	protein_codin	3.35477731	0	3.66537667	2.18065812
ENSG000001(	KIF27	protein_codin	0	0	0	0
ENSG000000	PTPRH	protein_codin	0	0	0	0
ENSG000001	GPR158	protein_codin	0	0	0	0
ENSG000001(	STXBP4	protein_codin	14.9195517	11.9642174	12.0518388	9.61670114
ENSG000001	CLYBL	protein_codin	0	0	1.69551102	0
ENSG000001	CLYBL	protein_codin	1.0842179	1.08564786	0	1.02383515
ENSG000001	THNSL1	protein_codin	2.39016052	7.14358611	3.15089635	0
ENSG000001	WDR45	protein_codin	2.46236888	0	0	0
ENSG000002	ENSG000002	protein_codin	0	10.3139928	0	0
ENSG000001(	GKAP1	protein_codin	0	0	1.63904411	0
ENSG000001	ENKUR	protein_codin	117.592267	175.852638	68.4941312	37.9720257
ENSG000001(	GKAP1	protein_codin	6.1012133	0	10.4617909	3.4768836
ENSG000001	WDR45	protein_codin	25.7873005	16.2537894	0	5.11117578
ENSG000001(	GKAP1	protein_codin	1.74838084	23.8867766	6.81664658	0
ENSG000001	WDR45	protein_codin	58.2435147	77.7095995	25.9693551	28.6734473
ENSG000000	PRTFDC1	protein_codin	1.15112138	1.14780372	0	2.05366821
ENSG000001	IER3	protein_codin	237.402517	200.601457	58.4994291	62.3076877
ENSG000000	PRTFDC1	protein_codin	3.17140309	11.2574127	9.5304125	3.1862013
ENSG000000	MBTD1	protein_codin	34.3889657	22.4728083	0	31.205967
ENSG000001	PAAF1	protein_codin	13.8362341	15.0737799	0	10.4563094
ENSG000002	PRAF2	protein_codin	0	0	0.47455614	0
ENSG000001	TM9SF2	protein_codin	604.516547	652.369259	282.352326	497.974402
ENSG000001	FLOT1	protein_codin	303.193798	419.797659	167.622317	231.668326
ENSG000000	NME1-NME2	protein_codin	0	0	0	0
ENSG000001	UBQLN1	protein_codin	366.765949	327.661535	201.497388	187.916495
ENSG000001	FAM182A	lncRNA	1.09643156	0	1.33553947	0
ENSG000002(	CFTRP3	processed_ps	0	0	0	0
ENSG000002(	ENSG000002(	transcribed_u	0	0	0	0
ENSG000001	FAM182B	lncRNA	0	0	0	0
ENSG000001	FAM182B	lncRNA	0	0	0	0
ENSG000001	MDC1	protein_codin	110.143018	127.661552	165.084511	47.3226622
ENSG000001(	ARHGAP21	protein_codin	342.376712	739.10637	228.420432	385.915113
ENSG000001(	GPR183	protein_codin	777.858253	155.853086	237.739399	419.882541
ENSG000001	IDNK	protein_codin	0	0	0	0
ENSG000001	IDNK	protein_codin	11.3302222	1.38098145	6.69412619	2.44335275
ENSG000001	NRM	protein_codin	29.8015085	0	11.6571888	0
ENSG000001	NRM	protein_codin	15.4894596	0	0	22.0778396
ENSG000000(	GRIPAP1	protein_codin	0	252.224804	0	0
ENSG000001	FRMD3	protein_codin	0	0	0	0
ENSG000001	ZNF337	protein_codin	55.8131853	66.5363015	50.9109834	28.015308
ENSG000002(	DHX16	protein_codin	6.68598851	0	2.37587173	0
ENSG000001	FRMD3	protein_codin	0	1.55084128	0	0

ENSG000001	RABL2A	protein_codin	0	2.88459398	7.94463716	0
ENSG000001	UBAC2	protein_codin	4.7699059	18.29841	1.32620275	17.6151335
ENSG000002	DHX16	protein_codin	186.303057	289.318779	109.482975	129.493913
ENSG000002	ENSG000002	lncRNA	0	0	0	0
ENSG000001	RASEF	protein_codin	0	0	0	0
ENSG000001	PDE2A	protein_codin	5.52104184	0	0	10.7312347
ENSG000001	KIAA1217	protein_codin	0	0	0	0
ENSG000001	KIAA1217	protein_codin	0	0	0	0
ENSG000001	KIAA1217	protein_codin	0	0	0	0
ENSG000001	KIAA1217	protein_codin	0	0	0	0
ENSG000002	SPATA31A7	protein_codin	0	0	0	0
ENSG000002	SPATA31D5P	transcribed_u	0	0	0	0
ENSG000000	DOCK9	protein_codin	0	0	0	0
ENSG000001	KIAA1217	protein_codin	0	0	0	0
ENSG000001	TLE1	protein_codin	21.3157592	5.73947175	6.51947478	2.13109276
ENSG000001	NPPB	protein_codin	0	0	0	0
ENSG000002	C6orf136	protein_codin	0	0	0	0
ENSG000002	C6orf136	protein_codin	12.5008348	19.9090292	1.50418777	10.7267064
ENSG000001	NPPA	protein_codin	0	0	0	0
ENSG000001	KCND1	protein_codin	6.11511511	0	0	0
ENSG000001	NPPA	protein_codin	0	0	0	0.75978453
ENSG000002	ENSG000002	lncRNA	0	0	0	0
ENSG000001	ATAT1	protein_codin	2.52789485	0	9.32401603	0
ENSG000001	TLE1	protein_codin	0	0	0	0
ENSG000001	ATAT1	protein_codin	0	0	0	3.75442255
ENSG000001	MTHFR	protein_codin	0	0	0	0
ENSG000000	OTUD5	protein_codin	100.16447	116.122625	25.6271059	37.7997466
ENSG000001	IL36G	protein_codin	0	0	1.08079701	0
ENSG000000	CLCN6	protein_codin	2.87394404	4.41160721	0	3.04065095
ENSG000000	CLCN6	protein_codin	0	0	0	0
ENSG000000	CLCN6	protein_codin	0	4.83621107	1.53292064	3.22177259
ENSG000000	SLC15A1	protein_codin	0	1.18702329	0	0
ENSG000001	OTUD1	protein_codin	925.639706	714.258285	358.439569	597.234667
ENSG000000	CLCN6	protein_codin	2.12626219	0	1.34640286	0
ENSG000000	CLCN6	protein_codin	22.3749777	3.43107562	6.05258405	4.85242723
ENSG000001	TLE1	protein_codin	97.1655665	79.8523854	24.2416093	19.3857327
ENSG000001	C10orf67	protein_codin	0	0	0	0
ENSG000001	C10orf67	protein_codin	0	0	0	0
ENSG000000	SLC15A1	protein_codin	0	0	0	0
ENSG000001	PTF1A	protein_codin	0	0	0	0
ENSG000001	PIM2	protein_codin	156.158532	104.796028	134.577964	42.6105483
ENSG000001	MSRB2	protein_codin	30.1801527	32.5059526	15.1200963	22.4479697
ENSG000002	PPP1R10	protein_codin	155.909797	309.404305	97.7884325	137.095311
ENSG000001	SLC35A2	protein_codin	0	0	0	0

ENSG000001	LENG8	protein_codin	218.120988	163.225444	206.028475	59.9570588
ENSG000001	SLC35A2	protein_codin	0	0	0	0
ENSG000001	SLC35A2	protein_codin	17.4958931	0	9.87055459	0
ENSG000001	TLE4	protein_codin	0	0	0	0
ENSG000001	TLE4	protein_codin	0	0	0	0
ENSG000001	ARMC3	protein_codin	0	0	0	0
ENSG000001	SLC35A2	protein_codin	0	5.7500236	3.00228192	0
ENSG000001	TTYH1	protein_codin	0	0	0	0
ENSG000001	TTYH1	protein_codin	0	0	0	0
ENSG000001	TLE4	protein_codin	0	0	0	0
ENSG000001	ABHD12	protein_codin	12.8345832	13.9816765	2.93911896	2.90190381
ENSG000001	TLE4	protein_codin	46.9365728	0	0	10.5993206
ENSG000002	ABCF1	protein_codin	136.908507	97.7357571	39.7596721	59.1613008
ENSG000001	STK24	protein_codin	83.6966292	187.756444	18.2886488	48.8100641
ENSG000000	XYLT2	protein_codin	0	13.7205686	0	0
ENSG000001	TLE4	protein_codin	156.661164	287.856533	143.346998	205.851291
ENSG000001	STK24	protein_codin	0	0	3.71502326	0
ENSG000002	PRR3	protein_codin	11.4455246	6.65664807	4.2468475	2.96594863
ENSG000002	PRR3	protein_codin	0	0	0	0
ENSG000000	CTTN	protein_codin	439.868625	1249.23663	604.275956	345.875249
ENSG000001	PQBP1	protein_codin	39.5688803	0	0	19.4846936
ENSG000001	PQBP1	protein_codin	10.3032272	11.1115125	0	1.89213384
ENSG000002	DDR1	protein_codin	0	0	0	0
ENSG000002	DDR1	protein_codin	0	0	0	0
ENSG000002	DDR1	protein_codin	0	0	4.35994699	0
ENSG000002	DDR1	protein_codin	0	0	0	0
ENSG000001	KIAA2013	protein_codin	186.264669	290.723609	94.0698026	148.266528
ENSG000001	PIP4K2A	protein_codin	873.019155	1632.78639	708.135541	727.691263
ENSG000002	DDR1	protein_codin	0	0	0	0
ENSG000001	KIAA2013	protein_codin	12.0359099	2.30604515	11.1249788	0
ENSG000001	FARP1	protein_codin	0	0	0	0
ENSG000001	TIMM17B	protein_codin	0	30.429666	10.1341137	8.38817426
ENSG000001	MTHFR	protein_codin	0	0	0	0
ENSG000001	MTHFR	protein_codin	0	0	0	0
ENSG000001	PSAT1	protein_codin	0	1.09814993	16.5683796	0
ENSG000001	MTHFR	protein_codin	80.3375823	198.493716	61.6868316	38.6947819
ENSG000001	MTHFR	protein_codin	317.081523	181.454091	69.4061296	142.470159
ENSG000002	ACOXL-AS1	lncRNA	0	0	1.1186837	0
ENSG000001	CEP78	protein_codin	11.8227334	0	0	0
ENSG000001	CEP78	protein_codin	0	0	0	0
ENSG000000	SPAG6	protein_codin	0	0	0	0
ENSG000002	LRCOL1	protein_codin	0	0	0	0
ENSG000002	ENSG000002	lncRNA	9.41869988	10.2116322	0	3.78928782
ENSG000000	HDAC6	protein_codin	9.51724633	0	0	0

ENSG000002	GALNT9-AS1	lncRNA	0	0	0	0
ENSG000001	CPT1A	protein_codin	0	4.15461261	0	3.54960112
ENSG000000	HDAC6	protein_codin	0	0	0	6.6428314
ENSG000001	C1orf167-AS1	lncRNA	0.95005692	0	0	0
ENSG000002	GNL1	protein_codin	226.531046	175.498973	105.432629	108.057862
ENSG000000	SPAG6	protein_codin	0	0	0	0
ENSG000001	VSTM1	protein_codin	0	0	0	0
ENSG000001	AGTRAP	protein_codin	12.0790423	8.68708102	10.5094694	9.86785436
ENSG000001	AGTRAP	protein_codin	131.389737	117.735663	67.88462	40.7231639
ENSG000002	HLA-E	protein_codin	8919.08953	15008.0028	8731.6216	6833.3418
ENSG000001	VPS13A	protein_codin	74.8689982	182.767654	146.178662	60.2321249
ENSG000001	AGTRAP	protein_codin	2.34107061	0	4.22677771	1.95541748
ENSG000000	HDAC6	protein_codin	0	0	0	0
ENSG000001	VPS13A	protein_codin	0	0	0	0
ENSG000001	PUS1	protein_codin	3.40893759	2.23952692	6.3432502	5.179819
ENSG000002	DPRX	protein_codin	0	0	0	0
ENSG000001	EDAR	protein_codin	0	0	0	0
ENSG000001	ENTPD6	protein_codin	3.91899504	23.415038	5.68767663	3.41207121
ENSG000001	MAD2L2	protein_codin	4.13184382	1.04498103	3.29650682	1.98714445
ENSG000002	TRIM39	protein_codin	7.89490437	0	0	0
ENSG000002	TRIM39	protein_codin	58.9001689	67.6189899	37.5765742	30.9763802
ENSG000001	BMI1	protein_codin	70.1645748	39.8815612	59.0135949	44.1444548
ENSG000001	MAD2L2	protein_codin	4.66217944	12.4588368	4.86408813	2.32531213
ENSG000001	GATA1	protein_codin	2.66987879	0	0	0
ENSG000001	ENTPD6	protein_codin	0	0	0	0
ENSG000001	MAD2L2	protein_codin	0	0	0	0
ENSG000001	MAD2L2	protein_codin	0	0	0	0
ENSG000001	GATA1	protein_codin	21.9007243	53.8168722	23.9978967	0
ENSG000001	MAD2L2	protein_codin	0	0	0	0
ENSG000001	MBNL2	protein_codin	0	0	30.5800752	0
ENSG000002	LINC01257	lncRNA	0	0	0	0
ENSG000001	TTLL6	protein_codin	0	0	0	0
ENSG000001	ADGRD1	protein_codin	0	0	0	0
ENSG000001	SUV39H1	protein_codin	12.7788029	24.706724	0	13.8857267
ENSG000001	MAD2L2	protein_codin	48.7964801	89.8809638	18.9559694	34.8856667
ENSG000001	NUDT8	protein_codin	0.91832925	0	0	0
ENSG000002	TRIM15	protein_codin	0	0	0	0
ENSG000000	WAS	protein_codin	623.208241	880.939591	426.375303	483.178171
ENSG000002	TRIM10	protein_codin	0	5.85765808	4.78379971	1.93705124
ENSG000001	HS6ST3	protein_codin	0	0	0	2.16858938
ENSG000001	VSX1	protein_codin	0	0	0	0
ENSG000002	FOXB2	protein_codin	0	0	0	0
ENSG000001	VSX1	protein_codin	0	0	0	0
ENSG000001	UGGT2	protein_codin	15.107831	9.46193795	11.2742583	8.43004072

ENSG000001	PRUNE2	protein_codin	0	0	0	0
ENSG000001	UGGT2	protein_codin	0	0	0	0
ENSG000001	ZNF615	protein_codin	0	0	21.2467824	0
ENSG000001	PRUNE2	protein_codin	16.6749841	15.2771412	0	0
ENSG000001	PRUNE2	protein_codin	0	0	0	0
ENSG000001	UGGT1	protein_codin	230.801306	281.482918	164.974138	169.209815
ENSG000002	TRIM40	protein_codin	19.8828942	21.7025902	21.1642437	8.68389744
ENSG000001	ACSS1	protein_codin	3.32223402	0	0	4.42733263
ENSG000001	WDR13	protein_codin	17.9100179	36.8817311	19.4465677	16.0915096
ENSG000001	GCNT1	protein_codin	12.8769373	68.6365463	0	0
ENSG000002	TRIM31	protein_codin	0	0	0	0
ENSG000001	RFK	protein_codin	49.9379829	24.7423396	14.0563721	38.0361022
ENSG000001	SLC15A4	protein_codin	9.54916747	1.95473135	1.33490183	2.10677612
ENSG000001	SP2	protein_codin	193.346927	242.815601	109.40815	112.987068
ENSG000001	SLC15A4	protein_codin	137.329916	0	0	0
ENSG000001	PPP1CA	protein_codin	352.345778	508.711212	197.014216	274.326107
ENSG000001	UGGT2	protein_codin	94.8795945	31.5878014	45.5713249	47.6912473
ENSG000002	RNF39	protein_codin	0	0	0	0
ENSG000000	PCSK5	protein_codin	68.9741526	87.6234416	67.786639	91.3531942
ENSG000001	FBXO6	protein_codin	58.1750819	80.5543016	12.6956894	33.927177
ENSG000001	RBM3	protein_codin	188.726103	265.539746	97.541818	205.607274
ENSG000001	SSH3	protein_codin	6.04514839	10.0992217	0	0
ENSG000002	PPP1R11	protein_codin	28.9854218	24.6906761	23.7196685	15.9711289
ENSG000001	RBM3	protein_codin	95.3354797	67.2179636	74.1447527	54.3225855
ENSG000001	FBXO44	protein_codin	0	0	0	0
ENSG000001	FBXO44	protein_codin	0	7.80007444	3.59697574	4.27495644
ENSG000002	PPP1R11	protein_codin	0	0	4.42650647	0
ENSG000002	PPP1R11	protein_codin	10.4190399	13.4747637	0	0
ENSG000000	PCSK5	protein_codin	55.2579166	26.4292391	42.9103673	49.6535351
ENSG000001	FBXO44	protein_codin	10.2061146	12.0011563	1.40451139	1.89918245
ENSG000002	PPP1R11	protein_codin	0	12.133898	8.11726534	3.28966061
ENSG000001	FBXO44	protein_codin	0	7.21808349	0	0
ENSG000000	TBC1D25	protein_codin	64.6873597	65.0689669	47.7568861	27.1244967
ENSG000002	PPP1R11	protein_codin	197.428909	249.135379	62.4621767	141.776823
ENSG000002	PPP1R11	protein_codin	0	0	0	0
ENSG000002	ENSG000002	lncRNA	0	3.20006762	0	2.11665135
ENSG000002	UBBP2	transcribed_p	0.90599678	0.81791646	0.980225	0
ENSG000000	POLR1H	protein_codin	7.70206015	0	0	16.4927183
ENSG000000	POLR1H	protein_codin	0	0	0	0
ENSG000001	DNAJC3	protein_codin	95.037687	155.433064	165.294658	91.455611
ENSG000002	POLR1HASP	lncRNA	0	2.7098771	2.40021841	0
ENSG000001	KLK13	protein_codin	0	0	0	0
ENSG000002	HCG9	lncRNA	0	0	2.5109276	0
ENSG000002	HLA-A	protein_codin	9.58776917	30.7210073	0	0

ENSG000001	UBIAD1	protein_codin	0	0	0	0
ENSG000002	HLA-A	protein_codin	0	0	0	0
ENSG000001	NMRK1	protein_codin	8.94556152	12.7705195	0	0
ENSG000002	HLA-A	protein_codin	5059.19048	3706.8824	533.348136	3050.43841
ENSG000001	UBIAD1	protein_codin	24.8701755	51.4508128	11.0205673	14.8758737
ENSG000001	NMRK1	protein_codin	18.1929222	0	7.09342754	14.6641072
ENSG000002	HLA-G	protein_codin	0	0	0	0
ENSG000002	RACK1	protein_codin	0	0	0	0
ENSG000002	HLA-G	protein_codin	0	0	0	0
ENSG000001	ANGPTL7	protein_codin	0	0	0	0
ENSG000002	HLA-G	protein_codin	0	0	0	0
ENSG000001	DZIP1	protein_codin	0	0	0	0
ENSG000001	CARNMT1	protein_codin	10.8063841	5.43021445	0	17.6156857
ENSG000001	CARNMT1	protein_codin	33.8545934	19.1618627	25.2602304	25.1603725
ENSG000001	COMMD3	protein_codin	72.4117102	107.874372	52.9836507	76.9609897
ENSG000001	MTOR	protein_codin	19.174496	7.79852283	4.89585006	12.8658675
ENSG000000	ACTN1	protein_codin	0	0	0	0
ENSG000002	TBC1D8	protein_codin	136.826543	75.9844622	50.9394775	137.282802
ENSG000001	BTNL9	protein_codin	0	0	0	0
ENSG000001	KLK6	protein_codin	0	0	0	0
ENSG000001	C9orf40	protein_codin	5.9912472	4.16168621	4.1582743	2.02695649
ENSG000001	CLDN10	protein_codin	0	0	0	0
ENSG000002	ARPP19P2	processed_ps	0	0	0	0
ENSG000002	HLA-F	protein_codin	14.6973469	15.2562099	2.38856141	0
ENSG000001	SYNDIG1	protein_codin	0	0	0	0
ENSG000002	NMS	protein_codin	0	0	0	0
ENSG000001	CLDN10	protein_codin	0	0	0	0
ENSG000001	RILPL1	protein_codin	2.20714966	18.1925325	2.30566985	5.98802077
ENSG000002	ZFP57	protein_codin	0	14.1824506	5.25455927	0
ENSG000002	MOG	protein_codin	0	0	0	0
ENSG000002	MOG	protein_codin	0	0	0	0
ENSG000002	MOG	protein_codin	0	0	0	0
ENSG000001	SSX3	protein_codin	0	0	0	0
ENSG000002	MOG	protein_codin	0	0	0	0.87960411
ENSG000001	SSX3	protein_codin	0	0	0	0
ENSG000001	RORB	protein_codin	0	0	0	0
ENSG000002	MOG	protein_codin	0	0	0	0
ENSG000001	YIF1A	protein_codin	0	3.39620497	2.48829387	0
ENSG000001	YIF1A	protein_codin	0	31.569398	8.40815127	0
ENSG000002	MOG	protein_codin	0	0	0	0
ENSG000002	SSX9P	transcribed_u	0	0	0	0
ENSG000001	ANXA1	protein_codin	514.863839	82.0363943	110.154879	322.284998
ENSG000002	ASPDH	protein_codin	0	0	0	0
ENSG000002	MOG	protein_codin	0	0	0	0

ENSG000001	EMC10	protein_codin	50.1641484	105.507213	0	52.2950532
ENSG000001	SSX1	protein_codin	0	0	0	0
ENSG000001	ARHGAP27	protein_codin	0	13.0969633	0	0
ENSG000001	CST3	protein_codin	1702.34814	3021.39062	729.841607	1440.32248
ENSG000000	MAP3K14	protein_codin	0	7.05340035	12.2417678	8.26249378
ENSG000001	MRNIP	protein_codin	0	0	0	0
ENSG000001	SSX6P	transcribed_u	0	0.59411975	0	0
ENSG000002	SMIM13	protein_codin	14.998629	0	6.27260201	7.14570039
ENSG000001	EXOSC10	protein_codin	264.98479	284.21401	153.524806	131.508176
ENSG000001	ALDH1A1	protein_codin	3.16292411	1.34110459	0	0
ENSG000001	SPACA5	protein_codin	0	0	0	0
ENSG000001	ZNF182	protein_codin	35.2610483	0	0	23.4613443
ENSG000001	SOX21	protein_codin	0	0.99526562	0	0.97552693
ENSG000001	DNAJC1	protein_codin	7.15197177	1.44909797	0.90971542	0
ENSG000001	ZNF81	protein_codin	1.13556241	3.05988968	0	2.70599398
ENSG000001	ZNF81	protein_codin	0	0	0	0
ENSG000001	ACBD4	protein_codin	6.7195349	0	0	0
ENSG000001	ZFAND5	protein_codin	94.655883	63.4280696	26.0386213	58.7169714
ENSG000001	SRM	protein_codin	37.5531093	58.2093025	82.0723821	49.0825218
ENSG000001	GPR180	protein_codin	17.1367244	12.26366	16.6839582	11.3137885
ENSG000001	KCNC3	protein_codin	0	0	0	0
ENSG000001	ZFAND5	protein_codin	929.862897	512.291513	183.206099	434.109542
ENSG000001	ZFAND5	protein_codin	519.36504	295.196872	134.408707	252.2748
ENSG000001	UXT	protein_codin	1.24262965	5.56798847	12.0753132	0
ENSG000001	MYH14	protein_codin	0	0	0	0
ENSG000001	CST9	protein_codin	0	0	0	0
ENSG000002	CBY3	protein_codin	0.8609527	0	0	0
ENSG000001	CST9L	protein_codin	0	0	0	0
ENSG000001	DNAJC1	protein_codin	192.43178	202.243066	142.055322	127.213787
ENSG000001	ELK1	protein_codin	35.5586801	40.940782	0	9.76038037
ENSG000001	GDA	protein_codin	0	0	0	0
ENSG000002	CST9LP1	transcribed_u	0	0	0	0
ENSG000001	GFAP	protein_codin	0	0	0	0
ENSG000001	DRAP1	protein_codin	74.2706374	149.854499	20.4500217	36.0942303
ENSG000002	CST13P	transcribed_u	0	0	0	0
ENSG000001	C1orf127	protein_codin	0	11.623922	5.16418235	0
ENSG000001	CFP	protein_codin	193.038346	184.105109	17.3912272	80.8888853
ENSG000001	CST11	protein_codin	0	0	0	0
ENSG000001	CST11	protein_codin	0	0	0	0
ENSG000001	VRK3	protein_codin	0	0	19.3496987	0
ENSG000002	GABBR1	protein_codin	172.491998	162.208613	70.5609031	50.7318253
ENSG000002	GABBR1	protein_codin	0	0	0	4.8399652
ENSG000001	TIMP1	protein_codin	26.1510388	20.3187798	35.7084506	0
ENSG000001	CASZ1	protein_codin	11.9318874	26.8634536	14.9281752	5.32017772

ENSG000002	C9orf57	protein_codin	0	0	0	0
ENSG000001	NAPB	protein_codin	47.2547052	39.6706823	0	33.1859228
ENSG000001	C9orf85	protein_codin	0	0	0	0
ENSG000000	DCT	protein_codin	0	0	0	0
ENSG000001	C9orf85	protein_codin	0	0	0	0
ENSG000002	IGKV1D-12	IG_V_gene	0	0	0	0
ENSG000002	GABBR1	protein_codin	0	0	0	0
ENSG000002	IL31	protein_codin	0	0	0	0
ENSG000001	DFFA	protein_codin	18.6580011	12.6032197	8.11447428	6.57279882
ENSG000001	DFFA	protein_codin	90.0820556	112.313911	58.5438678	39.345843
ENSG000000	ARAF	protein_codin	30.7742114	24.8463736	8.7682262	24.127676
ENSG000001	ABHD17B	protein_codin	0	16.1882555	0	0
ENSG000001	CEMIP2	protein_codin	0	0	0	1.56269391
ENSG000001	CEMIP2	protein_codin	240.66867	317.117692	180.651195	217.005098
ENSG000000	ARAF	protein_codin	200.034133	252.470117	115.414132	133.05725
ENSG000001	KAT5	protein_codin	0	0	10.3058518	28.5598972
ENSG000001	GPC6	protein_codin	0	0	0	0
ENSG000002	CORT	protein_codin	0	0	0	0
ENSG000002	UBD	protein_codin	0	0	0	0
ENSG000001	GZF1	protein_codin	0	0	0	0
ENSG000001	CEMIP2	protein_codin	0	0	1.26433725	3.91454022
ENSG000001	CEMIP2	protein_codin	4.02294731	2.27584629	0	0
ENSG000000	MLLT10	protein_codin	159.781127	118.759249	83.2865701	54.2777795
ENSG000001	CNNM3	protein_codin	0	3.60302484	0	0
ENSG000001	ZNF41	protein_codin	42.2102882	9.96094699	0	0
ENSG000001	CEMIP2	protein_codin	82.6445448	0	0	12.2082628
ENSG000001	GPC5	protein_codin	1.00914939	5.08785471	4.43150381	5.13384454
ENSG000000	KDM2B	protein_codin	42.9274472	75.5549116	18.5780642	36.3888521
ENSG000000	KDM2B	protein_codin	51.4224473	64.5717037	0	37.6816171
ENSG000000	MLLT10	protein_codin	30.6755355	0	19.5240443	19.6554308
ENSG000001	ZNF157	protein_codin	3.25349055	0	0	1.08511817
ENSG000001	CNNM4	protein_codin	74.9439424	88.842823	33.9947176	43.0940024
ENSG000001	USP11	protein_codin	0	0	0	0
ENSG000001	LMAN2L	protein_codin	0	0	0	0
ENSG000001	USP11	protein_codin	6.79164008	22.6606068	15.0304382	10.4954879
ENSG000000	KIF1B	protein_codin	0	71.6258263	0	0
ENSG000000	KIF1B	protein_codin	74.6788537	53.6375194	73.919421	80.4771853
ENSG000001	SLITRK1	protein_codin	0	1.09958145	0	0
ENSG000000	KIF1B	protein_codin	0	0	0	18.6918689
ENSG000001	DUSP18	protein_codin	0	0	7.1977974	0
ENSG000000	MLLT10	protein_codin	0	0	0	2.26989101
ENSG000000	FUZ	protein_codin	0	0	1.48105662	4.48593897
ENSG000000	KIF1B	protein_codin	171.806912	0	0	104.712288
ENSG000000	SLC25A39	protein_codin	33.0585986	54.4305378	17.5220951	15.184097

ENSG000000	TRPM3	protein_codin	0	0	0	0
ENSG000000	MLLT10	protein_codin	0	4.45784941	0	0
ENSG000000	TRPM3	protein_codin	0	0	0	0
ENSG000001	SPRY2	protein_codin	0	0	0	0
ENSG000001	THBD	protein_codin	157.389254	96.2164479	29.1529576	58.4235169
ENSG000001	SPRY2	protein_codin	4.10058627	5.15612636	8.18875353	9.49243892
ENSG000000	TRPM3	protein_codin	0	0	0	0
ENSG000001	USP11	protein_codin	34.7205314	71.7563367	42.3579908	25.8107151
ENSG000000	TRPM3	protein_codin	0	0	0	0
ENSG000000	TRPM3	protein_codin	0	0	0	0
ENSG000001	DOK3	protein_codin	0	3.89846425	0	0
ENSG000001	FOXA2	protein_codin	0	0	0	0
ENSG000000	NEBL	protein_codin	3.89398815	0	0	0
ENSG000002	ENSG000002	lncRNA	0	0	0	0
ENSG000000	NEBL	protein_codin	30.7301157	0	0	0
ENSG000001	KLF9	protein_codin	625.943949	296.427968	194.166591	250.409193
ENSG000002	MAS1L	protein_codin	0	0	0	0
ENSG000002	OR2H1	protein_codin	0	0	0	0
ENSG000002	OR2H1	protein_codin	0	0	0	0
ENSG000001	IRF3	protein_codin	5.93123049	4.48887487	0	2.96887268
ENSG000002	OR2H1	protein_codin	0	0	0	0
ENSG000001	IRF3	protein_codin	18.2575016	0	0	10.3180324
ENSG000001	NKX2-2	protein_codin	0	0	0	0
ENSG000002	OR11A1	protein_codin	0	0	0	0
ENSG000002	OR11A1	protein_codin	0	0	0	0
ENSG000001	UBE4B	protein_codin	0	1.59541822	0	1.44439617
ENSG000002	OR5V1	protein_codin	0	0	0	0
ENSG000002	OR14J1	protein_codin	0	0	0	0
ENSG000002	OR2U1P	unprocessed_	0	0	0	0
ENSG000002	OR2J2	protein_codin	0	0	0	0
ENSG000002	OR2J3	protein_codin	0	0	0	0
ENSG000002	OR2J1	protein_codin	0	0	0	0
ENSG000002	OR2B3	protein_codin	0	0	0	0
ENSG000002	OR2W1	protein_codin	0	0	0	0
ENSG000002	MAMDC2-AS1	lncRNA	0	0	0	0
ENSG000001	ZNF311	protein_codin	0	0	0	0.96178809
ENSG000001	KCNIP3	protein_codin	0	0	0	0
ENSG000001	MAMDC2	protein_codin	1.18887739	1.07216275	3.18950167	1.08258398
ENSG000001	MPP2	protein_codin	0	0	0	0
ENSG000002	SPDYC	protein_codin	3.96509601	33.0944454	3.02528393	2.84047616
ENSG000002	LINC01556	lncRNA	85.4874465	62.457126	39.8948372	108.911117
ENSG000001	SYVN1	protein_codin	31.372574	119.35778	46.9393404	0
ENSG000000	XRN2	protein_codin	1096.4217	1213.15543	619.571706	858.958527
ENSG000001	KNTC1	protein_codin	0	0	0	0

ENSG000002	TRIM27	protein_codin	0	79.877884	0	0
ENSG000002	CFAP95	protein_codin	0	0	0	0
ENSG000002	TRIM27	protein_codin	177.348298	185.957971	79.2381175	119.855461
ENSG000001	PTAR1	protein_codin	64.6539067	70.7999192	13.5089562	37.1046554
ENSG000001	CD300LG	protein_codin	0	0	0	0
ENSG000001	NMNAT1	protein_codin	14.1744071	50.0734817	7.17759662	20.203673
ENSG000001	POU4F1	protein_codin	0	0	0.92017135	1.11128187
ENSG000001	EDNRB	protein_codin	0	0	0	0
ENSG000001	LZIC	protein_codin	114.597105	129.840694	55.0658038	68.7551578
ENSG000000	TEAD2	protein_codin	0	0	0	0
ENSG000001	ENTREP1	protein_codin	0	0	0	0
ENSG000001	LZIC	protein_codin	81.2521454	88.9985917	36.4818009	46.0911128
ENSG000000	UIMC1	protein_codin	0	9.40110135	7.03540151	0
ENSG000001	SLAIN1	protein_codin	0	0	0.98498176	0
ENSG000001	PLXDC2	protein_codin	459.722476	279.477351	217.325286	158.46883
ENSG000001	PLXDC2	protein_codin	390.244058	378.314621	56.5652112	280.207702
ENSG000001	SNX15	protein_codin	68.4556118	89.6772066	36.7304696	47.0736276
ENSG000001	TJP2	protein_codin	122.075208	175.940975	192.707661	110.312329
ENSG000001	SCEL	protein_codin	0	0	0	0
ENSG000001	PLXDC2	protein_codin	1581.10687	806.098145	661.05416	1013.82656
ENSG000001	THNSL2	protein_codin	0	0	1.0611003	0
ENSG000001	ZKSCAN3	protein_codin	0	0	0	0
ENSG000001	CTNNBIP1	protein_codin	0	0	0	0
ENSG000001	CTNNBIP1	protein_codin	1.19785442	0	0	0
ENSG000001	TJP2	protein_codin	0	0	4.37254615	0
ENSG000001	CTNNBIP1	protein_codin	44.4742971	62.2901666	25.5645889	37.2982183
ENSG000001	ATG2A	protein_codin	270.68435	247.847376	104.57089	154.224083
ENSG000002	MALRD1	protein_codin	0	3.26017731	0	0
ENSG000002	MALRD1	protein_codin	2.00998993	0	2.21498698	0
ENSG000001	UBA1	protein_codin	28.3498323	30.4810136	28.219096	3.74960695
ENSG000001	FXN	protein_codin	9.8680473	0	0	0
ENSG000001	ARL5B	protein_codin	679.418215	177.055366	188.272046	232.75348
ENSG000001	PRKACG	protein_codin	0	0	0	0
ENSG000001	PIP5K1B	protein_codin	0	0	0	0
ENSG000001	HRH2	protein_codin	48.6168503	54.2839023	5.81067266	23.4005702
ENSG000000	CFAP61	protein_codin	0	0	0	0
ENSG000001	ZKSCAN4	protein_codin	6.96431976	21.3938321	4.46336338	5.99013448
ENSG000001	CLSTN1	protein_codin	0	19.1495543	0	0
ENSG000002	LINC01951	lncRNA	0	0	0	0
ENSG000000	CFAP61	protein_codin	0	0	0	0
ENSG000002	NSUN6	protein_codin	50.5285011	62.2958926	39.4652302	24.2649923
ENSG000000	CFAP61	protein_codin	0	0	0	0
ENSG000001	ARHGAP39	protein_codin	0	0	0	0
ENSG000000	CFAP61	protein_codin	0	0	0	0

ENSG000001	TMEM252	protein_codin	0	0	0	0
ENSG000001	MEN1	protein_codin	0	0	0	0
ENSG000001	CACNB2	protein_codin	0	0	0	0
ENSG000001	MEN1	protein_codin	4.73347183	0	0	0
ENSG000001	FOXH1	protein_codin	0	0.82882178	0	0
ENSG000001	CACNB2	protein_codin	0	0	0	0
ENSG000001	PIK3CD-AS1	lncRNA	1.96174159	2.96463447	0.91346839	5.90140363
ENSG000001	MEN1	protein_codin	0	0	0	0
ENSG000001	ZNF165	protein_codin	0	0	2.99067499	0
ENSG000001	MEN1	protein_codin	0	0	0	0
ENSG000001	CRNKL1	protein_codin	0	0	0	0
ENSG000001	CACNB2	protein_codin	0	0	0	0
ENSG000001	CACNB2	protein_codin	0	0	0	0
ENSG000001	CACNB2	protein_codin	0	0	0	0
ENSG000001	ST3GAL5	protein_codin	0	0	0	0
ENSG000001	CRNKL1	protein_codin	0	0	0	0
ENSG000001	ZNG1C	protein_codin	0	0	0	0
ENSG000001	ZNG1C	protein_codin	22.2744806	0	14.702072	0
ENSG000001	PIK3CD	protein_codin	504.05622	736.621663	277.701738	350.331746
ENSG000001	VPS28	protein_codin	0	10.7493032	0	0
ENSG000001	ZNG1C	protein_codin	0	0	0	0
ENSG000001	MAP4K2	protein_codin	100.759466	226.1051	129.258267	97.1074408
ENSG000001	UBA1	protein_codin	354.553101	579.860549	53.6241381	189.651654
ENSG000001	KCNIP1	protein_codin	0	0	0	0
ENSG000002	INSYN2B	protein_codin	1.98649459	2.94110308	1.06160933	0.9955184
ENSG000001	SLC39A12	protein_codin	0	0	0	0
ENSG000001	SLC39A12	protein_codin	0	0	0	0
ENSG000001	SLC39A12	protein_codin	0	0	0	0
ENSG000001	ZNG1E	protein_codin	0	0	0	0
ENSG000001	ZNG1E	protein_codin	32.1104406	15.9775807	18.3584606	8.95954003
ENSG000001	TGOLN2	protein_codin	1349.07417	1414.44794	557.756057	1111.14215
ENSG000001	SF1	protein_codin	0	39.4996331	0	0
ENSG000001	ZNG1E	protein_codin	0	0	0	0
ENSG000001	SF1	protein_codin	661.954656	725.404672	197.712606	388.348371
ENSG000002	ZNG1F	protein_codin	8.60766405	0	0	0
ENSG000001	ZNG1E	protein_codin	0	0	8.3465548	0
ENSG000001	SF1	protein_codin	0	0	27.4904476	0
ENSG000001	ZNG1E	protein_codin	0	0	0	0
ENSG000001	SPSB1	protein_codin	0	0	0	3.26465371
ENSG000001	ZNG1A	protein_codin	0	0	0	0
ENSG000001	H2BC13	protein_codin	2.94900319	4.03991929	30.3299805	5.68500838
ENSG000000	H6PD	protein_codin	467.795753	514.15574	166.910993	218.360864
ENSG000001	GPR157	protein_codin	30.0940809	24.8235792	6.99965758	8.01339558
ENSG000001	FOXD4L4	protein_codin	0.84532355	0	0.7521856	0

ENSG000001	SLC2A5	protein_codin	0	2.64157902	4.75527953	0
ENSG00000	ZNF184	protein_codin	21.5578721	0	0	11.2353923
ENSG000002	FOXD4L5	protein_codin	0	0	0.83179416	1.07747045
ENSG000001	SLC2A5	protein_codin	1.0556187	9.26997417	22.9663927	4.98293995
ENSG000001	AQP7	protein_codin	0.95600527	0	0	0
ENSG000001	SCP2D1	protein_codin	0	0	0	0
ENSG000001	TMEM143	protein_codin	0	0	0	0
ENSG000000	PYGM	protein_codin	3.49772879	0	4.60382575	4.00224575
ENSG000002	ENSG000002	lncRNA	0	0	0	0
ENSG000001	CA6	protein_codin	0	0	0	0
ENSG000001	CA6	protein_codin	0	0	0	0
ENSG000001	CA6	protein_codin	0	0	0	0
ENSG000001	ZNG1A	protein_codin	3.67756791	1.56631766	3.52378626	1.65203912
ENSG000001	DTD1	protein_codin	67.3050651	22.2938905	0	0
ENSG000001	CLN5	protein_codin	1.82347423	0	5.79358259	0
ENSG000001	PRSS16	protein_codin	0	0	0	0
ENSG000002	H2AC12	protein_codin	12.3344565	15.7800485	107.049222	12.5460474
ENSG000001	CARD8	protein_codin	70.2482939	12.3482239	9.23913408	14.457768
ENSG000001	ACOD1	protein_codin	2.02499521	1.98036594	0.98975842	2.0462585
ENSG000001	RERE	protein_codin	0	95.7987659	0	38.4642999
ENSG000001	SEC23B	protein_codin	47.6883746	36.8185739	28.1779838	28.3561916
ENSG000002	LINC01291	lncRNA	0	0	0	0
ENSG000001	SPATC1	protein_codin	0	0	2.8053005	6.03102104
ENSG000001	KCTD12	protein_codin	1777.0378	1509.78293	605.199456	1611.05952
ENSG000001	ERRFI1	protein_codin	4.29589803	6.39390178	0	0
ENSG000000	RASGRP2	protein_codin	0	0	0	0
ENSG000000	RASGRP2	protein_codin	0	0	0	0
ENSG000000	RASGRP2	protein_codin	0	0	0	0
ENSG000001	PARK7	protein_codin	0	0	0	0
ENSG000000	RASGRP2	protein_codin	20.3964578	0	19.6685742	0
ENSG000001	PARK7	protein_codin	3.99851399	0	0	0
ENSG000001	PARK7	protein_codin	0	2.86125203	0	0
ENSG000000	RASGRP2	protein_codin	0	0	0	0
ENSG000001	TMEM236	protein_codin	0	0	2.79613183	0
ENSG000000	RASGRP2	protein_codin	249.900126	313.631495	117.595325	153.980868
ENSG000001	LMO7	protein_codin	0	0	0	0
ENSG000001	STAM	protein_codin	0	0	0	6.6323871
ENSG000001	CCNJL	protein_codin	0	0	4.68292387	0
ENSG000001	CNTNAP4	protein_codin	0	0	0	0
ENSG000000	TNFRSF9	protein_codin	15.3143476	9.69669465	1.40150504	10.4207804
ENSG000000	UTS2	protein_codin	0	0	6.63058115	11.3291739
ENSG000002	FAM88C	lncRNA	0	0	0	0
ENSG000001	STAM	protein_codin	97.656112	99.9772678	45.8082563	95.5719094
ENSG000001	AUP1	protein_codin	199.092871	370.170116	105.384442	173.719185

ENSG000000	PER3	protein_codin	0	0	0	0
ENSG000001	SCRIB	protein_codin	0	0	0	0
ENSG000001	LMO7	protein_codin	0	0	0	0
ENSG000002	ODAD4	protein_codin	4.38994931	1.72400865	2.36363555	0
ENSG000000	PER3	protein_codin	0	0	0	0
ENSG000002	FAM27C	lncRNA	0	0	0	0
ENSG000001	METTL3	protein_codin	1.56169767	1.56329524	0	0
ENSG000002	ENSG000002	lncRNA	0	0	0	0
ENSG000002	ENSG000002	lncRNA	0	0	0	0
ENSG000001	NRXN2	protein_codin	0	0	0	0
ENSG000001	HECTD4	protein_codin	560.535269	180.50949	0	371.188122
ENSG000001	CNTNAP3B	protein_codin	0	0	0	0
ENSG000001	LBX2	protein_codin	0	0	0	0
ENSG000001	SLC22A12	protein_codin	0	0	0	0
ENSG000001	SLC22A12	protein_codin	0	0	0	0
ENSG000001	SLC22A12	protein_codin	0	0	0	0
ENSG000001	HMGN4	protein_codin	249.099805	218.346907	120.000488	131.343832
ENSG000000	DNAJC11	protein_codin	48.9375694	42.3709448	0	56.924075
ENSG000001	PYCR3	protein_codin	0	0	0	0
ENSG000001	SLC22A11	protein_codin	0	0	0	0
ENSG000001	SLC22A11	protein_codin	0	0	0	0
ENSG000001	UCHL3	protein_codin	50.779861	60.405741	27.578239	39.7691181
ENSG000002	ST8SIA6-AS1	lncRNA	0	0	1.98352938	0
ENSG000001	BTN2A1	protein_codin	22.0368689	54.5243986	0	19.814306
ENSG000001	ST8SIA6	protein_codin	21.1743336	11.8072972	22.0233352	14.0572937
ENSG000001	POLR3F	protein_codin	32.5754216	30.1796216	0	20.9759741
ENSG000001	RBM10	protein_codin	42.6127961	46.1450273	21.9403754	36.3932826
ENSG000001	COMMD6	protein_codin	0	0	0	0
ENSG000002	LINC01189	lncRNA	0	1.27248979	0	1.30281508
ENSG000001	COMMD6	protein_codin	34.6987674	50.906868	12.8632825	28.3897728
ENSG000001	COMMD6	protein_codin	2.09038298	0	0	0
ENSG000001	TBC1D4	protein_codin	82.950338	127.877427	34.3044134	54.4580781
ENSG000000	THAP3	protein_codin	4.06963837	2.05058053	1.18934761	2.30573007
ENSG000000	DZANK1	protein_codin	0	0	0	0
ENSG000001	SLC4A5	protein_codin	0	0	0	0
ENSG000001	VIPR2	protein_codin	0	0	0	0
ENSG000001	SLC4A5	protein_codin	0	0	0	0
ENSG000001	TBC1D4	protein_codin	10.6937091	0	0	0
ENSG000002	SPATA31A1	protein_codin	0	0	0	0
ENSG000001	PHF13	protein_codin	18.6928402	19.4844122	10.4906006	17.6308607
ENSG000002	ENSG000002	lncRNA	1.85656018	0	0.58755767	0.94579713
ENSG000001	CNTNAP3	protein_codin	0	0	0	0
ENSG000002	TCTN1	protein_codin	0	0	0	0
ENSG000001	CNTNAP3	protein_codin	0	0	0	0

ENSG000001	KLHL21	protein_codin	8.04164048	0	2.49466911	0
ENSG000001	CNTNAP3	protein_codin	0	0	0	0
ENSG000001	GALNT10	protein_codin	0	0	0	0
ENSG000001	BTN2A3P	transcribed_u	2.06786614	3.00209192	4.69527324	4.60793446
ENSG000001	KLHL21	protein_codin	187.728269	173.360148	60.3476299	73.539891
ENSG000001	KLF12	protein_codin	21.9277946	0	62.1422098	0
ENSG000001	HIF3A	protein_codin	0	0	0	0
ENSG000001	ZNF133	protein_codin	0	5.56633746	0	0
ENSG000002	FAM216A	protein_codin	6.99504976	5.88231332	6.65075106	9.21686909
ENSG000002	ZBTB48	protein_codin	51.720038	75.7037313	39.8397726	48.669218
ENSG000001	CYP11B1	protein_codin	0	0	0	0
ENSG000002	FAM201A	lncRNA	0	0	0	0
ENSG000001	KAT14	protein_codin	34.3213757	57.3794283	19.575249	12.4434562
ENSG000001	ATP2A2	protein_codin	348.75483	566.424053	332.056326	248.086822
ENSG000001	KLF5	protein_codin	5.20108309	12.7462986	0	0
ENSG000001	FAM220BP	processed_ps	0	0	0	0
ENSG000002	IGFL2	protein_codin	0	0	0	0
ENSG000001	IGFBPL1	protein_codin	0	0	0	0
ENSG000002	IGFL4	protein_codin	4.06764306	0	1.05922106	2.04497007
ENSG000001	ALDH1B1	protein_codin	8.89123548	15.6930217	5.03803385	8.93092095
ENSG000001	GPR137	protein_codin	13.1484183	28.3403575	7.62272616	10.4867137
ENSG000001	MGME1	protein_codin	12.7322152	0	0	0
ENSG000001	NOL9	protein_codin	127.947616	143.585518	66.2291097	82.6447848
ENSG000001	SHB	protein_codin	33.7097458	16.3759546	2.60198756	15.6234529
ENSG000001	BTN3A2	protein_codin	114.239884	186.391044	74.4692714	35.712128
ENSG000001	MGME1	protein_codin	0	7.10267515	5.78314963	0
ENSG000001	MGME1	protein_codin	31.5389007	45.3214663	18.7748333	26.1876431
ENSG000002	NAT8B	protein_codin	11.5861896	19.3366135	1.5690904	6.79278512
ENSG000001	SLC36A3	protein_codin	0	0	0	0
ENSG000001	SLC25A51	protein_codin	20.834246	8.92905484	5.36746815	7.27305572
ENSG000002	KRTAP9-2	protein_codin	0	0	0	0
ENSG000002	ENSG000002	lncRNA	0	0	0	0
ENSG000001	DCAF10	protein_codin	80.6697359	58.1508622	9.67026677	51.1519756
ENSG000001	PLEKHG5	protein_codin	0	0	0	0
ENSG000002	KRTAP4-2	protein_codin	0	0	0	0
ENSG000001	H4C8	protein_codin	48.4754698	103.625783	40.0208733	51.5561805
ENSG000001	PLEKHG5	protein_codin	0	0	0	0
ENSG000001	PLEKHG5	protein_codin	0	0	0	0
ENSG000002	H2BC10	protein_codin	10.7824408	25.3046754	44.4623818	9.52537635
ENSG000001	DMWD	protein_codin	7.92906851	18.1342596	18.1715781	0
ENSG000001	PLEKHG5	protein_codin	0	0	0	0
ENSG000002	GPR20	protein_codin	0	0.95373997	0	0
ENSG000002	H4C2	protein_codin	2.75579307	8.73636715	7.1402197	7.15010017
ENSG000002	KRTAP1-4	protein_codin	0	0	0	0

ENSG000001	PLEKHG5	protein_codin	0	0	0	0
ENSG000001	ANXA6	protein_codin	0	0	29.7138865	0
ENSG000001	TRMT10B	protein_codin	0	0	0	0
ENSG000001	TRMT10B	protein_codin	0	0	0	0
ENSG000002	KRT40	protein_codin	0	0	0	0
ENSG000000	SNX5	protein_codin	104.227176	78.9677699	19.0464158	64.7778515
ENSG000000	FRMPD1	protein_codin	0	0	0.94707857	0
ENSG000000	DIS3	protein_codin	40.7170078	27.9474104	24.4678759	9.37964497
ENSG000000	SNX5	protein_codin	0	19.938032	17.5594098	12.6395806
ENSG000001	DPP6	protein_codin	0	0	0	0
ENSG000001	TOMM5	protein_codin	4.40417232	8.77314229	3.7248776	3.40047359
ENSG000001	ACTR3B	protein_codin	17.3834505	0	0	0
ENSG000001	H2BC5	protein_codin	98.1762838	158.089709	88.4941938	82.656337
ENSG000000	DIS3	protein_codin	51.5336655	37.8971497	46.8782916	53.6267615
ENSG000002	TNFRSF25	protein_codin	0	0	0	0
ENSG000001	H2AC6	protein_codin	0	0	0	0
ENSG000001	POLR1E	protein_codin	0	2.44946656	1.97108672	3.46939806
ENSG000001	NAA40	protein_codin	0	21.3351445	0	0
ENSG000000	CD74	protein_codin	1391.64135	2837.8413	690.566158	1398.41592
ENSG000000	TCOF1	protein_codin	0	96.8750486	0	0
ENSG000001	POLR1E	protein_codin	23.4748766	34.4553631	21.5886161	10.0732063
ENSG000001	TRDMT1	protein_codin	54.8341553	12.2014248	28.7818106	6.12498864
ENSG000001	PLCXD3	protein_codin	0	0	0	0
ENSG000001	H4C3	protein_codin	29.5086531	69.3168263	56.3523144	26.2157649
ENSG000001	BANF2	protein_codin	0	0	0	0
ENSG000000	CEACAM1	protein_codin	0	0	0	0
ENSG000001	RRBP1	protein_codin	0	11.8937043	0	0
ENSG000000	SMARCE1	protein_codin	0	0	0	0
ENSG000000	MARK2	protein_codin	119.711164	114.433032	0	65.1427985
ENSG000001	NDUFB11	protein_codin	57.763624	122.472644	32.6812851	66.7062536
ENSG000001	RRBP1	protein_codin	389.030862	421.910996	424.522653	89.662169
ENSG000001	BORA	protein_codin	0	1.60390939	0	0
ENSG000002	MZT1	protein_codin	17.1841042	16.8409757	10.6940146	20.6408435
ENSG000001	RTN3	protein_codin	0	0	0	0
ENSG000001	CUBN	protein_codin	0	0	1.81094663	0
ENSG000001	GRHPR	protein_codin	0	0	0	0
ENSG000001	CUBN	protein_codin	12.425692	2.97560139	4.74867818	0
ENSG000000	HES2	protein_codin	0	0	0	0
ENSG000000	HES2	protein_codin	0	0	0	0
ENSG000000	HES2	protein_codin	0	0	0	0
ENSG000000	ZFAT	protein_codin	60.9249304	115.806206	49.125548	0
ENSG000001	PAX5	protein_codin	0	0	0	0
ENSG000000	ACOT7	protein_codin	1.55090721	5.150062	0	0
ENSG000001	CSNK1A1	protein_codin	282.375581	244.251909	122.186599	114.91697

ENSG000001	KLHL1	protein_codin	0	0	0	0
ENSG000000	ACOT7	protein_codin	0	0	0	0
ENSG000001	PAX5	protein_codin	0	0	0	0
ENSG000000	ACACB	protein_codin	36.4634801	0	45.8209967	2.77218815
ENSG000001	SLC17A2	protein_codin	0	0	0	0
ENSG000001	PAX5	protein_codin	2.16620672	0	0	0
ENSG000001	PAX5	protein_codin	0	0	0	0
ENSG000000	ACACB	protein_codin	0	36.7668979	0	30.9801575
ENSG000000	ACOT7	protein_codin	1.9955031	0	0	0
ENSG000000	ACOT7	protein_codin	0	0	0	0
ENSG000001	PCDH9	protein_codin	9.78759954	8.35455832	14.0073775	4.26244882
ENSG000001	CCN4	protein_codin	0	0	0	0
ENSG000001	PCDH9	protein_codin	11.0645941	19.1572211	0	7.61822539
ENSG000001	ASB10	protein_codin	0	0	0	0
ENSG000001	BFSP1	protein_codin	0	0	0	0
ENSG000001	SLC22A6	protein_codin	0	0	0	0
ENSG000001	BFSP1	protein_codin	0	1.58649234	0	0
ENSG000001	RNF38	protein_codin	0	14.6579683	0	0
ENSG000002	LINC01545	lncRNA	0	0	0	0
ENSG000000	TDRD3	protein_codin	73.0787632	33.6909666	33.9662704	16.4392116
ENSG000000	TDRD3	protein_codin	17.1304327	0	0	0
ENSG000001	USP30	protein_codin	6.73329984	0	0	0
ENSG000001	RNF38	protein_codin	0	154.636424	66.1312561	103.890035
ENSG000001	SLC17A1	protein_codin	0	0	0	0
ENSG000001	HTR4	protein_codin	0	0	0	0
ENSG000001	SLC3A2	protein_codin	0	0	3.39547376	0
ENSG000001	SLC3A2	protein_codin	0	0	2.23852186	0
ENSG000001	SLC3A2	protein_codin	0	0	0	0
ENSG000001	GPR153	protein_codin	0	4.01183857	2.51261318	0
ENSG000000	TDRD3	protein_codin	0	0	0	0
ENSG000001	STX5	protein_codin	0	0	0	34.2723534
ENSG000001	PCSK2	protein_codin	0	0.99706537	0.96903682	0
ENSG000001	TMEM71	protein_codin	26.2649121	4.72299042	0	0
ENSG000002	ASIC3	protein_codin	0	0	0	0
ENSG000001	SLC17A4	protein_codin	0	0	0	0
ENSG000002	SPINK9	protein_codin	0	0	0	0
ENSG000001	DIAPH3	protein_codin	0	0	11.3789961	0
ENSG000001	RSU1	protein_codin	268.366527	1207.09033	262.510152	403.004105
ENSG000001	PCDH17	protein_codin	0	0	5.76874406	0
ENSG000001	KRBOX4	protein_codin	0	1.37538707	0	0
ENSG000001	RSU1	protein_codin	41.286163	15.2845726	12.358399	3.66089166
ENSG000002	LRRC3C	protein_codin	0	0	0	0
ENSG000002	MFFP3	processed_ps	0	0	0	0
ENSG000001	ADCY8	protein_codin	0	0	0	0

ENSG000002	PRR20A	protein_codin	0	0	0	0
ENSG000001	DIPK2B	protein_codin	0	0	0	0
ENSG000001	GKN1	protein_codin	0	0	0	0
ENSG000001	RNF207	protein_codin	2.65628606	0	0	0
ENSG000001	ZPBP2	protein_codin	0	0	0	0
ENSG000001	PCDH8	protein_codin	0	0	0	0
ENSG000001	SNRPB2	protein_codin	149.196114	207.570684	77.3759516	116.947648
ENSG000001	IKZF3	protein_codin	0	0	0	0
ENSG000001	IKZF3	protein_codin	0	0	0	0
ENSG000001	LYPD5	protein_codin	0	0	0	0
ENSG000001	PPIAP26	transcribed_p	0	0	0	0
ENSG000001	IKZF3	protein_codin	0	0	0	0
ENSG000002	UQCC3	protein_codin	0	16.8597076	5.42972607	8.0512841
ENSG000001	LBHD1	protein_codin	0	2.59649764	0	0
ENSG000002	FBXO48	protein_codin	7.91024204	10.0488019	8.25194058	5.09966546
ENSG000001	IKZF3	protein_codin	0	0	0	0
ENSG000001	GLIPR2	protein_codin	0	6.01440802	0	5.27754246
ENSG000001	GLIPR2	protein_codin	610.876058	628.053067	212.73947	363.134484
ENSG000000	SCGN	protein_codin	0	0	0	0
ENSG000001	CNMD	protein_codin	0	0	0	0
ENSG000001	RECK	protein_codin	25.1787546	23.9000892	10.1824793	20.2981651
ENSG000001	KDM6A	protein_codin	0	0	4.56004335	0
ENSG000001	MYC	protein_codin	0	16.0943711	0	0
ENSG000001	CIR1	protein_codin	8.8419239	3.20288717	0	5.99715733
ENSG000002	GRXCR2	protein_codin	0	0	1.21564521	0
ENSG000002	LINC02934	lncRNA	0	0	0	0
ENSG000001	COX7A2	protein_codin	0	0	0	0
ENSG000001	OR13J1	protein_codin	0	0	0	0
ENSG000001	ACTR2	protein_codin	0	10.2919464	0	0
ENSG000002	FAM221B	protein_codin	0.82855625	0	2.2256156	0
ENSG000001	TMEM8B	protein_codin	9.15402122	15.0919381	0	0
ENSG000002	CMAHP	lncRNA	0	0	0	0
ENSG000000	CEP68	protein_codin	0	0	0	22.1070472
ENSG000001	TMEM8B	protein_codin	0	0	0	0
ENSG000001	CMAHP	transcribed_u	10.6724392	0	0	0
ENSG000001	TMEM8B	protein_codin	3.35184078	0	0	0
ENSG000001	CHD5	protein_codin	0	0	1.21816041	0
ENSG000001	PTER	protein_codin	52.5202663	40.5201854	28.2422007	17.2867319
ENSG000001	PGAP3	protein_codin	0	0	0	0
ENSG000002	CD177	protein_codin	0	0	0	0
ENSG000001	ARHGAP26	protein_codin	153.853598	54.0019591	53.0811656	67.8421228
ENSG000001	SSPOP	transcribed_u	46.4059726	29.7370902	11.4929288	18.3471181
ENSG000001	MTSS1	protein_codin	6.17252083	0	0	9.37208581
ENSG000001	RIPOR2	protein_codin	0	0	0	0

ENSG000001:	AHNAK	protein_codin	47657.9995	32512.5005	18578.4878	17932.5848
ENSG000001:	CKAP4	protein_codin	517.743393	484.186091	190.626797	441.680162
ENSG000001:	CKAP2	protein_codin	37.461558	28.2882899	0	45.4198367
ENSG000001	MINDY3	protein_codin	4.91060306	0	1.67696817	0
ENSG000001:	CKAP2	protein_codin	77.7113639	37.5757143	29.9990712	34.1921265
ENSG000001	BEST1	protein_codin	0	0	0	0
ENSG000000	FUNDC1	protein_codin	23.54388	28.4169384	8.80326331	7.38805586
ENSG000001	FGF1	protein_codin	0	0	0	0
ENSG000002	ZNF783	protein_codin	13.8349921	29.2666869	8.6293936	3.37556409
ENSG000001:	FLRT3	protein_codin	2.88495016	0	0	0
ENSG000001	GMNN	protein_codin	0	5.95753529	10.0502465	0
ENSG000001	GMNN	protein_codin	0	0	0	0
ENSG000001:	VPS36	protein_codin	192.374369	183.988063	141.984677	143.663489
ENSG000002	ZNF425	protein_codin	0.95868507	3.77273289	1.12348819	0.95382047
ENSG000000	MAOB	protein_codin	10.9572904	21.8650985	20.307524	8.13701317
ENSG000001	SEL1L2	protein_codin	0	0	0	0
ENSG000001	MEGF8	protein_codin	0	0	3.76436086	22.4078856
ENSG000002	LRRC10B	protein_codin	0	0	0	0
ENSG000000	ITGA8	protein_codin	0	0	0	1.05173319
ENSG000001	RGP1	protein_codin	94.2990239	115.614365	62.2129463	46.5332581
ENSG000002	FBXO47	protein_codin	0	0	0	0
ENSG000001	NDUFAF5	protein_codin	1.92039491	13.7969209	3.77748114	14.5770207
ENSG000000	KCNAB2	protein_codin	69.5267795	86.3844221	24.8481944	19.3616838
ENSG000000	GBA2	protein_codin	0	7.26338232	4.83513067	6.41973065
ENSG000002	UQCC6	protein_codin	0	0	0	0
ENSG000000	KCNAB2	protein_codin	0	3.43412572	0	2.50186646
ENSG000000	GBA2	protein_codin	0	55.2052469	0	0
ENSG000001:	RPL23	protein_codin	0	0	0	0
ENSG000000	KCNAB2	protein_codin	0	0	0	21.5226504
ENSG000001!	TPK1	protein_codin	0	0	0	13.099451
ENSG000001!	TPK1	protein_codin	0	0	0	0
ENSG000001	C6orf62	protein_codin	3.70765904	0	0	0
ENSG000000	GBA2	protein_codin	120.458159	154.568772	54.5810688	80.4043725
ENSG000002	PCDHGA1	protein_codin	0	0	0	0
ENSG000001	NDUFAF5	protein_codin	1.36118101	4.01330289	1.48066702	0
ENSG000000	KCNAB2	protein_codin	0	0	0	0
ENSG000001	C12orf42	protein_codin	0	0	0	0
ENSG000002	ARHGEF35	protein_codin	0	0	0	0
ENSG000001	FAM171A1	protein_codin	0	0	1.51499975	0
ENSG000001	C6orf62	protein_codin	145.065281	274.866414	202.407733	139.156158
ENSG000002	BLACE	lncRNA	0	0	0	0
ENSG000002	PCDHA9	protein_codin	0	0	0	0
ENSG000002	PCDHA8	protein_codin	0	0	0	0.98662749
ENSG000000	PCDHA6	protein_codin	0	0	0	0

ENSG000001	PPP1R2C	protein_codin	0	0	0	0
ENSG000002	PCDHA2	protein_codin	0	0	0	0
ENSG000002	PCDHA1	protein_codin	0	0	0	0
ENSG000001	GPR34	protein_codin	0	0	7.03749679	0
ENSG000002	ENSG000002	lncRNA	0	0	0	0
ENSG000001	GPR34	protein_codin	33.8040966	11.0576387	0	36.5500985
ENSG000002	PGA4	protein_codin	15.9230853	14.9647912	0	0
ENSG000001	NMT2	protein_codin	4.66617647	0	4.46209198	2.80687167
ENSG000000	ARHGEF1	protein_codin	49.764594	19.8651809	24.4849984	0
ENSG000001	CASK	protein_codin	0	0	0	0
ENSG000001	NPHP4	protein_codin	0	0	5.02792272	0
ENSG000001	CASK	protein_codin	17.6213437	29.6470657	0	0
ENSG000001	NPHP4	protein_codin	5.03716923	3.5878404	0	0
ENSG000001	CASK	protein_codin	0	6.5785773	0	0
ENSG000000	TAF2	protein_codin	0	0	0	0
ENSG000001	NMT2	protein_codin	2.02552744	8.76393038	5.92799946	0
ENSG000001	CASK	protein_codin	0	0	14.0351927	0
ENSG000001	CASK	protein_codin	0	0	0	0
ENSG000001	NPHP4	protein_codin	20.9581858	6.59618574	0	7.52390883
ENSG000001	CASK	protein_codin	3.0521927	0	0	0
ENSG000002	TRIM13	protein_codin	14.9121238	40.9427106	18.5244858	21.7424329
ENSG000002	TRIM13	protein_codin	0	0	0	0
ENSG000002	MS4A13	protein_codin	0	0	0	0
ENSG000002	MS4A13	protein_codin	0	0	0	0
ENSG000002	ERICH4	protein_codin	0	0	0	0
ENSG000001	AJAP1	protein_codin	0	0	0	0
ENSG000001	AJAP1	protein_codin	0	5.11288504	6.89005383	0
ENSG000001	TLN1	protein_codin	469.037564	1696.00138	263.849748	553.129848
ENSG000001	SPRYD7	protein_codin	0	0	0	0
ENSG000001	RPP38	protein_codin	0	7.82739663	0	0
ENSG000001	TDP2	protein_codin	32.0457683	51.8851018	47.1085172	69.0826952
ENSG000001	RPP38	protein_codin	0	0	0	3.82804645
ENSG000001	RPP38	protein_codin	14.8020257	26.4273387	9.59602534	17.9991067
ENSG000001	RPP38	protein_codin	0	0	0	0
ENSG000001	EXT1	protein_codin	168.19539	146.998047	64.2221832	106.90888
ENSG000001	DFFB	protein_codin	0	0	0	0
ENSG000001	DFFB	protein_codin	10.5198814	0	0	16.6383754
ENSG000001	KIAA0319	protein_codin	0	0	0	0
ENSG000001	HNRNPUL1	protein_codin	0	0	0	0
ENSG000001	OLAH	protein_codin	0	0	0	0
ENSG000001	NYX	protein_codin	0	0	0	0
ENSG000001	CEP104	protein_codin	0	0	0	0
ENSG000001	OLAH	protein_codin	0	0	0	0
ENSG000001	BTBD3	protein_codin	0	0	0	0

ENSG000001	OLAH	protein_codin	0	0	0	0
ENSG000001	CEP104	protein_codin	0	0	0	0
ENSG000001	FOXP2	protein_codin	0	0	0	0
ENSG000001	NRG2	protein_codin	0	0	1.39772791	2.9990134
ENSG000000	ERLEC1	protein_codin	0	0	0	0
ENSG000001	MEIG1	protein_codin	0	0	0	0
ENSG000001	DCLRE1C	protein_codin	28.1323688	14.5530911	4.03521271	29.249549
ENSG000001	DCLRE1C	protein_codin	0	1.66933153	0	0
ENSG000001	GPLD1	protein_codin	0	0	0	0
ENSG000001	DEPDC4	protein_codin	1.07048619	0	0	0
ENSG000001	DCLRE1C	protein_codin	0	56.5216256	0	13.6436723
ENSG000002	SPIRE2	protein_codin	3.58753373	0	0	0
ENSG000001	DCLRE1C	protein_codin	0	4.63889971	0	0
ENSG000001	LRRC47	protein_codin	105.3906	181.837551	47.9712899	69.0143697
ENSG000001	LINC02871	lncRNA	0	0	0	0
ENSG000001	DCLRE1C	protein_codin	17.8911091	0	0	0
ENSG000001	DCLRE1C	protein_codin	0	11.5126338	0	0
ENSG000002	CRTC1P1	processed_ps	0	0	0	0
ENSG000001	DCLRE1C	protein_codin	0	0	0	0
ENSG000001	NRXN1	protein_codin	0	0	0	1.08949743
ENSG000001	EBPL	protein_codin	3.25505213	1.59154487	0	0
ENSG000001	EBPL	protein_codin	0	0	0	0
ENSG000001	EBPL	protein_codin	0	0	0	12.7915411
ENSG000001	CDK10	protein_codin	0	0	0	1.60481812
ENSG000001	DCLRE1C	protein_codin	53.6544598	59.4910766	60.6583098	59.0042401
ENSG000002	AARD	protein_codin	0	0	0	0
ENSG000000	TP73	protein_codin	0	0	0	0
ENSG000001	EBPL	protein_codin	0	0	0	0
ENSG000000	TP73	protein_codin	0	0	0	0
ENSG000000	TP73	protein_codin	3.46410669	1.18366324	3.68076838	0
ENSG000001	DCLRE1C	protein_codin	0	7.90406866	0	0
ENSG000000	TP73	protein_codin	0	2.81082335	0	0
ENSG000001	TPM2	protein_codin	5.44971233	26.4462159	24.0073599	0
ENSG000000	TP73	protein_codin	0	0	0	0
ENSG000001	RCBTB1	protein_codin	119.817945	117.467297	42.9701384	40.2746758
ENSG000001	RAB4B	protein_codin	0	0	0	0
ENSG000001	USP9X	protein_codin	389.404149	239.527754	167.993171	281.959474
ENSG000002	TMX2	protein_codin	0	0	0	0
ENSG000001	ACTMAP	protein_codin	60.7143799	78.1188012	22.808199	36.0635729
ENSG000001	PHF11	protein_codin	354.298534	280.881702	98.7366956	243.445212
ENSG000001	WRAP73	protein_codin	13.0408531	42.8766262	7.78873992	19.3105168
ENSG000001	SERPING1	protein_codin	0	0	0	0
ENSG000001	SERPING1	protein_codin	0	55.2144872	0	0
ENSG000001	SUV39H2	protein_codin	0	0	0	0

ENSG000001	ANKRD11	protein_codin	175.575556	305.583753	75.4836836	0
ENSG000001	SUV39H2	protein_codin	6.25185923	0	0	0
ENSG000001	ANKRD11	protein_codin	33.358954	38.3147607	24.6966633	0
ENSG000002	ANKRD11P1	processed_ps	0	1.68743574	3.31399556	0
ENSG000001	REEP2	protein_codin	0	0	0	0
ENSG000002	LINC02138	lncRNA	0	0	0	0
ENSG000001	TPRG1L	protein_codin	61.7062449	110.326725	35.8312088	59.6453035
ENSG000001	ACSF3	protein_codin	0	0	19.1603949	0
ENSG000002	ENSG000002	lncRNA	1.90596257	1.87853966	0.7971062	6.53080401
ENSG000001	MRS2	protein_codin	0	10.6181891	0	4.15797773
ENSG000001	CA9	protein_codin	0	0	0	0
ENSG000001	GFRA3	protein_codin	0	0	1.00510718	0
ENSG000001	APRT	protein_codin	72.9946249	71.5339451	9.18989629	36.0847171
ENSG000002	ENSG000002	lncRNA	0	0	0	0
ENSG000001	ARHGEF16	protein_codin	0	0	0	0
ENSG000001	HSPA14	protein_codin	37.2080193	34.4315027	21.8460373	39.7452647
ENSG000001	ARHGEF16	protein_codin	0	0	0	0
ENSG000001	ARHGEF16	protein_codin	0	0	0	0
ENSG000001	ANKEF1	protein_codin	0	0	5.03885721	2.16805448
ENSG000001	FNDC3A	protein_codin	34.7996149	16.9055127	23.6525324	3.54792468
ENSG000001	MRS2	protein_codin	52.368066	54.2743366	0	23.3014709
ENSG000001	ARHGEF39	protein_codin	0	0	0	0
ENSG000001	PRDM16	protein_codin	0	0	0	0
ENSG000001	PRDM16	protein_codin	0	0	0	0
ENSG000001	ANKEF1	protein_codin	0	0	0	0
ENSG000002	OR5D16	protein_codin	0	0	0	0
ENSG000002	OR5L2	protein_codin	0	0	0	0
ENSG000002	PKHD1L1	protein_codin	200.542101	323.452314	369.396792	178.264985
ENSG000001	ACTRT2	protein_codin	0	0	0	0
ENSG000001	CCDC107	protein_codin	5.15055894	28.9427444	0	0
ENSG000001	CCDC107	protein_codin	0	0	7.62338168	0
ENSG000001	CCDC107	protein_codin	0	25.6139192	0	0
ENSG000001	MMEL1	protein_codin	0	0	0	0
ENSG000002	ENSG000002	lncRNA	2.6183356	0.83454564	3.46089882	0
ENSG000001	CXorf38	protein_codin	0	7.08941734	5.03022307	3.06413074
ENSG000001	PAK5	protein_codin	0	0	0	0
ENSG000001	PRXL2B	protein_codin	0	0	0	0
ENSG000001	PRXL2B	protein_codin	0	0	0	0
ENSG000001	CXorf38	protein_codin	8.58929678	8.87935285	2.55169407	10.4557491
ENSG000001	PRXL2B	protein_codin	0	0	8.51635253	15.2242082
ENSG000001	SLC13A4	protein_codin	0	0	0	0
ENSG000001	PAK5	protein_codin	0	0	0	0
ENSG000001	CD72	protein_codin	0	0	0	0
ENSG000001	CD72	protein_codin	0	0	0	0

ENSG000002	ENSG000002	lncRNA	1.00125287	6.65667636	3.37518987	0.95077745
ENSG000001	LPAR6	protein_codin	0	1.8832523	0	0
ENSG000001	ATP6AP2	protein_codin	111.077231	39.8397459	92.536744	87.658323
ENSG000001	CDNF	protein_codin	0	0	0	0
ENSG000001	CDNF	protein_codin	0	0	0	0
ENSG000001	BCOR	protein_codin	198.008567	250.331627	89.9761341	110.856216
ENSG000001	ZNF573	protein_codin	0	3.3349057	0	0
ENSG000001	DCDC2	protein_codin	0	0	0	1.35073504
ENSG000001	HES5	protein_codin	0	0	0	0
ENSG000001	DCDC2	protein_codin	0	1.14553187	0	0
ENSG000001	BCOR	protein_codin	0	0	0	5.12967048
ENSG000000	FAM107B	protein_codin	55.6485265	0	0	0
ENSG000000	TRPC7	protein_codin	0	0	0	0
ENSG000000	NUP160	protein_codin	58.1172183	89.7367428	56.0903413	48.5107467
ENSG000000	FAM107B	protein_codin	0	0	0	0
ENSG000001	BCOR	protein_codin	19.8397835	5.30246733	16.5276988	0
ENSG000000	FAM107B	protein_codin	57.2457831	145.774464	203.934949	73.3741453
ENSG000001	PANK4	protein_codin	0	123.379919	36.5622631	41.6825458
ENSG000000	FAM107B	protein_codin	165.802604	82.1025735	7.29630969	57.4710474
ENSG000000	FAM107B	protein_codin	29.0624069	82.334971	55.2028573	52.6384657
ENSG000001	PLCB4	protein_codin	0	0	0	0
ENSG000001	MID1IP1	protein_codin	66.6587357	215.948934	103.241586	68.2043924
ENSG000001	NRSN1	protein_codin	0	0	0	0
ENSG000001	NRSN1	protein_codin	0	0	0	0
ENSG000001	NRSN1	protein_codin	0	0	0	0
ENSG000002	LINC01121	lncRNA	0	0	0	0
ENSG000001	TSPAN7	protein_codin	0	0	0	0
ENSG000002	CLLU1	lncRNA	0	0	0	0
ENSG000001	PLCH2	protein_codin	0	0	0	0
ENSG000001	NRSN1	protein_codin	0	0	0	0
ENSG000001	PLCB4	protein_codin	0	0	0	0
ENSG000001	CAMKMT	protein_codin	8.34563934	11.1785835	0	1.86996399
ENSG000001	UNC13B	protein_codin	28.9971327	0	31.0524419	18.4168357
ENSG000001	PLCB4	protein_codin	0	0	0	0
ENSG000002	SLC35B4	protein_codin	25.8642647	46.4593907	33.1682768	18.1955452
ENSG000001	PREPL	protein_codin	3.63022543	0	0	0
ENSG000001	RER1	protein_codin	0	78.595326	10.8616138	52.3774135
ENSG000001	RER1	protein_codin	53.6731308	53.477033	23.8482981	0
ENSG000001	RER1	protein_codin	0	16.9525341	0	5.24842454
ENSG000001	PREPL	protein_codin	0	0	0	12.0993967
ENSG000001	MORN1	protein_codin	0	3.36708544	0	0
ENSG000000	LIG3	protein_codin	114.59552	125.421358	74.3554987	65.8532214
ENSG000001	MORN1	protein_codin	8.11950596	0	4.34553283	5.70493327
ENSG000001	MORN1	protein_codin	0	8.81982062	2.44836672	0

ENSG000001(	SRPX	protein_codin	0	0	0	0
ENSG000000	KITLG	protein_codin	0	25.9242257	12.9742954	0
ENSG000001	SKI	protein_codin	514.712345	539.678654	196.415961	295.780076
ENSG000000	SPI1	protein_codin	1783.6066	1977.37828	547.861049	1016.37209
ENSG000002	PLXNA4	protein_codin	0	0	0	0
ENSG000001	PPM1B	protein_codin	45.4233964	0	0	0
ENSG000001	FAAP20	protein_codin	0	0	0	0
ENSG000001	FAAP20	protein_codin	25.3845439	36.5840343	5.64744222	30.8901049
ENSG000001	ITM2B	protein_codin	1064.28369	1411.66028	666.847752	834.863355
ENSG000001	PPM1B	protein_codin	54.2442905	37.0535241	34.0138133	58.5925894
ENSG000001	DNAAF1	protein_codin	0.84142165	0	0	0
ENSG000001	PODXL	protein_codin	5.90153903	15.6696788	2.86668366	0
ENSG000000	ATOSB	protein_codin	24.395286	7.74318796	0	17.5088778
ENSG000000	TCF7	protein_codin	0	0	0	0
ENSG000000	ATOSB	protein_codin	5.67941361	7.58098706	16.0654435	3.6290636
ENSG000000	ATOSB	protein_codin	0	47.1324635	6.83589104	0
ENSG000000	PRKCZ	protein_codin	0	0	5.00804396	0
ENSG000001	CCL7	protein_codin	0	0	0	0
ENSG000001	PRPF18	protein_codin	172.098738	131.026816	89.2058733	102.180151
ENSG000002	DYNLT3P1	processed_ps	0	0	0	0
ENSG000001	MEST	protein_codin	0	0	0	0
ENSG000001	DYNLT3	protein_codin	39.134697	38.5215572	44.3429747	32.4580309
ENSG000001	DYNLT3	protein_codin	0	4.10520806	2.60197306	0
ENSG000001	GABRD	protein_codin	0	2.35888655	0	0
ENSG000001	MED4	protein_codin	44.6995407	25.4326925	39.944642	27.1369332
ENSG000001	DYNC2LI1	protein_codin	0	1.61769283	5.58306398	4.95761139
ENSG000001	CYBB	protein_codin	5032.5074	5487.89837	1763.27249	4323.31814
ENSG000001	CFAP74	protein_codin	0	0	0.85016646	0
ENSG000001	CFAP74	protein_codin	0.84215325	0	1.45974015	0
ENSG000000	AFF4	protein_codin	0	0	1.49716698	0
ENSG000000	AFF4	protein_codin	156.835168	145.955462	129.393591	153.814505
ENSG000001	TMEM52	protein_codin	0	0	0	0
ENSG000001	DDB2	protein_codin	0	0	0	0
ENSG000001	DDB2	protein_codin	0	0	0	2.74464386
ENSG000001	TMEM52	protein_codin	0	0	0	0
ENSG000001	DDB2	protein_codin	0	0	0	0
ENSG000001	CALML6	protein_codin	0	0	0	0
ENSG000001	BEND7	protein_codin	0	0	0	0
ENSG000000	GNB1	protein_codin	1359.58105	1674.06352	448.789303	752.656515
ENSG000001	CDKAL1	protein_codin	11.0715254	46.0425658	48.3053414	0
ENSG000001	C16orf46	protein_codin	0	1.12888772	0	0
ENSG000000	SEPHS1	protein_codin	0	24.1266838	0	0
ENSG000001	CSTPP1	protein_codin	0	0	0	0
ENSG000000	XK	protein_codin	104.343543	119.645241	88.2017729	81.9384415

ENSG000001(	PIGO	protein_codin	0	0	0	0
ENSG000001	CSTPP1	protein_codin	1.51154696	0	0	0
ENSG000001	LANCL3	protein_codin	44.65979	30.2602137	32.7275745	21.0505882
ENSG000001	LANCL3	protein_codin	0	0	0	15.4600535
ENSG000001	LRP4	protein_codin	0.93703427	1.00635979	5.27395639	0
ENSG000000	NADK	protein_codin	199.990205	306.976021	101.519904	114.151709
ENSG000002	LGALS7	protein_codin	0	0	0	0
ENSG000001	PRRG1	protein_codin	2.39016926	6.47607689	0	14.7005815
ENSG000000	CDK11A	protein_codin	0	0	0	0
ENSG000001	COPRS	protein_codin	0	0	0	0
ENSG000001	PLCB1	protein_codin	38.8890358	75.7014741	21.8443794	52.9205592
ENSG000000	CDK11A	protein_codin	0	0	0	0
ENSG000001	PLCB1	protein_codin	44.982148	39.1668575	118.308826	0
ENSG000002	FANCG	protein_codin	0	0	0	0
ENSG000002	SYCE1L	protein_codin	1.56190054	0	0	0
ENSG000001	CFAP47	protein_codin	0	0	0	0
ENSG000001	CFAP47	protein_codin	0	0	0	0
ENSG000001	OXER1	protein_codin	16.9161438	32.0670158	3.47826358	16.3484951
ENSG000001	UQCRQ	protein_codin	0	0	5.59100161	1.9591193
ENSG000001	UQCRQ	protein_codin	83.71528	122.797722	25.0549343	43.8058913
ENSG000001	COX7A2L	protein_codin	7.49469399	2.59678962	2.98282859	18.6667414
ENSG000001	UQCRQ	protein_codin	21.7898612	16.1878943	15.3534385	25.3387356
ENSG000001	GDF9	protein_codin	0	0	0	0
ENSG000001	MMP23B	protein_codin	2.3502466	0	0	0
ENSG000001	SHROOM1	protein_codin	0	0	0	0
ENSG000001	DMD	protein_codin	0	4.0348543	0	9.45241197
ENSG000001	SHROOM1	protein_codin	0	0	23.3337272	23.2125357
ENSG000001	DMD	protein_codin	0	0	0	0
ENSG000001	UCMA	protein_codin	0	0	0	0
ENSG000001	CCDC136	protein_codin	0	0	0	0
ENSG000001	GLIPR1L2	protein_codin	0	0	0	1.22301758
ENSG000001	SOWAHA	protein_codin	0	0	0	0
ENSG000000	MCM10	protein_codin	0	0	0	0
ENSG000001	GLIPR1L1	protein_codin	0	0	0	0
ENSG000001	ESD	protein_codin	0	0	3.03018618	5.99919768
ENSG000001	SEPTIN8	protein_codin	0	0	0	0
ENSG000001	ID4	protein_codin	0	0	1.16472106	0
ENSG000001	SEPTIN8	protein_codin	0	12.928501	0	0
ENSG000001	DMD	protein_codin	0	0	0	0
ENSG000001	DMD	protein_codin	0	0	0	0
ENSG000001	SEPTIN8	protein_codin	5.21644208	5.50078604	0	2.4559351
ENSG000001	DMD	protein_codin	0	0	0	0
ENSG000001	MIB2	protein_codin	0	0	0	0
ENSG000002	LINC02898	lncRNA	0	0	0	0

ENSG000001!	MIB2	protein_codin	2.24006086	0	3.91020735	0
ENSG000000	MCM10	protein_codin	0	0	4.0088649	0
ENSG000001	DGKZP1	processed_ps	7.59316539	6.28025047	1.73529712	1.29769097
ENSG000001	SEPTIN8	protein_codin	27.3704305	35.1149388	13.9259177	7.62039813
ENSG000001	ESD	protein_codin	120.854565	115.896407	60.3448963	103.615444
ENSG000001	SEPTIN8	protein_codin	0	0	0	0
ENSG000001!	DMD	protein_codin	0	0	0	0
ENSG000001	SSU72	protein_codin	1.58675723	1.85006499	0	0
ENSG000001	SSU72	protein_codin	9.74311146	13.3054455	1.4146973	6.63991732
ENSG000001	THUMPD2	protein_codin	3.97661492	0	0	0
ENSG000002	CCNI2	protein_codin	0	0	0	0
ENSG000002	TMEM240	protein_codin	0	0.88213379	0	0
ENSG000001	KIF3A	protein_codin	21.4030576	9.70218972	11.2997539	0
ENSG000001	ATAD3B	protein_codin	2.80878775	0	2.32257955	2.517906
ENSG000001	PAX4	protein_codin	0	0	0	0
ENSG000001	ZFC3H1	protein_codin	1132.50056	640.62323	606.609289	637.311945
ENSG000001	SPATA31G1	protein_codin	0	0	0	0
ENSG000001	KIF3A	protein_codin	0	5.49357877	0	0
ENSG000001	OPTN	protein_codin	54.358547	0	0	0
ENSG000001	OPTN	protein_codin	0	31.5670818	0	0
ENSG000001	PEX16	protein_codin	34.599091	47.9388297	23.8556349	18.3858545
ENSG000001	OPTN	protein_codin	0	0	0	20.2727208
ENSG000001!	ATAD3A	protein_codin	0	0	0	0
ENSG000001!	ATAD3A	protein_codin	55.4575077	97.4144221	0	26.2478628
ENSG000001	OPTN	protein_codin	0	19.760931	58.5419649	34.3522173
ENSG000002	FRG2	protein_codin	0	0	0	0
ENSG000001	OPTN	protein_codin	8.74999552	20.3428682	0	0
ENSG000002!	ENSG000002!	lncRNA	0	0	0	0
ENSG000002	COX17P1	processed_ps	0	0	0	0
ENSG000001	PTPRR	protein_codin	0	0	0	0
ENSG000002	LINC02716	lncRNA	0	0	0	0
ENSG000001	RUBCNL	protein_codin	0	0	0	0
ENSG000001	RUBCNL	protein_codin	0	0	0	0
ENSG000002	ATAD3C	protein_codin	0	0.9426788	0	0.9341181
ENSG000002!	ENSG000002!	lncRNA	0	0	0	0
ENSG000001	RUBCNL	protein_codin	0	3.05432107	3.64997243	0
ENSG000002	SPATA31F1	protein_codin	0	0	0	0
ENSG000001	HAO1	protein_codin	0	0	0	0
ENSG000001	CCL21	protein_codin	0	0	0	0
ENSG000001	NDUFA5	protein_codin	0	0	0	0
ENSG000001	RUBCNL	protein_codin	2.41592544	0	0	0
ENSG000001	IST1	protein_codin	13.9070894	66.9964954	64.286534	0
ENSG000001	IST1	protein_codin	0	0	0	5.17933306
ENSG000001	CCL19	protein_codin	0	0	1.18567213	0

ENSG000001	CYP4V2	protein_codin	71.9568432	31.3065475	22.8452595	27.3533421
ENSG000002	CDKL4	protein_codin	0	0	2.27512605	0
ENSG000001	LRRC63	protein_codin	0	0	0	0
ENSG000001	RAB3IP	protein_codin	26.0907388	0	0	0
ENSG000001	KIF13A	protein_codin	258.861324	271.246534	137.145853	197.45887
ENSG000001	RAB3IP	protein_codin	6.71608702	12.4656997	1.41433467	3.08379152
ENSG000001	TP53I13	protein_codin	0	0	0	0
ENSG000002	TMEM88B	protein_codin	0	0.8698814	0	0
ENSG000001	RAD50	protein_codin	6.70468089	6.46460808	14.6831183	14.8167208
ENSG000001	CCDC3	protein_codin	10.6350838	20.963803	3.73415941	0
ENSG000001	KIF13A	protein_codin	0	0	0	0
ENSG000001	BMP2	protein_codin	4.02853244	0	0.78929883	1.03153902
ENSG000002	ACCSL	protein_codin	0	0	1.04490875	0.99464694
ENSG000001	CCDC3	protein_codin	0	0	0	0
ENSG000002	GALT	protein_codin	9.19100942	13.9142216	8.64182891	12.3320693
ENSG000001	KIF13A	protein_codin	0	0	0	0
ENSG000001	FERMT1	protein_codin	0	0	0	0
ENSG000001	CAMK1D	protein_codin	24.6008229	46.5960849	0	8.98109357
ENSG000002	CFAP96	protein_codin	0	0	0	0
ENSG000001	API5	protein_codin	73.7586601	69.7246791	77.7484386	47.5595737
ENSG000001	AURKAIP1	protein_codin	0	4.10891363	0	0
ENSG000001	HYDIN	protein_codin	0	0	0	0
ENSG000001	LRRN4	protein_codin	0	0	0	0
ENSG000000	WDR62	protein_codin	0.8559773	0	0	0
ENSG000002	TRIQK	protein_codin	4.76588399	11.1408914	9.16581555	0
ENSG000000	CRLS1	protein_codin	0	60.2308184	17.5813637	37.9023832
ENSG000001	PRR5L	protein_codin	0	0	0	0
ENSG000000	CRLS1	protein_codin	8.63295815	37.3957556	5.59297351	0
ENSG000002	ALKBH6	protein_codin	10.8914744	0	0	0
ENSG000001	PHF12	protein_codin	15.7561956	0	0	0
ENSG000001	MCM8	protein_codin	0	0	10.2488496	0
ENSG000001	MCM8	protein_codin	0	0	0	0
ENSG000002	SDHAF1	protein_codin	11.9102761	34.657217	6.32290173	3.85950511
ENSG000001	DVL1	protein_codin	69.6348842	55.0379562	0	14.4830472
ENSG000001	DVL1	protein_codin	0	28.9300654	20.4735938	19.3412084
ENSG000001	SIGMAR1	protein_codin	0	0	0	0
ENSG000001	DHRS13	protein_codin	3.91861869	3.88305189	0	1.9031109
ENSG000001	MCM8	protein_codin	0	0	9.77189818	0
ENSG000001	ADAMTS17	protein_codin	1.67482827	0.81188523	0	0
ENSG000001	CDC123	protein_codin	0	0	0	0
ENSG000001	LYSMD4	protein_codin	0	0	0	1.07888145
ENSG000001	NUP107	protein_codin	56.1516195	18.8229451	24.2603728	50.8486629
ENSG000002	ARID3C	protein_codin	0	0.83701858	0	0
ENSG000001	NPHS1	protein_codin	0	0	0	0.83457031

ENSG000001:	DCTN3	protein_codin	0	0	0.95031733	1.61390164
ENSG000001:	FCSK	protein_codin	0	0	0	0
ENSG000001:	DCTN3	protein_codin	2.77547115	0	0	0
ENSG000001	HNRNPLL	protein_codin	0	0	0	0
ENSG000001:	DCTN3	protein_codin	12.5267939	2.56800004	5.27593759	11.5709378
ENSG000001	PGPEP1L	protein_codin	0	0	0	0
ENSG000001	NUDT5	protein_codin	3.85751831	0	0	2.94942409
ENSG000001	TAB3	protein_codin	0	0	0	0
ENSG000001	TAB3	protein_codin	31.3917921	84.8988325	94.4947417	0
ENSG000001	TAB3	protein_codin	0	32.6613378	0	7.54063956
ENSG000001	TAB3	protein_codin	0	0	0	0
ENSG000001	NUDT5	protein_codin	25.5595675	7.17337724	6.83793969	17.0776168
ENSG000001	GK	protein_codin	4.27239012	0	5.28371032	1.44463315
ENSG000001	NUDT5	protein_codin	0	20.1975572	18.0456504	0
ENSG000001	GK	protein_codin	17.466085	8.81340212	7.85339905	13.0995439
ENSG000001	GK	protein_codin	0	47.9688078	14.8847	38.1336322
ENSG000000	ARHGAP33	protein_codin	0	4.44538653	1.72118311	0
ENSG000001	GK	protein_codin	0	0	0	25.7448225
ENSG000001	GK	protein_codin	109.564366	78.0094989	50.3827098	59.8231357
ENSG000001	CARINH	lncRNA	7.89373815	7.92855447	5.3128064	4.6056983
ENSG000001	NUDT5	protein_codin	0	0	0	0
ENSG000001	CARINH	lncRNA	0	13.7640374	0	0
ENSG000001	ATL2	protein_codin	42.9999733	40.0573573	31.1066906	28.706312
ENSG000001	RPP25L	protein_codin	8.60139913	26.2035077	0	15.3324074
ENSG000000	CHGB	protein_codin	0.74357807	0	0	0
ENSG000001	TASL	protein_codin	144.223889	109.623148	132.662555	103.790045
ENSG000001	NR0B1	protein_codin	0	0	0	0
ENSG000001	CBY2	protein_codin	0	0	0	0
ENSG000001	NR0B1	protein_codin	0	0	0	0
ENSG000001	U2AF1L4	protein_codin	0	0	0	0
ENSG000000	SPAG5	protein_codin	0	0	0	0
ENSG000001	ERICH6B	protein_codin	0	1.47810005	1.22893119	0
ENSG000001	SHLD1	protein_codin	2.49391894	3.06230254	2.72661305	0
ENSG000001	CNTFR	protein_codin	0	0	0	0
ENSG000002	MAGEB1	protein_codin	0	0	0	0
ENSG000001	MAGEB4	protein_codin	0	0	0	0
ENSG000001	RAP1B	protein_codin	58.5982865	12.3728349	11.3699612	8.78030479
ENSG000000	MAGEB2	protein_codin	0	0	0.60112729	0
ENSG000001	CAP2	protein_codin	0	0	0	0
ENSG000001	IL1RAPL1	protein_codin	0	0	0	0
ENSG000002	ENSG000002	lncRNA	20.4747579	20.4965545	13.0981725	11.9516756
ENSG000002	C11orf91	protein_codin	0	0	0	0
ENSG000001	UPK1A	protein_codin	0	0	0	0
ENSG000001	HIPK3	protein_codin	0	0	0	0

ENSG000000	SEC61A2	protein_codin	0	0	0	3.27694214
ENSG000001	PDLIM4	protein_codin	0	0	0	0
ENSG000001	GPCPD1	protein_codin	606.011	334.492693	204.454238	356.444186
ENSG000000	SEC61A2	protein_codin	9.69049755	0	0	0
ENSG000001	ETV2	protein_codin	0	0.83423032	0.84018106	0
ENSG000000	NRCAM	protein_codin	0	0	0	0
ENSG000001	ETV2	protein_codin	0	0	0	0
ENSG000000	NRCAM	protein_codin	0	0	0	0
ENSG000001	PUSL1	protein_codin	5.49652468	38.5529305	4.83019097	8.68456222
ENSG000000	SEC61A2	protein_codin	0	7.32343102	0	0
ENSG000001	MAGEB6	protein_codin	0	0	0	0
ENSG000001	ACAP3	protein_codin	0	0	0	0
ENSG000000	ARX	protein_codin	0	0	0	0
ENSG000001	NQO1	protein_codin	0	2.67883974	0	0
ENSG000001	NQO1	protein_codin	0	0	3.66601054	4.33939071
ENSG000001	TPT1-AS1	lncRNA	1.96012731	1.23779304	0	2.8431967
ENSG000000	SEC61A2	protein_codin	0	0	0	0
ENSG000001	RBM24	protein_codin	0	0	0	0
ENSG000002	LINC00654	lncRNA	5.72552153	11.8932118	5.05958091	1.73273308
ENSG000001	TPT1	protein_codin	26.964627	37.2069849	9.16764607	23.8455602
ENSG000001	TPT1	protein_codin	1706.0392	3181.28192	235.555409	2078.75725
ENSG000001	POLA1	protein_codin	0	57.6445072	0	19.7265938
ENSG000001	TPT1	protein_codin	1707.27159	2916.28147	16.3401235	2153.94221
ENSG000000	SARM1	protein_codin	0	3.10444973	0	0
ENSG000002	SLC10A5P1	unprocessed_	0	0	0	0
ENSG000001	PRKD3	protein_codin	58.6295717	32.3185455	74.6858431	0
ENSG000001	POLA1	protein_codin	45.9497687	0	55.62927	0
ENSG000001	CDS2	protein_codin	77.9309575	79.8567377	33.7611751	39.8512743
ENSG000002	FABP9	protein_codin	0	0	0	0
ENSG000001	PMFBP1	protein_codin	0	0	0	0
ENSG000001	WT1	protein_codin	0	0	0	0
ENSG000001	FAM219A	protein_codin	0	0	0	0
ENSG000001	WT1	protein_codin	0	0	0	0
ENSG000001	FAM219A	protein_codin	33.5703944	0	0	0
ENSG000001	FAM219A	protein_codin	0	27.4306736	0	0
ENSG000000	SLC26A3	protein_codin	0	0	0	0
ENSG000001	FAM219A	protein_codin	0	0	0	0
ENSG000000	P4HA2	protein_codin	0	0	0.8870204	0
ENSG000001	FAM219A	protein_codin	0	38.5161736	14.2553829	0
ENSG000001	FAM219A	protein_codin	0	0	0	0
ENSG000001	ZNF774	protein_codin	1.17400463	1.16243268	0	0
ENSG000002	ZBTB10	protein_codin	70.6550899	50.643911	44.4668474	59.5083211
ENSG000001	NGRN	protein_codin	54.8465423	99.0684528	43.2953712	55.1351625
ENSG000000	TPD52	protein_codin	0	0	52.9189209	0

ENSG0000007	TPD52	protein_codin	14.8946131	20.1199653	0	6.72134185
ENSG0000010	SCNN1D	protein_codin	0	0	0.59662793	0
ENSG0000007	P4HA2	protein_codin	0	0.86129494	0	0
ENSG0000010	SCNN1D	protein_codin	0	4.15119178	0	0
ENSG0000022	LYRM9	protein_codin	9.33784863	19.8466594	7.57319817	1.22534553
ENSG0000022	LYRM9	protein_codin	3.31135091	3.68796885	0	0
ENSG0000007	P4HA2	protein_codin	0	0	0	0
ENSG0000000	PAX6	protein_codin	0	0	0	0
ENSG0000018	KCTD4	protein_codin	1.1523133	2.32842534	0	0
ENSG0000000	PAX6	protein_codin	0	0	0	0
ENSG0000000	PAX6	protein_codin	0	0	0	0
ENSG0000010	IL7	protein_codin	0	3.56068259	0	0
ENSG0000000	PAX6	protein_codin	0	0	0	0
ENSG0000010	SCNN1D	protein_codin	1.2213341	0	0	0
ENSG0000007	BCAP29	protein_codin	0	0	0	0
ENSG0000000	PAX6	protein_codin	0	0	0	0
ENSG0000010	SPMIP6	protein_codin	0	0	0	0
ENSG0000010	SPMIP6	protein_codin	0	0	0	0
ENSG0000010	SPMIP6	protein_codin	0	0	0	0
ENSG0000000	PAX6	protein_codin	0	0	0	0
ENSG0000000	PAX6	protein_codin	0	0	0	0
ENSG0000010	SPMIP6	protein_codin	0	0	0	0
ENSG0000010	USF2	protein_codin	0	25.6082878	65.9976896	38.9176482
ENSG0000013	LRIG3	protein_codin	0	0	0	0
ENSG0000010	MYORG	protein_codin	0	0	0	0
ENSG0000013	PCNA	protein_codin	36.9243844	59.3993362	50.3835061	29.7643682
ENSG0000010	PCYT1B	protein_codin	17.5963319	36.066924	39.8082889	22.1226657
ENSG0000010	PCYT1B	protein_codin	47.1593186	156.215159	58.4975668	64.5767605
ENSG0000013	GPALPP1	protein_codin	11.5726166	39.7938616	25.7329686	0
ENSG0000010	SLC7A6	protein_codin	0	0	0	0
ENSG000001	CD83	protein_codin	2788.22834	1495.87562	1558.54685	1355.88702
ENSG0000010	NUDT2	protein_codin	0	0	0	0
ENSG0000010	NUDT2	protein_codin	0	0	0	0
ENSG0000013	PCNA	protein_codin	0	0	0	0
ENSG0000008	NUFIP1	protein_codin	23.4433508	16.4203499	19.9991357	18.8748423
ENSG0000000	PDK3	protein_codin	16.6352053	32.6477789	12.5880682	10.3226404
ENSG0000010	ELP4	protein_codin	0	2.44663609	0	0
ENSG0000010	FSTL5	protein_codin	0	0	0	0
ENSG0000007	NFATC3	protein_codin	0	27.4654734	43.962129	8.96994478
ENSG0000003	MCUR1	protein_codin	160.68388	253.19147	187.952044	80.8560152
ENSG0000018	KIF24	protein_codin	0	0	0	0
ENSG0000000	ZFX	protein_codin	193.709766	153.668131	146.781372	234.540636
ENSG0000014	SERP2	protein_codin	0	2.04032325	0	0
ENSG0000010	UBAP1	protein_codin	13.015095	66.1945202	12.0904603	23.1432505

ENSG000000	ZFX	protein_codin	172.384421	0	0	0
ENSG000001	B3GALT6	protein_codin	41.0631118	58.739531	12.2618622	22.8950543
ENSG000001	PROSER2	protein_codin	0	0	0	0
ENSG000002	SCGB2B2	protein_codin	0	0	0	0
ENSG000002	C4orf46	protein_codin	40.6545348	45.9317993	20.7503836	0
ENSG000001	CXorf58	protein_codin	0	0	0	0
ENSG000002	LGR4	protein_codin	0	16.6683413	11.6827151	0
ENSG000001	ECHDC3	protein_codin	43.0928721	36.6541685	8.365856	21.9667738
ENSG000001	DNAJC15	protein_codin	85.3083093	41.4034226	68.714033	56.9903552
ENSG000001	ISG20	protein_codin	0	6.05514009	0	0
ENSG000001	UBAP2	protein_codin	0	1.77839577	0	0
ENSG000001	APOO	protein_codin	0	5.6550148	0	5.20897238
ENSG000001	UBAP2	protein_codin	0	0	0	1.24109436
ENSG000001	TNFRSF4	protein_codin	0	0	0	0
ENSG000001	USP6NL	protein_codin	0	0	0	0
ENSG000001	UBAP2	protein_codin	80.9364993	126.288324	0	51.596669
ENSG000001	UBAP2	protein_codin	6.04472892	0	0	14.3160524
ENSG000001	ACSL6	protein_codin	0	0	0	0
ENSG000002	VIT	protein_codin	0	0	0	0
ENSG000002	VIT	protein_codin	0	0	0	0
ENSG000001	ACSL6	protein_codin	0	0	0	0
ENSG000001	FEZ2	protein_codin	8.53126353	18.6218121	4.42004788	0
ENSG000001	ACSL6	protein_codin	0	0	0	0
ENSG000001	MAP9	protein_codin	0	0	0	0
ENSG000001	SIRT5	protein_codin	0	0	0	7.32088844
ENSG000001	SAT1	protein_codin	1073.67171	1044.34202	387.456781	650.799413
ENSG000001	SAT1	protein_codin	3299.85658	3649.79789	2484.22736	2890.95859
ENSG000001	SAT1	protein_codin	0	7.22265415	10.8867843	0
ENSG000001	ACSL6	protein_codin	0	0	0	0
ENSG000001	CELF2-AS1	lncRNA	3.24060514	0	5.5302262	0
ENSG000001	DNAJC2	protein_codin	14.6325251	0	0	0
ENSG000001	SIRT5	protein_codin	0	0	0	1.58142084
ENSG000001	DNAJC2	protein_codin	185.80433	248.273576	91.7275862	161.039049
ENSG000001	TNFRSF18	protein_codin	0	0	0	0
ENSG000001	TNFRSF18	protein_codin	0	1.35606374	0	0
ENSG000001	SAT1	protein_codin	3546.47167	4244.05955	2561.59718	2584.63819
ENSG000001	DGKH	protein_codin	0	0	0	6.20847115
ENSG000000	TMEM230	protein_codin	0	0	0	0
ENSG000000	TMEM230	protein_codin	0	0	0	18.3196423
ENSG000001	GFOD1	protein_codin	0	0	0	0
ENSG000000	TMEM230	protein_codin	0	6.24535478	3.56899295	0
ENSG000000	TMEM230	protein_codin	0	4.48899155	0	6.88284204
ENSG000001	GFOD1	protein_codin	0	0	0	9.38521708
ENSG000000	TMEM230	protein_codin	0	0	0	0

ENSG000001[illegible]	GFOD1	protein_codin	118.386904	108.490477	76.4553513	20.7955896
ENSG000001[illegible]	TTLL10	protein_codin	0.7945912	0.769752	0.88286864	0
ENSG000001[illegible]	TTLL10	protein_codin	0	0	0	0
ENSG000001[illegible]	TTLL10	protein_codin	0	0	0	0
ENSG000001[illegible]	TBC1D7	protein_codin	3.53448523	0	0	0
ENSG000001[illegible]	ACOT9	protein_codin	5.2883597	0	1.9653228	7.85338418
ENSG000001[illegible]	ACOT9	protein_codin	27.1038405	21.8845827	10.7397688	11.164234
ENSG000000[illegible]	TMEM230	protein_codin	0	5.10813262	0	0
ENSG000001[illegible]	TBC1D7	protein_codin	8.73232822	6.58446686	0	17.1291969
ENSG000001[illegible]	VWA8	protein_codin	0	0	0	6.82268792
ENSG000001[illegible]	ACOT9	protein_codin	142.574123	147.166932	36.2778385	53.7658421
ENSG000001[illegible]	FBXL13	protein_codin	0	0	0	0
ENSG000001[illegible]	TBC1D7	protein_codin	0	5.32879469	3.59881837	0
ENSG000001[illegible]	VWA8	protein_codin	124.852687	148.362068	109.675643	125.271199
ENSG000000[illegible]	RIPOR1	protein_codin	206.010636	46.2454508	15.481902	0
ENSG000001[illegible]	PDCD5	protein_codin	0	0	0	0
ENSG000002[illegible]	TTLL10-AS1	lncRNA	0.91236649	1.83519681	0	0
ENSG000001[illegible]	C1orf159	protein_codin	0	0	0	0
ENSG000001[illegible]	C1orf159	protein_codin	0	4.00728775	0	2.3308022
ENSG000001[illegible]	C1orf159	protein_codin	0	16.2991682	0	2.62268512
ENSG000001[illegible]	GATA3	protein_codin	8.42023076	0	0	0
ENSG000001	PHACTR1	protein_codin	56.96091	29.8007916	40.4573115	42.8677255
ENSG000001[illegible]	PRDX4	protein_codin	0	1.50704675	0	0
ENSG000000[illegible]	SLC23A2	protein_codin	0	12.1535127	0	19.5889009
ENSG000001	PHACTR1	protein_codin	25.0808068	17.1701494	24.4180284	22.0081262
ENSG000001[illegible]	C1orf159	protein_codin	0	0	0	0
ENSG000001[illegible]	POLR2J3	protein_codin	0	0	0	0
ENSG000001[illegible]	PRDX4	protein_codin	25.5474092	30.5190348	22.4436419	13.7185646
ENSG000001[illegible]	SLC30A6	protein_codin	7.55012421	0	0	0
ENSG000001[illegible]	PLEKHG4	protein_codin	0	0	0	0
ENSG000001	PHACTR1	protein_codin	0	0	0	0
ENSG000001[illegible]	PRDX4	protein_codin	0.93566125	0	0	0
ENSG000001	PHACTR1	protein_codin	216.607371	174.845172	160.573732	165.618443
ENSG000001[illegible]	ZSCAN2	protein_codin	0.92067147	1.97164393	0	1.16212399
ENSG000001[illegible]	RGCC	protein_codin	148.47691	74.6095064	67.8570072	80.7318383
ENSG000001[illegible]	PTCHD1	protein_codin	1.13709611	1.14410651	5.05147849	0
ENSG000001[illegible]	AGRN	protein_codin	22.9374674	28.6332056	6.92573108	7.28325237
ENSG000001[illegible]	PHEX	protein_codin	7.83228801	3.74367539	14.1975896	0
ENSG000000[illegible]	EDN1	protein_codin	2.94447181	1.02429238	2.62853955	5.20249774
ENSG000001[illegible]	RASSF2	protein_codin	0	0	0	49.2795679
ENSG000002[illegible]	E2F4	protein_codin	230.249472	315.055233	96.5812754	168.71474
ENSG000001[illegible]	LYRM7	protein_codin	47.929737	31.0668269	32.4881183	27.7288904
ENSG000001[illegible]	MEMO1	protein_codin	89.0202251	75.3480293	67.5575429	51.2540845
ENSG000001[illegible]	TM6SF1	protein_codin	0	14.3294364	0	0

ENSG000001:	UEVLD	protein_codin	0	0	0	0
ENSG00000(	HIVEP1	protein_codin	157.582112	96.2258965	47.0227236	88.0521398
ENSG000001:	TM6SF1	protein_codin	56.1739459	38.2502926	15.364377	20.639548
ENSG000001(	NEMP1	protein_codin	0	0	0	0
ENSG000001(	RASSF2	protein_codin	2435.52643	2947.8808	986.532315	1483.48724
ENSG00000(	BTBD1	protein_codin	0	0	1.44004406	22.0057133
ENSG000001(	SMS	protein_codin	7.85179105	2.49310485	0	0
ENSG000000	PRSS3	protein_codin	0	0	0	0
ENSG000001	NAA16	protein_codin	91.6475643	45.5230214	32.4199527	48.2059809
ENSG000001:	PLEKHN1	protein_codin	0	0	0	0
ENSG000001:	PLEKHN1	protein_codin	0	1.49155747	0	0
ENSG000001:	PLEKHN1	protein_codin	9.15174853	6.99238276	5.76478057	4.87356468
ENSG000001(	TAC3	protein_codin	0	0	0	0
ENSG000001:	LDHA	protein_codin	7.83783883	11.6281585	0	22.036134
ENSG000001	ADTRP	protein_codin	0	0	0	0
ENSG000001:	XDH	protein_codin	0	0	0.9858093	0
ENSG000002(	PDE7A	protein_codin	44.8433759	45.8546703	33.1791989	33.2512741
ENSG000001(	MOGAT3	protein_codin	0	0	0	0
ENSG00000(	TMEM170B	protein_codin	790.293625	518.930547	324.968989	734.066851
ENSG000001	NEDD9	protein_codin	16.3011333	29.5092367	16.846777	18.1748452
ENSG00000(	TRBV20OR9-2	TR_V_gene	0	0	0	0
ENSG00000(	MUC17	protein_codin	5.9397289	2.12954501	2.10558001	0
ENSG000001	PRNP	protein_codin	289.712145	460.672983	0	372.569964
ENSG000000	BAZ2A	protein_codin	43.3987652	0	83.6396115	0
ENSG00000(	MUC12	protein_codin	0	0	0	0
ENSG00000(	CTXN3	protein_codin	0	0	0	0
ENSG000001	NEDD9	protein_codin	0	0	0	0
ENSG000001:	ZNF827	protein_codin	0	0	0	0
ENSG000001!	ASPH	protein_codin	4.14873601	11.766401	8.26757679	1.88443374
ENSG000002	SUGT1P2	processed_ps	7.47105899	12.107349	2.23240393	2.11112367
ENSG000001	ADRA1D	protein_codin	0	0	0	0
ENSG000001!	ASPH	protein_codin	132.035354	69.2852205	66.8198914	79.5866715
ENSG00000(	MUC3A	protein_codin	0	0	0	0
ENSG00000(	SMOX	protein_codin	0	0	7.46756961	0
ENSG000002	CYB5AP2	processed_ps	0	0	0	0
ENSG000001:	NAE1	protein_codin	8.41607395	4.71146499	0	0
ENSG00000(	NOL6	protein_codin	0	3.52511867	0	0
ENSG00000(	NOL6	protein_codin	0	0	0	0
ENSG000001:	KCNC1	protein_codin	0	0	0	0
ENSG000001:	MTRF1	protein_codin	21.809748	13.2605871	11.8630382	26.2299233
ENSG000001:	MTRF1	protein_codin	0	3.48459462	0	0
ENSG000001:	KBTBD7	protein_codin	14.8823416	20.6641027	7.66219708	8.10166716
ENSG000000	MBTPS2	protein_codin	23.0216322	11.3101899	17.7590505	22.9700296
ENSG00000(	KBTBD6	protein_codin	5.23502291	7.28660522	5.8115473	4.03386262

ENSG000001	CMTM2	protein_codin	2.45760684	0	8.73282107	6.38382065
ENSG000001	WBP4	protein_codin	108.136133	54.882846	56.498188	62.9719741
ENSG000001	GCM2	protein_codin	0	0	0	0
ENSG000000	SMPX	protein_codin	0	0	0	0
ENSG000001	KLHL34	protein_codin	0	0	1.48521998	0
ENSG000000	CMTM1	protein_codin	0	0	0	0
ENSG000001	AQP7	protein_codin	0	0	0	0
ENSG000001	TOM1L2	protein_codin	111.474138	125.039356	63.8044567	0
ENSG000001	AQP7	protein_codin	0	0	0	0
ENSG000001	AQP7	protein_codin	0	0	0	0
ENSG000001	LCLAT1	protein_codin	25.8967065	23.4114819	15.5895847	11.1260942
ENSG000001	CNKSR2	protein_codin	0	0	0	0
ENSG000002	SUGT1P1	transcribed_u	0	0	0	0
ENSG000002	SUGT1P3	lncRNA	0	0	0	0
ENSG000002	SNX2	protein_codin	221.807007	90.5986603	58.9447867	118.292889
ENSG000001	YPEL5	protein_codin	253.218553	273.69459	29.7165771	149.159342
ENSG000001	YPEL5	protein_codin	101.996511	0	53.3036537	28.1987014
ENSG000000	NFX1	protein_codin	21.3590894	29.9987033	54.0864035	29.2341353
ENSG000002	ENSG000002	lncRNA	0.91232257	0	1.09271837	0
ENSG000001	MOSPD3	protein_codin	0	14.4536835	0	0
ENSG000001	TMEM14B	protein_codin	0	0	0	0
ENSG000001	MORF4L1	protein_codin	0	1.68855929	0	0
ENSG000000	NFX1	protein_codin	133.829973	129.426766	68.6716811	51.2150684
ENSG000001	TMEM14B	protein_codin	8.07827776	57.7640366	16.4740059	31.2149576
ENSG000001	ELF2	protein_codin	0	0	0	0
ENSG000001	ELF2	protein_codin	56.3937932	197.027468	176.480741	0
ENSG000001	PRR16	protein_codin	0	0	0	0
ENSG000001	INSC	protein_codin	0	0	0	0
ENSG000001	FAM170A	protein_codin	1.49893352	0	1.0680081	0
ENSG000001	INSC	protein_codin	0	0	0	0
ENSG000001	TOGARAM2	protein_codin	10.4912712	0	4.26402251	12.0617032
ENSG000001	FOXO1	protein_codin	275.778794	193.478519	78.9132862	167.949017
ENSG000001	KIN	protein_codin	70.8327603	55.7846073	30.3252808	50.909865
ENSG000001	RPS6KA3	protein_codin	509.200136	205.910404	201.467485	334.449408
ENSG000001	AP5S1	protein_codin	0	0	5.55659863	0
ENSG000001	PAK1IP1	protein_codin	38.3528422	48.1023582	26.2720921	34.6721736
ENSG000002	MAPK6P2	processed_ps	0	0	0	0
ENSG000001	AP5S1	protein_codin	6.21348546	0	0	0
ENSG000001	SPDYA	protein_codin	0	0	0	0
ENSG000001	C6orf52	protein_codin	0.94791519	0	0	0
ENSG000001	ITIH2	protein_codin	0	0	0	0
ENSG000001	LHFPL6	protein_codin	6.07851259	7.44719858	4.7758739	2.00318109
ENSG000002	GCNT2P1	processed_ps	2.1117282	1.07477949	0	1.06247294
ENSG000001	EIF1AX	protein_codin	44.5295773	0	0	5.61170342

ENSG000001	ATG12	protein_codin	3.24845159	0	0	10.6635942
ENSG000001	PSTPIP1	protein_codin	156.663463	246.380763	42.0142513	47.3478257
ENSG000001	GCNT2	protein_codin	71.1837359	61.4253957	38.1487024	43.2400949
ENSG000001	CDC25B	protein_codin	0	40.4912712	0	0
ENSG000001	NHLRC3	protein_codin	0	0	6.88718149	8.60492134
ENSG000001	NHLRC3	protein_codin	37.9046734	84.3905439	0	0
ENSG000001	EIF1AX	protein_codin	222.371705	188.395215	150.908086	134.274834
ENSG000001	TFAP2A	protein_codin	0	0	0	0
ENSG000001	CCDC112	protein_codin	121.450221	71.5972731	33.7915838	108.74156
ENSG000001	TFAP2A	protein_codin	0	0	0	0
ENSG000001	PGGT1B	protein_codin	16.4114237	27.5234085	10.2197084	0
ENSG000001	TRIM36	protein_codin	0	0	0	0
ENSG000001	TRIM36	protein_codin	0	0	0	0
ENSG000000	FOSL2	protein_codin	306.836142	314.171542	188.830729	77.4895005
ENSG000001	BABAM2	protein_codin	71.6524699	91.8465954	11.1318595	64.4268121
ENSG000001	BABAM2	protein_codin	4.95767219	11.1862005	0	0
ENSG000001	STOML3	protein_codin	0.96498693	0	0	0
ENSG000001	BABAM2	protein_codin	5.29236923	0	0	0
ENSG000001	REEP5	protein_codin	561.890768	476.374313	279.690416	450.592178
ENSG000002	ENSG000002	lncRNA	0	0	0	0
ENSG000001	MAP7D2	protein_codin	0	0	0	8.91750019
ENSG000001	HGSNAT	protein_codin	311.209063	345.020546	85.8700447	253.172219
ENSG000001	TRPC3	protein_codin	2.2547532	0	0.99342209	1.14603496
ENSG000001	UFM1	protein_codin	4.15862261	0	3.13846777	0
ENSG000001	MAP7D2	protein_codin	10.6722725	0	0	0
ENSG000001	KIFC3	protein_codin	0	0	7.30479632	0
ENSG000001	ARRDC2	protein_codin	293.517652	202.765462	29.6740743	33.7806033
ENSG000001	SLC35B3	protein_codin	5.94464662	4.62064808	0	0
ENSG000001	KATNB1	protein_codin	66.3291304	96.4501951	44.5483155	40.2583907
ENSG000001	EXOSC9	protein_codin	3.48677388	0	5.53581721	2.78679178
ENSG000002	OR6C65	protein_codin	0	0	0	0
ENSG000002	MRPL33	protein_codin	23.5795792	42.5513732	6.59897004	31.4679187
ENSG000002	OR6C1	protein_codin	0	0	0	0
ENSG000001	NREP	protein_codin	30.4984113	37.03287	7.66061987	41.9126073
ENSG000000	ARHGAP44	protein_codin	0	0	0	0
ENSG000001	TRPC4	protein_codin	0	0	0	0
ENSG000001	GOLGA6L4	protein_codin	0	0	0	0
ENSG000002	LINC01460	lncRNA	1.13671137	0	0	0
ENSG000001	TRPC4	protein_codin	0	2.07766423	0	0
ENSG000001	BCLAF3	protein_codin	0	10.0892694	87.1636383	23.3364139
ENSG000001	BCLAF3	protein_codin	92.4003175	64.7399197	0	26.4720477
ENSG000001	NDNF	protein_codin	0	0	0	0
ENSG000002	INSL3	protein_codin	0	0	0	0
ENSG000001	SH3KBP1	protein_codin	26.7935861	26.6636261	63.8705488	0

ENSG000001	SH3KBP1	protein_codin	311.231717	603.779274	226.243092	277.356775
ENSG000001	BAG1	protein_codin	17.1626785	16.142594	15.3344746	3.92329083
ENSG000001	BAG1	protein_codin	143.083059	221.169542	157.102435	112.611545
ENSG000001	TRPC4	protein_codin	0	0	0	0
ENSG000001	TSLP	protein_codin	1.24281687	0	0	0
ENSG000001	BAG1	protein_codin	24.6383683	76.1632161	16.3521777	9.38615752
ENSG000001	LMAN1L	protein_codin	0	0	0	0
ENSG000001	SFMBT2	protein_codin	0	0	0	0
ENSG000001	SFMBT2	protein_codin	8.1310865	1.44892659	3.91535832	4.80979495
ENSG000001	EEF1E1	protein_codin	6.22282347	12.2240243	11.2240222	12.4911938
ENSG000001	SH3KBP1	protein_codin	0	0	0	0
ENSG000002	ZNF512	protein_codin	76.1055786	125.70015	55.4862043	34.7043925
ENSG000002	IPO7	protein_codin	347.328315	283.024568	238.803936	283.301423
ENSG000001	SPINK4	protein_codin	0.83928247	0	0	0
ENSG000001	ZNF789	protein_codin	0	0	0	0
ENSG000001	SPINK4	protein_codin	0	0	0	0
ENSG000001	SH3KBP1	protein_codin	0	0	0	0
ENSG000001	CYP1A1	protein_codin	0	0	0	0
ENSG000001	IQGAP2	protein_codin	47.5080765	119.330787	139.513435	0
ENSG000000	B4GALT1	protein_codin	921.416786	725.249264	325.706053	551.467953
ENSG000001	POSTN	protein_codin	0	0	0	0
ENSG000001	POSTN	protein_codin	0	0	0	0
ENSG000001	POSTN	protein_codin	0	0	0	0
ENSG000001	POSTN	protein_codin	0	0	0	0
ENSG000001	CENPB	protein_codin	331.882047	550.507738	142.886542	255.21918
ENSG000002	SMU1P1	processed_ps	0	2.51393046	5.31910336	2.52388173
ENSG000001	SPEF1	protein_codin	0	0	0	0
ENSG000002	TXNDC5	protein_codin	498.103638	667.236367	2181.59935	212.472627
ENSG000001	ADISSP	protein_codin	79.0418971	135.093652	37.7446464	53.5847253
ENSG000001	CAMK2D	protein_codin	44.8440755	16.3306537	47.6814117	0
ENSG000001	ADPRM	protein_codin	0	13.5806682	10.479971	14.1929772
ENSG000001	PFKFB3	protein_codin	2232.0815	1066.91333	245.643143	967.438426
ENSG000000	CETP	protein_codin	0	0	0	6.70439184
ENSG000001	PFKFB3	protein_codin	0	0	0	0
ENSG000002	PRR13	protein_codin	0	61.6782356	0	0
ENSG000001	PFKFB3	protein_codin	119.478308	60.6932927	15.9932898	41.3003613
ENSG000001	AMHR2	protein_codin	0	0	0	0
ENSG000000	HERPUD1	protein_codin	84.4754794	124.657098	103.532621	34.9826026
ENSG000001	PAM	protein_codin	12.8347624	26.7452515	0	17.1321879
ENSG000001	CSNK1A1L	protein_codin	0	0	0	0
ENSG000000	DSP	protein_codin	0	0	0	0
ENSG000001	SIN3B	protein_codin	65.7727209	0	0	0
ENSG000001	PDHA1	protein_codin	42.4501953	29.0967226	52.252204	17.5790912
ENSG000001	PDHA1	protein_codin	0	37.3666083	44.3828287	54.6272245

ENSG000002	SLCO6A1	protein_codin	0	0	0	0
ENSG000001	MT1G	protein_codin	0	0	0	0
ENSG000001	APTX	protein_codin	0	0	0	0
ENSG000002	MT1IP	lncRNA	0	0	0	0
ENSG000001	APTX	protein_codin	0	0	7.54314196	0
ENSG000002	MT1M	protein_codin	0	0	0	0
ENSG000001	APTX	protein_codin	0	0	0	0
ENSG000001	APTX	protein_codin	0	0	0	0
ENSG000001	SMAD9	protein_codin	1.13863622	0	0	1.15196046
ENSG000001	OR10A4	protein_codin	0	0	0	0
ENSG000001	RIOK1	protein_codin	128.9684	31.3635461	30.3246676	43.6940019
ENSG000001	SFRP1	protein_codin	0	1.03476081	0	0
ENSG000001	CSAD	protein_codin	13.6764505	0	0	0
ENSG000001	NDUFB6	protein_codin	10.9705891	12.413692	7.76129108	6.94966949
ENSG000001	SPART-AS1	lncRNA	0	0	0	0
ENSG000001	CSAD	protein_codin	0	0	0	0
ENSG000001	NRBP1	protein_codin	346.905829	454.607363	186.148556	244.045657
ENSG000001	ADAM2	protein_codin	0	0	0	0
ENSG000001	TOPORS	protein_codin	173.334418	207.376743	126.068443	202.01982
ENSG000001	CIB3	protein_codin	0	0	0	0
ENSG000001	ADAM33	protein_codin	0	0	0	0
ENSG000002	CCDC169	protein_codin	0	0	0	0
ENSG000001	NRBP1	protein_codin	0	0	0	0
ENSG000002	CCDC169	protein_codin	0	0	0	0
ENSG000001	ADAM18	protein_codin	0	0	0	0
ENSG000001	RIGI	protein_codin	0	0	0	0
ENSG000001	ADGRG2	protein_codin	0	0	0	0
ENSG000001	ADGRG2	protein_codin	0	0	0	0
ENSG000001	ADGRG2	protein_codin	0	0	0	0
ENSG000001	ADGRG2	protein_codin	0	0	0	0
ENSG000001	SOHLH2	protein_codin	0	0	0	0
ENSG000001	RIGI	protein_codin	0	0	0	102.98531
ENSG000001	ARIH1	protein_codin	180.964687	140.019821	96.5589533	151.891791
ENSG000001	RBM17	protein_codin	214.78549	205.343701	91.1971477	123.344951
ENSG000001	DCLK1	protein_codin	0	0	0	0
ENSG000001	DCLK1	protein_codin	0	0	0	0
ENSG000001	TNS2	protein_codin	0	0	0	0
ENSG000001	ERAP2	protein_codin	0	0	0	0
ENSG000001	SLC6A2	protein_codin	0	0	0	0
ENSG000001	ADAM32	protein_codin	0	0	0	0
ENSG000002	ENSG000002	protein_codin	2.51537788	0	0	0
ENSG000001	ADAM9	protein_codin	41.9199369	0	0	0
ENSG000001	CAGE1	protein_codin	0	0	0	0
ENSG000001	MAB21L1	protein_codin	1.12811171	0	0.82499047	0

ENSG000001	NBEA	protein_codin	0	0	0	0
ENSG000001	ACO1	protein_codin	41.5302357	43.2760567	0	21.5719794
ENSG000002	ENSG000002	lncRNA	0	0	0	0
ENSG000001	RREB1	protein_codin	122.378259	204.243562	161.853613	84.7377213
ENSG000001	RBL2	protein_codin	58.4311347	17.7530986	0	0
ENSG000001	CNGA4	protein_codin	0.9285829	0	1.10928969	0.93561864
ENSG000001	BRF1	protein_codin	29.2301045	0	7.7561748	0
ENSG000001	RREB1	protein_codin	659.909032	727.790724	324.048131	251.298376
ENSG000001	NBEA	protein_codin	0	0	0	0
ENSG000000	PHKA2	protein_codin	168.023119	245.362881	81.6972366	139.491374
ENSG000002	OR52E6	protein_codin	0	0	0	0
ENSG000001	LEF1	protein_codin	0	0	0.76856273	2.00512581
ENSG000001	LY86	protein_codin	0	8.31998726	1.95855426	3.95217265
ENSG000001	IL2RA	protein_codin	0	0	0	0
ENSG000001	LETM2	protein_codin	0	4.12474539	0	0
ENSG000002	SAMD9	protein_codin	165.444467	130.70895	191.373748	230.07336
ENSG000001	IL2RA	protein_codin	0	3.60972217	0	0
ENSG000002	ITSN1	protein_codin	0	21.3244295	0	9.7910279
ENSG000000	PPEF1	protein_codin	0	0	2.00760068	1.09263765
ENSG000002	ENSG000002	lncRNA	0	0	0	0
ENSG000001	TRIM22	protein_codin	632.840351	781.888177	56.2009273	403.805009
ENSG000001	IL15RA	protein_codin	0	0	0	0
ENSG000001	IL15RA	protein_codin	0	0	0	0
ENSG000001	IL15RA	protein_codin	0	1.72134515	2.83326323	2.62984297
ENSG000001	IL15RA	protein_codin	27.1349126	0	14.6891794	0
ENSG000001	GLRX	protein_codin	15.3469225	16.0527845	0	6.54597692
ENSG000001	MYH10	protein_codin	0	0	0	0
ENSG000001	RHOBTB3	protein_codin	15.2380916	20.0056176	12.4420947	13.8841566
ENSG000001	UACA	protein_codin	0	0	104.511709	50.7677134
ENSG000001	RS1	protein_codin	0	1.03666987	0	1.05380376
ENSG000001	NPNT	protein_codin	0	0	1.10710337	0
ENSG000000	CDKL5	protein_codin	0	0	0	0
ENSG000001	SPATA9	protein_codin	0	0	0	0
ENSG000001	LINGO2	protein_codin	0	0	0	0
ENSG000001	C9orf72	protein_codin	291.948319	194.633005	187.155564	160.647758
ENSG000000	CDKL5	protein_codin	0	0	0	9.00747953
ENSG000001	C9orf72	protein_codin	296.412846	88.9359961	111.243825	216.859821
ENSG000001	FBH1	protein_codin	0	45.000743	6.70202047	0
ENSG000001	C9orf72	protein_codin	117.806484	40.8441569	61.4015695	127.309995
ENSG000001	RFESD	protein_codin	0	0	0	0
ENSG000001	SIAH1	protein_codin	63.3342674	41.6436566	18.8553501	8.90425488
ENSG000001	GPR150	protein_codin	0	0	0	0
ENSG000002	PCBP3-AS1	lncRNA	0	0.80507962	0	0
ENSG000001	ARSK	protein_codin	5.66112854	2.67885784	0	0

ENSG000001	SLC19A1	protein_codin	9.03500699	14.1157259	0	22.5780357
ENSG000001	TET2	protein_codin	245.935723	94.8070507	0	0
ENSG000001	NDEL1	protein_codin	0	12.0991372	0	0
ENSG000001	CENPE	protein_codin	30.5390672	6.62221575	0	26.3147199
ENSG000001	TRIM5	protein_codin	0	2.05272511	0	0
ENSG000001	BEND2	protein_codin	3.26626732	1.77271406	0	15.7260753
ENSG000001	EQTN	protein_codin	1.69279986	0	0	0
ENSG000001	EQTN	protein_codin	0	0	0	0
ENSG000001	BEND2	protein_codin	297.540894	307.50837	251.812538	151.352999
ENSG000001	TRIM5	protein_codin	23.2170676	66.0680607	6.44307497	47.9182291
ENSG000001	SKOR1	protein_codin	0	0	0	0
ENSG000001	TEK	protein_codin	0	0	0	0
ENSG000001	KLC1	protein_codin	52.6054854	0	15.7107471	43.6418229
ENSG000000	SCML1	protein_codin	0	0	0	0
ENSG000000	SCML1	protein_codin	0	0	0	0
ENSG000001	MPV17	protein_codin	19.13682	31.1731015	28.4541079	0
ENSG000000	SCML1	protein_codin	11.2401518	0	7.13545757	0
ENSG000000	CDK14	protein_codin	88.7494499	83.4670183	67.0448133	56.9158854
ENSG000001	RPP40	protein_codin	0	0	0	0
ENSG000001	LRRC19	protein_codin	0	0	1.32346211	0
ENSG000000	SLC4A11	protein_codin	0	0	0	0
ENSG000001	GTPBP10	protein_codin	15.0396473	0	0	0
ENSG000000	SLC4A11	protein_codin	0	0	0	0
ENSG000001	NHS	protein_codin	0	6.52478837	0	0
ENSG000000	IFT74	protein_codin	21.6182381	10.7475249	5.14215815	10.2321375
ENSG000001	SLC25A35	protein_codin	0	0	0	1.88017573
ENSG000002	EXOC3L4	protein_codin	0	0	0	0
ENSG000001	RFC3	protein_codin	16.9230269	18.4688062	20.342572	11.6510768
ENSG000001	TRIM54	protein_codin	0	0	0	0
ENSG000001	STEAP4	protein_codin	193.598669	187.248532	80.2682193	226.553777
ENSG000001	RBBP7	protein_codin	15.3455633	16.1889944	19.9985736	16.2164285
ENSG000001	RBBP7	protein_codin	109.964465	100.633537	64.531724	0
ENSG000001	ANKRD16	protein_codin	0	9.26087425	0	0
ENSG000001	ANKRD16	protein_codin	2.01753766	0	0	8.6511926
ENSG000002	KRTAP10-10	protein_codin	0	0	0	0
ENSG000001	TRIM6	protein_codin	0	0	0	0
ENSG000001	KL	protein_codin	11.7563855	5.54251967	1.87913997	0
ENSG000001	SMAGP	protein_codin	0	0	2.69283507	0
ENSG000002	KU-MEL-3	lncRNA	0	0	0	0
ENSG000001	TRIM6	protein_codin	3.27153906	0	0	0
ENSG000001	ELAVL2	protein_codin	0	0	0	0
ENSG000001	ITPA	protein_codin	23.8953892	40.8973557	15.7986011	7.44979918
ENSG000001	ELAVL2	protein_codin	0	0	0	0
ENSG000001	ECI2	protein_codin	0	0	13.706187	13.1295112

ENSG00000015	ECI2	protein_codin	7.96776725	2.40062194	0	4.81390129
ENSG00000024	N4BP2L2	protein_codin	0	0	0	0
ENSG00000008	TXLNG	protein_codin	105.117584	85.6470543	79.8298237	77.9385805
ENSG00000015	ECI2	protein_codin	6.28355475	0	0	0
ENSG00000003	GDI2	protein_codin	0	0	0	0
ENSG00000013	N4BP2L1	protein_codin	0	0	0	0
ENSG00000013	N4BP2L1	protein_codin	60.0498505	75.2705718	0	41.4183782
ENSG00000003	LETMD1	protein_codin	0	0	0	0
ENSG00000016	EDIL3	protein_codin	0	0	0	0
ENSG00000013	N4BP2L1	protein_codin	25.1503861	77.9176939	0	44.6472283
ENSG00000014	CDKN2B	protein_codin	9.4689237	5.55085496	9.405514	11.5351138
ENSG00000020	ENSG00000020	lncRNA	0	0	0	0
ENSG00000020	TP53TG3D	protein_codin	4.7030047	8.34013228	1.18889671	0
ENSG00000017	ALOXE3	protein_codin	0	0	0	0
ENSG00000014	CDKN2A	protein_codin	0	0	0	0
ENSG00000014	CDKN2A	protein_codin	0	0	0	0
ENSG00000013	BRCA2	protein_codin	0	0	0	0
ENSG00000010	UBXN8	protein_codin	0	0	0	11.294296
ENSG00000010	SYAP1	protein_codin	486.25371	303.813756	252.075772	246.444058
ENSG00000013	RAP1GDS1	protein_codin	0	48.7576936	0	0
ENSG00000025	ENSG00000025	lncRNA	2.09383671	1.37797787	0	0
ENSG00000013	ATRAID	protein_codin	14.8717582	22.3552375	38.4879155	46.2359632
ENSG00000017	ALOX15B	protein_codin	0	0	7.70232623	0
ENSG00000016	PDLIM5	protein_codin	0	0	0	0
ENSG00000016	PDLIM5	protein_codin	0	16.3900537	0	8.54784672
ENSG00000003	GDI2	protein_codin	154.017476	82.8453481	148.955592	0
ENSG00000017	ALOX15B	protein_codin	18.4104608	12.5574723	0	11.4522031
ENSG00000017	UBQLNL	protein_codin	0	0	0	0
ENSG00000014	FAM217A	protein_codin	0	0	0	0
ENSG00000013	CERS5	protein_codin	0	0	5.73709589	8.45887709
ENSG00000003	GDI2	protein_codin	1365.00065	1628.67119	526.375706	1135.96787
ENSG00000023	ENSG00000023	processed_ps	4.07667818	0	1.42316783	1.05978499
ENSG00000013	NABP2	protein_codin	3.42720204	3.90105486	2.01489366	2.94645234
ENSG00000013	ZCCHC9	protein_codin	31.3311486	43.1084003	0	15.3726306
ENSG00000016	S100G	protein_codin	0	0	1.02989434	0
ENSG00000015	DDRGK1	protein_codin	33.4881054	88.6043345	61.277189	41.5278786
ENSG00000011	IFNA8	protein_codin	0	0	0	0
ENSG00000018	IFNA2	protein_codin	0	0	0	0
ENSG00000012	IFNA6	protein_codin	0	0	0	0
ENSG00000016	OR51I1	protein_codin	0	1.6006539	0	0
ENSG00000014	IFNA16	protein_codin	0	0	0	0
ENSG00000007	FRY	protein_codin	0.79948159	0	0	0
ENSG00000017	OR51B6	protein_codin	0	0	0	0
ENSG00000016	TRAPPC10	protein_codin	40.0266095	18.9546921	17.6637236	29.4742458

ENSG000002	IFNA14	protein_codin	0	0	0	0
ENSG000001	OR51B4	protein_codin	0	0	0	0
ENSG000001	IFNA21	protein_codin	0	0	0	1.08288312
ENSG000000	CYP46A1	protein_codin	0	0	0	0
ENSG000001	IFNW1	protein_codin	0	1.07917195	0	0
ENSG000001	IFNB1	protein_codin	0	0	0	0
ENSG000002	HBE1	protein_codin	0	0	0	0
ENSG000000	CTPS2	protein_codin	24.3966002	46.6217405	0	15.1437596
ENSG000002	CCDC85C	protein_codin	7.11143399	3.03593285	5.75251106	4.03138942
ENSG000000	FUS	protein_codin	112.79717	105.741567	74.9452423	59.8173357
ENSG000001	FOCAD	protein_codin	4.66380506	24.0084116	0	0
ENSG000001	HBG2	protein_codin	0	0	0	0
ENSG000002	FBXO16	protein_codin	0	0	0	0
ENSG000001	PCLAF	protein_codin	0	0	6.61037535	0
ENSG000002	ENSG000002	protein_codin	0.96346926	0	21.4175851	5.8798776
ENSG000001	CNTROB	protein_codin	14.6075352	0	7.57103099	0
ENSG000001	HERC6	protein_codin	0	14.9132863	18.8319813	0
ENSG000002	FASTKD5	protein_codin	69.6215427	140.705205	0	14.5635662
ENSG000001	TASOR2	protein_codin	0	6.69627014	0	5.33839665
ENSG000001	FAM50B	protein_codin	0	7.83983444	0	0
ENSG000001	U2AF1	protein_codin	35.3421416	112.692091	6.81365054	27.678375
ENSG000001	PXDC1	protein_codin	0	0	0	0
ENSG000001	PRPF40B	protein_codin	2.29034693	0	0	0
ENSG000001	PXDC1	protein_codin	25.6949661	53.1959294	19.6498213	22.2961652
ENSG000000	SNX1	protein_codin	0	11.8552451	8.48352644	0
ENSG000001	GRPR	protein_codin	10.7167099	0	2.77029919	2.20294904
ENSG000001	CIAO2A	protein_codin	18.135775	32.22896	3.86940117	10.4538777
ENSG000001	AP1S2	protein_codin	0	0	7.98802688	38.2865235
ENSG000001	AVP	protein_codin	0.7262715	0	0	0
ENSG000001	SLC22A23	protein_codin	0	0	1.23518438	0
ENSG000001	SLC22A23	protein_codin	0	0	5.54988622	4.49493454
ENSG000001	PSMG4	protein_codin	0	6.63224174	3.16655681	5.49063909
ENSG000001	PSMG4	protein_codin	0	0	0	0
ENSG000001	ZRSR2	protein_codin	27.0802327	48.5983076	24.0210716	25.8192219
ENSG000001	CA5B	protein_codin	4.00237073	0	0	1.15452158
ENSG000001	RXFP2	protein_codin	1.33003967	0	0	0
ENSG000002	HBB	protein_codin	0	0	0	0
ENSG000000	BCL7C	protein_codin	0	1.79364557	1.22687107	6.29953653
ENSG000001	CA5B	protein_codin	0	1.87880983	3.1148426	0
ENSG000001	EMILIN1	protein_codin	13.0386062	33.3892534	29.7453674	10.3750635
ENSG000001	MLLT3	protein_codin	10.8592645	38.4236627	24.4099867	0
ENSG000001	USP3	protein_codin	532.019364	421.935957	163.339404	311.449923
ENSG000001	MRPS26	protein_codin	23.6891479	43.1710016	11.448906	12.2991518
ENSG000001	TROAP	protein_codin	0	0	0	0

ENSG000001	PDE9A	protein_codin	0	0	0	0
ENSG000002	CA5BP1	lncRNA	9.31787485	0	7.2405403	0
ENSG000001	CALML5	protein_codin	0	0	0	0
ENSG000002	CA5BP1	lncRNA	0	0	1.77328748	0
ENSG000002	CA5BP1	lncRNA	0	47.4042608	0	25.2440831
ENSG000002	CA5BP1	lncRNA	0	0	0	18.7255481
ENSG000001	MLLT3	protein_codin	17.83145	18.6253647	23.7912965	16.3606687
ENSG000001	APH1B	protein_codin	0	3.27138645	0	0
ENSG000001	CLTRN	protein_codin	0	0	3.14705893	0
ENSG000001	APH1B	protein_codin	3.38072728	7.16930763	3.68561002	0
ENSG000001	LHFPL2	protein_codin	0	30.1398958	11.875862	6.61582143
ENSG000001	GNRH2	protein_codin	0	0	0	0.6249422
ENSG000001	GNRH2	protein_codin	0	0.76358932	0	0
ENSG000001	ELP3	protein_codin	0	0	0	0
ENSG000001	CHD3	protein_codin	0	0	0	0
ENSG000001	NET1	protein_codin	47.3548547	76.6861742	66.6728224	43.9149367
ENSG000002	ENSG000002	protein_codin	183.551445	391.28756	174.110178	167.549533
ENSG000001	SERPINA9	protein_codin	0	0	0	0
ENSG000001	OR52A1	protein_codin	0	1.28491149	0	0
ENSG000001	BPHL	protein_codin	0	0	0	0
ENSG000002	OR52J3	protein_codin	0	0	0	0
ENSG000002	OR51A2	protein_codin	0	0	0	0
ENSG000001	BPHL	protein_codin	0	0	0	0
ENSG000001	TBCA	protein_codin	294.03951	489.437427	204.471217	216.943386
ENSG000001	OR51T1	protein_codin	0	0	0	0
ENSG000001	BPHL	protein_codin	0	7.85158745	4.03753974	0
ENSG000001	RPS6	protein_codin	37.5357262	51.0469364	18.4424206	23.4898177
ENSG000001	RPS6	protein_codin	8.31962997	1.90560711	6.39027238	2.03132944
ENSG000001	SCARA5	protein_codin	0	0	1.91037636	0
ENSG000002	MAPRE3-AS1	lncRNA	0	1.06612464	1.23173181	0
ENSG000001	MMP26	protein_codin	0	0	0	0
ENSG000002	C2CD4B	protein_codin	0	0.91236957	0	0
ENSG000001	RPS6	protein_codin	2239.82329	5161.33129	1395.24488	1853.45742
ENSG000001	HSPH1	protein_codin	24.6652079	0	40.1038083	0
ENSG000001	RIPK1	protein_codin	5.00264716	2.41101355	0	2.00818304
ENSG000002	PMS2P8	transcribed_u	0	0	0	0
ENSG000001	ZNF768	protein_codin	57.5447329	71.252727	32.1801399	31.4940684
ENSG000001	TUBAL3	protein_codin	0	0	0	0
ENSG000000	PIR	protein_codin	0	0.9674632	0	1.99550209
ENSG000000	PIR	protein_codin	0	0	0	0
ENSG000001	DENND4C	protein_codin	17.8157951	5.389694	2.4704149	7.99174424
ENSG000001	DENND4C	protein_codin	5.69378213	0	0	0
ENSG000001	NQO2	protein_codin	0	0	0	4.9888291
ENSG000001	UCN3	protein_codin	0	0	0	0

ENSG000001	DENND4C	protein_codin	13.8568145	0	0	13.2335598
ENSG000001	NQO2	protein_codin	5.60691892	0	0	5.77448934
ENSG000002	VPS16	protein_codin	25.9050327	21.7155649	8.90978943	2.6987934
ENSG000002	VPS16	protein_codin	95.3271822	176.633862	67.9014811	76.1636381
ENSG000001	AKR1C4	protein_codin	0	0	0	0
ENSG000001	SHBG	protein_codin	0	0	0	0
ENSG000001	NQO2	protein_codin	0	13.3646753	0	12.5739669
ENSG000001	NQO2	protein_codin	113.1585	110.30189	15.061056	104.173721
ENSG000002	RGL3	protein_codin	0	0	0	0
ENSG000001	PLIN2	protein_codin	0	14.44184	5.21549261	13.102268
ENSG000001	PLIN2	protein_codin	5.17326678	0	1.09599448	0
ENSG000001	SAT2	protein_codin	50.676928	72.7374003	24.7011903	27.5409618
ENSG000002	VPS16	protein_codin	55.924565	33.7969014	33.4256138	43.9828414
ENSG000001	ASB11	protein_codin	0	0	0	0
ENSG000001	NQO2	protein_codin	0	7.85244089	0	0
ENSG000001	TEX26	protein_codin	0	0	0	0
ENSG000001	EPHX2	protein_codin	0	0	0	0
ENSG000001	MEDAG	protein_codin	0	0	0	0
ENSG000001	ASB9	protein_codin	0	2.18181189	1.18457957	3.32347518
ENSG000001	ASB9	protein_codin	0	0	0	0
ENSG000001	C2CD2	protein_codin	48.7169412	46.2379987	26.1969135	32.340316
ENSG000001	ASB9	protein_codin	0	0	0	0
ENSG000001	ALOX5AP	protein_codin	90.2925113	141.991047	28.9382443	126.370665
ENSG000001	C12orf54	protein_codin	0	0	0	0
ENSG000001	MOSPD2	protein_codin	145.110192	65.534318	27.68279	74.7636895
ENSG000001	CERT1	protein_codin	53.2970348	41.5578064	64.5326072	0
ENSG000001	TLCD3B	protein_codin	0	0	0	0
ENSG000001	HAUS6	protein_codin	0	6.91887587	4.68502062	4.61358548
ENSG000001	CD68	protein_codin	276.192695	469.297295	117.234749	218.706584
ENSG000001	HAUS6	protein_codin	97.6608058	80.0824827	65.9601352	73.9555526
ENSG000001	INO80E	protein_codin	0	0	0	0
ENSG000001	SLTM	protein_codin	694.802185	282.281354	431.84572	475.030056
ENSG000001	COL2A1	protein_codin	0	0	0	0
ENSG000001	SERPINB6	protein_codin	0	11.5158084	0	0
ENSG000000	GEMIN8	protein_codin	0	0	2.41303642	0
ENSG000001	SERPINB6	protein_codin	0	10.7095737	6.00180797	0
ENSG000001	CARS1	protein_codin	11.8933811	0	22.0658684	0
ENSG000002	GEMIN8P4	transcribed_p	0	0.79134378	0.77016437	0.82042786
ENSG000001	RRAGA	protein_codin	103.335089	143.457503	33.6642362	50.5921351
ENSG000001	SERPINB6	protein_codin	0	61.0900316	0	14.3768459
ENSG000001	SAXO1	protein_codin	1.87023065	0	0	0
ENSG000001	SAXO1	protein_codin	0	0	0	0
ENSG000001	TNFSF13	protein_codin	7.46413122	16.8121725	4.51032803	2.93052292
ENSG000001	ADAMTSL1	protein_codin	0	0	0	0

ENSG000001:	SERPINB6	protein_codin	122.515583	207.941058	0	162.171034
ENSG000002(	NAP1L4	protein_codin	83.798819	104.09337	28.7069229	50.0477471
ENSG000001'	ADAMTSL1	protein_codin	0	0	0	0
ENSG000001:	SERPINB6	protein_codin	0	10.4653461	56.3178728	30.560181
ENSG000001'	ADAMTSL1	protein_codin	0	0	0	0
ENSG000000.	OFD1	protein_codin	0	0	32.8579049	33.4989112
ENSG000000.	ELN	protein_codin	0	0	0.72431897	0
ENSG000001!	AKR1C3	protein_codin	0	3.93838384	0	0
ENSG000002:	POLR2M	protein_codin	0	5.98623575	0	0
ENSG000001'	ADAMTSL1	protein_codin	0	0	0	0
ENSG000000.	ELN	protein_codin	0	0	0	0
ENSG000002(	MYZAP	protein_codin	8.29852491	5.67658727	0	0
ENSG000001'	ADAMTSL1	protein_codin	0	0	0	1.01736391
ENSG000000.	OFD1	protein_codin	0	28.0247766	0	0
ENSG000001:	GCOM1	protein_codin	23.0188564	0	65.4116104	0
ENSG000001:	GCOM1	protein_codin	98.1489148	0	0	0
ENSG000001'	ADAMTSL1	protein_codin	0	0	0	0
ENSG000001:	ADRA1A	protein_codin	0	0	0	0
ENSG000001:	ADRA1A	protein_codin	0	0	0	0
ENSG000001	SLC22A18	protein_codin	0	0	2.41125435	0
ENSG000000.	ELN	protein_codin	0	0	0	0
ENSG000000.	ELN	protein_codin	0	0	0	0
ENSG000001!	TRAPPC2	protein_codin	2.47365451	3.0258126	1.98522073	0
ENSG000001!	TRAPPC2	protein_codin	33.3794376	45.3747169	22.9628982	35.2901163
ENSG000001:	ADRA1A	protein_codin	0	0	0	0
ENSG000000.	ELN	protein_codin	0	0	0	0
ENSG000001!	TMEM239	protein_codin	0	0	0	0
ENSG000001:	ADRA1A	protein_codin	0	0	0	0
ENSG000001(	IGSF5	protein_codin	0	0	0	0
ENSG000002:	C20orf141	protein_codin	0	0	0	0
ENSG000001.	BTF3	protein_codin	43.4213839	36.3786842	30.9163796	7.21725804
ENSG000002.	ENSG000002.	protein_codin	0	0	0	0
ENSG000002(	BOLA2-SMG1	protein_codin	0	3.45162351	0	4.35000372
ENSG000002!	ENSG000002!	lncRNA	0	0	0	0
ENSG000001'	ZBTB4	protein_codin	159.883731	220.81716	131.745004	80.7826722
ENSG000001'	TCEANC	protein_codin	0	0	0	0
ENSG000002(	ENSG000002(	lncRNA	0.88472649	0	0	0.90169798
ENSG000001!	EGFL6	protein_codin	0	0	0	0
ENSG000001(	B3GALT5-AS1	lncRNA	0	0	0	0
ENSG00000(	CPXM1	protein_codin	7.87734756	13.4866759	2.87243057	0.96702544
ENSG000001(	SH3GL2	protein_codin	0	0	0	0
ENSG00000(	HDAC7	protein_codin	0	0	0	0
ENSG000001(	B3GALT5-AS1	lncRNA	0	0	0	0
ENSG000001(	KATNAL1	protein_codin	42.6016187	27.3767804	28.8731198	6.14442374

ENSG000001	KATNAL1	protein_codin	21.2897711	24.7558419	28.1706985	14.5589047
ENSG000001	B3GALT5	protein_codin	0	0	0	0
ENSG000001	B3GALT5	protein_codin	0	0	0	0
ENSG000001	ATXN3L	protein_codin	0	1.03839237	0	0
ENSG000001	FAM9C	protein_codin	0	0	1.37454433	0
ENSG000001	FGF5	protein_codin	0	0	0	0
ENSG000001	BNIP3L	protein_codin	803.224291	516.690861	374.942035	583.751475
ENSG000001	SH3BGR	protein_codin	0	0	0	0
ENSG000002	TMSB4X	protein_codin	143.092615	178.846036	247.904896	35.1798631
ENSG000001	SH3BGR	protein_codin	0	0	0	0
ENSG000002	TMSB4X	protein_codin	33.4985779	66.3669159	42.7077857	25.2456755
ENSG000002	TMSB4X	protein_codin	441.576984	544.407482	91.5757053	279.667414
ENSG000001	SH3BGR	protein_codin	0	0	0	0
ENSG000000	PTCD2	protein_codin	17.4331117	6.87831186	6.53524517	3.73706945
ENSG000000	CNTLN	protein_codin	36.0840869	15.4348816	9.69704437	31.1654063
ENSG000000	CNTLN	protein_codin	247.402179	286.367032	206.065193	140.161121
ENSG000000	EBF4	protein_codin	0	0	0	0
ENSG000000	HADHA	protein_codin	871.297407	1240.6851	243.7928	537.805397
ENSG000000	RPAP3	protein_codin	75.0274768	44.1598248	41.9010904	121.308791
ENSG000001	EBF1	protein_codin	0	0	0	0
ENSG000001	TTC8	protein_codin	0	0	0	0
ENSG000001	TLR7	protein_codin	143.441422	70.3864848	104.306738	97.5009326
ENSG000001	PRPS2	protein_codin	1.97243901	4.69759455	0	0
ENSG000001	EML5	protein_codin	3.13381624	4.57795536	9.16381165	2.16386901
ENSG000001	CDCA2	protein_codin	0	0	0	0
ENSG000001	BNC2	protein_codin	0	0	0	0
ENSG000001	BNC2	protein_codin	0	0	0	0.98211904
ENSG000001	PRPS2	protein_codin	60.5156084	64.3690292	35.6234735	49.4664427
ENSG000001	LCA5L	protein_codin	0	0	0	0
ENSG000001	BNC2	protein_codin	0	0	0	0
ENSG000001	UBL3	protein_codin	266.788875	154.555334	109.028559	198.79837
ENSG000001	FRMPD4	protein_codin	0	0	0	0
ENSG000002	LINC03041	lncRNA	0	0	0	0
ENSG000000	MSL3	protein_codin	0	0	0	0
ENSG000000	MSL3	protein_codin	0	0	0	0
ENSG000000	MSL3	protein_codin	0	0	0	0
ENSG000001	SERPINB9	protein_codin	52.0508014	270.520895	219.458837	0
ENSG000001	CCDC171	protein_codin	17.2148755	26.5596325	20.8906315	12.2342945
ENSG000000	PDE4A	protein_codin	128.038258	97.2100381	28.8686778	49.3104637
ENSG000001	SMN1	protein_codin	46.9602603	50.2441531	26.4206562	0
ENSG000001	GET1	protein_codin	0	0	0	5.24114061
ENSG000002	CHKB-DT	lncRNA	1.55803475	0	0.66714184	0
ENSG000001	AMELX	protein_codin	0	0	0	0
ENSG000001	GET1	protein_codin	0	0	0	0

ENSG000001	AMELX	protein_codin	0	0	0	0
ENSG000001	PSIP1	protein_codin	0	5.34847245	0	7.00551353
ENSG000001	PSIP1	protein_codin	0	0	0	0
ENSG000000	ARHGAP6	protein_codin	5.92652417	0	1.98446057	10.4113048
ENSG000000	ARHGAP6	protein_codin	0	0	0	0
ENSG000002	FLRT2-AS1	lncRNA	0	0	0	0
ENSG000001	CDKN1C	protein_codin	9.66956347	9.78187233	0	0
ENSG000001	GPS2	protein_codin	136.435962	111.724257	35.3728699	98.8923278
ENSG000001	GTF2H2C	protein_codin	43.5113128	28.5423466	27.0665918	0
ENSG000001	PSIP1	protein_codin	0	0	0	0
ENSG000000	ARHGAP6	protein_codin	291.425459	414.925749	266.508146	150.075954
ENSG000002	PPP2R2A	protein_codin	30.5569355	72.9085847	26.5394896	36.2344757
ENSG000001	PSIP1	protein_codin	234.089717	214.203186	210.440713	191.42109
ENSG000000	SERPINB1	protein_codin	1303.47261	1492.60344	468.045438	844.484235
ENSG000002	SMN2	protein_codin	0	0	0	0
ENSG000002	SMN2	protein_codin	0	0	0	0
ENSG000002	SMN2	protein_codin	0	0	0	0
ENSG000001	DNMT3A	protein_codin	0	0	59.7857023	0
ENSG000002	HMGN1	protein_codin	0	0	0	1.66807456
ENSG000002	HMGN1	protein_codin	0	0	5.94080755	0
ENSG000002	HMGN1	protein_codin	134.317082	319.244095	257.517355	171.210161
ENSG000002	SERF1B	protein_codin	0	0	7.44258484	0
ENSG000002	SERF1B	protein_codin	0	0	4.32447626	0
ENSG000001	SLC7A1	protein_codin	58.696969	97.2668158	70.3653625	30.7751007
ENSG000001	AKR1C2	protein_codin	0	0	0	0
ENSG000001	DNMT3A	protein_codin	38.809412	29.1732161	16.8026091	11.9483322
ENSG000002	POM121B	unprocessed_	9.7722551	9.95046148	0	8.44927897
ENSG000000	HCCS	protein_codin	20.068797	28.1114041	0	6.85518334
ENSG000000	HCCS	protein_codin	37.7599361	57.5004841	25.3445958	24.5095735
ENSG000001	WRNIP1	protein_codin	51.3164353	101.844153	18.098343	41.6343493
ENSG000002	SPDYE3	protein_codin	0	0	0	0
ENSG000001	WRNIP1	protein_codin	4.53058965	7.78946983	14.5767543	40.9138037
ENSG000001	ICAM4	protein_codin	15.7381447	7.77138155	0	5.56561727
ENSG000001	WRNIP1	protein_codin	0	0	48.7839498	37.2155684
ENSG000001	WRNIP1	protein_codin	0	0	0	0
ENSG000001	RAD17	protein_codin	15.7533366	24.8508035	0	0
ENSG000000	KCNQ1	protein_codin	0	0	0	0
ENSG000001	MID1	protein_codin	0	0	0	0
ENSG000001	MID1	protein_codin	0	0	0	0
ENSG000001	MID1	protein_codin	0	0	0	0
ENSG000001	MID1	protein_codin	0	0	0	0
ENSG000001	MID1	protein_codin	0	0	1.16066149	0
ENSG000000	ADAM7	protein_codin	0	0	0	0
ENSG000000	YAF2	protein_codin	0	0	0	0

ENSG000001	POMC	protein_codin	0	0	0	0
ENSG000001	SNAPC3	protein_codin	6.65103444	12.1328523	8.90955412	9.93979961
ENSG000001	BRWD1	protein_codin	166.414069	170.979559	0	59.6253528
ENSG000001	GMDS	protein_codin	3.93223605	0	0	0
ENSG000001	MTUS2	protein_codin	0	0	0	0
ENSG000001	DNAJC27	protein_codin	0	2.7199154	5.05037568	3.89491048
ENSG000001	SLC46A3	protein_codin	2.36457199	0	0	0
ENSG000001	GMDS	protein_codin	49.3499595	53.9868869	32.0539904	44.0543574
ENSG000001	SBF1	protein_codin	0	0	0	23.1757999
ENSG000000	AK6	protein_codin	9.68546541	7.15206948	13.2644321	8.12026408
ENSG000001	SNAPC3	protein_codin	15.8546028	21.6367654	5.24530902	19.0497011
ENSG000000	AK6	protein_codin	7.88281707	17.891381	0	6.48466634
ENSG000000	CLCN4	protein_codin	0	0	0	0
ENSG000000	CLCN4	protein_codin	62.721494	56.4360229	52.4366772	33.6452553
ENSG000001	CENPO	protein_codin	0	0	0	0
ENSG000001	NOXRED1	protein_codin	0	4.47726295	0	5.59825267
ENSG000001	THAP6	protein_codin	21.2935516	0	7.54910296	12.9843907
ENSG000001	ASL	protein_codin	0	0	0	0
ENSG000001	RCHY1	protein_codin	0	13.7619228	0	0
ENSG000001	POMP	protein_codin	45.693369	18.590716	15.0162646	51.7312859
ENSG000001	IDH3B	protein_codin	70.5410601	207.183003	0	64.270253
ENSG000000	TTC39A	protein_codin	0	0	0	0
ENSG000001	TTC39B	protein_codin	0	21.1415859	0	0
ENSG000001	IDH3B	protein_codin	0	0	0	41.8762791
ENSG000001	TTC39B	protein_codin	0	0	0	0
ENSG000001	SLC2A13	protein_codin	8.71910759	13.4293602	2.68621122	4.1609228
ENSG000001	AKR1C1	protein_codin	0	0	0	0
ENSG000001	SLC30A5	protein_codin	24.2309805	1.80388241	8.18269044	5.75059381
ENSG000000	WWC3	protein_codin	371.516319	451.769233	138.707539	189.943051
ENSG000001	ZNF738	protein_codin	0	0	0	0
ENSG000001	NKX3-1	protein_codin	7.0933615	2.00266109	5.82010751	5.93428188
ENSG000001	AKR1C1	protein_codin	3.36615702	6.70168914	1.19148195	0
ENSG000001	FREM1	protein_codin	0	0	0	4.49426823
ENSG000002	EIF3CL	protein_codin	0	21.5980864	0	0
ENSG000001	GABPB1	protein_codin	0	2.12338899	2.30625731	3.00522698
ENSG000001	FREM1	protein_codin	0	0	8.01765408	0
ENSG000001	ESRRB	protein_codin	0	0	0	0
ENSG000002	LINC01597	lncRNA	0	2.1453591	0	0
ENSG000001	PIN1	protein_codin	0	0	0	2.98811753
ENSG000001	FREM1	protein_codin	0	0	0	0
ENSG000001	GSG1L	protein_codin	0	0	0	0
ENSG000001	PSMG1	protein_codin	1.79029808	4.2260426	3.54697011	0
ENSG000001	SLC27A2	protein_codin	0	0	0	0
ENSG000000	SELENOO	protein_codin	116.478567	84.7820888	10.5986711	40.6655666

ENSG000001	HUS1B	protein_codin	3.74656154	0	0	1.83285061
ENSG000001	TRABD	protein_codin	145.815659	169.903761	7.82748214	62.7040764
ENSG000001	CER1	protein_codin	0	0	0	0
ENSG000001	SHROOM2	protein_codin	0	0	0.86820442	0
ENSG000001	ZDHHC21	protein_codin	36.2312293	21.4668792	28.6038538	29.3241031
ENSG000001	SREK1	protein_codin	160.280786	88.3518416	26.1218418	158.311883
ENSG000001	ASGR1	protein_codin	0	0	0	0
ENSG000001	NFIB	protein_codin	0	0	0	0
ENSG000001	NFIB	protein_codin	0	0	0	0
ENSG000001	SECISBP2L	protein_codin	0	0	0	0
ENSG000002	ETS2-AS1	lncRNA	0	0	0	0
ENSG000001	NFIB	protein_codin	0	0	0	0
ENSG000001	ERBIN	protein_codin	0	0	391.711006	0
ENSG000001	ERBIN	protein_codin	393.018445	0	0	216.886764
ENSG000000	KLF6	protein_codin	14.4055789	0	3.23412514	0
ENSG000001	CEP152	protein_codin	71.4335188	93.5307406	60.3508232	57.902387
ENSG000001	NFIB	protein_codin	0	0	0	0
ENSG000001	IRF4	protein_codin	85.3938263	139.89952	358.533922	38.2331451
ENSG000001	PAN3	protein_codin	326.420678	99.0821439	38.3555409	103.307227
ENSG000001	NFIB	protein_codin	0	0	0	0
ENSG000001	TBL1X	protein_codin	9.18325827	0	15.9555133	0
ENSG000002	LCMT1	protein_codin	0	15.8989247	0	0
ENSG000002	LCMT1	protein_codin	0	0	0	1.7155319
ENSG000001	MLH3	protein_codin	27.5793458	0	15.21464	0
ENSG000002	LINC01446	lncRNA	0	0	0	0
ENSG000002	LINC01310	lncRNA	0	0	0	0
ENSG000001	DDC	protein_codin	0	0	0	0
ENSG000001	NLN	protein_codin	40.9592744	96.9806903	74.1720132	29.7921295
ENSG000001	FKBP1B	protein_codin	7.64171445	16.6742941	8.97653919	13.9203212
ENSG000001	FLT3	protein_codin	66.8878894	0	22.2484354	32.5976413
ENSG000001	PITRM1	protein_codin	0	0	0	0
ENSG000002	LINC00898	lncRNA	0	0	0	0
ENSG000001	FKBP1B	protein_codin	10.1343852	3.2429307	0	0
ENSG000001	TSSC4	protein_codin	0	0	0	0
ENSG000000	SLC12A1	protein_codin	0	0	0	0
ENSG000001	PITRM1	protein_codin	0	0	0	0
ENSG000000	TBC1D22A	protein_codin	3.82976292	0	0	5.58153641
ENSG000001	TSSC4	protein_codin	8.64184694	12.5577449	0	0
ENSG000000	DNM1L	protein_codin	0	0	0	0
ENSG000001	FAM9A	protein_codin	0.63693097	0	0	0
ENSG000000	RUFY3	protein_codin	132.522139	92.7397108	45.3588077	58.5576135
ENSG000001	SGTB	protein_codin	248.357431	141.340673	125.142022	157.760165
ENSG000002	LINC00583	lncRNA	0	0	0	0
ENSG000001	MPDZ	protein_codin	0	0	0	0

ENSG000001	TRIM23	protein_codin	0	0	0	0
ENSG000001	TRMU	protein_codin	0	0	0	0
ENSG000001	CDX2	protein_codin	0	0	0	0
ENSG000001	TRMU	protein_codin	0	0	0	0
ENSG000001	ATAD2B	protein_codin	11.1386223	0	0	8.93611612
ENSG000001	ATP5F1EP2	processed_ps	0	0	0	0
ENSG000000	TTC38	protein_codin	23.0546945	43.2337937	15.5975614	10.0509149
ENSG000002	VCX3B	protein_codin	0	0	0	0
ENSG000001	PDX1	protein_codin	0	0	0	0
ENSG000000	MARCHF2	protein_codin	24.9690403	13.5211209	17.976273	26.8900641
ENSG000001	CD81	protein_codin	0	0	0	0
ENSG000002	CDPF1	protein_codin	0	0	0	0
ENSG000001	RBBP6	protein_codin	7.16765674	0	0	0
ENSG000000	PNPLA4	protein_codin	25.4517977	15.1689691	10.0431293	12.3376145
ENSG000001	MIRLET7BHG	lncRNA	0	0	0	0
ENSG000000	ADAMTS6	protein_codin	3.95958482	2.58350316	0	2.24441423
ENSG000001	RESF1	protein_codin	0	0	0	0
ENSG000000	ADAMTS6	protein_codin	0	0	1.25836369	0
ENSG000002	HTN3	protein_codin	0	0	0	0
ENSG000001	VCX	protein_codin	0	0	0	0
ENSG000001	STATH	protein_codin	0	0	0	0
ENSG000001	ATXN10	protein_codin	0	0	0	0
ENSG000001	CWC27	protein_codin	112.415027	66.151683	83.7189549	97.5981741
ENSG000000	PFKP	protein_codin	4.67172216	6.25167742	3.81050401	0
ENSG000001	SLC13A5	protein_codin	0	0	0	0
ENSG000000	PFKP	protein_codin	54.3887724	0	25.0901356	0
ENSG000001	PUDP	protein_codin	73.6937732	108.6094	44.2402045	65.5276912
ENSG000002	ENSG000002	lncRNA	0	0	0	0
ENSG000001	IGFBP3	protein_codin	0	0	0	0
ENSG000001	IGFBP3	protein_codin	0	7.68328215	8.98486791	0
ENSG000001	VCX3A	protein_codin	0	0	0	0
ENSG000001	LDAH	protein_codin	0	0	5.61833179	0
ENSG000001	NLGN4X	protein_codin	0	0	0	0
ENSG000001	NLGN4X	protein_codin	0	0	0	0
ENSG000001	NLGN4X	protein_codin	0	0	0	0
ENSG000000	KIF2A	protein_codin	42.2838757	51.0229015	64.0610702	39.6482654
ENSG000002	ENSG000002	lncRNA	0	0	0	0
ENSG000002	ENSG000002	lncRNA	0	0	0	0
ENSG000002	ENSG000002	lncRNA	4.09871834	0	0	0
ENSG000001	CCM2	protein_codin	0	70.1193176	0	39.5383473
ENSG000001	MTIF3	protein_codin	0	0	0	0
ENSG000000	TSPAN32	protein_codin	0	0	0	0
ENSG000000	ERCC8	protein_codin	6.54419497	2.54058112	8.88165998	2.01535048
ENSG000001	MTIF3	protein_codin	0	99.9457095	21.8408273	93.3100436

ENSG000000	TSPAN32	protein_codin	0	0	0	0
ENSG000001	H2AZ2	protein_codin	0	27.1383465	23.1677108	0
ENSG000000	PFKP	protein_codin	0	85.8638701	0	41.4774902
ENSG000000	ARSF	protein_codin	0	0	0	0
ENSG000001	AIPL1	protein_codin	0	0	0	0
ENSG000001	AIPL1	protein_codin	0	0	0	0
ENSG000002	ARSH	protein_codin	0	0	0	1.12760642
ENSG000001	RCAN1	protein_codin	0	0	0	0
ENSG000001	ARSL	protein_codin	0	0	0	0
ENSG000001	RCAN1	protein_codin	0	0	0	3.98829501
ENSG000001	TYRP1	protein_codin	0	0	0	0
ENSG000002	ACOT6	protein_codin	0	0	0	0
ENSG000001	GTF3A	protein_codin	168.995862	255.762322	69.2128653	120.745485
ENSG000001	TYRP1	protein_codin	0	0	0	0
ENSG000002	MRPS6P2	processed_ps	0	0	0	0
ENSG000001	SDC1	protein_codin	0	0	0	0
ENSG000001	SLC5A3	protein_codin	65.7389519	63.1637008	69.410166	60.4177666
ENSG000001	C11orf21	protein_codin	130.657915	230.146389	102.80105	62.7701965
ENSG000000	ARSD	protein_codin	222.132926	304.725602	88.4929296	149.434306
ENSG000000	GYG2	protein_codin	0	0	0	0
ENSG000001	RAB3C	protein_codin	0	0	0	0.88347858
ENSG000000	NPC1L1	protein_codin	0	0	0	0
ENSG000000	GYG2	protein_codin	0	0	0	0
ENSG000000	GYG2	protein_codin	0	0	0	0
ENSG000001	MIS12	protein_codin	30.4989296	0	13.8534286	12.6663727
ENSG000001	TH	protein_codin	0	0	0	0
ENSG000002	XGY1	transcribed_u	0	0	0	0
ENSG000001	XG	protein_codin	0	0	0	0
ENSG000001	TH	protein_codin	0	0	0	0
ENSG000001	SHISAL1	protein_codin	0	0	0	0
ENSG000000	CD99	protein_codin	10.9542185	5.55649373	0	1.38008472
ENSG000001	TH	protein_codin	0	0	0	0
ENSG000000	CD99	protein_codin	17.8935544	8.34701709	3.70223039	24.561226
ENSG000002	LINC00649	lncRNA	0	0	1.03708108	0
ENSG000000	CD99	protein_codin	0	0	0	0
ENSG000000	CD99	protein_codin	23.3693758	70.0377079	32.7683462	26.1488944
ENSG000000	CD99	protein_codin	574.787315	756.213066	276.03112	343.632941
ENSG000001	PTPRD	protein_codin	0	0	0	0
ENSG000001	PNPLA5	protein_codin	0	0	0	0
ENSG000001	MIER3	protein_codin	52.8751964	18.7294517	27.6123554	49.6074535
ENSG000001	RPAIN	protein_codin	0	0	0	0
ENSG000001	RPAIN	protein_codin	33.819775	42.0259757	17.7806488	36.7390836
ENSG000002	TECRL	protein_codin	0	0	0	7.50650276
ENSG000001	MIER3	protein_codin	0	19.2621651	10.011256	0

ENSG000001	MTHFSD	protein_codin	0	0	0	0
ENSG000001	ZNF605	protein_codin	5.74891771	3.1234304	0	0
ENSG000002	DPF3	protein_codin	2.88862763	5.57467875	0	2.80856528
ENSG000002	ENSG000002	lncRNA	4.13082989	1.01605366	0	3.10830999
ENSG000002	ZBED1	protein_codin	0	0	6.22373595	11.2631382
ENSG000002	ZBED1	protein_codin	49.1203227	63.9557931	0	158.665574
ENSG000002	ZBED1	protein_codin	195.519402	265.052601	107.308697	0
ENSG000001	MIER3	protein_codin	0	0	0	0
ENSG000000	POLR2B	protein_codin	25.5741936	36.8450071	0	17.4904223
ENSG000001	ASMT	protein_codin	0	0	0	0
ENSG000002	ENSG000002	lncRNA	0	0	0	0
ENSG000001	SIPA1L1	protein_codin	429.548969	219.464893	138.496405	180.20776
ENSG000001	ASMT	protein_codin	0	0	4.27685025	0
ENSG000001	SLC39A14	protein_codin	4.11987044	8.25490025	0	1.04013
ENSG000001	ASMT	protein_codin	0	0	0	0
ENSG000000	WDR76	protein_codin	0	0	20.4889567	0
ENSG000002	RDH14	protein_codin	25.7104052	33.8289513	18.9133595	22.5028547
ENSG000000	MAP3K9	protein_codin	0	0.78901755	0	0
ENSG000000	ZNF343	protein_codin	1.03372748	0	0	0
ENSG000001	GEN1	protein_codin	28.2554859	18.7871392	0	0
ENSG000001	HOPX	protein_codin	0	0	0	0
ENSG000001	CCDC91	protein_codin	52.2728623	0	29.2233688	0
ENSG000001	HOPX	protein_codin	0	0	0	0
ENSG000001	AKAP17A	protein_codin	47.3034018	107.796151	23.4610312	33.9024264
ENSG000001	SLC8A3	protein_codin	0	0	0	0
ENSG000000	KLHL42	protein_codin	59.636206	30.7125037	19.292393	31.2092432
ENSG000001	SMC6	protein_codin	74.8061628	21.2349834	65.0674508	59.4426656
ENSG000002	MANSC4	protein_codin	0	0	0	0
ENSG000001	A4GALT	protein_codin	0.79517238	0	0	0
ENSG000002	ENSG000002	processed_ps	0	0	0	0
ENSG000001	SMOC1	protein_codin	1.91410916	2.83556793	0	0
ENSG000002	ITSN1	protein_codin	0	0	0	0
ENSG000002	ITSN1	protein_codin	0	0	0	0
ENSG000002	ITSN1	protein_codin	5.12820347	0	0	0
ENSG000001	IL6ST	protein_codin	1.04267535	0	0	1.05971474
ENSG000002	ITSN1	protein_codin	0	0	0	0
ENSG000001	IL6ST	protein_codin	0	0	0	0
ENSG000001	IL6ST	protein_codin	0	95.3593216	0	0
ENSG000000	EXOC1	protein_codin	74.4561432	28.0450665	20.0014737	44.3629625
ENSG000001	P2RY8	protein_codin	294.700232	492.580409	165.264283	249.402739
ENSG000001	IL6ST	protein_codin	48.6145113	11.8180611	32.3589329	26.5352634
ENSG000002	ADARB2-AS1	lncRNA	0	0	0	0
ENSG000001	ADARB2	protein_codin	0	0	0	0
ENSG000001	KDM4C	protein_codin	12.8419213	13.272976	20.8426185	13.8935409

ENSG000001	KDM4C	protein_codin	226.256562	216.776691	158.810513	138.098286
ENSG000001	ADARB2	protein_codin	0	0	0	0
ENSG000001	CAMTA2	protein_codin	0	150.554496	0	23.4710405
ENSG000001	ADARB2	protein_codin	0	0	0.85545588	0
ENSG000001	ASMTL	protein_codin	64.7296234	54.8800428	42.4371667	40.5578314
ENSG000002	ITSN1	protein_codin	9.15106878	16.4799767	1.38923403	5.43762746
ENSG000001	CLOCK	protein_codin	30.5840765	15.4211147	24.6886131	17.5564541
ENSG000001	CYRIA	protein_codin	600.77974	498.029492	231.207518	342.833308
ENSG000001	RETN	protein_codin	0	0	0	0
ENSG000000	WDR37	protein_codin	65.3652729	84.2686466	29.9506766	43.6424418
ENSG000002	INS	protein_codin	0	0	0	0
ENSG000001	ASMTL	protein_codin	0	3.96406774	0	0
ENSG000001	TMEM165	protein_codin	177.736976	205.570344	33.6416255	123.852551
ENSG000000	THUMPD1	protein_codin	47.8067596	29.2419664	14.8926623	37.3650551
ENSG000001	ITPR2	protein_codin	1148.19515	629.871146	743.02674	801.965751
ENSG000000	DDX1	protein_codin	0	0	0	0
ENSG000001	SNRPB	protein_codin	216.835271	427.583688	209.999338	211.940064
ENSG000000	IDI1	protein_codin	29.349962	9.66349427	0	5.88210294
ENSG000000	RDH11	protein_codin	299.502525	372.226239	214.427343	230.23306
ENSG000002	SMIM45	protein_codin	0	0	0	0
ENSG000001	RASSF8	protein_codin	0	0	0	0
ENSG000002	ETFRF1	protein_codin	35.8658203	26.0888354	17.2147967	25.1612868
ENSG000002	ENSG000002	processed_ps	0	0	0	0
ENSG000001	SERF2	protein_codin	350.026058	936.804649	104.459384	296.680121
ENSG000001	GP2	protein_codin	0	0	0	0
ENSG000000	IGF2-AS	lncRNA	0	0	0	0
ENSG000001	GP2	protein_codin	0	0	0	0
ENSG000000	IGF2-AS	lncRNA	0	0	0	0
ENSG000002	C17orf107	protein_codin	209.810395	119.195487	72.3023441	100.999965
ENSG000001	UHRF2	protein_codin	4.44014101	0	0	10.2450702
ENSG000001	CDC20B	protein_codin	0	0	0	0
ENSG000002	ENSG000002	transcribed_p	0	0	0	0
ENSG000001	IGF2	protein_codin	0	0	0	0
ENSG000001	IGF2	protein_codin	0	0	0	0
ENSG000002	MBD3L2	protein_codin	0	0	0	0
ENSG000002	MBD3L4	protein_codin	0	0	0	0
ENSG000001	IGF2	protein_codin	0	0	0	0
ENSG000001	CCP110	protein_codin	0	30.6339816	84.5334726	8.78619355
ENSG000001	SLC25A6	protein_codin	1489.89189	2251.58155	414.33501	1220.17849
ENSG000001	ESM1	protein_codin	2.40864987	0	0	0
ENSG000001	ADGRE1	protein_codin	18.953345	0	13.8171851	0
ENSG000001	ESM1	protein_codin	0	0	0	0
ENSG000001	IGF2	protein_codin	0	2.79141068	0	0
ENSG000001	ADGRE1	protein_codin	0	0	0	65.1704804

ENSG000001	SNX18	protein_codin	592.054055	769.597179	379.097052	697.635856
ENSG000001	TMC5	protein_codin	0	0	0	0
ENSG000001	HR	protein_codin	0	0	0	0
ENSG000001	TGM6	protein_codin	0	0	0	0
ENSG000001	ST8SIA1	protein_codin	0	0	0	0
ENSG000001	TPD52L3	protein_codin	0	0	0	0
ENSG000001	SGCB	protein_codin	10.490312	14.2436587	12.4465739	1.12492701
ENSG000001	MCHR1	protein_codin	0	0	0	0
ENSG000001	IL33	protein_codin	0	0	0	0
ENSG000001	GPR12	protein_codin	0	0	0	0
ENSG000002	ITPRIPL2	protein_codin	138.972501	239.989633	59.782538	95.0255817
ENSG000001	DCUN1D4	protein_codin	0	23.4011901	16.5945866	0
ENSG000002	ENSG000002	lncRNA	0	0	0	0
ENSG000001	TGM3	protein_codin	0	0	0.86860238	0
ENSG000001	BRD10	protein_codin	50.5448362	86.6125919	115.967199	139.872174
ENSG000001	OCIAD2	protein_codin	0	0	0	0
ENSG000000	TRIB2	protein_codin	0	0	0	0
ENSG000002	ENSG000002	lncRNA	0	0	0	0
ENSG000001	IL3RA	protein_codin	16.1096319	8.99908897	14.1481145	17.7517305
ENSG000001	DMTN	protein_codin	0	0	0	0
ENSG000001	MLANA	protein_codin	0	0	0	0
ENSG000001	OCIAD1	protein_codin	24.7151202	11.8689933	0	18.5922449
ENSG000001	NOMO2	protein_codin	0	254.128333	123.958932	120.316174
ENSG000001	MLANA	protein_codin	0	0	0	0
ENSG000001	MLANA	protein_codin	1.3328066	0	1.32793284	0
ENSG000002	DENND1C	protein_codin	160.32327	137.040265	29.4117511	67.4160812
ENSG000001	STK35	protein_codin	77.8468652	67.0306963	47.3204232	27.7304005
ENSG000001	GREB1	protein_codin	0	0	0	0
ENSG000001	GREB1	protein_codin	0	0	0	0
ENSG000001	ARRB2	protein_codin	0	0	0	0
ENSG000001	DIP2C-AS1	lncRNA	0	0	0	0
ENSG000002	ENSG000002	lncRNA	0	0	0	0
ENSG000001	NPIPA6	protein_codin	80.5343578	74.3637559	0	0
ENSG000001	CSF2RA	protein_codin	12.0274452	31.4689143	0	18.9249669
ENSG000001	TEC	protein_codin	94.423509	75.6454897	47.0942533	46.5662598
ENSG000001	CSF2RA	protein_codin	44.0674378	36.1270869	11.2008288	25.6957825
ENSG000000	SMARCD1	protein_codin	0	0	0	0
ENSG000002	MRPL23	protein_codin	3.76082308	1.09520728	1.12786001	1.08635049
ENSG000002	MRPL23	protein_codin	17.0242508	29.2908399	7.37104453	9.84377053
ENSG000001	CSF2RA	protein_codin	196.344138	273.275612	50.5151572	101.671663
ENSG000001	E2F6	protein_codin	25.024432	33.0012353	8.75168638	0
ENSG000001	CDK8	protein_codin	50.1414479	46.98226	4.05231788	0
ENSG000001	CSF2RA	protein_codin	0	100.334263	39.8774472	54.2531403
ENSG000001	NPM2	protein_codin	0	0	0	0

ENSG000001	SYNGR1	protein_codin	18.2737477	0	2.33419222	0
ENSG000001	NFXL1	protein_codin	0	0	66.6354345	0
ENSG000001	NPSR1	protein_codin	0	0	0	0
ENSG000002	CRYZL1	protein_codin	0	0	0	4.14597348
ENSG000002	SLCO1B3-SL	protein_codin	0	0	0	0
ENSG000001	NPSR1	protein_codin	0	0	0	0
ENSG000001	NPSR1	protein_codin	0	0	0	0
ENSG000001	SLCO1B3	protein_codin	0	0	0	0
ENSG000001	GGT6	protein_codin	0	0	0	0
ENSG000001	PDGFB	protein_codin	12.26714	10.3871006	11.0978338	4.20898659
ENSG000001	NPSR1	protein_codin	0	0	0	0
ENSG000002	CRYZL1	protein_codin	20.7503137	0	0	33.857414
ENSG000001	MYBBP1A	protein_codin	77.9057849	114.099213	46.5257734	45.6173813
ENSG000001	TNNT3	protein_codin	0	0	0	0
ENSG000001	TNNT3	protein_codin	0	1.30430401	0	0
ENSG000001	TNNT3	protein_codin	1.18641383	0	0	0
ENSG000001	APOBEC3F	protein_codin	3.46992738	4.738599	1.33516263	0
ENSG000002	CRLF2	protein_codin	0	0	0	1.27132548
ENSG000002	ENSG000002	protein_codin	0	0	0	0
ENSG000000	LZTS1	protein_codin	0	0	0	0
ENSG000001	RNF6	protein_codin	0	0	0	9.14011954
ENSG000001	COMMD8	protein_codin	85.2963349	49.3284997	51.0998239	69.0400313
ENSG000001	CD274	protein_codin	0	0	0	0
ENSG000001	SHOX	protein_codin	0	0	0	0
ENSG000001	CD274	protein_codin	10.5773549	4.18207136	6.00819051	0
ENSG000001	SHOX	protein_codin	0	0.8450425	0	0
ENSG000001	TNNT3	protein_codin	0	0	0	0
ENSG000000	SIRPG	protein_codin	0	0	0	0
ENSG000001	GABRB1	protein_codin	0	0	0	0
ENSG000000	SIRPG	protein_codin	2.31373587	0	0	0
ENSG000000	ZMYND11	protein_codin	0	0	0	0
ENSG000001	C2orf50	protein_codin	1.06248225	0	0	0
ENSG000001	RNF6	protein_codin	104.699267	72.342461	61.7309449	132.952081
ENSG000001	TNNT3	protein_codin	0	0	0	0
ENSG000000	ZMYND11	protein_codin	11.8034645	58.057573	40.7834835	6.97314716
ENSG000001	SIRPB1	protein_codin	0	14.1969905	35.2375539	18.9187679
ENSG000001	SIRPB1	protein_codin	0	200.005509	20.1971285	0
ENSG000000	ZMYND11	protein_codin	0	0	0	69.6970198
ENSG000001	SIRPB1	protein_codin	368.720958	557.349975	194.533388	410.842432
ENSG000000	SLC18A1	protein_codin	1.15650448	0	0	0
ENSG000001	PDIA6	protein_codin	0	0	0	0
ENSG000001	GABRA2	protein_codin	7.43021982	0	0	0
ENSG000001	SIRPD	protein_codin	0	0	5.86395404	0
ENSG000001	SIRPD	protein_codin	39.2050771	41.2615337	0	26.9726221

ENSG000001	CATSPERD	protein_codin	0	0	0	0
ENSG000001	PPP2R3B	protein_codin	0	0	1.05913039	0.96064116
ENSG000001	RLN2	protein_codin	0	0	0	0
ENSG000001	SIRPB2	protein_codin	27.4345412	37.3271067	13.0299681	22.0349242
ENSG000000	NLRP2	protein_codin	0	0	0	0
ENSG000000	ZZEF1	protein_codin	798.102593	828.885065	492.261307	483.109693
ENSG000002	RP9P	lncRNA	0	0	0	3.28837012
ENSG000001	INSL6	protein_codin	0	0	0	0
ENSG000002	ENSG000002	lncRNA	3.65677035	4.01767825	1.95093114	5.36397596
ENSG000002	TPTEP2	transcribed_u	0	0	0	0
ENSG000002	RIMOC1	protein_codin	56.2302185	66.9667821	39.9161025	71.0521894
ENSG000001	ATP8A2	protein_codin	0	0	0	0
ENSG000000	JAK2	protein_codin	1276.12345	814.301342	643.827002	793.847395
ENSG000000	NSFL1C	protein_codin	33.016781	41.8166234	22.6134054	15.729783
ENSG000001	ATP8A2	protein_codin	0	0	0	0
ENSG000001	PLCXD1	protein_codin	0	0	27.8381257	26.4087172
ENSG000001	ATP6V1C2	protein_codin	0	0	0.98002401	0
ENSG000001	ATP8A1	protein_codin	0	0	0	0
ENSG000001	BAIAP2L2	protein_codin	0	0	0	0
ENSG000001	CARD6	protein_codin	25.3652262	21.0438334	24.125313	20.9407136
ENSG000001	SON	protein_codin	619.016185	472.668047	286.825657	355.360272
ENSG000002	CATSPER2P1	lncRNA	0	0	0	0
ENSG000001	EIF3L	protein_codin	0	0	0	0
ENSG000001	NOL10	protein_codin	0	0	0	0
ENSG000001	NUTM2A	protein_codin	0	1.40014276	0	0
ENSG000001	SON	protein_codin	0	0	0	0
ENSG000002	NUTM2D	protein_codin	0	0	5.08999741	4.95260558
ENSG000002	GAGE12E	protein_codin	0	0	0	0
ENSG000002	GAGE1	protein_codin	0	0	0	0
ENSG000001	NUTM2A	protein_codin	7.63256155	16.4713772	7.05088394	0
ENSG000002	GAGE1	protein_codin	0	0	0	0
ENSG000001	HMG20A	protein_codin	58.2799199	51.8106393	25.1506201	43.7735462
ENSG000000	FKBP1A	protein_codin	0	0	0	0
ENSG000001	NUP58	protein_codin	0	0	0	0
ENSG000000	FKBP1A	protein_codin	56.1639667	61.5212525	42.1650658	35.3487567
ENSG000002	GAGE12H	protein_codin	0	0	0	0
ENSG000000	FKBP1A	protein_codin	0	0	0	0
ENSG000001	RCL1	protein_codin	3.37358658	1.65048219	0	0
ENSG000001	RCL1	protein_codin	0	0	0	4.54568514
ENSG000001	RCL1	protein_codin	0	3.0418228	0	5.23443885
ENSG000001	ASAH1	protein_codin	0	0	0	0
ENSG000001	NUP58	protein_codin	0	57.549115	0	0
ENSG000001	NUP58	protein_codin	0	0	0	0
ENSG000001	RCL1	protein_codin	0	37.5634354	22.9361624	0

ENSG000000	LIMCH1	protein_codin	0	0	0	0
ENSG000001	GGA1	protein_codin	0	0	0	0
ENSG000001	KDM4B	protein_codin	43.7846006	38.6945072	34.8102515	30.9052741
ENSG000001	UCHL1	protein_codin	0	0	0	0
ENSG000001	CATSPER2	protein_codin	0	0	0	0
ENSG000001	HPCAL1	protein_codin	63.5550127	39.738646	20.6935285	24.5699446
ENSG000000	SIRT2	protein_codin	0	0	0	0
ENSG000000	CAMKK1	protein_codin	0	41.1854697	0	0
ENSG000001	CPPED1	protein_codin	1301.83267	999.957313	366.135226	664.992338
ENSG000001	LSP1	protein_codin	39.0503477	31.1233252	13.1141107	33.7155848
ENSG000001	NSUN7	protein_codin	0	0	0	0
ENSG000001	PAAF1	protein_codin	5.03146898	6.90351782	5.38183462	1.15890906
ENSG000001	RRM2	protein_codin	0	0	2.35703424	0
ENSG000001	TMEM138	protein_codin	15.3251602	17.5335473	5.61795162	0
ENSG000002	COPB2-DT	lncRNA	0	0	0	0
ENSG000001	TMPRSS6	protein_codin	0	0	0	0
ENSG000001	RBM47	protein_codin	89.0413858	143.976668	39.5603432	116.948769
ENSG000001	RBM47	protein_codin	361.801424	302.328749	89.043198	163.441403
ENSG000002	DPP9-AS1	lncRNA	3.34317395	0.83161056	0	0
ENSG000001	RHOH	protein_codin	6.64124083	2.05001409	14.0102584	1.02261942
ENSG000002	LOH12CR2	lncRNA	0.99612784	6.13121262	1.11936495	0
ENSG000001	MTMR6	protein_codin	183.676158	97.1454772	89.4889565	76.6055771
ENSG000001	SDCBP2	protein_codin	1.86091046	0	0	0
ENSG000001	AK3	protein_codin	63.9078216	27.8542274	18.0599894	39.8997
ENSG000002	ENSG000002	lncRNA	3.74415376	4.87510188	0	0
ENSG000001	SDCBP2	protein_codin	0	4.41882534	0	0
ENSG000002	CYS1	protein_codin	0.9660236	1.90928379	3.9858313	0
ENSG000001	GART	protein_codin	0	15.1237991	0	0
ENSG000001	CIMIP4	protein_codin	0	0	0	0
ENSG000001	GDNF	protein_codin	0.83001478	0	0	0
ENSG000001	GART	protein_codin	0	0	0	0
ENSG000001	CIITA	protein_codin	0	0	25.5861643	0
ENSG000001	GART	protein_codin	0	0	0	0
ENSG000001	FGL1	protein_codin	0	0	0	0
ENSG000001	FGL1	protein_codin	0	0	0	0
ENSG000001	NUP155	protein_codin	0	12.2693249	31.5039543	45.2048129
ENSG000001	YWHAQ	protein_codin	0	0	0	0
ENSG000001	LIAS	protein_codin	0	14.618599	0	6.33949877
ENSG000001	PRH2	protein_codin	0	0.67915591	0	0
ENSG000002	PLIN5	protein_codin	4.78306872	0.94971346	0	2.00398242
ENSG000002	TAS2R14	protein_codin	0	0	0	0
ENSG000001	CDC37L1	protein_codin	13.1718491	13.9882943	7.6988471	12.7423632
ENSG000001	CDC37L1	protein_codin	18.389579	22.1524776	16.6227465	6.52012201
ENSG000001	MTUS1	protein_codin	0	0	0	0

ENSG000001	SNPH	protein_codin	0	0	0	0
ENSG000001	MTUS1	protein_codin	0	0	0	0
ENSG000000	CTNS	protein_codin	0	0	0	9.50993479
ENSG000002	ENSG000002	lncRNA	0	0	0	0
ENSG000001	SNPH	protein_codin	24.0035539	6.8015015	13.2368488	8.44111491
ENSG000001	PPIP5K1	protein_codin	0	0	0	0
ENSG000002	PLPP6	protein_codin	6.06873987	21.523496	5.25312166	5.0745077
ENSG000001	CENPJ	protein_codin	21.2968873	0	22.3345669	0
ENSG000001	PPIP5K1	protein_codin	0	0	0	0
ENSG000001	USP7	protein_codin	86.0918168	118.286838	149.60341	46.1046426
ENSG000001	UBXN7	protein_codin	0	2.46255401	0	0
ENSG000001	SPATA6L	protein_codin	0	0	0.98634962	0
ENSG000001	TMEM74B	protein_codin	0	0	0	0
ENSG000000	RFC1	protein_codin	76.9038829	121.121101	0	12.8374447
ENSG000001	PSMF1	protein_codin	0	1.10428701	0.91531284	0
ENSG000001	PSMF1	protein_codin	0	0	0	0
ENSG000002	KLRC2	protein_codin	0	0	0	0
ENSG000002	KLRC2	protein_codin	0	0	0	0
ENSG000002	KLRC3	protein_codin	0	0	0	0
ENSG000001	TNNI2	protein_codin	25.9430978	11.58101	19.7323671	0
ENSG000001	TNNI2	protein_codin	0	0	0	0
ENSG000001	KLRD1	protein_codin	10.8480164	4.43919689	2.68645895	0
ENSG000001	TNNI2	protein_codin	0	0	0	0
ENSG000001	TRPV3	protein_codin	0	0	0	0
ENSG000000	TNK2	protein_codin	3.84481403	0	0	0
ENSG000000	SLC1A3	protein_codin	0	0	0	0
ENSG000000	METTL22	protein_codin	24.016853	34.052725	10.0314264	16.9007142
ENSG000001	ANGPT4	protein_codin	0	2.89555868	1.07500533	0.96096187
ENSG000001	TMEM52B	protein_codin	4.72235189	0	0	1.93471413
ENSG000001	KLHL5	protein_codin	28.546137	14.6156801	24.1104081	24.9930577
ENSG000001	NADK2	protein_codin	47.3047076	0	29.7489564	24.7306769
ENSG000001	TMEM156	protein_codin	9.57349002	0	35.1710666	6.74596309
ENSG000001	FAM110A	protein_codin	9.27439856	0	0	0
ENSG000001	FAM110A	protein_codin	164.471959	149.812289	36.3513285	129.714434
ENSG000001	SLC52A3	protein_codin	0	0	0	0
ENSG000000	ATP12A	protein_codin	0	0	0	0
ENSG000001	DNAJC28	protein_codin	0	0	0	0
ENSG000001	TLR6	protein_codin	5.07137312	21.0281239	2.69475892	19.1936101
ENSG000001	OR1A2	protein_codin	0	0	0	0
ENSG000001	NAGPA	protein_codin	0	0	12.4556642	0
ENSG000002	SRXN1	protein_codin	106.875917	175.846333	59.580416	91.4128054
ENSG000001	PGM2	protein_codin	56.2442276	55.8511324	14.997332	90.9173928
ENSG000001	SYT8	protein_codin	0	0	0	0
ENSG000001	GLIS3	protein_codin	0	0	0	0

ENSG000002	TPTE2P7	processed_ps	0	0	0	0
ENSG000001	TMEM44	protein_codin	0	0	0	0
ENSG000001	METTL16	protein_codin	0	0	0.77355836	0.81779691
ENSG000001	SYT8	protein_codin	0	0	0	0
ENSG000001	C4orf19	protein_codin	0	0	0	0
ENSG000001	SEC14L4	protein_codin	0	0	0	0
ENSG000000	RFX3	protein_codin	0	0	0	0
ENSG000001	PARP4	protein_codin	1211.91666	1171.60547	530.669923	769.433387
ENSG000001	GPNMB	protein_codin	0	0	0	0
ENSG000001	IFNGR2	protein_codin	27.5156797	18.7067251	8.80571331	13.575158
ENSG000000	MSR1	protein_codin	83.9786972	33.7319498	17.7567381	51.4155014
ENSG000000	RFX3	protein_codin	0	0	0	18.0115792
ENSG000002	ENSG000002	lncRNA	0	0	0	0
ENSG000001	MCM5	protein_codin	76.9848384	132.49157	121.937343	106.036785
ENSG000001	TUSC3	protein_codin	0	0	0	0
ENSG000001	DNAJC21	protein_codin	0	0	0	0
ENSG000001	ABHD12B	protein_codin	0	0	0	0
ENSG000001	MAP1A	protein_codin	0	56.5314422	0	0
ENSG000000	PUM3	protein_codin	0	0	0	0
ENSG000001	RAD1	protein_codin	13.3445837	11.0739817	5.92363243	13.2215525
ENSG000000	TP53BP1	protein_codin	50.9969657	62.9466707	29.0766289	0
ENSG000001	RSAD2	protein_codin	9.19810428	0	19.8136277	0
ENSG000001	NIN	protein_codin	553.990536	640.531241	1072.01192	628.887987
ENSG000001	NIN	protein_codin	90.0354817	0	0	0
ENSG000000	TP53BP1	protein_codin	54.1890246	55.2523512	52.5345199	19.1766213
ENSG000002	LINC00487	lncRNA	0	0	0	0
ENSG000001	SLC34A2	protein_codin	0	0	0	0
ENSG000001	SERPINF2	protein_codin	0	0	0	0
ENSG000001	COLEC11	protein_codin	0	0	0	0
ENSG000001	CLEC4D	protein_codin	0	0	0	0
ENSG000000	C1QTNF3	protein_codin	2.07994436	1.70948011	0	2.2448549
ENSG000002	ENSG000002	protein_codin	0	0	0	0
ENSG000000	CREBBP	protein_codin	1158.99559	1400.32556	882.141067	912.739754
ENSG000002	C1QTNF9	protein_codin	0	0	0	0
ENSG000002	AMACR	protein_codin	6.14764286	0	0	0
ENSG000002	CLEC6A	protein_codin	35.7955773	61.607827	7.87727212	21.8107896
ENSG000002	C1QTNF3-AM	protein_codin	0	3.61464887	0	0
ENSG000001	SGCZ	protein_codin	0	0	0	0
ENSG000001	KCNV2	protein_codin	0	0.82853074	0	0
ENSG000002	RFPL3S	lncRNA	0	0	0	0
ENSG000002	AMACR	protein_codin	8.22586441	13.6174695	4.19009651	0
ENSG000002	RFPL3S	lncRNA	0	0	0	0
ENSG000001	ADI1	protein_codin	18.8694015	28.8003356	18.5953685	10.0132385
ENSG000001	SPATA13	protein_codin	0	0	0	0

ENSG000001	VLDLR	protein_codin	0	0	0.87141398	0
ENSG000002	C22orf42	protein_codin	0	0	1.0997881	0
ENSG000001	VLDLR	protein_codin	0	0	0	0
ENSG000001	VLDLR	protein_codin	0	0	0	0
ENSG000001	SLC45A2	protein_codin	0	0	0	0
ENSG000001	SEPSECS	protein_codin	0	0	0	0
ENSG000001	SPATA13	protein_codin	0	0	21.6857348	1.7578963
ENSG000001	TRAPPC12	protein_codin	0	15.0231253	0	29.3624305
ENSG000001	DEPDC5	protein_codin	0	0	0	0
ENSG000001	DEPDC5	protein_codin	0	0	0	0
ENSG000001	LGI2	protein_codin	0	1.19127352	0	0
ENSG000002	NANOGNB	protein_codin	0	0	0	0
ENSG000001	SOD3	protein_codin	0	0	0	0.79114635
ENSG000002	IFITM10	protein_codin	0	9.352918	0	0
ENSG000002	LRRC72	protein_codin	0	0	0	0
ENSG000000	EIPR1	protein_codin	22.0353895	38.7469698	25.1723704	0
ENSG000002	PCOTH	lncRNA	0	0	0	0
ENSG000000	FETUB	protein_codin	0	0	0	0
ENSG000000	FETUB	protein_codin	0	0	0	0
ENSG000002	C1QTNF9B	protein_codin	0	0	0	0
ENSG000002	ENSG000002	protein_codin	1.99539828	0	0	0
ENSG000001	MTMR12	protein_codin	126.151709	218.769137	127.314968	142.307941
ENSG000002	C1QTNF9B	protein_codin	0	0	0	0
ENSG000001	KCNIP4	protein_codin	0	0	0	0
ENSG000001	KCNIP4	protein_codin	0	0	0	0
ENSG000001	KCNIP4	protein_codin	0	0	0	0
ENSG000002	PISD	protein_codin	85.804212	41.3132406	0	0
ENSG000001	KCNIP4	protein_codin	0	0	0	0
ENSG000001	GNG7	protein_codin	15.997523	24.4536003	23.1022119	3.88607555
ENSG000002	KRTAP5-6	protein_codin	0	0	0	0
ENSG000001	SFI1	protein_codin	51.0046078	49.411323	13.4905955	17.6037286
ENSG000000	DGKG	protein_codin	0	0	25.9255222	0
ENSG000002	FAM99B	lncRNA	0	0	0	0
ENSG000002	FAM99A	lncRNA	0	0	0	0
ENSG000002	KRTAP5-1	protein_codin	0	0	0	0
ENSG000000	MIPEP	protein_codin	8.01693023	8.98164863	9.92643334	0
ENSG000001	BRSK2	protein_codin	0	0	0	0
ENSG000001	RBCK1	protein_codin	0	0	0	0
ENSG000000	SMARCA2	protein_codin	0	0	0	0
ENSG000000	SMARCA2	protein_codin	0	0	0	0
ENSG000000	SMARCA2	protein_codin	0	0	0	0
ENSG000001	TRA2B	protein_codin	29.5767711	48.3143693	35.0559023	40.3563733
ENSG000001	ZNF205	protein_codin	7.27278392	11.050325	0	0
ENSG000000	SMARCA2	protein_codin	0	211.36135	0	0

ENSG000001	TPO	protein_codin	0	0	0	0
ENSG000000	IGF2BP2	protein_codin	117.721213	216.053297	136.514833	85.8581561
ENSG000001	TPO	protein_codin	0	0	0	0
ENSG000000	SMARCA2	protein_codin	0	14.8551439	53.9350934	0
ENSG000002	DEFB134	protein_codin	0	0	0	0
ENSG000002	DEFB135	protein_codin	0	0	0	0
ENSG000002	DEFB136	protein_codin	0	0	0	0
ENSG000000	IL32	protein_codin	0	0	0	0
ENSG000001	RBCK1	protein_codin	0	49.8446522	2.83557341	0
ENSG000002	C1RL-AS1	lncRNA	0	8.11242605	4.03933217	2.73838556
ENSG000001	LCORL	protein_codin	40.7108478	0	23.7571095	18.0297901
ENSG000002	ENSG000002	lncRNA	0	0	0	0
ENSG000001	LCORL	protein_codin	0	26.4662423	0	0
ENSG000001	TMEM41A	protein_codin	0.81679234	0	0	0
ENSG000001	IFNAR2	protein_codin	0	0	0	0
ENSG000001	PKMYT1	protein_codin	0	0	3.6554399	0
ENSG000002	ENSG000002	processed_ps	0	0	0	0
ENSG000002	BCRP3	transcribed_u	2.73879502	0.61053793	0	0
ENSG000001	DCAF16	protein_codin	123.351707	111.182837	53.6258388	58.3089112
ENSG000001	DMRT2	protein_codin	0	0	0	0
ENSG000002	ZNF316	protein_codin	226.191464	318.885571	76.3627607	175.938047
ENSG000001	CDH12	protein_codin	0	0	0	0
ENSG000001	DMRT2	protein_codin	0	0	0	0
ENSG000001	TNFRSF19	protein_codin	0.9777124	0	0	1.89842213
ENSG000001	TNFRSF19	protein_codin	0	3.41736185	1.05604878	0
ENSG000001	IFNAR2	protein_codin	39.8562463	78.3761274	0	28.4814025
ENSG000002	CCDST	lncRNA	0	0	0	0
ENSG000001	KDELR2	protein_codin	0	1.80813168	7.56951627	0
ENSG000001	TPO	protein_codin	0	0	0	0
ENSG000001	CDH18	protein_codin	0	0	0	0
ENSG000001	DMRT1	protein_codin	0	0	0	0
ENSG000001	ZG16B	protein_codin	1.99303146	9.21045829	2.72545024	7.70392429
ENSG000001	NRSN2	protein_codin	0	0	2.52675478	0
ENSG000001	KANK1	protein_codin	0	0	0	0
ENSG000001	KANK1	protein_codin	0	0	0	0
ENSG000001	NRSN2	protein_codin	0	0	0	0
ENSG000001	SACS	protein_codin	75.6767859	0	0	0
ENSG000001	KANK1	protein_codin	0	0	0	0
ENSG000002	DAZ4	protein_codin	0	0	0	0
ENSG000001	KANK1	protein_codin	0	0	0	0
ENSG000001	KANK1	protein_codin	1.55946553	0	0	0
ENSG000002	SRRM2-AS1	lncRNA	0.91795157	3.446814	1.06248238	0
ENSG000002	DAZ4	protein_codin	0	0	0	0
ENSG000002	GPR162	protein_codin	0	0	0	0

ENSG000001	TTC6	protein_codin	0	0	0	0
ENSG000001	PMS2	protein_codin	0	0	0	0
ENSG000002	ENSG000002	lncRNA	0	0	0	0
ENSG000001	DOCK8	protein_codin	295.384454	212.672224	179.21438	192.077378
ENSG000001	EIF4G1	protein_codin	0	0	0	0
ENSG000001	DOCK8	protein_codin	21.0930441	0	16.1565265	11.9974262
ENSG000001	FGFBP1	protein_codin	0	0	0	0
ENSG000001	DOCK8	protein_codin	0	0	0	41.8378546
ENSG000001	BST1	protein_codin	3.22588323	10.1831986	0	0
ENSG000001	OLIG1	protein_codin	65.6180474	92.2229218	10.8178047	25.2434869
ENSG000002	ONECUT3	protein_codin	1.73018597	0.86245023	0	0
ENSG000001	FGF9	protein_codin	0	1.36014485	6.68683367	0
ENSG000002	OLIG2	protein_codin	1.79678916	0	0	0
ENSG000000	FSCN1	protein_codin	33.5619278	85.6492217	13.3605812	28.2722115
ENSG000000	RNF215	protein_codin	16.0445344	18.6546128	0	8.34396532
ENSG000001	DAZ3	protein_codin	0	0	0	0
ENSG000001	RALGAPA1	protein_codin	0	0	65.3818373	26.9496313
ENSG000001	FBXL18	protein_codin	46.2777775	55.8511596	17.8580112	10.6462402
ENSG000001	C20orf96	protein_codin	0	7.1301628	9.74578727	4.57674568
ENSG000002	EPCIP	protein_codin	0	0	0	0
ENSG000001	MICU2	protein_codin	136.44371	96.4461206	64.0520564	106.636209
ENSG000002	EPCIP-AS1	lncRNA	0	0	0	0
ENSG000001	DEFB132	protein_codin	0	0	0	0
ENSG000002	EPCIP-AS1	lncRNA	0	0	0	0
ENSG000002	EPCIP-AS1	lncRNA	0	0	2.33616825	0
ENSG000001	ABCA3	protein_codin	15.0786948	0	0	17.4049123
ENSG000001	C1QTNF7	protein_codin	0	0	0	0
ENSG000001	WIPI2	protein_codin	107.362728	46.9648216	52.0613559	54.0544406
ENSG000001	DOCK8-AS1	lncRNA	7.07413603	7.15709988	0.73441456	1.02884039
ENSG000000	DEFB127	protein_codin	0	0	0	0
ENSG000001	ZNG1A	protein_codin	0	0	0	0
ENSG000001	ZNG1A	protein_codin	8.81333298	0	5.99771999	2.55405109
ENSG000001	CPEB2	protein_codin	126.301956	21.245931	0	0
ENSG000001	DEFB126	protein_codin	0	0	0	0
ENSG000001	CPEB2	protein_codin	0	0	15.6670854	0
ENSG000001	ZNG1E	protein_codin	0	0	0	1.85891647
ENSG000001	ZNG1E	protein_codin	0	0	0	0
ENSG000001	FAM177A1	protein_codin	58.0649807	40.2740673	45.7014382	44.9526368
ENSG000001	CDY1B	protein_codin	0	0	0	0
ENSG000001	DEAF1	protein_codin	42.3263669	102.254253	15.4556538	29.7172427
ENSG000001	DEFB125	protein_codin	0	0	0	0
ENSG000001	NOP2	protein_codin	0	0	0	0
ENSG000001	BAZ1A	protein_codin	317.420035	298.514246	146.981263	282.296925
ENSG000002	DAZ4	protein_codin	0	0	0	0

ENSG000002	DAZ2	protein_codin	0	0	0	0
ENSG000001	SLC35G5	protein_codin	0	0	0	0
ENSG000002	ZNG1F	protein_codin	0	0	0	0
ENSG000001	DNASE1L2	protein_codin	0	0	0	0
ENSG000001	NKX3-2	protein_codin	0	0	0	0
ENSG000002	DAZ2	protein_codin	0	0	0	0
ENSG000001	ZNG1A	protein_codin	127.829278	130.459429	91.9697979	60.5436347
ENSG000001	JMJD7-PLA2G	protein_codin	0	38.1263689	0	0
ENSG000002	DAZ2	protein_codin	0	0	0	0
ENSG000001	MLST8	protein_codin	0	0	0	2.59136781
ENSG000001	AP2M1	protein_codin	0	0	0	0
ENSG000000	NCAPD2	protein_codin	0	0	0	0
ENSG000001	XKR6	protein_codin	2.52010042	0	0	0
ENSG000001	HEATR5A	protein_codin	3.69901586	15.3387933	9.29718353	0
ENSG000001	ZDHHC20	protein_codin	136.801064	140.815179	44.3525031	105.351647
ENSG000002	USP17L6P	unprocessed_	0	0	0	0
ENSG000000	CBARP	protein_codin	0	0	0	0.75553066
ENSG000001	CPZ	protein_codin	0	0	0	0
ENSG000001	RP1L1	protein_codin	0	0	1.16773933	0
ENSG000001	GPR78	protein_codin	0	0	0	0
ENSG000002	ENSG000002	lncRNA	0.95707062	1.09117835	0.99828314	0
ENSG000001	HTR3D	protein_codin	0	0	0	0
ENSG000001	MSRA	protein_codin	22.4517415	0	9.93348993	0
ENSG000001	SYNJ1	protein_codin	14.9290938	21.4586335	15.8845809	35.1251694
ENSG000001	TAS2R1	protein_codin	0	1.2913571	0	0
ENSG000001	ABCC5	protein_codin	0	0	0	6.90201879
ENSG000001	SEMA5A	protein_codin	0	5.4183468	40.5083518	5.52671451
ENSG000001	SYNJ1	protein_codin	0	0	0	0
ENSG000001	FOXD4	protein_codin	0	0.91454776	0	2.35296196
ENSG000001	HTRA3	protein_codin	0	0	0	0
ENSG000000	CD9	protein_codin	0	0	0	0
ENSG000000	CD9	protein_codin	0	1.13110524	0	0
ENSG000000	CD9	protein_codin	24.4288964	0	36.811334	0
ENSG000001	SH3TC1	protein_codin	15.7535411	13.0475818	10.7207841	8.78257152
ENSG000002	LNCRI	lncRNA	0	0	0	0
ENSG000000	ADCY2	protein_codin	0	0	0	0
ENSG000001	SAP18	protein_codin	0	0	0	0
ENSG000001	TSC2	protein_codin	18.7149691	20.8968556	3.58801125	2.38505028
ENSG000001	GZMB	protein_codin	0	1.5203494	3.35517728	0
ENSG000001	AFAP1	protein_codin	6.13504415	7.36683169	0	0
ENSG000001	KCNA1	protein_codin	0	0	0	0
ENSG000001	GZMH	protein_codin	0	0	0	0
ENSG000001	CFAP298	protein_codin	0	7.66436958	7.96572949	3.57807613
ENSG000002	NYNRIN	protein_codin	0.99511725	17.471329	2.21012364	1.94102666

ENSG000002(	DNAJC19	protein_codin	6.57288538	17.3312173	0	5.95973693
ENSG000001	CHEK2	protein_codin	0	3.11453989	0	0
ENSG000001	MRFAP1	protein_codin	376.370973	598.202703	197.344619	326.012352
ENSG000001	MBP	protein_codin	0	0	0	0
ENSG000001	TTC14	protein_codin	12.3890257	12.0498819	5.36305759	23.9649091
ENSG000001	BPY2	protein_codin	0	0	0	0
ENSG000001	LATS2	protein_codin	394.041595	376.092566	187.55675	253.646463
ENSG000000	PPP2R2C	protein_codin	0	0	0	0
ENSG000001	IRX2	protein_codin	0	0	0	0
ENSG000002	IFITM5	protein_codin	0	0	0	0
ENSG000001	OR7E115P	unprocessed_	0	0	0	0
ENSG000001	PRMT8	protein_codin	0	0	0	0
ENSG000002	SMIM21	protein_codin	0	0	0	0
ENSG000002	RBMY1B	protein_codin	0	0	0	0
ENSG000002	ENSG000002	lncRNA	0	0	0	0
ENSG000000	RAD51	protein_codin	0	0	0	0
ENSG000001	MRPL36	protein_codin	14.4759173	0	0	14.2137049
ENSG000002	RBMY1D	protein_codin	0	0	0	0
ENSG000002	DEFB109B	protein_codin	0	0	0	0
ENSG000002	RBMY1E	protein_codin	0	0	0	0
ENSG000002	RBMY1E	protein_codin	0	0	0	0
ENSG000001	FAHD1	protein_codin	1.27372944	2.67132449	0	0
ENSG000001	FAHD1	protein_codin	0	0	0	1.2971239
ENSG000002	ENSG000002	lncRNA	0	0	0	0
ENSG000001	RBMY1A3P	transcribed_u	0	0	0	0
ENSG000001	PSMD13	protein_codin	36.9861378	0	76.7381053	0
ENSG000002	DEFA7P	unprocessed_	0	0	0	0
ENSG000002	RBMY1D	protein_codin	0	0	0	0
ENSG000000	MADCAM1	protein_codin	0	0	0	0
ENSG000001	ATXN2L	protein_codin	0	0	0	135.776304
ENSG000001	CCDC9B	protein_codin	0	41.8417481	0	54.3177801
ENSG000002	DEFA1B	protein_codin	0	0	2.57604708	0
ENSG000002	DEFA1	protein_codin	0	0	0	0
ENSG000001	CIMAP1D	protein_codin	0	0	0	0
ENSG000001	EVA1C	protein_codin	0	0	0	0
ENSG000002	RBMY1A1	protein_codin	0	0	0	0
ENSG000000	PSME1	protein_codin	0	0	0	0
ENSG000002	JPT2	protein_codin	0	0	0	0
ENSG000002	JPT2	protein_codin	0	0	0	0
ENSG000002	DOK6	protein_codin	27.0219671	10.3139685	17.135743	15.3066822
ENSG000001	CACNA2D4	protein_codin	0	0	8.53787331	0
ENSG000001	MSX1	protein_codin	0.86587641	0.9061667	0	0
ENSG000002	CRYBB2P1	lncRNA	0	23.3444657	17.7689059	1.37153816
ENSG000002	RPS17P14	processed_ps	0	0	0	0

ENSG000001	SIRT3	protein_codin	10.4419993	21.5649158	3.81172899	5.34586258
ENSG000001	CLCN7	protein_codin	189.622868	311.948718	18.5822083	131.786813
ENSG000002	SERPINB11	protein_codin	0	0	0	0
ENSG000001	URB1	protein_codin	63.2435789	107.601382	74.3078464	37.7933683
ENSG000001	TMEM128	protein_codin	10.9986244	11.6329096	2.50552945	0
ENSG000001	EEF1AKMT1	protein_codin	4.32650069	0	0	0
ENSG000000	UNKL	protein_codin	6.9875587	6.1464012	2.46372644	3.41097467
ENSG000001	EEF1AKMT1	protein_codin	0	6.88704569	4.22845847	2.99963483
ENSG000001	RBMY1HP	unprocessed_	0	0	0	0
ENSG000001	UPB1	protein_codin	0	4.51276943	0.72695497	2.16783482
ENSG000001	BET1L	protein_codin	61.0998662	105.459431	27.4785103	38.3817191
ENSG000001	PRORY	lncRNA	0	0	0	0
ENSG000001	SERPINB12	protein_codin	0	0	0	0
ENSG000002	SERPINB5	protein_codin	0	0	0	0
ENSG000001	EIF1AY	protein_codin	1.5066982	0	0	0
ENSG000001	HGFAC	protein_codin	0	0.79243336	0	0
ENSG000001	RGS12	protein_codin	17.907979	45.085611	20.6179684	13.1220775
ENSG000001	ARHGEF10	protein_codin	1.05596021	0	0	0
ENSG000000	KDM5D	protein_codin	0	0	0	0
ENSG000001	CMTM5	protein_codin	0	0	0	0
ENSG000001	CRYL1	protein_codin	0	0	0	0
ENSG000001	C22orf15	protein_codin	0	0	0	0
ENSG000002	KRTAP19-8	protein_codin	0	0	0	0
ENSG000001	FBXO25	protein_codin	0	0	0	0
ENSG000002	KRTAP20-3	protein_codin	0	0	0	0
ENSG000002	KRTAP20-4	protein_codin	0	0	0	0
ENSG000002	KRTAP22-2	protein_codin	0	0	0	0
ENSG000002	KRTAP27-1	protein_codin	0	0	0	0
ENSG000001	FAM193A	protein_codin	0	0	0	125.017057
ENSG000001	CD24P4	processed_ps	0	0	0	0
ENSG000001	IQSEC3	protein_codin	0	0.68273844	0	0
ENSG000001	GJB2	protein_codin	0	0	4.03816954	0
ENSG000001	GJB2	protein_codin	6.24538432	1.05317972	7.04200825	3.1520027
ENSG000002	CFAP99	protein_codin	0	0	0	1.79839111
ENSG000000	NEDD4L	protein_codin	0	0	0	1.8439876
ENSG000001	HSFY2	protein_codin	0	0	0	0
ENSG000001	HSFY1	protein_codin	0	0	0	0
ENSG000001	MSLN	protein_codin	0	0	0	0
ENSG000002	ENSG000002	lncRNA	0	0	0	0
ENSG000001	POLN	protein_codin	0	0	0	0
ENSG000001	CDY2B	protein_codin	0	0	0	0
ENSG000001	NLGN4Y	protein_codin	0	0	0	0
ENSG000001	ZMYM2	protein_codin	0	3.23739076	0	0
ENSG000001	ZMYM2	protein_codin	237.565391	117.969797	124.189141	127.777768

ENSG000001	NLGN4Y	protein_codin	0	0	0	0
ENSG000000	FECH	protein_codin	0	0	0	18.9204264
ENSG000001	ZMYM2	protein_codin	13.1377504	17.4007961	2.10185405	17.0378609
ENSG000001	ZMYM2	protein_codin	44.353804	0	13.6595904	19.2237157
ENSG000001	NELFA	protein_codin	58.4728393	75.718528	18.8862148	38.444188
ENSG000001	ZMYM2	protein_codin	86.864085	33.1331904	58.8178484	68.9262407
ENSG000001	NSD2	protein_codin	56.2774884	55.1861639	14.1075495	19.7573106
ENSG000001	NSD2	protein_codin	0	0	0	0
ENSG000001	NSD2	protein_codin	0	0	0	0
ENSG000001	UTY	protein_codin	0	0	0	0
ENSG000001	NSD2	protein_codin	0	0	0	0
ENSG000001	UTY	protein_codin	0	0	0	0
ENSG000002	LINC01539	lncRNA	0	0	0.94472999	0
ENSG000001	KDM6A	protein_codin	0	0	0	0
ENSG000001	ZMYM5	protein_codin	16.4594651	0	17.8233314	0
ENSG000001	ZMYM5	protein_codin	0	20.7739945	0	0
ENSG000001	ZMYM5	protein_codin	14.44091	0	0	0
ENSG000001	C18orf54	protein_codin	12.8524951	0	0	1.50291068
ENSG000000	ME2	protein_codin	0	0	27.4888394	0
ENSG000000	SLC7A4	protein_codin	0	0	0	0
ENSG000000	RPGRIP1	protein_codin	0	2.76487169	4.30861245	0
ENSG000001	TMEM129	protein_codin	27.8826988	44.4847561	0	31.5695463
ENSG000000	APBA2	protein_codin	0	0	0	0
ENSG000001	NME4	protein_codin	0	0	0	0
ENSG000001	MBD1	protein_codin	0	0	0	0
ENSG000001	GOLGA8G	protein_codin	0	0	0	0
ENSG000001	CTBP1	protein_codin	2.01247586	5.78230939	0	0
ENSG000002	RNASE13	protein_codin	2.76573705	0	0	0.90753896
ENSG000001	CTBP1	protein_codin	200.821611	448.031025	103.440541	162.563391
ENSG000002	ENSG000002	lncRNA	0	0	0	0
ENSG000002	GYG2P1	transcribed_u	0	0	0	0
ENSG000002	ENSG000002	lncRNA	0	0	0	0
ENSG000001	RNF212	protein_codin	3.92947629	0	1.13489772	0
ENSG000000	MED15	protein_codin	77.6190657	116.508322	125.606165	82.7785953
ENSG000001	TPTE2	protein_codin	0	0	0	0
ENSG000001	METTL17	protein_codin	47.3317579	0	10.0501427	19.7965345
ENSG000001	ENSG000001	transcribed_u	0	0	0	0
ENSG000001	MME	protein_codin	0	0	0	0
ENSG000002	C4orf46P1	transcribed_p	0	0	0	0
ENSG000001	CTIF	protein_codin	5.26358571	54.8259062	19.3690219	5.62041925
ENSG000002	TSPY8	protein_codin	0	0	0	0
ENSG000002	TSPY8	protein_codin	0	0	0	0
ENSG000002	TSPY4	protein_codin	0	0	0	0
ENSG000002	LINC02346	lncRNA	0	0	0	0

ENSG000002	RBMY1B	protein_codin	0	0	0	0
ENSG000001	PIGG	protein_codin	0	0	0	0
ENSG000001	TTC5	protein_codin	0	0	0	12.4516439
ENSG000000	TBL1Y	protein_codin	0	0	0	0
ENSG000000	AMELY	protein_codin	0	0	0	0
ENSG000002	DUXAP8	lncRNA	0	0	0	0
ENSG000001	TSPY2	protein_codin	0	0	0	0
ENSG000001	SEPTIN5	protein_codin	113.226581	290.487353	172.860303	100.120172
ENSG000000	ZFY	protein_codin	0	0	0	0
ENSG000001	TPGS2	protein_codin	15.9002509	0	0	0
ENSG000001	SRY	protein_codin	0	0	0	0
ENSG000001	ZIC4	protein_codin	0	0	0	0
ENSG000001	PLSCR4	protein_codin	0	0	0	0
ENSG000002	MICAL3	protein_codin	7.7205033	0	0	3.90360604
ENSG000001	CCDC178	protein_codin	0	0	0	0
ENSG000001	B4GALT6	protein_codin	0	0	0	0
ENSG000002	TPTEP1	lncRNA	0	0	0	0
ENSG000002	PRR23A	protein_codin	0	0	0	0
ENSG000002	FOXL2NB	protein_codin	0	1.11722196	0	0
ENSG000001	AQP4	protein_codin	0	0	0	0
ENSG000001	AQP4	protein_codin	0	0	0	0
ENSG000001	C2	protein_codin	0	0	0	0
ENSG000001	NME9	protein_codin	0	0	0	0
ENSG000001	STAG1	protein_codin	180.448687	116.578352	112.248444	50.7398483
ENSG000001	CEP63	protein_codin	0	0	0	0
ENSG000001	TMEM241	protein_codin	0	0	6.23889529	0
ENSG000002	LY6G5C	protein_codin	2.86552673	0	1.08196916	0
ENSG000001	CHRM5	protein_codin	0	2.12379415	0	1.07768419
ENSG000001	ESCO1	protein_codin	50.3840178	14.9433312	6.8658107	28.353524
ENSG000001	NPHP3	protein_codin	0	2.36411548	0	0
ENSG000001	RNMT	protein_codin	100.423872	59.5401542	53.458988	39.1872435
ENSG000002	HLA-C	protein_codin	417.281971	802.837747	30.450857	0
ENSG000002	HCG27	lncRNA	0	0	0	0
ENSG000001	NEK11	protein_codin	0	0	0	0
ENSG000001	CCDC125	protein_codin	0	18.3989044	13.9032563	11.8712288
ENSG000001	IMPA2	protein_codin	1.32718773	2.85056101	0	0
ENSG000001	PIEZO2	protein_codin	0	0	0	0
ENSG000000	RALBP1	protein_codin	468.178261	424.355684	40.3740249	355.480197
ENSG000002	H1-10-AS1	lncRNA	4.60030009	1.96237934	1.17324512	0
ENSG000001	HMCES	protein_codin	34.8670753	33.2171049	11.5441429	15.3113517
ENSG000002	LRRC30	protein_codin	0	0	0	0
ENSG000000	ARHGAP28	protein_codin	0	0	0	0
ENSG000002	TMEM200C	protein_codin	1.23089038	0	0	0
ENSG000001	TXNRD3	protein_codin	0	0	0	0

ENSG000001	ENOSF1	protein_codin	0	3.26608873	0	0
ENSG000001	UROC1	protein_codin	0	0	0	0
ENSG000001	USP14	protein_codin	0	30.8312581	26.2444685	0
ENSG000001	SLC41A3	protein_codin	0	0	0	0
ENSG000002	OR7E122P	transcribed_u	0	0	0	0
ENSG000002	HLA-H	transcribed_u	673.192973	407.66374	282.039755	520.252212
ENSG000002	OR2H2	protein_codin	0	0	0	0
ENSG000002	HACD2	protein_codin	91.7640584	70.0080096	62.5970364	78.9953673
ENSG000001	DTX3L	protein_codin	29.9367284	15.8638689	24.1082686	24.5699777
ENSG000001	CD80	protein_codin	0	0	1.5048194	0
ENSG000001	TIGIT	protein_codin	2.10407828	4.23127247	0	0
ENSG000001	DRD3	protein_codin	0	0	0	0
ENSG000001	NEPRO	protein_codin	0	31.3558444	0	0
ENSG000001	GTPBP8	protein_codin	0	4.43669145	0	0
ENSG000001	GTPBP8	protein_codin	13.2617242	16.6397612	0	11.1103662
ENSG000001	BTLA	protein_codin	3.95067619	2.91357633	4.08574522	0
ENSG000000	CD200	protein_codin	0	0	0	0
ENSG000002	NECTIN3-AS1	lncRNA	0	0	0	0
ENSG000002	LNP1	protein_codin	0	0	0	0
ENSG000001	FILIP1L	protein_codin	7.8187916	2.58134275	0	0
ENSG000002	OR5K3	protein_codin	0	0	0	0
ENSG000002	VGLL3	protein_codin	0	0	0	0
ENSG000001	CADM2	protein_codin	12.7786377	0	3.37317746	0
ENSG000001	EOGT	protein_codin	55.795548	34.307395	0	11.1438865
ENSG000001	LRIG1	protein_codin	0	0	0	0
ENSG000001	CADPS	protein_codin	0	0	0	0
ENSG000001	PDHB	protein_codin	2.78965791	6.72342358	0	4.77013208
ENSG000001	PXK	protein_codin	0	7.91809141	0	0
ENSG000001	PXK	protein_codin	0	0	0	0
ENSG000001	SLMAP	protein_codin	84.5306985	65.5342194	48.166423	46.6096683
ENSG000001	NEK4	protein_codin	66.5777227	92.2108706	74.4715241	88.6412256
ENSG000001	P4HTM	protein_codin	25.3887347	17.6834848	0	22.4503726
ENSG000002	SLC26A6	protein_codin	0	0	0	0
ENSG000001	PFKFB4	protein_codin	16.0241367	17.8679409	7.49633955	16.2337451
ENSG000000	MAP4	protein_codin	0	10.4435136	11.9401747	0
ENSG000001	CSPG5	protein_codin	1.7577129	0	0	0
ENSG000001	ALS2CL	protein_codin	0	0	0	0
ENSG000001	ZNF197	protein_codin	0	0	0	0
ENSG000001	ZNF197	protein_codin	7.07592827	18.2895897	0	0
ENSG000001	TCAIM	protein_codin	24.527339	23.5825738	7.89517556	10.1086277
ENSG000002	KRBOX1	protein_codin	0	0	0	0
ENSG000000	SEC22C	protein_codin	0	0	0	0
ENSG000001	MOBP	protein_codin	0	0	0.95537664	0
ENSG000001	VILL	protein_codin	0	47.1690148	17.396046	23.6199399

ENSG000002	TRIM71	protein_codin	0	3.29595424	0.67197149	0
ENSG000001	RBMS3	protein_codin	0	0	0	0
ENSG000001	RBMS3	protein_codin	0	0	1.26495803	0
ENSG000002	ZCWPW2	protein_codin	18.2426172	23.4283445	5.76694708	14.3091841
ENSG000001	NEK10	protein_codin	0	0	0	0
ENSG000000	RARB	protein_codin	0	0	0	0
ENSG000001	NR1D2	protein_codin	23.9911027	0	23.253153	0
ENSG000001	DPH3	protein_codin	64.051444	24.4358997	40.8083289	41.5904472
ENSG000001	HACL1	protein_codin	0	19.0837839	0	0
ENSG000002	COLQ	protein_codin	0	0	0	0
ENSG000002	COLQ	protein_codin	0	0	0	0.88064298
ENSG000002	COLQ	protein_codin	0	0	0	0
ENSG000002	METTL6	protein_codin	8.85502084	0	5.00991493	10.2607186
ENSG000002	METTL6	protein_codin	15.2201592	16.3165405	10.6155842	2.34714841
ENSG000001	SH3BP5	protein_codin	419.510714	240.231286	180.93836	151.583946
ENSG000001	CCDC174	protein_codin	78.0565859	90.5899168	72.9730302	71.7144578
ENSG000001	SEC13	protein_codin	0	0	0	4.98219174
ENSG000002	ENSG000002	lncRNA	1.0247522	4.54707583	0	0
ENSG000001	CRELD1	protein_codin	0	0	0	0
ENSG000001	IL17RC	protein_codin	14.5896152	8.03046035	0	0
ENSG000001	IL17RE	protein_codin	0	0	0	0
ENSG000001	IL17RE	protein_codin	0	0	0	0
ENSG000001	CIDEC	protein_codin	0	0	0	0
ENSG000001	RPUSD3	protein_codin	41.2879599	38.4663042	0	27.0634534
ENSG000001	OGG1	protein_codin	1.62331266	2.2991327	0	0
ENSG000001	OGG1	protein_codin	0	0	0	0
ENSG000002	TTLL3	protein_codin	85.4010483	53.2945832	15.3283475	22.9325339
ENSG000001	BRPF1	protein_codin	0	101.61176	0	0
ENSG000001	CPNE9	protein_codin	1.32193034	0	0	0
ENSG000001	CPNE9	protein_codin	0	1.65163133	0	0
ENSG000001	SRGAP3	protein_codin	14.0064669	27.7277042	8.92876079	0
ENSG000001	SUMF1	protein_codin	0	0	0	0
ENSG000000	IL5RA	protein_codin	0	0	0	0
ENSG000001	FAM131A	protein_codin	0	0	0	2.99257186
ENSG000002	Y_RNA	misc_RNA	0	0	0	0
ENSG000002	Y_RNA	misc_RNA	0	0	0	0
ENSG000002	RNU6-1292P	snRNA	0	0	0	0
ENSG000002	RNVU1-7	snRNA	8617.4188	9913.28856	3808.9533	6852.88168
ENSG000002	RNU6-46P	snRNA	0	0	0	0
ENSG000002	RNU6-132P	snRNA	0	0	0	0
ENSG000002	ENSG000002	snoRNA	0	0	0	0
ENSG000002	RNU6-47P	snRNA	19.6192902	36.5112865	16.6358104	36.2474846
ENSG000002	RNU6-877P	snRNA	0	0	0	0
ENSG000002	SNORA57	snoRNA	36.8639046	92.9148544	17.0559376	74.5427661

ENSG000002	RNU6-1286P	snRNA	0	0	0	0
ENSG000002	RNU6-841P	snRNA	5.43176997	1.00238172	0	3.62528369
ENSG000002	RNU6-25P	snRNA	3.50351931	6.57462186	0	0
ENSG000002	RNU6-431P	snRNA	0	0	0	0.92550869
ENSG000002	SNORD58A	snoRNA	65.7014277	173.081774	65.0844028	119.85751
ENSG000002	SNORA22B	snoRNA	0	0	0.88639392	0
ENSG000002	RNU6-425P	snRNA	0	0	0	0
ENSG000002	RNU6-946P	snRNA	0.90451589	0	0	0
ENSG000002	RNU6-1296P	snRNA	0	0	0	0
ENSG000002	SNORD116-1	snoRNA	0	0	0	0.91971121
ENSG000002	SNORD24	snoRNA	57.4002002	132.750099	50.8820563	125.626975
ENSG000002	SNORA2A	snoRNA	13.4326087	16.6717363	16.3827799	25.3226916
ENSG000002	RNU6-1336P	snRNA	0	0	0	0
ENSG000002	RNU6-477P	snRNA	0	0	0	0
ENSG000002	RNU6-338P	snRNA	0	0	0.91421483	0
ENSG000002	RNU6-741P	snRNA	0	0	0	0
ENSG000002	RNY1P5	misc_RNA	0	0	0	0
ENSG000002	RNU6-1264P	snRNA	0	0	0	0
ENSG000002	SNORD45C	snoRNA	28.9321569	72.3402316	14.0241496	42.1303214
ENSG000002	SNORD116-1	snoRNA	0	3.32866366	0	0
ENSG000002	SNORA69	snoRNA	12.942952	17.9603756	9.26818916	12.5319464
ENSG000002	RNU6-979P	snRNA	0	0	0.89346605	0
ENSG000002	RNU1-39P	snRNA	39.5388646	47.4175045	35.8358945	57.7156061
ENSG000002	RNU6-969P	snRNA	0	0	0	0
ENSG000002	RNU1-63P	snRNA	4.91428623	1.92518069	1.77997424	4.43853372
ENSG000002	SNORD60	snoRNA	16.3321298	63.775956	25.5247057	43.9670687
ENSG000002	RNU6-657P	snRNA	0	0	0	0.90568219
ENSG000002	SNORA80B	snoRNA	3.77080539	4.93525042	7.99972566	9.79504487
ENSG000002	SNORA22	snoRNA	50.4341907	131.791356	13.8372548	69.1014398
ENSG000002	RNU6-1062P	snRNA	1.61929708	0	5.56973937	0
ENSG000002	Y_RNA	misc_RNA	0	0	0	0
ENSG000002	SNORA70H	snoRNA	0	0	0	0
ENSG000002	RNU6-672P	snRNA	0	0	0	0
ENSG000002	Y_RNA	misc_RNA	0	0	0	0
ENSG000002	Y_RNA	misc_RNA	0	1.01052806	0	0
ENSG000002	RNU6-449P	snRNA	0	0	0	0
ENSG000002	RNU6-1279P	snRNA	0	0	0	0
ENSG000002	Y_RNA	misc_RNA	0	0	0	0
ENSG000002	Y_RNA	misc_RNA	0	0	0	0
ENSG000002	ENSG000002	snoRNA	0	0	0	0
ENSG000002	ENSG000002	snoRNA	0	0	0	0
ENSG000002	SNORA70G	snoRNA	0	0	0	0
ENSG000002	Y_RNA	misc_RNA	0	8.10159132	4.41555161	10.7588574
ENSG000002	RNU1-1	snRNA	34722.1095	54750.5229	16025.6704	34470.0588

ENSG000002	RNU6-608P	snRNA	0	0	0	0
ENSG000002	SNORD116-1	snoRNA	0	0	0	0
ENSG000002	RNU6-1039P	snRNA	0	0	0	0
ENSG000002	Y_RNA	misc_RNA	7.74554323	17.1401552	5.49354937	2.8564913
ENSG000002	Y_RNA	misc_RNA	0	0	0	0
ENSG000002	SNORA70	snoRNA	0	0	0	0
ENSG000002	Y_RNA	misc_RNA	0	0	0	0
ENSG000002	Y_RNA	misc_RNA	10.7117615	16.669535	17.8123008	9.24219085
ENSG000002	RNU6-573P	snRNA	0	0	0	0
ENSG000002	RNU6-1014P	snRNA	0	0	0	0
ENSG000002	Y_RNA	misc_RNA	18.9611099	15.8804985	4.80248487	6.25586352
ENSG000002	RNU6-471P	snRNA	0	0	0	0
ENSG000002	Y_RNA	misc_RNA	0	0	0	0
ENSG000002	RNU6-945P	snRNA	0	0	0	0
ENSG000002	RNU6-32P	snRNA	27.7603299	31.7922566	0	35.7765796
ENSG000002	Y_RNA	misc_RNA	0	0	0	0
ENSG000002	Y_RNA	misc_RNA	0	0	0	0
ENSG000002	RNU6-144P	snRNA	28.9445106	26.0569029	8.04119474	24.4504989
ENSG000002	Y_RNA	misc_RNA	0	0	0	0
ENSG000002	SNORD21	snoRNA	46.6319237	120.169456	54.034534	97.7904628
ENSG000002	RNU6-730P	snRNA	0	0	0	0
ENSG000002	Y_RNA	misc_RNA	0	0	0	0
ENSG000002	RNU6-157P	snRNA	0	0	0	0
ENSG000002	RNU6-1189P	snRNA	0	0	0	0.90557412
ENSG000002	RNU6-1036P	snRNA	0	0	0	0
ENSG000002	RNU1-109P	snRNA	0	0	0	0
ENSG000002	SNORD116-1	snoRNA	0	11.6681708	0.33988971	0
ENSG000002	Y_RNA	misc_RNA	0	0	0	0
ENSG000002	Y_RNA	misc_RNA	0	0	0	0
ENSG000002	SNORA56	snoRNA	0	0	0	0
ENSG000002	RNU6-442P	snRNA	0	0	0	0
ENSG000002	RNY1P8	misc_RNA	0	0	0	0
ENSG000002	RNU1-73P	snRNA	0	0	0	0
ENSG000002	Y_RNA	misc_RNA	0	0	0	0
ENSG000002	RNU6-723P	snRNA	0	0	0	0
ENSG000002	RNU6-1040P	snRNA	0	0	0	0
ENSG000002	RNU1-11P	snRNA	121.748049	176.953398	85.4406953	159.144629
ENSG000002	RNU6-128P	snRNA	0	1.00218871	0.89346605	0
ENSG000002	Y_RNA	misc_RNA	0	0	0	0
ENSG000002	Y_RNA	misc_RNA	0	1.03821701	0	0
ENSG000002	RNU6-1227P	snRNA	0	0	0	0
ENSG000002	RNU6-1080P	snRNA	0	0	0	0.90557412
ENSG000002	RNU6-147P	snRNA	0	0	0	0
ENSG000002	Y_RNA	misc_RNA	0	0	0	0

ENSG000002	RNU6-26P	snRNA	0	1.00232888	0	0.90557412
ENSG000002	Y_RNA	misc_RNA	0	0	0	0
ENSG000002	Y_RNA	misc_RNA	0	0	0	0
ENSG000002	RNU6-444P	snRNA	0	0	0	0
ENSG000002	RNU6-133P	snRNA	0	0	0	0
ENSG000002	Y_RNA	misc_RNA	0	0	0	0
ENSG000002	RNU6-546P	snRNA	0	0	0	0
ENSG000002	Y_RNA	misc_RNA	0	0	0	0
ENSG000002	Y_RNA	misc_RNA	2.67723753	1.03821701	0.88151049	0.92323821
ENSG000002	RNU6-1074P	snRNA	0	0	0	0.91949833
ENSG000002	RNU6-1056P	snRNA	0	0	0	0
ENSG000002	RNU6-756P	snRNA	0	0	0	0
ENSG000002	RNU6-1027P	snRNA	0	0	0	0
ENSG000002	RNU6-259P	snRNA	0	0	0	0
ENSG000002	Y_RNA	misc_RNA	0.88879741	0	0	0.91949833
ENSG000002	RNU6-1126P	snRNA	0	0	0	0
ENSG000002	RNU6-468P	snRNA	0	0	0	0
ENSG000002	ENSG000002	snoRNA	0	0	0	0
ENSG000002	RNU6-936P	snRNA	0.90451589	0	0	0
ENSG000002	RNU6-641P	snRNA	1.82736099	1.01480844	0.44673318	0
ENSG000002	Y_RNA	misc_RNA	0	0	0	0
ENSG000002	Y_RNA	misc_RNA	0	0	0	0
ENSG000002	Y_RNA	misc_RNA	0	0	0	0
ENSG000002	Y_RNA	misc_RNA	0	0	0	0
ENSG000002	RNU6-484P	snRNA	4.52257988	3.38644735	0.89346605	4.52787014
ENSG000002	RNU6-620P	snRNA	0.87856682	1.03539059	1.80484856	1.89028427
ENSG000002	RNU6-643P	snRNA	0	0	0	0
ENSG000002	RNU6-142P	snRNA	0	0	0	0
ENSG000002	RNU6-245P	snRNA	0	0	0	0
ENSG000002	RNU6-1083P	snRNA	0	0	0	0
ENSG000002	Y_RNA	misc_RNA	0	0	0	0
ENSG000002	RNU6-1323P	snRNA	25.8777271	19.9442666	17.0091384	32.2340064
ENSG000002	SNORD101	snoRNA	3.6335766	8.28193833	4.48648468	8.45794799
ENSG000002	SNORA30	snoRNA	0.93627601	2.95232148	4.39862002	2.69828993
ENSG000002	Y_RNA	misc_RNA	1.81893307	2.08950574	1.77638895	0
ENSG000002	RNU6-317P	snRNA	0	0	0	0.97873388
ENSG000002	RNU6-787P	snRNA	0	0	0	0
ENSG000002	SNORA6	snoRNA	74.0689101	134.832875	22.5386554	84.6986844
ENSG000002	U3	snoRNA	0	0	0	0
ENSG000002	RNU6-418P	snRNA	0	0	0	0
ENSG000002	RNU6-10P	snRNA	9.80191374	6.74951692	4.12014311	5.1418242
ENSG000002	RNU6-152P	snRNA	1.80903178	0	0	0
ENSG000002	RNU6-203P	snRNA	0	0	0	0
ENSG000002	RNU6-435P	snRNA	0	0	0	0

ENSG000002	RNU6-949P	snRNA	0	0	0	0
ENSG000002	Y_RNA	misc_RNA	2.21799083	2.46892132	0.88164333	0.92693649
ENSG000002	RNU6-116P	snRNA	2.95438287	1.02378407	1.03459994	1.21511957
ENSG000002	Y_RNA	misc_RNA	0	0	0	0
ENSG000002	RNU6-44P	snRNA	43.9400544	58.165473	4.99122263	34.4110669
ENSG000002	RNU6-211P	snRNA	0.912041	0	0	0
ENSG000002	SNORD37	snoRNA	7.67111319	3.32623348	2.39708709	5.30898877
[truncated: 429,914 more chars]
