# Supplementary material for: Integrative Transcriptomic and Network Analysis of Shared Osteo-Immune Regulatory Programs in Postmenopausal Osteoporosis and Osteosarcoma Within Central Mexican Cohorts
Source: Curr Issues Mol Biol. 2026 Jul 22;48(7):747. doi: 10.3390/cimb48070747 (PMC13409484; doi:10.3390/cimb48070747)
Supplement: Supplementary file 1 [file Table S2 processed OS RNA-seq expression matrix.pdf › Table S2 processed OS RNA-seq expression matrix.pdf]

Transcript	Gene_ID	Gene_name	ID_1	ID_2	ID_3	ID_4
ENSG000000	ARF5	protein_codin	2.69948663	11.1198092	13.397959	0
ENSG000000	M6PR	protein_codin	42.3421399	13.4114939	87.2284643	24.499496
ENSG000001	ESRRA	protein_codin	0	0	5.1988156	6.14650331
ENSG000000	FKBP4	protein_codin	34.4125064	110.056228	813.545826	25.8845762
ENSG000000	CYP26B1	protein_codin	6.56853129	28.056357	16.3491503	9.15341603
ENSG000000	NDUFAF7	protein_codin	9.94466139	12.7722843	14.7089189	8.03240408
ENSG000000	FUCA2	protein_codin	20.5842875	21.6684934	37.5642162	21.7508174
ENSG000000	DBNDD1	protein_codin	0	0	0	0
ENSG000000	HS3ST1	protein_codin	1.24114	6.48986427	10.9589064	7.11079547
ENSG000000	SEMA3F	protein_codin	2.03191676	15.3482097	0	0
ENSG000000	CFTR	protein_codin	0	0	0	0
ENSG000000	CYP51A1	protein_codin	0.73275335	33.1392281	45.3097964	0
ENSG000000	USP28	protein_codin	17.9791962	0	2.56544159	0
ENSG000000	STPG1	protein_codin	0	2.36639281	0	0
ENSG000000	NIPAL3	protein_codin	4.8958286	17.4226764	6.93456186	6.52848928
ENSG000000	TMEM176A	protein_codin	5.92474262	19.2348443	0	0.95589993
ENSG000000	SLC7A2	protein_codin	0	0	0	3.2033183
ENSG000000	HSPB6	protein_codin	10.6387581	2.65321729	0	3.28823357
ENSG000000	ZNF195	protein_codin	0	0	0	0
ENSG000000	PDK4	protein_codin	182.503245	115.142605	33.3323491	187.221425
ENSG000000	CCL26	protein_codin	0	0	0	0
ENSG000000	USH1C	protein_codin	0	1.12649327	0	0.69771146
ENSG000000	RALA	protein_codin	26.7353482	49.0323065	43.5792119	25.0799145
ENSG000000	BCAP29	protein_codin	29.0627601	38.4607233	27.9931381	22.6640705
ENSG000000	BAIAP2L1	protein_codin	2.83204687	14.8502869	84.5223741	4.7184376
ENSG000000	CACNG3	protein_codin	0.22512585	1.20662035	0	0
ENSG000000	TMEM132A	protein_codin	0	11.725625	13.527361	2.20280204
ENSG000000	DVL2	protein_codin	3.47586409	8.47353333	0	1.80122086
ENSG000000	GGCT	protein_codin	0	21.5991554	2.1489635	0
ENSG000000	RPAP3	protein_codin	14.3667525	30.7294892	46.1784872	5.00047518
ENSG000000	IFRD1	protein_codin	0	0	0	0
ENSG000000	UPP2	protein_codin	0	1.74045382	0	0
ENSG000000	PRSS21	protein_codin	0	0	0	0
ENSG000000	HOXA11	protein_codin	0.81183471	8.92326672	0	0
ENSG000000	CX3CL1	protein_codin	18.6411075	28.9404811	4.09386837	16.0333916
ENSG000001	PRR5	protein_codin	1.50583344	0	4.69849581	0
ENSG000000	TRAPPC6A	protein_codin	0	0	2.73514791	0
ENSG000000	SPATA20	protein_codin	8.23220526	29.1204933	34.1461262	15.6121935
ENSG000000	CEACAM7	protein_codin	0	0	0	0
ENSG000000	CD79B	protein_codin	0	0	0	0
ENSG000000	RHBDD2	protein_codin	0	0	0	0
ENSG000000	RPUSD1	protein_codin	0.68310254	0	1.02754918	1.74483167
ENSG000000	TSR3	protein_codin	22.0585223	52.6123073	57.3832166	14.4027973

ENSG000000	OSBPL7	protein_codin	4.48926655	13.3895217	3.40607962	0
ENSG000000	ARHGAP33	protein_codin	0	14.1110203	0	0
ENSG000000	NDUFAB1	protein_codin	11.8862708	11.0109532	31.6992337	4.90071916
ENSG000000	YBX2	protein_codin	0	0	3.84875283	0.51107609
ENSG000000	PDK2	protein_codin	0	2.07074267	0	0
ENSG000000	ITGA3	protein_codin	0	0	0	0
ENSG000000	KRT33A	protein_codin	0	0	0	0.9310865
ENSG000000	LRRC23	protein_codin	0	0	7.91370375	0.84228627
ENSG000000	IL32	protein_codin	0	12.6287643	0	3.21035615
ENSG000000	TFAP2D	protein_codin	0	1.98820883	0	1.12514632
ENSG000000	SPRTN	protein_codin	2.28405414	3.80744026	5.26459151	0
ENSG000000	CRY1	protein_codin	15.8830433	13.145478	12.1666947	15.7979096
ENSG000000	MAPK8IP2	protein_codin	0	0	0	0.81591219
ENSG000000	PGLYRP1	protein_codin	0	0	0	0
ENSG000000	STARD3NL	protein_codin	7.32347166	45.5821304	7.69813785	0
ENSG000000	CAMK1G	protein_codin	0	0	0	0.40542846
ENSG000000	CD9	protein_codin	12.2378619	34.1621001	89.595568	11.2340109
ENSG000000	CD74	protein_codin	115.491389	1807.69621	7.32681917	138.357525
ENSG000000	RPS20	protein_codin	159.807499	251.702913	286.223679	175.792113
ENSG000000	DYRK4	protein_codin	0	0	0	0
ENSG000000	FAM76A	protein_codin	0	0	0	0
ENSG000000	MGST1	protein_codin	0	5.25318208	0	0
ENSG000000	CPA1	protein_codin	0	0	0	0
ENSG000000	SYPL1	protein_codin	19.4811884	0	45.3410405	13.295712
ENSG000000	RANBP9	protein_codin	51.8958349	185.031731	147.474188	58.9278986
ENSG000000	CD4	protein_codin	31.4723217	106.82017	5.81023103	41.8995854
ENSG000000	PLEKHG6	protein_codin	0	0	0	0
ENSG000000	SS18L2	protein_codin	0	0	0	3.40571508
ENSG000000	VPS13D	protein_codin	0	5.66010437	0	0
ENSG000000	TSPAN9	protein_codin	0	48.5091481	36.9876502	20.0176677
ENSG000001	CYP4F2	protein_codin	0	0	0	1.11910698
ENSG000000	QPCTL	protein_codin	0	12.2458563	0	0
ENSG000000	HIVEP2	protein_codin	4.87065564	436.477547	0	0
ENSG000000	PPP5C	protein_codin	30.2419074	53.3179382	107.855395	17.5256484
ENSG000002	NME1	protein_codin	0	0	0	0.69221002
ENSG000000	UBR7	protein_codin	37.2696607	93.2052975	172.114611	32.841568
ENSG000000	MAP4K5	protein_codin	22.7793179	0	47.7914787	0
ENSG000002	INMT	protein_codin	8.80799253	7.58397361	0	2.57586829
ENSG000000	ERCC1	protein_codin	0	0	0	0
ENSG000000	ABHD5	protein_codin	0	4.47253303	0	0
ENSG000000	GPRC5A	protein_codin	0.25058246	0	3.58780229	12.8021384
ENSG000000	HEBP1	protein_codin	11.6317811	44.962238	22.4681474	13.8705931
ENSG000000	DNASE1L1	protein_codin	0	0	1.37136945	0
ENSG000000	COX15	protein_codin	4.87493142	2.71467799	3.50098397	3.82967046

ENSG000000	MS4A12	protein_codin	0	0	0	0
ENSG000000	RGPD5	protein_codin	0	0	0	154.254799
ENSG000000	XYLT2	protein_codin	10.7095697	41.5003963	30.1891254	0
ENSG000000	FTSJ1	protein_codin	0	0	0	8.66001245
ENSG000000	SCTR	protein_codin	0	0	0	0
ENSG000000	RALBP1	protein_codin	33.2825303	0	0	0
ENSG000000	PSD	protein_codin	0	0	0	0
ENSG000000	SYT13	protein_codin	0	4.15699994	5.75088932	0.43625104
ENSG000000	SNAI2	protein_codin	95.7322139	104.655557	91.6343692	52.0128357
ENSG000000	VDAC3	protein_codin	15.6434272	56.3229233	124.659147	8.95669578
ENSG000000	SLC7A9	protein_codin	0	0	0	0
ENSG000000	GABRA1	protein_codin	0	0	0	0.43986197
ENSG000000	RTF2	protein_codin	8.30314902	0	9.51221969	0
ENSG000000	SLC45A4	protein_codin	1.10508481	7.64278181	0	1.54748495
ENSG000000	RB1CC1	protein_codin	16.5985184	0	129.213135	0
ENSG000000	AKAP11	protein_codin	160.123184	244.714923	235.900951	91.5946626
ENSG000000	STRAP	protein_codin	16.0353143	55.3213096	40.5239889	0
ENSG000000	PIGQ	protein_codin	0	0	8.24511151	0
ENSG000000	CDH17	protein_codin	0	0	0	1.2860771
ENSG000000	RNASET2	protein_codin	0.92311073	55.4172811	10.7948608	0
ENSG000000	B4GALT7	protein_codin	3.84871341	0	17.483044	0
ENSG000000	SLC39A9	protein_codin	2.08821638	0	0	0
ENSG000000	FAM13B	protein_codin	45.0803273	149.059986	36.2156417	21.2108935
ENSG000000	RANBP3	protein_codin	6.60662081	15.9194652	24.9957074	4.3905484
ENSG000000	CHPF2	protein_codin	19.4348835	13.4804046	16.9490177	10.4227572
ENSG000000	GABARAPL2	protein_codin	30.9370403	61.4131072	42.7504466	26.3814071
ENSG000000	MYOC	protein_codin	0.70240524	0	0	3.1213596
ENSG000000	FAM136A	protein_codin	3.50922758	46.4510746	72.3987195	9.79577781
ENSG000000	NSMAF	protein_codin	23.6246844	44.0776703	26.4847889	6.75143273
ENSG000000	OTC	protein_codin	0	0	0	0
ENSG000000	TTC17	protein_codin	40.7786324	0	31.6431013	0
ENSG000000	HOXC8	protein_codin	25.6925856	94.8795439	37.6647225	5.89394668
ENSG000000	MRI1	protein_codin	0	0	0	6.03402123
ENSG000000	BOD1L1	protein_codin	139.70334	286.66591	199.84595	73.5938369
ENSG000000	TARBP1	protein_codin	21.7611448	45.1627491	83.8211682	18.2427138
ENSG000000	RIPOR1	protein_codin	0	15.7542388	8.52959745	0
ENSG000000	BEST2	protein_codin	0	0	0	0.85935166
ENSG000000	RTN4R	protein_codin	0	0	0	0
ENSG000000	PSMA4	protein_codin	0	4.67091131	3.18375089	4.47325715
ENSG000000	RIPOR3	protein_codin	2.19069933	15.262131	36.7775906	0
ENSG000000	SPATA7	protein_codin	0	0	0	0
ENSG000000	ZPBP	protein_codin	0	0	0	0
ENSG000000	CTNS	protein_codin	3.70854023	13.9653898	1.10272164	0.6604881
ENSG000000	LCP2	protein_codin	2.37292727	35.4045594	4.0380688	19.8129978

ENSG000000	GOPC	protein_codin	64.3202193	101.155659	162.4468	38.7339617
ENSG000000	DCN	protein_codin	11.6899082	35.4489267	2.37952687	5.41478632
ENSG000000	TNFRSF17	protein_codin	0	5.58729232	0	0
ENSG000000	MRPS10	protein_codin	28.3897316	24.1241382	28.5365382	7.24322273
ENSG000000	GRN	protein_codin	22.8286182	190.724556	8.57841898	67.2261851
ENSG000000	THAP3	protein_codin	5.80537994	14.606896	20.5996385	2.72773678
ENSG000000	VAMP3	protein_codin	26.5195714	74.3787584	30.6435714	28.6601323
ENSG000000	UTS2	protein_codin	0	0	2.29113882	0
ENSG000000	RCN1	protein_codin	134.937574	209.02278	149.726892	82.963945
ENSG000000	RFC2	protein_codin	0	0	9.1311036	1.69642604
ENSG000000	PPP1R3F	protein_codin	0	11.1804577	2.15337778	3.26258794
ENSG000000	NEXMIF	protein_codin	0	0	0	2.13845396
ENSG000000	ARHGEF5	protein_codin	11.0000151	22.1688775	23.5244466	14.9762336
ENSG000000	NFE2L3	protein_codin	3.86457021	43.0841593	16.3462962	3.98767777
ENSG000000	TNIP3	protein_codin	0	0	0	0
ENSG000000	SIKE1	protein_codin	43.1944217	44.0770135	0	18.7042291
ENSG000000	TLL1	protein_codin	2.12023392	2.14990965	1.42117314	11.9813805
ENSG000000	CBLN4	protein_codin	16.664401	29.0708646	0	4.64437489
ENSG000000	CLDN11	protein_codin	0	0	0	0
ENSG000000	FAM168A	protein_codin	0	0	0	6.07775572
ENSG000000	RELT	protein_codin	4.85005229	10.0537448	14.9865076	13.3919143
ENSG000000	CDC27	protein_codin	39.5129882	69.8042989	29.7294426	49.718056
ENSG000000	PKP2	protein_codin	0	0	0	0
ENSG000001	IL1RAP	protein_codin	6.89125251	0	5.60788606	2.91113061
ENSG000000	YIPF1	protein_codin	5.17510049	11.9238037	13.2693715	6.07345982
ENSG000001	ADCK2	protein_codin	0	10.1770289	0	2.1113535
ENSG000000	SLC2A3	protein_codin	0	0	11.7867779	0
ENSG000001	ENPP2	protein_codin	65.9199407	0	205.296539	24.2393043
ENSG000000	CTDP1	protein_codin	0	33.6230727	2.78657655	9.97442633
ENSG000000	STYK1	protein_codin	0	3.08752224	0.44736094	2.09663704
ENSG000000	GNA11	protein_codin	25.1489298	100.644426	96.6526058	18.6707207
ENSG000000	CREB3L3	protein_codin	0.30832301	1.52616308	0	0.41480058
ENSG000000	PIGV	protein_codin	0	0	0	0
ENSG000000	HDAC7	protein_codin	0	0	0	18.3599862
ENSG000000	MRPS35	protein_codin	40.4084251	60.0753981	138.753353	12.2660793
ENSG000000	BTN3A1	protein_codin	0	0	0	0
ENSG000000	APPBP2	protein_codin	24.418954	10.5725853	94.4349634	18.3587342
ENSG000000	RPL18	protein_codin	0	69.4801254	0	9.04200782
ENSG000000	CA11	protein_codin	1.10037809	18.5064946	10.1996592	2.45008463
ENSG000000	ISOC2	protein_codin	5.81495182	0	2.39798847	0
ENSG000000	EPN1	protein_codin	5.47727081	24.5383893	16.5830145	9.66624717
ENSG000000	CD22	protein_codin	0	0	0	3.64315889
ENSG000000	GSC2	protein_codin	0.26108507	4.01803382	0	1.2683489
ENSG000000	ZFY	protein_codin	5.35753425	0	0	0

ENSG000000	HDHD5	protein_codin	0	3.16226427	4.86885546	1.9953186
ENSG000000	KCNQ1	protein_codin	0	0	0	0
ENSG000000	TRIB2	protein_codin	58.481615	39.9168044	103.273139	36.3051614
ENSG000000	OTUD5	protein_codin	8.43905294	0	0	0
ENSG000000	GPKOW	protein_codin	20.3806122	47.203209	86.9231796	10.7173901
ENSG000000	AQR	protein_codin	32.4657826	46.992034	57.6798349	15.7739225
ENSG000001	KLK13	protein_codin	0	0	0	0
ENSG000001	KLK14	protein_codin	0	0	0	0
ENSG000000	ST6GALNAC1	protein_codin	0	0	0	0
ENSG000000	MBD3	protein_codin	5.05037988	20.0247754	16.2351413	6.04236736
ENSG000000	LMCD1	protein_codin	4.53190365	26.403718	4.8982142	25.3466514
ENSG000000	PSMC4	protein_codin	19.7945724	7.58607929	32.3547178	20.9226074
ENSG000000	FNDC8	protein_codin	0	0	0	1.95089577
ENSG000000	CAMKK1	protein_codin	0	2.37318632	0	0
ENSG000000	FAM50A	protein_codin	0	1.69340024	0	0
ENSG000000	ACAP1	protein_codin	0.33704929	32.2557203	0	0.826279
ENSG000000	DERL2	protein_codin	8.89420379	29.2158131	14.2684545	15.8008787
ENSG000000	NOX3	protein_codin	0	0	0	0.44126212
ENSG000000	ANO8	protein_codin	7.00283087	39.4267249	17.5250727	5.92764494
ENSG000001	KDM4B	protein_codin	3.41769336	15.4298978	1.81174235	0
ENSG000000	PANX2	protein_codin	0.48602713	0	2.85572399	0
ENSG000000	ICAM3	protein_codin	0	23.8229199	0	0
ENSG000000	CAMSAP3	protein_codin	0	0.75535675	0.46631355	0
ENSG000000	CTTNBP2	protein_codin	1.35099196	0	0	0
ENSG000000	ACTL6B	protein_codin	0	0	0	0
ENSG000001	FAM135B	protein_codin	0	0	0	0
ENSG000000	CIC	protein_codin	0	8.39645494	0	0
ENSG000000	KIF22	protein_codin	0	0	0	0
ENSG000000	PRSS22	protein_codin	0	0	0	0
ENSG000000	CEACAM1	protein_codin	0	0	0	0
ENSG000000	YTHDC2	protein_codin	18.5027571	70.769152	96.5100629	27.1486635
ENSG000000	TMEM161A	protein_codin	0	0	0	0
ENSG000000	BCAR1	protein_codin	6.48813173	5.90702757	0	1.42141943
ENSG000000	FOXJ2	protein_codin	22.668937	114.127695	96.2280082	35.572482
ENSG000000	TNFRSF1A	protein_codin	11.9095881	0	12.8287551	24.5249571
ENSG000000	GOLGA5	protein_codin	72.2945731	107.541562	188.962916	38.4380485
ENSG000000	METTL22	protein_codin	2.70595253	10.5551686	27.6393242	0
ENSG000000	CELSR3	protein_codin	1.08125284	13.8064408	4.13201844	2.43581182
ENSG000000	PPP2R5B	protein_codin	7.33579379	24.5284415	15.1416597	6.25164077
ENSG000000	PYGM	protein_codin	33.4859656	0	0	0
ENSG000000	BCL3	protein_codin	2.07902651	20.1426879	0	18.2117446
ENSG000000	KCNAB2	protein_codin	0	0	0	0
ENSG000000	PIGB	protein_codin	1.4305782	0	0	0
ENSG000000	PDZD4	protein_codin	6.94676109	11.2650541	0	0

ENSG000001	NPIPB4	protein_codin	0	0	0	0
ENSG000000	PRLH	protein_codin	0	0	0	0
ENSG000000	REEP1	protein_codin	6.25145002	0	0	0
ENSG000000	FSTL3	protein_codin	0	0	6.03701904	2.81372787
ENSG000000	EPHA8	protein_codin	0	0	1.05592747	0
ENSG000000	TRIP13	protein_codin	3.15267967	0	290.307079	2.16023743
ENSG000000	P4HA2	protein_codin	26.3688693	22.6391429	47.1818368	15.7218431
ENSG000000	VASH1	protein_codin	22.7054475	169.634009	42.535371	29.7762166
ENSG000000	PDCD2	protein_codin	0	0	0.83750028	0
ENSG000000	LLGL2	protein_codin	0	0	0	0
ENSG000001	KRT14	protein_codin	0	0	0.83169205	0
ENSG000001	KRT20	protein_codin	0.33169786	0	0	2.34342165
ENSG000000	ARHGEF10L	protein_codin	0	15.0135977	0	0
ENSG000000	SPP2	protein_codin	0	0	0	0
ENSG000000	HSD17B10	protein_codin	5.43219141	18.9718806	35.7728075	0
ENSG000000	FGF4	protein_codin	0.28241968	0	0	0.97179573
ENSG000000	RGS11	protein_codin	0.84733142	0	0	2.97466051
ENSG000000	NMRK2	protein_codin	0	0	0	0
ENSG000001	MASP1	protein_codin	0	0	0	1.05139636
ENSG000000	ST6GAL1	protein_codin	0	0	0	0
ENSG000000	TIMM21	protein_codin	6.73200685	0	29.4400119	3.24360755
ENSG000000	BPIFB2	protein_codin	0	0	0	0
ENSG000000	REXO1	protein_codin	18.4422418	73.625817	110.14268	20.9033948
ENSG000000	MKRN2	protein_codin	6.87044185	0	27.563057	0
ENSG000000	GPATCH1	protein_codin	24.8397604	30.8001034	55.3796971	7.93577681
ENSG000000	IL4R	protein_codin	0	0	4.1410523	33.7898315
ENSG000000	KEAP1	protein_codin	15.9201337	0	26.8953322	5.29288812
ENSG000000	P2RY10	protein_codin	3.83407607	37.6107184	0	4.54404024
ENSG000000	TNS1	protein_codin	28.9719801	70.4506901	0	56.5450723
ENSG000000	NGFR	protein_codin	13.6965259	43.768265	3.63557948	66.5480144
ENSG000000	NTN1	protein_codin	2.18392317	0.78188175	13.6998841	1.76932081
ENSG000000	ISOC1	protein_codin	2.65228733	7.87383208	21.4335536	4.95640823
ENSG000000	KLF6	protein_codin	0	0	0	0
ENSG000000	TRO	protein_codin	3.80217316	0	0	0
ENSG000000	MNT	protein_codin	15.742162	81.6697598	40.2721092	16.2502363
ENSG000000	LAPTM4A	protein_codin	65.8444617	103.450192	75.3469339	70.7527387
ENSG000000	ADAM7	protein_codin	0	0	0	0
ENSG000000	ASNS	protein_codin	2.73931802	0	0	0
ENSG000000	PTPN18	protein_codin	7.77467462	31.7635289	22.5928588	12.3022544
ENSG000000	DRD4	protein_codin	0	0	0	0.43984828
ENSG000000	SCT	protein_codin	0	0.36399537	0	0
ENSG000000	ALDH3A2	protein_codin	0	0	0	0
ENSG000000	STK10	protein_codin	58.1981514	471.095041	133.169902	90.2914892
ENSG000000	ALPK1	protein_codin	0	0	0	0

ENSG0000000	TBX21	protein_codin	0.22597506	14.4921167	0	5.09204374
ENSG0000000	MRPS34	protein_codin	4.66253089	14.8528772	46.1788956	0
ENSG0000000	CA12	protein_codin	0.44310977	1.80138804	28.5126177	5.76283783
ENSG0000001	MAP2K5	protein_codin	1.84042282	7.74499131	1.26184596	1.05277801
ENSG0000000	TIGAR	protein_codin	13.1180846	17.1454331	17.1029488	6.16062227
ENSG0000000	FGF20	protein_codin	0	0	0	0
ENSG0000000	MTMR7	protein_codin	0	0	0	1.53916973
ENSG0000000	CPB2	protein_codin	0	0	0	0
ENSG0000000	FAM107B	protein_codin	0	0	0	0
ENSG0000000	CDK13	protein_codin	12.902206	31.5971735	15.2988008	14.7844806
ENSG0000000	TSPAN32	protein_codin	0	0	0	0
ENSG0000000	FAR2	protein_codin	0	0	0	0
ENSG0000000	TRAM2	protein_codin	35.6635862	105.498873	48.782868	42.9485282
ENSG0000000	CLDN18	protein_codin	0	0	0	2.44922486
ENSG0000000	ROPN1	protein_codin	0.35050886	0	0	0
ENSG0000000	NDUFB4	protein_codin	4.19024526	0	15.9969724	7.95384587
ENSG0000000	HEATR6	protein_codin	10.1735206	24.3220658	99.0203249	7.45504076
ENSG0000000	ERLEC1	protein_codin	52.9054783	32.5148043	0	23.5360348
ENSG000001	CLIC5	protein_codin	0	0	0	0
ENSG0000000	TMEM131	protein_codin	63.7194053	332.156051	203.028234	60.263357
ENSG0000001	ARPP21	protein_codin	1.31426969	0	0	0
ENSG0000000	CEACAM21	protein_codin	0.70795077	0	0	0
ENSG0000000	TMEM38A	protein_codin	4.39912781	3.58150615	2.60076968	0.42467825
ENSG0000000	PVR	protein_codin	0	0	0	0
ENSG0000001	ZNF222	protein_codin	0	0	8.42310384	1.3086251
ENSG0000001	PSG6	protein_codin	0	0	0	0
ENSG0000000	ACTR6	protein_codin	6.429309	28.638316	30.6234145	0
ENSG0000000	SLC25A3	protein_codin	0	0	13.7398159	0
ENSG000000	NR1H4	protein_codin	0	0	0	0
ENSG0000000	FAP	protein_codin	83.0698774	39.1420294	27.826373	18.730713
ENSG0000000	NFKB2	protein_codin	0	0	4.46252844	0
ENSG0000000	MUSK	protein_codin	0	0	0	2.83819631
ENSG0000000	DMRT3	protein_codin	0.543235	0	7.85448538	1.22241499
ENSG0000000	OSBPL6	protein_codin	0	17.2718317	0	2.16965227
ENSG0000000	CCN5	protein_codin	3.46681395	0	0	0
ENSG0000000	CTSA	protein_codin	0	0	0	0
ENSG0000001	ANKRD16	protein_codin	0	0	0	0
ENSG0000001	GAL3ST2	protein_codin	0	0	0	1.12301636
ENSG0000000	BLTP3A	protein_codin	20.0722496	99.7057443	85.1467153	21.4398137
ENSG0000000	OSTM1	protein_codin	0	0	0	8.33345968
ENSG0000000	IMPG2	protein_codin	3.61687419	11.7845667	3.11888305	4.16570657
ENSG0000000	ACTN1	protein_codin	0	266.354026	39.2627971	57.7488979
ENSG0000002	NAIP	protein_codin	0	0	0	2.28258738
ENSG0000000	DELE1	protein_codin	0	0	12.1956783	5.14128463

ENSG000000	SLC13A1	protein_codin	0	0	0	0
ENSG000000	PCDHB4	protein_codin	3.86167457	8.15575698	4.47192923	5.75485809
ENSG000001	PCDHB2	protein_codin	13.8363838	14.9775396	0	4.10080998
ENSG000000	IFT25	protein_codin	8.30145391	11.9408528	19.6406494	3.33311036
ENSG000000	STRADB	protein_codin	0	2.38606164	14.1757851	13.2195276
ENSG000000	FAM135A	protein_codin	0	0	0	0
ENSG000000	DLG3	protein_codin	0	0	0	0
ENSG000000	SEMA5B	protein_codin	0.74601747	0	0	0
ENSG000000	SNAP91	protein_codin	0	0	0	0
ENSG000000	PLOD1	protein_codin	81.1405365	113.106212	243.535444	42.7381595
ENSG000000	TDRD3	protein_codin	0	5.99288933	0	0
ENSG000000	OXCT1	protein_codin	45.8506297	43.8016623	104.964807	28.7733046
ENSG000000	ZNF324	protein_codin	8.96910491	0	17.4697059	7.66079019
ENSG000000	ZNF416	protein_codin	2.00841538	11.5557974	4.37653841	3.01446035
ENSG000000	RPS5	protein_codin	85.5495577	84.1129368	265.1038	52.6539824
ENSG000000	FAM234B	protein_codin	4.25954054	32.1152384	22.4113948	5.9885145
ENSG000000	PILRA	protein_codin	1.59894252	11.6549597	2.78399744	0.44730621
ENSG000000	RRN3	protein_codin	30.717368	40.9504699	147.276652	40.377532
ENSG000001	MOGAT2	protein_codin	0	0	0	0
ENSG000000	CHERP	protein_codin	0	0	22.2678575	13.0680296
ENSG000001	AQP2	protein_codin	0	0	0	1.31059423
ENSG000000	DIMT1	protein_codin	0	0	1.27677649	1.31206337
ENSG000000	EIF2AK1	protein_codin	77.13589	176.236963	301.371107	39.0447871
ENSG000000	NME8	protein_codin	2.25749426	0	0	0
ENSG000000	EPDR1	protein_codin	31.3926129	49.1489492	61.586666	19.7148204
ENSG000000	MRPL28	protein_codin	2.46391902	2.14869015	14.1453992	0.7225748
ENSG000000	HBQ1	protein_codin	0	0	0.34061595	0
ENSG000000	CEACAM6	protein_codin	0	0	0	0.51552454
ENSG000000	RBM22	protein_codin	15.2121317	69.5423517	19.1206457	31.5902784
ENSG000000	HSD17B2	protein_codin	0	0	0	0.6727666
ENSG000000	MAP2	protein_codin	0	6.03668228	3.03401945	0
ENSG000000	ZW10	protein_codin	11.1214174	0	41.2057161	7.93649514
ENSG000001	ITGB4	protein_codin	0	10.8879495	0	0
ENSG000000	PPP1R15A	protein_codin	0	0	0	309.797498
ENSG000000	TRIP6	protein_codin	9.64829128	41.7156808	36.1136141	10.8626439
ENSG000000	ADAM11	protein_codin	0	0	2.58750104	0.85860128
ENSG000000	LAMP2	protein_codin	19.3239343	20.2816511	17.7328888	9.93141941
ENSG000001	SLC22A4	protein_codin	0.46422049	3.27741581	0.47675699	1.63148945
ENSG000000	CETP	protein_codin	0	0	0	0
ENSG000000	MT3	protein_codin	0	0	0	0
ENSG000000	PTHLH	protein_codin	0	0	0	0
ENSG000000	TFAP2C	protein_codin	0	1.06781667	1.97960451	1.27638401
ENSG000000	SULT2B1	protein_codin	0.17047282	0	0	0
ENSG000001	EPS8L1	protein_codin	0	0	0	0

ENSG000001	CACNB4	protein_codin	0	0	0	0
ENSG000001	NOL4L	protein_codin	0	0	0.90652711	0
ENSG000000	DNMT3B	protein_codin	0	0	0	0
ENSG000000	REM1	protein_codin	1.59112246	8.56969231	0.47381345	1.46837035
ENSG000000	PDRG1	protein_codin	7.16674636	7.39739811	21.573498	2.95451066
ENSG000000	EPB41L1	protein_codin	7.09806772	57.5980966	25.3703573	19.4133983
ENSG000000	PPP1R13B	protein_codin	4.4066241	18.5412875	20.7492788	11.3764788
ENSG000001	TGM6	protein_codin	0	1.10614559	0	0.81454929
ENSG000001	RALGAPA2	protein_codin	54.7745668	69.0218794	46.4228778	77.3552558
ENSG000000	RPL6	protein_codin	511.937073	586.145079	1452.92955	353.597856
ENSG000000	ESF1	protein_codin	0	0	0	0
ENSG000000	SLC8B1	protein_codin	0	10.187875	0	0
ENSG000000	TMEM230	protein_codin	26.45918	66.6717364	35.1421372	14.3968652
ENSG000000	OAS1	protein_codin	5.9515695	0	0	0
ENSG000000	SIRT4	protein_codin	0	0	0	0
ENSG000000	TRMT6	protein_codin	0	0	19.7877568	11.5840517
ENSG000002	HAUS5	protein_codin	0	16.1843629	5.66744034	5.45489208
ENSG000000	UQCRC1	protein_codin	0	0	22.557073	4.66063987
ENSG000000	GMIP	protein_codin	6.24252169	58.8731797	0	6.34039832
ENSG000000	LAG3	protein_codin	0	13.5876346	0	0
ENSG000000	MLF2	protein_codin	3.98027768	33.7764371	61.7567594	8.31157448
ENSG000000	OTUB2	protein_codin	1.92066141	1.45078324	6.25547062	1.73135613
ENSG000000	LTBP4	protein_codin	0	44.0146339	78.6604554	0
ENSG000000	TFAP4	protein_codin	6.26038334	9.69557743	16.187267	3.16081079
ENSG000000	PDCD7	protein_codin	29.8574262	55.6482239	71.3133525	11.7492954
ENSG000000	SPG21	protein_codin	3.26152377	33.5982588	10.5905002	3.32158191
ENSG000000	CHRD	protein_codin	0	0	0	0
ENSG000000	FLT3LG	protein_codin	0	0	0	0
ENSG000000	GNPTG	protein_codin	1.42083496	0	2.75653295	0
ENSG000000	GOLGA3	protein_codin	0	61.2949931	0	0
ENSG000000	CD209	protein_codin	5.51652827	40.2474093	4.52075741	9.56432628
ENSG000000	EFNB1	protein_codin	27.0356133	75.8968865	49.2405877	41.6242707
ENSG000000	GLG1	protein_codin	5.61137819	0	10.0982109	0
ENSG000000	PLEKHG2	protein_codin	0	0	2.80557	9.77636582
ENSG000000	DLL3	protein_codin	0	0	0	1.43228039
ENSG000000	NAT14	protein_codin	1.50514028	1.35673551	2.83143081	0
ENSG000001	AASDH	protein_codin	0	38.647985	0	0
ENSG000000	LAMB4	protein_codin	0	0	0	0
ENSG000000	DLD	protein_codin	14.0345625	11.4446834	16.9141445	5.04570855
ENSG000000	ABCC6	protein_codin	0.30848566	0	3.79332876	1.89128438
ENSG000000	CMTM6	protein_codin	41.0499223	102.155574	49.576031	29.745112
ENSG000000	APOH	protein_codin	0	0	0	0
ENSG000000	SPAG7	protein_codin	33.611556	58.7108437	67.2491034	24.7840171
ENSG000000	ESR1	protein_codin	0	0	0	0

ENSG00000(	RGS17	protein_codin	0	0	0	0
ENSG00000(	TMEM101	protein_codin	12.4090713	25.6332121	37.9836375	4.38158355
ENSG00000(	CCDC80	protein_codin	187.579714	67.6902367	39.3699555	45.8522262
ENSG00000(	CMA1	protein_codin	0	0	0	0
ENSG00000(	PSME1	protein_codin	19.7224468	149.547904	59.7055143	62.2705079
ENSG00000(	HAUS4	protein_codin	0	0	0	0
ENSG00000(	CEBPE	protein_codin	0	0	0	1.22916945
ENSG00000(	OSGEP	protein_codin	4.91225492	0	12.4944196	4.39842159
ENSG00000(	SLC22A17	protein_codin	0	0	0	0
ENSG00000(	G2E3	protein_codin	3.49046251	10.9991616	19.609632	0
ENSG00000(	TGM1	protein_codin	0	0	0.94322428	0
ENSG00000(	TBX15	protein_codin	17.9658993	0	0	0
ENSG00000(	TEKT2	protein_codin	0.69617925	0	0	0
ENSG00000(	UNC13D	protein_codin	1.90933514	14.195949	0	0
ENSG00000(	COMT	protein_codin	0	0	7.17302578	0
ENSG00000(	XYLB	protein_codin	1.6526477	26.2493979	19.0435734	0
ENSG00000(	OR1I1	protein_codin	0	0	0	0
ENSG00000(	ADH7	protein_codin	0	0	0	0
ENSG00000(	ADH1A	protein_codin	0	0	0	0
ENSG00000(	KRT23	protein_codin	0	0	0	0
ENSG00000(	CDC6	protein_codin	0.80687991	9.12556635	7.69204146	5.58049953
ENSG00000(	AAAS	protein_codin	0	0	10.1834499	0
ENSG00000(	CBX5	protein_codin	113.594962	288.10134	589.911204	68.2156443
ENSG00000(	KLHL20	protein_codin	30.6403969	54.1123311	30.0988328	9.73282816
ENSG00000(	FMO2	protein_codin	1.76904701	7.8112528	0	21.1245252
ENSG00000(	DHPS	protein_codin	4.8201455	0	17.0958132	5.78464817
ENSG00000(	RAB26	protein_codin	0	0	2.7642851	0
ENSG00000(	PTBP3	protein_codin	0	0	0	0
ENSG00000(	PSMD5	protein_codin	12.5565878	7.58466025	32.3459089	6.09539435
ENSG00000(	NANS	protein_codin	30.2296379	103.290463	85.3250382	33.3880979
ENSG00000(	SEMA4G	protein_codin	0	0	4.82624928	0
ENSG00000(	TPSD1	protein_codin	0	0	0	1.62382647
ENSG00000(	GSTA3	protein_codin	0	0	0	0
ENSG00000(	MAPK13	protein_codin	5.63371312	21.7476649	12.6745196	10.0657105
ENSG00000(	TMEM14A	protein_codin	4.37901986	12.8955699	20.8044873	1.91241548
ENSG00000(	BAG6	protein_codin	0	0	92.7737166	0
ENSG00000(	PRRT1	protein_codin	0	0	0	0
ENSG00000(	LST1	protein_codin	0	0	0	0
ENSG00000(	ZNF184	protein_codin	0	0	4.79043362	11.9053967
ENSG00000(	VCL	protein_codin	165.581088	238.948666	170.99082	93.1074227
ENSG00000(	SIRT1	protein_codin	27.4000708	67.2291103	65.5523649	39.1169046
ENSG00000(	TGFBR3	protein_codin	29.7889136	76.644507	30.6244447	42.4183308
ENSG00000(	TMED1	protein_codin	1.85510653	0	12.4555043	3.72798639
ENSG00000(	ERMP1	protein_codin	7.00810468	0	4.67112765	0

ENSG00000000	MZF1	protein_codin	0	5.4427047	0	0
ENSG00000000	OCEL1	protein_codin	0	0	2.91948171	0
ENSG00000000	PSMD8	protein_codin	0	0	0	0
ENSG00000000	STX1B	protein_codin	3.64457392	6.22200527	3.12045085	0.8738854
ENSG00000000	BCL7C	protein_codin	1.49505425	11.0964937	20.7338756	2.48776362
ENSG00000000	EFNA2	protein_codin	0.21106971	0	3.24094533	0
ENSG00000000	ATP5F1D	protein_codin	3.07463384	0	0	1.32722022
ENSG00000000	CBARP	protein_codin	0	0	0	0
ENSG00000000	PCDH11Y	protein_codin	0	0	0	0
ENSG00000000	FGF22	protein_codin	0	0	0	0
ENSG00000000	SMIM24	protein_codin	0	0	0	0
ENSG00000000	IGFALS	protein_codin	0	0	0	0
ENSG00000000	MARCHF2	protein_codin	3.06259356	1.25727195	0	4.09696343
ENSG00000000	NDUFB7	protein_codin	14.3637202	51.8858411	38.9123567	9.20237272
ENSG00000000	TECR	protein_codin	6.91450966	0	50.6148061	0
ENSG00000000	TIMM13	protein_codin	3.47319367	26.2027466	25.1731849	1.05143486
ENSG00000000	CDC34	protein_codin	9.85719812	17.6398231	43.3672502	8.03872972
ENSG00000000	MISP	protein_codin	0.56823577	2.71488129	0	1.71923126
ENSG00000000	POLR2E	protein_codin	3.16074989	31.6178509	25.9592129	13.2542659
ENSG00000000	GADD45B	protein_codin	15.7202054	48.7288417	63.632563	472.824898
ENSG00000000	MADCAM1	protein_codin	0	0	0	0
ENSG00000001	MAPK12	protein_codin	0	0	83.0590378	4.9502235
ENSG00000000	SERPIND1	protein_codin	0	0	0	0
ENSG00000000	SNAP29	protein_codin	37.6299918	46.3807269	93.0248848	17.3745963
ENSG00000001	THAP7	protein_codin	0	0	4.49251673	0
ENSG00000000	MMP11	protein_codin	15.9160558	0	15.7416736	4.7464466
ENSG00000002	MIF	protein_codin	15.2997619	20.0233994	386.174105	24.2830254
ENSG00000000	DDTL	protein_codin	0.59265457	1.15626834	3.03571692	0.47108877
ENSG00000000	OSM	protein_codin	0	0	0	0.8125194
ENSG00000000	TBC1D10A	protein_codin	0	0	11.0313643	3.29782912
ENSG00000000	SF3A1	protein_codin	69.9047603	371.292594	199.453749	69.2242161
ENSG00000001	USP18	protein_codin	3.09602628	80.7105085	0	2.41138196
ENSG00000000	RNF215	protein_codin	0	0	0	0
ENSG00000001	SEC14L3	protein_codin	0	0	0	0
ENSG00000001	SNRPD3	protein_codin	17.3241254	54.3643307	44.6388932	17.6665038
ENSG00000001	MAPK1	protein_codin	34.6461973	109.586438	54.4727952	20.7699299
ENSG00000001	TCN2	protein_codin	0	0	5.05232577	2.59685729
ENSG00000001	CRYBB3	protein_codin	0	0	0	0
ENSG00000001	MORC2	protein_codin	9.90548014	0	19.7228775	0
ENSG00000001	SLC25A1	protein_codin	7.01839466	30.4669149	23.2465528	5.9242183
ENSG00000001	PLA2G3	protein_codin	0	0	0	2.3216168
ENSG00000001	LGALS2	protein_codin	1.52816138	12.3983143	0	0
ENSG00000002	PDXP	protein_codin	1.31354981	4.1972909	9.01719839	0
ENSG00000001	ASPHD2	protein_codin	1.10132753	3.66508419	3.37620149	1.23653037

ENSG000001	LGALS1	protein_codin	56.9497183	54.1000603	69.5188011	31.2493112
ENSG000001	PIK3IP1	protein_codin	19.5263108	39.0109998	3.39172908	3.7389348
ENSG000001	SRRD	protein_codin	6.42481802	9.12201093	27.2976328	9.41324127
ENSG000001	PATZ1	protein_codin	0.76519206	2.00509232	6.07373622	1.33489531
ENSG000001	ANKRD54	protein_codin	2.48876278	0	4.50771031	1.93095511
ENSG000001	SNU13	protein_codin	0	0	0	0
ENSG000001	MICALL1	protein_codin	3.81468616	0	8.1115664	5.65473651
ENSG000001	CENPM	protein_codin	0	0	0	0
ENSG000001	KDELR3	protein_codin	14.7177006	15.7075855	24.8856875	18.7295702
ENSG000001	DDX17	protein_codin	199.267856	649.29862	603.365495	69.1238023
ENSG000001	DMC1	protein_codin	0	0	0	1.02622515
ENSG000001	HSCB	protein_codin	0	0	0	0
ENSG000001	CBY1	protein_codin	10.6878329	42.5845084	0	5.77000686
ENSG000001	TOMM22	protein_codin	20.8665696	88.4326667	78.8986716	17.8004003
ENSG000001	RSPH14	protein_codin	0	0	0	0
ENSG000001	XBP1	protein_codin	33.0582678	273.742281	81.381808	43.9730702
ENSG000001	RTCB	protein_codin	27.6316459	47.9462512	62.0147377	22.8259986
ENSG000001	JOSD1	protein_codin	0	37.1015644	0	0
ENSG000001	GTPBP1	protein_codin	17.5410738	64.4645808	50.3298228	29.0096116
ENSG000001	PPP6R2	protein_codin	0	69.832508	6.92325982	0
ENSG000001	DNAL4	protein_codin	3.13946954	7.16897607	14.4183175	3.24729599
ENSG000001	C22orf31	protein_codin	0.33709314	0	1.40853731	0
ENSG000001	MIOX	protein_codin	0	0	0	0
ENSG000001	LMF2	protein_codin	0	0	0	0
ENSG000001	CBX6	protein_codin	0	0	5.33987115	0
ENSG000001	RHBDD3	protein_codin	0	0	5.20891489	0
ENSG000002	APOBEC3D	protein_codin	6.80511826	11.1095742	0	0
ENSG000001	RASL10A	protein_codin	0.35617914	0	1.32374886	1.45174879
ENSG000001	HMGXB4	protein_codin	9.42689849	106.270281	47.9630315	0
ENSG000001	BIK	protein_codin	0	1.35451855	0	0.81822258
ENSG000001	HMOX1	protein_codin	15.472538	44.4362304	5.00933462	39.5198755
ENSG000001	NIPSNAP1	protein_codin	11.5835017	43.4646315	123.423583	5.55023138
ENSG000001	MCM5	protein_codin	11.3290147	35.0400104	59.1026734	1.33976764
ENSG000001	ARSA	protein_codin	1.26748251	19.6981768	2.58510178	5.92140324
ENSG000001	RASD2	protein_codin	1.39585913	2.8340265	1.09121946	0.88946642
ENSG000001	TTLL12	protein_codin	7.69513406	30.7129208	81.2036894	5.05937718
ENSG000001	CBX7	protein_codin	7.81673177	42.097883	3.04148723	7.18354403
ENSG000001	ACR	protein_codin	0	0	0.86548893	1.75572336
ENSG000001	CABP7	protein_codin	0.33229339	0.83547223	0.60003471	3.44678086
ENSG000001	RPL3	protein_codin	509.258275	894.508584	2029.16733	435.179608
ENSG000001	SYNGR1	protein_codin	0	0	0	0
ENSG000001	TAB1	protein_codin	9.74873084	34.3297431	25.1344349	6.42946269
ENSG000001	PNPLA5	protein_codin	0	0	0	0
ENSG000001	PNPLA3	protein_codin	1.52854714	5.37158417	7.06232599	0.86459144

ENSG000001	MYH9	protein_codin	1478.42176	0	1644.94165	1380.17605
ENSG000001	TXN2	protein_codin	12.8213494	38.7614249	15.1351782	8.95569659
ENSG000001	FOXRED2	protein_codin	0	0	0	0
ENSG000001	EIF3D	protein_codin	0	221.843517	45.3463296	26.227099
ENSG000002	ADSL	protein_codin	39.8615319	0	103.320056	13.6446535
ENSG000001	PVALB	protein_codin	0.34420235	0	0	0
ENSG000001	UPK3A	protein_codin	0	0	0	0.8454892
ENSG000001	FAM118A	protein_codin	0	0	0	0
ENSG000001	ST13	protein_codin	145.927844	345.792996	471.140051	116.409073
ENSG000001	IL2RB	protein_codin	0	0	0	0
ENSG000001	RBX1	protein_codin	2.4418325	4.99869876	13.7272361	0
ENSG000001	L3MBTL2	protein_codin	10.404035	30.9742385	37.6199839	6.42136916
ENSG000001	CHADL	protein_codin	3.62062666	4.01725185	0	0
ENSG000001	PHF5A	protein_codin	14.8858292	16.7119606	69.8916019	3.75717585
ENSG000001	ACO2	protein_codin	7.38320161	74.6604966	49.5767821	0
ENSG000001	PMM1	protein_codin	8.02853372	42.6093641	27.5472589	4.52438322
ENSG000001	CERK	protein_codin	20.292226	54.6239451	151.191191	15.1692212
ENSG000001	BRD1	protein_codin	0	0	0	3.70240576
ENSG000001	ZBED4	protein_codin	13.3767648	0	69.6251308	5.96955975
ENSG000001	HDAC10	protein_codin	0	0	0	0
ENSG000001	RIPK3	protein_codin	0	0	0	0
ENSG000000	PAPOLA	protein_codin	40.3933102	81.5309036	125.787252	40.5856042
ENSG000000	HSP90AA1	protein_codin	903.395658	1609.2155	6887.09572	1049.46931
ENSG000000	NID2	protein_codin	45.8535875	115.575733	44.4668609	45.360687
ENSG000000	MARK3	protein_codin	15.6599097	0	0	0
ENSG000000	SNAPC1	protein_codin	46.3280011	96.7392454	142.0128	48.0355635
ENSG000000	SUPT16H	protein_codin	263.374763	947.077456	1632.72441	95.2756372
ENSG000001	ABHD4	protein_codin	0	0	0	0
ENSG000001	FKBP3	protein_codin	0	0	0	0.92987919
ENSG000001	CTSG	protein_codin	2.26453065	4.77207068	0	19.3379139
ENSG000001	GZMH	protein_codin	0.89679873	13.8913317	0	0
ENSG000001	GZMB	protein_codin	0	0	0	0
ENSG000001	PRMT5	protein_codin	0	0	0	0
ENSG000001	COCH	protein_codin	0.34883834	0	0	0
ENSG000001	AP4S1	protein_codin	1.21551347	1.66679285	0	0.73988893
ENSG000001	POLE2	protein_codin	0	0	0	0
ENSG000001	SOS2	protein_codin	46.9203786	17.7921223	58.3100869	36.9736937
ENSG000001	CDKL1	protein_codin	2.3876793	4.8010428	3.17249493	0
ENSG000001	PYGL	protein_codin	15.5930168	34.4595634	63.6111507	17.1566403
ENSG000001	GNPNAT1	protein_codin	8.60491952	24.5967432	27.8132079	10.3506147
ENSG000001	CDKN3	protein_codin	0	0	0	0.54803804
ENSG000001	CNIH1	protein_codin	21.7819035	27.8049868	48.8101879	18.1512673
ENSG000001	CGRRF1	protein_codin	5.25616258	5.84779091	11.5879624	7.26760926
ENSG000001	ATP6V1D	protein_codin	0	0	5.88967921	4.82023092

ENSG000001	CCDC198	protein_codin	0	0	0	0
ENSG000001	PLEK2	protein_codin	0.56633233	0	1.95068344	0
ENSG000001	LRRC74A	protein_codin	0	0	0	0
ENSG000001	PIGH	protein_codin	5.76326677	9.37499759	10.1518045	4.7870518
ENSG000001	PSMA3	protein_codin	4.77850931	43.1137879	26.690957	13.0396774
ENSG000001	VTI1B	protein_codin	26.6166305	12.0379836	157.851139	9.81341874
ENSG000001	TIMM9	protein_codin	0	0	0	0
ENSG000001	GSTZ1	protein_codin	0	0	3.34418507	0
ENSG000001	TMED8	protein_codin	19.1209496	89.1055546	68.7782885	19.4325495
ENSG000001	SAMD15	protein_codin	3.1854733	6.5692216	24.2152016	1.77779277
ENSG000001	AHSA1	protein_codin	17.7908896	0	106.387636	16.1848112
ENSG000001	ISM2	protein_codin	0	0	0	0
ENSG000001	SPTLC2	protein_codin	30.9409885	170.646489	62.776505	34.8997367
ENSG000001	RIN3	protein_codin	16.8517862	79.9584115	18.7174908	25.4869292
ENSG000001	ALKBH1	protein_codin	1.479338	0	12.7563408	0
ENSG000001	CHGA	protein_codin	0	0	0	0
ENSG000001	DHRS7	protein_codin	0	0	0	0
ENSG000001	SIX4	protein_codin	15.0328759	6.04820835	73.3366696	5.82742993
ENSG000001	CEP128	protein_codin	3.92619455	21.6029305	0	2.17087518
ENSG000001	ERH	protein_codin	0	0	0	0
ENSG000001	SLC10A1	protein_codin	0	0	0	0.71806785
ENSG000001	EIF5	protein_codin	2.51227721	11.9287352	10.9427095	128.976714
ENSG000001	SLC8A3	protein_codin	0.49059152	10.7875358	4.44821326	0
ENSG000001	ZFYVE21	protein_codin	4.58332252	8.09755219	10.3628105	2.86299418
ENSG000001	BDKRB1	protein_codin	0	0	0	0
ENSG000001	VRK1	protein_codin	0	0	0	0
ENSG000001	PAPLN	protein_codin	0	0	0	0
ENSG000001	APEX1	protein_codin	38.7591208	0	130.78588	14.0722987
ENSG000001	PABPN1	protein_codin	50.4317479	301.124738	212.290062	38.2976903
ENSG000001	EFS	protein_codin	10.9981794	28.0202026	32.3202611	9.2133694
ENSG000001	ARHGAP5	protein_codin	2.37761831	1.96804759	11.236395	2.13587762
ENSG000001	CINP	protein_codin	0	3.12813164	13.6042595	0
ENSG000001	SRP54	protein_codin	0	0	0	0
ENSG000001	PCK2	protein_codin	2.33857955	22.3143724	19.0065848	2.05199491
ENSG000001	NFKBIA	protein_codin	24.9539556	173.982631	27.3934288	362.533292
ENSG000001	EMC9	protein_codin	0	0	0	0
ENSG000001	PSME2	protein_codin	12.8852511	82.1586378	93.736274	0
ENSG000001	BRMS1L	protein_codin	14.33227	39.8461593	23.2541026	6.19891828
ENSG000001	PNN	protein_codin	266.715222	860.276277	992.71194	249.463775
ENSG000001	RABGGTA	protein_codin	0	0	0	0
ENSG000000	CYP24A1	protein_codin	0	0	0	0
ENSG000001	PTPRA	protein_codin	10.3770358	0	0	0
ENSG000000	NSFL1C	protein_codin	7.74196417	0	0	0
ENSG000000	ZFP64	protein_codin	0	0	16.0138122	0

ENSG000000	SIRPG	protein_codin	0.26944271	0	0	0
ENSG000001	PYGB	protein_codin	30.9137773	88.4639215	26.4934608	19.0537494
ENSG000001	PROCR	protein_codin	9.93294927	5.40586633	8.10159705	8.03545233
ENSG000001	MYBL2	protein_codin	0	15.9774292	121.816148	3.46135942
ENSG000001	R3HDML	protein_codin	0	0	0	0
ENSG000001	PABPC1L	protein_codin	0	0	0	0
ENSG000001	PABPC1L	protein_codin	3.06120766	0	0	4.75607317
ENSG000001	PABPC1L	protein_codin	0	0	0	0
ENSG000001	SALL4	protein_codin	0.33520429	17.8298118	0.509406	2.88253474
ENSG000001	CSTF1	protein_codin	7.52696712	0	9.47259876	3.34317747
ENSG000001	TPD52L2	protein_codin	0	0	0	0
ENSG000001	CTSZ	protein_codin	93.7645672	6.01663302	111.044606	104.650116
ENSG000001	TUBB1	protein_codin	1.35855324	1.98579288	0	2.83645576
ENSG000001	SLCO4A1	protein_codin	0	0	0	0
ENSG000001	TCFL5	protein_codin	10.7822189	2.02796395	20.2313973	2.26329566
ENSG000001	BIRC7	protein_codin	0	0	0	0
ENSG000001	UBOX5	protein_codin	0	0	7.42196343	0
ENSG000001	EEF1A2	protein_codin	0.99294467	0	22.4348848	0
ENSG000001	PTK6	protein_codin	0	0.89337732	0	0
ENSG000001	SRMS	protein_codin	0	0	0	1.56895443
ENSG000001	ADISSP	protein_codin	0	0	0	0
ENSG000001	TRIB3	protein_codin	2.62066324	0	0	0
ENSG000001	CSNK2A1	protein_codin	2.95439588	25.6418012	11.4749432	0
ENSG000001	MACROD2	protein_codin	0	0	0	0
ENSG000001	SLC52A3	protein_codin	0	0	0	0
ENSG000001	RSPO4	protein_codin	0	1.00423043	1.58487209	0.80764027
ENSG000001	PROKR2	protein_codin	1.9297265	1.14595484	1.44469159	1.51142437
ENSG000001	FERMT1	protein_codin	3.18415965	0	0	0
ENSG000001	PDYN	protein_codin	0	0	0	0
ENSG000001	TM9SF4	protein_codin	0	0	0	0
ENSG000001	TLDC2	protein_codin	2.41839076	0	0	0
ENSG000001	SNTA1	protein_codin	11.7851837	23.7129935	27.3973877	8.56339843
ENSG000001	OXT	protein_codin	0.29139236	0	0	0
ENSG000001	PXMP4	protein_codin	0	0	0	0
ENSG000001	CHMP4B	protein_codin	158.345737	67.1278962	245.731934	146.551922
ENSG000001	LBP	protein_codin	0	2.44240984	0	1.57390484
ENSG000001	SLC32A1	protein_codin	0	0	0	0.75387879
ENSG000001	CST4	protein_codin	0	0	0	0
ENSG000001	WFDC2	protein_codin	0	0	0	0
ENSG000001	AHCY	protein_codin	18.5468129	35.338037	104.637593	6.86323597
ENSG000001	SPINT3	protein_codin	0	0	0	0
ENSG000001	PIGU	protein_codin	4.04099075	1.9613956	8.80830854	1.38680871
ENSG000001	ACOT8	protein_codin	2.62946504	0	4.92432584	0
ENSG000001	APMAP	protein_codin	43.0881809	0	64.3996303	13.7856716

ENSG00000009	TXNL1	protein_codin	12.733836	15.0570701	6.11622789	7.50265724
ENSG0000010	MYL12A	protein_codin	117.992636	737.889174	322.913741	152.425392
ENSG0000010	RNF125	protein_codin	4.44443464	17.8206512	2.78298244	15.3268622
ENSG0000010	POLI	protein_codin	6.28627868	0	0	10.458048
ENSG0000000	NOX1	protein_codin	0.30714894	0	0	0
ENSG0000000	ARSD	protein_codin	1.11921126	15.1583003	1.37766105	2.42081366
ENSG0000002	TAF9	protein_codin	1.78955494	0	12.2487802	3.50022448
ENSG0000000	IDH3G	protein_codin	6.87499674	8.07062273	26.7254287	15.4763213
ENSG0000000	SLC25A43	protein_codin	13.8789642	4.68349236	0	0
ENSG0000001	H2BW1	protein_codin	0	0	0	0
ENSG0000010	MXRA5	protein_codin	926.320212	1544.12046	95.5792577	325.53727
ENSG0000010	VSIG1	protein_codin	0	0	0	0
ENSG0000010	PSMD10	protein_codin	0	2.83142815	8.23552245	7.83423174
ENSG0000010	STS	protein_codin	0	0	0	0
ENSG0000010	PGRMC1	protein_codin	42.0809977	88.2818163	113.042544	27.7125589
ENSG0000010	RHOXF1	protein_codin	0	0	0	0
ENSG0000010	NXT2	protein_codin	0	0	0	0
ENSG0000010	GUCY2F	protein_codin	0.24487895	0	0	2.93383628
ENSG0000010	ATP1B4	protein_codin	0	0	0	0
ENSG0000010	TLR8	protein_codin	0	0	0	2.26098601
ENSG0000010	WDR13	protein_codin	2.33061982	3.68917776	16.122172	3.24704882
ENSG0000010	PAGE4	protein_codin	0	0	0	0
ENSG0000010	GLRA2	protein_codin	0	0	0	0
ENSG0000010	STAG2	protein_codin	1.10817279	21.1878516	0	2.48063632
ENSG0000010	F9	protein_codin	0	0	0	0
ENSG0000010	ABCD1	protein_codin	4.4579117	31.4904677	28.9003838	7.3510702
ENSG0000008	BCORL1	protein_codin	0	0	0	8.81347877
ENSG0000010	KCND1	protein_codin	2.11982679	7.19570871	2.96974344	5.47047406
ENSG0000010	SLC25A14	protein_codin	0	0	0	0
ENSG0000010	FMR1	protein_codin	43.6300689	49.9026083	0	0
ENSG0000010	PQBP1	protein_codin	0	10.4985101	6.16114549	8.24475821
ENSG0000010	PCSK1N	protein_codin	0	0.86755407	26.5217672	0.51678205
ENSG0000010	RAB40AL	protein_codin	0.46042824	0.81750508	0.5293467	0.44618292
ENSG0000010	GPR50	protein_codin	0	0	1.1518916	0
ENSG0000008	HUWE1	protein_codin	7.10639461	42.2709046	17.0071362	28.4282236
ENSG0000010	RP2	protein_codin	25.9145457	49.3991756	13.7893901	15.0043201
ENSG0000010	USP11	protein_codin	0	0	0	0
ENSG0000010	HTATSF1	protein_codin	211.107981	487.655171	270.602059	69.908014
ENSG0000010	TIMP1	protein_codin	5.62621926	57.9390334	1.66840913	75.5481979
ENSG0000010	PIN4	protein_codin	0	5.09613621	0	0
ENSG0000010	ITIH6	protein_codin	0	0	2.47057029	1.23399492
ENSG0000010	MAGED2	protein_codin	5.68632007	6.1172002	19.8513229	0
ENSG0000010	GLA	protein_codin	3.6379717	0	0	3.58150718
ENSG0000000	ATP12A	protein_codin	0.32881318	0	0	1.34272865

ENSG000001	NDFIP2	protein_codin	0	0	0	0
ENSG000001	MLNR	protein_codin	0	0	0	0
ENSG000001	ACP5	protein_codin	0	0	0	0
ENSG000001	ARHGEF7	protein_codin	0	11.6996759	0	5.13894708
ENSG000001	SGCG	protein_codin	5.83683797	2.50492315	16.6396543	4.8670819
ENSG000001	DHRS12	protein_codin	2.22058795	3.39294016	3.00015905	4.2867172
ENSG000001	OLFM4	protein_codin	0.50233134	0	0.94848461	0.88269625
ENSG000001	ACSM2A	protein_codin	0	0	0	0
ENSG000000	NTHL1	protein_codin	4.18325242	12.0299598	0	3.225415
ENSG000000	ZNF263	protein_codin	7.23322123	0	7.6588056	0
ENSG000000	MMP2	protein_codin	697.466314	428.300882	576.136054	216.472377
ENSG000001	CHD9	protein_codin	0	0	7.41269455	0
ENSG000001	ZNF205	protein_codin	0	3.46194971	10.7768706	4.56796149
ENSG000000	ORC6	protein_codin	7.12900857	0	32.8205794	0
ENSG000001	PHAF1	protein_codin	6.56209311	4.00766287	8.53086427	2.80895172
ENSG000001	CORO1A	protein_codin	4.06657635	0	0	6.64615059
ENSG000001	MT4	protein_codin	0	0	0	0
ENSG000001	LYRM1	protein_codin	2.44700081	7.34699605	9.4612611	3.42874446
ENSG000001	NUTF2	protein_codin	0	1.790556	5.47467466	0
ENSG000001	CBLN1	protein_codin	0	0	0	1.85497288
ENSG000001	ARL2BP	protein_codin	24.9791005	63.2168255	74.7680168	17.0955061
ENSG000001	PLLP	protein_codin	0	0	0	0
ENSG000001	CCL22	protein_codin	0.86030066	3.41007357	0.44215074	0.76233002
ENSG000001	DHODH	protein_codin	2.40382697	11.3486583	6.19526646	4.35813111
ENSG000001	CCL17	protein_codin	0	0	0	0
ENSG000001	ACD	protein_codin	0	0	0	0
ENSG000001	POLR2C	protein_codin	18.914685	72.4241937	138.694819	19.8173929
ENSG000001	PARD6A	protein_codin	0	0	0	0
ENSG000001	MMP15	protein_codin	4.87598598	20.2267048	118.178968	11.9913263
ENSG000001	USB1	protein_codin	0	0	0	0
ENSG000001	CFAP263	protein_codin	9.79375256	13.6813806	15.2627766	7.52678917
ENSG000001	PRSS54	protein_codin	0	0	0	0
ENSG000001	NME3	protein_codin	1.24980544	0	0	0
ENSG000001	PSMD7	protein_codin	69.2129484	155.316399	292.758189	46.0621222
ENSG000001	SETD6	protein_codin	5.08055625	8.99219799	4.00810201	0
ENSG000001	SLC38A7	protein_codin	0	0	13.3647485	0
ENSG000001	HAS3	protein_codin	0	0	0	0
ENSG000001	SMPD3	protein_codin	6.45587579	45.9915542	112.628839	45.3805569
ENSG000001	SLC7A6	protein_codin	4.84303386	0	21.4534577	15.6935732
ENSG000001	PLA2G15	protein_codin	4.61254757	24.8389922	11.8501946	5.43124416
ENSG000001	FA2H	protein_codin	0	0	0	0
ENSG000001	CMC2	protein_codin	2.66485047	12.6949652	14.4736852	4.09276381
ENSG000001	PDIA2	protein_codin	0	0	0	0
ENSG000002	ARHGDIG	protein_codin	0	0	0	0.36421956

ENSG000001	MPG	protein_codin	0.62483712	0	2.01788283	1.21860425
ENSG000001	HSDL1	protein_codin	11.2847923	14.7862939	30.1604573	9.2433862
ENSG000001	WFDC1	protein_codin	6.29778837	0	0.67661618	6.54550554
ENSG000001	USP10	protein_codin	15.020446	0	34.4762602	6.92233663
ENSG000001	TSC2	protein_codin	0	0	0	0
ENSG000001	ZNF500	protein_codin	7.21242035	5.00384232	4.51779582	3.35169376
ENSG000001	NME4	protein_codin	23.4852111	0	49.4834411	15.6037089
ENSG000002	DECR2	protein_codin	4.48521676	6.07409405	8.93575541	0
ENSG000001	ANTKMT	protein_codin	0	4.85896987	0	0
ENSG000001	METRN	protein_codin	0	0	1.71203905	2.33715093
ENSG000001	STUB1	protein_codin	21.8272221	85.6550613	57.7249603	13.1131745
ENSG000001	RHBDL1	protein_codin	0	0	0	0
ENSG000001	MEFV	protein_codin	1.17998263	3.31251158	1.52415415	0
ENSG000001	CRYM	protein_codin	0	0	0	0
ENSG000001	CAPN15	protein_codin	4.83638537	27.7720346	17.7071082	5.41653102
ENSG000001	AQP8	protein_codin	0	0	0	0
ENSG000001	USP31	protein_codin	27.7756904	74.5601768	108.356277	34.2403758
ENSG000001	HMOX2	protein_codin	0	0	0	0
ENSG000001	TOX3	protein_codin	1.19941827	0	3.65107283	0
ENSG000001	QPRT	protein_codin	0	0	0	0.52487721
ENSG000001	MAZ	protein_codin	5.66677584	0	83.6576089	5.1187699
ENSG000001	CDIPT	protein_codin	0	23.8320707	0	0
ENSG000001	BCKDK	protein_codin	7.66960793	0	0	1.56623124
ENSG000001	KAT8	protein_codin	0	37.2828461	12.2597531	4.21662871
ENSG000001	TMC5	protein_codin	0	0	0	0
ENSG000001	SLC6A2	protein_codin	0	0	0	0
ENSG000001	KNOP1	protein_codin	8.31452168	23.5080285	34.5338205	5.44898844
ENSG000001	MGA	protein_codin	95.6267267	191.129463	190.656134	0
ENSG000001	AQP9	protein_codin	0.63900879	0	0.53137312	2.72120598
ENSG000001	CSK	protein_codin	1.66021412	93.5306919	5.55926708	0
ENSG000001	MTFMT	protein_codin	3.52347584	1.57989256	5.16847074	6.47276835
ENSG000001	RASL12	protein_codin	3.2285376	7.86865201	2.19603407	4.46031822
ENSG000001	CTSH	protein_codin	0	0	0	0
ENSG000001	CEMIP	protein_codin	8.59687523	34.4276712	45.367451	13.9403438
ENSG000001	EHD4	protein_codin	35.2935503	153.048465	36.7006005	98.1047313
ENSG000001	TGM5	protein_codin	0	0	0	0
ENSG000001	GABPB1	protein_codin	7.46935803	25.1056837	28.5337907	12.4121637
ENSG000001	SCG3	protein_codin	0	0	0	1.98897166
ENSG000001	DNAJC17	protein_codin	2.58725352	8.64429435	3.85888397	5.47537167
ENSG000001	RHOV	protein_codin	0	0.81235242	0.52765186	2.68064113
ENSG000001	VPS18	protein_codin	11.2820264	19.0231661	11.1390809	7.62076255
ENSG000001	OIP5	protein_codin	1.6654689	0	6.29502956	0
ENSG000001	BLOC1S6	protein_codin	26.0091664	30.6519594	36.0174812	24.4485219
ENSG000000	EXTL3	protein_codin	0	0	0	0

ENSG0000007	FDFT1	protein_codin	18.2689424	0	12.9514104	0
ENSG000001	AGO2	protein_codin	44.0679258	129.087969	248.733286	35.0187226
ENSG0000007	PAG1	protein_codin	16.0492857	131.460935	18.8380493	25.1002138
ENSG000000	TG	protein_codin	0	14.5061962	1.79320752	1.68382535
ENSG000001	BRF2	protein_codin	3.38416906	15.0050617	34.8345735	4.32739689
ENSG000001	ZFAND1	protein_codin	0	0	0	0
ENSG000001	RP1	protein_codin	0.44450089	3.09875851	2.28007398	2.61518179
ENSG000001	RIPK2	protein_codin	5.71640348	4.25937543	4.18853479	10.0455208
ENSG000001	CPQ	protein_codin	34.1258874	82.2044974	17.0484567	26.1735275
ENSG000001	DECR1	protein_codin	5.74532055	8.40097071	4.04615307	0
ENSG000001	SFRP1	protein_codin	28.3705904	69.4873688	17.2587468	10.0708093
ENSG000001	PLAT	protein_codin	0.95317427	0	0	0
ENSG000001	DKK4	protein_codin	0.25995517	0	0	0.85138812
ENSG000001	GDAP1	protein_codin	0	0	0	0
ENSG000001	PHF20L1	protein_codin	45.7029793	112.38536	194.485079	24.7133264
ENSG000001	EIF3E	protein_codin	0	0	0	0
ENSG000001	EMC2	protein_codin	7.42496276	6.10988284	16.4715305	6.60867292
ENSG000001	CCN4	protein_codin	0	0	2.48513447	0.76308068
ENSG000001	STMN2	protein_codin	1.6888913	0	0	6.07675431
ENSG000001	TRPS1	protein_codin	0	0	51.5650502	0
ENSG000001	CHRAC1	protein_codin	8.30274676	19.9014118	34.6402178	10.5770517
ENSG000001	NCALD	protein_codin	10.3356918	44.3409465	0	9.85587862
ENSG000001	GML	protein_codin	0	0	0	2.71130758
ENSG000001	UBR5	protein_codin	28.6307391	332.769067	229.519803	50.4010119
ENSG000001	PYCR3	protein_codin	3.92661243	9.37934015	47.6164774	2.02202752
ENSG000001	MTMR9	protein_codin	22.9872988	43.0689993	38.4341535	11.6449576
ENSG000001	DCTN6	protein_codin	8.14016297	25.7347307	13.439218	5.77987944
ENSG000001	GSR	protein_codin	18.3443305	69.0163954	55.8790133	18.2317236
ENSG000001	TNFRSF10A	protein_codin	1.86487416	30.5343897	0.47730679	5.64798849
ENSG000001	PPP2CB	protein_codin	14.3390652	21.7234484	10.9365212	10.7811752
ENSG000001	NEFM	protein_codin	0	0	1.69949761	0
ENSG000001	KCTD9	protein_codin	40.0027926	44.0647892	24.7384016	21.1373607
ENSG000000	HOMER3	protein_codin	0	0	0	0
ENSG000000	CNOT3	protein_codin	7.49872805	0	11.7748503	0
ENSG0000007	EXOSC5	protein_codin	2.57436006	0	4.74614225	2.34445543
ENSG000000	PNPLA6	protein_codin	0	0	0	0
ENSG000000	PLAUR	protein_codin	1.57730519	7.05385265	0	14.965581
ENSG000000	PAF1	protein_codin	30.2745629	135.29669	44.2494007	39.9589525
ENSG0000007	STXBP2	protein_codin	0	0	0	0
ENSG000001	CYP4F3	protein_codin	0	0	0	0
ENSG000002	ZNF432	protein_codin	28.1255394	22.6512649	22.6711255	12.3679339
ENSG000001	ZNF180	protein_codin	0	0	0	0
ENSG000001	ZNF780B	protein_codin	0	28.2665293	7.06983517	0
ENSG000001	MAN2B1	protein_codin	2.32574235	0	8.8715939	4.48615069

ENSG000001	TULP2	protein_codin	0	0	0	0
ENSG000001	DHDH	protein_codin	0	0	0	0
ENSG000001	RUVBL2	protein_codin	0	0	0	0
ENSG000001	ECH1	protein_codin	17.4970992	28.4311221	18.3029307	18.17503
ENSG000001	HNRNPL	protein_codin	6.07366272	113.998662	67.4122558	13.3806248
ENSG000001	SARS2	protein_codin	0	0	0	0.57869223
ENSG000001	KCNA7	protein_codin	1.38696411	0	0	1.77880787
ENSG000001	SNRNP70	protein_codin	0	13.004795	20.8028934	18.3723395
ENSG000001	RELB	protein_codin	2.36244664	27.8671145	3.44682904	19.4788818
ENSG000001	CLASRP	protein_codin	0	0	0	0
ENSG000001	LIN7B	protein_codin	2.38976773	0	2.12446346	0
ENSG000001	PPP1R37	protein_codin	9.42286627	19.9439357	14.8549972	5.40271353
ENSG000001	FCGRT	protein_codin	0	171.506199	20.0161964	27.0213229
ENSG000001	CKM	protein_codin	182.808548	3.41124506	0.97480733	0.41549926
ENSG000001	PEX11G	protein_codin	0	0	7.39752449	0
ENSG000001	SLC17A7	protein_codin	1.98300768	0	4.49133051	0.93159207
ENSG000001	RNASEH2A	protein_codin	5.38077842	2.51333098	45.4120467	3.8563085
ENSG000001	SF3A2	protein_codin	7.52213805	67.5349873	36.8222723	8.78525421
ENSG000001	AMH	protein_codin	0	8.50870186	17.6978442	0
ENSG000001	DKKL1	protein_codin	0	0	0	0
ENSG000001	TRMT1	protein_codin	3.60321463	0	4.73376754	3.92371364
ENSG000001	RETN	protein_codin	0	0	0	0
ENSG000001	RSPH6A	protein_codin	0	3.26866835	0	0
ENSG000001	TBC1D17	protein_codin	1.12460775	5.33368375	1.01227589	1.09830904
ENSG000001	YJU2B	protein_codin	0.49467135	0	0	1.03043445
ENSG000001	TLE5	protein_codin	1.05122167	0	8.28011402	0.84065456
ENSG000001	SGTA	protein_codin	0	0	15.7541719	0
ENSG000001	SNAPC2	protein_codin	2.29975462	6.32245676	6.53281676	0
ENSG000001	C19orf53	protein_codin	0	0	0.63502301	0
ENSG000001	FIZ1	protein_codin	1.53924274	6.88403932	3.76367555	1.65618772
ENSG000001	C19orf44	protein_codin	1.53865666	0	0	0
ENSG000001	CYP4F2	protein_codin	0	0	0	0
ENSG000001	EPHX3	protein_codin	0	0	0	0
ENSG000001	ZNF419	protein_codin	0	0	1.529681	0
ENSG000001	SLC1A6	protein_codin	0	0	0	0
ENSG000001	POP4	protein_codin	0	0	0	0
ENSG000001	PDCD5	protein_codin	0.72638198	0	0	0
ENSG000001	LGALS13	protein_codin	0	0	0	0
ENSG000001	FBL	protein_codin	15.6068885	57.1110819	62.0963383	0
ENSG000001	CLC	protein_codin	0	0	0	0
ENSG000001	CCNP	protein_codin	0	1.15946446	0	0.80495412
ENSG000001	EBI3	protein_codin	0.51434914	3.77766694	0	0.4212558
ENSG000001	TBCB	protein_codin	8.33297135	0	11.1306714	7.93004925
ENSG000001	FSD1	protein_codin	0.34140608	0	2.21004652	0

ENSG000001	POLR2I	protein_codin	0	0	0	0
ENSG000001	APLP1	protein_codin	0	0	0	0
ENSG000001	CACTIN	protein_codin	0	0	32.7819909	0
ENSG000000	ARHGAP33	protein_codin	0	0	0	0
ENSG000001	CCDC9	protein_codin	17.7658965	73.7071973	0	20.651193
ENSG000001	TGFB1	protein_codin	17.0061413	230.917073	50.109925	95.1637258
ENSG000001	DMAC2	protein_codin	0	0	6.11834406	0.61931034
ENSG000001	CEACAM4	protein_codin	0	0	0	0
ENSG000001	PLIN3	protein_codin	29.1207132	35.4246062	40.1548313	16.4500566
ENSG000001	CD79A	protein_codin	0	0	0	0.66379582
ENSG000001	LIM2	protein_codin	0	0	0	0
ENSG000001	RPS19	protein_codin	183.615689	548.277887	426.721863	107.305
ENSG000001	NKG7	protein_codin	0.43986204	27.6542729	0	0
ENSG000001	ICAM5	protein_codin	0	0	11.9556063	0.44364379
ENSG000001	CEACAM5	protein_codin	0	2.85681034	0	0.50832455
ENSG000001	CRX	protein_codin	0	0	0	0
ENSG000001	SULT2A1	protein_codin	0	0	0	0.37137391
ENSG000001	CDC37	protein_codin	19.0006984	36.6645287	52.9440384	6.76491713
ENSG000001	RABAC1	protein_codin	0.76546062	13.6838233	10.443426	4.56089359
ENSG000001	CNFN	protein_codin	0	0	0	0
ENSG000001	ZNRF4	protein_codin	0	0	0.36718951	0
ENSG000001	HAS1	protein_codin	0	0	0	0
ENSG000001	RAB3D	protein_codin	2.54478745	17.6217034	4.09993256	7.03690818
ENSG000001	DBP	protein_codin	1.19841232	15.4717136	0	2.08069659
ENSG000001	EPOR	protein_codin	2.40951035	0	0	2.82996364
ENSG000001	RASIP1	protein_codin	3.91286697	19.5100159	0.59789426	8.75347141
ENSG000001	FGF21	protein_codin	0	0	0	0.34836566
ENSG000001	WDR83OS	protein_codin	0	0	0	0
ENSG000001	CACNG7	protein_codin	0	0	0	0.38248203
ENSG000001	GCDH	protein_codin	0	0	0	0
ENSG000001	DNASE2	protein_codin	7.29852273	26.7784791	7.83235912	0
ENSG000001	LENG1	protein_codin	5.27168399	32.9900971	10.1532412	7.04272536
ENSG000001	RPL18A	protein_codin	115.276494	259.215072	915.640534	189.027235
ENSG000001	SLC5A5	protein_codin	0	0	0	0.85028438
ENSG000001	KCNN1	protein_codin	0	0	0	0
ENSG000001	ARRDC2	protein_codin	9.78457011	21.8238476	0	13.5301498
ENSG000001	PIK3R2	protein_codin	20.8430641	0	142.903994	0
ENSG000001	RAB3A	protein_codin	0.64612379	5.4874804	0.44653278	0
ENSG000002	PSENEN	protein_codin	1.95741586	0	0	1.56565279
ENSG000001	COMP	protein_codin	124.385899	132.470221	2.61803541	4.19697771
ENSG000001	UPK1A	protein_codin	0	0	0	0
ENSG000001	TMEM147	protein_codin	5.17078378	0	9.28166861	2.60945881
ENSG000001	GAPDHS	protein_codin	0	0	0	0
ENSG000001	HAMP	protein_codin	0	0	0	0

ENSG000001	USF2	protein_codin	0	0	0	0
ENSG000001	KXD1	protein_codin	9.7692739	23.1693174	26.267889	0
ENSG000001	FKBP8	protein_codin	14.4235893	0	39.4149838	0
ENSG000001	ERF	protein_codin	5.71841269	18.5156439	0	0
ENSG000001	GSK3A	protein_codin	0	0	0	4.38763756
ENSG000001	ZNF574	protein_codin	0	0	0	0
ENSG000001	SIPA1L3	protein_codin	20.1864138	134.248	63.3062557	34.2955851
ENSG000001	CADM4	protein_codin	0	2.85462136	9.50685461	0.44302121
ENSG000000	PON1	protein_codin	0	3.77107362	0	0.5907002
ENSG000000	CYP3A43	protein_codin	0	0	0	0
ENSG000002	ABCF2-H2BK	protein_codin	45.6478948	27.1102263	113.529237	16.4652236
ENSG000000	HGF	protein_codin	6.46540701	107.560399	0.44205955	11.8237567
ENSG000000	LAMB1	protein_codin	161.657994	240.994891	354.03035	56.5769522
ENSG000000	UBE2D4	protein_codin	15.9929713	34.3747119	17.453504	10.8831917
ENSG000000	WNT16	protein_codin	7.06926215	1.12521825	1.79447473	0.50231769
ENSG000001	CPA2	protein_codin	0	0	0	0
ENSG000001	CPA4	protein_codin	0	0	0	0
ENSG000001	GTPBP10	protein_codin	20.3793018	27.0353263	23.9098463	0
ENSG000001	TFPI2	protein_codin	0	0	2.42808704	10.7937719
ENSG000001	BET1	protein_codin	6.00255953	8.88566691	15.3317522	2.39231665
ENSG000001	NAMPT	protein_codin	0	0	0	0
ENSG000001	POLR1F	protein_codin	41.7783385	56.7982633	338.249611	18.6476149
ENSG000001	PON2	protein_codin	2.23273328	6.79587887	0	6.56361729
ENSG000001	ITGB8	protein_codin	0.89989469	0	7.77983172	4.42462197
ENSG000001	HBP1	protein_codin	13.5350202	64.1674991	14.3902122	19.2758928
ENSG000001	SP4	protein_codin	8.03698282	23.5764316	30.7946028	0
ENSG000001	CBLL1	protein_codin	5.03310496	4.37353419	0	0
ENSG000001	PALS2	protein_codin	53.9914842	69.8642524	78.7229266	19.4406492
ENSG000001	OGDH	protein_codin	13.5910134	39.7125809	31.8437232	22.4692175
ENSG000001	NPVF	protein_codin	0.64165268	0	0	2.48138368
ENSG000001	H2AZ2	protein_codin	58.1701641	48.6718061	143.819336	53.3567726
ENSG000001	CAV2	protein_codin	27.3682728	37.9823825	19.3958066	26.9780286
ENSG000001	HOXA2	protein_codin	1.51287383	0	1.79694429	0
ENSG000001	LFNG	protein_codin	5.22811834	38.6899048	3.03393044	3.56368314
ENSG000001	HOXA5	protein_codin	3.51789387	3.5084193	5.0941402	0.43356374
ENSG000001	HOXA6	protein_codin	0.22581479	0	0	0.38934753
ENSG000001	TSPAN12	protein_codin	3.53702109	6.78038551	15.8733933	7.03732783
ENSG000001	EVX1	protein_codin	0	0	0	0
ENSG000001	CHN2	protein_codin	19.3466444	18.9720451	15.584557	3.76396598
ENSG000001	ABHD11	protein_codin	0	0	5.35274993	4.29028759
ENSG000001	FKBP14	protein_codin	51.5421422	53.5771698	58.984998	16.5913156
ENSG000001	STX1A	protein_codin	1.55252314	0	0	2.03484229
ENSG000001	NOD1	protein_codin	5.51483066	22.4602374	4.93135533	6.9670491
ENSG000001	CCL24	protein_codin	0.26389293	2.61916037	0.47871484	0.73670928

ENSG000001	BUD31	protein_codin	2.22100187	19.4681431	4.64732624	4.75536618
ENSG000001	CYP3A5	protein_codin	0	0	0	0
ENSG000001	SNX8	protein_codin	7.2930072	37.6157968	14.4013814	5.14310431
ENSG000001	WASL	protein_codin	87.1048567	166.969002	198.76533	52.6404619
ENSG000001	HYAL4	protein_codin	0	0	1.80012566	0
ENSG000001	SPAM1	protein_codin	0	0	0	0
ENSG000001	AIMP2	protein_codin	9.10149803	15.6677697	37.5503032	3.78768894
ENSG000001	TFR2	protein_codin	0	0	0	0
ENSG000001	MOSPD3	protein_codin	0	0	0	0
ENSG000001	PCOLCE	protein_codin	60.6059534	594.809696	176.688457	169.327062
ENSG000001	RBM28	protein_codin	31.6223118	119.581129	172.616705	38.8339508
ENSG000001	LSM5	protein_codin	0.39231213	3.79471251	3.68852633	0.54899843
ENSG000001	SERPINE1	protein_codin	32.2593817	34.2886902	11.5237894	31.9713099
ENSG000001	MOGAT3	protein_codin	0	0	0	0.43019371
ENSG000001	C1GALT1	protein_codin	9.39197922	0	15.2356927	0
ENSG000001	PLOD3	protein_codin	2.41085306	41.2003966	65.38271	7.25014792
ENSG000001	RPA3	protein_codin	0.9890127	1.94396146	4.75179601	0.73191457
ENSG000002	FIS1	protein_codin	8.60947978	28.1345715	19.3667248	9.03416936
ENSG000001	GLCCI1	protein_codin	3.81531656	52.5107206	14.0157551	6.01354968
ENSG000001	MYL10	protein_codin	0	0	0	0
ENSG000001	NRF1	protein_codin	9.27821056	37.9684783	31.5523985	7.89949581
ENSG000001	CEP41	protein_codin	0	0	13.5641583	0
ENSG000001	ZNF862	protein_codin	9.80285823	36.5958128	15.7169551	6.18787056
ENSG000001	MEST	protein_codin	8.76545043	7.60704818	0	1.38388844
ENSG000001	RARRES2	protein_codin	0.69971324	12.6308794	0	4.8233113
ENSG000002	YAE1	protein_codin	5.25191233	15.6566725	8.20002169	1.63630594
ENSG000001	GIMAP2	protein_codin	11.5678103	16.443099	0.9834399	4.91743388
ENSG000001	PSMA2	protein_codin	10.4836636	0	33.8687423	5.06959172
ENSG000001	MRPL32	protein_codin	22.5139186	45.0878935	55.3036919	9.94088135
ENSG000001	COA1	protein_codin	0	0	0	0
ENSG000001	AEBP1	protein_codin	397.040934	887.044005	278.361418	365.896794
ENSG000001	POLD2	protein_codin	0	0	29.2152219	0
ENSG000001	MYL7	protein_codin	0	0	0	0
ENSG000001	BCL7B	protein_codin	2.57016289	67.4912795	37.1163189	19.2356692
ENSG000001	YKT6	protein_codin	18.4390471	35.1334344	20.9316532	0
ENSG000001	CLIP2	protein_codin	0	9.20484402	12.812184	5.15310172
ENSG000000	PTGS1	protein_codin	0	24.3679557	0	0.67163117
ENSG000000	ZNF510	protein_codin	31.9039784	67.5246289	53.2407002	17.4982486
ENSG000002	ZNF688	protein_codin	0.4953952	2.63442269	12.4280005	0
ENSG000000	CHMP5	protein_codin	67.7370481	120.413523	225.794014	39.9604713
ENSG000001	SPATA6L	protein_codin	0.5323675	2.62032778	0	0
ENSG000001	FKTN	protein_codin	4.44486535	7.23046154	1.4282105	4.0418709
ENSG000001	SEC61B	protein_codin	11.2378223	17.2610729	29.8954955	5.22372735
ENSG000001	C5	protein_codin	4.16903119	12.1954066	16.8682331	0

ENSG000001	AKNA	protein_codin	0	0	0	0
ENSG000001	TNFSF8	protein_codin	6.85931216	45.9062499	0.45380418	8.29665663
ENSG000001	AK1	protein_codin	4.70166506	0	1.78009838	3.45835651
ENSG000001	RLN1	protein_codin	0	0	0	0
ENSG000001	PLGRKT	protein_codin	4.81080108	5.32045881	9.19259146	5.44617551
ENSG000001	TBC1D13	protein_codin	0	0	0	0
ENSG000001	ELAVL2	protein_codin	0	9.1213077	0	0
ENSG000001	EDF1	protein_codin	75.4048342	147.527526	216.267914	70.7746257
ENSG000001	SETX	protein_codin	247.468039	623.263682	440.491641	145.311264
ENSG000002	VIM	protein_codin	1710.91486	2405.70372	3358.6211	1280.13134
ENSG000000	BLNK	protein_codin	4.10535114	0	0	5.3714737
ENSG000000	CYP26A1	protein_codin	0	0	0	10.6725625
ENSG000001	ATE1	protein_codin	0	0	0	0
ENSG000001	CDH23	protein_codin	5.56927738	19.7467644	1.59733537	1.71628261
ENSG000001	CCSER2	protein_codin	35.725574	0	0	0
ENSG000001	ACTA2	protein_codin	72.9712901	252.592064	13.1518346	18.2472825
ENSG000001	PITRM1	protein_codin	9.01451026	0	0	0
ENSG000001	STN1	protein_codin	0	0	0	6.4356339
ENSG000001	DNAJC12	protein_codin	5.48577328	0	0	0
ENSG000001	PPIF	protein_codin	2.48218294	8.16726561	20.5355346	7.26873274
ENSG000001	TBC1D12	protein_codin	43.6855992	75.6595988	68.0433188	26.0539566
ENSG000000	MPO	protein_codin	0.23020275	0	0	1.67034599
ENSG000000	ST6GALNAC2	protein_codin	0	0	1.07205581	0
ENSG000000	DHX33	protein_codin	20.9396465	19.7262695	38.8978747	6.67908322
ENSG000000	UTP18	protein_codin	24.4921152	88.6677002	35.8091997	20.0199586
ENSG000000	SLC25A39	protein_codin	0	0	0	2.99698837
ENSG000000	P2RX5	protein_codin	0	0	0	0
ENSG000001	EPX	protein_codin	0	1.14268793	0	0
ENSG000001	CRYBA1	protein_codin	0	0	0	0
ENSG000001	NUFIP2	protein_codin	118.496997	246.06471	292.952918	169.203418
ENSG000001	GIT1	protein_codin	5.52763304	38.73081	11.3435062	0
ENSG000001	RPL19	protein_codin	863.711938	1120.28505	2765.35579	787.022548
ENSG000001	RUNDC3A	protein_codin	0	1.80895106	0	1.42108035
ENSG000001	CSF3	protein_codin	0	0	0	0
ENSG000002	SUPT4H1	protein_codin	11.3107257	0	22.5278336	8.39117104
ENSG000001	WNT3	protein_codin	0	0	0	2.9091821
ENSG000001	SHPK	protein_codin	6.10235367	8.02430695	18.615719	6.21815009
ENSG000002	TAX1BP3	protein_codin	21.3422425	12.8887396	9.340986	6.29600631
ENSG000001	P2RX1	protein_codin	0.15866192	2.04484409	0	3.59928542
ENSG000001	KRT37	protein_codin	0	0	0	0
ENSG000001	GOSR2	protein_codin	0	10.9642731	0	0
ENSG000001	PNPO	protein_codin	0	12.8124204	0	0
ENSG000001	TVP23C	protein_codin	4.24928909	106.080652	2.27002729	6.98164875
ENSG000001	RPS6KB1	protein_codin	16.0472404	21.3645295	76.0468525	22.9003584

ENSG000001	CBX1	protein_codin	50.2806323	68.5949502	96.6746711	20.967266
ENSG000001	PIGL	protein_codin	0	0	3.66012784	0
ENSG000001	GALK1	protein_codin	0	0	0	2.0782069
ENSG000001	HOXB6	protein_codin	2.89637599	22.5284508	5.39723154	0
ENSG000001	PFN1	protein_codin	33.8437273	167.454231	64.0335582	30.6536041
ENSG000001	SLC25A11	protein_codin	11.1014211	32.1575526	3.36122239	6.40604259
ENSG000001	RASD1	protein_codin	8.97307831	5.27500568	0	34.2395616
ENSG000001	NUP88	protein_codin	0	0	0	0
ENSG000001	C1QBP	protein_codin	19.2289788	4.0146969	36.898355	11.3449115
ENSG000001	CPD	protein_codin	123.058534	168.865869	241.341658	64.7846221
ENSG000001	GOSR1	protein_codin	0	4.37367524	30.3530498	0
ENSG000001	CCDC47	protein_codin	40.0372714	68.9366042	163.308726	23.0842872
ENSG000001	MED31	protein_codin	2.57344732	0	0	4.78646776
ENSG000001	DRG2	protein_codin	6.55405499	0	21.2378241	4.80828542
ENSG000001	AKAP10	protein_codin	25.6262354	104.865724	39.8672879	16.9615456
ENSG000001	ALDH3A1	protein_codin	0	0	0	0
ENSG000001	SMARCD2	protein_codin	41.9664601	0	70.716807	12.3922113
ENSG000002	PRR29	protein_codin	0.58837107	1.41954707	2.51998191	3.76649537
ENSG000001	SYNGR2	protein_codin	5.61254138	14.746262	9.05245244	10.0416805
ENSG000001	DDX5	protein_codin	0	0	0	0
ENSG000001	ASIC2	protein_codin	0	0	0	0
ENSG000001	CCL2	protein_codin	3.76205291	33.8973905	0.77316679	12.6200735
ENSG000001	CCL1	protein_codin	0	0	0	0.44088154
ENSG000001	CCL13	protein_codin	0	22.2206297	0	0.90901304
ENSG000001	PEX12	protein_codin	5.9957485	3.95704577	4.54393047	1.91013788
ENSG000001	KRT32	protein_codin	0.30452633	0	0	1.83128679
ENSG000001	KAT2A	protein_codin	1.01830196	9.39303069	11.1495472	0
ENSG000001	NAGLU	protein_codin	3.81255851	19.1549027	7.06170846	3.52560746
ENSG000001	HSD17B1	protein_codin	0.49242346	1.09685583	1.08443051	0
ENSG000001	ABI3	protein_codin	3.13199321	8.37473889	2.73457989	3.42601116
ENSG000001	COL1A1	protein_codin	16062.0756	110386.224	3295.28781	32867.4488
ENSG000001	MRPL27	protein_codin	4.59086023	0	7.97458712	0
ENSG000001	LRRC59	protein_codin	46.7187244	210.730364	123.29084	77.8661218
ENSG000001	HDAC5	protein_codin	0	0	0	0
ENSG000001	PPY	protein_codin	0	0	0	0
ENSG000001	DUSP3	protein_codin	23.7562281	49.9181797	32.6820333	18.375372
ENSG000001	CACNG1	protein_codin	0.22733218	0	0	0.79789049
ENSG000001	HLF	protein_codin	24.5697043	6.44100735	6.88434199	6.21713368
ENSG000001	EFNB3	protein_codin	0.47384002	9.13518111	8.46622102	3.27279663
ENSG000001	FAM20A	protein_codin	0.53283635	0	0	1.45783563
ENSG000001	RANGRF	protein_codin	0	0	2.39090082	0
ENSG000001	RCVRN	protein_codin	0.34434315	1.01489523	0	3.37203611
ENSG000001	MYH1	protein_codin	1319.12292	7.09267386	4.10663874	11.9638558
ENSG000001	VTN	protein_codin	0	0	0	0

ENSG000001	TNFAIP1	protein_codin	14.2912373	31.5495264	23.5670897	11.1370375
ENSG000001	TMEM97	protein_codin	1.44136542	0	27.5016623	0
ENSG000001	FOXN1	protein_codin	0	4.25004981	0	0
ENSG000001	ALDOC	protein_codin	1.71152719	0	4.85841417	0
ENSG000000	CD38	protein_codin	0.96843159	28.0019005	1.82738118	4.21742903
ENSG000000	IBSP	protein_codin	683.538779	5446.6468	12511.7143	3025.24251
ENSG000000	LAP3	protein_codin	0	0	0	0
ENSG000001	CXCL6	protein_codin	0	0	0	0
ENSG000000	JADE1	protein_codin	45.1185994	12.2668908	90.8187847	0
ENSG000000	RUFY3	protein_codin	31.9919072	68.3560137	30.3320955	13.3132867
ENSG000000	AFM	protein_codin	0	0	0	1.07676756
ENSG000000	AFP	protein_codin	0.28580405	0	0	0
ENSG000001	PHOX2B	protein_codin	0	0	0	1.65090215
ENSG000001	GNRHR	protein_codin	0	0	0	0.63912489
ENSG000001	CWH43	protein_codin	0	0	0	0
ENSG000001	SULT1E1	protein_codin	0	0	6.71969787	1.07167981
ENSG000001	SMR3A	protein_codin	0	0	0	0.81094164
ENSG000001	LAMTOR3	protein_codin	2.1339395	0	3.59551125	5.25398292
ENSG000001	PF4V1	protein_codin	0	0	0	0
ENSG000001	NFKB1	protein_codin	11.2069276	16.5199298	0	11.3082384
ENSG000001	KLHL2	protein_codin	11.277481	20.6054824	23.8106008	1.55406759
ENSG000001	IL2	protein_codin	0	0	0	0
ENSG000001	WFS1	protein_codin	5.54034206	0	0	0
ENSG000001	GAR1	protein_codin	3.13678539	0	19.0606822	5.41967008
ENSG000001	FRG1	protein_codin	30.8017576	134.915189	64.7284172	24.6025598
ENSG000001	CLNK	protein_codin	1.00415252	0	0	0
ENSG000001	FAM149A	protein_codin	7.89218328	0	0	0
ENSG000000	SPA17	protein_codin	0.57568838	2.53599987	3.33083222	0
ENSG000000	CD82	protein_codin	10.4965442	0	12.5459653	0
ENSG000001	LDHA	protein_codin	0	0	41.3419302	0
ENSG000000	SPI1	protein_codin	5.55064406	19.1620634	2.20711544	4.51329044
ENSG000000	PLEKHB1	protein_codin	0	4.36345385	0	0.35347808
ENSG000001	CRYAB	protein_codin	0	0	0	0
ENSG000001	CTSC	protein_codin	0	0	0	39.3236532
ENSG000001	ZPR1	protein_codin	1.53931943	0	3.88831682	23.538972
ENSG000001	CRTAM	protein_codin	1.53860345	7.75469385	0	0.83057595
ENSG000001	JHY	protein_codin	8.96728177	17.9114495	2.39453555	1.21665171
ENSG000001	HSPA8	protein_codin	0	0	546.934178	0
ENSG000001	DTX4	protein_codin	18.403208	82.4682656	42.5978259	15.1010829
ENSG000001	UNC93B1	protein_codin	5.52153378	24.9852495	11.1311189	12.6555271
ENSG000001	PUS3	protein_codin	9.41410577	0	0	0
ENSG000001	ST3GAL4	protein_codin	0	0	0	0
ENSG000001	SF1	protein_codin	0	0	0	4.45446676
ENSG000001	CCND1	protein_codin	224.502501	167.338072	176.202352	100.475509

ENSG000001	CCDC86	protein_codin	5.90559195	17.9564359	39.0681904	5.48259529
ENSG000001	PRPF19	protein_codin	10.3546306	46.704507	17.9674727	9.81069612
ENSG000001	TMEM109	protein_codin	13.4622969	28.6328545	36.8769299	9.11734693
ENSG000001	ANAPC15	protein_codin	0	0	18.3856178	2.2708858
ENSG000001	PANX1	protein_codin	19.1042906	24.2210699	41.8220993	15.4299187
ENSG000001	APOA5	protein_codin	0	0	0	0
ENSG000001	APOC3	protein_codin	0	0	0	0
ENSG000001	IL10RA	protein_codin	0	0	0	5.82136767
ENSG000001	GALNT18	protein_codin	2.18358575	19.8637309	18.0300959	13.0924652
ENSG000001	BIRC2	protein_codin	58.8408978	128.460981	152.823338	56.5158162
ENSG000001	PDHX	protein_codin	26.2055607	18.260951	37.6176083	18.3600826
ENSG000001	SLC15A3	protein_codin	0	0	0	0
ENSG000001	SCGB2A2	protein_codin	0	0	0	0
ENSG000000	DGAT2	protein_codin	0	18.3115855	13.0113083	0
ENSG000001	C11orf58	protein_codin	64.4013895	84.1594165	63.1995374	33.701044
ENSG000001	RPS13	protein_codin	0	0	0	0
ENSG000000	YBX3	protein_codin	15.5978458	21.4155904	82.5557296	34.2464802
ENSG000000	DDX11	protein_codin	7.13946315	10.009902	0	0
ENSG000000	SART3	protein_codin	2.39960826	0	0	3.3207907
ENSG000000	ZNF268	protein_codin	0	0	0	3.36287142
ENSG000000	RPLP0	protein_codin	33.5469401	28.122971	69.8359347	20.1179238
ENSG000000	PXN	protein_codin	4.23644444	0	0	0
ENSG000000	SLC25A3	protein_codin	0	0	0	0
ENSG000000	RAD52	protein_codin	5.29699422	0	0	0
ENSG000000	POLR3B	protein_codin	17.3054101	12.1988566	50.1850673	9.48347894
ENSG000001	PPFIBP1	protein_codin	34.6373089	75.7575259	73.6937537	47.7301794
ENSG000001	CD69	protein_codin	5.68612204	43.0583128	0.89310852	20.2729825
ENSG000001	PRDM4	protein_codin	24.815153	26.1727704	16.9886759	24.16551
ENSG000001	CLEC2B	protein_codin	31.2023394	25.9705658	0	27.0467425
ENSG000001	SELPLG	protein_codin	0	0	0	0.98162537
ENSG000001	ASIC1	protein_codin	2.64642988	0	0	0
ENSG000001	DAO	protein_codin	0	0	0	0
ENSG000001	KCTD10	protein_codin	20.2674746	81.1400049	11.1048579	27.8995728
ENSG000001	MLEC	protein_codin	63.2407237	301.345492	118.877082	40.8026434
ENSG000001	MVK	protein_codin	0.72701691	0	2.7333813	0
ENSG000001	CSRNP2	protein_codin	20.6154485	65.0209194	58.4253007	17.4220873
ENSG000001	IL23A	protein_codin	0.3751671	0	0	0
ENSG000001	SYT10	protein_codin	0	0	2.21086717	0
ENSG000001	CYP27B1	protein_codin	0	0	3.9171585	2.05753714
ENSG000001	MYF6	protein_codin	8.24158523	0.99321815	0	0.9109159
ENSG000001	MYF5	protein_codin	0	0	0	0.59714824
ENSG000001	GLI1	protein_codin	0.8715235	0	10.5658864	0
ENSG000001	PPM1H	protein_codin	14.2342015	58.9859948	20.1263236	14.479295
ENSG000001	LTA4H	protein_codin	5.71108473	51.09471	51.2395378	18.4287225

ENSG000001	ELK3	protein_codin	41.1802599	165.677925	40.0915594	52.8530491
ENSG000001	ITFG2	protein_codin	4.23440341	1.34331796	2.81943198	1.87238493
ENSG000001	PRR4	protein_codin	0	0	0	0
ENSG000001	PARP11	protein_codin	0	0	0	0
ENSG000001	ARPC3	protein_codin	87.8958236	440.597982	204.343965	146.964221
ENSG000001	GPN3	protein_codin	6.71499842	0	13.8353955	4.30436167
ENSG000001	FGF6	protein_codin	1.77199819	0	0	0.41141943
ENSG000001	MYL2	protein_codin	0	0	0	0
ENSG000001	RAD51AP1	protein_codin	0	0	17.1311194	1.16257686
ENSG000001	AKAP3	protein_codin	0	0	0	0
ENSG000001	DUSP16	protein_codin	2.71848404	0	20.9921783	4.43637991
ENSG000001	CREBL2	protein_codin	38.438088	183.894579	36.1008572	41.2898364
ENSG000001	CDKN1B	protein_codin	36.2512266	123.133204	58.675021	25.0539497
ENSG000001	GPRC5D	protein_codin	0	3.25716941	0	1.30721326
ENSG000001	SCNN1A	protein_codin	0	0	0	2.21377908
ENSG000001	LTBR	protein_codin	17.3866742	20.6891045	27.9863268	18.943915
ENSG000001	OGFOD2	protein_codin	0	0	0	0
ENSG000001	OAS3	protein_codin	0	0	0	0
ENSG000001	ART4	protein_codin	0	2.70255352	0	4.42167153
ENSG000001	MGP	protein_codin	159.064004	175.847723	28.9855007	55.2702203
ENSG000001	ARHGDIB	protein_codin	31.6559567	135.363742	3.76021785	51.5577813
ENSG000001	GTF2H3	protein_codin	0	0	0	0
ENSG000001	RERGL	protein_codin	0	0	0	0
ENSG000001	ENDOU	protein_codin	0.60171114	1.08875663	0	2.54757062
ENSG000001	VDR	protein_codin	0	0	0	0
ENSG000001	FZD10	protein_codin	1.05577508	0	8.29527155	4.44102517
ENSG000001	IL26	protein_codin	0	0	0	0
ENSG000001	IFNG	protein_codin	0	1.73483182	0	1.09879499
ENSG000001	NUP107	protein_codin	43.9322389	21.9752676	7.24220097	6.23797588
ENSG000001	CNOT2	protein_codin	400.886151	37.5726869	70.5178292	0
ENSG000001	TIMELESS	protein_codin	0	66.7253258	251.599818	9.82497901
ENSG000001	KRR1	protein_codin	33.1608002	46.9848367	64.3606375	43.1798331
ENSG000001	MRPL51	protein_codin	4.4985344	25.2602738	40.2918902	6.42313945
ENSG000001	GAPDH	protein_codin	281.003397	481.22884	1381.9475	166.204471
ENSG000001	ACRBP	protein_codin	0	3.48708548	5.57579148	2.4164815
ENSG000001	COPS7A	protein_codin	0	0	0	0
ENSG000001	GNB3	protein_codin	0	0	0	0
ENSG000001	CDCA3	protein_codin	0	0	6.62885719	0
ENSG000001	CHPT1	protein_codin	0.85873058	0	0	4.51916272
ENSG000001	USP5	protein_codin	25.4207172	68.3752657	0	0
ENSG000001	TPI1	protein_codin	0	0	0	1.40769435
ENSG000001	ENO2	protein_codin	0	10.7172185	0	0
ENSG000001	C12orf57	protein_codin	5.91895666	36.3707745	30.6374223	12.8130908
ENSG000001	APOBEC1	protein_codin	0	0	0	0

ENSG000001	NANOG	protein_codin	0	0	1.39819838	3.24100149
ENSG000001	GOLT1B	protein_codin	8.81135425	26.1053329	23.7826234	11.9489954
ENSG000001	PRKAB1	protein_codin	5.23618083	15.7954054	0	0
ENSG000001	CMAS	protein_codin	7.37267753	0	23.1133868	2.98570803
ENSG000001	HCFC2	protein_codin	49.3141326	29.8029363	30.858805	15.3391241
ENSG000001	CLEC4A	protein_codin	4.62134662	0	0	9.0582778
ENSG000001	AICDA	protein_codin	0	0	0	0
ENSG000001	RAB35	protein_codin	13.3200774	18.6332749	11.0286042	7.45086289
ENSG000001	COX6A1	protein_codin	21.0405906	15.043747	80.121239	18.4214649
ENSG000002	GATC	protein_codin	0	0	0	0
ENSG000001	RFX4	protein_codin	0	0	0	0
ENSG000001	SRSF9	protein_codin	8.34988161	74.2612203	41.3384375	15.2449362
ENSG000001	FGFR1OP2	protein_codin	5.01682143	97.6234033	19.4421018	13.0770998
ENSG000001	KLRB1	protein_codin	1.15741255	14.1406243	0.47884601	2.28886035
ENSG000000	ZNF76	protein_codin	0.71287628	2.64624278	10.7415367	4.9414487
ENSG000000	IYD	protein_codin	0	1.34095762	0	4.64751781
ENSG000000	FYN	protein_codin	1.64232284	43.3321976	0	0
ENSG000001	RSPH4A	protein_codin	0	12.2581043	7.51081549	0
ENSG000001	TMEM14C	protein_codin	18.0061499	20.9177682	42.7268458	21.3880892
ENSG000001	SMIM8	protein_codin	7.80212191	8.83175231	10.9204939	2.40823033
ENSG000001	ADTRP	protein_codin	0	4.99080476	0	0
ENSG000001	ASF1A	protein_codin	15.9361909	21.1811992	23.6704286	4.39027029
ENSG000001	HINT3	protein_codin	9.76252614	40.2379163	14.9619267	6.66496204
ENSG000001	NCOA7	protein_codin	14.5307067	26.2556292	32.774714	0
ENSG000001	ULBP1	protein_codin	0.28297078	0	0	0.50756035
ENSG000002	SLC44A4	protein_codin	0	0	0	0
ENSG000001	FBXO5	protein_codin	0	13.9816714	68.3296084	4.70146309
ENSG000001	OPRM1	protein_codin	0	0	0	0
ENSG000001	FANCE	protein_codin	0	0	0	0
ENSG000001	TULP1	protein_codin	0	0	0	0
ENSG000001	MAPK14	protein_codin	23.3004594	0	58.1350644	0
ENSG000001	MAPK14	protein_codin	7.70061884	102.930271	0	14.2179776
ENSG000001	STK38	protein_codin	59.0645365	277.901203	214.67592	35.9929821
ENSG000002	HLA-DOA	protein_codin	18.093404	88.2248676	1.03895412	6.87116364
ENSG000001	MCM3	protein_codin	0	0	0	0
ENSG000001	RNF8	protein_codin	19.4761185	73.7884194	79.0337527	9.24904263
ENSG000001	SAYSD1	protein_codin	4.49209686	41.0154088	26.3221141	0
ENSG000001	KIF6	protein_codin	0.78938355	0	3.25259019	0
ENSG000001	CAP2	protein_codin	3.46518384	0	0	0
ENSG000001	GPR63	protein_codin	1.12558299	1.60152182	6.76296195	1.50453744
ENSG000001	FBXL4	protein_codin	1.47059502	0	0	0
ENSG000001	GPLD1	protein_codin	0.77856348	2.34571051	3.45336075	2.63591799
ENSG000001	ACOT13	protein_codin	19.2921253	33.7772948	172.182698	7.67467167
ENSG000001	RPS12	protein_codin	244.297629	181.296039	396.291819	242.802188

ENSG000001	B3GAT2	protein_codin	0	0	0	0
ENSG000001	GMNN	protein_codin	0	8.35830287	20.5193078	0
ENSG000001	SNX3	protein_codin	69.9584563	327.338941	91.9834184	43.9296442
ENSG000001	ZBTB24	protein_codin	0	0	0	0
ENSG000001	FIG4	protein_codin	0	0	0	0
ENSG000001	ADGRG6	protein_codin	0	0	0	0
ENSG000001	UNC93A	protein_codin	0	0	0	0
ENSG000001	C6orf118	protein_codin	0	0	0	0
ENSG000001	MDFI	protein_codin	9.88299575	32.4415455	84.7345442	7.46300463
ENSG000001	TFEB	protein_codin	0	0	0	0
ENSG000001	BYSL	protein_codin	4.48042035	14.8413381	31.5163711	0.7903775
ENSG000001	TBP	protein_codin	0	0	20.250754	0
ENSG000001	GUCA1B	protein_codin	0.32172003	10.1028953	5.42155277	0.43068431
ENSG000001	PRPH2	protein_codin	1.75849808	0	0	1.2542985
ENSG000001	PPP2R5D	protein_codin	0	18.8203148	0	0
ENSG000001	MRPL2	protein_codin	0	0	0.62551655	3.00682554
ENSG000001	PTK7	protein_codin	0	0	0	0
ENSG000001	PTK7	protein_codin	8.57798257	0	0	9.42260358
ENSG000001	DNPH1	protein_codin	2.98497168	5.38849937	18.7012903	2.08554476
ENSG000001	EXOC2	protein_codin	35.6954449	77.8593304	134.825114	14.8785848
ENSG000001	TMEM30A	protein_codin	78.9086144	249.052581	120.846896	76.6824079
ENSG000001	VEGFA	protein_codin	0	0	1.19777395	0
ENSG000001	TTK	protein_codin	0	0	1.43649332	0
ENSG000001	CCN6	protein_codin	0	0	0	0
ENSG000001	LAMA4	protein_codin	1.93347893	7.16708039	14.1859732	25.304596
ENSG000001	ENPP5	protein_codin	0	1.27483385	0	0
ENSG000001	LY86	protein_codin	1.73946518	5.13465774	0.98794425	0
ENSG000001	PRSS16	protein_codin	0	0	0	1.55690549
ENSG000001	MEP1A	protein_codin	0	0	0	0
ENSG000000:	MTREX	protein_codin	58.3675052	90.3908816	245.16195	6.1344308
ENSG000000	ISL1	protein_codin	0.3263636	0	11.1639633	0.46792967
ENSG000000	SLC6A7	protein_codin	0	2.83415675	0	1.2596874
ENSG000001	HARS2	protein_codin	0	0	2.50322694	0
ENSG000001	NUDT12	protein_codin	7.25700328	13.8342688	36.8449838	8.15481983
ENSG000001	TENT4A	protein_codin	20.2398985	84.3779674	47.6452278	34.4584217
ENSG000001	GHR	protein_codin	2.13716891	8.65901224	20.1328493	7.48123568
ENSG000001	DAP	protein_codin	51.0260182	179.637864	397.826388	72.594434
ENSG000001	BRD8	protein_codin	0	99.6404302	0	17.6934374
ENSG000001	MRPS30	protein_codin	24.8560515	31.2530101	44.0836488	10.7259577
ENSG000001	HBEGF	protein_codin	3.75894063	26.7967599	4.15507665	71.7951692
ENSG000001	LOX	protein_codin	100.750953	105.45756	99.6720508	72.1496043
ENSG000001	GZMK	protein_codin	3.50820798	27.1726239	0	0.57484139
ENSG000001	CDH9	protein_codin	0	0	0	0
ENSG000001	SPARC	protein_codin	2233.54112	2248.82441	162.809666	2568.43761

ENSG000001	HAND1	protein_codin	0	0	1.83010296	0
ENSG000001	PCDHB3	protein_codin	3.31028231	8.66976681	7.31449185	5.8306981
ENSG000001	PCDHB5	protein_codin	6.5411116	25.1165295	25.1929537	4.0597628
ENSG000001	PCDHB6	protein_codin	1.16989265	9.7936649	6.15305274	4.3878748
ENSG000001	PCDHB7	protein_codin	0.75850416	16.3179936	18.7925211	6.63933765
ENSG000001	PCDHB15	protein_codin	1.89738495	15.8987652	4.88002233	4.09637687
ENSG000001	GRM6	protein_codin	0	1.23957541	0	0.72385871
ENSG000001	THG1L	protein_codin	5.14767471	8.11248652	12.4399394	5.88700362
ENSG000001	IL12B	protein_codin	0	8.348464	0	0
ENSG000001	BTNL8	protein_codin	0	0	0	0
ENSG000001	TTC1	protein_codin	0	0	0	16.1713515
ENSG000000	C1QTNF3	protein_codin	17.0419896	0	13.7388823	0
ENSG000001	IRX4	protein_codin	0	0	0	0
ENSG000001	LNPEP	protein_codin	113.163969	355.328796	379.676362	80.2690641
ENSG000001	AGXT2	protein_codin	0	0	0	0
ENSG000001	PRLR	protein_codin	0	0	0	0
ENSG000001	IL4	protein_codin	0	0	0	0
ENSG000001	IL5	protein_codin	0	0	0	0.75865084
ENSG000001	ST8SIA4	protein_codin	23.4639505	84.5955778	37.4214011	30.1443266
ENSG000001	PCDH12	protein_codin	8.58400317	47.2281409	21.1052091	19.6310651
ENSG000001	NUP155	protein_codin	14.5564038	29.7074219	0	12.1908535
ENSG000001	PPP2CA	protein_codin	2.59589201	6.93417708	9.69960526	3.83910843
ENSG000001	NR3C1	protein_codin	0	14.8618048	0	0
ENSG000001	C5orf15	protein_codin	36.3159746	52.863608	67.3585613	25.6490316
ENSG000001	TRIM23	protein_codin	14.0447321	21.7790162	23.1148421	10.9141346
ENSG000001	TRAPPC13	protein_codin	0	0	0	0
ENSG000001	RARS1	protein_codin	40.3392761	69.9179846	86.2058889	13.2314077
ENSG000001	CDX1	protein_codin	0	0	0	0
ENSG000001	BNIP1	protein_codin	0	1.5124703	0	0
ENSG000000	SLC7A14	protein_codin	0	0	0.50676353	8.1701864
ENSG000000	SEMA3G	protein_codin	26.5504021	37.9238321	7.54715644	14.1024122
ENSG000000	ZMYND10	protein_codin	0	0	0	0
ENSG000000	LTF	protein_codin	0	5.37657025	0	0
ENSG000000	MLH1	protein_codin	0	0	0	0
ENSG000001	EHHADH	protein_codin	4.81753994	3.94587723	21.6302965	0.80601597
ENSG000001	CRBN	protein_codin	0	0	0	0
ENSG000001	HRG	protein_codin	0	0	0	0
ENSG000001	BCL6	protein_codin	0	20.1095403	1.91960858	0
ENSG000001	FAM162A	protein_codin	4.92195015	9.26731922	19.3690285	2.11448018
ENSG000001	RBP2	protein_codin	0	0	0	0
ENSG000001	PFKFB4	protein_codin	0.71076258	1.43126092	0.66468619	1.59452147
ENSG000001	HES1	protein_codin	36.2357255	66.5558939	208.466774	43.9539805
ENSG000001	ECT2	protein_codin	21.6941454	36.5933812	0	0
ENSG000001	GNAT1	protein_codin	0.25326995	1.33433094	0	1.68081655

ENSG000001	TUSC2	protein_codin	2.51661957	2.81777724	2.76626406	1.2677578
ENSG000001	NPRL2	protein_codin	0	0	0	0
ENSG000001	CYB561D2	protein_codin	0	0	0	0
ENSG000001	CEP15	protein_codin	0	0	12.465916	0
ENSG000001	GNB4	protein_codin	69.2982352	116.83952	106.385585	29.6852396
ENSG000001	MORC1	protein_codin	0	0	0	0
ENSG000001	UMPS	protein_codin	3.68109195	6.49138753	7.5197684	4.61907613
ENSG000001	ABTB1	protein_codin	3.01138266	7.19755229	0	5.34441915
ENSG000001	KLHL18	protein_codin	8.8083908	35.221648	25.1729978	13.5960031
ENSG000001	HEMK1	protein_codin	3.65513975	25.2504496	8.50510307	8.40333125
ENSG000001	RRP9	protein_codin	3.34502241	3.70706776	12.4208361	1.96765795
ENSG000001	AADAC	protein_codin	0	0	0	0.81049167
ENSG000001	EIF1B	protein_codin	2.8514983	7.97025463	10.203159	8.63703958
ENSG000001	ZBTB47	protein_codin	0	0	0	0
ENSG000001	TNNC1	protein_codin	71.7278625	0.53673942	0	1.29817517
ENSG000001	NKTR	protein_codin	127.749783	0	420.382914	0
ENSG000001	SPCS1	protein_codin	15.3086674	0	30.4216741	12.9945905
ENSG000001	NEK4	protein_codin	7.61546541	12.8022753	14.406786	10.4544871
ENSG000000	LDAF1	protein_codin	0	0	0	0
ENSG000000	WDFY1	protein_codin	47.1703938	173.611005	51.9060204	63.8701779
ENSG000000	EIF2AK2	protein_codin	18.4631215	153.962701	0	29.0173207
ENSG000000	CPS1	protein_codin	0	0	0	0
ENSG000000	DAZAP1	protein_codin	9.90361595	27.5741629	22.9338641	16.0290788
ENSG000000	DDX1	protein_codin	93.9740303	0	290.579268	52.9549376
ENSG000000	FKBP7	protein_codin	9.98621079	12.9717115	0	0
ENSG000000	HEATR5B	protein_codin	27.1678792	75.8080586	102.77476	18.4921027
ENSG000000	MDH1	protein_codin	25.4247666	27.9010743	101.445517	12.0927169
ENSG000000	MAPRE3	protein_codin	1.64383073	2.29503624	0	3.53246739
ENSG000000	TMSB10	protein_codin	436.68921	3245.78675	878.134566	614.366384
ENSG000000	MSH2	protein_codin	0	0	0	0
ENSG000000	NCK2	protein_codin	26.3280467	64.8335972	45.0042781	23.1795611
ENSG000000	TFPI	protein_codin	0	0	47.3220824	23.6302352
ENSG000000	NDUFS1	protein_codin	60.9358135	66.9407998	143.824335	25.8103289
ENSG000000	SLC11A1	protein_codin	0.28933543	3.20212117	0	0
ENSG000000	APOB	protein_codin	0.44922681	4.53708958	0.42027218	8.05190017
ENSG000001	RTKN	protein_codin	10.4128039	12.0963319	17.2482328	4.6430852
ENSG000001	INO80B	protein_codin	2.75331957	20.8040773	0	5.11216596
ENSG000001	TTL	protein_codin	20.1995475	27.4405806	60.926527	19.106294
ENSG000001	FAHD2A	protein_codin	8.38265314	57.8858711	18.2985745	9.92866473
ENSG000001	SF3B6	protein_codin	32.7906637	24.8665171	144.502804	19.0163713
ENSG000001	CENPA	protein_codin	0	3.8514366	49.4275937	0
ENSG000001	SLC30A3	protein_codin	0	2.10310575	0	0
ENSG000001	MPV17	protein_codin	0	0	0	0
ENSG000001	NRBP1	protein_codin	19.9987114	60.2750868	33.2515911	6.91488307

ENSG000001	ITGA4	protein_codin	0	0	0	0
ENSG000001	SNX17	protein_codin	16.4269906	0	41.2264422	0
ENSG000001	REEP6	protein_codin	0	2.10895758	5.50469805	1.12181749
ENSG000001	APC2	protein_codin	0.66723202	0.82780442	0	0.85130045
ENSG000001	RPS15	protein_codin	2.24251555	0	47.7095233	12.6447979
ENSG000001	GCA	protein_codin	3.29053982	19.4045558	9.1108778	0
ENSG000002	WBP1	protein_codin	0	0	1.30317653	1.62123183
ENSG000001	TTC31	protein_codin	0	0	12.2182062	6.45797788
ENSG000001	NDUFS7	protein_codin	0	4.43318042	0	0
ENSG000001	PCGF1	protein_codin	4.08979359	0	9.66758226	2.2288228
ENSG000001	TLX2	protein_codin	0	0	1.01769697	1.65837155
ENSG000001	DOK1	protein_codin	4.65709025	15.8244495	0	0
ENSG000001	POLE4	protein_codin	0	0	5.9016283	1.42061167
ENSG000001	ACADL	protein_codin	2.06590834	0	12.6386265	0
ENSG000001	EVA1A	protein_codin	1.40014201	0	0	1.72259505
ENSG000001	LANCL1	protein_codin	0	0	0	0
ENSG000001	REG1A	protein_codin	0	0	0	0
ENSG000001	FANCL	protein_codin	0	0	0	0
ENSG000001	IGFBP2	protein_codin	4.52378512	4.4612165	116.461416	6.51264044
ENSG000001	IGFBP5	protein_codin	1040.15679	1908.97169	402.895869	379.297063
ENSG000001	KCNJ13	protein_codin	0	0	0	2.31223515
ENSG000001	GGCX	protein_codin	9.34585887	24.9403639	21.3138118	20.018846
ENSG000001	NEU2	protein_codin	0	0	0	0.37346644
ENSG000001	MOB4	protein_codin	0	0	0	0
ENSG000001	HSPE1	protein_codin	7.78660469	0	73.4147486	18.7358702
ENSG000001	WNT6	protein_codin	0.27974267	0	1.92174424	0
ENSG000001	IL1RL1	protein_codin	1.30610826	14.1605699	0	4.4175001
ENSG000001	IL18R1	protein_codin	0	8.98509608	0	16.2218725
ENSG000001	SLC9A2	protein_codin	2.18118747	6.71476211	4.82252005	7.97920423
ENSG000001	AZU1	protein_codin	0	0	0	0
ENSG000001	PPP1R7	protein_codin	1.96079955	0	1.55551253	0.79391545
ENSG000001	PASK	protein_codin	0	0	7.33688313	0
ENSG000001	PROC	protein_codin	0	0	0	0
ENSG000001	ID2	protein_codin	0	2.03841201	0	4.98942176
ENSG000001	ODC1	protein_codin	1.02500801	0	33.0178983	0
ENSG000001	PLEKHB2	protein_codin	0.56678655	0	0	1.3669016
ENSG000001	GREB1	protein_codin	10.0402116	6.81695053	0	3.94244683
ENSG000001	GORASP2	protein_codin	10.5619936	29.4207872	27.7762038	19.8601905
ENSG000001	CEBPZ	protein_codin	85.8196794	110.991231	280.177741	58.275419
ENSG000001	PRKD3	protein_codin	63.9116854	155.349771	161.691804	44.8063333
ENSG000001	RMDN2	protein_codin	0	0	0	0
ENSG000001	DLX2	protein_codin	0	0	11.5603722	1.2947867
ENSG000001	SLC1A4	protein_codin	0	32.9041131	11.6474112	4.13009084
ENSG000001	ORC2	protein_codin	18.0811439	42.5067822	58.0150016	6.79475384

ENSG000001	COX7A2L	protein_codin	4.34070192	11.4028454	11.8809016	8.9185475
ENSG000002	PPP3R1	protein_codin	41.2201006	88.1511305	94.461027	56.7264989
ENSG000001	PLEK	protein_codin	20.3293528	334.003551	15.2998309	60.8540702
ENSG000001	PRTN3	protein_codin	0	0	0.48394009	0.42324562
ENSG000001	KISS1R	protein_codin	0	0	0	0
ENSG000001	GRIN3B	protein_codin	0	0	0	0
ENSG000001	VAX2	protein_codin	0.88026296	0	3.17826152	0.4505366
ENSG000001	ATP6V1B1	protein_codin	0.2277302	0	2.83172429	0
ENSG000001	MSH6	protein_codin	0	0	0	0
ENSG000001	PLEKHA3	protein_codin	15.029719	34.6268617	14.387139	17.5505249
ENSG000001	SPR	protein_codin	0.90781964	0	13.4679771	2.30761402
ENSG000000	FAM76A	protein_codin	13.5132301	0	18.7934983	0
ENSG000000	ENO1	protein_codin	61.8370187	115.927198	354.660993	44.1106487
ENSG000000	CDC7	protein_codin	10.3264838	0	55.1413415	7.44146339
ENSG000000	SYDE2	protein_codin	5.94276909	7.79837592	9.86747701	3.41559486
ENSG000000	SARS1	protein_codin	65.1230221	143.940088	135.094733	29.8390352
ENSG000000	CLCA1	protein_codin	0	1.498826	0	0
ENSG000001	BCL9	protein_codin	0	70.5863891	50.0170326	0
ENSG000001	TPSG1	protein_codin	0.24874897	0	0	0
ENSG000001	ANGPTL1	protein_codin	17.884024	0	0	3.90414459
ENSG000001	TCEANC2	protein_codin	19.5353849	37.109288	52.4552141	12.501487
ENSG000001	TMEM59	protein_codin	24.7919463	31.9398514	32.427511	26.6298658
ENSG000001	RPL22	protein_codin	61.1297045	146.033434	112.00838	54.0107887
ENSG000001	OPRD1	protein_codin	0.29647617	3.35023207	3.09618425	3.22436428
ENSG000001	WDR77	protein_codin	4.58448855	16.7933787	26.1883444	1.43934088
ENSG000001	RNF19B	protein_codin	16.4457733	0	14.0713623	27.2255626
ENSG000001	DLGAP3	protein_codin	0	0.96840585	0.5505484	0.86894503
ENSG000001	C1orf21	protein_codin	26.559284	41.1191474	34.4894149	20.3630194
ENSG000001	MAD2L2	protein_codin	0	0	0	0
ENSG000001	MFN2	protein_codin	0	63.209527	51.4625483	27.6061278
ENSG000001	MIIP	protein_codin	2.23954062	11.1936393	21.9525345	6.41080842
ENSG000001	SLC35D1	protein_codin	19.9614979	26.8231992	51.5144037	9.4212843
ENSG000001	PRAMEF10	protein_codin	0	0	0	0
ENSG000002	PRAMEF4	protein_codin	0	0	0	0.77104261
ENSG000001	PRDM2	protein_codin	0	0	15.9594508	6.19212265
ENSG000001	RGS2	protein_codin	14.6567393	17.0256912	21.8149328	77.1283962
ENSG000002	DENND1B	protein_codin	0	0	0	0
ENSG000001	WARS2	protein_codin	0	3.93677408	26.9957795	8.90073159
ENSG000001	OSCP1	protein_codin	0	0	3.01398895	1.82879841
ENSG000001	NT5C1A	protein_codin	12.6796575	3.58944254	4.23349857	7.47676976
ENSG000001	KDM5B	protein_codin	0	8.52143715	2.25083416	0
ENSG000000	AKR7A2	protein_codin	6.09595891	3.06883845	30.4086664	5.2950447
ENSG000001	GBP3	protein_codin	2.13233954	19.733807	0	2.53144515
ENSG000001	RAB29	protein_codin	0.72104023	0	0	0

ENSG000001	CD160	protein_codin	0	6.06877718	0	1.91303716
ENSG000001	HMGCL	protein_codin	0	0	0	0
ENSG000001	APH1A	protein_codin	0.33605535	8.14827811	1.78396541	0
ENSG000001	P3H1	protein_codin	5.8853124	53.4231522	13.6177983	0
ENSG000001	EBNA1BP2	protein_codin	16.3742387	29.5534378	78.9925805	3.86824515
ENSG000001	ATP6V0B	protein_codin	0	0	0	0
ENSG000001	SLC19A2	protein_codin	2.43959211	0	0	0
ENSG000001	SELL	protein_codin	0	0	0	0
ENSG000001	VAMP4	protein_codin	19.4612364	34.0732514	16.4681359	7.61053804
ENSG000001	GRHL3	protein_codin	0.24133342	0	0	0
ENSG000001	SYF2	protein_codin	75.3440391	239.29593	187.401515	64.7343758
ENSG000001	DHDDS	protein_codin	7.68073847	0	0	7.63270447
ENSG000001	PPP1R8	protein_codin	0	0	3.79583637	0
ENSG000001	SLC5A9	protein_codin	0	0	0	0
ENSG000001	CTSD	protein_codin	0	146.697346	0	0
ENSG000001	COLEC11	protein_codin	0	0	0	0
ENSG000001	STAG1	protein_codin	1.62376128	0	0	0
ENSG000001	A4GNT	protein_codin	0	0	0	1.45984027
ENSG000001	MMP8	protein_codin	0.5687099	3.00274521	0	3.08330008
ENSG000001	APOA1	protein_codin	0	0	0	0
ENSG000001	SLC8A2	protein_codin	0	0	1.41355729	0
ENSG000001	NR5A2	protein_codin	0	0	0	0
ENSG000001	CAMSAP2	protein_codin	56.7095381	0	38.3505013	33.6178213
ENSG000000	FCGR2B	protein_codin	7.57963009	11.5027	0	4.63160574
ENSG000001	FCRLA	protein_codin	0	0	0	0
ENSG000001	EEF1B2	protein_codin	0	0	12.5315685	0
ENSG000001	ATIC	protein_codin	10.1982218	23.310229	56.114549	11.13543
ENSG000001	TNP1	protein_codin	0	0	0	0.99097691
ENSG000001	FASTKD2	protein_codin	10.5534446	27.3680787	26.4291391	8.35229292
ENSG000001	TTR	protein_codin	0	0	0	0.45964498
ENSG000001	B4GALT6	protein_codin	0	8.06457573	0	0
ENSG000000	DOP1A	protein_codin	23.5754014	48.4905798	19.8805668	23.0163871
ENSG000001	FILIP1	protein_codin	20.2520922	66.9424226	0	21.5981085
ENSG000001	CASP8AP2	protein_codin	6.01612492	28.8155483	16.2596122	4.93164151
ENSG000001	UBE3D	protein_codin	0	0	0	0
ENSG000001	SPACA1	protein_codin	0	0	0	0
ENSG000001	SGIP1	protein_codin	0	0	0	0
ENSG000000	TBPL1	protein_codin	1.25588773	4.67303742	5.31730466	2.77340334
ENSG000001	ZC2HC1B	protein_codin	0	0	0	0
ENSG000001	FBXO30	protein_codin	29.6756492	76.9144494	87.3141919	19.7576229
ENSG000001	ADAT2	protein_codin	4.75786376	10.2156143	23.0033442	8.07214955
ENSG000001	TNFAIP3	protein_codin	0	0	1.44774914	28.7536365
ENSG000001	SGK1	protein_codin	0	0	0	42.3244063
ENSG000001	TCF21	protein_codin	0	0	0	1.34509876

ENSG000001	PMFBP1	protein_codin	0.5731425	2.32338502	27.8572359	0
ENSG000001	MED28	protein_codin	34.9792773	158.765235	134.042367	28.5532568
ENSG000002	RNF103	protein_codin	22.8959886	25.0252237	48.8939168	16.67483
ENSG000001	MYL12B	protein_codin	131.979654	377.345049	261.531696	92.4398898
ENSG000001	ARMC2	protein_codin	1.24186153	8.96738227	3.64713321	2.53784845
ENSG000001	GHRH	protein_codin	0	0	0	0
ENSG000001	RPN2	protein_codin	133.66438	145.187625	339.686975	60.0418075
ENSG000001	MTCL2	protein_codin	93.5032878	188.956338	136.039376	73.5506295
ENSG000001	PKD2	protein_codin	46.1522616	152.180387	62.2946405	30.8358878
ENSG000001	ABCG2	protein_codin	0	0	4.57201961	0
ENSG000001	SPP1	protein_codin	17.8411937	398.939786	47.8054595	1336.95488
ENSG000001	STBD1	protein_codin	12.9219957	13.3520261	0	2.72137597
ENSG000001	ADH6	protein_codin	0	1.26288975	0	2.21663584
ENSG000001	CCNI	protein_codin	95.0336775	211.155416	122.131317	134.610013
ENSG000001	RARRES1	protein_codin	0.53630905	11.1293672	10.8707555	12.0012108
ENSG000001	RAB3GAP2	protein_codin	0	0	0	1.06236847
ENSG000001	LDAH	protein_codin	0	0	0	0
ENSG000001	FGF23	protein_codin	0.23741908	0	0	0.8990572
ENSG000001	ELL2	protein_codin	44.755022	194.093666	84.8335592	168.341841
ENSG000001	GLRX	protein_codin	8.95340344	15.1011614	0	15.4520187
ENSG000001	NDUFB3	protein_codin	11.1926689	12.2835899	23.4954616	7.5792513
ENSG000001	ZFAND5	protein_codin	39.9528507	0	0	51.3384034
ENSG000001	GDA	protein_codin	0	0	0	0
ENSG000001	ECRG4	protein_codin	2.22382605	9.6244818	0	0
ENSG000001	YWHAQ	protein_codin	47.4815721	38.3156142	124.176027	60.9702097
ENSG000001	ITGB1BP1	protein_codin	12.881817	0	15.132096	8.476491
ENSG000001	CPSF3	protein_codin	22.4781447	58.0072211	163.379864	14.901582
ENSG000001	PIGZ	protein_codin	0.82207339	0	2.17212691	1.07074519
ENSG000001	DDX55	protein_codin	5.127396	5.74991904	8.58432096	8.48460845
ENSG000001	CCDC92	protein_codin	6.58619224	17.1150481	4.31913668	5.27138529
ENSG000001	LGALS8	protein_codin	0.32334591	0	0	0.65661025
ENSG000001	FKBP15	protein_codin	10.5833127	45.8580822	0	0
ENSG000001	HDHD3	protein_codin	0	4.28188574	0	0.44079359
ENSG000001	ALG2	protein_codin	0	12.4582711	11.3363687	0
ENSG000001	APC2	protein_codin	0	0	0	0
ENSG000001	VPS4B	protein_codin	36.1274778	34.6489338	53.36329	23.1278418
ENSG000002	SERPINB10	protein_codin	0	1.97071944	0	1.42543702
ENSG000001	GSC	protein_codin	9.48641478	4.26210092	0	2.84650412
ENSG000000	ADCK1	protein_codin	0	3.2172322	0	3.98693969
ENSG000001	PGF	protein_codin	0	0	0	0
ENSG000001	IFI27L2	protein_codin	13.7373751	40.6905556	11.7247139	5.25102546
ENSG000001	NEK9	protein_codin	7.60301859	0	1.90022465	0
ENSG000001	ACYP1	protein_codin	3.97703362	8.98276974	21.1580138	0
ENSG000001	IFT43	protein_codin	0	0	0	0

ENSG000001	NPC2	protein_codin	0	0	0	0
ENSG000001	IRF2BPL	protein_codin	14.3807684	56.5173005	31.7762899	30.3880322
ENSG000001	ACOT2	protein_codin	0	0	0	0
ENSG000001	FLVCR2	protein_codin	3.10345559	28.0069224	6.25485886	2.8037561
ENSG000001	DLST	protein_codin	1.21635844	0	0	0
ENSG000001	TGFB3	protein_codin	221.74028	129.128276	115.653975	0
ENSG000001	ZC2HC1C	protein_codin	0	0	0	0
ENSG000001	SLIRP	protein_codin	0	0	0	15.4392012
ENSG000001	PAPOLG	protein_codin	11.978514	47.8749063	33.9123767	11.2058549
ENSG000001	TP53I3	protein_codin	0	0	0	1.88586933
ENSG000001	RHOQ	protein_codin	45.4724961	26.2972866	167.517236	22.7990637
ENSG000001	TMEM214	protein_codin	14.1437304	0	19.1855165	0
ENSG000001	ATAD2B	protein_codin	55.7489341	192.780606	223.621547	48.4327937
ENSG000001	FAM98A	protein_codin	7.98275007	43.2409003	32.9639312	0
ENSG000001	YIPF4	protein_codin	41.0495908	65.8955347	65.7704177	24.5978829
ENSG000001	AFTPH	protein_codin	11.2669668	9.14181541	22.344822	8.99878784
ENSG000001	LGALSL	protein_codin	13.2511138	0	0	2.3705676
ENSG000001	CRIPT	protein_codin	6.93506146	20.1740681	24.4633198	14.8410946
ENSG000001	ADGRB3	protein_codin	0.60533959	0	0	0
ENSG000001	MFSD13A	protein_codin	0	1.25458022	0	0.84890397
ENSG000001	SLF2	protein_codin	58.5803785	79.9810075	128.715294	32.2414914
ENSG000001	LIPF	protein_codin	0	0	0	0
ENSG000001	PPP1R3C	protein_codin	14.5114376	61.7470751	20.512813	2.87327559
ENSG000001	MXI1	protein_codin	0	0	0	0
ENSG000001	PRLHR	protein_codin	0	0	0	4.34047433
ENSG000001	KCNIP2	protein_codin	0	0	0.3904569	0
ENSG000001	HOXB8	protein_codin	0	0	0	0
ENSG000001	HOXB5	protein_codin	0	2.68802275	0.46956066	0
ENSG000002	HOXB7	protein_codin	0.68707544	4.73145172	7.57196062	0
ENSG000001	HOXB1	protein_codin	0	0	0	0
ENSG000001	DUSP1	protein_codin	70.854774	65.4762293	6.66996538	283.523485
ENSG000001	PANK3	protein_codin	115.702447	142.698954	235.007844	50.3211249
ENSG000001	MSX2	protein_codin	8.60973882	1.06562847	42.0721005	2.42841444
ENSG000001	INSL4	protein_codin	0	1.7402191	0	2.47539192
ENSG000002	IFNA7	protein_codin	0	0	0	2.42657298
ENSG000001	LRP11	protein_codin	30.4523091	43.6613248	72.9396853	18.1981148
ENSG000001	CCDC170	protein_codin	7.61470241	52.390301	4.74353138	12.635737
ENSG000001	ARAP3	protein_codin	0	4.31729301	11.4482372	1.8041481
ENSG000001	PCDHB8	protein_codin	0.54876332	7.70543885	0.57281681	1.50464251
ENSG000001	PCDHB10	protein_codin	5.13120536	11.8857989	16.0876	1.64289688
ENSG000001	PCDHB14	protein_codin	6.91725935	14.5318723	2.73312314	8.0938079
ENSG000001	PCDHB12	protein_codin	0.7867018	17.2652089	8.63304241	4.09721228
ENSG000001	SLC25A2	protein_codin	0	0	0.9636733	0.86484061
ENSG000001	PRRX1	protein_codin	605.087181	474.08277	485.785399	170.171317

ENSG000001:	TNN	protein_codin	68.3886448	43.0945736	0	64.8782982
ENSG000001:	TTLL2	protein_codin	0	0	0	0.46081719
ENSG000001:	MSANTD2	protein_codin	0	4.40125275	1.59123998	0
ENSG000001:	PDZD11	protein_codin	8.38056199	12.9110063	17.6620436	1.04374419
ENSG000001:	NUDCD1	protein_codin	10.3675457	0	0	8.44266948
ENSG000001:	CCDC77	protein_codin	0	0	0	0
ENSG000001:	MTRF1	protein_codin	0	0	0	0
ENSG000002	CCDC169	protein_codin	0	0	0	1.0280451
ENSG000002	CCDC169	protein_codin	0	0	0	0
ENSG000001:	UFM1	protein_codin	58.8949477	113.86822	144.1658	47.1613349
ENSG000001:	ELF1	protein_codin	29.6218131	91.9832053	9.05734506	55.1118339
ENSG000001:	ALG5	protein_codin	0	0	0	0
ENSG000001:	EXOSC8	protein_codin	0	0	0	0
ENSG000001:	FAM53C	protein_codin	12.6138873	26.8044495	25.7781291	14.5199557
ENSG000001:	MYOT	protein_codin	12.649504	0	0	0.95280362
ENSG000001:	EGR1	protein_codin	24.4093638	82.6201762	5.26018617	762.363605
ENSG000000	PFN2	protein_codin	11.8366861	0	12.0823133	0
ENSG000001:	SERP1	protein_codin	34.2652156	59.2840896	167.762375	44.9339208
ENSG000001:	MTERF2	protein_codin	0	0	1.07008143	0
ENSG000001:	NFYB	protein_codin	8.00406919	23.4971676	15.8563323	3.51454766
ENSG000001:	WASHC3	protein_codin	5.62829455	0	0	0
ENSG000001	FZD3	protein_codin	4.22991049	0	0	0
ENSG000001	SLC39A14	protein_codin	0	0	0	0
ENSG000001:	DUSP4	protein_codin	5.60212133	23.6633794	3.38248743	21.37656
ENSG000001:	DUSP4	protein_codin	0.45297946	5.76203249	0.68255216	0.58728105
ENSG000001:	SORBS3	protein_codin	5.53087467	0	0	2.88065144
ENSG000001:	CHRNA2	protein_codin	0	0	0.49964317	1.06209164
ENSG000001:	PPP3CC	protein_codin	0	29.4112379	0	1.43530225
ENSG000001:	RNF170	protein_codin	0	0	0	0
ENSG000001:	TARDBP	protein_codin	24.5816375	88.6442608	43.8264845	1.244963
ENSG000001:	PRAMEF2	protein_codin	0	1.4630062	0.51468837	0.40953453
ENSG000001:	RDH10	protein_codin	6.55215923	18.746721	22.7243447	6.52400717
ENSG000001	LUC7L3	protein_codin	233.320993	276.370843	656.37137	35.1177078
ENSG000001	DLX4	protein_codin	0	0	1.74030039	0
ENSG000001:	COIL	protein_codin	24.6497376	51.2836817	114.504379	12.1008357
ENSG000001:	TBX2	protein_codin	36.2318999	71.8859541	0	22.4961931
ENSG000001:	SLC35B1	protein_codin	0	0	0	0
ENSG000001:	TBX4	protein_codin	0	0	0	0
ENSG000001:	TEX14	protein_codin	0	0	0	0
ENSG000001:	FAM117A	protein_codin	4.20721661	27.0234193	7.99323766	11.8567394
ENSG000001:	NCAPH	protein_codin	0.39422713	1.99574735	11.092385	3.9558661
ENSG000001:	TMEM131L	protein_codin	19.603894	0	81.5634887	9.9151186
ENSG000001:	MND1	protein_codin	0	0	7.4885722	0.90091985
ENSG000001:	ZNF141	protein_codin	30.3226245	124.227274	97.254124	13.5679215

ENSG000001	TSHZ3	protein_codin	29.9578556	56.1311568	32.0252033	18.7785941
ENSG000001	TAS2R8	protein_codin	0	0	0	0
ENSG000001	PLBD1	protein_codin	9.41682194	35.2475424	14.1461861	14.2930402
ENSG000002	KLRK1	protein_codin	4.49190298	0	0	10.788679
ENSG000001	TAS2R10	protein_codin	0.67050339	0	6.56628038	0.54575223
ENSG000002	PRB1	protein_codin	0	0	0	0
ENSG000001	PYROXD1	protein_codin	8.39583247	4.41552657	11.5169379	4.65842935
ENSG000001	IAPP	protein_codin	0	0	0	0.67458142
ENSG000001	KCNJ8	protein_codin	0	0	0	0
ENSG000001	TAS2R7	protein_codin	0	1.53250703	0.45057229	0.46400429
ENSG000001	TAS2R9	protein_codin	0.20654596	2.12848679	0	0
ENSG000001	ZNF549	protein_codin	0	0	20.5006244	9.16233628
ENSG000001	ZSCAN18	protein_codin	0	0	0	0
ENSG000001	ZNF211	protein_codin	0.49414173	2.43267543	4.89607079	0
ENSG000001	TFG	protein_codin	4.26170473	0	14.2963173	0
ENSG000001	KALRN	protein_codin	0	0	0	0
ENSG000001	NAA50	protein_codin	32.2307557	42.9193589	94.9850104	33.142561
ENSG000000	PAX6	protein_codin	0	0	0	0
ENSG000001	MAPK8IP1	protein_codin	2.66725303	0	0.58143076	2.23703071
ENSG000001	PEX16	protein_codin	0	2.47968792	0	0
ENSG000001	DEPDC7	protein_codin	10.9871161	12.7000659	17.9306325	1.56604169
ENSG000001	CAT	protein_codin	58.2886727	94.0310424	41.3068126	40.9186322
ENSG000000	ACHE	protein_codin	0	0	0	0
ENSG000001	FBXO24	protein_codin	0	0	0	0
ENSG000001	GJB6	protein_codin	0	1.53555201	0	0
ENSG000001	GJA3	protein_codin	0.6196628	1.02099261	1.65487425	1.91420419
ENSG000001	GHSR	protein_codin	0	0	0	0
ENSG000001	TNFSF10	protein_codin	24.5098964	29.302739	0	19.8838607
ENSG000001	SLITRK3	protein_codin	0	0	0	0
ENSG000001	CPXM2	protein_codin	34.4190343	42.9685379	64.1336541	20.8393781
ENSG000002	GSTM2	protein_codin	1.45708473	19.635235	0	0
ENSG000002	ADORA3	protein_codin	0.41925389	3.81507914	0	0.62653049
ENSG000001	GTDC1	protein_codin	0	0	0	0
ENSG000001	CXCR4	protein_codin	18.1872996	65.005985	9.06863593	14.1180203
ENSG000001	ACVR2A	protein_codin	15.8555354	18.7770381	25.8277078	0
ENSG000001	POLK	protein_codin	33.3344774	107.514817	67.2141219	46.1392086
ENSG000001	FLT3	protein_codin	0	0	0	0.82958253
ENSG000001	RASL11A	protein_codin	1.61978062	8.30300592	3.03618458	7.61420156
ENSG000001	FYTTD1	protein_codin	68.486609	61.2302154	178.805754	35.3756196
ENSG000001	MTERF4	protein_codin	0	0	0	0
ENSG000001	MRPS2	protein_codin	3.44610669	2.03469908	8.06554816	3.32690829
ENSG000001	MYOG	protein_codin	0.84149251	0	0	0
ENSG000001	COPA	protein_codin	0	0	0	0
ENSG000001	PRM2	protein_codin	0	0.55301	0	0.82254577

ENSG000001	OPN4	protein_codin	0	1.24375026	0	0
ENSG000001	AHR	protein_codin	106.721079	63.0347777	73.553484	54.3189992
ENSG000001	SCRN1	protein_codin	25.3718929	47.3066449	163.448925	24.674427
ENSG000000	ETV1	protein_codin	0	0	0	0
ENSG000001	BBS9	protein_codin	0	0	0	2.36878956
ENSG000001	OCM	protein_codin	0	0	0	0
ENSG000001	EEPD1	protein_codin	6.09938143	18.2456057	10.5910061	6.5035848
ENSG000001	WIPF3	protein_codin	1.81728238	9.54761763	0	4.63631506
ENSG000001	NPY	protein_codin	0	0	0.67778227	0.93234787
ENSG000001	HOXA7	protein_codin	2.38836302	6.26948995	11.2198843	0.50228404
ENSG000001	INHBA	protein_codin	105.153903	153.021336	7.69452223	21.197346
ENSG000001	FKBP9	protein_codin	42.3619508	129.568987	46.5765371	57.6544748
ENSG000001	POLM	protein_codin	0	16.0639354	0	0
ENSG000001	RAMP3	protein_codin	1.46929717	3.39541251	0	1.28589853
ENSG000001	MRM2	protein_codin	15.4532232	44.5404717	35.7325236	12.6100587
ENSG000001	TWIST1	protein_codin	53.1894842	64.3898993	322.005198	41.0429718
ENSG000001	SLC25A51	protein_codin	4.59492679	0	0	1.87989353
ENSG000001	CLTA	protein_codin	0	0	0	0
ENSG000001	TAF1L	protein_codin	1.11302998	6.52840265	3.00774164	12.3022937
ENSG000001	PHF24	protein_codin	0.26478339	2.42537671	3.47770438	3.9837274
ENSG000001	DNAI1	protein_codin	0	0	0	0
ENSG000001	DCAF10	protein_codin	26.8842139	0	47.6148912	8.25761044
ENSG000001	ZC3HAV1	protein_codin	51.7148018	92.9047416	75.8659197	81.0667707
ENSG000001	AKR1D1	protein_codin	0	0	0	0
ENSG000001	NEUROG3	protein_codin	0	0	0	1.06802834
ENSG000001	SRGN	protein_codin	0	93.323485	24.276435	50.333156
ENSG000001	EGR2	protein_codin	0	0	0	5.36839459
ENSG000001	FAM149B1	protein_codin	6.11046897	26.3096535	17.6742142	3.35446203
ENSG000000	UNG	protein_codin	0	16.2508555	10.0053741	7.63517089
ENSG000000	DYNLL1	protein_codin	75.6328686	68.0761108	102.057109	34.1883083
ENSG000001	IFT81	protein_codin	0	0	3.13944476	0
ENSG000001	ACADS	protein_codin	4.73647435	6.18946803	5.28326609	4.55734375
ENSG000001	HVCN1	protein_codin	0	13.40948	0	2.73611272
ENSG000001	RNF11	protein_codin	50.8839113	89.3587536	51.7657686	43.7430015
ENSG000001	BHLHE41	protein_codin	72.7424978	85.0439715	11.8636403	24.1652957
ENSG000001	SSPN	protein_codin	52.8508078	9.59454928	0	37.0970225
ENSG000001	ITPR2	protein_codin	0	0	0	0
ENSG000001	STX10	protein_codin	3.2872121	0	0	0
ENSG000001	DDX39A	protein_codin	8.61884264	0	71.4013724	2.85980736
ENSG000001	PKN1	protein_codin	20.531675	23.4326072	0	30.3437629
ENSG000001	TRIR	protein_codin	27.7121753	142.351783	132.195036	29.3562292
ENSG000001	ADGRE5	protein_codin	0	0	28.050765	0
ENSG000001	ZNF442	protein_codin	1.14117859	13.6641731	5.21712577	4.37585846
ENSG000001	KLHL24	protein_codin	41.3639305	89.674745	61.6333782	38.5761886

ENSG000001:	CCDC70	protein_codin	0	0	0	0.78223501
ENSG000001:	EBPL	protein_codin	2.78108085	7.76213906	10.2693458	4.65552377
ENSG000001:	ATP7B	protein_codin	0	0	0	0
ENSG000001:	ZC3H13	protein_codin	61.834062	134.55557	51.3682847	18.5961624
ENSG000001:	CENPK	protein_codin	0	0	0	3.13361133
ENSG000001:	NEUROD4	protein_codin	0	1.47023479	0	0.90831439
ENSG000001:	PFDN5	protein_codin	0	0	0	1.36732459
ENSG000001:	ORMDL2	protein_codin	2.9703456	3.74970137	8.23505953	3.78959322
ENSG000001:	NR4A1	protein_codin	2.81378139	6.24755441	4.80093914	15.9662402
ENSG000001:	PDE1B	protein_codin	0	0	0	7.53862994
ENSG000001:	HOXC13	protein_codin	0	0	8.37909488	0
ENSG000001:	LRP1	protein_codin	1253.459	2751.51368	928.425548	819.067855
ENSG000001:	HOXC11	protein_codin	0	1.12763692	0	0
ENSG000001:	HOXC12	protein_codin	0.257564	1.01995949	0	2.07347872
ENSG000001!	HOXC6	protein_codin	12.1617187	69.2560279	0	1.79872838
ENSG000001:	SMUG1	protein_codin	0	0	0	0
ENSG000001	FAAH	protein_codin	0.82651856	2.54696887	1.49669366	0
ENSG000001	RSRP1	protein_codin	0	112.492999	0	0
ENSG000001:	IL13RA2	protein_codin	0.35124096	0	0	0
ENSG000001	LAMA4	protein_codin	0	0	0.87543003	0
ENSG000001:	COL10A1	protein_codin	18.1896405	31.514839	230.300348	14.0242316
ENSG000000:	SEC61A1	protein_codin	21.0825516	42.208216	0	15.8504759
ENSG000001!	TCEAL3	protein_codin	8.38513041	41.8619817	6.37615406	6.52754622
ENSG000001:	RAB9B	protein_codin	3.83724185	5.28158901	5.05926207	4.75325164
ENSG000001:	NRK	protein_codin	40.7771925	0	0	0.96725147
ENSG000001:	MAGEA9	protein_codin	0	3.83555082	0	0
ENSG000001:	RAB9A	protein_codin	0	2.94437733	0	0
ENSG000000:	RIF1	protein_codin	0	16.952735	36.5818329	0
ENSG000001:	TTC21B	protein_codin	0	0	22.9348925	0
ENSG000001:	NMI	protein_codin	9.41752806	119.31001	13.1740413	8.73415374
ENSG000001:	TNFAIP6	protein_codin	9.32272559	19.045454	0.98268626	8.33789841
ENSG000001:	ACVR1C	protein_codin	4.39079409	10.2350445	9.73160638	6.10054791
ENSG000001:	SLC36A1	protein_codin	6.56907276	21.1477482	14.15847	7.8780929
ENSG000001:	BATF3	protein_codin	2.44290712	10.2326313	31.352418	1.14199683
ENSG000001:	KCNJ2	protein_codin	25.8310201	6.1884405	63.8573971	5.87625164
ENSG000001:	EXOSC9	protein_codin	17.878668	45.9796991	53.9913829	13.0503724
ENSG000001:	PLA2G12A	protein_codin	15.3992129	17.3784507	28.8197113	13.4968198
ENSG000000!	LTBP4	protein_codin	0	0	0	0
ENSG000000	SNRPA	protein_codin	7.92786575	43.0189483	5.57489726	15.3974619
ENSG000001:	B9D2	protein_codin	0	0	0	0
ENSG000001:	COQ8B	protein_codin	0	0	0	0
ENSG000001:	C4BPB	protein_codin	0	0	0	0
ENSG000002	ZNF468	protein_codin	8.28600501	25.1945972	8.82177848	5.83785148
ENSG000001	ZNF415	protein_codin	2.36374233	0	0	0

ENSG000002	ZNF350	protein_codin	9.49195751	29.1626447	13.2954113	7.71778425
ENSG000001	RAB38	protein_codin	0.98687426	0	1.61107784	6.07713651
ENSG000001	GPR83	protein_codin	0	0	0.4989234	2.63467115
ENSG000002	HAUS3	protein_codin	13.0100103	30.3217337	51.4617805	7.57926345
ENSG000001	CHPF	protein_codin	51.6762016	26.7181039	31.2689522	11.3166586
ENSG000001	INHA	protein_codin	0	1.10722802	0.61809853	0.45510655
ENSG000001	FAM124B	protein_codin	0	0	0	0.58555458
ENSG000001	SP140L	protein_codin	0	80.8715239	0	0
ENSG000001	ENKD1	protein_codin	2.77548452	0	11.7396228	0
ENSG000001	UBE2C	protein_codin	0	0	0	0
ENSG000000	SLC35C2	protein_codin	0	0	0	0
ENSG000001	ACTR5	protein_codin	4.13283675	12.8616236	24.0668829	4.2859599
ENSG000001	MC3R	protein_codin	0	0	0	0.5152033
ENSG000001	GCNT7	protein_codin	0	0	0.46173255	0
ENSG000001	CTCFL	protein_codin	0.55610171	0	0	0
ENSG000002	SYS1	protein_codin	8.71096615	8.3495711	14.3549647	8.37011427
ENSG000001	PI3	protein_codin	0	2.21496348	0	0
ENSG000001	WFDC3	protein_codin	0	0	0	0
ENSG000001	SLC12A5	protein_codin	0	0	1.00933889	0
ENSG000001	ARHGAP40	protein_codin	0	0	0	0
ENSG000001	ATP5F1E	protein_codin	86.156264	162.872398	387.105954	60.4725281
ENSG000001	PLCG1	protein_codin	53.732076	178.80646	199.05978	28.1346284
ENSG000001	SRSF6	protein_codin	48.8369623	81.02002	83.710266	71.403519
ENSG000001	RAB22A	protein_codin	36.3053712	100.856315	52.570379	33.1482101
ENSG000001	PTGIS	protein_codin	20.7723025	73.4324736	44.7114658	36.0542021
ENSG000001	CDH26	protein_codin	0	0	0	0
ENSG000001	SNAI1	protein_codin	2.3473384	6.55227858	16.0703014	23.1706186
ENSG000001	MOCS3	protein_codin	4.06457264	12.6162443	10.8696076	3.76408012
ENSG000001	RNF114	protein_codin	4.6609778	0	0	0
ENSG000001	MAGEA10	protein_codin	0	0	0	0
ENSG000001	PEPD	protein_codin	11.9752421	0	13.2927224	15.3641724
ENSG000001	IL9R	protein_codin	0.54188648	3.73359834	0	0
ENSG000001	NAGK	protein_codin	0	2.2222505	1.89635623	7.10880151
ENSG000001	MCEE	protein_codin	7.58067121	16.8549628	9.49012649	5.33463834
ENSG000001	PAIP2B	protein_codin	5.4682254	17.5882215	44.0930819	10.1221116
ENSG000001	SNRNP27	protein_codin	17.3294942	53.865646	39.8733261	21.9297339
ENSG000001	MPHOSPH10	protein_codin	39.0282403	134.876654	180.990723	32.0974069
ENSG000001	IL17C	protein_codin	0	0	0	0
ENSG000000	LIPE	protein_codin	0.85989069	0	0	0
ENSG000001	PSG9	protein_codin	0	0	0	0
ENSG000002	PSG4	protein_codin	0	0	0	0
ENSG000002	PSG1	protein_codin	0	0	0	0.78786042
ENSG000001	HIF3A	protein_codin	1.94974953	0	0	0
ENSG000001	HIF3A	protein_codin	0	0	0	0

ENSG000001:	IRGC	protein_codin	0	0	0.53273119	0.42480849
ENSG000002:	PINLYP	protein_codin	0	0	0	0
ENSG000001:	LYPD3	protein_codin	0	0	0	0
ENSG000001:	CEACAM8	protein_codin	0.30677398	0	0	0.4684962
ENSG000002	RNF39	protein_codin	0	0	0	0
ENSG000001	DST	protein_codin	33.5979293	0	55.7863269	59.9042405
ENSG000001	FBXO9	protein_codin	0	0	0	0
ENSG000001:	PACSIN1	protein_codin	0	0	0	0
ENSG000001:	RRP36	protein_codin	22.5604308	69.1858077	80.2030438	9.26534121
ENSG000001:	BTN1A1	protein_codin	0.32344002	1.0896054	0	0
ENSG000001	BTN3A3	protein_codin	16.9888062	0	20.8572702	11.9460206
ENSG000001:	SNRPC	protein_codin	38.5736812	53.1013976	350.253345	16.6414522
ENSG000001:	SLC17A1	protein_codin	0	0	0	0
ENSG000001:	ABCC10	protein_codin	4.52090257	32.0091926	2.87323198	0
ENSG000001:	H1-3	protein_codin	21.1813854	137.904408	6.26790975	7.86942944
ENSG000002	H4C6	protein_codin	2.26094024	18.5538441	0.47265578	4.02310789
ENSG000001:	PEX6	protein_codin	0	0	0	0
ENSG000001:	UNC5CL	protein_codin	0	0	0	0
ENSG000001:	AARS2	protein_codin	16.2656498	41.1557946	69.586928	8.56311536
ENSG000001:	H1-1	protein_codin	2.49268283	3.08163112	0	0
ENSG000001:	ZNF391	protein_codin	3.49986638	8.77138458	15.9702874	2.09571123
ENSG000001:	OR2B6	protein_codin	0.45925268	0	3.04577524	0
ENSG000001	TCP11	protein_codin	0	0	0	0
ENSG000001:	APOBEC2	protein_codin	3.81133061	0	0	0.8299069
ENSG000001:	KLHDC3	protein_codin	0	0	0.83181574	0
ENSG000001:	TREM1	protein_codin	0.81105037	0	0.45175763	3.68620353
ENSG000001:	MEA1	protein_codin	0.37370865	1.71591686	0	1.05725904
ENSG000001:	COL21A1	protein_codin	0	76.8665191	0	15.8950864
ENSG000001:	CDKN1A	protein_codin	35.5745573	0	23.6497627	56.6055915
ENSG000001:	SOX4	protein_codin	74.2331511	229.494656	211.991529	113.166325
ENSG000001:	CPNE5	protein_codin	1.47421239	2.06348978	8.93436187	0
ENSG000001:	SSR1	protein_codin	41.050299	37.4950361	56.1733089	26.6354605
ENSG000001:	NRN1	protein_codin	5.28581954	30.8014918	2.00645192	17.9775116
ENSG000001:	ATXN1	protein_codin	0	0	0	0
ENSG000001:	DEK	protein_codin	5.5789389	8.70512922	40.314195	0
ENSG000001	BLOC1S5	protein_codin	9.80767444	0	42.8550897	10.9643117
ENSG000001:	OPN5	protein_codin	0	0	0	0
ENSG000001:	LRRFIP1	protein_codin	58.3404108	20.2913645	27.9779607	48.0902761
ENSG000001:	EREG	protein_codin	0	0	1.57013604	10.7388465
ENSG000001:	TRIM51	protein_codin	0	0	0	0
ENSG000001:	SCGB1D2	protein_codin	0	0	0	1.43217044
ENSG000001:	SCGB2A1	protein_codin	0	0	0	0.25146962
ENSG000001:	EMC3	protein_codin	11.9878895	0	20.5165631	9.29463824
ENSG000001:	SH3TC1	protein_codin	0	0	0	0

ENSG000001	KATNAL2	protein_codin	0	5.43094369	0	0
ENSG000001	BBS2	protein_codin	0	0	0	0
ENSG000001	MT2A	protein_codin	1.13881779	24.3499877	7.61527923	136.521973
ENSG000001	GOT2	protein_codin	26.5496011	38.5362642	1.95373341	7.15814221
ENSG000000	SPHK2	protein_codin	0	1.77956521	0	0
ENSG000001	PIWIL1	protein_codin	0	2.47484148	0	2.0826984
ENSG000001	RAP2A	protein_codin	52.5817041	84.863164	35.1483167	40.9443093
ENSG000001	SLC10A2	protein_codin	0	0	0	2.69844987
ENSG000001	HROB	protein_codin	0	0	0	0.72853881
ENSG000001	SLC22A5	protein_codin	0	0	2.36213014	0
ENSG000001	IRF1	protein_codin	7.44988729	53.1280254	0	62.476681
ENSG000001	DMAC2L	protein_codin	0	0	0	0
ENSG000001	BMP4	protein_codin	0	0	1.78564846	6.30968484
ENSG000001	PTGER2	protein_codin	1.05974167	1.91820465	0	0.91753857
ENSG000002	RPS29	protein_codin	46.6765498	81.913904	123.862679	42.2646304
ENSG000001	SOX9	protein_codin	29.7407321	42.3964279	284.021961	83.7048678
ENSG000001	MYH2	protein_codin	1766.61855	0	0	0.53728681
ENSG000001	MRPS7	protein_codin	7.80894379	9.86381337	25.5101336	6.31246479
ENSG000001	ARMC7	protein_codin	4.3726159	15.0361055	15.1189399	3.72962342
ENSG000001	NUP85	protein_codin	0	0	9.62283021	0
ENSG000001	MIF4GD	protein_codin	0	0	0	0
ENSG000001	NT5C	protein_codin	0	8.57746601	31.9945241	0
ENSG000001	MSTO1	protein_codin	8.78150092	0	0	0
ENSG000001	MBOAT7	protein_codin	0	0	4.52011011	0
ENSG000001	EPS8L1	protein_codin	0	0	3.53575452	0
ENSG000002	LILRB3	protein_codin	0	0	0	0
ENSG000002	LILRA6	protein_codin	0	10.8919544	0	0
ENSG000001	ZBTB46	protein_codin	5.52098604	31.8251272	9.60275074	25.9185364
ENSG000001	SLC35F5	protein_codin	72.6034006	134.666877	80.6561024	35.9340034
ENSG000001	INSIG2	protein_codin	16.783205	32.0479366	11.6383365	15.9559292
ENSG000001	PSD4	protein_codin	1.15224094	19.6933673	3.30000508	5.63105145
ENSG000001	PSPN	protein_codin	0	0	0	0
ENSG000001	ALKBH7	protein_codin	0	3.55295101	4.79125116	5.84765344
ENSG000001	CLPP	protein_codin	1.43525012	4.72311443	5.97209498	1.92807487
ENSG000001	TNFSF9	protein_codin	0	1.17759531	2.52403662	4.80166808
ENSG000001	THOC2	protein_codin	0	0	54.1249552	0
ENSG000001	RPL23	protein_codin	0	0	0	8.90664009
ENSG000002	STRADA	protein_codin	0	0	0	0
ENSG000001	CD70	protein_codin	0	0	0	0
ENSG000001	C3	protein_codin	59.5226532	62.0461143	0	13.4331163
ENSG000001	SH2D3A	protein_codin	0	5.41019028	0	0
ENSG000001	TNFSF14	protein_codin	0.54435211	0	0	4.27337422
ENSG000001	RTN2	protein_codin	0.84253239	0	0	0
ENSG000001	EML2	protein_codin	0	0	0	0

ENSG000001	VASP	protein_codin	4.17960331	53.7777818	27.4317939	34.8700538
ENSG000001	SYMPK	protein_codin	0	15.1851689	29.1181599	0
ENSG000000	CFAP61	protein_codin	0	0	0	0
ENSG000001	CDC25B	protein_codin	0	44.6106911	0	0
ENSG000001	GNRH2	protein_codin	0	0	0	0
ENSG000001	CD93	protein_codin	57.9208734	170.344415	61.7697127	197.010319
ENSG000001	CST8	protein_codin	0	0	0	0
ENSG000001	PSMF1	protein_codin	22.497528	51.3810199	30.3342999	12.8042703
ENSG000001	CSTL1	protein_codin	0	0	0	0
ENSG000001	TMX4	protein_codin	52.2147479	330.529335	73.3445965	38.6387531
ENSG000001	RRBP1	protein_codin	206.642529	282.891982	179.415303	80.431404
ENSG000001	DSTN	protein_codin	3.11551766	15.7875688	0.99711771	10.8991603
ENSG000001	LAMP5	protein_codin	36.0104212	4.99712181	2.79897396	0.40755706
ENSG000001	SNRPB2	protein_codin	3.02089682	23.6664487	1.55183042	0
ENSG000001	TCF15	protein_codin	1.33605569	1.60969132	1.21811323	0.43526927
ENSG000001	OTOR	protein_codin	0	0	0	0
ENSG000001	BANF2	protein_codin	0	0	0	0
ENSG000001	FAM110A	protein_codin	0	0	0	0
ENSG000002	SCRT2	protein_codin	0	4.46912492	0	2.65608593
ENSG000001	DEFB129	protein_codin	0	0.93969263	0	1.72386373
ENSG000002	RAD21L1	protein_codin	0	0	0	0.59296198
ENSG000001	TLE6	protein_codin	0	0	0	0
ENSG000001	S1PR4	protein_codin	0.2905154	1.79165168	0	0.4438245
ENSG000001	NCLN	protein_codin	9.35353102	9.69086452	45.5771597	9.12544486
ENSG000001	CITED1	protein_codin	0	0	0	0
ENSG000001	ANKHD1	protein_codin	0	0	1.15991653	0
ENSG000000	PITHD1	protein_codin	21.5754293	34.5773578	69.5166047	18.8015751
ENSG000001	MAX	protein_codin	0.67647218	6.71126007	0.62146026	0.45257319
ENSG000002	FNTB	protein_codin	4.58786587	6.45576449	25.0321474	0
ENSG000001	ARMCX5	protein_codin	0	0	0	0
ENSG000001	MMP24	protein_codin	0.68603638	0	0	1.63877554
ENSG000001	NECAB3	protein_codin	0	0	6.26594829	0
ENSG000001	RALY	protein_codin	49.9308142	117.639491	144.913978	64.5648074
ENSG000001	C20orf173	protein_codin	0	0	0	0.46392037
ENSG000001	PLAGL2	protein_codin	6.825545	29.7929814	24.827449	5.021099
ENSG000001	AGO3	protein_codin	8.50074442	22.6114403	37.0921177	3.63336879
ENSG000001	UROD	protein_codin	7.31803655	0	22.122896	0
ENSG000001	KLC1	protein_codin	0	13.5004622	0	0
ENSG000001	PCID2	protein_codin	0	0	0	0
ENSG000001	SLURP1	protein_codin	0	0	0	0.48636124
ENSG000001	LRFN3	protein_codin	0	0	0	0
ENSG000001	IGFLR1	protein_codin	0	0	0	0.38597128
ENSG000001	CAPNS1	protein_codin	11.5690993	55.8888112	10.4806933	18.0866352
ENSG000001	PDCD2L	protein_codin	0	0	4.11049703	0.75607664

ENSG000001	UBA2	protein_codin	4.48298766	55.0451823	5.35889875	11.9689923
ENSG000001	FFAR2	protein_codin	0	1.01291544	0	2.60278008
ENSG000001	HCST	protein_codin	0.88896856	0	0	2.48862276
ENSG000001	FFAR1	protein_codin	0	0.99369588	0	8.5074552
ENSG000001	KRT13	protein_codin	0	0	0	0
ENSG000001	KRT35	protein_codin	0	0	0	0.88436496
ENSG000001	KRT38	protein_codin	0.27987465	0	0	1.29095967
ENSG000001	CCR7	protein_codin	0	4.29267556	0	0.86567176
ENSG000001	KRT9	protein_codin	0	0.85623802	0	0
ENSG000001	NR1D1	protein_codin	5.0926969	7.4716113	2.33132587	13.2482913
ENSG000002	ARL2	protein_codin	6.16451784	44.3477612	20.6607237	8.08541858
ENSG000001	BCL2L12	protein_codin	0	0	8.74511259	0
ENSG000001	BCL2L12	protein_codin	0	0	0	0
ENSG000001	RRAS	protein_codin	9.66728278	20.0943298	7.77478351	3.61157876
ENSG000001	PRRG2	protein_codin	0	0	0.92904556	0
ENSG000001	TSKS	protein_codin	0.66694952	2.0756863	0	0
ENSG000001	NOP53	protein_codin	95.0146324	132.419204	223.648718	119.950762
ENSG000001	FLRT1	protein_codin	1.07139022	0	2.0566842	1.34683862
ENSG000001	SBDS	protein_codin	0	0	0	54.0634282
ENSG000001	CSN1S1	protein_codin	0	0	0	0
ENSG000001	STATH	protein_codin	0	0	0	0
ENSG000001	HTN1	protein_codin	0	0	0	0
ENSG000000	IFI35	protein_codin	0	0	0	3.52630038
ENSG000001	MLX	protein_codin	0	0	0	0
ENSG000001	WNK4	protein_codin	0	0	0	0
ENSG000002	DNASE1	protein_codin	0	0	0	0
ENSG000001	TRAP1	protein_codin	35.7271657	21.5245586	81.5470659	29.9417359
ENSG000001	SUGP1	protein_codin	0	0	0	5.55637509
ENSG000001	DDX49	protein_codin	3.80210342	37.0921529	26.011806	8.8198224
ENSG000001	GDF1	protein_codin	0	0	0	0
ENSG000001	SDF2	protein_codin	1.16935238	3.01790338	0	0
ENSG000001	NSRP1	protein_codin	64.6413839	213.045751	69.8125002	17.9657646
ENSG000001	AHDC1	protein_codin	0	0	0	11.0500148
ENSG000001	SLC35A2	protein_codin	1.40033441	0	2.9030503	0
ENSG000001	PQBP1	protein_codin	0	0	0	0
ENSG000001	CFP	protein_codin	0	0	0	0
ENSG000001	ELK1	protein_codin	11.7183897	33.9415281	52.1930697	10.0342877
ENSG000001	ATG14	protein_codin	31.2162817	57.9900658	57.9079962	16.0368536
ENSG000001	DLGAP5	protein_codin	4.01839986	21.9007706	201.299536	2.86773833
ENSG000001	L3HYPDH	protein_codin	4.61897827	5.81314621	14.4979283	0.56161
ENSG000001	HSPA2	protein_codin	107.001596	38.4387828	3.87496826	26.6161573
ENSG000001	TBPL2	protein_codin	0	0	0	0
ENSG000001	SGPP1	protein_codin	41.6971378	28.1710635	75.8316051	13.4515598
ENSG000001	PLEKHG3	protein_codin	2.73227057	28.1507266	0	8.86579018

ENSG000001:	EVI2A	protein_codin	7.67689477	15.8028294	0	0
ENSG000001:	OMG	protein_codin	0	0	0	0
ENSG000001:	AIF1L	protein_codin	6.95003846	0	11.6900679	13.0360925
ENSG000001:	FAM78A	protein_codin	0	14.5107722	0	0
ENSG000001:	CTAG2	protein_codin	0	0	0	0
ENSG000000	MAGEC2	protein_codin	0.28750936	0.80996619	0.49834491	0.83460435
ENSG000001:	CANX	protein_codin	8.39380705	0	0	0
ENSG000001	PYCARD	protein_codin	2.80547615	0	6.3434699	3.2770684
ENSG000001:	COX7C	protein_codin	104.61296	62.369202	740.827904	48.8255219
ENSG000000	PHPT1	protein_codin	12.6610219	19.8374117	33.3242615	6.45677626
ENSG000001:	TRAF2	protein_codin	6.72665116	24.4871749	20.0246733	4.58053341
ENSG000001:	ABHD8	protein_codin	1.72626494	5.69014232	6.06529737	0
ENSG000001:	HELB	protein_codin	0	26.4712287	0	2.86720556
ENSG000001:	TSPAN8	protein_codin	0	0	0	0
ENSG000001:	RAB3IP	protein_codin	12.800047	0	8.64054846	0
ENSG000001:	YEATS4	protein_codin	47.8498129	12.0549986	13.1445053	3.21137056
ENSG000000	NDUFB2	protein_codin	16.0836839	31.4581281	39.6935888	5.95978511
ENSG000001:	TAS2R3	protein_codin	0.28887075	2.45243394	2.62021715	0.41208538
ENSG000001:	TAS2R4	protein_codin	10.0769402	21.7596985	25.1178819	3.01626829
ENSG000001:	TAS2R5	protein_codin	0.75190326	1.05631157	2.83405968	0
ENSG000001	ZNF777	protein_codin	16.349364	39.2606403	46.7889654	14.9315104
ENSG000001:	IDUA	protein_codin	0	0	0	0
ENSG000001:	ZNF317	protein_codin	8.77148314	41.8872195	40.9198665	0
ENSG000001:	PIN1	protein_codin	11.0181477	31.2569801	49.7500435	7.66558042
ENSG000001:	FBXL12	protein_codin	0.63430776	0	4.19881587	7.08613648
ENSG000001	KIF17	protein_codin	0	0	0	0
ENSG000001	PLA2G2C	protein_codin	0	0	0	0
ENSG000001	CYP4F11	protein_codin	0	0	0	0
ENSG000001:	SIN3B	protein_codin	28.4069597	117.299792	59.2751613	12.1454426
ENSG000001:	EPS15L1	protein_codin	0	0	0	0
ENSG000001:	KLF2	protein_codin	19.4456167	64.2982976	23.7852617	74.8229139
ENSG000001:	OR7C2	protein_codin	0	0	0	0.41424429
ENSG000001:	OR7C1	protein_codin	0	0	0	0
ENSG000001:	F2RL3	protein_codin	1.97333394	22.9180663	2.20408149	5.93728328
ENSG000001	HCFC1R1	protein_codin	4.32962611	10.4293521	0	6.38515765
ENSG000002	JPT2	protein_codin	20.2497579	43.4267486	160.356206	12.2253626
ENSG000000	UNKL	protein_codin	0	0	11.0080416	0
ENSG000001:	GFER	protein_codin	1.42994163	9.53387566	5.89539408	0
ENSG000001:	SYNGR3	protein_codin	0	0	0	0
ENSG000001	RAB40C	protein_codin	0	9.77123723	7.13518508	3.98437718
ENSG000001:	WDR24	protein_codin	0	0	0	0
ENSG000001:	GNG13	protein_codin	0	0	0	0.40499674
ENSG000002	ZNF10	protein_codin	11.249855	0	4.27459402	7.25934507
ENSG000001:	TICAM1	protein_codin	4.89350818	15.5360818	8.27087201	6.91955769

ENSG000001	MON1B	protein_codin	22.0291791	27.9367949	50.4292647	20.1430543
ENSG000001	METTL25	protein_codin	8.38199312	0	18.5598179	2.56889501
ENSG000001	EIF3K	protein_codin	46.4309678	69.4802766	64.0853411	30.0135047
ENSG000001	EMC6	protein_codin	9.25010367	5.26874904	43.0697751	4.58062914
ENSG000001	OR1E2	protein_codin	0	0	0	0
ENSG000001	CACTIN	protein_codin	8.82629126	56.0659981	21.5116401	9.97243231
ENSG000001	TUBA4A	protein_codin	0	20.4054876	2.38024921	0
ENSG000001	VIL1	protein_codin	0	1.3996975	0	1.27068956
ENSG000001	AAMP	protein_codin	0	0	0	0
ENSG000001	PNKD	protein_codin	3.42000087	0	8.27980306	3.59654336
ENSG000001	TNFRSF19	protein_codin	54.1316363	10.1832776	2.27101475	23.6538475
ENSG000000	PHTF2	protein_codin	0	0	0	0
ENSG000001	HSPB1	protein_codin	29.6487208	252.567671	150.900839	80.1753096
ENSG000001	GNG11	protein_codin	22.8840075	115.633856	16.9808218	32.8009872
ENSG000001	SEM1	protein_codin	28.5774179	158.374056	125.308833	31.596563
ENSG000001	GNGT1	protein_codin	0	0	0	0
ENSG000001	PTPN12	protein_codin	73.0728282	166.91115	125.455873	122.696453
ENSG000001	FGL2	protein_codin	80.7845807	278.801406	16.9106576	53.4998213
ENSG000001	STYXL1	protein_codin	0	0	0	0
ENSG000001	PEX1	protein_codin	0	0	0	0
ENSG000001	LRFN1	protein_codin	0.55764304	5.08518698	3.06935756	0.43471253
ENSG000001	SPINK2	protein_codin	0	0	0	0
ENSG000001	RASL11B	protein_codin	3.25053177	0	1.86926336	5.14116227
ENSG000001	TUBGCP6	protein_codin	12.31411	72.3840295	41.5449455	18.0513087
ENSG000001	DGCR6L	protein_codin	17.8223869	12.9760285	105.835989	8.63820169
ENSG000001	NCF4	protein_codin	7.62357398	28.4471659	1.48077488	3.82293957
ENSG000001	CYTH4	protein_codin	7.84615409	61.5237473	0	17.7812381
ENSG000001	GCAT	protein_codin	6.07709396	5.8810695	16.2324439	0.9472575
ENSG000001	SGSM3	protein_codin	13.5719675	10.2874739	55.3421488	5.18173823
ENSG000001	SEZ6L	protein_codin	0	0	0	0
ENSG000001	VPREB3	protein_codin	0	0	0	0
ENSG000001	SDF2L1	protein_codin	0.30342564	8.73946416	19.5472134	1.64380862
ENSG000001	YWHAH	protein_codin	86.1767046	107.612986	112.902093	34.5069673
ENSG000001	RFPL2	protein_codin	0	0	0	0
ENSG000001	A4GALT	protein_codin	0	0	0	0
ENSG000001	RFPL3	protein_codin	0	0	0	0
ENSG000001	CDC42EP1	protein_codin	11.0340893	75.939091	4.86059186	13.0657483
ENSG000001	MCHR1	protein_codin	0	0	0	0.91478014
ENSG000001	GALR3	protein_codin	0	0	0	0
ENSG000001	TST	protein_codin	0	0	2.69759182	0
ENSG000001	APOL5	protein_codin	0.64881403	0.90377536	0	0.84093833
ENSG000001	IGLL1	protein_codin	0	8.53339759	0	0.42426345
ENSG000001	CCDC117	protein_codin	20.3689508	41.957457	68.7010882	9.18054198
ENSG000001	APOL2	protein_codin	2.60589757	0	0	11.3317136

ENSG000001	RAC2	protein_codin	0	40.627924	3.86439354	12.9432244
ENSG000001	LIF	protein_codin	4.33066776	12.6803334	1.63470933	14.889689
ENSG000001	C22orf23	protein_codin	0	0	0	1.05562228
ENSG000001	APOBEC3A	protein_codin	1.10897439	0	0	0
ENSG000001	EMC4	protein_codin	1.7508005	0	2.17273639	8.17117022
ENSG000001	PMPCB	protein_codin	0	0	0	0
ENSG000001	DNAJC2	protein_codin	19.9680336	62.713838	0	6.18355164
ENSG000001	TAS2R16	protein_codin	0	0	0	0
ENSG000001	ATP6V1F	protein_codin	51.3382417	147.960661	308.627454	65.7140338
ENSG000001	LSM8	protein_codin	24.7071646	26.0220168	47.1786429	8.21459888
ENSG000001	VGF	protein_codin	0	0	0	0
ENSG000001	STRIP2	protein_codin	9.83660907	3.02687186	15.1892299	16.6516553
ENSG000001	DNAJB9	protein_codin	22.2367053	47.7115713	40.1956346	34.3245956
ENSG000001	LRRC4	protein_codin	4.9417569	25.2717521	5.03475843	10.1164542
ENSG000001	CALU	protein_codin	0	0	0	0
ENSG000001	SMO	protein_codin	16.0635482	60.074574	74.5137002	2.03054313
ENSG000001	LRRC17	protein_codin	3.15320992	46.8190025	29.6525637	0
ENSG000001	OPN1SW	protein_codin	0	4.16068026	0	0
ENSG000000	SIRT2	protein_codin	10.2118679	13.7791939	0	6.51931322
ENSG000001	HOXD3	protein_codin	0.54122711	3.9324546	1.96601034	2.11175711
ENSG000001	MTX2	protein_codin	7.97722271	0	26.790513	8.1027141
ENSG000001	HOXD9	protein_codin	1.78925223	6.71021005	1.51576694	2.26858809
ENSG000001	HOXD10	protein_codin	1.79097421	17.1992298	5.25537234	2.60373305
ENSG000001	HOXD11	protein_codin	0.17658504	1.53092902	1.54580932	0
ENSG000001	ARHGAP22	protein_codin	2.04967797	28.1736417	0	0
ENSG000000	PIAS1	protein_codin	42.7882761	56.9741399	43.6023861	20.4166922
ENSG000000	SNAP23	protein_codin	28.6081435	107.505703	55.2244239	26.6210213
ENSG000001	TMOD2	protein_codin	19.5273228	45.8186879	27.6757828	22.3485736
ENSG000001	SLTM	protein_codin	35.7636696	53.4145211	97.4462777	29.1244655
ENSG000001	DLL4	protein_codin	8.40890207	18.0977382	14.276574	14.970279
ENSG000001	ALDH1A2	protein_codin	0	0	0	0
ENSG000001	KNSTRN	protein_codin	0	0	0	0
ENSG000001	SERF2	protein_codin	59.4386712	103.152141	158.4669	31.6830832
ENSG000001	CLN6	protein_codin	1.59544676	14.9293801	16.085251	4.28410256
ENSG000001	ARPP19	protein_codin	21.8276539	28.1116376	66.8567652	39.0966058
ENSG000001	VPS13C	protein_codin	80.8316974	351.598679	121.007073	98.205776
ENSG000001	ISLR	protein_codin	7.15495906	21.3693121	0	6.08570474
ENSG000001	THAP10	protein_codin	0.35537199	3.91246422	6.47755817	0
ENSG000001	AMOTL2	protein_codin	15.1180502	41.2263017	31.8870367	65.0287114
ENSG000001	ACKR4	protein_codin	1.09288406	0	0	1.23660315
ENSG000001	MBD4	protein_codin	0	0	0	0
ENSG000001	COPB1	protein_codin	83.1559247	233.113457	116.291627	0
ENSG000001	MYOD1	protein_codin	0.69959521	0	0	0
ENSG000001	TPH1	protein_codin	0	0	1.8525147	4.82843779

ENSG000001:	E2F8	protein_codin	0	0	0	0
ENSG000001:	SOX15	protein_codin	0	0	0	0
ENSG000001:	PIMREG	protein_codin	0	0	6.71901096	0
ENSG000001:	USP6	protein_codin	0	0	0	0
ENSG000001(	ZNF232	protein_codin	2.15471043	0	11.4626025	0
ENSG000001:	AIPL1	protein_codin	0	0	0	0.53006871
ENSG000001:	CD68	protein_codin	44.1854514	415.415294	8.17901975	143.043369
ENSG000001:	TXNDC17	protein_codin	2.7895168	6.88196601	6.60776153	0.99073641
ENSG000001:	ATP1B2	protein_codin	0.60006976	5.45784302	4.23093091	5.43653278
ENSG000001:	FXR2	protein_codin	9.39906511	38.3608137	19.0166978	7.15197774
ENSG000001:	MPDU1	protein_codin	0	0	3.34242339	0
ENSG000001(	CCN4	protein_codin	77.3350964	85.8446795	3.47851203	19.8565514
ENSG000001:	DNAAF11	protein_codin	0	0	0	0
ENSG000002	QTRT1	protein_codin	1.65934591	0	6.63182625	0
ENSG000001:	ILF3	protein_codin	0	0	0	0
ENSG000001:	AP1M2	protein_codin	0	0	0	0
ENSG000001(	ERI1	protein_codin	22.5041901	18.9398092	40.8959518	11.6666271
ENSG000001(	CLEC11A	protein_codin	12.6360909	215.467941	35.7654399	31.9440125
ENSG000001:	KLK12	protein_codin	0	0	0	0
ENSG000001:	SIGLEC9	protein_codin	0.68454657	1.07745257	0.50899901	0
ENSG000002(	ENSG000002(	protein_codin	0	0	0	0
ENSG000001(	NFATC4	protein_codin	4.2894928	0	0	0
ENSG000001(	PRORP	protein_codin	0	0	0	0
ENSG000000!	CMA1	protein_codin	1.38218943	5.06787003	0	17.503599
ENSG000000!	GEMIN2	protein_codin	0	0	4.65303858	0
ENSG000001(	DHRS2	protein_codin	0	0	0	0
ENSG000001:	BCL2L2	protein_codin	25.8083457	0	42.8345972	0
ENSG000001:	PARP2	protein_codin	19.4425112	58.7260934	54.6524431	14.8270298
ENSG000001:	FOXA1	protein_codin	0.18496739	0	0	0
ENSG000001:	EAPP	protein_codin	22.5208849	63.5402012	68.2934757	16.0044014
ENSG000001:	EGLN3	protein_codin	0.80631017	3.24155939	2.5409412	0.66507913
ENSG000001(	PIP4P1	protein_codin	0.63816832	9.1605535	5.27890843	1.14244745
ENSG000001:	NEDD8	protein_codin	43.6655615	139.743719	166.421222	39.1014211
ENSG000001:	DAD1	protein_codin	12.1589586	76.9472205	48.9093713	9.94991228
ENSG000001:	CDO1	protein_codin	5.34088545	0	0	8.55804792
ENSG000001:	RAP1B	protein_codin	167.823707	14.9435097	5.26259631	13.4854101
ENSG000001'	ADGRE1	protein_codin	0	0	0	0
ENSG000001:	AANAT	protein_codin	0	0	0.51839203	0
ENSG000001:	ARHGEF6	protein_codin	46.7648396	50.912173	0	61.8446188
ENSG000001:	ART1	protein_codin	0	0	0	0.39849895
ENSG000001:	CHRNA10	protein_codin	0	0	0.59798798	2.32210221
ENSG000001:	RPS4Y1	protein_codin	31.8033951	44.2955517	0	0
ENSG000001:	VCY1B	protein_codin	0	0	0	0
ENSG000001:	VCY	protein_codin	0	0	0	0

ENSG000002	RBMY1J	protein_codin	0	0	0	0
ENSG000001	CDY2A	protein_codin	0.34814874	2.00172552	0	0
ENSG000000	DAZL	protein_codin	0	0	0	0
ENSG000001	MAPK8IP3	protein_codin	0	49.5218707	0	43.4257141
ENSG000000	MKNK2	protein_codin	5.41654167	34.8115453	22.6838768	8.59212337
ENSG000001	KLF16	protein_codin	2.20226415	17.9902224	5.26261522	4.03524553
ENSG000001	PGAP6	protein_codin	0	0	19.2096461	3.53566106
ENSG000002	INS	protein_codin	0	0	0	0
ENSG000001	ABHD17A	protein_codin	0	3.42727531	8.29082085	0
ENSG000001	SALL1	protein_codin	0	0	11.4947026	0
ENSG000001	ZC3H14	protein_codin	5.508707	22.9493254	14.7019932	10.5771368
ENSG000000	MYO16	protein_codin	0	0	0	0.59955609
ENSG000000	LMAN1	protein_codin	126.851508	379.135767	542.599613	81.6236466
ENSG000000	NUP37	protein_codin	0	0	0	0
ENSG000001	DMXL2	protein_codin	41.9003356	0	63.7249952	9.12627307
ENSG000001	DSC2	protein_codin	1.00400343	0	1.69204142	0
ENSG000000	ANGEL1	protein_codin	4.69415433	0	0	0
ENSG000000	SAMD4A	protein_codin	20.9432952	0	53.3781277	0
ENSG000000	CNGB1	protein_codin	0	1.67319183	0	1.79970512
ENSG000001	CDADC1	protein_codin	6.8673223	20.0607762	7.05530244	3.58436385
ENSG000001	NALCN	protein_codin	0	0	0	0
ENSG000001	VPS35L	protein_codin	0	0	0	0
ENSG000001	DOCK7	protein_codin	0	0	0	0
ENSG000002	CORO7	protein_codin	0	0	0	0
ENSG000001	SEC14L5	protein_codin	0.22077459	0	0	5.22635775
ENSG000000	CEP170B	protein_codin	0	0	0.74182925	0
ENSG000000	CLSPN	protein_codin	2.13751509	6.17947743	50.81942	1.92522733
ENSG000001	PBX4	protein_codin	1.99409828	0	0	0
ENSG000001	DHX40	protein_codin	10.3801144	16.6046435	42.7786181	26.7438038
ENSG000001	DTWD1	protein_codin	0	0	0	0
ENSG000001	MEGF8	protein_codin	21.8774024	0	29.5178825	13.9500037
ENSG000001	ZNF221	protein_codin	0	0	0	0
ENSG000000	HCN2	protein_codin	1.04799457	10.1191722	2.47948216	2.36671688
ENSG000000	WDR18	protein_codin	0	0	0	0
ENSG000001	IGSF21	protein_codin	4.44460553	0	2.12689304	4.19117002
ENSG000001	INTS2	protein_codin	0	0	7.97456532	0
ENSG000001	KHNYN	protein_codin	0	0	54.4830471	23.1780946
ENSG000000	CERS4	protein_codin	1.49486002	0	7.44852691	0
ENSG000001	ESRP2	protein_codin	0	0	0	3.14428509
ENSG000001	LILRA1	protein_codin	0	0	0	0
ENSG000002	LILRA2	protein_codin	0.40138034	0	0	0.63243615
ENSG000002	LILRA2	protein_codin	0	0	0	0
ENSG000000	TUBG2	protein_codin	8.95040296	76.9247666	17.9555363	8.38251997
ENSG000001	TUBG1	protein_codin	11.7532763	0	0	0

ENSG000001[illegible]	CFHR4	protein_codin	0	0	0	0
ENSG000001[illegible]	RPS16	protein_codin	44.4954439	46.8018127	103.537764	56.4910004
ENSG000001[illegible]	MAST1	protein_codin	0	0.91757119	3.99385832	1.0320009
ENSG000001[illegible]	PLPPR2	protein_codin	7.78729371	0	0	0
ENSG000001[illegible]	SULT1C2	protein_codin	0	0	0	0
ENSG000001[illegible]	NCAPG	protein_codin	8.67318668	20.5515149	116.056654	5.31890566
ENSG000001[illegible]	RABGAP1L	protein_codin	26.4692072	209.9495	168.432969	24.2840195
ENSG000001	ESYT2	protein_codin	0	81.9272958	0	0
ENSG000001[illegible]	ALOX12	protein_codin	0.45719839	0	0.96048811	0
ENSG000001[illegible]	FBXO44	protein_codin	0	2.96582983	0	0
ENSG000001[illegible]	FBXO44	protein_codin	0	10.6999188	0	1.17478297
ENSG000001[illegible]	UGT2A3	protein_codin	0	0	0	2.68377171
ENSG000000[illegible]	ADAMTS2	protein_codin	175.357828	341.471647	14.757375	80.0997151
ENSG000001[illegible]	CIAO3	protein_codin	0	0	7.07060978	0
ENSG000001[illegible]	HBA2	protein_codin	7.95390874	16.7541821	0	6.38107256
ENSG000000[illegible]	TRNT1	protein_codin	0	0	0	0
ENSG000001[illegible]	PDGFRL	protein_codin	30.474215	26.3426529	7.74961131	4.07808298
ENSG000000[illegible]	DHX29	protein_codin	5.15269986	0	29.3793243	0
ENSG000001[illegible]	DHX58	protein_codin	2.91875878	11.7505094	0	1.07813971
ENSG000001[illegible]	KRT12	protein_codin	0	0	0	0
ENSG000000[illegible]	KRT31	protein_codin	0.81357878	1.00797744	0.4843051	0
ENSG000001[illegible]	KRT33B	protein_codin	0	0	0	0
ENSG000001	PCCB	protein_codin	5.43856891	12.9167456	34.3178144	2.93386969
ENSG000001	ARFGEF3	protein_codin	6.03394986	8.48927167	29.9046667	20.8154827
ENSG000000[illegible]	USP40	protein_codin	0	0	0	0
ENSG000000[illegible]	VPS50	protein_codin	2.64876418	0	0	0
ENSG000001	SNX4	protein_codin	31.1271872	37.8068218	109.493599	19.1715413
ENSG000001	ROPN1B	protein_codin	0	0	0	0
ENSG000001[illegible]	SPAG1	protein_codin	0	0	0	0
ENSG000000[illegible]	RRM2B	protein_codin	76.9090831	55.4804946	79.19256	26.8437727
ENSG000000[illegible]	RHOBTB2	protein_codin	0	48.176043	0	10.629465
ENSG000001[illegible]	RAF1	protein_codin	0	0	0	0
ENSG000000[illegible]	TDRD1	protein_codin	0	0	0	0
ENSG000000[illegible]	MED17	protein_codin	0	0	0	3.93246733
ENSG000001[illegible]	RIC1	protein_codin	1.6088644	5.99491901	3.49461287	0
ENSG000001[illegible]	SCML2	protein_codin	2.34804279	1.25415265	16.3666938	4.14289318
ENSG000000[illegible]	NXPE1	protein_codin	0.35833035	0	0	0
ENSG000000[illegible]	TSG101	protein_codin	8.20169807	43.7358152	21.3590039	4.48058917
ENSG000001[illegible]	CARD10	protein_codin	0	20.0381034	0	0
ENSG000001[illegible]	KIAA0930	protein_codin	0	50.2090636	0	0
ENSG000001[illegible]	DHX35	protein_codin	14.1139815	0	48.3835713	0
ENSG000001[illegible]	TRPC4AP	protein_codin	39.5009134	0	29.2737709	26.2617462
ENSG000000[illegible]	TYMP	protein_codin	0	2.69951817	0	0
ENSG000000[illegible]	DNAAF9	protein_codin	47.2495822	134.595624	54.4461041	27.9757806

ENSG000000[illegible]	ELN	protein_codin	70.1048789	0	0	0
ENSG000000[illegible]	FKBP6	protein_codin	0	0	0	0
ENSG000001	CUL9	protein_codin	10.6658152	0	56.5066303	10.8029796
ENSG000001[illegible]	ACTR3C	protein_codin	0	0	1.10656426	0.65998551
ENSG000002[illegible]	PCDHGA12	protein_codin	34.1345093	14.5070036	0	6.06680746
ENSG000002[illegible]	PCDHGC5	protein_codin	2.40810848	0	0	2.88882141
ENSG000001	TMCO6	protein_codin	0	0	0	0
ENSG000001[illegible]	NDUFA2	protein_codin	5.62918039	2.17070069	13.1468312	3.87157372
ENSG000001	UBE4A	protein_codin	88.5266108	150.46904	125.807634	49.4804435
ENSG000001[illegible]	POLDIP3	protein_codin	20.145285	0	25.1203367	0
ENSG000002[illegible]	MICAL3	protein_codin	3.15940775	17.7661492	4.65817879	5.00630879
ENSG000000[illegible]	TRMT2A	protein_codin	0	0	0	0
ENSG000001[illegible]	ESS2	protein_codin	2.32644249	6.07875629	4.86323388	1.25571006
ENSG000000[illegible]	MYH13	protein_codin	0	0	0	0
ENSG000001[illegible]	ZSCAN9	protein_codin	0	0	0	0
ENSG000001[illegible]	ZKSCAN3	protein_codin	1.11700581	6.45073336	9.29307671	2.16623334
ENSG000002[illegible]	MICB	protein_codin	0	0	0	0
ENSG000001[illegible]	KRT5	protein_codin	0	0	0	0
ENSG000001[illegible]	KRT1	protein_codin	0.29695695	3.39165317	0	2.68714149
ENSG000001[illegible]	KRT75	protein_codin	0.31720824	0.87725469	0.56870498	1.33498298
ENSG000001[illegible]	KRT6C	protein_codin	0	0	0.54005869	0
ENSG000001[illegible]	KRT6B	protein_codin	0.33467705	0	0	0
ENSG000001[illegible]	DPF2	protein_codin	0	14.8153403	7.3565641	0
ENSG000001[illegible]	GAMT	protein_codin	0.8237928	0	10.3216711	0.52747625
ENSG000001[illegible]	GALNT8	protein_codin	0.40573291	1.98644411	0	2.01359753
ENSG000001[illegible]	KCNA5	protein_codin	1.58619391	0.72993532	0.55104601	4.35621689
ENSG000001[illegible]	CRACR2A	protein_codin	3.03107117	57.3573651	21.65888	8.4639554
ENSG000001[illegible]	PSMG3	protein_codin	0	1.03624425	0	0
ENSG000001[illegible]	STARD8	protein_codin	0	0	0	0
ENSG000001[illegible]	NALF2	protein_codin	0	1.99780567	5.91762603	2.90749886
ENSG000001[illegible]	ECSIT	protein_codin	0	0	0	1.15712524
ENSG000001[illegible]	LDLR	protein_codin	0	38.9075848	6.35784222	4.34674937
ENSG000001[illegible]	ELOF1	protein_codin	4.76901197	4.77681772	15.8836393	5.3597483
ENSG000001[illegible]	ANGPTL8	protein_codin	0	0	0	0
ENSG000001[illegible]	CNN1	protein_codin	3.75597787	3.19176548	0	0.38872824
ENSG000001[illegible]	CDC16	protein_codin	0	0	0	0
ENSG000001[illegible]	CDC16	protein_codin	6.65651084	0	40.8234101	4.60519221
ENSG000001[illegible]	ZSCAN10	protein_codin	0	0	0	0
ENSG000001[illegible]	NECTIN2	protein_codin	2.65205323	14.252837	8.51789032	6.61586505
ENSG000001[illegible]	NECTIN2	protein_codin	6.94163402	64.3768151	17.6795862	13.3795846
ENSG000001[illegible]	APOE	protein_codin	74.3288198	848.788349	96.0736275	311.934742
ENSG000001[illegible]	TOMM40	protein_codin	4.70984361	8.08541964	6.45927477	0
ENSG000002[illegible]	APOC2	protein_codin	0	0	0	0
ENSG000001[illegible]	ALLC	protein_codin	0	0	0.2735122	0

ENSG000001	GADD45G	protein_codin	2.01964432	0	0	2.73106132
ENSG000001	XPO7	protein_codin	27.559011	79.3287587	72.6593255	29.7821166
ENSG000001	ACE2	protein_codin	0	0	0	0
ENSG000002	INF2	protein_codin	3.07679846	0	0	0
ENSG000001	FAM98C	protein_codin	2.18225629	0	3.41872926	1.33450345
ENSG000001	SAFB2	protein_codin	105.781209	236.942752	373.007775	100.05434
ENSG000001	NCAN	protein_codin	0	0	1.20685645	0.65276038
ENSG000001	PLVAP	protein_codin	35.604175	230.531045	49.5232088	207.398863
ENSG000001	BST2	protein_codin	16.7415184	186.127759	181.626166	39.4817594
ENSG000001	NSUN5	protein_codin	0	7.89376842	11.6852166	0
ENSG000001	SLC27A1	protein_codin	3.56434444	3.0506578	0	6.50827326
ENSG000001	USHBP1	protein_codin	4.23478465	4.9474522	0	5.3791273
ENSG000001	COLGALT1	protein_codin	80.2653955	70.8400379	272.303732	36.8529721
ENSG000001	MRPL34	protein_codin	4.60490148	0	0	0
ENSG000001	PGLS	protein_codin	3.484421	0	0	0
ENSG000001	LSM7	protein_codin	0	0	4.68585219	0
ENSG000001	MAS1	protein_codin	0.76692412	5.82623421	0.46265291	0
ENSG000001	MLLT1	protein_codin	39.6451575	150.508479	196.308825	39.7096118
ENSG000001	FUT5	protein_codin	0	0	0	0.47888543
ENSG000001	BMP15	protein_codin	0.07630969	4.83916814	0.30749177	0.34748061
ENSG000001	ACTN4	protein_codin	49.3811709	98.1594228	39.2051471	59.2873371
ENSG000001	NDUFA10	protein_codin	0	0	0	0
ENSG000001	ZNF655	protein_codin	35.1812194	0	89.7725859	25.4072906
ENSG000001	EPO	protein_codin	0	0	0.91026781	0.37890426
ENSG000001	CACNG6	protein_codin	0	0	0	0
ENSG000001	ZSWIM6	protein_codin	35.7607922	81.5366323	90.3192613	42.2120784
ENSG000002	SCO2	protein_codin	0	0	0	0
ENSG000001	ZNF764	protein_codin	0	0	0	0
ENSG000001	ZNF747	protein_codin	0	0	4.161455	1.42302732
ENSG000001	PXDN	protein_codin	47.830078	121.127987	277.522779	60.2030217
ENSG000001	GDF15	protein_codin	0.48745458	0	1.08613548	0
ENSG000001	PGPEP1	protein_codin	0	0	0	0
ENSG000001	LSM4	protein_codin	13.7843785	0	105.773843	6.57816027
ENSG000001	JUND	protein_codin	30.130684	102.19254	17.9154374	151.20209
ENSG000001	HRC	protein_codin	32.094583	27.5581424	2.11289565	5.39076406
ENSG000001	TRPM4	protein_codin	1.39769584	0	0	0
ENSG000001	ZNF557	protein_codin	13.9742422	30.5951598	28.676462	5.20308613
ENSG000001	OLFM1	protein_codin	6.6233263	0	0	0
ENSG000001	NUMBL	protein_codin	10.1011191	24.5218974	35.202871	5.7519801
ENSG000001	TNNI2	protein_codin	26.1104995	4.14161753	0.60968861	0.52485826
ENSG000001	ATXN10	protein_codin	25.8361124	80.0873795	98.2276204	15.9939713
ENSG000001	TUBGCP2	protein_codin	0	0	0	0
ENSG000001	CALY	protein_codin	0.79750202	0	0	1.2275776
ENSG000001	CYP2E1	protein_codin	0	0	0	0

ENSG000001	HBZ	protein_codin	0	0	0	0
ENSG000001	MNX1	protein_codin	0	0	0	0
ENSG000001	ZNF337	protein_codin	3.72504856	7.52509918	4.2168874	0
ENSG000002	ALKBH6	protein_codin	0.38540391	0	0	0
ENSG000001	CEP85	protein_codin	0	8.57444637	0	0
ENSG000001	TAF4	protein_codin	10.650572	24.4481823	22.2566765	5.24313072
ENSG000001	GATA5	protein_codin	0	0	0.49653728	0
ENSG000001	RBBP8NL	protein_codin	0	0	0	0.44140565
ENSG000001	LAMA5	protein_codin	29.646321	64.4678665	69.9245438	30.530269
ENSG000001	ADRM1	protein_codin	12.9543863	40.560139	0	7.3614058
ENSG000001	PRDM12	protein_codin	0	0.97380122	0	0.40039038
ENSG000001	UBE2M	protein_codin	6.23628021	49.2128261	19.8891883	7.08110506
ENSG000001	TRIM28	protein_codin	6.36515368	12.8447486	8.15924075	15.3807614
ENSG000001	YIPF2	protein_codin	2.28851125	0	11.7833073	3.64238043
ENSG000001	EIF2S3	protein_codin	86.0937127	180.099597	151.402908	106.490351
ENSG000001	TMEM160	protein_codin	1.30129702	11.256498	2.28173187	1.28525997
ENSG000001	ZC3H4	protein_codin	28.4397123	132.6489	79.1482967	31.8974411
ENSG000001	GMFG	protein_codin	0	8.40275217	0	1.49221545
ENSG000001	MAP3K10	protein_codin	0	24.2373396	4.53722079	3.29839657
ENSG000001	SESN2	protein_codin	12.5414273	20.0901046	19.3694968	14.6363981
ENSG000001	CCDC62	protein_codin	0	0	0	5.14068436
ENSG000001	HIP1R	protein_codin	4.7290972	12.9475738	41.0370507	16.2080488
ENSG000001	MRPL4	protein_codin	9.61403391	3.65619299	9.71449751	1.38437508
ENSG000001	PPAN	protein_codin	0	5.75735772	17.0176709	0
ENSG000001	EIF3G	protein_codin	9.64704133	75.1998046	68.0450655	24.0949375
ENSG000001	ANGPTL6	protein_codin	0	0	0	0
ENSG000001	SHFL	protein_codin	4.73480747	45.3715868	0	13.090654
ENSG000001	ZNF426	protein_codin	13.6313909	66.7663908	33.8951591	9.80836477
ENSG000001	SLC6A8	protein_codin	5.79117246	22.1470317	48.9632928	9.1754845
ENSG000001	ZNF331	protein_codin	0	8.99457295	22.0556913	2.1358099
ENSG000001	ZNF236	protein_codin	28.4193613	99.0957248	91.2745575	18.5715723
ENSG000001	SLC7A10	protein_codin	0	3.08758671	0	0
ENSG000001	LRP3	protein_codin	10.5010141	14.6232999	14.8565985	9.1691916
ENSG000001	MTRFR	protein_codin	11.5576128	45.4656265	0	0
ENSG000001	GRWD1	protein_codin	11.6042554	20.3638817	28.3935149	6.9787024
ENSG000001	NOL11	protein_codin	23.8045842	0	353.062033	24.1622189
ENSG000001	UBE4B	protein_codin	30.263511	59.3260946	181.769708	17.3186205
ENSG000001	PKDREJ	protein_codin	0.78068805	0	2.72472844	6.35814813
ENSG000001	NUTM2F	protein_codin	0	0	0	0.37327141
ENSG000001	SLC35D2	protein_codin	0	4.86520592	0	3.93545962
ENSG000001	PPIL4	protein_codin	110.984081	134.830432	204.696133	49.4191004
ENSG000001	AKAP12	protein_codin	113.283539	44.3690492	99.603391	106.034031
ENSG000001	LATS1	protein_codin	0	0	0	0
ENSG000001	BPIFB1	protein_codin	0.32449538	0	0	0

ENSG000001:	BPIFA2	protein_codin	0	0	0	0
ENSG000001:	RBM39	protein_codin	0	0	0	0
ENSG000001:	DEFB118	protein_codin	0	0	0	0.49999223
ENSG000001:	ACSS2	protein_codin	0	0	0	0
ENSG000001:	EDA2R	protein_codin	0	0	0	0
ENSG000001:	ARHGEF9	protein_codin	0	0	0	0
ENSG000001:	GFAP	protein_codin	0	0	0	0
ENSG000001:	HIGD1B	protein_codin	0	0	0	0
ENSG000001:	ATP6V1E1	protein_codin	14.2870512	63.8804836	9.59808242	32.5886334
ENSG000001:	CCL25	protein_codin	0	0	0	0
ENSG000001:	COX4I1	protein_codin	83.8645578	94.8519511	289.705255	86.9936116
ENSG000001:	EMC8	protein_codin	0	8.3420623	4.59489829	1.96403991
ENSG000001:	GSE1	protein_codin	26.9010923	80.9696618	72.9614269	28.0135191
ENSG000002(	C16orf95	protein_codin	0	1.45694241	1.86086431	0
ENSG000001:	GINS2	protein_codin	2.52723635	6.06863705	30.6860293	0
ENSG000001:	F12	protein_codin	0	0	0	0
ENSG000001:	NFATC1	protein_codin	12.055044	0	5.56397611	17.1187643
ENSG000001:	IDO1	protein_codin	5.70357187	0	0	0
ENSG000001:	ABCB7	protein_codin	0	18.7165099	0	0
ENSG000001:	HAUS8	protein_codin	4.24107642	16.4983757	18.8491417	0
ENSG000001:	ADGRE3	protein_codin	0	0	0	0
ENSG000001:	MRPS25	protein_codin	0	0	0	0
ENSG000001:	TBC1D5	protein_codin	18.8444749	9.63046316	16.8931039	5.3293357
ENSG000001:	CAPN7	protein_codin	56.3551169	78.6319833	42.5305598	23.4820406
ENSG000001:	C3orf20	protein_codin	0	0	0	0
ENSG000001:	RBSN	protein_codin	0	10.9749483	59.0961926	0
ENSG000001:	NAPSA	protein_codin	0	0	0	0
ENSG000001:	NR1H2	protein_codin	7.7600774	0	20.1711569	7.74068494
ENSG000001:	PDLIM4	protein_codin	0.56891239	13.166166	4.89901425	1.33354716
ENSG000001:	GFPT2	protein_codin	8.8148549	11.7012265	0	3.29658407
ENSG000001:	RPL27	protein_codin	240.796698	448.141378	430.636079	180.807122
ENSG000001:	PSMC3IP	protein_codin	0	0	0.82370046	0
ENSG000001:	VPS25	protein_codin	4.4734133	17.7045397	19.848839	4.66791022
ENSG000001:	RAMP2	protein_codin	2.53490499	5.13391265	6.5195036	7.4701178
ENSG000001:	AOC2	protein_codin	5.84642119	25.5978468	39.4459747	6.33678354
ENSG000001:	G6PC1	protein_codin	0	0	0.57048869	1.3815398
ENSG000002	PCDHAC1	protein_codin	0	3.02960956	0.7285281	0
ENSG000002:	PCDHGA3	protein_codin	0	0	5.3265901	0
ENSG000001:	NDFIP1	protein_codin	134.716013	176.895461	139.870605	69.1208994
ENSG000001:	EXOC4	protein_codin	20.036337	95.8844102	53.3213429	15.1759816
ENSG000001:	PPFIA1	protein_codin	45.595681	0	41.7513636	62.040728
ENSG000000:	FLYWCH1	protein_codin	0	11.1094191	21.4650325	0
ENSG000001:	TMEM204	protein_codin	0	0	0	0
ENSG000001:	THOC6	protein_codin	0	0	0	1.72010701

ENSG000001:	BARX1	protein_codin	0.26568795	21.7394121	18.4312921	0
ENSG000001:	WDR44	protein_codin	0	0	21.9244746	29.9940289
ENSG000001:	CKMT2	protein_codin	0	0	0	0
ENSG000001:	ZCCHC9	protein_codin	5.11122327	10.0202436	27.8330042	1.73445036
ENSG000001	KRT15	protein_codin	0	0	0	0
ENSG000001:	TNS4	protein_codin	0	2.06270531	0	2.42311738
ENSG000001:	RARA	protein_codin	3.44362434	23.0688756	0	17.6157731
ENSG000001	KRTAP9-8	protein_codin	0	0	0	0
ENSG000001:	PPP1R1B	protein_codin	2.40610843	5.92774876	2.94609781	0
ENSG000001:	FMO5	protein_codin	2.68914347	0	1.72335894	0
ENSG000001:	PRKAB2	protein_codin	84.2141946	56.0867018	72.6865012	11.9949748
ENSG000000	FUS	protein_codin	26.8224908	24.5130402	133.45711	0
ENSG000001:	FSHB	protein_codin	0	0	0	1.79705899
ENSG000001:	ZNF132	protein_codin	5.29614457	0	10.4967373	4.07246234
ENSG000001:	USP29	protein_codin	0	0	0	0
ENSG000001:	ZNF211	protein_codin	4.79704007	0	7.04231582	0
ENSG000001:	CHSY1	protein_codin	27.7662005	56.3877337	69.2950456	38.7146892
ENSG000001:	SNRPA1	protein_codin	12.8761707	0	51.1532715	9.01314413
ENSG000001:	NR0B2	protein_codin	0	0	0	0.78717771
ENSG000001:	LIN28A	protein_codin	0	0	0.55828815	2.65873136
ENSG000001:	ADCY7	protein_codin	0	0	0	17.0026219
ENSG000001	THAP1	protein_codin	16.3292026	27.3127185	23.6418569	6.44341951
ENSG000001:	RHPN2	protein_codin	0.53446592	3.54128248	3.1586684	0
ENSG000001:	FAAP24	protein_codin	0	0	0	0
ENSG000001:	LRRC9	protein_codin	0	0	0	0
ENSG000001:	ACTR10	protein_codin	0	0	8.658607	0
ENSG000001:	GCH1	protein_codin	0.89684953	19.9660889	2.94768691	12.4785026
ENSG000001:	LGALS3	protein_codin	28.0618173	77.1102509	11.5249425	28.682095
ENSG000001:	PODNL1	protein_codin	0	0	0	3.08887068
ENSG000001:	ZNF700	protein_codin	6.19788235	0	16.1033568	8.6171707
ENSG000001:	DNAJB1	protein_codin	35.470867	48.4526694	59.4579952	237.48658
ENSG000001:	ZSWIM4	protein_codin	0	16.3019589	0	0
ENSG000001:	RFX1	protein_codin	5.80665035	20.7294362	10.0139015	3.94150922
ENSG000001:	DCAF15	protein_codin	3.61563309	15.8144299	9.69514933	2.87392739
ENSG000001	SDC1	protein_codin	10.2829708	29.967904	64.7714172	0
ENSG000001:	TRIM21	protein_codin	30.1655549	133.760432	49.4630647	11.9092741
ENSG000000	WDR7	protein_codin	0	0	0	0
ENSG000001	SMARCC1	protein_codin	34.7947158	139.654189	33.6440392	29.8272663
ENSG000001:	SLC6A11	protein_codin	2.82860745	0	17.2703273	1.37797076
ENSG000001:	NUP210	protein_codin	2.79689124	70.5232627	38.0243119	2.66185592
ENSG000001:	HSD17B7	protein_codin	2.30820058	0	11.3036516	0
ENSG000001:	EMILIN2	protein_codin	44.7539316	98.0762819	29.6439354	57.3495245
ENSG000001	DNHD1	protein_codin	6.15944194	28.2169435	52.1637348	11.6657808
ENSG000001:	ARFIP2	protein_codin	0	0	4.60432738	0

ENSG000001	RRP8	protein_codin	8.30074174	26.7989538	27.666736	12.4320762
ENSG000001	TIMM10B	protein_codin	19.3310502	58.7974935	47.7113203	18.9513113
ENSG000001	EFR3A	protein_codin	66.190584	90.6215607	68.1579942	36.7109195
ENSG000002	OC90	protein_codin	0	0	0	0
ENSG000001	PTCD3	protein_codin	39.3257728	55.0744689	134.14595	18.4874762
ENSG000001	IMMT	protein_codin	8.01347674	0	0	0
ENSG000001	MRPL35	protein_codin	0	0	0	2.64579
ENSG000001	IQCA1	protein_codin	0	0	0	0
ENSG000001	ILKAP	protein_codin	9.46809786	15.3840253	0	0
ENSG000001	PER2	protein_codin	11.6283441	88.6128911	21.8433029	9.92618723
ENSG000001	RAMP1	protein_codin	0	4.45510525	7.23023277	0
ENSG000001	SCLY	protein_codin	0	0	0	0
ENSG000001	PTPRE	protein_codin	0.6627248	0	0	0
ENSG000001	CARD6	protein_codin	14.9836347	41.7186453	10.7434068	11.6982502
ENSG000001	RAP1GAP2	protein_codin	5.85837084	5.91451435	0	0
ENSG000001	MYBBP1A	protein_codin	7.60968104	17.7681332	0	0
ENSG000001	RPA1	protein_codin	21.5107065	51.8682659	54.2546298	11.0618251
ENSG000001	SERPINF1	protein_codin	190.860421	255.994093	19.9613335	707.351476
ENSG000001	EEFSEC	protein_codin	4.24538685	10.1847889	15.280748	4.92852913
ENSG000001	TMEM128	protein_codin	2.13224676	1.61057633	7.5778952	1.29805488
ENSG000001	COQ3	protein_codin	3.31833462	0	10.4965615	0.59406763
ENSG000001	POPDC3	protein_codin	0	0	0.71965143	0.46037232
ENSG000001	LANCL2	protein_codin	3.40476153	5.14407799	28.8464005	6.40881011
ENSG000001	GRSF1	protein_codin	17.3364242	16.1102635	24.1187495	8.76381422
ENSG000001	JCHAIN	protein_codin	2.12124382	830.786552	0	83.5314815
ENSG000001	UTP3	protein_codin	48.5781466	46.0832787	216.051452	25.1280572
ENSG000001	WBP2	protein_codin	6.26123183	0	0	6.53334927
ENSG000001	H3-3B	protein_codin	50.9246663	83.8262834	107.562193	198.483836
ENSG000001	TRIM47	protein_codin	3.28189281	6.08443179	3.78007225	0
ENSG000001	ZRANB2	protein_codin	0	59.1053636	40.1493004	18.4480418
ENSG000001	KDM6B	protein_codin	0	134.65214	32.6763321	73.624032
ENSG000001	ASGR2	protein_codin	0	0	0	0
ENSG000001	SLC52A1	protein_codin	0.35535851	0	0.71537458	0
ENSG000001	GUCY2D	protein_codin	0	0.87147597	0	0
ENSG000001	CLEC10A	protein_codin	0	20.7212455	0	1.92267422
ENSG000001	RIDA	protein_codin	4.9326813	14.093803	45.0114216	1.8119175
ENSG000001	MATN2	protein_codin	29.43128	26.2765257	0	0
ENSG000001	BRD8	protein_codin	0.37211792	2.28146625	1.55908981	0
ENSG000001	REEP2	protein_codin	0.95109563	0	0	0.42775612
ENSG000001	PCBD2	protein_codin	4.29920532	6.54637805	11.2345558	3.2436927
ENSG000001	ERAL1	protein_codin	8.34434836	0	26.8421139	4.9217164
ENSG000001	NIP7	protein_codin	0	12.3028665	35.5216057	0
ENSG000001	NIP7	protein_codin	6.42186942	0	0	7.59053508
ENSG000001	TERF2	protein_codin	23.8240349	61.7267425	59.552275	14.5958792

ENSG000001	VPS4A	protein_codin	0	0	0	0
ENSG000001	JAG1	protein_codin	75.8001147	170.634188	55.7791871	89.6843386
ENSG000001	HSPA12B	protein_codin	5.17853619	0	0	0
ENSG000001	SNAP25	protein_codin	2.5132349	0	2.96113865	0
ENSG000001	BTBD3	protein_codin	10.2271881	21.2476145	32.7203324	13.2821217
ENSG000001	NXT1	protein_codin	4.96437007	4.53690738	13.2942163	7.92949706
ENSG000001	RIN2	protein_codin	53.0400168	88.6886489	60.8560067	55.7675712
ENSG000001	SSTR4	protein_codin	1.59764015	0	0	1.89861839
ENSG000001	CRP	protein_codin	0	0	0	2.9239806
ENSG000001	HAPLN2	protein_codin	0	0	0	0
ENSG000001	APCS	protein_codin	0	0	0	1.01315653
ENSG000001	IGHMBP2	protein_codin	1.96241387	0	0	0
ENSG000001	ACY3	protein_codin	0	0	0	0
ENSG000001	TESMIN	protein_codin	0	0	5.87339379	0
ENSG000001	DPH2	protein_codin	0	0	0	0
ENSG000001	PABPC1L	protein_codin	0	0	1.06727684	2.08461526
ENSG000001	ZSWIM3	protein_codin	2.12537503	9.00136999	6.18839011	0
ENSG000001	OSER1	protein_codin	8.02793804	36.2814565	22.4223087	22.2945735
ENSG000001	SERINC3	protein_codin	0	43.9891061	0	0
ENSG000001	DMGDH	protein_codin	1.76269668	2.92744181	0	0
ENSG000001	BHMT2	protein_codin	13.1625637	6.24063234	0	4.16967735
ENSG000001	AP3B1	protein_codin	0	0	56.6388466	0
ENSG000001	ZBED3	protein_codin	7.05218763	28.2844942	15.1885231	13.1602988
ENSG000001	SYT4	protein_codin	0	0	0.53201001	3.05673447
ENSG000001	SLC14A2	protein_codin	0	0	0	1.43534161
ENSG000001	NMUR2	protein_codin	0	3.28244791	3.74525458	1.12772701
ENSG000001	PDE6A	protein_codin	0	0	0	0
ENSG000001	MTUS2	protein_codin	0	0	0	0
ENSG000001	USPL1	protein_codin	12.6214432	32.8330911	22.5084843	22.7420635
ENSG000001	XPO4	protein_codin	34.2253357	56.363395	76.7223672	25.4542496
ENSG000001	RNF17	protein_codin	0	0	0	1.67485787
ENSG000001	RNF17	protein_codin	0	0	0	0
ENSG000001	RNF17	protein_codin	0	0	0	0.87860362
ENSG000001	CHRM3	protein_codin	0	0	8.47845212	1.34965811
ENSG000002	MYH4	protein_codin	3.55528557	0	0	4.30214008
ENSG000001	PEMT	protein_codin	0	0	3.8066374	0
ENSG000001	SCO1	protein_codin	14.9060004	36.2196866	18.330888	9.78371282
ENSG000001	CHI3L1	protein_codin	3.5203552	0.94163842	0	0
ENSG000001	MYBPH	protein_codin	3.81194382	0	0	2.09267876
ENSG000001	CHIT1	protein_codin	0	0	0	0
ENSG000001	LGR6	protein_codin	5.56922765	0	0	0
ENSG000001	DCLK1	protein_codin	27.7192532	0	46.8258013	0
ENSG000001	CCNA1	protein_codin	0	0	0	0
ENSG000001	RFXAP	protein_codin	4.17876882	5.31257398	12.4972149	3.75706099

ENSG000001:	STARD13	protein_codin	3.87980127	0	11.4198214	3.60787157
ENSG000001:	MORC4	protein_codin	0	38.4645412	28.957002	0
ENSG000001:	RNF128	protein_codin	0	1.51385847	0	3.33191282
ENSG000001(	PCDH19	protein_codin	1.19869539	0	0	0
ENSG000001(	MTNAP1	protein_codin	1.82492744	0	0	0
ENSG000001:	SLC39A11	protein_codin	0	54.6003313	33.8678347	10.4132544
ENSG000001:	BTBD2	protein_codin	15.3287874	35.4018091	40.3977037	16.6970711
ENSG000001:	KMT5C	protein_codin	0.83011762	2.52287549	1.66048458	1.38362567
ENSG000001:	ZNF414	protein_codin	0.5161524	0	0.47493094	1.23108916
ENSG000001:	PDE6B	protein_codin	0	0	0	0
ENSG000001:	HSPBP1	protein_codin	0	0	0	0
ENSG000001:	CSNK1G2	protein_codin	7.26234789	25.1414966	61.6749737	16.8681807
ENSG000001'	RTTN	protein_codin	0	0	0	0
ENSG000001:	MACROD1	protein_codin	1.04717701	0	9.12645768	1.30847137
ENSG000001:	LGALS12	protein_codin	0.70107572	0	0	0
ENSG000001:	PLAAT4	protein_codin	4.20514492	33.0560489	0	4.46577652
ENSG000001:	PLAAT2	protein_codin	0.34546763	0	0	0.89624653
ENSG000001!	ZNF679	protein_codin	0	2.2248295	0	0
ENSG000001:	CEP20	protein_codin	18.9096918	0	65.5422605	12.9947974
ENSG000001:	MED10	protein_codin	7.45030254	24.3410618	12.1016723	3.96522589
ENSG000001(	CCDC134	protein_codin	7.69642504	42.674981	54.5074476	8.00172775
ENSG000001:	SLC2A11	protein_codin	0	0	0	0
ENSG000001:	SEC14L4	protein_codin	0	0	0	0.84649279
ENSG000002(	PI4KA	protein_codin	29.3098329	130.941686	137.632616	36.2144964
ENSG000001:	GIMAP4	protein_codin	22.7417845	287.587258	5.42804494	24.2614692
ENSG000001:	MKRN1	protein_codin	22.5705966	0	50.2450633	35.6131435
ENSG000001:	ACTR3B	protein_codin	4.91558791	0	0	2.20031342
ENSG000001:	NTS	protein_codin	0	0	0	0
ENSG000001:	BTG1	protein_codin	34.1810286	51.9066796	25.969603	130.272466
ENSG000001:	DYDC2	protein_codin	0	0	0	0
ENSG000001:	TMTC1	protein_codin	0	0	0	0
ENSG000001:	KRAS	protein_codin	0	6.19671827	0	0
ENSG000001:	IPO8	protein_codin	26.743304	97.7262171	140.816991	27.2805202
ENSG000001:	SPINK5	protein_codin	0	8.79745875	0	0
ENSG000001(	PMP2	protein_codin	0	0	0	2.23224825
ENSG000001'	FABP4	protein_codin	130.79074	35.7838415	1.07314386	197.919045
ENSG000001:	IMPA1	protein_codin	9.48393462	8.94494187	4.02432223	5.34605679
ENSG000001:	E2F5	protein_codin	0	0	4.27166981	1.05510504
ENSG000001:	CCDC59	protein_codin	0	0	0	2.66508227
ENSG000001:	LYVE1	protein_codin	5.98822051	6.95809782	1.98455096	16.7985142
ENSG000001:	SBF2	protein_codin	0	0	0	0
ENSG000001:	MICAL2	protein_codin	0	0	0	0
ENSG000001:	RRAS2	protein_codin	4.61431064	0	11.5495086	2.90481658
ENSG000001:	TEX15	protein_codin	0	0	34.7931327	4.7647426

ENSG000001:	SARAF	protein_codin	50.4147743	87.2274162	37.8750795	42.3432832
ENSG000001:	RNF122	protein_codin	0.78754725	2.23551727	4.57390077	9.93556206
ENSG000001:	DUSP26	protein_codin	4.93514501	0	1.07582804	0.91273079
ENSG000001:	ERG28	protein_codin	1.54510019	8.61083653	19.8424432	4.50344204
ENSG000001:	DGLUCY	protein_codin	1.62394676	0	25.6012721	3.92436929
ENSG000001:	UNC79	protein_codin	1.68651485	0	3.26794152	4.64782395
ENSG000001:	CATSPERB	protein_codin	0	2.13465936	0	1.75418109
ENSG000001:	VRTN	protein_codin	0.25334363	2.38410386	0.56031896	1.70919544
ENSG000002	SYNJ2BP	protein_codin	59.4253461	112.955837	105.202011	42.5008761
ENSG000001:	TTC9	protein_codin	1.69702819	15.6801921	4.85237706	3.81581262
ENSG000001:	MED6	protein_codin	0	19.4682761	12.8608177	6.22997235
ENSG000001:	EIF2S1	protein_codin	26.9796869	62.4193906	57.7966453	23.7155394
ENSG000001:	ADAM20	protein_codin	0	4.84605867	3.78033353	2.10613457
ENSG000001:	ELP3	protein_codin	22.7449879	52.8697816	10.268106	14.368836
ENSG000001:	PEBP4	protein_codin	0.86604565	0	0	0
ENSG000001:	ADAMDEC1	protein_codin	0	48.4676318	0	9.04449692
ENSG000001:	CTIF	protein_codin	16.1900351	24.2883909	45.7947268	14.4106788
ENSG000001:	MRO	protein_codin	4.21838842	0	5.28314697	4.85853981
ENSG000001:	MBD2	protein_codin	55.1026763	174.104489	98.2560394	53.3587614
ENSG000001:	IER3IP1	protein_codin	14.7231165	14.7858401	26.7559096	11.8648799
ENSG000001:	KGD4	protein_codin	2.4077898	6.09005002	10.8359777	2.40752154
ENSG000001:	CCNB1	protein_codin	3.68184938	7.27160971	5.17978263	0
ENSG000001:	CDK7	protein_codin	0	0	0	0
ENSG000001:	CD180	protein_codin	0.75605552	8.90596677	0.89606958	3.84404771
ENSG000000:	IL5RA	protein_codin	0.62754533	0	0	0
ENSG000001:	IRAK2	protein_codin	2.97867596	11.0343328	8.2152394	7.69643805
ENSG000001:	CAMK1	protein_codin	8.82525523	24.2701865	3.95758179	5.59478333
ENSG000001:	VHL	protein_codin	11.2949699	0	34.2750029	5.20690817
ENSG000001:	BHLHE40	protein_codin	22.9341185	88.1824789	97.5908963	298.313746
ENSG000001:	ARL8B	protein_codin	30.1397186	90.8493504	105.96291	45.4673128
ENSG000001:	EDEM1	protein_codin	27.3143811	88.8943512	49.6461116	23.6877011
ENSG000001:	CHL1	protein_codin	2.1358504	0	0	5.82530913
ENSG000001:	DPH6	protein_codin	2.50820185	9.79881812	3.38055635	0
ENSG000001:	KATNBL1	protein_codin	0.81975697	12.8266209	1.94417044	8.42298506
ENSG000001:	EMC7	protein_codin	14.0501445	62.8249383	45.3204806	15.9670272
ENSG000001:	TRPM1	protein_codin	0	0	0	0
ENSG000001	AMPD2	protein_codin	0	13.4997012	0	0
ENSG000001:	REG4	protein_codin	0	0	0	0
ENSG000001:	TSHB	protein_codin	0	0	0	0
ENSG000001:	GSTM5	protein_codin	0	0	0	0
ENSG000001:	GSTM3	protein_codin	17.6355461	8.4899146	8.06578267	13.4573248
ENSG000001:	SORT1	protein_codin	52.602864	123.960924	30.7362328	35.7860366
ENSG000001:	WNT2B	protein_codin	2.22459144	17.5253458	1.48944603	4.11784268
ENSG000001:	LAMTOR5	protein_codin	0.46002257	2.17593399	3.22828224	0.68028014

ENSG000001:	NOTCH2	protein_codin	325.874318	775.702037	503.498824	174.716431
ENSG000001:	TRIM45	protein_codin	4.02883527	23.8570954	20.5314375	3.86521745
ENSG000001:	CD101	protein_codin	2.02615691	7.28034404	1.56459261	1.24798346
ENSG000001:	AP4B1	protein_codin	0	27.0629332	0	0
ENSG000001:	PPHLN1	protein_codin	1.88794377	8.90084504	3.85410238	0
ENSG000001:	FKBP11	protein_codin	0	0	0	0
ENSG000001:	ARF3	protein_codin	21.1386041	57.4635681	103.888505	21.4447444
ENSG000001:	TMEM106C	protein_codin	5.87155516	0	0	0
ENSG000001:	SLC38A2	protein_codin	73.5199298	129.980518	253.683694	115.128251
ENSG000001:	KIDINS220	protein_codin	49.5709149	0	0	0
ENSG000001:	LPIN1	protein_codin	22.9461346	54.2733776	52.2231741	13.052668
ENSG000001:	CMPK2	protein_codin	4.4347715	25.142165	0	0
ENSG000001:	SAA2	protein_codin	0.45996742	0	0	0.40493483
ENSG000001:	ANO3	protein_codin	0	0	0	0
ENSG000001:	FST	protein_codin	21.969159	41.4629908	8.95666233	12.06438
ENSG000001:	CFHR5	protein_codin	0	0	0	0
ENSG000001:	ERN2	protein_codin	0	0	0	0
ENSG000000	NEDD4L	protein_codin	1.19967087	0	0	0
ENSG000001:	RAX	protein_codin	0	0	0.44245479	1.60278711
ENSG000001:	NARS1	protein_codin	39.0976094	53.1885888	102.319559	28.8221688
ENSG000001:	GRP	protein_codin	0	0	0	0
ENSG000001:	RELCH	protein_codin	28.6533914	36.3366299	36.0004348	6.33759872
ENSG000001:	IL2RA	protein_codin	0	0	0	0
ENSG000001:	CCNH	protein_codin	7.00385875	0	0	8.1300413
ENSG000001:	HRH4	protein_codin	0.17754079	0	1.00127846	0
ENSG000001:	CABLES1	protein_codin	3.63637584	11.0888351	5.6714969	4.273042
ENSG000001:	EMP1	protein_codin	154.555813	188.804922	12.7326282	249.016772
ENSG000001:	RERG	protein_codin	0	0	0	0
ENSG000001:	SLCO1B1	protein_codin	0	0	0	0
ENSG000001:	SPX	protein_codin	0	0	0	2.13572916
ENSG000001:	DDB2	protein_codin	12.8991592	15.2501612	7.72115804	3.56112844
ENSG000001:	ACP2	protein_codin	0	0	0	0
ENSG000000	FOLH1	protein_codin	0.80850029	9.23944852	1.07144436	2.74971421
ENSG000001:	RTL8C	protein_codin	19.9563441	26.1576372	34.065718	13.0130486
ENSG000001:	RAB33A	protein_codin	0.61861142	5.42946636	0	0
ENSG000001:	MTNR1B	protein_codin	0	0	0	0
ENSG000001:	PUM1	protein_codin	0	0	0	0
ENSG000001:	SPOCD1	protein_codin	0	0	0	0
ENSG000001:	PHC2	protein_codin	0	0	0	0
ENSG000001:	TUT4	protein_codin	11.5282974	39.7248826	23.453888	1.61080759
ENSG000001:	PRPF38A	protein_codin	74.3397274	77.5791258	143.311686	46.6393022
ENSG000001:	DSG3	protein_codin	0	1.58507593	0	2.32627589
ENSG000001:	RNF138	protein_codin	3.68963774	4.54255738	7.24967678	6.72564905
ENSG000001:	DSG1	protein_codin	0	0	0	0.76197969

ENSG000001	DSC1	protein_codin	0	0	0	0
ENSG000001	DSC1	protein_codin	0	0	0.49263777	0
ENSG000001	FHOD3	protein_codin	38.9032538	170.955944	135.481739	66.188252
ENSG000001	DAGLA	protein_codin	1.88108879	0	0	0
ENSG000001	TIMM10	protein_codin	7.57589682	3.13283855	4.55694973	4.28362749
ENSG000001	AHNAK	protein_codin	0	5.24554542	0	2.58333184
ENSG000001	CBLIF	protein_codin	0	0	0	0
ENSG000001	APLNR	protein_codin	0	0	0	0
ENSG000001	FADS2	protein_codin	11.0363798	7.58453081	1.52616173	0
ENSG000001	TMEM258	protein_codin	0	7.73546277	0	0
ENSG000001	TCN1	protein_codin	0	0	0	0
ENSG000001	CEP135	protein_codin	27.1854022	87.7394752	214.306371	26.9785268
ENSG000001	PDGFRA	protein_codin	105.869882	289.953638	432.845511	216.962804
ENSG000001	BIVM	protein_codin	11.1658599	7.30015378	15.9953179	5.70577589
ENSG000001	CARS2	protein_codin	6.11797594	27.3091499	17.362579	5.21648169
ENSG000001	ADAMTS8	protein_codin	0	1.98857172	0	0
ENSG000001	KLB	protein_codin	2.37210441	2.39924679	0.47177167	5.39573801
ENSG000000	SEMA6A	protein_codin	0	0	0	0
ENSG000001	APC	protein_codin	10.4790049	0	12.6520115	4.47513329
ENSG000001	NREP	protein_codin	33.9693594	52.4242758	76.3340094	9.04475265
ENSG000001	UBQLN1	protein_codin	0	54.8820694	27.812122	0
ENSG000001	ANXA1	protein_codin	237.814595	311.430375	158.921945	200.069264
ENSG000001	GOLM1	protein_codin	0.63353428	1.44011933	0	0
ENSG000001	ENTREP1	protein_codin	0	0	0	4.82595727
ENSG000001	ADAM19	protein_codin	4.0530834	91.489073	11.07456	14.8276004
ENSG000001	CCNJL	protein_codin	2.37817936	4.73078122	0	0
ENSG000001	USP30	protein_codin	6.65489023	0	7.90406783	7.3312971
ENSG000001	SDS	protein_codin	1.11831713	1.072351	0	0
ENSG000001	MSI1	protein_codin	0.28430691	1.89427456	2.84825308	1.13018032
ENSG000001	HNF1A	protein_codin	0	0	0	0
ENSG000001	TBX3	protein_codin	0.5199899	5.68235744	0	0
ENSG000001	OASL	protein_codin	0	0	0	0
ENSG000001	HRK	protein_codin	0.25148055	1.37168937	0.49008727	3.90751995
ENSG000001	RNFT2	protein_codin	1.11919465	5.26222862	2.51368159	1.55656225
ENSG000001	DTX1	protein_codin	0.93938113	1.0202668	1.54330937	0
ENSG000001	TRAFD1	protein_codin	3.72057709	4.3526526	1.40615208	0
ENSG000000	ASB4	protein_codin	0	0	0	0.52912226
ENSG000001	GSAP	protein_codin	8.83941878	42.4164058	10.4104661	14.0867176
ENSG000001	OCM2	protein_codin	0	0	0	0.33512555
ENSG000002	UPK3B	protein_codin	0	1.49653593	0	0
ENSG000001	TMEM243	protein_codin	8.18765941	5.31266669	0	3.7332554
ENSG000001	GTPBP10	protein_codin	0.78226374	0	3.81872272	0
ENSG000001	TMEM60	protein_codin	1.93892723	13.824494	4.84918652	1.12624905
ENSG000001	PNPLA8	protein_codin	1.40644051	15.7938324	0	3.90637978

ENSG000001	HILPDA	protein_codin	1.13563327	20.6579001	2.93042419	2.84744029
ENSG000001	RINT1	protein_codin	11.9279164	0	33.7469349	5.90790541
ENSG000000	KMT2E	protein_codin	59.4125886	212.913478	102.539296	79.2914907
ENSG000001	BACH2	protein_codin	10.4487614	6.81174954	14.2857866	8.72607393
ENSG000001	KHDC1	protein_codin	0	0	0	0
ENSG000001	CEP162	protein_codin	0	0	0	0
ENSG000001	NT5E	protein_codin	76.5703091	88.827839	14.7320631	28.2975726
ENSG000001	MRAP2	protein_codin	0.77989353	0	0	0
ENSG000001	AKIRIN2	protein_codin	30.8902519	63.146999	59.3730137	25.9947444
ENSG000001	ORC3	protein_codin	0	26.1815244	4.13127894	4.85190618
ENSG000001	LMO2	protein_codin	0.69826333	19.4416272	2.10430005	2.37325104
ENSG000001	NAT10	protein_codin	59.288105	163.892613	116.795451	20.0359396
ENSG000001	EHF	protein_codin	0.29223795	0	0	5.23457136
ENSG000001	ELF5	protein_codin	0	0	0	0
ENSG000001	PRRG4	protein_codin	1.48950979	2.28423846	0.49034297	1.75435717
ENSG000000	METTL1	protein_codin	22.5991381	3.81234544	11.8472111	0.64232926
ENSG000001	CD63	protein_codin	12.0902976	56.1297308	7.30667303	54.4143829
ENSG000001	PRPH	protein_codin	0	0	0	0
ENSG000001	AVIL	protein_codin	16.7139127	0	0	0
ENSG000001	AMHR2	protein_codin	0	0	0	0
ENSG000001	LACRT	protein_codin	0	0	0	0
ENSG000001	GDF11	protein_codin	39.479473	83.5394149	231.151571	18.9223759
ENSG000001	ITGA7	protein_codin	0	0	0	6.25199138
ENSG000001	FAM186B	protein_codin	2.31634839	2.11448059	2.96322406	4.33074749
ENSG000001	RDH5	protein_codin	0	0	0	0
ENSG000001	AGAP2	protein_codin	5.40065243	21.1705209	1.75025861	3.00782847
ENSG000002	ENSG000002	protein_codin	0.5142531	0	1.39484609	0
ENSG000001	KRT85	protein_codin	0	1.65179139	0	0.82062323
ENSG000001	CDK4	protein_codin	74.0362975	15.3058568	17.9474869	0.96452685
ENSG000001	PPP1R1A	protein_codin	19.2041899	5.50890359	1.01197779	3.14392667
ENSG000001	TROAP	protein_codin	0	1.58764591	21.7536633	0
ENSG000001	TSPAN31	protein_codin	44.8258237	3.34776121	4.56722133	4.02055426
ENSG000001	TFCP2	protein_codin	16.3004578	101.834932	0	12.3967325
ENSG000001	PAN2	protein_codin	0	0	0	0
ENSG000001	ESPL1	protein_codin	3.94559032	5.88338205	0	1.96063842
ENSG000001	ZC3H10	protein_codin	5.8889536	16.3434719	7.70238275	4.82341922
ENSG000001	KRT84	protein_codin	0.31559175	0	0	0
ENSG000001	ACVR1B	protein_codin	7.44529883	21.504488	12.1560697	6.79445826
ENSG000001	OS9	protein_codin	0	0	0	0
ENSG000001	KRT82	protein_codin	0.27779674	0	0.50183668	2.12657287
ENSG000001	KCNH3	protein_codin	0.45433209	0	16.2613629	0
ENSG000001	PKIB	protein_codin	0	0	0	0
ENSG000001	TAAR5	protein_codin	0	0	0	1.37630448
ENSG000001	NMBR	protein_codin	0	1.59574114	0	0.89201669

ENSG000001	SMPD2	protein_codin	1.46125626	3.54073785	2.55712267	1.91533705
ENSG000001	REPS1	protein_codin	5.15524343	24.0298219	11.0108191	0
ENSG000001	HTRA2	protein_codin	0	0	2.66079281	0
ENSG000001	PRADC1	protein_codin	3.59831867	5.03531096	7.7254949	0
ENSG000001	CCT7	protein_codin	54.2160607	82.6674579	256.620003	20.373447
ENSG000001	RAB11FIP5	protein_codin	9.5308113	18.0808118	11.6558985	13.6743574
ENSG000001	SMYD5	protein_codin	0	0	0	0
ENSG000001	DYSF	protein_codin	0	0	0	0
ENSG000002	MRPL53	protein_codin	3.39158659	7.71717224	19.308086	0
ENSG000001	EMX1	protein_codin	0	0	0	0
ENSG000001	KCNMB4	protein_codin	121.173723	33.1326514	12.6343617	8.64854382
ENSG000001	GNS	protein_codin	7.37976411	242.973453	28.3061585	74.5848746
ENSG000001	MDM2	protein_codin	9.84170605	0	0	0
ENSG000001	MDM2	protein_codin	1381.06504	165.619923	94.7812807	48.0167432
ENSG000001	KLHL36	protein_codin	0	0	0	0
ENSG000001	BCO1	protein_codin	0	0	0	0
ENSG000001	MPHOSPH6	protein_codin	18.4845279	51.1252617	64.2373793	8.46543823
ENSG000002	TMEM231	protein_codin	5.88821839	0	8.9192527	6.84306617
ENSG000001	NDRG4	protein_codin	0	0	0	0
ENSG000001	DYNC1LI2	protein_codin	144.976201	197.256654	180.603687	58.6358352
ENSG000001	FBXL8	protein_codin	0	0	0	0
ENSG000001	FHOD1	protein_codin	23.704943	66.9294099	36.5921386	8.31801853
ENSG000001	CCDC102A	protein_codin	8.73132096	16.7848403	45.0187787	5.0663552
ENSG000001	PCNX2	protein_codin	14.3554483	85.8685925	30.7049886	13.7759811
ENSG000001	URB2	protein_codin	9.78378709	18.359747	51.6603019	5.68350808
ENSG000001	TAF5L	protein_codin	4.92712904	31.6993954	26.7682457	11.0457446
ENSG000001	STX6	protein_codin	30.943685	53.406543	72.1819567	20.8739957
ENSG000001	RGS8	protein_codin	0	0	0.9365873	0
ENSG000001	NPL	protein_codin	0	0	0	0
ENSG000001	LAMC1	protein_codin	510.788009	522.053949	840.831042	204.614787
ENSG000001	PNKD	protein_codin	0	0	0	0
ENSG000001	SP110	protein_codin	0	45.5071274	13.0175013	0
ENSG000001	SP110	protein_codin	9.66236256	0	0	0
ENSG000001	MRPL44	protein_codin	12.6741303	18.8680734	20.4238256	15.6527516
ENSG000001	CHRND	protein_codin	0	0	0	0
ENSG000001	PAX3	protein_codin	0	0	0	0
ENSG000001	DOCK10	protein_codin	21.6161462	102.96421	18.1247632	33.7296287
ENSG000001	TTLL4	protein_codin	0	0	0	0
ENSG000001	USP37	protein_codin	29.5902112	20.4942242	38.3164348	31.0207066
ENSG000001	HTR2B	protein_codin	1.97729954	1.41388694	0.44578211	0.44865471
ENSG000001	SLC19A3	protein_codin	0	0	0	0.91142915
ENSG000001	SERPINE2	protein_codin	61.8328206	0	24.0130073	35.8887018
ENSG000001	WNT10A	protein_codin	0.49085258	10.3056989	0.4921838	0
ENSG000001	TMBIM1	protein_codin	24.2554603	19.4946211	7.32993096	13.1098785

ENSG000001:	CYP27A1	protein_codin	5.08424901	0	2.05975732	5.05586854
ENSG000001:	EIF4E2	protein_codin	29.6602125	212.122698	66.8091637	13.292891
ENSG000001:	CAB39	protein_codin	64.6981955	146.272173	52.9540645	27.567811
ENSG000001:	COX5B	protein_codin	21.7894938	179.705171	81.7693817	29.0992765
ENSG000001:	REV1	protein_codin	34.3044358	67.8417848	0	12.8579841
ENSG000001:	MFSD9	protein_codin	6.33936986	8.07545194	13.4146538	1.58518907
ENSG000001:	TMEM127	protein_codin	0	0	0	0
ENSG000001:	EDAR	protein_codin	0	1.3019821	0	0
ENSG000001:	MRPS9	protein_codin	30.7120403	46.57943	151.452264	18.7203028
ENSG000001:	GPR45	protein_codin	0	0	0	0
ENSG000001:	C2orf49	protein_codin	12.3598554	22.2015222	39.4400841	9.6433265
ENSG000001!	ANKRD36B	protein_codin	0	0	0	18.7041294
ENSG000001:	EPC2	protein_codin	18.205732	60.775865	124.00944	0
ENSG000001:	ALDH1L2	protein_codin	22.9226743	0	0	30.1013093
ENSG000001:	USP44	protein_codin	1.05024526	0	0	0
ENSG000001:	PLXNC1	protein_codin	30.9682747	91.0668343	12.4246954	9.07523584
ENSG000001:	APPL2	protein_codin	6.91970708	104.889369	29.3565999	11.9252768
ENSG000001:	DRAM1	protein_codin	5.88190256	27.5725804	3.48793755	12.5348522
ENSG000001:	SLC41A2	protein_codin	15.9476762	31.8776086	7.5205917	19.0374394
ENSG000001:	CKAP2	protein_codin	20.4878073	0	0	0
ENSG000001:	THSD1	protein_codin	2.01341942	34.8484503	2.93542825	12.5431945
ENSG000001:	RCBTB1	protein_codin	0	0	0	0
ENSG000001:	MED4	protein_codin	16.4403866	66.0894731	33.3266366	18.5824706
ENSG000001:	NUDT15	protein_codin	6.38074231	6.57063867	16.0310088	8.99101269
ENSG000001(	PLEKHA8	protein_codin	0.30719883	3.75094664	5.44073846	4.29620821
ENSG000000:	CAMK2B	protein_codin	0	0	0	0
ENSG000001:	SPDYE1	protein_codin	0	0	0	1.07190161
ENSG000001:	CHST12	protein_codin	0	0	0	0
ENSG000001:	IGF2BP3	protein_codin	0	0	87.7867122	3.08760003
ENSG000001:	GPNMB	protein_codin	43.7809254	263.144084	0	20.0940888
ENSG000000(	ABCB5	protein_codin	0	0	0.90062215	1.33120068
ENSG000001:	KDELR2	protein_codin	150.792916	179.676499	174.118941	59.9936197
ENSG000001:	NUP42	protein_codin	0	0	13.1744994	0
ENSG000001:	IL6	protein_codin	0	0	0	19.4868601
ENSG000001:	BZW2	protein_codin	13.3172492	5.68121504	61.9107865	8.63031467
ENSG000001:	TBRG4	protein_codin	0	0	18.2378512	0
ENSG000001:	DDX56	protein_codin	9.08097789	0	28.735723	9.9072834
ENSG000001:	HUS1	protein_codin	0	0	4.82320497	0.87497619
ENSG000001:	CCM2	protein_codin	9.77247316	17.5204845	22.8037306	16.3593643
ENSG000001:	MYO1G	protein_codin	0	0	0	0
ENSG000001:	TTYH3	protein_codin	20.2628882	0	52.0828446	26.5296511
ENSG000001:	CIDEB	protein_codin	1.54088412	5.68991559	3.15331228	2.09248342
ENSG000001:	TTC5	protein_codin	9.58369154	16.5192585	19.6906657	7.76449846
ENSG000001:	NKX2-8	protein_codin	0	0.73375531	0	0.44090408

ENSG000001	ACSBG1	protein_codin	0	0	0	0
ENSG000001	MTHFS	protein_codin	0.93584135	7.76266655	7.11385121	1.19936073
ENSG000001	ABHD17C	protein_codin	1.30067279	0	8.3666002	0.59901905
ENSG000001	IREB2	protein_codin	54.018797	91.0572773	121.722223	32.273339
ENSG000001	ALPK3	protein_codin	32.8370141	83.7688737	18.3658701	21.2462877
ENSG000001	TM6SF1	protein_codin	0	0	0	0
ENSG000001	CIB2	protein_codin	2.62668129	8.97286963	7.3957611	2.10140297
ENSG000001	CALCOCO2	protein_codin	9.97828253	70.3660531	0	20.086309
ENSG000001	RSAD1	protein_codin	13.4410654	27.6064149	21.6933287	7.12428942
ENSG000001	NMT1	protein_codin	0	0	0	0
ENSG000001	SRSF1	protein_codin	48.0427091	79.5956495	302.963391	60.6560076
ENSG000001	VEZF1	protein_codin	2.726432	39.9918639	44.5559431	21.9167025
ENSG000001	CHAD	protein_codin	6.27714675	28.2377579	4.01460561	108.101005
ENSG000001	TACO1	protein_codin	5.00076041	7.79112437	6.88380144	3.7361474
ENSG000001	TEX2	protein_codin	0	0	0	0
ENSG000002	CSHL1	protein_codin	0	0	0	0
ENSG000001	LIMD2	protein_codin	0	22.1993276	2.76481773	3.68152277
ENSG000001	BRIP1	protein_codin	0.78624698	0	22.1028569	3.92194494
ENSG000001	KAT7	protein_codin	6.87033672	26.5127603	10.2990791	7.17368367
ENSG000001	RTP4	protein_codin	1.21673342	12.4897237	1.28413336	1.17110778
ENSG000001	NDUFB5	protein_codin	4.91061396	0	4.73071774	3.68686474
ENSG000001	MRPL47	protein_codin	0	0	0	0
ENSG000001	TRA2B	protein_codin	0	0	0	0
ENSG000001	MARCHF7	protein_codin	36.9028538	71.2682469	65.6377609	16.6151288
ENSG000002	CD302	protein_codin	12.482512	0	9.93542375	33.4119433
ENSG000001	GALNT5	protein_codin	152.352223	193.131098	53.0801738	79.4512237
ENSG000001	TANK	protein_codin	0	0	0	47.6243448
ENSG000001	BLK	protein_codin	0	0	0	0
ENSG000001	SKIL	protein_codin	119.816254	54.7468838	93.589899	46.9628762
ENSG000001	KCTD3	protein_codin	33.405471	63.7005836	49.4819457	22.8023637
ENSG000001	ZNG1B	protein_codin	2.14296719	0	0	0
ENSG000001	IL36G	protein_codin	0	0	0	0
ENSG000001	IL1RN	protein_codin	0	0	0	0
ENSG000001	IL36A	protein_codin	0	0	0	0.56797839
ENSG000001	IL36B	protein_codin	0	0	0	1.19283896
ENSG000001	CFC1	protein_codin	0	0	0	0
ENSG000001	CCDC115	protein_codin	0	0	0	0
ENSG000001	SAP130	protein_codin	2.32830968	0	37.0953268	0
ENSG000001	SAP130	protein_codin	20.8057178	0	34.2125273	9.94208429
ENSG000001	BIN1	protein_codin	1.460189	0	0	0
ENSG000001	IMP4	protein_codin	1.52027289	0	6.40301878	1.7213495
ENSG000001	HS6ST1	protein_codin	9.23138395	41.0087834	26.9651349	21.0106325
ENSG000001	UGGT1	protein_codin	70.0881914	88.980669	77.217506	32.857569
ENSG000001	GYPC	protein_codin	15.7982692	35.8045173	0	18.6261531

ENSG000001	GAD2	protein_codin	0	0	0	0
ENSG000001	PALM2AKAP2	protein_codin	0.89253282	3.3811079	0	0
ENSG000001	LRRC8A	protein_codin	0	3.06401367	1.68847418	0
ENSG000001	ECPAS	protein_codin	0	0	0	0
ENSG000001	TOR1B	protein_codin	14.2644562	31.2183392	22.4989631	7.40626223
ENSG000001	RALGPS1	protein_codin	1.7047817	0	2.9381207	2.16173219
ENSG000001	OR1J1	protein_codin	0	0	0	0
ENSG000001	OR13C9	protein_codin	0.32987976	0	0	0.47967317
ENSG000001	TMOD1	protein_codin	17.214569	0	0	0
ENSG000001	DAB2IP	protein_codin	0	0	0	31.50048
ENSG000001	SLC31A2	protein_codin	2.5092058	7.55751618	0.93720341	5.88626273
ENSG000001	ZNF189	protein_codin	0.98690887	0	1.72936695	0
ENSG000002	ORM1	protein_codin	0.36283386	0.83112061	0	0
ENSG000001	STX17	protein_codin	18.0221028	61.1988704	35.034643	22.2771074
ENSG000001	BAAT	protein_codin	0	1.78320212	0	0
ENSG000001	GABBR2	protein_codin	0	0	0	3.96313043
ENSG000001	HEMGN	protein_codin	0	0	0	0
ENSG000001	PSMB7	protein_codin	20.5365698	28.9282013	66.8069699	11.6880915
ENSG000001	RABEPK	protein_codin	1.51520329	0	0	0
ENSG000001	OR1L4	protein_codin	0	1.62833467	0	0.44455286
ENSG000001	PDCL	protein_codin	21.1960009	130.767963	71.5160925	19.5997625
ENSG000001	CTSV	protein_codin	0	0	3.52605926	0
ENSG000001	ARPC5L	protein_codin	7.81423835	11.7239861	12.2531064	9.31501367
ENSG000001	ENPP2	protein_codin	83.5058792	43.3698434	92.3066998	81.9093049
ENSG000001	DERL1	protein_codin	10.340698	55.0664558	63.6163638	5.63032866
ENSG000001	MYC	protein_codin	0.79128966	0	2.08004997	4.19970137
ENSG000001	CCN3	protein_codin	8.53599035	5.21448827	10.4741202	6.33435251
ENSG000001	IFNA6	protein_codin	0	0	0	0
ENSG000001	RANBP6	protein_codin	33.141484	61.4549843	67.3261479	22.6063652
ENSG000001	RNF38	protein_codin	0	0	0	0
ENSG000001	CCL21	protein_codin	0	0	20.1502885	0.87072726
ENSG000001	SIT1	protein_codin	0	5.10423894	0	0
ENSG000001	DMRT2	protein_codin	0	0	0	0
ENSG000002	CCL27	protein_codin	0	0	0	0
ENSG000001	DCTN3	protein_codin	12.0506541	11.3438253	35.6481378	7.73508575
ENSG000001	CD72	protein_codin	0.33756305	28.0614325	0	6.39072997
ENSG000001	HINT2	protein_codin	0	14.6497635	5.58409194	0
ENSG000001	RIPOR2	protein_codin	0	0	0	0
ENSG000001	KLC4	protein_codin	0.70789405	0	0	3.41347702
ENSG000001	KIF13A	protein_codin	52.1271901	171.193687	126.277555	42.1177751
ENSG000001	GMPR	protein_codin	1.87238676	3.21794737	18.2325138	0
ENSG000001	TMEM63B	protein_codin	7.21596588	17.1335359	0	8.53523258
ENSG000001	FRS3	protein_codin	0	5.22388519	4.50329049	1.51928974
ENSG000001	TTBK1	protein_codin	0.33214418	0	0.54672548	3.40219006

ENSG000001	TJAP1	protein_codin	0	0	1.67373187	0
ENSG000001	TINAG	protein_codin	0	0	0	0
ENSG000001	GCM1	protein_codin	0.26624921	0	1.42153039	0.43163117
ENSG000001	RIPK1	protein_codin	10.8341754	56.1807551	0	0
ENSG000001	TUBB2B	protein_codin	13.9853281	10.3713801	102.881689	1.52060814
ENSG000002	PSORS1C2	protein_codin	0	2.96236942	0.37648358	3.17399429
ENSG000002	C6orf15	protein_codin	0	0	0	0
ENSG000002	MRPS18B	protein_codin	8.3858914	11.6137424	41.3437056	3.15379243
ENSG000001	IER3	protein_codin	4.56573025	34.0485304	5.48143568	53.0326412
ENSG000002	PSORS1C1	protein_codin	0	0	0	0
ENSG000001	PGBD1	protein_codin	15.3011011	0	0	0
ENSG000002	GTF2H4	protein_codin	1.46123504	0	10.7807158	0
ENSG000002	POU5F1	protein_codin	0	0	0	0
ENSG000001	CLPS	protein_codin	0	0	0	0
ENSG000001	RNF144B	protein_codin	24.5461875	43.79753	96.3589447	11.1137211
ENSG000002	HLA-F	protein_codin	0	0	2.72292433	0
ENSG000001	NRM	protein_codin	0	0	0	0
ENSG000001	FAM8A1	protein_codin	0	0	0	0
ENSG000001	C6orf52	protein_codin	0	0	0	0
ENSG000001	FGFBP2	protein_codin	17.6586468	2.00195634	137.853438	2.13992668
ENSG000001	TLR2	protein_codin	0.71698835	47.3863176	0.93791113	7.73177802
ENSG000001	THAP12	protein_codin	54.174647	103.542996	191.000747	42.9215604
ENSG000001	ANKRD42	protein_codin	0	13.0959822	0	4.77517263
ENSG000001	CCDC90B	protein_codin	1.00894145	4.82847515	6.0323374	0
ENSG000001	RAB30	protein_codin	3.3690427	14.0529434	5.98041736	0
ENSG000001	CREBZF	protein_codin	0	0	22.3203083	0
ENSG000001	LRRC32	protein_codin	0	29.7213489	1.98259016	16.2661165
ENSG000001	MRPL15	protein_codin	6.64867665	23.3260614	41.2180247	5.66717537
ENSG000001	PI15	protein_codin	11.9041288	3.09601722	575.240304	0.98695446
ENSG000001	TTPA	protein_codin	1.04927616	0	3.10637648	0
ENSG000001	GGH	protein_codin	14.9196539	16.6916659	113.930875	0
ENSG000001	SLCO5A1	protein_codin	4.70152185	13.8355656	3.80980125	5.9598293
ENSG000001	SULF1	protein_codin	60.6203109	29.5316916	0	36.4675764
ENSG000001	TGS1	protein_codin	26.8715392	27.7394665	46.1008689	4.75705548
ENSG000001	SDCBP	protein_codin	0	114.358749	0	28.4692398
ENSG000000	USP2	protein_codin	0.52876973	0	0	0
ENSG000001	HTR3B	protein_codin	0.31097469	0	0	1.37570587
ENSG000001	SORL1	protein_codin	25.3271191	209.62815	57.3292209	33.6376385
ENSG000001	BUD13	protein_codin	8.74291648	20.245405	4.19378562	9.01800949
ENSG000001	MMP7	protein_codin	0	0	6.76433253	0
ENSG000001	MMP20	protein_codin	1.41694412	0	0	0
ENSG000001	MMP27	protein_codin	0	0	0	0.5799723
ENSG000001	DCUN1D5	protein_codin	13.8849815	8.94603901	17.6471863	13.23687
ENSG000002	FDXACB1	protein_codin	1.9583935	0	0	0

ENSG000001:	POU2F3	protein_codin	0.32075872	0	0	0
ENSG000001:	FDX1	protein_codin	15.1814158	17.943749	34.6203505	10.4907527
ENSG000001:	CFAP68	protein_codin	1.7565787	3.38508193	1.49903591	5.06801667
ENSG000001:	FXYD6	protein_codin	0	0	0	1.69255925
ENSG000001:	ARHGAP20	protein_codin	0	0	0	0
ENSG000001:	FXYD2	protein_codin	0	0	0	0
ENSG000001:	MMP13	protein_codin	6.3937209	198.108068	175.523108	938.728132
ENSG000001:	CASP5	protein_codin	0	0	0	0
ENSG000001:	ALKBH8	protein_codin	0	0	0	0
ENSG000001:	UNC13C	protein_codin	0	24.6714898	0	0
ENSG000001:	SQOR	protein_codin	9.09213756	51.1226341	2.01664961	10.7240511
ENSG000001:	CTDSPL2	protein_codin	27.2630349	91.0669798	57.31621	21.0800489
ENSG000001:	THBS1	protein_codin	149.776082	1408.84089	75.8488682	552.478588
ENSG000001:	NUSAP1	protein_codin	0	0	102.914588	3.67124732
ENSG000001:	NDUFAF1	protein_codin	0	0	0	0
ENSG000001:	KIF23	protein_codin	0	0	0	0
ENSG000002:	SPESP1-NOX!	protein_codin	0	0	0	0
ENSG000001:	HAUS2	protein_codin	13.8421683	14.6396489	41.0313067	5.18905516
ENSG000001:	PARP6	protein_codin	0	0	0	0
ENSG000001:	RPLP1	protein_codin	513.065493	134.94359	1004.95892	268.032361
ENSG000001:	LRRC49	protein_codin	1.18719521	3.43359247	0	0
ENSG000001:	TUBGCP4	protein_codin	1.54428193	0	0	0
ENSG000001:	RMDN3	protein_codin	0	0	0	3.58714133
ENSG000001:	ITPKA	protein_codin	0	0	2.35721339	0.44914897
ENSG000001:	PLCB2	protein_codin	0	12.7069606	0	0
ENSG000001:	TMEM62	protein_codin	0	0	0	1.56546306
ENSG000001:	PAK6	protein_codin	0	0	0	0
ENSG000001:	ADAM10	protein_codin	56.1093271	125.003772	82.1293748	43.9595638
ENSG000001:	BCL2L10	protein_codin	0	2.39573744	0	0
ENSG000001:	RSL24D1	protein_codin	83.1080258	0	241.728472	34.8810861
ENSG000001:	GCHFR	protein_codin	0	3.12024523	6.43586192	0
ENSG000001:	MNS1	protein_codin	44.5003016	76.3195165	233.566368	19.1434303
ENSG000001:	BCAR3	protein_codin	9.96000275	44.7464855	10.223616	13.1350201
ENSG000001:	TTLL7	protein_codin	11.8823928	0	0	0
ENSG000001:	FNBP1L	protein_codin	26.611978	63.7870239	135.052871	34.0678118
ENSG000001:	KYAT3	protein_codin	12.5735988	0	50.9632719	13.2743649
ENSG000001:	ARHGAP29	protein_codin	15.3619347	116.649616	25.22904	31.9414101
ENSG000001:	RTCA	protein_codin	71.9265338	50.0664258	263.028279	25.6306045
ENSG000000	CEP68	protein_codin	0	0	0	0
ENSG000001:	IFT172	protein_codin	0	0	0	0
ENSG000001:	SELENOI	protein_codin	16.7966482	47.5092664	76.8544399	11.0864411
ENSG000001:	KHK	protein_codin	0	0	0	0
ENSG000001:	KHK	protein_codin	0.93630541	6.68293407	2.56942445	0
ENSG000001:	ADCY3	protein_codin	0	0	4.27903012	6.05816143

ENSG000001:	PNPT1	protein_codin	0	0	10.6696174	0
ENSG000001:	DYNC2LI1	protein_codin	8.89230314	17.1347062	7.16039808	0
ENSG000001:	ACTR2	protein_codin	179.895122	238.542749	258.398476	110.157017
ENSG000001:	PREB	protein_codin	5.13731172	0	1.69729092	10.6936733
ENSG000001:	PREPL	protein_codin	0	47.1479719	0	5.26192489
ENSG000001:	SLC3A1	protein_codin	0	0	0	0
ENSG000001:	SIX3	protein_codin	0	0	0.9419581	0.83395414
ENSG000001:	CENPO	protein_codin	3.0854993	0	14.3086728	0
ENSG000001:	LRPPRC	protein_codin	2.09739375	0	0	0
ENSG000001:	CYP2C9	protein_codin	0	0	0	2.35162132
ENSG000001:	LOXL4	protein_codin	7.35130368	10.2542324	44.2629953	11.8313965
ENSG000001:	BTBD16	protein_codin	0	2.96549445	0	0.43053873
ENSG000001:	KIF11	protein_codin	20.8084714	61.5631048	217.852492	6.59187115
ENSG000001:	TACC2	protein_codin	0	0	0	0
ENSG000001:	CALHM2	protein_codin	4.44476945	10.2746435	0	6.98772185
ENSG000001:	ARL3	protein_codin	21.2194304	36.1651594	56.9543007	24.2104982
ENSG000001:	KIF20B	protein_codin	31.9368218	43.7645607	100.271025	11.2975668
ENSG000001:	EXOC6	protein_codin	15.545355	0	0	0
ENSG000001	SHTN1	protein_codin	0	0	0	0
ENSG000000	FGFR3	protein_codin	0	0	1.15266391	0
ENSG000001:	DBR1	protein_codin	0	0	33.8828288	0
ENSG000001	TOPBP1	protein_codin	47.7262848	94.3935193	212.445137	25.2819387
ENSG000001:	DNAJC13	protein_codin	115.162654	198.248726	220.6471	73.8461264
ENSG000001:	GPR87	protein_codin	0	0	0	0
ENSG000002	SLFN12L	protein_codin	12.9319328	262.163319	0	19.7923294
ENSG000001	SATB2	protein_codin	0	0	0	42.2265497
ENSG000001:	AOX1	protein_codin	0	0	0	1.21519203
ENSG000001	ERBB4	protein_codin	0.60763055	3.30640342	0	0.97929413
ENSG000001:	BARD1	protein_codin	4.31611402	9.93295806	22.8244463	3.36998811
ENSG000001:	MSTN	protein_codin	2.29029321	0	2.64997942	0
ENSG000001:	ASNSD1	protein_codin	14.8402968	31.9055393	41.8430468	4.30189247
ENSG000001:	METTL5	protein_codin	0	0	4.22455279	0
ENSG000001:	SSB	protein_codin	101.080939	177.374405	285.133489	60.7364935
ENSG000001:	CDK15	protein_codin	0	0	0	0
ENSG000001:	PPIG	protein_codin	0	0	0	0
ENSG000001:	HECW2	protein_codin	0	0	0	13.6891731
ENSG000001	CRYGB	protein_codin	0	0	0	0
ENSG000001:	CHRNA1	protein_codin	0	0	0	0
ENSG000001:	WDR12	protein_codin	18.8018482	0	38.6779529	7.71524017
ENSG000001:	ABI2	protein_codin	0	0	0	0
ENSG000001:	ABI2	protein_codin	25.0986106	39.8007197	44.9099705	16.6638311
ENSG000001:	ABI2	protein_codin	0	0	0	0
ENSG000001:	ITGAV	protein_codin	72.4646666	0	85.1264292	24.8155807
ENSG000001:	SLC40A1	protein_codin	130.284991	563.10725	258.504864	144.295903

ENSG000001:	SLC35A5	protein_codin	6.05315938	11.37851	16.5466626	0
ENSG000001	COL8A1	protein_codin	93.4587054	375.912122	0	88.1164395
ENSG000001:	SLC49A4	protein_codin	6.16069788	10.6017416	11.686098	1.68895852
ENSG000001:	GUCA1C	protein_codin	0	0	0	0
ENSG000001:	CCDC54	protein_codin	0	0	0	0
ENSG000001:	COX17	protein_codin	21.4744327	49.6700752	55.6498371	15.3993558
ENSG000000	WBP11	protein_codin	36.1925281	72.0564985	149.520828	31.4729491
ENSG000001	ATF7IP	protein_codin	61.774976	147.440925	67.3683663	50.8825585
ENSG000000	LMO3	protein_codin	0	0	0	0
ENSG000000	GUCY2C	protein_codin	0	0	0	1.30089376
ENSG000000	EPYC	protein_codin	0	7.18073543	17.5823923	2.56021636
ENSG000001	TSPAN11	protein_codin	12.8836958	0	32.5041754	15.9075283
ENSG000000	BMAL2	protein_codin	2.91652631	0	25.5362809	0
ENSG000000	TRHDE	protein_codin	2.5712048	1.65626976	27.5283009	1.77234283
ENSG000000	OSBPL8	protein_codin	0	0	0	41.2486582
ENSG000001	SLC16A7	protein_codin	0	67.4658472	0	74.8443502
ENSG000000	INTS13	protein_codin	24.0009479	52.7757512	134.091903	9.1180499
ENSG000000	BCAT1	protein_codin	68.486079	92.7853529	286.230518	47.9427929
ENSG000001	GYS2	protein_codin	0	3.05614701	0	0
ENSG000001	SLCO1B3	protein_codin	0	0	0	0
ENSG000001	ST8SIA1	protein_codin	3.2960566	0	6.32972222	0
ENSG000000	ABCC9	protein_codin	21.3406722	37.0399436	18.5248645	42.4535448
ENSG000000	ABCC9	protein_codin	0	0	0	0
ENSG000001	LIN7A	protein_codin	1.19021177	4.07280112	4.77414066	0
ENSG000000	SYT1	protein_codin	0	0	0	1.31687663
ENSG000001	ACSS3	protein_codin	0	6.77991807	7.03791543	3.13592274
ENSG000000	PPP1R12A	protein_codin	0	0	0	0
ENSG000000	HAL	protein_codin	0	0	0	0
ENSG000001	TMPO	protein_codin	1.10969844	32.5887199	83.3347543	0
ENSG000000	CDK17	protein_codin	24.3410412	29.0888214	40.9331842	49.1585868
ENSG000001	METAP2	protein_codin	13.7894238	0	43.9183607	6.44127525
ENSG000000	TMCC3	protein_codin	11.1822147	26.6302277	14.8440033	7.7872319
ENSG000000	IRAK3	protein_codin	55.6350471	50.5343122	37.0050198	27.1330764
ENSG000001	RXYLT1	protein_codin	6.98657242	0	14.8027673	7.16818491
ENSG000001	KIAA0586	protein_codin	0.57815363	16.1761058	0.89925753	0
ENSG000000	MNAT1	protein_codin	0.54283196	0	0	0
ENSG000001	TRMT5	protein_codin	14.3596956	12.013727	45.9923691	14.3084382
ENSG000000	FERRY3	protein_codin	10.6220482	27.6956301	22.8792591	11.8939195
ENSG000001	PRMT8	protein_codin	0	0	0	0.89242975
ENSG000001	CCND2	protein_codin	86.9167251	297.545082	336.614407	9.31362936
ENSG000000	RAB21	protein_codin	50.8681906	100.641893	71.7091899	49.1796309
ENSG000001	PTPRB	protein_codin	20.2668295	34.2828409	0	6.29431799
ENSG000000	LYZ	protein_codin	50.1495648	136.069805	5.82528597	32.6946563
ENSG000001	ENTREP2	protein_codin	0	2.42384926	35.8879602	1.38763592

ENSG000001	LIPG	protein_codin	0.40570759	0	0	0.50533651
ENSG000000	TGDS	protein_codin	7.3674093	5.21177911	8.47956651	1.17219665
ENSG000000	FOXN3	protein_codin	0	46.1014524	0	0
ENSG000001	PSMC1	protein_codin	0.75779539	1.60603254	1.03920167	4.15840796
ENSG000000	GALC	protein_codin	13.6897196	34.3003797	27.627866	8.32732577
ENSG000001	PPWD1	protein_codin	19.8143117	0	60.4978253	10.5930001
ENSG000000	PEBP1	protein_codin	51.4254503	92.0994901	51.4154551	35.9067635
ENSG000001	SPRING1	protein_codin	13.5214817	40.9455665	32.0830943	17.0227947
ENSG000000	MOCOS	protein_codin	2.57177705	3.84277712	2.504678	2.46232901
ENSG000001	ZNF24	protein_codin	0	0	36.4534238	0
ENSG000001	ZNF397	protein_codin	0	0	0	0
ENSG000001	PZP	protein_codin	0	0	0	0
ENSG000000	CLEC2D	protein_codin	0	0	0	0
ENSG000000	CLEC2D	protein_codin	0	0	0	0
ENSG000000	LRP6	protein_codin	111.526882	105.455814	251.063169	43.4098637
ENSG000001	YPEL5	protein_codin	16.1880259	29.7784044	17.6938002	72.8475677
ENSG000001	LMNB1	protein_codin	5.17789246	101.139775	49.9031361	4.37765558
ENSG000000	SNCAIP	protein_codin	0.56917577	0	54.6715247	0
ENSG000000	SNCAIP	protein_codin	16.1243368	13.7145068	0	17.3814254
ENSG000000	SNX24	protein_codin	5.65277806	2.53433861	3.81870384	4.869474
ENSG000001	HS3ST2	protein_codin	0	5.65036149	0	0
ENSG000000	REXO5	protein_codin	0.63539431	0	0	0
ENSG000001	XYLT1	protein_codin	32.7557106	70.6911858	81.1814958	17.8371229
ENSG000001	DNAH3	protein_codin	0	0	0.90817399	0
ENSG000000	LDAF1	protein_codin	8.51473263	0	0	2.88886438
ENSG000000	NUP133	protein_codin	31.884383	58.6144457	71.4402113	18.3074699
ENSG000001	CORO1C	protein_codin	42.3042188	119.171829	39.2846343	45.7233376
ENSG000000	NUAK1	protein_codin	20.8331959	78.7143023	28.0207132	54.3165073
ENSG000001	VWF	protein_codin	64.4919402	636.8842	24.8904484	296.886176
ENSG000002	ENSG000002	protein_codin	0	6.1242294	6.88613648	0
ENSG000001	LPCAT3	protein_codin	1.1951039	6.46916906	6.42454103	0
ENSG000001	MRPS27	protein_codin	0	0	13.9266754	0
ENSG000001	CERT1	protein_codin	0	0	0	0
ENSG000000	HEXB	protein_codin	32.3497834	100.028935	30.0686781	19.9354975
ENSG000001	KLHL5	protein_codin	0	51.5984158	0	0
ENSG000001	KLHL5	protein_codin	0	0	0	0
ENSG000000	UBE2K	protein_codin	16.0386865	34.0415019	29.14937	7.23444803
ENSG000001	LIAS	protein_codin	0	0	0	0
ENSG000000	N4BP2	protein_codin	47.8554519	181.694424	219.577818	37.5377762
ENSG000001	KLF3	protein_codin	68.8075262	147.186577	110.181298	51.4877021
ENSG000000	TBC1D1	protein_codin	0	10.6149212	0	0
ENSG000000	RSBN1	protein_codin	0	0	0	0
ENSG000000	CSDE1	protein_codin	0	0	0	37.886294
ENSG000001	CASQ2	protein_codin	9.22407582	0	0	0

ENSG000000	PACC1	protein_codin	2.04374201	30.4499531	7.25238216	0
ENSG000000	HHAT	protein_codin	0	0	0	0
ENSG000000	PPP2R5A	protein_codin	11.1583191	29.2805527	11.2417004	17.9064888
ENSG000000	TRAF5	protein_codin	0	0	4.78884884	0
ENSG000001	HSD11B1	protein_codin	0	0	0	0
ENSG000000	PPP2R3C	protein_codin	5.97728981	47.9153267	0	18.4575892
ENSG000001	PSMA6	protein_codin	56.7083806	34.8651154	95.793002	27.6769992
ENSG000001	REEP5	protein_codin	0	0	0	0
ENSG000001	MAN2A1	protein_codin	70.8576614	89.6651259	108.943758	45.1071089
ENSG000001	EPB41L4A	protein_codin	5.6855938	13.2908471	3.66860994	8.2464516
ENSG000001	ENOX1	protein_codin	11.1082394	19.6343872	31.4141414	5.13201267
ENSG000001	TSC22D1	protein_codin	67.1822786	47.4780225	60.6699242	54.7896482
ENSG000001	DGKH	protein_codin	68.8995337	127.47213	299.523771	51.4769466
ENSG000001	USP22	protein_codin	110.877347	97.3666571	131.186312	60.0856122
ENSG000001	B9D1	protein_codin	0	0	0	0
ENSG000001	SPECC1	protein_codin	0	0	0	0
ENSG000000	MSMO1	protein_codin	4.8902725	0	13.8830929	0
ENSG000001	PALLD	protein_codin	12.6621498	0	2.29626925	0
ENSG000001	ICE2	protein_codin	0	0	0	0
ENSG000000	RORA	protein_codin	0	10.478092	2.77718436	7.9680752
ENSG000000	GPATCH2L	protein_codin	50.4270975	135.322649	162.630366	36.8175208
ENSG000001	SNW1	protein_codin	86.6037032	0	184.049753	65.4070104
ENSG000000	POMT2	protein_codin	0	0	0	0
ENSG000001	ROCK2	protein_codin	4.67944299	0	2.56009243	7.08502076
ENSG000001	MIB1	protein_codin	95.408626	77.0100187	207.48315	55.6634755
ENSG000000	TMEM260	protein_codin	0	14.0414614	38.8668646	7.63910195
ENSG000000	AP5M1	protein_codin	10.9677203	43.4445078	47.6400014	15.8360561
ENSG000001	ZNF430	protein_codin	9.95583162	35.0717601	14.2443578	6.78476259
ENSG000000	MAST4	protein_codin	8.31909125	9.56168459	10.625705	22.3549928
ENSG000000	IPO5	protein_codin	0	59.4596434	0	18.1526631
ENSG000000	PALB2	protein_codin	12.7887391	0	0	0
ENSG000000	KATNIP	protein_codin	21.8037791	44.9986114	59.2184437	19.300896
ENSG000000	DSG2	protein_codin	0	0	0	0
ENSG000001	NOL4	protein_codin	0	0	5.3987953	0
ENSG000001	RNF138	protein_codin	4.79036995	0	5.46232007	11.0803104
ENSG000001	LPIN2	protein_codin	0	22.4809727	55.9649121	0
ENSG000000	NDC80	protein_codin	15.1331104	52.3042243	218.729546	3.82099826
ENSG000000	THOC1	protein_codin	2.14699345	0	37.2303563	0
ENSG000001	USP14	protein_codin	31.5723586	33.2327498	31.6961054	28.0176062
ENSG000001	MYOM1	protein_codin	52.2095328	51.7050276	9.28847278	13.7364346
ENSG000001	HERC2	protein_codin	110.985339	407.325915	340.536356	70.5962531
ENSG000000	DPEP1	protein_codin	1.95372784	0	0	0
ENSG000001	SLC7A5	protein_codin	2.72304275	26.8322053	16.5791713	25.8272647
ENSG000000	CYBA	protein_codin	14.0154556	93.0776897	52.601475	24.9413021

ENSG000001:	ARL1	protein_codin	25.7178393	41.5152262	21.6772252	20.7235863
ENSG000001:	UTP20	protein_codin	77.6039382	181.352781	236.835908	33.5498923
ENSG000000	COX10	protein_codin	3.92681127	1.97972171	5.90501631	2.73438846
ENSG000002	FBXW10B	protein_codin	0	0	0	0.75496657
ENSG000000	SREBF1	protein_codin	0	4.03383965	0	7.84507762
ENSG000000	TTC19	protein_codin	0	31.4471315	15.6200965	10.9072895
ENSG000002	TNFRSF13B	protein_codin	0	16.138623	0	1.19426016
ENSG000001	STX2	protein_codin	13.7979473	53.7994086	21.637275	13.1942989
ENSG000001	ADGRD1	protein_codin	37.9665852	41.5279574	29.1622488	10.1337892
ENSG000000	RIMBP2	protein_codin	1.71940374	0	0	1.70524596
ENSG000000	CLEC16A	protein_codin	0	5.37454855	4.10505655	0
ENSG000001	BFAR	protein_codin	18.0893561	27.4824169	55.500155	10.7185026
ENSG000001	CPPED1	protein_codin	0	0	0	0
ENSG000001	KPNA3	protein_codin	66.4411453	142.00267	83.9347386	45.2977247
ENSG000000	SFSWAP	protein_codin	49.7662056	169.691715	106.570488	39.5484316
ENSG000000	PALS1	protein_codin	0	0	0	6.49781692
ENSG000001	CDK2AP1	protein_codin	3.00838244	18.2570124	6.03386236	3.08907378
ENSG000000	SCARB1	protein_codin	0	0	0	0
ENSG000000	L2HGDH	protein_codin	0	0	0	0
ENSG000000	RTRAF	protein_codin	7.01172058	18.6659496	42.9122896	21.0488307
ENSG000001	SLC6A4	protein_codin	0	0	0	0
ENSG000001	UTP6	protein_codin	3.65535452	11.8592545	38.5013766	8.68361205
ENSG000001	PSMD11	protein_codin	14.9022024	7.64973798	44.6902067	1.32970797
ENSG000000	TMEM98	protein_codin	0	0	0	0
ENSG000001	BLMH	protein_codin	25.4232411	49.8585266	46.2801122	11.8148904
ENSG000001	TAOK1	protein_codin	193.765264	310.698867	386.762766	126.68254
ENSG000000	BTBD1	protein_codin	49.4735441	38.5767237	52.4460956	23.4051291
ENSG000001	AP3B2	protein_codin	0	0	0	0
ENSG000001	CUX2	protein_codin	0	7.12190359	3.68192604	2.11369438
ENSG000001	RASAL1	protein_codin	0	0	0	0
ENSG000000	LHX5	protein_codin	0	0	0	2.23353441
ENSG000001	ALDH2	protein_codin	25.1472143	17.7716268	16.1078484	17.5370471
ENSG000000	ERP29	protein_codin	24.4101686	72.1634851	34.1923022	17.4273931
ENSG000000	ANKRD13A	protein_codin	5.96328317	23.9211414	0	10.3235154
ENSG000001	TRPV4	protein_codin	0	0	0	0
ENSG000001:	RBM19	protein_codin	39.1457653	77.0528628	109.389898	19.4565161
ENSG000001	NAA25	protein_codin	7.63637549	4.70986228	11.9885278	3.06720032
ENSG000000	ZFAND6	protein_codin	0	0	10.1267233	0
ENSG000001	CHRNB4	protein_codin	0	0	0	0.58890945
ENSG000001	FAH	protein_codin	0	0	0	0
ENSG000001	MESD	protein_codin	12.2831008	135.690155	82.7047314	19.5263791
ENSG000000	CDH1	protein_codin	0	0	0	0
ENSG000000	AARS1	protein_codin	0	0	0	0
ENSG000001	VAC14	protein_codin	10.4051207	63.8538848	44.0343299	0

ENSG000001[illegible]	TANGO6	protein_codin	15.4517586	45.6057367	43.1988862	8.17344925
ENSG000000[illegible]	ARG2	protein_codin	0.89817179	3.15369057	18.9567458	0.86669393
ENSG000001[illegible]	TM9SF1	protein_codin	9.39411091	0	0	0
ENSG000001[illegible]	IL17B	protein_codin	0	0	0	0
ENSG000000[illegible]	NDST1	protein_codin	29.2855661	116.240452	27.6850845	36.4408353
ENSG000001	CSNK1A1	protein_codin	0	0	0	0
ENSG000001	PDGFRB	protein_codin	125.486075	312.368073	32.8083498	117.837316
ENSG000000[illegible]	FAT2	protein_codin	1.13305786	7.68174803	0	12.6849969
ENSG000001[illegible]	CYSTM1	protein_codin	6.94033725	5.6769343	9.25609364	5.36989817
ENSG000001	PFDN1	protein_codin	19.3349933	30.7975404	54.2851114	19.2569937
ENSG000001	PSMD9	protein_codin	0	2.32152076	0	0.67938955
ENSG000000[illegible]	ANAPC5	protein_codin	33.5655778	0	9.29041676	3.4945384
ENSG000001	BCL7A	protein_codin	4.41053338	14.3487076	10.7749685	4.3607237
ENSG000000[illegible]	P2RX7	protein_codin	0	0	0	0
ENSG000001[illegible]	CIT	protein_codin	0	0	0	0
ENSG000000[illegible]	ATG2B	protein_codin	6.97149605	10.4294881	15.0303731	0
ENSG000000[illegible]	CYP46A1	protein_codin	0	0	0	0
ENSG000000[illegible]	GNB5	protein_codin	1.09927774	7.72407236	5.07546491	6.65656915
ENSG000001[illegible]	MYO5C	protein_codin	38.746924	40.398807	0	45.431711
ENSG000000[illegible]	AP4E1	protein_codin	38.0405246	60.1901251	36.0168646	19.7319299
ENSG000000[illegible]	ATOSA	protein_codin	0	0	0	0
ENSG000000[illegible]	MAPK6	protein_codin	53.8980854	14.9838605	87.1578154	71.9244133
ENSG000001[illegible]	SECISBP2L	protein_codin	20.9866885	20.3627268	0	27.9173212
ENSG000001[illegible]	SPPL2A	protein_codin	36.2448867	97.471605	43.5534126	24.8898747
ENSG000001[illegible]	GLCE	protein_codin	14.6278258	0	5.34088701	0
ENSG000001[illegible]	CORO2B	protein_codin	0	14.317508	8.29363547	6.76089373
ENSG000001[illegible]	THSD4	protein_codin	0	0	61.8140693	3.59152987
ENSG000001[illegible]	SPG11	protein_codin	0	0	0	0
ENSG000001[illegible]	SLC30A4	protein_codin	18.8289111	17.0522192	38.8737845	13.2849667
ENSG000001[illegible]	EIF3J	protein_codin	27.8546474	31.8770078	65.7684805	32.2301438
ENSG000000[illegible]	HACD3	protein_codin	56.3128252	53.1659339	112.451198	38.8213347
ENSG000001[illegible]	APH1B	protein_codin	17.8768709	28.6307914	28.2423342	8.80407281
ENSG000001[illegible]	AAGAB	protein_codin	11.7868668	17.494593	29.3584628	6.57790059
ENSG000000[illegible]	TIPIN	protein_codin	5.39852918	0	0	5.31500857
ENSG000001[illegible]	CILP	protein_codin	170.379027	3.09169871	0.96966612	2.06849478
ENSG000001[illegible]	TRIP4	protein_codin	18.2177373	21.6322475	41.8748053	8.78470082
ENSG000001[illegible]	PARP16	protein_codin	0	3.61708577	5.43604232	6.81193656
ENSG000000[illegible]	SNX1	protein_codin	0	34.2672235	6.92169929	0
ENSG000001[illegible]	RAB11A	protein_codin	26.3811335	38.4889629	28.2689525	17.9660117
ENSG000000[illegible]	DAPK2	protein_codin	0	0	0	0
ENSG000000[illegible]	SLC24A1	protein_codin	21.3032448	0	0	0
ENSG000001[illegible]	LACTB	protein_codin	7.49498205	9.72622303	7.43546039	25.6602059
ENSG000001	PRPF40B	protein_codin	0	5.95246316	0	0
ENSG000001[illegible]	CCNT1	protein_codin	36.5517788	0	134.619291	24.2563173

ENSG000000	NEO1	protein_codin	42.6296587	126.916438	72.4687357	28.1473513
ENSG000001	HCN4	protein_codin	0.26832049	1.02689903	0.52175039	2.58544772
ENSG000001	SEMA7A	protein_codin	10.3570191	0	0	6.08687668
ENSG000001	LOXL1	protein_codin	23.6426597	43.4119991	15.9627162	3.42416463
ENSG000000	FLT4	protein_codin	4.08069635	40.6583898	39.474507	21.5212511
ENSG000001	FAF2	protein_codin	37.2340388	108.914689	154.428658	28.6812832
ENSG000000	CDHR2	protein_codin	0	0	0	0
ENSG000001	RNF130	protein_codin	0	13.8026098	1.44702648	1.26404154
ENSG000001	CNOT6	protein_codin	11.5578984	40.3781666	70.2854424	19.5575299
ENSG000001	MCF2L	protein_codin	0	0	0	0
ENSG000001	TUBGCP3	protein_codin	14.1676249	58.6841402	38.5367528	15.9921356
ENSG000001	RBM25	protein_codin	64.5006383	65.6840777	122.784991	22.4723952
ENSG000001	LTBP2	protein_codin	173.649333	396.11826	43.8083993	105.333026
ENSG000001	VSX2	protein_codin	0.28250111	0.66599208	0	0.39482557
ENSG000001	SERPINA10	protein_codin	0	0	0.48472453	2.29460819
ENSG000000	SPAG9	protein_codin	198.271149	148.709603	81.0787278	96.4807286
ENSG000001	SGCA	protein_codin	0	0	0	0
ENSG000001	MARS1	protein_codin	146.632831	0	22.727113	13.717907
ENSG000001	ATP5F1B	protein_codin	75.7006752	38.3642146	152.089294	56.1153156
ENSG000000	RBMS2	protein_codin	28.3752562	82.292521	43.5739501	23.6451556
ENSG000001	IKZF4	protein_codin	4.51429167	0	0	0
ENSG000001	PTGES3	protein_codin	84.7080016	201.877683	424.717393	56.2792402
ENSG000000	PIK3C3	protein_codin	35.2846501	76.7445214	28.1822885	32.7967709
ENSG000001	MEOX2	protein_codin	57.9319014	30.4825411	0	15.842685
ENSG000001	PHF3	protein_codin	38.9566683	60.2262422	57.0299825	28.0620868
ENSG000001	SLC11A2	protein_codin	0	0	0	0
ENSG000001	ATF1	protein_codin	20.2068183	2.04410438	36.8755471	12.4236161
ENSG000000	LETMD1	protein_codin	0	0	0	5.37472311
ENSG000000	EIF4B	protein_codin	130.213469	186.851494	455.087574	164.478675
ENSG000000	CALCOCO1	protein_codin	0	14.2327679	0	0
ENSG000001	COPZ1	protein_codin	4.98929909	10.0309513	14.9461261	6.92435216
ENSG000001	MMD	protein_codin	5.66934644	4.97215399	8.51362065	7.11733641
ENSG000001	TSPAN13	protein_codin	2.93828235	2.71060987	7.31889947	6.57977686
ENSG000001	TTC23	protein_codin	0	0	0	0
ENSG000001	NUP153	protein_codin	42.7495834	128.324181	179.120382	57.581743
ENSG000000	FECH	protein_codin	1.78226856	0	1.38514209	0
ENSG000000	RAB27B	protein_codin	63.4466696	19.8905028	8.74976257	25.0821933
ENSG000001	ZDHHC2	protein_codin	23.6825151	29.5081604	55.3387942	15.3111174
ENSG000000	MSR1	protein_codin	19.6450732	0	0	0
ENSG000001	MTUS1	protein_codin	0	0	0	0
ENSG000000	CLXN	protein_codin	0.32834991	1.25049382	8.51257542	2.02783366
ENSG000001	MCM4	protein_codin	0	0	0	0
ENSG000001	ERICH1	protein_codin	33.979899	160.960892	30.7962642	21.9270607
ENSG000000	MYOM2	protein_codin	19.0551017	20.7179352	17.3235149	6.47610116

ENSG000001[illegible]	TWSG1	protein_codin	36.4350118	58.662385	53.6697437	24.3690873
ENSG000000[illegible]	SEH1L	protein_codin	20.722131	19.2861367	46.4448896	13.8096515
ENSG000001[illegible]	ANKRD12	protein_codin	20.0924094	117.928746	34.6686141	8.50736547
ENSG000001[illegible]	CEP76	protein_codin	4.38649958	0	7.37960582	0
ENSG000001[illegible]	RBL2	protein_codin	66.3482828	86.0460529	141.879544	43.8329411
ENSG000000[illegible]	LPCAT2	protein_codin	8.46812947	11.0380843	0	11.7201832
ENSG000001[illegible]	RPGRIP1L	protein_codin	11.10899	8.42026071	7.80917776	4.56236061
ENSG000000[illegible]	CACNG4	protein_codin	0.52926879	2.23768485	2.94389926	1.24797413
ENSG000000[illegible]	WIPI1	protein_codin	10.4888418	16.6039889	34.2656703	0
ENSG000001[illegible]	WDR59	protein_codin	5.10078021	10.2321939	11.5212794	7.26068476
ENSG000000[illegible]	MTFR1	protein_codin	0	14.9644386	11.9836645	10.4379152
ENSG000000[illegible]	CDH19	protein_codin	0.25937927	0	0	0.49955418
ENSG000001[illegible]	SMAD7	protein_codin	11.882665	17.0754141	34.2961461	43.4056897
ENSG000001[illegible]	SMAD2	protein_codin	30.7188638	84.1334076	66.9107165	25.6303447
ENSG000001[illegible]	RNMT	protein_codin	22.4912745	0	36.9281217	26.3909778
ENSG000001[illegible]	DNAJB6	protein_codin	13.7159188	28.4689533	25.5279073	10.9755414
ENSG000001[illegible]	VIPR2	protein_codin	1.68845448	1.0765981	0	0.81922691
ENSG000000[illegible]	KCNH2	protein_codin	0	0	0	0
ENSG000001[illegible]	RHEB	protein_codin	11.1814689	14.9366891	20.4822311	9.92393236
ENSG000000[illegible]	SMARCD3	protein_codin	8.86476251	34.7995725	21.4008086	6.58769886
ENSG000000[illegible]	KMT2C	protein_codin	130.486087	0	0	49.9235216
ENSG000000[illegible]	PSMB1	protein_codin	15.3249012	75.6378735	289.794643	14.2184349
ENSG000001[illegible]	RBFA	protein_codin	1.59373006	21.0879576	12.5823924	0
ENSG000001[illegible]	ADNP2	protein_codin	20.1617813	43.8337026	48.5979354	13.9675526
ENSG000001[illegible]	CRISPLD1	protein_codin	11.8354782	101.679673	325.617936	22.1889077
ENSG000001[illegible]	TRPA1	protein_codin	0.2723299	3.53492204	8.15514322	3.01903534
ENSG000001[illegible]	CSPP1	protein_codin	0	0	6.46532261	0
ENSG000000[illegible]	TRAM1	protein_codin	63.5471275	153.853307	53.0857749	65.4726686
ENSG000000[illegible]	ARFGEF1	protein_codin	106.82823	238.795097	187.534235	74.3030358
ENSG000001[illegible]	ANXA13	protein_codin	0	0	0	0
ENSG000000[illegible]	TMED2	protein_codin	191.850834	240.633452	265.463527	83.0341979
ENSG000000[illegible]	ARHGAP28	protein_codin	21.4961945	48.5501833	65.5182626	4.26141105
ENSG000001[illegible]	RIMS2	protein_codin	5.85494176	0	60.1214372	4.76040341
ENSG000000[illegible]	EML1	protein_codin	23.5630037	42.9915675	66.9670895	0
ENSG000001[illegible]	YY1	protein_codin	54.8218762	229.640058	91.1040026	53.2594891
ENSG000000[illegible]	RCOR1	protein_codin	28.2736912	78.9317848	111.282384	21.7940991
ENSG000001[illegible]	MOB3B	protein_codin	20.5526793	35.4224439	19.1821898	12.4618364
ENSG000001[illegible]	ZNF175	protein_codin	5.07297208	22.6014811	10.3809382	4.65797836
ENSG000001[illegible]	CD33	protein_codin	5.4511515	0	0	9.00937926
ENSG000001[illegible]	PIH1D1	protein_codin	5.89820961	0	5.98908227	3.68316274
ENSG000002[illegible]	ENSG000002[illegible]	protein_codin	0	0	0	1.08885872
ENSG000001[illegible]	SCPEP1	protein_codin	8.87123873	10.7645587	59.781489	9.46851071
ENSG000001[illegible]	LPO	protein_codin	0	0	0	0.48652896
ENSG000000[illegible]	VMP1	protein_codin	0	0	0	0

ENSG000000	PRR11	protein_codin	6.77637413	14.8404075	110.008488	3.75752064
ENSG000001	TRIM37	protein_codin	6.62304308	60.3092767	70.8435616	18.465815
ENSG000001	PKMYT1	protein_codin	0	0	4.02843352	0
ENSG000001	LMF1	protein_codin	1.42521603	0	0	2.57786061
ENSG000000	NUBP2	protein_codin	0	12.2238838	13.5886404	3.30421324
ENSG000000	PKD1	protein_codin	33.7206303	0	74.006635	6.71764173
ENSG000000	RAB11FIP3	protein_codin	2.40949865	29.3055735	0	0
ENSG000001	ELOB	protein_codin	0	0	18.9849732	0
ENSG000001	CHTF18	protein_codin	3.59632407	0	13.7949034	0
ENSG000000	RHBDF1	protein_codin	5.25284639	20.3802693	9.39962553	0
ENSG000001	CLCN7	protein_codin	0	10.2851666	0	11.1377458
ENSG000001	TELO2	protein_codin	4.51776602	31.995494	40.0321175	4.9524177
ENSG000001	AXIN1	protein_codin	8.78040203	51.7585197	9.61874748	2.31786814
ENSG000000	LIG3	protein_codin	0	20.5966164	7.75219738	0
ENSG000001	RPE65	protein_codin	0	0	0	0
ENSG000000	IL12RB2	protein_codin	0	0	5.36295952	0.81015097
ENSG000001	ANKRD13C	protein_codin	2.85199691	37.0603874	13.2256681	0
ENSG000001	WLS	protein_codin	29.7017155	70.5674411	83.0127983	23.0241936
ENSG000001	SLC1A1	protein_codin	3.18249247	0	11.352873	3.3548054
ENSG000001	FAM86B2	protein_codin	0	0	0	0
ENSG000001	GLIS2	protein_codin	0	0	0	0
ENSG000000	CREBBP	protein_codin	100.680612	237.572022	213.955484	60.7895613
ENSG000001	MGRN1	protein_codin	13.7717394	43.240303	22.6263624	19.9185823
ENSG000000	ALG1	protein_codin	1.93739305	0	0	0
ENSG000001	DNAJA3	protein_codin	1.64664663	3.19856897	0	0
ENSG000001	UBN1	protein_codin	14.2674738	116.893348	39.0791306	12.1584132
ENSG000001	N4BP1	protein_codin	8.3581149	45.1221166	14.4469966	12.4678599
ENSG000001	WSB1	protein_codin	12.5093156	25.2249703	41.7344064	20.7267042
ENSG000000	TRAF4	protein_codin	0.36979128	0	7.71119517	5.80805721
ENSG000000	TRAF4	protein_codin	0	2.17831554	0	0
ENSG000001	RGS9	protein_codin	0	11.6181849	5.75085064	0
ENSG000000	ITGA2B	protein_codin	0	0	0	0.45549161
ENSG000001	MAPT	protein_codin	1.7560585	0	0	0.70349585
ENSG000000	DHX8	protein_codin	12.3625562	30.2443168	23.3845192	8.56257747
ENSG000000	SLC4A1	protein_codin	0	0	0	2.45059054
ENSG000001	KANSL1	protein_codin	1.59588972	4.94375446	0	0
ENSG000001	CRISPLD2	protein_codin	52.7563022	68.2613217	7.71139902	36.2293363
ENSG000001	FOXF1	protein_codin	0.90860636	0	39.7023795	1.63524252
ENSG000001	COTL1	protein_codin	70.1917663	316.357251	182.097672	63.0672019
ENSG000000	ATP2C2	protein_codin	0	0	0	0
ENSG000001	MLYCD	protein_codin	32.2429916	54.4779844	80.0049807	18.231134
ENSG000001	METTL2B	protein_codin	9.05312905	20.7731578	17.681204	7.6760259
ENSG000001	SMURF2	protein_codin	17.9857607	16.1037003	5.13921476	16.2573411
ENSG000000	GLP2R	protein_codin	0	2.69812884	0	1.41720555

ENSG000000	DNAH9	protein_codin	0	0	2.04290073	7.78416261
ENSG000000	ARHGAP44	protein_codin	0	0	0	0
ENSG000001	CHD5	protein_codin	0	0	4.3580553	1.74987481
ENSG000002	ERP44	protein_codin	31.4093976	0	24.6479012	29.1847932
ENSG000001	INVS	protein_codin	11.5517838	18.3228857	35.4536412	0
ENSG000001	INVS	protein_codin	24.2592089	55.2827109	43.9695912	28.6569203
ENSG000001	GINS1	protein_codin	0	0	0	0
ENSG000000	SLC12A2	protein_codin	16.4104698	22.7763059	153.547179	14.004486
ENSG000001	SLC27A6	protein_codin	0	0	0	0.98220637
ENSG000001	FBN2	protein_codin	50.3115151	72.3149702	1698.2615	14.600627
ENSG000000	NLRP1	protein_codin	11.2386823	52.3533443	0	0
ENSG000001	RNF167	protein_codin	3.00482655	5.70342887	10.3824621	0
ENSG000000	PITPNM3	protein_codin	35.8016497	27.2311534	14.8596942	0
ENSG000001	ISM1	protein_codin	9.52492566	3.37977455	16.1413608	11.1046383
ENSG000000	GNAO1	protein_codin	0	0	8.7832766	0
ENSG000000	GNAO1	protein_codin	0	13.3503471	10.3018042	0
ENSG000000	CFAP20	protein_codin	4.46415436	17.5977162	46.7264261	6.55581133
ENSG000000	SLC12A3	protein_codin	0.28690976	1.20678934	0	0
ENSG000000	CSNK2A2	protein_codin	21.2296034	29.6669658	54.8601077	7.97540767
ENSG000000	COQ9	protein_codin	13.7319647	3.68427281	23.3704909	3.63250367
ENSG000001	NLRC5	protein_codin	0	147.450689	0	0
ENSG000000	SRCAP	protein_codin	0	0	0	0
ENSG000000	SETD1A	protein_codin	3.98798798	24.1119949	0	0
ENSG000000	HSD3B7	protein_codin	2.45504635	0	0	3.08669323
ENSG000001	ZNF629	protein_codin	24.1935444	46.7120345	101.404865	15.5973962
ENSG000000	PTPN3	protein_codin	0	0	0	4.97948421
ENSG000001	SEC23B	protein_codin	0	0	0	0
ENSG000001	PCSK2	protein_codin	0	0	0	0
ENSG000000	DZANK1	protein_codin	0	0	0	0
ENSG000001	OGN	protein_codin	17.5804149	36.8543477	0	6.35795789
ENSG000000	SPTLC1	protein_codin	49.797125	90.626597	61.6078903	18.6684682
ENSG000001	CHCHD3	protein_codin	48.6435065	37.9539289	157.5523	28.9647873
ENSG000000	ZC3H3	protein_codin	9.32709881	32.9482436	45.569135	8.24291379
ENSG000001	GSDMD	protein_codin	6.47631475	54.5793098	32.6801415	5.40247204
ENSG000001	RPL8	protein_codin	2.76214303	8.9866432	9.36219356	13.0461631
ENSG000001	DENND3	protein_codin	0	0	0	0
ENSG000001	DOK5	protein_codin	1.73375592	0	12.9389504	0
ENSG000001	TTPAL	protein_codin	0	21.5326002	0	8.12254382
ENSG000000	ADA2	protein_codin	0	42.9167591	0	0
ENSG000000	CECR2	protein_codin	0	0	9.62280113	0
ENSG000001	NHERF1	protein_codin	2.91120471	43.6666342	5.40971076	2.88291992
ENSG000001	CHST8	protein_codin	0	0	0	0
ENSG000001	ATP4A	protein_codin	0	0.98114412	0	0
ENSG000001	KIRREL2	protein_codin	0	0	0	0

ENSG000001	HPN	protein_codin	0	0	0	0
ENSG000000	TYROBP	protein_codin	0	22.8554095	2.77155062	2.91388788
ENSG000000	ZBTB32	protein_codin	0	0	0	0
ENSG000001	SCN1B	protein_codin	3.97269264	0	0	2.24878821
ENSG000001	RBM42	protein_codin	0	0	11.1262199	1.73157222
ENSG000001	VAMP7	protein_codin	0	0	0	0
ENSG000001	CCNE1	protein_codin	0	7.27152038	13.2439943	2.66075062
ENSG000001	BPNT2	protein_codin	111.03397	170.595691	103.491297	63.9496578
ENSG000001	RAB2A	protein_codin	47.7787779	113.328623	29.7210349	28.9834171
ENSG000000	ANOS1	protein_codin	22.0337079	21.2253514	38.6174938	2.15651132
ENSG000000	ITCH	protein_codin	0	0	0	0
ENSG000001	HCK	protein_codin	0	0	0	0
ENSG000001	CCM2L	protein_codin	0	0	3.59189999	0
ENSG000001	CDKN2C	protein_codin	0	0	1.07116428	0
ENSG000000	TTC39A	protein_codin	0	0	0	0
ENSG000000	TTC39A	protein_codin	0.53812677	2.1650814	0	4.31474767
ENSG000001	ACIN1	protein_codin	0	0	0	0
ENSG000001	AJUBA	protein_codin	1.84516546	16.5252963	14.1695122	0.61190714
ENSG000001	TEP1	protein_codin	22.4800726	136.922507	56.0098289	3.09764374
ENSG000001	CDH20	protein_codin	0	0	0	0
ENSG000000	PHLPP1	protein_codin	18.6936332	22.5209327	65.7023313	11.9380955
ENSG000000	FBLN1	protein_codin	25.3592574	38.2269398	98.4446061	2.38902331
ENSG000001	EFCAB6	protein_codin	0	0	19.23263	0
ENSG000000	CELSR1	protein_codin	0	12.1711764	0	0
ENSG000001	PIK3R3	protein_codin	0	40.1684532	18.7902691	0
ENSG000001	PRDX1	protein_codin	0	1.75835019	11.9173113	2.70139805
ENSG000000	RPS6KA6	protein_codin	0.99735647	17.6373565	99.3294177	6.59353832
ENSG000001	POF1B	protein_codin	0	0	0	1.02867008
ENSG000000	PGS1	protein_codin	5.70318786	10.0870964	16.7800845	5.16335124
ENSG000001	QRICH2	protein_codin	0	0	24.7585999	4.8571057
ENSG000000	TIMP2	protein_codin	254.453712	788.009544	200.019485	248.494849
ENSG000001	LGALS3BP	protein_codin	0	69.967425	59.706003	24.5527147
ENSG000000	MOV10L1	protein_codin	0	0	0	0
ENSG000002	SHANK3	protein_codin	0	0	0	0
ENSG000000	UPF1	protein_codin	35.131382	0	0	0
ENSG000001	PDE4C	protein_codin	0	0	0	0
ENSG000001	ELL	protein_codin	0	0	10.8776792	0
ENSG000000	MAST3	protein_codin	0	0	0	9.40276888
ENSG000001	COPE	protein_codin	15.1784729	45.9064667	79.0423334	24.8767827
ENSG000001	MAU2	protein_codin	12.2328554	29.283965	23.5264176	12.5989783
ENSG000001	MAU2	protein_codin	1.53747724	19.1938331	13.1798906	3.63425714
ENSG000001	TMEM59L	protein_codin	2.7841154	0	4.95297018	0.95965238
ENSG000000	KLHL13	protein_codin	1.38309436	3.02247969	4.76145342	0.87524661
ENSG000001	RBFOX2	protein_codin	0	0	0	0

ENSG0000007	PAK3	protein_codin	0	0	0	0
ENSG0000007	TRPC5	protein_codin	0	0	0.94485402	2.95313694
ENSG0000008	MID2	protein_codin	9.45314972	14.8381025	6.64651022	18.6357327
ENSG0000010	AMMECR1	protein_codin	8.31026347	0	23.3513269	2.76216417
ENSG0000018	PRKX	protein_codin	11.6064232	42.1114725	51.5058374	30.90828
ENSG0000008	RRAGB	protein_codin	0	0	18.5244236	3.53859893
ENSG0000008	HUWE1	protein_codin	197.282518	547.587903	1436.51216	34.0134022
ENSG000000	MAMLD1	protein_codin	0	0	0	0
ENSG0000010	BPI	protein_codin	0	0	0	0
ENSG0000010	SLA2	protein_codin	0	29.9781435	0	4.62933018
ENSG0000007	MYH7B	protein_codin	3.77722846	3.25999278	1.86822834	1.44327406
ENSG0000010	SAMHD1	protein_codin	0	0	0	0
ENSG0000017	RALGAPB	protein_codin	12.9126105	83.5596589	0	0
ENSG0000007	XRCC1	protein_codin	14.0920493	0	33.606074	15.4298052
ENSG0000007	PAFAH1B3	protein_codin	0	0	1.08623239	0.73687589
ENSG0000000	MARK4	protein_codin	1.77550706	35.6427555	9.63221957	7.2061615
ENSG0000002	ZNF225	protein_codin	2.00383845	26.5739843	6.08092175	1.80301571
ENSG0000010	GRIK5	protein_codin	0	0	0	0
ENSG000001	SIM1	protein_codin	2.08212173	1.23533245	0	0
ENSG0000008	HACE1	protein_codin	18.4831536	12.3567758	10.962632	10.20323
ENSG0000011	ST3GAL3	protein_codin	0	0	0	0
ENSG0000008	ATRN	protein_codin	55.8228706	104.698865	79.8345767	33.1962187
ENSG0000010	SIRPB1	protein_codin	0	0	0	0
ENSG0000010	CNPY4	protein_codin	37.5564933	52.2848081	112.248989	9.42357437
ENSG0000010	RASA4	protein_codin	6.14152699	31.947147	10.6778191	30.9928707
ENSG0000019	ZSCAN25	protein_codin	0	9.62939112	13.9001885	0
ENSG0000002	ARPC1A	protein_codin	16.486994	49.2756759	86.6724955	18.1856605
ENSG0000007	MYDGF	protein_codin	11.468546	24.6754863	31.1061092	10.0185351
ENSG0000011	MAP2K2	protein_codin	29.8713667	75.9000288	137.004852	26.3098449
ENSG0000000	HMG20B	protein_codin	0	0	3.42881553	2.26227872
ENSG0000000	TLE2	protein_codin	0	0	22.0582659	6.85879023
ENSG0000000	GNA15	protein_codin	6.80991931	27.1885829	1.84127561	15.5991086
ENSG0000014	DPP9	protein_codin	0	0	43.0777207	0
ENSG0000010	ZFR2	protein_codin	0	1.52689928	0.49562531	0.43024032
ENSG0000010	YJU2	protein_codin	4.19921519	10.8160762	26.1465853	10.146293
ENSG0000010	PTPRS	protein_codin	57.4421506	288.52233	229.844722	122.939087
ENSG0000007	TCF3	protein_codin	0	0	0	0
ENSG0000000	MPND	protein_codin	0	0	0	0
ENSG0000008	ANKRD24	protein_codin	0	4.4011719	0	0
ENSG0000010	PIAS4	protein_codin	4.15250155	25.1431647	23.6551524	4.03093393
ENSG0000010	ZMYND8	protein_codin	0	0	0	0
ENSG0000011	CSE1L	protein_codin	47.1352242	93.6332929	198.703918	28.9546049
ENSG0000010	ZNF330	protein_codin	21.9586544	19.2341548	44.3574956	22.6360801
ENSG0000010	INPP4B	protein_codin	28.0064826	36.0042041	15.5051821	24.1400806

ENSG000001	GAB1	protein_codin	22.8182066	132.902155	0	50.754225
ENSG000001	GAB1	protein_codin	0	0	73.9493157	10.2267442
ENSG000001	UCP1	protein_codin	0	1.25068806	0.5240453	1.35298586
ENSG000001	NOMO3	protein_codin	0	0	0	0
ENSG000001	MAPK3	protein_codin	0.60495597	11.7701474	0	1.69718979
ENSG000001	EEF2K	protein_codin	37.2574616	107.426206	90.075231	36.8837153
ENSG000001	SYTL4	protein_codin	3.14191039	8.02196954	10.9717916	0
ENSG000001	DHTKD1	protein_codin	19.0911697	41.7869616	33.2604913	13.9505072
ENSG000001	OPTN	protein_codin	0	0	0	0
ENSG000001	PHYH	protein_codin	17.5219405	35.4866243	24.8019097	0
ENSG000000	CRISP3	protein_codin	0	0	0	0
ENSG000001	AKAP7	protein_codin	7.96227514	0	0	0
ENSG000001	SORCS1	protein_codin	0	0	0	0.67338251
ENSG000001	MAP3K8	protein_codin	0	0	1.71867483	0
ENSG000001	EPC1	protein_codin	20.3326128	41.2269561	15.3191941	23.667643
ENSG000001	MTPAP	protein_codin	26.7092955	33.8098275	57.0315794	15.0779059
ENSG000001	LDB3	protein_codin	13.340575	0	0	0
ENSG000000	SCARF1	protein_codin	0	0	0	10.9398295
ENSG000000	SMG6	protein_codin	53.0264086	269.197484	119.507323	47.8636526
ENSG000001	ASPA	protein_codin	12.0442184	13.6721413	0	6.3304533
ENSG000001	DPH1	protein_codin	0	0	0	0
ENSG000001	DPH1	protein_codin	0	0	0	0
ENSG000000	ITGAE	protein_codin	0.61160352	15.7187201	0	1.34672322
ENSG000001	PLD2	protein_codin	0	22.8092856	0	12.4699144
ENSG000001	METTL16	protein_codin	10.9154687	0	21.3324536	11.4463175
ENSG000000	SLC27A5	protein_codin	0	0	0	0
ENSG000000	ABCA7	protein_codin	0	0	0	3.20325732
ENSG000000	ZNF264	protein_codin	37.0451177	94.2029465	43.8275527	32.3054384
ENSG000000	CNN2	protein_codin	44.0130159	13.061361	97.3329325	58.3441364
ENSG000001	A1BG	protein_codin	0	0	0	0
ENSG000001	CCDC6	protein_codin	59.8256146	163.801084	132.131429	48.3720735
ENSG000001	BICC1	protein_codin	0.54977637	6.64434418	2.63256549	0
ENSG000000	GGT5	protein_codin	8.6561813	0	0	0
ENSG000001	RAB36	protein_codin	2.33414441	2.95534971	0.53172582	2.51989474
ENSG000000	CABIN1	protein_codin	18.8830529	102.117099	140.167579	12.4785982
ENSG000000	SMARCB1	protein_codin	30.788418	0	153.519122	8.41763422
ENSG000000	PRKCQ	protein_codin	0	0	0	0
ENSG000001	AKR1C4	protein_codin	0	0	0	1.0301852
ENSG000000	WDR37	protein_codin	3.14006323	0	0	0
ENSG000000	SLC17A6	protein_codin	0.33558255	0	0	0.53731434
ENSG000001	CAPZA1	protein_codin	48.5042443	74.8060168	163.111098	53.1811991
ENSG000000	PALMD	protein_codin	47.3239459	68.1664987	18.4172052	49.6531996
ENSG000001	PLPPR5	protein_codin	0	0	0	0
ENSG000001	KIF18A	protein_codin	6.4929701	11.4585285	97.2683567	1.89424

ENSG000001:	BBOX1	protein_codin	0.66889618	0	0	2.79833633
ENSG000000	MSH4	protein_codin	0	0	0.95885317	1.00445959
ENSG000000	DGCR2	protein_codin	16.5930692	35.6602925	17.6834773	4.17236356
ENSG000000	CDC45	protein_codin	2.09502916	0	0	0
ENSG000000	UFD1	protein_codin	3.36768543	56.0049175	39.4344133	6.00803553
ENSG000000	MED15	protein_codin	14.0299133	12.3090539	35.1580943	12.4790813
ENSG000000	ARVCF	protein_codin	0	1.97321504	22.9638424	0
ENSG000001	HIRA	protein_codin	9.21592835	40.4730195	74.2810949	18.7666385
ENSG000001	PPM1F	protein_codin	18.980101	75.3011978	22.6717243	30.6481024
ENSG000001	UBE2R2	protein_codin	32.4044722	110.402181	65.390247	41.4463669
ENSG000001	SYP	protein_codin	0.88824592	4.06916047	0.61300265	0
ENSG000001	ACTR3	protein_codin	111.687407	31.732543	233.331697	103.977279
ENSG000000	DDX18	protein_codin	43.2743102	70.7321444	143.438183	32.7784641
ENSG000002	ARFGAP3	protein_codin	48.8867849	57.9170819	99.3240371	38.2941552
ENSG000001	PACSIN2	protein_codin	15.2312731	30.168729	46.7230358	11.5892687
ENSG000001	EP300	protein_codin	27.4694059	0	77.8513845	0
ENSG000001	SLC25A17	protein_codin	0	11.0861579	0	0
ENSG000001	DESI1	protein_codin	12.0255442	38.4650229	44.7492734	8.24337025
ENSG000001	NOVA2	protein_codin	0	0	4.37766223	5.24473579
ENSG000001	PLEKHA4	protein_codin	62.4577277	466.269971	15.7818567	73.1651873
ENSG000001	FAM83E	protein_codin	0	3.16657266	0	0.42479058
ENSG000001	MREG	protein_codin	1.01942258	7.48441813	3.61986848	3.03658608
ENSG000001	GRIN2D	protein_codin	1.52743589	1.81549202	0	0.90649971
ENSG000000	AP2S1	protein_codin	2.48642853	4.1906288	5.36907177	1.40390514
ENSG000001	LIG1	protein_codin	8.13163336	0	30.2356249	0
ENSG000001:	OPA3	protein_codin	4.5581475	36.1376645	11.3717905	7.38723879
ENSG000001	GYS1	protein_codin	0	0	0	0
ENSG000000	EHD2	protein_codin	38.5475888	166.161986	39.2803584	42.3161961
ENSG000000	HSD17B14	protein_codin	0	0	0	0
ENSG000000	STRN4	protein_codin	9.53938681	55.8850686	22.4314051	19.9344195
ENSG000000	GIPR	protein_codin	0	0	0	0.84332684
ENSG000001:	LY9	protein_codin	0	18.5384785	0	0
ENSG000000	CLNS1A	protein_codin	1.46101926	0	7.59919917	0
ENSG000001	P2RX3	protein_codin	0	0	0	0
ENSG000000	NOX4	protein_codin	0	0	0	0
ENSG000000	TYR	protein_codin	0	0	0	1.54035448
ENSG000001:	IL37	protein_codin	0	0	0	0
ENSG000001:	POLR1B	protein_codin	3.42402027	13.8362167	5.95987588	0
ENSG000001:	PAX8	protein_codin	0	0	0	0
ENSG000001:	PAX8	protein_codin	0	0	0	0
ENSG000001	IL1A	protein_codin	0	0	0	0
ENSG000001:	IL1B	protein_codin	0.47022648	1.47554244	0	2.31976196
ENSG000001	TCF25	protein_codin	0	0	0	0
ENSG000001	TCF25	protein_codin	14.1801226	33.2498557	25.81	12.263434

ENSG000001	ZNF541	protein_codin	0	0.77587606	0	0
ENSG000001	NAPA	protein_codin	5.76180546	15.1650382	17.7869753	8.26227513
ENSG000000	EED	protein_codin	0	0	0	0
ENSG000001	HNRNPUL1	protein_codin	1.49087189	3.83611512	0	0
ENSG000000	BLVRB	protein_codin	6.75830784	16.9039758	10.6368334	9.66646674
ENSG000002	MIA	protein_codin	0	0	19.5827571	0
ENSG000000	ITPKC	protein_codin	9.68621865	19.3335822	0	15.1260865
ENSG000000	KCNK6	protein_codin	15.8635953	476.824292	7.33895758	16.0743649
ENSG000001	BRD4	protein_codin	0	0	0	0
ENSG000001	IL27RA	protein_codin	5.14174313	30.1450329	16.837259	2.83850203
ENSG000000	WIZ	protein_codin	0	0	0	0
ENSG000001	ASF1B	protein_codin	0	3.09702498	4.62950662	0.74497911
ENSG000001	ILVBL	protein_codin	6.31033237	0	39.1773104	5.47389526
ENSG000001	FAM32A	protein_codin	44.3392752	108.416509	150.432887	50.9194329
ENSG000000	NOTCH3	protein_codin	44.7903542	334.720602	54.8315002	76.4279122
ENSG000001	MED26	protein_codin	2.93559353	0	5.82622505	0
ENSG000000	CD44	protein_codin	99.2614374	238.121604	20.6564605	160.004741
ENSG000001	COMMD9	protein_codin	2.01594109	10.7526222	4.98948207	6.42563331
ENSG000001	C9	protein_codin	0	0	0	0
ENSG000001	LIFR	protein_codin	0	113.574669	0	54.1484282
ENSG000000	C6	protein_codin	2.0881401	1.27927828	0	1.60087545
ENSG000001	PRKCG	protein_codin	0	0	0	0
ENSG000001	PPP1R12C	protein_codin	17.5178606	58.6699747	0	8.83675479
ENSG000000	PTPRH	protein_codin	0	0	0.91077492	0
ENSG000000	NLRP2	protein_codin	0	3.73315262	0	0
ENSG000000	ALG6	protein_codin	3.1760672	0	7.99309368	0
ENSG000001	DNAJC6	protein_codin	0	0	3.38977718	6.43137083
ENSG000001	WDR11	protein_codin	8.17171313	23.7785997	14.5730761	0
ENSG000000	PGR	protein_codin	0	0	0	0
ENSG000000	BIRC3	protein_codin	4.45925211	7.86448588	0	19.1964966
ENSG000001	CEP126	protein_codin	38.9373033	16.5335605	56.206146	14.83175
ENSG000001	SLC10A3	protein_codin	0	0	0	0
ENSG000000	ATP2B3	protein_codin	0	0	0	0
ENSG000001	LUC7L2	protein_codin	0	0	0	0
ENSG000000	PARP12	protein_codin	0	38.9538622	26.0029151	14.9735007
ENSG000001	P4HA1	protein_codin	4.35274992	0	0	18.0899493
ENSG000001	VPS26A	protein_codin	7.11073774	3.43643758	0	5.37248386
ENSG000001	PALD1	protein_codin	3.88093966	0	0	0
ENSG000000	APLP2	protein_codin	0	0	0	0
ENSG000001	DDX25	protein_codin	0	3.08083162	0	4.59590578
ENSG000000	CDON	protein_codin	7.52557644	0	14.0945314	0
ENSG000001	FOXRED1	protein_codin	0	0	0	0
ENSG000001	DCPS	protein_codin	5.65844772	22.7302552	6.64385956	7.76867268
ENSG000001	SIAE	protein_codin	0	4.79888752	0	0

ENSG000001	LCN1	protein_codin	0	0	0	0
ENSG000001	KCNT1	protein_codin	0	0	0	0
ENSG000001	BARHL1	protein_codin	0	0	0	0.40322491
ENSG000001	DBH	protein_codin	0	0	0	0.36540893
ENSG000001	ARID3A	protein_codin	11.4329565	66.4416881	19.9646805	8.93672765
ENSG000001	ELANE	protein_codin	0	2.07131185	0	0.40516119
ENSG000000	MTIF2	protein_codin	8.9885492	30.0624399	21.2872673	11.5906206
ENSG000001	CCDC88A	protein_codin	0	101.974156	0	0
ENSG000001	ASB3	protein_codin	9.29767371	0	26.0610035	7.60117449
ENSG000001	TANC1	protein_codin	173.738414	422.693008	405.483946	127.230658
ENSG000000	LY75	protein_codin	3.30732588	29.4207798	0	7.68193094
ENSG000001	ACVR1	protein_codin	0	0	0	0
ENSG000001	CD81	protein_codin	13.7193953	65.213149	6.20057868	23.8158469
ENSG000000	TOLLIP	protein_codin	6.12053439	31.3795377	25.0227919	0
ENSG000000	OSBPL5	protein_codin	0	0	0	0
ENSG000001	CNRIP1	protein_codin	5.09648133	6.33439988	0	10.3859814
ENSG000001	PNO1	protein_codin	8.26913426	16.647412	30.947463	10.0785304
ENSG000001	TAF1B	protein_codin	11.647864	16.1798906	51.6427394	4.74919576
ENSG000001	CNTN3	protein_codin	4.39019341	5.13800383	20.3469332	2.90792791
ENSG000001	PDZRN3	protein_codin	55.8040892	8.03799237	33.8024414	4.86532206
ENSG000000	CHRDL2	protein_codin	0	0	4.40659427	0
ENSG000001	SPCS2	protein_codin	8.8616859	38.0747173	13.9432524	6.53947194
ENSG000001	ARHGEF17	protein_codin	37.0017107	181.525881	61.9830413	66.7689206
ENSG000000	POLD3	protein_codin	16.6080462	21.5453984	46.1974371	10.7723342
ENSG000001	SELP	protein_codin	0	0	0	0
ENSG000000	SUCO	protein_codin	35.9603711	0	0	44.7964491
ENSG000000	SNRNP40	protein_codin	15.3173937	21.7380175	77.2314316	9.08881637
ENSG000001	DNAJC8	protein_codin	63.4120348	168.865862	243.971881	35.8244721
ENSG000001	MECR	protein_codin	0	0	1.80450443	1.15202816
ENSG000001	TFCP2L1	protein_codin	1.98611781	6.47431564	5.92190349	4.46214054
ENSG000000	PTPN4	protein_codin	57.7345057	97.4893457	87.8538101	44.036233
ENSG000000	CLASP1	protein_codin	0	0	0	0
ENSG000001	EPB41L5	protein_codin	10.6493843	0	9.06493437	8.03067341
ENSG000001	LHX4	protein_codin	0.54527503	0	0	3.33174947
ENSG000001	FAM20B	protein_codin	40.7269471	88.4184208	131.098486	30.1434786
ENSG000001	EPAS1	protein_codin	50.415062	420.872815	122.840387	317.070165
ENSG000001	EPCAM	protein_codin	0	0	7.63946137	0
ENSG000000	SRBD1	protein_codin	35.6395134	129.34989	233.027854	22.1175042
ENSG000000	SDF4	protein_codin	0	0	0	0
ENSG000001	SGO1	protein_codin	0	6.45451321	22.0820484	0
ENSG000001	KAT2B	protein_codin	63.2879099	84.916951	43.5178286	61.0823403
ENSG000001	FNBP4	protein_codin	65.2218113	385.286359	137.128925	52.934202
ENSG000002	NDUFS3	protein_codin	4.11120301	17.6289189	7.99879351	2.91074525
ENSG000001	ACCS	protein_codin	3.39598986	7.27251678	5.04821575	0.89534843

ENSG000000	CHMP2B	protein_codin	18.150315	69.9459375	77.9647895	23.224147
ENSG000001	EIF2AK4	protein_codin	112.733953	278.106466	262.801987	56.4338208
ENSG000000	WDR76	protein_codin	0	9.31367558	28.9432119	2.20098673
ENSG000000	TYRO3	protein_codin	4.05374481	3.48218362	6.91429176	4.1228462
ENSG000000	LTK	protein_codin	0	0	0	4.59920636
ENSG000000	TP53BP1	protein_codin	0	16.4459753	0	0
ENSG000001	ZNF106	protein_codin	103.963238	22.5653579	4.62204289	20.724432
ENSG000001	SLC25A12	protein_codin	13.7231286	0	69.7485707	3.19521489
ENSG000001	AKT3	protein_codin	0	49.6370286	0	0
ENSG000001	DESI2	protein_codin	0.3660916	1.33113749	0.57797404	0
ENSG000001	GREB1	protein_codin	0	2.30013984	0	0.58501159
ENSG000000	SYT7	protein_codin	0.27739813	3.7877079	12.1668265	0
ENSG000001	OSBP	protein_codin	90.8292105	187.945586	176.329573	66.8969281
ENSG000001	ZC2HC1A	protein_codin	0	0	16.3142351	0
ENSG000001	IL7	protein_codin	1.49864488	0	0	0
ENSG000001	CHMP3	protein_codin	39.3959266	107.453103	54.6971015	30.3904345
ENSG000000	POLR1A	protein_codin	21.3095488	37.5939018	73.4900054	31.2123998
ENSG000001	GNLY	protein_codin	0	0	0	1.26221575
ENSG000001	VAMP8	protein_codin	9.83799539	91.660569	7.13060408	13.3901091
ENSG000000	CAPG	protein_codin	0	0	0	0
ENSG000000	MAP4K3	protein_codin	20.4531826	23.565876	47.3287908	43.4625862
ENSG000001	RWDD3	protein_codin	1.23414378	5.99871869	0	0.96624549
ENSG000001	RND3	protein_codin	62.4158386	67.9687552	81.5039987	36.8700272
ENSG000000	USE1	protein_codin	10.8011983	18.1903691	31.8089934	6.21992449
ENSG000001	STAM2	protein_codin	38.3607907	86.6020801	63.867982	18.7813505
ENSG000001	GRB14	protein_codin	0	0	0	0
ENSG000001	STRN	protein_codin	64.794541	153.314951	193.475236	77.4198097
ENSG000001	CHIC2	protein_codin	14.4244124	40.9781816	47.1300314	15.2881099
ENSG000001	KDR	protein_codin	0	139.540598	47.8839166	61.7318257
ENSG000000	LNX1	protein_codin	10.5491001	0	0	3.09410231
ENSG000001	TNFRSF8	protein_codin	0	0	0	0
ENSG000000	KIF1B	protein_codin	6.91823639	45.6112515	19.8751183	0
ENSG000000	PKP1	protein_codin	1.05890482	0	0	0
ENSG000000	STK17B	protein_codin	24.770502	195.287776	0	45.4328182
ENSG000001	GTF3C3	protein_codin	5.53912067	29.5625998	0	2.60178357
ENSG000001	COQ10B	protein_codin	12.710436	2.18623259	9.46387197	13.537676
ENSG000001	PEX5L	protein_codin	0	0	0	0
ENSG000000	USP13	protein_codin	40.4918696	0	45.7299898	0
ENSG000001	PIK3CA	protein_codin	18.8295888	40.4388564	30.6998506	17.0718851
ENSG000001	MFN1	protein_codin	32.0437723	26.030806	24.5960347	27.2125458
ENSG000001	TAF12	protein_codin	0.95674568	10.4436242	13.4056808	0
ENSG000001	MAN1C1	protein_codin	6.88001587	21.2346824	9.77294367	0
ENSG000000	SLC9A1	protein_codin	5.25036795	59.7852422	11.5618906	10.5409023
ENSG000000	GATB	protein_codin	0	0	3.07096633	1.78024657

ENSG000001	EHBP1	protein_codin	12.9790504	0	0	4.97877284
ENSG000001	SLC7A6OS	protein_codin	9.24688667	20.4103757	26.334925	8.74882196
ENSG000002	CKLF	protein_codin	2.30073036	0	3.33585755	2.3151431
ENSG000002	LCAT	protein_codin	0.58673866	4.56143705	0	0
ENSG000001	CTCF	protein_codin	61.1023502	155.592481	154.700751	38.742397
ENSG000000	CDH3	protein_codin	0	0	0	2.89258317
ENSG000001	IFT46	protein_codin	9.71195831	0	15.2173437	2.70645334
ENSG000001	IFT46	protein_codin	0	0	0	0
ENSG000001	NECTIN1	protein_codin	7.03465205	28.7017485	24.4651683	6.46224199
ENSG000001	SC5D	protein_codin	19.5509194	18.6360554	37.0473093	7.12983184
ENSG000000	ARCN1	protein_codin	49.286086	192.696304	81.7197541	33.3847845
ENSG000001	TREH	protein_codin	4.66499928	1.96257971	4.80692374	2.43565956
ENSG000001	UPK2	protein_codin	0	0	0	0.35972979
ENSG000001	CBL	protein_codin	28.17277	78.6784921	30.4649039	26.9239729
ENSG000000	MCAM	protein_codin	35.549808	260.902212	65.1476608	86.586171
ENSG000001	TECTA	protein_codin	0	7.32933985	0	1.20463537
ENSG000000	GPC1	protein_codin	95.7693854	51.5023495	75.1823585	18.3193061
ENSG000000	FARP2	protein_codin	4.49875376	23.8365839	6.4136965	10.5582872
ENSG000001	SLC5A7	protein_codin	0	0	0	1.69506336
ENSG000000	NGEF	protein_codin	2.47752945	6.46172073	0.76833084	0
ENSG000000	SP100	protein_codin	25.1521119	28.6743216	1.23686219	9.65046581
ENSG000000	DGKD	protein_codin	12.9450468	39.0424631	31.5620259	3.89266806
ENSG000001	EFHD1	protein_codin	3.97246319	23.1871264	6.07201276	2.25567654
ENSG000000	DNAJC10	protein_codin	0	0	0	44.6505483
ENSG000001	TUBB4A	protein_codin	0	0	0.80512015	0
ENSG000000	MCOLN1	protein_codin	5.80512471	27.7028088	21.2022778	9.38218552
ENSG000001	GPR108	protein_codin	0	0	0	0
ENSG000001	SLC25A23	protein_codin	0	0	0	0
ENSG000001	DGUOK	protein_codin	0	0	0	0
ENSG000001	LOXL3	protein_codin	0	0	0	0
ENSG000000	ITGA6	protein_codin	0	43.5878352	0	35.558811
ENSG000001	HAT1	protein_codin	27.0750278	19.2298843	211.333702	6.55603896
ENSG000001	ATF2	protein_codin	10.6549306	0	0	15.4190733
ENSG000001	GPSM2	protein_codin	4.341593	0	30.7499076	6.1893878
ENSG000000	SLC25A24	protein_codin	36.7558449	58.7972659	54.6383394	16.2437259
ENSG000000	LAMC2	protein_codin	3.38165459	20.3493902	0	4.50458687
ENSG000001	OSGEPL1	protein_codin	0	0	0	0
ENSG000000	MCM6	protein_codin	44.9004817	66.4265156	123.164932	17.8556318
ENSG000000	CCNT2	protein_codin	0	0	30.9199192	0
ENSG000001	RAB3GAP1	protein_codin	0	0	0	6.98068905
ENSG000001	ZRANB3	protein_codin	7.52663106	9.61402738	9.02171167	6.87500819
ENSG000000	R3HDM1	protein_codin	0	0	0	0
ENSG000001	DARS1	protein_codin	47.3535461	102.508544	239.278823	28.4963451
ENSG000001	LCT	protein_codin	0	0	0	0

ENSG000000	AGPS	protein_codin	1.11453867	65.5751723	0	0
ENSG000001	ORC4	protein_codin	1.85833666	0	31.097425	2.34127029
ENSG000001	KYNU	protein_codin	0.94127265	17.7406529	0.48045303	11.7476279
ENSG000000	ERO1B	protein_codin	0	4.58523055	3.28537016	0.98797886
ENSG000000	ARID4B	protein_codin	0	0	0	0
ENSG000001	NID1	protein_codin	85.79378	327.835721	349.831589	62.7074144
ENSG000001	CYTIP	protein_codin	4.68939315	76.7237749	0	8.28088799
ENSG000000	MUL1	protein_codin	10.7236739	39.7493881	30.3284451	9.62168888
ENSG000000	CAPZB	protein_codin	31.6661589	134.703344	31.349004	25.1270996
ENSG000000	CAPZB	protein_codin	0	1.86658184	1.98098032	0
ENSG000001	ECE1	protein_codin	8.58891476	0	28.3297569	0
ENSG000000	EIF4G3	protein_codin	120.485429	211.998561	452.29352	82.5027016
ENSG000001	NMU	protein_codin	0	0	53.9646026	0
ENSG000001	PPAT	protein_codin	8.77884848	7.27376128	108.38136	0.69836504
ENSG000001	PAICS	protein_codin	0	0	47.3883726	0
ENSG000001	SRD5A3	protein_codin	0.72271069	0	12.7657873	1.63791124
ENSG000001	CRACD	protein_codin	0	0	0	0
ENSG000000	NOA1	protein_codin	10.4501536	14.1727817	37.5163489	9.24655061
ENSG000001	POPDC2	protein_codin	0	0	0	0
ENSG000000	POLQ	protein_codin	7.72313608	16.8238058	118.144054	5.23726376
ENSG000001	UPK1B	protein_codin	0	0	0	0
ENSG000000	GSK3B	protein_codin	0	68.155521	7.56599254	17.371416
ENSG000001	TIMMDC1	protein_codin	2.37544665	2.727832	5.43782168	0
ENSG000000	ARHGAP31	protein_codin	95.3038708	241.922217	255.367287	106.014195
ENSG000001	CD80	protein_codin	1.34723053	5.05220859	1.43784079	0
ENSG000001	CHST10	protein_codin	10.3623759	22.5478792	38.6616107	5.75584543
ENSG000001	PDCL3	protein_codin	10.5122952	47.7153572	38.452484	8.08418717
ENSG000001	TXNDC9	protein_codin	14.090684	11.2909604	11.063928	4.54802868
ENSG000001	IL1RL2	protein_codin	0	0	2.08589277	0
ENSG000000	RPL31	protein_codin	109.568299	43.4737874	356.735317	83.0901566
ENSG000001	IL18RAP	protein_codin	0	0	0	0
ENSG000001	GFM1	protein_codin	0	30.0103699	45.8304153	15.9391792
ENSG000000	LXN	protein_codin	16.5296596	35.4601512	123.887568	21.1655002
ENSG000001	MFSD1	protein_codin	0	0	0	0
ENSG000000	CASP8	protein_codin	0.83418114	0	0	0
ENSG000000	ALS2	protein_codin	0	0	0	1.56072136
ENSG000000	NOP58	protein_codin	65.1507224	102.613922	374.94085	69.1678522
ENSG000001	OCIAD1	protein_codin	0	0	0	14.2751802
ENSG000001	SLAIN2	protein_codin	27.8836673	86.8601959	31.6184536	18.4884102
ENSG000000	TXK	protein_codin	0	0	0	0
ENSG000001	GABRA4	protein_codin	0.23233278	2.78235291	0.92212706	6.57591472
ENSG000000	TOP2B	protein_codin	21.4091824	73.0683125	42.0030802	21.5860865
ENSG000001	YWHAE	protein_codin	2.66603989	269.515907	201.33734	62.2556511
ENSG000001	ARHGAP24	protein_codin	0.55908526	7.7702421	4.13921457	0

ENSG000001	FAM13A	protein_codin	12.5357562	26.8571949	40.0595841	0
ENSG000001	HERC6	protein_codin	6.38930183	68.8401094	15.3801391	0
ENSG000001	HERC5	protein_codin	7.55521832	76.4754546	33.288685	23.1133982
ENSG000001	PCDH10	protein_codin	2.98312273	28.7460434	1.63728017	1.11849159
ENSG000001	NDST4	protein_codin	0	0	0	0
ENSG000001	ANK2	protein_codin	7.82971035	0	26.0320476	0
ENSG000001	ZGRF1	protein_codin	4.74789536	0	18.9273536	0
ENSG000001	CRYGD	protein_codin	0	0	0	0
ENSG000001	ADAM23	protein_codin	9.51967066	0	55.8126656	7.44861311
ENSG000001	PIKFYVE	protein_codin	62.7472118	183.341167	155.631404	46.8357089
ENSG000001	BCHE	protein_codin	5.45542067	1.5182593	1.04950912	0.51938379
ENSG000000	SI	protein_codin	0	1.50488902	0	3.522811
ENSG000001	COPS4	protein_codin	19.3976174	26.4740914	72.479056	12.179246
ENSG000001	PRKG2	protein_codin	0	0	0	11.186554
ENSG000001	RASGEF1B	protein_codin	0	25.6897436	0	0
ENSG000001	SEC31A	protein_codin	1.96348477	0	5.61502428	1.3997461
ENSG000001	GPAT3	protein_codin	0	3.68681984	0	0
ENSG000000	CUL3	protein_codin	1.7713139	17.9726081	0	11.2763136
ENSG000000	GUCY1B1	protein_codin	17.7547812	29.5556154	0	12.2276075
ENSG000001	GRIA2	protein_codin	0	0	0	0
ENSG000001	GLRB	protein_codin	7.27793488	0	47.5147946	2.86897157
ENSG000001	RAPGEF2	protein_codin	9.00385066	0	13.6824975	21.8662265
ENSG000000	FNIP2	protein_codin	21.5798566	58.8330021	54.9765025	17.9868337
ENSG000001	C2orf42	protein_codin	4.06684117	9.44015131	0	4.81855666
ENSG000000	ADD2	protein_codin	1.84223467	6.17579262	49.9738765	13.0598491
ENSG000001	PCYOX1	protein_codin	9.44122203	0	38.3760212	0
ENSG000000	MXD1	protein_codin	13.3194992	23.7847016	32.0768525	36.9140462
ENSG000000	ZNF638	protein_codin	0	469.426447	392.112313	111.855469
ENSG000001	ATP8A1	protein_codin	13.4858794	78.1576908	25.398734	22.0128843
ENSG000000	SLC30A9	protein_codin	14.7989329	19.9054238	36.7485821	11.185528
ENSG000001	TMEM33	protein_codin	0.68550174	7.07371242	3.70885887	1.64820165
ENSG000000	SEC22C	protein_codin	19.0495814	0	31.4781335	13.7664923
ENSG000000	CDH10	protein_codin	1.85724833	0	0	0
ENSG000001	CD86	protein_codin	0	0	0	0
ENSG000001	CSTA	protein_codin	0.61396023	4.88120266	1.10056441	1.07481744
ENSG000000	SLC4A4	protein_codin	1.59501711	27.9453567	13.2066219	11.1830051
ENSG000001	FGF2	protein_codin	0	37.9727769	0	0
ENSG000001	BBS7	protein_codin	9.57005377	20.3761446	22.9109445	5.6074792
ENSG000001	BLTP1	protein_codin	9.55584079	122.89305	7.18011755	18.1540442
ENSG000001	RBBP5	protein_codin	28.6577839	102.909437	72.6399497	18.1940707
ENSG000001	IFT57	protein_codin	36.3640181	43.8953366	126.154885	22.1683401
ENSG000001	FRMD4A	protein_codin	23.1887754	39.5640376	44.6127328	4.61437985
ENSG000001	UBE2S	protein_codin	6.21439161	24.9153089	45.1167829	10.73778
ENSG000001	GZMM	protein_codin	0	4.48727457	0.55322378	0

ENSG000001:	SHC2	protein_codin	1.25037224	7.15763442	28.7147844	0
ENSG000000	PHRF1	protein_codin	43.3798836	0	224.806187	0
ENSG000000	PALM	protein_codin	0	0	0	0
ENSG000000	IL11	protein_codin	0	0	0	0
ENSG000001	BMPR1B	protein_codin	0	0	13.3861491	0
ENSG000001	RAP1GDS1	protein_codin	0	6.380855	15.2392232	0
ENSG000000	AGA	protein_codin	6.60654374	3.62304573	10.5870612	5.1697828
ENSG000001	NEIL3	protein_codin	4.46668386	8.8020222	27.9436974	2.4553661
ENSG000001	RAB17	protein_codin	0	0	0	0
ENSG000001	MLPH	protein_codin	2.16080518	2.13716928	0.45611525	0.6577718
ENSG000000	ASB1	protein_codin	7.10177709	29.6989807	12.4821485	10.0346397
ENSG000000	CP	protein_codin	4.81431394	13.5668211	0	25.9433628
ENSG000001	WNT5A	protein_codin	47.1756007	11.0967831	189.87185	12.8021145
ENSG000001	THRA	protein_codin	0	0	11.4308983	0
ENSG000001	CNTNAP1	protein_codin	19.6328057	92.3904435	3.59476161	2.00939649
ENSG000001	PSMD3	protein_codin	22.5142124	103.100591	68.5692726	25.6887545
ENSG000000	SMARCE1	protein_codin	0	0	0	0
ENSG000001	RAPGEFL1	protein_codin	0.83369481	0	0	2.89227326
ENSG000001	CASC3	protein_codin	17.9941038	57.4540868	0	29.3775826
ENSG000000	ATP6V0A1	protein_codin	0	0	1.17193719	0
ENSG000001	KRT24	protein_codin	0	0.86850873	0	4.58938095
ENSG000001	STAT3	protein_codin	0	0	0	0
ENSG000001	FBXL20	protein_codin	37.4116164	99.7173844	40.6768019	33.4602599
ENSG000000	KCNH4	protein_codin	0	0	0	0
ENSG000001	NNT	protein_codin	0	8.69393112	7.16674357	9.79282876
ENSG000000	FGF10	protein_codin	4.10335812	5.44220147	3.77275758	5.79063471
ENSG000001	MTRR	protein_codin	0	0	0	0
ENSG000001	FASTKD3	protein_codin	2.47482292	0	2.17317662	2.29344707
ENSG000000	NSUN2	protein_codin	36.0289582	0	57.8193794	15.9131031
ENSG000000	MECOM	protein_codin	0	0	7.78211219	0
ENSG000001	LRRC31	protein_codin	0	0	0	0
ENSG000001	SERPINI2	protein_codin	0	0	0	0
ENSG000001	UFSP2	protein_codin	8.93262489	27.9509522	3.75964146	7.10675633
ENSG000001	KLKB1	protein_codin	0	0	0	0
ENSG000000	F11	protein_codin	0	0	0	0
ENSG000001	SNX25	protein_codin	19.4882056	0	43.2480659	0
ENSG000001	FNDC4	protein_codin	1.97135445	5.00219029	3.10827189	5.10032238
ENSG000000	CAD	protein_codin	32.1375769	29.1954194	87.8793138	12.6996702
ENSG000001	POMC	protein_codin	0	0	0	0
ENSG000001	DNMT3A	protein_codin	0	0	0	0
ENSG000000	RAB10	protein_codin	35.8457395	112.919159	84.1825379	29.6969869
ENSG000001	DNAJC27	protein_codin	0	11.2108042	5.11338829	0
ENSG000000	KIF3C	protein_codin	4.86717784	23.555195	16.4374292	9.12586976
ENSG000000	FOSL2	protein_codin	67.7415235	284.352249	50.7731971	155.15263

ENSG00000[illegible]	GCKR	protein_codin	0	0	0	0.41581181
ENSG00000[illegible]	GPN1	protein_codin	0	10.8120091	4.78705494	0
ENSG00000[illegible]	EFR3B	protein_codin	1.38250736	0	0	0
ENSG000001	GTF3C2	protein_codin	10.6477611	76.434859	68.9666638	10.7184189
ENSG000001	CSPG5	protein_codin	0	0	0	0
ENSG00000[illegible]	TMEM40	protein_codin	0	0	0	0
ENSG00000[illegible]	TP63	protein_codin	5.71179171	14.0568153	0	0
ENSG000001	CLDN16	protein_codin	0	0	0	0
ENSG00000[illegible]	PLAAT1	protein_codin	0	0	0	0.42325204
ENSG00000[illegible]	ITGA9	protein_codin	21.4641814	183.198732	24.6928463	51.7682368
ENSG00000[illegible]	FGFRL1	protein_codin	10.521237	24.3115308	0	9.24805465
ENSG00000[illegible]	MAEA	protein_codin	14.1629926	0	0	0
ENSG00000[illegible]	ADD1	protein_codin	0	62.5080026	0	0
ENSG00000[illegible]	TMEM175	protein_codin	0	6.10435375	0	0
ENSG00000[illegible]	PLPP1	protein_codin	0.46360117	8.91685031	0	0
ENSG00000[illegible]	GPBP1	protein_codin	0	130.06533	0	19.2328704
ENSG00000[illegible]	SLC2A9	protein_codin	0	7.84501023	0.51875836	2.79153312
ENSG00000[illegible]	WDR1	protein_codin	0	0	59.2605492	0
ENSG00000[illegible]	MMRN1	protein_codin	3.59308008	36.6975844	1.35506853	17.0582939
ENSG00000[illegible]	PDE5A	protein_codin	8.63125605	32.721976	64.0837987	0
ENSG00000[illegible]	PRDM5	protein_codin	10.8760698	17.8744543	17.7699909	9.22692237
ENSG00000[illegible]	TRPC3	protein_codin	0	0	1.31163112	1.13594131
ENSG00000[illegible]	MIER2	protein_codin	6.64107162	12.8438129	33.4298298	7.04692484
ENSG00000[illegible]	LYL1	protein_codin	2.20310606	12.9173866	0.58793271	3.840744
ENSG00000[illegible]	HOOK2	protein_codin	10.1230461	0	31.8047749	8.89212427
ENSG00000[illegible]	COL5A3	protein_codin	80.9572507	263.081173	13.8058078	31.4971792
ENSG00000[illegible]	ICAM1	protein_codin	2.55290444	35.600223	2.64126924	55.9291177
ENSG00000[illegible]	OLFM2	protein_codin	0	8.35281374	1.69746388	0
ENSG00000[illegible]	KLF1	protein_codin	0	0	0	0.41927033
ENSG00000[illegible]	RIMS1	protein_codin	0.72632637	0	33.3933939	0
ENSG000001	C3orf52	protein_codin	7.14427576	0	25.5989637	0
ENSG00000[illegible]	SIDT1	protein_codin	0	7.59367186	0	0
ENSG00000[illegible]	PI4K2B	protein_codin	10.6093416	24.2050001	27.1084453	7.7304227
ENSG00000[illegible]	TBC1D19	protein_codin	8.88059282	11.982083	12.8822128	6.39678645
ENSG00000[illegible]	PPARGC1A	protein_codin	8.09238541	2.77006803	2.76308996	0.84831104
ENSG00000[illegible]	SEL1L3	protein_codin	0	145.501051	23.6048492	0
ENSG00000[illegible]	F13A1	protein_codin	102.798048	672.560081	28.2134433	421.394457
ENSG00000[illegible]	RIOK1	protein_codin	32.4505335	0	139.703853	10.6727361
ENSG00000[illegible]	NUP54	protein_codin	0	24.5357928	0	0
ENSG00000[illegible]	CXCL9	protein_codin	21.2775363	957.377131	0	8.74541014
ENSG00000[illegible]	SEPTIN11	protein_codin	233.83744	250.349903	381.249627	117.347315
ENSG00000[illegible]	SCARB2	protein_codin	17.6877917	81.1456814	75.6277231	56.5368215
ENSG00000[illegible]	USO1	protein_codin	35.822941	4.36912236	0	0
ENSG00000[illegible]	ANXA3	protein_codin	0	2.63981571	3.62602527	0

ENSG000001	ARSB	protein_codin	33.5977752	72.0097758	44.4731624	47.8021846
ENSG000001	PDE8B	protein_codin	3.11669106	0	0	2.72911721
ENSG000000	RAD18	protein_codin	8.6562705	19.9793202	56.5097071	7.14977275
ENSG000001	SLC12A7	protein_codin	15.586677	113.983765	226.9556	26.2426855
ENSG000000	SDHA	protein_codin	20.9911613	0	320.346954	20.3026824
ENSG000002	PDCD6	protein_codin	0	9.8764916	136.963125	0
ENSG000001	MTMR12	protein_codin	6.18733075	44.2477692	100.935326	13.4787823
ENSG000001	CEP72	protein_codin	2.16826283	22.1254172	143.346216	2.52433054
ENSG000000	SLC9A3	protein_codin	0	7.60376438	8.40048903	3.7829992
ENSG000001	XRN1	protein_codin	24.7535522	122.284732	6.22936484	8.89873289
ENSG000001	GRK7	protein_codin	0	0	0.48715226	0
ENSG000001	GRPEL1	protein_codin	5.21480775	0	30.7103498	6.53707294
ENSG000000	EVC	protein_codin	58.8000696	214.598105	111.451621	47.2698708
ENSG000001	LMAN2L	protein_codin	10.1780148	38.9861386	38.7453219	5.67943777
ENSG000000	MGAT4A	protein_codin	0	0	2.38622393	8.72397185
ENSG000001	ZAP70	protein_codin	0.7983824	0	2.89878155	0
ENSG000000	PPP2R3A	protein_codin	23.0297013	23.4970084	59.2901677	25.9549778
ENSG000001	CEP70	protein_codin	8.7387022	26.0975928	71.4982584	5.33733611
ENSG000002	ACAD11	protein_codin	0	32.5674892	40.7044334	10.544387
ENSG000000	UBA5	protein_codin	0	0	0	0
ENSG000000	ASTE1	protein_codin	0	0	0	0
ENSG000000	CDV3	protein_codin	4.37399289	130.708036	38.7265512	52.1635205
ENSG000001	MRPL3	protein_codin	33.3449784	44.3205752	118.147528	22.1259622
ENSG000001	GALNT7	protein_codin	7.31000859	0	25.3272224	5.55901996
ENSG000000	SOX30	protein_codin	0	0	0	0
ENSG000001	GCNT2	protein_codin	0	1.58836803	0.93481371	1.9983694
ENSG000001	BST1	protein_codin	0.43660588	3.57081175	0	1.35759097
ENSG000000	FAM184B	protein_codin	1.88127149	0	6.35709452	4.13193949
ENSG000000	DGKG	protein_codin	2.08180706	8.9342528	2.5712116	1.36079956
ENSG000000	MAP3K13	protein_codin	4.6396847	8.78386943	11.5899784	8.69312929
ENSG000000	DNAJB11	protein_codin	49.799869	73.869045	194.507648	41.9428212
ENSG000000	FETUB	protein_codin	0	0	0	0
ENSG000000	DEPDC1B	protein_codin	1.43825078	0	39.6679276	0
ENSG000000	ERCC8	protein_codin	0	0	0	0
ENSG000001	SSR3	protein_codin	79.2344902	137.414268	131.297514	66.4261615
ENSG000000	MGLL	protein_codin	3.90003833	23.788977	5.11376264	8.87539406
ENSG000000	MCM2	protein_codin	0	25.1131881	78.2057319	3.93723428
ENSG000000	RAB7A	protein_codin	33.7736474	72.9935872	60.2608639	0
ENSG000001	CFAP92	protein_codin	0.53247386	3.89511985	1.33551905	3.53268496
ENSG000000	ZFR	protein_codin	144.322037	249.327544	669.802938	90.596049
ENSG000001	GOLPH3	protein_codin	59.3654717	79.6780876	242.806694	39.4067663
ENSG000001	CDH6	protein_codin	7.69647219	21.8726297	3.26734584	27.6230831
ENSG000001	SUB1	protein_codin	57.6187515	43.5737803	183.388916	42.0062292
ENSG000001	NPR3	protein_codin	9.55142357	2.85732687	42.7110576	11.953441

ENSG000000	VCAN	protein_codin	1877.1204	2108.50519	579.646466	678.407484
ENSG000001	RASGRF2	protein_codin	10.2051369	49.3314591	5.83322892	34.8910986
ENSG000001	MSH3	protein_codin	43.5612948	0	177.951657	23.1065895
ENSG000001	CPEB4	protein_codin	30.9438682	331.176082	27.6081898	97.8908158
ENSG000001	STC2	protein_codin	9.16863865	9.66468902	5.11135459	10.8548671
ENSG000001	ATP6V0E1	protein_codin	45.8492931	116.727537	23.535351	29.5525359
ENSG000000	FBXW11	protein_codin	0	0	0	0
ENSG000000	THOC3	protein_codin	4.53333152	0	20.2289377	0
ENSG000000	RPL26L1	protein_codin	11.1211016	27.5189002	101.88918	5.54044888
ENSG000000	DNAH5	protein_codin	0	0	20.6700234	0
ENSG000000	WDR70	protein_codin	9.87996982	6.04856991	85.388324	10.0084228
ENSG000000	RAI14	protein_codin	23.4428224	145.234101	140.014741	25.4831878
ENSG000001	TARS1	protein_codin	24.6966969	0	268.104009	31.8811723
ENSG000000	SLC1A3	protein_codin	0	0	0	0
ENSG000001	AMBP	protein_codin	0	0	0	0.36498132
ENSG000000	WHRN	protein_codin	0	0	4.45291546	0
ENSG000001	COBL	protein_codin	0	0	0	0
ENSG000001	ARRDC3	protein_codin	75.5870745	355.673092	102.498158	195.773671
ENSG000001	SLF1	protein_codin	7.47200773	53.3155049	47.644376	4.92058849
ENSG000001	CENPE	protein_codin	3.45308118	20.646022	331.896647	0
ENSG000001	TET2	protein_codin	52.462345	90.1713277	169.117098	42.302672
ENSG000002	ARHGEF38	protein_codin	0	2.64697917	0	1.44230636
ENSG000001	ENPEP	protein_codin	71.9353699	193.424346	14.3635694	80.883396
ENSG000001	CASP6	protein_codin	2.63333867	9.32281349	0	0
ENSG000001	LEF1	protein_codin	12.3632417	36.4349704	0	0
ENSG000001	EGF	protein_codin	0	0	0	0
ENSG000001	PAPSS1	protein_codin	34.5906102	86.5204808	57.7287329	26.0658686
ENSG000001	SEC24B	protein_codin	34.8420419	32.5459078	91.1102716	26.9042837
ENSG000001	MCM8	protein_codin	9.0969411	34.5052174	15.4544703	0
ENSG000001	NME5	protein_codin	1.19436406	0	2.61821197	1.78948013
ENSG000001	PAIP2	protein_codin	99.7230229	0	203.079931	47.6689857
ENSG000001	SIL1	protein_codin	0	0	0	0
ENSG000000	IPCEF1	protein_codin	0	35.908566	0	8.61473008
ENSG000001	ANKRD7	protein_codin	0	0	0	0
ENSG000000	MRPL22	protein_codin	0	0	0	1.38036338
ENSG000001	IQCG	protein_codin	6.45572862	13.4951793	0	4.69098724
ENSG000000	LSG1	protein_codin	28.748782	63.0401936	99.8011286	30.8896107
ENSG000000	PCNP	protein_codin	19.5974173	100.481776	52.1440059	23.986495
ENSG000000	ST3GAL6	protein_codin	0	0	0	0
ENSG000000	RBM27	protein_codin	87.5489151	120.066354	133.945211	42.9197705
ENSG000001	JAKMIP2	protein_codin	22.0032911	5.72336314	99.9184675	5.28362514
ENSG000001	SHOC2	protein_codin	0	0	0	0
ENSG000001	TLE4	protein_codin	0	0	0	0
ENSG000001	WWC1	protein_codin	1.85976946	5.25218187	5.43794227	4.35921951

ENSG00000009	GABRP	protein_codin	0	0	0	1.34678208
ENSG00000004	SPDL1	protein_codin	20.3179159	16.798685	91.2067348	10.5597673
ENSG000001	MINDY4	protein_codin	7.58703693	13.7702638	5.27227656	0
ENSG000001	SSBP1	protein_codin	27.2456727	12.2348502	81.6958612	10.2907928
ENSG000001	TRPV5	protein_codin	0	0	0	0
ENSG000001	ABCB6	protein_codin	13.3607869	0	72.9729561	13.3844502
ENSG000001	PECR	protein_codin	4.43884755	0	0	1.70293869
ENSG000002	VDAC1	protein_codin	5.01528989	0	0	0
ENSG0000000	CDKL3	protein_codin	3.91714687	0	0	1.48050762
ENSG000001	UBE2B	protein_codin	8.69930376	8.86535559	8.22102897	10.4500139
ENSG0000000	PITX1	protein_codin	0	2.9657448	5.43287691	1.46666755
ENSG0000000	FSTL4	protein_codin	0.29117626	4.43641052	1.00707162	2.49849506
ENSG0000000	AFF4	protein_codin	149.532031	203.88986	148.400816	231.42971
ENSG000001	KCTD20	protein_codin	0	0	0	0
ENSG000001	ZMIZ2	protein_codin	0.80058323	42.8604107	0	0
ENSG0000000	CUL7	protein_codin	32.0990627	0	87.7838067	7.9138665
ENSG000001	MED20	protein_codin	12.605467	5.70572976	32.8649811	7.88108958
ENSG000001	XPO5	protein_codin	19.8555006	63.905576	135.967774	13.8038273
ENSG000001	SRF	protein_codin	18.7300152	117.671093	53.7092813	14.9531139
ENSG0000000	SEMA3C	protein_codin	80.4158366	65.9484601	87.4293019	35.3577467
ENSG0000000	SEMA3A	protein_codin	37.0555677	15.9848178	48.2960646	8.78036642
ENSG0000000	NRP1	protein_codin	0	0	0	0
ENSG000001	CCNY	protein_codin	0	0	1.36862545	0
ENSG000001	APBA1	protein_codin	7.37469026	18.9937871	34.3583549	9.48367254
ENSG000001	PIP5K1B	protein_codin	0	10.1908441	0	0
ENSG0000000	TNPO3	protein_codin	34.9380326	91.4533669	92.5537165	24.6408052
ENSG000001	TAX1BP1	protein_codin	82.6461955	176.324765	194.61445	48.9608237
ENSG000001	CPVL	protein_codin	12.8489825	156.440989	77.3267597	15.8822448
ENSG000001	HIBADH	protein_codin	13.6218533	25.8329058	44.2951029	10.3109727
ENSG000001	UGT2B10	protein_codin	0	0	0	0
ENSG0000000	STAP1	protein_codin	0	0	0	0
ENSG0000000	ADGRF5	protein_codin	23.9960844	40.3743298	25.5910566	34.9867502
ENSG0000000	POLB	protein_codin	1.4553931	5.66019091	0	0
ENSG000001	SLC17A2	protein_codin	0	0	0	0
ENSG000001	WWP1	protein_codin	29.4319264	55.7196393	45.4969253	49.799485
ENSG000001	CALB1	protein_codin	0	0	7.09975952	0
ENSG000001	NBN	protein_codin	0	0	0	0
ENSG0000000	ST7	protein_codin	0.75517735	0	4.14188173	2.49921377
ENSG000001	TFEC	protein_codin	23.8768259	12.5985554	0	13.1726435
ENSG000001	WNT2	protein_codin	0.84219986	0	1.06487658	1.31743124
ENSG000001	ANXA11	protein_codin	32.9509391	48.1154147	45.6060259	31.7179029
ENSG000001	TSPAN14	protein_codin	1.30752442	0	4.86990621	0
ENSG000001	NRXN2	protein_codin	0	0	0	0
ENSG000001	MYRF	protein_codin	0	0	7.15842473	0.85248736

ENSG000001	PRDX5	protein_codin	22.4596315	37.442642	78.174862	16.616575
ENSG000000	POLA2	protein_codin	0.71758275	2.50560023	0	0
ENSG000001	B3GAT3	protein_codin	5.07070283	10.5283631	10.2357508	3.02592195
ENSG000000	MGST2	protein_codin	2.41577336	0	7.53740979	2.93268511
ENSG000001	ADH4	protein_codin	0	1.39800995	0	1.68346646
ENSG000001	MTTP	protein_codin	0	0	0	0
ENSG000001	BLVRA	protein_codin	24.891375	118.482684	49.8825659	23.7811398
ENSG000000	KIF9	protein_codin	0	0	0	0
ENSG000000	LARS2	protein_codin	0	21.5177967	19.3085148	3.78346006
ENSG000000	RHOA	protein_codin	0	0	0	0
ENSG000001	USP4	protein_codin	12.9657294	33.4240726	6.58409181	5.43087828
ENSG000000	PTPN23	protein_codin	31.5110607	0	30.6203328	18.1315554
ENSG000001	PRKAR2A	protein_codin	14.1955546	30.5760555	41.6057514	11.6016024
ENSG000000	EXOSC7	protein_codin	0	0	4.47893986	0
ENSG000001	SCAP	protein_codin	3.78240955	12.5449226	0	8.11011766
ENSG000000	OPRK1	protein_codin	0	2.9308323	2.33972251	4.03641129
ENSG000000	ICA1	protein_codin	8.75929958	0	0	0
ENSG000001	ABCC5	protein_codin	1.02330694	15.4599764	38.8924336	14.1765966
ENSG000001	CLCN2	protein_codin	0	0	0	0
ENSG000000	MCCC1	protein_codin	0	0	33.0952076	0
ENSG000000	LAMP3	protein_codin	0.37225151	0	1.39940711	1.79373783
ENSG000001	WASF1	protein_codin	0	0	0	0
ENSG000001	AHI1	protein_codin	0	0	0	0
ENSG000001	ALDH8A1	protein_codin	0	8.52035016	1.26255251	0
ENSG000001	UBXN8	protein_codin	9.12271256	0	11.0709126	0
ENSG000001	VAPB	protein_codin	0	5.59977627	2.60312705	0
ENSG000000	GNAS	protein_codin	12.7217723	41.4895075	39.8171847	32.9172173
ENSG000001	PMEPA1	protein_codin	15.2857525	0	0	0
ENSG000001	PON3	protein_codin	0	0	0	0
ENSG000000	SLC25A13	protein_codin	0	10.0464556	38.2893048	13.3274511
ENSG000001	NPTX2	protein_codin	11.5003541	10.5691399	117.878473	10.5283089
ENSG000001	PPP6R3	protein_codin	0	0	0	0
ENSG000001	PPP6R3	protein_codin	0	0	0	0
ENSG000001	CPT1A	protein_codin	38.3603134	52.1173483	5.38291332	45.7721152
ENSG000000	GAL	protein_codin	0	0	4.52937986	0
ENSG000001	FBXO3	protein_codin	0	0	0	0
ENSG000001	KIAA1549L	protein_codin	10.4337503	12.6664284	0	0
ENSG000001	NSMF	protein_codin	0	22.7604065	26.2739755	2.05349904
ENSG000000	RPS6KA2	protein_codin	4.31079409	38.1853712	10.5216576	18.0161044
ENSG000001	TCIRG1	protein_codin	7.51472024	34.4284898	5.04879909	32.1563368
ENSG000001	CHKA	protein_codin	6.33818474	19.4067063	28.3363033	3.60347883
ENSG000001	ADAM18	protein_codin	0	0	0	0
ENSG000001	ADAM2	protein_codin	0	0	0	0
ENSG000000	ANK1	protein_codin	0	0	0	0

ENSG000000	KAT6A	protein_codin	34.3999358	65.728796	86.1795637	0
ENSG000000	PRKAR2B	protein_codin	7.30864107	0	0	7.89708512
ENSG000001	DUS4L	protein_codin	1.23844838	0	0	0
ENSG000000	ABCB4	protein_codin	5.89674259	1.29959334	8.44608681	6.60642128
ENSG000000	ABCB1	protein_codin	0	0	3.97790711	0
ENSG000000	ADAM22	protein_codin	0	6.34373938	0	0
ENSG000000	DBF4	protein_codin	0.78930557	4.69203104	0	7.92336004
ENSG000000	SRI	protein_codin	13.7793099	15.0658301	15.3611677	13.0471099
ENSG000001	RBM48	protein_codin	11.2168602	6.24385297	13.888042	8.20747989
ENSG000001	CDK6	protein_codin	83.1953659	90.0792892	0	37.0216968
ENSG000000	CDK14	protein_codin	1.19309512	0	0	0
ENSG000000	ANKIB1	protein_codin	33.525321	154.285529	162.635025	45.5318073
ENSG000000	VPS41	protein_codin	13.4833301	41.6440519	21.9976037	5.08318406
ENSG000000	ANLN	protein_codin	23.9655822	82.9531824	321.286925	8.85755462
ENSG000001	EIF4H	protein_codin	0	0	0	0
ENSG000000	GTF2IRD1	protein_codin	0	0	0	0
ENSG000000	BUD23	protein_codin	10.6609403	50.6565655	42.0067791	7.20766935
ENSG000000	ADAM28	protein_codin	2.32696156	14.3636179	0.62653073	10.2149171
ENSG000000	STMN4	protein_codin	0.4192379	0	0	0
ENSG000001	DMTN	protein_codin	0	0	0	0
ENSG000000	LZTS1	protein_codin	0	0	0	0
ENSG000001	R3HCC1	protein_codin	5.52039781	0	21.2320527	6.04150402
ENSG000001	SH2D4A	protein_codin	2.0770114	15.3822886	32.2731246	7.10823264
ENSG000000	SLC18A1	protein_codin	0	0	0	2.89943371
ENSG000001	PDLIM2	protein_codin	0.65821035	19.2167666	2.36146639	3.53329261
ENSG000001	FSCN3	protein_codin	0	0	0.90860411	0
ENSG000000	ZNF800	protein_codin	3.95166302	47.1638728	43.4500172	11.5152343
ENSG000000	ACAT1	protein_codin	28.293734	0	0	0
ENSG000001	ELMOD1	protein_codin	0	0	0	1.15846745
ENSG000001	EXPH5	protein_codin	30.1586486	9.12823592	21.6372648	21.9694288
ENSG000001	ADAP1	protein_codin	0	0	0	0
ENSG000001	PMS2	protein_codin	0	0	0	0
ENSG000000	MAD1L1	protein_codin	16.7768753	56.5000286	42.1304306	15.0687905
ENSG000002	GET4	protein_codin	4.36104328	14.2861894	24.437925	3.77548743
ENSG000002	RUFY2	protein_codin	3.89076457	13.4538198	9.78867621	2.8518695
ENSG000000	HNRNPH3	protein_codin	70.1526161	122.682908	134.051808	39.8477387
ENSG000001	SLC25A16	protein_codin	0.82203065	2.97073891	9.18852531	0.98426542
ENSG000000	CCAR1	protein_codin	0	53.4709303	0	15.9056932
ENSG000000	REXO2	protein_codin	43.1900183	108.213212	68.4305776	11.2339976
ENSG000001	SQLE	protein_codin	18.4802014	21.8033612	462.416396	6.62499704
ENSG000001	SNX19	protein_codin	84.7764276	238.14883	176.851901	60.3595549
ENSG000000	BRINP1	protein_codin	0.55437776	0	0	4.12258416
ENSG000001	MAPKAP1	protein_codin	0	36.1642323	24.1269887	14.5303485
ENSG000001	GTF2H1	protein_codin	17.9002931	40.2357499	34.4644849	6.71654501

ENSG000001:	SERGEF	protein_codin	0	0	7.51701585	0.5713665
ENSG000001:	CSRP3	protein_codin	10.4627774	0	0	0
ENSG000001:	KCNC1	protein_codin	0	0	0	0
ENSG000000	PIK3C2A	protein_codin	0	0	0	7.25142392
ENSG000000:	FHIP1B	protein_codin	3.16163786	0	6.9721987	2.93330178
ENSG000001	RNF141	protein_codin	5.45707012	11.0658624	10.8406994	7.43419283
ENSG000001	HPX	protein_codin	0.1727464	0	0	0
ENSG000001	IDE	protein_codin	4.16548268	0	0	0
ENSG000000!	BTAF1	protein_codin	107.423574	176.975129	255.778373	83.824411
ENSG000001	CCNJ	protein_codin	0	0	0	0
ENSG000001	TCTN3	protein_codin	0	0	0	0
ENSG000001	CPEB3	protein_codin	0	25.3558179	0	0
ENSG000002	DAXX	protein_codin	0	20.1107822	4.54949271	0
ENSG000000!	MLN	protein_codin	0	0	0	0
ENSG000000	GLT8D1	protein_codin	1.86964862	0	33.4678989	0
ENSG000000	RBM6	protein_codin	65.5153426	4.21444501	93.0373115	57.8930953
ENSG000001:	TMEM115	protein_codin	9.05167384	26.2738808	15.7338462	8.3224844
ENSG000001	GNAI2	protein_codin	1.21186973	20.4454372	7.86370702	3.89114656
ENSG000001	HYAL1	protein_codin	1.68397515	3.07987526	0	0
ENSG000000	DOCK3	protein_codin	1.54190111	7.29981183	51.0164935	7.15159766
ENSG000000	CACNA2D2	protein_codin	0	0	0	0
ENSG000000:	ITIH4	protein_codin	2.30945479	0	1.19424879	0
ENSG000001	SLIT1	protein_codin	0	0	0	0.85098554
ENSG000001:	SFRP5	protein_codin	0	2.23478581	0.51998601	1.28448375
ENSG000001	GMEB2	protein_codin	0	44.5549385	0	2.08511046
ENSG000001	GID8	protein_codin	31.5143628	55.4242096	56.6183376	31.3856433
ENSG000001	DIDO1	protein_codin	36.8591857	69.5236194	75.4842378	28.761618
ENSG000001:	SLC2A4RG	protein_codin	14.5915	58.7908389	83.1330841	15.1641572
ENSG000002	PCMTD2	protein_codin	5.5951416	32.4977971	9.56197029	4.33460895
ENSG000001	PRPF6	protein_codin	83.8961942	373.379452	241.395542	57.9395423
ENSG000001	TIMP3	protein_codin	521.86876	418.932341	353.385264	302.649089
ENSG000001	SLC5A4	protein_codin	0.8654648	6.16044133	3.2320995	2.18810478
ENSG000001	FBXO7	protein_codin	12.0342024	0	23.7039343	11.9490687
ENSG000001	SLC5A1	protein_codin	0	0	0	5.94456083
ENSG000002	PISD	protein_codin	0	5.72004395	0	0
ENSG000001	EIF2B2	protein_codin	0.75904719	3.17526202	3.39196998	2.30679833
ENSG000001:	RNF185	protein_codin	0	0	0	0
ENSG000001	TTLL1	protein_codin	4.27609795	10.2443793	10.8022317	4.03681803
ENSG000002	MTFP1	protein_codin	0	0	14.0119885	0
ENSG000001	PATZ1	protein_codin	0	15.1246892	0	0
ENSG000001	TEF	protein_codin	3.44089694	55.9045348	0	0
ENSG000001:	B4GALNT3	protein_codin	42.5450543	48.0683388	30.2811222	24.4597108
ENSG000001:	PDE6H	protein_codin	0	0	0	0
ENSG000001:	ERP27	protein_codin	0	2.46659475	1.4553943	2.78577252

ENSG000001	ETV6	protein_codin	0	0	0	0
ENSG000001	BCL2L14	protein_codin	0	0	0	0
ENSG000001	GABARAPL1	protein_codin	20.3755544	32.2868033	31.7387214	13.0401379
ENSG000000	DNM1L	protein_codin	0	0	0	0
ENSG000001	ALG10	protein_codin	3.08579505	0	12.5295819	3.02493868
ENSG000000	BMAL2	protein_codin	0	0	0	0
ENSG000001	PLCZ1	protein_codin	0	0	0	0
ENSG000001	AEBP2	protein_codin	8.76683099	118.593687	28.8433614	7.6513593
ENSG000001	SLCO1C1	protein_codin	0	0	0	0
ENSG000001	ETNK1	protein_codin	26.718863	47.586614	114.146239	21.4096939
ENSG000001	ZCRB1	protein_codin	0	0	0	0
ENSG000001	TMEM117	protein_codin	0	5.32232826	4.46292296	0
ENSG000001	C1RL	protein_codin	4.1998765	26.8569502	10.9945718	12.5318931
ENSG000001	NDUFA9	protein_codin	9.20994423	23.9362785	11.7177392	15.0023081
ENSG000001	CLSTN3	protein_codin	3.25842708	10.3344775	31.8076928	10.4274604
ENSG000001	KLRG1	protein_codin	0	0	0	0
ENSG000001	TAPBPL	protein_codin	1.69750597	17.7874185	0	2.18868588
ENSG000001	CD27	protein_codin	0.64806261	16.3424017	0	0
ENSG000001	RBP5	protein_codin	0	2.58398633	0	0
ENSG000001	PEX5	protein_codin	1.9667201	0	28.5933145	0
ENSG000001	PEX5	protein_codin	0	0	0	0
ENSG000001	SLC38A4	protein_codin	0	0	8.0093328	0
ENSG000001	AMIGO2	protein_codin	0.49832838	0	0	6.26739051
ENSG000001	SCAF11	protein_codin	78.6970479	4.58174155	15.3703126	12.4384025
ENSG000001	ANP32D	protein_codin	0.55042281	4.29565076	0.49208376	4.07339731
ENSG000001	LLPH	protein_codin	4.60800816	12.7078876	23.3527705	5.11508244
ENSG000001	MARCHF9	protein_codin	14.4396801	10.1513177	1.70711702	3.11953179
ENSG000001	INHBE	protein_codin	2.09840567	0	0	1.75140016
ENSG000001	GLIPR1	protein_codin	41.5963454	131.787827	14.5423234	44.6724333
ENSG000001	BEST3	protein_codin	2.08444601	0	0	1.55327689
ENSG000001	PHLDA1	protein_codin	0	0	0	0
ENSG000001	TMEM19	protein_codin	15.7114063	14.3300198	45.0714463	11.2069338
ENSG000001	LGR5	protein_codin	1.81021701	0	0	0
ENSG000001	CPSF6	protein_codin	0	0	7.13904012	0
ENSG000000	SLC6A15	protein_codin	0	0	0	0
ENSG000001	TMTC3	protein_codin	51.7216078	90.2955349	185.148282	20.3806501
ENSG000001	LUM	protein_codin	416.015992	739.750387	679.104884	243.183987
ENSG000001	KERA	protein_codin	0.69747694	60.5765948	0	0.93967496
ENSG000001	TMPO	protein_codin	16.2738793	25.9970669	119.379124	10.8873898
ENSG000001	SNRPF	protein_codin	18.9024514	34.5088155	52.1608642	12.6868759
ENSG000001	AMDHD1	protein_codin	0	0	4.4738434	0
ENSG000001	NEDD1	protein_codin	9.49552266	19.8262831	15.6507574	1.23330793
ENSG000001	SYCP3	protein_codin	0.6327357	0	1.00442197	0
ENSG000001	ASCL1	protein_codin	0	0	0.96015295	2.7015787

ENSG000001	GAS2L3	protein_codin	3.60010917	0	0	0
ENSG000001	SLC15A4	protein_codin	9.97542334	45.4352865	4.3718718	5.0523492
ENSG000001	TDG	protein_codin	0	4.17669902	0	0
ENSG000000	CHFR	protein_codin	4.25676924	27.295119	0.75790663	0
ENSG000001	SLC46A3	protein_codin	2.64768589	0	0	0
ENSG000001	CDK2	protein_codin	0	6.20794189	18.5009753	5.81290914
ENSG000001	SUOX	protein_codin	5.34023381	0	0	0
ENSG000001	SLC39A5	protein_codin	0	0	0	0
ENSG000001	CCDC65	protein_codin	0	0	0	0
ENSG000001	TARBP2	protein_codin	2.41642848	0	15.5115819	0
ENSG000001	GPR84	protein_codin	0	0	0.48385173	0
ENSG000001	NPFF	protein_codin	0	0	0	0
ENSG000001	NABP2	protein_codin	7.77608015	25.7998105	19.1547644	0
ENSG000001	SMARCC2	protein_codin	31.1608645	0	75.0691169	0
ENSG000002	N4BP2L2	protein_codin	28.37662	60.6430324	38.8512536	9.5684929
ENSG000001	MAP3K12	protein_codin	0	4.01824292	0	0
ENSG000001	ITGB7	protein_codin	0	7.80891952	0	3.77529995
ENSG000001	CSAD	protein_codin	0	0	0	3.02254059
ENSG000000	ERBB3	protein_codin	0	0	0	0
ENSG000001	LMBR1L	protein_codin	0	0	0	0
ENSG000001	MYG1	protein_codin	0	0	17.0001672	0
ENSG000001	ESYT1	protein_codin	7.71874331	50.0652747	2.71939187	0
ENSG000001	TMBIM6	protein_codin	102.078876	224.717821	140.768625	89.5119672
ENSG000001	ANKRD52	protein_codin	13.2303063	44.6023196	63.0257428	11.2596992
ENSG000001	KRT71	protein_codin	0	0	0	0.38383357
ENSG000001	RB1	protein_codin	0	0	0	6.84681277
ENSG000001	DIABLO	protein_codin	0	0	2.27108295	3.35729394
ENSG000001	SBNO1	protein_codin	33.5647778	0	180.175835	0
ENSG000001	VPS33A	protein_codin	5.87004415	15.2721095	0.75427439	4.11412709
ENSG000001	VPS37B	protein_codin	3.09226283	14.3539997	20.9228798	10.9775835
ENSG000001	RHOF	protein_codin	0.81493352	5.35508599	0.64096504	4.32186404
ENSG000001	DIAPH3	protein_codin	13.4295095	11.0763538	65.0673971	5.33722629
ENSG000001	SLAIN1	protein_codin	0	0	0	0
ENSG000001	RBM26	protein_codin	95.5638975	300.074739	256.497042	68.5745281
ENSG000000	PXN	protein_codin	0	0	0	6.33953857
ENSG000001	SRRM4	protein_codin	0.29259379	3.2149883	0	6.21405109
ENSG000001	METTL21C	protein_codin	0	0.83458685	0	0
ENSG000001	RNF113B	protein_codin	0	0	0	0.83654611
ENSG000001	ZIC5	protein_codin	0	2.3753631	65.1632316	0.94069217
ENSG000001	RAB20	protein_codin	5.78118857	17.5717288	3.69576259	8.77421973
ENSG000000	ANKRD10	protein_codin	16.1943322	27.2244008	34.2053411	10.9458525
ENSG000001	TTC6	protein_codin	0	3.36831893	0	0
ENSG000001	SSTR1	protein_codin	0	1.22680698	0.55487035	1.71305739
ENSG000001	CDH24	protein_codin	0	22.7935778	24.4709665	3.32034629

ENSG000001	REM2	protein_codin	0.28576991	2.62461412	1.60848016	0
ENSG000001	CBLN3	protein_codin	1.55550928	9.07845937	7.87543293	2.72800593
ENSG000000	TINF2	protein_codin	0	8.63427529	0	0
ENSG000001	NOP9	protein_codin	9.78347758	116.980993	47.1653925	12.6971043
ENSG000001	FITM1	protein_codin	0	1.39220454	1.13437588	0
ENSG000001	FANCM	protein_codin	0	0	0	0
ENSG000000	L2HGDH	protein_codin	8.43550128	9.10022751	24.9760774	4.20942991
ENSG000001	PELI2	protein_codin	15.4191378	48.8506565	53.6338774	32.1968492
ENSG000001	RTN1	protein_codin	10.4260472	21.8554171	29.043628	6.44494085
ENSG000001	ARMH4	protein_codin	33.6745299	178.523407	83.7359112	15.9405794
ENSG000001	SLC38A6	protein_codin	2.13906371	0	0	0
ENSG000001	RDH12	protein_codin	0	0.85675985	0	0.44663176
ENSG000001	RAB15	protein_codin	0	0	0	0
ENSG000001	WDR89	protein_codin	0	0	11.8445444	0
ENSG000001	ESR2	protein_codin	0	0	0	0
ENSG000001	GPR65	protein_codin	5.21525956	26.1306942	1.3695195	11.4994921
ENSG000001	PTGR2	protein_codin	2.80841748	14.5680089	16.4951595	0
ENSG000001	JDP2	protein_codin	11.2175882	61.5952724	18.2030482	9.55927416
ENSG000001	AK7	protein_codin	0	3.63728338	6.359942	3.26964751
ENSG000001	FAM181A	protein_codin	0	0	0	1.96132236
ENSG000001	ITPK1	protein_codin	2.42151589	13.6562069	4.71949563	0
ENSG000001	FBLN5	protein_codin	0	0	0	0
ENSG000001	TRIP11	protein_codin	116.238894	201.683079	156.999963	51.8837829
ENSG000001	EMC4	protein_codin	14.3165485	8.19759146	30.6170859	9.84293875
ENSG000001	DUOXA1	protein_codin	0	0	0	0
ENSG000001	ZNF280D	protein_codin	0	88.9882636	53.1791943	14.3909701
ENSG000001	TCF12	protein_codin	59.5695246	66.6182245	90.5342276	63.3365133
ENSG000001	MFAP1	protein_codin	99.9296243	332.14762	331.637614	47.713704
ENSG000001	SORD	protein_codin	0	0	0	0
ENSG000001	MYEF2	protein_codin	10.4069379	16.2164878	34.5418743	0
ENSG000001	LYSMD2	protein_codin	8.49884085	24.9248272	5.09376047	5.78884823
ENSG000001	SLC27A2	protein_codin	0	0	1.31476542	0
ENSG000001	FGF7	protein_codin	19.3098896	31.0033692	0	54.7003967
ENSG000001	HDC	protein_codin	0	0	0	0
ENSG000002	MYZAP	protein_codin	4.85946928	0	0	2.08163479
ENSG000000	RAD51	protein_codin	0	0	0	0
ENSG000001	GTF2A2	protein_codin	0	0	0	0
ENSG000001	SRP14	protein_codin	62.3860472	199.605988	173.143841	67.2131204
ENSG000001	DISP2	protein_codin	1.2133342	2.25910018	1.52428265	3.63607138
ENSG000001	TTBK2	protein_codin	24.1094279	105.407507	89.4687329	36.4841287
ENSG000001	ANP32A	protein_codin	40.0710109	321.801421	324.586219	20.6620937
ENSG000001	COMMD4	protein_codin	2.69035023	0	0	0
ENSG000001	UBE2Q2	protein_codin	63.9093174	0	63.2589313	19.201494
ENSG000001	ETFA	protein_codin	0	46.2280284	0	9.8002018

ENSG000001	BCL2A1	protein_codin	1.50517214	0	0	4.2420019
ENSG000001	TSPAN3	protein_codin	12.3862099	32.6531773	17.3463888	11.2382663
ENSG000001	SKIC8	protein_codin	4.37509171	0	0	9.90762162
ENSG000001	MAN2C1	protein_codin	7.12094028	0	21.3507515	0
ENSG000001	TLNRD1	protein_codin	6.18220304	40.418199	23.028182	11.5126045
ENSG000001	TPM1	protein_codin	0	0	0	36.3914136
ENSG000001	ARRDC4	protein_codin	26.5644462	59.432459	68.1914964	29.1671689
ENSG000001	PIF1	protein_codin	2.30212051	5.33775522	33.5895755	1.11019868
ENSG000001	USP3	protein_codin	0	0	0	0
ENSG000001	CYP11A1	protein_codin	0	0	0	0
ENSG000001	BBS4	protein_codin	8.45473665	0	0	3.98780186
ENSG000001	PML	protein_codin	0	64.7400253	0	0
ENSG000001	PML	protein_codin	2.9178093	0	0	0
ENSG000001	ADAMTS17	protein_codin	7.45551602	40.4740336	44.6052173	12.5589936
ENSG000001	CCDC33	protein_codin	0	0	0	0
ENSG000002	HEXA	protein_codin	2.92258015	33.214234	3.82094732	6.98037692
ENSG000001	SCAMP2	protein_codin	4.71380654	19.6836644	0	3.78623401
ENSG000001	RHCG	protein_codin	0	0	0	0
ENSG000001	POLG	protein_codin	0	0	0	0
ENSG000001	RLBP1	protein_codin	0	0	0	0
ENSG000001	WDR93	protein_codin	0	0	0	1.5774759
ENSG000001	TICRR	protein_codin	4.85868674	0	151.602025	0
ENSG000001	DET1	protein_codin	3.66051554	0	0	0
ENSG000001	MFGE8	protein_codin	3.6300067	26.6501751	1.77130714	0
ENSG000001	MFGE8	protein_codin	0	0	0	0
ENSG000001	ZNF710	protein_codin	5.25997182	96.1859956	12.3173908	11.5320919
ENSG000001	ST8SIA2	protein_codin	4.18359659	6.29318922	6.50939719	2.39148323
ENSG000001	FURIN	protein_codin	0	0	0	14.9202244
ENSG000001	IQGAP1	protein_codin	258.218067	523.137883	225.58264	181.067813
ENSG000001	CRTC3	protein_codin	16.5641341	56.269178	23.8307678	6.71307543
ENSG000001	EFL1	protein_codin	12.9218029	0	39.3265835	0
ENSG000001	SEC11A	protein_codin	31.063123	41.6953185	121.060569	55.9531105
ENSG000001	SEPTIN12	protein_codin	0	0	0	0
ENSG000001	ABAT	protein_codin	4.26008368	19.7891425	33.368364	10.1902248
ENSG000001	PMM2	protein_codin	16.9534601	0	20.9622479	11.0840726
ENSG000001	PRSS36	protein_codin	0	0	0	0
ENSG000001	ITGAX	protein_codin	7.45288102	0	0	0
ENSG000001	ARMC5	protein_codin	2.38911455	0	12.2607549	0
ENSG000001	FTO	protein_codin	28.4519489	0	19.0192235	0
ENSG000001	UQCRC2	protein_codin	37.1982123	52.9029671	43.6117007	23.067767
ENSG000001	CDR2	protein_codin	28.0697521	78.9459724	109.347845	21.7299635
ENSG000001	IGSF6	protein_codin	10.663743	73.1329642	7.91291916	13.2768715
ENSG000001	NKD1	protein_codin	34.1205863	28.7368497	90.4077912	12.8523015
ENSG000001	DHX38	protein_codin	48.1479942	215.029467	190.954175	42.6822814

ENSG000001	TXNL4B	protein_codin	2.92487878	5.32899542	19.2979095	4.5407222
ENSG000001	MARVELD3	protein_codin	0.65807852	2.18917856	1.11063171	0
ENSG000001	ZFHX3	protein_codin	205.444737	399.990705	328.210672	95.0031367
ENSG000001	NUDT7	protein_codin	0	0	0	0
ENSG000001	CMTM2	protein_codin	0	0	0	0
ENSG000001	CDH11	protein_codin	148.604628	272.091103	193.518956	149.268003
ENSG000001	MAP1LC3B	protein_codin	75.8470748	58.2892015	124.907351	133.362862
ENSG000001	CDH13	protein_codin	2.53619089	0	0	0
ENSG000001	ZCCHC14	protein_codin	27.628515	66.8822304	87.4030744	15.883696
ENSG000001	ADAD2	protein_codin	0	0	0	0
ENSG000001	IRF8	protein_codin	9.64066307	45.3365736	0	0
ENSG000001	ZNF174	protein_codin	5.70681475	9.59187633	14.5254879	0
ENSG000001	RPL3L	protein_codin	3.92961049	1.62241021	0	0
ENSG000001	NDUFB10	protein_codin	24.4831369	24.2113935	40.6124987	13.3130921
ENSG000001	PDPK1	protein_codin	0	0	0	0
ENSG000001	DEF8	protein_codin	3.61834077	0	0	0
ENSG000001	CBFA2T3	protein_codin	0	2.48742488	0	5.41351308
ENSG000001	GALNS	protein_codin	15.8369247	0	38.7019547	8.76925205
ENSG000001	GAS8	protein_codin	0	12.1541419	13.6240525	4.3462024
ENSG000001	SPG7	protein_codin	0	0	0	0
ENSG000001	MED9	protein_codin	7.96224206	20.3728406	13.6473004	8.68611688
ENSG000001	NCOR1	protein_codin	0	0	3.78047793	0
ENSG000001	COPS3	protein_codin	18.0734903	21.8173319	10.2884896	4.83157364
ENSG000001	GID4	protein_codin	6.73039684	12.9835208	9.43375283	8.25111463
ENSG000001	CPNE7	protein_codin	0	0	0	0
ENSG000001	PHF12	protein_codin	3.14815023	65.8584608	4.88930655	10.8894765
ENSG000000	PIGS	protein_codin	0	0	0	0
ENSG000001	NEK8	protein_codin	2.60179601	11.1870761	9.32612432	0.82338821
ENSG000001	DPEP3	protein_codin	0	0	0	0.87216704
ENSG000001	DPEP2	protein_codin	0	1.00878195	0	0
ENSG000001	GFOD2	protein_codin	3.68522036	0	0	2.79631219
ENSG000001	NOB1	protein_codin	14.5258881	54.040293	73.1597448	11.9353746
ENSG000001	PRPSAP2	protein_codin	9.69762597	0	6.06708611	3.53370916
ENSG000001	B9D1	protein_codin	0	0	0	0
ENSG000001	UNC45B	protein_codin	6.74090365	0	0	4.25643549
ENSG000000	TSPOAP1	protein_codin	14.2034525	84.9199083	51.8775407	0
ENSG000001	PCTP	protein_codin	9.94259781	18.3460356	19.8791194	0
ENSG000001	OR4D1	protein_codin	0	0	0	0
ENSG000001	KIF2B	protein_codin	0.64831106	0	0	4.33791205
ENSG000000	EPN3	protein_codin	0.41468121	0	0	1.64116976
ENSG000001	MTNAP1	protein_codin	0	21.0742957	0	0
ENSG000001	TOB1	protein_codin	0	0	0	12.8070419
ENSG000001	SPATA22	protein_codin	0	0	0	0
ENSG000001	SGSM2	protein_codin	11.2446978	53.7966812	31.2385579	25.0817122

ENSG000001	LRRC46	protein_codin	0.30972726	0	0	1.35878781
ENSG000001	SSH2	protein_codin	17.945894	0	18.7387928	18.8737749
ENSG000001	RHBDL3	protein_codin	0	0	0	0
ENSG000001	SPACA3	protein_codin	0	0	0	0
ENSG000001	ABCA8	protein_codin	0	41.1415994	0	0
ENSG000001	ABCA10	protein_codin	19.9073329	0	8.48604989	4.92426437
ENSG000001	MPP2	protein_codin	0	0	0	1.6081275
ENSG000001	G6PC3	protein_codin	2.05894934	29.9106562	0	0
ENSG000001	CLTC	protein_codin	4.2973336	7.64022117	19.0379834	13.2100422
ENSG000001	CHCT1	protein_codin	0.18798494	0	0.71548575	0
ENSG000001	SS18	protein_codin	0.81870419	8.50972823	0	1.07842778
ENSG000001	SS18	protein_codin	0	0	0	0
ENSG000001	CDH2	protein_codin	24.6660695	75.536349	33.1748679	85.650438
ENSG000001	TAF4B	protein_codin	0	0	0	11.0636098
ENSG000001	AFG3L2	protein_codin	36.9324856	17.369215	50.4657577	9.80696813
ENSG000001	IMPA2	protein_codin	0	0	0	0
ENSG000001	GNAL	protein_codin	0	0	0	0
ENSG000001	SLC39A6	protein_codin	44.9863867	82.7221635	104.402551	36.2700983
ENSG000001	DTNA	protein_codin	0	0	0	0
ENSG000001	C18orf21	protein_codin	1.78941678	0	0	0
ENSG000001	GALNT1	protein_codin	91.797044	134.813972	29.5642253	97.0486601
ENSG000001	ASXL3	protein_codin	0	0	0	0
ENSG000001	ADCYAP1	protein_codin	0.32323566	0	0	0
ENSG000001	MEP1B	protein_codin	0.33909303	0	0	0
ENSG000001	SLC25A52	protein_codin	0	0	0	0
ENSG000001	GAREM1	protein_codin	13.761367	18.4937327	17.8375464	0
ENSG000001	ESCO1	protein_codin	35.4296606	76.7713148	149.562529	20.7948291
ENSG000001	GATA6	protein_codin	0.8508718	0	3.5446782	2.63590507
ENSG000000	LAMA3	protein_codin	0	0	0	0
ENSG000001	GREB1L	protein_codin	0	0	8.35403905	0
ENSG000001	RMC1	protein_codin	9.55887975	0	0	0
ENSG000001	NPC1	protein_codin	15.2300269	43.2565306	41.0966903	15.9691561
ENSG000001	MYH10	protein_codin	0	0	120.374096	61.9349075
ENSG000001	ARRB2	protein_codin	0	55.5132991	0	13.7592822
ENSG000000	NLRP1	protein_codin	1.48958418	7.28553339	0	0
ENSG000001	ZMYND15	protein_codin	0	0	0	0
ENSG000001	SAT2	protein_codin	2.80557323	0	6.87641152	2.47830369
ENSG000001	ASGR1	protein_codin	0	0	0	0
ENSG000001	PIK3R5	protein_codin	0	0	2.49497205	8.44684651
ENSG000001	TP53	protein_codin	0	0	0	0
ENSG000001	CCDC40	protein_codin	0	0	0	1.59218
ENSG000001	ARHGDIA	protein_codin	25.4670871	64.1790913	152.009176	39.9273383
ENSG000001	TTYH2	protein_codin	13.7672568	15.1371902	24.4726449	13.7998957
ENSG000001	RAB40B	protein_codin	8.81031719	9.2757645	23.1322906	0

ENSG000001	CSNK1D	protein_codin	0	0	2.46933397	0
ENSG000001	FN3KRP	protein_codin	0	5.94459559	11.7945707	0
ENSG000001	TRIM65	protein_codin	10.7325276	9.74300312	21.8015148	2.77329453
ENSG000001	CBX8	protein_codin	2.37870218	13.2484639	25.0811412	3.65708631
ENSG000001	SECTM1	protein_codin	1.01088129	34.2528424	2.63982169	1.16994703
ENSG000001	RNF157	protein_codin	9.99247699	24.4555123	0	0
ENSG000001	CEP131	protein_codin	0	0	0	0
ENSG000001	ZNF750	protein_codin	0.22784446	2.04640725	0	0.44143046
ENSG000001	CBX4	protein_codin	23.6767722	128.06382	221.859591	48.428107
ENSG000001	CBX2	protein_codin	0	0	2.79600113	0.35097944
ENSG000001	ARK2C	protein_codin	0	6.78479259	4.47808331	0
ENSG000001	DYM	protein_codin	11.0142828	7.81841632	0	8.63710974
ENSG000001	ELAC1	protein_codin	3.74901508	15.2178933	10.1183277	3.44745393
ENSG000001	MBD1	protein_codin	0	0	0	0
ENSG000001	MBD1	protein_codin	0	0	0	0
ENSG000001	TNFRSF11A	protein_codin	0	16.2961546	3.38617898	13.7084011
ENSG000001	SERPINB13	protein_codin	0	0	18.9425671	0
ENSG000001	SERPINB12	protein_codin	0	0	0	1.43090297
ENSG000001	ZCCHC2	protein_codin	0	4.40581067	8.07209301	10.61468
ENSG000001	CBLN2	protein_codin	0	0	0	0
ENSG000001	PMAIP1	protein_codin	0	0	0	1.93076057
ENSG000001	ERBB2	protein_codin	0	0	0	0
ENSG000001	KRT10	protein_codin	9.00925645	15.1326645	32.874294	12.5734559
ENSG000001	PNMT	protein_codin	0	0	0	0
ENSG000001	ARL5C	protein_codin	0	0	0	0
ENSG000001	IGFBP4	protein_codin	115.325462	504.464466	42.7485112	80.8173225
ENSG000001	TXNL4A	protein_codin	9.42120608	8.2753356	20.298513	10.1033603
ENSG000001	AKAP8	protein_codin	1.09455004	0	0	0
ENSG000001	CYP4F22	protein_codin	0	0	0.91888956	0
ENSG000001	MISP3	protein_codin	0	0.71563644	0	0
ENSG000001	SAMD1	protein_codin	0	0	0	0
ENSG000001	SLC39A3	protein_codin	0.22115811	6.3390908	10.9407562	2.57440056
ENSG000001	PLPP2	protein_codin	0	1.32426589	0	0
ENSG000001	ZNF544	protein_codin	0	7.47206736	0	0
ENSG000001	ZIM3	protein_codin	0.28510524	1.34440348	0	2.14898708
ENSG000001	PRDM15	protein_codin	0	0	3.63066129	0
ENSG000001	FEM1A	protein_codin	22.5150776	40.2154757	45.3507605	11.2986268
ENSG000001	CIB3	protein_codin	0	0	0	0
ENSG000002	CALR3	protein_codin	0	0	0.73423902	0
ENSG000001	SH3GL1	protein_codin	31.3698183	65.2793933	118.734858	20.2386416
ENSG000001	PGPEP1	protein_codin	9.35003438	36.3833252	11.2483766	10.7099377
ENSG000001	ARMC6	protein_codin	0	0	0	0
ENSG000001	DMRTC2	protein_codin	0	0	0	0
ENSG000001	CCDC97	protein_codin	15.7641203	24.5717091	19.4998009	12.1240942

ENSG000001	ZNF45	protein_codin	7.12939387	35.9913808	10.5100468	9.91466546
ENSG000002	BCKDHA	protein_codin	7.77698354	17.7660327	25.7755088	4.00579712
ENSG000001	ZFP14	protein_codin	19.3265521	45.8198096	33.1041063	6.74692519
ENSG000002	ZNF155	protein_codin	5.62165704	0	2.38817512	0
ENSG000001	SSBP4	protein_codin	0	0	1.69110536	0
ENSG000001	SMG9	protein_codin	6.603315	13.6614023	20.1207446	5.51907432
ENSG000001	PSG9	protein_codin	0	0	0	0
ENSG000001	HUNK	protein_codin	7.40127593	22.7969572	116.932204	6.87450886
ENSG000001	LRRC56	protein_codin	2.09119854	2.10354965	4.04911218	0
ENSG000001	IFNAR1	protein_codin	22.6012223	0	0	9.0412975
ENSG000001	SOD1	protein_codin	45.5413253	91.6016155	249.46536	42.153417
ENSG000001	SIK1	protein_codin	0.72436387	0	0	1.55548042
ENSG000001	DNMT3L	protein_codin	0	0	0	0
ENSG000001	SCYL1	protein_codin	12.7378306	0	0	0
ENSG000001	IL19	protein_codin	0	0	0	0
ENSG000001	EMP3	protein_codin	8.38946693	0	3.61347456	0
ENSG000001	DMWD	protein_codin	12.638892	0	36.74116	14.9898206
ENSG000001	SAE1	protein_codin	8.43917742	105.622771	0	9.93620878
ENSG000001	BCAM	protein_codin	9.19308778	27.8757157	15.0353155	14.8985259
ENSG000001	NTN5	protein_codin	0	0	1.42908642	0.92767864
ENSG000001	GEMIN7	protein_codin	1.00766631	0	0.70457135	0.79048039
ENSG000000	WDR62	protein_codin	0	0	0	0
ENSG000002	FXYD7	protein_codin	0	0.96688119	0	1.32291612
ENSG000001	ADAMTS10	protein_codin	12.0525931	0	27.882798	0
ENSG000001	SLC6A3	protein_codin	0	1.95834693	0	0
ENSG000001	RNPEPL1	protein_codin	3.6756437	6.2765205	3.59468098	7.30220074
ENSG000001	CAPN10	protein_codin	0	0	0	0
ENSG000001	CAPN10	protein_codin	0	3.06909353	0	0
ENSG000002	KIR3DL2	protein_codin	0	0	0	1.87983844
ENSG000001	ZNF581	protein_codin	0	3.0206562	0	0
ENSG000001	LILRB4	protein_codin	0	0	0	0
ENSG000001	ZNF816	protein_codin	1.03470435	0	0.52650058	0
ENSG000001	CACNG8	protein_codin	0.95496013	2.95453322	43.3633814	12.7901206
ENSG000000	EPN1	protein_codin	4.80488863	30.9374447	9.61030632	13.0280329
ENSG000002	LILRA6	protein_codin	0	0	0	3.80361084
ENSG000001	TIMM29	protein_codin	2.55142283	10.3392484	5.39221168	0
ENSG000001	FBN3	protein_codin	0	0	0	0
ENSG000001	ECSIT	protein_codin	0	0	0	0
ENSG000001	EVI5L	protein_codin	6.0244596	29.0971993	39.0626319	0
ENSG000001	TIMM44	protein_codin	5.66584953	10.0135199	34.7563167	4.85898908
ENSG000001	TM4SF5	protein_codin	0	0	0	0
ENSG000001	SLC47A1	protein_codin	0	0	0	0
ENSG000001	KLHDC4	protein_codin	10.2622619	0	0	8.42914357
ENSG000001	PSMB6	protein_codin	10.8952302	40.7355792	28.0867851	9.08348915

ENSG000001	GPR32	protein_codin	0.20556303	0	0	0
ENSG000001	ACP4	protein_codin	0.25099532	0	0	0
ENSG000001	ZNF473	protein_codin	1.8307798	0	1.22924711	0
ENSG000001	RPS11	protein_codin	334.234081	319.658789	578.838634	275.616359
ENSG000001	PTH2	protein_codin	0	0	0	0
ENSG000002	SPIB	protein_codin	0	0	0	0
ENSG000001	IGLON5	protein_codin	0.32570364	0.91174627	2.31012598	0.40885705
ENSG000001	RCN3	protein_codin	70.4999617	135.610734	84.0755005	58.2084538
ENSG000001	ZNF614	protein_codin	23.7664632	14.0717365	34.0525406	8.23403209
ENSG000001	WRAP73	protein_codin	1.45147405	0	2.3678415	0
ENSG000001	CFAP74	protein_codin	0	0	0	0.40792324
ENSG000001	PRDM16	protein_codin	1.14413712	7.78185549	5.67482969	1.79795483
ENSG000001	ARHGEF19	protein_codin	14.8842548	17.5538655	2.80821157	4.15394697
ENSG000001	PGD	protein_codin	53.7726386	170.195929	250.897019	40.998648
ENSG000001	SH3BGRL3	protein_codin	11.9854702	48.7160365	35.9245475	11.5721833
ENSG000001	IL22RA1	protein_codin	0.60390234	2.46870471	2.12733479	2.30275957
ENSG000001	ZNF593	protein_codin	0	0	0	1.75332061
ENSG000001	C1orf216	protein_codin	15.9504138	33.1217017	32.146911	9.9975369
ENSG000001	EVA1B	protein_codin	0	0	0	0
ENSG000001	PLK4	protein_codin	0	0	0	0
ENSG000001	FCN3	protein_codin	0	0	0	0
ENSG000001	ITGB3BP	protein_codin	2.89329395	0	0	1.15377901
ENSG000001	TINAGL1	protein_codin	7.39801105	33.3815459	0	0
ENSG000001	MOB3C	protein_codin	0	0	0	0
ENSG000001	CYP4B1	protein_codin	0	0	0	0
ENSG000001	SLC44A3	protein_codin	0	0	1.74354223	4.53864302
ENSG000001	FNBP1L	protein_codin	0.62795447	0	1.65530499	1.43111418
ENSG000001	CTTNBP2NL	protein_codin	26.8941222	63.0551434	31.0889121	23.7865243
ENSG000001	PSMA5	protein_codin	5.6117942	3.07798752	0.92585484	8.43922904
ENSG000001	CD53	protein_codin	11.6643771	0	1.85844213	5.95767626
ENSG000001	PROK1	protein_codin	0	0	0	0
ENSG000001	CELSR2	protein_codin	5.0310511	17.8508824	14.7182518	6.82549032
ENSG000001	GPR161	protein_codin	0	0	0	0
ENSG000001	MPC2	protein_codin	0	0	0	0
ENSG000002	SFT2D2	protein_codin	50.4843484	186.84289	156.045613	54.5085317
ENSG000001	STYXL2	protein_codin	0	2.96110248	2.51095888	0
ENSG000001	POU2F1	protein_codin	0	0	0	0
ENSG000001	ILDR2	protein_codin	5.05387985	0	18.4112652	5.8893659
ENSG000001	FCGR2A	protein_codin	25.1048995	15.1914848	0	17.0320682
ENSG000001	NUF2	protein_codin	4.07775881	22.4034781	143.717951	0
ENSG000001	PRCC	protein_codin	58.1678298	54.5618711	107.864719	15.2079233
ENSG000001	FCRL4	protein_codin	0	0	0	0.92025658
ENSG000001	TOR1AIP1	protein_codin	10.6360984	27.5039425	0	0
ENSG000001	HMCN1	protein_codin	155.092928	526.422257	210.012269	28.86338

ENSG000001	PRUNE1	protein_codin	11.1686838	0	29.9495039	0
ENSG000001	SF3B4	protein_codin	12.7012298	15.2301231	28.2591169	6.22494288
ENSG000001	CGN	protein_codin	0	0	14.5202232	4.20252742
ENSG000001	S100A11	protein_codin	49.1541717	311.673818	16.6619276	46.4803359
ENSG000001	SETDB1	protein_codin	0	0	0	0
ENSG000001	ADAMTSL4	protein_codin	0	0	0	0
ENSG000001	CTSK	protein_codin	239.107062	222.40885	0	448.520598
ENSG000001	CERS2	protein_codin	0	16.8056684	0	0
ENSG000001	ENSA	protein_codin	68.0467267	0	121.244597	18.484021
ENSG000001	POGZ	protein_codin	2.65242612	31.4080782	6.82397185	0
ENSG000001	GOLPH3L	protein_codin	25.5515452	9.12519582	22.3024693	4.44267674
ENSG000001	KCNH1	protein_codin	0	0	0	0.62276222
ENSG000001	EIF2D	protein_codin	10.7053726	8.81419158	19.3978907	13.1077752
ENSG000001	VASH2	protein_codin	0.57830413	0	1.55825712	0
ENSG000001	CRNN	protein_codin	0	0	1.0498471	2.69527815
ENSG000001	ADAM15	protein_codin	0	0	0	0
ENSG000001	JTB	protein_codin	55.8110056	25.6173903	48.6105254	18.7770975
ENSG000001	TPM3	protein_codin	32.2266745	0	5.83851947	0
ENSG000001	NUP210L	protein_codin	0.83752786	8.16972171	1.38726088	4.60605633
ENSG000001	SLC27A3	protein_codin	1.41958956	0	6.12458805	3.66361588
ENSG000001	UBAP2L	protein_codin	16.9648849	49.6211782	47.0403203	4.40139635
ENSG000001	GON4L	protein_codin	42.8166523	233.065853	37.1277286	30.3271016
ENSG000001	CREB3L4	protein_codin	0	0	7.25902302	1.93253403
ENSG000001	KCNN3	protein_codin	19.436666	27.9968353	13.3397566	9.47039666
ENSG000001	CAPN9	protein_codin	0	0	0	0
ENSG000001	ACP1	protein_codin	18.8745193	56.8590534	52.8601242	12.4354717
ENSG000001	ACP1	protein_codin	42.0821224	0	149.152575	16.520667
ENSG000001	SDE2	protein_codin	31.4432663	85.0041978	81.0273694	54.7272741
ENSG000001	ARF1	protein_codin	99.9993324	52.7718629	292.29093	92.4700627
ENSG000001	ITPKB	protein_codin	32.6759825	0	0	0
ENSG000001	CNIH3	protein_codin	0.6048313	0	0.52063218	0.87854013
ENSG000002	LEFTY1	protein_codin	0	0	1.74068222	0
ENSG000001	C1orf35	protein_codin	34.0601881	81.7377039	126.317565	11.4034426
ENSG000001	LBR	protein_codin	29.8951769	65.735058	15.5351797	24.2376037
ENSG000001	WNT9A	protein_codin	0.62736607	0	0.63957281	0.85411458
ENSG000001	EPHX1	protein_codin	9.18587969	117.560166	33.5634688	29.809856
ENSG000001	REN	protein_codin	0	1.04559201	0	0
ENSG000001	SOX13	protein_codin	0	0	0	0
ENSG000001	PPFIA4	protein_codin	0	0	0	1.09196321
ENSG000001	PLEKHA6	protein_codin	0	0	0	0
ENSG000001	ARL8A	protein_codin	8.68927731	43.506021	10.4478815	5.38794704
ENSG000001	OSR1	protein_codin	3.56610228	2.43016846	1.08038564	0
ENSG000001	GDF7	protein_codin	0.76018073	0	13.6419357	3.47553193
ENSG000001	PDIA6	protein_codin	88.7269683	29.2936235	831.528952	98.5144857

ENSG000001	RHOB	protein_codin	107.277375	47.0787443	35.5832367	146.181502
ENSG000001	ATP6V1C2	protein_codin	0	0	0	0
ENSG000001	HNRNPLL	protein_codin	8.90493431	2.02016151	24.1825863	5.82883194
ENSG000001	GALM	protein_codin	10.872171	34.8783189	0	7.26043681
ENSG000001	RPL21	protein_codin	1.40502788	0	1.18018559	3.6085692
ENSG000001	ABCG8	protein_codin	0.33094911	1.32530729	0	1.88763291
ENSG000001	CALM2	protein_codin	4.02410876	0	0	0
ENSG000001	RPS27A	protein_codin	372.566455	566.315398	1406.68474	432.693995
ENSG000001	WDPCP	protein_codin	0	3.78886849	0	0.75994586
ENSG000001	VPS54	protein_codin	12.3989877	147.973522	112.73575	11.4851107
ENSG000001	REG3G	protein_codin	0	0	0	0
ENSG000001	ETAA1	protein_codin	26.2923504	44.8893911	147.950081	12.0490921
ENSG000001	SNRPG	protein_codin	13.5889011	17.1953059	39.7187313	3.98855875
ENSG000001	CLEC4F	protein_codin	0	0	4.09576462	0
ENSG000001	MEIS1	protein_codin	4.07372186	0	0	3.07219484
ENSG000001	OTOF	protein_codin	0	0	0	0.72682859
ENSG000001	TRIM43	protein_codin	0	0	0	0.80077926
ENSG000001	CIAO1	protein_codin	9.1753226	79.155542	51.507515	15.6896493
ENSG000001	MRPS5	protein_codin	0	26.8484238	0	0
ENSG000001	ANKRD53	protein_codin	0	0	0	0
ENSG000001	TPRKB	protein_codin	0	0	10.7811172	0
ENSG000001	NAT8	protein_codin	0	0	0	0
ENSG000001	EXOC6B	protein_codin	20.8459715	42.5550297	113.991947	35.4949813
ENSG000001	RTKN	protein_codin	0	0	0	1.81446363
ENSG000001	SFXN5	protein_codin	5.42026308	14.5573028	17.8287369	0
ENSG000001	TEX261	protein_codin	13.7751264	36.3944079	60.7300354	11.3607465
ENSG000001	DUSP11	protein_codin	10.7552548	0	5.86243209	0
ENSG000001	SULT1C4	protein_codin	0.35426472	10.7073816	0	3.60027598
ENSG000000	RGPD5	protein_codin	0	0	0	0
ENSG000001	MALL	protein_codin	0	0	0	0
ENSG000001	RALB	protein_codin	48.8393215	81.8023501	26.3436745	34.4609771
ENSG000001	C1QL2	protein_codin	0	0	0	0
ENSG000001	TMEM177	protein_codin	0	0	0	0
ENSG000001	SLC20A1	protein_codin	13.9141154	74.6794653	68.6056944	31.1589332
ENSG000001	FBLN7	protein_codin	1.56293887	12.5576518	18.3881491	0.99671516
ENSG000001	CNGA3	protein_codin	0	0	0	2.63192154
ENSG000001	FAHD2B	protein_codin	6.89136281	6.34056409	16.1085845	0
ENSG000001	UBXN4	protein_codin	164.250343	613.769262	272.354687	131.887174
ENSG000001	NXPH2	protein_codin	0.51928226	0	22.9274838	1.95581632
ENSG000001	GPR17	protein_codin	0	0	0	0
ENSG000001	POLR2D	protein_codin	13.2420951	29.2880217	26.4707028	8.63833305
ENSG000001	AMMECR1L	protein_codin	0	10.8268071	0	17.1844504
ENSG000001	SLC4A10	protein_codin	0	0	2.39264013	0
ENSG000001	SCRN3	protein_codin	8.70216763	0	3.6453982	0

ENSG000001	WIPF1	protein_codin	13.3116416	125.575476	50.7520753	0
ENSG000001	LNPK	protein_codin	28.3531892	40.4225003	73.4218801	16.092144
ENSG000001	TMEFF2	protein_codin	0	0	3.19165102	0.97063753
ENSG000001	UBR3	protein_codin	84.7963428	104.530565	374.291544	47.0888102
ENSG000002	KLHL23	protein_codin	0	0	1.9350745	1.09067071
ENSG000001	METTL21A	protein_codin	0	0	0	0
ENSG000001	UNC80	protein_codin	0	0	44.7784554	7.05709324
ENSG000001	PTH2R	protein_codin	0	0	0	0
ENSG000001	NRP2	protein_codin	28.0456867	70.1523465	82.9180421	26.0882601
ENSG000001	CPO	protein_codin	0	0	0	0.86898876
ENSG000000	CASP10	protein_codin	0	0	0	0
ENSG000001	ABCA12	protein_codin	1.6847026	0	0	10.3686249
ENSG000001	SUMF1	protein_codin	12.0694462	29.0372885	35.8933092	7.91513835
ENSG000001	NYAP2	protein_codin	3.14119644	7.676372	0.94005827	3.47649397
ENSG000001	ACKR3	protein_codin	4.97361069	31.2253733	2.25535748	26.3851683
ENSG000001	UBE2F	protein_codin	7.43301261	6.33650918	5.3805074	4.24756513
ENSG000001	HES6	protein_codin	0	0	2.02395062	0
ENSG000001	ANKMY1	protein_codin	0	0	0	0
ENSG000001	PPP1R7	protein_codin	0	2.39505435	0	2.41071612
ENSG000001	DIS3L2	protein_codin	3.38670289	26.6493868	18.4612519	10.3461328
ENSG000001	CPNE9	protein_codin	0	0	0	0
ENSG000001	TAMM41	protein_codin	0	0	7.64999943	0
ENSG000001	VGLL4	protein_codin	2.26661952	2.69608484	0	0
ENSG000001	RAB5A	protein_codin	30.0000035	70.4421125	25.2761164	32.9353328
ENSG000001	RETREG2	protein_codin	0	14.5939674	21.3853319	0
ENSG000001	CTDSP1	protein_codin	0	24.9736143	18.1981912	1.98797865
ENSG000001	SLC4A3	protein_codin	0	0	0	2.65353496
ENSG000001	CNOT9	protein_codin	16.4826717	27.6203741	60.8751498	12.7508249
ENSG000001	MARCHF4	protein_codin	0	1.88955813	0.94440258	0.9015114
ENSG000001	DNPEP	protein_codin	4.69801611	0	8.6101536	6.40807636
ENSG000001	PNKD	protein_codin	0	0	0	0
ENSG000001	DYNC1LI1	protein_codin	34.8086152	46.6512943	28.4938667	20.9547361
ENSG000001	RBMS3	protein_codin	0	0	0	0
ENSG000001	GASK1A	protein_codin	0	0	0	0.90263753
ENSG000001	CSRNP1	protein_codin	8.36474351	3.14384326	0	96.2533371
ENSG000000	SEC22C	protein_codin	2.09692214	3.26079515	0	0
ENSG000001	SLC22A14	protein_codin	0	0	0	0
ENSG000001	CTDSPL	protein_codin	3.73200137	46.5086733	18.8537815	12.9900926
ENSG000001	STAC	protein_codin	0	0	0.51155749	1.33972856
ENSG000001	IQSEC1	protein_codin	16.5165574	42.273543	31.7677677	14.8197652
ENSG000001	RPL32	protein_codin	113.391319	752.803212	614.467811	225.409222
ENSG000001	ARL6IP5	protein_codin	118.394032	170.33473	80.7862165	105.250939
ENSG000001	LRIG1	protein_codin	25.6231104	36.0339001	25.9539882	5.13868464
ENSG000000	ITIH1	protein_codin	0	0	0	0

ENSG000001	LRTM1	protein_codin	0	0	0	0.89361849
ENSG000002	CNPY2	protein_codin	9.21240934	5.78146344	18.2581764	9.39947854
ENSG000001	LIMD1	protein_codin	15.7672229	30.6180643	24.8539234	8.71558441
ENSG000001	ZKSCAN7	protein_codin	0	0	0	0
ENSG000001	COL8A1	protein_codin	0	112.328982	1.0416942	0
ENSG000001	NXPE3	protein_codin	9.13572878	0	0	4.5599
ENSG000001	ADGRG7	protein_codin	0	0	0	0
ENSG000001	MYH15	protein_codin	0	0	0	0
ENSG000001	ABHD10	protein_codin	30.253393	27.4607738	44.3130653	0
ENSG000001	TAGLN3	protein_codin	0	0	0	0
ENSG000001	PLA1A	protein_codin	0	0	0	0
ENSG000001	RABL3	protein_codin	0.53669912	13.5983257	1.07821659	10.7783028
ENSG000001	CFAP91	protein_codin	1.30138316	0	25.1217505	0
ENSG000001	BOC	protein_codin	0	0	0	0
ENSG000001	ATP6V1A	protein_codin	53.9910531	84.9052538	111.140423	85.5549149
ENSG000001	MED12L	protein_codin	5.68535557	6.35968652	42.931644	0
ENSG000001	EIF2A	protein_codin	43.614729	0	88.4501914	27.594403
ENSG000001	ALDH1L1	protein_codin	0	0	0	0
ENSG000001	RNF7	protein_codin	9.70565541	13.4980876	11.041759	8.38058815
ENSG000001	TRPC1	protein_codin	15.3480228	35.962328	36.9364202	0
ENSG000002	ISY1	protein_codin	17.0261242	28.9891904	27.2749969	6.65763365
ENSG000001	FTH1	protein_codin	10.5081878	29.3183355	19.1597926	62.9119476
ENSG000001	RUBCN	protein_codin	3.96991313	56.3524032	17.6869455	0
ENSG000001	AMT	protein_codin	0	0	0	0
ENSG000001	TCTA	protein_codin	11.9705631	4.75186882	4.42627481	6.44326737
ENSG000002	ABHD14A	protein_codin	1.74923541	1.62084382	0.64433663	3.46236644
ENSG000001	NICN1	protein_codin	1.26147949	0	0	6.60259602
ENSG000001	UCN2	protein_codin	0	4.80128265	0.59807666	0
ENSG000001	STXBP5L	protein_codin	0	0	0	0
ENSG000001	EAF2	protein_codin	0	13.5111707	0	0
ENSG000001	ILDR1	protein_codin	0	0	0	0
ENSG000001	TM4SF19	protein_codin	0	0	0	2.29185966
ENSG000001	SLIT2	protein_codin	4.06402988	48.3070196	0	3.44835129
ENSG000001	AHSG	protein_codin	0	0	0	0.89063147
ENSG000001	VWA5B2	protein_codin	0	0	0	0
ENSG000001	DGKQ	protein_codin	0	0	14.0864698	0
ENSG000001	CENPC	protein_codin	14.1290007	2.83778567	9.34634094	13.1052829
ENSG000001	EPHA5	protein_codin	0	0	0	1.52769247
ENSG000001	CORIN	protein_codin	9.60840531	6.06273353	0	0
ENSG000001	ATP10D	protein_codin	45.2542425	45.9843146	109.756446	26.0333294
ENSG000001	OCIAD2	protein_codin	4.52373249	25.8832837	52.3360392	4.97799895
ENSG000001	SLC10A4	protein_codin	0	1.40526232	0	0.58361881
ENSG000001	SLC10A6	protein_codin	0	1.1895028	0	0.42175058
ENSG000001	SCD5	protein_codin	2.27480802	1.9445304	0.98923871	2.07050996

ENSG000001	ENOPH1	protein_codin	8.53389887	39.2260911	28.4179794	14.0758649
ENSG000001	CABS1	protein_codin	0	0	0	1.87511008
ENSG000001	GC	protein_codin	0	0	0	0
ENSG000001	TRMT10A	protein_codin	0	0	0	1.45532227
ENSG000001	KLHL8	protein_codin	18.2731951	30.048187	48.6193554	1.81644504
ENSG000001	PYURF	protein_codin	55.1298812	100.738317	133.701099	30.2845094
ENSG000001	TBCK	protein_codin	0	0	0	0
ENSG000001	CISD2	protein_codin	4.48570358	1.95234574	25.2663308	7.11747118
ENSG000001	DDIT4L	protein_codin	3.27269204	3.6226765	0	0
ENSG000001	AP1AR	protein_codin	2.77618889	6.06114384	6.15474802	6.16604492
ENSG000001	AFG2A	protein_codin	10.96907	18.8818456	66.1056399	13.8146311
ENSG000001	FABP2	protein_codin	0.33620967	1.75768354	0	0
ENSG000001	CCNA2	protein_codin	9.76443125	20.623209	102.172692	0.42294627
ENSG000001	USP53	protein_codin	3.66097524	0	0	10.758126
ENSG000001	SETD7	protein_codin	108.906336	132.6495	113.431651	98.3686288
ENSG000001	NAF1	protein_codin	0	0	4.96850378	7.66894864
ENSG000001	MARCHF1	protein_codin	10.0791721	0	0.84779995	0
ENSG000001	SFRP2	protein_codin	278.869352	8.39759462	0	3.7104732
ENSG000001	RPS3A	protein_codin	855.391029	452.571277	1959.902	852.320166
ENSG000001	PDGFC	protein_codin	41.8470736	17.5738105	34.9697935	24.1473548
ENSG000001	GLRA3	protein_codin	9.11668518	4.1496605	0	1.06176253
ENSG000001	ROPN1L	protein_codin	0	0	0	0
ENSG000001	NDUFS6	protein_codin	13.8239441	17.4280673	173.286725	9.64285338
ENSG000001	MARCHF6	protein_codin	26.4126211	59.994006	138.705939	21.8148689
ENSG000001	NKD2	protein_codin	4.97014292	0	96.9509543	3.26807471
ENSG000001	CDH18	protein_codin	0	0	0	0
ENSG000001	ADAMTS16	protein_codin	8.39749716	3.48964244	0	1.25339744
ENSG000001	SRD5A1	protein_codin	11.2369739	0	22.4570714	4.85041826
ENSG000001	MYO10	protein_codin	0	0	54.5452954	18.9124146
ENSG000001	OTULINL	protein_codin	60.4603071	134.329188	282.299141	22.1265853
ENSG000001	RPL37	protein_codin	68.566186	68.3907072	674.622685	50.368892
ENSG000001	SKP2	protein_codin	0	0	2.64220028	1.18227323
ENSG000001	SKP2	protein_codin	0	0	0	0
ENSG000001	OSMR	protein_codin	13.2812874	43.928598	36.652568	70.9303889
ENSG000001	UGT3A1	protein_codin	0	0	0	1.89945554
ENSG000001	PLK2	protein_codin	83.884547	51.1824872	17.5187365	65.6659978
ENSG000001	GZMA	protein_codin	5.60001907	62.5639858	0	3.746436
ENSG000001	PELO	protein_codin	8.72383455	29.980283	26.5880585	10.5478722
ENSG000001	TRIM23	protein_codin	10.1953267	0	11.6704243	0
ENSG000001	HAPLN1	protein_codin	71.0318258	34.2582475	286.964409	10.6858721
ENSG000001	BHMT	protein_codin	1.22643779	0	0	0
ENSG000001	ANKRD31	protein_codin	2.26730202	6.29238275	31.9205991	3.6658607
ENSG000001	IQGAP2	protein_codin	54.1824383	240.873046	412.43885	60.8146059
ENSG000001	CRHBP	protein_codin	1.19027925	0	0	3.83592305

ENSG000001	RASA1	protein_codin	33.4978951	119.205334	102.12606	16.6387437
ENSG000001	LIX1	protein_codin	0	0	4.31878829	4.83530877
ENSG000001	GTF2H2	protein_codin	0	0	0	0
ENSG000001	SPATA9	protein_codin	1.00634572	0	0	0
ENSG000001	TNFAIP8	protein_codin	0	16.0308273	1.787509	2.98868738
ENSG000001	FEM1C	protein_codin	23.4113606	44.071855	33.1992782	47.872659
ENSG000001	COMMD10	protein_codin	3.50894594	0	35.5585208	7.81054982
ENSG000001	MEGF10	protein_codin	0	0	6.7521694	2.15769697
ENSG000001	ADAMTS19	protein_codin	0.76508788	0	5.83530048	1.55351421
ENSG000001	YIPF5	protein_codin	40.7420875	41.8957806	33.7629721	19.3716083
ENSG000001	ARHGAP26	protein_codin	0	91.5017046	0	0
ENSG000001	LECT2	protein_codin	0	0	0	0
ENSG000001	SLC25A48	protein_codin	0.30198337	1.07232241	1.30699765	0.43363186
ENSG000001	IL9	protein_codin	0	0	0	0
ENSG000001	TIMD4	protein_codin	0	0	0	0
ENSG000001	RNF145	protein_codin	0	0	0	0
ENSG000001	GABRA6	protein_codin	0	0	0	0
ENSG000001	GABRB2	protein_codin	0	0	0	0
ENSG000001	SPINK7	protein_codin	0	0	0	0
ENSG000001	PCYOX1L	protein_codin	3.8280808	0	0	0
ENSG000001	GLRA1	protein_codin	0	2.21730199	0	0
ENSG000001	ZNF300	protein_codin	2.91062519	0	15.5752029	1.11583235
ENSG000001	N4BP3	protein_codin	2.6413045	11.6300855	7.7061113	8.87176741
ENSG000001	NHP2	protein_codin	1.51707521	4.16544264	14.3374071	0
ENSG000000	ADAMTS2	protein_codin	5.94081516	0	0	1.46201177
ENSG000001	FGF18	protein_codin	4.90416212	0	5.42183291	0
ENSG000001	KCNMB1	protein_codin	9.04970329	4.66333148	0	3.77053064
ENSG000001	MYLK4	protein_codin	1.60567144	0	0	0
ENSG000001	FAM217A	protein_codin	0.2984495	1.25538982	0	0
ENSG000001	FARS2	protein_codin	9.67947859	28.2110789	21.1053144	7.50341426
ENSG000001	CDKAL1	protein_codin	23.8370877	64.6156413	129.879492	24.1692647
ENSG000001	PSD2	protein_codin	0	1.06792259	0	1.32231085
ENSG000001	LRRTM2	protein_codin	4.41609845	6.91248775	1.42128179	5.74793776
ENSG000001	ZMAT2	protein_codin	76.0419623	222.511874	129.400162	51.3901509
ENSG000001	GFRA3	protein_codin	0	0	0	0
ENSG000001	MRS2	protein_codin	0	0	0	0
ENSG000001	H2BC1	protein_codin	0	0	0	0
ENSG000001	TRIM7	protein_codin	0.34736665	0	1.43477429	0
ENSG000001	HIGD2A	protein_codin	3.51427869	20.9434527	4.35153385	2.3950741
ENSG000001	PLA2G7	protein_codin	0	38.854644	0	0
ENSG000001	RNF44	protein_codin	8.60073805	28.1981186	18.6985621	8.68889956
ENSG000001	MMUT	protein_codin	26.5009236	43.5355906	51.5816405	19.4212747
ENSG000001	ABT1	protein_codin	13.6398974	44.7922422	23.4371228	7.1768143
ENSG000001	PPP1R18	protein_codin	7.717186	212.295823	3.44351703	26.517526

ENSG000001	DAAM2	protein_codin	41.820433	259.621334	279.647679	34.1874497
ENSG000001	PRIM2	protein_codin	3.2785213	14.5849339	23.1966882	2.56109165
ENSG000001	MLIP	protein_codin	0	0	0	0
ENSG000001	HMGCLL1	protein_codin	0	3.73637579	3.85253094	0
ENSG000001	SCUBE3	protein_codin	46.2053241	12.2406844	23.7801367	15.6470931
ENSG000001	FGD2	protein_codin	4.48831246	40.6788605	0	4.97061831
ENSG000001	ANO7	protein_codin	0	0	0	0
ENSG000001	CRIP3	protein_codin	0	0	0	0
ENSG000001	NFKBIE	protein_codin	0	0	0	0
ENSG000001	CYP39A1	protein_codin	1.15835134	0	8.88804566	0
ENSG000001	PHIP	protein_codin	10.9758752	37.4005462	4.99532488	8.13409591
ENSG000001	HMGN3	protein_codin	38.3506368	55.4499451	64.4843594	10.0006376
ENSG000001	MMS22L	protein_codin	0	0	0	0
ENSG000001	PM20D2	protein_codin	27.4527391	28.0395349	85.1843131	9.93191289
ENSG000001	CD164	protein_codin	0	0	0	0
ENSG000001	TBC1D32	protein_codin	11.305847	21.6221943	0	0
ENSG000001	CLVS2	protein_codin	1.3911921	3.28261362	2.63421058	2.45968783
ENSG000001	GPR6	protein_codin	0	0	0	0
ENSG000001	TAAR2	protein_codin	0.37313854	0	0	0
ENSG000001	ARG1	protein_codin	0	0	0	0.37842771
ENSG000001	TAAR6	protein_codin	0	0	0	1.65998305
ENSG000001	TAAR8	protein_codin	0	0	0.69754893	2.39317152
ENSG000001	TAAR1	protein_codin	0	0	0	1.25245871
ENSG000001	SLC18B1	protein_codin	5.1967535	3.62110352	10.3761391	5.89169284
ENSG000001	SLC2A12	protein_codin	2.70871604	2.3928178	43.0145121	3.71953751
ENSG000001	SHPRH	protein_codin	1.1270706	0	0	4.18061175
ENSG000001	AIG1	protein_codin	0	0	0	2.08258565
ENSG000001	TIAM2	protein_codin	2.36805804	58.0831642	17.4066626	11.7032087
ENSG000001	QKI	protein_codin	0	0	0	0
ENSG000001	PNLDC1	protein_codin	0	0	0	0
ENSG000001	SLC22A3	protein_codin	3.71037173	6.25068466	11.3380224	29.3806318
ENSG000001	VWDE	protein_codin	0	0	0	2.93514755
ENSG000001	GNA12	protein_codin	17.2593594	62.4909598	37.3936993	13.0718909
ENSG000001	ESYT2	protein_codin	33.6458445	76.921973	12.91355	0
ENSG000000	GGCT	protein_codin	0	0	7.7173818	5.55633714
ENSG000001	FERD3L	protein_codin	0	2.5989063	0	1.27232796
ENSG000001	EGFR	protein_codin	51.8611051	146.042648	115.236381	0
ENSG000001	CDCA5	protein_codin	2.94557794	6.26838805	24.6207631	0
ENSG000001	IGFBP1	protein_codin	0	0	0	0
ENSG000002	KCTD7	protein_codin	12.8759625	0	0	0
ENSG000001	SSC4D	protein_codin	0.25062795	0	0	0.39097328
ENSG000001	POMZP3	protein_codin	5.35120357	0	11.6933192	1.7683175
ENSG000000	PHTF2	protein_codin	0	0	0	0
ENSG000001	CCT6A	protein_codin	74.6940483	166.534012	315.620306	40.3484621

ENSG000001·	PSPH	protein_codin	0	2.94235355	13.2894668	0
ENSG000001	SUMF2	protein_codin	0	0	5.96787778	0
ENSG000000	LAT2	protein_codin	0	0	0	7.44645296
ENSG000001·	ASB15	protein_codin	0.95107602	0	0	2.09784906
ENSG000001·	GIGYF1	protein_codin	0	0	0	0
ENSG000001·	AGBL3	protein_codin	0	0	2.66438211	3.41245032
ENSG000001·	STRA8	protein_codin	0	0	0	0
ENSG000001·	ZC3HAV1L	protein_codin	5.52477567	9.52926231	21.9953118	2.01478555
ENSG000001·	TMEM140	protein_codin	4.75470877	50.6272102	1.99018838	8.57375776
ENSG000001·	EPHA1	protein_codin	1.63243344	0	0	0
ENSG000001·	NOM1	protein_codin	25.2009791	93.9745799	77.1414884	11.9736319
ENSG000001·	ASB10	protein_codin	0	0	0	0
ENSG000001·	NLGN4X	protein_codin	2.19890289	0	1.32996695	10.7245799
ENSG000001·	DENND2A	protein_codin	0	0	0	0
ENSG000001·	TMEM47	protein_codin	28.4398832	26.7709766	2.53627224	20.041841
ENSG000001	SPIN2B	protein_codin	3.45217139	0	1.869474	1.37948552
ENSG000001·	CCNB3	protein_codin	1.08395737	2.82159597	0	9.15328588
ENSG000001	CDK16	protein_codin	0	0	0	0
ENSG000002	ZNF630	protein_codin	0	0	1.175706	0
ENSG000001·	CHST7	protein_codin	4.12965202	9.3526976	3.00295968	5.50613558
ENSG000001·	NDUFB11	protein_codin	0	0	0	0
ENSG000001·	RAB41	protein_codin	0	0	0	0
ENSG000001·	TAF1	protein_codin	0	0	0	0
ENSG000001·	NONO	protein_codin	0	0	0	0
ENSG000001·	EBP	protein_codin	6.33653408	0	11.3138677	2.92714386
ENSG000001·	AWAT2	protein_codin	0	0	0	0
ENSG000001·	SNX12	protein_codin	0	0	0	0
ENSG000001·	IL2RG	protein_codin	0	0	0	1.90946518
ENSG000001·	ZNF711	protein_codin	24.5307742	19.2133685	96.1072481	6.54482036
ENSG000001·	CPXCR1	protein_codin	0	0	0	0
ENSG000001	SYTL4	protein_codin	0	0	0	0
ENSG000001·	RIPPLY1	protein_codin	0	0	0	0
ENSG000001	TBC1D8B	protein_codin	15.3432225	0	0	0
ENSG000001·	FRMPD3	protein_codin	0	0	0	0
ENSG000001·	HTR2C	protein_codin	0	0	0	0
ENSG000001	UPF3B	protein_codin	28.3083071	0	86.3935157	0
ENSG000001·	DOCK11	protein_codin	79.1821553	288.512983	50.0822827	69.6978282
ENSG000001·	DOCK11	protein_codin	3.53675628	0	0	0
ENSG000001·	ARHGAP36	protein_codin	0	0	0	0
ENSG000001·	GPR119	protein_codin	0	1.09386157	0.48598506	0.57546987
ENSG000001	CT55	protein_codin	0	0	0	0
ENSG000001·	MFHAS1	protein_codin	9.01525287	29.5346343	15.8303286	16.0215446
ENSG000001	DLC1	protein_codin	54.0831292	163.399813	77.3322572	150.584361
ENSG000001·	FBXO25	protein_codin	0	0	0	0

ENSG000001	MAGEA4	protein_codin	0	0	0	0.89469093
ENSG000000	SLC18A1	protein_codin	0	3.50125971	0	0
ENSG000001	ATP6V1B2	protein_codin	43.5547928	36.2426837	27.5872644	60.4756692
ENSG000001	ADRA1A	protein_codin	0	0	0	0
ENSG000001	CHRNA6	protein_codin	0	0	0	1.05878117
ENSG000001	GNRH1	protein_codin	2.14161693	8.93134989	2.58724293	1.60027485
ENSG000001	BIN3	protein_codin	9.76015974	15.3541209	6.59128636	8.4771205
ENSG000001	DOK2	protein_codin	0	28.4847826	0	6.93636657
ENSG000001	TNFRSF10B	protein_codin	35.1176827	49.725123	25.249452	19.8621619
ENSG000001	DOCK5	protein_codin	31.7929206	122.865489	52.6777531	59.3711812
ENSG000001	STAR	protein_codin	0	0	0	0
ENSG000001	ST18	protein_codin	0	5.29282543	0	0
ENSG000001	RGS20	protein_codin	0	0	0	0
ENSG000001	TACC1	protein_codin	5.97510277	104.114001	10.9733661	25.4835644
ENSG000001	GINS4	protein_codin	2.40800995	3.67866209	45.9273789	2.75163786
ENSG000001	ARMC1	protein_codin	23.0320953	18.3586826	35.252122	0
ENSG000001	DNAJC5B	protein_codin	0.39425995	0	0	0.57424402
ENSG000001	CRH	protein_codin	0	0	0	0.44190075
ENSG000001	TRIM55	protein_codin	2.10942566	0.96904778	0	0
ENSG000001	ADHFE1	protein_codin	2.83041442	0	0	0
ENSG000001	MRPS28	protein_codin	3.28543863	0	8.89756585	3.35720207
ENSG000001	LACTB2	protein_codin	6.67686332	25.1795324	0	6.73960193
ENSG000001	PRDM14	protein_codin	0	0.96323322	0	2.47636907
ENSG000001	TERF1	protein_codin	0	26.5981722	0	3.50582652
ENSG000001	TERF1	protein_codin	0	0	0	2.3144401
ENSG000001	SLC26A7	protein_codin	0.9189026	9.8045345	0	0
ENSG000001	PSKH2	protein_codin	0	0	0	0.65887991
ENSG000001	SYBU	protein_codin	0	0	9.49299069	0
ENSG000001	LRP12	protein_codin	8.59589026	0	5.96078648	11.7921496
ENSG000001	RSPO2	protein_codin	0	0	0	0
ENSG000001	EIF3H	protein_codin	32.005325	151.662114	347.633377	55.9826339
ENSG000001	NDUFB9	protein_codin	30.3140296	65.6393333	398.788251	19.6775895
ENSG000001	TATDN1	protein_codin	0	0	10.9820725	7.75119954
ENSG000001	FAM83A	protein_codin	0	0	0	0
ENSG000001	C8orf76	protein_codin	7.49749899	13.9604037	68.3958393	0
ENSG000001	GSDMC	protein_codin	0	0	0.5434411	0.9372414
ENSG000001	FAM135B	protein_codin	0	5.01567114	0	0
ENSG000001	ZNF16	protein_codin	0	0	0	0
ENSG000001	ARHGAP39	protein_codin	3.35291229	0	0	2.55228368
ENSG000001	SLC39A4	protein_codin	0	0	0	0
ENSG000001	UHRF2	protein_codin	23.671707	83.6044169	46.6251898	10.3296546
ENSG000001	RIC1	protein_codin	9.74524857	14.7945078	11.3306787	9.42807985
ENSG000001	PLIN2	protein_codin	20.5549295	17.3628862	16.860141	51.0037528
ENSG000001	CDKN2B	protein_codin	9.71729389	20.6120709	61.3146397	5.11489143

ENSG000001	IFNA1	protein_codin	0	0	0	0
ENSG000001	ADAMTSL1	protein_codin	0	0	0	0.94639585
ENSG000001	IFNK	protein_codin	0	0	0	0.37419035
ENSG000001	FBXO10	protein_codin	0	0	0	0
ENSG000001	CNTNAP3B	protein_codin	1.65241255	0	9.89428404	3.19805103
ENSG000001	SIGMAR1	protein_codin	0	0	23.7383236	0
ENSG000001	CEP78	protein_codin	0	0	0	0
ENSG000001	NTRK2	protein_codin	0	0	0	0
ENSG000000	TUT7	protein_codin	0	0	82.9510239	0
ENSG000000	FAM120A	protein_codin	7.12725625	129.796397	12.0720619	0
ENSG000001	AOPEP	protein_codin	0	0	0	0
ENSG000001	OR13C4	protein_codin	0.26936195	0	0	0.48699442
ENSG000001	ZNF462	protein_codin	109.919898	316.761413	251.917748	80.883498
ENSG000001	OR1K1	protein_codin	0	0	0	0
ENSG000001	OLFM1	protein_codin	0	0	5.03608348	0
ENSG000001	ASB6	protein_codin	5.99520999	31.1439487	12.6982012	0
ENSG000001	ASB6	protein_codin	0	0	3.64158372	2.98941819
ENSG000001	PTGES2	protein_codin	0	4.50346994	0	0
ENSG000001	CIZ1	protein_codin	0	0	10.042483	0
ENSG000001	LCN2	protein_codin	0	0	0	0
ENSG000001	PAEP	protein_codin	0	0	0	0
ENSG000001	IDI2	protein_codin	6.7380925	0.79115326	0	1.82955736
ENSG000001	LCN9	protein_codin	0	0	0	0
ENSG000001	PNPLA7	protein_codin	0	0	0	2.9618753
ENSG000001	SEC16A	protein_codin	0	0	0	0
ENSG000001	DPH7	protein_codin	5.9337365	21.1426057	3.36396831	3.14730181
ENSG000001	CACNA1B	protein_codin	0.83586092	0.86138303	0	0
ENSG000001	NACC2	protein_codin	0	0	25.4640442	13.0447522
ENSG000001	PROSER2	protein_codin	0.88851421	6.87791971	3.77674019	11.6770734
ENSG000001	USP6NL	protein_codin	13.3473297	0	0	0
ENSG000001	MINDY3	protein_codin	11.8513027	39.5761879	25.6651541	13.3840075
ENSG000002	CCDC7	protein_codin	0	0	0	0
ENSG000001	NRBF2	protein_codin	26.8716134	0	62.539436	17.4684951
ENSG000001	TMEM26	protein_codin	0	0	0	0
ENSG000001	PBLD	protein_codin	0.70659232	2.22812437	3.89268417	2.90713568
ENSG000001	HERC4	protein_codin	9.9498043	0	12.7994249	0
ENSG000001	GLUD1	protein_codin	23.5941014	61.8165073	12.7119357	4.01349445
ENSG000001	HTR7	protein_codin	1.72863351	11.6637992	1.98831759	0.88669396
ENSG000001	RPP30	protein_codin	0	0	0	0
ENSG000001	ADD3	protein_codin	84.0131954	122.188262	114.686315	31.7474521
ENSG000001	VAX1	protein_codin	0.23998288	2.8612608	0.49112032	1.26247112
ENSG000001	NPFFR1	protein_codin	0.81310239	3.42813398	0.51715711	8.18101361
ENSG000001	TCF7L2	protein_codin	0	48.3027789	0	4.26516635
ENSG000000	SORBS1	protein_codin	0	0	0	0

ENSG000001	FUOM	protein_codin	1.90402243	0	0	0
ENSG000001	PAOX	protein_codin	0	0	0	0
ENSG000001	CFAP43	protein_codin	0	0	0	0
ENSG000001	PPRC1	protein_codin	10.4794963	28.1263114	22.6045746	22.0837034
ENSG000001	ITPRIP	protein_codin	0	0	0	0
ENSG000001	BCCIP	protein_codin	18.0841264	138.842509	59.6861794	9.68478703
ENSG000001	BTBD10	protein_codin	14.7983796	10.5740371	25.2646081	6.22636432
ENSG000001	ADM	protein_codin	0	14.3777154	3.05099735	25.4429362
ENSG000001	GAS2	protein_codin	0	0	0	0
ENSG000001	LIN7C	protein_codin	19.383758	58.0337613	32.736079	19.8242113
ENSG000001	LRRC4C	protein_codin	5.54638479	8.74125555	0	0
ENSG000001	IMMP1L	protein_codin	0	0	0	0
ENSG000001	SAA4	protein_codin	0.25149455	0	0	0
ENSG000001	CARS1	protein_codin	19.4370048	75.7142773	27.3897122	0
ENSG000001	PGAP2	protein_codin	0	0	0	0
ENSG000001	TUT1	protein_codin	0	0	0	0
ENSG000001	SCGB1A1	protein_codin	0	0	0	0
ENSG000001	TRIM6	protein_codin	0	0	4.16428224	1.18175396
ENSG000001	ZNF214	protein_codin	5.92398642	17.6803342	32.1625325	0
ENSG000001	TNNT3	protein_codin	32.3416536	0	1.15937754	0
ENSG000001	ZNF215	protein_codin	0	0	0	0
ENSG000001	HSD17B12	protein_codin	28.6963335	29.8910635	63.4066634	13.7740938
ENSG000001	SLC1A2	protein_codin	1.18226366	0	0	10.5662241
ENSG000000	CD44	protein_codin	2.37683869	0	0	0
ENSG000000	CD44	protein_codin	0	45.127844	1.83304287	4.81514606
ENSG000001	GLYAT	protein_codin	0	0	0	0
ENSG000001	SERPING1	protein_codin	41.9698117	333.413153	0	152.509166
ENSG000001	OR5F1	protein_codin	0	0	0	0
ENSG000001	SSRP1	protein_codin	53.5320894	213.180664	283.848483	30.9315532
ENSG000002	TMX2	protein_codin	0	0	29.4324767	0
ENSG000001	SLC43A1	protein_codin	0	0	0	0
ENSG000001	CSTPP1	protein_codin	1.10146685	0	0	0
ENSG000001	HIKESHI	protein_codin	0	0	9.29144982	2.29696995
ENSG000001	SESN3	protein_codin	0	58.9520681	8.22716429	0
ENSG000001	ENDOD1	protein_codin	33.2573963	40.8933794	87.7641208	19.8768251
ENSG000001	CCDC82	protein_codin	0	0	0	8.04528206
ENSG000000	ACER3	protein_codin	0	0	0	0
ENSG000001	TENM4	protein_codin	24.5112432	109.733053	106.594814	55.5453296
ENSG000001	PAK1	protein_codin	0	8.87764298	29.9038289	8.75175582
ENSG000001	RPS3	protein_codin	0	0	0	2.22247054
ENSG000001	ZC3H12C	protein_codin	0	17.2435075	24.8223565	0
ENSG000001	C11orf52	protein_codin	0	0	0.46949136	0
ENSG000001	NPAT	protein_codin	13.424296	0	20.8702859	9.61697628
ENSG000001	ATM	protein_codin	0	9.9499606	0	0

ENSG000001	AASDHPPT	protein_codin	12.9161874	14.4964508	17.6443241	0
ENSG000001	PLCB4	protein_codin	0	0	0	0
ENSG000001	LAMTOR1	protein_codin	10.5768384	19.3336198	42.8519933	11.3891185
ENSG000001	ST14	protein_codin	2.88697704	5.33584714	2.27794079	4.76146226
ENSG000000	APLP2	protein_codin	0	371.430646	0	0
ENSG000001	GGTLC1	protein_codin	0	0	0	0
ENSG000000	ZNF343	protein_codin	15.0361711	0	0	0
ENSG000001	OVOL2	protein_codin	0	0	0	0
ENSG000000	SMOX	protein_codin	0	0	0	0
ENSG000001	MTA2	protein_codin	6.30503722	69.3217385	46.6105579	26.3604749
ENSG000001	TMEM138	protein_codin	1.42963681	0	0	2.39746411
ENSG000002	FADS3	protein_codin	4.70400138	15.8179324	1.45771984	5.65686578
ENSG000001	ROM1	protein_codin	0	0	0	2.87130199
ENSG000001	MYRF	protein_codin	0	5.68994069	0	0
ENSG000001	FADS2	protein_codin	0	10.127967	21.3267108	10.0084466
ENSG000001	EML3	protein_codin	0	0	10.3210525	0
ENSG000001	INCENP	protein_codin	24.3904345	86.266767	141.887896	11.2716594
ENSG000001	ZP1	protein_codin	0	0	0	0.53067103
ENSG000001	OOSP2	protein_codin	0	0	0	0
ENSG000001	WDR74	protein_codin	0	0	23.9875484	0
ENSG000001	MS4A3	protein_codin	0	0	0	0
ENSG000001	PLCH2	protein_codin	0	0	0	0
ENSG000001	NINL	protein_codin	18.3761613	79.7506757	41.5568032	2.46852508
ENSG000001	MS4A2	protein_codin	0	18.9629094	0	44.436371
ENSG000001	BSCL2	protein_codin	0	0	0	0
ENSG000001	EI24	protein_codin	8.38773775	12.5766373	4.74373767	0
ENSG000001	CHEK1	protein_codin	0	6.49682633	0	0
ENSG000001	FEZ1	protein_codin	0.33384084	2.61155718	0	1.49311294
ENSG000001	ESAM	protein_codin	0	48.7070173	4.05513576	9.10711846
ENSG000001	CEP164	protein_codin	46.8819394	138.33394	34.904535	10.3211903
ENSG000001	MPZL2	protein_codin	2.66905504	17.9910685	2.11679107	4.5399727
ENSG000001	SCN2B	protein_codin	8.88099032	13.779017	14.169977	3.78503827
ENSG000001	MPZL3	protein_codin	4.82401823	8.00829037	4.97474062	2.36113134
ENSG000001	SIDT2	protein_codin	0	0	6.19919611	5.02664166
ENSG000001	TAGLN	protein_codin	13.7764319	32.2818814	0	0
ENSG000001	DUSP15	protein_codin	0	0	0	0
ENSG000001	COMMD7	protein_codin	11.9268665	33.5147993	0	0
ENSG000001	MYL9	protein_codin	31.9850611	83.1807616	25.5143134	18.6663732
ENSG000001	KIAA1755	protein_codin	1.71182467	0	0	1.76644526
ENSG000001	SLC13A3	protein_codin	0	0	0	0
ENSG000001	OCSTAMP	protein_codin	0	0	0	0.44959201
ENSG000001	MTCL2	protein_codin	25.1283484	56.0230078	46.1405203	15.4102268
ENSG000001	PIGT	protein_codin	0	0	0	6.07271885
ENSG000001	PIGT	protein_codin	0	0	44.6966095	0

ENSG000001	ERGIC3	protein_codin	4.03473768	13.0835144	5.83803115	3.71153825
ENSG000001	SPINT4	protein_codin	0	0	0	1.12058991
ENSG000001	LSM14B	protein_codin	20.2381292	22.6295047	53.7038628	10.8268956
ENSG000001	LSM14B	protein_codin	0	0	0	0
ENSG000001	CABLES2	protein_codin	1.79480267	10.6881048	6.53738258	2.62957189
ENSG000001	AIP	protein_codin	5.10813523	0	0	9.63505735
ENSG000001	LTO1	protein_codin	0	0	0	0
ENSG000001	GPHA2	protein_codin	0.34484971	0	0	0
ENSG000001	SLC22A9	protein_codin	0	1.34132128	0	0
ENSG000001	NUDT22	protein_codin	1.67915319	1.06563423	0	0
ENSG000001	FERMT3	protein_codin	0	65.5843855	0	0
ENSG000001	PLCB3	protein_codin	0	128.527453	39.3030057	25.778343
ENSG000001	MRPL49	protein_codin	0	0	9.80754349	0
ENSG000000	CAPN1	protein_codin	11.2189355	0	0	24.2979341
ENSG000001	CDC42EP2	protein_codin	0	0	2.44376264	0
ENSG000001	FAU	protein_codin	0	4.21306262	0	2.16124103
ENSG000001	TM7SF2	protein_codin	0	0	0	0
ENSG000001	VPS51	protein_codin	3.82094956	0	36.8934411	0
ENSG000001	TBX6	protein_codin	0.22901617	0	1.3356164	0.38530164
ENSG000001	PPP4C	protein_codin	2.13211523	48.0289733	6.94506	1.02981902
ENSG000001	TLCD3B	protein_codin	0	0	0	0
ENSG000001	HIRIP3	protein_codin	19.0258425	81.4787948	88.2031508	12.7321018
ENSG000001	TAOK2	protein_codin	15.2831563	7.20924792	64.1183634	16.8500305
ENSG000001	TMEM219	protein_codin	10.199277	6.7843066	5.83073796	4.10732289
ENSG000001	MMP10	protein_codin	0	1.04231414	0	0.50860261
ENSG000001	CNKSR2	protein_codin	0	0	45.2092514	0
ENSG000000	CD44	protein_codin	0	0	0	0
ENSG000001	CNTN5	protein_codin	0	0	0	4.07355144
ENSG000001	SIRPB1	protein_codin	0	0	0	3.08654341
ENSG000001	DUSP6	protein_codin	14.0106916	26.7796722	17.1190571	42.7070395
ENSG000001	KLRF1	protein_codin	0	0	0	0
ENSG000000	YBX3	protein_codin	65.55953	43.2898087	45.9723599	20.7614648
ENSG000002	PRB4	protein_codin	0	0	0	1.34448003
ENSG000001	OR8K1	protein_codin	0	0	0	0
ENSG000001	OR5M9	protein_codin	0	0	0	0
ENSG000001	CTF1	protein_codin	0.76284595	4.09990922	2.13304848	0
ENSG000001	CWC15	protein_codin	32.665028	7.8442518	75.1390014	22.4662374
ENSG000001	ARID5B	protein_codin	37.205176	281.638388	0	86.401684
ENSG000001	BLTP3B	protein_codin	56.5130264	102.269143	50.9105125	57.2667153
ENSG000001	TMEM218	protein_codin	0	0	0	0
ENSG000001	KIAA1328	protein_codin	10.1374078	0	10.9281446	6.38771737
ENSG000001	DHRS12	protein_codin	0	2.106909	0	0.57145315
ENSG000001	FAM124A	protein_codin	0	0	0.96048733	0
ENSG000001	MIA2	protein_codin	0	0	0	0

ENSG000001	MIA2	protein_codin	0	4.2336713	27.6583882	11.7451498
ENSG000001	HNMT	protein_codin	13.8348803	23.1925463	2.43385577	5.89389156
ENSG000001	HNMT	protein_codin	18.988409	33.6562573	2.1841479	10.1609002
ENSG000001	SPOPL	protein_codin	12.9530843	14.9013091	60.4433116	16.126041
ENSG000001	LYPD6B	protein_codin	0	0	1.1983288	0.55459175
ENSG000001	PDCD4	protein_codin	57.3377477	106.3226	63.4221476	40.6195427
ENSG000001	ADRA2A	protein_codin	3.91715771	2.05072819	0	3.39470221
ENSG000001	GPM6A	protein_codin	0	0	0	1.56698729
ENSG000001	WDR17	protein_codin	0	0	0	0
ENSG000001	SPATA4	protein_codin	0	0	0	0
ENSG000001	CD226	protein_codin	0	0	1.42106878	0
ENSG000001	DLG2	protein_codin	0	0	0	0
ENSG000001	CCDC83	protein_codin	0	0	0	0
ENSG000001	PRSS23	protein_codin	69.3219799	263.863604	9.15555323	75.7331747
ENSG000001	MTMR12	protein_codin	0	0	0	0
ENSG000001	PPP1R1C	protein_codin	0	0	0	0
ENSG000001	POU2AF2	protein_codin	0	0	0	0
ENSG000001	CCT5	protein_codin	7.75084741	5.97390814	45.5359894	3.36754922
ENSG000001	ATPSCKMT	protein_codin	2.26246295	0	0	0
ENSG000001	DOCK1	protein_codin	204.915366	476.550808	192.114548	108.34167
ENSG000001	DLAT	protein_codin	0	0	0	0
ENSG000001	PIH1D2	protein_codin	0	0	0	0.83180605
ENSG000001	NKAPD1	protein_codin	0	10.037557	0	0
ENSG000001	IL18	protein_codin	0	0	0	1.43111542
ENSG000001	TEX12	protein_codin	0.40437133	0	0	0
ENSG000001	PTS	protein_codin	1.73619628	0	11.6202225	2.51236737
ENSG000001	USP15	protein_codin	12.9978584	45.0531208	24.7053385	26.752349
ENSG000001	SLC5A12	protein_codin	0	0	0	0
ENSG000001	FREM2	protein_codin	4.17517745	0	184.597857	8.28469075
ENSG000001	CRIM1	protein_codin	79.692381	97.0488564	218.211453	103.241979
ENSG000001	SEC24D	protein_codin	43.8126508	0	0	42.8518448
ENSG000001	DENR	protein_codin	42.8189991	79.6171979	66.9965709	26.1632071
ENSG000001	ABCB9	protein_codin	0	0	0	0
ENSG000000	PITPNM2	protein_codin	0	12.120153	0	5.50268636
ENSG000001	RILPL2	protein_codin	9.61355953	53.3998445	7.27658735	13.6198524
ENSG000000	TRNT1	protein_codin	0	0	0	0
ENSG000001	TKTL2	protein_codin	0.32702593	0	0	0
ENSG000001	PRSS53	protein_codin	0	0	0	0
ENSG000001	SLC7A11	protein_codin	1.90371583	6.84426944	14.673869	5.13025746
ENSG000001	NOCT	protein_codin	4.20211111	9.69760603	5.60637094	29.7700453
ENSG000001	CACNA2D4	protein_codin	0	5.15500046	0	0
ENSG000001	DCP1B	protein_codin	7.99036216	0	19.1081943	6.70595225
ENSG000001	KCNA6	protein_codin	0.57222772	2.87659681	7.25959111	2.54190547
ENSG000001	THRB	protein_codin	0	0	0	0

ENSG000001	NGLY1	protein_codin	0	0	0	0
ENSG000001	NGLY1	protein_codin	0	0	0	0
ENSG000001	OXSM	protein_codin	0	3.59476647	4.87349018	0
ENSG000001	LDHC	protein_codin	0	0	0	0
ENSG000001	LDHAL6A	protein_codin	0	0	0	0
ENSG000001	TMEM86A	protein_codin	2.34858856	10.0613871	6.38733732	2.89674786
ENSG000001	NOPCHAP1	protein_codin	4.0709752	9.704771	9.59229393	2.64042969
ENSG000001	TMEM263	protein_codin	132.167594	106.993804	117.0763	52.0975579
ENSG000001	ABTB3	protein_codin	12.0266111	0	0	10.2535828
ENSG000001	ANK3	protein_codin	14.7135121	42.0201588	23.0790778	26.2757924
ENSG000001	FAM53B	protein_codin	0.45081975	19.0009196	0	1.17803963
ENSG000001	PLBD2	protein_codin	24.3098687	59.3542437	16.8839895	19.379963
ENSG000001	SLC2A13	protein_codin	34.7957272	29.6542811	44.0230581	12.8347921
ENSG000001	GXYLT1	protein_codin	1.30203228	0	10.0363057	0
ENSG000001	DIP2C	protein_codin	47.7499778	78.5955067	60.9026621	33.1696451
ENSG000001	EIF4E	protein_codin	0	0	1.53291401	0
ENSG000001	DSC2	protein_codin	0	0	0	9.07664327
ENSG000000	MAT2B	protein_codin	0	0	0	9.00441516
ENSG000001	AKAP6	protein_codin	23.9216994	50.8903595	22.4248232	66.2834806
ENSG000001	FAM177A1	protein_codin	36.3533685	17.1304347	26.6580613	24.0066213
ENSG000001	TMEM18	protein_codin	0	2.90051916	6.57296953	6.11601773
ENSG000001	THRSP	protein_codin	10.6563929	8.24224792	0	2.41489588
ENSG000001	NDUFC2	protein_codin	3.12039224	0	3.67813764	7.16397109
ENSG000001	NARS2	protein_codin	0	0	0	0
ENSG000001	MYCN	protein_codin	0.51774564	2.01263468	4.59185483	0
ENSG000001	MSGN1	protein_codin	0	1.10092013	0.59064381	0.83348397
ENSG000001	NUBPL	protein_codin	2.06698438	0	5.91589488	4.26754841
ENSG000001	ATP6V1G3	protein_codin	0	0	0	0
ENSG000001	FER	protein_codin	36.0151876	129.53696	125.784778	44.7422295
ENSG000000	NRXN3	protein_codin	0	2.69853067	0	1.31052397
ENSG000001	CEP128	protein_codin	17.9432492	106.318895	0	9.73103268
ENSG000001	CDC123	protein_codin	1.74703234	52.2179401	21.0739648	20.2293195
ENSG000001	SCLT1	protein_codin	32.8352399	79.2913555	106.911819	18.2902508
ENSG000001	C4orf33	protein_codin	5.6776118	0	0	0
ENSG000001	SLC25A31	protein_codin	0	3.63547563	0	1.90901535
ENSG000001	KHDRBS2	protein_codin	0	0	0	0
ENSG000001	PTPRO	protein_codin	0	0	0	0
ENSG000001	EPS8	protein_codin	0	0	0	0
ENSG000001	ACAD8	protein_codin	3.45256551	0	5.03583661	0
ENSG000001	VPS26B	protein_codin	7.79673883	67.469686	16.1579275	8.1805868
ENSG000001	QDPR	protein_codin	0	3.64606913	20.8602496	0
ENSG000001	MUC15	protein_codin	0	0	0	1.43443922
ENSG000001	QTRT2	protein_codin	0	28.2947176	0	0
ENSG000001	CGNL1	protein_codin	56.4539686	108.702455	67.7470305	8.21715636

ENSG000001	POU4F2	protein_codin	0	1.10386016	0	1.50664316
ENSG000001	PIGF	protein_codin	0	0	11.0419486	0
ENSG000001	PPP1R21	protein_codin	28.7317126	0	9.70287774	0
ENSG000001	WDR35	protein_codin	20.8988191	50.1856003	129.258868	14.2475925
ENSG000001	MFSD6	protein_codin	0	0	2.25024576	9.44406451
ENSG000001	ASAP2	protein_codin	0	0	0	0
ENSG000001	FLI1	protein_codin	3.68832536	97.9877428	10.1024398	15.3878697
ENSG000000	BARX2	protein_codin	0	1.09224556	0	2.23680846
ENSG000001	TMEM45B	protein_codin	0.39726689	0	0	0
ENSG000001	CENPU	protein_codin	1.44371569	21.5316126	43.4712353	4.46841773
ENSG000001	ACSL1	protein_codin	0	97.788818	0	19.9037371
ENSG000001	SLC25A4	protein_codin	4.52493779	12.0079884	3.08220115	2.90303821
ENSG000001	AMN1	protein_codin	4.2092947	0	0.72019816	0
ENSG000001	VWA8	protein_codin	7.87504733	15.4094891	46.9268183	8.42028644
ENSG000001	NBAS	protein_codin	32.613818	0	99.8530553	42.4702981
ENSG000001	ZNF385D	protein_codin	5.37232532	0	0	9.25634391
ENSG000001	GUF1	protein_codin	5.53045577	0	34.2933381	16.3501957
ENSG000001	CCDC175	protein_codin	0	1.47848973	7.96508096	0
ENSG000001	PABPC3	protein_codin	0	0	0	2.06303239
ENSG000001	ATP8A2	protein_codin	0	0	0	0
ENSG000001	FBXO4	protein_codin	3.5010288	3.27718596	7.4273872	1.42645988
ENSG000001	PARP8	protein_codin	5.15494243	11.1904266	0.91985315	8.34295935
ENSG000001	NVL	protein_codin	0	0	66.1741827	0
ENSG000001	GLT1D1	protein_codin	0.87680287	0	0	0
ENSG000001	FBXW7	protein_codin	0	56.5314708	1.8582608	1.6699928
ENSG000001	RBM46	protein_codin	0	0	0	0
ENSG000000	TXNDC16	protein_codin	15.2294906	43.3556022	39.5591906	14.0943851
ENSG000001	KANSL1L	protein_codin	9.84250331	53.54949	28.5871675	11.6341708
ENSG000001	MCHR2	protein_codin	0	0	0	1.30891559
ENSG000001	EPHA4	protein_codin	12.288755	42.901851	23.5183089	14.7317531
ENSG000001	FARSB	protein_codin	28.0263577	28.3573454	41.9009089	12.3703034
ENSG000001	KCNE4	protein_codin	9.96287971	17.5621042	0	17.2044514
ENSG000001	TNFSF4	protein_codin	1.19337799	2.40870501	0	0
ENSG000001	MZT2B	protein_codin	3.8386375	19.4155393	40.4360177	2.26499199
ENSG000001	ASTN1	protein_codin	0	0	0	1.97724207
ENSG000001	CFC1B	protein_codin	0	0	0	0
ENSG000001	MGAT5	protein_codin	0	0	13.9024866	0
ENSG000001	TMEM163	protein_codin	0.37066871	0	0	0
ENSG000001	MED13L	protein_codin	56.646439	315.656283	89.5232089	79.9556439
ENSG000001	GPATCH11	protein_codin	0	0	0	5.2345977
ENSG000001	HSPB8	protein_codin	19.2279842	0	7.49934374	0
ENSG000001	GEMIN6	protein_codin	1.88616236	11.0908555	12.7706897	0.97054273
ENSG000001	TMEM178A	protein_codin	0.5177702	8.40833684	2.43318701	0.84619936
ENSG000001	OBI1	protein_codin	24.0437253	34.8676791	39.3983596	14.5164531

ENSG000001	ZC3H13	protein_codin	283.922329	877.487776	309.121017	159.861687
ENSG000001	CYSLTR2	protein_codin	1.65734641	0	0	0
ENSG000001	GRID2	protein_codin	0.30688003	0	7.29197842	0
ENSG000001	ARL11	protein_codin	4.26759499	7.56374579	3.16466441	6.13502716
ENSG000001	ARL14EP	protein_codin	15.9445181	34.3551223	13.8872474	3.00343826
ENSG000001	EPG5	protein_codin	7.01036762	0	1.75848784	0
ENSG000001	ATP5F1A	protein_codin	0	0	0	0
ENSG000001	HAUS1	protein_codin	0	22.142565	7.21265244	0
ENSG000001	SPC25	protein_codin	2.47393301	0	43.5941511	3.68592279
ENSG000001	G6PC2	protein_codin	0	0	0	2.74197741
ENSG000001	PDK1	protein_codin	8.57385203	48.5615396	113.104425	27.3958282
ENSG000001	PTH	protein_codin	0	0	1.51227496	0
ENSG000001	PDE3B	protein_codin	19.0858411	24.1099541	39.9245647	12.3873216
ENSG000001	FASTKD3	protein_codin	0	0	0	0
ENSG000001	TCF7L1	protein_codin	18.0644627	77.5970104	110.844476	20.5316186
ENSG000001	CRYGC	protein_codin	0	0	0	0.4344331
ENSG000001	KCNK13	protein_codin	0	2.17684392	0.57656184	0.43275773
ENSG000001	UHMK1	protein_codin	4.37098566	22.3384873	25.208399	9.27406462
ENSG000001	ATG10	protein_codin	4.8344524	0	5.78702236	1.09692472
ENSG000001	FAM151B	protein_codin	1.47144362	0	1.88651399	0
ENSG000001	GUCY1A2	protein_codin	0	0	0	0
ENSG000001	CWF19L2	protein_codin	94.3737879	246.817796	142.813389	42.1250318
ENSG000001	HOMER1	protein_codin	0	0	3.43832219	0
ENSG000001	XRCC4	protein_codin	0	0	0	0
ENSG000000	ANKRD44	protein_codin	18.8941671	0	38.6798768	11.5024631
ENSG000002	MARS2	protein_codin	1.85027113	12.4397343	12.0516263	2.04090738
ENSG000001	BOLL	protein_codin	0	0	0	0
ENSG000001	ZNF547	protein_codin	3.52107763	3.84494341	2.43204254	0
ENSG000001	ZNF304	protein_codin	13.8651262	25.6970894	0	6.44085223
ENSG000001	ZNF773	protein_codin	0	5.41724707	0	0
ENSG000002	ZNF551	protein_codin	10.6273972	4.71040662	28.1904327	5.25563365
ENSG000001	ZNF256	protein_codin	1.72785636	0	0	0
ENSG000001	ZSCAN1	protein_codin	0	0	0	0.33532535
ENSG000001	CACNB2	protein_codin	0	0	0	0
ENSG000001	USP12	protein_codin	32.3016865	91.8859433	64.0688897	58.5823645
ENSG000001	CAMK4	protein_codin	6.31179667	48.2204391	24.4289491	7.99051407
ENSG000001	TRIM36	protein_codin	0.65103627	0	22.2945956	2.62723041
ENSG000002	TMED7-TICAM	protein_codin	0	43.6823195	1.85452096	0
ENSG000001	ZFP36L2	protein_codin	111.867508	181.339085	145.046645	157.666521
ENSG000001	FLT1	protein_codin	29.5983806	25.7917807	36.7256568	52.9471892
ENSG000001	PLEKHH2	protein_codin	16.7435818	87.0932136	76.2163221	54.3248061
ENSG000001	PPM1B	protein_codin	16.6622502	17.5185081	13.3260994	10.5265698
ENSG000001	YAP1	protein_codin	31.5915477	112.444255	101.587735	0
ENSG000001	IGSF10	protein_codin	26.5503675	26.5569443	22.8800303	9.38677235

ENSG000001	SPEF2	protein_codin	1.34500867	12.7841068	25.2573588	1.68340358
ENSG000001	SPARCL1	protein_codin	1032.5002	254.195932	6092.28086	740.135855
ENSG000001	DMP1	protein_codin	27.3624754	216.132421	12.7325728	137.279554
ENSG000001	MBNL1	protein_codin	3.80152652	0	42.9012137	0
ENSG000001	MBNL1	protein_codin	0	25.3902395	0	3.15362917
ENSG000001	GRIA4	protein_codin	0	0	2.97519355	0
ENSG000001	UGT3A2	protein_codin	0	0	0	0
ENSG000001	NADK2	protein_codin	8.72026417	19.3542832	22.2288931	4.7726164
ENSG000001	NIPBL	protein_codin	52.295377	93.220743	392.328645	43.3057116
ENSG000001	GADL1	protein_codin	1.40017505	4.28429423	0	1.68740727
ENSG000001	GPD1L	protein_codin	18.2096989	20.6569626	9.56404984	9.72555725
ENSG000001	OTX1	protein_codin	0	0	0	0
ENSG000001	GJA1	protein_codin	246.006407	1608.84192	553.010522	295.995202
ENSG000000	GMCL1	protein_codin	12.249712	15.8824427	41.183504	8.10027386
ENSG000001	CCNO	protein_codin	0.21724531	0	0	0
ENSG000001	TIA1	protein_codin	20.5485063	32.1736374	47.0484892	8.65376721
ENSG000001	SLC30A6	protein_codin	2.73487305	3.70099455	23.8732222	2.03940723
ENSG000002	ITGA1	protein_codin	10.6287802	14.201267	25.0785723	34.9176523
ENSG000000	JADE2	protein_codin	0	0	0	0
ENSG000001	CATSPER3	protein_codin	0.71673265	1.15293117	0	0
ENSG000000	WASHC2A	protein_codin	97.4934103	0	279.641285	15.9577808
ENSG000001	DYNLT5	protein_codin	0	0	1.22761597	0.57234447
ENSG000001	LIPA	protein_codin	4.12403255	0	5.16839835	0
ENSG000001	BMP3	protein_codin	19.233546	56.8425124	115.014248	62.7347327
ENSG000001	HHEX	protein_codin	1.81483491	32.1330384	0	5.6436464
ENSG000001	GRM1	protein_codin	0	0	0	0
ENSG000001	GGPS1	protein_codin	2.71672735	8.99083867	0	3.90803458
ENSG000001	ADAMTS18	protein_codin	18.8748777	4.562597	1.38086724	6.00046115
ENSG000001	ZNF117	protein_codin	10.2872335	58.9110554	0	0
ENSG000001	RAB3C	protein_codin	0.52589305	1.39876634	0	4.61294275
ENSG000001	LMNTD1	protein_codin	0	0	0	0
ENSG000001	RASSF8	protein_codin	0	0	1.47439786	0
ENSG000001	RAD17	protein_codin	6.08853031	46.0642263	0	9.52630647
ENSG000001	MED21	protein_codin	11.3641711	19.4220864	31.3557387	11.972034
ENSG000001	PLOD2	protein_codin	0	100.913172	0	0
ENSG000001	STK32B	protein_codin	2.88550007	19.984876	3.11539058	7.43867986
ENSG000001	JAKMIP1	protein_codin	0	0	12.8206852	0
ENSG000001	ZIC1	protein_codin	0.52555688	23.6404553	0	2.28719641
ENSG000001	ADGRA3	protein_codin	21.5552773	26.1999923	308.845676	10.6410053
ENSG000001	CPB1	protein_codin	0	0	0	0
ENSG000001	MR1	protein_codin	3.93555272	0	0	0
ENSG000001	SRP19	protein_codin	3.24168414	0	6.16418799	4.59450521
ENSG000001	CENPH	protein_codin	5.38639917	0	81.7099166	1.99726095
ENSG000001	TEKT5	protein_codin	0	2.86299176	0	0.94947034

ENSG000001	NUBP1	protein_codin	8.19715387	22.0948759	13.4787685	1.0482462
ENSG000001	TXNDC11	protein_codin	0	28.0110698	21.4661268	34.0013558
ENSG000001	NPIPA6	protein_codin	16.9718502	154.73575	225.329229	28.8476425
ENSG000000	RIOK2	protein_codin	0.48634678	49.9702396	9.91896747	3.29620939
ENSG000001	CETN3	protein_codin	1.29994478	4.64950998	3.18041425	6.05580543
ENSG000001	SMARCA5	protein_codin	216.44652	465.237276	590.812748	148.049908
ENSG000001	SYCP2L	protein_codin	2.58335538	2.65238521	35.7962303	0
ENSG000001	BMP6	protein_codin	6.39323694	36.6849438	50.1682275	8.6298307
ENSG000001	UXS1	protein_codin	19.8147229	32.5753456	57.5626074	17.2546667
ENSG000001	RASSF3	protein_codin	6.72057936	38.1665963	21.455762	0
ENSG000001	HNRNPU	protein_codin	0	0	0	0
ENSG000001	RANBP2	protein_codin	329.099904	508.93176	1027.76253	348.340213
ENSG000001	TMEM87B	protein_codin	27.3008372	66.6066006	31.2973866	0
ENSG000001	OR14K1	protein_codin	0.55413406	0	0	0
ENSG000001	PTPRR	protein_codin	2.0545805	0	1.76877151	0
ENSG000001	CCDC148	protein_codin	0	0	0	0
ENSG000001	PLA2R1	protein_codin	51.5527051	75.6938511	20.5051943	27.9599859
ENSG000001	ITGB6	protein_codin	0	0	0	0
ENSG000001	SCN3A	protein_codin	0	0	0	0
ENSG000001	SCN2A	protein_codin	0	0	0	0
ENSG000001	FEZF2	protein_codin	0	0	0	0
ENSG000001	CADPS	protein_codin	9.67146386	0	0	0
ENSG000001	CD96	protein_codin	0	0	0	0
ENSG000001	ATG3	protein_codin	44.5842968	35.0218465	71.1358462	33.5950183
ENSG000000	ADGRF5	protein_codin	27.9705352	68.0457758	0	64.1350771
ENSG000001	ADGRF1	protein_codin	0	3.30383734	0	0
ENSG000001	ADGRF4	protein_codin	0	0	0	0.67259476
ENSG000001	FRMD1	protein_codin	0	0	0	0
ENSG000001	TRAPPC8	protein_codin	3.02227466	7.34775864	8.73039912	2.56998059
ENSG000001	FAM81B	protein_codin	0	0	0	0
ENSG000001	DTNA	protein_codin	0	27.8380984	0	4.38817416
ENSG000001	INO80C	protein_codin	0	2.55251439	0.84370123	1.05264068
ENSG000001	LPCAT1	protein_codin	26.1715776	108.018675	192.287722	13.8940106
ENSG000001	PLEKHG4B	protein_codin	5.40538458	0	184.313484	0
ENSG000001	NMRAL1	protein_codin	4.52246695	0	0	6.80830817
ENSG000001	ZDHHC11	protein_codin	0	0	30.2586937	0
ENSG000001	UBALD1	protein_codin	1.52008488	0	6.37184229	0
ENSG000001	LYSET	protein_codin	5.06703446	0	5.89972767	1.551006
ENSG000001	TEX29	protein_codin	0.64052135	1.17607526	0	0
ENSG000001	SPACA7	protein_codin	0	0	0	0
ENSG000001	FBXL2	protein_codin	0	0	0	0
ENSG000001	UBP1	protein_codin	8.79845279	5.01941344	19.9075421	0
ENSG000001	UBP1	protein_codin	1.45302908	0	7.96731086	0
ENSG000001	RMND5A	protein_codin	57.0698672	61.5176052	146.677253	39.4436524

ENSG000001	CD8A	protein_codin	0	22.7085032	0	5.16525034
ENSG000001	RPIA	protein_codin	8.51788042	8.88585981	10.948732	3.85305379
ENSG000001	VILL	protein_codin	0	0	0	0
ENSG000000	SERPINB3	protein_codin	0	0	0	0
ENSG000001	HGD	protein_codin	0	0	0	0
ENSG000001	GTF2E1	protein_codin	19.1289038	49.8423091	45.9156352	6.90779876
ENSG000001	CFDP1	protein_codin	44.2858389	140.110417	201.201573	49.173462
ENSG000001	TGIF2LX	protein_codin	0	0	0	0
ENSG000001	SPMIP4	protein_codin	0	0	7.23870815	0
ENSG000001	TMPRSS11D	protein_codin	0	0	0	0
ENSG000002	HOXA10	protein_codin	8.69405906	20.5525067	9.48615044	3.95195758
ENSG000001	JAZF1	protein_codin	9.81110818	29.7971115	16.3106858	11.4987053
ENSG000001	KCNJ16	protein_codin	0	0	0	0
ENSG000001	TRIP12	protein_codin	0	0	0	4.16544312
ENSG000001	FBXO36	protein_codin	2.71778959	4.20449713	3.71134206	2.17337981
ENSG000000	PGM3	protein_codin	0	0	0	0
ENSG000001	CEBPG	protein_codin	16.6397911	5.85596094	0	15.0209476
ENSG000001	KCTD15	protein_codin	1.02738681	30.0005291	9.83443943	3.30557806
ENSG000001	MCOLN2	protein_codin	1.07459139	7.45182218	5.46552513	0
ENSG000001	DDAH1	protein_codin	21.3471731	38.5305696	28.4732374	3.5033562
ENSG000001	ERBIN	protein_codin	14.399751	48.051877	41.6187967	21.4062409
ENSG000001	SREK1	protein_codin	17.9022176	26.7094002	68.7978693	0
ENSG000001	DGKE	protein_codin	7.86001803	15.1348396	27.6108402	3.86345299
ENSG000001	MSI2	protein_codin	16.5778781	22.8961327	135.326709	10.2234511
ENSG000001	HS3ST3A1	protein_codin	3.41397992	11.1954458	34.2433302	1.9296219
ENSG000001	GDPD1	protein_codin	1.03679554	0	6.91120843	0
ENSG000001	SEMA3D	protein_codin	7.1613598	34.5409865	66.233374	46.5472324
ENSG000001	ASB17	protein_codin	0	0	0	0
ENSG000001	GRAP	protein_codin	2.0794534	8.37170359	3.04195693	1.38733772
ENSG000001	MAX	protein_codin	1.29681434	43.6552694	11.017882	0
ENSG000001	IMPACT	protein_codin	35.1786642	57.7110411	66.2174645	23.6591659
ENSG000001	THY1	protein_codin	44.126876	124.57635	21.8182672	21.6075239
ENSG000001	C16orf74	protein_codin	0	0	0	0
ENSG000001	TBCEL	protein_codin	1.90437108	0	0	1.19227299
ENSG000001	JPH3	protein_codin	0	0	12.1589457	1.55118641
ENSG000001	ANKH	protein_codin	23.7971973	136.360373	98.9460158	151.142801
ENSG000001	UBASH3B	protein_codin	3.28138098	23.9288372	56.6674741	11.0793022
ENSG000001	OTULIN	protein_codin	28.0840992	79.4910546	254.510707	52.2663904
ENSG000001	OR8A1	protein_codin	0	0	0	0
ENSG000001	PANX3	protein_codin	14.6762466	7.52957389	327.195935	0.4630626
ENSG000001	TBRG1	protein_codin	18.4374001	22.5443959	30.5000796	17.620125
ENSG000001	NRGN	protein_codin	0	9.65860835	8.29111717	0.44225822
ENSG000001	GPR15	protein_codin	0	13.1057389	0	1.36010613
ENSG000001	TOMM70	protein_codin	53.0620795	65.7233854	122.997274	30.4171564

ENSG000001	ABI3BP	protein_codin	34.693642	93.6172196	2.51195929	21.6083537
ENSG000001	CC2D1B	protein_codin	8.20691177	56.6155116	0	8.50294334
ENSG000001	CERS3	protein_codin	0	0	0	0
ENSG000001	PRKCA	protein_codin	0	27.9925969	26.5967306	0
ENSG000001	ABCA6	protein_codin	32.7429606	15.3543491	0	41.5446465
ENSG000001	C4orf19	protein_codin	0.32709456	0.90116023	4.06816356	0
ENSG000001	UCHL1	protein_codin	3.54484428	0	0	0
ENSG000001	DISP1	protein_codin	0	0	24.7547215	0
ENSG000001	TNIK	protein_codin	0	0	0	0
ENSG000001	FAM167A	protein_codin	6.12314801	6.7215365	6.09377206	5.33149711
ENSG000001	NEIL2	protein_codin	1.07328547	4.53134286	3.0266219	0
ENSG000001	ATP8B4	protein_codin	0	22.717018	8.83546845	24.0509324
ENSG000001	WNT3A	protein_codin	0	0	4.08208161	0
ENSG000001	OBSCN	protein_codin	38.6932555	24.6592515	49.0394241	0
ENSG000001	TRIM11	protein_codin	0	0	0	2.10132519
ENSG000001	ENAH	protein_codin	1.97784885	0	0.63516206	2.31258412
ENSG000001	PPP1R3A	protein_codin	28.6284848	1.48397989	0	5.5410617
ENSG000001	PPP1R3A	protein_codin	0	0	0	0
ENSG000001	CCSAP	protein_codin	0	36.9480564	0	0
ENSG000001	ASZ1	protein_codin	0	0	0	0
ENSG000001	SH3RF1	protein_codin	42.2468336	67.1440579	100.882073	33.3856875
ENSG000001	OSCAR	protein_codin	0	0	0	2.2811506
ENSG000002	KLHL41	protein_codin	100.960084	3.0519987	1.65301909	0.88561372
ENSG000001	GPR26	protein_codin	0.2749182	1.30257215	0	5.42710585
ENSG000000	DHX32	protein_codin	13.1450909	17.7832309	6.97241568	8.59451585
ENSG000001	C10orf90	protein_codin	0	0	0	0
ENSG000001	OLA1	protein_codin	7.26272805	9.17166217	15.2002884	1.72961014
ENSG000001	ATP5MC3	protein_codin	7.12101342	23.7186573	9.64482616	11.4426736
ENSG000001	PDLIM3	protein_codin	54.5132562	0	0	0
ENSG000001	PDLIM3	protein_codin	0	0	1.80345102	0
ENSG000001	PDLIM3	protein_codin	0	0	0	0
ENSG000001	SORBS2	protein_codin	0	0	0	0
ENSG000001	ELOC	protein_codin	1.0391517	0	4.28194454	0
ENSG000001	LY96	protein_codin	1.91563573	6.6172884	1.41344922	5.85589246
ENSG000001	TMSB4Y	protein_codin	13.8884655	432.699754	12.3317267	4.16148982
ENSG000001	CXADR	protein_codin	1.01481827	1.38212829	38.7873282	2.74538824
ENSG000001	C21orf91	protein_codin	7.4851054	24.1212972	11.3565416	4.56121871
ENSG000001	TMPRSS15	protein_codin	0	0	0	0.57027061
ENSG000001	NCAM2	protein_codin	4.3630822	16.6978173	41.5134715	3.66088683
ENSG000001	SEL1L2	protein_codin	0	0	0	0
ENSG000001	RABGEF1	protein_codin	19.2334165	58.5596512	37.9312741	29.3789131
ENSG000001	ATP5PF	protein_codin	27.7841012	79.2069797	91.1621362	20.8111753
ENSG000001	ADAMTS1	protein_codin	0	0	0	0
ENSG000001	ADAMTS5	protein_codin	26.5421237	62.2667862	53.1333753	20.1708642

ENSG000001	TSEN2	protein_codin	0	0	0	0
ENSG000001	SLFN13	protein_codin	0	9.49679299	0	5.11842924
ENSG000001	WNT7A	protein_codin	0	0	0	1.40422198
ENSG000001	XPC	protein_codin	21.8329165	0	25.1490329	0
ENSG000001	MYO5B	protein_codin	1.9486522	14.2956128	26.9165047	0
ENSG000001	FGD5	protein_codin	39.4668825	27.6647263	27.9022507	36.6161765
ENSG000001	FLCN	protein_codin	17.2031199	67.5809696	24.001021	4.24656039
ENSG000001	DPH3	protein_codin	0	0	0	0
ENSG000001	OXNAD1	protein_codin	5.73558423	18.4258835	14.7570409	9.39765669
ENSG000001	ACAA2	protein_codin	2.10686719	2.91284199	0.75861152	1.53112766
ENSG000001	CXXC1	protein_codin	0	0	18.1352893	0
ENSG000001	SKA1	protein_codin	1.56823196	0	11.2435151	0.62130992
ENSG000001	PPP4R1	protein_codin	14.5363278	20.8278469	47.1216979	7.75195279
ENSG000001	USP43	protein_codin	0	0	0	0
ENSG000001	SAMD14	protein_codin	0	0	0	0
ENSG000001	RAB6B	protein_codin	0	7.55274705	0	0
ENSG000001	ABCC3	protein_codin	0	5.03294115	0	3.90962259
ENSG000001	ANKRD40	protein_codin	75.3509953	125.882643	96.5643973	47.9765158
ENSG000001	CA10	protein_codin	0.32665227	0	0	0
ENSG000001	VOPP1	protein_codin	25.5007483	167.915969	69.9869736	0
ENSG000002	MRPS17	protein_codin	6.12465052	8.71004386	12.826723	6.03229419
ENSG000001	DKK2	protein_codin	34.805745	0	0.56869002	1.67819562
ENSG000001	CA2	protein_codin	7.00594915	9.97861345	10.639806	31.4463717
ENSG000001	CA3	protein_codin	490.824713	39.4526587	2.19546567	24.1302504
ENSG000001	ATP6V0D2	protein_codin	2.36263505	4.85242925	1.44996973	16.5496476
ENSG000001	AK9	protein_codin	2.78345511	12.7023367	8.13601381	1.37528152
ENSG000001	ERCC3	protein_codin	29.1681172	0	0	0
ENSG000001	ODF1	protein_codin	0	0	0.56483333	0
ENSG000001	KLF10	protein_codin	76.2127874	86.4465029	107.007638	323.633226
ENSG000001	PIP4P2	protein_codin	9.81572702	14.8451047	0	8.93024147
ENSG000001	OTUD6B	protein_codin	0	0	0	0
ENSG000001	AGPAT5	protein_codin	7.60371462	12.4250701	19.6437503	8.22462521
ENSG000000	MAN2B2	protein_codin	40.0194584	63.3927996	44.1859615	38.9577227
ENSG000001	OR4K1	protein_codin	0	0	0	0.46071646
ENSG000001	MARVELD1	protein_codin	31.2767587	57.6634398	64.522342	25.8513989
ENSG000001	HSPA13	protein_codin	16.0986888	60.9440063	40.5799003	8.68085355
ENSG000001	SAMSN1	protein_codin	0	0	0	0
ENSG000001	USP25	protein_codin	108.049482	301.30032	164.720562	75.1894162
ENSG000001	USP25	protein_codin	0	0	2.00438088	0
ENSG000001	GRAMD2B	protein_codin	1.90258142	0	0	0
ENSG000001	C16orf87	protein_codin	3.88930795	13.0856317	10.5576832	4.56564965
ENSG000001	RHOC	protein_codin	1.07489933	1.60388457	1.8203462	2.79269209
ENSG000001	LONP2	protein_codin	29.1340024	87.3450902	64.0907355	22.1325375
ENSG000001	TRIM74	protein_codin	0	3.76683695	0	0

ENSG000001	NIFK	protein_codin	11.8315783	15.6287269	63.0418266	3.3818635
ENSG000001	OXA1L	protein_codin	0	0	0	2.90079799
ENSG000001	SLC7A7	protein_codin	0	0	0	0
ENSG000001	CCDC146	protein_codin	15.0328321	89.3426253	23.9951256	10.7460311
ENSG000000	GEMIN5	protein_codin	25.1587548	42.3374595	89.8318377	14.4530226
ENSG000001	MAGEC1	protein_codin	0	1.49392101	75.4850731	4.38145651
ENSG000001	CNOT8	protein_codin	35.5720695	56.3284816	41.247071	17.1947024
ENSG000001	GRIA1	protein_codin	0	0	0	0
ENSG000001	BOD1	protein_codin	1.14433125	25.5087343	0	2.69240178
ENSG000001	LRGUK	protein_codin	0.87527929	1.39131459	8.81090272	0.89746428
ENSG000000	AKR1B1	protein_codin	13.7830174	28.907447	14.7780792	7.25939102
ENSG000001	SETD9	protein_codin	0	0	0	0
ENSG000001	NUP205	protein_codin	23.2275894	71.1792235	219.643349	27.3055661
ENSG000001	CYP2C18	protein_codin	0.60586107	1.3891852	0	3.23696033
ENSG000000	FIRRM	protein_codin	4.66050021	0	0	3.9174483
ENSG000001	XAGE2	protein_codin	0	0	0.86652921	0.42908678
ENSG000001	PDE11A	protein_codin	2.41831896	30.2654715	0	16.003697
ENSG000001	RBM45	protein_codin	4.75642723	5.0263365	0	4.48478214
ENSG000001	PDIA4	protein_codin	19.0807744	62.7898071	120.859688	14.3315551
ENSG000001	KDM8	protein_codin	0	5.3090976	6.68099143	0
ENSG000001	BANP	protein_codin	0	0	0	0
ENSG000001	OTOA	protein_codin	0	0	0	0
ENSG000002	PPIL3	protein_codin	0	0	0	0
ENSG000001	HYCC2	protein_codin	0	0	0	0
ENSG000000	CASP10	protein_codin	6.45407006	25.6480645	4.51675028	7.27128075
ENSG000001	FLACC1	protein_codin	5.21579211	0	0	0
ENSG000001	C2CD6	protein_codin	0	0	0	0
ENSG000001	TMEM237	protein_codin	0	5.20922033	11.6244064	5.38099914
ENSG000001	FZD7	protein_codin	19.0518581	31.4971596	74.7481408	19.7882689
ENSG000001	DEPTOR	protein_codin	23.9566863	42.1081479	79.625438	9.5587334
ENSG000001	SLC26A2	protein_codin	32.2243224	39.8005813	49.0026764	17.1621994
ENSG000001	CSF1R	protein_codin	37.4533435	246.517447	17.2808942	36.864712
ENSG000001	LSM11	protein_codin	8.20879731	6.64323184	9.14905036	3.00672065
ENSG000001	MED7	protein_codin	0	32.8424813	1.17495636	4.85524792
ENSG000001	SLC24A2	protein_codin	0.26164956	0	0	0
ENSG000001	TRIM42	protein_codin	0	0	0	0.43865465
ENSG000001	PXYLP1	protein_codin	11.5731214	16.9643293	16.9220666	0
ENSG000001	ADCY8	protein_codin	0	0	1.89143766	0
ENSG000001	RASA2	protein_codin	27.9335015	151.300904	62.8262813	27.4368951
ENSG000000	ATP1B3	protein_codin	36.4280088	60.1599822	72.7920625	31.4162833
ENSG000001	RAET1L	protein_codin	0	0	0	0
ENSG000001	SMC2	protein_codin	0	0	16.2865061	0
ENSG000001	TMBIM4	protein_codin	0	4.96955491	0	0
ENSG000001	VBP1	protein_codin	16.1431693	27.6421576	57.4594777	0

ENSG000001	AFF2	protein_codin	14.8801701	0	4.52771424	0
ENSG000001	VAMP7	protein_codin	23.1814798	32.79422	38.7361221	19.6631591
ENSG000001	KIF5A	protein_codin	0.41212022	0	0	0
ENSG000001	NAT2	protein_codin	0	0	0	3.16255675
ENSG000001	MAGEA8	protein_codin	0	0	22.4899407	0
ENSG000001	PSD3	protein_codin	0	0	0	0
ENSG000002	ARHGEF25	protein_codin	6.21294183	2.85557002	1.43870336	0
ENSG000001	MIDEAS	protein_codin	0	0	0	0
ENSG000001	FAM161B	protein_codin	9.42428601	24.3266393	13.6974031	7.0355619
ENSG000001	GNAQ	protein_codin	92.3591393	234.847449	132.617354	89.7144053
ENSG000001	WIF1	protein_codin	1.79541085	93.7281179	0	93.9995852
ENSG000001	UGT2A1	protein_codin	0	0	0	0
ENSG000001	MMP16	protein_codin	132.334214	186.265847	54.284566	146.202346
ENSG000001	ADK	protein_codin	0	0	0	0
ENSG000001	KCNMA1	protein_codin	58.5563378	122.282176	113.007102	22.8348143
ENSG000001	KCNMA1	protein_codin	0	0	0	0
ENSG000001	BATF	protein_codin	0	8.89955149	0	3.4378606
ENSG000001	DCK	protein_codin	20.6954279	54.1596788	39.7745367	14.7944174
ENSG000001	TTLL5	protein_codin	0	1.2649663	0	1.59873688
ENSG000001	ADAMTS3	protein_codin	10.0063758	9.88598257	45.7241103	5.17279075
ENSG000001	CFAP418	protein_codin	3.00308484	3.30761082	47.6584551	1.26017659
ENSG000001	DRAM2	protein_codin	0.65391359	2.02145429	5.78314124	4.81239224
ENSG000001	STOM	protein_codin	35.7754773	114.301379	39.0156155	51.8154525
ENSG000001	PPEF2	protein_codin	0	0	0	1.02329463
ENSG000001	CFAP161	protein_codin	0	1.68849377	0.50745582	0
ENSG000001	NAAA	protein_codin	2.1606229	0	0	1.36726096
ENSG000001	ADAMTSL3	protein_codin	8.78046283	14.2744081	0	6.95179283
ENSG000001	SLC28A1	protein_codin	0	0	0	0
ENSG000001	CXCL13	protein_codin	0	0	0	0
ENSG000001	WHAMM	protein_codin	21.77667	80.6251448	26.061567	36.6290756
ENSG000001	RWDD2B	protein_codin	18.5029092	18.7072151	34.8546013	10.6539575
ENSG000001	CCT8	protein_codin	53.0378912	77.3404619	338.477641	27.3589647
ENSG000001	NAA11	protein_codin	0	0	0	2.80674173
ENSG000001	BACH1	protein_codin	20.5196149	0	86.6619541	0
ENSG000001	CLDN17	protein_codin	0	0	0	0
ENSG000001	TSPAN7	protein_codin	5.08396761	0	0	0
ENSG000001	TIAM1	protein_codin	0	28.0249367	0	0
ENSG000001	SCAF4	protein_codin	35.0189377	0	82.4548792	42.913678
ENSG000001	GGTLC1	protein_codin	0	0	0	0.45409629
ENSG000001	ANKRD9	protein_codin	3.05294384	14.7620561	4.963053	2.04309697
ENSG000001	ATP5MJ	protein_codin	8.88629935	31.7524666	41.6590842	5.82319896
ENSG000001	FUT6	protein_codin	0	0	0	0
ENSG000001	PCDH1	protein_codin	2.16225025	43.7560067	6.35256474	12.5160345
ENSG000001	GDF6	protein_codin	0	0	72.700837	1.34309102

ENSG000001	UQCRB	protein_codin	6.31446556	22.4221582	12.9871087	9.65584135
ENSG000001	MTERF3	protein_codin	10.2320466	17.5244936	58.8454054	2.00166375
ENSG000001	RPL30	protein_codin	142.089461	182.945696	590.880447	97.4444038
ENSG000001	KCNS2	protein_codin	0	0	0	2.73701852
ENSG000001	TYSND1	protein_codin	0	0	0	0
ENSG000001	CD109	protein_codin	85.3914487	0	31.2869096	64.9979578
ENSG000001	NODAL	protein_codin	0	0	0	0.84417125
ENSG000001	PRG3	protein_codin	0	0	0	0
ENSG000001	KIF6	protein_codin	0	0	3.1071546	0
ENSG000001	UBE2L6	protein_codin	9.90687567	74.5448966	6.29825765	14.8025585
ENSG000001	ZDHHC5	protein_codin	38.2525865	67.3924207	95.8338491	33.9227347
ENSG000001	PARP6	protein_codin	0	0	0	0
ENSG000001	CELF6	protein_codin	0	0	5.38871528	2.63688961
ENSG000001	ZFAND3	protein_codin	26.6264752	39.3173888	46.1399451	27.2300263
ENSG000001	KAT6B	protein_codin	0	0	0	22.9171482
ENSG000001	RAB11FIP1	protein_codin	6.21266419	26.8065611	0	34.7204341
ENSG000001	UNC5D	protein_codin	3.34385048	0	0	0
ENSG000001	GLYATL2	protein_codin	1.18026065	0	0	0
ENSG000001	AIFM1	protein_codin	0	5.59964382	0	0
ENSG000001	BAG4	protein_codin	30.7857645	34.1101896	211.135759	19.0326459
ENSG000001	TBC1D31	protein_codin	0	0	0	0
ENSG000001	NTAQ1	protein_codin	0	0	20.5784869	0
ENSG000001	ATAD2	protein_codin	33.5702183	117.587532	1190.12962	28.2700456
ENSG000001	FBXO32	protein_codin	0.95907912	0	0	7.081761
ENSG000001	NSMCE2	protein_codin	0	9.89949198	44.4730131	1.60314121
ENSG000001	ZNF689	protein_codin	7.59156478	7.90013864	35.9733981	12.7083109
ENSG000001	PRR14	protein_codin	0	0	0	0
ENSG000001	FBRS	protein_codin	3.22496581	0	6.98561161	0
ENSG000001	FRRS1	protein_codin	1.69704442	0	16.917731	1.59104306
ENSG000001	SASS6	protein_codin	9.40869993	19.992947	111.106759	3.79233775
ENSG000001	COX6A2	protein_codin	0.74889358	0	0	0.72151295
ENSG000001	BCKDK	protein_codin	0	0	0	0
ENSG000001	ZIC3	protein_codin	0	0	4.5562538	0.91978018
ENSG000001	MALSU1	protein_codin	1.93895851	5.05463226	10.4363381	1.36421771
ENSG000001	VPS8	protein_codin	32.5484773	76.3051414	77.8778314	23.5929514
ENSG000001	LHFPL4	protein_codin	0.30004346	1.73648188	0.55994933	2.07479848
ENSG000001	B3GNT7	protein_codin	0.28057314	4.97532238	1.54024232	2.63324141
ENSG000001	MPV17L	protein_codin	4.75543495	0	0	5.73899896
ENSG000001	BUB1B	protein_codin	7.09378885	0	171.336566	0
ENSG000001	PDE6D	protein_codin	3.33899748	7.39872942	2.05695583	3.2075235
ENSG000001	KNG1	protein_codin	0	1.27731716	0	0
ENSG000001	SST	protein_codin	0	0	0	0
ENSG000001	FANCD2	protein_codin	0	4.72639493	12.2672732	0
ENSG000001	TATDN2	protein_codin	0	24.0119066	27.2391198	0

ENSG000001	GHRL	protein_codin	0	0	0	0.75378444
ENSG000001	NOMO1	protein_codin	69.2587083	169.303342	218.689281	31.2422918
ENSG000001	EXOG	protein_codin	15.657463	23.6783919	16.6165484	2.20621255
ENSG000001	HMBOX1	protein_codin	15.8210792	0	36.2171995	16.5962382
ENSG000001	NTAN1	protein_codin	15.0795466	8.488825	26.4864365	2.35097682
ENSG000001	NMNAT2	protein_codin	0	0	0.4838604	0
ENSG000002	TSPY8	protein_codin	0	0	0	0
ENSG000001	ZFYVE9	protein_codin	27.9201381	0	99.0562007	18.2042853
ENSG000001	LYZL4	protein_codin	0	0	0	0
ENSG000001	FCHO2	protein_codin	0	5.77188928	0	1.46349357
ENSG000001	SLC6A1	protein_codin	0	0	0	0
ENSG000001	RBPMS	protein_codin	0	0	0	0
ENSG000001	TMEM171	protein_codin	0.71967823	0	0	0
ENSG000001	KLHL40	protein_codin	6.3836639	0	0	1.93493355
ENSG000001	TIMP4	protein_codin	3.32286592	11.3385013	0	8.07251148
ENSG000001	PPARG	protein_codin	0	0	0	0
ENSG000001	NRG1	protein_codin	0	0	0	0
ENSG000000	ABCF2	protein_codin	8.60222359	22.1920419	51.9391705	5.44296847
ENSG000001	ODR4	protein_codin	67.3929209	75.8369922	136.082116	45.3985839
ENSG000001	PRKAG2	protein_codin	0	0	0	0
ENSG000002	CYB5RL	protein_codin	0	0	0	0
ENSG000001	HTR5A	protein_codin	0	0	0	3.03213958
ENSG000001	STEAP2	protein_codin	0	0	28.8558372	0
ENSG000001	RBM33	protein_codin	54.154632	351.722015	179.317307	55.5096826
ENSG000001	TSSK4	protein_codin	0	0	0	0
ENSG000001	CLDN12	protein_codin	0	32.6159864	106.687177	0
ENSG000001	FZD1	protein_codin	18.2170445	136.436205	40.3041253	23.6604955
ENSG000001	HMGCR	protein_codin	18.6549823	0	47.488782	0
ENSG000001	GATAD1	protein_codin	24.4809195	65.8143301	78.1196607	23.2961035
ENSG000001	IPPK	protein_codin	2.39579717	0	2.80477783	5.94600019
ENSG000002	THTPA	protein_codin	0	26.9194666	17.4086715	4.54773152
ENSG000002	PDF	protein_codin	0	3.53467222	3.19966827	0
ENSG000001	TMED6	protein_codin	0	0	0	2.08354008
ENSG000001	CLEC18A	protein_codin	0	0	0	0
ENSG000000	PDPR	protein_codin	31.2561119	80.2063392	53.8169872	15.4165888
ENSG000001	ARMC12	protein_codin	0	0	2.89985629	0
ENSG000001	DDX19B	protein_codin	15.3660458	10.1542819	11.9199196	6.17580879
ENSG000001	FCSK	protein_codin	1.1582434	0	1.51584451	0
ENSG000002	MDP1	protein_codin	0	2.92872135	1.46711747	0.84100791
ENSG000001	IL34	protein_codin	0	0	0	0
ENSG000001	DHRS1	protein_codin	0	6.88374992	0	0
ENSG000001	KIT	protein_codin	0	0	0	24.2958217
ENSG000001	CACNA1D	protein_codin	0	0	0	0
ENSG000000	IL17RB	protein_codin	0	0	5.47364633	0

ENSG000001	HYDIN	protein_codin	0.91128574	1.34157872	1.26318685	1.85532279
ENSG000001	ZNF19	protein_codin	0	0	0	1.11272433
ENSG000001	CCNB2	protein_codin	7.75476746	11.0033056	103.587765	0
ENSG000001	ERC2	protein_codin	0	0	1.66836763	0
ENSG000001	FAM81A	protein_codin	3.47056965	9.67282197	24.6470732	3.02437704
ENSG000001	MYO1E	protein_codin	54.9707141	100.490885	60.905421	107.835394
ENSG000001	APPL1	protein_codin	85.4329506	128.242552	96.1417306	49.1026782
ENSG000001	ERG	protein_codin	15.3554738	56.8219089	17.4961541	0
ENSG000002	HMGN1	protein_codin	0	0	0	0
ENSG000001	LCA5L	protein_codin	0	0	0	0
ENSG000000	PREX2	protein_codin	42.8704583	98.0711127	32.3161991	58.3124462
ENSG000001	TMEM164	protein_codin	0	0	0	0
ENSG000001	MX1	protein_codin	0	0	15.645298	0
ENSG000002	SPATS1	protein_codin	0	0	0	0
ENSG000001	TPM1	protein_codin	37.2292095	0	0	7.95143135
ENSG000001	TAB3	protein_codin	10.6442003	0	0	0
ENSG000001	SLC38A10	protein_codin	0	0	29.6510816	15.2520844
ENSG000001	DMD	protein_codin	0	0	0	0
ENSG000001	C9orf43	protein_codin	0	0	0	0
ENSG000001	ZNF618	protein_codin	19.2839949	68.2024469	73.7269408	22.7900184
ENSG000001	DGKI	protein_codin	5.54035295	0	0	4.42692707
ENSG000001	TMEM268	protein_codin	6.09384291	43.3118196	10.7862582	9.16538923
ENSG000001	SVOPL	protein_codin	0	0	0	0
ENSG000001	ASTN2	protein_codin	0	0	3.87447806	0
ENSG000001	COQ5	protein_codin	13.261416	34.0828169	13.9540912	2.86825908
ENSG000001	KLHL29	protein_codin	0	1.27217023	5.84450032	1.94460347
ENSG000000	NCOA1	protein_codin	0	0	0	17.5650908
ENSG000001	BRAF	protein_codin	0	0	0	0
ENSG000001	PSMG3	protein_codin	0.34059905	2.2097524	7.6371807	0.64889668
ENSG000001	CABP1	protein_codin	1.24429532	2.21739416	0	0
ENSG000001	DTNB	protein_codin	0	0	0	0
ENSG000001	FMNL2	protein_codin	87.1177359	200.383001	388.576938	52.9737344
ENSG000001	DPYSL5	protein_codin	0	0	0	0
ENSG000001	DRC1	protein_codin	0.3363783	0	0	0
ENSG000001	RAB28	protein_codin	1.05627321	12.7782854	16.9349014	0
ENSG000001	MEGF11	protein_codin	0	0	0.6435156	2.46814904
ENSG000001	C12orf43	protein_codin	0.67380991	0	0	1.75575452
ENSG000001	PEX10	protein_codin	0	0	7.47301519	0
ENSG000001	OLFML2A	protein_codin	0	0	0	0
ENSG000001	WIPI2	protein_codin	5.47441764	3.68767768	11.4643881	3.54331409
ENSG000002	SSX2B	protein_codin	0	0	0	0
ENSG000001	SMAD6	protein_codin	10.2165066	61.3524402	45.2220587	19.2055287
ENSG000001	CIB4	protein_codin	0	0	0	0
ENSG000001	KRTCAP3	protein_codin	0	0	0	0

ENSG000001	CFAP251	protein_codin	0	0	0	3.13619542
ENSG000001	MRPL17	protein_codin	7.50953166	42.3232731	36.9800789	5.36836928
ENSG000001	DUSP2	protein_codin	1.66392708	26.9943073	5.27299046	11.8386066
ENSG000001	PTPDC1	protein_codin	0	0	0	2.18342817
ENSG000001	ERCC6L2	protein_codin	1.78792924	0	6.35498562	5.03428484
ENSG000001	NCK1	protein_codin	24.3087868	27.0524404	39.7547973	7.13199683
ENSG000001	HPD	protein_codin	0	0	0	0
ENSG000001	RHPN1	protein_codin	0	0	23.3407083	0
ENSG000001	ZNF782	protein_codin	6.6591239	10.1359412	24.0516665	0
ENSG000001	FANCC	protein_codin	0	19.8784795	15.8180332	4.63705488
ENSG000001	MRAS	protein_codin	0	0	1.73232272	6.44465495
ENSG000001	ABHD3	protein_codin	1.62162773	0	0	2.37126957
ENSG000001	ESYT3	protein_codin	0	0	0	0
ENSG000000	PIK3CB	protein_codin	5.03022279	0	13.223436	1.19575866
ENSG000001	TENT5B	protein_codin	0	0	0.48257368	0
ENSG000001	LRRFIP1	protein_codin	48.4527351	509.239511	0	116.010809
ENSG000001	ACTR1B	protein_codin	11.7331264	16.3354225	51.8212396	9.49514447
ENSG000001	RHBDL2	protein_codin	0.41542092	0	1.15822818	1.52113909
ENSG000002	PCDHAC2	protein_codin	2.75685783	0	0	0
ENSG000002	PCDHA13	protein_codin	0	0	0	0
ENSG000001	PLS3	protein_codin	5.50752707	25.7770715	0	6.52595278
ENSG000001	SHROOM4	protein_codin	0	41.8809651	9.89627823	9.12898739
ENSG000001	H2BC5	protein_codin	5.94575142	19.5427637	18.4665724	0
ENSG000001	MITD1	protein_codin	32.3560085	77.0723868	23.7066903	14.5414184
ENSG000000	BTN3A1	protein_codin	15.5832554	90.6137399	15.8024662	33.3555724
ENSG000001	EIF5B	protein_codin	392.410215	1471.46526	1578.11791	206.790295
ENSG000001	TMSB15A	protein_codin	0	2.03284722	9.59790972	0
ENSG000001	CNOT11	protein_codin	9.81688509	31.5899356	18.768283	5.47234434
ENSG000001	CATIP	protein_codin	0	0	0	0
ENSG000001	NRG2	protein_codin	0	0	0	0
ENSG000000	ACSM3	protein_codin	0	0	0	6.02460964
ENSG000001	NRG2	protein_codin	0	0	0	0
ENSG000001	CD1A	protein_codin	0	0	0	0.4642449
ENSG000001	SPATA2	protein_codin	6.66956742	10.0426187	8.8636434	9.23683675
ENSG000001	HMHB1	protein_codin	0.39528159	0	0	0
ENSG000001	NCF1	protein_codin	2.95810748	20.2410774	0	2.93064234
ENSG000001	LRRC51	protein_codin	0	0	0	0
ENSG000001	PPP1R9A	protein_codin	0	5.08992091	12.4303952	5.47917241
ENSG000001	ZFAND2B	protein_codin	0	0	0	0
ENSG000000	NPC1L1	protein_codin	0	0	0	0
ENSG000001	SLCO2B1	protein_codin	3.78597735	5.93684683	0	0
ENSG000001	TMED4	protein_codin	1.57434181	2.78840242	1.40452585	1.97960681
ENSG000001	PAGE5	protein_codin	0	0	1.65291421	0.89072002
ENSG000001	OBSL1	protein_codin	0	0	0	0

ENSG000001	PKD1L1	protein_codin	0	0	0	0
ENSG000001	ELK4	protein_codin	0	0	0	0
ENSG000001	SLAMF8	protein_codin	5.2005248	56.9560205	0.46148405	1.46737492
ENSG000000	ANK1	protein_codin	0.88003517	0	0	0
ENSG000001	CDH15	protein_codin	2.89876856	32.1713184	30.1399549	28.1031139
ENSG000001	NBL1	protein_codin	0	0	0	0
ENSG000001	HTR6	protein_codin	0	1.14944806	0.54406428	0.40979403
ENSG000002	ENSG000002	protein_codin	0	0	0	0
ENSG000001	ZSCAN23	protein_codin	4.33692173	20.3571177	30.1990993	0.93056687
ENSG000001	SPATA2L	protein_codin	0.42612488	2.83097096	4.06476423	0
ENSG000001	VWA5B1	protein_codin	0	2.24817559	0	2.67521323
ENSG000001	ZNF276	protein_codin	13.1228773	0	108.325901	0
ENSG000001	NPM2	protein_codin	0	0	0	0
ENSG000001	USP21	protein_codin	0	0	0	0
ENSG000001	SLC45A1	protein_codin	0.79748939	0	0	0.36474286
ENSG000001	MACF1	protein_codin	251.146582	1054.10078	152.169119	205.443131
ENSG000001	FCER1G	protein_codin	9.55433698	23.3518444	0	7.39451414
ENSG000001	FHIP2B	protein_codin	5.81752428	35.9029534	19.762706	7.24148213
ENSG000001	SLC39A14	protein_codin	0	51.1878982	30.847496	0
ENSG000001	WFDC8	protein_codin	0	0	0	0
ENSG000001	CHRNB3	protein_codin	0	0	0.90088524	2.24673241
ENSG000001	PPP3CC	protein_codin	11.9430231	0	11.951083	18.188533
ENSG000001	ARHGAP17	protein_codin	5.40487827	29.7529317	13.9646457	20.6788214
ENSG000002	C8orf58	protein_codin	0	18.4431993	0	0
ENSG000001	WNT9B	protein_codin	0.5699552	5.77969869	0	2.77056561
ENSG000001	SEC16A	protein_codin	7.38881553	11.4624946	10.0256468	0
ENSG000001	CACHD1	protein_codin	0	0	0	0
ENSG000001	SLC25A37	protein_codin	5.2339214	0	13.9590791	0
ENSG000002	TMEM141	protein_codin	4.82282269	0	16.6988962	1.58751922
ENSG000001	CELA3A	protein_codin	0	0	0	0
ENSG000001	MIS18A	protein_codin	4.76142866	6.29549817	29.7349974	1.52064804
ENSG000001	CFAP298	protein_codin	7.19666483	0	3.98935339	8.29407797
ENSG000001	KPNB1	protein_codin	23.1008566	90.8318383	22.9152286	20.6228119
ENSG000001	WNT4	protein_codin	0	0	0.54948456	1.31382914
ENSG000001	PAXBP1	protein_codin	5.80224275	0	16.4120583	3.81661138
ENSG000002	IL10RB	protein_codin	5.96337903	28.4353648	10.6369856	3.10164126
ENSG000001	MRPL10	protein_codin	0	0	0	0
ENSG000001	SLC12A6	protein_codin	7.72978128	33.5511533	14.7588155	6.95333881
ENSG000001	SCRN2	protein_codin	6.64014148	11.1692493	0	4.85455904
ENSG000001	IFNGR2	protein_codin	30.7088671	66.2644047	31.194222	31.6353878
ENSG000001	NCOA5	protein_codin	19.5879958	46.7899149	50.6676073	15.1411318
ENSG000002	CRYZL1	protein_codin	5.02418198	0	7.20624748	2.47862664
ENSG000000	ELMO2	protein_codin	3.96952819	32.265164	0	0
ENSG000001	STC1	protein_codin	4.59216812	0	6.4425936	9.2201177

ENSG000000	OGFR	protein_codin	12.0327504	66.7596486	33.0472472	15.3694731
ENSG000001	PRAC1	protein_codin	0	0	0	0
ENSG000001	HOXB13	protein_codin	0.24920166	4.08253045	3.98847313	3.20185422
ENSG000002	ATP5PO	protein_codin	28.9279727	19.8370076	61.5190986	23.6625825
ENSG000001	KCNE2	protein_codin	0	0	1.42887395	0
ENSG000001	SLC13A3	protein_codin	0	0	0	2.1694749
ENSG000001	SNF8	protein_codin	8.21537733	0	13.5313255	4.15142977
ENSG000001	IGF2BP1	protein_codin	0	12.6339362	46.2252569	3.86926062
ENSG000001	CBR1	protein_codin	13.0872681	56.0397934	76.8201011	7.91539264
ENSG000001	CBR3	protein_codin	4.51329852	10.540271	7.80420844	5.55988413
ENSG000001	CIART	protein_codin	0	0.86535229	4.27326406	0
ENSG000001	GJD2	protein_codin	0	0	0	1.8737208
ENSG000001	ACTC1	protein_codin	16.0924147	0	0	0
ENSG000002	C2orf81	protein_codin	0	0	0	0
ENSG000001	SIM2	protein_codin	0.58659982	5.13457164	13.6515619	0.67587122
ENSG000001	GFRA4	protein_codin	0	0	0	0.41341208
ENSG000001	CCDC142	protein_codin	0	0	0	0
ENSG000000	PPP1R12B	protein_codin	0	0	0	0
ENSG000001	MCAT	protein_codin	0	0	5.52357376	0
ENSG000000	PKD2L2	protein_codin	0	0	0	0
ENSG000001	GOLGA6A	protein_codin	0	0	0	5.60834633
ENSG000001	SCUBE1	protein_codin	0.42188859	25.91616	66.885387	2.62242116
ENSG000001	ARHGAP27	protein_codin	0	0	0	0
ENSG000001	PLA2G4D	protein_codin	0	0	0	0
ENSG000001	PLA2G4F	protein_codin	0	0	0	0
ENSG000001	P3H3	protein_codin	4.37439493	15.6260559	6.8084981	8.8770797
ENSG000001	RFX5	protein_codin	17.4385483	0	6.05318627	0
ENSG000001	M1AP	protein_codin	0	0	0	0
ENSG000001	PSMB4	protein_codin	16.7747058	38.2545246	34.523947	4.26162467
ENSG000001	BTG2	protein_codin	18.8163162	104.124996	18.103359	92.5668037
ENSG000001	IRX6	protein_codin	0.52614943	6.11316296	12.4965105	2.83459359
ENSG000001	CES5A	protein_codin	0	0	0	0
ENSG000001	HK2	protein_codin	19.1223222	7.61763655	79.6804612	36.1813027
ENSG000001	CELF3	protein_codin	0	1.14373514	0	1.35266322
ENSG000001	CELF3	protein_codin	0	0	0	0
ENSG000001	ALDH4A1	protein_codin	0	0	0	0
ENSG000001	STARD9	protein_codin	73.859395	225.145215	94.7415924	60.9399828
ENSG000001	AMFR	protein_codin	25.7929481	17.791346	30.5334312	12.8805723
ENSG000001	UBR1	protein_codin	32.8461927	95.9067366	77.2540712	20.4443268
ENSG000001	MED8	protein_codin	11.0846584	29.7977368	9.35273657	0
ENSG000001	RGL4	protein_codin	0	0	0	0
ENSG000002	MT1A	protein_codin	0	0	0	1.65673756
ENSG000001	PGLYRP3	protein_codin	0	0	0	0
ENSG000000	DERL3	protein_codin	0	0	0	0.80380824

ENSG000001	ISL2	protein_codin	0	1.56938083	4.18712682	0
ENSG000001	GSTT2B	protein_codin	1.06942553	0	4.88907703	2.74293493
ENSG000001	CPNE2	protein_codin	2.50354438	0	7.53961986	0
ENSG000001	GPBP1L1	protein_codin	44.0428101	81.2567536	0	42.2229016
ENSG000001	NAE1	protein_codin	11.8239544	44.7387098	81.5329303	11.1580006
ENSG000000	CLEC2D	protein_codin	13.5761479	85.0190738	24.445158	17.0917374
ENSG000000	CBFB	protein_codin	7.25534895	0	0	0
ENSG000001	ACE	protein_codin	3.60841046	0	0	0
ENSG000001	ACE	protein_codin	0	26.2308114	0	7.16746631
ENSG000001	UROC1	protein_codin	0	0	0	0
ENSG000001	SPMIP8	protein_codin	0	1.13438707	0.46240266	0
ENSG000001	MATCAP1	protein_codin	0	43.626435	17.1521389	7.19671997
ENSG000001	SHF	protein_codin	0	0	0	1.53392195
ENSG000001	SPON2	protein_codin	2.34986027	7.25705807	0	0.83387514
ENSG000001	CHCHD6	protein_codin	0	2.63460541	0	0
ENSG000001	CTBP1	protein_codin	0	84.3110864	41.3734671	8.65309342
ENSG000001	TPPP3	protein_codin	0	0	0	0
ENSG000002	ANKRD18B	protein_codin	0	0	0	0
ENSG000001	ATP6V0D1	protein_codin	9.37155533	0	0	1.68449551
ENSG000001	AGRP	protein_codin	0	0	0	0.55205877
ENSG000001	ZFYVE28	protein_codin	2.59212951	10.6322974	20.6875629	3.44637768
ENSG000001	PIP	protein_codin	0	0	0	0
ENSG000001	PSKH1	protein_codin	3.93777418	9.58924645	9.30951645	5.67518154
ENSG000001	VPS53	protein_codin	0	3.37965864	0	3.32064655
ENSG000001	ABR	protein_codin	10.4897733	56.6321909	11.5930043	7.02623129
ENSG000001	ZNF235	protein_codin	1.1381208	0	0	0
ENSG000001	TNFRSF13C	protein_codin	0.29272904	3.59143762	10.269405	2.15169274
ENSG000001	DMPK	protein_codin	0	0	0	0
ENSG000001	PRKD2	protein_codin	4.94158312	30.2325173	0	10.3638437
ENSG000001	PTGIR	protein_codin	1.27327549	5.58796246	0.59541342	0.43051805
ENSG000001	CALM3	protein_codin	18.5171901	99.4542509	47.6274681	25.2413481
ENSG000001	HIF3A	protein_codin	0	0	0	0
ENSG000001	IQCC	protein_codin	2.73824163	0	0	2.7807181
ENSG000001	SSU72	protein_codin	30.5067318	118.296558	138.742928	28.0602452
ENSG000001	TRIM62	protein_codin	3.70387098	14.3908281	5.38904992	8.34250947
ENSG000000	AP1M1	protein_codin	9.76553494	17.8958646	35.6836257	7.52418019
ENSG000001	CPAMD8	protein_codin	1.82408457	0	0	1.74494641
ENSG000001	NR2F6	protein_codin	6.15162388	11.9888724	11.4240516	6.21311218
ENSG000001	MIX23	protein_codin	0	0	0	0
ENSG000001	KALRN	protein_codin	0	0	0	6.21290912
ENSG000001	HAPLN4	protein_codin	0.25590181	1.37677062	5.01272918	0
ENSG000001	CILP2	protein_codin	13.9126679	130.805862	27.3264054	5.70856285
ENSG000001	ATP13A1	protein_codin	0	0	0	0
ENSG000001	TFF2	protein_codin	0	0	0	0

ENSG000001	TFF1	protein_codin	0	0	0	0
ENSG000001	UBASH3A	protein_codin	0	0	0	0
ENSG000001	RSPH1	protein_codin	0.62897358	0	1.87652293	0
ENSG000001	PDE9A	protein_codin	3.72636385	0	0	11.0357597
ENSG000001	PKNOX1	protein_codin	13.5971441	12.6792935	18.8389908	0
ENSG000001	U2AF1	protein_codin	0	0	17.0985627	0
ENSG000001	CRYAA	protein_codin	0	0	0	0
ENSG000001	HSF2BP	protein_codin	0.53582254	15.1150767	0.86788795	0.89346835
ENSG000001	PDXK	protein_codin	0	0	50.8398047	17.1343642
ENSG000001	CSTB	protein_codin	14.6379372	45.8500248	20.3922944	39.7171023
ENSG000001	AGPAT3	protein_codin	12.2104116	7.59463112	16.036357	7.49741549
ENSG000001	TRAPPC10	protein_codin	21.7030381	48.920024	48.9972906	26.4372147
ENSG000002	PWP2	protein_codin	3.67067065	0	0	0
ENSG000001	GATD3	protein_codin	0	0	0	0
ENSG000001	AIRE	protein_codin	0	0	0	0
ENSG000001	LRRC3	protein_codin	5.59761643	15.1638863	21.9186271	3.18227498
ENSG000001	ZNF583	protein_codin	3.74525751	13.0460273	6.91159367	4.92943169
ENSG000001	KIR2DL1	protein_codin	0	0	0	0.45172899
ENSG000001	SLX9	protein_codin	1.37117329	10.9448595	15.5304485	0
ENSG000001	FTCD	protein_codin	0	0	0	0
ENSG000001	SPATC1L	protein_codin	1.45371547	1.64340792	1.93297404	3.07564344
ENSG000001	MCM3AP	protein_codin	85.1451753	522.040625	49.7897162	0
ENSG000001	C21orf58	protein_codin	0	10.7697667	14.1019476	0
ENSG000001	S100B	protein_codin	3.36916119	12.5213294	0	0.59145898
ENSG000001	PRMT2	protein_codin	16.521777	0	20.4472209	2.21247107
ENSG000002	SIGLEC12	protein_codin	0	0	0	1.98628094
ENSG000001	CLDND2	protein_codin	0	1.07514912	0	0
ENSG000001	CACFD1	protein_codin	0	3.88007321	0	0
ENSG000001	KLK8	protein_codin	0	0	0	0
ENSG000001	FCN2	protein_codin	0	0	0	0
ENSG000001	ZNF626	protein_codin	5.00816438	8.35635137	2.3920765	0
ENSG000001	PSG9	protein_codin	0	0	0	0
ENSG000002	LILRA4	protein_codin	0	8.19837744	0	0
ENSG000001	CYP2A7	protein_codin	0	0	0	0
ENSG000001	GPSM1	protein_codin	0.8460148	1.04298502	0.74393744	0
ENSG000001	HIPK4	protein_codin	0	2.07308679	0.5359597	0.42800142
ENSG000001	PRX	protein_codin	0	9.50792171	0	6.67062716
ENSG000001	ST6GALNAC6	protein_codin	0	0	0	0
ENSG000001	SHKBP1	protein_codin	0	25.9244686	0	7.74214498
ENSG000002	KIR3DL3	protein_codin	0.57950128	1.52819743	0	0.37686535
ENSG000001	NCR1	protein_codin	0	2.50714482	0	0
ENSG000001	RDH13	protein_codin	0.32598321	0	1.22213931	0.89446889
ENSG000001	ZER1	protein_codin	10.3839522	23.046266	24.7014976	7.15127234
ENSG000001	TNNT1	protein_codin	38.8749867	0	0	0

ENSG000001(	PKN3	protein_codin	2.74149845	14.3226484	8.65250508	6.54329356
ENSG000001(	TMEM190	protein_codin	0	0	0	0
ENSG000001'	NLRP8	protein_codin	0	0	0	0
ENSG000001(	MED27	protein_codin	1.2050465	7.86276398	5.10442105	0.98565635
ENSG000001(	JAML	protein_codin	0	33.9076789	0	0
ENSG000001!	ZNF667	protein_codin	12.0929076	9.06799202	0.89525754	0
ENSG000001:	FXYD2	protein_codin	0	0	0	0
ENSG000001(	TLCD1	protein_codin	0	0	1.57187063	0.4766259
ENSG000002	TMEM199	protein_codin	5.09221705	14.9817134	17.3089848	7.55618443
ENSG000001(	SAFB	protein_codin	2.91379519	114.695413	14.7090594	15.2927317
ENSG000001'	PSG6	protein_codin	0	0	0	0
ENSG000001'	PHLDB3	protein_codin	0	24.3584709	3.77708392	3.18783682
ENSG000001(	CD3G	protein_codin	0	0	0	0
ENSG000001(	ETHE1	protein_codin	2.13510826	0	3.09063714	0
ENSG000001(	S100A1	protein_codin	5.76851331	4.00609066	3.47265178	0
ENSG000001(	CXCR5	protein_codin	0	3.261569	0	3.43969024
ENSG000001(	ZBTB7B	protein_codin	25.2272473	15.6748733	25.0708606	4.77297914
ENSG000001(	FLAD1	protein_codin	3.40657877	17.1469709	34.7256603	3.2101688
ENSG000001(	NLRX1	protein_codin	0	0	0	0
ENSG000001(	UBE2Q1	protein_codin	0	0	0.83873656	3.35735171
ENSG000001(	ANO10	protein_codin	0	0	10.328406	0
ENSG000001(	RUSC1	protein_codin	0	0	0	0
ENSG000001(	PAQR6	protein_codin	0	0	0	0
ENSG000001(	CCR5	protein_codin	5.71097158	65.844527	0	8.00054766
ENSG000001(	CCDC12	protein_codin	0	0	0	0
ENSG000001(	MYL3	protein_codin	0	0	0	0
ENSG000001(	PPP1R35	protein_codin	1.9118569	1.1545252	1.46108367	0
ENSG000001	PEAR1	protein_codin	1.35655097	18.9793921	0	10.0105286
ENSG000000'	RNF126	protein_codin	0	0	9.05297091	2.47028418
ENSG000002	GPC2	protein_codin	0.36785741	0	2.31620543	0
ENSG000001	DBN1	protein_codin	92.1984546	5.45831929	403.818382	0
ENSG000001(	ZNF3	protein_codin	0	0	0	0
ENSG000001(	AZGP1	protein_codin	0.56487408	1.75555634	0	0
ENSG000001(	FGFR4	protein_codin	0	0	0	0
ENSG000001(	CYP11B1	protein_codin	0	0	0	0
ENSG000001(	LY6K	protein_codin	0	10.9604996	22.3717836	2.26584868
ENSG000001(	NACC1	protein_codin	11.2949313	31.5736067	71.4659751	8.77727716
ENSG000001(	HK3	protein_codin	0.92173491	13.3149146	0	0.42391661
ENSG000001(	IER2	protein_codin	17.7759096	105.154942	22.3253193	237.814685
ENSG000001(	ZSCAN21	protein_codin	15.0389916	0	30.5841357	0
ENSG000002	ATP5MF	protein_codin	6.47773813	3.22365394	10.8556577	7.33125388
ENSG000001(	CPSF4	protein_codin	0	0	6.43910904	2.73342503
ENSG000001(	PTCD1	protein_codin	2.61680622	22.5868199	9.22090109	7.04331075
ENSG000001(	LY6E	protein_codin	7.76425645	92.5916918	61.6549282	4.54389069

ENSG000001(	VPS28	protein_codin	0	0	0	1.80579564
ENSG000001(	PTGER1	protein_codin	0	0	0	0
ENSG000001(	LRRC14	protein_codin	7.48792919	32.0178984	20.9332706	4.94866444
ENSG000001(	ZNF333	protein_codin	8.03647364	0	8.70239639	11.3279445
ENSG000002:	CUX1	protein_codin	0	0	86.534169	0
ENSG000002:	CUX1	protein_codin	0	0	0	0
ENSG000001(	PPP1R16A	protein_codin	1.75645701	0	0	0
ENSG000001!	ZNF251	protein_codin	20.1799876	31.3668499	62.5214977	14.3448951
ENSG000001(	ALKBH4	protein_codin	1.88644511	0	7.52547829	0
ENSG000001(	TEKTL1	protein_codin	0	0	0	0
ENSG000001:	ABHD17A	protein_codin	4.38975623	0	0	5.18528241
ENSG000001!	ZNF250	protein_codin	0	0	0	0
ENSG000001(	MRNIP	protein_codin	0	0	0	0
ENSG000001(	MGAT4B	protein_codin	5.33231973	13.6274424	13.3605405	1.12279816
ENSG000002	LTC4S	protein_codin	0.85521502	1.52987314	0	0
ENSG000001(	MAML1	protein_codin	33.9583051	93.639368	121.01536	33.9344479
ENSG000001(	PGLYRP2	protein_codin	0	0	0	0.43595103
ENSG000000(	POLR2J	protein_codin	35.8595239	42.245766	169.319632	23.9150117
ENSG000001(	LRWD1	protein_codin	3.12385146	2.76218521	5.22124785	1.69794192
ENSG000001(	SCGB3A1	protein_codin	0	0	0	0.32384143
ENSG000001(	PSMC2	protein_codin	33.4801172	0	112.278543	21.1141103
ENSG000001(	CELF5	protein_codin	0	0	0	0
ENSG000000!	MED15	protein_codin	0	0	0	0
ENSG000002:	TUBA3FP	protein_codin	0	0	0.56360993	0
ENSG000001(	YDJC	protein_codin	0	0	0	0
ENSG000001(	CCDC116	protein_codin	0	1.76369987	1.07772301	1.70787318
ENSG000000:	DCUN1D1	protein_codin	23.3538895	65.4886053	55.6026093	17.1545719
ENSG000001(	AP2M1	protein_codin	0	70.7935517	48.0799249	0
ENSG000001(	ABCF3	protein_codin	0	16.4939016	4.29979568	0
ENSG000001(	PCYT1A	protein_codin	1.47890474	0	2.163153	16.5261929
ENSG000001!	ZNF585A	protein_codin	0	16.837769	7.67185556	2.10593682
ENSG000002(	FBXO17	protein_codin	4.29807147	8.06725745	12.5271568	1.2525742
ENSG000001(	FBXO27	protein_codin	1.39701226	2.26240496	0	0
ENSG000001(	U2AF1L4	protein_codin	0.60734358	0	0	0
ENSG000001(	THAP8	protein_codin	2.57379367	11.2186434	7.84481935	1.78818635
ENSG000002	HBE1	protein_codin	0	0	0	0
ENSG000002:	HBD	protein_codin	0	0	0	0
ENSG000001(	COX7A1	protein_codin	2.07806309	7.95224319	0	0.94174224
ENSG000001(	ZNF382	protein_codin	6.5236124	9.67381309	8.31945521	2.95674426
ENSG000001(	IKZF3	protein_codin	0	0	0	0
ENSG000001(	GRIN2C	protein_codin	0	0	2.54006661	0.48543267
ENSG000001(	FDXR	protein_codin	0.80451116	0	4.86920999	0
ENSG000001(	SAP30BP	protein_codin	0	10.1585391	0	0
ENSG000001(	ACOX1	protein_codin	47.1493804	0	43.149176	0

ENSG000001(	CYGB	protein_codin	9.10651325	58.8779208	4.41898312	17.9713095
ENSG000001(	CABP5	protein_codin	0.28070335	0	0	2.1524314
ENSG000001(	TMEM143	protein_codin	0	0	0	0
ENSG00000(	BAX	protein_codin	0.40217672	0	1.53224705	0.53548983
ENSG000001(	KLHL10	protein_codin	0	0	0	2.06261574
ENSG000001	KRT8	protein_codin	0	0	0	0
ENSG000001	STAT5B	protein_codin	0	0	0	0
ENSG000001(	HCRT	protein_codin	0	0	0	0
ENSG00000(	PLEKHH3	protein_codin	0	0	3.23952389	0
ENSG000001(	ALDH16A1	protein_codin	3.35770412	19.5554787	10.1363383	4.85926431
ENSG000001:	PSME3	protein_codin	0	0	0	0
ENSG000001(	DCD	protein_codin	0	0	0	0
ENSG000001:	NCKAP1L	protein_codin	9.70051832	135.9531	3.76314294	32.1739605
ENSG000001(	ITGA5	protein_codin	43.8997764	98.3916083	60.56036	42.1403898
ENSG000001(	CD300LG	protein_codin	0	0	0	0
ENSG000001(	NAGS	protein_codin	0	0	0	0
ENSG000001(	IZUMO2	protein_codin	0	0	0	0.49200376
ENSG000001(	LSM12	protein_codin	9.10712303	22.2737762	24.0139046	14.6303498
ENSG000001(	ASB16	protein_codin	0	0	0	0
ENSG00000(	MYL6	protein_codin	0	0	0	2.67781681
ENSG000001(	SHANK1	protein_codin	2.0717863	39.8775727	23.1249156	13.232363
ENSG000001(	FAM171A2	protein_codin	0	3.27882046	0.66266087	0
ENSG000001	ZNF613	protein_codin	6.17067801	8.06268041	5.6177181	1.77837688
ENSG000001	SDR9C7	protein_codin	0	0.99153618	0.55241341	0.43419664
ENSG000001	KRT86	protein_codin	0	0	0.57755151	0
ENSG000001:	WNT1	protein_codin	0	3.54162144	0	1.24809339
ENSG000001(	AQP5	protein_codin	0	0	0	0
ENSG000002(	C6orf136	protein_codin	0	0	0	1.88381551
ENSG000001(	LARP4	protein_codin	0	60.4227065	0	13.8121686
ENSG000001:	CELA1	protein_codin	0	0	0	0
ENSG000001	ZNF562	protein_codin	0	0	0	6.36427941
ENSG000001(	TAMALIN	protein_codin	4.46589126	9.7013511	0	14.753805
ENSG000001	KRT83	protein_codin	0	0	0	0.42978737
ENSG00000(	PDE4A	protein_codin	0	0	0	7.77385403
ENSG000001(	SYCE2	protein_codin	0.30603341	0	0.90119145	0.83338108
ENSG000001:	ZNF563	protein_codin	6.82390463	3.47451705	12.1511115	5.92000081
ENSG000001	KRT72	protein_codin	0	0	0	0
ENSG000001!	SYNGAP1	protein_codin	0	0	0	3.31405953
ENSG000001(	IP6K3	protein_codin	6.2387405	0	0	0
ENSG000001(	LEMD2	protein_codin	6.74500428	37.8564483	11.7195589	4.98611178
ENSG000001(	ALOX15	protein_codin	0	0	0	0
ENSG000001(	ZNF653	protein_codin	0	11.1628839	3.76244676	0
ENSG000001(	MED11	protein_codin	0.83153373	0	3.02344437	2.27172796
ENSG000001(	CXCL16	protein_codin	1.64888446	0	0	0

ENSG000001	SLC13A5	protein_codin	0	1.93095845	6.46589416	0
ENSG000001	BCL6B	protein_codin	7.32306847	30.3015414	9.6560926	15.5056302
ENSG000002	TNFSF12	protein_codin	5.16190293	4.25176107	0	0
ENSG000002	TNFSF12-TNF	protein_codin	0	39.7990654	2.16936925	5.70304431
ENSG000001	FGF11	protein_codin	0	0	0	2.04637737
ENSG000001	EIF4A1	protein_codin	66.9507227	0	236.118687	71.8781838
ENSG000001	CCDC42	protein_codin	0	0	0	0
ENSG000001	PRSS33	protein_codin	0	0	0	0
ENSG000001	POLR3K	protein_codin	3.10584917	2.63571288	23.257591	0.45833278
ENSG000001	SNRNP25	protein_codin	7.8709101	24.6282355	22.1554775	4.66122493
ENSG000000	LUC7L	protein_codin	0	0	13.609348	0
ENSG000000	PIGQ	protein_codin	0	0	0	0
ENSG000001	WDR90	protein_codin	1.21168438	0	0	0
ENSG000001	SSRP1	protein_codin	5.3507957	11.0435117	18.1296443	2.61258776
ENSG000001	WDR24	protein_codin	2.04968502	15.6044707	6.44026903	2.11773339
ENSG000001	CCDC78	protein_codin	0	0	0	0
ENSG000000	SOX8	protein_codin	0	0	7.19409804	1.76657361
ENSG000001	SSTR5	protein_codin	0	0	0	0
ENSG000002	PTX4	protein_codin	0	0	0.39769147	0
ENSG000000	CRAMP1	protein_codin	0	0	79.1222647	0
ENSG000001	CCNF	protein_codin	4.8393279	0	70.2935037	5.61585876
ENSG000001	AMDHD2	protein_codin	7.33588769	30.6075491	0	0
ENSG000001	NTN3	protein_codin	0.30012206	0	0.67106022	0
ENSG000001	PAQR4	protein_codin	1.56141943	0	0	4.83089156
ENSG000001	FLYWCH2	protein_codin	0	0	18.7091809	5.62229448
ENSG000001	SLX4	protein_codin	11.9402061	70.5214615	58.7493218	10.7932172
ENSG000001	ADCY9	protein_codin	48.8652976	97.9154053	115.845294	30.8135583
ENSG000001	CLPB	protein_codin	0	0	0	0
ENSG000001	NEU3	protein_codin	14.3316836	56.8717245	55.6090203	9.60272411
ENSG000001	MAP4K2	protein_codin	3.02163331	28.4185545	1.64115135	8.49690921
ENSG000001	CYB561A3	protein_codin	0	0	0	0
ENSG000001	GNG3	protein_codin	0.24287768	3.69730415	0	0.76337224
ENSG000001	UBXN1	protein_codin	0	0	0	1.20013433
ENSG000002	NCKIPSD	protein_codin	2.69874951	0	0	0
ENSG000001	TTC9C	protein_codin	0	4.96458082	0.5052291	0
ENSG000001	TAF6L	protein_codin	0.32289725	4.03224289	3.03964088	0
ENSG000001	NXF1	protein_codin	1.4704757	0	0	0
ENSG000001	STX5	protein_codin	4.68472935	49.6104831	4.02285236	0
ENSG000001	SLC25A45	protein_codin	0	0	0	0
ENSG000001	RPL29	protein_codin	176.320774	56.9898592	349.985673	158.873978
ENSG000002	DCP1A	protein_codin	0	0	2.44778233	8.58748233
ENSG000001	SPINDOC	protein_codin	5.15104894	19.0784346	21.6302821	4.95313763
ENSG000001	SYVN1	protein_codin	0	0	0	0
ENSG000001	ZFPL1	protein_codin	1.75453596	10.7156775	0	2.3781475

ENSG000001	CABP2	protein_codin	0	0	0	0
ENSG000001	LRP5	protein_codin	35.7799074	71.7440184	86.0555427	50.7742511
ENSG000001	TPCN2	protein_codin	3.93211672	22.7951953	5.23477882	0
ENSG000001	FGF19	protein_codin	0	0.94394502	0	0
ENSG000001	CYP4A22	protein_codin	0	0	0	0
ENSG000001	PDZK1IP1	protein_codin	0	0	0	0
ENSG000001	TAL1	protein_codin	0	11.5769223	0.70275086	0
ENSG000001	ZYG11B	protein_codin	51.8272123	76.2607744	72.0742566	28.6221376
ENSG000001	CZIB	protein_codin	5.62116213	12.4447691	10.3256591	3.5425734
ENSG000001	USP24	protein_codin	54.5804895	170.968281	139.376282	71.8949223
ENSG000000	DNAJC11	protein_codin	18.1674599	12.7573419	55.7632646	10.7959801
ENSG000001	GMEB1	protein_codin	8.15333537	8.23782963	19.9890861	13.0811313
ENSG000001	RHCE	protein_codin	0	0	0	0
ENSG000001	RAVER2	protein_codin	1.97184555	0	0	0
ENSG000001	RBP7	protein_codin	12.6675417	10.9225235	3.75425115	31.9084966
ENSG000001	KNCN	protein_codin	0	0	0	2.32334014
ENSG000001	SLC25A34	protein_codin	0.84654669	2.22993523	2.80274358	1.47377188
ENSG000002	DISP3	protein_codin	0	7.2414453	0	0.85589944
ENSG000001	DRAXIN	protein_codin	1.70545292	2.01884232	2.58448552	4.38530958
ENSG000001	PDPN	protein_codin	0	0	0	0
ENSG000001	LAPTM5	protein_codin	34.2271752	290.841571	12.0527525	93.2842433
ENSG000001	AZIN2	protein_codin	1.45669772	1.25956869	0	1.23541404
ENSG000001	NHSL3	protein_codin	1.80659331	0	0	0
ENSG000001	TMCO4	protein_codin	5.03762596	16.0509459	0	5.54681062
ENSG000001	MEGF6	protein_codin	0	0	0	0
ENSG000001	CCDC27	protein_codin	0	0	0	1.14267322
ENSG000001	DOCK6	protein_codin	17.9345921	114.641461	0	0
ENSG000001	FUBP1	protein_codin	19.0920281	110.867158	157.387532	29.9575754
ENSG000001	LRRC53	protein_codin	0	1.72833635	0	0.47756476
ENSG000001	LHX8	protein_codin	0	0	0	0
ENSG000001	NTNG1	protein_codin	6.49267024	7.88132682	3.9153007	2.85425499
ENSG000001	C1orf52	protein_codin	11.5138924	26.660178	22.2757927	4.07683326
ENSG000001	DNAI3	protein_codin	8.40490766	0	5.05306319	0
ENSG000002	GBP7	protein_codin	0	0	0	0.4603143
ENSG000001	ODF2L	protein_codin	0	0	0	0
ENSG000001	GFI1	protein_codin	0.38709139	0	20.7679719	3.94788077
ENSG000001	AGL	protein_codin	0	0	0	0
ENSG000001	KCNT2	protein_codin	0	0	0	0
ENSG000001	VCAM1	protein_codin	0	0	0	111.723946
ENSG000000	ASPM	protein_codin	0	0	0	0
ENSG000002	DENND1B	protein_codin	5.62316622	29.641575	2.91727429	3.74011762
ENSG000002	DENND1B	protein_codin	0	0	0.97554468	1.8279103
ENSG000001	ZNF281	protein_codin	0	0	0	0
ENSG000001	ARPC5	protein_codin	23.8737488	128.75891	31.1704444	13.3348912

ENSG000001	ZNF496	protein_codin	10.4831091	0	0	0
ENSG000001	NCSTN	protein_codin	10.3437242	0	18.430867	0
ENSG000001	OLFML2B	protein_codin	143.80211	947.601711	213.66743	205.361061
ENSG000001	FCRLA	protein_codin	0	0	0	0
ENSG000001	C1orf74	protein_codin	3.79919467	6.78153989	6.07807141	3.55805758
ENSG000001	LMX1A	protein_codin	0	0	3.67962139	0.51638858
ENSG000001	LRRC52	protein_codin	0	0	0	0
ENSG000001	GARIN4	protein_codin	0.35854075	0.92813667	0	3.02079142
ENSG000001	TDRD5	protein_codin	0	0	2.13941866	0
ENSG000001	RGSL1	protein_codin	0	0	0	0
ENSG000001	NMNAT2	protein_codin	1.44890517	14.3516944	0	2.03973155
ENSG000001	C1orf115	protein_codin	21.4155326	50.5604651	47.9534707	13.6122448
ENSG000001	BAZ2B	protein_codin	31.618421	15.7108935	82.3380001	15.6034165
ENSG000002	STPG4	protein_codin	0	0	0.68475411	0.41524002
ENSG000001	PPP1R21	protein_codin	0	84.8979421	92.6856432	22.9057906
ENSG000001	LHCGR	protein_codin	0	0	0	0
ENSG000001	PKDCC	protein_codin	0	0	4.17803742	0
ENSG000001	HAAO	protein_codin	2.30710014	7.90387645	2.16084898	1.57959568
ENSG000001	MAPKAPK2	protein_codin	3.89907113	7.08030779	6.74096477	8.72790589
ENSG000001	IL24	protein_codin	0	0	0	0
ENSG000001	CR1L	protein_codin	1.04392348	4.46401881	0	0
ENSG000001	CAPN2	protein_codin	34.4775033	229.298067	135.709432	61.47382
ENSG000001	MRPL55	protein_codin	0	0	2.91503831	0
ENSG000001	REL	protein_codin	2.75340391	0	4.25349558	0
ENSG000001	PEX13	protein_codin	22.6662482	46.6111334	48.8604666	10.4107862
ENSG000001	SANBR	protein_codin	0	0	1.61713063	0
ENSG000001	TRIM17	protein_codin	0	2.97354474	3.16427593	0
ENSG000001	RFTN2	protein_codin	31.3203631	38.2476502	1.78541482	30.4064759
ENSG000000	SPRTN	protein_codin	4.18300813	18.1777166	15.8786951	4.76006334
ENSG000001	DISC1	protein_codin	0	0	0	0
ENSG000001	CAPN13	protein_codin	0	0	1.56771771	0
ENSG000001	LRRTM1	protein_codin	0	0	0.63037573	0
ENSG000001	MEMO1	protein_codin	14.2707618	29.6148108	33.2645122	7.03253053
ENSG000001	DPY30	protein_codin	0	4.98884401	0	1.20390396
ENSG000001	MAIP1	protein_codin	0	0	0	0
ENSG000001	KCNF1	protein_codin	0	0	3.28128425	0
ENSG000001	SLC66A3	protein_codin	5.01495749	26.4631392	9.31208577	13.2655552
ENSG000001	ARL5A	protein_codin	60.65004	42.1449401	91.801957	26.8618184
ENSG000001	LRATD1	protein_codin	1.25252118	0	12.124873	3.47314771
ENSG000000	ARHGAP15	protein_codin	14.9154656	48.3105077	0	7.38904455
ENSG000001	KCNJ3	protein_codin	0	0	0	0
ENSG000001	NEUROD1	protein_codin	0	0	0	0
ENSG000001	FRZB	protein_codin	5.12757856	81.5356014	0.46239826	18.984263
ENSG000001	NUP35	protein_codin	4.79723041	0	9.05539261	6.60726826

ENSG000002	DNAAF10	protein_codin	5.69255653	7.0546598	0	3.26548514
ENSG000001	CCDC138	protein_codin	0	0	0	0
ENSG000001	ZSWIM2	protein_codin	0.31468735	0	0	0.55051125
ENSG000001	FBXO41	protein_codin	1.38125359	23.1145971	0	3.03621802
ENSG000001	WDCP	protein_codin	0	0	22.369451	1.22557156
ENSG000001	FAM228A	protein_codin	0.2950316	8.02698073	0	0.75750343
ENSG000001	VSNL1	protein_codin	2.19383205	0	14.3753158	0.56781439
ENSG000001	CCDC74A	protein_codin	0	0	0	0
ENSG000001	ANKRD30BL	protein_codin	0	0	0	0
ENSG000001	SLC16A14	protein_codin	1.87531345	0	2.09260944	2.64084021
ENSG000001	TEKT4	protein_codin	0.33164349	0	0	0
ENSG000001	EN1	protein_codin	20.4723731	30.3407925	19.4090055	11.8241529
ENSG000001	ZNF514	protein_codin	9.41103534	41.5970018	45.0766998	5.18501301
ENSG000001	SERPINB8	protein_codin	3.97470645	3.36567572	0	4.37415842
ENSG000001	SPATA18	protein_codin	12.9565152	21.2498318	0	5.55450852
ENSG000001	CFAP221	protein_codin	0	0	0	0
ENSG000001	KCNIP3	protein_codin	0	0	0	0
ENSG000001	INHBB	protein_codin	3.38788881	5.95009347	10.9301877	26.0673145
ENSG000000	CCNT2	protein_codin	10.9767535	46.7011015	0	8.1659005
ENSG000001	BBS5	protein_codin	1.71714631	0	5.92842683	0
ENSG000001	ANKRD36C	protein_codin	190.641581	417.778477	419.313035	73.908301
ENSG000001	HPGDS	protein_codin	2.06853946	4.9710173	2.60959733	8.18682692
ENSG000001	PDHA2	protein_codin	0	0	0	0
ENSG000001	STPG2	protein_codin	0.52722516	11.0373795	0.99039385	2.95664856
ENSG000001	SLC9A4	protein_codin	0.5721396	1.46998113	0	2.67738947
ENSG000001	PACRGL	protein_codin	0	1.07581188	0	0
ENSG000001	BNIPL	protein_codin	0	0	0	0
ENSG000001	C1QTNF7	protein_codin	7.665342	0	0	0
ENSG000001	CHAC2	protein_codin	0.50995175	1.36683866	13.5397737	0
ENSG000001	TMOD4	protein_codin	12.3164542	0	0	0
ENSG000001	RNF149	protein_codin	17.3496843	51.6517371	28.1413239	20.7913354
ENSG000001	IWS1	protein_codin	117.843277	327.977971	332.034292	57.8760571
ENSG000001	CDC42EP3	protein_codin	33.9429433	99.8178159	123.145826	141.177741
ENSG000001	BOLA3	protein_codin	1.47213125	0	0.76555025	0.58234631
ENSG000001	SPRR3	protein_codin	0	0	0	0.43143817
ENSG000001	BMP10	protein_codin	0	1.4450118	0	2.75694969
ENSG000001	TGFA	protein_codin	10.591057	12.3825332	21.1159395	4.41150075
ENSG000001	CPT1C	protein_codin	0	0	0	0.98054333
ENSG000001	MERTK	protein_codin	12.5376769	37.1582788	26.0259932	8.81009937
ENSG000001	CCNYL1	protein_codin	24.73784	30.7656316	58.1171554	27.7567825
ENSG000001	FZD5	protein_codin	6.91528733	96.8970047	40.4364004	36.8911618
ENSG000001	NPPC	protein_codin	0	0	0	0
ENSG000001	GNPDA2	protein_codin	0	8.488473	18.3432623	5.71585874
ENSG000001	GABRG1	protein_codin	1.81556813	1.70429266	0	5.30455308

ENSG000001(	ALPG	protein_codin	0	0.88333153	0	0
ENSG000001(	GABRB1	protein_codin	0	0	0	0.48835032
ENSG000001(	NIPAL1	protein_codin	0.29277672	0	1.57704252	1.07194578
ENSG000001(	ALPI	protein_codin	0	0	0	1.255035
ENSG000001:	HNRNPDL	protein_codin	6.7432315	5.17755238	0	5.1238396
ENSG000001(	HELQ	protein_codin	12.635316	8.52343304	0	0
ENSG000001(	MRPS18C	protein_codin	6.77951987	34.4419366	27.1843007	8.54654974
ENSG000001:	CHN1	protein_codin	13.4062706	0	0	0
ENSG000001(	GPR155	protein_codin	0	0	0.89139855	0
ENSG000001(	CLDN1	protein_codin	0.2952361	0	2.77739761	0.49585203
ENSG000001(	FLAD1	protein_codin	1.07320179	0	1.88897259	0
ENSG000001(	DCST1	protein_codin	0	0	0	0
ENSG000001:	ATP13A4	protein_codin	0	0	0	0
ENSG000001(	COL6A3	protein_codin	0	225.56523	21.2397486	91.4550798
ENSG000001(	YY1AP1	protein_codin	0	0	0	0
ENSG000001(	TAFA4	protein_codin	0	0	0	0
ENSG000001(	EOGT	protein_codin	0	6.28871784	0	0
ENSG000001(	POGLUT1	protein_codin	5.16955032	12.9840971	26.1321958	8.41226894
ENSG000001(	CCKAR	protein_codin	0	1.33160286	0.90260976	0.81117674
ENSG000001(	IGFN1	protein_codin	0	0	0	0
ENSG000001(	ATP1A1	protein_codin	164.703554	228.759578	307.1279	70.1322561
ENSG000001(	SLC15A2	protein_codin	0	0	0	0
ENSG000001(	EIF4E3	protein_codin	7.33569903	0	0	9.78040506
ENSG000001(	PROK2	protein_codin	5.30746294	0	0	0
ENSG000001(	TEX55	protein_codin	0	0	0	0
ENSG000001(	LRRC58	protein_codin	70.3788898	55.0713088	183.782256	45.9167177
ENSG000001(	FSTL1	protein_codin	18.7862849	194.317158	0	4.66737076
ENSG000001'	RNPEP	protein_codin	12.4119605	61.2852026	29.0853567	10.7394449
ENSG000001(	PDCL2	protein_codin	0	0	0.80138113	0.90140034
ENSG000001(	TMEM169	protein_codin	1.90132393	0	0	0
ENSG000001:	AMPD3	protein_codin	0	0	0	0
ENSG000001(	IGFBP7	protein_codin	236.746452	535.245331	207.378233	226.159124
ENSG000001(	KRTCAP2	protein_codin	32.3785041	16.7949859	66.1692662	6.79620256
ENSG000001(	CXCR1	protein_codin	0	0	0	2.74030869
ENSG000001(	ARPC2	protein_codin	1.31007454	10.3080206	4.52044349	1.10601441
ENSG000001(	CCT3	protein_codin	192.020829	122.865327	761.603359	34.6676844
ENSG000001(	TMEM79	protein_codin	1.0933522	8.15996765	1.83211426	1.3014361
ENSG000001.	CNOT9	protein_codin	10.1247032	0	20.1724322	4.91054629
ENSG000001(	SSR2	protein_codin	40.6127596	24.6515266	64.5378723	22.9889752
ENSG000001(	RNF25	protein_codin	8.44263515	15.2803427	0	4.80325563
ENSG000001.	PPFIA4	protein_codin	0	0	0	0
ENSG000001(	STK36	protein_codin	0	0	0	0
ENSG000000:	PTPRN	protein_codin	0	0	0	0
ENSG000001(	NEK10	protein_codin	0	0	0	2.99375866

ENSG000001	FEV	protein_codin	0.19211738	0	0	0.78142749
ENSG000001	CRYBA2	protein_codin	0	0	0	0
ENSG000001	CFAP65	protein_codin	0	0	0	0
ENSG000001	IHH	protein_codin	0	0	9.41996053	1.29047735
ENSG000000	SLC4A7	protein_codin	0	0	0	0
ENSG000002	SLC23A3	protein_codin	0	0	0.62042165	0
ENSG000001	EOMES	protein_codin	0	0	0	3.48288837
ENSG000001	CIP2A	protein_codin	16.0190177	0	100.487505	1.65170848
ENSG000001	AZI2	protein_codin	14.3739361	19.1202512	16.2368025	3.79092168
ENSG000001	ABCB6	protein_codin	13.6112077	0	0	0
ENSG000001	TGFBR2	protein_codin	38.9089201	94.6226234	8.20574643	40.3601358
ENSG000001	RETNLB	protein_codin	0	0	0	0.73464874
ENSG000001	TRAT1	protein_codin	0	0	0	0
ENSG000001	HDAC11	protein_codin	2.31422916	0	0	0
ENSG000001	GLB1L	protein_codin	0.90179554	0	2.01742391	0
ENSG000001	FBLN2	protein_codin	43.6558521	85.8905741	21.5261072	0
ENSG000001	FBLN2	protein_codin	0	56.6685687	0	0
ENSG000001	TUBA1A	protein_codin	0	0	0	3.79296539
ENSG000001	CHCHD4	protein_codin	0.59657758	0	1.03765712	0
ENSG000001	STT3B	protein_codin	30.1048138	85.1126175	75.565443	36.3934964
ENSG000001	SERPINI1	protein_codin	0	5.01782556	33.6091874	0.90533349
ENSG000001	PRKCI	protein_codin	25.524924	42.757203	70.0616292	12.5555252
ENSG000000	RETSAT	protein_codin	24.7879278	41.2389169	44.3879395	10.9804933
ENSG000001	IFI16	protein_codin	0	246.094734	0	0
ENSG000001	EIF5A2	protein_codin	7.39790275	5.44859816	17.150202	4.26391858
ENSG000001	EFHB	protein_codin	0	6.19335042	0	2.18863182
ENSG000001	RPL22L1	protein_codin	0.37226328	1.76854658	1.65655883	4.90521694
ENSG000001	FABP1	protein_codin	0	0	0	0.36480209
ENSG000001	PPM1L	protein_codin	0	0	0	0
ENSG000001	ABI2	protein_codin	4.35739627	27.5546573	16.7985181	11.025716
ENSG000001	CTLA4	protein_codin	0	0	0	0
ENSG000001	CD200R1	protein_codin	0	9.42262306	0.48770152	2.24409755
ENSG000001	GTPBP8	protein_codin	0	0	0	0
ENSG000002	CFAP44	protein_codin	0	20.2664232	0	0
ENSG000001	SPICE1	protein_codin	24.8021298	34.9398331	54.9953569	12.9246202
ENSG000001	PTPRG	protein_codin	23.7203699	171.735437	56.6438437	17.549414
ENSG000001	CCDC191	protein_codin	12.9052014	70.1191696	16.3491793	15.6518309
ENSG000001	DRD3	protein_codin	0	0	0	0
ENSG000001	NKX6-1	protein_codin	2.57455281	13.2382426	33.8294428	1.89957103
ENSG000001	CDS1	protein_codin	3.34953883	0	8.61908429	1.40743274
ENSG000001	WDFY3	protein_codin	93.2627009	403.351693	203.752189	105.009727
ENSG000001	COX18	protein_codin	0	10.5497532	0	0
ENSG000001	SYNPR	protein_codin	0	0	0	0
ENSG000001	C3orf49	protein_codin	0	0	0	0.45626061

ENSG000001(	ALB	protein_codin	0	0	0	0
ENSG000001(	C4orf36	protein_codin	0	0	0	0
ENSG000001(	THOC7	protein_codin	48.3299953	82.5477003	57.8178955	22.9324152
ENSG000001(	ATXN7	protein_codin	0	0	0	0
ENSG000001(	PSMD6	protein_codin	25.7375731	18.7546168	31.0150965	21.4311994
ENSG000001(	PRICKLE2	protein_codin	0	0	3.42529729	0
ENSG000001(	ADAMTS9	protein_codin	0	83.5167065	0	121.569235
ENSG000001(	PPM1K	protein_codin	8.67640835	9.86820868	10.7276785	0
ENSG000001(	ERICH6	protein_codin	0	0	0	0
ENSG000001(	CLRN1	protein_codin	0	0	0	0.49072571
ENSG000001(	GMPS	protein_codin	14.4483199	40.0880892	73.0609371	14.1499176
ENSG000001(	TIPARP	protein_codin	14.427042	14.3257695	5.61402499	62.9842958
ENSG000001(	CCNL1	protein_codin	0	0	0	0
ENSG000001(	CCNL1	protein_codin	16.6183718	0	22.6001674	63.0801955
ENSG000001(	PTX3	protein_codin	8.04615507	84.8685636	21.2645427	68.8658657
ENSG000001'	RSRC1	protein_codin	6.11345924	85.3390977	50.590709	22.2464006
ENSG000001(	HESX1	protein_codin	0	0	0	0
ENSG000001(	SLMAP	protein_codin	0	11.9542962	4.75848442	0
ENSG000001(	SLMAP	protein_codin	1.56348699	0	0	0
ENSG000001(	RPL9	protein_codin	5.10464795	0	0	0
ENSG000001:	FLNB	protein_codin	65.6730548	74.7398062	0	52.9132283
ENSG000001(	SMIM14	protein_codin	32.662879	64.5266171	21.2626562	22.2531632
ENSG000001(	RPP14	protein_codin	0	0	0	5.30274276
ENSG000001(	ABHD6	protein_codin	0	8.94676589	5.9790513	0
ENSG000001(	CFAP20DC	protein_codin	0	0	3.12512554	1.80631993
ENSG000001(	RBM47	protein_codin	0	0	0	13.794579
ENSG000001(	APBB2	protein_codin	14.9262064	56.7408728	40.2390681	0
ENSG000001(	IL17RC	protein_codin	0.40169854	0	0	0
ENSG000001(	PRRT3	protein_codin	0	0	0	0
ENSG000000(	SYN1	protein_codin	0	0	0	0
ENSG000001·	CAND2	protein_codin	0	0	0	0
ENSG000001(	PCOLCE2	protein_codin	8.25945916	0	54.7611826	0
ENSG000001(	MTMR14	protein_codin	7.47574242	1.59508854	0	15.3845051
ENSG000001(	TTC14	protein_codin	60.5333636	92.4732451	153.84502	18.7500888
ENSG000001(	CXCL3	protein_codin	0	0	0	0
ENSG000001(	CXCL5	protein_codin	0.30448138	0	0	1.51939367
ENSG000001(	PPBP	protein_codin	0	0	0	0
ENSG000001(	PF4	protein_codin	0	0	0	0.46486838
ENSG000000(	CXCL2	protein_codin	0	0	0	0
ENSG000001:	SHROOM3	protein_codin	22.9428838	26.5948858	12.7602332	9.9688639
ENSG000001(	CPA3	protein_codin	6.38511779	48.7326484	0	48.2709966
ENSG000001(	GYG1	protein_codin	8.87017066	19.1388682	25.2517188	5.51255742
ENSG000001(	HPS3	protein_codin	1.26555892	98.9963323	10.6830896	8.29984441
ENSG000001(	TM4SF18	protein_codin	4.05747038	37.8718904	5.74112003	19.1808537

ENSG000001	SNRK	protein_codin	0	25.2605394	14.0215248	61.0335682
ENSG000001	ZNF502	protein_codin	0	0	0	4.93393569
ENSG000001	ZNF35	protein_codin	0	0	0	2.56329458
ENSG000001	TCF23	protein_codin	0.74848316	0	0	2.24053229
ENSG000001	DNAJC5G	protein_codin	0	0	0	0
ENSG000001	TRIM54	protein_codin	0.94981152	0	0	0
ENSG000001	UCN	protein_codin	0	0.72855964	0	0
ENSG000002	MRPL33	protein_codin	6.98732162	0	7.04608623	5.49575847
ENSG000001	KIAA1143	protein_codin	26.172001	32.5367987	32.1007519	17.7525263
ENSG000002	PPP1CB	protein_codin	218.902704	0	485.516167	100.37185
ENSG000001	TGM4	protein_codin	0	0	0	0
ENSG000001	WDR43	protein_codin	0	0	1.30123336	0
ENSG000001	ZDHHC3	protein_codin	5.45732814	0	4.29628661	4.96476771
ENSG000001	CDCP1	protein_codin	1.01541018	9.23127	0	6.45969166
ENSG000001	CLEC3B	protein_codin	27.4821879	47.1852976	2.36340401	10.415698
ENSG000001	LZTFL1	protein_codin	0	0	12.7406554	0
ENSG000001	FYCO1	protein_codin	67.0036683	164.389861	33.7677863	51.8721298
ENSG000001	CCR1	protein_codin	12.9332756	84.826009	3.80563232	16.1998456
ENSG000001	RTP3	protein_codin	0	0	2.42526378	0.84094342
ENSG000001	LRRC2	protein_codin	0	0	0	2.08251999
ENSG000002	CRIPTO	protein_codin	0.8170334	0	0	2.01949549
ENSG000001	ELP6	protein_codin	4.34044808	0	0	4.5071408
ENSG000001	DTX3L	protein_codin	68.3541337	300.108701	158.846794	55.3520625
ENSG000000	ITGB5	protein_codin	115.744728	65.8486444	147.665786	61.4375108
ENSG000001	NMNAT3	protein_codin	0	0	0	0
ENSG000001	TPRA1	protein_codin	1.990711	15.7225491	5.07617264	0
ENSG000001	MEAF6	protein_codin	3.86782501	2.83967405	21.3904746	5.53673474
ENSG000001	SNIP1	protein_codin	12.5078958	58.9401191	14.3456213	7.22682423
ENSG000001	DNALI1	protein_codin	0	0	0	0
ENSG000001	OSBPL11	protein_codin	21.6942946	42.4608711	57.9282138	28.4805682
ENSG000001	KLF15	protein_codin	3.32730943	7.25075246	5.46243716	1.9965853
ENSG000001	CAMK2N2	protein_codin	0	0.68350556	2.59365094	0.4825689
ENSG000001	LIPH	protein_codin	0	2.46264559	0.53057405	2.15370937
ENSG000001	TMEM41A	protein_codin	0	0	4.49623571	0
ENSG000001	RPN1	protein_codin	127.248704	192.146228	382.613413	69.6609354
ENSG000001	SENP2	protein_codin	5.92654605	4.991756	20.5309869	8.21047504
ENSG000001	IFT122	protein_codin	0	0	0	0
ENSG000001	RHO	protein_codin	0	0	0	0.41849643
ENSG000001	RFC4	protein_codin	2.52291985	0	19.0092619	0
ENSG000001	RPL39L	protein_codin	0	2.84923552	26.4553794	0
ENSG000001	MASP1	protein_codin	0	0.90642115	0	0
ENSG000001	BAP1	protein_codin	0	0	0	0
ENSG000001	RFT1	protein_codin	2.55868563	11.3291809	6.95364856	5.57123595
ENSG000001	PBRM1	protein_codin	0	0	0	0

ENSG000001	ARHGEF3	protein_codin	0	82.1291041	0	9.91187981
ENSG000001	IL17RD	protein_codin	5.35500651	9.63488215	8.24274362	7.69342617
ENSG000001	LRPAP1	protein_codin	68.146891	116.073363	189.648501	19.3246279
ENSG000001	ZDHHC19	protein_codin	0	0	0	0.75907087
ENSG000001	SLC51A	protein_codin	0	0	0	0
ENSG000001	UBXN7	protein_codin	71.0478966	143.230433	161.164507	57.7777261
ENSG000001	PIGX	protein_codin	0	0	0	0
ENSG000001	RUBCN	protein_codin	12.6584544	46.0938328	21.4199557	19.1324287
ENSG000001	MELTF	protein_codin	1.53150014	27.313745	40.4294469	4.52999299
ENSG000001	MELTF	protein_codin	1.36690683	14.4884056	33.2166874	3.95289465
ENSG000001	OTOP1	protein_codin	0	2.34819534	0.50595846	0.42622912
ENSG000001	S100P	protein_codin	0	0	0	0
ENSG000001	EXO5	protein_codin	0	0	0	0
ENSG000001	CLDN19	protein_codin	0.65193309	0	0	1.95152369
ENSG000001	P3H1	protein_codin	43.5416368	25.1339646	92.5953731	14.1146814
ENSG000001	CAMK2D	protein_codin	0	49.2766462	29.561141	11.3955882
ENSG000001	METAP1	protein_codin	15.0806807	34.9268079	44.3510739	11.903315
ENSG000001	ADH5	protein_codin	9.667026	31.4582658	5.61273681	7.61748821
ENSG000000	DAPP1	protein_codin	0	0	0	0
ENSG000001	H2AZ1	protein_codin	10.9594692	29.578769	152.777434	0
ENSG000001	EMCN	protein_codin	11.5402066	68.7936932	4.36853742	31.8550603
ENSG000001	SLC9B1	protein_codin	0	0	0	0
ENSG000001	BDH2	protein_codin	9.62208273	12.2039775	5.82040694	8.75618497
ENSG000001	PGRMC2	protein_codin	11.1981814	82.5556533	40.4549273	17.0288906
ENSG000001	CAMP	protein_codin	0	0	0	0
ENSG000001	FBXW12	protein_codin	0	0	0	0
ENSG000001	PLXNB1	protein_codin	1.57395927	9.91489395	34.1577581	15.3764756
ENSG000001	SHISA5	protein_codin	0.92350263	0	0	0
ENSG000001	PRKAR2A	protein_codin	3.99106106	0	0	0
ENSG000001	IHO1	protein_codin	0	19.5552878	0	0
ENSG000001	BSN	protein_codin	6.00989083	6.0928992	6.52303977	9.87207364
ENSG000001	APEH	protein_codin	0	0	18.9229936	0
ENSG000001	HSPA4L	protein_codin	19.0245002	6.3384277	103.81311	7.64961269
ENSG000001	MFSD8	protein_codin	0	0	0	0
ENSG000001	CAMKV	protein_codin	0	0	0	0
ENSG000001	MON1A	protein_codin	4.24329248	0	0	2.33524221
ENSG000001	MST1R	protein_codin	0	0	0	0
ENSG000001	GRM2	protein_codin	0	0	0	0
ENSG000001	POC1A	protein_codin	0	0	8.19590259	0
ENSG000001	ETNPPL	protein_codin	0	0	0	0
ENSG000001	PPM1M	protein_codin	0.72623337	20.5304568	1.58633591	1.32647506
ENSG000001	WDR82	protein_codin	66.4437167	140.992011	43.6881692	40.6577374
ENSG000001	PRSS12	protein_codin	4.64092015	3.52245469	41.1077131	1.76102678
ENSG000001	NDST3	protein_codin	6.96912744	0	18.036822	3.6310124

ENSG000001	HMGB2	protein_codin	35.9857149	29.4248339	184.763759	13.4503232
ENSG000001	SAP30	protein_codin	29.3885909	39.3268174	47.6322231	14.633139
ENSG000001	SCRG1	protein_codin	5.1104392	4.72890976	0	3.454801
ENSG000001	MAD2L1	protein_codin	3.10353088	17.3013313	44.503827	3.48725161
ENSG000001	ANXA5	protein_codin	261.055251	405.982428	245.255403	129.816871
ENSG000001	ADAD1	protein_codin	0	0	0	0
ENSG000001	GUCY1A1	protein_codin	0	0	0	0
ENSG000001	CEP44	protein_codin	6.26483137	0	0	5.12648389
ENSG000001	HPGD	protein_codin	0	0	0	0
ENSG000001	HPGD	protein_codin	4.40993229	0	0	11.5727547
ENSG000001	ASB5	protein_codin	8.24427585	0	0	0
ENSG000001	GRIA2	protein_codin	0	0	0	0
ENSG000001	TMEM144	protein_codin	5.67715785	0	0	0
ENSG000001	GASK1B	protein_codin	6.63551952	163.553473	102.155977	0
ENSG000001	NPY1R	protein_codin	4.38520144	14.8267755	23.8597658	2.89902401
ENSG000001	NAA15	protein_codin	16.8464022	42.0011585	27.4641964	17.1996538
ENSG000001	IL15	protein_codin	0	11.6302461	0	1.75799276
ENSG000001	DCLK2	protein_codin	0	3.55225995	10.5100282	7.20148383
ENSG000001	FBXW7	protein_codin	0	13.0910433	33.4036846	0
ENSG000001	ICE1	protein_codin	73.7853336	140.621776	131.76048	56.8147535
ENSG000001	HHIP	protein_codin	45.1346578	1.49266535	37.489823	4.36736551
ENSG000001	ABCE1	protein_codin	30.6792555	45.6995515	162.820538	31.719177
ENSG000001	LSM6	protein_codin	0.63044542	2.77093756	8.52651883	2.11287082
ENSG000001	TMEM184C	protein_codin	32.1564321	28.8458135	60.5405	15.4380774
ENSG000001	ITGA2	protein_codin	4.31804882	20.3394838	115.14289	23.6103964
ENSG000001	SLC45A2	protein_codin	0	0	0	0
ENSG000001	EDIL3	protein_codin	86.3451834	113.068906	50.2954715	19.696396
ENSG000001	TMEM161B	protein_codin	10.5527568	0	0	0
ENSG000001	NDUFAF2	protein_codin	16.7911207	12.7483807	53.4637886	9.67554707
ENSG000001	ZNF474	protein_codin	0.26039579	0	0	0
ENSG000001	LMBRD2	protein_codin	23.2640657	40.0153029	77.0369343	14.9635891
ENSG000001	RANBP3L	protein_codin	15.0677753	39.7455965	106.398782	28.4064746
ENSG000001	RNF180	protein_codin	0	13.9342427	7.87368913	0
ENSG000001	STARD4	protein_codin	5.17648822	30.195348	45.5137214	9.99406068
ENSG000001	F2RL2	protein_codin	50.3055486	0	0	0.47975995
ENSG000001	PGGT1B	protein_codin	0	0	1.26306756	0
ENSG000001	ANKRD33B	protein_codin	3.62619893	32.0387018	33.4153391	6.93325207
ENSG000001	CMBL	protein_codin	15.9164428	12.0065835	235.111293	6.43429741
ENSG000001	C5orf63	protein_codin	0.73908626	0	3.54681753	0.47289653
ENSG000001	PRRC1	protein_codin	34.6743542	73.3066479	49.0371711	19.4891976
ENSG000001	RPS23	protein_codin	53.1590062	0	122.579688	32.8101401
ENSG000001	F2RL1	protein_codin	1.41735399	3.80601667	0.50905465	0.39540853
ENSG000001	WDR41	protein_codin	10.4365203	30.2088319	31.4171764	8.26125816
ENSG000001	PRDM9	protein_codin	0	0	0	2.04604828

ENSG000001	NDUFS4	protein_codin	62.6440067	62.113696	140.264222	42.0088791
ENSG000001	SCGB3A2	protein_codin	0	0	0	0
ENSG000001	SPINK1	protein_codin	0	0	0	0
ENSG000001	FBXO38	protein_codin	11.2076437	13.7931574	9.03022364	4.75667782
ENSG000001	TCERG1	protein_codin	2.18546815	0	0	8.43536357
ENSG000001	AFAP1L1	protein_codin	15.0849508	8.20331333	7.09163241	15.4280944
ENSG000001	CDC20B	protein_codin	0	0	0.51380577	0
ENSG000001	GPX8	protein_codin	0.90761211	0	0	1.60890901
ENSG000001	TIGD6	protein_codin	3.78523151	6.6027236	3.76811185	2.6081687
ENSG000001	SPZ1	protein_codin	0	0	0	0
ENSG000001	ENPP6	protein_codin	7.87197764	7.49766345	0.50591522	2.53237739
ENSG000001	CAGE1	protein_codin	0.26018293	0	0	1.81446809
ENSG000001	ERAP1	protein_codin	8.80126007	36.0202515	36.5350423	10.0679701
ENSG000001	MAP1B	protein_codin	2102.56782	3014.80999	1719.11425	704.838132
ENSG000001	TMEM174	protein_codin	0	0	0	0.75642871
ENSG000001	CARTPT	protein_codin	0	0	0	0
ENSG000001	RICTOR	protein_codin	0	0	15.2052043	0
ENSG000001	TENT2	protein_codin	0	39.3249793	13.746479	0
ENSG000001	ANKRA2	protein_codin	15.131901	22.8531013	18.6603411	9.64284989
ENSG000001	UBLCP1	protein_codin	36.3556422	58.6294575	43.0138701	17.5337945
ENSG000001	UTP15	protein_codin	0.75586135	0	4.25628994	0
ENSG000002	ARHGEF28	protein_codin	4.62677588	0	23.2886057	9.09306194
ENSG000001	TLR3	protein_codin	16.5344373	13.7539389	0	0
ENSG000002	ARHGEF28	protein_codin	0	0	0	0
ENSG000001	PRKAA1	protein_codin	1.85111114	8.86247556	5.28778104	2.68380238
ENSG000001	NSA2	protein_codin	69.1972889	13.1338063	299.123271	4.56470961
ENSG000001	GFM2	protein_codin	5.38094159	0	0	5.3123244
ENSG000001	FBXO4	protein_codin	0.72355525	0	1.51597929	0
ENSG000001	CCDC127	protein_codin	14.0920658	19.4880839	149.503342	12.6946451
ENSG000001	FOXQ1	protein_codin	0	0.95308898	0	0.9513191
ENSG000001	NKD2	protein_codin	0	11.6054277	82.3132024	0
ENSG000001	RAPGEF6	protein_codin	0	0	0	0
ENSG000001	TNFRSF21	protein_codin	10.268574	33.2307085	33.4699535	18.4737127
ENSG000001	ACSL6	protein_codin	0	0	0	0
ENSG000001	IL3	protein_codin	0	0	0	0
ENSG000001	CSF2	protein_codin	0	0	0	0
ENSG000001	SEPTIN8	protein_codin	0	93.5852543	0	0
ENSG000001	GDF9	protein_codin	0	0	0	0
ENSG000001	LEAP2	protein_codin	0	0	0.54128034	0
ENSG000002	CFAP206	protein_codin	0	1.5811698	0	6.88262806
ENSG000001	TLX3	protein_codin	0	0	0	0.82442491
ENSG000001	NPM1	protein_codin	0	0	0	0
ENSG000001	ADGRG6	protein_codin	0	0	0	0
ENSG000000	FBXW11	protein_codin	9.26350787	33.6908065	19.2637277	7.12821829

ENSG000001	TBXT	protein_codin	0	0	0	0
ENSG000001	CREBRF	protein_codin	38.0243766	195.473759	47.0312969	31.083565
ENSG000001	DCBLD1	protein_codin	5.86303209	0	6.50904671	0
ENSG000001	TMEM200A	protein_codin	15.2788923	18.4283193	11.8072207	4.46754901
ENSG000001	IL22RA2	protein_codin	0	0	0	0
ENSG000001	SPMIP7	protein_codin	0	0	0	1.33666588
ENSG000001	H2AC1	protein_codin	0	0	0	0
ENSG000001	IL31RA	protein_codin	0	0	0	0
ENSG000000	SCIN	protein_codin	1.91526435	4.24509763	56.1419833	6.85022103
ENSG000001	DAGLB	protein_codin	2.40953103	29.4975641	13.4862912	2.1195949
ENSG000001	MATCAP2	protein_codin	1.14324242	0	0	0
ENSG000001	TRA2A	protein_codin	2.71617062	0	12.1387289	7.06771388
ENSG000001	GALNT10	protein_codin	32.2556814	70.2839219	45.8625962	21.2839172
ENSG000001	SAP30L	protein_codin	19.1280548	45.4558969	11.816155	7.95600603
ENSG000001	MYOZ3	protein_codin	0	0	0	0
ENSG000001	COG5	protein_codin	39.6906838	0	77.918947	22.9097293
ENSG000001	NEUROD6	protein_codin	0	0	0	0.85670697
ENSG000001	BMT2	protein_codin	19.0024045	42.5131341	25.2745313	7.12820299
ENSG000001	GPR85	protein_codin	0	0	0	4.6050445
ENSG000001	SLU7	protein_codin	140.682783	358.698978	190.639662	61.4061766
ENSG000001	CAMLG	protein_codin	16.8705333	0	13.1286971	0
ENSG000001	RP9	protein_codin	45.6970094	55.9492417	75.2638785	8.41227807
ENSG000001	BMPER	protein_codin	0	0	0	3.50226997
ENSG000001	RELL2	protein_codin	0	3.85871852	0	0
ENSG000001	ANKHD1	protein_codin	0	0	34.9778013	23.1573894
ENSG000001	HSPA9	protein_codin	0	0	0	0
ENSG000001	RSPH10B2	protein_codin	0.40654276	0	0	0
ENSG000001	SLC29A4	protein_codin	0	0	0	0
ENSG000001	TEX47	protein_codin	0	0	0	0
ENSG000001	STEAP1	protein_codin	0	0	9.8879632	1.59429547
ENSG000001	FABP5	protein_codin	29.0754139	16.5424567	83.2202426	40.2775802
ENSG000001	SHH	protein_codin	0.64339851	0	0.60870307	2.68323322
ENSG000001	CHMP4C	protein_codin	3.46420892	37.37937	10.5302776	25.554623
ENSG000001	FNDC1	protein_codin	93.2280557	679.777222	301.072378	163.213581
ENSG000001	COL1A2	protein_codin	8206.21417	47196.6432	2726.88459	17612.5785
ENSG000001	CASD1	protein_codin	15.8788618	33.3840863	50.4741356	9.48814581
ENSG000001	PGAM2	protein_codin	3.24736442	0	0.59092391	0.42420825
ENSG000001	PLG	protein_codin	0	0	0	0
ENSG000001	BRI3	protein_codin	0	4.34281862	0	0
ENSG000001	LMTK2	protein_codin	24.467277	82.873101	85.2922854	28.1630676
ENSG000001	XKR6	protein_codin	0.65899996	7.98115059	3.80946097	0
ENSG000001	SLC35G3	protein_codin	0	0	0	0
ENSG000001	RGS20	protein_codin	0	0	0	0.45395264
ENSG000001	SOX17	protein_codin	1.41513984	7.55049474	0	6.16365908

ENSG000001	ADCY1	protein_codin	1.58521847	9.58281838	18.5385015	11.7269559
ENSG000001	C8orf48	protein_codin	2.81464494	3.33281287	5.93109893	1.87861007
ENSG000001	SUN3	protein_codin	0	0	0	0
ENSG000001	RAD21	protein_codin	91.4861292	82.1823933	268.913555	68.138636
ENSG000001	MED30	protein_codin	2.72228243	4.15569402	8.23387264	3.77317692
ENSG000001	TNFRSF11B	protein_codin	3.80672913	0	49.4570353	2.91642903
ENSG000001	SBSPON	protein_codin	0.69948638	0	0	1.66680708
ENSG000001	PHKG1	protein_codin	7.37891897	1.89641716	2.86905719	3.207248
ENSG000001	EN2	protein_codin	0.7483033	1.09577477	18.7519953	0.87100761
ENSG000001	KCNV1	protein_codin	0	0.85193118	0	0
ENSG000001	CSMD3	protein_codin	0	5.44515376	0	0
ENSG000001	SPIDR	protein_codin	1.92648359	5.28067056	1.7664187	13.740054
ENSG000001	ORC5	protein_codin	10.3206846	14.2892728	33.3762544	7.05528167
ENSG000001	DEFA4	protein_codin	0	0	0	0
ENSG000001	DEFA6	protein_codin	0	0	0	0
ENSG000001	OSGIN2	protein_codin	0	0	0	0
ENSG000001	DEFB1	protein_codin	0	0	0	0
ENSG000001	DNAAF5	protein_codin	7.34185725	17.398236	21.832112	0
ENSG000001	OXR1	protein_codin	3.47856125	0	6.92188496	1.71857935
ENSG000001	ANGPT1	protein_codin	7.80051793	63.2513916	20.5182876	35.2793614
ENSG000001	TMEM74	protein_codin	0	0	5.55550055	2.96922504
ENSG000001	GPER1	protein_codin	0	8.2798261	0	1.45626386
ENSG000001	TMEM184A	protein_codin	0	0	0	0
ENSG000001	MTUS1	protein_codin	15.6839853	28.1447443	2.67299707	23.5964845
ENSG000001	NOS3	protein_codin	1.89169859	0	0	6.40173833
ENSG000001	SPAG11B	protein_codin	0	0	0	0
ENSG000001	ABCB8	protein_codin	2.74764491	0	0	0
ENSG000001	MICALL2	protein_codin	0	14.5583596	0	6.67857817
ENSG000002	ASIC3	protein_codin	0	0	0	0
ENSG000001	CDK5	protein_codin	0	0	5.29072336	0
ENSG000001	SLC7A13	protein_codin	0	0	0	0
ENSG000001	FASTK	protein_codin	2.65743791	0	5.86001718	0
ENSG000001	TMUB1	protein_codin	0	9.06629458	0	0
ENSG000001	FMC1	protein_codin	1.45824715	0	0	2.01547534
ENSG000001	GBX1	protein_codin	0	0	0.64431077	1.36550322
ENSG000001	PHAX	protein_codin	16.2121416	17.0263279	32.2148619	8.18596672
ENSG000001	COX6C	protein_codin	16.6247213	36.3938244	0	0
ENSG000001	OSR2	protein_codin	1.20063151	0	0	10.4096104
ENSG000001	BAALC	protein_codin	0	0	0	0.58240881
ENSG000001	DCAF13	protein_codin	0	0	0	0
ENSG000001	DCSTAMP	protein_codin	0.2685921	1.94271998	0.49349442	2.71758652
ENSG000001	VIRMA	protein_codin	75.5987328	78.4535888	192.791841	37.2895388
ENSG000001	RAD54B	protein_codin	1.1708828	10.9942421	25.4156218	2.8660122
ENSG000001	GEM	protein_codin	3.12486751	0	0	5.23340122

ENSG000001(	PDP1	protein_codin	21.1396889	55.8332212	30.98422	20.413806
ENSG000001(	RPP25L	protein_codin	1.12811691	0	4.10335528	0
ENSG000001:	TTC39B	protein_codin	0	0	0	0
ENSG000001(	FAM219A	protein_codin	0	0	0	0
ENSG000001(	SPMIP6	protein_codin	0	0	0	0
ENSG000001(	MYORG	protein_codin	30.4125567	20.5789416	21.0735553	14.7814509
ENSG000001(	TMEM65	protein_codin	28.8515623	80.0120633	238.491097	24.0248471
ENSG000001(	UBAP1	protein_codin	11.9576729	0	21.2046487	9.77407448
ENSG000001(	CNTNAP3	protein_codin	3.76045608	8.0130026	20.1050062	7.70469693
ENSG00000(	HSD3B7	protein_codin	0	1.10043263	1.51720952	0
ENSG000001(	NFIL3	protein_codin	14.6155772	36.071719	62.3760881	113.441129
ENSG000001(	LETM2	protein_codin	0	0	0	0
ENSG000001(	ZMAT4	protein_codin	1.34623673	0	0	0
ENSG000001(	CPA6	protein_codin	0	0	0	1.28790717
ENSG000001(	TMC1	protein_codin	0	0	0	0
ENSG000001(	ALDH1A1	protein_codin	27.3882278	86.4115085	5.42017849	21.1422937
ENSG000001:	CCDC136	protein_codin	0	39.3545123	0	7.33587562
ENSG000001(	KDM1B	protein_codin	0	0	0	15.3038891
ENSG000001(	KIF27	protein_codin	7.7627352	36.9475374	15.741265	6.0808811
ENSG000001(	SSMEM1	protein_codin	0	0	0.48347785	0
ENSG000001:	COL14A1	protein_codin	173.101497	304.088988	4.5256764	38.6410834
ENSG000001(	ZHX1	protein_codin	0	0	0	48.6080615
ENSG000001(	CFAP47	protein_codin	0	0	0	0
ENSG000001(	METTL27	protein_codin	0.99706799	1.47694391	1.97537216	1.55994113
ENSG000001·	SYTL5	protein_codin	0	0	0	0
ENSG000001(	RNF183	protein_codin	0	0	0	0.48696464
ENSG000001(	VEGFD	protein_codin	0.28612051	1.51213182	0	4.04195303
ENSG000001·	MRRF	protein_codin	2.54606555	0	0	0
ENSG000001(	OR1Q1	protein_codin	0	0	0	0
ENSG000001(	GAPVD1	protein_codin	0.73950371	16.3505065	1.38172206	8.1753042
ENSG000001(	WNK2	protein_codin	3.86900425	0	0	0
ENSG000001(	HDX	protein_codin	4.27299126	0	5.45838965	0
ENSG000001·	AOPEP	protein_codin	0	0	11.0315516	3.36204581
ENSG000001(	AQP7	protein_codin	6.75052312	24.3569518	0	3.86011076
ENSG000001(	NOL6	protein_codin	2.94674903	0	18.6961066	4.85179625
ENSG000001(	AQP3	protein_codin	0.6201214	4.75133939	1.64222655	4.76822106
ENSG000001(	TRMT10B	protein_codin	6.56025633	0	0	0
ENSG000001(	PIGO	protein_codin	0	0	0	0
ENSG000001(	ARMC3	protein_codin	0	0	0	0
ENSG000001(	MELK	protein_codin	7.33962843	0	38.694616	0
ENSG000001(	EEF1A2	protein_codin	0	0	0	0
ENSG000001(	DEUP1	protein_codin	0	0	0	0
ENSG000001(	HECTD2	protein_codin	18.8790562	56.4860424	69.7373375	26.1169253
ENSG000001(	SLC7A3	protein_codin	0	0	1.19214717	0

ENSG000001	PABIR2	protein_codin	7.5473855	20.568868	14.2357757	0
ENSG000001	FBXO33	protein_codin	10.4911076	10.3864377	16.1312937	16.5568241
ENSG000002	METTL21EP	protein_codin	1.60668173	4.53752075	0	0
ENSG000001	LRFN5	protein_codin	0.63980516	21.5759541	20.6815226	4.76265933
ENSG000001	WDFY2	protein_codin	31.2730374	62.8631267	27.8722398	25.3691351
ENSG000001	SPTSSA	protein_codin	7.89296089	19.3114178	28.3091718	7.09515737
ENSG000001	WRN	protein_codin	16.0011921	25.0669678	16.5580782	15.0470808
ENSG000001	CFL2	protein_codin	0.69499405	4.883566	8.22157901	12.1252724
ENSG000001	TSHR	protein_codin	0	0	2.2170611	2.55599649
ENSG000001	GTF2A1	protein_codin	0	4.23857672	0	0
ENSG000001	SSX7	protein_codin	0	0	0	0
ENSG000001	KRBOX4	protein_codin	4.8245581	2.24288728	1.99283311	6.22891752
ENSG000001	PGM2L1	protein_codin	19.7237102	28.3259831	59.9586345	12.8287026
ENSG000001	FOLR2	protein_codin	4.5788387	24.874224	0.75857667	0
ENSG000001	INPPL1	protein_codin	36.7025234	117.061733	177.9667	41.823242
ENSG000001	PHOX2A	protein_codin	0	0	0	0
ENSG000001	CRYL1	protein_codin	11.1196275	16.2138356	0	9.41407851
ENSG000001	HEPACAM	protein_codin	0.55247824	1.11387398	0	0.84090009
ENSG000001	SKA3	protein_codin	0	0	4.23421287	0
ENSG000001	PCDH11X	protein_codin	0	2.99201888	39.4401249	2.17499638
ENSG000001	PCF11	protein_codin	4.38945622	14.3825643	0	3.19645204
ENSG000001	PKNOX2	protein_codin	0.51495163	0	0	0
ENSG000001	RPL10L	protein_codin	0	0	1.43924713	0.38139289
ENSG000001	LRR1	protein_codin	0.36644376	0	0	1.848268
ENSG000001	RPL36AL	protein_codin	63.9509905	154.425704	230.228326	77.2155983
ENSG000001	DNAAF2	protein_codin	4.48027681	14.8351668	20.7747664	4.29949891
ENSG000001	DEPP1	protein_codin	19.1714234	63.2351727	3.19211029	44.2201189
ENSG000001	MAGEC3	protein_codin	0	0	0	0
ENSG000001	ZNF22	protein_codin	14.0820459	26.5165267	74.4919529	12.3973255
ENSG000001	KLHDC2	protein_codin	0.37460557	4.17660385	3.79345407	5.14202157
ENSG000001	NEMF	protein_codin	19.6638432	13.1530636	31.3716925	9.78457723
ENSG000001	ARF6	protein_codin	30.072396	91.1146352	81.9272243	38.3668828
ENSG000001	RPUSD4	protein_codin	5.95751624	14.5273401	0	0
ENSG000001	TMEM63C	protein_codin	0.54393209	4.42926589	12.1951989	1.62769468
ENSG000001	NGB	protein_codin	0	0.82280665	0	0.36625803
ENSG000001	TRIM9	protein_codin	1.91032435	10.3475865	6.88427792	4.04074031
ENSG000001	AKR1E2	protein_codin	0	0	0	2.92678416
ENSG000001	RXFP2	protein_codin	0	0	0	0
ENSG000001	SSX3	protein_codin	0	0	0	0.4190955
ENSG000001	NAA30	protein_codin	6.82658885	0	0	10.1663448
ENSG000000	SEC61A2	protein_codin	0	0	0	2.89919691
ENSG000001	OXGR1	protein_codin	0.34855684	0	0	3.07666986
ENSG000001	VSTM4	protein_codin	0	7.96984898	3.28900536	1.04672134
ENSG000001	SOHLH1	protein_codin	0	0	0	0

ENSG000001	VDAC2	protein_codin	0	0	45.1749802	0
ENSG000001	ABRAXAS2	protein_codin	19.6596298	43.124075	17.7498585	13.4820557
ENSG000001	PRDX3	protein_codin	15.3981128	33.0551173	14.0507508	8.05366481
ENSG000001	CLEC7A	protein_codin	1.94343937	0	0	0
ENSG000001	CLEC1B	protein_codin	0	0	0	0
ENSG000001	TMEM52B	protein_codin	0	0	0	0
ENSG000001	SNAPC4	protein_codin	5.38348817	27.2921491	25.1633738	3.43999071
ENSG000001	ENTR1	protein_codin	1.24623185	2.83737646	22.3535401	0
ENSG000001	FRMD7	protein_codin	1.13943605	1.97368444	0	0
ENSG000001	AK8	protein_codin	0	0	0.92550461	0
ENSG000001	TSC1	protein_codin	0	0	0	0
ENSG000001	HPRT1	protein_codin	19.6415904	20.2651959	51.8413889	8.51760435
ENSG000000	TSPAN17	protein_codin	0.55032187	1.65977366	1.82588376	2.51038235
ENSG000001	BCL2L14	protein_codin	0	1.6783122	0	1.18724576
ENSG000001	KIAA1191	protein_codin	1.75924497	0	0	0
ENSG000001	BORCS5	protein_codin	0	0	0	0
ENSG000001	DUSP16	protein_codin	19.2331144	57.4927431	24.6278662	11.0992364
ENSG000001	ZMYND19	protein_codin	7.55392471	13.5272819	32.4738026	3.15301117
ENSG000001	STOX1	protein_codin	0	0	15.6036562	0
ENSG000001	JAKMIP3	protein_codin	0	0	0	0
ENSG000001	SARDH	protein_codin	0	0	0	0.92466822
ENSG000001	STK32C	protein_codin	1.8599296	8.76208943	3.297803	1.43890241
ENSG000001	OR4K2	protein_codin	0	0	0	0
ENSG000001	HK1	protein_codin	0	57.2261431	0	6.46835844
ENSG000001	SLC39A2	protein_codin	0	0	0	0
ENSG000001	NDRG2	protein_codin	0	0	0	1.2464012
ENSG000001	NDRG2	protein_codin	5.37890626	0	0	0
ENSG000001	RNASE7	protein_codin	0	0	0.338088	0
ENSG000001	ARHGEF40	protein_codin	32.2798074	29.7889227	6.25679262	11.2659166
ENSG000001	C12orf50	protein_codin	0	0	0	0
ENSG000001	PPP1R36	protein_codin	0	0	0	0
ENSG000001	VWA2	protein_codin	0	0	0	0
ENSG000001	METTL3	protein_codin	10.2859347	0	30.8704218	9.0986238
ENSG000001	ERICH6B	protein_codin	0	0	0	3.7404721
ENSG000001	GAS1	protein_codin	12.8163777	28.3117667	12.1627994	18.0389883
ENSG000001	TRUB1	protein_codin	18.4453428	12.8153598	33.0433665	4.64788666
ENSG000000	WAPL	protein_codin	161.516133	229.968947	246.299724	87.8051914
ENSG000001	SHLD2	protein_codin	54.863166	0	59.5631397	30.3669935
ENSG000001	SHLD2	protein_codin	13.3384029	65.6746495	39.2293996	0
ENSG000001	ANKRD2	protein_codin	0	0	0	0
ENSG000001	ARHGAP42	protein_codin	31.2720877	16.6419952	115.908258	11.7127127
ENSG000001	ISCA2	protein_codin	0.47098345	7.0308189	2.12888259	0
ENSG000001	OTOGL	protein_codin	1.36469374	0	2.08360023	0
ENSG000001	TTLL5	protein_codin	20.6745845	115.163543	54.2868471	14.6258748

ENSG000001(	PACSIN3	protein_codin	0	0	3.64328534	4.56814537
ENSG000001(	SERPINA4	protein_codin	0	0	0	0
ENSG0000001'	SERPINA9	protein_codin	0	0	0	0
ENSG000001(	PSMC3	protein_codin	0	0	0	5.77881283
ENSG000001(	RAPSN	protein_codin	0	0	0	0
ENSG000001:	DGLUCY	protein_codin	0	0	0	0
ENSG000001(	CPSF2	protein_codin	48.7284032	106.016014	169.341036	35.3431671
ENSG000001(	SMCO2	protein_codin	0	0	0	0
ENSG000001	CAPRIN2	protein_codin	0	0	0	0
ENSG000001(	MOAP1	protein_codin	11.2706499	0	21.3635206	25.4317445
ENSG000000	BTBD7	protein_codin	18.5054728	20.7512228	70.2184345	4.09364214
ENSG000001:	LRRK2	protein_codin	48.7282959	0	0	0
ENSG000001(	CLMN	protein_codin	23.0013212	74.4247407	31.7801812	23.0951792
ENSG000001(	PDZRN4	protein_codin	0	0	0	2.96904514
ENSG000001(	SLC6A5	protein_codin	0	0	0	0
ENSG000001(	NELL1	protein_codin	0	0	0	0
ENSG000001(	PTER	protein_codin	0	29.6409399	0	0
ENSG000001(	SMCO4	protein_codin	3.78562135	1.51861948	14.1182326	0
ENSG000001(	KCNC2	protein_codin	0	0	0	0
ENSG000001(	R3HCC1L	protein_codin	0	0	17.9305349	0
ENSG000001:	PIWIL4	protein_codin	0	5.47738114	0	2.73640359
ENSG000001(	AMOTL1	protein_codin	0.6729105	0	0	0.83870781
ENSG000001(	LIPC	protein_codin	0	0	0	0
ENSG000001(	TCP11L2	protein_codin	14.2855499	18.4595098	0	0
ENSG000001(	SPRED1	protein_codin	67.7068754	100.039467	76.7042601	70.5286158
ENSG000001(	C14orf93	protein_codin	0	0	0	0
ENSG000001(	GPR176	protein_codin	0.90634418	18.5148173	11.8203456	1.48738663
ENSG000001(	JAM3	protein_codin	73.212516	93.4492841	85.4287268	27.2175118
ENSG000001(	BCCIP	protein_codin	26.5646605	11.6588256	69.367022	21.0486238
ENSG000001.	WDR20	protein_codin	0	0	0	0
ENSG000000(	VPS35	protein_codin	8.58672223	20.6826387	14.2537756	7.92909654
ENSG000001(	SPATA19	protein_codin	0	0	0	0
ENSG000001(	AMN	protein_codin	0	0	0	0.37390348
ENSG000001(	IKBIP	protein_codin	19.3020752	32.5856227	141.313881	13.9411702
ENSG000001:	FOXN4	protein_codin	0	0	0	0
ENSG000001(	HIF1AN	protein_codin	19.1837306	88.4784241	70.3270915	15.3142299
ENSG000001(	ADAMTS15	protein_codin	1.921637	5.13026269	1.63816128	3.81932454
ENSG000001(	NDUFB8	protein_codin	0	0	0	0
ENSG000001(	PHKB	protein_codin	29.932293	61.7348437	79.9856733	31.2989562
ENSG000001(	ZFYVE19	protein_codin	0	0	0	0
ENSG000001(	PPP1R14D	protein_codin	0	0	0	0
ENSG000001(	AVPR1A	protein_codin	8.09037052	8.69213118	1.95497137	34.7690829
ENSG000000:	MRPL43	protein_codin	5.26869218	0	2.20645225	0
ENSG000001(	DEPDC4	protein_codin	0	0	3.83323311	0

ENSG000001	C16orf78	protein_codin	0	2.75083944	0	1.8052555
ENSG000001	HEATR3	protein_codin	7.59348801	9.73162732	15.6672678	0
ENSG000001	LRTM2	protein_codin	0	0	0	0
ENSG000001	TRMT61A	protein_codin	0	0	0	0
ENSG000001	BAG5	protein_codin	25.3738748	44.4733098	46.6692411	30.9168083
ENSG000001	POLL	protein_codin	0	0	0	0
ENSG000001	LARP6	protein_codin	13.8932087	48.0858973	12.6654334	10.5649076
ENSG000001	ZNF319	protein_codin	1.44372527	10.7492634	9.5650166	1.90028108
ENSG000001	HPS6	protein_codin	4.27714743	6.35447728	15.5775343	2.90553508
ENSG000000	TTC17	protein_codin	43.934954	132.234768	83.8507702	38.9200776
ENSG000001	MDM2	protein_codin	0	0	0	0
ENSG000001	COPS2	protein_codin	18.4546022	32.1938597	30.4012981	42.7038638
ENSG000001	GABRB3	protein_codin	0	0	0	0
ENSG000000	PLEKHA5	protein_codin	0	0	0	0
ENSG000001	TBATA	protein_codin	0	0	0	0
ENSG000001	CHODL	protein_codin	0	0	0	0
ENSG000001	SGPL1	protein_codin	0	0	0	0
ENSG000001	PCBD1	protein_codin	1.32831261	4.34179565	3.69653203	1.41681304
ENSG000001	CCT2	protein_codin	798.66116	0	273.142023	27.79812
ENSG000001	TMEM132B	protein_codin	6.10248569	0	0	0
ENSG000001	GNPTAB	protein_codin	75.0532857	162.94985	56.7919154	109.979795
ENSG000001	DNAAF8	protein_codin	0	2.0908787	0	0
ENSG000001	SCN3B	protein_codin	0	0	0	2.99801316
ENSG000001	COX11	protein_codin	9.92748826	12.8007615	29.5440853	0
ENSG000001	FAM227B	protein_codin	1.66570699	0	7.29785162	0
ENSG000001	CLDN10	protein_codin	0	0	0	1.26410765
ENSG000001	CYYR1	protein_codin	16.8311118	12.498958	0	20.9920905
ENSG000001	CDH8	protein_codin	0	0	0	0
ENSG000001	MYRFL	protein_codin	0	0	0	0
ENSG000002	BORCS7-ASM	protein_codin	0	12.7797189	0	2.9870931
ENSG000000	ACAT1	protein_codin	0	0	0	0.83594252
ENSG000001	CDH23	protein_codin	0	1.23150413	3.5243585	0.62766217
ENSG000001	C2	protein_codin	0	0	0	0
ENSG000001	ANAPC16	protein_codin	9.4366989	0	8.90693252	15.6376688
ENSG000001	APBB1	protein_codin	8.46012449	0	0	0
ENSG000001	TRIM44	protein_codin	170.754598	879.819359	271.647252	112.231356
ENSG000001	CCDC182	protein_codin	0	2.55246432	0.46032443	0.40034456
ENSG000001	ILK	protein_codin	0	0	19.7769198	11.5829662
ENSG000001	TAF10	protein_codin	0.46559272	10.0793056	0	0
ENSG000001	TPP1	protein_codin	0	0	31.5243741	42.3569232
ENSG000001	MSS51	protein_codin	13.9077631	4.49565944	3.70157007	1.96822703
ENSG000001	CYB5A	protein_codin	0	0	0	1.40772398
ENSG000001	RAG1	protein_codin	2.4023322	0	0	2.69657214
ENSG000001	DCHS1	protein_codin	48.0877617	91.4506984	100.609295	30.0403311

ENSG0000010	POTED	protein_codin	0.71470078	1.85947338	0	3.18962549
ENSG0000010	OR10A5	protein_codin	0	0	0	1.59738854
ENSG0000010	OR2D2	protein_codin	0	1.78905501	0	0
ENSG0000020	PCMTD2	protein_codin	2.77374607	0	5.9823597	3.94352071
ENSG0000011	NLRP14	protein_codin	0.31969041	1.07114054	5.02943926	2.63360085
ENSG0000010	PPFIBP2	protein_codin	12.2819828	22.826197	23.8954359	13.4234647
ENSG0000010	CYB5R2	protein_codin	0	0	6.6886503	0
ENSG0000019	SERPINB2	protein_codin	0	0	0	2.20620287
ENSG0000010	GARRE1	protein_codin	16.0988882	75.8348023	9.36121141	32.6594925
ENSG0000010	TUB	protein_codin	22.9127212	14.9137367	110.098832	0
ENSG0000010	IDH3A	protein_codin	1.46675694	0	0.75326741	0
ENSG0000010	CRABP1	protein_codin	0.28782865	0	1.77979	0
ENSG0000000	CTDP1	protein_codin	0	0	0	0
ENSG0000010	RNF169	protein_codin	57.2611321	119.759774	123.139804	38.5917596
ENSG0000010	CHRNA5	protein_codin	0	0	13.9585258	1.58300936
ENSG0000010	CENPN	protein_codin	0.74952082	3.66880195	13.5668594	4.49546066
ENSG0000010	ATMIN	protein_codin	17.7875094	35.9328597	8.58875051	21.1911187
ENSG0000010	AKIP1	protein_codin	2.9532619	6.04153347	2.78821044	0
ENSG0000010	C16orf46	protein_codin	1.16229619	0	0	0
ENSG0000010	TMEM41B	protein_codin	0	0	0	0
ENSG0000010	PKD1L2	protein_codin	0	0	0	2.09591203
ENSG0000010	LEO1	protein_codin	76.2499929	122.892525	140.425241	11.4549559
ENSG0000010	ZNF143	protein_codin	1.00931359	5.73446088	0	6.68266898
ENSG0000010	TMX3	protein_codin	42.5864139	46.592562	59.6520365	26.6985725
ENSG0000010	MFAP4	protein_codin	71.8263322	15.534807	13.0680754	0
ENSG0000010	MAPK7	protein_codin	4.06569583	0	0	0
ENSG0000010	WEE1	protein_codin	0	5.81140743	11.4873181	6.05495156
ENSG0000011	ALG8	protein_codin	0	0	0	1.13768602
ENSG0000010	HDGFL3	protein_codin	15.2406367	33.3868649	31.1721418	17.0035556
ENSG0000021	POLR2M	protein_codin	33.4008034	88.1661745	99.6996347	30.1587893
ENSG0000010	NDST2	protein_codin	0	0	0	7.30251378
ENSG0000010	CLEC3A	protein_codin	6.56118695	0	1.70465691	0
ENSG0000010	CLEC4E	protein_codin	0.85501184	0	0	0
ENSG0000010	CLEC4D	protein_codin	0.79106014	0	0	1.41852051
ENSG0000010	ZNF3	protein_codin	0	0	0	12.348027
ENSG0000010	RIMKLB	protein_codin	0	0	0	0
ENSG000002	ZNF407	protein_codin	1.67430344	0	29.4809464	5.79505019
ENSG0000010	BEAN1	protein_codin	2.03687814	0	0	1.35014644
ENSG0000010	TK2	protein_codin	4.17479562	19.7664712	0	0
ENSG0000010	A2ML1	protein_codin	0.29016193	0	0	0
ENSG0000010	TMED3	protein_codin	31.8656783	11.4358333	48.1316025	8.52511203
ENSG0000010	SLC38A8	protein_codin	0	0	0	0
ENSG0000010	SEC11C	protein_codin	0	0	0.68179067	0
ENSG0000010	CPLX4	protein_codin	0.29136724	0	0.46614607	2.37649313

ENSG000001(	GALR1	protein_codin	0.40574875	1.37671314	0	3.37834663
ENSG000001(	IQCD	protein_codin	0.55314276	0	1.88711831	0
ENSG000001(	CENPV	protein_codin	0	0	6.24805304	0
ENSG000001(	CDH16	protein_codin	0	0	0	0
ENSG000001(	RRAD	protein_codin	2.64734322	5.7649237	0	27.0392923
ENSG000001(	MC4R	protein_codin	0.26280903	1.53577326	0.47602685	1.42863407
ENSG000001(	HSP90B1	protein_codin	340.647409	0	854.177426	236.787234
ENSG000001:	SLC9A5	protein_codin	0	0	0	0
ENSG000000:	NCAPH2	protein_codin	0	0	23.3795764	5.8849049
ENSG000001(	PPP1R16B	protein_codin	3.86193547	78.3464603	0	6.24018093
ENSG000001'	VWA3A	protein_codin	0	3.68346931	1.78607308	0
ENSG000001(	CHRFAM7A	protein_codin	0	0	0	0
ENSG000000:	POLR3E	protein_codin	4.99893368	0	0	14.3383654
ENSG000001·	MMP3	protein_codin	0.29718673	0	0	0.53984962
ENSG000001(	TVP23A	protein_codin	0	0	0	0
ENSG000001:	ZNF211	protein_codin	0	0	0	0
ENSG000001(	TMPRSS5	protein_codin	0	0	0	0
ENSG000001(	COG1	protein_codin	11.4217432	53.2449612	41.6433221	7.76785452
ENSG000001:	FAM199X	protein_codin	0	9.90895316	0	0
ENSG000001(	ZNF592	protein_codin	0	0	0	0
ENSG000001·	MARVELD3	protein_codin	0	0	0	0
ENSG000001(	GOLM2	protein_codin	80.1777838	230.170647	117.028046	70.5539528
ENSG000001(	HTR3A	protein_codin	0	0	0	0
ENSG000001(	NNMT	protein_codin	2.12917368	0	0	0
ENSG000001:	SERINC4	protein_codin	0.30347652	0	0	0
ENSG000001(	SLFN5	protein_codin	159.340809	435.250828	53.4345511	92.5892222
ENSG000001(	AP1G1	protein_codin	24.0677184	55.5048115	77.9792337	23.5715488
ENSG000001!	ERI2	protein_codin	0	0	0.75672116	0
ENSG000001(	BMERB1	protein_codin	3.48380419	15.4152882	9.44360475	0
ENSG000001(	SAAL1	protein_codin	0	0	18.3344163	0
ENSG000001(	YPEL4	protein_codin	0	0	0	0
ENSG000001(	PPIB	protein_codin	86.8262512	373.941511	345.284158	76.6234414
ENSG000001·	FANCI	protein_codin	0	0	6.15112409	0
ENSG000001(	CIAO2A	protein_codin	18.1877515	57.9178803	38.1385281	18.3952987
ENSG000001(	PCLAF	protein_codin	2.25066561	0	6.71822278	0.69859562
ENSG000001:	MYH11	protein_codin	12.3086139	198.650911	0	72.067512
ENSG000001·	UEVLD	protein_codin	0	0	0	0.71334958
ENSG000001(	LDHD	protein_codin	0	0	0	0
ENSG000001(	PLIN1	protein_codin	48.0249923	100.755507	0	14.6155216
ENSG000001(	PEX11A	protein_codin	4.64298144	7.04368161	14.8028264	1.39218817
ENSG000001(	MESP1	protein_codin	0	0.69352459	0	0.50304804
ENSG000001(	ANPEP	protein_codin	19.6647374	0	1.50330572	0
ENSG000001(	SCNN1G	protein_codin	0	0	0	1.23792841
ENSG000001(	RBPMS2	protein_codin	0	3.58006267	4.28456473	0

ENSG000001(	GLYATL1	protein_codin	0	0	0	0
ENSG000001(	TERF2IP	protein_codin	77.1933247	126.250164	199.978557	52.3724136
ENSG000001(	DCTN5	protein_codin	25.3302721	42.6918728	69.2068006	13.5175702
ENSG000001(	C18orf54	protein_codin	6.8143812	0	29.3616176	1.09352173
ENSG000001(	PLK1	protein_codin	0.64791981	6.48056017	25.781309	0
ENSG000001(	GPR182	protein_codin	0.48743546	0	0.5195418	0.55740346
ENSG000001(	ZBTB39	protein_codin	7.82400945	42.5622377	28.264857	6.23909835
ENSG000001(	CACNG2	protein_codin	0	0	0	0.58346813
ENSG000001(	CLPX	protein_codin	14.7908201	26.9915374	21.2443581	10.285053
ENSG000001(	TAC3	protein_codin	0	0	0	0
ENSG000001(	CHP2	protein_codin	0	0	0	1.74322102
ENSG000001(	MYO1A	protein_codin	0	0	0.85130118	0
ENSG000001(	OR4D6	protein_codin	0	0	0	0
ENSG000001(	NEMP1	protein_codin	14.4088201	31.1872239	88.2449042	2.58559262
ENSG000001(	NAB2	protein_codin	7.51045876	36.8321765	6.20773268	0
ENSG000001(	STAT6	protein_codin	0	68.5934805	46.0333767	0
ENSG000000'	DPP8	protein_codin	29.7026155	53.3195005	47.5476391	0
ENSG000001(	ATP23	protein_codin	20.4665344	5.63127942	11.7311705	0
ENSG000001(	PATL1	protein_codin	27.0971967	115.53138	73.666384	40.0860113
ENSG000001(	MRPL16	protein_codin	26.5498612	73.8808868	67.7670878	8.99557571
ENSG000001(	SCG5	protein_codin	0	0	0	0
ENSG000001(	AGFG2	protein_codin	1.31061757	12.5561768	0	4.70845452
ENSG000001(	NYAP1	protein_codin	0.81415257	3.05721369	0	1.11247868
ENSG000001(	TSC22D4	protein_codin	6.42045184	17.8458138	6.4438609	3.92610407
ENSG000001(	MS4A6E	protein_codin	0.35322927	0	0	0
ENSG000001(	MS4A7	protein_codin	1.78728271	6.52094241	0	4.31279978
ENSG000001(	MS4A14	protein_codin	0	0	0	0
ENSG000001(	MS4A5	protein_codin	0	0	0	0
ENSG000001:	EEF1AKMT3	protein_codin	41.652897	15.4189365	16.7723984	4.77012034
ENSG000001(	CCNDBP1	protein_codin	0.43839575	3.27237462	0.87561479	0.97785954
ENSG000001(	MS4A8	protein_codin	0	0	0	0.85826354
ENSG000001(	CCDC178	protein_codin	0	0	0	0
ENSG000001(	MAP1A	protein_codin	335.211384	518.648648	63.6946273	203.603655
ENSG000001(	AKTIP	protein_codin	12.1541212	11.0122484	6.95686099	0
ENSG000001(	MAPRE2	protein_codin	13.6684903	69.0027505	30.1501816	24.5964576
ENSG000001(	EVA1C	protein_codin	0	0	0	0
ENSG000002·	TCP10L	protein_codin	0	0	0	0
ENSG000001·	CFAP298	protein_codin	6.31104285	8.85682504	76.6010518	0
ENSG000001·	SON	protein_codin	24.1727927	113.208694	28.5968731	95.2566709
ENSG000002:	CKMT1B	protein_codin	0	0	0	0.64500554
ENSG000001(	PDIA3	protein_codin	0	0	0	0
ENSG000001(	NUDT21	protein_codin	57.3083353	92.4009476	159.058782	40.26496
ENSG000000:	HERPUD1	protein_codin	0	28.6565053	9.77884855	4.54934499
ENSG000001(	NAT16	protein_codin	0	0	0	0.43404351

ENSG000001	RUNX1	protein_codin	0	27.5057726	3.75595561	0
ENSG000001	BPIFC	protein_codin	0	0	0	0
ENSG000000	TPX2	protein_codin	26.1596641	125.978269	537.090886	10.314775
ENSG000001	B4GALNT2	protein_codin	0.23742737	0	0	0
ENSG000001	GNGT2	protein_codin	0.33428765	0	0	0
ENSG000001	PHB1	protein_codin	0.80359357	0	0	0
ENSG000001	SNRPD1	protein_codin	12.1660522	28.2856555	41.5923182	11.9762565
ENSG000001	LRSAM1	protein_codin	0	0	0	0
ENSG000001	PIP5KL1	protein_codin	0	0	0	0
ENSG000001	TMEM92	protein_codin	0	0	0	0
ENSG000001	EEIG1	protein_codin	0	0	0	0
ENSG000001	ACSF2	protein_codin	6.13029954	7.93639915	4.27513024	3.65711498
ENSG000001	COQ4	protein_codin	2.80235499	0	4.23746754	0
ENSG000001	SLC27A4	protein_codin	5.50632463	14.9840943	26.2543437	2.35095536
ENSG000001	ANKRD40CL	protein_codin	0	0	0	0
ENSG000001	DYNLRB1	protein_codin	0	5.192721	0	2.29591057
ENSG000001	TRPM2	protein_codin	0	0	0	0
ENSG000001	TRPM2	protein_codin	0	0	0	0
ENSG000001	TBC1D21	protein_codin	0	0.94903554	0	0
ENSG000001	COL6A2	protein_codin	697.566216	1311.8339	391.432351	345.862413
ENSG000001	ZNF85	protein_codin	0	0	0	0
ENSG000001	PRR15L	protein_codin	0	0	0	1.83673771
ENSG000001	GPRC5B	protein_codin	7.86502504	35.9572012	50.880681	14.6315639
ENSG000001	CRK	protein_codin	5.2578171	6.60878198	32.5664226	17.86549
ENSG000001	C16orf92	protein_codin	0	1.13366571	0	0.3438956
ENSG000001	GOLGA6C	protein_codin	0.59600679	0	0.90883979	0
ENSG000001	TBC1D2B	protein_codin	66.5138988	146.493928	0	22.5764422
ENSG000001	NOD2	protein_codin	0	0	0	0
ENSG000001	SNX20	protein_codin	0.37885158	4.8236262	0	0
ENSG000001	LOXHD1	protein_codin	0	0.75802512	0	0
ENSG000001	HDHD2	protein_codin	9.08502002	14.1800611	19.3796088	7.5024423
ENSG000001	ZNF91	protein_codin	70.3289872	124.253597	179.745604	53.6815203
ENSG000000	GCN1	protein_codin	81.2795912	240.544209	183.319618	57.6484555
ENSG000001	RNF214	protein_codin	2.59351308	30.0741983	1.40698322	1.22799963
ENSG000001	MED1	protein_codin	70.2307256	124.419103	129.005356	49.3283443
ENSG000001	PGAP3	protein_codin	0	0	0	0
ENSG000001	ENGASE	protein_codin	0.55702981	0	1.09226738	0
ENSG000001	ATP5MG	protein_codin	18.9072394	14.1584464	17.3162765	19.2641714
ENSG000001	CD3D	protein_codin	2.00477626	17.8317858	0	0
ENSG000001	TEPSIN	protein_codin	0	0	0	0
ENSG000001	PGAP2	protein_codin	0	0	0	0
ENSG000001	STIM1	protein_codin	14.5870255	0	21.1985438	0
ENSG000001	RRM1	protein_codin	46.2621254	45.2087119	215.981282	14.5173158
ENSG000001	TRIM68	protein_codin	6.04767694	11.7184844	11.6736797	0

ENSG000001(	MMP26	protein_codin	0	1.25256519	0	1.16812755
ENSG000001(	OR51B5	protein_codin	0	0.83925919	0	3.00362394
ENSG000001(	OR51Q1	protein_codin	0	0	0	0.47334772
ENSG000001(	FN3K	protein_codin	0.40704973	6.04614687	0	0
ENSG000001(	PRRT2	protein_codin	0	3.17472716	0	0
ENSG000001:	ZNF428	protein_codin	7.86985443	18.9973511	19.649569	8.85082522
ENSG000001(	ZNF226	protein_codin	0	0	0	0
ENSG000001:	PRR14	protein_codin	1.78957795	0	8.32068768	0
ENSG000000(	MARK4	protein_codin	1.82775095	0	0	0
ENSG000001(	ZNF668	protein_codin	0	6.50927498	0.95652625	2.45467925
ENSG000001(	ZNF646	protein_codin	0	35.2042217	54.1379866	0
ENSG000001(	VKORC1	protein_codin	0	0	4.00165419	0
ENSG000000	ERCC1	protein_codin	0	6.66362236	7.16075196	0
ENSG000001:	HIF3A	protein_codin	0	0	3.94047639	0
ENSG000001	ZNF267	protein_codin	32.3252915	42.7302677	32.9193953	29.3051455
ENSG000001(	GNG8	protein_codin	0	0	0	0
ENSG000001!	DACT3	protein_codin	5.16674939	0	6.32858644	0
ENSG000001(	BBC3	protein_codin	7.50646261	0	0	0
ENSG000001	USP32	protein_codin	21.7290293	40.2831456	120.564648	27.1636379
ENSG000001(	CA4	protein_codin	0.99032757	0.99256843	2.14393909	2.07618816
ENSG000001(	SMG8	protein_codin	13.1885903	10.9643196	53.1007359	4.74850733
ENSG000001(	TPM4	protein_codin	0	0	0	0
ENSG000001(	RAB8A	protein_codin	35.0067345	140.200911	113.8567	38.6950999
ENSG000001(	MIDN	protein_codin	7.53366795	82.8713911	34.4435365	76.3675781
ENSG000001	PCSK4	protein_codin	0	0	2.32727923	0.87867877
ENSG000001(	JSRP1	protein_codin	1.86996828	10.533792	0.5333068	1.37339041
ENSG000001(	KLHL26	protein_codin	1.23155947	5.79899772	4.04560604	6.99258982
ENSG000001:	JPH3	protein_codin	0	0	0	0
ENSG000001:	ZC3H18	protein_codin	41.3259431	126.312274	87.6602961	21.1245526
ENSG000001(	MVD	protein_codin	0	0	10.2342207	0
ENSG000001(	PIEZO1	protein_codin	48.2474335	407.503402	239.257241	72.4018195
ENSG000001(	CDT1	protein_codin	4.21772829	7.38817158	25.2946772	0.93356878
ENSG000001(	TRAPPC2L	protein_codin	1.01326171	0	4.64785987	0
ENSG000001(	ANKRD11	protein_codin	152.5024	358.043398	412.311612	70.2132186
ENSG000001(	SPATA33	protein_codin	1.71101136	4.12634135	0	0.84333934
ENSG000001(	UNC119	protein_codin	0.65999574	0	0	3.74109549
ENSG000001(	RSKR	protein_codin	5.2121165	0	35.1226526	0
ENSG000001(	PROCA1	protein_codin	2.28207934	8.15865503	0	0
ENSG000001(	ZNF641	protein_codin	23.9294903	40.931388	37.409119	18.0234884
ENSG000001(	RAB34	protein_codin	0	0	0	0
ENSG000001(	LALBA	protein_codin	0	0	0	0
ENSG000001(	CACNB3	protein_codin	0	0	0	0
ENSG000001(	TP53I13	protein_codin	6.85504064	17.6513219	17.4344088	3.09262849
ENSG000001(	WNT10B	protein_codin	8.47906848	0	0	0

ENSG000001	KMT2D	protein_codin	0	269.204195	74.5070283	0
ENSG000001	RHEBL1	protein_codin	0	0	0	0
ENSG000001	TUBA1A	protein_codin	36.9921829	131.762576	53.2100961	16.9107413
ENSG000001	TUBA1C	protein_codin	0.89554842	0	1.97124942	1.25896559
ENSG000001	ZNF524	protein_codin	0	3.2664368	0	0
ENSG000001	ZNF534	protein_codin	0.53731418	0	0	0
ENSG000001	ZNF701	protein_codin	15.2581288	26.746095	26.6479732	3.90979936
ENSG000001	ZNF83	protein_codin	0	0	31.6382372	0
ENSG000002	CYP2A6	protein_codin	0	0	0	0
ENSG000001	CYP2A7	protein_codin	0	0	0	0
ENSG000001	GPD1	protein_codin	11.2535472	27.8863425	0	6.63059317
ENSG000002	LIN37	protein_codin	4.5838139	13.4564241	0	0
ENSG000001	PROSER3	protein_codin	0	0	0	0
ENSG000002	PRODH2	protein_codin	0	0	0	0.46797657
ENSG000001	AXL	protein_codin	16.4613469	14.2600753	0	9.3763895
ENSG000000	DIP2B	protein_codin	36.6460715	97.2270485	145.893545	37.8044373
ENSG000001	DMAC2	protein_codin	0	0	0	0
ENSG000001	ANKRD33	protein_codin	0	0	0	0
ENSG000001	TTYH1	protein_codin	0	0	0	0
ENSG000001	CDC42EP5	protein_codin	2.6103104	3.34318384	4.94846123	6.13837901
ENSG000001	LAIR2	protein_codin	0	0	0	0
ENSG000001	TMEM145	protein_codin	0.2044128	0	0	0
ENSG000001	ZNF526	protein_codin	13.9108735	0	27.294507	0
ENSG000001	GRIK5	protein_codin	0	2.37909626	0	0.58372753
ENSG000001	PPP1R14A	protein_codin	0	4.84167714	0	1.95695933
ENSG000001	SPINT2	protein_codin	0	0	0	1.25884817
ENSG000001	C19orf33	protein_codin	0.31748744	4.96981373	0	0
ENSG000001	PSCA	protein_codin	0	1.00255252	0	0.42036356
ENSG000001	LY6D	protein_codin	0	0	0	0
ENSG000001	DAPK3	protein_codin	9.91254992	38.2914946	34.9757596	7.33312042
ENSG000001	TMIGD2	protein_codin	0	0	0	0
ENSG000001	CHAF1A	protein_codin	4.78082539	70.2055965	94.1028631	19.6919121
ENSG000001	UBXN6	protein_codin	16.6182725	69.6549534	107.287709	25.7754515
ENSG000001	PLIN4	protein_codin	4.0412388	58.2038671	0	0
ENSG000001	NLRP4	protein_codin	0	0	0	0
ENSG000001	SLC39A4	protein_codin	0.27160759	0	0.71882606	1.08665007
ENSG000000	ZNF582	protein_codin	2.11218977	0	0	2.55634112
ENSG000001	ZFP28	protein_codin	5.30045213	16.4098483	0.97183217	4.54560157
ENSG000001	RECQL4	protein_codin	0	0	0	0
ENSG000001	TLCD3A	protein_codin	0	0	0	1.25885733
ENSG000001	MFSD3	protein_codin	0	0	0	0
ENSG000001	GLOD4	protein_codin	0	0	0	0
ENSG000001	GLOD4	protein_codin	12.4016322	38.6876929	12.789692	7.04429957
ENSG000001	KIFC2	protein_codin	2.42854813	10.3627184	8.21433299	3.57966539

ENSG000001(	SLC43A2	protein_codin	1.15383011	21.4913896	0.695167	3.40738586
ENSG000001(	RILP	protein_codin	0	1.75226579	0	0.71770676
ENSG000001(	TSR1	protein_codin	50.569797	95.5874234	201.029246	27.175354
ENSG000001(	TRPV3	protein_codin	0	0	0	0
ENSG000001(	HSD11B1L	protein_codin	0	0	0	0
ENSG000001(	CYB5D2	protein_codin	3.85388075	15.8938298	7.58106269	3.88139349
ENSG000001(	GGT6	protein_codin	0	0	0	0
ENSG000001	PELP1	protein_codin	4.09560522	0	0	0
ENSG000001(	ZNF577	protein_codin	12.027138	39.7065352	6.35720014	13.6995881
ENSG000002(	CGB1	protein_codin	0	0	0	0
ENSG000001	CGB5	protein_codin	0	0	0	0.4459812
ENSG000001(	KLK1	protein_codin	0	0	0	0
ENSG000001(	ACER1	protein_codin	0.23925261	0	0	0
ENSG000001(	OTUB1	protein_codin	0.32339656	7.72548186	4.79957807	0
ENSG000001	SLC25A23	protein_codin	12.3839201	13.3061732	77.1325183	4.11545554
ENSG000001(	ANGPTL4	protein_codin	12.0744236	0	103.995944	17.6738193
ENSG000002(	NDUFA7	protein_codin	0	0	23.7302695	2.32407773
ENSG000001(	CD320	protein_codin	3.10734435	2.3916818	37.829539	1.17941317
ENSG000001(	RCOR2	protein_codin	21.6652874	28.9904834	75.5939505	10.0677862
ENSG000001(	SPRYD3	protein_codin	19.0893895	51.0912893	26.7448655	16.7653368
ENSG000001(	IGFBP6	protein_codin	3.5051069	3.68382805	2.74386578	1.05453132
ENSG000001(	SOAT2	protein_codin	0	0	0	0
ENSG000001(	ZNF558	protein_codin	5.71525599	20.9055824	36.3788024	2.34863146
ENSG000001	ZNF560	protein_codin	0	4.19756873	1.9125731	1.7835127
ENSG000001(	CDK2AP2	protein_codin	4.3604435	8.0351462	13.3166573	0.67611422
ENSG000001(	NUDT8	protein_codin	0	0	0	0
ENSG000001(	PRDX2	protein_codin	13.0900842	34.7555661	6.14674307	9.56479074
ENSG000001(	OR5I1	protein_codin	0	0	0	0
ENSG000001	ZNF443	protein_codin	2.78420938	18.6777094	15.8390972	0.92919877
ENSG000001	CD300LF	protein_codin	0	0	0	0
ENSG000001(	MRPL58	protein_codin	11.1360669	15.980324	22.0197539	5.74699167
ENSG000001(	ATP5PD	protein_codin	40.1612147	58.2668545	180.560264	30.1918682
ENSG000001(	TMEM88	protein_codin	0.74990552	2.69122232	0.51260191	2.93494977
ENSG000001(	EVPL	protein_codin	0	0	0	0
ENSG000001(	ACOX1	protein_codin	0	50.8946925	32.8672575	22.2673518
ENSG000001(	MGAT5B	protein_codin	0	2.38561259	3.31003975	0
ENSG000000	BIRC5	protein_codin	2.15646437	0	0	0
ENSG000001(	TK1	protein_codin	1.73091626	15.7819769	34.2439504	0.86180353
ENSG000001(	CYP7A1	protein_codin	0.26654779	2.79146482	0	3.99975216
ENSG000001	KRT16	protein_codin	0.8919265	0	0	0
ENSG000001	KRT27	protein_codin	0	0	0	0
ENSG000001(	GSDMA	protein_codin	0	0	0	0
ENSG000001(	GHDC	protein_codin	1.13091248	0	6.42751718	2.00923671
ENSG000001(	FAM234A	protein_codin	7.15897359	0	0	0

ENSG000001	FAM234A	protein_codin	0.88785632	0	0	0
ENSG000001	METTL26	protein_codin	1.23578699	0	9.50655532	0
ENSG000001	SOST	protein_codin	2.21666139	44.5984399	5.5133811	11.1223881
ENSG000000	UNKL	protein_codin	0.28119792	0.86473255	1.66734551	1.20867285
ENSG000001	SPSB3	protein_codin	0	0	0	0
ENSG000001	MLST8	protein_codin	2.04275061	0	2.42080274	0
ENSG000001	E4F1	protein_codin	4.92820308	0	8.85099783	8.55431281
ENSG000001	ECI1	protein_codin	5.29171676	9.68451996	30.3102607	4.30440644
ENSG000002	RNPS1	protein_codin	0	0	0	0
ENSG000001	ABCA3	protein_codin	1.18433387	0	0	0
ENSG000001	KCTD5	protein_codin	10.1533568	21.9574177	55.2675779	3.23127503
ENSG000001	SRRM2	protein_codin	160.107546	918.117918	956.676528	433.306725
ENSG000001	ZNF597	protein_codin	4.53741714	18.4738941	6.21235957	5.48805985
ENSG000001	SDHAF2	protein_codin	7.43994288	0	0	0
ENSG000001	DDB1	protein_codin	0	0	83.1307267	0
ENSG000001	VPS37C	protein_codin	1.34247084	5.62712504	3.52137699	3.5408547
ENSG000001	VWCE	protein_codin	0.67823924	5.62130565	0	0
ENSG000001	RAB3IL1	protein_codin	0	0	0	0
ENSG000001	ASRGL1	protein_codin	0	0	4.80169092	0
ENSG000001	BSCL2	protein_codin	0	0	0	0
ENSG000002	HNRNPUL2	protein_codin	89.7957031	268.564669	199.021125	28.077294
ENSG000001	POLR2G	protein_codin	6.29035673	16.0538348	21.2033995	3.24346891
ENSG000001	PLAAT5	protein_codin	0	0	0	0
ENSG000000	ACAA1	protein_codin	0	0	0	0
ENSG000001	RPSA	protein_codin	1.95389399	0	173.474484	9.65575527
ENSG000001	ENTPD3	protein_codin	3.93796405	12.2468179	9.75270006	14.6738754
ENSG000001	ULK4	protein_codin	5.97271973	19.0517617	8.75737293	3.23486358
ENSG000001	FADD	protein_codin	19.3920899	39.2396556	49.1014207	5.43727394
ENSG000000	CTTN	protein_codin	41.277034	165.521329	48.3841741	43.4625021
ENSG000001	LTBP3	protein_codin	12.2064868	0	0	0
ENSG000002	ARL2-SNX15	protein_codin	6.85001874	14.3158432	10.5063152	4.130415
ENSG000001	BATF2	protein_codin	0	8.53004973	1.13118406	3.24631412
ENSG000001	SLC22A11	protein_codin	0	0	0	1.77682432
ENSG000001	NRXN2	protein_codin	2.32230825	16.2202563	9.36409054	0
ENSG000001	MAJIN	protein_codin	0	0	0.8464242	0
ENSG000001	CCDC88B	protein_codin	0	0	0	0
ENSG000001	SCARA3	protein_codin	57.0789621	32.7514729	100.3081	19.0512104
ENSG000001	PBK	protein_codin	2.76495984	0	24.8036563	0.55141536
ENSG000001	PNOC	protein_codin	0	0	0	6.03763713
ENSG000001	MSANTD4	protein_codin	22.3473093	55.6073467	29.3151336	6.19151129
ENSG000001	TNRC6B	protein_codin	62.3874998	181.329054	160.067751	23.8148909
ENSG000001	UBXN1	protein_codin	13.0935263	0	0	0
ENSG000001	FBXW10	protein_codin	0.75879651	0	0	0.99744806
ENSG000001	NXNL1	protein_codin	0	0	0	0

ENSG000001:	STEAP4	protein_codin	23.8057858	117.30043	0.86769322	70.9107742
ENSG000001	TADA3	protein_codin	10.8012571	0	59.6472241	7.89442169
ENSG000001	LRRC28	protein_codin	1.56637719	7.08724942	6.35206277	5.15119869
ENSG000001	B3GNT2	protein_codin	15.0533497	48.8730315	47.8018392	33.0178538
ENSG000001	PYGO1	protein_codin	26.3673149	0	69.9817341	0
ENSG000001	OGG1	protein_codin	0	0	0	0
ENSG000001	HSPB3	protein_codin	6.43241866	8.39097993	1.58574553	2.20142807
ENSG000001	IRX1	protein_codin	0.83599985	6.16329729	0	0
ENSG000001	OGG1	protein_codin	0	7.76334266	0	0
ENSG000001	CD14	protein_codin	0	0	0	0
ENSG000001	ZNF467	protein_codin	3.56590715	21.1766715	13.5756966	7.38483757
ENSG000001	SLAMF1	protein_codin	0.40532675	10.9039903	0	0.98034486
ENSG000001	OGG1	protein_codin	0	0	0	0
ENSG000001	OR5A2	protein_codin	0.31784038	0	0	0
ENSG000001	IRX2	protein_codin	3.49743911	0	23.948494	1.84593372
ENSG000001	DNAJC18	protein_codin	5.42082142	17.2898608	1.46764264	9.52479469
ENSG000001	FGB	protein_codin	0	0	0	0
ENSG000000	SESN1	protein_codin	0.61160499	0	0	0
ENSG000001	PLRG1	protein_codin	0	0	0	5.27349987
ENSG000001	PIEZO2	protein_codin	21.6211936	51.3726125	38.9713646	19.3421118
ENSG000001	SMAD1	protein_codin	7.90764118	21.9812261	17.0709031	11.5199363
ENSG000001	SPATA24	protein_codin	0	0	0	0
ENSG000001	LKAAEAR1	protein_codin	0	0	1.5560011	0
ENSG000001	ZNF280A	protein_codin	0	0	0.46059073	2.26267682
ENSG000001	NHLH1	protein_codin	0	1.20769421	0	0.78233083
ENSG000001	FUT9	protein_codin	0.26227605	0	2.07157184	3.34845721
ENSG000001	PCSK9	protein_codin	1.72768193	0	0	0
ENSG000001	OR8I2	protein_codin	0	0	0	0.73023986
ENSG000001	MZB1	protein_codin	0	45.0697799	0	3.11843321
ENSG000001	KCND3	protein_codin	0	0	7.63835397	0
ENSG000001	IRF2BP1	protein_codin	4.4106635	24.2186136	8.1721205	0.85289052
ENSG000001	NUDT9	protein_codin	3.34166977	22.847253	0	2.77674192
ENSG000001	DCLK2	protein_codin	2.2956508	0	0	0
ENSG000001	FOXA3	protein_codin	0	0	0	0
ENSG000001	UBB	protein_codin	283.160411	795.90769	615.749362	225.591918
ENSG000001	RBKS	protein_codin	0.5827482	0	0	0
ENSG000001	SDC2	protein_codin	90.9083974	87.8065301	142.402534	102.954757
ENSG000001	TPM3	protein_codin	111.044533	0	23.1663452	0
ENSG000001	ZC3H14	protein_codin	0	0	0	0
ENSG000001	HSD17B13	protein_codin	0.8667852	1.45223393	0	0.47787688
ENSG000001	LGALS9	protein_codin	0.48109071	0	0	0.65619522
ENSG000001	THAP9	protein_codin	2.85776974	6.79650079	8.73235063	0
ENSG000001	DDX19A	protein_codin	41.8825626	0	47.3906337	0
ENSG000001	DEFB4A	protein_codin	0	0	0	0

ENSG000001(	FAM151A	protein_codin	0	0	0	0.42634582
ENSG000001	PDGFD	protein_codin	15.629648	3.84235829	14.5729576	11.8493099
ENSG000001	DDI1	protein_codin	0	0	0	0.86071211
ENSG000001	GAA	protein_codin	0	12.7130668	8.17817835	0
ENSG000001	OR8U1	protein_codin	0	0	0	0
ENSG000001	HNRNPR	protein_codin	0	0	0	0
ENSG000001(	VPREB1	protein_codin	0	0	0	0
ENSG000001	ELOVL6	protein_codin	0	0	44.6521345	4.0224003
ENSG000001	ZNF804A	protein_codin	9.05479803	6.22351152	24.2479828	2.27371904
ENSG000001	ITGB1	protein_codin	35.7916323	1068.46752	0	0
ENSG000001(	FNTA	protein_codin	27.4771375	0	144.923328	20.1192514
ENSG000001	VSTM2A	protein_codin	1.23154907	0	0	0.93936541
ENSG000001(	LUZP1	protein_codin	10.354861	150.278162	87.6131107	50.5603603
ENSG000000	RFX3	protein_codin	0	0	0	0
ENSG000001(	AHSP	protein_codin	0	0	0	0
ENSG000001	WDR48	protein_codin	16.4134256	56.5802868	0	12.0649427
ENSG000002	CCDC7	protein_codin	0.56063472	0	3.81722525	0
ENSG000001(	MN1	protein_codin	8.13579704	39.8231541	22.774343	11.4074678
ENSG000001(	ING2	protein_codin	9.27776094	14.5723128	20.3630208	9.42408128
ENSG000001(	SCN11A	protein_codin	1.86141297	0	0	5.56543091
ENSG000001	BFSP2	protein_codin	0	0	0	0
ENSG000001	ZNF217	protein_codin	26.6945716	180.362754	80.5713761	0
ENSG000001	CANT1	protein_codin	13.3076056	29.6781815	25.5648642	17.1484023
ENSG000001(	ITGB2	protein_codin	0	0	0	0
ENSG000001(	CDKN2AIP	protein_codin	9.44102029	19.6725453	10.0033492	8.58514329
ENSG000001	ENC1	protein_codin	18.173419	23.9720858	22.2494651	3.42709441
ENSG000001	COPRS	protein_codin	7.59017753	37.6492507	47.7796765	4.89188675
ENSG000000	MPHOSPH9	protein_codin	36.2443675	36.5330795	64.7010635	17.5411942
ENSG000001	NME6	protein_codin	0	0	0	0
ENSG000001	GABARAP	protein_codin	2.8229835	46.4252348	7.50931369	22.5381929
ENSG000001(	TMEM42	protein_codin	2.8700863	8.65109891	0	3.51862741
ENSG000000	SMARCA2	protein_codin	0	0	0	1.40009243
ENSG000001	KIF5B	protein_codin	436.654	615.745297	1129.00416	251.024312
ENSG000002(	TMEM256	protein_codin	0	11.5114635	0.59192721	3.9622244
ENSG000001	TRIM8	protein_codin	3.54777987	17.3376425	1.71605821	11.6093874
ENSG000001	TRIAP1	protein_codin	0	0	0	0
ENSG000001	DMRT2	protein_codin	0	0	0	0
ENSG000000(	KARS1	protein_codin	39.1876862	98.3685926	193.057326	31.0073822
ENSG000001(	CKAP2L	protein_codin	20.4449966	7.31458476	258.654064	5.13374324
ENSG000001(	PDILT	protein_codin	0	0	0	1.73225497
ENSG000001	PTGER4	protein_codin	4.69266905	39.9848885	10.5997482	39.6564614
ENSG000001	MCC	protein_codin	0	0	42.6747338	18.6629029
ENSG000001(	KCNAB1	protein_codin	1.680676	0	0	0
ENSG000001(	HTRA4	protein_codin	0	1.513985	0	0

ENSG000001:	CLCC1	protein_codin	0	0	0	0
ENSG000001	MTCH2	protein_codin	15.5686371	4.85873942	21.896639	5.46420525
ENSG000001	CDC25A	protein_codin	3.807466	0	0	0
ENSG000001	C1QTNF4	protein_codin	0	0	0.55744901	0
ENSG000001	SF3B3	protein_codin	44.7633053	117.450541	199.887908	26.6778016
ENSG000001	CXXC5	protein_codin	17.3780284	52.8538418	25.1276421	27.0215066
ENSG000001:	CLIP1	protein_codin	0	0	0	0
ENSG000001	PYM1	protein_codin	8.46669796	9.17839944	29.8434106	0
ENSG000001	VAT1L	protein_codin	0.62914628	5.10570064	0.83972815	2.30030713
ENSG000001	ABR	protein_codin	26.6829568	2.0192111	17.8678834	3.68361161
ENSG000000	ABCC8	protein_codin	0	0	0	0
ENSG000001	MYRIP	protein_codin	0	0	0	0
ENSG000001	GPR82	protein_codin	1.82386429	1.27315763	0.47536464	2.63674724
ENSG000001	DESI2	protein_codin	28.6337946	23.9038787	63.3220676	14.9363409
ENSG000001	GP2	protein_codin	0	0	0	0
ENSG000001	RGPD8	protein_codin	38.6373229	0	0	0
ENSG000001	OR5B12	protein_codin	0	1.4497788	0	0
ENSG000001	OR5B2	protein_codin	0	0	0	0
ENSG000001	NEUROD2	protein_codin	0	0	0	1.15991214
ENSG000001	KYAT1	protein_codin	0	0	0	0
ENSG000001	ZNF25	protein_codin	37.2437812	49.0718527	27.3857289	26.1403985
ENSG000002	OR6Q1	protein_codin	0	0	0	0
ENSG000001	CDK5R2	protein_codin	0	0	0.46759411	0.81843784
ENSG000001	CALB2	protein_codin	0	0	0	0
ENSG000001	SCAMP3	protein_codin	0	0	0	0
ENSG000001	P2RY12	protein_codin	0.65761232	5.81966769	0.42613075	2.36119052
ENSG000001	ITPR1	protein_codin	0	0	11.282363	0
ENSG000001	PRSS27	protein_codin	0	0	0	0
ENSG000001	TRH	protein_codin	0	7.86439141	0	0.78316027
ENSG000001	OR2K2	protein_codin	0	0	0	1.60182842
ENSG000001	SLC25A33	protein_codin	3.10125364	7.26769196	10.0547619	7.4138023
ENSG000001	ALKBH3	protein_codin	0	10.1053608	3.35124059	0
ENSG000001	CLP1	protein_codin	2.5115602	9.29213552	1.81074753	0
ENSG000001	PDHB	protein_codin	3.91785979	62.51485	13.3087693	7.16470136
ENSG000001	JUNB	protein_codin	47.2988938	155.060638	19.320086	883.401854
ENSG000001	ANKRD49	protein_codin	0	0	0	0
ENSG000001	BUB1	protein_codin	9.58480517	0	128.328826	0
ENSG000000	CTNNA1	protein_codin	127.752854	152.082999	124.001705	102.095737
ENSG000001	NUDCD2	protein_codin	3.48445861	0	4.8079551	4.27628274
ENSG000001	PXK	protein_codin	0	35.1870083	0	0
ENSG000001	TTC36	protein_codin	0.34530139	0.95029097	0	0.503125
ENSG000001	LETM1	protein_codin	30.2755897	169.899793	257.673821	36.5663223
ENSG000001	MRGPRX1	protein_codin	0	0	0	0
ENSG000001	PALM2AKAP2	protein_codin	65.5463411	0	188.973942	34.4584168

ENSG000001	AURKC	protein_codin	0	0	0	0
ENSG000001	SPRY3	protein_codin	0	13.2928373	0	0
ENSG000000	FRYL	protein_codin	2.90060869	14.31995	26.9046885	5.87562528
ENSG000001	ACOX2	protein_codin	6.55676358	0	0	1.38902319
ENSG000001	STARD5	protein_codin	1.17456385	5.51960528	0	0.99769218
ENSG000001	INSR	protein_codin	19.5283587	6.95447406	42.4731438	5.80922347
ENSG000001	ZNF561	protein_codin	7.81927772	0	13.5495676	5.22503779
ENSG000001	ZCCHC4	protein_codin	6.61731902	23.5417485	12.528244	5.01898231
ENSG000001	UBTF	protein_codin	1.5043546	0	3.80050728	8.6652319
ENSG000001	RPS9	protein_codin	0	48.4881513	57.5755135	59.8386269
ENSG000001	KCNK3	protein_codin	0.57643651	5.28012653	0.52138063	3.794278
ENSG000000	ACHE	protein_codin	0	0	0	0
ENSG000001	TEX13B	protein_codin	0	0.54596435	0	0.4448409
ENSG000001	NLGN2	protein_codin	12.798593	57.7117342	59.4977958	20.9208218
ENSG000001	TSEN34	protein_codin	0	0	0	0
ENSG000001	GARIN3	protein_codin	0.35752925	0	0	1.31926003
ENSG000001	GSX1	protein_codin	0	0	0	0
ENSG000001	SLFNL1	protein_codin	0	0	0	0
ENSG000001	DLG4	protein_codin	4.35332837	28.1403964	25.0560709	18.7373856
ENSG000001	AMDHD2	protein_codin	0	0	0	0
ENSG000001	PYDC1	protein_codin	0	0	0	0
ENSG000001	POLR1D	protein_codin	7.11571398	30.8845204	13.9584713	9.37891211
ENSG000001	IL16	protein_codin	24.3504756	89.9158183	9.62416002	0
ENSG000001	ZBTB46	protein_codin	7.22752044	0	13.2494335	0
ENSG000001	CEBPB	protein_codin	15.2957335	53.9849075	18.9316447	118.489197
ENSG000001	KCNK9	protein_codin	0	0	0	0
ENSG000000	ARHGAP6	protein_codin	0	0	0	0
ENSG000001	CTRB2	protein_codin	0	0	0	0
ENSG000001	OR8J1	protein_codin	0	0	0	0.61896778
ENSG000001	COL22A1	protein_codin	5.6410539	231.597074	279.780722	99.4901313
ENSG000001	CSNK1G1	protein_codin	19.4542258	37.4871122	35.7412543	18.5360094
ENSG000001	ENTREP1	protein_codin	0	0	0	0
ENSG000001	DONSON	protein_codin	0	0	0	4.53751695
ENSG000001	SIX2	protein_codin	8.86122161	6.81022512	32.7200214	4.60766451
ENSG000000	RFXANK	protein_codin	0.72359804	0	7.72516051	0
ENSG000001	ARL13B	protein_codin	0	0	0	0
ENSG000001	DONSON	protein_codin	1.37917089	0	0	0.96969373
ENSG000001	IL7R	protein_codin	40.3342501	405.202607	6.66161386	30.0287479
ENSG000001	LMAN2	protein_codin	28.1558447	29.1543197	88.4760984	18.6274505
ENSG000001	ARHGAP42	protein_codin	0	0	0	0
ENSG000001	SHLD1	protein_codin	1.50848843	0	2.69061928	0
ENSG000001	COL8A2	protein_codin	27.1706426	0	0	0
ENSG000001	MDM1	protein_codin	0	0	0	15.2061525
ENSG000001	POP7	protein_codin	5.30462485	32.8918537	26.4710077	1.27429955

ENSG000001	NETO2	protein_codin	2.56169673	7.05121399	1.53714046	3.54048716
ENSG000001	NSG2	protein_codin	0	0	0	1.46197472
ENSG000001	PPDPFL	protein_codin	0	0	0	0
ENSG000001	PRELID1	protein_codin	18.9926018	7.29103544	73.705422	8.53135643
ENSG000001	GNB2	protein_codin	21.5004316	36.3234296	0	0
ENSG000001	NRTN	protein_codin	0	0	0.62117398	0.41861368
ENSG000001	EMB	protein_codin	13.138841	48.2950765	0	22.061564
ENSG000001	FUT3	protein_codin	0	0	0	0
ENSG000001	GLOD5	protein_codin	0	0	0	0
ENSG000001	HCN1	protein_codin	0	0	0	4.74215449
ENSG000001	EIF2AK3	protein_codin	29.3919029	126.469452	89.3089321	43.5619205
ENSG000001	RAB24	protein_codin	0	0	0	1.17767825
ENSG000001	SPMIP9	protein_codin	0	3.43627955	0	1.24291802
ENSG000001	RAB24	protein_codin	0	0	0	0
ENSG000001	TMEM129	protein_codin	0	0	0	1.96331607
ENSG000001	HIPK3	protein_codin	22.5794819	7.65066853	19.2106277	33.9978002
ENSG000001	PA2G4	protein_codin	24.4015514	100.289981	150.34932	18.8874898
ENSG000001	ZSCAN2	protein_codin	0	0	0	0
ENSG000001	MMADHC	protein_codin	24.1178724	15.3028229	34.5038172	15.867513
ENSG000001	OR2B2	protein_codin	0	0	0	0.97025062
ENSG000001	ARNT2	protein_codin	2.72282623	7.02633622	13.1378682	0.64731016
ENSG000001	CES3	protein_codin	0	0	0	0
ENSG000001	SLC50A1	protein_codin	6.52625127	0	2.85495675	3.73954884
ENSG000001	SCN9A	protein_codin	0	0	0	0
ENSG000001	TCTN2	protein_codin	3.94259341	0	0	0
ENSG000000	MRC2	protein_codin	217.384832	627.166306	1278.35493	233.275015
ENSG000001	SHCBP1	protein_codin	7.10715545	6.18420446	47.6084135	2.14181547
ENSG000001	MECP2	protein_codin	0	0	0	0
ENSG000001	SCN1A	protein_codin	0	1.65413091	0	2.3242447
ENSG000000	MAEA	protein_codin	0	44.2317244	51.7148517	10.8649688
ENSG000001	HOXC4	protein_codin	1.45602297	0	0	0
ENSG000001	BRD3	protein_codin	38.8137871	121.221474	100.683714	17.9735673
ENSG000000	SIRPG	protein_codin	0	0	0	0
ENSG000001	TRABD	protein_codin	0	0	0	0
ENSG000001	ARF4	protein_codin	66.218822	81.2822881	71.9548044	51.3359462
ENSG000001	HOXC9	protein_codin	3.99257179	8.83046347	7.26679687	2.50541635
ENSG000001	METTL15	protein_codin	11.0065945	0	30.7947949	0
ENSG000001	HOXC10	protein_codin	49.1485108	5.0241256	120.697887	19.8805921
ENSG000001	METTL18	protein_codin	4.69216893	0	0	0
ENSG000001	BTD	protein_codin	3.84426106	0	0	0
ENSG000001	SCAPER	protein_codin	17.7901087	0	42.3688998	2.34107772
ENSG000001	ACYP2	protein_codin	0	0	0	0
ENSG000001	PDS5A	protein_codin	159.590444	312.179141	523.411032	95.4685309
ENSG000001	RNF139	protein_codin	21.0510169	34.172656	159.658935	26.8978501

ENSG000001	NDUFA3	protein_codin	0	0	0	0.33611256
ENSG000001	FOS	protein_codin	47.3439707	112.975656	14.2326727	1155.45638
ENSG000001	CRTC2	protein_codin	0	0	0	0
ENSG000001	TMED10	protein_codin	85.4408986	96.6429194	199.67556	67.9623275
ENSG000001	PCBP1	protein_codin	89.6916535	180.541688	227.799191	84.7404472
ENSG000001	ZNF30	protein_codin	3.50518878	0	1.38416786	2.19074816
ENSG000001	KCNJ4	protein_codin	0	0	2.50912424	0.42214968
ENSG000001	THAP11	protein_codin	9.63169741	23.0958228	17.5216311	5.00816746
ENSG000001	AUH	protein_codin	0	0	0	0
ENSG000000	MARK3	protein_codin	0	0	0	0
ENSG000001	CAMTA1	protein_codin	8.9550366	4.81992803	16.9800339	6.18130735
ENSG000001	MRAP	protein_codin	0	0	0	1.340792
ENSG000000	RFX2	protein_codin	0	0	15.5107411	4.4101189
ENSG000001	DTNB	protein_codin	0	0	0	0
ENSG000001	ZEB2	protein_codin	16.7807196	24.0761143	0	31.2320455
ENSG000001	ARHGAP17	protein_codin	6.20218356	34.4740891	18.0913143	0
ENSG000001	CCDC174	protein_codin	0	0	0	0
ENSG000001	CHST11	protein_codin	9.02697542	54.7424619	32.7961011	30.5851176
ENSG000001	DCDC1	protein_codin	0	0	0	0.9546957
ENSG000001	NFU1	protein_codin	0.96260365	0	7.40327121	2.98228595
ENSG000001	ANTXR1	protein_codin	308.354134	535.267019	250.414792	152.564789
ENSG000001	FGGY	protein_codin	0	2.9364305	0	0
ENSG000001	PRY	protein_codin	0	0	0	0
ENSG000001	TRAPPC1	protein_codin	1.90634137	4.74446988	8.10753715	0.98058171
ENSG000001	SLC38A11	protein_codin	0	0	0	0
ENSG000001	KREMEN2	protein_codin	0	0	0	0
ENSG000001	SDR16C5	protein_codin	0	0	0	0
ENSG000002	LST1	protein_codin	0	0	0	0.62257467
ENSG000001	CHCHD7	protein_codin	0	0	0	0
ENSG000001	RBMY1F	protein_codin	0	0	0	0
ENSG000001	N6AMT1	protein_codin	0	0	0	7.01986955
ENSG000001	PROKR1	protein_codin	0.53411317	1.10269374	1.40549528	2.23540223
ENSG000001	KCNAB3	protein_codin	0	4.31974657	5.06098597	1.0309729
ENSG000001	APLF	protein_codin	12.2877246	20.3557447	18.8884004	0
ENSG000001	PRY2	protein_codin	0.28446159	6.78524993	0	0
ENSG000001	ZNF296	protein_codin	0	3.21171213	0	0.4025741
ENSG000001	EYA1	protein_codin	0	52.1589665	0	0
ENSG000001	REPS2	protein_codin	0	0	7.71832599	8.81553545
ENSG000001	SP7	protein_codin	0	0	0	0
ENSG000001	WDR87	protein_codin	0.62188846	2.62320693	0.44397009	16.5259212
ENSG000001	MCM7	protein_codin	19.3096257	64.0408436	99.6220728	0
ENSG000002	ATP6V1G2	protein_codin	0.38429566	0	0	1.35121328
ENSG000001	SYCE1	protein_codin	0	0.96286351	0	0
ENSG000001	COPS6	protein_codin	7.43549626	89.099021	42.2010415	4.75867125

ENSG000002	H2AC8	protein_codin	73.3205895	125.563709	18.6411469	38.9005744
ENSG000001	GPR37	protein_codin	0.77884837	0	6.29420619	1.96143514
ENSG000001	HAS2	protein_codin	7.67197169	6.13151093	40.904964	5.0307317
ENSG000001	CAVIN3	protein_codin	7.02079822	22.1108999	1.32994836	5.42866254
ENSG000001	ANKK1	protein_codin	0	0	0	0.89265618
ENSG000002	EGLN2	protein_codin	12.8973387	26.8857732	20.6708328	11.0884432
ENSG000000	ARAP2	protein_codin	16.8057372	72.7174521	16.8605204	20.7357299
ENSG000001	GPRIN1	protein_codin	2.12694203	5.03413235	20.7807428	3.24235229
ENSG000000	JMJD6	protein_codin	0	0	1.51291349	0
ENSG000001	POLR1C	protein_codin	0.94621262	10.8165116	1.41114186	0
ENSG000001	ZNF439	protein_codin	11.7695386	38.26477	27.3978233	2.74763957
ENSG000001	HS1BP3	protein_codin	5.41357395	20.9149465	16.4839261	0.78713106
ENSG000001	AGAP1	protein_codin	12.2991569	41.8141606	17.3355177	13.5392596
ENSG000001	HINT1	protein_codin	15.8797425	0	37.114435	8.08019064
ENSG000001	KLK7	protein_codin	0	0	0	0
ENSG000001	ORMDL3	protein_codin	24.997643	73.5135528	32.5607197	16.4363094
ENSG000001	NTRK2	protein_codin	0	0	0	0
ENSG000001	KBTBD2	protein_codin	46.3771931	78.5857774	125.232448	59.2146914
ENSG000001	DTWD2	protein_codin	4.92762848	7.80768708	6.0538078	2.72342955
ENSG000001	ZNF440	protein_codin	13.2688465	19.4892739	22.6469245	2.71829329
ENSG000001	RIOX1	protein_codin	3.9037532	19.7182531	11.8525327	2.9450301
ENSG000001	CPLX1	protein_codin	0	4.17938532	0	0
ENSG000001	VAV1	protein_codin	0	0	0	0
ENSG000001	NT5C1B	protein_codin	0	0	0	0
ENSG000001	CLEC7A	protein_codin	5.63188502	0	0	0
ENSG000001	KCNS3	protein_codin	6.69805593	18.5258772	8.7406242	6.78381328
ENSG000001	ZNF565	protein_codin	7.0311573	5.48155982	3.7889859	4.61937503
ENSG000001	AFAP1L2	protein_codin	15.3185937	26.0613698	0	0
ENSG000001	TTBK1	protein_codin	0	3.31950747	0	0
ENSG000001	CAVIN2	protein_codin	35.9810504	55.0094966	5.11204823	20.2571394
ENSG000001	MYO1B	protein_codin	0	11.8857876	0	3.15987601
ENSG000000	ADCYAP1R1	protein_codin	0	0	0	0
ENSG000001	C15orf40	protein_codin	9.65034077	25.6443633	25.5187378	19.8920789
ENSG000001	FAM110A	protein_codin	0	0	0	0
ENSG000001	RAMAC	protein_codin	6.59111088	4.47161264	13.7003237	5.11795196
ENSG000001	FRMD3	protein_codin	8.03218643	32.3981511	21.1709759	27.5145362
ENSG000000	GIPR	protein_codin	0	0	0	0
ENSG000001	H1-4	protein_codin	145.47241	328.974808	1371.94146	76.3597041
ENSG000001	ADORA2B	protein_codin	0.60947056	1.2981579	0	1.4329833
ENSG000001	HOMER2	protein_codin	4.09882086	19.9016163	3.09740292	0
ENSG000001	RAB40A	protein_codin	0	4.24168126	3.79130791	0.47058042
ENSG000001	ZNF420	protein_codin	0	0	0	0.61967376
ENSG000001	GPR25	protein_codin	0	1.05126671	0	0
ENSG000000	SEC61A2	protein_codin	3.32694281	0	0	0

ENSG000001	SPACA5B	protein_codin	0	0	0	1.08621545
ENSG000001	C11orf24	protein_codin	21.3146983	31.6877222	27.3121383	12.6613939
ENSG000001	ANKS3	protein_codin	0	0	6.84942111	0
ENSG000001	HSPB2	protein_codin	1.94631281	1.77604357	0	2.02183227
ENSG000001	NUDT16L1	protein_codin	0	0	7.85704009	2.07498233
ENSG000001	ATP5ME	protein_codin	40.4110951	239.103305	378.562083	37.451008
ENSG000001	RPAP1	protein_codin	0	0	0	0
ENSG000001	MACROH2A1	protein_codin	0	0	0	0
ENSG000001	TIGD4	protein_codin	2.48634929	1.08064754	8.7920704	3.39698893
ENSG000001	PPP4R4	protein_codin	0.55716868	0	28.0525298	3.76056627
ENSG000001	SPACA5	protein_codin	0	0	0	0
ENSG000001	CLEC12A	protein_codin	0	0	0	0
ENSG000001	KMT5B	protein_codin	13.8039794	32.9905075	17.466327	7.31723964
ENSG000001	KCTD19	protein_codin	0	0	0	0
ENSG000001	DRD5	protein_codin	0	2.34129669	0.45701108	2.22682922
ENSG000001	TMC7	protein_codin	0	0	19.12322	3.18053223
ENSG000001	TMEM154	protein_codin	1.26027985	0	0.77879594	8.23764402
ENSG000002	DISP3	protein_codin	0	0	1.07435781	0
ENSG000001	PAM	protein_codin	0	0	0	0
ENSG000001	GPR22	protein_codin	0.27001891	0	0	2.19851946
ENSG000001	GPR27	protein_codin	1.62570202	1.13147091	7.13586147	0.47464005
ENSG000001	ARL6IP1	protein_codin	30.010745	104.342428	135.461589	40.0230172
ENSG000001	OR5AU1	protein_codin	0	0	0	0
ENSG000001	FSHR	protein_codin	0	0	0	1.46440101
ENSG000001	NUDT6	protein_codin	4.8629838	4.6048765	0	0
ENSG000000	ELOVL5	protein_codin	39.2224792	129.750168	150.442599	32.0626429
ENSG000001	NKRF	protein_codin	0	0	0	0
ENSG000001	EXOSC10	protein_codin	0	0	45.4229756	0
ENSG000001	SLC6A19	protein_codin	0.25852397	2.07280521	0	2.17030847
ENSG000001	SRGAP2C	protein_codin	21.9605021	0	4.88373559	0
ENSG000001	UTF1	protein_codin	0	0	0	0
ENSG000001	MRM3	protein_codin	5.48595213	10.5812942	8.49988313	4.94034661
ENSG000001	CDKN2A	protein_codin	11.284409	0	0	0
ENSG000001	SOX7	protein_codin	1.79596853	13.3616886	3.0495504	14.3566436
ENSG000001	CRYGA	protein_codin	0	0	0	0
ENSG000001	IL13	protein_codin	0	0	0	0
ENSG000001	TMEM126A	protein_codin	3.37146047	5.41634275	6.56741995	3.45554104
ENSG000001	RGPD3	protein_codin	3.06865841	0	5.64804198	2.35393447
ENSG000001	INO80E	protein_codin	1.55836639	0	1.64524892	0
ENSG000001	C8orf74	protein_codin	0	0	0	0
ENSG000001	LDB2	protein_codin	8.16019419	0	12.3626029	5.71565192
ENSG000001	INSL5	protein_codin	0	0	0	0.89171212
ENSG000001	PRDM10	protein_codin	7.15132729	0	0	0
ENSG000000	ZFX	protein_codin	3.16216637	77.4087854	8.54973437	0

ENSG000001	ECEL1	protein_codin	0	0	0	0
ENSG000001	CXCR6	protein_codin	0	24.9740862	0	1.01718115
ENSG000001	RRM2	protein_codin	0	28.8057154	68.4573144	0
ENSG000001	TM4SF20	protein_codin	0	0	0	1.12575996
ENSG000000	PLEKHG6	protein_codin	0.53086191	0	0	0
ENSG000001	MFF	protein_codin	11.9855526	0	0	0
ENSG000001	PEX6	protein_codin	10.6107124	35.7923863	38.8237183	5.53844865
ENSG000001	KNDC1	protein_codin	0	0	0	1.1399988
ENSG000001	KRT78	protein_codin	0	0	0	0
ENSG000001	TTC39C	protein_codin	0	7.26859991	0	5.22277303
ENSG000001	CTNND2	protein_codin	0	0	21.9864328	0
ENSG000001	RNASE2	protein_codin	0	0	0.56369513	0
ENSG000001	COL3A1	protein_codin	63.7282326	4496.24912	160.649235	364.092531
ENSG000001	RNASE3	protein_codin	0	0	0	0
ENSG000001	OR1F1	protein_codin	0	0	0	0.47659826
ENSG000001	XPO6	protein_codin	10.3029191	3.87262694	68.5786972	3.3076368
ENSG000001	C1GALT1C1	protein_codin	8.52761113	14.1762133	13.0870556	4.76300654
ENSG000001	PTCRA	protein_codin	0	0	0	0.44054769
ENSG000001	RNASE6	protein_codin	4.01118892	31.1479641	1.23723278	7.23511019
ENSG000001	RGL1	protein_codin	0	14.3061091	5.49139115	1.52705456
ENSG000001	FAM171B	protein_codin	23.4126135	27.3195472	53.746417	9.11216233
ENSG000001	CST5	protein_codin	0	0	0.93139179	0
ENSG000000	AAMDC	protein_codin	0	2.46153652	0.74105424	0
ENSG000001	OR1L6	protein_codin	0	0	0	0.45801334
ENSG000001	CST2	protein_codin	0.31674639	0.80350409	0	0
ENSG000000	DBNDD1	protein_codin	0	0	0	0
ENSG000001	RPL7L1	protein_codin	0	0	0	0
ENSG000001	VASN	protein_codin	89.0560325	130.165676	193.851318	88.9700929
ENSG000001	PCNX1	protein_codin	49.126242	114.327583	74.5115838	27.4308021
ENSG000001	FPR1	protein_codin	0.84308848	7.60638318	0	0
ENSG000001	CST1	protein_codin	0	0	0	0
ENSG000001	AMOT	protein_codin	32.9479633	34.5482936	33.0624902	7.30875955
ENSG000001	SHE	protein_codin	12.1501112	53.0260787	9.90784351	27.0655987
ENSG000001	MAP6	protein_codin	0	0	2.84738903	0.64399622
ENSG000000	GLP2R	protein_codin	0	0	0	0
ENSG000001	DCC	protein_codin	0	0	0	0
ENSG000001	LONRF3	protein_codin	0	0	0	0
ENSG000001	SRP9	protein_codin	90.1450463	87.419346	173.172369	37.1217836
ENSG000001	NANP	protein_codin	3.789053	10.2889182	8.66849943	3.08620148
ENSG000001	HSFY2	protein_codin	0	0	0	0
ENSG000001	TMEM208	protein_codin	6.86986716	9.06449085	38.939084	3.12895013
ENSG000001	PGK2	protein_codin	0	1.29211932	0	1.95589016
ENSG000001	PAFAH1B2	protein_codin	6.72685871	15.2211185	9.24474269	3.9479213
ENSG000001	OR1L3	protein_codin	0	0	0	0.53957899

ENSG000001	JPT1	protein_codin	0	0	0	0
ENSG000001	TPST1	protein_codin	13.2084285	30.1835789	56.6323314	28.1426642
ENSG000001	HSPA4	protein_codin	118.11447	265.135806	419.29559	79.2473078
ENSG000001	UQCRFS1	protein_codin	17.3129975	18.7584232	23.9318108	19.6444566
ENSG000001	ASL	protein_codin	0	0	18.555516	0
ENSG000001	TPD52L1	protein_codin	0	0	0	0
ENSG000001	OR1N1	protein_codin	0	0	0	0.44192793
ENSG000001	TACR3	protein_codin	0.2865959	1.79518562	0.54248041	2.01641549
ENSG000001	SNAP25	protein_codin	0	0	0	0
ENSG000001	MUC7	protein_codin	0.26671536	0	0	0.59406406
ENSG000001	GUSB	protein_codin	3.58236703	0	29.328283	19.0035982
ENSG000001	SLFN12	protein_codin	0	2.83851735	0	0
ENSG000001	SMR3B	protein_codin	0	0	0	0
ENSG000001	LRATD2	protein_codin	4.56731598	0	47.8427705	3.532868
ENSG000001	IL17D	protein_codin	3.24269916	3.58500735	5.49134212	3.12775038
ENSG000001	ZSCAN32	protein_codin	4.19902548	0	0	3.47910774
ENSG000001	OR2C1	protein_codin	0	0	0.49766894	0.90942354
ENSG000001	HES4	protein_codin	1.93095532	7.50225745	7.03520722	0
ENSG000001	CSN3	protein_codin	0	0	0	0.53310845
ENSG000001	PPIH	protein_codin	0.23178176	22.8679376	0	0
ENSG000001	SIK2	protein_codin	65.0144425	88.4150327	50.2068798	39.9444417
ENSG000001	COL4A3	protein_codin	0	0	0	0
ENSG000001	PRPF8	protein_codin	165.973536	408.559175	274.052339	123.168683
ENSG000001	CHSY3	protein_codin	24.9837791	22.8084234	52.2037172	12.4489206
ENSG000001	ADH1B	protein_codin	18.74924	9.10835056	0	0
ENSG000001	OR4K15	protein_codin	0	0	0	1.67861073
ENSG000001	REG1B	protein_codin	0	1.53393962	0	0
ENSG000001	P2RY1	protein_codin	19.7604853	18.6775463	27.5722361	17.5505244
ENSG000001	COMMD5	protein_codin	0	0	2.67728905	1.35809235
ENSG000001	ZNHIT1	protein_codin	19.2448836	44.8509529	64.3512068	8.82060323
ENSG000001	UGT2B4	protein_codin	0	0	0	0
ENSG000001	NINJ2	protein_codin	0	0	0	0
ENSG000002	MUC12	protein_codin	0	0	0	0
ENSG000001	IRS1	protein_codin	72.1912685	51.4807566	110.126628	34.5370885
ENSG000001	GAP43	protein_codin	2.55451335	0	3.28498607	0
ENSG000001	YEATS2	protein_codin	56.3472804	200.274494	222.208306	36.2806042
ENSG000001	RAD17	protein_codin	3.3526617	0	24.0356375	7.96383016
ENSG000001	UGT1A6	protein_codin	0	0	0	0.99520808
ENSG000001	NMUR1	protein_codin	0.30688546	6.92643304	0	2.76974083
ENSG000001	OR2AT4	protein_codin	0	0	0	0
ENSG000001	REG3A	protein_codin	0	0	0	0
ENSG000001	ESCO2	protein_codin	0.87105519	10.6738156	22.2174646	2.94957439
ENSG000001	C22orf15	protein_codin	0	0	0	0
ENSG000002	UGT1A1	protein_codin	0	0	0	0

ENSG000001(	STIP1	protein_codin	54.2603828	144.38042	349.397561	31.8214885
ENSG000001'	UGT2B7	protein_codin	0	0	0	0
ENSG000000'	SIRT6	protein_codin	0	0	0	0
ENSG000001'	PWWP2B	protein_codin	2.78363095	21.9928035	1.48107306	7.56860778
ENSG000001(	ODAD2	protein_codin	0	0	0	0
ENSG000001	TACR1	protein_codin	1.02665006	0	0	5.39728503
ENSG000001(	TUB	protein_codin	0	30.1985978	0	12.9543754
ENSG000001'	HDAC3	protein_codin	9.55993665	21.5973915	22.4062763	0
ENSG000001'	EFCAB3	protein_codin	0	0	0	0
ENSG000001(	BOLA2B	protein_codin	0	0	0	0.48070399
ENSG000001(	HTR1E	protein_codin	0	0	0	1.94813044
ENSG000001'	S1PR1	protein_codin	17.3596553	75.4882871	0	36.0499126
ENSG000001(	TM4SF4	protein_codin	0	0	0	0
ENSG000000	TMPRSS11E	protein_codin	0	0	0	0
ENSG000001(	TRIM35	protein_codin	11.3543688	22.5597724	17.0013113	6.50374415
ENSG000001(	TM4SF1	protein_codin	21.8152245	88.7601973	6.23394609	76.1220216
ENSG000001	EPB41L4A	protein_codin	0.68518811	4.91568436	0.99470633	1.11968851
ENSG000001(	MGAT2	protein_codin	18.1818611	36.9703245	54.8595711	11.4472572
ENSG000001(	PTAFR	protein_codin	0	0	0	0
ENSG000001'	SCG2	protein_codin	0.54137907	4.35638858	1.28912471	0.92883623
ENSG000001	FGF13	protein_codin	0	0	0	0
ENSG000001(	MINAR1	protein_codin	7.52169306	12.9346789	15.8343616	0
ENSG000001(	GRM5	protein_codin	0	2.00203349	0	0
ENSG000001'	OR7G3	protein_codin	0	0	0	0
ENSG000001(	GRM5	protein_codin	0	0	0	0
ENSG000001(	VXN	protein_codin	0	0	0	0
ENSG000001(	SEMA4C	protein_codin	8.46957502	0	33.4007631	12.9396024
ENSG000001(	WFDC13	protein_codin	0	0	0	1.76143473
ENSG000001'	USP47	protein_codin	0	0	0	0
ENSG000001(	TMEM135	protein_codin	6.42882354	16.9164706	13.0466883	16.1788173
ENSG000001(	CNNM3	protein_codin	3.28080999	45.775819	20.4659262	4.44345196
ENSG000001	SMYD4	protein_codin	6.35170929	20.3690123	13.2095421	5.00667563
ENSG000001'	FRMPD2	protein_codin	0	0	0	0
ENSG000002	TWF2	protein_codin	21.4158578	53.8411424	22.4336209	12.5517795
ENSG000001	RBMX2	protein_codin	26.4903384	28.3377467	35.6709511	12.621789
ENSG000001'	LAMB2	protein_codin	61.6838965	168.703753	49.3242533	83.1655156
ENSG000001	RTKN	protein_codin	6.91624827	0	33.7381314	0
ENSG000001'	AFG2B	protein_codin	0	0	0	0
ENSG000001	ZNF100	protein_codin	0	13.4190827	0	2.91195782
ENSG000001(	NPNT	protein_codin	0	0	2.81325371	0
ENSG000001(	IL12A	protein_codin	0	0	0	0
ENSG000001	FXR1	protein_codin	6.97276698	0	30.9951815	0
ENSG000001(	CD2BP2	protein_codin	5.5604888	36.997187	36.6671284	12.4107822
ENSG000001'	KRT74	protein_codin	0	0	0	0.96409349

ENSG000001	MBD3L1	protein_codin	0	0	0	0.37805375
ENSG000001	RAB33B	protein_codin	20.5393841	52.7568231	3.15322233	9.35508715
ENSG000001	SIGLEC7	protein_codin	0	2.42979314	0.95902659	1.60240561
ENSG000001	DEGS2	protein_codin	0	0.93189281	0	0
ENSG000001	TBL2	protein_codin	4.65605573	7.63153654	16.2039887	11.8084872
ENSG000001	LCMT2	protein_codin	1.62942344	6.33939086	5.50918078	3.80563403
ENSG000001	GLYCTK	protein_codin	0	0	0	0
ENSG000001	FANCA	protein_codin	0	0	0	0
ENSG000001	TET2	protein_codin	21.5178408	65.6170503	88.376992	21.7731138
ENSG000001	PATE1	protein_codin	0	0	0	0
ENSG000001	CERS6	protein_codin	28.194977	107.812536	141.251322	30.1169761
ENSG000001	KRT73	protein_codin	0	1.1246115	0	0
ENSG000001	FAM98B	protein_codin	0	0	0	0
ENSG000001	CAMK2G	protein_codin	0	0	0	0
ENSG000001	PHACTR2	protein_codin	0	0	0	7.35700845
ENSG000001	CEP89	protein_codin	5.68957878	18.3952546	4.15998796	7.93233932
ENSG000001	RNFT1	protein_codin	0	0	3.79889807	0
ENSG000001	DTYMK	protein_codin	6.59930566	28.1861118	19.4024378	2.02707377
ENSG000001	CYCS	protein_codin	47.6016445	102.355846	155.447203	62.0816145
ENSG000001	TSPAN5	protein_codin	7.38809104	0	0	4.31844699
ENSG000001	TET3	protein_codin	0	0	0	0
ENSG000001	SLC9C1	protein_codin	0	0	0	0
ENSG000001	PRND	protein_codin	6.46213042	8.92445818	9.40070615	6.76133518
ENSG000001	CENPN	protein_codin	2.81066881	2.99741995	19.9304355	0
ENSG000001	FEZ2	protein_codin	0	0	0	0
ENSG000001	EMCN	protein_codin	0	0	0	0
ENSG000000	VPS50	protein_codin	0	0	9.35067289	0
ENSG000001	CCL11	protein_codin	0	0	0	0
ENSG000001	BCR	protein_codin	14.9034059	52.8824395	68.1879509	28.7105628
ENSG000001	GTSF1	protein_codin	0	0	13.5030527	0
ENSG000001	KLF11	protein_codin	7.19631952	23.2961976	0	0
ENSG000001	FEN1	protein_codin	6.05276342	27.0584984	35.9568604	2.83221265
ENSG000001	OR10H2	protein_codin	0	0	0	1.41100141
ENSG000001	ONECUT1	protein_codin	0.56420523	3.17266544	11.0957076	3.51087354
ENSG000001	DYNLRB2	protein_codin	0	0	0	0
ENSG000001	PPM1D	protein_codin	0	0	0	3.7189183
ENSG000001	RNF187	protein_codin	26.2707567	108.371522	245.510257	23.2381358
ENSG000001	MTBP	protein_codin	9.93257966	5.2211126	147.802297	6.22558759
ENSG000000	SMOX	protein_codin	0	0	0	13.1874482
ENSG000001	MMGT1	protein_codin	10.7937079	0	4.89152142	13.2685696
ENSG000001	SCAND1	protein_codin	5.9012445	9.9364487	23.2742938	2.98974441
ENSG000001	ADRB2	protein_codin	1.87881416	2.39339067	0.43759774	2.16048726
ENSG000001	SLN	protein_codin	23.4615575	1.60138538	0	0
ENSG000001	MBOAT2	protein_codin	15.3028483	26.4469803	38.3862047	6.04706624

ENSG000001	ZNF239	protein_codin	0	0	17.6236392	0
ENSG000001	MGMT	protein_codin	1.74227162	11.7666139	7.94364056	6.15866621
ENSG000001	ATOH1	protein_codin	0	0	0.52986543	0.95015842
ENSG000001	DDX54	protein_codin	27.3165434	0	37.5984613	18.8260244
ENSG000001	LSM3	protein_codin	36.9816238	67.3924076	213.980559	16.4672646
ENSG000001	USP19	protein_codin	8.11689051	0	35.2569769	0
ENSG000001	DPYD	protein_codin	1.62486085	22.7000041	1.63279934	0.9584439
ENSG000001	PTPRE	protein_codin	14.398642	89.7088505	7.22700971	27.3280957
ENSG000001	TEFM	protein_codin	0	0	25.9286704	2.24369989
ENSG000001	PTGDR	protein_codin	0.24279226	11.0772125	0.48518736	5.03638236
ENSG000001	LRP8	protein_codin	3.28087699	9.03658289	55.4845845	5.2166222
ENSG000001	ASXL1	protein_codin	0	0	0	9.88980736
ENSG000001	MT1E	protein_codin	1.43303098	6.25263338	0.56715614	41.4240444
ENSG000001	ANKRD27	protein_codin	22.7274138	92.1953323	34.4022618	13.6502853
ENSG000001	CHRNB1	protein_codin	0.6190415	0	0	1.12859411
ENSG000001	ISG20	protein_codin	0	0	2.67090531	1.67280317
ENSG000001	TMEM43	protein_codin	82.5109971	135.666263	174.803378	19.7247556
ENSG000001	KCNG3	protein_codin	0	0	0	0
ENSG000001	TXNDC2	protein_codin	0.18101127	0	0	0.77827663
ENSG000001	TRIM56	protein_codin	45.1150526	327.877418	52.3166249	33.4488555
ENSG000001	SOX14	protein_codin	0.22538412	1.0986515	0	1.28557413
ENSG000000	GNAS	protein_codin	0	0	0	0
ENSG000001	FSTL5	protein_codin	0	0	0	0
ENSG000001	HSPBAP1	protein_codin	2.74445783	0	12.1741723	0
ENSG000001	ALCAM	protein_codin	5.55253185	48.2301579	0	16.3453983
ENSG000001	TRMT61B	protein_codin	5.76521677	20.5411101	34.2199164	11.207449
ENSG000001	KCNS1	protein_codin	0	5.19679839	4.82641399	0
ENSG000000	GNAS	protein_codin	0	0	0	0
ENSG000001	SNX7	protein_codin	83.6472025	98.7999639	384.687109	36.5615529
ENSG000001	QARS1	protein_codin	36.705206	0	34.2231553	0
ENSG000002	TMEM276	protein_codin	0	0	11.0561471	0
ENSG000001	CSDC2	protein_codin	0.27347136	0	6.34138911	0
ENSG000001	MUC17	protein_codin	0.97178853	0	0	0
ENSG000001	PRKCE	protein_codin	13.7691014	23.6714523	34.55098	13.8712369
ENSG000001	MTM1	protein_codin	0	0	6.20409044	0
ENSG000001	ZPLD1	protein_codin	0.26731791	0	0	1.22354117
ENSG000001	USP42	protein_codin	5.19294976	17.8104126	15.8388985	17.9004734
ENSG000001	MRPL13	protein_codin	19.487175	0	99.0976119	7.57061527
ENSG000001	CBR4	protein_codin	17.1001981	18.2522605	17.1849721	6.17736561
ENSG000001	STX18	protein_codin	5.48372788	43.5382954	0	11.2005761
ENSG000001	SCGB1D1	protein_codin	0	0	0	0.62977416
ENSG000001	CHST14	protein_codin	0	10.2047484	8.59869071	2.09200783
ENSG000001	MLKL	protein_codin	0	4.13834979	4.65546428	0
ENSG000001	RER1	protein_codin	0	0	0	0

ENSG000001	ST3GAL5	protein_codin	0	0	0	0
ENSG000001	FOXI1	protein_codin	0	0	0	2.47780308
ENSG000000	IBTK	protein_codin	69.3616897	108.993035	75.6555961	50.0794707
ENSG000001	KRTAP1-1	protein_codin	0	0	0	0
ENSG000001	ATOH8	protein_codin	1.88988698	6.5383163	5.97182507	6.25652838
ENSG000001	STK32A	protein_codin	0.24712071	0	0	0.41373332
ENSG000001	REEP4	protein_codin	1.83263501	0	11.4981254	0
ENSG000001	SLC26A5	protein_codin	0	0	0	0
ENSG000001	ZNF608	protein_codin	4.29657052	0	6.02992084	2.72017379
ENSG000001	LGI3	protein_codin	0	0	0	0
ENSG000001	GBX2	protein_codin	0	0	0	0
ENSG000001	RETREG1	protein_codin	0	0	52.5994707	0
ENSG000002	PSG11	protein_codin	0	0	0	0
ENSG000001	HOXD4	protein_codin	1.8847286	6.76452143	5.82230334	2.7897109
ENSG000001	MTCL1	protein_codin	0	0	0	0
ENSG000001	C2orf68	protein_codin	14.9472977	39.9964598	36.9564215	13.0505553
ENSG000001	ZNF396	protein_codin	0	0	0	0
ENSG000001	BMP1	protein_codin	0	0	0	0
ENSG000001	TMEM150A	protein_codin	0	2.22430434	0	0
ENSG000001	STX8	protein_codin	14.9249072	17.3883051	16.2696869	10.0744877
ENSG000001	RNF181	protein_codin	7.82805511	4.62988804	23.363703	6.33648454
ENSG000002	ETV5	protein_codin	15.4792071	12.3519143	20.071267	14.4105928
ENSG000001	BPTF	protein_codin	6.07639354	108.362014	6.63652328	26.4439338
ENSG000001	VAMP5	protein_codin	6.65042945	23.6762149	1.46899236	4.61727231
ENSG000001	BMP1	protein_codin	22.2150683	6.11205369	9.14699027	6.35185828
ENSG000001	TBCA	protein_codin	0	18.8632265	0	1.18869061
ENSG000001	DPY19L2	protein_codin	0	1.72218634	2.6497337	0
ENSG000001	LRG1	protein_codin	0.18304333	0	0	1.63173781
ENSG000001	BAALC	protein_codin	0	0	0	0
ENSG000000	SORBS1	protein_codin	0	0	23.0179789	0
ENSG000001	TMEM37	protein_codin	6.08264843	17.3532192	19.8099004	3.27602657
ENSG000001	OTP	protein_codin	0.2657318	0.94553581	0	0
ENSG000001	POLR3D	protein_codin	16.8686149	12.5151286	23.0819988	5.40257022
ENSG000001	MAT2A	protein_codin	27.3950671	48.4395758	177.786934	66.7202247
ENSG000001	PPIC	protein_codin	40.7135731	93.6630635	93.8759378	44.0388024
ENSG000002	ATP6V1E2	protein_codin	0.57397887	2.10953274	1.06942048	0
ENSG000001	ARMC10	protein_codin	0	0	1.24477506	0.68853862
ENSG000001	PIGF	protein_codin	3.9769703	0	5.94817099	2.05554304
ENSG000001	CEP120	protein_codin	0	0	0	0
ENSG000001	TMEM192	protein_codin	7.83193227	21.3436629	31.7689931	9.47853769
ENSG000001	CEP120	protein_codin	17.0912343	0	0	0
ENSG000001	PRL	protein_codin	0	0	0	0
ENSG000001	SLC22A25	protein_codin	0.54035106	1.57393377	0	3.81215281
ENSG000001	ZNF778	protein_codin	3.54605586	0	0	0

ENSG000001	SOCS5	protein_codin	0	0	0	0
ENSG000001	FOXD4L1	protein_codin	0	1.08603449	1.82390728	0
ENSG000001	PSG8	protein_codin	0	0	0	0
ENSG000001	ROBO4	protein_codin	3.18075591	23.5992583	2.70469688	13.0497178
ENSG000001	PCDHB1	protein_codin	0.52030661	3.66712592	0.49132098	6.51594165
ENSG000001	HAS3	protein_codin	0	0	0	3.15047213
ENSG000001	ZNF672	protein_codin	4.50385921	11.9866131	7.35624159	6.0619493
ENSG000002	DERPC	protein_codin	0	0	0	0
ENSG000001	TMC6	protein_codin	0	0	0	0
ENSG000002	PCDHGC4	protein_codin	4.45118748	0	0	2.89051659
ENSG000001	ZNF692	protein_codin	0	0	0	0
ENSG000001	CXCL10	protein_codin	3.9991215	162.631374	1.33107717	2.8135152
ENSG000001	CDY1	protein_codin	0	0	0	0
ENSG000001	CXCL11	protein_codin	0	44.2216422	0	2.40010714
ENSG000001	UBE2E1	protein_codin	0	0	34.0151333	0
ENSG000001	CYBC1	protein_codin	5.9766983	0	0	0
ENSG000001	KRT28	protein_codin	0	0	0	0.55642355
ENSG000000	PTGER3	protein_codin	0.26537581	0	0.46855879	3.45422258
ENSG000001	ADRA1B	protein_codin	1.1456372	0	0	1.27005335
ENSG000001	RASA4B	protein_codin	0	0	8.32790605	0
ENSG000001	LRRC45	protein_codin	6.66105271	21.5974036	28.7572539	4.56576503
ENSG000001	SGO1	protein_codin	0	0	0	0.60317866
ENSG000001	CENPX	protein_codin	0	0	0	0
ENSG000001	LRRC8E	protein_codin	1.06994428	5.37489809	4.24898651	1.64444124
ENSG000001	CDCA7	protein_codin	0	0	23.2358312	0
ENSG000001	ASPSCR1	protein_codin	0	0	0	0
ENSG000001	AVEN	protein_codin	12.1300684	23.741198	24.8890027	4.48700039
ENSG000001	RBFA	protein_codin	5.21269386	19.9044082	14.7916054	4.96493742
ENSG000001	ASPSCR1	protein_codin	11.4426996	18.1432137	25.1385587	7.29727766
ENSG000001	FASN	protein_codin	38.2224906	126.85467	191.645467	0
ENSG000002	NFS1	protein_codin	0	0	0.78049843	0
ENSG000001	RCAN2	protein_codin	0	0	0	0
ENSG000001	NPTX1	protein_codin	0	0	0	0
ENSG000001	GFRA2	protein_codin	2.92379609	0	1.56496865	0
ENSG000001	DUS1L	protein_codin	0	16.1356584	50.8087332	8.83862993
ENSG000001	RNF150	protein_codin	0	0	0	0
ENSG000001	RPTOR	protein_codin	0	0	72.2900424	28.5373426
ENSG000001	BLTP1	protein_codin	0	0	0	0
ENSG000001	NEGR1	protein_codin	30.9719259	33.6248676	20.8317002	0
ENSG000001	GPS1	protein_codin	0	0	0	0
ENSG000001	OR8B12	protein_codin	0	0	0	0
ENSG000001	PAIP1	protein_codin	2.78868534	2.81847198	0	0
ENSG000001	B4GALT6	protein_codin	7.53961877	8.78352467	69.2584264	14.794823
ENSG000001	FAM83B	protein_codin	0.28448714	1.58829749	10.0583202	1.35886246

ENSG000001	C5orf34	protein_codin	6.06610709	4.59810769	20.4846528	2.8101727
ENSG000001	DCXR	protein_codin	0	0	0	1.69339385
ENSG000002	COG8	protein_codin	15.178059	19.0716016	31.1383925	6.79322507
ENSG000001	CDY1B	protein_codin	0	0	0	0
ENSG000000	LNX1	protein_codin	0	5.7029962	14.2820311	1.48965849
ENSG000001	RAC3	protein_codin	2.4298299	0	19.0045362	0
ENSG000001	CHRNA7	protein_codin	3.2631062	0	3.01750644	0
ENSG000001	RBMXL2	protein_codin	0	2.24877456	1.0525037	0.49412522
ENSG000001	ZNF57	protein_codin	0	0	16.0825164	2.64377012
ENSG000001	ZNF584	protein_codin	0	0	0	0
ENSG000001	FEM1B	protein_codin	48.2138037	85.4786751	82.4995521	58.5165879
ENSG000001	NTSR2	protein_codin	0	0	0	1.41813565
ENSG000001	FIP1L1	protein_codin	98.5389817	94.2959942	276.062478	45.9176194
ENSG000001	ZNF131	protein_codin	0	0	0	0
ENSG000001	GON7	protein_codin	5.15342259	10.8340611	25.2232014	1.80354559
ENSG000001	CHRM1	protein_codin	0	0	0	0
ENSG000001	MPLKIP	protein_codin	15.7441946	50.6078966	47.5845437	10.436282
ENSG000001	ANKMY2	protein_codin	15.1670148	61.3785171	46.8625834	9.09365167
ENSG000001	PAH	protein_codin	0	0	0	0
ENSG000001	OR10G6	protein_codin	0	0	0	0
ENSG000001	C7orf33	protein_codin	0	0	0	0.4303475
ENSG000001	USP38	protein_codin	0	0	0	20.4366453
ENSG000001	OR4D5	protein_codin	0	0	0	2.11067749
ENSG000000	IGF1	protein_codin	16.5383853	55.6266799	4.88534828	5.13248312
ENSG000001	OTUD7A	protein_codin	0.76435834	9.752212	2.09244385	6.79838079
ENSG000001	PWWP2A	protein_codin	6.11069128	60.977911	29.7756521	9.28448688
ENSG000001	SOSTDC1	protein_codin	1.36298788	2.04789186	33.9020935	0
ENSG000001	NT5DC2	protein_codin	0.60918845	0	0	0
ENSG000001	AXIN2	protein_codin	5.53207898	19.048683	98.9463112	0
ENSG000001	NSUN4	protein_codin	3.13130634	23.4723217	0	0
ENSG000001	SPRR1B	protein_codin	0	0	0	0
ENSG000001	MAP2K1	protein_codin	14.0087923	0	0	0
ENSG000001	UQCC5	protein_codin	0.87766042	0	0.6279936	0.49025767
ENSG000001	GTPBP2	protein_codin	0	0	0	0
ENSG000001	P4HA1	protein_codin	0	0	0	0
ENSG000001	GTPBP2	protein_codin	10.2655621	58.8676786	48.6860533	11.6534759
ENSG000001	MYOZ2	protein_codin	4.30780667	5.6876079	0	0.47653944
ENSG000001	RALGAPA1	protein_codin	0	20.8722845	8.69243349	0
ENSG000000	CACNG5	protein_codin	0	0	0	0
ENSG000001	SYNPO2	protein_codin	0	0	0	44.8532244
ENSG000001	LDHAL6B	protein_codin	0	0	0	0.98890381
ENSG000001	KLF13	protein_codin	64.4709166	147.620595	101.043116	52.9144109
ENSG000001	COG7	protein_codin	7.42585118	34.2698161	20.3628098	4.9327595
ENSG000001	MTNR1A	protein_codin	0	0	0	0

ENSG000001(	INSM2	protein_codin	0.22267305	3.16709027	0	2.45325198
ENSG000001:	USP8	protein_codin	12.6746797	0	27.4362936	19.5579263
ENSG000001	TSNARE1	protein_codin	0	0	0	0
ENSG000002	GIMAP1	protein_codin	3.58872424	37.8597911	0	9.81546394
ENSG000002	LRTOMT	protein_codin	0	1.23997621	0	0
ENSG000001(	ABHD15	protein_codin	7.62403295	22.4270271	23.2698849	6.19204162
ENSG000001(	TMEM87A	protein_codin	3.67087325	11.0237741	1.22280653	1.59499525
ENSG000001	DNAJB7	protein_codin	0	0	0.60853269	1.15814199
ENSG000001	SUCLG2	protein_codin	28.5754994	59.3832956	35.7962327	20.1405562
ENSG000001(	E2F6	protein_codin	0	0	0	0
ENSG000001(	JHY	protein_codin	0.46618096	0	4.01734943	2.01203238
ENSG000000(	PLPP1	protein_codin	17.9147223	30.2269499	17.5629879	27.4430754
ENSG000001	COPS9	protein_codin	0.39520325	0	0	0.91019791
ENSG000001	GIMAP8	protein_codin	31.8289343	128.315005	11.0299544	15.6344525
ENSG000001(	HEXIM2	protein_codin	0.41974344	0	0	0
ENSG000001(	SYVN1	protein_codin	0	0	0	5.37678644
ENSG000001	PDCD6IP	protein_codin	47.7325473	0	77.5759092	48.0872505
ENSG000001:	PATJ	protein_codin	0	0	0	0
ENSG000001:	NDUFA10	protein_codin	0.44010728	3.84967459	0.73410065	1.38266299
ENSG000001	MRPL39	protein_codin	0	20.0724244	1.37876195	2.1283567
ENSG000000(	PHTF2	protein_codin	10.1337882	33.9164637	60.3740267	17.0252458
ENSG000001(	SCNN1B	protein_codin	0	0	0	0
ENSG000001(	ANTXR2	protein_codin	0	14.1361328	0	0
ENSG000000	USH2A	protein_codin	0	1.4437121	0	9.87686431
ENSG000001!	ZNF138	protein_codin	0	0	14.7357018	4.34628391
ENSG000001	HTRA3	protein_codin	38.4812593	6.43233674	17.8399491	13.2174754
ENSG000002	PCDHA10	protein_codin	0	0	0	0
ENSG000001	GLB1	protein_codin	13.7588264	40.1263346	9.08111233	10.6716558
ENSG000002	SLC26A6	protein_codin	0	0	0	0
ENSG000001(	DDIT4	protein_codin	15.9154089	0	158.818238	124.748364
ENSG000001(	TMEM223	protein_codin	3.24990657	5.25601832	7.65198167	2.65382106
ENSG000001	CLCN5	protein_codin	0	0	1.28929548	0
ENSG000001	GLB1	protein_codin	0.74859984	0	0	0
ENSG000000	SLCO1A2	protein_codin	0	0	18.7623932	0
ENSG000001	OR52A5	protein_codin	0.31200387	0	1.23604943	0.60166278
ENSG000001	HSFY1	protein_codin	0	0	0	0
ENSG000001(	GP9	protein_codin	0.3095582	0	0	0
ENSG000001	RBM33	protein_codin	11.9980753	40.2679759	85.6740538	13.9658095
ENSG000001(	CXCL8	protein_codin	0	0	0	28.0831637
ENSG000001(	MRPL4	protein_codin	0	0	0	0
ENSG000001(	PARM1	protein_codin	16.1077481	94.7744201	16.2244288	39.0417013
ENSG000001	CNTNAP4	protein_codin	0	0	0	0
ENSG000001	FAM90A1	protein_codin	0	0	0.4034618	1.70563538
ENSG000001(	RASSF6	protein_codin	0	0	0	0.5805491

ENSG000001	ZNF33A	protein_codin	0	0	5.37246366	0
ENSG000001	MORN4	protein_codin	0	7.00184866	0	0
ENSG000001	CDKL2	protein_codin	0	0	0	0
ENSG000001	ZIK1	protein_codin	0	0	4.78366619	0
ENSG000001	CCDC126	protein_codin	8.2229681	0	11.8029125	1.4970454
ENSG000001	MAB21L4	protein_codin	0	0	0	0
ENSG000001	ACSM1	protein_codin	0	0	0	0
ENSG000001	AGXT	protein_codin	0	0	0	0
ENSG000001	FAM161A	protein_codin	0	0	0	0
ENSG000001	ANKRD2	protein_codin	0	0	0	0
ENSG000001	CMTM8	protein_codin	1.17563217	0	0	0
ENSG000001	ZBBX	protein_codin	0	0	0	0
ENSG000001	MRFAP1L2	protein_codin	0.42656273	0	1.02545043	1.96763945
ENSG000001	PANK1	protein_codin	6.97166007	8.77250929	42.8863247	1.71236244
ENSG000001	KIAA1217	protein_codin	13.06611	13.3109324	0	25.6400412
ENSG000000	MAGI3	protein_codin	5.1419077	0	31.8273957	5.78519911
ENSG000001	OR10V1	protein_codin	0	0	0	0
ENSG000001	AKNA	protein_codin	0	0	4.60914336	0
ENSG000001	CAVIN4	protein_codin	11.0768098	5.08051324	4.77666113	4.75366892
ENSG000001	CCDC110	protein_codin	5.54177289	0	0	0
ENSG000001	GOT1L1	protein_codin	0	0	0	0
ENSG000001	HOOK3	protein_codin	95.9298185	159.95286	493.243565	63.7757866
ENSG000001	ACOT12	protein_codin	0	0	0	0.92483892
ENSG000001	YWHAG	protein_codin	174.052167	527.547304	460.742023	112.367422
ENSG000001	HARS1	protein_codin	0	0	3.35993009	0
ENSG000001	ZNF556	protein_codin	0.63533721	0	7.01968034	0.44266582
ENSG000001	C3AR1	protein_codin	5.89820882	27.4901703	3.4898817	5.00959418
ENSG000001	NKIRAS2	protein_codin	0	0	0	1.21040998
ENSG000001	MCRIP2	protein_codin	0	3.25049923	0	0
ENSG000001	ZNF17	protein_codin	0	0	15.5233762	1.96389582
ENSG000001	KIAA0232	protein_codin	0	0	17.2367978	30.8923281
ENSG000001	SYNPO	protein_codin	36.4174717	99.143012	12.9426929	52.4392366
ENSG000001	ATP9B	protein_codin	0	32.6902379	0	0
ENSG000001	BCL2L1	protein_codin	2.82889889	0	2.01779456	4.57273693
ENSG000001	SEC23A	protein_codin	66.456411	120.971204	108.560494	50.7186969
ENSG000001	KHDRBS1	protein_codin	0	7.56458691	5.71196651	2.7235221
ENSG000001	NAT1	protein_codin	0	0	3.04533918	0
ENSG000001	PPID	protein_codin	29.4735732	80.0756711	63.8334022	22.7662242
ENSG000000	MLLT10	protein_codin	14.5722802	0	17.1025177	0
ENSG000001	CDC40	protein_codin	33.1172294	61.6429836	65.2008729	20.0058442
ENSG000001	ETFDH	protein_codin	0	10.4862672	12.2768207	6.55445421
ENSG000001	THOP1	protein_codin	2.22535541	8.41228661	3.21276093	6.35771653
ENSG000001	CYTL1	protein_codin	2.70270638	4.81636531	7.47224107	0.90562301
ENSG000001	ZBTB5	protein_codin	10.0385524	24.7036475	37.3125362	10.9053511

ENSG000001	LGALS4	protein_codin	0	0	0	0
ENSG000001	RXFP1	protein_codin	0	0	0	0
ENSG000001	TVP23B	protein_codin	16.8324389	0	0	0
ENSG000001	ZRSR2	protein_codin	35.6580114	126.839125	49.8540399	26.5475421
ENSG000001	CALML6	protein_codin	0.22551139	0	0	0.36474168
ENSG000001	ASCC2	protein_codin	4.65167856	23.9018914	25.2889443	10.2650867
ENSG000001	AFF1	protein_codin	98.8561753	226.70613	165.605709	0
ENSG000001	RBM23	protein_codin	3.10457373	0	0	2.39372331
ENSG000001	CARNS1	protein_codin	0	0	2.0865716	0
ENSG000001	MGAT1	protein_codin	2.14091165	74.6848649	4.79749166	12.3376901
ENSG000001	OR2Y1	protein_codin	0	0	0	0.49752696
ENSG000001	NABP1	protein_codin	0	0	0	0
ENSG000001	RPL15	protein_codin	94.8171486	84.8476717	38.0494805	113.158569
ENSG000001	STARD6	protein_codin	0	0	0	1.32220338
ENSG000001	HPCAL1	protein_codin	6.68819265	23.790928	32.5301031	18.5591714
ENSG000001	NABP1	protein_codin	11.1306565	0	57.7684533	5.69502126
ENSG000001	HAVCR2	protein_codin	0	0	0	0
ENSG000002	TRIM40	protein_codin	0.75182326	0	0	0
ENSG000000	AIFM2	protein_codin	7.25898838	11.0299346	0	0
ENSG000001	SRP68	protein_codin	28.2392755	0	36.780702	19.9517849
ENSG000001	ADCY6	protein_codin	0	0	40.5787228	3.44642126
ENSG000001	CTSW	protein_codin	0	0	0	0
ENSG000001	CYB5B	protein_codin	0	0	0	9.98279628
ENSG000000	THAP3	protein_codin	2.05688022	0	0	0
ENSG000001	ZWILCH	protein_codin	15.5309772	11.8839083	23.6812238	5.08195842
ENSG000000	ADAT1	protein_codin	4.14213389	0	29.0250399	0
ENSG000001	MCL1	protein_codin	39.2175568	0	19.7784977	107.349432
ENSG000002	CRYGS	protein_codin	0	1.723996	3.18544062	0
ENSG000002	FNIP1	protein_codin	0	0	0	0
ENSG000001	ARR3	protein_codin	0	0	0	0
ENSG000001	RPL4	protein_codin	42.0352053	46.8609074	81.8737195	48.3763907
ENSG000002	FNIP1	protein_codin	34.8210057	107.556104	50.5606723	31.4718641
ENSG000001	WFDC5	protein_codin	0	0	0	0
ENSG000001	FOXP4	protein_codin	0	31.6741911	0	0
ENSG000001	SNAPC5	protein_codin	0.54129172	9.49834508	3.98751626	0
ENSG000001	RAD9A	protein_codin	0	5.49050179	0	0
ENSG000001	EFEMP2	protein_codin	4.74340027	30.2616954	0	8.61395565
ENSG000001	RAPGEF6	protein_codin	11.2716163	59.9809734	56.8858515	16.6554013
ENSG000001	MRPS12	protein_codin	0	0	0	0
ENSG000001	PTDSS2	protein_codin	2.48679254	22.7684564	4.13240572	2.14536252
ENSG000001	TMEM134	protein_codin	0	0	0	1.43655151
ENSG000001	DDX23	protein_codin	51.4285743	79.1604015	146.264058	43.1852242
ENSG000001	SLC16A13	protein_codin	0	1.03574218	0.5415125	1.24862301
ENSG000001	ZNF471	protein_codin	18.7087547	35.484426	1.67845009	6.16784244

ENSG000000	DLEC1	protein_codin	3.76012449	5.31589437	0	4.33755247
ENSG000001	FRG2C	protein_codin	0.32015084	0	0	2.67818567
ENSG000001	CHST1	protein_codin	0	11.141805	0.52220604	0.89297713
ENSG000001	SLC37A2	protein_codin	0	0	0	0
ENSG000000	PTCD2	protein_codin	0	0	18.0929484	0
ENSG000001	EMILIN2	protein_codin	0.32104956	2.7338748	8.75029842	0
ENSG000001	THAP2	protein_codin	4.60406854	3.06143478	5.15240426	3.59647539
ENSG000001	RAPH1	protein_codin	0	13.2675016	0	2.22478633
ENSG000001	ZNF80	protein_codin	0.23391696	1.40338358	0	0
ENSG000001	METTL5	protein_codin	3.57978826	8.69820047	17.3981653	0
ENSG000001	SPMIP3	protein_codin	0	0	0	0
ENSG000001	CCNE2	protein_codin	0	2.34895095	38.9814079	0
ENSG000001	MUS81	protein_codin	7.36690725	36.3105457	11.5848974	0
ENSG000001	SSH3	protein_codin	2.47918323	0	0	3.53322858
ENSG000001	DSG4	protein_codin	0	0	0	1.07304798
ENSG000001	FAM53A	protein_codin	0	0	0	0.95888466
ENSG000001	H2AZ2	protein_codin	33.797044	20.4473237	45.2106265	16.0146646
ENSG000001	PHYKPL	protein_codin	0	0	1.13617646	0
ENSG000001	NUP93	protein_codin	0	1.94929244	0	0
ENSG000001	HMGCLL1	protein_codin	0	0	4.68398963	0
ENSG000001	CFL1	protein_codin	30.0885968	12.0907826	55.1875236	27.960483
ENSG000001	ELF4	protein_codin	0	38.6586763	1.77845988	4.74112753
ENSG000002	PCDHGC3	protein_codin	39.2978415	0	145.818079	67.1170266
ENSG000001	CRYBG2	protein_codin	0.27251749	0	0	0
ENSG000001	PDE7B	protein_codin	31.3860635	4.51900904	63.613863	21.9245762
ENSG000001	PLG	protein_codin	0	0.75063006	0	0
ENSG000001	COQ10A	protein_codin	0	0	0	0
ENSG000001	CCDC63	protein_codin	0	0	0	0
ENSG000001	TP53I11	protein_codin	0	0	0	0
ENSG000001	IQCK	protein_codin	0	7.52646834	0	0
ENSG000001	BRSK2	protein_codin	0	0	0	0
ENSG000001	RNASE8	protein_codin	0	0	0	0
ENSG000001	RGMB	protein_codin	0	14.8452412	0	0
ENSG000001	CRB3	protein_codin	0	0	0	0
ENSG000001	PPM1E	protein_codin	11.3528662	1.04306737	43.9425793	4.560308
ENSG000001	KCNG4	protein_codin	0.56652543	3.54171463	0	2.07450119
ENSG000001	MSRB3	protein_codin	3.61575663	124.595362	34.3578797	0
ENSG000001	PSMA8	protein_codin	0	0	0	0
ENSG000000	USP48	protein_codin	6.77272939	35.2658746	0	12.0894727
ENSG000001	FBXO22	protein_codin	43.1538747	47.1472567	68.0558325	11.6262684
ENSG000001	TLCD3A	protein_codin	4.63873636	5.18913889	6.43653862	0
ENSG000001	SEC16B	protein_codin	0	6.84200415	0	0
ENSG000001	MS4A10	protein_codin	0	0	0	1.54215463
ENSG000001	TENT5D	protein_codin	0	0	0	0.5305518

ENSG000001	SSH3	protein_codin	0	0	0	0
ENSG000001	GOLT1A	protein_codin	0	0	0	0.26471298
ENSG000001	PROP1	protein_codin	0	0	0	1.32490697
ENSG000000	GEMIN2	protein_codin	0	0	3.3664914	0
ENSG000001	LEMD3	protein_codin	27.4307872	45.4915867	73.0886936	26.4943201
ENSG000001	SNX32	protein_codin	0	0	3.15044245	0
ENSG000001	CLDN15	protein_codin	0	0	0	0
ENSG000001	PDLIM2	protein_codin	0	0	0	0
ENSG000000	PIGS	protein_codin	0	0	3.00732622	0
ENSG000001	DHX36	protein_codin	9.63341019	0	52.0321407	0
ENSG000001	PLA2G1B	protein_codin	0.44468893	0	0	0
ENSG000000	LTBP4	protein_codin	0	0	0	0
ENSG000001	GMPPB	protein_codin	0	6.02000178	0	0
ENSG000001	SLFN11	protein_codin	0	0	0	0
ENSG000001	WDR49	protein_codin	0	0	0	0
ENSG000001	DUSP6	protein_codin	0	0	0	0
ENSG000001	GMPPB	protein_codin	0	0	0	0
ENSG000001	CASP3	protein_codin	0	25.5735005	1.69089091	1.78280808
ENSG000001	MAPK7	protein_codin	0.42573175	4.65706113	1.8416692	0.5900001
ENSG000001	RNASEH2C	protein_codin	0	0	8.85622725	0
ENSG000001	AOC3	protein_codin	15.9334815	25.9528836	25.6754655	30.6080583
ENSG000001	TUT1	protein_codin	2.2224213	12.1533132	8.47571718	2.13229014
ENSG000001	ANKRD13D	protein_codin	0	0	0	0
ENSG000001	ATAD1	protein_codin	0.99614344	12.9894353	2.68541374	0.69815639
ENSG000001	SLC17A3	protein_codin	0	0	0	0
ENSG000001	MUC4	protein_codin	0	0	0	0
ENSG000002	DUSP13A	protein_codin	0	0	0	0
ENSG000001	LRRN3	protein_codin	13.8134931	9.796656	0	7.20137247
ENSG000001	LRRFIP1	protein_codin	17.4999113	21.3996915	14.4767788	16.9951504
ENSG000000	RSF1	protein_codin	44.506885	45.5883399	104.968606	19.1239892
ENSG000001	STOX2	protein_codin	41.994648	12.3217912	10.5158261	11.812902
ENSG000001	CSPG4	protein_codin	123.659794	117.962305	102.674258	50.5602353
ENSG000001	CCAR2	protein_codin	14.2685309	9.73941986	22.4147465	0
ENSG000001	APOBEC3F	protein_codin	3.39770961	36.3369638	11.1882202	4.26210706
ENSG000001	SNX33	protein_codin	24.0609552	129.873126	21.7083292	21.0272032
ENSG000001	LYG1	protein_codin	1.54603139	4.42085256	0	0.43870549
ENSG000001	PDZRN3	protein_codin	0.46125742	0	0	0
ENSG000001	LGR6	protein_codin	0	0	0.60575723	0
ENSG000001	TMOD3	protein_codin	78.5779635	184.4916	102.900116	56.6313194
ENSG000001	SNUPN	protein_codin	16.5096334	31.9958668	22.6253125	6.58108963
ENSG000001	GRK2	protein_codin	18.6737125	206.973271	58.4111856	36.2418411
ENSG000001	CD200R1	protein_codin	5.19976302	0	0	7.66434054
ENSG000001	EVX2	protein_codin	0	0	0	0.46022585
ENSG000001	SNTG2	protein_codin	0.7094491	0	0	0

ENSG000001	RELA	protein_codin	0	0	3.4374272	10.9860058
ENSG000001	APOBEC4	protein_codin	0	0	0	2.89574834
ENSG000001	ATAD3B	protein_codin	1.99131073	3.50227833	15.7693983	1.17126429
ENSG000001	ZNF699	protein_codin	1.69336949	9.38142569	9.92556943	1.8855366
ENSG000001	BCL2L11	protein_codin	0	0	5.1702391	0
ENSG000001	MARCHF3	protein_codin	0.46480961	8.81099473	1.95857573	2.65196022
ENSG000001	ATP2A2	protein_codin	34.4968811	214.888697	41.9996675	29.8883539
ENSG000001	ABCD2	protein_codin	24.4169619	24.2993306	2.70077866	4.74841402
ENSG000000	PRKACA	protein_codin	41.4940898	108.150942	62.6814888	45.8598169
ENSG000001	OR7D4	protein_codin	0	0	0	0
ENSG000001	ZNF622	protein_codin	31.2582251	93.6129959	230.125916	33.2465547
ENSG000001	PSMD1	protein_codin	41.0627008	0	0	0
ENSG000001	ISX	protein_codin	0	0	0	1.61846579
ENSG000001	NGLY1	protein_codin	0	0	12.1611522	0
ENSG000001	SEZ6L2	protein_codin	0	0	34.481196	2.50795337
ENSG000001	BCL2L14	protein_codin	0	0	0	0
ENSG000002	AP1G2	protein_codin	0	2.3030415	6.37231406	0
ENSG000000	BTK	protein_codin	0	0	0	0
ENSG000000	NFYC	protein_codin	0	0	0	5.6176441
ENSG000001	DHX37	protein_codin	5.09390997	19.9370085	20.0825126	10.1439593
ENSG000001	ALG10B	protein_codin	36.1662494	25.1985904	45.2536611	11.623931
ENSG000000	NPFFR2	protein_codin	0	0	0	0
ENSG000001	ASPHD1	protein_codin	0	0	2.16566496	1.20699005
ENSG000001	KCTD13	protein_codin	0	0	0	2.66048551
ENSG000001	COP1	protein_codin	0	0	0	0
ENSG000001	TRMT112	protein_codin	0	0	0	0
ENSG000001	DAG1	protein_codin	12.4341003	17.6291132	23.2320391	11.8937381
ENSG000001	KDM2A	protein_codin	7.40760203	105.201121	9.54277709	12.3827474
ENSG000001	MUCL1	protein_codin	0	0	0	0
ENSG000001	FBXW10	protein_codin	0	0	0	0
ENSG000001	MLKL	protein_codin	7.98789189	53.0378709	0	6.72169874
ENSG000001	ZNF596	protein_codin	1.78294072	0	0	0
ENSG000002	PCMTD2	protein_codin	31.443167	77.4003055	41.6425935	13.7957646
ENSG000001	RHOD	protein_codin	0.50584836	0	0.99660782	1.3206423
ENSG000001	ADCK5	protein_codin	0.38930442	0	0	0
ENSG000001	PIKFYVE	protein_codin	4.66220634	0	7.54888284	0
ENSG000001	LEP	protein_codin	5.85565685	9.90361187	0.4813045	4.20760206
ENSG000000	RUNX3	protein_codin	7.81269281	48.558269	0	18.1432029
ENSG000001	EGFL7	protein_codin	0	9.75044093	0	0
ENSG000001	TAOK2	protein_codin	15.5476944	196.567171	34.4254985	15.9205107
ENSG000001	LKAAEAR1	protein_codin	0	0	0	0
ENSG000001	GCSAM	protein_codin	0	2.77268346	0	2.42289308
ENSG000001	TUBB8B	protein_codin	0.29594776	0	0	0
ENSG000000	CYP2W1	protein_codin	0	0.81706347	2.50246155	0.82863729

ENSG000000	U2AF2	protein_codin	29.210411	57.9952607	130.247813	23.992149
ENSG000001	OR10H5	protein_codin	0	0	0	0
ENSG000001	PHYHD1	protein_codin	0	0	0	1.55823911
ENSG000001	SIPA1L2	protein_codin	0	0	0.85559192	1.71826456
ENSG000001	MYEOV	protein_codin	0	1.31009509	0	0
ENSG000001	NDUFA11	protein_codin	3.33688057	24.0981373	7.15328509	8.31547553
ENSG000001	C11orf86	protein_codin	0	0	0	0
ENSG000001	CCDC106	protein_codin	0	0	0.81005538	1.6705395
ENSG000001	TLR10	protein_codin	0	0	0	0
ENSG000001	TLR1	protein_codin	3.33526067	10.4091376	0	0
ENSG000001	ACAD9	protein_codin	0	0	0	0
ENSG000001	CKS1B	protein_codin	3.51226301	3.44253311	16.2585143	0
ENSG000001	DBN1	protein_codin	4.20074385	0	21.6363575	3.37619199
ENSG000001	PTPN7	protein_codin	0	0	0	0
ENSG000001	GOLIM4	protein_codin	561.465621	517.195983	1770.97332	204.195162
ENSG000000	BAD	protein_codin	2.90137964	13.1013523	7.97513162	3.39797148
ENSG000001	UBXN2A	protein_codin	9.02762361	42.8893251	19.650506	10.7931485
ENSG000001	CTBP2	protein_codin	0.6066701	17.1629136	8.79517006	3.23828308
ENSG000001	CEP290	protein_codin	0	0	0	0
ENSG000000	REST	protein_codin	73.0366631	26.8320306	75.8405788	7.44599967
ENSG000001	CENPS	protein_codin	0	0	16.9870569	0
ENSG000001	PBLD	protein_codin	4.59170354	5.50644842	0	0
ENSG000001	C12orf76	protein_codin	1.78132843	0	0	1.39669749
ENSG000001	LCLAT1	protein_codin	0	10.094542	0	0
ENSG000001	ZHX3	protein_codin	0	0	9.58372165	0
ENSG000001	DUS3L	protein_codin	3.00407269	23.0739801	16.0362613	0
ENSG000001	SLC2A9	protein_codin	0	0	0	0
ENSG000001	ZGRF1	protein_codin	0	0	4.82974803	2.88501768
ENSG000001	PTMS	protein_codin	109.353662	2001.47411	1237.68747	40.9472062
ENSG000000	GPBP1	protein_codin	2.93488034	21.4606947	12.0406394	3.7433009
ENSG000001	MRGPRF	protein_codin	1.49158518	0	0	0.79901976
ENSG000001	MAP3K11	protein_codin	12.3885857	20.5085752	17.976694	11.4064749
ENSG000001	MRGPRD	protein_codin	0	2.18360158	0	0
ENSG000001	VPS26C	protein_codin	3.8170719	0	14.1213501	0
ENSG000001	TMEM9B	protein_codin	0	0	0	0
ENSG000001	PSME3IP1	protein_codin	12.5216242	57.6600946	55.9261221	16.7388855
ENSG000001	OR6M1	protein_codin	0	0	0	0.48316461
ENSG000000	CRTAC1	protein_codin	0	0	0	0
ENSG000001	GRB7	protein_codin	0	0	0	0
ENSG000000	RORA	protein_codin	2.85726294	0	0	0
ENSG000001	CATSPERD	protein_codin	0.25152365	0	0.43357592	1.21498348
ENSG000001	NRIP3	protein_codin	4.58354885	5.17487007	6.89008428	12.8375837
ENSG000001	P2RY14	protein_codin	9.78002391	0	0	6.96503915
ENSG000001	GPR171	protein_codin	0	0	0	0

ENSG0000010	WDR81	protein_codin	16.8811422	0	20.5659151	0
ENSG000003	CAST	protein_codin	17.5585984	0	0	0
ENSG0000010	PTGR1	protein_codin	15.766986	25.8662985	29.7035794	13.2969868
ENSG0000010	MXRA8	protein_codin	41.2294979	117.687905	199.177533	49.3792998
ENSG000000	HFE	protein_codin	0	19.7591995	0	0
ENSG0000017	ZNF483	protein_codin	3.78321352	15.5076228	22.6968483	15.7720911
ENSG0000014	MED12L	protein_codin	0	0	0	0
ENSG0000015	PPARGC1B	protein_codin	6.35576679	41.0380737	13.5566941	13.5937056
ENSG0000010	ETFB	protein_codin	14.8904416	43.0970714	59.2725448	12.9469624
ENSG0000013	TPT1	protein_codin	1055.11302	587.119892	1243.45508	497.273778
ENSG0000017	SCUBE2	protein_codin	4.68952957	0	0	0
ENSG0000015	EEF1A1	protein_codin	0	0	0	0
ENSG0000015	PPM1J	protein_codin	0	0	0	0
ENSG0000007	SLC6A15	protein_codin	0	0	0	0
ENSG0000017	XCR1	protein_codin	0.92071593	0	0	1.25155788
ENSG0000017	EHBP1L1	protein_codin	43.9487894	258.794632	85.3968154	56.3170235
ENSG0000015	ATP6V1G3	protein_codin	0	0	0	0
ENSG0000010	EEF2	protein_codin	1528.27001	1416.81336	7213.12195	1285.17689
ENSG0000012	ZMIZ2	protein_codin	0	51.1326302	25.2664649	6.15067693
ENSG0000017	PPP1R14B	protein_codin	22.9692788	31.6783219	105.497649	18.0981004
ENSG0000017	MICOS13	protein_codin	1.97674614	4.62125145	13.9136572	3.47558032
ENSG0000017	ZNRD2	protein_codin	3.75029213	6.82997656	11.477992	2.4175681
ENSG0000015	ARID5B	protein_codin	31.736496	93.0793084	48.5832476	70.0877088
ENSG0000009	MKNK2	protein_codin	10.7994951	46.7914306	18.2004891	4.90834163
ENSG0000020	MRPL38	protein_codin	5.99607415	4.38630227	32.8187012	4.14694674
ENSG0000010	AKIP1	protein_codin	0	0	0	0
ENSG0000017	FKBP2	protein_codin	4.59926183	32.1016842	16.5758032	6.6883051
ENSG0000010	AKIP1	protein_codin	0	0	0	2.84402962
ENSG0000010	BRSK1	protein_codin	0	0	0	3.22705871
ENSG0000015	KLRC4	protein_codin	0	0	0	0
ENSG0000017	MOB1B	protein_codin	34.5773627	75.2702186	67.1862744	27.2513554
ENSG0000017	SH3RF3	protein_codin	27.4717938	74.0127936	44.0241366	26.2916976
ENSG0000017	VEGFB	protein_codin	2.99726646	41.7603978	2.54523385	7.38162125
ENSG000001	POLR1G	protein_codin	2.46044927	0	1.79762914	1.16073084
ENSG0000014	RETREG3	protein_codin	25.5963696	103.278	23.7813112	17.1410217
ENSG0000010	ANAPC10	protein_codin	10.5175922	15.8140961	23.518575	6.15144549
ENSG000001	KLF7	protein_codin	29.9472717	61.8144588	11.9604633	26.8276639
ENSG0000017	NWD2	protein_codin	0	0	0	4.79467908
ENSG0000017	MZT2A	protein_codin	1.42294294	3.16202279	8.52647319	3.0056938
ENSG0000013	ATG4D	protein_codin	2.16746469	0	0	0
ENSG0000007	GSDMB	protein_codin	2.01836141	0	0	0
ENSG0000010	ADORA1	protein_codin	0	0	0.51663801	0.40942189
ENSG0000013	IQCA1	protein_codin	0	0	1.60907224	6.70335266
ENSG000001	B4GALT2	protein_codin	0	0	11.8699563	5.16454173

ENSG000001	HADH	protein_codin	9.01269411	0	0	7.77208245
ENSG000001	CGGBP1	protein_codin	0	0	0	11.4890822
ENSG000001	SLC26A7	protein_codin	0	0	6.80367532	0
ENSG000001	OLR1	protein_codin	0.26234864	5.78996741	0	0
ENSG000002	LIME1	protein_codin	0	0	0	0
ENSG000001	SUSD5	protein_codin	11.3813502	42.7009135	56.1977978	11.7404734
ENSG000001	OR4B1	protein_codin	0	0	0	0
ENSG000001	CHST2	protein_codin	1.44534348	19.710872	17.8637336	10.7548185
ENSG000001	PPARG	protein_codin	0	0	0	7.24813625
ENSG000001	ATP5MK	protein_codin	20.7970649	20.7746486	31.1426184	11.2023826
ENSG000000	DNASE1L1	protein_codin	1.50514492	0	0	0
ENSG000001	MRPL57	protein_codin	8.75184198	15.5636204	19.2491701	5.94759378
ENSG000001	LRFN4	protein_codin	2.53036792	4.49236755	6.37968183	0.39814643
ENSG000001	FAM86B2	protein_codin	0.55294445	0	0	0
ENSG000001	APTX	protein_codin	0	0	0	0
ENSG000001	OR1L1	protein_codin	0	0	0	0
ENSG000001	PDE4D	protein_codin	0	0	0	0
ENSG000001	RCE1	protein_codin	0	0	0	0
ENSG000001	PTPN2	protein_codin	0	2.88229081	0	1.84868639
ENSG000001	LMAN1L	protein_codin	0	0	0	0
ENSG000001	INHBC	protein_codin	0.77280741	0	0	3.21610092
ENSG000001	OR10P1	protein_codin	0	0	0	0.47610879
ENSG000001	KRT2	protein_codin	0	1.84031572	0	1.45452235
ENSG000001	ZNF680	protein_codin	15.7247753	40.7532959	30.46023	9.31184797
ENSG000001	FCRLA	protein_codin	0	0	0	0
ENSG000001	AP1AR	protein_codin	9.47664276	20.8225625	31.6606757	12.1025851
ENSG000001	TMEM255A	protein_codin	0	0	0	0
ENSG000001	GRAMD2A	protein_codin	0	0	1.58522673	0
ENSG000001	FAM241A	protein_codin	5.84846015	165.10103	10.8657702	23.9370572
ENSG000001	RIC3	protein_codin	0	0	0	0
ENSG000001	RND1	protein_codin	0	0	0.44144925	5.07020422
ENSG000001	KLHL3	protein_codin	5.25389283	21.2475753	12.4570475	9.97274886
ENSG000001	HSPA6	protein_codin	5.10800824	12.6980078	8.22967255	133.940027
ENSG000001	TOPAZ1	protein_codin	0	0	0	2.43714642
ENSG000001	TMEM234	protein_codin	3.02780336	0	4.11413955	0
ENSG000002	TRIM59	protein_codin	9.9605119	12.9864149	7.66910401	1.05679477
ENSG000001	COL14A1	protein_codin	39.7128701	344.50303	8.4954401	17.2664711
ENSG000001	NARF	protein_codin	0	4.58827866	4.33680054	3.01117158
ENSG000001	UTP23	protein_codin	15.1663585	46.7021245	79.1876927	12.6203568
ENSG000001	HSFY1	protein_codin	0.21182848	0	0	0
ENSG000001	SPIRE1	protein_codin	0	0	0	0
ENSG000001	GSTM1	protein_codin	0	0	0	0
ENSG000001	GGA2	protein_codin	22.9507094	123.578191	67.6745421	33.7772652
ENSG000001	PGAP3	protein_codin	0	0	2.63775563	0

ENSG000001	GCC2	protein_codin	60.2205921	0	99.4970926	48.9294619
ENSG000001	ABLIM3	protein_codin	0	0	0	0
ENSG000001	IRF3	protein_codin	0	0	0	1.08711087
ENSG000001	ADCY5	protein_codin	0	4.60660618	3.91663737	0
ENSG000001	TIGD3	protein_codin	0	0	1.10696936	0.43158129
ENSG000001	CD36	protein_codin	0	0	0	0
ENSG000001	TCAP	protein_codin	52.7411306	0	1.49671379	1.40776451
ENSG000000	ATP2A3	protein_codin	0	0	0	0
ENSG000002	CSHL1	protein_codin	0	0	0	0
ENSG000001	HYDIN	protein_codin	4.82210408	13.2841322	4.56609631	7.31746227
ENSG000002	ZNF407	protein_codin	0	81.8669745	0	0
ENSG000001	FBXW8	protein_codin	5.03773812	13.9580605	8.87357627	3.90109764
ENSG000001	TRMT10C	protein_codin	5.69191624	0	18.5314848	8.37579625
ENSG000001	GPR148	protein_codin	0	1.04395302	0	0
ENSG000001	DAW1	protein_codin	0	0	0	0
ENSG000001	SEC22A	protein_codin	0	0	2.33999576	2.22678351
ENSG000001	DAPL1	protein_codin	0	0	0	1.78494117
ENSG000001	ACO1	protein_codin	8.94728928	11.6579673	8.90535898	9.18534558
ENSG000000	CFLAR	protein_codin	94.9482934	287.35673	83.9664703	94.8195694
ENSG000002	NAXD	protein_codin	0	0	0	0
ENSG000001	KLK10	protein_codin	0	0	0	0
ENSG000001	CLOCK	protein_codin	50.1491955	81.4085707	246.017288	32.138244
ENSG000001	GLE1	protein_codin	0	0	18.7168236	0
ENSG000001	MPPE1	protein_codin	1.85182799	0	0	0
ENSG000001	NDST2	protein_codin	2.38944352	0	4.61832754	0
ENSG000001	BAALC	protein_codin	0	23.3993726	0	3.33966307
ENSG000001	TPMT	protein_codin	14.5226924	41.822134	59.9929062	12.8035307
ENSG000001	MAL	protein_codin	0	0	0	0
ENSG000001	DAB2IP	protein_codin	20.8471543	58.7783932	14.6448453	8.86002918
ENSG000001	MSL2	protein_codin	11.9528158	29.3087683	17.5441308	16.574989
ENSG000001	SPTBN2	protein_codin	0	0	0	0
ENSG000001	CLEC7A	protein_codin	0	0	0.90439825	0
ENSG000001	SP3	protein_codin	24.1890742	40.5889777	23.4022672	22.7075849
ENSG000001	ATP6V0A4	protein_codin	0	0	0	0
ENSG000000	TSPAN17	protein_codin	0	7.44213287	0	0
ENSG000001	DSEL	protein_codin	43.266071	92.6381893	70.1636637	25.8412357
ENSG000001	RBM4B	protein_codin	2.77381024	13.8110558	7.22982919	0
ENSG000000	HLTF	protein_codin	79.2518683	144.802035	412.050776	18.233565
ENSG000001	CYP2S1	protein_codin	0.52960268	10.6166652	0	2.65713248
ENSG000001	TADA2B	protein_codin	8.64970543	52.4211149	17.2715096	8.60946882
ENSG000001	AGFG1	protein_codin	31.9002313	52.5038346	33.4336104	34.694093
ENSG000001	CFAP184	protein_codin	1.46318428	5.12420282	3.65989499	5.69893665
ENSG000001	RBM4	protein_codin	0	0	12.6422493	0
ENSG000001	PRLR	protein_codin	0	0	0	0

ENSG000001	LIPT2	protein_codin	1.12489674	1.24351212	2.20765648	0.52818757
ENSG000000	SNCB	protein_codin	0	0	0	0
ENSG000001	PSMD2	protein_codin	41.6286702	52.3965374	145.490234	12.123649
ENSG000001	LGI4	protein_codin	0	3.62578433	0	0
ENSG000001	TFDP3	protein_codin	0	0	0	0.46788475
ENSG000001	ATP8B3	protein_codin	0	2.60690173	0	1.53686993
ENSG000001	KCNE3	protein_codin	7.07532193	22.9520712	6.39845777	3.29972886
ENSG000000	MATK	protein_codin	0	21.4359603	6.50359392	2.46467889
ENSG000002	RBM14	protein_codin	16.7373121	19.038971	36.3044576	29.2700406
ENSG000000	PSMC5	protein_codin	28.4111301	113.376362	129.305443	12.8577666
ENSG000001	KLK6	protein_codin	0	0	0	0
ENSG000001	ZCCHC12	protein_codin	0	0	0	0
ENSG000001	CHRNA9	protein_codin	0.23068847	1.55157649	0.43201034	0.43676211
ENSG000001	CTDSPL	protein_codin	1.08247057	0	1.22561534	0.87405087
ENSG000001	CCS	protein_codin	0	0	0	0
ENSG000001	CYP7B1	protein_codin	25.5722828	39.5719643	20.7852918	14.2806506
ENSG000001	INSM1	protein_codin	0.3230081	0	0	1.09076769
ENSG000002	CCDC13	protein_codin	0	1.15858572	0	0
ENSG000001	OR10AD1	protein_codin	0	1.33552551	0	1.80745361
ENSG000002	TTLL3	protein_codin	0	0	0	0
ENSG000001	ARV1	protein_codin	1.85390276	0	2.19704578	4.78630836
ENSG000001	VANGL1	protein_codin	3.01201387	0	9.89562815	0
ENSG000000	ALAS1	protein_codin	0	12.6558679	4.78347111	0
ENSG000000	PDE8A	protein_codin	18.4364314	0	36.3846379	0
ENSG000000	LRCH4	protein_codin	0	0	9.73460003	5.26740086
ENSG000000	VPS41	protein_codin	30.5388749	156.41158	91.7284585	23.0943395
ENSG000001	DPP10	protein_codin	0	0	0	0
ENSG000001	CTSF	protein_codin	0	57.1653588	0	13.8099444
ENSG000001	NSUN5	protein_codin	0	0	1.48432677	1.24465979
ENSG000002	EIF4EBP3	protein_codin	0.9144748	6.46890621	3.22501736	1.93209463
ENSG000001	PIGG	protein_codin	0	0	0	0
ENSG000001	ARHGAP32	protein_codin	0	0	0	0
ENSG000000	TBX5	protein_codin	0	0	0	0
ENSG000001	CCDC14	protein_codin	0	0	0	0
ENSG000001	GPRC6A	protein_codin	0	0	0	0
ENSG000001	PARP15	protein_codin	0	0	0	10.9914657
ENSG000000	GP6	protein_codin	0	0	0	0
ENSG000001	XXYLT1	protein_codin	10.7613302	11.1012546	62.6601397	9.9279381
ENSG000001	ARL10	protein_codin	24.028507	41.5394722	61.0298229	12.4487752
ENSG000001	METTL18	protein_codin	0	11.1674301	12.5303788	0.92372259
ENSG000001	CPED1	protein_codin	51.3446782	68.5030378	59.9184533	21.5839066
ENSG000001	SNED1	protein_codin	6.67816236	104.979968	31.1796154	24.337496
ENSG000001	AGR3	protein_codin	0	0	0	0
ENSG000000	HHATL	protein_codin	0	0	0	0

ENSG000001	CLTB	protein_codin	0	0	2.80622676	0
ENSG000001	VCPIP1	protein_codin	44.4139916	116.663093	60.1325607	39.6605559
ENSG000001	TNKS	protein_codin	55.6737238	224.780645	94.8327205	38.0500869
ENSG000001	HCFC1	protein_codin	51.9183815	303.923539	216.523677	62.7939599
ENSG000001	ZDHHC24	protein_codin	4.56691519	10.6073089	5.20441252	3.51851148
ENSG000001	CASP2	protein_codin	4.19469532	31.3658802	33.6310992	6.5260951
ENSG000001	NAA20	protein_codin	1.27189606	3.69717618	0	1.35372465
ENSG000001	TBC1D8B	protein_codin	6.4405126	31.173434	9.27900289	2.7075134
ENSG000001	GPD2	protein_codin	0	12.1984734	3.42997507	0
ENSG000001	PPP1R3B	protein_codin	118.450961	79.2711375	17.1483584	42.4731753
ENSG000001	CCDC74B	protein_codin	0	0	0	0
ENSG000001	UCP2	protein_codin	0	0	0	0
ENSG000001	SLC39A1	protein_codin	0	0	0	2.90221622
ENSG000001	RFNG	protein_codin	0	4.27945299	0	2.30994916
ENSG000001	MEPCE	protein_codin	0	0	0	0
ENSG000001	CELF1	protein_codin	19.0320464	44.8616241	45.0773572	15.3022121
ENSG000001	CBY2	protein_codin	0	0	0	0
ENSG000001	TRPV1	protein_codin	0	0	0	0
ENSG000001	SUGT1	protein_codin	39.9594922	76.2226125	23.6873421	31.3860415
ENSG000001	DDX31	protein_codin	0.75692181	0	2.41749102	0.63072433
ENSG000001	PHOSPHO1	protein_codin	3.05494	5.45743397	11.7482394	22.065643
ENSG000001	SPSB4	protein_codin	1.9795375	2.88152969	10.4559232	0
ENSG000001	COA1	protein_codin	1.18839684	0	0	0
ENSG000001	PAAF1	protein_codin	4.40403966	31.5167107	8.87845371	6.62644684
ENSG000001	SLC5A6	protein_codin	0.94049325	9.81939441	21.4373023	0
ENSG000001	TERT	protein_codin	0	0	0	0
ENSG000001	FAM131A	protein_codin	0	8.01760721	13.1543798	0
ENSG000001	WNT5B	protein_codin	0	28.9553525	27.844223	5.86456309
ENSG000001	ZNHIT2	protein_codin	0.82686491	1.64261738	3.23770614	1.35639653
ENSG000001	DCAKD	protein_codin	0	0	0	0
ENSG000001	CYB561D1	protein_codin	0	23.8654946	0	0
ENSG000001	SULT1B1	protein_codin	2.25143688	15.5115608	1.81594733	23.6081246
ENSG000001	MRPL48	protein_codin	2.82568133	4.77061671	3.9155711	0
ENSG000001	NEFH	protein_codin	10.9268293	46.2017467	549.965223	14.6779488
ENSG000001	CYP4A11	protein_codin	0	0	0	0
ENSG000001	RAB6A	protein_codin	4.46754179	96.351413	30.623028	4.16649715
ENSG000002	SPESP1	protein_codin	0.90260598	8.87717526	31.8109502	7.33577508
ENSG000001	ADCY4	protein_codin	0	0	0	0
ENSG000001	SETD6	protein_codin	0	0	7.02192748	0
ENSG000001	JUP	protein_codin	11.169967	0	4.07911323	15.2783919
ENSG000001	CFAP70	protein_codin	2.02614974	12.7100476	17.8055963	0
ENSG000001	TRAM1L1	protein_codin	2.40951469	5.08239582	4.90296642	3.72556665
ENSG000001	ELMO1	protein_codin	4.06468817	35.9258466	7.12818465	19.244179
ENSG000001	FANCI	protein_codin	5.29233345	0	0	0

ENSG000001	MRPS22	protein_codin	7.76869283	0	0	5.95404011
ENSG000001	HAP1	protein_codin	0	0	0	1.55377582
ENSG000001	CD164	protein_codin	81.4290839	195.431065	90.3222349	53.7153791
ENSG000001	REP15	protein_codin	1.15687002	1.36979957	3.41963991	3.02860858
ENSG000001	MAPK14	protein_codin	0	4.07776058	0	0
ENSG000001	RASGRP1	protein_codin	0	0	0	0
ENSG000001	MIS18BP1	protein_codin	99.0940422	233.062015	315.139534	52.8192365
ENSG000001	MAPK10	protein_codin	0	0	0	0
ENSG000001	ADAM17	protein_codin	0	26.4946964	27.2214353	0
ENSG000001	ZBTB21	protein_codin	0	12.4818582	32.816959	15.1644719
ENSG000001	CD300A	protein_codin	0	0	0	0
ENSG000001	CD34	protein_codin	24.4263015	81.9800913	18.1429206	52.2204023
ENSG000001	UGT8	protein_codin	0.36727093	0	3.78965239	0.52148845
ENSG000001	EIF1	protein_codin	0	0	1.14191221	4.21964491
ENSG000001	POMZP3	protein_codin	0	9.08979327	0	0
ENSG000000	ANKRD10	protein_codin	48.124385	102.807208	119.046261	49.4847517
ENSG000001	DTD2	protein_codin	10.6024292	13.6457607	49.459429	8.10455221
ENSG000000	TNS1	protein_codin	1.80391369	4.82989178	3.03417085	0
ENSG000001	SLC25A30	protein_codin	0.62385611	0	2.56043437	0
ENSG000001	IQCB1	protein_codin	30.0123543	51.4036387	80.7186822	15.1190918
ENSG000001	NEURL3	protein_codin	0	0	0	0
ENSG000001	ZNF621	protein_codin	0	0	0	0
ENSG000001	MYO1H	protein_codin	0.20853058	0	0	2.00560397
ENSG000001	IQCF1	protein_codin	0	0	0	0
ENSG000001	EVC2	protein_codin	0	0	0	3.74266117
ENSG000001	TBC1D16	protein_codin	25.9063037	72.491633	24.375873	21.1107731
ENSG000001	SLCO2A1	protein_codin	2.98712952	15.9034455	19.3803536	6.46578333
ENSG000001	HDLBP	protein_codin	13.2713728	0	23.9286084	3.41597504
ENSG000001	CBX2	protein_codin	1.05438471	11.6338647	52.4888954	1.32853385
ENSG000001	SLCO4C1	protein_codin	0.73372804	2.84550065	3.39263592	2.83201154
ENSG000001	CDC20	protein_codin	0.92335754	8.6944271	34.9739742	0.39473933
ENSG000002	CCPG1	protein_codin	38.9608099	45.8323756	47.3354777	22.8821128
ENSG000000	LRRC7	protein_codin	0.60420927	0	0	0
ENSG000001	DPY19L1	protein_codin	0	39.3345183	0	15.4086573
ENSG000001	SFT2D3	protein_codin	4.86347376	7.1738973	10.3013818	2.99091748
ENSG000001	TMEM52	protein_codin	0	0	0	0
ENSG000001	TTLL9	protein_codin	0.14691758	0	0	0
ENSG000001	MRPL11	protein_codin	4.22339399	2.00674257	4.99645794	1.50621134
ENSG000000	BMAL2	protein_codin	0	0	6.60218463	1.827621
ENSG000001	SPNS1	protein_codin	0	0	2.20340428	0
ENSG000001	DNAI2	protein_codin	0	0	0	0
ENSG000001	ZNF738	protein_codin	6.7452082	5.7535702	6.51761248	2.03954901
ENSG000001	MADD	protein_codin	0	0	0	0
ENSG000001	NCALD	protein_codin	0	7.27795594	0	0

ENSG000001	NPAS4	protein_codin	0	0	0	0
ENSG000001	MOBP	protein_codin	0	0	0	0
ENSG000001	ZNF497	protein_codin	0	2.68758697	0	1.47523038
ENSG000001	ZNF431	protein_codin	22.3895895	36.6417805	39.4647492	13.5011252
ENSG000001	APBB1	protein_codin	0	25.208597	11.75758	2.41650833
ENSG000001	CARHSP1	protein_codin	0	6.59588014	0	6.37703491
ENSG000001	PRDM2	protein_codin	26.1756833	136.675766	44.9024424	16.4995488
ENSG000001	PCP2	protein_codin	0	0	0	0
ENSG000001	CSRP2	protein_codin	15.0280814	25.5239768	118.162291	16.180873
ENSG000001	DMXL1	protein_codin	23.7162857	119.504041	92.9882247	0
ENSG000001	BOD1	protein_codin	5.65957967	3.09712366	31.0054308	0
ENSG000001	DNAL1	protein_codin	1.14703618	0	2.73191832	1.98372049
ENSG000001	PARL	protein_codin	0	4.92832707	0	10.1837816
ENSG000001	GPR151	protein_codin	0	0	0.55460278	1.47496398
ENSG000001	PCSK1	protein_codin	0.66693798	1.57450078	0	0
ENSG000001	RPL38	protein_codin	58.1601731	31.477532	151.953706	71.0040553
ENSG000000	KMT2E	protein_codin	0	0	0	25.0250969
ENSG000001	ARMH3	protein_codin	1.72263666	10.5914729	5.00196335	0
ENSG000001	SLC19A1	protein_codin	1.27907668	5.61955011	23.991132	0
ENSG000001	HEG1	protein_codin	73.4288911	108.779741	53.5404392	48.6197266
ENSG000001	DENND6A	protein_codin	26.0273888	84.0435841	61.3267846	31.3320049
ENSG000001	PPP2R1B	protein_codin	2.82022081	18.7535476	3.35858644	1.49850048
ENSG000001	P2RY2	protein_codin	0	0	0	0
ENSG000001	ZFYVE21	protein_codin	4.39378276	13.47314	1.36576884	6.6882721
ENSG000001	ACOT1	protein_codin	0	3.75616172	0	0
ENSG000000	DPYSL2	protein_codin	122.56205	457.283232	166.401412	100.427301
ENSG000001	TNS3	protein_codin	57.0900388	247.871036	78.1737575	81.1944116
ENSG000001	SLC29A2	protein_codin	0	0	0	1.23250476
ENSG000002	VN1R4	protein_codin	0	0	0	0.56587336
ENSG000001	FCHSD2	protein_codin	0	61.1685058	10.3804638	0
ENSG000001	HOXB9	protein_codin	0.47518248	0	7.1195211	2.02276803
ENSG000001	PDE12	protein_codin	18.0116746	10.9023074	19.2006749	16.8588776
ENSG000001	B4GAT1	protein_codin	6.11113533	13.4580418	21.0878636	3.28155082
ENSG000000	CNGB1	protein_codin	0	0.85969447	0	0.58652085
ENSG000001	HRAS	protein_codin	3.589544	14.522446	37.8201757	5.11415262
ENSG000001	LRCH1	protein_codin	45.2593168	112.713411	70.2247866	43.5859926
ENSG000001	DNAH12	protein_codin	0	1.07648626	0	0
ENSG000001	KRT17	protein_codin	0	0	0	0
ENSG000001	ZNF706	protein_codin	17.051521	13.500699	47.6535033	7.85275087
ENSG000001	GLUL	protein_codin	1.33137413	0	0	1.09694959
ENSG000000	CD84	protein_codin	0	10.0669444	0	0
ENSG000000	TEAD2	protein_codin	0	16.0373005	0	7.35182202
ENSG000001	INTS6	protein_codin	7.19569635	4.41065992	0	0
ENSG000002	GPR162	protein_codin	0	4.83070675	0	0

ENSG000001	MARCHF10	protein_codin	0	0	0	0
ENSG000001	ZMYND8	protein_codin	0	0	0	0
ENSG000001	MAP9	protein_codin	0	0	0	0
ENSG000001	AKT2	protein_codin	7.23502514	0	0	0
ENSG000001	ABCC12	protein_codin	0	0	0	0.64975998
ENSG000001	TTC9B	protein_codin	0	0	0	0
ENSG000001	WASHC4	protein_codin	0.78029005	5.87441951	1.38367447	0
ENSG000001	RIN1	protein_codin	0.96000726	0	0	0.60096243
ENSG000001	IL37	protein_codin	0	0	0	0
ENSG000001	CD248	protein_codin	28.6419299	81.7388572	5.10740427	26.3270309
ENSG000001	ZNF624	protein_codin	16.3096773	27.7639624	7.40510534	7.5608384
ENSG000001	GNPDA1	protein_codin	11.3216542	0	15.3371719	0
ENSG000001	HERPUD2	protein_codin	14.9759983	91.2219343	14.2465834	8.20788416
ENSG000001	LSM1	protein_codin	21.0105919	24.8532772	133.507877	14.161657
ENSG000001	OR52W1	protein_codin	0	0	0	0
ENSG000001	RFESD	protein_codin	0	0	0	0
ENSG000000	SOX30	protein_codin	0	0	0	0
ENSG000001	WFIKKN2	protein_codin	0.89444218	2.77205165	0.54377106	0.88543196
ENSG000001	ARHGAP12	protein_codin	2.04203187	0	0	0
ENSG000001	MTRES1	protein_codin	12.3358085	28.8673305	14.0416507	6.41107224
ENSG000001	DEPDC7	protein_codin	0	0	0	0
ENSG000001	ZBTB4	protein_codin	1.8070501	0	9.79249198	3.419128
ENSG000001	HPSE	protein_codin	1.98463826	14.2710713	0	4.1247846
ENSG000001	RNF26	protein_codin	8.02584944	29.4803338	22.4928985	8.58949595
ENSG000001	ZMAT3	protein_codin	188.605591	119.542737	42.160388	33.4875128
ENSG000001	MDM2	protein_codin	3.32616967	75.7577141	3.45403157	0
ENSG000001	ZNF490	protein_codin	9.66699647	28.6210709	18.3116116	8.71737019
ENSG000001	SLC22A8	protein_codin	0	0	0	0
ENSG000001	CNIH2	protein_codin	0	0	1.14675505	0
ENSG000001	PLAC8L1	protein_codin	0.26826876	1.47009998	8.2482566	1.58447192
ENSG000001	DMAC2L	protein_codin	0	0	4.79985135	0
ENSG000001	COQ2	protein_codin	0	0	1.44359076	0
ENSG000001	COQ2	protein_codin	0	0	0	4.59954219
ENSG000001	RAB1B	protein_codin	24.8842867	68.5212054	39.7931815	16.620737
ENSG000001	RAG2	protein_codin	0	0	0	0
ENSG000001	HMGA1	protein_codin	1.94389331	21.4013334	12.8070172	6.72785515
ENSG000001	IMPA1	protein_codin	0	0	4.12297023	0
ENSG000001	RABL6	protein_codin	10.9905485	127.029473	0	0
ENSG000000	METTL2A	protein_codin	3.8818072	9.09950196	22.9760566	4.49815099
ENSG000001	PLAC8	protein_codin	0	0	0	0
ENSG000001	ADGRF3	protein_codin	0	2.12099569	0	0.41199001
ENSG000001	DBI	protein_codin	0	0	0	0
ENSG000001	RPL13	protein_codin	2.2605508	5.27384583	7.59297178	0
ENSG000001	ISCA1	protein_codin	13.9093307	0	20.3161003	8.59630557

ENSG000001	RPL21	protein_codin	87.9729627	296.878976	69.628717	92.0264693
ENSG000001	GABRB3	protein_codin	0	0	0	0
ENSG000000	CYLD	protein_codin	0	0	0	0
ENSG000001	PPARD	protein_codin	0	0	0	0
ENSG000001	YY1AP1	protein_codin	0	0	0	0
ENSG000001	FGFBP3	protein_codin	1.96690752	0	4.5975702	0.88502037
ENSG000001	EDN3	protein_codin	0	0	0	0
ENSG000001	ENGASE	protein_codin	9.13192089	47.9291554	0	0
ENSG000001	MLC1	protein_codin	0	4.35078523	42.310032	3.32179824
ENSG000001	SH3PXD2B	protein_codin	225.807035	208.407774	326.167182	123.713339
ENSG000001	LSP1	protein_codin	2.85249103	91.7717446	0	4.04318082
ENSG000001	ANKS4B	protein_codin	0	1.16702438	0	7.01128828
ENSG000001	ODAPH	protein_codin	0	0	0	0
ENSG000001	HOXB3	protein_codin	0.78664198	0	0	0
ENSG000001	FBXO45	protein_codin	3.05465692	8.28920607	7.02051096	7.40453235
ENSG000001	FBXO31	protein_codin	4.38797364	0	34.6288179	0
ENSG000000	ATP9A	protein_codin	0	0	0	0
ENSG000001	THAP6	protein_codin	0	0	0	0
ENSG000002	AGAP6	protein_codin	0	2.53728967	1.18894077	0
ENSG000001	UBQLN3	protein_codin	0	3.01976701	0	0.54435655
ENSG000001	C1QTNF1	protein_codin	0	0	0	1.49891258
ENSG000001	ADPGK	protein_codin	0	0	14.5760934	0
ENSG000001	UQCRH	protein_codin	30.2524336	62.3752711	93.4987234	32.6506319
ENSG000001	HECTD4	protein_codin	5.81682749	6.61480817	17.9067955	0
ENSG000001	WDR74	protein_codin	0	3.80695465	0	1.54883362
ENSG000001	IL1RL1	protein_codin	3.09120072	0	0	26.7696959
ENSG000002	PRSS1	protein_codin	0	0	0	0
ENSG000000	RBFOX1	protein_codin	0	0	0	0
ENSG000001	HIGD2B	protein_codin	0.25738545	1.47287679	0	0
ENSG000001	TRHR	protein_codin	0	0	0	0
ENSG000001	ZNF408	protein_codin	2.55878641	7.47713852	3.89979804	2.62106012
ENSG000001	PDP2	protein_codin	4.29273426	20.3879589	16.7903491	7.62680901
ENSG000001	PPP1R8	protein_codin	5.74216508	16.8903979	30.1602203	4.57552671
ENSG000001	SEC31A	protein_codin	247.425259	124.029814	303.971049	83.5556379
ENSG000001	FAM170B	protein_codin	0	0	0	1.21512928
ENSG000001	SSX5	protein_codin	0	0	0	0
ENSG000001	OXSR1	protein_codin	47.90328	87.4926837	63.9824799	38.7871972
ENSG000001	SNX31	protein_codin	0.42131736	0	0	0
ENSG000002	AQP1	protein_codin	42.9184975	36.1540761	0	64.1990201
ENSG000001	RNF32	protein_codin	0	0	0	0
ENSG000001	COMMD1	protein_codin	14.5564705	0	50.6823913	4.92054741
ENSG000002	ENSG000002	protein_codin	0	0	0	0
ENSG000001	MMP14	protein_codin	325.945279	512.846683	1345.65664	101.180423
ENSG000001	SLC22A13	protein_codin	0.65088914	0	0	0

ENSG000001	PRG2	protein_codin	0	0	0	0
ENSG000001	TCP11	protein_codin	0	0	0	0
ENSG000001	HSPB7	protein_codin	23.7176477	0	0	0
ENSG000001	MRPL52	protein_codin	0	0	0	0
ENSG000001	ISCU	protein_codin	71.2204488	113.425142	32.9663475	46.211736
ENSG000001	ERCC4	protein_codin	16.3513808	16.8372521	32.3014233	5.60834105
ENSG000001	F2	protein_codin	0	0	0	0
ENSG000001	TLR8	protein_codin	1.65381895	3.61361114	1.32686944	0
ENSG000001	KICS2	protein_codin	0	3.41480703	2.56297453	1.2188659
ENSG000001	TWNK	protein_codin	2.35932662	0	47.8712345	4.42192789
ENSG000001	ZNF507	protein_codin	15.8892225	16.9288482	10.9263903	0
ENSG000001	TRIB1	protein_codin	4.92224892	24.7325078	25.3828563	81.07029
ENSG000001	MOS	protein_codin	0	0	0	0.94743653
ENSG000001	CCL19	protein_codin	0	4.71443576	0	0
ENSG000001	KRAS	protein_codin	33.3842535	0	92.2711312	24.3758042
ENSG000000	SCFD1	protein_codin	0	0	0	0
ENSG000001	NIPAL4	protein_codin	0	2.7141663	0	3.81950537
ENSG000001	ABRA	protein_codin	14.7635478	1.09207586	0.53845275	1.52537754
ENSG000001	ARHGAP1	protein_codin	24.1458485	57.9368422	27.6849037	28.151988
ENSG000001	GLS2	protein_codin	0	0	0	0
ENSG000001	NRF1	protein_codin	0	0	0	0
ENSG000000	IL5RA	protein_codin	0	0	0	0
ENSG000001	GAL3ST3	protein_codin	0	0	0	0.99085375
ENSG000000	USP36	protein_codin	0	104.535525	0	0
ENSG000000	CYP3A43	protein_codin	0	0	0	0
ENSG000001	ZNF417	protein_codin	9.31247054	40.743175	14.5187198	5.29345693
ENSG000001	AKNA	protein_codin	16.0980379	21.3259366	4.28437567	23.7202629
ENSG000001	RPS14	protein_codin	1.58487258	2.96485018	18.9430892	1.23363654
ENSG000001	OXR1	protein_codin	41.9450427	77.7563357	94.8723023	34.8265975
ENSG000001	MEN1	protein_codin	0	0	0	0
ENSG000001	ADGRE1	protein_codin	0	0	0	0
ENSG000001	CKAP5	protein_codin	101.306254	258.615349	386.927335	55.6001661
ENSG000001	CTU2	protein_codin	2.42972144	0	0	0
ENSG000001	NSD2	protein_codin	0	0	0	0
ENSG000001	MED16	protein_codin	0	0	0	0
ENSG000001	CATSPER1	protein_codin	0	3.08859917	0	1.06861301
ENSG000002	CPT1B	protein_codin	2.01197561	0	0	0
ENSG000001	GAPVD1	protein_codin	0	31.3649721	35.9839077	0
ENSG000001	CST6	protein_codin	0	1.04298908	0	0.44354006
ENSG000001	CMKLR1	protein_codin	8.25770973	16.5579124	20.0332138	15.0214593
ENSG000002	KRT25	protein_codin	0	0	0	0.9783324
ENSG000001	NFE2	protein_codin	0	0	0	0
ENSG000001	NLRP6	protein_codin	0	0	0.5797048	0
ENSG000001	BANF1	protein_codin	3.87908708	34.2565431	20.1301258	2.94890728

ENSG000001	RSRC1	protein_codin	0	0	0	0
ENSG000001	TMEM70	protein_codin	8.7190982	8.54892423	12.1656164	9.57604584
ENSG000001	PHF19	protein_codin	3.35027952	0	0	0
ENSG000000	MSL3	protein_codin	0	0	0	0
ENSG000001	MINDY1	protein_codin	2.79337159	0	0	0
ENSG000001	MCTP1	protein_codin	0	0	0	0
ENSG000001	GLIS1	protein_codin	0	9.09597408	1.06294321	0
ENSG000001	EIF1AD	protein_codin	0	7.84406868	0	0
ENSG000001	HP1BP3	protein_codin	0	0	74.6876916	26.1399158
ENSG000001	NAGPA	protein_codin	0	0	0	0
ENSG000001	DCAF6	protein_codin	56.6375877	88.8395654	132.334002	30.8235531
ENSG000000	CATSPERG	protein_codin	0.29998638	0	0	0
ENSG000001	PMP22	protein_codin	0	0	0	0
ENSG000001	STX16	protein_codin	0	0	0	0
ENSG000001	SPATA31G1	protein_codin	0	0	0	0
ENSG000001	FOLR1	protein_codin	0	3.40162067	6.05799926	0
ENSG000001	RTP1	protein_codin	0	2.20967626	0	0.49042564
ENSG000001	OR5J2	protein_codin	0	0	0	0
ENSG000001	DNAJB5	protein_codin	10.9724306	39.665879	10.8388799	4.56923351
ENSG000001	ELF5	protein_codin	0	0	0	0
ENSG000001	OR10AG1	protein_codin	0	0	0	0.60037082
ENSG000001	FNDC9	protein_codin	0	0	0	0
ENSG000001	KCNK10	protein_codin	0	0	1.11470272	0
ENSG000001	PFKM	protein_codin	0	0	0	0
ENSG000000	SPEG	protein_codin	13.5694474	0	0	0
ENSG000001	AMZ1	protein_codin	0.2676669	2.02176222	0	0
ENSG000001	RUNDC3B	protein_codin	0	0	3.84586694	0
ENSG000001	RACGAP1	protein_codin	4.62229423	20.0954714	30.6477716	3.41982006
ENSG000001	TBC1D10C	protein_codin	0	0	0	1.37825194
ENSG000001	SART1	protein_codin	35.3945982	143.584882	160.851699	30.5795842
ENSG000002	PGA5	protein_codin	0	0	0	0
ENSG000001	CAMSAP1	protein_codin	19.1660796	0	0	0
ENSG000000	CLCN6	protein_codin	0	0	0	0
ENSG000001	POLD4	protein_codin	0	12.3439131	0	0
ENSG000001	SWSAP1	protein_codin	0.34564354	0	0	0.73290302
ENSG000001	DNAH7	protein_codin	1.50910839	10.9001442	6.8269802	2.1619516
ENSG000001	DST	protein_codin	312.548247	0	0	236.547295
ENSG000001	CLCF1	protein_codin	1.87158983	2.02944265	5.70381363	2.88085228
ENSG000002	PSG1	protein_codin	0	0	0	0
ENSG000001	GLIPR1L1	protein_codin	0	0	0	0.74192926
ENSG000001	GPR152	protein_codin	0.3024205	0	0	1.29034444
ENSG000001	FGF5	protein_codin	0	0	0	0
ENSG000001	MACROH2A1	protein_codin	19.6285493	0	22.1972864	7.24184152
ENSG000002	MRPL46	protein_codin	8.75631341	0	14.9567054	0

ENSG000001	COL6A5	protein_codin	0	0	0	0
ENSG000001	HOXC5	protein_codin	2.22668637	0	3.12479849	0
ENSG000001	PEAK1	protein_codin	80.8507262	0	224.50099	0
ENSG000001	RMI2	protein_codin	1.89542248	0	10.4996557	0
ENSG000001	VWC2L	protein_codin	0	0	0	0
ENSG000001	PRM1	protein_codin	0	0	0	0.55071349
ENSG000001	DRAP1	protein_codin	20.919098	31.0223155	62.8229776	19.445148
ENSG000001	NR1D2	protein_codin	46.5046984	48.5242779	0	32.5576239
ENSG000001	B3GAT1	protein_codin	0	0	0	0
ENSG000001	BTN2A1	protein_codin	0	0	0	0
ENSG000001	CHMP2A	protein_codin	28.0716909	181.867729	29.7108766	22.8345255
ENSG000001	PODN	protein_codin	19.7131947	34.6804103	0	0
ENSG000001	RESF1	protein_codin	131.412956	427.022606	137.222216	78.1742225
ENSG000001	FOSL1	protein_codin	0	0	0	14.2029423
ENSG000001	CCDC85B	protein_codin	5.20028679	100.235021	23.9537143	6.23110615
ENSG000001	TNFRSF10D	protein_codin	8.36147085	9.19705776	0.43625679	25.9428268
ENSG000001	PABPC5	protein_codin	4.0569532	2.69544288	5.43905391	1.27375579
ENSG000001	RPS6KB2	protein_codin	8.02728867	24.5032117	19.6664516	5.85035332
ENSG000001	USP15	protein_codin	2.53773692	7.24339831	2.31122742	8.69823951
ENSG000001	FAM174A	protein_codin	8.52980473	16.1057851	14.4822582	0
ENSG000001	CD7	protein_codin	0	3.65682635	0	0
ENSG000001	YPEL2	protein_codin	41.5862181	100.439174	38.0165607	35.4815802
ENSG000000	ENTPD2	protein_codin	0	0	0	0
ENSG000001	TNP2	protein_codin	0	0	0	0
ENSG000002	HS3ST5	protein_codin	0	0	0.55765701	1.44496905
ENSG000001	GUK1	protein_codin	12.9755106	3.80331488	75.0584954	12.8671867
ENSG000001	CGREF1	protein_codin	5.95582116	17.8795059	47.331917	9.64088826
ENSG000001	GFM1	protein_codin	0.30987002	2.04380245	0.5502579	1.86159626
ENSG000001	TCHP	protein_codin	0	0	0	0
ENSG000000	AURKA	protein_codin	0	0	4.05719308	0
ENSG000002	C1orf167	protein_codin	0	0	0	0
ENSG000001	TMEM168	protein_codin	19.6725136	16.2290315	26.6339422	6.60645549
ENSG000001	RNF152	protein_codin	16.9967742	58.9773806	16.7429024	43.7335868
ENSG000001	RNF166	protein_codin	3.94529502	14.4309728	6.82466765	4.97136339
ENSG000001	HR	protein_codin	9.18577237	12.2561142	12.1838483	0
ENSG000001	LSMEM1	protein_codin	3.78515175	0	0	2.39561391
ENSG000000	GPATCH2L	protein_codin	16.4413127	0	7.98896414	9.71273037
ENSG000001	MED25	protein_codin	0	0	0	0
ENSG000001	GJC3	protein_codin	0	0	0.52801019	1.2964185
ENSG000001	KDM3A	protein_codin	0	0	101.034875	0
ENSG000001	AGGF1	protein_codin	47.4072825	126.921934	148.173334	23.5598607
ENSG000001	PNPLA1	protein_codin	0.29034766	1.22362639	0	0
ENSG000000	ZBTB11	protein_codin	2.31862008	0	0	7.44060301
ENSG000001	KCNRG	protein_codin	0	0	1.94055208	0

ENSG000001	DOK3	protein_codin	0	0	0	0
ENSG000001	JAM2	protein_codin	0	33.8533441	6.15478897	0
ENSG000001	SIAH2	protein_codin	9.00300586	22.8422373	20.4676107	11.8474805
ENSG000001	KRI1	protein_codin	8.55165603	0	0	0
ENSG000001	TPM3	protein_codin	0	3.71473651	0	0
ENSG000001	TUBA3E	protein_codin	0	0	0	0
ENSG000001	PPP1CA	protein_codin	0	0	0	0
ENSG000001	CDK4	protein_codin	0.82978379	0	0	0
ENSG000000	EXD2	protein_codin	0	0	0	0
ENSG000001	BACE1	protein_codin	26.2507147	128.421852	70.0485824	2.90469083
ENSG000001	PRCP	protein_codin	0	0	0	2.66322129
ENSG000001	OR8H1	protein_codin	0	0	0	0.55793609
ENSG000001	PARP10	protein_codin	0	60.9082204	24.8469665	0
ENSG000001	OR5T3	protein_codin	0	0	0	0
ENSG000001	ZNF227	protein_codin	2.72903766	0	25.9211816	0
ENSG000001	PTH1R	protein_codin	7.50345617	0	28.9383155	22.081323
ENSG000001	SEC16A	protein_codin	36.5838043	0	63.9395712	31.1303347
ENSG000001	OGFOD3	protein_codin	1.76110783	8.92107879	13.3822677	4.50690537
ENSG000001	PARP10	protein_codin	0	0	0	0
ENSG000000	CADPS2	protein_codin	0.45441908	0	4.80253511	0
ENSG000001	FOXG1	protein_codin	0	0	0	0
ENSG000001	GPR137	protein_codin	0	0	0.72018374	0.46120387
ENSG000001	RHBDF2	protein_codin	1.54072127	0	0	0
ENSG000001	SEC16A	protein_codin	0	0	0	0
ENSG000001	ARHGAP45	protein_codin	8.93595726	126.960764	13.6447666	16.5140286
ENSG000000	RPLP0	protein_codin	54.0366433	0	50.0520291	55.8079715
ENSG000001	ATOX1	protein_codin	0	0	0	0
ENSG000001	ZNF41	protein_codin	0	0	14.7121283	7.25537432
ENSG000001	SRSF7	protein_codin	21.7847498	43.4716552	82.7270835	23.411309
ENSG000001	VDAC2	protein_codin	0	0	0	0
ENSG000001	UTS2R	protein_codin	0	4.71349362	1.07879012	0.43659187
ENSG000001	DVL3	protein_codin	34.12159	134.853052	90.9689695	39.4580923
ENSG000001	ISG20L2	protein_codin	7.29153743	23.192924	38.1147576	7.44803699
ENSG000001	CHST12	protein_codin	1.203905	0	0	0
ENSG000001	C7	protein_codin	0	0	0	8.98696292
ENSG000001	HOXD8	protein_codin	0.5285308	0	6.21360641	0
ENSG000001	INTS14	protein_codin	10.539404	16.787688	0	0
ENSG000001	PFN4	protein_codin	0.88726127	2.81646307	5.81472364	0.84263695
ENSG000001	CHMP7	protein_codin	0.72114067	0	1.47484872	8.25856054
ENSG000001	FBXL13	protein_codin	0	7.30364775	7.22717663	1.44700494
ENSG000001	KRT80	protein_codin	0	0	0	0
ENSG000001	TMEM25	protein_codin	0	0	0	0
ENSG000000	PHF21B	protein_codin	0	0	7.05879982	0
ENSG000001	MAK	protein_codin	0	0	0	0

ENSG000001	TRIP10	protein_codin	7.88779854	33.9861006	25.7021093	0
ENSG000001	DHRS4	protein_codin	0	18.9792881	3.05182635	0
ENSG000001	OR4D11	protein_codin	0	1.53263285	0	0.47144264
ENSG000001	GRINA	protein_codin	9.00481846	9.79630932	0	2.29114609
ENSG000001	TRIP10	protein_codin	38.8069567	21.7531936	59.3833588	20.3965174
ENSG000000	TACC3	protein_codin	11.9371539	48.3684697	165.565954	0
ENSG000002	TERB1	protein_codin	1.08495157	0	0	4.58818742
ENSG000000	SPAG6	protein_codin	2.24093822	0	0	0
ENSG000001	BTF3L4	protein_codin	3.47807337	20.942404	11.7812701	4.79672019
ENSG000001	NEUROG2	protein_codin	0	1.07735197	0.93074682	0.78760147
ENSG000001	SERTAD2	protein_codin	47.2278115	117.173727	247.399865	99.4760389
ENSG000001	CLASP2	protein_codin	0	0	0	0
ENSG000001	PUF60	protein_codin	0.84537552	0	2.74326476	0
ENSG000000	OSBPL3	protein_codin	24.8103371	49.36417	31.8636154	16.9778364
ENSG000001	TAF7	protein_codin	42.9486466	199.752164	113.527119	25.5249442
ENSG000000	MLXIPL	protein_codin	0	0	0	0
ENSG000001	RMND5B	protein_codin	6.04253092	8.96830119	11.8678182	0
ENSG000001	ASTN2	protein_codin	5.02618635	0	8.09609859	6.89964172
ENSG000001	CDK5R1	protein_codin	4.146585	8.30546763	4.64851851	1.62637544
ENSG000001	NDUFS7	protein_codin	0	0	3.00576782	0
ENSG000000	CD6	protein_codin	0.53961744	49.3405967	0	5.20065528
ENSG000001	PDE4DIP	protein_codin	0	9.41595412	32.2611412	0
ENSG000001	ATP2A2	protein_codin	3.02963802	2.76452	4.41539458	17.2222924
ENSG000001	RPA2	protein_codin	0	1.55556403	0	0
ENSG000001	ZNF135	protein_codin	1.68017153	18.9790731	0	0
ENSG000001	OR8K5	protein_codin	0	0	0	0
ENSG000001	SH2D4B	protein_codin	0	0	0	0
ENSG000002	C8orf82	protein_codin	0	5.03750236	0.97145168	0
ENSG000001	NDUFS8	protein_codin	1.46341812	8.37175284	11.6776664	3.45935838
ENSG000001	OR8H3	protein_codin	0	0	0	0
ENSG000001	MPRIP	protein_codin	0	0	20.062825	0
ENSG000001	PITPNA	protein_codin	17.9183405	90.8681062	13.2697438	13.466176
ENSG000001	OR8H2	protein_codin	0	0	0	0
ENSG000001	TUFM	protein_codin	42.5483393	74.1553609	177.656067	30.4250016
ENSG000001	THAP5	protein_codin	7.54806234	19.7143612	16.1114444	2.57005604
ENSG000001	SUV39H2	protein_codin	0	0	0	0
ENSG000000	DNAJC25	protein_codin	1.93388568	21.1801964	9.10763649	2.12062973
ENSG000001	GIMAP7	protein_codin	39.2412422	224.42466	6.24031671	26.3559154
ENSG000001	POC1B	protein_codin	6.10123789	0	5.03131854	5.82815091
ENSG000001	CFAP47	protein_codin	0	0	0	0
ENSG000001	ING5	protein_codin	6.35714744	38.1678045	8.70264594	0.94733783
ENSG000001	ZNF821	protein_codin	0	0	0	0
ENSG000001	AP4S1	protein_codin	0.86157369	0	0.51807751	0.49334401
ENSG000001	AQP11	protein_codin	0.3117359	0	0	1.09945054

ENSG000001	ANKAR	protein_codin	2.42321473	0	0	4.12492782
ENSG000001	NFKBIB	protein_codin	0	0	0	0.74790185
ENSG000001	GMPPA	protein_codin	7.18421451	16.0921549	0	0
ENSG000001	CD163L1	protein_codin	2.00801862	42.396912	0	4.83895351
ENSG000001	GNAI2	protein_codin	175.455107	667.885599	283.990433	181.414001
ENSG000001	MRPS23	protein_codin	10.2628816	11.2367487	50.364032	12.6790451
ENSG000001	MCMDC2	protein_codin	0	0	0	0
ENSG000001	EPSTI1	protein_codin	16.6852354	124.417048	13.2494738	17.1619437
ENSG000001	FZR1	protein_codin	0	0	0	0
ENSG000001	EPSTI1	protein_codin	0	0	0	0
ENSG000001	LGALS9	protein_codin	0	0	0	5.87803826
ENSG000000	LAMA3	protein_codin	14.9485237	0	0	10.7420083
ENSG000001	DSCC1	protein_codin	7.48953898	0	49.5382125	1.7722415
ENSG000001	C2CD3	protein_codin	0	0	0	0
ENSG000001	ARHGEF2	protein_codin	28.9130835	167.26042	26.9535249	24.0641252
ENSG000001	COL26A1	protein_codin	0	0	0	0
ENSG000001	PNMA8A	protein_codin	6.98623963	11.1111695	7.09519558	5.74607731
ENSG000001	ARHGEF2	protein_codin	22.4910713	0	0	0
ENSG000001	ACAD10	protein_codin	3.94399077	0	0	0
ENSG000001	EBF1	protein_codin	74.2034788	127.723786	110.529388	79.8737548
ENSG000001	DENND2B	protein_codin	11.113798	25.987681	29.5848976	17.1216643
ENSG000000	CASP10	protein_codin	5.12110745	0	0	0
ENSG000001	ZDHHC7	protein_codin	7.41757893	76.7412756	15.9034696	16.0457896
ENSG000001	OSBPL2	protein_codin	12.9784799	0	0	0
ENSG000000	NOS2	protein_codin	0	0	0	0
ENSG000001	PRODH	protein_codin	0	0	0	0
ENSG000001	FAM20C	protein_codin	21.3386761	59.435263	39.773106	64.2057491
ENSG000000	FAS	protein_codin	0	0	0	0.94612823
ENSG000000	FUZ	protein_codin	0	0	7.96072042	0
ENSG000001	DALRD3	protein_codin	0	0	0	0
ENSG000001	RCAN1	protein_codin	5.29256799	0	37.7804574	1.80508707
ENSG000001	FBXO34	protein_codin	14.4469489	28.077879	19.1831133	0
ENSG000001	GPR89A	protein_codin	0	0	4.69220174	4.52114053
ENSG000001	STX4	protein_codin	5.41765116	0	30.444809	6.39379878
ENSG000001	FAM216B	protein_codin	0	1.77895855	0	2.00686706
ENSG000000	LIMCH1	protein_codin	35.5814632	0	0	49.7937628
ENSG000000	MKS1	protein_codin	0	0	0	0
ENSG000001	AKAP17A	protein_codin	39.3107719	166.285439	57.7947329	24.445826
ENSG000001	HNRNPD	protein_codin	7.76385985	14.6548813	21.0184968	16.9520739
ENSG000001	TRDMT1	protein_codin	0	0	0	0
ENSG000001	OR5AN1	protein_codin	0	0	0	0
ENSG000000	GNAS	protein_codin	0	0	0	0
ENSG000001	RGS5	protein_codin	166.250818	670.990838	37.9394937	349.108905
ENSG000001	TEX44	protein_codin	0.3001472	0	0	0.40175464

ENSG000001(	B3GALNT2	protein_codin	0	0	0	0
ENSG000001:	ZNF436	protein_codin	9.87168503	0	13.7331199	12.8936506
ENSG000001{	BHLHA15	protein_codin	0	0	0	1.17784781
ENSG000001	CSNK1D	protein_codin	0	0	11.279542	19.267085
ENSG000001	UCP3	protein_codin	5.0004747	10.4583861	0	0.8691631
ENSG000001:	DDX54	protein_codin	0	89.3686514	29.3262475	0
ENSG000001	IFT43	protein_codin	6.12002768	14.0986214	0	0
ENSG000001:	SLAIN1	protein_codin	0	0	0	0
ENSG000001	BICRAL	protein_codin	37.0508481	116.107527	74.0363142	36.6590128
ENSG000001{	H2AC6	protein_codin	68.5381736	44.9022184	148.115649	8.7576152
ENSG000002:	CTAGE9	protein_codin	0	1.48447954	0	0.83783744
ENSG000001{	BTBD9	protein_codin	0	0	0	0
ENSG000001	CHAF1B	protein_codin	2.28934249	8.04028204	21.168923	6.04005977
ENSG000001(	CYP11B1	protein_codin	0	0	0	6.03994199
ENSG000001{	RELL1	protein_codin	2.47780079	0	6.56533437	7.44945839
ENSG000000{	TMEM40	protein_codin	0	0	0	0
ENSG000001:	AKAP1	protein_codin	0	33.987642	4.3486715	0
ENSG000001	STAT2	protein_codin	0	0	0	17.0359342
ENSG000001	COX8A	protein_codin	1.3082007	17.4555259	9.84781825	3.28334171
ENSG000001{	SLC35C1	protein_codin	10.5255855	0	13.0675424	4.76403468
ENSG000001(	RPL27A	protein_codin	9.66254534	8.83133713	23.6392461	23.4401876
ENSG000001	GPHB5	protein_codin	0	0	0	0
ENSG000001:	CCT6B	protein_codin	0	0	2.7679692	4.81256929
ENSG000001(	ASAH1	protein_codin	0	8.1510609	0	0
ENSG000001	CLEC18C	protein_codin	0	0	0	0
ENSG000001{	GPR89B	protein_codin	0	0	0	0
ENSG000001	GAK	protein_codin	24.1122574	158.086106	92.1455897	20.1563034
ENSG000001(	LUZP1	protein_codin	70.3007835	105.182483	157.183114	79.9297152
ENSG000001:	MEIS2	protein_codin	4.55566355	0	0	0
ENSG000002	PRKDC	protein_codin	0	0	7.63452436	0
ENSG000000:	ANK1	protein_codin	6.47820398	0	0	0
ENSG000001{	BBS12	protein_codin	0	15.3604135	8.43477197	3.39524639
ENSG000001{	PHLDA2	protein_codin	0.77070074	1.52321749	1.28538239	1.54631644
ENSG000001	ZNF575	protein_codin	0.87652373	0	0	0.74133822
ENSG000001	KCNMB3	protein_codin	0	2.75674325	0	5.61303675
ENSG000001	TNS2	protein_codin	26.1376998	0	0	0
ENSG000001(	SLC2A2	protein_codin	0	0	0	1.23182859
ENSG000001	MRGPRX4	protein_codin	0	0	0	0.73814682
ENSG000001	TMEM94	protein_codin	0	0	0	12.8696673
ENSG000001{	OR4C6	protein_codin	0	0	0	0
ENSG000001:	SENP7	protein_codin	65.1750594	61.0324799	10.4611247	0
ENSG000000{	NOP14	protein_codin	66.0638897	148.453783	380.409132	25.9229679
ENSG000001	DEFB104A	protein_codin	0	0	0	0
ENSG000001	TNS2	protein_codin	1.60549903	0	14.212613	0

ENSG000001	MRPL48	protein_codin	0	0	0	0.7472315
ENSG000000	RNF4	protein_codin	0	0	22.4070615	6.90916708
ENSG000000	ARHGAP28	protein_codin	0	0	0	15.3161675
ENSG000001	SPECC1L	protein_codin	0	97.0579032	0	0
ENSG000001	H2BC4	protein_codin	17.5537275	0	16.305371	5.55651622
ENSG000001	AGTRAP	protein_codin	2.33491488	0	3.01905872	0
ENSG000000	PPP2R2C	protein_codin	0	0	0	0
ENSG000000	ADAMTS6	protein_codin	0	4.77934	2.05730404	0
ENSG000001	CKS2	protein_codin	4.28925806	3.7849072	24.6189416	2.012456
ENSG000001	DEFB103A	protein_codin	0	1.46905665	0	0
ENSG000001	KDM3B	protein_codin	64.3203202	186.154103	138.456382	55.6068752
ENSG000001	ZNG1A	protein_codin	0	0	0	0
ENSG000001	C19orf18	protein_codin	0.87060902	6.60731702	3.16407219	2.85021949
ENSG000001	DPM2	protein_codin	0.86323295	0	2.39166	0
ENSG000001	ZHX2	protein_codin	19.1826244	53.0339927	23.2755709	16.2181665
ENSG000001	NHP2	protein_codin	0.59308526	0	0	0
ENSG000001	DNAJC28	protein_codin	0	0	1.67795029	0
ENSG000001	NEPRO	protein_codin	4.75934056	9.15726974	6.69020982	6.17802673
ENSG000001	CD300LB	protein_codin	0.33782936	0	0	0.87964329
ENSG000001	FUT7	protein_codin	0.38170546	1.45783593	0	0
ENSG000001	UTP4	protein_codin	11.926063	0	34.0555483	0
ENSG000001	ZMYM3	protein_codin	16.1999568	101.667539	89.8666701	11.0767868
ENSG000001	P2RX4	protein_codin	0.59735583	6.97448835	1.46576259	2.04707919
ENSG000001	LILRB2	protein_codin	0	0	0	0
ENSG000001	SAMD4B	protein_codin	0	25.589978	17.6975668	17.5812716
ENSG000001	TLCD5	protein_codin	0	0	0	0
ENSG000001	IGSF8	protein_codin	0	0	0	0
ENSG000001	CSRNP3	protein_codin	0	0	0	0
ENSG000001	MKRN3	protein_codin	0	5.3334844	15.2525963	6.60388313
ENSG000001	SUMO2	protein_codin	35.9665184	121.248829	95.798976	22.4841226
ENSG000001	PALM2AKAP2	protein_codin	19.6657194	17.3196183	60.9554763	16.2382846
ENSG000001	ZNF620	protein_codin	0	1.96636968	0.99828291	1.22247058
ENSG000001	ZNF77	protein_codin	5.24349554	9.24989419	18.4909315	2.09677425
ENSG000001	IQCF1	protein_codin	0	0	0	0
ENSG000001	CRHR1	protein_codin	0	0	0	0
ENSG000001	TPD52L3	protein_codin	0.32835695	0	0	4.35659324
ENSG000001	MITF	protein_codin	0	13.6876483	0	4.30012683
ENSG000001	BORCS5	protein_codin	6.05347903	23.1616362	9.18899172	8.86948118
ENSG000002	CDPF1	protein_codin	0	0	0	0
ENSG000001	YES1	protein_codin	96.9553361	69.5661133	133.519372	54.1644307
ENSG000001	HCLS1	protein_codin	0.95373606	113.544537	0	0
ENSG000001	EXOC3L1	protein_codin	0.97973968	4.44453257	0	0
ENSG000001	MITF	protein_codin	0	0	0	5.9763877
ENSG000001	R3HCC1L	protein_codin	25.0595618	0	24.3488727	21.2848599

ENSG000000	POLR2B	protein_codin	47.9064845	24.3600827	295.572504	8.44180497
ENSG000001	FARSA	protein_codin	0	27.2466756	14.4798637	2.50352104
ENSG000001	ZMYM4	protein_codin	72.4509178	166.318502	329.177118	64.3918483
ENSG000001	SUPT6H	protein_codin	135.375093	182.944065	249.479283	44.0328899
ENSG000001	NSUN3	protein_codin	7.99968911	17.4182496	19.8069323	6.08201892
ENSG000001	DHFR2	protein_codin	0	0	0	0
ENSG000001	BVES	protein_codin	13.8665773	16.3399094	13.8485523	7.95409579
ENSG000001	OR4C15	protein_codin	0	0	0	0
ENSG000000	WASHC2A	protein_codin	0	0	56.2223304	0
ENSG000001	PFAS	protein_codin	5.63510888	15.8155579	21.484724	10.0414956
ENSG000001	FHAD1	protein_codin	0	0	0	0
ENSG000000	SLC13A2	protein_codin	0	0	0	0
ENSG000001	KCNH6	protein_codin	0	3.15393555	0	0
ENSG000001	SNX14	protein_codin	0	0	0	0
ENSG000001	HBS1L	protein_codin	0	0	2.35009882	0
ENSG000001	UBXN11	protein_codin	0.31177955	1.89775781	3.22134928	0
ENSG000001	ZNF619	protein_codin	0	0	0	0
ENSG000001	QNG1	protein_codin	0	13.9985469	0	3.09800803
ENSG000001	OR4A16	protein_codin	0	0	0	3.03308236
ENSG000001	SAMD12	protein_codin	0.89257417	3.08583764	5.30733745	0.52966913
ENSG000000	EPN2	protein_codin	0	0	0	0
ENSG000000	ARHGAP33	protein_codin	0	0	2.78780221	0
ENSG000001	LINS1	protein_codin	1.07906848	10.7855294	0	2.47881753
ENSG000001	NEUROG1	protein_codin	0	0	0	0
ENSG000000	RECQL	protein_codin	0.52606642	0	0	0
ENSG000001	SULT1A1	protein_codin	3.04813294	9.92909287	0	0
ENSG000001	CD55	protein_codin	3.61827759	0	22.2892669	0
ENSG000001	TTC12	protein_codin	0	0	0	0
ENSG000001	SKA3	protein_codin	1.70309278	0	13.1691899	0
ENSG000001	WDR75	protein_codin	36.1142839	40.6430496	77.8502288	0
ENSG000002	PINX1	protein_codin	2.8932545	16.3817165	5.17088467	0
ENSG000001	COPG1	protein_codin	62.2606351	130.6733	218.871724	47.1024343
ENSG000000	SH2D3C	protein_codin	3.5617475	11.1045369	6.77558781	3.46399222
ENSG000001	PLD5	protein_codin	0	0	0	0
ENSG000001	AMBRA1	protein_codin	0	47.6588978	14.8192933	0
ENSG000001	IMP3	protein_codin	0	3.70553665	0.51850592	3.26964736
ENSG000001	SLIT1	protein_codin	0	0	0	0
ENSG000001	TLN1	protein_codin	376.246276	1801.42882	561.741032	306.191726
ENSG000001	ANXA2R	protein_codin	0.78227747	8.58905471	0.95452838	1.37881454
ENSG000001	LPCAT4	protein_codin	0	1.93277869	0.69379715	1.96516525
ENSG000001	BDNF	protein_codin	0	0	0	0.87331352
ENSG000001	PENK	protein_codin	2.55722992	0	0	0
ENSG000001	HNRNPA0	protein_codin	35.1430436	147.864576	108.604885	64.518446
ENSG000001	PRIMPOL	protein_codin	14.5930848	0	22.0109552	0

ENSG000001	LGALS7B	protein_codin	0	0	0	0
ENSG000001	EXD1	protein_codin	0	0	0	0
ENSG000001	DBF4B	protein_codin	0	0	0	0
ENSG000001	EXOC3	protein_codin	0	0	0	0
ENSG000000	OSBPL6	protein_codin	2.11382576	0	0	3.87094178
ENSG000001	GPR88	protein_codin	0.22089516	1.54826124	0.50414995	2.49342226
ENSG000001	TNIP1	protein_codin	0	0	0	0
ENSG000001	CNTD1	protein_codin	0.28830697	0	0	2.44873138
ENSG000001	TRIM41	protein_codin	3.84734272	0	19.8276906	0
ENSG000001	RHOT2	protein_codin	5.75964064	0	19.5102002	2.77866144
ENSG000001	ST8SIA5	protein_codin	0	0	0	0
ENSG000001	TARDBP	protein_codin	14.879972	153.975141	70.8551273	6.48239439
ENSG000001	STX19	protein_codin	3.62412138	0	0	0
ENSG000001	EDC3	protein_codin	10.7555687	0	13.0268631	3.18772903
ENSG000001	LEO1	protein_codin	0	0	0	19.7802008
ENSG000001	FLAD1	protein_codin	3.24998211	0	2.00931937	0
ENSG000002	NAIP	protein_codin	17.5791203	145.276056	57.8595151	16.957044
ENSG000001	RAB43	protein_codin	0	0	0	0
ENSG000002	PRSS50	protein_codin	0.50038412	0	0	0
ENSG000000	TNRC6A	protein_codin	0	0	271.424732	0
ENSG000001	GARIN1B	protein_codin	0	0	0	0
ENSG000001	SERTM1	protein_codin	0	1.63605044	0.53920297	1.37696523
ENSG000001	STK33	protein_codin	0	0	0	0
ENSG000001	RNASEH1	protein_codin	33.6541102	78.0119614	72.3002735	20.0043641
ENSG000000	ADGRA2	protein_codin	39.1951835	49.0237749	323.267272	18.8180116
ENSG000001	CALML3	protein_codin	0	0	0	0.75891341
ENSG000000	RFFL	protein_codin	0	0	0	3.52292983
ENSG000000	CHDH	protein_codin	2.28880294	6.50958503	8.6988562	2.95900355
ENSG000000	KIF26A	protein_codin	4.63104608	13.9321911	57.4531632	9.89458234
ENSG000001	GPHN	protein_codin	0	0	0	0
ENSG000001	ASAP2	protein_codin	28.7428881	96.1808209	120.594347	23.7210565
ENSG000001	MMP1	protein_codin	0	0	1.46326224	0.96215044
ENSG000000	SPAST	protein_codin	0	0	0	0
ENSG000001	CCDC43	protein_codin	21.5610657	48.4859537	38.4234747	11.1987792
ENSG000001	RTKN2	protein_codin	0	0	0.7817815	0
ENSG000001	IFT22	protein_codin	2.71436866	8.99489085	1.61172049	0.50616217
ENSG000001	FZD2	protein_codin	1.90968191	8.10720479	2.99950149	0.89711433
ENSG000001	CLEC1A	protein_codin	2.53184093	0	0	0.74462326
ENSG000001	DAZ3	protein_codin	0	0	0	0
ENSG000001	ARSJ	protein_codin	6.00039432	16.0606064	9.57606759	2.10048861
ENSG000001	PLEKHF2	protein_codin	26.8508673	0	20.3505643	0
ENSG000000	ANAPC4	protein_codin	21.7202324	75.5743045	97.4304721	17.5581015
ENSG000001	RPS6	protein_codin	737.601247	635.806179	1834.73884	401.482845
ENSG000001	PLXDC1	protein_codin	15.6301734	10.7996727	2.95826131	4.5637436

ENSG00000010	ODAD1	protein_codin	0	3.13029329	0	1.67633453
ENSG00000013	MEN1	protein_codin	0	0	17.4799572	3.0863736
ENSG00000010	TNIP2	protein_codin	55.0231162	86.2187806	246.400879	38.3710457
ENSG00000024	ZNF487	protein_codin	8.34933718	0	0	0
ENSG00000017	WSB2	protein_codin	20.2704496	10.2846516	9.75687496	9.80452592
ENSG00000018	OR2A4	protein_codin	0	0	0	0
ENSG00000013	DTNA	protein_codin	0	0	0	0.92200058
ENSG00000014	GCSH	protein_codin	0	13.592204	28.9337945	10.7250716
ENSG00000017	ZNF354C	protein_codin	26.1387816	32.9690597	102.551091	7.25401861
ENSG00000013	USP20	protein_codin	0	44.7616903	0	0
ENSG00000018	CIMAP1D	protein_codin	0	0	0	0
ENSG00000023	TUBB3	protein_codin	0	7.00520401	0	0
ENSG00000008	MPP4	protein_codin	0	0	0	1.27317035
ENSG00000017	OR11H6	protein_codin	0	0	0	0.89442031
ENSG00000008	AQP6	protein_codin	0.56542744	0	0	0
ENSG00000008	CNOT4	protein_codin	14.0171626	41.0204322	0	0
ENSG00000007	CDC42	protein_codin	5.9093493	14.4989787	7.57730826	5.56544597
ENSG00000005	RRP12	protein_codin	0	0	0	0
ENSG00000010	MRPL1	protein_codin	11.6994052	20.877259	31.7534221	4.68525908
ENSG00000017	LDLRAD3	protein_codin	25.0949301	9.89483878	27.1754712	6.65266869
ENSG00000012	ADGRE2	protein_codin	2.91135328	11.5964356	2.27748825	8.00235599
ENSG00000000	NCAPD2	protein_codin	0	73.4794689	87.3812766	13.6243104
ENSG00000018	ARL6IP4	protein_codin	55.8315884	0	58.8368429	57.0578685
ENSG00000007	CHFR	protein_codin	11.3095714	0	0	2.47824866
ENSG00000000	MED29	protein_codin	0	52.6241505	34.1134262	25.2576851
ENSG00000009	CD209	protein_codin	0	0	0	0
ENSG00000008	PDS5B	protein_codin	114.03314	151.71247	174.754568	69.3064438
ENSG00000009	CD209	protein_codin	2.48721258	16.4220478	1.76557481	18.2710313
ENSG00000020	ENSG00000020	protein_codin	0.46732391	0	0	0
ENSG00000013	ACRV1	protein_codin	0	0	0	0
ENSG000002	NEURL4	protein_codin	7.26899483	12.7385256	7.99201673	0
ENSG00000010	RPUSD2	protein_codin	1.07810596	15.1003472	7.17351058	1.24416411
ENSG00000013	CCL25	protein_codin	0	0	0	0
ENSG000001	ELMOD3	protein_codin	0	0	0	0
ENSG00000013	TSC22D3	protein_codin	0	0	0	0
ENSG00000017	DLGAP1	protein_codin	0	4.15594821	0	0
ENSG00000017	ZNF843	protein_codin	0	0	0	0
ENSG00000017	OR4L1	protein_codin	0	0	0	0
ENSG00000018	DIPK2A	protein_codin	11.8925333	30.3790172	22.2147859	8.95796331
ENSG00000009	CD200	protein_codin	0	0	0	0
ENSG000001	ABCD3	protein_codin	0.79976305	0	2.19947282	0
ENSG00000010	ARPC2	protein_codin	25.6710928	175.167251	50.9156585	50.660732
ENSG00000014	RPL7A	protein_codin	0	283.841245	27.4351573	0
ENSG00000018	LSM10	protein_codin	6.14587175	4.18885622	6.62926858	2.28094624

ENSG000001	RPL5	protein_codin	2.38118371	1.10694398	8.6268507	3.69404116
ENSG000001	ITGA11	protein_codin	212.959046	308.311419	0	79.1585711
ENSG000001	MDH2	protein_codin	13.6293512	49.652355	55.2362813	17.1214621
ENSG000001	WDR90	protein_codin	0	2.43248873	0	0
ENSG000001	HEPHL1	protein_codin	0.31108386	0	0.50999537	3.22846736
ENSG000001	ZMAT4	protein_codin	0	9.26323355	0	0.91161068
ENSG000001	SNAP47	protein_codin	0	0	0	0
ENSG000001	CPZ	protein_codin	0	35.5388136	3.59953607	0
ENSG000001	TCIM	protein_codin	35.4880885	38.2139475	10.6462389	13.3411158
ENSG000001	METAP1D	protein_codin	2.10758038	13.1191261	42.9267564	6.36460847
ENSG000001	PHF20L1	protein_codin	14.2235691	14.8432271	30.0805414	0
ENSG000001	ZNF114	protein_codin	0	0	0	0
ENSG000000	TFE3	protein_codin	19.5703517	77.2198669	0	0
ENSG000000	ING3	protein_codin	0.43689483	2.58847512	2.75386768	0.78427031
ENSG000001	ROCK2	protein_codin	151.011556	199.843797	167.67304	60.3967491
ENSG000001	ABHD14B	protein_codin	0	0	0	5.23515571
ENSG000001	SLC41A3	protein_codin	0	0	0	0
ENSG000001	RBP7	protein_codin	0	0	0	1.66124376
ENSG000001	ADAD2	protein_codin	0	0	0	0
ENSG000001	DMAP1	protein_codin	0	0	0	0
ENSG000001	OR4K5	protein_codin	0	0	0	0.60725929
ENSG000001	YOD1	protein_codin	0	0	13.1172144	16.8202022
ENSG000001	FGF13	protein_codin	6.05066468	7.17799471	15.6396183	0
ENSG000001	GDPD4	protein_codin	0	0	0	1.8908205
ENSG000000	WNK1	protein_codin	0	0	0	0
ENSG000001	OR4N2	protein_codin	0	0	0	0
ENSG000001	LYSMD3	protein_codin	17.0093447	24.7570659	52.0368699	12.6943443
ENSG000001	OR4M1	protein_codin	0	0	0	2.29045109
ENSG000001	TRIM55	protein_codin	1.01106976	0	0	0
ENSG000001	OS9	protein_codin	1382.26432	0	375.336477	94.4383556
ENSG000002	PPP2R2A	protein_codin	0	0	0	0
ENSG000001	KCND3	protein_codin	0	0	0	0
ENSG000001	ASB2	protein_codin	0	0	0	0
ENSG000001	SH3PXD2A	protein_codin	0	0	0	3.06002679
ENSG000001	C16orf89	protein_codin	0	0	0	0
ENSG000000	PKN2	protein_codin	5.13773619	38.7005374	21.1839317	0
ENSG000001	WRAP53	protein_codin	0.33652072	30.0433607	0	2.39872801
ENSG000001	TUBA8	protein_codin	0	0	0	0
ENSG000001	ZNF518A	protein_codin	11.0038107	31.3396378	2.96840846	0
ENSG000001	SMCO3	protein_codin	0	0	0	0
ENSG000001	EXOSC4	protein_codin	0	0	6.69990572	1.21002392
ENSG000001	ZFP91	protein_codin	74.5598848	187.323661	202.312033	77.4160096
ENSG000001	MCM9	protein_codin	2.46394653	6.47821489	6.2568086	0
ENSG000002	RPTN	protein_codin	0.90242243	4.15784666	0	3.79320443

ENSG000001	MAP7D3	protein_codin	28.4732913	57.5777051	47.65617	19.8201596
ENSG000001	TDRD6	protein_codin	0	0	8.56581669	0
ENSG000001	NKIRAS2	protein_codin	0	23.3541843	3.46219532	0
ENSG000001	RPS4X	protein_codin	398.754367	449.234557	1550.57474	485.910248
ENSG000001	SPATA9	protein_codin	0	0	0	0
ENSG000002	SCAMP4	protein_codin	0	0	0	2.740224
ENSG000001	HNF4A	protein_codin	0	0	0	0
ENSG000001	PCDHB9	protein_codin	2.3014307	10.7847062	28.5336	7.8239611
ENSG000001	LCN15	protein_codin	0	0	0	0
ENSG000001	NDUFAF4	protein_codin	9.49755709	13.6194559	6.67949631	5.21648899
ENSG000001	MAP7D1	protein_codin	31.9932564	163.40931	24.8753097	6.98781677
ENSG000001	LARP4B	protein_codin	6.69712343	96.0037957	44.0035469	19.1634545
ENSG000001	CYP8B1	protein_codin	0	0	0	0.84765816
ENSG000000	RGS11	protein_codin	0	0	1.87849615	0
ENSG000001	DEFB104B	protein_codin	0	0	0	0
ENSG000001	GCNT2	protein_codin	0.68452374	3.01432743	0	0
ENSG000001	SLC2A11	protein_codin	1.43939567	0	2.70620676	0
ENSG000001	CSH1	protein_codin	0	0	0	0
ENSG000001	AURKB	protein_codin	0	2.10270315	0	0
ENSG000000	PDIA5	protein_codin	18.9534936	14.4185384	48.679046	7.42922449
ENSG000001	LILRB5	protein_codin	13.5832362	0	0	0
ENSG000001	PRIMA1	protein_codin	0.61557544	0	0	0
ENSG000001	KCNG2	protein_codin	0	0	0	0
ENSG000001	BCAT2	protein_codin	0	0	0	0
ENSG000001	ZNF786	protein_codin	3.42476608	4.90334009	0	3.32202523
ENSG000001	EEF1A1	protein_codin	0	0	0	0
ENSG000001	ANO1	protein_codin	14.9844211	28.3329756	0	4.20092471
ENSG000001	PRKAG1	protein_codin	1.77426975	0	0	0
ENSG000001	LPA	protein_codin	0.28803326	0	1.81271624	13.5881676
ENSG000001	CLK4	protein_codin	41.1140614	0	21.0756092	18.4728065
ENSG000001	MCM9	protein_codin	0	25.1712526	0	16.774694
ENSG000001	UNCX	protein_codin	0	0	2.39074236	1.39668507
ENSG000001	LNX2	protein_codin	19.2700399	21.0740842	11.2034578	9.32466068
ENSG000001	SLC12A4	protein_codin	26.4468702	71.5069962	25.9756961	20.684449
ENSG000001	CCNG2	protein_codin	8.91001867	33.8832312	14.2245472	9.06310148
ENSG000001	SYTL2	protein_codin	6.2966891	0	21.7530368	11.7702114
ENSG000001	ZNF133	protein_codin	0	20.6836261	0	0
ENSG000001	KCNA2	protein_codin	0	0	0	5.74422587
ENSG000001	SEMA6D	protein_codin	0	0	34.9857142	53.2442579
ENSG000002	OR2AE1	protein_codin	0.31209523	0	0	0.48257235
ENSG000001	C11orf42	protein_codin	0	0	0	1.21815565
ENSG000001	ICOS	protein_codin	0	50.3017915	0	0
ENSG000001	CCDC89	protein_codin	3.02952691	6.86706621	3.5999106	2.76047138
ENSG000001	SOX6	protein_codin	0	0	0	0

ENSG000001	SIGLECL1	protein_codin	0	0	0	0
ENSG000001	CLU	protein_codin	43.5539314	43.3816633	60.8167547	12.6199764
ENSG000001	USF3	protein_codin	23.5447293	76.7502607	36.0057066	30.8268464
ENSG000001	FAM89B	protein_codin	0	0	0	0
ENSG000000	AHRR	protein_codin	0	0	0	1.82526138
ENSG000001	UGDH	protein_codin	19.3949462	40.8310409	41.2769452	8.24881527
ENSG000001	TMEM107	protein_codin	0	0	0	0
ENSG000001	LRRC31	protein_codin	0	0	0	0
ENSG000001	ZNF540	protein_codin	1.32020161	8.45998016	0	3.27563501
ENSG000001	LSMEM2	protein_codin	0.38459956	0	0	0.83531599
ENSG000001	CALR	protein_codin	157.1471	300.735977	628.66381	157.824342
ENSG000001	ZBTB8A	protein_codin	9.06839548	6.92783166	16.9428829	0
ENSG000001	TTC9C	protein_codin	0.30331436	0	1.97735796	1.95355938
ENSG000001	ABHD1	protein_codin	0	0	0	0
ENSG000001	KRBOX5	protein_codin	2.28019695	0	2.38587483	0
ENSG000001	ZFAND2A	protein_codin	6.15377519	0	7.75166327	5.74647675
ENSG000002	VAMP2	protein_codin	2.82680797	33.5656842	3.24867307	15.3691229
ENSG000000	AACS	protein_codin	3.39289765	48.6666593	16.8506784	10.1270586
ENSG000001	OR56B4	protein_codin	0	0	0	0
ENSG000001	NPHP1	protein_codin	0	8.81919629	0	1.06084604
ENSG000000	AACS	protein_codin	0.59782725	0	0	0
ENSG000001	SLC9A9	protein_codin	18.4015594	36.6177865	23.0781588	19.992233
ENSG000001	CCDC184	protein_codin	0.84954179	5.01897527	1.57223713	1.30297875
ENSG000001	PANK2	protein_codin	1.22307399	0	0	2.92563473
ENSG000001	TESPA1	protein_codin	0	0	0	0
ENSG000001	STK25	protein_codin	1.81969476	20.1838988	3.74421728	4.79185473
ENSG000001	HNRNPH2	protein_codin	100.340665	120.654911	125.143088	54.9025891
ENSG000001	DNAJC7	protein_codin	0	0	0	3.59616052
ENSG000001	ZSCAN26	protein_codin	2.2006506	0	0	0
ENSG000001	NSUN7	protein_codin	1.16786972	0	11.529195	1.76344879
ENSG000001	DLC1	protein_codin	7.46839959	0	0	0.73904949
ENSG000001	MBLAC2	protein_codin	7.18554309	3.32450899	59.317221	2.76496134
ENSG000001	GRB2	protein_codin	0	0	0	0
ENSG000001	FBN1	protein_codin	154.402718	17.4910328	59.4425419	90.464293
ENSG000000	GSK3B	protein_codin	0	0	0	2.58262727
ENSG000001	CDK1	protein_codin	11.3217709	5.50338458	92.4763655	2.67855093
ENSG000001	SNAPC5	protein_codin	4.91527039	12.9260165	6.29743809	8.8228671
ENSG000000	DEF6	protein_codin	0	0	0	6.58120341
ENSG000000	IL20RA	protein_codin	0	0	0	0
ENSG000001	PMAIP1	protein_codin	6.08881772	11.9361753	61.01768	29.935874
ENSG000001	HNF4A	protein_codin	0	0	0	0
ENSG000001	SHMT1	protein_codin	5.4738169	0	0	0
ENSG000001	PTPN13	protein_codin	0	138.835973	162.315161	31.3593492
ENSG000000	GPM6B	protein_codin	0	0	0	0

ENSG000001	MTX1	protein_codin	2.55412331	1.01976084	0	0
ENSG000001	BIN1	protein_codin	0	0	0	0
ENSG000001	CCZ1B	protein_codin	100.837887	47.4387517	50.4415673	20.6759343
ENSG000001	TSPAN16	protein_codin	0	0	0	0
ENSG000001	EFCAB11	protein_codin	0	1.8788629	0	4.78088132
ENSG000001	PUS10	protein_codin	0	0	12.8026956	2.67591997
ENSG000001	RHOJ	protein_codin	10.3673364	39.4195692	5.66410056	16.3070626
ENSG000000	APBA3	protein_codin	1.10376408	19.2997419	0	0.60860117
ENSG000001	VRK3	protein_codin	0	6.27213883	3.07196357	1.10015062
ENSG000001	OR10Q1	protein_codin	0	0	0.53324038	0
ENSG000001	AP3S1	protein_codin	18.9378816	69.008714	67.9672526	30.0460212
ENSG000001	OXTR	protein_codin	0.55291784	1.08792169	17.566065	1.26749016
ENSG000001	CABP1	protein_codin	0	1.30538821	1.19818068	0
ENSG000001	GRB2	protein_codin	32.5300148	226.860506	81.0579689	41.3817755
ENSG000001	ALX1	protein_codin	0	0	0	1.5151819
ENSG000001	PNMA1	protein_codin	29.3080098	38.0158355	55.9646542	33.5009956
ENSG000001	LLGL1	protein_codin	10.7577614	19.9253452	11.8039858	2.77158651
ENSG000001	MINDY2	protein_codin	0	7.97976112	0	7.39193308
ENSG000001	NCAM1	protein_codin	155.427249	228.486281	222.206324	276.825241
ENSG000001	KLHDC4	protein_codin	0	0	0	0
ENSG000001	RAD23A	protein_codin	3.77548996	11.9240024	19.9456282	3.32395638
ENSG000001	TRIM25	protein_codin	17.6666836	34.5906523	63.2894153	24.3077963
ENSG000000	TRIT1	protein_codin	0	0	0	0
ENSG000000	STOML1	protein_codin	0	0	0	0
ENSG000000	SLC7A8	protein_codin	9.55785346	0	18.7921605	0
ENSG000000	STOML1	protein_codin	0	0	2.30355767	0
ENSG000000	MAP2K3	protein_codin	0.34176751	0	0	0
ENSG000001	KLC2	protein_codin	0	0	0	0
ENSG000001	TRAPPC14	protein_codin	0	6.37021678	7.71920634	0
ENSG000001	GADD45GIP1	protein_codin	38.793178	45.207248	185.483049	13.3578836
ENSG000001	CACFD1	protein_codin	1.09170619	0	6.56264794	0.56729947
ENSG000001	ZNF569	protein_codin	0	0	3.0344879	5.03101223
ENSG000001	LYPLA1	protein_codin	0	22.5841281	20.3961024	21.8880564
ENSG000001	PLAG1	protein_codin	41.8037059	0	36.3810928	4.11477423
ENSG000001	NSD3	protein_codin	8.25917077	69.4621827	33.0190208	26.060888
ENSG000001	RBM44	protein_codin	3.27159131	0	0	0
ENSG000001	SMAP1	protein_codin	27.4373299	0	41.1252885	0
ENSG000001	FOXR1	protein_codin	0	0	0	0
ENSG000001	ZBED2	protein_codin	0.38164844	10.6734206	0	4.23940841
ENSG000001	MATCAP2	protein_codin	0	0	0	0
ENSG000001	NSD3	protein_codin	28.4030024	116.508305	199.767815	33.7794851
ENSG000001	OR52N2	protein_codin	0	1.59734628	0	0
ENSG000001	MVB12A	protein_codin	0	2.38088336	0	0.7834052
ENSG000001	SGF29	protein_codin	2.40905991	15.3752279	8.94243669	0

ENSG000001	DAND5	protein_codin	0	0	0	0
ENSG000001	CHTF18	protein_codin	0	0	0	0
ENSG000000	DNAJA2	protein_codin	69.0414248	65.0411658	223.10792	42.133946
ENSG000001	CES2	protein_codin	10.1950638	19.3731797	30.6751666	9.61805339
ENSG000001	OR52N5	protein_codin	0	0	0	0
ENSG000001	PARL	protein_codin	0	0	0	0
ENSG000001	VCX2	protein_codin	0	0	0	0
ENSG000001	PDE4D	protein_codin	0	9.48159539	14.7872276	2.38133125
ENSG000001	OR56B1	protein_codin	0	0	0	0.55902965
ENSG000001	ASCC1	protein_codin	27.2181889	0	63.0291368	15.3765494
ENSG000001	ARHGEF7	protein_codin	0	7.90230367	0	0.91311152
ENSG000001	LRCH2	protein_codin	23.2049111	25.0421257	82.9327243	5.14098178
ENSG000001	CNOT1	protein_codin	56.1866207	138.068398	154.78116	41.0666408
ENSG000001	SLC4A3	protein_codin	0.90579262	0	0	0
ENSG000001	ASCC1	protein_codin	1.29755242	0	0	0
ENSG000001	MSRA	protein_codin	0	0	0	0
ENSG000001	ZNF776	protein_codin	9.57327347	39.3165785	20.3287797	9.81414445
ENSG000001	EEF1D	protein_codin	0.7454346	44.4236375	83.0404095	28.55019
ENSG000001	HOXA3	protein_codin	0	0	3.17059161	2.24682821
ENSG000000	TOLLIP	protein_codin	5.7917119	9.93179302	11.249622	6.55620773
ENSG000001	EGR3	protein_codin	2.76625999	17.6333315	0	33.1442342
ENSG000001	ZNF559	protein_codin	0	0	13.3973196	0
ENSG000001	AFF3	protein_codin	0	0	0	11.1089931
ENSG000001	MPPE1	protein_codin	0	0	0	0
ENSG000001	ZNF554	protein_codin	1.23162523	5.68689185	0	0
ENSG000001	MPPE1	protein_codin	0	0	0	0
ENSG000001	SEPTIN4	protein_codin	0	0	0	0
ENSG000001	SETDB2	protein_codin	0	0	0	13.6861497
ENSG000001	SEPTIN4	protein_codin	0	12.9035211	0	0
ENSG000002	PVRIG	protein_codin	0	0	0	0
ENSG000001	PER1	protein_codin	11.7415138	0	23.9435422	12.1215897
ENSG000001	DNAJC14	protein_codin	0	0	0	6.11316596
ENSG000000	STAG3	protein_codin	0	0	0	0
ENSG000000	EYA2	protein_codin	0	8.93915636	0	0
ENSG000002	INSL3	protein_codin	0	0	0	0
ENSG000001	CD300LG	protein_codin	0	2.00565286	0	0
ENSG000001	ODF2L	protein_codin	12.1970243	0	48.0782707	31.7395044
ENSG000000	SEZ6	protein_codin	0	0	0	0
ENSG000001	PFKFB3	protein_codin	0	0	0	0
ENSG000001	SOBP	protein_codin	40.1334769	41.0327979	65.3959548	25.9246075
ENSG000000	BID	protein_codin	0	3.0190421	0	0
ENSG000000	ICA1	protein_codin	0	14.8612439	9.35311433	0
ENSG000001	AP1B1	protein_codin	20.0661066	74.6661518	44.1661024	23.0587395
ENSG000001	SLC2A4	protein_codin	0	0	3.49781182	0

ENSG000001	SSUH2	protein_codin	0	0	0	0
ENSG000001	SPOCK2	protein_codin	0	0	0	0
ENSG000001	TRAPPC5	protein_codin	0	0	0	1.76202939
ENSG000001	GLYATL1	protein_codin	0	0	0	0
ENSG000001	HRH3	protein_codin	0	0	0	0
ENSG000000	ZNF671	protein_codin	12.5500189	14.3849161	0	0
ENSG000001	GEN1	protein_codin	11.0387284	16.8385944	16.0583029	11.239927
ENSG000000	RABGAP1	protein_codin	1.46748137	3.01242344	4.0771472	2.66887347
ENSG000001	CEP112	protein_codin	0.39989504	1.12821943	0	0
ENSG000001	MAMDC4	protein_codin	0.59356109	3.20025396	0	0
ENSG000001	ACSF3	protein_codin	0.39251971	0	0	0.64351976
ENSG000001	LRRN4CL	protein_codin	4.03177871	1.1858855	0	0.44086968
ENSG000001	TRPT1	protein_codin	0	0	0	1.17818211
ENSG000001	KANK4	protein_codin	0	0	0	0
ENSG000001	PXMP2	protein_codin	0.4768925	2.94935893	3.80532458	0.82116152
ENSG000001	RAB23	protein_codin	80.8604381	0	19.3658816	6.34668969
ENSG000001	NTRK3	protein_codin	0	0	0	0
ENSG000001	MTG1	protein_codin	5.21151208	15.1630735	0.9397703	4.84662621
ENSG000001	RANBP10	protein_codin	9.27009562	47.1637425	33.7202997	10.5035863
ENSG000000	PRSS8	protein_codin	0	0.86906728	0	1.21305349
ENSG000001	TLE3	protein_codin	15.3031411	0	4.56038401	0
ENSG000001	TPM1	protein_codin	0	0	0	0
ENSG000000	MRPS24	protein_codin	4.36577223	0	7.03942089	2.1019424
ENSG000001	ZMYM6	protein_codin	0	0	0	0
ENSG000002	SLURP2	protein_codin	0	0	0	0
ENSG000001	CERS5	protein_codin	3.08649357	13.9429225	4.2640246	1.16318018
ENSG000001	MID1	protein_codin	0	0	0	0
ENSG000002	PGAM5	protein_codin	3.13772029	14.5478202	18.5927715	0
ENSG000001	PPHLN1	protein_codin	0.43585878	0	0	0.56806837
ENSG000001	FRMD8	protein_codin	10.1333042	26.2066386	20.3006045	9.75732574
ENSG000001	TTC39C	protein_codin	12.5503498	31.4758514	42.7480426	9.42317267
ENSG000001	SIX5	protein_codin	4.69612298	27.6292463	11.7485929	1.76336267
ENSG000000	BCL2L13	protein_codin	0	0	21.9062308	0
ENSG000001	DISC1	protein_codin	0.29149283	4.21567455	0	0
ENSG000001	S100Z	protein_codin	0	0	0	0
ENSG000001	FXYD2	protein_codin	0.69471168	0	0.55666494	1.34630499
ENSG000001	MAB21L2	protein_codin	5.15873719	4.75405218	0	5.14886752
ENSG000001	RTN4	protein_codin	27.8601946	458.825984	75.8689766	19.6447634
ENSG000001	RGS3	protein_codin	0	0	0	0
ENSG000001	PSMG2	protein_codin	3.64720059	0	0	0
ENSG000001	PROM2	protein_codin	0.48891774	4.76829468	0	2.98780745
ENSG000001	PCNX4	protein_codin	7.17568121	0	13.6958127	0
ENSG000001	C2orf73	protein_codin	0	0	0	0
ENSG000001	TMEM11	protein_codin	6.11456869	3.83450775	15.0045814	4.71990738

ENSG000001(	SIGLEC7	protein_codin	1.59921579	0	0	0
ENSG000001(	NOSTRIN	protein_codin	10.6553182	0	0	1.37297865
ENSG000001	CAPZA3	protein_codin	0	0	0	1.19629843
ENSG000001	PROM2	protein_codin	0	0	0	0
ENSG000001	DEFB124	protein_codin	0.23516853	1.52162742	0	1.32552687
ENSG000002	CPNE1	protein_codin	7.17570858	0	0	10.1441793
ENSG000001	FBXO46	protein_codin	4.68930731	9.00899712	11.4263387	3.62597083
ENSG000001	ASB8	protein_codin	16.121606	29.9169438	27.7180574	11.0513429
ENSG000001	TUBB6	protein_codin	53.5368494	87.9149598	95.7587644	48.9726155
ENSG000001	AK5	protein_codin	1.09057773	8.59047906	0	1.68792309
ENSG000001	CDHR3	protein_codin	0	0	0	0.68304073
ENSG000000	SLC35C2	protein_codin	3.89072311	12.4114138	0	4.50451343
ENSG000001	RRH	protein_codin	0	0	0.69663169	0
ENSG000001	WSCD1	protein_codin	0	0	0	5.59146251
ENSG000001	HOPX	protein_codin	0	3.32672333	0	0
ENSG000001	UGT2B17	protein_codin	0	0	0	0
ENSG000001	DRICH1	protein_codin	0.33181554	2.28882005	6.13308728	2.04509023
ENSG000001	IL1RAP	protein_codin	0	3.34655945	0	9.1952204
ENSG000001	SOHLH2	protein_codin	0.81934122	0	0	5.3260707
ENSG000000	NOS1	protein_codin	0.42631928	8.45105011	23.8802159	8.64912377
ENSG000001	HADHB	protein_codin	27.2136605	81.9751763	51.37831	0
ENSG000001	TSPYL6	protein_codin	0.84029632	0	0	1.74307144
ENSG000001	FJX1	protein_codin	2.83815039	2.24870821	3.43125435	6.64001374
ENSG000001	HES7	protein_codin	0	0	0	0
ENSG000001	TACC1	protein_codin	25.9230671	59.6249232	32.5685585	0
ENSG000001(	AMOTL1	protein_codin	0	186.906854	520.987532	0
ENSG000001	OR2D3	protein_codin	0	0	0	0
ENSG000001	OR14C36	protein_codin	0	0	0	0
ENSG000001	ATG4C	protein_codin	28.7146961	0	15.0356603	0
ENSG000001	HNRNPCL1	protein_codin	0	1.96521127	0.43849126	1.05983852
ENSG000001	NME9	protein_codin	0	1.36263523	0	0.82330275
ENSG000000	SLC25A5	protein_codin	15.0619751	42.4085236	56.3203182	24.8714284
ENSG000000	HFE	protein_codin	0	0	0	0
ENSG000001	MAGEF1	protein_codin	36.774239	39.7216254	171.221454	20.2782961
ENSG000001(	SPAG11B	protein_codin	0.3675613	0	0	0
ENSG000001(	RECQL5	protein_codin	0	0	35.4732034	0
ENSG000000	TTC27	protein_codin	13.4956998	24.2471214	57.5626121	7.14110951
ENSG000001	L3MBTL4	protein_codin	3.46716719	13.6764943	10.8620477	3.66480548
ENSG000001	KCTD1	protein_codin	0	0	0	0
ENSG000000	MYO3B	protein_codin	0	0	0	0
ENSG000001	COX14	protein_codin	0.644049	2.06899964	0	1.40716595
ENSG000001(	CCDC34	protein_codin	0.73308943	0	7.47652096	0
ENSG000001	NKPD1	protein_codin	0	0	0	0
ENSG000001(	RNF32	protein_codin	0	0	0	0

ENSG000000	KDM5D	protein_codin	8.68487526	0	0	0
ENSG000001	OR2M7	protein_codin	0	0	0	1.87058132
ENSG000000	RAP1GAP	protein_codin	0	0	0	0
ENSG000001	PDLIM5	protein_codin	68.1452646	152.420223	131.66255	34.842957
ENSG000001	SLC5A10	protein_codin	0	0	0	0
ENSG000001	FDCSP	protein_codin	0	0	0	0.77916231
ENSG000000	GRAMD1A	protein_codin	9.99200054	0	0	16.2196215
ENSG000001	CA8	protein_codin	0.38869295	1.09398292	0	0.49519916
ENSG000001	CC2D1A	protein_codin	9.45848859	48.9075692	35.1650633	7.52766931
ENSG000001	VPS39	protein_codin	30.7025984	133.042599	62.6529274	9.89162247
ENSG000001	PDLIM5	protein_codin	27.0064076	0	0	4.71230511
ENSG000000	LASP1	protein_codin	53.0953086	255.952368	82.3277776	58.6584022
ENSG000002	GANC	protein_codin	8.05801923	27.5689167	19.1488151	9.31993747
ENSG000001	OR2T33	protein_codin	0	0	0	0
ENSG000000	CAPN3	protein_codin	0	0	0	0
ENSG000000	USH1C	protein_codin	0	0	0	0
ENSG000001	RNF168	protein_codin	59.1866013	97.9847893	95.2439239	43.3756539
ENSG000001	IL11RA	protein_codin	8.46230943	3.27905386	9.29325481	0
ENSG000001	OR4F17	protein_codin	0.25557208	0	0	0
ENSG000002	PAM16	protein_codin	0	0	0	0
ENSG000001	MICU3	protein_codin	8.87758602	15.3430242	15.7559204	5.8932444
ENSG000001	NFATC1	protein_codin	0	0	0	0
ENSG000001	UBE2N	protein_codin	14.468269	40.9138342	34.4580864	15.3651055
ENSG000001	SYTL1	protein_codin	0	0	0	0
ENSG000000	CRAT	protein_codin	0	0	0	0
ENSG000001	MAMSTR	protein_codin	0	0	0.87363931	0
ENSG000001	TOM1L2	protein_codin	0	0	0	0
ENSG000002	RTEL1	protein_codin	0	0	0	0
ENSG000000	DERL3	protein_codin	0	1.65855189	0	0
ENSG000001	ZNF101	protein_codin	0.88587501	0	0	0
ENSG000000	CLSPN	protein_codin	4.09959141	6.13885366	35.5072685	10.4343203
ENSG000001	DEFB103B	protein_codin	0	0	0	1.39979945
ENSG000001	GINS3	protein_codin	2.85512234	0	31.2112293	1.92524129
ENSG000001	EDEM3	protein_codin	0	0	0	0
ENSG000001	DEFB4B	protein_codin	0	0	0	0
ENSG000001	GRHPR	protein_codin	11.2698228	23.3870613	15.6391888	10.926629
ENSG000001	GREM2	protein_codin	2.61732657	4.9814432	0	10.7055456
ENSG000001	OVCH1	protein_codin	0	1.86356718	0	0
ENSG000001	TPRKB	protein_codin	0	4.53778167	11.9169563	0
ENSG000001	PLCZ1	protein_codin	0	0	0	0
ENSG000001	DGKZ	protein_codin	0	0	0	0
ENSG000001	ZSCAN4	protein_codin	0	0	0	0
ENSG000001	RBM24	protein_codin	0	0	1.68796847	0
ENSG000001	MYO1D	protein_codin	35.3943077	145.074197	43.0244853	79.4500735

ENSG000001	NBPF3	protein_codin	0	0	18.2895827	6.98674156
ENSG000001	PKD2L1	protein_codin	0.31348657	0	0	0
ENSG000001	C3orf22	protein_codin	0	0	0	0
ENSG000001	ALOXE3	protein_codin	0.25571874	4.59814746	0	2.20100787
ENSG000001	SAMD9L	protein_codin	38.759622	230.869314	21.3846529	14.850388
ENSG000001	C18orf32	protein_codin	4.160455	1.783177	5.69003498	4.33084791
ENSG000001	OR2AJ1	protein_codin	0	5.04121544	0.59845291	2.84452435
ENSG000001	RORC	protein_codin	0	0	0	0
ENSG000001	NBPF3	protein_codin	0.49155299	0	0	0
ENSG000001	PTPRA	protein_codin	0	0	0	0
ENSG000001	MRTFB	protein_codin	5.24226633	0	0	0
ENSG000002	TSTD1	protein_codin	0	0	0	0
ENSG000001	PPFIBP1	protein_codin	0	0	0	0
ENSG000001	ZC3H14	protein_codin	0	0	0	0
ENSG000001	BBS1	protein_codin	6.27886271	0	16.3382798	5.44918916
ENSG000001	C5orf46	protein_codin	0	1.021904	0	0
ENSG000001	LRR1	protein_codin	1.27684094	1.00038401	4.58678099	0
ENSG000000	MRPL43	protein_codin	0.61728749	5.1742557	1.03844297	0.57136621
ENSG000001	FUT6	protein_codin	0	0	0	0
ENSG000001	GLTP	protein_codin	16.5577677	29.1191345	14.6675141	14.0612916
ENSG000001	HTR3C	protein_codin	0	1.30641159	0	0.39694943
ENSG000001	ANKRD23	protein_codin	0	0	0	0
ENSG000000	MRPL43	protein_codin	0	0	0	7.0239576
ENSG000001	SLBP	protein_codin	0	56.0736117	3.08624289	0
ENSG000001	ZWINT	protein_codin	0	0	0	0
ENSG000001	NDUFV2	protein_codin	35.7483236	40.6864404	66.8385371	19.4499574
ENSG000001	BOK	protein_codin	5.16645985	27.2060026	13.2313941	5.68163603
ENSG000001	WASHC5	protein_codin	36.5666585	54.206018	376.61218	2.58773003
ENSG000001	CD276	protein_codin	0	13.5408661	0	4.90940651
ENSG000001	TMC8	protein_codin	4.15350739	59.888103	0	9.18127859
ENSG000001	SYCN	protein_codin	0	0.76880103	0	0
ENSG000001	ZNF366	protein_codin	3.44075161	123.151148	3.24096045	10.5709457
ENSG000001	CD276	protein_codin	39.4766726	0	45.6493001	0
ENSG000001	SLCO3A1	protein_codin	18.2876522	47.9115026	4.689434	20.3980084
ENSG000001	GAPT	protein_codin	0	0	0	0
ENSG000001	PEX7	protein_codin	0	0	5.50731748	0
ENSG000001	MBIP	protein_codin	0	0	11.8303635	8.60435263
ENSG000001	MET	protein_codin	0	0	0	0
ENSG000001	ZNF185	protein_codin	0	0	0	0
ENSG000001	CXCR2	protein_codin	0.26439749	2.96979438	0.47680281	0.40787236
ENSG000001	EML4	protein_codin	28.44804	154.644001	156.055312	50.1316933
ENSG000000	NFX1	protein_codin	21.0255809	35.7277928	56.6350843	7.04041182
ENSG000001	ERICH5	protein_codin	0	1.10240688	1.26339422	2.77263119
ENSG000001	ZNF185	protein_codin	0	0	0	4.90204664

ENSG000000	ABL1	protein_codin	29.6458122	36.3207505	39.820448	25.7696185
ENSG000001	SFTPC	protein_codin	0	0	0	0
ENSG000001	EPS8L2	protein_codin	0	0	0	0
ENSG000001	HID1	protein_codin	0	0	0	0.58243032
ENSG000000	MEOX1	protein_codin	11.7151189	1.98270853	0	12.7359812
ENSG000001	FKRP	protein_codin	3.48565168	12.5386702	6.31942306	5.09123369
ENSG000001	SLC25A42	protein_codin	1.56128744	8.43504174	9.29027643	1.06095862
ENSG000001	A2M	protein_codin	1000.51201	2376.72815	3698.58346	797.808712
ENSG000000	PABPC1	protein_codin	0	56.0459608	18.2395546	0
ENSG000000	RHBDD2	protein_codin	4.27823339	17.4920323	8.34385293	0
ENSG000001	FIBIN	protein_codin	59.3191112	14.1417484	3.68134629	3.97528896
ENSG000001	MAP6D1	protein_codin	0.88711621	0	0	0
ENSG000001	CA5B	protein_codin	9.32105089	15.3139694	5.09877249	11.8496926
ENSG000001	ORAI3	protein_codin	2.01426079	15.8969927	3.07490136	0.81396405
ENSG000001	OR10D3	protein_codin	0	0	0	0.83919676
ENSG000001	SLC38A9	protein_codin	0	0	0	0
ENSG000002	ENSG000002	protein_codin	0	0	0.78350049	0
ENSG000001	EIF4E1B	protein_codin	0	0	0	0
ENSG000001	B3GNT3	protein_codin	0.27448993	1.43443115	0.49971507	1.57040662
ENSG000001	ANKFN1	protein_codin	0	0	0	0
ENSG000001	AVL9	protein_codin	6.41506461	91.2923412	22.1115785	20.4403422
ENSG000001	ATXN7L1	protein_codin	1.86919478	20.6127314	16.000972	0.64665955
ENSG000001	SHOC1	protein_codin	0	0	0.49644438	0.94315424
ENSG000001	LRRC75B	protein_codin	0	0	0	0
ENSG000001	FOXP1	protein_codin	0	0	0	0
ENSG000001	PAQR4	protein_codin	0	0	26.2298651	0
ENSG000001	HDDC2	protein_codin	1.45273494	0	0	3.36394557
ENSG000001	FOXP1	protein_codin	0	0	0	0
ENSG000001	SYNGR1	protein_codin	0	0	0	0
ENSG000001	ST6GALNAC5	protein_codin	0	0	0	5.69528637
ENSG000000	LTBP4	protein_codin	0	0	0	0
ENSG000001	ZFP3	protein_codin	15.4070048	30.4843894	13.4133268	4.2870119
ENSG000001	IGSF3	protein_codin	0	0	0	0
ENSG000001	ZFYVE1	protein_codin	0	0	0	6.05747804
ENSG000001	SYT9	protein_codin	0.23539359	0	5.7837949	0
ENSG000001	C3orf38	protein_codin	13.693747	12.275708	18.1170256	5.79143986
ENSG000001	C1orf131	protein_codin	6.73659863	11.4233554	25.464002	7.19193268
ENSG000001	CYC1	protein_codin	19.6871389	30.2702375	95.5152633	6.17529987
ENSG000000	ANKRD24	protein_codin	0.36728002	0	19.05302	3.14054688
ENSG000001	NRIP1	protein_codin	99.7964356	209.302638	130.050438	71.8094373
ENSG000001	SWAP70	protein_codin	51.2943198	121.108969	53.4198956	64.0720757
ENSG000001	ALS2CL	protein_codin	2.59574557	0	3.16665899	0
ENSG000001	INTS3	protein_codin	14.8782271	53.649045	16.6630514	2.80173263
ENSG000001	PTPN6	protein_codin	0	0	5.88722615	0

ENSG000001	CTXN1	protein_codin	0.1741863	2.48180895	3.14429411	0
ENSG000001	CTDNEP1	protein_codin	0	27.9997703	0	0
ENSG000001	ATAT1	protein_codin	0	9.20440578	0	1.79793608
ENSG000001	GEMIN4	protein_codin	6.14591666	0	12.1618632	3.83679077
ENSG000001	TALDO1	protein_codin	31.224581	49.6799308	96.7024439	24.0059897
ENSG000001	TMEM184A	protein_codin	0	0	0	0
ENSG000001	SLC25A20	protein_codin	4.60927519	27.1025336	20.9814001	3.74119291
ENSG000001	TMEM184A	protein_codin	0	0	0	0
ENSG000001	NADSYN1	protein_codin	3.27638337	42.9523978	24.5811736	8.33747535
ENSG000001	ATAT1	protein_codin	0	0	0	0
ENSG000001	ALG2	protein_codin	0	0	0	0
ENSG000001	SH3BGRL3	protein_codin	0	0	0	0.48463389
ENSG000001	WFIKKN1	protein_codin	0.29345482	0	0	0
ENSG000001	RNF170	protein_codin	0	0	0	0
ENSG000001	C14orf119	protein_codin	6.4482083	10.8213401	12.8977063	5.76729803
ENSG000001	MLXIP	protein_codin	35.6694233	122.887378	81.4287417	45.4996984
ENSG000002	ZBTB48	protein_codin	0	0	0	0
ENSG000001	PSAPL1	protein_codin	1.37816636	2.4950345	0	1.36018071
ENSG000001	RNF170	protein_codin	0.73229061	1.25993673	0.53955969	2.16322798
ENSG000001	TBC1D15	protein_codin	0	0	16.9082366	9.728943
ENSG000001	RALGPS1	protein_codin	0	3.19854719	0	0
ENSG000001	ZBTB34	protein_codin	7.91698024	18.3609817	0	16.3943642
ENSG000001	KLHL11	protein_codin	27.3418402	32.3108143	34.149149	22.6423397
ENSG000001	FBF1	protein_codin	0	33.2226176	4.65827388	4.79748002
ENSG000001	APOL1	protein_codin	0	0	0	0
ENSG000001	DNAJB8	protein_codin	0	0	0	0
ENSG000001	CES5A	protein_codin	0	0	0	0
ENSG000000	TRO	protein_codin	0	0	35.5875904	0
ENSG000001	RAPH1	protein_codin	98.2178603	147.0764	61.6215784	105.661444
ENSG000001	TMEM39A	protein_codin	11.2041784	52.3653497	72.6105444	17.0406921
ENSG000000	TTC7A	protein_codin	0	15.2724054	0	7.97423078
ENSG000001	DIS3L	protein_codin	0	0	0	0
ENSG000001	MPDZ	protein_codin	0	0	0	0
ENSG000001	F2R	protein_codin	66.1351643	41.2864331	19.4431395	29.5514359
ENSG000001	DIS3L	protein_codin	4.73401609	0	15.0297067	18.1916571
ENSG000001	MPDZ	protein_codin	0	90.4973006	5.63412318	0
ENSG000001	LRRC42	protein_codin	0	15.2231818	5.93523585	0
ENSG000001	KCNK10	protein_codin	0	0	0	0
ENSG000001	SHISA3	protein_codin	0.8403503	0	1.8362333	0.87715833
ENSG000001	GFRA4	protein_codin	0	0	0	0
ENSG000001	PRDX1	protein_codin	26.6733992	115.411889	141.729242	13.4224048
ENSG000001	LURAP1L	protein_codin	2.01211663	12.6629254	1.65952193	0.46233244
ENSG000000	TAC1	protein_codin	0.31998235	0	0	0
ENSG000001	GORASP1	protein_codin	0	0	0	0

ENSG000001	DCTPP1	protein_codin	0	5.2618558	0	0
ENSG000001	ZNF572	protein_codin	2.41414581	8.78355413	77.2984749	1.94378262
ENSG000001	UBASH3A	protein_codin	2.10815374	0	0	0
ENSG000001	ZNF771	protein_codin	3.3241101	6.78148524	5.16979094	3.27069522
ENSG000001	SERINC4	protein_codin	0	0	0	0
ENSG000001	LRRN1	protein_codin	59.3037182	11.3209718	502.998743	4.87528469
ENSG000000	P3H2	protein_codin	5.6742178	5.29281768	18.5223813	11.712468
ENSG000001	IGSF22	protein_codin	0	0	0	0
ENSG000001	CHST13	protein_codin	0.50315464	0.952872	0.581072	2.56977699
ENSG000001	ETV4	protein_codin	0	0	0	0
ENSG000001	STK17A	protein_codin	9.20740056	8.72036741	19.0665123	19.6774281
ENSG000001	ELL3	protein_codin	0.56752048	0	1.40223741	0
ENSG000001	IL17RA	protein_codin	11.7201586	67.075932	1.62672634	18.4543938
ENSG000001	ZDHHC22	protein_codin	0.50794385	1.23440837	0.96278176	1.17289844
ENSG000001	UBE2O	protein_codin	29.5799358	12.3056038	129.463582	24.4636746
ENSG000001	ITPRID1	protein_codin	0.629912	2.17599595	0.48991222	7.66193516
ENSG000001	WDTC1	protein_codin	0	0	0	0
ENSG000001	ETS1	protein_codin	0	63.42738	0	69.232404
ENSG000001	LCLAT1	protein_codin	5.52531551	7.35336242	14.060818	1.73532907
ENSG000000	KARS1	protein_codin	0	2.38968816	3.1073607	7.52229667
ENSG000002	TEX35	protein_codin	0	0	0	0
ENSG000001	SHISA2	protein_codin	4.73864135	4.72430016	5.14333875	14.4600216
ENSG000001	OR6B2	protein_codin	0	0	0	0
ENSG000001	SLC35E3	protein_codin	93.9932963	21.6668584	20.757338	8.36688218
ENSG000001	CCDC93	protein_codin	32.5430102	21.2454299	18.3278707	16.0290816
ENSG000001	PCK1	protein_codin	7.06435454	7.43821116	0.48644087	10.5540391
ENSG000001	RUFY1	protein_codin	6.55064624	30.2325214	13.1803138	9.79404438
ENSG000001	SORBS2	protein_codin	0	0	0	0
ENSG000001	OTOS	protein_codin	0	0.84692934	0	0
ENSG000001	MCFD2	protein_codin	28.1431609	48.9818268	91.2671175	28.795019
ENSG000001	SORBS2	protein_codin	0	0	0	0
ENSG000001	OSBPL1A	protein_codin	7.79320832	0	19.4650596	4.32691495
ENSG000001	KREMEN2	protein_codin	0	0	0	0
ENSG000001	TMUB2	protein_codin	4.42013998	13.6073912	10.233011	5.56497112
ENSG000001	TFAP2A	protein_codin	0	0	0	0
ENSG000001	ADGRL2	protein_codin	0	0	112.896851	0
ENSG000001	CPNE7	protein_codin	0	1.21976578	4.55021947	2.06913089
ENSG000001	RPP40	protein_codin	0	0	10.5171708	0
ENSG000001	SCD5	protein_codin	18.6624331	20.3073687	22.2764338	6.32552564
ENSG000000	MRI1	protein_codin	9.5949782	14.7292573	43.6797878	0
ENSG000001	NT5DC1	protein_codin	7.76339277	11.2479235	23.0416732	14.0595201
ENSG000001	KRBA1	protein_codin	0	3.84171434	12.3149021	0
ENSG000001	ZFPM1	protein_codin	1.47794263	14.6939398	11.7922071	4.15357539
ENSG000001	TMEM31	protein_codin	0	0	2.31833598	1.96690778

ENSG000001	FARP1	protein_codin	3.27021152	11.6609527	7.38212323	12.9297845
ENSG000001	ANKDD1A	protein_codin	3.8841927	0	0	0
ENSG000000	ARID1B	protein_codin	1.91786199	0	0	0
ENSG000001	KLK12	protein_codin	0	0	0	0
ENSG000001	HTR1F	protein_codin	1.82960925	10.2013592	2.34712515	6.08161762
ENSG000000	PKM	protein_codin	3.85902814	0	0	0
ENSG000001	ZNF479	protein_codin	0.31783416	1.29729293	0	0.5093887
ENSG000001	EPHA10	protein_codin	0	0	0	0.47070626
ENSG000001	LRRC37A3	protein_codin	1.87315323	15.4265257	14.118681	0
ENSG000001	FMN2	protein_codin	0.86720072	0	0	1.40362087
ENSG000001	TMCO5A	protein_codin	0	0	0	0.47445354
ENSG000001	ARHGEF18	protein_codin	18.8736818	83.6703852	0	0
ENSG000001	DCAF4L2	protein_codin	0	0	0	1.10951439
ENSG000001	TMTC1	protein_codin	1.91583326	7.30089347	7.50894068	0
ENSG000001	KIDINS220	protein_codin	6.54972022	2.77016104	14.8728165	0
ENSG000001	PRR15	protein_codin	0	0	0	1.20379281
ENSG000001	RBBP6	protein_codin	21.1376185	40.9355238	15.1880818	50.1036525
ENSG000001	KLK11	protein_codin	0	0	0	0
ENSG000001	FUBP3	protein_codin	17.846077	96.2785946	61.7058727	28.2849406
ENSG000001	KLK11	protein_codin	0	0	0	0
ENSG000002	OR4A5	protein_codin	0	0	0	0
ENSG000001	B4GALT3	protein_codin	3.26009185	0	12.2848595	0
ENSG000001	ZNF384	protein_codin	0	17.8564721	0	3.87684312
ENSG000001	EPC1	protein_codin	8.14031794	0	36.5904963	0
ENSG000002	ZNF611	protein_codin	0	10.9399225	0	0
ENSG000001	VKORC1	protein_codin	0	2.91987767	0	0
ENSG000001	NECTIN3	protein_codin	0	0	0	0
ENSG000001	OR4C5	protein_codin	0	0	0	1.78831106
ENSG000001	RPL21	protein_codin	1.52603017	3.60553066	0.9087026	0.72734554
ENSG000001	DYRK2	protein_codin	0.39665139	5.13328831	1.74169925	0
ENSG000001	MARCHF8	protein_codin	3.4024472	22.6068957	6.69923048	9.12773536
ENSG000001	GPR35	protein_codin	0	0	0	0
ENSG000001	ZNF140	protein_codin	0	0	0	0
ENSG000001	SHROOM1	protein_codin	0	0	0	0
ENSG000001	OR4C3	protein_codin	0	0	0	0.83234702
ENSG000001	GATD1	protein_codin	22.7528777	51.860957	73.9958913	11.918403
ENSG000001	METTL4	protein_codin	0	7.11952945	27.9556328	0
ENSG000001	AIFM1	protein_codin	1.50732094	0	0	0
ENSG000001	RNF213	protein_codin	45.2930636	696.906317	67.7847954	14.8263739
ENSG000001	MOB3C	protein_codin	13.6439553	34.7420765	25.4176976	6.84992041
ENSG000001	MACIR	protein_codin	2.60887925	0	13.7696537	2.50927093
ENSG000001	RABGGTB	protein_codin	0	0	1.50438258	1.64053501
ENSG000001	RNF157	protein_codin	0	0	6.73391462	1.15051712
ENSG000000	SLC4A8	protein_codin	0	0	0	0

ENSG000001	C2orf69	protein_codin	24.3015878	15.8689042	41.7785189	14.1600865
ENSG000001	HROB	protein_codin	0	10.8436791	0	0
ENSG000000	IFT88	protein_codin	0	0	10.1855293	0
ENSG000001	OR4S1	protein_codin	0	0	0	0
ENSG000001	OR2G3	protein_codin	0	0	0	1.26148978
ENSG000001	CNGB3	protein_codin	0	0	0	4.22199917
ENSG000001	SCAP	protein_codin	0	0	2.93935128	2.66997646
ENSG000001	HSPH1	protein_codin	28.6362254	19.9089407	32.4001956	30.4968604
ENSG000000	ITGA3	protein_codin	3.94478515	26.7856428	7.82699499	7.32628658
ENSG000001	ARL4D	protein_codin	5.66064893	21.5924995	2.56614588	7.3266987
ENSG000001	OR4X1	protein_codin	0	1.95990263	0	0.49642596
ENSG000001	PRPF18	protein_codin	14.1311538	48.4041946	55.4451814	5.94312971
ENSG000001	IL17RD	protein_codin	0	4.74014145	0	0
ENSG000001	GIT2	protein_codin	4.66714863	0	36.6677626	9.39422443
ENSG000001	OR2G2	protein_codin	0	0	0	0.53860303
ENSG000002	PSG11	protein_codin	0	0	0	0
ENSG000001	CLIP4	protein_codin	0	0	0	0
ENSG000001	METRNL	protein_codin	8.0304693	48.5817348	27.7522719	14.7543514
ENSG000000	LMO3	protein_codin	0	0	0	1.31569227
ENSG000001	ZNF454	protein_codin	10.2764742	0	7.05389182	2.61220438
ENSG000002	PMF1-BGLAP	protein_codin	0	1.58190921	0	0
ENSG000000	PRTFDC1	protein_codin	10.7971915	10.1155363	19.0209137	1.97334619
ENSG000001	DDI2	protein_codin	0	0	1.01507479	4.69813245
ENSG000001	ZNF48	protein_codin	0	0	7.35766967	0
ENSG000001	SGIP1	protein_codin	0	0	0	0
ENSG000001	TENT5A	protein_codin	69.563388	180.610402	82.2162923	136.81306
ENSG000001	FGF8	protein_codin	0	0	0	0
ENSG000000	PITPNM2	protein_codin	6.30040011	21.5704926	3.9093147	0
ENSG000001	RBPMS	protein_codin	0	0	0	3.47434553
ENSG000001	ATRIP	protein_codin	0	0	0	3.21814548
ENSG000001	ITGB2	protein_codin	11.9530463	171.480279	13.6973392	16.9472923
ENSG000001	ZDHHC20	protein_codin	0	0	11.2806796	0
ENSG000002	RNPS1	protein_codin	83.5812372	101.336307	387.341251	45.0387403
ENSG000001	SLC25A22	protein_codin	0	0	0	0
ENSG000001	NHLH2	protein_codin	0.92460035	0	0	0
ENSG000001	TFEC	protein_codin	0	0	5.88700908	0
ENSG000001	FOXL1	protein_codin	1.37055111	5.21449872	17.0339486	10.8794427
ENSG000001	NEK6	protein_codin	21.4556982	68.2140005	28.8253395	33.2902728
ENSG000001	KBTBD11	protein_codin	6.67374266	4.0815075	14.4439404	6.65310486
ENSG000001	LRRC37A	protein_codin	0	0	0	0
ENSG000002	ASPRV1	protein_codin	0.86812105	4.99092105	1.62892131	0.97098367
ENSG000001	DNAI7	protein_codin	0	0	0	0
ENSG000001	RRS1	protein_codin	8.22922968	25.8167929	26.1452539	6.92123112
ENSG000000	SCAMP1	protein_codin	12.6708009	23.1745827	43.6339877	0

ENSG000002	AGPAT4	protein_codin	12.2019641	0	21.5162754	17.61359
ENSG000001	HYAL1	protein_codin	0	0	0	0
ENSG000001	PCDH15	protein_codin	0	0	0	0
ENSG000001	TAGLN2	protein_codin	40.1884318	146.014104	83.3252078	18.4884485
ENSG000000	PHF23	protein_codin	0	52.2405886	0	0
ENSG000002	PAGR1	protein_codin	12.0781684	50.2078051	31.2366936	15.7986966
ENSG000001	OLFML3	protein_codin	86.5941862	314.424109	92.3272126	155.883321
ENSG000000	STEEP1	protein_codin	2.68383312	0	0	1.77556122
ENSG000001	INPP5K	protein_codin	0	0	1.40090222	0
ENSG000001	EZH2	protein_codin	0	0	10.3661124	0.84142442
ENSG000001	SLC25A19	protein_codin	0	0	0	0
ENSG000001	ITPRID2	protein_codin	23.8074395	108.044522	0	6.46581194
ENSG000001	KIF1A	protein_codin	0	0	0	0
ENSG000000	BCKDHB	protein_codin	10.786646	23.6515201	18.7122377	9.35735991
ENSG000001	IQCK	protein_codin	0	0	0	0
ENSG000000	ELN	protein_codin	2.82106368	5.53891902	0	0
ENSG000001	AMIGO3	protein_codin	1.18935265	0	0	0
ENSG000001	CARF	protein_codin	0	0	14.1005877	0
ENSG000001	ZNF121	protein_codin	49.9279467	109.601068	272.949934	21.2194554
ENSG000001	GLIPR1L2	protein_codin	0.4277388	0	1.42660615	0
ENSG000000	TP63	protein_codin	0	6.67379495	0	0
ENSG000001	B3GALNT1	protein_codin	0	0	0	0
ENSG000001	SCRIB	protein_codin	24.89583	97.0466813	209.966034	23.1543825
ENSG000001	CIDEA	protein_codin	0.58897737	0	0	0
ENSG000000	ELN	protein_codin	0	0	0	0
ENSG000002	CFHR1	protein_codin	0	0	0	0
ENSG000001	CCDC65	protein_codin	0	0	0	0
ENSG000001	SLC16A8	protein_codin	0	0	0	0
ENSG000001	TMEM270	protein_codin	0	0	0	0
ENSG000001	MBOAT4	protein_codin	0	0	0	0
ENSG000001	GPS1	protein_codin	0	0	9.39592632	6.75228491
ENSG000001	ZNF596	protein_codin	0	4.24083358	15.7704068	0
ENSG000001	ANO6	protein_codin	122.573954	135.371331	0	50.1121639
ENSG000001	KDF1	protein_codin	0	0	0	1.34333193
ENSG000001	POLE	protein_codin	4.47533085	0	0	14.4902055
ENSG000001	RAB39A	protein_codin	1.62408794	6.05643573	2.52781417	0.47415688
ENSG000001	PACS1	protein_codin	31.5076789	85.1484309	63.3004193	30.6967932
ENSG000001	ZNF655	protein_codin	0	5.38336232	0	0
ENSG000001	ANKRD30B	protein_codin	0	0	0	0
ENSG000001	CHORDC1	protein_codin	12.9535378	31.7188891	42.4309065	22.2588122
ENSG000001	PIANP	protein_codin	0.27579844	0	1.09783183	0
ENSG000000	ZDHHC8	protein_codin	3.63367781	23.6391541	19.203798	2.9138106
ENSG000001	ZNF236	protein_codin	10.128012	24.9800062	4.391068	21.218657
ENSG000001	ZNRF1	protein_codin	0	0	6.17113192	0

ENSG000001	NQO1	protein_codin	1.30220394	6.37376029	0	5.97942148
ENSG000001	EHD1	protein_codin	1.71937743	0	1.8248532	25.9354579
ENSG000001	FAIM2	protein_codin	0.54435129	0	0	1.56587959
ENSG000001	AKAP5	protein_codin	0	21.5122003	13.7925528	0
ENSG000001	ITFG1	protein_codin	26.85969	58.8087023	107.349977	16.0260452
ENSG000001	IL15	protein_codin	0	4.43833878	0	0
ENSG000001	CHRM2	protein_codin	0	0	1.53463461	0
ENSG000001	KYAT1	protein_codin	0	0	0	0
ENSG000001	SSBP2	protein_codin	11.7504277	0	9.28701557	11.9678165
ENSG000001	RBMX	protein_codin	43.6117764	90.3879618	126.090245	37.0812249
ENSG000001	INPPL1	protein_codin	0.70655573	0	0	0
ENSG000001	PIK3R1	protein_codin	1.71145443	2.32879449	7.39083695	1.17265365
ENSG000001	DUS3L	protein_codin	0	0	0	1.07552087
ENSG000001	DNASE1L2	protein_codin	0	0	0	0
ENSG000001	TSPY2	protein_codin	0	0	0	0
ENSG000001	OR6S1	protein_codin	0	0	0	0
ENSG000001	GLS	protein_codin	0	0	0	0
ENSG000001	PELI3	protein_codin	0	1.23026296	0	0
ENSG000001	ADIPOQ	protein_codin	49.0172171	50.6780334	0.48208714	22.9145987
ENSG000001	LRIG3	protein_codin	500.274372	34.9877874	35.4742865	16.1212241
ENSG000001	UEVLD	protein_codin	0	0	0	0
ENSG000001	COL9A1	protein_codin	0	0	0	0
ENSG000001	MAGOHB	protein_codin	3.477367	0	22.8062931	3.56283142
ENSG000001	ARL14	protein_codin	0	0	0	0
ENSG000001	BLOC1S4	protein_codin	1.68591602	15.2087386	5.20453048	0.83144448
ENSG000001	KIF1C	protein_codin	122.11844	395.696776	178.302544	78.9977085
ENSG000001	NFATC2IP	protein_codin	16.8687827	43.9760394	41.1277065	11.4242784
ENSG000001	DAB2	protein_codin	119.696098	88.6820122	40.856409	184.287647
ENSG000001	KLK8	protein_codin	0	0.71068044	0	0.50390082
ENSG000001	MRFAP1L1	protein_codin	80.486048	194.279989	338.618427	33.0630707
ENSG000001	AAR2	protein_codin	6.82793181	7.59249958	10.5775159	5.59390038
ENSG000001	SSR4	protein_codin	0	0	0.55855624	0.99468615
ENSG000001	B3GNTL1	protein_codin	0	14.67918	7.4213104	3.18256741
ENSG000002	HBA1	protein_codin	0.83115685	35.8441956	0	1.59852937
ENSG000001	SMCHD1	protein_codin	70.3232357	58.5038375	183.639721	42.4423066
ENSG000001	RNF144A	protein_codin	29.2615795	29.4115851	87.8713206	17.9224481
ENSG000000	CLPTM1L	protein_codin	8.48722212	33.7864451	187.287568	1.12964214
ENSG000001	OR4F21	protein_codin	0	0	0	0
ENSG000001	MRFAP1	protein_codin	5.204706	4.78988572	3.03214978	0
ENSG000001	MRGPRF	protein_codin	0	0.88886013	0	0
ENSG000002	FMN1	protein_codin	2.0302705	0	1.30487207	2.81149659
ENSG000001	PCSK7	protein_codin	0.93092876	10.5838817	0	9.50561188
ENSG000000	CIRBP	protein_codin	3.57326877	0	66.6126271	9.95115307
ENSG000001	CRTAP	protein_codin	108.398016	87.2472573	104.405771	50.9099064

ENSG000001	SPTBN5	protein_codin	3.70876861	21.3298375	11.0948098	11.5391124
ENSG000001	RCN2	protein_codin	28.9444135	70.7669843	66.9211203	20.2618277
ENSG000001	SEC14L4	protein_codin	0	0	0	0
ENSG000001	ZEB1	protein_codin	40.3495149	93.7609056	56.5999409	28.4026931
ENSG000001	IRX5	protein_codin	0	0	3.29407161	0
ENSG000001	PITPNB	protein_codin	0	64.3068242	0	0
ENSG000000	IGSF9B	protein_codin	0	2.83447554	0	0
ENSG000001	VWF	protein_codin	0	0	0	0
ENSG000000	GCFC2	protein_codin	5.21127202	0	0	0
ENSG000001	PRPF31	protein_codin	40.5391198	105.370661	105.846395	28.1652939
ENSG000000	ENPP4	protein_codin	10.3918018	17.0922978	20.3674912	9.46493268
ENSG000001	VN1R1	protein_codin	0.71528651	4.6961062	0.94647011	0.91921348
ENSG000000	NR1H4	protein_codin	0	0	0	0
ENSG000002	PLXNA4	protein_codin	26.4963225	30.5635433	74.9802187	0
ENSG000001	TOP3A	protein_codin	6.09230602	32.9542199	4.84929215	7.09731423
ENSG000001	RTL3	protein_codin	1.18690126	2.21973004	0	1.67001195
ENSG000001	DNMT3A	protein_codin	16.0940473	72.226165	52.0647579	19.8411565
ENSG000001	PRORP	protein_codin	0	0	0	0
ENSG000000	HCCS	protein_codin	0	0	0	0
ENSG000000	DPP8	protein_codin	3.1887041	7.57503907	12.0267893	2.58444281
ENSG000002	DNAAF4	protein_codin	0	0	0	0
ENSG000001	RPLP2	protein_codin	396.313424	300.176486	1836.8018	393.951392
ENSG000001	HACL1	protein_codin	3.81771083	0	15.5514196	0
ENSG000001	TMTC2	protein_codin	0	43.3404304	0	0
ENSG000001	SEC14L4	protein_codin	0	0	0	0
ENSG000001	TGIF2LY	protein_codin	0.22096752	0	0	0
ENSG000000	ZNF207	protein_codin	0	36.9389682	18.2485517	0
ENSG000001	LEPROTL1	protein_codin	11.0048777	37.9964469	15.9292321	4.85726219
ENSG000001	OR6T1	protein_codin	0	0	0	0
ENSG000000	TUBA3D	protein_codin	0	0	0	0
ENSG000001	NCBP2	protein_codin	13.2498422	10.0427776	16.4232031	12.5068777
ENSG000001	D2HGDH	protein_codin	0	0	0	0.78364602
ENSG000000	NUDC	protein_codin	33.8975059	202.984325	158.213604	33.3306658
ENSG000001	SGPP2	protein_codin	0	3.13925528	3.55384528	2.1201669
ENSG000001	BAIAP2	protein_codin	0	0	13.930957	0
ENSG000001	ZNF610	protein_codin	1.06672948	11.6544113	2.78484742	0
ENSG000001	ATG16L2	protein_codin	0	0	0	4.90670538
ENSG000001	BAIAP2	protein_codin	0	3.20212717	2.85589416	0
ENSG000001	TOMM5	protein_codin	54.0625026	42.8817648	207.430311	20.9977145
ENSG000001	DSCAML1	protein_codin	0	0	0	0
ENSG000001	FOLR2	protein_codin	0	0	1.51723052	5.95228479
ENSG000001	TMEM214	protein_codin	12.8930575	25.6826748	79.3342103	17.6694119
ENSG000001	HIGD1A	protein_codin	1.61432209	2.43002018	4.23676843	2.87907724
ENSG000001	SENP3	protein_codin	16.2293417	84.92101	49.3138458	14.9605528

ENSG000001	FTMT	protein_codin	0	1.6876513	0	1.1742017
ENSG000000	ME2	protein_codin	3.69415079	3.6654189	5.95718467	3.70690062
ENSG000000	CYBRD1	protein_codin	154.225369	233.816331	171.208131	96.5702478
ENSG000001	OR8D4	protein_codin	0	0	1.01241437	0
ENSG000000	CLK1	protein_codin	83.3276563	0	29.942351	0
ENSG000000	YBX1	protein_codin	294.779261	267.194211	806.910345	270.316213
ENSG000001	SEPTIN1	protein_codin	1.11761962	0	0	0
ENSG000001	CCDC33	protein_codin	0	0	0	0
ENSG000001	RNF151	protein_codin	0	0	0	0
ENSG000001	TCP1	protein_codin	8.9494335	0	13.9627681	10.1493804
ENSG000001	GCC1	protein_codin	14.6717453	46.1435076	39.4187221	16.227156
ENSG000001	AMER3	protein_codin	1.20137119	2.17955685	0.52298071	6.13553004
ENSG000001	SIGLEC8	protein_codin	0	0	0	1.16142925
ENSG000001	DUOX1	protein_codin	0	0	4.49486938	1.45271446
ENSG000001	RAB8B	protein_codin	17.4326903	66.4614623	21.554895	21.6449163
ENSG000001	SFXN1	protein_codin	10.2945987	28.4616822	43.8909694	3.10031608
ENSG000001	XRRA1	protein_codin	0	0	0	0
ENSG000001	PDE1C	protein_codin	1.28123429	0	0	0
ENSG000001	DNAJC4	protein_codin	0	0	3.26156317	1.92673901
ENSG000001	ZBTB38	protein_codin	22.1928548	43.3282404	15.9527034	0
ENSG000001	HYDIN	protein_codin	0.57940097	0	0	1.02066945
ENSG000001	KIAA1549L	protein_codin	0	0	16.813096	0
ENSG000001	SLC25A41	protein_codin	0	0	0	0
ENSG000001	PPIP5K2	protein_codin	7.84713929	13.9382837	31.9439803	9.01185765
ENSG000001	OR52E2	protein_codin	0	2.23365838	0	1.88203171
ENSG000001	OAZ3	protein_codin	0	4.31441575	0	0
ENSG000001	FBXO39	protein_codin	1.58108275	0	0.96663449	2.15143847
ENSG000001	ZNF543	protein_codin	7.28695919	24.3055808	17.3119152	19.643613
ENSG000001	PINK1	protein_codin	25.9227976	51.4844605	30.9424032	10.0883441
ENSG000001	PLD6	protein_codin	0.78337888	3.4824682	9.81914076	4.3785884
ENSG000001	FKBP10	protein_codin	24.8576135	196.603851	71.7998481	53.6664291
ENSG000001	TTLL11	protein_codin	10.4324763	14.3157546	4.50156362	6.70174571
ENSG000001	CATSPER2	protein_codin	0	0	0	0
ENSG000001	IP6K1	protein_codin	0	11.9567533	10.7975382	0
ENSG000001	DYNAP	protein_codin	0	0	0	0
ENSG000001	GLDC	protein_codin	0	0	0.96782795	0
ENSG000001	PHYHIP	protein_codin	0	0	0	0
ENSG000001	CASKIN2	protein_codin	10.2404171	27.2077948	17.0301925	31.6845054
ENSG000001	TMEM132E	protein_codin	0	5.30443516	0	4.96955036
ENSG000001	GJD4	protein_codin	0.20278747	0	0	0
ENSG000001	GPR137C	protein_codin	0.66909832	0	0	0
ENSG000001	THAP4	protein_codin	0	0	0	0
ENSG000001	STXBP5	protein_codin	10.8959915	72.7177818	18.0839631	22.9457907
ENSG000001	DNAJC4	protein_codin	0	8.3600426	0	0

ENSG000001	SEPTIN4	protein_codin	0	0	0	0
ENSG000001	VSIG10	protein_codin	0	0	0	0
ENSG000001	B3GNT8	protein_codin	0	0	0	0
ENSG000000	STAB1	protein_codin	75.1553641	320.232481	52.2726577	89.4873396
ENSG000001	PRKCB	protein_codin	3.71907604	0	6.73462229	0
ENSG000001	C14orf39	protein_codin	4.25934228	0	14.9743556	1.40875218
ENSG000001	CNPY1	protein_codin	0	0	0	0
ENSG000001	AURKAIP1	protein_codin	0	0	0	0
ENSG000001	PHETA2	protein_codin	8.50089092	9.97561569	19.5822655	1.09274436
ENSG000000	MAT2B	protein_codin	0	81.7052051	30.8020128	0
ENSG000001	TPPP2	protein_codin	0	0	0	0
ENSG000001	SPACA4	protein_codin	0	0	0	0
ENSG000001	CA13	protein_codin	1.88533053	11.2976341	1.61440553	3.87154736
ENSG000000	SPAG5	protein_codin	17.9857865	3.143574	202.018794	5.46433555
ENSG000001	RBIS	protein_codin	0	0	0	0
ENSG000002	RBMXL1	protein_codin	0	0	0	0
ENSG000001	LMO7	protein_codin	0	0	59.9831987	0
ENSG000001	BOLL	protein_codin	0	0	0	0
ENSG000001	UBE2D3	protein_codin	0	0	0	0
ENSG000002	P2RY11	protein_codin	1.46608331	4.14832952	13.6254117	0.77157382
ENSG000001	GVQW3	protein_codin	0	5.49621806	0	3.91220642
ENSG000001	ACBD4	protein_codin	0.36239624	14.5484378	0	0
ENSG000001	ULK1	protein_codin	8.09469456	60.7232571	4.96141044	8.633701
ENSG000001	BHLHE22	protein_codin	1.57432575	7.58645231	0	1.5069416
ENSG000001	TMEM108	protein_codin	2.66489731	0	0	0
ENSG000000	PIGQ	protein_codin	0	0	0	3.08660987
ENSG000001	BPTF	protein_codin	0	0	0	0
ENSG000001	CCDC71	protein_codin	9.11519777	14.9569908	15.6709295	4.94650786
ENSG000001	VARS2	protein_codin	0	34.2449473	38.5098565	0
ENSG000001	SPATS2	protein_codin	0	0	96.4913117	19.1084335
ENSG000001	GLYR1	protein_codin	11.7249425	59.8204196	68.5409355	10.1264118
ENSG000001	SLC14A1	protein_codin	0	0	0	0
ENSG000001	CLIC2	protein_codin	1.08607233	0	0	1.39531442
ENSG000001	FCRL6	protein_codin	0	0	0	0
ENSG000001	PAK4	protein_codin	0	0	0	0
ENSG000001	ABRAXAS1	protein_codin	5.70897542	0	0	4.28971651
ENSG000001	CRTC1	protein_codin	0	0	0	8.62739975
ENSG000000	NAALAD2	protein_codin	0	0	11.8241839	0
ENSG000001	ATAD5	protein_codin	9.20817135	21.1240741	48.6680086	9.06938281
ENSG000001	COQ7	protein_codin	1.95622941	11.0592659	7.89372604	1.7635463
ENSG000001	SOX11	protein_codin	9.41409834	3.56005685	17.4893587	3.73057352
ENSG000001	CD151	protein_codin	0	0	0	0
ENSG000001	TM6SF1	protein_codin	8.75357611	0	2.66418746	2.18800676
ENSG000001	POLR2L	protein_codin	5.14446654	4.9005209	14.929907	3.23310533

ENSG000001	SET	protein_codin	148.893099	240.598003	411.114132	76.3889632
ENSG000001	TSHZ1	protein_codin	0.96047906	54.4248811	5.43661691	0
ENSG000000	ROGDI	protein_codin	0	0	30.8314555	0
ENSG000001	OR51T1	protein_codin	0	0	0	1.21318837
ENSG000000	EHD3	protein_codin	16.4223775	34.1292931	44.8466943	11.2066921
ENSG000001	PUS1	protein_codin	2.49478792	0	10.3694882	0
ENSG000001	DEFB114	protein_codin	0	0	0	0
ENSG000001	BPNT1	protein_codin	3.92968613	0	18.8012106	0
ENSG000001	PPP2R1A	protein_codin	7.16088179	37.9197254	4.2240857	12.881915
ENSG000001	NIPSNAP2	protein_codin	34.9652	42.2444022	56.3935844	18.8874785
ENSG000000	PCBP4	protein_codin	0	0	13.1567297	0
ENSG000001	OR51S1	protein_codin	0	1.33084477	0	0.51313826
ENSG000001	OR10H4	protein_codin	0	0	0	0
ENSG000001	OR51F2	protein_codin	0	0	0	2.06145594
ENSG000001	TRIM72	protein_codin	6.2916634	1.94612791	0.56414446	5.97032244
ENSG000001	TSPYL5	protein_codin	11.7828183	28.6598368	159.883628	13.8270828
ENSG000001	FAM133A	protein_codin	0	0	0	0
ENSG000001	FHL2	protein_codin	0	0	0	0
ENSG000001	LTBP3	protein_codin	59.1365759	36.2831917	151.557171	55.750568
ENSG000000	IL12RB1	protein_codin	1.31411544	2.42924327	0	0.86696495
ENSG000001	NPEPPS	protein_codin	0	0	0	0
ENSG000000	HSD17B6	protein_codin	0.63413215	3.10985696	2.28519158	0
ENSG000001	NOP2	protein_codin	0	0	0	0
ENSG000001	TPRX1	protein_codin	0	0	0	0
ENSG000001	DNPEP	protein_codin	0	0	2.89126697	0
ENSG000001	RPP25	protein_codin	6.02783063	9.01131361	31.7918783	8.67654572
ENSG000001	PANK1	protein_codin	0	0	0	0
ENSG000001	RPL10A	protein_codin	257.97562	176.575064	1135.2684	120.321195
ENSG000001	NUDT4B	protein_codin	62.3963747	28.8492431	56.4012329	14.9965235
ENSG000000	SMC1A	protein_codin	83.7571372	433.621007	1030.06404	89.254619
ENSG000001	MYPOP	protein_codin	1.18110347	8.81695484	7.67399764	2.03403122
ENSG000001	SLC49A3	protein_codin	0	0	0	0
ENSG000001	XPNPEP1	protein_codin	0	0	0	0
ENSG000001	GPR62	protein_codin	0.37406759	1.14487421	0	0
ENSG000000	UBA6	protein_codin	42.3514018	125.611977	109.570935	38.813608
ENSG000001	FAM210A	protein_codin	0	0	0	0
ENSG000001	TULP1	protein_codin	0	0.70512244	0	0
ENSG000001	MAPK3	protein_codin	0	0	0	0
ENSG000000	FAM3A	protein_codin	0	0	0	0
ENSG000002	TNFSF12	protein_codin	0	0	0	0
ENSG000001	KLHL7	protein_codin	5.35047279	18.5027003	4.78190929	1.5413726
ENSG000001	SV2C	protein_codin	0	0	10.4682119	0
ENSG000001	OR7A5	protein_codin	0	0	0	0
ENSG000001	CD244	protein_codin	0	0	0	0.31883205

ENSG000001	SSNA1	protein_codin	4.32726225	5.20596819	13.4379126	2.81690126
ENSG000001	WDR33	protein_codin	53.2922317	155.797466	229.57467	32.0144439
ENSG000001	IFT20	protein_codin	1.49102747	0	0	0
ENSG000001	RPRML	protein_codin	0	0	0	0
ENSG000001	WDR20	protein_codin	2.09173724	7.24240909	8.71977406	2.33051635
ENSG000001	PTGR3	protein_codin	16.8930711	13.5936373	37.9858311	8.43933302
ENSG000001	HORMAD1	protein_codin	0	0	0	0
ENSG000000	PNKP	protein_codin	2.20967194	0	0	0
ENSG000001	COX5A	protein_codin	1.08978665	6.1912923	3.11796373	3.96365367
ENSG000001	GCNT4	protein_codin	0	0	0	0
ENSG000001	EEA1	protein_codin	363.173098	555.479523	393.442412	166.260525
ENSG000001	EGFLAM	protein_codin	1.17176954	5.64680144	0	0
ENSG000001	SERTAD3	protein_codin	2.9499064	15.0905833	2.03984162	6.90702953
ENSG000001	ZBTB7A	protein_codin	33.1559388	139.438367	47.9957765	33.2221665
ENSG000001	CDC20B	protein_codin	0	0	0	0
ENSG000001	PRMT9	protein_codin	9.18668197	22.6264468	17.3541078	15.6706754
ENSG000001	MAF1	protein_codin	13.814708	45.0768698	35.550989	16.31705
ENSG000001	ZNF354B	protein_codin	25.6103513	25.5959289	49.2464982	9.36987979
ENSG000001	KCTD2	protein_codin	0	33.1992226	0	12.8538651
ENSG000001	EIF2S3B	protein_codin	1.53069787	3.0213886	0	0
ENSG000001	FAM124A	protein_codin	1.53158165	4.38594158	0	7.41074204
ENSG000001	COL12A1	protein_codin	1716.20984	2436.19401	17.4440401	1401.40792
ENSG000001	INPP1	protein_codin	3.0584916	15.8456636	14.7457954	3.23434789
ENSG000001	CCDC168	protein_codin	0.9595229	1.43480133	8.1979123	15.9595893
ENSG000000	SF3B2	protein_codin	214.751386	439.141817	521.297857	120.732184
ENSG000001	DDX10	protein_codin	75.1656321	204.942626	232.14719	34.2377483
ENSG000000	WNT11	protein_codin	1.00880557	0	16.3815495	0
ENSG000001	PLEKHD1	protein_codin	0	1.02925887	0	0
ENSG000001	MMP19	protein_codin	0	0	0	0
ENSG000001	PRR18	protein_codin	0	0	0.59672247	0.49522325
ENSG000000	MFAP3	protein_codin	2.6793533	0	0	4.01136264
ENSG000001	OR1E1	protein_codin	0	0	0	0
ENSG000001	SH2B1	protein_codin	1.13654298	22.6659966	0	0
ENSG000001	BASP1	protein_codin	116.556294	96.9129183	1186.55217	24.8117345
ENSG000001	RUVBL1	protein_codin	25.1602514	53.0217061	218.91033	9.66212437
ENSG000001	CEP295NL	protein_codin	10.6247773	45.94401	18.0292096	15.4755429
ENSG000001	CAMK2G	protein_codin	0	0	32.1686308	0
ENSG000001	OR52D1	protein_codin	0	0	0	0.41606534
ENSG000001	SUZ12	protein_codin	38.4645653	66.4081947	172.012158	47.284669
ENSG000001	OR52H1	protein_codin	0	3.95197903	0	0
ENSG000001	PHF8	protein_codin	10.0159689	0	22.8843116	0
ENSG000002	DIO1	protein_codin	0	0	0	1.72185364
ENSG000001	CAMK2G	protein_codin	7.07492102	0	43.2947548	4.03887852
ENSG000001	MSI2	protein_codin	0	0	0	0

ENSG000001	NHERF4	protein_codin	0	0	0	0
ENSG000001	EPM2AIP1	protein_codin	30.8532222	57.8423321	97.0970298	21.4853931
ENSG000001	NCL	protein_codin	11.8547978	24.3611379	10.5400787	17.9278022
ENSG000001	ZNF660	protein_codin	24.6009616	16.0132423	49.7656152	9.01011447
ENSG000001	ZNF18	protein_codin	0	0	1.93855592	2.46624159
ENSG000001	MICOS10	protein_codin	2.87828036	8.53542128	7.55228243	0
ENSG000001	ZYX	protein_codin	16.9972583	46.8514611	9.05309798	19.8312944
ENSG000001	NDUFV1	protein_codin	22.6030588	44.0460724	32.4889836	10.1930319
ENSG000001	PLEC	protein_codin	53.8935744	28.0916908	50.0701302	51.7009181
ENSG000000	ATOSB	protein_codin	0	0	3.72408701	0
ENSG000001	ZCCHC7	protein_codin	48.4040692	88.7473552	92.9752266	26.5960229
ENSG000001	ZNF584	protein_codin	0	3.36945553	0	0
ENSG000001	B4GALNT3	protein_codin	2.93072664	15.6042167	0	5.49982247
ENSG000001	MYL11	protein_codin	36.4110668	0	0	0
ENSG000001	CD46	protein_codin	0	0	0	0
ENSG000001	E2F7	protein_codin	0	0	8.13599226	0
ENSG000001	SEC24A	protein_codin	4.74744211	7.85186022	8.22546409	3.36788458
ENSG000001	KCNH5	protein_codin	0.31656446	0	0	5.93931528
ENSG000001	NAPG	protein_codin	10.2539556	0	4.55136497	0
ENSG000000	ACADVL	protein_codin	7.82363996	27.7111906	4.00908812	13.1456337
ENSG000001	RTBDN	protein_codin	0	0	0	0
ENSG000001	TMC6	protein_codin	0	3.44150498	0	0
ENSG000001	CD46	protein_codin	0	0	0	0
ENSG000001	ZNF335	protein_codin	16.5706991	51.828813	33.1586534	16.6584618
ENSG000001	AMBN	protein_codin	0	0	0	0
ENSG000001	ABITRAM	protein_codin	5.48948432	11.5621633	11.6766966	2.21952602
ENSG000001	HIC1	protein_codin	0	15.6020202	7.19640085	0
ENSG000001	MAZ	protein_codin	0	46.1423687	0	0
ENSG000001	BANK1	protein_codin	0	6.92826717	0	14.0724776
ENSG000001	UACA	protein_codin	56.4456506	338.713888	28.1054838	81.1119166
ENSG000001	FOXJ1	protein_codin	0.22317413	0	0	0.41277706
ENSG000001	ANKRD29	protein_codin	4.62393851	0	4.15759302	3.50617727
ENSG000001	PODXL	protein_codin	10.8779098	7.93481876	10.1579445	11.9913035
ENSG000001	RPS15A	protein_codin	6.26736516	0	25.2753127	5.64550379
ENSG000001	WDR45	protein_codin	0	0	0	0
ENSG000000	CDH7	protein_codin	0	0	0	0
ENSG000001	RRP7A	protein_codin	4.54640054	35.8356175	41.1108818	7.81391495
ENSG000001	MIEF2	protein_codin	0	5.71602058	0	0
ENSG000001	CACNA1F	protein_codin	0	0	0	0
ENSG000001	DUOXA2	protein_codin	0	0	0	0.47539951
ENSG000001	ZNRF2	protein_codin	18.9770425	37.3086424	33.510262	14.7040534
ENSG000001	DYRK1B	protein_codin	0	5.02893436	0	1.73871081
ENSG000001	GPR4	protein_codin	0.25524603	17.1094528	3.30544637	9.69409745
ENSG000001	PPP3CA	protein_codin	28.7451072	0	55.7837616	37.9101306

ENSG000001	OPA3	protein_codin	0	1.23505892	0	0.37832687
ENSG000001	NAP1L5	protein_codin	6.16455313	9.53544927	7.8389842	6.95565205
ENSG000000	AGBL5	protein_codin	0	12.2201736	20.6304737	2.49982767
ENSG000001	LCP1	protein_codin	55.8397316	371.777004	19.9400223	86.40977
ENSG000001	LRRC61	protein_codin	0	0	0	0
ENSG000001	SPNS1	protein_codin	0	0	0	0
ENSG000001	TSPEAR	protein_codin	0	0	0	1.94122366
ENSG000000	SULT2B1	protein_codin	0	0	0	0.52872563
ENSG000002	SPATS1	protein_codin	0	0	0	0
ENSG000001	CYP11B2	protein_codin	0	4.46172081	0.56578104	0.83164758
ENSG000001	NTRK2	protein_codin	0	0	0	0
ENSG000000	ATP11B	protein_codin	42.2830621	152.293953	88.9659567	40.6346934
ENSG000001	TMEM67	protein_codin	0	0	0	0
ENSG000001	TPM3	protein_codin	0	0	0	0
ENSG000001	ZNF491	protein_codin	7.61266249	6.4770295	6.42592814	1.37426484
ENSG000001	PHF21A	protein_codin	6.60124641	0	0	0
ENSG000001	GCAT	protein_codin	0	0	0	2.3937164
ENSG000001	TMEM102	protein_codin	0	0	0	0.37791393
ENSG000001	EIF3M	protein_codin	0	0	8.13409438	9.36790394
ENSG000001	TYMS	protein_codin	0	20.7176285	0	0
ENSG000001	PRR7	protein_codin	0	0	0	0
ENSG000001	TYMS	protein_codin	0	3.12845124	20.5437003	0
ENSG000001	TMEM63B	protein_codin	0	0	7.65727889	0
ENSG000001	TYMS	protein_codin	18.7735901	0	59.7973895	7.03800025
ENSG000001	INTS11	protein_codin	0	0	1.68685616	0
ENSG000001	LRSAM1	protein_codin	0	0	0	0
ENSG000001	MOB4	protein_codin	14.6258047	8.11973169	10.7245353	15.5101732
ENSG000001	CLDN22	protein_codin	0	0	0	0
ENSG000002	GH1	protein_codin	0	0	0	0
ENSG000001	C10orf67	protein_codin	0	0	0	1.35775075
ENSG000001	SLC14A2	protein_codin	0	0	0	0.53233996
ENSG000001	RPL7A	protein_codin	157.016156	33.2284659	1199.97531	162.989751
ENSG000001	SLC17A8	protein_codin	0	0	0	4.2066236
ENSG000001	SMARCD2	protein_codin	0	28.1607634	4.35787106	0
ENSG000001	ANKZF1	protein_codin	0	0	0	0
ENSG000001	PIPOX	protein_codin	0	0	0	0
ENSG000001	NR2C2	protein_codin	0	0	0	0
ENSG000001	SPHK1	protein_codin	0	0	2.80643648	2.08045532
ENSG000001	OR3A1	protein_codin	0	0	1.65115247	0
ENSG000001	ME3	protein_codin	0.92193244	0	0	0
ENSG000001	RPH3AL	protein_codin	0	0	0	0
ENSG000001	HIF1A	protein_codin	0	0	0	0
ENSG000001	LRRC57	protein_codin	1.77584419	0	3.09990413	0
ENSG000001	CPT1C	protein_codin	0	0	0	0

ENSG000001	MTMR4	protein_codin	20.0017239	75.1288525	149.743312	13.5392706
ENSG000001	SENP5	protein_codin	21.69785	75.019076	54.9332097	24.6952154
ENSG000001	POGLUT3	protein_codin	0	0	0	30.2131894
ENSG000001	DIRAS1	protein_codin	8.82338575	9.38184618	18.4415606	4.85057034
ENSG000001	ACSS1	protein_codin	10.2746002	53.4759855	12.6362634	9.53871828
ENSG000000	CASP8	protein_codin	0	0	0	0
ENSG000001	TMEM45A	protein_codin	2.55585479	0	10.1329086	2.74144156
ENSG000001	RAP2B	protein_codin	45.645129	144.087133	92.7693893	43.4168122
ENSG000002	PLEKHO2	protein_codin	31.9286313	89.3208519	37.8602978	43.5878251
ENSG000001	FBXO9	protein_codin	18.8829144	31.0739526	30.469332	10.1898289
ENSG000001	MRPS31	protein_codin	20.7010205	44.2678523	31.4450123	5.32137258
ENSG000001	ELMOD2	protein_codin	24.5339909	30.7286858	42.3296666	8.30474911
ENSG000001	WIPF2	protein_codin	20.1998718	38.0806202	15.6530697	20.2419771
ENSG000001	CHID1	protein_codin	2.4008649	9.7222292	6.6171127	0
ENSG000001	PHKB	protein_codin	4.85876806	0	0	0
ENSG000001	PPM1M	protein_codin	9.07745513	0	0	0
ENSG000001	PHYKPL	protein_codin	0	0	0	0
ENSG000001	KMT5B	protein_codin	0	0	0	0
ENSG000001	MTMR3	protein_codin	14.287015	0	33.2943067	11.753547
ENSG000001	PLAAT3	protein_codin	6.02995886	13.141663	0	10.6366457
ENSG000001	GRIA2	protein_codin	0	0	0	0
ENSG000001	WDR97	protein_codin	0	2.5692799	4.25393335	0
ENSG000001	METAP2	protein_codin	22.5674647	7.70191077	31.8090034	27.5808042
ENSG000000	TCOF1	protein_codin	0	0	50.6793113	0
ENSG000001	DDHD1	protein_codin	0	0	2.94349726	11.1309245
ENSG000001	C19orf12	protein_codin	0.94671493	0	0	0
ENSG000001	SPEM1	protein_codin	0	0	0	1.04671078
ENSG000001	CBLL2	protein_codin	0.20878917	0	0	0.94656457
ENSG000000	NUCB2	protein_codin	122.262447	394.022614	270.380315	114.665809
ENSG000001	DEGS1	protein_codin	26.7203361	36.2405803	49.9327338	17.230668
ENSG000001	USP39	protein_codin	0	62.6990116	0	0
ENSG000000	LRRC23	protein_codin	3.18624256	0	4.02692725	0
ENSG000001	ZNF513	protein_codin	4.90751728	15.7827547	10.1598879	4.69980799
ENSG000001	ARMC10	protein_codin	0	0	21.7259078	6.91660051
ENSG000001	ARMC10	protein_codin	0	4.41689706	1.79771759	0
ENSG000001	MPI	protein_codin	2.09755415	0	0	0
ENSG000001	CDC25C	protein_codin	0.84165688	0	0	0
ENSG000001	HNRNPCL4	protein_codin	0	0	0	0
ENSG000001	MYCBPAP	protein_codin	4.02043505	18.045352	0	0
ENSG000001	HSF5	protein_codin	0.60352734	4.81231488	1.08262708	1.80470199
ENSG000001	COG4	protein_codin	0	0	0	0
ENSG000001	GYS1	protein_codin	19.2490002	51.1000148	19.6096286	9.51772578
ENSG000000	GAS7	protein_codin	0	0	0	0
ENSG000001	CDK19	protein_codin	0	0	0	0

ENSG000001	TRIM73	protein_codin	0	0	0	0
ENSG000001	SH3TC2	protein_codin	0	0	0	0
ENSG000001	CPN2	protein_codin	0	3.8352625	0	13.4509723
ENSG000001	TSFM	protein_codin	47.4009434	0	20.5287759	2.48756466
ENSG000001	SRRM1	protein_codin	0	0	0	0
ENSG000001	NDRG1	protein_codin	14.7737426	0	21.8886856	0
ENSG000001	SNRNP200	protein_codin	162.41662	567.907495	473.016994	107.693069
ENSG000001	ENDOV	protein_codin	0	0.76062009	0	0.5674258
ENSG000001	HTR1A	protein_codin	0	2.12171292	0.48232671	0
ENSG000000	PXN	protein_codin	8.69287134	39.5688128	0	0
ENSG000001	PIP4K2A	protein_codin	0	0	0	0
ENSG000001	CD37	protein_codin	1.12219519	47.7777066	0	0
ENSG000001	FN1	protein_codin	0	0	0	0
ENSG000001	ANAPC2	protein_codin	8.80251556	24.7550037	20.9594054	4.39783348
ENSG000000	MRE11	protein_codin	21.8203066	58.2597623	55.0299997	21.7301668
ENSG000001	LGALS8	protein_codin	0	0	0	0
ENSG000001	STRA6	protein_codin	0	0	0	0
ENSG000001	STXBP6	protein_codin	3.62730183	0	5.31787676	0
ENSG000001	CSTF3	protein_codin	24.210078	51.1924165	95.1167685	10.0582898
ENSG000001	EIF4A2	protein_codin	0	0	0	0
ENSG000000	MRE11	protein_codin	2.80245777	84.5404324	69.6672772	0
ENSG000001	TAF1D	protein_codin	293.890635	303.803552	883.502819	113.64611
ENSG000000	ST7	protein_codin	0	0	8.52573102	0
ENSG000001	ENO3	protein_codin	14.4649777	0	0	0
ENSG000001	ST8SIA3	protein_codin	0.5090821	2.81227275	0.45350727	5.30845674
ENSG000001	PRX	protein_codin	2.29708817	0	0	0
ENSG000001	SERPINF2	protein_codin	0	0	0	0
ENSG000001	GYPA	protein_codin	0	0	0	0
ENSG000001	KCNJ1	protein_codin	0	0	0	0
ENSG000001	RFLNA	protein_codin	0	2.63461795	1.49199719	0
ENSG000001	KLK4	protein_codin	0	0	0	1.81937496
ENSG000001	ZNF687	protein_codin	0	5.21909452	1.38614432	0
ENSG000000	CAPN6	protein_codin	7.90344051	1.38771869	9.42052486	2.80886108
ENSG000001	CYP2B6	protein_codin	0	0	0	0.55352236
ENSG000002	IRF9	protein_codin	0	44.8861421	0	0
ENSG000001	TDRP	protein_codin	8.15246032	9.80086597	11.6442927	5.55246642
ENSG000000	PLXND1	protein_codin	53.2251176	363.634094	72.3344128	108.491023
ENSG000001	MAP1S	protein_codin	5.45148374	27.4063326	33.0997022	7.89543919
ENSG000000	RNF31	protein_codin	0	0	0	0
ENSG000001	CD28	protein_codin	4.75466088	29.6408958	3.84347158	8.80017145
ENSG000001	DNMBP	protein_codin	25.8754531	56.3691578	45.0513547	20.6279659
ENSG000001	NLRP12	protein_codin	0	0	0	0
ENSG000001	FANCB	protein_codin	0	0	8.30740151	0
ENSG000001	PCNX2	protein_codin	0.59000555	2.14397819	0	1.55909553

ENSG000001	TH	protein_codin	0	0	0	0
ENSG000001	THNSL2	protein_codin	1.97757387	0	6.49466842	0
ENSG000001	ZCCHC10	protein_codin	10.697994	0	7.85341253	6.78315935
ENSG000001	SAMD3	protein_codin	0	0	0	0
ENSG000001	B3GNT4	protein_codin	0	0	0	0
ENSG000001	SLC25A36	protein_codin	4.45443278	0	1.81888461	6.46827419
ENSG000001	MBNL1	protein_codin	7.96859886	0	50.2526643	7.9814422
ENSG000001	GRK3	protein_codin	24.1807971	103.918676	54.9120553	26.2186354
ENSG000001	MBNL1	protein_codin	33.0545312	0	0	83.576835
ENSG000000	MAP4K4	protein_codin	10.8081811	265.869303	49.9599977	116.465727
ENSG000001	SIDT2	protein_codin	5.10785675	0	0	1.93941151
ENSG000001	CALCB	protein_codin	0	0	1.93825099	0
ENSG000001	CRLF3	protein_codin	10.0934775	81.5263875	16.4247797	15.2980445
ENSG000001	CNDP2	protein_codin	5.12179556	6.24779233	1.06342892	4.31565067
ENSG000001	TRAPPC12	protein_codin	18.6499425	47.8878091	54.8039651	0
ENSG000001	CIITA	protein_codin	21.5331111	176.332546	1.76429202	17.8666612
ENSG000001	LGALS9B	protein_codin	0	0	0	0.34894089
ENSG000001	EDNRA	protein_codin	0	0	0	0
ENSG000001	CNDP2	protein_codin	5.78662319	56.1667427	0	0
ENSG000001	ZKSCAN1	protein_codin	112.527568	307.256864	338.534137	61.6056691
ENSG000001	CCDC149	protein_codin	2.16677563	12.4840792	11.7352113	3.32596288
ENSG000001	MYEF2	protein_codin	4.28528253	1.87405787	9.2374528	5.58919141
ENSG000001	NIN	protein_codin	0	66.6546554	0	0
ENSG000001	FARS2	protein_codin	0	0	0	2.05109643
ENSG000001	GLIS3	protein_codin	0	0	0	0
ENSG000001	DDX39A	protein_codin	0	23.9761493	0	0
ENSG000001	GARIN6	protein_codin	0	0	0	1.02750588
ENSG000001	RNF128	protein_codin	0	0	0	0
ENSG000001	DCUN1D3	protein_codin	11.8522761	17.848701	20.1097378	12.4178629
ENSG000001	RNF227	protein_codin	0	0	4.25146834	0
ENSG000001	AGO3	protein_codin	1.57681365	7.78604711	8.55293292	4.66477477
ENSG000001	CCDC121	protein_codin	6.8087451	16.9854733	8.26036186	0
ENSG000001	PRMT5	protein_codin	8.25839559	0	64.4663394	11.4416906
ENSG000000	PPIE	protein_codin	2.49576256	16.0568678	8.97621272	7.41706067
ENSG000001	H1-8	protein_codin	0.22943394	0	0	0
ENSG000001	WFDC11	protein_codin	0	0	0	0
ENSG000000	BAIAP3	protein_codin	0	0	0	0
ENSG000001	ZNF875	protein_codin	0	0	2.28220647	0
ENSG000001	SLC34A1	protein_codin	0	0	0	0
ENSG000000	DTX2	protein_codin	2.64576704	0	0	0
ENSG000001	ACTL9	protein_codin	0	0	0	0.38552819
ENSG000001	RCHY1	protein_codin	4.23718753	8.53363857	6.48720716	7.2682716
ENSG000001	SYNE4	protein_codin	0.24304645	0	0	0
ENSG000001	SAGE1	protein_codin	0	0	0	3.10497679

ENSG000001	VEGFA	protein_codin	0	0	0	0
ENSG000001	MTURN	protein_codin	38.1441657	48.011219	26.4203634	0
ENSG000000	HSPA5	protein_codin	0	0	0	2.97008764
ENSG000001	ZNF283	protein_codin	0	0	0	0
ENSG000001	COQ8B	protein_codin	0	0	0	0
ENSG000001	DPY19L2	protein_codin	8.57190808	7.20067313	22.4832081	6.03154817
ENSG000001	MTURN	protein_codin	0	0	16.8639261	22.8835804
ENSG000000	PSEN1	protein_codin	0	0	0	0
ENSG000001	SH3GL3	protein_codin	0	0	0	0
ENSG000001	CIZ1	protein_codin	0	0	0	0
ENSG000001	USP9X	protein_codin	0	0	36.3745337	35.3630904
ENSG000001	USHBP1	protein_codin	2.65585366	0	0	0
ENSG000002	EEF1AKMT4	protein_codin	0.47515672	2.17827227	3.00804972	0.73585413
ENSG000001	ANO5	protein_codin	0	9.29894914	0	0
ENSG000001	ATF7IP2	protein_codin	0	0	0	0
ENSG000001	MYO5B	protein_codin	4.85231843	2.3391061	36.2709821	0
ENSG000001	GALNT14	protein_codin	0	0	0	0
ENSG000001	ZNF410	protein_codin	14.9760066	51.2511168	40.9471202	20.4274175
ENSG000001	LILRB1	protein_codin	11.901955	43.8177712	6.71794829	7.44343876
ENSG000001	MBOAT1	protein_codin	5.450751	16.2224716	35.8968725	4.94168632
ENSG000001	REDIC1	protein_codin	0.7670792	0	0	0
ENSG000001	CACNB2	protein_codin	2.05275002	2.9473205	8.42122572	0
ENSG000001	CYP4F12	protein_codin	0	1.05289691	0	0.79187556
ENSG000001	SLC6A18	protein_codin	0	0	0	0.39976772
ENSG000001	NLRC3	protein_codin	0	0	0	0
ENSG000001	CD19	protein_codin	0	0	0	0
ENSG000001	FAM193A	protein_codin	6.8771468	0	5.85138881	0
ENSG000001	FAM187B	protein_codin	0	0	0.48736377	0
ENSG000001	SSH2	protein_codin	0	0	0	9.48368157
ENSG000001	SAV1	protein_codin	28.0576172	63.1520048	37.9109787	28.1617955
ENSG000001	PPP1R42	protein_codin	0	0	0	0
ENSG000001	PRPSAP1	protein_codin	15.2173721	40.256442	90.9037878	13.3059641
ENSG000001	RNF40	protein_codin	0	0	44.9781694	14.2094566
ENSG000001	RNF135	protein_codin	0	20.8221212	0	4.39433166
ENSG000001	TRPM8	protein_codin	0	0	0	0
ENSG000001	ASCC3	protein_codin	10.9089188	5.7664311	0	0
ENSG000001	IQUB	protein_codin	0	0	7.21094594	0
ENSG000001	ASCC3	protein_codin	0.65686428	0	0	0
ENSG000001	SCN4B	protein_codin	0	0	13.2910106	13.5030067
ENSG000001	MC5R	protein_codin	0	0	0	0
ENSG000001	TAF7L	protein_codin	0	0	0.90628472	1.91143679
ENSG000001	DIAPH2	protein_codin	19.8352094	41.7147158	17.1817626	22.1100745
ENSG000001	SCAPER	protein_codin	0	72.9370086	0	17.5217619
ENSG000001	CREG2	protein_codin	0.81303146	0	1.51242547	4.66846559

ENSG000002	DDR1	protein_codin	0	0	0	0
ENSG000001	CAMKK2	protein_codin	0	0	0	0
ENSG000001	RALGPS2	protein_codin	1.3930751	0	0	0
ENSG000000	MRPS33	protein_codin	3.53307235	3.31373488	6.95211826	2.64377508
ENSG000000	PIAS2	protein_codin	10.7518243	46.7673492	21.8928271	4.99326139
ENSG000002	PPT2	protein_codin	0	6.09457428	0	1.47784415
ENSG000001	MED14	protein_codin	32.5097882	120.285676	92.3378916	27.2861146
ENSG000001	PLSCR3	protein_codin	0	0	0	0
ENSG000001	VPS37A	protein_codin	12.1496117	0	8.4528384	2.48510789
ENSG000001	FCAMR	protein_codin	0	0	0	2.06182219
ENSG000001	ARID1A	protein_codin	9.85987254	141.921363	63.1256123	9.04274954
ENSG000001	PRPF3	protein_codin	66.9259242	110.424439	117.014149	40.6950155
ENSG000001	FCAMR	protein_codin	0	0	0	0
ENSG000001	LRRC51	protein_codin	0	0	0	0.8847504
ENSG000001	YARS2	protein_codin	3.16859017	15.5833604	14.0510279	3.70284683
ENSG000000	METTL1	protein_codin	28.6822554	0	0	0
ENSG000001	NUPR1	protein_codin	11.5605084	65.5913936	10.9958053	16.1747498
ENSG000001	GTPBP3	protein_codin	0	13.4494855	0	0
ENSG000001	GRHL1	protein_codin	0	0	2.8199169	4.31307427
ENSG000001	CHCHD5	protein_codin	1.24988104	0	0	0.45738998
ENSG000001	SPATA31C2	protein_codin	0	0	0	3.12746009
ENSG000001	NUP98	protein_codin	0	0	0	0
ENSG000000	LIMS2	protein_codin	0	0	0	0
ENSG000001	VPS37D	protein_codin	0	0	2.48846454	0
ENSG000001	CD164	protein_codin	27.0797693	12.874798	20.8355417	0
ENSG000000	SMARCA2	protein_codin	0	0	0	0
ENSG000001	SLC22A1	protein_codin	0	0	0	0
ENSG000001	DYNC1I1	protein_codin	0	0	0	0
ENSG000001	AQP10	protein_codin	0	0	0	0
ENSG000001	PSMG4	protein_codin	2.2600679	0	0	0
ENSG000000	CRMP1	protein_codin	0	0	0	0
ENSG000001	AHCYL2	protein_codin	19.6915518	10.68741	31.1795644	0
ENSG000001	NKX2-6	protein_codin	0	0	0.44403459	1.16056295
ENSG000001	MTSS1	protein_codin	3.38341581	46.4569751	0	10.0654081
ENSG000000	PCM1	protein_codin	32.9096908	52.4110836	12.5035728	20.7219637
ENSG000001	SLITRK5	protein_codin	0.3550464	0	34.9430099	4.49043632
ENSG000001	MIF4GD	protein_codin	0	0	6.55027948	0
ENSG000001	HPS1	protein_codin	0	0	0	0
ENSG000001	TTC29	protein_codin	0	0	0	0
ENSG000001	HMGCS1	protein_codin	3.043257	0	1.16056011	0.89134273
ENSG000001	CIMAP1A	protein_codin	0	0	0	0
ENSG000001	VIPR1	protein_codin	0	0	0	4.58102305
ENSG000001	CYTH2	protein_codin	0	0	0	0
ENSG000001	ZBTB2	protein_codin	14.3003649	20.243905	17.7804548	30.3331228

ENSG000001	BET1L	protein_codin	0	0	1.49407367	0
ENSG000001	ENTHD1	protein_codin	0	0	0	1.28598673
ENSG000001	CHMP6	protein_codin	5.29244242	7.91487456	11.9196211	7.60936244
ENSG000001	GGA1	protein_codin	4.11471149	19.9195592	1.86345652	0
ENSG000001	C14orf28	protein_codin	0	2.5709326	5.82450831	1.20580415
ENSG000000	ANGPT2	protein_codin	7.17023215	0	10.8863945	30.8473763
ENSG000001	RIC8A	protein_codin	22.9435961	0	53.3627508	0
ENSG000001	CEP295	protein_codin	55.919785	130.381464	293.619229	19.408108
ENSG000001	PCTP	protein_codin	0	0	0	0
ENSG000001	ATXN2L	protein_codin	0	0	0	0
ENSG000001	ZNF7	protein_codin	0	0	0	0
ENSG000000	CUL1	protein_codin	0	0	0	0
ENSG000001	CFAP410	protein_codin	0.8446729	21.8475953	3.44622343	3.76411372
ENSG000001	C8orf34	protein_codin	0	0	1.10412255	0.92008291
ENSG000001	PLCB3	protein_codin	0	0	0	0
ENSG000001	WDFY4	protein_codin	21.5319546	323.339333	7.88168924	28.1629236
ENSG000001	MAGEB18	protein_codin	0	0	0	0.86385207
ENSG000001	MTHFD2L	protein_codin	0	0	0	0
ENSG000001	KLHL36	protein_codin	20.2595136	32.855034	0	17.1775301
ENSG000001	FBXW5	protein_codin	10.1547996	0	0	13.496655
ENSG000001	MIEF1	protein_codin	16.7890425	28.3688946	59.8453861	14.8178538
ENSG000001	CDK20	protein_codin	0	0	0	0
ENSG000000	HMGB3	protein_codin	12.0705407	18.7056489	56.168164	10.9445091
ENSG000002	DYNLT2B	protein_codin	0	0	4.7823497	0
ENSG000001	NELL1	protein_codin	0	0	0	0
ENSG000001	KLK2	protein_codin	0	0	0	0
ENSG000000	IPO11	protein_codin	18.9288416	0	54.8102334	5.0615546
ENSG000001	LMNB2	protein_codin	31.6793769	149.392502	320.173362	10.964706
ENSG000002	ZNF580	protein_codin	1.61303913	0	0	0
ENSG000001	STARD5	protein_codin	0	0	0	0
ENSG000001	ZNF784	protein_codin	1.38761251	3.19385562	1.19919688	0.45172459
ENSG000000	C5orf22	protein_codin	12.6961796	0	51.2699753	9.88747999
ENSG000001	DIS3L2	protein_codin	0	0	0	0
ENSG000001	SOX2	protein_codin	0	0	23.0853257	0.46741624
ENSG000001	SLC47A2	protein_codin	0	0	0	0
ENSG000001	HASPIN	protein_codin	1.64370114	6.78679893	23.7713303	2.9393323
ENSG000002	ZNF579	protein_codin	1.79138969	12.4058837	4.75070539	1.25661904
ENSG000001	SCNN1D	protein_codin	0.72325385	0	0	0
ENSG000001	UBE2I	protein_codin	0	0	0	0
ENSG000001	PACS2	protein_codin	0	0	0	0
ENSG000000	PGR	protein_codin	2.37804817	13.920925	30.4472436	7.12275585
ENSG000001	MED16	protein_codin	0	0	0	0
ENSG000001	LARGE2	protein_codin	0	0	0	0
ENSG000001	CEP57	protein_codin	0	0	67.4332211	13.2570699

ENSG000000!	HNRNPM	protein_codin	152.087167	269.415117	383.690889	101.210544
ENSG000001	MSC	protein_codin	2.67622779	8.62291004	0.96099356	5.14933818
ENSG000001	CEP57	protein_codin	13.5761106	0	0	0
ENSG000001	CTNNAL1	protein_codin	17.2844934	23.9693676	43.465574	45.5029277
ENSG000002	PGA3	protein_codin	0	0	0	0
ENSG000000	IL32	protein_codin	0	0	0	0
ENSG000001	RAD1	protein_codin	4.67420566	0	57.3111124	0
ENSG000001	RABGAP1L	protein_codin	0	0	0	0
ENSG000000	AMPH	protein_codin	7.33609391	15.329824	25.3240259	1.00920082
ENSG000001	SHQ1	protein_codin	6.2490038	7.78966517	17.4636082	0
ENSG000001	P2RY13	protein_codin	5.58767755	4.06564676	0.95940245	4.47079626
ENSG000001	CLGN	protein_codin	1.37547624	0	53.2291872	0.51395227
ENSG000001	SPINK6	protein_codin	0	0	0	0
ENSG000001	MARVELD2	protein_codin	0	1.16441473	18.9385503	0
ENSG000001	C11orf71	protein_codin	0	0	0	0
ENSG000001	PPM1A	protein_codin	0	0	23.6451085	0
ENSG000001	SPATA31E1	protein_codin	0	5.68873297	0	3.27626339
ENSG000001	CABP4	protein_codin	2.18555362	9.54962207	2.05006382	2.6506879
ENSG000001	PPM1A	protein_codin	14.1310153	10.2088536	19.1051506	18.3463595
ENSG000003	CAST	protein_codin	0	0	0	0
ENSG000001	YLPM1	protein_codin	74.4992136	224.519995	184.421337	65.5592677
ENSG000001	LACC1	protein_codin	50.4145351	56.9526395	32.520083	29.5763896
ENSG000001	OR52K2	protein_codin	0	0	0	1.67324181
ENSG000001	KIAA0319L	protein_codin	0	0	7.26133356	5.22357076
ENSG000001	CYP4F8	protein_codin	0	0	0	0
ENSG000001	CEP152	protein_codin	24.7860916	32.4943163	149.109466	3.93390703
ENSG000001	PRKRA	protein_codin	17.1845212	0	6.60233186	0
ENSG000001	TUBD1	protein_codin	4.14552245	15.9678433	39.8128381	2.20700286
ENSG000001	ORMDL1	protein_codin	0	0	6.01045728	1.15911892
ENSG000001	BBX	protein_codin	65.6163735	51.9439459	171.25401	37.9193546
ENSG000001	MRPS11	protein_codin	6.42466922	11.8647163	22.8406403	7.63468933
ENSG000001	DMRTA1	protein_codin	4.18972381	9.60700912	34.3654522	1.79588376
ENSG000001	RMI1	protein_codin	0	0	5.75605968	0
ENSG000001	SNX22	protein_codin	0	0	0	0
ENSG000000	ASB4	protein_codin	3.27903596	0	0	0
ENSG000001	FLNC	protein_codin	22.5390277	0	0	0
ENSG000001	CLVS1	protein_codin	0	0	0	0
ENSG000001	PRDM7	protein_codin	0.70881223	0	0	0
ENSG000001	RPRM	protein_codin	0	0.86534362	7.93647595	0
ENSG000002	NEU4	protein_codin	0	0	0	0
ENSG000001	FRAS1	protein_codin	0	6.80573256	0	0.95569019
ENSG000001	HAO2	protein_codin	0	0	0	0
ENSG000000	RNF10	protein_codin	20.1943833	51.4096492	29.1588388	22.3093595
ENSG000000	CLEC2D	protein_codin	1.91398406	0	0.93470537	7.46661655

ENSG000001	MEIOB	protein_codin	0	0	11.7532019	0
ENSG000001	CEP192	protein_codin	19.094041	84.4555826	108.927399	10.0557685
ENSG000001	CCZ1	protein_codin	40.887069	46.0329597	203.467943	14.3452983
ENSG000001	IQCE	protein_codin	0	0	0	0
ENSG000001	VENTX	protein_codin	0.61982938	1.02257353	0	0.85956126
ENSG000001	KLHL38	protein_codin	1.09689523	1.22980381	0	0
ENSG000001	IQCE	protein_codin	13.6991886	0	18.1503723	0
ENSG000001	WFDC9	protein_codin	0	0	0	0
ENSG000001	KLK3	protein_codin	0	0	0	0
ENSG000001	HMGB1	protein_codin	18.6573828	98.170162	92.4778344	41.1690425
ENSG000001	OAZ2	protein_codin	39.4757638	35.786225	91.644605	22.5715061
ENSG000001	DCAF11	protein_codin	0	0	0	0
ENSG000001	UPB1	protein_codin	0	2.25658551	0	0
ENSG000001	ARHGEF4	protein_codin	0	0	0	0
ENSG000001	SLC4A1AP	protein_codin	52.2061537	88.5400914	122.342226	29.062956
ENSG000001	NIM1K	protein_codin	0	0	0	2.11077965
ENSG000001	GRTP1	protein_codin	0.60088003	0	2.140552	0
ENSG000001	MAF	protein_codin	18.4213193	37.0514004	16.926516	9.96350534
ENSG000001	ZNF561	protein_codin	1.99965888	0	10.0868099	0
ENSG000001	KIF15	protein_codin	11.3381922	50.6776889	123.435663	3.73003873
ENSG000001	C11orf16	protein_codin	0	0	0	0
ENSG000001	NOL4L	protein_codin	0	1.1819389	2.66761441	0
ENSG000001	FAXDC2	protein_codin	6.76778176	30.3793174	2.87301042	14.6604706
ENSG000001	RPL21	protein_codin	0.87991701	0	1.22911759	0
ENSG000001	ISCA1	protein_codin	0.83176715	0	0	0
ENSG000001	RNF185	protein_codin	11.5773834	0	0	0
ENSG000001	MROH1	protein_codin	0	0	0	0
ENSG000001	GHRHR	protein_codin	0	0	0	0
ENSG000001	NFKBIZ	protein_codin	18.5534897	0	11.7188997	0
ENSG000001	HSD11B2	protein_codin	0.59980787	6.66426778	0.57824468	1.32123082
ENSG000001	GTPBP6	protein_codin	4.24897315	126.947553	27.2399297	8.05439884
ENSG000001	CD300LF	protein_codin	0	0	0	0
ENSG000001	NFKBIZ	protein_codin	0	11.2580934	12.0186378	206.410621
ENSG000001	TRAF7	protein_codin	11.8801634	15.7980377	33.838734	4.68405483
ENSG000001	SLC22A24	protein_codin	0	0	0	0
ENSG000001	PORCN	protein_codin	0	0	0	0
ENSG000002	ABCF1	protein_codin	3.32842505	0	57.9553399	0
ENSG000000	SCMH1	protein_codin	0	0	4.52448366	0
ENSG000001	TAC4	protein_codin	0	0	0	0
ENSG000001	AHCTF1	protein_codin	31.969025	134.352224	144.014842	87.8434576
ENSG000001	SLC37A3	protein_codin	10.5998	0	31.3663536	9.77263892
ENSG000001	LCE1D	protein_codin	0	0	0	0
ENSG000001	ITLN1	protein_codin	0	0	0	0
ENSG000001	OR10A7	protein_codin	0	0	0	0.52561162

ENSG000001	THOC6	protein_codin	0	0	21.4455822	0
ENSG000001	TLK2	protein_codin	6.6909648	30.7800407	34.2413064	7.75968313
ENSG000001	VGLL2	protein_codin	0	0	6.29088074	0
ENSG000001	SNX18	protein_codin	1.05650558	0	0	0
ENSG000001	LIN28A	protein_codin	0.33639269	0	0	0
ENSG000001	EID2B	protein_codin	2.37170651	3.50497337	5.98842531	3.02754275
ENSG000000	PIBF1	protein_codin	63.7376433	0	104.98343	20.5448569
ENSG000002	PTPRCAP	protein_codin	5.3745903	75.3134971	0	1.75415798
ENSG000001	CCNC	protein_codin	0	0	0	0
ENSG000001	ACOT4	protein_codin	0	0	0	0
ENSG000001	CCR8	protein_codin	0	2.91629977	0	0
ENSG000001	SGSH	protein_codin	1.46662118	0	0	2.71588917
ENSG000002	KIR3DL2	protein_codin	0	2.34789799	0	0
ENSG000000	FGFR1	protein_codin	0	0	0	32.5293057
ENSG000001	CUL4A	protein_codin	0	0	292.592432	39.5809688
ENSG000001	WASHC3	protein_codin	0	4.51050865	0	0
ENSG000001	ZNF639	protein_codin	1.90049986	12.1488132	0	10.7699785
ENSG000001	RWDD4	protein_codin	18.3922527	4.10188707	43.5679863	2.31642806
ENSG000001	SLC36A4	protein_codin	9.46211858	9.46893494	24.0642992	7.55163998
ENSG000001	ITM2C	protein_codin	0	0	4.85671485	0
ENSG000001	RIIAD1	protein_codin	0	0	0	1.32033863
ENSG000001	ITM2C	protein_codin	29.4082172	125.454029	80.3660572	18.2938543
ENSG000001	CRELD1	protein_codin	0	0	0	0
ENSG000001	PEG3	protein_codin	3.18917432	0	0	0
ENSG000001	TMEM176B	protein_codin	10.3575139	124.267339	0	14.5384575
ENSG000001	ARL6IP6	protein_codin	1.94516553	0	3.3948112	3.97531729
ENSG000000	IFT80	protein_codin	0	12.67449	0	16.3070196
ENSG000000	SSH1	protein_codin	0	4.18479202	1.34686722	4.74922219
ENSG000001	C3orf80	protein_codin	2.3219544	3.29229132	0	0.89311896
ENSG000000	SSH1	protein_codin	18.9411677	25.3074868	8.95067874	36.3250203
ENSG000001	VNN2	protein_codin	0	0	0	2.95895724
ENSG000001	B3GNT5	protein_codin	5.15704741	1.49588875	16.5283002	82.6213083
ENSG000001	GDNF	protein_codin	0	0	0	2.59343315
ENSG000001	COX6B2	protein_codin	0	0	0	0.37327771
ENSG000001	KIR3DL1	protein_codin	0	1.27320113	0	0.43019078
ENSG000001	SPAG11A	protein_codin	0	0	0	0
ENSG000001	GPR139	protein_codin	0	0	0	0
ENSG000001	KDSR	protein_codin	0	0	0	1.82917405
ENSG000000	TNFRSF12A	protein_codin	0.48644164	0	0	0
ENSG000001	C4orf17	protein_codin	0.67344439	0	0	2.91063234
ENSG000001	MAGED1	protein_codin	33.3408974	299.517772	320.988863	52.2832522
ENSG000001	CCDC66	protein_codin	3.41899196	0	0	0
ENSG000001	GGA1	protein_codin	2.64975758	16.9201739	4.37168815	8.75050814
ENSG000000	VSIG2	protein_codin	0	0	0	0.79102863

ENSG000001	ZBTB33	protein_codin	7.98702286	39.8529647	31.2693247	0
ENSG000001	SPAG11A	protein_codin	0	0	0	0
ENSG000001	PLEKHJ1	protein_codin	3.34544218	0	13.7858392	0
ENSG000001	TNNI3K	protein_codin	1.92420578	0	2.32853335	0
ENSG000001	LARP1B	protein_codin	0	0	9.42801923	0
ENSG000001	ZNF609	protein_codin	47.5444305	223.908302	176.079166	74.3884308
ENSG000001	TMEM150B	protein_codin	0	0	0	0
ENSG000001	ERGIC1	protein_codin	2.26657915	0	0	0
ENSG000001	PTPMT1	protein_codin	5.31278214	10.1350264	6.99164659	4.6178308
ENSG000001	ERICH3	protein_codin	2.6499908	4.85980672	1.38959275	0
ENSG000001	SUMO4	protein_codin	4.70396481	23.1565791	11.1944855	2.40877965
ENSG000001	PTPMT1	protein_codin	0	6.41896392	0.63345128	6.76439222
ENSG000000	ABCC9	protein_codin	0	0	0	0
ENSG000001	ABLIM3	protein_codin	17.8131247	47.1699853	36.0776233	20.6018
ENSG000001	RIT2	protein_codin	0	0	0	0
ENSG000001	VWDE	protein_codin	0	0	7.29721608	0
ENSG000001	AATK	protein_codin	0	0	0	0
ENSG000001	GSTM4	protein_codin	4.72192379	1.96607826	0	3.57644663
ENSG000001	ATP13A2	protein_codin	0	0	0	0
ENSG000001	HARBI1	protein_codin	1.52436614	6.18776015	1.79554348	2.05157912
ENSG000001	IMPDH2	protein_codin	52.2070625	0	176.518576	29.8390185
ENSG000001	CYP4F11	protein_codin	0	0	0	0.65255742
ENSG000001	TRIML2	protein_codin	0	0	0	0
ENSG000001	ZNF518B	protein_codin	23.9229447	66.4120618	111.40705	15.385063
ENSG000001	LENG8	protein_codin	31.0832064	30.0959881	73.5593581	53.3720869
ENSG000001	APOLD1	protein_codin	0	21.9651477	0	0
ENSG000001	FHL5	protein_codin	0	0	0	0
ENSG000001	ZKSCAN5	protein_codin	0.95621559	0	0	0
ENSG000001	EDDM3B	protein_codin	0	0	0	0
ENSG000001	ETV3	protein_codin	1.35145801	10.3293061	2.60736968	11.7761589
ENSG000001	ACAP2	protein_codin	50.3986529	94.0713877	74.4255511	43.4966326
ENSG000001	YME1L1	protein_codin	15.6230579	47.2077356	21.6460514	0
ENSG000001	ZNF274	protein_codin	0	0	0	0
ENSG000001	DNAI3	protein_codin	0	1.72068321	0	0
ENSG000000	CHRNA3	protein_codin	0	0	1.81345991	5.64459108
ENSG000001	ATP10B	protein_codin	0	0	0	1.36140373
ENSG000001	DCAF8	protein_codin	9.43677664	43.7897234	5.74966947	7.03690682
ENSG000000	DCBLD2	protein_codin	28.7002275	80.238207	117.214486	21.7526178
ENSG000001	EDDM3A	protein_codin	0	0	0.52258227	0.56089324
ENSG000001	BRSK1	protein_codin	0	1.15656903	2.34505712	0
ENSG000001	SULT1C2	protein_codin	0	0	0	1.03171613
ENSG000001	OR5AK2	protein_codin	0	0	0	0
ENSG000001	KLK15	protein_codin	0	0	0	0
ENSG000000	DCBLD2	protein_codin	0	3.25562501	3.96841835	0

ENSG000001	ZFP42	protein_codin	0	0	0	0
ENSG000001	STK11	protein_codin	1.73585782	0	0	0
ENSG000001	LCORL	protein_codin	0	0	0	7.9646119
ENSG000001	GSX2	protein_codin	0.28338907	0	0	0
ENSG000001	NDUFAF3	protein_codin	0	0	2.09655396	0
ENSG000001	ADGB	protein_codin	0	2.01889137	0	5.14558563
ENSG000001	NDUFAF3	protein_codin	2.84702028	3.19070903	0	4.43453516
ENSG000001	ADGB	protein_codin	0	0	0	0
ENSG000000	DKK3	protein_codin	38.0262661	0	48.0365416	12.1015583
ENSG000001	SSBP3	protein_codin	0	0	0	0
ENSG000001	NPR3	protein_codin	4.59153096	15.6128693	12.1030657	0
ENSG000001	HACD1	protein_codin	0	0	0.86748086	0.53567321
ENSG000001	TAGAP	protein_codin	0	0	0	0
ENSG000001	KLHDC3	protein_codin	19.5725245	21.5724085	41.5987739	13.3769743
ENSG000001	CCDC57	protein_codin	12.4396411	39.3576001	90.7277374	7.97008839
ENSG000001	VIPAS39	protein_codin	0	0	1.53148545	0
ENSG000001	FLII	protein_codin	50.9038242	144.308255	79.4373222	32.7369692
ENSG000001	AHI1	protein_codin	0	0	27.0473982	4.22943531
ENSG000001	SEC16B	protein_codin	0	0	0	0
ENSG000001	PSD3	protein_codin	34.2458654	229.39228	98.0345093	52.8874307
ENSG000001	TMEM86B	protein_codin	0	3.89318156	0	0
ENSG000001	NOC2L	protein_codin	9.33797584	61.8779104	104.808719	3.52454938
ENSG000001	CLRN1	protein_codin	0	0	0	1.20331748
ENSG000001	CARM1	protein_codin	8.95334686	38.6257175	42.9856579	6.41873469
ENSG000000	MARCO	protein_codin	3.36957871	3.12854559	0	1.23248395
ENSG000001	TBC1D31	protein_codin	0	7.41330332	0	4.89356794
ENSG000001	NR2F1	protein_codin	28.5470087	23.0580221	6.67285766	9.16207752
ENSG000001	PAK2	protein_codin	90.0626754	287.444374	220.493861	89.9722156
ENSG000001	TLE5	protein_codin	44.6337918	60.4838023	87.5211752	22.6820773
ENSG000001	RPAIN	protein_codin	0	0	0	0
ENSG000001	RBBP8	protein_codin	23.8181677	59.8918579	97.5898126	15.0182622
ENSG000001	PRM3	protein_codin	0	0	0	0
ENSG000001	TIMM22	protein_codin	16.2181746	28.8544486	21.8977978	9.94628983
ENSG000001	GALK2	protein_codin	0	0	0	0
ENSG000001	ZSCAN2	protein_codin	2.78527439	4.95788563	0	2.87681128
ENSG000001	CABYR	protein_codin	0	0	0	0
ENSG000001	FGFR1OP2	protein_codin	24.2269744	38.0370686	41.5195594	24.2381217
ENSG000001	OR5M8	protein_codin	0.3761694	0	0	0
ENSG000001	FAM9B	protein_codin	0	0	0	0
ENSG000001	CETN1	protein_codin	0	0	0	0.47159796
ENSG000001	RUSF1	protein_codin	0	38.9637423	0	0
ENSG000001	MMS19	protein_codin	0	0	0	0
ENSG000001	ATP10B	protein_codin	0	0	0	0
ENSG000001	GBA1	protein_codin	0	0	0	0

ENSG000001	TMEM151A	protein_codin	0	0	1.162992	0.43081931
ENSG000001	SLC16A6	protein_codin	0.97472843	0	0	0
ENSG000001	GOLGA8R	protein_codin	21.0395183	19.0993158	21.9591823	16.4117388
ENSG000001	PTPN2	protein_codin	38.9925965	47.0852283	24.3353146	0
ENSG000001	SENP6	protein_codin	0	0	0	0
ENSG000001	AK4	protein_codin	1.21329011	4.66899784	0	6.98649847
ENSG000001	KHDRBS1	protein_codin	111.693509	255.78357	352.279441	86.4359763
ENSG000001	EXOSC3	protein_codin	3.88238258	0	22.0110047	2.55455696
ENSG000001	NETO1	protein_codin	0	0	1.10502393	0
ENSG000000	RRN3	protein_codin	0	0	0	0
ENSG000000	EED	protein_codin	23.1395878	0	40.9904569	5.43004902
ENSG000001	CLEC4M	protein_codin	0	0	0	0
ENSG000001	CDCA8	protein_codin	0	13.9432842	45.3710739	3.67470306
ENSG000001	OR6C70	protein_codin	0	0	0	0.57410928
ENSG000001	FAM186A	protein_codin	0.6795989	4.21748087	0	0
ENSG000000	SEPHS1	protein_codin	12.0390461	30.3662812	61.0171976	12.4780449
ENSG000001	CCDC107	protein_codin	0	0	1.47614736	0
ENSG000001	FAM174B	protein_codin	3.99160792	7.63704126	2.04299564	3.05529927
ENSG000001	BRF1	protein_codin	0	0	0	0
ENSG000000	GGT5	protein_codin	0	34.74309	0	26.7244764
ENSG000001	SMAD3	protein_codin	34.820137	155.137245	277.310667	77.2021196
ENSG000001	TANGO2	protein_codin	1.65752601	26.477398	3.30878756	4.67471749
ENSG000002	XKR4	protein_codin	2.75797203	7.1682917	1.76656108	6.38229232
ENSG000001	PIEZO1	protein_codin	0	0	0	0
ENSG000001	IL36B	protein_codin	0	0	0	0.8997408
ENSG000001	C11orf87	protein_codin	0	3.21242947	0	2.82880007
ENSG000001	PRR14L	protein_codin	94.0630364	204.976472	226.186117	56.6148745
ENSG000001	BOLA3	protein_codin	5.0641601	9.70048705	16.7538538	5.28321972
ENSG000001	ADI1	protein_codin	33.2730483	73.9161434	140.094789	26.3900455
ENSG000001	MIPOL1	protein_codin	2.18650043	8.11636168	7.98190216	4.73762623
ENSG000001	KPNA7	protein_codin	0	0.47660896	1.19921561	0.49729167
ENSG000001	SP1	protein_codin	0	20.6310018	0	9.99329622
ENSG000001	SLC25A18	protein_codin	0	0	0	0
ENSG000001	FANCF	protein_codin	3.35610761	14.6019914	5.53908861	3.03338501
ENSG000001	TNFAIP8L1	protein_codin	5.08020275	0	22.3269044	10.1654655
ENSG000001	DNAH8	protein_codin	0	0	0	2.28698122
ENSG000001	CBFA2T3	protein_codin	0	3.5945192	15.1433343	0
ENSG000001	UBALD2	protein_codin	6.40599812	28.0992817	19.3784953	22.652131
ENSG000001	TOB2	protein_codin	57.963325	97.223997	94.010382	66.008665
ENSG000002	PSG3	protein_codin	0	0	0	0
ENSG000001	AGPAT3	protein_codin	0	0	0	0
ENSG000001	JMJD1C	protein_codin	27.6413038	39.9123225	43.2202827	37.7851668
ENSG000001	DZIP1L	protein_codin	7.46721003	17.6072591	33.8525851	4.1927382
ENSG000001	IFNLR1	protein_codin	3.04584083	9.41786906	17.7623918	2.01259278

ENSG000001	TNFAIP8L3	protein_codin	0	7.04646376	0	0
ENSG000000	BRAP	protein_codin	22.5091834	9.30939015	28.3465058	14.1618173
ENSG000001	CCDC60	protein_codin	0	0	0	1.77152421
ENSG000001	MCAT	protein_codin	1.49669438	1.93045176	0	0.9827938
ENSG000001	ATP11C	protein_codin	0	0	0	0
ENSG000001	RWDD4	protein_codin	0	0	0	0
ENSG000001	PDXK	protein_codin	0	1.25548948	0	2.26943385
ENSG000001	IFNLR1	protein_codin	0	0	2.48320247	0
ENSG000001	NT5DC4	protein_codin	0	0	0	2.13567558
ENSG000001	OXCT2	protein_codin	1.0028785	12.456958	12.2650206	13.6443646
ENSG000001	FOXP1	protein_codin	0	11.5737803	0	2.58165241
ENSG000001	MC2R	protein_codin	0.28224336	0	0.47949261	1.38206135
ENSG000000	EYA2	protein_codin	20.1158155	23.2238864	0	11.8169626
ENSG000001	TRAK1	protein_codin	0	0	0	0
ENSG000001	ZNF84	protein_codin	0	0	4.08017629	0
ENSG000001	FAM110C	protein_codin	0.89873422	0	0.86613106	0.45629085
ENSG000001	FUT10	protein_codin	14.7044501	23.0752179	36.1062088	11.7355683
ENSG000001	COL10A1	protein_codin	0	0	0	0
ENSG000001	SERPINA7	protein_codin	0	0	0	0
ENSG000001	SERHL2	protein_codin	0	0	0	0
ENSG000001	PARPBP	protein_codin	0	0	0	0
ENSG000001	USP45	protein_codin	2.17063767	0	0	0
ENSG000001	RNF123	protein_codin	5.35550694	0	0	0
ENSG000001	CACNA1C	protein_codin	0	0	0	0
ENSG000001	BTNL9	protein_codin	21.5119666	29.255049	26.0585802	51.5575952
ENSG000001	SIX6	protein_codin	0.78807566	0	0	1.96321922
ENSG000001	CFD	protein_codin	12.5426468	51.0480797	0	32.6272352
ENSG000001	ACSM6	protein_codin	0	0	0	2.06861679
ENSG000002	KRT81	protein_codin	0	0.61508038	0	0.65178934
ENSG000001	ADGRF4	protein_codin	0	0	0	0
ENSG000001	PLB1	protein_codin	2.17765889	0	0	1.83338453
ENSG000000	RASSF1	protein_codin	7.63526793	35.3006022	8.82696564	6.99895513
ENSG000001	NDUFA12	protein_codin	1.48574194	6.7825286	12.8708862	7.94836067
ENSG000001	GAS6	protein_codin	38.2921899	161.344518	25.0141581	52.4780504
ENSG000001	GRIK1	protein_codin	0	0	0	0
ENSG000001	FAAP100	protein_codin	5.14374157	9.45799184	18.5100826	2.55533969
ENSG000001	PLPP2	protein_codin	0	0	0	0
ENSG000000	YAF2	protein_codin	3.52290253	0	0	1.83850378
ENSG000001	DNAH14	protein_codin	0.35638272	0	2.8931507	0.62626942
ENSG000001	FFAR3	protein_codin	0	0	0	0
ENSG000001	DOLPP1	protein_codin	4.42716929	0	0	0
ENSG000001	KREMEN1	protein_codin	0	2.4721845	0	2.44342156
ENSG000001	HAPSTR1	protein_codin	32.9538716	170.554537	75.8595518	51.3715226
ENSG000001	ZNF704	protein_codin	0	0	0	0

ENSG000002	DEFA3	protein_codin	0	0	0	0
ENSG000000	FBLN1	protein_codin	222.036378	49.6243271	130.347625	13.6473041
ENSG000001	KCNK12	protein_codin	0.87701097	3.41880458	0	10.7606079
ENSG000001	CXorf38	protein_codin	8.29164099	40.1643358	6.8735862	0
ENSG000001	ADAMTSL1	protein_codin	3.50105691	3.53639873	0	0
ENSG000001	TUBB	protein_codin	228.268679	651.134474	1452.48841	109.578121
ENSG000001	AFMID	protein_codin	0	0	0	0
ENSG000000	PHF7	protein_codin	1.23972227	6.59892524	3.06138424	3.57780983
ENSG000001	LRIT3	protein_codin	0	0	0	0
ENSG000001	ZNF610	protein_codin	4.02047714	0	10.4353114	5.17280239
ENSG000000	EML1	protein_codin	0	0	0	0
ENSG000001	OTOL1	protein_codin	0	0	0	0.73363064
ENSG000002	OR6X1	protein_codin	0.22785234	0	0	0
ENSG000001	ZAR1	protein_codin	0	0	0	0
ENSG000001	GRID1	protein_codin	0.5401683	1.98434546	5.76835136	7.25547723
ENSG000001	HEXD	protein_codin	1.42726896	0	18.8666515	0
ENSG000001	SCN5A	protein_codin	0	0	0	4.52885475
ENSG000001	DDX53	protein_codin	0	3.67002541	0	0.48051954
ENSG000000	RIPOR3	protein_codin	0	0	0	2.88246819
ENSG000001	IGDCC3	protein_codin	2.34733155	6.6202499	5.48395788	1.37962015
ENSG000000	CMTM1	protein_codin	0	0	0	0
ENSG000001	RAB11B	protein_codin	12.0861111	54.9254003	79.375551	29.6863879
ENSG000001	KCNH7	protein_codin	0	0	1.1623418	1.7642706
ENSG000001	SLC24A3	protein_codin	2.71810247	2.37143868	10.3975937	1.27052676
ENSG000001	KLHL15	protein_codin	0	27.4048412	0	0
ENSG000001	SPRR4	protein_codin	0	1.61102026	0	0.3535951
ENSG000001	OR8B8	protein_codin	0	0	0	0
ENSG000001	ZNF71	protein_codin	1.77262045	14.8969895	0	3.54134608
ENSG000001	GLUD2	protein_codin	0.69154267	1.87348241	41.015533	1.28685502
ENSG000001	NPIPA1	protein_codin	3.99703453	0	14.7010499	0
ENSG000001	ZKSCAN2	protein_codin	15.1123754	27.6160025	37.5313713	10.7141155
ENSG000001	IL22	protein_codin	0	0	0	0
ENSG000001	SPSB1	protein_codin	2.61318262	2.65216381	0.78629662	3.40054323
ENSG000001	TASOR2	protein_codin	0	88.0380674	0	0
ENSG000001	NLRP7	protein_codin	0	0	0	0
ENSG000000	DNMT3B	protein_codin	0	0	0	0
ENSG000001	LGALS9C	protein_codin	0	0	0	0
ENSG000001	KRT36	protein_codin	0	0	0	0.82549238
ENSG000001	ATAD1	protein_codin	36.2685863	55.3270966	76.2174666	19.7758538
ENSG000001	TPM3	protein_codin	0	0	1.87883236	17.5348012
ENSG000001	ZNF85	protein_codin	8.47634729	27.1703995	0	9.95867188
ENSG000001	OR4C46	protein_codin	0	0	0	0.54481976
ENSG000001	VTCN1	protein_codin	0	0	0	0.48290894
ENSG000001	DENND5A	protein_codin	10.3262767	25.8998693	15.0973719	0

ENSG000001	PLPBP	protein_codin	19.3415142	48.0528125	89.8946762	16.0791463
ENSG000001	ZNF662	protein_codin	7.63375797	4.30692625	0	0
ENSG000001	LIN9	protein_codin	0	3.36662158	0	0
ENSG000001	OR5BS1P	protein_codin	0	1.48587512	0	0
ENSG000001	IFITM1	protein_codin	6.67903318	0	29.4107691	3.25080258
ENSG000001	KCNA4	protein_codin	0.27307975	7.03491935	14.8983657	3.38938271
ENSG000001	FAM120C	protein_codin	3.15976259	35.4442551	4.57348026	0
ENSG000001	CEP120	protein_codin	0	0	0	0
ENSG000001	ODF4	protein_codin	0	0	0	0
ENSG000001	ERMAP	protein_codin	0.56573305	0	3.88687901	5.54707551
ENSG000001	PAPPA	protein_codin	45.4529328	55.6850847	10.5833397	25.5009007
ENSG000002	PPME1	protein_codin	12.5394719	34.2677631	30.9096781	9.40661028
ENSG000001	CRELD2	protein_codin	14.947829	0	73.3238277	7.73959321
ENSG000001	TNRC18	protein_codin	0	0	0	4.60246342
ENSG000001	PHKG2	protein_codin	0	6.38642258	6.94373233	0
ENSG000001	LRRC14B	protein_codin	0	0.92165093	0.56546655	0.88647874
ENSG000001	ST6GALNAC3	protein_codin	3.22656316	7.96775339	22.7393398	7.54839771
ENSG000001	COL4A5	protein_codin	0	0	6.08669476	0
ENSG000000	SLC9A7	protein_codin	8.10397341	0	9.819353	3.54415222
ENSG000001	ENPP7	protein_codin	0	0	0	0
ENSG000001	OR6C76	protein_codin	0	0	0	0
ENSG000001	COL7A1	protein_codin	0	0	0	0
ENSG000001	POU3F2	protein_codin	0.27923307	0	43.0998322	2.46589807
ENSG000001	PTRHD1	protein_codin	1.99244287	5.25486345	13.6563894	2.69307415
ENSG000001	RNF135	protein_codin	5.85176471	0	8.51332537	1.58742242
ENSG000001	SKP1	protein_codin	0	52.3798725	0	4.74809374
ENSG000001	BTBD9	protein_codin	6.19438954	11.4618022	5.50039253	2.94525924
ENSG000002	CATSPERZ	protein_codin	0	0	1.3910736	0
ENSG000001	KCNH8	protein_codin	0	0	7.72117518	0
ENSG000001	COA3	protein_codin	2.19096111	5.80074303	4.64614693	2.11626298
ENSG000001	CTAG1B	protein_codin	0	0	0	0
ENSG000001	MYT1	protein_codin	1.16750076	0.92843006	4.12789705	3.05656487
ENSG000001	RNASE10	protein_codin	0	0	0	0
ENSG000001	C1QTNF8	protein_codin	0.23551196	1.8676106	1.06899121	0.47649787
ENSG000001	IZUMO1R	protein_codin	0	0	0	0
ENSG000001	TTC7B	protein_codin	10.6753379	17.4007365	26.8015474	16.7894476
ENSG000001	CCDC144A	protein_codin	0	0	3.52039376	0
ENSG000001	GINS3	protein_codin	0.45199723	4.42118054	2.51023523	0
ENSG000001	BRICD5	protein_codin	0	2.79814648	0	0
ENSG000001	HSD17B13	protein_codin	0	0	2.39323116	0
ENSG000002	RTL10	protein_codin	0	0	44.9748731	0
ENSG000001	DNAH14	protein_codin	0	0	0	0
ENSG000001	NRROS	protein_codin	0.91483391	32.4335813	0.81745148	5.46828102
ENSG000001	LRP2BP	protein_codin	0	3.18296805	0	0

ENSG000000	ATP2C1	protein_codin	0	0	0	0
ENSG000001	AGAP2	protein_codin	0	0	15.861569	0
ENSG000001	OR2T29	protein_codin	0	1.97271535	0	0
ENSG000001	TNFRSF18	protein_codin	1.08421635	0	0	0
ENSG000001	TRMT12	protein_codin	8.99205857	18.5764748	137.073542	5.4032527
ENSG000001	NLRP10	protein_codin	0	4.57542443	0	0.95545358
ENSG000001	OR51M1	protein_codin	0.23994576	0	0	0
ENSG000001	PCP4	protein_codin	0	0	0.86948476	0
ENSG000000	IP6K2	protein_codin	50.7430799	42.1975824	76.5401766	23.1116616
ENSG000001	SYNM	protein_codin	0	0	0	0
ENSG000001	CIB1	protein_codin	0	15.6102303	0	0
ENSG000001	ZNF26	protein_codin	9.41444491	36.6535627	11.9885939	14.2531717
ENSG000001	PRSS55	protein_codin	0	0	0	0.42969711
ENSG000001	KCNJ15	protein_codin	1.10888039	5.81313879	8.6208432	11.1187516
ENSG000002	TAF9	protein_codin	0	0	0	0
ENSG000001	RHD	protein_codin	0	0	0	1.09258766
ENSG000001	SIRT7	protein_codin	0.8017056	5.26837031	13.375525	0
ENSG000001	ARSI	protein_codin	1.6904727	1.70484361	1.75242902	2.11134601
ENSG000001	CCDC34	protein_codin	26.2597112	60.4755154	302.358247	8.84905455
ENSG000001	DEFB108B	protein_codin	0	0	0	0
ENSG000001	HAX1	protein_codin	40.5740919	0	54.6412961	3.94886598
ENSG000001	CAPS2	protein_codin	0	0	0	2.74591167
ENSG000001	COA5	protein_codin	12.7674272	11.9629685	35.7220937	6.50572733
ENSG000000	PPP2R5C	protein_codin	17.8454306	5.84619059	67.3194309	19.0584109
ENSG000001	BACE2	protein_codin	14.1902009	0	36.768086	14.2100926
ENSG000000	PTPN21	protein_codin	27.3298567	0	25.6399549	14.2962483
ENSG000000	ANKRD44	protein_codin	0	3.68254606	4.42120597	2.52531503
ENSG000001	VMO1	protein_codin	0	0	0	1.05193418
ENSG000001	NXPH3	protein_codin	0	4.24222567	0	0
ENSG000001	ZNF92	protein_codin	14.4112389	37.5005564	28.4390356	20.0720755
ENSG000001	ZSWIM9	protein_codin	2.09136021	0	0	0
ENSG000001	ARMCX2	protein_codin	0	0	0	0
ENSG000001	TMTC4	protein_codin	0	0	0	0
ENSG000001	DHX34	protein_codin	3.80643966	20.0151661	23.0016264	0
ENSG000001	OR2T34	protein_codin	0	0	0	1.05974256
ENSG000001	FRMD3	protein_codin	0	0	0	0
ENSG000001	SPDYE4	protein_codin	0	0	0	0
ENSG000001	OR4N4	protein_codin	0	0	0	0
ENSG000001	CLDN6	protein_codin	0	2.35521134	0	0.40613768
ENSG000001	OTOP3	protein_codin	0	0	0.60572854	2.43262649
ENSG000001	WBP2NL	protein_codin	2.22515723	3.99571518	0	0
ENSG000001	PAWR	protein_codin	16.5916398	79.2202754	70.3627486	24.1283928
ENSG000001	SH2D7	protein_codin	0	0	0	1.66268852
ENSG000001	CNOT10	protein_codin	10.2044506	32.4775792	12.5585391	0

ENSG000001	PPP4R4	protein_codin	0	0	6.379685	0
ENSG000001	NOP10	protein_codin	3.27835571	3.16454219	3.96357552	7.35701393
ENSG000001	FES	protein_codin	6.10405496	7.20072698	0	8.96063912
ENSG000001	CLEC4G	protein_codin	0	0	0	0
ENSG000001	PDE9A	protein_codin	0	0	0	0
ENSG000001	CLRN1	protein_codin	0	0	0	0
ENSG000001	CAPN12	protein_codin	0	0	0	0
ENSG000000	KLHL22	protein_codin	7.93419652	24.8326825	43.4552594	10.0380174
ENSG000001	HCAR2	protein_codin	1.20767183	1.22930416	0	1.61055994
ENSG000001	OR4F6	protein_codin	0	0	0	0
ENSG000001	TMIGD1	protein_codin	0	0	0	0
ENSG000001	OR1G1	protein_codin	0	0	0	1.70145433
ENSG000001	GKN2	protein_codin	0	0	0	0
ENSG000001	GIMAP6	protein_codin	15.7413588	124.383539	6.82740904	16.7059458
ENSG000001	CDYL	protein_codin	0	0	0	0
ENSG000000	MCF2L2	protein_codin	1.16323416	0	0	0
ENSG000001	FOXK1	protein_codin	48.408369	161.980731	96.5146156	37.0347183
ENSG000001	C1S	protein_codin	0	0	0	8.94923115
ENSG000001	SHMT2	protein_codin	0	0	8.79547915	6.96966209
ENSG000001	THEMIS2	protein_codin	0	0	0	0
ENSG000001	SYNGR1	protein_codin	3.99222452	9.24288168	14.9971042	2.27638642
ENSG000001	KCNIP1	protein_codin	0	0	0	0
ENSG000001	SDR42E1	protein_codin	0.52014184	6.23846601	8.48755076	10.8657296
ENSG000001	GALNT9	protein_codin	0	0	0	0
ENSG000000	P2RX7	protein_codin	12.5212665	34.8975204	1.8642896	4.21827738
ENSG000001	OAF	protein_codin	35.407895	27.0934705	48.041362	24.9558246
ENSG000001	ZGPAT	protein_codin	0	0	0	0
ENSG000001	GALR2	protein_codin	0	0	0	0
ENSG000001	MANEAL	protein_codin	0.6584632	2.62240163	0	0
ENSG000001	CALN1	protein_codin	0	0	0	0
ENSG000002	NFAM1	protein_codin	2.55241704	25.9047661	1.30239063	2.83286125
ENSG000001	TCEAL2	protein_codin	0	0	0	1.29141445
ENSG000001	NFXL1	protein_codin	33.2243203	19.292599	238.635341	0
ENSG000001	SEPTIN9	protein_codin	85.2477	155.451392	9.54070645	49.8225637
ENSG000001	TPO	protein_codin	0	0	0	0
ENSG000001	DDX51	protein_codin	0.34219919	4.63634532	3.8275055	0.46319076
ENSG000001	SPNS2	protein_codin	8.4791736	12.356241	6.41497332	16.582226
ENSG000001	RGMA	protein_codin	0.68247521	2.58213569	2.79313787	3.91639903
ENSG000001	RPL35A	protein_codin	6.79437068	12.6739817	0	0
ENSG000001	RFLNB	protein_codin	24.0782224	48.8320302	34.2792535	25.0752096
ENSG000001	NFATC1	protein_codin	0	14.1420887	0	0
ENSG000001	SULT1C3	protein_codin	0	0	0	1.2997963
ENSG000001	BCAN	protein_codin	0	0	0	0
ENSG000001	KRTAP6-1	protein_codin	0	0	0	0

ENSG000001	GP1BA	protein_codin	0	3.50619204	0	0
ENSG000001	UTY	protein_codin	0	0	0	0
ENSG000001	HGS	protein_codin	0	0	0	0
ENSG000001	DDIAS	protein_codin	1.03291429	0	0	0
ENSG000001	CRIP2	protein_codin	12.3451161	13.2844464	10.8970328	8.30104977
ENSG000001	TMEM54	protein_codin	1.79995399	0	1.53185589	0
ENSG000000	LZTS3	protein_codin	0	0	0	1.52946259
ENSG000000	PLEKHH1	protein_codin	3.42476149	0	27.4212668	5.63518662
ENSG000001	SPPL2C	protein_codin	0	1.00984434	0	1.73259976
ENSG000001	OGFOD3	protein_codin	0	0	5.75922186	0
ENSG000001	NKX2-5	protein_codin	0	0	0.49431946	0
ENSG000001	FAM181B	protein_codin	0	0	3.03993987	2.59309686
ENSG000001	RIBC1	protein_codin	0	0	0	0.36724914
ENSG000001	CCDC137	protein_codin	11.121115	0	50.7902126	12.6223836
ENSG000001	ATPAF1	protein_codin	7.85431413	20.7964223	7.41502288	0
ENSG000001	AP1S2	protein_codin	19.5725189	1.83984933	20.7482807	11.9796696
ENSG000001	RBM10	protein_codin	20.626563	38.1531048	24.7267606	0
ENSG000001	ARL17A	protein_codin	0	0	0	0
ENSG000001	TOP1MT	protein_codin	16.8147597	36.485666	108.620744	11.5408963
ENSG000002	EEF1G	protein_codin	357.028961	794.218961	1129.57717	394.485087
ENSG000001	S100A7A	protein_codin	0	0	0	0.47472056
ENSG000001	PRSS57	protein_codin	0	0	0	0
ENSG000001	GRPEL2	protein_codin	13.7872858	24.9465156	19.8478356	6.37799643
ENSG000001	NOP56	protein_codin	14.0645355	18.7313429	81.2301839	14.9091218
ENSG000001	BLZF1	protein_codin	0	0	9.13851868	0
ENSG000001	ZNF599	protein_codin	1.30088714	0	3.58671208	0.44429337
ENSG000001	PGBD2	protein_codin	4.30329663	13.4085265	17.2288441	3.01385273
ENSG000001	OLFML1	protein_codin	42.8992218	81.4709832	0	6.16179789
ENSG000001	TPM2	protein_codin	332.386628	158.303496	237.785623	54.2678335
ENSG000001	NUPR2	protein_codin	0	0	0	0
ENSG000001	CYLC1	protein_codin	0	0	0.40089031	0
ENSG000001	TRIM61	protein_codin	0	0	0	0
ENSG000001	YBEY	protein_codin	0	0	0	0
ENSG000001	GPR39	protein_codin	0	0	0	0.60844936
ENSG000002	OR52E6	protein_codin	0	0	0	0
ENSG000001	SETD3	protein_codin	2.00150257	16.6089182	3.11059054	1.62843817
ENSG000001	RP1L1	protein_codin	0	0	0	0
ENSG000001	MAGEA6	protein_codin	0	3.87127461	61.1556969	1.70858207
ENSG000001	FAM47B	protein_codin	0	0	0	0.38265476
ENSG000001	CIMAP1B	protein_codin	0	0	0	0
ENSG000001	HNMT	protein_codin	5.45478692	19.4398124	0	2.50729737
ENSG000001	KIAA0825	protein_codin	2.80540717	0	0	2.05570818
ENSG000001	PTP4A3	protein_codin	0	0	0	1.61149945
ENSG000001	PDLIM1	protein_codin	11.3906183	20.5496305	28.113939	19.9684936

ENSG000001	GAST	protein_codin	0.39642508	0	0	0
ENSG000001	PMCH	protein_codin	0.27274311	0	0	0.4713819
ENSG000001	C16orf54	protein_codin	1.27574467	17.2291626	0.54556963	4.04359146
ENSG000001	CRISP1	protein_codin	0	0	0	0
ENSG000001	YIF1B	protein_codin	0	7.26792799	0	0
ENSG000001	MARCKSL1	protein_codin	18.6108414	78.1887648	317.018345	15.7769227
ENSG000001	ASAH2	protein_codin	0	0	0	0
ENSG000001	HNRNPH1	protein_codin	24.6620938	0	22.5581698	18.6787041
ENSG000001	OR5P2	protein_codin	0	0	0	0.57723827
ENSG000001	C10orf120	protein_codin	0	0	0	1.1713161
ENSG000001	PRR23B	protein_codin	0	0	0	0
ENSG000001	LMNTD2	protein_codin	0	0	0	0.37487466
ENSG000001	SRARP	protein_codin	0	0	0	0.93574868
ENSG000001	DHX36	protein_codin	32.6837921	129.494661	99.1752364	27.2670324
ENSG000001	TRIM69	protein_codin	0	0	0	0
ENSG000001	NPAP1	protein_codin	0.25424803	1.217354	1.42553793	4.60890628
ENSG000000	CARMIL1	protein_codin	14.7069728	5.24463684	89.5940925	16.2561844
ENSG000001	NPY2R	protein_codin	0	0	0	0
ENSG000002	ADAT3	protein_codin	0	1.10067805	2.22248339	0
ENSG000000	MAPK8IP2	protein_codin	0.51920593	1.72257314	3.78743035	0
ENSG000001	TEKTIP1	protein_codin	0	0	0	0
ENSG000000	DZANK1	protein_codin	0	0	0	0
ENSG000002	PSG2	protein_codin	0	0	0	0
ENSG000001	RGPD6	protein_codin	75.9394285	156.215256	114.466674	0
ENSG000001	GNB1L	protein_codin	0	2.02001334	4.42297452	4.27114113
ENSG000000	NFATC3	protein_codin	0	0	43.0942743	0
ENSG000001	UNC5A	protein_codin	0.5207054	0	0	2.19962024
ENSG000001	MFSD5	protein_codin	2.55557315	1.96891038	7.74034242	1.19737496
ENSG000001	RIPPLY3	protein_codin	0	0	0	0
ENSG000001	NDUFB1	protein_codin	0	0	0	2.38612429
ENSG000001	TSPO	protein_codin	0.60895714	0	0	0
ENSG000001	SNN	protein_codin	4.10461039	45.796139	12.1542489	10.3744302
ENSG000001	IL20RB	protein_codin	0.69844427	0	0	0
ENSG000001	SERPINA5	protein_codin	0	0	0	0
ENSG000001	GDPGP1	protein_codin	0.48607891	8.82783263	2.87654667	0
ENSG000001	CSF1	protein_codin	24.8538061	85.5527195	8.57306833	138.031059
ENSG000001	CIMIP2C	protein_codin	0	0	0	0
ENSG000001	WBP2NL	protein_codin	0	0	0	1.56031528
ENSG000001	KRTAP8-1	protein_codin	0	0	0	0
ENSG000001	C2CD2	protein_codin	17.0799891	0	0	0
ENSG000001	DAOA	protein_codin	0	0	0	0
ENSG000002	PEX26	protein_codin	23.0428316	0	0	17.5322941
ENSG000001	FNDC1	protein_codin	0	119.790795	0	35.2345835
ENSG000001	ZNF775	protein_codin	2.49013372	9.36033133	7.13171439	0.61653849

ENSG000001[illegible]	PDE4B	protein_codin	0	59.1477028	2.7655026	0
ENSG000001[illegible]	ZSCAN22	protein_codin	0	17.9932866	0	4.7968948
ENSG000001[illegible]	PIGP	protein_codin	1.77834504	0	0	0
ENSG000001[illegible]	FBXL7	protein_codin	0	0	0	0
ENSG000001[illegible]	MFSD10	protein_codin	0	4.46960723	5.05661998	3.25912265
ENSG000000[illegible]	ACSM2B	protein_codin	0	0	0	3.55243395
ENSG000001[illegible]	TBX1	protein_codin	0	0	0.85746962	0
ENSG000001[illegible]	MEX3B	protein_codin	17.1634395	6.40381958	75.4398462	13.4363511
ENSG000001[illegible]	IL25	protein_codin	0	0	0	0
ENSG000001[illegible]	C6orf58	protein_codin	0	0	0	1.54606198
ENSG000001[illegible]	IRX3	protein_codin	10.7837174	47.6288423	102.030559	13.6498824
ENSG000002[illegible]	MBD3L5	protein_codin	0	0	0	0
ENSG000001[illegible]	TRIM49	protein_codin	0	0	0	0
ENSG000001[illegible]	FAM43A	protein_codin	11.3402483	40.0214235	22.6656569	25.9868608
ENSG000001[illegible]	MRGPRX2	protein_codin	0	0	0	2.32002652
ENSG000001[illegible]	SLC16A5	protein_codin	0	0	0	0
ENSG000001[illegible]	GPR132	protein_codin	2.11157286	0	0	0
ENSG000001[illegible]	FREM3	protein_codin	0.30933035	1.06251189	3.87412619	3.45406067
ENSG000001[illegible]	TMCC2	protein_codin	0	0	0	0
ENSG000001[illegible]	MFSD6L	protein_codin	0	0.94557092	0	0
ENSG000001[illegible]	MRPL11	protein_codin	0	0	16.8943222	0
ENSG000001[illegible]	ALDH1A3	protein_codin	2.45550836	32.615747	39.0096708	0
ENSG000001[illegible]	LPAR5	protein_codin	4.14293612	14.5589652	1.03947897	1.25659699
ENSG000001[illegible]	TRIM64B	protein_codin	0	0	0	0
ENSG000001[illegible]	PYCR1	protein_codin	0	0	0	0
ENSG000001[illegible]	CSH1	protein_codin	0	0	0	0
ENSG000001[illegible]	TEDC1	protein_codin	0	0	0	0
ENSG000001[illegible]	CNGA2	protein_codin	0.30696396	3.28128858	1.4895024	1.26595712
ENSG000001[illegible]	CALHM1	protein_codin	0	1.969148	0	0.84874967
ENSG000001[illegible]	IST1	protein_codin	0	0	0	0
ENSG000001[illegible]	GDF3	protein_codin	0.18725552	0	0	0.72847506
ENSG000001[illegible]	LRRC36	protein_codin	0	0	0	0
ENSG000001[illegible]	MOB2	protein_codin	1.3489713	15.7431075	7.42271756	1.77307704
ENSG000001[illegible]	B4GALNT4	protein_codin	0.90765135	0	26.8450031	1.31087479
ENSG000001[illegible]	USP45	protein_codin	9.53506551	0	10.4941677	3.94734124
ENSG000001[illegible]	SIVA1	protein_codin	0	0	8.18206274	0
ENSG000001[illegible]	GTF3C6	protein_codin	47.281525	36.4459773	136.458798	23.5180565
ENSG000001[illegible]	ATAT1	protein_codin	0	0	0	0
ENSG000001[illegible]	SLC52A2	protein_codin	0	0	0	0
ENSG000001[illegible]	NEXN	protein_codin	0	0	0	0
ENSG000001[illegible]	DLG2	protein_codin	0	0	0	0
ENSG000002[illegible]	ZNF722	protein_codin	0	0	0	3.02355285
ENSG000001[illegible]	SETD2	protein_codin	41.0682542	16.7089965	131.752988	28.4106866
ENSG000001[illegible]	UQCR10	protein_codin	9.73059892	40.1376362	30.0356867	8.05613728

ENSG000001	BRCC3	protein_codin	9.43938883	0	72.6634749	21.7521524
ENSG000001	ADRA2C	protein_codin	1.53648362	4.09720816	0	0
ENSG000001	IDH2	protein_codin	22.4990105	5.50177177	18.287042	19.9025328
ENSG000001	HOXB2	protein_codin	0.9116827	14.9558689	0	2.77561186
ENSG000000	AKR7A2	protein_codin	3.59923883	4.61649831	13.4195436	4.39445851
ENSG000001	ATAT1	protein_codin	0	0	0	0
ENSG000001	CEND1	protein_codin	0	0	0	0
ENSG000001	TMEM44	protein_codin	0.70146902	2.43581135	2.36558969	0.95715624
ENSG000001	RXFP3	protein_codin	0.23746414	0	0	1.23382405
ENSG000002	ZNF616	protein_codin	5.8009987	0	8.3034154	1.92307937
ENSG000001	EIF4ENIF1	protein_codin	20.9290221	54.4016505	46.8077949	20.3297237
ENSG000001	MRPL54	protein_codin	4.62077179	14.1775536	10.1146346	2.97157676
ENSG000001	SKA2	protein_codin	22.8776252	63.6936581	128.549802	14.3449813
ENSG000001	ZNF696	protein_codin	4.33525088	6.7632023	26.0964353	7.24804766
ENSG000001	TRAPPC6B	protein_codin	0	30.8861294	24.2065855	8.22259828
ENSG000001	ARMCX2	protein_codin	2.53942177	0	27.2436246	0
ENSG000001	DMBT1	protein_codin	0	0	0	0
ENSG000002	ENSG000002	protein_codin	0	0	2.12111063	0
ENSG000001	ZNF570	protein_codin	16.1350237	27.4519108	13.4824876	9.38570204
ENSG000001	SAMD14	protein_codin	0.58725695	1.48883116	0	0.79627715
ENSG000001	SLX1B	protein_codin	0	0	3.8807971	0
ENSG000001	TPM3	protein_codin	51.5496615	0	33.9708225	30.1860898
ENSG000001	CABCOCO1	protein_codin	0	0	0	1.0756316
ENSG000001	LMLN	protein_codin	0	0	0	5.89498941
ENSG000001	SPATC1L	protein_codin	0	6.29515284	8.77579375	0
ENSG000001	ST3GAL3	protein_codin	0	0	0	0
ENSG000001	ZDHHC23	protein_codin	0	0	10.4275933	0
ENSG000000	ADA2	protein_codin	0	0	0	11.3259296
ENSG000002	CRIP1	protein_codin	0.87121655	0.83247083	0	2.75748488
ENSG000001	ZKSCAN8	protein_codin	28.5995812	55.7498253	89.803764	23.1129803
ENSG000001	IRF7	protein_codin	0	19.5738803	3.52660674	0
ENSG000001	DHRS7C	protein_codin	0.19761102	0	0	0
ENSG000001	PPP1R27	protein_codin	2.73308948	0	0	0
ENSG000000	MRTO4	protein_codin	14.7731077	32.21642	117.020618	9.75607849
ENSG000001	SV2B	protein_codin	1.9865349	8.72741459	0	12.6567232
ENSG000001	GTF2H2	protein_codin	2.99870421	0	0	0
ENSG000001	F8	protein_codin	2.35143382	0	12.6997313	0
ENSG000001	SMIM10	protein_codin	26.6416677	34.5346515	13.5037109	21.0947369
ENSG000000	SLC12A1	protein_codin	0.26220654	0	0	0.45380104
ENSG000001	CTHRC1	protein_codin	11.7390031	31.3665438	17.5085143	12.3615603
ENSG000001	CTNNA3	protein_codin	0	0	0	0
ENSG000001	WDR4	protein_codin	3.71416733	1.69773259	2.34119534	0
ENSG000001	RGPD6	protein_codin	0	0	0	0
ENSG000001	BACE2	protein_codin	18.7857172	39.378291	45.6688894	8.19734491

ENSG000001	HDDC3	protein_codin	0	0	6.82001113	0
ENSG000001	ATP6V0A2	protein_codin	15.566977	43.3839856	10.1781761	18.1017118
ENSG000001	ACTRT3	protein_codin	3.27520255	0.81044431	0.56223245	0
ENSG000001	VMA21	protein_codin	13.5832208	23.6413599	82.1594149	11.65797
ENSG000001	IGLL1	protein_codin	0	0	0	0
ENSG000001	EP400	protein_codin	0	0	0	0
ENSG000001	CREB3L2	protein_codin	70.1391131	185.952224	198.022445	61.9968006
ENSG000001	C1QTNF12	protein_codin	0	0	0	0
ENSG000001	EPHB3	protein_codin	15.465678	37.2642044	28.02758	4.17173139
ENSG000001	ATP6V0C	protein_codin	9.90538174	35.7712576	56.973293	30.2175449
ENSG000001	TUBA8	protein_codin	0	3.15909228	0	0
ENSG000001	RCAN2	protein_codin	8.0812565	0	0	3.81351753
ENSG000001	OPRM1	protein_codin	0.25718088	5.1064185	0	6.20521652
ENSG000001	CYP2A13	protein_codin	0	0	0	0.41583128
ENSG000001	MT1E	protein_codin	0.71547065	0	0	18.0881918
ENSG000001	PRKCD	protein_codin	16.7712353	0	14.5254211	0
ENSG000001	KPNA2	protein_codin	10.4378436	0	462.657491	0
ENSG000001	TBX18	protein_codin	0.71361436	0	0	0
ENSG000001	ADAMTSL5	protein_codin	0	0	2.08022079	0
ENSG000001	CHD3	protein_codin	29.5947856	17.9905627	0	18.4611722
ENSG000001	PRR14L	protein_codin	0	0	0.48038742	0.71932926
ENSG000001	SLC5A2	protein_codin	0	0	0	0
ENSG000001	OR2T10	protein_codin	0	0	0	0.74444827
ENSG000001	ZNF397	protein_codin	27.7143881	65.5510145	66.4151335	13.5580526
ENSG000001	DDX41	protein_codin	10.8261788	30.5376495	36.649503	13.2307015
ENSG000001	TGIF1	protein_codin	0.76112146	2.34296509	0	0
ENSG000001	GJC1	protein_codin	0	0	5.40161296	4.6365516
ENSG000001	WFDC10B	protein_codin	0	0	0	1.05810425
ENSG000001	NOMO2	protein_codin	0	0	0	9.19677048
ENSG000001	CD86	protein_codin	17.8521062	97.9113258	5.00918383	22.2484192
ENSG000000	SLC22A16	protein_codin	0	0	0	2.83551349
ENSG000001	KRT79	protein_codin	0	0	0	2.6998342
ENSG000001	CDCA2	protein_codin	2.0083336	20.510794	22.3821079	2.19016052
ENSG000001	INTS5	protein_codin	4.62612706	11.7587727	6.67420149	1.54531246
ENSG000001	RGPD8	protein_codin	32.3797924	0	160.380619	30.2652078
ENSG000001	NOC4L	protein_codin	1.40583577	6.51296321	12.7695939	0
ENSG000001	DEFA5	protein_codin	0	0	0	0
ENSG000002	HBG1	protein_codin	0	0	0	0
ENSG000001	ZNF470	protein_codin	12.3803131	23.2508571	0	8.72727372
ENSG000001	FBXO21	protein_codin	6.65832473	16.5133757	17.7650766	3.42319558
ENSG000002	INF2	protein_codin	3.16198749	17.519903	14.3458623	5.7570066
ENSG000002	LYRM4	protein_codin	5.6206021	10.0918701	22.4236008	5.37552692
ENSG000001	MAPK11	protein_codin	0	15.6274825	11.6654704	4.16018983
ENSG000001	TMCC2	protein_codin	0	0	0	0

ENSG000001	TLCD2	protein_codin	59.3912549	64.2394174	22.3293437	37.7776314
ENSG000001	NR2C1	protein_codin	0	0	0	2.48892943
ENSG000001	GRIN2A	protein_codin	0	0	0	10.8440031
ENSG000001	CMTM4	protein_codin	1.98382364	0	11.7581658	0
ENSG000000	RARB	protein_codin	3.03002659	0	22.4522614	13.7790834
ENSG000001	WDR5B	protein_codin	5.62695881	6.40905718	22.1064911	3.28590592
ENSG000000	ZNF195	protein_codin	0	0	0	0
ENSG000001	ZFP41	protein_codin	5.26674277	43.9805762	35.562363	2.92265113
ENSG000001	HHIPL1	protein_codin	11.4582598	13.7207715	0.75884322	4.12336446
ENSG000001	MX2	protein_codin	7.62372322	9.66008339	1.07890418	4.79067663
ENSG000001	KDELR1	protein_codin	8.09881013	70.224795	17.0269153	26.8614951
ENSG000002	KRT6A	protein_codin	0	3.57975288	0	1.9586461
ENSG000001	OR56A4	protein_codin	0	0	0	0
ENSG000001	RBBP7	protein_codin	8.8749563	27.624517	6.89277758	4.7006116
ENSG000001	ANKRD11	protein_codin	55.0403324	381.132218	207.47908	63.2961826
ENSG000001	HNRNPA1	protein_codin	261.743514	333.820917	863.545321	345.049569
ENSG000001	FLRT2	protein_codin	9.68270877	18.2749024	14.4117238	19.7770833
ENSG000001	TMEM95	protein_codin	0	0	0	0
ENSG000001	SLC37A4	protein_codin	0	0	0	2.70278965
ENSG000001	CD300C	protein_codin	0.54740722	2.07257281	0	2.38153142
ENSG000001	STING1	protein_codin	3.43517072	0	1.40698216	0
ENSG000001	PRMT3	protein_codin	0	0	5.17435401	0
ENSG000001	KRTAP20-2	protein_codin	0	0	0	0
ENSG000001	EYS	protein_codin	0	0	0	0
ENSG000001	ALG12	protein_codin	7.06958845	14.140959	14.3512821	2.91629752
ENSG000001	C12orf60	protein_codin	0	0	3.13277308	0.56044713
ENSG000001	ANKRD17	protein_codin	7.93594562	55.8722223	49.5579668	22.7323313
ENSG000001	RAB11FIP1	protein_codin	23.5508001	77.7043042	57.3984555	9.07878661
ENSG000001	NR4A3	protein_codin	5.00753533	0	7.46063095	0
ENSG000001	RAB28	protein_codin	0	14.3791392	18.0915347	5.5023659
ENSG000000	SUGP2	protein_codin	0	0	0	0
ENSG000001	TMEM89	protein_codin	0	0	0	0
ENSG000001	SOCS3	protein_codin	13.9164545	59.8094293	3.76262553	907.637462
ENSG000001	ADSS1	protein_codin	0	0	0	0
ENSG000002	PRAMEF7	protein_codin	0	0	0	0
ENSG000000	KCNH2	protein_codin	0	0	0	0
ENSG000001	SULT4A1	protein_codin	0	0	0	0
ENSG000001	ADAP2	protein_codin	16.1525833	64.1386319	9.25504031	18.9158833
ENSG000001	BEST3	protein_codin	0	0	0	0
ENSG000000	DNAJA1	protein_codin	138.21169	544.277451	676.153733	241.467848
ENSG000001	MAGI1	protein_codin	0	0	0	0
ENSG000001	TREX2	protein_codin	0	0	0	0
ENSG000001	TUBB	protein_codin	0	0	0	0
ENSG000001	KANK3	protein_codin	2.42781072	0	7.61008219	7.39816889

ENSG000001	EVI2B	protein_codin	10.1992364	79.7800166	3.69018602	36.3713343
ENSG000001	PTTG1IP	protein_codin	53.7424332	155.581973	119.225491	53.8798376
ENSG000001	UBE2G2	protein_codin	3.05847476	42.6946231	20.4724573	0
ENSG000001	SNX20	protein_codin	1.82878207	24.9489204	1.58127884	2.36296732
ENSG000001	LRRC8B	protein_codin	0	0	0	0
ENSG000001	SOX1	protein_codin	0	1.27219159	6.83563389	0.44484179
ENSG000001	CCR4	protein_codin	1.85979753	14.3626709	0	4.06658971
ENSG000001	BAALC	protein_codin	0	0	0	0
ENSG000001	RPS27L	protein_codin	0.96678076	9.71391265	1.77432995	2.08196486
ENSG000001	BOLA2	protein_codin	3.50473494	0	17.1501572	0
ENSG000001	COPG2	protein_codin	0	0	4.44792869	0
ENSG000001	ANKRD23	protein_codin	45.8383379	0	0	0
ENSG000001	CDK10	protein_codin	2.05558163	1.49846896	0.72214223	0
ENSG000001	NTF3	protein_codin	1.16300406	0	3.42478488	2.31534629
ENSG000001	TTLL1	protein_codin	0	0	0	0
ENSG000001	EWSR1	protein_codin	0	0	0	0
ENSG000001	IL3RA	protein_codin	1.83140155	1.6222693	0	1.77571178
ENSG000001	NRXN1	protein_codin	0	0	0	2.5476839
ENSG000001	PDE6G	protein_codin	0	3.8803927	0	0
ENSG000001	MOSMO	protein_codin	0	0	0	0
ENSG000001	BPY2	protein_codin	0	0	0	0
ENSG000001	INPP5J	protein_codin	0	2.85261729	0	0
ENSG000001	PRMT3	protein_codin	9.22654318	13.3993027	64.782861	5.92593518
ENSG000001	REC114	protein_codin	0	0	0	0
ENSG000001	TRIOBP	protein_codin	0	0.98485644	0.61536416	0
ENSG000001	CYP2F1	protein_codin	0	0	0	1.20855489
ENSG000001	KCND2	protein_codin	1.7342855	2.74177183	41.6326036	8.19360798
ENSG000001	H2AC21	protein_codin	26.3526261	55.3798684	344.134789	15.1449384
ENSG000001	ID2	protein_codin	0	5.72651687	5.75902904	1.05024371
ENSG000001	NPLOC4	protein_codin	21.3733451	39.1559896	40.0160506	26.1183891
ENSG000001	CLSTN3	protein_codin	0	0	0	0
ENSG000001	ENKUR	protein_codin	0	0	0.85776789	0
ENSG000001	ZNF479	protein_codin	0	0	0	0
ENSG000001	PDGFB	protein_codin	4.05330849	37.8721132	3.8817113	7.92891345
ENSG000001	CSTF2T	protein_codin	48.9250408	98.9263196	98.4392106	26.3037677
ENSG000001	DUT	protein_codin	3.07128085	13.7112159	18.5449227	2.46924463
ENSG000001	FBLN7	protein_codin	0	0	0	0
ENSG000001	MAGEA2B	protein_codin	0	0	36.1871383	0
ENSG000001	CLN8	protein_codin	8.03972957	16.1342762	3.51362486	7.57177219
ENSG000001	DLK1	protein_codin	0.87859094	0	0	0
ENSG000001	RTN4RL1	protein_codin	6.01207637	15.6696969	10.3022557	3.95901787
ENSG000001	C11orf54	protein_codin	13.9930871	0	0	3.19329248
ENSG000001	DMTF1	protein_codin	0	12.7249739	51.4803013	15.2188082
ENSG000001	LRATD1	protein_codin	0	5.40199181	0	0

ENSG000001	GLRX3	protein_codin	7.82293115	38.1606436	10.0360089	7.42351707
ENSG000001	ACP7	protein_codin	0	0	0	0
ENSG000001	GLTPD2	protein_codin	0	0	0	0
ENSG000001	MORF4L1	protein_codin	0	3.64828318	14.0891763	13.7306108
ENSG000001	FBXW4	protein_codin	2.33913775	106.737868	7.28316749	1.72358567
ENSG000001	PCYT2	protein_codin	0	0	0	0
ENSG000001	ASCL2	protein_codin	0.46862144	3.47601601	0	0
ENSG000001	SLC25A21	protein_codin	1.1377381	0	1.16042419	0.51460481
ENSG000001	RPH3AL	protein_codin	1.43653884	0	0	0
ENSG000001	DDX59	protein_codin	21.8006375	34.0986012	47.9570824	8.65751909
ENSG000001	MTA1	protein_codin	0	43.3289774	61.7206455	0
ENSG000001	PURA	protein_codin	75.7732651	168.126987	93.5198715	53.4246932
ENSG000001	GLRX5	protein_codin	4.80832729	34.9185064	11.7312317	1.46959142
ENSG000001	FILIP1L	protein_codin	0	0	0	0
ENSG000001	IKZF1	protein_codin	0	0	0	0
ENSG000001	SNORC	protein_codin	0	0	0	0
ENSG000001	POFUT2	protein_codin	1.19743846	18.7450365	8.35865842	2.79407327
ENSG000001	HS3ST4	protein_codin	0.58190036	3.0078918	10.0139979	2.27374094
ENSG000001	CPNE8	protein_codin	25.6098202	42.4104233	101.426232	21.3070767
ENSG000001	POMK	protein_codin	7.57236384	6.22404711	63.8772236	1.7358283
ENSG000001	H2AC20	protein_codin	63.5555305	77.516932	165.940166	52.2419107
ENSG000001	EPB41L5	protein_codin	2.65542261	11.8788621	0	0
ENSG000001	UTY	protein_codin	0	0	0	0
ENSG000001	ZNF789	protein_codin	1.45585721	1.6073506	4.806905	1.24388235
ENSG000001	RBM43	protein_codin	16.8345268	38.8392671	14.7921775	11.3411439
ENSG000001	OTOP2	protein_codin	0	0	0	0.85491207
ENSG000001	XKR3	protein_codin	0	0	0	0
ENSG000001	CLCNKA	protein_codin	0	0	0	0
ENSG000001	C8orf33	protein_codin	15.9022666	27.6740698	85.4686677	0
ENSG000001	NPIPA6	protein_codin	0	0	6.91227438	6.34624412
ENSG000001	TMEM121B	protein_codin	0	0	0	3.04199104
ENSG000001	H1-5	protein_codin	59.3626249	58.8233129	7.74773162	7.64164852
ENSG000001	DGCR6	protein_codin	4.38481337	5.88331414	13.493349	0
ENSG000001	LRRC28	protein_codin	0	0	0	0
ENSG000001	TAB1	protein_codin	0	0	0	2.89859841
ENSG000001	TRAIP	protein_codin	0	1.82384026	9.14972912	0
ENSG000001	DRG1	protein_codin	17.7268563	26.5571601	55.8974861	8.60042011
ENSG000001	HOXD1	protein_codin	1.76401857	1.16080599	31.038556	0.42475225
ENSG000001	CD8B	protein_codin	0	8.80944337	0	0
ENSG000001	BEST3	protein_codin	0	0	0	0
ENSG000001	SMDT1	protein_codin	1.4037712	16.3008556	11.6600835	5.83267093
ENSG000001	P4HB	protein_codin	0	0	0	0
ENSG000001	H3C13	protein_codin	1.13019293	2.73918471	2.68372048	0.56487646
ENSG000001	EFCAB13	protein_codin	1.93046492	0	0	14.1204323

ENSG000001	FMNL1	protein_codin	0	0	0	0
ENSG000001	NGRN	protein_codin	12.9269561	25.225096	40.5651056	17.5225534
ENSG000001	RAI2	protein_codin	0	0	0	0
ENSG000001	EEF1A1	protein_codin	3.96698731	16.0184899	7.68779027	6.53990778
ENSG000001	SLC25A10	protein_codin	0	0	0	0
ENSG000000	CROT	protein_codin	9.55205878	42.3404345	13.4087276	9.38184092
ENSG000001	NR2C2AP	protein_codin	1.06785484	0	0	0
ENSG000002	RHEX	protein_codin	0	0	0	0
ENSG000001	MEIS3	protein_codin	0	0	0	0
ENSG000001	PKP3	protein_codin	0	0	0	0
ENSG000001	SLC6A17	protein_codin	0	2.94555212	0	1.68837113
ENSG000001	ZNF703	protein_codin	5.4595475	12.6586879	25.7325494	10.5646414
ENSG000001	PSMG1	protein_codin	6.80985506	7.04065452	22.4306318	1.71123178
ENSG000001	CADM1	protein_codin	0	0	0	0
ENSG000001	CALCA	protein_codin	0	0	0	3.1799501
ENSG000001	DUSP8	protein_codin	0.91048893	2.82471423	0.60824127	0
ENSG000001	BMP8A	protein_codin	12.1139014	46.3662521	5.19172722	77.4359522
ENSG000001	BGN	protein_codin	298.369455	716.666053	293.801443	419.586734
ENSG000001	P4HA3	protein_codin	22.3162443	0	0	0
ENSG000001	SYNDIG1L	protein_codin	0	0	0	0
ENSG000001	PCARE	protein_codin	0	1.08216875	0	2.96308924
ENSG000001	EIF3C	protein_codin	332.415653	828.959397	1353.62298	192.007105
ENSG000001	DSE	protein_codin	2.04934177	39.4964062	26.3047007	0
ENSG000001	SPAG16	protein_codin	0	0	0	2.35241531
ENSG000001	TBK1	protein_codin	0	0	0	0
ENSG000001	ZNF605	protein_codin	6.4023751	4.65016589	3.51727067	0
ENSG000001	OLFML2A	protein_codin	3.64419953	0	3.71764575	0
ENSG000001	KCNJ12	protein_codin	0.70037861	0	0	0
ENSG000001	LIMK2	protein_codin	5.53326409	0	0	0
ENSG000002	FAM187A	protein_codin	0	6.08188608	0	0
ENSG000001	ZNF438	protein_codin	4.20742994	0	0	0
ENSG000001	RSRC2	protein_codin	13.7019739	68.7585354	20.6960743	7.88695235
ENSG000001	SS18L1	protein_codin	11.2943627	20.9511408	28.2119615	4.20699295
ENSG000001	IMMP2L	protein_codin	3.56515201	5.16723636	5.87588325	3.70744088
ENSG000001	SETD3	protein_codin	39.4905877	99.1609594	74.8723532	37.6295818
ENSG000001	CSF3	protein_codin	0	0	0	0
ENSG000001	RNLS	protein_codin	1.38692225	7.94861308	4.1553529	0
ENSG000001	SPATA32	protein_codin	0	0	0	0
ENSG000001	JAG2	protein_codin	0	15.6166307	20.0173962	7.08204139
ENSG000002	ZNF345	protein_codin	0	0	6.8236922	2.40267335
ENSG000001	UPP1	protein_codin	0	0	0	2.83489706
ENSG000001	DEXI	protein_codin	5.16352411	3.55899012	11.7781088	2.0405496
ENSG000001	KRT7	protein_codin	0	0	0	0
ENSG000000	RANBP1	protein_codin	39.374268	75.6725576	317.34836	30.9541479

ENSG000000	FTL	protein_codin	226.328924	1371.29427	94.7980574	275.143524
ENSG000001	PCSK6	protein_codin	0	0	0	0
ENSG000001	CNTN2	protein_codin	2.25062807	0	0	5.95411429
ENSG000001	SELENOF	protein_codin	44.0033113	11.9038868	105.390721	25.3174929
ENSG000001	ACSM5	protein_codin	4.14793932	0	4.49594317	2.91247052
ENSG000001	SLC35D3	protein_codin	0	0	2.25034248	0.4628744
ENSG000001	SPRR3	protein_codin	0	0	0	0
ENSG000001	GLUL	protein_codin	1.90337371	0	0	7.98878682
ENSG000000	DGKA	protein_codin	0	0	0	0
ENSG000001	CNOT10	protein_codin	11.9101955	0	34.6142041	12.7176944
ENSG000001	FBXL6	protein_codin	0	0	0	0
ENSG000001	OPCML	protein_codin	0	0	0.4660577	0
ENSG000001	PTCH1	protein_codin	26.1682692	18.9411608	102.412315	1.55010817
ENSG000001	PAXBP1	protein_codin	0	0	0	0
ENSG000001	C14orf180	protein_codin	0.37764357	3.9784518	0	1.30613647
ENSG000001	PRKD1	protein_codin	0	0	0	1.93326571
ENSG000001	IFNL2	protein_codin	0	0	0	1.37874139
ENSG000001	DPP6	protein_codin	0	0	0	0
ENSG000002	C1QTNF9	protein_codin	0.3027579	0	0	2.04283075
ENSG000001	SOCS1	protein_codin	1.37317689	0	2.19833711	6.07971167
ENSG000001	EWSR1	protein_codin	0	0	0	5.29563204
ENSG000001	CEP63	protein_codin	0	0	0	0
ENSG000001	EWSR1	protein_codin	0	24.8796032	0	0
ENSG000001	EXOC7	protein_codin	39.6839274	10.5318808	67.7492416	15.1189454
ENSG000001	PHF6	protein_codin	13.2084854	0	16.6091281	0
ENSG000000	WSCD2	protein_codin	0	6.67798458	0	4.48546182
ENSG000001	DAB1	protein_codin	0	0	9.79302901	0
ENSG000001	EPGN	protein_codin	0	0	0	0
ENSG000001	SRPRA	protein_codin	41.0270383	71.2825472	63.7534734	28.5304038
ENSG000000	ZC3H11A	protein_codin	0	0	0	0
ENSG000001	KIF21B	protein_codin	0	0	0	0
ENSG000001	SETD4	protein_codin	1.49654395	11.743986	0	0.88183848
ENSG000001	VCF2	protein_codin	0	0	1.94090207	0
ENSG000001	KCNH7	protein_codin	0	0	0	1.06501362
ENSG000001	CIMAP1C	protein_codin	0.29378555	0	0	0.8173346
ENSG000001	TMPRSS2	protein_codin	0	0	0	0
ENSG000001	TMT1A	protein_codin	10.6678109	77.6009729	21.7863488	24.3801906
ENSG000001	PTPRM	protein_codin	0	36.6074472	0	30.5177241
ENSG000001	WASHC4	protein_codin	101.947635	86.8677997	157.124999	53.2402105
ENSG000001	ROBO2	protein_codin	0	0	0	0
ENSG000001	VDAC2	protein_codin	18.8106592	53.60185	75.095509	16.6098666
ENSG000000	YBX1	protein_codin	14.3263823	38.9236485	12.1149176	8.73691891
ENSG000001	AP2A2	protein_codin	0	0	0	9.76775137
ENSG000001	C2CD4C	protein_codin	0	0.58132569	1.18907515	0

ENSG000001	OR4F15	protein_codin	0	0	0	0.55212311
ENSG000001	CSGALNACT1	protein_codin	0	0	0	0
ENSG000001	OR52L1	protein_codin	0	1.9895092	0	0.52050789
ENSG000001	GPRASP2	protein_codin	5.12339242	0	0	0
ENSG000001	PIWIL3	protein_codin	0	0	0	0
ENSG000001	CHST6	protein_codin	35.5417427	37.6775909	51.6393169	13.5513678
ENSG000001	SNAI3	protein_codin	0.45817575	0.71794011	0	0.78228837
ENSG000001	C6orf120	protein_codin	17.0393324	37.4121148	76.9292767	13.6210318
ENSG000001	PRAMEF1	protein_codin	0	0	0	2.12976735
ENSG000001	RGS19	protein_codin	0	5.05148167	0	7.76700245
ENSG000001	ZNF816	protein_codin	0	0	0	0
ENSG000001	NSMCE3	protein_codin	7.3349384	36.1489229	65.7004937	6.51048616
ENSG000001	FBLIM1	protein_codin	0	0	0	0
ENSG000001	ZFP1	protein_codin	6.85790075	0	33.1132838	6.47324866
ENSG000001	EMILIN3	protein_codin	5.84942393	2.04148724	0	2.95329693
ENSG000001	MRGPRG	protein_codin	0	0	0	0.3907049
ENSG000001	ZNF534	protein_codin	0	0	0	0
ENSG000001	DGAT1	protein_codin	0	5.39507297	0	0
ENSG000001	LIMS1	protein_codin	5.70798174	4.1827619	1.60497984	3.66354333
ENSG000001	JRKL	protein_codin	41.8837913	71.9924698	101.828972	11.3192646
ENSG000001	WT1	protein_codin	0	0	0	0.51970427
ENSG000001	FIGLA	protein_codin	0.35501847	0	0.55109107	0.42700934
ENSG000002	MT1H	protein_codin	0	1.86105406	0	0.97578592
ENSG000001	SPACDR	protein_codin	0	0	0	0.36478009
ENSG000001	KRTAP11-1	protein_codin	0	0	0	0
ENSG000001	NIBAN3	protein_codin	0	0	0	0
ENSG000001	DDX28	protein_codin	2.25867383	0	8.81291973	0.86152287
ENSG000002	SPDYE3	protein_codin	0	0	0	2.59627328
ENSG000001	SHC4	protein_codin	2.62639585	3.29923372	0.76953066	0
ENSG000001	KRT76	protein_codin	0	0.93284811	0	1.40400353
ENSG000001	GPR19	protein_codin	0	0	0	1.65679446
ENSG000001	TCEAL7	protein_codin	0.39552674	0	0	0
ENSG000001	MARCHF11	protein_codin	0	0	0	0
ENSG000000	POLR1H	protein_codin	2.7813756	0	0	0
ENSG000001	CCR10	protein_codin	0	1.01615157	0	0
ENSG000001	CYB5D1	protein_codin	14.3488074	18.5304752	14.2246672	10.7840097
ENSG000000	RNF215	protein_codin	0.36121688	2.06231554	3.12445995	4.12562501
ENSG000001	EP400	protein_codin	4.91426724	26.726519	16.2646123	2.99089285
ENSG000001	SEMA4B	protein_codin	0	0	0	0
ENSG000001	HEXIM1	protein_codin	16.9577007	51.7673698	17.4013805	33.1827336
ENSG000001	HOXB4	protein_codin	8.75922065	13.2934205	2.67624137	3.22003804
ENSG000001	PLA2G6	protein_codin	0	0	0	0
ENSG000001	RIPK4	protein_codin	0	0.99840596	0	0
ENSG000001	TRIML1	protein_codin	0	0	0	0.98798212

ENSG000001:	BAIAP2L2	protein_codin	0.20866277	0	2.07200489	0
ENSG000001	PTGDR2	protein_codin	0.16258385	1.41697507	0.46888208	2.25099667
ENSG000001	IL1R2	protein_codin	0	0	0	2.86940864
ENSG000001	LAMP1	protein_codin	113.455083	205.313641	368.189316	62.4889519
ENSG000002	ELOA2	protein_codin	0.54760272	0.88168695	0	2.13586452
ENSG000001	GPR173	protein_codin	6.88408308	32.7554778	32.5309237	3.18289278
ENSG000001	OSBP2	protein_codin	0	0	2.92088594	0
ENSG000001:	ZNF484	protein_codin	24.7253172	46.3544189	33.9588675	0
ENSG000001	DCUN1D2	protein_codin	0	0	13.5720786	2.83854419
ENSG000001	TMED9	protein_codin	55.2374894	70.9976471	129.043412	21.4377519
ENSG000001	TRAK2	protein_codin	97.3363616	194.024936	109.740276	72.2851749
ENSG000001:	ST3GAL3	protein_codin	0	0	0	0
ENSG000001	WDR53	protein_codin	2.39021913	3.60224958	9.57063626	1.12158204
ENSG000001	LMLN	protein_codin	0	0	0	0
ENSG000001	TMPRSS11B	protein_codin	0	0	0	1.19845804
ENSG000001	FAM133A	protein_codin	6.43620412	68.5774181	8.1721216	0.95039065
ENSG000002	OR5K1	protein_codin	0.35019122	0	0	0
ENSG000001	OR4M2B	protein_codin	0	2.0758082	0.53583236	1.03516956
ENSG000001	MXI1	protein_codin	0	0	20.4592415	4.36787683
ENSG000001	ANXA2	protein_codin	105.955217	0	96.0904442	0
ENSG000001	TRIM49B	protein_codin	0	0	0	0
ENSG000001	RLIM	protein_codin	65.9324146	105.57067	145.244714	87.4724319
ENSG000001	TAS2R60	protein_codin	0	0	0	0
ENSG000000	CMTM1	protein_codin	0	0	0	0
ENSG000001	DBX2	protein_codin	2.36451178	0	0	0.46157906
ENSG000001	TBL3	protein_codin	0	7.66354516	0	0
ENSG000001	XPOT	protein_codin	81.6734893	120.152137	195.128385	48.2075957
ENSG000001	TBX1	protein_codin	0	0	0	0
ENSG000001	URAD	protein_codin	0	0	0	0
ENSG000001	SIGIRR	protein_codin	0	0	0	0
ENSG000001	LYSMD4	protein_codin	0	0	0	0
ENSG000001	SIMC1	protein_codin	0.72451208	0	0	2.16813679
ENSG000001	KLHDC8B	protein_codin	1.90955747	9.65278738	3.03581133	2.92130823
ENSG000001	STAC3	protein_codin	26.01753	0	6.4643691	7.54649715
ENSG000001	ASB7	protein_codin	16.2255027	18.1046361	23.3839393	4.69992672
ENSG000001	SLC22A10	protein_codin	0	0	0	0
ENSG000001	GH2	protein_codin	0	0	0	0
ENSG000001	AEN	protein_codin	9.76661677	9.73526393	13.940575	4.1539647
ENSG000001	HOATZ	protein_codin	0	0	0	0
ENSG000000	SERPINB3	protein_codin	0	0	0	1.40385038
ENSG000001	NOG	protein_codin	0.30970814	1.26746673	27.5626728	3.15140331
ENSG000001	ANO9	protein_codin	0	1.32912803	0	0
ENSG000001	PHF12	protein_codin	21.9121579	0	39.277622	14.4486226
ENSG000001	NLRP9	protein_codin	0	0	0	1.52353448

ENSG000001	SLC8A1	protein_codin	4.43653039	19.3946463	0.55353329	10.7886206
ENSG000001	COLEC10	protein_codin	0.28682447	0	2.06358362	3.20143791
ENSG000001	VSTM4	protein_codin	47.7917967	22.4828459	28.6287087	25.0421677
ENSG000001	ZNF530	protein_codin	0	6.87503186	0	6.32538633
ENSG000001	SPATA31A6	protein_codin	0	2.34313615	0.43845343	0
ENSG000001	TUBA1B	protein_codin	0.35641647	0	10.5740415	14.923523
ENSG000001	SUMO3	protein_codin	23.1117794	98.9022227	93.6867549	30.5717656
ENSG000001	BET1L	protein_codin	1.8406171	0	0	0
ENSG000001	MACC1	protein_codin	0	0	0	0
ENSG000001	CYP2U1	protein_codin	12.3918464	27.5578898	37.0268978	17.1665191
ENSG000000	GEMIN8	protein_codin	0	10.1957396	0	2.34266823
ENSG000001	CRADD	protein_codin	5.58027251	12.1569671	8.70266895	4.78272466
ENSG000001	CDHR1	protein_codin	0	0	0.59358793	1.69074566
ENSG000001	SPATA45	protein_codin	0.28703757	0	0	0
ENSG000000	LMO3	protein_codin	0.2461849	0	0	0
ENSG000001	FAM43B	protein_codin	7.05534437	0	2.20790295	1.63904192
ENSG000001	PLEKHA7	protein_codin	0	0	0	0
ENSG000001	IZUMO1	protein_codin	0	0	0	0.34847076
ENSG000001	RFX6	protein_codin	0	1.32168179	0	2.65774995
ENSG000001	SLIT3	protein_codin	54.1189154	34.7360292	180.329597	0
ENSG000002	RPL17-C18orf	protein_codin	0	0	0	0
ENSG000001	ADSS1	protein_codin	0	0	0	0
ENSG000000	PRKCH	protein_codin	6.90746466	115.203391	15.8097462	20.4092856
ENSG000001	APOL4	protein_codin	0	0	1.56600171	4.9703766
ENSG000001	YIPF7	protein_codin	0	0	0	1.0493191
ENSG000001	PHACTR1	protein_codin	0	0	0	0
ENSG000000	CMTM1	protein_codin	0	0	0	0
ENSG000001	NR2C1	protein_codin	7.21504701	8.38980104	2.36600652	6.81635107
ENSG000001	TNFAIP2	protein_codin	0	0	0	40.384563
ENSG000001	JAKMIP2	protein_codin	0	10.3691047	11.813249	0
ENSG000001	EEF1AKMT3	protein_codin	45.1135775	0	0	0
ENSG000001	LYG2	protein_codin	0	0	0	0
ENSG000001	DGAT2L6	protein_codin	0	0	0	1.84558085
ENSG000001	MTMR3	protein_codin	14.5427776	0	0	18.9585792
ENSG000001	TPRN	protein_codin	1.25740987	0	0	1.85893528
ENSG000001	MAD2L1	protein_codin	5.47520847	12.6685435	89.1449727	0
ENSG000001	TMEM186	protein_codin	1.11432254	0.95189509	15.7932416	1.6151895
ENSG000001	MGAT1	protein_codin	0	0	0	0
ENSG000001	LCK	protein_codin	1.48514253	0	0	1.86641054
ENSG000001	PSMF1	protein_codin	12.0995546	0	17.8923552	9.31034598
ENSG000001	PP2D1	protein_codin	0	0	0	0
ENSG000001	TSKU	protein_codin	8.10919762	16.3720984	11.7958784	14.1036314
ENSG000001	MAGEA11	protein_codin	0	0	0	0
ENSG000001	UXT	protein_codin	5.86874408	7.60653662	10.3539978	6.45969713

ENSG000001	IQCF2	protein_codin	0	0	0	0
ENSG000001	FIGN	protein_codin	12.863633	12.4444949	55.180335	6.49142595
ENSG000001	MRPL40	protein_codin	19.4368782	51.3382865	234.339813	11.8483622
ENSG000001	SMTN	protein_codin	48.5028146	51.0796298	13.1771469	0
ENSG000001	DCAF4L1	protein_codin	1.17069525	1.28525919	2.45333374	3.63415065
ENSG000001	TRIM50	protein_codin	0	0	0	0
ENSG000002	H2AC14	protein_codin	30.1779329	31.8886714	21.1110968	6.35777151
ENSG000000	ACAA1	protein_codin	6.74433974	9.27395727	0	4.95196598
ENSG000001	TAS1R1	protein_codin	0	0	0	0.91004429
ENSG000001	TMEM259	protein_codin	0	85.2885895	0	0
ENSG000001	COPB2	protein_codin	70.6027541	171.753667	634.208538	67.1683345
ENSG000001	ZNF804B	protein_codin	0	0	1.00825045	2.5164269
ENSG000001	PDE8B	protein_codin	0	0.98998621	0	0
ENSG000001	ZNF583	protein_codin	3.35657447	9.47650989	0	1.01446013
ENSG000001	TM2D3	protein_codin	0	0	0	0
ENSG000001	ZSCAN30	protein_codin	0	31.0429055	21.2495312	0
ENSG000001	GPRIN3	protein_codin	0	68.1655372	11.3699322	5.58636722
ENSG000001	TSEN54	protein_codin	0	0	0	0
ENSG000001	ING1	protein_codin	2.35295239	20.2667045	19.1134187	9.03134688
ENSG000001	BRWD1	protein_codin	14.8615035	26.1418567	14.3522026	20.5487566
ENSG000001	C18orf21	protein_codin	0	0	0	0
ENSG000001	AHNAK2	protein_codin	0	54.0942643	85.7920937	37.0823445
ENSG000001	CCDC172	protein_codin	0	0	0	0.55284526
ENSG000001	TSSC4	protein_codin	4.34196708	0	0	0
ENSG000001	EFNA5	protein_codin	22.4836923	62.4706492	45.0252601	14.2895752
ENSG000001	ANKRD45	protein_codin	1.66568093	6.71573686	0.89039194	1.29141374
ENSG000001	IGIP	protein_codin	8.55671476	13.6865173	8.58322775	4.52381323
ENSG000002	TMED7-TICAM	protein_codin	0	0	0	0
ENSG000001	INKA1	protein_codin	3.3527919	2.32508018	0	1.61579271
ENSG000002	OLIG2	protein_codin	0	0	0	0
ENSG000000	SELE	protein_codin	0	0	0	8.20838297
ENSG000001	VPS33B	protein_codin	1.21762924	6.8536713	2.60606433	0
ENSG000001	NPB	protein_codin	0	0	0	0
ENSG000002	SPDYE8	protein_codin	0	0	0	0
ENSG000001	EWSR1	protein_codin	0	0	0	0
ENSG000001	RIOX2	protein_codin	0	0	12.5903672	0
ENSG000001	FAM83F	protein_codin	0.58431199	3.42984907	0	8.59517294
ENSG000001	POLR2F	protein_codin	0	0	0	0
ENSG000001	ABHD17B	protein_codin	4.36912145	30.7156545	14.9287026	6.62009036
ENSG000001	PIF1	protein_codin	0	0	4.24186563	0
ENSG000001	S1PR5	protein_codin	0	0	0	0
ENSG000002	POLR2J2	protein_codin	2.37785388	30.3881943	10.6993599	0
ENSG000001	TEX19	protein_codin	0	1.14521856	0	2.77573259
ENSG000001	PPFIA2	protein_codin	8.42499983	0	3.85335757	0

ENSG000001	TMEM179B	protein_codin	1.06320565	10.3397938	5.3744009	5.95186641
ENSG000001	STAC2	protein_codin	0	0	0	1.85578139
ENSG000001	APOBEC3B	protein_codin	0	4.66845881	0	0
ENSG000001	ADGRF3	protein_codin	0	0	0	0
ENSG000001	KNTC1	protein_codin	17.0635812	51.8116267	116.114428	10.7892714
ENSG000001	MAFA	protein_codin	0.68998412	2.22655654	4.62896485	0.42548489
ENSG000000	METTL2A	protein_codin	4.30877739	14.7772784	22.2756321	3.82543467
ENSG000001	UBA7	protein_codin	21.0615287	190.38347	9.49635224	15.9950677
ENSG000001	ADGRG3	protein_codin	0	0	0	0
ENSG000001	CLN3	protein_codin	0	0	0	0
ENSG000001	PGP	protein_codin	1.60054269	5.71637191	9.90170651	2.00720435
ENSG000001	BSG	protein_codin	0	0	5.86701595	1.1675007
ENSG000001	RESP18	protein_codin	0	2.35127099	0	0
ENSG000001	SCN5A	protein_codin	0	0	0	0
ENSG000001	GALNT17	protein_codin	2.64688501	0	17.7987342	0
ENSG000001	RALY	protein_codin	0	36.2236633	0	0
ENSG000001	EP400	protein_codin	3.86532279	0	0	0
ENSG000001	PIGA	protein_codin	1.52326842	0	0	0
ENSG000001	MCEMP1	protein_codin	0	0	0	0
ENSG000000	TNK2	protein_codin	0	80.8598223	0	0
ENSG000001	TTC32	protein_codin	0.31885163	4.08375575	3.4562868	1.11842342
ENSG000001	LIMK2	protein_codin	5.24178932	0	21.0583765	3.93909924
ENSG000001	LSAMP	protein_codin	0	0	0	0
ENSG000001	IFNL1	protein_codin	0	0	0	0
ENSG000001	TUBB2A	protein_codin	49.8963663	9.17708402	102.745301	17.7219665
ENSG000001	SH3BGR	protein_codin	20.1243187	0	0	0
ENSG000001	POU6F1	protein_codin	0	0	0	4.05669238
ENSG000001	BEX5	protein_codin	0.3331247	0	6.42760157	2.38641596
ENSG000001	MED12	protein_codin	0	0	0	0
ENSG000000	HMG20B	protein_codin	20.3462764	62.5603954	129.421637	15.4929732
ENSG000001	RNF212	protein_codin	0	0	0.756803	0
ENSG000002	MRPL12	protein_codin	10.337412	22.4930781	59.1192488	3.41216603
ENSG000001	ARHGEF37	protein_codin	0.31981826	4.42207194	6.46623777	3.28602239
ENSG000001	BCL2	protein_codin	15.9634991	4.47221765	38.4991409	4.85440532
ENSG000001	TH	protein_codin	0	0	0	0.42177487
ENSG000000	PCDH11Y	protein_codin	8.47923852	0	0	0
ENSG000001	C12orf56	protein_codin	0.27048762	0	0	2.38927166
ENSG000001	TCF12	protein_codin	0	0	0	0
ENSG000001	ANKS1B	protein_codin	0	0	0	0
ENSG000001	GABRG3	protein_codin	0	0	0	0
ENSG000001	CRACR2A	protein_codin	1.0309877	36.2960714	1.18044224	5.62178442
ENSG000002	KCTD11	protein_codin	3.30937083	48.2004042	4.17610813	3.35917013
ENSG000001	NUTM1	protein_codin	0	0	0	0
ENSG000001	H1-10	protein_codin	31.7440345	222.736381	221.282062	64.5071158

ENSG000001	NAA38	protein_codin	0.84366324	4.40119381	6.5317932	2.77955834
ENSG000001	CLASP2	protein_codin	1.54276517	0	0	2.17045154
ENSG000002	USP17L2	protein_codin	0	1.04904027	0	0.83758446
ENSG000001	UGT3A1	protein_codin	0	0	0	0
ENSG000001	TRARG1	protein_codin	8.42398233	5.32182015	0	2.94404358
ENSG000002	KRTAP4-8	protein_codin	0	0	0	0
ENSG000001	NELL2	protein_codin	0	0	0	0
ENSG000001	MBD3L3	protein_codin	0	0	0	0
ENSG000002	EGFL8	protein_codin	0	0	6.21099973	0
ENSG000001	TPH2	protein_codin	0.26480697	0	0	1.42989746
ENSG000001	CCDC87	protein_codin	0	0	2.3900571	0
ENSG000001	CASP9	protein_codin	0	10.0381305	0	0
ENSG000001	SPEM2	protein_codin	0	0	0	0
ENSG000001	NELFA	protein_codin	0	0	0	0
ENSG000002	LCE3C	protein_codin	0	0	0	0
ENSG000000	GP6	protein_codin	0	2.74694679	0	1.79719238
ENSG000001	PCLO	protein_codin	0	0	9.34167404	6.6790682
ENSG000001	KRTAP21-2	protein_codin	0	0	0	0
ENSG000001	SPTBN1	protein_codin	522.452407	495.018075	766.643799	319.931123
ENSG000001	UNC80	protein_codin	0.47677156	1.22498043	3.6578313	0
ENSG000001	NME9	protein_codin	0	0	0	0
ENSG000001	CAAP1	protein_codin	15.5373765	0	44.7168296	13.6323536
ENSG000001	BCDIN3D	protein_codin	0.79441982	6.83802057	5.80112602	2.26092293
ENSG000001	CISD1	protein_codin	6.51614702	3.99352846	10.7452196	5.24357726
ENSG000001	SPIN2B	protein_codin	0.62369627	0	24.3674672	0
ENSG000001	RHOT1	protein_codin	35.3535192	0	104.034864	13.4613651
ENSG000001	ZNF677	protein_codin	0	0	6.58289829	0
ENSG000001	C2CD5	protein_codin	0	0	0	0
ENSG000002	ARHGEF25	protein_codin	65.3880346	44.3840692	0	0
ENSG000001	OR5D18	protein_codin	0	0	0	0
ENSG000001	ZNF568	protein_codin	9.60716693	13.7058704	10.1932124	0
ENSG000001	POLR1B	protein_codin	0	0	0	0
ENSG000001	APAF1	protein_codin	0	0	0	0
ENSG000001	FAM9C	protein_codin	0	0	0	0
ENSG000001	ACTL7A	protein_codin	0	0	0	1.23948682
ENSG000001	GSAP	protein_codin	0	9.82874336	6.3030716	0
ENSG000001	OIT3	protein_codin	0.83550187	0	0	0.90243325
ENSG000001	EMID1	protein_codin	6.26524425	15.0238926	6.41744798	0
ENSG000001	AKR1E2	protein_codin	0	0	0	0
ENSG000001	RGS7BP	protein_codin	0.75477815	5.83439653	5.19344754	4.32026413
ENSG000001	PRODH	protein_codin	0.75229434	0	1.73416134	0
ENSG000001	CPEB4	protein_codin	23.0764736	0	29.6011857	45.3714877
ENSG000001	DMRTC1B	protein_codin	0	0	0	0
ENSG000001	KRTAP19-6	protein_codin	0	0	0	0

ENSG000001	F3	protein_codin	23.0375501	27.0798974	16.4254648	13.9300562
ENSG000001	GNAL	protein_codin	0	0	2.84040857	0
ENSG000001	KRTAP19-2	protein_codin	0	0	0	0
ENSG000001	SPDYA	protein_codin	0	0	0	0
ENSG000001	KRTAP19-4	protein_codin	0	0	0	1.23336892
ENSG000001	SHOX	protein_codin	6.84319195	6.27299045	0	0
ENSG000001	RASA3	protein_codin	56.4550441	375.002585	88.2822049	52.4796414
ENSG000002	KRTAP19-3	protein_codin	0	0	0	0
ENSG000001	KRTAP15-1	protein_codin	0	1.95136224	0	0
ENSG000001	KRTAP13-4	protein_codin	0	0	0	0
ENSG000001	ZNF197	protein_codin	0	0	0	0
ENSG000001	TAL2	protein_codin	0	0	0	0
ENSG000001	HOMER1	protein_codin	17.7939853	14.3683328	86.9863111	14.4098702
ENSG000001	ZNF761	protein_codin	0	0	0	0
ENSG000001	AZI2	protein_codin	27.5162098	0	25.5034853	8.53660188
ENSG000002	KRTAP9-4	protein_codin	0	0	0	0
ENSG000001	ZNF529	protein_codin	12.4081927	6.55447012	17.4348291	10.6848545
ENSG000001	SREK1	protein_codin	10.0001645	16.3185269	29.025158	21.8854208
ENSG000001	TEX38	protein_codin	0	0	0	0
ENSG000001	C2CD3	protein_codin	0	0	0	0
ENSG000001	HTR3D	protein_codin	0	0	0	0
ENSG000001	RFTN1	protein_codin	30.2977758	248.721607	3.8490144	54.6798434
ENSG000001	FGF3	protein_codin	0	0	0	0
ENSG000000	HDAC6	protein_codin	0	0	9.08672976	0
ENSG000001	ZSCAN2	protein_codin	0	0	0	1.36764741
ENSG000001	TMEM52B	protein_codin	0	0	0	0
ENSG000001	KRTAP19-5	protein_codin	0	0	0	1.13805905
ENSG000001	KRTAP23-1	protein_codin	0	0	0	0
ENSG000001	POLR3C	protein_codin	9.89461223	0	0	0
ENSG000002	TCP10L	protein_codin	0	0	0	0
ENSG000001	LYPD6	protein_codin	0	0	7.5492672	0
ENSG000001	RGS9BP	protein_codin	0.51522742	0	0.50878692	0
ENSG000001	UBL4B	protein_codin	0	0.93943979	0	0.42795641
ENSG000001	ZNF749	protein_codin	5.66335229	10.9241485	4.56290073	1.73327091
ENSG000001	PPFIA3	protein_codin	0	4.50427311	0	0
ENSG000000	EML1	protein_codin	11.8951797	0	0	13.8077021
ENSG000001	CYP4Z1	protein_codin	0	0	1.08265452	0
ENSG000001	ZNF347	protein_codin	12.8800805	14.5369917	1.31387925	7.09107716
ENSG000001	KRTAP17-1	protein_codin	0	0	0	0
ENSG000001	KIF27	protein_codin	8.16973346	36.8707761	19.4266582	0
ENSG000001	RPS6KA4	protein_codin	9.24435925	8.51681017	0	7.83015194
ENSG000001	MPPED1	protein_codin	0	0	0	0
ENSG000001	ARAP1	protein_codin	49.5715738	168.899137	36.0547961	35.4458388
ENSG000001	ZNF20	protein_codin	0	14.898911	2.26544919	0

ENSG000001	CDC42BPA	protein_codin	54.330297	0	62.2608422	29.5297307
ENSG000001	DLST	protein_codin	30.3242482	57.4867825	64.3225632	19.0271558
ENSG000001	C1QL4	protein_codin	0	0.93461144	33.9105064	0
ENSG000001	DNAJB14	protein_codin	0	0	0	2.26932079
ENSG000001	EDARADD	protein_codin	0	3.93805024	0	1.95054882
ENSG000001	MAPT	protein_codin	0	0	0	0
ENSG000001	ZNF555	protein_codin	3.73247677	9.96801011	3.46227849	4.39693386
ENSG000001	KPNA4	protein_codin	19.8188121	33.1611257	21.1102071	18.6950005
ENSG000001	SYNE3	protein_codin	0	0	0	0
ENSG000001	TAS2R42	protein_codin	0	0	0	0
ENSG000001	DYNC2H1	protein_codin	0	0	0	0
ENSG000001	TRDN	protein_codin	134.107586	0	0.9583356	1.66986182
ENSG000001	LCE5A	protein_codin	0	0	0	1.00331485
ENSG000001	TTF1	protein_codin	16.5723011	24.1021062	56.6076941	10.3810883
ENSG000001	AP1S3	protein_codin	0.66390426	0	0	0
ENSG000001	TCP11L1	protein_codin	12.3852568	40.1010805	21.4683268	0
ENSG000001	SLC51B	protein_codin	0	0	0	0.40916831
ENSG000001	ANGPTL5	protein_codin	2.75075131	1.68369378	0	0
ENSG000001	CELF5	protein_codin	0	0	0	0
ENSG000001	TPGS2	protein_codin	4.3921064	28.856017	15.4693657	0.65927845
ENSG000001	ADGRA3	protein_codin	12.1825263	0	124.077948	1.52248605
ENSG000001	SOWAHB	protein_codin	0.60795057	4.79506687	0	3.10883977
ENSG000001	IFTAP	protein_codin	7.04817853	0	17.9404737	11.5658697
ENSG000001	TEAD1	protein_codin	18.8598983	17.8764856	60.8584945	8.76466476
ENSG000001	PTBP3	protein_codin	0	9.97559139	2.66937538	0
ENSG000001	SPATA12	protein_codin	0	0	0	1.30466675
ENSG000001	ARMH4	protein_codin	5.54715533	0	0	0
ENSG000001	ARID2	protein_codin	291.656147	58.6602701	166.154213	34.0307466
ENSG000001	MT1B	protein_codin	0	0	0	0
ENSG000002	UPK3B	protein_codin	0	0	0	0
ENSG000001	MT1F	protein_codin	0.95178529	0	0	4.2537748
ENSG000001	PNRC2	protein_codin	68.7731407	121.288668	125.914786	50.743911
ENSG000001	USP16	protein_codin	0	0	0	0
ENSG000001	AKAP14	protein_codin	0	0	0	0
ENSG000001	TXNRD2	protein_codin	0	11.9450636	0	1.2183184
ENSG000001	MEGF8	protein_codin	0	16.3981185	0	0
ENSG000002	LCE1F	protein_codin	0	0	0	0
ENSG000000	TRMT11	protein_codin	6.56721262	0	32.0327087	4.86937936
ENSG000001	QRFPR	protein_codin	0	0	0	0
ENSG000001	ARGFX	protein_codin	0	0	0	0
ENSG000001	DEFB128	protein_codin	0	0	0	0
ENSG000001	TMLHE	protein_codin	18.7621724	26.5103364	8.66162384	7.99239844
ENSG000001	PDCD1	protein_codin	0	0	0	0
ENSG000001	PTPRB	protein_codin	27.7322685	49.7890326	6.35633172	28.6290383

ENSG000001	CENATAC	protein_codin	2.67987393	15.5425192	0	0.69318708
ENSG000001	TRIM7	protein_codin	3.39733565	0	22.0035339	0
ENSG000001	GPR141	protein_codin	1.63238082	2.94248922	0.48529699	1.1738066
ENSG000001	TACC2	protein_codin	9.58947244	0	4.24929206	0
ENSG000001	OR10T2	protein_codin	0	0	0	0
ENSG000001	ABCC5	protein_codin	0	80.2440369	0	0
ENSG000000	CCDC88C	protein_codin	0	0	0	0.8268418
ENSG000001	CEP41	protein_codin	0	0	0	0
ENSG000001	PSMB5	protein_codin	0	0	1.78235863	1.41377931
ENSG000001	PDE2A	protein_codin	0	0	0	0
ENSG000001	C17orf58	protein_codin	0	0	25.2137446	0
ENSG000001	TMEM150A	protein_codin	0.99418351	0	0	0
ENSG000001	ERCC6L	protein_codin	1.62818774	0	28.7667215	3.44508195
ENSG000001	PDZD8	protein_codin	57.0138041	85.892347	160.513221	84.2311909
ENSG000001	TREM1	protein_codin	0	0	0	0
ENSG000001	PFDN5	protein_codin	83.1180251	86.341763	132.543508	52.296383
ENSG000001	PRDX2	protein_codin	0	0	0	0
ENSG000002	S1PR3	protein_codin	1.1246638	0	5.71368236	5.18336272
ENSG000001	WDR86	protein_codin	0	0	11.003332	0
ENSG000001	PRMT2	protein_codin	3.56752342	36.8812014	6.08981092	0.89110436
ENSG000001	TREX2	protein_codin	0	0	0	0
ENSG000001	COL4A6	protein_codin	0	0	0	0
ENSG000001	SLC25A23	protein_codin	0	0	0.81672903	0
ENSG000001	ZMIZ1	protein_codin	45.413524	144.51675	100.249286	71.4932834
ENSG000001	NUDT17	protein_codin	1.57135094	3.14359357	1.05385642	0.84834963
ENSG000001	ZNF410	protein_codin	0	0	0	0
ENSG000001	NDEL1	protein_codin	4.23394092	53.6727989	0	12.4591015
ENSG000002	FMN1	protein_codin	15.012967	49.7012522	26.2681807	29.060664
ENSG000001	BTLA	protein_codin	0	7.56565491	0.55037134	0.4938955
ENSG000001	REEP4	protein_codin	0	5.77664363	0	0
ENSG000001	SLX4IP	protein_codin	14.9086638	42.9983314	42.3328456	7.09155026
ENSG000001	SPNS1	protein_codin	0	0	0	0
ENSG000001	POFUT2	protein_codin	0	67.3502825	0	0
ENSG000000	PPP2R3A	protein_codin	6.00365898	0	8.62733525	0.96946662
ENSG000001	KCNK18	protein_codin	0	0	0	0
ENSG000001	KLC1	protein_codin	0	0	0	0
ENSG000000	ZDHHC8	protein_codin	0.60756355	6.4459848	6.11915727	0
ENSG000001	FMR1	protein_codin	0	0	0	0
ENSG000001	ZNF480	protein_codin	0	0	0	0
ENSG000001	TMEM212	protein_codin	0	0	0	0
ENSG000001	TAC4	protein_codin	0	0	0	0
ENSG000001	COQ6	protein_codin	0	8.08286716	0	0
ENSG000001	FSD2	protein_codin	1.63560583	0	0	2.23799905
ENSG000002	GSTA1	protein_codin	0	0	5.17576967	0

ENSG000001	CARMIL2	protein_codin	0	10.8610685	0	0
ENSG000001	ZACN	protein_codin	0	0	0	0
ENSG000002	ZNF726	protein_codin	0	0	0.96120665	0
ENSG000001	INO80C	protein_codin	0	0	3.51387566	0
ENSG000002	PRAMEF14	protein_codin	0	0	0	0
ENSG000001	TERT	protein_codin	0	0	3.96855206	0
ENSG000001	CCKBR	protein_codin	0	0	1.48310478	0
ENSG000001	TRIM46	protein_codin	0	0	0	0
ENSG000001	DCUN1D4	protein_codin	0	0	0	0
ENSG000001	CYP2R1	protein_codin	0	0	0.73928305	0
ENSG000001	DHRSX	protein_codin	7.24502691	20.5341499	4.45479075	5.98714536
ENSG000001	CHGA	protein_codin	0	0	0	0
ENSG000001	CHP1	protein_codin	18.1637617	34.6479956	43.7307323	20.0347376
ENSG000001	PLCD1	protein_codin	19.2644006	37.3350421	6.68506953	11.7988402
ENSG000001	KRTAP10-8	protein_codin	0	0	0	0
ENSG000002	KRTAP20-1	protein_codin	0	0	0	0
ENSG000001	TMEM68	protein_codin	0	0	1.53517474	0
ENSG000002	HLA-F	protein_codin	0.69167835	0	0	1.36298187
ENSG000002	KRTAP10-11	protein_codin	0	0	0	0
ENSG000001	RPS19BP1	protein_codin	24.1452914	69.8980679	98.109608	13.8935169
ENSG000001	DUSP18	protein_codin	0	13.1878372	0	0
ENSG000001	KRTAP22-1	protein_codin	0	0	0	0
ENSG000001	CCK	protein_codin	0.55720615	0	0	0
ENSG000001	PLCH1	protein_codin	0.39900038	0	0	3.67267562
ENSG000001	TRAPPC11	protein_codin	58.6629478	0	0	17.7238856
ENSG000001	ENTPD5	protein_codin	7.51175066	8.09971363	21.2778442	5.73254382
ENSG000000	HSP90AA1	protein_codin	2.05309671	0	53.7883948	0
ENSG000001	FAM91A1	protein_codin	20.0856208	41.1386627	129.246169	21.5854764
ENSG000001	SERPINA11	protein_codin	0	2.40358747	1.73149851	1.51770125
ENSG000001	ZSCAN25	protein_codin	7.34446966	0	0	2.61411055
ENSG000001	AP4S1	protein_codin	0	0	0	0
ENSG000001	OR13F1	protein_codin	0.53278859	0	0	0
ENSG000001	C9orf85	protein_codin	0.64967704	0	1.68723594	1.55131116
ENSG000001	EMSY	protein_codin	0	0	0	0
ENSG000000	PPP2R5C	protein_codin	0	0	0	0
ENSG000001	PRELID2	protein_codin	0	0	0	0
ENSG000000	BTBD7	protein_codin	0	0	33.5222463	16.2370511
ENSG000001	PLK5	protein_codin	0	0	0	0
ENSG000001	DEFB105A	protein_codin	0	0	0	0
ENSG000001	NEXN	protein_codin	59.3168052	0	0	0
ENSG000001	FOXD2	protein_codin	0.46210526	1.89802794	1.57537051	0.42593595
ENSG000001	BCL9L	protein_codin	0	6.6136805	3.28700667	3.24041147
ENSG000002	STARD10	protein_codin	0.65319718	0	2.91501181	0
ENSG000001	CTBP2	protein_codin	16.1007129	0	22.0494171	0

ENSG000001	HPDL	protein_codin	0.25375713	1.08924458	0	0
ENSG000001	C2orf76	protein_codin	1.67753	0	2.83846237	0
ENSG000001	TMEM262	protein_codin	0	0	0	0
ENSG000001	PGAM1	protein_codin	65.6988256	82.5665216	170.465606	54.7557212
ENSG000001	TMPRSS11A	protein_codin	0	0	0	0
ENSG000001	CMC1	protein_codin	0	0	0	0
ENSG000002	KRTAP19-7	protein_codin	0	0	0	0
ENSG000001	FSCN2	protein_codin	0	0	0	0.37439781
ENSG000001	OR10J5	protein_codin	0	0	0	0.45360596
ENSG000001	LRCH3	protein_codin	15.4325236	0	59.6915792	5.5235176
ENSG000001	LGMN	protein_codin	0	95.9550145	38.2588225	37.3392252
ENSG000001	DEFB131A	protein_codin	0	2.14011782	0	0.81992902
ENSG000000	KMT2E	protein_codin	14.1103831	74.2568095	0	0
ENSG000001	TMEM216	protein_codin	3.02032535	0	0	0
ENSG000001	RAX	protein_codin	0	0	0	0
ENSG000001	TPM1	protein_codin	0	0	0	0
ENSG000001	KRTAP6-2	protein_codin	0	0	0	0
ENSG000001	CELF4	protein_codin	0	0	0	1.95938006
ENSG000001	OR10H1	protein_codin	0	0	0	0
ENSG000001	GGN	protein_codin	0.20688507	2.10462233	0	0
ENSG000001	PPIP5K1	protein_codin	0	0	0	0
ENSG000001	ZNF81	protein_codin	0	1.34597033	0	0
ENSG000001	SF1	protein_codin	0	0	0	0
ENSG000001	RSBN1L	protein_codin	50.6859692	184.818255	192.509584	22.0301998
ENSG000001	PARVA	protein_codin	63.9032004	124.13829	73.7888128	52.1218308
ENSG000001	NF2	protein_codin	0	0	0	0
ENSG000001	LRRC37A3	protein_codin	0	8.84912454	11.5873709	0
ENSG000001	KTN1	protein_codin	3.37083671	3.94818814	7.29840641	1.2590672
ENSG000001	EMC10	protein_codin	7.87758612	254.376899	20.9809391	20.0722986
ENSG000001	NAA20	protein_codin	7.94428387	0	18.9099411	5.91343103
ENSG000001	CMTM7	protein_codin	1.54191327	9.75200906	8.49463581	2.82604237
ENSG000001	RREB1	protein_codin	0	0	0	0
ENSG000001	HEATR4	protein_codin	0	4.17108935	0	5.73487397
ENSG000001	LRRC70	protein_codin	2.36131228	3.78097213	5.41927431	4.58498843
ENSG000001	ITM2C	protein_codin	6.31985349	0	60.5794879	0
ENSG000001	PPP1CC	protein_codin	27.8925865	17.5618338	12.1373565	46.6342823
ENSG000001	C3orf70	protein_codin	12.7594699	7.35544794	32.8090413	6.19783729
ENSG000001	H1-7	protein_codin	0	0	0	3.65556201
ENSG000001	DEFB107A	protein_codin	0	0	0	0
ENSG000001	PPIL2	protein_codin	2.38468346	0	3.3899473	1.50603292
ENSG000001	OR13C8	protein_codin	0	0	0	0
ENSG000001	UBE2C	protein_codin	0	0	1.90580465	0
ENSG000001	RASSF6	protein_codin	0.2590289	0	0	0
ENSG000001	FOXE3	protein_codin	0	0	0	0.97663779

ENSG000001	KDM4D	protein_codin	0.9424575	0	0	0
ENSG000001	ZNF480	protein_codin	0	0	0	0
ENSG000001	KRTAP21-1	protein_codin	0	0	0	0
ENSG000001	RTN4RL2	protein_codin	0.80149283	2.8571544	2.46726008	1.39233315
ENSG000000	MARK3	protein_codin	0	0	0	0
ENSG000001	FAT4	protein_codin	139.915853	130.541459	291.151405	52.6622224
ENSG000002	ZNF486	protein_codin	11.2686949	19.8096802	10.6374558	4.66574204
ENSG000001	CCIN	protein_codin	0.54792029	0	0.88803455	2.10759918
ENSG000001	LCE1A	protein_codin	0	0	0	0
ENSG000001	DHRS4L2	protein_codin	0	0	0	4.58895167
ENSG000001	GATA4	protein_codin	0	0	0	0
ENSG000001	EXOC7	protein_codin	39.5405145	11.2754889	131.171086	24.43738
ENSG000001	ETNK1	protein_codin	0	0	0	0
ENSG000001	FMNL3	protein_codin	168.131664	384.026796	172.471253	143.998433
ENSG000001	ERLIN2	protein_codin	2.70035052	0	0	0
ENSG000001	ANKRD37	protein_codin	1.11238709	13.332722	2.95659956	0
ENSG000000	PKM	protein_codin	66.3827694	196.180383	143.142309	49.0680254
ENSG000001	CDKN3	protein_codin	3.64626296	14.0455604	20.0866449	0
ENSG000001	CCDC73	protein_codin	0	0	0	0.54549946
ENSG000001	DEFB106A	protein_codin	0	0	0	0
ENSG000001	POLM	protein_codin	0	0	0	0
ENSG000000	TESC	protein_codin	0.47632173	0	0	0
ENSG000001	IGFN1	protein_codin	122.938686	24.0371578	1.08262491	9.40278418
ENSG000001	PES1	protein_codin	0	9.44436258	0	0
ENSG000001	PTRH1	protein_codin	0	0	0	0
ENSG000001	SLC36A3	protein_codin	0	0	0	0.88186425
ENSG000002	OR4C12	protein_codin	0	0	0	0
ENSG000001	SLC36A2	protein_codin	8.43985707	17.3154877	0.93158466	11.1203624
ENSG000001	CYP27C1	protein_codin	1.72054126	0	0	2.28376827
ENSG000001	INTU	protein_codin	10.5131875	11.8709856	28.6115645	11.7390143
ENSG000001	FOXK2	protein_codin	15.4067728	67.7559982	107.676648	9.80682866
ENSG000001	ZFAND4	protein_codin	1.16270492	2.82022424	4.72471137	0
ENSG000001	WDR20	protein_codin	2.09868661	0	1.17523553	0
ENSG000000	MAP4	protein_codin	0	21.9945991	0	0
ENSG000001	PITPNB	protein_codin	45.3194348	0	102.506373	31.0693815
ENSG000001	GNG2	protein_codin	2.76321211	4.4427222	0	5.92108909
ENSG000001	CFAP144	protein_codin	0	0	0	0
ENSG000001	FAM170A	protein_codin	0	0	0	0
ENSG000001	ATP4B	protein_codin	0.21979325	0	0	0.81260654
ENSG000001	SESTD1	protein_codin	8.41817702	0	7.95749929	2.1320161
ENSG000001	WDR25	protein_codin	0	0	0	0
ENSG000002	HBB	protein_codin	20.2144914	30.0122317	1.44490815	10.9146762
ENSG000001	OR1J2	protein_codin	0	0	0	0.43845035
ENSG000001	HTR3E	protein_codin	0	2.28093746	0	0.71064409

ENSG000001	PIP5K1C	protein_codin	0	51.9639638	79.0846127	28.4709488
ENSG000001	ZNRF1	protein_codin	1.38143069	12.7628022	0	0
ENSG000001	WASF3	protein_codin	1.44357511	0	17.8813239	6.88111467
ENSG000001	UNC5B	protein_codin	115.384824	300.952468	266.029017	132.519181
ENSG000001	TCFL5	protein_codin	0	6.4088171	52.8397694	5.62339228
ENSG000001	GIT1	protein_codin	0	0	0	0
ENSG000001	SPATA2L	protein_codin	0.70997343	0	0	3.29439344
ENSG000001	AGAP3	protein_codin	0	0	1.32000846	0
ENSG000001	TBX10	protein_codin	0	0	0	0
ENSG000000	RC3H2	protein_codin	2.77472821	17.163516	8.8104608	0
ENSG000001	TMEM17	protein_codin	1.45360201	0	3.41054506	0.99580523
ENSG000001	NIBAN3	protein_codin	0	0	0	0
ENSG000001	OR9Q1	protein_codin	0	0	0	0
ENSG000001	KLHDC10	protein_codin	26.2041815	40.8680365	69.1462711	19.3389469
ENSG000001	KLK13	protein_codin	0.31412231	1.14507264	0	0
ENSG000001	RIC3	protein_codin	0	0	1.96074316	1.24737289
ENSG000001	SELENOV	protein_codin	0	0	0.27654236	0
ENSG000001	ST3GAL3	protein_codin	0	0	0	0
ENSG000001	ARL13B	protein_codin	0	0	2.78498973	0
ENSG000001	PDE9A	protein_codin	0	0	0	0
ENSG000001	GLDN	protein_codin	4.70614637	6.34161186	10.2682996	36.1185412
ENSG000001	ACVR1C	protein_codin	0	0	0	0.60442529
ENSG000001	TRABD2A	protein_codin	0	0	0	0
ENSG000001	TMEM104	protein_codin	0	0	0	0
ENSG000001	SLC10A7	protein_codin	0	0	0	0
ENSG000001	MYO18B	protein_codin	2.81362595	0	0	4.10039981
ENSG000000	KCNQ1	protein_codin	2.27622476	19.8241088	0	2.49040253
ENSG000001	DEFB106B	protein_codin	0.28291499	0	0	0
ENSG000001	ADGRD1	protein_codin	1.24437632	0	0	0
ENSG000001	TYSND1	protein_codin	0.63078813	12.2490337	0	0
ENSG000001	SPATA21	protein_codin	0	0	0	1.72067756
ENSG000001	RNF220	protein_codin	0	2.17938711	14.6702696	26.4665208
ENSG000001	GPATCH8	protein_codin	19.136269	13.3962487	12.8347331	7.17354151
ENSG000001	CCT6A	protein_codin	2.61790177	5.32782397	6.57142242	2.70044048
ENSG000001	MAGED4B	protein_codin	0	0	0	5.5551939
ENSG000001	SF3B1	protein_codin	349.994907	767.651237	556.546355	299.811198
ENSG000001	TPCN1	protein_codin	0	45.1280798	0	19.6108663
ENSG000001	DEFB105B	protein_codin	0	0	0	0.3441626
ENSG000001	PDE9A	protein_codin	0	0	0	0
ENSG000001	ATP5IF1	protein_codin	82.2974497	189.060504	283.820273	48.6034001
ENSG000001	ENSG000001	protein_codin	0.65339557	6.44051212	1.52903623	0.81546508
ENSG000001	VSTM2B	protein_codin	0	0	0.64055131	0.94300252
ENSG000001	PRR30	protein_codin	0.24509359	0	0	0
ENSG000001	PLA2G6	protein_codin	0	0	0	0

ENSG000001	GHRL	protein_codin	0	0	0	0
ENSG000001	CSMD1	protein_codin	0	6.49191681	0	0
ENSG000001	KRT26	protein_codin	0	0	0.51408244	1.50251396
ENSG000001	SAMD7	protein_codin	0.25497047	0	0	0.81172982
ENSG000001	SLITRK2	protein_codin	0.54351033	0	0	4.25678454
ENSG000001	UGT2B28	protein_codin	0	0	0	0
ENSG000000	PPP2R2C	protein_codin	0	0	1.35002319	2.60307292
ENSG000001	OR5D14	protein_codin	0	0	0	0
ENSG000002	AMACR	protein_codin	2.33987631	20.3174996	8.54193518	5.83139072
ENSG000001	VWCE	protein_codin	0	0	13.7285596	0
ENSG000001	CFAP73	protein_codin	0	0	0	0
ENSG000001	VSIG10L	protein_codin	0	0	0	0.91233654
ENSG000001	GABRA5	protein_codin	1.71792681	0	0	0
ENSG000001	TCF20	protein_codin	20.707256	77.387237	110.109769	27.5142583
ENSG000001	LCE3B	protein_codin	0	0	0	0
ENSG000001	KATNA1	protein_codin	0	0	0	0
ENSG000001	KATNA1	protein_codin	5.35287615	0	0	0
ENSG000000	ANK1	protein_codin	0	0	0	0
ENSG000001	DPPA4	protein_codin	0	1.77917319	0	1.89218133
ENSG000001	ANKRD46	protein_codin	12.5760788	17.5197413	34.2681573	0
ENSG000001	BCL2A1	protein_codin	0	0	0	0
ENSG000002	GPR142	protein_codin	0	0	0	0
ENSG000000	RORA	protein_codin	34.0187095	72.9945801	33.9801753	44.2138505
ENSG000001	LCE3A	protein_codin	0	0	0	0.46458919
ENSG000001	GRK1	protein_codin	0	0	0	0.67569458
ENSG000001	NPAS2	protein_codin	5.05380305	19.3412164	11.5322493	4.00870712
ENSG000001	SUGCT	protein_codin	4.16768137	0	0	0
ENSG000001	GGTLC1	protein_codin	0	0	0	0
ENSG000001	ATP5F1C	protein_codin	19.1469907	28.2131824	39.4009053	12.8172541
ENSG000001	BCL11A	protein_codin	0	0	33.6058925	6.75461626
ENSG000001	SULT1A2	protein_codin	0	0	0	0
ENSG000000	TDP1	protein_codin	2.81820716	8.54109926	14.5687784	2.35423166
ENSG000001	TNRC6B	protein_codin	72.6277924	178.607287	0	59.3883617
ENSG000001	LOXHD1	protein_codin	3.56693056	12.1448679	2.85152901	0
ENSG000000	NRXN3	protein_codin	0	0	3.89215628	18.1530997
ENSG000000	SLK	protein_codin	263.151871	343.940797	714.16283	279.902887
ENSG000001	ACTR8	protein_codin	11.4252009	35.6387905	24.1107219	6.23216569
ENSG000001	CENPA	protein_codin	2.0865203	0	0	0
ENSG000001	SLC44A2	protein_codin	0	0	0	0
ENSG000001	APOBEC3B	protein_codin	1.03952763	0	0	0
ENSG000001	CACNA1C	protein_codin	0	18.525146	0	0
ENSG000001	UNC119	protein_codin	6.14281002	13.5380174	4.12550549	0
ENSG000001	CDKN2D	protein_codin	4.35530923	0	1.2505595	2.53821236
ENSG000001	WFDC10B	protein_codin	0	0	0	0

ENSG000001:	F11R	protein_codin	0	5.2984966	0	0
ENSG00000:	CENPQ	protein_codin	6.61605792	13.3214961	33.9433033	3.09092629
ENSG000001(	LMO1	protein_codin	0	0	0	0
ENSG000001:	ST6GALNAC4	protein_codin	0	22.2948266	3.04827766	1.83972535
ENSG000001'	CDC42EP4	protein_codin	13.4050221	26.1849226	36.3917024	68.3680449
ENSG000001:	UBE2E2	protein_codin	1.81934192	0	0	0
ENSG000001(	ZNF354A	protein_codin	14.1967638	18.7533027	34.3352279	9.80581968
ENSG00000:	ZNF446	protein_codin	0	0	0	0
ENSG000001:	CRISP1	protein_codin	0	0	0	0
ENSG000001(	PAQR6	protein_codin	0	0	0	0
ENSG000001:	ALAS2	protein_codin	0	0	0.87046913	0
ENSG000001:	RAD51D	protein_codin	0	0	0	0
ENSG000001(	GRB10	protein_codin	12.5596449	35.5710317	41.0764112	13.583448
ENSG000001(	DACT1	protein_codin	0	0	3.38657945	0.85815615
ENSG000001'	ZNF212	protein_codin	6.69173386	19.9612681	10.9701047	3.52052998
ENSG000000(	SLC6A16	protein_codin	0	15.9717454	4.07870067	0
ENSG000001:	PSMF1	protein_codin	3.53368228	20.4180496	2.91979491	4.19962311
ENSG000001:	SERHL2	protein_codin	0	0	3.02442798	0
ENSG000001:	UXT	protein_codin	0	2.6609775	0	0.67931881
ENSG000001(	IQCH	protein_codin	0	0	0	2.75333064
ENSG000001:	BTF3	protein_codin	52.1535729	98.0765826	174.501162	91.4829798
ENSG00000'	FGFR1	protein_codin	0	0	3.10977357	2.35629536
ENSG000001:	RASGEF1B	protein_codin	0	56.4335698	0	0
ENSG000001	TP53I3	protein_codin	3.7932474	4.18200827	32.0195827	0
ENSG000002:	ENSG000002:	protein_codin	0	0	0	0
ENSG000001(	ZBTB16	protein_codin	38.1870416	26.8483715	38.2655983	7.8405422
ENSG000001:	ZDHHC4	protein_codin	0	0	0	0
ENSG000001:	TARS3	protein_codin	17.8187448	35.1195477	51.3132282	0
ENSG000001:	UBA1	protein_codin	38.1203828	37.1201095	88.0567512	33.6415281
ENSG000001	ARL6	protein_codin	0	0	1.89811716	0
ENSG000001'	OVOL1	protein_codin	0	0	0	0.37249877
ENSG000001(	ELF4	protein_codin	18.5909299	98.5890671	26.8703145	20.0536025
ENSG000001(	NCKAP5L	protein_codin	17.5948826	68.1974345	53.0541028	16.3479503
ENSG000001:	TUBA1B	protein_codin	122.269328	621.100258	541.890147	106.224047
ENSG000001:	PNRC1	protein_codin	163.622738	547.057428	102.709843	349.447141
ENSG000001(	DEDD2	protein_codin	0.47171285	0	1.5432714	1.15175018
ENSG00000!	HNRNPC	protein_codin	17.1561423	47.8554479	23.6323872	4.76880558
ENSG000001(	HDAC5	protein_codin	0	4.96759467	0	5.27640127
ENSG000001:	PANK2	protein_codin	24.4612621	27.1330695	43.1984508	12.8254541
ENSG000001(	TOR2A	protein_codin	0	0	0	0
ENSG000001:	FRMD1	protein_codin	0	0	0	0
ENSG000001:	KIR2DL1	protein_codin	0	0	0	0
ENSG00000'	ZNF532	protein_codin	0	0	2.25650973	0
ENSG000000(	DDX3Y	protein_codin	30.518627	178.391249	0	0

ENSG0000017	RAB6A	protein_codin	82.1044338	47.2114609	93.3404078	54.6587565
ENSG0000010	URGCP	protein_codin	1.3905555	31.9934074	8.34529185	2.81175854
ENSG000001:	TNNI1	protein_codin	5.83583078	0	0	0
ENSG0000010	RNF24	protein_codin	0	21.3579631	0	0
ENSG000002:	NME1	protein_codin	3.01912954	0	7.93121726	0
ENSG0000017	FGG	protein_codin	0	0	0	0
ENSG0000010	NINL	protein_codin	1.12504257	0	0	0
ENSG000000:	OGFOD1	protein_codin	9.43616836	10.5167904	0	6.87028297
ENSG000001	ASXL2	protein_codin	70.6321474	215.211288	265.536592	50.1612059
ENSG0000010	NLRP3	protein_codin	0	0	0	0
ENSG000001	IL17F	protein_codin	0	0	0	0.37744148
ENSG000001:	ARL17A	protein_codin	0	48.2828924	11.9668643	1.9766287
ENSG000001:	PCGF5	protein_codin	0	30.4659612	7.48899898	0
ENSG000001:	ZNF548	protein_codin	11.5946102	22.8062006	0	11.8313558
ENSG0000020	SARNP	protein_codin	64.515765	109.476443	158.438896	27.2181713
ENSG000001:	THEM6	protein_codin	1.7858573	7.74956886	3.45514971	1.19547127
ENSG0000010	ZNF202	protein_codin	1.09982788	5.82215784	0	0
ENSG000001!	RAD54B	protein_codin	0.9779482	7.34603804	25.2153771	1.86046102
ENSG000001	HTR7	protein_codin	0	0	0	0
ENSG0000010	KIAA0930	protein_codin	25.0219108	35.3652539	74.6254152	20.9615776
ENSG000001:	KLRD1	protein_codin	0	27.9897693	0	0
ENSG000001:	RGN	protein_codin	0.46705788	1.10921763	0	0.52390217
ENSG0000020	STRADA	protein_codin	0	0	0	0
ENSG0000010	LIMK1	protein_codin	16.7109211	63.3135325	8.61393971	7.77947508
ENSG000000:	TRAF5	protein_codin	11.0457443	0	0	0
ENSG000001	AKT3	protein_codin	0	0	0	0
ENSG000001	HMG20A	protein_codin	9.71290805	56.0995044	0	15.7080513
ENSG0000017	CDCA4	protein_codin	3.87431112	19.8342494	31.36788	5.85978399
ENSG000001	MRPL37	protein_codin	0	0	0	0
ENSG000001	SLC22A8	protein_codin	0	0	0	0
ENSG0000010	LIPA	protein_codin	18.5158361	60.0303221	30.4275056	20.517134
ENSG0000000	NDUFAF7	protein_codin	0	0	0	0
ENSG000001:	CHST5	protein_codin	0	1.3128605	0	0.45478777
ENSG000001	MTDH	protein_codin	79.3515483	114.877748	218.125574	72.0900387
ENSG0000010	SNTB2	protein_codin	66.4559786	171.016355	162.800337	54.0074714
ENSG000000:	HEPH	protein_codin	0	0	0	0
ENSG000002	SRA1	protein_codin	7.75681665	0	11.496216	2.89893093
ENSG000001:	SYNM	protein_codin	25.0943772	112.267424	11.5542216	28.7697142
ENSG000001:	TMEM39B	protein_codin	0	0	0	0
ENSG0000010	MRPL55	protein_codin	0	0	0	0
ENSG000001:	HYAL3	protein_codin	2.50044583	0	0	0
ENSG000001:	STARD3	protein_codin	0.87268329	1.98903807	0.95641223	2.12860492
ENSG000001	LARP1	protein_codin	0	11.1858595	0	0
ENSG000000:	CMTM1	protein_codin	0	0	0	0

ENSG0000007	ZXDC	protein_codin	6.85163142	20.0718042	16.014955	11.7955737
ENSG0000010	KLK5	protein_codin	0	0	0	0
ENSG0000013	SPAG17	protein_codin	3.74723575	28.5228578	13.1877066	5.24597757
ENSG0000017	SPTY2D1	protein_codin	54.9216504	99.6235804	180.873617	94.4035845
ENSG0000012	LRAT	protein_codin	0.44726891	0	2.71576295	0
ENSG0000010	CYP3A7	protein_codin	0	0	0	0.92404374
ENSG000000	ACP3	protein_codin	1.42192653	3.91781187	2.40203995	0.86989257
ENSG0000008	DNAAF6	protein_codin	0	0	0	0
ENSG0000010	TESK1	protein_codin	4.57942703	0	19.6846372	14.262092
ENSG0000010	CYP3A4	protein_codin	0	0	0	0
ENSG0000014	CEP170	protein_codin	50.9927284	0	125.002384	73.3500242
ENSG0000015	AP3S2	protein_codin	10.8474131	16.312146	12.9363759	7.26319171
ENSG0000005	TBXAS1	protein_codin	0	0	0	0
ENSG0000010	SERPINB7	protein_codin	0	0	0	0
ENSG0000012	ACOT9	protein_codin	12.992769	34.9604536	9.74004964	2.46761694
ENSG0000013	GGT7	protein_codin	3.9918008	24.9216387	19.1926998	5.37366596
ENSG0000018	ZFP36L1	protein_codin	0.64955103	13.7141323	3.42091342	12.7034722
ENSG0000010	EPPIN	protein_codin	0	0	0	0
ENSG0000015	RMND1	protein_codin	0.48043321	0	0	0
ENSG0000013	EIF5A	protein_codin	4.14316043	0	1.3513511	0
ENSG0000007	CDC14A	protein_codin	6.38064591	11.8410687	44.5300491	20.1483581
ENSG0000010	ZFYVE19	protein_codin	0	0	0	0
ENSG0000013	EIF5A	protein_codin	10.2581473	7.96599091	75.3662193	19.1895548
ENSG0000019	SLC22A12	protein_codin	0	0	0	0
ENSG0000013	GNL3L	protein_codin	0	0	0	0
ENSG0000014	PIK3R1	protein_codin	25.871673	37.532528	3.37549398	6.42415841
ENSG0000007	NFKB2	protein_codin	0	0	0	0
ENSG0000007	ARHGAP10	protein_codin	61.6045402	101.958477	132.094327	40.0030737
ENSG0000017	SCAI	protein_codin	10.702327	39.4023685	22.510847	6.83462001
ENSG0000009	MFSD11	protein_codin	0	0	0	0
ENSG0000018	ZP3	protein_codin	0	0	3.6425123	0
ENSG0000010	MRPL55	protein_codin	0	6.51191461	2.00555135	0
ENSG0000006	FGFR2	protein_codin	0	9.63338273	0	0
ENSG0000013	CIDEB	protein_codin	0	0	0	0
ENSG0000012	ZNF576	protein_codin	5.2882511	8.03008077	8.03429915	3.67602297
ENSG0000013	DNAJB2	protein_codin	3.9468791	11.6437191	0	2.88625439
ENSG0000000	MMP25	protein_codin	0	0	2.46743671	0
ENSG0000010	PBLD	protein_codin	0	0	0	2.59292768
ENSG0000015	CUL4B	protein_codin	24.3385442	0	0	0
ENSG0000004	EPHA3	protein_codin	100.308527	92.9815934	62.3894769	35.1585253
ENSG0000018	LINC03040	protein_codin	0.65599102	1.99976675	0	0
ENSG000000	IFFO1	protein_codin	0	22.1617247	0	0
ENSG0000024	ZNF324B	protein_codin	0.72740425	3.40012819	2.70229038	2.52519868
ENSG0000017	PNPLA2	protein_codin	11.332571	19.7366353	9.42519268	13.6103511

ENSG000001	RNASEH2B	protein_codin	0	3.67740444	0	0
ENSG000000	HFE	protein_codin	0	0	0	0
ENSG000001	PPP2R2B	protein_codin	0	0	0	0
ENSG000001	GCK	protein_codin	0	0	0	2.26286973
ENSG000000	SLC39A9	protein_codin	46.9745043	71.4938253	69.860041	24.8506749
ENSG000001	HLCS	protein_codin	4.5355484	0	25.6667811	0
ENSG000001	CDK20	protein_codin	0	0	1.67473862	0
ENSG000001	TMEM217	protein_codin	0	0	0	0
ENSG000001	AGAP1	protein_codin	47.5584971	0	55.9244968	15.8399807
ENSG000001	PLSCR2	protein_codin	0	0	0	0
ENSG000000	LRRFIP2	protein_codin	9.66436232	7.20647462	0	0.84897074
ENSG000001	TMEM97	protein_codin	0	0	0	1.95958254
ENSG000000	ASAP3	protein_codin	23.617282	66.8597248	56.865063	0
ENSG000001	ZC3H14	protein_codin	0	0	0	0
ENSG000001	ROMO1	protein_codin	0.1928551	0	0.35126779	0.63919313
ENSG000001	C2CD2L	protein_codin	3.52558596	4.44352376	1.66409712	2.37993762
ENSG000002	TRIM16	protein_codin	0	17.4264362	0	16.1368123
ENSG000001	SEC23B	protein_codin	0	0	0	0
ENSG000001	ZNF687	protein_codin	13.2900916	37.8192134	15.2643924	0
ENSG000001	HORMAD2	protein_codin	0	0	0	0
ENSG000001	RGS12	protein_codin	3.16516685	0	11.102427	0
ENSG000001	SLC25A26	protein_codin	0	0	0	0
ENSG000000	SEL1L	protein_codin	93.5267822	223.307834	164.458188	58.9800011
ENSG000000	HDHD5	protein_codin	2.81693435	0	0	0
ENSG000001	EGFLAM	protein_codin	1.52566752	0	1.65737601	0.94063257
ENSG000000	MOXD1	protein_codin	1.15521881	0	3.89754892	1.9400831
ENSG000001	HJV	protein_codin	0	0	0	0
ENSG000001	ZCCHC7	protein_codin	17.2752908	47.600173	42.7205132	17.0501114
ENSG000000	DAZAP1	protein_codin	0	76.0605921	0	0
ENSG000001	BRIX1	protein_codin	17.6359165	44.2183711	117.9408	13.1033857
ENSG000002	TAFA5	protein_codin	0	0	1.80309381	0
ENSG000001	BVES	protein_codin	0	1.50494737	0	0
ENSG000002	SSX2	protein_codin	0	6.47939562	71.0318298	0
ENSG000001	ATXN2L	protein_codin	0	0	0	0
ENSG000000	RAB27A	protein_codin	2.45932436	10.0732357	1.41463425	5.35825934
ENSG000001	SDC3	protein_codin	0.71189915	18.158195	6.98218298	1.02393864
ENSG000001	CLDN2	protein_codin	0	0	0	0
ENSG000002	ANG	protein_codin	0	1.37193591	0	0
ENSG000001	DHX15	protein_codin	60.6746481	249.089057	241.767828	47.1957378
ENSG000001	CAPS2	protein_codin	1.88385248	0	2.55422958	0
ENSG000000	FNDC3B	protein_codin	0	0	36.1820234	18.3686796
ENSG000001	DRC7	protein_codin	0	0	0	0
ENSG000001	FCRLB	protein_codin	0	0	0	0
ENSG000001	CIDEC	protein_codin	1.89766442	0	0	2.56175173

ENSG000001	CCDC88A	protein_codin	0	0	0	0
ENSG000001	PAX3	protein_codin	0	0	0	0
ENSG000002	CSH2	protein_codin	0	0	0	0
ENSG000001	ATG101	protein_codin	3.02575636	24.8715124	13.0252315	3.34024837
ENSG000001	KRT13	protein_codin	0	0	0	0
ENSG000000	UNG	protein_codin	0	0	0	0
ENSG000001	OPRL1	protein_codin	0	0	0	0
ENSG000001	NXN	protein_codin	13.0726879	33.5753318	49.5883544	7.40214649
ENSG000001	HPS4	protein_codin	0	0	0	0
ENSG000001	LCK	protein_codin	0	27.3687332	0	0
ENSG000001	GDPD5	protein_codin	1.700308	9.25709293	0	5.39233727
ENSG000001	SNCA	protein_codin	0	0	0	0
ENSG000001	HBG2	protein_codin	0	1.32499368	0	0
ENSG000001	SKAP1	protein_codin	5.46147938	35.9313865	0	9.62156109
ENSG000001	FN1	protein_codin	33.4629527	0	59.7511634	0
ENSG000001	ZNF385B	protein_codin	0.6353463	0	26.5711645	0
ENSG000001	HIP1	protein_codin	30.7628066	99.433445	43.2050983	33.8779329
ENSG000001	LUZP2	protein_codin	2.8609167	0	3.71783081	0
ENSG000001	STARD13	protein_codin	15.0769614	0	0	0
ENSG000001	ATF3	protein_codin	0	0	0	0
ENSG000001	MIER3	protein_codin	0	0	0	0
ENSG000001	MID1IP1	protein_codin	5.60769449	3.53881812	0	9.9097419
ENSG000002	ARHGAP8	protein_codin	0	0	0	0
ENSG000001	MYADM	protein_codin	0	0	0	0
ENSG000002	ZNF224	protein_codin	0	0	0	0
ENSG000002	AGPAT1	protein_codin	0	0	0	0
ENSG000001	PRR5	protein_codin	0	0	0	0
ENSG000001	PRELID3A	protein_codin	0	0	5.48031295	0
ENSG000002	SLC26A6	protein_codin	1.44570133	19.9107728	15.606769	1.95310618
ENSG000000	SEC62	protein_codin	47.3626151	61.910173	89.3697301	32.7818189
ENSG000001	ATP5MK	protein_codin	0	0	0	0
ENSG000001	PTDSS1	protein_codin	21.1934799	57.4303455	87.38454	18.375499
ENSG000001	AGTPBP1	protein_codin	0	0	0	4.45274136
ENSG000001	HEXD	protein_codin	0	14.7141972	0	0
ENSG000000	SUGP2	protein_codin	12.7028376	44.891093	88.1728271	0
ENSG000001	PACRG	protein_codin	0	0	0	0
ENSG000001	OPRM1	protein_codin	0	0	0	0
ENSG000000	EPB41L2	protein_codin	143.245928	180.607864	223.659739	121.463543
ENSG000001	ZNF444	protein_codin	0	0	0	0
ENSG000001	C8orf34	protein_codin	0.95112369	0	0	1.45326629
ENSG000002	CELA3B	protein_codin	0	0	0	0
ENSG000001	MRPL35	protein_codin	9.44406535	21.6058624	34.9018967	6.41107141
ENSG000001	MFF	protein_codin	0	0	3.36452629	0
ENSG000001	SH2B1	protein_codin	5.4660596	5.67252212	7.23844686	0

ENSG000001[illegible]	UGP2	protein_codin	9.13941966	0	16.8688248	5.51195695
ENSG000001[illegible]	IL18BP	protein_codin	0	0	0	0
ENSG000001[illegible]	NECAP2	protein_codin	10.6022647	45.0455754	38.5270199	0
ENSG000000[illegible]	TBC1D22A	protein_codin	0	11.1736698	7.32793095	4.02806912
ENSG000001[illegible]	HIF1A	protein_codin	55.0335074	149.798084	118.709136	109.806299
ENSG000000[illegible]	EZR	protein_codin	0	123.327325	17.956329	205.659963
ENSG000001	CAMKK2	protein_codin	4.08031915	25.7470502	0	0
ENSG000001[illegible]	NUDT4	protein_codin	0.64308418	26.4829301	0	1.84339184
ENSG000001[illegible]	MXD4	protein_codin	14.4870759	91.8629454	36.6875298	19.2422723
ENSG000001[illegible]	CTBP2	protein_codin	6.89634529	0	5.96910148	4.74348507
ENSG000001[illegible]	AZIN1	protein_codin	69.5625158	64.9803747	127.41717	69.4238175
ENSG000001[illegible]	WFDC3	protein_codin	0	0	0	0
ENSG000001[illegible]	PCGF6	protein_codin	9.99620882	0	0	0
ENSG000001[illegible]	SCARA3	protein_codin	0.78957036	1.70207494	24.7099124	0
ENSG000001[illegible]	CXCL14	protein_codin	121.453577	23.7689134	107.303426	8.68777417
ENSG000000[illegible]	RBBP9	protein_codin	14.9968547	26.9728105	27.7459818	14.7893727
ENSG000001[illegible]	CDR2L	protein_codin	9.07503658	19.8766612	30.9458302	7.01738132
ENSG000001[illegible]	P2RX4	protein_codin	0	0	0	11.7287095
ENSG000000[illegible]	STPG1	protein_codin	2.43595471	0	4.3550906	0
ENSG000000[illegible]	TNPO1	protein_codin	85.4766071	81.9090254	187.983993	19.8188599
ENSG000000[illegible]	STARD7	protein_codin	47.5350554	175.869433	161.072959	39.8144845
ENSG000001[illegible]	COL2A1	protein_codin	0	0	121.316829	0
ENSG000001[illegible]	PBRM1	protein_codin	26.298081	0	29.4197366	0
ENSG000001[illegible]	ATF4	protein_codin	2.66139879	22.4349295	6.93296556	16.2455282
ENSG000001[illegible]	FAM53B	protein_codin	10.5835449	55.1673128	23.837575	13.4575789
ENSG000001[illegible]	BAG5	protein_codin	22.1506561	54.0520971	63.8225562	17.2861086
ENSG000001[illegible]	CRYGN	protein_codin	0.30822449	0	0	1.26711669
ENSG000001	NPHP3	protein_codin	29.1355764	137.226326	65.9901684	18.7843444
ENSG000001[illegible]	ABHD12B	protein_codin	0	0	0	1.48173895
ENSG000001[illegible]	LRRC8D	protein_codin	16.2652617	5.67917849	45.294914	10.1209139
ENSG000000[illegible]	MSL3	protein_codin	7.94771523	0	20.0766682	0
ENSG000001[illegible]	DGKH	protein_codin	8.49123001	0	22.0160132	9.91308003
ENSG000001[illegible]	PCID2	protein_codin	0	0	5.15529698	0
ENSG000000[illegible]	LUC7L	protein_codin	4.42237108	7.9368949	6.48542933	0
ENSG000001[illegible]	FGD3	protein_codin	0	0	0	0
ENSG000001[illegible]	SEC13	protein_codin	0	0	0	0
ENSG000001[illegible]	CCDC68	protein_codin	8.9975778	7.62516009	3.39382789	5.34564006
ENSG000001[illegible]	KCNE1	protein_codin	0.70609303	0	0	0
ENSG000001[illegible]	NAB1	protein_codin	14.0048273	45.1098222	85.7732303	19.0461746
ENSG000001[illegible]	WTAP	protein_codin	4.95156256	0	7.15239226	9.25967917
ENSG000001[illegible]	CLPTM1	protein_codin	19.7219763	63.1129499	46.4824829	25.910287
ENSG000001[illegible]	ZNF644	protein_codin	154.54555	229.504882	395.05174	94.0560526
ENSG000001	PPARD	protein_codin	5.5005225	17.5523322	4.27393474	6.88379522
ENSG000000[illegible]	ZNF112	protein_codin	0	16.5192395	5.03256861	2.46781859

ENSG000002	SOD2	protein_codin	9.26760541	15.6426286	11.9673461	20.96706
ENSG000000	ARHGAP6	protein_codin	0	0	11.5514779	0
ENSG000001	SERPINA9	protein_codin	0	0	0	0
ENSG000001	LRRC71	protein_codin	0	0	0	0
ENSG000001	RAD51C	protein_codin	0	0	0	0
ENSG000001	ZNF226	protein_codin	18.6872399	0	0	0
ENSG000001	NIPA1	protein_codin	4.2790519	10.7649398	13.1173617	6.1460012
ENSG000001	NIPA2	protein_codin	2.38353248	0	0	0
ENSG000001	AVPR2	protein_codin	0	0	0	0
ENSG000001	ITPRIP	protein_codin	5.15052167	18.9572972	8.70917699	65.0820917
ENSG000001	FIP1L1	protein_codin	0	0	0	0
ENSG000000	SIRT6	protein_codin	0	8.35723149	2.58978392	0
ENSG000000	SCMH1	protein_codin	0	0	0	0
ENSG000002	SSX2	protein_codin	0	0	22.2387022	0
ENSG000000	NRIP2	protein_codin	1.56652353	12.4239056	0	2.51514972
ENSG000000	IGF1	protein_codin	65.3649612	81.7573298	6.26954742	41.6934558
ENSG000002	DXO	protein_codin	0.74706771	0	2.82517914	0
ENSG000001	RTN4	protein_codin	0	83.1431214	0	79.9619983
ENSG000001	KIAA1958	protein_codin	0.70701424	0	5.15068434	7.89484818
ENSG000001	MPP7	protein_codin	0	0	0	0
ENSG000001	PPP2R5E	protein_codin	18.9695437	68.5150598	27.1167743	21.327256
ENSG000001	ADORA2A	protein_codin	0	9.15844345	0	0
ENSG000001	ZFYVE27	protein_codin	0	0	4.64190865	7.42354359
ENSG000001	TSPO	protein_codin	4.77956039	5.61768016	5.64220307	2.22058805
ENSG000001	POLR3H	protein_codin	0	0	0	0
ENSG000000	CAPN3	protein_codin	0	0	0	0
ENSG000001	EBAG9	protein_codin	0	0	0	0
ENSG000000	LZTS3	protein_codin	0	0	0	0
ENSG000001	RSPH10B	protein_codin	0	0	0	1.50884579
ENSG000001	SMUG1	protein_codin	0	0	0	0
ENSG000001	MUC1	protein_codin	2.58049446	0	0	0
ENSG000000	BCL2L13	protein_codin	0	0	0	0
ENSG000001	AP1S1	protein_codin	13.1452933	7.77209849	13.591412	3.05792735
ENSG000001	CBX3	protein_codin	2.30111215	34.3213079	8.44501721	4.39117317
ENSG000001	EDRF1	protein_codin	0	34.943565	0	0
ENSG000001	GSG1	protein_codin	0	0	0	0
ENSG000000	CHAT	protein_codin	0	1.10672712	0.5130156	1.2967387
ENSG000000	CREM	protein_codin	0	0	0.83484945	0
ENSG000001	PRP4K	protein_codin	32.0858186	34.0420576	175.667883	26.6372421
ENSG000001	AADAT	protein_codin	7.52566143	11.4417265	0	1.48930207
ENSG000000	ARHGEF1	protein_codin	0	0	0	0
ENSG000001	MED19	protein_codin	2.44159815	0	3.94549918	3.24146318
ENSG000001	ZNF394	protein_codin	15.9498714	34.2616273	10.3441238	18.684329
ENSG000001	YIF1B	protein_codin	0	4.50894535	0	2.94001528

ENSG000001:	SINHCAF	protein_codin	5.80239477	7.99796641	37.2647043	6.69414474
ENSG000001:	PWWP3B	protein_codin	0	0	0	1.93423391
ENSG000001	TTC33	protein_codin	8.45265923	15.8729429	54.8131298	6.86035201
ENSG000001	FGF1	protein_codin	0	0	0	0
ENSG000001:	AKAP1	protein_codin	11.3234131	7.10229792	76.6950176	0
ENSG000001	DTX3	protein_codin	44.8412573	0	13.1290439	0
ENSG000001	PTPA	protein_codin	0	0	0	0
ENSG000000	TASP1	protein_codin	12.2773205	13.6962332	27.0960075	0
ENSG000001	GHRHR	protein_codin	0	1.33653664	0	0.93101759
ENSG000001	MGAT4B	protein_codin	0.60870039	8.94399453	14.9549478	5.47464665
ENSG000001	SUPT7L	protein_codin	6.72089942	13.8448956	9.9117494	9.24905805
ENSG000001:	MASP1	protein_codin	0.36931209	1.08004451	0	1.86845179
ENSG000001:	PLS1	protein_codin	0	0	8.4666707	0
ENSG000001	AQP12A	protein_codin	0	0	0	0
ENSG000001	ZNF664	protein_codin	0	0	116.592331	0
ENSG000001:	STIL	protein_codin	0	3.39144519	4.24793949	0
ENSG000001	GALNT16	protein_codin	2.00831641	0	0	0.4542753
ENSG000000:	C6	protein_codin	0	0	0	0
ENSG000000	SPTLC1	protein_codin	0.76564126	1.77982439	0	0
ENSG000001:	C1QTNF6	protein_codin	5.91182357	15.2317396	0.5270653	13.5035776
ENSG000001	SGCD	protein_codin	151.778504	0	50.9711949	20.1488793
ENSG000001	SUV39H1	protein_codin	0	0	0	0
ENSG000000	ZC3H15	protein_codin	117.097502	249.767286	417.764015	116.43804
ENSG000001	ZBTB49	protein_codin	0	0	0	0
ENSG000001	HOPX	protein_codin	0.99265169	0	0	0
ENSG000001	ADORA1	protein_codin	0	0	0	0
ENSG000001:	PPHLN1	protein_codin	4.36257549	35.0028675	20.8299599	4.42419761
ENSG000001	RERE	protein_codin	32.1331349	41.401577	52.7757348	23.1098342
ENSG000001	MS4A4A	protein_codin	7.7185306	0	0	0
ENSG000001	AIRE	protein_codin	0	0	0	0
ENSG000000	RHOBTB1	protein_codin	34.1408667	31.3989659	93.7342664	53.5657165
ENSG000001	MS4A15	protein_codin	0	0	0	0
ENSG000002	AIF1	protein_codin	4.52443248	92.184486	0	19.3541376
ENSG000001	HEY1	protein_codin	16.5733314	0	24.7607548	0
ENSG000001:	PHF20L1	protein_codin	0	0	0	0
ENSG000001	IL21R	protein_codin	0	29.4259868	0.49120515	9.6959196
ENSG000001:	EDN3	protein_codin	0	0	0	0
ENSG000001	NR1I2	protein_codin	0	0	0	0
ENSG000001	PYCR1	protein_codin	0.91947947	0	10.6295191	0
ENSG000001	PACSIN2	protein_codin	0	0	0	0
ENSG000001:	ZMYM5	protein_codin	0	0	0	5.30286559
ENSG000001	HNRNPF	protein_codin	86.5895926	161.522513	287.449016	78.9623969
ENSG000001	KLHL25	protein_codin	3.68135966	8.42137497	8.95419116	1.91627819
ENSG000001	STX3	protein_codin	1.82718503	14.2053088	4.52119682	6.62686789

ENSG00000(	CABIN1	protein_codin	2.98341669	2.47433521	0	4.29404479
ENSG00000	ZC4H2	protein_codin	0	0	0	1.22052491
ENSG00000	TSPAN16	protein_codin	0	0	0	0.38092793
ENSG00000	ZNF420	protein_codin	7.22023331	13.6441149	16.3177241	0
ENSG000002	CCDC183	protein_codin	0.91583676	4.70070526	1.25013859	0.97031937
ENSG00000	ZFYVE16	protein_codin	22.2117438	44.9230596	63.9853686	25.2130233
ENSG00000	ZNF385A	protein_codin	0.42045609	0	0	0
ENSG00000	SLITRK4	protein_codin	6.54573017	4.27428755	2.34593634	4.31397851
ENSG00000	MAPK15	protein_codin	0	0	0	0
ENSG00000	ELAC2	protein_codin	5.82409686	0	27.2956198	9.08477115
ENSG00000	PLCB1	protein_codin	41.7323355	0	29.7960273	20.4062253
ENSG00000	FBXO40	protein_codin	14.689498	3.73952645	0.45569095	5.46291716
ENSG00000	MEF2A	protein_codin	5.81463518	0	39.7381712	0
ENSG00000	LIMS1	protein_codin	11.8673864	0	0	0.96889366
ENSG00000	C5orf24	protein_codin	20.9424459	23.7627077	31.0209476	0
ENSG00000	RUNDC3B	protein_codin	0	0	0	0
ENSG00000	EPB41L1	protein_codin	0	0	0	0
ENSG00000	PUM2	protein_codin	25.3243025	0	0	0
ENSG00000	SLA	protein_codin	17.8825354	96.6519989	3.18673497	7.24410129
ENSG00000	CMTR2	protein_codin	0	0	0	7.076797
ENSG00000	NOS1	protein_codin	1.02907147	0	6.18213797	0
ENSG00000	TTC8	protein_codin	0	0	0	0
ENSG00000	ENSG00000	protein_codin	0	0	2.409362	0
ENSG00000	GZF1	protein_codin	23.5269192	52.9334984	29.2753135	20.042096
ENSG00000	ISYNA1	protein_codin	0	0	6.78040349	0
ENSG00000	NQO2	protein_codin	1.55200908	2.53265461	2.1226399	4.34410145
ENSG00000	KPTN	protein_codin	0	0	0	0
ENSG00000	ENOX2	protein_codin	0	0	0	0
ENSG00000	UBE2D3	protein_codin	0	0	0	2.94946388
ENSG00000	SPRYD4	protein_codin	4.76643524	22.9089696	22.1744623	7.24886578
ENSG00000	MRPL30	protein_codin	4.60597785	25.2057271	20.9386817	4.87644271
ENSG00000	CAGE1	protein_codin	0	0	0	0
ENSG00000	PHF8	protein_codin	0	13.3745104	1.20746263	0.79490332
ENSG00000	RBM39	protein_codin	0	0	0	0
ENSG00000	EME1	protein_codin	1.07761395	0	15.6197447	0
ENSG00000	APLP2	protein_codin	95.3322752	0	109.325274	75.1043574
ENSG00000	RAB28	protein_codin	5.02486826	0	8.48657965	0
ENSG00000	LBR	protein_codin	4.10993186	0	0	0
ENSG00000	CDIN1	protein_codin	0	6.25606563	0	0
ENSG00000	PRIM1	protein_codin	5.64255795	21.1648358	59.0067879	0
ENSG00000	ECPAS	protein_codin	83.2116445	0	222.685674	56.9439602
ENSG00000	UGT2B15	protein_codin	0	0	0	0
ENSG00000	UBQLN2	protein_codin	16.8147489	41.7899833	69.8513665	16.8165455
ENSG00000	ZNF600	protein_codin	0	0	0	2.15405999

ENSG00000(	SLC23A2	protein_codin	21.8726365	67.3160244	56.9967967	29.7690647
ENSG000003	CAST	protein_codin	0	0	0	0
ENSG000001	TRIM69	protein_codin	0	0	0	0
ENSG000001	ERAS	protein_codin	0	0	0	0
ENSG000002	TMEM167B	protein_codin	20.0345254	35.5496472	31.6924991	16.6281959
ENSG000001	PEAR1	protein_codin	0	0	0	0
ENSG000001	LRFN2	protein_codin	0	0	1.36370542	0
ENSG000001	CCDC157	protein_codin	0.60840219	1.48788298	2.05931973	1.97342575
ENSG00000(	ANGPT2	protein_codin	1.60111131	13.8395023	8.35626305	3.85808939
ENSG000001	TAGAP	protein_codin	1.58896404	0	0	3.60645289
ENSG000002	MFSD2B	protein_codin	0	0	0	0
ENSG00000(	ADCY2	protein_codin	12.2018479	8.09035242	17.3260696	2.50514565
ENSG000001	CSMD2	protein_codin	0.26050221	0	0.57428333	1.5914798
ENSG000001	CHODL	protein_codin	0	0	0	0
ENSG000002	SNURF	protein_codin	0	0	0	0
ENSG000001	FBLL1	protein_codin	0.33889623	0	0	0.50622944
ENSG000001	AURKAIP1	protein_codin	11.5294672	28.3485668	120.666855	13.106979
ENSG00000(	FBXL19	protein_codin	1.98455854	18.6743244	22.1200759	3.17258143
ENSG000001	KCNJ5	protein_codin	0	0	0	0
ENSG000001	OTUD6A	protein_codin	0	0	0	0.76279519
ENSG000001	FAM78B	protein_codin	0	0	0	0
ENSG000001	DMBT1	protein_codin	0	0	0	0
ENSG000001	CPM	protein_codin	0	0	0	0
ENSG000001	RFLNA	protein_codin	0	8.13860989	0	0
ENSG000001	TAF13	protein_codin	19.4662637	69.1820909	68.0856168	24.0648146
ENSG000002	PRKDC	protein_codin	259.22339	692.410118	3656.50616	147.304477
ENSG000001	FIBP	protein_codin	0.56256691	0	3.84102776	0
ENSG000001	AURKAIP1	protein_codin	0	1.64062021	0	0
ENSG000001	VSTM1	protein_codin	0	0	0	0
ENSG000001	RMDN3	protein_codin	0	0	13.5696529	0
ENSG000001	H1-6	protein_codin	0.78331942	0	4.13502341	0.38117989
ENSG000001	SLPI	protein_codin	0.46988941	1.77675817	131.880017	0.34092092
ENSG000001	FOCAD	protein_codin	19.3030319	37.4025279	39.1715131	9.94365548
ENSG00000(	CLUL1	protein_codin	0	0	0	0
ENSG000001	CDK5RAP3	protein_codin	12.9209266	0	39.4346202	14.414635
ENSG000001	METTL26	protein_codin	0	0	0	0
ENSG000001	QPCT	protein_codin	1.36028225	0	0.92941109	1.05040587
ENSG000000	NUDCD3	protein_codin	0.75313769	0	0	0
ENSG00000(	ACACB	protein_codin	0	0	0	0
ENSG000001	GLS	protein_codin	69.9073803	108.620044	124.085761	42.5276454
ENSG000001	CA7	protein_codin	0	0	0	0
ENSG000001	FAIM	protein_codin	1.79600795	0	0	0
ENSG000001	NAA40	protein_codin	0	21.717884	7.36962049	7.18048294
ENSG00000(	PALM	protein_codin	11.5045893	0	21.8143014	2.86370046

ENSG000001	ING1	protein_codin	6.07808497	0	0	0
ENSG000001	ARHGEF3	protein_codin	16.0980696	0	18.4720305	4.7696047
ENSG000001	USP37	protein_codin	0	0	1.75741743	0.92100985
ENSG000001	PRKN	protein_codin	0	0	0	0
ENSG000000	TREM2	protein_codin	0	0	0	0
ENSG000001	CHST4	protein_codin	0	0	0	0
ENSG000001	MAFF	protein_codin	1.57883414	0	0	40.2177211
ENSG000001	DUSP29	protein_codin	0.26267716	0	0	0
ENSG000001	NR4A3	protein_codin	0.39445859	2.09119039	1.13181575	0.82476799
ENSG000001	DPYSL4	protein_codin	0	3.46430214	1.97999369	1.6834089
ENSG000001	ZFTA	protein_codin	0	0	0	0
ENSG000001	SPRED3	protein_codin	0.26184029	13.7464736	2.29389915	0
ENSG000001	SHANK2	protein_codin	0	0	0	0
ENSG000001	GJB5	protein_codin	0	0	0	0.41925834
ENSG000001	SCOC	protein_codin	23.1647019	0	0	0
ENSG000000	SNX10	protein_codin	0	17.1683085	0	12.9239657
ENSG000001	TREX2	protein_codin	0	0	0	0
ENSG000001	MLPH	protein_codin	0	0	0	0
ENSG000001	MATCAP2	protein_codin	1.57683942	2.64379872	0	1.84520918
ENSG000001	DYNLRB2	protein_codin	0	0	0	0
ENSG000001	HPS1	protein_codin	0	0	0	0
ENSG000001	PSMD10	protein_codin	1.15195206	0	1.01580182	1.83923199
ENSG000001	SCNN1D	protein_codin	0	1.43783435	5.19027444	0.74922293
ENSG000001	TRPV2	protein_codin	1.75753781	24.3492127	1.61528469	4.65665278
ENSG000001	RARG	protein_codin	15.4657881	11.8592947	34.8618509	5.80247651
ENSG000001	MEIS2	protein_codin	0	0	4.16857651	3.68712078
ENSG000000	ZFX	protein_codin	17.8021681	22.5285624	16.1655638	13.9455931
ENSG000001	NPY5R	protein_codin	0	0	6.53888741	0
ENSG000001	OTOF	protein_codin	0	0	0	2.32724785
ENSG000001	MCF2	protein_codin	0	0	1.71863224	0
ENSG000001	FAM180A	protein_codin	3.0720655	0	0	0
ENSG000001	KLHL17	protein_codin	0	4.84780483	8.23881248	0.81763313
ENSG000002	CRCP	protein_codin	1.68497867	17.3467323	17.8308171	0
ENSG000000	GSTO2	protein_codin	0	0	0	0
ENSG000001	SRSF10	protein_codin	4.67341302	0	45.5981896	0
ENSG000001	SLC28A1	protein_codin	0	0	0	1.91526076
ENSG000001	SAXO4	protein_codin	0	0	0	0
ENSG000001	OR13H1	protein_codin	0	0	0	0
ENSG000001	MBOAT7	protein_codin	0	0	0	0
ENSG000001	SLC25A15	protein_codin	2.44574749	8.2795545	0	0
ENSG000001	ACIN1	protein_codin	0	0	5.97787807	0
ENSG000001	XRCC4	protein_codin	0	13.2602634	0	2.37416257
ENSG000001	ZNF81	protein_codin	26.3830658	84.6696732	29.9914624	0
ENSG000001	PARK7	protein_codin	16.6122738	50.7610931	32.2255385	19.8924012

ENSG000001	NF2	protein_codin	0	70.056381	0	0
ENSG000001	ZFP92	protein_codin	5.70451204	8.50333651	1.5403493	4.8611941
ENSG000001	VWA1	protein_codin	8.52835427	57.9670391	35.5457741	13.3387727
ENSG000001	ATP5MC2	protein_codin	3.90728332	0.90377769	0	0
ENSG000001	SLC3A2	protein_codin	0	0	0	0
ENSG000001	ANKRD34B	protein_codin	0	1.60152652	0.9872547	1.37076041
ENSG000001	MUC1	protein_codin	0	0	0	0
ENSG000001	TEKT1	protein_codin	0	0	0	1.74343296
ENSG000001	TEKT3	protein_codin	0	2.78515728	0	0
ENSG000001	TRIM2	protein_codin	0	0	0	0
ENSG000001	MAOA	protein_codin	14.4871005	16.7490724	22.3909907	0
ENSG000001	DCBLD1	protein_codin	5.68016077	12.3826999	8.69212127	6.06979794
ENSG000001	LFNG	protein_codin	0	0	0	0
ENSG000001	TBCCD1	protein_codin	10.0466903	0	20.3648843	4.71267575
ENSG000001	SATB1	protein_codin	12.6552446	20.7081641	2.57261343	6.76851898
ENSG000001	SCNN1A	protein_codin	0	0	1.69879403	0
ENSG000001	TPST2	protein_codin	2.71855491	36.2056715	3.12953852	2.57352601
ENSG000001	PARVB	protein_codin	2.63595385	39.7632548	2.82418012	4.90948017
ENSG000001	MTSS2	protein_codin	48.3074641	137.470324	183.862134	46.6459178
ENSG000000	GNAS	protein_codin	5.97057519	4.89938038	17.9318197	6.75117342
ENSG000001	TNFSF13	protein_codin	1.04639773	0	2.30561361	0
ENSG000001	DYRK1A	protein_codin	17.3765624	60.4185975	9.67012001	0
ENSG000001	IMPDH1	protein_codin	0	0	0	0
ENSG000001	CRTC1	protein_codin	10.8153707	35.4016522	89.1095418	0
ENSG000000	ESR1	protein_codin	0	2.13853186	0.96678158	0.51471563
ENSG000001	RGS12	protein_codin	0	0	0	0.66873403
ENSG000001	DNAJB12	protein_codin	1.16746407	16.2196998	0	1.63254848
ENSG000000	ATP9A	protein_codin	32.8622194	144.480497	121.763218	40.8914304
ENSG000001	EIF4EBP1	protein_codin	12.4436314	17.4071201	150.13977	3.154201
ENSG000001	PLET1	protein_codin	0	0	0	0.76778314
ENSG000001	NCR1	protein_codin	0	0	0	0
ENSG000001	TPSAB1	protein_codin	0	8.11177466	0	12.7637489
ENSG000001	RTN3	protein_codin	0	1.04197921	0	0
ENSG000001	CATSPER4	protein_codin	0	0	0	0
ENSG000001	OLFM3	protein_codin	0	0	0	0
ENSG000001	SMTNL2	protein_codin	1.82302974	0	0.47518291	0.38769122
ENSG000001	PCDH8	protein_codin	0	0	0	0.97594044
ENSG000000	TEAD3	protein_codin	4.27983245	22.3342655	17.4096849	1.67874402
ENSG000001	HELT	protein_codin	0	0	0	0
ENSG000001	HSFY1	protein_codin	0	0	0	0
ENSG000000	METTL24	protein_codin	6.28345341	0	1.92077325	2.23768488
ENSG000001	MAP3K15	protein_codin	0	0	0	0
ENSG000000	RUNX3	protein_codin	0	0	2.17490165	4.54756395
ENSG000001	FAM184A	protein_codin	0	0	0	0

ENSG000001(	DFFB	protein_codin	0	0	0	0
ENSG000002:	CLEC12B	protein_codin	0	0	0	0
ENSG000001:	KRTDAP	protein_codin	0	0	0	0
ENSG000001:	EDA	protein_codin	0	0	0	0
ENSG000001:	RNASE9	protein_codin	0	0	0	0
ENSG000001.	MYOM3	protein_codin	0	0	0	2.5605441
ENSG000001:	PSPC1	protein_codin	39.5285521	58.4538934	50.8237296	0
ENSG000001:	GAL3ST1	protein_codin	0	0	0	0
ENSG000002	SYT3	protein_codin	0	0	0	0
ENSG000001	PRRC2C	protein_codin	0	0	0	0
ENSG000001'	PHF8	protein_codin	5.58329494	0	10.350976	4.78137054
ENSG000001:	TRPC4	protein_codin	0	0	0	0
ENSG000001:	CUZD1	protein_codin	0	0	0	0
ENSG000000.	DTNBP1	protein_codin	0.38560455	13.0912435	0	4.86307273
ENSG000001(	ZC3H12B	protein_codin	1.60903308	9.75867453	0.93904434	2.9477948
ENSG000001.	PTGES2	protein_codin	0	0	12.4405415	0
ENSG000001:	LRP1	protein_codin	3.93989322	26.6939295	5.70021666	8.67250514
ENSG000000(	NEDD4	protein_codin	0	0	0	0
ENSG000001:	NCR3LG1	protein_codin	7.96495439	7.16439701	15.2708593	13.3951149
ENSG000001(	TRIM9	protein_codin	0.22165613	0	0	0
ENSG000001:	RPL14	protein_codin	34.8240073	47.8220716	29.4452931	34.1900596
ENSG000002(	SULT1A3	protein_codin	0	0	0	0
ENSG000001'	PAIP1	protein_codin	0	73.0751184	0	0
ENSG000001:	ZNF721	protein_codin	51.5556108	0	228.153356	0
ENSG000001	USP9Y	protein_codin	45.8641478	63.6639037	0	0
ENSG000000!	MAP3K20	protein_codin	87.7998314	38.4644072	84.2476966	28.2857
ENSG000001.	COMMD4	protein_codin	0	0	0	3.82820707
ENSG000001'	LRRC25	protein_codin	0	19.2773101	0	0
ENSG000001(	CFAP36	protein_codin	0	4.00118571	0	0
ENSG000001:	SMIM15	protein_codin	8.90438614	15.7841097	30.2517277	13.5911284
ENSG000001(	CYP2C18	protein_codin	0	0	0.53645499	0
ENSG000001:	MAP3K7CL	protein_codin	0	0	0	0
ENSG000001:	DUSP21	protein_codin	0	0	0	0
ENSG000000(	TSPAN32	protein_codin	0	0	0	2.26506589
ENSG000001:	CRHR1	protein_codin	0	0	0	0
ENSG000001:	KLHL7	protein_codin	0	3.14124697	0	1.13620353
ENSG000000	PLAUR	protein_codin	2.61252485	0	0	1.46859441
ENSG000001:	RHOC	protein_codin	25.6292037	0	22.2065001	10.5717933
ENSG000000:	PHF20	protein_codin	0	0	0	0
ENSG000000(	TFPI	protein_codin	19.2578698	79.4440157	36.1129639	9.12556868
ENSG000001.	NOSIP	protein_codin	16.6070366	30.9629565	18.006445	0
ENSG000001(	CERKL	protein_codin	0	0	0	0
ENSG000001:	CLYBL	protein_codin	1.35977412	0	3.8045996	1.58824254
ENSG000001!	ATAD3A	protein_codin	0	0	0	6.20967935

ENSG0000007	ING3	protein_codin	2.18594406	4.53916379	31.9943647	3.10686275
ENSG000000	SMOX	protein_codin	2.53955931	3.32043659	2.02863941	0
ENSG000001	NANOS3	protein_codin	0	0	0	0
ENSG000001	C9orf153	protein_codin	0	0	0	0
ENSG000001	CRISP2	protein_codin	0	0	0	0
ENSG000001	FOXR2	protein_codin	0	1.62510188	0	2.27663765
ENSG000001	C1QTNF1	protein_codin	0	0	1.33097543	0
ENSG000001	DEFB119	protein_codin	0	0	0	0
ENSG000001	IFI6	protein_codin	0	28.8571976	1.31021174	0
ENSG000001	NUDT6	protein_codin	0	0	3.65629796	1.65904294
ENSG000001	ABHD12	protein_codin	3.008608	44.4453749	3.54104232	4.65480742
ENSG000001	PDLIM2	protein_codin	0	0	0	1.35612051
ENSG000001	NLGN4Y	protein_codin	0	0	0	0
ENSG000001	NRN1L	protein_codin	0	0	0	0
ENSG000001	CMTM5	protein_codin	0	0	0	0
ENSG000001	YBEY	protein_codin	0	0	0.90588305	0
ENSG000001	PHF10	protein_codin	0	133.419052	0	0
ENSG000001	TCEA2	protein_codin	0	0	0	0
ENSG000001	FPR3	protein_codin	30.4833237	77.5952707	6.85591638	10.8394647
ENSG000001	SLC25A14	protein_codin	0	0	0	0
ENSG000001	FBXL14	protein_codin	2.2953472	27.9062821	14.5722989	2.78795329
ENSG000001	SPRY1	protein_codin	0	0	0	10.0195009
ENSG000001	ZNF449	protein_codin	16.0163937	52.9831791	14.2737332	9.60790816
ENSG000001	MUC4	protein_codin	0	0	0	0
ENSG000001	HAVCR1	protein_codin	0	0	0	0.52020472
ENSG000000	NIPAL3	protein_codin	0	0	0.84385791	0.60190518
ENSG000000	DLGAP4	protein_codin	0	0	0	2.97769871
ENSG000001	CDK5RAP1	protein_codin	0	0	0	0
ENSG000001	OASL	protein_codin	0	0	0	0
ENSG000001	SFN	protein_codin	0.79185359	0	0	0.47553937
ENSG000002	ZNF564	protein_codin	24.7892368	49.6098542	47.8440355	15.2600578
ENSG000001	SH2D4B	protein_codin	0	0	0	0
ENSG000001	MYB	protein_codin	0	0	0	0
ENSG000001	ZNF621	protein_codin	30.8836973	59.499021	45.8540682	14.2524819
ENSG000001	SOCS4	protein_codin	0	0	5.26719151	2.83324047
ENSG000001	ITGA4	protein_codin	0.25204265	8.18496705	1.80936298	1.11173568
ENSG000001	POLL	protein_codin	0	0	0.65824559	1.62027547
ENSG000001	SIGLEC10	protein_codin	0	0	0	0
ENSG000001	AMTN	protein_codin	0	0	0	1.55291817
ENSG000001	FCRL6	protein_codin	0	0	0	0
ENSG000001	DFFB	protein_codin	0	26.8181403	0	0
ENSG000001	DYNLT4	protein_codin	0	0	0	0
ENSG000001	RAP1GDS1	protein_codin	0	0	0	0
ENSG000000	NEO1	protein_codin	0	0	0	0

ENSG000001	RPGR	protein_codin	11.2715659	25.2560273	5.06060332	14.2965621
ENSG000001	PIK3AP1	protein_codin	17.3461966	76.2075551	0	21.9807649
ENSG000001	SEC24C	protein_codin	9.47413644	0	0	0
ENSG000001	METTL17	protein_codin	0	0	0	2.27922998
ENSG000001	TMEM130	protein_codin	0	0	0	1.34500096
ENSG000001	TAS1R3	protein_codin	0	0	0	0.43747989
ENSG000001	PURG	protein_codin	1.79914068	0	3.33721588	1.76345548
ENSG000001	SDC3	protein_codin	79.6099015	204.811042	209.332681	68.7247617
ENSG000001	SRFBP1	protein_codin	45.2677714	73.4345323	203.777845	28.950735
ENSG000001	ANP32B	protein_codin	400.221664	2732.76891	1604.94462	225.72943
ENSG000000	CASP8	protein_codin	0	9.34913076	0	9.22210476
ENSG000000	ZNF76	protein_codin	3.25786439	0	23.7626711	0
ENSG000001	YIF1B	protein_codin	0	3.07740428	0	0.60419454
ENSG000000	DOCK9	protein_codin	6.55244418	0	0	0
ENSG000001	NUDT14	protein_codin	0.54464354	0	1.99551531	0
ENSG000001	ZDHHC3	protein_codin	5.54853022	22.2373271	0	5.08750589
ENSG000001	HSD11B1L	protein_codin	0	0	0	0
ENSG000001	LITAF	protein_codin	0	7.33141958	21.2329839	2.92519433
ENSG000001	LRRC17	protein_codin	19.0591685	0	81.007265	17.2535334
ENSG000001	SRSF3	protein_codin	0	0	0	0
ENSG000000	TRNT1	protein_codin	0	0	0	0
ENSG000000	CSDE1	protein_codin	81.0129479	0	0	15.6136922
ENSG000001	SLC26A5	protein_codin	0	0	0	0
ENSG000001	ABCB7	protein_codin	0	0	0	5.99075155
ENSG000000	ERMP1	protein_codin	0	0	0	0
ENSG000001	DCHS2	protein_codin	0	1.0062043	0	0
ENSG000001	GFI1B	protein_codin	0	0	0	1.26514457
ENSG000001	WNT7B	protein_codin	0.99463812	2.6411394	33.4308527	0.89040806
ENSG000001	SAXO2	protein_codin	0	0	0.85759201	1.24311725
ENSG000001	YPEL1	protein_codin	1.25031954	31.1100729	2.64861089	2.29305665
ENSG000001	RPS16	protein_codin	0	15.3779341	3.07534859	0.95584798
ENSG000001	LRRC37A3	protein_codin	1.09754226	0	27.9380589	0
ENSG000001	OTX2	protein_codin	0	0	0	0.51236887
ENSG000001	GJB4	protein_codin	0	0	0	0.46580997
ENSG000001	VMAC	protein_codin	4.06783395	14.0472209	5.6205871	3.21532923
ENSG000001	RIOK3	protein_codin	37.8622601	0	24.22892	14.1209808
ENSG000002	PTCHD4	protein_codin	45.5392945	35.000484	10.6035859	16.3926896
ENSG000000	PHKA1	protein_codin	0	0	22.7224642	0
ENSG000001	CCNY	protein_codin	0	36.1337784	5.1381457	0
ENSG000001	ZNF573	protein_codin	0	0	0	0
ENSG000001	DCAF17	protein_codin	1.85226559	0	0.67443883	0
ENSG000001	PRMT7	protein_codin	0	0	3.82168527	0
ENSG000001	OGG1	protein_codin	1.42549058	0	1.47120663	1.13420615
ENSG000001	MYO1B	protein_codin	25.6607773	78.3664476	6.53807162	119.102237

ENSG000001	RNF17	protein_codin	0	0	0	0
ENSG000001	GLUL	protein_codin	29.1214995	120.405348	19.4882531	37.1156203
ENSG000002	LST1	protein_codin	0	0	0	0
ENSG000001	ZCCHC13	protein_codin	0	0	0	1.97655034
ENSG000001	MROH7	protein_codin	0	0	0	0
ENSG000001	S100A13	protein_codin	0	0	0	0
ENSG000001	PODNL1	protein_codin	0	0	0	0
ENSG000001	CLIC5	protein_codin	17.2855523	5.7183684	0.605751	5.57534137
ENSG000001	NR4A2	protein_codin	5.26270419	0	2.0544746	94.5918113
ENSG000001	STAMBP	protein_codin	0	0	0	0
ENSG000001	MSL1	protein_codin	42.8329461	22.2649645	24.3884428	5.55384066
ENSG000000	SCARB1	protein_codin	0	0	0	0
ENSG000001	GRM8	protein_codin	0	0	0	0
ENSG000000	BAZ1B	protein_codin	101.679553	987.940921	319.865205	91.8677281
ENSG000001	OTOF	protein_codin	0.2524928	7.71715481	0.4464556	0
ENSG000001	NDUFA4	protein_codin	8.58319454	16.4469313	24.7095414	16.5174956
ENSG000001	IL22RA2	protein_codin	0	0	0	1.67995988
ENSG000002	TTLL13	protein_codin	0	0	0	0
ENSG000000	ALDH3A2	protein_codin	12.0630319	0	0	0
ENSG000001	SERF2	protein_codin	5.60756431	46.0132595	4.33985385	3.47108074
ENSG000002	ZNF254	protein_codin	0	0	0	0
ENSG000001	ENSA	protein_codin	0	0	0	0
ENSG000001	UBC	protein_codin	1452.84324	1861.32317	2544.52631	2132.71721
ENSG000001	ZNF587	protein_codin	23.7398325	58.6396814	46.2321876	10.177031
ENSG000001	ZNF189	protein_codin	10.1161044	0	0	0
ENSG000001	DMP1	protein_codin	2.04130081	0	0	89.9560659
ENSG000001	POLDIP3	protein_codin	0	0	0	0
ENSG000001	DMKN	protein_codin	0	0	0	1.5633528
ENSG000001	SERINC1	protein_codin	147.544613	161.654065	185.909718	131.9486
ENSG000001	CSMD3	protein_codin	0	0	0	0
ENSG000000	PDE8A	protein_codin	0	0	0	0
ENSG000001	PRDM8	protein_codin	0	0	0	0
ENSG000000	BRPF3	protein_codin	0	48.6642763	0	0
ENSG000001	OLFM1	protein_codin	0	0	0	0
ENSG000001	ARL4C	protein_codin	16.6771007	127.204117	0	33.4694452
ENSG000001	GALNT15	protein_codin	3.31602477	33.8024956	2.75299006	8.97333008
ENSG000000	SLC7A8	protein_codin	0	42.4757598	0	0.87718211
ENSG000001	NUDT1	protein_codin	0	0	0	0
ENSG000001	DUSP15	protein_codin	0	0	1.04359057	0
ENSG000001	ANKRD6	protein_codin	0	0	0	0
ENSG000000	NECAP1	protein_codin	0	0	18.1926078	0
ENSG000000	NLRP2	protein_codin	0	0	0	1.12445336
ENSG000001	DNAJC12	protein_codin	0	0	1.46723867	0
ENSG000001	SLC35F4	protein_codin	0	0	0	0

ENSG000001	DNAJB13	protein_codin	0	0	0	0
ENSG000001	CLEC2A	protein_codin	0	0	0	0
ENSG000001	SEMA4F	protein_codin	0	0	3.61745655	0
ENSG000001	BTG3	protein_codin	0	0	0	0
ENSG000001	LRRC43	protein_codin	0	0	0	0
ENSG000001	DAB2	protein_codin	0	73.3349832	31.3974052	4.51668917
ENSG000000	ETV7	protein_codin	0	0	0	0
ENSG000000	CHAT	protein_codin	0	0	0	0
ENSG000000	ICA1	protein_codin	0	0	0	0
ENSG000001	H2BC11	protein_codin	8.01529525	31.3932907	54.1061939	4.61606948
ENSG000001	CFAP410	protein_codin	0	1.72982898	0	2.07445646
ENSG000001	KSR2	protein_codin	0.54532248	8.60820443	0.54935588	10.4352149
ENSG000001	BORCS7	protein_codin	19.4127324	35.2172411	20.0048976	12.372227
ENSG000001	NMNAT3	protein_codin	0	0	0	0
ENSG000001	CEP83	protein_codin	8.80104522	0	70.5493988	0
ENSG000001	ELSPBP1	protein_codin	0	0	0	0
ENSG000001	ZNF28	protein_codin	0	0	0	0
ENSG000001	CLEC17A	protein_codin	0	0	0	0
ENSG000001	NCCRP1	protein_codin	0	1.3714629	0	0.43932073
ENSG000001	MEFV	protein_codin	0	0	0	0
ENSG000001	USP54	protein_codin	0	16.1782536	0	0
ENSG000001	SEMA4D	protein_codin	0.49253424	0	0	1.89670297
ENSG000001	USP47	protein_codin	3.56171687	5.65807814	17.3672161	0
ENSG000000	SLC24A1	protein_codin	0	15.5799664	9.88319147	10.4353265
ENSG000001	HMGB1	protein_codin	96.3823839	168.591382	162.96082	64.1156011
ENSG000001	MARCHF1	protein_codin	1.61801762	8.43316401	2.22788364	0
ENSG000001	NFASC	protein_codin	0	11.3259742	0	0
ENSG000001	RBPMS	protein_codin	0.15825056	17.9874117	0	0
ENSG000001	CCNYL1	protein_codin	3.62525251	4.58843093	8.55840524	0
ENSG000001	SECISBP2	protein_codin	0	82.5094485	89.2383612	0
ENSG000001	ZNF292	protein_codin	0	0	64.4730218	19.4870763
ENSG000001	ZNF493	protein_codin	3.22310658	0	1.64069384	0
ENSG000001	TSSK4	protein_codin	0	0	0	0
ENSG000001	VHLL	protein_codin	0	0	0	1.4920418
ENSG000002	KIR2DS4	protein_codin	0	1.4126534	0	0.39989924
ENSG000001	ENY2	protein_codin	2.64664491	3.44682674	14.5092099	1.84531856
ENSG000001	MRAP	protein_codin	0	0	0	0
ENSG000001	WFDC2	protein_codin	0	0	0	0
ENSG000001	ZNF648	protein_codin	0	0	4.91254293	0.86963868
ENSG000001	USP1	protein_codin	1.09417209	25.7088339	28.1982123	2.10450894
ENSG000001	CLEC18B	protein_codin	0	0	0	0
ENSG000001	OLR1	protein_codin	0	0	0	0
ENSG000001	ZNF695	protein_codin	1.09155268	0	7.40047958	2.03805079
ENSG000001	SDCBP2	protein_codin	0	0	0	1.52955224

ENSG000001	HMX2	protein_codin	0	0	2.05483922	0
ENSG000001	KCNJ11	protein_codin	0	0	0	0
ENSG000001	EIF4G2	protein_codin	9.09244383	0	38.3651431	0
ENSG000001	MYMK	protein_codin	0	0	0	0
ENSG000001	RTN3	protein_codin	16.2249736	23.6628132	83.5718999	13.0654473
ENSG000001	MBD1	protein_codin	0	0	0	3.62179467
ENSG000001	ABCA9	protein_codin	8.16453877	0	0	0
ENSG000001	DOK1	protein_codin	0	0	0	0
ENSG000001	CSRP1	protein_codin	34.7398765	40.1316442	13.5019929	22.0043231
ENSG000000	SLC26A3	protein_codin	0	0	0	0
ENSG000001	SPAM1	protein_codin	0	0	0	0
ENSG000001	TMC1	protein_codin	0	0	0.81030185	0
ENSG000001	AIDA	protein_codin	58.8468092	55.7554011	94.9862039	22.852743
ENSG000000	KRIT1	protein_codin	0	0	0	0
ENSG000001	FPR2	protein_codin	0	0	0	0
ENSG000000	TRAF3IP2	protein_codin	0.78558038	0	0	0
ENSG000002	NCR3	protein_codin	0	0	0	0
ENSG000001	FASLG	protein_codin	0	0	0	0
ENSG000002	GLI4	protein_codin	0	0	2.43828043	0
ENSG000001	RET	protein_codin	0	0	0	0
ENSG000001	PLCH1	protein_codin	0	0	0	0
ENSG000001	STYXL1	protein_codin	0	0	0	1.12659285
ENSG000001	KCTD21	protein_codin	6.47320446	9.0502499	4.77699634	4.43670556
ENSG000001	UBE3B	protein_codin	0	0	0	0
ENSG000001	PARD3	protein_codin	0	47.1822877	0	50.8152441
ENSG000001	MIOS	protein_codin	5.84807815	3.74041433	4.57634549	0
ENSG000001	DOK7	protein_codin	0	0	0	0
ENSG000001	NANOS1	protein_codin	0	0	0	0
ENSG000000	PLAUR	protein_codin	0	0	0	11.3711888
ENSG000000	OFD1	protein_codin	49.6664232	94.0942229	66.1616911	35.7842936
ENSG000001	DOK4	protein_codin	2.14297995	9.0771601	2.5127117	0
ENSG000000	FGFR3	protein_codin	4.43753338	0	57.8241685	0
ENSG000001	RNF133	protein_codin	0.69176277	1.56956373	12.0708963	1.43456758
ENSG000001	ENOSF1	protein_codin	0	0	0	0
ENSG000001	GPT2	protein_codin	3.21498232	24.1494208	21.8264272	1.4681564
ENSG000000	SP100	protein_codin	94.9932347	86.8439111	22.6751597	100.575799
ENSG000001	WASH6P	protein_codin	0	17.8800305	0	0
ENSG000002	IFITM10	protein_codin	0	23.4549333	0.65915792	3.11427249
ENSG000001	INTS12	protein_codin	7.20062194	11.8764891	10.6482888	0.48650007
ENSG000001	C5orf47	protein_codin	0	0	0	0
ENSG000000	GAB2	protein_codin	35.0020173	93.8795488	9.16002709	36.7221257
ENSG000000	CYP2W1	protein_codin	0	0	0	0
ENSG000000	VRK2	protein_codin	2.37576731	0	0	5.86974054
ENSG000001	TSPAN18	protein_codin	23.311208	0	0	0

ENSG000001	LIAS	protein_codin	0	0	0	0
ENSG000001	HIRA	protein_codin	0	0	24.3799463	0
ENSG000001	C3orf33	protein_codin	0	0	0	0
ENSG000001	HRH3	protein_codin	0.3010171	0	0	0
ENSG000001	ZNF69	protein_codin	2.11434746	1.24809615	3.62926514	1.17472385
ENSG000000	ETV7	protein_codin	0	8.94594106	0	0
ENSG000001	PAQR6	protein_codin	0	0	0	0
ENSG000001	BTNL8	protein_codin	0	0	0	0
ENSG000000	ERCC1	protein_codin	5.70233723	0	11.011867	3.97916951
ENSG000001	PSMD10	protein_codin	4.9852799	0	0	0
ENSG000000	MEF2C	protein_codin	0	13.9423261	0	0
ENSG000001	GLRA3	protein_codin	4.20868003	0	0	0
ENSG000001	SERHL2	protein_codin	0	0	0	0
ENSG000001	LGALS12	protein_codin	0	0	0	0.41117758
ENSG000001	NAALADL1	protein_codin	0	1.92997888	0	0
ENSG000001	FBXO38	protein_codin	18.8057649	46.7582798	53.6329353	15.0312489
ENSG000001	RASSF4	protein_codin	1.34505943	14.5505723	0	5.39906017
ENSG000001	MMP13	protein_codin	0	0	1.53144816	0
ENSG000001	ZNF326	protein_codin	30.8396152	62.8022983	70.6039775	26.8900317
ENSG000001	SPAG8	protein_codin	0	0	0	0
ENSG000000	DYNC1I2	protein_codin	11.9999568	6.35330596	130.212236	0
ENSG000001	TMEM201	protein_codin	1.63172098	9.50090466	15.0345782	0
ENSG000001	SLC37A3	protein_codin	0	0	0	0
ENSG000002	UBE2V1	protein_codin	0	10.98087	0	0
ENSG000001	KCNK7	protein_codin	0	0	0	0
ENSG000001	SH2D6	protein_codin	0	0	0	0
ENSG000001	CFAP70	protein_codin	0	0	0	0
ENSG000000	FOLH1	protein_codin	5.52178594	0	6.95235925	0
ENSG000001	ADGRG5	protein_codin	0	0	0	0
ENSG000001	CTSL	protein_codin	2.67394831	0	0	0
ENSG000001	NDUFV3	protein_codin	0.95393491	0	2.55261424	0
ENSG000001	KRTAP24-1	protein_codin	0	1.48839027	0	0
ENSG000001	ZDHHC14	protein_codin	0	0	0	0
ENSG000000	CFAP61	protein_codin	0	1.5111811	0	0
ENSG000001	TMEM135	protein_codin	10.2590635	23.4370259	4.21766169	6.46881023
ENSG000002	SOX18	protein_codin	6.86100456	16.2405484	1.65129006	10.0201273
ENSG000001	XRRA1	protein_codin	1.31615937	0	11.3803904	6.05466667
ENSG000001	C17orf99	protein_codin	0	0	0	0
ENSG000001	INSIG1	protein_codin	30.9067127	17.5492871	68.5878978	24.0444486
ENSG000001	XIRP1	protein_codin	9.76865519	0	0	0
ENSG000001	DOCK7	protein_codin	5.89384886	0	0	11.2389061
ENSG000001	TMEM201	protein_codin	0	0	4.25748495	0
ENSG000001	TUBB4B	protein_codin	22.796676	80.7210237	219.981853	67.4472085
ENSG000001	PRDX6	protein_codin	50.6431594	80.0624932	66.4401668	36.3966962

ENSG000001	NRG2	protein_codin	0	5.47123642	1.56234599	0
ENSG000001	ATXN2L	protein_codin	0	0	0	0
ENSG000001	H3-5	protein_codin	0	1.74013345	1.14230956	1.05739641
ENSG000001	NUP43	protein_codin	26.2600892	21.4338261	59.5134961	5.61302912
ENSG000001	RAB37	protein_codin	0	0	0	0
ENSG000001	LIN54	protein_codin	19.8639199	8.08782603	7.87351931	13.6650306
ENSG000001	RTL4	protein_codin	0	0	0	1.46453471
ENSG000001	PTAR1	protein_codin	57.2217632	140.68051	163.724356	19.2142035
ENSG000001	CPSF7	protein_codin	0	0	0	0
ENSG000001	GSPT2	protein_codin	12.4960687	49.2930585	45.9104372	5.26693625
ENSG000001	FSCB	protein_codin	0	0	0	0.85923636
ENSG000000	DOCK9	protein_codin	1.49362619	1.37651554	2.03594776	1.44636773
ENSG000001	CAP1	protein_codin	0	0	0	0
ENSG000002	UPK3BL1	protein_codin	3.05904222	6.18872382	12.8253698	0
ENSG000001	GFRAL	protein_codin	0	0	0	0
ENSG000001	GTF2F2	protein_codin	7.51032302	42.2589261	27.7326645	8.71944258
ENSG000001	SYNE4	protein_codin	0	0	0	0
ENSG000001	HIPK1	protein_codin	0	52.3321236	0	0
ENSG000001	ZSCAN16	protein_codin	1.36041306	10.8999035	0.97260156	0
ENSG000001	NAPRT	protein_codin	0	0	0	0
ENSG000000	DLGAP4	protein_codin	44.9864404	22.3131987	35.4238968	18.3875695
ENSG000000	TPX2	protein_codin	0	0	0	0
ENSG000001	UTS2B	protein_codin	0.33421263	3.06582319	0.51831073	0.45338965
ENSG000001	CYB5A	protein_codin	0	4.474478	0	0.64876788
ENSG000001	MIA3	protein_codin	65.1100459	255.227912	389.340082	18.9434778
ENSG000001	FAM86B1	protein_codin	0	0	0	1.66750472
ENSG000001	VAPA	protein_codin	4.05817819	5.46352167	20.4691882	27.5167637
ENSG000001	MEIS2	protein_codin	1.3522521	0	0	0
ENSG000001	SIGLEC8	protein_codin	0.22412079	1.21517527	0	0
ENSG000001	LIMK2	protein_codin	0	0	5.61597158	0
ENSG000001	FOXN2	protein_codin	20.4651682	60.3078681	44.2462355	25.0290319
ENSG000001	ACOXL	protein_codin	0	1.32112286	0	0
ENSG000001	UBE2L6	protein_codin	0.47121766	5.52647719	0	0
ENSG000000	RANBP3	protein_codin	0	26.4497895	25.6224876	8.93051276
ENSG000001	ADGRG2	protein_codin	0	0	1.80171908	2.58570357
ENSG000001	LHX6	protein_codin	0	0	0	0
ENSG000001	GRIA1	protein_codin	7.60944354	0	0	0
ENSG000000	SLC4A4	protein_codin	0	0	1.37608566	0
ENSG000001	SOCS2	protein_codin	2.704066	0	0	0
ENSG000001	PTGES	protein_codin	12.1603198	9.95928772	5.98563609	2.83145303
ENSG000001	BRAT1	protein_codin	6.21294635	35.1034862	15.1118522	5.25179478
ENSG000001	CCDC30	protein_codin	0	0	0	0
ENSG000001	CCDC144A	protein_codin	0	0	2.68288308	0
ENSG000001	ZNF746	protein_codin	0	0	0	7.07546884

ENSG000001	C2orf88	protein_codin	8.09094049	1.48669405	6.10535928	1.65715294
ENSG000001	SHBG	protein_codin	0	0	0	0
ENSG000001	BCLAF3	protein_codin	1.71492237	8.58136114	0	5.92943457
ENSG000001	IFT27	protein_codin	0	0	0	0
ENSG000001	PAQR9	protein_codin	0	2.68892094	15.212447	2.7095136
ENSG000001	PDE4D	protein_codin	0	0	8.91710435	0
ENSG000001	GOLGB1	protein_codin	0	0	0	0
ENSG000001	CPED1	protein_codin	6.75251277	0	10.1482669	0
ENSG000001	BRCC3	protein_codin	7.61382758	6.85771508	46.950735	0
ENSG000001	RRP1B	protein_codin	59.7470842	179.50855	252.798105	29.5416895
ENSG000001	NHLRC1	protein_codin	0	4.20928732	4.35082838	0.80662983
ENSG000001	VWC2	protein_codin	2.8756895	0	1.44415006	2.92872441
ENSG000000	ATXN3	protein_codin	11.1231694	0	0	0
ENSG000000	SYN1	protein_codin	1.12022522	2.26892534	0	0
ENSG000002	CDK11B	protein_codin	26.9900797	84.2058949	162.655413	0
ENSG000001	ZNF124	protein_codin	8.77172805	16.8301392	7.75247638	1.94885367
ENSG000001	SERPING1	protein_codin	9.6855538	0	0	0
ENSG000001	MOCS1	protein_codin	8.37687333	25.3348148	12.776667	11.5881191
ENSG000001	PPP1R9A	protein_codin	0	0	0	0.76718896
ENSG000001	PBX1	protein_codin	8.18245269	1.51603074	38.0938639	0
ENSG000001	KCNK10	protein_codin	0	4.81143641	0.83066331	3.03404484
ENSG000001	RASEF	protein_codin	0.27088331	1.70231221	1.76232652	0.49484922
ENSG000002	TCL1B	protein_codin	0	0	0	0
ENSG000001	RILPL1	protein_codin	0.92167512	10.3000239	2.20219742	0.62066979
ENSG000001	EYA1	protein_codin	13.9005509	0	14.1460268	0
ENSG000001	CLPB	protein_codin	0	0	0	0
ENSG000000	ADGRL1	protein_codin	0	0	25.0241328	0
ENSG000001	PAPLN	protein_codin	7.57579874	0	4.9025085	1.73563978
ENSG000001	DNMT1	protein_codin	16.6649065	0	115.534872	13.6620483
ENSG000001	SLC2A14	protein_codin	0	0	0	0
ENSG000002	OR1J4	protein_codin	0	0	0	0.44508587
ENSG000001	IL19	protein_codin	0	0	0	0.54614653
ENSG000001	PPP1CC	protein_codin	17.1172154	19.2736514	14.0420492	0
ENSG000001	OBP2A	protein_codin	0	0	0	0
ENSG000001	SLC26A9	protein_codin	0	0	0	0
ENSG000001	SPATA31F3	protein_codin	0	0	0	0.90155373
ENSG000001	PALM3	protein_codin	0.91097925	1.60065671	17.5427732	5.03675795
ENSG000000	ACSL4	protein_codin	0	0	0	0
ENSG000001	PFKM	protein_codin	0	0	0	0
ENSG000001	LRRIQ4	protein_codin	0	0	0	0
ENSG000001	GPR18	protein_codin	0	0	0	0
ENSG000000	PKP2	protein_codin	0	0	0	0
ENSG000000	ARHGAP44	protein_codin	0	0	0	0
ENSG000001	TERB2	protein_codin	0	0	0	0

ENSG000001	CCNG1	protein_codin	44.9431163	21.827131	45.1007735	33.3960421
ENSG000000	CDK13	protein_codin	2.32949106	17.0752575	7.735719	11.0548472
ENSG000001	RECQL5	protein_codin	0	0	0	0
ENSG000001	CDC25B	protein_codin	0	0	29.3145497	0
ENSG000001	DST	protein_codin	115.060205	117.270442	146.002267	93.6579352
ENSG000001	TRERF1	protein_codin	0	0	0	0
ENSG000001	NLRP7	protein_codin	0	0	0	0
ENSG000001	TBC1D16	protein_codin	0	0	15.3299466	0
ENSG000001	RHCE	protein_codin	0	0	0	0
ENSG000001	PLEKHG5	protein_codin	0	0	0	0
ENSG000001	HM13	protein_codin	11.7533178	43.956691	55.3189013	19.7169336
ENSG000000	IDS	protein_codin	16.3524374	25.1004072	14.1075643	31.6308858
ENSG000001	H1-0	protein_codin	82.997519	77.3469705	162.323078	45.2101406
ENSG000001	CRYZ	protein_codin	16.592076	17.6381461	31.2402459	0
ENSG000001	NVL	protein_codin	0.87850656	0	0	0
ENSG000000	IP6K2	protein_codin	0	0	0	0
ENSG000001	PGLYRP2	protein_codin	0	0	0	0
ENSG000001	PPIL4	protein_codin	71.9141254	0	88.3782894	0
ENSG000001	NECTIN1	protein_codin	0	0	0	0
ENSG000001	PNCK	protein_codin	0	0	0	0
ENSG000001	TC2N	protein_codin	0	3.36003942	0	0
ENSG000001	RNASE1	protein_codin	9.29905859	0	11.6138792	14.0786086
ENSG000000	WNK1	protein_codin	0	11.8453746	0	0
ENSG000001	ZNF169	protein_codin	0	4.88169684	7.01578413	0
ENSG000001	SENP8	protein_codin	1.96185107	0	7.94668241	0.57843863
ENSG000001	HNRNPA1	protein_codin	14.3152234	6.99872041	51.558877	30.9662897
ENSG000000	EXOC5	protein_codin	0	29.9791825	0	6.80323998
ENSG000000	FBLN1	protein_codin	0	0	8.70071415	0
ENSG000001	SUN1	protein_codin	4.11976425	25.0103063	8.75105955	9.14226191
ENSG000001	PTK2	protein_codin	0	8.58883946	0	33.39521
ENSG000000	SPHK2	protein_codin	0	0	0	0
ENSG000001	BROX	protein_codin	5.17721413	46.905586	13.7788127	0
ENSG000001	KLRG2	protein_codin	0	0	0.53394213	0.43714371
ENSG000001	MCCC2	protein_codin	0	0	0	0
ENSG000001	EXD3	protein_codin	0	0	0	0.71373667
ENSG000001	STAU1	protein_codin	0	0	22.3300694	0
ENSG000001	FAM131A	protein_codin	2.66552733	0	0	0
ENSG000001	CLDN4	protein_codin	0	0	0	0
ENSG000001	ZNF780A	protein_codin	0	0	0	0
ENSG000001	PAQR5	protein_codin	0	0	1.23055857	0
ENSG000001	ACER2	protein_codin	3.06089835	5.94374164	4.28150224	4.18191637
ENSG000001	ANKRD33	protein_codin	0	0	0	0
ENSG000001	MAP2K5	protein_codin	1.40955837	3.39735982	5.56615955	0.73107781
ENSG000001	PDE8B	protein_codin	0	0	0	0

ENSG000001	ADIPOR1	protein_codin	27.0448454	44.0223957	27.6077022	25.4082652
ENSG000001	ICAM4	protein_codin	0	0	0.54881998	0
ENSG000001	TUBD1	protein_codin	0	0	0	0
ENSG000001	PKHD1	protein_codin	0	3.89947138	2.85301208	4.68597743
ENSG000001	IL1F10	protein_codin	0	0	0	0
ENSG000001	POMT1	protein_codin	0	0	0	0
ENSG000001	H2AP	protein_codin	0	1.50291585	0	0.39931809
ENSG000001	H2AC7	protein_codin	23.3240168	46.2044001	27.3546958	5.02873118
ENSG000002	CDK11B	protein_codin	42.4077824	192.777197	113.655137	16.1555896
ENSG000001	ART3	protein_codin	0	0	0	0
ENSG000000	TNC	protein_codin	91.7670628	189.106026	0	153.008382
ENSG000001	POP5	protein_codin	0	0	0	2.74876171
ENSG000001	LCN10	protein_codin	0.76475178	1.01236483	0	0
ENSG000001	SYCP2L	protein_codin	0	0	4.04612371	3.50760087
ENSG000001	ANKRD55	protein_codin	0	0	0	0
ENSG000001	CAV1	protein_codin	21.0535413	48.0104093	4.74920845	38.3865332
ENSG000001	TDP2	protein_codin	5.92988605	11.5461149	0	0
ENSG000001	TRIM60	protein_codin	0	0	0	0
ENSG000001	SAMD11	protein_codin	0	16.2643624	0	0
ENSG000001	ANAPC1	protein_codin	22.0014807	24.8997611	111.070145	21.2692742
ENSG000001	BSDC1	protein_codin	6.77952065	19.7425236	11.691277	4.29147487
ENSG000002	SERPINB4	protein_codin	0	0	0	0
ENSG000001	BEND7	protein_codin	0	0	0	0.68847678
ENSG000000	RNF19A	protein_codin	83.8739825	75.5706089	170.381113	52.649182
ENSG000001	ESR2	protein_codin	0	5.01456075	0.97922655	1.26142846
ENSG000001	GATA2	protein_codin	4.42416818	14.5628414	0	28.3674103
ENSG000001	IL4I1	protein_codin	0	0	0	0
ENSG000000	MCOLN3	protein_codin	0	0	0	0
ENSG000001	MPST	protein_codin	0	0	0	0
ENSG000000	SLC25A40	protein_codin	12.2466745	16.0727059	35.2147929	8.64137551
ENSG000001	POPDC2	protein_codin	0	0	1.55988521	0
ENSG000001	TMEM221	protein_codin	0.110909	0	0	0
ENSG000001	GMPPA	protein_codin	0	0	0	0
ENSG000001	RBM33	protein_codin	0	0	0	0
ENSG000001	SRSF10	protein_codin	2.60708351	10.3504905	0	7.44294691
ENSG000001	B4GALNT1	protein_codin	4.09218757	3.75913057	31.9869627	0
ENSG000001	FCF1	protein_codin	11.2542459	24.8619082	30.8178839	2.95626677
ENSG000001	ZNF606	protein_codin	0	4.31674068	0	0
ENSG000001	NBR1	protein_codin	6.35610788	0	0	0
ENSG000001	NIPAL2	protein_codin	1.13019294	0	0	0
ENSG000001	TSTD2	protein_codin	19.5868148	36.3879779	37.7425997	7.5044398
ENSG000001	DNM1	protein_codin	0	0	0	0
ENSG000000	CLDND1	protein_codin	31.7653249	13.3942632	26.2664303	3.10060059
ENSG000000	MKNK1	protein_codin	9.33317108	0	23.3014726	0

ENSG000001:	MGAT3	protein_codin	0.54113505	8.90716972	5.61080841	3.43365995
ENSG000001	ARMCX3	protein_codin	14.2124412	7.13122396	21.3456971	8.77956508
ENSG000001!	ZNF823	protein_codin	4.77372162	0	7.58040109	4.20697531
ENSG000001	HAP1	protein_codin	0.28248298	0	1.56645034	0
ENSG000001:	TOX2	protein_codin	0	18.7552681	8.66178226	0
ENSG000001	SIMC1	protein_codin	0	0	0	0
ENSG000002	LCN6	protein_codin	0	0	0	0
ENSG000001!	FAM72A	protein_codin	0	0	5.90570933	2.91170158
ENSG000000	ADCK1	protein_codin	0.5013036	25.1709147	2.02167694	0
ENSG000001	FAM222B	protein_codin	2.34471486	0	0	0
ENSG000000	CFLAR	protein_codin	0	0	0.86849484	0
ENSG000001	CFL2	protein_codin	13.5080903	2.04334234	12.1590239	0
ENSG000001	SERPINA12	protein_codin	0	0	0	0.4754576
ENSG000000:	LIMA1	protein_codin	12.7824466	26.3440791	1.32017833	16.5300281
ENSG000001	METTL23	protein_codin	0	0	7.73226839	2.02319769
ENSG000001	RTL6	protein_codin	21.4067307	19.3721917	99.800197	16.3245666
ENSG000001	SH2B3	protein_codin	30.859573	145.374315	35.3564971	61.7997203
ENSG000001	DLK1	protein_codin	0	1.43957817	1.32659773	0.83219722
ENSG000001	ENO4	protein_codin	0	0	0	0
ENSG000001	FBXL5	protein_codin	10.1167992	8.49439099	8.19690354	10.7524629
ENSG000001	C2orf80	protein_codin	0	0	0	0
ENSG000001	PERM1	protein_codin	0.64926603	1.77665568	0	0
ENSG000001	NANOS2	protein_codin	0	1.59552608	0	2.75666796
ENSG000001	DPM3	protein_codin	0	0.91488924	0	2.42897252
ENSG000002	TP53TG3B	protein_codin	0	0	0	0
ENSG000001:	RTN3	protein_codin	3.18900601	0	0	0
ENSG000000	GLI2	protein_codin	0	0	0	5.3657957
ENSG000001!	SPG7	protein_codin	0	0	7.36154547	1.57346733
ENSG000001	KAT5	protein_codin	0	0	0	0
ENSG000001	KLHL6	protein_codin	0	44.3977532	1.97059909	8.61491757
ENSG000001	BRWD1	protein_codin	7.74966994	32.8383574	20.0055187	5.03519882
ENSG000001	RHBDD1	protein_codin	9.85808114	0	0	0
ENSG000001	TEX264	protein_codin	5.39350466	0	0	0
ENSG000001	NOXA1	protein_codin	0	0	0	0.92587403
ENSG000001:	RAP1B	protein_codin	10.7066717	0	0	0
ENSG000001	CORO1B	protein_codin	0	26.3220991	6.88997258	3.11737906
ENSG000000:	ATP2B4	protein_codin	0	0	0	0
ENSG000001	PTMA	protein_codin	168.403912	1173.69164	302.452127	160.681151
ENSG000001	TPM3	protein_codin	0	0	0	0
ENSG000001	ZDHHC14	protein_codin	0	0	0	0
ENSG000000	NFYA	protein_codin	18.3514767	62.9557622	166.540995	35.2639526
ENSG000001:	CAPRIN1	protein_codin	33.1251389	0	29.5809593	27.7168854
ENSG000001	LBX2	protein_codin	0	0	0.52304042	0
ENSG000001	NECTIN1	protein_codin	0	0	0	0

ENSG000001	NPIPB4	protein_codin	0	0	0	0
ENSG000001	UBXN8	protein_codin	0.3894495	6.95423385	0	0.63887512
ENSG000001	VCX	protein_codin	0	0	0	0
ENSG000001	SLCO2B1	protein_codin	2.30660853	14.4847419	0	6.63038611
ENSG000001	HAGHL	protein_codin	1.44871617	0	0	0
ENSG000001	IGFL3	protein_codin	0	0	0	0
ENSG000001	SKOR1	protein_codin	0	0	1.4759642	2.01703149
ENSG000001	FLRT3	protein_codin	0	0	4.64899767	0
ENSG000001	TRAK1	protein_codin	18.1957906	35.8365753	8.63139287	0
ENSG000001	HMGB1	protein_codin	6.46018168	30.7051737	22.6403691	13.7497689
ENSG000000	NADK	protein_codin	28.0741146	178.198233	145.282239	9.47176674
ENSG000001	NEK10	protein_codin	1.06620886	4.65510288	0	1.75828889
ENSG000001	MEST	protein_codin	0	0	0	0
ENSG000001	BRI3BP	protein_codin	5.17172552	9.12528706	9.25853541	3.23914728
ENSG000001	CCDC66	protein_codin	13.9045174	12.5794479	0	10.5856745
ENSG000001	SGK2	protein_codin	0.74917473	3.3514693	0	0.41215024
ENSG000001	SLC24A5	protein_codin	0	0	0	0.94585967
ENSG000000	SYDE2	protein_codin	25.380143	0	16.3073475	0
ENSG000000	FGFR1	protein_codin	0	0	0	17.9264103
ENSG000001	NABP2	protein_codin	0	0	0	5.27389247
ENSG000001	ZKSCAN3	protein_codin	5.13476743	6.40406641	6.18251461	0
ENSG000001	TRIM14	protein_codin	6.57588024	87.2894127	25.3952005	8.27551804
ENSG000001	C14orf93	protein_codin	0	0	0	0
ENSG000000	SYNE2	protein_codin	0.40572315	8.82368363	2.62353016	0
ENSG000001	PARN	protein_codin	6.29679776	0	32.1081536	5.16525376
ENSG000001	TPM3	protein_codin	0	0	0	0
ENSG000001	ATF3	protein_codin	11.2762021	0	0	42.3469716
ENSG000001	TREML4	protein_codin	0	0	0	0
ENSG000001	INSR	protein_codin	29.3970747	117.966774	125.503925	41.6250327
ENSG000001	MIA2	protein_codin	4.83041498	2.75898249	5.71681954	1.37134587
ENSG000001	CBLC	protein_codin	0	0	0	0.36787484
ENSG000001	LCTL	protein_codin	0	0	0	2.64214263
ENSG000001	ABCA2	protein_codin	0	61.9845542	15.3198332	26.4023592
ENSG000001	ATP7A	protein_codin	0	0	0	0
ENSG000001	DCAF5	protein_codin	57.909434	144.469728	164.321046	39.2196667
ENSG000001	PDE4B	protein_codin	0	0	0	0
ENSG000000	KCNAB2	protein_codin	0	0	0	0
ENSG000001	CDH5	protein_codin	14.619717	79.4826049	19.5675451	37.0981957
ENSG000001	NAPEPLD	protein_codin	15.0366452	13.4581513	16.7645766	0
ENSG000001	THYN1	protein_codin	0	39.7813247	0	0
ENSG000001	LMO7	protein_codin	9.27955713	4.5517716	17.3147594	19.2276057
ENSG000001	ING4	protein_codin	0	7.86967234	0	0
ENSG000001	CFAP65	protein_codin	0	0	0	0
ENSG000001	FOXO4	protein_codin	0	0	0	0

ENSG000001(	TMEM248	protein_codin	64.8210725	138.75879	114.796839	30.7420923
ENSG000000(	CFLAR	protein_codin	0	0	0	0
ENSG000001	SERPINA6	protein_codin	0	1.13322456	0	0.45968728
ENSG000000	FERMT2	protein_codin	56.7322668	54.8608534	62.3204596	59.1782422
ENSG000002:	TMEM179	protein_codin	0	0	0.58133255	0.41981756
ENSG000001	GRM8	protein_codin	0	0	0	0
ENSG000001:	MAP3K7CL	protein_codin	0	0	0	0
ENSG000000(	TNFRSF12A	protein_codin	0	0	0	0
ENSG000001(	CLUAP1	protein_codin	1.21472489	0.76048041	0	0
ENSG000001(	PAX4	protein_codin	0	0	0	0.49666695
ENSG000001(	GP2	protein_codin	0	1.19453888	0	0.85187979
ENSG000001:	MAX	protein_codin	0	0	0	0
ENSG000002(	SPDYE2	protein_codin	0	0	0	0
ENSG000001:	ANKFY1	protein_codin	34.7364233	128.148174	35.1351615	17.6963092
ENSG000001:	LRRC37B	protein_codin	0	0	0	0
ENSG000001	ATP13A2	protein_codin	3.85719803	0	0	0
ENSG000000	MAP4K3	protein_codin	23.6412009	92.5725432	67.9260241	0
ENSG000001(	MYL1	protein_codin	104.341762	0	0	1.40560467
ENSG000001:	TMEM202	protein_codin	0	0	0	4.48435646
ENSG000001(	TAF1C	protein_codin	1.41533631	0	6.31303301	0
ENSG000001:	DCTN3	protein_codin	0	0	0	0
ENSG000001:	PPA2	protein_codin	0	0	0	0
ENSG000001	SEMA6C	protein_codin	4.96143518	3.84200838	7.11290885	5.44121102
ENSG000001:	PEDS1-UBE2\	protein_codin	0	0	0	0
ENSG000001	GNA14	protein_codin	3.94689787	12.0133611	0	8.73117348
ENSG000001!	VN1R2	protein_codin	0.37092563	0	0.58104771	0
ENSG000001:	MPRIP	protein_codin	134.76334	349.064724	197.673958	71.8053941
ENSG000001:	HEPACAM2	protein_codin	0	0	0	0
ENSG000001	ASTN2	protein_codin	1.18347149	0	8.08889802	0
ENSG000001:	MESP2	protein_codin	0	0	0	0
ENSG000001:	PMEPA1	protein_codin	56.79106	82.2490185	72.5993678	30.0826624
ENSG000001:	PRR19	protein_codin	0	0	0	0
ENSG000001	MIA2	protein_codin	0	36.8887532	0	0
ENSG000002:	RFPL4AL1	protein_codin	0	0	0	0.68461507
ENSG000001:	ANKS1B	protein_codin	0.35129155	0	0	0
ENSG000001	TRIM28	protein_codin	11.4661536	45.2778106	48.9829178	0
ENSG000001	RAD1	protein_codin	0.71479998	14.3407353	14.5475103	4.02268054
ENSG000000(	SCIN	protein_codin	1.24716325	16.9735717	83.298034	9.91577497
ENSG000001	WDR31	protein_codin	0	0	3.5927877	4.70962498
ENSG000001:	DNER	protein_codin	0.42105104	0	4.08921675	0
ENSG000000	CD22	protein_codin	0.54849	0	0	0
ENSG000000(	JARID2	protein_codin	33.6215402	298.960393	120.472931	58.0034189
ENSG000001	FAM163A	protein_codin	0	3.12406415	0.53949251	2.18578068
ENSG000001:	SSUH2	protein_codin	0	0	0	0

ENSG00000010	COL22A1	protein_codin	0	0	3.18277547	0
ENSG00000018	KRT77	protein_codin	0	0	0.51049877	0
ENSG000002	CDK11B	protein_codin	0	0	0	0
ENSG00000019	ZKSCAN7	protein_codin	0	0	0	0
ENSG00000010	CRHR2	protein_codin	0	0	0	0
ENSG00000013	TRIM29	protein_codin	0	0	0	0
ENSG00000013	TNIK	protein_codin	0	62.4618027	64.3793998	0
ENSG0000007	DPP8	protein_codin	6.05726689	3.71939957	24.7601055	12.4604273
ENSG00000010	TSPAN14	protein_codin	0	7.9441597	0	0
ENSG00000018	TAF9B	protein_codin	10.6190787	41.9965481	17.7002567	9.95947488
ENSG00000010	C6orf132	protein_codin	0.6198162	0	0	0
ENSG00000018	TMEM220	protein_codin	5.00476086	3.4263846	4.31604941	5.47761174
ENSG000001	LGALS8	protein_codin	11.3153022	0	20.8451805	10.2378712
ENSG0000017	ZBTB8OS	protein_codin	2.63163406	0	5.22795333	0
ENSG00000010	CEP97	protein_codin	0	0	0	0
ENSG00000014	DLX1	protein_codin	0	7.25867943	0	0
ENSG00000018	SBK1	protein_codin	0.53944065	10.8014836	8.01645387	1.389617
ENSG000001	MYB	protein_codin	0	0	0	0
ENSG00000018	CXCL17	protein_codin	0	0	0	0.41222659
ENSG00000014	NFIC	protein_codin	0	0	0	0
ENSG0000003	RABEP1	protein_codin	80.0258724	82.4806902	74.2394255	47.1975571
ENSG000003	CAST	protein_codin	0	9.680284	0	16.3294718
ENSG0000008	EPB41L3	protein_codin	2.126157	10.3779898	8.39613105	0.97634439
ENSG000001	CCR6	protein_codin	0	0	0	0
ENSG00000010	ABLIM2	protein_codin	0	0	0	0
ENSG00000018	LAMTOR4	protein_codin	10.7351932	12.8679371	26.4023801	7.31207807
ENSG0000020	COL11A2	protein_codin	25.8750966	131.145746	942.587523	68.0951977
ENSG00000018	PCDHB13	protein_codin	11.3777465	27.0385792	12.6635853	4.65550923
ENSG0000017	DALRD3	protein_codin	0	0	0	0
ENSG00000013	CCDC62	protein_codin	0	3.62165615	0	0
ENSG00000014	SYT8	protein_codin	0	0	0	0
ENSG0000027	OR2S2	protein_codin	0	1.11047896	0	0.45023255
ENSG00000018	ZNF70	protein_codin	26.7460051	62.9679857	119.960186	20.7134674
ENSG00000013	PPP2R1B	protein_codin	0	0	8.88402961	0
ENSG00000010	BBC3	protein_codin	0	9.82720186	6.79943562	0
ENSG00000010	RAB36	protein_codin	0.69049086	0	0	0.8525455
ENSG00000013	CNTNAP3B	protein_codin	0	0	0	0
ENSG0000000	NADK	protein_codin	0	0	0	0
ENSG00000013	SLC24A2	protein_codin	0	0	0	9.89204193
ENSG0000003	IKZF2	protein_codin	0	0	0	0
ENSG00000010	BMX	protein_codin	0	4.2563655	0	1.80286144
ENSG00000013	CIR1	protein_codin	95.6532979	197.700669	247.979195	63.4856367
ENSG00000014	GTF3C5	protein_codin	0	0	0	0
ENSG00000013	CTSL	protein_codin	0	0	0	13.743882

ENSG000001	CCDC30	protein_codin	0	0	0	4.62953024
ENSG000001	PPP1R17	protein_codin	0	0	0	2.17951258
ENSG000001	TRIM14	protein_codin	0	0	7.88588761	0
ENSG000001	BABAM2	protein_codin	0	0	0	0
ENSG000001	RXFP1	protein_codin	0	0	0	0
ENSG000001	RHD	protein_codin	0	0	0	0
ENSG000001	KCNQ5	protein_codin	0	0	0	0
ENSG000001	FBLN5	protein_codin	22.7047183	0	15.4395933	0
ENSG000001	PEAK3	protein_codin	0.27078144	3.0732434	0	1.57677528
ENSG000001	SAMD11	protein_codin	0	0	0	0
ENSG000000	MRPL43	protein_codin	1.11606594	0	0	0
ENSG000000	PMS1	protein_codin	0	0	54.1515527	10.7626038
ENSG000001	RASSF6	protein_codin	0	2.78201908	0.5607566	0
ENSG000001	PTPRR	protein_codin	0.7821217	0	0	1.336881
ENSG000001	PDPK1	protein_codin	19.0618179	90.4423453	66.6862901	24.0841481
ENSG000001	IFNAR2	protein_codin	0	0	0	0
ENSG000001	NBPF3	protein_codin	0	20.1567515	0	2.13554441
ENSG000000	CREM	protein_codin	0	0	0	0
ENSG000001	CLDN14	protein_codin	0	0	0	0
ENSG000000	BID	protein_codin	0	0	0	0
ENSG000000	TBC1D2	protein_codin	0	0	0	8.03248132
ENSG000001	OBP2A	protein_codin	0	0	0	0.27251667
ENSG000001	AMPD2	protein_codin	0	25.3390762	37.2948619	0
ENSG000001	IFNAR2	protein_codin	11.9322366	0	0	0
ENSG000001	COX8C	protein_codin	0	0	0	0
ENSG000002	EMP2	protein_codin	1.78708964	2.33289561	8.12294216	8.04507562
ENSG000001	JMJD7-PLA2G	protein_codin	0	0	0	0
ENSG000000	HUWE1	protein_codin	0	324.114239	0	172.79073
ENSG000001	DPY30	protein_codin	17.20107	0	30.7685759	6.6538529
ENSG000001	DPH5	protein_codin	0	0	4.18964588	5.98620089
ENSG000001	TTN	protein_codin	0	0	0	0
ENSG000001	DPY19L3	protein_codin	42.7497611	38.7109963	36.1207527	18.7280531
ENSG000001	NRXN1	protein_codin	0	0	0	0
ENSG000001	TMEM91	protein_codin	0	0	0	0
ENSG000001	SUMF2	protein_codin	25.2780277	0	40.0655237	0
ENSG000001	UBE2L3	protein_codin	10.4415062	0	8.02781891	5.72450747
ENSG000000	COBLL1	protein_codin	13.4352427	9.74549421	23.2929474	16.6874675
ENSG000001	KCNK7	protein_codin	0	0	0	0
ENSG000001	SSX2IP	protein_codin	8.30616131	39.5019915	27.0667176	7.46297554
ENSG000000	IGBP1	protein_codin	0	0	4.29217948	0
ENSG000001	CLEC14A	protein_codin	5.42944184	49.9694803	5.38306738	19.0841231
ENSG000001	PKN1	protein_codin	0	109.496692	70.2708577	0
ENSG000001	ISM2	protein_codin	0	0.92495585	7.03650154	0
ENSG000001	COL18A1	protein_codin	0.7438896	24.9715305	0	0

ENSG000001	IKBKB	protein_codin	0	0	2.08566461	0
ENSG000001	SLC20A2	protein_codin	0	0	0	0
ENSG000001	JPH1	protein_codin	17.8231734	0	82.6459235	2.02921378
ENSG000001	SP6	protein_codin	0	5.74810832	0.99194856	1.28995027
ENSG000001	SMPD1	protein_codin	9.64851928	25.0446067	13.1531559	7.08520601
ENSG000000	CECR2	protein_codin	0	0	9.2841552	0
ENSG000001	AFF2	protein_codin	23.5209231	16.4848282	15.7653833	0
ENSG000001	NHSL1	protein_codin	1.59313765	0	23.9682539	0
ENSG000001	AKAP7	protein_codin	0.47652613	11.0785886	4.62128439	1.83449306
ENSG000001	JPH2	protein_codin	0.52955342	0	0	1.39751024
ENSG000001	BCOR	protein_codin	0	0	0	0
ENSG000001	GJA4	protein_codin	3.09380973	6.53304268	0	10.2790487
ENSG000001	PBX3	protein_codin	16.8687909	0	49.7425716	6.13399083
ENSG000001	KCNJ14	protein_codin	0.31831871	1.08658273	0	2.48815557
ENSG000001	RBPJ	protein_codin	0	0	0	1.18629311
ENSG000000	POU2F2	protein_codin	0	0	1.7827968	0
ENSG000000	TMEM230	protein_codin	0	0	0	0
ENSG000001	LMX1A	protein_codin	2.80919304	0	0	0
ENSG000002	ADSL	protein_codin	3.74894742	7.33288679	40.4856783	0
ENSG000002	GOLGA8B	protein_codin	0	0	0	26.1929358
ENSG000001	OAS2	protein_codin	0	2.32923648	0	0
ENSG000001	CSRNP3	protein_codin	0	0	14.4471477	32.1277761
ENSG000001	RBPJ	protein_codin	0	0	0	0
ENSG000001	ASCL4	protein_codin	0.22217654	0	0	0.91335904
ENSG000001	PDE8B	protein_codin	0	0	3.54824023	0
ENSG000000	NADK	protein_codin	0	4.71914895	0	0
ENSG000001	DCAKD	protein_codin	0	0	0	4.68390623
ENSG000001	DCDC1	protein_codin	0	0	2.96656063	3.60900477
ENSG000001	ATP13A5	protein_codin	0	2.25289754	0	0.94608594
ENSG000002	GH1	protein_codin	0	0	0	0
ENSG000001	SERINC3	protein_codin	114.933645	197.154539	100.193296	82.6205552
ENSG000002	KIR2DL3	protein_codin	0	1.48692169	0	2.14704536
ENSG000001	ASTL	protein_codin	0	0	0.54932288	3.53287703
ENSG000001	MASTL	protein_codin	6.20170992	0	14.2465091	5.68572653
ENSG000001	DIABLO	protein_codin	1.00997009	0	2.34706351	0
ENSG000001	CCDC83	protein_codin	0	0	0	1.06775321
ENSG000001	INSIG1	protein_codin	0	0	0	0
ENSG000001	FRMD4A	protein_codin	0	0	35.9315147	0
ENSG000001	BIRC7	protein_codin	0	3.0513288	0	0.80622098
ENSG000001	SNRNP48	protein_codin	11.9115848	64.6118809	63.5458498	16.5503006
ENSG000001	EYS	protein_codin	0	0	0	0
ENSG000001	RAB5IF	protein_codin	0	0	3.80684445	0
ENSG000001	ZNF341	protein_codin	0	0	0	0
ENSG000001	PLSCR1	protein_codin	10.1417464	31.2312447	15.6854423	14.7358254

ENSG000001:	NR3C2	protein_codin	0	0	0	0
ENSG000001(	TSHR	protein_codin	0	0	0	0
ENSG000001:	ASCC1	protein_codin	0	0	0	0
ENSG000001(	BRWD1	protein_codin	76.0397014	197.353541	285.441561	49.215939
ENSG000000!	HAUS4	protein_codin	0	0	4.3707537	0
ENSG000000(	ALDH3B1	protein_codin	0	0	0	0
ENSG000001:	TMPPE	protein_codin	1.82651121	0	1.75258335	1.29060893
ENSG000001(	CMTM5	protein_codin	0	0	0	0
ENSG000001(	DUSP18	protein_codin	0	0	0	0
ENSG000001	PODXL2	protein_codin	20.2202605	14.8684408	117.684392	5.88942364
ENSG000001:	MUC1	protein_codin	0	0	0	0
ENSG000001!	ZNF79	protein_codin	8.59656154	0	3.15159216	0
ENSG000001:	UBE3B	protein_codin	19.9873433	51.3960457	29.080931	0
ENSG000001(	IKBIP	protein_codin	0	4.4301467	3.256062	9.70389989
ENSG000001:	RTN1	protein_codin	0	2.98666238	0	0
ENSG000001(	JAK1	protein_codin	0	0	0	56.2084848
ENSG000001(	C2orf66	protein_codin	0	0	0.56175987	2.21421288
ENSG000001:	PANK1	protein_codin	0.46582468	0	3.58302576	4.58569723
ENSG000001:	AGMO	protein_codin	0.77694213	0	0.51695466	2.3140096
ENSG000001(	OTOG	protein_codin	0	0	0	0.89851143
ENSG000001'	ZBTB42	protein_codin	0.55221654	6.10733171	1.69074261	0.40764467
ENSG000001!	IL1RAP	protein_codin	0	0	0	0
ENSG000000	ZNF207	protein_codin	0	0	0	2.8092107
ENSG000001(	NAB2	protein_codin	0	8.06914279	6.99541309	6.2789465
ENSG000001(	PPP3CB	protein_codin	19.497063	30.6177325	27.0201621	10.2923827
ENSG000001:	GDAP1L1	protein_codin	0	0	0	0
ENSG000000'	MKNK1	protein_codin	0	0	0	0
ENSG000001'	CYBC1	protein_codin	0	0	0	0
ENSG000001'	BPTF	protein_codin	0	0	0	0
ENSG000001:	NYX	protein_codin	0	0	0	0
ENSG000001:	TMEM120B	protein_codin	1.80820395	37.5216296	0	0
ENSG000002(	RUFY2	protein_codin	0	3.50455777	10.8696904	0
ENSG000001(	DUSP19	protein_codin	0	0	0	0
ENSG000001:	RGS3	protein_codin	0.86860113	0	0	0
ENSG000001(	OR14I1	protein_codin	0	0	0	0
ENSG000001:	TMTC4	protein_codin	0	8.95732451	6.43690906	4.94274724
ENSG000001	FOXM1	protein_codin	0	0	0	0
ENSG000001(	SPMAP2	protein_codin	0	0	0	0
ENSG000001:	SNX21	protein_codin	0	0	0	0
ENSG000000(	HIPK2	protein_codin	6.53723224	27.6995058	55.2205153	7.43538314
ENSG000001'	GPRC5C	protein_codin	3.87650404	1.62791478	0	6.13368715
ENSG000001'	TCAIM	protein_codin	7.52759052	13.2406822	16.2490063	5.81026442
ENSG000001(	ZNF12	protein_codin	20.3073665	60.3400948	31.0131471	9.88378038
ENSG000000(	INPP5A	protein_codin	0	0	0	0

ENSG000001	SOX12	protein_codin	10.3024798	61.795027	61.6513194	5.18960335
ENSG000001	CAMK2D	protein_codin	0	0	0	0
ENSG000001	SNRPD2	protein_codin	22.4961396	36.1719436	65.280316	17.732307
ENSG000000	FUT8	protein_codin	24.9749894	67.8502274	145.748852	38.6270211
ENSG000000	MAP2K3	protein_codin	12.5439967	26.1628278	0	29.2101614
ENSG000001	C19orf12	protein_codin	0	0	0.71255582	0
ENSG000001	C15orf61	protein_codin	0.96457257	3.65594453	0	1.04423761
ENSG000001	RBM38	protein_codin	0	0	0	0
ENSG000001	NPR2	protein_codin	0.64903721	23.8934131	3.91042874	0
ENSG000001	ATP13A4	protein_codin	0.33886131	0	0	2.76497671
ENSG000001	TP53INP1	protein_codin	8.51593632	144.431946	0	20.3810102
ENSG000001	WDR20	protein_codin	0	0	43.4308501	0
ENSG000000	CBFA2T2	protein_codin	0	78.155243	0	18.4487523
ENSG000001	CDC42BPG	protein_codin	0.64966029	10.2655674	0.62324825	7.10825758
ENSG000001	CARMIL3	protein_codin	0	0	3.74650205	0.39393227
ENSG000001	PKLR	protein_codin	0	0	0	0
ENSG000001	TMEM215	protein_codin	0	0	0	4.63121379
ENSG000001	SUSD6	protein_codin	41.6749525	116.915168	67.470432	88.6279012
ENSG000001	SDHC	protein_codin	6.3180805	0	0	0
ENSG000001	LY6H	protein_codin	0	0	0	0
ENSG000001	MXRA8	protein_codin	1.44898449	9.75754155	7.05907832	0
ENSG000001	NEXN	protein_codin	48.7703056	88.9922294	109.319001	17.8448017
ENSG000001	AUTS2	protein_codin	29.7467441	0	0	0
ENSG000001	DUSP9	protein_codin	0	0	22.3257475	0
ENSG000001	PROZ	protein_codin	0	0	0	0
ENSG000001	SYDE1	protein_codin	12.0646232	0	0	7.70025113
ENSG000000	VCAN	protein_codin	0	0	0	0
ENSG000001	ERBB4	protein_codin	0	0	0	0
ENSG000001	ZDHHC3	protein_codin	4.85745679	0	9.15881787	0
ENSG000000	CFLAR	protein_codin	2.86896501	5.49052111	0	0
ENSG000001	BTBD8	protein_codin	0	0	0	0
ENSG000001	PAQR3	protein_codin	0	0	1.55948762	0
ENSG000001	FOXD4L3	protein_codin	0	0	0	0
ENSG000000	SPTB	protein_codin	0	0	0.33585506	0
ENSG000000	PRSS3	protein_codin	0	0	0	0
ENSG000000	TCF7	protein_codin	6.05309309	16.0666671	34.6460269	7.20004339
ENSG000001	BTNL3	protein_codin	0	0	0	0.91281794
ENSG000001	WFDC2	protein_codin	0	0	0	0
ENSG000001	SCGB1C1	protein_codin	0	0	0	0.4188893
ENSG000000	FXYD5	protein_codin	0.9242282	3.47556565	0	0.68667854
ENSG000001	CFAP54	protein_codin	0.93706205	0	0	0
ENSG000001	LRRC39	protein_codin	0	0	0	1.8285633
ENSG000000	NLRC4	protein_codin	1.82104074	0	0	0
ENSG000001	ST3GAL2	protein_codin	19.6000646	45.2539055	46.6594486	12.815873

ENSG000001	EGFR	protein_codin	0	0	0	0
ENSG000001	MADD	protein_codin	9.83015276	21.5200406	18.50009	0
ENSG000001	NLRP13	protein_codin	0.29115262	2.43366979	0	3.81512785
ENSG000001	PPCDC	protein_codin	0	4.34146025	6.45532248	0
ENSG000000	EPB41L3	protein_codin	0	0	0	0
ENSG000000	CD82	protein_codin	0	4.24854126	0	3.60112099
ENSG000001	SUSD4	protein_codin	0	0	0	0
ENSG000000	BCAT1	protein_codin	0	34.6334133	23.3066492	0
ENSG000001	GSDME	protein_codin	0	0	0	0
ENSG000002	PSG5	protein_codin	0	0	0	0
ENSG000001	HSD11B1L	protein_codin	0	0	0	0
ENSG000001	MAGEH1	protein_codin	10.9386611	16.6326581	65.2360592	8.49981588
ENSG000001	EIF4G1	protein_codin	0	0	16.383384	0
ENSG000001	RAP2C	protein_codin	23.6739145	24.3213158	22.0234885	13.9048601
ENSG000001	SMAD4	protein_codin	25.6730201	0	0	0
ENSG000001	TTN	protein_codin	16958.8189	229.391397	37.772395	71.1398327
ENSG000001	EZHIP	protein_codin	0	0	56.1700183	0.42026359
ENSG000000	HEPH	protein_codin	6.01727904	0	0	0
ENSG000001	SPATA13	protein_codin	7.15774487	0	1.92375961	37.1405822
ENSG000001	PABIR2	protein_codin	1.99569629	27.7904017	15.2095177	7.32079423
ENSG000001	C3orf62	protein_codin	3.3831979	0	1.70136806	6.09239354
ENSG000001	SNX18	protein_codin	1.46446114	4.06201479	5.19648158	4.68457307
ENSG000001	MCM7	protein_codin	0.32718798	0	2.65551958	0
ENSG000000	TM7SF3	protein_codin	14.5690232	27.7570703	22.3902196	9.08388757
ENSG000001	BSX	protein_codin	0	0	0	0
ENSG000001	CFAP77	protein_codin	0	0	0	0
ENSG000001	DAP3	protein_codin	25.8889984	48.0538482	32.2723915	7.75272481
ENSG000001	NELFB	protein_codin	15.8255829	61.8902124	53.2826559	11.5232336
ENSG000002	LYPD4	protein_codin	0	0	0	0.82889583
ENSG000001	ESPNL	protein_codin	0	0	0	0
ENSG000001	EPB41	protein_codin	0	0	0	0
ENSG000001	SCNN1B	protein_codin	0	4.4320695	0	1.16748534
ENSG000001	ALKBH2	protein_codin	0	0	8.09596659	0
ENSG000001	QRFP	protein_codin	0	0	0	0
ENSG000001	UBE4B	protein_codin	15.9367787	56.608538	49.6822659	25.4625515
ENSG000001	NEMP2	protein_codin	0	0	0	0
ENSG000001	UBE2D3	protein_codin	0	0	1.86481665	6.57120059
ENSG000001	PRELP	protein_codin	26.8060836	91.5027363	12.9337775	10.8301505
ENSG000000	MAPK9	protein_codin	0	0	0	0
ENSG000000	CTNNA2	protein_codin	0	0	0	0
ENSG000001	B3GALT5	protein_codin	0	0	0	0
ENSG000001	BACH2	protein_codin	0	0	0	0
ENSG000001	CD300LF	protein_codin	1.01044355	3.53310513	0	0
ENSG000001	SYCE1	protein_codin	0	0	0	0

ENSG000001	PRDM2	protein_codin	12.3697929	0	0	0
ENSG000001!	HLA-DQA1	protein_codin	10.236195	100.759135	1.92633207	0
ENSG000001	TBC1D7	protein_codin	0	0	0	0
ENSG000001	CTSL	protein_codin	15.2649448	9.00500342	13.6996719	0
ENSG000001	INTS8	protein_codin	0	63.4395936	30.3056697	0
ENSG000000	SLC6A13	protein_codin	0	0	0	0
ENSG000001	IFT56	protein_codin	0	0	0	0
ENSG000001	KCNIP2	protein_codin	0	0	0	0
ENSG000000	VCAN	protein_codin	401.892069	333.425477	0	431.424033
ENSG000001	RIC3	protein_codin	0.90579952	0	2.51272722	0
ENSG000000	ST7L	protein_codin	0	0	14.3175417	0
ENSG000001	CAV2	protein_codin	0	0	0	0
ENSG000001	RTP5	protein_codin	0	0	0	0
ENSG000001	DUSP28	protein_codin	0	0	0	0
ENSG000001	DPYSL3	protein_codin	188.059298	163.544493	93.7492208	52.94305
ENSG000000	SLC12A2	protein_codin	0	45.2876742	68.7223974	5.41326383
ENSG000001	LYPLA1	protein_codin	6.20375324	0	14.8822709	0
ENSG000001!	MAFK	protein_codin	2.07269381	38.9645246	5.89511977	12.8922496
ENSG000000	ATG5	protein_codin	0	0	2.66745572	0
ENSG000001!	KRTAP4-5	protein_codin	0	0	0	0
ENSG000001	CCDC18	protein_codin	9.5274413	0	37.5876325	3.85778016
ENSG000001	SRSF10	protein_codin	18.3072	33.8393403	15.7406092	9.03501338
ENSG000001	MUC1	protein_codin	0	0	0	0
ENSG000001	CLCN1	protein_codin	0	0	0	0
ENSG000001	VAV3	protein_codin	3.07827768	0	0	0
ENSG000001	RPS2	protein_codin	29.6344563	128.225745	406.935416	110.958219
ENSG000001	APOD	protein_codin	37.4476969	31.267602	0	8.68725001
ENSG000001	ASB7	protein_codin	0.81896492	0	9.09904835	0
ENSG000000	FERMT2	protein_codin	0	0	0	0
ENSG000001!	ZNF772	protein_codin	0	2.0539806	0	0
ENSG000001	N4BP2L1	protein_codin	0	14.7541436	0	0
ENSG000000	NT5C2	protein_codin	0	58.2016712	43.1169385	0
ENSG000001	TRIP12	protein_codin	0.72417019	0	0	0
ENSG000001	IDO2	protein_codin	0	2.60295655	0.53214078	1.85314888
ENSG000001	SIGLEC6	protein_codin	0	0	0	0
ENSG000001	LRRC41	protein_codin	6.13903432	0	0	0
ENSG000001	B3GLCT	protein_codin	20.1120425	26.1803516	22.0992583	13.5942178
ENSG000001	TMPO	protein_codin	0	0	39.8009147	0
ENSG000001	TFEB	protein_codin	1.20707083	0	4.33914094	0
ENSG000002	LRRD1	protein_codin	0	2.31940187	0	0
ENSG000001	CHIA	protein_codin	0	0	0	0
ENSG000001	ZNF791	protein_codin	29.3161887	52.904263	75.8281125	25.2250754
ENSG000001	PTBP3	protein_codin	3.82731099	0	0	1.73709573
ENSG000000	ZNF195	protein_codin	0	0	0	0

ENSG000001	MYOCD	protein_codin	0	0	0	0
ENSG000000	SEMA6A	protein_codin	13.6564998	26.7473825	65.7335682	21.830273
ENSG000001	FAM98C	protein_codin	0	8.22950501	0	0
ENSG000001	CDHR4	protein_codin	0	0	0	0
ENSG000001	DMPK	protein_codin	3.79551067	0	0	0
ENSG000002	FDX2	protein_codin	0	0	0	0
ENSG000001	E2F1	protein_codin	4.05210401	4.89579453	6.6536085	2.50101327
ENSG000001	ZNF669	protein_codin	0	0	0	0
ENSG000001	TLK2	protein_codin	10.4355898	62.2781304	78.6155215	7.76068175
ENSG000001	OR6C74	protein_codin	0	4.06480654	0	2.47707181
ENSG000001	HHIPL2	protein_codin	0	0	32.6411151	2.54615762
ENSG000001	MBTPS1	protein_codin	90.017087	121.191334	210.356614	49.9969452
ENSG000001	CD55	protein_codin	0	33.5935071	0	0
ENSG000001	ZFAND5	protein_codin	0	0	10.2734821	5.28025856
ENSG000001	FYB2	protein_codin	0	0	0	0
ENSG000001	TADA3	protein_codin	0.54056507	3.60036869	5.74901909	0
ENSG000001	DICER1	protein_codin	4.57741597	99.9028491	0	0
ENSG000001	LRRC66	protein_codin	0	0	0	2.51845449
ENSG000001	ZNF34	protein_codin	0	0	0	0
ENSG000001	LYAR	protein_codin	26.6960283	102.458301	146.319336	0
ENSG000001	SHISA6	protein_codin	0	1.22525446	0	2.0797266
ENSG000000	HOXA9	protein_codin	0	0	0	0
ENSG000001	TCEA2	protein_codin	7.17835262	11.1965385	26.4689895	6.01992809
ENSG000001	ZNF177	protein_codin	1.71958982	0	0	0
ENSG000001	NHSL1	protein_codin	0	59.5074317	54.0925813	36.0408632
ENSG000001	CSMD3	protein_codin	0	0	0	0
ENSG000001	CASKIN1	protein_codin	0	2.21748631	5.95697059	0.95374647
ENSG000001	POTEH	protein_codin	0	0	0	3.03055565
ENSG000001	DMD	protein_codin	0	0	0	0
ENSG000001	ATG4A	protein_codin	3.90527419	13.554339	0	0
ENSG000001	TRIM24	protein_codin	8.69428649	17.2380874	21.1487508	19.661872
ENSG000001	PDXK	protein_codin	0	0	0	0
ENSG000001	RELN	protein_codin	3.56456022	0	28.2218555	0
ENSG000001	ATP7A	protein_codin	0	0	0	0
ENSG000001	C1orf162	protein_codin	3.37519102	19.9412869	0.70034297	2.66900039
ENSG000001	TP53BP2	protein_codin	42.3613921	48.0233295	144.97965	62.8160834
ENSG000001	RXFP1	protein_codin	0	0	0	0
ENSG000001	THNSL2	protein_codin	0	0	0	0
ENSG000001	P4HTM	protein_codin	0	0	0	0
ENSG000001	USF2	protein_codin	21.4856171	28.290147	13.6271207	10.3773262
ENSG000001	EFHC2	protein_codin	0	0	0	0
ENSG000002	DOC2B	protein_codin	0.87805648	2.17499109	0	2.17217515
ENSG000001	CXCL12	protein_codin	21.6018898	111.276406	2.4465808	80.2190851
ENSG000001	STX10	protein_codin	0	0	7.47139504	0

ENSG000001	FAM111B	protein_codin	0	3.19142801	5.18943142	2.31192806
ENSG000001	ZNF540	protein_codin	0	0	0	0
ENSG000001	MBNL2	protein_codin	74.0300242	160.742186	27.8020526	37.4137267
ENSG000001	SLC35E4	protein_codin	1.21425041	1.87884695	1.84320036	2.36262017
ENSG000000	ATP6V0A1	protein_codin	0	0	0	0
ENSG000001	RASAL3	protein_codin	5.29942079	55.0890613	5.25281123	6.28397575
ENSG000001	MEAK7	protein_codin	6.00277869	12.718688	7.65045136	4.64975657
ENSG000001	EXT2	protein_codin	0	0	3.62945093	0
ENSG000001	GGA1	protein_codin	0	12.7789293	7.73399602	7.21953289
ENSG000001	UBTF	protein_codin	0	0	7.2704893	0
ENSG000001	TMEM203	protein_codin	15.8958399	3.16101703	31.4939015	4.88526001
ENSG000001	DGKZ	protein_codin	0	0	7.61455022	0
ENSG000001	H1-2	protein_codin	174.581496	476.985309	1719.88945	66.3093084
ENSG000001	NKAPL	protein_codin	4.38870098	3.37992708	1.33348449	4.11671815
ENSG000001	ABCG1	protein_codin	0	3.89511282	0	1.36706576
ENSG000001	RBPJL	protein_codin	0	0	0	0
ENSG000000	NTN4	protein_codin	0	23.9954611	0	6.15295997
ENSG000001	PDCD1	protein_codin	0.26335626	0	0	0.42299886
ENSG000001	SEZ6L	protein_codin	0	0	0.40122605	1.34015562
ENSG000002	LRRC69	protein_codin	0.48177169	0	0	1.24844121
ENSG000000	NOX4	protein_codin	0	0	0	0
ENSG000001	MED22	protein_codin	2.95001799	0	14.3017108	1.63267924
ENSG000000	CLDN18	protein_codin	0	0	0	0
ENSG000000	TSPOAP1	protein_codin	0	0	0	19.9191548
ENSG000000	WNT8B	protein_codin	0	1.6318723	0	1.25203756
ENSG000001	LRRK2	protein_codin	6.35539901	0	0	0
ENSG000001	ACOT11	protein_codin	0	0	0	0
ENSG000001	DAPL1	protein_codin	0	0	0	0
ENSG000001	CDYL	protein_codin	0	0	1.59483243	0
ENSG000001	VIPAS39	protein_codin	0	0	0	0
ENSG000001	ZNF93	protein_codin	5.08733354	8.0469837	19.5423511	0.859288
ENSG000001	ZNF678	protein_codin	15.0299856	0	38.8814176	7.28674746
ENSG000001	SUGT1	protein_codin	35.4018122	71.3191024	76.464041	19.4880994
ENSG000001	DNAJA4	protein_codin	0	0	0	0
ENSG000001	NR3C1	protein_codin	0	17.2221949	0	6.50372004
ENSG000000	SP140	protein_codin	0	0	0.53379181	0
ENSG000001	MCRS1	protein_codin	2.5448691	53.7082341	0	0
ENSG000001	MROH8	protein_codin	0	0	0	0
ENSG000001	ICMT	protein_codin	16.4639081	49.1081352	29.1401774	15.1450537
ENSG000001	UBAP2L	protein_codin	0	0	0	0
ENSG000001	RGS3	protein_codin	0	20.2896821	0	0
ENSG000001	PYCR2	protein_codin	8.09614677	44.9263031	54.4128136	11.1209417
ENSG000001	TGIF1	protein_codin	0	10.3984071	5.94263986	4.44670083
ENSG000001	ASH2L	protein_codin	9.48093466	36.6618781	43.7549386	15.0951504

ENSG000001	TCF12	protein_codin	34.7992598	0	60.8671924	14.5343424
ENSG000001	SNTN	protein_codin	0	0	0	1.08794086
ENSG000001	SUSD4	protein_codin	0	0	0	0
ENSG000001	PSMA8	protein_codin	0	0	0	0
ENSG000001	CAV3	protein_codin	0.6021784	0	0	0
ENSG000001	RGS6	protein_codin	0	0	0	0
ENSG000001	CCDC141	protein_codin	4.94920009	21.1031305	0	11.5372464
ENSG000001	FOXO3	protein_codin	80.9035813	69.3263639	73.6182764	87.1113826
ENSG000001	PLCH2	protein_codin	0	8.86341168	0	1.86967388
ENSG000001	ELK1	protein_codin	0	0	0	0
ENSG000001	IL18BP	protein_codin	1.43054875	45.0096261	3.54812693	0
ENSG000001	SOWAHD	protein_codin	0.72849073	4.43243149	0	0.46330829
ENSG000001	CYP1A2	protein_codin	0.25993901	1.286653	0	0.41325261
ENSG000001	CORO2A	protein_codin	0	8.64134839	0	4.38733654
ENSG000002	CPTP	protein_codin	2.82228414	4.25002051	9.39580098	4.52242883
ENSG000001	OSGIN1	protein_codin	0	0	0	3.10446111
ENSG000001	KBTBD12	protein_codin	0	0	0	0
ENSG000001	C19orf67	protein_codin	0	0	0	0
ENSG000001	P2RX2	protein_codin	0	0	0	0
ENSG000001	ZNF763	protein_codin	1.14893198	5.66926265	3.45776501	0
ENSG000001	FGD6	protein_codin	7.56019165	19.7884589	5.59646108	16.8006732
ENSG000001	FAM25A	protein_codin	0	0	0	0
ENSG000001	MS4A4A	protein_codin	0	0	0	11.0439734
ENSG000001	CEP41	protein_codin	0	0	0	0
ENSG000001	HMGCR	protein_codin	0	31.5954579	38.2228998	0
ENSG000001	ZNF136	protein_codin	19.5017904	52.9402347	50.8084242	5.29592753
ENSG000001	SEPTIN6	protein_codin	3.97494216	0	0	0
ENSG000001	NUDT1	protein_codin	0	0	0	0
ENSG000001	RPS21	protein_codin	111.195793	56.4744609	288.590425	64.6514345
ENSG000001	PLEKHM3	protein_codin	0	15.1408902	0	0
ENSG000001	DIPK1C	protein_codin	30.1590492	7.07101282	9.98253047	4.2545386
ENSG000001	RNF222	protein_codin	0.30561474	0	0	0
ENSG000001	CASZ1	protein_codin	0	0	0.96540361	0
ENSG000000	FXYD3	protein_codin	0	0	0	0
ENSG000001	PXMP4	protein_codin	0	0	0	0
ENSG000001	SUSD4	protein_codin	2.23799714	1.13417901	0	0.97627866
ENSG000001	PPM1G	protein_codin	97.9883635	572.023288	404.758705	47.8118948
ENSG000001	VDAC2	protein_codin	0	0	0	0
ENSG000001	RAPGEF5	protein_codin	8.65368716	0	0	0
ENSG000001	HM13	protein_codin	3.84352615	0	0	0
ENSG000001	SPINT1	protein_codin	0	0	0	0
ENSG000001	RPL28	protein_codin	3.43475544	9.34734979	8.71696194	7.49316161
ENSG000001	LRRC27	protein_codin	0	0	0	0
ENSG000002	ZNF880	protein_codin	0	9.98271071	0	0

ENSG000001(	IL6R	protein_codin	2.58950694	27.3910902	0	2.40400814
ENSG000002	NPIPA7	protein_codin	0	0	0	0
ENSG000001(	TAF6	protein_codin	0	0	0	0
ENSG000001:	DYRK2	protein_codin	55.1910477	91.0681509	161.335639	21.4479482
ENSG000001(	ZNF14	protein_codin	7.21630502	43.2505515	12.286367	11.2612183
ENSG000001:	CACNA1C	protein_codin	0	0	7.6099225	0
ENSG000001	WARS1	protein_codin	14.8229768	116.910619	41.2000244	11.8816751
ENSG000000:	SIRPG	protein_codin	0	7.93231033	0	1.58173285
ENSG000002	COX19	protein_codin	7.54891337	43.9086034	21.3229587	12.1292069
ENSG000000:	SYNE2	protein_codin	114.01447	735.837568	644.527295	252.006301
ENSG000000:	HERPUD1	protein_codin	0	0	0	0
ENSG000001:	ENTPD8	protein_codin	0	0	0.54988836	0.80935168
ENSG000001:	SPRY4	protein_codin	0	0	0	0
ENSG000001(	CT55	protein_codin	0	0	0	2.82238639
ENSG000000:	TRIO	protein_codin	0.56322541	0	0	0
ENSG000001(	GRAP2	protein_codin	1.77938163	49.6597265	0	3.6840728
ENSG000000(	CACNB1	protein_codin	6.4125283	0	0	0
ENSG000001:	ZCCHC17	protein_codin	15.8918984	0	69.1202056	0
ENSG000001	CARM1	protein_codin	0.50645537	0	0	0.92054942
ENSG000000:	YTHDC1	protein_codin	3.9409558	3.32470855	0	5.41441806
ENSG000001:	SBK2	protein_codin	0.25087954	0	0	0.42721333
ENSG000002(	AKT1S1	protein_codin	2.41216443	11.2224931	0	5.88460618
ENSG000001:	AFDN	protein_codin	0	0	10.5058637	0
ENSG000000	CBFA2T2	protein_codin	1.59319339	11.1245645	1.2905686	2.01195098
ENSG000000:	TRIO	protein_codin	38.61344	112.787099	248.560947	0
ENSG000001(	MST1R	protein_codin	0	0	0	0
ENSG000001	ILDR1	protein_codin	0	0	0	0
ENSG000001	FHL2	protein_codin	0	0	0	0
ENSG000002:	ZNF550	protein_codin	7.37968576	0	18.353224	2.32708327
ENSG000001	CARD14	protein_codin	0	0	0	0
ENSG000000:	RNF13	protein_codin	0	0	4.69308162	0
ENSG000001	ERFE	protein_codin	0	0	0	0
ENSG000001(	OR7D2	protein_codin	0	0	0	0
ENSG000001(	FGF8	protein_codin	0	0	0	0
ENSG000001(	CDC25B	protein_codin	17.8156133	0	0	0
ENSG000001:	GAD1	protein_codin	0	0	0	0
ENSG000000(	POU1F1	protein_codin	0	0	0	0
ENSG000001	STX2	protein_codin	0.55629027	0	0	0
ENSG000001:	ABCB9	protein_codin	0.57296836	3.03291354	0	0
ENSG000001	RGS20	protein_codin	0	0	0	0
ENSG000002:	ZSCAN31	protein_codin	7.25150812	0	30.0208857	0
ENSG000001(	CCDC38	protein_codin	0.18962584	0	0	0.40441004
ENSG000001	ESR2	protein_codin	0	0	0	0
ENSG000001:	MAPT	protein_codin	2.75509348	0	0	0.76524032

ENSG000001(	TAF3	protein_codin	27.5639875	57.8319465	54.1720699	24.2483439
ENSG000001(	C15orf48	protein_codin	0	0	0	0
ENSG000001	KRBOX4	protein_codin	3.94116706	8.9682878	2.0568083	0
ENSG000001	TEX11	protein_codin	0	0	0	0
ENSG000001	STARD13	protein_codin	0.73101493	4.37333772	1.56602622	3.00166267
ENSG000001(	ZMAT5	protein_codin	1.72780655	2.31992856	4.37356344	0.86322681
ENSG000001	C15orf62	protein_codin	0.28125287	0	0.54092978	0.8897397
ENSG000001	RUFY4	protein_codin	2.72896323	14.9047995	1.52071894	0
ENSG000001	TRPC6	protein_codin	3.70422572	3.13369385	9.28852878	7.28394515
ENSG000001(	ICOSLG	protein_codin	0	18.1892436	0	0
ENSG000001	KPNA1	protein_codin	38.3680694	49.4554009	39.1702739	18.0448697
ENSG000001	DMBT1	protein_codin	0	0	0	0
ENSG000001(	XBP1	protein_codin	0	21.7061617	0	0
ENSG000000	CREM	protein_codin	0	0	3.41526113	0
ENSG000001(	C1orf52	protein_codin	0	0	6.28642834	0
ENSG000001	OLA1	protein_codin	0	0	0	0
ENSG000000	RBL1	protein_codin	7.71043895	12.5123318	40.3196912	3.03283811
ENSG000002	KRTAP1-3	protein_codin	0	0	0	0.32820118
ENSG000000	CA12	protein_codin	0	0	28.0771831	0
ENSG000001	ADGRE3	protein_codin	0	0	0	0
ENSG000001(	ATG4B	protein_codin	0	0	0	0
ENSG000001(	ASB11	protein_codin	1.39311275	0	0	0
ENSG000001	CC2D2B	protein_codin	0	0	0	2.32435172
ENSG000001	ZNF197	protein_codin	16.9144805	22.3907773	15.50549	11.853221
ENSG000000	CEACAM1	protein_codin	0	1.60788063	0	0.44322119
ENSG000001(	TRIOBP	protein_codin	0	0	0	0
ENSG000001	EVC2	protein_codin	28.7056112	61.9027413	84.7155131	13.1681458
ENSG000000	NPFFR2	protein_codin	0	0	0	0
ENSG000001	ALKAL2	protein_codin	0	0	0	0
ENSG000001	GRAPL	protein_codin	0	0	0	0.60746922
ENSG000001	INTS15	protein_codin	3.12995552	21.5666877	4.40305826	7.83633206
ENSG000000(	IYD	protein_codin	0	0	0	0
ENSG000002	SLC35F6	protein_codin	13.7815198	41.3508657	9.58987458	0
ENSG000001	ZNF395	protein_codin	12.2945086	58.3295452	36.7184194	21.9946588
ENSG000000	KCNQ2	protein_codin	0	0	0	0
ENSG000001	NOVA1	protein_codin	1.0894436	1.15762536	6.64813312	0
ENSG000001	LARP7	protein_codin	0	0	112.009139	0
ENSG000001	DUSP22	protein_codin	1.78955406	0	1.29850709	1.16925258
ENSG000001	TMEM198	protein_codin	0	0	0	0
ENSG000001(	TMEM234	protein_codin	0	0	0	0
ENSG000000	KCNQ2	protein_codin	0	0	0	0
ENSG000000	DGKG	protein_codin	0	0	0	0
ENSG000001	PAX3	protein_codin	0	0	0	0
ENSG000001	OR5W2	protein_codin	0	0.90680185	0	0

ENSG000001	ABCB10	protein_codin	9.60796539	16.1332582	23.0109841	3.91551652
ENSG000001	ZNF66	protein_codin	17.1546421	43.9647716	13.9102896	10.425996
ENSG000001	NR6A1	protein_codin	0	0	6.88241381	0
ENSG000001	PRR27	protein_codin	0	0	0	0
ENSG000001	SH3BP4	protein_codin	0	0	0	3.14907966
ENSG000001	PIGC	protein_codin	4.02569241	13.8370191	19.1154357	5.94245325
ENSG000001	RCBTB2	protein_codin	18.2047906	19.1423356	14.8917289	0
ENSG000000	DTNBP1	protein_codin	0	0	15.4457381	9.15140393
ENSG000001	TPD52L3	protein_codin	0	0	0	1.39313357
ENSG000001	ATP5PD	protein_codin	1.28307124	0	2.47685853	0
ENSG000000	CDC42	protein_codin	7.75295436	20.8564526	17.3578829	0
ENSG000001	CEACAM3	protein_codin	0	0	0	0
ENSG000001	FZD9	protein_codin	0	1.11091853	8.17700317	1.33883029
ENSG000001	EGFR	protein_codin	0	0	0	2.30497796
ENSG000001	LIPI	protein_codin	0	0	0	0
ENSG000001	TNNT3	protein_codin	1.69137954	0	0	0
ENSG000001	TPRG1L	protein_codin	0	0	0	0
ENSG000001	KLHL33	protein_codin	0	0	0.5529019	0
ENSG000001	NKX2-3	protein_codin	0	0.87129694	0	0.83589447
ENSG000001	RSRC2	protein_codin	0	0	0	36.0280334
ENSG000000	FLYWCH1	protein_codin	0	0	12.2563283	0
ENSG000001	SRR	protein_codin	3.96767318	0	2.04077645	1.87987162
ENSG000001	ECM2	protein_codin	85.127301	452.755827	4.1567696	65.2796343
ENSG000001	TMEM156	protein_codin	0	13.5846903	0	1.86844829
ENSG000001	LEPR	protein_codin	0	0	0	0
ENSG000001	S100A14	protein_codin	0	0	0	0
ENSG000001	GDNF	protein_codin	0	0	0	0
ENSG000001	DROSHA	protein_codin	5.29218025	0	0	0
ENSG000001	SMARCA4	protein_codin	0	0	0	0
ENSG000001	SGCA	protein_codin	0	0	0	1.25865264
ENSG000001	OGG1	protein_codin	0	10.458162	7.39367666	3.18285971
ENSG000001	PLEKHG7	protein_codin	0	0	3.68897836	1.72235177
ENSG000001	MRRF	protein_codin	2.71740299	22.3680403	9.47327046	7.52845774
ENSG000001	LDLRAD2	protein_codin	0.69277156	0	0	1.9519179
ENSG000002	UGT1A10	protein_codin	0	0	0	0
ENSG000001	ZFAND4	protein_codin	0	0	4.05192888	4.64599505
ENSG000001	UNC119B	protein_codin	19.9345141	0	34.1782829	11.792297
ENSG000001	SPHKAP	protein_codin	0.83836922	2.347127	12.6440542	2.64948799
ENSG000001	RTN2	protein_codin	0	1.92889778	0.96169462	0
ENSG000001	MCPH1	protein_codin	11.1898923	7.74202638	2.17116911	7.99916419
ENSG000000	MAP3K14	protein_codin	11.3153248	28.0703564	0	0
ENSG000001	RUNX1	protein_codin	61.6164084	0	41.8881095	60.7853596
ENSG000002	GLI4	protein_codin	0	0	0	0
ENSG000001	POMGNT2	protein_codin	0	0	6.32641555	0

ENSG000001	PCNX2	protein_codin	3.69063192	2.98306073	0.64077361	3.45430613
ENSG000001	RPS10	protein_codin	0	0	2.36740963	0
ENSG000001	EIF4ENIF1	protein_codin	0	0	0	0
ENSG000001	AK5	protein_codin	0	4.60632604	0.52913168	5.22011934
ENSG000001	NR3C2	protein_codin	0	0	0	1.41530126
ENSG000001	SMC4	protein_codin	0	0	0	0
ENSG000001	HMGN3	protein_codin	35.6417589	32.3812023	58.7216269	19.017101
ENSG000001	ABL2	protein_codin	20.1957422	23.182822	56.9192353	54.2436168
ENSG000001	SERPINB13	protein_codin	0	0	0	0
ENSG000001	RGS12	protein_codin	1.202661	0	0	1.3031584
ENSG000001	GLYAT	protein_codin	0	0	0	0
ENSG000001	RPL10	protein_codin	0	0	0	0
ENSG000000	SIGLEC1	protein_codin	7.01908054	130.927334	9.52376874	28.8556818
ENSG000001	INSIG1	protein_codin	6.17823668	25.0227208	2.50989325	19.7625041
ENSG000001	CCDC15	protein_codin	0	24.0102266	28.0637647	6.61765249
ENSG000001	FRMD6	protein_codin	0	85.5589823	0	115.435423
ENSG000001	PDZK1	protein_codin	1.78132758	0	0	0
ENSG000001	BABAM2	protein_codin	0	0	2.54368391	0
ENSG000001	CIMIP2A	protein_codin	0	0	0.51458624	0.96210014
ENSG000001	DHRS2	protein_codin	0	0	0	0
ENSG000001	SNX21	protein_codin	7.64914185	28.854132	0	2.75985603
ENSG000001	RBM38	protein_codin	0	2.16030064	8.72657435	8.58343433
ENSG000000	TFAP2B	protein_codin	0	0	0	0
ENSG000001	LYSMD4	protein_codin	0	0	0	0
ENSG000001	RAB5IF	protein_codin	7.00520372	0	14.4748518	7.87948594
ENSG000002	SPATA31D1	protein_codin	0	1.52057891	0	3.01659458
ENSG000001	ZNF174	protein_codin	0	0	0	0
ENSG000001	TPM4	protein_codin	2.72732482	20.8830313	8.4788538	0
ENSG000001	KLRD1	protein_codin	0	0	0	0
ENSG000001	USP7	protein_codin	1.97781471	84.4251883	12.3899078	22.4593729
ENSG000001	EFHB	protein_codin	0.29687434	0	0	0
ENSG000001	BSPH1	protein_codin	0	0	0	0
ENSG000002	MRPL20	protein_codin	13.858656	9.77342426	52.2708993	7.57678174
ENSG000001	SYNGR4	protein_codin	0	0	0	0
ENSG000001	ENG	protein_codin	0	30.3524186	0	0
ENSG000001	GTDC1	protein_codin	7.02157309	0	0	6.42880853
ENSG000001	ZDHHC7	protein_codin	5.86574627	0	0	0
ENSG000001	PUS7L	protein_codin	147.16438	53.622592	191.54107	23.2158987
ENSG000001	ASPSCR1	protein_codin	0	0	25.8391016	0
ENSG000001	LARP6	protein_codin	1.19733111	3.13395049	2.0592912	0
ENSG000001	PCDH18	protein_codin	12.4149423	9.2796677	9.34467366	35.4609196
ENSG000001	ANAPC11	protein_codin	0	2.0479465	8.3881707	6.3013331
ENSG000001	TNNI3	protein_codin	0	0	1.04474383	0
ENSG000001	TSLP	protein_codin	0	0	0	2.56666043

ENSG0000010	MAP3K2	protein_codin	0	0	0	0
ENSG0000007	NTN4	protein_codin	17.4966061	0	50.1854465	11.457436
ENSG000001	NNT	protein_codin	13.1458012	0	0	0
ENSG0000009	SMARCB1	protein_codin	0	0	0	0
ENSG0000001	MIA3	protein_codin	193.258927	100.113163	368.121659	73.2389358
ENSG0000001	BPGM	protein_codin	9.98687674	21.4580368	21.2598578	0
ENSG0000001	ZNF107	protein_codin	10.6760949	0	49.2324604	0
ENSG000000	CD6	protein_codin	0	0	0	0
ENSG0000001	CPSF4L	protein_codin	0	0	0.70765133	0.30511553
ENSG0000001	ARHGAP12	protein_codin	0	0	0	25.2156816
ENSG000001	CLCN2	protein_codin	1.80803261	0	0	0
ENSG0000001	SLC15A5	protein_codin	0	2.22668657	0	1.77093946
ENSG0000000	TBC1D23	protein_codin	0	0	0	0
ENSG0000000	CUL3	protein_codin	12.8078391	70.7752721	20.8005952	7.13843851
ENSG0000001	FLI1	protein_codin	0	0	0	0
ENSG0000000	PVR	protein_codin	0	0	0	8.06457352
ENSG0000001	FAM221A	protein_codin	0	0	0	0
ENSG0000000	PDE4A	protein_codin	0	0	0	0
ENSG0000001	ZNF433	protein_codin	2.32367316	0	0	0
ENSG0000001	MPPE1	protein_codin	0	0	0	0
ENSG0000001	SRSF10	protein_codin	0	0	43.7407333	0
ENSG0000000	LAT2	protein_codin	0	0	0	0
ENSG0000001	GYG1	protein_codin	1.58065969	0	0.61401862	4.8205749
ENSG0000001	CLK3	protein_codin	0	0	4.38235072	0
ENSG0000001	LYPD1	protein_codin	0	0	0	0
ENSG0000001	SNCA	protein_codin	0	0	0	0
ENSG0000000	KCNQ1	protein_codin	0	0	0	0
ENSG0000000	AP3D1	protein_codin	0	108.1098	0	0
ENSG0000001	ZNF85	protein_codin	0	0	1.38104628	0
ENSG000002	RSC1A1	protein_codin	0	18.2563041	29.2479061	3.65110203
ENSG0000001	HSPD1	protein_codin	0	0	0	0
ENSG0000001	OR52B6	protein_codin	0	1.60003131	1.21906339	2.07145709
ENSG0000001	SLC2A11	protein_codin	0	0	0	0
ENSG0000001	BCAP31	protein_codin	11.0621627	382.370501	200.945721	22.5454951
ENSG0000001	TRADD	protein_codin	0	8.66937438	2.59548196	0.84894613
ENSG0000001	ADRB3	protein_codin	0	0	0	0
ENSG0000001	TPRG1	protein_codin	2.72008866	9.95337194	0	4.37590325
ENSG0000001	CORO6	protein_codin	0	0	0	0
ENSG0000001	LIN28B	protein_codin	0	0	0.62757433	0
ENSG0000001	MRPS5	protein_codin	0	5.11816065	0	1.17447292
ENSG0000001	DPPA3	protein_codin	0	0	0	4.24677823
ENSG0000001	RNF41	protein_codin	0	0	0	8.35710071
ENSG0000001	THUMPD3	protein_codin	2.58924922	0	71.7518303	0
ENSG0000001	TBC1D28	protein_codin	0	0	0	0.74186505

ENSG000000	FOXN3	protein_codin	67.612169	146.905115	68.5939902	23.236318
ENSG000001	ZAR1L	protein_codin	0	0	0	0.44267089
ENSG000000	MLXIPL	protein_codin	0.90880949	2.52206833	6.43783933	0
ENSG000001	THAP1	protein_codin	0	0	6.66684448	0
ENSG000001	ARHGAP5	protein_codin	44.812135	171.529038	45.0554108	38.3501858
ENSG000001	CTSB	protein_codin	0	0	0	0
ENSG000001	PRICKLE1	protein_codin	38.7267233	0	13.2594838	0
ENSG000001	TGIF1	protein_codin	0	0	0	0
ENSG000001	PLEC	protein_codin	81.04748	314.255401	442.988076	74.3568196
ENSG000001	IDH1	protein_codin	28.1438953	59.6272215	39.2471328	17.6803309
ENSG000001	CCDC78	protein_codin	0	0	0	0
ENSG000001	GRK4	protein_codin	1.36087432	3.6468088	0	0
ENSG000001	PTK7	protein_codin	0	32.7184366	0	0
ENSG000001	GFM2	protein_codin	0	4.0604576	26.227716	0
ENSG000001	PPM1B	protein_codin	0	0	0	0.8047733
ENSG000001	AKR1E2	protein_codin	0	0	1.22499466	0
ENSG000001	SEC24C	protein_codin	25.8002306	40.0718702	73.3842085	9.16979396
ENSG000001	RSU1	protein_codin	36.8872184	63.7926604	37.1673492	28.89714
ENSG000001	RAD17	protein_codin	0	0	0	0
ENSG000000	SKAP2	protein_codin	47.4170126	113.128171	99.366495	45.4014835
ENSG000001	NPTN	protein_codin	15.6983369	13.5551317	7.43172292	13.4222428
ENSG000001	TM7SF2	protein_codin	0	0	0	0
ENSG000001	COL12A1	protein_codin	18.2843747	20.2013261	0	0
ENSG000000	BAX	protein_codin	2.23643396	20.5392221	1.62777558	2.81673623
ENSG000002	LTB4R	protein_codin	0	0	0	0
ENSG000001	RAD51D	protein_codin	1.39857428	0	1.60307703	0
ENSG000002	CSH2	protein_codin	0	0	0	0
ENSG000001	GCK	protein_codin	0	0	0	0
ENSG000001	BNC1	protein_codin	0.54371185	0	4.80929083	0
ENSG000001	VHL	protein_codin	5.25990494	0	18.0650998	0
ENSG000001	NARF	protein_codin	4.88163893	3.34564463	32.1568441	0
ENSG000001	GIPC1	protein_codin	0	0	0	0
ENSG000001	MBNL2	protein_codin	0	0	8.27328684	0
ENSG000002	CKLF	protein_codin	0	0	0	0
ENSG000001	CPEB2	protein_codin	0	0	0	0
ENSG000000	CREM	protein_codin	2.52739806	2.32886219	0	0
ENSG000001	UBE2G2	protein_codin	11.1336349	0	0	23.5610312
ENSG000001	STAT5A	protein_codin	0	4.65266961	0	0
ENSG000000	PTPRU	protein_codin	0	5.65219397	0	4.21620018
ENSG000001	BCL11B	protein_codin	0	0	0	0
ENSG000001	ACTG2	protein_codin	4.24365217	13.2895079	3.99260331	0
ENSG000001	CLTA	protein_codin	82.1409548	202.685711	184.612186	64.1134332
ENSG000001	WDR35	protein_codin	0	0	0	0
ENSG000001	SLX1A	protein_codin	0	0	0	0

ENSG000001	KIR2DL4	protein_codin	0	0	0	0
ENSG000000	WAC	protein_codin	18.7376416	50.9930196	52.8846616	17.2440521
ENSG000001	TMEM130	protein_codin	0.4022599	0	0	0
ENSG000000	APLP2	protein_codin	0	0	0	0
ENSG000000	HDAC4	protein_codin	40.1277822	0	0	0
ENSG000001	CASP7	protein_codin	0	0	0	0
ENSG000001	SDSL	protein_codin	0	0	0	0
ENSG000001	CENPL	protein_codin	0	0	0	0
ENSG000001	SGK3	protein_codin	0	32.8503404	0	6.3939908
ENSG000000	NISCH	protein_codin	22.8461574	94.8540735	29.9186443	0
ENSG000001	PAM	protein_codin	1.39600671	25.62582	0	2.86392243
ENSG000001	MALT1	protein_codin	0	0	0	0
ENSG000001	FERMT3	protein_codin	0	0	0	0
ENSG000001	MS4A1	protein_codin	0	0	0	0
ENSG000001	ATG4A	protein_codin	0	0	0	0
ENSG000001	ATF2	protein_codin	17.3928828	32.9886281	65.7727605	3.57655631
ENSG000001	ZDHHC16	protein_codin	7.99898313	0	19.7333531	0
ENSG000001	NLRP12	protein_codin	0	0	0	0
ENSG000001	RBM10	protein_codin	31.2311252	0	87.7596482	0
ENSG000001	GOLM2	protein_codin	48.092059	0	0	0
ENSG000001	CLTB	protein_codin	47.8619227	56.6328423	109.474763	37.1885107
ENSG000001	ZNF274	protein_codin	0	0	5.79167029	0
ENSG000001	MAGEA8	protein_codin	0	0	0	0
ENSG000001	RBPJ	protein_codin	0	0	0	0
ENSG000001	KRTAP4-6	protein_codin	0	0	0	0
ENSG000001	TRIM3	protein_codin	4.11023005	7.27625223	7.95176181	1.47563397
ENSG000001	UPF3B	protein_codin	36.0771787	186.767147	103.047008	36.9466894
ENSG000001	LILRB5	protein_codin	0	0	0	0
ENSG000000	SPO11	protein_codin	0	0	0	0
ENSG000001	YAP1	protein_codin	13.0116988	0	9.72634063	30.5639941
ENSG000001	CERS2	protein_codin	0	0	0	9.09503106
ENSG000000	P2RX5	protein_codin	0	0	0	0
ENSG000001	TPO	protein_codin	0	0	0	0
ENSG000001	FCAR	protein_codin	0	0	0	0
ENSG000001	LPAR6	protein_codin	0	20.7938249	0	0
ENSG000001	SNX16	protein_codin	0	0	25.8996262	0
ENSG000001	NOL10	protein_codin	0	0	0	0
ENSG000001	PPL	protein_codin	0	0	0	3.77809679
ENSG000001	CSNK1G3	protein_codin	0	10.1696075	0	0
ENSG000001	CLEC4A	protein_codin	0	10.2502188	0	0
ENSG000001	TLK2	protein_codin	6.99794432	36.0802011	17.9665089	7.17943343
ENSG000001	PDE8B	protein_codin	0	0	0	0
ENSG000001	PTK2B	protein_codin	8.27292738	0	11.4204315	0
ENSG000000	ARID1B	protein_codin	2.7505167	0	33.8611552	15.0819471

ENSG000001	TJP1	protein_codin	0	33.7614939	5.67315977	3.80859278
ENSG000000	EXOC1	protein_codin	0	0	0	0
ENSG000001	TUBD1	protein_codin	0	0	0	0
ENSG000000	MADCAM1	protein_codin	0	0	0	0
ENSG000001	MUC4	protein_codin	0	0	0	0
ENSG000000	SRPK1	protein_codin	0	0	10.6881316	0
ENSG000001	RNF6	protein_codin	37.4288443	32.4914534	33.2185685	28.0098104
ENSG000001	EIF4G1	protein_codin	0	0	0	0
ENSG000001	FLNC	protein_codin	101.354436	0	0	0
ENSG000000	GANAB	protein_codin	3.77232168	0	0	0
ENSG000000	NFATC3	protein_codin	40.1420631	41.3986436	54.8616904	30.816031
ENSG000000	IGF2BP2	protein_codin	0	0	72.1929526	0
ENSG000001	FAM47A	protein_codin	0	1.35548019	0	2.4918523
ENSG000001	TCF7L2	protein_codin	1.3753114	17.0773007	0	0
ENSG000000	NNAT	protein_codin	0	1.93331653	0	0
ENSG000001	GATA3	protein_codin	1.26626812	4.25099709	0	0
ENSG000001	RAB5C	protein_codin	14.3063817	58.9066071	16.7634394	28.6645549
ENSG000000	DLEC1	protein_codin	0	0	1.06163746	0
ENSG000001	BIN1	protein_codin	0	0	0	0
ENSG000001	OSTF1	protein_codin	12.9656828	63.6579833	11.7026974	22.329912
ENSG000001	IKZF3	protein_codin	0	0	0	0
ENSG000001	ARID3B	protein_codin	9.13755558	31.9887131	12.7662487	4.82350263
ENSG000001	CHST15	protein_codin	0	52.1893503	0	26.9116862
ENSG000001	TPD52L2	protein_codin	27.8445632	24.7999522	86.6508715	12.9844204
ENSG000001	XAGE3	protein_codin	0	0	0	0
ENSG000000	MTMR2	protein_codin	0	0	0	0
ENSG000001	TTC8	protein_codin	9.84516441	10.6997479	42.7487994	0
ENSG000000	MYLK	protein_codin	53.328401	0	0	0
ENSG000000	CTTN	protein_codin	0	4.8441235	6.79717454	8.10323728
ENSG000000	UBE2A	protein_codin	0	0	0	0
ENSG000001	ARRB2	protein_codin	0	0	8.23669189	0
ENSG000000	F7	protein_codin	0	0	0	0
ENSG000001	SNX14	protein_codin	0	0	0	0
ENSG000001	NUDT2	protein_codin	5.73850276	0	17.4341613	8.20586763
ENSG000002	LILRB3	protein_codin	1.76788742	0	0	0
ENSG000001	SNRPN	protein_codin	5.49645516	141.559707	32.3235983	12.3429828
ENSG000001	CDK5RAP1	protein_codin	9.8752224	41.7880346	19.4152522	0
ENSG000001	AIFM1	protein_codin	0	9.6700471	0	0
ENSG000000	TBL1Y	protein_codin	0	0	0	0
ENSG000000	CLCN6	protein_codin	12.7491894	30.5843706	39.7381994	6.48253823
ENSG000000	FXYD3	protein_codin	0	0	0	1.47525913
ENSG000001	RHCE	protein_codin	0	0	0.67908933	0
ENSG000001	DRD2	protein_codin	0	0	0	1.20762545
ENSG000001	DDIT3	protein_codin	108.656291	0	0	0

ENSG000001	SIGLEC6	protein_codin	0	0	0	0
ENSG000001	TSPAN3	protein_codin	8.06869835	0	30.0881189	0
ENSG000002	FBXO16	protein_codin	0.97206396	0	0	0
ENSG000002	TSPAN4	protein_codin	0	5.5459335	0	0
ENSG000001	RBM23	protein_codin	0	0	0	10.6719924
ENSG000001	ABCB9	protein_codin	0	0	0	0
ENSG000000	CBFA2T2	protein_codin	0	0	0	0
ENSG000001	NPAS3	protein_codin	0	0	6.92241203	0
ENSG000001	ECM1	protein_codin	3.60216429	0	0	0
ENSG000001	KIR2DL4	protein_codin	0	0	0	1.41600539
ENSG000000	SMOX	protein_codin	0	0	0	0
ENSG000001	DHRS7B	protein_codin	0	3.27725641	1.41859402	5.05776726
ENSG000002	CSHL1	protein_codin	0	0	0	0
ENSG000000	ZFP64	protein_codin	1.09395415	0	0	0
ENSG000001	E2F3	protein_codin	4.11128711	10.6684269	56.3534169	7.56624901
ENSG000001	ALDH1A3	protein_codin	5.13131046	0	0	3.65232093
ENSG000001	ANTXR2	protein_codin	0.73693709	4.89082676	1.13197367	0
ENSG000001	FCER2	protein_codin	0	0	0	2.52593055
ENSG000001	IKZF1	protein_codin	0	0	0	0
ENSG000001	ATRIP	protein_codin	1.97818496	0	12.2453521	0
ENSG000001	CD99L2	protein_codin	3.31437797	0	5.73456496	3.80208606
ENSG000001	FGF8	protein_codin	0	0	0	0
ENSG000001	BRME1	protein_codin	0.38691258	2.69696987	7.05375804	0
ENSG000001	XAF1	protein_codin	0	0	0	0
ENSG000001	TMPRSS6	protein_codin	0	0	0	0
ENSG000001	SLC41A3	protein_codin	0	0	0	0
ENSG000001	MYL9	protein_codin	103.988698	154.213543	47.2589106	43.3223777
ENSG000001	APP	protein_codin	0	448.549031	0	0
ENSG000001	CTH	protein_codin	0.9377392	0	3.11246166	0
ENSG000001	IL36RN	protein_codin	0	2.50548739	0	0
ENSG000000	CASP10	protein_codin	0	0	0	2.13809662
ENSG000001	ABI1	protein_codin	0	0	0	0
ENSG000001	MLX	protein_codin	2.09815416	15.5893683	0	1.9359902
ENSG000001	SLC4A5	protein_codin	0	0	0	0
ENSG000001	UBE2E1	protein_codin	31.1860817	13.5691505	35.3449619	20.2319816
ENSG000000	TAC1	protein_codin	0	0	0	0
ENSG000001	IKZF3	protein_codin	6.13618545	83.9383067	9.22181883	0
ENSG000001	PARD3	protein_codin	0	0	0	0
ENSG000001	SPMAP2	protein_codin	0	2.255391	0	0.61910298
ENSG000001	BSG	protein_codin	0	0	0	0
ENSG000001	PAM	protein_codin	1.5407838	16.2641583	0	0
ENSG000001	SEZ6L2	protein_codin	0	0	0	0
ENSG000001	TPO	protein_codin	0	0	0	0
ENSG000000	DLG1	protein_codin	0	0	0	12.6531273

ENSG000001:	CACNG6	protein_codin	0	0	0	0
ENSG000000:	CAMK2B	protein_codin	0	0	0	0
ENSG000001:	KNL1	protein_codin	0	23.128546	62.6419471	0
ENSG000000	FGFR2	protein_codin	0	0	0	0
ENSG000001:	JAG2	protein_codin	1.94930125	0	0	0
ENSG000000	PHF7	protein_codin	0	0	0	0
ENSG000001:	ITGA7	protein_codin	0	0	0	0
ENSG000001	CAMKK2	protein_codin	0	0	0	0
ENSG000001:	SYNE1	protein_codin	4.83323101	0	4.02182191	0
ENSG000001	PTPA	protein_codin	0	0	0	0
ENSG000001	COG5	protein_codin	0	66.6092645	0	0
ENSG000001	KRCC1	protein_codin	64.6472855	49.6435183	95.9531089	26.8796474
ENSG000001	RNF175	protein_codin	1.11015436	0	20.5406167	0
ENSG000001	SIVA1	protein_codin	1.49014224	0	0	0.4579471
ENSG000001	ZNF546	protein_codin	5.84049257	6.93078187	6.76114299	3.49686125
ENSG000001	YY1AP1	protein_codin	0	0	0	0
ENSG000001:	DZIP1	protein_codin	0	0	0	18.6901795
ENSG000001	KCNQ4	protein_codin	0	0	0	0
ENSG000001	GPR107	protein_codin	67.3161536	181.69126	69.2770409	24.2916369
ENSG000001	R3HDM2	protein_codin	0	0	0	0
ENSG000001	TMEM44	protein_codin	0	0	0	0
ENSG000001:	PSAT1	protein_codin	0	7.70725842	0	0
ENSG000001:	KLC4	protein_codin	8.71006374	0	21.1398219	0
ENSG000000:	CAMK2B	protein_codin	0	0	0	0
ENSG000001:	CRHR1	protein_codin	0	0	0	0
ENSG000001	IFTAP	protein_codin	0	12.8255356	0	0
ENSG000001:	PMEPA1	protein_codin	7.54945845	0	0	15.0004181
ENSG000000	ZFYVE26	protein_codin	0	0	24.0703605	0
ENSG000001	RABGAP1L	protein_codin	13.3483042	16.4399599	9.77130674	10.7401405
ENSG000001	PPP1R14A	protein_codin	0	0	0	0
ENSG000001:	ZNF644	protein_codin	0	0	0	0
ENSG000001	IFT122	protein_codin	0	0	0	0
ENSG000001:	ZNF211	protein_codin	1.11765307	0	0	0
ENSG000001	IL23R	protein_codin	0	0	0	0
ENSG000001	LARP4	protein_codin	17.256772	0	67.5390647	12.7894715
ENSG000000	AURKA	protein_codin	0	0	3.24529846	0
ENSG000001	STOM	protein_codin	15.9002365	60.9564097	8.27380902	19.1912365
ENSG000001:	MKKS	protein_codin	0	0	0	0
ENSG000001	UBE2J2	protein_codin	0	0	0	0
ENSG000001:	CSTL1	protein_codin	0	0	0	0
ENSG000001	COL6A3	protein_codin	0	0	0	0
ENSG000000	KITLG	protein_codin	0	0	0	1.22524556
ENSG000001	EIF2B4	protein_codin	22.694983	150.937746	80.0949928	12.9718545
ENSG000001:	STAU1	protein_codin	0	0	0	0

ENSG000000	ATG16L1	protein_codin	0	0	0	0
ENSG000001	ZNF419	protein_codin	0.58612191	0	0	0
ENSG000000	MAPK9	protein_codin	0	0	0	0
ENSG000001	SMARCC2	protein_codin	57.069358	343.935513	0	78.0479036
ENSG000001	NOVA1	protein_codin	0	0.84182869	0	0
ENSG000001	SUPT6H	protein_codin	29.3948285	344.957453	205.20454	105.069555
ENSG000001	RPL36	protein_codin	61.5270202	208.442281	347.674661	53.4197669
ENSG000002	CHMP4A	protein_codin	18.1101641	0	39.9846332	19.4480038
ENSG000000	ANK1	protein_codin	0	0	9.76109858	0
ENSG000001	EPB41	protein_codin	0	0	0	0
ENSG000000	ARHGEF1	protein_codin	0	0	19.4708694	0
ENSG000001	MAGED2	protein_codin	5.83520554	28.8492832	52.1391038	4.71614043
ENSG000001	LRP8	protein_codin	0	0	0	0
ENSG000001	SMTN	protein_codin	0	0	35.3908927	0
ENSG000001	ADAM2	protein_codin	0	0	0	0
ENSG000001	ALDH1A2	protein_codin	0	0	0	3.49061842
ENSG000001	GUCY1A2	protein_codin	0.74229815	0	0	0
ENSG000001	CACNA1C	protein_codin	0	0	15.1067454	12.1222832
ENSG000001	ZNF334	protein_codin	1.7000435	0	6.61712572	0
ENSG000001	LSR	protein_codin	0.34372891	0	0	0.94055132
ENSG000001	GRIN2C	protein_codin	0.86081655	0	0	0.52856136
ENSG000001	CLCN3	protein_codin	0	0	9.24738855	6.95645835
ENSG000001	SMG7	protein_codin	24.3787407	41.8361003	105.31528	0
ENSG000000	CNTN1	protein_codin	0	29.1325891	0	8.45687946
ENSG000001	HMGA1	protein_codin	0	0	0	0
ENSG000001	KLK8	protein_codin	0	0	0	0
ENSG000001	LRRC15	protein_codin	234.380843	310.723079	4.15393455	176.98503
ENSG000001	SPOP	protein_codin	0	0	0	0
ENSG000001	ST3GAL3	protein_codin	0	0	0	0
ENSG000000	NUP50	protein_codin	38.9975091	94.2399412	125.312107	27.9310485
ENSG000000	RNF14	protein_codin	7.1610714	5.27083929	0	0
ENSG000001	SLC28A2	protein_codin	0.31856651	2.91696773	0	2.18770433
ENSG000001	VCAM1	protein_codin	22.6945077	423.106249	0	46.1191714
ENSG000001	BACE2	protein_codin	0	0	0	0
ENSG000001	TRAPPC6B	protein_codin	16.9506021	0	0	0
ENSG000000	EPN2	protein_codin	4.58472337	9.50465934	8.48531187	7.16621811
ENSG000000	MAP4K4	protein_codin	151.566405	21.29041	472.654533	167.808672
ENSG000001	CDCA7	protein_codin	5.91042309	0	78.9398777	0
ENSG000002	HGH1	protein_codin	1.82319764	11.9396107	10.1277678	0
ENSG000000	ERC1	protein_codin	0	0	0	0
ENSG000001	TNFRSF10B	protein_codin	0	30.124767	16.2522817	0
ENSG000001	PIDD1	protein_codin	0	0	0	0
ENSG000001	SSX5	protein_codin	0	0	0	0
ENSG000000	HAUS4	protein_codin	0	0	0	0

ENSG000001[illegible]	AZIN1	protein_codin	0	70.8831733	36.3114409	18.9658488
ENSG000001[illegible]	CAB39L	protein_codin	0	5.96692569	1.1349199	0
ENSG000001	CD5	protein_codin	1.96452339	24.2966843	0	2.03742773
ENSG000001[illegible]	ABCG1	protein_codin	0	0	3.34786018	0
ENSG000001[illegible]	KLRC1	protein_codin	0.57870974	0	0	0
ENSG000001[illegible]	KLC1	protein_codin	2.40101319	6.42157577	0	4.02738654
ENSG000000[illegible]	ASIC4	protein_codin	0	0	0	2.91365529
ENSG000000[illegible]	PTPN18	protein_codin	9.79454785	0	0	0
ENSG000000[illegible]	RBM5	protein_codin	30.1032051	337.512342	80.6067444	45.831268
ENSG000001[illegible]	SLC5A11	protein_codin	0	0	0	1.66214497
ENSG000001[illegible]	KIRREL2	protein_codin	0	0	0	0
ENSG000001[illegible]	HAP1	protein_codin	0	0	0	0
ENSG000001[illegible]	KLHDC4	protein_codin	0	56.6002521	8.7101855	0
ENSG000000[illegible]	WAC	protein_codin	31.2796735	96.2825907	37.2697483	32.0510484
ENSG000001	SLC22A18	protein_codin	0	0	0	0.5706675
ENSG000001[illegible]	PRDX5	protein_codin	1.30997629	0	1.91295913	0
ENSG000001[illegible]	SLC49A3	protein_codin	0	3.46556523	0	0
ENSG000001[illegible]	MBD1	protein_codin	0	0	0	0
ENSG000001[illegible]	TM2D3	protein_codin	3.43626315	0	0	3.90402024
ENSG000001[illegible]	FGF8	protein_codin	0	0	0	0
ENSG000001[illegible]	NSD1	protein_codin	27.0175103	0	0	0
ENSG000001[illegible]	MINK1	protein_codin	0	0	0	0
ENSG000001[illegible]	MIA2	protein_codin	56.7532756	99.84225	108.664	11.3382783
ENSG000001[illegible]	RBBP6	protein_codin	24.8000316	194.948253	162.674825	98.7590839
ENSG000001[illegible]	UBOX5	protein_codin	6.4201983	14.4456316	0	5.0742585
ENSG000001[illegible]	RAC1	protein_codin	57.9426607	83.172187	75.8473477	53.3524884
ENSG000000[illegible]	ANK1	protein_codin	0	3.28881405	0	0
ENSG000000[illegible]	OSBPL5	protein_codin	4.42106708	0	0	9.54462955
ENSG000001[illegible]	CAMTA2	protein_codin	4.72422245	50.0162949	0	7.48860031
ENSG000001[illegible]	NLRP3	protein_codin	0	0	0	0
ENSG000000[illegible]	MYL6	protein_codin	0	0	0	0
ENSG000001[illegible]	RGS7	protein_codin	0	0	0	0
ENSG000001[illegible]	TRAF6	protein_codin	0	12.2945773	3.63440617	6.48214444
ENSG000001[illegible]	PAM	protein_codin	0	82.9266099	30.3777538	29.8755246
ENSG000001[illegible]	IMPDH1	protein_codin	12.9485346	0	28.8654035	12.6605731
ENSG000001	PTPA	protein_codin	0	0	0	0
ENSG000001	MEF2D	protein_codin	7.6115091	0	0	3.79816797
ENSG000001[illegible]	RBPJ	protein_codin	2.24681273	17.8711783	1.13267939	0
ENSG000001[illegible]	TOM1L1	protein_codin	0	0	0	0
ENSG000000[illegible]	UBE3C	protein_codin	52.8685645	91.2259541	99.7916424	28.1763066
ENSG000001	TJP2	protein_codin	0	0	0	0
ENSG000001	DGUOK	protein_codin	0.58839334	0	2.83996472	0
ENSG000001[illegible]	PTN	protein_codin	473.00414	695.258987	183.286812	67.4507105
ENSG000000[illegible]	WDR54	protein_codin	0	0	7.04761016	0

ENSG000001	LAIR1	protein_codin	0	0	0	0
ENSG000001	TPD52L2	protein_codin	0	0	0	0
ENSG000001	MRPL55	protein_codin	2.65670711	7.31173661	3.66278624	0.93818841
ENSG000001	CACNA1H	protein_codin	0	0	0	0
ENSG000001	PRLR	protein_codin	0	0	0	2.97736183
ENSG000001	CLCC1	protein_codin	0	0	0	0
ENSG000000	DNMT3B	protein_codin	0	0	21.2407972	1.75365019
ENSG000001	CHEK2	protein_codin	0	0	0	0
ENSG000001	ACVR1C	protein_codin	0	0	0	0
ENSG000000	NCOA1	protein_codin	71.090615	163.003593	105.63856	24.2363039
ENSG000002	TNFRSF25	protein_codin	0	0	0	0
ENSG000000	CAMKK1	protein_codin	0	0	0	3.39715237
ENSG000001	C8orf34	protein_codin	0	0	1.6552172	0
ENSG000001	BMX	protein_codin	2.30100605	0	0	0
ENSG000001	BTG3	protein_codin	9.40386775	21.5296941	17.8856556	5.62871402
ENSG000001	GPAM	protein_codin	63.7120007	53.595148	20.8266802	6.6706073
ENSG000001	RNF111	protein_codin	5.38433639	55.5699279	17.7172299	0
ENSG000003	CAST	protein_codin	0	0	0	0
ENSG000001	STRBP	protein_codin	0	19.4703256	11.3644314	0
ENSG000001	SEC31A	protein_codin	0	0	0	0
ENSG000000	FTSJ1	protein_codin	7.98466816	24.096836	69.3213029	0
ENSG000001	IFT122	protein_codin	0	0	0	0
ENSG000000	CNN2	protein_codin	5.27052508	0	5.33445794	7.92535073
ENSG000001	TRPC6	protein_codin	0	0	6.67588997	10.1953686
ENSG000001	IKZF3	protein_codin	0	0	0	0
ENSG000000	REXO5	protein_codin	0	0	0	0
ENSG000001	CRHR2	protein_codin	0	0	0	0
ENSG000001	HELLS	protein_codin	19.6391173	0	35.9214033	5.54288173
ENSG000001	RPL35	protein_codin	223.486435	666.254082	890.639238	218.787901
ENSG000000	ARID4A	protein_codin	4.68003032	0	0	0
ENSG000001	SSBP4	protein_codin	5.30522613	4.35020754	28.5051581	8.71781237
ENSG000001	GATD3	protein_codin	0	0	0	0
ENSG000000	ACSL4	protein_codin	0	0	0	4.94210211
ENSG000000	SMARCE1	protein_codin	0	0	0	1.88543945
ENSG000002	DNAAF4	protein_codin	0	0	18.2285129	0.97360929
ENSG000001	KLC1	protein_codin	10.2159731	36.1571227	19.2795374	9.05544447
ENSG000001	VPS39	protein_codin	8.20525515	0	5.04941665	11.3985127
ENSG000001	ERGIC3	protein_codin	0	0	53.3678083	20.5145612
ENSG000001	CASP9	protein_codin	0	3.72823291	1.49594288	4.99381217
ENSG000000	PTPRC	protein_codin	5.23396477	6.80448999	0	1.3235353
ENSG000001	ZDHHC1	protein_codin	0	5.03940067	0.91538525	0.67219083
ENSG000001	EXO1	protein_codin	3.27275994	0	12.9532282	3.23282898
ENSG000001	CCNB3	protein_codin	0	0	0	0
ENSG000001	SENP7	protein_codin	0	42.0253703	30.5812589	0

ENSG000001:	CDH26	protein_codin	0	3.86552973	2.74294478	3.23872024
ENSG000000	CAMK2A	protein_codin	0	0	0	0
ENSG000000	CHRNA3	protein_codin	0	0	0	0
ENSG000001	IRF7	protein_codin	0	0	0	0
ENSG000001	POLDIP3	protein_codin	0	0	41.8897092	12.5480736
ENSG000001	CLEC1B	protein_codin	0	0	0	0
ENSG000001:	PPA2	protein_codin	0	0	1.32322488	0
ENSG000001:	PAX8	protein_codin	1.44851905	0	0	0
ENSG000001	CISH	protein_codin	2.39673336	15.1529814	5.16968632	0
ENSG000001	SLC23A1	protein_codin	0	0	0	0.87568438
ENSG000001:	CHRNA1	protein_codin	3.89193007	0	0.47004558	2.30994927
ENSG000001:	BIN1	protein_codin	0	63.9986277	22.1863466	0
ENSG000000	CNTN1	protein_codin	0	0	0	0
ENSG000000	CREM	protein_codin	0	0	0	0
ENSG000001	SNCG	protein_codin	0	0	0	0
ENSG000001	P2RX2	protein_codin	0	0	0	0
ENSG000001:	MDM2	protein_codin	430.157204	0	0	0
ENSG000001	WSB1	protein_codin	0	0	4.22581762	0
ENSG000001	DYRK1B	protein_codin	3.2480679	0	0	0
ENSG000000	MAP3K4	protein_codin	12.435367	99.2521542	78.8812982	0
ENSG000001	ADARB1	protein_codin	16.8888329	118.641748	38.9030855	32.3937417
ENSG000001	RBMS1	protein_codin	23.7191769	73.5981466	52.1401975	31.6169869
ENSG000001:	KCNIP2	protein_codin	2.28181677	0	0.54609539	2.28670676
ENSG000001	C7orf57	protein_codin	0	0	0	0
ENSG000001	SBF1	protein_codin	0	89.1749924	0	15.5303222
ENSG000001:	AMELX	protein_codin	0	0	0	0.45278368
ENSG000001	ALDH5A1	protein_codin	0	0	0	0
ENSG000001:	ZPBP2	protein_codin	0	0	0	0
ENSG000000	HNRNPM	protein_codin	0	10.5053474	0	0
ENSG000001	APOBEC3H	protein_codin	0	4.33465922	0	0
ENSG000001	CKB	protein_codin	20.0783337	37.3182603	69.1788354	102.101749
ENSG000001	PTPRO	protein_codin	0	0	0	0
ENSG000001:	DHX30	protein_codin	0	0	0	5.01017951
ENSG000001	PROS1	protein_codin	0	0	0	0
ENSG000000	CHRD	protein_codin	0	0	0	0
ENSG000001	APP	protein_codin	278.946659	40.1238107	1416.67094	148.11602
ENSG000001:	DTNA	protein_codin	0.42404809	4.0131664	2.35809608	0.64489651
ENSG000001	NDRG3	protein_codin	10.5452161	0	35.2091006	11.541488
ENSG000001	ADNP	protein_codin	83.7065696	141.833978	208.438805	28.8799726
ENSG000001:	ALDH3B2	protein_codin	0	0	0	0
ENSG000001:	TEX14	protein_codin	1.44844627	4.72697092	2.69054428	0
ENSG000000	GNAS	protein_codin	0	0	0	0
ENSG000000	PTBP1	protein_codin	0	0	0	33.9793406
ENSG000001	PFKL	protein_codin	44.1567494	115.12819	191.799628	30.3405994

ENSG000001	NUDT13	protein_codin	0	1.28141912	0	0
ENSG000001	TRIM4	protein_codin	17.7558557	33.8337304	41.3914439	0
ENSG000002	ASIC3	protein_codin	0	1.93457961	2.5665374	0
ENSG000001	COLEC11	protein_codin	0	0	0	0
ENSG000001	PDE9A	protein_codin	0	0	0	0
ENSG000001	TGM5	protein_codin	0	0	0	1.28274104
ENSG000001	PTP4A3	protein_codin	0	0	0	1.34366399
ENSG000000	DOP1A	protein_codin	43.4573023	96.1061838	33.9518482	4.00561832
ENSG000000	WDR3	protein_codin	47.4180519	104.277593	266.602248	26.0352907
ENSG000001	TBX3	protein_codin	23.1023582	0	70.3947641	8.98429018
ENSG000001	PUF60	protein_codin	13.6103641	96.9581065	196.793954	15.3025854
ENSG000001	IL22RA2	protein_codin	0	0	0	0
ENSG000000	ARID4B	protein_codin	0	0	0	0
ENSG000001	HPS5	protein_codin	11.3044659	0	28.1562768	11.5254232
ENSG000000	NFATC3	protein_codin	3.6885033	0	0	0
ENSG000001	RLIM	protein_codin	52.900913	91.15595	100.386145	58.5959242
ENSG000001	TNFSF13	protein_codin	0	0	0	1.61406555
ENSG000001	MADD	protein_codin	0	0	0	0
ENSG000001	PTK7	protein_codin	0	0	0	0
ENSG000001	AGTR1	protein_codin	0	0	0	4.24012727
ENSG000001	THSD1	protein_codin	0	0	0	0
ENSG000001	B2M	protein_codin	0	0	0	0
ENSG000001	H2AZ2	protein_codin	8.29001027	0	24.8810105	5.93138561
ENSG000001	ALDH8A1	protein_codin	0	0	0	0
ENSG000001	AKT1	protein_codin	0	9.07633845	0	0
ENSG000001	UBE2D3	protein_codin	0	0	0	0
ENSG000001	APOL3	protein_codin	1.85824809	41.9494801	0	0
ENSG000001	RHCE	protein_codin	1.19317899	0	0	0
ENSG000001	ART3	protein_codin	0	0	0	0
ENSG000001	GSTM1	protein_codin	0	18.2762977	0	0
ENSG000001	CNBD2	protein_codin	0	0	0	0
ENSG000001	SNX3	protein_codin	23.8470509	0	95.3386829	27.5237872
ENSG000001	RREB1	protein_codin	3.45900971	0	28.0475086	31.8052705
ENSG000001	NXPH4	protein_codin	0.44899353	0	3.76838683	0.80747949
ENSG000001	UBE2J2	protein_codin	0.59020763	0	0	0
ENSG000001	RHCE	protein_codin	0	0	0	0
ENSG000001	IFT122	protein_codin	0	0	0	0
ENSG000001	OPRL1	protein_codin	0	0	0	0
ENSG000001	CD8B	protein_codin	0	0	0	0
ENSG000001	CFAP36	protein_codin	47.8937577	52.3857452	132.71891	21.3467887
ENSG000001	ADGRG5	protein_codin	0	7.27432428	0	0
ENSG000001	PELI3	protein_codin	0	0	0	0
ENSG000001	EPB41	protein_codin	0	0	0	0
ENSG000000	ATP2B3	protein_codin	0	0	0	0

ENSG000001	POFUT2	protein_codin	5.49785941	0	0	0
ENSG000001	C8orf34	protein_codin	0	2.72879593	0	0
ENSG000001	CTNNB1	protein_codin	0	0	0	0
ENSG000001	CLIC6	protein_codin	0	4.2112299	6.97390186	2.12043294
ENSG000001	HDAC10	protein_codin	0	0	0	0
ENSG000001	UBA3	protein_codin	21.0593876	19.6990497	0	17.9962867
ENSG000001	FCRLA	protein_codin	0	0	0	0
ENSG000001	LEPR	protein_codin	0.28113532	15.0988363	2.06015919	5.33117792
ENSG000001	BPIFB6	protein_codin	0	0	0	0.3290522
ENSG000001	CALB2	protein_codin	0	0	0	1.2421748
ENSG000001	GSTZ1	protein_codin	0	0	0	0
ENSG000002	CEP43	protein_codin	0	0	0	0
ENSG000000	CDHR5	protein_codin	0	0	0	0
ENSG000000	EXOC1	protein_codin	37.6841515	62.1560779	124.208559	19.1880722
ENSG000000	MYLIP	protein_codin	0	46.2164859	0	6.32627808
ENSG000001	MUC4	protein_codin	0	0	0	0
ENSG000001	ZNF683	protein_codin	0	3.14036667	0	0
ENSG000001	HNRNPDL	protein_codin	24.7800128	22.7664656	24.6348851	23.5025893
ENSG000001	KDM5C	protein_codin	0	0	0	0
ENSG000001	POP1	protein_codin	0	10.0575684	60.6438356	3.77801834
ENSG000001	KCNMB3	protein_codin	0	0	0	0
ENSG000000	RFC1	protein_codin	38.8131297	31.9376783	57.7636075	15.841479
ENSG000001	UQCC1	protein_codin	0	0	0	0
ENSG000000	TBX5	protein_codin	0	2.80030193	0	3.23693569
ENSG000001	CMTM7	protein_codin	0	0	0	0
ENSG000000	SMARCA2	protein_codin	0	0	0	0
ENSG000001	CSNK2A1	protein_codin	12.5946452	0	12.3889877	0
ENSG000000	CAPN3	protein_codin	0	0	0	0
ENSG000001	GALNT14	protein_codin	0	4.01773762	37.7321596	5.9780167
ENSG000001	P2RY6	protein_codin	0	0	0	0
ENSG000000	SNAP23	protein_codin	0.45465141	3.52201797	0	1.09076623
ENSG000001	CDK5RAP2	protein_codin	0	0	0	0
ENSG000001	PIP5K1A	protein_codin	40.8834699	0	61.8484106	0
ENSG000001	IL37	protein_codin	0	0	0	0
ENSG000001	MAL	protein_codin	0.37720792	0	0	0
ENSG000000	CD79B	protein_codin	0	0	0	0
ENSG000001	IQCB1	protein_codin	0	0	0.90252093	0
ENSG000001	ARHGEF10	protein_codin	5.53224418	43.9657377	46.3797907	23.1099476
ENSG000000	MYNN	protein_codin	9.77957617	15.5839771	21.847401	8.66208337
ENSG000001	SCEL	protein_codin	0	0	0	0
ENSG000000	PPEF1	protein_codin	0	0	0	0
ENSG000001	NAV2	protein_codin	0	4.60181627	0	13.8654986
ENSG000001	COPE	protein_codin	2.42148435	0	0	0
ENSG000001	MFF	protein_codin	0	0	0	0

ENSG000001	CLEC7A	protein_codin	0	0	0	0
ENSG000001	TCF7L2	protein_codin	9.39416948	0	3.64847281	1.33058927
ENSG000001	ARMC9	protein_codin	0	0	0	0
ENSG000001	NFAT5	protein_codin	0	120.358963	4.29919836	0
ENSG000001	PARD3B	protein_codin	4.6271954	80.4657572	0	0
ENSG000001	PKIG	protein_codin	0	0	0	0
ENSG000001	CCR6	protein_codin	0	0	0	0
ENSG000001	COG3	protein_codin	27.1792318	41.8891242	38.5115854	16.0813629
ENSG000001	DTNB	protein_codin	0	0	0	0
ENSG000000	HFE	protein_codin	0	34.4871075	0	0
ENSG000001	ADAM33	protein_codin	4.57034011	0	0	3.28179701
ENSG000001	NDUFB6	protein_codin	1.95764488	12.4679026	0	0
ENSG000001	IL4	protein_codin	0	0	0	0
ENSG000000	ARID1B	protein_codin	0	62.3074682	70.2499569	0
ENSG000001	TAF1A	protein_codin	1.40485823	0	4.38497492	0
ENSG000001	SAMM50	protein_codin	10.882613	36.7110092	38.1567841	8.75069952
ENSG000001	NASP	protein_codin	0	213.791401	29.8594257	2.50432008
ENSG000001	COPS7B	protein_codin	0	0	3.23445772	0
ENSG000001	TRIM55	protein_codin	0	0	0	0
ENSG000001	P2RX2	protein_codin	0	0	0	0
ENSG000000	BIRC5	protein_codin	3.49113338	5.83412913	84.7563849	0
ENSG000001	MDM2	protein_codin	0	0	38.1912397	0
ENSG000000	ARHGAP4	protein_codin	11.1407329	36.1618763	0	11.9436523
ENSG000001	CACNA1D	protein_codin	0	0	0	0
ENSG000001	CILK1	protein_codin	0	29.4479248	0	16.2125824
ENSG000000	PTBP1	protein_codin	0	0	0	0
ENSG000001	CNTN6	protein_codin	0	0	0	7.21960518
ENSG000001	DOC2A	protein_codin	0	0	0	0
ENSG000001	SMAD9	protein_codin	11.998911	0	25.5761343	0
ENSG000001	RNF38	protein_codin	1.53670712	0	0	0
ENSG000001	ENTREP3	protein_codin	0	0	0	0
ENSG000001	FSIP1	protein_codin	1.39879994	0	5.72677871	1.41129073
ENSG000001	KCNC2	protein_codin	0	0	0	0
ENSG000001	DUOXA2	protein_codin	0	0	0	0
ENSG000000	PPP2R5C	protein_codin	6.0322736	0	0	0
ENSG000001	ALDH6A1	protein_codin	0	0	0	7.92454632
ENSG000001	KLRD1	protein_codin	0	0	0	0
ENSG000001	FBXO25	protein_codin	10.628318	15.4131753	7.83422729	8.6806389
ENSG000000	ACADVL	protein_codin	1.74721467	0	2.94472809	0
ENSG000001	SEPTIN7	protein_codin	38.3139832	12.0299703	59.210756	27.4138697
ENSG000001	FCN2	protein_codin	0	0	0	0.36870653
ENSG000000	PRKACA	protein_codin	0	0	0	0
ENSG000000	POU1F1	protein_codin	0	0	0	0
ENSG000001	C7orf25	protein_codin	2.02520892	0	6.84760144	0

ENSG000001	UBE2D3	protein_codin	0	11.7342144	0	0
ENSG000000	DDX11	protein_codin	0.72251455	0	11.8667199	2.99393909
ENSG000001	ANO10	protein_codin	2.42860842	0	7.11818632	9.12939231
ENSG000001	EIF4G1	protein_codin	0	0	0	0
ENSG000000	TAC1	protein_codin	0	0	0	0
ENSG000000	MARK2	protein_codin	0	0	0	0
ENSG000001	ELP2	protein_codin	0	0	0	0
ENSG000001	PDAP1	protein_codin	44.0307695	118.223806	183.158511	32.5845628
ENSG000001	SPACA9	protein_codin	0	0	0	0
ENSG000001	GRHL3	protein_codin	0	0	0	0
ENSG000001	OR9A2	protein_codin	0.63451136	0	0.52118069	1.90769961
ENSG000001	PAX3	protein_codin	0	0	1.99262901	0.47299695
ENSG000001	SEZ6L2	protein_codin	0.87991358	0	0	0
ENSG000001	IKZF3	protein_codin	0	0	0	0
ENSG000001	PARD3	protein_codin	0	0	0	0
ENSG000001	PRSS37	protein_codin	0	0	0	1.38009624
ENSG000000	PILRA	protein_codin	1.03289009	0	0	0.4349319
ENSG000001	C1orf43	protein_codin	0	98.6439415	27.7250898	5.5618803
ENSG000001	PYCARD	protein_codin	2.47053572	15.1038157	0	2.3729363
ENSG000000	DDT	protein_codin	0	0	1.98452956	0
ENSG000001	SUPT20H	protein_codin	0	32.695795	23.7216611	0
ENSG000001	CASP2	protein_codin	3.36747235	0	17.3291905	0
ENSG000001	ELP4	protein_codin	0	0	0	0
ENSG000001	SLC47A2	protein_codin	0	0	0	0
ENSG000001	CLEC12A	protein_codin	0	0	0	0.6295127
ENSG000001	LDHB	protein_codin	29.7173347	21.8644291	287.750327	28.1176879
ENSG000001	SLC25A10	protein_codin	0	0	15.4919327	0
ENSG000001	RGS3	protein_codin	0	0	0	0
ENSG000001	SEC13	protein_codin	74.6842784	90.2096572	365.526731	31.8679748
ENSG000001	FCRLA	protein_codin	0	0	0	0
ENSG000001	ATR	protein_codin	0	0	0	0
ENSG000001	INPP5K	protein_codin	0	0	0	0
ENSG000000	TNC	protein_codin	99.6692926	348.69134	160.900217	133.301724
ENSG000001	MAPKAP1	protein_codin	0	4.0564547	14.2616847	0
ENSG000001	H2AZ2	protein_codin	0	0	0	0
ENSG000001	TSC2	protein_codin	0	0	0	0
ENSG000001	HINFP	protein_codin	1.09880493	7.00597191	0	1.89788626
ENSG000001	NCR1	protein_codin	0	0	0.42079965	0
ENSG000001	NDRG2	protein_codin	0	0	7.98421513	0
ENSG000000	CYTH3	protein_codin	63.9203441	90.7754595	73.3138216	26.111676
ENSG000000	CAMK2B	protein_codin	0	0	0	0
ENSG000001	CDH26	protein_codin	0.37528696	0	0	0
ENSG000000	MAP4K4	protein_codin	180.603368	441.919734	349.1429	101.259203
ENSG000001	THBS4	protein_codin	284.042393	1.67367411	9.36114687	25.4239621

ENSG000001	SNRNP35	protein_codin	0	0	4.89441296	0
ENSG000000	STMN4	protein_codin	0	0	0	0
ENSG000000	MSR1	protein_codin	9.0884138	34.4645453	11.2773854	6.39386012
ENSG000001	GRIN1	protein_codin	0	0	0	0
ENSG000001	FOXP2	protein_codin	3.90918787	0	31.1908829	0
ENSG000001	BBS9	protein_codin	0	0	0	0
ENSG000001	EZH2	protein_codin	4.6710463	53.3938067	13.6245284	0
ENSG000001	FADS1	protein_codin	42.5297213	51.1000074	51.2633024	19.1360945
ENSG000001	GNAI1	protein_codin	0	0	12.654865	0
ENSG000001	SPATA16	protein_codin	0	0	0	0
ENSG000000	CAMK2A	protein_codin	1.22348017	0	0	0
ENSG000001	AFDN	protein_codin	0	0	0	0
ENSG000001	RHOG	protein_codin	0	14.0389845	0	6.47920011
ENSG000001	ODF2	protein_codin	0	0	3.97184643	0
ENSG000001	ST3GAL3	protein_codin	0	0	0	0
ENSG000001	GNAT2	protein_codin	0	0	0	0.45485906
ENSG000000	WASHC2A	protein_codin	36.9760462	139.573797	0	59.4998192
ENSG000001	COPE	protein_codin	0	0	1.16331465	0
ENSG000001	USP25	protein_codin	0	0	0	0
ENSG000001	MRPL10	protein_codin	0	0	4.25127719	0
ENSG000001	TAS1R1	protein_codin	0.5070255	0	0	0
ENSG000002	CKLF	protein_codin	0	0	0	0
ENSG000001	PARD3B	protein_codin	10.4448584	0	0	0
ENSG000001	NMD3	protein_codin	68.4842052	66.777197	144.305837	43.5222129
ENSG000001	CABP1	protein_codin	0	0	0	0
ENSG000001	SMN1	protein_codin	0	0	0	0
ENSG000001	NPTN	protein_codin	6.68699978	0	0	3.59595607
ENSG000001	ZFP30	protein_codin	0	0	1.62924235	0
ENSG000001	P2RX2	protein_codin	0	0	0	0
ENSG000001	NASP	protein_codin	39.5574028	87.7259697	229.738316	41.8630214
ENSG000001	CDC25A	protein_codin	0	5.27824431	14.7494762	0.45181159
ENSG000001	MTMR14	protein_codin	10.3355434	0	18.8170119	0
ENSG000001	CNTFR	protein_codin	0	0	0	0
ENSG000001	HABP2	protein_codin	0	0	0.97923642	0
ENSG000000	ACP3	protein_codin	0.62598777	0	3.53652476	0
ENSG000001	SMPD4	protein_codin	0	0	0	0
ENSG000001	CAMK2G	protein_codin	37.7679047	77.1307741	35.6572965	17.0685369
ENSG000000	PLD1	protein_codin	4.14789348	0	7.34821535	34.8373535
ENSG000002	FXYD1	protein_codin	1.7862249	0	0	0.93322956
ENSG000000	CS	protein_codin	2.07137033	24.6474415	9.35793037	0
ENSG000001	EFS	protein_codin	0	0	0	0
ENSG000001	SLC66A2	protein_codin	0	15.0500811	0	0
ENSG000002	GH1	protein_codin	0	0	0	0
ENSG000001	ELP2	protein_codin	7.21263289	0	0	10.793124

ENSG000001	TPD52L2	protein_codin	0	0	0	0
ENSG000001	N6AMT1	protein_codin	11.6525682	26.3843034	47.2781737	0
ENSG000001	SPAG11A	protein_codin	0	0	0	0
ENSG000001	PDE1A	protein_codin	19.978145	28.4450843	22.4150599	6.06618757
ENSG000001	PPA2	protein_codin	38.8708911	34.7351443	132.579307	0
ENSG000001	BNIP1	protein_codin	7.35791403	14.494769	12.9922376	4.03917635
ENSG000001	UPF3A	protein_codin	0	0	0	0
ENSG000001	MTMR3	protein_codin	0	34.6425399	57.535878	0
ENSG000001	PFDN5	protein_codin	0	3.86886416	0	4.96754551
ENSG000001	DPYS	protein_codin	0.78143867	0	2.45405067	1.55115658
ENSG000001	SLAIN1	protein_codin	0	0	0	3.00406244
ENSG000000	CD59	protein_codin	3.98320354	47.7212147	1.31894523	3.36913191
ENSG000000	CHAT	protein_codin	0	0	0	0
ENSG000001	MAPT	protein_codin	0	0	0.76405047	0
ENSG000000	FYB1	protein_codin	0	0	0	0
ENSG000001	SLX1B	protein_codin	0.77971654	5.47974744	0	3.76261064
ENSG000001	GSG1	protein_codin	0	0	0	0
ENSG000000	EED	protein_codin	0	0	0	0
ENSG000001	BIN1	protein_codin	1.03176798	0	0	0
ENSG000000	DHPS	protein_codin	0	0	0	0
ENSG000001	PTPN11	protein_codin	105.901293	0	126.694333	50.7587052
ENSG000001	IKZF3	protein_codin	0	0	0	1.57230924
ENSG000001	TRAF3	protein_codin	0	0	0	0
ENSG000001	TOR1A	protein_codin	30.4233022	0	40.6624451	17.72499
ENSG000000	NRCAM	protein_codin	5.18391355	0	42.6846463	5.90607496
ENSG000001	DNAH12	protein_codin	0	1.79145833	0	1.61555495
ENSG000002	TNFRSF25	protein_codin	0.48116453	0	0	0
ENSG000001	IAH1	protein_codin	4.52267143	0	0	0
ENSG000000	ZNF37A	protein_codin	34.7040771	38.6466697	23.5250466	5.89526616
ENSG000000	FAM114A2	protein_codin	8.49859051	36.5569055	16.2387145	4.97601224
ENSG000001	OSCAR	protein_codin	0	0	0	0
ENSG000000	IFT88	protein_codin	21.2204442	23.8716617	26.2413091	7.53310681
ENSG000001	NKX2-4	protein_codin	0	0	0	0
ENSG000001	AKR1A1	protein_codin	0	0	0	0
ENSG000001	HNRNPK	protein_codin	0	0	0	0
ENSG000001	LAIR2	protein_codin	0	4.84670407	0	1.31850561
ENSG000001	USP4	protein_codin	28.5303945	71.290316	45.5959022	17.5736206
ENSG000001	MTERF1	protein_codin	4.71279161	20.8413723	17.8664323	0
ENSG000000	SLC4A4	protein_codin	0	33.7374751	14.5647678	0
ENSG000001	PATZ1	protein_codin	3.06403075	8.27593669	26.0087015	6.95491846
ENSG000000	FGFR2	protein_codin	1.37496374	0	0	0
ENSG000001	TRIM41	protein_codin	0	14.4081492	0	5.75640003
ENSG000001	SMC6	protein_codin	50.774849	53.9786193	226.009981	41.9839872
ENSG000002	TNFRSF25	protein_codin	3.51602886	0	0	3.1581615

ENSG000001:	NUMA1	protein_codin	0	0	0	0
ENSG000001:	NPM1	protein_codin	0	0	0	0
ENSG000001:	DGCR8	protein_codin	23.6526827	60.4728114	37.4016307	11.2171552
ENSG000000	CD6	protein_codin	0	0	0	0
ENSG000000	ATP2A3	protein_codin	0	20.7834931	0	0
ENSG000001:	ACLY	protein_codin	0	0	30.9610802	44.1947646
ENSG000001:	ADAMTS12	protein_codin	0	0	14.8768429	0
ENSG000001	PLAT	protein_codin	17.2580639	54.7234843	0	12.5131863
ENSG000001	TCF7L2	protein_codin	0	0	0	0
ENSG000002	NUP62	protein_codin	12.4640227	0	26.8656348	6.43301033
ENSG000001	SNX15	protein_codin	0	0	0	0
ENSG000000	ELMO2	protein_codin	0	0	0	0
ENSG000001:	RGN	protein_codin	0	0	0	0
ENSG000001	ACAN	protein_codin	240.845012	62.0922073	24.607111	0
ENSG000001	CACNB2	protein_codin	0	0	0	0
ENSG000001:	XRCC3	protein_codin	0	0	0	0
ENSG000000	RFC2	protein_codin	1.01782779	0	0	0
ENSG000001	SLC37A1	protein_codin	2.6023567	6.99432333	1.57763418	1.58008305
ENSG000001	FMNL3	protein_codin	32.3442872	320.360538	30.750775	0
ENSG000000	NRDC	protein_codin	15.5227023	106.927401	83.769161	44.5091394
ENSG000001	CBS	protein_codin	0	16.8301468	0	3.29701549
ENSG000001:	IL37	protein_codin	0	0	0	0
ENSG000001:	SLC43A3	protein_codin	0	0	0	0
ENSG000000	TRPC7	protein_codin	0	0	0	0
ENSG000000	AP2S1	protein_codin	0	0	0	0
ENSG000001	PPOX	protein_codin	0	0	0	0
ENSG000001	HTRA2	protein_codin	0	4.77681438	0	0
ENSG000001	LGALS8	protein_codin	0	0	0	0
ENSG000001:	MITF	protein_codin	0	0	0	0
ENSG000001:	PROSER1	protein_codin	5.64749746	0	12.2046036	0
ENSG000001	SPNS1	protein_codin	0	0	0	0
ENSG000001	ZNF385A	protein_codin	0	0	0	0
ENSG000000	MTMR2	protein_codin	0	0	46.8592723	8.16558803
ENSG000001:	HNRNPD	protein_codin	16.1313693	81.8982066	152.2646	19.5466206
ENSG000001:	PSMD13	protein_codin	0	20.7572869	0	0
ENSG000001	CFAP100	protein_codin	0	0	0	0
ENSG000001	UTP4	protein_codin	0	0	2.34565203	0
ENSG000001:	THYN1	protein_codin	0	0	0	0
ENSG000001:	KIF23	protein_codin	0	0	51.3720307	0
ENSG000001	APOL4	protein_codin	0	0	0	0
ENSG000001	IGDCC4	protein_codin	16.1023923	26.6767963	59.5879321	0
ENSG000000	HFE	protein_codin	0	0	0	0
ENSG000002	CPNE1	protein_codin	0	0	0	0
ENSG000001	CYB5R3	protein_codin	2.16809323	45.5826479	9.61227076	7.49874746

ENSG000001	MPI	protein_codin	4.54161828	0	7.45519109	1.68270444
ENSG000001	P2RX2	protein_codin	0	0	0	0
ENSG000001	ZMYND8	protein_codin	0	51.2544677	0	0
ENSG000001	ATP2B2	protein_codin	0	0	0	0
ENSG000001	PTTG1	protein_codin	3.09571439	16.4219201	47.9580557	0
ENSG000001	PRDX5	protein_codin	0	0	3.23428194	0
ENSG000001	TOR3A	protein_codin	0	0	0	1.43819026
ENSG000001	MYL1	protein_codin	126.429457	0	0	0
ENSG000001	HNRNPUL1	protein_codin	19.6842382	0	18.668032	8.46956272
ENSG000001	HPS5	protein_codin	0	0	0	0.59521931
ENSG000001	ASS1	protein_codin	4.33941417	0	1.97629586	26.181167
ENSG000001	TPD52L2	protein_codin	0	10.4645099	0	0
ENSG000001	RIPK4	protein_codin	0	0	0	0
ENSG000001	RAPSN	protein_codin	0	0	0	0
ENSG000001	ACVR2B	protein_codin	8.9231783	53.8799801	17.9831629	14.9699207
ENSG000001	BNIP1	protein_codin	0	0	0	0
ENSG000000	KCNAB2	protein_codin	0	20.3306406	0	0
ENSG000001	CACNG6	protein_codin	0	0	0	1.29925767
ENSG000001	VGLL2	protein_codin	1.19122696	0	0	0.42605922
ENSG000001	UBE2C	protein_codin	0	0	0	0
ENSG000001	CD8A	protein_codin	0	0	0	0
ENSG000000	CEACAM1	protein_codin	0	7.79881386	0	3.21179281
ENSG000001	CHIA	protein_codin	0	0	0	0
ENSG000001	RAD51AP1	protein_codin	0.90808862	0	0	1.11282167
ENSG000001	CLEC4A	protein_codin	0	0	0	0
ENSG000001	SPTBN4	protein_codin	0	0	0	0
ENSG000001	ZDHHC16	protein_codin	0	0	0	0
ENSG000001	ZC3H7B	protein_codin	41.6718274	156.782922	130.351518	32.4711288
ENSG000001	CFAP52	protein_codin	0	0	0	0
ENSG000001	RHBDL1	protein_codin	0	0	0.61171784	0
ENSG000001	FBXO25	protein_codin	4.07524191	23.8411039	14.1624172	6.1037435
ENSG000001	MKLN1	protein_codin	52.6114722	124.009984	107.461372	41.3136327
ENSG000001	CD96	protein_codin	1.61488959	22.0282834	0	4.62262236
ENSG000001	ABHD2	protein_codin	51.6995041	211.045259	64.214187	50.9419302
ENSG000002	PRR5-ARHGA	protein_codin	0.31543961	1.29482873	0	0
ENSG000001	EIF4G1	protein_codin	2.31269523	0	5.05208519	13.6115728
ENSG000001	TAC4	protein_codin	0	0	0	0
ENSG000001	TBL1XR1	protein_codin	0	11.3923366	6.30163985	13.0859468
ENSG000000	CD44	protein_codin	3.9253377	0	0	0
ENSG000000	CACNA1G	protein_codin	0	0	0	0
ENSG000000	PKP1	protein_codin	0	0	0	0
ENSG000001	BIN1	protein_codin	39.9237421	0	0	0
ENSG000001	CRHR1	protein_codin	0	0	0	0
ENSG000001	SEC61G	protein_codin	11.3191877	66.3678305	60.2361434	14.3885622

ENSG000001:	BTN2A2	protein_codin	0.90964638	0	1.18836724	0
ENSG000001	CAPN10	protein_codin	0	0	0	0
ENSG000001	FAM184A	protein_codin	0	0	0	0
ENSG000001	TEX9	protein_codin	2.27307229	0	5.13358976	0
ENSG000000	FGFR3	protein_codin	0	32.2500338	0	0
ENSG000001	TH	protein_codin	0	0	0	0
ENSG000001	PTK7	protein_codin	0	75.183823	19.162268	0
ENSG000001	OGG1	protein_codin	4.44164702	0	11.1204926	3.08321656
ENSG000002	DDX47	protein_codin	0	0	0	0
ENSG000001	MRPL39	protein_codin	11.3481919	12.3667809	43.9370749	4.13387802
ENSG000001	PKIA	protein_codin	10.2863855	0	32.0774753	0
ENSG000001	TAF1A	protein_codin	6.26006806	4.64694002	57.3173828	0
ENSG000001	KAT5	protein_codin	0	0	19.5369314	0
ENSG000001	CASP6	protein_codin	0	0	0	0
ENSG000001	RPL7	protein_codin	289.519491	417.774951	964.142901	198.814927
ENSG000000	BRCA1	protein_codin	0	0	0	0
ENSG000001	ZNF383	protein_codin	0	16.6930369	0	0
ENSG000001	MAL	protein_codin	0	0	0	0
ENSG000001	CTSB	protein_codin	55.1353546	0	0	0
ENSG000001	PROK2	protein_codin	0	2.5648729	0	2.10621737
ENSG000001	KCNIP2	protein_codin	0	0	0	0
ENSG000000	NSFL1C	protein_codin	0	0	0	0
ENSG000001	POLR2K	protein_codin	8.08556567	13.3090697	69.4758629	5.97150478
ENSG000001	ST3GAL3	protein_codin	0	0	0	0
ENSG000001	ABHD12B	protein_codin	0	0	0	0
ENSG000000	HFE	protein_codin	0	0	1.05384524	0
ENSG000001	CSN2	protein_codin	0	1.39410976	0.49739742	1.18040821
ENSG000001	NOL6	protein_codin	8.72391668	28.5130548	18.8464167	2.86936337
ENSG000001	KLHDC4	protein_codin	0	0	9.84159932	0
ENSG000001	KCTD14	protein_codin	0	1.09356896	3.23942576	1.73780434
ENSG000001	PHYHD1	protein_codin	0	0	0	0
ENSG000000	CAMK2B	protein_codin	0	0	0	0
ENSG000001	AADAT	protein_codin	0	0	0	0
ENSG000001	ACLY	protein_codin	4.23076324	14.1356332	26.5996634	2.86156042
ENSG000000	NFYA	protein_codin	15.6820552	15.2172432	64.272417	22.1905523
ENSG000001	ARPC5L	protein_codin	0.3616875	8.48934784	0.70537479	2.18635784
ENSG000001	PAK5	protein_codin	0	0	0	0
ENSG000001	IL37	protein_codin	0	0	0	0
ENSG000001	CLEC7A	protein_codin	0	0	0	0
ENSG000001	ANKS6	protein_codin	7.19330866	59.2362175	40.6058162	15.2146784
ENSG000001	YWHAZ	protein_codin	11.4751215	0	0	65.2132384
ENSG000000	GDE1	protein_codin	7.98939925	24.1293222	37.9340592	17.3044147
ENSG000001	PARD6G	protein_codin	15.3765189	46.3542576	36.66583	23.2468968
ENSG000001	CREB1	protein_codin	1.68351623	4.01131562	21.0797072	1.20838014

ENSG000001	NSD2	protein_codin	1.24864788	0	0	0
ENSG000001	SLC6A20	protein_codin	0	0	2.63460361	1.56976631
ENSG000001	TRIM55	protein_codin	0	0	0	2.33935574
ENSG000001	PTPN2	protein_codin	0	0	0	0
ENSG000002	BCLAF1	protein_codin	0	0	0	0
ENSG000001	MTMR14	protein_codin	0	13.8395395	14.5222142	0
ENSG000000	CD74	protein_codin	596.282358	5156.4788	89.1067327	420.333911
ENSG000001	MFF	protein_codin	0	0	0	0
ENSG000001	HNRNPD	protein_codin	50.4707211	306.194243	213.871052	30.7398672
ENSG000001	USP15	protein_codin	5.08893776	0	7.85264725	0
ENSG000001	CDK10	protein_codin	0	0	0	0
ENSG000001	RAI1	protein_codin	24.1524498	87.2945911	55.7115951	30.313821
ENSG000001	SKP1	protein_codin	12.3747081	35.1487662	21.9734557	125.87574
ENSG000000	COL11A1	protein_codin	0	0	19.446508	2.96830546
ENSG000001	GABRB2	protein_codin	0	9.25011953	0	0
ENSG000001	LMBR1	protein_codin	7.09009857	41.3265715	14.5372775	11.3498337
ENSG000001	SIGMAR1	protein_codin	12.1645149	0	0	0
ENSG000001	SPPL3	protein_codin	4.78023871	26.605385	12.9180842	6.30677555
ENSG000001	ATP6V0A4	protein_codin	0	0	0	0
ENSG000001	DDX4	protein_codin	0	0	0	0
ENSG000000	MAP2K4	protein_codin	21.842154	29.9402121	9.82420679	6.94843419
ENSG000001	ARFGAP1	protein_codin	0	0	0	0
ENSG000001	DIABLO	protein_codin	0	2.36949902	0	0
ENSG000001	BSG	protein_codin	22.2086375	119.675865	162.033317	29.853236
ENSG000001	COL6A3	protein_codin	1362.48943	916.610921	29.4582153	166.28651
ENSG000001	MRPS11	protein_codin	0	0	0.63437996	0
ENSG000001	FOSB	protein_codin	4.07780327	0	0	532.192738
ENSG000001	ARFIP1	protein_codin	13.913705	20.7780799	17.8290646	10.9002647
ENSG000000	CAMK2B	protein_codin	0	0	0	0
ENSG000001	NPHS1	protein_codin	0	0	0	0
ENSG000000	GYG2	protein_codin	0	21.5838974	0	0
ENSG000001	RBCK1	protein_codin	0	0	0	0
ENSG000001	ACAP3	protein_codin	0	0	7.97940524	0
ENSG000001	CHIA	protein_codin	0	0	0	0
ENSG000001	RAB34	protein_codin	0	0	0	0
ENSG000001	CCNB1IP1	protein_codin	0	0	0	0
ENSG000001	YWHAB	protein_codin	45.4480826	65.5143929	0	39.1117995
ENSG000001	CREB3	protein_codin	13.7440046	10.7027846	16.1125784	1.0057197
ENSG000001	SERPINB8	protein_codin	0	0	0	8.05235235
ENSG000001	RNF38	protein_codin	9.98344715	67.4597324	15.2251229	19.9033732
ENSG000001	FCAR	protein_codin	0	0	0	0
ENSG000001	ESR2	protein_codin	0	0	0	0
ENSG000001	DCAF4	protein_codin	3.67168018	0	2.98671912	0
ENSG000001	ABCC11	protein_codin	0	0	0.45934714	0

ENSG000001	SNX16	protein_codin	14.7444964	0	0	0
ENSG000001	RBAK	protein_codin	0	0	17.5247849	0
ENSG000000	HSP90AB1	protein_codin	128.953625	57.0404686	654.641119	254.928616
ENSG000001	OCA2	protein_codin	1.21856658	0	7.79769054	2.68899144
ENSG000001	SIGLEC10	protein_codin	0	0	0	0
ENSG000001	FASTK	protein_codin	0	0	0	1.9437409
ENSG000000	DNMT3B	protein_codin	0	0	0	0
ENSG000000	HMMR	protein_codin	1.36629709	0	0	4.72530016
ENSG000001	NRF1	protein_codin	0	0	0	0
ENSG000001	NF2	protein_codin	0	0	0	0
ENSG000001	CABP2	protein_codin	0	0	0	0
ENSG000001	MBD1	protein_codin	0	16.7117451	0	0
ENSG000001	MATN4	protein_codin	0	0	0	0
ENSG000001	SLC23A1	protein_codin	0	0	0	0
ENSG000001	ZDHHC16	protein_codin	0	0	0	0
ENSG000001	EIF4H	protein_codin	69.4150927	217.96043	324.385082	44.508229
ENSG000000	RASGRP2	protein_codin	0	0	0	0
ENSG000001	PML	protein_codin	0	0	0	0
ENSG000001	SLC13A4	protein_codin	0	0	0	0
ENSG000001	PLTP	protein_codin	0	0	0	0
ENSG000001	CDK2	protein_codin	0	0	0	0
ENSG000001	TMEM25	protein_codin	0	3.59940569	0	0
ENSG000001	PID1	protein_codin	0	21.1960917	2.03866186	0
ENSG000000	BRCA1	protein_codin	8.73284341	21.9122194	78.2434353	8.53473327
ENSG000001	MAL	protein_codin	0	0	0	0
ENSG000001	CAPN10	protein_codin	0.45893386	0	1.1174825	0
ENSG000001	SHMT1	protein_codin	3.44545041	0	12.2927081	4.75799866
ENSG000000	CWF19L1	protein_codin	0	0	5.19441205	0
ENSG000000	SORBS1	protein_codin	1.72790958	0	0.7541122	5.02406593
ENSG000001	IL1RN	protein_codin	0	0	0	0
ENSG000001	C1QTNF1	protein_codin	2.03266936	0	21.1751159	23.9020536
ENSG000001	PPA2	protein_codin	0	0	2.49418274	0
ENSG000001	SLC12A9	protein_codin	0	1.05230949	0	0
ENSG000001	GPX4	protein_codin	0	0	0	9.30684583
ENSG000001	SELENON	protein_codin	0	24.8308555	0	0
ENSG000001	NSD1	protein_codin	0	0	22.0511877	0
ENSG000001	SLC12A6	protein_codin	0	0	0	3.82239725
ENSG000001	DDX21	protein_codin	45.8268893	145.033582	145.414547	54.4268052
ENSG000001	KALRN	protein_codin	10.2758964	0	44.070436	0
ENSG000001	DNAI7	protein_codin	0	0	0	3.02675589
ENSG000000	PLEKHB1	protein_codin	1.20004021	0	0.90826322	0
ENSG000000	BORCS8-MEF	protein_codin	0	0	1.24801239	0
ENSG000001	APP	protein_codin	0	0	15.6398344	0
ENSG000001	SVIP	protein_codin	6.32120379	46.1510192	14.1014337	6.31154194

ENSG000001	VMO1	protein_codin	0.35535422	0	0	0
ENSG000001	TBC1D20	protein_codin	0	0	0	0
ENSG000001	DPAGT1	protein_codin	0	0	0	0
ENSG000001	KANSL3	protein_codin	6.53852684	0	26.732777	19.4295168
ENSG000001	PRKAA1	protein_codin	56.6480878	46.4931585	373.684189	21.9455806
ENSG000001	MAGI2	protein_codin	32.7012935	48.6606329	40.9267788	0
ENSG000001	UCKL1	protein_codin	7.72233252	17.3860954	8.33289834	8.95516482
ENSG000001	REG4	protein_codin	0	0	0	1.89722636
ENSG000001	DUSP19	protein_codin	9.51779124	6.56198678	12.7279334	4.02778621
ENSG000000	HGF	protein_codin	0	0	0	0.70698895
ENSG000001	DMPK	protein_codin	5.19364853	0	10.7515284	8.18864834
ENSG000001	SEPTIN6	protein_codin	26.0178563	81.9575026	60.7218536	18.9221205
ENSG000001	ETFB	protein_codin	0.9213883	9.00726225	7.10845841	3.67971651
ENSG000001	SETDB2	protein_codin	15.6634389	28.2667265	36.4810432	3.14401987
ENSG000001	TRIM4	protein_codin	28.3797915	29.5641006	161.172486	5.87309995
ENSG000001	NOXO1	protein_codin	0	1.40582337	0	0
ENSG000001	NDUFV3	protein_codin	28.9158903	106.691953	68.4952933	24.616008
ENSG000001	TAP1	protein_codin	0	0	0	0
ENSG000001	RHOT1	protein_codin	0	0	0	0
ENSG000001	OSCP1	protein_codin	0.76317155	0	0	0
ENSG000001	SMARCAD1	protein_codin	19.6069065	0	33.5592466	4.13555036
ENSG000001	IMPDH1	protein_codin	0	0	0	0
ENSG000001	ACSL5	protein_codin	9.94021146	0	1.44084095	0
ENSG000001	PKIB	protein_codin	0	0	0	0
ENSG000001	PLA2G4C	protein_codin	0	0	0	0
ENSG000001	EPPIN	protein_codin	0	0	0	1.2536899
ENSG000001	DDX46	protein_codin	79.1176407	221.938527	285.087977	48.7875008
ENSG000001	TMEM25	protein_codin	1.35923369	0	1.79190832	0
ENSG000001	DENND5B	protein_codin	0	3.08613919	1.40746143	0
ENSG000001	GATD1	protein_codin	0	0	0	0
ENSG000001	FBXO2	protein_codin	0	0	0	0
ENSG000001	NAMPT	protein_codin	0	6.855738	11.7721182	0
ENSG000001	DDX55	protein_codin	0	0	0	0.57909001
ENSG000001	AP2A1	protein_codin	18.5365843	81.2536935	30.0404194	16.9178604
ENSG000001	BPIFA1	protein_codin	0	0	0	0
ENSG000001	PPTC7	protein_codin	8.60215744	40.7602298	9.65488329	27.4358151
ENSG000001	SYT5	protein_codin	0	0	0	0
ENSG000002	ZNF805	protein_codin	32.1755411	16.6057235	35.1220775	4.83882127
ENSG000001	KRT72	protein_codin	0.27838328	0	0	0
ENSG000001	RAD17	protein_codin	8.80030529	10.3179274	34.8148931	0
ENSG000001	ITPK1	protein_codin	0	0	0	0
ENSG000001	DUS1L	protein_codin	1.91728999	0	0	0
ENSG000001	HRCT1	protein_codin	1.02144548	0	0	1.25972603
ENSG000001	TRERF1	protein_codin	10.0088004	0	0	6.14953674

ENSG000001	S100A4	protein_codin	0	0	0	0
ENSG000000	HDAC7	protein_codin	15.190876	0	21.5497442	0
ENSG000000	CRKL	protein_codin	62.9789557	82.8861139	324.142028	35.0938579
ENSG000000	ZNF112	protein_codin	9.8961836	0	5.1149108	0
ENSG000000	SLC11A1	protein_codin	0	0	0	0
ENSG000001	KCNMA1	protein_codin	7.40210449	0	0	3.55501918
ENSG000000	GNAS	protein_codin	19.2580401	5.57150294	52.4294109	5.2715494
ENSG000001	SYF2	protein_codin	0.37828161	0	0.7750626	0.55155867
ENSG000001	PRPF40A	protein_codin	2.68146136	0	1.16143165	2.44610336
ENSG000001	NRDE2	protein_codin	16.8346851	64.8251247	32.7333842	20.7862279
ENSG000001	HNF4G	protein_codin	2.14222402	0	8.68286127	1.87032308
ENSG000001	COPS8	protein_codin	7.08508188	18.261781	5.81858015	21.4576865
ENSG000001	RFPL1	protein_codin	0	0	0	0
ENSG000001	ZNF774	protein_codin	1.00587546	0	2.72397681	0.83555058
ENSG000000	LRRFIP2	protein_codin	0	23.7019596	0	1.59385436
ENSG000001	KANK4	protein_codin	1.0110429	0.93886206	0	0
ENSG000001	MUTYH	protein_codin	0	0	0	0
ENSG000002	STMND1	protein_codin	0	0	0.76886956	3.22440008
ENSG000001	KIAA0586	protein_codin	2.01271181	0	14.5183368	0
ENSG000001	MYPN	protein_codin	17.2341779	6.34926079	0	0
ENSG000001	ARNT	protein_codin	10.1456887	62.4930366	11.338303	0
ENSG000000	CD209	protein_codin	0	0	0	0
ENSG000001	APBB3	protein_codin	0	0	0	0
ENSG000000	MEF2A	protein_codin	0	12.4239339	22.624062	20.4332545
ENSG000000	NLRP1	protein_codin	0	0	0	0
ENSG000001	LRP8	protein_codin	0	0	3.92923467	0
ENSG000001	SEPTIN6	protein_codin	0	0	0	0
ENSG000000	RNH1	protein_codin	0	0	0	0
ENSG000001	C11orf54	protein_codin	2.17268093	20.1719047	9.26379521	3.01699721
ENSG000001	FAM78B	protein_codin	0	4.77870835	0	4.67362268
ENSG000001	ELP5	protein_codin	0	0	0	0
ENSG000001	LRRIQ3	protein_codin	0	0	0	0
ENSG000002	FBXW10B	protein_codin	0	0	0	0.88607951
ENSG000001	ZYX	protein_codin	0	0	0	0
ENSG000001	NFAT5	protein_codin	0	0	0	0
ENSG000000	TP73	protein_codin	0	0	0	0
ENSG000001	TTC8	protein_codin	0	0	0	0
ENSG000001	CEP63	protein_codin	0	0	0	3.79425724
ENSG000001	ATG7	protein_codin	0	0	0	0
ENSG000001	TCF4	protein_codin	0	29.5629593	0	27.5002152
ENSG000001	IRAG2	protein_codin	0	0	0	0
ENSG000001	IPO4	protein_codin	9.73810548	0	106.319403	0
ENSG000000	BAX	protein_codin	0	0	0	0
ENSG000001	VPS72	protein_codin	2.74280258	0	0	3.42226036

ENSG000001	WDR91	protein_codin	0	0	18.8469064	0
ENSG000001	SPATA31G1	protein_codin	0	1.2927451	0	1.32887104
ENSG000001	RBM3	protein_codin	16.6847345	49.8804341	22.4387583	32.6296946
ENSG000001	ADAMTSL2	protein_codin	0	0	0	0
ENSG000001	KANK1	protein_codin	10.523392	37.6603196	26.7726523	4.8431133
ENSG000001	DDRGK1	protein_codin	3.58498382	13.8063085	22.6369806	2.38610142
ENSG000001	MAK	protein_codin	0	0	0	0
ENSG000001	RTN3	protein_codin	0	61.7945557	0	0
ENSG000001	MAP2K5	protein_codin	0	0	0	5.10747287
ENSG000000	TRPM3	protein_codin	1.42899857	6.59795229	1.95252332	6.81683728
ENSG000001	MFF	protein_codin	4.91799373	9.47843189	12.6517184	5.30124291
ENSG000001	VPS54	protein_codin	4.033639	2.61778181	24.5815241	6.61554621
ENSG000001	RPE	protein_codin	21.8672774	21.2251428	0	4.28561421
ENSG000001	PDGFA	protein_codin	0	3.0642936	0	0
ENSG000002	H2AB2	protein_codin	0	0	0.43900435	0
ENSG000000	ARHGEF1	protein_codin	17.3016512	210.31031	0	0
ENSG000001	SCN8A	protein_codin	0	0	0	0
ENSG000001	SMOC2	protein_codin	10.6119503	19.9334054	2.27011028	1.40182571
ENSG000001	CAPN9	protein_codin	0	0	0	0
ENSG000001	MBP	protein_codin	0	0	0	0
ENSG000001	RMDN2	protein_codin	13.7402124	20.5394828	38.350535	4.41038981
ENSG000001	ANXA6	protein_codin	0	0	2.46809264	12.8264531
ENSG000001	COL13A1	protein_codin	9.18085117	0	0	19.0030504
ENSG000001	ADRA1A	protein_codin	0	0	1.15446192	0
ENSG000001	FILIP1L	protein_codin	4.28431542	0	0	0
ENSG000001	TP53I11	protein_codin	0	0	0	1.12109899
ENSG000001	CKAP5	protein_codin	0	0	0	0
ENSG000001	TEDC1	protein_codin	0	11.2461101	0	0
ENSG000001	AK5	protein_codin	14.5213507	40.9648184	0	21.7473773
ENSG000001	MAP7	protein_codin	0	0	0	0
ENSG000001	TWIST1	protein_codin	2.75989458	0	2.65725621	2.43025754
ENSG000001	MED23	protein_codin	0	0	3.35131158	0
ENSG000001	ITPR1	protein_codin	0	0	0	0
ENSG000001	STYX	protein_codin	7.90276578	101.364273	34.4304446	19.2252668
ENSG000001	LBHD1	protein_codin	0.88446719	11.8314434	11.7694408	0
ENSG000001	PLEC	protein_codin	75.3013637	143.29776	160.082712	12.6742235
ENSG000001	ZBTB45	protein_codin	2.93177545	0	0	0
ENSG000001	CYP3A4	protein_codin	0	0	0	0
ENSG000000	ZNF195	protein_codin	24.9228148	27.5199724	55.2046085	4.94848348
ENSG000000	TP63	protein_codin	0	0	0	1.29729517
ENSG000001	TYW5	protein_codin	5.17306468	2.04762649	0	6.71606652
ENSG000000	MLXIPL	protein_codin	0	0	0	0
ENSG000001	SLC37A2	protein_codin	0	29.8126042	0	0
ENSG000000	THRAP3	protein_codin	85.7090483	107.563476	85.0009185	25.1988862

ENSG000000	ERO1B	protein_codin	0	5.24210137	4.89040632	3.38845282
ENSG000000	CRISP3	protein_codin	0	0	0	0
ENSG000001	HKDC1	protein_codin	0.30356276	2.02276124	0	1.69626001
ENSG000001	RPS6KL1	protein_codin	0	4.33138469	0.60022127	0
ENSG000001	CLEC4C	protein_codin	0	0	0	0
ENSG000001	TRDMT1	protein_codin	11.5431026	0	1.89699123	4.43444175
ENSG000001	HMGA2	protein_codin	1.36872678	0	0	0
ENSG000001	OCA2	protein_codin	0	0	0	0
ENSG000001	STK31	protein_codin	0	0	0	0
ENSG000001	WNK3	protein_codin	0	0	0	0
ENSG000001	NUTM2G	protein_codin	0	1.42956846	1.66081946	4.88590491
ENSG000000	FYN	protein_codin	32.7561798	63.9451421	35.9663563	33.2205845
ENSG000001	ACSL5	protein_codin	0	0	0	0
ENSG000001	ZNF44	protein_codin	0	0	0	0.3746076
ENSG000000	LMO3	protein_codin	0	0	0	0
ENSG000001	DIDO1	protein_codin	9.37341437	0	0	0
ENSG000001	ELOVL2	protein_codin	3.40198509	8.23992623	17.3205598	2.34449349
ENSG000001	HNRNPA2B1	protein_codin	0	0	0	0
ENSG000001	BMF	protein_codin	11.0547168	62.5366275	38.3159585	7.91242728
ENSG000001	IGSF11	protein_codin	1.20981835	0	0	2.28357178
ENSG000001	SYNE1	protein_codin	0	0	0	0
ENSG000001	AKAP12	protein_codin	325.25533	40.3613649	250.718047	72.0495856
ENSG000001	CALML4	protein_codin	0	0.85769737	0.54726198	0.69213564
ENSG000001	HCFC1R1	protein_codin	0	0	0	0
ENSG000001	DNHD1	protein_codin	1.9582981	12.6752934	14.4019745	1.7429827
ENSG000001	YY1AP1	protein_codin	0	0	0	0
ENSG000001	PES1	protein_codin	14.1248599	17.1922127	97.2139941	6.84675564
ENSG000000	HNRNPH3	protein_codin	34.8760712	25.9617415	112.493438	0
ENSG000001	ZNF589	protein_codin	15.132496	13.8100988	21.6900694	5.87864739
ENSG000001	ACAP3	protein_codin	4.88580163	50.9360483	27.6599224	6.29056441
ENSG000001	ENSA	protein_codin	0	0	1.01027165	0
ENSG000001	STK26	protein_codin	0	19.4387482	0	0
ENSG000001	HEY1	protein_codin	8.39823814	0	11.2342546	0
ENSG000001	SND1	protein_codin	92.1561216	204.749391	369.890764	86.4787968
ENSG000002	UGT1A9	protein_codin	0.34175517	1.5159404	0.55660677	1.47978751
ENSG000001	SEMA6D	protein_codin	0	0	0	0
ENSG000001	TTC3	protein_codin	39.2344518	138.274009	0	0
ENSG000001	GPSM1	protein_codin	0	3.89511872	35.2278467	0
ENSG000001	CCDC81	protein_codin	0	0	0	0
ENSG000001	PCDHB11	protein_codin	1.88644735	26.0883326	20.8597553	7.92839185
ENSG000000	CREM	protein_codin	0	0	0	0
ENSG000001	CRYBA4	protein_codin	0	1.14394312	0	0
ENSG000001	PGAP1	protein_codin	14.1324104	0	19.4913707	3.22351696
ENSG000001	PLAGL1	protein_codin	0	0	0	0

ENSG000001(	GPT	protein_codin	0	0	0.65270847	0
ENSG000000!	SLC22A17	protein_codin	0	0	0	4.1823503
ENSG000001	ALDH9A1	protein_codin	26.1046418	31.2461096	45.0443832	19.4687931
ENSG000001	WLS	protein_codin	3.48169383	3.89273842	8.63715271	4.3943172
ENSG000001	FN1	protein_codin	574.681357	682.186077	835.450107	0
ENSG000001	ADGRG2	protein_codin	0	0	0	0
ENSG000001(	BPNT1	protein_codin	0	0	0	0
ENSG000001	RPL15	protein_codin	2.33486316	15.5691597	4.74952828	3.61215302
ENSG000001!	FAR1	protein_codin	17.0313479	15.7548949	0	10.1442619
ENSG000001	NKX2-1	protein_codin	0	0	0	0
ENSG000001	GABPA	protein_codin	43.3801269	29.8783137	88.3128634	33.3518048
ENSG000000	CYP3A43	protein_codin	0	0	0	0
ENSG000000	NRDC	protein_codin	0	0	0	0
ENSG000001	SERF1A	protein_codin	0	27.3831981	32.4202668	0
ENSG000001	EPHA5	protein_codin	0	0	14.5434205	0
ENSG000000	FMO1	protein_codin	0	0	0	0
ENSG000001!	ARMCX4	protein_codin	6.63714117	0	7.40043147	0
ENSG000001	CD46	protein_codin	0	59.336004	0	0
ENSG000002	TRIM6-TRIM3	protein_codin	2.73014102	0	0	3.80358601
ENSG000000	MOV10L1	protein_codin	0	0	0	1.25909463
ENSG000001	KLRF1	protein_codin	0	0	0	0
ENSG000000(	AK2	protein_codin	5.75252322	0	0	0
ENSG000002	NAA80	protein_codin	0	0	0	0
ENSG000001(	AXIN1	protein_codin	0	21.8123315	21.7236032	8.76137072
ENSG000001	RAD17	protein_codin	0	0	0	0
ENSG000000	RABL2B	protein_codin	0	0	0	0
ENSG000001(	BMP1	protein_codin	0	0	0	0
ENSG000000(	CTSA	protein_codin	19.4764644	0	4.44125592	0
ENSG000001(	TMEM205	protein_codin	1.45859532	0	1.68611426	0
ENSG000001	SLC25A26	protein_codin	0	0	0	0
ENSG000001(	SLC39A13	protein_codin	25.8044672	19.9707481	36.7465308	7.38267749
ENSG000001	SLC38A6	protein_codin	2.38496522	9.06642681	2.89645794	0
ENSG000001(	EGFLAM	protein_codin	0	0	0	0
ENSG000001(	VKORC1	protein_codin	0	0	0	0
ENSG000000	CELF2	protein_codin	0	18.4554639	0	2.62385781
ENSG000001(	LSR	protein_codin	0	0	0	0
ENSG000000	SMG6	protein_codin	0	0	0	0
ENSG000001	PER1	protein_codin	0	1.82361392	9.56552811	0
ENSG000001!	TMEM207	protein_codin	0.30354702	0	0	0.89813787
ENSG000001	MIA3	protein_codin	4.16794829	14.9911478	4.28716022	16.609271
ENSG000001	ANAPC13	protein_codin	10.4023457	32.0161844	50.1228667	7.31637438
ENSG000000!	WAC	protein_codin	2.07053713	66.7070522	0	14.5596716
ENSG000001(	SCARA5	protein_codin	20.8317949	0	0	0
ENSG000001	SUV39H2	protein_codin	0	7.9778332	33.5101424	0

ENSG000001	PNRC1	protein_codin	2.77481192	0	1.24252716	4.8802203
ENSG000001	OR5K4	protein_codin	0.30941337	0	0	1.05177158
ENSG000001	PITX2	protein_codin	0.69485463	0	0	0.84889596
ENSG000001	LUC7L2	protein_codin	17.4204683	84.8167237	30.4861347	10.9292328
ENSG000001	BPIFA2	protein_codin	0	0	0	0
ENSG000001	PIP4K2C	protein_codin	50.9700646	0	4.49654998	10.4454953
ENSG000001	PLSCR4	protein_codin	15.6840039	0	21.7029677	24.0585389
ENSG000001	FMOD	protein_codin	45.5711003	110.966784	0	15.9385468
ENSG000001	ATG7	protein_codin	11.1656034	0	9.71348003	0
ENSG000001	ZNF649	protein_codin	7.28951377	17.6773651	8.77760105	0
ENSG000001	PLEC	protein_codin	61.3359163	72.6206148	88.9158517	69.4958205
ENSG000001	ZNF43	protein_codin	1.00402469	34.1986796	0	11.5944455
ENSG000001	PDE5A	protein_codin	36.7121046	39.4673487	245.312027	12.886429
ENSG000001	IL31RA	protein_codin	0	0	0	0
ENSG000001	MYL4	protein_codin	0	0	0	0
ENSG000001	ACAP3	protein_codin	3.69612561	0	3.58776717	0
ENSG000000	KIF16B	protein_codin	41.0746407	125.526808	55.0691047	27.5742429
ENSG000001	FCN3	protein_codin	0	0	0	0
ENSG000000	CACNA1G	protein_codin	8.31069816	0	0	3.36521566
ENSG000001	ENTPD6	protein_codin	0.74590543	0	0	0
ENSG000001	DDX4	protein_codin	0	0	0	0
ENSG000001	LARGE1	protein_codin	4.22090124	0	6.58710828	0
ENSG000001	PTPA	protein_codin	0	0	0	0
ENSG000001	BRDT	protein_codin	0	0	0	0
ENSG000001	SEMA4A	protein_codin	0	10.3118806	0	0
ENSG000001	H2BW2	protein_codin	0	0	0	0.47351288
ENSG000001	GLRA2	protein_codin	0	0	0	0
ENSG000001	BANP	protein_codin	0	0	0	0
ENSG000001	INCA1	protein_codin	0	0	0	0
ENSG000000	BCL2L13	protein_codin	18.0141714	0	0	3.24995655
ENSG000001	NET1	protein_codin	12.4528784	25.1841052	52.9614207	4.71261951
ENSG000001	ASGR2	protein_codin	1.42538343	9.02716728	0	2.2748814
ENSG000001	DNAJC4	protein_codin	0	0	0.37517829	0
ENSG000001	ATRNL1	protein_codin	2.1268171	0	8.84338442	5.07496606
ENSG000001	TSGA10	protein_codin	0	0	10.2133309	0
ENSG000001	H4C11	protein_codin	0	39.0145161	21.7678207	0
ENSG000001	NUMB	protein_codin	0	0	0	0
ENSG000001	NEIL1	protein_codin	0	0	0	0.41401053
ENSG000001	EIF5A	protein_codin	0	0	0.51136198	0.36577862
ENSG000001	BBS9	protein_codin	7.03244289	0	0	0
ENSG000001	HTT	protein_codin	23.7945274	126.386066	115.44567	0
ENSG000001	KCTD6	protein_codin	0.82605914	8.64791159	0	0
ENSG000001	PITX2	protein_codin	0	6.66810609	1.88756649	0
ENSG000001	KLHL28	protein_codin	0	6.89732632	1.92604396	1.40897635

ENSG000000	EIF3I	protein_codin	0	0	0	0
ENSG000002	STIMATE	protein_codin	16.2058244	49.7750438	16.7046876	8.10360209
ENSG000001	C5AR1	protein_codin	3.67323485	1.69111298	1.8186678	5.77965967
ENSG000001	SRGAP1	protein_codin	5.66847088	42.1005675	7.4391472	38.7919319
ENSG000001	GPAA1	protein_codin	0	0	0	0
ENSG000002	ZNF736	protein_codin	6.18898124	0	28.9628857	2.86860406
ENSG000000	ENTPD2	protein_codin	0	0.93206205	0	0
ENSG000001	GPBP1L1	protein_codin	23.8207237	0	117.351453	0
ENSG000001	BLM	protein_codin	0	0	0	0
ENSG000001	ZNF565	protein_codin	0	0	0	0
ENSG000000	LIMS2	protein_codin	0	4.83637034	0	0
ENSG000001	ITSN2	protein_codin	0	0	0	0
ENSG000000	BTBD7	protein_codin	16.05309	80.6685277	16.1699941	0
ENSG000001	SCN8A	protein_codin	0	0	17.4582097	0
ENSG000000	GPM6B	protein_codin	8.44130525	0	0	0
ENSG000002	MTFP1	protein_codin	0	0	0	1.1148095
ENSG000001	ZNF160	protein_codin	0	0	1.01209937	0.53054521
ENSG000001	CD99L2	protein_codin	0.97824806	0	0	0
ENSG000001	MRPL52	protein_codin	1.27935289	3.3158897	2.45404531	1.79472699
ENSG000001	ITGB2	protein_codin	0	8.32429114	0	4.21142809
ENSG000001	VPS13B	protein_codin	0	0	1.16687283	0
ENSG000000	TBL1Y	protein_codin	0	0	0	0
ENSG000000	BCAT1	protein_codin	0	0	0	1.13119602
ENSG000000	LTK	protein_codin	0	6.52857983	0	0
ENSG000001	EYA4	protein_codin	14.4249485	13.7635232	0.64600785	5.33449883
ENSG000001	BEGAIN	protein_codin	0	0	0	0
ENSG000001	ATG10	protein_codin	0	1.63795536	0	0
ENSG000001	SPART	protein_codin	0	0	0	0
ENSG000001	C6orf89	protein_codin	7.68050086	2.87774001	10.0949109	1.7527936
ENSG000001	MSRB3	protein_codin	27.6150711	16.6034594	34.0987013	8.8313923
ENSG000001	KCNQ5	protein_codin	3.47977454	0	17.9447058	0
ENSG000001	SEC31A	protein_codin	0.67382841	0	0	0
ENSG000001	AQP10	protein_codin	0	0	0	0
ENSG000001	ZNF607	protein_codin	0	0	0	0
ENSG000001	EOLA2	protein_codin	0	0	0	0
ENSG000001	POLR3H	protein_codin	0	33.6889869	0	8.427343
ENSG000001	FOXN4	protein_codin	0	0	0	0
ENSG000000	CECR2	protein_codin	0	0	0	0
ENSG000001	MAGEA11	protein_codin	0	0	39.430317	0
ENSG000000	ATP6V1H	protein_codin	0	0	0	0
ENSG000001	FAM72B	protein_codin	0	0	5.17525813	0
ENSG000001	HMBOX1	protein_codin	0	19.2770212	0	1.9927255
ENSG000001	PTPRD	protein_codin	0	0	0	9.78485252
ENSG000001	TBC1D9B	protein_codin	92.6900841	264.895532	170.38049	53.4864568

ENSG000001	OCLN	protein_codin	0	0	4.53962795	0
ENSG000001	SYNCRIP	protein_codin	62.3959256	59.2121215	56.7394203	49.1548104
ENSG000000	PAX2	protein_codin	0	0	0	0
ENSG000001	NUP98	protein_codin	1.5574257	0	0	0
ENSG000001	AP3M1	protein_codin	6.90242204	16.5074393	14.1753864	9.76295419
ENSG000001	KEL	protein_codin	0	2.37235271	0.79619878	0
ENSG000001	OR5H2	protein_codin	0.2818918	5.30124715	0	1.34739756
ENSG000000	FAS	protein_codin	0	38.9758747	7.22651979	0
ENSG000001	MINK1	protein_codin	12.1711229	154.812371	0	24.9987083
ENSG000001	OR2L5	protein_codin	0	0	0.80687879	0
ENSG000000	MSR1	protein_codin	0	30.4937217	0	0
ENSG000001	AK9	protein_codin	0	0	0	0
ENSG000001	APCDD1	protein_codin	47.1177231	14.5870206	112.315118	16.9405882
ENSG000001	EYA4	protein_codin	6.48491723	0	0	0
ENSG000001	ANK3	protein_codin	3.65619178	0	0	0
ENSG000001	GRM1	protein_codin	0	0	0	0
ENSG000001	TDRD7	protein_codin	11.6402301	45.6389002	9.66060764	12.9854601
ENSG000001	DNAJA3	protein_codin	13.66209	10.378895	56.4708593	6.60911977
ENSG000001	GMPR2	protein_codin	0	0	0	0
ENSG000001	LINGO1	protein_codin	6.9503319	9.94535197	12.4893718	7.15478927
ENSG000001	NPHP1	protein_codin	0	0	1.06727747	0
ENSG000001	ANO1	protein_codin	14.5427304	45.812506	8.61352909	14.0851173
ENSG000001	DNM3	protein_codin	0	0	0	0
ENSG000001	EBF3	protein_codin	14.8605628	256.17136	0	10.9259773
ENSG000001	GIT2	protein_codin	31.9624541	159.82233	32.2053673	13.8390756
ENSG000001	CHCHD7	protein_codin	2.8295597	23.967649	0	0
ENSG000001	THSD4	protein_codin	8.70225542	28.9340836	0	0
ENSG000001	MORN3	protein_codin	0	1.36824674	1.47216669	2.78087611
ENSG000001	WARS1	protein_codin	0	0	0	0
ENSG000001	ZFYVE19	protein_codin	0	0	0	0
ENSG000001	ITGB1BP1	protein_codin	0	0	0	0
ENSG000000	MYH7	protein_codin	341.98548	0	0	0
ENSG000001	CD47	protein_codin	35.2415824	39.1771644	64.1058877	14.9235083
ENSG000001	MIER1	protein_codin	64.8811388	108.835712	61.2084128	35.0777095
ENSG000001	PGBD2	protein_codin	2.55951972	3.07761719	0.91425958	0
ENSG000001	SHTN1	protein_codin	0	0	4.94473182	5.07701333
ENSG000001	ZCCHC10	protein_codin	0.829316	21.8700444	0	0
ENSG000001	SYT17	protein_codin	0.71566663	1.14979069	20.9111247	0
ENSG000001	SCAMP3	protein_codin	0	0	0	0
ENSG000001	SPATA22	protein_codin	1.10304979	0	0	0
ENSG000001	BOC	protein_codin	0	0	0	3.95910965
ENSG000001	RNF220	protein_codin	0	0	0	0
ENSG000001	GABRA5	protein_codin	0	0	2.40663461	1.32847264
ENSG000001	SUSD1	protein_codin	0	0	0	0

ENSG000001	MFSD12	protein_codin	2.50435665	15.8947588	7.88957874	7.0581054
ENSG000001	CCDC69	protein_codin	14.3774037	62.5648871	0	7.02666318
ENSG000001	GFRA1	protein_codin	0	0	0	0
ENSG000001	SAP30BP	protein_codin	0	0	0	0
ENSG000001	EFEMP1	protein_codin	22.1594236	22.0724087	342.564172	14.0079052
ENSG000000	ARID4A	protein_codin	85.3917082	56.4702023	186.839959	61.9796237
ENSG000001	CSF2RA	protein_codin	0	0	0	0
ENSG000001	MFSD10	protein_codin	0	0	10.0660395	0
ENSG000001	TMEM116	protein_codin	0	0	0	0
ENSG000000	ERC1	protein_codin	0	0	0	0
ENSG000000	NUDCD3	protein_codin	37.5283525	64.2360654	80.8012911	21.3517664
ENSG000000	LAMC3	protein_codin	0	0	0.85221441	0
ENSG000001	KRTAP13-1	protein_codin	0	0	0	1.12072607
ENSG000001	MBNL1	protein_codin	0	0	0	0
ENSG000002	ZNF512	protein_codin	0	0	0	0
ENSG000001	P3H4	protein_codin	0.76793528	18.6282825	4.07762643	1.60667532
ENSG000002	NFAM1	protein_codin	0	0	0	0
ENSG000001	GRK6	protein_codin	2.45041824	29.1315172	0	0
ENSG000001	RBPJ	protein_codin	55.6035454	113.295014	130.035799	27.3689476
ENSG000001	CLN3	protein_codin	0	0	0	0
ENSG000001	RIC8B	protein_codin	0	30.654899	13.0471263	0
ENSG000001	COL18A1	protein_codin	1.70843888	0	0	0
ENSG000001	RYR1	protein_codin	0	0	0	0
ENSG000001	FADS2	protein_codin	0	0	0.81173602	0
ENSG000001	VANGL1	protein_codin	13.2548441	12.9244634	16.3401841	12.5486291
ENSG000001	LTO1	protein_codin	0	0	0	0
ENSG000001	TXNL4A	protein_codin	0	8.8100643	0	0
ENSG000001	PLEKHA4	protein_codin	0	3.4959104	0	1.68160466
ENSG000001	LMX1B	protein_codin	0	0	0	0
ENSG000001	MUTYH	protein_codin	0	0	0	0
ENSG000001	YY1AP1	protein_codin	0	0	0	0
ENSG000001	COMMD9	protein_codin	0.63855145	3.14709033	1.95985083	6.40346848
ENSG000002	PDE4C	protein_codin	0	0	0	0
ENSG000001	ZNF493	protein_codin	0	0	0	12.1799092
ENSG000001	CARD11	protein_codin	0	0	0.34300431	0
ENSG000001	RGS6	protein_codin	0	1.36062319	14.1347934	0
ENSG000000	OPHN1	protein_codin	16.2557636	35.6392657	27.6295071	50.3843457
ENSG000001	C2CD4A	protein_codin	0	0	0	0.48604351
ENSG000001	FCAR	protein_codin	0	0	0	3.39694791
ENSG000000	DPF1	protein_codin	0	0	0	0
ENSG000001	DHCR7	protein_codin	0.75166445	0	0	0
ENSG000001	TBCD	protein_codin	0	0	0	0
ENSG000001	SPNS3	protein_codin	0	0	0	0
ENSG000001	ZNF536	protein_codin	0	0	0	0

ENSG000001!	TRRAP	protein_codin	25.8413168	0	257.176003	0
ENSG000001	NHERF4	protein_codin	0	0	0	0
ENSG000001	TPRA1	protein_codin	6.08193385	9.63980497	0	8.61335624
ENSG000001	HTR3A	protein_codin	0	0	0	0
ENSG000001!	ZNF140	protein_codin	12.0846909	0	8.31232922	0
ENSG000001	CLK2	protein_codin	0	11.016271	0	0
ENSG000001!	NEK5	protein_codin	1.30397284	5.21305254	0.98239507	2.94727911
ENSG000001!	PDLIM7	protein_codin	4.41532817	8.36671677	5.91134065	0.5402374
ENSG000001	SLC26A8	protein_codin	0	0	0	0
ENSG000001	CFAP70	protein_codin	0	0	5.73065744	0
ENSG000000	SYNJ2	protein_codin	16.0927152	28.8952061	51.9569695	0
ENSG000001	TRIM17	protein_codin	0	0	0.97715238	0
ENSG000001	COX7B2	protein_codin	0	1.95169269	0	0.9915979
ENSG000001!	ANKRD35	protein_codin	0	0	0	0
ENSG000001!	DEFB107B	protein_codin	0	0	0	0
ENSG000001	STIP1	protein_codin	0.4913577	0	2.4044588	0.59660691
ENSG000001	IFFO2	protein_codin	0	0	0	0
ENSG000001	MORC4	protein_codin	44.4783382	53.4909172	18.387095	14.2543198
ENSG000001!	KRT39	protein_codin	0	0	0	0
ENSG000000	FBXL3	protein_codin	88.468891	157.804083	91.5140606	47.6534125
ENSG000001	UBE2H	protein_codin	59.2452854	167.047099	226.5635	42.9401883
ENSG000001	TLR4	protein_codin	19.5221336	114.968104	7.67007066	34.4534792
ENSG000001	RAB11FIP2	protein_codin	0	59.2583756	0	0
ENSG000001	TASOR	protein_codin	0	6.34580965	51.5403115	0
ENSG000001!	MRTFA	protein_codin	11.0985817	88.3812538	0	16.2032461
ENSG000001	OPRL1	protein_codin	1.17102893	5.94458248	2.87133735	1.22272367
ENSG000001	ZNF397	protein_codin	1.46694932	0	4.67853274	0.94919648
ENSG000001	HOXA1	protein_codin	0	4.42173166	1.72400524	0
ENSG000001	SORBS2	protein_codin	0	0	0	0
ENSG000001	PMFBP1	protein_codin	0	0	1.17306658	0
ENSG000000	RBFOX1	protein_codin	0	0	0	0
ENSG000000	ADAM11	protein_codin	0	1.74943084	2.86745501	0
ENSG000001	XIAP	protein_codin	1.28588358	8.09086814	0	0
ENSG000000	PHACTR3	protein_codin	0	0	0	0
ENSG000002	ZNF253	protein_codin	2.35889128	0	8.24637461	2.26364423
ENSG000001	VAT1	protein_codin	13.3073468	66.7001875	28.2098096	21.8988864
ENSG000001	TMEM18	protein_codin	1.11765752	0	0	3.06290419
ENSG000001	KCNIP2	protein_codin	0	0	0	0
ENSG000001	PLEKHA7	protein_codin	5.37468947	11.210233	0	0
ENSG000000	YTHDC1	protein_codin	112.465001	599.551469	231.3301	131.194971
ENSG000000	DNM2	protein_codin	0	0	0	0
ENSG000001	MBD6	protein_codin	50.8327799	56.9409841	27.2623695	14.7445157
ENSG000001	RSL1D1	protein_codin	18.4874371	36.7475222	89.6517175	9.46535894
ENSG000001	PRMT2	protein_codin	37.1837079	96.0535946	89.3262467	20.5490541

ENSG000001:	FNBP1	protein_codin	0	178.225501	16.0697252	0
ENSG000001:	STRN3	protein_codin	25.9359627	61.8068206	55.8680151	15.5147363
ENSG000001:	ZNF44	protein_codin	0	8.89769333	11.6994094	9.28378746
ENSG000001:	IFT70A	protein_codin	4.97730359	15.7360744	18.6204835	9.4428378
ENSG000001	CLEC12A	protein_codin	2.55516631	0	0	1.76733299
ENSG000001:	COA4	protein_codin	3.94573665	0	24.026174	2.22576774
ENSG000001:	SFXN4	protein_codin	2.17857846	0	4.79691707	0
ENSG000001	ADAMTS13	protein_codin	0	0	0	0
ENSG000001:	UBAC2	protein_codin	0	37.7497668	0	0
ENSG000001:	PCNX3	protein_codin	13.4712833	65.0180853	40.3011939	15.3762849
ENSG000000:	TBC1D22A	protein_codin	0	0	6.32851682	0
ENSG000001:	PLGLB1	protein_codin	5.92522706	14.9017618	0	2.13332816
ENSG000001	RET	protein_codin	0	4.49723394	0	0
ENSG000001:	TNFRSF14	protein_codin	2.73733125	61.7168336	0	11.9067552
ENSG000001.	TCF7L2	protein_codin	0	0	0.91363563	0
ENSG000001	NAALADL1	protein_codin	0	0	0	0
ENSG000001.	TRPM8	protein_codin	0	0	0	0
ENSG000001:	THOC2	protein_codin	139.561212	333.704026	62.001134	86.5279733
ENSG000001:	AIDA	protein_codin	0	56.4166937	0	0
ENSG000001:	RPL23A	protein_codin	176.452261	0	2221.93052	0
ENSG000000	ADD2	protein_codin	0	0	1.54814463	0
ENSG000000:	FAS	protein_codin	0	0	0	0
ENSG000001:	NDUFA5	protein_codin	0.67326354	3.75335569	5.9286459	6.88522218
ENSG000001	GBP4	protein_codin	19.7571717	294.773917	1.86267623	28.4982432
ENSG000001:	ZC3H7A	protein_codin	36.54781	37.7769404	42.2152286	29.4038463
ENSG000001:	PRPF39	protein_codin	0	0	10.4966806	2.35018489
ENSG000001:	VPS13A	protein_codin	1.64125216	0	12.3136317	0
ENSG000001:	PER2	protein_codin	0.24383008	0	0	0
ENSG000001:	ARHGEF4	protein_codin	0	2.52743091	0	0
ENSG000001:	ZNF384	protein_codin	9.06075655	0	28.2251819	5.42858151
ENSG000001	SLC17A5	protein_codin	16.9525981	23.6156228	9.9220424	7.66477039
ENSG000001	MLH3	protein_codin	5.70995539	42.127839	26.2653652	9.54829263
ENSG000001:	TRPC4	protein_codin	0	0	7.76979159	0
ENSG000000.	STAU2	protein_codin	14.9963548	19.8793484	6.84059791	0
ENSG000001:	OR11L1	protein_codin	0	0	0	0.41951934
ENSG000001	LRRC20	protein_codin	1.50852859	0	0	2.43855075
ENSG000001:	MXRA7	protein_codin	10.5304818	21.7515823	4.9716588	9.96312656
ENSG000001:	COMMD6	protein_codin	13.112552	32.6734411	7.52806054	5.17845446
ENSG000001	UBE2I	protein_codin	0	0	0	0
ENSG000001:	CSF2RA	protein_codin	0	19.7959569	0	0
ENSG000001:	PDHA1	protein_codin	0	4.48361361	0	0
ENSG000001:	TUT4	protein_codin	0.6859883	0	5.57585409	0
ENSG000001.	ART3	protein_codin	0	0	0	0
ENSG000001:	SERPINA1	protein_codin	0	2.5466462	0	0

ENSG000000	SREBF1	protein_codin	7.79659927	7.83638189	0	0
ENSG000001	CLEC9A	protein_codin	0.49231221	1.45069953	0	0.86786895
ENSG000001	ZNF483	protein_codin	1.75141112	4.7290706	5.08455525	2.26563996
ENSG000001	MIB2	protein_codin	0	0	14.018658	5.58447533
ENSG000001	FAM227A	protein_codin	0	0	0	0
ENSG000002	ERCC6	protein_codin	0	0	0	0
ENSG000001	HNRNPAB	protein_codin	0	0	61.7745599	0
ENSG000001	MMS19	protein_codin	6.42961507	0	0	6.71256318
ENSG000001	PDLIM7	protein_codin	18.2611177	0	97.6643077	0
ENSG000000	ADD1	protein_codin	0	0	0	0
ENSG000001	LIPF	protein_codin	0	0	0	0
ENSG000001	C1D	protein_codin	0	0	1.41072484	0
ENSG000000	PCBP4	protein_codin	0	0	0	0
ENSG000001	CAB39L	protein_codin	1.76275467	0	0	0
ENSG000001	DBI	protein_codin	126.722847	169.102335	301.865206	51.0577698
ENSG000001	WWOX	protein_codin	0	0	2.54182894	0
ENSG000001	SVIL	protein_codin	0	0	0	0
ENSG000001	WDR88	protein_codin	0	0	0	0
ENSG000001	STK31	protein_codin	0	0	0	0
ENSG000001	MLF1	protein_codin	4.73372398	0	22.7966733	8.36071999
ENSG000001	GPR160	protein_codin	1.90949593	1.64614716	2.55228082	1.81661217
ENSG000001	ZNF507	protein_codin	0	36.90387	0	1.27981005
ENSG000001	PLS3	protein_codin	161.556791	134.317248	59.1240273	96.0037057
ENSG000001	GTF2E2	protein_codin	13.9507862	35.5131622	17.6414493	6.4859412
ENSG000001	NLGN4Y	protein_codin	0	0	0	0.44261629
ENSG000002	HP	protein_codin	0	0	0	0
ENSG000000	DTNBP1	protein_codin	6.12258424	0	0	0
ENSG000001	LCK	protein_codin	0	0	0	0
ENSG000001	PRELID3B	protein_codin	13.5839065	11.8971577	18.2987257	10.2790146
ENSG000001	ATP5MC1	protein_codin	4.66966154	8.69336063	10.6389334	6.22832016
ENSG000001	SLC25A46	protein_codin	10.2326746	18.7681689	7.45027909	5.17022622
ENSG000001	SH3PXD2A	protein_codin	17.0597791	118.452122	52.4946615	0
ENSG000001	TRIM4	protein_codin	0	27.6078686	0	6.03633684
ENSG000001	TCAF1	protein_codin	0	0	0	0
ENSG000000	MFSD11	protein_codin	0	0	0	0
ENSG000001	ADAM15	protein_codin	0	0	0	0
ENSG000001	STX16	protein_codin	0	14.9750605	16.5499365	22.9965219
ENSG000001	GRK6	protein_codin	2.93933243	20.6640918	0	3.42538746
ENSG000001	PORCN	protein_codin	0	0	0	0
ENSG000001	TAT	protein_codin	0	0	0	0.85866162
ENSG000001	PPIA	protein_codin	117.24587	127.846963	549.736177	66.7003773
ENSG000001	ZGPAT	protein_codin	0	0	12.5369262	15.4734887
ENSG000001	ZMYND8	protein_codin	0	0	0	0
ENSG000002	GABBR1	protein_codin	0	107.894881	1.53144017	4.03446663

ENSG000001!	MIER1	protein_codin	1.99522436	0	2.25085566	0
ENSG000001	FRMD8	protein_codin	0	0	0	0
ENSG000001	DDX19B	protein_codin	36.0301322	39.5655634	37.847808	27.8615694
ENSG000001!	MBP	protein_codin	0.53181742	0	0	0
ENSG000001	TCF7L2	protein_codin	0	0	0	3.67609942
ENSG000001	SEMA6D	protein_codin	0	0	0	0
ENSG000001!	STK39	protein_codin	3.23844835	32.4915084	48.1895551	13.3027642
ENSG000001	RTN3	protein_codin	0.80959402	0	0	0
ENSG000001!	WWP2	protein_codin	5.05909606	0	0	2.55474906
ENSG000001	FN1	protein_codin	0	72.4673927	0	0
ENSG000001	MAPK8IP3	protein_codin	15.7206481	4.60640361	93.7496738	0
ENSG000001!	LCOR	protein_codin	0	0	0	0
ENSG000002	TRIM13	protein_codin	0	0	0	4.33829267
ENSG000001	BTG4	protein_codin	0	0	0	0
ENSG000002	NPIPB2	protein_codin	0	6.56837286	7.06252094	3.38441492
ENSG000001!	SIRPA	protein_codin	23.4203326	59.7644695	21.5479907	11.0504047
ENSG000001	MMP23B	protein_codin	0	0	0	0
ENSG000001	SPEF2	protein_codin	1.0419798	10.6597947	10.5187419	1.07577832
ENSG000001	JPT1	protein_codin	0	0	0	0
ENSG000001	GRHL3	protein_codin	0	0	0	0
ENSG000001	HNRNPF	protein_codin	25.966728	0	9.9641176	5.6547301
ENSG000000	MYO9A	protein_codin	104.208035	274.668741	108.244965	51.3015014
ENSG000000	JKAMP	protein_codin	2.07779163	0	0	0
ENSG000000	PHLDB1	protein_codin	46.5717083	0	87.5403118	43.237857
ENSG000001	ARFIP1	protein_codin	4.82289421	8.5295716	5.19788457	2.05616643
ENSG000001!	TCF4	protein_codin	0	0	0	0
ENSG000001	CYP20A1	protein_codin	36.8269522	66.8186156	27.1662819	22.5786164
ENSG000001	ADD3	protein_codin	0	90.7160816	12.6250902	24.0248003
ENSG000001!	LAMB3	protein_codin	4.57658214	0	0	4.57437099
ENSG000001!	COL27A1	protein_codin	27.1334576	166.163892	67.6944793	25.9669858
ENSG000001	EFCAB6	protein_codin	0.69210478	1.55737757	0	0
ENSG000000	NCDN	protein_codin	0	0	16.9289608	1.45363804
ENSG000002	NPEPL1	protein_codin	10.1107573	13.6753261	9.07736033	3.24224078
ENSG000001	ARSA	protein_codin	0	0	0	2.31199232
ENSG000002	ARHGAP8	protein_codin	0	0	0	0
ENSG000001!	PTPRT	protein_codin	0	0	0	5.75931907
ENSG000001	TJP1	protein_codin	23.4999253	0	46.4685973	66.4840354
ENSG000001!	ZNF44	protein_codin	8.07966954	0	0	0
ENSG000001	LIPC	protein_codin	2.52854128	6.80195747	0	0
ENSG000001!	ACSL5	protein_codin	0	58.2589324	0	0
ENSG000001!	NOXO1	protein_codin	0	0	0	0
ENSG000001	SUOX	protein_codin	0	7.87787654	0	0
ENSG000001!	PSMD12	protein_codin	20.6224426	20.280017	39.774885	15.8003107
ENSG000001	TRAPPC3L	protein_codin	0.44322858	1.71225465	0	0

ENSG000002	OR8B4	protein_codin	0	0	0	0
ENSG000001	ST3GAL4	protein_codin	0	0	0	0
ENSG000000	ANO2	protein_codin	0	0	0	0
ENSG000001	CHKA	protein_codin	0	0	0	11.4452067
ENSG000001	UVRAG	protein_codin	11.1322208	20.2840809	12.0434378	12.3612739
ENSG000001	ACMSD	protein_codin	0	0	0	0
ENSG000001	NPAS3	protein_codin	0	18.4035709	19.8342042	2.08388337
ENSG000001	RAC1	protein_codin	2.02252711	0	6.37360044	0
ENSG000000	RNF14	protein_codin	0	0	7.73632327	0
ENSG000001	CDKL1	protein_codin	0	0.97570229	0.53181204	0
ENSG000001	PTPRA	protein_codin	0	0	6.09487754	8.66143736
ENSG000000	SH3YL1	protein_codin	0	0	0	0
ENSG000001	PXK	protein_codin	34.2515	8.51933665	3.3463146	9.23103801
ENSG000002	NPIPB5	protein_codin	1.74338173	1.83523425	3.92701474	3.02953568
ENSG000001	KATNAL2	protein_codin	0	0	0	0
ENSG000001	FBRS	protein_codin	17.0588667	135.245633	55.2532713	24.6194587
ENSG000001	SUN5	protein_codin	0	0	0	0
ENSG000001	NF1	protein_codin	0	0	0	0
ENSG000001	ZNF527	protein_codin	0	0	6.85577643	0
ENSG000000	GTF3C1	protein_codin	91.5070573	63.9680907	32.4691496	72.5287311
ENSG000001	SUPT20H	protein_codin	0	0	0	0
ENSG000000	RNH1	protein_codin	0	0	2.90333612	7.80739275
ENSG000001	ACBD7	protein_codin	2.00198393	4.10697605	6.1937572	0.48227919
ENSG000001	CDCA7L	protein_codin	0	0	0	0
ENSG000001	CENPL	protein_codin	1.12452645	0	0	0
ENSG000000	CDK11A	protein_codin	0	0	0	0
ENSG000001	SLC39A1	protein_codin	53.4723698	42.4134045	32.1812486	22.5855279
ENSG000001	ENTPD4	protein_codin	4.22380614	0	4.01601686	3.39806773
ENSG000000	FGFR1	protein_codin	0	78.236995	0	17.3422537
ENSG000001	RBM38	protein_codin	2.40206584	0	0	0
ENSG000001	FRMD6	protein_codin	0	0	0	0
ENSG000000	DCX	protein_codin	0	0	0	0
ENSG000001	CDC16	protein_codin	0	0	0	5.83254977
ENSG000000	FGFR2	protein_codin	12.7551226	0	21.4128754	0
ENSG000001	CDAN1	protein_codin	6.42528401	6.92635873	12.8550395	5.5211216
ENSG000000	UBA5	protein_codin	0	2.17667087	0	0
ENSG000001	AKAP9	protein_codin	0	0	0	0
ENSG000001	RANGAP1	protein_codin	21.2162607	159.500328	149.749083	7.08594889
ENSG000001	G3BP1	protein_codin	0	0	0	0
ENSG000001	CEMIP	protein_codin	0	0	4.13341767	0
ENSG000001	SDAD1	protein_codin	0	99.1041831	2.99663355	0
ENSG000001	LHX8	protein_codin	0	0	0	0
ENSG000000	AMPH	protein_codin	0	0	0	0
ENSG000000	MED24	protein_codin	0	0	0	0

ENSG000001	CXADR	protein_codin	0	0	0	0
ENSG000001	SMOC2	protein_codin	253.824454	273.947698	83.169677	60.3133301
ENSG000001	RBCK1	protein_codin	4.99500936	13.8100941	9.39959935	0
ENSG000001	JAML	protein_codin	5.68539494	0	0	0.79078206
ENSG000001	TMPRSS11F	protein_codin	0.33262265	0	0	2.45476706
ENSG000001	NUMB	protein_codin	0	3.02741747	0	2.33635707
ENSG000001	PXDNL	protein_codin	1.25251936	6.46301837	0.97250979	1.25757828
ENSG000000	JPH4	protein_codin	0.522823	1.01673064	1.02318907	1.7643683
ENSG000001	EEF1A1	protein_codin	0	0	0	0
ENSG000001	PAOX	protein_codin	0	0	0	0
ENSG000001	NCAPG2	protein_codin	21.0285266	0	82.8218808	5.71936085
ENSG000001	SPACA9	protein_codin	1.18539484	2.68799246	1.50484192	2.08188271
ENSG000001	GALNT6	protein_codin	0	0	0	0
ENSG000001	ZNF770	protein_codin	49.0988662	49.3136954	85.123312	28.0746166
ENSG000001	ZNF614	protein_codin	3.73707696	9.37468289	0	0
ENSG000000	PLD1	protein_codin	89.7847397	94.6207793	34.4831745	0
ENSG000000	SH3BP2	protein_codin	0	0	0	0
ENSG000001	MYO5A	protein_codin	0	0	0	0
ENSG000001	PAK1	protein_codin	2.16943406	0	0	1.77587467
ENSG000001	PLEC	protein_codin	23.6289261	25.3932055	9.71213231	22.10694
ENSG000001	KPNA5	protein_codin	10.3120944	18.4406412	24.1402942	0
ENSG000001	UPF2	protein_codin	0	6.582749	0	2.96382034
ENSG000001	AREL1	protein_codin	0	0	40.4110691	0
ENSG000000	PICALM	protein_codin	14.4316339	0	0	0
ENSG000000	PUS7	protein_codin	0	0	45.2729227	0
ENSG000001	RXRA	protein_codin	3.59314497	11.6525394	3.81320138	0
ENSG000001	TMEM91	protein_codin	0	0	0	0
ENSG000001	BTN3A2	protein_codin	0	0	13.8224293	12.1794879
ENSG000001	ORAI2	protein_codin	12.5459331	0	0	0
ENSG000001	SPRED2	protein_codin	11.7310855	37.6085456	29.5361858	24.4617926
ENSG000001	ODAD3	protein_codin	0	0	0	0
ENSG000001	LSS	protein_codin	0	0	1.29104621	3.36286581
ENSG000001	ARIH2	protein_codin	16.730416	75.8868516	20.721581	21.3910847
ENSG000001	PITPNM1	protein_codin	0	0	0	0
ENSG000000	ACOX3	protein_codin	6.27515432	27.958298	5.21765686	2.09300541
ENSG000001	RAB19	protein_codin	0	0	0	0
ENSG000001	CARD11	protein_codin	0	0	1.21827738	0
ENSG000000	IGBP1	protein_codin	49.2638171	131.432613	141.179596	40.2019101
ENSG000001	RAP1A	protein_codin	24.9229609	43.7718409	30.2038433	31.0588626
ENSG000001	ATF7IP2	protein_codin	5.21171422	14.4012797	0	0
ENSG000001	MPG	protein_codin	6.2910444	0	10.5888009	2.87761163
ENSG000000	DLL3	protein_codin	0	0	0	0
ENSG000001	PTPRD	protein_codin	193.649708	140.982601	166.917681	79.8984947
ENSG000001	TBC1D7	protein_codin	0	0	0	0

ENSG000001!	HYLS1	protein_codin	0	6.78622867	0	1.19654932
ENSG000001	NRBP1	protein_codin	0	130.58974	2.2972927	0
ENSG000001	MYOM1	protein_codin	6.44823011	10.9150961	0	1.82567952
ENSG000001	SEMA4D	protein_codin	0	0	0	0
ENSG000001	THRB	protein_codin	9.65190819	0	0	0
ENSG000001	ARHGEF26	protein_codin	0	0	0	0
ENSG000001	NUDT10	protein_codin	3.61236954	6.58976784	24.3211247	4.95211579
ENSG000001!	SOWAHC	protein_codin	7.38400141	9.63637004	15.1353769	7.3717184
ENSG000001	UBE2C	protein_codin	2.30451853	0	39.754415	0
ENSG000001!	ZNF140	protein_codin	0	0	0	0
ENSG000001!	ASPH	protein_codin	0	0	0	0
ENSG000002	EML6	protein_codin	1.57522834	14.7757668	5.0400354	4.9233618
ENSG000000	NEDD4L	protein_codin	0	0	0	0
ENSG000001!	WDR45	protein_codin	0	0	2.76387423	0
ENSG000001!	RPS26	protein_codin	0	0	0	0
ENSG000000	FAM168A	protein_codin	86.4697721	220.047464	156.425993	54.8114608
ENSG000001!	H3C4	protein_codin	0	19.5927764	19.6749219	4.27394026
ENSG000000	BAX	protein_codin	0	0	0	0
ENSG000001	GPI	protein_codin	8.25898167	10.5678593	10.5243395	26.7648449
ENSG000000	SPATA20	protein_codin	0	0	0	0
ENSG000000	BCKDHB	protein_codin	2.30929256	0	10.9459391	0
ENSG000001	STOML2	protein_codin	13.6710917	17.3165005	56.0342301	6.91263937
ENSG000001	PIGR	protein_codin	0	0.95950403	0	1.74847545
ENSG000001	ADPRHL1	protein_codin	0	0	3.1600417	0
ENSG000001	GABRA2	protein_codin	0	0	0	0
ENSG000001	PLD3	protein_codin	1.58188338	0	0	0
ENSG000001	GMNN	protein_codin	3.62774487	13.4769986	15.683753	3.89727585
ENSG000000	PPIE	protein_codin	0	15.3217325	0	0
ENSG000001!	AADACL2	protein_codin	0	0	0	0
ENSG000001	ADAM33	protein_codin	0	5.98911651	0	0
ENSG000001	ZNG1A	protein_codin	0	0	0	0
ENSG000001	CD34	protein_codin	11.5024358	33.549587	2.40238598	39.9150097
ENSG000001	SAA1	protein_codin	0.5052826	0	0	0
ENSG000001	ENSA	protein_codin	0	77.653815	0	0
ENSG000002	H2BC7	protein_codin	4.77477344	21.5736	6.28949761	3.05628379
ENSG000001	OSCAR	protein_codin	1.35990043	0	0	0
ENSG000000	CHRD	protein_codin	0	0	0	0
ENSG000001	FABP7	protein_codin	0	0	0	0
ENSG000001	CCDC25	protein_codin	0	18.9835905	24.2206506	0
ENSG000001!	LEKR1	protein_codin	0	0	0.4997555	8.5044019
ENSG000001	C6orf132	protein_codin	1.12900348	0	0	0
ENSG000001	RSPO1	protein_codin	0.95121915	8.02014515	0	0
ENSG000001!	MAN1A2	protein_codin	193.054372	633.010234	458.160063	131.980743
ENSG000001	WFDC11	protein_codin	0	0	0	0

ENSG000001(	MEGF6	protein_codin	0	0	0	0
ENSG000001:	COG6	protein_codin	0	0	0	0
ENSG000001:	SON	protein_codin	432.989075	862.555397	1607.88474	282.274618
ENSG000001:	INS-IGF2	protein_codin	0	0	0	0
ENSG000000	SPATA7	protein_codin	6.82192533	0	16.4526192	0
ENSG000001!	ZNF772	protein_codin	3.71537227	11.0499515	3.54697524	5.03199308
ENSG000002	TSGA13	protein_codin	0	0	0	0
ENSG000001(	ADAMTS13	protein_codin	0	0	0	0
ENSG000001	APOLD1	protein_codin	32.9689625	34.9940514	56.4705969	79.8835197
ENSG000001	GABRG2	protein_codin	0	0	0	0
ENSG000000:	PTGER3	protein_codin	0	0	0	0
ENSG000001:	DTNB	protein_codin	0	24.6221951	12.1866779	0
ENSG000001	ADGRG2	protein_codin	1.762032	0	0	0
ENSG000001	PEX14	protein_codin	5.17489879	21.5050385	35.6958937	8.88279582
ENSG000001:	ABCC11	protein_codin	0	0	0	0.94320525
ENSG000001!	SLC30A10	protein_codin	0	0	0	0
ENSG000001!	ARC	protein_codin	0.94689883	0.77241064	1.17206013	8.51366601
ENSG000001!	NRARP	protein_codin	2.74840674	19.4550854	5.2778317	9.33447638
ENSG000001(	NAALADL1	protein_codin	0	0	0	0
ENSG000000(	KDM1A	protein_codin	4.23418083	0	0	0
ENSG000001	OSCP1	protein_codin	0	0	0	0
ENSG000000	GANAB	protein_codin	42.921028	198.23808	156.216948	47.997175
ENSG000001:	KCNIP2	protein_codin	0	4.53525568	0	0
ENSG000001!	ARHGEF12	protein_codin	307.96345	203.653253	314.540562	127.621034
ENSG000000	SESN1	protein_codin	13.2723464	0	0.94279663	2.93844026
ENSG000001	JTB	protein_codin	31.7442231	52.8451437	29.3810354	8.92173099
ENSG000001	ATN1	protein_codin	0	117.324384	0	0
ENSG000001(	FDPS	protein_codin	0	0	17.8513629	0
ENSG000001	BDNF	protein_codin	0	0	0	0
ENSG000001!	MAGEA1	protein_codin	0	0.91735427	0	0.89208491
ENSG000001	TMEM259	protein_codin	0	0	0	22.666737
ENSG000000!	PGC	protein_codin	0	0	0	0
ENSG000001:	HNRNPA2B1	protein_codin	0	0	0	0
ENSG000001	ELP5	protein_codin	0	0	2.53018181	0
ENSG000001:	SEM1	protein_codin	0	0	0	0
ENSG000001	SEPTIN10	protein_codin	0	5.67007972	0	0
ENSG000001	S100PBP	protein_codin	1.51461686	16.696693	0.99408586	2.36237272
ENSG000001(	PPP4R2	protein_codin	62.472922	149.103566	84.8976146	29.1488936
ENSG000000	FOLH1	protein_codin	4.74182522	0	0	0
ENSG000001	RSPO3	protein_codin	7.17619973	29.1120844	32.068658	11.3084129
ENSG000001(	ATP5F1C	protein_codin	6.69798998	16.6461831	0	12.3743
ENSG000001:	BTN2A2	protein_codin	0	0	0	2.48424468
ENSG000000	MYO16	protein_codin	0	3.09257903	5.77965774	3.00236456
ENSG000001	CNNM1	protein_codin	0.26419089	0	7.84622157	2.19972517

ENSG000001	NUDT1	protein_codin	3.75038709	16.559011	21.784214	2.98953846
ENSG000000	MYNN	protein_codin	4.56948913	14.3422533	31.6886065	0
ENSG000001	SANBR	protein_codin	0	0	0	0
ENSG000001	SIAH1	protein_codin	2.39262911	17.8168367	15.0511878	9.9015066
ENSG000001	ZNF790	protein_codin	6.62721331	0	0	0
ENSG000001	RORC	protein_codin	0	0	0	0
ENSG000000	ATP2B4	protein_codin	0.40660234	2.16569714	3.88496785	0.44674221
ENSG000001	HNRNPH1	protein_codin	2.37895638	20.6851504	11.6540066	7.20993311
ENSG000001	SLC39A8	protein_codin	16.2548996	0	0	18.1345644
ENSG000001	APBB3	protein_codin	0	0	0	0
ENSG000001	XXYLT1	protein_codin	0	0	1.81288415	0
ENSG000001	HVCN1	protein_codin	0	4.74457842	0	1.00476188
ENSG000001	MAMSTR	protein_codin	0	0	0	0
ENSG000001	NDRG4	protein_codin	0	0	0	0
ENSG000001	TMEM217	protein_codin	0	0	0	0
ENSG000001	CRB3	protein_codin	0	0	0	0.3675266
ENSG000001	PIK3R4	protein_codin	28.3166377	72.0780947	82.5201168	16.5077735
ENSG000000	PPP1R12B	protein_codin	0	22.55938	0	0
ENSG000001	PIWIL2	protein_codin	1.32137233	3.61223977	0	3.16377696
ENSG000001	SLC26A5	protein_codin	0	0	0	0
ENSG000001	PCYT1B	protein_codin	0	0	0	0
ENSG000001	NACA	protein_codin	1.01740312	7.14951802	5.12198186	0
ENSG000001	PBRM1	protein_codin	7.83077659	9.51207013	0	26.4886608
ENSG000001	TNNT1	protein_codin	8.3572895	0	0	0
ENSG000001	TAF7L	protein_codin	0	0	1.9395182	0
ENSG000000	APBB1IP	protein_codin	1.60838725	17.5473706	0.5982533	5.79447104
ENSG000001	CCDC88B	protein_codin	5.51116873	140.369366	0	7.77596493
ENSG000001	MAGEB10	protein_codin	0	1.41510955	0	2.95027846
ENSG000001	EDRF1	protein_codin	31.2558011	0	32.3563496	0
ENSG000001	ARL4A	protein_codin	3.83821087	0	10.7703956	5.87723846
ENSG000000	ITGAL	protein_codin	0	74.8036547	0	4.0885471
ENSG000001	RNF146	protein_codin	0	0	0	0
ENSG000000	SMARCD3	protein_codin	0	0	1.8565529	0
ENSG000001	SPTBN1	protein_codin	860.09455	1174.97947	948.148325	496.896514
ENSG000002	RTEL1	protein_codin	5.67206704	11.9499023	22.666313	0
ENSG000002	HBM	protein_codin	0	0	0	0
ENSG000001	PPP1R26	protein_codin	0	10.5113757	0	0
ENSG000001	NRG1	protein_codin	0	0	3.70736397	0
ENSG000001	ARMCX2	protein_codin	8.16537983	42.5310034	0	13.7996357
ENSG000001	SMAD2	protein_codin	42.3981766	46.8711607	97.2468315	26.9901171
ENSG000001	BLTP3B	protein_codin	13.3316273	0	9.97513341	0
ENSG000001	TBC1D9B	protein_codin	4.19270058	0	2.45232388	0
ENSG000001	B4GALT2	protein_codin	0.45592114	0	0	0
ENSG000001	TMEM71	protein_codin	0	0	0	0

ENSG000000	ACADVL	protein_codin	34.7140666	0	84.5319521	22.9225574
ENSG000000	MYLIP	protein_codin	23.0906994	0	37.0598076	44.3793379
ENSG000001	BCL11A	protein_codin	0	0	2.17769897	0
ENSG000001	GOLGA4	protein_codin	0	0	0	0
ENSG000001	NHEJ1	protein_codin	6.84225071	10.7406366	19.488466	9.64476824
ENSG000001	C10orf90	protein_codin	0	1.39590237	0	1.54367239
ENSG000000	VEZT	protein_codin	2.54512099	13.3696258	5.02955658	0
ENSG000001	CACNA2D1	protein_codin	17.0956114	0	71.4744814	12.6441948
ENSG000001	TNPO2	protein_codin	7.64748706	0	0	9.69615236
ENSG000001	TNFRSF10C	protein_codin	0	0	0	2.28510964
ENSG000002	ATP10A	protein_codin	0.85813885	32.448848	0	0
ENSG000001	PCED1A	protein_codin	0	0	0	0
ENSG000002	TNFRSF25	protein_codin	0	2.05764201	0	1.6473111
ENSG000002	PCDHA7	protein_codin	0	1.44745646	0.64813095	0.46377553
ENSG000001	BICD2	protein_codin	9.53389326	22.7906923	26.8037909	6.52629094
ENSG000001	GYPC	protein_codin	0.32869073	0	0	2.76583847
ENSG000001	FIGNL1	protein_codin	9.44747157	0	0	0
ENSG000001	RLIG1	protein_codin	0.74214859	0	0	2.46067711
ENSG000001	SASH3	protein_codin	10.8690129	75.5229044	4.09485057	12.2442666
ENSG000000	IFFO1	protein_codin	2.87597475	0	0	7.5246543
ENSG000001	IL27	protein_codin	0	1.59847298	0	0.41099847
ENSG000001	PARVG	protein_codin	1.79102849	22.9617267	5.27550581	0
ENSG000000	EIF4G3	protein_codin	0.80160558	0	0	0
ENSG000000	CREM	protein_codin	0	0	11.6400928	5.43599043
ENSG000001	NEK11	protein_codin	0	0	15.0171243	0
ENSG000001	POTEE	protein_codin	2.07865518	0	28.0494123	3.42915631
ENSG000001	SLC8A3	protein_codin	0	0	0	3.27866313
ENSG000001	ABCD4	protein_codin	2.45545852	0	0	3.13815803
ENSG000001	SLITRK4	protein_codin	2.55131324	0	0	0
ENSG000001	ZNF708	protein_codin	9.83925969	9.98218364	16.9817983	8.25780953
ENSG000001	NCL	protein_codin	534.911291	0	1677.58986	237.023201
ENSG000000	CDK11A	protein_codin	0	0	0	0
ENSG000000	RAB18	protein_codin	0	0	0	0
ENSG000000	GPM6B	protein_codin	1.66471101	38.1243966	2.26299412	5.00846754
ENSG000000	PTBP1	protein_codin	13.8365739	0	107.047783	0
ENSG000001	H2BC12	protein_codin	14.6904669	117.035784	174.08233	19.7411819
ENSG000001	ADAM15	protein_codin	0	0	0	0
ENSG000001	IGF2R	protein_codin	57.2094407	406.061017	334.486622	41.0155148
ENSG000001	TXNDC11	protein_codin	11.1372531	0	0	0
ENSG000001	ZNF585A	protein_codin	6.42857465	0	0	3.56406671
ENSG000001	ARG1	protein_codin	0	0	0	0
ENSG000001	CLCC1	protein_codin	0	0	0	0
ENSG000001	CILK1	protein_codin	0	9.43226139	2.84600444	0
ENSG000001	SPINK14	protein_codin	0	0	0	0

ENSG000001	PICK1	protein_codin	0	0	7.76078892	0
ENSG000001	CALM1	protein_codin	60.4216384	192.163422	52.1762565	54.2824269
ENSG000001	PAQR6	protein_codin	0.513739	1.42218646	1.20222908	0.57598326
ENSG000001	KLRG1	protein_codin	1.16485343	11.5506721	0	3.17608553
ENSG000001	RNF19B	protein_codin	0	50.8124462	0	14.4581777
ENSG000001	SCRIB	protein_codin	0	10.8880108	20.1689936	0
ENSG000001	MFNG	protein_codin	0	0	0	1.28043135
ENSG000001	GUCA2A	protein_codin	0	0	0.50115834	0.40191836
ENSG000002	ZNF254	protein_codin	22.0520543	0	4.63485283	0
ENSG000001	ADPRH	protein_codin	6.58646064	28.2021736	3.70887067	6.64755565
ENSG000001	ZBTB14	protein_codin	0	0	0	0
ENSG000001	CHD4	protein_codin	0	204.578411	0	0
ENSG000001	MED27	protein_codin	0	0	0.95575419	0
ENSG000001	DMD	protein_codin	131.425493	215.453403	0	0
ENSG000000	ELN	protein_codin	0	0	0	0
ENSG000001	CAVIN1	protein_codin	283.515611	862.482052	249.502836	174.520026
ENSG000001	PEX2	protein_codin	11.8517067	8.68480533	32.039943	5.0308948
ENSG000001	OSBPL1A	protein_codin	0	3.10778734	0	0
ENSG000000	RASSF1	protein_codin	0	18.7531366	0	0
ENSG000001	CAPN10	protein_codin	0	7.00246756	0	2.04107532
ENSG000001	RAB4B	protein_codin	0	0	0	0
ENSG000001	SLC30A6	protein_codin	0	0	0	0
ENSG000001	RNF38	protein_codin	0	0	0	0
ENSG000001	CFAP43	protein_codin	0	0	1.76815236	0
ENSG000001	LMO7	protein_codin	0	0	0	0
ENSG000001	HNRNPF	protein_codin	3.72749599	0	71.7203682	4.06601679
ENSG000001	MOB3A	protein_codin	10.2564513	82.1152317	30.2032295	25.9676513
ENSG000001	DCTD	protein_codin	5.73023783	18.9125225	0	14.1093443
ENSG000001	PRODH	protein_codin	0	0	0	0
ENSG000001	ECE1	protein_codin	2.62572572	69.1030845	2.38387414	0
ENSG000001	ANK2	protein_codin	0	0	0	0
ENSG000000	OSBPL6	protein_codin	0	0	13.1124318	0
ENSG000001	AGTPBP1	protein_codin	25.1937464	59.717283	59.2719707	18.9996885
ENSG000002	ZHX1-C8orf76	protein_codin	0	2.41867915	2.49898205	3.36136343
ENSG000001	ELAVL4	protein_codin	0	0	0	0
ENSG000001	ATP2A1	protein_codin	5.40354882	1.01854877	0	0
ENSG000001	TRIM38	protein_codin	58.8795788	230.732371	40.1119554	43.3702515
ENSG000001	ITPR1	protein_codin	0	27.3438909	83.9400465	0
ENSG000001	UBXN11	protein_codin	0	0	0	0
ENSG000001	ACSL6	protein_codin	0	0	0	0
ENSG000000	ADIPOR2	protein_codin	21.4254602	32.5574114	74.5503742	18.8651986
ENSG000001	LRBA	protein_codin	9.25457098	61.5080359	36.8257992	3.39371758
ENSG000000	KITLG	protein_codin	0	0	0	0
ENSG000001	NRP2	protein_codin	0	0	3.26508586	1.14395432

ENSG000001	ZGPAT	protein_codin	0	0	0	0
ENSG000001	OCRL	protein_codin	26.4496426	31.8687955	36.331797	10.4361385
ENSG000000	SPAG9	protein_codin	20.3945753	0	125.415011	80.7821218
ENSG000001	MCRS1	protein_codin	0	0	9.03990766	0
ENSG000001	NELL1	protein_codin	1.28990039	0	0	5.29766923
ENSG000001	XPNPEP3	protein_codin	18.9111386	49.989946	40.8771843	9.47044732
ENSG000001	PSMD12	protein_codin	23.2344675	47.7576894	47.8368251	14.5517025
ENSG000001	SPMIP10	protein_codin	0	0	0	0
ENSG000001	SPOCK3	protein_codin	0	0	19.8175499	0
ENSG000002	MYBPHL	protein_codin	0	0	0	0.93394819
ENSG000001	DYNLRB1	protein_codin	8.28441057	139.79342	31.0811322	13.450619
ENSG000001	MCU	protein_codin	0	0	0	0
ENSG000001	VPS13B	protein_codin	52.4436508	0	246.646439	45.0963236
ENSG000001	GM2A	protein_codin	20.2729751	110.760842	27.7057413	33.342117
ENSG000001	ZDHHC9	protein_codin	5.38600038	27.9627879	10.8319863	15.2099301
ENSG000001	ABTB3	protein_codin	4.05972004	0	4.47936437	0
ENSG000001	CEPT1	protein_codin	16.699715	0	18.6339566	2.39352573
ENSG000001	PWWP3B	protein_codin	3.83201892	0	26.95362	0
ENSG000001	TOP3B	protein_codin	0	13.3036793	0	1.7165856
ENSG000001	ZMYM6	protein_codin	10.5564183	42.343829	20.6818012	8.30290713
ENSG000001	RAET1E	protein_codin	0	5.71294618	0.52226256	5.65078764
ENSG000001	MPV17	protein_codin	0.58017401	4.06794016	0.5403445	1.01710707
ENSG000001	UBE2D3	protein_codin	0	0	0	0
ENSG000001	BCL11B	protein_codin	11.5726266	130.573664	147.404256	14.6253608
ENSG000001	PTPA	protein_codin	0	0	15.3449573	0
ENSG000001	DOK3	protein_codin	0	0	0	0
ENSG000001	WFDC8	protein_codin	0.37172371	1.92014061	0	0
ENSG000000	C3orf18	protein_codin	0	9.05542987	0	0
ENSG000001	ZFYVE9	protein_codin	21.8385231	41.6236559	0	0
ENSG000001	TRAPPC11	protein_codin	0	81.2328814	84.670123	0
ENSG000001	SFPQ	protein_codin	225.41543	453.229398	1063.51264	336.712576
ENSG000001	TMEM217	protein_codin	0	0	0	0
ENSG000001	HHIPL1	protein_codin	0	0	1.15208618	0
ENSG000001	CDK16	protein_codin	0	9.64643464	0	0
ENSG000001	EXOC7	protein_codin	0	0	0	0
ENSG000001	DCHS2	protein_codin	0	0	12.967199	4.54321513
ENSG000001	IRF5	protein_codin	0	0	0	0
ENSG000001	CDC42SE1	protein_codin	34.9665126	67.4602803	71.0467514	27.9019414
ENSG000000	DZANK1	protein_codin	0	0	0	0.88110523
ENSG000001	TBC1D8B	protein_codin	12.4840292	13.2686243	15.2491414	8.90182497
ENSG000000	RC3H2	protein_codin	8.35023095	0	136.386037	43.561721
ENSG000001	MAP1LC3C	protein_codin	0.28651348	4.13453153	0	0.43708915
ENSG000000	SMARCA2	protein_codin	0	0	0	0
ENSG000000	KCNQ2	protein_codin	0	0	0	0

ENSG000001	COL9A1	protein_codin	0	0	15.4302242	0
ENSG000001	ZNF23	protein_codin	0	0	0	0
ENSG000001	ZBTB20	protein_codin	298.463763	0	27.8229798	181.429477
ENSG000001	INKA2	protein_codin	9.82170844	4.63883007	14.3333742	6.49181119
ENSG000000	FKBP5	protein_codin	215.053836	422.098067	363.141191	53.9164732
ENSG000001	GRB10	protein_codin	0	0	0	0
ENSG000002	DBNDD2	protein_codin	0	0	2.6096621	0
ENSG000001	SMARCAL1	protein_codin	0	0	0	0
ENSG000001	REPS2	protein_codin	3.98459952	18.3681015	0	0
ENSG000001	PAOX	protein_codin	0	0	0	0
ENSG000001	CSF1	protein_codin	2.28805071	2.81503079	0	21.8095658
ENSG000001	ERFE	protein_codin	0	0	1.11653471	1.36850899
ENSG000001	GFPT1	protein_codin	5.22840351	0	0	0
ENSG000001	APAF1	protein_codin	0	0	0	10.9214204
ENSG000001	SRPK2	protein_codin	0	8.66634198	18.6941662	0
ENSG000001	NUDT13	protein_codin	0	0	2.25123968	0.45678341
ENSG000001	ATP13A1	protein_codin	13.4295406	71.0229627	47.142444	8.91773631
ENSG000001	HDGF	protein_codin	111.636187	0	544.553969	36.616067
ENSG000001	TNIK	protein_codin	0	0	0	9.85499609
ENSG000001	ZNF248	protein_codin	9.03917009	13.59295	53.5825078	0
ENSG000001	GALP	protein_codin	0	0	0	0
ENSG000001	GET3	protein_codin	22.279806	8.29280942	103.708983	0
ENSG000000	MYCBP2	protein_codin	25.5935212	0	0	0
ENSG000001	DLK2	protein_codin	0	0	0	1.31621678
ENSG000000	FAS	protein_codin	0	0	0	0
ENSG000001	TCAF2	protein_codin	0	0	0	0
ENSG000001	SYNJ1	protein_codin	0	0	0	0
ENSG000000	RTF2	protein_codin	1.3177816	0	17.0155969	1.20679126
ENSG000001	ADGRE5	protein_codin	19.2667994	61.036101	0	36.3466403
ENSG000001	LAGE3	protein_codin	0.52887775	4.01907988	7.49420579	0.40214497
ENSG000001	MDGA2	protein_codin	0	0	7.33296319	0
ENSG000001	ENTR1	protein_codin	1.92830798	0	21.9149226	0
ENSG000001	PRAMEF8	protein_codin	0	0	0	0
ENSG000001	IFNA10	protein_codin	0	2.74038815	0	0.64366553
ENSG000001	SIGLEC10	protein_codin	0	0	0	0
ENSG000001	RTN4	protein_codin	0	3.46899948	0	134.827924
ENSG000001	OR5B17	protein_codin	0	0	0	0
ENSG000001	CGB3	protein_codin	0	0	0	0.45409119
ENSG000001	RPRD1A	protein_codin	4.06694331	0	0	0
ENSG000001	ANAPC11	protein_codin	2.02898724	0	2.9128975	0.45037058
ENSG000001	RICTOR	protein_codin	75.0947928	128.887423	92.7836922	51.8814054
ENSG000001	SMC4	protein_codin	29.6831494	0	0	0
ENSG000001	MFN1	protein_codin	9.53590192	60.1423454	39.8062022	0
ENSG000001	CCRL2	protein_codin	1.2221048	5.59501845	0	0

ENSG000001	AKNAD1	protein_codin	0	0	0	0
ENSG000001	ERGIC3	protein_codin	6.49369678	45.0346609	24.6674969	4.88004696
ENSG000001	CEACAM3	protein_codin	0	0	0	0
ENSG000001	NCR1	protein_codin	0	0	0	0.68139818
ENSG000000	MVP	protein_codin	0	159.271121	22.1308872	31.8296639
ENSG000000	EYA2	protein_codin	11.848267	0	25.2939654	0
ENSG000001	ABHD11	protein_codin	0	0	0	0
ENSG000001	CCR3	protein_codin	0	0	0	0
ENSG000001	GIP	protein_codin	0	0	0	0.68477273
ENSG000000	USP33	protein_codin	0	47.0888554	0	0
ENSG000001	C7orf50	protein_codin	1.28062738	0	3.92346004	0
ENSG000001	ACSL3	protein_codin	0	0	7.50967013	0
ENSG000001	CEP350	protein_codin	1.62238771	5.13965912	2.4327317	1.06749458
ENSG000001	SLC29A2	protein_codin	0	0	0	0
ENSG000001	MOV10	protein_codin	0	0	0	0
ENSG000001	RABGAP1L	protein_codin	1.82178824	79.3214725	6.6097492	4.47760443
ENSG000001	FRMD4A	protein_codin	40.5666807	56.6323006	81.0718894	51.8678579
ENSG000000	SMC1B	protein_codin	0.62170956	0	2.86417602	0
ENSG000001	CERT1	protein_codin	0	0	0	0
ENSG000002	HOMEZ	protein_codin	6.84022508	26.952718	7.8045753	5.69921022
ENSG000001	TMEM229B	protein_codin	2.51604818	4.54212787	0	0
ENSG000001	TENT4B	protein_codin	0	0	0	0
ENSG000001	RABL6	protein_codin	0	0	0	0
ENSG000001	MBNL1	protein_codin	0	18.2803915	0	0
ENSG000001	ECE2	protein_codin	0	0	0	0
ENSG000001	SSBP3	protein_codin	3.08380351	0	0	0
ENSG000001	STRN3	protein_codin	0	7.57685479	23.0912184	5.33741533
ENSG000000	NFE2L1	protein_codin	0	0	0	0
ENSG000001	ACIN1	protein_codin	1.77372029	0.53053158	10.7400152	1.64841548
ENSG000001	USP6	protein_codin	0.36199232	1.01427609	0	0.90794024
ENSG000002	ARPIN	protein_codin	20.1273849	80.4043837	19.4524422	14.4448892
ENSG000001	ARID5A	protein_codin	5.08449078	0	0	0
ENSG000001	ZNF451	protein_codin	2.31865268	0	28.8673835	12.4863527
ENSG000001	COPS7B	protein_codin	0	0	0	0
ENSG000001	ZNF43	protein_codin	16.9690934	0	0	0
ENSG000001	KIR2DL4	protein_codin	0	0	0	0
ENSG000001	HNRNPA1L2	protein_codin	10.3646221	18.2031857	22.3228586	7.1233301
ENSG000001	QRICH1	protein_codin	0	0	20.1208716	24.7497202
ENSG000001	POP5	protein_codin	2.08212204	0	0	1.18018259
ENSG000001	TMEM79	protein_codin	2.0124736	0	0	0
ENSG000001	ZNF74	protein_codin	0	0	0	2.39040425
ENSG000001	TOR4A	protein_codin	0.79544618	23.5221784	0.55710221	5.1509001
ENSG000001	DTHD1	protein_codin	0	0	0	0
ENSG000002	N4BP2L2	protein_codin	0	0	0	0

ENSG000001	ARMH1	protein_codin	0	0	0	0
ENSG000001	ZNF92	protein_codin	0	0	1.85419974	2.08603787
ENSG000001	PCDH1	protein_codin	5.22089784	0	4.6855011	11.5400696
ENSG000001	FIBP	protein_codin	8.448288	0	26.5649665	5.36292586
ENSG000001	BET1	protein_codin	0.86925715	1.87571189	1.59274039	2.26699033
ENSG000001	PLN	protein_codin	5.30040081	2.55266565	0	2.74210945
ENSG000001	RIMKLB	protein_codin	0	28.3101636	24.7043086	0
ENSG000000	TRPM3	protein_codin	0	0	0	0
ENSG000001	GPALPP1	protein_codin	1.45420666	0	0.78857434	0.64126158
ENSG000001	ZFYVE27	protein_codin	3.46855092	0	0	0
ENSG000001	RHD	protein_codin	0	0	0	0
ENSG000001	ADGRG2	protein_codin	0	0	0	0
ENSG000001	SPOCK3	protein_codin	0	0	0	0
ENSG000000	DHRS9	protein_codin	0	0	0	0
ENSG000001	SYCP2	protein_codin	0	0	0	0
ENSG000000	FGFR2	protein_codin	13.5684962	0	22.6172934	0
ENSG000001	CIZ1	protein_codin	0	0	0	0
ENSG000001	FXR1	protein_codin	8.02929491	14.496475	11.0399643	10.18918
ENSG000001	APBB3	protein_codin	4.86732909	0	0	0
ENSG000001	SETD3	protein_codin	0	0	0	0
ENSG000001	POC5	protein_codin	0	0	0	0
ENSG000000	CAPN3	protein_codin	0	5.90421565	0	2.58715569
ENSG000001	RABEP2	protein_codin	0.56908417	0	0	0
ENSG000000	WDR7	protein_codin	45.2818518	108.56277	106.683757	38.076596
ENSG000001	SLC66A2	protein_codin	0	0	0	0.91564557
ENSG000001	ALDH5A1	protein_codin	7.67879543	0	0	0
ENSG000001	MAP3K6	protein_codin	2.22995285	0	4.29143285	0
ENSG000001	SSTR2	protein_codin	12.7328567	26.4043626	48.0455847	9.59259303
ENSG000001	AP1B1	protein_codin	11.6612371	45.7268685	0	8.98157278
ENSG000001	SLC37A4	protein_codin	0	0	0	0
ENSG000001	PHF11	protein_codin	0	0	0	2.49543567
ENSG000000	SEMA5B	protein_codin	0	0	0	0
ENSG000000	IPO5	protein_codin	6.23332646	33.643248	34.7699207	3.89731401
ENSG000001	UPF2	protein_codin	141.710315	318.136576	314.100068	86.8120196
ENSG000001	OR51D1	protein_codin	0	0	0	0
ENSG000001	DNAJC14	protein_codin	0	9.54201164	2.59828167	0
ENSG000001	BMX	protein_codin	0	0	0	0
ENSG000001	TMEM170A	protein_codin	0	24.141721	18.3256882	0
ENSG000001	TAC3	protein_codin	0	0	0	0
ENSG000000	HFE	protein_codin	0	0	0	3.83970889
ENSG000001	POT1	protein_codin	8.74242166	19.6353154	28.4826895	9.09412983
ENSG000001	LRRC20	protein_codin	2.327183	0	0	0
ENSG000001	CCR9	protein_codin	0	0	0	0
ENSG000001	FAM219B	protein_codin	15.9764235	12.2887209	19.3873966	7.38487768

ENSG00000015	PIGN	protein_codin	0	0	0	0
ENSG0000013	ENPP3	protein_codin	0	0	0	0
ENSG0000010	DENND2D	protein_codin	1.57802639	42.707337	4.9431403	4.34596387
ENSG0000009	BRPF3	protein_codin	11.491836	11.2887797	39.9535078	0
ENSG0000017	PLEC	protein_codin	0	58.0580825	46.2921902	18.832819
ENSG0000010	SLC30A7	protein_codin	24.9497736	70.5511702	119.898769	28.9871954
ENSG000000	BRCA1	protein_codin	0	0	0	0
ENSG0000019	SZT2	protein_codin	0.48914111	2.67992838	0	0
ENSG0000013	ZNF816	protein_codin	0	0	0	0
ENSG0000015	ASAP1	protein_codin	0	154.406491	0	0
ENSG0000007	DLG1	protein_codin	0	0	0	0
ENSG0000005	ATP2B4	protein_codin	269.284921	301.207403	348.4799	131.798324
ENSG0000019	BCO2	protein_codin	0.54086916	0	2.65743794	0
ENSG0000019	MIER1	protein_codin	5.74936788	0	42.5956214	8.11282443
ENSG0000019	ASB13	protein_codin	4.17485671	3.21451599	10.8060977	1.97317073
ENSG0000008	MYBPC2	protein_codin	73.7428865	6.83359712	0.45063924	0.44986727
ENSG0000013	SAP130	protein_codin	0	0	0	0
ENSG000001	GHR	protein_codin	30.1336925	0	26.3642032	24.4669501
ENSG0000008	PSEN1	protein_codin	20.1138874	0	63.7309018	0
ENSG000001	CD46	protein_codin	0	0	0	0
ENSG0000019	GRM7	protein_codin	1.66414089	0	0	0
ENSG0000015	DCLRE1C	protein_codin	0	0	0	0
ENSG0000010	TRMT1	protein_codin	0	17.7616405	32.1578173	0
ENSG0000013	ETFBKMT	protein_codin	4.42217301	10.1389362	5.1612002	3.90284354
ENSG0000014	NTRK3	protein_codin	0	0	0	0
ENSG000001	PRAMEF12	protein_codin	0.30155303	1.31490559	0	0.84479226
ENSG0000014	CREB5	protein_codin	21.0540757	9.52549852	12.44952	7.29028934
ENSG0000019	SLC6A9	protein_codin	0	0	0	0
ENSG0000017	NEGR1	protein_codin	2.66876401	7.61830468	4.8935854	13.0969383
ENSG000001	RTN4	protein_codin	0	0	0	0
ENSG0000019	MAFG	protein_codin	17.2626586	32.4111243	23.4401545	14.1802053
ENSG0000014	MCTP2	protein_codin	20.2063364	20.8015198	0.94161525	13.3615503
ENSG0000014	GOLGA7	protein_codin	0	0	6.85483289	0
ENSG0000018	CHM	protein_codin	35.0397131	38.3287565	65.9672972	19.1440554
ENSG0000000	HYAL2	protein_codin	11.6992014	0	0	14.8412677
ENSG0000010	DDHD1	protein_codin	0	0	0	4.02031229
ENSG000001	HHLA2	protein_codin	0	0	0	0
ENSG0000000	CDK11A	protein_codin	0	0	0	0
ENSG0000023	HP	protein_codin	0	0	0	0
ENSG000001	UNC50	protein_codin	7.28926133	51.233524	31.0320721	8.42068804
ENSG0000018	THSD4	protein_codin	0	0	0	0
ENSG0000013	GJA9	protein_codin	0	0	0	0
ENSG0000019	ZNF98	protein_codin	0.38767181	1.9774126	0.49704752	0.5239693
ENSG0000010	TXNDC2	protein_codin	0	3.96286198	0.34970627	1.6068549

ENSG000001(	CCDC103	protein_codin	0	0	0	0
ENSG000001	APOA4	protein_codin	0	0	0	1.34801138
ENSG000001	PHTF1	protein_codin	6.52070134	15.2491603	27.6713292	12.3610071
ENSG000001	NRP2	protein_codin	116.974648	74.1401427	359.590308	86.4700677
ENSG000001:	RPLP1	protein_codin	265.228274	746.172837	1638.92061	160.092462
ENSG000001!	NPIPB9	protein_codin	0	0	0	4.25836482
ENSG000001!	OGA	protein_codin	0	0	0	0
ENSG000001	NPAS3	protein_codin	0	0	0	0
ENSG000001(	SGO2	protein_codin	39.1915086	36.4278676	402.857359	7.75915313
ENSG000001	CLN3	protein_codin	0	0	0	0
ENSG000001!	COL13A1	protein_codin	0	0	0	4.11744329
ENSG000001(	NAT9	protein_codin	7.10750106	0	0	0
ENSG000001(	AMER2	protein_codin	0	0	1.54420247	0
ENSG000001!	OR8D1	protein_codin	0	0	0	0.60332006
ENSG000001(	HJV	protein_codin	0.30404678	0	0	0.69542836
ENSG000001	AIG1	protein_codin	9.46545334	20.2389922	26.3145518	11.0921084
ENSG000001	COPS5	protein_codin	20.6829233	33.6412911	34.2724922	10.0759452
ENSG000001!	SFI1	protein_codin	0	0	0	0
ENSG000001	G3BP2	protein_codin	41.6919559	88.5117202	48.7598622	40.3169947
ENSG000001	CLN3	protein_codin	0	0	0	0
ENSG000001!	ZNF655	protein_codin	0	0	0	0
ENSG000001	STMN1	protein_codin	0	0	0	0
ENSG000001	ARL6IP4	protein_codin	0	0	0	0
ENSG000001	FN1	protein_codin	0	0	0	89.9230445
ENSG000001	ADCY6	protein_codin	4.82383506	40.85541	0	0
ENSG000001	LVRN	protein_codin	9.85823551	8.47510737	5.47376086	1.8443142
ENSG000001	MDM1	protein_codin	0	0	0	0
ENSG000001	SEMA4F	protein_codin	0	0	4.10413918	0
ENSG000001	HMX3	protein_codin	0	0.76865467	1.02606748	0.91809903
ENSG000001	RFX4	protein_codin	0	3.68458391	0.41142284	0
ENSG000001(	C19orf47	protein_codin	0	0	0	0
ENSG000001(	CDK5RAP1	protein_codin	0	0	0	0
ENSG000001(	RNF40	protein_codin	0	0	0	0
ENSG000000	NEDD4L	protein_codin	0	0	0	0
ENSG000001(	IFT20	protein_codin	0	0	0	0
ENSG000001	SPSB1	protein_codin	1.43178543	3.26500353	4.25664202	1.67964888
ENSG000001!	ZBTB44	protein_codin	0	0	0	0
ENSG000001!	ZNF441	protein_codin	24.199676	40.7797445	19.3147706	11.3269396
ENSG000001	APP	protein_codin	120.032777	76.6427812	726.924908	117.644502
ENSG000002	MAGEA12	protein_codin	0	0	0	0
ENSG000000	RHOBTB1	protein_codin	0	0	0	0
ENSG000002	ASIC3	protein_codin	0	0	0	0
ENSG000002	ANKRD18B	protein_codin	0	0	0	1.8127477
ENSG000001	DDC	protein_codin	0	0	0	0

ENSG000001	CCNE1	protein_codin	0	0	0	0
ENSG000000	TLL2	protein_codin	0.27597936	3.11872119	2.01351471	3.56044286
ENSG000001	CADPS	protein_codin	0	0	0	0
ENSG000001	SERTAD1	protein_codin	1.94291673	10.5115151	0.97142195	27.4167973
ENSG000001	MAPKAPK3	protein_codin	6.98654545	25.2726002	14.5282208	5.75807866
ENSG000001	ERI2	protein_codin	4.01892385	0	0	5.91682125
ENSG000001	BIN1	protein_codin	0	0	0	0
ENSG000001	ACYP1	protein_codin	3.23092998	0	0	4.05515562
ENSG000001	NFIA	protein_codin	0	0	0	0
ENSG000001	TPM1	protein_codin	0	0	0	0
ENSG000001	TMUB2	protein_codin	0	0	0	0
ENSG000001	FAM3B	protein_codin	0	0	0	0.47454555
ENSG000001	PHF8	protein_codin	0	0	0	0
ENSG000001	ADGRG2	protein_codin	0	0	0	0
ENSG000001	ELK4	protein_codin	78.4860284	220.589972	71.6012126	61.8246501
ENSG000001	FEZ2	protein_codin	5.75792554	0	7.39379349	5.60062723
ENSG000001	ANKLE2	protein_codin	37.3920317	64.0336363	78.7331131	19.5960184
ENSG000001	FKTN	protein_codin	0	0	0	0
ENSG000001	CCDC32	protein_codin	0	0	0	0
ENSG000002	DDX47	protein_codin	23.7012522	32.0777967	117.640872	14.075339
ENSG000001	AIMP1	protein_codin	0	0	0	1.08429386
ENSG000001	TACC2	protein_codin	0	0	0	0
ENSG000001	AIRIM	protein_codin	0	0	13.039947	0
ENSG000001	RAD23B	protein_codin	129.677955	88.6353474	348.658367	109.236848
ENSG000001	TUSC1	protein_codin	3.9689642	9.30312148	8.6548254	3.26862339
ENSG000001	TMCC2	protein_codin	2.04614954	11.0413371	0	6.81105098
ENSG000000	SYNE2	protein_codin	152.979045	277.492438	195.915763	0
ENSG000000	NIPAL3	protein_codin	0.69549931	0	0.82029777	0
ENSG000001	STX16	protein_codin	0	0	0	0
ENSG000001	RAD17	protein_codin	0	0	0	0
ENSG000001	KCNIP2	protein_codin	0	0	0	0
ENSG000000	ST7L	protein_codin	4.22741368	0	0	6.41289787
ENSG000001	OXA1L	protein_codin	0	0	9.86862135	0
ENSG000001	FAM47C	protein_codin	0.24246008	1.20900781	0	3.65471969
ENSG000001	KLK2	protein_codin	0	0	0	0
ENSG000001	OSBPL2	protein_codin	0	0	5.25330598	4.23796414
ENSG000001	SLC4A3	protein_codin	0	0	0	0
ENSG000001	GMFB	protein_codin	21.5648994	44.6203221	46.7304229	12.4168295
ENSG000001	DMD	protein_codin	0	0	0	0
ENSG000001	H4C16	protein_codin	1.15946942	1.81767492	3.40222205	1.4505692
ENSG000001	SETMAR	protein_codin	2.16730342	0	27.4720934	0
ENSG000001	SEMA6D	protein_codin	5.57468086	0	10.1430376	0
ENSG000001	ZNF329	protein_codin	5.89071242	4.84171713	0	3.22053281
ENSG000000	DCX	protein_codin	0	0	0	0

ENSG000001	RAD9B	protein_codin	0	0	0	0
ENSG000001	MAGT1	protein_codin	2.93042121	0	0	0
ENSG000001	DAPK1	protein_codin	0	0	0	0
ENSG000000	ASIC4	protein_codin	0	0	0	0
ENSG000001	THOC5	protein_codin	0	0	0.8533462	0.94400143
ENSG000000	TRPM3	protein_codin	0	0	0	0
ENSG000001	POTEE	protein_codin	0	0	280.169208	0
ENSG000001	KLF8	protein_codin	0	0	7.37632996	0
ENSG000001	KLHL14	protein_codin	0	0	0	0.82983696
ENSG000002	CYP2D7	protein_codin	0	0	0	0.64260753
ENSG000001	NR3C2	protein_codin	10.0347458	23.1449643	15.6135648	8.72870029
ENSG000001	CFAP299	protein_codin	0.35388625	0	0	0
ENSG000001	RGR	protein_codin	0	0	0	0
ENSG000000	MPPED2	protein_codin	20.0242305	4.73093712	20.3423644	2.68873568
ENSG000001	OR2L13	protein_codin	0	0	0	0
ENSG000001	PAX5	protein_codin	0	0	0	0
ENSG000001	FHL2	protein_codin	2.87435859	0	5.00820549	0
ENSG000001	HMGN5	protein_codin	59.6989619	165.681806	686.83507	17.9899355
ENSG000001	TOX2	protein_codin	0	0	0	0
ENSG000000	PLOD1	protein_codin	0	0	0	0
ENSG000001	PDE1A	protein_codin	0.57113735	0	0.85398604	0
ENSG000001	LRRC20	protein_codin	0	0	3.64177912	0
ENSG000001	CNTNAP3	protein_codin	1.56535242	12.3719197	2.12037345	0
ENSG000001	DMRT2	protein_codin	0.84305353	0	0	0
ENSG000001	WDSUB1	protein_codin	0	0	0	0
ENSG000001	ZNF346	protein_codin	7.49436749	34.1639981	30.0289141	2.83621787
ENSG000001	ZNF483	protein_codin	0	0	1.11298347	2.10378831
ENSG000001	MS4A3	protein_codin	0	0	0	0
ENSG000001	METTL9	protein_codin	7.91657473	0	16.3226438	3.29119038
ENSG000001	SRSF2	protein_codin	0	0	0	4.9007384
ENSG000002	S1PR3	protein_codin	37.2064416	77.7499117	43.3050879	56.221867
ENSG000001	GRAMD1C	protein_codin	0	14.0498856	10.1967321	1.90026504
ENSG000001	SPTAN1	protein_codin	163.844406	220.125963	290.778658	187.923007
ENSG000000	ITGAL	protein_codin	0	104.516684	0	0
ENSG000000	RNASET2	protein_codin	0	5.27335757	0	1.50800314
ENSG000001	CD46	protein_codin	0	0	0	10.612916
ENSG000001	SERPINH1	protein_codin	81.7309289	126.85782	255.792018	80.7877521
ENSG000001	EPHB4	protein_codin	5.8902578	34.4394048	35.56355	8.20727146
ENSG000001	KIR3DL1	protein_codin	0	0	0	0
ENSG000001	CHD3	protein_codin	0	0	76.2058317	0
ENSG000001	CTSE	protein_codin	0	0	0	0
ENSG000001	BDH1	protein_codin	0.827361	2.90885311	2.69251852	1.47082633
ENSG000001	ITPRIP	protein_codin	5.12948724	30.1782278	9.53180391	0
ENSG000001	CIMAP2	protein_codin	0	0	0	0

ENSG000001:	GAD1	protein_codin	0.64907808	0	0	0
ENSG000001	RABEP2	protein_codin	6.76844587	25.6704216	16.1988175	0
ENSG000001	TES	protein_codin	17.958081	7.56646725	58.029824	14.2512913
ENSG000001	SMARCAL1	protein_codin	0	0	0	0
ENSG000001!	SRC	protein_codin	0	0	0	0
ENSG000000	DNM1L	protein_codin	0	0	0	0
ENSG000001	GMPPA	protein_codin	0	0	0	0
ENSG000000	WDR37	protein_codin	0	0	0	0
ENSG000001	DMAC1	protein_codin	22.4129985	12.3240655	53.9589969	9.37932252
ENSG000001	ENPP3	protein_codin	0.55801776	0	0	2.71650933
ENSG000001	TMBIM4	protein_codin	0.54299569	20.8407517	0	4.83942741
ENSG000001	ELP2	protein_codin	20.9166194	46.3670242	18.6444679	10.2244899
ENSG000001	PPP1CA	protein_codin	1.24645402	0	0	0
ENSG000001!	RTP2	protein_codin	0	0	0	0
ENSG000001	DMTN	protein_codin	0	0	0	0
ENSG000000	CACNA1G	protein_codin	0	0	0	0
ENSG000001	MS4A7	protein_codin	48.2353219	65.457989	6.31881711	27.7688125
ENSG000001	TNKS1BP1	protein_codin	35.2910096	0	67.087327	33.2822282
ENSG000001	LCA5L	protein_codin	0	0	0	0
ENSG000001!	NF1	protein_codin	0	73.615989	135.935177	15.8232701
ENSG000001	TPM1	protein_codin	230.722604	0	0	77.1332184
ENSG000001!	HSD17B11	protein_codin	12.0188941	19.8042435	35.0409097	13.1131376
ENSG000001	FCRL1	protein_codin	0	3.86102731	0	0
ENSG000001!	FAM217B	protein_codin	0	0	0	0
ENSG000002	TAFA5	protein_codin	0	0	24.923351	0
ENSG000001!	ZNF100	protein_codin	6.95047077	20.0858728	34.0304382	5.11571924
ENSG000001	SUV39H2	protein_codin	0	0	0	2.52319215
ENSG000001	ICA1L	protein_codin	0.71891825	0	1.28615836	0
ENSG000001	PAK4	protein_codin	5.140665	0	0	0
ENSG000000!	ZC3HC1	protein_codin	0	0	0	0
ENSG000000	FUT8	protein_codin	1.1040177	0	0	0
ENSG000002	ARHGAP19	protein_codin	0	0	11.0420625	0
ENSG000001	CX3CR1	protein_codin	0	0	0	0
ENSG000001	PPHLN1	protein_codin	2.07143706	4.99020781	0	0
ENSG000001!	KCNMB2	protein_codin	0	0	0	0
ENSG000000!	SUSD2	protein_codin	5.76016375	2.06782407	2.65028678	0
ENSG000001	MYOF	protein_codin	67.7909194	0	0	0
ENSG000001	AKT2	protein_codin	0.30774639	0	1.1967334	0
ENSG000001	WDR55	protein_codin	9.92127122	30.6304596	19.30082	10.8870745
ENSG000001!	HNRNPAB	protein_codin	50.930842	91.2555489	260.804909	34.8938562
ENSG000000	FRYL	protein_codin	5.18608367	92.2107798	73.6054512	0
ENSG000000!	CDHR5	protein_codin	0	0	0	0
ENSG000001	USP20	protein_codin	0	0	0	0
ENSG000001	RUNX1	protein_codin	15.2812841	104.242696	40.8805489	57.7368313

ENSG000001	ECHDC2	protein_codin	0	0	0	0
ENSG000001	PPIP5K2	protein_codin	0	0	0	0
ENSG000001	GRIK2	protein_codin	0	1.67863194	1.33602586	0
ENSG000001	RHOT1	protein_codin	0	0	3.24206697	0
ENSG000001	HNRNPLL	protein_codin	0	10.7530107	0	0
ENSG000000	XAB2	protein_codin	18.1530772	56.6307952	69.0925122	11.6348516
ENSG000001	MIGA2	protein_codin	0	0	0	0
ENSG000001	MAP3K19	protein_codin	0	0	0	0
ENSG000001	WTAP	protein_codin	0	0	0.91412809	3.62191557
ENSG000001	GRM8	protein_codin	0	0	0	0
ENSG000001	OSCAR	protein_codin	0	0	0	0
ENSG000001	DCAF4	protein_codin	0	7.3914529	0	0
ENSG000001	KIF13A	protein_codin	0	0	7.74336373	3.99860789
ENSG000001	ATL1	protein_codin	3.38560784	0	0	0
ENSG000001	TXNDC15	protein_codin	7.61674211	69.6992925	32.9254745	0
ENSG000000	CNOT3	protein_codin	1.74270566	9.90481265	0	4.02876629
ENSG000000	COL11A1	protein_codin	0	0	3.78069932	0
ENSG000001	C20orf204	protein_codin	0	0	0	0
ENSG000000	CEACAM1	protein_codin	0	0	0	0
ENSG000001	RNF24	protein_codin	20.5711242	0	8.88111034	9.55000102
ENSG000001	ESRP1	protein_codin	0	0	0	0
ENSG000001	GDA	protein_codin	9.41292914	0	0	2.6165911
ENSG000001	MAX	protein_codin	0	0	0	10.7243893
ENSG000001	GSTK1	protein_codin	18.0968861	96.2283073	7.52864071	15.1012314
ENSG000001	TEAD4	protein_codin	0	0	0	0
ENSG000001	DNA2	protein_codin	2.70380441	0	24.9619085	0
ENSG000000	ZNF343	protein_codin	0	0	0	0
ENSG000001	ITIH2	protein_codin	0	0	0	0
ENSG000001	TXLNB	protein_codin	38.5528413	23.6699265	3.55356621	9.62955067
ENSG000001	KIAA1671	protein_codin	28.3104051	0	54.0889824	28.2024925
ENSG000001	EPHA2	protein_codin	25.7214303	46.2708039	41.9044352	50.4495684
ENSG000001	OR6C6	protein_codin	0	0	0	0
ENSG000001	TOMM7	protein_codin	27.8488756	59.6806857	30.6869524	19.2719336
ENSG000002	POLR2J2	protein_codin	1.74223976	0	3.97675367	5.05347147
ENSG000001	ARMC8	protein_codin	0	0	27.514462	0
ENSG000001	PDE11A	protein_codin	0	0	0	0
ENSG000001	VCF2	protein_codin	0	8.05580322	0	1.4303436
ENSG000001	AFAP1	protein_codin	46.3577084	102.294607	45.8572252	26.891321
ENSG000001	ZFHX3	protein_codin	0	1.84217349	0.87753638	0
ENSG000001	TRIM33	protein_codin	40.5138265	58.4224147	77.9506646	31.8327196
ENSG000001	STAT4	protein_codin	0	0	6.7697289	0
ENSG000001	ZSCAN2	protein_codin	3.86082188	0	0	0
ENSG000001	DMAC2L	protein_codin	0	0	0	0
ENSG000001	POGZ	protein_codin	19.3564392	11.594147	4.25174911	0

ENSG000001	TRPC4	protein_codin	0	0	0	0
ENSG000000	CASP8	protein_codin	0	31.4078325	0	1.67888739
ENSG000000	FGFR2	protein_codin	0	0	0	0
ENSG000001	ZNF429	protein_codin	11.3472955	23.2291732	14.8204085	8.0207523
ENSG000000	RAD52	protein_codin	0	1.78919275	0	0
ENSG000001	APOL2	protein_codin	20.3198239	170.644878	40.4605083	0
ENSG000001	KCNN3	protein_codin	0	0	4.20075855	0.73096402
ENSG000001	BCL11A	protein_codin	0	0	0	0
ENSG000002	COL6A6	protein_codin	8.39132738	1.37333907	7.96911816	4.69109829
ENSG000002	PSMB10	protein_codin	3.51653852	36.5774227	3.03771668	5.1531788
ENSG000001	PLEKHG1	protein_codin	0	8.55064849	0	0
ENSG000001	NRG2	protein_codin	0	0	0	0
ENSG000001	ZNF682	protein_codin	0	0	0	0
ENSG000001	PATE2	protein_codin	0.70508681	0.9993899	9.09362572	0.88040465
ENSG000001	SLC6A20	protein_codin	0	0	0	0
ENSG000001	AKAP4	protein_codin	0	0	0	0
ENSG000001	EXO5	protein_codin	0	0	0	1.45570044
ENSG000000	CSDE1	protein_codin	0	0	0	0
ENSG000002	ARHGAP19	protein_codin	3.70600707	0	0	15.1819553
ENSG000001	PLXNB1	protein_codin	0	0	0	0
ENSG000001	FLNB	protein_codin	113.230872	577.045649	304.107987	108.532305
ENSG000001	PTPN5	protein_codin	2.25884664	0	0	0
ENSG000001	ALKAL1	protein_codin	0	0	0	0
ENSG000001	VPS13B	protein_codin	0	0	0	0
ENSG000001	TNFSF11	protein_codin	0	0	0	1.92575564
ENSG000000	RAB21	protein_codin	29.2303032	52.711601	41.0263308	21.4679009
ENSG000001	TPD52L2	protein_codin	0	0	0	0
ENSG000001	SIPA1L1	protein_codin	21.8401586	19.9867179	100.890655	18.1036822
ENSG000000	NFIX	protein_codin	0	0	0	0
ENSG000001	EDNRA	protein_codin	7.40632431	0	11.9147939	15.4183879
ENSG000001	PFN3	protein_codin	0	0	0	0
ENSG000001	TMA16	protein_codin	8.77330097	4.81223655	19.4081881	0.51003107
ENSG000001	FIP1L1	protein_codin	0	0	0	0
ENSG000001	MICAL1	protein_codin	0	0	28.3262377	13.447323
ENSG000001	APBB3	protein_codin	0	0	0	0
ENSG000001	SCGB1D4	protein_codin	0	5.03870599	0	2.03610435
ENSG000001	FRMD1	protein_codin	0.62614603	6.94512687	0	0.77267867
ENSG000001	ARNT	protein_codin	29.7066771	67.3819866	20.432779	31.7885995
ENSG000001	CELF1	protein_codin	10.7118576	0	20.1750784	9.69797121
ENSG000001	PRKAR1A	protein_codin	108.492337	0	137.078827	62.1171713
ENSG000001	ESR2	protein_codin	0	0	0	0
ENSG000001	ADGRE5	protein_codin	0	29.1177398	0	3.19775996
ENSG000001	ANKRD17	protein_codin	23.0294836	112.795029	46.9024325	30.5037019
ENSG000001	ZNG1B	protein_codin	2.23846472	4.38837403	22.3567629	0

ENSG000000	REX1BD	protein_codin	1.50898152	0	22.0687203	5.16460265
ENSG000001	GYPE	protein_codin	0	0	1.33958522	0
ENSG000001	WDR5	protein_codin	14.2022535	18.1838798	54.933096	8.31167695
ENSG000001	CYP11A1	protein_codin	0	0	0	1.27581137
ENSG000001	ELF2	protein_codin	2.04295636	9.74932113	7.14308385	6.82536418
ENSG000001	ASTN2	protein_codin	0.99226286	2.49810797	0.73720476	4.52263497
ENSG000001	OR5AC2	protein_codin	0	0	0	0
ENSG000001	GIMAP5	protein_codin	14.0327673	83.0772294	0	13.845305
ENSG000001	PASK	protein_codin	2.01121095	14.9804802	0	0
ENSG000002	SPECC1L-AD	protein_codin	0	0	5.48362364	9.02976892
ENSG000001	WARS1	protein_codin	20.6809795	0	0	12.6621915
ENSG000001	ZNF707	protein_codin	0	0	2.7699916	0
ENSG000000	SLC4A8	protein_codin	0	0	8.7183415	6.30920551
ENSG000001	NAALADL1	protein_codin	0	0	0	0
ENSG000001	NTRK1	protein_codin	0.63628762	0	0	0.89698303
ENSG000001	SP110	protein_codin	5.08988189	0	0	8.71341428
ENSG000001	MAX	protein_codin	13.6846427	79.0837517	29.5400732	9.77732612
ENSG000001	UBL5	protein_codin	0.38795381	0	4.36219521	0
ENSG000000	FCGR2B	protein_codin	1.27988362	8.41439282	0	4.45323011
ENSG000001	ENAH	protein_codin	161.310236	216.237691	152.678057	62.2009857
ENSG000001	SPATS2L	protein_codin	0	0	0	0
ENSG000001	MRPL42	protein_codin	0	0	0	0
ENSG000001	SLAIN1	protein_codin	0	0	0	0
ENSG000001	EXT2	protein_codin	35.4750469	0	68.7135094	0
ENSG000001	SLC4A5	protein_codin	0	0	0	0
ENSG000001	ENTPD4	protein_codin	28.4554632	126.168661	40.8376419	22.0418441
ENSG000001	HELZ	protein_codin	69.4150335	212.506939	274.428156	75.940956
ENSG000001	CTNND1	protein_codin	0	0	0	0
ENSG000001	ZXDA	protein_codin	7.87234028	17.1298927	33.7100135	10.0860693
ENSG000001	ZNF23	protein_codin	0	0	3.66439778	0
ENSG000001	QSOX2	protein_codin	9.1704364	20.7019057	34.5875536	7.13742116
ENSG000001	ZBTB18	protein_codin	0	0	0	0
ENSG000000	CAMK2B	protein_codin	0	0	0	0
ENSG000001	UCKL1	protein_codin	0	0	10.9091282	0
ENSG000001	GATAD2A	protein_codin	1.80520543	2.78796039	0	4.14409194
ENSG000000	HMMR	protein_codin	0	0	0	0
ENSG000001	BAZ1A	protein_codin	0	0	0	3.55183779
ENSG000001	IPO8	protein_codin	0.60975468	1.44259881	0	0.81590801
ENSG000002	SMIM7	protein_codin	2.80088546	1.14107118	12.0341392	0
ENSG000001	AMPD2	protein_codin	0	30.6746139	0	0
ENSG000000	RFX3	protein_codin	0.38989048	0	3.7676661	3.93464094
ENSG000001	BDP1	protein_codin	130.563117	137.53545	367.035441	76.9195295
ENSG000001	ZBTB1	protein_codin	16.6322883	55.8759758	85.2353427	19.8319866
ENSG000001	H2AC13	protein_codin	10.8370863	43.4279762	232.327717	0.94444491

ENSG000001!	NLGN3	protein_codin	0	0	0	0
ENSG000001	SMTN	protein_codin	0	0	0	24.6760724
ENSG000001	ZNF571	protein_codin	8.40805373	5.87134357	0	0
ENSG000001!	SKIC3	protein_codin	0	0	0	0
ENSG000002	SLC26A6	protein_codin	0	0	0	0
ENSG000000	NPFFR2	protein_codin	0	0	0	0
ENSG000001!	FUT4	protein_codin	8.7524189	22.0945673	21.5101263	7.47052354
ENSG000001	FZD6	protein_codin	8.23292365	0	23.4886555	0
ENSG000002	ENSG000002	protein_codin	0.78416822	0	0	0
ENSG000001	PRRT2	protein_codin	0	0	0	0
ENSG000001	SYN3	protein_codin	1.60136292	0	0.99288614	3.33650858
ENSG000001	IQCH	protein_codin	0	2.80388525	0	1.40782669
ENSG000001	PARD3B	protein_codin	23.0219441	0	28.0602583	15.1284231
ENSG000001	PBLD	protein_codin	4.12276456	0	19.3201912	4.30879389
ENSG000001!	SIRPA	protein_codin	0	9.55189918	6.08812408	1.92033017
ENSG000001!	ACADSB	protein_codin	18.3870451	21.4461497	14.2582453	22.7120792
ENSG000001	CEACAM19	protein_codin	0	0	0	0
ENSG000000	CDK11A	protein_codin	0	8.34107045	0	0
ENSG000000	FAM76B	protein_codin	0.76842685	8.83301895	23.7745977	8.94442447
ENSG000001!	FAM3D	protein_codin	0	0	0	0
ENSG000000	GNB5	protein_codin	2.83755306	1.95319309	5.13502455	0
ENSG000001	MRPL19	protein_codin	0	0	0	0
ENSG000001	KLK10	protein_codin	0	0	0	4.29746446
ENSG000001	BCAS4	protein_codin	0	0	1.64373778	0
ENSG000001	GTPBP3	protein_codin	3.37865173	9.4407254	17.1921864	0
ENSG000001	STIP1	protein_codin	0	0	0	0
ENSG000001	MICAL1	protein_codin	14.7137766	112.055916	0	0
ENSG000001	CLIP1	protein_codin	0	0	0	0
ENSG000001	MANEA	protein_codin	19.9291725	0	0	8.91895895
ENSG000001	CCL20	protein_codin	0	0	0	0
ENSG000001	CNDP1	protein_codin	0	0	0	0
ENSG000001	CAMSAP2	protein_codin	26.0006075	72.3311153	61.4650653	13.2683496
ENSG000001	PRDM10	protein_codin	0	0	0	0
ENSG000001	TSKS	protein_codin	0	0	0	0
ENSG000001	PNLIPRP1	protein_codin	0	0	0	0
ENSG000000	REV3L	protein_codin	0	0	0	0
ENSG000001!	GK2	protein_codin	0	0	0	0.49187227
ENSG000001!	ABCB8	protein_codin	1.22270538	0	14.709597	5.56676867
ENSG000001!	NOL8	protein_codin	1.02325879	0	1.37849358	0
ENSG000001!	CCER1	protein_codin	0	4.49493034	0	0.52576877
ENSG000001	TMC2	protein_codin	0	0	0	0.42543361
ENSG000000	DZANK1	protein_codin	0	0	0	0
ENSG000001	TMEM126B	protein_codin	0	0	7.17305516	3.6680013
ENSG000001!	FAM114A1	protein_codin	39.7908287	46.6476847	26.0758246	15.0487431

ENSG000001	TFEB	protein_codin	0	0	0	0
ENSG000001	STK33	protein_codin	0	0	0	0
ENSG000001	LPAR1	protein_codin	0	0	0	0
ENSG000002	RPL41	protein_codin	12.5089336	83.1891115	59.3697457	8.84379918
ENSG000000	MAMLD1	protein_codin	0	6.89019311	0.39172663	0
ENSG000001	COL20A1	protein_codin	0	1.58858002	0	2.24317816
ENSG000001	DUS2	protein_codin	2.02108293	8.46304996	5.71352321	0
ENSG000001	FHAD1	protein_codin	0	0	0	0
ENSG000001	VCP	protein_codin	55.8253316	0	34.5750382	0
ENSG000001	FAM222A	protein_codin	1.24886598	1.45232452	0	1.90951109
ENSG000001	R3HDM2	protein_codin	0	0	0	0
ENSG000001	PELI1	protein_codin	27.8133419	74.4010799	73.2266605	87.0389024
ENSG000001	MYPN	protein_codin	3.19379578	0	0	0
ENSG000001	APP	protein_codin	35.2949821	201.216123	42.2777646	22.857572
ENSG000001	DLC1	protein_codin	0	0	0	5.07550933
ENSG000001	PDE4D	protein_codin	0	14.8493027	0	14.2024653
ENSG000000	SIRT2	protein_codin	0	0	0	0
ENSG000001	CCNB1IP1	protein_codin	16.4421028	6.47061936	35.9089064	10.2602489
ENSG000000	EDC4	protein_codin	11.1811738	24.1893311	36.7520293	13.805894
ENSG000001	MARCHF5	protein_codin	14.4385535	17.0464331	21.2385443	13.0997369
ENSG000000	DPP8	protein_codin	24.7863597	33.5814727	61.1928431	17.4560198
ENSG000002	RBMY1E	protein_codin	0	0	0	0
ENSG000001	SETMAR	protein_codin	0	0	0	0
ENSG000001	RAB3GAP2	protein_codin	31.6224652	13.5668898	49.5706584	11.9278427
ENSG000000	AASS	protein_codin	0	0	0	0
ENSG000001	NUMA1	protein_codin	0	0	0	0
ENSG000001	GGPS1	protein_codin	0	0	0	0
ENSG000001	POTEC	protein_codin	0	0	1.42050705	0
ENSG000001	SEPSECS	protein_codin	0	0	0	0
ENSG000001	ANKRD30B	protein_codin	0	0	0	0
ENSG000001	ZNF763	protein_codin	0	0	0	1.81630502
ENSG000001	ANKRD46	protein_codin	0	0	0	0
ENSG000001	NCKAP5	protein_codin	0	0	0	0
ENSG000001	SPANXC	protein_codin	0	0	0	0
ENSG000001	PTPA	protein_codin	0	0	23.9465123	0
ENSG000001	NCOA6	protein_codin	41.5568185	129.433577	59.3400563	22.0485317
ENSG000001	TRAPPC4	protein_codin	1.98893476	0	0	0
ENSG000001	VTCN1	protein_codin	0	0	0	0
ENSG000001	TGFBR2	protein_codin	3.86658657	54.2447726	0	39.583398
ENSG000001	MAP3K5	protein_codin	0	32.3594966	0	14.0906538
ENSG000001	AKAP9	protein_codin	42.1433573	0	83.8898588	122.003283
ENSG000001	GABPB1	protein_codin	0.54610321	0	0	0
ENSG000001	AP2A1	protein_codin	0	0	0	0
ENSG000001	CYP2D6	protein_codin	0	0	0	0

ENSG000001	ZNF511	protein_codin	4.92496173	12.3875688	9.96079886	0
ENSG000001	KLHL9	protein_codin	45.9587965	32.739504	45.9195031	13.8272967
ENSG000001	IL31RA	protein_codin	0	0	0	0
ENSG000001	MTNAP1	protein_codin	3.83811351	0	0	0
ENSG000001	ZNF574	protein_codin	6.47162448	18.3688654	9.36013053	4.57042139
ENSG000001	TRPM6	protein_codin	0	0	0	1.66479962
ENSG000001	HYAL3	protein_codin	0	0	0	2.33905187
ENSG000001	SMARCAD1	protein_codin	0	115.209523	34.3917182	0
ENSG000001	CLEC4M	protein_codin	0	0	0	0
ENSG000001	SSU72	protein_codin	8.52102161	16.9940806	40.6551272	10.386804
ENSG000001	PDE3A	protein_codin	20.5609813	37.195209	4.07915352	20.9326927
ENSG000001	INTS15	protein_codin	0	0	0	0
ENSG000001	SGMS2	protein_codin	0.71611596	0	12.4277081	2.67427426
ENSG000001	SHANK1	protein_codin	1.98051721	0	9.0797129	0
ENSG000001	KIR2DL4	protein_codin	0	0	0	0
ENSG000001	SFTA2	protein_codin	0	0	0	0
ENSG000001	NAE1	protein_codin	0	0	0	0
ENSG000000	ST6GALNAC1	protein_codin	0	0	0	0
ENSG000001	AXL	protein_codin	0	36.934775	7.19982113	7.67465231
ENSG000001	MAP2K6	protein_codin	1.4437261	0	6.23488571	0.66414933
ENSG000001	GNRH2	protein_codin	0	0	0	0
ENSG000000	CACNA1G	protein_codin	0	0	13.5067154	0
ENSG000001	MTHFD2L	protein_codin	2.36242264	0	0	0
ENSG000002	NOL12	protein_codin	2.21733336	20.6793934	4.26912848	1.05261282
ENSG000001	MLF1	protein_codin	0	6.92566846	0	0
ENSG000001	SH3RF2	protein_codin	0	0	0	0
ENSG000000	KCNQ2	protein_codin	0	0	0	0
ENSG000001	NLRC3	protein_codin	3.53323271	56.3341984	0	0
ENSG000001	PIGK	protein_codin	4.90674558	6.67228044	10.4167933	3.8476881
ENSG000001	GULP1	protein_codin	0	0	0	4.11241811
ENSG000001	PPP6R2	protein_codin	0	0	0	0
ENSG000001	ECE2	protein_codin	0	0	0	0
ENSG000000	ATP2B1	protein_codin	0	0	0	0
ENSG000000	ATP2B3	protein_codin	0	0	0	0
ENSG000001	KLRC1	protein_codin	0	0	0	0
ENSG000001	SYTL2	protein_codin	18.5280343	143.237923	128.291328	17.2766377
ENSG000001	WWP2	protein_codin	0	97.4029486	29.4393089	0
ENSG000001	CD163	protein_codin	5.4629685	82.7625369	0	24.3102244
ENSG000001	ANKRD12	protein_codin	101.569273	293.409566	281.458122	99.1961339
ENSG000000	RFX2	protein_codin	1.48379263	4.33224564	0	0
ENSG000001	ESRRG	protein_codin	0	0	0	0
ENSG000001	NUP98	protein_codin	0	0	0	0
ENSG000001	AHCYL1	protein_codin	1.22354617	3.03896717	4.76540493	2.34467508
ENSG000001	MAP3K15	protein_codin	0	0	1.05345699	1.34926164

ENSG000001	SPTSSB	protein_codin	0	0	0	1.3736484
ENSG000001	GOLGA8A	protein_codin	33.2504973	230.441444	311.775361	55.3176877
ENSG000001	ABI1	protein_codin	0	0	0	0
ENSG000001	SUPT5H	protein_codin	0	15.4691059	5.14209394	0
ENSG000001	H2AC11	protein_codin	45.4747644	9.90146144	430.70265	9.07944406
ENSG000001	PIK3CG	protein_codin	0	0	5.49912659	0
ENSG000001	IKZF1	protein_codin	0	0	0	0
ENSG000002	PRICKLE4	protein_codin	0	0	0	0
ENSG000001	FTHL17	protein_codin	0	0	0	0.324872
ENSG000000	RRAGD	protein_codin	8.99924085	20.4261095	6.66821766	0
ENSG000001	FRA10AC1	protein_codin	17.8543944	70.1091088	55.9095804	14.0184669
ENSG000001	YY1AP1	protein_codin	0	0	0	0
ENSG000000	ABCB4	protein_codin	2.31035418	1.30469822	27.9944283	0
ENSG000000	DNTTIP2	protein_codin	76.6702283	136.162216	251.909729	114.030467
ENSG000001	KIRREL1	protein_codin	136.656108	121.532298	146.360954	68.1548454
ENSG000000	POLR3E	protein_codin	7.40334542	0	0	0
ENSG000001	B4GALT4	protein_codin	9.58182192	3.04044196	26.3925035	0
ENSG000001	TTN	protein_codin	0	0	0	0
ENSG000001	MAPK10	protein_codin	0	0	0	0
ENSG000001	UQCC1	protein_codin	8.81627224	8.27189874	0	0
ENSG000001	ELAVL3	protein_codin	0	1.8877729	0.46913601	1.19595883
ENSG000001	LYPD2	protein_codin	0.26038661	0	0	0
ENSG000001	SLC25A29	protein_codin	0	0	0	1.23133093
ENSG000001	VSIG10	protein_codin	7.35399004	23.7285848	12.1549216	10.3093689
ENSG000001	PLAC1	protein_codin	0.28932431	0	1.01118658	0
ENSG000000	SNX11	protein_codin	1.38163583	0	0.93586848	1.99558871
ENSG000001	ADAM29	protein_codin	0	0	0	0
ENSG000001	ODF2L	protein_codin	20.9489739	65.3237701	30.9611671	9.86399599
ENSG000001	PHF2	protein_codin	41.2232854	161.917867	104.039326	43.5107252
ENSG000001	FHOD3	protein_codin	0	0	0	0
ENSG000001	CCDC197	protein_codin	0	0	0	0
ENSG000001	MYOF	protein_codin	106.345886	344.930974	187.73265	126.332037
ENSG000001	PDLIM5	protein_codin	0.99049507	1.52460775	10.520832	7.62733215
ENSG000001	SLC2A10	protein_codin	16.8118534	18.594727	36.1379313	14.8741332
ENSG000001	FCAR	protein_codin	0	0	0	0
ENSG000000	BCS1L	protein_codin	0	0	0	2.12930209
ENSG000001	PLD3	protein_codin	0	0	0	0
ENSG000000	CTPS2	protein_codin	16.9885115	0	16.6491577	7.57023495
ENSG000001	ADAM15	protein_codin	0	0	0	0
ENSG000001	PCBP2	protein_codin	14.464831	75.8001007	22.7815264	0
ENSG000001	SH2B1	protein_codin	0	20.9421047	0	0
ENSG000001	ZNF705A	protein_codin	0	0	0	0
ENSG000001	EOLA1	protein_codin	0	0	0	0
ENSG000001	STK40	protein_codin	0.4540298	0	0	3.20134482

ENSG000001	ANXA10	protein_codin	0	0	0	0
ENSG000001	H3C12	protein_codin	0.73065354	1.82931602	0.62117119	0
ENSG000001	XRCC6	protein_codin	4.88478628	38.3224838	27.1127711	2.92752738
ENSG000001	CD2AP	protein_codin	51.0909815	190.318539	112.483259	53.2815668
ENSG000001	TPGS1	protein_codin	0.7326861	3.21335654	2.54424763	0.81507213
ENSG000001	MTARC2	protein_codin	0	2.3176342	0	0
ENSG000001	AADACL3	protein_codin	0	0	0	0
ENSG000001	CMTM5	protein_codin	0	0	0	0
ENSG000001	XRCC2	protein_codin	7.5783335	0	86.1671776	0
ENSG000001	MYOZ1	protein_codin	21.8997191	1.09587948	0	0
ENSG000000	FIRRM	protein_codin	0	0	0	0
ENSG000000	SLAMF7	protein_codin	2.88009468	7.04862083	0	0
ENSG000001	KLHDC1	protein_codin	4.43039723	3.15412447	2.76800643	4.32104842
ENSG000001	TMEM139	protein_codin	0	0	0	0
ENSG000001	PLXNB2	protein_codin	94.0147353	0	218.77565	0
ENSG000001	CGB2	protein_codin	0	0	0	0
ENSG000001	SLFNL1	protein_codin	0	1.8112408	1.20777538	0
ENSG000001	OR51A7	protein_codin	0	0	0	0
ENSG000001	DDX42	protein_codin	49.5937464	224.10033	205.252295	57.5652587
ENSG000000	FGFR2	protein_codin	8.42150145	32.4316558	40.1689321	4.59914814
ENSG000001	DNAH8	protein_codin	2.3468809	0	0	0
ENSG000001	KLHL14	protein_codin	0.90255005	1.7068454	3.09161672	2.83010968
ENSG000001	C6orf89	protein_codin	0	0	15.2048174	0
ENSG000000	ARSF	protein_codin	0	0	0	0.90338772
ENSG000001	EDARADD	protein_codin	0	16.8559755	11.1871932	0
ENSG000000	RASSF1	protein_codin	0	0	0	2.86756709
ENSG000000	ATXN3	protein_codin	5.63423172	0	0	0
ENSG000001	RBFOX2	protein_codin	10.1749549	0	22.9199727	17.2004667
ENSG000001	FGF1	protein_codin	1.40145905	0	5.69413375	1.2282855
ENSG000001	ARAP1	protein_codin	0	0	0	9.11721982
ENSG000000	POLR1H	protein_codin	3.01317993	0	16.5956017	4.53134953
ENSG000001	DYNC1I1	protein_codin	5.20645878	0	0	6.65646918
ENSG000001	EHD1	protein_codin	0	0	0	0
ENSG000001	TRPV6	protein_codin	0	0	0	0
ENSG000000	ARCN1	protein_codin	0	0	0	4.95741437
ENSG000001	CIBAR1	protein_codin	0	0	3.75080988	0
ENSG000001	HK1	protein_codin	0	0	0	6.20102409
ENSG000001	NUP214	protein_codin	0	0	0	0
ENSG000001	RPE	protein_codin	0	0	54.9090051	11.8584154
ENSG000001	BABAM1	protein_codin	1.76695808	23.2250662	5.22706069	4.96242031
ENSG000001	TMC3	protein_codin	0	0	0	0
ENSG000001	FGF17	protein_codin	0	0	0.90634257	0
ENSG000001	EFL1	protein_codin	0	0	0	0
ENSG000001	LDLRAD4	protein_codin	47.5819349	57.0670674	56.2849463	0

ENSG000001[illegible]	MYO18A	protein_codin	5.81139313	23.122502	1.49326167	5.6774718
ENSG000001	WASF1	protein_codin	0	0	2.07288835	3.89910436
ENSG000001[illegible]	RGR	protein_codin	0	0	0	0
ENSG000001[illegible]	CCDC196	protein_codin	0	0	0	0
ENSG000001[illegible]	YIF1A	protein_codin	2.44154989	0	9.21218858	0
ENSG000001[illegible]	PCBP2	protein_codin	25.1592384	0	64.0612328	17.258184
ENSG000001[illegible]	IFT172	protein_codin	0	8.37323849	9.0202943	0
ENSG000001[illegible]	ZNF33B	protein_codin	32.6701486	15.4877311	125.719176	15.3299797
ENSG000001[illegible]	C1orf116	protein_codin	0.26894067	4.50355148	0.49633894	4.54685342
ENSG000001[illegible]	MFAP5	protein_codin	26.567821	0	0	0
ENSG000001[illegible]	SLC33A1	protein_codin	2.30725671	12.6312368	4.22717644	1.78455126
ENSG000001[illegible]	PLGLB2	protein_codin	0	0	4.43101846	0
ENSG000001[illegible]	TCF20	protein_codin	0	0	0	0
ENSG000001	APOA1	protein_codin	0	0	0	0
ENSG000001[illegible]	KRT78	protein_codin	0	0	0	3.01134759
ENSG000001[illegible]	TBX1	protein_codin	0	0	0	0
ENSG000001[illegible]	SLC25A44	protein_codin	19.1856545	15.5667258	17.1934798	0
ENSG000001[illegible]	WASH6P	protein_codin	4.69058387	22.1093997	5.23875098	0
ENSG000001[illegible]	ATG13	protein_codin	0	0	0	2.46321058
ENSG000001	TRIM3	protein_codin	0	0	0	0
ENSG000001[illegible]	TECPR2	protein_codin	8.25898308	39.7952397	34.2875067	12.4330858
ENSG000001[illegible]	MPZL1	protein_codin	2.22972342	48.0848392	10.94182	5.36398751
ENSG000001[illegible]	RUNX2	protein_codin	0	115.313242	0	0
ENSG000001[illegible]	LIN9	protein_codin	0	0	0	0
ENSG000001[illegible]	DNMT1	protein_codin	11.2381736	24.7165124	0	0
ENSG000001[illegible]	MBIP	protein_codin	0	0	0	0
ENSG000000[illegible]	ATP6V1H	protein_codin	0	43.6456297	8.07351977	12.2644291
ENSG000001[illegible]	KCNK5	protein_codin	0.26218035	7.51697349	6.42214372	3.65623643
ENSG000001[illegible]	BCR	protein_codin	0	21.5408903	19.0193732	0
ENSG000001	GTF3C2	protein_codin	0	0	0	0
ENSG000002	EMP2	protein_codin	26.2044558	95.7717076	55.8178949	51.3164161
ENSG000001[illegible]	UBAP1	protein_codin	0	0	0	0
ENSG000001[illegible]	CPLX2	protein_codin	0	0	0	0
ENSG000001[illegible]	GPAT2	protein_codin	0	0	0	0
ENSG000001[illegible]	SHARPIN	protein_codin	0	1.84818945	0	0
ENSG000001[illegible]	NUMB	protein_codin	0	0	0	0
ENSG000001[illegible]	HAND2	protein_codin	2.91921154	0	6.47939834	0
ENSG000001	DSE	protein_codin	36.8564608	0	66.1740312	19.2921803
ENSG000001[illegible]	PCNT	protein_codin	104.574899	325.54228	537.269161	77.3702991
ENSG000001[illegible]	INPP5D	protein_codin	2.04012707	192.988334	3.31247533	36.1648405
ENSG000001[illegible]	LFNG	protein_codin	0.80524722	3.6619594	0	0
ENSG000001[illegible]	AKR1B10	protein_codin	0	0	0	0
ENSG000001[illegible]	LRP10	protein_codin	26.5962555	140.66177	51.9776313	33.7673595
ENSG000002[illegible]	AP4M1	protein_codin	0	0	0	0

ENSG000001!	OR2T3	protein_codin	0	0	0	0
ENSG000001	HAPLN3	protein_codin	2.47614794	12.8189275	0.53331661	0
ENSG000001!	RYR1	protein_codin	0	0	0	0
ENSG000001	TP53	protein_codin	0	0	0	0
ENSG000001	ZSWIM5	protein_codin	2.63390592	2.36419931	18.9286343	2.07932734
ENSG000000	CBFA2T2	protein_codin	0	0	0	0
ENSG000001!	OR6K2	protein_codin	0	0	0	0.57537882
ENSG000002	H2AC17	protein_codin	13.1683225	130.746937	191.277335	9.88502073
ENSG000001	SDHAF2	protein_codin	0	0	0	0
ENSG000001	STX16	protein_codin	0	0	0	2.78866492
ENSG000001	CELA2A	protein_codin	0	0	0	0
ENSG000001	LRRC61	protein_codin	0	0	8.5125392	0.6318885
ENSG000001	CBS	protein_codin	0	0	9.74559907	0
ENSG000001!	TYW1	protein_codin	28.290687	0	109.352444	0
ENSG000001	BCL11A	protein_codin	0	0	0	0
ENSG000001!	SLC39A10	protein_codin	24.2739127	18.1833927	74.9343793	17.8848388
ENSG000000	CFH	protein_codin	0	0	0	0.70010616
ENSG000000	ATP2C1	protein_codin	0	0	64.02147	0
ENSG000001!	MBP	protein_codin	0	0	0	0
ENSG000002	RBM12	protein_codin	0	0	99.1960501	24.9804159
ENSG000001	OSCAR	protein_codin	0	0	0	0
ENSG000001	PGLYRP4	protein_codin	0	0	0	0
ENSG000001	ELF3	protein_codin	0.3090818	2.88797574	0	2.12133406
ENSG000000	ZRANB1	protein_codin	50.2796573	107.123576	60.1116409	45.4099948
ENSG000001	SUPV3L1	protein_codin	7.41025638	27.2424246	23.109998	4.93855143
ENSG000001	FN1	protein_codin	0	0	0	0
ENSG000001	SLC6A12	protein_codin	0	0	0	0.79818832
ENSG000001	NDRG3	protein_codin	6.08285582	13.0964859	0	0
ENSG000001!	NOL4L	protein_codin	21.30488	0	33.6629466	29.8259128
ENSG000001!	HIBCH	protein_codin	10.9648627	0	0	0
ENSG000001!	TRAPPC2	protein_codin	0	0	0	0
ENSG000001!	RPL37A	protein_codin	0	2.92694227	20.8280324	5.19494065
ENSG000001!	NIF3L1	protein_codin	0	12.5612422	24.3688066	4.67331302
ENSG000000	OSBPL6	protein_codin	9.27519742	0	4.1691678	0
ENSG000000	DNM2	protein_codin	0	0	67.0324793	0
ENSG000001	STYXL1	protein_codin	0	3.99839326	11.0361911	0
ENSG000001	RNF217	protein_codin	0.36624362	0	0	0
ENSG000001	G3BP2	protein_codin	0	0	0	0
ENSG000001	IFI16	protein_codin	94.9267162	0	66.0338132	67.7732581
ENSG000001	ITGB1BP1	protein_codin	9.96737484	15.6445103	44.7992463	2.81687207
ENSG000000	MGST1	protein_codin	0.2714925	3.08009673	2.37453638	1.73769667
ENSG000001	APP	protein_codin	0	0	0	0
ENSG000001	NIPA2	protein_codin	0	0	5.0273422	0
ENSG000001!	ZNF138	protein_codin	1.82378464	0	0	0

ENSG000000	RGS11	protein_codin	0	0	0	0
ENSG000001	SLC39A14	protein_codin	0	0	55.501959	0
ENSG000001	GRIP1	protein_codin	0	0	0	0
ENSG000001	PHOSPHO2	protein_codin	2.32815131	0	7.41232907	0
ENSG000001	DSG4	protein_codin	0.31024796	0	0	0
ENSG000000	STOML1	protein_codin	0.45621751	0	0	0
ENSG000002	EFNA4	protein_codin	0	0	0	0.80490004
ENSG000001	AKAP12	protein_codin	0	284.277573	84.9477346	0
ENSG000001	SPAG11B	protein_codin	0	0	0	0
ENSG000001	COL18A1	protein_codin	0	44.4908435	9.81545931	4.68912659
ENSG000001	WIPF1	protein_codin	0	0	0	0
ENSG000001	TLK1	protein_codin	60.8891475	29.5669132	201.370991	17.6595987
ENSG000001	WDSUB1	protein_codin	1.463505	6.66099989	7.17430706	3.90844106
ENSG000001	ZDHHC14	protein_codin	8.40582511	46.620572	35.3559366	17.1981464
ENSG000001	SIRT5	protein_codin	0	0	24.2714447	0
ENSG000001	AMZ2	protein_codin	0	0	0	0
ENSG000001	PTPN22	protein_codin	8.30207863	0	0	17.5687994
ENSG000001	MB	protein_codin	0	0	0	0
ENSG000001	ZNF675	protein_codin	12.7890249	14.6001226	36.0894213	2.81669998
ENSG000001	NPSR1	protein_codin	0	0	0	0
ENSG000001	CEP57L1	protein_codin	0	0	0	0
ENSG000001	PFKM	protein_codin	0	0	0	0
ENSG000001	ZNF808	protein_codin	6.92065145	49.7845091	10.1768442	4.03066604
ENSG000001	SIRPB2	protein_codin	1.31233426	8.92028265	0	0.81010959
ENSG000001	MDK	protein_codin	0	0	0	0
ENSG000001	MPDU1	protein_codin	0	0	0	0
ENSG000002	PLXNA4	protein_codin	3.70811131	0	0	16.02352
ENSG000000	TRAF3IP2	protein_codin	0	0	0	0
ENSG000002	ATP5MF	protein_codin	0	0	0	0
ENSG000001	DMD	protein_codin	0	0	0	0
ENSG000001	RXRG	protein_codin	1.7741572	1.16065002	0	1.279542
ENSG000001	FOXM1	protein_codin	0	12.9417832	10.4503561	0
ENSG000001	NT5C1B	protein_codin	0	0	0	1.48388996
ENSG000001	NTRK2	protein_codin	18.7509832	0	0	0
ENSG000001	ARPC5	protein_codin	8.33119202	77.4479841	15.1221335	14.6053318
ENSG000001	ZMYM1	protein_codin	8.40049645	0	7.48494128	0
ENSG000001	WASHC2C	protein_codin	0	0	0	0
ENSG000001	TMEM25	protein_codin	0	0	0	0
ENSG000001	TRRAP	protein_codin	0	0	0	0
ENSG000001	TEAD4	protein_codin	1.16533799	0	14.6348004	1.4305846
ENSG000001	MTCL1	protein_codin	0.70791997	0	0	0
ENSG000001	DDA1	protein_codin	1.75462813	3.85649317	8.17639334	2.63523272
ENSG000002	CSNK1E	protein_codin	3.46834582	0	0	0
ENSG000001	ASIC2	protein_codin	0.6212574	2.43830853	0.54542729	0

ENSG000001	SPINK5	protein_codin	0	0	0	0
ENSG000001	KCTD18	protein_codin	1.83938139	0	5.33789731	0
ENSG000001	PORCN	protein_codin	0	0	0	0
ENSG000001	AK3	protein_codin	10.1510957	7.36838384	11.4562011	9.05887118
ENSG000001	CTAG1B	protein_codin	0	0	0	1.43988228
ENSG000000	FAM3A	protein_codin	0	0	0	0
ENSG000001	RBM23	protein_codin	3.13926839	12.1083119	5.49964464	9.61953784
ENSG000000	BZW1	protein_codin	0	0	0	0
ENSG000001	PLPPR3	protein_codin	0.2487321	0	0	0
ENSG000001	PDLIM7	protein_codin	15.2495065	23.7512175	0	8.16344564
ENSG000001	ANKRD36B	protein_codin	127.503743	284.059808	318.528471	53.3808025
ENSG000001	CCDC88B	protein_codin	0	0	0.79816639	0
ENSG000001	AMZ2	protein_codin	10.9459656	0	21.3113372	5.69764261
ENSG000001	ART5	protein_codin	0	0	0	0
ENSG000001	ARHGEF18	protein_codin	0	0	0	0
ENSG000000	PHACTR3	protein_codin	0	0	0	0
ENSG000000	FRMPD1	protein_codin	0.27623173	1.1564889	0	0
ENSG000001	PML	protein_codin	0	0	0	0
ENSG000001	ADGRL2	protein_codin	0	27.3636421	0	15.640837
ENSG000001	SULF2	protein_codin	9.77641094	10.6842693	0	1.1037272
ENSG000000	ATG2B	protein_codin	36.2362561	154.589857	100.794645	38.6735537
ENSG000000	MPND	protein_codin	0	0	0	0
ENSG000001	KIF19	protein_codin	0	0	0	0
ENSG000001	IPP	protein_codin	0	0	6.24357667	0
ENSG000001	FAM3C	protein_codin	82.4759007	59.8960566	111.007501	22.3544133
ENSG000001	NDUFB5	protein_codin	0	0	10.5712664	6.52413916
ENSG000001	PTPRF	protein_codin	14.8111255	30.6380306	0	25.8923065
ENSG000001	P2RX4	protein_codin	0	0	0	0
ENSG000001	BRMS1	protein_codin	3.27814406	0	10.9797682	0
ENSG000001	OR2L3	protein_codin	0	0	0	0.70772888
ENSG000000	MPP4	protein_codin	0	0	0	0
ENSG000001	CCT8L2	protein_codin	0	0	0	1.31501339
ENSG000001	ZNF517	protein_codin	1.46370901	0	3.39912739	0
ENSG000001	APAF1	protein_codin	8.53207031	0	0	0
ENSG000001	ZNF135	protein_codin	0	0	0	0
ENSG000001	CCSER2	protein_codin	5.6153827	52.3284893	49.3850013	18.0688048
ENSG000001	ZFYVE27	protein_codin	4.10914537	20.7700894	0	0
ENSG000001	SIGLEC6	protein_codin	0	0	0	0
ENSG000000	ATP2A3	protein_codin	0	0	0	0
ENSG000001	CLN3	protein_codin	0	0	0	0
ENSG000001	NRAP	protein_codin	0	0	0	0
ENSG000001	SRSF2	protein_codin	16.1382717	0	82.4609246	28.5360243
ENSG000001	CRB2	protein_codin	0	0	0	0
ENSG000001	TCEAL8	protein_codin	8.5157309	49.9022327	13.4092827	5.81126377

ENSG0000007	SDF4	protein_codin	50.4307701	155.963319	183.212837	36.1462751
ENSG000001	MCMBP	protein_codin	15.2792012	0	0	0
ENSG000001	HLA-DRB1	protein_codin	22.7798672	131.671931	0	5.28483661
ENSG000001	DHDDS	protein_codin	0	12.3027828	20.2462072	0
ENSG000001	ZNF133	protein_codin	0	0	0	0
ENSG000001	RAI2	protein_codin	4.14661389	0	0	0
ENSG000001	ESRRG	protein_codin	0	0	0	4.88482619
ENSG000001	KALRN	protein_codin	0	0	0	0
ENSG000001	BRD4	protein_codin	33.6882781	69.4088284	93.6022592	35.9550248
ENSG000001	CLN3	protein_codin	0	0	0	0
ENSG000001	ARRB1	protein_codin	9.75803285	72.5178938	8.8384428	6.1278015
ENSG000001	SH2D1A	protein_codin	0	0	0	0
ENSG000001	DCAF12L2	protein_codin	0	1.06976425	3.73946447	0.90362176
ENSG000001	KCTD8	protein_codin	0.26796617	0	17.3584859	0.5223467
ENSG000001	ENTPD6	protein_codin	0	0	0	0
ENSG000001	DMBX1	protein_codin	0.24461348	0	2.08302144	3.69473037
ENSG000001	GAL3ST4	protein_codin	9.24256562	0	0	0
ENSG000001	HOXA4	protein_codin	0.49784056	0	1.02933767	3.2999519
ENSG000001	PDE4D	protein_codin	11.1918287	24.2516101	2.06389518	1.80459887
ENSG000001	SEPTIN2	protein_codin	0	0	9.64318029	0
ENSG000000	CD84	protein_codin	1.66415681	0	0	1.82756088
ENSG000001	TPK1	protein_codin	0	0	0	0
ENSG000001	GTPBP4	protein_codin	0	1.76449928	0	0
ENSG000001	PLOD2	protein_codin	28.5532829	0	115.029907	0
ENSG000001	CDK18	protein_codin	0	0	0	0
ENSG000001	FCER2	protein_codin	0.70521219	4.20508641	0	0
ENSG000001	XRCC6	protein_codin	102.506513	189.985717	254.869971	49.9701664
ENSG000001	PYY	protein_codin	0	0	0	0
ENSG000001	KIRREL1	protein_codin	8.97540987	9.2831677	12.6091165	6.61143721
ENSG000001	LCE1B	protein_codin	0	0	0	0.44132688
ENSG000001	EWSR1	protein_codin	0	540.365133	5.01516603	0
ENSG000001	ARL9	protein_codin	0	0	0	0.54131708
ENSG000000	KMT2C	protein_codin	6.56853997	77.1256093	19.6514992	0
ENSG000000	NFIX	protein_codin	170.817154	189.615298	296.096103	65.9538283
ENSG000001	CACNA1E	protein_codin	0	0	1.33121691	0
ENSG0000007	TFRC	protein_codin	0	0	199.88226	0
ENSG000001	MACF1	protein_codin	0	0	0	0
ENSG000001	ZNF107	protein_codin	0	0	0	0
ENSG000001	SPN	protein_codin	0	0	0	9.17872806
ENSG000001	CYP2D6	protein_codin	0	0	0	0
ENSG000001	LIAT1	protein_codin	0	0	0.54181707	0.63056216
ENSG000001	MAK16	protein_codin	26.2029032	19.8850484	69.127723	15.4280117
ENSG000000	AGBL5	protein_codin	1.16246187	2.76409001	5.8001728	1.6456314
ENSG000001	MAGED4B	protein_codin	0	38.3661609	41.6275291	0

ENSG000001	PPIP5K1	protein_codin	0	0	0	0
ENSG000001	CD300A	protein_codin	4.01587123	18.4245187	3.72382526	3.0933356
ENSG000000	FGFR2	protein_codin	4.66239459	0	10.2183368	3.66783898
ENSG000001	MYT1	protein_codin	0	0	0	0
ENSG000000	ERGIC2	protein_codin	50.94155	46.0517373	65.2571817	17.6199518
ENSG000001	NPIPA5	protein_codin	0	0	0	6.95114049
ENSG000001	SEPTIN6	protein_codin	1.97076411	10.0632743	0	2.30105557
ENSG000000	DDX3Y	protein_codin	23.1676121	10.8741763	0.45935193	0.85071519
ENSG000001	ADD3	protein_codin	34.3080322	34.4826301	2.41313463	22.2550411
ENSG000000	ZMYND10	protein_codin	0	0	0	0
ENSG000002	MFRP	protein_codin	0	0	0	0
ENSG000001	SCNN1A	protein_codin	0	0	0	0
ENSG000001	ZNG1C	protein_codin	0	0	0	0
ENSG000001	DYNC1H1	protein_codin	0	0	0	12.8487687
ENSG000001	ZNF605	protein_codin	4.06747852	0	1.15390245	5.08737436
ENSG000001	CDK5RAP2	protein_codin	0	147.512797	13.7843859	24.1445988
ENSG000001	FAM118B	protein_codin	0	1.17467674	1.7684038	2.16633599
ENSG000001	KIRREL2	protein_codin	0.52402787	10.8662125	0	8.08192033
ENSG000002	RTEL1	protein_codin	0	0	18.3916159	0
ENSG000001	ZNF66	protein_codin	1.48176919	2.63382546	1.25918509	0
ENSG000001	CD46	protein_codin	18.8576476	0	23.8005836	0
ENSG000001	OR52M1	protein_codin	0	0	0.67654713	1.04170847
ENSG000001	ETS2	protein_codin	0	0	0	0
ENSG000001	LHFPL5	protein_codin	0	0	0	0
ENSG000001	CTSE	protein_codin	0	0	0	0
ENSG000001	PTGES3L-AAR	protein_codin	0	0.90021249	0	0
ENSG000001	ADGRE2	protein_codin	3.60609711	21.0294112	0	4.73734081
ENSG000001	CACNA1A	protein_codin	0	0	0	0
ENSG000001	FOXP2	protein_codin	0.34728209	0	0	0
ENSG000001	CUEDC1	protein_codin	0	0	0	0
ENSG000000	MAP4	protein_codin	52.1136314	0	40.0363048	45.3771095
ENSG000001	CCDC102B	protein_codin	7.95009734	57.5144158	8.24499736	12.499639
ENSG000001	APH1A	protein_codin	9.04655308	62.7100898	14.8116025	8.30281762
ENSG000001	SUPT20H	protein_codin	21.5304355	0	17.1532618	0
ENSG000001	DISP1	protein_codin	0	0	4.11555578	1.30819144
ENSG000001	F8	protein_codin	11.3572341	14.6574138	7.61271043	54.8735245
ENSG000001	RIOX2	protein_codin	0	0	43.9357119	8.61883775
ENSG000002	CUX1	protein_codin	39.9862534	67.6241325	0	0
ENSG000001	AFAP1	protein_codin	0	24.9245391	0	0
ENSG000001	MSN	protein_codin	342.689366	668.769936	548.825527	189.613876
ENSG000001	UAP1L1	protein_codin	2.16611059	11.2691815	7.97706473	0
ENSG000001	ATP2B2	protein_codin	0	0	0	0
ENSG000001	PRKAR1B	protein_codin	0	0	0	3.91226467
ENSG000001	ADGRG2	protein_codin	0	0	0	0

ENSG000001	VPS13A	protein_codin	11.4695397	23.1533592	42.5280277	14.1839647
ENSG000001	CACNB4	protein_codin	0	0	1.13032876	1.82508318
ENSG000001	NR4A1	protein_codin	0	0	0	0
ENSG000001	SUDS3	protein_codin	0	0	0	0.49463861
ENSG000001	CEP85L	protein_codin	0	0	0	0
ENSG000000	SEZ6	protein_codin	0	0	0	0
ENSG000001	RAB5B	protein_codin	8.22211799	7.5548493	3.85810626	1.12663978
ENSG000001	PPIP5K1	protein_codin	10.0611895	0	0	0
ENSG000000	MYLK	protein_codin	0	0	4.76287103	25.584858
ENSG000001	BAZ1A	protein_codin	23.1637724	547.038676	8.25795155	89.0463523
ENSG000001	ZNF860	protein_codin	3.0785019	4.61714633	7.01994565	1.75980402
ENSG000000	CASS4	protein_codin	0	0	0	0
ENSG000001	GATAD2A	protein_codin	0	12.5416881	4.08386786	0
ENSG000000	GSDMB	protein_codin	0	5.0973789	4.91273069	1.16884785
ENSG000001	FLNA	protein_codin	207.442391	1102.83098	474.791791	193.319675
ENSG000001	C20orf96	protein_codin	0	0	13.6856981	4.34191619
ENSG000002	HLA-G	protein_codin	0	0	0	0
ENSG000001	CLDN7	protein_codin	0	0	0	0
ENSG000001	MAPK8	protein_codin	0	0	0	0
ENSG000001	ZNF460	protein_codin	88.0331157	132.195174	114.228528	79.7348827
ENSG000000	LZTS3	protein_codin	0.92683621	11.6496952	6.12645759	0
ENSG000001	CLEC4C	protein_codin	0	0	0	0
ENSG000000	RUNX1T1	protein_codin	0	0	0	0
ENSG000000	MAP2	protein_codin	0	0	0	0
ENSG000001	PARP9	protein_codin	0	33.1375647	7.61466345	18.9450282
ENSG000001	ZNF461	protein_codin	0	0	0	0
ENSG000002	OR2W3	protein_codin	0	0	0	0.82571882
ENSG000000	ANKS1A	protein_codin	23.0778912	48.8540972	57.6401124	16.5536852
ENSG000001	TIAM2	protein_codin	0	0	3.39463984	0
ENSG000001	SLC41A3	protein_codin	4.41980355	0	0	0
ENSG000001	OR5B21	protein_codin	0	0	0	0.52665558
ENSG000001	LRRCC1	protein_codin	69.8902783	67.2422183	124.981164	19.8932052
ENSG000001	SPRR2D	protein_codin	0	2.96292613	0	0
ENSG000001	STIL	protein_codin	0	0	0	3.28974339
ENSG000001	SDHAF3	protein_codin	0	0	2.99448943	2.29178964
ENSG000001	CDC16	protein_codin	0	0	0	0
ENSG000001	HNRNPK	protein_codin	88.9208765	177.339519	175.68901	63.1588548
ENSG000001	ZNF317	protein_codin	0	0	0	21.7555668
ENSG000001	SLC35F1	protein_codin	2.29735591	0	0	4.03025683
ENSG000001	TRIM9	protein_codin	0	4.03452289	0.57409723	0.54813645
ENSG000000	EIF2B3	protein_codin	4.19632457	26.5608828	20.2007659	3.43015518
ENSG000001	NRP2	protein_codin	9.34929426	119.747878	86.0994699	12.4239145
ENSG000001	SRGAP3	protein_codin	0	49.7388789	0	0
ENSG000002	CRCP	protein_codin	2.24853624	10.9454689	1.50656656	1.46120953

ENSG000001:	MYH10	protein_codin	0	0	0	0
ENSG0000024	UGT1A1	protein_codin	0	0	0	1.48167548
ENSG000001!	SLC22A6	protein_codin	0	0	0	1.21741868
ENSG000001	OPRM1	protein_codin	0	0	0	0
ENSG000002	SULT1A4	protein_codin	0	0	0	0
ENSG000001:	STAU1	protein_codin	32.0540796	159.34661	78.3527083	0
ENSG000001:	DSC3	protein_codin	0	0	8.2959073	0
ENSG000001:	MDM2	protein_codin	0	0	0	0
ENSG000001(	SIN3A	protein_codin	0	0	53.0680989	0
ENSG000001:	PAK4	protein_codin	2.06246263	8.43088623	15.3240447	5.05167158
ENSG000001(	SYTL3	protein_codin	0	14.2064291	0	0.69036386
ENSG000001:	CPNE8	protein_codin	0	0	0	0
ENSG000001	PPARGC1B	protein_codin	1.23850454	0	0	0
ENSG000001!	CLPSL2	protein_codin	0	0	0	0
ENSG000001:	MORF4L2	protein_codin	13.1909962	0	88.8249607	11.4444175
ENSG000001!	PLEKHG4	protein_codin	0	0	0	1.61514724
ENSG000001(	NDRG2	protein_codin	0	0	12.2318263	0
ENSG000001!	FABP12	protein_codin	0	0	0	0
ENSG000001(	ZNF528	protein_codin	5.96416072	46.2546469	27.8733681	0
ENSG000001(	UBE2J2	protein_codin	0	0	0	0
ENSG000001:	COL4A2	protein_codin	40.4200003	0	62.8789589	0
ENSG000001!	MAN2A2	protein_codin	19.4052293	0	0	0
ENSG000001(	PEA15	protein_codin	49.5804986	159.717927	42.9256327	37.4176122
ENSG000001!	KCNRG	protein_codin	0	0	0	0
ENSG000001!	NMB	protein_codin	0	0	1.96017874	1.55495242
ENSG000001!	NRAP	protein_codin	67.3685398	0	0	1.81047693
ENSG0000007	KCNQ2	protein_codin	0	0	0	0
ENSG000001:	SPOCD1	protein_codin	0	0	0	0
ENSG000001	SLC17A2	protein_codin	0.28242465	1.72429159	0	1.94893398
ENSG000001!	MME	protein_codin	12.5184034	28.8786393	0	2.38527567
ENSG000001	CCDC144A	protein_codin	0	0	0	0
ENSG000001(	SNTB2	protein_codin	0	2.40411551	0	0
ENSG000001:	TRPC6	protein_codin	0	10.764425	11.1603004	0
ENSG000001	CLEC4A	protein_codin	0	16.3498583	2.15538454	0
ENSG000001:	GSTCD	protein_codin	0	0	6.57036447	0
ENSG000001	ANGEL2	protein_codin	5.08232287	9.67502421	6.88568941	0
ENSG000001	CNPPD1	protein_codin	5.19878212	31.8137221	0	5.95719042
ENSG000001(	WDR72	protein_codin	0	40.4069876	0	0
ENSG000001:	CDC42SE2	protein_codin	26.9749655	80.5834225	55.0594687	32.5467083
ENSG000001	PFKFB3	protein_codin	0	0	0	0
ENSG000001	CCDC144A	protein_codin	0	0	0	0
ENSG000001:	PIGP	protein_codin	0.97735663	0	0	0
ENSG000001!	CES1	protein_codin	2.62899288	2.19010467	0	0
ENSG000001	KIF21B	protein_codin	3.77602366	135.570844	7.00598748	6.84808275

ENSG000001!	DPP4	protein_codin	0	0	0	0
ENSG000001(	CLIP3	protein_codin	14.745287	42.7289016	8.47509571	12.5713326
ENSG000001	PLAGL1	protein_codin	7.64379429	34.4052122	14.9079722	10.3572632
ENSG000001!	TOPORS	protein_codin	7.53337289	0	3.51134317	2.7020356
ENSG000001(	PCMTD1	protein_codin	0	0	34.2477918	4.67904285
ENSG000001:	ETF1	protein_codin	60.9800678	95.1334462	140.004584	81.2285739
ENSG000001!	KRTAP26-1	protein_codin	0	0	0	4.41805733
ENSG000001'	IL16	protein_codin	0	0	0	1.34879275
ENSG000001:	TACC2	protein_codin	0	0	0	0
ENSG000001!	MMP17	protein_codin	0	0	9.10698305	0
ENSG000001:	SCAF1	protein_codin	40.1472895	347.459906	95.9914145	33.7819901
ENSG000001'	RRM2	protein_codin	5.3038443	0	3.65032758	0
ENSG000000'	P4HA2	protein_codin	17.8136036	7.9789039	17.5333669	0
ENSG000001	FAIM	protein_codin	2.75307045	1.67305204	10.61611	4.77546336
ENSG000001'	TPPP	protein_codin	1.64511695	2.62296764	42.1075242	4.62467267
ENSG000001	VPS29	protein_codin	7.68526897	0	4.97970205	8.08629985
ENSG000001:	NPSR1	protein_codin	0	0	0	0
ENSG000001!	SLC6A9	protein_codin	0	0	0	0
ENSG000001!	WDHD1	protein_codin	13.2166523	21.5925868	107.328296	8.27836377
ENSG000001!	FBXL22	protein_codin	1.018294	1.01528502	2.30789096	0.4474932
ENSG000001	ANKRD53	protein_codin	0	0	0	1.93372156
ENSG000001:	STYXL1	protein_codin	0	0	3.74984967	0
ENSG000001(	TC2N	protein_codin	0	32.5401252	0	0
ENSG000001	MEF2D	protein_codin	11.0114086	22.7065539	3.23065236	29.0870562
ENSG000001:	ACSS2	protein_codin	7.59190404	17.3474734	14.4158839	3.13443004
ENSG000001	MACROH2A1	protein_codin	0	0	0	0
ENSG000001(	URI1	protein_codin	61.266955	144.727866	88.0865913	30.822532
ENSG000001:	MATN4	protein_codin	0	0	65.6236722	0
ENSG000001!	PIM3	protein_codin	3.38659051	27.0218129	16.7873573	21.7981193
ENSG000001!	LONP1	protein_codin	0	0	74.3779146	4.58084572
ENSG000001	KLK3	protein_codin	0	0	0	0
ENSG000001!	EPHB4	protein_codin	0	0	0	0
ENSG000001:	ARGLU1	protein_codin	63.4128985	0	65.1538018	58.0885774
ENSG000001:	DCLK1	protein_codin	7.18480686	47.7524213	19.6900454	9.85650894
ENSG000000	WWTR1	protein_codin	118.091957	121.622529	205.034833	91.5984264
ENSG000001	ITGB1BP1	protein_codin	5.18501983	7.5953811	1.90435874	3.42600152
ENSG000001(	C15orf39	protein_codin	0	0	0	0
ENSG000001(	CLCN3	protein_codin	0	26.1533398	42.2309435	8.68124952
ENSG000001(	ZNF148	protein_codin	97.1062305	71.0684843	147.008354	50.2418604
ENSG000000'	PAK3	protein_codin	0	0	0	0
ENSG000001:	PCED1A	protein_codin	5.02351824	21.3063159	15.992641	8.38353425
ENSG000001(	NAV2	protein_codin	50.286137	211.454046	150.735231	59.5841541
ENSG000001:	SLC17A3	protein_codin	0	0	0	0
ENSG000002:	TLR9	protein_codin	0	0	0	0

ENSG000001	PPP3CB	protein_codin	14.9530435	0	3.73879012	0
ENSG000000	ATG5	protein_codin	0	0	0	0
ENSG000001	MAP1LC3A	protein_codin	0	0	0	0
ENSG000001	FAM153A	protein_codin	0	0	0	0
ENSG000001	SLA2	protein_codin	0.27970654	0	0.93624423	0
ENSG000001	ADAM15	protein_codin	0	3.60369035	0	0
ENSG000000	LGALS14	protein_codin	0	0	0	0
ENSG000001	CSNK1G3	protein_codin	20.7308452	0	39.8716624	8.52736799
ENSG000001	GDPD3	protein_codin	2.12819558	0	0	0
ENSG000000	FUT8	protein_codin	0	0	0	0
ENSG000001	HTR4	protein_codin	0	0	0	0.66994184
ENSG000001	PPARD	protein_codin	6.71376495	120.027991	43.3533333	0
ENSG000001	ADARB1	protein_codin	0	0	0	0
ENSG000001	ZNF711	protein_codin	0	0	26.7218133	4.00953927
ENSG000000	ZC3HC1	protein_codin	0	0	0	0
ENSG000001	DRC7	protein_codin	0	0	0	0
ENSG000001	SQSTM1	protein_codin	83.9322355	335.187269	145.379577	63.3678685
ENSG000001	PAQR8	protein_codin	4.05357062	0	0	4.79707521
ENSG000001	ZNF567	protein_codin	0	0	0	0
ENSG000001	CLIC6	protein_codin	1.53485821	0	0	0
ENSG000001	TTI2	protein_codin	2.45159747	0	0	0
ENSG000000	ST7L	protein_codin	0	5.0792739	0	0
ENSG000001	RDH16	protein_codin	0	0	1.1322151	3.33501924
ENSG000001	SPATS2L	protein_codin	13.2231864	72.6533961	0	0
ENSG000000	CACNA1G	protein_codin	0	0	0	0
ENSG000001	VKORC1L1	protein_codin	0	0	0	0
ENSG000002	KRTAP12-2	protein_codin	0	0	0	1.02749732
ENSG000001	GYPA	protein_codin	0	0	0	0
ENSG000000	MYLK	protein_codin	24.0713079	7.02061582	0	0
ENSG000001	TRPM6	protein_codin	0	0	0	0
ENSG000001	SDCBP2	protein_codin	0	0	0	0
ENSG000001	C20orf203	protein_codin	0	1.41925389	0	0
ENSG000001	GJA9	protein_codin	2.64617541	6.66989589	2.08399246	5.85436013
ENSG000001	HNRNPA2B1	protein_codin	106.50749	972.233258	431.24197	71.2427921
ENSG000001	RBBP8	protein_codin	0.97925896	0	0	0
ENSG000000	CYB561	protein_codin	2.21373478	12.1787509	0	0
ENSG000001	BSCL2	protein_codin	0	0	0	0
ENSG000001	LSR	protein_codin	0	2.05799027	0	0
ENSG000001	UBAP2	protein_codin	0	0	0	0
ENSG000001	GTPBP4	protein_codin	46.4197477	48.2055263	197.264863	40.2055672
ENSG000001	SPP1	protein_codin	1.05369272	0	2.22242603	163.292144
ENSG000001	RRBP1	protein_codin	0	689.504143	0	0
ENSG000001	GLT8D2	protein_codin	110.275114	77.0738727	74.5245357	13.1837468
ENSG000001	FAM217B	protein_codin	9.29730998	54.895124	32.4755207	5.6426204

ENSG000001	C1S	protein_codin	125.069361	549.04709	0	82.6073652
ENSG000001	SCYL2	protein_codin	36.3244233	40.5289516	76.7591985	22.9076506
ENSG000001	CDK5RAP2	protein_codin	0	0	46.0476095	0
ENSG000000	TRPM3	protein_codin	0	0	0	0
ENSG000001	FAM177B	protein_codin	0	0	0	0
ENSG000001	RPS24	protein_codin	0.70301655	0	2.05959608	0
ENSG000000	NLE1	protein_codin	0	0	4.84288989	0
ENSG000001	ELMO3	protein_codin	0	0	0	0
ENSG000001	SCUBE1	protein_codin	3.51743199	100.282538	484.73435	24.248392
ENSG000001	ANKHD1	protein_codin	0	0	0	42.2585177
ENSG000001	MRPL37	protein_codin	13.3136058	10.2834671	29.4863122	3.2569563
ENSG000001	TLK1	protein_codin	0	0	8.66692121	0
ENSG000002	SIGLEC14	protein_codin	2.86451652	33.0953263	1.50798504	2.82935551
ENSG000001	GNL3L	protein_codin	35.9728183	126.748814	197.241111	23.6108519
ENSG000001	RGL1	protein_codin	79.1176445	249.430968	112.760218	90.9727731
ENSG000001	COL9A1	protein_codin	0	0	0	0
ENSG000001	NAA60	protein_codin	0	6.59006711	0	0
ENSG000001	RGS22	protein_codin	0	18.5494174	0	0
ENSG000001	DNAJC5	protein_codin	8.80784139	67.9990853	24.7903046	9.66122194
ENSG000001	NOL8	protein_codin	20.1045046	55.7347949	15.7293577	13.9260365
ENSG000001	TTN	protein_codin	204.540912	20.5161524	3.78624205	22.1500174
ENSG000001	PRDM10	protein_codin	0	0	0	0
ENSG000002	LAT	protein_codin	0	0	0	0
ENSG000000	GLI2	protein_codin	0	0	0	0
ENSG000001	EIF3B	protein_codin	0	88.3959118	0	24.4992291
ENSG000001	SOX10	protein_codin	0.28850955	0	0	0.88194574
ENSG000001	WDFY4	protein_codin	0	16.6535342	0	0
ENSG000001	MTHFSD	protein_codin	2.89583529	0	13.1753242	2.0071165
ENSG000001	TRRAP	protein_codin	0	19.6767487	29.9752406	4.48560387
ENSG000000	ERC1	protein_codin	26.2859188	0	42.5623525	38.4185472
ENSG000000	NLRC4	protein_codin	0	2.033579	1.60766358	0
ENSG000001	DAAM1	protein_codin	45.5716857	132.666512	168.324105	18.7758291
ENSG000001	ZMYND8	protein_codin	15.2782686	82.5053375	30.4371584	0
ENSG000001	PACRGL	protein_codin	0	0	0	0
ENSG000001	SEZ6L	protein_codin	2.52714887	0	0	1.90888414
ENSG000000	AOC1	protein_codin	0.64722581	0	0	0
ENSG000001	ETS2	protein_codin	21.7519858	54.2701396	35.1140808	30.0706302
ENSG000001	UBE2Z	protein_codin	21.64799	89.1625094	28.4921562	14.5028356
ENSG000001	TOMM20L	protein_codin	0	0	0	0
ENSG000001	ZNF219	protein_codin	0	0	0	0
ENSG000000	ZCWPW1	protein_codin	2.0327212	0	0	0
ENSG000001	HS3ST3B1	protein_codin	8.34732973	2.40889229	44.3676644	3.372464
ENSG000001	PPP1R13L	protein_codin	0.41531079	0	0	2.57236209
ENSG000000	MPC1	protein_codin	14.6960328	0	16.1351017	14.8419155

ENSG000000	CACNA2D2	protein_codin	0	0	4.95075767	4.34107634
ENSG000001	FGF1	protein_codin	0	0	0	0.95764821
ENSG000002	DBNDD2	protein_codin	8.79572202	12.6095923	0	9.63184723
ENSG000000	NCKAP1	protein_codin	124.654284	3.08249486	389.093495	66.9284349
ENSG000001	CPZ	protein_codin	5.9374182	0	0	9.95902651
ENSG000001	KCNIP3	protein_codin	0	0	0	0
ENSG000001	AEBP2	protein_codin	15.475621	0	9.52220118	6.47436554
ENSG000001	FAM107A	protein_codin	3.46372064	6.73391367	0	0
ENSG000001	STRBP	protein_codin	4.60186404	0	34.1046988	4.78770063
ENSG000000	PRSS3	protein_codin	0	0	0	0
ENSG000001	GIT2	protein_codin	12.07989	0	0	27.1799127
ENSG000001	MYO1C	protein_codin	8.51507595	71.3142822	32.2904937	0
ENSG000001	CTRB1	protein_codin	0	0	0	0.67936395
ENSG000001	DENND4C	protein_codin	0	0	0	0
ENSG000001	ZSCAN12	protein_codin	0.76101035	23.0680609	5.05331931	3.58043778
ENSG000001	TRDN	protein_codin	0	0	0	0
ENSG000001	TMEM239	protein_codin	0.25714539	1.32155741	0	1.28647797
ENSG000000	GRAMD4	protein_codin	0	0	0	10.7182036
ENSG000001	HAO2	protein_codin	0	1.36454887	0	0
ENSG000001	GPAA1	protein_codin	0	0	0	0
ENSG000001	GON4L	protein_codin	17.8126775	0	23.4653129	0
ENSG000001	WASF3	protein_codin	5.21953435	48.2963545	0	7.62119615
ENSG000002	RBMY1A1	protein_codin	0	0	0	0
ENSG000001	PLEKHS1	protein_codin	0	0	0	0
ENSG000001	MPEG1	protein_codin	64.0927839	404.596595	14.5772558	93.6602197
ENSG000001	BCO2	protein_codin	0	0	0	0
ENSG000000	WDR47	protein_codin	0	0	0	0
ENSG000001	UBA3	protein_codin	17.6272905	6.64246777	37.6257251	0
ENSG000001	MEPE	protein_codin	0	147.782835	11.9539384	133.828234
ENSG000002	MATR3	protein_codin	73.7024683	0	124.101716	68.4502998
ENSG000001	GFPT1	protein_codin	40.9711391	89.5851647	96.6608388	59.5347379
ENSG000001	GSTM3	protein_codin	0	0	0	0
ENSG000000	LAMC3	protein_codin	6.25973454	7.25977654	40.054207	3.29363127
ENSG000001	RFWD3	protein_codin	13.2791478	38.0769584	77.9594158	11.2738961
ENSG000000	PUM2	protein_codin	22.4560764	19.3492861	121.604805	18.2382611
ENSG000000	LAT2	protein_codin	0	0	0	0
ENSG000001	RAB25	protein_codin	0	0	0	0
ENSG000000	ZNF37A	protein_codin	18.619084	15.2101427	17.1517769	0
ENSG000001	NMRK1	protein_codin	7.93781066	0	3.93549997	0
ENSG000001	GART	protein_codin	0	0	10.1484257	0
ENSG000001	STAT1	protein_codin	44.0523961	315.898263	0	0
ENSG000001	PIK3CD	protein_codin	0	15.3011372	0	0
ENSG000001	SPAG11B	protein_codin	0	0	0	0
ENSG000001	ZNF627	protein_codin	18.2932465	20.2403592	38.4844454	0

ENSG000001	MICU1	protein_codin	0	19.2718897	0	9.35132091
ENSG000001	CCL28	protein_codin	1.62675096	0	4.77837445	6.68900247
ENSG000001	GPALPP1	protein_codin	0	0	0	0
ENSG000001	ITPRIPL1	protein_codin	0	22.5498906	19.8232028	3.23038528
ENSG000001	SMURF1	protein_codin	0	0	0	0
ENSG000001	LRIG2	protein_codin	25.7904806	86.6095019	40.9165805	14.0069567
ENSG000000	MARK2	protein_codin	0	0	0	0
ENSG000001	PPP1R14C	protein_codin	10.5166994	2.28798433	0.54881516	1.41865055
ENSG000001	RASGEF1C	protein_codin	0.2121077	1.78061119	0.86001889	2.53156381
ENSG000001	SLC34A3	protein_codin	0	0	0	0
ENSG000001	SMC5	protein_codin	164.784567	454.786811	435.576487	154.408164
ENSG000001	YY1AP1	protein_codin	0	5.30208737	0	0
ENSG000001	ABHD14B	protein_codin	2.49131697	0	7.79956545	10.8844886
ENSG000001	KAZN	protein_codin	1.88970236	34.2482583	18.3275981	0
ENSG000001	KCNN3	protein_codin	1.83170549	0	0	0
ENSG000001	ZWINT	protein_codin	0	1.3377141	18.7769789	1.22069084
ENSG000001	OPA1	protein_codin	0	0	0	0
ENSG000001	DZIP1	protein_codin	0	50.0616579	36.8197539	0
ENSG000001	IFI6	protein_codin	16.5442222	168.903776	145.011041	19.8465167
ENSG000001	RBM39	protein_codin	46.6598078	42.2012147	0	24.3056261
ENSG000001	POTEF	protein_codin	0.66727101	5.85657136	115.878019	13.8613566
ENSG000001	NF2	protein_codin	0	0	20.1052779	0
ENSG000001	CLK2	protein_codin	35.5617302	0	57.2075853	0
ENSG000001	IQGAP3	protein_codin	15.6093061	22.1033332	121.659153	0
ENSG000001	MVB12B	protein_codin	8.53083634	31.5043699	21.9032557	10.788967
ENSG000001	PAPSS2	protein_codin	24.0084292	16.7450744	40.1235952	34.1761745
ENSG000001	CAPZA2	protein_codin	34.6979385	65.119353	71.776017	39.3758314
ENSG000001	PRC1	protein_codin	0	0	2.56422511	3.66508329
ENSG000001	PJA2	protein_codin	224.197518	279.190794	576.855646	183.988853
ENSG000001	SLC26A11	protein_codin	0	0	0	0
ENSG000001	QKI	protein_codin	14.5510265	85.0729497	131.757297	0
ENSG000001	LDB1	protein_codin	4.01556447	51.1137463	1.96192323	0
ENSG000001	STYXL2	protein_codin	50.6875892	0	0	0
ENSG000001	DST	protein_codin	255.991827	771.610435	169.365326	288.551264
ENSG000001	SREBF2	protein_codin	0	0	88.8356804	0
ENSG000001	ASTN2	protein_codin	0	0	0	0
ENSG000000	PTGER3	protein_codin	0	0	0	0
ENSG000001	CSAG1	protein_codin	0	0	0.3358692	0
ENSG000000	ATP1A2	protein_codin	65.9419796	28.6252435	0	6.93429505
ENSG000001	DENND4B	protein_codin	12.839943	48.7474681	38.8249127	8.96544572
ENSG000001	SERBP1	protein_codin	35.3952712	82.2784976	0	19.5679759
ENSG000000	ARHGEF10L	protein_codin	7.45218874	40.9543693	22.5106543	15.5324937
ENSG000001	RUSC2	protein_codin	0	82.856401	26.036841	20.7188904
ENSG000001	MT-ND3	protein_codin	27.0902739	290.213412	89.8378853	160.23883

ENSG00000015	RASSF9	protein_codin	13.1044354	11.4464777	22.6362435	7.0747246
ENSG00000015	GPRASP3	protein_codin	5.10428463	9.21882913	0	0
ENSG000001	BTN3A3	protein_codin	0	86.735463	28.3540302	0
ENSG00000017	RNF34	protein_codin	13.6440115	58.2954981	0	0
ENSG00000013	TMEM123	protein_codin	0	5.9239519	0	0
ENSG00000015	ATG9A	protein_codin	13.4621041	7.71144681	0	4.39458137
ENSG00000017	AKAP13	protein_codin	115.4666	480.177865	133.34573	69.229167
ENSG00000015	CDC42BPB	protein_codin	48.7186117	126.565658	118.411987	27.0767075
ENSG000001	ARHGEF2	protein_codin	0	3.79691364	26.4979495	9.32747701
ENSG000001	MXI1	protein_codin	0	0	0	0
ENSG00000015	C8A	protein_codin	0	0	0	0
ENSG00000016	CD300LF	protein_codin	0	0	0	0
ENSG000001	TRPM6	protein_codin	0	0	18.3970458	0
ENSG00000015	SPOUT1	protein_codin	9.2911716	0	55.2150467	10.1233908
ENSG00000015	DCAF12	protein_codin	15.324927	74.2465294	30.2703834	16.0281568
ENSG00000010	HACD1	protein_codin	1.69653224	0	3.65055077	5.56091144
ENSG00000015	CNOT7	protein_codin	20.7062637	32.0734552	23.9455896	20.2388622
ENSG00000010	BNIPL	protein_codin	0	0	0	0
ENSG00000013	TBRG4	protein_codin	1.13652785	0	0	0
ENSG00000015	CHAMP1	protein_codin	11.4721401	0	33.6016982	0
ENSG0000000	TAF11	protein_codin	2.18846829	0	1.09483617	0
ENSG0000000	CTNNA2	protein_codin	0	0	0	0
ENSG00000013	CHD1L	protein_codin	0	32.5441645	0	0
ENSG0000008	MAST2	protein_codin	30.311923	45.193047	64.2153746	18.9456291
ENSG00000010	BEX3	protein_codin	37.9184575	76.3586915	124.657076	29.302092
ENSG0000008	PHACTR3	protein_codin	0	0	4.04853655	0.43870169
ENSG0000000	WNT16	protein_codin	0	0	0	0
ENSG00000010	AGL	protein_codin	0	0	0	0
ENSG00000010	LDLRAD4	protein_codin	0	0	0	0
ENSG00000015	EGFL6	protein_codin	0	0	0	0
ENSG00000010	LMNA	protein_codin	0	0	0	0
ENSG00000015	CD47	protein_codin	6.62957488	18.5883032	15.7041531	11.7298705
ENSG00000014	ZNF438	protein_codin	0	21.1083648	4.62696354	0
ENSG00000017	CLSTN1	protein_codin	31.4037465	0	270.345337	35.5715809
ENSG000001	GPX7	protein_codin	6.75458873	13.8947706	21.741133	5.68093809
ENSG00000017	TCEA2	protein_codin	0	0	0	0
ENSG00000010	ATP1B4	protein_codin	0	0	0	4.33945975
ENSG00000015	LRRTM3	protein_codin	0.26629774	1.51294331	3.4978704	0.9032695
ENSG00000012	ZNF644	protein_codin	1.5466208	1.63381035	4.8126663	3.40264358
ENSG0000000	CAMK1G	protein_codin	0	0.87229632	0	0
ENSG00000012	ZSCAN20	protein_codin	4.86412522	0	13.5956341	0
ENSG00000015	CTNND1	protein_codin	0	0	0	0
ENSG000002	MT-ND4L	protein_codin	51.1827934	77.2524186	165.091094	130.94192
ENSG00000015	TOP1	protein_codin	229.741306	559.780866	869.317951	196.779705

ENSG000001	IFT140	protein_codin	0	0	0	0
ENSG000001	FOXJ3	protein_codin	40.8491015	59.6319214	83.1627405	25.8866494
ENSG000001	UBL7	protein_codin	2.5651605	10.6215885	0.70388477	3.54973293
ENSG000001	VWA5A	protein_codin	5.61500978	12.6652587	0	10.1720086
ENSG000000	NCKAP1	protein_codin	29.3071991	17.768502	54.6125957	13.9464459
ENSG000002	AMY2B	protein_codin	2.08904869	28.4360221	0	7.48271391
ENSG000001	MAP3K3	protein_codin	0	69.365937	0	11.9482503
ENSG000001	POU3F3	protein_codin	6.48271303	0	114.231199	0
ENSG000001	ENTREP3	protein_codin	0.85478308	7.34370887	9.901633	1.55354569
ENSG000001	ZFP2	protein_codin	0	0	0	0
ENSG000001	EIF1AY	protein_codin	8.05636029	28.0325629	0	0
ENSG000001	CTR9	protein_codin	73.8875008	117.652609	325.485227	56.2886424
ENSG000001	SMURF1	protein_codin	15.6642396	62.8371042	56.1031466	24.1806717
ENSG000001	LTN1	protein_codin	58.9975935	81.9328139	238.749985	30.0631726
ENSG000001	LDB3	protein_codin	0	0	0	0
ENSG000001	MOCS2	protein_codin	0	0	0	0
ENSG000001	TNNI1	protein_codin	6.52924565	0	0	2.26655762
ENSG000001	MT-ND4	protein_codin	936.306746	4779.40512	3016.40363	3106.01959
ENSG000001	CTNNBL1	protein_codin	26.5617059	101.66182	58.5695605	25.9043276
ENSG000001	DCLRE1A	protein_codin	2.75731294	0	0	0
ENSG000000	ZFP64	protein_codin	3.19809483	5.59924166	14.9851034	3.64036807
ENSG000001	GSTZ1	protein_codin	0	0	0.85662096	0
ENSG000001	MT-ND1	protein_codin	310.558828	1692.14106	731.413366	1602.65534
ENSG000001	CTNND1	protein_codin	0	0	0	0
ENSG000001	ST3GAL3	protein_codin	0	0	0	0
ENSG000001	ESRRG	protein_codin	0	0	0	0
ENSG000001	DZIP1	protein_codin	0	10.535838	21.9605819	0
ENSG000001	ST3GAL3	protein_codin	0	0	0	0
ENSG000001	ARHGEF11	protein_codin	0	0	1.10081202	1.40349882
ENSG000001	KIAA0753	protein_codin	9.32700227	38.7120727	34.9339567	13.9150198
ENSG000000	PHLDB1	protein_codin	0	72.2529464	0	0
ENSG000001	KIF21A	protein_codin	15.094235	0	6.64348091	0
ENSG000001	CERS2	protein_codin	0	22.9687892	15.6569053	0
ENSG000001	IGSF1	protein_codin	0	0	0	0
ENSG000001	TOX	protein_codin	3.5420334	48.4656993	23.5079979	5.87852552
ENSG000001	TLR10	protein_codin	0	6.7333512	0	0
ENSG000001	HMGN2	protein_codin	56.1003194	118.048453	266.088247	51.6588156
ENSG000001	ZNF318	protein_codin	49.6209847	207.409028	238.351691	52.1216415
ENSG000001	DENND10	protein_codin	14.8573196	12.8810746	9.4183164	10.6685586
ENSG000001	IRAG2	protein_codin	0	0	0	0
ENSG000000	ADGRL1	protein_codin	11.5148745	47.7157272	37.853084	7.6413819
ENSG000001	RPL12	protein_codin	437.438347	444.018384	983.483684	465.261146
ENSG000001	NSMCE1	protein_codin	8.71403641	26.3282675	30.8482494	5.91009285
ENSG000002	APOBEC3C	protein_codin	18.480319	54.0657059	12.530444	12.1052983

ENSG000001(	SP8	protein_codin	0	0	0	0
ENSG000001:	ST6GALNAC4	protein_codin	2.58601656	0	2.86424765	0
ENSG000001!	MTOR	protein_codin	0	0	105.727806	0
ENSG000002:	GAGE12B	protein_codin	0	0	0	0
ENSG000001:	NF2	protein_codin	0	0	0	16.1640437
ENSG000001!	MT-ND2	protein_codin	306.990647	1345.88774	903.438251	1534.07821
ENSG000001!	TOGARAM1	protein_codin	14.6988348	57.1455421	69.7491038	0
ENSG000001!	OGA	protein_codin	177.917869	160.381521	402.346515	110.355967
ENSG000001!	MYBPC1	protein_codin	7.61646473	0	0	0
ENSG000001!	MAGEE1	protein_codin	1.42267051	8.5496531	4.00896156	3.75315645
ENSG000001!	DMD	protein_codin	0	0	0	0
ENSG000002·	PRR5-ARHGA	protein_codin	0	0	0	0.38436492
ENSG000001:	NRG2	protein_codin	1.00626531	0	0	0
ENSG000001!	TGM2	protein_codin	5.48637375	0	7.18435529	13.0778023
ENSG000001·	ASTN2	protein_codin	0	3.40956362	0	0
ENSG000001:	PJA1	protein_codin	3.48848578	2.34064715	17.1376669	0
ENSG000001!	MPHOSPH8	protein_codin	120.08627	453.166425	124.114254	60.1679747
ENSG000001!	PHETA1	protein_codin	0	0	0	0
ENSG000001!	LRRC10	protein_codin	1.02918579	0	0	3.42184791
ENSG000001	PSMD9	protein_codin	0	2.13223408	0	0
ENSG000001!	PAX9	protein_codin	3.19789754	0	13.6056825	0
ENSG000001(	FAM110B	protein_codin	0	0	0	0
ENSG000001(	HPS1	protein_codin	0	0	0	0
ENSG000000'	GLI2	protein_codin	24.5984265	18.6520218	56.8597162	8.78569334
ENSG000001:	BCL2L11	protein_codin	0	0	0	0
ENSG000001!	RCSD1	protein_codin	0	0	0	0
ENSG000000:	FAM135A	protein_codin	8.46552221	0	5.71156679	18.6394491
ENSG000001!	CES1	protein_codin	0	0	0	0
ENSG000001!	PNP	protein_codin	12.737253	22.632471	34.1093123	85.0247695
ENSG000000:	GAB2	protein_codin	0	16.6664115	0	0
ENSG000000:	IGSF9	protein_codin	0	0	0	0
ENSG000001!	OPA1	protein_codin	0	0	0	0
ENSG000000:	PPEF1	protein_codin	0	19.4065392	0	0
ENSG000001:	FCRL2	protein_codin	0	0	0	0
ENSG000001!	ZNF511	protein_codin	0	0	0	0
ENSG000000!	ACOT7	protein_codin	0	2.4963743	0	0
ENSG000001:	BECN1	protein_codin	13.4255044	0	30.1590607	7.66089915
ENSG000001!	ZNF521	protein_codin	81.8645402	100.936542	115.847314	18.1246827
ENSG000001!	ESRRG	protein_codin	0	0	0	0
ENSG000000:	CNOT4	protein_codin	0	0	0	0
ENSG000001·	SLC25A47	protein_codin	0	0	0	0
ENSG000001·	CNKSR1	protein_codin	0	0	0	0
ENSG000001·	MRPL24	protein_codin	7.83812861	7.1434413	51.4796838	2.25651736
ENSG000001·	ENSA	protein_codin	3.95848291	6.52627776	6.46575482	2.1822734

ENSG000002	CRYZL1	protein_codin	0.50841045	0	2.77747502	0
ENSG000001	ECI2	protein_codin	8.96127633	0	0	0
ENSG000001	BRINP2	protein_codin	0	0	0	0
ENSG000001	SGMS1	protein_codin	0.81735453	7.97398124	3.61997524	1.3820238
ENSG000000	CDC14A	protein_codin	2.41053864	26.3829027	11.1366816	10.2457866
ENSG000001	CLIP2	protein_codin	14.4254848	140.750589	38.7395091	24.0219695
ENSG000001	UBAP2L	protein_codin	0	0	0	0
ENSG000001	SELENON	protein_codin	0	0	2.64738361	0
ENSG000001	GRHL3	protein_codin	0	0	3.97097654	0
ENSG000001	FICD	protein_codin	0	0	0	0
ENSG000001	OSBPL9	protein_codin	0	0	0	0
ENSG000001	PJA2	protein_codin	7.66814269	186.532238	7.76460458	0
ENSG000001	MUC2	protein_codin	0	1.88998127	0	4.78738088
ENSG000000	MAP2	protein_codin	0	0	0	0
ENSG000000	WNT8A	protein_codin	0	0	0	0
ENSG000001	OSTC	protein_codin	125.568118	133.807043	429.663404	55.59108
ENSG000001	IPO9	protein_codin	64.3019269	115.234132	141.817895	29.0116874
ENSG000001	KRT19	protein_codin	0	0	0	0.73572185
ENSG000001	MT-ND5	protein_codin	572.442782	3800.4139	1295.3147	2377.09686
ENSG000002	PPT2	protein_codin	0	0	7.65118866	0
ENSG000001	MAPK10	protein_codin	0	0	0	0
ENSG000001	CAMTA2	protein_codin	1.89093773	0	6.61731033	0
ENSG000001	RBPJ	protein_codin	0	0	0	0
ENSG000001	SLC9A8	protein_codin	8.5444476	37.1639855	21.4835518	0
ENSG000001	R3HDM4	protein_codin	9.55344509	2.83273626	23.835112	0
ENSG000001	RPL39	protein_codin	233.95703	228.413419	505.834334	167.671762
ENSG000001	TOGARAM1	protein_codin	0	0	0	8.12307805
ENSG000000	KIFAP3	protein_codin	64.7339978	58.9589213	18.7243061	15.8792372
ENSG000001	ABLIM2	protein_codin	0	0	0	0
ENSG000001	DZIP3	protein_codin	7.09894714	0	17.239119	4.65952532
ENSG000001	ZFP69B	protein_codin	0	0	0	0
ENSG000001	HIPK1	protein_codin	0	0	0	0
ENSG000001	BCAN	protein_codin	0	0	0	2.05264754
ENSG000001	NLGN1	protein_codin	1.33611093	0	0	0
ENSG000000	CREM	protein_codin	0	0	0	0
ENSG000001	GPRASP1	protein_codin	12.8753253	43.6981579	49.0213908	31.6450843
ENSG000001	COL4A5	protein_codin	2.11479127	0	48.004928	0
ENSG000001	C1orf174	protein_codin	8.45034058	5.50237404	14.6596002	0
ENSG000001	DLX1	protein_codin	0	0	3.61189726	1.37532597
ENSG000001	PSMB5	protein_codin	12.2048688	16.4054194	50.5365553	8.83417078
ENSG000001	MFAP3L	protein_codin	10.3963953	0	9.41599936	6.94354318
ENSG000001	GTPBP3	protein_codin	0	0	0	3.76996667
ENSG000001	MT-CO1	protein_codin	5531.72787	9094.7071	14332.0141	8049.70057
ENSG000001	ZFYVE9	protein_codin	0	0	34.3814505	10.5753348

ENSG000001	ARHGAP11A	protein_codin	12.653786	9.16885494	29.1885214	5.24858791
ENSG000001	ENSA	protein_codin	0	0	0	0
ENSG000001	AKR7A3	protein_codin	0	2.02713305	0.57012199	0
ENSG000001	TSEN15	protein_codin	0	0	0	0
ENSG000001	MAGEB3	protein_codin	0	1.30759144	0	0
ENSG000001	ATP11C	protein_codin	20.7631138	0	73.09405	14.2498308
ENSG000001	CLIP1	protein_codin	67.9436708	0	63.886434	62.612259
ENSG000001	PCDH11X	protein_codin	0	0	11.1322537	0
ENSG000000	ULK2	protein_codin	5.88772836	31.7356554	3.94451614	4.19747999
ENSG000001	TYW1	protein_codin	0	195.651666	0	15.4356066
ENSG000001	SLC26A1	protein_codin	0	0	0	0
ENSG000001	SAXO5	protein_codin	0	0	0	0
ENSG000000	NFE2L1	protein_codin	36.9961564	115.473923	0	56.9185341
ENSG000001	GRM3	protein_codin	0	0	0	2.2466444
ENSG000001	LSM14B	protein_codin	0.78501891	23.8556126	4.75114132	0.75760189
ENSG000001	NAA35	protein_codin	30.6209526	41.5499671	82.5346243	18.2302107
ENSG000000	MSL3	protein_codin	0	0	0	0
ENSG000001	ALPK2	protein_codin	24.0374197	3.09319139	0	4.96336743
ENSG000001	CALD1	protein_codin	341.574382	2390.01739	343.004245	357.195127
ENSG000001	NF2	protein_codin	0	0	0	13.3584715
ENSG000001	RUNDC1	protein_codin	11.8676487	32.7219399	23.8221795	7.37640059
ENSG000001	ANKMY1	protein_codin	0	0	0	0
ENSG000001	WDR88	protein_codin	0	0	0	0
ENSG000001	MT-ND6	protein_codin	35.3275067	143.457477	39.7603143	38.0346497
ENSG000000	COMT	protein_codin	0	0	0	0
ENSG000001	TMEM184B	protein_codin	0	0	0	0
ENSG000001	MYBPC1	protein_codin	47.2657069	0	0	0
ENSG000001	ST6GAL2	protein_codin	0	0	0	0
ENSG000001	TBCK	protein_codin	0	10.4122228	8.87398699	0
ENSG000001	MACF1	protein_codin	0	0	0	0
ENSG000000	SRPK1	protein_codin	0	0	0	0
ENSG000001	TIA1	protein_codin	3.98677354	21.9194416	20.8469918	0
ENSG000000	UTS2	protein_codin	0	2.36069292	31.3749079	1.22981283
ENSG000001	L1CAM	protein_codin	1.18037488	0	1.51874224	0
ENSG000001	SLC17A3	protein_codin	0	0	0	0.43115296
ENSG000001	BABAM2	protein_codin	1.64364231	0	10.8125227	2.91607974
ENSG000001	APOL3	protein_codin	0	0	0	5.79943025
ENSG000001	OSGIN1	protein_codin	0	0	0.97713445	0
ENSG000001	ANKRD30A	protein_codin	0	1.1432099	0.69854106	3.25348782
ENSG000001	OPA1	protein_codin	0	0	0	0
ENSG000001	VAMP1	protein_codin	0	0	9.59662527	4.17156985
ENSG000001	TIFA	protein_codin	2.99696195	22.1531853	24.8515314	10.0923661
ENSG000001	GRM1	protein_codin	0	0	0	6.11242587
ENSG000001	GPATCH3	protein_codin	6.86072754	35.6794553	19.4967994	11.0767988

ENSG000001	TBKBP1	protein_codin	0	2.18864209	0	5.93955091
ENSG000001	FAM111A	protein_codin	0	0	0	16.6791734
ENSG000001	PCDH11X	protein_codin	0.71186673	0	69.8373787	0
ENSG000001	DLX1	protein_codin	0	0	18.2472643	0
ENSG000001	GJB1	protein_codin	0	0	0	0
ENSG000001	CNTNAP2	protein_codin	1.25321956	21.0745422	0	0
ENSG000000	E2F2	protein_codin	0	4.2809396	13.5653685	3.10149916
ENSG000001	SFT2D1	protein_codin	3.89629573	9.50833542	13.6341798	3.77266185
ENSG000001	RAD17	protein_codin	0	0	0	0
ENSG000001	MAP3K3	protein_codin	34.5643209	0	48.1860656	14.0503734
ENSG000000	METTL13	protein_codin	0	8.28605551	0	1.78892804
ENSG000001	ABLIM2	protein_codin	0	0	0	0
ENSG000001	MINDY1	protein_codin	11.2491167	0	0	2.02785961
ENSG000001	MT-CO2	protein_codin	184.277371	1377.02495	926.962589	623.733992
ENSG000001	CYB5R3	protein_codin	0	0	0	0
ENSG000001	ISLR2	protein_codin	0	0	0	0
ENSG000001	DMAP1	protein_codin	0	0	23.1216129	0
ENSG000001	ST3GAL3	protein_codin	0	0	0	0
ENSG000001	QKI	protein_codin	53.4353614	97.4126697	60.1073114	23.8121619
ENSG000001	RNF121	protein_codin	7.32718686	16.5357742	20.2540734	0
ENSG000000	SBNO2	protein_codin	3.15099466	97.8370641	27.2333739	0
ENSG000001	QKI	protein_codin	46.7748419	74.2547817	135.734705	64.2589057
ENSG000001	PCDH7	protein_codin	21.2659639	6.29428431	0.61143787	12.1205716
ENSG000001	CD3E	protein_codin	2.06949592	55.9063516	0	0
ENSG000001	WDTC1	protein_codin	4.4372441	24.6959602	15.3098336	7.29694893
ENSG000001	TGFB1I1	protein_codin	0	22.2684152	13.94237	6.28354285
ENSG000001	FOXJ3	protein_codin	0	0	0	0
ENSG000001	PIGO	protein_codin	0	14.9172002	0	0
ENSG000001	IL1RN	protein_codin	0	0	0	0
ENSG000001	SGMS1	protein_codin	18.729939	5.93325772	0	0
ENSG000000	RNF13	protein_codin	2.96028505	0	0	0
ENSG000001	CIPC	protein_codin	15.6106704	16.0826323	36.0486935	9.89438323
ENSG000001	MT-CYB	protein_codin	468.971951	1481.94268	1174.06004	1187.72067
ENSG000001	LSR	protein_codin	0	0	0	0
ENSG000000	PAX2	protein_codin	0	0	0	0
ENSG000001	L3MBTL3	protein_codin	9.83715169	50.2467781	14.0966698	16.3425109
ENSG000001	CELF4	protein_codin	0	0	0	0
ENSG000001	CTNND1	protein_codin	0	0	0	0
ENSG000001	RNF220	protein_codin	0	22.6047268	2.36867346	5.92270482
ENSG000001	ABCG1	protein_codin	6.31346224	53.6464492	5.39140655	31.4668592
ENSG000001	ST6GAL2	protein_codin	0	0	0	0
ENSG000001	SMC3	protein_codin	91.3327719	295.93079	277.48957	59.125403
ENSG000001	ZNF485	protein_codin	5.668552	12.0951839	29.9848222	3.4172191
ENSG000001	ST3GAL3	protein_codin	0	0	0	0

ENSG000001	SMG5	protein_codin	66.8184782	122.03841	98.0532527	24.2817765
ENSG000001	PSMD10	protein_codin	2.80215527	24.7306225	4.18190318	0
ENSG000000	MAP2K3	protein_codin	0	0	0	0
ENSG000001	GRIN3A	protein_codin	0.53441192	6.92986089	3.15676087	4.46360981
ENSG000001	ZNF277	protein_codin	7.84103178	4.94578424	3.6534939	6.46208389
ENSG000000	TRPM3	protein_codin	0	0	0	0
ENSG000001	HORMAD1	protein_codin	5.27749071	0	0	0
ENSG000001	OPA1	protein_codin	0	0	0	0
ENSG000001	YY1AP1	protein_codin	0	0	0	0.70173955
ENSG000001	BLOC1S2	protein_codin	0	0	0	1.55322217
ENSG000001	ASTN1	protein_codin	0	0	5.13730363	3.47197531
ENSG000001	FCRL5	protein_codin	0	22.6473541	0	0
ENSG000001	TEDC2	protein_codin	0	0	8.09129722	0
ENSG000001	SPRYD7	protein_codin	8.09425363	12.3930776	9.23812236	8.30043505
ENSG000001	XAF1	protein_codin	127.512904	223.934738	22.3043278	35.4371823
ENSG000000	ST7L	protein_codin	0	0	13.600486	0
ENSG000000	MOK	protein_codin	0	0	0	0
ENSG000001	PCDH15	protein_codin	0	0	0	0
ENSG000002	MT-ATP8	protein_codin	76.9573403	262.944137	311.026566	349.667565
ENSG000001	EPS8L3	protein_codin	0	0	0	0
ENSG000001	COL6A1	protein_codin	889.165928	2614.81107	543.902682	345.014811
ENSG000001	MSRB1	protein_codin	3.93999758	3.03028854	7.4769034	1.60422818
ENSG000001	GMEB1	protein_codin	0	26.3107253	0	0
ENSG000002	DCTN1	protein_codin	0	0	0	0
ENSG000001	TSC22D2	protein_codin	0	0	60.6909708	79.4417479
ENSG000002	KRTAP1-5	protein_codin	0	0	0	0.65090398
ENSG000001	TAFA3	protein_codin	0.78402585	0.9803998	0	1.22299925
ENSG000001	PNMA5	protein_codin	0	0	0	3.62520286
ENSG000001	ILF2	protein_codin	145.358473	80.8303313	359.952536	29.8653398
ENSG000000	JADE2	protein_codin	0	0	0	0
ENSG000001	NOS1AP	protein_codin	2.25830132	0	16.3316436	3.93612239
ENSG000001	MT-ATP6	protein_codin	476.017418	2080.87239	1085.45771	1570.42066
ENSG000001	SCAMP5	protein_codin	0	0	0	0
ENSG000001	CALD1	protein_codin	0	0	0	0
ENSG000001	GPX6	protein_codin	0	1.54876319	0	0.43775084
ENSG000001	CELF1	protein_codin	0	0	0	0
ENSG000001	TMEM184B	protein_codin	8.3048644	35.4788286	24.4738807	13.4397361
ENSG000001	OPA1	protein_codin	0	0	69.4756972	0
ENSG000001	CMTM3	protein_codin	0.77722702	0	0	0
ENSG000001	ARMCX6	protein_codin	12.5791512	42.1101875	23.6798775	7.59379737
ENSG000001	ZNF326	protein_codin	2.38858467	6.05892438	7.01506095	1.55769542
ENSG000001	AGL	protein_codin	72.6949755	72.5135132	245.338028	25.7466168
ENSG000002	COL11A2	protein_codin	0	0	0	0
ENSG000002	DIO1	protein_codin	0	0	0	0

ENSG0000010	MAG	protein_codin	0	0	0	0
ENSG0000004	PER3	protein_codin	33.3925326	0	160.136702	0
ENSG0000014	GOLGA4	protein_codin	327.774111	544.34702	843.908362	292.519927
ENSG000001	GABRG2	protein_codin	0	0	0	0
ENSG0000015	ARHGEF15	protein_codin	4.15807034	32.6952106	0	4.16383728
ENSG0000015	COLGALT2	protein_codin	20.936404	10.8200108	15.6435625	2.80794866
ENSG0000010	CD300A	protein_codin	0	0	0	0
ENSG0000014	MINDY1	protein_codin	5.17650099	3.19292932	8.75890242	1.39841581
ENSG0000009	SORBS1	protein_codin	0	0	0	0
ENSG0000015	ZNF277	protein_codin	12.2922584	32.5163231	31.6568478	7.35096521
ENSG0000011	IFT81	protein_codin	2.72001672	7.27207574	3.68045781	0.71121197
ENSG0000015	ZNF830	protein_codin	11.6168891	49.794591	19.703209	6.78243274
ENSG000001	FOXM1	protein_codin	0	0	0	0
ENSG0000015	SMOC1	protein_codin	13.5803172	26.0466166	0	6.40347625
ENSG0000011	ZNF384	protein_codin	0	0	0	5.44363934
ENSG0000013	KIF21A	protein_codin	27.8991427	14.2610256	86.0331311	0
ENSG0000017	CDY1	protein_codin	0	0	0	0
ENSG0000015	EPS8L3	protein_codin	0	0	0	0
ENSG0000015	CCDC152	protein_codin	2.4824356	4.8256229	0	5.54489688
ENSG0000015	PLXNB3	protein_codin	0	7.6676108	0	0
ENSG0000015	SFMBT2	protein_codin	0	0	0	0
ENSG0000010	SLC25A14	protein_codin	0	0	0	0
ENSG0000015	L1CAM	protein_codin	0	0	0	0
ENSG0000015	KIFBP	protein_codin	18.0176516	46.0675186	20.8163935	0
ENSG0000024	CNTF	protein_codin	0	0	3.29244336	2.41142071
ENSG0000010	PORCN	protein_codin	1.47060113	0	0	0
ENSG0000015	CSNK1G3	protein_codin	13.9898562	8.39401262	50.704489	0
ENSG0000013	PHF20L1	protein_codin	11.1005435	2.45363237	15.5335623	1.73494684
ENSG0000015	ITSN2	protein_codin	38.2748417	442.458323	109.763966	60.9785228
ENSG0000011	ASL	protein_codin	0	0	0	0
ENSG0000015	ASB12	protein_codin	0	0	0	1.02811126
ENSG0000018	PCGF3	protein_codin	4.56240129	62.6361682	28.8492598	18.0854261
ENSG0000011	BRDT	protein_codin	0	0	0	0
ENSG000002	CCDC7	protein_codin	5.60979978	6.1948242	20.1969335	0
ENSG0000015	GLMP	protein_codin	0	0	0	0
ENSG0000015	VEPH1	protein_codin	0	0	0	0
ENSG0000015	SHISA4	protein_codin	5.10748906	9.25725424	0	2.02273745
ENSG0000009	PTGS1	protein_codin	1.31926597	12.4267711	0.68608411	8.54673735
ENSG000002	MTCP1	protein_codin	0	0	0	0
ENSG000000	METTL13	protein_codin	11.1747238	28.6444745	22.1119878	8.35857307
ENSG0000012	IFI6	protein_codin	3.08493518	38.7980341	47.9399237	2.55633677
ENSG0000010	SLC39A13	protein_codin	0	0	0	0
ENSG0000012	RAB3IP	protein_codin	0	0	0	0
ENSG0000010	SLC9B2	protein_codin	0	0	0	5.0415915

ENSG000001:	SNX6	protein_codin	2.37386233	0	3.4265617	0
ENSG000001!	SUCNR1	protein_codin	3.88709071	5.13211768	0.48598343	15.3207239
ENSG000001!	MRPL21	protein_codin	4.33733556	0	12.7374318	1.14068619
ENSG00000:	NFE2L1	protein_codin	112.792277	306.316448	43.0053451	29.2435646
ENSG000002	BAG6	protein_codin	0	27.1449061	0	0
ENSG000001	ENSA	protein_codin	2.55537985	3.26623148	7.25940196	6.07042967
ENSG00000!	WHRN	protein_codin	0	0	0	0
ENSG00000:	CACNA1S	protein_codin	0	0	0	0
ENSG000001!	ZNF652	protein_codin	26.974515	141.361899	54.5337315	0
ENSG000001!	FAN1	protein_codin	0	0	0	0
ENSG000001	FAM9B	protein_codin	0	0	0	0.43188039
ENSG000001	DRD2	protein_codin	0	0	0	0
ENSG000002	NOTCH2NLA	protein_codin	123.703291	103.649962	166.375054	40.06327
ENSG000001	C1orf43	protein_codin	5.04709301	0	10.2915993	0
ENSG000002	MRPS6	protein_codin	0	0	0	0
ENSG000001!	MT-CO3	protein_codin	682.843333	1350.23537	1971.16695	1253.9831
ENSG000001!	CD247	protein_codin	0	0	0	0
ENSG000001:	FBH1	protein_codin	0	0	0	0
ENSG000002	CKLF	protein_codin	0	0	0	0
ENSG000001	CNR1	protein_codin	6.42694389	0	51.6347813	0
ENSG00000!	PCDH11Y	protein_codin	0	1.68953926	0	0
ENSG000001	UTY	protein_codin	1.08350253	0	0	0
ENSG000001	GAGE2A	protein_codin	0	0	43.1046965	1.53926799
ENSG000000	MBTPS2	protein_codin	1.37993891	9.91518953	4.57077175	1.17150678
ENSG000001:	SENP7	protein_codin	0	0	0	0
ENSG000001	ECT2	protein_codin	0	0	0	0
ENSG000001	GART	protein_codin	1.80272743	0	0	0
ENSG00000:	RNF10	protein_codin	0.84785686	0	0.59167264	1.77521596
ENSG000001!	SPATS2L	protein_codin	0	0	0	0.73943565
ENSG000001	SCNN1A	protein_codin	0	0	0	0
ENSG00000:	MTIF2	protein_codin	0	0	1.68260302	0
ENSG000001	FEZ1	protein_codin	1.79097931	3.6516883	0	0
ENSG00000	TNFRSF1A	protein_codin	0	76.2608924	3.01280386	2.54652682
ENSG000001	VSTM1	protein_codin	0	0	0	0
ENSG000002	PSG5	protein_codin	0	0	0	0
ENSG000002:	KANTR	protein_codin	0.37727215	2.77908614	1.98139845	1.45189321
ENSG000001	ZNF548	protein_codin	0	0	7.8904528	2.51626209
ENSG000001	NDRG2	protein_codin	0	0	0	0
ENSG000001!	STRN3	protein_codin	2.02533801	1.42956023	1.00075236	0.44743295
ENSG000001	SEPTIN2	protein_codin	0	1.77853648	0	0.6906499
ENSG000001	SAXO4	protein_codin	0	0	0	0
ENSG000002:	STMND1	protein_codin	0	1.68208263	0	1.70731815
ENSG000002	CSNK1E	protein_codin	1.82653299	8.90860913	8.22497455	0.47577633
ENSG000001	ECT2	protein_codin	0	0	0	0

ENSG000001	MLXIP	protein_codin	0.55927896	0	2.61905253	2.20047748
ENSG000001	RHNO1	protein_codin	0	5.78973236	0	0
ENSG000000	YBX3	protein_codin	0	0	0	0
ENSG000000	YBX3	protein_codin	0	0	0	0
ENSG000001	SLC15A4	protein_codin	0	0	1.04497283	1.01387929
ENSG000001	MTRFR	protein_codin	0	5.18895697	1.75950765	0
ENSG000000	ANAPC5	protein_codin	0.43420333	0	1.23610949	0.60088194
ENSG000001	C2CD3	protein_codin	0	0	0	0.53794285
ENSG000002	DPF3	protein_codin	0	0	0	0
ENSG000001	AKAP7	protein_codin	0	4.84713991	0	0
ENSG000001	FOXM1	protein_codin	0	1.23422	1.64012599	0
ENSG000000	QPCTL	protein_codin	2.20717224	0	0	0
ENSG000001	SH3BP5	protein_codin	0	0	0	0
ENSG000001	NUMA1	protein_codin	0	0	0	0
ENSG000001	RAP1GAP2	protein_codin	0	0	8.26087327	5.77650168
ENSG000001	ZPR1	protein_codin	0	0	0	0
ENSG000002	GP1BB	protein_codin	1.34402835	7.36389332	6.42906155	2.37525723
ENSG000001	IHO1	protein_codin	3.60639471	3.52146076	4.2527651	1.43120269
ENSG000001	ATAD2B	protein_codin	1.79497123	5.555789	0	0
ENSG000001	SHC1	protein_codin	0	1.70961411	0	0
ENSG000001	FOXRED2	protein_codin	2.24643227	12.1196679	16.9622366	2.03630693
ENSG000001	NDUFB6	protein_codin	0	0	0	0
ENSG000001	ZNF692	protein_codin	0	0	0	0
ENSG000001	SH3BP5L	protein_codin	15.500895	64.6580609	34.1537447	7.55956942
ENSG000001	OR2T4	protein_codin	0	0	0	0
ENSG000001	OR2M5	protein_codin	0.30919863	0	0	0.90526061
ENSG000002	OR2L2	protein_codin	0	0	0	0
ENSG000001	OR2AK2	protein_codin	0	0	0	0
ENSG000001	TRIM58	protein_codin	0.95020211	9.1324627	3.10084221	5.29095893
ENSG000002	OR14A2	protein_codin	0	0	0	0
ENSG000001	OR2C3	protein_codin	0	0	0	3.78053813
ENSG000001	GCSAML	protein_codin	0	0	0.57510206	12.4932348
ENSG000001	GCSAML	protein_codin	0	0	0	0
ENSG000001	GCSAML	protein_codin	0	0	0	0
ENSG000001	NLRP3	protein_codin	2.89913414	30.2791311	0	0
ENSG000001	ZNF669	protein_codin	0	0	0.60168968	0
ENSG000001	ZNF669	protein_codin	0	0	0	0
ENSG000002	ZNF670	protein_codin	2.14934582	2.0084701	7.06570118	0.39714503
ENSG000001	ZNF695	protein_codin	0	0	0	0
ENSG000001	ZNF141	protein_codin	0	0	0	0
ENSG000001	AHCTF1	protein_codin	0	0	32.4600707	14.1908583
ENSG000001	SCCPDH	protein_codin	10.5269469	25.1745092	37.2046832	5.95662637
ENSG000001	CNST	protein_codin	0.38815369	0	0	0
ENSG000001	CNST	protein_codin	3.74403683	0	12.5984941	10.6241662

ENSG000001	CNST	protein_codin	19.8450156	63.6917012	59.0047231	13.2488001
ENSG000001	TFB2M	protein_codin	4.26533426	5.80880866	21.8878723	2.99381384
ENSG000001	SMYD3	protein_codin	0.92260358	0	1.11946371	1.72406198
ENSG000001	SMYD3	protein_codin	3.10717204	0	1.93742128	0
ENSG000001	KIF26B	protein_codin	0	0	0	6.48376462
ENSG000002	EFCAB2	protein_codin	3.52377386	0	17.320038	3.00796149
ENSG000002	EFCAB2	protein_codin	0.64295064	5.34862266	18.3925753	3.96252994
ENSG000002	EFCAB2	protein_codin	3.75756212	0	4.10716426	2.08819814
ENSG000001	HNRNPU	protein_codin	42.4044467	121.747104	76.6570888	125.55886
ENSG000001	HNRNPU	protein_codin	0	8.04197651	44.9141692	17.9640008
ENSG000002	COX20	protein_codin	5.18059578	4.56735362	14.8606484	2.97283865
ENSG000001	CATSPERE	protein_codin	0	0	0	0
ENSG000001	CATSPERE	protein_codin	2.02146288	0	0.9654903	0
ENSG000001	CATSPERE	protein_codin	0	0	0	0
ENSG000000	ADSS2	protein_codin	26.1092951	50.7760059	27.9590445	20.6174669
ENSG000001	SPMIP3	protein_codin	0	0	0	0
ENSG000001	AKT3	protein_codin	25.8883766	0	0	0
ENSG000001	AKT3	protein_codin	0	0	0	25.3159243
ENSG000000	SDCCAG8	protein_codin	71.7294037	119.481141	125.254567	57.1370409
ENSG000001	CEP170	protein_codin	22.1252283	84.6183968	87.8010664	17.1027699
ENSG000001	CEP170	protein_codin	0	24.2143729	0	14.1495669
ENSG000001	CEP170	protein_codin	16.6060792	55.2103177	2.20733178	5.25641826
ENSG000001	PLD5	protein_codin	0	0	0	0
ENSG000001	EXO1	protein_codin	0	0	45.9312363	0
ENSG000001	WDR64	protein_codin	0	0	0	0
ENSG000002	CHML	protein_codin	51.2760043	58.1885058	194.598169	42.2854714
ENSG000000	OPN3	protein_codin	1.94799408	12.9011064	3.78082233	3.32184762
ENSG000001	KMO	protein_codin	0.25713422	2.72600265	0	0
ENSG000001	KMO	protein_codin	0	4.52902092	0	1.51941975
ENSG000001	KMO	protein_codin	0	0	0	0
ENSG000001	KMO	protein_codin	0	0	0	0
ENSG000000	FH	protein_codin	10.6355812	0	22.7534922	0
ENSG000001	RGS7	protein_codin	0	0	0	0
ENSG000001	RGS7	protein_codin	0	0	0	0
ENSG000001	RGS7	protein_codin	0	0	0	0
ENSG000001	ZP4	protein_codin	0	0	0	0
ENSG000001	RYR2	protein_codin	0	0	0	0
ENSG000001	MTR	protein_codin	24.4621875	37.2315585	56.6468367	0
ENSG000001	MTR	protein_codin	28.4003382	0	135.990927	0
ENSG000000	ACTN2	protein_codin	52.2359548	0	0	0
ENSG000001	HEATR1	protein_codin	0	0	0	0
ENSG000001	HEATR1	protein_codin	0	0	0	0
ENSG000001	HEATR1	protein_codin	75.8830716	133.433659	269.135149	40.8234515
ENSG000001	LGALS8	protein_codin	1.40343986	1.76745848	2.81395008	2.62226096

ENSG000001	LGALS8	protein_codin	38.7192067	13.1888943	0	41.9156177
ENSG000000	ERO1B	protein_codin	1.06959943	4.63786791	0	1.19479419
ENSG000000	GPR137B	protein_codin	0	0	0	0
ENSG000000	GPR137B	protein_codin	4.80614091	26.4691056	4.44427388	5.68350166
ENSG000001	NID1	protein_codin	105.317953	275.516309	343.909716	110.310744
ENSG000001	GNG4	protein_codin	0	0	0	2.97829155
ENSG000001	GNG4	protein_codin	0	0	0	0
ENSG000001	B3GALNT2	protein_codin	20.0123808	17.8157118	38.1978997	0
ENSG000002	TBCE	protein_codin	0	0	0	0
ENSG000000	ARID4B	protein_codin	132.594447	206.996296	421.885863	71.2043574
ENSG000001	TOMM20	protein_codin	48.4577078	50.257874	116.70744	49.0538284
ENSG000001	IRF2BP2	protein_codin	133.321769	141.550553	77.0008341	56.077317
ENSG000001	IRF2BP2	protein_codin	28.4576765	62.0795045	19.8184709	174.845632
ENSG000001	COA6	protein_codin	2.71598196	2.31668364	0.74023102	0
ENSG000001	COA6	protein_codin	6.77510309	0	38.2649179	6.27510532
ENSG000001	COA6	protein_codin	0.59679386	2.38657148	0	4.81295413
ENSG000001	SLC35F3	protein_codin	0.22138128	2.75349426	1.51960266	1.25124828
ENSG000001	SLC35F3	protein_codin	0	0	0	0
ENSG000001	KCNK1	protein_codin	0	0	5.87275795	4.71507298
ENSG000001	KCNK1	protein_codin	0.54122832	4.37273532	2.20352541	1.45254471
ENSG000001	MAP3K21	protein_codin	0	9.70723322	0	3.95329391
ENSG000001	MAP3K21	protein_codin	0.84287657	0	3.137223	2.3394301
ENSG000001	MAP3K21	protein_codin	1.07054831	0	12.1662318	0
ENSG000001	NTPCR	protein_codin	0.96893678	0.95812943	3.64945707	0.49518625
ENSG000001	NTPCR	protein_codin	10.8052879	4.37410011	10.5548956	8.81485744
ENSG000001	SIPA1L2	protein_codin	0	0	0	0
ENSG000001	DISC1	protein_codin	0	0	0	0
ENSG000001	DISC1	protein_codin	0	0	4.24828865	12.9885105
ENSG000001	DISC1	protein_codin	0	39.8316667	17.5448312	0
ENSG000001	DISC1	protein_codin	6.8523727	0	0	0
ENSG000001	TSNAX	protein_codin	22.328884	81.2865351	29.0984719	18.2959094
ENSG000001	EGLN1	protein_codin	33.57282	23.842195	79.0809153	29.1058116
ENSG000000	SPRTN	protein_codin	0	0	0	0
ENSG000001	EXOC8	protein_codin	16.987962	43.3112905	22.9107326	16.6913514
ENSG000001	GNPAT	protein_codin	0	55.5576479	58.94374	17.8126948
ENSG000001	C1orf131	protein_codin	0	0	15.6722933	0
ENSG000001	C1orf131	protein_codin	2.90234574	23.4357319	22.133845	2.81485051
ENSG000001	TRIM67	protein_codin	0	0	0	0
ENSG000001	FAM89A	protein_codin	0	4.73672678	15.8487874	2.89679817
ENSG000001	ARV1	protein_codin	14.5546568	21.2864996	38.4258804	0
ENSG000001	TTC13	protein_codin	10.5503474	21.172083	17.0648309	2.89292146
ENSG000001	TTC13	protein_codin	12.5421491	36.8843062	12.1646858	17.986292
ENSG000001	C1orf198	protein_codin	47.7015545	231.433412	108.384936	70.4413797
ENSG000001	CAPN9	protein_codin	0.20978985	1.16784042	0.38142618	0.40626502

ENSG000001	AGT	protein_codin	2.8495518	15.0252076	0.87536244	0
ENSG000001	COG2	protein_codin	0	0	0	0
ENSG000001	COG2	protein_codin	25.9591628	29.662324	51.8414601	11.1306249
ENSG000001	OTX1	protein_codin	0	0	2.90444412	0.81290923
ENSG000001	GALNT2	protein_codin	68.8116147	145.385095	170.214805	27.7153096
ENSG000001	TAF5L	protein_codin	0	1.1475469	0.72122021	0.93613053
ENSG000001	TAF5L	protein_codin	6.27072509	2.00348968	11.983893	19.2634077
ENSG000000	NUP133	protein_codin	0	0	0	0
ENSG000001	ACTA1	protein_codin	325.345384	0	0.97412707	0
ENSG000001	ACTA1	protein_codin	298.49305	3.14425261	0	2.2043604
ENSG000001	CCSAP	protein_codin	0.81336767	0	0	6.5233643
ENSG000001	CCSAP	protein_codin	12.9755427	0	56.3090694	5.13319344
ENSG000001	RAB4A	protein_codin	9.5563243	12.6607715	20.7208371	4.58567849
ENSG000001	RHOU	protein_codin	10.0457862	8.07426208	21.6815509	15.5582985
ENSG000001	H2AC25	protein_codin	2.48740992	0	0	1.2288176
ENSG000001	H3-4	protein_codin	0.34388786	0	0	0
ENSG000001	TRIM17	protein_codin	0	0	1.94938416	1.29754853
ENSG000001	TRIM17	protein_codin	0	0	0	1.3285195
ENSG000001	TRIM11	protein_codin	4.26883946	24.5892953	18.8111933	5.42349649
ENSG000001	OBSCN	protein_codin	4.86988206	17.0800414	20.1047941	9.99924631
ENSG000001	OBSCN	protein_codin	0	0	0	0
ENSG000001	IBA57	protein_codin	12.2468289	42.6096633	22.6963228	7.68998168
ENSG000001	GJC2	protein_codin	0.34391146	1.50491892	0.70199903	1.00251158
ENSG000001	GUK1	protein_codin	0	0	0	1.19931573
ENSG000001	GUK1	protein_codin	0	0	0	1.53417143
ENSG000001	GUK1	protein_codin	0	0	0	0.74643237
ENSG000001	GUK1	protein_codin	0	2.66643424	2.06255008	0
ENSG000001	GUK1	protein_codin	0	0	2.35184239	0
ENSG000001	GUK1	protein_codin	0	0	0	0
ENSG000001	GUK1	protein_codin	0	0	0	15.338639
ENSG000001	GUK1	protein_codin	2.16412078	3.85785724	1.92460887	0
ENSG000001	MRPL55	protein_codin	0	0	0	0
ENSG000001	MRPL55	protein_codin	0	0	0	0
ENSG000001	MRPL55	protein_codin	0	0	0	0
ENSG000001	MRPL55	protein_codin	0	0	0	0
ENSG000001	MRPL55	protein_codin	0	0	0	0
ENSG000001	MRPL55	protein_codin	0	0	0	0
ENSG000001	MRPL55	protein_codin	0	0	0	0
ENSG000001	MRPL55	protein_codin	0	0	0	0
ENSG000001	MRPL55	protein_codin	4.28043325	0	0	0
ENSG000001	MRPL55	protein_codin	0	0	0	0
ENSG000001	MRPL55	protein_codin	0	0	0	0
ENSG000001	MRPL55	protein_codin	0	0	0	0
ENSG000001	MRPL55	protein_codin	0	0	0	4.12531411

ENSG000001(	MRPL55	protein_codin	0	0	0	0
ENSG000001!	DLL1	protein_codin	1.63098151	10.9457321	89.4742921	11.4996719
ENSG000001;	PRSS38	protein_codin	0	0	0	0.85324249
ENSG000001·	SNAP47	protein_codin	0	0	0	0
ENSG000001·	SNAP47	protein_codin	0	0	10.0350638	0
ENSG000001·	CDC42BPA	protein_codin	171.922704	148.981828	407.342271	93.8667401
ENSG000001·	CDC42BPA	protein_codin	21.9907203	0	48.1938885	0
ENSG000001·	CDC42BPA	protein_codin	29.1035903	0	28.0722341	3.45298856
ENSG000001·	CDC42BPA	protein_codin	37.4202487	145.727344	100.115004	78.6292747
ENSG000001:	ERMARD	protein_codin	3.87528662	23.0397081	25.0413661	2.45146532
ENSG000001:	ERMARD	protein_codin	0	0	5.04276892	0
ENSG000001:	ERMARD	protein_codin	0	0	0	0
ENSG000001;	DYNLT2	protein_codin	0	0	0	1.25680898
ENSG000001(	COQ8A	protein_codin	0	24.9012783	24.2118067	11.4417025
ENSG000001(	COQ8A	protein_codin	17.3293665	0	0	0
ENSG000002;	ENSG000002;	protein_codin	0	0	3.6083915	11.3649001
ENSG000001:	PHF10	protein_codin	11.5865301	22.3595172	211.779168	2.98392698
ENSG000001·	PSEN2	protein_codin	0	0	0	0
ENSG000001·	PSEN2	protein_codin	0	0	0	0
ENSG000001·	ITPKB	protein_codin	0.55667226	2.35319765	0	13.6315232
ENSG000001;	THBS2	protein_codin	225.432024	0	0	63.4128364
ENSG000002(	STUM	protein_codin	2.03275722	13.2544809	1.54795099	2.0176966
ENSG000002(	STUM	protein_codin	0	9.39173112	0	0
ENSG000001·	PARP1	protein_codin	0.29480666	8.78710337	1.01526803	1.72873682
ENSG000001·	PARP1	protein_codin	0	0	0.56878416	0
ENSG000001·	PARP1	protein_codin	29.1405925	193.048847	151.704761	0
ENSG000001(	DACT2	protein_codin	0	0	0	0
ENSG000001(	DACT2	protein_codin	0	0	0	1.44427282
ENSG000001:	AFDN	protein_codin	0	20.8706372	22.1610288	2.54413337
ENSG000001;	LIN9	protein_codin	0	0	0	0
ENSG000001:	AFDN	protein_codin	0	0	2.75724623	0
ENSG000001;	MIXL1	protein_codin	0	0	0	0
ENSG000001;	ACBD3	protein_codin	183.455595	504.198802	391.618212	135.714835
ENSG000001(	H3-3A	protein_codin	1.79874225	2.53411716	5.56186572	2.88583481
ENSG000001(	H3-3A	protein_codin	2.98368439	13.6493375	13.571609	12.9304891
ENSG000001(	H3-3A	protein_codin	31.7493019	102.064969	154.429454	44.3783073
ENSG000001(	H3-3A	protein_codin	0.64704966	0	0	0
ENSG000001·	LEFTY2	protein_codin	0.23748226	0	0	1.29809005
ENSG000001	UNC93A	protein_codin	0	0	0	0
ENSG000001	UNC93A	protein_codin	0	0	0	0
ENSG000001:	GPR31	protein_codin	0.52491441	0	0	0.4464037
ENSG000001!	TMEM63A	protein_codin	14.7209098	42.6960835	49.4169238	15.2227465
ENSG000001·	EPHX1	protein_codin	0	9.82742904	2.9028153	0
ENSG000001·	SRP9	protein_codin	0.78082744	10.3249869	0	0

ENSG000001	SRP9	protein_codin	0	1.31784367	0	14.7691286
ENSG000001	ENAH	protein_codin	85.3231538	163.628296	113.826676	51.4061256
ENSG000001	ENAH	protein_codin	0	33.9305501	0	0
ENSG000002	CEP43	protein_codin	1.7171674	32.4554131	8.09796179	12.1590108
ENSG000001	DNAH14	protein_codin	0	0	0	0
ENSG000001	DNAH14	protein_codin	0	0	1.64854623	0
ENSG000001	DNAH14	protein_codin	0	0	9.6490654	0
ENSG000000	RNASET2	protein_codin	1.39307754	9.54909964	1.27420528	4.98982425
ENSG000001	CNIH4	protein_codin	1.09418754	0	5.80656178	0
ENSG000001	CNIH4	protein_codin	0	0	1.88969425	0
ENSG000001	CNIH4	protein_codin	0	0	1.31354116	0
ENSG000001	CNIH4	protein_codin	4.55239126	5.67232507	15.6254407	2.89894231
ENSG000001	FBXO28	protein_codin	13.4388877	9.0684918	44.4258208	10.2038785
ENSG000000	RPS6KA2	protein_codin	0	0	0	0
ENSG000001	RPS6KA1	protein_codin	0	0	0	0
ENSG000000	MPC1	protein_codin	1.36313431	0	0	0
ENSG000001	TBXT	protein_codin	0	0	0	0
ENSG000002	CAPN8	protein_codin	0	0	0.4380871	0.41817635
ENSG000002	CAPN8	protein_codin	0	0	0	0.46420524
ENSG000001	CCDC185	protein_codin	0	0.8784728	1.59816768	3.16213584
ENSG000001	TBXT	protein_codin	0	0	0	0.87173248
ENSG000001	SUSD4	protein_codin	0	0	0	0
ENSG000001	PDE10A	protein_codin	0	0	0	0
ENSG000001	PACRG	protein_codin	0	0	0.39343532	0
ENSG000001	PACRG	protein_codin	0	0	0	0
ENSG000001	TAF1A	protein_codin	0	0	0	2.32464938
ENSG000001	PRKN	protein_codin	0	0	0	0.7490592
ENSG000001	PRKN	protein_codin	0	0	0	0
ENSG000001	PRKN	protein_codin	0.97246714	0	4.44602579	0
ENSG000001	PRKN	protein_codin	0	5.27721287	0	2.42610423
ENSG000001	PRKN	protein_codin	0	0	0	0
ENSG000001	DUSP10	protein_codin	8.20175147	6.83619436	0.64555063	8.82225039
ENSG000001	HLX	protein_codin	2.23054674	8.31349058	16.4739995	6.73449938
ENSG000000	AGPAT4	protein_codin	0.66840636	0	1.7111335	0
ENSG000001	MTARC1	protein_codin	1.98204605	0	1.29015545	7.10646779
ENSG000000	AGPAT4	protein_codin	0	29.6328559	0	0
ENSG000001	MTARC2	protein_codin	6.86892725	17.9015472	13.4853327	4.57872085
ENSG000001	MARK1	protein_codin	0	0	0	0
ENSG000001	MARK1	protein_codin	0	0	3.16234779	0
ENSG000000	MAP3K4	protein_codin	0	0	0	19.9855651
ENSG000000	MAP3K4	protein_codin	0	0	0	0
ENSG000000	IARS2	protein_codin	35.7776499	70.1613233	92.602159	35.550566
ENSG000001	EPRS1	protein_codin	123.566475	286.495717	330.928175	97.0720822
ENSG000001	PLG	protein_codin	0	0	0	0

ENSG000001!	SLC30A10	protein_codin	0	0	0	0
ENSG000001	LYPLAL1	protein_codin	0	0	0	0
ENSG000001	LYPLAL1	protein_codin	0	0	0	0
ENSG000000!	TGFB2	protein_codin	2.15925851	0	0	1.39430968
ENSG000000!	TGFB2	protein_codin	60.9892841	73.5907812	68.4173	31.7572508
ENSG000000	RRP15	protein_codin	18.0672722	64.9521678	37.9846695	20.2274873
ENSG000001	SPATA17	protein_codin	1.2071041	0	7.35723629	1.05899029
ENSG000000!	GPATCH2	protein_codin	37.0581431	64.1540107	53.6158384	8.04346908
ENSG000000!	GPATCH2	protein_codin	9.46314093	14.6549551	9.60789125	14.7280959
ENSG000001!	ESRRG	protein_codin	0	0	0	0
ENSG000001!	ESRRG	protein_codin	0	0	0	0
ENSG000001!	ESRRG	protein_codin	0	0	0	0
ENSG000000	USH2A	protein_codin	0.32980758	0	0	0
ENSG000001	SLC22A2	protein_codin	0	2.22132827	0	2.59850143
ENSG000001	SLC22A2	protein_codin	0	0	0	0
ENSG000001	CENPF	protein_codin	147.860545	301.55591	1729.17588	0
ENSG000001	PTPN14	protein_codin	140.491803	252.83099	220.096409	108.000896
ENSG000001	SMYD2	protein_codin	11.2453234	29.827708	49.9382358	17.2522507
ENSG000001	PROX1	protein_codin	1.71596785	0	10.3854419	3.60629191
ENSG000001	RPS6KC1	protein_codin	0	0	0	0
ENSG000001	RPS6KC1	protein_codin	7.54133028	20.0239592	14.1901129	2.80699005
ENSG000001	ANGEL2	protein_codin	5.36337961	0	0	7.02076489
ENSG000001	SLC22A1	protein_codin	0	0	0	0.58679291
ENSG000001	VASH2	protein_codin	0	2.58495836	1.7472241	0.93586528
ENSG000001	VASH2	protein_codin	0	1.7177876	9.97449062	2.30282135
ENSG000001	VASH2	protein_codin	0	0	0	0
ENSG000001	VASH2	protein_codin	0	0	0	0
ENSG000001	VASH2	protein_codin	11.0769404	0	14.8007448	0
ENSG000001	VASH2	protein_codin	0	2.02513706	0	0
ENSG000001	FLVCR1	protein_codin	6.03390017	14.445532	16.8814951	0.50969926
ENSG000002	TATDN3	protein_codin	0	21.1499705	6.60939915	4.79577298
ENSG000002	TATDN3	protein_codin	0	0	5.58279672	1.00856221
ENSG000001	NSL1	protein_codin	0	0	2.80501734	8.31628314
ENSG000001	NSL1	protein_codin	29.5023915	39.1221963	36.4584839	16.8994284
ENSG000001	NSL1	protein_codin	0	0	0	0
ENSG000001	ATF3	protein_codin	0	0	0	0
ENSG000001	ATF3	protein_codin	0.45231037	12.6725679	0	6.05285188
ENSG000001	ATF3	protein_codin	0	0	0	7.03404695
ENSG000001	ATF3	protein_codin	0	11.9056745	5.04861468	0
ENSG000001	NENF	protein_codin	25.2932421	20.0308654	9.19788615	9.22273307
ENSG000001	DTL	protein_codin	5.0122645	9.90963748	31.9079367	1.61473304
ENSG000001	INTS7	protein_codin	0	0	8.60358288	0
ENSG000001	INTS7	protein_codin	0	0	0	0
ENSG000001	INTS7	protein_codin	11.4389834	48.622212	7.54403234	6.69767379

ENSG000001	LPGAT1	protein_codin	0	0	0	0
ENSG000001	LPGAT1	protein_codin	79.4337161	105.3417	109.0387	43.8115621
ENSG000001	NEK2	protein_codin	0	0	0	0
ENSG000001	NEK2	protein_codin	2.39031813	8.09444023	30.0588178	0
ENSG000001	SLC30A1	protein_codin	23.0849941	63.1337621	12.7561259	162.19074
ENSG000001	RD3	protein_codin	0	0	0	0
ENSG000000	TRAF5	protein_codin	11.4349077	39.3443188	20.1882649	8.59033549
ENSG000001	RCOR3	protein_codin	65.0322665	56.7916563	56.2628347	17.8630018
ENSG000001	RCOR3	protein_codin	0	7.62615459	0	1.07894324
ENSG000001	KCNH1	protein_codin	0	0	0	0
ENSG000000	HHAT	protein_codin	1.27468463	0	0	3.28190446
ENSG000000	HHAT	protein_codin	0	0	0	0
ENSG000000	SERTAD4	protein_codin	44.1928308	10.4411482	22.8203533	7.85830582
ENSG000001	SYT14	protein_codin	0	0	0	0
ENSG000001	SYT14	protein_codin	0	0	0	0
ENSG000001	IRF6	protein_codin	0	0	0	0
ENSG000000	TRAF3IP3	protein_codin	1.33781353	29.7301112	0	1.64561032
ENSG000000	TRAF3IP3	protein_codin	0	6.06252148	0	5.02623135
ENSG000000	TRAF3IP3	protein_codin	0	39.985052	0	0
ENSG000000	TRAF3IP3	protein_codin	0.69411707	0	0	0
ENSG000001	HSD11B1	protein_codin	2.13038784	0	0	0
ENSG000001	HSD11B1	protein_codin	0	0	0	0
ENSG000001	G0S2	protein_codin	57.3908736	108.883626	3.83833785	32.6357434
ENSG000001	LAMB3	protein_codin	0	6.67521168	2.02881902	0
ENSG000000	PLXNA2	protein_codin	83.9428054	133.091814	40.1933398	42.5148505
ENSG000001	MRPL18	protein_codin	16.0044352	33.8543968	44.8662053	4.85916703
ENSG000001	CD34	protein_codin	0.23274571	16.5451434	0	1.73454972
ENSG000001	CD46	protein_codin	14.8110914	0	43.518011	25.0683105
ENSG000001	CD46	protein_codin	32.947105	26.3138406	0	4.85014021
ENSG000001	CD46	protein_codin	0	0	0	0
ENSG000001	ACAT2	protein_codin	5.79893134	19.8945882	52.7175158	2.42578482
ENSG000002	CR1	protein_codin	0	0	0	0
ENSG000002	CR1	protein_codin	0	0	0	1.38390969
ENSG000002	CR1	protein_codin	0	0	0	0
ENSG000002	CR1	protein_codin	0	37.3250995	0	0
ENSG000002	CR1	protein_codin	0	0	0	8.24895991
ENSG000002	SOD2	protein_codin	0	0	0	0
ENSG000002	SOD2	protein_codin	0	0	0	18.3180934
ENSG000001	CR2	protein_codin	0	0	0	5.47805381
ENSG000001	CR2	protein_codin	0	0	0	0
ENSG000001	CR2	protein_codin	0	0	0	0
ENSG000001	CD55	protein_codin	0	0	0	0
ENSG000001	CD55	protein_codin	6.88772592	0	0	1.95052083
ENSG000001	TAGAP	protein_codin	2.97849459	39.4494587	0	7.52514704

ENSG000001:	RSPH3	protein_codin	24.8065337	48.9387725	118.668741	15.6118913
ENSG000001:	C4BPA	protein_codin	0	0	0	0
ENSG000001(	S100B	protein_codin	0	4.06171351	0	0.43029472
ENSG000000!	EZR	protein_codin	144.239135	328.766171	705.009315	0
ENSG000001:	C4BPB	protein_codin	0	0	0	0
ENSG000001:	C4BPB	protein_codin	0	0	0	0
ENSG000001:	PFKFB2	protein_codin	3.92964212	22.9885674	4.76805268	3.74368261
ENSG000001:	PFKFB2	protein_codin	5.8385737	36.4606245	10.9825721	8.61258836
ENSG000001(	SYTL3	protein_codin	3.0282997	12.8913625	0	6.62978464
ENSG000001(	YOD1	protein_codin	4.68675579	16.2490311	0	0
ENSG000001.	DYNLT1	protein_codin	4.15576211	0	0	0
ENSG000001.	DYNLT1	protein_codin	2.54970345	17.4913463	7.53939888	2.14100238
ENSG000001.	DYNLT1	protein_codin	11.7461911	68.5525753	16.4813612	6.54967005
ENSG000001.	TMEM181	protein_codin	0	0	0	0
ENSG000001(	FCMR	protein_codin	2.24591747	49.4378839	0	7.92968588
ENSG000001(	IL24	protein_codin	0	0	0	0
ENSG000001:	TULP4	protein_codin	16.9285841	31.0502746	19.8630532	7.75489107
ENSG000001(	IL24	protein_codin	0	0	0	0
ENSG000001(	IL20	protein_codin	0	0	0	0
ENSG000001:	TULP4	protein_codin	42.2562079	142.346396	155.240824	30.1390223
ENSG000001(	IL20	protein_codin	0	0	0	0
ENSG000001:	IL10	protein_codin	0	6.62636205	0	0.79876298
ENSG000001:	SERAC1	protein_codin	0	0	8.3192785	0
ENSG000001(	MAPKAPK2	protein_codin	57.0983563	124.559704	48.6975637	51.7238103
ENSG000001.	DYRK3	protein_codin	0	1.80007664	2.86896969	1.45309531
ENSG000001.	DYRK3	protein_codin	0	0	0	7.05730461
ENSG000001.	DYRK3	protein_codin	6.01870236	25.0932952	12.8360993	24.4827371
ENSG000000'	SYNJ2	protein_codin	1.20152119	6.45333511	15.8252493	2.69845203
ENSG000000'	SYNJ2	protein_codin	0.52514188	0	1.7262414	1.60078051
ENSG000001.	EIF2D	protein_codin	0	0	0	0
ENSG000000'	SYNJ2	protein_codin	0.37305407	0	0	0
ENSG000001'	SRGAP2C	protein_codin	104.279572	114.733651	87.0461209	64.4828434
ENSG000001!	AVPR1B	protein_codin	0.91767611	5.41954896	0	0.86195181
ENSG000001!	FAM72A	protein_codin	1.45148762	11.0959629	0	2.15952894
ENSG000001!	FAM72A	protein_codin	0	0	0	0
ENSG000001'	SLC26A9	protein_codin	0	0	0	0
ENSG000001'	SLC26A9	protein_codin	0	0	0	0
ENSG000001(	PM20D1	protein_codin	0	0	0	0
ENSG000001:	SLC41A1	protein_codin	7.72720044	69.5854147	19.7482614	20.3685752
ENSG000001	RAB29	protein_codin	11.1981087	17.7339368	6.17800438	10.3283374
ENSG000000(	NUCKS1	protein_codin	564.884579	1294.72959	1137.50653	330.81969
ENSG000002	TMEM242	protein_codin	0.30039006	3.98964292	1.80076041	0.43624147
ENSG000001:	SLC45A3	protein_codin	0.63532782	3.77244249	0.562325	3.00460683
ENSG000001'	MFSD4A	protein_codin	0	0	0	0

ENSG000001	LEMD1	protein_codin	0	1.16630759	0	0.98219941
ENSG000001	LEMD1	protein_codin	0	0	0	0
ENSG000001	LEMD1	protein_codin	0	0	0	0
ENSG000001	LEMD1	protein_codin	0	0	0	0
ENSG000001	LEMD1	protein_codin	0	0	0	0
ENSG000001	KLHDC8A	protein_codin	0	0	0	0
ENSG000001	KLHDC8A	protein_codin	0	0	0	0
ENSG000001	NUAK2	protein_codin	1.12418478	19.6263553	0.52538108	5.35924496
ENSG000001	TMCC2	protein_codin	0	0	0	0
ENSG000001	DSTYK	protein_codin	41.4176368	0	22.0079866	18.671036
ENSG000001	DSTYK	protein_codin	2.04197205	69.8655774	18.7725847	3.59600072
ENSG000001	RBBP5	protein_codin	6.54861644	0	0	0
ENSG000001	CLDN20	protein_codin	0	1.71230017	0	0
ENSG000000	TFB1M	protein_codin	6.64355002	12.9811386	23.002244	3.37698437
ENSG000001	TMEM81	protein_codin	1.66316249	1.30929756	0.99996025	0.42126772
ENSG000001	NFASC	protein_codin	0	0	0	0
ENSG000001	LRRN2	protein_codin	0	0	0.64208159	0.96111382
ENSG000001	LRRN2	protein_codin	0	0	0	0
ENSG000001	LRRN2	protein_codin	0.85816685	4.35820628	5.95438695	0
ENSG000002	SCAF8	protein_codin	7.80304479	177.700083	28.1528077	91.706744
ENSG000001	MDM4	protein_codin	6.43646103	31.0936319	3.36860419	3.37444397
ENSG000001	MDM4	protein_codin	1.35605851	0	1.96993482	2.407403
ENSG000001	MDM4	protein_codin	47.7681109	250.377924	88.8591168	62.3087101
ENSG000001	MDM4	protein_codin	0	0	0	0
ENSG000001	PIK3C2B	protein_codin	0	0	0	1.94439793
ENSG000002	SCAF8	protein_codin	11.356556	79.1343002	0	0
ENSG000001	PIK3C2B	protein_codin	1.24053102	0	21.8199699	0
ENSG000001	PPP1R15B	protein_codin	43.0609882	136.402608	67.3122214	177.664144
ENSG000001	KISS1	protein_codin	0	0	0	0
ENSG000001	CTSD	protein_codin	0	0	0	6.32111395
ENSG000001	ETNK2	protein_codin	0	0	0	0
ENSG000001	ETNK2	protein_codin	0.56463596	0	1.69071777	0.43912243
ENSG000001	ETNK2	protein_codin	0	0	0	0
ENSG000001	SOX13	protein_codin	3.55478166	0	2.37820592	3.14765992
ENSG000001	SOX13	protein_codin	9.00941361	27.0619032	7.90804019	6.26997589
ENSG000001	SOX5	protein_codin	0	0	12.0493515	0
ENSG000001	SNRPE	protein_codin	0	0	15.7718419	0
ENSG000000	ZC3H11A	protein_codin	137.901483	93.4980507	176.41566	62.1164509
ENSG000000	ZC3H11A	protein_codin	0	0	18.9345882	4.66471361
ENSG000000	ZC3H11A	protein_codin	0	0	9.36109804	0
ENSG000001	LAX1	protein_codin	0	0	0	0
ENSG000001	LAX1	protein_codin	2.60922545	27.1502303	0.50253246	4.5627435
ENSG000000	IPCEF1	protein_codin	2.10301157	0	0	0
ENSG000001	OPTC	protein_codin	0	0	0	0

ENSG000000	RGS17	protein_codin	5.68489119	4.5638942	12.4930822	3.01843364
ENSG000001	CHIT1	protein_codin	0	0	0	1.27275678
ENSG000001	MTRF1L	protein_codin	0	0	0	0
ENSG000001	MTRF1L	protein_codin	0	0	0	0
ENSG000001	MTRF1L	protein_codin	2.87161156	18.8572222	0	2.85116226
ENSG000001	ADORA1	protein_codin	0	0	0	0
ENSG000001	ADORA1	protein_codin	0	0	0	0
ENSG000001	PPFIA4	protein_codin	0	11.4052654	23.863492	1.69920409
ENSG000001	FBXO5	protein_codin	6.52153125	3.99704608	17.6271404	1.86803909
ENSG000001	TMEM183A	protein_codin	18.8810959	51.9492492	29.6145309	16.5595746
ENSG000001	VIP	protein_codin	0	0	0	0
ENSG000001	VIP	protein_codin	0	2.09670516	0	0
ENSG000001	MYCT1	protein_codin	5.07987637	60.7183054	4.65859803	22.7671626
ENSG000001	SYNE1	protein_codin	0	0	0	0
ENSG000001	CYB5R1	protein_codin	9.21245244	0	6.88653798	12.7279069
ENSG000001	SYNE1	protein_codin	0	0	0	0
ENSG000001	SYNE1	protein_codin	0	0	0	0
ENSG000001	ADIPOR1	protein_codin	0	35.3880734	29.2642943	0
ENSG000001	SYNE1	protein_codin	179.017182	341.0319	223.768834	94.2575032
ENSG000001	SYNE1	protein_codin	0	0	0	0
ENSG000001	SYNE1	protein_codin	0	0	17.1713397	0
ENSG000001	KLHL12	protein_codin	0	24.1393371	0	0
ENSG000001	KLHL12	protein_codin	0	0	0	0
ENSG000001	KLHL12	protein_codin	16.8456412	44.4533845	44.6261489	12.3337238
ENSG000001	RABIF	protein_codin	6.15795364	6.87067676	4.33687914	8.17828102
ENSG000001	KDM5B	protein_codin	0	0	6.78285919	6.56369243
ENSG000001	KDM5B	protein_codin	1.98657873	0	2.70027759	0
ENSG000001	SYT2	protein_codin	8.90553843	8.73145924	0	4.17042818
ENSG000001	SYT2	protein_codin	0	0	43.6819856	0
ENSG000000	PPP1R12B	protein_codin	0	0	0	0
ENSG000001	LGR6	protein_codin	0	5.19693562	0	0
ENSG000001	PTPN7	protein_codin	0	0	0	4.05119548
ENSG000001	GPR37L1	protein_codin	0	0	0	0.78634939
ENSG000001	ELF3	protein_codin	0	0	0	0
ENSG000001	ELF3	protein_codin	0	0	0	0.44555254
ENSG000001	RNPEP	protein_codin	0	0	0	0
ENSG000001	TIMM17A	protein_codin	7.99104585	0	18.6484042	3.33962858
ENSG000001	LMOD1	protein_codin	38.7160196	144.065683	3.7963704	10.2632674
ENSG000001	ARMT1	protein_codin	15.6931721	15.1456524	30.0824401	5.16415061
ENSG000001	NAV1	protein_codin	16.4932678	28.9107344	55.5396382	15.813538
ENSG000001	NAV1	protein_codin	0	0	0	0
ENSG000001	NAV1	protein_codin	75.7632008	146.279021	159.324943	56.3660792
ENSG000001	CSRP1	protein_codin	0	2.86102799	2.1753855	1.58791831
ENSG000001	MTHFD1L	protein_codin	0	0	0	0

ENSG000001:	MTHFD1L	protein_codin	0	0	0	5.21525514
ENSG000001	PHLDA3	protein_codin	10.7651236	14.8464786	4.70056428	5.24397665
ENSG000001	PHLDA3	protein_codin	10.1186143	30.2156305	2.96862794	3.43684933
ENSG000001	TNNI1	protein_codin	44.7551577	0	0	0.56221924
ENSG000001	LAD1	protein_codin	0	0	0	0.79454448
ENSG000001	TNNT2	protein_codin	0	0	0	0
ENSG000001	TNNT2	protein_codin	0	0	0	0
ENSG000001:	MTHFD1L	protein_codin	18.5518603	52.8457265	47.2152822	0
ENSG000001	TNNT2	protein_codin	0	0	0	0
ENSG000000	PKP1	protein_codin	0	2.12268972	0	2.02390081
ENSG000001:	PLEKHG1	protein_codin	0	0	0.49172845	0.5239274
ENSG000001	TMEM9	protein_codin	0	0	0	0
ENSG000001	TMEM9	protein_codin	15.868915	0	0	7.47197104
ENSG000001	TMEM9	protein_codin	0	0	0	0
ENSG000001	TMEM9	protein_codin	0	33.7978429	39.5460654	0
ENSG000000	IYD	protein_codin	0	0	0	0
ENSG000000	CACNA1S	protein_codin	0	0	0	0
ENSG000001	ULBP3	protein_codin	8.70675409	15.0033979	16.3559285	8.10391771
ENSG000001	RAET1L	protein_codin	0	0	0	0
ENSG000001	INAVA	protein_codin	0	0	0	0
ENSG000001	KIF14	protein_codin	0	27.3433367	96.0059746	0
ENSG000001	ULBP2	protein_codin	1.29119689	5.30208475	4.30316565	2.46220394
ENSG000001	ZNF281	protein_codin	21.4440908	0	0	13.2176256
ENSG000001	ZNF281	protein_codin	12.6397861	70.0324579	89.3050077	14.058838
ENSG000001	NR5A2	protein_codin	0	0	0	1.04548412
ENSG000002	RAET1G	protein_codin	0	0	0	0
ENSG000002	RAET1G	protein_codin	0.2605213	1.2119854	0.46086738	0.41437495
ENSG000001	NR5A2	protein_codin	5.51496401	9.98038671	3.25582875	7.40854321
ENSG000001	RAET1E	protein_codin	0	0	0	0
ENSG000000	PTPRC	protein_codin	3.53100344	10.7759428	1.53678292	1.35931139
ENSG000000	PTPRC	protein_codin	10.1977289	0	0	0
ENSG000001	LRP11	protein_codin	3.29734706	12.4753349	4.20574876	1.76234286
ENSG000001	WDR45	protein_codin	0.962368	0	0	0
ENSG000001:	PCMT1	protein_codin	4.50471339	1.37676704	32.802735	6.26273429
ENSG000000	PTPRC	protein_codin	0	0	0	0
ENSG000001:	PCMT1	protein_codin	0	0	0	0
ENSG000001	ATP6V1G3	protein_codin	0	0	0	0
ENSG000001	NEK7	protein_codin	0.43054621	0	1.43491987	0
ENSG000001:	PCMT1	protein_codin	20.5635128	28.8291963	26.3601605	7.34375892
ENSG000001	NEK7	protein_codin	70.9053559	86.4318446	52.3613031	33.0959132
ENSG000001	LHX9	protein_codin	2.96290737	0	9.62917612	0
ENSG000001	LHX9	protein_codin	0	0	0.68982724	0.51438153
ENSG000001	LHX9	protein_codin	0	0	0	2.25205378
ENSG000001	LHX9	protein_codin	0	0	0	0

ENSG000002	C1orf53	protein_codin	0.1835907	0	3.04684707	0
ENSG000002	DENND1B	protein_codin	1.24686341	0	0	0
ENSG000001	CRB1	protein_codin	0	0	0	2.64574547
ENSG000001	CRB1	protein_codin	0	0	0	0
ENSG000001	CRB1	protein_codin	0	0	0	0
ENSG000001	NUP43	protein_codin	0	0	7.49804857	0
ENSG000001	NUP43	protein_codin	0	0	0	0
ENSG000001	ZBTB41	protein_codin	42.1232722	39.6557583	52.8813487	19.3723751
ENSG000000	ASPM	protein_codin	2.48853228	16.953798	39.9716967	2.88491561
ENSG000000	ASPM	protein_codin	5.75291329	72.2331089	235.580668	0
ENSG000001	KATNA1	protein_codin	0	24.6916651	6.1543494	4.6567334
ENSG000001	F13B	protein_codin	0	0	0	0
ENSG000000	CFHR2	protein_codin	0	0	0	0
ENSG000001	CFHR4	protein_codin	0	0	0	0
ENSG000000	GINM1	protein_codin	72.1478148	105.327021	78.2502217	40.1339838
ENSG000000	CFHR2	protein_codin	0	1.8730727	0	0
ENSG000002	CFHR1	protein_codin	0	0	0	0
ENSG000001	CFHR3	protein_codin	0	0	0	0
ENSG000001	CFHR3	protein_codin	0	0	0	0
ENSG000000	CFH	protein_codin	357.543254	576.366359	213.947372	111.599857
ENSG000001	KCNT2	protein_codin	25.7142189	25.8379556	0	7.74861883
ENSG000001	B3GALT2	protein_codin	1.73910829	10.1176541	0.99590736	3.94124087
ENSG000001	CDC73	protein_codin	38.6863044	12.9527808	57.4581566	39.1226573
ENSG000000	GLRX2	protein_codin	1.39344176	6.38488054	1.8790955	0
ENSG000000	GLRX2	protein_codin	0	0	0	0
ENSG000001	RO60	protein_codin	40.3689482	67.3533299	100.602157	24.1405392
ENSG000001	RO60	protein_codin	0	0	0	0
ENSG000001	RO60	protein_codin	0	11.9145726	0	0
ENSG000001	RO60	protein_codin	6.78594223	0	0	0
ENSG000001	RO60	protein_codin	5.73405445	0	7.09546091	5.50787995
ENSG000001	UCHL5	protein_codin	0	0	0	0
ENSG000001	UCHL5	protein_codin	2.22754612	19.6014633	6.63205002	2.30089511
ENSG000001	UCHL5	protein_codin	0	0	9.59797222	4.85359033
ENSG000001	UCHL5	protein_codin	4.36534155	0	0	0
ENSG000001	UCHL5	protein_codin	11.5765878	0	47.7312423	12.4154071
ENSG000001	UCHL5	protein_codin	6.69186079	30.6694925	4.82532964	0
ENSG000000	TAB2	protein_codin	34.5607109	0	19.3444596	35.0751939
ENSG000000	RGS1	protein_codin	23.7990483	67.4031418	0	21.8346719
ENSG000001	RGS18	protein_codin	4.25777832	8.14181381	0	6.9791594
ENSG000001	BRINP3	protein_codin	0.23344932	1.39893528	0	0.89813298
ENSG000001	UST	protein_codin	20.6219395	55.6134024	26.9488666	40.1906769
ENSG000001	PLA2G4A	protein_codin	20.014912	22.447457	42.231375	7.55196204
ENSG000001	SASH1	protein_codin	125.6876	375.148701	156.200354	182.848955
ENSG000000	PTGS2	protein_codin	2.24299174	0	1.99669124	75.2818015

ENSG000001	SASH1	protein_codin	0.36752095	3.17851782	1.91136934	3.40601337
ENSG000001	ODR4	protein_codin	0	0	11.305492	0
ENSG000002	SAMD5	protein_codin	4.2677888	19.5142861	115.812043	4.62731986
ENSG000001	STXBP5	protein_codin	0	0	0	0
ENSG000000	TPR	protein_codin	457.662282	1010.95781	1316.16063	268.870925
ENSG000001	STXBP5	protein_codin	4.55591196	0	11.6572137	0
ENSG000001	STXBP5	protein_codin	0	0	0	0
ENSG000001	PRG4	protein_codin	0	0	0	0
ENSG000001	PRG4	protein_codin	0	0	0	0
ENSG000001	PRG4	protein_codin	22.2922773	5.70588993	0	1.38880297
ENSG000001	ADGB	protein_codin	0	0	0	0
ENSG000001	RAB32	protein_codin	14.410201	11.3602316	16.7934682	9.89948866
ENSG000001	IVNS1ABP	protein_codin	1.39676136	0	0	0
ENSG000001	IVNS1ABP	protein_codin	32.1684688	0	19.8778215	0
ENSG000001	SWT1	protein_codin	2.58647185	4.48866534	0	13.1407297
ENSG000001	SWT1	protein_codin	40.4595805	71.1424479	47.1492014	0
ENSG000001	SHPRH	protein_codin	56.4195742	100.589759	222.939995	38.7512478
ENSG000001	TRMT1L	protein_codin	39.998259	25.1744108	39.1550118	6.53771458
ENSG000001	RNF2	protein_codin	1.7015024	0	0	0
ENSG000001	RNF2	protein_codin	0	0	15.7676964	4.19482345
ENSG000001	NIBAN1	protein_codin	112.037089	285.214533	120.384559	74.8558915
ENSG000001	EDEM3	protein_codin	0	0	0	0
ENSG000001	C1orf21	protein_codin	9.89004558	10.0754208	6.11259276	0
ENSG000001	TSEN15	protein_codin	1.19647432	0	1.36305345	0.659048
ENSG000001	EPM2A	protein_codin	0	0	0	0
ENSG000001	COLGALT2	protein_codin	3.15318386	0	3.25872121	2.91849678
ENSG000001	COLGALT2	protein_codin	0.67730993	0	0	0
ENSG000001	UTRN	protein_codin	0	0	0	0
ENSG000001	UTRN	protein_codin	0.58631084	5.2137048	0.8748828	0.48753623
ENSG000001	UTRN	protein_codin	0	1.98325921	7.69611934	0
ENSG000001	ARPC5	protein_codin	17.2205647	31.4228128	25.4661986	10.0016568
ENSG000001	NCF2	protein_codin	0	24.2615941	0	0
ENSG000001	NCF2	protein_codin	0	41.56864	4.80691255	7.89034797
ENSG000001	SMG7	protein_codin	6.27913025	92.1319649	5.28948764	7.40966559
ENSG000001	SMG7	protein_codin	1.15740354	4.17197046	0.61855658	0.48759312
ENSG000001	UTRN	protein_codin	475.419734	2583.50441	1481.5456	529.141225
ENSG000001	SHCBP1L	protein_codin	0	0	0	0
ENSG000001	DHX9	protein_codin	105.425966	258.513017	497.181446	91.2630263
ENSG000001	NPL	protein_codin	0	21.702307	0	0
ENSG000001	NPL	protein_codin	0	0	0	0
ENSG000001	NPL	protein_codin	1.2371471	1.40021562	4.67606066	7.31158655
ENSG000001	NPL	protein_codin	2.82477323	12.0057239	0	2.57848815
ENSG000001	RGS8	protein_codin	0	0	0	0
ENSG000001	RGS8	protein_codin	0	0	0	0

ENSG000001	RGS16	protein_codin	1.35215942	16.1044809	4.17324454	27.9049727
ENSG000001	RNASEL	protein_codin	12.1659514	27.3282988	6.51822258	11.4840879
ENSG000001	RGSL1	protein_codin	0	0	0	0
ENSG000002	TEDDM1	protein_codin	0	0	0	3.04323149
ENSG000001	STX11	protein_codin	0.76703081	8.2762998	0	1.48332594
ENSG000001	SF3B5	protein_codin	5.27702747	17.1471844	22.627598	7.11174466
ENSG000001	CACNA1E	protein_codin	0	0	0	0
ENSG000001	PLAGL1	protein_codin	0	0	3.49751275	1.10101342
ENSG000001	PLAGL1	protein_codin	17.0839803	0	0	0
ENSG000001	CACNA1E	protein_codin	0	0	0	10.4031214
ENSG000001	PORCN	protein_codin	0	0	0	0
ENSG000001	LTV1	protein_codin	74.0550524	101.292452	248.622321	33.3944281
ENSG000001	IER5	protein_codin	31.2007584	74.6691202	30.6802769	121.009699
ENSG000001	MR1	protein_codin	2.74553519	2.2430166	0.58900985	0
ENSG000001	MR1	protein_codin	0	0	0	0
ENSG000001	MR1	protein_codin	20.4457427	58.1680622	11.4144507	16.4835649
ENSG000001	PHACTR2	protein_codin	42.2929971	65.0192153	110.525186	26.2290095
ENSG000001	PHACTR2	protein_codin	5.70499829	15.0424367	18.1366507	0
ENSG000000	FUCA2	protein_codin	0	0	0	0
ENSG000001	KIAA1614	protein_codin	0	0	0	0
ENSG000001	KIAA1614	protein_codin	7.72948648	52.6718282	24.5426823	14.0516868
ENSG000001	XPR1	protein_codin	10.1054267	14.1316799	166.867791	42.4594398
ENSG000001	XPR1	protein_codin	15.0739554	58.6933092	143.083307	34.1968995
ENSG000000	PEX3	protein_codin	10.9162686	38.7311339	24.5101758	5.41555546
ENSG000000	PEX3	protein_codin	0	0	0	0
ENSG000001	ADAT2	protein_codin	0	3.21915953	2.13610457	0.61782197
ENSG000002	ACBD6	protein_codin	31.599749	52.3518756	87.9064975	22.8404541
ENSG000001	AIG1	protein_codin	0.25219684	0	1.02368203	0
ENSG000001	QSOX1	protein_codin	7.37638389	34.7621254	19.5436409	11.1011679
ENSG000001	AIG1	protein_codin	0	0	0	0
ENSG000001	QSOX1	protein_codin	4.31426071	16.7745793	6.10148039	11.1125614
ENSG000000	HIVEP2	protein_codin	0	0	0	0
ENSG000000	HIVEP2	protein_codin	8.88355307	10.6732152	3.56872676	72.3874908
ENSG000001	METTL3	protein_codin	0	0	0	0
ENSG000001	CEP350	protein_codin	0	0	0	52.313099
ENSG000001	ADGRG6	protein_codin	0	0	0	0
ENSG000001	ADGRG6	protein_codin	8.05142593	31.379353	25.1018434	12.6148447
ENSG000001	TOR1AIP2	protein_codin	0	0	116.762409	0
ENSG000001	TDRD5	protein_codin	0	0	0	2.41346111
ENSG000001	NPHS2	protein_codin	0	1.34239139	0	0
ENSG000001	NPHS2	protein_codin	0	0	0	0
ENSG000001	AXDND1	protein_codin	0	0	1.33919883	0
ENSG000000	SOAT1	protein_codin	16.5883784	0	29.160041	14.9670914
ENSG000000	VTA1	protein_codin	10.9870245	0	28.3119022	0

ENSG000001	ABL2	protein_codin	0	0	0	0
ENSG000001	TOR3A	protein_codin	0	0	0	0
ENSG000001	TOR3A	protein_codin	10.8152459	52.1133446	35.4414351	5.7287727
ENSG000001	ANGPTL1	protein_codin	28.8007552	7.18831418	0	2.53308116
ENSG000000	VTA1	protein_codin	1.63328821	1.53269505	0.66794762	13.6988951
ENSG000001	RALGPS2	protein_codin	0	0	0	0
ENSG000001	RALGPS2	protein_codin	11.346077	0	39.3816716	19.2504229
ENSG000001	RALGPS2	protein_codin	0	48.0513827	0	0
ENSG000002	TEX35	protein_codin	0	1.17942368	0	0
ENSG000002	TEX35	protein_codin	0	0	0	0
ENSG000002	TEX35	protein_codin	0	0	0	0
ENSG000002	TEX35	protein_codin	0	0	0	0
ENSG000000	RASAL2	protein_codin	69.4868909	167.564577	34.7124049	34.6978781
ENSG000001	CITED2	protein_codin	24.2263447	45.8026419	29.7100551	70.9576884
ENSG000001	TXLNB	protein_codin	0	0	0	0
ENSG000001	ASTN1	protein_codin	1.94483595	0	0	0
ENSG000001	HECA	protein_codin	46.6142611	134.975365	50.6001359	74.875526
ENSG000001	ABRACL	protein_codin	5.72787793	20.2457559	17.0626891	6.87219577
ENSG000001	PAPPA2	protein_codin	0	0	0	0
ENSG000001	PAPPA2	protein_codin	4.44443746	8.23425297	0.95452385	5.58258937
ENSG000001	REPS1	protein_codin	0	0	29.2233735	0
ENSG000001	COP1	protein_codin	0	0	0	2.30664063
ENSG000001	COP1	protein_codin	21.2775547	0	9.29235941	0
ENSG000001	COP1	protein_codin	0	45.7533225	34.2121184	9.85970146
ENSG000001	TNR	protein_codin	0	0	0	0
ENSG000001	MRPS14	protein_codin	0.69516536	0	0.65837655	0
ENSG000001	CACYBP	protein_codin	13.4472704	32.5089618	36.7240502	16.6283529
ENSG000002	ECT2L	protein_codin	0	1.75485909	0.99534139	1.44197865
ENSG000002	GPR52	protein_codin	0.99325279	3.18758997	3.85756078	1.54152003
ENSG000001	RABGAP1L	protein_codin	0	0	0	0
ENSG000001	RABGAP1L	protein_codin	9.69713642	27.3457045	28.1227754	6.65968982
ENSG000001	RABGAP1L	protein_codin	0	8.30104794	7.21091565	0.63230953
ENSG000001	RC3H1	protein_codin	28.5258365	38.4436265	62.9802192	22.6202004
ENSG000001	RC3H1	protein_codin	53.0093314	147.110934	101.51565	58.8337653
ENSG000000	HEBP2	protein_codin	15.7126966	9.34624537	78.7361695	3.59389107
ENSG000001	SERPINC1	protein_codin	0	0	0.80375233	0
ENSG000001	ZBTB37	protein_codin	30.9205063	112.960359	61.4585947	23.3477548
ENSG000001	ZBTB37	protein_codin	0	0	0	0
ENSG000001	ZBTB37	protein_codin	0.99716448	0	3.02610347	3.14272616
ENSG000001	CENPL	protein_codin	3.87541995	5.60714847	5.32895234	1.84394578
ENSG000001	ANKRD45	protein_codin	0	0	0	0
ENSG000001	SLC9C2	protein_codin	0	0	0	1.36739816
ENSG000001	TNFSF4	protein_codin	3.17301611	0	0	0
ENSG000001	FASLG	protein_codin	0.56094396	14.4790947	0.47277564	1.32243403

ENSG00000	SUCO	protein_codin	15.2783594	62.0500028	124.444992	8.2297899
ENSG000001	C1orf105	protein_codin	0	0	0	0
ENSG000001	C1orf105	protein_codin	0	0	0	0
ENSG000001	C1orf105	protein_codin	0	0	0	0
ENSG000001	PIGC	protein_codin	0.9193559	0	3.27616436	0
ENSG000001	DNM3	protein_codin	2.60688146	0	12.8616599	4.36761613
ENSG000001	DNM3	protein_codin	1.82627878	2.65184812	6.98925282	9.6029394
ENSG000001	OLIG3	protein_codin	0.30603613	0	0	0
ENSG000000	METTL13	protein_codin	0	0	0	0
ENSG000000	IFNGR1	protein_codin	0	0	0	22.3933262
ENSG000001	VAMP4	protein_codin	1.23497927	32.2903788	0	1.69531754
ENSG000001	PRRC2C	protein_codin	0	0	0	0
ENSG000000	IL20RA	protein_codin	0.66062904	0	0	0
ENSG000000	IL20RA	protein_codin	0	0	5.30345505	0
ENSG000000	FMO4	protein_codin	1.39596229	7.92049376	8.34457394	2.40863468
ENSG000000	FMO1	protein_codin	0	0	0	0
ENSG000000	FMO3	protein_codin	0.74550038	55.4340241	0.41638952	5.72958104
ENSG000001	PEX7	protein_codin	0	0	0	0
ENSG000001	MROH9	protein_codin	0	0	0	4.1638091
ENSG000001	MROH9	protein_codin	0.63859756	0	0	0
ENSG000001	PRRX1	protein_codin	29.2362736	121.761646	13.9601115	16.4455559
ENSG000001	GORAB	protein_codin	2.31414909	38.8387512	12.2895506	0
ENSG000001	GORAB	protein_codin	0	0	6.27851406	0
ENSG000002	NTMT2	protein_codin	1.12644454	0	0	0.40615542
ENSG000000	KIFAP3	protein_codin	2.60811003	0	1.74600055	3.45321681
ENSG000000	KIFAP3	protein_codin	0	0	0	0
ENSG000000	SCYL3	protein_codin	10.0552722	0	0	2.55989662
ENSG000000	SCYL3	protein_codin	2.10347476	19.7699268	2.82752521	3.03278648
ENSG000000	SCYL3	protein_codin	0	37.789907	32.3536859	0
ENSG000000	SELE	protein_codin	0	0	0	0
ENSG000000	SELE	protein_codin	0	10.1523063	0	0
ENSG000000	SELE	protein_codin	0	0	0	0
ENSG000000	SELE	protein_codin	3.10598991	0	0	0
ENSG000001	MTFR2	protein_codin	0	0	8.07801696	1.82555056
ENSG000001	SELP	protein_codin	0	0	0	0
ENSG000001	SELP	protein_codin	0	0	0	2.11944968
ENSG000001	SELP	protein_codin	0	11.2400835	1.13391222	0
ENSG000001	F5	protein_codin	3.92054933	34.2222639	9.91501102	40.4563159
ENSG000001	F5	protein_codin	0.92628444	0	0	10.4419742
ENSG000001	AHI1	protein_codin	10.3394965	40.4472781	11.5455609	9.2239122
ENSG000001	AHI1	protein_codin	0	0	0	1.31359372
ENSG000001	SLC19A2	protein_codin	0	0	0	0
ENSG000001	CCDC181	protein_codin	0	0	8.80813418	0
ENSG000001	CCDC181	protein_codin	2.79442897	0	3.1991143	0

ENSG000001	BLZF1	protein_codin	4.40670402	22.6853644	9.53146106	0
ENSG000001	BLZF1	protein_codin	17.9711186	61.8084395	47.019364	13.6600023
ENSG000001	NME7	protein_codin	6.56764813	10.3786459	21.0575572	0
ENSG000001	MYB	protein_codin	0	0	0	0
ENSG000001	MYB	protein_codin	0	0	1.50351404	0
ENSG000001	ATP1B1	protein_codin	55.8652916	0	37.4937816	0
ENSG000001	ATP1B1	protein_codin	1.25457733	0	0	0
ENSG000001	DPT	protein_codin	267.36067	148.369607	36.3545761	54.8345449
ENSG000001	XCL1	protein_codin	0.57837157	3.50018138	0	1.41271729
ENSG000001	XCL2	protein_codin	0	1.60055001	0	0
ENSG000001	HBS1L	protein_codin	0	0	0	0
ENSG000001	TBX19	protein_codin	1.12697919	23.5232573	15.3086262	3.8627546
ENSG000001	HBS1L	protein_codin	7.5812158	15.5608761	39.9325115	16.0938651
ENSG000001	HBS1L	protein_codin	25.3148891	11.2623254	143.105274	9.59287465
ENSG000002	SFT2D2	protein_codin	0	0	0	0
ENSG000001	TIPRL	protein_codin	4.25450013	5.93670542	27.6216341	5.46619278
ENSG000001	TIPRL	protein_codin	38.0928982	45.8136123	74.0461209	14.4298851
ENSG000001	GPR161	protein_codin	0	0	0	0
ENSG000001	GPR161	protein_codin	0	2.67373229	0	0
ENSG000001	HBS1L	protein_codin	2.30465297	6.32219134	23.5165277	13.6347999
ENSG000001	GPR161	protein_codin	0	0	0	0
ENSG000001	DCAF6	protein_codin	6.32900343	0	0	15.4924085
ENSG000001	DCAF6	protein_codin	0	0	37.29824	0
ENSG000001	ALDH8A1	protein_codin	0	0	0	1.22258652
ENSG000001	MPC2	protein_codin	10.6303501	14.6659422	25.6177744	7.07665786
ENSG000001	ALDH8A1	protein_codin	0	0	0	0
ENSG000001	ADCY10	protein_codin	0	0	1.03999129	0
ENSG000001	ADCY10	protein_codin	0	0	0	0
ENSG000001	MPZL1	protein_codin	0.85306071	0	18.8445825	4.08520935
ENSG000001	RCSD1	protein_codin	21.144983	49.3130525	0	4.34642386
ENSG000001	SGK1	protein_codin	0	0	5.76073537	0
ENSG000001	SGK1	protein_codin	11.2382457	27.7064145	7.84187445	3.68910228
ENSG000001	SGK1	protein_codin	0.58227171	7.1503477	7.41023048	0
ENSG000001	POU2F1	protein_codin	0	0	0	0
ENSG000001	POU2F1	protein_codin	8.12243043	0	26.3447431	0
ENSG000001	POU2F1	protein_codin	27.6043788	0	88.9151442	34.0502437
ENSG000001	GPA33	protein_codin	0.33283886	2.54655447	0	0
ENSG000000	TBPL1	protein_codin	0	0	12.4587381	0
ENSG000001	MAEL	protein_codin	0	0	0	0
ENSG000000	TBPL1	protein_codin	7.38077801	7.01872161	15.4433454	1.9292056
ENSG000001	MAEL	protein_codin	0	0	0	0
ENSG000001	TADA1	protein_codin	6.96295191	3.46979055	15.0809962	3.5345593
ENSG000001	POGK	protein_codin	5.17326543	43.9994844	31.8933738	6.42954699
ENSG000001	POGK	protein_codin	23.5485401	146.603396	57.9354785	14.0610904

ENSG000001	UCK2	protein_codin	0	0	16.3828956	6.58664969
ENSG000001	TMCO1	protein_codin	43.0877332	120.595931	285.779029	23.9455992
ENSG000001	TCF21	protein_codin	0.22725844	0	0	0
ENSG000001	MGST3	protein_codin	0	0	0	0
ENSG000001	MGST3	protein_codin	3.1216158	0	0	0
ENSG000001	MGST3	protein_codin	0	16.044775	15.1554786	0
ENSG000001	MGST3	protein_codin	2.27179211	0	3.83629937	4.25536319
ENSG000001	MGST3	protein_codin	8.58112101	12.3818732	0	7.00665992
ENSG000001	LMX1A	protein_codin	0	1.13205651	0	0
ENSG000001	PBX1	protein_codin	2.59630589	0	38.9071473	0
ENSG000001	NUF2	protein_codin	1.27811469	0	13.58677	1.79010659
ENSG000001	RGS5	protein_codin	0	0	0	0
ENSG000001	RGS4	protein_codin	0	0	0	0
ENSG000001	RGS4	protein_codin	0	0	2.26468548	2.37573155
ENSG000001	RGS4	protein_codin	1.96320548	3.58082435	0	0
ENSG000001	CCDC190	protein_codin	0	0	0	0
ENSG000001	CCDC190	protein_codin	0	0	0	0
ENSG000001	CCDC190	protein_codin	1.69667671	1.42425218	0.55081262	5.98655616
ENSG000001	HSD17B7	protein_codin	0	0	0	0
ENSG000001	SLC18B1	protein_codin	0	0	0	0
ENSG000001	DDR2	protein_codin	448.6108	892.61229	751.041071	251.369842
ENSG000001	DDR2	protein_codin	0	0	0	0
ENSG000001	UAP1	protein_codin	27.2044234	22.1903949	23.6451542	0
ENSG000001	UAP1	protein_codin	0	16.2582678	47.3393714	36.0133166
ENSG000001	VNN1	protein_codin	2.29478622	7.57439929	0.88682607	9.86861613
ENSG000001	SH2D1B	protein_codin	0.71025908	0	0	1.5576647
ENSG000001	TAAR2	protein_codin	0	0	0	2.59869673
ENSG000002	ENSG000002	protein_codin	0	0	0	0
ENSG000001	SPATA46	protein_codin	0	0	0	0
ENSG000000	STX7	protein_codin	20.4340589	19.9131931	10.2073387	2.4280561
ENSG000001	OLFML2B	protein_codin	3.00823909	55.327617	9.50644403	0
ENSG000001	OLFML2B	protein_codin	271.497751	94.5488566	361.769436	174.239487
ENSG000000	STX7	protein_codin	73.5106464	134.246709	66.7478945	38.2920548
ENSG000001	ATF6	protein_codin	28.3367895	54.694783	34.2608274	26.1340176
ENSG000000	DUSP12	protein_codin	2.52133823	8.82742408	11.9902406	0.9179768
ENSG000001	FCRLB	protein_codin	0	0	0	0
ENSG000001	FCRLB	protein_codin	0.24031784	2.66524877	0	0
ENSG000001	FCRLB	protein_codin	0	0	0	0
ENSG000001	FCRLB	protein_codin	0	0	0	0
ENSG000001	FCRLA	protein_codin	0	0	0	1.43859607
ENSG000001	FCRLA	protein_codin	0	0	0	0
ENSG000001	FCRLA	protein_codin	0	0	0	0
ENSG000001	FCRLA	protein_codin	0	0	0	0
ENSG000001	FCRLA	protein_codin	0	0	0	0

ENSG000000	FCGR2B	protein_codin	2.36250729	0	0	0
ENSG000000	MOXD1	protein_codin	10.2845514	13.2702111	16.1301791	0
ENSG000001	FCGR3B	protein_codin	0	0	0	0
ENSG000002	FCGR3A	protein_codin	0	0	0	0
ENSG000001	FCGR2A	protein_codin	9.23528174	0	0	9.04118535
ENSG000001	CFAP126	protein_codin	0	0	0	0
ENSG000001	SDHC	protein_codin	13.2423096	41.1948899	46.4040042	7.18094124
ENSG000001	CCN2	protein_codin	81.1389992	293.676628	70.9565475	321.506564
ENSG000001	NR1I3	protein_codin	0	0	0	0
ENSG000001	NR1I3	protein_codin	0	0	0	0
ENSG000001	NR1I3	protein_codin	0	0	0	0
ENSG000001	NR1I3	protein_codin	0	0	0	0
ENSG000001	NR1I3	protein_codin	0	0	0	0
ENSG000001	NR1I3	protein_codin	0	0	1.02342971	0
ENSG000001	NR1I3	protein_codin	0	0	0	0
ENSG000001	TOMM40L	protein_codin	0	7.44952097	0	0
ENSG000001	TOMM40L	protein_codin	4.0541993	10.363358	14.5562611	4.79848043
ENSG000001	APOA2	protein_codin	0	0	0	0
ENSG000001	FCER1G	protein_codin	2.742395	0	0	3.03675291
ENSG000001	NDUFS2	protein_codin	0	0	0	0
ENSG000001	ADAMTS4	protein_codin	6.3147883	9.20490171	4.89206205	24.9061569
ENSG000001	ADAMTS4	protein_codin	8.56211982	27.5103617	4.69851701	49.204876
ENSG000001	B4GALT3	protein_codin	0	17.4090386	0	0
ENSG000001	PPOX	protein_codin	0	0	0	0
ENSG000001	USP21	protein_codin	0	0	7.51396394	0
ENSG000001	USP21	protein_codin	2.9602067	0	0	0
ENSG000001	UFC1	protein_codin	12.0732883	33.795293	34.0775523	11.266188
ENSG000001	DEDD	protein_codin	0	1.70908646	0	1.00562716
ENSG000001	DEDD	protein_codin	0	25.9205084	0	4.13245873
ENSG000001	NIT1	protein_codin	0	0	0	2.6594442
ENSG000001	NIT1	protein_codin	0	4.2056574	1.50201312	3.03754177
ENSG000001	NIT1	protein_codin	8.07589745	11.3438101	15.8259628	0
ENSG000001	PFDN2	protein_codin	45.0609293	65.9338013	230.674845	36.5270489
ENSG000001	KLHDC9	protein_codin	0	0	0	0
ENSG000001	NECTIN4	protein_codin	0	0	0	1.74956225
ENSG000001	ARHGAP30	protein_codin	40.6257763	705.105732	5.0995841	42.4881639
ENSG000001	ARHGAP30	protein_codin	0	0	5.46285567	0
ENSG000001	ARHGAP30	protein_codin	0	0	0	0
ENSG000001	ARHGAP30	protein_codin	0	0	0	0
ENSG000001	USF1	protein_codin	0	0	0.70266695	0
ENSG000001	USF1	protein_codin	0	0	0	0
ENSG000001	USF1	protein_codin	6.73317148	48.1223871	0	3.12777403
ENSG000002	TSTD1	protein_codin	0	0	0	0
ENSG000002	TSTD1	protein_codin	0	1.53319807	0	1.27107948

ENSG000001	F11R	protein_codin	6.52393692	24.3846367	12.1622501	13.6392912
ENSG000001	ITLN2	protein_codin	0	0	0	0
ENSG000001	CD244	protein_codin	0	0	0	0
ENSG000001	CD244	protein_codin	1.14497007	8.99515822	0	0
ENSG000001	LY9	protein_codin	0	0	0	0
ENSG000001	LY9	protein_codin	0	0	0	0
ENSG000001	LY9	protein_codin	0.3525008	3.73057549	0	1.23942405
ENSG000000	SLAMF7	protein_codin	3.33475977	103.893664	0	0
ENSG000000	SLAMF7	protein_codin	0	48.8029462	0	0
ENSG000001	CD48	protein_codin	0	3.72849764	0	0.97192358
ENSG000001	CD48	protein_codin	7.29258933	91.887001	0.9454457	7.85832335
ENSG000000	CD84	protein_codin	1.78582642	30.2734055	1.53122495	10.4210989
ENSG000000	CD84	protein_codin	0	0	0	1.38017692
ENSG000000	CD84	protein_codin	0	0	0	0
ENSG000001	MED23	protein_codin	0	44.1883918	14.6285043	8.11097921
ENSG000000	CD84	protein_codin	40.7774702	237.498072	4.74609124	153.639241
ENSG000001	SLAMF6	protein_codin	0	2.12477126	0	0
ENSG000001	SLAMF6	protein_codin	0	96.688569	0	0
ENSG000001	MED23	protein_codin	0	0	0	0
ENSG000001	SLAMF6	protein_codin	6.03733345	0	0.44130737	7.05857476
ENSG000001	MED23	protein_codin	0	0	0	0
ENSG000001	VANGL2	protein_codin	3.20357739	0	16.0319464	0
ENSG000001	NCSTN	protein_codin	0	0	1.92227768	0
ENSG000001	MED23	protein_codin	25.3681851	47.1050042	57.1751422	13.3762299
ENSG000001	COPA	protein_codin	67.6195249	0	0	0
ENSG000001	PEX19	protein_codin	14.3090713	0	7.5614753	0
ENSG000001	DCAF8	protein_codin	8.21637826	8.6122716	8.3684641	0
ENSG000001	DCAF8	protein_codin	17.662719	110.207385	61.3512881	18.2403935
ENSG000001	PEA15	protein_codin	1.97433298	16.1899255	0	5.80220514
ENSG000001	PEA15	protein_codin	14.8073206	36.4863752	6.62781338	3.33646584
ENSG000001	CASQ1	protein_codin	68.8225747	0	0	0
ENSG000001	ATP1A4	protein_codin	0	0	0	0
ENSG000001	IGSF8	protein_codin	0	4.84970608	0	0
ENSG000001	ARG1	protein_codin	0	0	0	0
ENSG000001	KCNJ9	protein_codin	0	2.5699784	0	3.55014681
ENSG000001	PIGM	protein_codin	6.84319639	25.3769383	39.2267244	6.67968802
ENSG000001	SLAMF9	protein_codin	0	0	0	0
ENSG000001	SLAMF9	protein_codin	0	0	0	0
ENSG000000	IGSF9	protein_codin	0	1.17307559	0	0
ENSG000001	TAGLN2	protein_codin	0	0	1.84846046	3.37187535
ENSG000001	TAGLN2	protein_codin	25.9737216	37.8822764	46.7273758	18.9205306
ENSG000002	CFAP45	protein_codin	2.26274118	6.60355004	4.40991905	5.55692829
ENSG000002	VSIG8	protein_codin	0	0	0	0
ENSG000002	ENSG000002	protein_codin	0	0	0	0

ENSG000001:	SLAMF8	protein_codin	0	2.62076121	0	0
ENSG000001	FCRL6	protein_codin	0	2.9604531	0	0
ENSG000001	DUSP23	protein_codin	0	14.0006945	0	0
ENSG000001	DUSP23	protein_codin	4.0210934	0	0	3.79579542
ENSG000001	DUSP23	protein_codin	0	0	0	0
ENSG000001	CRP	protein_codin	0	0	0	0
ENSG000001	CRP	protein_codin	0	0	0	0
ENSG000001	CRP	protein_codin	0	0	0	0
ENSG000001	FCER1A	protein_codin	0	8.64261474	0	0
ENSG000001	FCER1A	protein_codin	4.04223075	0	0	3.64743976
ENSG000002	ACKR1	protein_codin	0	0	0	0
ENSG000002	ACKR1	protein_codin	2.97486306	4.26413312	0	2.6291011
ENSG000001	CADM3	protein_codin	2.75900974	0	1.48896834	0
ENSG000001	CADM3	protein_codin	0	30.5084417	0	2.04902751
ENSG000000	EPB41L2	protein_codin	22.667148	0	20.7980856	0
ENSG000000	EPB41L2	protein_codin	0	0	0	0
ENSG000001	AIM2	protein_codin	0	16.7766237	0	0
ENSG000001	AIM2	protein_codin	0	35.7741741	0	1.00094602
ENSG000001	IFI16	protein_codin	121.377656	100.343001	0	0
ENSG000001	IFI16	protein_codin	119.77773	458.85093	37.5805391	103.773628
ENSG000001	SAMD3	protein_codin	1.1318412	0	0	6.32339253
ENSG000001	PYHIN1	protein_codin	0	0	0	0.57325653
ENSG000001	L3MBTL3	protein_codin	0	16.5736757	0	0
ENSG000001	PYHIN1	protein_codin	0	0	0	0
ENSG000001	L3MBTL3	protein_codin	0	0	3.61661166	0
ENSG000001	PYHIN1	protein_codin	3.0534529	64.3936594	0	6.84445723
ENSG000001	MNDA	protein_codin	17.3200969	67.5738765	2.11327035	12.3664096
ENSG000002	TMEM244	protein_codin	0	0	0	0
ENSG000002	OR6K3	protein_codin	0	0	0	0
ENSG000002	OR6K3	protein_codin	0	0	0	0.56131605
ENSG000001	ARHGAP18	protein_codin	73.5114206	166.533528	67.9848894	73.9541786
ENSG000001	CD1E	protein_codin	0	6.43913396	0	0
ENSG000001	CD1E	protein_codin	0	0	0	0
ENSG000001	CD1E	protein_codin	0	0	0	0
ENSG000001	CD1E	protein_codin	0	0	0	0.30883996
ENSG000001	CD1E	protein_codin	0	0	0	0
ENSG000001	CD1E	protein_codin	0	0	0	0
ENSG000001	CD1E	protein_codin	2.0337984	0	0	0
ENSG000001	CD1E	protein_codin	0	0	0	0
ENSG000001	CD1E	protein_codin	0	0	0	0
ENSG000001	CD1E	protein_codin	0	0	0	0
ENSG000001	CD1E	protein_codin	0	5.21225697	0	0
ENSG000001	CD1E	protein_codin	0	0	0	0
ENSG000001	CD1B	protein_codin	0	1.11200856	0	0.46953856

ENSG000001	CD1C	protein_codin	1.54052253	10.8375716	0	4.14448897
ENSG000001	CD1D	protein_codin	0	0	0	0
ENSG000001	KIRREL1	protein_codin	21.4350514	0	19.7146914	23.5476657
ENSG000001	KIRREL1	protein_codin	26.3755647	301.745074	0	0
ENSG000000	CD5L	protein_codin	0	0	0	3.14053457
ENSG000001	FCRL1	protein_codin	0.71933663	0	0	0
ENSG000001	FCRL1	protein_codin	0	0	0	0
ENSG000001	FCRL2	protein_codin	0	0	0	0
ENSG000001	FCRL2	protein_codin	0	0	0	0
ENSG000001	FCRL3	protein_codin	0	27.1110485	0	0
ENSG000001	FCRL3	protein_codin	0	0	0	0
ENSG000001	FCRL5	protein_codin	0.46230417	27.101923	0	3.23097879
ENSG000001	FCRL5	protein_codin	0	11.9090966	0	0
ENSG000001	ETV3	protein_codin	106.852892	130.743277	132.436229	75.2113574
ENSG000001	ARHGEF11	protein_codin	157.636062	324.128987	154.328477	42.3711122
ENSG000000	INSRR	protein_codin	3.59314784	3.8465433	1.10204282	2.44524655
ENSG000001	NTRK1	protein_codin	0	0	0	0
ENSG000000	SH2D2A	protein_codin	0.89280659	0	1.37303681	2.12348264
ENSG000000	SH2D2A	protein_codin	0	18.2276225	0	0
ENSG000001	PTPRK	protein_codin	0	0.98945362	0	0
ENSG000001	PTPRK	protein_codin	0	0	0	0.43367928
ENSG000001	HDGF	protein_codin	1.54653062	66.1465842	0	0
ENSG000001	PTPRK	protein_codin	0	0	0	0
ENSG000001	HDGF	protein_codin	3.62657814	0	0	0
ENSG000001	PTPRK	protein_codin	0	0	0	0
ENSG000001	MRPL24	protein_codin	37.732332	29.8607329	35.2762929	11.6027303
ENSG000001	PTPRK	protein_codin	0	74.4865763	37.0630482	53.3068463
ENSG000001	PTPRK	protein_codin	12.610128	0	24.5681996	0
ENSG000001	METTL25B	protein_codin	2.4164348	17.5086079	5.83715866	1.11963968
ENSG000001	METTL25B	protein_codin	0	0	0.75790845	2.59688535
ENSG000001	ISG20L2	protein_codin	31.3695815	0	33.6668112	5.55026715
ENSG000001	CRABP2	protein_codin	29.8788623	18.4401904	16.4199925	22.2268969
ENSG000001	CRABP2	protein_codin	0	22.2249028	0	0
ENSG000001	CRABP2	protein_codin	43.0572871	6.93182755	19.7697565	16.7948198
ENSG000001	NES	protein_codin	456.153776	296.979684	96.8013131	71.6245716
ENSG000001	PTPRK	protein_codin	36.2681116	33.6141567	0	0
ENSG000001	GPATCH4	protein_codin	9.633595	26.1153647	24.9439899	7.51030258
ENSG000001	NAXE	protein_codin	0	0	0	1.34676084
ENSG000001	NAXE	protein_codin	0	0	0	0
ENSG000001	NAXE	protein_codin	10.6335359	4.94437532	8.20429803	0
ENSG000001	TTC24	protein_codin	0	3.50125233	0	2.21646069
ENSG000001	THEMIS	protein_codin	0	0	0	0
ENSG000001	THEMIS	protein_codin	0	0	0	1.81212474
ENSG000001	TSACC	protein_codin	0	0	0.55149911	0.49411479

ENSG000001(	TSACC	protein_codin	0	0	0	0
ENSG000001(	TSACC	protein_codin	0	0	0	0
ENSG000001(	TSACC	protein_codin	0	0	0	0
ENSG000001(	TSACC	protein_codin	0	0	0	0
ENSG000001(	CCT3	protein_codin	4.57758627	0	13.8139543	0
ENSG000001(	CCT3	protein_codin	0	0	3.46735383	0
ENSG000001(	CCT3	protein_codin	3.46577668	0	14.6615698	0
ENSG000001(	CCT3	protein_codin	0	0	0	0
ENSG000001(	CCT3	protein_codin	0	0	0	0
ENSG000001!	GLMP	protein_codin	0	3.16932731	0	0
ENSG000001(	PAQR6	protein_codin	0	0	0	0
ENSG000002	BGLAP	protein_codin	69.5740845	200.613523	0	284.720403
ENSG000001(	PMF1	protein_codin	3.17299725	9.76351025	0	0
ENSG000002(	PMF1-BGLAP	protein_codin	0	0	0	0
ENSG000001(	PMF1	protein_codin	11.7249417	0	6.29550644	0
ENSG000001(	PMF1	protein_codin	0.79329358	0	0	0
ENSG000001	KIAA0408	protein_codin	0	0	0	5.68553706
ENSG000001!	SEMA4A	protein_codin	0	0	0	7.90712734
ENSG000001!	SEMA4A	protein_codin	0.74628112	0	0.49090644	0
ENSG000001!	SEMA4A	protein_codin	0	0	0	0
ENSG000000!	ECHDC1	protein_codin	0	0	0	0
ENSG000000!	ECHDC1	protein_codin	0	0	22.2254607	0
ENSG000000!	ECHDC1	protein_codin	15.1016474	71.8825389	44.3247684	0
ENSG000000!	ECHDC1	protein_codin	0	0	0	0
ENSG000000!	ECHDC1	protein_codin	0	0	0	0
ENSG000001(	LMNA	protein_codin	8.2164694	0	0	0
ENSG000001(	LMNA	protein_codin	5.68793585	3.60749115	2.85944502	20.9112502
ENSG000001(	LMNA	protein_codin	0	0	0	0
ENSG000001(	LMNA	protein_codin	114.949241	146.815307	138.844638	53.0895408
ENSG000001(	LMNA	protein_codin	0	0	0	0
ENSG000001	LAMTOR2	protein_codin	0	0	7.72992113	0
ENSG000001	LAMTOR2	protein_codin	0	9.36243578	0	0
ENSG000001	LAMTOR2	protein_codin	6.15279653	0	0	3.55945191
ENSG000001(	UBQLN4	protein_codin	35.0328413	55.7781907	66.2624373	8.1254376
ENSG000001(	SSR2	protein_codin	0.95383268	6.41861514	6.7418393	1.53357867
ENSG000001	RNF146	protein_codin	17.9800906	0	0	18.0154244
ENSG000001	RSPO3	protein_codin	2.3355199	28.5046995	22.7321527	8.0636692
ENSG000001	RXFP4	protein_codin	0	0	0	0
ENSG000001	KHDC4	protein_codin	0	0	0	0
ENSG000001	KHDC4	protein_codin	43.4709832	42.4974379	28.7785716	8.89533166
ENSG000001	KHDC4	protein_codin	17.5686046	22.8146194	37.1232944	0
ENSG000001	RIT1	protein_codin	31.3425274	12.5948861	38.7054894	6.54264244
ENSG000001	RIT1	protein_codin	28.9971186	16.7279097	13.5691553	25.3808458
ENSG000001	SYT11	protein_codin	53.754863	50.1953977	6.81126694	14.7117656

ENSG000002(	CENPW	protein_codin	2.91149779	4.66066432	8.24430311	0
ENSG000002(	CENPW	protein_codin	0.5155141	0	0	0
ENSG000002(	CENPW	protein_codin	2.33529381	0	31.6504937	2.4219359
ENSG000001(	YY1AP1	protein_codin	0	3.09048394	2.98928207	0
ENSG000001	GON4L	protein_codin	0	0	0	12.6828415
ENSG00000(	TRMT11	protein_codin	0	0	0	0
ENSG000001:	DAP3	protein_codin	1.66890549	0	8.20233389	5.213101
ENSG000001(	YY1AP1	protein_codin	0	0	0	0
ENSG000001(	YY1AP1	protein_codin	0	0	0	0
ENSG000001:	MSTO1	protein_codin	0	0	13.5964219	0
ENSG000001	ASH1L	protein_codin	0	0	0	7.19872463
ENSG000001(	RUSC1	protein_codin	0.39939894	0	0	0
ENSG000001(	RUSC1	protein_codin	0	0	5.12628292	0
ENSG000001(	RUSC1	protein_codin	4.36086316	0	0	5.1953129
ENSG000001(	RUSC1	protein_codin	0	12.0829652	0	0
ENSG000001(	FDPS	protein_codin	2.05735484	0	0	1.7127865
ENSG000001	NCOA7	protein_codin	26.7139554	55.7776735	25.3326344	42.1073645
ENSG000001	HCN3	protein_codin	1.16728839	3.12935206	0	2.19047755
ENSG000001:	HEY2	protein_codin	20.1381304	80.0362197	14.6673316	13.104885
ENSG000001:	HEY2	protein_codin	0	0	0	0
ENSG000001(	ENTREP3	protein_codin	0	0	2.59515603	0
ENSG000001(	ENTREP3	protein_codin	0	0	0	0
ENSG000001	GBA1	protein_codin	27.8717574	42.2284638	35.3949834	6.57195132
ENSG000001	MTX1	protein_codin	0	0	12.2308888	4.25254143
ENSG000001(	THBS3	protein_codin	49.8662253	46.5443575	27.2206324	5.72328249
ENSG000001(	TRIM46	protein_codin	0	0	0	0
ENSG000001(	TRIM46	protein_codin	0	1.74326261	0	0
ENSG000001(	TRIM46	protein_codin	0	0	0	0
ENSG000001	TPD52L1	protein_codin	0	0	0	0
ENSG000001	MUC1	protein_codin	0	0	0	0
ENSG000001	MUC1	protein_codin	0	0	4.2528681	0
ENSG000001	MUC1	protein_codin	0	0	0	0
ENSG000001	MUC1	protein_codin	0	0	0	0
ENSG000001	MUC1	protein_codin	0	0	0	0
ENSG000001	MUC1	protein_codin	0	0	0	0
ENSG000001	DPM3	protein_codin	0	0	0.78373569	0
ENSG000001	DPM3	protein_codin	10.0600768	1.86469349	15.7100434	4.45756819
ENSG000001(	SLC50A1	protein_codin	0	15.8457213	0	0
ENSG000001	TPD52L1	protein_codin	0	4.13507436	87.8251969	2.56353683
ENSG000001(	SLC50A1	protein_codin	0	0	0	0
ENSG000001(	SLC50A1	protein_codin	0	0	0	0
ENSG000001(	EFNA1	protein_codin	3.04971887	0	0	1.65428009
ENSG000001(	EFNA1	protein_codin	27.90823	33.0077544	30.9711699	10.8004083
ENSG000001	EFNA3	protein_codin	0.38808268	1.76863752	7.46938063	1.14315465

ENSG000002	EFNA4	protein_codin	3.57228757	7.20128656	16.7418895	0
ENSG000001	ADAM15	protein_codin	0	0	0	0
ENSG000001	ADAM15	protein_codin	0	0	0	0
ENSG000001	RNF217	protein_codin	0	0	4.81162995	1.96970554
ENSG000001	RNF217	protein_codin	0	0	0	0
ENSG000001	NKAIN2	protein_codin	0	0	0.57315775	0
ENSG000001	NKAIN2	protein_codin	0	3.24583213	9.5172627	1.95738324
ENSG000001	DCST1	protein_codin	0	0	0	0
ENSG000001	DCST1	protein_codin	0	0	0	0.36305158
ENSG000001	DCST2	protein_codin	0	0	2.13514483	1.19108931
ENSG000001	DCST2	protein_codin	0	0	0	0
ENSG000001	ZBTB7B	protein_codin	3.82316711	41.014132	5.77832953	4.00746943
ENSG000001	LENEP	protein_codin	0	1.23930913	0	0
ENSG000001	FLAD1	protein_codin	1.90377568	0	0	0
ENSG000001	FLAD1	protein_codin	0	0	2.57166679	0.84056857
ENSG000001	FLAD1	protein_codin	0	0	0	1.93987448
ENSG000001	FLAD1	protein_codin	0	0	3.62638871	0
ENSG000001	CKS1B	protein_codin	0.43171063	0	3.34379318	0
ENSG000001	CLVS2	protein_codin	0	0	0	0
ENSG000001	CKS1B	protein_codin	4.55811527	2.06114566	9.76842545	0
ENSG000001	SMPDL3A	protein_codin	5.49787818	0	1.0843978	5.5921194
ENSG000001	FABP7	protein_codin	0.9459172	0	0	0
ENSG000001	SHC1	protein_codin	0	0	0	0
ENSG000001	PKIB	protein_codin	0	2.35218278	0	0
ENSG000001	PKIB	protein_codin	1.01108771	0	0	0
ENSG000001	SHC1	protein_codin	14.6704844	0	0	0
ENSG000001	SHC1	protein_codin	0	0	0	12.1798461
ENSG000001	PKIB	protein_codin	0	0	0	0
ENSG000001	SHC1	protein_codin	45.6015086	36.5327008	105.718706	19.1310094
ENSG000000	HSF2	protein_codin	15.3065766	20.607426	7.25746422	9.01635563
ENSG000001	PYGO2	protein_codin	0.64489037	0	0	0
ENSG000001	PYGO2	protein_codin	27.0014835	60.9306387	39.7498995	6.59016312
ENSG000001	PBXIP1	protein_codin	0	0	0	0
ENSG000001	PBXIP1	protein_codin	149.332732	269.842347	63.2978318	56.6486488
ENSG000001	TBC1D32	protein_codin	0	0	0.90595306	1.00535214
ENSG000001	PBXIP1	protein_codin	0	131.168361	19.3877935	0
ENSG000001	PMVK	protein_codin	17.2289184	32.4818082	37.5664508	3.78376746
ENSG000001	MAN1A1	protein_codin	86.7975756	380.618914	146.363308	80.7474779
ENSG000001	ADAR	protein_codin	82.7192332	0	29.5837541	0
ENSG000001	FAM184A	protein_codin	0	0	0	0
ENSG000001	ADAR	protein_codin	0	0	106.564943	0
ENSG000001	FAM184A	protein_codin	9.60019354	0	0	0
ENSG000001	CHRNB2	protein_codin	0	0	0.49933573	4.9743803
ENSG000001	MCM9	protein_codin	0.57371055	0	0.9563284	0

ENSG000001(	TDRD10	protein_codin	0.59050764	0	0.52001874	0
ENSG000001(	TDRD10	protein_codin	0	3.80654848	0	0.89573896
ENSG000001(	IL6R	protein_codin	9.41993174	36.9660311	1.4889254	12.8598396
ENSG000001	ATP8B2	protein_codin	0.79601392	3.11206585	3.65306414	0
ENSG000001	CEP85L	protein_codin	0	93.3406405	0	27.6239292
ENSG000001	ATP8B2	protein_codin	35.0521103	210.76191	40.2734945	26.0348547
ENSG000001	ATP8B2	protein_codin	0	0	0	0
ENSG000001	CEP85L	protein_codin	45.74022	10.8684332	87.06388	4.09514433
ENSG000001	NUS1	protein_codin	24.7490627	56.57849	94.0471897	42.7917644
ENSG000000	GOPC	protein_codin	0	14.8720194	3.64238377	0
ENSG000001	UBAP2L	protein_codin	3.47982195	0	4.17276512	0
ENSG000000	ROS1	protein_codin	0	2.75728017	0	1.4385157
ENSG000000	ROS1	protein_codin	0.27222378	2.8856234	0	0
ENSG000001	C1orf43	protein_codin	0	0	2.85121908	0
ENSG000001	SYCE1	protein_codin	0	0	0	0
ENSG000001	C1orf43	protein_codin	2.38155684	11.7987457	1.42763237	0.60896139
ENSG000001	C1orf43	protein_codin	0	0	0	0
ENSG000001	CYP2E1	protein_codin	0	0	0.52744162	4.37430287
ENSG000001	C1orf43	protein_codin	34.3911804	0	60.7096688	17.648535
ENSG000001(	CFAP141	protein_codin	0	0	0	0
ENSG000001	TPM3	protein_codin	0.77540422	0	0	4.38486052
ENSG000001	TPM3	protein_codin	176.856661	0	0	0
ENSG000001	TPM3	protein_codin	48.5956989	288.13337	351.39898	80.7892922
ENSG000001	TPM3	protein_codin	2.36614693	10.1940435	6.71671541	0
ENSG000001	PAOX	protein_codin	0.53108183	0	0	0.47209801
ENSG000001	TPM3	protein_codin	0	30.8174434	10.5699113	5.87888009
ENSG000001	ECHS1	protein_codin	42.3169383	44.0759402	52.8859883	33.5733669
ENSG000001	GPRC6A	protein_codin	0	0	0	0
ENSG000001	FUOM	protein_codin	0	0	1.17016493	0
ENSG000001	FUOM	protein_codin	0	0	0	0
ENSG000001	NUP210L	protein_codin	0	0	0	0
ENSG000002	ZNF511-PRAP	protein_codin	0.74074056	0	0	0.80487719
ENSG000001	CALY	protein_codin	0	0.95680257	0	0
ENSG000001	FAM162B	protein_codin	1.52757769	1.43386561	0	0.51569913
ENSG000001	CALY	protein_codin	0	0	0	0
ENSG000001	NUP210L	protein_codin	0	0	0	0
ENSG000001	TUBGCP2	protein_codin	1.36563485	24.9193935	0	0.74749292
ENSG000001	KPNA5	protein_codin	4.37915029	2.1482654	27.1106976	14.7727678
ENSG000001	KNDC1	protein_codin	0	1.56219411	0	1.2715291
ENSG000001	ZUP1	protein_codin	1.64397148	0	15.308279	0
ENSG000001	RAB13	protein_codin	229.670732	313.487681	259.849448	78.1591984
ENSG000001	ZUP1	protein_codin	8.54934957	12.1987689	18.3170291	4.1862577
ENSG000001	RSPH4A	protein_codin	0	0	0	0
ENSG000001	RSPH4A	protein_codin	1.08346483	0	0	5.24331543

ENSG000001	CFAP46	protein_codin	0	0	0	2.34983737
ENSG000001	CFAP46	protein_codin	0.77265026	0	0.61042827	0.45070866
ENSG000001	JTB	protein_codin	2.67322536	0	0	0
ENSG000001	RWDD1	protein_codin	0	3.02088123	0	6.05099312
ENSG000001	NKX6-2	protein_codin	0	0	0	1.48824688
ENSG000000	INPP5A	protein_codin	3.80928136	3.94504267	0	0
ENSG000000	INPP5A	protein_codin	7.93311588	8.56049921	7.28304548	5.86259196
ENSG000001	CALHM4	protein_codin	0	0	0	0
ENSG000001	CALHM4	protein_codin	0	0	0	0
ENSG000001	CALHM5	protein_codin	7.69292112	21.1370358	0.97178088	7.03941171
ENSG000001	CREB3L4	protein_codin	0	0	0	0
ENSG000001	CREB3L4	protein_codin	0	0	0	0
ENSG000001	TRAPPC3L	protein_codin	0	0	0	0
ENSG000001	CREB3L4	protein_codin	0.58179296	0	0	0
ENSG000001	CALHM6	protein_codin	5.05757226	0	0	3.63525539
ENSG000001	CALHM6	protein_codin	0	15.0377673	0	0.72213948
ENSG000001	CALHM6	protein_codin	0	0	0	0
ENSG000001	CREB3L4	protein_codin	0	0	0	0
ENSG000001	TSPYL1	protein_codin	61.6446034	190.835987	126.887953	63.0329895
ENSG000001	LRRC27	protein_codin	5.14586657	0	4.6903984	0
ENSG000001	LRRC27	protein_codin	0	13.2850116	0	0
ENSG000001	LRRC27	protein_codin	0.3884708	0	0	2.10048654
ENSG000001	STK32C	protein_codin	0.60011968	1.3786261	0	1.05465899
ENSG000001	STK32C	protein_codin	0	1.21467236	0	0
ENSG000001	SLC39A1	protein_codin	0	0	0	1.05236847
ENSG000001	STK32C	protein_codin	0	0	0	0
ENSG000001	SLC39A1	protein_codin	3.09011587	0	0	0
ENSG000001	DPYSL4	protein_codin	0	0	0	0
ENSG000001	CRTC2	protein_codin	2.99782482	0	3.24857503	0
ENSG000001	HDAC2	protein_codin	0	15.187812	111.136245	28.7778156
ENSG000001	CRTC2	protein_codin	4.61456299	41.3387142	4.64391417	6.15667233
ENSG000001	BNIP3	protein_codin	18.6975352	22.4102202	27.5937792	13.1680301
ENSG000001	LAMA4	protein_codin	1.08527339	21.7547888	11.5363733	6.19519904
ENSG000001	LAMA4	protein_codin	0	0	24.3498008	0
ENSG000001	TCERG1L	protein_codin	0	0	0	0
ENSG000001	GLRX3	protein_codin	4.26629989	0	1.46326852	3.42703333
ENSG000001	DENND4B	protein_codin	7.01497944	29.0262389	0	4.7127516
ENSG000001	EBF3	protein_codin	19.1625191	43.6756052	9.74187333	31.7752822
ENSG000001	MKI67	protein_codin	97.7772564	217.278958	708.741292	34.1202214
ENSG000001	MKI67	protein_codin	58.5157331	173.605696	383.405895	19.2614391
ENSG000001	GATAD2B	protein_codin	99.0528256	151.072509	103.96264	32.986469
ENSG000002	FAM229B	protein_codin	3.8302318	0	3.07955318	1.9118908
ENSG000000	TUBE1	protein_codin	7.13268172	25.6331244	12.6910169	4.45636124
ENSG000001	SLC27A3	protein_codin	0	0	0	0

ENSG000001	SLC27A3	protein_codin	0	0	0	0
ENSG000000	TUBE1	protein_codin	3.42582518	0	3.8151927	1.90345992
ENSG000001	CCN6	protein_codin	0	0	0	0
ENSG000001	CCN6	protein_codin	0	0	0	0
ENSG000000	FYN	protein_codin	0	23.8253211	24.8489512	2.02868105
ENSG000001	INTS3	protein_codin	0	8.94683532	9.19131986	0
ENSG000001	INTS3	protein_codin	2.13611514	0	0	0
ENSG000001	CLRN3	protein_codin	0	0	0	2.08696533
ENSG000001	C10orf90	protein_codin	0	0	0	0
ENSG000001	ADAM12	protein_codin	54.1130349	116.168582	13.4576221	11.718676
ENSG000001	NPR1	protein_codin	2.43929598	3.26037531	7.11562084	0
ENSG000000	FYN	protein_codin	0	0	0	0
ENSG000001	ADAM12	protein_codin	0	57.2693678	0	19.4859649
ENSG000001	NPR1	protein_codin	9.8303644	23.6637882	6.07688225	19.4142619
ENSG000001	ILF2	protein_codin	0	0	0	0
ENSG000000	FYN	protein_codin	11.3816853	65.8944473	14.4866125	15.5196359
ENSG000001	ILF2	protein_codin	0	18.7723736	3.69207138	0
ENSG000001	SNAPIN	protein_codin	8.50836938	17.1857822	14.4830569	5.45790533
ENSG000001	CHTOP	protein_codin	8.29887567	12.8903496	15.0701415	3.31271179
ENSG000001	CHTOP	protein_codin	2.88024436	0	13.260751	0
ENSG000002	FANK1	protein_codin	0	0	0	0
ENSG000001	CHTOP	protein_codin	17.9656263	24.0150954	0	7.73650118
ENSG000002	FANK1	protein_codin	0	0	0	0
ENSG000002	FANK1	protein_codin	0.74529074	0	1.07249176	0
ENSG000001	CHTOP	protein_codin	0	0	0	0
ENSG000001	S100A1	protein_codin	0	0	0	0
ENSG000001	S100A1	protein_codin	0.40382863	0	0	0.37964221
ENSG000001	S100A14	protein_codin	0	0	0	0
ENSG000001	S100A14	protein_codin	0	0	0	0
ENSG000001	S100A14	protein_codin	0	0	0	0
ENSG000001	S100A16	protein_codin	0	0	0	0
ENSG000001	S100A16	protein_codin	0	0	0	0
ENSG000001	S100A16	protein_codin	2.5366751	4.52324012	5.2129058	3.90789069
ENSG000001	S100A16	protein_codin	17.4040821	23.6922844	13.8420874	6.64391088
ENSG000001	S100A2	protein_codin	0	0	0	0
ENSG000001	S100A2	protein_codin	0.68107293	0	0	0.99800922
ENSG000001	S100A2	protein_codin	0	0	0	1.06378133
ENSG000001	S100A2	protein_codin	0	0	0.58113069	0
ENSG000001	S100A3	protein_codin	0	0	0	0
ENSG000001	S100A3	protein_codin	0.39643379	0	0	0.48359074
ENSG000001	S100A4	protein_codin	0	128.652909	8.1324253	0
ENSG000001	S100A4	protein_codin	12.8074545	0	0	0
ENSG000001	S100A4	protein_codin	12.7070869	0	0	14.9440908
ENSG000001	S100A5	protein_codin	0	0	0	0

ENSG000001!	S100A5	protein_codin	0.38831258	1.64592471	0	0.46065092
ENSG000001!	S100A6	protein_codin	300.51031	190.620241	467.565453	109.862674
ENSG000001!	S100A6	protein_codin	1.03507389	4.77881099	0.90392546	0.78786561
ENSG000000	DHX32	protein_codin	4.08616753	3.86093072	11.438418	3.42105057
ENSG000001	S100A7	protein_codin	0	0	0	0
ENSG000001	S100A7	protein_codin	0	0	0	0.33816992
ENSG000001	S100A7A	protein_codin	0	0	0	0
ENSG000001	S100A7A	protein_codin	0.49776179	0	0	1.26368044
ENSG000000	TRAF3IP2	protein_codin	0	0	0	0
ENSG000000	TRAF3IP2	protein_codin	0	0	0	1.77992232
ENSG000001	S100A8	protein_codin	0	0	0	0
ENSG000001	S100A8	protein_codin	0	0	0	0.40753607
ENSG000000	TRAF3IP2	protein_codin	0	0	0	0
ENSG000000	TRAF3IP2	protein_codin	0	0	0	0
ENSG000001	S100A12	protein_codin	0	2.04887614	0	0.70515332
ENSG000001	S100A9	protein_codin	0.35020258	2.20871824	0.96565412	2.05400109
ENSG000001	PGLYRP4	protein_codin	0	0	0	0
ENSG000002	LORICRIN	protein_codin	0	0	0.59786682	0.76387934
ENSG000002	PRR9	protein_codin	1.11596227	0	0	0
ENSG000002	LELP1	protein_codin	0	1.69117153	0	0
ENSG000001	SPRR2G	protein_codin	0	2.90723083	0.53966479	0.8528429
ENSG000002	SPRR2E	protein_codin	0.3244352	0	0	0
ENSG000002	SPRR2E	protein_codin	0	0	0	0
ENSG000001!	SPRR2B	protein_codin	0.38348473	0	0	0
ENSG000001	SPRR2D	protein_codin	0	0	0	1.31581943
ENSG000001	SPRR2D	protein_codin	0	0	0	0
ENSG000001	SPRR2D	protein_codin	0	0	0	0
ENSG000001	BCCIP	protein_codin	3.12779532	23.4492965	13.3774754	7.39572695
ENSG000000	TRAF3IP2	protein_codin	1.82352432	0	0	4.13814326
ENSG000001	SPRR1A	protein_codin	0	0	0	0
ENSG000001	IVL	protein_codin	0	1.77986228	1.1355266	0.92033037
ENSG000001	SMCP	protein_codin	0	0	0	0
ENSG000001	LCE1E	protein_codin	0	0	0.53876002	1.01701489
ENSG000001	LCE1E	protein_codin	0	0	0	0
ENSG000001	UROS	protein_codin	0	1.17494977	11.3510205	0
ENSG000001!	KPLCE	protein_codin	0	0	0	0.43688277
ENSG000001	LCE4A	protein_codin	0	0	0	0
ENSG000001	UROS	protein_codin	0.8888804	3.26168936	0	0
ENSG000001	LCE2A	protein_codin	0	0	0	0
ENSG000001	LCE2B	protein_codin	0	0	0	0
ENSG000001	LCE2C	protein_codin	0	0	0	0.47465635
ENSG000001	LCE2D	protein_codin	0	0	0	0
ENSG000001	UROS	protein_codin	0	0	0	0
ENSG000001	LCE3D	protein_codin	0	0	0	0.41765424

ENSG000001[illegible]	LCE3E	protein_codin	0	0	0	0
ENSG000001[illegible]	CRCT1	protein_codin	0	0	0	0.44710857
ENSG000001[illegible]	UROS	protein_codin	0	5.26902867	0	0
ENSG000001[illegible]	FLG	protein_codin	5.04965094	1.93596451	0.47206258	18.9043083
ENSG000001[illegible]	HRNR	protein_codin	1.06343523	4.13374094	0.87194438	11.9198046
ENSG000000[illegible]	REV3L	protein_codin	104.372673	143.331966	148.529392	62.1110415
ENSG000001[illegible]	TCHHL1	protein_codin	0.64911466	0	1.87054111	8.51705132
ENSG000001[illegible]	MMP21	protein_codin	0	0	0	0
ENSG000001[illegible]	S100A10	protein_codin	0	93.0247973	0	0
ENSG000001[illegible]	S100A10	protein_codin	314.156331	34.1643856	144.817257	69.1713284
ENSG000001[illegible]	EDRF1	protein_codin	0	0	0	0
ENSG000001[illegible]	EDRF1	protein_codin	0	0	20.6909699	0
ENSG000001[illegible]	THEM4	protein_codin	10.2566277	6.36103524	16.651532	5.38037333
ENSG000001[illegible]	EDRF1	protein_codin	0	0	0	7.39586306
ENSG000001[illegible]	THEM5	protein_codin	0	0	0	0.35973059
ENSG000002	LINGO4	protein_codin	0	1.218149	0	0.41595217
ENSG000001[illegible]	TEX36	protein_codin	0	0	0	0
ENSG000001[illegible]	TDRKH	protein_codin	0	0	0	0
ENSG000001[illegible]	TDRKH	protein_codin	0	0	12.1927798	0
ENSG000001[illegible]	TDRKH	protein_codin	0	21.4684162	0	0
ENSG000001[illegible]	TDRKH	protein_codin	0	0	0	0
ENSG000001[illegible]	TDRKH	protein_codin	0	15.2438795	0	0
ENSG000001[illegible]	MRPL9	protein_codin	4.19735769	0	1.773614	0
ENSG000001[illegible]	MRPL9	protein_codin	28.5496113	0	35.0003035	4.45531037
ENSG000002[illegible]	EEF1AKMT2	protein_codin	0	0	0	0
ENSG000001[illegible]	SNX27	protein_codin	8.06701904	0	0	0
ENSG000001[illegible]	LHPP	protein_codin	0	0	0	2.68884941
ENSG000001[illegible]	SNX27	protein_codin	0	2.448189	0	0
ENSG000001[illegible]	LHPP	protein_codin	2.54598587	4.68525067	6.86241885	0
ENSG000001[illegible]	SNX27	protein_codin	15.1295628	20.6097013	0	0
ENSG000000[illegible]	OAT	protein_codin	17.47997	15.3790585	42.6857752	26.8324152
ENSG000001[illegible]	MFSD4B	protein_codin	0	8.75296265	7.50709639	0
ENSG000001[illegible]	TUFT1	protein_codin	0	3.62723232	8.02442137	0
ENSG000001[illegible]	TUFT1	protein_codin	0	0	0	0
ENSG000001	SLC16A10	protein_codin	0	0	7.74401041	3.79035217
ENSG000001	SLC16A10	protein_codin	6.73843211	1.39851383	0	0
ENSG000001[illegible]	CPXM2	protein_codin	0	0	0	0
ENSG000001[illegible]	BUB3	protein_codin	0	0	9.94799929	0
ENSG000001[illegible]	BUB3	protein_codin	0	0	0	0
ENSG000001[illegible]	POGZ	protein_codin	85.9090807	140.187249	128.835523	37.7620193
ENSG000001[illegible]	RPF2	protein_codin	14.1375446	22.0645771	36.3967619	3.70069094
ENSG000001[illegible]	BUB3	protein_codin	11.853767	31.1154061	44.6238867	8.13784356
ENSG000001[illegible]	SELENBP1	protein_codin	4.2442596	0	0	0
ENSG000001[illegible]	ACADSB	protein_codin	0	3.72051876	0	0

ENSG000001	RFX5	protein_codin	3.64450197	27.1286305	0	0
ENSG000001	PI4KB	protein_codin	36.8199121	43.5954471	40.8319544	12.1956172
ENSG000001	PI4KB	protein_codin	12.5322463	17.4738463	14.1351455	0
ENSG000001	PI4KB	protein_codin	2.58793294	0	12.7688716	10.2046613
ENSG000001	PI4KB	protein_codin	12.36531	0	0	0
ENSG000001	AMD1	protein_codin	0	0	0	0
ENSG000001	AMD1	protein_codin	7.81760999	42.5800166	18.2463894	10.0439247
ENSG000001	PSMD4	protein_codin	17.0329162	50.9826267	36.728582	0
ENSG000001	PSMD4	protein_codin	155.495319	40.2564994	272.80101	41.3831814
ENSG000001	AMD1	protein_codin	12.2703279	23.2457539	14.5046492	11.7087228
ENSG000000	IKZF5	protein_codin	23.0754303	16.0071197	21.1419185	23.4688208
ENSG000001	PSTK	protein_codin	1.02826315	1.33414239	0	0
ENSG000001	PIP5K1A	protein_codin	3.99397358	0	0	15.8420277
ENSG000001	PIP5K1A	protein_codin	0	0	0	0
ENSG000001	C10orf88	protein_codin	0	0	1.91553609	0
ENSG000001	VPS72	protein_codin	40.5257256	12.4251898	86.6868251	12.8129001
ENSG000002	FAM24A	protein_codin	0	0	0	0.65060818
ENSG000002	FAM24B	protein_codin	0	0	0	0
ENSG000002	FAM24B	protein_codin	0	0	0	0
ENSG000001	CUZD1	protein_codin	0.50060305	1.54684754	1.23501233	1.74679224
ENSG000001	CUZD1	protein_codin	0	0	0	0
ENSG000001	CUZD1	protein_codin	0	0	0	0
ENSG000001	SCNM1	protein_codin	13.2599315	8.23278359	12.4542266	2.89515797
ENSG000002	ENSG000002	protein_codin	0	0	0	0
ENSG000001	SCNM1	protein_codin	0	0	0.86536702	2.12572413
ENSG000001	LYSMD1	protein_codin	10.8800407	14.2942969	16.665156	4.01504319
ENSG000001	DMBT1	protein_codin	0	0	0	0
ENSG000001	TNFAIP8L2	protein_codin	1.54670259	10.3960089	0.94959407	1.52634202
ENSG000001	CDK19	protein_codin	0	52.4380478	0	21.2141869
ENSG000001	SEMA6C	protein_codin	0	0	4.77737184	0
ENSG000001	SEMA6C	protein_codin	0	0	0	0
ENSG000001	SEMA6C	protein_codin	4.40501994	16.2266053	0	0
ENSG000001	GABPB2	protein_codin	32.650389	49.7653359	28.3068495	11.3866264
ENSG000001	GABPB2	protein_codin	49.0047698	47.7159459	18.1170674	16.8654461
ENSG000000	SLC22A16	protein_codin	0	0	0	0
ENSG000002	MLLT11	protein_codin	15.8402532	9.6076009	20.7304201	3.77882096
ENSG000002	DDO	protein_codin	0	0	0	0
ENSG000002	DDO	protein_codin	0	0	0	3.13078356
ENSG000001	C1orf56	protein_codin	2.37496501	5.47486247	7.25350228	3.19002976
ENSG000001	CDC40	protein_codin	13.3920777	18.3688936	13.3888089	0
ENSG000001	BNIPL	protein_codin	0	0	0.60942435	0
ENSG000001	CDC40	protein_codin	0	0	0	0
ENSG000001	PRUNE1	protein_codin	0	0	15.3003671	3.4377419
ENSG000001	PRUNE1	protein_codin	1.21336047	0	0	0

ENSG000001	PRUNE1	protein_codin	30.3196703	5.51767476	0	0
ENSG000001	PRUNE1	protein_codin	3.35543642	3.8197604	0	0
ENSG000001	WASF1	protein_codin	0	0	0	0
ENSG000001	FIG4	protein_codin	3.20111157	0	0	0
ENSG000001	ANXA9	protein_codin	0	0.88253122	0	0.4361924
ENSG000001	AK9	protein_codin	0	0	0	0
ENSG000001	CERS2	protein_codin	0	0	9.05709608	0
ENSG000001	CERS2	protein_codin	28.5072078	103.27661	48.0774565	24.3961195
ENSG000001	NUMA1	protein_codin	0	0	0	0
ENSG000001	SETDB1	protein_codin	0	6.24261852	0	4.43979341
ENSG000001	SETDB1	protein_codin	15.8874615	0	22.5178209	2.99159054
ENSG000001	SETDB1	protein_codin	0	0	0	0.96091183
ENSG000001	CEP57L1	protein_codin	0	0	0	4.34286659
ENSG000001	SETDB1	protein_codin	0	0	0	0
ENSG000001	CEP57L1	protein_codin	0	0	0	0
ENSG000000	SESN1	protein_codin	47.5208548	49.9607012	25.8920067	10.0413634
ENSG000001	ARMC2	protein_codin	0	0	0	3.46271733
ENSG000001	AFG1L	protein_codin	3.75328697	9.23559137	8.59099168	6.72604612
ENSG000001	SNX3	protein_codin	0.98127351	0	0	4.46694709
ENSG000001	NR2E1	protein_codin	0	0	0	2.10105236
ENSG000001	HTRA1	protein_codin	295.951443	71.5095439	107.165422	102.319634
ENSG000001	CTSS	protein_codin	19.7599596	0	6.22872923	27.2168501
ENSG000001	NR2E1	protein_codin	0	0	0	0
ENSG000001	HORMAD1	protein_codin	1.59249673	0	0	0
ENSG000001	PLEKHA1	protein_codin	0	0	0	0
ENSG000001	PLEKHA1	protein_codin	21.6435908	0	26.191304	0
ENSG000001	PLEKHA1	protein_codin	0	36.9789464	0	0
ENSG000001	HORMAD1	protein_codin	0	0	0.52982774	0.55557184
ENSG000001	TACC2	protein_codin	12.5172082	0	22.7966567	4.1123855
ENSG000001	TACC2	protein_codin	0	0	0	0
ENSG000001	TACC2	protein_codin	0	13.0848006	1.54676314	0
ENSG000001	TACC2	protein_codin	0.57208595	0	1.14014481	0
ENSG000000	SEC63	protein_codin	116.097816	206.335049	149.645774	82.5708085
ENSG000001	TACC2	protein_codin	0	0	0	0
ENSG000001	TACC2	protein_codin	0	0	0	0
ENSG000001	ENSA	protein_codin	45.1490772	53.4449158	83.1172727	9.50817256
ENSG000001	ENSA	protein_codin	0	0	0	0
ENSG000001	NSMCE4A	protein_codin	3.64683626	4.46545035	0	1.03049697
ENSG000001	SCML4	protein_codin	1.19547794	5.30236874	0.55409961	2.88773534
ENSG000001	SCML4	protein_codin	0	8.93173385	0	1.12359673
ENSG000001	NSMCE4A	protein_codin	23.0191559	4.73613426	16.1569057	5.91301256
ENSG000001	SCML4	protein_codin	0	2.19088277	0	0
ENSG000001	MCL1	protein_codin	12.5917934	0	0	456.486274
ENSG000001	PDSS2	protein_codin	0.56084444	3.05149535	0	0.87240429

ENSG000001	PDSS2	protein_codin	8.31061632	37.4693209	16.6104522	10.126501
ENSG000001	ADAMTSL4	protein_codin	0	0	9.31937537	0
ENSG000001	ADAMTSL4	protein_codin	0	10.9331744	0	0
ENSG000001	ATE1	protein_codin	0	0	0	0
ENSG000001	ADAMTSL4	protein_codin	0	0	0	0
ENSG000001	BEND3	protein_codin	1.07815898	0	19.1983202	4.32731079
ENSG000001	ATE1	protein_codin	0	0	0	0
ENSG000001	QRSL1	protein_codin	1.3438554	10.4907767	9.39862225	1.09487051
ENSG000001	QRSL1	protein_codin	12.0441068	24.7364155	8.48658648	9.88380603
ENSG000001	ECM1	protein_codin	3.18659355	0	0	4.93806849
ENSG000001	ECM1	protein_codin	0	8.16975587	0	0
ENSG000001	TARS2	protein_codin	7.38836171	13.4608725	5.9677645	1.25840441
ENSG000001	TARS2	protein_codin	0	0	1.64945773	0
ENSG000001	TARS2	protein_codin	0.55375482	0	0	0
ENSG000000	FGFR2	protein_codin	0	0	0	0
ENSG000000	FGFR2	protein_codin	0	0	0	0
ENSG000000	FGFR2	protein_codin	0	0	0	0
ENSG000000	FGFR2	protein_codin	143.436622	51.9088962	310.085344	33.6852779
ENSG000000	FGFR2	protein_codin	53.7977832	0	0	0
ENSG000001	RTN4IP1	protein_codin	7.46171803	9.60690111	10.0173894	2.86096093
ENSG000001	TARS2	protein_codin	7.41110601	6.86257265	0	6.99373745
ENSG000001	RPRD2	protein_codin	0	0	0.95707287	0
ENSG000001	RPRD2	protein_codin	89.6916081	99.1565079	99.7826925	34.2430898
ENSG000000	ATG5	protein_codin	0	0	0	0
ENSG000002	PLPP4	protein_codin	0	0	0	0
ENSG000001	SEC23IP	protein_codin	6.7649453	10.8288485	13.6753772	8.5901834
ENSG000000	ATG5	protein_codin	7.1886785	37.3340841	26.7926485	10.8516132
ENSG000001	MCMBP	protein_codin	6.49230939	61.004063	17.5915515	9.58708141
ENSG000001	INPP5F	protein_codin	0	0	0.58442584	0
ENSG000001	BAG3	protein_codin	63.5510203	76.9045404	97.2720091	193.977812
ENSG000001	TIAL1	protein_codin	5.43748898	12.5416841	9.92829831	2.71006932
ENSG000001	TIAL1	protein_codin	7.16438845	8.57997646	15.2974346	1.3442334
ENSG000000	PRDM1	protein_codin	0	9.23319267	0	0
ENSG000000	PRDM1	protein_codin	0	0	0	1.55649164
ENSG000001	TIAL1	protein_codin	22.2685325	20.1064655	18.3546167	11.4811
ENSG000001	CIART	protein_codin	0	0	0	0
ENSG000001	CIART	protein_codin	1.99455398	0	0	0
ENSG000000	PRDM1	protein_codin	19.2725367	170.878556	4.82341375	62.3922596
ENSG000001	C1orf54	protein_codin	23.0327917	5.67823988	2.29955232	4.37226063
ENSG000001	C1orf54	protein_codin	34.8428077	58.9289356	1.01811352	11.5955576
ENSG000001	RGS10	protein_codin	16.2425598	0	10.2091537	8.68760894
ENSG000001	C1orf54	protein_codin	0	1.13858653	0	1.15514259
ENSG000001	RGS10	protein_codin	16.6230111	95.0382725	9.65180055	69.0828222
ENSG000001	GRK5	protein_codin	0	0	0	0

ENSG000001	APH1A	protein_codin	23.7647599	0	24.0008811	6.27957317
ENSG000000	PREP	protein_codin	12.4847631	55.9114152	23.1775754	3.10026572
ENSG000001	CA14	protein_codin	0	0	0	0.38037226
ENSG000001	ANP32E	protein_codin	2.81077341	51.5454846	9.17564417	0
ENSG000001	ANP32E	protein_codin	139.352668	225.480281	272.020994	23.1107354
ENSG000000	PLEKHO1	protein_codin	45.7524922	206.82962	60.9768626	27.6750543
ENSG000000	HACE1	protein_codin	0	0	13.1572405	0
ENSG000000	PLEKHO1	protein_codin	1.20385395	28.3806134	1.94853874	0
ENSG000000	HACE1	protein_codin	2.51981287	2.64209602	10.0168062	1.46693719
ENSG000001	VPS45	protein_codin	0	0	0	0
ENSG000001	SFXN4	protein_codin	0	0	0	0
ENSG000001	GRIK2	protein_codin	3.91002685	0	0	0
ENSG000001	GRIK2	protein_codin	0	0	0	0
ENSG000001	GRIK2	protein_codin	0	0	10.1292747	0
ENSG000000	MTMR11	protein_codin	0	10.6299685	0	0
ENSG000001	ASCC3	protein_codin	21.8656466	23.1204358	28.2953456	11.422568
ENSG000001	EIF3A	protein_codin	496.1102	2115.54307	1105.51542	433.907464
ENSG000001	SV2A	protein_codin	0	5.24360528	0	2.17467964
ENSG000001	SV2A	protein_codin	15.4707754	12.1261896	30.4400282	0
ENSG000001	BOLA1	protein_codin	1.75062606	0	0	1.18789784
ENSG000001	CACUL1	protein_codin	30.9390163	45.4888673	36.7990194	25.2316652
ENSG000001	BOLA1	protein_codin	0	0.9848211	0	0
ENSG000001	BOLA1	protein_codin	0.76794257	0	1.96556694	1.37640484
ENSG000001	H2BC21	protein_codin	63.0123276	51.0425736	190.71657	11.4691063
ENSG000002	H3C14	protein_codin	0	0	0	0
ENSG000002	H2AC18	protein_codin	321.765167	190.681888	1289.97576	52.1974455
ENSG000001	ASCC3	protein_codin	81.9490751	145.588563	119.09456	46.4448119
ENSG000002	H3C10	protein_codin	0.92945826	0	6.38294822	0
ENSG000002	H2BC18	protein_codin	30.5156886	67.8354819	16.9502194	9.83612745
ENSG000001	FCGR1A	protein_codin	2.22287867	15.1201372	0	2.82853486
ENSG000001	FAM204A	protein_codin	0.50045453	0	0	0
ENSG000001	FAM204A	protein_codin	16.398921	69.3132685	20.1350618	11.7644715
ENSG000002	FAM72C	protein_codin	0	0	5.61357601	0.8808847
ENSG000001	FAM204A	protein_codin	10.3612921	28.4625728	11.682278	14.3238528
ENSG000001	RAB11FIP2	protein_codin	24.2073325	0	72.5051379	18.6003219
ENSG000001	VAX1	protein_codin	0	0	0	1.24498869
ENSG000001	ENO4	protein_codin	0	2.58638846	0.65065727	0
ENSG000001	SIM1	protein_codin	1.84989924	0	0	3.8136158
ENSG000001	HSPA12A	protein_codin	0	0	0	0
ENSG000001	SPMIP5	protein_codin	0	0	0	0
ENSG000001	MCHR2	protein_codin	0.48072853	0	0	0
ENSG000001	PRDM13	protein_codin	0	0	0	0
ENSG000001	PRDM13	protein_codin	0	0	9.61899325	0.91228162
ENSG000001	CCNC	protein_codin	1.19973151	3.36627023	2.85056147	0.94654213

ENSG000001	CCNC	protein_codin	0	0	0	0
ENSG000001	PNLIP	protein_codin	0	0	0	1.34392743
ENSG000002	NBPF19	protein_codin	0	65.8126233	0	0
ENSG000002	PNLIPRP3	protein_codin	0	0	0	0.56696069
ENSG000001	USP45	protein_codin	0	0	0	0
ENSG000001	USP45	protein_codin	2.95599042	0	12.5328071	0
ENSG000001	GFRA1	protein_codin	0	0	0	0
ENSG000001	GJA8	protein_codin	0	0	0	1.39685587
ENSG000001	GFRA1	protein_codin	9.53241577	61.6228318	7.42687054	39.2858544
ENSG000001	PNISR	protein_codin	0	0	0	0
ENSG000001	COQ3	protein_codin	0	0	0	1.78387814
ENSG000001	FBXL4	protein_codin	23.9178245	30.307756	31.2946648	17.348525
ENSG000001	FHIP2A	protein_codin	0.44828054	4.53480562	0.72228198	0.59163673
ENSG000001	FHIP2A	protein_codin	70.6581408	118.95325	67.8424383	35.7238103
ENSG000001	FHIP2A	protein_codin	0	0	0	0.94903672
ENSG000001	MMS22L	protein_codin	4.3262667	0	65.9059685	0
ENSG000000	ABLIM1	protein_codin	12.4035186	18.2924406	0	0
ENSG000001	KLHL32	protein_codin	0	0	0	0
ENSG000000	ABLIM1	protein_codin	0.72713213	28.7875188	7.05756593	1.45289119
ENSG000001	CHD1L	protein_codin	0	0	0	0
ENSG000001	CHD1L	protein_codin	43.5109424	0	0	0
ENSG000001	KLHL32	protein_codin	0	0	0	0
ENSG000001	AFAP1L2	protein_codin	0	0	28.3491041	14.5195699
ENSG000001	FMO5	protein_codin	3.32967282	0	0	3.32908707
ENSG000000	UFL1	protein_codin	117.529807	171.701152	172.565534	47.8423897
ENSG000000	TDRD1	protein_codin	0	0	0	0
ENSG000000	TDRD1	protein_codin	0	0	0	0
ENSG000001	CCDC186	protein_codin	0	0	0	0
ENSG000001	CCDC186	protein_codin	4.53463929	21.5793833	0	0
ENSG000001	CCDC186	protein_codin	129.797823	30.5227805	166.514679	65.1909369
ENSG000001	CD160	protein_codin	0	0	0	0
ENSG000001	MANEA	protein_codin	2.32905888	2.38590288	0.54782471	0.49590143
ENSG000001	POLR3C	protein_codin	4.88037348	0	14.3401011	0
ENSG000000	ADRB1	protein_codin	1.0482474	0.94491997	2.15112443	0.93823131
ENSG000001	EPHA7	protein_codin	0.55658249	0	1.72285596	0
ENSG000001	PIAS3	protein_codin	0	0	0	0
ENSG000001	PIAS3	protein_codin	0	23.7194009	2.84846364	1.72076404
ENSG000001	NHLRC2	protein_codin	55.6862767	67.0935242	85.1657365	29.5474994
ENSG000001	EPHA7	protein_codin	0	0	2.40745761	0
ENSG000001	ITGA10	protein_codin	18.2468586	21.0029123	15.517282	53.6838656
ENSG000001	DCLRE1A	protein_codin	7.11116653	15.807446	24.7302096	4.14074974
ENSG000001	PEX11B	protein_codin	8.03513597	10.2261986	10.7263723	3.0155337
ENSG000002	RBM8A	protein_codin	37.8454692	0	127.520974	16.281609
ENSG000001	PLEKHS1	protein_codin	0	0	0	0

ENSG000001	PLEKHS1	protein_codin	0	6.6947465	0	0.84033318
ENSG000001	PLEKHS1	protein_codin	0	0	0	0
ENSG000001	POLR3GL	protein_codin	39.9890057	265.031352	43.7994995	0
ENSG000001	POLR3GL	protein_codin	84.219101	112.555405	150.768747	43.0170724
ENSG000001	CASP7	protein_codin	0	0	0	0
ENSG000001	CASP7	protein_codin	13.2333905	0	17.5739724	15.4936114
ENSG000001	MAP3K7	protein_codin	5.87175178	11.0209401	2.62933439	2.56795461
ENSG000001	CASP7	protein_codin	0	0	0	4.59071303
ENSG000001	MAP3K7	protein_codin	0	0	0	0
ENSG000001	MAP3K7	protein_codin	0	0	0	0
ENSG000001	MAP3K7	protein_codin	0	0	0	0
ENSG000001	CASP7	protein_codin	0	0	0	0
ENSG000001	MAP3K7	protein_codin	25.1604667	12.6613412	23.0448257	12.908685
ENSG000001	PDE4DIP	protein_codin	0	0	0	0
ENSG000001	PDE4DIP	protein_codin	16.7730936	0	0	2.92504839
ENSG000001	PDE4DIP	protein_codin	0	0	3.70661782	0
ENSG000001	GJA10	protein_codin	0	0	0	0
ENSG000001	PDE4DIP	protein_codin	58.9439475	0	0	0
ENSG000001	PDE4DIP	protein_codin	7.46917919	41.9102629	0	0
ENSG000001	NRAP	protein_codin	146.68627	0	0	0
ENSG000001	NRAP	protein_codin	0	0	0	0
ENSG000001	SCAF11	protein_codin	971.021382	355.447844	158.816183	65.6936088
ENSG000001	NBPF20	protein_codin	0	0	0	0
ENSG000001	TCF7L2	protein_codin	0	0	0	0
ENSG000001	TCF7L2	protein_codin	0	0	0	2.13563051
ENSG000001	FAM72B	protein_codin	0	3.52247115	0	0
ENSG000001	MDN1	protein_codin	83.7735209	180.795688	224.453241	76.7054597
ENSG000001	TCF7L2	protein_codin	3.26386216	0	1.33481456	0
ENSG000001	TCF7L2	protein_codin	5.18508825	0	0	0
ENSG000001	ADAM30	protein_codin	0.28052907	0	0	4.477068
ENSG000001	REG4	protein_codin	0.25417834	0	0	1.95663584
ENSG000000	ZDHHC6	protein_codin	10.6046415	0	16.987921	0
ENSG000000	ZDHHC6	protein_codin	0	0	0	0
ENSG000001	HMGCS2	protein_codin	0	0	0	0
ENSG000000	PHGDH	protein_codin	0.61337228	2.83552538	0	0
ENSG000001	ANKRD6	protein_codin	0	0	0	11.0808166
ENSG000000	PHGDH	protein_codin	0	0	0	0
ENSG000002	HSD3B1	protein_codin	0	0	0	0
ENSG000000	RRAGD	protein_codin	0	0	31.108252	12.1320528
ENSG000002	HSD3B2	protein_codin	0	0	0	0
ENSG000001	TECTB	protein_codin	0	0	0	0
ENSG000001	IL9R	protein_codin	0.32528582	3.55306877	0	2.09079404
ENSG000001	GPAM	protein_codin	2.04000534	10.8359876	0	3.50935931
ENSG000001	WARS2	protein_codin	11.6689941	4.59365306	0	0

ENSG000000	TBX15	protein_codin	66.9013786	164.189662	31.8983567	23.908263
ENSG000001	TMLHE	protein_codin	14.7144271	25.6307415	17.157715	5.27748187
ENSG000000	WDR3	protein_codin	3.19381552	0	0	0.51417227
ENSG000001	GDAP2	protein_codin	9.264611	0	16.4737763	3.96409097
ENSG000001	GDAP2	protein_codin	25.6875387	69.8487825	38.3754152	16.5447506
ENSG000001	TENT5C	protein_codin	15.7556186	173.254904	40.1517387	22.5116026
ENSG000001	CLIC2	protein_codin	11.2298027	30.705395	1.75258285	11.1844423
ENSG000001	GABRR1	protein_codin	0	0	0	0
ENSG000001	SHOC2	protein_codin	0	69.4868455	0	21.3545597
ENSG000001	RAB39B	protein_codin	1.24038746	2.05295625	7.54660804	2.43886916
ENSG000001	VTCN1	protein_codin	0	0	8.35224018	0
ENSG000001	BRCC3	protein_codin	0	0	0	0
ENSG000001	TRIM45	protein_codin	0	0	0	0
ENSG000001	BRCC3	protein_codin	2.93973527	18.2018577	9.65050304	0
ENSG000001	TRIM45	protein_codin	0	0	11.9057649	0
ENSG000001	TTF2	protein_codin	20.3153557	58.0078974	114.162261	8.12973058
ENSG000001	CD101	protein_codin	0	0	0	0
ENSG000001	PNRC1	protein_codin	0	0	4.13780723	1.41212542
ENSG000001	RNGTT	protein_codin	3.16884294	0	0	0
ENSG000002	MTCP1	protein_codin	0	0	3.39268544	0
ENSG000001	CD2	protein_codin	0	40.4462865	0	0
ENSG000001	CD2	protein_codin	8.43746811	106.627799	0	3.05020941
ENSG000001	CMC4	protein_codin	0.33477516	2.25580847	3.08451828	0
ENSG000001	IGSF3	protein_codin	0	105.959946	0	0
ENSG000001	CMC4	protein_codin	3.56488424	10.1891255	20.8482494	0.63772606
ENSG000001	RNGTT	protein_codin	19.805312	32.9367248	13.8896753	17.1113387
ENSG000001	IGSF3	protein_codin	28.6843909	0	100.584358	58.2909114
ENSG000001	CD58	protein_codin	0	0	0	0
ENSG000001	CD58	protein_codin	14.9591246	32.2208489	14.5232653	10.4348321
ENSG000001	ATP1A1	protein_codin	2.6890436	3.82415604	5.3918386	2.47372837
ENSG000001	ATP1A1	protein_codin	5.97440798	0	9.49584735	22.1148519
ENSG000001	FUNDC2	protein_codin	4.02591021	6.81068466	3.18218796	6.25524828
ENSG000001	CNR1	protein_codin	0	0	0	0.75304981
ENSG000001	MAB21L3	protein_codin	0	0	13.4125615	0
ENSG000001	CNR1	protein_codin	0	0	0	0
ENSG000001	SLC22A15	protein_codin	0	0	0	0
ENSG000001	SLC22A15	protein_codin	13.3172546	38.6132942	35.8628967	10.9632385
ENSG000002	F8A2	protein_codin	2.15846155	0	9.99931826	0
ENSG000001	NHLH2	protein_codin	0.31012841	0	0	4.1269001
ENSG000001	VANGL1	protein_codin	0	6.53299178	0	0
ENSG000001	VANGL1	protein_codin	0	0	0	0
ENSG000001	NGF	protein_codin	0	0	0	1.39195518
ENSG000001	TSPAN2	protein_codin	0	0	0	0
ENSG000001	TSPAN2	protein_codin	4.3524162	12.6000094	5.02660164	0.82936496

ENSG000001!	SYCP1	protein_codin	0	0	0	0
ENSG000002	RBM20	protein_codin	9.96202814	10.0060782	20.3236531	9.52282285
ENSG000001!	SYCP1	protein_codin	0	0	4.86352819	0
ENSG000000	SIKE1	protein_codin	0	7.31328331	0	0
ENSG000000	SIKE1	protein_codin	0	12.4628618	82.491796	0
ENSG000002	SMIM9	protein_codin	0	0	0	0
ENSG000000	CSDE1	protein_codin	77.7041604	0	3.98002085	27.7430828
ENSG000001	MPP1	protein_codin	0	0	0	0
ENSG000001	MPP1	protein_codin	3.39953163	0	0	8.72192884
ENSG000002	NRAS	protein_codin	41.4587605	41.642381	116.593664	39.3262759
ENSG000001	RARS2	protein_codin	0	0	0	0
ENSG000001	AMPD1	protein_codin	0	0	0	0.43187957
ENSG000001	BCAS2	protein_codin	8.40530859	30.4973756	19.6539215	24.2052294
ENSG000001!	TRIM33	protein_codin	27.9224109	40.5719722	94.3973787	18.0957947
ENSG000001	SYT6	protein_codin	0	0	0	0
ENSG000001	DKC1	protein_codin	0	0	0	0
ENSG000001	OLFML3	protein_codin	0	0	8.97563647	6.6803113
ENSG000001	SLC35A1	protein_codin	5.92971769	2.90962668	16.4839792	0
ENSG000001	HIPK1	protein_codin	0	0	7.33454621	0
ENSG000001	HIPK1	protein_codin	39.6213888	0	78.1617022	38.4382083
ENSG000001	SLC35A1	protein_codin	0	0	0	0
ENSG000001	SLC35A1	protein_codin	0	0	0	0
ENSG000001	HIPK1	protein_codin	10.2698143	116.292479	159.250068	73.7828268
ENSG000001	HIPK1	protein_codin	11.1805487	34.1138475	104.396601	0
ENSG000001	HIPK1	protein_codin	0	0	0	0
ENSG000002	CFAP206	protein_codin	0	0	0	0
ENSG000001	AP4B1	protein_codin	0	0	0	0
ENSG000001	AP4B1	protein_codin	0	0	0	0
ENSG000001	GAB3	protein_codin	5.72245118	0	6.23953124	7.58799416
ENSG000001	AP4B1	protein_codin	0	0	0	0
ENSG000001	SMIM8	protein_codin	1.07030149	8.64819543	2.65357958	0.42691943
ENSG000001	AP4B1	protein_codin	0	0	0	0
ENSG000001	SMIM8	protein_codin	1.20056283	0	3.48304769	0
ENSG000002	C6orf163	protein_codin	0	4.10339234	5.71347894	0
ENSG000001	GAB3	protein_codin	7.13517174	0	0	0
ENSG000001	GJB7	protein_codin	0	0	0	0
ENSG000001	ZNF292	protein_codin	29.8158594	107.288625	41.3230138	59.9168239
ENSG000001	ZNF292	protein_codin	2.41620396	56.7382409	8.37408196	0.54256795
ENSG000001	BCL2L15	protein_codin	0	0	1.22220636	0
ENSG000000	RSBN1	protein_codin	0	0	0	0
ENSG000001	CGA	protein_codin	0	0	0	0
ENSG000001	DUSP5	protein_codin	2.58262315	20.1498624	2.91112695	37.9945267
ENSG000001	CTAG2	protein_codin	0	0.56582341	0	0
ENSG000001	SMNDC1	protein_codin	16.3464734	27.9974726	33.9736892	5.84629186

ENSG000001	PHTF1	protein_codin	0	0	0	0
ENSG000001	PHTF1	protein_codin	7.1772863	4.70616186	5.6975916	0
ENSG000001	SMNDC1	protein_codin	7.84006071	25.682695	11.334404	7.22185507
ENSG000001	PHTF1	protein_codin	0	0	0	0
ENSG000000	MAGI3	protein_codin	0	0	0	0
ENSG000001	MXI1	protein_codin	0	0	0	0
ENSG000000	MAGI3	protein_codin	36.4579108	52.5315075	120.342473	17.7187889
ENSG000000	MAGI3	protein_codin	0	4.71436633	1.66771451	1.43065347
ENSG000001	G6PD	protein_codin	1.25644473	0	0	2.30701514
ENSG000001	SYNCRIP	protein_codin	0	0	0	0
ENSG000001	SLC16A1	protein_codin	34.5310379	59.9032896	247.982461	7.6412608
ENSG000001	SNX14	protein_codin	0	0	0	0
ENSG000001	TAFA3	protein_codin	0	0	0	0
ENSG000001	RHOC	protein_codin	0	0	0	0
ENSG000001	RHOC	protein_codin	0	0	0	0
ENSG000001	SNX14	protein_codin	0	0	0	0
ENSG000001	RHOC	protein_codin	2.85832796	0	7.27861205	0
ENSG000001	RHOC	protein_codin	12.5670853	70.3150541	0	0
ENSG000001	RHOC	protein_codin	0	7.83722634	0	0
ENSG000000	FAM3A	protein_codin	1.86615149	5.36764851	9.12348328	0.9616821
ENSG000001	RHOC	protein_codin	0	21.0686031	4.32505746	12.5149533
ENSG000000	FAM3A	protein_codin	0.58384849	0	0	0
ENSG000001	MOV10	protein_codin	0	0	0	0
ENSG000001	MOV10	protein_codin	1.49792722	35.4773858	10.3086155	0
ENSG000001	NT5E	protein_codin	0	0	1.15763053	0
ENSG000001	SLC10A3	protein_codin	0	0	1.87876836	0
ENSG000001	NT5E	protein_codin	0.40175851	5.37885024	0	0
ENSG000001	UBL4A	protein_codin	0	0	0	0
ENSG000001	XPNPEP1	protein_codin	0	4.52797567	0	0
ENSG000001	UBL4A	protein_codin	16.1636755	31.4372218	91.7191133	4.62566206
ENSG000001	TBX18	protein_codin	57.5319426	48.5339057	71.0899471	20.4560402
ENSG000000	ST7L	protein_codin	0	5.31030069	0	0
ENSG000000	ST7L	protein_codin	0	0	0	0
ENSG000000	ST7L	protein_codin	3.15756143	0	0	0
ENSG000000	CYB5R4	protein_codin	0	0	2.71808654	2.38465565
ENSG000000	CYB5R4	protein_codin	15.64174	45.1214126	10.5049702	11.0066822
ENSG000001	PLXNA3	protein_codin	17.9406999	109.553917	90.7661759	30.9150557
ENSG000001	XPNPEP1	protein_codin	0	0	0	0
ENSG000001	WNT2B	protein_codin	0	0	0	0
ENSG000001	WNT2B	protein_codin	2.26264571	2.80917258	1.7741639	7.077751
ENSG000002	RIPPLY2	protein_codin	0	0	0	0
ENSG000002	RIPPLY2	protein_codin	0	0	0	0
ENSG000000	SNAP91	protein_codin	0	0	0	0
ENSG000000	SNAP91	protein_codin	0	0	0	0

ENSG000000	SNAP91	protein_codin	0	0	0	0
ENSG000001	KCND3	protein_codin	1.60622446	2.14465295	0	2.21246302
ENSG000001	SORCS1	protein_codin	0	2.36547012	0	2.05832919
ENSG000001	PRSS35	protein_codin	72.0034572	188.292973	4.8208757	157.054125
ENSG000001	SORCS3	protein_codin	0.31664596	0.8885542	11.9007292	3.70712299
ENSG000000	DDX20	protein_codin	0	0	0	0
ENSG000001	CFAP58	protein_codin	0.37506465	0	0	0
ENSG000001	CFAP58	protein_codin	1.7000462	1.22587575	9.53904636	1.8221173
ENSG000000	ME1	protein_codin	28.4101897	50.887665	24.1426413	38.4924976
ENSG000000	GSTO2	protein_codin	0	2.88839605	5.03916195	4.41823644
ENSG000001	RAP1A	protein_codin	21.0995019	32.7849681	21.7073212	7.053683
ENSG000001	GSTO1	protein_codin	0.90844295	0	0	0
ENSG000001	GSTO1	protein_codin	5.78002975	26.0793324	24.0745734	6.82557344
ENSG000001	TMIGD3	protein_codin	0	0	0	0
ENSG000001	TMIGD3	protein_codin	0	0	0	0
ENSG000001	C1orf162	protein_codin	2.7754133	3.64174893	1.37692405	2.88332392
ENSG000001	CFAP43	protein_codin	0.65111822	0	5.71527181	0
ENSG000001	CFAP43	protein_codin	0	0	2.70895005	3.44516022
ENSG000001	ATP5PB	protein_codin	0	0	0	26.4920415
ENSG000001	ATP5PB	protein_codin	6.1353366	13.4897804	12.1436675	4.57068131
ENSG000000	RWDD2A	protein_codin	9.39953823	8.48277314	7.31926218	5.60629545
ENSG000001	SFR1	protein_codin	1.98841278	3.28472006	17.500861	6.21161127
ENSG000001	SFR1	protein_codin	1.15971833	0	0	0
ENSG000000	OVGP1	protein_codin	1.22325391	5.0946192	3.96947399	2.39515167
ENSG000000	COL17A1	protein_codin	0	0	0.53603444	0
ENSG000001	CIMAP3	protein_codin	0	0	0	0
ENSG000001	CIMAP3	protein_codin	0	0	0	0
ENSG000000	DOP1A	protein_codin	0	0	0	0
ENSG000001	CHIA	protein_codin	0	0	0	0
ENSG000000	CHI3L2	protein_codin	0	8.14807025	0	0
ENSG000001	UBE3D	protein_codin	4.09219821	5.13387614	7.21535165	3.87526899
ENSG000001	UBE3D	protein_codin	1.06377988	8.3322041	0	0
ENSG000000	CHI3L2	protein_codin	0	21.2572229	0	11.8314802
ENSG000001	TPBG	protein_codin	0	24.6695165	21.2045853	0
ENSG000000	IBTK	protein_codin	0	0	27.2460204	5.79314294
ENSG000001	DENND2D	protein_codin	0.9593323	0	1.03468523	0
ENSG000001	TENT5A	protein_codin	0	0	10.0466436	7.00235132
ENSG000000	SLK	protein_codin	7.90805386	91.4185731	66.3879788	19.5009553
ENSG000001	TENT5A	protein_codin	0	0	0	0
ENSG000002	PCMTD2	protein_codin	0	0	15.4459529	16.6760942
ENSG000000	BCKDHB	protein_codin	2.83359096	0	0	2.98297227
ENSG000000	ATP6AP1	protein_codin	18.1174464	19.0705381	63.451415	15.4939026
ENSG000001	LRIF1	protein_codin	7.81032359	9.98785786	19.1837345	5.58597003
ENSG000001	STN1	protein_codin	3.96773649	6.60297048	0.92519285	1.83911974

ENSG000001:	NPBWR2	protein_codin	0	1.27952879	0	0.3942769
ENSG000001	KCNA3	protein_codin	0	0	0	0
ENSG000001	KCNA2	protein_codin	0	0	0	0
ENSG000001	KCNA10	protein_codin	0	0	0	0
ENSG000001	SH3PXD2A	protein_codin	688.018985	627.875048	333.426156	318.330046
ENSG000001	TAFAZZIN	protein_codin	0	0	1.14366023	0
ENSG000001	SLC16A4	protein_codin	0	0	0	0
ENSG000001	NEURL1	protein_codin	0	0.80658648	0	0.86358109
ENSG000001	SLC16A4	protein_codin	0	0	0	0
ENSG000001	CALHM3	protein_codin	0	0	0	0.46798162
ENSG000001	RBM15	protein_codin	0	101.360604	0	0
ENSG000001	KCNC4	protein_codin	5.26653773	54.0050489	13.5544839	15.9692328
ENSG000001	CALHM2	protein_codin	5.45547485	0	4.68815295	0
ENSG000001	STRIP1	protein_codin	18.4586804	39.6042368	43.8785507	11.3176326
ENSG000001	STRIP1	protein_codin	0	0	0	0
ENSG000001	PDCD11	protein_codin	52.2063048	71.3413735	187.226681	5.11867477
ENSG000001	TTK	protein_codin	5.08798058	5.83551406	52.0693991	0
ENSG000001	AHCYL1	protein_codin	102.509496	79.7127077	260.624582	44.7314422
ENSG000002	NEURL1B	protein_codin	22.3962898	47.3809562	65.0783173	28.0571038
ENSG000001	CSF1	protein_codin	0	0	0	0
ENSG000001	CSF1	protein_codin	1.46720229	76.5464197	0	14.7416745
ENSG000001	EPS8L3	protein_codin	0	0	0	0
ENSG000000	DNASE1L1	protein_codin	0	0	0	1.32233046
ENSG000000	DNASE1L1	protein_codin	4.28469757	45.4108952	9.08372728	9.2528318
ENSG000000	DNASE1L1	protein_codin	0	0	0	0
ENSG000001	ATP5MK	protein_codin	0	0	0	0.56638911
ENSG000001	GSTM5	protein_codin	0	0	0	0
ENSG000001	GSTM5	protein_codin	1.28974454	0	0	2.74140993
ENSG000001	ATP5MK	protein_codin	0	13.4641667	5.25363758	0
ENSG000001	ELOVL4	protein_codin	10.4191654	1.46625588	4.48442647	2.15796787
ENSG000001	RPL10	protein_codin	0.55424776	1.58431392	0.79612801	0
ENSG000001	GSTM1	protein_codin	0	0	0	0
ENSG000001	GSTM1	protein_codin	0	16.8116748	0	0
ENSG000001	ATP5MK	protein_codin	1.6406805	0	4.6995329	0
ENSG000002	GSTM2	protein_codin	0	0	4.55032517	2.43890881
ENSG000002	GSTM2	protein_codin	6.2694388	2.77489935	0	0
ENSG000002	GSTM2	protein_codin	0	0	0	0
ENSG000001	GSTM4	protein_codin	1.73563871	28.3049961	6.49501562	7.39314639
ENSG000001	EMD	protein_codin	0	8.74124127	2.5060541	2.19017254
ENSG000001	GSTM4	protein_codin	0	3.05874763	2.04875726	0
ENSG000001	SH3BGRL2	protein_codin	7.07305195	6.44193989	31.2768222	22.8396179
ENSG000001	TAF5	protein_codin	5.48368102	0	14.6629422	0
ENSG000001	AMPD2	protein_codin	0	0	0	0
ENSG000001	EMD	protein_codin	3.46600936	0	13.526741	0

ENSG000001:	LCA5	protein_codin	28.8002406	42.9919048	30.1999873	11.4530633
ENSG000001:	PCGF6	protein_codin	2.7996507	0	8.29097046	7.04308179
ENSG000001	INA	protein_codin	4.07836254	0.97703062	1.46973763	2.30567176
ENSG000001!	FLNA	protein_codin	53.3752724	662.571923	309.447465	196.573781
ENSG000000	GNAI3	protein_codin	100.881977	103.271009	162.358361	73.3885306
ENSG000001!	FLNA	protein_codin	48.1685244	106.291083	317.762032	48.5452474
ENSG000000	NT5C2	protein_codin	0	0	0	0
ENSG000001	AMIGO1	protein_codin	0	2.55277038	0	1.04050581
ENSG000001	AMIGO1	protein_codin	1.46928985	2.17126154	6.73662402	0.62355001
ENSG000000:	MPHOSPH9	protein_codin	0	0	0	0
ENSG000001	CYB561D1	protein_codin	0	0	0	0
ENSG000001	ATXN7L2	protein_codin	0	0	0	0
ENSG000001	ATXN7L2	protein_codin	0	0	0	1.59842446
ENSG000001	SYPL2	protein_codin	3.95109819	0	2.48573739	1.57892405
ENSG000001	CNNM2	protein_codin	5.1825894	8.22073209	18.3397637	3.62297311
ENSG000001	CNNM2	protein_codin	3.58644895	22.5561657	6.91946781	8.82550153
ENSG000002	AS3MT	protein_codin	3.1150806	0	19.4035579	0
ENSG000001	BORCS7	protein_codin	0	0	7.65803564	0
ENSG000001	WBP1L	protein_codin	0.24701861	0	0.43023488	0.34477084
ENSG000001:	SAMD10	protein_codin	0	0	0	0.44787193
ENSG000001	CYP17A1	protein_codin	0	0	0	0
ENSG000001!	ZNF512B	protein_codin	24.6957169	76.7094599	87.9304728	15.9453093
ENSG000001	WBP1L	protein_codin	0	92.5827507	0	31.9969785
ENSG000001:	SFXN2	protein_codin	7.06312803	18.3746379	30.5850406	4.182172
ENSG000001	SUFU	protein_codin	0	0	0	0
ENSG000001	SUFU	protein_codin	13.3133401	22.0854791	26.138787	8.46034812
ENSG000001:	PSRC1	protein_codin	0	2.91610525	0	0
ENSG000001:	PSRC1	protein_codin	3.02042253	0	31.773234	0
ENSG000001:	ACTR1A	protein_codin	41.7413603	103.369401	86.1820969	29.4206556
ENSG000001:	PSRC1	protein_codin	0	0	0	0
ENSG000001!	UCKL1	protein_codin	0	0	1.91080562	0
ENSG000001:	PSRC1	protein_codin	0	3.0483984	20.3691989	0.72683703
ENSG000000	TKTL1	protein_codin	0	0	0	0
ENSG000000	TKTL1	protein_codin	0.57149567	0	1.24668217	1.39853992
ENSG000001	ABHD16B	protein_codin	0	0	0	0
ENSG000000:	SARS1	protein_codin	0.98708799	0	0	0
ENSG000001	TPD52L2	protein_codin	8.87144181	10.8682507	16.7534388	4.24815079
ENSG000001	OPN1MW2	protein_codin	0	0	0	0
ENSG000001:	MFSD13A	protein_codin	0	0	0.67987939	0
ENSG000001	ELAPOR1	protein_codin	1.59026746	5.58653181	0	3.70220072
ENSG000001	CUEDC2	protein_codin	19.6215526	19.8917261	39.3317284	11.5501079
ENSG000001	ELAPOR1	protein_codin	0.37909636	4.16792558	0	0
ENSG000001	ELAPOR1	protein_codin	0	0	3.37366012	0
ENSG000001	IRAK1BP1	protein_codin	5.38978462	6.20761901	6.40362724	3.72113337

ENSG000001	CFAP276	protein_codin	0	0	0	0
ENSG000001	CFAP276	protein_codin	0	0	0	0
ENSG000001	HTR1B	protein_codin	0	0	0	0
ENSG000001	CFAP276	protein_codin	0	0	0	0
ENSG000001	CFAP276	protein_codin	0	0	0	0
ENSG000001	IMPG1	protein_codin	0	0	0	0
ENSG000001	OPN1LW	protein_codin	0	0	0	0
ENSG000001	IMPG1	protein_codin	0	0	0	0.48086883
ENSG000001	FBXL15	protein_codin	0.97962072	0	1.80977513	1.60916793
ENSG000001	MECP2	protein_codin	0	0	0	0
ENSG000000	WDR47	protein_codin	0	0	59.925553	16.9172526
ENSG000001	IMPG1	protein_codin	0	0	0	0
ENSG000000	WDR47	protein_codin	19.8133987	36.8992963	3.4383915	0
ENSG000000	NFKB2	protein_codin	2.94598179	23.3422734	0	13.6945391
ENSG000001	ZGPAT	protein_codin	17.9961822	0	8.21582517	0
ENSG000001	CLCC1	protein_codin	0	0	0	0
ENSG000001	CLCC1	protein_codin	0	0	0	0
ENSG000001	CLCC1	protein_codin	0	0	0	0
ENSG000001	IRAK1	protein_codin	0	37.1694419	0	3.23246197
ENSG000001	IRAK1	protein_codin	0	0	0	0
ENSG000001	MYO6	protein_codin	0	0	0	0
ENSG000001	CLCC1	protein_codin	0	0	21.8047311	0
ENSG000001	MYO6	protein_codin	0	137.203133	0	52.870367
ENSG000001	IRAK1	protein_codin	6.15236232	44.5293338	18.9213551	11.6508582
ENSG000001	TMEM187	protein_codin	1.1681637	2.37793757	5.35469448	0.40560759
ENSG000001	GBF1	protein_codin	0	0	11.6523175	0
ENSG000001	HCFC1	protein_codin	1.61176049	0	11.5269552	0
ENSG000001	MYO6	protein_codin	63.6114883	0	60.3454911	0
ENSG000001	AKNAD1	protein_codin	0	0	0	0
ENSG000001	AKNAD1	protein_codin	0	0	0	0
ENSG000002	TNFRSF6B	protein_codin	0	0	0	0
ENSG000001	RENBP	protein_codin	0.67223097	22.1303334	0	0
ENSG000001	AKNAD1	protein_codin	0	0	0	0
ENSG000001	PITX3	protein_codin	0.19442767	0	0.48560614	0
ENSG000002	RTEL1	protein_codin	0	0	0	0
ENSG000001	ELOVL3	protein_codin	0	0.9699928	0	1.24014123
ENSG000001	NOLC1	protein_codin	17.2434259	32.7209177	61.0408461	22.8155845
ENSG000001	STXBP3	protein_codin	44.3609258	69.714723	101.075364	24.5911315
ENSG000001	NAA10	protein_codin	11.1678369	0	72.0325488	0
ENSG000001	SENP6	protein_codin	19.28889	136.250407	121.036794	0
ENSG000001	NAA10	protein_codin	0	0	0	0
ENSG000001	PPRC1	protein_codin	0	0	0	0
ENSG000001	NAA10	protein_codin	0	0	0	0
ENSG000000	ARHGAP4	protein_codin	0	0	6.47217804	0

ENSG000001	FNDC7	protein_codin	0	0	0	0
ENSG000002	RTEL1	protein_codin	0	0	0	0
ENSG000001	FILIP1	protein_codin	26.0071082	0	14.9588884	19.1281916
ENSG000001	PRPF38B	protein_codin	120.672148	965.58699	485.874246	95.1983152
ENSG000001	PRPF38B	protein_codin	0	0	0	3.76624462
ENSG000001	PRPF38B	protein_codin	29.0687017	121.713909	83.5894432	20.2573649
ENSG000000	ARHGAP4	protein_codin	0	52.9973796	0	0
ENSG000001	HENMT1	protein_codin	0	0	0	0
ENSG000001	HENMT1	protein_codin	0	21.9189547	22.0767836	0
ENSG000001	ARMH3	protein_codin	20.5544512	51.7589292	34.4464906	15.7759129
ENSG000001	EEIG2	protein_codin	46.3327861	67.2343216	25.0544862	20.564145
ENSG000001	NBPF6	protein_codin	0	0	0	0
ENSG000000	SLC25A24	protein_codin	0	0	0	0
ENSG000001	KCNIP2	protein_codin	0	0	0	0
ENSG000001	AVPR2	protein_codin	0.89166399	0	0	0
ENSG000001	TMEM30A	protein_codin	13.2526094	0	28.4715766	5.66178456
ENSG000001	STMN3	protein_codin	13.4239581	3.68064408	30.4265554	2.31063808
ENSG000001	L1CAM	protein_codin	0	0	0	1.20005662
ENSG000001	VAV3	protein_codin	4.99207354	6.01194058	55.0404647	12.451021
ENSG000001	L1CAM	protein_codin	0	1.78352257	0	0
ENSG000001	L1CAM	protein_codin	0	0	0	0
ENSG000001	NTNG1	protein_codin	0	0	0	0
ENSG000001	NTNG1	protein_codin	0	0	0	0
ENSG000001	NTNG1	protein_codin	0	0	1.12077622	0
ENSG000001	NTNG1	protein_codin	2.00192498	1.37121967	0	1.54756422
ENSG000001	GMEB2	protein_codin	0	0	0	0
ENSG000001	NTNG1	protein_codin	0	0	0	0
ENSG000001	NTNG1	protein_codin	11.5947841	3.13126948	8.44322507	0
ENSG000001	NTNG1	protein_codin	0	0	0	0.67849914
ENSG000001	GMEB2	protein_codin	6.93869956	0	31.1815239	10.1210856
ENSG000001	PRMT6	protein_codin	4.30583404	14.5843865	14.7182961	1.46091514
ENSG000001	AMY1C	protein_codin	0.33469346	0	0	0
ENSG000001	AMY1B	protein_codin	0	0	0	0
ENSG000001	COX7A2	protein_codin	0	0	0	0
ENSG000001	HELZ2	protein_codin	1.55220997	0	0	0
ENSG000002	AMY1A	protein_codin	0	0	0	4.44872314
ENSG000001	SSR4	protein_codin	0	0	0	0
ENSG000001	SSR4	protein_codin	6.3057105	98.4404489	32.6219371	7.87598436
ENSG000001	SSR4	protein_codin	2.43389987	0	5.96638248	0
ENSG000001	COX7A2	protein_codin	0.39121911	0	0	0
ENSG000000	IDH3G	protein_codin	0	0	0	0
ENSG000000	IDH3G	protein_codin	0	4.32892266	1.75023949	0
ENSG000001	OGA	protein_codin	19.5690503	145.60949	91.0727668	63.3732405
ENSG000000	COL11A1	protein_codin	502.312426	937.136977	1856.62903	210.995799

ENSG000001:	FNDC11	protein_codin	0	0	0	0
ENSG000001:	FNDC11	protein_codin	0	0	0	0
ENSG000001	SRPK3	protein_codin	0	0	0	0
ENSG000001	SRPK3	protein_codin	0	0	2.05855886	0
ENSG000001	OLFM3	protein_codin	0	0	3.47256866	0.86232018
ENSG000001	SRPK3	protein_codin	2.20359793	0	9.92151843	0
ENSG000001	DPH5	protein_codin	0	0	0	0
ENSG000001	SRPK3	protein_codin	0	0	0	0
ENSG000001	DPH5	protein_codin	5.08720537	0	13.223019	0
ENSG000001	NPM3	protein_codin	9.60411887	0	43.4690576	4.19232022
ENSG000001	SLC30A7	protein_codin	0	0	2.57896844	0
ENSG000001	SLC30A7	protein_codin	9.87227654	0	8.18736168	0
ENSG000001	EXTL2	protein_codin	10.7279653	23.0582048	0	7.5267142
ENSG000001	EXTL2	protein_codin	26.6333812	0	162.237151	0
ENSG000001	VCAM1	protein_codin	0	0	0	0
ENSG000001	VCAM1	protein_codin	24.4572656	503.763807	3.95653117	40.4885749
ENSG000000	CDC14A	protein_codin	1.48462297	0	10.2387406	0
ENSG000001:	RTCA	protein_codin	0	0.80171404	1.6429077	0
ENSG000001:	RTCA	protein_codin	2.34055635	8.13389426	20.4192925	8.42257541
ENSG000001	ABCD1	protein_codin	1.08982777	0	0	0
ENSG000001:	DBT	protein_codin	5.15414721	0	5.69163243	0
ENSG000001:	DBT	protein_codin	30.0783396	47.7811975	94.8807904	17.201476
ENSG000001	LRRC39	protein_codin	5.12260832	0	0	0
ENSG000001	LRRC39	protein_codin	8.87927174	11.865847	6.24700657	0
ENSG000001	TRMT13	protein_codin	1.64232761	0	24.8949441	0
ENSG000001	TRMT13	protein_codin	36.8443558	68.7947516	269.516056	15.0587864
ENSG000001:	PNCK	protein_codin	0	0	0	0
ENSG000001	TRMT13	protein_codin	5.106221	8.23975215	9.23926772	0
ENSG000001:	PNCK	protein_codin	0	0	0	0
ENSG000001	DPCD	protein_codin	3.18253101	0	0	0
ENSG000001:	PNCK	protein_codin	0	0	0	0
ENSG000001	DPCD	protein_codin	0	1.24763985	7.79140424	2.64571612
ENSG000001	MFSD14A	protein_codin	13.291438	86.3916344	92.3915685	32.8446663
ENSG000001	SLC35A3	protein_codin	0	0	0	0
ENSG000001	SLC35A3	protein_codin	1.73704254	0	0	0
ENSG000001	AGL	protein_codin	0	0	0	0
ENSG000001	POLL	protein_codin	0.47521628	0	0	2.18221728
ENSG000001	AGL	protein_codin	0	0	0	0
ENSG000001	AGL	protein_codin	0	0	0	0
ENSG000001:	DUSP9	protein_codin	0	1.70514635	0	0.89887382
ENSG000001	POLL	protein_codin	0	3.73452206	2.24137251	1.08086597
ENSG000001	POLL	protein_codin	0	0	11.2746407	0
ENSG000001	POLL	protein_codin	0	0	0	0
ENSG000001	FRRS1	protein_codin	0	0	4.00844296	0

ENSG000001:	PPDPF	protein_codin	2.11106375	14.2671617	2.85441558	3.6831841
ENSG000001:	PPDPF	protein_codin	20.6636461	42.4832486	35.5398443	14.201215
ENSG000001	BTRC	protein_codin	0	0	0.52054186	0
ENSG000001	PLPPR4	protein_codin	12.1667672	0	22.2821595	6.20727146
ENSG000001	PLPPR4	protein_codin	0	28.977703	0	1.78149623
ENSG000000	ATP2B3	protein_codin	0	0	0	0
ENSG000001	BTRC	protein_codin	12.1609894	29.1765055	25.315195	8.61487925
ENSG000001	PLPPR5	protein_codin	0	0	0	0
ENSG000001	DPYD	protein_codin	40.1656118	178.310816	39.2885241	29.0588429
ENSG000001:	LBX1	protein_codin	0	0	0	0.75632177
ENSG000001	TLX1	protein_codin	0	2.15240862	1.8465374	1.17310322
ENSG000001	PTBP2	protein_codin	17.5012435	0	46.1420994	0
ENSG000001	PTBP2	protein_codin	0	0	0	0
ENSG000001	KAZALD1	protein_codin	8.67278021	14.3252704	28.9094721	12.2516873
ENSG000001:	RWDD3	protein_codin	8.73449444	7.13948721	22.1808176	0
ENSG000001	TLCD4	protein_codin	4.53446658	2.14474436	3.81620772	3.31971978
ENSG000001	ALG14	protein_codin	4.52865906	4.74016221	5.51378936	3.04992177
ENSG000001	CNN3	protein_codin	163.858825	110.638922	209.977301	83.267052
ENSG000001	F3	protein_codin	0	0	0	0
ENSG000002	HAUS7	protein_codin	3.75236427	28.0584542	11.8851539	3.30874107
ENSG000002	HAUS7	protein_codin	0	0	44.5853813	0
ENSG000001	TREX2	protein_codin	0	0	0	0.80485846
ENSG000001	ABCD3	protein_codin	24.8575798	61.730367	30.6477181	14.0319696
ENSG000001	PDZD7	protein_codin	1.14721018	0	0	0
ENSG000001:	ARHGAP29	protein_codin	4.30919447	43.2995431	0	1.99119666
ENSG000001	LZTS2	protein_codin	1.08939959	13.4255541	22.2641189	7.91071605
ENSG000000	KCNQ2	protein_codin	0	0	0	0
ENSG000001	LZTS2	protein_codin	0	18.6836103	0	0
ENSG000000	KCNQ2	protein_codin	0	0	0	0
ENSG000001	ABCA4	protein_codin	0	3.54678916	0.44225743	0.61673036
ENSG000001	TWNK	protein_codin	0	18.4307764	0	0
ENSG000001	TREX2	protein_codin	0	0	0	0
ENSG000001	TREX2	protein_codin	0	1.19441304	1.26723114	0
ENSG000000	MRPL43	protein_codin	0	0	1.29869022	0
ENSG000000	MRPL43	protein_codin	0	0	2.27072903	0
ENSG000000:	GCLM	protein_codin	4.9282917	5.74125188	8.06191245	9.70023031
ENSG000000	MRPL43	protein_codin	0	0	0	1.35989975
ENSG000000	MRPL43	protein_codin	0	0	0	0
ENSG000001:	BCAR3	protein_codin	0	0	0	0
ENSG000001:	BCAR3	protein_codin	0	0	0	0
ENSG000001:	BCAR3	protein_codin	2.22670157	0	0	0
ENSG000000	ZNF275	protein_codin	7.97142566	28.7752128	0	0
ENSG000000	SEMA4G	protein_codin	0	0	0	0.91300095
ENSG000000	ZNF275	protein_codin	0	0	0	0

ENSG000001	FNBP1L	protein_codin	33.4909929	27.0571328	126.01937	28.1646497
ENSG000001	CHRNA4	protein_codin	0	0	0	0
ENSG000001	DR1	protein_codin	0	25.6990032	11.4713571	4.25966314
ENSG000001	ZNF185	protein_codin	3.43837485	12.0162137	0	0
ENSG000001	SLF2	protein_codin	65.133996	127.212225	218.292453	21.5175119
ENSG000001	SLF2	protein_codin	2.88209593	15.8634038	18.661163	4.87509035
ENSG000001	DR1	protein_codin	66.3519047	47.8849461	151.609653	32.1227692
ENSG000001	NSDHL	protein_codin	4.21626953	8.46520748	16.0228133	4.63680343
ENSG000001	ARFGAP1	protein_codin	0	0	0	0
ENSG000001	CCDC18	protein_codin	0	0	0	0
ENSG000001	CETN2	protein_codin	18.7997028	41.1333225	87.3781051	14.9140757
ENSG000002	MAGEA3	protein_codin	0	5.0231266	34.0621428	0.52935913
ENSG000001	TMED5	protein_codin	39.7504152	41.2569853	169.640959	43.8008143
ENSG000001	TMED5	protein_codin	11.4719619	21.2900007	35.0779363	25.9067488
ENSG000001	ARFGAP1	protein_codin	14.6382433	36.9776998	0	3.43919962
ENSG000001	CSAG1	protein_codin	0	0	0	0
ENSG000001	TMED5	protein_codin	20.1222418	40.1918237	34.8214779	32.6019056
ENSG000001	CSAG1	protein_codin	0	0	0	0.33729638
ENSG000001	MAGEA2B	protein_codin	0	0	0	0
ENSG000000	PAX2	protein_codin	0	0	0	0
ENSG000001	MTF2	protein_codin	0	61.0843831	0	0
ENSG000001	MTO1	protein_codin	0	0	0	0
ENSG000001	MTF2	protein_codin	7.4859894	0	30.6567834	0
ENSG000001	MTO1	protein_codin	15.495541	0	17.9598763	6.91067081
ENSG000001	NKAIN4	protein_codin	0	0	0	0
ENSG000001	MTO1	protein_codin	0	33.9979715	0	0
ENSG000001	DIPK1A	protein_codin	1.93909361	13.4138975	5.25993627	7.59369617
ENSG000001	NKAIN4	protein_codin	0	0	0	0
ENSG000000	GABRA3	protein_codin	0.545829	2.5174875	22.9328304	3.18362983
ENSG000001	CGAS	protein_codin	5.42506458	29.2715527	3.99110269	11.3604781
ENSG000001	NKAIN4	protein_codin	0	0	0	0.46728331
ENSG000001	NKAIN4	protein_codin	0	0	0	0
ENSG000001	CGAS	protein_codin	0	0	0	0
ENSG000001	NDUFB8	protein_codin	0	0	0	0
ENSG000001	RPL5	protein_codin	713.995198	636.916673	1691.26314	650.321906
ENSG000001	NDUFB8	protein_codin	1.10545195	0	0	0
ENSG000001	MAGEA10	protein_codin	0	5.33354142	0	0.92941193
ENSG000001	GABRE	protein_codin	0	0	1.42506869	0
ENSG000000	EVI5	protein_codin	20.2225005	0	42.6218316	0
ENSG000001	GFI1	protein_codin	0	0	11.7046715	0
ENSG000001	YTHDF1	protein_codin	4.54820748	0	8.09205338	0
ENSG000000	DDX43	protein_codin	0	7.46429355	0	3.25082309
ENSG000001	YTHDF1	protein_codin	13.9317771	36.2230707	30.2624159	14.5474938
ENSG000001	MAGEA4	protein_codin	0	0	0	0

ENSG000000	SEC31B	protein_codin	3.27287563	0	8.0210705	0
ENSG000001	BHLHE23	protein_codin	0	0	0	0
ENSG000001	SLC17A9	protein_codin	1.08878002	0	0	0
ENSG000001	FATE1	protein_codin	0	0	0	1.51609712
ENSG000001	SLC17A9	protein_codin	0	10.6901329	0	2.09056083
ENSG000001	PRRG3	protein_codin	0	0	0	7.35965431
ENSG000001	PRRG3	protein_codin	0	0	0.65502528	2.95053814
ENSG000000	SCD	protein_codin	250.929527	694.08284	206.931161	170.423176
ENSG000001	PASD1	protein_codin	0	0	0	2.61014048
ENSG000002	OOEP	protein_codin	0	0	0	0
ENSG000001	GLMN	protein_codin	11.0818239	14.4725176	52.9026688	1.00834171
ENSG000001	VMA21	protein_codin	0	0	0	0
ENSG000002	OOEP	protein_codin	0	0	0	0
ENSG000001	DIDO1	protein_codin	33.2545022	91.8498813	74.9131492	14.9862061
ENSG000002	KHDC3L	protein_codin	0	0	0	0
ENSG000001	DIDO1	protein_codin	0	0	0	0
ENSG000002	DPPA5	protein_codin	0	0	0	0.56598807
ENSG000001	DIDO1	protein_codin	0	36.350115	14.8914625	18.3770346
ENSG000001	BLOC1S2	protein_codin	0.97953817	8.72943249	0	3.51728286
ENSG000002	C1orf146	protein_codin	0	0	0	0
ENSG000002	C1orf146	protein_codin	0	0	0	0
ENSG000001	CD99L2	protein_codin	22.4379692	0	11.6051336	9.74548455
ENSG000001	BTBD8	protein_codin	0	0	0	0
ENSG000001	BTBD8	protein_codin	0	10.0017872	3.05445901	0
ENSG000001	EPHX4	protein_codin	0	0	0	0.55245244
ENSG000001	KHDC1	protein_codin	0	0	1.47693106	0
ENSG000000	MTMR1	protein_codin	2.80405121	18.1023642	2.04933377	3.40662186
ENSG000002	KHDC1L	protein_codin	0	0	0	0
ENSG000001	BRDT	protein_codin	0	0	0	0
ENSG000000	MTMR1	protein_codin	0	0	0	4.00441
ENSG000001	KCNQ5	protein_codin	0	0	0.72832887	0
ENSG000001	MTM1	protein_codin	1.00885315	0	0.51437238	0
ENSG000001	MTM1	protein_codin	0	0	0	0
ENSG000002	CHUK	protein_codin	21.7872016	29.7285428	40.2989218	9.56390748
ENSG000001	KCNQ5	protein_codin	0	0	0	0
ENSG000000	TGFBR3	protein_codin	0	0	0	0
ENSG000000	MAMLD1	protein_codin	0	5.48186002	0	3.04480218
ENSG000001	EOLA2	protein_codin	3.99856669	0	0	0
ENSG000001	EOLA2	protein_codin	3.79963386	0	13.803014	3.93847078
ENSG000001	ERLIN1	protein_codin	7.17890752	0	0	0
ENSG000001	EOLA2	protein_codin	0.93732979	10.4775778	0	2.1201425
ENSG000001	HSFX1	protein_codin	0.50851558	3.64408208	1.38261939	0.84128931
ENSG000001	CPN1	protein_codin	0	0	0	0
ENSG000000	RIMS1	protein_codin	0.34467352	0	12.256198	0

ENSG000000	RIMS1	protein_codin	0	3.03630304	28.0134098	0
ENSG000001	HFM1	protein_codin	5.79872272	0	3.40640163	0
ENSG000000	ABCC2	protein_codin	0	2.57897752	0	1.06160249
ENSG000001	OGFRL1	protein_codin	22.5518885	57.3750251	37.5089387	32.5558341
ENSG000001	ZNF644	protein_codin	3.2355375	0	4.60644838	4.64906834
ENSG000000	IDS	protein_codin	0	23.0042899	0	0
ENSG000001	BARHL2	protein_codin	0	0	0	0.47235402
ENSG000001	ZNF326	protein_codin	0	0	0	0
ENSG000001	SMAP1	protein_codin	0	35.8075676	0	23.7898285
ENSG000002	ENSG000002	protein_codin	0	0	0	0
ENSG000001	LRRC8C	protein_codin	17.7775839	169.162142	45.4003738	32.7040452
ENSG000001	SMAP1	protein_codin	0	0	6.25594422	0
ENSG000001	GBP6	protein_codin	1.33051636	6.89582611	2.63303477	4.17506882
ENSG000001	AFF2	protein_codin	16.0911285	24.7538439	13.7406004	10.523423
ENSG000001	AFF2	protein_codin	0	0	0	0
ENSG000001	GBP5	protein_codin	12.2471576	342.982243	0	13.6034133
ENSG000001	AFF2	protein_codin	0	2.53947063	0	9.591867
ENSG000000	OGFR	protein_codin	1.26214191	29.5276754	13.6816656	2.84807803
ENSG000001	GBP2	protein_codin	45.5415877	339.131936	4.85230966	49.8825155
ENSG000001	FMR1NB	protein_codin	0	0	0	0.99255642
ENSG000001	FMR1	protein_codin	0	0	0	0
ENSG000001	FMR1	protein_codin	0	0	0	0
ENSG000001	CUTC	protein_codin	0	0	0	0
ENSG000001	GBP1	protein_codin	19.0696168	428.414803	5.64891458	21.0950024
ENSG000001	SDHAF4	protein_codin	3.23306933	2.2641972	7.54875155	0.91315451
ENSG000001	FMR1	protein_codin	2.06558094	20.995549	13.2627046	0
ENSG000001	CUTC	protein_codin	8.23755046	18.7032711	6.07941073	6.37178471
ENSG000001	FMR1	protein_codin	12.4637335	53.7056419	0	0
ENSG000000	FAM135A	protein_codin	0	0	0	0
ENSG000001	GBP3	protein_codin	0	0	0	13.2997883
ENSG000001	GBP3	protein_codin	1.49925815	4.76174511	0	0
ENSG000000	COX15	protein_codin	8.50816559	22.1413528	19.1666777	4.42512112
ENSG000001	KYAT3	protein_codin	0	2.34198944	0	0
ENSG000001	MRGBP	protein_codin	8.01013343	27.6144461	32.0979015	7.28864638
ENSG000001	ENTPD7	protein_codin	31.7668274	43.4668911	45.5006841	24.3299244
ENSG000001	SLITRK2	protein_codin	0.71148243	2.54371858	0.45744775	4.30540254
ENSG000001	KYAT3	protein_codin	1.75967042	35.3981793	0	0
ENSG000002	SPANXN1	protein_codin	0	0	0	0.80251619
ENSG000001	SLC25A28	protein_codin	0	8.88093033	0	0
ENSG000001	COL9A1	protein_codin	0	0	0	0
ENSG000001	GTF2B	protein_codin	5.62509673	29.6898615	13.5696234	7.87299641
ENSG000001	NTSR1	protein_codin	0.47840965	0	0	0
ENSG000001	SPANXN3	protein_codin	0	1.01020665	0	0
ENSG000001	SPANXN4	protein_codin	0	0	0	0

ENSG000001(	SLCO4A1	protein_codin	1.48527812	0	0.78230841	0.4154642
ENSG000001:	GOT1	protein_codin	6.07110965	19.9955049	15.6710867	3.39927203
ENSG000001.	CREG1	protein_codin	66.2324917	105.994855	74.3151273	56.0565942
ENSG00000(	PKN2	protein_codin	52.2697998	73.0233624	122.601283	20.2332302
ENSG000001!	SPANXD	protein_codin	0	0	0	0.43925863
ENSG000002(	SPANXA2	protein_codin	0	0	0	0
ENSG000001!	SPANXA1	protein_codin	0	0	0	0
ENSG00000(	PKN2	protein_codin	31.1998102	18.9620986	88.4931822	54.5830773
ENSG000001(	LDOC1	protein_codin	14.9002137	12.6636108	4.41246635	3.62354346
ENSG000001:	SOX3	protein_codin	0	0	0	0.45719083
ENSG000002(	CXorf66	protein_codin	0	0	0	1.84692518
ENSG000001.	LMO4	protein_codin	27.8635666	35.3591937	94.3523947	15.0041351
ENSG000001.	LMO4	protein_codin	31.1839068	21.5568587	44.1653318	7.3812587
ENSG000001	HPSE2	protein_codin	0	0	0	0
ENSG000002(	ENSG000002(	protein_codin	0	0	0	0
ENSG000001	HPSE2	protein_codin	0	0	0	0
ENSG000001(	HS2ST1	protein_codin	3.49085709	13.728429	6.50746285	0.93181529
ENSG000001(	HS2ST1	protein_codin	4.69047005	22.2230904	24.4996573	0
ENSG000001	HPSE2	protein_codin	0	0	0	0
ENSG000001(	SELENOF	protein_codin	18.853379	3.66309774	0	11.0839662
ENSG000001(	ATP11C	protein_codin	8.73022476	24.3891992	0	0
ENSG00000(	SH3GLB1	protein_codin	59.9939121	81.6192622	33.205284	46.4951041
ENSG000001	RPS21	protein_codin	0	1.85006583	4.384843	0.52259208
ENSG000000	CLCA4	protein_codin	0	0	0	0
ENSG000001:	CLCA2	protein_codin	0	0	0	1.50790076
ENSG000001:	ODF2L	protein_codin	0	0	0	0
ENSG000001(	LMBRD1	protein_codin	5.64308737	0	0	0
ENSG000001	COL24A1	protein_codin	26.8557996	163.102906	264.993165	80.157084
ENSG000001(	MCF2	protein_codin	0	0	0	0
ENSG000001	ZNHIT6	protein_codin	60.766076	179.263754	159.864594	31.1236524
ENSG000001	PYROXD2	protein_codin	4.6464139	8.85379581	2.64777424	1.59816872
ENSG000001(	MCF2	protein_codin	0	0	0	3.76252725
ENSG000001(	R3HCC1L	protein_codin	2.28235178	71.9596343	5.27683396	0
ENSG000001(	R3HCC1L	protein_codin	0	0	0	0
ENSG00000(	MCOLN3	protein_codin	0	0	5.31351363	0.82301336
ENSG00000(	MCOLN3	protein_codin	8.81390984	16.4126337	60.5325001	4.00399018
ENSG00000(	CRTAC1	protein_codin	0	0	0	0
ENSG000001	RPS21	protein_codin	0	0	1.78567819	0
ENSG000001(	DNAI3	protein_codin	0	0	0	0
ENSG00000(	CRTAC1	protein_codin	3.47130668	0	4.52513462	0
ENSG000001:	ADGRB3	protein_codin	24.6478	7.85329326	1.48260567	1.4954815
ENSG000001.	GOLGA7B	protein_codin	0.57925788	2.95489447	0.51682992	2.5524856
ENSG000001:	ZIC3	protein_codin	0	0	0	0
ENSG000001.	MCOLN2	protein_codin	0.68980533	0	0	0

ENSG000001	ZFYVE27	protein_codin	0	4.35000356	0	0
ENSG000001	LPAR3	protein_codin	0	0	64.5236001	13.630892
ENSG000001	ZFYVE27	protein_codin	0	0	0	0
ENSG000001	EYS	protein_codin	0	0	0.66928648	0
ENSG000001	ARHGEF6	protein_codin	59.6384164	222.971171	60.5610108	36.4087476
ENSG000001	EYS	protein_codin	0	0	6.09831548	0
ENSG000001	ARHGEF6	protein_codin	0	0	0	0
ENSG000001	CTBS	protein_codin	4.1701471	9.90702495	10.4509267	3.26526712
ENSG000001	AVPI1	protein_codin	3.92264942	6.06746183	2.65382416	4.5379107
ENSG000001	CD40LG	protein_codin	0	0	0	0
ENSG000001	CD40LG	protein_codin	0.89809762	25.122062	0	0
ENSG000001	CTBS	protein_codin	7.20215722	8.97750101	15.1409026	6.88003738
ENSG000001	PI4K2A	protein_codin	10.6270463	45.6432825	16.1099591	27.6718532
ENSG000001	VGLL1	protein_codin	0	0	0	0
ENSG000001	MORN4	protein_codin	0.36995623	0	0	0.78864276
ENSG000002	C10orf62	protein_codin	0.75991594	1.00130108	0	0.44311404
ENSG000001	GNG5	protein_codin	0	0	0	10.0344593
ENSG000002	HOGA1	protein_codin	0	0	0	0
ENSG000001	GNG5	protein_codin	2.89262346	18.2344983	8.70868169	1.66623817
ENSG000002	HOGA1	protein_codin	0	0	0	0
ENSG000002	HOGA1	protein_codin	0.59316814	0	1.70900715	0
ENSG000001	BRS3	protein_codin	0	0	0	0
ENSG000002	ENSG000002	protein_codin	4.92760683	23.1684623	0	0
ENSG000001	ADGRG4	protein_codin	0	0	0	1.80121581
ENSG000001	RPF1	protein_codin	11.6787258	25.0834348	30.8676873	12.7096978
ENSG000001	ANKRD2	protein_codin	6.52127692	0	0	0
ENSG000001	RPF1	protein_codin	9.64627245	6.68438298	14.7418472	5.12261226
ENSG000001	LGSN	protein_codin	0	0	0	0.72767471
ENSG000001	LGSN	protein_codin	0	1.64094042	0	0
ENSG000001	FKBP1C	protein_codin	0.33777142	0	0	0.49976582
ENSG000001	MAP7D3	protein_codin	57.1451899	94.7576532	132.503263	25.5467422
ENSG000001	MAP7D3	protein_codin	1.64598136	0	0	3.95763237
ENSG000001	DNASE2B	protein_codin	0	0	0	0
ENSG000001	MAP7D3	protein_codin	4.24823515	0	0	0
ENSG000001	UBTD1	protein_codin	8.87646634	9.2971818	14.0550945	13.5415631
ENSG000001	DNASE2B	protein_codin	0	2.33988354	0	2.4330531
ENSG000002	SAMD13	protein_codin	1.14243607	3.92344352	0	0.79096313
ENSG000002	SAMD13	protein_codin	0	0	0	0
ENSG000002	SAMD13	protein_codin	0	0	0	0
ENSG000002	SAMD13	protein_codin	0	0	0	0
ENSG000002	SAMD13	protein_codin	0	0	1.46059938	0
ENSG000000	FHL1	protein_codin	4.07236324	0	7.39454569	2.66661553
ENSG000000	FHL1	protein_codin	0	0	0	0
ENSG000001	LAMA5	protein_codin	2.79244304	0	0	0

ENSG000001	PRKACB	protein_codin	0.58287534	0	0	0
ENSG000000	FHL1	protein_codin	124.574318	0	0	11.9009955
ENSG000001	PRKACB	protein_codin	0	0	0	0
ENSG000001	PRKACB	protein_codin	33.1845022	88.5143155	7.32788699	28.8350844
ENSG000001	PRIM2	protein_codin	0	0	0	0
ENSG000001	PRKACB	protein_codin	16.0492824	37.3910793	5.56255096	9.31670338
ENSG000001	PRKACB	protein_codin	0	7.54357226	69.9308214	0
ENSG000001	LAMA5	protein_codin	1.29131168	0	2.84424437	0
ENSG000001	BAG2	protein_codin	39.6129785	23.2539306	62.4108793	9.02374173
ENSG000001	SLC9A6	protein_codin	13.9169169	8.56617469	9.54880623	5.36014945
ENSG000001	SLC9A6	protein_codin	0	0	0	0
ENSG000001	SLC9A6	protein_codin	0	4.68314687	0	0
ENSG000001	ZNF451	protein_codin	0	0	0	0
ENSG000001	ZNF451	protein_codin	18.8711022	50.8828118	46.0374714	0
ENSG000001	ZNF451	protein_codin	50.3572967	64.7519096	94.4524356	55.9337894
ENSG000001	SAGE1	protein_codin	0	0	8.77499877	0
ENSG000001	ZNF451	protein_codin	0	4.43232967	0	0.75949801
ENSG000001	ZNF451	protein_codin	9.61039485	27.3358404	0	0
ENSG000001	ADGRL2	protein_codin	0	0	0	0
ENSG000001	ADGRL2	protein_codin	0	0	0	0
ENSG000001	ADGRL2	protein_codin	0	0	0	0
ENSG000001	ADGRL2	protein_codin	0	0	0	0
ENSG000001	ADGRL2	protein_codin	0	0	0	0
ENSG000001	ADGRL2	protein_codin	0	0	0	0
ENSG000001	ADGRL2	protein_codin	0	0	0	0
ENSG000001	ADGRL2	protein_codin	0	0	0	0
ENSG000001	ADGRL2	protein_codin	0	0	0	0
ENSG000001	KIAA1586	protein_codin	9.65999845	0	52.6959781	12.8733487
ENSG000001	ADGRL4	protein_codin	18.5477058	60.3931443	13.6666728	23.0183926
ENSG000001	BEND6	protein_codin	3.09536029	19.0366143	0	6.53824042
ENSG000001	BEND6	protein_codin	3.6306339	8.28913908	11.553663	3.96739875
ENSG000001	IFI44	protein_codin	31.2810408	174.025486	107.833927	40.4605074
ENSG000001	BEND6	protein_codin	8.35138831	4.22691219	34.9978043	6.02278397
ENSG000001	IFI44L	protein_codin	24.8659199	707.717377	48.5343384	51.5064798
ENSG000001	INTS6L	protein_codin	8.20887216	50.3146414	0	0
ENSG000001	PTGFR	protein_codin	0	0	0	0
ENSG000001	PTGFR	protein_codin	0	32.04457	0	2.18080146
ENSG000001	PTGFR	protein_codin	6.42500941	0	1.87039599	6.0561527
ENSG000001	GIPC2	protein_codin	1.10682167	5.07976076	0.491882	5.0282642
ENSG000001	ZNF449	protein_codin	0.44820264	6.9755768	0	0
ENSG000001	DNAJB4	protein_codin	23.9320549	49.785318	30.0304544	41.2902293
ENSG000001	ZNF75D	protein_codin	0	0	14.3136949	4.49411094
ENSG000001	DST	protein_codin	88.9350054	148.254144	508.511708	90.3345015
ENSG000001	ZNF75D	protein_codin	12.3830388	33.2115747	4.997159	8.76916674

ENSG0000010	FUBP1	protein_codin	62.2362142	124.562756	201.920829	27.4573679
ENSG0000010	FUBP1	protein_codin	84.8713017	78.0595536	245.249998	53.9558658
ENSG0000020	RTL8A	protein_codin	0.81020813	0	0	6.59423933
ENSG0000010	MOSPD1	protein_codin	0	0	0	0
ENSG0000010	MOSPD1	protein_codin	0	0	0	0
ENSG000001	MMS19	protein_codin	0	0	0	0
ENSG0000010	MOSPD1	protein_codin	3.98508119	2.41577083	2.89985054	4.35497902
ENSG000001	PABIR3	protein_codin	0	0	0	0
ENSG000001	PABIR3	protein_codin	0	0	0	0
ENSG000001	DST	protein_codin	9.74546926	11.513841	0	0
ENSG000001	PABIR2	protein_codin	0	13.0047632	0	4.01521833
ENSG000001	MIGA1	protein_codin	10.6738614	0	0	19.9671978
ENSG000000	USP33	protein_codin	28.3725647	205.148888	71.9657688	25.6532241
ENSG000000	USP33	protein_codin	0	0	50.0834343	32.3214418
ENSG000000	USP33	protein_codin	15.5643346	0	0	0
ENSG000000	ZZZ3	protein_codin	11.5759942	18.2232137	15.1980463	3.38441226
ENSG000001	PHF6	protein_codin	1.33023605	8.32525322	2.02983779	7.95940179
ENSG000001	PHF6	protein_codin	3.26779649	0	12.4635419	0
ENSG000000	ZZZ3	protein_codin	101.043931	115.082132	275.1127	30.6147567
ENSG000001	PHF6	protein_codin	0	0	0	12.7840586
ENSG0000020	CCDC160	protein_codin	0	0	0	2.27975934
ENSG000001	PIGK	protein_codin	17.7100235	65.7153048	56.3221963	26.3132943
ENSG000001	COL21A1	protein_codin	0	0	0	0
ENSG000001	GPC3	protein_codin	0	0	4.0590331	0
ENSG000001	COL21A1	protein_codin	10.1475177	0	0.91310485	23.4010976
ENSG0000010	MTG2	protein_codin	5.02865049	18.9436361	13.7991707	2.97902843
ENSG000001	RABGGTB	protein_codin	0	0	0	0
ENSG000000	GPC4	protein_codin	59.3974909	129.300361	108.063039	58.0057202
ENSG000001	BMP5	protein_codin	19.9813327	62.8619739	190.683728	1.82853039
ENSG000001	USP26	protein_codin	0	0	0	1.83611054
ENSG000001	HS6ST2	protein_codin	0	0	5.83470315	0
ENSG000001	ACADM	protein_codin	0	0	0	0
ENSG000001	HS6ST2	protein_codin	0.90548962	0	0	0
ENSG000000	MBNL3	protein_codin	0	0	0	0
ENSG000001	ACADM	protein_codin	22.2808717	0	0	0
ENSG000001	ZDHHC16	protein_codin	0.68105491	0	0	0
ENSG000000	MBNL3	protein_codin	0	0	0.56954026	0
ENSG000001	ZDHHC16	protein_codin	0	0	0	0
ENSG000001	SS18L1	protein_codin	0	0	0	0
ENSG000000	MBNL3	protein_codin	0	0	0	0
ENSG000001	HMGCLL1	protein_codin	0	0	1.69884422	0
ENSG000001	HMGCLL1	protein_codin	1.99356684	0	0	1.55555217
ENSG000000	MBNL3	protein_codin	5.70353281	0	11.193057	8.18175286
ENSG000001	ZDHHC16	protein_codin	0	10.560791	0	0

ENSG000001:	SLC44A5	protein_codin	18.3042004	12.0140029	26.7224046	6.00176958
ENSG000000	MBNL3	protein_codin	14.6545071	35.3655064	28.3059339	13.2551096
ENSG000001	PSMA7	protein_codin	1.48998056	0	1.36861979	0.72722165
ENSG000001:	SLC44A5	protein_codin	8.06977632	18.5373441	8.69637693	10.8590843
ENSG000001	PSMA7	protein_codin	0.5050114	18.5641857	0.64857648	4.79205168
ENSG000001:	HCRTR2	protein_codin	0	1.52134352	0	0
ENSG000001:	TINAG	protein_codin	0	0	0	0
ENSG000001	TYW3	protein_codin	16.4749604	36.5950758	32.4925078	12.5959638
ENSG000001:	TINAG	protein_codin	0	0	0	0
ENSG000001	CRYZ	protein_codin	0	3.18202781	0	0
ENSG000001	CRYZ	protein_codin	0	0	0	0
ENSG000001	CRYZ	protein_codin	0	0	0	0
ENSG000001	PSMA7	protein_codin	40.8357866	164.986583	164.829701	19.5771584
ENSG000001:	RAP2C	protein_codin	0	0	0	0
ENSG000001	MLIP	protein_codin	1.04189638	0	0	0
ENSG000001	MLIP	protein_codin	0	0	0	0
ENSG000001	FRMD7	protein_codin	27.4311562	8.99292642	3.38428521	5.85467781
ENSG000001:	LRRC1	protein_codin	2.11026745	0	2.99628149	0
ENSG000001	EXOSC1	protein_codin	2.65071649	0	0	0
ENSG000001	EXOSC1	protein_codin	0	0	0.93715629	0
ENSG000001	EXOSC1	protein_codin	0	0	1.45485391	0
ENSG000001:	LRRC1	protein_codin	26.2424075	52.5004904	79.7527509	16.4293703
ENSG000001	TNNI3K	protein_codin	0.36984545	0	0	0
ENSG000002	FPGT-TNNI3K	protein_codin	3.71222089	6.3201482	1.44041247	0.65195994
ENSG000002	FPGT	protein_codin	0	0	0	0
ENSG000002	FPGT-TNNI3K	protein_codin	8.61863888	19.2343572	24.249483	9.65899522
ENSG000002	FPGT	protein_codin	9.53457005	22.4150199	13.2891912	4.30864438
ENSG000002	FPGT-TNNI3K	protein_codin	1.47887993	14.1419823	0	1.3942717
ENSG000001	IGSF1	protein_codin	0	0	0	0
ENSG000001	IGSF1	protein_codin	1.47530449	0	0	0
ENSG000001	EXOSC1	protein_codin	2.05555243	7.3771915	0	3.96778193
ENSG000001	IGSF1	protein_codin	0	0	0	0
ENSG000001	IGSF1	protein_codin	0	0	1.16756351	5.8315585
ENSG000001:	KLHL31	protein_codin	19.4101729	3.89070254	21.0968574	1.77585681
ENSG000001	LRRIQ3	protein_codin	0	4.20176046	5.31234692	0
ENSG000001	IGSF1	protein_codin	0	0	8.13053044	0
ENSG000001	LRRIQ3	protein_codin	0	0	3.38336986	0
ENSG000000	ELOVL5	protein_codin	1.7097541	4.2159941	8.98841481	3.84425203
ENSG000001	LSM14B	protein_codin	0	5.51697016	1.28709704	0
ENSG000000	ELOVL5	protein_codin	0	0	0	0
ENSG000001:	ZRANB2	protein_codin	61.2656347	36.8612399	32.362458	15.1496922
ENSG000001	ARHGAP36	protein_codin	0.18755485	0	0	0.39280015
ENSG000001	ARHGAP36	protein_codin	0	0	0	0
ENSG000000:	PTGER3	protein_codin	6.14353655	8.1159631	5.01490962	5.43652739

ENSG000001(	ENOX2	protein_codin	0	0	0	0
ENSG00000(	PTGER3	protein_codin	0	0	0	0.58692405
ENSG000001(	ENOX2	protein_codin	0	0	2.27849778	0
ENSG000001	CTH	protein_codin	0	0	0	1.93546894
ENSG000001	FBXO9	protein_codin	16.4355088	20.4103538	50.0148589	9.03908975
ENSG000001	ANKRD13C	protein_codin	16.6942161	34.3745101	18.4005461	12.9334644
ENSG000001:	RBMX2	protein_codin	0.3966418	0	0.58222006	0
ENSG000001	SRSF11	protein_codin	0	0	0	0
ENSG000001	SRSF11	protein_codin	14.6634819	0	13.6874951	1.72846743
ENSG000001	SRSF11	protein_codin	13.5246773	182.696479	77.5511289	10.2238537
ENSG00000(	LRRC40	protein_codin	8.9166779	26.2944136	19.9725695	9.48403878
ENSG00000:	LRRC7	protein_codin	0	0	0	0
ENSG000001'	GSTA4	protein_codin	0	0	0	4.19448696
ENSG000001'	GSTA4	protein_codin	0	0	1.12690117	2.22579308
ENSG000001'	GSTA4	protein_codin	7.23905227	0	28.6245426	0
ENSG00000:	DEPDC1	protein_codin	11.7758717	4.09233956	55.7098856	0
ENSG000001'	GSTA3	protein_codin	0	0	0	0
ENSG000001	WLS	protein_codin	2.44529691	0	53.0123024	2.19984112
ENSG000001	WLS	protein_codin	0	0	0	0
ENSG000001	WLS	protein_codin	1.03347518	0	5.39329616	0
ENSG00000:	ZNF280C	protein_codin	2.86503422	6.2062515	8.07287829	13.5027843
ENSG000001(	DIRAS3	protein_codin	0	0	0	0
ENSG000001'	GNG12	protein_codin	51.3888217	46.331073	59.5501972	27.1684334
ENSG000001	GADD45A	protein_codin	0	0	0	0.78872603
ENSG000001	GADD45A	protein_codin	11.9848399	7.86461389	7.5761252	78.2199795
ENSG000001:	GSTA5	protein_codin	0	0	0.42986808	0
ENSG000001.	SERBP1	protein_codin	175.911331	214.325034	1162.48794	125.677033
ENSG000001:	CDH26	protein_codin	0	0	2.16667024	3.70667624
ENSG00000:	RRP12	protein_codin	5.13529317	9.25615588	29.4500966	7.18960603
ENSG000001.	SERBP1	protein_codin	0	24.6771385	68.0522169	25.8826346
ENSG000001.	SERBP1	protein_codin	70.27151	44.255967	186.781501	35.2185231
ENSG000001:	PPP1R3D	protein_codin	1.52436735	14.459127	2.53333975	3.91992554
ENSG00000(	IL12RB2	protein_codin	0	0	0	0
ENSG000001!	SYCP2	protein_codin	15.4280611	34.3124947	92.0407027	12.9198535
ENSG000002(	C1orf141	protein_codin	0	0	0	0
ENSG000002(	C1orf141	protein_codin	0	0	0	0
ENSG000002(	C1orf141	protein_codin	0	0	0	0
ENSG000001!	MIER1	protein_codin	2.29011564	20.3319751	3.99100063	5.32834656
ENSG000001!	MIER1	protein_codin	25.7164741	65.2350395	72.8563483	0
ENSG00000:	PHACTR3	protein_codin	0.3916963	0	0	0
ENSG000001!	MIER1	protein_codin	0	0	0	0
ENSG000001!	MIER1	protein_codin	0	4.41318894	0	0
ENSG000001:	FRAT2	protein_codin	0.83094115	4.14551519	2.11641723	3.3288362
ENSG000001(	FRAT1	protein_codin	1.78124969	0.82598687	1.22485136	2.54738702

ENSG000001	DNAI4	protein_codin	0	0	11.8201927	0
ENSG000001	DNAI4	protein_codin	10.1106356	19.0053558	8.43869811	3.45494213
ENSG000001	EDN3	protein_codin	0	0	0	0
ENSG000001	DNAI4	protein_codin	3.00514479	0	0	0
ENSG000002	ARHGAP19	protein_codin	0	0	0	0
ENSG000001	EDN3	protein_codin	1.01212616	0	0	0
ENSG000001	ZNF831	protein_codin	0.76099399	57.8541081	2.50519788	10.5756685
ENSG000001	PRELID3B	protein_codin	0	13.327442	13.0168134	5.63701978
ENSG000001	SGIP1	protein_codin	1.34783906	0	0	0
ENSG000001	SGIP1	protein_codin	0	0	0	0
ENSG000001	SLIT1	protein_codin	0	0	0	0
ENSG000001	PDE4B	protein_codin	18.7565235	77.3942079	3.38974665	47.9096905
ENSG000001	PDE4B	protein_codin	0.57442774	0	1.30824309	0
ENSG000001	LEPR	protein_codin	0	0	0	0
ENSG000001	LEPR	protein_codin	1.61392119	0	3.83214399	1.53727653
ENSG000001	LEPR	protein_codin	4.03236163	27.7079425	0	0
ENSG000001	ZDHHC9	protein_codin	18.8945862	0	16.9088538	2.82124689
ENSG000002	LEPROT	protein_codin	34.8882356	84.6753967	32.3833437	42.2946397
ENSG000000	EFHC1	protein_codin	1.26776898	6.14352522	4.16575055	7.49782568
ENSG000001	DNAJC6	protein_codin	1.7963685	0	0	0
ENSG000001	SLIT1	protein_codin	0	0	0	0
ENSG000001	RAVER2	protein_codin	9.49154423	14.684177	46.6640145	5.80262834
ENSG000000	GNAS	protein_codin	0	0	15.889049	0
ENSG000001	UBE2U	protein_codin	0	0	0	0
ENSG000001	UBE2U	protein_codin	0	0	0	0
ENSG000001	ROR1	protein_codin	26.9896919	62.8374004	44.8355054	8.0644707
ENSG000001	ROR1	protein_codin	0.36623021	0	1.21195348	0.53411611
ENSG000000	GNAS	protein_codin	104.582186	54.7758761	393.744358	34.7416209
ENSG000000	PGM1	protein_codin	5.57838127	4.47422788	21.4434122	0
ENSG000000	PGM1	protein_codin	24.173162	53.7793249	28.6913609	8.87176705
ENSG000000	GNAS	protein_codin	56.8683712	0	211.722879	23.058719
ENSG000002	EFCAB7	protein_codin	1.50597128	22.1596232	8.3101359	2.34163071
ENSG000001	ITGB3BP	protein_codin	0	0	16.3977998	0
ENSG000000	GNAS	protein_codin	93.2754955	313.627985	299.928416	182.765429
ENSG000001	LCOR	protein_codin	0	0	0	0
ENSG000000	GNAS	protein_codin	0	0	0	0
ENSG000000	GNAS	protein_codin	0	3.73420639	2.36303021	0
ENSG000000	GNAS	protein_codin	0	0	0	0
ENSG000000	GNAS	protein_codin	0	0	0	0
ENSG000001	LCOR	protein_codin	0	0	0	0
ENSG000001	XPNPEP2	protein_codin	0	0	0	0
ENSG000001	XPNPEP2	protein_codin	5.01041218	3.25089375	0	0.49223324
ENSG000001	PIK3AP1	protein_codin	8.27147621	26.3329026	0	11.3723573
ENSG000001	PIK3AP1	protein_codin	0	0	7.10955908	0

ENSG000001	OCRL	protein_codin	0	0	0	0
ENSG000001	FOXD3	protein_codin	0	0	0	0.44612966
ENSG000001	PKHD1	protein_codin	0	0	0	7.53332295
ENSG000001	ATG4C	protein_codin	0	0	0	0
ENSG000001	ATG4C	protein_codin	0	45.169149	33.713036	16.3253921
ENSG000001	SMARCA1	protein_codin	0	0.00748915	49.652944	88.53052
ENSG000001	SMARCA1	protein_codin	47.6617355	207.441452	79.5074348	0
ENSG000001	SMARCA1	protein_codin	136.963506	0	156.49706	0
ENSG000001	ACTRT1	protein_codin	0	0	0	0.4616723
ENSG000001	PRR32	protein_codin	0	0	0	0.42352846
ENSG000001	DCAF12L1	protein_codin	0	0	0.62110349	0.9923814
ENSG000001	ANGPTL3	protein_codin	0	0	0	0
ENSG000000	TENM1	protein_codin	15.2731041	12.5334379	51.709936	0
ENSG000001	STX16	protein_codin	0	0	58.771774	0
ENSG000001	SH2D1A	protein_codin	0	0	0	0
ENSG000001	STX16	protein_codin	17.3740212	74.0031473	0	0
ENSG000000	TM9SF3	protein_codin	130.759932	460.961539	144.023071	113.465206
ENSG000001	STAG2	protein_codin	122.406414	156.501394	220.557078	79.2118267
ENSG000001	STAG2	protein_codin	0	0	0	0
ENSG000001	USP1	protein_codin	64.1047117	108.844248	183.40969	27.3529043
ENSG000002	DEFB110	protein_codin	0	0	0	0
ENSG000001	APCDD1L	protein_codin	0.27465003	0	0	0
ENSG000001	KANK4	protein_codin	0	0	0	0
ENSG000001	KANK4	protein_codin	0	0	2.01051758	4.4631908
ENSG000001	STAG2	protein_codin	0	0	0	0
ENSG000001	PATJ	protein_codin	8.21443879	0	77.791077	0
ENSG000000	CRISP3	protein_codin	0	0	0	1.8677524
ENSG000001	STAG2	protein_codin	8.9646517	0	0	0
ENSG000001	CIMIP1	protein_codin	0	2.93625939	0	1.78108617
ENSG000001	OPALIN	protein_codin	0	0	0	0
ENSG000001	ZBP1	protein_codin	0	0	0	0
ENSG000001	DNTT	protein_codin	0	1.00264836	0	0.41913614
ENSG000001	RHAG	protein_codin	0	0	0	0
ENSG000000	BLNK	protein_codin	0	0	0	0
ENSG000001	TM2D1	protein_codin	5.21410972	12.3069235	12.3505735	2.4729912
ENSG000001	TM2D1	protein_codin	1.36230303	0	1.48370936	0
ENSG000001	TM2D1	protein_codin	1.18646772	8.84751805	0	2.47380733
ENSG000001	NFIA	protein_codin	0	0	0	0
ENSG000001	NFIA	protein_codin	0	0	0	0
ENSG000001	NFIA	protein_codin	0	0	0	0
ENSG000001	NFIA	protein_codin	0	0	0	0.78671752
ENSG000001	NFIA	protein_codin	0	0	0	0
ENSG000001	C6orf141	protein_codin	0	0	0	0.89110962
ENSG000001	CTCFL	protein_codin	0	0	0	0

ENSG000002	GLYATL3	protein_codin	0	0	0	0
ENSG000001	XIAP	protein_codin	36.1204952	106.151251	35.6535914	25.913284
ENSG000001	C1orf87	protein_codin	0	0	0	0
ENSG000001	CYP2J2	protein_codin	0	0	2.75844862	0
ENSG000001	ENTPD1	protein_codin	16.4844199	56.5272047	9.44871381	10.3223865
ENSG000001	ENTPD1	protein_codin	1.67820688	3.57781573	0	0.8655849
ENSG000001	ENTPD1	protein_codin	0	7.82834285	0	0
ENSG000001	HOOK1	protein_codin	0	0	1.03967817	6.20704453
ENSG000001	TCTN3	protein_codin	0	5.98128014	0	0
ENSG000001	FGGY	protein_codin	0	0	0	0
ENSG000001	OPN5	protein_codin	0	0	0	0.61812593
ENSG000001	FGGY	protein_codin	0	0	0	0
ENSG000001	TCTN3	protein_codin	0	0	5.96363313	0
ENSG000001	FGGY	protein_codin	0	0	0	0
ENSG000001	RBM38	protein_codin	0	2.06693453	0.55948812	0.43993302
ENSG000001	ADGRF4	protein_codin	0	0	0	3.10625926
ENSG000000	ALDH18A1	protein_codin	0	0	0	20.3805013
ENSG000001	JUN	protein_codin	432.144213	311.785141	185.767155	1330.60536
ENSG000000	ALDH18A1	protein_codin	24.6498613	81.1829788	50.9844066	0
ENSG000001	TACSTD2	protein_codin	0.21887438	3.90903622	0	3.17633534
ENSG000001	OMA1	protein_codin	12.7441712	14.6745614	21.1099855	4.11813797
ENSG000000	SORBS1	protein_codin	10.993775	0	0	0
ENSG000000	SORBS1	protein_codin	0	0	0	0
ENSG000001	DAB1	protein_codin	0	0	0	0
ENSG000001	DAB1	protein_codin	0	0	0	0
ENSG000001	DAB1	protein_codin	0	0	0.58630261	0
ENSG000001	DAB1	protein_codin	0	0	33.4695141	0
ENSG000000	C8B	protein_codin	0	0	0	0
ENSG000000	SORBS1	protein_codin	0	12.5990854	8.41172678	0
ENSG000001	FYB2	protein_codin	0	0	0	0
ENSG000000	SORBS1	protein_codin	0	0	0	0
ENSG000001	RAE1	protein_codin	0	0	0	0
ENSG000001	ADGRF1	protein_codin	0	0	0	0
ENSG000001	PRKAA2	protein_codin	10.0562715	3.79109422	25.8632857	5.79348491
ENSG000000	SORBS1	protein_codin	0	0	0	0
ENSG000000	SORBS1	protein_codin	0	0	0	0
ENSG000000	SORBS1	protein_codin	0	0	0	0
ENSG000001	VPS37B	protein_codin	3.47636093	20.9615673	7.20638218	10.3759048
ENSG000000	SORBS1	protein_codin	0	0	0	0
ENSG000001	PLPP3	protein_codin	21.6501604	89.5068643	36.3480808	31.2999737
ENSG000001	ADGRF1	protein_codin	0	0	0	2.76911413
ENSG000000	SPO11	protein_codin	0	0	0	0
ENSG000000	SPO11	protein_codin	0	0	0	0
ENSG000001	TMEM61	protein_codin	0	0	0	0

ENSG000001	DHCR24	protein_codin	5.62344854	0	36.6180029	18.3322965
ENSG000001	CYP2C8	protein_codin	0	0	0	0
ENSG000001	CIMAP2	protein_codin	0.23557497	0	0.44217896	0.72255799
ENSG000000	TTC22	protein_codin	0	0	0	0.47133059
ENSG000000	TTC22	protein_codin	0	1.60404811	1.81932233	0.53601909
ENSG000001	PARS2	protein_codin	1.83363996	2.77731645	6.11387989	1.17175507
ENSG000002	TTC4	protein_codin	9.5579847	25.8041528	24.4140255	7.60593594
ENSG000002	TTC4	protein_codin	0	0	0	0
ENSG000001	MROH7	protein_codin	1.16604929	2.60983548	0	2.30935628
ENSG000001	FAM151A	protein_codin	0.2112885	0	0.51410882	0
ENSG000002	CT47B1	protein_codin	0	0	0	0
ENSG000001	C1GALT1C1	protein_codin	0	0	1.45577946	0
ENSG000002	MCTS1	protein_codin	1.00852762	0	3.26249996	0
ENSG000001	ACOT11	protein_codin	27.4145809	28.0211008	4.88922109	20.0628133
ENSG000002	MCTS1	protein_codin	15.1176759	20.7498961	20.744017	6.56380483
ENSG000001	SSBP3	protein_codin	4.13016635	0	4.03494983	0
ENSG000001	SSBP3	protein_codin	0	0	0	6.83494475
ENSG000001	CYP2C19	protein_codin	0	0	0	0
ENSG000001	CUL4B	protein_codin	0	0	0	0
ENSG000001	CUL4B	protein_codin	0	0	0	0
ENSG000002	FAM209B	protein_codin	0	0	0.43928245	1.00926166
ENSG000001	HELLS	protein_codin	10.6367761	24.4646876	73.0118169	5.79684003
ENSG000001	FAM209A	protein_codin	0	0.42401108	0	1.0895763
ENSG000001	CDCP2	protein_codin	0	2.86842728	0	0.3768724
ENSG000001	TCEANC2	protein_codin	0	0	0	0
ENSG000001	HELLS	protein_codin	0	0	4.39473747	0
ENSG000000	LAMP2	protein_codin	52.860849	95.8736677	31.428878	22.3106942
ENSG000001	TMEM59	protein_codin	12.7896611	18.2012366	28.3051494	6.98616781
ENSG000001	TMEM59	protein_codin	6.30928566	0	0	0
ENSG000001	TMEM59	protein_codin	1.88950336	0	8.26681849	2.42994694
ENSG000001	SLC25A27	protein_codin	13.4865502	20.4440611	58.5598005	6.60081662
ENSG000001	TMEM59	protein_codin	8.00884647	14.8881059	14.3086713	17.2148921
ENSG000001	TMEM255A	protein_codin	1.28918499	0	0	2.88027309
ENSG000000	AURKA	protein_codin	0	0	0	0
ENSG000001	CACNA1B	protein_codin	0	0	0	3.42057128
ENSG000002	LDLRAD1	protein_codin	0	0	0	3.03096106
ENSG000001	NOC3L	protein_codin	49.753319	108.713285	264.050851	22.4926445
ENSG000002	LDLRAD1	protein_codin	0	0	0	0
ENSG000001	CACNA1B	protein_codin	0	0	0	0
ENSG000001	LRRC42	protein_codin	0	2.12296359	2.18206167	0.78962616
ENSG000001	TMEM255A	protein_codin	0	0	0	0
ENSG000001	LRRC42	protein_codin	8.97450909	6.96709504	42.3338486	4.75607196
ENSG000001	CACNA1B	protein_codin	0	0	0	0
ENSG000001	RCAN2	protein_codin	7.48087008	36.566443	7.37172226	8.06050102

ENSG000001	PLCE1	protein_codin	6.07112143	9.28316049	13.3725322	5.39503661
ENSG000000	IFT25	protein_codin	0.93485556	6.41287256	2.82358725	0
ENSG000000	IFT25	protein_codin	3.12274825	13.6889041	14.7290933	5.42962476
ENSG000000	IFT25	protein_codin	3.34508165	16.6773192	6.9577183	2.19629553
ENSG000001	PLCE1	protein_codin	2.5625839	0	0	5.01853071
ENSG000001	ENPP5	protein_codin	0.6584881	0	3.90869389	6.79173285
ENSG000001	FAM210B	protein_codin	26.8258368	33.9718575	40.0872989	18.6166523
ENSG000001	PLCE1	protein_codin	0	0	2.13801854	0
ENSG000001	RHOXF2	protein_codin	0.29480676	0	0	1.840942
ENSG000001	EHMT1	protein_codin	13.6242852	2.9350976	17.5744788	0
ENSG000000	YIPF1	protein_codin	0	0	0	0
ENSG000002	RHOXF2B	protein_codin	0	0	0	0
ENSG000001	SLC35G1	protein_codin	4.32451744	0	14.3145858	3.29240802
ENSG000001	NKAP	protein_codin	11.82716	26.4905793	4.72770175	9.46765837
ENSG000001	LGI1	protein_codin	0	0	0	0
ENSG000001	LGI1	protein_codin	0	0	0	0
ENSG000001	PFDN4	protein_codin	18.4815133	47.8492346	81.4977161	17.4774642
ENSG000001	ARRDC1	protein_codin	1.42666802	0	0	0.92152415
ENSG000001	AKAP14	protein_codin	0	0	0	0
ENSG000001	AKAP14	protein_codin	0	0	0	0
ENSG000000	NDC1	protein_codin	9.28222857	16.4690038	31.4383436	6.074689
ENSG000001	AKAP14	protein_codin	0	0	0	0
ENSG000001	RUNX2	protein_codin	17.4283203	0	130.14685	50.7161839
ENSG000000	BCAS1	protein_codin	1.41299039	1.2952467	0	0
ENSG000001	RUNX2	protein_codin	0	0	0	0
ENSG000001	NDUFA1	protein_codin	12.3715615	52.9452481	10.5745364	15.8441705
ENSG000001	RUNX2	protein_codin	17.0526236	0	5.41206469	39.7314348
ENSG000001	RNF113A	protein_codin	4.51117583	43.413427	9.81007615	10.7856856
ENSG000001	MRPL41	protein_codin	9.27871631	26.9220241	18.8702877	1.81968285
ENSG000001	DMRTB1	protein_codin	0	0	0	1.26048886
ENSG000000	PDE6C	protein_codin	0	0	0	0.49214316
ENSG000001	LRP8	protein_codin	0	0	0	0
ENSG000001	SUPT3H	protein_codin	0	0	0	0
ENSG000001	SUPT3H	protein_codin	3.46897164	19.1214994	1.37842898	1.46450888
ENSG000001	SUPT3H	protein_codin	1.17268961	0	2.90877895	2.62486813
ENSG000001	RBP4	protein_codin	5.74516719	9.30113997	20.4528466	3.30500415
ENSG000001	MAGOH	protein_codin	1.22262276	0	0	0
ENSG000001	RBP4	protein_codin	0	0	2.56066166	0
ENSG000001	NSMF	protein_codin	0.27984616	3.3053242	0.49575643	0
ENSG000001	RBP4	protein_codin	0	0	0	0
ENSG000001	MAGOH	protein_codin	13.5129744	29.8998637	28.9090613	7.14206552
ENSG000001	ZNF217	protein_codin	14.9452664	0	0	26.9002076
ENSG000001	NSMF	protein_codin	7.13792918	9.12279034	25.3446366	5.0527482
ENSG000001	NSMF	protein_codin	0	0	0	0

ENSG000001	NSMF	protein_codin	0	0	0	0
ENSG000001	NSMF	protein_codin	1.76401057	0	0	0
ENSG000000	CDC5L	protein_codin	121.033415	246.437748	593.284559	70.1265849
ENSG000001	SEPTIN8	protein_codin	0	0	0	0
ENSG000001	FFAR4	protein_codin	1.77060072	8.11515696	0	3.20200112
ENSG000001	NSMF	protein_codin	0	6.93461037	0	0
ENSG000001	FFAR4	protein_codin	0	0	0	0
ENSG000001	CEP55	protein_codin	14.0067327	27.222156	214.465872	3.40118134
ENSG000001	CPT2	protein_codin	3.27610818	14.216568	12.5728863	0
ENSG000001	MYOF	protein_codin	0	1.45747673	2.75315403	1.02809215
ENSG000001	MYOF	protein_codin	0	39.6568631	0	2.38977118
ENSG000001	SLC1A7	protein_codin	0	0	0	1.31621854
ENSG000001	SEPTIN8	protein_codin	0	0	0	0.68637947
ENSG000001	SLC1A7	protein_codin	0	0	0	0
ENSG000001	TSHZ2	protein_codin	61.43166	46.3427206	19.1004658	17.5318698
ENSG000001	PODN	protein_codin	0	6.35886578	0	0
ENSG000001	TCTE1	protein_codin	0	0	0	0
ENSG000001	TCTE1	protein_codin	0	1.15223322	3.02542153	0.83632457
ENSG000001	ENTPD8	protein_codin	0	0	0	0
ENSG000001	SCP2	protein_codin	13.0012077	0	0	0
ENSG000001	SCP2	protein_codin	0	0	3.62146348	0
ENSG000001	SCP2	protein_codin	1.81248893	35.4462619	0	10.7455364
ENSG000000	ZFP64	protein_codin	5.93615311	9.47848495	0	1.73314339
ENSG000000	ZFP64	protein_codin	0	0	0	0
ENSG000001	ECHDC2	protein_codin	0	4.80666299	0	0
ENSG000001	NDOR1	protein_codin	0	0	0	2.00798331
ENSG000001	ECHDC2	protein_codin	0	0	0	0
ENSG000000	ZFP64	protein_codin	0	0	0	0
ENSG000002	ZYG11A	protein_codin	0	0	0.56079391	2.05815477
ENSG000000	CYP26A1	protein_codin	0	0	0	1.20145402
ENSG000002	ZYG11A	protein_codin	0	0	0	0
ENSG000001	COA7	protein_codin	9.7596652	32.4677354	37.6138705	5.64681971
ENSG000001	SALL4	protein_codin	0.82473779	0	0	0
ENSG000001	TUT4	protein_codin	12.8189212	40.827571	32.4776199	11.5238716
ENSG000001	LRRC26	protein_codin	0	0	0	0
ENSG000001	EXOC6	protein_codin	1.5853436	6.90184251	0.92850421	2.53332494
ENSG000001	TUT4	protein_codin	2.03415339	58.9163064	10.8400457	1.5982648
ENSG000001	GRIN1	protein_codin	0	0	0	0
ENSG000001	GRIN1	protein_codin	0	0	0.4338136	0
ENSG000001	EXOC6	protein_codin	0	0	0	0
ENSG000001	GRIN1	protein_codin	0	0	0	0
ENSG000000	HSP90AB1	protein_codin	2.41899868	0	31.6711306	46.9943688
ENSG000001	GRIN1	protein_codin	0	0	0	0
ENSG000000	UBE2A	protein_codin	24.1137085	0	16.6409738	0

ENSG000001	GRIN1	protein_codin	0	0	0	0
ENSG000001	GRIN1	protein_codin	0	0.88411802	0	0
ENSG000001	GRIN1	protein_codin	0	0	0	0
ENSG000001	NFATC2	protein_codin	0	0	11.0524085	0
ENSG000000	ORC1	protein_codin	0	6.69106847	8.13835535	6.37843377
ENSG000000	ORC1	protein_codin	4.63155274	0	30.3464705	0
ENSG000000	UBE2A	protein_codin	2.43695896	9.10538	10.5904052	10.4660531
ENSG000000	KCNG1	protein_codin	2.44281272	0	8.71870093	1.44451007
ENSG000001	DPP7	protein_codin	0	0	0	0
ENSG000001	IDE	protein_codin	0	0	0	0
ENSG000000	DPM1	protein_codin	0	0	0	0
ENSG000000	DPM1	protein_codin	0	0	0	0
ENSG000001	CC2D1B	protein_codin	0	0	8.83818439	0
ENSG000001	MAN1B1	protein_codin	0	0	0	0
ENSG000000	DPM1	protein_codin	13.2648372	0	6.78835248	6.09776079
ENSG000001	MAN1B1	protein_codin	17.6503568	0	0	10.6697878
ENSG000001	ZFYVE9	protein_codin	6.70678821	23.577169	17.7683849	2.7616159
ENSG000001	NPDC1	protein_codin	9.93770392	9.55554838	13.4768912	5.84793025
ENSG000001	NPDC1	protein_codin	1.7806712	15.7678654	0	9.04343614
ENSG000001	ADNP	protein_codin	56.4380876	56.7652249	153.196879	19.7176184
ENSG000001	ABCA2	protein_codin	12.0076289	0	0	0
ENSG000001	BCAS4	protein_codin	0	0	3.20700271	0.44848756
ENSG000001	PARD6B	protein_codin	0.26222703	3.4704416	15.3534868	1.25362349
ENSG000001	KTI12	protein_codin	1.70034932	6.19385327	3.72798357	4.52520659
ENSG000001	PAXX	protein_codin	0.91253637	0	0	3.47645396
ENSG000001	PTPN1	protein_codin	14.1930691	91.4737037	63.9375336	35.9379171
ENSG000001	PTGDS	protein_codin	0	4.44931531	0	0
ENSG000001	PTGDS	protein_codin	0.91214202	85.3937246	5.79586216	42.1775604
ENSG000001	TXNDC12	protein_codin	10.8854405	33.4471502	33.9067332	6.37237417
ENSG000001	TNKS2	protein_codin	70.3963425	166.726397	154.67641	62.6835461
ENSG000001	LONRF3	protein_codin	1.68270327	0	0	4.36889231
ENSG000001	LCN12	protein_codin	0	0	0	0
ENSG000001	LCN12	protein_codin	0	0	0	0
ENSG000001	C8G	protein_codin	0	0	0	0
ENSG000001	IL13RA1	protein_codin	0	15.9508275	0	4.22865663
ENSG000000	HSP90AB1	protein_codin	1744.05847	4298.8788	11726.6658	1320.79166
ENSG000001	EDF1	protein_codin	0	5.61529175	0	0.49229115
ENSG000001	EDF1	protein_codin	1.8650341	4.04895337	5.65847974	1.71978534
ENSG000002	PEDS1	protein_codin	0	0	0	0
ENSG000002	PEDS1	protein_codin	6.81733275	14.7250733	4.65880236	8.62736404
ENSG000001	RAB3B	protein_codin	0.52789465	7.44761258	2.77397016	8.03669269
ENSG000002	PEDS1	protein_codin	0	10.0219838	0	0
ENSG000002	UBE2V1	protein_codin	0	0	2.6047713	0
ENSG000002	PEDS1	protein_codin	0	0	0	1.34405028

ENSG000000	PHPT1	protein_codin	0	0	1.81784149	2.8846739
ENSG000001	RABL6	protein_codin	55.9946269	15.2110796	386.759057	39.9269232
ENSG000001	IL13RA1	protein_codin	21.8680292	84.4526829	15.6062453	34.2658163
ENSG000001	HECTD2	protein_codin	0	0	0	0
ENSG000001	RABL6	protein_codin	0	2.32223522	0	0
ENSG000002	UBE2V1	protein_codin	33.286464	100.745871	71.0175491	34.0573218
ENSG000002	UBE2V1	protein_codin	0	0	12.8864667	0
ENSG000001	HECTD2	protein_codin	5.71048846	17.3741995	69.1933488	1.1512344
ENSG000002	CCDC183	protein_codin	0	0	0	0
ENSG000002	LCN8	protein_codin	0	0	0	0
ENSG000001	DIPK1B	protein_codin	0	4.97954975	12.8104911	4.65947483
ENSG000001	DIPK1B	protein_codin	1.23432216	9.42976362	10.8487913	0
ENSG000001	AGPAT2	protein_codin	0	0	0	0
ENSG000001	AGPAT2	protein_codin	5.75909508	13.4500185	14.8482184	4.85298428
ENSG000001	ANKRD1	protein_codin	179.032302	0	2.7473685	1.92343725
ENSG000001	EGFL7	protein_codin	0	0	5.50018824	0
ENSG000001	EGFL7	protein_codin	0	0	0	0.63474723
ENSG000001	RPP30	protein_codin	1.74316477	2.44402282	0.97311754	0.7944729
ENSG000001	SEC16A	protein_codin	0	0	0	0
ENSG000001	SLC29A1	protein_codin	0	10.519297	2.81744586	0
ENSG000001	B4GALT5	protein_codin	20.296478	43.4998388	46.4115181	23.1203297
ENSG000001	INPP5E	protein_codin	0	20.5440804	16.2118449	0
ENSG000001	SLC29A1	protein_codin	0.83573889	4.93965884	0	0
ENSG000001	OSBPL9	protein_codin	0	0	4.83062392	0
ENSG000001	PMPCA	protein_codin	7.98523574	0	30.3239638	6.83921557
ENSG000001	HTR7	protein_codin	0	0	0	0
ENSG000001	PMPCA	protein_codin	0	0	0	0
ENSG000001	ENTR1	protein_codin	1.73257235	5.78123898	15.2153087	3.54534795
ENSG000001	SLC29A1	protein_codin	0	0	0	0
ENSG000001	ENTR1	protein_codin	6.85477345	57.1613052	28.6462436	6.60143081
ENSG000000	EPS15	protein_codin	0.30673741	0	3.65622131	4.03899701
ENSG000001	KIF20B	protein_codin	8.18655982	0	72.1540007	0
ENSG000000	EPS15	protein_codin	0	0	0	0
ENSG000001	SLC29A1	protein_codin	0	0	0	0
ENSG000001	CARD9	protein_codin	0	0	0	0
ENSG000000	EPS15	protein_codin	0	0	0	0
ENSG000001	CARD9	protein_codin	2.66582201	0	0	0
ENSG000002	DNLZ	protein_codin	0.62521229	12.9804865	5.02770862	2.02969269
ENSG000002	DNLZ	protein_codin	0	0	0	0
ENSG000001	SLC29A1	protein_codin	3.86749741	11.1524389	16.835485	3.32092684
ENSG000001	KCNB1	protein_codin	19.709844	34.469364	4.86707474	16.8342464
ENSG000001	ZNFX1	protein_codin	0	112.35662	19.1287953	0
ENSG000001	LHX3	protein_codin	0	0	0	0
ENSG000000	TTC39A	protein_codin	0	0	0	0.90594838

ENSG000001(	LHX3	protein_codin	0	4.1660558	0.56047382	0
ENSG000000	TTC39A	protein_codin	0	0	0	0
ENSG000001	ZNFX1	protein_codin	0	71.4658716	22.2955718	28.5294692
ENSG000001	NACC2	protein_codin	9.69428477	25.0264223	0	0
ENSG000001	ZNFX1	protein_codin	1.2927136	25.4502303	8.6166156	3.11159585
ENSG000001	SLC29A1	protein_codin	0	0	0	6.5889718
ENSG000001	UBAC1	protein_codin	18.7272608	43.2445435	17.1948291	14.6929822
ENSG000001(	KCNT1	protein_codin	0	0	0	0
ENSG000002(	C1orf185	protein_codin	0	0	0	0
ENSG000001	CDKN2C	protein_codin	5.10162462	1.42972185	2.71979549	0
ENSG000002(	GLT6D1	protein_codin	0	0	0	1.26270589
ENSG000001	PAEP	protein_codin	0	0	0	0
ENSG000001	PAEP	protein_codin	0	0	0	0.72001269
ENSG000001	OBP2A	protein_codin	0	1.45044179	0	0
ENSG000001(	LCN1	protein_codin	0	0	0	0
ENSG000001	MRPS2	protein_codin	0	0	7.82772616	0
ENSG000001(	PIERCE1	protein_codin	0	0	0	0
ENSG000001(	PIERCE1	protein_codin	0	0	0	0
ENSG000001	SLC16A12	protein_codin	0.31787229	1.26383878	0.53549299	0.89796942
ENSG000001(	PIERCE1	protein_codin	0	0	0	0
ENSG000001	STAU1	protein_codin	0	0	0	0
ENSG000001	OLFM1	protein_codin	0	5.70064872	0	0
ENSG000001	IFIT5	protein_codin	12.3909755	29.6450943	22.8090247	2.88823324
ENSG000001	OLFM1	protein_codin	0	0	13.5588854	3.81694182
ENSG000001	OLFM1	protein_codin	0	0	0.54725793	0.82974052
ENSG000001	OLFM1	protein_codin	0	0	0	0
ENSG000001	STAU1	protein_codin	0	0	0	0
ENSG000001	IFIT1	protein_codin	18.2293075	118.726601	65.7281239	5.21223701
ENSG000000	FCN1	protein_codin	7.64114285	140.555348	1.73331634	10.3404552
ENSG000002(	IFIT1B	protein_codin	0	0	0	1.40723545
ENSG000001	IFIT3	protein_codin	0	71.7370091	2.60378901	0
ENSG000001	COL5A1	protein_codin	1520.01329	5415.44932	523.348368	1244.45045
ENSG000001	IFIT3	protein_codin	40.7612308	80.3703201	16.9731785	27.9590611
ENSG000001(	ELAVL4	protein_codin	0	0	0	0
ENSG000001	COL5A1	protein_codin	129.766592	101.423285	0	0
ENSG000001(	ELAVL4	protein_codin	0	0	0	0
ENSG000001	WDR44	protein_codin	0	0	0	0
ENSG000001(	ELAVL4	protein_codin	0	0	0	0
ENSG000001(	ELAVL4	protein_codin	0	0	0	0
ENSG000001	WDR44	protein_codin	14.2804549	66.3743168	0	0
ENSG000001	IFIT2	protein_codin	0	67.3316473	0	0
ENSG000001(	ELAVL4	protein_codin	0	0	3.57827511	0.66337794
ENSG000001	STAU1	protein_codin	48.3614228	20.1574191	27.312232	37.9849982
ENSG000001(	BEND5	protein_codin	2.6399557	6.85847485	6.56174536	0.72237828

ENSG000001(	BRD3	protein_codin	10.5612089	69.4698808	40.6736633	17.8866588
ENSG000001	BRD4	protein_codin	6.22878352	37.7234957	19.6941284	6.13482365
ENSG000001	AGBL4	protein_codin	0	0	0	0
ENSG000001(	LIPA	protein_codin	0.84723744	0	0	0
ENSG000001	AGBL4	protein_codin	0	0	0	0.44267117
ENSG000001	AGBL4	protein_codin	0	1.20260349	0.87328233	1.77413332
ENSG000001	SPATA6	protein_codin	0	3.75845841	0	0
ENSG000001(	BRD3	protein_codin	0	0	0	0
ENSG000001	SPATA6	protein_codin	0	0	0	0
ENSG000001	SPATA6	protein_codin	7.45494464	13.5068897	8.36456319	0
ENSG000001	WDR44	protein_codin	30.5420275	47.3979303	19.0383074	0
ENSG000001(	VAV2	protein_codin	0	0	0	0
ENSG000001(	VAV2	protein_codin	0	90.7050056	0	4.49367436
ENSG000001	CH25H	protein_codin	0.29144693	1.56350636	0	0.90791989
ENSG000001	STAU1	protein_codin	0	0	55.2711156	28.449432
ENSG000000	FAS	protein_codin	0	30.957632	0	0
ENSG000001	SARDH	protein_codin	0.33661162	11.4509407	0.62375549	0
ENSG000001	SARDH	protein_codin	0	0	3.11186505	1.62027685
ENSG000001(	CMPK1	protein_codin	11.6866194	37.6224427	40.5938867	0
ENSG000001(	CMPK1	protein_codin	0	0	0	0
ENSG000001	SARDH	protein_codin	0	0	0	0
ENSG000001(	CMPK1	protein_codin	5.27441046	66.1773075	0	62.5670966
ENSG000000(	KLHL13	protein_codin	0.59091467	0	0.77474422	4.86524181
ENSG000001	STIL	protein_codin	0	0	0	0
ENSG000000(	KLHL13	protein_codin	0	0	0	0
ENSG000000(	KLHL13	protein_codin	3.35870058	0	20.014888	2.28351974
ENSG000001(	TAL1	protein_codin	0	0	0	0
ENSG000001(	PDZK1IP1	protein_codin	0	0	0	0
ENSG000001(	CYP4A22	protein_codin	0	0	0	0
ENSG000001(	CYP4A22	protein_codin	0	0	0	1.19727203
ENSG000001	TMEM63B	protein_codin	0	0	16.2519043	0
ENSG000002(	CT83	protein_codin	0	0	0	0
ENSG000001(	SLC2A6	protein_codin	0	5.73388667	0	0
ENSG000001(	SLC2A6	protein_codin	0	0	6.60236239	0
ENSG000001	CYP4X1	protein_codin	0	2.06381311	5.07712423	2.53080177
ENSG000001	CYP4A11	protein_codin	0	0	0	0
ENSG000001	AGTR2	protein_codin	0	0	0	0
ENSG000001(	ADAMTS13	protein_codin	0.69223169	0	2.60509604	0
ENSG000001(	ADAMTS13	protein_codin	0	0	0	0
ENSG000001(	ADAMTS13	protein_codin	0	6.11597367	3.34493378	2.17475116
ENSG000001	ARFGEF2	protein_codin	67.0219535	205.297828	61.5802433	48.1517645
ENSG000001	CYP4B1	protein_codin	0	0	0	0
ENSG000001(	LUZP4	protein_codin	0	0	0	0
ENSG000001(	LUZP4	protein_codin	0.34654452	0	0	2.82447737

ENSG000001:	STAMBPL1	protein_codin	0.69789648	13.1354036	0	3.13627112
ENSG000001	CYP4B1	protein_codin	0	0	0	0
ENSG000001:	STAMBPL1	protein_codin	0	6.02771581	3.00243392	4.486858
ENSG000001:	STAMBPL1	protein_codin	3.11858984	18.1441118	3.3196892	0
ENSG000001:	STAMBPL1	protein_codin	0.37205147	0	0.82686235	2.71253529
ENSG000001	ADAMTS13	protein_codin	0	0	0	0
ENSG000001	ANKRD22	protein_codin	0.29203639	5.1501223	0	0
ENSG000001	EFCAB14	protein_codin	0	0	0	0
ENSG000001	REXO4	protein_codin	0.41735438	0	0	0
ENSG000001	IL13RA2	protein_codin	0	0	0	0
ENSG000002	LIPJ	protein_codin	0	0	0	0
ENSG000001	MOB3C	protein_codin	2.48204077	20.7020822	9.10814609	3.80391502
ENSG000001	PREX1	protein_codin	87.7762875	502.638136	170.122439	129.383617
ENSG000001	REXO4	protein_codin	20.1369828	53.967058	70.2868939	11.553388
ENSG000000	MKNK1	protein_codin	0	54.4779083	0	6.19020882
ENSG000000	MKNK1	protein_codin	0	0	0	0
ENSG000001	RNLS	protein_codin	0	0	1.98228936	3.2429812
ENSG000001	HTR2C	protein_codin	0	0	2.36792388	6.59826413
ENSG000001	HTR2C	protein_codin	0	1.6372046	0	0
ENSG000001	PTEN	protein_codin	23.2794819	0	0	25.0397901
ENSG000001	STKLD1	protein_codin	0	0	0	0.77288517
ENSG000001	AMOT	protein_codin	1.498079	0	1.51251662	7.93935747
ENSG000001	AMOT	protein_codin	58.1866879	42.8180906	49.969532	13.6639119
ENSG000001	AMOT	protein_codin	0	15.1960378	0	0
ENSG000001	SURF2	protein_codin	8.53592744	15.6622836	33.3326187	6.45879843
ENSG000001	LHFPL1	protein_codin	0.23677255	0	0	0.38139824
ENSG000002	TRPC5OS	protein_codin	0	0	0	0
ENSG000001	SURF1	protein_codin	0	5.61658805	0	0
ENSG000000	RAD54L	protein_codin	0	5.31665947	8.33659728	0.82566786
ENSG000001	ALG13	protein_codin	7.14360706	21.0256907	4.10418325	4.99865803
ENSG000001	LURAP1	protein_codin	3.14277297	4.77275819	2.29836572	1.32765382
ENSG000001	SURF4	protein_codin	0	0	0	0
ENSG000000	POMGNT1	protein_codin	0	0	0	0
ENSG000001	SURF4	protein_codin	53.6448458	0	152.864459	74.3796754
ENSG000001	SURF4	protein_codin	0	33.5817094	15.249616	0
ENSG000000	POMGNT1	protein_codin	0	0	0	0
ENSG000000	DCX	protein_codin	1.24338459	0	0	9.54415752
ENSG000001	MINPP1	protein_codin	12.276718	43.2142818	14.1370355	0
ENSG000001	MINPP1	protein_codin	0	0	0	4.74101797
ENSG000001	NCOA3	protein_codin	36.3792954	0	66.8477799	6.72349383
ENSG000001	NCOA3	protein_codin	0	0	0	0
ENSG000001	MED22	protein_codin	0	0	0	0
ENSG000001	TSPAN1	protein_codin	0	0	0	0
ENSG000001	NCOA3	protein_codin	36.3208971	207.493432	66.5926212	48.5794643

ENSG000001	PIK3R3	protein_codin	15.8763516	0	0	0
ENSG000000	PAK3	protein_codin	4.02934382	11.4425158	2.4652693	8.96865537
ENSG000000	MAST2	protein_codin	1.61258659	0	0	0
ENSG000000	PAK3	protein_codin	3.26868128	0	0	0
ENSG000001	ADIRF	protein_codin	6.88977139	21.2505111	0.49278061	16.0789321
ENSG000001	MRPL14	protein_codin	5.47800119	17.5885972	21.7777418	2.65056653
ENSG000001	SNCG	protein_codin	4.17098058	7.88457381	0	0
ENSG000001	SURF6	protein_codin	32.9718922	184.738489	129.700598	25.7848119
ENSG000001	ZMYND8	protein_codin	0	0	0	0
ENSG000001	TMEM69	protein_codin	4.78908151	10.9008231	6.05463349	3.54068548
ENSG000001	MMRN2	protein_codin	12.6546171	108.863054	14.0550788	51.5259973
ENSG000001	OBP2B	protein_codin	0	0	0	0
ENSG000001	GBGT1	protein_codin	0	0	0	0
ENSG000001	BMPR1A	protein_codin	19.0741441	0	0	7.02725654
ENSG000001	GBGT1	protein_codin	0	0	0	0
ENSG000001	GBGT1	protein_codin	0	0	0	0
ENSG000001	CHRDL1	protein_codin	37.6651511	15.8057565	2.4053659	9.6790178
ENSG000001	GBGT1	protein_codin	0	0	0	0
ENSG000001	CCDC17	protein_codin	0	0	0	0
ENSG000001	CHRDL1	protein_codin	0	0	0	0
ENSG000001	RALGDS	protein_codin	0	0	0	0
ENSG000001	RALGDS	protein_codin	0.71607651	0	8.28866261	14.8534186
ENSG000001	NASP	protein_codin	0	12.0767501	0	0
ENSG000001	GNG5B	protein_codin	0	0	0	0
ENSG000001	VEGFA	protein_codin	0	0	0	0
ENSG000001	LDB3	protein_codin	0	0	0	0
ENSG000001	AMMECR1	protein_codin	0	0	0	0
ENSG000001	AMMECR1	protein_codin	0	7.45850826	0	0
ENSG000001	RALGDS	protein_codin	1.72682804	10.3836141	0.97522357	10.4419588
ENSG000001	VEGFA	protein_codin	0	0	0	0
ENSG000001	LDB3	protein_codin	0	0	0	0
ENSG000001	VEGFA	protein_codin	19.0839558	0	154.736725	145.707063
ENSG000001	TMEM164	protein_codin	0	0	0	0
ENSG000001	AKR1A1	protein_codin	0	0	0	0
ENSG000001	OPN4	protein_codin	0	0	0	0
ENSG000001	TMEM164	protein_codin	6.31891207	30.7535002	20.2724118	8.33364712
ENSG000001	TMEM164	protein_codin	0	0	0	0
ENSG000000	WAPL	protein_codin	0	0	0	0
ENSG000001	VEGFA	protein_codin	0	0	35.1567085	86.8411084
ENSG000001	PRDX1	protein_codin	0	0	1.55944606	0
ENSG000001	CEL	protein_codin	0.30197049	0	1.02632863	2.65612645
ENSG000000	TESK2	protein_codin	0	0	0	0
ENSG000000	TESK2	protein_codin	4.49939864	18.1453287	0	0
ENSG000001	CCSER2	protein_codin	19.1970415	54.3957681	0	26.9205123

ENSG000001:	TOE1	protein_codin	2.40949306	0	6.31140326	0
ENSG000001	RGR	protein_codin	0	0	0	0
ENSG000001	GTF3C5	protein_codin	3.85575913	10.5902839	28.5725478	0
ENSG000001	MUTYH	protein_codin	0	0	0	0
ENSG000001	GTF3C5	protein_codin	1.44030123	25.2845516	0	1.5624758
ENSG000001	KCNE5	protein_codin	0	0	0	1.49097814
ENSG000001	TP53RK	protein_codin	5.90139071	0	16.5725041	0
ENSG000001	NXT2	protein_codin	0	0	2.42865386	0
ENSG000001	MUTYH	protein_codin	0	0	0	0
ENSG000001	LRIT1	protein_codin	0	0	0	1.37249137
ENSG000001	NXT2	protein_codin	2.53330101	5.8161244	2.4265296	3.64010613
ENSG000001	NXT2	protein_codin	0	0	0	0
ENSG000001	GTF3C5	protein_codin	0	0	0	0
ENSG000001	MUTYH	protein_codin	0	0	0	0
ENSG000002	LRIT2	protein_codin	0	1.26920262	0	0
ENSG000001	TP53RK	protein_codin	7.4470903	7.07634033	9.00954912	4.02443978
ENSG000001	MUTYH	protein_codin	0	0	0	0
ENSG000000	MRPS18A	protein_codin	5.47938577	0	38.8312948	0
ENSG000001	CDHR1	protein_codin	0	0	2.89971568	0
ENSG000001	SLC13A3	protein_codin	0	0	7.57778738	1.12170948
ENSG000001	GFI1B	protein_codin	0	0	0	0
ENSG000001	GFI1B	protein_codin	0	0	0	0
ENSG000001	GPR15LG	protein_codin	0	0	0	0
ENSG000001	IRS4	protein_codin	0	0.99861861	0	0
ENSG000000	MRPS18A	protein_codin	3.79743609	16.6360376	11.7872941	0.9222096
ENSG000001	GHITM	protein_codin	25.1672436	58.3577781	62.0206061	22.7468444
ENSG000001	SPACA9	protein_codin	0	0	0	0
ENSG000001	NRG3	protein_codin	0	0	0	2.66072229
ENSG000001	NRG3	protein_codin	0	0	0	0
ENSG000001	GTF3C4	protein_codin	31.9127545	62.1065516	70.6482181	21.6810167
ENSG000001	SH2D4B	protein_codin	0	0	0	0
ENSG000001	DDX31	protein_codin	0	11.288171	6.18753026	0
ENSG000001	TSPAN14	protein_codin	1.40748477	15.6330758	1.46483824	0
ENSG000001	TSPAN14	protein_codin	0	0	0	0
ENSG000001	TSPAN14	protein_codin	4.21849969	31.5825343	4.08796938	20.9125699
ENSG000001	DDX31	protein_codin	16.3281672	26.5314322	83.2647	6.41890032
ENSG000001	RSPH9	protein_codin	0.3086739	0	0.5316052	1.20822907
ENSG000001	TSPAN14	protein_codin	6.62115117	0	2.87562247	0
ENSG000001	RSPH9	protein_codin	0	0	0	0
ENSG000001	HECTD3	protein_codin	0	0	0	1.52862186
ENSG000001	MAD2L1BP	protein_codin	3.58966612	10.4417856	9.44224017	3.0871089
ENSG000001	HECTD3	protein_codin	14.5328949	58.1120248	43.957569	10.7258817
ENSG000000	ELMO2	protein_codin	0	0	0	0
ENSG000001	PRXL2A	protein_codin	1.46886214	3.35301109	0	0

ENSG0000007	EIF2B3	protein_codin	0	0	0	0
ENSG0000007	EIF2B3	protein_codin	15.4885951	9.68253465	45.3651373	4.0712512
ENSG000001:	PRXL2A	protein_codin	28.3059663	17.9768451	0	0
ENSG000001:	PRXL2A	protein_codin	0	0	0	6.80805176
ENSG000001:	PRXL2A	protein_codin	0	50.0788344	30.8567531	0
ENSG000001(	RAPGEF1	protein_codin	1.42369802	0	0	0
ENSG000001(	RAPGEF1	protein_codin	1.1822333	0	5.18074377	0
ENSG000001	PTCH2	protein_codin	1.45797678	2.82814497	3.15444433	0
ENSG000001(	RAPGEF1	protein_codin	62.9103493	392.267788	123.230473	64.1319711
ENSG000001:	DYDC2	protein_codin	0	0	0	0
ENSG000001:	DYDC2	protein_codin	0	0	0	0
ENSG000001:	DYDC2	protein_codin	0	0	0	0.46257178
ENSG0000017	PLK3	protein_codin	9.16408729	5.82443041	6.53692876	10.7281749
ENSG0000017	DYDC1	protein_codin	0	0	0	0
ENSG0000017	DYDC1	protein_codin	0	0	0	0
ENSG000001	BEST4	protein_codin	0	2.96510526	0.48150901	1.5582949
ENSG000001:	UCK1	protein_codin	0	0	0	0
ENSG000001	RPS8	protein_codin	91.4017776	0	641.519874	9.58028354
ENSG000001:	UCK1	protein_codin	0	3.80530435	0	0
ENSG000001:	UCK1	protein_codin	3.53394328	8.71627177	3.3211913	2.03616484
ENSG000001:	MAT1A	protein_codin	0	0	0	0
ENSG000001:	UCK1	protein_codin	0	0	6.96835436	7.2692426
ENSG000001!	COL4A6	protein_codin	0	1.06345836	0	0
ENSG000001	KIF2C	protein_codin	0	0	0	5.12674112
ENSG000001:	POMT1	protein_codin	0	0	0	0
ENSG000001	KIF2C	protein_codin	3.25355922	19.6211663	123.743179	0
ENSG0000017	POLH	protein_codin	16.2353162	26.1032892	10.5753509	2.36594333
ENSG000000:	SLC35C2	protein_codin	0	0	0	3.0551227
ENSG000001:	POMT1	protein_codin	0	0	0	0
ENSG000000:	SLC35C2	protein_codin	1.52277166	3.46159457	13.6956981	5.91961184
ENSG000001:	ANXA11	protein_codin	1.59296866	0	1.6817376	0
ENSG000001(	ATG4A	protein_codin	0	0	0	5.64408306
ENSG000001:	TMEM53	protein_codin	0	2.67366245	0	0
ENSG0000017	POLH	protein_codin	16.6503669	5.21634831	24.9572688	6.40704359
ENSG000001:	TMEM53	protein_codin	0	0	0	0
ENSG000001:	TMEM53	protein_codin	0	0	2.0734877	0
ENSG000001:	TMEM53	protein_codin	0	1.591694	0	0
ENSG000001:	TMEM53	protein_codin	0.28860543	0	2.39781827	0.40876672
ENSG000001(	ATG4A	protein_codin	2.32796697	0	8.88157301	0
ENSG000001	ERI3	protein_codin	3.61519571	0	16.7051282	0
ENSG000001	ERI3	protein_codin	0	7.26985665	5.97404768	0
ENSG000001(	PLPP7	protein_codin	1.03887613	1.112446	1.50008342	0.90490497
ENSG000001	PLAC9	protein_codin	3.55001326	0	0	0.50534443
ENSG000001(	PLPP7	protein_codin	3.54754098	0	6.25616308	1.15305984

ENSG000001	PLAC9	protein_codin	26.1204332	4.37427034	2.57007331	2.96475135
ENSG000001	FAM78A	protein_codin	0	10.0140984	0	0
ENSG000001	PLAC9	protein_codin	3.98726271	5.89607318	0	0
ENSG000001	FAM78A	protein_codin	4.24737046	22.3617605	1.22028372	12.6850058
ENSG000001	TMEM254	protein_codin	0	0	0	0
ENSG000001	TMEM254	protein_codin	0	0	0	0
ENSG000001	TMEM254	protein_codin	0	0	0	0
ENSG000001	CD40	protein_codin	0	0	5.84782982	5.16599947
ENSG000001	TMEM254	protein_codin	0	0	0	0
ENSG000001	TMEM254	protein_codin	0	0	4.22380742	0
ENSG000001	CD40	protein_codin	3.38051183	20.6819843	0	0
ENSG000001	DMAP1	protein_codin	1.10470257	16.8057099	0	0
ENSG000001	DMAP1	protein_codin	0	0	0	0
ENSG000001	NCOA5	protein_codin	6.93932833	16.3842008	13.1902003	0
ENSG000001	SFTPD	protein_codin	0	0	0	0
ENSG000001	PSMD10	protein_codin	0	11.4839588	12.2151244	0
ENSG000001	PSMD10	protein_codin	3.98510671	0	0	0
ENSG000001	AIF1L	protein_codin	0	0	0	0
ENSG000001	AIF1L	protein_codin	0	9.7333664	0	0
ENSG000001	KLF17	protein_codin	0	1.07456108	0	2.65537371
ENSG000001	AIF1L	protein_codin	0	14.8533477	0	0
ENSG000001	AIF1L	protein_codin	0	0	0	0
ENSG000001	AIF1L	protein_codin	0	0	1.89440117	0
ENSG000001	SLC6A9	protein_codin	0	0	0	0
ENSG000001	SLC6A9	protein_codin	0	1.47172344	0	0
ENSG000001	AIF1L	protein_codin	0	0	0	0
ENSG000001	SLC6A9	protein_codin	0	0	3.28731772	0.91380333
ENSG000001	AIF1L	protein_codin	1.15197769	0	0	0
ENSG000001	SLC12A5	protein_codin	0	0	0	0
ENSG000001	CALCOCO2	protein_codin	0	0	0	0
ENSG000001	CCDC24	protein_codin	0	0	0	0
ENSG000001	NUTM2B	protein_codin	0	10.3131412	0	2.31376713
ENSG000001	NUTM2G	protein_codin	0	1.18663767	2.15624433	0
ENSG000001	B4GALT2	protein_codin	0	12.0495689	0	0
ENSG000001	SFTPA2	protein_codin	0.35039712	0	0	1.20563905
ENSG000001	SFTPA2	protein_codin	0	0	0	0
ENSG000001	MMP9	protein_codin	166.885709	844.560969	153.85257	967.826528
ENSG000001	ZCCHC24	protein_codin	8.47481943	12.6901169	14.3931829	0
ENSG000001	ZCCHC24	protein_codin	51.991851	41.9083417	67.7127284	36.3931849
ENSG000001	FIBCD1	protein_codin	0.17781205	0	0	0
ENSG000001	FIBCD1	protein_codin	0	0	0	0
ENSG000001	IPO13	protein_codin	0	7.36327226	1.64370316	0.41200645
ENSG000001	IPO13	protein_codin	14.1453766	27.6037558	21.341831	3.57309879
ENSG000001	POLR1C	protein_codin	0	0	0	0

ENSG00000[illegible]	ABL1	protein_codin	21.9592247	157.760732	55.5950333	29.2362679
ENSG00001[illegible]	EXOSC2	protein_codin	3.8250367	0	0	3.66022188
ENSG00001[illegible]	EXOSC2	protein_codin	0	0	7.50310853	0
ENSG00001[illegible]	EXOSC2	protein_codin	0	3.62977073	0	0
ENSG000001	ARTN	protein_codin	0	0	0	0
ENSG00001[illegible]	EXOSC2	protein_codin	0	0	13.5150624	0
ENSG000001	ARTN	protein_codin	0	0	0	0
ENSG00001[illegible]	RPS24	protein_codin	446.938952	716.140026	884.427405	357.406471
ENSG00001[illegible]	ST3GAL3	protein_codin	0	0	0	0
ENSG00001[illegible]	ST3GAL3	protein_codin	0	0	0	0
ENSG00001[illegible]	ST3GAL3	protein_codin	0	0	0	0
ENSG00001[illegible]	ST3GAL3	protein_codin	0	0	0	0
ENSG00001[illegible]	ST3GAL3	protein_codin	0	0	0	0
ENSG00001[illegible]	ST3GAL3	protein_codin	0	0	0	0
ENSG00001[illegible]	POLR3A	protein_codin	0	0	0	0
ENSG00001[illegible]	ST3GAL3	protein_codin	0	2.21680997	0	0
ENSG00001[illegible]	ST3GAL3	protein_codin	0	0	0	0
ENSG00001[illegible]	TSC22D3	protein_codin	0.45914723	9.96386565	0	0
ENSG00001[illegible]	TSC22D3	protein_codin	0.81444519	2.23844515	2.52580399	0.78735422
ENSG00001[illegible]	TSC22D3	protein_codin	0	0	0	0
ENSG00001[illegible]	ASS1	protein_codin	0	0	0	0
ENSG00001[illegible]	TSC22D3	protein_codin	8.47617566	2.38604791	2.16018759	2.25427287
ENSG00001[illegible]	DLG5	protein_codin	72.0318314	51.9399536	148.12819	53.7734268
ENSG00001[illegible]	ASS1	protein_codin	0	72.5562289	0	0
ENSG00001[illegible]	ASS1	protein_codin	0	2.35358505	0	0
ENSG00000[illegible]	KDM4A	protein_codin	43.0743891	142.638996	112.957581	24.9284461
ENSG00001[illegible]	TSC22D3	protein_codin	85.4352605	130.593746	2.98220172	73.3164011
ENSG00001[illegible]	NCS1	protein_codin	13.5094716	42.4463152	11.6190919	14.2928849
ENSG00001[illegible]	KCNMA1	protein_codin	0	0	0	0
ENSG00001[illegible]	PTPRF	protein_codin	0	0	0	0
ENSG00001[illegible]	GPR107	protein_codin	0	0	0	0
ENSG00001[illegible]	PTPRF	protein_codin	18.9943734	0	52.6314155	11.3062598
ENSG00001[illegible]	KCNMA1	protein_codin	0	0	0	0
ENSG00001[illegible]	PCIF1	protein_codin	13.5190623	108.520684	44.4490559	9.81061181
ENSG00001[illegible]	GPR107	protein_codin	5.77121526	16.2220134	39.5266267	13.1136246
ENSG00001[illegible]	YIPF3	protein_codin	2.45893363	0	0	0
ENSG00001[illegible]	PRPS1	protein_codin	1.37970255	0	1.28778998	1.55660578
ENSG00001[illegible]	PRPS1	protein_codin	0	0	0	2.95847681
ENSG00001[illegible]	PLTP	protein_codin	1.06596165	3.97841006	0	0
ENSG00001[illegible]	KCNMA1	protein_codin	0	0	0	0
ENSG00001[illegible]	YIPF3	protein_codin	13.195397	0	66.851042	3.76624725
ENSG00001[illegible]	HYI	protein_codin	2.06758545	28.650854	11.9907387	5.12995927
ENSG00001[illegible]	PRPS1	protein_codin	0	0	0	0
ENSG00001[illegible]	USP20	protein_codin	8.05412142	24.6057816	14.6136401	9.45228479

ENSG000001	HYI	protein_codin	0	0	3.36980311	0
ENSG000001	PLTP	protein_codin	16.7755484	166.16828	20.5255622	37.414895
ENSG000001	HYI	protein_codin	0	0	0	0
ENSG000001	HYI	protein_codin	1.27997515	0	0	0
ENSG000001	HYI	protein_codin	0	0	0	0
ENSG000001	PRPS1	protein_codin	0.96549555	5.97934651	0	4.0983726
ENSG000001	KCNMA1	protein_codin	0	0	0	0
ENSG000001	KCNMA1	protein_codin	0	0	0	0
ENSG000002	LRRC73	protein_codin	0	0.75050801	0.64283656	1.8166732
ENSG000001	KCNMA1	protein_codin	0	0	1.01255683	0
ENSG000001	TJAP1	protein_codin	0	0	3.78273352	0
ENSG000001	TJAP1	protein_codin	0	0	0	3.27028913
ENSG000001	C9orf78	protein_codin	42.7051731	152.499613	81.8787464	30.2725026
ENSG000001	TJAP1	protein_codin	0	0	0	0
ENSG000001	SZT2	protein_codin	0.81004957	0	6.74394445	0.46251432
ENSG000001	TJAP1	protein_codin	5.28625491	48.1463333	8.74741586	5.55993795
ENSG000000	DNAAF6	protein_codin	0	0	0	0
ENSG000001	TJAP1	protein_codin	2.17917308	0	0	0
ENSG000001	MED8	protein_codin	0	0	18.6155541	0
ENSG000001	MED8	protein_codin	8.70478673	0	3.32179281	6.58429719
ENSG000000	ELOVL1	protein_codin	0	18.6070408	0	0
ENSG000000	CTSA	protein_codin	0	0	0	0
ENSG000001	CDC20	protein_codin	0	0	2.40873035	0
ENSG000001	NUP62CL	protein_codin	0	0	9.24561948	1.91111129
ENSG000001	PRRX2	protein_codin	6.71089333	17.2349861	52.0292818	3.50982929
ENSG000001	MPL	protein_codin	0	1.70793859	0	0
ENSG000000	TIE1	protein_codin	0	28.4222472	0	11.1842661
ENSG000001	C9orf50	protein_codin	0.28211635	0	0	0
ENSG000000	RBM41	protein_codin	4.01079603	0	6.22455134	10.1481302
ENSG000001	NTMT1	protein_codin	0	0	0	0
ENSG000001	NTMT1	protein_codin	1.93708316	6.05125446	5.09659282	0
ENSG000001	NTMT1	protein_codin	0.33454229	14.5571901	0	0
ENSG000000	CTSA	protein_codin	0	0	0	0
ENSG000001	DLK2	protein_codin	0	0	0	0
ENSG000001	NTMT1	protein_codin	0	0	0	0
ENSG000000	RBM41	protein_codin	0	0	0	0
ENSG000001	DLK2	protein_codin	0	0	0	0
ENSG000001	TMEM156	protein_codin	0	0	0	0
ENSG000001	IER5L	protein_codin	3.01008498	40.6344995	3.80834565	16.2095435
ENSG000002	CFAP57	protein_codin	0.53709076	1.37285973	0.40893575	0.46165345
ENSG000001	LRMDA	protein_codin	1.25693557	7.66891504	2.41111509	0
ENSG000001	SLC2A1	protein_codin	0	0	0	0
ENSG000001	ZNF691	protein_codin	0	0	0	0
ENSG000001	ZNF691	protein_codin	0.58410149	4.01710293	0	0

ENSG000001(	ZNF691	protein_codin	0	0	0	0
ENSG000001(	ZNF691	protein_codin	0	0	0	0
ENSG000001(	ZNF691	protein_codin	0	4.05919485	0	4.00919415
ENSG000001(	ZNF691	protein_codin	5.38746225	2.82427133	11.0894086	0
ENSG000001:	ABCC10	protein_codin	0	1.18737932	0.80361613	0
ENSG000001(	ERMAP	protein_codin	10.5933083	0	9.84944228	0
ENSG000001:	ABCC10	protein_codin	1.09600152	18.6788055	6.38171997	4.58204457
ENSG000001(	ERMAP	protein_codin	0	13.3092687	0	0
ENSG000001:	NEURL2	protein_codin	0	0.98830551	3.60934956	0
ENSG000001·	SPATA25	protein_codin	0	2.9015037	3.0839912	0.84197352
ENSG000001'	SVBP	protein_codin	11.4440836	14.7048744	22.95079	5.57817797
ENSG000001'	SVBP	protein_codin	13.271132	0	0	0
ENSG000001(	ZSWIM1	protein_codin	11.1065623	9.97392973	26.4860246	2.81495661
ENSG000001(	ZNF503	protein_codin	21.4755179	25.039022	51.1674598	34.3134427
ENSG000001(	C1orf50	protein_codin	6.11144367	12.9772245	13.9542081	4.74982262
ENSG000001	P3H1	protein_codin	0	0	0	0
ENSG000001:	ABCC10	protein_codin	2.76767468	6.75274715	3.78290591	4.84592528
ENSG000001(	COMTD1	protein_codin	0	0	0	1.34396753
ENSG000001(	CLDN19	protein_codin	0	1.08958424	0	0
ENSG000001:	SNX21	protein_codin	0	0	0	0.52549521
ENSG000001:	SNX21	protein_codin	1.26091415	0	0	0
ENSG000001·	RADX	protein_codin	2.24171407	0	0	1.26032911
ENSG000001(	DOLPP1	protein_codin	0	14.1722173	7.37195586	0.68829513
ENSG000001:	SNX21	protein_codin	0	0	0	0
ENSG000001·	RADX	protein_codin	0	0	28.94925	0
ENSG000001'	PPIH	protein_codin	0	0.8278776	0	0
ENSG000001'	PPIH	protein_codin	0	0	0	0
ENSG000001	PWWP3B	protein_codin	9.28716926	1.54631165	19.5136727	0
ENSG000001·	SH3GLB2	protein_codin	1.65513701	0	0	0
ENSG000001(	TNNC2	protein_codin	171.684614	1.7350807	1.30119047	1.29897804
ENSG000001:	PPCS	protein_codin	19.8750899	63.337689	29.077113	19.9552363
ENSG000001(	TNNC2	protein_codin	0	0	0	0
ENSG000001·	SH3GLB2	protein_codin	0	0	0	3.70300922
ENSG000001:	PPCS	protein_codin	0	0	0	0
ENSG000001:	PPCS	protein_codin	0	10.6650162	2.42858465	3.15081233
ENSG000001:	PPCS	protein_codin	0.40707406	2.56996522	0	0
ENSG000001:	SERPINA7	protein_codin	0	0	0	0.90602947
ENSG000001·	SH3GLB2	protein_codin	0	7.44624011	1.76624322	0
ENSG000000(	ZMYND12	protein_codin	0	0	0	0
ENSG000001'	UBE2C	protein_codin	0	11.7150755	12.8077298	0
ENSG000001·	CRIP3	protein_codin	0	0	0	0
ENSG000001!	FOXJ3	protein_codin	6.79450546	2.36619132	7.86033411	7.33496214
ENSG000001!	FOXJ3	protein_codin	0	0	6.51837965	0
ENSG000001!	FOXJ3	protein_codin	0	0	0	3.93906203

ENSG000001:	SLC22A7	protein_codin	0	0	0	0
ENSG00000(	NUP188	protein_codin	35.8670662	95.5804303	175.424306	20.8925277
ENSG00000(	GUCA2B	protein_codin	0	0	0	0
ENSG000001	IL1RAPL2	protein_codin	3.46831464	0	6.91189443	1.7244058
ENSG000001	HIVEP3	protein_codin	0	153.605701	14.8001053	0
ENSG000001	HIVEP3	protein_codin	0	0	0	0
ENSG000001	SLC22A7	protein_codin	0	0	0	0
ENSG000001	DOLK	protein_codin	3.94482643	2.40792215	7.4653802	3.10419139
ENSG000001	EDN2	protein_codin	0	0	0.54537341	0
ENSG000001	ESX1	protein_codin	0	0	0	2.38381787
ENSG000001	SLC22A7	protein_codin	0	0	0	0
ENSG000002	FOXO6	protein_codin	3.00392414	0	1.45741335	0.82996096
ENSG000001	PHYHD1	protein_codin	0	0	0	0
ENSG000000	SCMH1	protein_codin	4.87335255	12.8645411	23.765175	5.93447542
ENSG000000	SCMH1	protein_codin	2.80686806	0	3.10204878	0
ENSG000000	SCMH1	protein_codin	0	0	4.53143714	0
ENSG000001	LRRC8A	protein_codin	0	0	0	34.281539
ENSG000001	LRRC8A	protein_codin	16.0038059	97.104959	65.6815276	0
ENSG000001	SLFNL1	protein_codin	0.30807762	0	0	0
ENSG000001	SLFNL1	protein_codin	0	0	0	0.50676991
ENSG000001	CTPS1	protein_codin	0	1.62134864	0	0
ENSG000001	DNTTIP1	protein_codin	14.2254322	6.99508531	37.8537508	0
ENSG000001	TCEAL1	protein_codin	5.25928224	2.58539739	0	0
ENSG000001	TCEAL1	protein_codin	5.12875884	12.180218	7.20508894	3.14046461
ENSG000001	TCEAL1	protein_codin	12.6255781	9.7273322	11.2112783	12.1698681
ENSG000001	TCEAL3	protein_codin	26.2883126	101.001079	9.86127695	14.5025328
ENSG000001	TCEAL3	protein_codin	0.72159009	7.66577643	3.63118664	3.84758691
ENSG000001	TCEAL4	protein_codin	0	0	3.34045631	0
ENSG000001	WFDC3	protein_codin	0	0	0	0
ENSG000001	WFDC3	protein_codin	0	0	0	0
ENSG000001	RAB40A	protein_codin	0.56547201	0	1.10092078	1.42624959
ENSG000001	BEX3	protein_codin	0	0	2.04892886	0
ENSG000001	BEX3	protein_codin	0	4.17095086	1.32866861	0
ENSG000001	CITED4	protein_codin	0.69585259	3.0572481	0	1.32464736
ENSG000001	ENDOG	protein_codin	2.42073046	0.75230402	3.8701552	1.43442737
ENSG000001	WFDC10A	protein_codin	0	0	0	0
ENSG000001	BEX3	protein_codin	18.2051701	13.8175127	74.1523724	13.3241365
ENSG000001	CUL9	protein_codin	0	0	0	0
ENSG000001	TBC1D13	protein_codin	9.71606255	53.8245783	26.6269185	6.17758162
ENSG00000(	NFYC	protein_codin	0	0	0	0
ENSG00000(	NFYC	protein_codin	0	0	4.16774142	0
ENSG00000(	NFYC	protein_codin	0	2.21130701	1.16693634	0
ENSG00000(	NFYC	protein_codin	0	0	0	0
ENSG000001	TCEAL9	protein_codin	20.145093	117.087001	22.2932919	33.248247

ENSG000001	TCEAL9	protein_codin	93.0555436	133.336636	200.034375	53.1746332
ENSG000001	ZDHHC12	protein_codin	4.35387331	4.07201985	5.53676171	1.19858893
ENSG000001	TCEAL7	protein_codin	4.44680346	0	0.5009486	0.44171211
ENSG000001	ZDHHC12	protein_codin	0	0	0	0
ENSG000000	NFYC	protein_codin	0	0	0	0
ENSG000002	WFDC6	protein_codin	0	0	0	0
ENSG000001	BEX2	protein_codin	0.44558041	0	4.85388522	0
ENSG000001	WFDC2	protein_codin	0	0	0	0
ENSG000001	BEX2	protein_codin	0	3.92265661	0	1.8935845
ENSG000002	TCEAL5	protein_codin	0.35978156	0.93521887	4.07039358	0.46326179
ENSG000001	RIMS3	protein_codin	0	0	0	0
ENSG000001	RIMS3	protein_codin	0.91615821	5.3776665	1.04508869	2.84644848
ENSG000001	TCEAL8	protein_codin	53.1133673	44.0186613	121.498586	19.5735769
ENSG000001	SET	protein_codin	253.742681	0	480.923756	152.592533
ENSG000001	SAMD8	protein_codin	9.72606807	31.5884163	24.0333923	18.0822732
ENSG000001	SET	protein_codin	64.9576043	433.616835	367.064858	44.9007178
ENSG000001	PIGT	protein_codin	7.68616395	0	0	0
ENSG000001	BEX4	protein_codin	0	34.2859214	4.42375165	0
ENSG000001	SET	protein_codin	0	9.22382153	0	0
ENSG000001	BEX4	protein_codin	7.71902742	20.3821968	0	7.33164856
ENSG000001	ZNF684	protein_codin	0.4017754	0	0	0
ENSG000001	ZNF684	protein_codin	0	0	0	0
ENSG000001	ZNF684	protein_codin	5.12233865	3.82077533	10.5124514	0
ENSG000002	DUSP13A	protein_codin	0	0	0	0
ENSG000001	EXO5	protein_codin	2.11806427	9.5493318	2.56570339	3.89825229
ENSG000001	ZFP69	protein_codin	5.93354319	0	7.41667177	4.89588399
ENSG000001	ZFP69	protein_codin	0.65980626	8.08524814	0	0
ENSG000000	SMAP2	protein_codin	0	0	0	0
ENSG000002	DBNDD2	protein_codin	0	0	0	1.70467996
ENSG000001	KAT6B	protein_codin	0	0	0	0
ENSG000002	DBNDD2	protein_codin	0	4.28594636	0	1.79304615
ENSG000001	KAT6B	protein_codin	42.2409233	401.167394	210.79214	47.9158413
ENSG000001	DYNC2I2	protein_codin	2.24463008	3.14564313	28.098302	0.57883529
ENSG000002	DBNDD2	protein_codin	0	0	0	0
ENSG000000	SMAP2	protein_codin	29.1044973	123.324487	29.5651606	22.7577336
ENSG000002	DBNDD2	protein_codin	0	0	0	0
ENSG000002	DBNDD2	protein_codin	0	0	0	0
ENSG000002	DBNDD2	protein_codin	0	0	0	0
ENSG000001	KAT6B	protein_codin	0	0	0	0
ENSG000001	KAT6B	protein_codin	61.4619115	0	0	0
ENSG000001	TP53TG5	protein_codin	0	0	0	0.44856664
ENSG000002	SYS1	protein_codin	0	0	0	0.46410899
ENSG000001	BEX1	protein_codin	4.63774175	0	46.6777934	0
ENSG000001	SPTAN1	protein_codin	425.683283	772.838661	232.915666	96.6344455

ENSG000001	SDC4	protein_codin	27.6991229	58.0632398	132.23261	13.7562206
ENSG000001	ADK	protein_codin	3.84157086	0	20.383156	7.42650485
ENSG000001	GPRASP3	protein_codin	0	0	5.91324687	4.75588859
ENSG000000	COL9A2	protein_codin	0	0	0	0
ENSG000001	SPTAN1	protein_codin	0	0	0	0
ENSG000001	RBPJL	protein_codin	0	0	0	0
ENSG000001	ARMCX5	protein_codin	4.39805836	0	7.90024602	0
ENSG000001	RBPJL	protein_codin	0	0	0.94789982	0
ENSG000001	AP3M1	protein_codin	8.41607646	18.4382349	20.7584437	2.73792816
ENSG000000	COL9A2	protein_codin	7.61302114	10.9795456	127.704507	1.26363686
ENSG000001	MATN4	protein_codin	0	0	1.23687082	0
ENSG000000	VCL	protein_codin	7.56728467	0	0	24.8033335
ENSG000001	MATN4	protein_codin	0	0	0	0
ENSG000000	ZMPSTE24	protein_codin	27.3889487	0	65.5979581	16.6488844
ENSG000001	PLAU	protein_codin	15.7628739	38.4863381	5.90697002	0
ENSG000001	CAMK2G	protein_codin	0	0	0	0
ENSG000001	TMCO2	protein_codin	0	0	0.31427794	0
ENSG000001	SEMG2	protein_codin	0.20929295	0	0	2.29466732
ENSG000001	GLE1	protein_codin	0	0	0	0
ENSG000001	RLF	protein_codin	61.5463212	113.157869	161.835675	96.870406
ENSG000002	TCEAL6	protein_codin	0	0	0	0
ENSG000002	TCEAL6	protein_codin	0	0	0	0
ENSG000001	PPT1	protein_codin	0	3.33318694	0.48895062	0
ENSG000001	PPT1	protein_codin	0	36.3532024	0	0
ENSG000001	TCEAL2	protein_codin	0	3.50523096	15.5881043	0
ENSG000001	SEMG1	protein_codin	0	1.49781547	0	2.70243358
ENSG000001	ZMAT1	protein_codin	0	0	0	0
ENSG000001	WFDC12	protein_codin	0	0	0	1.13538847
ENSG000001	WFDC5	protein_codin	0	0	0	0
ENSG000001	ODF2	protein_codin	0	0	0	11.9209056
ENSG000001	CAP1	protein_codin	12.1691588	18.7898131	1.98031231	17.338738
ENSG000001	CAP1	protein_codin	7.10032169	41.3826319	16.8549773	11.3662885
ENSG000001	CAP1	protein_codin	11.7247081	76.8308267	16.2784276	15.3013118
ENSG000001	STK4	protein_codin	0	0	0	14.7018324
ENSG000001	CAP1	protein_codin	0	0	0	2.00526302
ENSG000001	CAP1	protein_codin	54.0278691	118.839126	69.8005668	45.5475825
ENSG000001	STK4	protein_codin	23.5901312	200.694305	42.8477634	35.4079734
ENSG000001	ODF2	protein_codin	52.7203192	81.4682497	134.412408	17.4402951
ENSG000001	GNMT	protein_codin	0	0	0	0.99534671
ENSG000001	MFSD2A	protein_codin	0	0	0	0
ENSG000001	MFSD2A	protein_codin	0	0	0	17.6163199
ENSG000000	TOMM34	protein_codin	10.2172797	30.2781683	87.54678	8.2692006
ENSG000001	ODF2	protein_codin	9.73231763	0	64.1032126	0
ENSG000001	MYCL	protein_codin	0	0	0	0

ENSG000001	MYCL	protein_codin	0	12.6097099	5.75000845	0
ENSG000000	TRIT1	protein_codin	0	0	13.2841553	0
ENSG000001	PABPC1L	protein_codin	1.50103376	1.99233508	6.46718391	1.38728694
ENSG000001	PABPC1L	protein_codin	0	0	0	0
ENSG000001	PABPC1L	protein_codin	5.66106176	15.0294537	32.9529088	4.36340266
ENSG000001	PABPC1L	protein_codin	0	6.1802094	6.41082319	0
ENSG000001	BMP8B	protein_codin	8.89664774	39.6872223	24.3883517	71.6097184
ENSG000001	ARMCX1	protein_codin	20.7414986	65.804493	9.81301028	11.1084545
ENSG000000	PPIE	protein_codin	1.3437037	0	2.09049759	1.37702864
ENSG000001	CHCHD1	protein_codin	2.42330722	4.10808483	9.13600443	1.52191788
ENSG000000	PPIE	protein_codin	0	0	0	0
ENSG000001	CNPY3	protein_codin	25.7479552	216.083042	123.94796	26.7006458
ENSG000001	CHCHD1	protein_codin	3.97850798	5.96727443	1.20608502	1.23488095
ENSG000001	CERCAM	protein_codin	80.8652354	13.2245047	49.360742	51.1442417
ENSG000001	YWHAB	protein_codin	72.8590903	121.310964	172.710776	29.7427595
ENSG000001	FUT11	protein_codin	10.0515487	24.6192244	25.8562181	6.8183928
ENSG000001	CERCAM	protein_codin	6.80949562	21.1751041	14.0314657	3.09856405
ENSG000001	HPCAL4	protein_codin	0	0	0	0
ENSG000001	URM1	protein_codin	0	0	1.69516282	0
ENSG000002	RPL36A	protein_codin	0	0	0	0
ENSG000001	URM1	protein_codin	3.31765875	22.6002698	1.26480189	2.14096019
ENSG000001	RIMS4	protein_codin	12.6621747	0	15.9675939	1.16243682
ENSG000001	HEYL	protein_codin	18.3329315	54.8884687	14.1780102	13.5330999
ENSG000001	URM1	protein_codin	0	3.29551926	9.54337871	3.14384925
ENSG000000	PABPC4	protein_codin	0	0	0	0
ENSG000000	PABPC4	protein_codin	0	0	0	0
ENSG000000	PABPC4	protein_codin	12.7828405	46.1241852	22.2126644	21.0327753
ENSG000001	KCNK15	protein_codin	0	0	0.52825091	0.42100688
ENSG000000	PABPC4	protein_codin	0.67142029	0	0	0
ENSG000000	CCN5	protein_codin	0	0	0	7.73688344
ENSG000000	CCN5	protein_codin	0	0	0	0
ENSG000001	SLC27A4	protein_codin	0	0	0	0
ENSG000001	SYNPO2L	protein_codin	1.84569295	7.51285879	0	0
ENSG000001	ADA	protein_codin	0	0	0	0
ENSG000001	COQ4	protein_codin	0.98892178	4.84098786	10.0188679	0.87269231
ENSG000001	TBCC	protein_codin	3.33845002	41.8317556	11.0378348	6.46008051
ENSG000001	TOMM7	protein_codin	0	0	0.86365019	0.58856105
ENSG000001	PKIG	protein_codin	14.666308	0	38.3138079	5.38524939
ENSG000001	PKIG	protein_codin	0	5.47197274	0	0.83120603
ENSG000001	PKIG	protein_codin	0	0	0	0.79595588
ENSG000001	TRUB2	protein_codin	13.0144193	24.3508277	45.5923137	4.75364623
ENSG000001	PKIG	protein_codin	0	2.40944708	0	0.58734455
ENSG000001	PKIG	protein_codin	0	4.97627657	0	0
ENSG000001	PKIG	protein_codin	6.32620553	16.6476472	5.1238276	6.3220687

ENSG000001	SWI5	protein_codin	0	0	0	0
ENSG000000	UBR2	protein_codin	82.2271054	100.661135	186.715839	11.5990717
ENSG000000	UBR2	protein_codin	34.7991082	216.196817	92.8488955	51.0107784
ENSG000001	TIMM8A	protein_codin	2.23232311	5.10091143	14.1554969	2.8310108
ENSG000000	UBR2	protein_codin	0.59454482	2.18710542	6.0113033	1.62584756
ENSG000001	TTPAL	protein_codin	3.63957203	0	20.8099326	0
ENSG000001	TTPAL	protein_codin	0	0	0	0
ENSG000001	TAF7L	protein_codin	0.64610868	0	0	0.92323741
ENSG000001	MSS51	protein_codin	0	0	0	0
ENSG000001	MACF1	protein_codin	0	0	0	0
ENSG000001	DRP2	protein_codin	1.97557648	0	0.7657678	3.46090205
ENSG000001	TRERF1	protein_codin	0	30.7992214	46.8105859	0
ENSG000001	ANXA7	protein_codin	4.53665743	48.5768381	0	0
ENSG000001	HNF4A	protein_codin	0	0	0	0
ENSG000001	ANXA7	protein_codin	29.375307	0	55.693871	26.9875286
ENSG000001	TRERF1	protein_codin	0	35.2883372	0	0
ENSG000001	DNM1	protein_codin	0	0	21.0253638	0
ENSG000001	MACF1	protein_codin	226.543812	429.039392	254.772272	116.383714
ENSG000001	CENPI	protein_codin	0.38717232	0	0	0
ENSG000001	CENPI	protein_codin	0	0	0	0
ENSG000001	TMEM35A	protein_codin	1.26238002	3.95223015	0	2.0178998
ENSG000001	TRMT2B	protein_codin	0	0	0	2.91168919
ENSG000001	TRMT2B	protein_codin	0	32.6145631	0	0.91205903
ENSG000001	CIZ1	protein_codin	47.2621742	0	56.558447	10.40758
ENSG000001	TRMT2B	protein_codin	0	0	7.51343555	0
ENSG000001	MRPS16	protein_codin	3.41678513	2.83439015	5.20474161	0
ENSG000001	MRPS16	protein_codin	13.8085165	0	14.8249204	10.0385945
ENSG000001	CIZ1	protein_codin	0	0	0	0
ENSG000002	DNAJC9	protein_codin	17.2885802	37.5973086	117.41798	14.5507814
ENSG000001	GDAP1L1	protein_codin	0	0	0	0
ENSG000001	ARL13A	protein_codin	0	0	0	0
ENSG000001	CIZ1	protein_codin	1.20617191	35.8150688	25.4202909	0
ENSG000001	FAM149B1	protein_codin	3.02480844	0	7.90229583	0
ENSG000001	XKRX	protein_codin	0.28202137	0	0	0
ENSG000000	GUCA1A	protein_codin	0	0	0	0.79011314
ENSG000000	NOX1	protein_codin	0	0	0	0
ENSG000002	CIMIP3	protein_codin	0	0	0	0
ENSG000000	NOX1	protein_codin	0	0	0	0
ENSG000000	NOX1	protein_codin	0	0	0	0
ENSG000001	NDUFS5	protein_codin	67.1884304	61.1275191	80.2000154	57.4128105
ENSG000001	NDUFS5	protein_codin	31.0275806	68.7579118	119.111875	8.82726707
ENSG000001	OSER1	protein_codin	6.72293765	0	0	0
ENSG000001	CSTF2	protein_codin	6.27079699	12.1207	23.0283965	7.85020141
ENSG000001	TAF8	protein_codin	0	42.0007248	24.6900379	15.1191268

ENSG000001[illegible]	TAF8	protein_codin	2.20047991	1.71023201	2.98262432	0
ENSG000001[illegible]	ECD	protein_codin	4.98238407	46.4944319	12.7524671	15.4115388
ENSG000001[illegible]	JPH2	protein_codin	4.51578518	13.1341768	2.06344127	3.98653048
ENSG000001[illegible]	SYTL4	protein_codin	1.06961227	22.1816046	10.4479138	1.78518931
ENSG000001[illegible]	TAF8	protein_codin	0	0	0	0
ENSG000001[illegible]	AKIRIN1	protein_codin	4.20500412	0	0	1.01998959
ENSG000001	CCND3	protein_codin	0	0	0	0.88759881
ENSG000001	CCND3	protein_codin	0	0	0	0
ENSG000001[illegible]	SYTL4	protein_codin	7.34139201	0	6.09908485	0
ENSG000001[illegible]	RHBDL2	protein_codin	16.3058634	89.4422716	11.4543703	25.8406097
ENSG000001	CCND3	protein_codin	12.5585847	26.812918	8.97581183	8.24040896
ENSG000001[illegible]	BBLN	protein_codin	6.89726978	9.02999561	7.81167053	2.41686179
ENSG000001	BYSL	protein_codin	0	0	0	1.69962722
ENSG000001[illegible]	NUDT13	protein_codin	1.58375592	0	0	0
ENSG000001[illegible]	LCN2	protein_codin	0	0	0.34587706	0
ENSG000001[illegible]	TOX2	protein_codin	0	0	0	0
ENSG000001	RRAGC	protein_codin	43.2726676	70.5340174	58.6351182	28.4310391
ENSG000001[illegible]	GTSF1L	protein_codin	0.2314527	0	0	0.74691852
ENSG000001[illegible]	SRPX2	protein_codin	65.583141	47.3922708	1.34808439	22.5703802
ENSG000001[illegible]	GTSF1L	protein_codin	0	0	0	0
ENSG000001[illegible]	USP49	protein_codin	0.3522926	2.07690868	2.86142071	1.39531947
ENSG000001[illegible]	P4HA1	protein_codin	55.6822923	51.1993391	162.815575	40.7984692
ENSG000001[illegible]	USP49	protein_codin	0.38550728	0	0.61566661	1.06408699
ENSG000001[illegible]	POU3F1	protein_codin	0	0	0	0.4395036
ENSG000001[illegible]	UTP11	protein_codin	17.0756217	0	92.4958929	14.0687451
ENSG000001[illegible]	FHL3	protein_codin	5.65106876	4.09674887	0	0
ENSG000001[illegible]	LCN2	protein_codin	0	0	0	0
ENSG000001[illegible]	FRS3	protein_codin	0	0	0	0
ENSG000001[illegible]	SF3A3	protein_codin	69.7080246	48.6310643	167.400809	29.9095479
ENSG000000[illegible]	TSPAN6	protein_codin	22.6634094	39.6469516	51.9305156	10.8757023
ENSG000002[illegible]	INPP5B	protein_codin	1.34754343	4.39227434	3.61651437	2.28608134
ENSG000002[illegible]	INPP5B	protein_codin	2.31264748	64.6363938	14.7452545	6.05229657
ENSG000000[illegible]	PGC	protein_codin	0	0	0	0
ENSG000002[illegible]	INPP5B	protein_codin	0	22.155028	0	0
ENSG000002[illegible]	INPP5B	protein_codin	25.5973508	0	29.4637455	11.2010094
ENSG000001[illegible]	IFT52	protein_codin	11.1389851	46.0413968	7.50080854	5.85970965
ENSG000000[illegible]	TNMD	protein_codin	9.1350607	1.22244265	0.4291785	1.70978971
ENSG000001[illegible]	PLA2G12B	protein_codin	0	1.40719337	0.4228239	0.74625537
ENSG000001	TFEB	protein_codin	4.29705458	4.35761939	9.15369494	4.0267641
ENSG000001[illegible]	PCDH19	protein_codin	4.53181982	6.81660791	1.2163057	12.8453551
ENSG000001[illegible]	MTF1	protein_codin	16.5255968	28.611464	20.7145195	13.6318519
ENSG000001[illegible]	IFT52	protein_codin	3.34238838	0	4.26814498	0
ENSG000002[illegible]	RPA4	protein_codin	2.54680092	13.5301522	8.11668998	3.81750642
ENSG000001[illegible]	C1orf122	protein_codin	3.80699825	0	2.77047756	0

ENSG000001!	C1orf122	protein_codin	0	5.22751403	0	0
ENSG000001!	YRDC	protein_codin	2.73859392	2.27030835	9.62262094	2.82411933
ENSG000001	MANEAL	protein_codin	0	0	0	0
ENSG000001	EPHA10	protein_codin	0	1.89476088	0.56882078	0.4180356
ENSG000001	DIAPH2	protein_codin	39.8685918	95.8225101	93.589256	21.9692719
ENSG000001	MDFI	protein_codin	3.91091461	18.6423479	73.9912496	8.51350924
ENSG000001	MDFI	protein_codin	0	0	0	1.97934063
ENSG000001	MCU	protein_codin	0	28.870011	31.4811948	6.64730789
ENSG000001	CDCA8	protein_codin	4.36517809	0	9.28134251	0
ENSG000001	FOXP4	protein_codin	0	0	0	0
ENSG000001	GNL2	protein_codin	78.7110759	147.556215	406.585521	35.2403354
ENSG000001	FOXP4	protein_codin	3.55730449	0	13.0616262	1.84076903
ENSG000001	SLC25A25	protein_codin	0.55030161	0	0	0
ENSG000001	SLC25A25	protein_codin	0	0	0	0
ENSG000001	SLC25A25	protein_codin	2.10739711	4.64451736	5.12231137	9.45960169
ENSG000001	SLC25A25	protein_codin	0	0	0	0
ENSG000001	MEAF6	protein_codin	11.0146673	0	9.55954401	8.31368147
ENSG000001	MEAF6	protein_codin	11.1575978	17.275259	21.6224454	4.30580018
ENSG000001	MEAF6	protein_codin	15.3203717	36.2522923	0	0
ENSG000001	SGK2	protein_codin	0	0	0	0
ENSG000001	NAIF1	protein_codin	0.48761714	6.56809275	4.14407204	1.75748925
ENSG000001	NAP1L3	protein_codin	22.2520559	27.1681186	42.9019701	0
ENSG000000!	NCR2	protein_codin	0	0	0	0
ENSG000001	EEIG1	protein_codin	6.84056119	0	13.55858	5.14429933
ENSG000000!	NCR2	protein_codin	0	0	0	0
ENSG000001	ZC3H12A	protein_codin	1.03716919	0	2.33780039	19.8851733
ENSG000001	PCDH11X	protein_codin	0	0	0	0
ENSG000000!	NCR2	protein_codin	0	0	0	0.8406277
ENSG000001	GRIK3	protein_codin	2.91648519	7.73974482	5.35518172	14.4727081
ENSG000001	GRIK3	protein_codin	0	0	0	0
ENSG000001	PCDH11X	protein_codin	0	0	0	0
ENSG000001	EEIG1	protein_codin	4.55032292	88.3635562	12.2880724	12.725681
ENSG000001	PCDH11X	protein_codin	0	0	0	5.48043701
ENSG000001	SGK2	protein_codin	0	0	0	0
ENSG000001	CSF3R	protein_codin	0	0	0	0
ENSG000001	CSF3R	protein_codin	0	2.97036612	0	0
ENSG000001	PABPC5	protein_codin	0	0	3.63376002	0
ENSG000001	CSF3R	protein_codin	0	0	0	0
ENSG000001	SPOCK2	protein_codin	5.68898005	129.020842	4.34348374	23.5804014
ENSG000001	DPM2	protein_codin	0	6.09496295	0.57612711	0
ENSG000001	CPXCR1	protein_codin	0	0	0	0
ENSG000000!	TREM2	protein_codin	3.44472885	2.94320501	0.5788748	0.82378302
ENSG000001	KLHL4	protein_codin	0	0	2.29216515	0
ENSG000001	CHST3	protein_codin	34.4264003	50.7899662	56.0102768	46.6167292

ENSG000001	MRPS15	protein_codin	22.2524095	31.1550586	51.7089696	20.5281238
ENSG000001	KLHL4	protein_codin	6.16810343	0	0	3.1879894
ENSG000000	TREM2	protein_codin	0	0	0	0
ENSG000001	DACH2	protein_codin	0	0	0	0
ENSG000001	TREML1	protein_codin	0	0	0	0
ENSG000001	STK40	protein_codin	0	0	0	0
ENSG000001	STK40	protein_codin	0	0	0	6.26476599
ENSG000001	DACH2	protein_codin	0	0	0	0
ENSG000001	STK40	protein_codin	14.6791172	52.469753	26.9463693	0
ENSG000001	L3MBTL1	protein_codin	0	0	0	0
ENSG000001	L3MBTL1	protein_codin	0	0	0	0
ENSG000001	L3MBTL1	protein_codin	0	0	0	0
ENSG000002	SH3D21	protein_codin	3.11576031	8.7107829	2.53070494	0
ENSG000001	ST6GALNAC6	protein_codin	0	0	0	0
ENSG000001	ST6GALNAC6	protein_codin	2.90359286	9.70237567	3.39178886	4.23618418
ENSG000001	ST6GALNAC6	protein_codin	8.99575327	45.3156713	8.35669653	5.6510753
ENSG000001	POF1B	protein_codin	0.19408132	0	0.45654426	0
ENSG000001	ST6GALNAC6	protein_codin	0	0	0	0
ENSG000001	MAP7D1	protein_codin	117.505213	358.572395	157.347886	46.9573127
ENSG000001	MAP7D1	protein_codin	17.5485144	236.705877	25.0456747	24.9527179
ENSG000001	MAP7D1	protein_codin	25.6657929	0	38.8600313	0
ENSG000001	OARD1	protein_codin	0	11.2076959	0	0
ENSG000001	AK1	protein_codin	12.6464228	0	2.40636785	2.59640407
ENSG000001	COL6A5	protein_codin	0	0	0	4.52410634
ENSG000001	TSPO2	protein_codin	0	0	0	0
ENSG000000	TRAPPC3	protein_codin	0	0	8.41915036	0
ENSG000001	TSPO2	protein_codin	0	0	0	2.03966005
ENSG000000	TRAPPC3	protein_codin	0	2.35262889	5.44830898	0
ENSG000000	TRAPPC3	protein_codin	0	0	0	0
ENSG000001	UNC5CL	protein_codin	0	0	0	0
ENSG000001	ZNF711	protein_codin	7.28116792	4.68175726	86.9120663	8.85269979
ENSG000000	TRAPPC3	protein_codin	9.21238138	0	13.295697	0
ENSG000001	APOOL	protein_codin	12.1321322	30.7343809	44.3470776	13.1103303
ENSG000001	HDX	protein_codin	0	0	0	0
ENSG000001	ADPRS	protein_codin	8.25798523	50.0641387	22.0273079	4.00756892
ENSG000001	MOCS1	protein_codin	0	0	0	0
ENSG000001	PTPRT	protein_codin	1.24665107	0	0	0
ENSG000001	PTPRT	protein_codin	0	0	0	0
ENSG000001	MOCS1	protein_codin	0	0	0	0
ENSG000001	SLC29A3	protein_codin	0	0	0	0
ENSG000001	PTPRT	protein_codin	1.28624114	0	0	0
ENSG000001	AGO3	protein_codin	79.8050086	92.5835741	110.864091	43.38112
ENSG000001	UNC5B	protein_codin	0	0	0	0
ENSG000001	PTPRT	protein_codin	0	0	0	0

ENSG000001:	MOCS1	protein_codin	0	0	0	0
ENSG000001!	PTPRT	protein_codin	0	0	0	0
ENSG000001!	PTPRT	protein_codin	0	0	0	0
ENSG000001(	SGPL1	protein_codin	26.4686674	73.083874	72.2535651	29.4470017
ENSG000001(	ENG	protein_codin	12.8017877	0	6.12212251	0
ENSG000000!	AGO1	protein_codin	35.7701617	125.753179	84.0679919	30.6552135
ENSG000000!	AGO1	protein_codin	0	0	7.13273579	0
ENSG000001:	ADAMTS14	protein_codin	0	0	0	0
ENSG000001:	ADAMTS14	protein_codin	3.18565569	9.03380585	5.03813368	4.35308689
ENSG000001(	PRF1	protein_codin	3.07729234	70.467505	0	3.35937049
ENSG000001:	AGO4	protein_codin	23.4489824	108.834583	45.7389099	28.9927367
ENSG000001:	SH3BGRL	protein_codin	106.117955	103.442722	171.943635	70.6093913
ENSG000001/	EIF4EBP2	protein_codin	101.132694	217.406214	231.486118	84.0842329
ENSG000000!	CLSPN	protein_codin	2.32002203	42.3575563	4.09512126	0
ENSG000001:	CHD6	protein_codin	4.11593825	0	34.3612952	7.4837066
ENSG000001'	LRRC20	protein_codin	0	0	2.08357058	0
ENSG000001:	FPGS	protein_codin	0	3.81641623	0	2.98981277
ENSG000000!	KCNK16	protein_codin	0	0	0	0
ENSG000001:	FPGS	protein_codin	0	0	0	2.02612267
ENSG000000!	KCNK16	protein_codin	0	0	0	0
ENSG000001(	PPA1	protein_codin	0.58524924	0	0	3.35510224
ENSG000001:	KCNK17	protein_codin	0	1.18342622	0	3.69839995
ENSG000001(	PPA1	protein_codin	27.3358729	16.1937062	64.2688386	17.2062178
ENSG000001:	CHD6	protein_codin	109.646587	287.451939	292.563921	85.6877647
ENSG000001	TFAP2E	protein_codin	0	1.17641177	0	1.0736116
ENSG000000'	SAR1A	protein_codin	12.2325123	25.3182957	11.1781879	4.16887443
ENSG000001:	PSMB2	protein_codin	12.5004078	37.4231356	33.9326256	12.9557644
ENSG000000'	SAR1A	protein_codin	62.2480488	68.4271601	75.8099803	40.2311822
ENSG000000'	SAR1A	protein_codin	0	0	0	0
ENSG000000'	SAR1A	protein_codin	7.90354599	27.2998134	28.0245404	12.2408592
ENSG000000'	SAR1A	protein_codin	0	21.259734	4.37004443	1.43538559
ENSG000000:	NCDN	protein_codin	4.03908638	15.7585847	14.8573412	3.03478292
ENSG000001:	FPGS	protein_codin	0	0	0	0
ENSG000000/	AIFM2	protein_codin	0.4495401	0	1.88292016	0
ENSG000001	SAYSD1	protein_codin	0	0	0	6.85191144
ENSG000001!	HMGN5	protein_codin	0	1.23305566	9.99518521	0
ENSG000000:	NCDN	protein_codin	0	4.22370511	5.52840561	0
ENSG000000!	MACROH2A2	protein_codin	4.76886996	0	43.8829638	0
ENSG000001	GLP1R	protein_codin	0	2.60662298	2.36523091	2.06846224
ENSG000001:	LPIN3	protein_codin	6.34904454	30.8573864	0	4.80245983
ENSG000001'	ZHX3	protein_codin	12.4545855	0	5.64949856	0
ENSG000001:	CDK9	protein_codin	13.3282298	109.456897	21.6491639	14.6447279
ENSG000001/	KIAA0319L	protein_codin	0	0	0	0
ENSG000001:	PLCG1	protein_codin	3.32809823	40.9745061	16.2497417	24.7560861

ENSG000001	BRWD3	protein_codin	59.4582971	210.26132	275.079914	56.5904196
ENSG000000	SH2D3C	protein_codin	0	2.03563289	0.91882512	0
ENSG000000	SH2D3C	protein_codin	0.41446522	7.4415056	0	0
ENSG000001	DNAH8	protein_codin	0	0	0	0
ENSG000001	FAM241B	protein_codin	0	0	0	0
ENSG000001	TOR2A	protein_codin	0	0	0	0
ENSG000001	TOR2A	protein_codin	1.97026232	0	0	0.97165123
ENSG000000	FARP2	protein_codin	3.17914945	3.62144307	2.32459355	5.2031442
ENSG000001	TTC16	protein_codin	0	0	0	0
ENSG000000	TSPAN15	protein_codin	6.82733556	2.6837072	0	0.74260341
ENSG000001	HDLBP	protein_codin	183.17889	557.367916	369.738426	125.020799
ENSG000001	CFAP157	protein_codin	0	0	0	0
ENSG000001	TBX22	protein_codin	0	0	4.73101675	0
ENSG000001	CFAP157	protein_codin	0	0	0	0
ENSG000001	TBX22	protein_codin	0	0	0	0
ENSG000000	ITM2A	protein_codin	74.4880649	27.2988163	0.80285249	3.37109612
ENSG000001	STXBP1	protein_codin	0	0	0	0
ENSG000001	STXBP1	protein_codin	0	0	0	0
ENSG000001	CYSLTR1	protein_codin	3.69845519	11.0532989	0	3.19917774
ENSG000000	TACR2	protein_codin	0	2.8762949	0	2.6630189
ENSG000000	TACR2	protein_codin	0	0	0	0
ENSG000001	NIBAN2	protein_codin	61.6361628	125.450913	85.5128501	39.1608279
ENSG000002	MAFB	protein_codin	62.373606	402.894942	56.233823	203.24484
ENSG000001	NIBAN2	protein_codin	0	21.5824693	0	0
ENSG000001	PGK1	protein_codin	25.1955701	58.0532804	69.3322146	39.9502051
ENSG000001	ANKMY1	protein_codin	0	0	0.73023518	0
ENSG000001	LRSAM1	protein_codin	0	0	0	0
ENSG000001	DHX35	protein_codin	0	2.73969147	0	0
ENSG000001	LRSAM1	protein_codin	0	0	0	0
ENSG000001	DHX35	protein_codin	0	25.9936666	0	2.85294503
ENSG000002	TRAF3IP1	protein_codin	1.20565313	0	0	0
ENSG000001	ZMYM1	protein_codin	0	0	31.6323164	0
ENSG000001	ZMYM1	protein_codin	0	0	0	0
ENSG000001	PPP1R16B	protein_codin	0	0	6.02608423	10.6129867
ENSG000001	ZMYM6	protein_codin	1.16912625	1.90969542	0	0
ENSG000001	COX7B	protein_codin	9.56213905	0	0	1.46025783
ENSG000001	MAGT1	protein_codin	0	29.0542089	0	0.71998676
ENSG000002	TMEM35B	protein_codin	8.36984721	23.4849171	8.04560729	6.82314088
ENSG000000	ATRX	protein_codin	46.1556772	84.8372622	86.8696991	53.3236082
ENSG000001	ARHGAP40	protein_codin	0	0	0	0.37718235
ENSG000001	DLGAP3	protein_codin	0	0	0	0
ENSG000001	ADIG	protein_codin	0	0	0	0.56117297
ENSG000001	SLC2A8	protein_codin	0	0	0	0
ENSG000001	PBDC1	protein_codin	6.10330611	140.070187	8.22134009	3.81713663

ENSG000001	PBDC1	protein_codin	24.3839631	25.9920322	50.4789306	17.2836296
ENSG000001	MAGEE2	protein_codin	0.84238581	0	0	1.46250246
ENSG000001	SLC2A8	protein_codin	0	0	3.51049803	0
ENSG000001	GJB3	protein_codin	0.3300281	0	0	0.46131128
ENSG000001	GLO1	protein_codin	12.9680131	74.4940775	125.721535	27.3107337
ENSG000001	GJB3	protein_codin	0	0	0	0
ENSG000001	ZDHHC15	protein_codin	3.16605455	4.80066151	0.49030535	2.70303794
ENSG000000	SPP2	protein_codin	0	0	0	0
ENSG000001	SLC2A8	protein_codin	0	5.29612307	0	0
ENSG000000	UPRT	protein_codin	0	0	0.99234451	0
ENSG000001	C1orf94	protein_codin	0	0	0	0
ENSG000001	CSMD2	protein_codin	0	0	0	0
ENSG000000	UPRT	protein_codin	0	0	0	0
ENSG000001	CSMD2	protein_codin	0	0	0	0
ENSG000001	CSMD2	protein_codin	5.5924677	0	5.12312845	0
ENSG000001	VPS26A	protein_codin	0	2.42915334	0	1.85249792
ENSG000000	UPRT	protein_codin	8.30660584	39.4788889	6.90609283	10.5798175
ENSG000001	GARNL3	protein_codin	0	7.49280398	0	0
ENSG000001	GARNL3	protein_codin	4.11190706	0	10.3403838	6.1263047
ENSG000001	CSMD2	protein_codin	0	7.4130719	0	6.77223832
ENSG000001	ZFAND3	protein_codin	0	3.99090509	0	3.55520291
ENSG000001	ZFAND3	protein_codin	0	0	0	0
ENSG000001	ABCB7	protein_codin	8.94169548	0	8.31727794	0
ENSG000001	HJURP	protein_codin	0	0	1.01209644	0
ENSG000001	MDGA1	protein_codin	2.21565391	8.24462439	6.06231764	1.59198859
ENSG000001	TGM2	protein_codin	0	0	0	0
ENSG000001	CCDC167	protein_codin	9.63426699	23.3545368	99.8480135	6.05002452
ENSG000002	UGT1A4	protein_codin	0	0	0	0
ENSG000001	ZSCAN20	protein_codin	0.94535977	4.29069384	0	0.50768868
ENSG000002	UGT1A5	protein_codin	0	0	0	1.35958908
ENSG000001	ANGPTL2	protein_codin	0	22.1024857	0	0
ENSG000001	PHC2	protein_codin	17.803902	62.3503341	25.926558	14.6793565
ENSG000001	PHC2	protein_codin	0	13.8681106	6.59779822	2.75610821
ENSG000001	UGT1A6	protein_codin	0	0	0	0
ENSG000001	ANGPTL2	protein_codin	87.652151	82.051908	39.433668	24.4221117
ENSG000002	UGT1A7	protein_codin	0	0	0	0
ENSG000001	ZNF362	protein_codin	0	0	0	14.4741344
ENSG000001	RPRD1B	protein_codin	15.3229957	34.1816221	37.2339275	4.8629484
ENSG000001	RALGPS1	protein_codin	0	0	0	0
ENSG000001	RALGPS1	protein_codin	0	0	0	0.62156747
ENSG000001	RALGPS1	protein_codin	0.54754018	3.13700165	0.64808782	1.1374006
ENSG000001	AZIN2	protein_codin	2.48715143	1.63122203	0	0
ENSG000001	AZIN2	protein_codin	0	0	0	0
ENSG000002	UGT1A10	protein_codin	0.33288816	0	0	1.00651831

ENSG000001	TTI1	protein_codin	17.3706659	30.5181832	71.8674723	7.70347732
ENSG000001	TTI1	protein_codin	0	4.13813744	3.03632987	1.92132433
ENSG000000	AK2	protein_codin	0	0	28.2850283	0
ENSG000002	UGT1A8	protein_codin	0	0	0	2.20552642
ENSG000001	CMTR1	protein_codin	18.027262	123.406466	89.1456245	11.7522106
ENSG000001	RNF19B	protein_codin	4.81419522	0	0	5.93157717
ENSG000001	ZBTB43	protein_codin	0	39.0121169	0	49.5435391
ENSG000001	VSTM2L	protein_codin	0	0	0	0
ENSG000001	VSTM2L	protein_codin	0.27036766	0	0	0
ENSG000001	TMEM54	protein_codin	0	1.76744101	0	0
ENSG000001	ZBTB43	protein_codin	14.7815044	0	0	29.6972975
ENSG000001	HPCA	protein_codin	0	1.66801629	0	0
ENSG000001	CTNNBL1	protein_codin	0	0	0	0
ENSG000001	FNDC5	protein_codin	0	0	0	0
ENSG000001	CTNNBL1	protein_codin	0	0	0	0
ENSG000001	LMX1B	protein_codin	0	0	23.5223074	0
ENSG000001	S100PBP	protein_codin	10.5457746	20.1307954	23.129892	10.6512781
ENSG000001	S100PBP	protein_codin	0	0	0	0
ENSG000001	YARS1	protein_codin	17.343679	155.530968	51.9298569	14.8461579
ENSG000001	RNF8	protein_codin	0	0	0	2.59274454
ENSG000001	NHSL3	protein_codin	0	0	0	0
ENSG000001	NHSL3	protein_codin	0	0	0	0
ENSG000001	PBX3	protein_codin	0	0	0	0
ENSG000001	SYNC	protein_codin	13.2354211	12.9902521	0	0
ENSG000001	RBBP4	protein_codin	0	0	0	0
ENSG000001	PBX3	protein_codin	0	0	6.71302188	0
ENSG000001	PBX3	protein_codin	0	33.7535709	0	0
ENSG000000	TBC1D22B	protein_codin	7.76407431	35.2012514	24.8698023	14.2915128
ENSG000001	PBX3	protein_codin	6.9619729	55.7684112	9.21138017	8.38456936
ENSG000001	RBBP4	protein_codin	31.3398551	64.3776957	131.134857	29.9177892
ENSG000001	MAPKAP1	protein_codin	0	0	0	0
ENSG000001	MAPKAP1	protein_codin	15.1070627	0	0	0
ENSG000001	ZBTB8OS	protein_codin	0	0	1.15700477	0
ENSG000002	CHIC1	protein_codin	0	22.5585419	0	0
ENSG000001	MAPKAP1	protein_codin	0	0	11.1672657	0
ENSG000002	CHIC1	protein_codin	10.0196999	0	12.2367774	5.03335692
ENSG000001	ZBTB8OS	protein_codin	0	0	1.66823819	0
ENSG000001	PIM1	protein_codin	6.47022386	51.6166351	18.7471215	39.4143561
ENSG000001	ZBTB8A	protein_codin	13.8254837	26.8894795	17.1914921	8.40211068
ENSG000001	MAPKAP1	protein_codin	0	0	3.82995857	0
ENSG000001	CDX4	protein_codin	0	0	0	0.54377842
ENSG000001	NAP1L2	protein_codin	8.53623375	3.37925401	34.0169554	2.57545928
ENSG000001	PABPC1L2A	protein_codin	0.27182887	1.79615526	0	0
ENSG000001	BSDC1	protein_codin	0	0	0	0

ENSG000001	PABPC1L2B	protein_codin	0	0	1.11854925	4.09384576
ENSG000000	ATG16L1	protein_codin	0	0	0	0
ENSG000001	DMRTC1B	protein_codin	0	0	0	0
ENSG000001	DMRTC1B	protein_codin	0	0	0	0
ENSG000001	TSSK3	protein_codin	0	0	0.88065937	0
ENSG000001	FGD2	protein_codin	0	0	1.78077466	0
ENSG000001	BLCAP	protein_codin	13.1364895	51.7694299	20.1099379	13.5363878
ENSG000001	RABEPK	protein_codin	0	0	1.69266138	0
ENSG000000	PHKA1	protein_codin	0	0	0	6.04771125
ENSG000000	PHKA1	protein_codin	0	0	0	0
ENSG000001	RABEPK	protein_codin	0.33188133	8.86663425	2.45269461	0.58658157
ENSG000000	PHKA1	protein_codin	0	0	0	0
ENSG000001	PPP6C	protein_codin	17.1868928	36.7400405	29.7109428	16.4137819
ENSG000001	HDAC1	protein_codin	13.8354897	66.3856498	77.4254785	0
ENSG000001	SCAI	protein_codin	0.44492514	6.2558469	3.20843487	0
ENSG000001	GOLGA1	protein_codin	0.4751911	0	0	0
ENSG000000	NGEF	protein_codin	3.95560226	0	1.63136184	2.00060487
ENSG000001	HDAC8	protein_codin	0	2.33263291	12.9960445	2.95747322
ENSG000001	GOLGA1	protein_codin	24.5029084	95.4638935	73.7104474	17.2483211
ENSG000001	HDAC8	protein_codin	0	4.89631504	0	0
ENSG000001	LCK	protein_codin	0	0	0	0
ENSG000001	SRC	protein_codin	0	0	0	0
ENSG000001	HDAC8	protein_codin	0	0	0	0
ENSG000001	HDAC8	protein_codin	0	0	0	0
ENSG000001	LCK	protein_codin	0	0	0	0
ENSG000002	GIGYF2	protein_codin	0	0	0	0
ENSG000001	SRC	protein_codin	0	0	0	0
ENSG000001	HDAC8	protein_codin	0	0	0	0
ENSG000001	RPL35	protein_codin	4.64063852	0	4.39269028	0
ENSG000001	HDAC8	protein_codin	0.98775707	3.86993834	1.60386784	5.21548798
ENSG000001	HDAC8	protein_codin	0	0	0	0
ENSG000001	WDR38	protein_codin	0	0	0	0
ENSG000001	SRC	protein_codin	6.86473985	25.7777438	19.5288054	25.2209225
ENSG000001	OLFML2A	protein_codin	43.2600406	186.248212	33.5450398	60.930287
ENSG000001	FAM167B	protein_codin	0	0	0	0.94047799
ENSG000001	HDAC8	protein_codin	0	0	0	0
ENSG000001	NR6A1	protein_codin	0	0	0	0
ENSG000001	DDX50	protein_codin	43.9898175	74.1880553	95.5015187	18.2179974
ENSG000000	EIF3I	protein_codin	0	0	0	0
ENSG000001	NR5A1	protein_codin	0	0	0	0
ENSG000001	NR5A1	protein_codin	0	0	0	0
ENSG000001	HDAC8	protein_codin	0	0	0	0
ENSG000001	TMEM234	protein_codin	0	0	0	2.32685437
ENSG000001	NEK6	protein_codin	6.31016723	0	0	0.89728552

ENSG000001	NEK6	protein_codin	0	7.32465003	0	3.30404535
ENSG000001	CCDC28B	protein_codin	4.91408616	10.6558752	9.28358287	0
ENSG000001	NEK6	protein_codin	0	3.73218489	0	0
ENSG000001	MANBAL	protein_codin	0	0	12.4898379	4.13844394
ENSG000001	MANBAL	protein_codin	0	0	4.39793611	0
ENSG000001	COPS7B	protein_codin	0	0	0	0
ENSG000000	TXLNA	protein_codin	13.1609727	55.2596379	22.7743865	22.1190247
ENSG000000	TXLNA	protein_codin	132.110848	187.970611	334.339407	46.2959729
ENSG000001	GHRH	protein_codin	0	0	0	0
ENSG000001	LHX2	protein_codin	0	0	0	0
ENSG000001	MTCH1	protein_codin	0	0	0	0
ENSG000001	DENND1A	protein_codin	0	9.25603027	5.33322399	1.39309305
ENSG000001	CITED1	protein_codin	0	0	0	0
ENSG000001	DENND1A	protein_codin	0	7.01410379	7.18844119	2.94805961
ENSG000001	RPN2	protein_codin	5.70247518	17.4588422	28.7195531	4.52125541
ENSG000001	DENND1A	protein_codin	9.34499171	37.7497526	32.7328927	6.23653655
ENSG000000	KPNA6	protein_codin	52.690033	71.4634094	161.54718	45.4381119
ENSG000001	RPS4X	protein_codin	0	0	295.620386	0
ENSG000001	MTCH1	protein_codin	17.3724156	0	61.6339947	0
ENSG000001	CRB2	protein_codin	0.28422197	0	0	0
ENSG000001	RPN2	protein_codin	0	0	0	1.37706814
ENSG000001	PSMD1	protein_codin	0	0	2.74317763	0
ENSG000002	C2orf72	protein_codin	0	0	0	0
ENSG000001	TET1	protein_codin	63.7759308	69.5971547	190.919631	27.2941383
ENSG000000	SP140	protein_codin	0	12.9833265	0	0
ENSG000000	RABGAP1	protein_codin	66.9320079	187.15906	157.540787	61.7999908
ENSG000001	FBXO36	protein_codin	1.69255329	0	0	0
ENSG000001	ZBTB26	protein_codin	0	7.11491562	1.26342006	1.93642803
ENSG000001	ADGRB2	protein_codin	0	0	0	0
ENSG000001	ZBTB26	protein_codin	10.1911174	8.27932527	25.3319585	1.95064538
ENSG000001	ERCC6L	protein_codin	0	16.1235201	0	0
ENSG000001	ADGRB2	protein_codin	0	0	2.58716774	0
ENSG000001	ZBTB6	protein_codin	23.8536413	18.4172946	26.1106606	5.3560328
ENSG000001	PIN4	protein_codin	0	0	1.02773729	0
ENSG000000	RBL1	protein_codin	11.1532983	38.2668444	16.4395698	3.50874337
ENSG000001	DAW1	protein_codin	0	0	0	1.56042929
ENSG000000	COL16A1	protein_codin	15.7972126	0	22.1870298	0
ENSG000000	COL16A1	protein_codin	54.7743799	0	25.403854	12.427726
ENSG000001	PIN4	protein_codin	10.2270344	7.61145305	21.81112	5.4012018
ENSG000000	RC3H2	protein_codin	73.8888272	129.162497	0	0
ENSG000001	AGFG1	protein_codin	0	0	0	0
ENSG000000	COL16A1	protein_codin	305.809331	663.241999	372.646137	98.4688636
ENSG000001	ATOH7	protein_codin	0	0	0	0.4532506
ENSG000001	PI16	protein_codin	0	0	0.39929943	1.43029359

ENSG000001	MYPN	protein_codin	0	0	0	0
ENSG000002	NHSL2	protein_codin	0.48375456	0	0	2.81691746
ENSG000001	OR5C1	protein_codin	0.20691815	0	0	0.88935395
ENSG000001	OR1L6	protein_codin	0	0	0	0
ENSG000001	C6orf89	protein_codin	0	0	0	0
ENSG000001	OR1L1	protein_codin	0	0	0	0
ENSG000001	OR1N2	protein_codin	0	0	0	0.9723232
ENSG000001	CXCR3	protein_codin	0	7.80243645	0	2.44020281
ENSG000001	CXCR3	protein_codin	0	0	0	0
ENSG000001	GCNA	protein_codin	0	13.7410432	0	4.68504165
ENSG000001	GCNA	protein_codin	3.92054919	0	10.4296956	0
ENSG000000	PTGS1	protein_codin	0	0	0	0
ENSG000001	PPIL1	protein_codin	2.09392509	4.39100051	19.2833592	1.22241831
ENSG000001	HERC4	protein_codin	17.2591704	22.0063419	73.8666498	0
ENSG000001	OGT	protein_codin	0	0	0	0
ENSG000001	PEF1	protein_codin	10.9999501	13.0186965	23.5242454	5.82106429
ENSG000001	HCRTR1	protein_codin	0	0	0	0
ENSG000001	HCRTR1	protein_codin	0	0	0	0
ENSG000001	SERINC2	protein_codin	0	0	2.00379533	0
ENSG000001	SERINC2	protein_codin	0	0	0	0
ENSG000001	CDKN1A	protein_codin	0	37.729349	3.51139506	0
ENSG000001	FABP3	protein_codin	3.71582782	1.76541592	0	0.47737567
ENSG000001	ZCCHC17	protein_codin	11.9405214	41.5861893	25.6295961	0
ENSG000001	SRSF3	protein_codin	11.0508914	41.9871948	82.737572	0
ENSG000001	OGT	protein_codin	65.6591412	0	41.1988431	20.5670062
ENSG000000	SNRNP40	protein_codin	5.30348361	0	11.6722164	1.68469742
ENSG000001	MRRF	protein_codin	0	0	1.01361662	0
ENSG000001	MRRF	protein_codin	0	0	1.64305171	0
ENSG000001	MRRF	protein_codin	1.93945618	19.3601228	11.6735615	0.97913743
ENSG000001	MRRF	protein_codin	3.97306016	9.06419109	9.54636981	0
ENSG000001	MRRF	protein_codin	5.21819816	15.7836241	18.1893951	3.77571096
ENSG000001	KCTD20	protein_codin	76.8489224	112.832416	155.474166	49.3671561
ENSG000001	DSN1	protein_codin	0	0	0	0
ENSG000001	CTNNA3	protein_codin	0	0	0	0.81447814
ENSG000000	NKAIN1	protein_codin	0	0	0	0
ENSG000000	ETV7	protein_codin	0.58869138	0	0	1.74449847
ENSG000000	ETV7	protein_codin	0	0	0	0
ENSG000001	DSN1	protein_codin	5.22225626	0	0	0
ENSG000001	PUM1	protein_codin	0	0	0	0
ENSG000001	PUM1	protein_codin	7.33156982	13.5213161	12.9710642	1.3426273
ENSG000001	PUM1	protein_codin	32.7416969	0	0	22.6075574
ENSG000001	DSN1	protein_codin	0	0	0	1.42699681
ENSG000001	LHX6	protein_codin	4.37690315	10.0093021	0	7.11841271
ENSG000001	LHX6	protein_codin	0	0	10.2844579	0

ENSG000001[illegible]	REEP3	protein_codin	38.8836396	44.4637141	40.3823719	30.3681967
ENSG000001[illegible]	MAPK13	protein_codin	0	9.17065065	0	0
ENSG000001	SLC4A3	protein_codin	0	13.752052	4.72383713	0
ENSG000001[illegible]	MORN5	protein_codin	0	0	0	0
ENSG000001[illegible]	MATN1	protein_codin	0.44138011	3.77456735	0	1.69394345
ENSG000001[illegible]	MAPK13	protein_codin	0	0	0	0
ENSG000001	NDUFA8	protein_codin	8.39886954	18.7963493	20.1004397	2.91060862
ENSG000001[illegible]	NDRG3	protein_codin	0	12.95801	0	0
ENSG000001[illegible]	TAF1	protein_codin	0	0	0	0
ENSG000001[illegible]	TTLL11	protein_codin	0	0	0	0
ENSG000001[illegible]	TTLL11	protein_codin	0	0	0	0
ENSG000000[illegible]	PTPRU	protein_codin	0	0	12.7769437	0
ENSG000001[illegible]	ADO	protein_codin	20.2757737	32.7200893	46.0114819	7.2201052
ENSG000001[illegible]	RTKN2	protein_codin	2.91997492	10.0105409	36.892148	1.47667583
ENSG000001[illegible]	TAF1	protein_codin	5.50357393	0	0	0
ENSG000001	MECR	protein_codin	2.63284268	14.152664	7.1143811	0
ENSG000002[illegible]	GGTA1	protein_codin	1.34652896	0	0	5.94318024
ENSG000001	SRSF4	protein_codin	61.6032055	257.771427	109.26845	62.6601712
ENSG000001[illegible]	EPB41	protein_codin	13.0435924	52.2264606	122.29779	0
ENSG000001[illegible]	EPB41	protein_codin	2.46460292	0	0	0
ENSG000001[illegible]	EPB41	protein_codin	5.44998714	16.0794928	29.4335872	2.5037237
ENSG000001[illegible]	NDRG3	protein_codin	0	0	0	0
ENSG000001[illegible]	GSN	protein_codin	17.5921014	0	11.4355928	3.53097633
ENSG000001[illegible]	GSN	protein_codin	2.92627078	2.11384284	1.70708834	4.15852937
ENSG000001[illegible]	GSN	protein_codin	76.225042	42.5549321	146.034863	41.7452212
ENSG000001[illegible]	CDK1	protein_codin	0	0	0	0
ENSG000001[illegible]	YTHDF2	protein_codin	52.3492989	0	131.585689	38.2162121
ENSG000001[illegible]	ANK3	protein_codin	0	0	0	0
ENSG000001[illegible]	GMEB1	protein_codin	12.0953534	41.4270362	41.2267298	12.8001448
ENSG000001[illegible]	GSN	protein_codin	381.438715	158.047434	0	110.892645
ENSG000001[illegible]	ANK3	protein_codin	0	0	5.74789022	5.86823599
ENSG000000[illegible]	SRPK1	protein_codin	0	0	0	0
ENSG000001[illegible]	GSN	protein_codin	6.10023733	8.8670439	14.2174652	1.12949692
ENSG000001[illegible]	TAF12	protein_codin	6.09235051	0	7.04342294	7.96834242
ENSG000000[illegible]	SRPK1	protein_codin	0	86.9232924	274.486658	12.9548077
ENSG000001[illegible]	RAB42	protein_codin	1.75280082	0	3.99248863	0
ENSG000001[illegible]	ANK3	protein_codin	13.2477479	69.947755	22.4691717	24.0148782
ENSG000001[illegible]	ITGB1BP2	protein_codin	0	5.08693835	3.13792266	2.58675052
ENSG000001[illegible]	TRNAU1AP	protein_codin	4.22345024	6.32415714	7.22849954	5.79159114
ENSG000001[illegible]	RCC1	protein_codin	0	0	0	0
ENSG000001[illegible]	RCC1	protein_codin	0	11.9341174	34.2372896	1.48390205
ENSG000001[illegible]	RCC1	protein_codin	0	0	10.3696295	0
ENSG000002[illegible]	PHACTR4	protein_codin	29.5829056	88.4204046	98.7143341	22.3260467
ENSG000002[illegible]	PHACTR4	protein_codin	8.38351794	45.9515522	81.7699317	20.2796274

ENSG000001	RAB14	protein_codin	2.28684305	19.4968502	3.99153488	3.26225652
ENSG000001	NONO	protein_codin	4.51407285	0	2.10854209	10.307602
ENSG000001	MED18	protein_codin	2.00612087	2.31221108	5.6366906	3.165397
ENSG000001	CNTRL	protein_codin	3.96857783	25.2835935	10.8628308	6.77065465
ENSG000001	CNTRL	protein_codin	15.9715624	79.3850445	0	29.2956477
ENSG000001	CNTRL	protein_codin	23.6476202	0	113.80021	3.18585167
ENSG000001	CNTRL	protein_codin	0	0	0	0
ENSG000001	RAB5IF	protein_codin	0	0	0	0
ENSG000001	CNTRL	protein_codin	0	0	0	0
ENSG000001	NONO	protein_codin	49.8949984	0	78.6204397	69.5885679
ENSG000001	PTAFR	protein_codin	2.17045004	23.9793034	0	3.16482468
ENSG000002	CLPSL1	protein_codin	0	0	0	0
ENSG000001	EYA3	protein_codin	0	0	75.5330433	0
ENSG000001	EYA3	protein_codin	0	0	0	0
ENSG000001	CNTRL	protein_codin	0	0	0	0
ENSG000001	ARMC12	protein_codin	0	0	0	0
ENSG000001	ARMC12	protein_codin	0.33990905	1.30499079	0	0.93017787
ENSG000001	EYA3	protein_codin	0	0	0	0
ENSG000001	TGIF2	protein_codin	0	20.6258127	28.282034	0
ENSG000001	OBSL1	protein_codin	19.0165816	51.7905425	128.552631	9.16984938
ENSG000001	TGIF2	protein_codin	0	0	0	0
ENSG000001	OBSL1	protein_codin	8.02698559	15.9998568	36.8709516	0
ENSG000001	PHYHIPL	protein_codin	0	0	0	0
ENSG000001	PHYHIPL	protein_codin	0	0	2.92375027	0
ENSG000001	TMEM198	protein_codin	2.39922273	0	1.25326312	1.8428961
ENSG000001	XKR8	protein_codin	0	0	0	0
ENSG000001	BICC1	protein_codin	138.599352	168.27041	197.479859	95.5483898
ENSG000000	TRAF1	protein_codin	7.07485968	120.951278	14.5545976	11.3299423
ENSG000001	SMPDL3B	protein_codin	0.92908591	1.99224494	0.53176932	0
ENSG000001	CHPF	protein_codin	0	0	0	0
ENSG000001	TULP1	protein_codin	0	0	0	0
ENSG000001	SMPDL3B	protein_codin	0	0	0	0
ENSG000001	TFAM	protein_codin	0	10.0617181	4.98041327	4.14536906
ENSG000001	PHF19	protein_codin	0	76.2939028	80.500173	0
ENSG000001	TFAM	protein_codin	0	0	1.15519517	1.96113307
ENSG000000	PSMD5	protein_codin	0	0	0	0
ENSG000000	PSMD5	protein_codin	2.73118373	22.138504	1.86523407	3.77497642
ENSG000000	DLGAP4	protein_codin	0.43736989	0	0.9675016	0
ENSG000001	GMPPA	protein_codin	0	0	0	0
ENSG000001	RPA2	protein_codin	0	0	60.1868742	0
ENSG000000	UBE2D1	protein_codin	0	7.88407922	10.5417265	10.0391665
ENSG000001	RPA2	protein_codin	27.5281118	46.4529131	45.0820232	13.6629878
ENSG000000	DLGAP4	protein_codin	0	0	0	0
ENSG000001	GMPPA	protein_codin	0	0	0	0

ENSG000000	PSMD5	protein_codin	0	0	0	0
ENSG000001	THEMIS2	protein_codin	8.6133033	155.46329	6.25716104	12.1545013
ENSG000001	THEMIS2	protein_codin	0	0	0	0
ENSG000001	THEMIS2	protein_codin	0	0	0	0.72814837
ENSG000001	MEGF9	protein_codin	22.4398834	42.3085017	25.4981837	18.2039262
ENSG000001	PPP1R8	protein_codin	0	0	0	0
ENSG000001	AAR2	protein_codin	0	0	0	0
ENSG000001	CDCA7L	protein_codin	6.13727609	0	1.34223363	0
ENSG000001	IPMK	protein_codin	11.2186981	10.3857314	12.650879	16.628529
ENSG000000	EPB41L1	protein_codin	0	0	0	0
ENSG000001	STX12	protein_codin	41.0278073	62.7673559	35.8435479	23.8471287
ENSG000001	ZWINT	protein_codin	0	0	0	0
ENSG000000	EPB41L1	protein_codin	0.42074303	3.51787017	2.06930946	0
ENSG000000	EPB41L1	protein_codin	0	0	0	0
ENSG000000	FAM76A	protein_codin	0	0	4.90185616	0
ENSG000000	EPB41L1	protein_codin	6.6531139	0	0	2.28397295
ENSG000000	ZNF76	protein_codin	1.19989744	31.9939517	9.5373038	0
ENSG000000	FAM76A	protein_codin	4.85962026	19.6214482	6.64356332	1.09694298
ENSG000001	PCDH15	protein_codin	0	0	0	0
ENSG000001	PCDH15	protein_codin	0	0	0	0
ENSG000001	PCDH15	protein_codin	0	0	0	0
ENSG000001	DES	protein_codin	289.139057	0	0	2.19855182
ENSG000001	DNPEP	protein_codin	0	0	0	0
ENSG000000	BRINP1	protein_codin	0	0.92830421	0	0
ENSG000001	PCDH15	protein_codin	0.53568091	1.30066783	0	4.54374373
ENSG000001	DNPEP	protein_codin	0	7.11850068	3.94546164	1.63557407
ENSG000001	MBL2	protein_codin	0	0	0	0
ENSG000001	DKK1	protein_codin	0.39536134	6.74845341	3.7290026	0
ENSG000001	DNPEP	protein_codin	0	0	0	0
ENSG000001	CNBD2	protein_codin	0	0	0	0
ENSG000001	TCP11	protein_codin	0	0	0	0.44723602
ENSG000001	PRKG1	protein_codin	2.15351748	0	0	0
ENSG000001	PRKG1	protein_codin	0	15.8112273	1.27269633	2.1020576
ENSG000001	ZMYM3	protein_codin	0	0	0	0
ENSG000001	TCP11	protein_codin	0	0	0	0
ENSG000001	PRKG1	protein_codin	13.6116026	0	45.4422381	0
ENSG000001	ZMYM3	protein_codin	0	18.4555712	0	0
ENSG000001	ZMYM3	protein_codin	0	0	0	0
ENSG000001	TRIM32	protein_codin	4.78161791	13.4319349	19.2853136	6.55852892
ENSG000001	ZMYM3	protein_codin	0	0	0	0
ENSG000001	ASTN2	protein_codin	0	0	0	0
ENSG000001	ZMYM3	protein_codin	0	0	0	0
ENSG000001	SCAND1	protein_codin	2.16739305	1.55063323	2.90585435	2.51424883
ENSG000001	A1CF	protein_codin	0	0	0	0

ENSG000001	A1CF	protein_codin	0	0	0	0
ENSG000001	A1CF	protein_codin	0	1.35566041	0	4.41586145
ENSG000001	ZMYM3	protein_codin	0	0	2.1512609	0
ENSG000000	PHF20	protein_codin	8.85534932	23.2545447	0	10.6614575
ENSG000001	A1CF	protein_codin	0	0	0	0
ENSG000000	FGR	protein_codin	0	0	0	2.32633733
ENSG000000	FGR	protein_codin	2.18096896	0	0	0
ENSG000000	FGR	protein_codin	0	12.4601354	0	0
ENSG000002	ASAH2B	protein_codin	0	0	0	0
ENSG000002	ASAH2B	protein_codin	0.71372388	0	1.02089407	1.97004876
ENSG000001	AHDC1	protein_codin	0	0	0	0
ENSG000000	PHF20	protein_codin	35.2955697	85.7807659	62.2279598	15.6414131
ENSG000001	SNRPC	protein_codin	0.48801046	5.16111528	6.34720941	2.64344494
ENSG000001	SNRPC	protein_codin	0.85871415	6.61932582	12.8840212	0
ENSG000001	ILRUN	protein_codin	2.55509779	23.8123859	0	0
ENSG000001	ILRUN	protein_codin	32.0469867	83.2985556	99.3701702	29.7639064
ENSG000001	GPR3	protein_codin	0.25858757	0	0.48610196	0.84931779
ENSG000001	CD164L2	protein_codin	0.36243517	0	0.59443261	3.2260715
ENSG000001	ILRUN	protein_codin	2.85894128	0	0	0
ENSG000001	CD164L2	protein_codin	0	0	0	0
ENSG000001	GJB1	protein_codin	0	0	0	0
ENSG000001	CD164L2	protein_codin	0	0	0	0
ENSG000001	SPDEF	protein_codin	0	0	0	0
ENSG000001	RBM39	protein_codin	54.5400949	63.3351793	189.866176	85.3950376
ENSG000001	MAP3K6	protein_codin	0	0	0	1.17247234
ENSG000001	PACSIN1	protein_codin	0	7.18862445	7.51603155	1.68216069
ENSG000001	TNFSF15	protein_codin	0	0	0	1.82740846
ENSG000001	TNFSF15	protein_codin	1.19481826	3.03479148	0.43337014	3.9278288
ENSG000001	TMEM268	protein_codin	2.12630423	0	0	0
ENSG000001	ATP6V1G1	protein_codin	41.1763101	112.018634	54.0866728	44.4129807
ENSG000001	NLGN3	protein_codin	0	0	0	0
ENSG000002	AGAP6	protein_codin	0	0	0	0
ENSG000000	WHRN	protein_codin	0.51188951	0	1.08046907	7.36520924
ENSG000001	ROMO1	protein_codin	0	0	0.85537522	0
ENSG000001	AKNA	protein_codin	0	8.5009717	0	0
ENSG000001	TMEM222	protein_codin	0	0	0	0
ENSG000001	ROMO1	protein_codin	2.85196196	11.3289518	14.6654086	3.62096149
ENSG000001	ROMO1	protein_codin	0.24199	2.17249264	2.17996947	0
ENSG000001	AKNA	protein_codin	0	0	0	0
ENSG000001	MED12	protein_codin	0	0	0	0
ENSG000000	SLC9A1	protein_codin	0	0	0	0
ENSG000002	NFS1	protein_codin	0	0	0	0
ENSG000000	SLC9A1	protein_codin	0	4.90204169	0.77127342	0.63936866
ENSG000001	AKNA	protein_codin	21.3356559	473.617683	0	22.4866064

ENSG000000!	SLC9A1	protein_codin	0.92049815	2.29324201	8.36917705	2.25629222
ENSG000002	NFS1	protein_codin	0	0	3.9402987	3.54884732
ENSG000001	AGAP5	protein_codin	0	0	0	0
ENSG000001	MED12	protein_codin	0	0	0	0
ENSG000001	OGDHL	protein_codin	0	0	0	0
ENSG000002	RBM12	protein_codin	15.4830196	70.3771612	0	0
ENSG000001	C10orf53	protein_codin	0	0	0	2.19548057
ENSG000001	C10orf53	protein_codin	0.26590388	1.70765643	0	0.43965316
ENSG000001	C10orf53	protein_codin	0	0	0	0
ENSG000002	RBM12	protein_codin	25.9941131	38.728252	79.7994104	18.4644044
ENSG000001	SLC18A3	protein_codin	0.26676741	1.03660501	0	0
ENSG000001	HMGA1	protein_codin	0	0	0	1.08351385
ENSG000001	ZNF618	protein_codin	0	0	8.46333249	9.50400028
ENSG000001	ZNF618	protein_codin	0	9.10338034	13.0630967	10.4091282
ENSG000001	GPN2	protein_codin	1.74785226	0	3.17478382	1.79793355
ENSG000001	RGS3	protein_codin	0	0	0	0
ENSG000001	GPN2	protein_codin	7.03820994	18.4569657	13.0651052	8.88801502
ENSG000001	RGS3	protein_codin	1.2548636	17.3903694	0	4.09245289
ENSG000001	DRGX	protein_codin	0.23811185	0	0	0.41214927
ENSG000001	RGS3	protein_codin	5.64960928	3.24143526	12.859858	4.58513402
ENSG000002	ZDHHC18	protein_codin	1.93420101	0	0	0
ENSG000002	ZDHHC18	protein_codin	3.09411304	17.5387579	14.180967	6.29505527
ENSG000001	C10orf71	protein_codin	31.1568161	0	0.47634722	3.11384728
ENSG000000	PIGV	protein_codin	0	0	0	0
ENSG000002	TMEM273	protein_codin	1.56954479	7.92150945	0	1.41421401
ENSG000002	TMEM273	protein_codin	0.69441734	13.4691359	0	0
ENSG000001	ARID1A	protein_codin	5.92065298	43.4692486	37.3285958	0
ENSG000002	TMEM273	protein_codin	0	0	0	0
ENSG000001	RUFY4	protein_codin	2.87520308	36.061567	0	3.7225509
ENSG000002	TMEM273	protein_codin	0.49136142	0	0	2.87996856
ENSG000001	LRRC18	protein_codin	1.71621784	2.54684659	0	1.28643116
ENSG000001	WDFY4	protein_codin	0	0	0	0
ENSG000001	RPS6KA1	protein_codin	0	0	0	0
ENSG000001	C9orf43	protein_codin	0.85544767	2.65449007	1.29593248	1.77532374
ENSG000001	RPS6KA1	protein_codin	0	0	0	5.1215888
ENSG000001	RPS6KA1	protein_codin	0	0	31.8673402	0
ENSG000001	POLE3	protein_codin	48.0337489	307.23027	332.352317	35.3765455
ENSG000001	ARHGAP22	protein_codin	0	0	0	0
ENSG000001	POLE3	protein_codin	0	0	36.5392806	0
ENSG000001	ARHGAP22	protein_codin	0.58634409	8.35196638	0	6.86236379
ENSG000001	MAPK8	protein_codin	0	0	8.42251133	0
ENSG000001	MAPK8	protein_codin	0	0	0	0
ENSG000001	GRM4	protein_codin	0	0	0	0
ENSG000001	MAPK8	protein_codin	10.4326125	0	0	7.29087477

ENSG000001	HDHD3	protein_codin	0.49951038	2.407668	2.36734984	0
ENSG000001	GRM4	protein_codin	0	0	0	0
ENSG000001	MAPK8	protein_codin	0	0	0	0
ENSG000001	BSPRY	protein_codin	0.75416987	0	0	0
ENSG000001	DHDDS	protein_codin	1.24408818	0	1.54761581	1.17892915
ENSG000001	DHDDS	protein_codin	0	0	0	1.47972614
ENSG000001	MAPK8	protein_codin	0	0	5.1868045	0
ENSG000001	WDR31	protein_codin	2.09478403	0	0	0
ENSG000001	PRPF4	protein_codin	19.0039138	69.4996136	66.1122789	10.0129444
ENSG000001	PRPF4	protein_codin	0.56287111	7.81994976	13.5303155	0.91060243
ENSG000001	FRMPD2	protein_codin	0	0	0	0
ENSG000001	IL2RG	protein_codin	2.17383257	41.6375205	0	0
ENSG000001	CDC26	protein_codin	14.4541759	22.8403115	32.5234732	4.98841571
ENSG000001	CRYBG2	protein_codin	0	1.78275134	0	0
ENSG000001	CRYBG2	protein_codin	0	0	0	0
ENSG000001	CRYBG2	protein_codin	0	0	0	0
ENSG000001	SLC31A1	protein_codin	13.0430924	44.3888611	28.5184942	16.5429429
ENSG000001	CD52	protein_codin	2.42080632	21.3532852	0	2.68661034
ENSG000001	UQCC2	protein_codin	2.12682915	15.2949248	9.62941879	1.65223178
ENSG000001	UBXN11	protein_codin	0	0	0	0
ENSG000001	UBXN11	protein_codin	0	0	0	1.10278017
ENSG000002	PTPN20	protein_codin	1.23201809	0	3.78365251	0
ENSG000001	SLC31A2	protein_codin	0	0	0	0
ENSG000001	UBXN11	protein_codin	0	0	0	0
ENSG000001	UBXN11	protein_codin	0	17.8610331	0	0
ENSG000001	UBXN11	protein_codin	0	0	0	0
ENSG000001	ZFP37	protein_codin	2.30048014	11.7404038	4.50170721	2.18799963
ENSG000001	SLC46A2	protein_codin	0	0	0	2.35607706
ENSG000001	UQCC2	protein_codin	29.4494639	19.6646327	131.112946	3.42215082
ENSG000001	SNX30	protein_codin	36.8377699	124.895954	57.3785853	46.4322214
ENSG000001	INIP	protein_codin	0	0	0	0
ENSG000001	INIP	protein_codin	0	0	0	0
ENSG000001	INIP	protein_codin	14.7138424	45.5382063	50.7355922	9.07125436
ENSG000001	KIAA1958	protein_codin	3.43249979	5.60692452	23.5040977	3.25028578
ENSG000002	CXorf65	protein_codin	0	0	0	0
ENSG000001	CNKSR1	protein_codin	0	0	0	0
ENSG000001	PTBP3	protein_codin	0.41857068	0	0	0
ENSG000001	PTBP3	protein_codin	51.8136879	206.870139	76.135218	62.0549295
ENSG000001	FOXO4	protein_codin	7.03200362	19.2852459	12.8103465	9.88820443
ENSG000001	SUSD1	protein_codin	0	152.315909	0	0
ENSG000001	SUSD1	protein_codin	0	0	0	0
ENSG000001	ZNF593	protein_codin	8.06009274	5.42725098	32.3237506	0
ENSG000001	FAM110D	protein_codin	0	3.61520222	0.48433625	5.12411629
ENSG000001	PDIK1L	protein_codin	5.39493742	0	30.3891192	4.35326945

ENSG000001	SUSD1	protein_codin	20.3066564	0	22.8321116	24.6981106
ENSG000001	PDIK1L	protein_codin	1.39886013	0	0	0
ENSG000001	TRIM63	protein_codin	33.7685317	2.2752935	0	3.77718032
ENSG000000	SPAG4	protein_codin	0	0	0	0
ENSG000001	SNX12	protein_codin	10.49082	20.6723586	0	0
ENSG000001	SLC30A2	protein_codin	0	0	0	0
ENSG000001	SLC30A2	protein_codin	1.07395646	6.08924349	0	1.24511486
ENSG000001	UGCG	protein_codin	10.4449953	48.2872839	13.0921812	59.0680087
ENSG000001	EXTL1	protein_codin	1.92407662	10.4257357	4.69934807	2.66184519
ENSG000000	ADD1	protein_codin	0	0	0	0
ENSG000001	PAFAH2	protein_codin	13.0887109	4.3748172	17.7537274	6.77927111
ENSG000001	SHOC1	protein_codin	0	0	0	0
ENSG000001	PAFAH2	protein_codin	0	10.2044528	0	0
ENSG000001	SHOC1	protein_codin	0	0	0	0
ENSG000001	STMN1	protein_codin	167.708036	126.669419	2146.49878	38.0329874
ENSG000002	GNG10	protein_codin	29.6799717	24.1992689	32.4249303	16.7205982
ENSG000002	DNAJC25-GN	protein_codin	2.44068502	8.79937241	20.6458655	4.51323417
ENSG000001	PAQR7	protein_codin	0	0	1.76813557	0
ENSG000001	AUNIP	protein_codin	1.51301171	0	20.0749536	0
ENSG000001	SLC7A3	protein_codin	0	0	0	1.28143812
ENSG000001	MTFR1L	protein_codin	4.66855075	23.4835225	2.32588901	0
ENSG000001	MTFR1L	protein_codin	0	0	0	0
ENSG000001	MTFR1L	protein_codin	7.60842872	0	0	3.57343172
ENSG000001	MTFR1L	protein_codin	0	0	0	0
ENSG000001	PTGR1	protein_codin	0.8735691	5.3105242	2.91169342	1.38238404
ENSG000002	NPY4R	protein_codin	0	0	0	4.02398653
ENSG000001	PTGR1	protein_codin	0	0	0	0
ENSG000002	GPRIN2	protein_codin	0	0	0	0
ENSG000001	SELENON	protein_codin	56.2455931	80.2093829	93.0515658	38.286969
ENSG000000	ITPR3	protein_codin	9.44228519	39.2614251	0	0
ENSG000002	GPRIN2	protein_codin	0	2.43254471	0	3.99081698
ENSG000000	IKZF2	protein_codin	0	22.58053	12.5557209	0
ENSG000001	TEX11	protein_codin	0.23301316	0	0	0.46617076
ENSG000002	SYT15	protein_codin	0	8.89880689	7.94106634	3.77827491
ENSG000002	SYT15	protein_codin	0	0	0	0
ENSG000001	PTGR1	protein_codin	0.42198002	1.63065543	0.66029506	1.77532217
ENSG000000	IKZF2	protein_codin	1.7608095	0	0	0
ENSG000002	SYT15	protein_codin	0	0	0	0
ENSG000001	MAN1C1	protein_codin	0	0	0	4.92359485
ENSG000001	MAN1C1	protein_codin	2.30109343	2.75052325	0	1.98887278
ENSG000001	TEX11	protein_codin	0	0	0	0
ENSG000001	LDLRAP1	protein_codin	8.3402452	11.5062839	22.014887	3.70755868
ENSG000002	PTPN20	protein_codin	0	0	22.396393	0
ENSG000002	PTPN20	protein_codin	0	0	1.64381742	1.41799837

ENSG000002	MACO1	protein_codin	15.7631595	26.490834	56.3241513	23.2502564
ENSG000001	C20orf173	protein_codin	0	0	0	0
ENSG000002	PTPN20	protein_codin	0	0	0	0
ENSG000000	DLG3	protein_codin	8.0967742	19.8550977	9.57153061	4.1007729
ENSG000001	TMEM50A	protein_codin	11.6699379	76.0439491	13.6307508	22.5771459
ENSG000001	WASHC2C	protein_codin	1.34040052	0	0	0
ENSG000000	DLG3	protein_codin	0	0	0	0
ENSG000001	WASHC2C	protein_codin	36.3200918	0	0	0
ENSG000001	ZFAND4	protein_codin	1.86743534	0	0	0
ENSG000001	GDF5	protein_codin	0.78525494	0	0	1.20266891
ENSG000001	ZFAND4	protein_codin	4.51586876	15.0786187	19.9324498	4.19993463
ENSG000001	ZFAND4	protein_codin	0	0	0	0
ENSG000001	GDF5	protein_codin	0	0	0	0
ENSG000001	ZNF483	protein_codin	0	1.07053126	0	1.53407236
ENSG000001	CLIC4	protein_codin	302.872282	458.117871	349.045411	175.069599
ENSG000001	UQCC1	protein_codin	0	0	0	0
ENSG000001	GDPD2	protein_codin	0	0	0	0
ENSG000001	ECPAS	protein_codin	0	0	0	0
ENSG000001	UQCC1	protein_codin	0	0	0	0
ENSG000001	UQCC1	protein_codin	0	0	13.8934891	4.06432635
ENSG000001	SRRM1	protein_codin	35.3344471	0	0	55.1667028
ENSG000000	ALOX5	protein_codin	6.22855921	0	1.49402825	7.44293484
ENSG000001	NCMAP	protein_codin	0	0	6.06249156	2.87100022
ENSG000001	RCAN3	protein_codin	3.19703347	31.1221823	2.0541608	0
ENSG000001	UQCC1	protein_codin	0	0	0	0
ENSG000001	RCAN3	protein_codin	20.6892288	21.6239721	68.7364837	13.6177462
ENSG000000	NIPAL3	protein_codin	16.6469793	54.0993728	53.332947	10.9968079
ENSG000002	OR13A1	protein_codin	0	0	0	0
ENSG000000	KIF4A	protein_codin	11.7553957	25.8823146	265.557018	5.08250657
ENSG000001	FAM83C	protein_codin	0	1.94698005	0	1.22472112
ENSG000000	STPG1	protein_codin	0	0	0	0
ENSG000001	MDH1B	protein_codin	0	0	0	0
ENSG000001	ADAM23	protein_codin	20.0631231	7.12860957	305.763407	0
ENSG000001	IFNLR1	protein_codin	0	0	0	0
ENSG000001	IFNLR1	protein_codin	0	0	0	0
ENSG000001	IFNLR1	protein_codin	0	0	0	0
ENSG000002	ZDBF2	protein_codin	0	0	0	0
ENSG000001	CXCL12	protein_codin	3.32970327	100.968532	1.02588524	17.598308
ENSG000001	OR2K2	protein_codin	0	0	0	0
ENSG000001	CXCL12	protein_codin	16.3589178	396.696279	14.0945993	253.253691
ENSG000001	LPAR1	protein_codin	0	0	0	0
ENSG000001	LPAR1	protein_codin	48.6792909	75.4423363	54.3951988	24.6047968
ENSG000001	ZNF32	protein_codin	5.24388493	8.53892401	72.716116	6.19107321
ENSG000001	MYOM3	protein_codin	12.3698401	4.91838569	1.83920702	1.20838083

ENSG000001!	ZNF485	protein_codin	0	0	0	0
ENSG000002	EIF6	protein_codin	0	0	13.8732261	0
ENSG000000:	MUSK	protein_codin	0	0	0	0
ENSG000000:	MUSK	protein_codin	0	0	0	0
ENSG000002	EIF6	protein_codin	0	0	0	0
ENSG000001!	ZNF239	protein_codin	0	0	13.1065423	0
ENSG000000:	MUSK	protein_codin	0.9664637	0	0.56119776	0
ENSG000002	EIF6	protein_codin	7.59172355	14.5722365	13.6349516	0.84979261
ENSG000001	SRSF10	protein_codin	0	0	0	0
ENSG000001	SRSF10	protein_codin	11.1579133	9.18528927	75.3640736	0
ENSG000001:	PDZD11	protein_codin	0	2.25007368	0	0
ENSG000001!	RASGEF1A	protein_codin	0	0	0	0
ENSG000001!	RASGEF1A	protein_codin	0.59674804	3.66791879	2.82814776	1.25037515
ENSG000001	SVEP1	protein_codin	1.96575028	23.1989316	4.42570023	0.93201034
ENSG000001	CSGALNACT2	protein_codin	65.7409979	190.421212	120.172992	89.1677707
ENSG000000:	BAK1	protein_codin	2.51419798	8.58108224	9.10895897	3.28860037
ENSG000001	PNRC2	protein_codin	9.34884629	29.9327791	0	3.37880488
ENSG000001	SVEP1	protein_codin	92.794316	280.683113	70.0265413	114.888239
ENSG000001	CNR2	protein_codin	0.28420811	2.49568263	0.50651198	0.82013571
ENSG000001	RAB41	protein_codin	0	0	0	0
ENSG000001	FUCA1	protein_codin	16.2734789	95.1298508	27.0892603	23.9956019
ENSG000001	CD28	protein_codin	1.91131943	8.62737687	0	12.5555475
ENSG000001	CUTA	protein_codin	0	0	0	0
ENSG000001	HMGCL	protein_codin	0	0	1.36546989	0
ENSG000001	HMGCL	protein_codin	3.78558774	13.1591022	11.1707755	4.16561771
ENSG000000	EDEM2	protein_codin	5.4526748	0	7.16061843	0
ENSG000000	EDEM2	protein_codin	0	16.3030227	0	5.75870855
ENSG000001	RAPH1	protein_codin	0	0	0	0
ENSG000001:	ARR3	protein_codin	0	0	0	0
ENSG000001	CUTA	protein_codin	0	0	0	0
ENSG000001	GALE	protein_codin	0	0	0	0
ENSG000001	CUTA	protein_codin	2.63800724	0	1.89111965	0
ENSG000000	LYPLA2	protein_codin	0	0	0	0
ENSG000000	LYPLA2	protein_codin	0	0	0	3.31627118
ENSG000000	LYPLA2	protein_codin	0	1.3043721	0	0.69822317
ENSG000000	LYPLA2	protein_codin	2.32618961	0	0	0
ENSG000002	TXNDC8	protein_codin	0	0	0	0.50606162
ENSG000002	TXNDC8	protein_codin	0	0	0	0
ENSG000002	TXNDC8	protein_codin	0	0	0	0
ENSG000001	PHF1	protein_codin	1.40639499	9.32370833	0	0
ENSG000000	LYPLA2	protein_codin	1.93448401	11.6949078	17.8364337	0
ENSG000001:	TXN	protein_codin	0	0	0	0
ENSG000001	PHF1	protein_codin	0	0	6.08037794	13.8667046
ENSG000001:	TXN	protein_codin	10.1245584	49.337716	62.5599111	17.1821192

ENSG000001(	BMS1	protein_codin	156.89292	410.018827	685.938476	68.0002591
ENSG000001	P2RY4	protein_codin	0.59426906	4.16006084	1.12885627	0
ENSG000002	AWAT1	protein_codin	0	0	0	0
ENSG000000	PITHD1	protein_codin	0	0	0	0.69522913
ENSG000001	PALM2AKAP2	protein_codin	116.516186	370.508437	147.432407	249.171274
ENSG000001	PALM2AKAP2	protein_codin	6.744185	0	0	0
ENSG000001	PALM2AKAP2	protein_codin	0	0	0	0
ENSG000000	PTPN3	protein_codin	4.32592652	27.7228885	32.8499974	0
ENSG000002	DAXX	protein_codin	11.226585	0	37.6161343	7.34259615
ENSG000001	EDA	protein_codin	0	0	0	0
ENSG000001	RPL11	protein_codin	109.053412	0	425.250518	67.9513972
ENSG000001	EDA	protein_codin	0	0	0	4.87830577
ENSG000001	EDA	protein_codin	0	0	10.7962785	0
ENSG000000	EPB41L4B	protein_codin	4.30780191	3.62079882	27.5951711	3.42838646
ENSG000001	ID3	protein_codin	9.61466399	19.6014851	33.0015556	23.2969307
ENSG000000	EPB41L4B	protein_codin	0.35195883	1.94302304	1.35572456	4.4285196
ENSG000001	PJA1	protein_codin	23.1605552	49.7065726	49.0523365	17.2226256
ENSG000002	BMPR2	protein_codin	0	0	1.221151	0
ENSG000002	BMPR2	protein_codin	202.737918	358.923144	211.936062	168.858939
ENSG000001	PJA1	protein_codin	0	0	0	0
ENSG000001	PJA1	protein_codin	0	0	1.08132167	0
ENSG000001	TMEM245	protein_codin	15.344452	102.034985	62.7090601	27.5041552
ENSG000001	CTNNAL1	protein_codin	1.27941024	0	0	0
ENSG000001	CTNNAL1	protein_codin	0	0	0	0
ENSG000001	CTNNAL1	protein_codin	2.14517552	38.5995798	42.7168001	0
ENSG000001	STARD8	protein_codin	5.20375793	40.7004797	13.7951529	11.4521163
ENSG000001	STARD8	protein_codin	8.93898192	0	6.67766264	6.46308821
ENSG000002	TCEA3	protein_codin	1.09078279	0	0	0
ENSG000002	TCEA3	protein_codin	0	0	0	0
ENSG000002	PFDN6	protein_codin	0	0	4.94829774	1.13980251
ENSG000002	PFDN6	protein_codin	6.3473566	0	17.0571507	3.20323383
ENSG000001	ZNF436	protein_codin	13.9081685	20.9280158	41.7710753	0
ENSG000002	PFDN6	protein_codin	0	16.6447105	11.1379625	0
ENSG000001	NARF	protein_codin	0	17.5374073	2.52055774	1.35995579
ENSG000001	HNRNPR	protein_codin	120.409578	312.619335	347.702676	85.2617941
ENSG000001	HNRNPR	protein_codin	51.8383301	0	195.201558	34.5845939
ENSG000002	WDR46	protein_codin	36.1772974	114.24761	111.328626	7.53207202
ENSG000001	ZNF33A	protein_codin	12.5324091	0	25.4098849	6.68213388
ENSG000001	HTR1D	protein_codin	0.28540082	1.32246214	0	1.73072281
ENSG000001	YIPF6	protein_codin	0	0	0	0
ENSG000001	ABITRAM	protein_codin	0	0	0	0
ENSG000001	EPHB2	protein_codin	0	0	12.6650826	0
ENSG000001	EPHB2	protein_codin	0	22.8827712	10.3804882	7.12286521
ENSG000001	EPHB2	protein_codin	23.2714268	8.82768139	83.4237478	0

ENSG000001[illegible]	ZNF25	protein_codin	16.9818459	0	0	0
ENSG000001[illegible]	C1QC	protein_codin	0	118.824595	12.4008117	0
ENSG000001[illegible]	C1QC	protein_codin	0	0	0	0
ENSG000001[illegible]	C1QC	protein_codin	25.9582344	252.036873	0	48.12996
ENSG000000[illegible]	ZNF510	protein_codin	0	0	0	0
ENSG000001[illegible]	C1QA	protein_codin	6.23547023	127.429184	12.5201105	25.6743532
ENSG000001[illegible]	YIPF6	protein_codin	0	0	5.10576219	0
ENSG000000[illegible]	EPHA8	protein_codin	0	0	0	2.46363848
ENSG000000[illegible]	ELP1	protein_codin	0	0	0	0
ENSG000001[illegible]	ZNF248	protein_codin	0	0	0	0
ENSG000000[illegible]	CASP10	protein_codin	0	3.65696929	0	2.46745429
ENSG000001[illegible]	ZBTB40	protein_codin	0	0	0	0
ENSG000002[illegible]	RING1	protein_codin	16.971975	38.7875646	77.705	8.13422736
ENSG000001[illegible]	ANKRD30A	protein_codin	0	0	0	0
ENSG000002[illegible]	HSD17B8	protein_codin	0	2.80671826	4.75009396	0
ENSG000001[illegible]	CELA3A	protein_codin	0	0	0	0
ENSG000002	CELA3B	protein_codin	0	0	0	0
ENSG000001[illegible]	ACTL7B	protein_codin	0.28975962	0	0	0.8757823
ENSG000001[illegible]	KLF4	protein_codin	8.25661347	0	15.6593777	163.934274
ENSG000001[illegible]	HSPG2	protein_codin	0	16.2235058	0	0
ENSG000001	SLC39A7	protein_codin	13.9843226	0	0	23.942424
ENSG000001[illegible]	HSPG2	protein_codin	0	0	0	0
ENSG000001	SLC39A7	protein_codin	16.2909343	0	64.7008914	11.5783866
ENSG000002[illegible]	RXRB	protein_codin	2.56176495	0	14.4654261	3.42078898
ENSG000002[illegible]	RXRB	protein_codin	4.17715188	42.7880614	14.3168549	0
ENSG000001[illegible]	ZNF462	protein_codin	0	13.8504076	0	5.35950298
ENSG000000[illegible]	TMEM38B	protein_codin	2.11021751	0	1.1452406	3.68082658
ENSG000001[illegible]	AR	protein_codin	55.8637389	29.7766117	25.6795456	6.7170196
ENSG000000[illegible]	TMEM38B	protein_codin	3.68574389	15.4363891	19.7070807	3.49621515
ENSG000001[illegible]	FZD8	protein_codin	14.0285101	149.574397	34.9202012	26.8603035
ENSG000001[illegible]	HSPG2	protein_codin	417.61472	1667.71359	764.376273	660.354611
ENSG000001[illegible]	AOX1	protein_codin	11.6556111	0	2.68451706	1.97029963
ENSG000001[illegible]	CCNY	protein_codin	25.2545493	7.60308953	47.8823731	20.6714531
ENSG000001[illegible]	FKTN	protein_codin	0	0	0	0
ENSG000001[illegible]	CCNY	protein_codin	0	0	4.03565162	9.66411992
ENSG000001[illegible]	FSD1L	protein_codin	4.41202156	0	12.2197606	0
ENSG000002[illegible]	COL11A2	protein_codin	0	147.033453	251.352534	0
ENSG000000[illegible]	CREM	protein_codin	0	0	0.86799054	0
ENSG000001[illegible]	EDA2R	protein_codin	0	0	0	0
ENSG000000[illegible]	SLC44A1	protein_codin	113.116792	136.028857	100.82266	50.8779951
ENSG000000[illegible]	CREM	protein_codin	0	0	0	0
ENSG000000[illegible]	SLC44A1	protein_codin	0	0	0	0
ENSG000000[illegible]	SLC44A1	protein_codin	5.07812564	13.4266699	10.6097182	2.12653915
ENSG000000[illegible]	CREM	protein_codin	2.30950372	0	15.2145183	0

ENSG000000	USP48	protein_codin	16.6489248	52.6089965	35.8732106	9.30147944
ENSG000000	USP48	protein_codin	23.7378251	33.9681025	67.1846169	14.3850008
ENSG000001	ABCA1	protein_codin	0	0	0.91809733	4.64874544
ENSG000000	CREM	protein_codin	0	0	6.60573967	0
ENSG000001	ABCA1	protein_codin	70.7630714	302.733313	132.310013	88.7628469
ENSG000001	VSIG4	protein_codin	0	19.4613689	9.32118763	0
ENSG000001	PGAP1	protein_codin	0	4.91352097	0	0
ENSG000002	OXLD1	protein_codin	2.51462477	0	17.8726174	0
ENSG000001	CUL2	protein_codin	0	0	0	1.79996758
ENSG000001	NPLOC4	protein_codin	3.99270829	29.314136	8.80592287	6.89232256
ENSG000001	CUL2	protein_codin	0	0	0	0
ENSG000001	CUL2	protein_codin	0	0	0	0
ENSG000001	CUL2	protein_codin	0	8.64590029	2.97449931	0
ENSG000001	ACAD8	protein_codin	0	0	0	0
ENSG000001	CUL2	protein_codin	0	0	7.72443674	0
ENSG000000	RAP1GAP	protein_codin	1.50162974	0	2.33298	0
ENSG000001	SLC38A10	protein_codin	43.0425685	70.9748065	55.1759851	19.9635009
ENSG000000	RAP1GAP	protein_codin	0	5.44771625	22.8872442	0
ENSG000001	NIPSNAP3B	protein_codin	6.15715214	7.15502293	11.6752024	8.48349589
ENSG000000	RAP1GAP	protein_codin	0	0	1.19706446	0
ENSG000000	RAP1GAP	protein_codin	0	0	0	0
ENSG000001	NIPSNAP3A	protein_codin	6.66694214	23.5668864	19.4521864	6.09583463
ENSG000001	PARD3	protein_codin	1.61881373	0	0	3.0205619
ENSG000001	PARD3	protein_codin	38.9585517	0	74.7716963	0
ENSG000001	PARD3	protein_codin	0	66.4809695	65.659488	0
ENSG000001	OPCML	protein_codin	0	0	0	0
ENSG000002	OR13C5	protein_codin	0	0	0	0
ENSG000001	CEP131	protein_codin	0	0	0	0
ENSG000001	NTM	protein_codin	1.36998677	5.28031915	0	0
ENSG000001	NTM	protein_codin	0.42474076	0	0	0
ENSG000001	SMC2	protein_codin	66.2969338	62.9772755	584.526716	26.6137604
ENSG000001	PARD3	protein_codin	0	0	0	0
ENSG000001	PARD3	protein_codin	0	0	0	0
ENSG000001	PARD3	protein_codin	0	0	0	0
ENSG000001	NTM	protein_codin	2.36356086	0	0	3.18565119
ENSG000001	AATK	protein_codin	0	5.69812079	0	0
ENSG000001	SMC2	protein_codin	17.3826729	67.1313585	181.134628	2.7703176
ENSG000001	PARD3	protein_codin	21.1173687	20.5912892	0	10.0493492
ENSG000001	NCOA6	protein_codin	5.02285877	0	27.2044058	16.2715379
ENSG000001	CYLC2	protein_codin	0	0	0	0
ENSG000000	LAS1L	protein_codin	10.5709799	0	0	0
ENSG000001	PPP3R2	protein_codin	0	7.06204	0	2.19861279
ENSG000000	LAS1L	protein_codin	0	0	0	0
ENSG000000	TP53INP2	protein_codin	17.5163325	0	10.2545496	26.1335309

ENSG0000007	TP53INP2	protein_codin	0	31.8701058	8.56777567	0
ENSG0000000	LAS1L	protein_codin	0	0	0	0
ENSG0000002	HLA-DOA	protein_codin	0	0	0	0
ENSG0000009	NRP1	protein_codin	2.07745954	33.2021944	5.03302252	2.54354886
ENSG0000001	RNF20	protein_codin	0	0	0	0
ENSG0000001	PIGU	protein_codin	0	0	0	2.38216978
ENSG0000009	NRP1	protein_codin	0	0	0	0
ENSG0000009	NRP1	protein_codin	1.03513736	14.3809077	1.66485963	0
ENSG0000009	NRP1	protein_codin	2.70667544	15.7449767	3.43661411	8.34559753
ENSG0000002	BRD2	protein_codin	0.78475383	12.0564964	2.72558609	1.1585395
ENSG0000000	PMS1	protein_codin	0	0	2.66508535	0
ENSG0000001	ALPL	protein_codin	0	1.46806785	0	0.96478148
ENSG0000001	ALPL	protein_codin	0	0	1.57120145	0
ENSG0000002	BRD2	protein_codin	0	15.9844864	0	0
ENSG0000001	ALPL	protein_codin	77.5824593	0	352.148833	29.3661878
ENSG0000001	MAP1LC3A	protein_codin	0	0	0	0
ENSG0000001	ZC4H2	protein_codin	0	0	0	1.21554705
ENSG0000001	ALPL	protein_codin	33.1738914	316.911728	180.73805	44.65121
ENSG0000002	HLA-DMA	protein_codin	0	9.04962304	1.87305763	6.71746706
ENSG0000001	DYNLRB1	protein_codin	0	0	0	0
ENSG0000001	PGAP4	protein_codin	1.00097024	0	19.8089803	0
ENSG0000001	PGAP4	protein_codin	0	16.5146225	0	2.97632275
ENSG0000001	PGAP4	protein_codin	0	0	0	0
ENSG0000001	MTMR8	protein_codin	0	0	0	1.48261724
ENSG0000002	PSMB9	protein_codin	0	18.3350759	2.02263568	3.17442499
ENSG0000001	ZNF189	protein_codin	9.33351621	79.2555168	33.6811325	15.6726408
ENSG0000007	ITCH	protein_codin	1.64160889	12.2803633	2.26124761	0
ENSG0000001	MRPL50	protein_codin	32.5200523	56.3943508	127.323116	10.657019
ENSG0000002	COL5A2	protein_codin	850.248598	2393.41524	1054.0204	835.330732
ENSG0000009	NRP1	protein_codin	158.385918	397.352408	163.763492	264.113414
ENSG0000001	AMER1	protein_codin	14.4802201	58.6198476	38.7507638	11.3740016
ENSG0000001	ARHGEF9	protein_codin	0	0	0	0
ENSG0000001	ARHGEF9	protein_codin	3.32619152	11.5440266	15.5611471	0
ENSG0000001	PLPPR1	protein_codin	0	0	0	0
ENSG0000009	NRP1	protein_codin	18.9260454	32.3472217	41.1187191	8.73981143
ENSG0000001	CCDC40	protein_codin	0.62472858	0	1.0074599	0
ENSG0000001	CCDC40	protein_codin	3.12929115	23.9494983	25.6838787	0
ENSG0000001	ARHGEF9	protein_codin	0	0	0	2.73964815
ENSG0000002	TMEFF1	protein_codin	0	6.03672646	20.8401022	0
ENSG0000002	PSMB8	protein_codin	0.70944219	0	0	0
ENSG0000002	PSMB8	protein_codin	4.8651697	0	0	6.34756513
ENSG0000001	SPIN4	protein_codin	8.3379136	30.8999795	49.7362737	2.31842229
ENSG0000000	MSANTD3	protein_codin	0	0	0	0
ENSG0000000	MSANTD3	protein_codin	0	0	0	0

ENSG000001[illegible]	ZXDB	protein_codin	5.1031479	24.4323641	12.1784603	8.37781494
ENSG000001	ECE1	protein_codin	0	0	0	0
ENSG000002[illegible]	TAP2	protein_codin	0	156.748291	0	12.0511783
ENSG000001[illegible]	FAAH2	protein_codin	0.3046817	7.72667636	1.01843157	0.87229102
ENSG000001[illegible]	TEX10	protein_codin	6.27992867	28.6880347	23.0774267	12.0298569
ENSG000001[illegible]	SPIN2A	protein_codin	0	0	0	0.97245095
ENSG000001[illegible]	ITGAV	protein_codin	10.622266	0	4.66732198	0
ENSG000001[illegible]	SPIN2A	protein_codin	1.45013107	6.76214145	17.2215345	7.59120026
ENSG000001[illegible]	SPIN2B	protein_codin	0	0	0	0.91438251
ENSG000002[illegible]	SPIN3	protein_codin	0	0	0	6.71346881
ENSG000001	INVS	protein_codin	3.49005818	16.1363497	20.0311648	2.10260195
ENSG000002[illegible]	NBDY	protein_codin	6.99769849	16.1269122	38.9956247	6.37931181
ENSG000001[illegible]	KLF8	protein_codin	0	0	0	0
ENSG000001[illegible]	NUP35	protein_codin	6.05481993	0	0	0
ENSG000000[illegible]	EIF4G3	protein_codin	2.73763679	0	8.6052729	2.25361382
ENSG000000[illegible]	EIF4G3	protein_codin	3.32370946	5.56583537	18.6509399	0
ENSG000002[illegible]	HLA-DQA2	protein_codin	3.46259055	234.989595	0	13.5220654
ENSG000000[illegible]	RRAGB	protein_codin	11.230306	34.0393743	0	0
ENSG000001[illegible]	HLA-DQB1	protein_codin	24.7925288	0	0	0
ENSG000000[illegible]	BIRC5	protein_codin	0	0	4.14229784	2.86961025
ENSG000001[illegible]	HLA-DQA1	protein_codin	0	0	0	0
ENSG000002[illegible]	PAGE3	protein_codin	0	0	0	0
ENSG000001[illegible]	PAGE5	protein_codin	0	0	0	0
ENSG000001[illegible]	ASIP	protein_codin	0	0	0	0
ENSG000001[illegible]	PAGE5	protein_codin	0	4.88809317	98.5331156	0
ENSG000002[illegible]	PAGE2	protein_codin	0	0	0	0
ENSG000001[illegible]	CERKL	protein_codin	0	0	0	0
ENSG000002[illegible]	PAGE2	protein_codin	0	0	0	0
ENSG000001[illegible]	CERKL	protein_codin	0	2.77821269	0	0
ENSG000001[illegible]	CERKL	protein_codin	0	0	0	0
ENSG000002[illegible]	PAGE2B	protein_codin	0	0	0	0.42447824
ENSG000002[illegible]	PAGE2B	protein_codin	0	0	0	0
ENSG000001[illegible]	HLA-DRB5	protein_codin	31.5522007	487.559593	1.85593387	48.3057846
ENSG000001[illegible]	MSANTD2	protein_codin	0.8151713	3.22136871	1.158922	0
ENSG000001[illegible]	EIF2S2	protein_codin	130.710039	294.629763	311.683406	54.8633407
ENSG000002[illegible]	HLA-DRA	protein_codin	19.5068042	11.7389905	0	33.9595167
ENSG000001[illegible]	APEX2	protein_codin	6.71933814	29.3556798	21.1645149	5.56868095
ENSG000001[illegible]	TGFBR1	protein_codin	0	88.1547402	63.4759527	0
ENSG000001[illegible]	PFKFB1	protein_codin	0	0	0	0
ENSG000002[illegible]	BTNL2	protein_codin	0	0	0.74463764	0
ENSG000001[illegible]	TGFBR1	protein_codin	0	0	0	0
ENSG000001[illegible]	MGAT5B	protein_codin	0	0	0.67247979	0
ENSG000001[illegible]	HP1BP3	protein_codin	0	3.02587607	16.3943654	2.34366925
ENSG000002[illegible]	COL15A1	protein_codin	126.955335	426.025012	42.7356971	281.434313

ENSG000001	HP1BP3	protein_codin	23.9356438	149.259989	111.440713	15.0869017
ENSG000001	PFKFB1	protein_codin	0.55151814	9.14643816	1.69635455	3.68543192
ENSG000001	GALNT12	protein_codin	2.16514487	0	4.45470511	4.32654708
ENSG000002	TSBP1	protein_codin	0	0	0	0
ENSG000001	ANKS6	protein_codin	0	0	3.0634342	0
ENSG000000	TRO	protein_codin	0	0	0	0
ENSG000002	NOTCH4	protein_codin	5.66762851	43.1647891	8.21459671	7.83257748
ENSG000002	OR10G9	protein_codin	0	0	0.58655499	1.16072
ENSG000002	CCDC7	protein_codin	13.092341	0	5.9173337	2.23642365
ENSG000002	TMEM225	protein_codin	0	0	0	0
ENSG000002	CCDC7	protein_codin	0.2880297	0	0	0
ENSG000001	SH2D5	protein_codin	0	0	0	0
ENSG000001	MXRA7	protein_codin	6.34244389	50.484413	5.78607243	4.9795651
ENSG000002	GPSM3	protein_codin	0	22.2494117	0	9.23600178
ENSG000000	TRO	protein_codin	0	0	0	0
ENSG000002	GPSM3	protein_codin	0	0	0	2.73077917
ENSG000001	KIF17	protein_codin	0	0	1.38819853	0
ENSG000002	PBX2	protein_codin	39.2112346	43.386525	88.053534	25.1214153
ENSG000001	MAGED2	protein_codin	20.8102098	6.04004532	0	22.9477226
ENSG000002	AGER	protein_codin	0	0	0	0
ENSG000002	AGER	protein_codin	0.64131639	0	0	0
ENSG000001	MAGED2	protein_codin	0	0	0	0
ENSG000001	MAGED2	protein_codin	35.5291362	30.7693673	156.893219	17.7691738
ENSG000000	TBC1D2	protein_codin	3.04721393	0	1.68104153	0.68769909
ENSG000000	TBC1D2	protein_codin	0	16.6379522	1.9396585	0
ENSG000000	TBC1D2	protein_codin	0	0	0	0
ENSG000002	AGER	protein_codin	0	0	0	0
ENSG000001	MAGED2	protein_codin	0	0	185.077248	20.8505105
ENSG000002	AGER	protein_codin	0	0	0	0
ENSG000002	AGER	protein_codin	0	0	0	0
ENSG000001	CDA	protein_codin	0.2505331	1.40944762	0	0
ENSG000002	AGER	protein_codin	0	0	0	0
ENSG000001	CORO2A	protein_codin	1.22764995	0	6.25867872	0
ENSG000001	CAMK2N1	protein_codin	9.60526652	7.04149637	13.6225361	4.45218378
ENSG000001	VWA5B1	protein_codin	0	0	0	0
ENSG000001	GRIK4	protein_codin	0	0	0	0
ENSG000002	RNF5	protein_codin	9.19131886	12.6961191	30.6980354	8.64025541
ENSG000001	TLCD5	protein_codin	8.739899	0	0	0
ENSG000001	TRIM14	protein_codin	0	0	0	0
ENSG000001	UBXN10	protein_codin	0.57749949	0	0.95084696	4.639147
ENSG000001	PLA2G2F	protein_codin	0	0	0	0.79510246
ENSG000002	AGPAT1	protein_codin	0	0	0	0
ENSG000001	PLA2G2D	protein_codin	0	0	0	0
ENSG000002	AGPAT1	protein_codin	21.3450042	54.8087417	49.8020199	12.6061402

ENSG000001	PLA2G5	protein_codin	1.84296999	3.71129049	0	3.22094131
ENSG000001	EPC1	protein_codin	10.2118009	14.0828402	5.63297442	17.0217607
ENSG000001	PLA2G2A	protein_codin	0	0	0	0
ENSG000001	RALY	protein_codin	0	0	3.64452333	0
ENSG000001	PLA2G2E	protein_codin	0.30516552	0	0	0.36571841
ENSG000001	TRMO	protein_codin	0	0	0	0
ENSG000001	TRMO	protein_codin	0.46337777	5.27209437	0.7677509	4.53277043
ENSG000001	TRMO	protein_codin	5.43363913	0	7.4153341	0
ENSG000001	OTUD3	protein_codin	26.6910338	29.7656716	48.4602381	9.29355847
ENSG000001	RNF186	protein_codin	0.30818069	0	0	0
ENSG000001	FOXE1	protein_codin	0	0	2.41307447	0.50209549
ENSG000001	TMCO4	protein_codin	1.89598035	0	14.0807283	0
ENSG000001	XPA	protein_codin	31.2667221	44.7581653	44.984556	17.9411975
ENSG000002	PJVK	protein_codin	0	4.3896953	0	0
ENSG000001	NCBP1	protein_codin	0	53.5354561	0	0
ENSG000001	FGD1	protein_codin	12.8248057	36.6050002	77.689524	11.2008616
ENSG000001	NBL1	protein_codin	40.1356632	34.1597573	30.4144968	7.83100666
ENSG000002	PPT2	protein_codin	0.93123174	0	0	0
ENSG000002	DRC12	protein_codin	0	0	0	0
ENSG000000	CAPZB	protein_codin	2.453599	0	0	0
ENSG000002	PPT2	protein_codin	0	0	0	0
ENSG000000	CAPZB	protein_codin	6.78574397	24.6691155	0	0
ENSG000001	NCBP1	protein_codin	37.0444845	73.2060476	102.756118	19.2169208
ENSG000002	PRRT1	protein_codin	0	0	0	0
ENSG000001	TSR2	protein_codin	25.8546978	102.403919	66.1338612	18.1460513
ENSG000000	SLC66A1	protein_codin	2.14306708	0	0	0
ENSG000000	SLC66A1	protein_codin	0	0	2.96757926	0
ENSG000002	FKBPL	protein_codin	1.24726619	0.94604874	3.29478776	1.61668661
ENSG000001	TSTD2	protein_codin	4.40113238	11.9119354	7.93324385	0
ENSG000001	TSTD2	protein_codin	1.06054796	0	5.71564328	1.45906272
ENSG000001	H2AX	protein_codin	3.31957876	3.52888865	29.8667664	3.51206914
ENSG000001	WNK3	protein_codin	8.748355	34.4110505	76.0484503	9.33920211
ENSG000001	TSTD2	protein_codin	1.58435562	0	0	0.99748059
ENSG000001	TSTD2	protein_codin	1.71387246	0	2.88617681	2.85884164
ENSG000001	TMOD1	protein_codin	0	0	0	0
ENSG000001	FAM120C	protein_codin	17.2798952	32.8837438	43.1463855	8.94488605
ENSG000000	TBXA2R	protein_codin	0.64583483	0	0	0
ENSG000001	EMC1	protein_codin	0	5.14202777	19.8198538	0
ENSG000001	ZNF341	protein_codin	0	0	0	0
ENSG000002	ATF6B	protein_codin	0	13.7373175	0	0
ENSG000002	ATF6B	protein_codin	25.5164149	62.9657328	64.1908445	15.1137615
ENSG000001	EMC1	protein_codin	14.2211925	0	86.6628185	8.81677647
ENSG000000	MAP3K20	protein_codin	2.69538584	0	18.8346516	1.6309385
ENSG000002	SMIM5	protein_codin	0.36504041	0.8497689	0.59986081	0

ENSG000001:	UBR4	protein_codin	5.72553544	19.7123143	26.0942901	5.2059486
ENSG000001·	C20orf144	protein_codin	0	0	0	0
ENSG000001:	UBR4	protein_codin	0	13.6862118	12.1440443	6.46717486
ENSG000001:	UBR4	protein_codin	2.70070706	26.8619184	16.4304889	3.32932925
ENSG000000'	LLGL2	protein_codin	0	6.10636326	2.33029689	0
ENSG000000:	ZNF510	protein_codin	3.15215558	0	25.3094336	0
ENSG000001:	PRXL2C	protein_codin	12.2887457	0	7.75117946	10.0097617
ENSG000001:	NECAB3	protein_codin	0	1.36699432	0	1.32057057
ENSG000000:	CDC14B	protein_codin	0	0	2.32211413	0
ENSG000000:	CDC14B	protein_codin	15.0971224	0	21.1363109	15.0732098
ENSG000000:	CDC14B	protein_codin	0	0	0	4.88675499
ENSG000001(	TNXB	protein_codin	0	0	0	6.80621661
ENSG000001(	ARHGAP12	protein_codin	12.207898	14.8789918	37.954914	3.74582133
ENSG000001'	TMEM94	protein_codin	0	0	0	0
ENSG000001:	HABP4	protein_codin	22.1227467	124.519193	42.7263159	20.2787319
ENSG000001(	ARHGAP12	protein_codin	38.5966003	77.6976451	29.8741231	19.7905542
ENSG000001:	HABP4	protein_codin	0	0	0	0
ENSG000000'	CYBRD1	protein_codin	0	0	0	0
ENSG000001:	UBR4	protein_codin	177.646934	568.07904	579.264503	161.246069
ENSG000001	DCAF17	protein_codin	0	0	75.7818482	6.04127423
ENSG000001(	ZNF367	protein_codin	11.7308014	7.4128292	72.4824122	1.34384275
ENSG000001:	SLC35D2	protein_codin	0.42168878	1.41225106	0	0.59741772
ENSG000001:	METTL8	protein_codin	0	0	47.7658448	0
ENSG000001:	SLC35D2	protein_codin	4.47360318	0	5.5179452	0
ENSG000001:	SLC25A19	protein_codin	0	5.14218698	0	0
ENSG000001:	HSD17B3	protein_codin	0	0	0	0
ENSG000001:	HSD17B3	protein_codin	0	0	0	0
ENSG000001:	PYROXD1	protein_codin	0	0	0	3.35067247
ENSG000001:	PTCH1	protein_codin	0	0	0	0
ENSG000001:	GAD1	protein_codin	0	0	0	0
ENSG000000'	CBFA2T2	protein_codin	20.454399	0	0	0
ENSG000002(	SP5	protein_codin	0.21863952	0.84678874	2.81452548	0
ENSG000001:	KCTD2	protein_codin	20.7688489	21.4615458	53.1217634	0
ENSG000001:	PTCH1	protein_codin	3.37017298	21.1579459	48.8691619	7.19980033
ENSG000000'	HSD17B10	protein_codin	0	0	0	0
ENSG000001(	UPF3A	protein_codin	30.0564847	27.4243949	69.2111298	19.3131369
ENSG000001(	SIK3	protein_codin	38.8644489	118.211579	0	24.5616099
ENSG000000'	HSD17B10	protein_codin	0	0	0	0
ENSG000001:	FANCC	protein_codin	3.58915579	0	0	0
ENSG000001:	CDC16	protein_codin	6.4660095	5.47044569	9.7422848	0
ENSG000001:	ZNF438	protein_codin	2.81587798	26.4724734	0	0
ENSG000001·	AOPEP	protein_codin	11.5962684	14.1207114	11.0414552	0
ENSG000001·	LYZL2	protein_codin	0	0	0	0
ENSG000001	APOA1	protein_codin	0	0	0	0

ENSG000001	MAP3K8	protein_codin	4.04356247	9.88099857	1.79534277	31.2317137
ENSG000001	MAP3K8	protein_codin	0.99454259	3.79326015	0	10.0568284
ENSG000001	APOA1	protein_codin	0	0	0	0
ENSG000001	FBP1	protein_codin	0	0	0	0
ENSG000001	RIBC1	protein_codin	0	1.93499348	4.95719129	0
ENSG000001	APOA1	protein_codin	0	0	0	0
ENSG000002	STK19	protein_codin	0	0	0	0
ENSG000002	STK19	protein_codin	0	0	1.45069623	0
ENSG000001	FBP2	protein_codin	4.20801133	0	0	0
ENSG000000	SMC1A	protein_codin	0	0	0	0
ENSG000001	ALDH4A1	protein_codin	3.01348077	5.62783912	4.39273724	0.60808406
ENSG000001	MFSD14B	protein_codin	20.2706163	64.5959876	35.9989149	17.0383563
ENSG000001	APOC3	protein_codin	0	0	0	0
ENSG000001	TMEM255B	protein_codin	1.04778507	1.08774718	0	0
ENSG000002	DXO	protein_codin	0	0	0	0
ENSG000001	NSD1	protein_codin	0	0	0	0
ENSG000002	CD300LD	protein_codin	0	0	0	0.34602744
ENSG000001	TMEM255B	protein_codin	0	5.94425628	2.24924278	5.25525903
ENSG000002	DXO	protein_codin	0	0	0	0
ENSG000001	PTPDC1	protein_codin	22.2470485	42.4431309	29.4887275	11.1332455
ENSG000001	G6PC2	protein_codin	0	0	0	0
ENSG000001	IQSEC2	protein_codin	3.29439	0	0	0
ENSG000002	BTBD17	protein_codin	0	1.06102981	0	0
ENSG000001	TFDP1	protein_codin	36.2842615	58.2318073	76.5519473	14.8320394
ENSG000001	TAS1R2	protein_codin	0.30251183	0	0	0.88018196
ENSG000000	PAX7	protein_codin	0	0	0	0
ENSG000001	JCAD	protein_codin	94.1066092	288.261657	57.4252541	89.0114417
ENSG000001	KDM5C	protein_codin	0	0	108.665766	12.3403791
ENSG000001	KDM5C	protein_codin	24.2841571	32.2462393	0	0
ENSG000000	FAM120A	protein_codin	12.5756513	2.95711212	37.234308	3.65783412
ENSG000001	TMCO3	protein_codin	0	0	0	0
ENSG000002	SKIC2	protein_codin	0	0	40.3532774	0
ENSG000001	SVIL	protein_codin	186.026837	54.3267611	75.6294195	37.0455831
ENSG000001	SVIL	protein_codin	27.319729	99.0274393	109.520423	51.9269614
ENSG000001	KDM5C	protein_codin	5.43398794	152.410159	14.493698	24.6372541
ENSG000001	DCUN1D2	protein_codin	4.08142006	16.1683352	0	0
ENSG000001	SCN1A	protein_codin	0	0	0	0
ENSG000001	ACTL8	protein_codin	0	0	0	0
ENSG000000	ARHGEF10L	protein_codin	1.15402447	0	0	0
ENSG000001	FAM120AOS	protein_codin	11.2135487	35.0157819	35.4087199	9.20870138
ENSG000001	BPIFA1	protein_codin	0	0	0	0.49369255
ENSG000000	ARHGEF10L	protein_codin	5.75942763	21.3603592	0	0
ENSG000001	ADPRHL1	protein_codin	7.32076333	0	0	0
ENSG000001	BPIFA1	protein_codin	0	0	0	0

ENSG000002(	NELFE	protein_codin	0	0	0	0
ENSG000002(	NELFE	protein_codin	18.038927	59.4756979	53.8528059	12.0906283
ENSG000001:	GRTP1	protein_codin	0	0	0	0
ENSG000001:	GRTP1	protein_codin	0	0	12.1394135	0
ENSG000001'	RCC2	protein_codin	17.4513919	82.160732	158.404661	7.69750657
ENSG000001'	RCC2	protein_codin	29.6625833	105.703869	104.251544	27.0643947
ENSG000001:	SCN2A	protein_codin	0	0	0	0
ENSG000001:	CUL4A	protein_codin	5.37705742	0	0	1.29393633
ENSG000001:	CUL4A	protein_codin	1.7529646	0	0	3.3037257
ENSG000001(	TSPYL2	protein_codin	37.5845755	213.058671	98.3616776	127.2335
ENSG000001:	BUD13	protein_codin	17.841982	41.5079343	25.1381183	7.78649857
ENSG000001:	NINJ1	protein_codin	14.0061644	22.4521438	12.9371361	20.9654404
ENSG000001	PADI4	protein_codin	0	1.30490134	0	0.40650086
ENSG000001:	BPIFA3	protein_codin	0	0	0	0
ENSG000001	PADI4	protein_codin	0	0	0	0
ENSG000001:	BPIFA3	protein_codin	0	0	0	0
ENSG000001:	PCID2	protein_codin	0.55868431	6.96156517	1.05319394	1.11490101
ENSG000000	COBLL1	protein_codin	0	0	14.1822974	20.3499187
ENSG000001:	PCID2	protein_codin	0	0	0	0
ENSG000001	PADI3	protein_codin	0	1.27503929	0	1.27405907
ENSG000001(	CARD19	protein_codin	4.49723285	7.04878179	6.49585083	3.12275049
ENSG000001	GPR173	protein_codin	0	0	1.20288502	0
ENSG000001	SUSD3	protein_codin	1.20415432	0	0	0
ENSG000001	PADI1	protein_codin	0	1.21967554	0	0.77414446
ENSG000001	SUSD3	protein_codin	0	0	0	0.97075096
ENSG000001:	PCID2	protein_codin	0	0	0	0
ENSG000001:	NXPE4	protein_codin	0	0	0	0
ENSG000001:	PCID2	protein_codin	0	0	0	0
ENSG000001	PADI2	protein_codin	0	0	0	0
ENSG000001:	FGD3	protein_codin	2.22065649	0	7.36667071	0
ENSG000001	BPIFB4	protein_codin	0.29455559	1.8398814	0	0
ENSG000001	PADI2	protein_codin	17.0706005	7.55342388	4.83762599	3.614779
ENSG000000'	RBM7	protein_codin	2.61048103	0	3.28571015	9.7400038
ENSG000001'	XAGE3	protein_codin	0	0	0	0
ENSG000001	BPIFB3	protein_codin	0	0	0	0.43066048
ENSG000001:	ZNF484	protein_codin	14.3428193	0	28.84185	0
ENSG000001	GCG	protein_codin	0	0	0	0.47872046
ENSG000001(	HTR3A	protein_codin	0	0	0	2.88814654
ENSG000001	SDHB	protein_codin	0	0	21.6046221	0
ENSG000001:	LYZL1	protein_codin	0	0	0	0
ENSG000001'	XAGE5	protein_codin	0	0	0.36382993	0
ENSG000001'	XAGE5	protein_codin	0	0	0	1.27158188
ENSG000002(	SPANXN5	protein_codin	0	0	0	0.38086873
ENSG000001	BICD2	protein_codin	48.6036542	105.918808	87.8545745	37.0833936

ENSG000001[illegible]	SLC4A10	protein_codin	0	0	0	3.55721868
ENSG000001[illegible]	SUN5	protein_codin	0	0	0	0
ENSG000001[illegible]	IPPK	protein_codin	7.90619495	38.1167688	32.1640268	0
ENSG000001[illegible]	SUN5	protein_codin	0	0	0	0.37675188
ENSG000002[illegible]	ZBTB12	protein_codin	1.24412759	7.48576275	4.96265483	0.88332289
ENSG000002[illegible]	EHMT2	protein_codin	0	0	0	0
ENSG000002[illegible]	EHMT2	protein_codin	12.9007321	0	114.840416	6.97764851
ENSG000000[illegible]	BAMBI	protein_codin	14.6610147	27.6385972	26.6000389	31.4569877
ENSG000001	MFAP2	protein_codin	0	1.79079827	1.17697771	0.83204624
ENSG000001	MFAP2	protein_codin	33.0743937	12.4751258	71.9471203	5.2394956
ENSG000002[illegible]	EHMT2	protein_codin	27.3894641	287.646853	88.7999938	19.1922223
ENSG000001[illegible]	ECM2	protein_codin	10.4508436	13.2488907	4.14018345	6.81356271
ENSG000000[illegible]	CROCC	protein_codin	13.6021214	83.1981217	61.6847084	26.7964006
ENSG000001[illegible]	ASPN	protein_codin	9.19197146	0	0	0
ENSG000001[illegible]	ASPN	protein_codin	451.128618	309.327923	23.1047737	35.6241014
ENSG000001[illegible]	PROZ	protein_codin	0	1.28950327	0	0
ENSG000002[illegible]	SDHD	protein_codin	9.82042963	10.6895695	15.7585376	5.02281513
ENSG000001[illegible]	OMD	protein_codin	546.263171	472.775836	165.339932	207.130315
ENSG000001[illegible]	F10	protein_codin	0	0	0	0
ENSG000001[illegible]	FAM20A	protein_codin	5.01431107	46.3551631	0.96187661	4.12136111
ENSG000001[illegible]	F10	protein_codin	0.73150795	0	0	0
ENSG000001[illegible]	OGN	protein_codin	23.4430164	15.1123627	4.92665268	7.58326538
ENSG000002[illegible]	SLC44A4	protein_codin	0	0	0	0
ENSG000001[illegible]	MAPRE1	protein_codin	55.4076238	172.123452	159.460915	68.4876579
ENSG000001[illegible]	CENPP	protein_codin	0.76891296	0	1.70940064	1.49181955
ENSG000001[illegible]	SPATA21	protein_codin	0	0	0	0.60972425
ENSG000001[illegible]	CENPP	protein_codin	0	0	5.7750332	0
ENSG000000[illegible]	F7	protein_codin	0	0	0	0
ENSG000001[illegible]	CENPP	protein_codin	23.5625966	65.9182093	80.8874135	12.6083659
ENSG000000[illegible]	FBXO42	protein_codin	17.6588675	52.9296103	22.1806259	17.3654014
ENSG000002[illegible]	XAGE1A	protein_codin	0	0	37.7193386	0.53038866
ENSG000001[illegible]	MCF2L	protein_codin	0	0	0	0
ENSG000001[illegible]	CPLANE2	protein_codin	0.50469629	3.10895691	0.97390397	3.66479997
ENSG000002[illegible]	XAGE1A	protein_codin	0	0	65.129974	0
ENSG000002[illegible]	XAGE1A	protein_codin	0	0	697.816318	0
ENSG000001[illegible]	MCF2L	protein_codin	0	0	0	0
ENSG000001[illegible]	MCF2L	protein_codin	0	15.5312117	10.6083231	0
ENSG000002[illegible]	LAYN	protein_codin	4.71127863	0	0	0
ENSG000002[illegible]	LAYN	protein_codin	5.07402942	7.45875393	8.70338859	0
ENSG000002[illegible]	XAGE1B	protein_codin	0	0	0	0.72263333
ENSG000001[illegible]	HOATZ	protein_codin	0	0	0	0
ENSG000001[illegible]	MAGED4	protein_codin	0	0	0	0
ENSG000001[illegible]	IARS1	protein_codin	0.41488612	0	0	0
ENSG000000[illegible]	ATP11A	protein_codin	16.1272415	0	0	4.83446792

ENSG000002	NEU1	protein_codin	0	0	0	0
ENSG000001	IARS1	protein_codin	7.04192456	95.9007066	0	0
ENSG000000	ATP11A	protein_codin	23.4405615	92.7729442	71.9082894	29.3537494
ENSG000000	WAC	protein_codin	0	0	0.89113891	0
ENSG000001	ZBTB40	protein_codin	50.0644611	179.719684	105.49611	35.486017
ENSG000002	HSPA1B	protein_codin	116.348495	116.481226	638.354798	1260.51569
ENSG000002	HSPA1A	protein_codin	236.998993	330.605462	1410.32154	2254.81544
ENSG000002	HSPA1L	protein_codin	1.96861313	1.84578708	6.5550468	4.8879918
ENSG000002	LSM2	protein_codin	0	0	0	0
ENSG000001	FAM131C	protein_codin	0.78026009	2.00590696	0	1.25245091
ENSG000002	VARS1	protein_codin	15.3383822	34.2601368	111.560863	11.0441672
ENSG000000	WAC	protein_codin	0	0	0	0
ENSG000001	CLCNKB	protein_codin	0.76534252	0	0	0
ENSG000001	TUBGCP3	protein_codin	17.3839652	0	44.684534	6.2850018
ENSG000001	NOL4L	protein_codin	0.67593793	0	0	3.38799508
ENSG000001	NOL4L	protein_codin	0	0	0	0
ENSG000001	CLCNKB	protein_codin	0	0	0	0
ENSG000001	SLC35F2	protein_codin	0	0	0	0
ENSG000001	ASXL1	protein_codin	17.3958146	56.0171907	63.5239095	34.077768
ENSG000002	VWA7	protein_codin	0	0	0	0
ENSG000001	ASXL1	protein_codin	1.24874323	0	3.67785993	0
ENSG000001	CLCNKA	protein_codin	0	0	0	0
ENSG000001	MAGED1	protein_codin	0	0	0	0
ENSG000001	SPACA7	protein_codin	0	0	0	0
ENSG000001	AXIN2	protein_codin	0	0	0	0
ENSG000002	MSH5	protein_codin	0	0	8.36182225	0
ENSG000001	ROR2	protein_codin	10.3156376	53.4459747	68.9176639	9.70661817
ENSG000001	LDHA	protein_codin	1.45443289	2.63203461	25.3243781	10.220742
ENSG000001	KIF3B	protein_codin	48.2706408	134.748995	77.6098108	33.394745
ENSG000001	ROR2	protein_codin	2.69813416	0	2.01353346	0.63639813
ENSG000001	HSPB7	protein_codin	0	0	0	3.29335125
ENSG000001	MPP7	protein_codin	3.27445222	0	13.955971	0
ENSG000001	MAGED1	protein_codin	18.9984571	65.1553207	7.89872334	0
ENSG000001	ARHGEF7	protein_codin	5.40552758	17.890418	10.2574131	9.73788357
ENSG000002	CASP12	protein_codin	0	0	0	0
ENSG000001	POFUT1	protein_codin	0.85809224	0	0.85094041	0
ENSG000001	AUH	protein_codin	3.71488401	17.3222156	19.1711889	5.94244034
ENSG000001	MPP7	protein_codin	0	0	0	0
ENSG000001	ZBTB17	protein_codin	0	0	0	0
ENSG000001	RND3	protein_codin	54.4849333	43.5816067	82.6877258	72.6896585
ENSG000001	DYNC2H1	protein_codin	95.4823988	123.301064	423.036732	92.8412139
ENSG000001	ARHGEF7	protein_codin	19.9614299	55.9626243	0	0
ENSG000001	ARHGEF7	protein_codin	0	0	7.88108057	0
ENSG000002	MSH5	protein_codin	0	17.9856802	0	0

ENSG000001	ARHGEF7	protein_codin	0	0	0	0
ENSG000001	ZBTB17	protein_codin	10.614355	33.8498128	20.9533261	4.97739658
ENSG000001	SYK	protein_codin	0	0	0	0
ENSG000001	SYK	protein_codin	0	15.1171314	0	0
ENSG000001	POFUT1	protein_codin	25.7961309	0	0	22.478424
ENSG000002	MSH5	protein_codin	0	0	0	0
ENSG000001	SYK	protein_codin	38.6235647	73.0116222	0	0
ENSG000001	SYK	protein_codin	7.6486986	41.8567527	20.1839056	41.9699768
ENSG000002	MSH5	protein_codin	0	0	0	0
ENSG000000	SPEN	protein_codin	189.808696	605.779439	527.252436	132.94873
ENSG000001	DIRAS2	protein_codin	2.58693351	7.31434174	0.49323265	9.4397672
ENSG000001	FBLIM1	protein_codin	5.40249701	30.13708	6.9954993	9.11765862
ENSG000001	GADD45G	protein_codin	0	0	0	0
ENSG000001	FBLIM1	protein_codin	0	0	0	0
ENSG000001	MAGED1	protein_codin	6.25784	0	3.52185219	0
ENSG000001	KYNU	protein_codin	1.77068124	5.5354038	0.95888382	0
ENSG000001	ING1	protein_codin	1.06661057	5.41459337	5.6338015	4.75265849
ENSG000001	ING1	protein_codin	0	0	23.39264	15.0222309
ENSG000002	CLIC1	protein_codin	0	0	0	0
ENSG000002	CLIC1	protein_codin	0	0	0	0
ENSG000001	CARS2	protein_codin	2.10076319	0	3.98546476	0
ENSG000001	TMEM82	protein_codin	0	1.33074492	0	0
ENSG000002	CLIC1	protein_codin	30.0174271	185.228534	66.7897834	32.9129569
ENSG000002	DDAH2	protein_codin	12.9882446	0	0	3.48846524
ENSG000002	DDAH2	protein_codin	0	0	31.9822306	0
ENSG000001	MKX	protein_codin	0	0	0	2.39306862
ENSG000002	DDAH2	protein_codin	0	25.5858822	0	0
ENSG000001	PLEKHM2	protein_codin	21.7782404	81.4650323	40.4420645	0
ENSG000001	PLEKHM2	protein_codin	0	131.820541	0	47.9821627
ENSG000000	RAB18	protein_codin	0	9.48893192	0	7.03744519
ENSG000002	MPIG6B	protein_codin	0	0	0	0
ENSG000002	MPIG6B	protein_codin	0	0	0	0
ENSG000001	SECISBP2	protein_codin	35.4001657	94.6889754	0	16.4440276
ENSG000002	MPIG6B	protein_codin	0	2.36607031	0	0
ENSG000002	MPIG6B	protein_codin	0	0	0	0
ENSG000000	PSMC5	protein_codin	0	0	0	0
ENSG000002	MPIG6B	protein_codin	0	0	0	0
ENSG000002	LY6G6C	protein_codin	0	0	0	0.4237754
ENSG000001	COL4A1	protein_codin	223.897208	210.972958	315.922339	239.408534
ENSG000002	LY6G6D	protein_codin	0	0	0	0
ENSG000002	LY6G6D	protein_codin	0	0	0	0
ENSG000001	AGMAT	protein_codin	2.83042726	9.02429695	5.06196818	0.85427773
ENSG000001	SHC3	protein_codin	1.08347394	1.77760443	0	0
ENSG000002	LY6G6F	protein_codin	0	0	0	0

ENSG000001	SHC3	protein_codin	5.29915205	5.39478727	4.34241691	4.54385298
ENSG000001	DNAJC16	protein_codin	1.58291573	3.8892254	4.80212572	0
ENSG000002	STRADA	protein_codin	0	0	11.22862	0
ENSG000001	MAP3K19	protein_codin	0	0	0	0
ENSG000001	MAP3K19	protein_codin	0	0	0	0
ENSG000002	S1PR3	protein_codin	1.43408137	10.2702689	16.8748854	11.9729737
ENSG000001	DNAJC16	protein_codin	32.351924	57.2631062	77.2735604	9.10276753
ENSG000001	DNAJC16	protein_codin	0.7144719	0	2.12951239	0.63849116
ENSG000002	S1PR3	protein_codin	0	0	0	0
ENSG000001	HCK	protein_codin	5.54127146	18.7280256	5.95970247	3.66519567
ENSG000001	NXNL2	protein_codin	0	0	0	0.62173597
ENSG000001	NXNL2	protein_codin	0	1.19275022	0.61906018	2.56608124
ENSG000001	IRS2	protein_codin	12.4360879	11.3145015	39.5736269	44.9792417
ENSG000000	MYO16	protein_codin	0.2706547	0	0	0
ENSG000001	SPIN1	protein_codin	41.4521402	71.4944296	73.1530214	19.3653684
ENSG000001	HCK	protein_codin	0	0	0	5.67816819
ENSG000002	LY6G5C	protein_codin	0	2.77616027	1.32541573	1.94541517
ENSG000002	LY6G5B	protein_codin	1.90042481	19.9048639	8.16729196	2.72384037
ENSG000002	CSNK2B	protein_codin	15.5624511	0	0	15.5220859
ENSG000002	CSNK2B	protein_codin	0	0	30.2001038	0
ENSG000001	CDK20	protein_codin	1.02505136	0	0	0
ENSG000002	ENSG000002	protein_codin	0	0	0	0
ENSG000002	CSNK2B	protein_codin	0	67.9873193	23.3346023	0
ENSG000001	CDK20	protein_codin	0	0	0	0
ENSG000002	CSNK2B	protein_codin	0	0	0	0
ENSG000001	TNFSF13B	protein_codin	0	0	0	4.84970232
ENSG000001	ACBD5	protein_codin	2.75333285	25.1130556	0	0
ENSG000001	CASP9	protein_codin	0	0	0	0
ENSG000002	GPANK1	protein_codin	3.64054835	25.755195	6.28611229	0
ENSG000001	CTSL	protein_codin	1.51621409	27.0408343	2.3678364	18.8011176
ENSG000002	GPANK1	protein_codin	0	0	0	0.76160551
ENSG000002	GPANK1	protein_codin	0	0	0	2.12735125
ENSG000001	ACBD5	protein_codin	0	0	0	0
ENSG000001	ABHD13	protein_codin	17.9546857	42.9202938	28.9602763	11.4482911
ENSG000002	GPANK1	protein_codin	0	4.30600738	0	0
ENSG000001	ACBD5	protein_codin	0	0	28.4159286	15.1936541
ENSG000001	ACBD5	protein_codin	0	0	0	0
ENSG000002	GPANK1	protein_codin	0	4.032357	5.39499019	0
ENSG000002	CELA2B	protein_codin	0	0	0	0
ENSG000002	C6orf47	protein_codin	7.90490123	36.0758242	21.8836894	7.16437958
ENSG000002	NALF1	protein_codin	13.8370972	32.3581507	5.4521007	8.79477772
ENSG000002	APOM	protein_codin	0	0	0	0
ENSG000002	APOM	protein_codin	0.48734944	9.53386206	0	0
ENSG000002	APOM	protein_codin	0	0	0.64338365	0.37868678

ENSG000001	TTLL9	protein_codin	0	0	0	0
ENSG000001	TTLL9	protein_codin	0	0	0	0
ENSG000001	ARGLU1	protein_codin	9.74098891	14.2386097	0	14.7898852
ENSG000001	TTLL9	protein_codin	0	0	0.55782107	0.32725472
ENSG000001	DAOA	protein_codin	0	0	0	0.52407545
ENSG000001	TTLL9	protein_codin	0	0	0	0
ENSG000001	MASTL	protein_codin	0	14.6250997	0	2.94604683
ENSG000001	CTRC	protein_codin	0	2.02669588	0	2.57074934
ENSG000000	NAALAD2	protein_codin	0	5.52115259	0	0
ENSG000001	MASTL	protein_codin	2.50323126	0	6.15526766	4.56513539
ENSG000000	TUT7	protein_codin	16.259362	18.7384829	0	0
ENSG000000	TUT7	protein_codin	2.90227723	0	1.5794198	1.02649021
ENSG000001	CTRC	protein_codin	0	0	0	0
ENSG000001	ERCC5	protein_codin	1.27632887	55.3459327	9.65579494	9.87378625
ENSG000000	TUT7	protein_codin	29.671022	0	61.309305	0
ENSG000001	ERCC5	protein_codin	0	0	0	0
ENSG000000	TUT7	protein_codin	75.4978459	524.254198	188.518065	54.9550781
ENSG000000	TUT7	protein_codin	37.5133354	129.451726	0	43.4572435
ENSG000002	BAG6	protein_codin	0	0	0	0
ENSG000001	DUSP15	protein_codin	0	0	0	0
ENSG000002	BAG6	protein_codin	6.26570128	0	0	0
ENSG000001	FOXS1	protein_codin	1.79621803	0	1.0986092	3.34682393
ENSG000000	NOX4	protein_codin	0	0	0	0
ENSG000001	EFHD2	protein_codin	32.2828172	134.898762	114.154351	66.7545229
ENSG000001	MYLK2	protein_codin	10.981987	2.88955658	1.96197779	0.78846866
ENSG000001	ISCA1	protein_codin	7.98468601	34.568156	18.8431929	11.4888706
ENSG000001	NUDT11	protein_codin	6.54589809	2.93597125	40.2608372	0.86692975
ENSG000001	MYLK2	protein_codin	0	0	0	0
ENSG000001	FHAD1	protein_codin	0	0	0	0
ENSG000001	FHAD1	protein_codin	0	0	0	0
ENSG000001	FHAD1	protein_codin	6.8021974	2.03438132	5.2549114	6.06117853
ENSG000001	FHAD1	protein_codin	3.0070485	27.0155088	7.6374597	0.96819694
ENSG000001	C9orf153	protein_codin	0	0	0	0
ENSG000001	POGLUT2	protein_codin	0	14.0124518	0	1.71222049
ENSG000001	NUDT10	protein_codin	0	0	0	0
ENSG000002	PRRC2A	protein_codin	45.7019704	450.646755	363.590568	82.201099
ENSG000001	TMEM51	protein_codin	0	2.59000345	0	0
ENSG000001	TMEM51	protein_codin	0	0	15.968181	1.88392054
ENSG000001	YME1L1	protein_codin	33.29403	106.513718	58.5725505	49.1084121
ENSG000001	TEX30	protein_codin	0	0	0	1.04193863
ENSG000001	SHROOM4	protein_codin	10.6662015	65.5047756	17.4651989	23.1018466
ENSG000001	TEX30	protein_codin	3.32565669	0	0	0
ENSG000001	TEX30	protein_codin	0.63198302	0	0	0
ENSG000001	TEX30	protein_codin	0	0	0	0

ENSG000001[illegible]	KAZN	protein_codin	0	0	0.66643265	0
ENSG000001[illegible]	TEX30	protein_codin	0	0	4.34591115	0
ENSG000001[illegible]	KAZN	protein_codin	2.40016788	26.9756691	33.8400477	0
ENSG000001[illegible]	TEX30	protein_codin	0	5.21457305	4.73196792	5.98361265
ENSG000002[illegible]	PRRC2A	protein_codin	41.4822429	34.8086868	62.0449509	25.88878
ENSG000001[illegible]	CCNB3	protein_codin	0	0	0	0
ENSG000001[illegible]	NAA35	protein_codin	6.1886306	15.4126832	16.9984405	1.34643463
ENSG000001[illegible]	CCNB3	protein_codin	0	0	0	0
ENSG000001	PRDM2	protein_codin	0	0	0	0
ENSG000002[illegible]	AIF1	protein_codin	27.141105	0	9.45842934	0
ENSG000001[illegible]	TPP2	protein_codin	4.52986489	14.5439005	8.86044082	11.7127066
ENSG000001[illegible]	BCL2L1	protein_codin	0	0	0	0
ENSG000001[illegible]	PDPN	protein_codin	0	0	13.2878491	0
ENSG000002[illegible]	AIF1	protein_codin	11.4886383	215.440158	5.70287961	13.8568596
ENSG000001[illegible]	PDPN	protein_codin	0	0	0	0
ENSG000001[illegible]	BCL2L1	protein_codin	0	0	17.83762	4.25864224
ENSG000001[illegible]	AKAP4	protein_codin	0	0	0	0
ENSG000001[illegible]	TPP2	protein_codin	0	0	0	0
ENSG000002[illegible]	NCR3	protein_codin	0	0	0	0
ENSG000002[illegible]	NCR3	protein_codin	0	1.14003408	0	0
ENSG000002[illegible]	NCR3	protein_codin	0	0	0	0
ENSG000001[illegible]	COX4I2	protein_codin	0.97462407	2.18143854	0	5.91373052
ENSG000001[illegible]	AGTPBP1	protein_codin	0	0	2.7365189	1.71007763
ENSG000001[illegible]	AGTPBP1	protein_codin	3.75925186	0	0	0
ENSG000001[illegible]	AGTPBP1	protein_codin	0	0	0	0
ENSG000001[illegible]	LRRC38	protein_codin	0	1.33849866	0.9282605	0.77818835
ENSG000002[illegible]	LST1	protein_codin	0	0	0	0
ENSG000001[illegible]	ANKRD26	protein_codin	5.44685047	0	9.42103532	12.0657491
ENSG000001[illegible]	CLCN5	protein_codin	11.9525866	0	30.6095886	14.4577959
ENSG000002[illegible]	LST1	protein_codin	0	1.16933484	0	0.72963384
ENSG000002[illegible]	LST1	protein_codin	1.32988286	0	0	0
ENSG000001[illegible]	CLCN5	protein_codin	0	33.8096324	47.5998822	0
ENSG000002[illegible]	LST1	protein_codin	0	0	0	0
ENSG000002[illegible]	LST1	protein_codin	0	0	0	0
ENSG000001[illegible]	TUBD1	protein_codin	0	0	0	0
ENSG000002[illegible]	LST1	protein_codin	0.38095106	0	0	1.4211182
ENSG000002[illegible]	PRAMEF17	protein_codin	0	2.86713858	0.44872661	1.73819498
ENSG000002[illegible]	LST1	protein_codin	0	0	0	0
ENSG000002[illegible]	PRAMEF19	protein_codin	0	0	0	0.52668322
ENSG000001[illegible]	DLG2	protein_codin	0	0	0	0
ENSG000001[illegible]	ID1	protein_codin	0.82270781	3.01185735	2.5513162	4.71840394
ENSG000001[illegible]	CLCN5	protein_codin	0	0	2.65881861	0
ENSG000002[illegible]	LST1	protein_codin	0	0	0	0
ENSG000001[illegible]	ID1	protein_codin	0	22.6918004	11.5103199	19.4008553

ENSG000001:	BIN1	protein_codin	0	0	0	6.5379564
ENSG000001(	FGF14	protein_codin	0	0	0	9.49682331
ENSG000001:	ABI2	protein_codin	1.48498827	0	9.89561352	1.43971427
ENSG000001:	ABI1	protein_codin	43.4362201	20.0533158	52.8476115	27.8224254
ENSG000001:	ABI1	protein_codin	0	0	0	5.34900646
ENSG000001:	ABI1	protein_codin	4.01439395	15.2077093	13.2649926	0
ENSG000001:	ABI1	protein_codin	0	0	0	0
ENSG000001(	PAGE4	protein_codin	0	0	5.77447149	0
ENSG000001:	ABI1	protein_codin	0	0	0	0
ENSG000001(	FGF14	protein_codin	0	1.70570243	0	0
ENSG000002(	NFKBIL1	protein_codin	0	0	2.09098463	0
ENSG000002(	NFKBIL1	protein_codin	7.65038615	26.7010284	30.7493599	6.10232356
ENSG000002(	NFKBIL1	protein_codin	0	4.23278314	0	0
ENSG000000(	PAGE1	protein_codin	0	0	0	0
ENSG000002	ATP6V1G2	protein_codin	0	1.96983052	0	0
ENSG000002(	PRAMEF15	protein_codin	0	0	0	0
ENSG000001:	ALG8	protein_codin	0	0	0	0
ENSG000001:	ABI1	protein_codin	0	0	4.83911865	0
ENSG000001!	ITGBL1	protein_codin	96.0580654	139.588503	9.63103337	56.3199666
ENSG000001:	ABI1	protein_codin	0	47.5538075	0	12.914242
ENSG000001:	ABI1	protein_codin	0	0	0	0
ENSG000001!	DDX39B	protein_codin	7.54137301	0	8.55449165	0
ENSG000001!	ITGBL1	protein_codin	25.8631344	103.325613	0	31.7607848
ENSG000002:	ATP6V1G2-DI	protein_codin	4.33629582	21.7146694	0	0
ENSG000000.	PPP1R3F	protein_codin	0	1.46365998	0.80164399	0
ENSG000002:	PRAMEF6	protein_codin	0.38005712	0	0	0.65986601
ENSG000002(	MCCD1	protein_codin	0	1.28632812	0	0.71010767
ENSG000000.	FOXP3	protein_codin	0	0	0	0
ENSG000000.	FOXP3	protein_codin	0	0	0	0
ENSG000001(	NALCN	protein_codin	0	0	0	0
ENSG000000.	FOXP3	protein_codin	0	14.1978718	0.47745991	0
ENSG000001.	NTRK2	protein_codin	0	0	0	0
ENSG000001.	CFAP107	protein_codin	0	0	0	0
ENSG000001.	NTRK2	protein_codin	0	0	0	0
ENSG000001.	PDSS1	protein_codin	5.67607134	2.58479192	21.4721357	0
ENSG000001'	GDPD4	protein_codin	0	0	0	0
ENSG000002(	AADACL4	protein_codin	0	1.51219702	0	1.9085764
ENSG000001(	CCDC22	protein_codin	10.0136594	44.2810173	27.2597976	4.95385415
ENSG000002(	HLA-C	protein_codin	94.6926798	2918.32535	581.569003	363.461639
ENSG000002:	ZNF550	protein_codin	0	0	1.55317099	0
ENSG000001:	ZNF549	protein_codin	18.0459733	27.9059859	0	0
ENSG000001:	TMTC4	protein_codin	8.6635161	0	0	0
ENSG000000'	APBB1IP	protein_codin	15.8626253	133.12224	5.76979175	64.1852238
ENSG000002(	HLA-C	protein_codin	0	73.2492068	22.0057574	0

ENSG000001	SLC28A3	protein_codin	0	0	0	1.46309239
ENSG000001	GAD2	protein_codin	0	0	0	0
ENSG000001	GGACT	protein_codin	0	0	0	0
ENSG000001	CACNA1F	protein_codin	0.24885181	10.0517352	0	0.46271313
ENSG000001	TCF19	protein_codin	0	0	0	0
ENSG000001	HNRNPK	protein_codin	3.12730162	1.18911399	15.3803422	7.10816078
ENSG000001	TCF19	protein_codin	0	0	17.3777492	0
ENSG000002	TNFRSF1B	protein_codin	9.21104142	118.861785	0	21.1038468
ENSG000001	GAD2	protein_codin	0.58924602	0	0.51788009	4.14014366
ENSG000000	DGAT2	protein_codin	4.12131255	0	0	2.39084433
ENSG000001	HNRNPK	protein_codin	29.8723822	0	0	0
ENSG000001	CACNA1F	protein_codin	0	0	0	0
ENSG000002	CCHCR1	protein_codin	4.76297646	0	21.30643	0
ENSG000001	PCCA	protein_codin	7.3043719	0	0	14.0672017
ENSG000001	HNRNPK	protein_codin	0	0	0	0
ENSG000001	PCCA	protein_codin	13.1891962	13.3285287	11.7863516	0
ENSG000001	PCCA	protein_codin	0	0	0	0
ENSG000002	CDSN	protein_codin	0.24176226	3.28033687	0	2.77234777
ENSG000001	KLHL35	protein_codin	1.43144989	1.4929368	0.69535983	0
ENSG000002	MUC21	protein_codin	0	10.3721284	0	5.07434247
ENSG000001	CCDC93	protein_codin	34.122583	201.81563	127.304445	37.3248414
ENSG000000	MYO3A	protein_codin	0	0	0	0
ENSG000000	MYO3A	protein_codin	0.3038311	1.69284629	4.03466419	0.52701435
ENSG000001	SYP	protein_codin	0	0	0	0
ENSG000001	DEFB123	protein_codin	0	1.81212702	0	0
ENSG000000	PRICKLE3	protein_codin	1.55218971	4.56717122	2.27181929	1.39881827
ENSG000002	DEFB121	protein_codin	0	0	0	0
ENSG000002	DEFB121	protein_codin	0	0	0	0
ENSG000001	DEFB119	protein_codin	0	0	0	0
ENSG000002	GTF2H4	protein_codin	0	0	0	0
ENSG000000	PRICKLE3	protein_codin	0	0	0	0
ENSG000001	DEFB119	protein_codin	0	0	0	0
ENSG000001	PLP2	protein_codin	0	0	1.98769983	0.46999365
ENSG000000	CHRDL2	protein_codin	0	0	0	0
ENSG000001	SHISA7	protein_codin	0.49166923	2.37139652	2.46659579	0
ENSG000001	PLP2	protein_codin	4.98404203	10.484343	8.64398809	7.63638893
ENSG000000	CHRDL2	protein_codin	0	0	1.24939761	0
ENSG000000	ZIC2	protein_codin	0	0	7.43812497	0.54616849
ENSG000001	QNG1	protein_codin	0	0	0	0
ENSG000001	QNG1	protein_codin	10.5371876	6.53968402	18.4927361	2.86470845
ENSG000001	GID4	protein_codin	0	0	11.6459907	0
ENSG000001	KIF27	protein_codin	2.50127317	0	8.6329549	2.30353841
ENSG000000	PTPRH	protein_codin	0	1.16295929	0	0
ENSG000001	GPR158	protein_codin	2.26167263	0	0	1.53465756

ENSG000001	STXBP4	protein_codin	22.5338958	69.9430701	147.351513	20.8810003
ENSG000001	CLYBL	protein_codin	0	0	0	0
ENSG000001	CLYBL	protein_codin	0	4.68654236	0	2.73964889
ENSG000001	THNSL1	protein_codin	11.8781309	18.7115905	18.448934	3.73018539
ENSG000001	WDR45	protein_codin	0	0	0	0
ENSG000002	ENSG000002	protein_codin	0	0	0	0
ENSG000001	GKAP1	protein_codin	0.34073604	0	2.05516788	0.54679061
ENSG000001	ENKUR	protein_codin	1.09919831	6.4745239	1.00802884	0.61740214
ENSG000001	GKAP1	protein_codin	8.25212595	16.9073684	23.0501178	0
ENSG000001	WDR45	protein_codin	0	0	0	0
ENSG000001	GKAP1	protein_codin	3.0334306	0	11.6883024	3.48559372
ENSG000001	WDR45	protein_codin	0	0	0	0
ENSG000000	PRTFDC1	protein_codin	1.10268418	0	1.82814523	1.05801986
ENSG000001	IER3	protein_codin	0	9.18710819	0	4.9992794
ENSG000000	PRTFDC1	protein_codin	3.02397465	0	12.9800677	3.32576998
ENSG000000	MBTD1	protein_codin	9.36678526	0	5.39498232	1.48549443
ENSG000001	PAAF1	protein_codin	3.21919669	0	20.3922171	0
ENSG000002	PRAF2	protein_codin	0.22017978	0	0	0
ENSG000001	TM9SF2	protein_codin	79.4723039	161.046096	115.274074	66.8141977
ENSG000001	FLOT1	protein_codin	34.9710376	39.4693804	58.9681369	17.7732236
ENSG000000	NME1-NME2	protein_codin	0	0	0	0
ENSG000001	UBQLN1	protein_codin	39.8194039	43.1922585	59.5986135	45.6379903
ENSG000001	MDC1	protein_codin	76.627586	224.033902	432.366647	56.8180958
ENSG000001	ARHGAP21	protein_codin	40.9724788	0	0	0
ENSG000001	GPR183	protein_codin	6.74944483	25.9333703	0.77529198	40.3034716
ENSG000001	IDNK	protein_codin	0	0	0	0
ENSG000001	IDNK	protein_codin	1.67052478	0	0	0
ENSG000001	NRM	protein_codin	0	0	0	1.5597195
ENSG000001	NRM	protein_codin	0	0	0	0
ENSG000000	GRIPAP1	protein_codin	0	0	0	0
ENSG000001	FRMD3	protein_codin	0	0	0	0
ENSG000001	ZNF337	protein_codin	25.3198102	52.5762749	28.5219581	19.3350032
ENSG000002	DHX16	protein_codin	0	0	5.60781698	0
ENSG000001	FRMD3	protein_codin	2.77734021	21.0223185	0	1.68068176
ENSG000001	RABL2A	protein_codin	0.40378341	0	0	0.79409915
ENSG000001	UBAC2	protein_codin	0	3.30700858	0.95233754	0
ENSG000002	DHX16	protein_codin	28.6502644	93.3087442	62.9583001	20.5262242
ENSG000001	RASEF	protein_codin	0	1.23915771	3.7285769	1.43314773
ENSG000001	PDE2A	protein_codin	2.45970369	0	0	7.93164859
ENSG000001	KIAA1217	protein_codin	46.9266343	170.153645	17.8872008	45.9607694
ENSG000001	KIAA1217	protein_codin	0	109.866462	0	0
ENSG000001	KIAA1217	protein_codin	94.5989162	0	72.7048454	36.1206544
ENSG000001	KIAA1217	protein_codin	0	0	0	0
ENSG000002	SPATA31A7	protein_codin	0	0	0	0

ENSG000000	DOCK9	protein_codin	0	0	0	0
ENSG000001	KIAA1217	protein_codin	0	0	0	0
ENSG000001	TLE1	protein_codin	1.96441045	0	1.42572364	2.30323673
ENSG000001	NPPB	protein_codin	0	0	0	0
ENSG000002	C6orf136	protein_codin	0.41163706	0	0	0
ENSG000002	C6orf136	protein_codin	0	0	0	0
ENSG000001	NPPA	protein_codin	0	0	0	0
ENSG000001	KCND1	protein_codin	0	0	0	0.67753905
ENSG000001	NPPA	protein_codin	0	0	0	0
ENSG000001	ATAT1	protein_codin	3.90473803	0	9.89315255	0
ENSG000001	TLE1	protein_codin	0	0	0	0
ENSG000001	ATAT1	protein_codin	0	0	0	0
ENSG000001	MTHFR	protein_codin	0	1.06353039	0	0
ENSG000000	OTUD5	protein_codin	0	31.7751867	21.9929902	17.4032624
ENSG000001	IL36G	protein_codin	0	0	0	0.44528693
ENSG000000	CLCN6	protein_codin	0	0	0	0
ENSG000000	CLCN6	protein_codin	0	0	1.56916821	1.73047755
ENSG000000	CLCN6	protein_codin	0	9.20631436	1.5602137	0
ENSG000000	SLC15A1	protein_codin	0	0	0	0.48898504
ENSG000001	OTUD1	protein_codin	18.2850519	27.5286704	5.16225463	81.8305665
ENSG000000	CLCN6	protein_codin	0	0	0.83983812	0
ENSG000000	CLCN6	protein_codin	0.57417742	21.7229127	0	1.58045546
ENSG000001	TLE1	protein_codin	16.0030878	125.238418	61.3717282	24.8761556
ENSG000001	C10orf67	protein_codin	0	0	0	0
ENSG000001	C10orf67	protein_codin	0	0	0	0.45867657
ENSG000000	SLC15A1	protein_codin	0	0	0	2.35010899
ENSG000001	PTF1A	protein_codin	0	0	0	0
ENSG000001	PIM2	protein_codin	0.5178017	59.736963	1.89773485	11.5296233
ENSG000001	MSRB2	protein_codin	2.7442751	3.94129577	8.22579018	3.72773318
ENSG000002	PPP1R10	protein_codin	61.685514	212.116536	132.048242	26.2792168
ENSG000001	SLC35A2	protein_codin	0	0	0	0
ENSG000001	LENG8	protein_codin	50.6278355	94.0395212	80.1930676	71.0066262
ENSG000001	SLC35A2	protein_codin	0	4.98242656	0	0
ENSG000001	SLC35A2	protein_codin	0	0.77086811	0	1.39932532
ENSG000001	TLE4	protein_codin	0	0	0	0
ENSG000001	TLE4	protein_codin	0	14.5757454	0.44528278	0
ENSG000001	ARMC3	protein_codin	0	0	0	0
ENSG000001	SLC35A2	protein_codin	0	0	0	0
ENSG000001	TTYH1	protein_codin	0	0	0	0
ENSG000001	TTYH1	protein_codin	0	0	0	0
ENSG000001	TLE4	protein_codin	0	0	0	0
ENSG000001	ABHD12	protein_codin	0	0	9.85340232	1.32904657
ENSG000001	TLE4	protein_codin	0	54.9226875	10.2619996	9.71619095
ENSG000002	ABCF1	protein_codin	0	45.6153446	0	0

ENSG000001	STK24	protein_codin	0	12.9238002	0	6.23904973
ENSG000000	XYLT2	protein_codin	0	0	0	10.1086796
ENSG000001	TLE4	protein_codin	16.3200888	0	16.4411098	0
ENSG000001	STK24	protein_codin	0	0	0	0
ENSG000002	PRR3	protein_codin	0.94032948	7.45582514	3.50172206	0
ENSG000002	PRR3	protein_codin	0	0	0	0
ENSG000000	CTTN	protein_codin	57.7240224	0	77.28378	59.2570231
ENSG000001	PQBP1	protein_codin	3.35667575	0	0	0
ENSG000001	PQBP1	protein_codin	0	0	0	0
ENSG000002	DDR1	protein_codin	0	2.05039079	0	0
ENSG000002	DDR1	protein_codin	0	11.3519045	0	0
ENSG000002	DDR1	protein_codin	5.29151876	0	0	0
ENSG000002	DDR1	protein_codin	0	0	0	0
ENSG000001	KIAA2013	protein_codin	12.7532452	23.839385	49.5357674	11.9558128
ENSG000001	PIP4K2A	protein_codin	46.612007	126.542768	42.4588539	42.4933092
ENSG000002	DDR1	protein_codin	0	0	0	0
ENSG000001	KIAA2013	protein_codin	2.35645884	5.52047837	2.5869051	0
ENSG000001	FARP1	protein_codin	1.36441125	0	4.18446998	0.42487977
ENSG000001	TIMM17B	protein_codin	1.33456683	2.78984229	4.22994692	0
ENSG000001	MTHFR	protein_codin	0	0	0	0
ENSG000001	MTHFR	protein_codin	0	0	2.40241535	0
ENSG000001	PSAT1	protein_codin	1.37742365	0	7.39246774	1.65775193
ENSG000001	MTHFR	protein_codin	0	35.7132628	4.02305008	12.9106033
ENSG000001	MTHFR	protein_codin	3.43975366	9.04070033	0	0
ENSG000001	CEP78	protein_codin	0	0	0	0
ENSG000001	CEP78	protein_codin	0	0	0	0
ENSG000000	SPAG6	protein_codin	0	0	0	0
ENSG000002	LRCOL1	protein_codin	0	0	0	0
ENSG000000	HDAC6	protein_codin	0	0	3.61876846	0
ENSG000001	CPT1A	protein_codin	0	0	0	0
ENSG000000	HDAC6	protein_codin	0	0	0	0
ENSG000002	GNL1	protein_codin	24.2685522	140.795354	92.7334609	42.1666252
ENSG000000	SPAG6	protein_codin	0	0	0	0
ENSG000001	VSTM1	protein_codin	0	0	0	0
ENSG000001	AGTRAP	protein_codin	0	0	0	0
ENSG000001	AGTRAP	protein_codin	0	1.86896374	0	0.4304753
ENSG000002	HLA-E	protein_codin	253.648898	2297.5808	124.685361	560.439698
ENSG000001	VPS13A	protein_codin	12.062278	135.523464	79.1908114	14.2323561
ENSG000001	AGTRAP	protein_codin	0	0	0	0.4274219
ENSG000000	HDAC6	protein_codin	0	0	0	0
ENSG000001	VPS13A	protein_codin	0	0	0	0
ENSG000001	PUS1	protein_codin	0.89054689	11.5000429	5.8014354	2.27511708
ENSG000002	DPRX	protein_codin	0	0	0	1.03660135
ENSG000001	EDAR	protein_codin	0	0	0	1.29854982

ENSG000001!	ENTPD6	protein_codin	2.30468056	13.0333025	1.10647581	0.67880549
ENSG000001	MAD2L2	protein_codin	0	0	0	0
ENSG000002	TRIM39	protein_codin	2.12343612	32.4245712	0	0
ENSG000002	TRIM39	protein_codin	9.12954964	7.05824337	33.1635706	17.0788397
ENSG000001	BMI1	protein_codin	47.0452987	33.8795846	101.638964	30.1085426
ENSG000001	MAD2L2	protein_codin	1.0197758	0	1.63204636	1.21744867
ENSG000001	GATA1	protein_codin	0	0	0	0
ENSG000001!	ENTPD6	protein_codin	1.76702072	0	0	0
ENSG000001	MAD2L2	protein_codin	0	0	37.0482865	4.04261977
ENSG000001	MAD2L2	protein_codin	5.0004551	22.2685568	0	0
ENSG000001	GATA1	protein_codin	0	0	0	0
ENSG000001	MAD2L2	protein_codin	0	0	0	0
ENSG000001	MBNL2	protein_codin	0	0	0	0
ENSG000001	TTLL6	protein_codin	0	0	0.47000905	3.04547217
ENSG000001	ADGRD1	protein_codin	0	0	1.59567101	0
ENSG000001	SUV39H1	protein_codin	1.45102894	14.657135	14.5830158	1.66392172
ENSG000001	MAD2L2	protein_codin	2.33961954	4.03358224	0	0
ENSG000001	NUDT8	protein_codin	0.9397156	0.95683648	0.69589985	0
ENSG000002	TRIM15	protein_codin	0	0	0.9230454	0.39726384
ENSG000000	WAS	protein_codin	8.37940044	56.7061802	0	4.70165915
ENSG000002	TRIM10	protein_codin	0	0	0.52178891	0.85751471
ENSG000001	HS6ST3	protein_codin	0	2.03640827	0.51872838	3.67042748
ENSG000001	VSX1	protein_codin	0.55932059	0	0	0
ENSG000002	FOXB2	protein_codin	0	0	0	0.39031752
ENSG000001	VSX1	protein_codin	0	0	0	0
ENSG000001	UGGT2	protein_codin	7.33912539	6.53789916	20.4339509	3.41188526
ENSG000001	PRUNE2	protein_codin	0	0	0	0
ENSG000001	UGGT2	protein_codin	0.66831671	0.64417133	6.17572178	0
ENSG000001!	ZNF615	protein_codin	4.24516811	0	0	3.60084926
ENSG000001	PRUNE2	protein_codin	0	0	0	0
ENSG000001	PRUNE2	protein_codin	0	55.836062	9.38687151	0
ENSG000001	UGGT1	protein_codin	39.9246314	195.629881	170.198888	42.0818224
ENSG000002	TRIM40	protein_codin	0	0	0	0.8569242
ENSG000001	ACSS1	protein_codin	1.94607596	3.59433904	0.900174	0.60272759
ENSG000001	WDR13	protein_codin	4.24058093	9.21059405	3.62631384	1.65317162
ENSG000001	GCNT1	protein_codin	0	0	35.0254264	0
ENSG000002	TRIM31	protein_codin	0	0	0	0.82027846
ENSG000001	RFK	protein_codin	0	10.5072837	11.975796	4.80677
ENSG000001	SLC15A4	protein_codin	0.36235127	0	1.9899473	1.73271938
ENSG000001	SP2	protein_codin	15.7436534	51.2699986	21.6129253	9.47819161
ENSG000001	SLC15A4	protein_codin	1.46649026	0	0	0
ENSG000001	PPP1CA	protein_codin	14.8726021	69.8978266	47.1695158	13.2755495
ENSG000001	UGGT2	protein_codin	83.3730589	63.2772915	134.595148	27.7948639
ENSG000002	RNF39	protein_codin	0	0	0	0.81486988

ENSG000000	PCSK5	protein_codin	15.2918822	100.549389	50.8498098	32.8830456
ENSG000001	FBXO6	protein_codin	0	10.9311016	0	0
ENSG000001	RBM3	protein_codin	22.3041001	85.1458083	52.2779785	37.7287861
ENSG000001	SSH3	protein_codin	0.47499206	4.4512719	4.82221037	0
ENSG000002	PPP1R11	protein_codin	0.6354491	1.23279375	3.47819493	1.25282692
ENSG000001	RBM3	protein_codin	9.01207575	17.2678352	26.0876942	15.1407745
ENSG000001	FBXO44	protein_codin	0	9.12141137	0	1.4444579
ENSG000001	FBXO44	protein_codin	2.3120406	0	0	0
ENSG000002	PPP1R11	protein_codin	0	0	0	0
ENSG000002	PPP1R11	protein_codin	0	1.28277716	0	0
ENSG000000	PCSK5	protein_codin	18.2259531	43.9994488	82.2261899	16.0511108
ENSG000001	FBXO44	protein_codin	1.26388204	0	0	0
ENSG000002	PPP1R11	protein_codin	0.56102653	0	0	1.08370664
ENSG000001	FBXO44	protein_codin	0	0	3.29986969	0
ENSG000000	TBC1D25	protein_codin	1.2029337	6.41098462	2.52006446	3.82988128
ENSG000002	PPP1R11	protein_codin	20.1304024	59.4298834	62.3903887	8.64886306
ENSG000002	PPP1R11	protein_codin	0	0	0	0
ENSG000000	POLR1H	protein_codin	2.31527091	6.24096854	0	0
ENSG000000	POLR1H	protein_codin	0	0	0	0
ENSG000001	DNAJC3	protein_codin	18.85561	71.3696719	55.7809999	16.5275421
ENSG000001	KLK13	protein_codin	0	0	0	0
ENSG000002	HLA-A	protein_codin	0	0	1.39130628	0
ENSG000001	UBIAD1	protein_codin	0	3.56578063	0	2.85855657
ENSG000002	HLA-A	protein_codin	0	0	0	0
ENSG000001	NMRK1	protein_codin	10.269018	29.1562444	16.6239644	2.2177416
ENSG000002	HLA-A	protein_codin	29.5995655	0	0	109.48191
ENSG000001	UBIAD1	protein_codin	6.84353434	4.93018353	14.8980863	3.00517902
ENSG000001	NMRK1	protein_codin	0	16.5203697	1.5186679	3.09543908
ENSG000002	HLA-G	protein_codin	0	0	0	0
ENSG000002	RACK1	protein_codin	14.4895146	0	0	0
ENSG000002	HLA-G	protein_codin	0	0	0	0
ENSG000001	ANGPTL7	protein_codin	0.69530213	0	0	1.95376451
ENSG000002	HLA-G	protein_codin	0	0	0	0
ENSG000001	DZIP1	protein_codin	30.3354655	0	0	0
ENSG000001	CARNMT1	protein_codin	10.0081664	26.7979479	33.2570138	4.60292476
ENSG000001	CARNMT1	protein_codin	10.3163582	14.3187629	31.1583312	7.88919626
ENSG000001	COMMD3	protein_codin	6.27394694	24.0674681	14.4046241	8.14178986
ENSG000001	MTOR	protein_codin	0.64312394	13.088944	0	0
ENSG000000	ACTN1	protein_codin	2.82889774	0	6.82415391	0
ENSG000002	TBC1D8	protein_codin	0	68.4790617	18.1486253	15.5830214
ENSG000001	BTNL9	protein_codin	0	0	0	0
ENSG000001	KLK6	protein_codin	0	0	0	0
ENSG000001	C9orf40	protein_codin	2.43566059	2.8405304	23.2015214	2.07538437
ENSG000001	CLDN10	protein_codin	0	0	0	0

ENSG000002	HLA-F	protein_codin	0	33.3869879	0.83419839	0
ENSG000001	SYNDIG1	protein_codin	2.96759026	0.80775102	0.53432521	2.43450362
ENSG000002	NMS	protein_codin	0	0	0	0
ENSG000001	CLDN10	protein_codin	0	0	0	0
ENSG000001	RILPL1	protein_codin	9.72626453	5.09359032	3.68510888	7.23473762
ENSG000002	ZFP57	protein_codin	0	0	0	0
ENSG000002	MOG	protein_codin	0	0	0	0
ENSG000002	MOG	protein_codin	0	0	0	0
ENSG000002	MOG	protein_codin	0	0	0	0
ENSG000001	SSX3	protein_codin	0	0	3.09309746	0
ENSG000002	MOG	protein_codin	0	1.06991663	0	0.44260821
ENSG000001	SSX3	protein_codin	0	0	0	0
ENSG000001	RORB	protein_codin	9.38526609	11.7671132	4.04630732	5.8893655
ENSG000002	MOG	protein_codin	0	0	0	0
ENSG000001	YIF1A	protein_codin	0.98672424	4.62915152	3.85894962	2.21612527
ENSG000001	YIF1A	protein_codin	0	0	4.77207115	0
ENSG000002	MOG	protein_codin	0	0	0	0
ENSG000001	ANXA1	protein_codin	24.3624857	2.61096806	7.58060492	57.6274442
ENSG000002	ASPDH	protein_codin	0	0	0	0
ENSG000002	MOG	protein_codin	0	0	0	0
ENSG000001	EMC10	protein_codin	0	0	0	0
ENSG000001	SSX1	protein_codin	0	3.43438556	0	0.52160565
ENSG000001	ARHGAP27	protein_codin	0	0	0	6.4531978
ENSG000001	CST3	protein_codin	132.150262	271.564833	39.7295191	424.87905
ENSG000000	MAP3K14	protein_codin	0	0	0	0
ENSG000001	MRNIP	protein_codin	0	0	0	0
ENSG000002	SMIM13	protein_codin	0	0	0	0
ENSG000001	EXOSC10	protein_codin	43.8713927	67.3964853	122.830636	43.3370226
ENSG000001	ALDH1A1	protein_codin	0.60973641	0	0	0
ENSG000001	SPACA5	protein_codin	0	0	0	1.5110148
ENSG000001	ZNF182	protein_codin	11.7552292	32.9584493	18.7851062	4.35114048
ENSG000001	SOX21	protein_codin	0	0	0.52013399	0.94473037
ENSG000001	DNAJC1	protein_codin	0.48026304	0	0	0.74896252
ENSG000001	ZNF81	protein_codin	0	0	0	0
ENSG000001	ZNF81	protein_codin	0	0	0	11.5867912
ENSG000001	ACBD4	protein_codin	0	0	0	2.92836647
ENSG000001	ZFAND5	protein_codin	0	24.1263253	10.6529544	0
ENSG000001	SRM	protein_codin	11.9912747	25.653426	57.0038701	14.7968757
ENSG000001	GPR180	protein_codin	31.8901698	25.3138338	24.9413718	9.89696294
ENSG000001	KCNC3	protein_codin	1.22635097	0	4.52043481	0
ENSG000001	ZFAND5	protein_codin	64.2475252	17.7169849	102.678756	74.3070145
ENSG000001	ZFAND5	protein_codin	5.62811896	35.4494952	31.5313705	12.3861214
ENSG000001	UXT	protein_codin	0	0	0	0
ENSG000001	MYH14	protein_codin	1.8624897	0	0	0

ENSG000001	CST9	protein_codin	0	0	0	0.45202501
ENSG000002	CBY3	protein_codin	0	0	0	0
ENSG000001	CST9L	protein_codin	0	0	0	0
ENSG000001	DNAJC1	protein_codin	76.2260429	46.715831	115.431966	46.3341911
ENSG000001	ELK1	protein_codin	0	0	0	0
ENSG000001	GDA	protein_codin	0	0	0	0
ENSG000001	GFAP	protein_codin	0	0	0	0
ENSG000001	DRAP1	protein_codin	0	27.6016128	0	4.47090942
ENSG000001	C1orf127	protein_codin	0	0	0	0
ENSG000001	CFP	protein_codin	0	0	0	0
ENSG000001	CST11	protein_codin	0	0	0	0.45161594
ENSG000001	CST11	protein_codin	0	0	0	0
ENSG000001	VRK3	protein_codin	0	0	0	0
ENSG000002	GABBR1	protein_codin	12.5592175	0	27.4736295	7.97846318
ENSG000002	GABBR1	protein_codin	0	0	0	0
ENSG000001	TIMP1	protein_codin	11.4012318	170.643943	5.1509027	0
ENSG000001	CASZ1	protein_codin	5.06413119	7.80130659	0	1.43572586
ENSG000002	C9orf57	protein_codin	0	0	0	0
ENSG000001	NAPB	protein_codin	0	0	0	0
ENSG000001	C9orf85	protein_codin	14.6136873	13.816014	17.1421997	2.81138746
ENSG000000	DCT	protein_codin	0.23374501	0	0	0.97542277
ENSG000001	C9orf85	protein_codin	0	0	0	0.84252096
ENSG000002	GABBR1	protein_codin	0	11.624723	0	0
ENSG000002	IL31	protein_codin	0.28263985	0	0	1.3931322
ENSG000001	DFFA	protein_codin	1.95264774	0	20.2019506	0.61040663
ENSG000001	DFFA	protein_codin	22.4958759	38.7255443	92.3244709	14.157628
ENSG000000	ARAF	protein_codin	1.17013981	0	0.82948855	0.65332656
ENSG000001	ABHD17B	protein_codin	0	0	0	0.7909171
ENSG000001	CEMIP2	protein_codin	0	0	0.56956316	1.6757943
ENSG000001	CEMIP2	protein_codin	39.1787585	0	172.255155	83.3267602
ENSG000000	ARAF	protein_codin	12.8194131	69.8938027	25.118098	14.4622259
ENSG000001	KAT5	protein_codin	7.60712391	17.3973203	0	0
ENSG000001	GPC6	protein_codin	14.9754339	105.67584	59.5573728	24.0107449
ENSG000002	CORT	protein_codin	2.36516126	2.62366961	1.84289886	0.41096505
ENSG000002	UBD	protein_codin	2.93040696	227.891523	0	0
ENSG000001	GZF1	protein_codin	0	0	0	22.7107969
ENSG000001	CEMIP2	protein_codin	0	0	0	0.46901625
ENSG000001	CEMIP2	protein_codin	0	0	1.34228687	0.77242165
ENSG000000	MLLT10	protein_codin	0	0	0	14.7609749
ENSG000001	CNNM3	protein_codin	0.64858185	0	0	0
ENSG000001	ZNF41	protein_codin	6.87903718	0	0	0
ENSG000001	CEMIP2	protein_codin	6.29499025	0	0	9.55155032
ENSG000001	GPC5	protein_codin	0	7.74646895	1.76498466	0
ENSG000000	KDM2B	protein_codin	12.437377	22.029214	1.57143845	0

ENSG000000	KDM2B	protein_codin	0	0	0	0
ENSG000000	MLLT10	protein_codin	0	0	0	0
ENSG000001	ZNF157	protein_codin	1.03348326	0	0.53795493	0
ENSG000001	CNNM4	protein_codin	5.28919868	28.4756344	10.8520068	6.77665541
ENSG000001	USP11	protein_codin	5.63801927	0	0	0
ENSG000001	LMAN2L	protein_codin	0	0	0	0
ENSG000001	USP11	protein_codin	17.0305455	20.6131583	49.1780131	0
ENSG000000	KIF1B	protein_codin	27.1053645	0	0	0
ENSG000000	KIF1B	protein_codin	93.6750267	89.3181765	220.171885	41.2950104
ENSG000001	SLITRK1	protein_codin	0	0	0	0
ENSG000000	KIF1B	protein_codin	0	0	0	0
ENSG000001	DUSP18	protein_codin	4.30914609	0	0	0
ENSG000000	MLLT10	protein_codin	0.63174543	0	0.77718219	0
ENSG000000	FUZ	protein_codin	0	0	0	0
ENSG000000	KIF1B	protein_codin	31.7824457	38.8506013	63.3050346	0
ENSG000000	SLC25A39	protein_codin	0	0	6.77165944	0
ENSG000000	TRPM3	protein_codin	0	0	0	0
ENSG000000	MLLT10	protein_codin	0	0	0	0
ENSG000000	TRPM3	protein_codin	0	0	0	1.07373081
ENSG000001	SPRY2	protein_codin	1.7753966	3.39753137	0	4.28707919
ENSG000001	THBD	protein_codin	24.368119	18.3220716	3.1409336	108.80418
ENSG000001	SPRY2	protein_codin	12.4340459	10.231911	11.114947	13.5567651
ENSG000000	TRPM3	protein_codin	0	10.4855004	0	0
ENSG000001	USP11	protein_codin	26.1205465	79.118344	69.6683484	30.4151644
ENSG000000	TRPM3	protein_codin	0	2.61278245	0	11.1511472
ENSG000000	TRPM3	protein_codin	0	0	0	0
ENSG000001	DOK3	protein_codin	0	0	0	0
ENSG000001	FOXA2	protein_codin	0.28920796	0.86948759	0	0.92609908
ENSG000000	NEBL	protein_codin	0.66456826	0	0	1.62685039
ENSG000000	NEBL	protein_codin	0	0	12.2914015	0
ENSG000001	KLF9	protein_codin	82.4541627	60.0676379	65.3009089	149.273541
ENSG000002	MAS1L	protein_codin	0.35478614	1.69521025	0	1.00744126
ENSG000002	OR2H1	protein_codin	0	0	0	0
ENSG000002	OR2H1	protein_codin	0	0	0	0
ENSG000001	IRF3	protein_codin	0	0	0	0
ENSG000002	OR2H1	protein_codin	0	0	0	2.1215593
ENSG000001	IRF3	protein_codin	0	0	0	0
ENSG000001	NKX2-2	protein_codin	0.20058382	0	5.04876625	1.29138484
ENSG000002	OR11A1	protein_codin	0	0	0	0
ENSG000002	OR11A1	protein_codin	0	0	0	3.86736561
ENSG000001	UBE4B	protein_codin	0.52898325	0	6.10149192	0
ENSG000002	OR5V1	protein_codin	0	0	0	0
ENSG000002	OR14J1	protein_codin	0	0	0	0
ENSG000002	OR2J2	protein_codin	0	0	0.55435418	0.50573917

ENSG000002	OR2J3	protein_codin	0	0	0	0
ENSG000002	OR2J1	protein_codin	0	0	0	0
ENSG000002	OR2B3	protein_codin	0	0	0	0
ENSG000002	OR2W1	protein_codin	0	0	0	0
ENSG000001	ZNF311	protein_codin	2.08567088	0	10.9769443	0
ENSG000001	KCNIP3	protein_codin	0	0	0	0
ENSG000001	MAMDC2	protein_codin	145.757983	67.0761641	11.1578674	78.1557445
ENSG000001	MPP2	protein_codin	0	0	0	0
ENSG000002	SPDYC	protein_codin	0	0	0	0
ENSG000001	SYVN1	protein_codin	5.24113621	40.3409492	27.5452198	0
ENSG000000	XRN2	protein_codin	100.49998	265.221208	160.296953	107.074509
ENSG000001	KNTC1	protein_codin	0	3.5859552	1.25603532	0
ENSG000002	TRIM27	protein_codin	0	0	0	0
ENSG000002	CFAP95	protein_codin	0	0	0	0
ENSG000002	TRIM27	protein_codin	22.0422808	56.2361442	110.740176	10.8728416
ENSG000001	PTAR1	protein_codin	40.4679196	0	155.368328	20.4978949
ENSG000001	CD300LG	protein_codin	0	0	0	0
ENSG000001	NMNAT1	protein_codin	7.55844583	8.70186518	13.4864405	2.78904992
ENSG000001	POU4F1	protein_codin	0.58865957	2.99306782	22.487622	1.71557707
ENSG000001	EDNRB	protein_codin	1.71225145	18.8019708	0	5.92175776
ENSG000001	LZIC	protein_codin	21.7308653	53.0613268	38.1954925	13.4096953
ENSG000000	TEAD2	protein_codin	0.62941182	2.15934918	3.67379965	1.12251732
ENSG000001	ENTREP1	protein_codin	1.12437844	0	0	0
ENSG000001	LZIC	protein_codin	18.4032564	27.5640073	21.3519971	8.04302602
ENSG000000	UIMC1	protein_codin	0	6.89939818	0	0
ENSG000001	SLAIN1	protein_codin	0	0	0.68504112	0
ENSG000001	PLXDC2	protein_codin	35.0518993	49.3505694	5.96720299	22.4161189
ENSG000001	PLXDC2	protein_codin	45.4099066	69.0655783	128.011843	0
ENSG000001	SNX15	protein_codin	0	0	0	0
ENSG000001	TJP2	protein_codin	11.701754	0	31.0776798	0
ENSG000001	SCEL	protein_codin	0	0	0	0
ENSG000001	PLXDC2	protein_codin	158.465237	116.513996	62.839086	75.2359741
ENSG000001	THNSL2	protein_codin	0	0	0	0
ENSG000001	ZKSCAN3	protein_codin	0	0	5.77628123	0
ENSG000001	CTNNBIP1	protein_codin	0	12.1505337	3.26043123	0
ENSG000001	CTNNBIP1	protein_codin	0	0	0	1.27940081
ENSG000001	TJP2	protein_codin	0	0	0	0
ENSG000001	CTNNBIP1	protein_codin	1.99369123	7.19558267	18.519487	7.11086448
ENSG000001	ATG2A	protein_codin	2.45535895	51.7562344	24.8752656	12.2054885
ENSG000002	MALRD1	protein_codin	0	0	0	0
ENSG000002	MALRD1	protein_codin	0.64202836	2.85287557	0	0.57224615
ENSG000001	UBA1	protein_codin	9.2899175	3.24087291	13.7962827	9.67841972
ENSG000001	FXN	protein_codin	0	0	0	0
ENSG000001	ARL5B	protein_codin	19.0755132	26.8376067	54.0162587	27.8160609

ENSG000001(	PRKACG	protein_codin	0.25692346	0	0	1.77603298
ENSG000001(	PIP5K1B	protein_codin	0	0	0	0
ENSG000001	HRH2	protein_codin	1.27827875	0	0	0
ENSG00000(	CFAP61	protein_codin	0	0	0	0
ENSG000001	ZKSCAN4	protein_codin	7.10175884	11.6501899	16.5632632	4.55519016
ENSG000001	CLSTN1	protein_codin	90.8741206	155.660667	119.425458	21.7562639
ENSG00000(	CFAP61	protein_codin	0	0	0	0
ENSG000002	NSUN6	protein_codin	15.1061839	31.16016	75.2379346	8.68626387
ENSG00000(	CFAP61	protein_codin	0	0	0	0
ENSG000001	ARHGAP39	protein_codin	0	27.0786377	17.5581749	0
ENSG00000(	CFAP61	protein_codin	0	0	0	0
ENSG000001	TMEM252	protein_codin	0	0	0	0.86845071
ENSG000001	MEN1	protein_codin	0	0	0	0
ENSG000001(	CACNB2	protein_codin	0	0	0	0
ENSG000001	MEN1	protein_codin	0	5.74980149	0	0
ENSG000001(	FOXH1	protein_codin	0.64934593	0	0	0.4011068
ENSG000001(	CACNB2	protein_codin	0	0	0.99965886	0
ENSG000001	MEN1	protein_codin	0	0	5.46138663	0
ENSG000001!	ZNF165	protein_codin	0	0	1.86561154	0
ENSG000001	MEN1	protein_codin	0	0	0	0
ENSG000001(	CRNKL1	protein_codin	0	0	0	0
ENSG000001(	CACNB2	protein_codin	0	0	0	0.81919843
ENSG000001(	CACNB2	protein_codin	0	0	0	4.27815947
ENSG000001(	CACNB2	protein_codin	0	0	0	0
ENSG000001	ST3GAL5	protein_codin	0	0	0	0
ENSG000001(	CRNKL1	protein_codin	0	0	0	0
ENSG000001!	ZNG1C	protein_codin	0	0	0	0
ENSG000001!	ZNG1C	protein_codin	0	0	0	0
ENSG000001	PIK3CD	protein_codin	3.146546	79.4972407	0	11.6598445
ENSG000001(	VPS28	protein_codin	0	0	0	0
ENSG000001!	ZNG1C	protein_codin	0.56931824	0	0	0
ENSG000001(	MAP4K2	protein_codin	3.60363988	0	7.72121731	0
ENSG000001	UBA1	protein_codin	10.6777552	112.913967	0	0
ENSG000001	KCNIP1	protein_codin	0	0	0	0
ENSG000002(	INSYN2B	protein_codin	1.96355897	1.62481368	0.85662078	3.89484761
ENSG000001	SLC39A12	protein_codin	0	0	0	0
ENSG000001	SLC39A12	protein_codin	0	0	0	0
ENSG000001	SLC39A12	protein_codin	0	0	0	0.51707943
ENSG000001	ZNG1E	protein_codin	0	0	0	0
ENSG000001	ZNG1E	protein_codin	2.53609098	0	6.9193755	1.26705521
ENSG000001	TGOLN2	protein_codin	0	127.184669	0	0
ENSG000001(	SF1	protein_codin	0	0	0	0
ENSG000001	ZNG1E	protein_codin	0	0	6.14971749	0
ENSG000001(	SF1	protein_codin	18.3297074	131.620784	91.866707	43.0239726

ENSG000002	ZNG1F	protein_codin	0	0	0	0
ENSG000001	ZNG1E	protein_codin	0	0	0	3.27096527
ENSG000001	SF1	protein_codin	0	0	0	0
ENSG000001	ZNG1E	protein_codin	0	0	0	0
ENSG000001	SPSB1	protein_codin	0	0	0	0
ENSG000001	ZNG1A	protein_codin	0	0	0	0
ENSG000001	H2BC13	protein_codin	5.95709197	21.2219261	86.7483425	0.4641653
ENSG000000	H6PD	protein_codin	66.8884485	133.811564	75.0110426	41.9111256
ENSG000001	GPR157	protein_codin	2.33885363	15.0851675	16.2940416	5.68222093
ENSG000001	FOXD4L4	protein_codin	0	0	0	0.7537992
ENSG000001	SLC2A5	protein_codin	0	0	0.95097808	0
ENSG000000	ZNF184	protein_codin	19.6700947	24.8204414	63.5658859	0
ENSG000002	FOXD4L5	protein_codin	0.2872147	4.51353815	1.84892729	4.30306317
ENSG000001	SLC2A5	protein_codin	1.08200841	2.65859575	0	3.66593585
ENSG000001	AQP7	protein_codin	0	0	0	0
ENSG000001	SCP2D1	protein_codin	0	2.779686	0	0
ENSG000001	TMEM143	protein_codin	1.11573918	8.20572941	0.58989673	1.81008364
ENSG000000	PYGM	protein_codin	0	8.21572108	1.44451793	4.14680782
ENSG000001	CA6	protein_codin	0	0	0	0
ENSG000001	CA6	protein_codin	0	0	0	0
ENSG000001	CA6	protein_codin	0	0	0	0
ENSG000001	ZNG1A	protein_codin	8.37852432	15.0497462	28.2650696	1.98107063
ENSG000001	DTD1	protein_codin	0	5.12361525	0	10.2137591
ENSG000001	CLN5	protein_codin	1.71236294	0	0.91232012	0
ENSG000001	PRSS16	protein_codin	0	0	0	0
ENSG000002	H2AC12	protein_codin	13.8934232	7.36506373	181.079651	4.37052959
ENSG000001	CARD8	protein_codin	5.23711961	26.9150355	1.62147668	0
ENSG000001	ACOD1	protein_codin	0	0	0	0
ENSG000001	RERE	protein_codin	0	0	1.56310719	15.4632128
ENSG000001	SEC23B	protein_codin	0	0	4.66032062	1.61517369
ENSG000001	SPATC1	protein_codin	0.22353148	5.06032826	0	0
ENSG000001	KCTD12	protein_codin	303.666037	792.650845	729.787482	238.379656
ENSG000001	ERRFI1	protein_codin	46.3871948	71.4487404	251.886938	90.2481779
ENSG000000	RASGRP2	protein_codin	0	0	0	0
ENSG000000	RASGRP2	protein_codin	0	0	0	0
ENSG000000	RASGRP2	protein_codin	0	0	0	0
ENSG000001	PARK7	protein_codin	0	0	0	0
ENSG000000	RASGRP2	protein_codin	0	0	0	0
ENSG000001	PARK7	protein_codin	51.380609	0	140.10056	0
ENSG000001	PARK7	protein_codin	0	7.42366599	0	0
ENSG000000	RASGRP2	protein_codin	0	0	0	0
ENSG000001	TMEM236	protein_codin	0.73844784	4.35864255	1.46233994	2.14705903
ENSG000000	RASGRP2	protein_codin	1.32970058	0	1.67680817	0
ENSG000001	LMO7	protein_codin	16.7935584	226.533286	29.0572323	8.79986311

ENSG000001	STAM	protein_codin	5.29237858	0	2.10238793	4.87268949
ENSG000001	CCNJL	protein_codin	1.45905799	0	0	1.30558088
ENSG000001	CNTNAP4	protein_codin	0	5.94767067	0	3.53040841
ENSG000000	TNFRSF9	protein_codin	4.19683339	26.7746894	1.59373614	5.40926034
ENSG000000	UTS2	protein_codin	0.28364185	5.03100059	74.8039508	3.79699294
ENSG000001	STAM	protein_codin	8.61718532	22.9751411	23.0536018	9.90824505
ENSG000001	AUP1	protein_codin	16.7382659	62.711312	122.361967	16.798279
ENSG000000	PER3	protein_codin	45.2715028	171.119506	82.002324	0
ENSG000001	SCRIB	protein_codin	0	0	0	0
ENSG000001	LMO7	protein_codin	0	0	0	0
ENSG000002	ODAD4	protein_codin	3.36464528	0	2.95065725	2.51426243
ENSG000000	PER3	protein_codin	24.8559835	181.698469	56.9081816	4.0789555
ENSG000001	METTL3	protein_codin	0	0	0	0
ENSG000001	NRXN2	protein_codin	4.43540173	0	1.19891144	0
ENSG000001	HECTD4	protein_codin	74.333651	91.3891618	0	74.048357
ENSG000001	CNTNAP3B	protein_codin	0	0	3.6302494	1.67050185
ENSG000001	LBX2	protein_codin	0	0	0	0
ENSG000001	SLC22A12	protein_codin	0	0	0	0
ENSG000001	SLC22A12	protein_codin	0	0	0	0
ENSG000001	SLC22A12	protein_codin	0	0	0	0
ENSG000001	HMGN4	protein_codin	39.7649123	98.7510373	153.90735	14.7638659
ENSG000000	DNAJC11	protein_codin	0	0	3.78805575	0
ENSG000001	PYCR3	protein_codin	0	0	0	0
ENSG000001	SLC22A11	protein_codin	0	0	0	0
ENSG000001	SLC22A11	protein_codin	0	0	0	0
ENSG000001	UCHL3	protein_codin	4.33711157	14.8240689	14.9020556	0
ENSG000001	BTN2A1	protein_codin	0	94.8188498	6.6748648	10.8869481
ENSG000001	ST8SIA6	protein_codin	1.8489347	2.53224345	4.42825656	5.06936156
ENSG000001	POLR3F	protein_codin	0	0	0	0
ENSG000001	RBM10	protein_codin	0	0	0	23.4190494
ENSG000001	COMMD6	protein_codin	0	7.978926	0	0
ENSG000001	COMMD6	protein_codin	2.06650648	0	1.20597685	4.79498751
ENSG000001	COMMD6	protein_codin	1.0475095	6.87360035	0	0
ENSG000001	TBC1D4	protein_codin	139.757064	197.68021	144.538563	51.3752411
ENSG000000	THAP3	protein_codin	1.12420043	6.11076853	2.09705409	1.38175542
ENSG000000	DZANK1	protein_codin	0	0	0	0
ENSG000001	SLC4A5	protein_codin	0	0	0	0
ENSG000001	VIPR2	protein_codin	0	0	0	0.78353022
ENSG000001	SLC4A5	protein_codin	0	0	0	0
ENSG000001	TBC1D4	protein_codin	4.56192724	4.37534191	0	0
ENSG000002	SPATA31A1	protein_codin	0.79271546	4.83354189	0	3.78562105
ENSG000001	PHF13	protein_codin	11.5649677	41.1126025	33.0778058	20.3281658
ENSG000001	CNTNAP3	protein_codin	0	0	0.58862145	0
ENSG000002	TCTN1	protein_codin	0	0	0	0

ENSG000001	CNTNAP3	protein_codin	0	10.7135334	0	0
ENSG000001	KLHL21	protein_codin	11.149425	23.5167193	0	0
ENSG000001	CNTNAP3	protein_codin	0.19714079	0	0	0
ENSG000001	GALNT10	protein_codin	0	0	0	0
ENSG000001	KLHL21	protein_codin	2.16901018	14.0643632	32.7285642	55.891429
ENSG000001	KLF12	protein_codin	50.9024079	130.74096	0	17.6461501
ENSG000001	HIF3A	protein_codin	4.27358898	2.83001957	0.62257301	2.43291157
ENSG000001	ZNF133	protein_codin	4.21656747	0	0	0
ENSG000002	FAM216A	protein_codin	3.26713918	13.0195375	0	0
ENSG000002	ZBTB48	protein_codin	3.95943548	0	9.49643144	2.49551873
ENSG000001	CYP11B1	protein_codin	0	0	0	0
ENSG000001	KAT14	protein_codin	18.2723359	0	20.5950164	5.75071748
ENSG000001	ATP2A2	protein_codin	91.728031	80.3090365	102.649635	69.9306547
ENSG000001	KLF5	protein_codin	10.3871729	11.0614317	11.9186784	2.43516516
ENSG000002	IGFL2	protein_codin	0	0	0	0
ENSG000001	IGFBPL1	protein_codin	0.28510022	1.32476135	4.30970977	0.86240017
ENSG000002	IGFL4	protein_codin	0	0	1.87158377	1.96911275
ENSG000001	ALDH1B1	protein_codin	7.50997156	8.73653826	19.5199904	6.00755304
ENSG000001	GPR137	protein_codin	0	4.63513506	5.64025885	2.16177926
ENSG000001	MGME1	protein_codin	0	0	1.14208988	0
ENSG000001	NOL9	protein_codin	29.7485943	67.3172921	75.1426528	24.7922448
ENSG000001	SHB	protein_codin	2.03656525	22.673181	21.4418037	28.341493
ENSG000001	BTN3A2	protein_codin	10.8985935	152.46047	0	23.0903803
ENSG000001	MGME1	protein_codin	0	0	0	0
ENSG000001	MGME1	protein_codin	14.239664	26.6087587	36.7160611	8.98129011
ENSG000002	NAT8B	protein_codin	0	0	0	0
ENSG000001	SLC36A3	protein_codin	0	0	0	0
ENSG000001	SLC25A51	protein_codin	2.79428603	14.2992715	9.28842834	2.35746731
ENSG000002	KRTAP9-2	protein_codin	0.55098546	0	0	0
ENSG000001	DCAF10	protein_codin	7.39205834	40.4264183	11.6084468	10.4475174
ENSG000001	PLEKHG5	protein_codin	0	0	0	0
ENSG000002	KRTAP4-2	protein_codin	0	0	0	0.6253265
ENSG000001	H4C8	protein_codin	14.2069618	16.9281085	29.8254522	4.10027911
ENSG000001	PLEKHG5	protein_codin	0	0	0	0
ENSG000001	PLEKHG5	protein_codin	24.8343879	36.5278694	4.44990365	4.98034859
ENSG000002	H2BC10	protein_codin	5.53249901	81.2048031	0	2.50726724
ENSG000001	DMWD	protein_codin	0	93.0201423	0	0
ENSG000001	PLEKHG5	protein_codin	0	4.5119506	0	1.94602154
ENSG000002	GPR20	protein_codin	0.62318678	1.21156053	0	0.88321915
ENSG000002	H4C2	protein_codin	2.13335	15.8612016	19.5145058	0.81325334
ENSG000002	KRTAP1-4	protein_codin	0	0	0	0
ENSG000001	PLEKHG5	protein_codin	0	0	0	0
ENSG000001	ANXA6	protein_codin	57.7119017	0	109.289298	0
ENSG000001	TRMT10B	protein_codin	0	0	0	0

ENSG000001	TRMT10B	protein_codin	0	0	0	0
ENSG000002	KRT40	protein_codin	0	0	0	0
ENSG000000	SNX5	protein_codin	12.4467489	7.91099273	18.7664597	10.9732679
ENSG000000	FRMPD1	protein_codin	0.33109544	2.71197934	2.34263852	10.2031333
ENSG000000	DIS3	protein_codin	7.82560857	11.5780925	4.96542015	12.0167407
ENSG000000	SNX5	protein_codin	1.77754599	3.39923147	3.75669806	0
ENSG000001	DPP6	protein_codin	0	0	0	1.72526314
ENSG000001	TOMM5	protein_codin	0.73712816	0	6.13860825	0
ENSG000001	ACTR3B	protein_codin	0	9.6520221	0	0
ENSG000001	H2BC5	protein_codin	17.4216522	93.3238687	247.497879	21.8502048
ENSG000000	DIS3	protein_codin	19.1672414	34.3308438	16.0526546	18.7867637
ENSG000002	TNFRSF25	protein_codin	0	0	0	0
ENSG000001	H2AC6	protein_codin	0	88.4819586	33.6385737	19.1398998
ENSG000001	POLR1E	protein_codin	0	0	0	0
ENSG000001	NAA40	protein_codin	11.8951171	16.9845043	37.9007814	0
ENSG000000	CD74	protein_codin	124.685302	0	27.669832	25.5231132
ENSG000000	TCOF1	protein_codin	0	0	0	0
ENSG000001	POLR1E	protein_codin	12.5086348	31.4838402	48.4907753	0
ENSG000001	TRDMT1	protein_codin	5.9356494	37.1465023	29.433934	9.45437947
ENSG000001	PLCXD3	protein_codin	2.90252183	0	0	1.90719629
ENSG000001	H4C3	protein_codin	11.6941858	15.6717614	59.7797809	8.15917211
ENSG000001	BANF2	protein_codin	0	0	0	0
ENSG000000	CEACAM1	protein_codin	0	0	0	0
ENSG000001	RRBP1	protein_codin	12.5881984	193.903215	20.9191449	6.31636759
ENSG000000	SMARCE1	protein_codin	0	0	0	0
ENSG000000	MARK2	protein_codin	0	4.09276368	14.1787927	4.23981399
ENSG000001	NDUFB11	protein_codin	2.89279	53.8716	8.2993267	8.30973746
ENSG000001	RRBP1	protein_codin	186.237174	0	223.614156	117.724238
ENSG000001	BORA	protein_codin	0	0	0	0
ENSG000002	MZT1	protein_codin	12.0526684	16.4508282	12.4914799	4.59736149
ENSG000001	RTN3	protein_codin	0	0	0	0
ENSG000001	CUBN	protein_codin	0.78466365	4.4577668	1.39165441	1.42028868
ENSG000001	GRHPR	protein_codin	2.59601717	9.68915972	0	0
ENSG000001	CUBN	protein_codin	7.90915987	26.1471658	19.7946998	9.15164675
ENSG000000	HES2	protein_codin	0	0	0.56613294	0.45006445
ENSG000000	HES2	protein_codin	0	0	0	0
ENSG000000	HES2	protein_codin	0	1.28159108	0	0
ENSG000000	ZFAT	protein_codin	0	0	0	0
ENSG000001	PAX5	protein_codin	0	0	0	0
ENSG000000	ACOT7	protein_codin	0.56984644	0	0	3.01409304
ENSG000001	CSNK1A1	protein_codin	68.8605146	58.6929416	28.3842905	42.4909467
ENSG000001	KLHL1	protein_codin	0	0	0	0
ENSG000000	ACOT7	protein_codin	0	0	0	0
ENSG000001	PAX5	protein_codin	0	0	0	0

ENSG0000007	ACACB	protein_codin	39.7722708	160.462936	10.7323487	22.8275012
ENSG000001	SLC17A2	protein_codin	0	0	0	0
ENSG000001	PAX5	protein_codin	0	0	0	0
ENSG000001	PAX5	protein_codin	0	0	0	0
ENSG0000007	ACACB	protein_codin	16.8296056	64.321609	0	17.6304435
ENSG000000	ACOT7	protein_codin	0	0	0	0
ENSG000000	ACOT7	protein_codin	0	0	0	0
ENSG000001	PCDH9	protein_codin	217.358135	258.292897	154.96965	50.7049068
ENSG000001	CCN4	protein_codin	0	0	0	0
ENSG000001	PCDH9	protein_codin	0	0	0	22.2182408
ENSG000001	ASB10	protein_codin	0	0	0	0
ENSG000001	BFSP1	protein_codin	0	0	0	3.63150175
ENSG000001	SLC22A6	protein_codin	0	0	0	0
ENSG000001	BFSP1	protein_codin	9.36567046	0	0	0
ENSG000001	RNF38	protein_codin	0	15.3138436	3.41554374	1.26758258
ENSG000000	TDRD3	protein_codin	27.8754303	0	31.48764	13.952364
ENSG000000	TDRD3	protein_codin	0	0	0	0
ENSG000001	USP30	protein_codin	0	3.19193012	0	0
ENSG000001	RNF38	protein_codin	11.0975936	0	0	0
ENSG000001	SLC17A1	protein_codin	0	0	0	0
ENSG000001	HTR4	protein_codin	0	0	0	0
ENSG000001	SLC3A2	protein_codin	0	0	0	0
ENSG000001	SLC3A2	protein_codin	1.20725666	0	0	0
ENSG000001	SLC3A2	protein_codin	0	0	0	0
ENSG000001	GPR153	protein_codin	21.6519182	19.9228423	18.6490907	10.8218346
ENSG000000	TDRD3	protein_codin	0	55.758499	0	28.1549767
ENSG000001	STX5	protein_codin	0	0	0	0
ENSG000001	PCSK2	protein_codin	0.46288121	4.07325856	0	3.60072955
ENSG000001	TMEM71	protein_codin	1.56808941	2.57063505	0	1.57347638
ENSG000002	ASIC3	protein_codin	0	0	0	2.1822181
ENSG000001	SLC17A4	protein_codin	0	1.54709424	0	5.61273985
ENSG000002	SPINK9	protein_codin	0.78716656	0	0	0
ENSG000001	DIAPH3	protein_codin	0	0	44.8906955	0
ENSG000001	RSU1	protein_codin	31.5729765	52.657616	54.8429977	17.5963155
ENSG000001	PCDH17	protein_codin	7.99274639	69.4117239	22.6494496	45.6594937
ENSG000001	KRBOX4	protein_codin	0.58680285	0	1.46509882	2.09660682
ENSG000001	RSU1	protein_codin	0	4.44611309	0	0
ENSG000002	LRRC3C	protein_codin	0	0	0	0
ENSG000001	ADCY8	protein_codin	0	0	2.28670433	2.49633256
ENSG000002	PRR20A	protein_codin	0	0	0	2.10379519
ENSG000001	DIPK2B	protein_codin	2.20574668	4.9269062	1.87417593	3.71715107
ENSG000001	GKN1	protein_codin	0	0	0	0.44389264
ENSG000001	RNF207	protein_codin	0.42785965	6.29618027	8.27443377	2.0527268
ENSG000001	ZPBP2	protein_codin	0	0	0	0

ENSG000001:	PCDH8	protein_codin	0	0.89669134	1.15218376	0
ENSG000001:	SNRPB2	protein_codin	29.5612467	38.7430433	48.2229596	23.4617741
ENSG000001	IKZF3	protein_codin	0	0	0	0
ENSG000001	IKZF3	protein_codin	0	0	0	0
ENSG000001	LYPD5	protein_codin	0	0	0	0
ENSG000001	IKZF3	protein_codin	0	0	0	0
ENSG000002	UQCC3	protein_codin	1.37801816	0	6.69482451	0
ENSG000001	LBHD1	protein_codin	0	0	0	0
ENSG000002	FBXO48	protein_codin	1.43374334	1.43657529	3.6701795	1.94881649
ENSG000001	IKZF3	protein_codin	0	0	0	5.11776639
ENSG000001:	GLIPR2	protein_codin	0	7.32870311	0	0
ENSG000001:	GLIPR2	protein_codin	6.50163638	9.47279043	0	0
ENSG000000	SCGN	protein_codin	0	0	0	0
ENSG000001:	CNMD	protein_codin	0	0	0	0
ENSG000001:	RECK	protein_codin	24.9297501	37.6114893	34.5564339	11.1126444
ENSG000001	KDM6A	protein_codin	1.3852008	0	0	0
ENSG000001:	MYC	protein_codin	5.15226842	31.5607984	72.8967595	30.2901115
ENSG000001:	CIR1	protein_codin	1.25737454	1.57305998	0	1.54254063
ENSG000002	GRXCR2	protein_codin	0	0	0	0.52005648
ENSG000001	COX7A2	protein_codin	0	0	0	0
ENSG000001	OR13J1	protein_codin	0	1.34160772	0	3.05327664
ENSG000001:	ACTR2	protein_codin	0	0	0	0
ENSG000002	FAM221B	protein_codin	0	0	0	0
ENSG000001:	TMEM8B	protein_codin	0	0	0	0
ENSG000000	CEP68	protein_codin	0	0	0	2.41453214
ENSG000001:	TMEM8B	protein_codin	12.2360832	41.2127162	15.7625121	0
ENSG000001:	TMEM8B	protein_codin	0	0	0	0
ENSG000001	CHD5	protein_codin	0	0	0	0
ENSG000001	PTER	protein_codin	0	0	0	0
ENSG000001	PGAP3	protein_codin	0	0	0	0
ENSG000002	CD177	protein_codin	0	0	0	0
ENSG000001	ARHGAP26	protein_codin	0	0	0	0
ENSG000001	MTSS1	protein_codin	23.835989	0	94.4571062	0
ENSG000001	RIPOR2	protein_codin	0	0	0	0
ENSG000001:	AHNAK	protein_codin	4127.19026	6523.1555	4275.12999	2524.65374
ENSG000001:	CKAP4	protein_codin	76.3711649	173.734883	119.963023	68.6716452
ENSG000001:	CKAP2	protein_codin	2.54681973	62.30669	44.5131147	8.63384093
ENSG000001	MINDY3	protein_codin	0.97034701	0	0	0
ENSG000001:	CKAP2	protein_codin	18.5872132	44.978949	105.856976	16.7101995
ENSG000001	BEST1	protein_codin	0	0	0	0
ENSG000000	FUNDC1	protein_codin	5.59931015	11.0543191	9.06515999	6.28839942
ENSG000001	FGF1	protein_codin	0	4.54367928	0	3.63683009
ENSG000002	ZNF783	protein_codin	2.28482294	18.1023527	0	3.88662781
ENSG000001:	FLRT3	protein_codin	3.76346888	10.5331748	0	2.66575295

ENSG000001	GMNN	protein_codin	7.27488256	12.7711865	61.876108	0
ENSG000001	GMNN	protein_codin	0	0	0	0
ENSG000001	VPS36	protein_codin	74.3239174	112.148709	65.1874113	19.7273854
ENSG000002	ZNF425	protein_codin	6.66380412	8.03027793	11.0631327	3.28155865
ENSG000000	MAOB	protein_codin	4.47523751	16.1877745	0	7.17699029
ENSG000001	SEL1L2	protein_codin	0	0	0	0
ENSG000001	MEGF8	protein_codin	0	65.4085472	0	0
ENSG000002	LRRC10B	protein_codin	0	0	1.18025787	0.45386363
ENSG000000	ITGA8	protein_codin	6.05680352	19.6064684	84.3497073	8.86021786
ENSG000001	RGP1	protein_codin	18.049256	92.1580958	43.3621948	21.1614912
ENSG000002	FBXO47	protein_codin	0	1.81761513	0	1.11561195
ENSG000001	NDUFAF5	protein_codin	1.34397127	0	0	0
ENSG000000	KCNAB2	protein_codin	0	0	0	0
ENSG000000	GBA2	protein_codin	0	6.36594029	0	3.03228723
ENSG000002	UQCC6	protein_codin	0	0	0	0
ENSG000000	KCNAB2	protein_codin	0	9.30651665	0	0
ENSG000000	GBA2	protein_codin	0	0	0	0
ENSG000001	RPL23	protein_codin	0	0	0	0
ENSG000000	KCNAB2	protein_codin	3.80416356	0	3.23228236	4.71791885
ENSG000001	TPK1	protein_codin	0	3.4457596	0	0
ENSG000001	TPK1	protein_codin	0	0	0	0
ENSG000001	C6orf62	protein_codin	0	15.1663489	8.88433252	0
ENSG000000	GBA2	protein_codin	13.8071723	19.3226381	24.8687351	5.72346316
ENSG000002	PCDHGA1	protein_codin	0	12.6705993	1.57868935	3.17530678
ENSG000001	NDUFAF5	protein_codin	3.29888593	24.8099833	2.7876457	4.34605102
ENSG000000	KCNAB2	protein_codin	0	0	0.73569217	0
ENSG000001	C12orf42	protein_codin	0	0	0	0
ENSG000002	ARHGEF35	protein_codin	0	0	5.49725375	0
ENSG000001	FAM171A1	protein_codin	28.2817235	144.643002	54.3601619	20.8986506
ENSG000001	C6orf62	protein_codin	39.4465861	0	100.110244	36.2266687
ENSG000002	PCDHA9	protein_codin	1.10406332	7.58233296	5.25989322	0.97593629
ENSG000002	PCDHA8	protein_codin	0.57484134	9.13741229	3.62221621	0.53303739
ENSG000000	PCDHA6	protein_codin	0	6.72253079	8.20932921	0
ENSG000001	PPP1R2C	protein_codin	0	0	0	0.37640233
ENSG000002	PCDHA2	protein_codin	0.36644276	2.72841282	0	0.63075053
ENSG000002	PCDHA1	protein_codin	0	1.12530913	0	0
ENSG000001	GPR34	protein_codin	1.14403316	0	0	0
ENSG000001	GPR34	protein_codin	26.4365197	39.6212875	9.6874827	28.1540215
ENSG000002	PGA4	protein_codin	0	0	0	0
ENSG000001	NMT2	protein_codin	14.115679	41.0479475	63.7857373	14.8584972
ENSG000000	ARHGEF1	protein_codin	5.30994715	25.0238567	0	5.55772987
ENSG000001	CASK	protein_codin	0	0	0	0
ENSG000001	NPHP4	protein_codin	0	0	0	2.39575954
ENSG000001	CASK	protein_codin	0	0	0	0

ENSG000001:	NPHP4	protein_codin	0.4403578	0	0	0.44054382
ENSG000001	CASK	protein_codin	7.88226392	34.1282978	0	0
ENSG000000	TAF2	protein_codin	0	0	0	0
ENSG000001	NMT2	protein_codin	21.7608609	62.1246819	29.3597821	8.04128327
ENSG000001	CASK	protein_codin	40.2559718	54.8396848	3.27923022	10.2426363
ENSG000001	CASK	protein_codin	0	0	0	0
ENSG000001:	NPHP4	protein_codin	5.74823829	22.2183508	9.59972637	0
ENSG000001	CASK	protein_codin	0.99534375	0	2.35999781	1.53222716
ENSG000002	TRIM13	protein_codin	17.3459043	25.3296213	18.2190105	5.6727349
ENSG000002	TRIM13	protein_codin	0	0	0	0
ENSG000002	MS4A13	protein_codin	0	0	0	0
ENSG000002	MS4A13	protein_codin	0	0	0	0
ENSG000002	ERICH4	protein_codin	0.59287207	1.56833721	0	2.37980674
ENSG000001	AJAP1	protein_codin	0.56814975	0	0	0
ENSG000001	AJAP1	protein_codin	8.89842441	11.1432161	1.48368508	6.8921805
ENSG000001	TLN1	protein_codin	65.2211108	60.1961774	129.436447	56.8284636
ENSG000001	SPRYD7	protein_codin	0.4704159	1.58959624	0	1.07204635
ENSG000001	RPP38	protein_codin	0	0	0	0
ENSG000001	TDP2	protein_codin	14.9845834	40.7755392	58.2142567	13.6937612
ENSG000001	RPP38	protein_codin	4.79221675	0	0	0
ENSG000001	RPP38	protein_codin	2.43409254	15.1477056	7.33653311	2.11429721
ENSG000001	RPP38	protein_codin	0	0	0	0
ENSG000001	EXT1	protein_codin	30.6629837	91.4081477	82.4539707	88.782624
ENSG000001	DFFB	protein_codin	0	0	17.4643001	0
ENSG000001	DFFB	protein_codin	0	0	0	0
ENSG000001	KIAA0319	protein_codin	0	0	0	0
ENSG000001	HNRNPUL1	protein_codin	0	0	0	0
ENSG000001	OLAH	protein_codin	0	0	0	0
ENSG000001	NYX	protein_codin	0	0	0	0
ENSG000001	CEP104	protein_codin	0.76229095	0	0.63686176	0
ENSG000001	OLAH	protein_codin	0	0	0	0
ENSG000001	BTBD3	protein_codin	5.04422396	10.7473879	0	6.41025921
ENSG000001	OLAH	protein_codin	0	0	0	0.58685028
ENSG000001	CEP104	protein_codin	0	0	0	0
ENSG000001	FOXP2	protein_codin	0	2.19474999	8.09634515	0.57069009
ENSG000001	NRG2	protein_codin	1.0887053	6.10807676	0	0
ENSG000000	ERLEC1	protein_codin	29.465515	0	0	0
ENSG000001	MEIG1	protein_codin	0.3140254	0.99576556	0.49821901	0
ENSG000001	DCLRE1C	protein_codin	0.9142235	0	0	0
ENSG000001	DCLRE1C	protein_codin	0.4644477	0	0.88582389	0
ENSG000001	GPLD1	protein_codin	0	0	0	0
ENSG000001	DEPDC4	protein_codin	0	0	0	0.46692061
ENSG000001	DCLRE1C	protein_codin	0	0	0	0
ENSG000002	SPIRE2	protein_codin	0	0	2.37859268	0

ENSG000001	DCLRE1C	protein_codin	0	0	0	0
ENSG000001	LRRC47	protein_codin	9.28643104	58.0908109	43.0295194	13.335109
ENSG000001	DCLRE1C	protein_codin	0	0	0	0
ENSG000001	DCLRE1C	protein_codin	0	0	0	0
ENSG000001	DCLRE1C	protein_codin	0	0	0	0
ENSG000001	NRXN1	protein_codin	0	0	0	0
ENSG000001	EBPL	protein_codin	0	2.00052048	0	0
ENSG000001	EBPL	protein_codin	3.03413616	0	0	0
ENSG000001	EBPL	protein_codin	0	0	8.46576412	0
ENSG000001	CDK10	protein_codin	1.08685449	7.72776131	0	0.54988258
ENSG000001	DCLRE1C	protein_codin	15.2073113	28.137977	17.1978309	11.2883007
ENSG000002	AARD	protein_codin	0.76351855	0	2.54319088	1.09082022
ENSG000000	TP73	protein_codin	0	0	0	0
ENSG000001	EBPL	protein_codin	0	0	0	0
ENSG000000	TP73	protein_codin	0	0	0	0
ENSG000000	TP73	protein_codin	0	0	10.883117	1.5830386
ENSG000001	DCLRE1C	protein_codin	1.70874959	0	0	0.99910264
ENSG000000	TP73	protein_codin	0	0	11.4529531	0
ENSG000001	TPM2	protein_codin	3.84102846	420.478591	31.13572	8.00976732
ENSG000000	TP73	protein_codin	0	0	0	0
ENSG000001	RCBTB1	protein_codin	15.7112288	31.5808639	21.1532043	12.6500593
ENSG000001	RAB4B	protein_codin	0	0	0	0
ENSG000001	USP9X	protein_codin	18.92167	0	0	0
ENSG000002	TMX2	protein_codin	0	0	0	0
ENSG000001	ACTMAP	protein_codin	0.81031799	0	5.49491418	3.68450253
ENSG000001	PHF11	protein_codin	0	318.896968	0	45.9207294
ENSG000001	WRAP73	protein_codin	2.50414598	8.65762011	9.41597653	2.97869722
ENSG000001	SERPING1	protein_codin	0.80248561	14.2918781	0	0
ENSG000001	SERPING1	protein_codin	0	0	15.1423597	0
ENSG000001	SUV39H2	protein_codin	0	0	0	0
ENSG000001	ANKRD11	protein_codin	0	97.3100488	21.4332479	0
ENSG000001	SUV39H2	protein_codin	9.38244554	0	0	2.76702607
ENSG000001	ANKRD11	protein_codin	0	1.30520258	14.043299	0
ENSG000001	REEP2	protein_codin	0	0	0	0
ENSG000001	TPRG1L	protein_codin	9.00424422	34.7706375	15.313058	6.66744391
ENSG000001	ACSF3	protein_codin	0	13.191416	0	0
ENSG000001	MRS2	protein_codin	0	12.9974137	3.51951153	1.04222461
ENSG000001	CA9	protein_codin	0	0	0	0
ENSG000001	GFRA3	protein_codin	0.3572464	0	0	0
ENSG000001	APRT	protein_codin	2.79700847	11.9472457	7.8043199	0
ENSG000001	ARHGEF16	protein_codin	0	0	0	0
ENSG000001	HSPA14	protein_codin	5.15037056	4.77647267	19.3201286	11.7451508
ENSG000001	ARHGEF16	protein_codin	0	0	0.94212719	0.40173281
ENSG000001	ARHGEF16	protein_codin	0	0	0	0

ENSG000001	ANKEF1	protein_codin	4.99314796	6.31597102	16.7953258	5.94300038
ENSG000001	FNDC3A	protein_codin	21.0193336	52.4741208	11.4362469	0
ENSG000001	MRS2	protein_codin	15.2482783	23.658541	38.237132	9.47345335
ENSG000001	ARHGEF39	protein_codin	0	0	1.69855181	0
ENSG000001	PRDM16	protein_codin	0.3323325	0	0	0.98255858
ENSG000001	PRDM16	protein_codin	0	0	6.55011999	0
ENSG000001	ANKEF1	protein_codin	0	0	0	0
ENSG000002	OR5D16	protein_codin	0	0	0	0
ENSG000002	OR5L2	protein_codin	0	0	0	0
ENSG000002	PKHD1L1	protein_codin	1.19297619	10.5400113	0.88042031	6.62431059
ENSG000001	ACTRT2	protein_codin	0.32169757	0	0	1.69600558
ENSG000001	CCDC107	protein_codin	0	8.46140902	0	0
ENSG000001	CCDC107	protein_codin	0	0	0	0
ENSG000001	CCDC107	protein_codin	1.60757665	10.6738156	0	0
ENSG000001	MMEL1	protein_codin	0	0	0	0
ENSG000001	CXorf38	protein_codin	0.98272192	7.51360997	9.92689916	4.5810659
ENSG000001	PAK5	protein_codin	0.35047649	0	2.22308694	0
ENSG000001	PRXL2B	protein_codin	0	0	0	0
ENSG000001	PRXL2B	protein_codin	0	15.7909843	0	3.96733247
ENSG000001	CXorf38	protein_codin	1.19283321	8.19441998	3.62823643	8.62474053
ENSG000001	PRXL2B	protein_codin	0	0	0	0
ENSG000001	SLC13A4	protein_codin	0	0	0	0
ENSG000001	PAK5	protein_codin	0	0	0	2.96683011
ENSG000001	CD72	protein_codin	0	0	0	0
ENSG000001	CD72	protein_codin	0	0	1.38079406	0
ENSG000001	LPAR6	protein_codin	1.70229396	0	0	3.9514598
ENSG000001	ATP6AP2	protein_codin	0	0	39.1225719	22.4805038
ENSG000001	CDNF	protein_codin	0	0	0	0
ENSG000001	CDNF	protein_codin	0	0	0	0
ENSG000001	BCOR	protein_codin	0	53.9362789	7.75915086	15.0593914
ENSG000001	ZNF573	protein_codin	0	0	0	0
ENSG000001	DCDC2	protein_codin	0.30847044	3.68320331	0	0
ENSG000001	HES5	protein_codin	0	0	0	0.37161778
ENSG000001	DCDC2	protein_codin	0	0	3.15023883	3.35248882
ENSG000001	BCOR	protein_codin	12.833528	3.82367635	0	0
ENSG000000	FAM107B	protein_codin	29.7071549	0	0	11.7948565
ENSG000000	TRPC7	protein_codin	0	0	0	0
ENSG000000	NUP160	protein_codin	38.6629573	73.4427585	61.3636005	11.881363
ENSG000000	FAM107B	protein_codin	0	0	0	0
ENSG000001	BCOR	protein_codin	12.1111445	0	0	0
ENSG000000	FAM107B	protein_codin	0	86.9344764	67.9171319	0
ENSG000001	PANK4	protein_codin	3.02273468	13.472517	27.9372841	7.10035361
ENSG000000	FAM107B	protein_codin	2.59122091	40.2981082	2.10196949	5.05548446
ENSG000000	FAM107B	protein_codin	5.77846348	23.6928967	1.27100928	0

ENSG000001	PLCB4	protein_codin	0	0	0	0
ENSG000001	MID1IP1	protein_codin	0	27.2185872	0	7.98577789
ENSG000001	NRSN1	protein_codin	0	0	0	0
ENSG000001	NRSN1	protein_codin	0	0	0	0
ENSG000001	NRSN1	protein_codin	0	0	0.43533769	0
ENSG000001	TSPAN7	protein_codin	8.30549989	0	0	12.0801741
ENSG000001	PLCH2	protein_codin	0	0	0	0
ENSG000001	NRSN1	protein_codin	0	4.04670342	0	2.04170816
ENSG000001	PLCB4	protein_codin	0	0	0	0
ENSG000001	CAMKMT	protein_codin	2.9018409	0	10.4560515	0
ENSG000001	UNC13B	protein_codin	40.0745883	0	33.6754328	0
ENSG000001	PLCB4	protein_codin	0	0	0	0
ENSG000002	SLC35B4	protein_codin	20.908587	18.1961669	68.0243728	11.8381485
ENSG000001	PREPL	protein_codin	0	0	0	0
ENSG000001	RER1	protein_codin	0	19.9221121	0	0
ENSG000001	RER1	protein_codin	10.3123888	34.2007036	48.9296249	16.4432538
ENSG000001	RER1	protein_codin	0	0	0	0
ENSG000001	PREPL	protein_codin	14.0981104	0	68.6107984	6.89503802
ENSG000001	MORN1	protein_codin	0	1.55503849	0	0
ENSG000000	LIG3	protein_codin	18.3733323	47.7462466	51.2901677	8.26453065
ENSG000001	MORN1	protein_codin	0	5.67572148	3.2043816	0
ENSG000001	MORN1	protein_codin	0	2.05308509	3.03618711	0
ENSG000001	SRPX	protein_codin	30.4495327	105.536258	0	85.4639092
ENSG000000	KITLG	protein_codin	25.5131723	0	46.2631252	29.1531601
ENSG000001	SKI	protein_codin	103.205959	512.437402	295.345455	131.936675
ENSG000000	SPI1	protein_codin	12.633566	38.3390335	0	15.5382708
ENSG000002	PLXNA4	protein_codin	0	0	0.92086384	0.62430884
ENSG000001	PPM1B	protein_codin	0	0	12.8195969	0
ENSG000001	FAAP20	protein_codin	0	0	0	0
ENSG000001	FAAP20	protein_codin	0	14.5881986	3.47953317	0
ENSG000001	ITM2B	protein_codin	66.6562122	301.322615	63.9196977	103.345954
ENSG000001	PPM1B	protein_codin	11.4567778	17.2545205	16.8052042	5.44890811
ENSG000001	DNAAF1	protein_codin	0	0	4.46510743	0
ENSG000001	PODXL	protein_codin	16.9946852	121.261845	39.0427685	35.5302875
ENSG000000	ATOSB	protein_codin	0	0	0	3.41351949
ENSG000000	TCF7	protein_codin	0	0	0	0
ENSG000000	ATOSB	protein_codin	6.1863022	17.7924149	2.25489395	6.49505767
ENSG000000	ATOSB	protein_codin	0	0	0	0
ENSG000000	PRKCZ	protein_codin	0.71580569	0	7.14569442	0
ENSG000001	CCL7	protein_codin	0	0	0	0
ENSG000001	PRPF18	protein_codin	35.9847653	55.0424672	74.2411351	30.8078772
ENSG000001	MEST	protein_codin	0	0	0	0
ENSG000001	DYNLT3	protein_codin	17.0751416	39.9203147	2.64443807	14.1245088
ENSG000001	DYNLT3	protein_codin	0.34636047	0	0	0

ENSG000001	GABRD	protein_codin	0	0	0	0
ENSG000001	MED4	protein_codin	24.2968583	32.9769554	26.2399516	13.6005749
ENSG000001	DYNC2LI1	protein_codin	0.97908155	0	6.62215112	1.24209976
ENSG000001	CYBB	protein_codin	39.7610509	66.3102049	7.77721437	38.3993467
ENSG000001	CFAP74	protein_codin	0	0	0	0
ENSG000001	CFAP74	protein_codin	0	1.07689651	0	0
ENSG000000	AFF4	protein_codin	0	0	0	0
ENSG000000	AFF4	protein_codin	31.6448361	148.209512	117.920198	54.7019412
ENSG000001	TMEM52	protein_codin	0	0	1.19608078	0
ENSG000001	DDB2	protein_codin	1.03758417	0	0	0
ENSG000001	DDB2	protein_codin	0	0	0	0
ENSG000001	TMEM52	protein_codin	0	0	0	0
ENSG000001	DDB2	protein_codin	0	0	0	0
ENSG000001	CALML6	protein_codin	0	0	0	0
ENSG000001	BEND7	protein_codin	0	0	0	0
ENSG000000	GNB1	protein_codin	74.1480625	244.595281	240.889476	71.0720692
ENSG000001	CDKAL1	protein_codin	0	0	0	0
ENSG000001	C16orf46	protein_codin	0	1.08075432	0	0
ENSG000000	SEPHS1	protein_codin	8.52823715	2.75559368	27.5982264	5.34616522
ENSG000001	CSTPP1	protein_codin	0	0	0	0
ENSG000000	XK	protein_codin	0.86977168	0	0	1.40198661
ENSG000001	PIGO	protein_codin	0	0	0	0
ENSG000001	CSTPP1	protein_codin	0.54523653	0	0	0
ENSG000001	LANCL3	protein_codin	5.12445378	3.60991771	0	3.08331711
ENSG000001	LANCL3	protein_codin	0	0	0	0
ENSG000001	LRP4	protein_codin	110.792415	84.6733035	325.484051	70.278705
ENSG000000	NADK	protein_codin	0.70768463	3.63164296	0	4.64499799
ENSG000002	LGALS7	protein_codin	0	0.59339676	0	0.33680823
ENSG000001	PRRG1	protein_codin	0	35.542913	0	13.9184516
ENSG000000	CDK11A	protein_codin	0	0	0	18.1080551
ENSG000001	COPRS	protein_codin	0	0	0	0
ENSG000001	PLCB1	protein_codin	1.47286116	27.2389841	0	0
ENSG000000	CDK11A	protein_codin	0	36.1904214	0	0
ENSG000001	PLCB1	protein_codin	0	21.6480684	0	0
ENSG000002	FANCG	protein_codin	0	0	0	0
ENSG000002	SYCE1L	protein_codin	0.88520392	2.31241237	0	2.07704227
ENSG000001	CFAP47	protein_codin	1.24209043	0	2.38894781	6.41276395
ENSG000001	CFAP47	protein_codin	0	0	0	0
ENSG000001	OXER1	protein_codin	0.6273494	5.28982003	1.89651285	0.40809911
ENSG000001	UQCRQ	protein_codin	0	2.94642324	0	0
ENSG000001	UQCRQ	protein_codin	29.7807702	38.6074294	81.0610133	14.3626208
ENSG000001	COX7A2L	protein_codin	0	0	0	0
ENSG000001	UQCRQ	protein_codin	4.19019598	5.85107878	19.5144493	0.61911946
ENSG000001	GDF9	protein_codin	0	0	0	0

ENSG000001:	MMP23B	protein_codin	0.28589087	0	0	0
ENSG000001(	SHROOM1	protein_codin	0	0	0	1.2223867
ENSG000001!	DMD	protein_codin	0	0	0	12.7940707
ENSG000001(	SHROOM1	protein_codin	0	0	0	0
ENSG000001!	DMD	protein_codin	2.78107165	5.33397338	1.78900625	0
ENSG000001(	UCMA	protein_codin	0	0	0	0
ENSG000001:	CCDC136	protein_codin	4.78509268	10.592849	16.4206929	0
ENSG000001(	GLIPR1L2	protein_codin	0	0	0	0
ENSG000001!	SOWAHA	protein_codin	0.26542784	0	0	0.50699174
ENSG000000(	MCM10	protein_codin	0	9.83099833	9.27985591	6.12664948
ENSG000001'	GLIPR1L1	protein_codin	0	0	0	0
ENSG000001:	ESD	protein_codin	5.23951927	7.20650181	18.418259	0
ENSG000001(	SEPTIN8	protein_codin	0	0	0	0
ENSG000001'	ID4	protein_codin	23.8129391	183.661727	156.964891	175.442951
ENSG000001(	SEPTIN8	protein_codin	0	0	0	0
ENSG000001!	DMD	protein_codin	0	0	0	0
ENSG000001!	DMD	protein_codin	0	0	0	0
ENSG000001(	SEPTIN8	protein_codin	0	0	7.57534525	0
ENSG000001!	DMD	protein_codin	0	0	0	0
ENSG000001!	MIB2	protein_codin	0	0	0	0
ENSG000001!	MIB2	protein_codin	2.16767319	0	0	0
ENSG000000(	MCM10	protein_codin	1.20414953	0	41.8793779	0
ENSG000001(	SEPTIN8	protein_codin	7.03390528	0	2.50083379	15.2054971
ENSG000001:	ESD	protein_codin	16.5484724	11.2122847	5.86443312	6.71226831
ENSG000001(	SEPTIN8	protein_codin	36.6740361	0	35.4344269	6.38140998
ENSG000001!	DMD	protein_codin	0	0	0	0
ENSG000001(	SSU72	protein_codin	0	0	0	0
ENSG000001(	SSU72	protein_codin	0	5.56978576	2.46519479	0
ENSG000001:	THUMPD2	protein_codin	0	6.55193501	15.8319577	0
ENSG000002(	CCNI2	protein_codin	0	0	0	0.98467986
ENSG000002(	TMEM240	protein_codin	0	0	0	0.44409131
ENSG000001:	KIF3A	protein_codin	10.7203522	67.5721757	17.7215233	3.81299351
ENSG000001(	ATAD3B	protein_codin	1.51210639	8.54348445	17.5706021	0
ENSG000001(	PAX4	protein_codin	0	0	0	0
ENSG000001:	ZFC3H1	protein_codin	130.94587	269.730079	227.46683	109.446843
ENSG000001'	SPATA31G1	protein_codin	0	0	0	0
ENSG000001:	KIF3A	protein_codin	18.4935331	29.9830144	27.7006251	11.0457324
ENSG000001:	OPTN	protein_codin	0	214.924413	0	0
ENSG000001:	OPTN	protein_codin	0	0	0	13.8799015
ENSG000001:	PEX16	protein_codin	0.93526084	0	0	0
ENSG000001:	OPTN	protein_codin	0	188.388363	0	0
ENSG000001!	ATAD3A	protein_codin	0	0	0	0
ENSG000001!	ATAD3A	protein_codin	0	25.0764734	130.780082	0
ENSG000001:	OPTN	protein_codin	184.718575	120.654012	73.0142983	67.705211

ENSG000002	FRG2	protein_codin	0	0	0.53714372	1.40259731
ENSG000001	OPTN	protein_codin	26.0643006	0	48.7068344	27.9444339
ENSG000001	PTPRR	protein_codin	0	0	0	0
ENSG000001	RUBCNL	protein_codin	0	0	0	0
ENSG000001	RUBCNL	protein_codin	0	0	0	0
ENSG000002	ATAD3C	protein_codin	0	6.65395252	0	1.68259352
ENSG000001	RUBCNL	protein_codin	0	0	0	0
ENSG000002	SPATA31F1	protein_codin	0	0	0	2.57648612
ENSG000001	HAO1	protein_codin	0	0	0	0.45771175
ENSG000001	CCL21	protein_codin	0	0	0	0
ENSG000001	NDUFA5	protein_codin	0	0	11.4052289	0
ENSG000001	RUBCNL	protein_codin	0	0	0	0
ENSG000001	IST1	protein_codin	5.70909446	30.6296246	0	0
ENSG000001	IST1	protein_codin	0	0	0	0
ENSG000001	CCL19	protein_codin	0	0	0	0.52868985
ENSG000001	CYP4V2	protein_codin	13.3209958	34.1156913	14.6241892	8.57320147
ENSG000002	CDKL4	protein_codin	0	0	0	2.48135597
ENSG000001	LRRC63	protein_codin	0	0	0	0
ENSG000001	RAB3IP	protein_codin	0	15.8809698	2.58176946	0
ENSG000001	KIF13A	protein_codin	30.6395373	73.71647	153.054761	22.4002028
ENSG000001	RAB3IP	protein_codin	3.65578003	8.01356583	0	0
ENSG000001	TP53I13	protein_codin	0	0	0	0
ENSG000002	TMEM88B	protein_codin	0.60269349	0	0	1.78024616
ENSG000001	RAD50	protein_codin	0	0	0	0
ENSG000001	CCDC3	protein_codin	4.89173142	28.081159	124.600111	13.3730842
ENSG000001	KIF13A	protein_codin	0	0	0	0
ENSG000001	BMP2	protein_codin	28.1829169	21.6955441	39.7202359	30.47142
ENSG000002	ACCSL	protein_codin	0	0	0	0
ENSG000001	CCDC3	protein_codin	0.36311471	0	0	0
ENSG000002	GALT	protein_codin	0	0	0	0
ENSG000001	KIF13A	protein_codin	0	0	0	0
ENSG000001	FERMT1	protein_codin	5.16608139	0	9.08688921	0
ENSG000001	CAMK1D	protein_codin	10.1397688	0	10.9480393	6.02291498
ENSG000002	CFAP96	protein_codin	0	0	4.69118872	0
ENSG000001	API5	protein_codin	33.6761678	0	41.1770887	11.2555711
ENSG000001	AURKAIP1	protein_codin	0	17.371411	0	1.69385599
ENSG000001	HYDIN	protein_codin	2.26871131	1.86191298	0.88911466	0.95741688
ENSG000001	LRRN4	protein_codin	0	0	0	2.23186863
ENSG000000	WDR62	protein_codin	0	0	1.9102909	0
ENSG000002	TRIQK	protein_codin	0	12.6339554	4.27960701	0
ENSG000000	CRLS1	protein_codin	11.2995422	0	0	8.25486968
ENSG000001	PRR5L	protein_codin	0	0	0	0.93460289
ENSG000000	CRLS1	protein_codin	0	0	0	0
ENSG000002	ALKBH6	protein_codin	0	0	0	0

ENSG000001(	PHF12	protein_codin	0	32.7369036	0	0
ENSG000001:	MCM8	protein_codin	0	0	6.72191459	0
ENSG000001:	MCM8	protein_codin	0	3.30144066	0	4.25752224
ENSG000002(	SDHAF1	protein_codin	3.26941864	6.81283303	2.32450656	2.47157277
ENSG000001(	DVL1	protein_codin	9.05920748	61.2609627	71.7469331	10.5403193
ENSG000001(	DVL1	protein_codin	0	4.92747361	30.6181078	0
ENSG000001	SIGMAR1	protein_codin	0	0	0	0
ENSG000001(	DHRS13	protein_codin	0	0	0	0
ENSG000001:	MCM8	protein_codin	0	0	31.3266013	0
ENSG000001	ADAMTS17	protein_codin	2.83682863	0	21.9247009	7.74036073
ENSG000001	CDC123	protein_codin	0.33350212	0	0	0
ENSG000001	LYSMD4	protein_codin	0	2.07909319	0	0
ENSG000001	NUP107	protein_codin	509.624101	0	108.071282	0
ENSG000002(	ARID3C	protein_codin	0	0	0.53472311	0.39256433
ENSG000001(	NPHS1	protein_codin	0.59795277	3.09313638	0	8.57387911
ENSG000001:	DCTN3	protein_codin	0	0	0	0
ENSG000001	FCSK	protein_codin	0	10.7180591	0	0
ENSG000001:	DCTN3	protein_codin	0	0	0	0
ENSG000001	HNRNPLL	protein_codin	0	0	0	0
ENSG000001:	DCTN3	protein_codin	0	20.1143449	0	1.50548313
ENSG000001	PGPEP1L	protein_codin	0	0	0	0
ENSG000001(	NUDT5	protein_codin	1.14473228	0	2.47309102	0
ENSG000001	TAB3	protein_codin	1.63199719	9.21341262	0.35423046	1.22734913
ENSG000001	TAB3	protein_codin	0	21.4880479	1.39606194	7.44498968
ENSG000001	TAB3	protein_codin	4.26807327	21.3166017	0	4.00861532
ENSG000001	TAB3	protein_codin	0	0	0	0
ENSG000001(	NUDT5	protein_codin	1.29212579	0	0	1.69859946
ENSG000001!	GK	protein_codin	0	5.99651111	0	0
ENSG000001(	NUDT5	protein_codin	6.01204292	5.65427979	21.4545905	0
ENSG000001!	GK	protein_codin	0	0	0	0
ENSG000001!	GK	protein_codin	0	0	0	0
ENSG000000(	ARHGAP33	protein_codin	1.88847428	0	0	1.68184502
ENSG000001!	GK	protein_codin	0	0	0	0
ENSG000001!	GK	protein_codin	3.75769977	4.04159715	0	6.07626522
ENSG000001(	NUDT5	protein_codin	0	0	0	0
ENSG000001	ATL2	protein_codin	3.92614613	5.29002044	12.8841372	3.13641643
ENSG000001(	RPP25L	protein_codin	1.69857911	6.27518912	14.3911678	0.40651667
ENSG000000	CHGB	protein_codin	1.96674811	9.75079766	16.3741197	9.4891647
ENSG000001:	TASL	protein_codin	8.6045116	24.2846565	1.95181138	3.00588355
ENSG000001(	NR0B1	protein_codin	0	0	0	0
ENSG000001	CBY2	protein_codin	0	0	0	0.85328337
ENSG000001(	NR0B1	protein_codin	0	0	0	0
ENSG000001(	U2AF1L4	protein_codin	0	0	0	1.21606274
ENSG000000	SPAG5	protein_codin	0	9.88523238	24.9390073	0

ENSG000001	ERICH6B	protein_codin	0.70677356	10.629947	0	17.2057498
ENSG000001	SHLD1	protein_codin	0.67230879	1.27739414	0	0
ENSG000001	CNTFR	protein_codin	1.7307812	0	10.423864	0
ENSG000002	MAGEB1	protein_codin	0	0	0	0
ENSG000001	MAGEB4	protein_codin	0	0	0	1.45591778
ENSG000001	RAP1B	protein_codin	11.0097227	0	2.0745505	1.94488416
ENSG000000	MAGEB2	protein_codin	0	0	0.39636999	0.80153728
ENSG000001	CAP2	protein_codin	6.10341424	0	6.25017843	0
ENSG000001	IL1RAPL1	protein_codin	2.10308824	0	0.50700082	0.92087541
ENSG000002	C11orf91	protein_codin	0	0	0	0
ENSG000001	UPK1A	protein_codin	0	0	0	0
ENSG000001	HIPK3	protein_codin	59.1737811	107.641323	23.6959487	16.0202122
ENSG000000	SEC61A2	protein_codin	0	0	0	0
ENSG000001	PDLIM4	protein_codin	1.95091237	0	18.5122441	0
ENSG000001	GPCPD1	protein_codin	15.1751772	29.5567123	9.18645019	11.2002134
ENSG000000	SEC61A2	protein_codin	0	0	0	0
ENSG000001	ETV2	protein_codin	0.74439303	0	0	0
ENSG000000	NRCAM	protein_codin	0	2.2678846	2.4080949	0
ENSG000001	ETV2	protein_codin	0	0	0	0
ENSG000000	NRCAM	protein_codin	0	0	0	0
ENSG000001	PUSL1	protein_codin	1.32868783	26.538089	10.2065624	1.73338803
ENSG000000	SEC61A2	protein_codin	3.64694069	2.37786479	5.43322788	0
ENSG000001	MAGEB6	protein_codin	0	0	0	0.41609221
ENSG000001	ACAP3	protein_codin	0	0	0	0
ENSG000000	ARX	protein_codin	0.2885364	5.77605	0	0.51614117
ENSG000001	NQO1	protein_codin	6.10843829	0	9.61594734	4.39549295
ENSG000001	NQO1	protein_codin	0	0	5.7615032	0
ENSG000000	SEC61A2	protein_codin	0	0	0	0
ENSG000001	RBM24	protein_codin	12.5768459	0	0	1.19329578
ENSG000001	TPT1	protein_codin	0.93485751	0	2.43471411	5.85441128
ENSG000001	TPT1	protein_codin	381.817223	422.873401	552.423537	309.558623
ENSG000001	POLA1	protein_codin	0	0	56.0926033	0
ENSG000001	TPT1	protein_codin	64.2838846	721.293303	0	207.350175
ENSG000000	SARM1	protein_codin	2.80767769	0	0	0
ENSG000001	PRKD3	protein_codin	25.989608	0	79.666847	0
ENSG000001	POLA1	protein_codin	18.0418534	0	0	9.8490406
ENSG000001	CDS2	protein_codin	4.09374539	9.53098192	5.11765871	0
ENSG000002	FABP9	protein_codin	0.19812993	0	0	0
ENSG000001	PMFBP1	protein_codin	0	0	0	0
ENSG000001	WT1	protein_codin	0	0	0	0.50182035
ENSG000001	FAM219A	protein_codin	0	0	0	0
ENSG000001	WT1	protein_codin	0	0	0	0
ENSG000001	FAM219A	protein_codin	0	0	0	0
ENSG000001	FAM219A	protein_codin	0	0	0	0

ENSG000000!	SLC26A3	protein_codin	0	0	0	0
ENSG000001(	FAM219A	protein_codin	0	0	1.75627405	0
ENSG000000'	P4HA2	protein_codin	11.2330001	19.8572888	0	12.898424
ENSG000001(	FAM219A	protein_codin	4.9556731	42.0438025	0	0
ENSG000001(	FAM219A	protein_codin	0	0	0	0
ENSG000001!	ZNF774	protein_codin	1.42939014	3.05309123	1.85382876	0.86187901
ENSG000002(	ZBTB10	protein_codin	17.8595921	27.0545234	55.0237178	15.9660391
ENSG000001(	NGRN	protein_codin	89.1141786	84.5435976	197.984917	35.2881296
ENSG000000'	TPD52	protein_codin	0	0	24.5702282	0
ENSG000000'	TPD52	protein_codin	0	0	10.6035208	5.32941587
ENSG000001(	SCNN1D	protein_codin	0	0	0	0
ENSG000000'	P4HA2	protein_codin	4.44272017	0	12.4315904	0
ENSG000001(	SCNN1D	protein_codin	0	4.31831422	0	0
ENSG000002:	LYRM9	protein_codin	3.14897864	0	5.71128434	1.50070974
ENSG000002:	LYRM9	protein_codin	0	10.5973657	0	0
ENSG000000'	P4HA2	protein_codin	0	0	0	5.25226801
ENSG000000(	PAX6	protein_codin	0	0	0	0
ENSG000001(	KCTD4	protein_codin	0	0	0	0.55301548
ENSG000000(	PAX6	protein_codin	0	0	0	0
ENSG000000(	PAX6	protein_codin	0	0	0	0
ENSG000001(	IL7	protein_codin	0	6.41729091	0	1.37766686
ENSG000000(	PAX6	protein_codin	0	0	0	0
ENSG000001(	SCNN1D	protein_codin	0	0	4.40732827	0
ENSG000000'	BCAP29	protein_codin	12.3844982	0	8.92838854	8.05968873
ENSG000000(	PAX6	protein_codin	0	0	0	0
ENSG000001(	SPMIP6	protein_codin	0	0	0	0
ENSG000001(	SPMIP6	protein_codin	0	0	0	0
ENSG000001(	SPMIP6	protein_codin	0.26583592	0	0	0
ENSG000000(	PAX6	protein_codin	0	0	0	0
ENSG000000(	PAX6	protein_codin	0	0	0	0
ENSG000001(	SPMIP6	protein_codin	0	0	0	0
ENSG000001(	USF2	protein_codin	10.0692336	20.1664996	8.63285748	15.1672157
ENSG000001:	LRIG3	protein_codin	454.998	26.8621741	52.9273674	0
ENSG000001(	MYORG	protein_codin	0	0	0	0.73693886
ENSG000001:	PCNA	protein_codin	36.6445445	35.4686886	65.611048	16.2131963
ENSG000001(	PCYT1B	protein_codin	0	3.82517352	0	6.98849865
ENSG000001(	PCYT1B	protein_codin	0.31567283	0	0.45636035	0
ENSG000001:	GPALPP1	protein_codin	19.2437481	44.5699732	16.8702568	14.7437659
ENSG000001(	SLC7A6	protein_codin	0	0	0	0
ENSG000001	CD83	protein_codin	15.0993987	68.412778	33.4944045	122.272169
ENSG000001(	NUDT2	protein_codin	0	0	0	0
ENSG000001(	NUDT2	protein_codin	0	18.6094653	0	0
ENSG000001:	PCNA	protein_codin	0	0	7.58146488	0
ENSG000000(	NUFIP1	protein_codin	15.5926994	23.3891792	40.0063262	11.4550173

ENSG0000000	PDK3	protein_codin	0	0	0	0
ENSG000001	ELP4	protein_codin	0	0	0	1.13299319
ENSG000001	FSTL5	protein_codin	3.07022263	0	0.59841439	1.62079589
ENSG000000	NFATC3	protein_codin	1.67269921	0	0	0
ENSG000000	MCUR1	protein_codin	14.6063052	40.7057049	31.7898543	15.0943142
ENSG000001	KIF24	protein_codin	0	11.9912571	0	3.84609455
ENSG000000	ZFX	protein_codin	0	0	30.4666634	17.0891316
ENSG000001	SERP2	protein_codin	0	0	0	0.48378457
ENSG000001	UBAP1	protein_codin	4.11899302	20.4001923	0	3.71748526
ENSG000000	ZFX	protein_codin	58.0264647	0	52.876166	27.6508996
ENSG000001	B3GALT6	protein_codin	3.0578827	26.8381809	24.5070688	5.13429325
ENSG000001	PROSER2	protein_codin	0	0	0	0
ENSG000002	SCGB2B2	protein_codin	0	0	0	0
ENSG000002	C4orf46	protein_codin	3.66288166	8.45240519	26.7611068	7.47635797
ENSG000001	CXorf58	protein_codin	0	0	0	0
ENSG000002	LGR4	protein_codin	16.7629121	43.2376021	105.41112	13.5891668
ENSG000001	ECHDC3	protein_codin	2.91013669	6.40229147	0	0.83066263
ENSG000001	DNAJC15	protein_codin	24.5370101	37.8345509	3.16822907	10.9421357
ENSG000001	ISG20	protein_codin	0	0	0	0
ENSG000001	UBAP2	protein_codin	0	1.92723938	2.03329271	1.15217852
ENSG000001	APOO	protein_codin	0	0	0	0
ENSG000001	UBAP2	protein_codin	0.39978607	0	0	0
ENSG000001	TNFRSF4	protein_codin	0	0	0	0
ENSG000001	USP6NL	protein_codin	0	46.8408192	0	0
ENSG000001	UBAP2	protein_codin	15.6664603	0	48.1008385	17.6811315
ENSG000001	UBAP2	protein_codin	0	0	0	0
ENSG000001	ACSL6	protein_codin	0	0	0	0
ENSG000002	VIT	protein_codin	0.84136403	8.18653484	1.62167119	2.09862696
ENSG000002	VIT	protein_codin	32.2035759	15.8091525	4.7196622	9.78991951
ENSG000001	ACSL6	protein_codin	0	0	0	0
ENSG000001	FEZ2	protein_codin	4.08763916	4.56220921	0	0
ENSG000001	ACSL6	protein_codin	0	0	0	0
ENSG000001	MAP9	protein_codin	0	1.48325639	3.63178183	0.57436151
ENSG000001	SIRT5	protein_codin	6.86961157	0	0	3.89494168
ENSG000001	SAT1	protein_codin	3.03269626	15.0462691	5.84188872	25.4499408
ENSG000001	SAT1	protein_codin	24.408814	269.858736	65.1915213	59.0548811
ENSG000001	SAT1	protein_codin	0	0	0	2.5577479
ENSG000001	ACSL6	protein_codin	0	0	0	0
ENSG000001	DNAJC2	protein_codin	0	0	0	0
ENSG000001	SIRT5	protein_codin	0.36101733	0	5.63707086	0
ENSG000001	DNAJC2	protein_codin	62.6973224	94.2397567	337.513496	39.3050916
ENSG000001	TNFRSF18	protein_codin	0	0	0	0
ENSG000001	TNFRSF18	protein_codin	0	3.1886914	0	0
ENSG000001	SAT1	protein_codin	149.351772	230.970887	185.666351	228.959183

ENSG000001	DGKH	protein_codin	0	20.3366305	16.0386212	0
ENSG000000	TMEM230	protein_codin	0	0	0	0
ENSG000000	TMEM230	protein_codin	0	0	2.37902814	0
ENSG000001	GFOD1	protein_codin	0	0	0	0
ENSG000000	TMEM230	protein_codin	0	10.6366336	0	3.28311892
ENSG000000	TMEM230	protein_codin	0	16.5282569	0	0
ENSG000001	GFOD1	protein_codin	17.0372381	0	0	0
ENSG000000	TMEM230	protein_codin	0	0	0	0
ENSG000001	GFOD1	protein_codin	0	27.5153349	23.0499867	15.8623273
ENSG000001	TTLL10	protein_codin	0	0.96192294	0	0.76120452
ENSG000001	TTLL10	protein_codin	0	0	0.45862314	0
ENSG000001	TTLL10	protein_codin	0	0	0	0
ENSG000001	TBC1D7	protein_codin	0	3.97426885	0	0.80614348
ENSG000001	ACOT9	protein_codin	1.49321739	4.89517628	0.84343233	2.14268393
ENSG000001	ACOT9	protein_codin	0.87208033	0	0.70959198	3.6601982
ENSG000000	TMEM230	protein_codin	1.78072179	0	6.94765125	0
ENSG000001	TBC1D7	protein_codin	0	0	0	0
ENSG000001	VWA8	protein_codin	0	0	0	0
ENSG000001	ACOT9	protein_codin	10.6596009	25.8054815	6.56641743	9.2111106
ENSG000001	FBXL13	protein_codin	0	0	0	0.78623009
ENSG000001	TBC1D7	protein_codin	1.19925271	0	6.18736026	0
ENSG000001	VWA8	protein_codin	58.7870712	69.7175563	91.4923949	24.2540701
ENSG000000	RIPOR1	protein_codin	2.73731054	0	0	2.19798508
ENSG000001	PDCD5	protein_codin	0	0	0	0
ENSG000001	C1orf159	protein_codin	0.43376449	1.50557265	0	0
ENSG000001	C1orf159	protein_codin	0	0	0	0
ENSG000001	C1orf159	protein_codin	0	0	4.65321493	0
ENSG000001	GATA3	protein_codin	0	11.9970225	5.55163275	2.5850823
ENSG000001	PHACTR1	protein_codin	0.43138047	2.95959422	0.543807	0
ENSG000001	PRDX4	protein_codin	0	3.21021208	1.45937909	0
ENSG000000	SLC23A2	protein_codin	0	0	0	0
ENSG000001	PHACTR1	protein_codin	0	0	0	3.10489037
ENSG000001	C1orf159	protein_codin	0	0	0	0
ENSG000001	POLR2J3	protein_codin	0	0	0	0
ENSG000001	PRDX4	protein_codin	31.8207406	54.0046588	57.5245901	20.9810556
ENSG000001	SLC30A6	protein_codin	0	0	0	0
ENSG000001	PLEKHG4	protein_codin	11.3206649	0	0	0
ENSG000001	PHACTR1	protein_codin	0	0	0	0
ENSG000001	PRDX4	protein_codin	0	0	0	2.21852916
ENSG000001	PHACTR1	protein_codin	1.01427499	0	2.33881721	4.82579233
ENSG000001	ZSCAN2	protein_codin	0	0	0	1.4112766
ENSG000001	RGCC	protein_codin	13.0669316	20.9449617	14.2150915	26.2461889
ENSG000001	PTCHD1	protein_codin	0	1.55757872	0.9332185	6.1261082
ENSG000001	AGRN	protein_codin	21.063654	109.636007	182.508725	31.5018838

ENSG000001	PHEX	protein_codin	0.35804398	42.8627527	9.38453823	11.170785
ENSG000000	EDN1	protein_codin	4.8639134	23.2801572	0.89270422	2.77224928
ENSG000001	RASSF2	protein_codin	0	0	0	0
ENSG000002	E2F4	protein_codin	9.96588579	36.26423	45.822419	8.68022713
ENSG000001	LYRM7	protein_codin	31.1464703	46.7932787	40.0904894	23.601645
ENSG000001	MEMO1	protein_codin	7.07335228	34.5265258	27.668642	10.9286509
ENSG000001	TM6SF1	protein_codin	0	0	0	0
ENSG000001	UEVLD	protein_codin	0	0	0	0
ENSG000000	HIVEP1	protein_codin	32.9911456	88.5855656	33.2083875	22.1645406
ENSG000001	TM6SF1	protein_codin	0	0	0	0
ENSG000001	NEMP1	protein_codin	0	0	0	9.96276301
ENSG000001	RASSF2	protein_codin	53.0946399	133.229682	18.300148	47.1950249
ENSG000000	BTBD1	protein_codin	0.78573026	0	0	0
ENSG000001	SMS	protein_codin	0	0	0	0
ENSG000000	PRSS3	protein_codin	0	0	0	0
ENSG000001	NAA16	protein_codin	12.7419776	7.648284	35.3250718	9.08197782
ENSG000001	PLEKHN1	protein_codin	0	0	0	0
ENSG000001	PLEKHN1	protein_codin	0	0	0	0
ENSG000001	PLEKHN1	protein_codin	1.04300898	10.9222004	1.33154595	5.64209831
ENSG000001	TAC3	protein_codin	0.33095285	0	0	0
ENSG000001	LDHA	protein_codin	0	11.889766	9.85479672	8.11804412
ENSG000001	ADTRP	protein_codin	0	0	0	0
ENSG000001	XDH	protein_codin	0	2.3209924	0.43217152	2.11115129
ENSG000002	PDE7A	protein_codin	6.5194994	6.69095915	5.05648964	7.84430183
ENSG000001	MOGAT3	protein_codin	0	0	0	0
ENSG000002	TMEM170B	protein_codin	13.8463021	17.1319481	33.778497	15.8112594
ENSG000001	NEDD9	protein_codin	7.16178683	43.7980638	20.6305362	12.8306309
ENSG000001	MUC17	protein_codin	0	0	0.53178622	16.7368171
ENSG000001	PRNP	protein_codin	0	112.209805	0	0
ENSG000000	BAZ2A	protein_codin	4.3114631	40.003508	0	0
ENSG000002	MUC12	protein_codin	1.71230862	0	0	0
ENSG000002	CTXN3	protein_codin	0	0	1.31337016	0
ENSG000001	NEDD9	protein_codin	24.3325206	0	35.1845726	52.2398952
ENSG000001	ZNF827	protein_codin	0	0	0	0
ENSG000001	ASPH	protein_codin	12.8356773	24.3299145	13.1508222	7.06089088
ENSG000001	ADRA1D	protein_codin	0.27761956	1.36615963	8.5308171	1.71963746
ENSG000001	ASPH	protein_codin	195.674336	202.540551	158.265362	135.467108
ENSG000001	MUC3A	protein_codin	0	21.20698	0	1.48445309
ENSG000000	SMOX	protein_codin	0	0	0	1.75387631
ENSG000001	NAE1	protein_codin	4.47632192	0	0	6.52637994
ENSG000001	NOL6	protein_codin	0	2.36783438	0	3.86896829
ENSG000001	NOL6	protein_codin	0	18.6934881	4.67008657	0
ENSG000001	KCNC1	protein_codin	0	3.91222688	2.83595426	1.34623693
ENSG000001	MTRF1	protein_codin	7.78765125	24.4022765	10.8460629	4.64153276

ENSG000001:	MTRF1	protein_codin	0	7.09297369	0	0
ENSG000001:	KBTBD7	protein_codin	8.72059233	25.8735305	17.972418	5.11611941
ENSG000000	MBTPS2	protein_codin	15.8952089	34.0408853	20.4436209	5.43513231
ENSG000001	KBTBD6	protein_codin	14.1036217	18.6548752	33.6386518	6.32546887
ENSG000001	CMTM2	protein_codin	0	0	0	0
ENSG000001:	WBP4	protein_codin	28.0912198	43.3954393	34.8049176	22.9247241
ENSG000001:	GCM2	protein_codin	0	1.20581659	0	0.82965004
ENSG000000	SMPX	protein_codin	10.9103899	0	0	0
ENSG000001	KLHL34	protein_codin	0.51205558	2.01820175	0	1.37210787
ENSG000000	CMTM1	protein_codin	0	0	0	0
ENSG000001	AQP7	protein_codin	0	0	0	0
ENSG000001	TOM1L2	protein_codin	13.6666637	0	14.4180474	17.5755635
ENSG000001	AQP7	protein_codin	0	0	0	0
ENSG000001	AQP7	protein_codin	0	0	0	0
ENSG000001	LCLAT1	protein_codin	14.3939195	0	43.8585066	6.71885197
ENSG000001	CNKSR2	protein_codin	3.73883701	0	1.86662618	0
ENSG000002	SNX2	protein_codin	5.04936176	40.507397	6.82871143	13.4791257
ENSG000001	YPEL5	protein_codin	6.79454474	43.7273061	12.1478006	21.7447526
ENSG000001	YPEL5	protein_codin	0	0	1.67070792	26.2014409
ENSG000000	NFX1	protein_codin	5.22841594	17.6181547	5.68066955	0.94050957
ENSG000001	MOSPD3	protein_codin	0	0	4.43473182	0
ENSG000001:	TMEM14B	protein_codin	17.7904082	0	32.9641239	0
ENSG000001	MORF4L1	protein_codin	0	0	0	0
ENSG000000	NFX1	protein_codin	28.7036138	45.9354617	38.838236	10.9143849
ENSG000001:	TMEM14B	protein_codin	2.42966942	0	0	10.1796344
ENSG000001	ELF2	protein_codin	0	0	0	0
ENSG000001	ELF2	protein_codin	44.0564028	54.676777	43.531151	13.0537046
ENSG000001	PRR16	protein_codin	0	0	0	0
ENSG000001	INSC	protein_codin	0	0	0	0
ENSG000001	FAM170A	protein_codin	0.31751506	0	0	4.29567457
ENSG000001	INSC	protein_codin	19.8628464	22.2948474	2.07504842	26.538928
ENSG000001	TOGARAM2	protein_codin	0	10.943254	0	0
ENSG000001	FOXO1	protein_codin	18.5301676	82.6820477	15.1582746	52.4734158
ENSG000001	KIN	protein_codin	23.1003473	52.3749945	42.4488627	13.9635467
ENSG000001	RPS6KA3	protein_codin	54.1702913	132.807924	36.0005367	58.4283957
ENSG000001:	AP5S1	protein_codin	0	0	0	0
ENSG000001	PAK1IP1	protein_codin	14.8031105	43.9524986	78.6004562	5.06665194
ENSG000001:	AP5S1	protein_codin	0	3.4248701	0	0
ENSG000001	SPDYA	protein_codin	0	0	0	0
ENSG000001	C6orf52	protein_codin	0	0	0	0
ENSG000001	ITIH2	protein_codin	0	0	0	0.41437526
ENSG000001	LHFPL6	protein_codin	46.8208411	114.918974	35.5996387	78.6085673
ENSG000001	EIF1AX	protein_codin	3.96710552	0	8.27500539	12.5670979
ENSG000001	ATG12	protein_codin	0.22771948	8.87025663	0	0

ENSG000001	PSTPIP1	protein_codin	3.12366632	13.4474171	0	3.78517183
ENSG000001	GCNT2	protein_codin	0.89699654	13.569224	0	4.03300221
ENSG000001	CDC25B	protein_codin	0	0	55.208148	6.43834814
ENSG000001	NHLRC3	protein_codin	4.21506562	8.83344763	1.92892742	5.38390817
ENSG000001	NHLRC3	protein_codin	0	29.4821827	0	0
ENSG000001	EIF1AX	protein_codin	48.8361577	70.5787006	75.378601	57.6554989
ENSG000001	TFAP2A	protein_codin	0	0	0	1.36815804
ENSG000001	CCDC112	protein_codin	13.8146944	38.7081496	0	0
ENSG000001	TFAP2A	protein_codin	0	0	18.4657948	0
ENSG000001	PGGT1B	protein_codin	0	0	0	0
ENSG000001	TRIM36	protein_codin	0	0	0	0
ENSG000001	TRIM36	protein_codin	0	0	0	0
ENSG000000	FOSL2	protein_codin	47.0106411	27.3507906	9.6274685	25.3859873
ENSG000001	BABAM2	protein_codin	7.28987942	13.3056127	10.0506013	8.99050151
ENSG000001	BABAM2	protein_codin	0	14.7120371	0	0
ENSG000001	STOML3	protein_codin	0.25864738	0	0.93481637	1.64490734
ENSG000001	BABAM2	protein_codin	0	0	0	0
ENSG000001	REEP5	protein_codin	60.3933406	193.977664	123.440771	69.4099659
ENSG000001	MAP7D2	protein_codin	0	0	0	0
ENSG000001	HGSNAT	protein_codin	40.5321049	111.646691	105.644104	35.3658482
ENSG000001	TRPC3	protein_codin	0.76483288	8.19628194	4.52489163	1.93037217
ENSG000001	UFM1	protein_codin	0	0	0	0
ENSG000001	MAP7D2	protein_codin	0	3.72667051	3.28691175	1.26892513
ENSG000001	KIFC3	protein_codin	0	0	10.4122828	0
ENSG000001	ARRDC2	protein_codin	5.37543827	3.71802498	0	5.27368354
ENSG000001	SLC35B3	protein_codin	0	44.1359332	0	1.21329382
ENSG000001	KATNB1	protein_codin	5.55044944	21.7097063	25.267626	4.65063176
ENSG000001	EXOSC9	protein_codin	0	0	2.32496406	0
ENSG000002	OR6C65	protein_codin	0	0	0	0.54319746
ENSG000002	MRPL33	protein_codin	3.76734236	28.1545916	13.7030336	6.35933089
ENSG000002	OR6C1	protein_codin	0	0	0	0.44409479
ENSG000001	NREP	protein_codin	13.3143862	60.5022158	54.0038927	4.43586669
ENSG000000	ARHGAP44	protein_codin	0	0	0	0
ENSG000001	TRPC4	protein_codin	0	0	0	0
ENSG000001	GOLGA6L4	protein_codin	0	0	9.89223631	0
ENSG000001	TRPC4	protein_codin	0	4.4539185	0	0
ENSG000001	BCLAF3	protein_codin	6.66788963	0	32.9374671	0
ENSG000001	BCLAF3	protein_codin	31.981921	102.864249	0	19.3267888
ENSG000001	NDNF	protein_codin	64.3566862	375.302705	879.747678	187.950512
ENSG000002	INSL3	protein_codin	0	0	0	0
ENSG000001	SH3KBP1	protein_codin	8.23729768	3.93744065	13.405663	0
ENSG000001	SH3KBP1	protein_codin	30.2531952	223.008835	56.8292873	28.0535037
ENSG000001	BAG1	protein_codin	0	7.21232093	1.36574246	4.69853001
ENSG000001	BAG1	protein_codin	37.5774066	0	72.9819433	26.4525396

ENSG000001[illegible]	TRPC4	protein_codin	4.43121808	0	3.00145357	2.01409342
ENSG000001[illegible]	TSLP	protein_codin	0.74308501	0	7.00759315	0
ENSG000001[illegible]	BAG1	protein_codin	7.25999577	18.7385439	6.9574194	10.6563276
ENSG000001[illegible]	LMAN1L	protein_codin	0	0	0	1.52880466
ENSG000001[illegible]	SFMBT2	protein_codin	0.39907946	7.04628188	0.7151624	0
ENSG000001[illegible]	SFMBT2	protein_codin	2.69179869	2.39472693	1.24826495	1.27305442
ENSG000001[illegible]	EEF1E1	protein_codin	4.53233545	14.0600506	19.3593847	2.22762133
ENSG000001[illegible]	SH3KBP1	protein_codin	0	0	1.39545365	0
ENSG000002[illegible]	ZNF512	protein_codin	45.3170165	89.9623935	122.125924	0
ENSG000002[illegible]	IPO7	protein_codin	180.55485	386.990992	538.170411	119.78592
ENSG000001[illegible]	SPINK4	protein_codin	0	0	0	0
ENSG000001[illegible]	ZNF789	protein_codin	0	0	0	0
ENSG000001[illegible]	SPINK4	protein_codin	0	0	0	0
ENSG000001[illegible]	SH3KBP1	protein_codin	0	0	0	0
ENSG000001[illegible]	CYP1A1	protein_codin	0	0	0	0
ENSG000001[illegible]	IQGAP2	protein_codin	0	0	13.4716734	0
ENSG000000[illegible]	B4GALT1	protein_codin	68.0025322	130.874441	49.4648249	72.9147906
ENSG000001[illegible]	POSTN	protein_codin	761.295915	290.832316	70.3365905	99.845545
ENSG000001[illegible]	POSTN	protein_codin	617.015056	428.991243	269.425391	187.65037
ENSG000001[illegible]	POSTN	protein_codin	42.4699227	0	373.745113	0
ENSG000001[illegible]	POSTN	protein_codin	220.259533	113.099519	0	75.9346732
ENSG000001[illegible]	CENPB	protein_codin	56.1266819	305.344142	76.50018	36.4276208
ENSG000001[illegible]	SPEF1	protein_codin	0	0.96275444	0.50953898	0.76004743
ENSG000002[illegible]	TXNDC5	protein_codin	169.868736	830.867023	298.986692	90.8286804
ENSG000001[illegible]	ADISSP	protein_codin	3.5305152	11.8722881	10.5828979	1.37963849
ENSG000001[illegible]	CAMK2D	protein_codin	86.5190873	128.643654	457.391707	21.6124152
ENSG000001[illegible]	ADPRM	protein_codin	9.54145731	0	0	1.73945934
ENSG000001[illegible]	PFKFB3	protein_codin	0	134.968781	0	60.6767947
ENSG000000[illegible]	CETP	protein_codin	0.2435133	0	0	0.84264471
ENSG000001[illegible]	PFKFB3	protein_codin	0	0	0	1.56643035
ENSG000002[illegible]	PRR13	protein_codin	0	0	0	0
ENSG000001[illegible]	PFKFB3	protein_codin	22.5905254	0	34.4893815	7.78704612
ENSG000001[illegible]	AMHR2	protein_codin	0	0	0	1.18136238
ENSG000000[illegible]	HERPUD1	protein_codin	15.5180082	0	15.8543429	18.121609
ENSG000001[illegible]	PAM	protein_codin	34.2076813	82.9542749	151.657163	31.6933968
ENSG000001[illegible]	CSNK1A1L	protein_codin	0.6585475	1.27342239	0	2.33493916
ENSG000000[illegible]	DSP	protein_codin	0	0	286.929895	0
ENSG000001[illegible]	SIN3B	protein_codin	0	0	18.702835	0
ENSG000001[illegible]	PDHA1	protein_codin	14.0139716	0	31.2696763	4.49918616
ENSG000001[illegible]	PDHA1	protein_codin	12.295686	51.5781094	26.3071888	12.9243311
ENSG000002[illegible]	SLCO6A1	protein_codin	0	0	0	0
ENSG000001[illegible]	MT1G	protein_codin	0	0	0	1.16306661
ENSG000001[illegible]	APTX	protein_codin	6.19133547	8.71895556	0	0
ENSG000001[illegible]	APTX	protein_codin	0	0	0	5.59670042

ENSG0000020	MT1M	protein_codin	0.94833736	1.97232428	1.97659751	7.84850464
ENSG000001	APTX	protein_codin	0	0	0	0
ENSG000001	APTX	protein_codin	0	0	0	0
ENSG000001	SMAD9	protein_codin	19.7891686	11.7055509	29.4276316	15.6225969
ENSG000001	OR10A4	protein_codin	0	0	0	0.41601538
ENSG000001	RIOK1	protein_codin	19.8794355	92.5714806	44.7993913	3.33727275
ENSG000001	SFRP1	protein_codin	0	0	17.564174	0
ENSG000001	CSAD	protein_codin	0	0	0	0
ENSG000001	NDUFB6	protein_codin	1.88367919	2.17652846	8.90554606	4.30732828
ENSG000001	CSAD	protein_codin	0	6.70825074	0	0
ENSG000001	NRBP1	protein_codin	58.6747343	130.757041	169.800264	41.4831668
ENSG000001	ADAM2	protein_codin	0	0	0	0.51540335
ENSG000001	TOPORS	protein_codin	92.2870629	84.3404626	232.59685	83.944791
ENSG000001	CIB3	protein_codin	0	0	0	0
ENSG000001	ADAM33	protein_codin	0	0	4.90438461	0
ENSG000002	CCDC169	protein_codin	0	0	0	0
ENSG000001	NRBP1	protein_codin	0	0	0	0
ENSG000002	CCDC169	protein_codin	0.27993573	4.82724674	2.17189205	1.28922778
ENSG000001	ADAM18	protein_codin	0	0	0	0
ENSG000001	RIGI	protein_codin	2.40205816	0	0	0
ENSG000001	ADGRG2	protein_codin	0	0	0	0
ENSG000001	ADGRG2	protein_codin	0	0	0	0
ENSG000001	ADGRG2	protein_codin	0	0	0	0
ENSG000001	ADGRG2	protein_codin	0	0	0	0
ENSG000001	SOHLH2	protein_codin	0	0	0	0
ENSG000001	RIGI	protein_codin	17.6125755	60.7228613	0	0
ENSG000001	ARIH1	protein_codin	25.1235454	57.332692	25.1969801	35.3099617
ENSG000001	RBM17	protein_codin	27.8540784	64.5406386	61.5674596	22.0702153
ENSG000001	DCLK1	protein_codin	0	12.489494	0	0
ENSG000001	DCLK1	protein_codin	11.4684234	0	17.396131	0
ENSG000001	TNS2	protein_codin	0	65.1866737	0	0
ENSG000001	ERAP2	protein_codin	0	0	3.10448436	0
ENSG000001	SLC6A2	protein_codin	0	0	0	0
ENSG000001	ADAM32	protein_codin	2.64874532	0	13.9436131	0
ENSG0000020	ENSG0000020	protein_codin	0.64923377	17.8992096	1.05576192	0
ENSG000001	ADAM9	protein_codin	0	0	0	0
ENSG000001	CAGE1	protein_codin	0	0	0	0
ENSG000001	MAB21L1	protein_codin	0	0	3.83524684	0
ENSG000001	NBEA	protein_codin	0	0	0	0
ENSG000001	ACO1	protein_codin	21.9528969	52.5953401	0	17.8719642
ENSG000001	RREB1	protein_codin	0	141.448202	11.5314984	0
ENSG000001	RBL2	protein_codin	1.24087986	0	0	3.15758869
ENSG000001	CNGA4	protein_codin	0	0	1.73520176	0.95560243
ENSG000001	BRF1	protein_codin	0	0	0	0

ENSG000001:	RREB1	protein_codin	48.6093376	193.291351	136.538832	50.9910175
ENSG000001	NBEA	protein_codin	0	0	0	0
ENSG000000	PHKA2	protein_codin	17.4998394	54.6502384	47.424873	16.5840254
ENSG000002	OR52E6	protein_codin	0	0	0	0.63211374
ENSG000001:	LEF1	protein_codin	0	0	46.5594922	11.8367104
ENSG000001	LY86	protein_codin	0	0	0	3.46758527
ENSG000001:	IL2RA	protein_codin	0	0	0	0
ENSG000001	LETM2	protein_codin	0	0	2.27524878	0
ENSG000002	SAMD9	protein_codin	20.165838	183.785452	43.4022272	0
ENSG000001:	IL2RA	protein_codin	7.08919023	40.823186	3.46859598	2.47708559
ENSG000002	ITSN1	protein_codin	7.43532629	73.922491	14.4148537	3.18438573
ENSG000000	PPEF1	protein_codin	0.49430689	0	0	3.76346278
ENSG000001:	TRIM22	protein_codin	10.6288228	0	0	31.0340059
ENSG000001:	IL15RA	protein_codin	0	0	0	0
ENSG000001:	IL15RA	protein_codin	0	0	0	0
ENSG000001:	IL15RA	protein_codin	0.86236152	0.7073425	0	0.94560421
ENSG000001:	IL15RA	protein_codin	0	0	0	0
ENSG000001	GLRX	protein_codin	1.94287367	8.52742632	0	0.71163734
ENSG000001:	MYH10	protein_codin	0	0	0	0
ENSG000001	RHOBTB3	protein_codin	82.1550575	115.737471	182.265979	35.4683363
ENSG000001:	UACA	protein_codin	226.224773	286.672159	0	147.242815
ENSG000001	RS1	protein_codin	0.28541904	0	0	3.63977229
ENSG000001	NPNT	protein_codin	33.7793767	91.1784796	23.3768425	35.3581071
ENSG000000	CDKL5	protein_codin	0	0	0	0
ENSG000001	SPATA9	protein_codin	0	0	0	0
ENSG000001	LINGO2	protein_codin	0	0	4.28881605	0
ENSG000001	C9orf72	protein_codin	5.93832607	40.1311394	10.5304372	0
ENSG000000	CDKL5	protein_codin	0	0	0	0
ENSG000001	C9orf72	protein_codin	5.82430392	5.8984258	13.6394209	4.84003885
ENSG000001:	FBH1	protein_codin	0	19.575346	3.36979198	11.1708366
ENSG000001	C9orf72	protein_codin	0	0	6.3336013	6.40289843
ENSG000001	RFESD	protein_codin	0	0	0	0
ENSG000001	SIAH1	protein_codin	0.39890308	9.61093435	3.444849	5.49775662
ENSG000001	GPR150	protein_codin	0.46572472	1.25188659	0.58333976	0.42152572
ENSG000001	ARSK	protein_codin	4.00965501	10.3964629	12.4847025	4.70745933
ENSG000001	SLC19A1	protein_codin	0	0	0	0.86573721
ENSG000001	TET2	protein_codin	8.79351665	38.569271	0	0
ENSG000001	NDEL1	protein_codin	0	0	0	0
ENSG000001:	CENPE	protein_codin	54.1796737	115.245223	621.103915	18.428252
ENSG000001:	TRIM5	protein_codin	0	0	0	1.74391074
ENSG000001	BEND2	protein_codin	0	0	0	0
ENSG000001:	EQTN	protein_codin	0	0	0	1.32686618
ENSG000001:	EQTN	protein_codin	0.52650002	0	0	0
ENSG000001	BEND2	protein_codin	0	0	0.60938555	2.29730197

ENSG000001:	TRIM5	protein_codin	4.9849698	0	4.2041914	1.50999827
ENSG000001	SKOR1	protein_codin	0	0	0	0
ENSG000001:	TEK	protein_codin	3.75310986	30.4010193	2.16647406	16.9552206
ENSG000001:	KLC1	protein_codin	11.3987482	13.1391184	0	7.10693671
ENSG000000	SCML1	protein_codin	0	0	0	6.04387055
ENSG000000	SCML1	protein_codin	4.93561349	0	0	0
ENSG000001	MPV17	protein_codin	2.399061	0	6.7422682	6.52732043
ENSG000000	SCML1	protein_codin	0	12.6177795	11.9128595	4.44076309
ENSG000000	CDK14	protein_codin	81.2139571	193.863446	148.44162	61.8140681
ENSG000001:	RPP40	protein_codin	0	0	0	1.00495166
ENSG000001	LRRC19	protein_codin	0	0	0	0
ENSG000000	SLC4A11	protein_codin	0	0	0	0
ENSG000001	GTPBP10	protein_codin	0	0	0	11.2852665
ENSG000000	SLC4A11	protein_codin	0	0	0	0
ENSG000001	NHS	protein_codin	0	0	0	0
ENSG000000	IFT74	protein_codin	2.34169224	2.968602	7.01311526	12.4400108
ENSG000001:	SLC25A35	protein_codin	0.72744026	0	0	0.94820449
ENSG000002	EXOC3L4	protein_codin	0	7.55775371	0	0.94547578
ENSG000001:	RFC3	protein_codin	5.11047385	16.5381646	35.2982224	5.05416446
ENSG000001:	TRIM54	protein_codin	6.76841699	0	0.72673648	1.20136028
ENSG000001:	STEAP4	protein_codin	33.4245693	67.0100172	0	67.8036717
ENSG000001	RBBP7	protein_codin	1.76054713	5.67007064	1.19940429	0
ENSG000001	RBBP7	protein_codin	28.7172925	51.8548778	73.7004601	0
ENSG000001:	ANKRD16	protein_codin	0	0	0	0
ENSG000001:	ANKRD16	protein_codin	0.72387957	11.6168863	2.20552319	1.22183041
ENSG000002:	KRTAP10-10	protein_codin	0	0	0	0.45494983
ENSG000001:	TRIM6	protein_codin	0	0	0	0
ENSG000001:	KL	protein_codin	5.3774627	2.54871584	0.95159779	3.87728374
ENSG000001	SMAGP	protein_codin	0	0	0	0
ENSG000001:	TRIM6	protein_codin	0	0	0	0
ENSG000001	ELAVL2	protein_codin	0	0	16.8199428	0
ENSG000001:	ITPA	protein_codin	4.07839726	20.3125594	8.83112503	2.79817518
ENSG000001	ELAVL2	protein_codin	1.45686046	0	0	0
ENSG000001	ECI2	protein_codin	0	23.1090764	0	11.8663873
ENSG000001	ECI2	protein_codin	5.04536444	0	0	0
ENSG000002	N4BP2L2	protein_codin	0	0	22.4760092	0
ENSG000000	TXLNG	protein_codin	25.3639809	65.8019939	78.4089945	35.9645584
ENSG000001	ECI2	protein_codin	0	0	0	0
ENSG000000	GDI2	protein_codin	0	0	0	0
ENSG000001:	N4BP2L1	protein_codin	0	0	0	0
ENSG000001:	N4BP2L1	protein_codin	0	8.98179422	0	0
ENSG000000	LETMD1	protein_codin	0	0	0	0
ENSG000001	EDIL3	protein_codin	141.393707	121.932829	79.3087424	35.1317874
ENSG000001:	N4BP2L1	protein_codin	2.51719671	8.63789289	0	0

ENSG000001	CDKN2B	protein_codin	4.23751177	0	21.0804787	0
ENSG000002	TP53TG3D	protein_codin	0	0	0	0
ENSG000001	ALOXE3	protein_codin	0	0	0	0
ENSG000001	CDKN2A	protein_codin	0	0	0	0
ENSG000001	CDKN2A	protein_codin	0	3.89110688	0	0.44537034
ENSG000001	BRCA2	protein_codin	0	0	0	0
ENSG000001	UBXN8	protein_codin	0	0	0	0
ENSG000001	SYAP1	protein_codin	61.145854	105.229409	95.9639953	47.5479768
ENSG000001	RAP1GDS1	protein_codin	4.98863559	0	14.5183513	0
ENSG000001	ATRAID	protein_codin	3.86684153	1.51674796	9.81172732	1.90217949
ENSG000001	ALOX15B	protein_codin	0	0	0	0
ENSG000001	PDLIM5	protein_codin	0.76104584	0	5.46438565	1.9563022
ENSG000001	PDLIM5	protein_codin	0	0	0	0
ENSG000000	GDI2	protein_codin	0	0	22.2718193	5.92111314
ENSG000001	ALOX15B	protein_codin	0	0	0	0
ENSG000001	UBQLNL	protein_codin	1.95435715	0	0.52013317	0
ENSG000001	FAM217A	protein_codin	0	0	0	0
ENSG000001	CERS5	protein_codin	0	0	0	0
ENSG000000	GDI2	protein_codin	185.052118	218.040313	273.097817	118.69538
ENSG000001	NABP2	protein_codin	1.31600134	0	7.73806506	1.24389603
ENSG000001	ZCCHC9	protein_codin	15.1065013	50.3486873	45.021897	5.83196063
ENSG000001	S100G	protein_codin	0	0	0	0.74028017
ENSG000001	DDRGK1	protein_codin	13.0683027	96.7725145	0	14.4722421
ENSG000001	IFNA8	protein_codin	0	0	0	1.12976041
ENSG000001	IFNA2	protein_codin	0	0	0	0
ENSG000001	IFNA6	protein_codin	0	0	0	0
ENSG000001	OR51I1	protein_codin	0	0	0	0.61134022
ENSG000001	IFNA16	protein_codin	0	1.5389556	0	0
ENSG000000	FRY	protein_codin	0	0	0	0
ENSG000001	OR51B6	protein_codin	0	1.35166473	0	0
ENSG000001	TRAPPC10	protein_codin	4.02065576	16.2970431	9.00329681	3.43973813
ENSG000002	IFNA14	protein_codin	0	0	0	0.52842799
ENSG000001	OR51B4	protein_codin	0	0	0	0.70374279
ENSG000001	IFNA21	protein_codin	0	0	0	0.45463477
ENSG000000	CYP46A1	protein_codin	0.53117778	0	0	0
ENSG000001	IFNW1	protein_codin	0	0	0	0.85875194
ENSG000001	IFNB1	protein_codin	0	0	0	0
ENSG000002	HBE1	protein_codin	0	2.27209179	0	0
ENSG000000	CTPS2	protein_codin	0	26.3965082	0	0
ENSG000002	CCDC85C	protein_codin	1.55185578	10.0562949	13.679389	7.0952861
ENSG000000	FUS	protein_codin	3.56383759	14.1913607	17.0151328	47.6450242
ENSG000001	FOCAD	protein_codin	7.96011755	0	0	3.11900683
ENSG000001	HBG2	protein_codin	0	0	0	0
ENSG000002	FBXO16	protein_codin	4.34839402	0	3.85725685	0

ENSG000001(	PCLAF	protein_codin	0	0	4.88170118	0
ENSG000002:	ENSG000002:	protein_codin	1.35760845	1.29395539	0.49129635	0.45577061
ENSG000001'	CNTROB	protein_codin	0	26.806589	5.01415736	0
ENSG000001:	HERC6	protein_codin	0	0	0	5.87909152
ENSG000002	FASTKD5	protein_codin	4.24059507	0	0	0
ENSG000001(	TASOR2	protein_codin	4.49636709	11.0368887	4.67831929	2.79656314
ENSG000001.	FAM50B	protein_codin	0	0	0	1.93959196
ENSG000001(	U2AF1	protein_codin	27.9316967	14.4213493	127.727365	4.33607528
ENSG000001(	PXDC1	protein_codin	0	0	0	4.15946111
ENSG000001	PRPF40B	protein_codin	7.26263616	0	22.5882491	0
ENSG000001(	PXDC1	protein_codin	49.3267899	63.9327647	76.5285694	42.6323716
ENSG000000:	SNX1	protein_codin	0	0	0	0
ENSG000001:	GRPR	protein_codin	0.19397002	1.87973035	0.4407292	1.01480554
ENSG000001(	CIAO2A	protein_codin	0	0	0	0
ENSG000001.	AP1S2	protein_codin	0	19.2499928	0	0
ENSG000001(	AVP	protein_codin	0	0	0	0
ENSG000001:	SLC22A23	protein_codin	0.65766245	0	0	0
ENSG000001:	SLC22A23	protein_codin	0	10.228615	0	3.83576808
ENSG000001:	PSMG4	protein_codin	0	0	7.91326571	0
ENSG000001.	PSMG4	protein_codin	1.10900153	0	0	0
ENSG000001(	ZRSR2	protein_codin	4.66594113	0	3.21828173	0
ENSG000001(	CA5B	protein_codin	0.20092215	0	0	1.036163
ENSG000001:	RXFP2	protein_codin	0	0	0	0.49700302
ENSG000002.	HBB	protein_codin	0	0	0	0
ENSG000000!	BCL7C	protein_codin	1.51749348	0	2.53094002	0
ENSG000001(	CA5B	protein_codin	0.20033836	0	0	0
ENSG000001:	EMILIN1	protein_codin	119.938899	414.201736	582.537068	79.3066952
ENSG000001'	MLLT3	protein_codin	0	0	0	0
ENSG000001.	USP3	protein_codin	11.1160569	19.1295112	15.6615471	16.9102586
ENSG000001:	MRPS26	protein_codin	9.04765789	17.9067831	17.0529367	3.62439996
ENSG000001:	TROAP	protein_codin	0	0	0	0
ENSG000001(	PDE9A	protein_codin	0	0	0	2.77993706
ENSG000001'	CALML5	protein_codin	0	0	0	0
ENSG000001'	MLLT3	protein_codin	7.97381261	14.4148664	15.5083781	4.2183471
ENSG000001:	APH1B	protein_codin	0	0	0	0
ENSG000001.	CLTRN	protein_codin	0	0	1.17863934	0
ENSG000001:	APH1B	protein_codin	0	0	0	0
ENSG000001.	LHFPL2	protein_codin	6.19363978	0	19.2895956	7.71086226
ENSG000001:	GNRH2	protein_codin	0	0	0	0
ENSG000001:	GNRH2	protein_codin	0.3119845	1.95123413	0	0.41139823
ENSG000001:	ELP3	protein_codin	0	0	0	0
ENSG000001'	CHD3	protein_codin	88.7944862	892.653078	0	0
ENSG000001'	NET1	protein_codin	259.09433	172.538836	137.099348	102.423454
ENSG000002:	ENSG000002:	protein_codin	44.3570118	198.887933	62.0877853	30.1200138

ENSG000001	SERPINA9	protein_codin	0	0	0	0
ENSG000001	OR52A1	protein_codin	0	0	0	2.33728706
ENSG000001	BPHL	protein_codin	0	0	0	0
ENSG000002	OR52J3	protein_codin	0	0	0	0
ENSG000002	OR51A2	protein_codin	0	0	0	0
ENSG000001	BPHL	protein_codin	0	0	1.75755433	0
ENSG000001	TBCA	protein_codin	156.687335	284.023703	524.349112	66.9057858
ENSG000001	OR51T1	protein_codin	0	0	0	0
ENSG000001	BPHL	protein_codin	3.28969076	3.69611764	1.55963164	1.6055064
ENSG000001	RPS6	protein_codin	5.7450788	5.85480326	17.1559647	3.12068053
ENSG000001	RPS6	protein_codin	0	0	2.0250859	0.80680919
ENSG000001	SCARA5	protein_codin	0	0	3.85785614	2.06259882
ENSG000001	MMP26	protein_codin	0	0	0	0
ENSG000002	C2CD4B	protein_codin	0.48481308	6.95481314	0	4.26916053
ENSG000001	RPS6	protein_codin	182.159822	133.861297	228.973934	261.37301
ENSG000001	HSPH1	protein_codin	24.388118	54.5606913	99.591162	9.29768313
ENSG000001	RIPK1	protein_codin	1.41374477	11.8068799	5.47349182	1.16855247
ENSG000001	ZNF768	protein_codin	20.6586916	60.2561387	54.1060265	7.5094895
ENSG000001	TUBAL3	protein_codin	0	0	0	0
ENSG000000	PIR	protein_codin	2.79664592	11.3972957	11.284653	4.0262444
ENSG000000	PIR	protein_codin	0	0	0	0
ENSG000001	DENND4C	protein_codin	1.02789802	0	0	0.95806071
ENSG000001	DENND4C	protein_codin	0	0	0	0
ENSG000001	NQO2	protein_codin	0	0	0	0
ENSG000001	UCN3	protein_codin	0	0	0	0.67697
ENSG000001	DENND4C	protein_codin	6.50032289	0	0	5.21758314
ENSG000001	NQO2	protein_codin	0	0	0	0
ENSG000002	VPS16	protein_codin	18.1999	4.04984722	26.6907685	0
ENSG000002	VPS16	protein_codin	10.1606997	35.023416	12.7751581	8.24880713
ENSG000001	AKR1C4	protein_codin	0	0	0	0
ENSG000001	SHBG	protein_codin	0	0	0	0
ENSG000001	NQO2	protein_codin	0	0	0	4.30147508
ENSG000001	NQO2	protein_codin	12.0103337	17.5432429	14.0743275	2.29553916
ENSG000002	RGL3	protein_codin	0	0	0	0
ENSG000001	PLIN2	protein_codin	0.61119417	0	0	6.86391483
ENSG000001	PLIN2	protein_codin	0.65211811	0	0	1.98601494
ENSG000001	SAT2	protein_codin	12.0339748	27.9574982	0	4.82402476
ENSG000002	VPS16	protein_codin	0	7.36444104	0	2.95191301
ENSG000001	ASB11	protein_codin	2.36234646	0	0	2.47668804
ENSG000001	NQO2	protein_codin	0	0	0	0
ENSG000001	TEX26	protein_codin	0	0	0	0.47995919
ENSG000001	EPHX2	protein_codin	0	0	0	0
ENSG000001	MEDAG	protein_codin	5.82234083	4.17304696	0	4.98081333
ENSG000001	ASB9	protein_codin	1.02348237	0	0	0

ENSG000001(	ASB9	protein_codin	0	0	0	0
ENSG000001	C2CD2	protein_codin	3.33726277	39.7657289	7.39307505	7.72008114
ENSG000001(	ASB9	protein_codin	0	0	0	0
ENSG000001	ALOX5AP	protein_codin	2.35790043	33.5418289	0	2.28356167
ENSG000001	C12orf54	protein_codin	0	0	0	0
ENSG000001	MOSPD2	protein_codin	7.12412811	25.058108	4.63977029	8.01288063
ENSG000001	CERT1	protein_codin	15.2318966	0	12.2517821	3.78968972
ENSG000001	TLCD3B	protein_codin	0	0	0	0.38924543
ENSG000001	HAUS6	protein_codin	1.03519803	0	5.98265508	0.93538067
ENSG000001	CD68	protein_codin	3.28402485	36.0058883	0	11.3086957
ENSG000001	HAUS6	protein_codin	23.2157453	75.8710232	116.299737	21.5036225
ENSG000001(	INO80E	protein_codin	0	0	0	0.94977055
ENSG000001	SLTM	protein_codin	159.08431	249.664333	369.054472	73.1975368
ENSG000001	COL2A1	protein_codin	0	13.1193924	942.884737	0
ENSG000001	SERPINB6	protein_codin	0	84.6920546	0	0
ENSG000000	GEMIN8	protein_codin	2.02221709	6.37047506	4.84162872	0
ENSG000001	SERPINB6	protein_codin	0	6.68794946	7.5242448	0
ENSG000001	CARS1	protein_codin	2.05341818	16.9044576	19.1961692	0
ENSG000001	RRAGA	protein_codin	16.3859334	71.2889093	48.6367007	14.3925493
ENSG000001	SERPINB6	protein_codin	0	17.7260419	0	0
ENSG000001	SAXO1	protein_codin	0	0	0	0
ENSG000001	SAXO1	protein_codin	0	0	0.62334053	0
ENSG000001(	TNFSF13	protein_codin	0	0	0.74987575	0
ENSG000001	ADAMTSL1	protein_codin	0	0	0	0
ENSG000001	SERPINB6	protein_codin	27.4766381	0	0	0
ENSG000002(	NAP1L4	protein_codin	5.63250257	0	9.58106566	0
ENSG000001	ADAMTSL1	protein_codin	0	0	0	0
ENSG000001	SERPINB6	protein_codin	0	0	31.8796143	0
ENSG000001	ADAMTSL1	protein_codin	21.2901702	0	0	0
ENSG000000	OFD1	protein_codin	0	34.6022698	0	2.39016525
ENSG000000	ELN	protein_codin	3.10825639	0	0	1.48282582
ENSG000001	AKR1C3	protein_codin	22.335803	0	0	4.96600505
ENSG000002	POLR2M	protein_codin	0	0	0	0
ENSG000001	ADAMTSL1	protein_codin	0	8.69120651	1.3588419	0
ENSG000000	ELN	protein_codin	0	0	15.6143053	0
ENSG000002(	MYZAP	protein_codin	0	0	0	0
ENSG000001	ADAMTSL1	protein_codin	3.4231658	0	0.94505751	0.88308168
ENSG000000	OFD1	protein_codin	0	0	0	0
ENSG000001	GCOM1	protein_codin	0	0	0	0
ENSG000001	GCOM1	protein_codin	0	0	0	0
ENSG000001	ADAMTSL1	protein_codin	0	0	0	0.45199717
ENSG000001	ADRA1A	protein_codin	0	0	1.8326275	0
ENSG000001	ADRA1A	protein_codin	1.29974008	0	57.9143984	4.35063388
ENSG000001	SLC22A18	protein_codin	0	0	0	0

ENSG000000	ELN	protein_codin	0	0	0	2.81447518
ENSG000000	ELN	protein_codin	0	44.6097361	0	0
ENSG000001	TRAPPC2	protein_codin	1.10295032	4.24422329	0.96574782	1.26347282
ENSG000001	TRAPPC2	protein_codin	0	0	0	0
ENSG000001	ADRA1A	protein_codin	0.51702742	0	0	0
ENSG000000	ELN	protein_codin	28.1362335	0	0	0
ENSG000001	TMEM239	protein_codin	0	0	0	0
ENSG000001	ADRA1A	protein_codin	0	0	0	0
ENSG000001	IGSF5	protein_codin	0.2063819	0	0	1.19571432
ENSG000002	C20orf141	protein_codin	0	0	0	0
ENSG000001	BTF3	protein_codin	10.637646	13.5772574	41.2571459	6.77097785
ENSG000002	ENSG000002	protein_codin	0	0	0	0
ENSG000002	BOLA2-SMG1	protein_codin	0	0	0	0
ENSG000001	ZBTB4	protein_codin	41.1374187	224.802452	16.5513323	43.1337712
ENSG000001	TCEANC	protein_codin	0	0	0	3.59328601
ENSG000001	EGFL6	protein_codin	1.84644553	14.5479833	0	3.11892186
ENSG000000	CPXM1	protein_codin	33.7050492	112.003502	236.563135	31.5366479
ENSG000001	SH3GL2	protein_codin	2.64825478	1.25898897	2.47017839	7.48944533
ENSG000000	HDAC7	protein_codin	0	0	0	0
ENSG000001	KATNAL1	protein_codin	20.9673724	41.0182946	24.6479658	12.5829256
ENSG000001	KATNAL1	protein_codin	12.8079091	37.3308528	28.8446219	5.17932506
ENSG000001	B3GALT5	protein_codin	0	0	0	0
ENSG000001	B3GALT5	protein_codin	0	0	0	0
ENSG000001	ATXN3L	protein_codin	0	0	0	2.00634392
ENSG000001	FAM9C	protein_codin	0	0	0	0
ENSG000001	FGF5	protein_codin	0	0	0	0
ENSG000001	BNIP3L	protein_codin	71.4906909	68.3423149	77.3371161	94.1828996
ENSG000001	SH3BGR	protein_codin	0	0	1.08028712	0
ENSG000002	TMSB4X	protein_codin	23.0822696	123.478422	8.77739802	15.7720045
ENSG000001	SH3BGR	protein_codin	0	0	1.44148146	0
ENSG000002	TMSB4X	protein_codin	9.52127788	0	1.96097233	4.65988723
ENSG000002	TMSB4X	protein_codin	7.92732514	78.6086102	2.4506364	7.48308016
ENSG000001	SH3BGR	protein_codin	1.00666693	0	1.21814353	0
ENSG000000	PTCD2	protein_codin	13.4272028	34.0578118	29.9491977	9.33049904
ENSG000000	CNTLN	protein_codin	7.02605888	20.9595332	29.6961344	3.87344189
ENSG000000	CNTLN	protein_codin	111.448447	229.334322	395.987235	41.0097338
ENSG000000	EBF4	protein_codin	0	0	0	0
ENSG000000	HADHA	protein_codin	24.8848547	0	67.3902892	20.6809076
ENSG000000	RPAP3	protein_codin	25.3207394	0	106.018425	23.6242655
ENSG000001	EBF1	protein_codin	0.62682881	0	2.06282678	0
ENSG000001	TTC8	protein_codin	2.15654228	0	0	5.9016799
ENSG000001	TLR7	protein_codin	8.12363899	46.3619162	2.01543532	5.33172176
ENSG000001	PRPS2	protein_codin	0	18.5930445	0	0
ENSG000001	EML5	protein_codin	0	2.47732766	10.4839226	0

ENSG000001	CDCA2	protein_codin	1.4339776	0	19.4895178	0
ENSG000001	BNC2	protein_codin	0.52653324	0	2.71275064	0
ENSG000001	BNC2	protein_codin	18.6794397	0	33.6318429	0
ENSG000001	PRPS2	protein_codin	8.59481603	49.5163319	37.9973243	4.47431009
ENSG000001	LCA5L	protein_codin	1.08207837	0	0	0
ENSG000001	BNC2	protein_codin	67.824581	48.4844561	65.9230841	17.0024015
ENSG000001	UBL3	protein_codin	37.7843884	86.6185193	13.8720834	37.7070821
ENSG000001	FRMPD4	protein_codin	0	25.5217015	0	0
ENSG000000	MSL3	protein_codin	0	0	0	0
ENSG000000	MSL3	protein_codin	0	0	2.51201663	0
ENSG000000	MSL3	protein_codin	0	0	0	0
ENSG000001	SERPINB9	protein_codin	8.64565145	58.1706771	4.86607141	10.5090608
ENSG000001	CCDC171	protein_codin	19.8721498	55.9710803	123.183966	17.4988072
ENSG000000	PDE4A	protein_codin	5.57850753	51.9158617	11.7389465	6.56569286
ENSG000001	SMN1	protein_codin	6.47385604	0	41.8067043	6.0376616
ENSG000001	GET1	protein_codin	10.4375875	4.49716471	31.5739689	0
ENSG000001	AMELX	protein_codin	0	0	0	0
ENSG000001	GET1	protein_codin	0	0	0	0
ENSG000001	AMELX	protein_codin	0	0	0	0
ENSG000001	PSIP1	protein_codin	5.95243364	4.61909708	5.09169039	0
ENSG000001	PSIP1	protein_codin	0	0	0	0
ENSG000000	ARHGAP6	protein_codin	0	6.11393929	0	0.95034623
ENSG000000	ARHGAP6	protein_codin	0	0	0	0
ENSG000001	CDKN1C	protein_codin	2.70680746	4.79920381	0	3.21450173
ENSG000001	GPS2	protein_codin	0	0	0	0
ENSG000001	GTF2H2C	protein_codin	10.5724123	24.1078807	0	8.36289444
ENSG000001	PSIP1	protein_codin	90.9596347	0	489.147705	69.7986312
ENSG000000	ARHGAP6	protein_codin	9.35934697	15.5007167	5.94315945	7.18118227
ENSG000002	PPP2R2A	protein_codin	17.0537914	24.2978143	12.0829845	11.0930583
ENSG000001	PSIP1	protein_codin	0	326.179874	0	0
ENSG000000	SERPINB1	protein_codin	18.5737678	46.2409938	10.0429994	27.3058051
ENSG000002	SMN2	protein_codin	0	0	0	0
ENSG000002	SMN2	protein_codin	9.79687452	0	13.1738707	0
ENSG000002	SMN2	protein_codin	2.92037599	0	0	0
ENSG000001	DNMT3A	protein_codin	5.59745201	6.34720595	0	0
ENSG000002	HMGN1	protein_codin	0	0	0	0
ENSG000002	HMGN1	protein_codin	0	0	0	0
ENSG000002	HMGN1	protein_codin	65.9008018	143.450018	228.419839	40.161089
ENSG000002	SERF1B	protein_codin	7.49144535	0	0	5.47438375
ENSG000002	SERF1B	protein_codin	7.59070273	0.35369681	26.6431834	0
ENSG000001	SLC7A1	protein_codin	14.1073358	36.3943062	36.2510909	14.194931
ENSG000001	AKR1C2	protein_codin	2.94181548	21.9363433	0	0
ENSG000001	DNMT3A	protein_codin	0	67.9054015	22.9891531	13.8035248
ENSG000000	HCCS	protein_codin	3.14924464	11.4754524	6.45839521	7.79041341

ENSG000000	HCCS	protein_codin	4.16256875	5.36211622	14.7083673	0
ENSG000001	WRNIP1	protein_codin	4.36019612	0	0	0
ENSG000002	SPDYE3	protein_codin	0	0	1.07880008	0.75283009
ENSG000001	WRNIP1	protein_codin	0	0	4.82130468	10.9422852
ENSG000001	ICAM4	protein_codin	0	0	0	0
ENSG000001	WRNIP1	protein_codin	12.1146809	42.1838823	47.4830869	0
ENSG000001	WRNIP1	protein_codin	0	0	0	0
ENSG000001	RAD17	protein_codin	0	0	0	0
ENSG000000	KCNQ1	protein_codin	0	0	0	0
ENSG000001	MID1	protein_codin	0	0	0	0
ENSG000001	MID1	protein_codin	0	0	0	0
ENSG000001	MID1	protein_codin	0	0	0	0
ENSG000001	MID1	protein_codin	0	0	0	0
ENSG000001	MID1	protein_codin	16.8237182	16.6102547	10.198607	0
ENSG000000	ADAM7	protein_codin	0	0	0	1.81746019
ENSG000000	YAF2	protein_codin	1.18980523	0	0	0.75549754
ENSG000001	POMC	protein_codin	0	0	0	0
ENSG000001	SNAPC3	protein_codin	5.0208859	0	6.84322042	0
ENSG000001	BRWD1	protein_codin	103.637818	34.674262	537.63195	32.5803715
ENSG000001	GMDS	protein_codin	0	9.99776144	4.53501928	1.1503818
ENSG000001	MTUS2	protein_codin	0	0	0	0
ENSG000001	DNAJC27	protein_codin	1.24325571	0	3.59942858	0
ENSG000001	SLC46A3	protein_codin	0	9.67372728	0.47658999	2.60959411
ENSG000001	GMDS	protein_codin	4.8805872	34.8735645	63.0760616	13.7974389
ENSG000001	SBF1	protein_codin	0	0	0	0
ENSG000000	AK6	protein_codin	0	0	19.8838441	6.35014968
ENSG000001	SNAPC3	protein_codin	12.8192223	14.6680304	12.0660586	4.66084196
ENSG000000	AK6	protein_codin	7.01986871	8.93804297	13.8306289	1.42131808
ENSG000000	CLCN4	protein_codin	0	0	0	0
ENSG000000	CLCN4	protein_codin	13.4052916	31.7843567	4.23806654	4.6267918
ENSG000001	CENPO	protein_codin	0	0	0	0
ENSG000001	NOXRED1	protein_codin	0.31054937	0	1.05679531	0.4036107
ENSG000001	THAP6	protein_codin	7.42420886	11.771308	15.0395048	4.356045
ENSG000001	ASL	protein_codin	1.53976307	0	0	0
ENSG000001	RCHY1	protein_codin	1.03556511	0	0	0
ENSG000001	POMP	protein_codin	5.9603143	80.3934049	15.2070099	10.0661751
ENSG000001	IDH3B	protein_codin	5.01379734	0	9.85373742	4.31360103
ENSG000000	TTC39A	protein_codin	0	0	0	0.43639523
ENSG000001	TTC39B	protein_codin	0	0	0	0
ENSG000001	IDH3B	protein_codin	0	0	0	0
ENSG000001	TTC39B	protein_codin	0	3.35251977	0	0
ENSG000001	SLC2A13	protein_codin	4.39229654	1.73200631	23.3235994	7.85150474
ENSG000001	AKR1C1	protein_codin	1.37211707	0	0	0
ENSG000001	SLC30A5	protein_codin	0.51481711	2.73445703	2.77917878	0.51332809

ENSG000000	WWC3	protein_codin	46.463706	196.199507	138.707073	31.5946187
ENSG000001	ZNF738	protein_codin	0	0	0	0
ENSG000001	NKX3-1	protein_codin	1.72481119	6.06348208	2.97595971	6.57134891
ENSG000001	AKR1C1	protein_codin	4.93034546	13.1317236	3.64737217	14.2605937
ENSG000001	FREM1	protein_codin	0	0	2.84063264	1.16229773
ENSG000002	EIF3CL	protein_codin	0	0	0	0
ENSG000001	GABPB1	protein_codin	1.81221007	2.72054376	0	0
ENSG000001	FREM1	protein_codin	20.7651567	13.1704871	15.8312453	7.80258313
ENSG000001	ESRRB	protein_codin	0	0	0	0
ENSG000001	PIN1	protein_codin	0	0	0	0
ENSG000001	FREM1	protein_codin	0	0	0	0
ENSG000001	GSG1L	protein_codin	0	0	0	0
ENSG000001	PSMG1	protein_codin	1.50511225	19.6841407	14.7049243	3.98647583
ENSG000001	SLC27A2	protein_codin	0	0	0	0
ENSG000000	SELENOO	protein_codin	2.97677511	6.53627779	7.67305668	0.97061024
ENSG000001	HUS1B	protein_codin	0.27080112	8.31414451	3.36628077	0.427849
ENSG000001	TRABD	protein_codin	0	0	0	5.23352935
ENSG000001	CER1	protein_codin	0	1.13507825	0	1.30358773
ENSG000001	SHROOM2	protein_codin	2.88236544	22.0721588	11.7200026	14.7006377
ENSG000001	ZDHHC21	protein_codin	33.1481473	59.3841068	34.9073275	9.28232478
ENSG000001	SREK1	protein_codin	3.6578523	0	17.43909	24.1605121
ENSG000001	ASGR1	protein_codin	0	0	0	0
ENSG000001	NFIB	protein_codin	2.9062622	0	3.92896097	0
ENSG000001	NFIB	protein_codin	0	0	0	0
ENSG000001	SECISBP2L	protein_codin	0	0	0	0
ENSG000001	NFIB	protein_codin	0	0	0	0
ENSG000001	ERBIN	protein_codin	93.1132631	0	292.225068	0
ENSG000001	ERBIN	protein_codin	8.0121125	0	0	0
ENSG000000	KLF6	protein_codin	4.21256493	0	0	1.17867185
ENSG000001	CEP152	protein_codin	27.4309991	96.7908262	176.994567	13.5210438
ENSG000001	NFIB	protein_codin	13.1880424	0	48.9233202	0
ENSG000001	IRF4	protein_codin	2.46846162	78.3069561	0	11.0362578
ENSG000001	PAN3	protein_codin	4.77398459	46.3972531	13.8187411	0
ENSG000001	NFIB	protein_codin	178.26405	78.5947138	55.8920285	51.8213121
ENSG000001	TBL1X	protein_codin	0	36.447519	0	1.89260742
ENSG000002	LCMT1	protein_codin	1.41937626	0	0	4.30987321
ENSG000002	LCMT1	protein_codin	0	9.19524283	0	0
ENSG000001	MLH3	protein_codin	3.54924252	0	5.08054981	0
ENSG000001	DDC	protein_codin	0	0	0	0.78560385
ENSG000001	NLN	protein_codin	9.72205021	31.2192025	26.5175379	8.18662197
ENSG000001	FKBP1B	protein_codin	1.92507527	0	0	0
ENSG000001	FLT3	protein_codin	1.15815074	19.1875537	0	0
ENSG000001	PITRM1	protein_codin	0	0	0	1.88732888
ENSG000001	FKBP1B	protein_codin	0	2.79752336	4.43280828	0

ENSG000001	TSSC4	protein_codin	0	0	0	0
ENSG000000	SLC12A1	protein_codin	0	0	0	0
ENSG000001	PITRM1	protein_codin	0	0	0	0
ENSG000000	TBC1D22A	protein_codin	1.84493277	0	0	0
ENSG000001	TSSC4	protein_codin	0	0	0	0
ENSG000000	DNM1L	protein_codin	0	0	0	0
ENSG000001	FAM9A	protein_codin	0.28935142	0	0	0
ENSG000000	RUFY3	protein_codin	14.0532352	40.6236557	9.68639408	12.4815471
ENSG000001	SGTB	protein_codin	17.9553646	24.1136196	36.0308312	23.6962338
ENSG000001	MPDZ	protein_codin	6.42173331	2.98713248	8.19077102	3.39928286
ENSG000001	TRIM23	protein_codin	0	30.4786427	0	0
ENSG000001	TRMU	protein_codin	0	0	0	0
ENSG000001	CDX2	protein_codin	0	4.41181747	5.04839014	1.14007788
ENSG000001	TRMU	protein_codin	0	0	0	0
ENSG000001	ATAD2B	protein_codin	2.17809316	0	0	0
ENSG000000	TTC38	protein_codin	2.41251413	7.39613066	7.44944226	1.72785095
ENSG000002	VCX3B	protein_codin	0	0	0	0
ENSG000001	PDX1	protein_codin	0	1.02016512	2.39069724	4.5379332
ENSG000000	MARCHF2	protein_codin	0	0	0.85909816	0
ENSG000001	CD81	protein_codin	0	0	0	0
ENSG000002	CDPF1	protein_codin	0	0	0	0
ENSG000001	RBBP6	protein_codin	0	40.8682259	0	0
ENSG000000	PNPLA4	protein_codin	0	7.57022033	0	0
ENSG000000	ADAMTS6	protein_codin	1.40411529	0	0	0
ENSG000001	RESF1	protein_codin	0	0	0	0
ENSG000000	ADAMTS6	protein_codin	7.32656297	17.8525081	8.19381663	7.25886011
ENSG000002	HTN3	protein_codin	0	0	0	0
ENSG000001	VCX	protein_codin	0	0	0	0
ENSG000001	STATH	protein_codin	0	0	0	0
ENSG000001	ATXN10	protein_codin	0	0	0	2.51783035
ENSG000001	CWC27	protein_codin	18.5835072	0	53.305261	9.13938757
ENSG000000	PFKP	protein_codin	1.19331763	10.8047692	3.95040322	4.68306647
ENSG000001	SLC13A5	protein_codin	0.71482829	7.56879059	12.1310834	0
ENSG000000	PFKP	protein_codin	0	0	0	7.93000994
ENSG000001	PUDP	protein_codin	3.71358848	20.721254	0	0.55366908
ENSG000001	IGFBP3	protein_codin	0	0	0	11.0352398
ENSG000001	IGFBP3	protein_codin	0	0	0	25.1637232
ENSG000001	VCX3A	protein_codin	0	0	0	0
ENSG000001	LDAH	protein_codin	0	0	0	0
ENSG000001	NLGN4X	protein_codin	0	0	0	0
ENSG000001	NLGN4X	protein_codin	0	0	0	0
ENSG000001	NLGN4X	protein_codin	0	0	0	3.13093208
ENSG000000	KIF2A	protein_codin	3.33217689	29.3723353	27.0083216	0
ENSG000001	CCM2	protein_codin	0	0	0	0

ENSG000001:	MTIF3	protein_codin	1.36260704	0	0	0
ENSG000000	TSPAN32	protein_codin	0	0	0	0
ENSG000004	ERCC8	protein_codin	3.92178942	0	14.0592343	0
ENSG000001:	MTIF3	protein_codin	6.10552053	0	4.96821326	22.9633979
ENSG000000	TSPAN32	protein_codin	0	0	0	0
ENSG000001	H2AZ2	protein_codin	0	55.0066738	13.9012874	0
ENSG000000	PFKP	protein_codin	11.5284179	16.1748738	15.0989788	0
ENSG000000	ARSF	protein_codin	0	0	0	0
ENSG000001:	AIPL1	protein_codin	0	0	0	0
ENSG000001:	AIPL1	protein_codin	0	0	0	0
ENSG000002	ARSH	protein_codin	0.31535361	0	0	0
ENSG000001	RCAN1	protein_codin	8.48603809	15.8315916	0	2.4518339
ENSG000001	ARSL	protein_codin	0	0	0	0
ENSG000001	RCAN1	protein_codin	0	0	0	0
ENSG000001	TYRP1	protein_codin	0	0	0	0
ENSG000002	ACOT6	protein_codin	0	0	0	0
ENSG000001:	GTF3A	protein_codin	18.4986632	10.903593	54.346172	11.8959862
ENSG000001	TYRP1	protein_codin	0	0	0	0
ENSG000001	SDC1	protein_codin	0	13.8385334	7.5104192	17.3926716
ENSG000001	SLC5A3	protein_codin	93.2611609	190.621266	417.387982	138.266064
ENSG000001	C11orf21	protein_codin	1.87659808	2.13113348	2.65833135	2.537411
ENSG000000	ARSD	protein_codin	8.26452462	48.5375943	12.0629603	8.18765287
ENSG000000	GYG2	protein_codin	0	0	0	0
ENSG000001	RAB3C	protein_codin	0	0	0	0
ENSG000000	NPC1L1	protein_codin	0	0	0	0
ENSG000000	GYG2	protein_codin	0	7.98036287	0	0
ENSG000000	GYG2	protein_codin	0	0	0	0
ENSG000001	MIS12	protein_codin	17.4982284	0	0	6.93271205
ENSG000001	TH	protein_codin	0	0	0	0
ENSG000001:	XG	protein_codin	5.09254425	0	0	1.85178645
ENSG000001	TH	protein_codin	0	0	0	0
ENSG000001	SHISAL1	protein_codin	10.7461314	7.05292612	82.0888883	6.89401862
ENSG000000	CD99	protein_codin	0	1.33273098	0.74134874	1.40976614
ENSG000001	TH	protein_codin	0	0	0	0
ENSG000000	CD99	protein_codin	0	4.20307172	0.95621195	0.35503472
ENSG000000	CD99	protein_codin	1.00278133	5.29203328	0	0
ENSG000000	CD99	protein_codin	51.3962578	62.950393	35.646762	3.7235063
ENSG000000	CD99	protein_codin	4.45171962	136.783354	5.50243692	50.2391793
ENSG000001	PTPRD	protein_codin	1.552655	0	15.2081293	4.00565913
ENSG000001	PNPLA5	protein_codin	0	0.81989519	0	0.37799585
ENSG000001	MIER3	protein_codin	10.673713	37.5336579	77.6801219	15.8560216
ENSG000001:	RPAIN	protein_codin	0	0	0	0
ENSG000001:	RPAIN	protein_codin	0	0	41.2443195	11.1828996
ENSG000002	TECRL	protein_codin	1.60022982	0	0	0.70052199

ENSG000001	MIER3	protein_codin	24.196157	0	18.2447662	0
ENSG000001	MTHFSD	protein_codin	0	0	0	0
ENSG000001	ZNF605	protein_codin	5.02849524	8.62783289	1.14104447	1.47889052
ENSG000002	DPF3	protein_codin	0.44934221	0	8.06971866	0
ENSG000002	ZBED1	protein_codin	1.88465202	0	0	2.02073441
ENSG000002	ZBED1	protein_codin	33.3235003	16.9250789	21.9819009	0
ENSG000002	ZBED1	protein_codin	0	0	0	0
ENSG000001	MIER3	protein_codin	0	0	0	0
ENSG000000	POLR2B	protein_codin	4.45775789	0	37.7218018	3.87942853
ENSG000001	ASMT	protein_codin	0	0	0	0
ENSG000001	SIPA1L1	protein_codin	3.82719523	142.748578	4.5106014	26.2989935
ENSG000001	ASMT	protein_codin	0	0	0	0
ENSG000001	SLC39A14	protein_codin	35.3434066	98.7933476	0	110.897443
ENSG000001	ASMT	protein_codin	0	0	0	0
ENSG000000	WDR76	protein_codin	4.27463998	8.88577103	0	0
ENSG000002	RDH14	protein_codin	20.0556884	14.7954182	45.2839578	7.38440511
ENSG000000	MAP3K9	protein_codin	0	0	6.41137987	0
ENSG000000	ZNF343	protein_codin	0	0	0	0
ENSG000001	GEN1	protein_codin	0	0	45.5826313	0
ENSG000001	HOPX	protein_codin	0	0	0	0
ENSG000001	CCDC91	protein_codin	0	0	0	4.87458087
ENSG000001	HOPX	protein_codin	0	0	0	0
ENSG000001	AKAP17A	protein_codin	2.7303122	34.7817977	5.01457767	4.26896888
ENSG000001	SLC8A3	protein_codin	0	3.7151871	1.82980696	0
ENSG000000	KLHL42	protein_codin	15.8044929	52.308505	51.3843666	9.87591163
ENSG000001	SMC6	protein_codin	26.387019	107.482799	207.468362	7.73074907
ENSG000002	MANSC4	protein_codin	0	2.66432361	0	0.45159527
ENSG000001	A4GALT	protein_codin	0	0	5.35778387	4.62345883
ENSG000001	SMOC1	protein_codin	9.22750169	30.4777978	7.84102015	8.42257396
ENSG000002	ITSN1	protein_codin	0	0	0	0
ENSG000002	ITSN1	protein_codin	0	0	0	0
ENSG000002	ITSN1	protein_codin	0	0	0	0
ENSG000001	IL6ST	protein_codin	0	0	0	0
ENSG000002	ITSN1	protein_codin	0	0	0	0
ENSG000001	IL6ST	protein_codin	0	0	0	0
ENSG000001	IL6ST	protein_codin	0	0	0	0
ENSG000000	EXOC1	protein_codin	4.04005825	45.5703345	9.78385366	2.62242863
ENSG000001	P2RY8	protein_codin	3.4121481	60.886036	4.56055039	11.9855443
ENSG000001	IL6ST	protein_codin	16.5860239	14.5489277	0	7.19056234
ENSG000001	ADARB2	protein_codin	0	0	0	0
ENSG000001	KDM4C	protein_codin	0.90289338	8.56289836	4.56576574	2.40396449
ENSG000001	KDM4C	protein_codin	35.6876093	53.3273103	83.7431077	22.4660342
ENSG000001	ADARB2	protein_codin	0	0	0	0
ENSG000001	CAMTA2	protein_codin	0	0	0	0

ENSG000001	ADARB2	protein_codin	0.77789528	5.02869159	0	5.78867898
ENSG000001	ASMTL	protein_codin	5.41171804	46.1503019	6.65223455	10.614225
ENSG000002	ITSN1	protein_codin	8.49481065	19.3197536	6.21792343	10.311431
ENSG000001	CLOCK	protein_codin	1.57237745	4.8575132	45.704967	11.8599302
ENSG000001	CYRIA	protein_codin	10.7197814	48.0704563	6.39987141	9.17987926
ENSG000001	RETN	protein_codin	0	0	0	0
ENSG000000	WDR37	protein_codin	2.64695385	5.19985673	2.75524663	5.56646658
ENSG000002	INS	protein_codin	0	0	0	0
ENSG000001	ASMTL	protein_codin	0	0	3.15752402	0
ENSG000001	TMEM165	protein_codin	26.5476751	29.5776454	64.9654821	8.38363259
ENSG000000	THUMPD1	protein_codin	17.1645527	32.901668	59.7755041	1.7748665
ENSG000001	ITPR2	protein_codin	212.973961	494.257566	454.233827	122.39449
ENSG000000	DDX1	protein_codin	0	0	0	0
ENSG000001	SNRPB	protein_codin	31.2207318	118.803153	153.662581	24.754926
ENSG000000	IDI1	protein_codin	0	0	0	2.92621992
ENSG000000	RDH11	protein_codin	12.0748537	53.3777241	31.0642747	17.6731446
ENSG000002	SMIM45	protein_codin	0	0	0	0.78747565
ENSG000001	RASSF8	protein_codin	0	0	1.0937868	1.01812133
ENSG000002	ETFRF1	protein_codin	5.01278134	27.3859873	28.9499268	7.58649137
ENSG000001	SERF2	protein_codin	24.6480296	90.9503927	55.6833844	36.9226254
ENSG000001	GP2	protein_codin	0	0	0.52683411	0
ENSG000001	GP2	protein_codin	0	0	0	0
ENSG000002	C17orf107	protein_codin	0.56757032	5.0611458	0.48821355	7.43161299
ENSG000001	UHRF2	protein_codin	0	0	2.85471349	2.90764813
ENSG000001	CDC20B	protein_codin	0	0	0	1.84208953
ENSG000001	IGF2	protein_codin	0	0	0	0
ENSG000001	IGF2	protein_codin	0	0	0	0
ENSG000002	MBD3L2	protein_codin	0	0	0	0.88032571
ENSG000002	MBD3L4	protein_codin	0	0.71005262	1.03140428	0
ENSG000001	IGF2	protein_codin	106.387939	1171.37059	387.94651	249.137052
ENSG000001	CCP110	protein_codin	25.7582207	0	0	0
ENSG000001	SLC25A6	protein_codin	80.8774779	201.060884	139.392961	104.755643
ENSG000001	ESM1	protein_codin	0	0	9.52528508	14.8388145
ENSG000001	ADGRE1	protein_codin	0	3.29501641	0	1.65326359
ENSG000001	ESM1	protein_codin	1.49912435	29.3878285	1.75744645	45.1317654
ENSG000001	IGF2	protein_codin	4.02216892	91.8666595	21.9089449	45.2140646
ENSG000001	ADGRE1	protein_codin	0	0	0	0
ENSG000001	SNX18	protein_codin	66.7185304	133.417893	118.649887	44.0647085
ENSG000001	TMC5	protein_codin	0	0	0	0
ENSG000001	HR	protein_codin	0	0	0	1.47441935
ENSG000001	TGM6	protein_codin	0	0	0	0
ENSG000001	ST8SIA1	protein_codin	0	0	0.75480356	0.68066371
ENSG000001	TPD52L3	protein_codin	0	0	0	0
ENSG000001	SGCB	protein_codin	63.4769299	178.586512	123.660258	49.5775308

ENSG000001:	MCHR1	protein_codin	0	0	0	0
ENSG000001:	IL33	protein_codin	0	26.9818904	0	0
ENSG000001:	GPR12	protein_codin	0	1.60008631	0	0
ENSG000002	ITPRIPL2	protein_codin	69.9261889	197.951572	77.9324752	38.9985965
ENSG000001	DCUN1D4	protein_codin	0	29.0149057	71.301465	15.4464413
ENSG000001:	TGM3	protein_codin	0.78897888	0	0	0
ENSG000001	BRD10	protein_codin	31.563627	0	45.0483049	31.1865419
ENSG000001	OCIAD2	protein_codin	0	0	0.57529384	0.568943
ENSG000000	TRIB2	protein_codin	24.2223014	21.0540743	0	0
ENSG000001	IL3RA	protein_codin	0	21.7589137	0.4022826	2.76853195
ENSG000001	DMTN	protein_codin	0	0	0	0
ENSG000001:	MLANA	protein_codin	0	0	0	0
ENSG000001	OCIAD1	protein_codin	16.7570006	25.0454441	33.7359362	3.54446536
ENSG000001	NOMO2	protein_codin	50.6073776	185.434755	222.011828	34.900782
ENSG000001:	MLANA	protein_codin	0	0	0	0
ENSG000001:	MLANA	protein_codin	0	0	0	0
ENSG000002	DENND1C	protein_codin	0	0	0	0
ENSG000001:	STK35	protein_codin	18.634648	44.039895	38.6832606	11.674744
ENSG000001	GREB1	protein_codin	0	0	0	0
ENSG000001	GREB1	protein_codin	0	0	0	0
ENSG000001	ARRB2	protein_codin	0	0	0	0
ENSG000001	NPIPA6	protein_codin	0	0	26.6906889	29.4866649
ENSG000001	CSF2RA	protein_codin	0	0	0	0
ENSG000001:	TEC	protein_codin	10.7137666	43.1626161	11.0559954	15.6174847
ENSG000001	CSF2RA	protein_codin	0	18.757503	0	1.69505914
ENSG000000	SMARCD1	protein_codin	0	10.085214	4.878138	0
ENSG000002	MRPL23	protein_codin	0	0	0	0
ENSG000002	MRPL23	protein_codin	0	0	13.2800624	9.4755855
ENSG000001	CSF2RA	protein_codin	4.9356509	12.1329926	0	1.69103017
ENSG000001	E2F6	protein_codin	3.81056266	0	9.99058719	0
ENSG000001:	CDK8	protein_codin	8.63463531	0	20.3597295	0
ENSG000001	CSF2RA	protein_codin	0	0	0	0
ENSG000001	NPM2	protein_codin	0	0	0	0
ENSG000001	SYNGR1	protein_codin	0	0	4.01677586	0
ENSG000001	NFXL1	protein_codin	0	15.4894271	32.5524823	0
ENSG000001	NPSR1	protein_codin	0	0	0	0.76499081
ENSG000002	CRYZL1	protein_codin	0	0	14.9755073	0
ENSG000002	SLCO1B3-SL(	protein_codin	0	0	0	0
ENSG000001	NPSR1	protein_codin	0	1.01861537	0	0
ENSG000001	NPSR1	protein_codin	0	0	0	0
ENSG000001	SLCO1B3	protein_codin	0	0	0	0
ENSG000001	GGT6	protein_codin	0	0	0	0
ENSG000001	PDGFB	protein_codin	6.5423349	10.66724	3.71442167	5.7815394
ENSG000001	NPSR1	protein_codin	0	0	0	0

ENSG0000020	CRYZL1	protein_codin	3.58580999	5.06775221	0	0
ENSG0000013	MYBBP1A	protein_codin	17.2647182	0	89.2253473	16.3013585
ENSG0000013	TNNT3	protein_codin	2.83461651	0	0	0
ENSG0000013	TNNT3	protein_codin	14.2917383	0	0	0.94923777
ENSG0000013	TNNT3	protein_codin	20.9774297	0	0	0
ENSG0000012	APOBEC3F	protein_codin	1.00636881	0	3.89888825	0
ENSG0000020	CRLF2	protein_codin	0	0	0	0
ENSG0000028	ENSG0000028	protein_codin	0	0	0	0
ENSG0000000	LZTS1	protein_codin	6.07743026	38.3336216	9.03187099	13.0988429
ENSG0000012	RNF6	protein_codin	0	11.377925	0	0
ENSG0000016	COMMD8	protein_codin	4.78678231	11.2354173	25.1690083	2.96762573
ENSG0000012	CD274	protein_codin	0	0	0	0
ENSG0000018	SHOX	protein_codin	0	0	0	0
ENSG0000012	CD274	protein_codin	2.05221682	38.3416656	0	5.08974294
ENSG0000018	SHOX	protein_codin	0	5.09948782	1.30590145	7.07150227
ENSG0000013	TNNT3	protein_codin	12.5795622	0	0	0
ENSG0000008	SIRPG	protein_codin	0	6.54416108	0	0
ENSG0000016	GABRB1	protein_codin	0	0	0	0
ENSG0000008	SIRPG	protein_codin	0	0	0	0
ENSG000000	ZMYND11	protein_codin	0	0	0	0
ENSG0000013	C2orf50	protein_codin	1.15114732	5.07408948	0	4.36423061
ENSG0000012	RNF6	protein_codin	4.3748743	9.42520241	1.84338333	4.83438883
ENSG0000013	TNNT3	protein_codin	62.1956453	0	3.75320897	0
ENSG000000	ZMYND11	protein_codin	16.5595173	6.4712841	48.7489702	0
ENSG0000010	SIRPB1	protein_codin	0.23647919	0	0.430757	0
ENSG0000010	SIRPB1	protein_codin	0	0	0	0
ENSG000000	ZMYND11	protein_codin	0	92.8542014	57.2592253	35.6243017
ENSG0000010	SIRPB1	protein_codin	1.28448359	7.75245131	1.45978809	6.09988448
ENSG0000003	SLC18A1	protein_codin	0	0	0	0
ENSG0000014	PDIA6	protein_codin	0	0	0	0
ENSG0000015	GABRA2	protein_codin	0.9130947	0	0	0.65364505
ENSG0000012	SIRPD	protein_codin	0	0	0	0
ENSG0000012	SIRPD	protein_codin	0.27020302	4.08131203	0	0
ENSG0000017	CATSPERD	protein_codin	0	1.43706342	0	0
ENSG0000010	PPP2R3B	protein_codin	3.44893083	6.18095718	22.5685796	0
ENSG0000010	RLN2	protein_codin	0	0	0	0
ENSG0000019	SIRPB2	protein_codin	0	6.49740583	0	0.42082293
ENSG0000002	NLRP2	protein_codin	0	0	0	0
ENSG0000007	ZZEF1	protein_codin	33.6797426	151.710048	60.6846332	47.9429376
ENSG0000012	INSL6	protein_codin	0	0	0	0.38328879
ENSG0000020	RIMOC1	protein_codin	16.2823093	40.1327335	41.9582458	17.1111822
ENSG0000013	ATP8A2	protein_codin	0	0	0	0
ENSG0000009	JAK2	protein_codin	52.7191172	168.68775	78.7276027	29.4545433
ENSG0000008	NSFL1C	protein_codin	0	2.14479083	3.21356563	0

ENSG000001	ATP8A2	protein_codin	0	0	0	0
ENSG000001	PLCXD1	protein_codin	4.06826049	0	0	0
ENSG000001	ATP6V1C2	protein_codin	0.26322193	0	0	2.80269735
ENSG000001	ATP8A1	protein_codin	9.05534388	0	0	0
ENSG000001	BAIAP2L2	protein_codin	0	0	0	0
ENSG000001	CARD6	protein_codin	22.066334	18.4748475	16.1300291	10.5687899
ENSG000001	SON	protein_codin	32.0795661	58.965597	93.3414448	82.2274408
ENSG000001	EIF3L	protein_codin	13.834778	102.256399	0	0
ENSG000001	NOL10	protein_codin	0	0	0	0
ENSG000001	NUTM2A	protein_codin	2.03021958	4.19904679	0	2.2428794
ENSG000001	SON	protein_codin	0	0	0	0
ENSG000002	NUTM2D	protein_codin	0	0	0	0
ENSG000002	GAGE12E	protein_codin	0	0	45.5946791	0
ENSG000002	GAGE1	protein_codin	0	6.06610818	0	0.98139465
ENSG000001	NUTM2A	protein_codin	0	17.8750587	3.32645652	0
ENSG000002	GAGE1	protein_codin	0	0	0	0
ENSG000001	HMG20A	protein_codin	32.8218339	42.4313286	76.5162347	19.5020138
ENSG000000	FKBP1A	protein_codin	0	0	0	0
ENSG000001	NUP58	protein_codin	0	0	0	0
ENSG000000	FKBP1A	protein_codin	12.4362758	22.8669601	26.1974256	14.7302154
ENSG000002	GAGE12H	protein_codin	0	0	0	0
ENSG000000	FKBP1A	protein_codin	10.2685662	0	0	0
ENSG000001	RCL1	protein_codin	0	0	13.4300731	0
ENSG000001	RCL1	protein_codin	5.76182609	0	0	0
ENSG000001	RCL1	protein_codin	0	0	1.544447	1.29408725
ENSG000001	ASAH1	protein_codin	1.05346592	0	0	0
ENSG000001	NUP58	protein_codin	0	0	0	0
ENSG000001	NUP58	protein_codin	0	0	0	0
ENSG000001	RCL1	protein_codin	1.35440011	15.1002518	5.94490965	4.5955765
ENSG000000	LIMCH1	protein_codin	0	311.342878	0	0
ENSG000001	GGA1	protein_codin	0	0	0	0
ENSG000001	KDM4B	protein_codin	2.02396995	8.40198333	5.44545057	4.31571016
ENSG000001	UCHL1	protein_codin	0.75505038	0	0	0
ENSG000001	CATSPER2	protein_codin	0	0	0	0
ENSG000001	HPCAL1	protein_codin	0.59468151	2.48081922	1.63135897	4.86911507
ENSG000000	SIRT2	protein_codin	0	0	0	0
ENSG000000	CAMKK1	protein_codin	9.13241524	5.51218805	1.57840197	0
ENSG000001	CPPED1	protein_codin	24.4651609	24.8159431	28.7905635	14.5851259
ENSG000001	LSP1	protein_codin	0.36155564	8.98147118	2.79852603	2.96184021
ENSG000001	NSUN7	protein_codin	0	0	1.56946625	0
ENSG000001	PAAF1	protein_codin	0.91222489	4.17134501	2.69132161	1.24395863
ENSG000001	RRM2	protein_codin	0.30969203	8.85349246	3.4380488	0.875833
ENSG000001	TMEM138	protein_codin	0	0	1.12770997	0
ENSG000001	TMPRSS6	protein_codin	0	0	0	0

ENSG000001(	RBM47	protein_codin	10.3379656	75.9396203	0	4.51036732
ENSG000001(	RBM47	protein_codin	0	0	3.5585737	1.31615013
ENSG000001(	RHOH	protein_codin	0.54824876	9.81466929	0	10.4313119
ENSG000001:	MTMR6	protein_codin	6.8917684	50.2868905	0	12.914767
ENSG000001:	SDCBP2	protein_codin	2.38018955	1.09884631	0.55474448	0
ENSG000001	AK3	protein_codin	24.3485008	32.1941078	25.5824077	11.9917762
ENSG000001:	SDCBP2	protein_codin	0	0	0	0
ENSG000002(	CYS1	protein_codin	21.5098028	15.7830791	35.7225603	5.87664029
ENSG000001	GART	protein_codin	8.87576746	11.8591997	137.580952	2.08520588
ENSG000001(	CIMIP4	protein_codin	0	0	0	0
ENSG000001(	GDNF	protein_codin	0	0	0	0
ENSG000001	GART	protein_codin	0	0	6.34527809	0
ENSG000001	CIITA	protein_codin	0	0	1.59081297	0.83902408
ENSG000001	GART	protein_codin	0	56.5558987	77.7368696	0
ENSG000001(	FGL1	protein_codin	0	0	0	0
ENSG000001(	FGL1	protein_codin	0	0	0	0
ENSG000001	NUP155	protein_codin	17.579012	0	102.330979	0
ENSG000001:	YWHAQ	protein_codin	0	0	10.0678582	0
ENSG000001:	LIAS	protein_codin	0	0	0	0
ENSG000001:	PRH2	protein_codin	0.24914303	0	0	0
ENSG000002	PLIN5	protein_codin	1.18003292	0.76315725	0	1.03305754
ENSG000002	TAS2R14	protein_codin	0	0	7.49422331	0
ENSG000001(	CDC37L1	protein_codin	13.151847	50.5005207	10.0653535	10.9494535
ENSG000001(	CDC37L1	protein_codin	33.5440623	0	50.7271602	0
ENSG000001:	MTUS1	protein_codin	0	0	0	0
ENSG000001(	SNPH	protein_codin	0.686203	0	0	0
ENSG000001:	MTUS1	protein_codin	0	0	0	12.1830946
ENSG000000	CTNS	protein_codin	0	0	0	1.92210808
ENSG000001(	SNPH	protein_codin	8.02043872	1.8438951	2.79410543	0
ENSG000001(	PPIP5K1	protein_codin	0	0	0	0
ENSG000002(	PLPP6	protein_codin	1.72941876	10.2372584	4.62052314	5.06614904
ENSG000001	CENPJ	protein_codin	0	0	58.9219362	0
ENSG000001(	PPIP5K1	protein_codin	0	0	0	0
ENSG000001	USP7	protein_codin	48.2528023	0	159.694479	8.20741108
ENSG000001(	UBXN7	protein_codin	1.56489669	4.429481	7.21151006	0
ENSG000001(	SPATA6L	protein_codin	0	0	0	0
ENSG000001:	TMEM74B	protein_codin	0.93623554	0.94698806	2.18803876	3.39128286
ENSG000000:	RFC1	protein_codin	20.3740889	114.301545	40.3993052	11.8813845
ENSG000001:	PSMF1	protein_codin	0	0	0	0
ENSG000001:	PSMF1	protein_codin	0	0	0	0
ENSG000002(	KLRC2	protein_codin	0	0	0	0
ENSG000002(	KLRC2	protein_codin	0	5.96828084	0	0
ENSG000002(	KLRC3	protein_codin	0	0	0	0
ENSG000001:	TNNI2	protein_codin	0	0	0	0

ENSG000001:	TNNI2	protein_codin	0.5176418	0	0	0
ENSG000001:	KLRD1	protein_codin	2.44019024	0	1.9196961	8.96571156
ENSG000001:	TNNI2	protein_codin	32.4444166	0	0	0
ENSG000001(	TRPV3	protein_codin	0	1.63352041	3.83618661	0
ENSG000000(	TNK2	protein_codin	0	2.039755	1.94989759	0
ENSG000000'	SLC1A3	protein_codin	0	0	0	0
ENSG000000(	METTL22	protein_codin	5.68702265	9.52887601	18.6420926	3.70434623
ENSG000001(	ANGPT4	protein_codin	3.91765116	10.2564567	0	15.3793017
ENSG000001(	TMEM52B	protein_codin	0	1.76086761	0	1.54410787
ENSG000001(	KLHL5	protein_codin	5.84158638	19.4623884	7.44891805	2.18123852
ENSG000001:	NADK2	protein_codin	7.2081171	2.49673819	0	0
ENSG000001:	TMEM156	protein_codin	0.25958161	10.8052472	0	2.34130894
ENSG000001:	FAM110A	protein_codin	0	0	0	0
ENSG000001:	FAM110A	protein_codin	0	0	0	0
ENSG000001(	SLC52A3	protein_codin	0	0	0	0
ENSG000000'	ATP12A	protein_codin	0	0	0	0
ENSG000001'	DNAJC28	protein_codin	1.80191695	4.87101846	4.51776865	0.47189525
ENSG000001'	TLR6	protein_codin	0	3.89788662	0	0
ENSG000001'	OR1A2	protein_codin	0	0	0	0
ENSG000001(	NAGPA	protein_codin	0	0	0	0
ENSG000002'	SRXN1	protein_codin	8.24135167	10.7840825	20.4868661	3.77960116
ENSG000001(	PGM2	protein_codin	3.04257838	0	24.4209378	7.4098095
ENSG000001·	SYT8	protein_codin	0	0	0	0
ENSG000001(	GLIS3	protein_codin	0	0	0	1.29050287
ENSG000001·	TMEM44	protein_codin	0	0	0	0
ENSG000001:	METTL16	protein_codin	0	0	0.82313491	0
ENSG000001·	SYT8	protein_codin	0	0	0	0
ENSG000001:	C4orf19	protein_codin	0	0	1.75730629	0.93100904
ENSG000001:	SEC14L4	protein_codin	0	0	0.49755171	0
ENSG000000:	RFX3	protein_codin	0	0	0	0.76078531
ENSG000001(	PARP4	protein_codin	87.9793208	294.909931	91.8302362	62.1013595
ENSG000001:	GPNMB	protein_codin	16.7138457	40.4800898	7.20409819	18.7829717
ENSG000001:	IFNGR2	protein_codin	0	0	0	0
ENSG000000:	MSR1	protein_codin	34.4512923	34.453764	5.06937841	18.4983444
ENSG000000:	RFX3	protein_codin	21.411762	0	45.1619702	0
ENSG000001(	MCM5	protein_codin	12.2118603	20.6314006	37.9931132	7.58755121
ENSG000001(	TUSC3	protein_codin	7.48037377	0	9.00372861	4.14475089
ENSG000001(	DNAJC21	protein_codin	0	0	0	19.5103178
ENSG000001:	ABHD12B	protein_codin	0	0	0	0
ENSG000001(	MAP1A	protein_codin	19.4330451	75.7506332	62.5237139	7.25177513
ENSG000000:	PUM3	protein_codin	0	0	0	0
ENSG000001	RAD1	protein_codin	13.2629464	13.6042651	40.1404478	7.89783484
ENSG000000(	TP53BP1	protein_codin	0	0	125.888312	44.6020371
ENSG000001:	RSAD2	protein_codin	0	153.275468	10.7183841	0

ENSG000001	NIN	protein_codin	115.615905	209.617896	372.716256	55.849274
ENSG000001	NIN	protein_codin	0	0	0	0
ENSG000000	TP53BP1	protein_codin	62.0025432	100.24314	86.0865085	49.3494788
ENSG000001	SLC34A2	protein_codin	0.24772574	0	0	4.37778865
ENSG000001	SERPINF2	protein_codin	0	0	0	0
ENSG000001	COLEC11	protein_codin	0	0	0	0
ENSG000001	CLEC4D	protein_codin	0	0	0	0
ENSG000000	C1QTNF3	protein_codin	6.64384004	19.0648569	11.3299192	2.74832827
ENSG000002	ENSG000002	protein_codin	0	1.34517766	0	0
ENSG000000	CREBBP	protein_codin	50.66508	117.535432	152.971799	89.8912818
ENSG000002	C1QTNF9	protein_codin	0	0	0	0
ENSG000002	AMACR	protein_codin	0	0	27.6679078	0
ENSG000002	CLEC6A	protein_codin	0	0	0	0.47646371
ENSG000002	C1QTNF3-AM	protein_codin	6.63769049	0	0	0
ENSG000001	SGCZ	protein_codin	0.91463904	0	1.66007736	2.02433796
ENSG000001	KCNV2	protein_codin	0	0	0	1.46719441
ENSG000002	AMACR	protein_codin	8.33081169	12.5451564	54.9225498	0
ENSG000001	ADI1	protein_codin	3.42325723	2.03875207	15.5928871	13.3124982
ENSG000001	SPATA13	protein_codin	3.81128871	0	0	0
ENSG000001	VLDLR	protein_codin	0	0	0	0
ENSG000002	C22orf42	protein_codin	0	0	0	0
ENSG000001	VLDLR	protein_codin	0	0	0	0
ENSG000001	VLDLR	protein_codin	0.85240824	5.25656711	0	5.93758038
ENSG000001	SLC45A2	protein_codin	0	0	0	0
ENSG000001	SEPSECS	protein_codin	3.51368548	0	0	0
ENSG000001	SPATA13	protein_codin	7.99068197	76.8581771	28.8543713	22.8577943
ENSG000001	TRAPPC12	protein_codin	0	9.23777996	0	19.52823
ENSG000001	DEPDC5	protein_codin	0	0	0	0
ENSG000001	DEPDC5	protein_codin	0	0	0	0
ENSG000001	LGI2	protein_codin	10.2927054	6.74261685	5.32182212	6.37714689
ENSG000002	NANOGNB	protein_codin	0	0	0	0
ENSG000001	SOD3	protein_codin	3.99805	21.4159826	0	5.12180197
ENSG000002	IFITM10	protein_codin	5.88709074	9.82604781	0	7.55720156
ENSG000002	LRRC72	protein_codin	0	0	0	0
ENSG000000	EIPR1	protein_codin	0	0	12.5305866	0
ENSG000000	FETUB	protein_codin	0	0	0	0
ENSG000000	FETUB	protein_codin	0	0	0	0
ENSG000002	C1QTNF9B	protein_codin	0.30430823	0	0	1.65828029
ENSG000002	ENSG000002	protein_codin	0	0	0	0
ENSG000001	MTMR12	protein_codin	12.7549971	24.9945321	91.7146629	14.4087688
ENSG000002	C1QTNF9B	protein_codin	0	0	0	0
ENSG000001	KCNIP4	protein_codin	0	0	3.16860391	0
ENSG000001	KCNIP4	protein_codin	0	0	0	0
ENSG000001	KCNIP4	protein_codin	0	0	0	0

ENSG000002	PISD	protein_codin	0	0	5.88531653	0
ENSG000001	KCNIP4	protein_codin	0	0	0	0
ENSG000001	GNG7	protein_codin	1.80880781	10.8363138	9.44376068	2.69133858
ENSG000002	KRTAP5-6	protein_codin	0	0	0	0.95505229
ENSG000001	SFI1	protein_codin	0.81910908	7.6712535	1.54699537	0.77082009
ENSG000000	DGKG	protein_codin	0	0	0	0
ENSG000002	KRTAP5-1	protein_codin	0	0	0	0
ENSG000000	MIPEP	protein_codin	2.98802163	13.1250191	12.7991498	2.58856019
ENSG000001	BRSK2	protein_codin	0	0	0	0
ENSG000001	RBCK1	protein_codin	0	0	0	0
ENSG000000	SMARCA2	protein_codin	2.42070405	0	2.00915314	0
ENSG000000	SMARCA2	protein_codin	3.85066736	0	0	0
ENSG000000	SMARCA2	protein_codin	0	0	0	0
ENSG000001	TRA2B	protein_codin	19.2869862	0	99.222605	16.0696729
ENSG000001	ZNF205	protein_codin	0	0	2.04960451	0
ENSG000000	SMARCA2	protein_codin	0	0	0	0
ENSG000001	TPO	protein_codin	0	0	0	0
ENSG000000	IGF2BP2	protein_codin	44.6379612	66.0811824	51.0491592	18.9051812
ENSG000001	TPO	protein_codin	0	0	0	0
ENSG000000	SMARCA2	protein_codin	21.4074362	0.71839348	5.93294767	3.80890785
ENSG000002	DEFB134	protein_codin	0	0	0	0
ENSG000002	DEFB135	protein_codin	0	0	0	0
ENSG000002	DEFB136	protein_codin	0	0	0	0
ENSG000000	IL32	protein_codin	0	0	0	0
ENSG000001	RBCK1	protein_codin	0	0	0	0
ENSG000001	LCORL	protein_codin	3.88252272	6.87772099	14.5785199	0
ENSG000001	LCORL	protein_codin	3.55752087	0	37.3470383	3.42504294
ENSG000001	TMEM41A	protein_codin	0	0	0	0
ENSG000001	IFNAR2	protein_codin	0	0	0	0
ENSG000001	PKMYT1	protein_codin	0	0	6.27029249	2.06711169
ENSG000001	DCAF16	protein_codin	24.337852	62.8665708	73.1728415	21.908319
ENSG000001	DMRT2	protein_codin	0	0	0	1.8325547
ENSG000002	ZNF316	protein_codin	4.85955244	111.97128	22.8817852	7.95404944
ENSG000001	CDH12	protein_codin	0	0	12.410224	0
ENSG000001	DMRT2	protein_codin	0	0	0	0
ENSG000001	TNFRSF19	protein_codin	9.8981091	4.40485082	3.45852117	0
ENSG000001	TNFRSF19	protein_codin	0	77.8523968	45.3420174	10.7342118
ENSG000001	IFNAR2	protein_codin	2.53338051	0	12.7758403	3.08958222
ENSG000001	KDELR2	protein_codin	0.26508466	2.76280518	0.6619484	0.4111804
ENSG000001	TPO	protein_codin	1.68239882	0	0	0
ENSG000001	CDH18	protein_codin	0	1.30257553	4.58661504	0
ENSG000001	DMRT1	protein_codin	0	0	2.26180547	0
ENSG000001	ZG16B	protein_codin	0.36745921	0	0	0.42659145
ENSG000001	NRSN2	protein_codin	0	0	1.12301823	0

ENSG000001	KANK1	protein_codin	0	0	0	0
ENSG000001	KANK1	protein_codin	0	0	0	0
ENSG000001	NRSN2	protein_codin	0	0	0	0
ENSG000001	SACS	protein_codin	48.4699888	125.880574	186.511326	50.0062414
ENSG000001	KANK1	protein_codin	0	0	0	0
ENSG000002	DAZ4	protein_codin	0	0	0	0
ENSG000001	KANK1	protein_codin	0	0	27.0365705	0
ENSG000001	KANK1	protein_codin	0	0	0	0
ENSG000002	DAZ4	protein_codin	0	0	0	0
ENSG000002	GPR162	protein_codin	0	0	0.43746962	0
ENSG000001	TTC6	protein_codin	0	0	0	0
ENSG000001	PMS2	protein_codin	0	0	0	0
ENSG000001	DOCK8	protein_codin	0	22.0465529	6.12498204	4.31430587
ENSG000001	EIF4G1	protein_codin	0	0	0	0
ENSG000001	DOCK8	protein_codin	0	0	0	0
ENSG000001	FGFBP1	protein_codin	0	0	0	0.42623999
ENSG000001	DOCK8	protein_codin	0	0	2.00669764	0
ENSG000001	BST1	protein_codin	0	0	0	0
ENSG000001	OLIG1	protein_codin	0.20467963	0	0.50074307	0.79246951
ENSG000002	ONECUT3	protein_codin	0.24752533	0.9363078	0	4.29780137
ENSG000001	FGF9	protein_codin	0	4.76430371	6.68791526	4.17963125
ENSG000002	OLIG2	protein_codin	0	0	3.55223781	0
ENSG000000	FSCN1	protein_codin	41.8549497	139.058192	171.905992	40.0378536
ENSG000000	RNF215	protein_codin	3.02276849	6.2884482	9.83011308	1.23993924
ENSG000001	DAZ3	protein_codin	0	0	0	0
ENSG000001	RALGAPA1	protein_codin	0	0	0	0
ENSG000001	FBXL18	protein_codin	4.31245422	19.0931728	6.63182487	5.09095921
ENSG000001	C20orf96	protein_codin	1.72360356	18.9651493	0	0
ENSG000002	EPCIP	protein_codin	0	0	0	0
ENSG000001	MICU2	protein_codin	26.34526	28.0417693	17.0437383	13.5285365
ENSG000001	DEFB132	protein_codin	0	0	0	3.35760091
ENSG000001	ABCA3	protein_codin	0	6.99811327	15.7033551	8.27674583
ENSG000001	C1QTNF7	protein_codin	0	0	0	2.27362085
ENSG000001	WIPI2	protein_codin	7.09105362	0	0	6.92322323
ENSG000000	DEFB127	protein_codin	0	0	0	0
ENSG000001	ZNG1A	protein_codin	3.89369928	0	0	0
ENSG000001	ZNG1A	protein_codin	0	0	1.33351476	0
ENSG000001	CPEB2	protein_codin	3.72733045	0	0	7.79687419
ENSG000001	DEFB126	protein_codin	0	0	0	0
ENSG000001	CPEB2	protein_codin	4.06826363	0	0	0
ENSG000001	ZNG1E	protein_codin	4.4653341	12.6849996	9.26909908	0.48796362
ENSG000001	ZNG1E	protein_codin	0	0	0	0
ENSG000001	FAM177A1	protein_codin	69.8069482	92.9619269	155.362578	19.9804542
ENSG000001	CDY1B	protein_codin	0	0	0	0

ENSG000001	DEAF1	protein_codin	8.19319665	29.5124625	7.76520872	8.16607924
ENSG000001	DEFB125	protein_codin	0	0	0	0.56822031
ENSG000001	NOP2	protein_codin	0	0	0	0
ENSG000001	BAZ1A	protein_codin	94.3566055	0	356.152831	0
ENSG000002	DAZ4	protein_codin	0	0	0	0
ENSG000002	DAZ2	protein_codin	0	0	0	0
ENSG000001	SLC35G5	protein_codin	0.40182057	2.9415665	0	0
ENSG000002	ZNG1F	protein_codin	5.67479809	0	0	0
ENSG000001	DNASE1L2	protein_codin	0	0	0	0
ENSG000001	NKX3-2	protein_codin	1.54403771	4.71734809	8.83923998	2.28146852
ENSG000002	DAZ2	protein_codin	0	0	0	0
ENSG000001	ZNG1A	protein_codin	25.6677376	26.2594095	69.0216101	9.00130799
ENSG000001	JMJD7-PLA2G	protein_codin	0	0	0	0
ENSG000002	DAZ2	protein_codin	0	0	0	0
ENSG000001	MLST8	protein_codin	0	0	0	0
ENSG000001	AP2M1	protein_codin	0	0	0	0
ENSG000000	NCAPD2	protein_codin	0	0	0	5.92276862
ENSG000001	XKR6	protein_codin	0	0	0	1.05151889
ENSG000001	HEATR5A	protein_codin	3.49531872	3.29788371	5.30933519	0
ENSG000001	ZDHHC20	protein_codin	11.7007664	0	6.2839809	21.4217145
ENSG000000	CBARP	protein_codin	0.85226248	0	0	0
ENSG000001	CPZ	protein_codin	0	0	0	0
ENSG000001	RP1L1	protein_codin	1.42369885	8.78571267	0	10.1011963
ENSG000001	GPR78	protein_codin	0	0	0	1.76081393
ENSG000001	HTR3D	protein_codin	0	0	0	0
ENSG000001	MSRA	protein_codin	0	0	0	0
ENSG000001	SYNJ1	protein_codin	0	51.7554525	21.1844491	18.4525471
ENSG000001	TAS2R1	protein_codin	0.28552727	0	0	1.79563035
ENSG000001	ABCC5	protein_codin	0	0	0	0
ENSG000001	SEMA5A	protein_codin	78.5356208	106.96312	579.970634	150.621617
ENSG000001	SYNJ1	protein_codin	0	0	0	0
ENSG000001	FOXD4	protein_codin	0	0	0	0.89973152
ENSG000001	HTRA3	protein_codin	44.6758474	0	26.0538006	6.74102661
ENSG000000	CD9	protein_codin	0.56182723	0	0	0.95168219
ENSG000000	CD9	protein_codin	0	0	0.70897526	0
ENSG000000	CD9	protein_codin	0	0	0	0
ENSG000001	SH3TC1	protein_codin	0.30891178	0	0.58190793	0.39997826
ENSG000000	ADCY2	protein_codin	0	0	0	0
ENSG000001	SAP18	protein_codin	0	0	0	0
ENSG000001	TSC2	protein_codin	0	8.45344381	5.124362	5.02908178
ENSG000001	GZMB	protein_codin	0	0	0	0
ENSG000001	AFAP1	protein_codin	0	0	0	2.72534579
ENSG000001	KCNA1	protein_codin	2.74304304	2.14262593	1.47488155	2.85669743
ENSG000001	GZMH	protein_codin	0	0	0	0

ENSG000001	CFAP298	protein_codin	1.28734642	6.03500573	19.3258828	0.77026421
ENSG000002	NYNRIN	protein_codin	17.2278951	75.558535	107.916175	19.9301411
ENSG000002	DNAJC19	protein_codin	0	0	0	0
ENSG000001	CHEK2	protein_codin	0	0	0	0
ENSG000001	MRFAP1	protein_codin	125.547997	296.132988	608.677848	92.615689
ENSG000001	MBP	protein_codin	0	0	0	0
ENSG000001	TTC14	protein_codin	5.51108598	10.7639495	14.8978856	3.56862009
ENSG000001	BPY2	protein_codin	0	0	0	0
ENSG000001	LATS2	protein_codin	66.1634679	119.660906	71.9214127	75.2052834
ENSG000000	PPP2R2C	protein_codin	0	0	0	0
ENSG000001	IRX2	protein_codin	0.67404053	3.146356	0	0
ENSG000002	IFITM5	protein_codin	1.99889885	27.8155448	19.4504914	9.85951947
ENSG000001	PRMT8	protein_codin	0	0	0	0
ENSG000002	SMIM21	protein_codin	0	1.28484557	0	0
ENSG000002	RBMY1B	protein_codin	0	0	0	0
ENSG000000	RAD51	protein_codin	0	0	0	0
ENSG000001	MRPL36	protein_codin	2.56064137	7.47504048	0	3.10645016
ENSG000002	RBMY1D	protein_codin	0	0	0	0
ENSG000002	DEFB109B	protein_codin	0	0	0	0
ENSG000002	RBMY1E	protein_codin	0	0	0	0
ENSG000002	RBMY1E	protein_codin	0	0	0	0
ENSG000001	FAHD1	protein_codin	0	0	0	0
ENSG000001	FAHD1	protein_codin	0.45120668	0	1.54902248	0
ENSG000001	PSMD13	protein_codin	50.2375626	0	141.063966	0
ENSG000002	RBMY1D	protein_codin	0	0	0	0
ENSG000000	MADCAM1	protein_codin	0	0	0	0
ENSG000001	ATXN2L	protein_codin	0	0	0	0
ENSG000001	CCDC9B	protein_codin	0	0	0	10.8709703
ENSG000002	DEFA1B	protein_codin	0	0	0	0
ENSG000002	DEFA1	protein_codin	0	0	0	0
ENSG000001	CIMAP1D	protein_codin	0	0	0	0
ENSG000001	EVA1C	protein_codin	0	0	0	0
ENSG000002	RBMY1A1	protein_codin	0	0	0	0
ENSG000000	PSME1	protein_codin	0	0	4.92697735	0
ENSG000002	JPT2	protein_codin	0	0	0	0
ENSG000002	JPT2	protein_codin	0	0	0	0
ENSG000002	DOK6	protein_codin	10.541662	28.8417675	32.0796549	14.1437062
ENSG000001	CACNA2D4	protein_codin	0	0	0	0
ENSG000001	MSX1	protein_codin	3.80348654	6.17016744	14.0183986	5.10357066
ENSG000001	SIRT3	protein_codin	0.56588005	0	0	2.69437492
ENSG000001	CLCN7	protein_codin	5.15404905	31.5396021	0	0
ENSG000002	SERPINB11	protein_codin	0	0	0	0
ENSG000001	URB1	protein_codin	44.0154918	93.0069045	347.291791	21.5767575
ENSG000001	TMEM128	protein_codin	0	3.18921845	0	0

ENSG000001[illegible]	EEF1AKMT1	protein_codin	0.85490805	0	0	0
ENSG000000[illegible]	UNKL	protein_codin	0	0	0	0
ENSG000001[illegible]	EEF1AKMT1	protein_codin	6.57881595	4.51177194	4.28571559	5.88323645
ENSG000001[illegible]	UPB1	protein_codin	0.32958529	0	0	0.42842932
ENSG000001[illegible]	BET1L	protein_codin	11.5541063	49.5954518	33.219232	8.66827002
ENSG000001[illegible]	SERPINB12	protein_codin	0	0	0	0
ENSG000002[illegible]	SERPINB5	protein_codin	0	0	0	2.59359458
ENSG000001[illegible]	EIF1AY	protein_codin	13.8536078	14.7735157	0	0
ENSG000001[illegible]	HGFAC	protein_codin	0	0	0	0.41291962
ENSG000001[illegible]	RGS12	protein_codin	13.0769294	95.6012962	59.1746612	10.2946525
ENSG000001[illegible]	ARHGEF10	protein_codin	0	0	0	0
ENSG000000	KDM5D	protein_codin	13.7610201	30.1033379	0	0
ENSG000001[illegible]	CMTM5	protein_codin	0	0	0	0
ENSG000001[illegible]	CRYL1	protein_codin	0	0	0	0
ENSG000001[illegible]	C22orf15	protein_codin	0	0	0	0
ENSG000002[illegible]	KRTAP19-8	protein_codin	0	0	0	0
ENSG000001[illegible]	FBXO25	protein_codin	0	0	0	0
ENSG000002[illegible]	KRTAP20-3	protein_codin	0	0	0	0
ENSG000002[illegible]	KRTAP20-4	protein_codin	0	0	0	0
ENSG000002[illegible]	KRTAP22-2	protein_codin	0	0	0	0
ENSG000002[illegible]	KRTAP27-1	protein_codin	0	0	0	0
ENSG000001[illegible]	FAM193A	protein_codin	0	0	0	0
ENSG000001[illegible]	IQSEC3	protein_codin	2.23001601	0	1.3085571	0
ENSG000001[illegible]	GJB2	protein_codin	0	0	0	0
ENSG000001[illegible]	GJB2	protein_codin	4.64249232	0.76097372	12.7317511	5.06409789
ENSG000002[illegible]	CFAP99	protein_codin	0	2.38577669	0	0.8331054
ENSG000000[illegible]	NEDD4L	protein_codin	9.93411444	0	8.16800094	2.6439803
ENSG000001[illegible]	HSFY2	protein_codin	0	0	0	0
ENSG000001[illegible]	HSFY1	protein_codin	0	0	0	0
ENSG000001[illegible]	MSLN	protein_codin	0	0	0	0
ENSG000001[illegible]	POLN	protein_codin	0	6.25922065	0	0
ENSG000001[illegible]	CDY2B	protein_codin	0	0	0	0
ENSG000001[illegible]	NLGN4Y	protein_codin	15.611042	10.3049645	0	0
ENSG000001[illegible]	ZMYM2	protein_codin	0	0	3.89419371	0
ENSG000001[illegible]	ZMYM2	protein_codin	36.7431157	99.5325001	64.4875451	25.3750298
ENSG000001[illegible]	NLGN4Y	protein_codin	0	0	0	0
ENSG000000[illegible]	FECH	protein_codin	9.8408834	0	8.34809044	8.93892767
ENSG000001[illegible]	ZMYM2	protein_codin	17.3633453	27.171382	14.7485749	10.5796378
ENSG000001[illegible]	ZMYM2	protein_codin	7.44434442	0	18.2298058	6.12337278
ENSG000001[illegible]	NELFA	protein_codin	4.78968278	5.0769228	28.2228161	0
ENSG000001[illegible]	ZMYM2	protein_codin	21.4259019	48.4303164	34.5325515	10.252081
ENSG000001[illegible]	NSD2	protein_codin	0	65.8890852	17.3426976	11.0649964
ENSG000001[illegible]	NSD2	protein_codin	0	0	0	0
ENSG000001[illegible]	NSD2	protein_codin	0	0	0	0

ENSG000001	UTY	protein_codin	5.86881995	29.2757835	0	0
ENSG000001	NSD2	protein_codin	0	0	0	0
ENSG000001	UTY	protein_codin	0	0	0	0
ENSG000001	KDM6A	protein_codin	0	0	0	0
ENSG000001	ZMYM5	protein_codin	7.75021348	9.92766359	10.2314524	12.2965235
ENSG000001	ZMYM5	protein_codin	0	0	0	0
ENSG000001	ZMYM5	protein_codin	0	0	8.39939824	0
ENSG000001	C18orf54	protein_codin	0	0	0	4.21000394
ENSG000000	ME2	protein_codin	0	0	0	0
ENSG000000	SLC7A4	protein_codin	0	0	0	0
ENSG000000	RPGRIP1	protein_codin	0	0	0	0
ENSG000001	TMEM129	protein_codin	2.73720629	17.4849479	6.91144895	0
ENSG000000	APBA2	protein_codin	0.70243163	0	3.79974071	0.90054409
ENSG000001	NME4	protein_codin	0	0	0	0
ENSG000001	MBD1	protein_codin	0	0	0	0
ENSG000001	GOLGA8G	protein_codin	0	0	0	0
ENSG000001	CTBP1	protein_codin	0.84386507	0	0.82332131	0
ENSG000002	RNASE13	protein_codin	0.61266866	0	0	0.72497214
ENSG000001	CTBP1	protein_codin	26.8204687	28.1940568	66.9906058	10.0029531
ENSG000001	RNF212	protein_codin	0	0	5.31072621	1.13199476
ENSG000000	MED15	protein_codin	0	36.8722658	0	17.9148434
ENSG000001	TPTE2	protein_codin	0	0	0	0
ENSG000001	METTL17	protein_codin	3.78092488	8.25079193	0	0
ENSG000001	MME	protein_codin	0.43482784	1.18371255	5.29326534	0
ENSG000001	CTIF	protein_codin	8.51904208	10.8989878	23.0544276	10.2004434
ENSG000002	TSPY8	protein_codin	0	0	0	0
ENSG000002	TSPY8	protein_codin	0	0	0	0
ENSG000002	TSPY4	protein_codin	0	0	0	0
ENSG000002	RBMY1B	protein_codin	0	0	0	0
ENSG000001	PIGG	protein_codin	0	0	0	0
ENSG000001	TTC5	protein_codin	0	0	0	0
ENSG000000	TBL1Y	protein_codin	0	0	0	0
ENSG000000	AMELY	protein_codin	0	0	0	0
ENSG000001	TSPY2	protein_codin	0	0	0	0
ENSG000001	SEPTIN5	protein_codin	9.34331011	0	50.2648121	6.74228795
ENSG000000	ZFY	protein_codin	28.4839554	23.9947939	0	0
ENSG000001	TPGS2	protein_codin	7.413094	0	0	7.68879317
ENSG000001	SRY	protein_codin	0.19468445	0	0	0
ENSG000001	ZIC4	protein_codin	0	0	0	0
ENSG000001	PLSCR4	protein_codin	0	0	8.8074098	0
ENSG000002	MICAL3	protein_codin	17.3657521	0	40.5624348	20.6186497
ENSG000001	CCDC178	protein_codin	0	0	0	0
ENSG000001	B4GALT6	protein_codin	0	0	0	0
ENSG000002	PRR23A	protein_codin	0	0	0	1.28849274

ENSG000002	FOXL2NB	protein_codin	0.33616841	0	0	0
ENSG000001	AQP4	protein_codin	1.98849058	0	0.49984205	2.85842354
ENSG000001	AQP4	protein_codin	0	0	0	0
ENSG000001	C2	protein_codin	0	58.5150968	0	6.44889155
ENSG000001	NME9	protein_codin	0	0	0	0
ENSG000001	STAG1	protein_codin	43.6667287	141.964185	46.2253242	20.4044558
ENSG000001	CEP63	protein_codin	0	0	0	0
ENSG000001	TMEM241	protein_codin	0	0	0	0
ENSG000002	LY6G5C	protein_codin	0	0	0	0
ENSG000001	CHRM5	protein_codin	0	1.38662703	0	1.02134793
ENSG000001	ESCO1	protein_codin	8.40493084	44.0767712	10.1648399	2.02742057
ENSG000001	NPHP3	protein_codin	0	0	0	0
ENSG000001	RNMT	protein_codin	0	49.5093624	0	0
ENSG000002	HLA-C	protein_codin	0	0	19.1964201	0
ENSG000001	NEK11	protein_codin	0	0	0	0
ENSG000001	CCDC125	protein_codin	0.96097932	0	2.84458086	3.09415719
ENSG000001	IMPA2	protein_codin	0	0	1.26329386	0.57483571
ENSG000001	PIEZO2	protein_codin	0	0	0	0
ENSG000000	RALBP1	protein_codin	39.3667437	252.997812	295.446057	49.064381
ENSG000001	HMCES	protein_codin	9.22837645	25.1327934	10.9087917	9.07635346
ENSG000002	LRRC30	protein_codin	0	0	0	1.77500243
ENSG000000	ARHGAP28	protein_codin	39.9584875	31.6550762	77.3848886	0
ENSG000002	TMEM200C	protein_codin	0	5.7159311	0	2.76221907
ENSG000001	TXNRD3	protein_codin	0	1.39866445	0.42306586	0.6700311
ENSG000001	ENOSF1	protein_codin	1.05069105	0	0	13.7484935
ENSG000001	UROC1	protein_codin	0	0.90843161	0.53589247	0
ENSG000001	USP14	protein_codin	32.7540872	25.0031131	80.7849392	0
ENSG000001	SLC41A3	protein_codin	0	0	0	4.8320168
ENSG000002	OR2H2	protein_codin	0	0	0	0.43558166
ENSG000002	HACD2	protein_codin	25.2830038	38.7918273	94.8979249	14.8434965
ENSG000001	DTX3L	protein_codin	0	80.1567054	52.0219865	7.7313889
ENSG000001	CD80	protein_codin	0	0	0	0
ENSG000001	TIGIT	protein_codin	2.01028578	0	0.56770985	2.20366353
ENSG000001	DRD3	protein_codin	0	0	0	0
ENSG000001	NEPRO	protein_codin	0	0	0	0
ENSG000001	GTPBP8	protein_codin	3.63733708	10.8432981	19.183826	0
ENSG000001	GTPBP8	protein_codin	5.93653125	0	9.71708128	4.50430776
ENSG000001	BTLA	protein_codin	0.38126677	14.4311069	0	1.18122717
ENSG000000	CD200	protein_codin	8.83156653	57.3567701	0	0
ENSG000002	LNP1	protein_codin	0	0	16.4492825	5.41138191
ENSG000001	FILIP1L	protein_codin	58.0658481	53.1755098	0	57.465679
ENSG000002	OR5K3	protein_codin	0	0	0	1.76148266
ENSG000002	VGLL3	protein_codin	0	0	0	2.40823552
ENSG000001	CADM2	protein_codin	1.64061681	0	0.6426543	1.18772112

ENSG000001	EOGT	protein_codin	11.0475985	27.6612745	2.359165	22.2811046
ENSG000001	LRIG1	protein_codin	0	0	0	0
ENSG000001	CADPS	protein_codin	0	0	0	0
ENSG000001	PDHB	protein_codin	0.72599246	0	0	0
ENSG000001	PXK	protein_codin	0	0	0	0
ENSG000001	PXK	protein_codin	0	0	0	0
ENSG000001	SLMAP	protein_codin	10.8673852	49.5235556	19.7872473	5.13085722
ENSG000001	NEK4	protein_codin	15.5224431	0	19.0953708	0
ENSG000001	P4HTM	protein_codin	5.89971306	22.3352967	0	2.9497572
ENSG000002	SLC26A6	protein_codin	0	0	0	0
ENSG000001	PFKFB4	protein_codin	1.19282504	5.53574433	1.20658711	0
ENSG000000	MAP4	protein_codin	9.93460751	6.84006382	13.9443101	5.05046377
ENSG000001	CSPG5	protein_codin	0.28185521	3.76342799	0	3.93364548
ENSG000001	ALS2CL	protein_codin	0	0	0	0
ENSG000001	ZNF197	protein_codin	1.47295896	0	0	0
ENSG000001	ZNF197	protein_codin	0.73006385	0	4.99964109	2.11116358
ENSG000001	TCAIM	protein_codin	0	0	1.30800364	0
ENSG000002	KRBOX1	protein_codin	1.70571596	0	0	0
ENSG000000	SEC22C	protein_codin	0	0	0	0
ENSG000001	MOBP	protein_codin	0	0	0.81414627	0.88252447
ENSG000001	VILL	protein_codin	0.56352349	23.7503438	0.88208697	0
ENSG000002	TRIM71	protein_codin	0	1.31735897	2.5261357	3.4751478
ENSG000001	RBMS3	protein_codin	0	0	0	9.25321394
ENSG000001	RBMS3	protein_codin	43.2730679	0	11.6246633	0
ENSG000002	ZCWPW2	protein_codin	9.70868215	1.14585956	2.4656622	3.94794249
ENSG000001	NEK10	protein_codin	0	0	0	0
ENSG000000	RARB	protein_codin	0	0	0	0
ENSG000001	NR1D2	protein_codin	0	20.6122512	0	5.25487792
ENSG000001	DPH3	protein_codin	16.019149	34.4886756	19.2151765	13.1995852
ENSG000001	HACL1	protein_codin	0	5.22464908	0	5.90900033
ENSG000002	COLQ	protein_codin	0	0	0	0
ENSG000002	COLQ	protein_codin	0.94663685	0	0	0
ENSG000002	COLQ	protein_codin	0	0	0	0
ENSG000002	METTL6	protein_codin	1.19994883	2.40665094	0	0.57564449
ENSG000002	METTL6	protein_codin	6.93407327	0	9.08266434	2.65917274
ENSG000001	SH3BP5	protein_codin	27.312972	135.691248	39.6226738	42.0100326
ENSG000001	CCDC174	protein_codin	35.5646943	127.112231	43.859601	23.9273079
ENSG000001	SEC13	protein_codin	0	0	3.91033791	0
ENSG000001	CRELD1	protein_codin	0	0	0	0
ENSG000001	IL17RC	protein_codin	0	0	0	0
ENSG000001	IL17RE	protein_codin	0	0	0	0
ENSG000001	IL17RE	protein_codin	0	0	0	0
ENSG000001	CIDEC	protein_codin	0	0	0	0
ENSG000001	RPUSD3	protein_codin	3.03749517	0	7.36294337	0

ENSG000001	OGG1	protein_codin	0	0	0	0
ENSG000001	OGG1	protein_codin	0	0	0	0
ENSG000002	TTLL3	protein_codin	0	23.8360184	16.603966	0
ENSG000001	BRPF1	protein_codin	0	0	0	0
ENSG000001	CPNE9	protein_codin	0	0	0.43912766	0
ENSG000001	CPNE9	protein_codin	0	0	0	0
ENSG000001	SRGAP3	protein_codin	18.1642092	0	57.5868876	45.7651277
ENSG000001	SUMF1	protein_codin	0	0	0	0
ENSG000000	IL5RA	protein_codin	0	0	0	0
ENSG000001	FAM131A	protein_codin	0	0	0	0
ENSG000001	ADAMTSL1	protein_codin	0	0	0	0
ENSG000001	GOLM1	protein_codin	4.31996986	8.22013228	0	0
ENSG000001	GOLM1	protein_codin	95.7324816	297.134284	418.620635	44.0644056
ENSG000001	PNPLA1	protein_codin	0	0	0	0.39190608
ENSG000001	FLG2	protein_codin	1.8816911	1.26170361	0	4.77568043
ENSG000001	ADAD1	protein_codin	0	3.60973001	0	2.40554194
ENSG000001	ADAD1	protein_codin	0	0	0	0
ENSG000001	BLTP1	protein_codin	77.2669622	376.82875	86.0367667	92.6092177
ENSG000001	EYA1	protein_codin	0	0	0	0
ENSG000001	EYA1	protein_codin	0	0	0	0
ENSG000001	EYA1	protein_codin	0	0	0	18.2184354
ENSG000001	EYA1	protein_codin	0	0	0	4.43078873
ENSG000001	PIP5KL1	protein_codin	0	0	0	0
ENSG000001	HNRNPL	protein_codin	1.47072016	0	1.90414223	3.40469924
ENSG000001	MRPL2	protein_codin	1.51488258	2.0878488	3.07160458	0
ENSG000001	NKIRAS1	protein_codin	0	0	0	0
ENSG000001	UFSP1	protein_codin	0	0.92841081	0.65321421	0
ENSG000001	CORO6	protein_codin	9.14077787	0	0	0
ENSG000002	RUFY2	protein_codin	0	2.431913	0	0
ENSG000000	LAMB4	protein_codin	0	0	0	0
ENSG000001	GKAP1	protein_codin	0.94926834	2.80591239	6.81688513	0
ENSG000001	ABHD18	protein_codin	0	0	3.16312152	3.56086896
ENSG000001	SPAG1	protein_codin	2.38039396	17.8415744	14.0163676	6.01040074
ENSG000002	ADGRG1	protein_codin	0	0	0	0
ENSG000001	ADAMTS7	protein_codin	6.08632791	25.2212344	13.70158	8.0863493
ENSG000001	METTL14	protein_codin	43.0868508	91.2855822	67.1530915	14.4183094
ENSG000002	GPX3	protein_codin	17.6751295	28.6136352	0	28.3825049
ENSG000001	CCDC152	protein_codin	0	0	0.63389548	0
ENSG000001	TSNAXIP1	protein_codin	0	0	0	0
ENSG000001	KRT18	protein_codin	0	7.7504301	0	0
ENSG000001	KRT18	protein_codin	1.11932284	0	12.9369852	0
ENSG000001	FLNC	protein_codin	0	0	0	0
ENSG000002	SELENOH	protein_codin	0	8.55466663	13.7710339	0
ENSG000002	NOX5	protein_codin	0	0	0	0

ENSG000002	DIO1	protein_codin	0	0	0	0
ENSG000001	TNFAIP8	protein_codin	0	0	0	0
ENSG000001	STAB2	protein_codin	0	11.3443235	5.70505425	1.30376035
ENSG000001	COPS2	protein_codin	20.7582936	31.4177029	27.5687171	38.1866485
ENSG000001	FAM83H	protein_codin	0	1.82267237	6.66143062	4.25709043
ENSG000001	CCDC158	protein_codin	0	0	0	0
ENSG000001	TYRP1	protein_codin	0.17624084	0	0	0
ENSG000001	FOXI2	protein_codin	1.55360454	0	0	0
ENSG000001	ACVRL1	protein_codin	0	29.3510388	0	0
ENSG000002	C6orf163	protein_codin	2.24121939	6.66852137	3.74824346	1.59538079
ENSG000001	STPG3	protein_codin	0	0	0	0
ENSG000001	MAB21L4	protein_codin	0	0	0	0
ENSG000001	SCFD2	protein_codin	11.097019	29.398362	77.0002422	8.38121365
ENSG000001	TMEM266	protein_codin	0	0	0	1.36573964
ENSG000001	LRRK1	protein_codin	4.81748395	91.8658221	14.8801463	32.0773778
ENSG000002	FAM221B	protein_codin	0	0	0	0
ENSG000001	OTOA	protein_codin	0	0	0	0
ENSG000001	OTOA	protein_codin	0.39051988	0	0	1.31148543
ENSG000001	OTOA	protein_codin	0	0	0	0
ENSG000001	SELPLG	protein_codin	0	47.5437332	0	6.07636793
ENSG000001	HSPD1	protein_codin	43.2720495	65.4423133	276.378463	0
ENSG000001	SEPTIN14	protein_codin	0.75426478	0	1.25871903	1.17989196
ENSG000001	HYKK	protein_codin	0	0	0	0
ENSG000001	FAM83G	protein_codin	0	5.12248324	1.13554592	1.83590245
ENSG000001	KCNQ3	protein_codin	4.36790708	0	8.97085926	7.51610782
ENSG000000	BLTP2	protein_codin	20.8520127	48.6295597	159.495589	0
ENSG000000	NCBP3	protein_codin	71.3739157	244.996153	146.151828	48.9828198
ENSG000001	BMP2K	protein_codin	0	0	2.97745727	2.17347609
ENSG000001	BORCS6	protein_codin	1.40479913	40.5145282	7.54513209	3.88637895
ENSG000002	SLCO6A1	protein_codin	0	0	0	0
ENSG000002	NT5M	protein_codin	0	0	0	0
ENSG000002	STK38L	protein_codin	31.1200449	46.4278564	39.2288575	25.6656875
ENSG000001	DUOX1	protein_codin	0	0	0	0
ENSG000001	DUOX2	protein_codin	0	0	3.11702662	3.09358531
ENSG000001	VPS53	protein_codin	0	0	0	0
ENSG000001	TRIP12	protein_codin	0	0	0	0
ENSG000001	TRIP12	protein_codin	4.52086303	0	0	0
ENSG000001	ALK	protein_codin	0	0	0.68867433	2.99533011
ENSG000001	PP2D1	protein_codin	0	0	0	0
ENSG000001	DIAPH1	protein_codin	85.8464219	314.229086	131.639096	39.4654307
ENSG000002	SACM1L	protein_codin	0	0	0	0
ENSG000001	DCP2	protein_codin	32.8734648	25.5133809	48.2271004	0
ENSG000001	SHISAL2B	protein_codin	0	0	0	0
ENSG000002	H2AJ	protein_codin	17.0660952	0	0	4.9249885

ENSG000001	DENND5B	protein_codin	27.6062179	24.0478546	21.1397223	19.7823175
ENSG000000	PKM	protein_codin	6.40193462	12.5122441	0	0
ENSG000001	RNF180	protein_codin	26.8501164	53.0520546	12.9326792	12.7460093
ENSG000000	UBE3C	protein_codin	0.50297604	23.4213725	15.013668	0
ENSG000002	RANBP17	protein_codin	0	0	0	0
ENSG000001	RNF20	protein_codin	134.518495	395.565257	292.414622	62.7715172
ENSG000001	GRIK1	protein_codin	0	0	0	0
ENSG000001	GRIK1	protein_codin	0	0	6.45478345	0
ENSG000001	LOXL2	protein_codin	33.9204774	69.9509008	78.9769482	17.7118236
ENSG000002	CNEP1R1	protein_codin	0	0	0	0
ENSG000001	ERCC4	protein_codin	7.79542521	30.6210015	13.2359642	0
ENSG000001	OS9	protein_codin	0	0	0	0
ENSG000001	OS9	protein_codin	0	0	0	0
ENSG000002	ATXN2	protein_codin	0	0	0	0
ENSG000002	ATXN2	protein_codin	0	0	0	0
ENSG000001	FAM169A	protein_codin	0	0	70.9711279	0
ENSG000001	GPS2	protein_codin	12.1383272	39.5219728	9.13172619	10.4657281
ENSG000001	FLCN	protein_codin	1.48440625	8.60217148	0.82899867	0
ENSG000001	FLCN	protein_codin	0	0	0	0
ENSG000001	FLCN	protein_codin	4.07537626	14.5500912	3.91600003	1.91511772
ENSG000001	DNAH2	protein_codin	0.22135449	5.9084158	1.35597003	0
ENSG000001	CCDC150	protein_codin	4.71559195	0	20.530836	0
ENSG000001	CCDC154	protein_codin	0	0	3.14355698	0.90721453
ENSG000001	LTN1	protein_codin	0	0	0	0
ENSG000001	LTN1	protein_codin	8.5907741	13.9844737	7.7506914	0.95160413
ENSG000001	LRRC4B	protein_codin	0.28692982	2.1142774	0	0.85176261
ENSG000001	ITGAD	protein_codin	0.30742528	8.83929984	0	0.40893358
ENSG000001	ASPH	protein_codin	19.1031032	12.0360672	12.7306315	4.47693412
ENSG000002	ASPDH	protein_codin	0	0	0	0
ENSG000000	RAPGEF3	protein_codin	0	0	0	0
ENSG000000	UNKL	protein_codin	7.12693069	24.1426202	13.6058177	3.23212292
ENSG000001	PDPK1	protein_codin	0	0	0	2.42765014
ENSG000001	USP5	protein_codin	4.80031531	0	131.37613	17.0665585
ENSG000001	RYR3	protein_codin	5.02893695	0	1.55766046	0
ENSG000000	DNM2	protein_codin	17.3420936	18.3248211	24.8274405	12.2429835
ENSG000001	EFHB	protein_codin	0	0	0	0
ENSG000000	DGCR2	protein_codin	1.92692749	8.01881723	10.7751424	0
ENSG000001	GARS1	protein_codin	60.2762605	0	83.5823481	0
ENSG000001	STAT3	protein_codin	0	0	0	2.91020456
ENSG000001	CCAR2	protein_codin	5.35556318	0	2.80729088	13.4673487
ENSG000000	WIZ	protein_codin	0	17.053047	0	0
ENSG000001	PRTG	protein_codin	9.51930232	24.8783349	13.6046664	25.3819978
ENSG000001	CFAP69	protein_codin	0.96552869	7.84387113	1.97337399	5.48406456
ENSG000001	FANCA	protein_codin	0	7.55282895	0	2.86810228

ENSG000001	FANCA	protein_codin	0	0	0	0
ENSG000001	SMTNL2	protein_codin	0	0	0	0
ENSG000001	GRM7	protein_codin	0	0	0	0
ENSG000001	GRM7	protein_codin	0	0	0	0
ENSG000000	POU2F2	protein_codin	1.39302393	37.5975782	0	6.26184577
ENSG000001	BICDL2	protein_codin	0	0	0	0
ENSG000001	FAM149A	protein_codin	0	0	0.73385805	1.26316303
ENSG000001	PRB2	protein_codin	1.35829956	1.27842821	0	1.7051109
ENSG000002	TM6SF2	protein_codin	0	0	0	0
ENSG000000	IFT88	protein_codin	1.2940115	0	0	1.49516054
ENSG000001	TNIP1	protein_codin	0	0	34.6199608	30.7743856
ENSG000000	ATXN7L3	protein_codin	9.6277329	0	8.60203082	10.9608305
ENSG000000	RPH3A	protein_codin	0	20.9726778	2.84005373	4.86623821
ENSG000000	VPS9D1	protein_codin	3.1789159	0	5.42639501	5.27609349
ENSG000001	DNAH6	protein_codin	2.33152106	2.69724644	25.0538399	6.22147661
ENSG000001	VWA3A	protein_codin	0	0	0	0
ENSG000001	PTPRN2	protein_codin	0	0	0	0
ENSG000001	PTPRN2	protein_codin	1.85553922	0	1.97283063	4.31111827
ENSG000001	PTPRN2	protein_codin	0	0	0	0
ENSG000001	ADAMTS20	protein_codin	1.39109575	0	14.6071949	4.81973307
ENSG000001	SEMA6D	protein_codin	2.24521476	6.54286764	4.23728724	6.16168605
ENSG000001	SEMA6D	protein_codin	0	0	0	0
ENSG000001	TMEM132D	protein_codin	0.24937783	0	0	1.23136065
ENSG000001	PTMS	protein_codin	55.9606335	80.7111146	163.294817	34.1145241
ENSG000001	LAMA4	protein_codin	154.468456	0	292.707806	43.6260872
ENSG000001	SIGLEC15	protein_codin	4.98953803	11.6717516	0	35.9440281
ENSG000001	LRP1B	protein_codin	8.19820669	3.09965301	186.056658	10.190083
ENSG000001	CHRNG	protein_codin	0	0	0	0
ENSG000001	SMYD5	protein_codin	5.17198685	39.7606272	34.414164	7.24169423
ENSG000001	KMT2A	protein_codin	49.6874601	0	149.99532	28.1228254
ENSG000001	TANC2	protein_codin	0	29.7232416	0	13.5754598
ENSG000001	SDK1	protein_codin	2.95082204	0	18.1482594	0
ENSG000001	CAMSAP1	protein_codin	14.8367689	21.437422	49.3958741	20.5255708
ENSG000001	ZNF841	protein_codin	0	65.090446	0	0
ENSG000001	DNAH12	protein_codin	0	0	0	0
ENSG000001	PPFIA1	protein_codin	54.4353658	151.292409	215.758148	0
ENSG000001	TBR1	protein_codin	0.26608647	0	0	1.46033786
ENSG000001	EP400	protein_codin	83.9505336	202.136168	181.156632	76.2941014
ENSG000001	ESYT3	protein_codin	0	0	1.53040042	0
ENSG000001	ALKBH8	protein_codin	0	0	0	0
ENSG000001	SUN1	protein_codin	0	3.97257569	0	0
ENSG000001	LPO	protein_codin	0	0	0	0
ENSG000001	TMEM72	protein_codin	0	0	0	2.52250402
ENSG000002	NXPE2	protein_codin	0.59138068	0	0	1.83090959

ENSG000001[illegible]	SHOX2	protein_codin	87.0997754	6.60755265	93.1658306	6.74546011
ENSG000001[illegible]	CCDC196	protein_codin	0	0	0	0
ENSG000001[illegible]	MYRF	protein_codin	0.25845733	0	0	0
ENSG000001[illegible]	CCDC149	protein_codin	3.91090458	35.2149423	6.48185872	5.08931253
ENSG000001[illegible]	GPX2	protein_codin	0	0	0	0
ENSG000001[illegible]	GXYLT2	protein_codin	109.295911	125.345771	92.4114994	140.568713
ENSG00000[illegible]	CRYBG3	protein_codin	165.779231	238.959673	537.423259	101.648141
ENSG000001[illegible]	SSC5D	protein_codin	33.7153389	37.1690762	1.59017396	6.83726088
ENSG000001[illegible]	RTF1	protein_codin	126.716214	401.804688	302.299623	88.2724685
ENSG00000[illegible]	KCNAB2	protein_codin	0	21.7286035	0	0
ENSG000001[illegible]	KCNAB1	protein_codin	0	0	0.57471057	0
ENSG000001[illegible]	KCNAB1	protein_codin	2.43306073	0	0	4.41556301
ENSG000001[illegible]	CABCOCO1	protein_codin	0	0	0	0
ENSG000001[illegible]	CCDC57	protein_codin	12.385424	60.303793	0	0
ENSG000001[illegible]	CAPRIN1	protein_codin	23.8818694	81.957761	92.8379691	27.9024721
ENSG000001[illegible]	ADAL	protein_codin	2.9900961	0	3.17696596	0
ENSG00000[illegible]	CC2D2A	protein_codin	0	0	0	0
ENSG00000[illegible]	CFAP61	protein_codin	0	0	0	0
ENSG000001[illegible]	LAMA1	protein_codin	1.59645689	5.49318501	143.722925	0
ENSG000001[illegible]	ABCA12	protein_codin	0	3.19190683	0.78825314	0
ENSG00000[illegible]	EPHA6	protein_codin	1.51083827	0	20.0884063	4.59749329
ENSG00000[illegible]	MRPL28	protein_codin	4.88814582	0	27.9981462	0
ENSG000001[illegible]	FAXC	protein_codin	0.55169345	9.46207634	1.62146664	10.0008506
ENSG000002	TSN	protein_codin	16.9409749	0	15.2581177	0
ENSG000001[illegible]	PDE11A	protein_codin	0	0	0	0
ENSG000001[illegible]	GATD3	protein_codin	0	0	0	0
ENSG000001[illegible]	PRR5L	protein_codin	0	12.8590243	0	3.9559581
ENSG000001[illegible]	RALGAPA1	protein_codin	0	0	0	0
ENSG000001[illegible]	HAGHL	protein_codin	0	0	6.07500672	1.18259463
ENSG000001[illegible]	HAGHL	protein_codin	0	0	0	0
ENSG000001[illegible]	EXOSC8	protein_codin	6.15730496	8.08224372	8.87215607	4.51113974
ENSG000001[illegible]	BMAL1	protein_codin	0	0	0	0
ENSG00000[illegible]	ZXDC	protein_codin	4.09809787	14.7830443	11.5583866	1.51892056
ENSG00000[illegible]	SPTB	protein_codin	16.5216776	4.73231488	2.31215514	2.42997516
ENSG00000[illegible]	SPTB	protein_codin	1.89575868	4.31648815	0	5.10786586
ENSG000001[illegible]	RFLNA	protein_codin	0	0	0	0
ENSG000001[illegible]	HMCES	protein_codin	0	0	8.01423563	0
ENSG000001[illegible]	TRMT44	protein_codin	6.54880268	13.8205228	11.7637357	7.07754509
ENSG000001[illegible]	GPR149	protein_codin	0	0	1.02561216	4.87079318
ENSG000001[illegible]	KLC1	protein_codin	64.9590896	166.296315	155.297699	33.1252835
ENSG000001[illegible]	TRMT61A	protein_codin	2.60644573	4.34150755	11.2081757	2.91005814
ENSG000001[illegible]	PKP4	protein_codin	3.3961908	6.02149732	15.8123813	16.4552167
ENSG000001[illegible]	MROH2A	protein_codin	0	0	0	1.8613082
ENSG000001[illegible]	PKP4	protein_codin	24.3001186	40.8804678	117.206769	23.4873892

ENSG000002	ARHGAP8	protein_codin	0	0	1.42464438	0
ENSG000002	ARHGAP8	protein_codin	0	0	0	0
ENSG000001	LYST	protein_codin	57.2551996	136.776377	76.6959412	66.3570964
ENSG000001	LRCH1	protein_codin	5.96632461	25.6392574	17.2385439	8.01299678
ENSG000001	LRCH1	protein_codin	0	0	0	0
ENSG000001	SQSTM1	protein_codin	0	65.8338931	36.2695414	16.0469996
ENSG000001	ACOXL	protein_codin	0	0	0	0
ENSG000000	ABCC8	protein_codin	0	0	0	0
ENSG000001	GREB1	protein_codin	0	0	0	0
ENSG000001	EIF4E3	protein_codin	0	17.4038968	13.2762464	0
ENSG000001	MRGPRE	protein_codin	0.48624188	1.12585004	0	2.33684109
ENSG000001	TMEM87A	protein_codin	44.750256	0	59.8663354	19.1216889
ENSG000001	DNAH17	protein_codin	0	0	0	1.05758051
ENSG000001	UVSSA	protein_codin	0	0	0	2.79361961
ENSG000000	CCDC88C	protein_codin	0	9.95294924	0.47017943	0
ENSG000000	CCDC88C	protein_codin	27.8545999	244.886538	11.0840021	48.4835693
ENSG000002	LGR4	protein_codin	0	0	0	0
ENSG000001	ADARB1	protein_codin	1.53822315	38.8918335	22.6392416	11.9427013
ENSG000001	ADARB1	protein_codin	5.51976435	88.1129424	12.8975206	12.7096046
ENSG000001	NIN	protein_codin	0	0	0	0
ENSG000001	FAM124B	protein_codin	0	0	0	0
ENSG000000	CCNK	protein_codin	1.92996506	10.7924959	7.81358705	3.55040206
ENSG000001	GLB1L3	protein_codin	0	0	0	0.6183599
ENSG000001	POTEC	protein_codin	0	0	0	0
ENSG000000	RNF216	protein_codin	49.9102196	93.9902116	85.1421773	33.6908827
ENSG000000	RNF216	protein_codin	43.7837168	76.705619	100.423427	29.1132748
ENSG000001	RUBCNL	protein_codin	0	0	0	0
ENSG000001	COX16	protein_codin	7.56839939	17.8116026	15.046	4.68600892
ENSG000001	FAM168B	protein_codin	14.921027	57.7891763	72.9812154	9.50142713
ENSG000001	KIF19	protein_codin	0.31383428	3.77318936	0	0
ENSG000002	ZFP91-CNTF	protein_codin	78.3031231	0	178.535496	44.0186446
ENSG000001	DDX42	protein_codin	52.3634493	36.8361316	148.250754	13.8484282
ENSG000001	TEX14	protein_codin	0	0	0	0
ENSG000001	SH2D6	protein_codin	0	1.65665706	0	0
ENSG000001	MS4A1	protein_codin	0	0	0	0
ENSG000001	SYTL2	protein_codin	0	0	0	0
ENSG000001	SYTL2	protein_codin	7.8846415	38.826353	8.31702874	2.03813851
ENSG000001	SYTL2	protein_codin	39.2112799	90.0419942	105.0475	29.6168102
ENSG000001	CLBA1	protein_codin	3.2200389	0	6.39155649	0.73203348
ENSG000002	ATP10A	protein_codin	0	0	0	0
ENSG000002	VIT	protein_codin	45.4842735	12.1177041	0	0
ENSG000001	SPTBN1	protein_codin	0.26352426	2.43558553	0	0
ENSG000001	TMEM95	protein_codin	0	0	0	0
ENSG000001	IL17REL	protein_codin	0.32371122	6.1999399	0.59186486	1.22770451

ENSG000000	OSBPL5	protein_codin	0	0	0	0
ENSG000000	PSME4	protein_codin	0	0	0	0
ENSG000001	SOD1	protein_codin	1.69384279	5.70224623	0	1.54539466
ENSG000001	DCAF5	protein_codin	11.1186993	1.49842568	27.4334898	7.15130306
ENSG000001	DIS3L2	protein_codin	0	0	0	2.6959966
ENSG000001	NARF	protein_codin	0	0	7.21503065	0
ENSG000001	ACTN4	protein_codin	0	0	58.9775852	0
ENSG000001	GAA	protein_codin	7.27918468	0	35.4722004	17.3557708
ENSG000001	GLS2	protein_codin	0.69470966	0	0	0
ENSG000001	POGLUT1	protein_codin	0	4.72891825	1.03313948	0.82942351
ENSG000001	ARL4C	protein_codin	18.8705559	92.8766561	16.5522308	56.8455941
ENSG000001	NLRP5	protein_codin	0	2.51844247	0	0.80873475
ENSG000001	BCAS3	protein_codin	0	0	0	0
ENSG000000	COL23A1	protein_codin	0	6.0763005	2.17706162	1.17566713
ENSG000001	CD8B	protein_codin	1.23248772	12.1761792	0	3.4662838
ENSG000001	EID2	protein_codin	3.32111004	4.67275919	13.5779205	4.37938827
ENSG000001	KRTAP4-4	protein_codin	0	0	0	0
ENSG000001	CHST6	protein_codin	0	0	0	0
ENSG000001	PPP2R3B	protein_codin	6.98704396	20.3089436	32.9266242	5.12246593
ENSG000002	TSSK1B	protein_codin	2.66169806	1.04738836	12.2583139	0.94574607
ENSG000001	BORA	protein_codin	0	0	14.99456	0
ENSG000001	FOXP2	protein_codin	0	0	0	0
ENSG000001	CCL25	protein_codin	0	0	0	0
ENSG000002	PRR22	protein_codin	0	0	0	0.32527855
ENSG000002	TAS2R19	protein_codin	1.84593473	8.54608566	11.1513238	2.85456036
ENSG000002	TAS2R31	protein_codin	0.7990948	6.51959778	9.67159924	0.75572016
ENSG000002	TAS2R13	protein_codin	4.2099165	7.14538652	12.0909859	0.98463409
ENSG000001	TPTE2	protein_codin	0	0	0	0
ENSG000001	SNRPN	protein_codin	0	0	18.1427319	0
ENSG000001	KRTAP19-1	protein_codin	0	0	0	0
ENSG000002	KRTAP13-3	protein_codin	0	0	0	0
ENSG000001	SRP19	protein_codin	0	0	5.72716283	0
ENSG000002	KRTAP16-1	protein_codin	0	0	0	2.85548622
ENSG000002	KRTAP29-1	protein_codin	0	2.72522992	0	0
ENSG000001	KRTAP9-7	protein_codin	0	0	0	0
ENSG000002	KRTAP9-6	protein_codin	0	0	0	0
ENSG000001	KRTAP4-3	protein_codin	0	0	0	1.14607073
ENSG000001	TCEANC2	protein_codin	0	0	0	0
ENSG000002	CTAGE1	protein_codin	0	0	0	3.15547246
ENSG000002	KRTAP4-11	protein_codin	0	0	0	0
ENSG000002	KRTAP4-9	protein_codin	0	2.26734192	0	1.11903101
ENSG000002	KRTAP4-7	protein_codin	0	0	0.71667976	4.80494763
ENSG000002	KRTAP2-3	protein_codin	0	0	0	1.33220319
ENSG000002	KRTAP2-1	protein_codin	0	0	0	0

ENSG000002	BHLHA9	protein_codin	0	0	0	0
ENSG000002	RTL8B	protein_codin	3.97991866	11.1977216	4.05102836	4.04230642
ENSG000002	RNF208	protein_codin	0	0	0	0
ENSG000002	KRTAP3-3	protein_codin	0	0	0	0
ENSG000002	KRTAP3-2	protein_codin	0	0	1.07859501	0
ENSG000002	KRTAP3-1	protein_codin	0	0	0	0.53238668
ENSG000001	PCNX4	protein_codin	8.66927637	36.8542836	61.1510633	13.4161815
ENSG000001	JCHAIN	protein_codin	0	18.2972731	0	0.95615746
ENSG000001	KRTAP12-1	protein_codin	0	0	0	0.4848174
ENSG000002	KRTAP12-4	protein_codin	0	0	0	0
ENSG000002	KRTAP10-3	protein_codin	0	0	0	0
ENSG000002	KRTAP10-2	protein_codin	0	1.78440421	0	0
ENSG000001	HAUS2	protein_codin	0	0	0.76751772	0
ENSG000002	KRTAP6-3	protein_codin	0	0	0	0
ENSG000001	KIAA0930	protein_codin	10.1847184	0	81.3268664	0
ENSG000000	ZNF446	protein_codin	0	0	0	0
ENSG000001	ZSCAN1	protein_codin	0	0	0	0
ENSG000001	ZNF552	protein_codin	4.97410407	6.02754188	12.3063246	0
ENSG000000	ZNF586	protein_codin	0	0	0	0
ENSG000001	ZNF211	protein_codin	0	0	0	4.63966639
ENSG000001	ZNF304	protein_codin	0	0	20.4466359	0
ENSG000001	ZNF470	protein_codin	0	0	1.44993737	0
ENSG000001	ZNF829	protein_codin	0	0	0	0
ENSG000001	ZSCAN5A	protein_codin	0	7.62627945	0	3.6166248
ENSG000001	DNAAF3	protein_codin	0	0	0	0
ENSG000000	NLRP2	protein_codin	0	0	0	0
ENSG000001	FCAR	protein_codin	0	2.60207209	0	0
ENSG000001	FCAR	protein_codin	0	0	0	0
ENSG000001	FCAR	protein_codin	0	0	0	0
ENSG000001	FCAR	protein_codin	0	0	0	0
ENSG000001	KIR3DL1	protein_codin	0	0	0	0
ENSG000002	KIR2DS4	protein_codin	0.19971926	0	0	0
ENSG000001	LILRB4	protein_codin	2.0895331	0	0	5.35256201
ENSG000001	LILRB4	protein_codin	0	0	0	0
ENSG000001	LILRB4	protein_codin	1.26556576	0	1.34346615	1.01735258
ENSG000002	LILRA2	protein_codin	0.97243222	0	0	1.51103551
ENSG000002	LILRA2	protein_codin	0	0	0	0
ENSG000001	TTYH1	protein_codin	0	2.05436951	1.82954044	1.18657931
ENSG000001	LAIR1	protein_codin	0	0	0	0
ENSG000001	LAIR1	protein_codin	7.26666639	26.2762502	2.68968423	9.03369494
ENSG000001	LAIR1	protein_codin	0	81.7908896	0	10.6094193
ENSG000001	LILRB2	protein_codin	0	0	0	0
ENSG000001	LILRB2	protein_codin	10.2116028	28.990226	3.57481543	0
ENSG000001	LILRB2	protein_codin	0	0	0	0

ENSG000002	LILRB3	protein_codin	0	9.22834434	0	0
ENSG000001	RPS9	protein_codin	0.36178055	108.260457	0	0
ENSG000001	RPS9	protein_codin	2.02750705	0	0	0
ENSG000001	RPS9	protein_codin	49.5998118	231.666165	53.8398849	0
ENSG000001	MBOAT7	protein_codin	0	0	0	2.77633451
ENSG000001	PRPF31	protein_codin	0	0	0	0
ENSG000001	TFPT	protein_codin	0	0	0.58903475	0
ENSG000001	TFPT	protein_codin	4.6581408	0	0	2.71589042
ENSG000001	TFPT	protein_codin	4.05027951	9.13491055	22.5227822	1.95195292
ENSG000001	OSCAR	protein_codin	0	0	0	0
ENSG000001	OSCAR	protein_codin	0	0	0	0
ENSG000001	NDUFA3	protein_codin	0	0	0	1.87642904
ENSG000001	NDUFA3	protein_codin	0	7.29195366	0	0
ENSG000001	NDUFA3	protein_codin	0	0	0	0
ENSG000001	CACNG7	protein_codin	0	0	0	0
ENSG000001	CACNG7	protein_codin	0	0	0	0
ENSG000001	MYADM	protein_codin	0	0	0	0.52522435
ENSG000001	MYADM	protein_codin	0	5.17145174	0.71129834	12.6114542
ENSG000001	MYADM	protein_codin	0	0	21.95909	32.7965076
ENSG000001	MYADM	protein_codin	25.1396749	68.3151326	0	0
ENSG000001	NLRP12	protein_codin	1.17283515	0	0	0
ENSG000001	NLRP12	protein_codin	0	1.82402152	0.57085411	3.70863477
ENSG000001	NLRP12	protein_codin	0	0	0	0
ENSG000002	ZNF816-ZNF3	protein_codin	0	5.81164896	0	0
ENSG000001	ZNF583	protein_codin	0	0	0	0
ENSG000001	ZNF320	protein_codin	17.7323241	22.7590238	31.7945874	10.8872649
ENSG000001	ZNF28	protein_codin	7.57910568	17.2965388	7.95314978	3.31319937
ENSG000001	ZNF701	protein_codin	1.11590722	11.6452153	1.572605	2.31663316
ENSG000001	ZNF816	protein_codin	0	0	0	0
ENSG000001	ZNF528	protein_codin	12.0131712	37.2169094	10.0793609	3.81289514
ENSG000001	ZNF528	protein_codin	5.13401782	4.11268811	3.45691448	5.46625578
ENSG000001	PPP2R1A	protein_codin	0	0	1.09258595	1.06400756
ENSG000001	ZNF613	protein_codin	0	0	0	1.76044852
ENSG000001	ZNF615	protein_codin	0	0	10.5016159	2.19196631
ENSG000001	CD33	protein_codin	0	0	1.11823598	0
ENSG000001	SIGLEC6	protein_codin	0	0	0	0
ENSG000001	KLK14	protein_codin	0	0	0	0
ENSG000001	KLK11	protein_codin	0	0	0	0
ENSG000001	KLK10	protein_codin	0	0	0	0
ENSG000001	KLK8	protein_codin	0	0	0	0
ENSG000001	KLK7	protein_codin	0	0	0	0
ENSG000001	KLK6	protein_codin	0	0	0	0
ENSG000001	KLK5	protein_codin	0	0	0	0
ENSG000001	KLK2	protein_codin	0	2.65286572	0	0

ENSG000001(	SHANK1	protein_codin	0	0	0	0
ENSG000001(	SHANK1	protein_codin	0	1.34345968	5.95846168	0
ENSG000001	ZNF473	protein_codin	0	9.06841093	0	3.91818875
ENSG000001(	IL4I1	protein_codin	3.3112371	25.0370385	3.40111396	3.41860552
ENSG000001(	NLRP3	protein_codin	0	0	0	15.0217138
ENSG000001(	NLRP3	protein_codin	0	0	0	0
ENSG000002(	AKT1S1	protein_codin	0	0	0	0
ENSG000002(	AKT1S1	protein_codin	0.49479617	0	3.22239335	0
ENSG000002(	AKT1S1	protein_codin	0	2.18597184	0	0
ENSG000002(	AKT1S1	protein_codin	2.49984606	5.11349141	4.77534251	0.67028072
ENSG000002(	AKT1S1	protein_codin	0	0	0	0
ENSG000002(	AKT1S1	protein_codin	5.08460794	49.9024663	7.39157053	8.06668631
ENSG000001	SMYD3	protein_codin	3.14286855	18.4790752	16.8138293	0
ENSG000002(	EFCAB2	protein_codin	0	0	0	0
ENSG000002(	COX20	protein_codin	6.08862513	48.5346713	4.95093816	6.12212462
ENSG000001(	PTOV1	protein_codin	5.43293714	2.25304195	2.15292215	0
ENSG000001(	AKT2	protein_codin	3.32772121	0	0	0
ENSG000001(	AKT2	protein_codin	0	0	0.48256181	0
ENSG000001	PRMT1	protein_codin	7.80309127	6.49681253	60.7487936	5.6172659
ENSG000001(	GNG4	protein_codin	7.28008302	0	6.89112072	13.7546925
ENSG000001	GGPS1	protein_codin	9.75252265	15.6977945	12.3131652	2.51547085
ENSG000001	RPL13A	protein_codin	133.463923	0	370.106102	158.631484
ENSG000000	SPRTN	protein_codin	0.56610469	7.14781733	2.17541255	3.34452618
ENSG000001(	CD37	protein_codin	0	8.5088677	0	0.56582103
ENSG000001	PGBD5	protein_codin	3.35622396	12.3222111	2.47697756	8.56277788
ENSG000001(	LIN7B	protein_codin	0	0	0	0
ENSG000001(	MRPL55	protein_codin	0	0	0	0
ENSG000001	ENAH	protein_codin	0	52.4303327	0	7.73991727
ENSG000001	NVL	protein_codin	0	0	0	0
ENSG000001	TP53BP2	protein_codin	0	8.62520683	5.56048119	0
ENSG000001	FAM177B	protein_codin	0	0	0	0
ENSG000001(	CYTH2	protein_codin	2.81554947	32.6805947	7.62867654	6.86593936
ENSG000001	TAF1A	protein_codin	0	0	0	0
ENSG000001	KCNJ14	protein_codin	0	0	2.25337686	0
ENSG000001	ESRRG	protein_codin	0	0	0	0
ENSG000000	KCNK2	protein_codin	0	0	2.60378127	0
ENSG000000	KCNK2	protein_codin	0	0	0	0
ENSG000001(	CARD8	protein_codin	0	0	0	0
ENSG000001	ZNF541	protein_codin	1.60782696	0	1.51555038	3.76467099
ENSG000001	FKRP	protein_codin	0	18.2308308	0	0
ENSG000000	STRN4	protein_codin	3.67550868	0	0	2.7015239
ENSG000001	LAMB3	protein_codin	0	0	0	0
ENSG000001	DACT3	protein_codin	0	12.3432367	0	1.00497805
ENSG000001(	CALM3	protein_codin	0.40002318	156.065778	0	1.4255962

ENSG000000	PPP5C	protein_codin	0	0	41.7477425	0
ENSG000001	CD55	protein_codin	18.2143951	12.8963792	38.3662863	5.32186333
ENSG000001	C4BPB	protein_codin	0	0	0	0
ENSG000001	IL24	protein_codin	0	0	0	0.40906785
ENSG000001	IL20	protein_codin	0	0	0	0
ENSG000001	SNRPD2	protein_codin	0.79460572	10.7251251	0.48156803	0
ENSG000001	LEMD1	protein_codin	0	0	0	0
ENSG000001	ERCC2	protein_codin	0	0	0	0
ENSG000001	ERCC2	protein_codin	2.17474977	4.59511209	10.7873703	0
ENSG000001	ERCC2	protein_codin	0.72920373	1.9768185	0	2.24723238
ENSG000001	ERCC2	protein_codin	0	0	0	0
ENSG000001	KLC3	protein_codin	0	0	0	0
ENSG000001	MDM4	protein_codin	0	0	0	0
ENSG000001	GEMIN7	protein_codin	0	0	0	0
ENSG000001	CLASRP	protein_codin	8.00441164	19.5580229	23.8797036	15.6878697
ENSG000001	CLASRP	protein_codin	0	0	0	0
ENSG000001	ZNF180	protein_codin	7.67800374	0	0	0
ENSG000001	ZNF233	protein_codin	0	0	0	0
ENSG000000	PPP1R12B	protein_codin	8.7143259	230.762993	16.368787	14.8514542
ENSG000001	ZNF222	protein_codin	5.93495528	9.75608634	0	0
ENSG000001	ZNF227	protein_codin	0	0	0	0
ENSG000001	ZNF576	protein_codin	0	0	0	0
ENSG000001	LAD1	protein_codin	0	0	0	0
ENSG000002	NEU4	protein_codin	0	0	0	0
ENSG000000	PTPRC	protein_codin	0	0	0	0
ENSG000001	SEPTIN2	protein_codin	211.201614	310.606568	210.156047	99.7741843
ENSG000001	SEPTIN2	protein_codin	0	0	0	1.74668613
ENSG000001	NEK7	protein_codin	0.58785009	5.25564932	1.04712322	0.57549672
ENSG000001	HDLBP	protein_codin	19.7934094	61.2744199	83.6199993	42.8210507
ENSG000001	HDLBP	protein_codin	309.031124	136.226388	474.948031	247.917565
ENSG000001	MTERF4	protein_codin	12.9656112	8.80799484	15.1505485	0
ENSG000001	CAPN10	protein_codin	0	3.89811348	0	0
ENSG000001	CAPN10	protein_codin	0	0	0	0
ENSG000001	CFHR3	protein_codin	1.39142164	0	0	2.28257637
ENSG000001	ANKMY1	protein_codin	0.32755619	2.65587289	0	0
ENSG000001	OTOS	protein_codin	0	0	0	0
ENSG000002	TRAF3IP1	protein_codin	13.0252279	144.031889	21.5550209	16.2170968
ENSG000001	RGS13	protein_codin	0	0	0	0
ENSG000001	PDC	protein_codin	0	0	0	0
ENSG000001	LRRFIP1	protein_codin	94.3924785	85.8905628	93.1095286	23.9541382
ENSG000001	RAB17	protein_codin	0	0	0	0
ENSG000001	TMEM91	protein_codin	0.65110003	0	0	0
ENSG000001	COL6A3	protein_codin	71.5106963	0	0	0
ENSG000001	COL6A3	protein_codin	0	0	0	0

ENSG000001	HNRNPUL1	protein_codin	81.2161942	191.956209	192.152886	83.4375154
ENSG000001	COPS8	protein_codin	12.7702617	0	19.2157978	1.39966998
ENSG000001	SH3BP4	protein_codin	29.845441	50.5853845	55.3351483	26.784236
ENSG000000	ATG16L1	protein_codin	12.4898207	0	0	0
ENSG000000	ATG16L1	protein_codin	0	0	0	0
ENSG000000	ATG16L1	protein_codin	0	80.4481415	16.4528382	0
ENSG000000	ATG16L1	protein_codin	0	0	0	0
ENSG000001	SPTBN4	protein_codin	0	1.07668973	0	0.56032805
ENSG000001	ALPP	protein_codin	0	0.83698907	0	0
ENSG000001	SERTAD3	protein_codin	0	0	0	0
ENSG000001	QSOX1	protein_codin	0	1.24257195	0	0
ENSG000001	PLD3	protein_codin	0.45761617	0	0	0
ENSG000001	C19orf47	protein_codin	0	0	3.70927506	0
ENSG000001	AKT2	protein_codin	0	0	0	0
ENSG000001	AKT2	protein_codin	6.13166484	25.3355468	3.78076268	8.38254377
ENSG000001	GPR55	protein_codin	0	0	0	0
ENSG000001	GPR55	protein_codin	0.28692368	12.0603705	0	2.87992972
ENSG000001	ZNF140	protein_codin	0	0	0	0
ENSG000000	SP140	protein_codin	2.82212887	167.491956	0	7.61450579
ENSG000001	SP110	protein_codin	0	103.232067	0	0
ENSG000002	LGALS16	protein_codin	0	0	0	0
ENSG000000	LGALS14	protein_codin	0	0	0	0
ENSG000001	PID1	protein_codin	1.83876107	6.01023346	29.4424497	19.3604988
ENSG000001	PID1	protein_codin	24.7848422	0	29.2015961	0
ENSG000001	SPHKAP	protein_codin	0	0	3.63014174	0
ENSG000001	MFF	protein_codin	0	4.47939724	6.16045679	0
ENSG000002	CEBPZOS	protein_codin	0	0	0	0
ENSG000001	RHBDD1	protein_codin	0	23.1807489	22.4001881	11.3713779
ENSG000001	RABGAP1L	protein_codin	0	0	0	0
ENSG000001	ACSL3	protein_codin	15.3180534	18.016587	39.3754524	0
ENSG000000	SAMD4A	protein_codin	41.8071998	0	23.6292189	0
ENSG000001	PAX3	protein_codin	0	11.681216	0	3.65279263
ENSG000001	PAX3	protein_codin	0	0	18.0970755	0
ENSG000001	NFKBIB	protein_codin	0.3219144	0	0	0
ENSG000000	SIRT2	protein_codin	5.91788972	9.5678765	7.74831286	0
ENSG000001	RESP18	protein_codin	0	0	0	0
ENSG000001	DNAJB2	protein_codin	4.39127832	0	11.0384829	6.07494135
ENSG000001	DNAJB2	protein_codin	0	0	0	0
ENSG000001	TUBA4A	protein_codin	0.87897502	0	0	3.23285535
ENSG000001	GLB1L	protein_codin	0	0	0	2.0688629
ENSG000000	PDCD2	protein_codin	4.3182218	0	0	0
ENSG000001	TBP	protein_codin	14.1390637	19.776837	91.2508692	7.54095427
ENSG000001	ERMARD	protein_codin	0	0	0	0
ENSG000001	CRYBA2	protein_codin	0	0	0	0

ENSG000001	SMOC2	protein_codin	0.75913587	0	0.33803795	0
ENSG000001	TTLL4	protein_codin	1.13487361	19.5592189	0	7.33304831
ENSG000001	STK36	protein_codin	2.30250607	0	0	0
ENSG000001	AFDN	protein_codin	0	0	0	0
ENSG000000	BCS1L	protein_codin	0	0	0	0
ENSG000000	BCS1L	protein_codin	0	0	0	0
ENSG000000	BCS1L	protein_codin	0	0	0	0
ENSG000001	AFDN	protein_codin	54.307116	0	725.556463	45.3787848
ENSG000001	VIL1	protein_codin	0	0	0	0
ENSG000001	MPZL1	protein_codin	42.1960188	21.2683988	57.1313448	17.2834731
ENSG000001	CD247	protein_codin	0	0	0	0
ENSG000001	YIF1B	protein_codin	0	0	0	1.92852075
ENSG000001	QKI	protein_codin	45.7639682	81.1345994	94.5296423	41.0767033
ENSG000001	SMARCAL1	protein_codin	0	40.929154	0	0
ENSG000000	XRCC5	protein_codin	105.181358	311.257059	373.428118	97.2659071
ENSG000000	XRCC5	protein_codin	0	8.10642687	4.84485117	1.84316273
ENSG000001	ZNF573	protein_codin	0	0	0	0
ENSG000000	MAP3K4	protein_codin	0	0	0	0
ENSG000001	ZNF569	protein_codin	11.9605036	10.1383662	17.1719722	0
ENSG000001	ZNF569	protein_codin	0	0	0	0
ENSG000001	CHP1	protein_codin	0	0	0	0
ENSG000001	ZNF875	protein_codin	7.63168068	30.4428801	0	0
ENSG000002	DDX47	protein_codin	0	0	0	2.0431473
ENSG000002	ZNF585B	protein_codin	0	0	0	0.51231442
ENSG000001	ZNF585A	protein_codin	5.8782981	7.81283001	11.6408494	0
ENSG000001	ZFP82	protein_codin	9.85732609	30.8738366	10.2072551	6.61835194
ENSG000001	PNLDC1	protein_codin	0	0	0	0.39370501
ENSG000001	TCP1	protein_codin	0	10.4786683	130.445356	11.2470854
ENSG000001	SDHC	protein_codin	0	0	0	0
ENSG000001	ZNF566	protein_codin	0	0	0	0
ENSG000001	ZNF565	protein_codin	0	0	0	0
ENSG000001	TBCB	protein_codin	0	3.38279908	0	0
ENSG000001	NDUFS2	protein_codin	0	0	0	0
ENSG000002	ALKBH6	protein_codin	1.29656536	0	0	0
ENSG000001	SNX9	protein_codin	8.18360752	43.1974264	2.00474972	4.64083508
ENSG000001	NIT1	protein_codin	0	0	0	0
ENSG000001	KLHDC9	protein_codin	0	0	0	0
ENSG000001	KLHDC9	protein_codin	0	0	0	0
ENSG000000	MAP2	protein_codin	0.47421192	0	8.90878799	0
ENSG000000	MAP2	protein_codin	7.38256318	0	30.3218582	4.63052219
ENSG000000	ZBTB32	protein_codin	0	0	0	0
ENSG000001	COX6B1	protein_codin	0	0	0	0
ENSG000001	PIKFYVE	protein_codin	0	0	3.59420416	0
ENSG000001	LY9	protein_codin	1.08646904	0	0	2.26519019

ENSG000001(	TMEM147	protein_codin	0	6.26378174	0	0
ENSG000001(	TMEM147	protein_codin	0	0	0	0
ENSG000001(	DMKN	protein_codin	0	0	0	0
ENSG000001(	CCNYL1	protein_codin	0	0	0	0
ENSG000001'	PPP1R14B	protein_codin	0	0	0	0
ENSG000001(	MAG	protein_codin	0	0	0	0
ENSG000000:	FXYD5	protein_codin	0	0	0	0
ENSG000000:	FXYD5	protein_codin	0	0	0	0.50164687
ENSG000000:	FXYD5	protein_codin	58.3641095	107.554381	49.8418759	57.8942344
ENSG000001(	PEX19	protein_codin	9.31327292	0	14.3230245	0
ENSG000001	EEF1B2	protein_codin	280.993203	27.2766464	982.355778	211.905034
ENSG000001:	LGI4	protein_codin	22.5344591	64.1766531	1.61940938	18.7021048
ENSG000001(	HPN	protein_codin	0	0	0	0
ENSG000001!	ZNF181	protein_codin	3.9928188	0	5.66308889	0
ENSG000000	ATP1A2	protein_codin	0	0	0	0
ENSG000001:	FCRL6	protein_codin	0.21715817	0	0.43866977	0
ENSG000001(	ICA1L	protein_codin	7.70358763	11.0260191	10.1858243	7.06285723
ENSG000001:	FAM117B	protein_codin	13.9735333	40.2263629	47.1294458	22.0627015
ENSG000001	SUMO1	protein_codin	0	0	0	0
ENSG000001	SUMO1	protein_codin	6.53550515	65.3227654	38.6510731	12.7792022
ENSG000001	SUMO1	protein_codin	32.0125963	25.9856732	42.6842379	20.0914975
ENSG000000:	STRADB	protein_codin	8.94251537	0	0	0
ENSG000001'	DPY19L3	protein_codin	0	0	0	0
ENSG000001(	PYHIN1	protein_codin	0	0	0	0
ENSG000001(	PYHIN1	protein_codin	0	0	0	0
ENSG000000(	IYD	protein_codin	0	0	0	0
ENSG000000(	IYD	protein_codin	0	0	0	0
ENSG000001:	FLACC1	protein_codin	0	0	0	0
ENSG000000(	CASP8	protein_codin	0	0	0	0.78204126
ENSG000000(	CASP8	protein_codin	0	0	0	0
ENSG000001(	RAET1E	protein_codin	0	0	0	0
ENSG000001(	URI1	protein_codin	5.85381571	7.40135239	10.3813783	9.95906998
ENSG000001:	LATS1	protein_codin	0	0	0	1.0395276
ENSG000001:	C19orf12	protein_codin	1.12020907	3.8005661	1.45727601	0.59032227
ENSG000001:	C19orf12	protein_codin	0	8.66236589	11.899075	3.69041153
ENSG000002	PPIL3	protein_codin	0	0	0	4.25127291
ENSG000001!	ZNF493	protein_codin	13.3654398	29.7003205	31.7736969	0
ENSG000001(	MAIP1	protein_codin	3.99739671	2.66063386	18.387603	1.60884992
ENSG000001(	STXBP5	protein_codin	2.23541436	0	0	0
ENSG000001!	NTRK1	protein_codin	0	0	0	0
ENSG000000:	SH2D2A	protein_codin	0	0	0	0
ENSG000001	TMEFF2	protein_codin	0	0	0	0
ENSG000001:	MYO1B	protein_codin	7.89291108	9.88845186	0	13.5191391
ENSG000001:	MYO1B	protein_codin	141.128948	156.711499	257.69906	178.178203

ENSG000001!	ZNF84	protein_codin	0	0	3.12567484	0
ENSG000001:	STAT4	protein_codin	8.87039593	0	0	0
ENSG000001!	ZNF605	protein_codin	49.5694041	83.0330353	20.642312	12.7813077
ENSG000001	STAT1	protein_codin	0	0	0	0
ENSG000001	STAT1	protein_codin	0	0	0	0
ENSG000000	RFXANK	protein_codin	0	2.301771	7.89816679	2.13622762
ENSG000001	WDR45B	protein_codin	11.7467749	14.5113819	51.3443818	27.5054992
ENSG000001	MFSD6	protein_codin	7.84318973	38.6375919	17.3788402	7.3365187
ENSG000001	INPP1	protein_codin	2.60738901	0	2.78573987	0
ENSG000001!	HIBCH	protein_codin	4.95291781	17.3471817	39.5639391	5.95269193
ENSG000001!	HIBCH	protein_codin	0	0	0	0
ENSG000001	CSNK1D	protein_codin	16.7913606	49.3342899	33.3251485	0
ENSG000001	ARMC6	protein_codin	1.00117254	0	6.39277075	2.98859614
ENSG000001	ARMC6	protein_codin	0	0	4.03009349	0
ENSG000001	SLC16A3	protein_codin	0	0	0	0
ENSG000001	SLC16A3	protein_codin	0	0	0	0
ENSG000001	CCDC57	protein_codin	0	0	124.133547	10.8397778
ENSG000001	CCDC57	protein_codin	0	0	0	0
ENSG000001	CCDC57	protein_codin	2.59160262	10.8310368	8.45306271	0
ENSG000002	BCLAF1	protein_codin	93.317457	203.263727	571.96482	88.6826945
ENSG000001	ORMDL1	protein_codin	0	0	0	0
ENSG000001	ORMDL1	protein_codin	3.61796474	4.51223761	6.53197134	3.27172681
ENSG000000	HOMER3	protein_codin	9.31478324	18.9560202	18.4888374	7.1128812
ENSG000001	GPS1	protein_codin	1.39054205	13.6961588	3.78433416	1.59193216
ENSG000001	GPS1	protein_codin	2.04409007	16.8982709	17.1615343	1.27134939
ENSG000001	CENPX	protein_codin	1.0213523	0	7.53349043	0.42703052
ENSG000000	TFPI	protein_codin	23.1169898	47.0408234	0	0
ENSG000001!	MAFG	protein_codin	0	30.8347112	13.4077444	5.75532726
ENSG000001	RAN	protein_codin	0	0	30.3526445	0
ENSG000001	RAN	protein_codin	0.61012089	0	0	0
ENSG000000	CALCRL	protein_codin	24.5436638	24.4409025	0	26.6917801
ENSG000001	STX2	protein_codin	0	0	32.3793842	0
ENSG000001	ANAPC11	protein_codin	0	0	0	0
ENSG000001	BDH1	protein_codin	0	0	0	0
ENSG000001	BDH1	protein_codin	0	0	0	0
ENSG000000	DLG1	protein_codin	0	0	0	0
ENSG000000	DLG1	protein_codin	11.9206935	14.9837082	35.6065912	11.1440545
ENSG000000	DLG1	protein_codin	0	0	0	0
ENSG000000	CRLF1	protein_codin	0	0	7.88089738	0.93017968
ENSG000001	VNN2	protein_codin	0	0	0	0
ENSG000001	PIGX	protein_codin	9.08453318	16.9052221	11.203657	6.17136856
ENSG000000	TFRC	protein_codin	0	0	0	0
ENSG000000	MOXD1	protein_codin	0.79610708	0	1.48462009	1.18091617
ENSG000001	ASH1L	protein_codin	21.5426513	73.0694769	44.8129063	0

ENSG000001	ZNF664	protein_codin	64.1237396	32.2737952	134.764214	22.6282232
ENSG000001	MUC4	protein_codin	0	0	0	0
ENSG000001	IQCN	protein_codin	0.8507292	0	0	2.5135901
ENSG000001	PKLR	protein_codin	0	1.83824429	0	1.22082016
ENSG000001	UBE2E3	protein_codin	39.1111313	0	111.113117	12.9825653
ENSG000000	EPB41L2	protein_codin	0	0	0	0
ENSG000001	TMEM200A	protein_codin	1.99454128	26.8917277	1.43742766	7.45351615
ENSG000001	TMEM44	protein_codin	0	0	0	0
ENSG000001	ARL6IP4	protein_codin	2.89981811	0	2.498937	0
ENSG000001	ARL6IP4	protein_codin	20.0378101	21.7181318	20.0105545	2.75610664
ENSG000001	OPA1	protein_codin	2.12444743	0	3.63012849	0
ENSG000001	OPA1	protein_codin	9.47128327	47.4615536	38.7132469	27.2914847
ENSG000001	ABCB9	protein_codin	0	0	0	0
ENSG000001	CCDC62	protein_codin	1.67329894	0	0.86572915	0
ENSG000001	CCDC62	protein_codin	0	0	0	0
ENSG000001	RSRC2	protein_codin	2.69459967	32.1073438	13.741537	8.93841742
ENSG000001	ATP13A4	protein_codin	0	0	0	0
ENSG000001	C1QTNF1	protein_codin	23.5296207	60.4477659	0	10.4330532
ENSG000001	CANT1	protein_codin	0	39.4230822	3.73489516	0
ENSG000001	PTPRK	protein_codin	0	0	0	0.93573123
ENSG000001	MB21D2	protein_codin	3.69049205	6.56727086	12.6573922	6.30541534
ENSG000001	CCDC50	protein_codin	71.2638162	230.761101	74.4095801	61.4260618
ENSG000001	CCDC50	protein_codin	67.3858155	74.3021609	85.9972686	41.2247861
ENSG000000	TP63	protein_codin	0	0	0.47201043	0
ENSG000000	TP63	protein_codin	0	0	0	0
ENSG000001	MORN3	protein_codin	0	0	0	0
ENSG000000	TP63	protein_codin	0	0	0	0
ENSG000001	RNF34	protein_codin	0	61.0389325	6.10770349	4.41023344
ENSG000001	RNF34	protein_codin	9.70337185	0	14.8861472	0
ENSG000001	TMC6	protein_codin	0	0	4.5120114	0
ENSG000001	MASP1	protein_codin	1.01711222	0	0	0
ENSG000001	MASP1	protein_codin	1.6076175	0	0.55763528	0
ENSG000001	CAMKK2	protein_codin	0	8.25364693	0	0.71031438
ENSG000001	CAMKK2	protein_codin	0	0	0	0
ENSG000001	MASP1	protein_codin	0	0	0	0
ENSG000001	SEC14L1	protein_codin	0	0	0	0
ENSG000001	NCOA7	protein_codin	0	0	0	6.93465587
ENSG000001	RFC4	protein_codin	0	0	8.02744577	0
ENSG000001	TPD52L1	protein_codin	0	0	0	0
ENSG000001	TPD52L1	protein_codin	4.05378173	8.270833	54.9350249	0
ENSG000001	SRSF2	protein_codin	0.56984678	65.6446981	9.26134002	12.7558583
ENSG000001	LENEP	protein_codin	0	0	0	0
ENSG000001	PKIB	protein_codin	0	0	0	0
ENSG000001	PKIB	protein_codin	0	0	0	0

ENSG000001	AANAT	protein_codin	0	0	0	0
ENSG000001	SPPL3	protein_codin	2.64792779	14.4403878	4.33705326	6.14397967
ENSG000001	SPHK1	protein_codin	0.82865558	0	1.39362215	0
ENSG000002	CRYGS	protein_codin	0	0	0	0
ENSG000001	CEP85L	protein_codin	0	0	0	0
ENSG000001	TRA2A	protein_codin	3.94128001	66.4376352	40.9184002	6.54928605
ENSG000000	OSBPL6	protein_codin	0	0	0	0
ENSG000000	DYNLL1	protein_codin	17.4331584	7.37558361	29.8232518	11.7836952
ENSG000000	DYNLL1	protein_codin	6.11503449	0	8.54345995	1.74640156
ENSG000000	RPLP0	protein_codin	303.263682	401.641808	790.515242	373.10854
ENSG000001	CPT1C	protein_codin	0	0	0	0
ENSG000001	TMEM121	protein_codin	0	0	1.55548826	0
ENSG000001	CIT	protein_codin	0	0	0	0
ENSG000001	CIT	protein_codin	18.8377467	17.9762821	58.0349441	4.43509608
ENSG000001	TEDC1	protein_codin	0	0	12.6812224	0
ENSG000001	TEDC1	protein_codin	2.0565941	0	0	0
ENSG000001	HNRNPA3	protein_codin	0	0	0	0
ENSG000001	TEDC1	protein_codin	0	0	0	0.97356685
ENSG000002	CRIP1	protein_codin	7.71236296	53.6376883	14.5065959	7.5840505
ENSG000001	TAOK3	protein_codin	36.5333987	116.70677	31.60655	58.0979886
ENSG000001	EIF4G1	protein_codin	0	0	434.424459	0
ENSG000001	HOXD13	protein_codin	0	0	12.370588	1.37708453
ENSG000001	LNPK	protein_codin	0.43651758	0	1.02967038	0
ENSG000001	ATP5MC3	protein_codin	0	2.8500904	0	0
ENSG000001	RFC5	protein_codin	0	1.94358069	2.31138417	0
ENSG000001	ATF2	protein_codin	0	16.8589598	0	0
ENSG000001	WIPF1	protein_codin	0	0	1.72643612	3.75729899
ENSG000001	WIPF1	protein_codin	31.3017513	93.8537007	0	37.0754008
ENSG000001	RNFT2	protein_codin	0	0	0	0
ENSG000000	LLGL2	protein_codin	0	0	0	0
ENSG000001	GPR155	protein_codin	0	0	0	0
ENSG000001	GPR155	protein_codin	0	53.0982568	0	12.3046523
ENSG000001	BTBD6	protein_codin	0.48827585	0	0	0
ENSG000001	BTBD6	protein_codin	0	0	0	0
ENSG000001	BRF1	protein_codin	2.37582933	12.288826	33.3234031	9.21175833
ENSG000001	RPS27	protein_codin	1.18907602	6.14545464	3.42762442	5.51304
ENSG000001	OLA1	protein_codin	13.7540888	30.7059168	60.1307238	14.9757177
ENSG000001	RBM19	protein_codin	16.3245923	0	0	0
ENSG000001	GRB2	protein_codin	0.55867916	1.50910257	0	1.08685072
ENSG000001	GRB2	protein_codin	0	0	0	0
ENSG000001	GRB2	protein_codin	6.80809753	0	0	0
ENSG000001	NUDT14	protein_codin	1.29219419	3.49232238	1.38065297	0
ENSG000001	TPCN1	protein_codin	0	0	0	0
ENSG000001	PDK1	protein_codin	0.54831901	0	0	1.94853773

ENSG000001	ABCC5	protein_codin	0	11.5962547	0	0
ENSG000001	B3GAT1	protein_codin	0	0	2.72434297	0
ENSG000001	METAP1D	protein_codin	0	0	0	0
ENSG000001	OAS2	protein_codin	9.23028801	95.4956795	10.4205959	0
ENSG000001	HAT1	protein_codin	1.87750538	4.24181383	11.8437381	2.21534839
ENSG000001	GPR132	protein_codin	0	0	0	0
ENSG000001	WASF1	protein_codin	0	5.054443	11.2565809	0
ENSG000001	WASF1	protein_codin	5.16303488	18.007658	32.1008143	0
ENSG000001	WASF1	protein_codin	0	0	0	0
ENSG000001	WASF1	protein_codin	1.76409697	0	0	0
ENSG000001	CDCA4	protein_codin	3.38850643	0	15.3745388	0
ENSG000001	PLD4	protein_codin	0	0	0	0
ENSG000001	THYN1	protein_codin	8.9712769	0	7.44747874	7.29717579
ENSG000001	THYN1	protein_codin	0	0	0	0
ENSG000001	PTPN11	protein_codin	0	0	1.37024692	1.28362832
ENSG000001	METTL8	protein_codin	0	0	3.97382712	0
ENSG000001	METTL8	protein_codin	0	0	0	0
ENSG000001	ADGRA1	protein_codin	0.58985659	0.8820164	0	0
ENSG000001	ADGRA1	protein_codin	0	0	0	0
ENSG000001	RAB37	protein_codin	0	0	0	0
ENSG000001	RAB37	protein_codin	0	0	0	0
ENSG000001	RAB37	protein_codin	0	0	0	0
ENSG000001	RAB37	protein_codin	0	0	0	0
ENSG000001	RAB37	protein_codin	0	0	0	0
ENSG000001	RAB37	protein_codin	0	0	0	0
ENSG000001	CD300E	protein_codin	4.49938247	34.1967316	4.97243142	0
ENSG000001	CD300LB	protein_codin	0	0	0	0
ENSG000001	S100A13	protein_codin	1.69541201	68.2411563	0	0
ENSG000001	S100A13	protein_codin	0	0	1.2454494	0
ENSG000001	CD300A	protein_codin	2.47281153	0	0	0
ENSG000001	GPRC5C	protein_codin	0	0	14.1283429	0
ENSG000001	GPRC5C	protein_codin	0	0	0	0
ENSG000001	GPRC5C	protein_codin	0	0	0	0
ENSG000001	UBR3	protein_codin	9.2887154	0	55.9744244	4.98474025
ENSG000002	INF2	protein_codin	0	0	0	0
ENSG000001	METTL5	protein_codin	0	0	15.3475571	5.61026668
ENSG000001	ARMC2	protein_codin	1.8883747	8.34982359	8.7537902	0
ENSG000002	ATXN2	protein_codin	0	7.32694289	1.46510211	3.92320268
ENSG000002	KLHL23	protein_codin	7.32059834	6.71312651	50.5431777	3.92093883
ENSG000001	PEX5L	protein_codin	1.158015	0	0	6.2483943
ENSG000000	SDK2	protein_codin	40.4578075	54.3979647	347.190041	37.0603659
ENSG000002	SPRR2A	protein_codin	0	0	0	1.92677021
ENSG000001	ARHGAP32	protein_codin	0.94640294	0	0	0
ENSG000001	MRPL47	protein_codin	5.60093761	0	8.71466168	3.40274923

ENSG000001	ACTL6A	protein_codin	0	0	0	0
ENSG000001	BBS5	protein_codin	0	0	0	2.15713533
ENSG000001	KCNJ1	protein_codin	0	0	0	0.43503327
ENSG000001	KCNJ1	protein_codin	0	1.33265316	0	0
ENSG000001	KCNJ1	protein_codin	0	0	0	0
ENSG000001	ETS1	protein_codin	30.1595293	0	26.4947899	35.259265
ENSG000001	ST3GAL4	protein_codin	0	0	0	0
ENSG000001	KCNJ16	protein_codin	0	0	0	3.40274864
ENSG000001	KCNJ16	protein_codin	0	0	0	0
ENSG000001	RAD9B	protein_codin	0	0	0	0
ENSG000001	ABCA5	protein_codin	21.633742	38.7237441	44.1572917	9.67644691
ENSG000001	TIRAP	protein_codin	0	0	0	0
ENSG000001	TIRAP	protein_codin	0	0	0	4.36552101
ENSG000001	TIRAP	protein_codin	0.80871469	0	0	0
ENSG000001	KCNMB3	protein_codin	1.78546889	0	0.78924064	0
ENSG000001	KCNMB3	protein_codin	0	8.16519909	11.8814316	0
ENSG000001	CERS6	protein_codin	0	0	11.4993211	0
ENSG000001	B3GALT1	protein_codin	6.4969652	3.42829181	21.8157463	10.5634762
ENSG000001	ECT2	protein_codin	0	0	99.5093478	3.81535365
ENSG000000	CDON	protein_codin	0	0	0	6.38250562
ENSG000001	TTC21B	protein_codin	0	0	0.98757106	0
ENSG000001	GALNT3	protein_codin	5.33176641	24.5725606	13.2269774	6.2561958
ENSG000001	STT3A	protein_codin	6.42533429	0	12.2630837	5.38017902
ENSG000001	FEZ1	protein_codin	7.30732428	0	0.71846942	6.38575779
ENSG000001	PRKAR1A	protein_codin	20.7106762	244.844299	17.2860403	29.9158414
ENSG000001	PRKAR1A	protein_codin	0	30.5785608	1.52836379	3.61311188
ENSG000001	TUFT1	protein_codin	3.82875902	0	0	1.35373582
ENSG000001	EIF5	protein_codin	60.1790009	29.0784998	154.563716	21.6607155
ENSG000001	AMZ2	protein_codin	0	0	0	9.30226269
ENSG000001	POGZ	protein_codin	0	0	0	0
ENSG000001	MVK	protein_codin	0	0	0	0
ENSG000001	RNF32	protein_codin	0.27045432	0	0	0.47052163
ENSG000001	RNF32	protein_codin	0	0	0	0
ENSG000001	VWA5A	protein_codin	0.51965188	3.3455502	0	2.07883321
ENSG000001	TRAF3	protein_codin	6.88332138	77.5804704	9.20417515	4.09537786
ENSG000001	RFX5	protein_codin	0	0	0	3.13937847
ENSG000001	RNF32	protein_codin	0	0	0	0
ENSG000001	VWA5A	protein_codin	9.79346077	12.2193901	5.92053951	9.55815861
ENSG000001	TANK	protein_codin	30.2026314	61.6400967	54.2321062	0
ENSG000001	PDCD10	protein_codin	1.7078526	1.97783901	1.78417805	1.65691583
ENSG000001	FAM53B	protein_codin	0.55360037	11.3102408	1.82885659	1.33698851
ENSG000001	RBM33	protein_codin	0	0	0	0
ENSG000001	LHPP	protein_codin	2.4576085	0	8.85164834	0
ENSG000001	RBM33	protein_codin	3.27297227	39.9641326	12.0194126	8.84273026

ENSG000001	RBM33	protein_codin	7.05640763	0	9.37211022	7.02205472
ENSG000001	ZBBX	protein_codin	0.60808787	0	8.15874723	0.95251975
ENSG000001	ZBBX	protein_codin	0	0	0	0
ENSG000001	ZBBX	protein_codin	0	0	0	0
ENSG000001	CEP112	protein_codin	0	0	0	0
ENSG000001	SCN3B	protein_codin	3.5678188	0	0	0
ENSG000001	PLA2R1	protein_codin	32.6023094	78.8018154	21.7041536	21.3861764
ENSG000001	SAE1	protein_codin	0	0	0	0
ENSG000001	B3GALNT1	protein_codin	0	0	37.405041	4.08784616
ENSG000001	B3GALNT1	protein_codin	0	0	0	0
ENSG000001	BAZ2B	protein_codin	75.7998584	0	325.740997	0
ENSG000001	BAZ2B	protein_codin	112.284304	342.060251	257.585243	118.192415
ENSG000001	USP30	protein_codin	0	0	0	0
ENSG000001	SC5D	protein_codin	7.7456903	20.8160003	0	0
ENSG000001	CUZD1	protein_codin	0	0	0	0
ENSG000001	TECTA	protein_codin	0	0	0	0
ENSG000000	CD79B	protein_codin	0	4.97163743	0	3.48352744
ENSG000001	WDSUB1	protein_codin	0	0	0	0
ENSG000001	PLEKHA1	protein_codin	1.17538954	0	0	0
ENSG000001	GALNTL5	protein_codin	0	0	0	0
ENSG000001	PRKAG2	protein_codin	0	0	0	0
ENSG000001	BNIPL	protein_codin	0	0	0.55557695	0
ENSG000001	TMEM119	protein_codin	42.0409878	294.381363	30.6117177	86.7130965
ENSG000001	ISCU	protein_codin	0	5.37236373	0	5.2440086
ENSG000000	SMARCD3	protein_codin	0	0	0	0
ENSG000001	MFSD1	protein_codin	0	0	0	0
ENSG000001	NOXA1	protein_codin	0	0	0	0
ENSG000001	TMUB1	protein_codin	0	3.67065562	6.14618508	0
ENSG000001	GALNT13	protein_codin	0	0	27.8273495	0
ENSG000001	SLC4A2	protein_codin	1.43735857	0	0	0
ENSG000002	RNF208	protein_codin	0.48909835	0	0.55356948	0
ENSG000001	MTERF2	protein_codin	3.27177315	2.85808913	0	0
ENSG000001	VEPH1	protein_codin	0	2.67467226	0	0
ENSG000001	VEPH1	protein_codin	2.00380177	0	0.68785856	3.52599183
ENSG000001	DPAGT1	protein_codin	0	0	0	0
ENSG000001	RIC8B	protein_codin	12.726243	5.35757479	16.5215425	2.61473886
ENSG000001	RIC8B	protein_codin	2.93185868	0	7.25312134	11.3825981
ENSG000002	HMBS	protein_codin	0	0	3.78593583	0
ENSG000001	RFX4	protein_codin	0	0	0	0
ENSG000001	ORC3	protein_codin	0	0	0	0
ENSG000001	CYP4F2	protein_codin	0	0	0	0
ENSG000001	DLK1	protein_codin	0	0	0	0
ENSG000000	PHLDB1	protein_codin	1.94144572	7.4004416	1.25425885	0
ENSG000001	LYPD6	protein_codin	0	0	0	0

ENSG000001	ORC4	protein_codin	12.9293894	21.6580388	19.6613187	0
ENSG00000	ARCN1	protein_codin	102.896621	171.089461	123.052886	42.2356247
ENSG000001	ZEB2	protein_codin	57.9169485	0	0	30.685723
ENSG000001	SMIM8	protein_codin	0	0	0	0
ENSG000001	RGS10	protein_codin	3.2922975	52.3406487	0	4.49587558
ENSG000001	GTDC1	protein_codin	0	0	0	0
ENSG000001	GTDC1	protein_codin	0	0	0	0
ENSG000001	GRK5	protein_codin	4.42804835	39.2073675	5.43991271	7.7844662
ENSG000001	GTDC1	protein_codin	0	37.9662483	0	0
ENSG000001	TDG	protein_codin	17.5129617	25.1990452	23.4609772	15.1558822
ENSG000001	KMT2A	protein_codin	0.68815462	6.1237704	6.11705794	1.54940004
ENSG000001	NT5DC3	protein_codin	5.25799314	24.8058634	22.7362339	7.92586697
ENSG000001	WARS1	protein_codin	0	118.384919	34.1035308	0
ENSG000001	CD3G	protein_codin	2.05466479	0	0	1.80987244
ENSG000001	CD3D	protein_codin	0.73782396	1.32000847	0	2.40621889
ENSG000002	CSH2	protein_codin	0	0	0	0
ENSG00000	RNF13	protein_codin	25.7402121	100.213395	49.1770717	7.34046594
ENSG000001	TCAF1	protein_codin	0	0	0	0
ENSG000001	SHTN1	protein_codin	0	0	0	0
ENSG000001	SHTN1	protein_codin	32.4212624	79.7694937	32.9393389	24.0433853
ENSG000000	IGF1	protein_codin	0	0	0	0
ENSG000000	IGF1	protein_codin	0	0	0	0.75932204
ENSG000001	SLC25A29	protein_codin	0	3.35889807	0	0
ENSG000001	PARPBP	protein_codin	0	0	0	0
ENSG000001	ZYX	protein_codin	0	21.9318537	29.0491292	0
ENSG000001	PARPBP	protein_codin	0	0	5.57008997	1.46536215
ENSG000000	HLTF	protein_codin	1.01918996	0	1.24525971	1.15767216
ENSG000001	PARPBP	protein_codin	0.35995243	5.13538032	2.9151546	0
ENSG000001	MAP3K19	protein_codin	0	0	0	0
ENSG000001	MAP3K19	protein_codin	0	0	0	0
ENSG000001	MAP3K19	protein_codin	0	0	0	0
ENSG000001	GNPTAB	protein_codin	0	0	0	0.45761434
ENSG000001	EVL	protein_codin	14.5279168	130.832282	0	9.04019333
ENSG000001	SYCP3	protein_codin	0	0	0	0
ENSG000001	CASP2	protein_codin	2.09801832	0	0	0
ENSG000001	SYCP3	protein_codin	0	0	0	0
ENSG000001	ACMSD	protein_codin	0	0	0	0.84325271
ENSG000001	MYBPC1	protein_codin	0	0	0	0
ENSG000001	BACE1	protein_codin	32.0168971	0	0	0
ENSG000001	GPSM1	protein_codin	0	4.72756946	0	0
ENSG000002	H3-7	protein_codin	0.45309789	0	0	0
ENSG000002	STRADA	protein_codin	0	0	0	2.32519131
ENSG000001	TAGLN	protein_codin	57.8273206	30.4094668	4.94519243	15.7804897
ENSG00000	ABLIM1	protein_codin	0	75.9403436	19.4771362	48.6285029

ENSG000001	ARHGEF4	protein_codin	0	0	0	0
ENSG000000	ABLIM1	protein_codin	28.3893187	0	31.7001514	70.5462865
ENSG000001	LCA5	protein_codin	0	0	7.12485438	0
ENSG000001	ADGRE2	protein_codin	0	0	0	0
ENSG000002	NBPF1	protein_codin	23.4817079	63.6538219	152.148621	30.105207
ENSG000001	ADGRE2	protein_codin	0	0	0	0
ENSG000000	DBNDD1	protein_codin	0	0	0	0.47691028
ENSG000000	CYB561	protein_codin	0	0	23.1325548	1.45550061
ENSG000000	CYB561	protein_codin	0	0	0	1.49869844
ENSG000001	ANO4	protein_codin	0	0	18.1646681	0
ENSG000001	ANO4	protein_codin	3.96617827	0	12.8546408	0
ENSG000001	XRN1	protein_codin	53.5503999	0	108.464701	36.6798257
ENSG000001	VWA2	protein_codin	0.58617808	2.35639552	0	0.89554428
ENSG000001	CCDC74B	protein_codin	0	0	0	0
ENSG000001	ZNF292	protein_codin	0.41657686	5.11848528	0.95841425	1.42198193
ENSG000000	NR1H4	protein_codin	0	0	0	0
ENSG000001	ZNF333	protein_codin	0.38155558	3.12017687	4.45620355	1.18080781
ENSG000001	ACP6	protein_codin	0	0	0	0
ENSG000001	SLC17A8	protein_codin	0	2.02959003	1.04644588	0
ENSG000000	PAPOLA	protein_codin	8.61135793	0	25.4814788	2.62782224
ENSG000001	OLFM1	protein_codin	0	0	0	0
ENSG000001	GK5	protein_codin	11.1261806	40.7002229	30.8052859	16.4493186
ENSG000002	RPL36A	protein_codin	25.7325629	16.5032342	52.1416578	62.0879955
ENSG000001	PPM1D	protein_codin	13.6621254	11.1413552	3.26440951	0
ENSG000001	ZBTB16	protein_codin	17.26993	18.4148716	28.7501022	0
ENSG000001	RNF7	protein_codin	7.95545629	0	44.7201537	0
ENSG000001	AMMECR1L	protein_codin	0	0	0	0
ENSG000001	USP32	protein_codin	2.78436755	18.5073809	43.4626378	0
ENSG000001	FILIP1	protein_codin	31.4682592	52.2066012	6.18436158	15.827042
ENSG000001	WDR33	protein_codin	5.42825665	39.3944694	15.2190953	3.54402695
ENSG000000	MRPS33	protein_codin	0.67935184	0	2.34876	1.11979093
ENSG000001	PXYLP1	protein_codin	0	0	0	3.74981698
ENSG000001	SLC25A36	protein_codin	0	0	0	0
ENSG000001	GPR17	protein_codin	0	0	0	0
ENSG000001	TTC12	protein_codin	0	0	0	0
ENSG000001	RPS6KB1	protein_codin	0	0	0	2.88550906
ENSG000001	GIPC1	protein_codin	0	1.80315147	3.69635425	0
ENSG000001	GIPC1	protein_codin	1.57005784	9.50246385	4.86666695	0
ENSG000001	FAIM	protein_codin	0	0	24.0469368	15.0315479
ENSG000001	FAIM	protein_codin	10.5193864	7.6842266	50.6056713	1.64315164
ENSG000001	PTRH2	protein_codin	13.6341728	12.9163452	123.795493	7.97820427
ENSG000001	KLRG2	protein_codin	0	0	0	0
ENSG000001	BIN1	protein_codin	0	28.7651582	0	4.50250806
ENSG000001	BIN1	protein_codin	0	0	0	0

ENSG000001	IKBIP	protein_codin	10.1419718	13.5112782	22.2394441	0
ENSG000001	CLTC	protein_codin	0	0	18.8279221	0
ENSG000001	PIAS3	protein_codin	17.6131396	55.2634267	96.8603432	9.62305256
ENSG000001	PIAS3	protein_codin	12.0414293	0	20.6473916	4.62008059
ENSG000001	NKAPD1	protein_codin	15.64273	0	48.2689796	11.6377627
ENSG000001	DLAT	protein_codin	0	0	0	0
ENSG000001	TMPO	protein_codin	3.23571995	67.4597614	18.0348151	7.30127126
ENSG000001	ATP6V0A4	protein_codin	0	0	0	0
ENSG000001	PPP2R1B	protein_codin	5.85179588	0	0	0
ENSG000001	DBH	protein_codin	0	0	0	0
ENSG000001	ADAMTSL2	protein_codin	0	17.8313347	0	1.72591521
ENSG000001	ADAMTSL2	protein_codin	0	0	0	0
ENSG000002	SPIRE2	protein_codin	0.30732695	0	0	0
ENSG000001	DICER1	protein_codin	22.2275893	0	0	0
ENSG000001	TRIM37	protein_codin	33.6315492	22.6893855	223.125772	0
ENSG000001	TRIM37	protein_codin	0	0	0	0
ENSG000001	POU2AF1	protein_codin	0	39.4145451	1.11026489	0
ENSG000001	VTI1A	protein_codin	15.901943	0	19.1003887	15.2666652
ENSG000001	SERPINA3	protein_codin	1.16652595	0	0	0
ENSG000001	SLC35G2	protein_codin	3.27680233	0	2.31519722	3.53235668
ENSG000001	SERPINA3	protein_codin	0	0	0	0
ENSG000001	ACSL5	protein_codin	0	0	0	2.12470736
ENSG000001	PTN	protein_codin	5.06745555	6.73447913	0.5838166	0
ENSG000001	C11orf65	protein_codin	0	0	0.97464589	0
ENSG000001	MTPN	protein_codin	70.9504191	119.346316	271.37091	73.1343292
ENSG000001	SEPTIN4	protein_codin	0	0	6.21621193	28.8201866
ENSG000001	SERPINA1	protein_codin	0	0	0	0
ENSG000001	SERPINA1	protein_codin	0	0	0	0
ENSG000001	USP44	protein_codin	0	0	0	0
ENSG000000	DPEP1	protein_codin	0	3.47799344	0	0.68916146
ENSG000001	CUL5	protein_codin	42.6389732	48.4067849	39.0702353	19.6306986
ENSG000001	SERPINA10	protein_codin	0	0	0	0
ENSG000001	RPL13	protein_codin	0.79059661	0	2.91800415	4.25851982
ENSG000001	ALKBH8	protein_codin	0	0	0	0
ENSG000001	NR2C1	protein_codin	0	0	2.78361789	0
ENSG000001	NR2C2	protein_codin	0	0	4.9473085	0
ENSG000001	DBI	protein_codin	0	0	0	0
ENSG000001	PDCD4	protein_codin	20.2978167	19.8343152	4.26982248	17.2606358
ENSG000001	STEAP3	protein_codin	12.6224201	15.0965091	41.9538436	13.7091645
ENSG000001	STEAP3	protein_codin	0	19.3757316	29.4839239	3.66354016
ENSG000001	STEAP3	protein_codin	7.09020855	0	21.6812714	22.8128722
ENSG000001	CYREN	protein_codin	0	0	0	0
ENSG000001	IFI27L1	protein_codin	0	0	0	0
ENSG000001	CALD1	protein_codin	0	0	0	2.32988904

ENSG000000	MKS1	protein_codin	0	0	0	0
ENSG000000	MKS1	protein_codin	0	0	0	0
ENSG000001	GRIA4	protein_codin	0	2.97444493	0	0
ENSG000001	MRPL42	protein_codin	5.32997746	0	7.59434805	0
ENSG000001	TMEM108	protein_codin	3.18592508	9.20565213	5.61720799	0
ENSG000001	BPGM	protein_codin	0	0	0	7.19913349
ENSG000001	MXI1	protein_codin	3.79355765	0	0	0
ENSG000001	PRIMA1	protein_codin	0	0	0	0
ENSG000001	CASP5	protein_codin	0	0	0	0
ENSG000001	PRIMA1	protein_codin	0.99266259	1.21579927	3.68926054	4.58930183
ENSG000001	ACSF3	protein_codin	3.72813124	29.7984139	35.8117315	4.37594656
ENSG000001	DPP10	protein_codin	0	0	0	0
ENSG000001	DPP10	protein_codin	0	0	0	0
ENSG000001	CASP4	protein_codin	0	0	2.55812641	0
ENSG000001	UNC79	protein_codin	0	9.09449833	0	0
ENSG000000	DCN	protein_codin	90.9130023	116.109395	57.7231888	70.6308112
ENSG000001	RALGDS	protein_codin	0	0	0	0
ENSG000001	PDGFD	protein_codin	70.922682	57.47655	40.6959279	47.4920632
ENSG000001	RALGDS	protein_codin	8.23935186	18.3258319	14.963609	3.64206941
ENSG000001	EXOC4	protein_codin	1.11356178	0	12.7494423	4.47994091
ENSG000001	RABL2A	protein_codin	0	0	0	0
ENSG000001	RABL2A	protein_codin	0	0	0	0
ENSG000001	RABL2A	protein_codin	0	0	0	0
ENSG000001	SORCS3	protein_codin	0	0	0	0
ENSG000001	POC1B	protein_codin	0	0	0	0
ENSG000002	NME2	protein_codin	1.98617696	79.5781704	1.02517696	4.04025319
ENSG000001	MEST	protein_codin	0	0	28.6733261	2.24994154
ENSG000001	CPNE5	protein_codin	0	0	0	1.97137423
ENSG000002	NME2	protein_codin	3.02334098	6.07314111	7.55689359	3.82607808
ENSG000000	NME1-NME2	protein_codin	0	0	0	0
ENSG000002	NME1	protein_codin	11.3535217	19.8689572	24.6411544	3.04178763
ENSG000001	IL1F10	protein_codin	0	0	0	0.85435734
ENSG000001	IL36RN	protein_codin	0.25159633	0	0.44490309	2.16608754
ENSG000001	PTGFRN	protein_codin	194.830634	73.2628489	188.142429	33.1109491
ENSG000001	BANP	protein_codin	0	0	35.2170707	0
ENSG000001	BANP	protein_codin	0	0	0	0
ENSG000000	COL17A1	protein_codin	0	0	0	0
ENSG000001	LRRIQ1	protein_codin	0	0	0	0
ENSG000001	CPA5	protein_codin	0	0	0	0
ENSG000001	CFAP77	protein_codin	0	0.89157392	0	0
ENSG000001	CFAP77	protein_codin	0	0	0	0.3621979
ENSG000001	LRRIQ1	protein_codin	0.88075608	0	32.6073281	0
ENSG000001	LGMN	protein_codin	8.91096507	11.5024139	0	7.0142102
ENSG000000	MTMR2	protein_codin	0	0	0	0

ENSG000001	LUC7L3	protein_codin	187.842532	436.153788	173.462816	42.3510673
ENSG000001	NTNG2	protein_codin	1.05551646	5.58165781	1.51355402	1.67888076
ENSG000001	NRF1	protein_codin	0	0	5.50848662	0.71738361
ENSG000001	NRF1	protein_codin	4.20007669	5.9562483	0	0
ENSG000001	RTBDN	protein_codin	0	0	0	0
ENSG000001	TMCC1	protein_codin	21.8281742	61.0355649	58.443228	23.3811652
ENSG000000	SYT1	protein_codin	0	0	0	2.8882707
ENSG000000	MRE11	protein_codin	0	0	0	0
ENSG000001	GSE1	protein_codin	0	0	0	0
ENSG000000	OSBPL8	protein_codin	153.225249	211.489114	322.950111	55.6433271
ENSG000000	OSBPL8	protein_codin	21.9104823	56.1735677	26.9413195	34.3241523
ENSG000001	BCL2L11	protein_codin	9.20701068	35.0094793	25.726491	0
ENSG000001	BCL2L11	protein_codin	14.3268266	0	0	40.7583204
ENSG000001	TAF1D	protein_codin	0	0	0	0
ENSG000001	FBXW9	protein_codin	1.31438907	0	4.01553844	0
ENSG000001	NAP1L1	protein_codin	46.9142442	348.144943	117.785075	130.750067
ENSG000001	SLC24A4	protein_codin	0	0	0	0
ENSG000001	EME1	protein_codin	0	0	0	0
ENSG000001	NPHP1	protein_codin	0	0	14.1116571	0
ENSG000001	DENND2C	protein_codin	10.9616754	0	13.3423209	3.39626468
ENSG000001	DENND2C	protein_codin	1.23901088	25.8252442	7.98433733	2.01203445
ENSG000001	MBD4	protein_codin	0	0	0	0
ENSG000000	NOX4	protein_codin	0	0	0	0
ENSG000000	RGPD5	protein_codin	0	3.09749746	4.37350325	1.47290973
ENSG000001	CAPS2	protein_codin	0	0	0	0
ENSG000000	ATXN3	protein_codin	17.4820717	34.6875444	12.639001	13.4574018
ENSG000001	KCNC2	protein_codin	0	0	0	0
ENSG000002	ISY1	protein_codin	8.58252005	58.9949841	24.2253879	4.40160482
ENSG000000	ABL1	protein_codin	0	0	0	0
ENSG000001	GRM5	protein_codin	0	0	0	0
ENSG000002	ISY1	protein_codin	5.1882806	11.0614216	9.6580714	5.17574391
ENSG000000	FAM135A	protein_codin	2.57202611	0	7.82687111	3.74679906
ENSG000001	OLFML3	protein_codin	7.06373751	0	8.3839788	8.28051107
ENSG000001	CTSC	protein_codin	0	0	0	0
ENSG000001	RAB43	protein_codin	0	16.0416862	0	0
ENSG000001	RAB43	protein_codin	0	0	0	0
ENSG000001	OSGIN1	protein_codin	0	0.81340054	0	0
ENSG000001	RAB43	protein_codin	0	23.3063258	0	0
ENSG000001	RAB43	protein_codin	0	0	8.64963643	0
ENSG000001	LIMS1	protein_codin	5.83063524	22.5278943	1.89236486	12.8204788
ENSG000000	ZNF800	protein_codin	20.8137349	37.9231063	76.7828545	14.5695527
ENSG000000	ZNF800	protein_codin	10.1810273	0	0	0
ENSG000001	TRIM7	protein_codin	0	0	0	0
ENSG000001	BCL2L15	protein_codin	0	0	0	2.19800608

ENSG000001	TRIM7	protein_codin	0	0	1.17665226	1.13337093
ENSG000001	BCL2L15	protein_codin	0	0	0	0
ENSG000001	GCC2	protein_codin	6.84933834	14.0138984	10.420191	0
ENSG000001	ME3	protein_codin	0	8.18502046	0	2.88068352
ENSG000001	SPOP	protein_codin	3.01198395	0	9.84986843	0
ENSG000001	POT1	protein_codin	4.60863508	0	7.40858372	0
ENSG000001	TSPAN8	protein_codin	24.9796097	0	0	0
ENSG000001	CENPN	protein_codin	0	0	0	0
ENSG000001	ZNF44	protein_codin	0	0	12.3128101	5.61306797
ENSG000001	MGAT1	protein_codin	53.7559228	50.7402139	66.2730739	30.4182689
ENSG000001	PHB1	protein_codin	0	0	0	0
ENSG000000	PICALM	protein_codin	14.1550564	20.262368	8.11116407	8.77337183
ENSG000000	FLT4	protein_codin	0	0	12.2386602	0
ENSG000000	NCK2	protein_codin	0	13.6015459	0	0
ENSG000000	NCK2	protein_codin	0	0	0	0
ENSG000001	MAF	protein_codin	91.1428016	302.724625	92.1995702	95.4340741
ENSG000001	FHL2	protein_codin	0	0	0	0
ENSG000001	FHL2	protein_codin	3.22316674	11.9256409	0	0
ENSG000001	B4GALNT2	protein_codin	0.86477284	1.58912484	0.46544671	5.93951312
ENSG000001	CNOT6	protein_codin	7.31182509	0	0	0
ENSG000001	PHTF1	protein_codin	9.63901491	0	10.8756615	0
ENSG000001	TGFBRAP1	protein_codin	0	35.1155931	77.0201029	21.5024023
ENSG000000	MAPK9	protein_codin	0	0	16.5934627	12.6127987
ENSG000000	MAPK9	protein_codin	0	0	0	0
ENSG000001	ATP5MC1	protein_codin	0	0	0	0
ENSG000001	PTPA	protein_codin	12.0096571	46.6758456	0	9.29024436
ENSG000001	RASGEF1C	protein_codin	0	0	0	0
ENSG000001	TMEM126B	protein_codin	12.1681155	10.6823121	0	10.697579
ENSG000000	AASS	protein_codin	5.11515529	0	0	0
ENSG000001	EYS	protein_codin	0	0	7.16247368	0
ENSG000000	HPF1	protein_codin	9.34018212	20.2142149	22.6366335	7.76975642
ENSG000001	TTLL6	protein_codin	0	0	0	0
ENSG000000	CRAT	protein_codin	0.39344935	1.61842038	2.48085845	1.70153131
ENSG000001	PTPRZ1	protein_codin	0	0	47.1317753	33.731802
ENSG000001	PHF3	protein_codin	154.76862	720.529091	792.92098	201.764879
ENSG000001	ANKRD42	protein_codin	0.44043454	7.11477657	0	8.73879437
ENSG000001	ANKRD42	protein_codin	0	0	0	0.83843224
ENSG000001	PRCP	protein_codin	0	33.7955806	0	0
ENSG000001	TPRA1	protein_codin	0	0	7.98417459	0
ENSG000000	SNX11	protein_codin	0	12.0003519	0	0
ENSG000001	CBX1	protein_codin	5.93179161	7.00239874	4.93109587	3.69299114
ENSG000001	PLXNA1	protein_codin	65.4013012	148.635678	309.993684	28.9695903
ENSG000001	MDM2	protein_codin	0	0	0	0
ENSG000001	MDM2	protein_codin	0.44509259	0	0	0

ENSG000001	MDM2	protein_codin	0	0	0	0
ENSG000001	IL1R2	protein_codin	0	0	9.03583851	0
ENSG000001	MDM2	protein_codin	1009.25454	0	52.9040862	12.0551303
ENSG000001	MDM2	protein_codin	24.654692	0	0	2.52316162
ENSG000001	MDM2	protein_codin	238.689671	0	11.5267992	0
ENSG000000	BCAR1	protein_codin	0	18.9977608	0	0
ENSG000000	BCAR1	protein_codin	0	0	0	0
ENSG000002	RGL3	protein_codin	0	2.30734141	0	0
ENSG000000	AAMDC	protein_codin	1.17822115	0	5.60154201	2.61050179
ENSG000001	ZFP1	protein_codin	0	10.3579629	10.1145251	0
ENSG000001	ALDH1L1	protein_codin	0.9229254	1.40649669	0	0
ENSG000001	HNRNPH1	protein_codin	28.0475061	0	124.99723	51.2335394
ENSG000001	ALDH1L1	protein_codin	0	0	0	0
ENSG000001	RAP1B	protein_codin	528.679715	109.709821	69.9104266	42.4424649
ENSG000001	LONRF2	protein_codin	7.03784939	13.7695729	14.162101	9.33039689
ENSG000001	RUFY1	protein_codin	1.63649241	70.6516111	0	0
ENSG000001	BTRC	protein_codin	9.69751943	0	33.2138503	0
ENSG000000	ST7	protein_codin	0	0	2.60173998	0
ENSG000000	ST7	protein_codin	0	0	0	5.78480721
ENSG000001	REV1	protein_codin	0	0	65.9283342	0
ENSG000000	ST7	protein_codin	0	0	0	1.01728272
ENSG000000	ST7	protein_codin	0	0	0	0
ENSG000001	RUFY1	protein_codin	2.04857811	16.1737945	7.9216301	1.41397287
ENSG000000	ST7	protein_codin	0.98234736	0	0	0
ENSG000001	MYL4	protein_codin	0	0	0	0
ENSG000000	ST7	protein_codin	3.56291748	0	0	0
ENSG000000	TDP1	protein_codin	0	0	0	0
ENSG000000	TDP1	protein_codin	0	0	0	0
ENSG000001	GOSR2	protein_codin	0	0	0.87709068	0
ENSG000001	SFXN3	protein_codin	16.8792828	45.194235	11.5157855	8.93398561
ENSG000001	WNT9B	protein_codin	0.27560356	0	0	0
ENSG000001	LRRC37A	protein_codin	0	0	0.93139038	0
ENSG000001	CAV1	protein_codin	2.44265497	18.1156635	0.81049156	0.89711453
ENSG000001	CAV1	protein_codin	0.86486175	0	0	1.01824706
ENSG000002	SLC12A8	protein_codin	0	0	0	0
ENSG000001	CAV1	protein_codin	4.23309983	0	0	8.54969172
ENSG000001	LIPT1	protein_codin	0	0	0	0
ENSG000001	LIPT1	protein_codin	0	0	0	3.56953685
ENSG000001	CAV2	protein_codin	0.57723622	1.2712316	2.45090522	0
ENSG000001	TES	protein_codin	0	0	0	0
ENSG000001	TSGA10	protein_codin	0	0	0	0
ENSG000001	TSGA10	protein_codin	2.46390732	0	4.89376923	0
ENSG000001	TES	protein_codin	0.44510662	0	0	0
ENSG000001	TFEC	protein_codin	4.23339092	2.73168798	0	2.12834568

ENSG00000013	MDFIC	protein_codin	81.9756127	141.601558	246.746124	57.3473245
ENSG0000007	MGAT4A	protein_codin	22.6871272	94.8559971	21.4211097	0
ENSG0000017	PHYKPL	protein_codin	2.46804817	0	0	0
ENSG0000012	FOXP2	protein_codin	0	0	0	0
ENSG000001	UNC50	protein_codin	0	0	0	0
ENSG0000012	FOXP2	protein_codin	0.56360193	0	0.941147	1.0374224
ENSG0000012	FOXP2	protein_codin	0	0	0	0
ENSG0000016	KALRN	protein_codin	11.0152791	4.3813631	99.1178163	19.3541786
ENSG0000012	FOXP2	protein_codin	0	0	1.36243046	0
ENSG0000016	KALRN	protein_codin	1.46995999	0	18.3725434	5.47249871
ENSG0000014	CNGA3	protein_codin	0	0	0	0.38519563
ENSG0000016	AP1G1	protein_codin	9.7184632	0	0	0
ENSG0000016	ZC3H14	protein_codin	5.09821146	0	0	6.83432172
ENSG0000015	SORBS2	protein_codin	0	0	0	0
ENSG0000004	PHLPP2	protein_codin	0	0	10.4687098	8.94244655
ENSG0000016	FAM178B	protein_codin	0	0	0	0
ENSG0000013	ODF2	protein_codin	2.25233092	56.8431915	57.0493896	12.529786
ENSG0000015	SORBS2	protein_codin	0	0	0	0
ENSG0000013	MPP1	protein_codin	0	14.9570247	3.86734446	0
ENSG0000013	MPP1	protein_codin	7.69035419	0	4.08171026	0
ENSG0000013	ODF2	protein_codin	0	37.5047606	0	0
ENSG000002	ANKRD39	protein_codin	4.54300766	2.90012407	3.64501498	5.611733
ENSG0000016	ZNF23	protein_codin	0	0	0	0
ENSG0000016	CCDC110	protein_codin	0	0	0	0
ENSG000001	MDM1	protein_codin	0	21.4383528	9.55773256	1.17415245
ENSG0000004	SPATA7	protein_codin	0	0	0	0
ENSG0000019	PDLIM7	protein_codin	0	0	0	0
ENSG0000016	DBF4B	protein_codin	2.0483384	17.174882	1.22759126	2.79593344
ENSG0000019	PDLIM7	protein_codin	2.96652699	4.58998756	0	2.51990396
ENSG0000015	HYDIN	protein_codin	0.61547671	0	0	1.01105998
ENSG0000012	DYRK2	protein_codin	7.53806702	14.7541783	24.0104583	5.67959044
ENSG0000009	LAMB1	protein_codin	0	3.78930814	0	0
ENSG0000009	LAMB1	protein_codin	0.42408144	0	0	0
ENSG0000009	LAMB1	protein_codin	0	0	0	0
ENSG0000016	G6PD	protein_codin	0	0	0	0
ENSG0000016	G6PD	protein_codin	0	0	28.4679549	0
ENSG000001	DBN1	protein_codin	0	5.45909146	0	0
ENSG0000015	HYDIN	protein_codin	7.72976607	2.37384528	5.95116573	8.92671007
ENSG0000005	GALC	protein_codin	0	0	0	0
ENSG0000005	GALC	protein_codin	0	0	0.98988575	1.21611988
ENSG0000007	FAM3A	protein_codin	11.4105192	28.8693927	49.7644563	6.09058646
ENSG0000019	GRK6	protein_codin	0	0	7.727362	0
ENSG0000014	HMGA2	protein_codin	56.1580117	0	0	0
ENSG0000014	HMGA2	protein_codin	109.358336	1.4224307	13.1712553	0

ENSG000000	RELT	protein_codin	0	14.9479628	0	0
ENSG000000	SEMA5B	protein_codin	0	0	1.53967974	0
ENSG000001	CASP3	protein_codin	4.49590276	34.9711103	11.5150906	4.2031086
ENSG000001	SLC10A3	protein_codin	3.29517867	0	6.81847239	5.82479959
ENSG000001	SLC10A3	protein_codin	0	0	0	0
ENSG000001	CASP3	protein_codin	0	6.17255721	0	0
ENSG000001	P2RY6	protein_codin	0	0	0	0
ENSG000001	P2RY6	protein_codin	0	0	0	0
ENSG000001	P2RY6	protein_codin	0	0	0	0
ENSG000001	IRF2	protein_codin	13.6978097	107.217875	0	10.9851268
ENSG000001	DNM1	protein_codin	0	0	0	0
ENSG000001	P2RY2	protein_codin	0	0	0	0
ENSG000001	P2RY2	protein_codin	0.86666184	2.43231571	1.59645025	4.80765902
ENSG000001	CDKN2D	protein_codin	2.59118702	6.20040338	0	0
ENSG000000	FAM50A	protein_codin	34.0255463	86.8902334	64.7676538	27.5994932
ENSG000001	COG5	protein_codin	6.3551192	31.3764852	10.5282801	4.15277582
ENSG000001	ARAP1	protein_codin	0	42.7847061	13.5019177	0
ENSG000001	UBTF	protein_codin	0	47.3334343	55.0148022	0
ENSG000001	ARAP1	protein_codin	0	0	0	0
ENSG000001	RAB24	protein_codin	0	0	0	0
ENSG000001	RAB24	protein_codin	3.50692475	0	5.47827633	2.44757491
ENSG000001	COG4	protein_codin	0	0	0	0
ENSG000000	PRKAR2B	protein_codin	0	0	0	0
ENSG000001	AHCYL1	protein_codin	0	0	0	5.19753426
ENSG000001	NAMPT	protein_codin	0	0	18.2162918	48.0118247
ENSG000000	KEAP1	protein_codin	0	11.7255924	34.1021432	0
ENSG000001	CD86	protein_codin	0	0	0	0
ENSG000000	MON2	protein_codin	0	0	0	0
ENSG000000	MON2	protein_codin	15.982893	4.12863587	19.2021393	19.1267166
ENSG000001	ILDR1	protein_codin	0	1.60845283	0	0
ENSG000001	FGFR4	protein_codin	0	0	0	0
ENSG000000	DNASE1L1	protein_codin	0	0	0	0
ENSG000000	ADCK1	protein_codin	0.3575937	0	2.48870654	0
ENSG000001	ST3GAL2	protein_codin	0	14.5820029	6.51401443	0
ENSG000001	FGFR4	protein_codin	0	0	0	0
ENSG000001	IQCB1	protein_codin	0	0	9.58144058	0
ENSG000001	SRPK2	protein_codin	0	16.9835296	34.6654939	29.3650862
ENSG000000	TFAP2B	protein_codin	0	1.18066381	0	3.39261532
ENSG000001	DDX19B	protein_codin	0	0	2.28912378	0
ENSG000001	GPM6A	protein_codin	0	0	0	0
ENSG000002	DEFB110	protein_codin	0	0	0	0
ENSG000000	MEOX1	protein_codin	3.84202398	0	0	3.27083271
ENSG000001	ETV4	protein_codin	0	0	0	0
ENSG000001	GOLGB1	protein_codin	0	0	0	0

ENSG000001	FBXO8	protein_codin	6.92569285	0	14.623893	10.6906262
ENSG000001	FOLR1	protein_codin	0.35769755	0	0	0
ENSG000001	ZFYVE27	protein_codin	0	0	0	0
ENSG000001	FOLR1	protein_codin	0	0	0	1.31089774
ENSG000001	FOLR1	protein_codin	0	0	3.47363187	0
ENSG000001	IRAK1	protein_codin	4.28705759	0	0	0
ENSG000000	SNCB	protein_codin	0	0	0	0
ENSG000001	NUMA1	protein_codin	318.347213	848.944645	716.180976	249.030079
ENSG000001	OPN5	protein_codin	0	0	0	0
ENSG000001	RENBP	protein_codin	0	0	0	0
ENSG000001	CLEC18A	protein_codin	0	0	0	0
ENSG000001	MFAP3L	protein_codin	0.32220198	1.89004666	0.52319589	0
ENSG000001	IL18BP	protein_codin	0	0	0	2.80188842
ENSG000001	MFAP3L	protein_codin	0	20.1636747	0	0
ENSG000001	IL18BP	protein_codin	0	0	0	0
ENSG000001	FPGS	protein_codin	0	3.65979992	14.3341545	0
ENSG000001	IL18BP	protein_codin	0	0	0	0
ENSG000002	FDX2	protein_codin	1.5700946	0	6.19910386	3.09657158
ENSG000001	CYB561D1	protein_codin	0	0	0	0
ENSG000001	NAA10	protein_codin	0	0	0	0
ENSG000001	RNF121	protein_codin	0	0	0	0
ENSG000001	NAA10	protein_codin	2.79416014	3.71785168	23.1852802	0
ENSG000001	RNF121	protein_codin	0	0	0	0
ENSG000001	NR1I2	protein_codin	0	0	0	0
ENSG000001	ICAM4	protein_codin	0	0	0	0
ENSG000000	ARHGAP4	protein_codin	0	3.46302094	0	0
ENSG000001	SLC26A5	protein_codin	0	0	0	0
ENSG000001	KIAA1191	protein_codin	0	32.7071912	0	0
ENSG000001	PALLD	protein_codin	0	0	0	0
ENSG000001	SLC26A5	protein_codin	0	0	0	0
ENSG000001	KIAA1191	protein_codin	0	0	0	0
ENSG000001	SLC26A5	protein_codin	0	0	0	0
ENSG000001	SLC26A5	protein_codin	0	0	0	0
ENSG000001	MRPL4	protein_codin	0	0	1.95782667	0
ENSG000001	GSTZ1	protein_codin	0	0	0	0
ENSG000001	SLC26A5	protein_codin	0	0	0	0
ENSG000001	RERG	protein_codin	0	0	0	0
ENSG000001	NFAT5	protein_codin	102.309108	106.721273	272.473503	95.0155678
ENSG000001	DDX60	protein_codin	52.6372613	185.448399	79.7855128	35.3351323
ENSG000001	CPLX2	protein_codin	0	0.92836497	0.57240752	1.24561727
ENSG000000	CTTN	protein_codin	0	0	0	0
ENSG000001	FABP1	protein_codin	0	0	0	1.84066054
ENSG000001	DRD1	protein_codin	0.31547072	0	0.53136767	0.87054997
ENSG000000	PDZD4	protein_codin	0	0	0	0

ENSG000001	CD8B	protein_codin	0	0	0	0.51380758
ENSG000001	ZDHHC16	protein_codin	0	3.23315078	4.81610488	0
ENSG000001	CD8B	protein_codin	0	0	0	0
ENSG000001	B4GALT4	protein_codin	4.67087155	0	31.8420742	4.47525777
ENSG000000	MSMO1	protein_codin	0	17.1157628	13.2867416	0
ENSG000001	BNIP1	protein_codin	0	0	0	0
ENSG000001	CHMP3	protein_codin	3.03022839	0	0	0
ENSG000001	LRRC74A	protein_codin	0	0	0	0
ENSG000001	IGSF11	protein_codin	0	0	0	0
ENSG000001	GAP43	protein_codin	0	0	0	0
ENSG000001	ERGIC1	protein_codin	38.2135468	65.4438137	98.9561822	18.0968409
ENSG000001	SRPK3	protein_codin	0	0	0	0
ENSG000001	ARHGAP9	protein_codin	0	8.62031231	0	0
ENSG000001	UBTD2	protein_codin	67.2598811	100.749798	114.964505	29.304007
ENSG000001	PPAN	protein_codin	3.13161858	0	13.8746771	0
ENSG000001	PSMC3IP	protein_codin	4.39058097	23.4608997	19.030815	1.00554677
ENSG000002	PPAN-P2RY11	protein_codin	0	0	0	0
ENSG000001	ARHGAP9	protein_codin	0.96792981	0	0	0
ENSG000001	PPP6R3	protein_codin	15.3281982	50.411005	30.3336379	35.263908
ENSG000001	PPP6R3	protein_codin	0	0	0	0
ENSG000000	FBXW11	protein_codin	6.21924581	9.1078219	5.86095749	0
ENSG000001	ST3GAL5	protein_codin	0	0	0	0
ENSG000001	GASK1B	protein_codin	11.44884	0	0	0
ENSG000001	ST3GAL5	protein_codin	0	0	0	0
ENSG000001	SLC35B2	protein_codin	0	0	0	0
ENSG000001	R3HDM2	protein_codin	10.2281693	2.16728038	3.67501013	0
ENSG000001	SLC35B2	protein_codin	0.76109141	30.4465323	22.0509973	3.73795109
ENSG000001	GRIA2	protein_codin	0	0	0	0
ENSG000000	COASY	protein_codin	4.92108068	0	13.825417	0
ENSG000001	NPM1	protein_codin	21.2580149	4.84156451	480.394166	24.7603594
ENSG000001	SFTPB	protein_codin	0	0	0	0
ENSG000002	CUX1	protein_codin	6.74270413	23.3682813	26.8806177	0
ENSG000001	NDUFA4L2	protein_codin	2.20851687	0	0	4.09618545
ENSG000000	ATP6V0A1	protein_codin	13.2649888	20.5601149	0	10.7650947
ENSG000000	SIDT1	protein_codin	2.13819021	0	1.49865492	3.06578497
ENSG000001	GUCY1A1	protein_codin	0	0	0	0
ENSG000001	SLC29A1	protein_codin	0	0	3.79644262	0
ENSG000000	ATP2B3	protein_codin	0.62858962	0	0	1.06716173
ENSG000001	SLC29A1	protein_codin	2.68981307	53.0354266	0	14.4893858
ENSG000002	CFAP44	protein_codin	25.9533402	113.451883	160.48735	32.8727002
ENSG000001	FGG	protein_codin	0	0	0	0
ENSG000001	DPEP2	protein_codin	2.06111509	18.4336116	0	0
ENSG000001	ELMOD3	protein_codin	0	0	4.63044723	0
ENSG000001	RAB5C	protein_codin	0	0	0	0

ENSG000001	TREX2	protein_codin	0	0	0	0
ENSG000001	TAC3	protein_codin	0	0	0	0
ENSG000001	SUCLG1	protein_codin	16.96428	29.911914	52.0330027	12.0283856
ENSG000002	MAGEA12	protein_codin	0	0	0	0
ENSG000001	OPALIN	protein_codin	0	0	0	0
ENSG000001	OPALIN	protein_codin	0	0	0	0
ENSG000001	TMEM134	protein_codin	0	0	0	0
ENSG000001	REG3A	protein_codin	0	0	0	0
ENSG000001	NKIRAS2	protein_codin	0	0	0.71093568	0.76446127
ENSG000001	NKIRAS2	protein_codin	0	0	1.34001107	3.32485408
ENSG000001	NKIRAS2	protein_codin	0	0	0	0
ENSG000001	ZNF559	protein_codin	0.9000357	0	10.0375065	1.38058257
ENSG000001	NKIRAS2	protein_codin	7.31297152	0	8.91180771	6.6099944
ENSG000001	CNP	protein_codin	7.85064953	34.065158	27.4142131	0
ENSG000001	NACA	protein_codin	4.79215012	0	144.185761	24.8745184
ENSG000001	CNP	protein_codin	9.22643234	34.8971861	37.6307093	15.8782838
ENSG000001	CORO1B	protein_codin	15.9115853	0	27.8580124	5.76486374
ENSG000001	WWC1	protein_codin	6.24856642	0	6.23576799	0
ENSG000001	ACLY	protein_codin	13.9164702	0	0	0
ENSG000001	REG3G	protein_codin	0	1.51363913	0	0
ENSG000002	MAGEA12	protein_codin	0	0.78949284	0.49247549	0.84653449
ENSG000000	TBC1D10A	protein_codin	0	0	0	0
ENSG000001	MRPL19	protein_codin	21.9069581	62.2667214	41.1750006	13.3289202
ENSG000001	NT5C3B	protein_codin	0	0	6.58066557	0
ENSG000001	EVA1A	protein_codin	0	0	0	0
ENSG000000	HMMR	protein_codin	6.47835823	0	48.1373422	0
ENSG000001	TAGLN3	protein_codin	0	0	0	0
ENSG000001	MAGEA4	protein_codin	0	0	0	0
ENSG000001	MAGEA4	protein_codin	0	0	0	0
ENSG000001	PHLDB2	protein_codin	0	0	0	0
ENSG000001	GNB2	protein_codin	0	0	59.5597029	11.6047381
ENSG000001	PHLDB2	protein_codin	76.2021927	0	35.2536842	0
ENSG000001	GNB2	protein_codin	0	4.50772605	0	0
ENSG000001	ZNF414	protein_codin	0.69856175	2.94761456	1.73525065	0
ENSG000001	P3H4	protein_codin	25.838741	0	20.95298	0
ENSG000001	JUP	protein_codin	0	16.6660858	0	0
ENSG000001	JUP	protein_codin	0	10.4774042	0	0
ENSG000002	PLCXD2	protein_codin	0	2.57421016	0	0
ENSG000001	LOXL3	protein_codin	9.19784199	0	0	0
ENSG000001	HAP1	protein_codin	0	1.06385655	0	0.89581266
ENSG000000	GABRA1	protein_codin	0	0	0	0
ENSG000001	SYT12	protein_codin	0	0	0	0
ENSG000001	MOSPD3	protein_codin	0	7.66152665	2.90075221	0
ENSG000001	DQX1	protein_codin	0	0	0	0

ENSG000001	LRFN4	protein_codin	0	0	0	0
ENSG000001	PC	protein_codin	0	0	0	0
ENSG000001	FBXW7	protein_codin	29.4257145	45.2832198	0	13.1876965
ENSG000001	TPPP3	protein_codin	12.1796745	18.7736661	5.51451961	3.68570561
ENSG000001	PC	protein_codin	0	0	0	0
ENSG000001	GABRB2	protein_codin	12.3216371	5.61919242	1.39116956	17.0312286
ENSG000001	PC	protein_codin	0	0	0	1.94087639
ENSG000001	ANGPTL4	protein_codin	0	8.56653395	0	10.1276482
ENSG000001	GUCA1C	protein_codin	0	0	0	0
ENSG000001	PTTG1	protein_codin	0	0	0	0
ENSG000001	CCDC142	protein_codin	6.36559806	12.182531	15.9753632	2.01209918
ENSG000001	PLEKHG4	protein_codin	2.64316798	3.83343461	4.99974853	0
ENSG000002	WBP1	protein_codin	1.80391681	13.9625578	4.54818536	0
ENSG000001	KRT15	protein_codin	0	0.94324817	0	0
ENSG000001	C1QTNF2	protein_codin	0	0	0	0
ENSG000001	KRT15	protein_codin	0	0	0	0
ENSG000001	CCNJL	protein_codin	0	24.0858834	2.12564932	2.34703732
ENSG000002	RBM14	protein_codin	0	18.3894064	19.4460377	0
ENSG000001	FABP6	protein_codin	0	0	0	1.24893398
ENSG000001	EOLA1	protein_codin	0.56466114	0	0	1.44130874
ENSG000001	KRT36	protein_codin	0	0	0	0
ENSG000001	DNPH1	protein_codin	0	2.79997868	6.65406831	0.93306022
ENSG000001	TSC22D4	protein_codin	0	27.9300255	9.09481708	4.82306556
ENSG000001	BBS1	protein_codin	0	0	0	0
ENSG000001	ELMO3	protein_codin	0	0	0	0
ENSG000000	PILRA	protein_codin	0	0	0	0
ENSG000001	KRT34	protein_codin	0	0	0.61310463	1.45274973
ENSG000002	DCTN1	protein_codin	0	0	0	0
ENSG000001	ACSM6	protein_codin	0	0	0	0
ENSG000001	KRTAP9-9	protein_codin	0	0	0	0.74398626
ENSG000001	BBOF1	protein_codin	8.79009852	19.9692603	19.7378723	4.3762029
ENSG000001	RALGPS1	protein_codin	0	0	0	0
ENSG000001	RNF41	protein_codin	4.79863983	0	0	0
ENSG000002	KRTAP4-12	protein_codin	0	2.21246033	0	0.45237761
ENSG000002	KRTAP2-4	protein_codin	0	1.73503963	0	1.45848736
ENSG000000	STAG3	protein_codin	0	0	0	0
ENSG000001	SLC4A5	protein_codin	0	0	0	0
ENSG000001	RALGPS1	protein_codin	0	0	0	0
ENSG000001	SMARCC2	protein_codin	0	0	0	0
ENSG000001	COQ6	protein_codin	0	0	1.61833997	0
ENSG000001	CBLB	protein_codin	26.0451068	164.465202	28.1301617	69.0911587
ENSG000001	TRAPPC14	protein_codin	0	5.98817607	0	0
ENSG000001	HELLS	protein_codin	0	11.080609	0	0
ENSG000001	CES3	protein_codin	0.35614013	0.96330115	0	0

ENSG000001	HELLS	protein_codin	0	0	0	0
ENSG000001	ESYT1	protein_codin	23.6326906	88.8042908	72.7742534	27.7246354
ENSG000002	KRT222	protein_codin	0	0	0	0
ENSG000002	KRT222	protein_codin	2.31166409	0	0	1.2736866
ENSG000000	MTHFD2	protein_codin	3.16479605	8.32464662	26.591108	7.73670165
ENSG000001	NFKBIZ	protein_codin	21.136168	16.2863448	0	69.890535
ENSG000001	CDH16	protein_codin	0	0	0	0
ENSG000001	KLC4	protein_codin	0	0	1.11041024	0
ENSG000001	SLC10A7	protein_codin	0.53391171	2.35824961	5.90671603	0
ENSG000001	MAPKAP1	protein_codin	24.1980997	29.1054607	71.0169386	8.18486311
ENSG000002	AP4M1	protein_codin	0	0	0	0
ENSG000001	MAPKAP1	protein_codin	0	0	0	0
ENSG000001	KLC2	protein_codin	0.66293063	7.26890382	10.4812309	6.73524399
ENSG000001	KLC2	protein_codin	0	0	0	0
ENSG000001	KLC2	protein_codin	5.18119652	17.8355695	22.6094337	0
ENSG000001	CA7	protein_codin	0	0	0	0
ENSG000001	STAMBP	protein_codin	0	0	0	0
ENSG000001	MIDEAS	protein_codin	26.8743544	108.159358	40.7948886	38.4454427
ENSG000001	TNS4	protein_codin	0	0	0	0
ENSG000001	STAMBP	protein_codin	0	0	0	0
ENSG000001	NAE1	protein_codin	8.04456782	0	0	0
ENSG000001	RPL24	protein_codin	181.497303	65.7250951	934.125052	192.296034
ENSG000001	KLC2	protein_codin	0	0	0	0
ENSG000001	RARA	protein_codin	2.07068864	10.1886109	3.38996712	1.00845323
ENSG000001	SNRPA1	protein_codin	0	0	0	0
ENSG000001	GAPVD1	protein_codin	0	0	0	0
ENSG000001	GAPVD1	protein_codin	1.15510354	4.98590067	9.78929084	2.72198045
ENSG000001	SENP7	protein_codin	2.0407842	9.03810373	8.26970859	0
ENSG000001	RARA	protein_codin	0	0	0	0
ENSG000001	RARA	protein_codin	13.5043566	0	0	10.7329736
ENSG000001	F9	protein_codin	0	0	0	0.44612963
ENSG000001	SENP7	protein_codin	4.25597928	18.751876	2.46220919	0
ENSG000001	SMAD1	protein_codin	0	0	0	0
ENSG000001	SENP7	protein_codin	0	17.4087458	8.92185044	0
ENSG000001	SENP7	protein_codin	45.224725	0	78.5390376	39.6593576
ENSG000001	WIPF2	protein_codin	0	7.770432	3.25202172	0
ENSG000001	GAPVD1	protein_codin	44.1618903	59.7326637	39.6833216	35.2375766
ENSG000001	GAPVD1	protein_codin	14.138358	0	41.8631536	11.7553406
ENSG000001	CMTM4	protein_codin	18.0748028	0	5.66195952	3.94209802
ENSG000001	SUOX	protein_codin	0	9.35426565	10.3760546	4.71228963
ENSG000001	PPP2R5D	protein_codin	0	10.4473817	0	9.89173418
ENSG000001	EMX1	protein_codin	0	0	0	0
ENSG000001	CERS3	protein_codin	0	0	0	0
ENSG000001	CASC3	protein_codin	3.74504708	13.1226868	4.11114325	0

ENSG000001	SUOX	protein_codin	0	0	0	0
ENSG000001	DYSF	protein_codin	0	0	0	0
ENSG000001	THRA	protein_codin	2.18442616	4.33960833	0	4.38267525
ENSG000001	CLEC4M	protein_codin	0	0	0	0
ENSG000001	G3BP1	protein_codin	0	0	0	0
ENSG000001	RABEPK	protein_codin	4.74372304	5.31049405	11.5056082	0
ENSG000000	MED24	protein_codin	0	0	0	0
ENSG000000	MED24	protein_codin	0	0	0	0
ENSG000000	MED24	protein_codin	13.4946155	0	0	0
ENSG000001	TTC23	protein_codin	3.50287741	0	2.47592581	5.18912204
ENSG000001	TTC23	protein_codin	20.6985632	0	26.6181834	10.007108
ENSG000001	TTC23	protein_codin	4.63812909	32.1638588	38.2747251	3.76932832
ENSG000001	NIT2	protein_codin	2.71525637	11.9246516	22.8486846	9.91558693
ENSG000001	ADGRG4	protein_codin	0.22574632	1.8307424	0	3.75710828
ENSG000001	ADGRG4	protein_codin	0	0	0	0
ENSG000000	TBC1D23	protein_codin	49.4593599	36.4169261	97.0374787	17.1135439
ENSG000000	DGKA	protein_codin	0	0	0	0
ENSG000001	CSF3	protein_codin	0.34011406	0.54285378	0	1.21280435
ENSG000001	CSF3	protein_codin	0	0	0	0
ENSG000001	ZSCAN25	protein_codin	0	1.7691188	0	0
ENSG000001	ZSCAN25	protein_codin	0	11.8792384	9.45990115	0
ENSG000000	FHL1	protein_codin	0	0	0	0
ENSG000000	FHL1	protein_codin	0	0	0	0
ENSG000001	CDH11	protein_codin	2.44193783	0	20.3766032	0
ENSG000000	PSEN1	protein_codin	0	0	0	0
ENSG000001	IL15	protein_codin	0	0	0	0
ENSG000000	CD209	protein_codin	0	0	0	0
ENSG000000	ST3GAL6	protein_codin	6.85834921	15.2320608	12.620311	0
ENSG000001	ZNF655	protein_codin	0	37.9716443	0	5.90005942
ENSG000000	PSEN1	protein_codin	0	6.39110354	2.17086012	9.34223143
ENSG000001	NR2F2	protein_codin	6.28886755	55.6674068	10.1323152	19.473394
ENSG000001	BICRAL	protein_codin	0	0	0	0
ENSG000001	ORMDL3	protein_codin	0	0	0	0.6271183
ENSG000001	ZKSCAN5	protein_codin	0	0	2.00349534	0
ENSG000001	NR2F2	protein_codin	7.41080724	23.0004215	0	0
ENSG000000	CD209	protein_codin	0	4.22036317	0	2.6967568
ENSG000000	GSDMB	protein_codin	0	0	0	0
ENSG000001	ZNF394	protein_codin	0	0	0	0.62593971
ENSG000000	GSDMB	protein_codin	0	0	0	0
ENSG000000	CLDND1	protein_codin	0.73340469	0	2.09931534	0
ENSG000000	CLDND1	protein_codin	0	0	0	0
ENSG000000	CLDND1	protein_codin	0	0	14.0052738	0
ENSG000002	ATP5MF	protein_codin	22.6755687	25.7229	73.2753744	13.3552525
ENSG000001	IKZF3	protein_codin	0	0	0	0

ENSG000002	OR5H8	protein_codin	0	0	0	0
ENSG000001	CHD2	protein_codin	136.39734	294.665323	256.542669	166.232434
ENSG000001	RIOX2	protein_codin	9.71124462	13.7004218	10.0594844	7.11356415
ENSG000001	NEK6	protein_codin	0	0	0	0
ENSG000001	CNN3	protein_codin	25.5318196	116.280622	64.3362233	1.75686175
ENSG000001	SCOC	protein_codin	0	0	13.3154078	10.7956579
ENSG000001	GRB7	protein_codin	0	0	0	0
ENSG000001	SCOC	protein_codin	0	0	0	0
ENSG000001	ZFYVE1	protein_codin	0	0	0	0
ENSG000001	GRB7	protein_codin	0	0	0	1.6142818
ENSG000001	GRB7	protein_codin	0	0	0	0
ENSG000001	DENND1A	protein_codin	0	10.141279	0	1.78496617
ENSG000001	KCNK7	protein_codin	0	1.23242422	0	1.50197809
ENSG000001	KCNK7	protein_codin	0	0	0	0
ENSG000001	DHFR2	protein_codin	9.98991949	10.1010679	29.1399919	5.84485703
ENSG000001	ARL13B	protein_codin	0	0	0	0
ENSG000001	NDUFC1	protein_codin	0	23.5993802	0	0
ENSG000002	SIPA1	protein_codin	0	0	0	0
ENSG000001	NDUFC1	protein_codin	6.70726396	0	0	3.03955552
ENSG000001	NDUFC1	protein_codin	0	0	0	0
ENSG000001	MIEN1	protein_codin	3.82922927	11.0373823	0	2.90473452
ENSG000001	SV2B	protein_codin	0	0	3.39182373	0
ENSG000001	DCAF4	protein_codin	0	0	0	0
ENSG000001	ELF2	protein_codin	0	0	0	2.81802781
ENSG000001	PROS1	protein_codin	17.949749	0	0	0
ENSG000001	TUBD1	protein_codin	0	0	0	0
ENSG000001	TGFA	protein_codin	0	0	0	0
ENSG000001	SYNPO	protein_codin	0.91301708	17.7007967	10.582651	1.66296169
ENSG000001	PNMT	protein_codin	0	0	0	0
ENSG000001	PRC1	protein_codin	13.6012105	0	113.888868	3.82942594
ENSG000001	STARD3	protein_codin	5.85992846	0	0	0
ENSG000001	MED20	protein_codin	0	0	0	0
ENSG000001	TMT1B	protein_codin	0	5.0162339	2.99375014	2.12483988
ENSG000001	USP49	protein_codin	0	19.6488037	31.7510074	0
ENSG000001	OR6C4	protein_codin	0	0	0	0
ENSG000001	RCCD1	protein_codin	1.9849274	11.5756223	0	0
ENSG000002	PRICKLE4	protein_codin	0	0	0	0
ENSG000002	PRICKLE4	protein_codin	0	0	0	0
ENSG000001	CNTN6	protein_codin	0.55686811	4.13206853	0.54371751	0
ENSG000001	PABIR1	protein_codin	8.24456845	40.4690055	20.9143514	14.6854313
ENSG000001	PPP1R1B	protein_codin	0	0	0	0
ENSG000001	SETD6	protein_codin	0	0	0	3.14261106
ENSG000001	PPP1R1B	protein_codin	0	0	0	0.89454741
ENSG000000	TCOF1	protein_codin	3.1813276	0	0	0

ENSG000001	HDDC3	protein_codin	0.77840877	3.78341885	2.20992274	0
ENSG000001	UNC45A	protein_codin	0	0	0	0
ENSG000001	PGRMC2	protein_codin	3.19763644	0	14.9511495	0
ENSG000001	PDE1B	protein_codin	0	0	0	0
ENSG000001	NDRG4	protein_codin	0	0	0	0
ENSG000001	NDRG4	protein_codin	2.56557836	0	0	0
ENSG000001	TFEB	protein_codin	0.90115821	0	0	0
ENSG000001	PDCL	protein_codin	4.12175829	14.9674752	8.30572862	0
ENSG000001	MED1	protein_codin	0	0	0	0
ENSG000001	LARP1B	protein_codin	0	34.8486398	0	7.18297978
ENSG000001	FBXL20	protein_codin	0	14.215086	0	0
ENSG000001	ANXA4	protein_codin	3.0652487	19.5050644	3.96366446	9.00861561
ENSG000001	GPC3	protein_codin	0	0	0	0
ENSG000001	FES	protein_codin	0	0	0	0
ENSG000001	FES	protein_codin	0	0	0	3.81193675
ENSG000000	CACNB1	protein_codin	7.78316481	26.3498352	14.2481031	0
ENSG000001	NFU1	protein_codin	0	0	0	0
ENSG000000	ASNS	protein_codin	0	0	0	0
ENSG000000	ASNS	protein_codin	0	0	6.61234085	0
ENSG000000	CACNB1	protein_codin	0	11.7499384	11.2139542	4.45876794
ENSG000000	MBNL3	protein_codin	0	0	0	0
ENSG000001	ZNF385A	protein_codin	0	0	0	2.90172033
ENSG000001	MRRF	protein_codin	0	0	0	0
ENSG000001	PLXDC1	protein_codin	15.7437396	8.05677839	0	0
ENSG000001	LHX6	protein_codin	0	0	0	0
ENSG000001	PPARGC1B	protein_codin	1.85168621	7.89375646	2.57252067	3.49507559
ENSG000000	MCOLN1	protein_codin	0	0	0	0
ENSG000002	ERV3-1	protein_codin	0	0	0	0
ENSG000001	BHLHE41	protein_codin	0.35508605	0	0.6563317	0
ENSG000001	NUP58	protein_codin	0	3.15898386	9.59721082	0
ENSG000001	FAT4	protein_codin	131.446707	267.388109	388.92888	67.4814905
ENSG000001	SLC8A3	protein_codin	0	0	0	0
ENSG000001	HOXC6	protein_codin	6.38740747	25.775766	13.3072278	0
ENSG000001	RPL23	protein_codin	14.0424442	0	172.623657	0
ENSG000001	RPL23	protein_codin	0	0	0	0
ENSG000001	STK26	protein_codin	15.3265097	0	43.4276903	0
ENSG000001	STK26	protein_codin	0	0	14.1630809	7.51110156
ENSG000001	DRC7	protein_codin	0	0	0.87251463	0.42341593
ENSG000001	SPRY1	protein_codin	5.50165506	0	0	15.830838
ENSG000001	MITF	protein_codin	0	0	1.21335562	0
ENSG000001	ATP5MC2	protein_codin	20.6036758	56.3258931	74.8889861	25.4392271
ENSG000001	MITF	protein_codin	0	14.7894975	0	0
ENSG000001	GSN	protein_codin	0	0	0	0
ENSG000001	TARBP2	protein_codin	0	0	0	0

ENSG000001	ADGRG5	protein_codin	0	0	0	0
ENSG000001	KIF6	protein_codin	0	0	0	0
ENSG000001	ENOX2	protein_codin	0.65394242	6.87343545	0	2.68908992
ENSG000001	SRSF5	protein_codin	4.67152334	2.65838539	9.43946301	0
ENSG000001	FBXO38	protein_codin	3.27042425	28.0888717	38.9845781	0
ENSG000001	MEN1	protein_codin	0	0	0	0
ENSG000001	LIPF	protein_codin	0	0	0	0
ENSG000001	MEN1	protein_codin	0	0	0	0
ENSG000000	AAAS	protein_codin	2.14519865	0	15.4333094	0
ENSG000000	CIAPIN1	protein_codin	0	0	22.4233147	5.56457265
ENSG000001	DNAH8	protein_codin	0	0	0	0
ENSG000001	SMIM43	protein_codin	0.28286719	0	0	0
ENSG000001	SMIM43	protein_codin	0	0	0	1.08361652
ENSG000001	PPP2R2B	protein_codin	0.2821798	1.56670826	0	6.05527024
ENSG000001	PPP2R2B	protein_codin	0	0	0	0
ENSG000001	KIF7	protein_codin	37.6813993	92.0890806	117.710133	21.7362476
ENSG000001	PPP2R2B	protein_codin	0	0	0	0
ENSG000001	PPP2R2B	protein_codin	0	0	0	0
ENSG000001	UGP2	protein_codin	41.4690749	0	49.7587747	26.2401494
ENSG000000	ACTN1	protein_codin	0	0	0	0
ENSG000001	RSPRY1	protein_codin	17.5470223	32.7877782	107.651621	12.5270778
ENSG000001	TCERG1	protein_codin	67.5519866	97.4615538	342.915253	58.8981694
ENSG000001	UTP14A	protein_codin	30.320865	60.5855243	75.6039165	0
ENSG000001	RARG	protein_codin	0	0	10.0459122	9.4272869
ENSG000001	QRFPR	protein_codin	0	1.16942716	3.22855897	0
ENSG000000	RASGRP2	protein_codin	0	11.9143078	0	0
ENSG000000	RASGRP2	protein_codin	0	0	0	0
ENSG000000	RASGRP2	protein_codin	0	12.2994895	0	0.93067053
ENSG000001	PSMD6	protein_codin	0	0	0	0
ENSG000000	RASGRP2	protein_codin	0	0	0	2.46940439
ENSG000001	LARS1	protein_codin	0	0	0	0
ENSG000001	PRDM5	protein_codin	4.02252825	2.64579726	18.5927297	2.21607387
ENSG000001	PDE5A	protein_codin	0	0	0	14.8145683
ENSG000001	CCT4	protein_codin	18.449	63.6623529	165.685376	30.4282448
ENSG000000	CALCR	protein_codin	0.30319331	0	0	0.77406024
ENSG000000	TBC1D22A	protein_codin	2.88483983	0	10.8044597	4.1581514
ENSG000001	PRELID2	protein_codin	0.54860191	0	0	0.84492197
ENSG000000	ZFYVE26	protein_codin	1.25019442	3.80966138	4.95798278	1.71182251
ENSG000001	GTF2F1	protein_codin	28.4795285	0	74.8133461	46.2179349
ENSG000001	ANKLE1	protein_codin	0	0	0	0
ENSG000001	TRIML2	protein_codin	0	0	0	0
ENSG000001	NR3C1	protein_codin	72.5419941	91.673547	55.7130543	42.1204041
ENSG000001	NR3C1	protein_codin	8.68599676	0	24.6375075	0
ENSG000001	HEPACAM2	protein_codin	0	0	0	0

ENSG000001	STAG2	protein_codin	0	0	0	0
ENSG000001	STAG2	protein_codin	0	0	0	0
ENSG000001	REL	protein_codin	12.9217068	74.5779077	52.7504635	119.195202
ENSG000001	NTRK3	protein_codin	1.47171807	3.93329918	3.71048964	0
ENSG000001	FAM107A	protein_codin	0	0	0	2.51264246
ENSG000001	MT1X	protein_codin	2.37062271	2.95775831	11.1070784	14.5516078
ENSG000001	TLR4	protein_codin	3.55255365	0	0	6.25541482
ENSG000001	NDST3	protein_codin	2.20727203	2.54631907	11.1715082	0
ENSG000001	FGF1	protein_codin	0	0	0	0
ENSG000000	KRIT1	protein_codin	0	47.1405354	5.46757857	0
ENSG000000	KRIT1	protein_codin	0	0	0	0
ENSG000000	KRIT1	protein_codin	0	0	0	0
ENSG000001	AKAP13	protein_codin	48.2470631	91.2059741	32.2376238	0
ENSG000001	UGT8	protein_codin	1.72274322	0	2.90179432	17.8589818
ENSG000000	RNF14	protein_codin	37.9098184	49.4071695	55.4146307	13.8049931
ENSG000001	AKAP13	protein_codin	0	37.3731621	0	108.52062
ENSG000000	RNF14	protein_codin	0	0	0	0
ENSG000000	RNF14	protein_codin	0	0	0	0
ENSG000001	CAMK2D	protein_codin	0	0	0	0
ENSG000001	CAMK2D	protein_codin	0	0	0	0
ENSG000001	KCNK4	protein_codin	0	0	0	0
ENSG000001	BRDT	protein_codin	0	0	0	0
ENSG000000	BAD	protein_codin	0	0	0	0
ENSG000000	BAD	protein_codin	0.83015061	0	0	0
ENSG000001	AKAP9	protein_codin	0	0	0	0
ENSG000001	PCDH1	protein_codin	0	0	0	0
ENSG000001	ANK2	protein_codin	0	0	0	0
ENSG000001	TRPT1	protein_codin	0	0	0	0
ENSG000001	TRPT1	protein_codin	2.48066452	0	0	0
ENSG000001	DNASE1L3	protein_codin	1.6016583	13.2480255	0	7.10666213
ENSG000001	MCTS2	protein_codin	2.01609113	14.577747	2.54489257	0
ENSG000000	PDE8A	protein_codin	0	55.0256884	0	17.7977977
ENSG000001	EFEMP1	protein_codin	2.35614246	0	24.4548588	1.4723799
ENSG000001	SLFN12	protein_codin	2.52210193	0	0	0
ENSG000001	AKAP9	protein_codin	22.8635266	110.847003	139.279267	12.2370706
ENSG000001	SLFN11	protein_codin	42.9842202	132.476182	41.8928009	28.1874154
ENSG000001	UNC45B	protein_codin	0	2.27762077	0	0
ENSG000001	PNPLA1	protein_codin	0	0	0	0
ENSG000001	SLC28A1	protein_codin	0	0	0	0
ENSG000000	PCDHGA2	protein_codin	0	0	0	0
ENSG000001	RPL36	protein_codin	1.74532119	2.86039445	20.2193957	3.59237283
ENSG000001	ZNF326	protein_codin	38.5737632	334.766395	143.452869	34.2008352
ENSG000000	FUT8	protein_codin	0	0	0	0
ENSG000001	NMB	protein_codin	0.32889714	1.68009436	1.47024936	0

ENSG000001	RAD51D	protein_codin	0	0	0	0
ENSG000001	LRRC8D	protein_codin	0	36.312617	0	4.19669169
ENSG000001	PITX2	protein_codin	0	0	0	0
ENSG000000	RFFL	protein_codin	6.20661353	32.506559	5.54191078	0
ENSG000000	MTIF2	protein_codin	16.1308702	0	99.6250465	12.5931559
ENSG000000	PTBP1	protein_codin	0	0	0	0
ENSG000001	SLC26A8	protein_codin	0	0	0	0
ENSG000001	CLDN12	protein_codin	0	0	0	0
ENSG000001	CLDN12	protein_codin	20.5640182	0	0	10.7902867
ENSG000001	MAX	protein_codin	0	6.29635616	0	0
ENSG000001	ELOVL6	protein_codin	15.5974252	24.8203271	14.9398709	6.98328748
ENSG000001	RTN4	protein_codin	42.0436681	6.55754589	37.6834046	43.0573597
ENSG000001	SEPTIN6	protein_codin	6.29564932	53.147492	16.4508305	11.2465809
ENSG000001	RTN4	protein_codin	31.8952243	0	0	17.4219561
ENSG000001	PLAAT3	protein_codin	8.37664787	9.81359189	0.61970171	0
ENSG000001	PLAAT5	protein_codin	0	3.52711722	0	0
ENSG000001	LGALS12	protein_codin	0	0	0	0
ENSG000001	CCL8	protein_codin	1.68184334	30.8635041	0	7.15596426
ENSG000001	STEAP2	protein_codin	6.01914532	23.8741434	3.02762933	0
ENSG000001	STEAP2	protein_codin	0	0	38.0583532	4.02591608
ENSG000001	STEAP2	protein_codin	0	0	0	0
ENSG000001	CCL7	protein_codin	0	0	0	0
ENSG000001	STEAP2	protein_codin	0	0	0	0
ENSG000001	CCL7	protein_codin	0	0	0	0
ENSG000001	GAR1	protein_codin	0	10.1049188	0	0
ENSG000001	STEAP2	protein_codin	0	0	0	0
ENSG000002	PCDHA1	protein_codin	0	0	0	1.16136193
ENSG000002	CFI	protein_codin	0	2.00973478	0	0
ENSG000002	CFI	protein_codin	0	0	0	0
ENSG000001	IRX5	protein_codin	14.6168966	71.2154389	77.3897687	22.3619638
ENSG000001	SPACA3	protein_codin	0	0	0	0
ENSG000001	SPACA3	protein_codin	0	0	0	0
ENSG000000	SRI	protein_codin	0	0	0	0
ENSG000000	TMEM98	protein_codin	14.0108104	29.0363279	6.79954306	7.97984377
ENSG000001	RGS3	protein_codin	0	0	0	0.67255832
ENSG000001	MYO1D	protein_codin	14.7201032	0	6.01196618	6.59083623
ENSG000000	MCUB	protein_codin	1.22368126	45.8443317	7.93490579	2.90147736
ENSG000001	IL16	protein_codin	11.7439346	44.7943154	0	4.70362013
ENSG000001	RUNDC3B	protein_codin	1.67996011	6.1960969	2.93847214	0
ENSG000001	AKTIP	protein_codin	0.95839236	3.64942519	0	8.7127701
ENSG000001	IL16	protein_codin	0	0	0	0
ENSG000001	RPL34	protein_codin	2.12171749	0	8.40673405	0
ENSG000001	ACYP2	protein_codin	2.35543728	0	4.59801751	0
ENSG000001	RPL34	protein_codin	169.933827	0	418.201947	0

ENSG000001	RPL34	protein_codin	4.01863734	5.70646042	14.8431951	13.98701
ENSG000001	TMCO6	protein_codin	0	0	0	0
ENSG000000	ZNF207	protein_codin	82.9764673	213.046523	79.3330028	77.4920025
ENSG000001	TMCO6	protein_codin	0	0	0	0
ENSG000001	CCDC66	protein_codin	16.3872354	32.7130594	41.1737673	0
ENSG000000	ZNF207	protein_codin	19.7836441	0	33.2474122	18.7298173
ENSG000000	ZNF207	protein_codin	0	0	0	0
ENSG000001	SGMS2	protein_codin	16.1803108	229.463428	103.461773	107.88964
ENSG000001	CEMIP	protein_codin	0	0	0	0
ENSG000001	SGMS2	protein_codin	0	8.32132069	3.36160439	1.28115331
ENSG000001	BRD7	protein_codin	10.7917112	123.921086	0	0
ENSG000001	BRD7	protein_codin	64.7110102	87.6888131	182.802589	28.8071703
ENSG000001	STX5	protein_codin	12.5942459	0	12.1770172	6.24999198
ENSG000001	PLEKHG3	protein_codin	0	0	11.3103656	0
ENSG000001	RHOT1	protein_codin	0	0	0	0
ENSG000001	TCP11	protein_codin	0	0	0	0
ENSG000001	ADCY7	protein_codin	0	82.6171808	0	0
ENSG000001	AIMP1	protein_codin	0	0	0	0
ENSG000001	DMTF1	protein_codin	0	0	0	0
ENSG000001	GPR75	protein_codin	0.8419465	8.46550917	0.96124819	1.2333259
ENSG000001	TBCK	protein_codin	0	0	0	1.78639362
ENSG000001	TBCK	protein_codin	9.63540719	27.2017978	11.420535	9.72991274
ENSG000001	HSPA2	protein_codin	1.13534542	0	0	2.00731207
ENSG000000	CLCA1	protein_codin	0	0	0	0.47371766
ENSG000001	ELAPOR2	protein_codin	0	0	34.7873465	0
ENSG000000	ZBTB25	protein_codin	0.65279631	17.0249483	10.2437905	0
ENSG000001	ASB3	protein_codin	16.220502	0	36.1516069	1.07392536
ENSG000001	AKAP5	protein_codin	5.63812311	40.6209441	15.4021281	4.83952246
ENSG000001	ANKHD1	protein_codin	0.64735728	0	4.60947081	9.7333884
ENSG000001	ANKHD1	protein_codin	0	0	0	0
ENSG000001	SIAH1	protein_codin	10.3991332	0	7.89056528	0
ENSG000001	GSTCD	protein_codin	0	0	0	0
ENSG000001	PRKCD	protein_codin	0	0	0	0
ENSG000001	GSTCD	protein_codin	7.04450027	12.3107766	16.6599348	3.65087366
ENSG000001	ODF2L	protein_codin	0	0	0	0
ENSG000001	INTS12	protein_codin	0	0	0	0
ENSG000001	RFT1	protein_codin	5.28024293	4.32396354	2.87600645	0
ENSG000001	RAB11FIP4	protein_codin	2.20658037	0	40.0312794	3.91816979
ENSG000000	RASGRF1	protein_codin	0	4.83344693	0	0
ENSG000001	ABCC11	protein_codin	0	0	0	1.1969753
ENSG000001	ABCC11	protein_codin	0	0	0	0
ENSG000000	STON1-GTF2/	protein_codin	0	38.2257199	46.6730256	0
ENSG000001	SFMBT1	protein_codin	18.7360376	49.6603834	37.0794087	13.9398124
ENSG000000	STON1-GTF2/	protein_codin	0	0	0	0

ENSG000001	CAMK2G	protein_codin	0	0	0	0
ENSG000001	UBXN6	protein_codin	15.9630564	0	0	0
ENSG000001	CXXC4	protein_codin	5.77970055	0	34.3910751	1.40347527
ENSG000000	SYNE2	protein_codin	0	0	2.2311823	2.27993601
ENSG000001	EML3	protein_codin	0	14.5373104	16.9625831	0
ENSG000001	CCDC121	protein_codin	0	0	15.0876589	7.51770993
ENSG000001	EML3	protein_codin	0	0	0	0
ENSG000001	SHOC1	protein_codin	0	0	0	0
ENSG000001	SHOC1	protein_codin	0	0	0	0
ENSG000001	ALG13	protein_codin	0	0	0	8.74123797
ENSG000000	GLT8D1	protein_codin	0	0	0	0
ENSG000001	SLC9B2	protein_codin	10.0575558	9.90313847	9.50452807	36.8944733
ENSG000001	CD36	protein_codin	0	0	0	0
ENSG000001	SLC9B1	protein_codin	0	0	0	0
ENSG000001	FUT11	protein_codin	1.6147258	1.65556485	1.21371567	2.62782569
ENSG000002	ENSG000002	protein_codin	0	2.76073213	0	0
ENSG000001	PAIP2	protein_codin	1.49223669	7.59074336	3.52755981	1.6494536
ENSG000001	CHRDL1	protein_codin	2.47924411	0	0	0
ENSG000001	GNL3	protein_codin	0	0	0	0
ENSG000002	MATR3	protein_codin	0	0	0	0
ENSG000001	UBE2D3	protein_codin	2.17594277	41.9179432	10.6303139	0
ENSG000001	CHRNA5	protein_codin	0	1.48255699	7.7489527	0
ENSG000001	UBE2D3	protein_codin	29.8615189	12.6758928	65.7153683	18.1992231
ENSG000001	UBE2D3	protein_codin	0	0	1.33242881	3.95514786
ENSG000000	MATR3	protein_codin	21.5570243	25.2582887	35.7949062	18.2632497
ENSG000001	C16orf87	protein_codin	0	0	0	0
ENSG000001	ZBTB3	protein_codin	4.21875323	3.65050399	5.78306972	2.13121075
ENSG000001	MYLK3	protein_codin	0.9404808	0	0	3.51546424
ENSG000001	SYNPO2L	protein_codin	9.56969314	0	0	2.89129418
ENSG000001	KRT80	protein_codin	0	0	0	0
ENSG000001	SIL1	protein_codin	21.8793049	43.7000804	41.9878561	16.1986466
ENSG000001	INCENP	protein_codin	37.2587348	202.84568	304.064604	8.4805602
ENSG000001	NFKB1	protein_codin	6.21170189	14.5253562	0	16.3250541
ENSG000001	SLC6A4	protein_codin	0.53580063	0	0.4346853	0
ENSG000001	NR4A1	protein_codin	0	0	0	5.42735301
ENSG000001	NR4A1	protein_codin	3.61649534	0	0	37.4516784
ENSG000001	NSRP1	protein_codin	113.100406	310.923251	224.436758	87.581612
ENSG000001	PPP3CB	protein_codin	0	70.7543762	46.0932721	0
ENSG000001	PPP3CB	protein_codin	50.267533	0	0	18.0620319
ENSG000001	PBRM1	protein_codin	27.148827	140.369942	15.1492495	0
ENSG000001	SLC39A8	protein_codin	0.89486305	27.8805667	0	0
ENSG000002	SAMD13	protein_codin	0	0	0	0
ENSG000001	EFCAB5	protein_codin	0	0	0	0
ENSG000001	RAB3IL1	protein_codin	14.3292105	47.7265853	10.1603508	9.8270446

ENSG000001	PRKACB	protein_codin	0	0	0	2.32760573
ENSG000001	ZNF267	protein_codin	1.31056675	34.137296	0	2.16194517
ENSG000001	ANXA7	protein_codin	0	0	0	0.45098177
ENSG000001	SSH2	protein_codin	7.30676272	27.7929876	14.3289638	0
ENSG000002	UPK3B	protein_codin	0	0	0.54544589	0
ENSG000000	TTC7A	protein_codin	0	0	0	0
ENSG000001	DNAJA4	protein_codin	4.39427126	0	15.9365673	14.9828869
ENSG000001	PPP3CA	protein_codin	0	65.7076126	15.7341446	0
ENSG000001	PPP3CA	protein_codin	14.733335	66.2375123	0	41.6012874
ENSG000001	DNAJA4	protein_codin	0	0	11.2643664	0
ENSG000001	ZP3	protein_codin	0	1.69404506	0	0
ENSG000001	TGFB1I1	protein_codin	0	0	0	0
ENSG000001	ANKRD13B	protein_codin	0	3.69506707	0	0
ENSG000001	ZP3	protein_codin	0	0	0	0
ENSG000001	SOCS5	protein_codin	26.8934028	40.8638533	52.0290826	24.7175747
ENSG000001	TGFB1I1	protein_codin	17.8312844	0	0	0
ENSG000001	CFAP70	protein_codin	0	0	0	0
ENSG000001	MAP2K2	protein_codin	0	11.9146432	0	0
ENSG000001	GIT1	protein_codin	0	0	0	0
ENSG000001	COL4A6	protein_codin	0	0	0	0
ENSG000001	PRKCE	protein_codin	4.35733708	6.32316644	11.8463709	1.81298966
ENSG000001	TRMT10A	protein_codin	4.32756592	0	22.5327892	0
ENSG000001	TRMT10A	protein_codin	3.62034807	8.28081547	0	0
ENSG000001	RCN2	protein_codin	0	0	13.4722619	0
ENSG000000	CDC23	protein_codin	0.82234639	8.46867291	4.59116647	0
ENSG000001	RCN2	protein_codin	1.05513907	2.10010679	3.83113974	0.59343445
ENSG000000	CDC23	protein_codin	18.0459233	37.4150742	31.9729304	9.8477714
ENSG000001	CPSF7	protein_codin	25.6610911	70.2867604	51.6883076	24.4332247
ENSG000001	P4HA1	protein_codin	32.690966	94.9949596	102.109194	43.2495224
ENSG000001	ATG4A	protein_codin	0	0	0	0
ENSG000001	POR	protein_codin	0	3.74694782	0	0
ENSG000001	KIF20A	protein_codin	8.98073866	26.4623925	208.982121	0
ENSG000001	ADH6	protein_codin	0	0	0	0
ENSG000001	ADH6	protein_codin	0	1.61550042	0	0
ENSG000001	TKFC	protein_codin	3.4036168	2.14160241	5.48945492	4.19090331
ENSG000001	DHRS13	protein_codin	0	0	0	0
ENSG000001	DNAJB12	protein_codin	0	0	0	0
ENSG000001	SLC11A2	protein_codin	0	0	0	0
ENSG000000	CCL26	protein_codin	0	0	0	0
ENSG000001	FLOT2	protein_codin	0	0	0	0
ENSG000001	NRG4	protein_codin	0	0	0	0
ENSG000001	FLOT2	protein_codin	5.91934358	39.6423739	12.59232	5.45104554
ENSG000001	ZNF16	protein_codin	4.50308184	6.78542067	10.9813696	3.61285958
ENSG000001	ASCC1	protein_codin	0	0	0	0

ENSG000001(	ZNF12	protein_codin	0	1.18581439	0	0
ENSG000001(	RPL8	protein_codin	1.0761279	0	77.5837351	3.54390391
ENSG0000007	TRAF4	protein_codin	0	0	0.64322998	0.81446775
ENSG000001(	FSD1L	protein_codin	0	16.3056276	7.46181615	0
ENSG000001:	BMPR1B	protein_codin	0	0	0	0
ENSG000001(	TLCD1	protein_codin	0	0	0	0
ENSG000001!	RPL23A	protein_codin	24.4001942	0	0	15.1761948
ENSG000001!	PSAP	protein_codin	460.501588	1366.26547	388.448866	413.015743
ENSG000001!	RPL23A	protein_codin	0	0	7.46354239	0
ENSG000001	WDR89	protein_codin	11.0913969	0	0	7.93955298
ENSG000000:	LIMA1	protein_codin	0	0	0	0
ENSG000001:	SPOCK1	protein_codin	12.3163799	20.2440946	2.92996275	9.99313688
ENSG000001(	SIN3A	protein_codin	9.24854676	14.7800161	5.66277098	32.7629617
[truncated: 496,671 more chars]
